# dRHP-PseRA: detecting remote homology proteins using profile-based pseudo protein sequence and rank aggregation

**DOI:** 10.1038/srep32333

**Published:** 2016-09-01

**Authors:** Junjie Chen, Ren Long, Xiao-long Wang, Bin Liu, Kuo-Chen Chou

**Affiliations:** 1 School of Computer Science and Technology, Harbin Institute of Technology Shenzhen Graduate School, Shenzhen, Guangdong 518055, China; 2 Key Laboratory of Network Oriented Intelligent Computation, Harbin Institute of Technology Shenzhen Graduate School, Shenzhen, Guangdong 518055, China; 3 Gordon Life Science Institute, Boston, MA 02478, USA; 4 Center of Excellence in Genomic Medicine Research (CEGMR), King Abdulaziz University, Jeddah 21589, Saudi Arabia

## Abstract

Protein remote homology detection is an important task in computational proteomics. Some computational methods have been proposed, which detect remote homology proteins based on different features and algorithms. As noted in previous studies, their predictive results are complementary to each other. Therefore, it is intriguing to explore whether these methods can be combined into one package so as to further enhance the performance power and application convenience. In view of this, we introduced a protein representation called profile-based pseudo protein sequence to extract the evolutionary information from the relevant profiles. Based on the concept of pseudo proteins, a new predictor, called “dRHP-PseRA”, was developed by combining four state-of-the-art predictors (PSI-BLAST, HHblits, Hmmer, and Coma) via the rank aggregation approach. Cross-validation tests on a SCOP benchmark dataset have demonstrated that the new predictor has remarkably outperformed any of the existing methods for the same purpose on ROC50 scores. Accordingly, it is anticipated that dRHP-PseRA holds very high potential to become a useful high throughput tool for detecting remote homology proteins. For the convenience of most experimental scientists, a web-server for dRHP-PseRA has been established at http://bioinformatics.hitsz.edu.cn/dRHP-PseRA/.

In the post-genomic age, protein sequence database (such as UniProtKB^1^) has been greatly enriched benefited from the rapid development of sequencing technology, while protein structure and function data in PDB^2^ is growing relatively much slower. Such a gap is increasingly getting enlarged^3^. To deal with this situation, it is critical to use the sequence data to infer the structures and functions of proteins^4^. Because protein structure is more conserved than sequences, proteins sharing low sequence similarities might have similar structures, known as remote homologs. Protein remote homology detection is aimed at finding the remote homologs with known structures and functions^4^. Unfortunately, it remains a challenging task in computational biology due to the low sequence identities.

Protein remote homology detection has been studied for a long time, and many researchers have proposed various approaches to address this task. They can be categorized into three groups^4^,^5^,^6^: (1) alignment method, (2) discriminative method, and (3) ranking method.

The alignment method is the traditional detection one, which identifies the remote homology relationships by using the pairwise alignment scores via a specified threshold. The early approaches were based on the sequence alignment tools, such as Blast^7^ and FASTA^8^. Owing to the low similarity among remote homologous proteins, their performance was quite limited. By considering the information from the multiple sequence alignments (MSA), the profile alignment approaches were proposed to improve the detection sensitivity. For examples, PSI-BLAST^9^ and IMPALA^10^ are two sequence-alignment methods, while COMPASS^11^, FFAS^12^,^13^,^14^, SPARK-X^15^ and COMA^16^ are the methods based on profile-profile alignment. The latter have achieved much better results than the former. In comparison with the sequence/profile alignment, however, the profile Hidden Markov Model (profile-HMM) alignment approaches (such as Hmmer^17^ and HHblits^18^) can further take into account the position-specific probabilities for insertions and deletions, and hence can achieve even better performance.

The discriminative method refers to classification models based on machine learning techniques. It can be used to classify a new protein into one of the superfamilies. Many machine learning techniques (such as RF^19^, NN^20^, SVM^21^) were used to train the models, in which SVM achieved the state-of-the-art performance^22^, such as SVM-fisher^23^, SVM-DR^24^, SVM-LA^25^, SVM-LSA^26^, SVM-pairwise^27^, and SVM-PDT^28^. Most of them can construct the kernel-based feature vectors by using the pairwise score output by the alignment approaches. Unfortunately, since these approaches require the labelled samples for training the models, they cannot work for those proteins whose superfamilies or families are still unknown. Besides, it is often difficult to construct useful web-servers or standalone tools for these classification models.

The ranking method is with the idea to build a ranking model to detect the remote homologs relationships. Similar to the alignment method, the ranking approach is also based on the estimated score over a specific threshold. But the ranking method is featured by training the ranking model according to the ranking list returned from the basic method to construct a kernel-based feature space, and measure the homology relationship by using the distance in the feature space. Based on the PageRank algorithm of Google^29^, an unsupervised graph diffusion-based method called RankProp^30^ was proposed, which built a protein similarity network. Motivated by the techniques in the field of natural language processing, ProtEmbed^31^ employs a large-scale semantic embedding method to learn a semantic embedding of protein sequences. Recently, ProtDec-L2R^6^ is proposed, which combines various ranking approaches via a learning to rank algorithm.

The aforementioned computational methods have considerably stimulated the development of protein remote homology detection. However, there is still some further work needed to do because of the following reasons. (1) Since remote homologous proteins share very low sequence similarity (<30%), a more accurate protein representation by incorporating the evolutionary information into the profiles is needed. (2) The outcomes of homology detection methods based on different techniques and models are complementary with each other; hence, it would be much more efficient to develop a new framework by which to combine them into one. (3) Although several tools or web-servers have been proposed, most of them are not suitable for large scale analysis due to the high computational cost; in this sence an easy-to-use web-servers or stand-alone tools will be certainly welcome.

To address the aforemention three points, we construct a profile-based pseudo protein sequence to replace the original protein sequence. This protein representation approach can transform the evolutionary information of profiles into a pseduo protein sequence. Furthermore, the new approch is featured by combining a rank aggregation method. The newly proposed predictor thus formed is called dRHP-PseRA. Finally, a web-server for dRHP-PseRA is established, and it is avaliable at http://bioinformatics.hitsz.edu.cn/dRHP-PseRA/. The detailed usage about this webserver can be found in the ReadMe page.

## Results and Discussion

### Performance of different predictors can be improved by profile-based pseudo protein sequences

The four state-of-the-art predictors, namely PSI-BLAST^9^, HHblits^18^, Hmmer^17^, and Coma^16^, are selected to verify whether the proposed pseudo protein representation can improve their performance or not. The corresponding results were listed in Table 1. As we can see, the pseudo protein representation can improve the performance of PSI-BLAST, Hmmer, and Coma, as reflecting by both the ROC1 and ROC50 scores. Such outcomes are not surprising at all since the pseudo proteins contain the evolutionary information from the relevant profiles. Consequently, they are more smart and accurate than the original sequence representation in detecting remote homology proteins. One exception is that the pseudo protein representation cannot improve the performance of HHblits. This is because HMM model has already incorporated the evolutionary information via the position-specific probabilities for insertions and deletion.

### Combining complementary predictors via the rank aggregation approach

**Figure 1:**
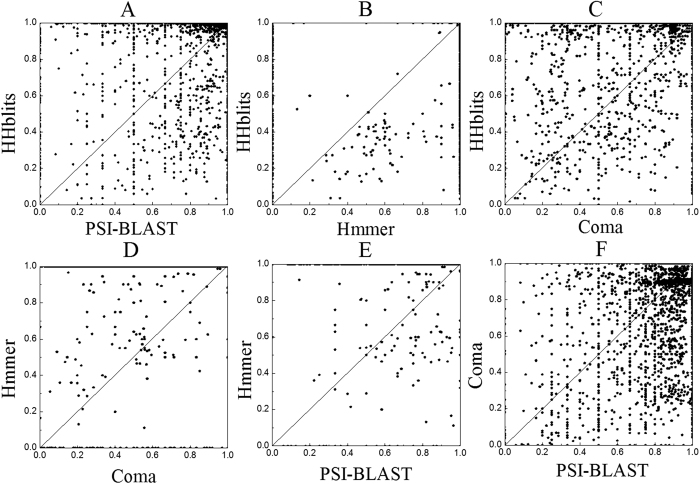
Pairwise comparison results of the four methods. The coordinates of the points in the plot represent the ROC1 scores obtained by the two methods labeled near the axis.

**Figure 2:**
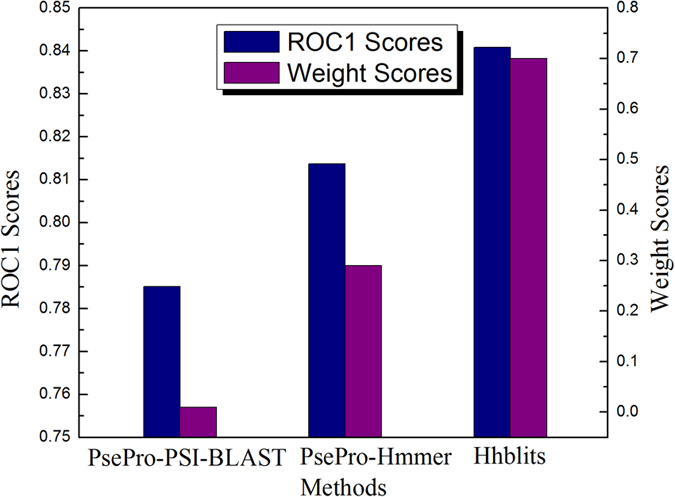
The correlation between weight values and performance of different methods.

**Figure 3:**
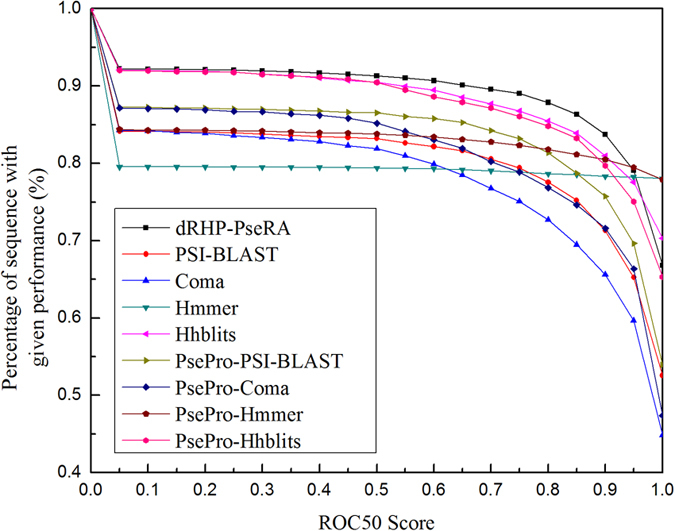
Comparisons of various methods. The graph plots the percentage of sequences for which the method exceeds a given performance. The higher curve means the method performs better.

As shown in Table 1, the performances of various predictors on the same benchmark dataset are quite uneven. They can be combined together to improve the performance. The pairwise comparison results of the four basic predictors are shown in Fig. 1, from which we can see that for each sub-figure most of the points are located at the both sides of diagonal line and only a few points are located on the diagonal line, indicating that their predictive results are complementary to each other. Various combinations of these predictors are combined via the proposed linear weighting rank aggregation approach (see the Method section later). The dRHP-PseRA predictor shows the best performance when combining the three methods PsePro-PSI-BLAST, PsePro-Hummer, and HHblits, with the corresponding weights being 0.01, 0.29 and 0.7, respectively (Table 1). The correlations between the weight values and the ROC1 scores of the three methods are plotted in Fig. 2, from which we can see that the method with higher performance is assigned higher weight value, indicating that the rank aggregation approach is able to reflect the different importance of the three predictors. The performance of each method is plotted in Fig. 3, where a larger area under the curve means a better performance. As we can see from the figure, dRHP-PseRA obviously outperforms other predictors on ROC50 score, indicating that combining different predictors via a rank aggregation approach is indeed a quite promising strategy, and that dRHP-PseRA is a more powerful predictor for protein remote homology detection.

## Discussion

Protein remote homology detection is a key technique for studying protein structures and functions. However, it is still a big challenging task since remote homologous proteins usually share very low sequence similarities (<30%). Although several computational methods have been proposed, their performances are still too low for many practical applications. In this paper, we introduced the profile-based pseudo protein sequence formulation derived from protein profile, and found that it was quite useful to improve the performance compared with their individual approaches. Based on such interesting findings, a novel predictor called dRHP-PseRA is proposed by combining the aforementioned four state-of-the-art predictors into one framework through the pseudo protein approach. Experimental results show that dRHP-PseRA outperforms each of the individual predictors based on ROC50 scores. Furthermore, a user-friendly web-server for dRHP-PseRA has been established at http://bioinformatics.hitsz.edu.cn/dRHP-PseRA/.

It is instructive to point out that, in addition to the four basic predictors selected in the current study, there are some other methods as well in the area of protein remote homology detection, such as FFAS^12^,^13^,^14^, SPARK-X^15^. It would be intriguing to extend the current study by exploring whether these methods can also be incorporated into the proposed ensemble learning framework, and to further improve the performance. We will address this interesting problem in our future study.

## Materials and Methods

### Benchmark Datasets

**Figure 4:**
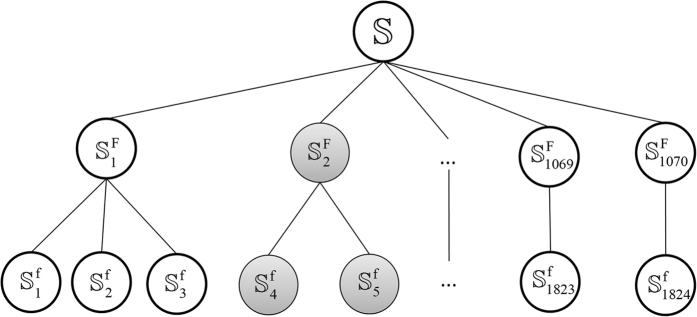
A schematic drawing to show the dataset for protein remote homology detection. For a query protein **P** in family 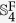, the aim is to find the proteins in the superfamily 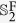 (gray circles).

In this study, we adopted a commonly used benchmark dataset^31^, which was constructed based on the SCOP database and the sequences were extracted from Astral^32^. Because this benchmark data is used to evaluate the performance of the un-supervised methods (training set is not required), a higher similarity threshold score of 95% was used to exclude the redundancy. Therefore, the similarity between any two sequences must be lower than 95%. The benchmark dataset contains 7329 protein sequences from 1824 families and 1070 superfamilies (Fig. 4), which can be defined as


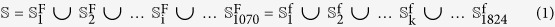


where 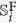 (*i* = 1, 2,…, 1070) represents the *i*-th superfamily; 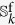 (*k* = 1, 2,…, 1824) represents the *k*-th family, and the symbol ∪ represents the ‘union’ in the set theory. The benchmark dataset is given in the Supplementary Information S1.

First, for a given query protein **P**, we search its potential homologues against 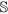. According to the searched results, we can form a rank vector **R** with its components in a decending order


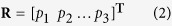


where *p*_*i*_ (*i* = 1, 2, …, n) represents the *i*-th homologous sequence with **P** in **R**; *n* is the total number of potential proteins in the ranking list of **R**; and **T** denotes the traspose operator. If all the query protein’s homologous proteins are ranked before the non-homologous ones, then the prediction is perfect.

### Descriptions of four predictors

For the reason of diversity and mutually complementary, here we selected the following four state-of-the-art ranking methods as the basic predictors: PSI-BLAST^9^, HHblits^18^, Hmmer^17^ and Coma^16^.

PSI-BLAST is a profile-sequence alignment method, which uses the query proteins to construct profiles and iteratively searches the sequence database. In this study, the PSI-BLAST version 2.2.30 was employed with the iterations times set at 3.

HHblits is a HMM-HMM alignment method, which constructs a HMM model for both the sequence of the query protein **P** and the sequences in the database, and then iteratively searches the query HMM profile against the database of HMM profiles. HHblits version 2.0.16 was employed with the default parameters except that the maximum time of iterations was set at 2.

Hmmer is a method based on probabilistic inference and HMM model. In this study, the Hmmer version 3.1b2 with default parameters was used.

Coma is a profile-profile alignment method adopting position-dependent gap penalties and a global score system. The multiple sequence alignments generated by using PSI-BLAST version 2.2.30 are fed into the Coma for calculation. In this study, the Coma version 1.10 with default parameters was employed.

### Profile-based pseudo protein sequence

Remote homology proteins have very low sequence similarities (<30%), therefore only based on sequence information is not enough for accurate homology detection. As demonstrated in previous studies^33^, evolutionary information extracted from profiles is useful for improving protein remote homology detection. Here, we construct the protein representation by using the proposed method in these studies^5^,^33^. The main steps of generating the profile-based pseudo protein sequence representation are simply descripted as following.

Firstly, for a protein sequence **P**, it is searched against the NCBI’s nrdb90 database by running PSI-BLAST^9^ with parameters (-j 10, -e 0.001) to generate a MSA. Then the frequency profile of sequence **P**, a matrix **M** of size 20*L (20 is the number of native amino acids and *L* is the length of sequence **P**), can be calculated based on the frequency of each amino acid at each site in generated MSA.

Secondly, for each column in **M**, we sort the amino acids in the descending order according to their frequency values, and then select the amino acids with the maximal frequency value in each column. These selected amino acids are combined to form a new pseudo protein sequence, which is called profile-based pseudo protein sequence. The higher scores in **M** represent more conserved sites in protein sequence **P**. Such representation of proteins defined by frequency profiles would be more sensitivity than raw protein sequences for detecting remote homologs.

The profile-based pseudo protein sequences were used to replace the raw protein sequences as inputs for the aforementioned four predictors without the need to modify the programs.

### Rank aggregation

The aim of rank aggregation is to combine different ranking lists (Eq. 2) so as to obtain more accurate ranking results^34^. In this study, a rank aggregation method based on the linear weighting method was introduced to combine various methods, as described below.

Given *k* ranking lists (Eq. 2) generated by *k* predictors, the rank aggregation calculates a rank aggregation score *S*(*p*_*i*_) between a query protein **P** and a potential homologous protein *p*_*i*_ in the database 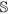, which can be defined as


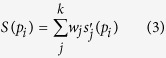


where *w*_*j*_ (*j* = 1, 2,…*k*) is the weight of *j*-th predictor; 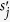
(*p*_*i*_) (*i* = 1, 2, … *n*) is the normalized alignment score between protein **P** and protein *p*_*i*_ calculated by the *j*-th predictor; and 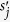
(*p*_*i*_) can be calculated by


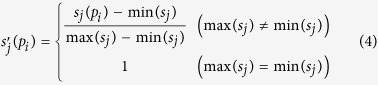


where *s*_*j*_(*p*_*i*_) (*i* = 1, 2, …, *n*) is the aligned score between query protein **P** and *p*_*i*_ given by the *j*-th predictor, max(*s*_*j*_) and min(*s*_*j*_) present the maximum and minimum aligned scores returned by the *j*-th predictor for the query protein **P**, respectively.

**Figure 5:**
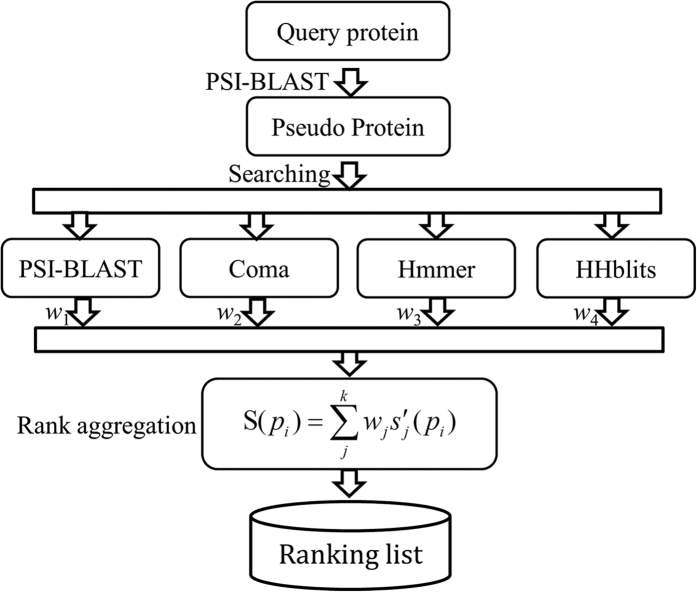
The flowchart of dRHP-PseRA. Proteins are replaced by their corresponding pseudo proteins, and then fed into predictors for protein remote homology detection. Finally, the ranking lists generated by these predictors are combined via a linear weighting rank aggregation approach.

Larger rank aggregation score *S*(*p*_*i*_) means the query protein **P** and protein *p*_*i*_ has closer homologous relationship. Consequently, the rank aggregation approach will automatically sort the proteins in 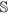 in a descending order according to their rank aggregation scores. By means of such an approach, various ranking lists generated by different predictors can be combined into a framework to produce a more accurate ranking list. Figure 5 is a flowchart of the proposed dRHP-PseRA predictor based on the rank aggregation approach.

### Evaluation method of performance

The jackknife or leave-out-out test was employed in remote homology detection. The jackknife test is deemed the most objective cross-validation approach^3^. ROC1 and ROC50 scores are used to evaluate the performance of various predictors^4^. ROC1 and ROC50 represent the area under the ROC curve^35^ when first false positive and fiftieth false positives appear, respectively. The larger score means a better performance.

## Additional Information

**How to cite this article**: Chen, J. *et al*. dRHP-PseRA: detecting remote homology proteins using profile-based pseudo protein sequence and rank aggregation. *Sci. Rep*. **6**, 32333; doi: 10.1038/srep32333 (2016).

## Supplementary Material

Supplementary Information

## Figures and Tables

**Table 1:** The performance of various predictors on the benchmark dataset 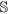.

Methods	ROC1	ROC50
PSI-BLAST	0.7506	0.8008
HHblits	0.8409	0.8827
Hmmer	0.7894	0.7915
Coma	0.6989	0.7785
PsePro-PSI-BLAST^a^	0.7851	0.8361
PsePro-HHblits^b^	0.8238	0.8781
PsePro-Hmmer^c^	0.8137	0.8302
PsePro-Coma^d^	0.7345	0.8152
dRHP-PseRA^e^	0.8314	0.8924

^a^Represents the PSI-BLAST predictor combined with pseudo proteins.

^b^Represents the HHblits predictor combined with pseudo proteins.

^c^Represents the Hmmer predictor combined with pseudo proteins.

^d^Represents the Coma predictor combined with pseudo proteins.

^e^Represents the dRHP-PseRA method combining three predictors (PsePro-PSI-BLAST, PsePro-Hmmer, and HHblits) via a linear weighting rank aggregation approach.
