# Supplementary material for: dRHP-PseRA: detecting remote homology proteins using profile-based pseudo protein sequence and rank aggregation
Source: Sci Rep. 2016 Sep 1;6:32333. doi: 10.1038/srep32333 (PMC5007510; doi:10.1038/srep32333)
Supplement: Supplementary Information [file srep32333-s1.pdf]

# Supplementary Information for

# dRHP-PseRA: detecting remote homology proteins using profile-based pseudo protein sequence and rank aggregation

Junjie Chen, Ren Long, Xiao-long Wang, Bin Liu, Kuo-Chen Chou

**Supplementary Information S1.** The benchmark dataset 𝕊 contains 7329 sequences from 1824 families $\mathbb{S}^f$ and 1070 superfamilies $\mathbb{S}^F$, which were extracted from the Astral database. None of the sequences included has ≥ 95% pairwise sequence identity to any other. See the main text for further explanation.

---

```
>d1dlwa_ a.1.1.1 (A:) Truncated hemoglobin {Ciliate (Paramecium
caudatum)}
SLFEQLGGQAAVQAVTAQFYANIQADATVATFFNGIDMPNQTNKTAAFLCAALGGPNAWTGR
NLKEVHANMGVSNAQFTTVIGHLRSALTGAGVAAALVEQTVAVAETVRGDVVTV
>d1dlya_ a.1.1.1 (A:) Truncated hemoglobin {Green alga
(Chlamydomonas eugametos)}
SLFAKLGGREAVEAAVDKFYNKIVADPTVSTYFSNTDMKVQRSKQFAFLAYALGGASEWKGK
DMRTAHKDLVPHLSDVHFQAVARHLSDTLTELGVPPEDITDAMAVVASTRTEVLNMPQQ
>d1idra_ a.1.1.1 (A:) Truncated hemoglobin {Mycobacterium
tuberculosis}
GLLSRLRKREPISIYDKIGGHEAIEVVVEDFYVRVLADDQLSAFFSGTNMSRLKGKQVEFFA
AALGGPEPYTGAPMKQVHQGRGITMHHFSLVAGHLADALTAAGVPSETITEILGVIAPLAVD
VTS
>d1scta_ a.1.1.2 (A:) Hemoglobin I {Ark clam (Scapharca
inaequivalvis)}
VDAAVAKVCGSEAIKANLRRSWGVLSADIEATGLMLMSNLFTLRPDTKTYFTRLGDVQKGKA
NSKLRGHAITLTYALNNFVDSLDDPSRLKCVVEKFAVNHINRKISGDAFGAIVEPMKETLKA
RMGNYYSDDVAGAWAALVGVVQAAL
>d1sctb_ a.1.1.2 (B:) Hemoglobin I {Ark clam (Scapharca
inaequivalvis)}
KVAELANAVVSNADQKDLLRMSWGVLSVDMEGTGLMLMANLFKTSPSAKGKFARLGDVSAGK
DNSKLRGHSITLMYALQNFVDALDDVERLKCVVEKFAVNHINRQISADEFGEIVGPLRQTLK
ARMGNYFDEDTVAAWASLVAVVQASL
>d3sdha_ a.1.1.2 (A:) Hemoglobin I {Ark clam (Scapharca
inaequivalvis)}
SVYDAAAQLTADVKKDLRDSWKVIGSDKKGNGVALMTTLFADNQETIGYFKRLGNVSQGMAN
DKLRGHSITLMYALQNFIDQLDNPDDLVCVVEKFAVNHITRKISAAEFGKINGPIKKVLASK
NFGDKYANAWAKLVAVVQAAL
>d1b0b__ a.1.1.2 (-) Hemoglobin I {Clam (Lucina pectinata)}
SLSAAQKDNVKSSWAKASAAWGTAGPEFFMALFDAHDDVFAKFSGLFSGAAKGTVKNTPEMA
AQAQSFKGLVSNWVDNLDNAGALEGQCKTFAANHKARGISAGQLEAAFKVLAGFMKSYGGDE
GAWTAVAGALMGMIRPDM
>d1h97a_ a.1.1.2 (A:) Trematode hemoglobin/myoglobin
{Paramphistomum epiclitum}
TLTKHEQDILLKELGPHVDTPAHIVETGLGAYHALFTAHPQYISHFSRLEGHTIENVMQSEG
IKHYARTLTEAIVHMLKEISNDAEVKKIAAQYGKDHTSRKVTKDEFMSGEPIFTKYFQNLVK
DAEGKAAVEKFLKHVFPMMAAEI
>d1vrea_ a.1.1.2 (A:) Glycera globin {Marine bloodworm (Glycera
dibranchiata)}
```

GLSAAQRQVVASTWKDIAGSDNGAGVGKECFTKFLSAHHDMAAVFGFSGASDPGVADLGAKV
LAQIGVAVSHLGDEGKMVAEMKAVGVRHKGYGNKHIKAEYFEPLGASLLSAMEHRIGGKMNA
AAKDAWAAAYADISGALISGLQS
>d2hbg__ a.1.1.2 (-) Glycera globin {Marine bloodworm (Glycera dibranchiata)}
GLSAAQRQVIAATWKDIAGADNGAGVGKKCLIKFLSAHPQMAAVFGFSGASDPGVAALGAKV
LAQIGVAVSHLGDEGKMVAQMKAVGVRHKGYGNKHIKAQYFEPLGASLLSAMEHRIGGKMNA
AAKDAWAAAYADISGALISGLQS
>d1a6m__ a.1.1.2 (-) Myoglobin {Sperm whale (Physeter catodon)}
VLSEGEWQLVLHVWAKVEADVAGHGQDILIRLFKSHPETLEKFDRFKHLKTEAEMKASEDLK
KHGVTVLTALGAILKKKGHHEAELKPLAQSHATKHKIPIKYLEFISEAIIHVLHSRHPGDFG
ADAQGAMNKALELFRKDIAAKYKELGY
>d1mba__ a.1.1.2 (-) Myoglobin {Sea hare (Aplysia limacina)}
SLSAAEADLAGKSWAPVFANKNANGLDFLVALFEKFPDSANFFADFKGKSVADIKASPKLRD
VSSRIFTRLNEFVNNAANAGKMSAMLSQFAKEHVGFGVGSAQFENVRSMFPGFVASVAAPPA
GADAAWTKLFGLIIDALKAAGA
>d1mbs__ a.1.1.2 (-) Myoglobin {Common seal (Phoca vitulina)}
GLSDGEWHLVLNVWGKVETDLAGHGQEVLIRLFKSHPETLEKFDKFKHLKSEDDMRRSEDLR
KHGNTVLTALGGILKKKGHHEAELKPLAQSHATKHKIPIKYLEFISEAIIHVLHSKHPAEFG
ADAQAAMKKALELFRNDIAAKYKELGFHG
>d1mwca_ a.1.1.2 (A:) Myoglobin {Pig (Sus scrofa)}
GLSDGEWQLVLNVWGKVEADVAGHGQEVLIRLFKGHPETLEKFDKFKHLKSEDEMKASEDLK
KHGNTVLTALGGILKKKGHHEAELTPLAQSHATKHKIPVKYLEFISEAIIQVLQSKHPGDFG
ADAQGAMSKALELFRNDMAAKYKELGFQG
>d1dwta_ a.1.1.2 (A:) Myoglobin {Horse (Equus caballus)}
GLSDGEWQQVLNVWGKVEADIAGHGQEVLIRLFTGHPETLEKFDKFKHLKTEAEMKASEDLK
KHGTVVLTALGGILKKKGHHEAELKPLAQSHATKHKIPIKYLEFISDAIIHVLHSKHPGDFG
ADAQGAMTKALELFRNDIAAKYKELGFQ
>d2mm1__ a.1.1.2 (-) Myoglobin {Human (Homo sapiens)}
GLSDGEWQLVLNVWGKVEADIPGHGQEVLIRLFKGHPETLEKFDRFKHLKSEDEMKASEDLK
KHGATVLTALGGILKKKGHHEAEIKPLAQSHATKHKIPVKYLEFISEAIIQVLQSKHPGDFG
ADAQGAMNKALELFRKDMASNYKELGFQG
>d1emy__ a.1.1.2 (-) Myoglobin {Asian elephant (Elephas maximus)}
GLSDGEWELVLKTWGKVEADIPGHGETVFVRLFTGHPETLEKFDKFKHLKTEGEMKASEDLK
KQGVTVLTALGGILKKKGHHEAEIQPLAQSHATKHKIPIKYLEFISDAIIHVLQSKHPAEFG
ADAQGAMKKALELFRNDIAAKYKELGFQG
>d1lht__ a.1.1.2 (-) Myoglobin {Loggerhead sea turtle (Caretta caretta)}
GLSDDEWNHVLGIWAKVEPDLSAHGQEVIIRLFQLHPETQERFAKFKNLTTIDALKSSEEVK
KHGTTVLTALGRILKQKNNHEQELKPLAESHATKHKIPVKYLEFICEIIVKVIAEKHPSDFG
ADSQAAMKKALELFRNDMASKYKEFGFQG
>d1myt__ a.1.1.2 (-) Myoglobin {Yellowfin tuna (Thunnus albacares)}
ADFDAVLKCWGPVEADYTTMGGLVLTRLFKEHPETQKLFPKFAGIAQADIAGNAAISAHGAT

VLKKLGELLKAKGSHAAILKPLANSHATKHKIPINNFKLISEVLVKVMHEKAGLDAGGQTAL
RNVMGIIIADLEANYKELGFSG
>d1eco__ a.1.1.2 (-) Erythrocruorin {Midge (Chironomus thummi thummi), fraction III}
LSADQISTVQASFDKVKGDPVGILYAVFKADPSIMAKFTQFAGKDLESIKGTAPFETHANRI
VGFFSKIIGELPNIEADVNTFVASHKPRGVTHDQLNNFRAGFVSYMKAHTDFAGAEAAWGAT
LDTFFGMIFSKM
>d2gdm__ a.1.1.2 (-) Leghemoglobin {Yellow lupin (Lupinus luteus)}
GALTESQAALVKSSWEEFNANIPKHTHRFFILVLEIAPAAKDLFSFLKGTSEVPQNNPELQA
HAGKVFKLVYEAAIQLEVTGVVVTDATLKNLGSVHVSKGVADAHFPVVKEAILKTIKEVVGA
KWSEELNSAWTIAYDELAIVIKKEMDDAA
>d1fsla_ a.1.1.2 (A:) Leghemoglobin {Soybean (Glycine max), isoform A}
VAFTEKQDALVSSSFEAFKANIPQYSVVFYTSILEKAPAAKDLFSFLANGVDPTNPKLTGHA
EKLFALVRDSAGQLKASGTVVADAALGSVHAQKAVTDPQFVVVKEALLKTIKAAVGDKWSDE
LSRAWEVAYDELAAAIKKA
>d1d8ua_ a.1.1.2 (A:) Non-symbiotic plant hemoglobin {Rice (Oryza sativa)}
ALVEDNNAVAVSFSEEQEALVLKSWAILKKDSANIALRFFLKIFEVAPSASQMFSFLRNSDV
PLEKNPKLKTHAMSVFVMTCEAAAQLRKAGKVTVRDTTLKRLGATHLKYGVGDAHFEVVKFA
LLDTIKEEVPADMWSPAMKSAWSEAYDHLVAAIKQEMKPAE
>d1i3da_ a.1.1.2 (A:) Hemoglobin, alpha-chain {Human (Homo sapiens)}
GHFTEEDKATITSLWGKVNVEDAGGETLGRLLVVYPWTQRFFDSFGNLSSASAIMGNPKVKA
HGKKVLTSLGDAIKHLDDLKGTFAQLSELHCDKLHVDPENFKLLGNVLVTVLAIHFGKEFTP
EVQASWQKMVTAVASALSSRYH
>d1irda_ a.1.1.2 (A:) Hemoglobin, alpha-chain {Human (Homo sapiens)}
VLSPADKTNVKAAWGKVGAHAGEYGAEALERMFLSFPTTKTYFPHFDLSHGSAQVKGHGKKV
ADALTNAVAHVDDMPNALSALSDLHAHKLRVDPVNFKLLSHCLLVTLAAHLPAEFTPAVHAS
LDKFLASVSTVLTSKYR
>d1jeba_ a.1.1.2 (A:) Hemoglobin, alpha-chain {Human (Homo sapiens), zeta isoform}
SLTKTERTIIVSMWAKISTQADTIGTETLERLFLSHPQTKTYFPHFDLHPGSAQLRAHGSKV
VAAVGDAVKSIDDIGGALSKLSELHAYILRVDPVNFKLLSHCLLVTLAARFPADFTAEAHAA
WDKFLSVVSSVLTEKYR
>d1ibea_ a.1.1.2 (A:) Hemoglobin, alpha-chain {Horse (Equus caballus)}
VLSAADKTNVKAAWSKVGGHAGEFGAEALERMFLGFPTTKTYFPHFDLSHGSAQVKAHGKKV
GDALTLAVGHLDDLPGALSDLSNLHAHKLRVDPVNFKLLSHCLLSTLAVHLPNDFTPAVHAS
LDKFLSSVSTVLTSKYR
>d1hdsa_ a.1.1.2 (A:) Hemoglobin, alpha-chain {Deer (Odocoileus virginianus)}
VLSAANKSNVKAAWGKVGGNAPAYGAQALQRMFLSFPTTKTYFPHFDLSHGSAQQKAHGQKV

ANALTKAQGHLNDLPGTLSNLSNLHAHKLRVNPVNFKLLSHSLLVTLASHLPTNFTPAVHAN
LNKFLANDSTVLTSKYR
>d1g08a_ a.1.1.2 (A:) Hemoglobin, alpha-chain {Cow (Bos taurus)}
VLSAADKGNVKAAWGKVGGHAAEYGAEALERMFLSFPTTKTYFPHFDLSHGSAQVKGHGAKV
AAALTKAVEHLDDLPGALSELSDLHAHKLRVDPVNFKLLSHSLLVTLASHLPSDFTPAVHAS
LDKFLANVSTVLTSKYR
>d1qpwa_ a.1.1.2 (A:) Hemoglobin, alpha-chain {Pig (Sus scrofa)}
VLSAADKANVKAAWGKVGGQAGAHGAEALERMFLGFPTTKTYFPHFNLSHGSDQVKAHGQKV
ADALTKAVGHLDDLPGALSALSDLHAHKLRVDPVNFKLLSHCLLVTLAAHHPDDFNPSVHAS
LDKFLANVSTVLTSKYR
>d1fhja_ a.1.1.2 (A:) Hemoglobin, alpha-chain {Maned wolf (Chrysocyon brachyurus)}
VLSPADKTNIKSTWDKIGGHAGDYGGEALDRTFQSFPTTKTYFPHFDLSPGSAQVKAHGKKV
ADALTTAVAHLDDLPGALSALSDLHAYKLRVDPVNFKLLSHCLLVTLACHHPTEFTPAVHAS
LDKFFTAVSTVLTSKYR
>d1hbra_ a.1.1.2 (A:) Hemoglobin, alpha-chain {Chicken (Gallus gallus)}
MLTAEDKKLIQQAWEKAASHQEEFGAEALTRMFTTYPQTKTYFPHFDLSPGSDQVRGHGKKV
LGALGNAVKNVDNLSQAMAELSNLHAYNLRVDPVNFKLLSQCIQVVLAVHMGKDYTPEVHAA
FDKFLSAVSAVLAEKYR
>d1a4fa_ a.1.1.2 (A:) Hemoglobin, alpha-chain {Bar-headed goose (Anser indicus)}
VLSAADKTNVKGVFSKISGHAEEYGAETLERMFTAYPQTKTYFPHFDLQHGSAQIKAHGKKV
VAALVEAVNHIDDIAGALSKLSDLHAQKLRVDPVNFKFLGHCFLVVVAIHHPSALTAEVHAS
LDKFLCAVGTVLTAKYR
>d1outa_ a.1.1.2 (A:) Hemoglobin, alpha-chain {Trout (Oncorhynchus mykiss)}
SLTAKDKSVVKAFWGKISGKADVVGAEALGRMLTAYPQTKTYFSHWADLSPGSGPVKKHGGI
IMGAIGKAVGLMDDLVGGMSALSDLHAFKLRVDPGNFKILSHNILVTLAIHFPSDFTPEVHI
AVDKFLAAVSAALADKYR
>d1cg5a_ a.1.1.2 (A:) Hemoglobin, alpha-chain {Cartilaginous fish akaei (Dasyatis akajei)}
VLSSQNKKAIEELGNLIKANAEAWGADALARLFELHPQTKTYFSKFSGFEACNEQVKKHGKR
VMNALADATHHLDNLHLHLEDLARKHGENLLVDPHNFHLFADCIVVTLAVNLQAFTPVTHCA
VDKFLELVAYELSSCYR
>d1t1na_ a.1.1.2 (A:) Hemoglobin, alpha-chain {Fish (Trematomus newnesi)}
SLSDKDKAAVRALWSKIGKSSDAIGNDALSRMIVVYPQTKIYFSHWPDVTPGSPNIKAHGKK
VMGGIALAVSKIDDLKTGLMELSEQHAYKLRVDPSNFKILNHCILVVISTMFPKEFTPEAHV
SLDKFLSGVALALAERYR
>d1spga_ a.1.1.2 (A:) Hemoglobin, alpha-chain {Teleost fish (Leiostomus xanthurus)}
SLSATDKARVKALWDKIEGKSAELGAEALGRMLVSFPQTKIYFSEWGQDLGPQTPQVRNHGA
VIMAAVGKAVKSIDNLVGGLSQLSELHAFKLRVDPANFKILAHNIILVISMYFPGDFTPEVH

LSVDKFLACLALALSEKYR
>d1gcva_ a.1.1.2 (A:) Hemoglobin, alpha-chain {Houndshark (Mustelus griseus)}
AFTACEKQTIGKIAQVLAKSPEAYGAECLARLFVTHPGSKSYFEYKDYSAAGAKVQVHGGKV
IRAVVKAAEHVDDLHSHLETLALTHGKKLLVDPQNFPMLSECIIVTLATHLTEFSPDTHCAV
DKLLSAICQELSSRYR
>d1irdb_ a.1.1.2 (B:) Hemoglobin, beta-chain {Human (Homo sapiens)}
VHLTPEEKSAVTALWGKVNVDEVGGEALGRLLVVYPWTQRFFESFGDLSTPDAVMGNPKVKA
HGKKVLGAFSDGLAHLDNLKGTFATLSELHCDKLHVDPENFRLLGNVLVCVLAHHFGKEFTP
PVQAAYQKVVAGVANALAHKYH
>d1a9we_ a.1.1.2 (E:) Hemoglobin, beta-chain {Human (Homo sapiens), embryonic gower II}
VHFTAEEKAAVTSLWSKMNVEEAGGEALGRLLVVYPWTQRFFDSFGNLSSPSAILGNPKVKA
HGKKVLTSFGDAIKNMDNLKPAFAKLSELHCDKLHVDPENFKLLGNVMVIILATHFGKEFTP
EVQAAWQKLVSAVAIALAHKY
>d1ibeb_ a.1.1.2 (B:) Hemoglobin, beta-chain {Horse (Equus caballus)}
VQLSGEEKAAVLALWDKVNEEEVGGEALGRLLVVYPWTQRFFDSFGDLSNPGAVMGNPKVKA
HGKKVLHSFGEGVHHLDNLKGTFAALSELHCDKLHVDPENFRLLGNVLVVVLARHFGKDFTP
ELQASYQKVVAGVANALAHKYH
>d1hdsb_ a.1.1.2 (B:) Hemoglobin, beta-chain {Deer (Odocoileus virginianus)}
MLTAEEKAAVTGFWGKVDVDVVGAQALGRLLVVYPWTQRFFQHFGNLSSAGAVMNNPKVKAH
GKRVLDAFTQGLKHLDDLKGAFAQLSGLHCNKLHVNPQNFRLLGNVLALVVARNFGGQFTPN
VQALFQKVVAGVANALAHKYH
>d1g08b_ a.1.1.2 (B:) Hemoglobin, beta-chain {Cow (Bos taurus)}
MLTAEEKAAVTAFWGKVKVDEVGGEALGRLLVVYPWTQRFFESFGDLSTADAVMNNPKVKAH
GKKVLDSFSNGMKHLDDLKGTFAALSELHCDKLHVDPENFKLLGNVLVVVLARNFGKEFTPV
LQADFQKVVAGVANALAHRYH
>d1qpwb_ a.1.1.2 (B:) Hemoglobin, beta-chain {Pig (Sus scrofa)}
VHLSAEEKEAVLGLWGKVNVDEVGGEALGRLLVVYPWTQRFFESFGDLSNADAVMGNPKVKA
HGKKVLQSFSDGLKHLDNLKGTFAKLSELHCDQLHVDPENFRLLGNVIVVVLARRLGHDFNP
DVQAAFQKVVAGVANALAHKYH
>d1fhjb_ a.1.1.2 (B:) Hemoglobin, beta-chain {Maned wolf (Chrysocyon brachyurus)}
VHLTAEEKSLVSGLWGKVNVDEVGGEALGRLLIVYPWTQRFFDSFGDLSTPDAVMSNAKVKA
HGKKVLNSFSDGLKNLDNLKGTFAKLSELHCDKLHVDPENFKLLGNVLVCVLAHHFGKEFTP
QVQAAYQKVVAGVANALAHKYH
>d1jebb_ a.1.1.2 (B:) Hemoglobin, beta-chain {Mouse (Mus musculus)}
VHLTDAEKAAVSGLWGKVNADEVGGEALGRLLVVYPWTQRYFDSFGDLSSASAIMGNAKVKA
HGKKVITAFNDGLNHLDSLKGTFASLSELHCDKLHVDPENFRLLGNMIVIVLGHHLGKDFTP
AAQAAFQKVVAGVAAALAH

>d1hbrb_ a.1.1.2 (B:) Hemoglobin, beta-chain {Chicken (Gallus gallus)}
VHWTAEEKQLITGLWGKVNVAECGAEALARLLIVYPWTQRFFASFGNLSSPTAILGNPMVRA
HGKKVLTSFGDAVKNLDNIKNTFSQLSELHCDKLHVDPENFRLLGDILIIVLAAHFSKDFTP
ECQAAWQKLVRVVAHALARK
>d1a4fb_ a.1.1.2 (B:) Hemoglobin, beta-chain {Bar-headed goose (Anser indicus)}
VHWSAEEKQLITGLWGKVNVADCGAEALARLLIVYPWTQRFFSSFGNLSSPTAILGNPMVRA
HGKKVLTSFGDAVKNLDNIKNTFAQLSELHCDKLHVDPENFRLLGDILIIVLAAHFAKEFTP
DCQAAWQKLVRVVAHALARKYH
>d1outb_ a.1.1.2 (B:) Hemoglobin, beta-chain {Trout (Oncorhynchus mykiss)}
VEWTDAEKSTISAVWGKVNIDEIGPLALARVLIVYPWTQRYFGSFGNVSTPAAIMGNPKVAA
HGKVVCGALDKAVKNMGNILATYKSLSETHANKLFVDPDNFRVLADVLTIVIAAKFGASFTP
EIQATWQKFMKVVVAAMGSRYF
>d1pbxb_ a.1.1.2 (B:) Hemoglobin, beta-chain {Antarctic fish (Pagothenia bernacchii)}
VEWTDKERSIISDIFSHMDYDDIGPKALSRCLIVYPWTQRHFSGFGNLYNAEAIIGNANVAA
HGIKVLHGLDRGVKNMDNIAATYADLSTLHSEKLHVDPDNFKLLSDCITIVLAAKMGHAFTA
ETQGAFQKFLAVVVSALGKQYH
>d1cg5b_ a.1.1.2 (B:) Hemoglobin, beta-chain {Cartilaginous fish akaei (Dasyatis akajei)}
VKLSEDQEHYIKGVWKDVDHKQITAKALERVFVVYPWTTRLFSKLQGLFSANDIGVQQHADK
VQRALGEAIDDLKKVEINFQNLSGKHQEIGVDTQNFKLLGQTFMVELALHYKKTFRPKEHAA
AYKFFRLVAEALSSNYH
>d1t1nb_ a.1.1.2 (B:) Hemoglobin, beta-chain {Fish (Trematomus newnesi)}
VEWTDKERSIISDIFSHMDYDDIGPKALSRCLVVYPWTQRYFSGFGNLYNAEGIMSNANVAA
HGIKVLHGLDRGMKNMDNIADAYTDLSTLHSEKLHVDPDNFKLLSDCITIVLAAKMGHAFTA
ETQGAFQKFLAAVVSALGKQYH
>d1spgb_ a.1.1.2 (B:) Hemoglobin, beta-chain {Teleost fish (Leiostomus xanthurus)}
VDWTDAERAAIKALWGKIDVGEIGPQALSRLLIVYPWTQRHFKGFGNISTNAAILGNAKVAE
HGKTVMGGLDRAVQNMDNIKNVYKQLSIKHSEKIHVDPDNFRLLGEIITMCVGAKFGPSAFT
PEIHEAWQKFLAVVVSALGRQYH
>d1gcvb_ a.1.1.2 (B:) Hemoglobin, beta-chain {Houndshark (Mustelus griseus)}
VHWTQEERDEISKTFQGTDMKTVVTQALDRMFKVYPWTNRYFQKRTDFRSSIHAGIVVGALQ
DAVKHMDDVKTLFKDLSKKHADDLHVDPGSFHLLTDCIIVELAYLRKDCFTPHIQGIWDKFF
EVVIDAISKQYH
>d1ch4a_ a.1.1.2 (A:) Chimeric hemoglobin beta-alpha {Synthetic, based on Homo sapiens sequence}
VHLTPEEKSAVTALWGKVNVDEVGGEALGRLLVVYPWTQRFFESFGDLSTPDAVMGNPKVKA
HGKKVLGAFSDGLAHLDNLKGTFATLSELHCDKLRVDPVNFKLLSHCLLVTLAAHLPAEFTP

AVHASLDKVLASVSTVLTSKYR
>d1it2a_ a.1.1.2 (A:) Hagfish hemoglobin {Inshore hagfish (Eptatretus burgeri)}
PIIDQGPLPTLTDGDKKAINKIWPKIYKEYEQYSLNILLRFLKCFPQAQASFPKFSTKKSNL
EQDPEVKHQAVVIFNKVNEIINSMDNQEEIIKSLKDLSQKHKTVFKVDSIWFKELSSIFVST
IDGGAEFEKLFSIICILLRSAY
>d2lhb__ a.1.1.2 (-) Lamprey globin {Sea lamprey (Petromyzon marinus)}
PIVDTGSVAPLSAAEKTKIRSAWAPVYSTYETSGVDILVKFFTSTPAAQEFFPKFKGLTTAD
ELKKSADVRWHAERIINAVDDAVASMDDTEKMSMKLRNLSGKHAKSFQVDPEYFKVLAAVIA
DTVAAGDAGFEKLMSMICILLRSAY
>d1ash__ a.1.1.2 (-) Ascaris hemoglobin, domain 1 {Pig roundworm (Ascaris suum)}
ANKTRELCMKSLEHAKVDTSNEARQDGIDLYKHMFENYPPLRKYFKSREEYTAEDVQNDPFF
AKQGQKILLACHVLCATYDDRETFNAYTRELLDRHARDHVHMPPEVWTDFWKLFEEYLGKKT
TLDEPTKQAWHEIGREFAKEINK
>d1itha_ a.1.1.2 (A:) Hemoglobin {Innkeeper worm (Urechis caupo)}
GLTAAQIKAIQDHWFLNIKGCLQAAADSIFFKYLTAYPGDLAFFHKFSSVPLYGLRSNPAYK
AQTLTVINYLDKVVDALGGNAGALMKAKVPSHDAMGITPKHFGQLLKLVGGVFQEEFSADPT
TVAAWGDAAGVLVAAMK
>d1hlb__ a.1.1.2 (-) Hemoglobin, different isoforms {Sea cucumber (Caudina (Molpadia) arenicola)}
GGTLAIQAQGDLTLAQKKIVRKTWHQLMRNKTSFVTDVFIRIFAYDPSAQNKFPQMAGMSAS
QLRSSRQMQAHAIRVSSIMSEYVEELDSDILPELLATLARTHDLNKVGADHYNLFAKVLMEA
LQAELGSDFNEKTRDAWAKAFSVVQAVLLVKHG
>d1hlm__ a.1.1.2 (-) Hemoglobin, different isoforms {Sea cucumber (Caudina (Molpadia) arenicola)}
GATQSFQSVGDLTPAEKDLIRSTWDQLMTHRTGFVADVFIRIFHNDPTAQRKFPQMAGLSPA
ELRTSRQMHAHAIRVSALMTTYIDEMDTEVLPELLATLTRTHDKNHVGKKNYDLFGKVLMEA
IKAELGVGFTKQVHDAWAKTFAIVQGVLITKHAS
>d1vhba_ a.1.1.2 (A:) Bacterial dimeric hemoglobin {Vitreoscilla stercoraria}
LDQQTINIIKATVPVLKEHGVTITTTFYKNLFAKHPEVRPLFDMGRQESLEQPKALAMTVLA
AAQNIENLPAILPAVKKIAVKHCQAGVAAAHYPIVGQELLGAIKEVLGDAATDDILDAWGKA
YGVIADVFIQVEADLYAQAV
>d1cqxa1 a.1.1.2 (A:1-150) Flavohemoglobin, N-terminal domain {Alcaligenes eutrophus}
MLTQKTKDIVKATAPVLAEHGYDIIKCFYQRMFEAHPELKNVFNMAHQEQGQQQQALARAVY
AYAENIEDPNSLMAVLKNIANKHASLGVKPEQYPIVGEHLLAAIKEVLGNAATDDIISAWAQ
AYGNLADVLMGMESELYERSAEQPGG
>d1ew6a_ a.1.1.2 (A:) Dehaloperoxidase {Marine worm (Amphitrite ornata)}
GFKQDIATIRGDLRTYAQDIFLAFLNKYPDERRYFKNYVGKSDQELKSMAKFGDHTEKVFNL
MMEVADRATDCVPLASDANTLVQMKQHSSLTTGNFEKLFVALVEYMRASGQSFDSQSWDRFG

KNLVSALSSAGMK
>d1phna_ a.1.1.3 (A:) Phycocyanin {Red alga (Cyanidium caldarium)}
MKTPITEAIAAADNQGRFLSNTELQAVNGRYQRAAASLEAARSLTSNAERLINGAAQAVYSK
FPYTSQMPGPQYASSAVGKAKCARDIGYYLRMVTYCLVVGGTGPMDEYLIAGLEEINRTFDL
SPSWYVEALNYIKANHGLSGQAANEANTYIDYAINALS
>d1phnb_ a.1.1.3 (B:) Phycocyanin {Red alga (Cyanidium caldarium)}
MLDAFAKVVAQADARGEFLSNTQLDALSKMVSEGNKRLDVVNRITSNASAIVTNAARALFSE
QPQLIQPGGNAYTNRRMAACLRDMEIILRYVSYAIIAGDSSILDDRCLNGLRETYQALGVPG
ASVAVGIEKMKDSAIAIANDPSGITTGDCSALMAEVGTYFDRAATAVQ
>d1f99a_ a.1.1.3 (A:) Phycocyanin {Red alga (Polysiphonia urceolata)}
MKTPLTEAIAAADSQGRFLSNTELQVVNGRYNRATSSLEAAKALTANADRLISGAANAVYSK
FPYTTQMPGPNYSSTAIGKAKCARDIGYYLRMVTYCLVVGGTGPMDDYLVAGLEEINRTFEL
SPSWYIEALKYIKNNHGLSGDVANEANTYIDYAINTLS
>d1f99b_ a.1.1.3 (B:) Phycocyanin {Red alga (Polysiphonia urceolata)}
MLDAFAKVVAQADARGEFLSNTQIDALLAIVSEGNKRLDVVNKITNNASAIVTNAARALFAE
QPQLISPGGNAYTSRRMAACLRDMEIVLRYVSYAMIAGDASVLDDRCLNGLRETYQALGTPG
ASVAVAIQKMKDAALALVNDTTGTPAGDCASLVAEIATYFDRAAAAVA
>d1cpca_ a.1.1.3 (A:) Phycocyanin {Cyanobacterium (Fremyella diplosiphon)}
MKTPLTEAVAAADSQGRFLSSTEIQTAFGRFRQASASLAAAKALTEKASSLASGAANAVYSK
FPYTTSQNGPNFASTQTGKDKCVRDIGYYLRMVTYCLVVGGTGPLDDYLIGGIAEINRTFDL
SPSWYVEALKYIKANHGLSGDPAVEANSYIDYAINALS
>d1cpcb_ a.1.1.3 (B:) Phycocyanin {Cyanobacterium (Fremyella diplosiphon)}
MLDAFAKVVSQADARGEYLSGSQIDALSALVADGNKRMDVVNRITGNSSTIVANAARSLFAE
QPQLIAPGGNAYTSRRMAACLRDMEIILRYVTYAIFAGDASVLDDRCLNGLKETYLALGTPG
SSVAVGVQKMKDAALAIAGDTNGITRGDCASLMAEVASYFDKAASAVA
>d1i7ya_ a.1.1.3 (A:) Phycocyanin {Synechococcus vulcanus}
MKTPITEAIAAADTQGRFLSNTELQAVDGRFKRAVASMEAARALTNNAQSLIDGAAQAVYQK
FPYTTTMQGSQYASTPEGKAKCARDIGYYLRMITYCLVAGGTGPMDEYLIAGLSEINSTFDL
SPSWYIEALKYIKANHGLTGQAAVEANAYIDYAINALS
>d1i7yb_ a.1.1.3 (B:) Phycocyanin {Synechococcus vulcanus}
MLDAFAKVVAQADARGEFLTNAQFDALSNLVKEGNKRLDAVNRITSNASTIVANAARALFAE
QPQLIQPGGNAYTNRRMAACLRDMEIILRYVTYAILAGDSSVLDDRCLNGLRETYQALGTPG
SSVAVAIQKMKDAAIAIANDPNGITPGDCSALMSEIAGYFDRAAAAVA
>d1gh0a_ a.1.1.3 (A:) Phycocyanin {Spirulina platensis}
MKTPLTEAVSVADSQGRFLSSTEIQVAFGRFRQAKAGLEAAKALTSKADSLISGAAQAVYNK
FPYTTQMQGPNYAADQRGKDKCARDIGYYLRMVTYCLIAGGTGPMDEYLIAGIDEINRTFEL
SPSWYIEALKYIKANHGLSGDAAVEANSYLDYAINALS
>d1gh0b_ a.1.1.3 (B:) Phycocyanin {Spirulina platensis}
MFDAFTKVVSQADTRGEMLSTAQIDALSQMVAESNKRLDVVNRITSNASTIVSNAARSLFAE
QPQLIAPGGNAYTSRRMAACLRDMEIILRYVTYAVFAGDASVLEDRCLNGLRETYLALGTPG

SSVAVGVGKMKEAALAIVNDPAGITPGDCSALASEIAGYFDRAAAAVS
>d1alla_ a.1.1.3 (A:) Allophycocyanin {Spirulina platensis}
SIVTKSIVNADAEARYLSPGELDRIKSFVTSGERRVRIAETMTGARERIIKQAGDQLFGKRP
DVVSPGGNAYGADMTATCLRDLDYYLRLITYGIVAGDVTPIEEIGVVGVREMYKSLGTPIEA
IAEGVRAMKSVATSLLSGADAAEAGSYFDYLIGAMS
>d1allb_ a.1.1.3 (B:) Allophycocyanin {Spirulina platensis}
MQDAITSVINSSDVQGKYLDASAIQKLKAYFATGELRVRAATTISANAANIVKEAVAKSLLY
SDVTRPGGNMYTTRRYAACIRDLDYYLRYATYAMLAGDPSILDERVLNGLKETYNSLGVPIG
ATVQAIQAMKEVTAGLVGGGAGKEMGIYFDYICSGLS
>d1b33a_ a.1.1.3 (A:) Allophycocyanin {Cyanobacterium (Mastigocladus laminosus)}
SIVTKSIVNADAEARYLSPGELDRIKSFVSSGEKRLRIAQILTDNRERIVKQAGDQLFQKRP
DVVSPGGNAYGQEMTATCLRDLDYYLRLITYGIVAGDVTPIEEIGIVGVREMYKSLGTPIDA
VAAGVSAMKNVASSILSAEDAAEAGAYFDYVAGALA
>d1b33b_ a.1.1.3 (B:) Allophycocyanin {Cyanobacterium (Mastigocladus laminosus)}
MQDAITAVINSSDVQGKYLDTAALEKLKSYFSTGELRVRAATTIAANAAAIVKEAVAKSLLY
SDITRPGGNMYTTRRYAACIRDLDYYLRYATYAMLAGDPSILDERVLNGLKETYNSLGVPIS
ATVQAIQAMKEVTASLVGPDAGKEMGVYFDYICSGLS
>d1liaa_ a.1.1.3 (A:) Phycoerythrin {Red alga (Polysiphonia urceolata)}
MKSVITTTISAADAAGRYPSTSDLQSVQGNIQRAAARLEAAEKLGSNHEAVVKEAGDACFSK
YGYNKNPGEAGENQEKINKCYRDIDHYMRLINYTLVVGGTGPLDEWGIAGAREVYRTLNLPS
AAYIAAFVFTRDRLCIPRDMSAQAGVEFCTALDYLINSLS
>d1liab_ a.1.1.3 (B:) Phycoerythrin {Red alga (Polysiphonia urceolata)}
MLDAFSRVVVNSDSKAAYVSGSDLQALKTFINDGNKRLDAVNYIVSNSSCIVSDAISGMICE
NPGLITPGGNCYTNRRMAACLRDGEIILRYVSYALLAGDASVLEDRCLNGLKETYIALGVPT
NSTVRAVSIMKAAAVCFISNTASQRKVEVIEGDCSALASEVASYCDRVVAAVS
>d1b8da_ a.1.1.3 (A:) Phycoerythrin {Red alga (Griffithsia monilis)}
MKSVITTTISAADAAGRFPSSSDLESIQGNIQRAAARLEAAQKLSGNHEAVVKEAGDACFAK
YSYLKNAGEAGDSPEKINKCYRDIDHYMRLINYSLVVGGTGPVDEWGIAGSREVYRALNLPG
SAYIAAFTFTRDRLCVPRDMSSQAGVEFTSALDYVINSLC
>d1b8db_ a.1.1.3 (B:) Phycoerythrin {Red alga (Griffithsia monilis)}
MLDAFSRVVVTSDAKAAYVGGSDLQSLKSFINDGNKRLDAVNYIVSNASCIVSDAVSGMICE
NPGLIAPGGNCYTNRRMAACLRDGEIILRYVSYALLAGDSSVLDDRCLNGLKETYIALGVPT
ASSSRAVSIMKATATAFITNTASGRKVEVAAGDCQALQAEAASYFDKVGSSID
>d1eyxa_ a.1.1.3 (A:) Phycoerythrin {Red algae (Gracilaria chilensis)}
MKSVITTVISAADSAGRFPSSSDLESVQGNIQRASARLEAAEKLASNHEAVVKEAGDACFGK
YGYLKNPGEAGENQEKINKCYRDIDHYMRLVNYSLVIGGTGPLDEWGIAGAREVYRTLNLPT
SAYIAAFAFTRDRLCGPRDMSAQAGVEYSTALDYIINSLS

>d1eyxb_ a.1.1.3 (B:) Phycoerythrin {Red algae (Gracilaria chilensis)}
MLDAFSRVISNADAKAAYVGGSDLQALRTFISDGNKRLDAVNYIVSNSSCIVSDAISGMICE
NPGLITPGGNCYTNRRMAACLRDGEIILRYISYALLAGDSSVLEDRCLNGLKETYIALGVPT
NSTVRAVSIMKAAVGAFISNTASQRKGEVIEGDCSALAAEIASYCDRISAAVS
>d1qgwc_ a.1.1.3 (C:) Phycoerythrin {Cryptophite (Rhodomonas sp.), cs24}
DAFSRVVTNADSKAAYVGGADLQALKKFISEGNKRLDSVNSIVSNASCIVSDAVSGMICENP
SLISPSGNCYTNRRMAACLRDGEIILRYVSYALLSGDASVLEDRCLNGLKETYSSLGVPANS
NARAVSIMKACAVAFVNNTASQKKLSTPQGDCSGLASEVGGYFDKVTAAIS
>d1fumb1 a.1.2.1 (B:106-243) Fumarate reductase iron-sulfur protein, C-terminal domain {Escherichia coli}
MTHFIESLEAIKPYIIGNSRTADQGTNIQTPAQMAKYHQFSGCINCGLCYAACPQFGLNPEF
IGPAAITLAHRYNEDSRDHGKKERMAQLNSQNGVWSCTFVGYCSEVCPKHVDPAAAIQQGKV
ESSKDFLIATLKPR
>d1qlab1 a.1.2.1 (B:107-239) Fumarate reductase iron-sulfur protein, C-terminal domain {Wolinella succinogenes}
TGNWFNGMSQRVESWIHAQKEHDISKLEERIEPEVAQEVFELDRCIECGCCIAACGTKIMRE
DFVGAAGLNRVVRFMIDPHDERTDEDYYELIGDDDGVFGCMTLLACHDVCPKNLPLQSKIAY
LRRKMVSVN
>d1h7wa1 a.1.2.2 (A:2-183) Dihydropyrimidine dehydrogenase, N-terminal domain {Pig (Sus scrofa)}
APVLSKDVADIESILALNPRTQSHAALHSTLAKKLDKKHWKRNPDKNCFHCEKLENNFDDIK
HTTLGERGALREAMRCLKCADAPCQKSCPTHLDIKSFITSISNKNYYGAAKMIFSDNPLGLT
CGMVCPTSDLCVGGCNLYATEEGSINIGGLQQFASEVFKAMNIPQIRNPCLPSQEKMP
>d1grj_1 a.2.1.1 (2-79) GreA transcript cleavage protein, N-terminal domain {Escherichia coli}
QAIPMTLRGAEKLREELDFLKSVRRPEIIAAIAEAREHGDLKENAEYHAAREQQGFCEGRIK
DIEAKLSNAQVIDVTK
>d1jj2u_ a.2.2.1 (U:) Ribosomal protein L29 (L29p) {Archaeon Haloarcula marismortui}
TVLHVQEIRDMTPAEREAELDDLKTELLNARAVQAAGGAPENPGRIKELRKAIARIKTIQGE
EGD
>d1fpoa1 a.2.3.1 (A:1-76) HSC20 (HSCB), N-terminal (J) domain {Escherichia coli}
MDYFTLFGLPARYQLDTQALSLRFQDLQRQYHPDKFASGSQAEQLAAVQQSATINQAWQTLR
HPLMRAEYLLSLHG
>d1hdj__ a.2.3.1 (-) HSP40 {Human (Homo sapiens)}
MGKDYYQTLGLARGASDEEIKRAYRRQALRYHPDKNKEPGAEEKFKEIAEAYDVLSDPRKRE
IFDRYGEEGLKGSGC
>d1xbl__ a.2.3.1 (-) DnaJ chaperone, N-terminal (J) domain {Escherichia coli}
AKQDYYEILGVSKTAEEREIRKAYKRLAMKYHPDRNQGDKEAEAKFKEIKEAYEVLTDSQKR
AAYDQYGHAAFEQ

>d1fafa_ a.2.3.1 (A:) Large T antigen, the N-terminal J domain {Murine polyomavirus}
MDRVLSRADKERLLELLKLPRQLWGDFGRMQQAYKQQSLLLHPDKGGSHALMQELNSLWGTF
KTEVYNLRMNLGGTGFQ
>d1gh6a_ a.2.3.1 (A:) Large T antigen, the N-terminal J domain {Simian virus 40, Sv40}
SHMREESLQLMDLLGLERSAWGNIPLMRKAYLKKCKEFHPDKGGDEEKMKKMNTLYKKMEDG
VKYAHQPDFGGFWDATEIPTYGTDEWEQWWNAFNEENLFCSEEMPSSDDEAT
>d1du2a_ a.2.4.1 (A:) Theta subunit of DNA polymerase III {Escherichia coli}
MLKNLAKLDQTEMDKVNVDLAAAGVAFKERYNMPVIAEAVEREQPEHLRSWFRERLIAHRLA
SVNLSRLPYEPKLK
>d1fxkc_ a.2.5.1 (C:) Prefoldin alpha subunit {Archaeon Methanobacterium thermoautotrophicum}
AALAEIVAQLNIYQSQVELIQQQMEAVRATISELEILEKTLSDIQGKDGSETLVPVGAGSFI
KAELKDTSEVIMSVGAGVAIKKNFEDAMESIKSQKNELESTLQKMGENLRAITDIMMKLSPQ
AEELLAAVA
>d1fxka_ a.2.5.1 (A:) Prefoldin beta subunit {Archaeon Methanobacterium thermoautotrophicum}
QNVQHQLAQFQQLQQQAQAISVQKQTVEMQINETQKALEELSRAADDAEVYKSSGNILIRVA
KDELTEELQEKLETLQLREKTIERQEERVMKKLQEMQVNIQEAMK
>d1cxzb_ a.2.6.1 (B:) Effector domain of the protein kinase pkn/prk1 {Human (Homo sapiens)}
WSLLEQLGLAGADLAAPGVQQQLELERERLRREIRKELKLKEGAENLRRATTDLGRSLGPVE
LLLRGSSRRLDLLHQQLQELHAHV
>d1seta1 a.2.7.1 (A:1-110) Seryl-tRNA synthetase (SerRS) {Thermus thermophilus, strain hb27}
MVDLKRLRQEPEVFHRAIREKGVALDLEALLALDREVQELKKRLQEVQTERNQVAKRVPKAP
PEEKEALIARGKALGEEAKRLEEALREKEARLEALLLQVPLPPWPGAP
>d1eiya1 a.2.7.2 (A:6-84) Phenylalanyl-tRNA synthetase (PheRS) {Thermus thermophilus}
LAAIQNARDLEELKALKARYLGKKGLLTQEMKGLSALPLEERRKRGQELNAIKAALEAALEA
REKALEEAALKEALERE
>d1a36a1 a.2.8.1 (A:641-712) Eukaryotic DNA topoisomerase I, dispensable insert domain {Human (Homo sapiens)}
EKSMMNLQTKIDAKKEQLADARRDLKSAKADAKVMKDAKTKKVVESKKKAVQRLEEQLMKLE
VQATDREENK
>d1e52a_ a.2.9.1 (A:) C-terminal, UvrC-binding domain of UvrB {Escherichia coli}
LEPDNVPMDMSPKALQQKIHELEGLMMQHAQNLEFEEAAQIRDQLHQLRELFIAAS
>d1qoja_ a.2.9.1 (A:) C-terminal, UvrC-binding domain of UvrB {Escherichia coli}
SPKALQQKIHELEGLMMQHAQNLEFEEAAQIRDQLHQLRELFIAAS
>d1aqt_1 a.2.10.1 (87-136) Epsilon subunit of F1F0-ATP synthase

C-terminal domain {Escherichia coli}
QDLDEARAMEAKRKAEEHISSSHGDVDYAQASAELAKAIAQLRVIELTKK
>d1e79h1 a.2.10.1 (H:101-145) Epsilon subunit of F1F0-ATP synthase C-terminal domain {Cow (Bos taurus)}
DMLDLGAAKANLEKAQSELLGAADEATRAEIQIRIEANEALVKAL
>d1idsa1 a.2.11.1 (A:2-85) Fe superoxide dismutase (FeSOD) {Mycobacterium tuberculosis}
AEYTLPDLDWDYGALEPHISGQINELHHSKHHATYVKGANDAVAKLEEARAKEDHSAILLNE
KNLAFNLAGHVNHTIWWKNLSP
>d1dt0a1 a.2.11.1 (A:1-83) Fe superoxide dismutase (FeSOD) {Pseudomonas ovalis}
AFELPPLPYAHDALQPHISKETLEFHHDKHHNTYVVNLNNLVPGTEFEGKTLEEIVKTSSGG
IFNNAAQVWNHTFYWNCLSPN
>d3sdpa1 a.2.11.1 (A:5-83) Fe superoxide dismutase (FeSOD) {Pseudomonas ovalis}
PPLPYAHDALQPHISKETLEYHHDKHHNTYVVNLNNLVPGTPEFEGKTLEEIVKSSSGGIFN
NAAQVWNHTFYWNCLSP
>d1isaa1 a.2.11.1 (A:1-82) Fe superoxide dismutase (FeSOD) {Escherichia coli}
SFELPALPYAKDALAPHISAETIEYHYGKHHQTYVTNLNNLIKGTAFEGKSLEEIIRSSEGG
VFNNAAQVWNHTFYWNCLAP
>d1coja1 a.2.11.1 (A:2-90) Fe superoxide dismutase (FeSOD) {Aquifex pyrophilus}
VHKLEPKDHLKPQNLEGISNEQIEPHFEAHYKGYVAKYNEIQEKLADQNFADRSKANQNYSE
YRELKVEETFNYMGVVLHELYFGMLTP
>d1sssa1 a.2.11.1 (A:4-92) Fe superoxide dismutase (FeSOD) {Archaeon Sulfolobus solfataricus}
IQFKKYELPPLPYKIDALEPYISKDIIDVHYNGHHKGYVNGANSLLERLEKVVKGDLQTGQY
DIQGIIRGLTFNINGHKLHALYWENMA
>d1b06a1 a.2.11.1 (A:3-92) Fe superoxide dismutase (FeSOD) {Archaeon Sulfolobus acidocaldarius}
VIQLKRYEFPQLPYKVDALEPYISKDIIDVHYNGHHKGYVNGANSLLDRLEKLIKGDLPQGQ
YDLQGILRGLTFNINGHKLHAIYWNNMA
>d1ap6a1 a.2.11.1 (A:1-83) Mn superoxide dismutase (MnSOD) {Human (Homo sapiens)}
KHSLPDLPYDYGALEPHINAQIMQLHHSKHHAAFVNNLNVTEEKYQEALAKGDVTAQIALQP
ALKFNGGGHINHSIFWTNLSP
>d1kkca1 a.2.11.1 (A:14-97) Mn superoxide dismutase (MnSOD) {Aspergillus fumigatus}
QQYTLPPLPYPYDALQPYISQQIMELHHKKHHQTYVNGLNAALEAQKKAAEATDVPKLVSVQ
QAIKFNGGGHINHSLFWKNLAP
>d1i0ha1 a.2.11.1 (A:1-90) Mn superoxide dismutase (MnSOD) {Escherichia coli}
SYTLPSLPYAYDALEPHFDKQTMEIHHTKHHQTYVNNANAALESLPEFANLPVEELITKLDQ

LPADKKTVLRNNAGGHANHSLFWKGLKK
>d1mnga1 a.2.11.1 (A:1-92) Mn superoxide dismutase (MnSOD) {Thermus thermophilus}
PYPFKLPDLGYPYEALEPHIDAKTMEIHHQKHHGAYVTNLNAALEKYPYLHGVEVEVLLRHL
AALPQDIQTAVRNNGGGHLNHSLFWRLLTP
>d1bsma1 a.2.11.1 (A:1-86) Cambialistic superoxide dismutase {Propionibacterium shermanii}
AVYTLPELPYDYSALEPYISGEIMELHHDKHHKAYVDGANTALDKLAEARDKADFGAINKLE
KDLAFNLAGHVNHSVFWKNMAPKG
>d1qnna1 a.2.11.1 (A:1-84) Cambialistic superoxide dismutase {Porphyromonas gingivalis}
MTHELISLPYAVDALAPVISKETVEFHHGKHLKTYVDNLNKLIIGTEFENADLNTIVQKSEG
GIFNNAGQTLNHNLYFTQFRPG
>d1dava_ a.139.1.1 (A:) Cellulosome endoglucanase SS {Clostridium thermocellum}
MSTKLYGDVNDDGKVNSTDAVALKRYVLRSGISINTDNADLNEDGRVNSTDLGILKRYILKE
IDTLPYKNG
>d1h9ea_ a.140.1.1 (A:) Thymopoietin, LAP2 {Human (Homo sapiens)}
PEFLEDPSVLTKDKLKSELVANNVTLPAGEQRKDVYVQLYLQHLTARNRPPLPAGT
>d1h9fa_ a.140.1.1 (A:) Thymopoietin, LAP2 {Human (Homo sapiens)}
RQEDKDDLDVTELTNEDLLDQLVKYGVNPGPIVGTTRKLYEKKLLKLREQGTESRSS
>d1jeia_ a.140.1.1 (A:) Inner nuclear membrane protein emerin {Human (Homo sapiens)}
DNYADLSDTELTTLLRRYNIPHGPVVGSTRRLYEKKIFEYETQRRRLSPPSSS
>d1jeqa1 a.140.2.1 (A:559-609) DNA binding C-terminal domain of ku70 {Human (Homo sapiens)}
YSEEELKTHISKGTLGKFTVPMLKEACRAYGLKSGLKKQELLEALTKHFQD
>d1kcfa1 a.140.2.1 (A:3-38) Mitochondrial resolvase ydc2 N-terminal domain {Fission yeast (Schizosaccharomyces pombe)}
TVKLSFLQHICKLTGLSRSGRKDELLRRIVDSPIYP
>d1a62_1 a.140.3.1 (1-47) Rho termination factor, N-terminal domain {Escherichia coli}
MNLTELKNTPVSELITLGENMGLENLARMRKQDIIFAILKQHAKSGE
>d1e7la1 a.140.4.1 (A:104-157) Recombination endonuclease VII, C-terminal and dimerization domains {Bacteriophage T4}
IHPNFVGDKSKEFSRLGKEEMMAEMLQRGFEYNESDTKTQLIASFKKQLRKSLK
>d1c75a_ a.3.1.1 (A:) Cytochrome c6 (synonym: cytochrome c553) {Bacillus pasteurii}
VDAEAVVQQKCISCHGGDLTGASAPAIDKAGANYSEEEILDIILNGQGGMPGGIAKGAEAEA
VAAWLAEKK
>d1ctj__ a.3.1.1 (-) Cytochrome c6 (synonym: cytochrome c553) {Monoraphidium braunii}
EADLALGKAVFDGNCAACHAGGGNNVIPDHTLQKAAIEQFLDGGFNIEAIVYQIENGKGAMP
AWDGRLDEDEIAGVAAYVYDQAAGNKW

>d1c53__ a.3.1.1 (-) Cytochrome c6 (synonym: cytochrome c553) {Desulfovibrio vulgaris, different strains}
ADGAALYKSCVGCHGADGSKQAMGVGHAVKGQKADELFKKLKGYADGSYGGEKKAVMTNLVK
RYSDEEMKAMADYMSKL
>d2dvh__ a.3.1.1 (-) Cytochrome c6 (synonym: cytochrome c553) {Desulfovibrio desulfuricans}
ADGAALYKSCIGCHGADGSKAAMGSAKPVKGQGAEELYKKMKGYADGSYGGERKAMMTNAVK
KASDEELKALADYMSKL
>d1cyi__ a.3.1.1 (-) Cytochrome c6 (synonym: cytochrome c553) {Chlamydomonas reinhardtii}
ADLALGAQVFNGNCAACHMGGRNSVMPEKTLDKAALEQYLDGGFKVESIIYQVENGKGAMPA
WADRLSEEEIQAVAEYVFKQATDAAWK
>d1c6s__ a.3.1.1 (-) Cytochrome c6 (synonym: cytochrome c553) {Cyanobacterium (Synechococcus elongatus)}
ADLANGAKVFSGNCAACHMGGGNVVMANKTLKKEALEQFGMYSEDAIIYQVQHGKNAMPAFA
GRLTDEQIQDVAAYVLDQAAKGWAG
>d1f1fa_ a.3.1.1 (A:) Cytochrome c6 (synonym: cytochrome c553) {Arthrospira maxima}
DVAAGASVFSANCAACHMGGRNVIVANKTLSKSDLAKYLKGFDDDAVAAVAYQVTNGKNAMP
GFNGRLSPLQIEDVAAYVVDQAEKGW
>d1c6ra_ a.3.1.1 (A:) Cytochrome c6 (synonym: cytochrome c553) {Green alga (Scenedesmus obliquus)}
ADLALGKQTFEANCAACHAGGNNSVIPDHTLRKAAMEQFLQGGFNLEAITYQVENGKGAMPA
WSGTLDDDEIAAVAAYVYDQASGDKW
>d1gdva_ a.3.1.1 (A:) Cytochrome c6 (synonym: cytochrome c553) {Red alga (Porphyra yezoensis)}
ADLDNGEKVFSANCAACHAGGNNAIMPDKTLKKDVLEANSMNTIDAITYQVQNGKNAMPAFG
GRLVDEDIEDAANYVLSQSEKGW
>d1c52__ a.3.1.1 (-) Cytochrome c552 {Thermus thermophilus}
QADGAKIYAQCAGCHQQNGQGIPGAFPPLAGHVAEILAKEGGREYLILVLLYGLQGQIEVKG
MKYNGVMSSFAQLKDEEIAAVLNHIATAWGDAKKVKGFKPFTAEEVKKLRAKKLTPQQVLAE
RKKLGLK
>d1cnoa_ a.3.1.1 (A:) Cytochrome c552 {Pseudomonas nautica}
AGDIEAGKAKAAVCAACHGQNGISQVPIYPNLAGQKEQYLVAALKAYKAGQRQGGQAPVMQG
QATALSDADIANLAAYYASNPAAA
>d1ql3a_ a.3.1.1 (A:) Cytochrome c552 {Paracoccus denitrificans}
ADPAAGEKVFGKCKACHKLDGNDGVGPHLNGVVGRTVAGVDGFNYSDPMKAHGGDWTPEALQ
EFLTNPKAVVKGTKMAFAGLPKIEDRANLIAYLEGQQ
>d1ayg__ a.3.1.1 (-) Cytochrome c552 {Hydrogenobacter thermophilus}
NEQLAKQKGCMACHDLKAKKVGPAYADVAKKYAGRKDAVDYLAGKIKKGGSGVWGSVPMPPQ
NVTDAEAKQLAQWILSIK
>d1a56__ a.3.1.1 (-) Cytochrome c552 {Nitrosomonas europaea}
DADLAKKNNCIACHQVETKVVGPALKDIAAKYADKDDAATYLAGKIKGGSSGVWGQIPMPPN

VNVSDADAKALADWILTLK
>d1e29a_ a.3.1.1 (A:) Photosystem II associated cytochrome c549 {Synechocystis sp., pcc 6803}
VELTESTRTIPLDEAGGTTTLTARQFTNGQKIFVDTCTQCHLQGKTKTNNNVSLGLADLAGA
EPRRDNVLALVEFLKNPKSYDGEDDYSELHPNISRPDIYPEMRNYTEDDIFDVAGYTLIAPK
LDERWGGTIYF
>d1f1ca_ a.3.1.1 (A:) Photosystem II associated cytochrome c549 {Arthrospira maxima}
LTEELRTFPINAQGDTAVLSLKEIKKGQQVFNAACAQCHALGVTRTNPDVNLSPEALALATP
PRDNIAALVDYIKNPTTYDGFVEISELHPSLKSSDIFPKMRNISEDDLYNVAGYILLQPKVR
GEQWG
>d1ycc__ a.3.1.1 (-) Mitochondrial cytochrome c {Baker's yeast (Saccharomyces cerevisiae)}
TEFKAGSAKKGATLFKTRCLQCHTVEKGGPHKVGPNLHGIFGRHSGQAEGYSYTDANIKKNV
LWDENNMSEYLTNPKKYIPGTKMAFGGLKKEKDRNDLITYLKKACE
>d1yeb__ a.3.1.1 (-) Mitochondrial cytochrome c {Baker's yeast (Saccharomyces cerevisiae)}
TEFKAGSAKKGATLFKTRCQQCHTIEEGGPNKVGPNLHGIFGRHSGQVKGYSYTDANINKNV
KWDEDSMSEYLTNPKKYIPGTKMAFGGLKKEKDRNDLITYLKKACE
>d1ytc__ a.3.1.1 (-) Mitochondrial cytochrome c {Baker's yeast (Saccharomyces cerevisiae)}
AKESTGFKPGSAKKGATLFKTRCQQCHTIEEGGPNKVGPNLHGIFGRHSGQVKGYSYTDAII
NKNVKWDEDSMSEYLTNPKKYIPGTKMAFAGLKKEKDRNDLITYMTKAAK
>d1wejf_ a.3.1.1 (F:) Mitochondrial cytochrome c {Horse (Equus caballus)}
GDVEKGKKIFVQKCAQCHTVEKGGKHKTGPNLHGLFGRKTGQAPGFTYTDANKNKGITWKEE
TLMEYLENPKKYIPGTKMIFAGIKKKTEREDLIAYLKKATNE
>d1ccr__ a.3.1.1 (-) Mitochondrial cytochrome c {Rice embryos (Oryza sativa)}
ASFSEAPPGNPKAGEKIFKTKCAQCHTVDKGAGHKQGPNLNGLFGRQSGTTPGYSYSTADKN
MAVIWEENTLYDYLLNPKKYIPGTKMVFPGLKKPQERADLISYLKEATS
>d5cytr_ a.3.1.1 (R:) Mitochondrial cytochrome c {Tuna (Thunnus alalunga and Thunnus thynnus)}
GDVAKGKKTFVQKCAQCHTVENGGKHKVGPNLWGLFGRKTGQAEGYSYTDANKSKGIVWNND
TLMEYLENPKKYIPGTKMIFAGIKKKGERQDLVAYLKSATS
>d1qn2a_ a.3.1.1 (A:) Cytochrome ch {Methylobacterium extorquens}
EGDAAAGEKAFAPCKACHNFEKNGVGPTLKGVVGAKAGEGADGYAFSDALKKSGLTWDQADL
KQWLADPKKKVPGTKMVFPGISDPKKVDDIIAYLKTK
>d3c2c__ a.3.1.1 (-) Cytochrome c2 {Rhodospirillum rubrum}
EGDAAAGEKVSKKCLACHTFDQGGANKVGPNLFGVFENTAAHKDNYAYSESYTEMKAKGLTW
TEANLAAYVKNPKAFVLEKSGDPKAKSKMTFKLTKDDEIENVIAYLKTLK
>d1c2ra_ a.3.1.1 (A:) Cytochrome c2 {Rhodobacter capsulatus}
GDAAKGEKEFNKCKTCHSIIAPDGTEIVKGAKTGPNLYGVVGRTAGTYPEFKYKDSIVALGA
SGFAWTEEDIATYVKDPGAFLKEKLDDKKAKTGMAFKLAKGGEDVAAYLASVVK

>d1cxc__ a.3.1.1 (-) Cytochrome c2 {Rhodobacter sphaeroides}
QEGDPEAGAKAFNQCQTCHVIVDDSGTTIAGRNAKTGPNLYGVVGRTAGTQADFKGYGEGMK
EAGAKGLAWDEEHFVQYVQDPTKFLKEYTGDAKAKGKMTFKLKKEADAHNIWAYLQQVAVRP
>d1co6a_ a.3.1.1 (A:) Cytochrome c2 {Rhodopseudomonas viridis}
QDAASGEQVFKQCLVCHSIGPGAKNKVGPVLNGLFGRHSGTIEGFAYSDANKNSGITWTEEV
FREYIRDPKAKIPGTKMIFAGVKDEQKVSDLIAYIKQFNADGSKK
>d1i8oa_ a.3.1.1 (A:) Cytochrome c2 {Rhodopseudomonas palustris}
EDAKAGEAVFKQCMTCHRADKNMVGPALAGVVGRKAGTAAGFTYSPLNHNSGEAGLVWTADN
IVPYLADPNAFLKKFLTEKGKADQAVGVTKMTFKLANEQQRKDVVAYLATLK
>d1hroa_ a.3.1.1 (A:) Cytochrome c2 {Rhodopila globiformis}
SAPPGDPVEGKHLFHTICITCHTDIKGANKVGPSLYGVVGRHSGIEPGYNYSEANIKSGIVW
TPDVLFKYIEHPQKIVPGTKMGYPGQPDPQKRADIIAYLETLK
>d155c__ a.3.1.1 (-) Cytochrome c2 {Paracoccus denitrificans}
NEGDAAKGEKEFNKCKACHMIQAPDGTDIKGGKTGPNLYGVVGRKIASEEGFKYGEGILEVA
EKNPDLTWTEANLIEYVTDPKPLVKKMTDDKGAKTKMTFKMGKNQADVVAFLAQDDPDAXXX
XXXXXXXXXX
>d1cot__ a.3.1.1 (-) Cytochrome c2 {Paracoccus denitrificans}
DGDAAKGEKEFNKCKACHMIQAPDGTDIIKGGKTGPNLYGVVGRKIASEEGFKYGEGILEVA
EKNPDLTWTEADLIEYVTDPKPWLVKMTDDKGAKTKMTFKMGKNQADVVAFLAQNSPDA
>d1jdla_ a.3.1.1 (A:) Cytochrome c2 {Rhodospirillum centenum}
GDPAKGEAVFKKCMACHRVGPDAKNLVGPALTGVIDRQAGTAPGFNYSAINHAAGEAGLHWT
PENIIAYLPDPNAFLRKFLADAGHAEQAKGSTKMVFKLPDEQERKDVVAYLKQFSP
>d1cc5__ a.3.1.1 (-) Cytochrome c5 {Azotobacter vinelandii}
GGGARSGDDVVAKYCNACHGTGLLNAPKVGDSAAWKTRADAKGGLDGLLAQSLSGLNAMPPK
GTCADCSDDELKAAIGKMSGL
>d1kx2a_ a.3.1.1 (A:) Mono-heme c-type cytochrome ScyA {Shewanella putrefaciens}
ADLQDAEAIYNKACTVCHSMGVAGAPKSHNTADWEPRLAKGVDNLVKSVKTGLNAMPPGGMC
TDCTDEDYKAAIEFMSKAK
>d1cch__ a.3.1.1 (-) Cytochrome c551 {Pseudomonas stutzeri}
QDGEALFKSKPCAACHSVDTKMVGPALKEVAAKNAGVEGAADTLALHIKNGSQGVWGPIPMP
PNPVTEEEAKILAEWVLSLK
>d1cor__ a.3.1.1 (-) Cytochrome c551 {Pseudomonas stutzeri}
EDGEALFKSKPCAACHSIDAKLVGPAFKEVAAKYAGQDGAADLLAGHIKNGSQGVWGPIPMP
PNPVTEEEAKILAEWILSQK
>d1dvva_ a.3.1.1 (A:) Cytochrome c551 {Pseudomonas aeruginosa}
EDPEVLAKNKGCMACHAIDTKMVGPAYKDVAAKYAGQAGAEAYLAQRIKNGSQGVWGPIPMP
PNAVSDDEAQTLAKWILSQK
>d451c__ a.3.1.1 (-) Cytochrome c551 {Pseudomonas aeruginosa}
EDPEVLFKNKGCVACHAIDTKMVGPAYKDVAAKFAGQAGAEAELAQRIKNGSQGVWGPIPMP
PNAVSDDEAQTLAKWVLSQK
>d2mtac_ a.3.1.1 (C:) Cytochrome c551 {Paracoccus denitrificans}
APQFFNIIDGSPLNFDDAMEEGRDTEAVKHFLETGENVYNEDPEILPEAEELYAGMCSGCHG
HYAEGKIGPGLNDAYWTYPGNETDVGLFSTLYGGATGQMGPMWGSLTLDEMLRTMAWVRHLY

TGDPKDASWLTDEQKAGFTPFQP
>d1gks__ a.3.1.1 (-) Cytochrome c551 {Ectothiorhodospira halophila}
DGESIYINGTAPTCSSCHDRGVAGAPELNAPEDWADRPSSVDELVESTLAGKGAMPAYDGRA
DREDLVKAIEYMLSTL
>d05c1__ a.3.1.1 (-) Cytochrome c555 {Chlorobium thiosulfatophilum}
YDAAAGKATYDASCAMCHKTGMMGAPKVGDKAAWAPHIAKGMNVMVANSIKGYKGTKGMMPA
KGGNPKLTDAQVGNAVAYMVGQSK
>d1dw0a_ a.3.1.1 (A:) SHP, an oxygen binding cytochrome c {Rhodobacter sphaeroides}
GDTSPAQLIAGYEAAAGAPADAERGRALFLSTQTGGKPDTPSCTTCHGADVTRAGQTRTGKE
IAPLAPSATPDRFTDSARVEKWLGRNCNSVIGRDCTPGEKADLLAWLAAQ
>d1e8ea_ a.3.1.1 (A:) Cytochrome c'' {Methylophilus methylotrophus, strain w3a1}
DVTNAEKLVYKYTNIAHSANPMYEAPSITDGKIFFNRKFKTPSGKEAACASCHTNNPANVGK
NIVTGKEIPPLAPRVNTKRFTDIDKVEDEFTKHCNDILGADCSPSEKANFIAYLLTETKPTK
>d1diqc_ a.3.1.1 (C:) p-Cresol methylhydroxylase, cytochrome c subunit {Pseudomonas putida}
SQWGSGKNLYDKVCGHCHKPEVGVGPVLEGRGLPEAYIKDIVRNGFRAMPAFPASYVDDESL
TQVAEYLSSLPA
>d1dy7b1 a.3.1.2 (B:32-135) N-terminal (heme c) domain of cytochrome cd1-nitrite reductase {Paracoccus pantotrophus}
LAQQDVAAPGAPEGVSALSDAQYNEANKIYFERCAGCHGVLRKGATGKALTPDLTRDLGFDY
LQSFITYGSPAGMPNWGTSGELSAEQVDLMANYLLLDPAAPP
>d1h9xa1 a.3.1.2 (A:42-133) N-terminal (heme c) domain of cytochrome cd1-nitrite reductase {Paracoccus pantotrophus}
APEGVSALSDAQYNEANKIYFERCAGCHGVLRKGATGKALTPDLTRDLGFDYLQSFITYGSP
AGMPNWGTSGELSAEQVDLMANYLLLDPAA
>d1hj3a1 a.3.1.2 (A:17-133) N-terminal (heme c) domain of cytochrome cd1-nitrite reductase {Paracoccus pantotrophus}
HKTRTDNRYEPSLDNLAQQDVAAPGAPEGVSALSDAQYNEANKIYFERCAGCHGVLRKGATG
KALTPDLTRDLGFDYLQSFITYGSPAGMPNWGTSGELSAEQVDLMANYLLLDPAA
>d1hzua1 a.3.1.2 (A:23-117) N-terminal (heme c) domain of cytochrome cd1-nitrite reductase {Pseudomonas aeruginosa}
VRTNGAPDMSESEFNEAKQIYFQRCAGCHGVLRKGATGKPLTPDITQQRGQQYLEALITYGT
PLGMPNWGSSGELSKEQITLMAKYIQHTPPQPP
>d1nira1 a.3.1.2 (A:6-117) N-terminal (heme c) domain of cytochrome cd1-nitrite reductase {Pseudomonas aeruginosa}
AAEQYQGAASAVDPAHVVRTNGAPDMSESEFNEAKQIYFQRCAGCHGVLRKGATGKPLTPDI
TQQRGQQYLEALITYGTPLGMPNWGSSGELSKEQITLMAKYIQHTPPQPP
>d1e2rb1 a.3.1.2 (B:25-135) N-terminal (heme c) domain of cytochrome cd1-nitrite reductase {Paracoccus denitrificans}
YEPSLDNLAQQDVAAPGAPEGVTALSDAQYNEANKIYFERCAGCHGVLRKGATGKALTPDLT

RDLGFDYLQSFITYASPAGMPNWGTSGELSAEQVDLMANYLLLDPAAPP
>d1qksa1 a.3.1.2 (A:9-135) N-terminal (heme c) domain of cytochrome cd1-nitrite reductase {Paracoccus denitrificans}
DPAAALEDHKTRTDNRYEPSLDNLAQQDVAAPGAPEGVTALSDAQYNEANKIYFERCAGCHG
VLRKGATGKALTPDLTRDLGFDYLQSFITYASPAGMPNWGTSGELSAEQVDLMANYLLLDPA
APP
>d1kb0a1 a.3.1.6 (A:579-675) Quinoprotein alcohol dehydrogenase, C-terminal domain {Comamonas testosteroni}
TGQLLQGVKYDPAKVEAGTMLYVANCVFCHGVPGVDRGGNIPNLGYMDASYIENLPNFVFKG
PAMVRGMPDFTGKLSGDDVESLKAFIQGTADAIRP
>d1qcrd2 a.3.1.3 (D:167-195) Cytochrome bc1 domain {Cow (Bos taurus)}
EVLEFDDGTPATMSQVAKDVCTFLRWAAE
>d1bccd2 a.3.1.3 (D:1-195) Cytochrome bc1 domain {Chicken (Gallus gallus)}
SDLELHPPSYPWSHRGPLSSLDHTSIRRGFQVYKQVCSSCHSMDYVAYRHLVGVCYTEDEAK
ALAEEVEVQDGPNEDGEMFMRPGKLSDYFPKPYPNPEAARAANNGALPPDLSYIVRARHGGE
DYVFSLLTGYCEPPTGVSVREGLYFNPYFPGQAIGMAPPIYNDVLEFDDGTPATMSQVAKDV
CTFLRWAAE
>d1ezvd1 a.3.1.3 (D:62-260) Cytochrome bc1 domain {Baker's yeast (Saccharomyces cerevisiae)}
MTAAEHGLHAPAYAWSHNGPFETFDHASIRRGYQVYREVCAACHSLDRVAWRTLVGVSHTNE
EVRNMAEEFEYDDEPDEQGNPKKRPGKLSDYIPGPYPNEQAARAANQGALPPDLSLIVKARH
GGCDYIFSLLTGYPDEPPAGVALPPGSNYNPYFPGGSIAMARVLFDDMVEYEDGTPATTSQM
AKDVTTFLNWCAE
>d1etpa1 a.3.1.4 (A:1-92) Cytochrome c4 {Pseudomonas stutzeri}
AGDAEAGQGKVAVCGACHGVDGNSPAPNFPKLAGQGERYLLKQLQDIKAGSTPGAPEGVGRK
VLEMTGMLDPLSDQDLEDIAAYFSSQKGSV
>d1etpa2 a.3.1.4 (A:93-190) Cytochrome c4 {Pseudomonas stutzeri}
GYADPALAKQGEKLFRGGKLDQGMPACTGCHAPNGVGNDLAGFPKLGGQHAAYTAKQLTDFR
EGNRTNDGDTMIMRGVAAKLSNKDIEALSSYIQGLH
>d1fcdc1 a.3.1.4 (C:1-80) Flavocytochrome c sulfide dehydrogenase, FCSD, cytochrome subunit {Purple phototrophic bacterium (Chromatium vinosum)}
EPTAEMLTNNCAGCHGTHGNSVGPASPSIAQMDPMVFVEVMEGFKSGEIASTIMGRIAKGYS
TADFEKMAGYFKQQTYQP
>d1fcdc2 a.3.1.4 (C:81-174) Flavocytochrome c sulfide dehydrogenase, FCSD, cytochrome subunit {Purple phototrophic bacterium (Chromatium vinosum)}
AKQSFDTALADTGAKLHDKYCEKCHVEGGKPLADEEDYHILAGQWTPYLQYAMSDFREERRP
MEKKMASKLRELLKAEGDAGLDALFAFYASQQ
>d1eb7a1 a.3.1.5 (A:1-164) Di-haem cytochrome c peroxidase {Pseudomonas aeruginosa}
DALHDQASALFKPIPEQVTELRGQPISEQQRELGKKLFFDPRLSRSHVLSCNTCHNVGTGGA

DNVPTSVGHGWQKGPRNSPTVFNAVFNAAQFWDGRAKDLGEQAKGPIQNSVEMHSTPQLVEQ
TLGSIPEYVDAFRKAFPKAGKPVSFDNMALAIEAYEATLV
>d1eb7a2 a.3.1.5 (A:165-323) Di-haem cytochrome c peroxidase {Pseudomonas aeruginosa}
TPDSPFDLYLKGDDKALDAQQKKGLKAFMDSGCSACHNGINLGGQAYFPFGLVKKPDASVLP
SGDKGRFAVTKTQSDEYVFRAAPLRNVALTAPYFHSGQVWELKDAVAIMGNAQLGKQLAPDD
VENIVAFLHSLSGKQPRVEYPLLPASTETTPRPAE
>d1iqca1 a.3.1.5 (A:1-150) Di-haem cytochrome c peroxidase {Nitrosomonas europaea}
ANEPIQPIKAVTPENADMAELGKMLFFDPRLSKSGFISCNSCHNLSMGGTDNITTSIGHKWQ
QGPINAPTVLNSSMNLAQFWDGRAKDLKEQAAGPIANPKEMASTHEIAEKVVASMPQYRERF
KKVFGSDEVTIDRITTAIAQFEETLV
>d1iqca2 a.3.1.5 (A:151-308) Di-haem cytochrome c peroxidase {Nitrosomonas europaea}
TPGSKFDKWLEGDKNALNQDELEGYNLFKGSGCVQCHNGPAVGGSSYQKMGVFKPYETKNPA
AGRMDVTGNEADRNVFKVPTLRNIELTYPYFHDGGAATLEQAVETMGRIQLNREFNKDEVSK
IVAFLKTLTGDQPDFKLPILPPSNNDTPRSQPYE
>d1jjua1 a.3.1.7 (A:1-85) Quinohemoprotein amine dehydrogenase A chain, domains 1 and 2 {Paracoccus denitrificans}
VTGEEVLQNACAACHVQHEDGRWERIDAARKTPEGWDMTVTRMMRNHGVALEPEERAAIVRH
LSDTRGLSLAETEERRYILEREP
>d1jjua2 a.3.1.7 (A:86-165) Quinohemoprotein amine dehydrogenase A chain, domains 1 and 2 {Paracoccus denitrificans}
VAWDEGPDTSMTQTCGRCHSYARVALQRRTPEDWKHLVNFHLGQFPTLEYQALARDRDWWGI
AQAEIIPFLARTYPLGEA
>d1jmxa1 a.3.1.7 (A:2-85) Quinohemoprotein amine dehydrogenase A chain, domains 1 and 2 {Pseudomonas putida}
EQGPSLLQNKCMGCHIPEGNDTYSRISHQRKTPEGWLMSIARMQVMHGLQISDDDRRTLVKY
LADKQGLAPSETDGVRYAMERR
>d1jmxa2 a.3.1.7 (A:86-162) Quinohemoprotein amine dehydrogenase A chain, domains 1 and 2 {Pseudomonas putida}
LNTVEQFDTQLSETCGRCHSGARVALQRRPAKEWEHLVNFHLGQWPSLEYQAQARDRDWLPI
ALQQVVPDLAKRYPL
>d1enh__ a.4.1.1 (-) Engrailed Homeodomain {Drosophila melanogaster}
RPRTAFSSEQLARLKREFNENRYLTERRRQQLSSELGLNEAQIKIWFQNKRAKI
>d2hdda_ a.4.1.1 (A:) Engrailed Homeodomain {Drosophila melanogaster}
RTAFSSEQLARLKREFNENRYLTERRRQQLSSELGLNEAQIKIWFKNKRAKIKKS
>d1akha_ a.4.1.1 (A:) Mating type protein A1 Homeodomain {Baker's yeast (Saccharomyces cerevisiae)}
ISPQARAFLEEVFRRKQSLNSKEKEEVAKKCGITPLQVRVWFINKRMRS
>d1f43a_ a.4.1.1 (A:) Mating type protein A1 Homeodomain {Baker's yeast (Saccharomyces cerevisiae)}

KKEKSPKGKSSISPQARAFLEQVFRRKQSLNSKEKEEVAKKCGITPLQVRVWFINKRMRSK
>d1akhb_ a.4.1.1 (B:) mat alpha2 Homeodomain {Baker's yeast (Saccharomyces cerevisiae)}
TKPYRGHRFTKENVRILESWFAKNIENPYLDTKGLENLMKNTSLSRIQIKNWVSNRRRKEKT
ITIAPELADLLSGEPL
>d1aplc_ a.4.1.1 (C:) mat alpha2 Homeodomain {Baker's yeast (Saccharomyces cerevisiae)}
YRGHRFTKENVRILESWFAKNIENPYLDTKGLENLMKNTSLSRIQIKNWVSNRRRKEKT
>d1mnmc_ a.4.1.1 (C:) mat alpha2 Homeodomain {Baker's yeast (Saccharomyces cerevisiae)}
GLVFNVVTQDMINKSTKPYRGHRFTKENVRILESWFAKNIENPYLDTKGLENLMKNTSLSRI
QIKNWVSNRRRKEKT
>d1lfb__ a.4.1.1 (-) Transcription factor LFB1 {Rat (Rattus rattus)}
RFKWGPASQQILFQAYERQKNPSKEERETLVEECNRAECIQRGVSPSQAQGLGSNLVTEVRV
YNWFANRRKEEAFRHK
>d2lfb__ a.4.1.1 (-) Transcription factor LFB1 {Rat (Rattus rattus)}
MARIDPTKKGRRNRFKWGPASQQILFQAYERQKNPSKEERETLVEECNRAECIQRGVSPSQA
QGLGSNLVTEVRVYNWFANRRKEEAFRHKLAMDTYKLN
>d1e3oc1 a.4.1.1 (C:104-160) Oct-1 POU Homeodomain {Human (Homo sapiens)}
KRTSIETNIRVALEKSFMENQKPTSEDITLIAEQLNMEKEVIRVWFSNRRQKEKRIN
>d1hf0a1 a.4.1.1 (A:102-159) Oct-1 POU Homeodomain {Human (Homo sapiens)}
RKKRTSIETNIRVALEKSFLENQKPTSEEITMIADQLNMEKEVIRVWFSNRRQKEKRI
>d1au7a1 a.4.1.1 (A:103-160) Pit-1 POU homeodomain {Rat (Rattus norvegicus)}
KRRTTISIAAKDALERHFGEHSKPSSQEIMRMAEELNLEKEVVRVWFCNRRQREKRVK
>d1ftt__ a.4.1.1 (-) Thyroid transcription factor 1 homeodomain {Rat (Rattus norvegicus)}
MRRKRRVLFSQAQVYELERRFKQQKYLSAPEREHLASMIHLTPTQVKIWFQNHRYKMKRQAK
DKAAQQ
>d1hdp__ a.4.1.1 (-) Oct-2 POU Homeodomain {Human (Homo sapiens)}
RRKKRTSIETNVRFALEKSFLANQKPTSEEILLIAEQLHMEKEVIRVWFCNRRQKEKRINPC
S
>d1ocp__ a.4.1.1 (-) Oct-3 POU Homeodomain {Mouse (Mus musculus)}
METLVQARKRKRTSIENRVRWSLETMFLKCPKPSLQQITHIANQLGLEKDVVRVWFCNRRQK
GKRSS
>d1b72a_ a.4.1.1 (A:) Homeobox protein hox-b1 {Human (Homo sapiens)}
ARTFDWMKVKRNPPKTAKVSEPGLGSPSGLRTNFTTRQLTELEKEFHFNKYLSRARRVEIAA
TLELNETQVKIWFQNRRMKQKKRERE
>d1b72b_ a.4.1.1 (B:) pbx1 {Human (Homo sapiens)}

RKRRNFNKQATEILNEYFYSHLSNPYPSEEAKEELAKKCGITVSQVSNWFGNKRIRYKKNIG
KFQEEANIYAA
>d1du6a_ a.4.1.1 (A:) pbx1 {Mouse (Mus musculus)}
SSGHIEGRHMNKQATEILNEYFYSHLSNPYPSEEAKEELAKKCGITVSQVSNWFGNKRIRYK
KN
>d1bw5__ a.4.1.1 (-) Insulin gene enhancer protein isl-1 {Rat
(Rattus norvegicus)}
MKTTRVRTVLNEKQLHTLRTCYAANPRPDALMKEQLVEMTGLSPRVIRVWFQNKRCKDKKRS
IMMK
>d1ig7a_ a.4.1.1 (A:) Msx-1 homeodomain {Mouse (Mus musculus)}
RKPRTPFTTAQLLALERKFRQKQYLSIAERAEFSSSLSLTETQVKIWFQNRRAKAKRL
>d1ahdp_ a.4.1.1 (P:) Antennapedia Homeodomain {Drosophila
melanogaster}
MRKRGRQTYTRYQTLELEKEFHFNRYLTRRRRIEIAHALSLTERQIKIWFQNRRMKWKKENK
TKGEPG
>d1san__ a.4.1.1 (-) Antennapedia Homeodomain {Drosophila
melanogaster}
MTYTRYQTLELEKEFHFNRYLTRRRRIEIAHALSLTERQIKIWFQNRRMKWKKENKTKGEPG
>d9anta_ a.4.1.1 (A:) Antennapedia Homeodomain {Drosophila
melanogaster}
RQTYTRYQTLELEKEFHFNRYLTRRRRIEIAHALSLTERQIKIWFQNRRMKWKKEN
>d1b8ia_ a.4.1.1 (A:) Ultrabithorax (ubx) homeodomain {Drosophila
melanogaster}
FYPWMAIAGTNGLRRRGRQTYTRYQTLELEKEFHTNHYLTRRRRIEMAHALSLTERQIKIWF
QNRRMKLKKEI
>d1b8ib_ a.4.1.1 (B:) Extradenticle (exd) homeodomain {Drosophila
melanogaster}
RRNFSKQASEILNEYFYSHLSNPYPSEEAKEELARKCGITVSQVSNWFGNKRIRYKKN
>d1jgga_ a.4.1.1 (A:) Even-skipped homeodomain {Fruit fly
(Drosophila melanogaster)}
RYRTAFTRDQLGRLEKEFYKENYVSRPRRCELAAQLNLPESTIKVWFQNRRMKDKRQ
>d1ftz__ a.4.1.1 (-) Fushi Tarazu protein {Fruit fly (Drosophila
melanogaster)}
MDSKRTRQTYTRYQTLELEKEFHFNRYITRRRRIDIANALSLSERQIKIWFQNRRMKSKKDR
TLDSSPEH
>d1nk3p_ a.4.1.1 (P:) VND/NK-2 protein {Fruit fly (Drosophila
melanogaster)}
KKRKRRVLFTKAQTYELERRFRQQRYLSAPEREHLASLIRLTPTQVKIWFQNHRYKTKRAQN
E
>d1vnd__ a.4.1.1 (-) VND/NK-2 protein {Fruit fly (Drosophila
melanogaster)}
ASDGLPNKKRKRRVLFTKAQTYELERRFRQQRYLSAPEREHLASLIRLTPTQVKIWFQNHRY
KTKRAQNEKGYEGHP
>d1fjla_ a.4.1.1 (A:) Paired protein {Fruit fly (Drosophila

melanogaster)}
KQRRSRTTFSASQLDELERAFERTQYPDIYTREELAQRTNLTEARIQVWFQNRRARLRKQHT
SVS
>d1fjlb_ a.4.1.1 (B:) Paired protein {Fruit fly (Drosophila
melanogaster)}
QRRSRTTFSASQLDELERAFERTQYPDIYTREELAQRTNLTEARIQVWFQNRRARLRK
>d1hcra_ a.4.1.2 (A:) HIN recombinase (DNA-binding domain)
{Synthetic}
GRPRAINKHEQEQISRLLEKGHPRQQLAIIFGIGVSTLYRYFPASSIKKRMN
>d1ijwc_ a.4.1.2 (C:) HIN recombinase (DNA-binding domain)
{Synthetic}
GRPRAINKHEQEQISRLLEKGHPRQQLAIIFGIGVSTLYRYFPASSI
>d1gdta1 a.4.1.2 (A:141-183) gamma,delta resolvase (C-terminal
domain) {Escherichia coli}
GRKRKIDRDAVLNMWQQGLGASHISKTMNIARSTVYKVINESN
>d1tc3c_ a.4.1.2 (C:) Transposase tc3a1-65 {Caenorhabditis
elegans}
PRGSALSDTERAQLDVMKLLNVSLHEMSRKISRSRHCIRVYLKDPVSYGTS
>d2ezl__ a.4.1.2 (-) Ibeta subdomain of the mu end DNA-binding
domain of phage mu transposase {Bacteriophage mu}
MIARPTLEAHDYDREALWSKWDNASDSQRRLAEKWLPAVQAADEMLNQGISTKTAFATVAGH
YQVSASTLRDKYYQVQKFAKPDWAAALVDGRGASRRN
>d2ezh__ a.4.1.2 (-) Transposase {Bacteriophage mu}
SEFDEDAWQFLIADYLRPEKPAFRKCYERLELAAREHGWSIPSRATAFRRIQQLDEAMVVAC
REG
>d2ezi__ a.4.1.2 (-) Transposase {Bacteriophage mu}
MNVHKSEFDEDAWQFLIADYLRPEKPAFRKCYERLELAAREHGWSIPSRATAFRRIQQLDEA
MVVACREGEHALM
>d1h88c1 a.4.1.3 (C:39-88) c-Myb, DNA-binding domain {Mouse (Mus
musculus)}
GKTRWTREEDEKLKKLVEQNGTDDWKVIANYLPNRTDVQCQHRWQKVLNP
>d1h88c2 a.4.1.3 (C:89-143) c-Myb, DNA-binding domain {Mouse (Mus
musculus)}
ELIKGPWTKEEDQRVIKLVQKYGPKRWSVIAKHLKGRIGKQCRERWHNHLNPEVK
>d1idz__ a.4.1.3 (-) c-Myb, DNA-binding domain {Mouse (Mus
musculus)}
MEVKKTSWTEEEDRILYQAHKRLGNRWAEIAKLLPGRTDNAIKNHWNSTMRRKV
>d1a5j_1 a.4.1.3 (1-55) b-Myb DNA binding domain {Chicken (Gallus
gallus)}
GIPDLVKGPWTKEEDQKVIELVKKYGTKQWTLIAKHLKGRLGKQCRERWHNHLNP
>d1a5j_2 a.4.1.3 (56-110) b-Myb DNA binding domain {Chicken (Gallus
gallus)}
EVKKSSWTEEEDRIIFEAHKVLGNRWAEIAKLLPGRTDNAVKNHWNSTIKRKVDT
>d1h8ac1 a.4.1.3 (C:87-143) v-Myb {Avian myeloblastosis virus}

NPELNKGPWTKEEDQRVIEHVQKYGPKRWSDIAKHLKGRIGKQCRERWHNHLNPEVK
>d1h8ac2 a.4.1.3 (C:144-191) v-Myb {Avian myeloblastosis virus}
KTSWTEEEDRIIYQAHKRLGNRWAEIAKLLPGRTDNAVKNHWNSTMRR
>d1fexa_ a.4.1.3 (A:) Rap1 {Human (Homo sapiens)}
GRIAFTDADDVAILTYVKENARSPSSVTGNALWKAMEKSSLTQHSWQSLKDRYLKHLRG
>d1ba5__ a.4.1.4 (-) DNA-binding domain of human telomeric protein, htrf1 {Human (Homo sapiens)}
RKRQAWLWEEDKNLRSGVRKYGEGNWSKILLHYKFNNRTSVMLKDRWRTMKKL
>d1k78a1 a.4.1.5 (A:19-81) Pax-5 {Human (Homo sapiens)}
GVNQLGGVFVNGRPLPDVVRQRIVELAHQGVRPCDISRQLRVSHGCVSKILGRYYETGSIKP
G
>d1k78a2 a.4.1.5 (A:82-142) Pax-5 {Human (Homo sapiens)}
VIGGSKPKVATPKVVEKIAEYKRQNPTMFAWEIRDRLLAERVCDNDTVPSVSSINRIIRTK
>d6paxa1 a.4.1.5 (A:1-68) Pax-6 {Human (Homo sapiens)}
SHSGVNQLGGVFVNGRPLPDSTRQRIVELAHSGARPCDISRILQVSNGCVSKILGRYYATGS
IRPRAI
>d6paxa2 a.4.1.5 (A:69-133) Pax-6 {Human (Homo sapiens)}
GGSKPRVATPEVVSKIAQYKQECPSIFAWEIRDRLLSEGVCTNDNIPSVSSINRVLRNLASE
KQQ
>d1pdnc_ a.4.1.5 (C:) Paired protein (prd) {Fruit fly (Drosophila melanogaster)}
QGRVNQLGGVFINGRPLPNNIRLKIVEMAADGIRPCVISRQLRVSHGCVSKILNRYQETGSI
RPGVIGGSKPRIATPEIENRIEEYKRSSPGMFSWEIREKLIREGVCDRSTAPSVSAISRLV
>d1igna1 a.4.1.6 (A:360-445) DNA-binding domain of rap1 {Baker's yeast (Saccharomyces cerevisiae)}
KASFTDEEDEFILDVVRKNPTRRTTHTLYDEISHYVPNHTGNSIRHRFRVYLSKRLEYVYEV
DKFGKLVRDDDGNLIKTKVLPPSI
>d1igna2 a.4.1.6 (A:446-594) DNA-binding domain of rap1 {Baker's yeast (Saccharomyces cerevisiae)}
KRKFSADEDYTLAIAVKKQFYRDLFQIDPDTGRSLITDEDTPTAIARRNMTMDPNHVPGSEP
NFAAYRTQSRRGPIAREFFKHFAEEHAAHTENAWRDRFRKFLLAYGIDDYISYYEAEKAQNR
EPEPMKNLTNRPKRPGVPTPGNYNS
>d1bw6a_ a.4.1.7 (A:) DNA-binding domain of centromere binding protein B (CENP-B) {Human (Homo sapiens)}
MGPKRRQLTFREKSRIIQEVEENPDLRKGEIARRFNIPPSTLSTILKNKRAILASE
>d1hlva1 a.4.1.7 (A:1-66) DNA-binding domain of centromere binding protein B (CENP-B) {Human (Homo sapiens)}
MGPKRRQLTFREKSRIIQEVEENPDLRKGEIARRFNIPPSTLSTILKNKRAILASERKYGVA
STCR
>d1hlva2 a.4.1.7 (A:67-131) DNA-binding domain of centromere binding protein B (CENP-B) {Human (Homo sapiens)}
KTNKLSPYDKLEGLLIAWFQQIRAAGLPVKGIILKEKALRIAEELGMDDFTASNGWLDRFRR
RRS
>d1g2ha_ a.4.1.10 (A:) Transcriptional regulator TyrR, C-terminal

domain {Haemophilus influenzae}
SAVISLDEFENKTLDEIIGFYEAQVLKLFYAEYPSTRKLAQRLGVSHTAIANKLKQYGIGK
>d1bl0a1 a.4.1.8 (A:9-62) MarA {Escherichia coli}
DAITIHSILDWIEDNLESPLSLEKVSERSGYSKWHLQRMFKKETGHSLGQYIRS
>d1bl0a2 a.4.1.8 (A:63-124) MarA {Escherichia coli}
RKMTEIAQKLKESNEPILYLAERYGFESQQTLTRTFKNYFDVPPHKYRMTNMQGESRFLHPL
>d1d5ya1 a.4.1.8 (A:3-56) Rob transcription factor, N-terminal domain {Escherichia coli}
QAGIIRDLLIWLEGHLDQPLSLDNVAAKAGYSKWHLQRMFKDVTGHAIGAYIRA
>d1d5ya2 a.4.1.8 (A:57-121) Rob transcription factor, N-terminal domain {Escherichia coli}
RRLSKSAVALRLTARPILDIALQYRFDSQQTFTRAFKKQFAQTPALYRRSPEWSAFGIRPPL
RLG
>d1a6i_1 a.4.1.9 (2-67) Tetracyclin repressor (Tet-repressor, TetR) {Escherichia coli}
SRLDKSKVINSALELLNEVGIEGLTTRKLAQKLGVEQPTLYWHVKNKRALLDALAVEILARH
HDYS
>d2tct_1 a.4.1.9 (2-67) Tetracyclin repressor (Tet-repressor, TetR) {Escherichia coli}
ARLNRESVIDAALELLNETGIDGLTTRKLAQKLGIEQPTLYWHVKNKRALLDALAVEILARH
HDYS
>d1jt6a1 a.4.1.9 (A:2-72) Multidrug binding protein QacR {Staphylococcus aureus}
NLKDKILGVAKELFIKNGYNATTTGEIVKLSESSKGNLYYHFKTKENLFLEILNIEESKWQE
QWKKEQIKA
>d1sfe_1 a.4.2.1 (93-176) Ada DNA repair protein {Escherichia coli}
GTAFQQQVWQALRTIPCGETVSYQQLANAIGKPKAVRAVASACAANKLAIVIPCHRVVRGDG
SLSGYRWGVSRKAQLLRREAEN
>d1qnta1 a.4.2.1 (A:92-176) O6-alkylguanine-DNA alkyltransferase {Human (Homo sapiens)}
ESFTRQVLWKLLKVVKFGEVISYQQLAALAGNPKAARAVGGAMRGNPVPILIPCHRVVCSSG
AVGNYSGGLAVKEWLLAHEGHRL
>d1mgta1 a.4.2.1 (A:89-169) O6-alkylguanine-DNA alkyltransferase {Archaeon Pyrococcus kodakaraensis}
VTPFEKKVYEWLTKNVKRGSVITYGDLAKALNTSPRAVGGAMKRNPYPIVVPCHRVVAHDGI
GYYSSGIEEKKFLLEIEGV
>d1c20a_ a.4.3.1 (A:) DNA-binding domain from the dead ringer protein {Fruit fly (Drosophila melanogaster)}
GWSFEEQFKQVRQLYEINDDPKRKEFLDDLFSFMQKRGTPINRLPIMAKSVLDLYELYNLVI
ARGGLVDVINKKLWQEIIKGLHLPSSITSAAFTLRTQYMKYLYPYECEKKNLSTPAELQAAI
DGNR
>d1ig6a_ a.4.3.2 (A:) MRF-2 DNA-binding domain {Human (Homo sapiens)}
RADEQAFLVALYKYMKERKTPIERIPYLGFKQINLWTMFQAAQKLGGYETITARRQWKHIYD

ELGGNPGSTSAATCTRRHYERLILPYERFIKGEEDKPLPPIKPRK
>d1bia_1 a.4.5.1 (1-63) Biotin repressor, N-terminal domain {Escherichia coli}
MKDNTVPLKLIALLANGEFHSGEQLGETLGMSRAAINKHIQTLRDWGVDVFTVPGKGYSLPE
P
>d1jhfa1 a.4.5.2 (A:2-72) LexA repressor, N-terminal DNA-binding domain {Escherichia coli}
KALTARQQEVFDLIRDHISQTGMPPTRAEIAQRLGFRSPNAAEEHLKALARKGVIEIVSGAS
RGIRLLQEE
>d1aoy__ a.4.5.3 (-) Arginine repressor (ArgR), N-terminal DNA-binding domain {Escherichia coli}
MRSSAKQEELVKAFKALLKEEKFSSQGEIVAALQEQGFDNINQSKVSRMLTKFGAVRTRNAK
MEMVYCLPAELGVPTT
>d1b4aa1 a.4.5.3 (A:4-78) Arginine repressor (ArgR), N-terminal DNA-binding domain {Bacillus stearothermophilus}
GQRHIKIREIIMSNDIETQDELVDRLREAGFNVTQATVSRDIKEMQLVKVPMANGRYKYSLP
SDQRFNPLQKLKR
>d1f9na1 a.4.5.3 (A:3-78) Arginine repressor (ArgR), N-terminal DNA-binding domain {Bacillus subtilis}
KGQRHIKIREIITSNEIETQDELVDMLKQDGYKVTQATVSRDIKELHLVKVPTNNGSYKYSL
PADQRFNPLSKLKR
>d1hw5a1 a.4.5.4 (A:138-208) Catabolite gene activator protein (CAP), C-terminal domain {Escherichia coli}
DVTGRIAQTLLNLAKQPDAMTHPDGMQIKITRQEIGQIVGCSRETVGRILKMLEDQNLISAH
GKTIVVYGT
>d1ft9a1 a.4.5.4 (A:134-213) CO-sensing protein CooA, C-terminal domain {Rhodospirillum rubrum}
DIKQRIAGFFIDHANTTGRQTQGGVIVSVDFTVEEIANLIGSSRQTTSTALNSLIKEGYISR
QGRGHYTIPNLVRLKAAA
>d1i1ga1 a.4.5.32 (A:2-61) LprA {Archaeon Pyrococcus furiosus}
IDERDKIILEILEKDARTPFTEIAKKLGISETAVRKRVKALEEKGIIEGYTIKINPKKLG
>d1smta_ a.4.5.5 (A:) SmtB repressor {Cyanobacteria (Synechococcus), pcc7942}
ELQAIAPEVAQSLAEFFAVLADPNRLRLLSLLARSELCVGDLAQAIGVSESAVSHQLRSLRN
LRLVSYRKQGRHVYYQLQDHHIVALYQNALDHLQEC
>d1jgsa_ a.4.5.28 (A:) Multiple antibiotic resistance repressor, MarR {Escherichia coli}
LFNEIIPLGRLIHMVNQKKDRLLNEYLSPLDITAAQFKVLCSIRCAACITPVELKKVLSVDL
GALTRMLDRLVCKGWVERLPNPNDKRGVLVKLTTGGAAICEQCHQLVGQDLHQELTKNLTAD
EVATLEYLLKKVLP
>d1hsja1 a.4.5.28 (A:373-487) staphylococcal accessory regulator A homolog, SarR {Staphylococcus aureus}
MSKINDINDLVNATFQVKKFFRDTKKKFNLNYEEIYILNHILRSESNEISSKEIAKCSEFKP
YYLTKALQKLKDLKLLSKKRSLQDERTVIVYVTDTQKANIQKLISELEEYIKN

>d1fzpb_ a.4.5.28 (B:) Pleiotropic regulator of virulence genes, SarA {Staphylococcus aureus}
AITKINDCFELLSMVTYADKLKSLIKKEFSISFEEFAVLTYISENKEKEYYLKDIINHLNYK
QPQVVKAVKILSQEDYFDKKRNEHDERTVLILVNAQQRKKIESLLSRVNKRIT
>d1hw1a1 a.4.5.6 (A:5-78) Fatty acid responsive transcription factor FadR, N-terminal domain {Escherichia coli}
AQSPAGFAEEYIIESIWNNRFPPGTILPAERELSELIGVTRTTLREVLQRLARDGWLTIQHG
KPTKVNNFWETS
>d1bm9a_ a.4.5.7 (A:) Replication terminator protein (RTP) {Bacillus subtilis}
EEKRSSTGFLVKQRAFLKLYMITMTEQERLYGLKLLEVLRSEFKEIGFKPNHTEVYRSLHEL
LDDGILKQIKVKKEGAKLQEVVLYQFKDYEAAKLYKKQLKVELDRCKKLIEKALSDNF
>d1b9ma1 a.4.5.8 (A:-1-126) N-terminal domain of molybdate-dependent transcriptional regulator ModE {Escherichia coli}
HMQAEILLTLKLQQKLFADPRRISLLKHIALSGSISQGAKDAGISYKSAWDAINEMNQLSEH
ILVERATGGKGGGGAVLTRYGQRLIQLYDLLAQIQQKAFDVLSDDDALPLNSLLAAISRFSL
QTS
>d1bjaa_ a.4.5.9 (A:) Transcription factor MotA, activation domain {Bacteriophage T4}
SKVTYIIKASNDVLNEKTATILITIAKKDFITAAEVREVHPDLGNAVVNSNIGVLIKKGLVE
KSGDGLIITGEAQDIISNAATLYAQENAPELLK
>d1repc1 a.4.5.10 (C:15-143) RepE54 {Escherichia coli, mini-F plasmid}
SPRIVQSNDLTEAAYSLSRDQKRMLYLFVDQIRKSDGTLQEHDGICEIHVAKYAEIFGLTSA
EASKDIRQALKSFAGKEVVFYRPEEDAGDEKGYESFPWFIKPAHSPSRGLYSVHINPYLIPF
FIGLQ
>d1repc2 a.4.5.10 (C:144-246) RepE54 {Escherichia coli, mini-F plasmid}
NRFTQFRLSETKEITNPYAMRLYESLCQYRKPDGSGIVSLKIDWIIERYQLPQSYQRMPDFR
RRFLQVCVNEINSRTPMRLSYIEKKKGRQTTHIVFSFRDIT
>d1hqca1 a.4.5.11 (A:243-318) Holliday junction helicase RuvB {Thermus thermophilus}
LGLEKRDREILEVLILRFGGGPVGLATLATALSEDPGTLEEVHEPYLIRQGLLKRTPRGRVP
TELAYRHLGYPPPV
>d1in4a1 a.4.5.11 (A:255-329) Holliday junction helicase RuvB {Thermotoga maritima}
EGLDEFDRKILKTIIEIYRGGPVGLNALAASLGVEADTLSEVYEPYLLQAGFLARTPRGRIV
TEKAYKHLKYEVP
>d1fnna1 a.4.5.11 (A:277-388) CDC6, C-terminal domain {Archaeon Pyrobaculum aerophilum}
ISEEVLIGLPLHEKLFLLAIVRSLKISHTPYITFGDAEESYKIVCEEYGERPRVHSQLWSYL
NDLREKGIVETRQNKRGEGVRGRTTLISIGTEPLDTLEAVITKLIKEELR
>d1foka3 a.4.5.12 (A:287-386) Restriction endonuclease FokI,

N-terminal (recognition) domain {Flavobacterium okeanokoites}
VPKRVYWEMLATNLTDKEYVRTRRALILEILIKAGSLKIEQIQDNLKKLGFDEVIETIENDI
KGLINTGIFIEIKGRFYQLKDHILQFVIPNRGVTKQLV
>d2foka1 a.4.5.12 (A:5-143) Restriction endonuclease FokI,
N-terminal (recognition) domain {Flavobacterium okeanokoites}
IRTFGWVQNPGKFENLKRVVQVFDRNSKVHNEVKNIKIPTLVKESKIQKELVAIMNQHDLIY
TYKELVGTGTSIRSEAPCDAIIQATIADQGNKKGYIDNWSSDGFLRWAHALGFIEYINKSDS
FVITDVGLAYSKSAD
>d2foka2 a.4.5.12 (A:144-286) Restriction endonuclease FokI,
N-terminal (recognition) domain {Flavobacterium okeanokoites}
GSAIEKEILIEAISSYPPAIRILTLLEDGQHLTKFDLGKNLGFSGESGFTSLPEGILLDTLA
NAMPKDKGEIRNNWEGSSDKYARMIGGWLDKLGLVKQGKKEFIIPTLGKPDNKEFISHAFKI
TGEGLKVLRRAKGSTKFTR
>d2foka3 a.4.5.12 (A:287-386) Restriction endonuclease FokI,
N-terminal (recognition) domain {Flavobacterium okeanokoites}
VPKRVYWEMLATNLTDKEYVRTRRALILEILIKAGSLKIEQIQDNLKKLGFDEVIETIENDI
KGLINTGIFIEIKGRFYQLKDHILQFVIPNRLGKPDLV
>d1f1za1 a.4.5.27 (A:169-267) TnsA endonuclease, C-terminal domain
{Escherichia coli}
NPVVKENIEWLYSVKTEEVSAELLAQLSPLAHILQEKGDENIINVCKQVDIAYDLELGKTLS
EIRALTANGFIKFNIYKSFRANKCADLCISQVVNMEE
>d1fp1d1 a.4.5.29 (D:19-128) Chalcone O-methyltransferase
{Alfalfa (Medicago sativa)}
QTEDSACLSAMVLTTNLVYPAVLNAAIDLNLFEIIAKATPPGAFMSPSEIASKLPASTQHSD
LPNRLDRMLRLLASYSVLTSTTRTIEDGGAERVYGLSMVGKYLVPDES
>d1fp2a1 a.4.5.29 (A:8-108) Isoflavone O-methyltransferase
{Alfalfa (Medicago sativa)}
RKPSEIFKAQALLYKHIYAFIDSMSLKWAVEMNIPNIIQNHGKPISLSNLVSILQVPSSKIG
NVRRLMRYLAHNGFFEIITKEEESYALTVASELLVRGSD
>d1hsta_ a.4.5.13 (A:) Histone H5, globular domain {Chicken (Gallus
gallus)}
SHPTYSEMIAAAIRAEKSRGGSSRQSIQKYIKSHYKVGHNADLQIKLSIRRLLAAGVLKQTK
GVGASGSFRLAK
>d1ghc__ a.4.5.13 (-) Histone H1, globular domain {Chicken (Gallus
gallus)}
MAGPSVTELITKAVSASKERKGLSLAALKKALAAGGYDVEKNNSRIKLGLKSLVSKGTLVQT
KGTGASGSFRLSK
>d1e17a_ a.4.5.14 (A:) Afx (Foxo4) {Human (Homo sapiens)}
SRRNAWGNQSYAELISQAIESAPEKRLTLAQIYEWMVRTVPYFKDKGDSNSSAGWKNSIRHN
LSLHSKFIKVHNEATGKSSWWMLNPEGG
>d1d5va_ a.4.5.14 (A:) Adipocyte-transcription factor FREAC-11
(s12, fkh-14) {Human (Homo sapiens)}
MLVKPPYSYIALITMAIQNAPEKKITLNGIYQFIMDRFPFYRENKQGWQNSIRHNLSLNECF
VKVPRDDKKPGKGSYWTLDPDSYNMFENGSFL

>d2hdca_ a.4.5.14 (A:) Genesis {Rat (Rattus norvegicus)}
VKPPYSYIALITMAILQSPQKKLTLSGICEFISNRFPYYREKFPAWQNSIRHNLSLNDCFVK
IPREPGNPGKGNYWTLDPQSEDMFDNGSFLRRRKR
>d1kq8a_ a.4.5.14 (A:) HFH-1 (HNF-3 forkhead homolog-1) {Rat (Rattus norvegicus)}
YIALITMAIRDSAGGRLTLAEINEYLMGKFPFFRGSYTGWRNSVRHNLSLNDCFVKVLRDPS
RPWGKDNYWMLNP
>d2bby__ a.4.5.15 (-) DNA-binding domain from rap30 {Human (Homo sapiens)}
RARADKQHVLDMLFSAFEKHQYYNLKDLVDITKQPVVYLKEILKEIGVQNVKGIHKNTWELK
PEYRHYQ
>d1i27a_ a.4.5.30 (A:) C-terminal domain of the rap74 subunit of TFIIF {Human (Homo sapiens)}
GPLGSGDVQVTEDAVRRYLTRKPMTTKDLLKKFQTKKTGLSSEQTVNVLAQILKRLNPERKM
INDKMHFSLKE
>d1dpua_ a.4.5.16 (A:) C-terminal domain of RPA32 {Human (Homo sapiens)}
ANGLTVAQNQVLNLIKACPRPEGLNFQDLKNQLKHMSVSSIKQAVDFLSNEGHIYSTVDDDH
FKSTDAE
>d1fsha_ a.4.5.31 (A:) Segment polarity protein Dishevelled-1 {Mouse (Mus musculus)}
EAPLTVKSDMSAIVRVMQLPDSGLEIRDRMWLKITIANAVIGADVVDWLYTHVEGFKERREA
RKYASSMLKHGFLRHTVNKITFSEQCYYVFGD
>d1cf7a_ a.4.5.17 (A:) Cell cycle transcription factor e2f-dp {Human (Homo sapiens)}
SRHEKSLGLLTTKFVSLLQEAKDGVLDLKLAADTLAVRQKRRIYDITNVLEGIGLIEKKSKN
SIQWK
>d1cf7b_ a.4.5.17 (B:) Cell cycle transcription factor e2f-dp {Human (Homo sapiens)}
GKGLRHFSMKVCEKVQRKGTTSYNEVADELVSEFTNSNNHLAADSAYDQKNIRRRVYDALNV
LMAMNIISKEKKEIKWIGLP
>d1d8ja_ a.4.5.18 (A:) The central core domain of TFIIE beta {Human (Homo sapiens)}
ALSGSSGYKFGVLAKIVNYMKTRHQRGDTHPLTLDEILDETQHLDIGLKQKQWLMTEALVNN
PKIEVIDGKYAFKPKYNVR
>d1qbja_ a.4.5.19 (A:) Z-alpha domain of dsRNA-specific adenosine deaminase, ADAR1 {Human (Homo sapiens)}
SIYQDQEQRILKFLEELGEGKATTAHDLSGKLGTPKKEINRVLYSLAKKGKLQKEAGTPPLW
KIA
>d1qgpa_ a.4.5.19 (A:) Z-alpha domain of dsRNA-specific adenosine deaminase, ADAR1 {Human (Homo sapiens)}
LSSHFQELSIYQDQEQRILKFLEELGEGKATTAHDLSGKLGTPKKEINRVLYSLAKKGKLQK
EAGTPPLWKIAVSD
>d1j75a_ a.4.5.19 (A:) Dlm-1 {Mouse (Mus musculus)}

NLEQKILQVLSDDGGPVKIGQLVKKCQVPKKTLNQVLYRLKKEDRVSSPEPATWSIG
>d1dp7p_ a.4.5.20 (P:) MHC class II transcription factor RFX1 {Human (Homo sapiens)}
TVQWLLDNYETAEGVSLPRSTLYNHYLLHSQEQKLEPVNAASFGKLIRSVFMGLRTRRLGTR
GNSKYHYYGLRIKA
>d1flia_ a.4.5.21 (A:) Fli-1 {Human (Homo sapiens)}
PGSGQIQLWQFLLELLSDSANASCITWEGTNGEFKMTDPDEVARRWGERKSKPNMNYDKLSR
ALRYYYDKNIMTKVHGKRYAYKFDFHGIAQALQPHP
>d1k78b_ a.4.5.21 (B:) ETS-1 transcription factor, residues 331-440 {Mouse (Mus musculus)}
IQLWQFLLELLTDKSCQSFISWTGDGWEFKLSDPDEVARRWGKRKNKPKMNYEKLSRGLRYY
YDKNIIHKTAGKRYVYRFVCDLQSLLGYTPEELHAMLDVK
>d2stta_ a.4.5.21 (A:) ETS-1 transcription factor, residues 331-440 {Human (Homo sapiens)}
VIPAAALAGYTGSGPIQLWQFLLELLTDKSCQSFISWTGDGWEFKLSDPDEVARRWGKRKNK
PKMNYEKLSRGLRYYYDKNIIHKTAGKRYVYRFV
>d1puee_ a.4.5.21 (E:) Transcription factor PU.1, residues 171-259 {Mouse (Mus musculus)}
KIRLYQFLLDLLRSGDMKDSIWWVDKDKGTFQFSSKHKEALAHRWGIQKGNRKKMTYEKMAR
ALRNYGKTGEVKKVKKKLTYQFSGEV
>d1awca_ a.4.5.21 (A:) GA binding protein (GABP) alpha {Mouse (Mus musculus)}
IQLWQFLLELLTDKDARDCISWVGDEGEFKLNQPELVAQKWGQRKNKPTMNYEKLSRALRYY
YDGDMICKVQGKRFVYKFVCDLKTLIGYSAAELNRLVIECEQKKLARM
>d1bc8c_ a.4.5.21 (C:) Serum responce factor accessory protein 1a, SAP-1 {Human (Homo sapiens)}
MDSAITLWQFLLQLLQKPQNKHMICWTSNDGQFKLLQAEEVARLWGIRKNKPNMNYDKLSRA
LRYYYVKNIIKKVNGQKFVYKFVSYPEILNM
>d1hbxg_ a.4.5.21 (G:) Serum responce factor accessory protein 1a, SAP-1 {Human (Homo sapiens)}
DSAITLWQFLLQLLQKPQNKHMICWTSNDGQFKLLQAEEVARLWGIRKNKPNMNYDKLSRAL
RYYYVKNIIKKVNGQKFVYKFVSYPEILNMDPMTVGRIEGDCESLNFSEVSSSSKDVENGGK
DKPPQPGAKTSSRNDYIHSGLYSSFTLNSLN
>d1duxc_ a.4.5.21 (C:) Elk-1 {Human (Homo sapiens)}
VTLWQFLLQLLREQGNGHIISWTSRDGGEFKLVDAEEVARLWGLRKNKTNMNYDKLSRALRY
YYDKNIIRKVSGQKFVYKFVSYPE
>d1hks__ a.4.5.22 (-) Heat-shock transcription factor {Drosophila melanogaster}
GSGVPAFLAKLWRLVDDADTNRLICWTKDGQSFVIQNQAQFAKELLPLNYKHNNMASFIRQL
NMYGFHKITSIDNGGLRFDRDEIEFSHPFFKRNSPFLLDQIKRK
>d1fbqa_ a.4.5.22 (A:) Heat-shock transcription factor {Milk yeast (Kluyveromyces lactis)}
PAFVNKLWSMVNDKSNEKFIHWSTSGESIVVPNRERFVQEVLKKYFKHSNFASFVRQLNMYG
WHKVQDVKSGSMLSNNDSRWEFENERH

>d2hts__ a.4.5.22 (-) Heat-shock transcription factor {Milk yeast (Kluyveromyces lactis)}
ARPAFVNKLWSMVNDKSNEKFIHWSTSGESIVVPNRERFVQEVLPKYFKHSNFASFVRQLNM
YGWHKVQDVKSGSNDSRWEFENERHA
>d1if1a_ a.4.5.23 (A:) Interferon regulatory factor 1 (IRF-1) {Mouse (Mus musculus)}
RMRPWLEMQINSNQIPGLIWINKEEMIFQIPWKHAAKHGWDINKDACLFRSWAIHTGRYKAG
EKEPDPKTWKANFRCAMNSLPDIEEVKDQSRNKGSSAVRVYRM
>d2irfg_ a.4.5.23 (G:) Interferon regulatory factor-2, IRF-2 {Mouse (Mus musculus)}
RMRMRPWLEEQINSNTIPGLKWLNKEKKIFQIPWMHAARHGWDVEKDAPLFRNWAIHTGKHQ
PGIDKPDPKTWKANFRCAMNSLPDIEEVKDRSIKKGNNAFRVYRMLP
>d1g3sa1 a.4.5.24 (A:4-64) Diphtheria toxin repressor (DtxR) {Corynebacterium diphtheriae}
LVDTTEMYLRTIYELEEEGVTPLRARIAERLEQSGPTVSQTVARMERDGLVVVASDRSLQM
>d1fx7a1 a.4.5.24 (A:1-64) Iron-dependent regulator IdeR {Mycobacterium tuberculosis}
MNELVDTTEMYLRTIYDLEEEGVTPLRARIAERLDQSGPTVSQTVSRMERDGLLRVAGDRHL
EL
>d1xgsa1 a.4.5.25 (A:195-271) Methionine aminopeptidase, insert domain {Archaeon Pyrococcus furiosus}
GQVIEVPPTLIYMYVRDVPVRVAQARFLLAKIKREYGTLPFAYRWLQNDMPEGQLKLALKTL
EKAGAIYGYPVLKEI
>d1b6a_1 a.4.5.25 (375-448) Methionine aminopeptidase, insert domain {Human (Homo sapiens)}
HDDMECSHYMKNFDVGHVPIRLPRTKHLLNVINENFGTLAFCRRWLDRLGESKYLMALKNLC
DLGIVDPYPPLC
>d1g4da_ a.4.5.26 (A:) mu transposase, DNA-binding domain {Bacteriophage mu}
KSIWCSPQEIMAADGMPGSVAGVHYRANVQGWTKRKKEGVKGGKAVEYDVMSMPTKEREQVI
AHLGLST
>d1tns__ a.4.5.26 (-) mu transposase, DNA-binding domain {Bacteriophage mu}
MELWVSPKELANLPGLPKTSAGVIYVAKKQGWQNRTRAGVKGGKAIEYNANSLPVEAKAALL
LRQGEIETSLGYFE
>d1opc__ a.4.6.1 (-) OmpR {Escherichia coli}
VIAFGKFKLNLGTREMFREDEPMPLTSGEFAVLKALVSHPREPLSRDKLMNLARGREYSAME
RSIDVQISRLRRMVEEDPAHPRYIQTVWGLGYVFVPD
>d1kgsa1 a.4.6.1 (A:124-225) PhoB {Thermotoga maritima}
SKSTKLVCGDLILDTATKKAYRGSKEIDLTKKEYQILEYLVMNKNRVVTKEELQEHLWSFDD
EVFSDVLRSHIKNLRKKVDKGFKKKIIHTVRGIGYVARDE
>d1qqia_ a.4.6.1 (A:) PhoB {Escherichia coli}
MAVEEVIEMQGLSLDPTSHRVMAGEEPLEMGPTEFKLLHFFMTHPERVYSREQLLNHVWGTN
VYVEDRTVDVHIRRLRKALEPGGHDRMVQTVRGTGYRFSTRF

>d1fsea_ a.4.6.2 (A:) Germination protein GerE {Bacillus subtilis}
SKPLLTKREREVFELLVQDKTTKEIASELFISEKTVRNHISNAMQKLGVKGRSQAVVELLRM
GELEL
>d1a04a1 a.4.6.2 (A:150-216) Nitrate/nitrite response regulator (NarL) {Escherichia coli}
ERDVNQLTPRERDILKLIAQGLPNKMIARRLDITESTVKVHVKHMLKKMKLKSRVEAAVWVH
QERIF
>d1fc3a_ a.4.6.3 (A:) SpoOA {Bacillus stearothermophilus}
NKPKNLDASITSIIHEIGVPAHIKGYLYLREAIAMVYHDIELLGSITKVLYPDIAKKYNTTA
SRVERAIRHAIEVAWSRGNLESISSLFGYTVSVSKAKPTNSEFIAMVADKLRLEHKA
>d1fox__ a.4.7.1 (-) Ribosomal protein L11, C-terminal domain {Bacillus stearothermophilus}
MTFITKTPPAAVLLKKAAGIESGSGEPNRNKVATIKRDKVREIAELKMPDLNAASIEAAMRM
IEGTARSMGIVVED
>d1qa6a_ a.4.7.1 (A:) Ribosomal protein L11, C-terminal domain {Bacillus stearothermophilus}
KTPPAAVLLKKAAGIESGSGEPNRNKVATIKRDKVREIAELKMPDLNAASIEAAMRMIEGTA
RSMGI
>d1mmsa1 a.4.7.1 (A:71-140) Ribosomal protein L11, C-terminal domain {Thermotoga maritima}
KTPPASFLLKKAAGIEKGSSEPKRKIVGKVTRKQIEEIAKTKMPDLNANSLEAAMKIIEGTA
KSMGIEVV
>d1fjgr_ a.4.8.1 (R:) Ribosomal protein S18 {Thermus thermophilus}
PSRKAKVKATLGEFDLRDYRNVEVLKRFLSETGKILPRRRTGLSGKEQRILAKTIKRARILG
LLPFTEKLVRK
>d1g1xc_ a.4.8.1 (C:) Ribosomal protein S18 {Thermus thermophilus}
DLRDYRNVEVLKRFLSETGKILPRRRTGLSGKEQRILAKTIKRARILGLLPFT
>d1i94r_ a.4.8.1 (R:) Ribosomal protein S18 {Thermus thermophilus}
KPKKEAQRRPSRKAKVKATLGEFDLRDYRNVEVLKRFLSETGKILPRRRTGLSGKEQRILAK
TIKRARILGLLPFTEKLVRK
>d1e3ha1 a.4.9.1 (A:263-345) Polynucleotide phosphorylase/guanosine pentaphosphate synthase (PNPase/GPSI), domain 3 {Streptomyces antibioticus}
YQDDVLEALSAAVRPELSAALTIAGKQDREAELDRVKALAAEKLLPEFEGREKEISAAYRAL
TKSLVRERVIAEKKRIDGRGV
>d1k6ya1 a.4.10.1 (A:1-46) N-terminal Zn binding domain of HIV integrase {Human immunodeficiency virus type 1}
FLDGIDKAQEEHEKYHSNWRAMASDFNLPPVVAKEIVASCDKCQLK
>d1wjba_ a.4.10.1 (A:) N-terminal Zn binding domain of HIV integrase {Human immunodeficiency virus type 1}
FLDGIDKAQEEHEKYHSNWRAMASDFNLPPVVAKEIVASCDKCQLKGEAMHGQVD
>d1e0ea_ a.4.10.1 (A:) N-terminal Zn binding domain of HIV integrase {Human immunodeficiency virus type 2}
FLEKIEPAQEEHEKYHSNVKELSHKFGIPNLVARQIVNSCAQCQQK

>d1ef4a_ a.4.11.1 (A:) RNA polymerase subunit RPB10 {Archaeon Methanobacterium thermoautotrophicum}
MIPVRCLSCGKPVSAYFNEYQRRVADGEDPKDVLDDLGLKRYCCRRMLISHVETW
>d1i50j_ a.4.11.1 (J:) RNA polymerase subunit RPB10 {Baker's yeast (Saccharomyces cerevisiae)}
MIVPVRCFSCGKVVGDKWESYLNLLQEDELDEGTALSRLGLKRYCCRRMILTHVDLIEKFLR
YNP
>d1cuk_1 a.5.1.1 (156-203) DNA helicase RuvA subunit, C-terminal domain {Escherichia coli}
TDDAEQEAVARLVALGYKPQEASRMVSKIARPDASSETLIREALRAAL
>d1bvsa1 a.5.1.1 (A:148-203) DNA helicase RuvA subunit, C-terminal domain {Mycobacterium leprae}
NAVRGSVVEALVGLGFAAKQAEEATDQVLDGELGKDGAVATSSALRAALSLLGKTR
>d1f4ia_ a.5.2.1 (A:) C-terminal UBA domain of the hhr23a DNA repair protein {Human (Homo sapiens)}
QEKEAIERLKALGFEESLVIQAYFACEKNENLAANFLLSQNFDDE
>d1efub3 a.5.2.2 (B:1-54) Elongation factor Ts (EF-Ts), N-terminal domain {Escherichia coli}
AEITASLVKELRERTGAGMMDCKKALTEANGDIELAIENMRKSGAIKAAKKAGN
>d1aipc1 a.5.2.2 (C:2-53) Elongation factor Ts (EF-Ts), N-terminal domain {Thermus thermophilus}
SQMELIKKLREATGAGMMDVKRALEDAGWDEEKAVQLLRERGAMKAAKKADR
>d1go5a_ a.5.2.3 (A:) FG-binding, C-terminal domain of TAP {Human (Homo sapiens)}
PAPTPSSSPVPTLSPEQQEMLQAFSTQSGMNLEWSQKCLQDNNWDYTRSAQAFTHLKAKGEI
PEVAFMK
>d1aua_1 a.5.3.1 (4-96) N-terminal domain of phosphatidylinositol transfer protein sec14p {Baker's yeast (Saccharomyces cerevisiae)}
QQEKEFLESYPQNCPPDALPGTPGNLDSAQEKALAELRKLLEDAGFIERLDDSTLLRFLRAR
KFDVQLAKEMFENCEKWRKDYGTDTILQDFH
>d1enwa_ a.5.4.1 (A:) Elongation factor TFIIS domain 2 {Baker's yeast (Saccharomyces cerevisiae)}
GSHMPRNSKNDGVDTAIYHHKLRDQVLKALYDVLAKESEHPPQSILHTAKAIESEMNKVNNC
DTNEAAYKARYRIIYSNVISKNNPDLKHKIANGDITPEFLATCDAKDLAPAP
>d1fjgm_ a.5.5.1 (M:) Ribosomal protein S13 {Thermus thermophilus}
ARIAGVEIPRNKRVDVALTYIYGIGKARAKEALEKTGINPATRVKDLTEAEVVRLREYVENT
WKLEGELRAEVAANIKRLMDIGCYRGLRHRRGLPVRGQRTRTNARTRKGPRKTVAGKKKAPR
K
>d1i94m_ a.5.5.1 (M:) Ribosomal protein S13 {Thermus thermophilus}
ARIAGVEIPRNKRVDVALTYIYGIGKARAKEALEKTGINPATRVKDLTEAEVVRLREYVENT
WKLEGELRAEVAANIKRLMDIGCYRGLRHRR
>d1eija_ a.5.6.1 (A:) Hypothetical protein MTH1615 {Archaeon Methanobacterium thermoautotrophicum}
MRQQLEMQKKQIMMQILTPEARSRLANLRLTRPDFVEQIELQLIQLAQMGRVRSKITDEQLK

ELLKRVAGKK
>d1jjcb1 a.6.1.1 (B:1-38,B:152-190) Domains B1 and B5 of PheRS-beta, PheT {Thermus thermophilus (Thermus aquaticus)}
MRVPFSWLKAYVPELESPEVLEERLAGLGFETDRIERVXEEVVLDLEVTPNRPDALGLLGLA
RDLHALGYALVEPEAA
>d1jjcb2 a.6.1.1 (B:400-474) Domains B1 and B5 of PheRS-beta, PheT {Thermus thermophilus (Thermus aquaticus)}
PPEAIPFRPEYANRLLGTSYPEAEQIAILKRLGCRVEGEGPTYRVTPPSHRLDLRLEEDLVE
EVARIQGYETIPL
>d1d4ua1 a.6.1.2 (A:37-111) DNA repair factor XPA DNA- and RPA-binding domain, C-terminal subdomain {Human (Homo sapiens)}
DKHKLITKTEAKQEYLLKDCDLEKREPPLKFIVKKNPHHSQWGDMKLYLKLQIVKRSLEVWG
SQEALEEAKEVRQ
>d1exja1 a.6.1.3 (A:3-120) Transcription activator BmrR {Bacillus subtilis}
ESYYSIGEVSKLANVSIKALRYYDKIDLFKPAYVDPDTSYRYYTDSQLIHLDLIKSLKYIGT
PLEEMKKAQDLEMEELFAFYTEQERQIREKLDFLSALEQTISLVKKRMKRQMEYPA
>d1jbga_ a.6.1.3 (A:) Multidrug transporter activator MtaN {Bacillus subtilis}
KYQVKQVAEISGVSIRTLHHYDNIELLNPSALTDAGYRLYSDADLERLQQILFFKEIGFRLD
EIKEMLDHPNFDRKAALQSQKEILMKKKQRMDEMIQTIDRTLLS
>d2spca_ a.7.1.1 (A:) Spectrin {Fruit fly (Drosophila sp.)}
QNLDLQLYMRDCELAESWMSAREAFLNADDDANAGGNVEALIKKHEDFDKAINGHEQKIAAL
QTVADQLIAQNHYASNLVDEKRKQVLERWRHLKEGLIEKRSRLGD
>d1aj3__ a.7.1.1 (-) Spectrin {Chicken (Gallus gallus)}
HQFFRDMDDEESWIKEKKLLVSSEDYGRDLTGVQNLRKKHKRLEAELAAHEPAIQGVLDTGK
KLSDDNTIGKEEIQQRLAQFVDHWKELKQLAAARGQ
>d1cuna1 a.7.1.1 (A:7-115) Spectrin {Chicken (Gallus gallus)}
MVHQFFRDMDDEESWIKEKKLLVSSEDYGRDLTGVQNLRKKHKRLEAELAAHEPAIQSVLDT
GKKLSDDNTIGKEEIQQRLAQFVDHWKELKQLAAARGQRLEESLEYQ
>d1cuna2 a.7.1.1 (A:116-219) Spectrin {Chicken (Gallus gallus)}
QFVANVEEEEAWINEKMTLVASEDYGDTLAAIQGLLKKHEAFETDFTVHKDRVNDVCANGED
LIKKNNHHVENITAKMKGLKGKVSDLEKAAAQRKAKLDENSA
>d1hcia1 a.7.1.1 (A:272-396) alpha-actinin {Human (Homo sapiens)}
SSAVNQENERLMEEYERLASELLEWIRRTIPWLENRTPEKTMQAMQKKLEDFRDYRRKHKPP
KVQEKCQLEINFNTLQTKLRISNRPAFMPSEGKMVSDIAGAWQRLEQAEKGYEEWLLNEIRR
L
>d1hcia4 a.7.1.1 (A:633-746) alpha-actinin {Human (Homo sapiens)}
HANERLRRQFAAQANAIGPWIQNKMEEIARSSIQITGALEDQMNQLKQYEHNIINYKNNIDK
LEGDHQLIQEALVFDNKHTNYTMEHIRVGWELLLTTIARTINEVETQILTRD
>d1quua1 a.7.1.1 (A:1-124) alpha-actinin {Human (Homo sapiens)}
GSSNEIRRLERLEHLAEKFRQKASTHETWAYGKEQILLQKDYESASLTEVRALLRKHEAFES
DLAAHQDRVEQIAAIAQELNELDYHDAVNVNDRCQKICDQWDRLGTLTQKRREALERMEKLL
>d1quua2 a.7.1.1 (A:125-248) alpha-actinin {Human (Homo sapiens)}

ETIDQLHLEFAKRAAPFNNWMEGAMEDLQDMFIVHSIEEIQSLITAHEQFKATLPEADGERQ
SIMAIQNEVEKVIQSYNIRISSSNPYSTVTMDELRTKWDKVKQLVPIRDQSLQEELARQHAN
>d1e2aa_ a.7.2.1 (A:) Enzyme IIa from lactose specific PTS, IIa-lac {Lactococcus lactis}
MNREEMTLLGFEIVAYAGDARSKLLEALKAAENGDFAKADSLVVEAGSCIAEAHSSQTGMLA
REASGEELPYSVTMMHGQDHLMTTILLKDVIHHLIELYKR
>d1chua1 a.7.3.1 (A:423-533) L-aspartate oxidase {Escherichia coli}
DESRVENPDERVVIQHNWHELRLFMWDYVGIVRTTKRLERALRRITMLQQEIDEYYAHFRVS
NNLLELRNLVQVAELIVRCAMMRKESRGLHFTLDYPELLTHSGPSILSP
>d1fuma1 a.7.3.1 (A:443-575) Fumarate reductase flavoprotein subunit {Escherichia coli}
DGGENWAKIRDEMGLAMEEGCGIYRTPELMQKTIDKLAELQERFKRVRITDTSSVFNTDLLY
TIELGHGLNVAECMAHSAMARKESRGAHQRLDEGCTERDDVNFLKHTLAFRDADGTTRLEYS
DVKITTLPP
>d1qlaa1 a.7.3.1 (A:458-655) Fumarate reductase flavoprotein subunit {Wolinella succinogenes}
KGTEDVFKIKNRMKDVMDDNVGIFRDGPHLEKSVKELEELYKKSKNVGIKNKRLHANPELEE
AYRVPMMLKVALCVAKGALDRTESRGAHNREDYPKRDDINWLNRTLASWPNPEQTLPTLEYE
ALDVNEMEIAPRYRGYGAKGNYIENPLSVKRQEEIDKIQSELEAAGKDRHAIQEALMPYELP
AKYKARNERLGD
>d1jnra1 a.7.3.1 (A:503-643) Adenylylsulfate reductase A subunit {Archaeon Archaeoglobus fulgidus}
TADDVNPEYILPWQGLVRLQKIMDEYAAGIATIYKTNEKMLQRALELLAFLKEDLEKLAARD
LHELMRAWELVHRVWTAEAHVRHMLFRKETRWPGYYYRTDYPELNDEEWKCFVCSKYDAEKD
EWTFEKVPYVQVIEWSF
>d1fewa_ a.7.4.1 (A:) Smac/diablo {Human (Homo sapiens)}
SLSSEALMRRAVSLVTDSTSTFLSQTTYALIEAITEYTKAVYTLTSLYRQYTSLLGKMNSEE
EDEVWQVIIGARAEMTSKHQEYLKLETTWMTAVGLSEMAAEAAYQTGADQASITARNHIQLV
KLQVEEVHQLSRKAETKLAEAQIEELKQKTQEEGEERAESEQEAYLRED
>d1g73a_ a.7.4.1 (A:) Smac/diablo {Human (Homo sapiens)}
AVPIAQKSEPHSLSSEALMRRAVSLVTDSTSTDLSQTTYALIEAITEYTKAVYTLTSLYRQY
TSLLGKMNSEEEDEVWQVIIGARAEMTSKHQEYLKLETTWMTAVGLSEMAAEAAYQTGADQA
SITARNHIQLVKLQVEEVHQLSRKAETKLAEAQ
>d1hx1b_ a.7.7.1 (B:) BAG-family molecular chaperon regulator-1, BAG1 {Human (Homo sapiens)}
GNSPQEEVELKKLKHLEKSVEKIADQLEELNKELTGIQQGFLPKDLQAEALCKLDRRVKATI
EQFMKILEEIDTLILPENFKDSRLKRKGLVKKVQAFLAECDTVEQNICQE
>d1i6za_ a.7.7.1 (A:) BAG-family molecular chaperon regulator-1, BAG1 {Mouse (Mus musculus)}
GSPEFMLIGEKSNPEEEVELKKLKDLEVSAEKIANHLQELNKELSGIQQGFLAKELQAEALC
KLDRKVKATIEQFMKILEEIDTMVLPEQFKDSRLKRKNLVKKVQVFLAECDTVEQYICQETE
RLQSTNLALAE
>d1qsda_ a.7.5.1 (A:) beta-Tubulin binding post-chaperonin

cofactor Rbl2p {Baker's yeast (Saccharomyces cerevisiae)}
TQLDIKVKALKRLTKEEGYYQQELKDQEAHVAKLKEDKSVDPYDLKKQEEVLDDTKRLLPTL
YEKIREFKEDLEQFLKTYQGTEDVSDARSAITSAQELLDS
>d1fjgt_ a.7.6.1 (T:) Ribosomal protein S20 {Thermus thermophilus}
RNLSALKRHRQSLKRRLRNKAKKSAIKTLSKKAVQLAQEGKAEEALKIMRKAESLIDKAAKG
STLHKNAAARRKSRLMRKVRQLLEAAGAPLIGGGLSA
>d1bdc__ a.8.1.1 (-) Immunoglobulin-binding protein A modules
{Staphylococcus aureus}
TADNKFNKEQQNAFYEILHLPNLNEEQRNGFIQSLKDDPSQSANLLAEAKKLNDAQAPKA
>d1deeg_ a.8.1.1 (G:) Immunoglobulin-binding protein A modules
{Staphylococcus aureus}
DQQSAFYEILNMPNLNEAQRNGFIQSLKDDPSQSTNVLGEAKKLNESQAPK
>d1edj__ a.8.1.1 (-) Immunoglobulin-binding protein A modules
{Staphylococcus aureus}
AQHDEAQQNAFYQVLNMPNLNADQRNGFIQSLKDDPSQSANVLGEAQKLNDSQAPK
>d1fc2c_ a.8.1.1 (C:) Immunoglobulin-binding protein A modules
{Staphylococcus aureus}
FNKEQQNAFYEILHLPNLNEEQRNGFIQSLKDDPSQSANLLAEA
>d2spza_ a.8.1.1 (A:) Immunoglobulin-binding protein A modules
{Staphylococcus aureus}
VDNKFNKEQQNAFYEILHLPNLNEEQRNAFIQSLKDDPSQSANLLAEAKKLNDAQAPK
>d1gab__ a.8.1.2 (-) PAB {Peptostreptococcus magnus}
TIDQWLLKNAKEDAIAELKKAGITSDFYFNAINKAKTVEEVNALKNEILKAHA
>d1gjta_ a.8.1.2 (A:) IgG binding protein G {Streptococcus sp.,
group G}
MKAIFVLNAQHDEAVDANSLAEAKVLANRELDKYGVSDYYKNLINNAKTVEGVKALIDEILA
ALP
>d1ebdc_ a.9.1.1 (C:) E3-binding domain of dihydrolipoamide
acetyltransferase {Bacillus stearothermophilus}
IAMPSVRKYAREKGVDIRLVQGTGKNGRVLKEDIDAFLAGG
>d1bal__ a.9.1.1 (-) E3-binding domain of dihydrolipoamide
succinyltransferase {Escherichia coli}
YASLEEQNNDALSPAIRRLLAEHNLDASAIKGTGVGGRLTREDVEKHLAKA
>d1bbl__ a.9.1.1 (-) E3-binding domain of dihydrolipoamide
succinyltransferase {Escherichia coli}
LSPAIRRLLAEHNLDASAIKGTGVGGRLTREDVEKHL
>d2erl__ a.10.1.1 (-) ER-1 {Euplotes raikovi}
DACEQAAIQCVESACESLCTEGEDRTGCYMYIYSNCPPYV
>d1erd__ a.10.1.1 (-) ER-2 {Euplotes raikovi}
DPMTCEQAMASCEHTMCGYCQGPLYMTCIGITTDPECGLP
>d1erp__ a.10.1.1 (-) ER-10 {Euplotes raikovi}
DLCEQSALQCNEQGCHNFCSPEDKPGCLGMVWNPELCP
>d1ery__ a.10.1.1 (-) ER-11 {Euplotes raikovi}
DECANAAAQCSITLCNLYCGPLIEICELTVMQNCEPPFS

>d1hd6a_ a.10.1.1 (A:) ER-22 {Euplotes raikovi}
DICDIAIAQCSLTLCQDCENTPICELAVKGSCPPPWS
>d1aca__ a.11.1.1 (-) Acyl-CoA binding protein {Cow (Bos taurus)}
SQAEFDKAAEEVKHLKTKPADEEMLFIYSHYKQATVGDINTERPGMLDFKGKAKWDAWNELK
GTSKEDAMKAYIDKVEELKKKYGI
>d1hbka_ a.11.1.1 (A:) Acyl-CoA binding protein {Plasmodium falciparum}
HMAQQVFEEECVSFINGLPRTINNLPNELKLDLYKYYKQSTIGNCNIKEPSAHKYIDRKKYE
EAWKSVENLNREDAQKKRYVDIVSEIFPYWQD
>d1ef1a1 a.11.2.1 (A:88-198) Moesin {Human (Homo sapiens)}
DVSEELIQDITQRLFFLQVKEGILNDDIYCPPETAVLLASYAVQSKYGDFNKEVHKSGYLAG
DKLLPQRVLEQHKLNKDQWEERIQVWHEEHRGMLREDAVLEYLKIAQDL
>d1gc7a1 a.11.2.1 (A:88-198) Radixin {Mouse (Mus musculus)}
DVSEELIQEITQRLFFLQVKEAILNDEIYCPPETAVLLASYAVQAKYGDYNKEIHKPGYLAN
DRLLPQRVLEQHKLTKEQWEERIQNWHEEHRGMLREDSMMEYLKIAQDL
>d1gg3a1 a.11.2.1 (A:82-187) Erythroid membrane protein 4.1R {Human (Homo sapiens)}
PDPAQLTEDITRYYLCLQLRQDIVAGRLPCSFATLALLGSYTIQSELGDYDPELHGVDYVSD
FKLAPNQTKELEEKVMELHKSYRSMTPAQADLEFLENAKKLSMY
>d1h4ra1 a.11.2.1 (A:104-214) Merlin {Human (Homo sapiens)}
NAEEELVQEITQHLFFLQVKKQILDEKIYCPPEASVLLASYAVQAKYGDYDPSVHKRGFLAQ
EELLPKRVINLYQMTPEMWEERITAWYAEHRGRARDEAEMEYLKIAQDL
>d1kdxa_ a.12.1.1 (A:) Kix domain of CBP (creb binding protein) {Mouse (Mus musculus)}
GVRKGWHEHVTQDLRSHLVHKLVQAIFPTPDPAALKDRRMENLVAYAKKVEGDMYESANSRD
EYYHLLAEKIYKIQKELEE
>d1lre__ a.13.1.1 (-) alpha-2-Macroglobulin receptor associated protein (RAP) domain 1 {Human (Homo sapiens)}
GEEFRMEKLNQLWEKAQRLHLPPVRLAELHADLKIQERDELAWKKLKLDGLDEDGEKEARLI
RNLNVILAKYGLDGKKDAR
>d1qqva_ a.14.1.1 (A:) Thermostable subdomain from chicken villin headpiece {Chicken (Gallus gallus)}
PTKLETFPLDVLVNTAAEDLPRGVDPSRKENHLSDEDFKAVFGMTRSAFANLPLWKQQNLKK
EKGLF
>d1vii__ a.14.1.1 (-) Thermostable subdomain from chicken villin headpiece {Chicken (Gallus gallus)}
MLSDEDFKAVFGMTRSAFANLPLWKQQNLKKEKGLF
>d1tbaa_ a.15.1.1 (A:) TAF(II)230 TBP-binding fragment {Fruit fly (Drosophila melanogaster)}
EGSIGNGLDLTGILFGNIDSEGRLLQDDDGEGRGGTGFDAELRENIGSLSKLGLDSMLLEVI
DLKEA
>d1ail__ a.16.1.1 (-) N-terminal, RNA-binding domain of nonstructural protein NS1 {Influenza A virus}
MDSNTVSSFQVDCFLWHVRKQVVDQELGDAPFLDRLRRDQKSLRGRGSTLGLNIEAATHVGK

QIVEKILK
>d1a32__ a.16.1.2 (-) Ribosomal protein S15 {Bacillus stearothermophilus}
LTQERKREIIEQFKVHENDTGSPEVQIAILTEQINNLNEHLRVHKKDHHSRRGLLKMVGKRR
RLLAYLRNKDVARYREIVEKLGL
>d1dk1a_ a.16.1.2 (A:) Ribosomal protein S15 {Thermus thermophilus}
PITKEEKQKVMQEFARFPGDTGSTEVQVALLTLRINRLSEHLKVHKKDHHSHRGLLMMVGQR
RRLLRYLQREDPERYRMLIEKLGI
>d1g1xb_ a.16.1.2 (B:) Ribosomal protein S15 {Thermus thermophilus}
PITKEEKQKVIQEFARFPGDTGSTEVQVALLTLRINRLSEHLKVHKKDHHSHRGLLMMVGQR
RRLLRYLQREDPERYREIVEKLGLRG
>d1d2da_ a.16.1.3 (A:) Multifunctional Glu-Pro-tRNA synthase (EPRS) second repeated element {Chinese hamster (Cricetulus griseus)}
MVYDKIAAQGEVVRKLKAEKAPKAKVTEAVECLLSLKAEYKEKTGKEYVPGLEHHH
>d1fyja_ a.16.1.3 (A:) Multifunctional Glu-Pro-tRNA synthase (EPRS) second repeated element {Human (Homo sapiens)}
DSLVLYNRVAVQGDVVRELKAKKAPKEDVDAAVKQLLSLKAEYKEKTGQEYKPGNPP
>d2hp8__ a.17.1.1 (-) p8-MTCP1 {Human (Homo sapiens)}
MPQKDPCQKQACEIQKCLQANSYMESKCQAVIQELRKCCAQYPKGRSVVCSGFEKEEEENLT
RKSASK
>d1ijxa_ a.141.1.1 (A:) Secreted Frizzled-related protein 3 (SFRP-3;fzb) {Mouse (Mus musculus)}
AACEPVRIPLCKSLPWEMTKMPNHLHHSTQANAILAMEQFEGLLGTHCSPDLLFFLCAMYAP
ICTIDFQHEPIKPCKSVCERARQGCEPILIKYRHSWPESLACDELPVYDRGVCISPEAIVTA
D
>d1ijya_ a.141.1.1 (A:) Frizzled 8 (FZ8) {Mouse (Mus musculus)}
ELACQEITVPLCKGIGYEYTYMPNQFNHDTQDEAGLEVHQFWPLVEIQCSPDLKFFLCSMYT
PICLEDYKKPLPPCRSVCERAKAGCAPLMRQYGFAWPDRMRCDRLPEQGNPDTLCMDYER
>d2end__ a.18.1.1 (-) T4 endonuclease V {Bacteriophage T4 (Escherichia coli)}
TRINLTLVSELADQHLMAEYRELPRVFGAVRKHVANGKRVRDFKISPTFILGAGHVTFFYDK
LEFLRKRQIELIAECLKRGFNIKDTTVQDISDIPQEFRGDYIPHEASIAISQARLDEKIAQR
PTWYKYYGKAIYA
>d2lisa_ a.19.1.1 (A:) Lysin {Red abalone (Haliotis rufescens)}
HYVEPKFLNKAFEVALKVQIIAGFDRGLVKWLRVHGRTLSTVQKKALYFVNRRYMQTHWANY
MLWINKKIDALGRTPVVGDYTRLGAEIGRRIDMAYFYDFLKDKNMIPKYLPYMEEINRMRPA
DVPVKYM
>d3lyna_ a.19.1.1 (A:) Lysin {Green abalone (Haliotis fulgens)}
INKAYEVTMKIQIISGFDRQLTAWLRVHGRRLTNNQKKTLFFVNRRYMQTHWQNYMLWVKRK
IKALGRPAAVGDYTRLGAEIGRRVDMVFFYNFLSGRKMIPPYSAYMAKLNALRPADVPVK
>d1gaka_ a.19.1.1 (A:) SP18 {Abalone (Haliotis fulgens)}
FDDVVVSRQEQSYVQRGMVNFLDEEMHKLVKRFRDMRWNLGPGFVFLLKKVNRERMMRYCMD

YARYSKKILQLKHLPVNKKTLTKMGRFVGYRNYGVIRELYADVFRDVQGFRGPKMTAAMRKY
SSKDPGTFPCKNE
>d1lbu_1 a.20.1.1 (1-83) Zn2+ DD-carboxypeptidase, N-terminal domain {Streptomyces albus G}
DGCYTWSGTLSEGSSGEAVRQLQIRVAGYPGTGAQLAIDGQFGPATKAAVQRFQSAYGLAAD
GIAGPATFNKIYQLQDDDCTP
>d1ck7a6 a.20.1.2 (A:31-107) Gelatinase A {Human (Homo sapiens)}
PSPIIKFPGDVAPKTDKELAVQYLNTFYGCPKESCNLFVLKDTLKKMQKFFGLPQTGDLDQN
TIETMRKPRCGNPDV
>d1slm_1 a.20.1.2 (16-80) Stromelysin-1 (MMP-3) {Human (Homo sapiens), fibroblast}
LVQKYLENYYDLKKDVKQFVRRKDSGPVVKKIREMQKFLGLEVTGKLDSDTLEVMRKPRCGV
PDV
>d1aab__ a.21.1.1 (-) HMG1, domains A and B {Rat (Rattus norvegicus)}
GKGDPKKPRGKMSSYAFFVQTSREEHKKKHPDASVNFSEFSKKCSERWKTMSAKEKGKFEDM
AKADKARYEREMKTYIPPKGE
>d1ckta_ a.21.1.1 (A:) HMG1, domains A and B {Rat (Rattus norvegicus)}
KPRGKMSSYAFFVQTCREEHKKKHPDASVNFSEFSKKCSERWKTMSAKEKGKFEDMAKADKA
RYEREMKTY
>d1hme__ a.21.1.1 (-) HMG1, domains A and B {Rat (Rattus norvegicus)}
FKDPNAPKRPPSAFFLFCSEYRPKIKGEHPGLSIGDVAKKLGEMWNNTAADDKQPYEKKAAK
LKEKYEKDIAAYRAK
>d1hsm__ a.21.1.1 (-) HMG1, domains A and B {Hamster (Cricetulus griseus)}
NAPKRPPSAFFLFCSEYRPKIKGEHPGLSIGDVAKKLGEMWNNTAADDKQPYEKKAAKLKEK
YEKDIAAYRAKGKPDAA
>d1qrva_ a.21.1.1 (A:) HMG-D {Drosophila melanogaster}
SDKPKRPLSAYMLWLNSARESIKRENPGIKVTEVAKRGGELWRAMKDKSEWEAKAAKAKDDY
DRAVKEFEANG
>d1cg7a_ a.21.1.1 (A:) NHP6a {Baker's yeast (Saccharomyces cerevisiae)}
MVTPREPKKRTTRKKKDPNAPKRALSAYMFFANENRDIVRSENPDITFGQVGKKLGEKWKAL
TPEEKQPYEAKAQADKKRYESEKELYNATLA
>d1hrza_ a.21.1.1 (A:) SRY {Human (Homo sapiens)}
DRVKRPMNAFIVWSRDQRRKMALENPRMRNSEISKQLGYQWKMLTEAEKWPFFQEAQKLQAM
HREKYPNYKYR
>d1j46a_ a.21.1.1 (A:) SRY {Human (Homo sapiens)}
MQDRVKRPMNAFIVWSRDQRRKMALENPRMRNSEISKQLGYQWKMLTEAEKWPFFQEAQKLQ
AMHREKYPNYKYRPRRKAKMLPK
>d1i11a_ a.21.1.1 (A:) Sox-5 {Mouse (Mus musculus)}
PHIKRPMNAFMVWAKDERRKILQAFPDMHNSNISKILGSRWKAMTNLEKQPYYEEQARLSKQ

HLEKYPDY
>d2lefa_ a.21.1.1 (A:) Lymphoid enhancer-binding factor, LEF1 {Mouse (Mus musculus)}
MHIKKPLNAFMLYMKEMRANVVAESTLKESAAINQILGRRWHALSREEQAKYYELARKERQL
HMQLYPGWSARDNYGKKKKRKREK
>d1k99a_ a.21.1.1 (A:) Upstream binding factor, the first HMG box {Human (Homo sapiens)}
MKKLKKHPDFPKKPLTPYFRFFMEKRAKYAKLHPEMSNLDLTKILSKKYKELPEKKKMKYIQ
DFQREKQEFERNLARFREDHPDLIQNAKK
>d1eqza_ a.22.1.1 (A:) Histone H2A {Chicken (Gallus gallus), erythrocytes}
SGRGKQGGKARAKAKSRSSRAGLQFPVGRVHRLLRKGNYAERVGAGAPVYLAAVLEYLTAEI
LELAGNAARDNKKTRIIPRHLQLAIRNDEELNKLLGKVTIAQGGVLPNIQAVLLPKKTDSHK
A
>d1hioa_ a.22.1.1 (A:) Histone H2A {Chicken (Gallus gallus), erythrocytes}
KSRSSRAGLQFPVGRVHRLLRKGNYAERVGAGAPVYLAAVLEYLTAEILELAGNAARDNKKT
RIIPRHLQLAIRNDEELNKLLGKVTIAQGGVLP
>d1hq3a_ a.22.1.1 (A:) Histone H2A {Chicken (Gallus gallus), erythrocytes}
KAKSRSSRAGLQFPVGRVHRLLRKGNYAERVGAGAPVYLAAVLEYLTAEILELAGNAARDNK
KTRIIPRHLQLAIRNDEELNKLLGKVTIAQGGVLPNIQAVLLPK
>d1aoic_ a.22.1.1 (C:) Histone H2A {African clawed frog (Xenopus laevis)}
GKQGGKTRAKAKTRSSRAGLQFPVGRVHRLLRKGNYAERVGAGAPVYLAAVLEYLTAEILEL
AGNAARDNKKTRIIPRHLQLAVRNDEELNKLLGRVTIAQGGVLPNIQSVLLPK
>d1f66c_ a.22.1.1 (C:) Histone H2A {Human (Homo sapiens), variant H2A.Z}
AVSRSQRAGLQFPVGRIHRHLKSRTTSHGRVGATAAVYSAAILEYLTAEVLELAGNASKDLK
VKRITPRHLQLAIRGDEELDSLIKATIAGGGVIPHIHKSLI
>d1id3c_ a.22.1.1 (C:) Histone H2A {Baker's yeast (Saccharomyces cerevisiae), H2A.1}
QSRSAKAGLTFPVGRVHRLLRRGNYAQRIGSGAPVYLTAVLEYLAAEILELAGNAARDNKKT
RIIPRHLQLAIRNDDELNKLLGNVTIAQGGVLPNIHQNLLPKKSAKAT
>d1eqzb_ a.22.1.1 (B:) Histone H2B {Chicken (Gallus gallus), erythrocytes}
VTKTQKKGDKKRKKSRKESYSIYVYKVLKQVHPDTGISSKAMGIMNSFVNDIFERIAGEASR
LAHYNKRSTITSREIQTAVRLLLPGELAKHAVSEGTKAVTKYTSSK
>d1hiob_ a.22.1.1 (B:) Histone H2B {Chicken (Gallus gallus), erythrocytes}
SYSIYVYKVLKQVHPDTGISSKAMGSMNSFVNDIFERIAGLASRLAHYNKRSTITSREIQTA
VRLLLPGELAKHAVSEGTKAVTKHTSSK
>d1hq3b_ a.22.1.1 (B:) Histone H2B {Chicken (Gallus gallus), erythrocytes}

RKESYSIYVYKVLKQVHPDTGISSKAMGIMNSFVNDIFERIAGEASRLAHYNKRSTITSREI
QTAVRLLLPGELAKHAVSEGTKAVTKYTSS
>d1aoid_ a.22.1.1 (D:) Histone H2B {African clawed frog (Xenopus laevis)}
KKRRKTRKESYAIYVYKVLKQVHPDTGISSKAMSIMNSFVNDVFERIAGEASRLAHYNKRST
ITSREIQTAVRLLLPGELAKHAVSEGTKAVTKYTSAK
>d1id3d_ a.22.1.1 (D:) Histone H2B {Baker's yeast (Saccharomyces cerevisiae), H2B.2}
RKETYSSYIYKVLKQTHPDTGISQKSMSILNSFVNDIFERIATEASKLAAYNKKSTISAREI
QTAVRLILPGELAKHAVSEGTRAVTKYSSST
>d1eqzc_ a.22.1.1 (C:) Histone H3 {Chicken (Gallus gallus), erythrocytes}
APATGGVKKPHRYRPGTVALREIRRYQKSTELLIRKLPFQRLVREIAQDFKTDLRFQSSAVM
ALQEASEAYLVGLFEDTNLCAIHAKRVTIMPKDIQLARRIRGERA
>d1eqzg_ a.22.1.1 (G:) Histone H3 {Chicken (Gallus gallus), erythrocytes}
PRKQLATKAARKSAPATGGVKKPHRYRPGTVALREIRRYQKSTELLIRKLPFQRLVREIAQD
FKTDLRFQSSAVMALQEASEAYLVGLFEDTNLCAIHAKRVTIMPKDIQLARRIRGERA
>d1hq3c_ a.22.1.1 (C:) Histone H3 {Chicken (Gallus gallus), erythrocytes}
YRPGTVALREIRRYQKSTELLIRKLPFQRLVREIAQDFKTDLRFQSSAVMALQEASEAYLVG
LFEDTNLCAIHAKRVTIMPKDIQLARRIRGERA
>d1id3a_ a.22.1.1 (A:) Histone H3 {Baker's yeast (Saccharomyces cerevisiae)}
PHRYKPGTVALREIRRFQKSTELLIRKLPFQRLVREIAQDFKTDLRFQSSAIGALQESVEAY
LVSLFEDTNLAAIHAKRVTIQKKEIKLARRLRGER
>d1eqzh_ a.22.1.1 (H:) Histone H4 {Chicken (Gallus gallus), erythrocytes}
KGLGKGGAKRHRKVLRDNIQGITKPAIRRLARRGGVKRISGLIYEETRGVLKVFLENVIRDA
VTYTEHAKRKTVTAMDVVYALKRQGRTLYGFGG
>d1hq3d_ a.22.1.1 (D:) Histone H4 {Chicken (Gallus gallus), erythrocytes}
KVLRDNIQGITKPAIRRLARRGGVKRISGLIYEETRGVLKVFLENVIRDAVTYTEHAKRKTV
TAMDVVYALKRQGRTLYGFGG
>d1id3b_ a.22.1.1 (B:) Histone H4 {Baker's yeast (Saccharomyces cerevisiae)}
DNIQGITKPAIRRLARRGGVKRISGLIYEEVRAVLKSFLESVIRDSVTYTEHAKRKTVTSLD
VVYALKRQGRTLYGFGG
>d1b67a_ a.22.1.2 (A:) Archaeal histone {Archaeon Methanothermus fervidus, histone A}
GELPIAPIGRIIKNAGAERVSDDARIALAKVLEEMGEEIASEAVKLAKHAGRKTIKAEDIEL
ARKMFK
>d1a7w__ a.22.1.2 (-) Archaeal histone {Archaeon Methanothermus fervidus, histone B}

MELPIAPIGRIIKDAGAERVSDDARITLAKILEEMGRDIASEAIKLARHAGRKTIKAEDIEL
AVRRFK
>d1f1ea_ a.22.1.2 (A:) Archaeal histone {Archaeon Methanopyrus kandleri}
ELPKAAIERIFRQGIGERRLSQDAKDTIYDFVPTMAEYVANAAKSVLDASGKKTLMEEHLKA
LADVLMVEGVEDYDGELFGRATVRRILKRAGIERASSDAVDLYNKLICRATEELGEKAAEYA
DEDGRKTVQGEDVEKAITYSMPKGGEL
>d1tafa_ a.22.1.3 (A:) TAF(II)42 {Fruit fly (Drosophila melanogaster)}
PKDAQVIMSILKELNVQEYEPRVVNQLLEFTFRYVTSILDDAKVYANHARKKTIDLDDVRLA
TEVTLD
>d1tafb_ a.22.1.3 (B:) TAF(II)62 {Fruit fly (Drosophila melanogaster)}
MLYGSSISAESMKVIAESIGVGSLSDDAAKELAEDVSIKLKRIVQDAAKFMNHAKRQKLSVR
DIDMSLKV
>d1bh9a_ a.22.1.3 (A:) TAF(II)18 {Human (Homo sapiens)}
LFSKELRCMMYGFGDDQNPYTESVDILEDLVIEFITEMTHKAMSI
>d1bh9b_ a.22.1.3 (B:) TAF(II)28 {Human (Homo sapiens)}
FSEEQLNRYEMYRRSAFPKAAIKRLIQSITGTSVSQNVVIAMSGISKVFVGEVVEEALDVCE
KWGEMPPLQPKHMREAVRRLKSKGQIP
>d1jfia_ a.22.1.3 (A:) Negative cofactor 2, NC2, alpha chain {Human (Homo sapiens)}
ARFPPARIKKIMQTDEEIGKVAAAVPVIISRALELFLESLLKKACQVTQSRNAKTMTTSHLK
QCIE
>d1jfib_ a.22.1.3 (B:) Negative cofactor 2, NC2, beta chain {Human (Homo sapiens)}
DDLTIPRAAINKMIKETLPNVRVANDARELVVNCCTEFIHLISSEANEICNKSEKKTISPEH
VIQALESLGFGSYISEVKEVLQECKTVALKRRKASSRLENLGIPEEELLRQQQELFAKARQQ
QAELAQQEWLQ
>d1fpoa2 a.23.1.1 (A:77-171) HSC20 (HSCB), C-terminal oligomerisation domain {Escherichia coli}
FDLASEQHTVRDTAFLMEQLELREELDEIEQAKDEARLESFIKRVKKMFDTRHQLMVEQLDN
ETWDAAADTCRKLRFLDKLRSSAEQLEEKLLDF
>d1eexg_ a.23.2.1 (G:) Diol dehydratase, gamma subunit {Klebsiella oxytoca}
SARVSDYPLANKHPEWVKTATNKTLDDFTLENVLSNKVTAQDMRITPETLRLQASIAKDAGR
DRLAMNFERAAELTAVPDDRILEIYNALRPYRSTKEELLAIADDLESRYQAKICAAFVREAA
TLYVERKKLKGDD
>d1mtyg_ a.23.3.1 (G:) Methane monooxygenase hydrolase, gamma subunit {Methylococcus capsulatus}
LGIHSNDTRDAWVNKIAHVNTLEKAAEMLKQFRMDHTTPFRNSYELDNDYLWIEAKLEEKVA
VLKARAFNEVDFRHKTAFGEDAKSVLDGTVAKMNAAKDKWEAEKIHIGFRQAYKPPIMPVNY
FLDGERQLGTRLMELRNLNYYDTPLEELRKQRGVRVVH
>d1mhyg_ a.23.3.1 (G:) Methane monooxygenase hydrolase, gamma

subunit {Methylosinus trichosporium}
AKREPIHDNSIRTEWEAKIAKLTSVDQATKFIQDFRLAYTSPFRKSYDIDVDYQYIERKIEE
KLSVLKTEKLPVADLITKATTGEDAAAVEATWIAKIKAAKSKYEAEAIHIEFRQLYKPPVLP
VNVFLRTDAALGTVLMEIRNTDYYGTPLEGLRKERGVKVLHLQ
>d1om2a_ a.23.4.1 (A:) Mitochondrial import receptor subunit Tom20
{Rat (Rattus norvegicus)}
RAGLSKLPDLKDAEAVQKFFLEEIQLGEELLAQGDYEKGVDHLTNAIAVCGQPQQLLQVLQQ
TLPPPVFQMLLTKLPTISQRIVSAQSLGEDDVE
>d1jw2a_ a.23.5.1 (A:) Hemolysin expression modulating protein HHA
{Escherichia coli}
MSEKPLTKTDYLMRLRRCQTIDTLERVIEKNKYELSDNELAVFYSAADHRLAELTMNKLYDK
IPSSVWKFIR
>d1nfn__ a.24.1.1 (-) Apolipoprotein E3 {Human (Homo sapiens)}
GQRWELALGRFWDYLRWVQTLSEQVQEELLSSQVTQELRALMDETMKELKAYKSELEEQLTP
VAEETRARLSKELQAAQARLGADMEDVCGRLVQYRGEVQAMLGQSTEELRVRLASHLRKLRK
RLLRDADDLQKRLAVYQA
>d2asr__ a.24.2.1 (-) Aspartate receptor, ligand-binding domain
{Escherichia coli}
KSFVVSNQLREQQGELTSTWDLMLQTRINLSRSAVRMMMDSSNQQSNAKVELLDSARKTLAQ
AATHYKKFKSMAPLPEMVATSRNIDEKYKNYYTALTELIDYLDYGNTGAYFAQPTQGMQNAM
GERFAQYALSSEKLYRDI
>d1vlta_ a.24.2.1 (A:) Aspartate receptor, ligand-binding domain
{Salmonella typhimurium}
GFVISNELRQQQSELTSTWDLMLQTRINLSRSAARMMMDASNQQSSAKTDLLQNAKTTLAQA
AAHYANFKNMTPLPAMAEASANVDEKYQRYQAALAELIQFLDNGNMDAYFAQPTQGMQNALG
EALGNYARVSENLYRQTF
>d2liga_ a.24.2.1 (A:) Aspartate receptor, ligand-binding domain
{Salmonella typhimurium}
MGGLLFSSLQHCQQGFVISNELRQQQSELTSTWDLMLQTRINLSRSAARMMMDASNQQSSAK
TDLLQNAKTTLAQAAAHYANFKNMTPLPAMAEASANVDEKYQRYQAALAELIQFLDNGNMDA
YFAQPTQGMQNALGEALGNYARVSENLYRQTFD
>d256ba_ a.24.3.1 (A:) Cytochrome b562 {Escherichia coli}
ADLEDNMETLNDNLKVIEKADNAAQVKDALTKMRAAALDAQKATPPKLEDKSPDSPEMKDFR
HGFDILVGQIDDALKLANEGKVKEAQAAAEQLKTTRNAYHQKYR
>d2ccya_ a.24.3.2 (A:) Cytochrome c' {Rhodospirillum molischianum}
QSKPEDLLKLRQGLMQTLKSQWVPIAGFAAGKADLPADAAQRAENMAMVAKLAPIGWAKGTE
ALPNGETKPEAFGSKSAEFLEGWKALATESTKLAAAAKAGPDALKAQAAATGKVCKACHEEF
KQD
>d1bbha_ a.24.3.2 (A:) Cytochrome c' {Chromatium vinosum}
AGLSPEEQIETRQAGYEFMGWNMGKIKANLEGEYNAAQVEAAANVIAAIANSGMGALYGPGT
DKNVGDVKTRVKPEFFQNMEDVGKIAREFVGAANTLAEVAATGEAEAVKTAFGDVGAACKSC
HEKYRAK
>d1e85a_ a.24.3.2 (A:) Cytochrome c' {Alcaligenes sp.}
EFAKPEDAVKYRQSALTLMASHFGRMTPVVKGQAPYDAAQIKANVEVLKTLSALPWAAFGPG

TEGGDARPEIWSDAASFKQKQQAFQDNIVKLSAAADAGDLDKLRAAFGDVGASCKACHDAYR
K
>d1jafa_ a.24.3.2 (A:) Cytochrome c' {Rhodocyclus gelatinosus}
QFQKPGDAIEYRQSAFTLIANHFGRVAAMAQGKAPFDAKVAAENIALVSTLSKLPLTAFGPG
TDKGHGTEAKPAVWSDAAGFKAAADKFAAAVDKLDAAGKTGDFAQIKAAVGETGGACKGCHD
KFKE
>d1cpq__ a.24.3.2 (-) Cytochrome c' {Rhodobacter capsulatus}
ADTKEVLEAREAYFKSLGGSMKAMTGVAKAFDAEAAKVEAAKLEKILATDVAPLFPAGTSST
DLPGQTEAKAAIWANMDDFGAKGKAMHEAGGAVIAAANAGDGAAFGAALQKLGGTCKACHDD
YREED
>d1a7va_ a.24.3.2 (A:) Cytochrome c' {Rhodopseudomonas palustris}
QTDVIAQRKAILKQMGEATKPIAAMLKGEAKFDQAVVQKSLAAIADDSKKLPALFPADSKTG
GDTAALPKIWEDKAKFDDLFAKLAAAATAAQGTIKDEASLKANIGGVLGNCKSCHDDFRAKK
S
>d2hmza_ a.24.4.1 (A:) Hemerythrin {Sipunculid worm (Themiste dyscrita)}
GFPIPDPYCWDISFRTFYTIVDDEHKTLFNGILLLSQADNADHLNELRRCTGKHFLNEQQLM
QASQYAGYAEHKKAHDDFIHKLDTWDGDVTYAKNWLVNHIKTIDFKYRGKI
>d1hrb__ a.24.4.1 (-) Hemerythrin {Phascolopsis gouldii}
GFPIPDPYVWDPSFRTFYSIIDDEHKTLFNGIFHLAIDDNADNLGELRRCTGKHFLNQEVLM
EASQYQFYDEHKKEHDGFINALDNWKGDVKWAKAWLVNHIKTIDFKYKGKI
>d1i4ya_ a.24.4.1 (A:) Hemerythrin {Phascolopsis gouldii}
GFPIPDPYVWDPSFRTFYSIIDDEHKTLFNGIFHLAIDDNADNLGELRRCTGKHFLNEQVLM
QASQYQFYDEHKKEHETFIHALDNWKGDVKWAKSWLVNHIKTIDFKYKGKI
>d2mhr__ a.24.4.1 (-) Myohemerythin {Sipunculan worm (Themiste zostericola)}
GWEIPEPYVWDESFRVFYEQLDEEHKKIFKGIFDCIRDNSAPNLATLVKVTTNHFTHEEAMM
DAAKYSEVVPHKKMHKDFLEKIGGLSAPVDAKNVDYCKEWLVNHIKGTDFKYKGKL
>d1ei7a_ a.24.5.1 (A:) Tobacco mosaic virus coat protein {Tobacco mosaic virus, vulgare strain}
SYSITTPSQFVFLSSAWADPIELINLCTNALGNQFQTQQARTVVQRQFSEVWKPSPQVTVRF
PDSDFKVYRYNAVLDPLVTALLGAFDTRNRIIEVENQANPTTAETLDATRRVDDATVAIRSA
INNLIVELIRGTGSYNRSSFESSSGLVWTSGPAT
>d1vtmp_ a.24.5.1 (P:) Tobacco mosaic virus coat protein {Tobacco mosaic virus, vulgare strain}
PYTINSPSQFVYLSSAYADPVELINLCTNALGNQFQTQQARTTVQQQFADAWKPSPVMTVRF
PASDFYVYRYNSTLDPLITALLNSFDTRNRIIEVNNQPAPNTTEIVNATQRVDDATVAIRAS
INNLANELVRGTGMFNQAGFETASGLVWTTTPAT
>d1cgme_ a.24.5.1 (E:) Cucumber green mottle mosaic virus {Cucumber green mottle mosaic virus, strain watermelon}
AYNPITPSKLIAFSASYVPVRTLLNFLVASQGTAFQTQAGRDSFRESLSALPSSVVDINSRF
PDAGFYAFLNGPVLRPIFVSLLSSTDTRNRVIEVVDPSNPTTAESLNAVKRTDDASTAARAE
IDNLIESISKGFDVYDRASFEAAFSVVWSEATTSKA
>d1rmva_ a.24.5.1 (A:) Ribgrass mosaic virus {Ribgrass mosaic

virus}
SYNITNSNQYQYFAAVWAEPTPMLNQCVSALSQSYQTQAGRDTVRQQFANLLSTIVAPNQRF
PDTGFRVYVNSAVIKPLYEALMKSFDTRNRIIETEEESRPSASEVANATQRVDDATVAIRSQ
IQLLLNELSNGHGYMNRAEFEAILPWTTAPAT
>d3fapb_ a.24.7.1 (B:) FKBP12-rapamycin-binding domain of
FKBP-rapamycin-associated protein (FRAP) {Human (Homo sapiens)}
VAILWHEMWHEGLEEASRLYFGERNVKGMFEVLEPLHAMMERGPQTLKETSFNQAYGRDLME
AQEWCRKYMKSGNVKDLTQAWDLYYHVFRRIS
>g1avo.1 a.24.8.1 (A:,B:) Proteasome activator reg(alpha) {Human
(Homo sapiens)}
LRVQPEAQAKVDVFREDLCTKTENLLGSYFPKKISELDAFLKEPALNEANLSNLKAPLDIXA
VNCNEKIVVLLQRLKPEIKDVIEQLNLVTTWLQLQIPRIEDGNNFGVAVQEKVFELMTSLHT
KLEGFHTQISKYFSERGDAVTKAAKQPHVGDYRQLVHELDEAEYRDIRLMVMEIRNAYAVLY
DIILKNFEKLKKPRG
>d1dova_ a.24.9.1 (A:) alpha-catenin {Mouse (Mus musculus)}
ESQFLKEELVVAVEDVRKQGDLMKSAAGEFADDPCSSVKRGNMVRAARALLSAVTRLLILAD
MADVYKLLVQLKVVEDGILKLRNAGNEQDLGIQYKALKPEVDKLNIMAAKRQQELKDVGNRD
QMAAARGILQKNVPILYTASQACLQHPDVAAYKANRDLIYKQLQQAVTGISNAAQAT
>d1dowa_ a.24.9.1 (A:) alpha-catenin {Mouse (Mus musculus)}
KAHVLAASVEQATENFLEKGDKIAKESQFLKEELVVAVEDVRKQGDLMKSAAGEFADDPCSS
VKRGNMVRAARALLSAVTRLLILADMADVYKLLVQLKVVEDGILKLRNAGNEQDLGIQYKAL
KPEVDKLNIMAAKRQQELKDVGNRDQMAAARGILQKNVPILYTASQACLQHPDVAAYKANRD
LIYKQLQQAVTGISNAAQA
>d1h6ga1 a.24.9.1 (A:377-507) alpha-catenin {Human (Homo sapiens)}
DLRRQLRKAVMDHVSDSFLETNVPLLVLIEAAKNGNEKEVKEYAQVFREHANKLIEVANLAC
SISNNEEGVKLVRMSASQLEALCPQVINAALALAAKPQSKLAQENMDLFKEQWEKQVRVLTD
AVDDITS
>d1h6ga2 a.24.9.1 (A:508-631) alpha-catenin {Human (Homo sapiens)}
IDDFLAVSENHILEDVNKCVIALQEKDVDGLDRTAGAIRGRAARVIHVVTSEMDNYEPGVYT
EKVLEATKLLSNTVMPRFTEQVEAAVEALSSDPAQPMDENEFIDASRLVYDGIRDIRKAVLM
>d1h6gb1 a.24.9.1 (B:392-507) alpha-catenin {Human (Homo sapiens)}
DSFLETNVPLLVLIEAAKNGNEKEVKEYAQVFREHANKLIEVANLACSISNNEEGVKLVRMS
ASQLEALCPQVINAALALAAKPQSKLAQENMDLFKEQWEKQVRVLTDAVDDITS
>d1qkra_ a.24.9.1 (A:) Vinculin {Chicken (Gallus gallus)}
KDEEFPEQKAGEAINQPMMMAARQLHDEARKWSSKGNDIIAAAKRMALLMAEMSRLVRGGSG
NKRALIQCAKDIAKASDEVTRLAKEVAKQCTDKRIRTNLLQVCERIPTISTQLKILSTVKAT
MLGRTNISDEESEQATEMLVHNAQNLMQSVKETVREAEAASIKIRTDAGFTLRWVRK
>d1k04a_ a.24.14.1 (A:) FAT domain of focal adhesion kinase {Human
(Homo sapiens)}
EISPPPTANLDRSNDKVYENVTGLVKAVIEMSSKIQPAPPEEYVPMVKEVGLALRTLLATVD
ETIPLLPASTHREIEMAQKLLNSDLGELINKMKLAQQYVMTSLQQEYKKQMLTAAHALAVDA
KNLLDVIDQARLKMLGQT
>d1k40a_ a.24.14.1 (A:) FAT domain of focal adhesion kinase {Mouse
(Mus musculus)}

NDKVYENVTGLVKAVIEMSSKIQPAPPEEYVPMVKEVGLALRTLLATVDETIPALPASTHRE
IEMAQKLLNSDLGELISKMKLAQQYVMTSLQQEYKKQMLTAAHALAVDAKNLLDVIDQARLK
ML
>d2a0b__ a.24.10.1 (-) Aerobic respiration control sensor protein, ArcB {Escherichia coli}
SKSEALLDIPMLEQYLELVGPKLITDGLAVFEKMMPGYVSVLESNLTAQDKKGIVEEGHKIK
GAAGSVGLRHLQQLGQQIQSPDLPAWEDNVGEWIEEMKEEWRHDVEVLKAWVAKAT
>d1c02a_ a.24.10.2 (A:) Phosphorelay protein ypd1 {Baker's yeast (Saccharomyces cerevisiae)}
STIPSEIINWTILNEIISMDDDDSDFSKGLIIQFIDQAQTTFAQMQRQLDGEKNLTELDNLG
HFLKGSSAALGLQRIAWVCERIQNLGRKMQHFFPNKTELVNTLSDKSIINGINIDEDDEEIK
IQVDDKDENSIYLILIAKALNQSRLEFKLARIELSKYYNTNL
>d1i5na_ a.24.10.3 (A:) Chemotaxis protein CheA P1 domain {Salmonella typhimurium}
DISDFYQTFFDEADELLADMEQHLLDLVPESPDAEQLNAIFRAAHSIKGGAGTFGFTILQET
THLMENLLDEARRGEMQLNTDIINLFLETKDIMQEQLDAYKNSEEPDAASFEYICNALRQLA
LEAK
>d1he1a_ a.24.11.1 (A:) ExoS toxin {Pseudomonas aeruginosa}
ASSAVVFKQMVLQQALPMTLKGLDKASELATLTPEGLAREHSRLASGDGALRSLSTALAGIR
AGSQVEESRIQAGRLLERSIGGIALQQWGTTGGAASQLVLDASPELRREITDQLHQVMSEVA
LLRQAVESEVS
>d1g4us1 a.24.11.1 (S:167-296) SptP tyrosine phosphatase {Salmonella typhimurium}
SKQPLLDIALKGLKRTLPQLEQMDGNSLRENFQEMASGNGPLRSLMTNLQNLNKIPEAKQLN
DYVTTLTNIQVGVARFSQWGTCGGEVERWVDKASTHELTQAVKKIHVIAKELKNVTAELEKI
EAGAPM
>d1hy5a_ a.24.11.1 (A:) YopE {Yersinia pestis}
TSFSDSIKQLAAETLPKYMQQLNSLDAEMLQKNHDQFATGSGPLRGSITQCQGLMQFCGGEL
QAEASAILNTPVCGIPFSQWGTIGGAASAYVASGVDLTQAANEIKGLAQQMQKLLSLM
>d1f1ma_ a.24.12.1 (A:) Outer surface protein C (OspC) {Lyme disease spirochete (Borrelia burgdorferi), different strains?}
PNLTEISKKITESNAVVLAVKEVETLLTSIDELAKAIGKKIKSDVSLDNEADHNGSLMSGAY
LISTLITKKISAIKDSGELKAEIEKAKKCSEEFTAKLKGEHTDLGKEGVTDDNAKKAILKTN
NDKTKGADELEKLFESVKNLSKAAKEMLTNSVKELTSP
>d1g5za_ a.24.12.1 (A:) Outer surface protein C (OspC) {Lyme disease spirochete (Borrelia burgdorferi), different strains?}
PNLTEISKKITESNAVVLAVKEVETLLASIDELATKAIGKKIGNNGLEANQSKNTSLLSGAY
AISDLIAEKLNVLKNEELKEKIDTAKQCSTEFTNKLKSEHAVLGLDNLTDDNAQRAILKKHA
NKDKGAAELEKLFKAVENLSKAAQDTLKNAVKELTSPIVA
>d1ggqa_ a.24.12.1 (A:) Outer surface protein C (OspC) {Lyme disease spirochete (Borrelia burgdorferi), different strains?}
GPNLTEISKKITDSNAVLLAVKEVEALLSSIDEIAAKAIGKKIHQNNGLDTENNHNGSLLAG
AYAISTLIKQKLDGLKNEGLKEKIDAAKKCSETFTNKLKEKHTDLGKEGVTDADAKEAILKT
NGTKTKGAEELGKLFESVEVLSKAAKEMLANSVKELTS

>d1jpna1 a.24.13.1 (A:1-88) Signal sequence recognition protein Ffh {Thermus aquaticus}
MFQQLSARLQEAIGRLRGRGRITEEDLKATLREIRRALMDADVNLEVARDFVERVREEALGK
QVLESLTPAEVILATVYEALKEALGG
>d1j8mf1 a.24.13.1 (F:3-86) Signal sequence recognition protein Ffh {Archaeon Acidianus ambivalens}
LLDNLRDTVRKFLTGSSSYDKAVEDFIKELQKSLISADVNVKLVFSLTNKIKERLKNEKPPT
YIERREWFIKIVYDELSNLFGG
>d1fts_1 a.24.13.1 (201-284) Signal recognition particle receptor, FtsY {Escherichia coli}
RSLLKTKENLGSGFISLFRGKKIDDDLFEELEEQLLIADVGVETTRKIITNLTEGASRKQLR
DAEALYGLLKEEMGEILAKVDE
>d1jr8a_ a.24.15.1 (A:) Thiol oxidase Erv2p {Baker's yeast (Saccharomyces cerevisiae)}
DDKVKKEVGRASWKYFHTLLARFPDEPTPEEREKLHTFIGLYAELYPCGECSYHFVKLIEKY
PVQTSSRTAAAMWGCHIHNKVNEYLKKDIYDCATILEDYDCGC
>d1h99a1 a.142.1.1 (A:54-168) Transcriptional antiterminator LicT {Bacillus subtilis}
GAMEKFKTLLYDIPIECMEVSEEIISYAKLQLGKKLNDSIYVSLTDHINFAIQRNQKGLDIK
NALLWETKRLYKDEFAIGKEALVMVKNKTGVSLPEDEAGFIALHIVNAELNEE
>d1h99a2 a.142.1.1 (A:169-275) Transcriptional antiterminator LicT {Bacillus subtilis}
MPNIINITKVMEEILSIVKYHFKIEFNEESLHYYRFVTDLKFFAQRLFNGTHMESEDDFLLD
TVKEKYHRAYECTKKIQTYIEREYEHKLTSDELLYLTIDIERVVK
>d1dvba1 a.25.1.1 (A:1-147) Rubrerythrin, N-terminal domain {Desulfovibrio vulgaris}
MKSLKGSRTEKNILTAFAGESQARNRYNYFGGQAKKDGFVQISDIFAETADQEREHAKRLFK
FLEGGDLEIVAAFPAGIIADTHANLIASAAGEHHEYTEMYPSFARIAREEGYEEIARVFASI
AVAEEFHEKRFLDFARNIKEGRV
>d1bcfa_ a.25.1.1 (A:) Bacterioferritin (cytochrome b1) {Escherichia coli}
MKGDTKVINYLNKLLGNELVAINQYFLHARMFKNWGLKRLNDVEYHESIDEMKHADRYIERI
LFLEGLPNLQDLGKLNIGEDVEEMLRSDLALELDGAKNLREAIGYADSVHDYVSRDMMIEIL
RDEEGHIDWLETELDLIQKMGLQNYLQAQIREEG
>d1jgca_ a.25.1.1 (A:) Bacterioferritin (cytochrome b1) {Rhodobacter capsulatus}
MKGDAKVIEFLNAALRSELTAISQYWVHFRLQEDWGLAKMAKKSREESIEEMGHADKIIARI
LFLEGHPNLQKLDPLRIGEGPRETLECDLAGEHDALKLYREARDYCAEVGDIVSKNIFESLI
TDEEGHVDFLETQISLYDRLGPQGFALLNAAPMDAA
>d1euma_ a.25.1.1 (A:) Non-hem ferritin {Escherichia coli, ecFtnA}
LKPEMIEKLNEQMNLELYSSLLYQQMSAWCSYHTFEGAAAFLRRHAQEEMTHMQRLFDYLTD
TGNLPRINTVESPFAEYSSLDELFQETYKHEQLITQKINELAHAAMTNQDYPTFNFLQWYVS
EQHEEEKLFKSIIDKLSLAGKSGEGLYFIDKELSTLD
>d1krqa_ a.25.1.1 (A:) Non-hem ferritin {Campylobacter jejuni}

MLSKEVVKLLNEQINKEMYAANLYLSMSSWCYENSLDGAGAFLFAHASEESDHAKKLITYLN
ETDSHVELQEVKQPEQNFKSLLDVFEKTYEHEQFITKSINTLVEHMLTHKDYSTFNFLQWYV
SEQHEEEALFRGIVDKIKLIGEHGNGLYLADQYIKNIALSR
>d1dpsa_ a.25.1.1 (A:) Dodecameric ferritin homolog DPS {Escherichia coli}
SKATNLLYTRNDVSDSEKKATVELLNRQVIQFIDLSLITKQAHWNMRGANFIAVHEMLDGFR
TALIDHLDTMAERAVQLGGVALGTTQVINSKTPLKSYPLDIHNVQDHLKELADRYAIVANDV
RKAIGEAKDDDTADILTAASRDLDKFLWFIECNIE
>d1qgha_ a.25.1.1 (A:) Dodecameric ferritin homolog DPS {Listeria innocua}
VDTKEFLNHQVANLNVFTVKIHQIHWYMRGHNFFTLHEKMDDLYSEFGEQMDEVAERLLAIG
GSPFSTLKEFLENASVEEAPYTKPKTMDQLMEDLVGTLELLRDEYKQGIELTDKEGDDVTND
MLIAFKASIDKHIWMFKAFLGKAPLE
>d2fha__ a.25.1.1 (-) (Apo)ferritin {Human (Homo sapiens), H chain}
TSQVRQNYHQDSEAAINRQINLELYASYVYLSMSYYFDRDDVALKNFAKYFLHQSHEEREHA
EKLMKLQNQRGGRIFLQDIQKPDCDDWESGLNAMECALHLEKNVNQSLLELHKLATDKNDPH
LCDFIETHYLNEQVKAIKELGDHVTNLRKMGAPESGLAEYLFDKHTLG
>d1aew__ a.25.1.1 (-) (Apo)ferritin {Horse (Equus caballus), L chain}
SQIRQNYSTEVEAAVNRLVNLYLRASYTYLSLGFYFDRDDVALEGVCHFFRELAEEKREGAE
RLLKMQNQRGGRALFQDLQKPSQDEWGTTPDAMKAAIVLEKSLNQALLDLHALGSAQADPHL
CDFLESHFLDEEVKLIKKMGDHLTNIQRLVGSQAGLGEYLFERLTL
>d1h96a_ a.25.1.1 (A:) (Apo)ferritin {Mouse (Mus musculus)}
TSQIRQNYSTEVEAAVNRLVNLHLRASYTYLSLGFFFDRDDVALEGVGHFFRELAEEKREGA
ERLLEFQNDRGGRALFQDVQKPSQDEWGKTQEAMEAALAMEKNLNQALLDLHALGSARADPH
LCDFLESHYLDKEVKLIKKMGNHLTNLRRVAGPQPAQTGAPQGSLGEYLFERLTLK
>d1bg7__ a.25.1.1 (-) (Apo)ferritin {Bullfrog (Rana catesbeiana)}
DSQVRQNFHRDCEAAINRMVNMELYASYTYLSMAFYFDRDDIALHNVAKFFKEQSHEEREHA
EKLMKDQNKRGGRIVLQDVQKPERDEWGNTLEAMQAALQLEKTVNQALLDLHKVGSDKVDPH
LCDFLETEYPEEQVKSIKQLGDYITNLKRLGLPQNGMGEYLFDKHTMGE
>d1mfra_ a.25.1.1 (A:) (Apo)ferritin {Bullfrog (Rana catesbeiana)}
VSQVRQNYHSDCEAAVNRMLNLELYASYTYSSMYAFFDRDDVALHNVAEFFKEHSHEEREHA
EKFMKYQNKRGGRVVLQDIKKPERDEWGNTLEAMQAALQLEKTVNQALLDLHKLATDKVDPH
LCDFLESEYLEEQVKDIKRIGDFITNLKRLGLPENGMGEYLFDKHSV
>d1rcd__ a.25.1.1 (-) (Apo)ferritin {Bullfrog (Rana catesbeiana)}
SQVRQNFHQDCEAGLNRTVNLKFHSSYVYLSMASYFNRDDVALSNFAKFFRERSEEEKEHAE
KLIEYQNQRGGRVFLQSVEKPERDDWANGLEALQTALKLQKSVNQALLDLHAVAADKSDPHM
TDFLESPYLSESVETIKKLGDHITSLKKLWSSHPGMAEYLFNKHTLG
>d1mtyb_ a.25.1.2 (B:) Methane monooxygenase hydrolase, beta and alpha subunits {Methylococcus capsulatus}
ERRRGLTDPEMAAVILKALPEAPLDGNNKMGYFVTPRWKRLTEYEALTVYAQPNADWIAGGL
DWGDWTQKFHGGRPSWGNETTELRTVDWFKHRDPLRRWHAPYVKDKAEEWRYTDRFLQGYSA
DGQIRAMNPTWRDEFINRYWGAFLFNEYGLFNAHSQGAREALSDVTRVSLAFWGFDKIDIAQ
MIQLERGFLAKIVPGFDESTAVPKAEWTNGEVYKSARLAVEGLWQEVFDWNESAFSVHAVYD

ALFGQFVRREFFQRLAPRFGDNLTPFFINQAQTYFQIAKQGVQDLYYNCLGDDPEFSDYNRT
VMRNWTGKWLEPTIAALRDFMGLFAKLPAGTTDKEEITASLYRVVDDWIEDYASRIDFKADR
DQIVKAVLAGLK
>d1mtyd_ a.25.1.2 (D:) Methane monooxygenase hydrolase, beta and alpha subunits {Methylococcus capsulatus}
AANRAPTSVNAQEVHRWLQSFNWDFKNNRTKYATKYKMANETKEQFKLIAKEYARMEAVKDE
RQFGSLQVALTRLNAGVRVHPKWNETMKVVSNFLEVGEYNAIAATGMLWDSAQAAEQKNGYL
AQVLDEIRHTHQCAYVNYYFAKNGQDPAGHNDARRTRTIGPLWKGMKRVFSDGFISGDAVEC
SLNLQLVGEACFTNPLIVAVTEWAAANGDEITPTVFLSIETDELRHMANGYQTVVSIANDPA
SAKYLNTDLNNAFWTQQKYFTPVLGMLFEYGSKFKVEPWVKTWDRWVYEDWGGIWIGRLGKY
GVESPRSLKDAKQDAYWAHHDLYLLAYALWPTGFFRLALPDQEEMEWFEANYPGWYDHYGKI
YEEWRARGCEDPSSGFIPLMWFIENNHPIYIDRVSQVPFCPSLAKGASTLRVHEYNGEMHTF
SDQWGERMWLAEPERYECQNIFEQYEGRELSEVIAELHGLRSDGKTLIAQPHVRGDKLWTLD
DIKRLNCVFKNPVKAF
>d1mhyb_ a.25.1.2 (B:) Methane monooxygenase hydrolase, beta and alpha subunits {Methylosinus trichosporium}
KRGLTDPERAAIIAAAVPDHALDTQRKYHYFIQPRWKPLSEYEQLSCYAQPNPDWIAGGLDW
GDWTQKFHGGRPSWGNESTELRTTDWYRHRDPARRWHHPYVKDKSEEARYTQRFLAAYSSEG
SIRTIDPYWRDEILNKYFGALLYSEYGLFNAHSSVGRDCLSDTIRQTAVFAALDKVDNAQMI
QMERLFIAKLVPGFDASTDVPKKIWTTDPIYSGARATVQEIWQGVQDWNEILWAGHAVYDAT
FGQFARREFFQRLATVYGDTLTPFFTAQSQTYFQTTRGAIDDLFVYCLANDSEFGAHNRTFL
NAWTEHYLASSVAALKDFVGLYAKVEKVAGATDSAGVSEALQRVFGDWKIDYADKIGFRVDV
DQKVDAVLAGY
>d1mhyd_ a.25.1.2 (D:) Methane monooxygenase hydrolase, beta and alpha subunits {Methylosinus trichosporium}
NRAPVGVEPQEVHKWLQSFNWDFKENRTKYPTKYHMANETKEQFKVIAKEYARMEAAKDERQ
FGTLLDGLTRLGAGNKVHPRWGETMKVISNFLEVGEYNAIAASAMLWDSATAAEQKNGYLAQ
VLDEIRHTHQCAFINHYYSKHYHDPAGHNDARRTRAIGPLWKGMKRVFADGFISGDAVECSV
NLQLVGEACFTNPLIVAVTEWASANGDEITPTVFLSVETDELRHMANGYQTVVSIANDPASA
KFLNTDLNNAFWTQQKYFTPVLGYLFEYGSKFKVEPWVKTWNRWVSEDWGGIWIGRLGKYGV
ESPRSLRDAKRDAYWAHHDLALAAYAMWPLGFARLALPDEEDQAWFEANYPGWADHYGKIFN
EWKKLGYEDPKSGFIPYQWLLANGHDVYIDRVSQVPFIPSLAKGTGSLRVHEFNGKKHSLTD
DWGERQWLIEPERYECHNVFEQYEGRELSEVIAEGHGVRSDGKTLIAQPHTRGDNLWTLEDI
KRAGCVFPDPLAKF
>d1jqca_ a.25.1.2 (A:) Ribonucleotide reductase R2 {Escherichia coli}
AYTTFSQTKNDQLKEPMFFGQPVNVARYDQQKYDIFEKLIEKQLSFFWRPEEVDVSRDRIDY
QALPEHEKHIFISNLKYQTLLDSIQGRSPNVALLPLISIPELETWVETWAFSETIHSRSYTH
IIRNIVNDPSVVFDDIVTNEQIQKRAEGISSYYDELIEMTSYWHLLGEGTHTVNGKTVTVSL
RELKKKLYLCLMSVNALEAIRFYVSFACSFAFAERELMEGNAKIIRLIARDEALHLTGTQHM
LNLLRSGADDPEMAEIAEECKQECYDLFVQAAQQEKDWADYLFRDGSMIGLNKDILCQYVEY
ITNIRMQAVGLDLPFQTRSNPIPWINTWLV
>d1r2fa_ a.25.1.2 (A:) Ribonucleotide reductase R2 {Salmonella typhimurium}

ISAINWNKIQDDKDLEVWNRLTSNFWLPEKVPLSNDIPAWQTLSAAEQQLTIRVFTGLTLLD
TIQNIAGAPSLMADAITPHEEAVLSNISFMEAVHARSYSSIFSTLCQTKEVDAAYAWSEENP
PLQRKAQIILAHYVSDEPLKKKIASVFLESFLFYSGFWLPMYFSSRGKLTNTADLIRLIIRD
EAVHGYYIGYKYQIALQKLSAIEREELKLFALDLLMELYDNEIRYTEALYAETGWVNDVKAF
LCYNANKALMNLGYEALFPPEMADVNPAILAALSP
>d1kgna_ a.25.1.2 (A:) Ribonucleotide reductase R2
{Corynebacterium ammoniagenes}
SNEYDEYIANHTDPVKAINWNVIPDEKDLEVWDRLTGNFWLPEKIPVSNDIQSWNKMTPQEQ
LATMRVFTGLTLLDTIQGTVGAISLLPDAETMHEEAVYTNIAFMESVHAKSYSNIFMTLAST
PQINEAFRWSEENENLQRKAKIIMSYYNGDDPLKKKVASTLLESFLFYSGFYLPMYLSSRAK
LTNTADIIRLIIRDESVHGYYIGYKYQQGVKKLSEAEQEEYKAYTFDLMYDLYENEIEYTED
IYDDLGWTEDVKRFLRYNANKALNNLGYEGLFPTDETKVSPAILSSLS
>d1xsm__ a.25.1.2 (-) Ribonucleotide reductase R2 {Mouse (Mus
musculus)}
NPSVEDEPLLRENPRRFVVFPIEYHDIWQMYKKAEASFWTAEEVDLSKDIQHWEALKPDERH
FISHVLAFFAASDGIVNENLVERFSQEVQVTEARCFYGFQIAMENIHSEMYSLLIDTYIKDP
KEREYLFNAIETMPCVKKKADWALRWIGDKEATYGERVVAFAAVEGIFFSGSFASIFWLKKR
GLMPGLTFSNELISRDEGLHCDFACLMFKHLVHKPAEQRVREIITNAVRIEQEFLTEALPVK
LIGMNCTLMKQYIEFVADRLMLELGFNKIFRVENPFDFME
>d1jk0a_ a.25.1.2 (A:) Ribonucleotide reductase R2 {Baker's yeast
(Saccharomyces cerevisiae)}
LNKELETLREENRVKSDMLKEKLSKDAENHKAYLKSHQVHRHKLKEMEKEEPLLNEDKERTV
LFPIKYHEIWQAYKRAEASFWTAEEIDLSKDIHDWNNRMNENERFFISRVLAFFAASDGIVN
ENLVENFSTEVQIPEAKSFYGFQIMIENIHSETYSLLIDTYIKDPKESEFLFNAIHTIPEIG
EKAEWALRWIQDADALFGERLVAFASIEGVFFSGSFASIFWLKKRGMMPGLTFSNELICRDE
GLHTDFACLLFAHLKNKPDPAIVEKIVTEAVEIEQRYFLDALPVALLGMNADLMNQYVEFVA
DRLLVAFGNKKYYKVENPFDFMEN
>d1jk0b_ a.25.1.2 (B:) Ribonucleotide reductase R2 {Baker's yeast
(Saccharomyces cerevisiae)}
FQKERHDMKEAEKDEILLMENSRRFVMFPIKYHEIWAAYKKVEASFWTAEEIELAKDTEDFQ
KLTDDQKTYIGNLLALSISSDNLVNKYLIENFSAQLQNPEGKSFYGFQIMMENIYSEVYSMM
VDAFFKDPKNIPLFKEIANLPEVKHKAAFIERWISNDDSLYAERLVAFAAKEGIFQAGNYAS
MFWLTDKKIMPGLAMANRNICRDRGAYTDFSCLLFAHLRTKPNPKIIEKIITEAVEIEKEYY
SNSLPVEKFGMDLKSIHTYIEFVADGLLQGFGNEKYY
>d1afra_ a.25.1.2 (A:) delta 9-stearoyl-acyl carrier protein
desaturase {Castor bean (Ricinus communis)}
MPPREVHVQVTHSMPPQKIEIFKSLDNWAEENILVHLKPVEKCWQPQDFLPDPASDGFDEQV
RELRERAKEIPDDYFVVLVGDMITEEALPTYQTMLNTLDGVRDETGASPTSWAIWTRAWTAE
ENRHGDLLNKYLYLSGRVDMRQIEKTIQYLIGSGMDPRTENSPYLGFIYTSFQERATFISHG
NTARQAKEHGDIKLAQICGTIAADEKRHETAYTKIVEKLFEIDPDGTVLAFADMMRKKISMP
AHLMYDGRDDNLFDHFSAVAQRLGVYTAKDYADILEFLVGRWKVDKLTGLSAEGQKAQDYVC
RLPPRIRRLEERAQGRAKEAPTMPFSWIFDRQVKL
>d1rhga_ a.26.1.1 (A:) Granulocyte-colony stimulating factor
(G-CSF) {Human (Homo sapiens)}

LPQSFLLKCLEQVRKIQGDGAALQEKLCATYKLCHPEELVLLGHSLGIPWAPLSSCPSQALQ
LAGCLSQLHSGLFLYQGLLQALEGISPELGPTLDTLQLDVADFATTIWQQMEELGMAPALQP
TQGAMPAFASAFQRRAGGVLVASHLQSFLEVSYRVLRHLA
>d1bgc__ a.26.1.1 (-) Granulocyte-colony stimulating factor (G-CSF) {Cow (Bos taurus)}
SLPQSFLLKCLEQVRKIQADGAELQERLCAAHKLCHPEELMLLRHSLGIPQAPLSSCSSQSL
QLRGCLNQLHGGLFLYQGLLQALAGISPELAPTLDTLQLDVTDFATNIWLQMEDLGAAPAVQ
PTQGAMPTFTSAFQRRAGGVLVASQLHRFLELAYRGLRYLA
>d1bgea_ a.26.1.1 (A:) Granulocyte-colony stimulating factor (G-CSF) {Dog (Canis familiaris)}
PLPQSFLLKCLEQMRKVQADGTALQETLCATHQLCHPEELVLLGHALGIPQPPLSSCSSQAL
QLMGCLRQLHSGLFLYQGLLQALAGISPELAPTLDTLQLDTTDFAINIWQQMEDLGMAPAVP
PTQGTMPAFTSAFQRRAGGVLVASNLQSFLELAYRALRHFAK
>d1alu__ a.26.1.1 (-) Interleukin-6 {Human (Homo sapiens)}
LTSSERIDKQIRYILDGISALRKETCNKSNMCESSKEALAENNLNLPKMAEKDGCFQSGFNE
ETCLVKIITGLLEFEVYLEYLQNRFESSEEQARAVQMSTKVLIQFLQKKAKNLDAITTPDPT
TNASLLTKLQAQNQWLQDMTTHLILRSFKEFLQSSLRALRQM
>d1i1rb_ a.26.1.1 (B:) Interleukin-6 {Human herpesvirus 8, Kaposi's sarcoma herpes-virus}
EFEKDLLIQRLNWMLWVIDECFRDLCYRTGICKGILEPAAIFHLKLPAINDTDHCGLIGFNE
TSCLKKLADGFFEFEVLFKFLTTEFGKSVINVDVMELLTKTLGWDIQEELNKLTKTHYSPPK
FDRGLLGRLQGLKYWVRHFASFYVLSAMEKFAGQAVRVLDSIP
>d1a7m__ a.26.1.1 (-) Leukemia inhibitory factor (LIF) {Mouse (Mus musculus)}
SPLPITPVNATCAIRHPCHGNLMNQIKNQLAQLNGSANALFISYYTAQGEPFPNNLDKLCGP
NVTDFPPFHANGTEKAKLVELYRMVAYLSASLTNITRDQKVLNPSAVSLHSKLNATIDVMRG
LLSNVLCRLCNKYRVGHVDVPPVPDHSDKEVFQKKKLGCQLLGTYKQVISVVVQAF
>d1lki__ a.26.1.1 (-) Leukemia inhibitory factor (LIF) {Mouse (Mus musculus)}
NATCAIRHPCHGNLMNQIKNQLAQLNGSANALFISYYTAQGEPFPNNVEKLCAPNMTDFPSF
HGNGTEKTKLVELYRMVAYLSASLTNITRDQKVLNPTAVSLQVKLNATIDVMRGLLSNVLCR
LCNKYRVGHVDVPPVPDHSDKEAFQRKKLGCQLLGTYKQVISVVVQAF
>d1emra_ a.26.1.1 (A:) Leukemia inhibitory factor (LIF) {Human (Homo sapiens)}
LMNQIRSQLAQLNGSANALFILYYTAQGEPFPNNLEKLCGPNVTDFPPFHANGTEKAKLVEL
YRIVVYLGTSLGNITRDQKILNPSALSLHSKLNATADILRGLLSNVLCRLCSKYHVGHVDVT
YGPDTSGKDVFQKKKLGCQLLGKYKQVISVLAQAF
>d1a22a_ a.26.1.1 (A:) Growth hormone, somatotropin {Human (Homo sapiens)}
FPTIPLSRLFDNAMLRAHRLHQLAFDTYQEFEEAYIPKEQKYSFLQNPQTSLCFSESIPTPS
NREETQQKSNLELLRISLLLIQSWLEPVQFLRSVFANSLVYGASDSNVYDLLKDLEERIQTL
MGRLEGQIFKQTYSKFDTDALLKNYGLLYCFRKDMDKVETFLRIVQCRSVEGSCGF
>d1axia_ a.26.1.1 (A:) Growth hormone, somatotropin {Human (Homo sapiens)}

TIPLSRLFDNAMLRAHRLHQLAFDTYQEFEEAYIPKEQKYSFLQNPQTSLCFSESIPTPSNR
EETQQKSNLELLRISLLLIQSWLEPVQFLRSVFANSLVYGASDSNVYDLLKDLEERIQTLMG
RLTDGSPRTGQIFKQTYSKFDTNSHNDDALLKNYGLLYCFRRDMTYVATYLRIVQCRSVEGS
CGF
>d1hgu__ a.26.1.1 (-) Growth hormone, somatotropin {Human (Homo sapiens)}
PTIPLSRLFQNAMLRAHRLHQLAFDTYEEFEEAYIPKEQKYSFLQAPQASLCFSESIPTPSN
REQAQQKSNLQLLRISLLLIQSWLEPVGFLRSVFANSLVYGASDSDVYDLLKDLEEGIQTLM
GRLEDGSPRTGQAFKQTYAKFDANSHNDDALLKNYGLLYCFRKDMDKVETFLRIVQCRSVEG
SCG
>d1huw__ a.26.1.1 (-) Growth hormone, somatotropin {Human (Homo sapiens)}
FPTIPLSRLADNAWLRADRLNQLAFDTYQEFEEAYIPKEQIHSFWWNPQTSLCPSESIPTPS
NKEETQQKSNLELLRISLLLIQSWLEPVQFLRSVFANSLVYGASDSNVYDLLKDLEEGIQTL
MGRLEALLKNYGLLYCFNKDMSKVSTYLRTVQCRSVEGSCGF
>d1f6fa_ a.26.1.1 (A:) Placental lactogen {Sheep (Ovis aries)}
AQHPPYCRNQPGKCQIPLQSLFDRATTVANYNSKLAGEMVNRFDEQYGQGINSESKVINCHT
SSITTPNSKAEAINTEDKILFKLVISLLHSWDEPLHHAVTELANSKGTSPALLTKAQEIKEK
AKVLVDGVEVIQKRIHPGEKNEPYPVWSEQSSLTSQDENVRRVAFYRLFHCLHRDSSKIYTY
LRILKCRLTSC
>d1cnt1_ a.26.1.1 (1:) Ciliary neurotrophic factor (CNTF) {Human (Homo sapiens)}
PHRRDLCSRSIWLARKIRSDLTALTESYVKHQGLNKNINLDSADGMPVASTDQWSELTEAER
LQENLQAYRTFHVLLARLLEDQQVHFTPTEGDFHQAIHTLLLQVAAFAYQIEELMILLEYKI
PRNEADGMPINVGDGGLFEKKLWGLKVLQELSQWTVRSIHDLRFISSHQTGIP
>d1ax8__ a.26.1.1 (-) Leptin (obesity protein) {Human (Homo sapiens)}
IQKVQDDTKTLIKTIVTRINDISHTQSVSSKQKVTGLDFIPGLHPILTLSKMDQTLAVYQQI
LTSMPSRNVIQISNDLENLRDLLHVLAFSKSCHLPEASGLETLDSLGGVLEASGYSTEVVAL
SRLQGSLQDMLWQLDLSPGC
>d1evsa_ a.26.1.1 (A:) Oncostatin M {Human (Homo sapiens)}
GSCSKEYRVLLGQLQKQTDLMQDTSRLLDPYIRIQGLDVPKLREHCRERPGAFPSEETLRGL
GRRGFLQTLNATLGCVLHRLADLEQRLPKAQDLERSGLNIEDLEKLQMARPNILGLRNNIYC
MAQLLDNSDTAEPTKAGRGASQPPTPTPASDAFQRKLEGCRFLHGYHRFMHSVGRVFSKW
>d1f45b_ a.26.1.1 (B:) Heterodimeric interleukin-12 alpha chain {Human (Homo sapiens)}
QNLLRAVSNMLQKARQTLEFYPCTSEEIDHEDITKDKTSTVEACLPLELTKNESCLNSRETS
FITNGSCLASRKTSFMMALCLSSIYEDLKMYQVEFKTMNAKLLMDPKRQIFLDQNMLAVIDE
LMQALNFNSETVPQKSSLEEPDFYKTKIKLCILLHAFRIRAVTIDRVMSYLNAS
>d1eera_ a.26.1.2 (A:) Erythropoietin {Human (Homo sapiens)}
APPRLICDSRVLERYLLEAKEAEKITTGCAEHCSLNEKITVPDTKVNFYAWKRMEVGQQAVE
VWQGLALLSEAVLRGQALLVKSSQPWEPLQLHVDKAVSGLRSLTTLLRALGAQKEAISNSDA
ASAAPLRTITADTFRKLFRVYSNFLRGKLKLYTGEACRTGDR
>d2gmfa_ a.26.1.2 (A:) Granulocyte-macrophage colony-stimulating

factor (GM-CSF) {Human (Homo sapiens)}
RSPSPSTQPWEHVNAIQEARRLLNLSRDTAAEMNETVEVISEMFDLQEPTCLQTRLELYKQG
LRGSLTKLKGPLTMMASHYKQHCPPTPETSCATQIITFESFKENLKDFLLVIPFDCWEP
>d1hzia_ a.26.1.2 (A:) Interleukin-4 (IL-4) {Human (Homo sapiens)}
HKCDITLQAIIKTLNSLTEQKTLCTELTVTDIFAASKNTTEKETFCRAATVLRQFYSHHEKD
TRCLGATAQQFHRHKQLIRFLKRLDRNLWGLAGLNSCPVKEANQSTLENFLERLKTIMREKY
SKCSS
>d1hula_ a.26.1.2 (A:) Interleukin-5 {Human (Homo sapiens)}
IPTSALVKETLALLSTHRTLLIANETLRIPVPVHKNHQLCTEEIFQGIGTLESQTVQGGTVE
RLFKNLSLIKKYIDGQKKKCGEERRRVNQFLDYLQEFLGVMNTEWI
>d1hmca_ a.26.1.2 (A:) Macrophage colony-stimulating factor (M-CSF)
{Human (Homo sapiens)}
SEYCSHMIGSGHLQSLQRLIDSQMETSCQITFEFVDQEQLKDPVCYLKKAFLLVQDIMEDTM
RFRDNTPNAIAIVQLQELSLRLKSCFTKDYEEHDKACVRTFYETPLQLLEKVKNVFNETKNL
LDKDWNIFSKNCNNSFAECSSQGH
>d1etea_ a.26.1.2 (A:) Flt3 ligand {Human (Homo sapiens)}
TQDCSFQHSPISSDFAVKIRELSDYLLQDYPVTVASNLQDDELCGGLWRLVLAQRWMERLKT
VAGSKMQGLLERVNTEIHFVTKCAFQPPPSCLRFVQTNISRLLQETSEQLVALKPWITRQNF
SRCLELQCQP
>d1scfa_ a.26.1.2 (A:) Stem cell factor, SCF {Human (Homo sapiens)}
NVKDVTKLVANLPKDYMITLKYVPGMDVLPSHCWISEMVVQLSDSLTDLLDKFSNISEGLSN
YSIIDKLVNIVDDLVECVKENSSKDLKKSFKSPEPRLFTPEEFFRIFNRSIDAFKDFVVASE
TSDCVVS
>d1scfc_ a.26.1.2 (C:) Stem cell factor, SCF {Human (Homo sapiens)}
NVKDVTKLVANLPKDYMITLKYVPGMDVLPSHCWISEMVVQLSDSLTDLLDKFSNISEGLSN
YSIIDKLVNIVDDLVECVKENSSKDLKKSFKSPEPRLFTPEEFFRIFNRSIDAF
>d3inkc_ a.26.1.2 (C:) Interleukin-2 (IL-2) {Human (Homo sapiens)}
STKKTQLQLEHLLLDLQMILNGINNYKNPKLTRMLTFKFYMPKKATELKHLQCLEEELKPLE
EVLNLAQSKNFHLRPRDLISNINVIVLELKGSETTFMCEYADETATIVEFLNRWITFAQSII
STLT
>d1jli__ a.26.1.2 (-) Interleukin-3 (IL-3) {Human (Homo sapiens)}
ANCSIMIDEIIHHLKRPPNPLLDPNNLNSEDMDILMERNLRTPNLLAFVRAVKHLENASAIE
SILKNLLPCLPLATAAPTRHPIHIKDGDWNEFRRKLTFYLKTLENAQAQQ
>d1ga3a_ a.26.1.2 (A:) Interleukin-13 (IL-13) {Human (Homo
sapiens)}
GGPVPPSTALRELIEELVNITQNQKAPLCNGSMVWSINLTAGMYCAALESLINVSGCSAIEK
TQRMLSGFCPHKVSAGQFSSLHVRDTKIEVAQFVKDLLLHLKKLFREGRFN
>d2ilk__ a.26.1.3 (-) Interleukin-10 (cytokine synthesis
inhibitory factor, CSIF) {Human (Homo sapiens)}
TQSENSCTHFPGNLPNMLRDLRDAFSRVKTFFQMKDQLDNLLLKESLLEDFKGYLGCQALSE
MIQFYLEEVMPQAENQDPDIKAHVNSLGENLKTLRLRLRRCHRFLPCENKSKAVEQVKNAFN
KLQEKGIYKAMSEFDIFINYIEAYMTMKIRN
>d1vlk__ a.26.1.3 (-) Interleukin-10 (cytokine synthesis
inhibitory factor, CSIF) {Epstein-Barr virus}

CDNFPQMLRDLRDAFSRVKTFFQTKDEVDNLLLKESLLEDFKGYLGCQALSEMIQFYLEEVM
PQAENQDPEAKDHVNSLGENLKTLRLRLRRCHRFLPCENKSKAVEQIKNAFNKLQEKGIYKA
MSEFDIFINYIEAYMTIK
>d1au1a_ a.26.1.3 (A:) Interferon-beta {Human (Homo sapiens)}
MSYNLLGFLQRSSNFQCQKLLWQLNGRLEYCLKDRMNFDIPEEIKQLQQFQKEDAALTIYEM
LQNIFAIFRQDSSSTGWNETIVENLLANVYHQINHLKTVLEEKLEKEDFTRGKLMSSLHLKR
YYGRILHYLKAKEYSHCAWTIVRVEILRNFYFINRLTGYLRN
>d1rmi__ a.26.1.3 (-) Interferon-beta {Mouse (Mus musculus)}
INYKQLQLQERTNIRKCQELLEQLNGKINLTYRADFKIPMEMTEKMQKSYTAFAIQEMLQNV
FLVFRNNFSSTGWNETIVVRLLDELHQQTVFLKTVLEEKQEERLTWEMSSTALHLKSYYWRV
QRYLKLMKYNSYAWMVVRAEIFRNFLIIRRLTRNFQ
>d1rh2a_ a.26.1.3 (A:) Interferon-alpha 2b {Human (Homo sapiens)}
THSLGSRRTLMLLAQMRRISLFSCLKDRHDFGFPQEEFGNQFQKAETIPVLHEMIQQIFNLF
STKDSSAAWDETLLDKFYTELYQQLNDLEACVIQGVGVTETPLMNEDSILAVRKYFQRITLY
LKEKKYSPCAWEVVRAEIMRSFSLSTNLQE
>d1b5l__ a.26.1.3 (-) Interferon-tau {Sheep (Ovis aries)}
CYLSRKLMLDARENLKLLDRMNRLSPHSCLQDRKDFGLPQEMVEGDQLQKDQAFPVLYEMLQ
QSFNLFYTEHSSAAWDTTLLEQLCTGLQQQLDHLDTCRGQVMGEEDSELGNMDPIVTVKKYF
QGIYDYLQEKGYSDCAWEIVRVEMMRALTVSTTLQKRLTK
>d1d9ca_ a.26.1.3 (A:) Interferon-gamma {Cow (Bos taurus)}
QGQFFREIENLKEYFNASSPDVAKGGPLFSEILKNWKDESDKKIIQSQIVSFYFKLFENLKD
NQVIQRSMDIIKQDMFQKFLNGSSEKLEDFKKLIQIPVDDLQIQRKAINELIKVMNDLS
>d1fyha1 a.26.1.3 (A:0-124) Interferon-gamma {Human (Homo
sapiens)}
MQDPYVKEAENLKKYFNAGHSDVADNGTLFLGILKNWKEESDRKIMQSQIVSFYFKLFKNFK
DDQSIQKSVETIKEDMNVKFFNSNKKKRDDFEKLTNYSVTDLNVQRKAIDELIQVMAELGAN
V
>d1fyha2 a.26.1.3 (A:201-324) Interferon-gamma {Human (Homo
sapiens)}
SGEFVKEAENLKKYFNAGHSDVADNGTLFLGILKNWKEESDRKIMQSQIVSFYFKLFKNFKD
DQSIQKSVETIKEDMNVKFFNSNKKKRDDFEKLTNYSVTDLNVQRKAIHELIQVMAELSPAA
>d2rig__ a.26.1.3 (-) Interferon-gamma {Rabbit (Oryctolagus
cuniculus)}
QDTLTRETEHLKAYLKANTSDVANGGPLFLNILRNWKEESDNKIIQSQIVSFYFKLFDNLKD
HEVIKKSMESIKEDIFVKFFNSNLTKMDDFQNLTRISVDDRLVQRKAVSELSNVLNF
>d1a8h_1 a.27.1.1 (349-500) Methionyl-tRNA synthetase (MetRS)
{Thermus thermophilus}
LADDLGNLVQRTRAMLFRFAEGRIPEPVAGEELAEGTGLAGRLRPLVRELKFHVALEEAMAY
VKALNRYINEKKPWELFKKEPEEARAVLYRVVEGLRIASILLTPAMPDKMAELRRALGLKEE
VRLEEAERWGLAEPRPIPEEAPVLFPKK
>d1f4la1 a.27.1.1 (A:389-548) Methionyl-tRNA synthetase (MetRS)
{Escherichia coli}
VVNLASRNAGFINKRFDGVLASELADPQLYKTFTDAAEVIGEAWESREFGKAVREIMALADL
ANRYVDEQAPWVVAKQEGRDADLQAICSMGINLFRVLMTYLKPVLPKLTERAEAFLNTELTW

DGIQQPLLGHKVNPFKALYNRIDMRQVEALVEASKE
>d1ile_1 a.27.1.1 (642-821) Isoleucyl-tRNA synthetase (IleRS) {Thermus thermophilus}
YFLTLWNVYSFFVTYANLDRPDLKNPPPPEKRPEMDRWLLARMQDLIQRVTEALEAYDPTTS
ARALRDFVVEDLSQWYVRRNRRRFWKNEDALDREAAYATLYEALVLVATLAAPFTPFLAEVL
WQNLVRSVRLEAKESVHLADWPEADPALADEALVAQMRAVLKVVDLARAARAKSGV
>d1ffya1 a.27.1.1 (A:645-917) Isoleucyl-tRNA synthetase (IleRS) {Staphylococcus aureus}
YRKIRNTLRFMLGNINDFNPDTDSIPESELLEVDRYLLNRLREFTASTINNYENFDYLNIYQ
EVQNFINVELSNFYLDYGKDILYIEQRDSHIRRSMQTVLYQILVDMTKLLAPILVHTAEEVW
SHTPHVKEESVHLADMPKVVEVDQALLDKWRTFMNLRDDVNRALETARNEKVIGKSLEAKVT
IASNDKFNASEFLTSFDALHQLFIVSQVKVVDKLDDQATAYEHGDIVIEHADGEKCERCWNY
SEDLGAVDELTHLCPRCQQVVKSLV
>d1qu3a1 a.27.1.1 (A:645-881) Isoleucyl-tRNA synthetase (IleRS) {Staphylococcus aureus}
YRKIRNTLRFMLGNINDFNPDTDSIPESELLEVDRYLLNRLREFTASTINNYENFDYLNIYQ
EVQNFINVELSNFYLDYGKDILYIEQRDSHIRRSMQTVLYQILVDMTKLLAPILVHTAEEVW
SHTPHVKEESVHLADMPKVVEVDQALLDKWRTFMNLRDDVNRALETARNEKVIGKSLEAKVT
IASNDKFNASEFLTSFDALHQLFIVSQVKVVDKLDDQATAYEHGDIVIEHA
>d1gaxa1 a.27.1.1 (A:579-862) Valyl-tRNA synthetase (ValRS) {Thermus thermophilus}
ANKLYNAARFVLLSREGFQAKEDTPTLADRFMRSRLSRGVEEITALYEALDLAQAAREVYEL
VWSEFCDWYLEAAKPALKAGNAHTLRTLEEVLAVLLKLLHPMMPFLTSELYQALTGKEELAL
EAWPEPGGRDEEAERAFEALKQAVTAVRALKAEAGLPPAQEVRVYLEGETAPVEENLEVFRF
LSRADLLPERPAKALVKAMPRVTARMPLEGLLDVEEWRRRQEKRLKELLALAERSQRKLASP
GFREKAPKEVVEAEEARLKENLEQAERIREALSQIG
>d1f7ua1 a.27.1.1 (A:484-607) Arginyl-tRNA synthetase (ArgRS) {Baker's yeast (Saccharomyces cerevisiae)}
DTGPYLQYAHSRLRSVERNASGITQEKWINADFSLLKEPAAKLLIRLLGQYPDVLRNAIKTH
EPTTVVTYLFKLTHQVSSCYDVLWVAGQTEELATARLALYGAARQVLYNGMRLLGLTPVERM
>d1iq0a1 a.27.1.1 (A:467-592) Arginyl-tRNA synthetase (ArgRS) {Thermus thermophilus}
GDTGPYVQYAHARAHSILRKAGEWGAPDLSQATPYERALALDLLDFEEAVLEAAEERTPHVL
AQYLLDLAASWNAYYNARENGQPATPVLTAPEGLRELRLSLVQSLQRTLATGLDLLGIPAPE
VM
>d1acp__ a.28.1.1 (-) Acyl carrier protein {Escherichia coli}
STIEERVKKIIGEQLGVKQEEVTNNASFVEDLGADSLDTVELVMALEEEFDTEIPDEEAEKI
TTVQAAIDYINGHQA
>d1f80d_ a.28.1.1 (D:) Acyl carrier protein {Bacillis subtilis}
SADTLERVTKIIVDRLGVDEADVKLEASFKEDLGADXLDVVELVMELEDEFDMEISDEDAEK
IATVGDAVNYIQ
>d1af8__ a.28.1.1 (-) Actinorhodin polyketide synthase acyl carrier protein, ACT ACP {Streptomyces coelicolor, A3(2)}
MATLLTTDDLRRALVECAGETDGTDLSGDFLDLRFEDIGYDSLALMETAARLESRYGVSIPD

DVAGRVDTPRELLDLINGALAEAA
>d1dnya_ a.28.1.2 (A:) Peptidyl carrier protein (PCP), thioester domain {Bacillus brevis}
YVAPTNAVESKLAEIWERVLGVSGIGILDNFFQIGGHSLKAMAVAAQVHREYQVELPLKVLF
AQPTIKALAQYVAT
>d1dv5a_ a.28.1.3 (A:) apo-D-alanyl carrier protein {Lactobacillus casei}
ADEAIKNGVLDILADLTGSDDVKKNLDLNLFETGLLDSMGTVQLLLELQSQFGVDAPVSEFD
RKEWDTPNKIIAKVEQAQ
>d1unka_ a.28.2.1 (A:) ImmE7 protein (Im7) {Escherichia coli}
MELKNSISDYTEAEFVQLLKEIEKENVAATDDVLDVLLEHFVKITEHPDGTDLIYYPSDNRD
DSPEGIVKEIKEWRAANGKPGFKQG
>d1imy__ a.28.2.1 (-) ImmE8 (Im8) {Escherichia coli}
MELKNSISDYTETEFKKIIEDIINCEGDEKKQDDNLEHFISVTEHPSGSDLIYYPEGNNDGS
PEAVIKEIKEWRAANGKSGFKQG
>d1emva_ a.28.2.1 (A:) ImmE9 protein (Im9) {Escherichia coli}
LKHSISDYTEAEFLQLVTTICNADTSSEEELVKLVTHFEEMTEHPSGSDLIYYPKEGDDDSP
SGIVNTVKQWRAANGKSGFKQ
>d2eiaa1 a.28.3.1 (A:148-222) EIAV capsid protein p26 {Equine infectious anemia virus}
PKAQNIRQGAKEPYPEFVDRLLSQIKSEGHPQEISKFLTDTLTIQNANEECRNAMRHLRPED
TLEEKMYACRDIG
>d1qrjb1 a.28.3.1 (B:131-214) HTLV-I capsid protein {Human T-cell leukemia virus type 1}
PSWASILQGLEEPYHAFVERLNIALDNGLPEGTPKDPILRSLAYSNANKECQKLLQARGHTN
SPLGDMLRACQTWTPKDKTKVL
>d1a8o__ a.28.3.1 (-) HIV capsid protein, dimerisation domain {Human immunodeficiency virus type 1}
MDIRQGPKEPFRDYVDRFYKTLRAEQASQEVKNWMTETLLVQNANPDCKTILKALGPGATLE
EMMTACQG
>d1d1da1 a.28.3.1 (A:151-230) RSV capsid protein {Rous sarcoma virus}
GPWADITQGPSESFVDFANRLIKAVEGSDLPPSARAPVIIDCFRQKSQPDIQQLIRAAPSTL
TTPGEIIKYVLDRQKIAP
>d1eoqa_ a.28.3.1 (A:) RSV capsid protein {Rous sarcoma virus}
MDIMQGPSESFVDFANRLIKAVEGSDLPPSARAPVIIDCFRQKSQPDIQQLIRTAPSTLTTP
GEIIKYVLDRQKTAP
>d1e6ia_ a.29.2.1 (A:) GCN5 {Baker's yeast (Saccharomyces cerevisiae)}
RGPHDAAIQNILTELQNHAAAWPFLQPVNKEEVPDYYDFIKEPMDLSTMEIKLESNKYQKME
DFIYDARLVFNNCRMYNGENTSYYKYANRLEKFFNNKVKEIPEYSHLID
>d1f68a_ a.29.2.1 (A:) GCN5 {Human (Homo sapiens)}
GDQLYTTLKNLLAQIKSHPSAWPFMEPVKKSEAPDYYEVIRFPIDLKTMTERLRSRYYVTRK
LFVADLQRVIANCREYNPPDSEYCRCASALEKFFYFKLKEG

>d1b91a_ a.29.2.1 (A:) P300/CAF histone acetyltransferase bromodomain {Human (Homo sapiens)}
GSHMSKEPRDPDQLYSTLKSILQQVKSHQSAWPFMEPVKRTEAPGYYEVIRSPMDLKTMSER
LKNRYYVSKKLFMADLQRVFTNCKEYNAPESEYYKCANILEKFFFSKIKEAGLIDK
>d1eqfa1 a.29.2.1 (A:1359-1497) TAFII250 double bromodomain module {Human (Homo sapiens)}
GTTVHCDYLNRPHKSIHRRRTDPMVTLSSILESIINDMRDLPNTYPFHTPVNAKVVKDYYKI
ITRPMDLQTLRENVRKRLYPSREEFREHLELIVKNSATYNGPKHSLTQISQSMLDLCDEKLK
EKEDKLARLEKAINP
>d1eqfa2 a.29.2.1 (A:1498-1625) TAFII250 double bromodomain module {Human (Homo sapiens)}
LLDDDDQVAFSFILDNIVTQKMMAVPDSWPFHHPVNKKFVPDYYKVIVNPMDLETIRKNISK
HKYQSRESFLDDVNLILANSVKYNGPESQYTKTAQEIVNVCYQTLTEYDEHLTQLEKDICTA
KEAA
>d1gm5a1 a.29.4.1 (A:7-105) RecG, N-terminal domain {Thermotoga maritima}
FTSSLFLWGEALPTLLEEFLNEVEKMLKNQVNTRRIHQLLKELDDPLLENKDLEEKLQAFLD
YVKEIPNLPEARKRYRIQKSLEMIEKLRSWFLIDYLE
>d1gkza1 a.29.5.1 (A:38-185) Branched-chain alpha-ketoacid dehydrogenase kinase (BCK) {Rat (Rattus norvegicus)}
VRLTPTMMLYSGRSQDGSHLLKSGRYLQQELPVRIAHRIKGFRSLPFIIGCNPTILHVHELY
IRAFQKLTDFPPIKDQADEAQYCQLVRQLLDDHKDVVTLLAEGLRESRKHIEDEKLVRYFLD
KTLTSRLGIRMLATHHLALHEDKP
>d1jm6a1 a.29.5.1 (A:1003-1169) Pyruvate dehydrogenase kinase {Rat (Rattus norvegicus), isozyme 2}
ASLAGAPKYIEHFSKFSPSPLSMKQFLDFGSSNACEKTSFTFLRQELPVRLANIMKEINLLP
DRVLSTPSVQLVQSWYVQSLLDIMEFLDKDPEDHRTLSQFTDALVTIRNRHNDVVPTMAQGV
LEYKDTYGDDPVSNQNIQYFLDRFYLSRISIRMLINQHTLIFD
>d1buca1 a.29.3.1 (A:233-383) Butyryl-CoA dehydrogenase {Megasphaera elsdenii}
GKGFKIAMMTLDGGRIGVAAQALGIAEAALADAVEYSKQRVQFGKPLCKFQSISFKLADMKM
QIEAARNLVYKAACKKQEGKPFTVDAAIAKRVASDVAMRVTTEAVQIFGGYGYSEEYPVARH
MRDAKITQIYEGTNEVQLMVTGGALLR
>d1jqia1 a.29.3.1 (A:235-387) Butyryl-CoA dehydrogenase {Rat (Rattus norvegicus)}
MGFKIAMQTLDMGRIGIASQALGIAQASLDCAVKYAENRHAFGAPLTKLQNIQFKLADMALA
LESARLLTWRAAMLKDNKKPFTKESAMAKLAASEAATAISHQAIQILGGMGYVTEMPAERYY
RDARITEIYEGTSEIQRLVIAGHLLRSYR
>d3mdda1 a.29.3.1 (A:242-395) Medium chain acyl-CoA dehydrogenase {Pig (Sus scrofa)}
GAGFKIAMGTFDKTRPPVAAGAVGLAQRALDEATKYALERKTFGKLLAEHQGISFLLADMAM
KVELARLSYQRAAWEIDSGRRNTYYASIAKAYAADIANQLATDAVQVFGGNGFNTEYPVEKL
MRDAKIYQIYEGTAQIQRIIIAREHIGRYK
>d1egda1 a.29.3.1 (A:242-396) Medium chain acyl-CoA dehydrogenase

{Human (Homo sapiens)}
GAGFKVAMGAFDKERPVVAAGAVGLAQRALDEATKYALERKTFGKLLVEHQAISFMLAEMAM
KVELARMSYQRAAWEVDSGRRNTYYASIAKAFAGDIANQLATDAVQILGGNGFNTEYPVEKL
MRDAKIYQIYGGTSQIQRLIVAREHIDKYKN
>d1ivha1 a.29.3.1 (A:242-392) Isovaleryl-CoA dehydrogenase {Human (Homo sapiens)}
KGVYVLMSGLDLERLVLAGGPLGLMQAVLDHTIPYLHVREAFGQKIGHFQLMQGKMADMYTR
LMACRQYVYNVAKACDEGHCTAKDCAGVILYSAECATQVALDGIQCFGGNGYINDFPMGRFL
RDAKLYEIGAGTSEVRRLVIGRAFNAD
>d1b6q__ a.30.1.1 (-) ROP protein {Escherichia coli}
MTKQEKTALNMARFIRSQTLTLLEKLNELDPDEQADICESLHDHADELYRSCLARF
>d1f4na_ a.30.1.1 (A:) ROP protein {Escherichia coli}
GTKQEKTILNMARFIRSQALTILEKANELDADEIADIAESIHDHADEIYRSALARFGDDG
>d1f4nb_ a.30.1.1 (B:) ROP protein {Escherichia coli}
EKTILNMARFIRSQALTILEKANELDADEIADIAESIHDHADEIYRSALAR
>d1gtoa_ a.30.1.1 (A:) ROP protein {Escherichia coli}
GTKQEKTALNMARFIRSQTLTLLEKLNELGADEQADICESLHDHADELYRSCLARFGDDGEN
>d1nkd__ a.30.1.1 (-) ROP protein {Escherichia coli}
MTKQEKTALNMARFIRSQTLTLLEKLNELADAADEQADICESLHDHADELYRSCLARFG
>d1joya_ a.30.2.1 (A:) EnvZ histidine kinase {Escherichia coli}
MAAGVKQLADDRTLLMAGVSHDLRTPLTRIRLATEMMSEQDGYLAESINKDIEECNAIIEQF
IDYLR
>d1b3qa1 a.30.2.1 (A:293-354) Histidine kinase CheA {Thermotoga maritima}
SQTVRVDIEKLDNLMDLMGELVIARSRILETLKKYNIKELDESLSHLSRITLDLQNVVMKIR
>d1r2aa_ a.31.1.1 (A:) Dimerization-anchoring domain of cAMP-dependent type II PK regulatory subunit {Mouse (Mus musculus)}
HMGHIQIPPGLTELLQGYTVEVLRQQPPDLVDFAVEYFTRLREARR
>d1ytfb1 a.32.1.1 (B:) Transcription factor IIA (TFIIA), N-terminal domain {Baker's yeast (Saccharomyces cerevisiae)}
SNAEASRVYEIIVESVVNEVREDFENAGIDEQTLQDLKNIWQKKLT
>d1ytfd1 a.32.1.1 (D:5-54) Transcription factor IIA (TFIIA), N-terminal domain {Baker's yeast (Saccharomyces cerevisiae)}
GYYELYRRSTIGNSLVDALDTLISDGRIEASLAMRVLETFDKVVAETLKD
>d1ecia_ a.33.1.1 (A:) Ectatomin, A & B chains {Ant (Ectatomma tuberculatum), venom}
GVIPKKIWETVCPTVEPWAKKCSGDIATYIKRECGKL
>d1ecib_ a.33.1.1 (B:) Ectatomin, A & B chains {Ant (Ectatomma tuberculatum), venom}
WSTIVKLTICPTLKSMAKKCEGSIATMIKKKCDK
>d1b0na1 a.34.1.1 (A:74-108) SinR repressor (dimerisation domain)-SinI anti-repressor complex {Bacillus subtilis}
LDSEWEKLVRDAMTSGVSKKQFREFLDYQKWRKSQ
>d1b0nb1 a.34.1.1 (B:) SinR repressor (dimerisation domain)-SinI

anti-repressor complex {Bacillus subtilis}
FELDQEWVELMVEAKEANISPEEIRKYLLLN
>d1f93f_ a.34.1.1 (F:) Dimerization cofactor of HNF-1 alpha {Mouse (Mus musculus)}
LSQLQTELLAALLESGLSKEALIQALG
>d1g2ya_ a.34.1.1 (A:) Dimerization cofactor of HNF-1 alpha {Mouse (Mus musculus)}
MVSKLSQLQTEMLAALLESGLSKEALIQALG
>d1g2za_ a.34.1.1 (A:) Dimerization cofactor of HNF-1 alpha {Mouse (Mus musculus)}
MVSKLSQLQTELMAALLESGLSKEALIQALGE
>d1g39a_ a.34.1.1 (A:) Dimerization cofactor of HNF-1 alpha {Mouse (Mus musculus)}
MVSKLSQLQTELLAALLESGLSKEALIQ
>d1jb6b_ a.34.1.1 (B:) Dimerization cofactor of HNF-1 alpha {Mouse (Mus musculus)}
SKLSQLQTELMAALLESGLSKEALIQAL
>d1e3oc2 a.35.1.1 (C:1-75) Oct-1 {Human (Homo sapiens)}
EEPSDLEELEQFAKTFKQRRIKLGFTQGDVGLAMGKLYGNDFSQTTISRFEALNLSFKNMSK
LKPLLEKWLNDAE
>d1au7a2 a.35.1.1 (A:5-76) Pit-1 {Rat (Rattus norvegicus)}
GMRALEQFANEFKVRRIKLGYTQTNVGEALAAVHGSEFSQTTICRFENLQLSFKNACKLKAI
LSKWLEEAEQ
>d1llib_ a.35.1.2 (B:) lambda C1 repressor, DNA-binding domain {Bacteriophage lambda (Escherichia coli)}
STKKKPLTQEQLEDARRLKAIYEKKKNELGLSQESLADKLGMGQSGIGALFNGINALNAYNA
ALLAKILKVSVEEFSPSIAREIYEMYEAVS
>d1lmb3_ a.35.1.2 (3:) lambda C1 repressor, DNA-binding domain {Bacteriophage lambda (Escherichia coli)}
PLTQEQLEDARRLKAIYEKKKNELGLSQESVADKMGMGQSGVGALFNGINALNAYNAALLAK
ILKVSVEEFSPSIAREIYEMYEAVS
>d1r69__ a.35.1.2 (-) 434 C1 repressor, DNA-binding domain {Bacteriophage 434 (Escherichia coli)}
SISSRVKSKRIQLGLNQAELAQKVGTTQQSIEQLENGKTKRPRFLPELASALGVSVDWLLNG
T
>d2cro__ a.35.1.2 (-) cro 434 {Bacteriophage 434}
MQTLSERLKKRRIALKMTQTELATKAGVKQQSIQLIEAGVTKRPRFLFEIAMALNCDPVWLQ
YGT
>d1adr__ a.35.1.2 (-) P22 C2 repressor, DNA-binding domain {Salmonella bacteriophage P22}
MNTQLMGERIRARRKKLKIRQAALGKMVGVSNVAISQWERSETEPNGENLLALSKALQCSPD
YLLKGDLSQTNVAY
>d1copd_ a.35.1.2 (D:) cro lambda repressor {Bacteriophage lambda (Escherichia coli)}

MEQRITLKDYAMRFGQTKTAKDLGVYQSAINKAIHAGRKIFLTINADGSVYAEEVKPFPSNK
KTTA
>d1d1la_ a.35.1.2 (A:) cro lambda repressor {Bacteriophage lambda (Escherichia coli)}
MEQRITLKDYAMRFGQTKTAKDLGVYQSAINKAIHAGRKIFLTINADGSVYAEEVKPWPSN
>d3orca_ a.35.1.2 (A:) cro lambda repressor {Bacteriophage lambda (Escherichia coli)}
EQRITLKDYAMRFGQTKTAKDLGVYQSAINKAIHAGRKIFLTINADGSVYAEEVKDGEVKPF
PSN
>d1ner__ a.35.1.2 (-) Ner {Bacteriophage mu}
CSNEKARDWHRADVIAGLKKRKLSLSALSRQFGYAPTTLANALERHWPKGEQIIANALETKP
EVIWPSRYQAGE
>d1b0na2 a.35.1.3 (A:1-68) SinR repressor, DNA-binding domain {Bacillus subtilis}
MIGQRIKQYRKEKGYSLSELAEKAGVAKSYLSSIERNLQTNPSIQFLEKVSAVLDVSVHTLL
DEKHET
>d1dw9a1 a.35.1.4 (A:1-86) Cyanase N-terminal domain {Escherichia coli}
MIQSQINRNIRLDLADAILLSKAKKDLSFAEIADGTGLAEAFVTAALLGQQALPADAARLVG
AKLDLDEDSILLLQMIPLRGCIDD
>d1pru__ a.35.1.5 (-) Purine repressor (PurR), N-terminal domain {Escherichia coli}
MATIKDVAKRANVSTTTVSHVINKTRFVAEETRNAVWAAIKELHYSPSAVARSLKV
>d1vpwa1 a.35.1.5 (A:3-58) Purine repressor (PurR), N-terminal domain {Escherichia coli}
TIKDVAKRANVSTTTVSHVINKTRFVAEETRNAVWAAIKELHYSPSAVARSMKVNH
>d1efaa1 a.35.1.5 (A:2-60) Lac repressor (LacR), N-terminal domain {Escherichia coli}
KPVTLYDVAEYAGVSYQTVSRVVNQASHVSAKTREKVEAAMAELNYIPNRVAQQLAGKQ
>d1lcca_ a.35.1.5 (A:) Lac repressor (LacR), N-terminal domain {Escherichia coli}
MKPVTLYDVAEYAGVSYQTVSRVVNQASHVSAKTREKVEAAMAELNYIPNR
>d1uxc__ a.35.1.5 (-) Fructose repressor (FruR), N-terminal domain {Escherichia coli}
MKLDEIARLAGVSRTTASYVINGKAKQYRVSDKTVEKVMAVVREHNYHPN
>d1uxd__ a.35.1.5 (-) Fructose repressor (FruR), N-terminal domain {Escherichia coli}
MKLDEIARLAGVSRTTASYVINGKAKQYRVSDKTVEKVMAVVREHNYHPNAVAAGLRLQ
>d1dula_ a.36.1.1 (A:) Signal sequence binding protein Ffh {Escherichia coli}
FDLNDFLEQKVLVRMEAIINSMTMKERAKPEIIKGSRKRRIAAGSGMQVQDVNRLLKQFDDM
QRMMKKM
>d1hq1a_ a.36.1.1 (A:) Signal sequence binding protein Ffh {Escherichia coli}

GFDLNDFLEQLRQMKNMGGMASLMGKLPGMGQIPDNVKSQMDDKVLVRMEAIINSMTMKERA
KPEIIKGSRKRRIAAGSGMQVQDVNRLLKQFDDMQRMMKKMK
>d2ffha2 a.36.1.1 (A:319-418) Signal sequence binding protein Ffh {Thermus aquaticus}
ELSLEDFLKQMQNLKRLGPFSEILGLLPGVPQGLKVDEKAIKRLEAIVLSMTPEERKDPRIL
NGSRRKRIAKGSGTSVQEVNRFIKAFEEMKALMKSLEK
>d1qb2a_ a.36.1.1 (A:) SRP54M {Human (Homo sapiens)}
QFTLRDMYEQFQNIMKMGPFSQILGMIPGFGTDFMSKGNEQESMARLKKLMTIMDSMNDQEL
DSTDGAKVFSKQPGRIQRVARGSGVSTRDVQELLTQYTKFAQMVK
>d1sknp_ a.37.1.1 (P:) Binding domain of Skn-1 {Caenorhabditis elegans}
GRQSKDEQLASDNELPVSAFQISEMSLSELQQVLKNESLSEYQRQLIRKIRRRGKNKVAART
CRQRRTDRHDKM
>d1hloa_ a.38.1.1 (A:) Max protein {Human (Homo sapiens)}
NDDIEVESDADKRAHHNALERKRRDHIKDSFHSLRDSVPSLQGEKASRAQILDKATEYIQYM
RRKNHTHQQDIDDLKRQN
>d1an2a_ a.38.1.1 (A:) Max protein {Mouse (Mus musculus)}
ADKRAHHNALERKRRDHIKDSFHSLRDSVPSLQGEKASRAQILDKATEYIQYMRRKNHTHQQ
DIDDLKRQNALLEQQVRALEKARS
>d1mdya_ a.38.1.1 (A:) Myod B/HLH domain {Mouse (Mus musculus)}
MELKRKTTNADRRKAATMRERRRLSKVNEAFETLKRSTSSNPNQRLPKVEILRNAIRYIEGL
QALLRD
>d1an4a_ a.38.1.1 (A:) Usf B/HLH domain {Human (Homo sapiens)}
MDEKRRAQHNEVERRRRDKINNWIVQLSKIIPDSSMESTKSGQSKGGILSKASDYIQELRQS
NHR
>d1a0aa_ a.38.1.1 (A:) Pho4 B/HLH domain {Baker's yeast (Saccharomyces cerevisiae)}
MKRESHKHAEQARRNRLAVALHELASLIPAEWKQQNVSAAPSKATTVEAACRYIRHLQQNGS
T
>d1am9a_ a.38.1.1 (A:) SREBP-1a {Human (Homo sapiens)}
QSRGEKRTAHNAIEKRYRSSINDKIIELKDLVVGTEAKLNKSAVLRKAIDYIRFLQHSNQKL
KQENLSLRTAVHKSKSLK
>d1bod__ a.39.1.1 (-) Calbindin D9K {Cow (Bos taurus)}
MKSPEELKGIFEKYDKEGDGQLSKEELKLLLQTEFPSLLKGMSTLDELFEELDKNGDGEVSF
EEFQVLVKKISQ
>d1ig5a_ a.39.1.1 (A:) Calbindin D9K {Cow (Bos taurus)}
KSPEELKGIFEKYAAKEGDPNQLSKEELKLLLQTEFPSLLKGPSTLDELFEELDKNGDGEVS
FEEFQVLVKKISQ
>d1cb1__ a.39.1.1 (-) Calbindin D9K {Pig (Sus scrofa)}
SAQKSPAELKSIFEKYAAKEGDPNQLSKEELKQLIQAEFPSLLKGPRTLDDLFQELDKNGDG
EVSFEEFQVLVKKISQ
>d1a03a_ a.39.1.2 (A:) Calcyclin (S100) {Rabbit (Oryctolagus cuniculus)}
MASPLDQAIGLLIGIFHKYSGKEGDKHTLSKKELKELIQKELTIGSKLQDAEIVKLMDDLDR

NKDQEVNFQEYITFLGALAMIYNEALKG
>d1k2ha_ a.39.1.2 (A:) Calcyclin (S100) {Rat (Rattus norvegicus), s100a1}
GSELETAMETLINVFHAHSGKEGDKYKLSKKELKDLLQTELSSFLDVQKDADAVDKIMKELD
ENGDGEVDFQEFVVLVAALTVACNNFFWENS
>d1qlka_ a.39.1.2 (A:) Calcyclin (S100) {Rat (Rattus norvegicus), s100b}
MSELEKAMVALIDVFHQYSGREGDKHKLKKSELKELINNELSHFLEEIKEQEVVDKVMETLD
EDGDGECDFQEFMAFVSMVTTACHEFFEHE
>d1mho__ a.39.1.2 (-) Calcyclin (S100) {Cow (Bos taurus), s100b}
SELEKAVVALIDVFHQYSGREGDKHKLKKSELKELINNELSHFLEEIKEQEVVDKVMETLDS
DGDGECDFQEFMAFVAMITTACHEFF
>d1a4pa_ a.39.1.2 (A:) Calcyclin (S100) {Human (Homo sapiens), P11 s100a10, calpactin}
PSQMEHAMETMMFTFHKFAGDKGYLTKEDLRVLMEKEFPGFLENQKDPLAVDKIMKDLDQCR
DGKVGFQSFFSLIAGLTIACNDYFVVHMKQ
>d1psra_ a.39.1.2 (A:) Calcyclin (S100) {Human (Homo sapiens), psoriasin s100a7}
SNTQAERSIIGMIDMFHKYTRRDDKIDKPSLLTMMKENFPNFLSACDKKGTNYLADVFEKKD
KNEDKKIDFSEFLSLLGDIATDYHKQSHGAAPCSGGSQ
>d1qlsa_ a.39.1.2 (A:) Calcyclin (S100) {Pig (Sus scrofa), calgizzarin s100c (s100a11)}
PTETERCIESLIAIFQKHAGRDGNNTKISKTEFLIFMNTELAAFTQNQKDPGVLDRMMKKLD
LDSDGQLDFQEFLNLIGGLAIACHDSFIKSTQK
>d1mr8a_ a.39.1.2 (A:) Calcyclin (S100) {Human (Homo sapiens), calgranulin s100a8, MRP8}
MLTELEKALNSIIDVYHKYSLIKGNFHAVYRDDLKKLLETECPQYIRKKGADVWFKELDINT
DGAVNFQEFLILVIKMGVAAHKKSHEES
>d1e8aa_ a.39.1.2 (A:) Calcyclin (S100) {Human (Homo sapiens), calgranulin C, s100a12}
TKLEEHLEGIVNIFHQYSVRKGHFDTLSKGELKQLLTKELANTIKNIKDKAVIDEIFQGLDA
NQDEQVDFQEFISLVAIALKAAHYH
>d1irja_ a.39.1.2 (A:) Calcyclin (S100) {Human (Homo sapiens), s100a9 (mrp14)}
TCKMSQLERNIETIINTFHQYSVKLGHPDTLNQGEFKELVRKDLQNFLKKENKNEKVIEHIM
EDLDTNADKQLSFEEFIMLMARL
>d1sra__ a.39.1.3 (-) C-terminal (EC) domain of BM-40/SPARC/osteonectin {Human (Homo sapiens)}
PPCLDSELTEFPLRMRDWLKNVLVTLYERDEDNNLLTEKQKLRVKKIHENEKRLEAGDHPVE
LLARDFEKNYNMYIFPVHWQFGQLDQHPIDGYLSHTELAPLRAPLIPMEHCTTRFFETCDLD
NDKYIALDEWAGCFGIKQKDIDKDLVI
>d1rro__ a.39.1.4 (-) Oncomodulin {Rat (Rattus norvegicus)}
SITDILSAEDIAAALQECQDPDTFEPQKFFQTSGLSKMSASQVKDIFRFIDNDQSGYLDGDE
LKYFLQKFQSDARELTESETKSLMDAADNDGDGKIGADEFQEMVHS

>d1cdp__ a.39.1.4 (-) Parvalbumin {Carp (Cyprinus carpio)}
AFAGVLNDADIAAALEACKAADSFNHKAFFAKVGLTSKSADDVKKAFAIIDQDKSGFIEEDE
LKLFLQNFKADARALTDGETKTFLKAGDSDGDGKIGVDEFTALVKA
>d1pvaa_ a.39.1.4 (A:) Parvalbumin {Pike (Esox lucius)}
AAKDLLKADDIKKALDAVKAEGSFNHKKFFALVGLKAMSANDVKKVFKAIDADASGFIEEEE
LKFVLKSFAADGRDLTDAETKAFLKAADKDGDGKIGIDEFETLVHEA
>d2pvba_ a.39.1.4 (A:) Parvalbumin {Pike (Esox lucius)}
SFAGLKDADVAAALAACSAADSFKHKEFFAKVGLASKSLDDVKKAFYVIDQDKSGFIEEDEL
KLFLQNFSPSARALTDAETKAFLADGDKDGDGMIGVDEFAAMIKA
>d5pal__ a.39.1.4 (-) Parvalbumin {Leopard shark (Triakis semifasciata)}
PMTKVLKADDINKAISAFKDPGTFDYKRFFHLVGLKGKTDAQVKEVFEILDKDQSGFIEEEE
LKGVLKGFSAHGRDLNDTETKALLAAGDSDHDGKIGADEFAKMVAQA
>d1a75a_ a.39.1.4 (A:) Parvalbumin {Whiting (Merlangius merlangus)}
AGILADADCAAAVKACEAADSFSYKAFFAKCGLSGKSADDIKKAFVFIDQDKSGFIEEDELK
LFLQVFKAGARALTDAETKAFLKAGDSDGDGAIGVEEWVALVKA
>d1bu3__ a.39.1.4 (-) Parvalbumin {Silver hake (Merluccius bilinearis)}
AFSGILADADVAAALKACEAADSFNYKAFFAKVGLTAKSADDIKKAFFVIDQDKSGFIEEDE
LKLFLQVFSAGARALTDAETKAFLKAGDSDGDGAIGVDEWAALVKA
>d1g33a_ a.39.1.4 (A:) Parvalbumin {Rat (Rattus rattus)}
MKSADDVKKVFHILDKDKSGFIEEDELGSILKGFSSDARDLSAKETKTLMAAGDKDGDGKIG
VEEFSTLVAES
>d1rtp1_ a.39.1.4 (1:) Parvalbumin {Rat (Rattus rattus)}
SMTDLLSAEDIKKAIGAFTAADSFDHKKFFQMVGLKKKSADDVKKVFHILDKDKSGFIEEDE
LGSILKGFSSDARDLSAKETKTLMAAGDKDGDGKIGVEEFSTLVAES
>d1avsa_ a.39.1.5 (A:) Troponin C {Chicken (Gallus gallus)}
QAEARAFLSEEMIAEFKAAFDMFDADGGGDISTKELGTVMRMLGQNPTKEELDAIIEEVDED
GSGTIDFEEFLVMMVRQMK
>d1ctda_ a.39.1.5 (A:) Troponin C {Chicken (Gallus gallus)}
KSEEELANAFRIFDKNADGYIDIEELGEILRATG
>d1dtla_ a.39.1.5 (A:) Troponin C {Chicken (Gallus gallus)}
YKAAVEQLTEEQKNEFKAAFDIFVLGAEDGSISTKELGKVMRMLGQNPTPEELQEMIDEVDE
DGSGTVDFDEFLVMMVRSMKDDSKGKSEEELSDLFRMFDKNADGYIDLEELKIMLQATGETI
TEDDIEELMKDGDKNNDGRIDYDEFLEFMKGV
>d1jc2a_ a.39.1.5 (A:) Troponin C {Chicken (Gallus gallus)}
EDAKGKSEEELANCFRIFDKNADGFIDIEELGEILRATGEHVIEEDIEDLMKDSDKNNDGRI
DFDEFLKMMEGVQ
>d1ncx__ a.39.1.5 (-) Troponin C {Chicken (Gallus gallus)}
ASMTDQQAEARAFLSEEMIAEFKAAFDMFDADGGGDISTKELGTVMRMLGQNPTKEELDAII
EEVDEDGSGTIDFEEFLVMMVRQMKEDAKGKSEEELANCFRIFDKNADGFIDIEELGEILRA
TGEHVTEEDIEDLMKDSDKNNDGRIDFDEFLKMMEGVQ
>d1smg__ a.39.1.5 (-) Troponin C {Chicken (Gallus gallus)}

ASMTDQQAEARAFLSEEMIAEFKAAFDMFDADGGGDISTKALGTVMRMLGQNPTKEELDAII
EEVDEDGSGTIDFEEFLVMMVRQMKEDA
>g1pon.1 a.39.1.5 (A:,B:) Troponin C {Chicken (Gallus gallus)}
KSEEELANAFRIFDKNADGYIDIEELGEILRATGXVTEEDIEDLMKDSDKNNDGRIDFDEFL
KMMEGVQ
>d1tn4__ a.39.1.5 (-) Troponin C {Rabbit (Oryctolagus cuniculus)}
TDQQAEARSYLSEEMIAEFKAAFDMFDADGGGDISVKELGTVMRMLGQTPTKEELDAIIEEV
DEDGSGTIDFEEFLVMMVRQMKEDAKGKSEEELAELFRIFDRNADGYIDAEELAEIFRASGE
HVTDEEIESLMKDGDKNNDGRIDFDEFLKMMEG
>d1fi5a_ a.39.1.5 (A:) Troponin C {Chicken (Gallus gallus), cardiac isoform}
MVRCMKDDSKGKTEEELSDLFRMFDKNADGYIDLEELKIMLQATGETITEDDIEELMKDGDK
NNDGRIDYDEFLEFMKGVE
>d1ap4__ a.39.1.5 (-) Troponin C {Human (Homo sapiens), cardiac isoform}
MDDIYKAAVEQLTEEQKNEFKAAFDIFVLGAEDGCISTKELGKVMRMLGQNPTPEELQEMID
EVDEDGSGTVDFDEFLVMMVRCMKDDS
>d1ih0a_ a.39.1.5 (A:) Troponin C {Human (Homo sapiens), cardiac isoform}
GKSEEELSDLFRMFDKNADGYIDLEELKIMLQATGETITEDDIEELMKDGDKNNDGRIDYDE
FLEFMKGVE
>d2scpa_ a.39.1.5 (A:) Sarcoplasmic calcium-binding protein {Sandworm (Nereis diversicolor)}
SDLWVQKMKTYFNRIDFDKDGAITRMDFESMAERFAKESEMKAEHAKVLMDSLTGVWDNFLT
AVAGGKGIDETTFINSMKEMVKNPEAKSVVEGPLPLFFRAVDTNEDNNISRDEYGIFFGMLG
LDKTMAPASFDAIDTNNDGLLSLEEFVIAGSDFFMNDGDSTNKVFWGPLV
>d2sas__ a.39.1.5 (-) Sarcoplasmic calcium-binding protein {Amphioxus (Branchiostoma lanceolatum)}
GLNDFQKQKIKFTFDFFLDMNHDGSIQDNDFEDMMTRYKEVNKGSLSDADYKSMQASLEDEW
RDLKGRADINKDDVVSWEEYLAMWEKTIATCKSVADLPAWCQNRIPFLFKGMDVSGDGIVDL
EEFQNYCKNFQLQCADVPAVYNVITDGGKVTFDLNRYKELYYRLLTSPAADAGNTLMGQKP
>d1c7va_ a.39.1.5 (A:) Calcium vector protein {Amphioxus (Branchiostoma lanceolatum)}
EEEILRAFKVFDANGDGVIDFDEFKFIMQKVGEEPLTDAEVEEAMKEADEDGNGVIDIPEFM
DLIKKS
>d1j7qa_ a.39.1.5 (A:) Calcium vector protein {Amphioxus (Branchiostoma lanceolatum)}
AAPKARALGPEEKDECMKIFDIFDRNAENIAPVSDTMDMLTKLGQTYTKRETEAIMKEARGP
KGDKKNIGPEEWLTLCSKWVRQDD
>d1j7ra_ a.39.1.5 (A:) Calcium vector protein {Amphioxus (Branchiostoma lanceolatum)}
LGPEEKDECMKIFDIFDRNAENIAPVSDTMDMLTKLGQTYTKRETEAIMKEARGPKGDKKNI
GPEEWLTLCSKWVRQ
>d1ej3a_ a.39.1.5 (A:) Calcium-regulated photoprotein {Jellyfish

(Aequorea aequorea), aequorin}
LTSDFDNPRWIGRHKHMFNFLDVNHNGKISLDEMVYKASDIVINNLGATPEQAKRHKDAVEA
FFGGAGMKYGVETDWPAYIEGWKKLATDELEKYAKNEPTLIRIWGDALFDIVDKDQNGAITL
DEWKAYTKAAGIIQSSEDCEETFRVCDIDESGQLDVDEMTRQHLGFWYTMDPACEKLYGGAV
P
>d1el4a_ a.39.1.5 (A:) Calcium-regulated photoprotein {Hydrozoa
(Obelia longissima), obelin}
SSKYAVKLKTDFDNPRWIKRHKHMFDFLDINGNGKITLDEIVSKASDDICAKLEATPEQTKR
HQVCVEAFFRGCGMEYGKEIAFPQFLDGWKQLATSELKKWARNEPTLIREWGDAVFDIFDKD
GSGTITLDEWKAYGKISGISPSQEDCEATFRHCDLDNSGDLDVDEMTRQHLGFWYTLDPEAD
GLYGNGVP
>d1jf0a_ a.39.1.5 (A:) Calcium-regulated photoprotein {Hydrozoa
(Obelia geniculata), obelin}
KYAVKLQTDFDNPKWIKRHKFMFDYLDINGNGQITLDEIVSKASDDICKNLGATPAQTQRHQ
DCVEAFFRGCGLEYGKETKFPEFLEGWKNLANADLAKWARNEPTLIREWGDAVFDIFDKDGS
GTITLDEWKAYGRISGISPSEEDCEKTFQHCDLDNSGELDVDEMTRQHLGFWYTLDPEADGL
YGNGVP
>d1jfja_ a.39.1.5 (A:) EHCABP {Entamoeba (Entamoeba histolytica)}
MAEALFKEIDVNGDGAVSYEEVKAFVSKKRAIKNEQLLQLIFKSIDADGNGEIDQNEFAKFY
GSIQGQDLSDDKIGLKVLYKLMDVDGDGKLTKEEVTSFFKKHGIEKVAEQVMKADANGDGYI
TLEEFLEFSL
>d1cmg__ a.39.1.5 (-) Calmodulin {Cow (Bos taurus)}
MKDTDSEEEIREAFRVFDKDGNGYISAAELRHVMTNLGEKLTDEEVDEMIREADIDGDGQVN
YEEFVQMMTAK
>d1fw4a_ a.39.1.5 (A:) Calmodulin {Cow (Bos taurus)}
SEEEIREAFRVFDKDGNGYISAAELRHVMTNLGEKLTDEEVDEMIREADIDGDGQVNYEEFV
QMM
>d1g4yr_ a.39.1.5 (R:) Calmodulin {Rat (Rattus rattus)}
ADQLTEEQIAEFKEAFSLFDKDGDGTITTKELGTVMRSLGQNPTEAELQDMINEVDADGNGT
IDFPEFLTMMARKMKDTDSEEEIREAFRVFDKDGNGYISAAELRHVMTNLGEKLTDEEVDEM
IREADIDGDGQVNYEEFVQMMTA
>d1f70a_ a.39.1.5 (A:) Calmodulin {African frog (Xenopus laevis)}
ADQLTEEQIAEFKEAFSLFDKDGDGTITTKELGTVMRSLGQNPTEAELQDMINEVDADGNGT
IDFPEFLTMMARKM
>d1exra_ a.39.1.5 (A:) Calmodulin {Ciliate (Paramecium
tetraurelia)}
EQLTEEQIAEFKEAFALFDKDGDGTITTKELGTVMRSLGQNPTEAELQDMINEVDADGNGTI
DFPEFLSLMARKMKEQDSEEELIEAFKVFDRDGNGLISAAELRHVMTNLGEKLTDDEVDEMI
READIDGDGHINYEEFVRMMVS
>d1ggwa_ a.39.1.5 (A:) Cdc4p {Fission yeast (Schizosaccharomyces
pombe)}
STDDSPYKQAFSLFDRHGTGRIPKTSIGDLLRACGQNPTLAEITEIESTLPAEVDMEQFLQV
LNRPNGFDMPGDPEEFVKGFQVFDKDATGMIGVGELRYVLTSLGEKLSNEEMDELLKGVPVK
DGMVNYHDFVQMILAN

>d1wdcb_ a.39.1.5 (B:) Myosin Essential Chain {Bay scallop (Aequipecten irradians)}
LPQKQIQEMKEAFSMIDVDRDGFVSKEDIKAISEQLGRAPDDKELTAMLKEAPGPLNFTMFL
SIFSDKLSGTDSEETIRNAFAMFDEQETKKLNIEYIKDLLENMGDNFNKDEMRMTFKEAPVE
GGKFDYVKFTAMIKGSGE
>d1br1b_ a.39.1.5 (B:) Myosin Essential Chain {Chicken (Gallus gallus)}
FSEEQTAEFKEAFQLFDRTGDGKILYSQCGDVMRALGQNPTNAEVMKVLGNPKSDEMNLKTL
KFEQFLPMMQTIAKNKDQGCFEDYVEGLRVFDKEGNGTVMGAEIRHVLVTLGEKMTEEEVEQ
LVAGHEDSNGCINYEELVRMVLSG
>d2mysb_ a.39.1.5 (B:) Myosin Essential Chain {Chicken (Gallus gallus)}
FDETEIEDFKEAFTVIDQNADGIIDKDDLRETFAAMGRLNVKNEELDAMIKEASGPINFTVF
LTMFGEKLKGADPEDVIMGAFKVLDPDGKGSIKKSFLEELLTTGGGRFTPEEIKNMWAAFPP
DVAGNVDYKNICYVITHGEDA
>d1wdcc_ a.39.1.5 (C:) Myosin Regulatory Chain {Bay scallop (Aequipecten irradians)}
LSQDEIDDLKDVFELFDFWDGRDGAVDAFKLGDVCRCLGINPRNEDVFAVGGTHKMGEKSLP
FEEFLPAYEGLMDCEQGTFADYMEAFKTFDREGQGFISGAELRHVLTALGERLSDEDVDEII
KLTDLQEDLEGNVKYEDFVKKVMAGPYP
>d2mysc_ a.39.1.5 (C:) Myosin Regulatory Chain {Chicken (Gallus gallus)}
AAADDFKEAFLLFDRTGDAKITASQVGDIARALGQNPTNAEINKILGNPSKEEMNAAAITFE
EFLPMLQAAANNKDQGTFEDFVEGLRVFDKEGNGTVMGAELRHVLATLGEKMTEEEVEELMK
GQEDSNGCINYEAFVKHIMSV
>d1auib_ a.39.1.5 (B:) Calcineurin regulatory subunit (B-chain) {Human (Homo sapiens)}
SYPLEMCSHFDADEIKRLGKRFKKLDLDNSGSLSVEEFMSLPELQQNPLVQRVIDIFDTDGN
GEVDFKEFIEGVSQFSVKGDKEQKLRFAFRIYDMDKDGYISNGELFQVLKMMVGNNLKDTQL
QQIVDKTIINADKDGDGRISFEEFCAVVGGLDIHKKMVVDV
>d1rec__ a.39.1.5 (-) Recoverin {Cow (Bos taurus)}
LSKEILEELQLNTKFTEEELSSWYQSFLKECPSGRITRQEFQTIYSKFFPEADPKAYAQHVF
RSFDANSDGTLDFKEYVIALHMTSAGKTNQKLEWAFSLYDVDGNGTISKNEVLEIVTAIFKM
ISPEDTKHLPEDENTPEKRAEKIWGFFGKKDDDKLTEKEFIEGTLANKEILRLIQFEPQKVK
EKLK
>d1g8ia_ a.39.1.5 (A:) Frequenin (neuronal calcium sensor 1) {Human (Homo sapiens)}
SNSKLKPEVVEELTRKTYFTEKEVQQWYKGFIKDCPSGQLDAAGFQKIYKQFFPFGDPTKFA
TFVFNVFDENKDGRIEFSEFIQALSVTSRGTLDEKLRWAFKLYDLDNDGYITRNEMLDIVDA
IYQMVGNTVELPEEENTPEKRVDRIFAMMDKNADGKLTLQEFQEGSKADPSIVQALSLYDGL
V
>d1fpwa_ a.39.1.5 (A:) Frequenin (neuronal calcium sensor 1) {Baker's yeast (Saccharomyces cerevisiae)}
MGAKTSKLSKDDLTCLKQSTYFDRREIQQWHKGFLRDCPSGQLAREDFVKIYKQFFPFGSPE

DFANHLFTVFDKDNNGFIHFEEFITVLSTTSRGTLEEKLSWAFELYDLNHDGYITFDEMLTI
VASVYKMMGSMVTLNEDEATPEMRVKKIFKLMDKNEDGYITLDEFREGSKVDPSIIGALNLY
DGLI
>d1jbaa_ a.39.1.5 (A:) Guanylate cyclase activating protein 2, GCAP-2 {Cow (Bos taurus)}
GQQFSWEEAEENGAVGAADAAQLQEWYKKFLEECPSGTLFMHEFKRFFKVPDNEEATQYVEA
MFRAFDTNGDNTIDFLEYVAALNLVLRGTLEHKLKWTFKIYDKDRNGCIDRQELLDIVESIY
KLKKACSVEVEAEQQGKLLTPEEVVDRIFLLVDENGDGQLSLNEFVEGARRDKWVMKMLQMD
LNP
>d1bjfa_ a.39.1.5 (A:) Neurocalcin {Cow (Bos taurus)}
NSKLRPEVMQDLLESTDFTEHEIQEWYKGFLRDCPSGHLSMEEFKKIYGNFFPYGDASKFAE
HVFRTFDANGDGTIDFREFIIALSVTSRGKLEQKLKWAFSMYDLDGNGYISKAEMLEIVQAI
YKMVSSVMKMPEDESTPEKRTEKIFRQMDTNRDGKLSLEEFIRGAKSDPSIVRLLQC
>d1dgua_ a.39.1.5 (A:) Calcium- and integrin-binding protein, CIB {Human (Homo sapiens)}
SKELLAEYQDLTFLTKQEILLAHRRFCELLPQEQRSVESSLRAQVPFEQILSLPELKANPFK
ERICRVFSTSPAKDSLSFEDFLDLLSVFSDTATPDIKSHYAFRIFDFDDDGTLNREDLSRLV
NCLTGEGEDTRLSASEMKQLIDNILEESDIDRDGTINLSEFQHVISRSPDFASSFKIVL
>d1qjta_ a.39.1.6 (A:) Eps15 {Mouse (Mus musculus)}
LSLTQLSSGNPVYEKYYRQVEAGNTGRVLALDAAAFLKKSGLPDLILGKIWDLADTDGKGVL
SKQEFFVALRLVACAQNGLEVSLSSLSLAVPPPRFHD
>d1c07a_ a.39.1.6 (A:) Eps15 {Human (Homo sapiens)}
TWVVSPAEKAKYDEIFLKTDKDMDGFVSGLEVREIFLKTGLPSTLLAHIWSLCDTKDCGKLS
KDQFALAFHLISQKLIKGIDPPHVLTPEMIPPS
>d1f8ha_ a.39.1.6 (A:) Eps15 {Human (Homo sapiens)}
PWAVKPEDKAKYDAIFDSLSPVNGFLSGDKVKPVLLNSKLPVDILGRVWELSDIDHDGMLDR
DEFAVAMFLVYCALEKEPVPMSLPPALVPPSKR
>d1iq3a_ a.39.1.6 (A:) Pob1 {Human (Homo sapiens)}
GSLQDNSSYPDEPWRITEEQREYYVNQFRSLQPDPSSFISGSVAKNFFTKSKLSIPELSYIW
ELSDADCDGALTLPEFCAAFHLIVARKNGYPLPEGLPPTLQPEFIVTD
>d1fi6a_ a.39.1.6 (A:) Reps1 {Mouse (Mus musculus)}
WKITDEQRQYYVNQFKTIQPDLNGFIPGSAAKEFFTKSKLPILELSHIWELSDFDKDGALTL
DEFCAAFHLVVARKNGYDLPEKLPESLMPK
>d1hqva_ a.39.1.8 (A:) Apoptosis-linked protein alg-2 {Mouse (Mus musculus)}
PGPGGGPGPAAGAALPDQSFLWNVFQRVDKDRSGVISDNELQQALSNGTWTPFNPVTVRSII
SMFDRENKAGVNFSEFTGVWKYITDWQNVFRTYDRDNSGMIDKNELKQALSGFGYRLSDQFH
DILIRKFDRQGRGQIAFDDFIQGCIVLQRLTDIFRRYDTDQDGWIQVSYEQYLSMVF
>d1juoa_ a.39.1.8 (A:) Sorcin {Human (Homo sapiens)}
FPGQTQDPLYGYFAAVAGQDGQIDADELQRCLTQSGIAGGYKPFNLETCRLMVSMLDRDMSG
TMGFNEFKELWAVLNGWRQHFISFDTDRSGTVDPQELQKALTTMGFRLSPQAVNSIAKRYST
NGKITFDDYIACCVKLRALTDSFRRRDTAQQGVVNFPYDDFIQCVMSV
>d1djxb1 a.39.1.7 (B:158-298) Phosphoinositide-specific phospholipase C, isozyme D1 (PLC-D!) {Rat (Rattus norvegicus)}

NKMNFKELKDFLKELNIQVDDGYARKIFRECDHSQTDSLEDEEIETFYKMLTQRAEIDRAFE
EAAGSAETLSVERLVTFLQHQQREEEAGPALALSLIERYEPSETAKAQRQMTKDGFLMYLLS
ADGNAFSLAHRRVYQDM
>d1qasa1 a.39.1.7 (A:205-298) Phosphoinositide-specific
phospholipase C, isozyme D1 (PLC-D!) {Rat (Rattus norvegicus)}
YKMLTQRAEIDRAFEEAAGSAETLSVERLVTFLQHQQREEEAGPALALSLIERYEPSETAKA
QRQMTKDGFLMYLLSADGNAFSLAHRRVYQDM
>d1k94a_ a.39.1.7 (A:) Grancalcin {Human (Homo sapiens)}
SVYTYFSAVAGQDGEVDAEELQRCLTQSGINGTYSPFSLETCRIMIAMLDRDHTGKMGFNAF
KELWAALNAWKENFMTVDQDGSGTVEHHELRQAIGLMGYRLSPQTLTTIVKRYSKNGRIFFD
DYVACCVKLRALTDFFRKRDHLQQGSANFIYDDFLQGTMAI
>d1kfus_ a.39.1.7 (S:) Calpain small (regulatory) subunit (domain
VI) {Human (Homo sapiens)}
THYSNIEANESEEVRQFRRLFAQLAGDDMEVSATELMNILNKVVTRHPDLKTDGFGIDTCRS
MVAVMDSDTTGKLGFEEFKYLWNNIKRWQAIYKQFDTDRSGTICSSELPGAFEAAGFHLNEH
LYNMIIRRYSDESGNMDFDNFISCLVRLDAMFRAFKSLDKDGTGQIQVNIQEWLQLTMYS
>d1dvia_ a.39.1.7 (A:) Calpain small (regulatory) subunit (domain
VI) {Rat (Rattus norvegicus)}
EEERQFRKLFVQLAGDDMEVSATELMNILNKVVTRHPDLKTDGFGIDTSRSMVAVMDSDTTG
KLGFEEFKYLWNNIKKWQGIYKRFDTDRSGTIGSNELPGAFEAAGFHLNQHIYSMIIRRYSD
ETGNMDFDNFISCLVRLDAMFRAFRSLDKNGTGQIQVNIQEWLQLTMYS
>d1alva_ a.39.1.7 (A:) Calpain small (regulatory) subunit (domain
VI) {Pig (Sus scrofa)}
EEVRQFRRLFAQLAGDDMEVSATELMNILNKVVTRHPDLKTDGFGIDTCRSMVAVMDSDTTG
KLGFEEFKYLWNNIKKWQAIYKQFDVDRSGTIGSSELPGAFEAAGFHLNEHLYSMIIRRYSD
EGGNMDFDNFISCLVRLDAMFRAFKSLDKDGTGQIQVNIQEWLQLTMYS
>d1kful1 a.39.1.7 (L:515-700) Calpain large subunit, C-terminal
domain (domain IV) {Human (Homo sapiens)}
EIEANLEEFDISEDDIDDGVRRLFAQLAGEDAEISAFELQTILRRVLAKRQDIKSDGFSIET
CKIMVDMLDSDGSGKLGLKEFYILWTKIQKYQKIYREIDVDRSGTMNSYEMRKALEEAGFKM
PCQLHQVIVARFADDQLIIDFDNFVRCLVRLETLFKIFKQLDPENTGTIELDLISWLCFSVL
>d1df0a1 a.39.1.7 (A:515-700) Calpain large subunit, C-terminal
domain (domain IV) {Rat (Rattus norvegicus)}
EIEANIEEIEANEEDIGDGFRRLFAQLAGEDAEISAFELQTILRRVLAKREDIKSDGFSIET
CKIMVDMLDEDGSGKLGLKEFYILWTKIQKYQKIYREIDVDRSGTMNSYEMRKALEEAGFKL
PCQLHQVIVARFADDELIIDFDNFVRCLVRLEILFKIFKQLDPENTGTIQLDLISWLSFSVL
>d1eg3a1 a.39.1.7 (A:85-209) Dystrophin {Human (Homo sapiens)}
HPKMTELYQSLADLNNVRFSAYRTAMKLRRLQKALCLDLLSLSAACDALDQHNLKQNDQPMD
ILQIINCLTTIYDRLEQEHNNLVNVPLCVDMCLNWLLNVYDTGRTGRIRVLSFKTGIISLCK
A
>d1eg3a2 a.39.1.7 (A:210-306) Dystrophin {Human (Homo sapiens)}
HLEDKYRYLFKQVASSTGFCDQRRLGLLLHDSIQIPRQLGEVASFGGSNIEPSVRSCFQFAN
NKPEIEAALFLDWMRLEPQSMVWLPVLHRVAAAET
>d2cbla1 a.39.1.7 (A:178-263) Cbl {Human (Homo sapiens)}

TFRITKADAAEFWRKAFGEKTIVPWKSFRQALHEVHPISSGLEAMALKSTIDLTCNDYISVF
EFDIFTRLFQPWSSLLRNWNSLAV
>d1h8ba_ a.39.1.7 (A:) alpha-Actinin {Human (Homo sapiens)}
MADTDTAEQVIASFRILASDKPYILAEELRRELPPDQAQYCIKRMPAYSGPGSVPGALDYAA
FSSALYGESDL
>d1c3za_ a.39.2.1 (A:) Thp12-carrier protein {Yellow mealworm (Tenebrio molitor)}
ETPREKLKQHSDACKAESGVSEESLNKVRNREEVDDPKLKEHAFCILKRAGFIDASGEFQLD
HIKTKFKENSEHPEKVDDLVAKCAVKKDTPQHSSADFFKCVHDNRS
>d1dqea_ a.39.2.1 (A:) Pheromone binding protein {Silkworm (Bombyx mori)}
SQEVMKNLSLNFGKALDECKKEMTLTDAINEDFYNFWKEGYEIKNRETGCAIMCLSTKLNML
DPEGNLHHGNAMEFAKKHGADETMAQQLIDIVHGCEKSTPANDDKCIWTLGVATCFKAEIHK
LNWAPSMDVAVGE
>d1iioa_ a.39.4.1 (A:) Hypothetical protein MTH865 {Archaeon Methanobacterium thermoautotrophicum}
GSHMKMGVKEDIRGQIIGALAGADFPINSPEELMAALPNGPDTTCKSGDVELKASDAGQVLT
ADDFPFKSAEEVADTIVNKAGL
>d1cpo_1 a.39.3.1 (0-119) Cloroperoxidase {Fungus (Caldariomyces fumago)}
EEPGSGIGYPYDNNTLPYVAPGPTDSRAPCPALNALANHGYIPHDGRAISRETLQNAFLNHM
GIANSVIELALTNAFVVCEYVTGSDCGDSLVNLTLLAEPHAFEHDHSFSRKDYKQGVA
>d1cpo_2 a.39.3.1 (120-298) Cloroperoxidase {Fungus (Caldariomyces fumago)}
NSNDFIDNRNFDAETFQTSLDVVAGKTHFDYADMNEIRLQRESLSNELDFPGWFTESKPIQN
VESGFIFALVSDFNLPDNDENPLVRIDWWKYWFTNESFPYHLGWHPPSPAREIEFVTSASSA
VLAASVTSTPSSLPSGAIGPGAEAVPLSFASTMTPFLLATNAPYYAQDPTLGPND
>d1h67a_ a.40.1.1 (A:) Calponin {Chicken (Gallus gallus)}
MPQTERQLRVWIEGATGRRIGDNFMDGLKDGVILCELINKLQPGSVQKVNDPVQNWHKLENI
GNFLRAIKHYGVKPHDIFEANDLFENTNHTQVQSTLIALASQAKTK
>d1bkra_ a.40.1.1 (A:) beta-spectrin {Human (Homo sapiens)}
KSAKDALLLWCQMKTAGYPNVNIHNFTTSWRDGMAFNALIHKHRPDLIDFDKLKKSNAHYNL
QNAFNLAEQHLGLTKLLDPEDISVDHPDEKSIITYVVTYYHYFSKM
>d1aoa_1 a.40.1.1 (121-251) N-terminal actin-crosslinking domain from fimbrin {Human (Homo sapiens)}
YSEEEKYAFVNWINKALENDPDCRHVIPMNPNTDDLFKAVGDGIVLCKMINLSVPDTIDERA
INKKKLTPFIIQENLNLALNSASAIGCHVVNIGAEDLRAGKPHLVLGLLWQIIKIGLFADIE
LSRNEAL
>d1aoa_2 a.40.1.1 (260-375) N-terminal actin-crosslinking domain from fimbrin {Human (Homo sapiens)}
TLEELMKLSPEELLLRWANFHLENSGWQKINNFSADIKDSKAYFHLLNQIAPKGQKEGEPRI
DINMSGFNETDDLKRAESMLQQADKLGCRQFVTPADVVSGNPKLNLAFVANLFN
>d1bhda_ a.40.1.1 (A:) Utrophin {Human (Homo sapiens)}
LQQTNSEKILLSWVRQTTRPYSQVNVLNFTTSWTDGLAFNAVLHRHKPDLFSWDKVVKMSPI

ERLEHAFSKAQTYLGIEKLLDPEDVAVRLPDKKSIIMYLTSLFEVL
>d1qaga1 a.40.1.1 (A:31-151) Utrophin {Human (Homo sapiens)}
DVQKKTFTKWINARFSKSGKPPINDMFTDLKDGRKLLDLLEGLTGTSLPKERGSTRVHALNN
VNRVLQVLHQNNVELVNIGGTDIVDGNHKLTLGLLWSIILHWQVKDVMKDVMSDLQQTN
>d1dxxa1 a.40.1.1 (A:9-119) Dystrophin {Human (Homo sapiens)}
DSYEREDVQKKTFTKWVNAQFSKFGKQHIENLFSDLQDGRRLLDLLEGLTGQKLPKEKGSTR
VHALNNVNKALRVLQNNNVDLVNIGSTDIVDGNHKLTLGLIWNIILHWQ
>d1dxxa2 a.40.1.1 (A:120-246) Dystrophin {Human (Homo sapiens)}
VKNVMKNIMAGLQQTNSEKILLSWVRQSTRNYPQVNVINFTTSWSDGLALNALIHSHRPDLF
DWNSVVSQQSATQRLEHAFNIARYQLGIEKLLDPEDVDTTYPDKKSILMYITSLFQVLPQQV
SIE
>d1a26_1 a.41.1.1 (662-796) Domain of poly(ADP-ribose) polymerase {Chicken (Gallus gallus)}
KSKLAKPIQDLIKMIFDVESMKKAMVEFEIDLQKMPLGKLSKRQIQSAYSILNEVQQAVSDG
GSESQILDLSNRFYTLIPHDFGMKKPPLLSNLEYIQAKVQMLDNLLDIEVAYSLLRGGNEDG
DKDPIDINYEK
>d1ycqa_ a.42.1.1 (A:) MDM2 {African clawed frog (Xenopus laevis)}
EKLVQPTPLLLSLLKSAGAQKETFTMKEVIYHLGQYIMAKQLYDEKQQHIVHCSNDPLGELF
GVQEFSVKEPRRLYAMISRNLVSANV
>d1ycra_ a.42.1.1 (A:) MDM2 {Human (Homo sapiens)}
ETLVRPKPLLLKLLKSVGAQKDTYTMKEVLFYLGQYIMTKRLYDEKQQHIVYCSNDLLGDLF
GVPSFSVKEHRKIYTMIYRNLVV
>d1b28a_ a.43.1.1 (A:) Arc repressor {Salmonella bacteriophage P22}
MKGMSKMPQFNLRWPREVLDLVRKVAEENGMSVNSYIYQLVMESFKKEGRIGA
>d1baza_ a.43.1.1 (A:) Arc repressor {Salmonella bacteriophage P22}
SKMPQVNLRWPREVLDLVRKVAEENGRSVNSEIYQRVMESFKKEGRIGA
>d1bazb_ a.43.1.1 (B:) Arc repressor {Salmonella bacteriophage P22}
KMPQVNLRWPREVLDLVRKVAEENGRSVNSEIYQRVMESFK
>d1bdta_ a.43.1.1 (A:) Arc repressor {Salmonella bacteriophage P22}
MKGMSKMPQFNLRWPREVLDLVRKVAEENGRSVNSEIYQRVMESFKKEGRIG
>d1myka_ a.43.1.1 (A:) Arc repressor {Salmonella bacteriophage P22}
KMLQFNLRWPREVLDLVRKVAEENGRSVNSEIYQRVMESFKKEGRIG
>d1myla_ a.43.1.1 (A:) Arc repressor {Salmonella bacteriophage P22}
KMPQFNLRWPREVLDLVRKVAEENGMSVNSYIYQLVMESFKKEGR
>d1mylb_ a.43.1.1 (B:) Arc repressor {Salmonella bacteriophage P22}
MPQFNLRWPREVLDLVRKVAEENGMSVNSYIYQLVMESFK
>d1mnta_ a.43.1.1 (A:) Mnt repressor {Salmonella bacteriophage

P22}
ARDDPHFNFRMPMEVREKLKFRAEANGRSMNSELLQIVQDALSKPSPVTGYRNDAERLADEQ
SELV
>d2cpga_ a.43.1.2 (A:) Transcriptional repressor CopG
{Streptococcus agalactiae}
MKKRLTITLSESVLENLEKMAREMGLSKSAMISVALENYKKGQ
>d1irqa_ a.43.1.2 (A:) Omega transcriptional repressor
{Streptococcus pyogenes}
IMGDKTVRVRADLHHIIKIETAKNGGNVKEVMDQALEEYIRKYLPDKL
>d1cmba_ a.43.1.2 (A:) Met repressor, MetR {Escherichia coli}
AEWSGEYISPYAEHGKKSEQVKKITVSIPLKVLKILTDERTRRQVNNLRHATNSELLCEAFL
HAFTGQPLPDDADLRKERSDEIPEAAKEIMREMGINPETWEY
>d1fvka1 a.44.1.1 (A:65-128) Disulphide-bond formation
facilitator (DSBA), insertion domain {Escherichia coli}
GGDLGKDLTQAWAVAMALGVEDKVTVPLFEGVQKTQTIRSASDIRDVFINAGIKGEEYDAAW
NS
>d1bed_1 a.44.1.1 (63-126) Disulphide-bond formation facilitator
(DSBA), insertion domain {Vibrio cholerae}
GNMGQAMSKAYATMIALEVEDKMVPVMFNRIHTLRKPPKDEQELRQIFLDEGIDAAKFDAAY
NG
>d1aqwa1 a.45.1.1 (A:77-209) Glutathione S-transferase {Human
(Homo sapiens), class pi}
GLYGKDQQEAALVDMVNDGVEDLRCKYISLIYTNYEAGKDDYVKALPGQLKPFETLLSQNQG
GKTFIVGDQISFADYNLLDLLLIHEVLAPGCLDAFPLLSAYVGRLSARPKLKAFLASPEYVN
LPINGNGKQ
>d2gsra1 a.45.1.1 (A:77-207) Glutathione S-transferase {Pig (Sus
scrofa), class pi}
YGKDQKEAALVDMVNDGVEDLRCKYATLIYTNYEAGKEKYVKELPEHLKPFETLLSQNQGGQ
AFVVGSQISFADYNLLDLLRIHQVLNPSCLDAFPLLSAYVARLSARPKIKAFLASPEHVNRP
INGNGKQ
>d1glqa1 a.45.1.1 (A:79-209) Glutathione S-transferase {Mouse (Mus
musculus), class pi}
YGKNQREAAQMDMVNDGVEDLRGKYVTLIYTNYENGKNDYVKALPGHLKPFETLLSQNQGGK
AFIVGDQISFADYNLLDLLLIHQVLAPGCLDNFPLLSAYVARLSARPKIKAFLSSPEHVNRP
INGNGKQ
>d1gtua1 a.45.1.1 (A:85-217) Glutathione S-transferase {Human
(Homo sapiens), class mu}
LCGETEEEKIRVDILENQTMDNHMQLGMICYNPEFEKLKPKYLEELPEKLKLYSEFLGKRPW
FAGNKITFVDFLVYDVLDLHRIFEPKCLDAFPNLKDFISRFEGLEKISAYMKSSRFLPRPVF
SKMAVWGNK
>d1hna_1 a.45.1.1 (85-217) Glutathione S-transferase {Human (Homo
sapiens), class mu}
LCGESEKEQIREDILENQFMDSRMQLAKLCYDPDFEKLKPEYLQALPEMLKLYSQFLGKQPW
FLGDKITFVDFIAYDVLERNQVFEPSCLDAFPNLKDFISRFEGLEKISAYMKSSRFLPRPVF

TKMAVFGNK
>d3gtub1 a.45.1.1 (B:85-224) Glutathione S-transferase {Human (Homo sapiens), class mu}
RKHNMCGETEEEKIRVDIIENQVMDFRTQLIRLCYSSDHEKLKPQYLEELPGQLKQFSMFLG
KFSWFAGEKLTFVDFLTYDILDQNRIFDPKCLDEFPNLKAFMCRFEALEKIAAYLQSDQFCK
MPINNKMAQWGNKPVC
>d4gtua1 a.45.1.1 (A:85-217) Glutathione S-transferase {Human (Homo sapiens), class mu}
LCGETEEEKIRVDILENQAMDVSNQLARVCYSPDFEKLKPEYLEELPTMMQHFSQFLGKRPW
FVGDKITFVDFLAYDVLDLHRIFEPNCLDAFPNLKDFISRFEGLEKISAYMKSSRFLPKPLY
TRVAVWGNK
>d2gsta1 a.45.1.1 (A:85-217) Glutathione S-transferase {Rat (Rattus norvegicus), class mu}
LCGETEEERIRADIVENQVMDNRMQLIMLCYNPDFEKQKPEFLKTIPEKMKLYSEFLGKRPW
FAGDKVTYVDFLAYDILDQYHIFEPKCLDAFPNLKDFLARFEGLKKISAYMKSSRYLSTPIF
SKLAQWSNK
>d1gsua1 a.45.1.1 (A:85-217) Glutathione S-transferase {Chicken (Gallus gallus), class mu}
MCGETEVEKQRVDVLENHLMDLRMAFARLCYSPDFEKLKPAYLEQLPGKLRQLSRFLGSRSW
FVGDKLTFVDFLAYDVLDQQRMFVPDCPELQGNLSQFLQRFEALEKISAYMRSGRFMKAPIF
WYTALWNNK
>d1gsea1 a.45.1.1 (A:81-222) Glutathione S-transferase {Human (Homo sapiens), class alpha (a1-1)}
LYGKDIKERALIDMYIEGIADLGEMILLLPVCPPEEKDAKLALIKEKIKNRYFPAFEKVLKS
HGQDYLVGNKLSRADIHLVELLYYVEELDSSLISSFPLLKALKTRISNLPTVKKFLQPGSPR
KPPMDEKSLEEARKIFRF
>d1gula1 a.45.1.1 (A:81-220) Glutathione S-transferase {Human (Homo sapiens), class alpha (a1-1)}
LFGKNLKERTLIDMYVEGTLDLLELLIMHPFLKPDDQQKEVVNMAQKAIIRYFPVFEKILRG
HGQSFLVGNQLSLADVILLQTILALEEKIPNILSAFPFLQEYTVKLSNIPTIKRFLEPGSKK
KPPPDEIYVRTVYNIF
>d1ev4a1 a.45.1.1 (A:80-222) Glutathione S-transferase {Rat (Rattus norvegicus), class alpha (a1-1)}
DLYGKDMKERALIDMYSEGILDLTEMIMQLVICPPDQKEAKTALAKDRTKNRYLPAFEKVLK
SHGQDYLVGNKLTRVDIHLLELLLYVEEFDASLLTSFPLLKAFKSRISSLPNVKKFLQPGSQ
RKLPMDAKQIEEARKIYKF
>d1ev4c1 a.45.1.1 (C:80-208) Glutathione S-transferase {Rat (Rattus norvegicus), class alpha (a1-1)}
DLYGKDMKERALIDMYSEGILDLTEMIMQLVICPPDQKEAKTALAKDRTKNRYLPAFEKVLK
SHGQDYLVGNKLTRVDIHLLELLLYVEEFDASLLTSFPLLKAFKSRISSLPNVKKFLQPGSQ
RKLPM
>d1f3aa1 a.45.1.1 (A:80-221) Glutathione S-transferase {Mouse (Mus musculus), class alpha (a1-1)}
LYGKDMKERALIDMYSEGILDLTEMIGQLVLCPPDQREAKTALAKDRTKNRYLPAFEKVLKS

HGQDYLVGNRLTRVDIHLLEVLLYVEEFDASLLTPFPLLKAFKSRISSLPNVKKFLQPGSQR
KPPMDAKQIQEARKAFKI
>d1b48a1 a.45.1.1 (A:80-222) Glutathione S-transferase {Mouse (Mus musculus), class alpha (a1-4)}
NLYGKDLKERVRIDMYADGTQDLMMMIAVAPFKTPKEKEESYDLILSRAKTRYFPVFEKILK
DHGEAFLVGNQLSWADIQLLEAILMVEELSAPVLSDFPLLQAFKTRISNIPTIKKFLQPGSQ
RKPPPDGPYVEVVRIVLKF
>d1ljra1 a.45.1.1 (A:80-244) Glutathione S-transferase {Human (Homo sapiens), class theta}
TPDHWYPSDLQARARVHEYLGWHADCIRGTFGIPLWVQVLGPLIGVQVPEEKVERNRTAMDQ
ALQWLEDKFLGDRPFLAGQQVTLADLMALEELMQPVALGYELFEGRPRLAAWRGRVEAFLGA
ELCQEAHSIILSILEQAAKKTLPTPSPEAYQAMLLRIARIP
>d1pd211 a.45.1.1 (1:76-199) Glutathione S-transferase {Rat (Rattus norvegicus), class sigma}
DLAGKTELEQCQVDAVVDTLDDFMSLFPWAEENQDLKERTFNDLLTRQAPHLLKDLDTYLGD
KEWFIGNYVTWADFYWDICSTTLLVLKPDLLGIYPRLVSLRNKVQAIPAISAWILKRPQTKL
>d2gsq_1 a.45.1.1 (76-202) Glutathione S-transferase {Squid (Ommastrephes sloani pacificus), class sigma}
LDGKTSLEKYRVDEITETLQDIFNDVVKIKFAPEAAKEAVQQNYEKSCKRLAPFLEGLLVSN
GGGDGFFVGNSMTLADLHCYVALEVPLKHTPELLKDCPKIVALRKRVAECPKIAAYLKKRPV
RDF
>d1eema1 a.45.1.1 (A:103-241) Glutathione S-transferase {Human (Homo sapiens), class omega}
LPDDPYEKACQKMILELFSKVPSLVGSFIRSQNKEDYAGLKEEFRKEFTKLEEVLTNKKTTF
FGGNSISMIDYLIWPWFERLEAMKLNECVDHTPKLKLWMAAMKEDPTVSALLTSEKDWQGFL
ELYLQNSPEACDYGL
>d1fw1a1 a.45.1.1 (A:88-212) Glutathione S-transferase {Human (Homo sapiens), class zeta}
LLPQDPKKRASVRMISDLIAGGIQPLQNLSVLKQVGEEMQLTWAQNAITCGFNALEQILQST
AGIYCVGDEVTMADLCLVPQVANAERFKVDLTPYPTISSINKRLLVLEAFQVSHPCRQPDTP
T
>d1bg5_1 a.45.1.1 (81-254) Glutathione S-transferase {Schistosoma japonicum}
MLGGCPKERAEISMLEGAVLDIRYGVSRIAYSKDFETLKVDFLSKLPEMLKMFEDRLCHKTY
LNGDHVTHPDFMLYDALDVVLYMDPMCLDAFPKLVCFKKRIEAIPQIDKYLKSSKYIAWPLQ
GWQATFGGGDHPPKSDLVPRGSSYYQEAKSSKIMESFKNMVPQQALVNSS
>d1duga1 a.45.1.1 (A:81-220) Glutathione S-transferase {Schistosoma japonicum}
LGGCPKERAEISMLEGAVLDIRYGVSRIAYSKDFETLKVDFLSKLPEMLKMFEDRLCHKTYL
NGDHVTHPDFMLYDALDVVLYMDPMCLDAFPKLVCFKKRIEAIPQIDKYLKSSKYIAWPLQG
WQATFGGGDHPPKSDP
>d1gne_1 a.45.1.1 (80-232) Glutathione S-transferase {Schistosoma japonicum}
MLGGCPKERAEISMLEGAVLDIRYGVSRIAYSKDFETLKVDFLSKLPEMLKMFEDRLCHKTY

LNGDHVTHPDFMLYDALDVVLYMDPMCLDAFPKLVCFKKRIEAIPQIDKYLKSSKYIAWPLQ
GWQATFGGGDHPPKSDLVPRGSMELDKWA
>d1fhe_1 a.45.1.1 (81-214) Glutathione S-transferase {Fasciola hepatica}
LGTTPEERARISMIEGAAMDLRIGFGRVCYNPKFEEVKEEYVKELPKTLKMWSDFLGDRHYL
TGSSVSHVDFMLYETLDSIRYLAPHCLDEFPKLKEFKSRIEALPKIKAYMESKRFIKWPLNG
WAASFGAGDA
>d2fhea1 a.45.1.1 (A:81-216) Glutathione S-transferase {Fasciola hepatica}
IGTTSEERARVSMIEGAAVDLRQGISRISYQPKFEQLKEGYLKDLPTTMKMWSDFLGKNPYL
RGTSVSHVDFMVYEALDAIRYLEPHCLDHFPNLQQFMSRIEALPSIKAYMESNRFIKWPLNG
WHAQFGGGDAPP
>d1gnwa1 a.45.1.1 (A:86-211) Glutathione S-transferase {Mouse-ear cress (Arabidopsis thaliana)}
LQTDSKNISQYAIMAIGMQVEDHQFDPVASKLAFEQIFKSIYGLTTDEAVVAEEEAKLAKVL
DVYEARLKEFKYLAGETFTLTDLHHIPAIQYLLGTPTKKLFTERPRVNEWVAEITKRPASEK
VQ
>d1axda1 a.45.1.1 (A:81-210) Glutathione S-transferase {Maize (Zea mays), type I}
ELLREGNLEEAAMVDVWIEVEANQYTAALNPILFQVLISPMLGGTTDQKVVDENLEKLKKVL
EVYEARLTKCKYLAGDFLSLADLNHVSVTLCLFATPYASVLDAYPHVKAWWSGLMERPSVQK
VAALM
>d1aw9_1 a.45.1.1 (83-217) Glutathione S-transferase {Maize (Zea mays), type III}
GTDLLPATASAAKLEVWLEVESHHFYPNASPLVFQLLVRPLLGGAPDAAVVDKHAEQLAKVL
DVYEAHLARNKYLAGDEFTLADANHASYLLYLSKTPKAGLVAARPHVKAWWEAIVARPAFQK
TVAAIPLPPPP
>d1e6ba1 a.45.1.1 (A:88-220) Glutathione S-transferase {Mouse-ear cress (Arabidopsis thaliana), class zeta}
PLLPRDLHKRAVNYQAMSIVLSGIQPHQNLAVIRYIEEKINVEEKTAWVNNAITKGFTALEK
LLVNCAGKHATGDEIYLADLFLAPQIHGAINRFQINMEPYPTLAKCYESYNELPAFQNALPE
KQPDAPSST
>d1a0fa1 a.45.1.1 (A:81-201) Glutathione S-transferase {Escherichia coli}
QLLAPVNSISRYKTIEWLNYIATELHKGFTPLFRPDTPEEYKPTVRAQLEKKLQYVNEALKD
EHWICGQRFTIADAYLFTVLRWAYAVKLNLEGLEHIAAFMQRMAERPEVQDALSAEGLK
>d1b8xa1 a.45.1.1 (A:81-260) Glutathione S-transferase {Escherichia coli}
LGGCPKERAEISMLEGAVLDIRYGVSRIAYSKDFETLKVDFLSKLPEMLKMFEDRLCHKTYL
NGDHVTHPDFMLYDALDVVLYMDPMCLDAFPKLVCFKKRIEAIPQIDKYLKSSKYIAWPLQG
WQATFGGGDHPPKSDLVPRGSRRASVGSRMHYPGAFTYSPTPVTSGIGIGMSAMGS
>d1pmt_1 a.45.1.1 (81-201) Glutathione S-transferase {Proteus mirabilis}
NLIAPPKALERYHQIEWLNFLASEVHKGYSPLFSSDTPESYLPVVKNKLKSKFVYINDVLSK

QKCVCGDHFTVADAYLFTLSQWAPHVALDLTDLSHLQDYLARIAQRPNVHSALVTEGLI
>d1f2ea1 a.45.1.1 (A:81-201) Glutathione S-transferase {Sphingomonas paucimobilis}
GLAPAEGSLDRYRLLSRLSFLGSEFHKAFVPLFAPATSDEAKAAAAESVKNHLAALDKELAG
RDHYAGNAFSVADIYLYVMLGWPAYVGIDMAAYPALGAYAGKIAQRPAVGAALKAEGLA
>d1g7oa1 a.45.1.1 (A:76-215) Glutaredoxin 2 {Escherichia coli}
PLLTGKRSPAIEEWLRKVNGYANKLLLPRFAKSAFDEFSTPAARKYFVDKKEASAGNFADLL
AHSDGLIKNISDDLRALDKLIVKPNAVNGELSEDDIQLFPLLRNLTLVAGINWPSRVADYRD
NMAKQTQINLLSSMAI
>d1k0da1 a.45.1.1 (A:201-351) Yeast prion protein ure2p, nitrogen regulation fragment {Baker's yeast (Saccharomyces cerevisiae)}
LWSDDLADQSQINAWLFFQTSGHAPMIGQALHFRYFHSQKIASAVERYTDEVRRVYGVVEMA
LAERREALVMELDTENAAAYSAGTTPMSQSRFFDYPVWLVGDKLTIADLAFVPWNNVVDRIG
INIKIEFPEVYKWTKHMMRRPAVIKAL
>d1k0ma1 a.45.1.1 (A:92-240) Chloride intracellular channel 1 (clic1) {Human (Homo sapiens)}
RYPKLAALNPESNTAGLDIFAKFSAYIKNSNPALNDNLEKGLLKALKVLDNYLTSPLPEGVD
ETSAEDEGVSQRKFLDGNELTLADCNLLPKLHIVQVVCKKYRGFTIPEAFRGVHRYLSNAYA
REEFASTCPDDEEIELAYEQVAKAL
>d1bmta1 a.46.1.1 (A:651-740) Methionine synthase domain {Escherichia coli}
QAEWRSWEVNKRLEYSLVKGITEFIEQDTEEARQQATRPIEVIEGPLMDGMNVVGDLFGEGK
MFLPQVVKSARVMKQAVAYLEPFIEASK
>d2tpt_1 a.46.2.1 (1-70) Thymidine phosphorylase {Escherichia coli}
LFLAQEIIRKKRDGHALSDEEIRFFINGIRDNTISEGQIAALAMTIFFHDMTMPERVSLTMA
MRDSGTVL
>d1brwa1 a.46.2.1 (A:1-70) Pyrimidine nucleoside phosphorylase {Bacillus stearothermophilus}
MRMVDLIAKKRDGKALTKEEIEWIVRGYTNGDIPDYQMSALAMAIYFRGMTEEETAALTMAM
VQSGEMLD
>d1bf5a1 a.47.1.1 (A:136-316) STAT-1, coiled coil domain {Human (Homo sapiens)}
LDKQKELDSKVRNVKDKVMCIEHEIKSLEDLQDEYDFKCKTLQNREHLLLKKMYLMLDNKRK
EVVHKIIELLNVTELTQNALINDELVEWKRRQQSACIGGPPNACLDQLQNWFTIVAESLQQV
RQQLKKLEELEQKYTYEHDPITKNKQVLWDRTFSLFQQLIQSS
>d1bg1a1 a.47.1.1 (A:136-321) STAT3b {Mouse (Mus musculus)}
VVTEKQQMLEQHLQDVRKRVQDLEQKMKVVENLQDDFDFNYKTLKSQGDMQDLNGNNQSVTR
QKMQQLEQMLTALDQMRRSIVSELAGLLSAMEYVQKTLTDEELADWKRRQQIACIGGPPNIC
LDRLENWITSLAESQLQTRQQIKKLEELQQKVSYKGDPIVQHRPMLEERIVELFRNLMKSAF
>d1dn1b_ a.47.2.1 (B:) Syntaxin 1A N-terminal domain {Rat (Rattus norvegicus)}
DRFMDEFFEQVEEIRGFIDKIAENVEEVKRKHSAILASPNPDEKTKEELEELMSDIKKTANK
VRSKLKSIEQSIEQEEGLNRSSADLRIRKTQHSTLSRKFVEVMSEYNATQSDYRERCKGRIQ

RQLEITGRTTTSEELEDMLESGNPAIFASGIIMDSSISKQALSEIETRHSEIIKLENSIREL
HDMFMDMAMLVESQGEMIDRIEYNVEHAVDYVERAV
>d1ez3a_ a.47.2.1 (A:) Syntaxin 1A N-terminal domain {Rat (Rattus norvegicus)}
RDRFMDEFFEQVEEIRGFIDKIAENVEEVKRKHSAILASPNPDEKTKEELEELMSDIKKTAN
KVRSKLKSIEQSIEQEEGLNRSSADLRIRKTQHSTLSRKFVEVMSEYNATQSDYRERCKGRI
>d1fioa_ a.47.2.1 (A:) Sso1 {Baker's yeast (Saccharomyces cerevisiae)}
MHDFVGFMNKISQINRDLDKYDHTINQVDSLHKRLLTEVNEEQASHLRHSLDNFVAQATDLQ
FKLKNEIKSAQRDGIHDTNKQAQAENSRQRFLKLIQDYRIVDSNYKEENKEQAKRQYMIIQP
EATEDEVEAAISDVGGQQIFSQALLNANRRGEAKTALAEVQARHQELLKLEKSMAELTQLFN
DMEELVIEQQ
>d1hs7a_ a.47.2.1 (A:) Vam3p N-terminal domain {Baker's yeast (Saccharomyces cerevisiae)}
TNQKTKELSNLIETFAEQSRVLEKECTKIGSKRDSKELRYKIETELIPNCTSVRDKIESNIL
IHQNGKLSADFKNLKTKYQSLQQSYNQRKSLFPLK
>d2cbla2 a.48.1.1 (A:47-177) N-terminal domain of cbl (N-cbl) {Human (Homo sapiens)}
PPGTVDKKMVEKCWKLMDKVVRLCQNPKLALKNSPPYILDLLPDTYQHLRTILSRYEGKMET
LGENEYFRVFMENLMKKTKQTISLFKEGKERMYEENSQPRRNLTKLSLIFSHMLAELKGIFP
SGLFQGD
>d1de4c1 a.48.2.1 (C:609-756) Transferrin receptor ectodomain, C-terminal domain {Human (Homo sapiens)}
LDYERYNSQLLSFVRDLNQYRADIKEMGLSLQWLYSARGDFFRATSRLTTDFGNAEKTDRFV
MKKLNDRVMRVEYHFLSPYVSPKESPFRHVFWGSGSHTLPALLENLKLRKQNNGAFNETLFR
NQLALATWTIQGAANALSGDVWDI
>d1eo0a_ a.48.3.1 (A:) Transcription elongation factor TFIIS N-domain {Baker's yeast (Saccharomyces cerevisiae)}
MDSKEVLVHVKNLEKNKSNDAAVLEILHVLDKEFVPTEKLLRETKVGVEVNKFKKSTNVEIS
KLVKKMISSWKDAIN
>d1f6va_ a.49.1.1 (A:) C-terminal domain of B transposition protein {Bacteriophage mu}
GSRIAKRTAINKTKKADVKAIADAWQINGEKELELLQQIAQKPGALRILNHSLRLAAMTAHG
KGERVNEDYLRQAFRELDLDVDISTLLRN
>d1i6ve_ a.143.1.1 (E:) RNA polymerase omega subunit {Thermus aquaticus}
MAEPGIDKLFGMVDSKYRLTVVVAKRAQQLLRHRFKNTVLEPEERPKMRTLEGLYDDPNAVT
WAMKELLTGRLFFGENLVPEDRLQKEMERLYPTEEE
>d1cfaa_ a.50.1.1 (A:) C5a anaphylotoxin {Human (Homo sapiens)}
MLQKKIEEIAAKYKHSVVKKCCYDGASVNNDETCEQRAARISLGPRCIKAFTECCVVASQLR
ANISHKDMC
>d1c5a__ a.50.1.1 (-) C5a anaphylotoxin {Pig (Sus scrofa domestica)}
MLQKKIEEEAAKYKYAMLKKCCYDGAYRNDDETCEERAARIKIGPKCVKAFKDCCYIANQVR

AEQS
>d0c3a__ a.50.1.1 (-) C3a anaphylotoxin {Human (Homo sapiens)}
SVQLTEKRMNKVGKYPKELRKCCEDGMRQNPMRFSCQRRTRFISLGEACKKVFLDCCNYITE
LRRQHARASHLGLAR
>d1ocrh_ a.51.1.1 (H:) Cytochrome c oxidase subunit h {Cow (Bos taurus)}
KIKNYQTAPFDSRFPNQNQTRNCWQNYLDFHRCEKAMTAKGGDVSVCEWYRRVYKSLCPISW
VSTWDDRRAEGTFPGKI
>d1hyp__ a.52.1.1 (-) Soybean hydrophobic protein {Soybean (Glycine max)}
PSCPDLSICLNILGGSLGTVDDCCALIGGLGDIEAIVCLCIQLRALGILNLNRNLQLILNSC
GRSYPSNATCPRT
>d1bwoa_ a.52.1.1 (A:) Plant non-specific lipid-transfer protein (ns-LTP) {Wheat (Triticum aestivum), L. seeds}
IDCGHVDSLVRPCLSYVQGGPGPSGQCCDGVKNLHNQARSQSDRQSACNCLKGIARGIHNLN
EDNARSIPPKCGVNLPYTISLNIDCSRV
>d1be2__ a.52.1.1 (-) Plant non-specific lipid-transfer protein (ns-LTP) {Barley (Hordeum vulgare)}
LNCGQVDSKMKPCLTYVQGGPGPSGECCNGVRDLHNQAQSSGDRQTVCNCLKGIARGIHNLN
LNNAASIPSKCNVNVPYTISPDIDCSRIY
>d1fk5a_ a.52.1.1 (A:) Plant non-specific lipid-transfer protein (ns-LTP) {Maize (Zea mays)}
AISCGQVASAIAPCISYARGQGSGPSAGCCSGVRSLNNAARTTADRRAACNCLKNAAAGVSG
LNAGNAASIPSKCGVSIPYTISTSTDCSRVN
>d1rzl__ a.52.1.1 (-) Plant non-specific lipid-transfer protein (ns-LTP) {Rice (Oryza sativa)}
ITCGQVNSAVGPCLTYARGGAGPSAACCSGVRSLKAAASTTADRRTACNCLKNAARGIKGLN
AGNAASIPSKCGVSVPYTISASIDCSRVS
>d1hssa_ a.52.1.2 (A:) 0.19 alpha-amylase inhibitor {Wheat (Triticum aestivum)}
MCYPGQAFQVPALPACRPLLRLQCNGSQVPEAVLRDCCQQLAHISEWCRCGALYSMLDSMYK
EHGAQEGQAGTGAFPRCRREVVKLTAASITAVCRLPIVVDASGDGAYVCKDVAAYPDA
>d1tmqb_ a.52.1.2 (B:) Trypsin/alpha-amylase inhibitor RBI {Ragi (Elucine coracana gaertneri), seeds}
SVGTSCIPGMAIPHNPLDSCRWYVSTRTCGVGPRLATQEMKARCCRQLEAIPAYCRCEAVRI
LMDGVVTSSGQHEGRLLQDLPGCPRQVQRAFAPKLVTEVECNLATIHGGPFCLSL
>d1bea__ a.52.1.2 (-) Hageman factor/amylase inhibitor {Maize (Zea mays)}
SCVPGWAIPHNPLPSCRWYVTSRTCGIGPRLPWPELKRRCCRELADIPAYCRCTALSILMDG
AIPPGPDAQLEGRLEDLPGCPREVQRGFAATLVTEAECNLATISGVAECPWILG
>g1pnb.1 a.52.1.3 (A:,B:) Napin BNIb {Rape (Brassica napus)}
QPQKCQREFQQEQHLRACQQWIRQQLAGSPFXQSGPQQGPWLREQCCNELYQEDQVCVCPTL
KQAAKSVRVQGQHGPFQSTRIYQIAKNLPNVCNMKQIGTCPFIAI
>d1a1ua_ a.53.1.1 (A:) p53 tetramerization domain {Human (Homo

sapiens)}
EYFTLQIRGRERFEKIREYNEALELKDAQ
>d1aie__ a.53.1.1 (-) p53 tetramerization domain {Human (Homo sapiens)}
EYFTLQIRGRERFEMFRELNEALELKDAQAG
>d1hs5a_ a.53.1.1 (A:) p53 tetramerization domain {Human (Homo sapiens)}
DGEYFTLQIRGRERFEQFRERNEALELKDAQAGK
>d1saia_ a.53.1.1 (A:) p53 tetramerization domain {Human (Homo sapiens)}
KKKPLDGEYFTLQIRGRERFEMFRELNEALELKDAQAGKEPG
>d1k1fa_ a.147.1.1 (A:) Bcr-Abl oncoprotein oligomerization domain {Human (Homo sapiens)}
MVDPVGFAEAWKAQFPDSEPPRMELRSVGDIEQELERAKASIRRLEQEVNQERFRMIYLQTL
LAKEK
>d1adt_1 a.54.1.1 (176-265) Domain of early E2A DNA-binding protein, ADDBP {Human adenovirus type 5}
PIVSAWEKGMEAARALMDKYHVDNDLKANFKLLPDQVEALAAVCKTWLNEEHRGLQLTFTSN
KTFVTMMGRFLQAYLQSFAEVTYKHHEP
>d1ihfa_ a.55.1.1 (A:) Integration host factor (IHF) {Escherichia coli}
ALTKAEMSEYLFDKLGLSKRDAKELVELFFEEIRRALENGEQVKLSGFGNFDLRDKNQRPGR
NPKTGEDIPITARRVVTFRPGQKLKSRVENASPK
>d1ihfb_ a.55.1.1 (B:) Integration host factor (IHF) {Escherichia coli}
MTKSELIERLATQQSHIPAKTVEDAVKEMLEHMASTLAQGERIEIRGFGSFSLHYRAPRTGR
NPKTGDKVELEGKYVPHFKPGKELRDRANIYG
>d1hns__ a.55.1.1 (-) DNA-binding domain of H1 protein, (H-NS) {Escherichia coli}
AQRPAKYSYVDENGETKTWTGQGRTPAVIKKAMDEQGKSLDDFLIKQ
>d1huua_ a.55.1.1 (A:) HU protein {Bacillus stearothermophilus}
MNKTELINAVAETSGLSKKDATKAVDAVFDSITEALRKGDKVQLIGFGNFEVRERAARKGRN
PQTGEEMEIPASKVPAFKPGKALKDAVK
>d1b8za_ a.55.1.1 (A:) HU protein {Thermotoga maritima}
MNKKELIDRVAKKAGAKKKDVKLILDTILETITEALAKGEKVQIVGFGSFEVRKAAARKGVN
PQTRKPITIPERKVPKFKPGKALKEKVK
>d1exea_ a.55.1.1 (A:) Transcription factor 1, TF1 {Bacteriophage SPO1 (Bacillus subtilis)}
MNKTELIKAIAQDTGLTQVSVSKMLASFEKIITETVAKGDKVQLTGFLNIKPVARQARKGFN
PQTQEALEIAPSVGVSVKPGESLKKAAEGLKYEDFAK
>d1dp3a_ a.55.1.2 (A:) DNA-binding domain (fragment?) of the TraM protein {Escherichia coli}
AKVQAYVSDEIVYKINKIVERRRAEGAKSTDVSFSSISTMLLELGLRVYEAQMER
>d1hlra1 a.56.1.1 (A:81-193) Aldehyde oxidoreductase, domain 2

{Desulfovibrio gigas}
QPENLHPLQKAWVLHGGAQCGFCSPGFIVSAKGLLDTNADPSREDVRDWFQKHRNACRCTGY
KPLVDAVMDAAAVINGKKPETDLEFKMPADGRIWGSKYPRPTAVAKVTGTL
>d1dgja1 a.56.1.1 (A:81-193) Aldehyde oxidoreductase, domain 2
{Desulfovibrio desulfuricans}
APDCLHPLQHAWIQHGAAQCGFCTPGFIVSAKALLDENVAPSREDVRDWFQKHHNICRCTGY
KPLVDAVMDAAAILRGEKTVEEISFKMPADGRIWGSSIPRPSAVAKVTGLA
>d1fo4a1 a.56.1.1 (A:93-165) Xanthine oxidase, domain 2 {Cow (Bos
taurus)}
STKTRLHPVQERIAKSHGSQCGFCTPGIVMSMYTLLRNQPEPTVEEIEDAFQGNLCRCTGYR
PILQGFRTFAK
>d1jroa1 a.56.1.1 (A:85-166) Xanthine dehydrogenase chain A,
domain 2 {Rhodobacter capsulatus}
DGRLHPVQQAMIDHHGSQCGFCTPGFIVSMAAAHDRDRKDYDDLLAGNLCRCTGYAPILRAA
EAAAGEPPADWLQADAAFTL
>d1qj2a1 a.56.1.1 (A:82-161) Carbon monoxide (CO) dehydrogenase
iron-sulfur protein, C-domain {Pseudomonas carboxydovorans}
APDGTLSALQEGFRMMHGLQCGYCTPGMIMRSHRLLQENPSPTEAEIRFGIGGNLCRCTGYQ
NIVKAIQYAAAKINGVPF
>d1ffva1 a.56.1.1 (A:82-157) Carbon monoxide (CO) dehydrogenase
iron-sulfur protein, C-domain {Hydrogenophaga pseudoflava}
NKGVLHAVQEGFYKEHGLQCGFCTPGMLMRAYRFLQENPNPTEAEIRMGMTGNLCRCTGYQN
IVKAVQYAARKLQE
>d1dj8a_ a.57.1.1 (A:) Protein HNS-dependent expression A; HdeA
{Escherichia coli}
NKKPVNSWTCEDFLAVDESFQPTAVGFAEALNNKDKPEDAVLDVQGIATVTPAIVQACTQDK
QANFKDKVKGEWDKIKK
>d1g9la_ a.144.1.1 (A:) poly(A) binding protein {Human (Homo
sapiens)}
GPLGSAAAATPAVRTVPQYKYAAGVRNPQQHLNAQPQVTMQQPAVHVQGQEPLTASMLASAP
PQEQKQMLGERLFPLIQAMHPTLAGKITGMLLEIDNSELLHMLESPESLRSKVDEAVAVLQA
HQAKEAAQKAVNSATGVPTV
>d1i2ta_ a.144.1.1 (A:) hyperplastic discs protein {Human (Homo
sapiens)}
HRQALGERLYPRVQAMQPAFASKITGMLLELSPAQLLLLLASEDSLRARVDEAMELIIAHG
>d1af7_1 a.58.1.1 (11-91) Chemotaxis receptor methyltransferase
CheR, N-terminal domain {Salmonella typhimurium}
SVLLQMTQRLALSDAHFRRICQLIYQRAGIVLADHKRDMVYNRLVRRLRALGLDDFGRYLSM
LEANQNSAEWQAFINALTT
>d1e91a_ a.59.1.1 (A:) Sin3B {Mouse (Mus musculus)}
ESDSVEFNNAISYVNKIKTRFLDHPEIYRSFLEILHTYQKEQLHTKGRPFRGMSEEEVFTEV
ANLFRGQEDLLSEFGQFLPEAKR
>d1g1eb_ a.59.1.1 (B:) Sin3A {Mouse (Mus musculus)}
SLQNNQPVEFNHAINYVNKIKNRFQGQPDIYKAFLEILHTYQKEQRNAKEAGGNYTPALTEQ

EVYAQVARLFKNQEDLLSEFGQFLPDA
>d1bqv__ a.60.1.1 (-) Ets-1 transcription factor pointed domain {Mouse (Mus musculus)}
MECADVPLLTPSSKEMMSQALKATFSGFTKEQQRLGIPKDPRQWTETHVRDWVMWAVNEFSL
KGVDFQKFCMSGAALCALGKECFLELAPDFVGDILWEHLEILQKEDVK
>d1b0xa_ a.60.1.2 (A:) EphA4 receptor tyrosine kinases {Mouse (Mus musculus)}
FSAVVSVGDWLQAIKMDRYKDNFTAAGYTTLEAVVHMSQDDLARIGITAITHQNKILSSVQA
MRTQMQQMHG
>d1b4fa_ a.60.1.2 (A:) EphB2 receptor {Human (Homo sapiens)}
PDYTSFNTVDEWLEAIKMGQYKESFANAGFTSFDVVSQMMMEDILRVGVTLAGHQKKILNSI
QVMRAQMNQIQS
>d1sgg__ a.60.1.2 (-) EphB2 receptor {Chicken (Gallus gallus)}
YTSFNTVDEWLDAIKMSQYKESFASAGFTTFDIVSQMTVEDILRVGVTLAGHQKKILNSIQV
MRAQM
>d1coka_ a.60.1.2 (A:) C-terminal domain of p73 {Human (Homo sapiens)}
YHADPSLVSFLTGLGCPNCIEYFTSQGLQSIYHLQNLTIEDLGALKIPEQYRMTIWRGLQDL
KQGHDY
>d1dxsa_ a.60.1.2 (A:) C-terminal domain of p73 {Human (Homo sapiens)}
SLVSFLTGLGCPNCIEYFTSQGLQSIYHLQNLTIEDLGALKIPEQYRMTIWRGLQDL
>d1cuk_2 a.60.2.1 (65-142) DNA helicase RuvA subunit, middle domain {Escherichia coli}
NKQERTLFKELIKTNGVGPKLALAILSGMSAQQFVNAVEREEVGALVKLPGIGKKTAERLIV
EMKDRFKGLHGDLFTP
>d1bvsa2 a.60.2.1 (A:64-134) DNA helicase RuvA subunit, middle domain {Mycobacterium leprae}
DAENRDLFLALLSVSGVGPRLAMATLAVHDAAALRQALADSDVASLTRVPGIGRRGAERIVL
ELADKVGPV
>d1dgsa1 a.60.2.2 (A:401-581) NAD+-dependent DNA ligase, domain 3 {Thermus filiformis}
RWPEACPECGHRLVKEGKVHRCPNPLCPAKRFEAIRHYASRKAMDIEGLGEKLIERLLEKGL
VRDVADLYHLRKEDLLGLERMGEKSAQNLLRQIEESKHRGLERLLYALGLPGVGEVLARNLA
RRFGTMDRLLEASLEELIEVEEVGELTARAILETLKDPAFRDLVRRLKEAGVSMESK
>d1coo__ a.60.3.1 (-) C-terminal domain of RNA polymerase alpha subunit {Escherichia coli}
FDPILLRPVDDLELTVRSANCLKAEAIHYIGDLVQRTEVELLKTPNLGKKSLTEIKDVLASR
GLSLGMRLENWPPASIADE
>d1doqa_ a.60.3.1 (A:) C-terminal domain of RNA polymerase alpha subunit {Thermus thermophilus}
EQEEELDLPLEELGLSTRVLHSLKEEGIESVRALLALNLKDLKNIPGIGERSLEEIKEALEK
KGFTLKE
>d1b22a_ a.60.4.1 (A:) DNA repair protein Rad51, N-terminal domain

{Human (Homo sapiens)}
EEESFGPQPISRLEQCGINANDVKKLEEAGFHTVEAVAYAPKKELINIKGISEAKADKILAE
AAKLVPMG
>d1ci4a_ a.60.5.1 (A:) Barrier-to-autointegration factor, BAF
{Human (Homo sapiens)}
MTTSQKHRDFVAEPMGEKPVGSLAGIGEVLGKKLEERGFDKAYVVLGQFLVLKKDEDLFREW
LKDTCGANAKQSRDCFGCLREWCDAFL
>d1bpya1 a.60.6.1 (A:10-91) DNA polymerase beta, N-terminal (8
kD)-domain {Human (Homo sapiens)}
TLNGGITDMLTELANFEKNVSQAIHKYNAYRKAASVIAKYPHKIKSGAEAKKLPGVGTKIAE
KIDEFLATGKLRKLEKIRQD
>d1dk2a_ a.60.6.1 (A:) DNA polymerase beta, N-terminal (8
kD)-domain {Rat (Rattus norvegicus)}
SKRKAPQETLNGGITDMLVELANFEKNVSQAIHKYNAYRKAASVIAKYPHKIKSGAEAKKLP
GVGTKIAEKIDEFLATGKLRKLEK
>d1jmsa1 a.60.6.1 (A:148-242) Terminal deoxynucleotidyl
transferase {Mouse (Mus musculus)}
KKISQYACQRRTTLNNYNQLFTDALDILAENDELRENEGSCLAFMRASSVLKSLPFPITSMK
DTEGIPCLGDKVKSIIEGIIEDGESSEAKAVLN
>d1tfr_1 a.60.7.1 (183-305) T4 RNase H {Bacteriophage T4}
GSAEIDCMTKILKGDKKDNVASVKVRSDFWFTRVEGERTPSMKTSIVEAIANDREQAKVLLT
ESEYNRYKENLVLIDFDYIPDNIASNIVNYYNSYKLPPRGKIYSYFVKAGLSKLTNSINEF
>d1bgxt1 a.60.7.1 (T:174-289) 5' to 3' exonuclease domain of DNA
polymerase Taq {Thermus aquaticus}
LRPDQWADYRALTGDESDNLPGVKGIGEKTARKLLEEWGSLEALLKNLDRLKPAIREKILAH
MDDLKLSWDLAKVRTDLPLEVDFAKRREPDRERLRAFLERLEFGSLLHEFGLLE
>d1xo1a1 a.60.7.1 (A:186-290) T5 5'-exonuclease {Bacteriophage T5}
VDDVEQFISLKAIMGDLGDNIRGVEGIGAKRGYNIIREFGNVLDIIDQLPLPGKQKYIQNLN
ASEELLFRNLILVDLPTYCVDAIAAVGQDVLDKFTKDILEIAE
>d1a77_1 a.60.7.1 (209-316) Flap endonuclease-1 {Archaeon
Methanococcus jannaschii}
ISLDDLIDIAIFMGTDYNPGGVKGIGFKRAYELVRSGVAKDVLKKEVEYYDEIKRIFKEPKV
TDNYSLSLKLPDKEGIIKFLVDENDFNYDRVKKHVDKLYNLIANKT
>d1b43a1 a.60.7.1 (A:220-339) Fen-1 nuclease {Archaeon Pyrococcus
furiosus}
LTREKLIELAILVGTDYNPGGIKGIGLKKALEIVRHSKDPLAKFQKQSDVDLYAIKEFFLNP
PVTDNYNLVWRDPDEEGILKFLCDEHDFSEERVKNGLERLKKAIKSGKQSTLESWFKR
>d1d8ba_ a.60.8.1 (A:) HRDC domain from RecQ helicase {Baker's yeast
(Saccharomyces cerevisiae)}
ELNNLRMTYERLRELSLNLGNRMVPPVGNFMPDSILKKMAAILPMNDSAFATLGTVEDKYRR
RFKYFKATIADLSKKRSSE
>d1go3f_ a.60.8.2 (F:) RNA polymerase II subunit RBP7 (RpoF)
{Archaeon Methanococcus jannaschii}
MIGKKILGERYVTVSEAAEIMYNRAQIGELSYEQGCALDYLQKFAKLDKEEAKKLVEELISL

GIDEKTAVKIADILPEDLDDLRAIYYKRELPENAEEILEIVRKYI
>d1f44a1 a.60.9.1 (A:20-129) Cre recombinase {Bacteriophage P1}
SDEVRKNLMDMFRDRQAFSEHTWKMLLSVCRSWAAWCKLNNRKWFPAEPEDVRDYLLYLQAR
GLAVKTIQQHLGQLNMLHRRSGLPRPSDSNAVSLVMRRIRKENVDAGE
>d1a0p_1 a.60.9.1 (3-100) Recombinase XerD {Escherichia coli}
QDLARIEQFLDALWLEKNLAENTLNAYRRDLSMMVEWLHHRGLTLATAQSDDLQALLAERLE
GGYKATSSARLLSAVRRLFQYLYREKFREDDPSAHL
>d1floa1 a.60.9.1 (A:2-129) Flp recombinase {Baker's yeast (Saccharomyces cerevisiae)}
PQFDILCKTPPKVLVRQFVERFERPSGEKIALCAAELTYLCWMITHNGTAIKRATFMSYNTI
ISNSLSFDIVNKSLQFKYKTQKATILEASLKKLIPAWEFTIIPYYGQKHQSDITDIVSSLQL
QFES
>d1zyma1 a.60.10.1 (A:22-144) Enzyme I of the PEP:sugar phosphotransferase system HPr-binding (sub)domain {Escherichia coli}
DEIVIDRKKISADQVDQEVERFLSGRAKASAQLETIKTKAGETFGEEKEAIFEGHIMLLEDE
ELEQEIIALIKDKHMTADAAAHEVIEGQASALEELDDEYLKERAADVRDIGKRLLRNILGL
>d1jyga_ a.60.11.1 (A:) Hypothetical protein YjbJ {Escherichia coli}
MNKDEAGGNWKQFKGKVKEQWGKLTDDDMTIIEGKRDQLVGKIQERYGYQKDQAEKEVVDWE
TRNEYRW
>d1hiwa_ a.61.1.1 (A:) HIV-1 matrix protein (HIV-1 MA, HIVp17, p17, MA) {Human immunodeficiency virus type 1}
VLSGGELDKWEKIRLRPGGKKQYKLKHIVWASRELERFAVNPGLLETSEGCRQILGQLQPSL
QTGSEELRSLYNTIAVLYCVHQRIDVKDTKEALDKIEEEQNKSKKKAQQAAAD
>d1hiws_ a.61.1.1 (S:) HIV-1 matrix protein (HIV-1 MA, HIVp17, p17, MA) {Human immunodeficiency virus type 1}
VLSGGELDKWEKIRLRPGGKKQYKLKHIVWASRELERFAVNPGLLETSEGCRQILGQLQPSL
QTGSEELRSLYNTIAVLYCVHQRIDVKDTKEALDKIEEEQN
>d1tam__ a.61.1.1 (-) HIV-1 matrix protein (HIV-1 MA, HIVp17, p17, MA) {Human immunodeficiency virus type 1}
MGARASVLSGGELDRWEKIRLRPGGKKKYKLKHIVWASRELERFAVNPGLLETSEGCRQILG
QLQPSLQTGSEELRSLYNTVATLYCVHQRIEIKDTKEALDKIEEEQNKSKKKAQQAAA
>d2hmx__ a.61.1.1 (-) HIV-1 matrix protein (HIV-1 MA, HIVp17, p17, MA) {Human immunodeficiency virus type 1}
HMGARASVLSGGELDKWEKIRLRPGGKKQYKLKHIVWASRELERFAVNPGLLETSEGCRQIL
GQLQPSLQTGSEELRSLYNTIAVLYCVHQRIDVKDTKEALDKIEEEQNKSKKKAQQAAADTG
NNSQVSQNY
>d1ed1a_ a.61.1.1 (A:) SIV matrix antigen {Simian immunodeficiency virus}
SVLSGKKADELEKIRLRPGGKKKYMLKHVVWAANELDRFGLAESLLENKEGCQKILSVLAPL
VPTGSENLKSLYNTVCVIWCIHAEEKVKHTEEAKQIVQRHLVVETGTAETMP
>d1jvr__ a.61.1.2 (-) HTLV-II matrix protein {Human T-cell leukemia virus type 2}

HMGQIHGLSPTPIPKAPRGLSTHHWLNFLQAAYRLQPGPSDFDFQQLRRFLKLALKTPIWLN
PIDYSLLASLIPKGYPGRVVEIINILVKNQVSPSAPAAPVPTPICPTTTPPPPPPPSPEAHV
PPPYVEPTTTQCF
>d1bax__ a.61.1.3 (-) Mason-pfizer monkey virus matrix protein {Simian mason-pfizer virus}
MGQELSQHERYVEQLKQALKTRGVKVKYADLLKFFDFVKDTCPWFPQEGTIDIKRWRRVGDC
FQDYYNTFGPEKVPVTAFSYWNLIKELIDKKE
>d1a6s__ a.61.1.4 (-) GAG polyprotein M-domain {Rous sarcoma virus}
GEAVIKVISSACKTYCGKTSPSKKEIGAMLSLLQKEGLLMSPSDLYSPGSWDPITAALSQRA
MILGKSGELKTWGLVLGALKAAREE
>d1heka_ a.61.1.5 (A:) EIAV matrix antigen {Equine infectious anemia virus, EIAV}
AMADIGSMGDPLTWSKALKKLEKVTVQGSQKLTTGNCNWALSLVDLFHDTNFVKEKDWQLRD
VIPLLEDVTQTLSGQEREAFERTWWAISAVKMGLQINNVVDGKASFQLLRAKYE
>d1qgta_ a.62.1.1 (A:) Hepatitis B viral capsid (hbcag) {Hepatitis B virus}
MDIDPYKEFGATVELLSFLPSDFFPSVRDLLDTASALYREALESPEHCSPHHTALRQAILCW
GELMTLATWVGNNLEDPASRDLVVNYVNTNMGLKIRQLLWFHISCLTFGRETVLEYLVSFGV
WIRTPPAYRPPNAPILST
>d1aep__ a.63.1.1 (-) Apolipophorin-III {African locust (Locusta migratoria)}
NIAEAVQQLNHTIVNAAHELHETLGLPTPDEALNLLTEQANAFKTKIAEVTTSLKQEAEKHQ
GSVAEQLNAFARNLNNSIHDAATSLNLQDQLNSLQSALTNVGHQWQDIATKTQASAQEAWAP
VQSALQEAAEKTKEAAANLQNSIQSAVQK
>d1eq1a_ a.63.1.1 (A:) Apolipophorin-III {Manduca sexta}
DAPAGGNAFEEMEKHAKEFQKTFSEQFNSLVNSKNTQDFNKALKDGSDSVLQQLSAFSSSLQ
GAISDANGKAKEALEQARQNVEKTAEELRKAHPDVEKEANAFKDKLQAAVQTTVQESQKLAK
EVASNMEETNKKLAPKIKQAYDDFVKHAEEVQKKLHEAATKQ
>d1nkl__ a.64.1.1 (-) NK-lysin {Pig (Sus scrofa)}
GYFCESCRKIIQKLEDMVGPQPNEDTVTQAASQVCDKLKILRGLCKKIMRSFLRRISWDILT
GKKPQAICVDIKICKE
>d1qdma1 a.64.1.2 (A:1S-104S) (Pro)phytepsin {Barley (Hordeum vulgare)}
VVSQECKTIVSQYGQQILDLLLAETQPKKICSQVGLCTFDGTRGVSAGIRSVVDDEPVKSNG
LRADPMCSACEMAVVWMQNQLAQNKTQDLILDYVNQLCNRLP
>d1e68a_ a.64.2.1 (A:) Bacteriocin AS-48 {Enterococcus faecalis}
MAKEFGIPAAVAGTVLNVVEAGGWVTTIVSILTAVGSGGLSLLAAAGRESIKAYLKKEIKKK
GKRAVIAW
>d1ain__ a.65.1.1 (-) Annexin I {Human (Homo sapiens)}
GSAVSPYPTFNPSSDVAALHKAIMVKGVDEATIIDILTKRNNAQRQQIKAAYLQETGKPLDE
TLKKALTGHLEEVVLALLKTPAQFDADELRAAMKGLGTDEDTLIEILASRTNKEIRDINRVY
REELKRDLAKDITSDTSGDFRNALLSLAKGDRSEDFGVNEDLADSDARALYEAGERRKGTDV
NVFNTILTTRSYPQLRRVFQKYTKYSKHDMNKVLDLELKGDIEKCLTAIVKCATSKPAFFAE
KLHQAMKGVGTRHKALIRIMVSRSEIDMNDIKAFYQKMYGISLCQAILDETKGDYEKILVAL

CGGN
>d1bo9a_ a.65.1.1 (A:) Annexin I {Human (Homo sapiens)}
TFNPSSDVAALHKAIMVKGVDEATIIDILTKRNNAQRQQIKAAYLQETGKPLDETLKKALTG
HLEEVVLALLK
>d1hm6a_ a.65.1.1 (A:) Annexin I {Pig (Sus scrofa)}
AMVSEFLKQAWFIDNEEQEYIKTVKGSKGGPGSAVSPYPTFNPSSDVEALHKAITVKGVDEA
TIIEILTKRTNAQRQQIKAAYLQEKGKPLDEALKKALTGHLEEVALALLKTPAQFDADELRA
AMKGLGTDEDTLNEILASRTNREIREINRVYKEELKRDLAKDITSDTSGDYQKALLSLAKGD
RSEDLAINDDLADTDARALYEAGERRKGTDLNVFITILTTRSYPHLRRVFQKYSKYSKHDMN
KVLDLELKGDIENCLTVVVKCATSKPMFFAEKLHQAMKGIGTRHKTLIRIMVSRSEIDMNDI
KACYQKLYGISLCQAILDETKGDYEKILVALCG
>d1axn__ a.65.1.1 (-) Annexin III {Human (Homo sapiens)}
SASIWVGHRGTVRDYPDFSPSVDAEAIQKAIRGIGTDEKMLISILTERSNAQRQLIVKEYQA
AYGKELKDDLKGDLSGHFEHLMVALVTPPAVFDAKQLKKSMKGAGTNEDALIEILTTRTSRQ
MKDISQAYYTVYKKSLGDDISSETSGDFRKALLTLADGRRDESLKVDEHLAKQDAQILYKAG
ENRWGTDEDKFTEILCLRSFPQLKLTFDEYRNISQKDIVDSIKGELSGHFEDLLLAIVNCVR
NTPAFLAERLHRALKGIGTDEFTLNRIMVSRSEIDLLDIRTEFKKHYGYSLYSAIKSDTSGD
YEITLLKICGGDD
>d1i4aa_ a.65.1.1 (A:) Annexin IV {Cow (Bos taurus)}
ASGFNAAEDAQTLRKAMKGLGTDEDAIINVLAYRSTAQRQEIRTAYKTTIGRDLMDDLKSEL
SGNFEQVILGMMTPTVLYDVQELRKAMKGAGTDEGCLIEILASRTPEEIRRINQTYQLQYGR
SLEDDIRSDTSFMFQRVLVSLSAGGRDESNYLDDALMRQDAQDLYEAGEKKWGTDEVKFLTV
LCSRNRNHLLHVFDEYKRIAQKDIEQSIKSETSGSFEDALLAIVKCMRNKSAYFAERLYKSM
KGLGTDDDTLIRVMVSRAEIDMLDIRANFKRLYGKSLYSFIKGDTSGDYRKVLLILCGGDD
>d1ala__ a.65.1.1 (-) Annexin V {Chicken (Gallus gallus)}
KYTRGTVTAFSPFDARADAEALRKAMKGMGTDEETILKILTSRNNAQRQEIASAFKTLFGRD
LVDDLKSELTGKFETLMVSLMRPARIFDAHALKHAIKGAGTNEKVLTEILASRTPAEVQNIK
QVYMQEYEANLEDKITGETSGHFQRLLVVLLQANRDPDGRVEEALVEKDAQVLFRAGELKWG
TDEETFITILGTRSVSHLRRVFDKYMTISGFQIEETIDRETSGDLEKLLLAVVKCIRSVPAY
FAETLYYSMKGAGTDDDTLIRVMVSRSEIDLLDIRHEFRKNFAKSLYQMIQKDTSGDYRKAL
LLLCGG
>d1hvd__ a.65.1.1 (-) Annexin V {Human (Homo sapiens)}
VLRGTVTDFPGFDGRADAETLRKAMKGLGTDEESILTLLTSRSNAQRQEISAAFKTLFGRDL
LDDLKSELTGKFEKLIVALMKPSRLYDAYELKHALKGAGTNEKVLTEIIASRTPEELRAIKQ
VYEEEYGSSLEDDVVGDTSGYYQRMLVVLLQANRDPDAGIDEAQVEQDAQALFQAGELKWGT
DEEKFITIFGTRSVSHLRKVFDKYMTISGFQIEETIDRETSGNLEQLLLAVVKSIRSIPAYL
AETLYYAMKGAGTDDHTLIRVMVSRSEIDLFNIRKEFRKNFATSLYSMIKGDTSGDYKKALL
LLC
>d1g5na_ a.65.1.1 (A:) Annexin V {Rat (Rattus norvegicus)}
ALRGTVTDFSGFDGRADAEVLRKAMKGLGTDEDSILNLLTARSNAQRQQIAEEFKTLFGRDL
VNDMKSELTGKFEKLIVALMKPSRLYDAYELKHALKGAGTDEKVLTEIIASRTPEELRAIKQ
AYEEEYGSNLEDDVVGDTSGYYQRMLVVLLQANRDPDTAIDDAQVELDAQALFQAGELKWGT
DEEKFITILGTRSVSHLRRVFDKYMTISGFQIEETIDRETSGNLENLLLAVVKSIRSIPAYL
AETLYYAMKGAGTDDHTLIRVIVSRSEIDLFNIRKEFRKNFATSLYSMIKGDTSGDYKKALL

LLCGGEDD
>d1avc_1 a.65.1.1 (10-350) Annexin VI {Cow (Bos taurus)}
YRGSIRDFPDFNPSQDAETLYNAMKGFGSDKEAIINLITSRSNKQRQEICQNYKSLYGKDLI
ADLKYELTGKFERLIVGLMRPPAYADAKEIKDAISGIGTDEKCLIEILASRTNEQIHQLVAA
YKDAYERDLEADITGDTSGHFRKMLVVLLQGTREEDDVVSEDLVQQDVQDLYEAGELKWGTD
EAQFIYILGNRSKQHLRLVFDEYLKTTGKPIEASIRGELSGDFEKLMLAVVKCIRSTAEYFA
ERLFKAMKGLGTRDNTLIRIMVSRSELDMLDIREIFRTKYEKSLYSMIKNDTSGEYKKTLLK
LCGGDDDAAGQFFPEAAQVAYQMWELSAVAR
>d1avc_2 a.65.1.1 (351-671) Annexin VI {Cow (Bos taurus)}
VELKGTVRPAGDFNPDADAKALRKAMKGLGTDEDTIIDIITHRSNAQRQQIRQTFKSHFGRD
LMADLKSELSGDLARLILGLMMPPAHYDAKQLKKAMEGAGTDEKALIEILATRTNAEIQAIN
KAYKEDYHKTLEDALSSDTSGHFKRILISLATGNREEGGEDRERAREDAQVAAEILEIADTT
SGDKSSLETRFMMILCTRSYPDLRRVFQEFVKMTNYDVEHTIKKEMSGDVRDVFVAIVQSVK
NKPLFFADKLYKSMKGAGTEEKTLTRIMVSRSEIDLLNIRREFIEKYDKSLHQAIEGDTSGH
FLKALLAICGG
>d1dm5a_ a.65.1.1 (A:) Annexin XII {Hydra vulgaris}
VVQGTVKPHASFNSREDAETLRKAMKGIGTDEKSITHILATRSNAQRQQIKTDYTTLFGKHL
EDELKSELSGNYEAAALALLRKPDEFLAEQLHAAMKGLGTDKNALIDILCTQSNAQIHAIKA
AFKLLYKEDLEKEIISETSGNFQRLLVSMLQGGRKEDEPVNAAHAAEDAAAIYQAGEGQIGT
DESRFNAVLATRSYPQLHQIFHEYSKISNKTILQAIENEFSGDIKNGLLAIVKSVENRFAYF
AERLHHAMKGLGTSDKTLIRILVSRSEIDLANIKETFQAMYGKSLYEFIADDCSGDYKDLLL
QITGH
>d1dk5a_ a.65.1.1 (A:) Annexin 24(ca32) {Bell pepper (Capsicum annuum)}
HHHHMASLTVPAHVPSAAEDCEQLRSAFKGWGTNEKLIISILAHRTAAQRKLIRQTYAETFG
EDLLKELDRELTHDFEKLVLVWTLDPSERDAHLAKEATKRWTKSNFVLVELACTRSPKELVL
AREAYHARYKKSLEEDVAYHTTGDHRKLLVPLVSSYRYGGEEVDLRLAKAESKILHEKISDK
AYSDDEVIRILATRSKAQLNATLNHYKDEHGEDILKQLEDGDEFVALLRATIKGLVYPEHYF
VEVLRDAINRRGTEEDHLTRVIATRAEVDLKIIADEYQKRDSIPLGRAIAKDTRGDYESMLL
ALLGQE
>d1azsc1 a.66.1.1 (C:86-201) Transducin (alpha subunit), insertion domain {Cow (Bos taurus)}
GEKATKVQDIKNNLKEAIETIVAAMSNLVPPVELANPENQFRVDYILSVMNVPDFDFPPEFY
EHAKALWEDEGVRACYERSNEYQLIDCAQYFLDKIDVIKQDDYVPSDQDLLRCR
>d1tada1 a.66.1.1 (A:57-177) Transducin (alpha subunit), insertion domain {Cow (Bos taurus)}
YSLEECLEFIAIIYGNTLQSILAIVRAMTTLNIQYGDSARQDDARKLMHMADTIEEGTMPKE
MSDIIQRLWKDSGIQACFDRASEYQLNDSAGYYLSDLERLVTPGYVPTEQDVLRSRVKT
>d1cipa1 a.66.1.1 (A:61-181) Transducin (alpha subunit), insertion domain {Rat (Rattus norvegicus)}
YSEEECKQYKAVVYSNTIQSIIAIIRAMGRLKIDFGDAARADDARQLFVLAGAAEEGFMTAE
LAGVIKRLWKDSGVQACFNRSRSREYQLNDSAAYYLNDLDRIAQPNYIPTQQDVLRTRVKT
>d1ej5a_ a.68.1.1 (A:) Wiscott-Aldrich syndrome protein, WASP, C-terminal domain {Human (Homo sapiens)}

SGFKHVSHVGWDPQNGFDVNNLDPDLRSLFSRAGISEAQLTDAETSKLIYDFIEDQGGLEAV
RQEMRRQGGSGGSQSSEGLVGALMHVMQKRSRAIHSSDEGEDQAG
>d1e79a1 a.69.1.1 (A:380-510) C-terminal domain of alpha and beta subunits of F1 ATP synthase {Cow (Bos taurus)}
TRAMKQVAGTMKLELAQYREVAAFAQFGSDLDAATQQLLSRGVRLTELLKQGQYSPMAIEEQ
VAVIYAGVRGYLDKLEPSKITKFENAFLSHVISQHQALLGKIRTDGKISEESDAKLKEIVTN
FLAGFEA
>d1e79d1 a.69.1.1 (D:358-475) C-terminal domain of alpha and beta subunits of F1 ATP synthase {Cow (Bos taurus)}
MDPNIVGSEHYDVARGVQKILQDYKSLQDIIAILGMDELSEEDKLTVSRARKIQRFLSQPFQ
VAEVFTGHLGKLVPLKETIKGFQQILAGEYDHLPEQAFYMVGPIEEAVAKADKLAE
>d1skyb1 a.69.1.1 (B:372-502) C-terminal domain of alpha and beta subunits of F1 ATP synthase {Bacillus sp., strain ps3}
IKAMKKVAGTLRLDLAAYRELEAFAQFGSDLDKATQANVARGARTVEVLKQDLHQPIPVEKQ
VLIIYALTRGFLDDIPVEDVRRFEKEFYLWLDQNGQHLLEHIRTTKDLPNEDDLNQAIEAFK
KTFVVSQ
>d1skye1 a.69.1.1 (E:357-470) C-terminal domain of alpha and beta subunits of F1 ATP synthase {Bacillus sp., strain ps3}
EIVGEEHYQVARKVQQTLERYKELQDIIAILGMDELSDEDKLVVHRARRIQFFLSQNFHVAE
QFTGQPGSYVPVKETVRGFKEILEGKYDHLPEDRFRLVGRIEEVVEKAKAMG
>d1fx0a1 a.69.1.1 (A:373-501) C-terminal domain of alpha and beta subunits of F1 ATP synthase {Spinach (Spinacia oleracea), chloroplast}
IKAMKKVAGKLKLELAQFAELEAFAQFASDLDKATQNQLARGQRLRELLKQPQSAPLTVEEQ
VMTIYTGTNGYLDSLELDQVRKYLVELRTYVKTNKPEFQEIISSTKTFTEEAEALLKEAIQE
QMERF
>d1fx0b1 a.69.1.1 (B:378-485) C-terminal domain of alpha and beta subunits of F1 ATP synthase {Spinach (Spinacia oleracea), chloroplast}
RIVGEEHYEIAQRVKETLQRYKELQDIIAILGLDELSEEDRLTVARARKIERFLSQPFFVAE
VFTGSPGKYVGLAETIRGFQLILSGELDSLPEQAFYLVGNIDEATA
>d1fkma1 a.69.2.1 (A:249-442) Ypt/Rab-GAP domain of gyp1p {Baker's yeast (Saccharomyces cerevisiae)}
NSIIQRISKFDNILKDKTIINQQDLRQISWNGIPKIHRPVVWKLLIGYLPVNTKRQEGFLQR
KRKEYRDSLKHTFSDQHSRDIPTWHQIEIDIPRTNPHIPLYQFKSVQNSLQRILYLWAIRHP
ASGYVQGINDLVTPFFETFLTEYLPPSQIDDVEIKDPSTYMVDEQITDLEADTFWCLTKLLE
QITDNYIH
>d1fkma2 a.69.2.1 (A:443-630) Ypt/Rab-GAP domain of gyp1p {Baker's yeast (Saccharomyces cerevisiae)}
GQPGILRQVKNLSQLVKRIDADLYNHFQNEHVEFIQFAFRWMNCLLMREFQMGTVIRMWDTY
LSETSQEVTSSYSMSSNDIKPPVTPTEPRVASFVTPTKDFQSPTTALSNMTPNNAVEDSGKM
RQSSLNEFHVFVCAAFLIKWSDQLMEMDFQETITFLQNPPTKDWTETDIEMLLSEAFIWQSL
YK
>d1k5ha1 a.69.3.1 (A:301-398) 1-deoxy-D-xylulose-5-phosphate

reductoisomerase, C-terminal domain {Escherichia coli}
KLSALTFAAPDYDRYPCLKLAMEAFEQGQAATTALNAANEITVAAFLAQQIRFTDIAALNLS
VLEKMDMREPQCVDDVLSVDANAREVARKEVMRLAS
>d1abv__ a.70.1.1 (-) N-terminal domain of the delta subunit of the F1F0-ATP synthase {Escherichia coli}
SEFITVARPYAKAAFDFAVEHQSVERWQDMLAFAAEVTKNEQMAELLSGALAPETLAESFIA
VCGEQLDENGQNLIRVMAENGRLNALPDVLEQFIHLRAVSEAT
>d1g7da_ a.71.1.1 (A:) Endoplasmic reticulum protein ERP29, C-domain {Rat (Rattus norvegicus)}
PGCLPAYDALAGQFIEASSREARQAILKQGQDGLSGVKETDKKWASQYLKIMGKILDQGEDF
PASELARISKLIENKMSEGKKEELQRSLNILTAFRKKGAEKEEL
>d1dvka_ a.72.1.1 (A:) Functional domain of the splicing factor Prp18 {Baker's yeast (Saccharomyces cerevisiae)}
MRIQEAIAQDKTISVIIDPSQIGSTEGKPLLSMKCNLYIHEILSRWKASLEAYHPELFLDTK
KALFPLLLQLRRNQLAPDLLISLATVLYHLQQPKEINLAVQSYMKLSIGNVAWPIGVTSVGI
HARSAHSKIQGGRNAANIMIDERTRLWITSIKRLITFEEWYTSNH
>d1ak4c_ a.73.1.1 (C:) HIV-1 capsid protein {Human immunodeficiency virus type 1}
PIVQNLQGQMVHQAISPRTLNAWVKVVEEKAFSPEVIPMFSALSEGATPQDLNTMLNTVGGH
QAAMQMLKETINEEAAEWDRLHPVHAGPIAPGQMREPRGSDIAGTTSTLQEQIGWMTHNPPI
PVGEIYKRWIILGLNKIVRMY
>d1e6jp2 a.73.1.1 (P:11-147) HIV-1 capsid protein {Human immunodeficiency virus type 1}
VHQAISPRTLNAWVKVVEEKAFSPEVIPMFSALSEGATPQDLNTMLNTVGGHQAAMQMLKET
INEEAAEWDRVHPVHAGPIAPGQMREPRGSDIAGTTSTLQEQIGWMTNNPPIPVGEIYKRWI
ILGLNKIVRMYSP
>d2eiaa2 a.73.1.1 (A:17-147) EIAV capsid protein p26 {Equine infectious anemia virus}
PRGYTTWVNTIQTNGLLNEASQNLFGILSVDCTSEEMNAFLDVVPGQAGQKQILLDAIDKIA
DDWDNRHPLPNAPLVAPPQGPIPMTARFIRGLGVPRERQMEPAFDQFRQTYRQWIIEAMSEG
IKVMIGK
>d1g03a_ a.73.1.1 (A:) HTLV-I capsid protein {Human T-cell leukemia virus type 1}
PVMHPHGAPPNHRPWQMKDLQAIKQEVSQAAPGSPQFMQTIRLAVQQFDPTAKDLQDLLQYL
CSSLVASLHHQQLDSLISEAETRGITSYNPLAGPLRVQANNPQQQGLRREYQQLWLAAFAAL
PGSAKDPSWA
>g1qrj.1 a.73.1.1 (A:,B:16-130) HTLV-I capsid protein {Human T-cell leukemia virus type 1}
HHHHHSSGHIEGRHMXQMKDLQAIKQEVSQAAPGSPQFMQTIRLAVQQFDPTAKDLQDLLQY
LCSSLVASLHHQQLDSLISEAETRGITGYNPLAGPLRVQANNPQQQGLRREYQQLWLAAFAA
LPGSAKD
>d1d1da2 a.73.1.1 (A:11-150) RSV capsid protein {Rous sarcoma virus}
WTPLEPKLITRLADTVRTKGLRSPITMAEVEALMSSPLLPHDVTNLMRVILGPAPYALWMDA

WGVQLQTVIAAATRDPRHPANGQGRGERTNLDRLKGLADGMVGNPQGQAALLRPGELVAITA
SALQAFREVARLAEPA
>d1em9a_ a.73.1.1 (A:) RSV capsid protein {Rous sarcoma virus}
PVVIKTEGPAWTPLEPKLITRLADTVRTKGLRSPITMAEVEALMSSPLLPHDVTNLMRVILG
PAPYALWMDAWGVQLQTVIAAATRDPRHPANGQGRGERTNLNRLKGLADGMVGNPQGQAALL
RPGELVAITASALQAFREVARLA
>d1jsub2 a.74.1.1 (B:310-432) Cyclin A {Human (Homo sapiens)}
TVNQFLTQYFLHQQPANCKVESLAMFLGELSLIDADPYLKYLPSVIAGAAFHLALYTVTGQS
WPESLIRKTGYTLESLKPCLMDLHQTYLKAPQHAQQSIREKYKNSKYHGVSLLNPPETLNL
>d1vin_1 a.74.1.1 (181-308) Cyclin A {Cow (Bos taurus)}
DIHTYLREMEVKCKPKVGYMKKQPDITNSMRAILVDWLVEVGEEYKLQNETLHLAVNYIDRF
LSSMSVLRGKLQLVGTAAMLLASKFEEIYPPEVAEFVYITDDTYTKKQVLRMEHLVLKVLAF
DLAA
>d1vin_2 a.74.1.1 (309-432) Cyclin A {Cow (Bos taurus)}
PTINQFLTQYFLHQQPANCKVESLAMFLGELSLIDADPYLKYLPSVIAAAAFHLALYTVTGQ
SWPESLVQKTGYTLETLKPCLLDLHQTYLRAPQHAQQSIREKYKNSKYHGVSLLNPPETLNL
>d1jkw_1 a.74.1.1 (11-161) Cyclin H (mcs2) {Human (Homo sapiens)}
WTFSSEEQLARLRADANRKFRCKAVANGKVLPNDPVFLEPHEEMTLCKYYEKRLLEFCSVFK
PAMPRSVVGTACMYFKRFYLNNSVMEYHPRIIMLTCAFLACKVDEFNVSSPQFVGNLRESPL
GQEKALEQILEYELLLIQQLNFHLIVH
>d1jkw_2 a.74.1.1 (162-287) Cyclin H (mcs2) {Human (Homo sapiens)}
NPYRPFEGFLIDLKTRYPILENPEILRKTADDFLNRIALTDAYLLYTPSQIALTAILSSASR
AGITMESYLSESLMLKENRTCLSQLLDIMKSMRNLVKKYEPPRSEEVAVLKQKLDRCHSAEL
AL
>d1bu2a1 a.74.1.1 (A:22-148) Viral cyclin {Herpes virus saimiri}
RVLNNLKLRELLLPKFTSLWEIQTEVTVDNRTILLTWMHLLCESFELDKSVFPLSVSILDRY
LCKKQGTKKTLQKIGAACVLIGSKIRTVKPMTVSKLTYLSCDCFTNLELINQEKDILEALKW
DTE
>d1bu2a2 a.74.1.1 (A:149-250) Viral cyclin {Herpes virus saimiri}
AVLATDFLIPLCNALKIPEDLWPQLYEAASTTICKALIQPNIALLSPGLICAGGLLTTIETD
NTNCRPWTCYLEDLSSILNFSTNTVRTVKDQVSEAFSLYD
>d1f5qb1 a.74.1.1 (B:6-146) Viral cyclin {Murine herpes virus gamma
68}
FQGFLDSSLLNEEDCRQMIYRSEREHDARMVGVNVDQHFTSQYRKVLTTWMFCVCKDLRQDN
NVFPLAVALLDELFLSTRIDRENYQSTAAVALHIAGKVRAYMPIKATQLAYLCGGATTADKL
LTLEVKSLDTLSWVADR
>d1f5qb2 a.74.1.1 (B:147-252) Viral cyclin {Murine herpes virus
gamma 68}
CLSTDLICYILHIMHAPREDYLNIYNLCRPKIFCALCDGRSAMKRPVLITLACMHLTMNQKY
DYYENRIDGVCKSLYITKEELHQCCDLVDIAIVSFDENYFKINA
>d1g3nc1 a.74.1.1 (C:16-147) Viral cyclin {Kaposi
sarcoma-associated virus}
LCEDRIFYNILEIEPRFLTSDSVFGTFQQSLTSHMRKLLGTWMFSVCQEYNLEPNVVALALN
LLDRLLLIKQVSKEHFQKTGSACLLVASKLRSLTPISTSSLCYAADSFSRQELIDQEKELL

EKLAWRTE
>d1g3nc2 a.74.1.1 (C:148-253) Viral cyclin {Kaposi sarcoma-associated virus}
AVLATDVTSFLLLKLVGGSQHLDFWHHEVNTLITKALVDPLTGSLPASIISAAGCALLVPAN
VIPQDTHSGGVVPQLASILGCDVSVLQAAVEQILTSVSDFDLRI
>d1vola1 a.74.1.2 (A:113-207) Transcription factor IIB (TFIIB), core domain {Human (Homo sapiens)}
AMMNAFKEITTMADRINLPRNKVDRTNNLFRQAYEQKSLKGRANDAIASACLYIACRQEGVP
RTFKEICAVSRISKKEIGRCFKLILKALETSVD
>d1vola2 a.74.1.2 (A:208-316) Transcription factor IIB (TFIIB), core domain {Human (Homo sapiens)}
LITTGDFMSRFCSNLCLPKQVQMAATHIARKAVELDLVPGRSPISVAAAAIYMASQASAEKR
TQKEIGDIAGVADVTIRQSYRLIYPRAPDLFPTDFKFDTPVDKLPQL
>d1aisb1 a.74.1.2 (B:1108-1205) Transcription factor IIB (TFIIB), core domain {Archaeon Pyrococcus woesei}
NLAFALSELDRITAQLKLPRHVEEEAARLYREAVRKGLIRGRSIESVMAACVYAACRLLKVP
RTLDEIADIARVDKKEIGRSYRFIARNLNLTPKKLF
>d1aisb2 a.74.1.2 (B:1206-1300) Transcription factor IIB (TFIIB), core domain {Archaeon Pyrococcus woesei}
VKPTDYVNKFADELGLSEKVRRRAIEILDEAYKRGLTSGKSPAGLVAAALYIASLLEGEKRT
QREVAEVARVTEVTVRNRYKELVEKLKIKVPIA
>d1guxa_ a.74.1.3 (A:) Retinoblastoma tumor suppressor domains {Human (Homo sapiens)}
NTIQQLMMILNSASDQPSENLISYFNNCTVNPKESILKRVKDIGYIFKEKFAKAVGQGCVEI
GSQRYKLGVRLYYRVMESMLKSEEERLSIQNFSKLLNDNIFHMSLLACALEVVMATYSRSTS
QNLDSGTDLSFPWILNVLNLKAFDFYKVIESFIKAEGNLTREMIKHLERCEHRIMESLAWLS
DSPLFDLIKQSK
>d1guxb_ a.74.1.3 (B:) Retinoblastoma tumor suppressor domains {Human (Homo sapiens)}
TSLSLFYKKVYRLAYLRLNTLCERLLSEHPELEHIIWTLFQHTLQNEYELMRDRHLDQIMMC
SMYGICKVKNIDLKFKIIVTAYKDLPHAVQETFKRVLIKEEEYDSIIVFYNSVFMQRLKTNI
LQYASTRPPTLSPIPHI
>d1k8ke_ a.148.1.1 (E:) Arp2/3 complex 21 kDa subunit ARPC3 {Cow (Bos taurus)}
PAYHSSLMDPDTKLIGNMALLPIRSQFKGPAPRETKDTDIVDEAIYYFKANVFFKNYEIKNE
ADRTLIYITLYISECLKKLQKCNSKSQGEKEMYTLGITNFPIPGEPGFPLNAIYAKPANKQE
DEVMRAYLQQLRQETGLRLCEKVFDPQNDKPSKWWTCFVKRQFMNKSLSG
>d1jfza_ a.149.1.1 (A:) RNase III endonuclease domain {Aquifex aeolicus}
GMKMLEQLEKKLGYTFKDKSLLEKALTHVSYSKKEHYETLEFLGDALVNFFIVDLLVQYSPN
KREGFLSPLKAYLISEEFFNLLAQKLELHKFIRIKRGKINETIIGDVFEALWAAVYIDSGRD
ANFTRELFYKLFKEDILSAIKEGR
>d1hus__ a.75.1.1 (-) Ribosomal protein S7 {Bacillus stearothermophilus}

RDVLPDPIYNSKLVTRLINKIMIDGKKSKAQKILYTAFDIIRERTGKDPMEVFEQALKNVMP
VLEVRARRVGGANYQVPVEVRPDRRVSLGLRWLVQYARLRNEKTMEERLANEIMDAANNTGA
AVKKREDTHKMAEAN
>d1rss__ a.75.1.1 (-) Ribosomal protein S7 {Thermus thermophilus}
LQPDLVYGDVLVTAFINKIMRDGKKNLAARIFYDACKIIQEKTGQEPLKVFKQAVENVKPRM
EVRSRRVGGANYQVPMEVSPRRQQSLALRWLVQAANQRPERRAAVRIAHELMDAAEGKGGAV
KKKEDVERMAEANRAYAHYRW
>d1iqva_ a.75.1.1 (A:) Ribosomal protein S7 {Archaeon Pyrococcus horikoshii}
IKVMGRWSTEDVEVKDPSLKPYINLEPRLLPHTHGRHAKKHFGKANVHIVERLINKVMRSGG
SHYKVAGHFMRREHRSLNSKKVRAYEVVKEAFKIIEKRTGKNPIQVLVWAIENAAPREDTTS
VMFGGIRYHVAVDISPLRRLDVALRNIALGASAKCYRTKMSFAEALAEEIILAANKDPKSYA
YSKKLEIERIAESSR
>d1jr3a1 a.80.1.1 (A:243-368) gamma subunit {Escherichia coli}
TLDDDQALSLVEAMVEANGERVMALINEAAARGIEWEALLVEMLGLLHRIAMVQLSPAALGN
DMAAIELRMRELARTIPPTDIQLYYQTLLIGRKELPYAPDRRMGVEMTLLRALAFHPRMPLP
EP
>d1a5t_1 a.80.1.1 (208-330) delta prime subunit {Escherichia coli}
DNWQARETLCQALAYSVPSGDWYSLLAALNHEQAPARLHWLATLLMDALKRHHGAAQVTNVD
VPGLVAELANHLSPSRLQAILGDVCHIREQLMSVTGINRELLITDLLLRIEHYLQPGVVLP
>d1jr3d1 a.80.1.1 (D:212-338) delta subunit {Escherichia coli}
FTPFHWVDALLMGKSKRALHILQQLRLEGSEPVILLRTLQRELLLLVNLKRQSAHTPLRALF
DKHRVWQNRRGMMGEALNRLSQTQLRQAVQLLTRTELTLKQDYGQSVWAELEGLSLLLCHKP
LAD
>d1iqpa1 a.80.1.1 (A:233-327) Replication factor C {Archaeon Pyrococcus furiosus}
RARPEDIREMMLLALKGNFLKAREKLREILLKQGLSGEDVLVQMHKEVFNLPIEEPKKVLLA
DKIGEYNFRLVEGANEIIQLEALLAQFTLIGKK
>d1jr5a_ a.150.1.1 (A:) Anti-sigma factor Asia {Bacteriophage T4}
MNKNIDTVREIITVASILIKFSREDIVENRANFIAFLNEIGVTHEGRKLNQNSFRKIVSELT
QEDKKTLIDEFNEGFEGVYRYLEMYTNK
>d1f5ta2 a.76.1.1 (A:1065-1121) Diphtheria toxin repressor (DtxR) {Corynebacterium diphtheriae}
TPTGRTLATAVMRKHRLAERLLTDIIGLDINKVHDEADRWEHVMSDEVERRLVKVLK
>d1g3sa2 a.76.1.1 (A:65-140) Diphtheria toxin repressor (DtxR) {Corynebacterium diphtheriae}
TPTGRTLATAVMRKHRLAERLLTDIIGLDINKVHDEASRWEHVMSDEVERRLVKVLKDVSRS
PFGNPIPGLDELGV
>d1fx7a2 a.76.1.1 (A:65-144) Iron-dependent regulator {Mycobacterium tuberculosis}
TEKGRALAIAVMRKHRLAERLLVDVIGLPWEEVHAEACRWEHVMSEDVERRLVKVLNNPTTS
PFGNPIPGLDELGVGPEP
>d1gpja1 a.151.1.1 (A:303-404) Glutamyl tRNA-reductase dimerization domain {Archaeon Methanopyrus kandleri}

EIPKVEKLIEEELSTVEEELEKLKERRLVADVAKSLHEIKDRELERALRRLKTGDPENVLQD
FAEAYTKRLINVLTSAIMELPDEYRRAASRALRRASELNG
>d1ngr__ a.77.1.1 (-) p75 low affinity neurotrophin receptor {Rat (Rattus norvegicus)}
GNLYSSLPLTKREEVEKLLNGDTWRHLAGELGYQPEHIDSFTHEACPVRALLASWGAQDSAT
LDALLAALRRIQRADIVESLCSE
>d1ddf__ a.77.1.1 (-) Fas {Human (Homo sapiens)}
METVAINLSDVDLSKYITTIAGVMTLSQVKGFVRKNGVNEAKIDEIKNDNVQDTAEQKVQLL
RNWHQLHGKKEAYDTLIKDLKKANLCTLAEKIQTIILKDITSDSENSNFRNEIQSLVLEHHH
HHH
>d1a1w__ a.77.1.1 (-) FADD (Mort1) {Human (Homo sapiens)}
MDPFLVLLHSVSSSLSSSELTELKYLCLGRVGKRKLERVQSGLDLFSMLLEQNDLEPGHTEL
LRELLASLRRHDLLRRVDDFE
>d1e41a_ a.77.1.1 (A:) FADD (Mort1) {Human (Homo sapiens)}
GSHMGEEDLCAAFNVICDNVGKDWRRLARQLKVSDTKIDSIEDRYPRNLTERVRESLRIWKN
TEKENATVAHLVGALRSCQMNLVADLVQEVQQARDLQNRSGA
>d1fada_ a.77.1.1 (A:) FADD (Mort1) {Mouse (Mus musculus)}
AAPPGEAYLQVAFDIVCDNVGRDWKRLARELKVSEAKMDGIEEKYPRSLSERVRESLKVWKN
AEKKNASVAGLVKALRTCRLNLVADLVEEAQES
>d3crd__ a.77.1.1 (-) Raidd CARD domain {Human (Homo sapiens)}
MEARDKQVLRSLRLELGAEVLVEGLVLQYLYQEGILTENHIQEINAQTTGLRKTMLLLDILP
SRGPKAFDTFLDSLQEFPWVREKLKKAREEAMTDLPAG
>d1cy5a_ a.77.1.1 (A:) Apoptotic protease activating factor 1, APAF-1 {Human (Homo sapiens)}
MDAKARNCLLQHREALEKDIKTSYIMDHMISDGFLTISEEEKVRNEPTQQQRAAMLIKMILK
KDNDSYVSFYNALLHEGYKDLAALLHDGIPV
>d3ygsp_ a.77.1.1 (P:) Procaspase 9 prodomain {Human (Homo sapiens)}
SMDEADRRLLRRCRLRLVEELQVDQLWDVLLSRELFRPHMIEDIQRAGSGSRRDQARQLIID
LETRGSQALPLFISCLEDTGQDMLASFLRTNRQAG
>d1dgna_ a.77.1.1 (A:) Iceberg {Human (Homo sapiens)}
ADQLLRKKRRIFIHSVGAGTINALLDCLLEDEVISQEDMNKVRDENDTVMDKARVLIDLVTG
KGPKSCCKFIKHLCEEDPQLASKMGLH
>d1d2za_ a.77.1.1 (A:) Pelle death domain {Drosophila melanogaster}
LDNTMAIRLLPLPVRAQLCAHLDALDVWQQLATAVKLYPDQVEQISSQKQRGRSASNEFLNI
WGGQYNHTVQTLFALFKKLKLHNAMRLIKDYVSEDLHKYI
>d1d2zb_ a.77.1.1 (B:) Tube death domain {Drosophila melanogaster}
LSSKYSRNTELRRVEDNDIYRLAKILDENSCWRKLMSIIPKGMDVQACSGAGCLNFPAEIKK
GFKYTAQDVFQIDEAANRLPPDQSKSQMMIDEWKTSGKLNERPTVGVLLQLLVQAELFSAAD
FVALDFLNESTPARPVDGPGALISLE
>d1hw1a2 a.78.1.1 (A:79-230) Fatty acid responsive transcription factor FadR, C-terminal domain {Escherichia coli}
GLNILETLARLDHESVPQLIDNLLSVRTNISTIFIRTAFRQHPDKAQEVLATANEVADHADA

FAELDYNIFRGLAFASGNPIYGLILNGMKGLYTRIGRHYFANPEARSLALGFYHKLSALCSE
GAHDQVYETVRRYGHESGEIWHRMQKNL
>d1eyva_ a.79.1.1 (A:) Antitermination factor NusB {Mycobacterium tuberculosis}
GRHQARKRAVALLFEAEVRGISAAEVVDTRAALAEAKPDIARLHPYTAAVARGVSEHAAHID
DLITAHLRGWTLDRLPAVDRAILRVSVWELLHAADVPEPVVVDEAVQLAKELSTDDSPGFVN
GVLGQVM
>d1ey1a_ a.79.1.1 (A:) Antitermination factor NusB {Escherichia coli}
MKPAARRRARECAVQALYSWQLSQNDIADVEYQFLAEQDVKDVDVLYFRELLAGVATNTAYL
DGLMKPYLSRLLEELGQVEKAVLRIALYELSKRSDVPYKVAINEAIELAKSFGAEDSHKFVN
GVLDKAAPVIRPNKK
>d1b79a_ a.81.1.1 (A:) N-terminal domain of DnaB helicase {Escherichia coli}
PPHSIEAEQSVLGGLMLDNERWDDVAERVVADDFYTRPHRHIFTEMARLQESGSPIDLITLA
ESLERQGQLDSVGGFAYLAELSKNTPSAANISAYADIVRE
>d1jwea_ a.81.1.1 (A:) N-terminal domain of DnaB helicase {Escherichia coli}
MKVPPHSIEAEQSVLGGLMLDNERWDDVAERVVADDFYTRPHRHIFTEMARLQESGSPIDLI
TLAESLERQGQLDSVGGFAYLAELSKNTPSAANISAYADIVRERAVVREMIS
>d1qc7a_ a.82.1.1 (A:) FliG C-terminal domain {Thermotoga maritima}
MFVFEDILKLDDRSIQLVLREVDTRDLALALKGASDELKEKIFKNMSKRAAALLKDELEYMG
PVRLKDVEEAQQKIINIIRRLEEAGEIVIARGGGEELIM
>d1qc7b_ a.82.1.1 (B:) FliG C-terminal domain {Thermotoga maritima}
MFVFEDILKLDDRSIQLVLREVDTRDLALALKGASDELKEKIFKNMSKRAAALLKDELEYMG
PVRLKDVEEAQQKIINIIRRLEEAGEIV
>d1crka1 a.83.1.1 (A:1-98) Creatine kinase, N-terminal domain {Chicken (Gallus gallus), mitochondria}
TVHEKRKLFPPSADYPDLRKHNNCMAECLTPAIYAKLRDKLTPNGYSLDQCIQTGVDNPGHP
FIKTVGMVAGDEESYEVFAEIFDPVIKARHNGYDPR
>d1qh4a1 a.83.1.1 (A:2-102) Creatine kinase, N-terminal domain {Chicken (Gallus gallus), brain-type}
PFSNSHNLLKMKYSVDDEYPDLSVHNNHMAKVLTLDLYKKLRDRQTSSGFTLDDVIQTGVDN
PGHPFIMTVGCVAGDEESYEVFKELFDPVIEDRHGGYKP
>d1qk1a1 a.83.1.1 (A:1-102) Creatine kinase, N-terminal domain {Human (Homo sapiens), mitochondria}
AASERRRLYPPSAEYPDLRKHNNCMASHLTPAVYARLCDKTTPTGWTLDQCIQTGVDNPGHP
FIKTVGMVAGDEETYEVFADLFDPVIQERHNGYDPRTMKH
>d2crka1 a.83.1.1 (A:8-102) Creatine kinase, N-terminal domain {Rabbit (Oryctolagus cuniculus)}
NKYKLNYKSEEEYPDLSKHNNHMAKVLTPDLYKKLRDKETPSGFTLDDVIQTGVDNPGHPFI
MTVGCVAGDEESYTVFKDLFDPIIQDRHGGFKP

>d1g0wa1 a.83.1.1 (A:2-102) Creatine kinase, N-terminal domain {Cow (Bos taurus), retinal isoform}
PFSNSHNTLKLRFPAEDEFPDLSGHNNHMAKVLTPELYAELRAKSTPSGFTVDDVIQTGVDN
PGHPYIMTVGCVAGDEESYDVFKELFDPIIEDRHGGYKP
>d1bg0_1 a.83.1.1 (2-95) Arginine kinase {Horseshoe crab (Limulus polyphemus)}
VDQATLDKLEAGFKKLQEASDCKSLLKKHLTKDVFDSIKNKKTGMGATLLDVIQSGVENLDS
GVGIYAPDAESYRTFGPLFDPIIDDYHGGFKL
>d1al01_ a.84.1.1 (1:) Scaffolding protein gpD of bacteriophage procapsid {Bacteriophage phi-X174}
EQSVRFQTALASIKLIQASAVLDLTEDDFDFLTSNKVWIATDRSRARRCVEACVYGTLDFVG
YPRFPAPVEFIAAVIAYYVHPVNIQTACLIMEGAEFTENIINGVERPVKAAELFAFTLRVRA
GNTDVLTDAEENVRQKLRA
>d1ll1_1 a.85.1.1 (1-109) Hemocyanin, N-terminal domain {Horseshoe crab (Limulus polyphemus)}
TLHDKQIRVCHLFEQLSSATVIRLKNVGKLQPGAIFSCFHPDHLEEARHLYEVFWEAGDFND
FIEIAKEARTFVNEGLFAFAAEVAVLHRDDCKGLYVP
>d1lla_1 a.85.1.1 (2-109) Hemocyanin, N-terminal domain {Horseshoe crab (Limulus polyphemus)}
LHDKQIRICHLFEQLSSATVIGDGDKHKHSDRLKNVGKLQPGAIFSCFHPDHLEEARHLYEV
FWEAGDFNDFIEIAKEARTFVNEGLFAFAAEVAVLHRDDCKGLYVP
>d1hc2_1 a.85.1.1 (5-135) Hemocyanin, N-terminal domain {Spiny lobster (Panulirus interruptus)}
TGNAQKQQDINHLLDKIYEPTKYPDLKDIAENFNPLGDTSIYNDHGAAVETLMKELNDHRLL
EQRHWYSLFNTRQRKEALMLFAVLNQCKEWYCFRSNAAYFRERMNEGEFVYALYVSVIHSKL
GDGIVLP
>d1lla_2 a.86.1.1 (110-379) Hemocyanin {Horseshoe crab (Limulus polyphemus)}
PVQEIFPDKFIPSAAINEAFKKAHVRPEFDESPILVDVQDTGNILDPEYRLAYYREDVGINA
HHWHWHLVYPSTWNPKYFGKKKDRKGELFYYMHQQMCARYDCERLSNGMHRMLPFNNFDEPL
AGYAPHLTHVASGKYYSPRPDGLKLRDLGDIEISEMVRMRERILDSIHLGYVISEDGSHKTL
DELHGTDILGALVESSYESVNHEYYGNLHNWGHVTMARIHDPDGRFHEEPGVMSDTSTSLRD
PIFYNWHRFIDNIFHEYKNTLK
>d1hc2_2 a.86.1.1 (136-398) Hemocyanin {Spiny lobster (Panulirus interruptus)}
PLYQITPHMFTNSEVIDKAYSAKMTQKPGTFNVSFTGTKKNREQRVAYFGEDIGMNIHHVTW
HMDFPFWWEDSYGYHLDRKGELFFWVHHQLTARFDFERLSNWLDPVDELHWDRIIREGFAPL
TSYKYGGEFPVRPDNIHFEDVDGVAHVHDLEITESRIHEAIDHGYITDSDGHTIDIRQPKGI
ELLGDIIESSKYSSNVQYYGSLHNTAHVMLGRQGDPHGKFNLPPGVMEHFETATRDPSFFRL
HKYMDNIFKKHTDSF
>d1js8a1 a.86.1.1 (A:2503-2791) Functional unit from octopus hemocyanin {Giant octopus (Octopus dofleini)}
AIIRKNVNSLTPSDIKELRDAMAKVQADTSDNGYQKIASYHGIPLSCHYENGTAYACCQHGM
VTFPNWHRLLTKQMEDALVAKGSHVGIPYWDWTTTFANLPVLVTEEKDNSFHHAHIDVANTD

TTRSPRAQLFDDPDKGDKSFFYRQIALALEQTDFCDFEIQFEIGHNAIHSWVGGSSPYGMST
LHYTSYDPLFYLHHSNTDRIWSVWQALQKYRGLPYNTANCEINKLVKPLKPFNLDTNPNAVT
KAHSTGATSFDYHKLGYDYDNLNFHGMTIPELEEHLKEIQH
>d1bt3a_ a.86.1.2 (A:) Catechol oxidase {Sweet potato (Ipomoea batatas)}
APIQAPEISKCVVPPADLPPGAVVDNCCPPVASNIVDYKLPAVTTMKVRPAAHTMDKDAIAK
FAKAVELMKALPADDPRNFYQQALVHCAYCNGGYDQVNFPDQEIQVHNSWLFFPFHRWYLYF
YERILGKLIGDPSFGLPFWNWDNPGGMVLPDFLNDSTSSLYDSNRNQSHLPPVVVDLGYNGA
DTDVTDQQRITDNLALMYKQMVTNAGTAELFLGKAYRAGDAPSPGAGSIETSPHIPIHRWVG
DPRNTNNEDMGNFYSAGRDIAFYCHHSNVDRMWTIWQQLAGKPRKRDYTDSDWLNATFLFYD
ENGQAVKVRIGDSLDNQKMGYKYAKTPLPWL
>d1dbha1 a.87.1.1 (A:198-404) Son of sevenless-1 (sos-1) {Human (Homo sapiens)}
EQTYYDLVKAFMAEIRQYIRELNLIIKVFREPFVSNSKLFSANDVENIFSRIVDIHELSVKL
LGHIEDTVEMTDEGSPHPLVGSCFEDLAEELAFDPYESYARDILRPGFHDRFLSQLSKPGAA
LYLQSIGEGFKEAVQYVLPRLLLAPVYHCLHYFELLKQLEEKSEDQEDKECLKQAITALLNV
QSGMEKICSKSLAKRRLSESA
>d1by1a_ a.87.1.1 (A:) beta-pix {Human (Homo sapiens)}
MKGFDTTAINKSYYNVVLQNILETENEYSKELQTVLSTYLRPLQTSEKLSSANISYLMGNLE
EICSFQQMLVQSLEECTKLPEAQQRVGGCFLNLMPQMKTLYLTYCANHPSAVNVLTEHSEEL
GEFMETKGASSPGILVLTTGLSKPFMRLDKYPTLLKELERHMEDYHTDRQDIQKSMAAFKNL
SAQCQEVRKRKELELQILTEAIR
>d1f5xa_ a.87.1.1 (A:) RhoGEF Vav {Mouse (Mus musculus)}
MKGDEIYEDLMRLESVPTPPKMTEYDKRCCCLREIQQTEEKYTDTLGSIQQHFMKPLQRFLK
PQDMETIFVNIEELFSVHTHFLKELKDALAGPGATTLYQVFIKYKERFLVYGRYCSQVESAS
KHLDQVATAREDVQMKLEECSQRANNGRFTLRDLLMVPMQRVLKYHLLLQELVKHTQDATEK
ENLRLALDAMRDLAQCVNEVKR
>d1foea1 a.87.1.1 (A:1034-1239) GEF of TIAM1 (T-Lymphoma invasion and metastasis indusing protein 1) {Mouse (Mus musculus)}
QLSDADKLRKVICELLETERTYVKDLNCLMERYLKPLQKETFLTQDELDVLFGNLTEMVEFQ
VEFLKTLEDGVRLVPDLEKLEKVDQFKKVLFSLGGSFLYYADRFKLYSAFCASHTKVPKVLV
KAKTDTAFKAFLDAQNPRQQHSSTLESYLIKPIQRVLKYPLLLRELFALTDAESEEHYHLDV
AIKTMNKVASHINEMQKIHE
>d1boua_ a.88.1.1 (A:) LigA subunit of an aromatic-ring-opening dioxygenase LigAB {Sphingomonas paucimobilis, formerly Pseudomonas paucimobilis}
IDVHAYLAEFDDIPGTRVFTAQRARKGYNLNQFAMSLMKAENRERFKADESAYLDEWNLTPA
AKAAVLARDYNAMIDEGGNVYFLSKLFSTDGKSFQFAAGSMTGMTQEEYAQMMIDGGRSPAG
VRSIKGGY
>d1hbna1 a.89.1.1 (A:270-549) Alpha chain {Archaeon Methanobacterium thermoautotrophicum}
RRARGENEPGGVPFGYLADICQSSRVNYEDPVRVSLDVVATGAMLYDQIWLGSYMSGGVGFT
QYATAAYTDNILDDFTYFGKEYVEDKYGLCEAPNNMDTVLDVATEVTFYGLEQYEEYPALLE
DQFGGSQRAAVVAAAAGCSTAFATGNAQTGLSGWYLSMYLHKEQHSRLGFYGYDLQDQCGAS

NVFSIRGDEGLPLELRGPNYPNYAMNVGHQGEYAGISQAPHAARGDAFVFNPLVKIAFADDN
LVFDFTNVRGEFAKGALREFEPAGERALITPA
>d1e6va1 a.89.1.1 (A:273-552) Alpha chain {Archaeon Methanopyrus kandleri}
RRARGENEPGGVPFGVLADCVQTMRKYPDDPAKVALEVIAAGAMLYDQIWLGSYMSGGVGFT
QYATAVYPDNILDDYVYYGLEYVEDKYGIAEAEPSMDVVKDVATEVTLYGLEQYERYPAAME
THFGGSQRAAVCAAAAGCSTAFATGHAQAGLNGWYLSQILHKEGQGRLGFYGYALQDQCGAA
NSLSVRSDEGLPLELRGPNYPNYAMNVGHLGEYAGIVQAAHAARGDAFCVHPVIKVAFADEN
LVFDFTEPRKEFAKGALREFEPAGERDLIVPA
>d1e6ya1 a.89.1.1 (A:1284-1569) Alpha chain {Archaeon Methanosarcina barkeri}
RRARGPNEPGGLSFGHLSDIVQTSRVSEDPAKIALEVVGAGCMLYDQIWLGSYMSGGVGFTQ
YATAAYTDDILDNNTYYDVDYINDKYNGAATVGKDNKVKASLEVVKDIATESTLYGIETYEK
FPTALEDHFGGSQRATVLAAAAGVACSLATGNANAGLSGWYLSMYLHKEAWGRLGFFGFDLQ
DQCGATNVLSYQGDEGLPDELRGPNYPNYAMNVGHQGGYAGIAQAAHSGRGDAFTVNPLLKV
CFADDLLPFNFAEPRREFGRGAIREFVPAGERSLVIPA
>d1hbnb1 a.89.1.1 (B:189-443) Beta chain {Archaeon Methanobacterium thermoautotrophicum}
GYALRNIMVNHVVAATLKNTLQAAALSTILEQTAMFEMGDAVGAFERMHLLGLAYQGMNADN
LVFDLVKANGKEGTVGSVIADLVERALEDGVIKVEKELTDYKVYGTDDLAMWNAYAAAGLMA
ATMVNQGAARAAQGVSSTLLYYNDLIEFETGLPSVDFGKVEGTAVGFSFFSHSIYGGGGPGI
FNGNHIVTRHSKGFAIPCVAAAMALDAGTQMFSPEATSGLIKEVFSQVDEFREPLKYVVEAA
AEIKNEI
>d1e6vb1 a.89.1.1 (B:190-442) Beta chain {Archaeon Methanopyrus kandleri}
GYALRNIMVNHIVAATRKNTMQAVCLAATLQQTAMFEMGDALGPFERLHLLGYAYQGLNADN
MVYDIVKKHGKEGTVGTVVREVVERALEDGVIEVKEELPSFKVYKANDMDLWNAYAAAGLVA
AVMVNQGAARAAQGVSATILYYNDLLEYETGLPGVDFGRAEGTAVGFSFFSHSIYGGGGPGI
FHGNHIVTRHSKGFAIPPVAAAMALDAGTQMFSPEVTSKLIGDVFGEIDEFREPMKYITEAA
AEEAK
>d1e6yb1 a.89.1.1 (B:2186-2433) Beta chain {Archaeon Methanosarcina barkeri}
GFSLRNIMANHVAAISNRNAMNASALSSIYEQSGIFEMGGAVGMFERHQLLGLAYQGLNANN
LLYDIVKENGKDGTIGTVIESVVRRAIEAGIISVDKTAPSGYNFYKANDVPKWNACAAVGTL
AATLVNCGAGRAAQNVSSTLLYFNDILEKETGLPGCDYGKVEGTAVGFSFFSHSIYGGGGPG
VFNGNHVVTRHSRGFAIPCVCAAVALDAGTQMFSIESTSGLIGDVFGAIPEFREPIKAVAGV
>d1bgf__ a.90.1.1 (-) Transcription factor STAT-4 N-domain {Mouse (Mus musculus)}
GGSQWNQVQQLEIKFLEQVDQFYDDNFPMEIRHLLAQWIETQDWEVASNNETMATILLQNLL
IQLDEQLGRVSKEKNLLLIHNLKRIRKVLQGKFHGNPMHVAVVISNCLREERRILAAANMPI
>d1agre_ a.91.1.1 (E:) Regulator of G-protein signalling 4, RGS4 {Rat (Rattus norvegicus)}
VSQEEVKKWAESLENLINHECGLAAFKAFLKSEYSEENIDFWISCEEYKKIKSPSKLSPKAK
KIYNEFISVQATKEVNLDSCTREETSRNMLEPTITCFDEAQKKIFNLMEKDSYRRFLKSRFY

LDLT
>d1fqia_ a.91.1.1 (A:) RGS9, RGS domain {Cow (Bos taurus)}
KLVDIPTKMRVERWAFNFSELIRDPKGRQSFQHFLRKEFSGENLGFWEACEDLKYGDQSKVK
EKAEEIYKLFLAPGARRWINIDGKTMDITVKGLKHPHRYVLDAAQTHIYMLMKKDSYARYLK
SPIYKEMLAKAIEP
>d1cmza_ a.91.1.1 (A:) Galpha interacting protein, GaIP {Human (Homo sapiens)}
PSPEEVQSWAQSFDKLMHSPAGRSVFRAFLRTEYSEENMLFWLACEELKAEANQHVVDEKAR
LIYEDYVSILSPKEVSLDSRVREGINKKMQEPSAHTFDDAQLQIYTLMHRDSYPRFLSSPTY
RALL
>d1dk8a_ a.91.1.1 (A:) Axin RGS-homologous domain {Human (Homo sapiens)}
GSASPTPPYLKWAESLHSLLDDQDGISLFRTFLKQEGCADLLDFWFACTGFRKLEPCDSNEE
KRLKLARAIYRKYILDNNGIVSRQTKPATKSFIKGCIMKQLIDPAMFDQAQTEIQATMEENT
YPSFLKSDIYLEYTRTGSESPKV
>d1emua_ a.91.1.1 (A:) Axin RGS-homologous domain {Human (Homo sapiens)}
PPYLKWAESLHSLLDDQDGISLFRTFLKQEGCADLLDFWFACTGFRKLEPCDSNEEKRLKLA
RAIYRKYILDNNGIVSRQTKPATKSFIKGCIMKQLIDPAMFDQAQTEIQATMEENTYPSFLK
SDIYLEYT
>d1iapa_ a.91.1.1 (A:) p115RhoGEF {Human (Homo sapiens)}
SQFQSLEQVKRRPAHLMALLQHVALQFEPGPLLCCLHADMLGSLGPKEAKKAFLDFYHSFLE
KTAVLRVPVPPNVAFELDRTRADLISEDVQRRFVQEVVQSQQVAVGRQLEDFRSKRLMGMTP
WEQELAQLEAWVGRDRASYEARERHVAERLLMHLEEMQHTISTDEEKSAAVVNAIGLYMRHL
GVRT
>d1htjf_ a.91.1.1 (F:) Pdz-RhoGEF RGS-like domain {Human (Homo sapiens)}
ESDIIFQDLEKLKSRPAHLGVFLRYIFSQADPSPLLFYLCAEVYQQASPKDSRSLGKDIWNI
FLEKNAPLRVKIPEMLQAEIDSRLRNSEDARGVLCEAQEAAMPEIQEQIHDYRTKRTLGLGS
LYGENDLLDLDGDPLRERQVAEKQLAALGDILSAYAADRSAPMDFALNTYMSHAGIRL
>d1a9xa1 a.92.1.1 (A:403-555) Carbamoyl phosphate synthetase, large subunit connection domain {Escherichia coli}
EVGATGFDPKVSLDDPEALTKIRRELKDAGADRIWYIADAFRAGLSVDGVFNLTNIDRWFLV
QIEELVRLEEKVAEVGITGLNADFLRQLKRKGFADARLAKLAGVREAEIRKLRDQYDLHPVY
KRVDTCAAEFATDTAYMYSTYEEECEANP
>d1b80a_ a.93.1.1 (A:) Lignin peroxidase {White rot basidiomycete (Phanerochaete chrysosporium)}
VIEKRATCSNGKTVGDASCCAWFDVLDDIQQNLFHGGQCGAEAHESIRLVFHDSIAISPAME
AQGKFGGGGADGSIMIFDDIETAFHPNIGLDEIVKLQKPFVQKHGVTPGDFIAFAGAVALSN
CPGAPQMNFFTGRAPATQPAPDGLVPEPFHTVDQIINRVNDAGEFDELELVWMLSAHSVAAV
NDVDPTVQGLPFDSTPGIFDSQFFVETQLRGTAFPGSGGNQGEVESPLPGEIRIQSDHTIAR
DSRTACEWQSFVNNQSKLVDDFQFIFLALTQLGQDPNAMTDCSDVIPQSKPIPGNLPFSFFP
AGKTIKDVEQACAETPFPTLTTLPGPETSVQRIPPPPGA
>d1llp__ a.93.1.1 (-) Lignin peroxidase {White rot basidiomycete

(Phanerochaete chrysosporium)}
ATCANGKTVGDASCCAWFDVLDDIQANMFHGGQCGAEAHESIRLVFHDSIAISPAMEAKGKF
GGGGADGSIMIFDTIETAFHPNIGLDEVVAMQKPFVQKHGVTPGDFIAFAGAVALSNCPGAP
QMNFFTGRKPATQPAPDGLVPEPFHTVDQIIARVNDAGEFDELELVWMLSAHSVAAVNDVDP
TVQGLPFDSTPGIFDSQFFVETQFRGTLFPGSGGNQGEVESGMAGEIRIQTDHTLARDSRTA
CEWQSFVGNQSKLVDDFQFIFLALTQLGQDPNAMTDCSDVIPLSKPIPGNGPFSFFPPGKSH
SDIEQACAETPFPSLVTLPGPATSVARIPPHKA
>d1qpaa_ a.93.1.1 (A:) Lignin peroxidase {White rot basidiomycete
(Phanerochaete chrysosporium)}
VACPDGVHTASNAACCAWFPVLDDIQQNLFHGGQCGAEAHEALRMVFHDSIAISPKLQSQGK
FGGGGADGSIITFSSIETTYHPNIGLDEVVAIQKPFIAKHGVTPGDFIAFAGAVGVSNCPGA
PQMQFFLGRPEATQAAPDGLVPEPFHTIDQVLARMLDAGGFDEIETVWLLSAHSIAAANDVD
PTISGLPFDSTPGQFDSQFFVETQLRGTAFPGKTGIQGTVMSPLKGEMRLQTDHLFARDSRT
ACEWQSFVNNQTKLQEDFQFIFTALSTLGHDMNAMIDCSEVIPAPKPVNFGPSFFPAGKTHA
DIEQACASTPFPTLITAPGPSASVARIPPPPSPN
>d1aru__ a.93.1.1 (-) Peroxidase {Arthromyces ramosus}
SVTCPGGQSTSNSQCCVWFDVLDDLQTNFYQGSKCESPVRKILRIVFHDAIGFSPALTAAGQ
FGGGGADGSIIAHSNIELAFPANGGLTDTIEALRAVGINHGVSFGDLIQFATAVGMSNCPGS
PRLEFLTGRSNSSQPSPPSLIPGPGNTVTAILDRMGDAGFSPDEVVDLLAAHSLASQEGLNS
AIFRSPLDSTPQVFDTQFYIETLLKGTTQPGPSLGFAEELSPFPGEFRMRSDALLARDSRTA
CRWQSMTSSNEVMGQRYRAAMAKMSVLGFDRNALTDCSDVIPSAVSNNAAPVIPGGLTVDDI
EVSCPSEPFPEIATASGPLPSLAPAP
>d1jdra_ a.93.1.1 (A:) Cytochrome c peroxidase, CCP {Baker's yeast
(Saccharomyces cerevisiae)}
TTPLVHVASVEKGRSYEDFQKVYNAIALKLREDDEYDNYIGYGPVLVRLAWHTSGTWDKHDN
TGGSYGGTYRFKKEFNDPSNAGLQNGFKFLEPIHKEFPWISSGDLFSLGGVTAVQEMQGPKI
PWRCGRVDTPEDTTPDNGRLPDADKDADYVRTFFQRLNMNDREVVALMGAHTLGKTHLKNSG
YEGPWTANNNVFDNSFYLNLLNEDWKLEKNDANNEQWDSKSGYMMLPTDYSLIQDPKYLSIV
KEYANDQDKFFKDFSKAFEKLLENGITFPKDAPSPFIFKTLEEQGL
>d1mn2__ a.93.1.1 (-) Manganese peroxidase {Basidomycetos fungus
(Phanerochaete chrysosporium)}
AVCPDGTRVSHAACCAFIPLAQDLQETIFQNECGQDAHEVIRLTFHDAIAISRSQGPKAGGG
ADGSMLLFPTVEPNFSANNGIDDSVNNLIPFMQKHNTISAADLVQFAGAVALSNCPGAPRLE
FLAGRPNKTIAAVDGLIPEPQDSVTKILQRFEDAGGFTPFEVVSLLASHSVARANKVDQTID
AAPFDSTPFTFDTQVFLEVLLKGVGFPGSANNTGEVASPLPLGSGSDTGEMRLQSDFALAHD
PRTACIWQGFVNEQAFMAASFRAAMSKLAVLGHNRNSLIDCSDVVPVPKPATGQPAMFPAST
GPQDLELSCPSERFPTLTTQPGASQSLIAHCPDGSMSCPGVQFNGPA
>d1apxa_ a.93.1.1 (A:) Ascorbate peroxidase {Pea (Pisum sativum)}
GKSYPTVSPDYQKAIEKAKRKLRGFIAEKKCAPLILRLAWHSAGTFDSKTKTGGPFGTIKHQ
AELAHGANNGLDIAVRLLEPIKEQFPIVSYADFYQLAGVVAVEITGGPEVPFHPGREDKPEP
PPEGRLPDATKGSDHLRDVFGKAMGLSDQDIVALSGGHTIGAAHKERSGFEGPWTSNPLIFD
NSYFTELLTGEKDGLLQLPSDKALLTDSVFRPLVEKYAADEDVFFADYAEAHLKLSELGFAE
A
>d7atja_ a.93.1.1 (A:) Plant peroxidase {Horseradish (Armoracia

rusticana)}
QLTPTFYDNSCPNVSNIVRDTIVNELRSDPRIAASILRLHFHDCFVNGCDASILLDNTTSFR
TEKDAFGNANSARGFPVIDRMKAAVESACPRTVSCADLLTIAAQQSVTLAGGPSWRVPLGRR
DSLQAFLDLANANLPAPFFTLPQLKDSFRNVGLNRSSDLVALSGGHTFGKNQCRFIMDRLYN
FSNTGLPDPTLNTTYLQTLRGLCPLNGNLSALVDFDLRTPTIFDNKYYVNLEEQKGLIQSDQ
ELFSSPNATDTIPLVRSFANSTQTFFNAFVEAMDRMGNITPLTGTQGQIRLNCRVVN
>d1scha_ a.93.1.1 (A:) Plant peroxidase {Peanut (Arachis
hypogaea)}
ELSSNFYATKCPNALSTIKSAVNSAVAKEARMGASLLRLHFHDCFVQGCDASVLLDDTSNFT
GEKTAGPNANSIRGFEVIDTIKSQVESLCPGVVSCADILAVAARDSVVALGGASWNVLLGRR
DSTTASLSSANSDLPAPFFNLSGLISAFSNKGFTTKELVTLSGAHTIGQAQCTAFRTRIYNE
SNIDPTYAKSLQANCPSVGGDTNLSPFDVTTPNKFDNAYYINLRNKKGLLHSDQQLFNGVST
DSQVTAYSNNAATFNTDFGNAMIKMGNLSPLTGTSGQIRTNCRKTN
>d1fhfa_ a.93.1.1 (A:) Plant peroxidase {Soybean (Glycine max)}
QLTPTFYRETCPNLFPIVFGVIFDASFTDPRIGASLMRLHFHDCFVQGCDGSVLLNNTDTIE
SEQDALPNINSIRGLDVVNDIKTAVENSCPDTVSCADILAIAAEIASVLGGGPGWPVPLGRR
DSLTANRTLANQNLPAPFFNLTQLKASFAVQGLNTLDLVTLSGGHTFGRARCSTFINRLYNF
SNTGNPDPTLNTTYLEVLRARCPQNATGDNLTNLDLSTPDQFDNRYYSNLLQLNGLLQSDQE
LFSTPGADTIPIVNSFSSNQNTFFSNFRVSMIKMGNIGVLTGDEGEIRLQCNFVNG
>d1bgp__ a.93.1.1 (-) Plant peroxidase {Barley (Hordeum vulgare),
peroxidase 1}
AEPPVAPGLSFDFYWQTCPRAESIVREFVQEAVRKDIGLAAGLLRLHFHDCFVQGCDASVLL
DGSATGPGEQQAPPNLTLRPSAFKAVNDIRDRLERECRGAVVSCSDILALAARDSVVVSGGP
DYRVPLGRRDSRSFASTQDVLSDLPGPSSNVQSLLALLGRLGLDATDLVTISGGHTIGLAHC
SSFEDRLFPRPDPTISPTFLSRLKRTCPAKGTDRRTVLDVRTPNVFDNKYYIDLVNREGLFV
SDQDLFTNAITRPIVERFAQSQQDFFEQFGVSIGKMGQMRVRTSDQGEVRRNCSVRNPGPG
>d1qgja_ a.93.1.1 (A:) Plant peroxidase {Mouse-ear cress
(Arabidopsis thaliana), peroxidase N}
QLSPDIYAKSCPNLVQIVRKQVAIALKAEIRMAASLIRLHFHDCFVNGCDASLLLDGADSEK
LAIPNINSARGFEVIDTIKAAVENACPGVVSCADILTLAARDSVVLSGGPGWRVALGRKDGL
VANQNSANNLPSPFEPLDAIIAKFVAVNLNITDVVALSGAHTFGQAKCAVFSNRLFNFTGAG
NPDATLETSLLSNLQTVCPLGGNSNITAPLDRSTTDTFDNNYFKNLLEGKGLLSSDQILFSS
DLAVNTTKKLVEAYSRSQSLFFRDFTCAMIRMGNISNGASGEVRTNCRVINN
>d1pa2a_ a.93.1.1 (A:) Plant peroxidase {Mouse-ear cress
(Arabidopsis thaliana), peroxidase A2}
MQLNATFYSGTCPNASAIVRSTIQQALQSDTRIGASLIRLHFHDCFVNGCDASILLDDTGSI
QSEKNAGPNVNSARGFNVVDNIKTALENACPGVVSCSDVLALASEASVSLAGGPSWTVLLGR
RDSLTANLAGANSSIPSPIESLSNITFKFSAVGLNTNDLVALSGAHTFGRARCGVFNNRLFN
FSGTGNPDPTLNSTLLSTLQQLCPQNGSASTITNLDLSTPDAFDNNYFANLQSNDGLLQSDQ
ELFSTTGSSTIAIVTSFASNQTLFFQAFAQSMINMGNISPLTGSNGEIRLDCKKVNGS
>g1cxp.1 a.93.1.2 (A:,C:) Myeloperoxidase {Human (Homo sapiens)}
CPEQDKYRTITGMCNNRRSPTLGASNRAFVRWLPAEYEDGFSLPYGWTPGVKRNGFPVALAR
AVSNEIVRFPTDQLTPDQERSLMFMQWGQLLDHDLDFTPEPAXVNCETSCVQQPPCFPLKIP
PNDPRIKNQADCIPFFRSCPACPGSNITIRNQINALTSFVDASMVYGSEEPLARNLRNMSNQ

LGLLAVNQRFQDNGRALLPFDNLHDDPCLLTNRSARIPCFLAGDTRSSEMPELTSMHTLLLR
EHNRLATELKSLNPRWDGERLYQEARKIVGAMVQIITYRDYLPLVLGPTAMRKYLPTYRSYN
DSVDPRIANVFTNAFRYGHTLIQPFMFRLDNRYQPMEPNPRVPLSRVFFASWRVVLEGGIDP
ILRGLMATPAKLNRQNQIAVDEIRERLFEQVMRIGLDLPALNMQRSRDHGLPGYNAWRRFCG
LPQPETVGQLGTVLRNLKLARKLMEQYGTPNNIDIWMGGVSEPLKRKGRVGPLLACIIGTQF
RKLRDGDRFWWENEGVFSMQQRQALAQISLPRIICDNTGITTVSKNNIFMSNSYPRDFVNCS
TLPALNLASWREA
>d1eqga1 a.93.1.2 (A:74-583) Prostaglandin H2 synthase {Sheep (Ovis aries)}
IWTWLRTTLRPSPSFIHFLLTHGRWLWDFVNATFIRDTLMRLVLTVRSNLIPSPPTYNIAHD
YISWESFSNVSYYTRILPSVPRDCPTPMGTKGKKQLPDAEFLSRRFLLRRKFIPDPQGTNLM
FAFFAQHFTHQFFKTSGKMGPGFTKALGHGVDLGHIYGDNLERQYQLRLFKDGKLKYQMLNG
EVYPPSVEEAPVLMHYPRGIPPQSQMAVGQEVFGLLPGLMLYATIWLREHNRVCDLLKAEHP
TWGDEQLFQTARLILIGETIKIVIEEYVQQLSGYFLQLKFDPELLFGAQFQYRNRIAMEFNQ
LYHWHPLMPDSFRVGPQDYSYEQFLFNTSMLVDYGVEALVDAFSRQPAGRIGGGRNIDHHIL
HVAVDVIKESRVLRLQPFNEYRKRFGMKPYTSFQELTGEKEMAAELEELYGDIDALEFYPGL
LLEKCHPNSIFGESMIEMGAPFSLKGLLGNPICSPEYWKASTFGGEVGFNLVKTATLKKLVC
LNTKTCPYVSFHVP
>d1cvua1 a.93.1.2 (A:74-583) Prostaglandin H2 synthase {Mouse (Mus musculus)}
FLTRIKLLLKPTPNTVHYILTHFKGVWNIVNNIPFLRSLIMKYVLTSRSYLIDSPPTYNVHY
GYKSWEAFSNLSYYTRALPPVADDCPTPMGVKGNKELPDSKEVLEKVLLRREFIPDPQGSNM
MFAFFAQHFTAQFFKTDHKRGPGFTRGLGHGVDLNHIYGETLDRQHKLRLFKDGKLKYQVIG
GEVYPPTVKDTQVEMIYPPHIPENLQFAVGQEVFGLVPGLMMYATIWLREHQRVCDILKQEH
PEWGDEQLFQTSKLILIGETIKIVIEDYVQHLSGYHFKLKFDPELLFNQQFQYQNRIASEFN
TLYHWHPLLPDTFNIEDQEYSFKQFLYNNSILLEHGLTQFVESFTRQIAGRVAGGRNVPIAV
QAVAKASIDQSREMKYQSLNEYRKRFSLKPYTSFEELTGEKEMAAELKALYSDIDVMELYPA
LLVEKPRPDAIFGETMVELGAPFSLKGLMGNPICSPQYWKPSTFGGEVGFKIINTASIQSLI
CNNVKGCPFTSFNVQ
>d1jj2o_ a.94.1.1 (O:) Ribosomal protein L19 (L19e) {Archaeon Haloarcula marismortui}
TDLSAQKRLAADVLDVGKNRVWFNPERQGDIADAITREDVRELVDEGAIQAKDKKGNSRGRA
RERQKKRAKGHQKGAGSRKGKAGARQNSKEDWESRIRAQRTKLRELRDEGTLSSSQYRDLYD
KAGGGEFDSVADLERYIDA
>d1aa7a_ a.95.1.1 (A:) Influenza virus matrix protein M1 {Influenza virus}
MSLLTEVETYVLSIIPSGPLKAEIAQRLEDVFAGKNTDLEVLMEWLKTRPILSPLTKGILGF
VFTLTVPSERGLQRRRFVQNALNGNGDPNNMDKAVKLYRKLKREITFHGAKEISLSYSAGAL
ASCMGLIYNRMGAVTTEVAFGLVCATCEQIADSQ
>d2abk__ a.96.1.1 (-) Endonuclease III {Escherichia coli}
MNKAKRLEILTRLRENNPHPTTELNFSSPFELLIAVLLSAQATDVSVNKATAKLYPVANTPA
AMLELGVEGVKTYIKTIGLYNSKAENIIKTCRILLEQHNGEVPEDRAALEALPGVGRKTANV
VLNTAFGWPTIAVDTHIFRVCNRTQFAPGKNVEQVEEKLLKVVPAEFKVDCHHWLILHGRYT
CIARKPRCGSCIIEDLCEYKEKVDI

>d1mun__ a.96.1.2 (-) Catalytic domain of MutY {Escherichia coli}
MQASQFSAQVLDWYDKYGRKTLPWQIDKTPYKVWLSEVMLQQTQVATVIPYFERFMARFPTV
TDLANAPLDEVLHLWTGLGYYARARNLHKAAQQVATLHGGKFPETFEEVAALPGVGRSTAGA
ILSLSLGKHFPILNGNVKRVLARCYAVSGWPGKKEVENKLWSLSEQVTPAVGVERFNQAMMD
LGAMICTRSKPKCSLCPLQNGCIAAANNSWALYPGKKPK
>d1keaa_ a.96.1.2 (A:) Thymine-DNA glycosylase {Archaeon Methanobacterium thermoformicicum}
DATNKKRKVFVSTILTFWNTDRRDFPWRHTRDPYVILITEILLRRTTAGHVKKIYDKFFVKY
KCFEDILKTPKSEIAKDIKEIGLSNQRAEQLKELARVVINDYGGRVPRNRKAILDLPGVGKY
TCAAVMCLAFGKKAAMVDANFVRVINRYFGGSYENLNYNHKALWELAETLVPGGKCRDFNLG
LMDFSAIICAPRKPKCEKCGMSKLCSYYEKC
>d1mpga1 a.96.1.3 (A:100-282) 3-Methyladenine DNA glycosylase II (gene alkA or aidA) {Escherichia coli}
AARPGLRLPGCVDAFEQGVRAILGQLVSVAMAAKLTARVAQLYGERLDDFPEYICFPTPQRL
AAADPQALKALGMPLKRAEALIHLANAALEGTLPMTIPGDVEQAMKTLQTFPGIGRWTANYF
ALRGWQAKDVFLPDDYLIKQRFPGMTPAQIRRYAERWKPWRSYALLHIWYTEGWQPDEA
>d1ko9a1 a.96.1.3 (A:136-323) 8-oxoguanine glycosylase {Human (Homo sapiens)}
DPIECLFSFICSSNNNIARITGMVERLCQAFGPRLIQLDDVTYHGFPSLQALAGPEVEAHLR
KLGLGYRARYVSASARAILEEQGGLAWLQQLRESSYEEAHKALCILPGVGTKVADCICLMAL
DKPQAVPVDVHMWHIAQRDYSWHPTTSQAKGPSPQTNKELGNFFRSLWGPYAGWAQAVLFSA
DL
>d1gln_1 a.97.1.1 (306-468) Anticodon-binding (C-terminal) domain of glutamyl-tRNA synthetase (GluRS) {Thermus thermophilus}
DLEKLRWMNGKYIREVLSLEEVAERVKPFLREAGLSWESEAYLRRAVELMRPRFDTLKEFPE
KARYLFTEDYPVSEKAQRKLEEGLPLLKELYPRLRAQEEWTEAALEALLRGFAAEKGVKLGQ
VAQPLRAALTGSLETPGLFEILALLGKERALRRLERALA
>d1rlr_1 a.98.1.1 (10-221) R1 subunit of ribonucleotide reductase, N-terminal domain {Escherichia coli}
RDGSTERINLDKIHRVLDWAAEGLHNVSISQVELRSHIQFYDGIKTSDIHETIIKAAADLIS
RDAPDYQYLAARLAIFHLRKKAYGQFEPPALYDHVVKMVEMGKYDNHLLEDYTEEEFKQMDT
FIDHDRDMTFSYAAVKQLEGKYLVQNRVTGEIYESAQFLYILVAACLFSNYPRETRLQYVKR
FYDAVSTFKISLPTPIMSGVRTPTRQ
>d1dnpa1 a.99.1.1 (A:201-469) FAD-binding (C-terminal) domain of DNA photolyase {Escherichia coli}
PVEEKAAIAQLRQFCQNGAGEYEQQRDFPAVEGTSRLSASLATGGLSPRQCLHRLLAEQPQA
LDGGAGSVWLNELIWREFYRHLITYHPSLCKHRPFIAWTDRVQWQSNPAHLQAWQEGKTGYP
IVDAAMRQLNSTGWMHNRLRMITASFLVKDLLIDWREGERYFMSQLIDGDLAANNGGWQWAA
STGTDAAPYFRIFNPTTQGEKFDHEGEFIRQWLPELRDVPGKVVHEPWKWAQKAGVTLDYPQ
PIVEHKEARVQTLAAYEAARK
>d1iqra1 a.99.1.1 (A:172-416) FAD-binding (C-terminal) domain of DNA photolyase {Thermus thermophilus}
LPLPEPGEEAALAGLRAFLEAKLPRYAEERDRLDGEGGSRLSPYFALGVLSPRLAAWEAERR
GGEGARKWVAELLWRDFSYHLLYHFPWMAERPLDPRFQAFPWQEDEALFQAWYEGKTGVPLV

DAAMRELHATGFLSNRARMNAAQFAVKHLLLPWKRCEEAFRHLLLDGDRAVNLQGWQWAGGL
GVDAAPYFRVFNPVLQGERHDPEGRWLKRWAPEYPSYAPKDPVVDLEEARRRYLRLARD
>d1qnf_1 a.99.1.1 (205-475) FAD-binding (C-terminal) domain of DNA photolyase {Anacystis nidulans}
PVEPGETAAIARLQEFCDRAIADYDPQRNFPAEAGTSGLSPALKFGAIGIRQAWQAASAAHA
LSRSDEARNSIRVWQQELAWREFYQHALYHFPSLADGPYRSLWQQFPWENREALFTAWTQAQ
TGYPIVDAAMRQLTETGWMHNRCRMIVASFLTKDLIIDWRRGEQFFMQHLVDGDLAANNGGW
QWSASSGMDPKPLRIFNPASQAKKFDATATYIKRWLPELRHVHPKDLISGEITPIERRGYPA
PIVNHNLRQKQFKALYNQLKAAI
>d2pgd_1 a.100.1.1 (177-473) 6-phosphogluconate dehydrogenase (6PGD) {Sheep (Ovis orientalis aries)}
GAGHFVKMVHNGIEYGDMQLICEAYHLMKDVLGLGHKEMAKAFEEWNKTELDSFLIEITASI
LKFQDADGKHLLPKIRDSAGQKGTGKWTAISALEYGVPVTLIGEAVFARCLSSLKDERIQAS
KKLKGPQNIPFEGDKKSFLEDIRKALYASKIISYAQGFMLLRQAATEFGWTLNYGGIALMWR
GGCIIRSVFLGKIKDAFDRNPGLQNLLLDDFFKSAVENCQDSWRRAISTGVQAGIPMPCFTT
ALSFYDGYRHAMLPANLIQAQRDYFGAHTYELLAKPGQFIHTNWTGHGG
>d1pgja1 a.100.1.1 (A:179-478) 6-phosphogluconate dehydrogenase (6PGD) {Trypanosoma brucei}
GAGSCVKMYHNSGEYAILQIWGEVFDILRAMGLNNDEVAAVLEDWKSKNFLKSYMLDISIAA
ARAKDKDGSYLTEHVMDRIGSKGTGLWSAQEALEIGVPAPSLNMAVVSRQFTMYKTERQANA
SNAPGITQSPGYTLKNKSPSGPEIKQLYDSVCIAIISCYAQMFQCLREMDKVHNFGLNLPAT
IATFRAGCILQGYLLKPMTEAFEKNPNISNLMCAFQTEIRAGLQNYRDMVALITSKLEVSIP
VLSASLNYVTAMFTPTLKYGQLVSLQRDVFGRHGYERVDKDGRESFQWPELQ
>d1qmga1 a.100.1.2 (A:308-595) Acetohydroxy acid isomeroreductase, ketoacid reductoisomerase (KARI) {Spinach (Spinacia oleracea)}
LEQEYKSDIFGERGILLGAVHGIVECLFRRYTESGMSEDLAYKNTVECITGVISKTISTKGM
LALYNSLSEEGKKDFQAAYSASYYPSMDILYECYEDVASGSEIRSVVLAGRRFYEKEGLPAF
PMGKIDQTRMWKVGEKVRSVRPAGDLGPLYPFTAGVYYALMMAQIEILRKKGHSYSEIINES
VIEAVDSLNPFMHARGVSFMVDNCSTTARLGSRKWAPRFDYILSQQALVAVDNGAPINQDLI
SNFLSDPVHEAIGVCAQLRPSVDISVTADADFVRPELRQA
>d1f0ya1 a.100.1.3 (A:204-302) Short chain L-3-hydroxyacyl CoA dehydrogenase {Human (Homo sapiens)}
GFIVNRLLVPYLMEAIRLYERGDASKEDIDTAMKLGAGYPMGPFELLDYVGLDTTKFIVDGW
HEMDAENPLHQPSPSLNKLVAENKFGKKTGEGFYKYK
>d3hdha1 a.100.1.3 (A:204-302) Short chain L-3-hydroxyacyl CoA dehydrogenase {Pig (Sus scrofa)}
GFIVNRLLVPYLIEAVRLYERGDASKEDIDTAMKLGAGYPMGPFELLDYVGLDTTKFIIDGW
HEMDSQNPLFQPSPAMNKLVAENKFGKKTGEGFYKYK
>d1dlja1 a.100.1.4 (A:197-294) UDP-glucose dehydrogenase (UDPGDH), middle domain {Streptococcus pyogenes}
ASEAEAVKLFANTYLALRVAYFNELDTYAESRKLNSHMIIQGISYDDRIGMHYNNPSFGYGG
YSLPKDTKQLLANYNNIPQTLIEAIVSSNNVRKSYI
>d1bg6_1 a.100.1.5 (188-359) N-(1-D-carboxylethyl)-L-norvaline dehydrogenase {Arthrobacter, strain 1c}

NVLHTSLTNVNAVMHPLPTLLNAARCESGTPFQYYLEGITPSVGSLAEKVDAERIAIAKAFD
LNVPSVCEWYKESYGQSPATIYEAVQGNPAYRGIAGPINLNTRYFFEDVSTGLVPLSELGRA
VNVPTPLIDAVLDLISSLIDTDFRKEGRTLEKLGLSGLTAAGIRSAVE
>d1evya1 a.100.1.6 (A:189-357) Glycerol-3-phosphate dehydrogenase {Trypanosome (Leishmania mexicana)}
DRSFVCWATTDTVGCEVASAVKNVLAIGSGVANGLGMGLNARAALIMRGLLEIRDLTAALGG
DGSAVFGLAGLGDLQLTCSSELSRNFTVGKKLGKGLPIEEIQRTSKAVAEGVATADPLMRLA
KQLKVKMPLCHQIYEIVYKKKNPRDALADLLSCGLQDEGLPPLFK
>d1ks9a1 a.100.1.7 (A:168-291) Ketopantoate reductase PanE {Escherichia coli}
NIRAELWRKLAVNCVINPLTAIWNCPNGELRHHPQEIMQICEEVAAVIEREGHHTSAEDLRD
YVMQVIDATAENISSMLQDIRALRHTEIDYINGFLLRRARAHGIAVPENTRLFEMVKRKESE
>d1utg__ a.101.1.1 (-) Uteroglobin {Rabbit (Oryctolagus cuniculus)}
GICPRFAHVIENLLLGTPSSYETSLKEFEPDDTMKDAGMQMKKVLDSLPQTTRENIMKLTEK
IVKSPLCM
>d1ccd__ a.101.1.1 (-) Clara cell 17kDa protein {Rat (Rattus norvegicus)}
SSDICPGFLQVLEALLLGSESNYEAALKPFNPASDLQNAGTQLKRLVDTLPQETRINIVKLT
EKILTSPLCEQDLRV
>d1utra_ a.101.1.1 (A:) Clara cell 17kDa protein {Rat (Rattus norvegicus)}
ICPGFLQVLEALLLGSESNYEAALKPFNPASDLQNAGTQLKRLVDTLPQETRINIVKLTEKI
LTSPLC
>d1gai__ a.102.1.1 (-) Glucoamylase {Aspergillus awamori, variant x100}
ATLDSWLSNEATVARTAILNNIGADGAWVSGADSGIVVASPSTDNPDYFYTWTRDSGLVIKT
LVDLFRNGDTDLLSTIEHYISSQAIIQGVSNPSGDLSSGGLGEPKFNVDETAYTGSWGRPQR
DGPALRATAMIGFGQWLLDNGYTSAATEIVWPLVRNDLSYVAQYWNQTGYDLWEEVNGSSFF
TIAVQHRALVEGSAFATAVGSSCSWCDSQAPQILCYLQSFWTGSYILANFDSSRSGKDTNTL
LGSIHTFDPEAGCDDSTFQPCSPRALANHKEVVDSFRSIYTLNDGLSDSEAVAVGRYPEDSY
YNGNPWFLCTLAAAEQLYDALYQWDKQGSLEITDVSLDFFKALYSGAATGTYSSSSSTYSSI
VSAVKTFADGFVSIVETHAASNGSLSEQFDKSDGDELSARDLTWSYAALLTANNRRNSVVPP
SWGETSASSVPGTCAATSASGTYSSVTVTSWPSIVATG
>d1ayx__ a.102.1.1 (-) Glucoamylase {Baker's yeast (Saccharomycopsis fibuligera)}
AYPSFEAYSNYKVDRTDLETFLDKQKEVSLYYLLQNIAYPEGQFNNGVPGTVIASPSTSNPD
YYYQWTRDSAITFLTVLSELEDNNFNTTLAKAVEYYINTSYNLQRTSNPSGSFDDENHKGLG
EPKFNTDGSAYTGAWGRPQNDGPALRAYAISRYLNDVNSLNEGKLVLTDSGDINFSSTEDIY
KNIIKPDLEYVIGYWDSTGFDLWEENQGRHFFTSLVQQKALAYAVDIAKSFDDGDFANTLSS
TASTLESYLSGSDGGFVNTDVNHIVENPDLLQQNSRQGLDSATYIGPLLTHDIGESSSTPFD
VDNEYVLQSYYLLLEDNKDRYSVNSAYSAGAAIGRYPEDVYNGDGSSEGNPWFLATAYAAQV
PYKLAYDAKSASNDITINKINYDFFNKYIVDLSTINSAYQSSDSVTIKSGSDEFNTVADNLV
TFGDSFLQVILDHINDDGSLNEQLNRYTGYSTGAYSLTWSSGALLEAIRLRNKVKALA

>d1cem__ a.102.1.2 (-) CelA cellulase {Clostridium thermocellum}
AGVPFNTKYPYGPTSIADNQSEVTAMLKAEWEDWKSKRITSNGAGGYKRVQRDASTNYDTVS
EGMGYGLLLAVCFNEQALFDDLYRYVKSHFNGNGLMHWHIDANNNVTSHDGGDGAATDADED
IALALIFADKQWGSSGAINYGQEARTLINNLYNHCVEHGSYVLKPGDRWGGSSVTNPSYFAP
AWYKVYAQYTGDTRWNQVADKCYQIVEEVKKYNNGTGLVPDWCTASGTPASGQSYDYKYDAT
RYGWRTAVDYSWFGDQRAKANCDMLTKFFARDGAKGIVDGYTIQGSKISNNHNASFIGPVAA
ASMTGYDLNFAKELYRETVAVKDSEYYGYYGNSLRLLTLLYITGNFPNPLSDL
>d1tf4a1 a.102.1.2 (A:1-460) Endo/exocellulase:cellobiose E-4, N-terminal domain {Thermomonospora fusca}
EPAFNYAEALQKSMFFYEAQRSGKLPENNRVSWRGDSGLNDGADVGLDLTGGWYDAGDHVKF
GFPMAFTATMLAWGAIESPEGYIRSGQMPYLKDNLRWVNDYFIKAHPSPNVLYVQVGDGDAD
HKWWGPAEVMPMERPSFKVDPSCPGSDVAAETAAAMAASSIVFADDDPAYAATLVQHAKQLY
TFADTYRGVYSDCVPAGAFYNSWSGYQDELVWGAYWLYKATGDDSYLAKAEYEYDFLSTEQQ
TDLRSYRWTIAWDDKSYGTYVLLAKETGKQKYIDDANRWLDYWTVGVNGQRVPYSPGGMAVL
DTWGALRYAANTAFVALVYAKVIDDPVRKQRYHDFAVRQINYALGDNPRNSSYVVGFGNNPP
RNPHHRTAHGSWTDSIASPAENRHVLYGALVGGPGSPNDAYTDDRQDYVANEVATDYNAGFS
SALAMLVEEYGGTPLADFPPTEEPDG
>d1clc_1 a.102.1.2 (135-575) CelD cellulase {Clostridium thermocellum}
AMNVYEDAFKTAMLGMYLLRCGTSVSATYNGIHYSHGPCHTNDAYLDYINGQHTKKDSTKGW
HDAGDYNKYVVNAGITVGSMFLAWEHFKDQLEPVALEIPEKNNSIPDFLDELKYEIDWILTM
QYPDGSGRVAHKVSTRNFGGFIMPENEHDERFFVPWSSAATADFVAMTAMAARIFRPYDPQY
AEKCINAAKVSYEFLKNNPANVFANQSGFSTGEYATVSDADDRLWAAAEMWETLGDEEYLRD
FENRAAQFSKKIEADFDWDNVANLGMFTYLLSERPGKNPALVQSIKDSLLSTADSIVRTSQN
HGYGRTLGTTYYWGCNGTVVRQTMILQVANKISPNNDYVNAALDAISHVFGRNYYNRSYVTG
LGINPPMNPHDRRSGADGIWEPWPGYLVGGGWPGPKDWVDIQDSYQTNEIAINWNAALIYAL
AGFVNYN
>d1faea_ a.102.1.2 (A:) Processive endocellulase CelF (Cel48F) {Clostridium cellulolyticum}
ASSPANKVYQDRFESMYSKIKDPANGYFSEQGIPYHSIETLMVEAPDYGHVTTSEAMSYYMW
LEAMHGRFSGDFTGFDKSWSVTEQYLIPTEKDQPNTSMSRYDANKPATYAPEFQDPSKYPSP
LDTSQPVGRDPINSQLTSAYGTSMLYGMHWILDVDNWYGFGARADGTSKPSYINTFQRGEQE
STWETIPQPCWDEHKFGGQYGFLDLFTKDTGTPAKQFKYTNAPDADARAVQATYWADQWAKE
QGKSVSTSVGKATKMGDYLRYSFFDKYFRKIGQPSQAGTGYDAAHYLLSWYYAWGGGIDSTW
SWIIGSSHNHFGYQNPFAAWVLSTDANFKPKSSNGASDWAKSLDRQLEFYQWLQSAEGAIAG
GATNSWNGRYEAVPSGTSTFYGMGYVENPVYADPGSNTWFGMQVWSMQRVAELYYKTGDARA
KKLLDKWAKWINGEIKFNADGTFQIPSTIDWEGQPDTWNPTQGYTGNANLHVKVVNYGTDLG
CASSLANTLTYYAAKSGDETSRQNAQKLLDAMWNNYSDSKGISTVEQRGDYHRFLDQEVFVP
AGWTGKMPNGDVIKSGVKFIDIRSKYKQDPEWQTMVAALQAGQVPTQRLHRFWAQSEFAVAN
GVYAILFPD
>d1fp3a_ a.102.1.3 (A:) N-acyl-D-glucosamine 2-epimerase {Pig (Sus scrofa)}
MEKERETLQAWKERVGQELDRVMAFWLEHSHDREHGGFFTCLGRDGRVYDDLKYVWLQGRQV
WMYCRLYRKLERFHRPELLDAAKAGGEFLLRHARVAPPEKKCAFVLTRDGRPVKVQRSIFSE

CFYTMAMNELWRVTAEARYQSEAVDMMDQIVHWVREDPSGLGRPQLPGAVASESMAVPMMLL
CLVEQLGEEDEELAGRYAQLGHWCARRILQHVQRDGQAVLENVSEDGEELSGCLGRHQNPGH
ALEAGWFLLRHSSRSGDAKLRAHVIDTFLLLPFRSGWDADHGGLFYFQDADGLCPTQLEWAM
KLWWPHSEAMIAFLMGYSESGDPALLRLFYQVAEYTFRQFRDPEYGEWFGYLNREGKVALTI
KGGPFKGCFHVPRCLAMCEEMLSALLSRLA
>d1h54a1 a.102.1.4 (A:269-753) Lactobacillus maltose phosphorylase, central domain {Lactobacillus brevis}
DTQESLTAAMHQLSDKVAQSSYEDLLNAHTAIWAQRWEKSDVVIKGDDESQQGIRFNLFQLF
STYYGEDARLNIGPKGFTGEKYGGATYWDTEAFAFPVYLGITDPKVTRNLLMYRYKQLDGAY
INAQEQGLKGALFPMVTFDGIECHNEWEITFEEIHRNGDIAFAIYNYTRYTGDDSYVLHEGA
KVLTEISRFWADRVHFSKRNNQYMIHGVTGADEYENNVDNNWDTNMLAQWTLKYTLEILGKV
DQDTAKQLDVSDEEKTKWQDIVDRMYLPYDKDLNIFVQHDGFLDKDIEPVSSIPADQRPINQ
NWSWDKILRSPYIKQGDVLQGIWDFIDDYTPEQKKANFDFYEPLTVHESSLSPAIHSVLAAD
LHYEDKAVELYSRTARLDLDNYNNDTTDGLHITSMTGAWIAVVQGFAGMRVRDGQLHYAPFL
PKTWTSYTFRQVFRDRLIEVSVHADGPHFKLLSGEPLTIDVAGAAAAAAAA
>d1dl2a_ a.102.2.1 (A:) Class I alpha-1;2-mannosidase, catalytic domain {Baker's yeast (Saccharomyces cerevisiae)}
GAGEMRDRIESMFLESWRDYSKHGWGYDVYGPIEHTSHNMPRGNQPLGWIIVDSVDTLMLMY
NSSTLYKSEFEAEIQRSEHWINDVLDFDIDAEVNVFETTIRMLGGLLSAYHLSDVLEVGNKT
VYLNKAIDLGDRLALAFLSTQTGIPYSSINLHSGQAVKNHADGGASSTAEFTTLQMEFKYLA
YLTGNRTYWELVERVYEPLYKNNDLLNTYDGLVPIYTFPDTGKFGASTIRFGSRGDSFYEYL
LKQYLLTHETLYYDLYRKSMEGMKKHLLAQSKPSSLWYIGEREQGLHGQLSPKMDHLVCFMG
GLLASGSTEGLSIHEARRRPFFSKSDWDLAKGITDTCYQMYKQSSSGLAPEIVVFNDGNIKQ
DGWWRSSVGDFFVKPLDRHNLQRPETVESIMFMYHLSHDHKYREWGAEIATSFFENTCVDCN
DPKLRRFTSLSDCITLPTKKSNNMESFWLAETLKYLYILFLDEFDLTKVVFNTEAHPFPVLD
EEILKSQSLTTGWSL
>d1hcua_ a.102.2.1 (A:) Class I alpha-1;2-mannosidase, catalytic domain {Trichoderma reesei}
KRGSPNPTRAAAVKAAFQTSWNAYHHFAFPHDDLHPVSNSFDDERNGWGSSAIDGLDTAILM
GDADIVNTILQYVPQINFTTTAVANQGSSVFETNIRYLGGLLSAYDLLRGPFSSLATNQTLV
NSLLRQAQTLANGLKVAFTTPSGVPDPTVFFNPTVRRSGASSNNVAEIGSLVLEWTRLSDLT
GNPQYAQLAQKGESYLLNPKGSPEAWPGLIGTFVSTSNGTFQDSSGSWSGLMDSFYEYLIKM
YLYDPVAFAHYKDRWVLGADSTIGHLGSHPSTRKDLTFLSSYNGQSTSPNSGHLASFGGGNF
ILGGILLNEQKYIDFGIKLASSYFGTYTQTASGIGPEGFAWVDSVTGAGGSPPSSQSGFYSS
AGFWVTAPYYILRPETLESLYYAYRVTGDSKWQDLAWEALSAIEDACRAGSAYSSINDVTQA
NGGGASDDMESFWFAEALKYAYLIFAEESDVQVQATGGNKFVFNTEAHPFSIRS
>d1krea_ a.102.2.1 (A:) Class I alpha-1;2-mannosidase, catalytic domain {Fungus (Penicillium citrinum)}
SNQAKADAVKEAFQHAWNGYMKYAFPHDELTPVSNGHADSRNGWGASAVDALSTAVIMGKAD
VVNAILEHVADIDFSKTSDTVSLFETTIRYLAGMLSGYDLLQGPAKNLVDNQDLIDGLLDQS
RNLADVLKFAFDTPSGVPYNNINITSHGNDGATTNGLAVTGTLVLEWTRLSDLTGDEEYAKL
SQKAESYLLKPQPSSSEPFPGLVGSSININDGQFADSRVSWNGGDDSFYEYLIKMYVYDPKR
FETYKDRWVLAAESTIKHLKSHPKSRPDLTFLSSYSNRNYDLSSQHLTCFDGGSFLLGGTVL
DRQDFIDFGLELVDGCEATYNSTLTKIGPDSWGWDPKKVPSDQKEFYEKAGFYISSGSYVLR

PEVIESFYYAHRVTGKEIYRDWVWNAFVAINSTCRTDSGFAAVSDVNKANGGSKYDNQESFL
FAEVMKYSYLAHSEDAAWQVQKGGKNTFVYNTEAHPISVAR
>d1fo3a_ a.102.2.1 (A:) Class I alpha-1;2-mannosidase, catalytic domain {Human (Homo sapiens)}
QGPVHLNYRQKGVIDVFLHAWKGYRKFAWGHDELKPVSRSFSEWFGLGLTLIDALDTMWILG
LRKEFEEARKWVSKKLHFEKDVDVNLFESTIRILGGLLSAYHLSGDSLFLRKAEDFGNRLMP
AFRTPSKIPYSDVNIGTGVAHPPRWTSDSTVAEVTSIQLEFRELSRLTGDKKFQEAVEKVTQ
HIHGLSGKKDGLVPMFINTHSGLFTHLGVFTLGARADSYYEYLLKQWIQGGKQETQLLEDYV
EAIEGVRTHLLRHSEPSKLTFVGELAHGRFSAKMDHLVCFLPGTLALGVYHGLPASHMELAQ
ELMETCYQMNRQMETGLSPEIVHFNLYPQPGRRDVEVKPADRHNLLRPETVESLFYLYRVTG
DRKYQDWGWEILQSFSRFTRVPSGGYSSINNVQDPQKPEPRDKMESFFLGETLKYLFLLFSD
DPNLLSLDAYVFNTEAHPLPIWT
>d1qaza_ a.102.3.1 (A:) Alginate lyase A1-III {Sphingomonas sp., A1}
GSHPFDQAVVKDPTASYVDVKARRTFLQSGQLDDRLKAALPKEYDCTTEATPNPQQGEMVIP
RRYLSGNHGPVNPDYEPVVTLYRDFEKISATLGNLYVATGKPVYATCLLNMLDKWAKADALL
NYDPKSQSWYQVEWSAATAAFALSTMMAEPNVDTAQRERVVKWLNRVARHQTSFPGGDTSCC
NNHSYWRGQEATIIGVISKDDELFRWGLGRYVQAMGLINEDGSFVHEMTRHEQSLHYQNYAM
LPLTMIAETASRQGIDLYAYKENGRDIHSARKFVFAAVKNPDLIKKYASEPQDTRAFKPGRG
DLNWIEYQRARFGFADELGFMTVPIFDPRTGGSATLLAYKP
>d1cb8a1 a.102.3.2 (A:26-335) Chondroitinase AC {Pedobacter heparinus (Flavobacterium heparinum)}
GTAELIMKRVMLDLKKPLRNMDKVAEKNLNTLQPDGSWKDVPYKDDAMTNWLPNNHLLQLET
IIQAYIEKDSHYYGDDKVFDQISKAFKYWYDSDPKSRNWWHNEIATPQALGEMLILMRYGKK
PLDEALVHKLTERMKRGEPEKKTGANKTDIALHYFYRALLTSDEALLSFAVKELFYPVQFVH
YEEGLQYDYSYLQHGPQLQISSYGAVFITGVLKLANYVRDTPYALSTEKLAIFSKYYRDSYL
KAIRGSYMDFNVEGRGVSRPDILNKKAEKKRLLVAKMIDLKHTEEWADAIARTDSTVAAGYK
>d1egua1 a.102.3.2 (A:171-540) Hyaluronate lyase {Streptococcus pneumoniae}
KDTYTDRLDDWNGIIAGNQYYDSKNDQMAKLNQELEGKVADSLSSISSQADRIYLWEKFSNY
KTSANLTATYRKLEEMAKQVTNPSSRYYQDETVVRTVRDSMEWMHKHVYNSEKSIVGNWWDY
EIGTPRAINNTLSLMKEYFSDEEIKKYTDVIEKFVPDPEHFRKTTDNPFKALGGNLVDMGRV
KVIAGLLRKDDQEISSTIRSIEQVFKLVDQGEGFYQDGSYIDHTNVAYTGAYGNVLIDGLSQ
LLPVIQKTKNPIDKDKMQTMYHWIDKSFAPLLVNGELMDMSRGRSISRANSEGHVAAVEVLR
GIHRIADMSEGETKQRLQSLVKTIVQSDSYYDVFKNLKTYKDISLMQSLLSDAGVASVPR
>d1f1sa1 a.102.3.2 (A:249-619) Hyaluronate lyase {Streptococcus agalactiae}
EDNFTKLLDKWNDVTIGNYVYDTNDSNMQKLNQKLDETNAKNIEAIKLDSNRTFLWKDLDNL
NNSAQLTATYRRLEDLAKQITNPHSTIYKNEKAIRTVKESLAWLHQNFYNVNKDIEGSANWW
DFEIGVPRSITGTLSLMNNYFTDAEIKTYTDPIEHFVPDAEYFRKTLVNPFKALGGNLVDMG
RVKIIEGLLRKDNTIIEKTSHSLKNLFTTATKAEGFYADGSYIDHTNVAYTGAYGNVLIDGL
TQLLPIIQETDYKISNQELDMVYKWINQSFLPLIVKGELMDMSRGRSISREAASSHAAAVEV
LRGFLRLANMSNEERNLDLKSTIKTIITSNKFYNVFNNLKSYSDIANMNKLLNDSTVATKP
>d5eau_1 a.102.4.1 (21-220) 5-Epi-aristolochene synthase,

N-terminal domain {Tobacco (Nicotiana tabacum)}
SPSLWGDQFLSFSIDNQVAEKYAKEIEALKEQTRNMLLATGMKLADTLNLIDTIERLGISYH
FEKEIDDILDQIYNQNSNCNDLCTSALQFRLLRQHGFNISPEIFSKFQDENGKFKESLASDV
LGLLNLYEASHVRTHADDILEDALAFSTIHLESAAPHLKSPLREQVTHALEQCLHKGVPRVE
TRFFISSIYDKEQS
>d2sqca1 a.102.4.2 (A:8-36,A:308-630) Squalene-hopene cyclase
{Alicyclobacillus acidocaldarius}
APAYARTLDRAVEYLLSCQKDEGYWWGPLXISPVWDTGLAVLALRAAGLPADHDRLVKAGEW
LLDRQITVPGDWAVKRPNLKPGGFAFQFDNVYYPDVCDTAVVVWALNTLRLPDERRRRDAMT
KGFRWIVGMQSSNGGWGAYDVDNTSDLPNHIPFSDFGEVTDPPSEDVTAHVLECFGSFGYDD
AWKVIRRAVEYLKREQKPDGSWFGRWGVNYLYGTGAVVSALKAVGIDTREPYIQKALDWVEQ
HQNPDGGWGEDCRSYEDPAYAGKGASTPSQTAWALMALIAGGRAESEAARRGVQYLVETQRP
DGGWDEPYYTGTGFPGDFYLGYTMYRHVFPTLALGRYKQAIER
>d2sqca2 a.102.4.2 (A:37-307) Squalene-hopene cyclase
{Alicyclobacillus acidocaldarius}
LSNVTMEAEYVLLCHILDRVDRDRMEKIRRYLLHEQREDGTWALYPGGPPDLDTTIEAYVAL
KYIGMSRDEEPMQKALRFIQSQGGIESSRVFTRMWLALVGEYPWEKVPMVPPEIMFLGKRMP
LNIYEFGSWARATVVALSIVMSRQPVFPLPERARVPELYETDVPPRRRGAKGGGGWIFDALD
RALHGYQKLSVHPFRRAAEIRALDWLLERQAGDGSWGGIQPPWFYALIALKILDMTQHPAFI
KGWEGLELYGVELDYGGWMFQAS
>d1d8db_ a.102.4.3 (B:) Protein farnesyltransferase, beta-subunit
{Rat (Rattus norvegicus)}
PVWSEPLYSLRPEHARERLQDDSVETVTSIEQAKVEEKIQEVFSSYKFNHLVPRLVLQREKH
FHYLKRGLRQLTDAYECLDASRPWLCYWILHSLELLDEPIPQIVATDVCQFLELCQSPDGGF
GGGPGQYPHLAPTYAAVNALCIIGTEEAYNVINREKLLQYLYSLKQPDGSFLMHVGGEVDVR
SAYCAASVASLTNIITPDLFEGTAEWIARCQNWEGGIGGVPGMEAHGGYTFCGLAALVILKK
ERSLNLKSLLQWVTSRQMRFEGGFQGRCNKLVDGCYSFWQAGLLPLLHRALHAQGDPALSMS
HWMFHQQALQEYILMCCQCPAGGLLDKPGKSRDFYHTCYCLSGLSIAQHFGSGAMLHDVVMG
VPENVLQPTHPVYNIGPDKVIQATTHFLQKPVPGF
>d1dceb_ a.102.4.3 (B:) Rab geranylgeranyltransferase, beta
subunit {Rat (Rattus norvegicus)}
TQQKDVTIKSDAPDTLLLEKHADYIASYGSKKDDYEYCMSEYLRMSGVYWGLTVMDLMGQLH
RMNKEEILVFIKSCQHECGGVSASIGHDPHLLYTLSAVQILTLYDSIHVINVDKVVAYVQSL
QKEDGSFAGDIWGEIDTRFSFCAVATLALLGKLDAINVEKAIEFVLSCMNFDGGFGCRPGSE
SHAGQIYCCTGFLAITSQLHQVNSDLLGWWLCERQLPSGGLNGRPEKLPDVCYSWWVLASLK
IIGRLHWIDREKLRSFILACQDEETGGFADRPGDMVDPFHTLFGIAGLSLLGEEQIKPVSPV
FCMPEEVLQRVNVQPELVS
>d1c3d__ a.102.4.4 (-) C3D, a C3 fragment and ligand for complement
receptor 2 {Human (Homo sapiens)}
MLDAERLKHLIVTPSGAGEQNMIGMTPTVIAVHYLDETEQWEKFGLEKRQGALELIKKGYTQ
QLAFRQPSSAFAAFVKRAPSTWLTAYVVKVFSLAVNLIAIDSQVLCGAVKWLILEKQKPDGV
FQEDAPVIHQEMIGGLRNNNEKDMALTAFVLISLQEAKDICEEQVNSLPGSITKAGDFLEAN
YMNLQRSYTVAIAGYALAQMGRLKGPLLNKFLTTAKDKNRWEDPGKQLYNVEATSYALLALL
QLKDFDFVPPVVRWLNEQRYYGGGYGSTQATFMVFQALAQYQKDAP

>d1qqfa_ a.102.4.4 (A:) C3D, a C3 fragment and ligand for complement receptor 2 {Rat (Rattus norvegicus)}
CGEQNMIGMTPTVIAVHYLDQTEQWEKFGLEKRQEALELIKKGYTQQLAFKQPISAYAAFNN
RPPSTWLTAYVSRVFSLAANLIAIDSQVLCGAVKWLILEKQKPDGVFQEDGPVIHQEMIGGF
RNTKEADVSLTAFVLIALQEARDICEGQVNSLPGSINKAGEYLEASYLNLQRPYTVAIAGYA
LALMNKLEEPYLTKFLNTAKDRNRWEEPGQQLYNVEATSYALLALLLLKDFDSVPPVVRWLN
DERYYGGGYGSTQATFMVFQALAQYRADV
>d1csc__ a.103.1.1 (-) Citrate synthase {Chicken (Gallus gallus)}
ASSTNLKDVLAALIPKEQARIKTFRQQHGGTALGQITVDMSYGGMRGMKGLVYETSVLDPDE
GIRFRGFSIPECQKLLPKGGXGGEPLPEGLFWLLVTGQIPTGAQVSWLSKEWAKRAALPSHV
VTMLDNFPTNLHPMSQLSAAITALNSESNFARAYAEGILRTKYWEMVYESAMDLIAKLPCVA
AKIYRNLYRAGSSIGAIDSKLDWSHNFTNMLGYTDAQFTELMRLYLTIHSDHEGGNVSAHTS
HLVGSALSDPYLSFAAAMNGLAGPLHGLANQEVLGWLAQLQKAXXXAGADASLRDYIWNTLN
SGRVVPGYGHAVLRKTDPRYTCQREFALKHLPGDPMFKLVAQLYKIVPNVLLEQGAAANPWP
NVDAHSGVLLQYYGMTEMNYYTVLFGVSRALGVLAQLIWSRALGFPLERPKSMSTDGLIAL
>d1csh__ a.103.1.1 (-) Citrate synthase {Chicken (Gallus gallus)}
STNLKDVLASLIPKEQARIKTFRQQHGNTAVGQITVDMSYGGMRGMKGLIYETSVLDPDEGI
RFRGFSIPECQKLLPKAGGGEEPLPEGLFWLLVTGQIPTPEQVSWVSKEWAKRAALPSHVVT
MLDNFPTNLHPMSQLSAAITALNSESNFARAYAEGINRTKYWEFVYEDAMDLIAKLPCVAAK
IYRNLYRAGSSIGAIDSKLDWSHNFTNMLGYTDPQFTELMRLYLTIHSDHEGGNVSAHTSHL
VGSALSDPYLSFAAAMNGLAGPLHGLANQEVLLWLSQLQKDLGADASDEKLRDYIWNTLNSG
RVVPGYGHAVLRKTDPRYTCQREFALKHLPSDPMFKLVAQLYKIVPNVLLEQGKAKNPWPNV
DAHSGVLLQYYGMTEMNYYTVLFGVSRALGVLAQLIWSRALGFPLERPKSMSTAGLEKLSAG
G
>d2cts__ a.103.1.1 (-) Citrate synthase {Pig (Sus scrofa)}
ASSTNLKDILADLIPKEQARIKTFRQQHGNTAVGQITVDMMYGGMRGMKGLVYETSVLDPDE
GIRFRGYSIPECQKMLPKAKGGEEPLPEGLFWLLVTGQIPTEEQVSWLSKEWAKRAALPSHV
VTMLDNFPTNLHPMSQLSAAITALNSESNFARAYAEGIHRTKYWELIYEDCMDLIAKLPCVA
AKIYRNLYREGSSIGAIDSKLDWSHNFTNMLGYTDAQFTELMRLYLTIHSDHEGGNVSAHTS
HLVGSALSDPYLSFAAAMNGLAGPLHGLANQEVLVWLTQLQKEVGKDVSDEKLRDYIWNTLN
SGRVVPGYGHAVLRKTDPRYTCQREFALKHLPHDPMFKLVAQLYKIVPNVLLEQGKAKNPWP
NVDAHSGVLLQYYGMTEMNYYTVLFGVSRALGVLAQLIWSRALGFPLERPKSMSTDGLIKLV
DSK
>d1aj8a_ a.103.1.1 (A:) Citrate synthase {Archaeon Pyrococcus furiosus}
LAKGLEDVYIDQTNICYIDGKEGKLYYRGYSVEELAELSTFEEVVYLLWWGKLPSLSELENF
KKELAKSRGLPKEVIEIMEALPKNTHPMGALRTIISYLGNIDDSGDIPVTPEEVYRIGISVT
AKIPTIVANWYRIKNGLEYVPPKEKLSHAANFLYMLHGEEPPKEWEKAMDVALILYAEHEIN
ASTLAVMTVGSTLSDYYSAILAGIGALKGPIHGGAVEEAIKQFMEIGSPEKVEEWFFKALQQ
KRKIMGAGHRVYKTYDPRARIFKKYASKLGDKKLFEIAERLERLVEEYLSKKGISINVDYWS
GLVFYGMKIPIELYTTIFAMGRIAGWTAHLAEYVSHNRIIRPRLQYVGEIGKKYLPIELRR
>d1a59__ a.103.1.1 (-) Citrate synthase {Antarctic bacterium DS2-3R}
EPTIHKGLAGVTADVTAISKVNSDTNSLLYRGYPVQELAAKCSFEQVAYLLWNSELPNDSEL

KAFVNFERSHRKLDENVKGAIDLLSTACHPMDVARTAVSVLGANHARAQDSSPEANLEKAMS
LLATFPSVVAYDQRRRRGEELIEPREDLDYSANFLWMTFGEEAAPEVVEAFNVSMILYAEHS
FNASTFTARVITSTLADLHSAVTGAIGALKGPLHGGANEAVMHTFEEIGIRKDESLDEAATR
SKAWMVDALAQKKKVMGFGHRVYKNGDSRVPTMKSALDAMIKHYDRPEMLGLYNGLEAAMEE
AKQIKPNLDYPAGPTYNLMGFDTEMFTPLFIAARITGWTAHIMEQVADNALIRPLSEYNGPE
QRQVP
>d1dz4a_ a.104.1.1 (A:) Cytochrome P450-CAM {Pseudomonas putida}
LAPLPPHVPEHLVFDFDMYNPSNLSAGVQEAWAVLQESNVPDLVWTRCNGGHWIATRGQLIR
EAYEDYRHFSSECPFIPREAGEAYDFIPTSMDPPEQRQFRALANQVVGMPVVDKLENRIQEL
ACSLIESLRPQGQCNFTEDYAEPFPIRIFMLLAGLPEEDIPHLKYLTDQMTRPDGSMTFAEA
KEALYDYLIPIIEQRRQKPGTDAISIVANGQVNGRPITSDEAKRMCGLLLVGGLDTVVNFLS
FSMEFLAKSPEHRQELIQRPERIPAACEELLRRFSLVADGRILTSDYEFHGVQLKKGDQILL
PQMLSGLDERENACPMHVDFSRQKVSHTTFGHGSHLCLGQHLARREIIVTLKEWLTRIPDFS
IAPGAQIQHKSGIVSGVQALPLVWDPATTKAV
>d1jpza_ a.104.1.1 (A:) Cytochrome P450 bm-3 {Bacillus megaterium}
TIKEMPQPKTFGELKNLPLLNTDKPVQALMKIADELGEIFKFEAPGRVTRYLSSQRLIKEAC
DESRFDKNLSQALKFVRDFAGDGLFTSWTHEKNWKKAHNILLPSFSQQAMKGYHAMMVDIAV
QLVQKWERLNADEHIEVPEDMTRLTLDTIGLCGFNYRFNSFYRDQPHPFITSMVRALDEAMN
KLQRANPDDPAYDENKRQFQEDIKVMNDLVDKIIADRKASGEQSDDLLTHMLNGKDPETGEP
LDDENIRYQIITFLIAGHETTSGLLSFALYFLVKNPHVLQKAAEEAARVLVDPVPSYKQVKQ
LKYVGMVLNEALRLWPTAPAFSLYAKEDTVLGGEYPLEKGDELMVLIPQLHRDKTIWGDDVE
EFRPERFENPSAIPQHAFKPFGNGQRACIGQQFALHEATLVLGMMLKHFDFEDHTNYELDIK
ETLTLKPEGFVVKAKSKKIPLGGI
>d1jfba_ a.104.1.1 (A:) Cytochrome P450-NOR, nitric reductase {Fungus (Fusarium oxysporum)}
APSFPFSRASGPEPPAEFAKLRATNPVSQVKLFDGSLAWLVTKHKDVCFVATSEKLSKVRTR
QGFPELSASGKQAAKAKPTFVDMDPPEHMHQRSMVEPTFTPEAVKNLQPYIQRTVDDLLEQM
KQKGCANGPVDLVKEFALPVPSYIIYTLLGVPFNDLEYLTQQNAIRTNGSSTAREASAANQE
LLDYLAILVEQRLVEPKDDIISKLCTEQVKPGNIDKSDAVQIAFLLLVAGNATMVNMIALGV
ATLAQHPDQLAQLKANPSLAPQFVEELCRYHTASALAIKRTAKEDVMIGDKLVRANEGIIAS
NQSANRDEEVFENPDEFNMNRKWPPQDPLGFGFGDHRCIAEHLAKAELTTVFSTLYQKFPDL
KVAVPLGKINYTPLNRDVGIVDLPVIF
>d1jipa_ a.104.1.1 (A:) Cytochrome P450-ERYF {Saccaropolyspora erythraea}
TTVPDLESDSFHVDWYRTYAELRETAPVTPVRFLGQDAWLVTGYDEAKAALSDLRLSSDPKK
KYPGVEVEFPAYLGFPEDVRNYFATNMGTSDPPTHTRLRKLVSQEFTVRRVEAMRPRVEQIT
AELLDEVGDSGVVDIVDRFAHPLPIKVICELLGVDEKYRGEFGRWSSEILVMDPERAEQRGQ
AAREVVNFILDLVERRRTEPGDDLLSALIRVQDDDDGRLSADELTSIALVLLLAGFESSVSL
IGIGTYLLLTHPDQLALVRRDPSALPNAVEEILRYIAPPETTTRFAAEEVEIGGVAIPQYST
VLVANGAANRDPKQFPDPHRFDVTRDTRGHLSFGQGIHFCMGRPLAKLEGEVALRALFGRFP
ALSLGIDADDVVWRRSLLLRGIDHLPVRLDG
>d1cpt__ a.104.1.1 (-) Cytochrome P450-TERP {Pseudomonas sp.}
MDARATIPEHIARTVILPQGYADDEVIYPAFKWLRDEQPLAMAHIEGYDPMWIATKHADVMQ
IGKQPGLFSNAEGSEILYDQNNEAFMRSISGGCPHVIDSLTSMDPPTHTAYRGLTLNWFQPA

SIRKLEENIRRIAQASVQRLLDFDGECDFMTDCALYYPLHVVMTALGVPEDDEPLMLKLTQD
FFGVEAARRFHETIATFYDYFNGFTVDRRSCPKDDVMSLLANSKLDGNYIDDKYINAYYVAI
ATAGHDTTSSSSGGAIIGLSRNPEQLALAKSDPALIPRLVDEAVRWTAPVKSFMRTALADTE
VRGQNIKRGDRIMLSYPSANRDEEVFSNPDEFDITRFPNRHLGFGWGAHMCLGQHLAKLEMK
IFFEELLPKLKSVELSGPPRLVATNFVGGPKNVPIRFTKA
>d1e9xa_ a.104.1.1 (A:) Cytochrome p450 14 alpha-sterol demethylase (cyp51) {Mycobacterium tuberculosis}
MSAVALPRVSGGHDEHGHLEEFRTDPIGLMQRVRDECGDVGTFQLAGKQVVLLSGSHANEFF
FRAGDDDLDQAKAYPFMTPIFGEGVVFDASPERRKEMLHNAALRGEQMKGHAATIEDQVRRM
IADWGEAGEIDLLDFFAELTIYTSSACLIGKKFRDQLDGRFAKLYHELERGTDPLAYVDPYL
PIESFRRRDEARNGLVALVADIMNGRIANPPTDKSDRDMLDVLIAVKAETGTPRFSADEITG
MFISMMFAGHHTSSGTASWTLIELMRHRDAYAAVIDELDELYGDGRSVSFHALRQIPQLENV
LKETLRLHPPLIILMRVAKGEFEVQGHRIHEGDLVAASPAISNRIPEDFPDPHDFVPARYEQ
PRQEDLLNRWTWIPFGAGRHRCVGAAFAIMQIKAIFSVLLREYEFEMAQPPESYRNDHSKMV
VQLAQPACVRYRRRT
>d1io7a_ a.104.1.1 (A:) CYP119 {Archaeon Sulfolobus solfataricus}
MYDWFSEMRKKDPVYYDGNIWQVFSYRYTKEVLNNFSKFSSDLTGYHERLEDLRNGKIRFDI
PTRYTMLTSDPPLHDELRSMSADIFSPQKLQTLETFIRETTRSLLDSIDPREDDIVKKLAVP
LPIIVISKILGLPIEDKEKFKEWSDLVAFRLGKPGEIFELGKKYLELIGYVKDHLNSGTEVV
SRVVNSNLSDIEKLGYIILLLIAGNETTTNLISNSVIDFTRFNLWQRIREENLYLKAIEEAL
RYSPPVMRTVRKTKERVKLGDQTIEEGEYVRVWIASANRDEEVFHDGEKFIPDRNPNPHLSF
GSGIHLCLGAPLARLEARIAIEEFSKRFRHIEILDTEKVPNEVLNGYKRLVVRLKS
>d1dt6a_ a.104.1.1 (A:) Mammalian cytochrome p450 2c5 {Rabbit (Oryctolagus cuniculus)}
PPGPTPFPIIGNILQIDAKDISKSLTKFSECYGPVFTVYLGMKPTVVLHGYEAVKEALVDLG
EEFAGRGSVPILEKVSKGLGIAFSNAKTWKEMRRFSLMTLRNFGMGKRSIEDRIQEEARCLV
EELRKTNASPCDPTFILGCAPCNVICSVIFHNRFDYKDEEFLKLMESLHENVELLGTPWLQV
YNNFPALLDYFPGIHKTLLKNADYIKNFIMEKVKEHQKLLDVNNPRDFIDCFLIKMEQENNL
EFTLESLVIAVSDLFGAGTETTSTTLRYSLLLLLKHPEVAARVQEEIERVIGRHRSPCMQDR
SRMPYTDAVIHEIQRFIDLLPTNLPHAVTRDVRFRNYFIPKGTDIITSLTSVLHDEKAFPNP
KVFDPGHFLDESGNFKKSDYFMPFSAGKRMCVGEGLARMELFLFLTSILQNFKLQSLVEPKD
LDITAVVNGFVSVPPSYQLCFIPIHH
>d1etob_ a.105.1.1 (B:) FIS protein {Escherichia coli}
MFEQRVNSDVLTVSTVNSQDQVTQKPLRDSVKQALKNYFAQLNGQDVNDLYELVLAEVEQPL
LDMVMQYTLGNQTRAALMMGINRGTLRKKLKKYGMN
>d1etxa_ a.105.1.1 (A:) FIS protein {Escherichia coli}
VLTVSTVNSQDQVTQKPLRDSVKQALKNYFAQLNGQDVNDLYELVLAEVEQPLLDMVMQYTR
GNATRAALMMGINRGTLRKKLKKYGMN
>d1fipa_ a.105.1.1 (A:) FIS protein {Escherichia coli}
PLRDSVKQALKNYFAQLNGQDVNDLYELVLAEVEQALLDMVMQYTRGNQTRAALMMGINRGT
LRKKLKKYGMN
>d1ntca_ a.105.1.1 (A:) DNA-binding domain of NTRC {Salmonella typhimurium}
MDLPGELFEASTPDSPSHLPPDSWATLLAQWADRALRSGHQNLLSEAQPELERTLLTTALRH

TQGHKQEAARLLGWGAATLTAKLKELGME
>d1g8ea_ a.145.1.1 (A:) Flagellar transcriptional activator FlhD {Escherichia coli}
MHTSELLKHIYDINLSYLLLAQRLIVQDKASAMFRLGINEEMATTLAALTLPQMVKLAETNQ
LVCHFRFDSHQTITQLTQDSRVDDLQQIHTGIMLST
>d1g8eb_ a.145.1.1 (B:) Flagellar transcriptional activator FlhD {Escherichia coli}
TSELLKHIYDINLSYLLLAQRLIVQDKASAMFRLGINEEMATTLAALTLPQMVKLAETNQLV
CHFRFDSHQTITQLTQDS
>d1jhga_ a.107.1.1 (A:) Trp repressor {Escherichia coli}
SAAMAEQRHQEWLRFVDLLKNAYQNDLHLPLLNLMLTPDEREALGTRVRIIEELLRGEMSQR
ELKNELGAGIATITRGSNSLKAAPVELRQWLEEVLLKSD
>d1trra_ a.107.1.1 (A:) Trp repressor {Escherichia coli}
AQQSPYSAAMAEQRHEEWLRFVDLLKNAYQNDLHLPLLNLMLTPDEREALGTRVRIVEELLR
GEMSQRELKNELGAGIATITRGSNSLKAAPVELRQWLEEVLLK
>d1dd3a1 a.108.1.1 (A:1-57) Ribosomal protein L7/12, oligomerisation (N-terminal) domain {Thermotoga maritima}
MTIDEIIEAIEKLTVSELAELVKKLEDKFGVTAAAPVAVAAAPVAGAAAGAAQEEKT
>d1dd3c1 a.108.1.1 (C:) Ribosomal protein L7/12, oligomerisation (N-terminal) domain {Thermotoga maritima}
MTIDEIIEAIEKLTVSELAELVKKLEDKFGVT
>d1dd4d1 a.108.1.1 (D:) Ribosomal protein L7/12, oligomerisation (N-terminal) domain {Thermotoga maritima}
MTIDEIIEAIEKLTVSELAELVKKLEDKFGVTAAAPVAVA
>d1iiea_ a.109.1.1 (A:) MHC class II-associated invariant chain ectoplasmic trimerization domain {Human (Homo sapiens)}
YGNMTEDHVMHLLQNADPLKVYPPLKGSFPENLRHLKNTMETIDWKVFESWMHHWLLFEMSR
HSLEQKPTDAPPK
>d1aora1 a.110.1.1 (A:211-605) Aldehyde ferredoxin oxidoreductase {Archaeon Pyrococcus furiosus}
IADKQKFMLVVREKVNKLRNDPVAGGGLPKYGTAVLVNIINENGLYPVKNFQTGVYPYAYEQ
SGEAMAAKYLVRNKPCYACPIGCGRVNRLPTVGETEGPEYESVWALGANLGINDLASIIEAN
HMCDELGLDTISTGGTLATAMELYEKGHIKDEELGDAPPFRWGNTEVLHYYIEKIAKREGFG
DKLAEGSYRLAESYGHPELSMTVKKLELPAYDPRGAEGHGLGYATNNRGGCHIKNYMISPEI
LGYPYKMDPHDVSDDKIKMLILFQDLTALIDSAGLCLFTTFGLGADDYRDLLNAALGWDFTT
EDYLKIGERIWNAERLFNLKAGLDPARDDTLPKRFLEEPMPEGPNKGHTVRLKEMLPRYYKL
RGWTEDGKIPKEKLEELGIAEFY
>d1b25a1 a.110.1.1 (A:211-619) Formaldehyde ferredoxin oxidoreductase {Archaeon Pyrococcus furiosus}
DKEELKKLSQEAYNEILNSPGYPFWKRQGTMAAVEWCNTNYALPTRNFSDGYFEFARSIDGY
TMEGMKVQQRGCPYCNMPCGNVVLDAEGQESELDYENVALLGSNLGIGKLNEVSVLNRIADE
MGMDTISLGVSIAHVMEAVERGILKEGPTFGDFKGAKQLALDIAYRKGELGNLAAEGVKAMA
EKLGTHDFAMHVKGLEVSGYNCYIYPAMALAYGTSAIGAHHKEAWVIAWEIGTAPIEGEKAE
KVEYKISYDPIKAQKVVELQRLRGGLFEMLTACRLPWVEVGLSLDYYPKLLKAITGVTYTWD

DLYKAADRVYSLIRAYWVREFNGKWDRKMDYPPKRWFTEGLKSGPHKGEHLDEKKYDELLSE
YYRIRGWDERGIPKKETLKELDLDFVIPELEKVTNLE
>d1d2ta_ a.111.1.1 (A:) Acid phosphatase {Escherichia blattae}
GNDTTTKPDLYYLKNSEAINSLALLPPPPAVGSIAFLNDQAMYEQGRLLRNTERGKLAAEDA
NLSSGGVANAFSGAFGSPITEKDAPALHKLLTNMIEDAGDLATRSAKDHYMRIRPFAFYGVS
TCNTTEQDKLSKNGSYPSGHTSIGWATALVLAEINPQRQNEILKRGYELGQSRVICGYHWQS
DVDAARVVGSAVVATLHTNPAFQQQLQKAKAEFAQHQK
>d1qi9a_ a.111.1.2 (A:) Haloperoxidase (bromoperoxidase)
{Ascophyllum nodosum}
TCSTSDDADDPTPPNERDDEAFASRVAAAKRELEGTGTVCQINNGETDLAAKFHKSLPHDDL
GQVDADAFAALEDCILNGDLSICEDVPVGNSEGDPVGRLVNPTAAFAIDISGPAFSATTIPP
VPTLPSPELAAQLAEVYWMALARDVPFMQYGTDDITVTAAANLAGMEGFPNLDAVSIGSDGT
VDPLSQLFRATFVGVETGPFISQLLVNSFTIDSITVEPKQETFAPDVNYMVDFDEWLNIQNG
GPPAGPELLDDELRFVRNARDLARVTFTDNINTEAYRGALILLGLDAFNRAGVNGPFIDIDR
QAGFVNFGISHYFRLIGAAELAQRSSWYQKWQVHRFARPEALGGTLHLTIKGELNADFDLSL
LENAELLKRVAAINAAQNPNNEVTYLLPQAIQEGSPTHPSYPSGHATQNGAFATVLKALIGL
DRGGDCYPDPVYPDDDGLKLIDFRGSCLTFEGEINKLAVNVAFGRQMLGIHYRFDGIQGLLL
GETITVRTLHQELMTFAEESTFEFRLFTGEVIKLFQDGTFTIDGFKCPGLVYTGVENCV
>d1qhba_ a.111.1.2 (A:) Haloperoxidase (bromoperoxidase) {Red
algae (Corallina officinalis)}
GIPADNLQSRAKASFDTRVAAAELALARGAVPSFANGEELLYRNSETGDPSFIGSFTKGLPH
DDNGAIIDPDDFLAFVRAINSGDEKEIAALTLGPARDPETGLPIWRSDLANSLDLEVRGWEN
SSAGLTFDLEGPDAQSVAMPPAPVLTSPELIAEMAELYLMALGRDIEFSEFDSPKNAAFIRS
AIERLNGLEWFNTPAKLGDPPAEIRRRRGEVTVGNLFRGILPGSEVGPYLSQFIIVGSKQIG
SATVGNKTLVSPNAADEFDGEIAYGSITISQRVRIATPGRDFMTDLKVFLDVQDAADFRGFE
SYEPGARLIRTIRDLATWVHFDSLYEAYLNACLILLANGVPFDPNLPFQQEDKLDNQDVFVN
FGSAHVLSLVTEVATRALKAVRYQKFNIHRRLRPEATGGLISVNKNAFLKSESVFPEVDVLV
EELSSILDDSASSNEKQNIADGDVSPGKSFLLPMAFAEGSPFHPSYGSGHAVVAGACVTILK
AFFDANFQIDQVFEVDTDEDKLVKSSFPGPLTVAGELNKLADNVAIGRNMAGVHYFSDQFES
LLLGEQIAIGILEEQSLTYGENFFFNLPKFDGTTIQI
>d1vns__ a.111.1.3 (-) Chloroperoxidase {Curvularia inaequalis}
VTPIPLPKIDEPEEYNTNYILFWNHVGLELNRVTHTVGGPLTGPPLSARALGMLHLAIHDAY
FSICPPTDFTTFLSPDTENAAYRLPSPNGANDARQAVAGAALKMLSSLYMKPVEQPNPNPGA
NISDNAYAQLGLVLDRSVLEAPGGVDRESASFMFGEDVADVFFALLNDPRGASQEGYHPTPG
RYKFDDEPTHPVVLIPVDPNNPNGPKMPFRQYHAPFYGKTTKRFATQSEHFLADPPGLRSNA
DETAEYDDAVRVAIAMGGAQALNSTKRSPWQTAQGLYWAYDGSNLIGTPPRFYNQIVRRIAV
TYKKEEDLANSEVNNADFARLFALVDVACTDAGIFSWKEKWEFEFWRPLSGVRDDGRPDHGD
PFWLTLGAPATNTNDIPFKPPFPAYPSGHATFGGAVFQMVRRYYNGRVGTWKDDEPDNIAID
MMISEELNGVNRDLRQPYDPTAPIEDQPGIVRTRIVRHFDSAWELMFENAISRIFLGVHWRF
DAAAARDILIPTTTKDVYAVDNNGATVFQNVEDIRYTTRGTREDEEGLFPIGGVPLGIEIAD
EIFNNGLKPTPPEIQP
>d1sig__ a.112.1.1 (-) sigma70 subunit fragment from RNA polymerase
{Escherichia coli}
MEGEIDIAKRIEDGINQVQCSVAEYPEAITYLLEQYNRVEAEEARLSDLITGFVDPNAEEDL

APTATHVGSELSQEDLDDDEDEDEEDGDDDSADDDNSIDPELAREKFAELRAQYVVTRDTIK
AKGRSHATAQEEILKLSEVFKQFRLVPKQFDYLVNSMRVMMDRVRTQERLIMKLCVEQCKMP
KKNFITLFTGNETSDTWFNAAIAMNKPWSEKLHDVSEEVHRALQKLQQIEEETGLTIEQVKD
INRRMSIGEAKARRAKKEMVEANLRLVISIAKKYTNRGLQFLDLIQEGNIGLMKAVDKFEYR
RGYKFSTYATWWIRQAITRSIADQ
>d1ewqa1 a.113.1.1 (A:267-541) DNA repair protein MutS, domain III {Thermus aquaticus}
RGQDTLFSVLDETRTAPGRRLLQSWLRHPLLDRGPLEARLDRVEGFVREGALREGVRRLLYR
LADLERLATRLELGRASPKDLGALRRSLQILPELRALLGEEVGLPDLSPLKEELEAALVEDP
PLKVSEGGLIREGYDPDLDALRAAHREGVAYFLELEERERERTGIPTLKVGYNAVFGYYLEV
TRPYYERVPKEYRPVQTLKDRQRYTLPEMKEKEREVYRLEALIRRREEEVFLEVRERAKRQA
EALREAARILAELDVYAALAEVAVRYG
>d1e3ma1 a.113.1.1 (A:270-566) DNA repair protein MutS, domain III {Escherichia coli}
DAATRRNLEITQNLAGGAENTLASVLDCTVTPMGSRMLKRWLHMPVRDTRVLLERQQTIGAL
QDFTAGLQPVLRQVGDLERILARLALRTARPRDLARMRHAFQQLPELRAQLETVDSAPVQAL
REKMGEFAELRDLLERAIIDTPPVLVRDGGVIASGYNEELDEWRALADGATDYLERLEVRER
ERTGLDTLKVGFNAVHGYYIQISRGQSHLAPINYMRRQTLKNAERYIIPELKEYEDKVLTSK
GKALALEKQLYEELFDLLLPHLEALQQSASALAELDVLVNLAERAYTLN
>d1f5na1 a.114.1.1 (A:284-583) Interferon-induced guanylate-binding protein 1 (GBP1), C-terminal domain {Human (Homo sapiens)}
GGIQVNGPRLESLVLTYVNAISSGDLPCMENAVLALAQIENSAAVQKAIAHYEQQMGQKVQL
PTESLQELLDLHRDSEREAIEVFIRSSFKDVDHLFQKELAAQLEKKRDDFCKQNQEASSDRC
SGLLQVIFSPLEEEVKAGIYSKPGGYRLFVQKLQDLKKKYYEEPRKGIQAEEILQTYLKSKE
SMTDAILQTDQTLTEKEKEIEVERVKAESAQASAKMLHEMQRKNEQMMEQKERSYQEHLKQL
TEKMENDRVQLLKEQERTLALKLQEQEQLLKEGFQKESRIMKNEIQDLQTKM
>d1bvp11 a.115.1.1 (1:1-120,1:255-349) Bluetongue virus capsid protein vp7 (BTV-10 vp7) {Bluetongue virus}
MDTIAARALTVMRACATLQEARIVLEANVMEILGIAINRYNGLTLRGVTMRPTSLAQRNEMF
FMCLDMMLSAAGINVGPISPDYTQHMATIGVLATPEIPFTTEAANEIARVTGETSTWGXKTL
NQYPALTAEIFNVYSFRDHTWHGLRTAILNRTTLPNMLPPIFPPNDRDSILTLLLLSTLADV
YTVLRPEFAIHGVNPMPGPLTRAIARAAYV
>d1qhda1 a.115.1.2 (A:1-148,A:333-397) vp6, the major capsid protein of group A rotavirus {Bovine rotavirus}
MDVLYSLSKTLKDARDKIVEGTLYSNVSDLIQQFNQMIITMNGNEFQTGGIGNLPIRNWNFD
FGLLGTTLLNLDANYVETARNTIDYFVDFVDNVCMDEMVRESQRNGIAPQSDSLIKLSGIKF
KRINFDNSSEYIENWNLQNRRQRTXSVLADASETMLANVTSVRQEYAIPVGPVFPPGMNWTD
LITNYSPSREDNLQRVFTVASIRSMLVK
>d1tx4a_ a.116.1.1 (A:) p50 RhoGAP domain {Human (Homo sapiens)}
PLPNQQFGVSLQHLQEKNPEQEPIPIVLRETVAYLQAHALTTEGIFRRSANTQVVREVQQKY
NMGLPVDFDQYNALHLPAVILKTFLRELPEPLLTFDLYPHVVGFLNIDESQRVPATLQVLQT
LPEENYQVLRFLTAFLVQISAHSDQNKMTNTNLAVVFGPNLLWAKDAAITLKAINPINTFTK
FLLDHQGELF

>d1pbwa_ a.116.1.1 (A:) p85 alpha subunit RhoGAP domain {Human (Homo sapiens)}
LPDLAEQFAPPDIAPPLLIKLVEAIEKKGLECSTLYRTQSSSNLAELRQLLDCDTPSVDLEM
IDVHVLADAFKRYLLDLPNPVIPAAVYSEMISLAPEVQSSEEYIQLLKKLIRSPSIPHQYWL
TLQYLLKHFFKLSQTSSKNLLNARVLSEIFSPMLFRFSAASSDNTENLIKVIEILISTEW
>d1f7ca_ a.116.1.1 (A:) Graf {Chicken (Gallus gallus)}
AQLDSIGFSIIKKCIHAVETRGINEQGLYRIVGVNSRVQKLLSILMDPKTATETETEICAEW
EIKTITSALKTYLRMLPGPLMMYQFQRSFIKAAKLENQESRVSEIHSLVHRLPEKNRQMLHL
LMNHLAKVADNHKQNLMTVANLGVVFGPTLLRPQEETVAAIMDIKFQNIVIEILIENHEKIF
NTVPE
>d1wer__ a.116.1.2 (-) p120GAP domain {Human (Homo sapiens)}
MPEEEYSEFKELILQKELHVVYALSHVCGQDRTLLASILLRIFLHEKLESLLLCTLNDREIS
MEDEATTLFRATTLASTLMEQYMKATATQFVHHALKDSILKIMESKQSCELSPSKLEKNEDV
NTNLTHLLNILSELVEKIFMASEILPPTLRYIYGCLQKSVQHKWPTNTTMRTRVVSGFVFLR
LICPAILNPRMFNIISDSPSPIAARTLILVAKSVQNLANLVEFGAKEPYMEGVNPFIKSNKH
RMIMFLDELGNVPELPDTTEHSRTDLSRDLAALHEICVAHSDELRTLSNERGAQQHVLKKLL
AITELLQQKQNQYT
>d1nf1a_ a.116.1.2 (A:) GAP related domain of neurofibromin {Human (Homo sapiens)}
ERLVELVTMMGDQGELPIAMALANVVPCSQWDELARVLVTLFDSRHLLYQLLWNMFSKEVEL
ADSMQTLFRGNSLASKIMTFCFKVYGATYLQKLLDPLLRIVITSSDWQHVSFEVDPTRLEPS
ESLEENQRNLLQMTEKFFHAIISSSSEFPPQLRSVCHCLYQVVSQRFPQNSIGAVGSAMFLR
FINPAIVSPYEAGILDKKPPPIIERGLKLMSKILQSIANHVLFTKEEHMRPFNDFVKSNFDA
ARRFFLDIASDCPTSDAVNHSLSFISDGNVLALHRLLWNNQEKIGQYLSSNRDHKAVGRRPF
DKMATLLAYLGPPE
>d1bkds_ a.117.1.1 (S:) Son of sevenless-1 (sos-1) {Human (Homo sapiens)}
RLPSADVYRFAEPDSEENIIFEEGIPIIKAGTVIKLIERLTYHMYADPNFVRTFLTTYRSFC
KPQELLSLIIERFEIPEPRFRKEYIQPVQLRVLNVCRHWVEHHFYDFERDAYLLQRMEEFIG
TVRGKAMKKWVESITKIIQRKKITFQSSPPTVEWHISRPGHIETFDLLTLHPIEIARQLTLL
ESDLYRAVQPSELVGSVWTKEDKEINSPNLLKMIRHTTNLTLWFEKCIVETENLEERVAVVS
RIIEILQVFQELNNFNGVLEVVSAMNSSPVYRLDHTFEQIPSRQKKILEEAHELSEDHYKKY
LAKLRSINPPCVPFFGIYLTNILKTEEGNPEVLKRHGKELINFSKRRKVAEITGEIQQYQNQ
PYCLRVESDIKRFFENLNPMGNSMEKEFTDYLFNKSLEIEPRNPKPLPRFPKKYSYPLKSPG
VRPSN
>d1h6oa_ a.146.1.1 (A:) TRF1 {Human (Homo sapiens)}
EDAGLVAEAEAVAAGWMLDFLCLSLCRAFRDGRSEDFRRTRNSAEAIIHGLSSLTACQLRTI
YICQFLTRIAAGKTLDAQFENDERITPLESALMIWGSIEKEHDKLHEEIQNLIKIQAIAVCM
ENGNFKEAEEVFERIFGDPNSHMPFKSKLLMIISQKDTFHSFFQHFSYNHMMEKIKSYVNYV
LSEKSSTFLMKAAAKVVE
>d1h6pa_ a.146.1.1 (A:) TRF2 {Human (Homo sapiens)}
AGEARLEEAVNRWVLKFYFHEALRAFRGSRYGDFRQIRDIMQALLVRPLGKEHTVSRLLRVM
QCLSRIEEGENLDCSFDMEAELTPLESAINVLEMIKTEFTLTEAVVESSRKLVKEAAVIICI
KNKEFEKASKILKKHMSKDPTTQKLRNDLLNIIREKNLAHPVIQNFSYETFQQKMLRFLESH

LDDAEPYLLTMAKKALK
>d1g3jc_ a.118.1.1 (C:) beta-Catenin {Human (Homo sapiens)}
HHREGLLAIFKSGGIPALVKMLGSPVDSVLFYAITTLHNLLLHQEGAKMAVRLAGGLQKMVA
LLNKTNVKFLAITTDCLQILAYGNQESKLIILASGGPQALVNIMRTYTYEKLLWTTSRVLKV
LSVCSSNKPAIVEAGGMQALGLHLTDPSQRLVQNCLWTLRNLSDAATKQEGMEGLLGTLVQL
LGSDDINVVTCAAGILSNLTCNNYKNKMMVCQVGGIEALVRTVLRAGDREDITEPAICALRH
LTSRHQEAEMAQNAVRLHYGLPVVVKLLHPPSHWPLIKATVGLIRNLALCPANHAPLREQGA
IPRLVQLLVRAHQDTQRRTSMGGTQQQFVEGVRMEEIVEGCTGALHILARDVHNRIVIRGLN
TIPLFVQLLYSPIENIQRVAAGVLCELAQDKEAAEAIEAEGATAPLTELLHSRNEGVATYAA
AVLFRMSE
>d1jdha_ a.118.1.1 (A:) beta-Catenin {Human (Homo sapiens)}
AVVNLINYQDDAELATRAIPELTKLLNDEDQVVVNKAAVMVHQLSKKEASRHAIMRSPQMVS
AIVRTMQNTNDVETARCTAGTLHNLSHHREGLLAIFKSGGIPALVKMLGSPVDSVLFYAITT
LHNLLLHQEGAKMAVRLAGGLQKMVALLNKTNVKFLAITTDCLQILAYGNQESKLIILASGG
PQALVNIMRTYTYEKLLWTTSRVLKVLSVCSSNKPAIVEAGGMQALGLHLTDPSQRLVQNCL
WTLRNLSDAATKQEGMEGLLGTLVQLLGSDDINVVTCAAGILSNLTCNNYKNKMMVCQVGGI
EALVRTVLRAGDREDITEPAICALRHLTSRHQEAEMAQNAVRLHYGLPVVVKLLHPPSHWPL
IKATVGLIRNLALCPANHAPLREQGAIPRLVQLLVRAHQDTQRRTSMGGTQQQFVEGVRMEE
IVEGCTGALHILARDVHNRIVIRGLNTIPLFVQLLYSPIENIQRVAAGVLCELAQDKEAAEA
IEAEGATAPLTELLHSRNEGVATYAAAVLFRMS
>d1iala_ a.118.1.1 (A:) Importin alpha {Mouse (Mus musculus)}
DEQMLKRRNVSSFPDDATSPLQENRNNQGTVNWSVEDIVKGINSNNLESQLQATQAARKLLS
REKQPPIDNIIRAGLIPKFVSFLGKTDCSPIQFESAWALTNIASGTSEQTKAVVDGGAIPAF
ISLLASPHAHISEQAVWALGNIAGDGSAFRDLVIKHGAIDPLLALLAVPDLSTLACGYLRNL
TWTLSNLCRNKNPAPPLDAVEQILPTLVRLLHHNDPEVLADSCWAISYLTDGPNERIEMVVK
KGVVPQLVKLLGATELPIVTPALRAIGNIVTGTDEQTQKVIDAGALAVFPSLLTNPKTNIQK
EATWTMSNITAGRQDQIQQVVNHGLVPFLVGVLSKADFKTQKEAAWAITNYTSGGTVEQIVY
LVHCGIIEPLMNLLSAKDTKIIQVILDAISNIFQAAEKLGETEKLSIMIEECGGLDKIEALQ
RHENESVYKASLNLIEKYF
>d1ibrb_ a.118.1.1 (B:) Importin beta {Human (Homo sapiens)}
ELITILEKTVSPDRLELEAAQKFLERAAVENLPTFLVELSRVLANPGNSQVARVAAGLQIKN
SLTSKDPDIKAQYQQRWLAIDANARREVKNYVLQTLGTETYRPSSASQCVAGIACAEIPVNQ
WPELIPQLVANVTNPNSTEHMKESTLEAIGYICQDIDPEQLQDKSNEILTAIIQGMRKEEPS
NNVKLAATNALLNSLEFTKANFDKESERHFIMQVVCEATQCPDTRVRVAALQNLVKIMSLYY
QYMETYMGPALFAITIEAMKSDIDEVALQGIEFWSNVCDEEMDLAIEASEAAEQGRPPEHTS
KFYAKGALQYLVPILTQTLTKQDENDDDDDWNPCKAAGVCLMLLATCCEDDIVPHVLPFIKE
HIKNPDWRYRDAAVMAFGCILEGPEPSQLKPLVIQAMPTLIELMKDPSVVVRDTAAWTVGRI
CELLPEAAINDVYLAPLLQCLIEG
>d1qgra_ a.118.1.1 (A:) Importin beta {Human (Homo sapiens)}
MELITILEKTVSPDRLELEAAQKFLERAAVENLPTFLVELSRVLANPGNSQVARVAAGLQIK
NSLTSKDPDIKAQYQQRWLAIDANARREVKNYVLHTLGTETYRPSSASQCVAGIACAEIPVN
QWPELIPQLVANVTNPNSTEHMKESTLEAIGYICQDIDPEQLQDKSNEILTAIIQGMRKEEP
SNNVKLAATNALLNSLEFTKANFDKESERHFIMQVVCEATQCPDTRVRVAALQNLVKIMSLY
YQYMETYMGPALFAITIEAMKSDIDEVALQGIEFWSNVCDEEMDLAIEASEAAEQGRPPEHT

SKFYAKGALQYLVPILTQTLTKQDENDDDDDWNPCKAAGVCLMLLATCCEDDIVPHVLPFIK
EHIKNPDWRYRDAAVMAFGCILEGPEPSQLKPLVIQAMPTLIELMKDPSVVVRDTAAWTVGR
ICELLPEAAINDVYLAPLLQCLIEGLSAEPRVASNVCWAFSSLAEAAYEAADVADDQEEPAT
YCLSSSFELIVQKLLETTDRPDGHQNNLRSSAYESLMEIVKNSAKDCYPAVQKTTLVIMERL
QQVLQMESHIQSTSDRIQFNDLQSLLCATLQNVLRKVQHQDALQISDVVMASLLRMFQSTAG
SGGVQEDALMAVSTLVEVLGGEFLKYMEAFKPFLGIGLKNYAEYQVCLAAVGLVGDLCRALQ
SNIIPFCDEVMQLLLENLGNENVHRSVKPQILSVFGDIALAIGGEFKKYLEVVLNTLQQASQ
AQVDKSDYDMVDYLNELRESCLEAYTGIVQGLKGDQENVHPDVMLVQPRVEFILSFIDHIAG
DEDHTDGVVACAAGLIGDLCTAFGKDVLKLVEARPMIHELLTEGRRSKTNKAKTLARWATKE
LRKLKNQA
>d1qbkb_ a.118.1.1 (B:) Karyopherin beta2 {Human (Homo sapiens)}
YEWKPDEQGLQQILQLLKESQSPDTTIQRTVQQKLEQLNQYPDFNNYLIFVLTKLKSEDEPT
RSLSGLILKNNVKAHFQNFPNGVTDFIKSECLNNIGDSSPLIRATVGILITTIASKGELQNW
PDLLPKLCSLLDSEDYNTCEGAFGALQKICEDSAEILDSDVLDRPLNIMIPKFLQFFKHSSP
KIRSHAVACVNQFIISRTQALMLHIDSFTENLFALAGDEEPEVRKNVCRALVMLLEVRMDRL
LPHMHNIVEYMLQRTQDQDENVALEACEFWLTLAEQPICKDVLVRHLPKLIPVLVNGMKYSD
IDIILLKGDVEEDETIPDSEQDIRPRFHRSRTVAQQHDEDGIEEEDDDDDEIDDDDTISDWN
LRKCSAAALDVLANVYRDELLPHILPLLKELLFHHEWVVKESGILVLGAIAEGCMQGMIPYL
PELIPHLIQCLSDKKALVRSITCWTLSRYAHWVVSQPPDTYLKPLMTELLKRILDSNKRVQE
AACSAFATLEEEACTELVPYLAYILDTLVFAFSKYQHKNLLILYDAIGTLADSVGHHLNKPE
YIQMLMPPLIQKWNMLKDEDKDLFPLLECLSSVATALQSGFLPYCEPVYQRCVNLVQKTLAQ
AMLNNAQPDQYEAPDKDFMIVALDLLSGLAEGLGGNIEQLVARSNILTLMYQCMQDKMPEVR
QSSFALLGDLTKACFQHVKPCIADFMPILGTNLNPEFISVCNNATWAIGEISIQMGIEMQPY
IPMVLHQLVEIINRPNTPKTLLENTAITIGRLGYVCPQEVAPMLQQFIRPWCTSLRNIRDNE
EKDSAFRGICTMISVNPSGVIQDFIFFCDAVASWINPKDDLRDMFCKILHGFKNQVGDENWR
RFSDQFPLPLKERLAAFYGV
>d1ee4a_ a.118.1.1 (A:) Karyopherin alpha {Baker's yeast
(Saccharomyces cerevisiae)}
QELPQMTQQLNSDDMQEQLSATVKFRQILSREHRPPIDVVIQAGVVPRLVEFMRENQPEMLQ
LEAAWALTNIASGTSAQTKVVVDADAVPLFIQLLYTGSVEVKEQAIWALGNVAGDSTDYRDY
VLQCNAMEPILGLFNSNKPSLIRTATWTLSNLCRGKKPQPDWSVVSQALPTLAKLIYSMDTE
TLVDACWAISYLSDGPQEAIQAVIDVRIPKRLVELLSHESTLVQTPALRAVGNIVTGNDLQT
QVVINAGVLPALRLLLSSPKENIKKEACWTISNITAGNTEQIQAVIDANLIPPLVKLLEVAE
DKTKKEACWAISNASSGGLQRPDIIRYLVSQGCIKPLCDLLEIADNRIIEVTLDALENILKM
GEADKEARGLNINENADFIEKAGGMEKIFNCQQNENDKIYEKAYKIIETYF
>d1b3ua_ a.118.1.2 (A:) Constant regulatory domain of protein
phosphatase 2a, pr65alpha {Human (Homo sapiens)}
AAADGDDSLYPIAVLIDELRNEDVQLRLNSIKKLSTIALALGVERTRSELLPFLTDTIYDED
EVLLALAEQLGTFTTLVGGPEYVHCLLPPLESLATVEETVVRDKAVESLRAISHEHSPSDLE
AHFVPLVKRLAGGDWFTSRTSACGLFSVCYPRVSSAVKAELRQYFRNLCSDDTPMVRRAAAS
KLGEFAKVLELDNVKSEIIPMFSNLASDEQDSVRLLAVEACVNIAQLLPQEDLEALVMPTLR
QAAEDKSWRVRYMVADKFTELQKAVGPEITKTDLVPAFQNLMKDCEAEVRAAASHKVKEFCE
NLSADCRENVIMSQILPCIKELVSDANQHVKSALASVIMGLSPILGKDNTIEHLLPLFLAQL
KDECPEVRLNIISNLDCVNEVIGIRQLSQSLLPAIVELAEDAKWRVRLAIIEYMPLLAGQLG

VEFFDEKLNSLCMAWLVDHVYAIREAATSNLKKLVEKFGKEWAHATIIPKVLAMSGDPNYLH
RMTTLFCINVLSEVCGQDITTKHMLPTVLRMAGDPVANVRFNVAKSLQKIGPILDNSTLQSE
VKPILEKLTQDQDVDVKYFAQEALTVLSLA
>d1hu3a_ a.118.1.2 (A:) Eukaryotic initiation factor eIF4G {Human (Homo sapiens)}
SDPENIKTQELFRKVRSILNKLTPQMFNQLMKQVSGLTVDTEERLKGVIDLVFEKAIDEPSF
SVAYANMCRCLVTLKVPMADKPGNTVNFRKLLLNRCQKEFEKDKADDDVFEKKQKELEAASA
PEERTRLHDELEEAKDKARRRSIGNIKFIGELFKLKMLTEAIMHDCVVKLLKNHDEESLECL
CRLLTTIGKDLDFEKAKPRMDQYFNQMEKIVKERKTSSRIRFMLQDVIDLRLCNWVS
>d1h6ka1 a.118.1.2 (A:27-290) CBP80, 80KDa nuclear cap-binding protein {Human (Homo sapiens)}
TEDHLESLICKVGEKSACSLESNLEGLAGVLEADLPNYKSKILRLLCTVARLLPEKLTIYTT
LVGLLNARNYNFGGEFVEAMIRQLKESLKANNYNEAVYLVRFLSDLVNCHVIAAPSMVAMFE
NFVSVTQEEDVPQVRRDWYVYAFLSSLPWVGKELYEKKDAEMDRIFANTESYLKRRQKTHVP
MLQVWTADKPHPQEEYLDCLWAQIQKLKKDRWQERHILRPYLAFDSILCEALQHNLPPFTPP
PHTEDSVYPMPRVIFR
>d1h6ka2 a.118.1.2 (A:291-480) CBP80, 80KDa nuclear cap-binding protein {Human (Homo sapiens)}
MFDYTDDPEGPVMPGSHSVERFVIEENLHCIIKSHWKERKTCAAQLVSYPGKNKIPLNYHIV
EVIFAELFQLPAPPHIDVMYTTLLIELCKLQPGSLPQVLAQATEMLYMRLDTMNTTCVDRFI
NWFSHHLSNFQFRWSWEDWSDCLSQDPESPKPKFVREVLEKCMRLSYHQRILDIVPPTFSAL
CPSN
>d1h6ka3 a.118.1.2 (A:481-790) CBP80, 80KDa nuclear cap-binding protein {Human (Homo sapiens)}
PTCIYKYGDESSNSLPGHSVALCLAVAFKSKATNDEIFSILKDVPNPNQDDDDDEGFSFNPL
KIEVFVQTLLHLAAKSFSHSFSALAKFHEVFKTLAESDEGKLHVLRVMFEVWRNHPQMIAVL
VDKMIRTQIVDCAAVANWIFSSELSRDFTRLFVWEILHSTIRKMNKHVLKIQKELEEAKEKL
ARQHDGVLEEQIERLQEKVESAQSEQKNLFLVIFQRFIMILTEHLVRCETDGTSVLTPWYKN
CIERLQQIFLQHHQIIQQYMVTLENLLFTAELDPHILAVFQQFCALQA
>d1hs6a1 a.118.1.7 (A:461-610) Leukotriene A4 hydrolase C-terminal domain {Human (Homo sapiens)}
DMTLTNACIALSQRWITAKEDDLNSFNATDLKDLSSHQLNEFLAQTLQRAPLPLGHIKRMQE
VYNFNAINNSEIRFRWLRLCIQSKWEDAIPLALKMATEQGRMKFTRPLFKDLAAFDKSHDQA
VRTYQEHKASMHPVTAMLVGKDLKVD
>d1b89a_ a.118.1.3 (A:) Clathrin heavy chain proximal leg segment {Cow (Bos taurus)}
RLAELEEFINGPNNAHIQQVGDRCYDEKMYDAAKLLYNNVSNFGRLASTLVHLGEYQAAVDG
ARKANSTRTWKEVCFACVDGKEFRLAQMCGLHIVVHADELEELINYYQDRGYFEELITMLEA
ALGLERAHMGMFTELAILYSKFKPQKMREHLELFWSRVNIPKVLRAAEQAHLWAELVFLYDK
YEEYDNAIITMMNHPTDAWKEGQFKDIITKVANVELYYRAIQFYLEFKPLLLNDLLMVLSPR
LDHTRAVNYFSKVKQLPLVKPYLRSVQNHNNKSVNESLNNLFITEEDYQALRTSIDAYDNFD
NISLAQRLEKHELIEFRRIAAYLFKG
>d1bpoa1 a.118.1.4 (A:331-487) Clathrin heavy-chain linker domain {Rat (Rattus norvegicus)}

EENIIPYITNVLQNPDLALRMAVRNNLAGAEELFARKFNALFAQGNYSEAAKVAANAPKGIL
RTPDTIRRFQSVPAQPGQTSPLLQYFGILLDQGQLNKYESLELCRPVLQQGRKQLLEKWLKE
DKLECSEELGDLVKSVDPTLALSVYLRANVPNK
>d1c9la1 a.118.1.4 (A:331-359) Clathrin heavy-chain linker domain {Rat (Rattus norvegicus)}
EENIIPYITNVLQNPDLALRMAVRNNLAG
>d1lrv__ a.118.1.5 (-) Leucine-rich repeat variant {Azotobacter vinelandii}
TPIGDCRVCSFRMSLLLTGRCTPGDACVAVESGRQIDRFFRNNPHLAVQYLADPFWERRAIA
VRYSPVEALTPLIRDSDEVVRRAVAYRLPREQLSALMFDEDREVRITVADRLPLEQLEQMAA
DRDYLVRAYVVQRIPPGRLFRFMRDEDRQVRKLVAKRLPEESLGLMTQDPEPEVRRIVASRL
RGDDLLELLHDPDWTVRLAAVEHASLEALRELDEPDPEVRLAIAGRL
>d1e8xa1 a.118.1.6 (A:525-725) Phoshoinositide 3-kinase (PI3K) helical domain {Pig (Sus scrofa)}
HPIALPKHRPTPDPEGDRVRAEMPNQLRKQLEAIIATDPLNPLTAEDKELLWHFRYESLKDP
KAYPKLFSSVKWGQQEIVAKTYQLLAKREVWDQSALDVGLTMQLLDCNFSDENVRAIAVQKL
ESLEDDDVLHYLLQLVQAVKFEPYHDSALARFLLKRGLRNKRIGHFLFWFLRSEIAQSRHYQ
QRFAVILEAYLRGCG
>d1ib2a_ a.118.1.8 (A:) Pumilio 1 {Human (Homo sapiens)}
GRSRLLEDFRNNRYPNLQLREIAGHIMEFSQDQHGSRFIQLKLERATPAERQLVFNEILQAA
YQLMVDVFGNYVIQKFFEFGSLEQKLALAERIRGHVLSLALQMYGCRVIQKALEFIPSDQQN
EMVRELDGHVLKCVKDQNGNHVVQKCIECVQPQSLQFIIDAFKGQVFALSTHPYGCRVIQRI
LEHCLPDQTLPILEELHQHTEQLVQDQYGNYVIQHVLEHGRPEDKSKIVAEIRGNVLVLSQH
KFASNVVEKCVTHASRTERAVLIDEVCTMNDGPHSALYTMMKDQYANYVVQKMIDVAEPGQR
KIVMHKIRPHIA
>d1ho8a_ a.118.1.9 (A:) Regulatory subunit H of the V-type ATPase {Baker's yeast (Saccharomyces cerevisiae)}
GATKILMDSTHFNEIRSIIRSRSVAWDALARSEELSEIDASTAKALESILVKKNIGDGLSSS
NNAHSGFKVNGKTLIPLIHLLSTSDNEDCKKSVQNLIAELLSSDKYGDDTVKFFQEDPKQLE
QLFDVSLKGDFQTVLISGFNVVSLLVQNGLHNVKLVEKLLKNNNLINILQNIEQMDTCYVCI
RLLQELAVIPEYRDVIWLHEKKFMPTLFKILQRATDSQLATRIVATNSNHLGIQLQYHSLLL
IWLLTFNPVFANELVQKYLSDFLDLLKLVKITIKEKVSRLCISIILQCCSTRVKQHKKVIKQ
LLLLGNALPTVQSLSERKYSDEELRQDISNLKEILENEYQELTSFDEYVAELDSKLLCWSPP
HVDNGFWSDNIDEFKKDNYKIFRQLIELLQAKVRNGDVNAKQEKIIIQVALNDITHVVELLP
ESIDVLDKTGGKADIMELLNHSDSRVKYEALKATQAIIGYTFK
>d1ycsb1 a.118.2.1 (B:327-456) 53BP2 {Human (Homo sapiens)}
PLALLLDSSLEGEFDLVQRIIYEVDDPSLPNDEGITALHNAVCAGHTEIVKFLVQFGVNVNA
ADSDGWTPLHCAASCNNVQVCKFLVESGAAVFAMTYSDMQTAADKCEEMEEGYTQCSQFLYG
VQEKMG
>d1awcb_ a.118.2.1 (B:) GA bindinig protein (GABP) beta 1 {Mouse (Mus musculus)}
DLGKKLLEAARAGQDDEVRILMANGAPFTTDWLGTSPLHLAAQYGHFSTTEVLLRAGVSRDA
RTKVDRTPLHMAASEGHANIVEVLLKHGADVNAKDMLKMTALHWATEHNHQEVVELLIKYGA
DVHTQSKFCKTAFDISIDNGNEDLAEILQ

>d1bd8__ a.118.2.1 (-) Cell cycle inhibitor p19ink4D {Human (Homo sapiens)}
RAGDRLSGAAARGDVQEVRRLLHRELVHPDALNRFGKTALQVMMFGSTAIALELLKQGASPN
VQDTSGTSPVHDAARTGFLDTLKVLVEHGADVNVPDGTGALPIHLAVQEGHTAVVSFLAAES
DLHRRDARGLTPLELALQRGAQDLVDILQGHM
>d1blxb_ a.118.2.1 (B:) Cell cycle inhibitor p19ink4D {Mouse (Mus musculus)}
VCVGDRLSGAAARGDVQEVRRLLHRELVHPDALNRFGKTALQVMMFGSPAVALELLKQGASP
NVQDASGTSPVHDAARTGFLDTLKVLVEHGADVNALDSTGSLPIHLAIREGHSSVVSFLAPE
SDLHHRDASGLTPLELARQRGAQNLMDILQGHMMIP
>d1ihba_ a.118.2.1 (A:) p18ink4C(ink6) {Human (Homo sapiens)}
WGNELASAAARGDLEQLTSLLQNNVNVNAQNGFGRTALQVMKLGNPEIARRLLLRGANPDLK
DRTGFAVIHDAARAGFLDTLQTLLEFQADVNIEDNEGNLPLHLAAKEGHLRVVEFLVKHTAS
NVGHRNHKGDTACDLARLYGRNEVVSLMQANG
>d1a5e__ a.118.2.1 (-) Cell cycle inhibitor p16ink4A {Human (Homo sapiens)}
MEPAAGSSMEPSADWLATAAARGRVEEVRALLEAGALPNAPNSYGRRPIQVMMMGSARVAEL
LLLHGAEPNCADPATLTRPVHDAAREGFLDTLVVLHRAGARLDVRDAWGRLPVDLAEELGHR
DVARYLRAAAGGTRGSNHARIDAAEGPSDIPD
>d1bi7b_ a.118.2.1 (B:) Cell cycle inhibitor p16ink4A {Human (Homo sapiens)}
EPSADWLATAAARGRVEEVRALLEAGANPNAPNSYGRRPIQVMMMGSARVAELLLLHGAEPN
CADPATLTRPVHDAAREGFLDTLVVLHRAGARLDVRDAWGRLPVDLAEELGHRDVARYLRAA
A
>d1iknd_ a.118.2.1 (D:) I-kappa-B-alpha {Human (Homo sapiens)}
DGDSFLHLAIIHEEKALTMEVIRQVKGDLAFLNFQNNLQQTPLHLAVITNQPEIAEALLGAG
CDPELRDFRGNTPLHLACEQGCLASVGVLTQSCTTPHLHSILKATNYNGHTCLHLASIHGYL
GIVELLVSLGADVNAQEPCNGRTALHLAVDLQNPDLVSLLLKCGADVNRVTYQGYSPYQLTW
GRPSTRIQQQLGQLTLENLQMLPESEDEESYDTES
>d1k1aa_ a.118.2.1 (A:) bcl-3 {Human (Homo sapiens)}
EDGDTPLHIAVVQGNLPAVHRLVNLFQQGGRELDIYNNLRQTPLHLAVITTLPSVVRLLVTA
GASPMALDRHGQTAAHLACEHRSPTCLRALLDSAAPGTLDLEARNYDGLTALHVAVNTECQE
TVQLLLERGADIDAVDIKSGRSPLIHAVENNSLSMVQLLLQHGANVNAQMYSGSSALHSASG
RGLLPLVRTLVRSGADSSLKNCHNDTPLMVARSRRVIDILRG
>d1myo__ a.118.2.1 (-) Myotrophin {Rat (Rattus norvegicus)}
MCDKEFMWALKNGDLDEVKDYVAKGEDVNRTLEGGRKPLHYAADCGQLEILEFLLLKGADIN
APDKHHITPLLSAVYEGHVSCVKLLLSKGADKTVKGPDGLTALEATDNQAIKALLQ
>d1sw6a_ a.118.2.1 (A:) Swi6 ankyrin-repeat fragment {Baker's yeast (Saccharomyces cerevisiae)}
GPIITFTHDLTSDFLSSPLKIMKALPSPVVNDNEQKMKLEAFLQRLLFPEIQEMPTSLNNDS
SNRNSEGGSSNQQQQHVSFDSLLQEVNDAFPNTQLNLNIPVDEHGNTPLHWLTSIANLELVK
HLVKHGSNRLYGDNMGESCLVKAVKSVNNYDSGTFEALLDYLYPCLILEDSMNRTILHHIII
TSGMTGCSAAAKYYLDILMGWIVKKQNRPIQSGTNEKESKPNDKNGERKDSILENLDLKWII
ANMLNAQDSNGDTCLNIAARLGNISIVDALLDYGADPFIANKSGLRPVDFGAG

>d1dcqa1 a.118.2.1 (A:369-522) Pyk2-associated protein beta {Mouse (Mus musculus)}
ADTAAKLHSLCEAVKTRDIFGLLQAYADGVDLTEKIPLANGHEPDETALHLAVRSVDRTSLH
IVDFLVQNSGNLDKQTGKGSTALHYCCLTDNAECLKLLLRGKASIEIANESGETPLDIAKRL
KHEHCEELLTQALSGRFNSHVHVEYEWRLL
>d1pbv__ a.118.3.1 (-) Exchange factor ARNO {Human (Homo sapiens)}
ANEGSKTLQRNRKMAMGRKKFNMDPKKGIQFLVENELLQNTPEEIARFLYKGEGLNKTAIGD
YLGEREELNLAVLHAFVDLHEFTDLNLVQALRQFLWSFRLPGEAQKIDRMMEAFAQRYCLCN
PGVFQSTDTCYVLSFAVIMLNTSLHNPNVRDKPGLERFVAMNRGINEGGDLPEELLRNLYDS
IRNEPFKIP
>d1bc9__ a.118.3.1 (-) Cytohesin-1/b2-1 {Human (Homo sapiens)}
MKNMQRNKQVAMGRKKFNMDPKKGIQFLIENDLLKNTCEDIAQFLYKGEGLNKTAIGDYLGE
RDEFNIQVLHAFVELHEFTDLNLVQALRQFLWSFRLPGEAQKIDRMMEAFAQRYCQCNNGVF
QSTDTCYVLSFAIIMLNTSLHNPNVKDKPTVERFIAMNRGINDGGDLPEELLRNLYESIKNE
PFKIPELEHHHHHH
>d1qsaa1 a.118.5.1 (A:1-450) 70 KDa soluble lytic transglycosylase (SLT70), superhelical domain {Escherichia coli}
DSLDEQRSRYAQIKQAWDNRQMDVVEQMMPGLKDYPLYPYLEYRQITDDLMNQPAVTVTNFV
RANPTLPPARTLQSRFVNELARREDWRGLLAFSPEKPGTTEAQCNYYYAKWNTGQSEEAWQG
AKELWLTGKSQPNACDKLFSVWRASGKQDPLAYLERIRLAMKAGNTGLVTVLAGQMPADYQT
IASAIISLANNPNTVLTFARTTGATDFTRQMAAVAFASVARQDAENARLMIPSLAQAQQLNE
DQIQELRDIVAWRLMGNDVTDEQAKWRDDAIMRSQSTSLIERRVRMALGTGDRRGLNTWLAR
LPMEAKEKDEWRYWQADLLLERGREAEAKEILHQLMQQRGFYPMVAAQRIGEEYELKIDKAP
QNVDSALTQGPEMARVRELMYWNLDNTARSEWANLVKSKSKTEQAQLARYAFNNQWWDLSVQ
ATIAGKLWDHLEERFP
>d1d8da_ a.118.6.1 (A:) Protein farnesyltransferase alpha-subunit {Rat (Rattus norvegicus)}
FLSLDSPTYVLYRDRAEWADIDPVPQNDGPSPVVQIIYSEKFRDVYDYFRAVLQRDERSERA
FKLTRDAIELNAANYTVWHFRRVLLRSLQKDLQEEMNYIIAIIEEQPKNYQVWHHRRVLVEW
LKDPSQELEFIADILNQDAKNYHAWQHRQWVIQEFRLWDNELQYVDQLLKEDVRNNSVWNQR
HFVISNTTGYSDRAVLEREVQYTLEMIKLVPHNESAWNYLKGILQDRGLSRYPNLLNQLLDL
QPSHSSPYLIAFLVDIYEDMLENQCDNKEDILNKALELCEILAKEKDTIRKEYWRYIGRSLQ
SKHSRESDIPASV
>d1jcqa_ a.118.6.1 (A:) Protein farnesyltransferase alpha-subunit {Human (Homo sapiens)}
FVSLDSPSYVLYRDRAEWADIDPVPQNDGPNPVVQIIYSDKFRDVYDYFRAVLQRDERSERA
FKLTRDAIELNAANYTVWHFRRVLLKSLQKDLHEEMNYITAIIEEQPKNYQVWHHRRVLVEW
LRDPSQELEFIADILNQDAKNYHAWQHRQWVIQEFKLWDNELQYVDQLLKEDVRNNSVWNQR
YFVISNTTGYNDRAVLEREVQYTLEMIKLVPHNESAWNYLKGILQDRGLSKYPNLLNQLLDL
QPSHSSPYLIAFLVDIYEDMLENQCDNKEDILNKALELCEILAKEKDTIRKEYWRYIGRSLQ
SKH
>d1dcea1 a.118.6.1 (A:1-240,A:351-443) Rab geranylgeranyltransferase alpha-subunit, N-terminal domain {Rat (Rattus norvegicus)}

MHGRLKVKTSEEQAEAKRLEREQKLKLYQSATQAVFQKRQAGELDESVLELTSQILGANPDF
ATLWNCRREVLQHLETEKSPEESAALVKAELGFLESCLRVNPKSYGTWHHRCWLLSRLPEPN
WARELELCARFLEADERNFHCWDYRRFVAAQAAVAPAEELAFTDSLITRNFSNYSSWHYRSC
LLPQLHPQPDSGPQGRLPENVLLKELELVQNAFFTDPNDQSAWFYHRWLLGRAEXLFRCELS
VEKSTVLQSELESCKELQELEPENKWCLLTIILLMRALDPLLYEKETLQYFSTLKAVDPMRA
AYLDDLRSKFLLENSVLKMEYADV
>d1qjba_ a.118.7.1 (A:) zeta isoform {Human (Homo sapiens)}
MDKNELVQKAKLAEQAERYDDMAACMKSVTEQGAELSNEERNLLSVAYKNVVGARRSSWRVV
SSIEQKTEGAEKKQQMAREYREKIETELRDICNDVLSLLEKFLIPNASQAESKVFYLKMKGD
YYRYLAEVAAGDDKKGIVDQSQQAYQEAFEISKKEMQPTHPIRLGLALNFSVFYYEILNSPE
KACSLAKTAFDEAIAELDTLSEESYKDSTLIMQLLRDNLTLWTSDT
>d1a17__ a.118.8.1 (-) Protein phosphatase 5 {Human (Homo sapiens)}
PPADGALKRAEELKTQANDYFKAKDYENAIKFYSQAIELNPSNAIYYGNRSLAYLRTECYGY
ALGDATRAIELDKKYIKGYYRRAASNMALGKFRAALRDYETVVKVKPHDKDAKMKYQECNKI
VKQKAFERAIAGDEHKRSVVDSLDIESMTIEDEYS
>d1elra_ a.118.8.1 (A:) Hop {Human (Homo sapiens)}
GKQALKEKELGNDAYKKKDFDTALKHYDKAKELDPTNMTYITNQAAVYFEKGDYNKCRELCE
KAIEVGRENREDYRQIAKAYARIGNSYFKEEKYKDAIHFYNKSLAEHRTPDVLKKCQQAEKI
LKEQ
>d1elwa_ a.118.8.1 (A:) Hop {Human (Homo sapiens)}
EQVNELKEKGNKALSVGNIDDALQCYSEAIKLDPHNHVLYSNRSAAYAKKGDYQKAYEDGCK
TVDLKPDWGKGYSRKAAALEFLNRFEEAKRTYEEGLKHEANNPQLKEGLQNMEAR
>d1qqea_ a.118.8.1 (A:) Vesicular transport protein sec17 {Baker's yeast (Saccharomyces cerevisiae)}
ISDPVELLKRAEKKGVPSSGFMKLFSGSDSYKFEEAADLCVQAATIYRLRKELNLAGDSFLK
AADYQKKAGNEDEAGNTYVEAYKCFKSGGNSVNAVDSLENAIQIFTHRGQFRRGANFKFELG
EILENDLHDYAKAIDCYELAGEWYAQDQSVALSNKCFIKCADLKALDGQYIEASDIYSKLIK
SSMGNRLSQWSLKDYFLKKGLCQLAATDAVAAARTLQEGQSEDPNFADSRESNFLKSLIDAV
NEGDSEQLSEHCKEFDNFMRLDKWKITILNKIKESIQQQEDD
>d1hh8a_ a.118.8.1 (A:) Neutrophil cytosolic factor 2 (NCF-2, p67-phox) {Human (Homo sapiens)}
SLVEAISLWNEGVLAADKKDWKGALDAFSAVQDPHSRICFNIGCMYTILKNMTEAEKAFTRS
INRDKHLAVAYFQRGMLYYQTEKYDLAIKDLKEALIQLRGNQLIDYKILGLQFKLFACEVLY
NIAFMYAKKEEWKKAEEQLALATSMKSEPRHSKIDKAMECVWKQKLYEPVVIPVGRLFRPNE
RQVAQL
>d1fcha_ a.118.8.1 (A:) Peroxin pex5 (peroxisomal targeting signal 1 (PTS1) receptor) {Human (Homo sapiens)}
SATYDKGYQFEEENPLRDHPQPFEEGLRRLQEGDLPNAVLLFEAAVQQDPKHMEAWQYLGTT
QAENEQELLAISALRRCLELKPDNQTALMALAVSFTNESLQRQACEILRDWLRYTPAYAHLV
TPAEEGAGGAGLGPSKRILGSLLSDSLFLEVKELFLAAVRLDPTSIDPDVQCGLGVLFNLSG
EYDKAVDCFTAALSVRPNDYLLWNKLGATLANGNQSEEAVAAYRRALELQPGYIRSRYNLGI
SCINLGAHREAVEHFLEALNMQRKSRGPRGEGGAMSENIWSTLRLALSMLGQSDAYGAADAR
DLSTLLTMFGLPQ
>d1hxia_ a.118.8.1 (A:) Peroxin pex5 (peroxisomal targeting signal

1 (PTS1) receptor) {Trypanosoma brucei}
NNTDYPFEANNPYMYHENPMEEGLSMLKLANLAEAALAFEAVCQKEPEREEAWRSLGLTQAE
NEKDGLAIIALNHARMLDPKDIAVHAALAVSHTNEHNANAALASLRAWLL
>d1ihga1 a.118.8.1 (A:197-365) Cyclophilin 40 {Cow (Bos taurus)}
GSGDSHPDFPEDADVDLKDVDKILLISEDLKNIGNTFFKSQNWEMAIKKYTKVLRYVEGSRA
AAEDADGAKLQPVALSCVLNIGACKLKMSDWQGAVDSCLEALEIDPSNTKALYRRAQGWQGL
KEYDQALADLKKAQEIAPEDKAIQAELLKVKQKIKAQKDKEKAAY
>d1iipa1 a.118.8.1 (A:197-298) Cyclophilin 40 {Cow (Bos taurus)}
GSGDSHPDFPEDADVDLKDVDKILLISEDLKNIGNTFFKSQNWEMAIKKYTKVLRYVEGSRA
AAEDADGAKLQPVALSCVLNIGACKLKMSDWQGAVDSCLE
>d1hz4a_ a.118.8.2 (A:) Transcription factor MalT domain III
{Escherichia coli}
EIKDIREDTMHAEFNALRAQVAINDGNPDEAERLAKLALEELPPGWFYSRIVATSVLGEVLH
CKGELTRSLALMQQTEQMARQHDVWHYALWSLIQQSEILFAQGFLQTAWETQEKAFQLINEQ
HLEQLPMHEFLVRIRAQLLWAWARLDEAEASARSGIEVLSSYQPQQQLQCLAMLIQCSLARG
DLDNARSQLNRLENLLGNGKYHSDWISNANKVRVIYWQMTGDKAAAANWLRHTAKPEFANNH
FLQGQWRNIARAQILLGEFEPAEIVLEELNENARSLRLMSDLNRNLLLLNQLYWQAGRKSDA
QRVLLDALKLANRTGFISHFVIEGEAMAQQLRQLIQLNTLPELEQHRAQRILREIN
>d1eyha_ a.118.9.1 (A:) Epsin 1 {Rat (Rattus norvegicus)}
HNYSEAEIKVREATSNDPWGPSSSLMSEIADLTYNVVAFSEIMSMIWKRLNDHGKNWRHVYK
AMTLMEYLIKTGSERVSQQCKENMYAVQTLKDFQYVDRDGKDQGVNVREKAKQLVALLRDED
RLREERAHALKTKEKLAQTA
>d1inza_ a.118.9.1 (A:) Epsin 1 {Human (Homo sapiens)}
GSSRMSTSSLRRQMKNIVHNYSEAEIKVREATSNDPWGPSSSLMSEIADLTYNVVAFSEIMS
MIWKRLNDHGKNWRHVYKAMTLMEYLIKTGSERVSQQCKENMYAVQTLKDFQYVDRDGKDQG
VNVREKAKQLVALLRDEDRLREER
>d1dvpa1 a.118.9.2 (A:1-145) Hrs {Fruit fly (Drosophila
melanogaster)}
MFRSSFCKNLENATSHLRLEPDWPSILLICDEINQKDVTPKNAFAAIKKKMNSPNPHSSCYS
LLVLESIVKNCGAPVHEEVFTKENCEMFSSFLESTPHENVRQKMLELVQTWAYAFRSSDKYQ
AIKDTMTILKAKGHTFPELRE
>d1elka_ a.118.9.2 (A:) Tom1 protein {Human (Homo sapiens)}
SDFLLGNPFSSPVGQRIEKATDGSLQSEDWALNMEICDIINETEEGPKDALRAVKKRIVGNK
NFHEVMLALTVLETCVKNCGHRFHVLVASQDFVESVLVRTILPKNNPPTIVHDKVLNLIQSW
ADAFRSSPDLTGVVTIYEDLRRKGLEFPM
>d1juqa_ a.118.9.2 (A:) Gga3 {Human (Homo sapiens)}
ESLESWLNKATNPSNRQEDWEYIIGFCDQINKELEGPQIAVRLLAHKIQSPQEWEALQALTV
LEACMKNCGRRFHNEVGKFRFLNELIKVVSPKYLGDRVSEKVKTKVIELLYSWTMALPEEAK
IKDAYHMLKRQGIVQSDPPIPVDRTLI
>d1hf8a_ a.118.10.1 (A:) Clathrin assembly lymphoid myeloid
leukaemia protein, Calm {Rat (Rattus norvegicus)}
GSAVSKTVCKATTHEIMGPKKKHLDYLIQCTNEMNVNIPQLADSLFERTTNSSWVVVFKSLI
TTHHLMVYGNERFIQYLASRNTLFNLSNFLDKSGLQGYDMSTFIRRYSRYLNEKAVSYRQVA
FDFTKVKRGADGVMRTMNTEKLLKTVPIIQNQMDALLDFNVNSNELTNGVINAAFMLLFKDA

IRLFAAYNEGIINLLEKYFDMKKNQCKEGLDIYKKFLTRMTRISEFLKVAEQVGIDRGDIPD
LSQAPSSLLDALEQH
>d1hx8a_ a.118.10.1 (A:) AP180 (Lap) {Fruit fly (Drosophila melanogaster)}
QGLAKSVCKATTEECIGPKKKHLDYLVHCANEPNVSIPHLANLLIERSQNANWVVVYKSLIT
THHLMAYGNERFMQYLASSNSTFNLSSFLDKGTVQDGGMGVPGGRMGYDMSPFIRRYAKYLN
EKSLSYRAMAFDFCKVKRGKEEGSLRSMNAEKLLKTLPVLQAQLDALLEFDCQSNDLSNGVI
NMSFMLLFRDLIRLFACYNDGIINLLEKYFDMNKKHARDALDLYKKFLVRMDRVGEFLKVAE
NVGIDKGDIPDLTKAPSSLLDALEQHLATL
>d1kpsb_ a.118.12.1 (B:) Ran-GTPase activating protein 1 (RanGAP1), C-terminal domain {Mouse (Mus musculus)}
TDLSTFLSFPSPEKLLRLGPKVSVLIVQQTDTSDPEKVVSAFLKVASVFRDDASVKTAVLDA
IDALMKKAFSCSSFNSNTFLTRLLIHMGLLKSEDKIKAIPSLHGPLMVLNHVVRQDYFPKAL
APLLLAFVTKPNGALETCSFARHNLLQTLYNI
>d1k8kg_ a.118.13.1 (G:) Arp2/3 complex 16 kDa subunit ARPC5 {Cow (Bos taurus)}
ARFRKVDVDEYDENKFVDEDDGGDGQAGPDEGEVDSCLRQGNMTAALQAALKNPPINTKSQA
VKDRAGSIVLKVLISFKANDIEKAVQSLDKNGVDLLMKYIYKGFESPSDNSSAVLLQWHEKA
LAAGGVGSIVRVLTARKTV
>d1ocre_ a.118.11.1 (E:) Cytochrome c oxidase subunit E {Cow (Bos taurus)}
SHGSHETDEEFDARWVTYFNKPDIDAWELRKGMNTLVGYDLVPEPKIIDAALRACRRLNDFA
SAVRILEVVKDKAGPHKEIYPYVIQELRPTLNELGISTPEELGLDKV
>d1f8na1 a.119.1.1 (A:150-839) Lipoxigenase, C-terminal domain {Soybean (Glycine max), isozyme L1}
VPSETPAPLVSYREEELKSLRGNGTGERKEYDRIYDYDVYNDLGNPDKSEKLARPVLGGSST
FPYPRRGRTGRGPTVTDPNTEKQGEVFYVPRDENLGHLKSKDALEIGTKSLSQIVQPAFESA
FDLKSTPIEFHSFQDVHDLYEGGIKLPRDVISTIIPLPVIKELYRTDGQHILKFPQPHVVQV
SQSAWMTDEEFAREMIAGVNPCVIRGLEEFPPKSNLDPAIYGDQSSKITADSLDLDGYTMDE
ALGSRRLFMLDYHDIFMPYVRQINQLNSAKTYATRTILFLREDGTLKPVAIELSLPHSAGDL
SAAVSQVVLPAKEGVESTIWLLAKAYVIVNDSCYHQLMSHWLNTHAAMEPFVIATHRHLSVL
HPIYKLLTPHYRNNMNINALARQSLINANGIIETTFLPSKYSVEMSSAVYKNWVFTDQALPA
DLIKRGVAIKDPSTPHGVRLLIEDYPYAADGLEIWAAIKTWVQEYVPLYYARDDDVKNDSEL
QHWWKEAVEKGHGDLKDKPWWPKLQTLEDLVEVCLIIIWIASALHAAVNFGQYPYGGLIMNR
PTASRRLLPEKGTPEYEEMINNHEKAYLRTITSKLPTLISLSVIEILSTHASDEVYLGQRDN
PHWTSDSKALQAFQKFGNKLKEIEEKLVRRNNDPSLQGNRLGPVQLPYTLLYPSSEEGLTFR
GIPNSISI
>d1ik3a1 a.119.1.1 (A:168-857) Lipoxigenase, C-terminal domain {Soybean (Glycine max), isozyme L3}
LPSETPAPLVKYREEELHNLRGDGTGERKEWERIYDYDVYNDLGDPDKGENHARPVLGGNDT
FPYPRRGRTGRKPTRKDPNSESRSNDVYLPRDEAFGHLKSSDFLTYGLKSVSQNVLPLLQSA
FDLNFTPREFDSFDEVHGLYSGGIKLPTDIISKISPLPVLKEIFRTDGEQALKFPPPKVIQV
SKSAWMTDEEFAREMLAGVNPNLIRCLKDFPPRSKLDSQVYGDHTSQITKEHLEPNLEGLTV
DEAIQNKRLFLLDHHDPIMPYLRRINATSTKAYATRTILFLKNDGTLRPLAIELSLPHPQGD

QSGAFSQVFLPADEGVESSIWLLAKAYVVVNDSCYHQLVSHWLNTHAVVEPFIIATNRHLSV
VHPIYKLLHPHYRDTMNINGLARLSLVNDGGVIEQTFLWGRYSVEMSAVVYKDWVFTDQALP
ADLIKRGMAIEDPSCPHGIRLVIEDYPYTVDGLEIWDAIKTWVHEYVFLYYKSDDTLREDPE
LQACWKELVEVGHGDKKNEPWWPKMQTREELVEACAIIIWTASALHAAVNFGQYPYGGLILN
RPTLSRRFMPEKGSAEYEELRKNPQKAYLKTITPKFQTLIDLSVIEILSRHASDEVYLGERD
NPNWTSDTRALEAFKRFGNKLAQIENKLSERNNDEKLRNRCGPVQMPYTLLLPSSKEGLTFR
GIPNSISI
>d1lox_1 a.119.1.2 (113-663) 15-Lipoxygenase {Rabbit (Oryctolagus cuniculus)}
TGCTTVGDPQGLFQKHREQELEERRKLYQWGSWKEGLILNVAGSKLTDLPVDERFLEDKKID
FEASLAWGLAELALKNSLNILAPWKTLDDFNRIFWCGRSKLARRVRDSWQEDSLFGYQFLNG
ANPMLLRRSVQLPARLVFPPGMEELQAQLEKELKAGTLFEADFALLDNIKANVILYCQQYLA
APLVMLKLQPDGKLMPMVIQLHLPKIGSSPPPLFLPTDPPMVWLLAKCWVRSSDFQVHELNS
HLLRGHLMAEVFTVATMRCLPSIHPVFKLIVPHLRYTLEINVRARNGLVSDFGIFDQIMSTG
GGGHVQLLQQAGAFLTYRSFCPPDDLADRGLLGVESSFYAQDALRLWEIISRYVQGIMGLYY
KTDEAVRDDLELQSWCREITEIGLQGAQKQGFPTSLQSVAQACHFVTMCIFTCTGQHSSIHL
GQLDWFTWVPNAPCTMRLPPPTTKDATLETVMATLPNLKQSSLQMSIVWQLGRDQPIMVPLG
QHQEEYFSGPEPRAVLEKFREELAIMDKEIEVRNEKLDIPYEYLRPSIVENSVAI
>d1c1ka_ a.120.1.1 (A:) gene 59 helicase assembly protein {Bacteriophage T4}
MIKLRMPAGGERYIDGKSVYKLYLMIKQHMNGKYDVIKYNWCMRVSDAAYQKRRDKYFFQKL
SEKYKLKELALIFISNLVANQDAWIGDISDADALVFYREYIGRLKQIKFKFEEDIRNIYYFS
KKVEVSAFKEIFEYNPKVQSSYIFKLLQSNIISFETFILLDSFLNIIDKHDEQTDNLVWNNY
SIKLKAYRKILNIDSQKAKNVFIETVKSCKY
>d2tct_2 a.121.1.1 (68-208) Tetracyclin repressor (Tet-repressor, TetR) {Escherichia coli}
LPAAGESWQSFLRNNAMSFRRALLRYRDGAKVHLGTRPDEKQYDTVETQLRFMTENGFSLRD
GLYAISAVSHFTLGAVLEQQEHTAALTDRPAAPDENLPPLLREALQIMDSDDGEQAFLHGLE
SLIRGFEVQLTALLQIV
>d1jt6a2 a.121.1.1 (A:73-187) Multidrug binding protein QacR {Staphylococcus aureus}
KTNREKFYLYNELSLTTEYYYPLQNAIIEFYTEYYKTNSINEKMNKLENKYIDAYHVIFKEG
NLNGEWSINDVNAVSKIAANAVNGIVTFTHEQNINERIKLMNKFSQIFLNGLS
>d1fqva1 a.122.1.1 (A:107-145) Skp1-Skp2 dimerisation domains {Human (Homo sapiens)}
VSWDSLPDELLLGIFSCLCLPELLKVSGVCKRWYRLASD
>d1fqvb1 a.122.1.1 (B:85-160) Skp1-Skp2 dimerisation domains {Human (Homo sapiens)}
IPVWDQEFLKVDQGTLFELILAANYLDIKGLLDVTCKTVANMIKGKTPEEIRKTFNIKNDFT
EEEEAQVRKENQWC
>d1fs1a1 a.122.1.1 (A:109-149) Skp1-Skp2 dimerisation domains {Human (Homo sapiens)}
WDSLPDELLLGIFSCLCLPELLKVSGVCKRWYRLASDESLW
>d1fs1b1 a.122.1.1 (B:86-140) Skp1-Skp2 dimerisation domains

{Human (Homo sapiens)}
PVWDQEFLKVDQGTLFELILAANYLDIKGLLDVTCKTVANMIKGKTPEEIRKTFN
>d1fs2b1 a.122.1.1 (B:80-146) Skp1-Skp2 dimerisation domains {Human (Homo sapiens)}
KRTDDIPVWDQEFLKVDQGTLFELILAANYLDIKGLLDVTCKTVANMIKGKTPEEIRKTFNI
KNDFT
>d1g5ya_ a.123.1.1 (A:) Retinoid-X receptor (RXR-alpha) {Human (Homo sapiens)}
PVERILEAELAVEPKTETYVEANMGLNPSSPNDPVTNICQAADKQLFTLVEWAKRIPHFSEL
PLDDQVILLRAGWNELLIASFSHRSIAVKDGILLATGLHVHRNSAHSAGVGAIFDRVLTELV
SKMRDMQMDKTELGCLRAIVLFNPDSKGLSNPAEVEALREKVYASLEAYCKHKYPEQPGRFA
KLLLRLPALRSIGLKCLEHLFFFKLIGDTPIDTFLMEMLEAP
>d1dkfb_ a.123.1.1 (B:) Retinoic acid receptor alpha (RAR-alpha) {Human (Homo sapiens)}
PEVGELIEKVRKAHQETFPALCQLGKYTTNNSSEQRVSLDIDLWDKFSELSTKCIIKTVEFA
KQLPGFTTLTIADQITLLKAACLDILILRICTRYTPEQDTMTFSDGLTLNRTQMHNAGFGPL
TDLVFAFANQLLPLEMDDAETGLLSAICLICGDRQDLEQPDRVDMLQEPLLEALKVYVRKRR
PSRPHMFPKMLMKITDLRSISAKGAERVITLKMEIPGSMPPLIQEMLEN
>d1fcya_ a.123.1.1 (A:) Retinoic acid receptor gamma (RAR-gamma) {Human (Homo sapiens)}
ASPQLEELITKVSKAHQETFPSLCQLGKYTTNSSADHRVQLDLGLWDKFSELATKCIIKIVE
FAKRLPGFTGLSIADQITLLKAACLDILMLRICTRYTPEQDTMTFSDGLTLNRTQMHNAGFG
PLTDLVFAFAGQLLPLEMDDTETGLLSAICLICGDRMDLEEPEKVDKLQEPLLEALRLYARR
RRPSQPYMFPRMLMKITDLRGISTKGAERAITLKMEIPGPMPPLIREMLE
>d1a28a_ a.123.1.1 (A:) Progesterone receptor {Human (Homo sapiens)}
QLIPPLINLLMSIEPDVIYAGHDNTKPDTSSSLLTSLNQLGERQLLSVVKWSKSLPGFRNLH
IDDQITLIQYSWMSLMVFGLGWRSYKHVSGQMLYFAPDLILNEQRMKESSFYSLCLTMWQIP
QEFVKLQVSQEEFLCMKVLLLLNTIPLEGLRSQTQFEEMRSSYIRELIKAIGLRQKGVVSSS
QRFYQLTKLLDNLHDLVKQLHLYCLNTFIQSRALSVEFPEMMSEVIAAQLPKILAGMVKPLL
FHK
>d3erda_ a.123.1.1 (A:) Estrogen receptor alpha {Human (Homo sapiens)}
SLALSLTADQMVSALLDAEPPILYSEYDPTRPFSEASMMGLLTNLADRELVHMINWAKRVPG
FVDLTLHDQVHLLECAWLEILMIGLVWRSMEHPGKLLFAPNLLLDRNQGKCVEGMVEIFDML
LATSSRFRMMNLQGEEFVCLKSIILLNSGVYTFLSSTLKSLEEKDHIHRVLDKITDTLIHLM
AKAGLTLQQQHQRLAQLLLILSHIRHMSNKGMEHLYSMKCKNVVPLYDLLLEMLDAHRLH
>d1qkma_ a.123.1.1 (A:) Estrogen receptor beta {Human (Homo sapiens)}
LDALSPEQLVLTLLEAEPPHVLISRPSAPFTEASMMMSLTKLADKELVHMISWAKKIPGFVE
LSLFDQVRLLESCWMEVLMMGLMWRSIDHPGKLIFAPDLVLDRDEGKCVEGILEIFDMLLAT
TSRFRELKLQHKEYLCVKAMILLNSSMYPLVTATQDADSSRKLAHLLNAVTDALVWVIAKSG
ISSQQQSMRLANLLMLLSHVRHASNKGMEHLLNMKCKNVVPVYDLLLEMLNAHVL
>d1qkna_ a.123.1.1 (A:) Estrogen receptor beta {Rat (Rattus

norvegicus)}
TLSPEQLVLTLLEAEPPNVLVSRPSMPFTEASMMMSLTKLADKELVHMIGWAKKIPGFVELS
LLDQVRLLESCWMEVLMVGLMWRSIDHPGKLIFAPDLVLDRDEGKCVEGILEIFDMLLATTS
RFRELKLQHKEYLCVKAMILLNSSMYPLASANQEAESSRKLTHLLNAVTDALVWVIAKSGIS
SQQQSVRLANLLMLLSHVRHISNKGMEHLLSMKCKNVVPVYDLLLEMLNA
>d1i37a_ a.123.1.1 (A:) Androgen receptor {Rat (Rattus
norvegicus)}
IFLNVLEAIEPGVVCAGHDNNQPDSFAALLSSLNELGERQLVHVVKWAKALPGFRNLHVDDQ
MAVIQYSWMGLMVFAMGWRSFTNVNSRMLYFAPDLVFNEYRMHKSRMYSQCVRMRHLSQEFG
WLQITPQEFLCMKALLLFSIIPVDGLKNQKFFDELRMNYIKELDRIIACKRKNPTSCSRRFY
QLTKLLDSVQPIARELHQFTFDLLIKSHMVSVDFPEMMAEIISVQVPKILSGKVKPIYFH
>d1k7la_ a.123.1.1 (A:) Peroxisome proliferator activated receptor
alpha, PPAR-alpha {Human (Homo sapiens)}
DLKSLAKRIYEAYLKNFNMNKVKARVILSGKASNNPPFVIHDMETLCMAEKTLVAKLVANGI
QNKEAEVRIFHCCQCTSVETVTELTEFAKAIPGFANLDLNDQVTLLKYGVYEAIFAMLSSVM
NKDGMLVAYGNGFITREFLKSLRKPFCDIMEPKFDFAMKFNALELDDSDISLFVAAIICCGD
RPGLLNVGHIEKMQEGIVHVLRLHLQSNHPDDIFLFPKLLQKMADLRQLVTEHAQLVQIIKK
TESDAALHPLLQEIYRDMY
>d2prga_ a.123.1.1 (A:) Peroxisome proliferator activated receptor
gamma, PPAR-gamma {Human (Homo sapiens)}
ESADLRALAKHLYDSYIKSFPLTKAKARAILTGKTTDKSPFVIYDMNSLMMGEDKIKFKHIT
PLQEQSKEVAIRIFQGCQFRSVEAVQEITEYAKSIPGFVNLDLNDQVTLLKYGVHEIIYTML
ASLMNKDGVLISEGQGFMTREFLKSLRKPFGDFMEPKFEFAVKFNALELDDSDLAIFIAVII
LSGDRPGLLNVKPIEDIQDNLLQALELQLKLNHPESSQLFAKLLQKMTDLRQIVTEHVQLLQ
VIKKTETDMSLHPLLQEIYKDLY
>d2gwxa_ a.123.1.1 (A:) Peroxisome proliferator-activated
receptor delta, PPAR-DELTA {Human (Homo sapiens)}
LKAFSKHIYNAYLKNFNMTKKKARSILTGKASHTAPFVIHDIETLWQAEKGLVWKQLVNGLP
PYKEISVHVFYRCQCTTVETVRELTEFAKSIPSFSSLFLNDQVTLLKYGVHEAIFAMLASIV
NKDGLLVANGSGFVTREFLRSLRKPFSDIIEPKFEFAVKFNALELDDSDLALFIAAIILCGD
RPGLMNVPRVEAIQDTILRALEFHLQANHPDAQQLFPKLLQKMADLRQLVTEHAQMMQRIKK
TETETSLHPLLQEIYKDM
>d1ilga_ a.123.1.1 (A:) Pregnane x receptor, PXR {Human (Homo
sapiens)}
GLTEEQRMMIRELMDAQMKTFDTTFSHFKNFRLPGVLSSGCELPESLQAPSREEAAKWSQVR
KDLCSLKVSLQLRGEDGSVWNYKPPADSGGKEIFSLLPHMADMSTYMFKGIISFAKVISYFR
DLPIEDQISLLKGAAFELCQLRFNTVFNAETGTWECGRLSYCLEDTAGGFQQLLLEPMLKFH
YMLKKLQLHEEEYVLMQAISLFSPDRPGVLQHRVVDQLQEQFAITLKSYIECNRPQPAHRFL
FLKIMAMLTELRSINAQHTQRLLRIQDIHPFATPLMQELFGI
>d1ie9a_ a.123.1.1 (A:) Vitamin D nuclear receptor {Human (Homo
sapiens)}
DSLRPKLSEEQQRIIAILLDAHHKTYDPTYSDFCQFRPPVRVNDGGGSVTLELSQLSMLPHL
ADLVSYSIQKVIGFAKMIPGFRDLTSEDQIVLLKSSAIEVIMLRSNESFTMDDMSWTCGNQD
YKYRVSDVTKAGHSLELIEPLIKFQVGLKKLNLHEEEHVLLMAICIVSPDRPGVQDAALIEA

IQDRLSNTLQTYIRCRHPPPGSHLLYAKMIQKLADLRSLNEEHSKQYRCLSFQPECSMKLTP
LVLEVFG
>d1bsxa_ a.123.1.1 (A:) Thyroid hormone receptor beta (TR-beta) {Human (Homo sapiens)}
KPEPTDEEWELIKTVTEAHVATNAQGSHWKQKRKFLPEDIGQAPIVNAPEGGKVDLEAFSHF
TKIITPAITRVVDFAKKLPMFCELPCEDQIILLKGCCMEIMSLRAAVRYDPESETLTLNGEM
AVTRGQLKNGGLGVVSDAIFDLGMSLSSFNLDDTEVALLQAVLLMSSDRPGLACVERIEKYQ
DSFLLAFEHYINYRKHHVTHFWPKLLMKVTDLRMIGACHASRFLHMKVECPTELFPPLFLEV
FED
>d1hg4a_ a.123.1.1 (A:) Ultraspiracle protein, usp {Drosophila melanogaster}
FSIERIIEAEQRAETQCGDRALTFLRVGPYSTVQPDYKGAVSALCQVVNKQLFQMVEYARMM
PHFAQVPLDDQVILLKAAWIELLIANVAWCSIVSLDDGGAGGGGGGLGHDGSFERRSPGLQP
QQLFLNQSFSYHRNSAIKAGVSAIFDRILSELSVKMKRLNLDRRELSCLKAIILYNPDIRGI
KSRAEIEMCREKVYACLDEHCRLEHPGDDGRFAQLLLRLPALRSISLKCQDHLFLFRITSDR
PLEELFLEQLEAPPPPG
>d1g2na_ a.123.1.1 (A:) Ultraspiracle protein, usp {Heliothis virescens}
AAVQELSIERLLEMESLVADPSEEFQFLRVGPDSNVPPKFRAPVSSLCQIGNKQIAALVVWA
RDIPHFSQLEMEDQILLIKGSWNELLLFAIAWRSMEFLTEERDGVDGTGNRTTSPPQLMCLM
PGMTLHRNSALQAGVGQIFDRVLSELSLKMRTLRVDQAEYVALKAIILLNPDVKGLKNRQEV
EVLREKMFLCLDEYCRRSRSSEEGRFAALLLRLPALRSISLKSFEHLFFFHLVADTSIAGYI
RDALRNHA
>d1ah7__ a.124.1.1 (-) Bacterial phosholipase C {Bacillus cereus}
WSAEDKHKEGVNSHLWIVNRAIDIMSRNTTLVKQDRVAQLNEWRTELENGIYAADYENPYYD
NSTFASHFYDPDNGKTYIPFAKQAKETGAKYFKLAGESYKNKDMKQAFFYLGLSLHYLGDVN
QPMHAANFTNLSYPQGFHSKYENFVDTIKDNYKVTDGNGYWNWKGTNPEEWIHGAAVVAKQD
YSGIVNDNTKDWFVKAAVSQEYADKWRAEVTPMTGKRLMDAQRVTAGYIQLWFDTYGDR
>d1ca1_1 a.124.1.1 (1-249) Alpha-toxin, N-terminal domain {Clostridium perfringens}
WDGKIDGTGTHAMIVTQGVSILENDLSKNEPESVRKNLEILKENMHELQLGSTYPDYDKNAY
DLYQDHFWDPDTDNNFSKDNSWYLAYSIPDTGESQIRKFSALARYEWQRGNYKQATFYLGEA
MHYFGDIDTPYHPANVTAVDSAGHVKFETFAEERKEQYKINTVGCKTNEDFYADILKNKDFN
AWSKEYARGFAKTGKSIYYSHASMSHSWDDWDYAAKVTLANSQKGTAGYIYRFLHDVSEGND
P
>d1ak0__ a.124.1.2 (-) P1 nuclease {Penicillium citrinum}
WGALGHATVAYVAQHYVSPEAASWAQGILGSSSSSYLASIASWADEYRLTSAGKWSASLHFI
DAEDNPPTNCNVDYERDCGSSGCSISAIANYTQRVSDSSLSSENHAEALRFLVHFIGDMTQP
LHDEAYAVGGNKINVTFDGYHDNLHSDWDTYMPQKLIGGHALSDAESWAKTLVQNIESGNYT
AQAIGWIKGDNISEPITTATRWASDANALVCTVVMPHGAAALQTGDLYPTYYDSVIDTIELQ
IAKGGYRLANWINEIH
>d1f0ja_ a.125.1.1 (A:) Catalytic domain of cyclic nucleotide phosphodiesterase 4b2b {Human (Homo sapiens)}
SISRFGVNTENEDHLAKELEDLNKWGLNIFNVAGYSHNRPLTCIMYAIFQERDLLKTFRISS

DTFITYMMTLEDHYHSDVAYHNSLHAADVAQSTHVLLSTPALDAVFTDLEILAAIFAAAIHD
VDHPGVSNQFLINTNSELALMYNDESVLENHHLAVGFKLLQEEHCDIFMNLTKKQRQTLRKM
VIDMVLATDMSKHMSLLADLKTMVETKKVTSSGVLLLDNYTDRIQVLRNMVHCADLSNPTKS
LELYRQWTDRIMEEFFQQGDKERERGMEISPMCDKHTASVEKSQVGFIDYIVHPLWETWADL
VQPDAQDILDTLEDNRNWYQSMIPQAPAPPLDEQNRDCQGLMEKFQF
>d1gnia1 a.126.1.1 (A:3-196) Serum albumin {Human (Homo sapiens)}
HKSEVAHRFKDLGEENFKALVLIAFAQYLQQCPFEDHVKLVNEVTEFAKTCVADESAENCDK
SLHTLFGDKLCTVATLRETYGEMADCCAKQEPERNECFLQHKDDNPNLPRLVRPEVDVMCTA
FHDNEETFLKKYLYEIARRHPYFYAPELLFFAKRYKAAFTECCQAADKAACLLPKLDELRDE
GKASSAKQ
>d1gnia2 a.126.1.1 (A:197-388) Serum albumin {Human (Homo sapiens)}
RLKCASLQKFGERAFKAWAVARLSQRFPKAEFAEVSKLVTDLTKVHTECCHGDLLECADDRA
DLAKYICENQDSISSKLKECCEKPLLEKSHCIAEVENDEMPADLPSLAADFVESKDVCKNYA
EAKDVFLGMFLYEYARRHPDYSVVLLLRLAKTYETTLEKCCAAADPHECYAKVFDEFKPLVE
EPQNLI
>d1gnia3 a.126.1.1 (A:389-584) Serum albumin {Human (Homo sapiens)}
KQNCELFEQLGEYKFQNALLVRYTKKVPQVSTPTLVEVSRNLGKVGSKCCKHPEAKRMPCAE
DYLSVVLNQLCVLHEKTPVSDRVTKCCTESLVNRRPCFSALEVDETYVPKEFNAETFTFHAD
ICTLSEKERQIKKQTALVELVKHKPKATKEQLKAVMDDFAAFVEKCCKADDKETCFAEEGKK
LVAASQAALG
>d1j78a1 a.126.1.1 (A:13-198) Vitamin D binding protein {Human (Homo sapiens)}
CKEFSHLGKEDFTSLSLVLYSRKFPSGTFEQVSQLVKEVVSLTEACCAEGADPDCYDTRTSA
LSAKSCESNSPFPVHPGTAECCTKEGLERKLCMAALKHQPQEFPTYVEPTNDEICEAFRKDP
KEYANQFMWEYSTNYGQAPLSLLVSYTKSYLSMVGSCCTSASPTVCFLKERLQLKHLSLLTT
>d1j78a2 a.126.1.1 (A:199-386) Vitamin D binding protein {Human (Homo sapiens)}
LSNRVCSQYAAYGEKKSRLSNLIKLAQKVPTADLEDVLPLAEDITNILSKCCESASEDCMAK
ELPEHTVKLCDNLSTKNSKFEDCCQEKTAMDVFVCTYFMPAAQLPELPDVELPTNKDVCDPG
NTKVMDKYTFELSRRTHLPEVFLSKVLEPTLKSLGECCDVEDSTTCFNAKGPLLKKELSSFI
DK
>d1j78a3 a.126.1.1 (A:387-457) Vitamin D binding protein {Human (Homo sapiens)}
GQELCADYSENTFTEYKKKLAERLKAKLPDATPTELAKLVNKRSDFASNCCSINSPPLYCDS
EIDAELKNI
>d1jswa_ a.127.1.1 (A:) L-aspartate ammonia lyase {Escherichia coli}
MSNNIRIEEDLLGTREVPADAYYGVHTLRAIENFYISNNKISDIPEFVRGMVMVKKAAAMAN
KELQTIPKSVANAIIAACDEVLNNGKCMDQFPVDVYQGGAGTSVNMNTNEVLANIGLELMGH
QKGEYQYLNPNDHVNKCQSTNDAYPTGFRIAVYSSLIKLVDAINQLREGFERKAVEFQDILK
MGRTQLQDAVPMTLGQEFRAFSILLKEEVKNIQRTAELLLEVNLGATAIGTGLNTPKEYSPL
AVKKLAEVTGFPCVPAEDLIEATSDCGAYVMVHGALKRLAVKMSKICNDLRLLSSGPRAGLN

EINLPELQAGSSIMPAKVNPVVPEVVNQVCFKVIGNDTTVTMAAEAGQLQLNVMEPVIGQAM
FESVHILTNACYNLLEKCINGITANKEVCEGYVYNSIGIVTYLNPFIGHHNGDIVGKICAET
GKSVREVVLERGLLTEAELDDIFSV
>d1jswc_ a.127.1.1 (C:) L-aspartate ammonia lyase {Escherichia coli}
IRIEEDLLGTREVPADAYYGVHTLRAIENFYISNNKISDIPEFVRGMVMVKKAAAMANKELQ
TIPKSVANAIIAACDEVLNNGKCMDQFPVDVYQGGAGTSVNMNTNEVLANIGLELMGHQKGE
YQYLNPNDHVNKCQSTNDAYPTGFRIAVYSSLIKLVDAINQLREGFERKAVEFQDILKMGRT
QLQDAVPMTLGQEFRAFSILLKEEVKNIQRTAELLLEVNLGATAIGTGLNTPKEYSPLAVKK
LAEVTGFPCVPAEDLIEATSDCGAYVMVHGALKRLAVKMSKICNDLRLLSSGPRAGLNEINL
PELQAGSSIMPAKVNPVVPEVVNQVCFKVIGNDTTVTMAAEAGQLQLNVMEPVIGQAMFESV
HILTNACYNLLEKCINGITANKEVCEGYVYNSIGIVTYLNP
>d1fura_ a.127.1.1 (A:) Fumarase {Escherichia coli}
VRSEKDSMGAIDVPADKLWGAQTQRSLEHFRISTEKMPTSLIHALALTKRAAAKVNEDLGLL
SEEKASAIRQAADEVLAGQHDDEFPLAIWQTGSGTQSNMNMNEVLANRASELLGGVRGMERK
VHPNDDVNKSQSSNDVFPTAMHVAALLALRKQLIPQLKTLTQTLNEKSRAFADIVKIGRTNL
QDATPLTLGQEISGWVAMLEHNLKHIEYSLPHVAELALGGTAVGTGLNTHPEYARRVADELA
VITCAPFVTAPNKFEALATCDALVQAHGALKGLAASLMKIANDVRWLASGPRCGIGEISIPE
NEPGSSIMPGKVNPTQCEALTMLCCQVMGNDVAINMGGASGNFELNVFRPMVIHNFLQSVRL
LADGMESFNKHCAVGIEPNRERINQLLNESLMLVTALNTHIGYDKAAEIAKKAHKEGLTLKA
AALALGYLSEAEFDSWVRPEQM
>d1yfm__ a.127.1.1 (-) Fumarase {Baker's yeast (Saccharomyces cerevisiae)}
SFRTETDAFGEIHVPADKYWGAQTQRSFQNFKIGGARERMPLPLVHAFGVLKKSAAIVNESL
GGLDPKISKAIQQAADEVASGKLDDHFPLVVFQTGSGTQSNMNANEVISNRAIEILGGKIGS
KQVHPNNHCNQSQSSNDTFPTVMHIAASLQIQNELIPELTNLKNALEAKSKEFDHIVKIGRT
HLQDATPLTLGQEFSGYVQQVENGIQRVAHSLKTLSFLAQGGTAVGTGLNTKPGFDVKIAEQ
ISKETGLKFQTAPNRFEALAAHDAIVECSGALNTLACSLFKIAQDIRYLGSGPRCGYHELML
PENEPGSSIMPGKVNPTQNEALTQVCVQVMGNNAAITFAGSQGQFELNVFKPVMIANLLNSI
RLITDAAYSFRVHCVEGIKANEPRIHELLTKSLMLVTALNPKIGYDAASKVAKNAHKKGITL
KESALELGVLTEKEFDEWVVPEHML
>d1k62a_ a.127.1.1 (A:) Argininosuccinate lyase/delta-crystallin {Human (Homo sapiens)}
GAVDPIMEKFNASIAYDRHLWEVDVQGSKAYSRGLEKAGLLTKAEMDQILHGLDKVAEEWAQ
GTFKLNSNDEDIHTANERRLKELIGATAGKLHTGRSRNDQVVTDLRLWMRQTCSTLSGLLWE
LIRTMVDRAEAERDVLFPGYTHLQRAQPIRWSHWILSHAVALTRDSERLLEVRKRINVLPLG
SGAIAGNPLGVDRELLRAELNFGAITLNSMDATSERDFVAEFLFWRSLCMTHLSRMAEDLIL
YCTKEFSFVQLSDAYSTGSSLMPRKKNPDSLELIRSKAGRVFGRCAGLLMTLKGLPSTYNKD
LQEDKEAVFEVSDTMSAVLQVATGVISTLQIHQENMGQALSPDMLATDLAYYLVRKGMPFRQ
AHEASGKAVFMAETKGVALNQLSLQELQTISPLFSGDVICVWDYRHSVEQYGALGGTARSSV
DWQIRQVRALLQAQQA
>d1auwa_ a.127.1.1 (A:) Argininosuccinate lyase/delta-crystallin {Domestic duck (Anas platyrhynchos), delta-crystallin}
TDPIMEKLNSSIAYDQRLSEVDIQGSMAYAKALEKAGILTKTELEKILSGLEKISEEWSKGV

FVVKQSDEDINTANERRLKELIGDIAGKLHTGRSRNDQVVTDLKLFMKNSLSIISTHLLQLI
KTLVERAAIEIDVILPGYTHLQKAQPIRWSQFLLSHAVALTRDSERLGEVKKRINVLPLGSG
ALAGNPLDIDREMLRSELEFASISLNSMDAISERDFVVEFLSFATLLMIHLSKMAEDLIIYS
TSEFGFLTDSDAFSTGSSLMPQKKNPDSLELIRSKAGRVFGRLASILMVLKGLPSTYNKDLQ
EDKEAVFDVVDTLTAVLQVATGVISTLQISKENMEKALTPEMLATDLALYLVRKGVPFRQAH
TASGKAVHLAETKGITINKLSLEDLKSISPQFSSDVSQVFNFVNSVEQYTALGGTAKSSVTT
QIEQLRELMKKQK
>d1dcnb_ a.127.1.1 (B:) Argininosuccinate lyase/delta-crystallin
{Domestic duck (Anas platyrhynchos), delta-crystallin}
DPIMAKLNSSIAYDQRLSEVDIQGSMAYAKALEKAGILTKTELAKILSGLEKISEEDIHTAN
ERRLKELIGDIAGKLNTGRSRNDQVVTDLKLFMKNSLSIISTHLLQLIKTLVERAAIEIDVI
LPGYTNLQKAQPIRWSQFLLSHAVALTRDSERLGEVKKRINVLPLGSGALAGNPLDIDREML
RSELEFASISLNSMDAISERDFVVEFLSFATLLMIHLSKMAEDLIIYSTSEFGFLTLSPDSL
ELIRSKSGRVFGRLASILMVLKGLPSTYNKDLQEDKEAVFDVVDTLTAVLQVATGVISTLQI
SKENMEKALTPEMLATDLALYLVRKGVPFRQAHTASGKAVHLAETKGITINNLSLEDLKSIS
PQFSSDVSQVFNFVNSVEQYTALGGTAKSSVTTQIEQLRELMKKQK
>d1hy0a_ a.127.1.1 (A:) Argininosuccinate lyase/delta-crystallin
{Domestic duck (Anas platyrhynchos), delta-crystallin}
DPIMQMLSTSISTEQRLSEVDIQASIAYAKALEKAGILTKTELEKILSGLEKISEELSKGVI
VVTQSDEDIQTANERRLKELIGDIAGKLHTGRSRNEQVVTDLKLFMKNSLSIISTHLLQLIK
TLVERAAIEIDVILPGYTHLQKAQPIRWSQFLLSHAVALTRDSERLGEVKKRINVLPLGSGA
LAGNPLDIDREMLRSELEFASISLNSMDAISERDFVVEFLSVATLLLIHLSKMAEDLIIYST
SEFGFLTLSDAFSTGSSLMPQKKNPDSLELIRSKSGRVFGRLASILMVLKGLPSTYNKDLQE
DKEAVIDVVDTLTAVLQVATGVISTLQISKENMEKALTPEMLATDLALYLVRKGMPFRQAHT
ASGKAVHLAETKGIAINNLTLEDLKSISPLFSSDVSQVFNFVNSVEQYTALGGTAKSSVTTQ
IEQLRELMKKQKE
>d1i0aa_ a.127.1.1 (A:) Argininosuccinate lyase/delta-crystallin
{Turkey (Meleagris gallopavo), delta-crystallin}
GRFVGSVDPIMEILSSSISTEQRLTEVDIQASMAYAKALEKASILTKTELEKILSGLEKISE
ESSKGVLVMTQSDEDIQTAIERRLKELIGDIAGKLQTGRSRNEQVVTDLKLLLKSSISVIST
HLLQLIKTLVERAAIEIDIIMPGYTHLQKALPIRWSQFLLSHAVALTRDSERLGEVKKRITV
LPLGSGVLAGNPLEIDRELLRSELDMTSITLNSIDAISERDFVVELISVATLLMIHLSKLAE
DLIIFSTTEFGFVTLSDAYSTGSSLLPQKKNPDSLELIRSKAGRVFGRLAAILMVLKGIPST
FSKDLQEDKEAVLDVVDTLTAVLQVATGVISTLQINKENMEKALTPELLSTDLALYLVRKGM
PIRQAQTASGKAVHLAETKGITINNLTLEDLKSISPLFASDVSQVFSVVNSVEQYTAVGGTA
KSSVTAQIEQLRELLKKQK
>d1c3ca_ a.127.1.1 (A:) Adenylosuccinate lyase {Thermotoga
maritima}
VERYSLSPMKDLWTEEAKYRRWLEVELAVTRAYEELGMIPKGVTERIRNNAKIDVELFKKIE
EKTNHDVVAFVEGIGSMIGEDSRFFHYGLTSSDVLDTANSLALVEAGKILLESLKEFCDVLW
EVANRYKHTPTIGRTHGVHAEPTSFGLKVLGWYSEMKRNVQRLERAIEEVSYGKISGAVGNY
ANVPPEVEEKALSYLGLKPEPVSTQVVPRDRHAFYLSTLAIVAAGIERIAVEIRHLQRTEVL
EVEEPFRKGQRGSSAMPHKKNPITCERLTGLSRMMRAYVDPSLENIALWHERDISHSSVERY
VFPDATQTLYYMIVTATNVVRNMKVNEERMKKNIDLTKGLVFSQRVLLKLIEKGLTRKEAYD

IVQRNALKTWNSEKHFLEYLLEDEEVKKLVTKEELEELFDISYYLKHVDHIFERFEK
>d1dofa_ a.127.1.1 (A:) Adenylosuccinate lyase {Archaeon Pyrobaculum aerophilum}
HVSPFDWRYGSEEIRRLFTNEAIINAYLEVERALVCALEELGVAERGCCEKVNKASVSADEV
YRLERETGHDILSLVLLLEQKSGCRYVHYGATSNDIIDTAWALLIRRALAAVKEKARAVGDQ
LASMARKYKTLEMVGRTHGQWAEPITLGFKFANYYYELYIACRQLALAEEFIRAKIGGAVGT
MASWGELGLEVRRRVAERLGLPHHVITTQVAPRESFAVLASALALMAAVFERLAVEIRELSR
PEIGEVVEGGGGSSAMPHKANPTASERIVSLARYVRALTHVAFENVALWHERDLTNSANERV
WIPEALLALDEILTSALRVLKNVYIDEERITENLQKALPYILTEFHMNRMIKEGASRAEAYK
KAKEVKALTFEYQKWPVERLIEDALSLKLC
>d1f1oa_ a.127.1.1 (A:) Adenylosuccinate lyase {Bacillus subtilis}
EMSAIWTDENRFQAWLEVEILACEAWAELGVIPKEDVKVMRENASFDINRILEIEKDTRHDV
VAFTRAVSESLGEERKWVHYGLTSTDVVDTALSYLLKQANDILLKDLERFVDIIKEKAKEHK
YTVMMGRTHGVHAEPTTFGLKLALWHEEMKRNLERFKQAKAGIEVGKISGAVGTYANIDPFV
EQYVCEKLGLKAAPISTQTLQRDRHADYMATLALIATSIEKFAVEIRGLQKSETREVEEFFA
KGQKGSSAMPHKRNPIGSENMTGMARVIRGYMMTAYENVPLWHERDISHSSAERIILPDATI
ALNYMLNRFSNIVKNLTVFPENMKRNMDRTLGLIYSQRVLLALIDTGLTREEAYDTVQPKAM
EAWEKQVPFRELVEAEEKITSRLSPEKIADCFDYNY
>d1gk2a_ a.127.1.2 (A:) Histidine ammonia-lyase (HAL) {Pseudomonas putida}
TELTLKPGTLTLAQLRAIHAAPVRLQLDASAAPAIDASVACVEQIIAEDRTAYGINTGFGLL
ASTRIASHDLENLQRSLVLSHAAGIGAPLDDDLVRLIMVLKINSLSRGFSGIRRKVIDALIA
LVNAEVYPHIPLKGSVGASGDLAPLAHMSLVLLGEGKARYKGQWLSATEALAVAGLEPLTLA
AKEGLALLNGTQASTAYALRGLFYAEDLYAAAIACGGLSVEAVLGSRSPFDARIHEARGQRG
QIDTAACFRDLLGDSSEVSLSHKNADKVQDPYSLRCQPQVMGACLTQLRQAAEVLGIEANAV
SDNPLVFAAEGDVISGGNGHAEPVAMAADNLALAIAEIGSLSERRISLMMDKHMSQLPPFLV
ENGGVNSGFMIAQVTAAALASENKALSHPHSVDSLPTSANQEDHVSMAPAAGKRLWEMAENT
RGVLAIEWLGACQGLDLRKGLKTSAKLEKARQALRSEVAHYDRDRFFAPDIEKAVELLAKGS
LTGLLPAGVLPSL
>d1uby__ a.128.1.1 (-) Farnesyl diphosphate synthase {Chicken (Gallus gallus)}
SPVVVEREREEFVGFFPQIVRDLTEDGIGHPEVGDAVARLKEVLQYNAPGGKCNRGLTVVAA
YRELSGPGQKDAESLRCALAVGWCIELFQAASLVADDIMDQSLTRRGQLCWYKKEGVGLDAI
NDSFLLESSVYRVLKKYCRQRPYYVHLLELFLQTAYQTELGQMLDLITAPVSKVDLSHFSEE
RYKAIVKYKTAFYSFYLPVAAAMYMVGIDSKEEHENAKAILLEMGEYFQIQDDYLDCFGDPA
LTGAVGTDIQDNKCSWLVVQCLQRVTPEQRQLLEDNYGRKEPEKVAKVKELYEAVGMRAAFQ
QYEESSYRRLQELIEKHSNRLPKEIFLGLAQKIYKRQK
>d1ezfa_ a.128.1.2 (A:) Squalene synthase {Human (Homo sapiens)}
NSLKTCYKYLNQTSRSFAAVIQALDGEMRNAVCIFYLVLRALDTLEDDMTISVEKKVPLLHN
FHSFLYQPDWRFMESKEKDRQVLEDFPTISLEFRNLAEKYQTVIADICRRMGIGMAEFLDKH
VTSEQEWDKYCHYVAGLVGIGLSRLFSASEFEDPLVGEDTERANSMGLFLQKTNIIRDYLED
QQGGREFWPQEVWSRYVKKLGDFAKPENIDLAVQCLNELITNALHHIPDVITYLSRLRNQSV
FNFCAIPQVMAIATLAACYNNQQVFKGAVKIRKGQAVTLMMDATNMPAVKAIIYQYMEEIYH
RIPDSDPSSSKTRQIISTIRTQN

>d5eau_2 a.128.1.3 (221-548) 5-Epi-aristolochene synthase, C-terminal domain {Tobacco (Nicotiana tabacum)}
KNNVLLRFAKLDFNLLQMLHKQELAQVSRWWKDLDFVTTLPYARDRVVECYFWALGVYFEPQ
YSQARVMLVKTISMISIVDDTFDAYGTVKELEAYTDAIQRWDINEIDRLPDYMKISYKAILD
LYKDYEKELSSAGRSHIVCHAIERMKEVVRNYNVESTWFIEGYTPPVSEYLSNALATTTYYY
LATTSYLGMKSATEQDFEWLSKNPKILEASVIICRVIDDTATYEVEKSRGQIATGIECCMRD
YGISTKEAMAKFQNMAETAWKDINEGLLRPTPVSTEFLTPILNLARIVEVTYIHNLDGYTHP
EKVLKPHIINLLVDSIKI
>d1di1a_ a.128.1.4 (A:) Aristolochene synthase {Fungus (Penicillium roqueforti)}
TPPPTQWSYLCHPRVKEVQDEVDGYFLENWKFPSFKAVRTFLDAKFSEVTCLYFPLALDDRI
HFACRLLTVLFLIDDVLEHMSFADGEAYNNRLIPISRGDVLPDRTKPEEFILYDLWESMRAH
DAELANEVLEPTFVFMRAQTDRARLSIHELGHYLEYREKDVGKALLSALMRFSMGLRLSADE
LQDMKALEANCAKQLSVVNDIYSYDKEEEASRTGHKEGAFLCSAVKVLAEESKLGIPATKRV
LWSMTREWETVHDEIVAEKIASPDGCSEAAKAYMKGLEYQMSGNEQWSKTTR
>d1ps1a_ a.128.1.4 (A:) Pentalenene synthase {Streptomyces sp., UC5319}
QDVDFHIPLPGRQSPDHARAEAEQLAWPRSLGLIRSDAAAERHLRGGYADLASRFYPHATGA
DLDLGVDLMSWFFLFDDLFDGPRGENPEDTKQLTDQVAAALDGPLPDTAPPIAHGFADIWRR
TCEGMTPAWCARSARHWRNYFDGYVDEAESRFWNAPCDSAAQYLAMRRHTIGVQPTVDLAER
AGRFEVPHRVFDSAVMSAMLQIAVDVNLLLNDIASLEKEEARGEQNNMVMILRREHGWSKSR
SVSHMQNEVRARLEQYLLLESCLPKVGEIYQLDTAEREALERYRTDAVRTVIRGSYDWHRSS
G
>d1jfaa_ a.128.1.5 (A:) Trichodiene synthase {Fusarium sporotrichioides}
MENFPTEYFLNTTVRLLEYIRYRDSNYTREERIENLHYAYNKAAHHFAQPRQQQLLKVDPKR
LQASLQTIVGMVVYSWAKVSKECMADLSIHYTYTLVLDDSKDDPYPTMVNYFDDLQAGREQA
HPWWALVNEHFPNVLRHFGPFCSLNLIRSTLDFFEGCWIEQYNFGGFPGSHDYPQFLRRMNG
LGHCVGASLWPKEQFNERSLFLEITSAIAQMENWMVWVNDLMSFYKEFDDERDQISLVKNYV
VSDEISLHEALEKLTQDTLHSSKQMVAVFSDKDPQVMDTIECFMHGYVTWHLCDRRYRLSEI
YEKVKEEKTEDAQKFCKFYEQAANVGAVSPSEWAYPPVAQLANV
>d1oela1 a.129.1.1 (A:2-136,A:410-525) GroEL {Escherichia coli}
AAKDVKFGNDAGVKMLRGVNVLADAVKVTLGPKGRNVVLDKSFGAPTITKDGVSVAREIELE
DKFENMGAQMVKEVASKANDAAGDGTTTATVLAQAIITEGLKAVAAGMNPMDLKRGIDKAVT
VAVEELKALSVXGVVAGGGVALIRVASKLADLRGQNEDQNVGIKVALRAMEAPLRQIVLNCG
EEPSVVANTVKGGDGNYGYNAATEEYGNMIDMGILDPTKVTRSALQYAASVAGLMITTECMV
TDLP
>d1ioka1 a.129.1.1 (A:2-136,A:410-526) GroEL {Paracoccus denitrificans}
AAKEVKFNSDARDRMLKGVNILADAVKVTLGPKGRNVVIDKSFGAPRITKDGVSVAKEIELS
DKFENMGAQMVREVASRTNDEAGDGTTTATVLAQAIVREGLKAVAAGMNPMDLKRGIDVATA
KVVEAIKSAARXGIVVGGGVALVQGAKVLEGLSGANSDQDAGIAIIRRALEAPMRQIAENAG
VDGAVVAGKVRESSDKAFGFNAQTEEYGDMFKFGVIDPAKVVRTALEDAASVAGLLITTEAM
IAEKP

>d1a6da1 a.129.1.2 (A:17-145,A:404-519) Thermosome {Archaeon Thermoplasma acidophilum}
REQGKNAQRNNIEAAKAIADAVRTTLGPKGMDKMLVDSIGDIIISNDGATILKEMDVEHPTA
KMIVEVSKAQDTAVGDGTTTAVVLSGELLKQAETLLDQGVHPTVISNGYRLAVNEARKIIDE
IAEKSXFLWGGGAVEAELAMRLAKYANSVGGREQLAIEAFAKALEIIPRTLAENAGIDPINT
LIKLKADDEKGRISVGVDLDNNGVGDMKAKGVVDPLRVKTHALESAVEVATMILRIDDVI
>d1a6db1 a.129.1.2 (B:20-144,B:404-521) Thermosome {Archaeon Thermoplasma acidophilum}
KDAMKENIEAAIAISNSVRSSLGPRGMDKMLVDSLGDIVITNDGVTILKEMDVEHPAAKMMV
EVSKTQDSFVGDGTTTAVIIAGGLLQQAQGLINQNVHPTVISEGYRMASEEAKRVIDEISTK
IXAYAAGGGATAAEIAFRLRSYAQKIGGRQQLAIEKFADAIEEIPRALAENAGLDPIDILLK
LRAEHAKGNKTYGINVFTGEIEDMVKNGVIEPIRVGKQAIESATEAAIMILRIDDVIA
>d1ecma_ a.130.1.1 (A:) Chorismate mutase domain of P-protein {Escherichia coli}
NPLLALREKISALDEKLLALLAERRELAVEVGKAKLLSHRPVRDIDRERDLLERLITLGKAH
HLDAHYITRLFQLIIEDSVLTQQALLQQH
>d5csma_ a.130.1.2 (A:) Allosteric chorismate mutase {Baker's yeast (Saccharomyces cerevisiae)}
MDFTKPETVLNLQNIRDELVRMEDSIIFKFIERSHFATCPSVYEANHPGLEIPNFKGSFLDW
ALSNLEIAHSRIRRFESPDETPFFPDKIQKSFLPSINYPQILAPYAPEVNYNDKIKKVYIEK
IIPLISKRDGDDKNNFGSVATRDIECLQSLSRRIHFGKFVAEAKFQSDIPLYTKLIKSKDVE
GIMKNITNSAVEEKILERLTKKAEVYGVDPTERRIERRISPEYLVKIYKEIVIPITKEVEVE
YLLRRLEE
>d1pprm1 a.131.1.1 (M:1-156) Peridinin-chlorophyll protein {Dinoflagellate (Amphidinium carterae)}
DEIGDAAKKLGDASYAFAKEVDWNNGIFLQAPGKLQPLEALKAIDKMIVMGAAADPKLLKAA
AEAHHKAIGSISGPNGVTSRADWDNVNAALGRVIASVPENMVMDVYDSVSKITDPKVPAYMK
SLVNGADAEKAYEGFLAFKDVVKKSQVTSAAG
>d1pprm2 a.131.1.1 (M:157-312) Peridinin-chlorophyll protein {Dinoflagellate (Amphidinium carterae)}
PATVPSGDKIGVAAQQLSEASYPFLKEIDWLSDVYMKPLPGVSAQQSLKAIDKMIVMGAQAD
GNALKAAAEAHHKAIGSIDATGVTSAADYAAVNAALGRVIASVPKSTVMDVYNAMAGVTDTS
IPLNMFSKVNPLDANAAAKAFYTFKDVVQAAQ
>d1qq8a_ a.132.1.1 (A:) Heme oxygenase-1 (HO-1) {Human (Homo sapiens)}
PQDLSEALKEATKEVHTQAENAEFMRNFQKGQVTRDGFKLVMASLYHIYVALEEEIERNKES
PVFAPVYFPEELHRKAALEQDLAFWYGPRWQEVIPYTPAMQRYVKRLHEVGRTEPELLVAHA
YTRYLGDLSGGQVLKKIAQKALDLPSSGEGLAFFTFPNIASATKFKQLYRSRMNSLEMTPAV
RQRVIEEAKTAFLLNIQLFEELQELLTH
>d1dvga_ a.132.1.1 (A:) Heme oxygenase-1 (HO-1) {Rat (Rattus norvegicus)}
SQDLSEALKEATKEVHIRAENSEFMRNFQKGQVSREGFKLVTASLYHIYTALEEEIERNKQN
PVYAPLYFPEELHRRAALEQDLAFWYGPHWQEAIPYTPATQHYVKRLHEVGGTHPELLVAHA
YTRYLGDLSGGQVLKKIAQKALALPSSGEGLASFTFPSIDNPTKFKQLYRARMNTLELTPEV

KHRVTEEAKTAFLLNIELFEELQALLTE
>d1j77a_ a.132.1.2 (A:) Gram-negative bacterial heme oxygenase {Neisseria meningitidis}
ALTFAKRLKADTTAVHDSVDNLVMSVQPFVSKENYIKFLKLQSVFHKAVDHIYKDAELNKAI
PELEYMARYDAVTQDLKDLGEEPYKFDKELPYEAGNKAIGWLYCAEGSNLGAAFLFKHAQKL
DYNGEHGARHLAPHPDGRGKHWRAFVEHLNALNLTPEAEAEAIQGAREAFAFYKVVLRETFG
LAADAEAPEGMMPH
>d1knca_ a.152.1.1 (A:) Antioxidant defence protein AhpD {Mycobacterium tuberculosis}
SIEKLKAALPEYAKDIKLNLSSITRSSVLDQEQLWGTLLASAAATRNPQVLADIGAEATDHL
SAAARHAALGAAAIMGMNNVFYRGRGFLEGRYDDLRPGLRMNIIANPGIPKANFELWSFAVS
AINGCSHCLVAHEHTLRTVGVDREAIFEALKAAAIVSGVAQALATIEALS
>d1poa__ a.133.1.2 (-) Snake phospholipase A2 {Taiwan cobra (Naja naja atra)}
NLYQFKNMIQCTVPSRSWWDFADYGCYCGRGGSGTPVDDLDRCCQVHDNCYNEAEKISGCWP
YFKTYSYECSQGTLTCKGGNNACAAAVCDCDRLAAICFAGAPYNDNDYNINLKARC
>d1pp2l_ a.133.1.2 (L:) Snake phospholipase A2 {Western diamondback rattlesnake (Crotalus atrox)}
SLVQFETLIMKIAGRSGLLWYSAYGCYCGWGGHGLPQDATDRCCFVHDCCYGKATDCNPKTV
SYTYSEENGEIICGGDDPCGTQICECDKAAAICFRDNIPSYDNKYWLFPPKDCREEPEPC
>d1bjja_ a.133.1.2 (A:) Snake phospholipase A2 {Chinese water moccasin (Agkistrodon halys pallas), different isoforms}
NLLQFNKMIKEETGKNAIPFYAFYGCYCGWGGQGKPKDGTDRCCFVHDCCYGRLVNCNTKSD
IYSYSLKEGYITCGKGTNCEEQICECDRVAAECFRRNLDTYNNGYMFYRDSKCTETSEEC
>d1jiaa_ a.133.1.2 (A:) Snake phospholipase A2 {Chinese water moccasin (Agkistrodon halys pallas), different isoforms}
HLLQFRKMIKKMTGKEPVVSYAFYGCYCGSGGRGKPKDATDRCCFVHDCCYEKVTGCDPKWD
DYTYSWKNGTIVCGGDDPCKKEVCECDKAAAICFRDNLKTYKKRYMAYPDILCSSKSEKC
>d1psj__ a.133.1.2 (-) Snake phospholipase A2 {Chinese water moccasin (Agkistrodon halys pallas), different isoforms}
SLIQFETLIMKVAKKSGMFWYSNYGCYCGWGGQGRPQDATDRCCFVHDCCYGKVTGCDPKMD
VYSFSEENGDIVCGGDDPCKKEICECDRAAAICFRDNLTLYNDKKYWAFGAKNCPQEESEPC
>d1ppa__ a.133.1.2 (-) Snake phospholipase A2 {Eastern cottonmouth snake (Agkistridon piscivorus)}
SVLELGKMILQETGKNAITSYGSYGCNCGWGHRGQPKDATDRCCFVHKCCYKKLTDCNHKTD
RYSYSWKNKAIICEEKNPCLKEMCECDKAVAICLRENLDTYNKKYKAYFKLKCKKPDTC
>d1vapa_ a.133.1.2 (A:) Snake phospholipase A2 {Eastern cottonmouth snake (Agkistridon piscivorus)}
NLFQFEKLIKKMTGKSGMLWYSAYGCYCGWGGQGRPKDATDRCCFVHDCCYGKVTGCNPKMD
IYTYSVDNGNIVCGGTNPCKKQICECDRAAAICFRDNLKTYDSKTYWKYPKKNCKEESEPC
>d1ijla_ a.133.1.2 (A:) Snake phospholipase A2 {Viper (Deinagkistrodon acutus)}
SLIQFETLIMKVVKKSGMFWYSAYGCYCGWGGHGRPQDATDRCCFVHDCCYGKVTGCDPKMD
SYTYSEENGDIVCGGDDPCKREICECDRVAADCFRDNLDTYNSDTYWRYPRQDCEESPEPC

>d1fe7a_ a.133.1.2 (A:) Snake phospholipase A2 {Snake (Daboia russelli pulchella)}
SLLEFGKMILEETGKLAIPSYSSYGCYCGWGGKGTPKDATDRCCFVHDCCYGNLPDCNPKSD
RYKYKRVNGAIVCEKGTSCENRICECDKAAAICFRQNLNTYSKKYMLYPDFLCKGELKC
>d1ae7__ a.133.1.2 (-) Snake phospholipase A2 {Mainland tiger snake (Notechis scutatus scutatus), notexin}
NLVQFSYLIQCANHGKRPTWHYMDYGCYCGAGGSGTPVDELDRCCKIHDDCYDEAGKKGCFP
KMSAYDYYCGENGPYCRNIKKKCLRFVCDCDVEAAFCFAKAPYNNANWNIDTKKRCQ
>d2nota_ a.133.1.2 (A:) Snake phospholipase A2 {Mainland tiger snake (Notechis scutatus scutatus), notechis II-5}
NLVQFSYLIQCANHGRRPTRHYMDYGCYCGWGGSGTPVDELDRCCKIHDDCYSDAEKKGCSP
KMSAYDYYCGENGPYCRNIKKKCLRFVCDCDVEAAFCFAKAPYNNANWNIDTKKRCQ
>d1qlla_ a.133.1.2 (A:) Snake phospholipase A2 {Bothrops pirajai, Piratoxin-II (PRTX-II)}
SLFELGKMILQETGKNPAKSYGAYGCNCGVLGRGKPKDATDRCCYVHKCCYKKLTGCNPKKD
RYSYSWKDKTIVCGENNPCLKELCECDKAVAICLRENLGTYNKKYRYHLKPFCKKADKC
>d1vip__ a.133.1.2 (-) Snake phospholipase A2 {Russell's viper (Vipera russelli)}
NLFQFAEMIVKMTGKNPLSSYSDYGCYCGWGGKGKPQDATDRCCFVHDCCYEKVKSCKPKLS
LYSYSFQNGGIVCGDNHSCKRAVCECDRVAATCFRDNLNTYDKKYHNYPPSQCTGTEQC
>d1jlta_ a.133.1.2 (A:) Snake phospholipase A2 {Sand viper (Vipera ammodytes meridionalis), vipoxin}
NLFQFGDMILQKTGKEAVHSYAIYGCYCGWGGQARAQDATDRCCFAQDCCYGRVNDCNPKTA
TYTYSFENGDIVCGDNDLCLRAVCECDRAAAICLGENVNTYDKNYEYYSISHCTEESEQC
>d1jltb_ a.133.1.2 (B:) Snake phospholipase A2 {Sand viper (Vipera ammodytes meridionalis), vipoxin}
NLFQFAKMINGKLGAFSVWNYISYGCYCGWGGQGTPKDATDRCCFVHDCCYGRVRGCNPKLA
IYSYSFKKGNIVCGKNNGCLRDICECDRVAANCFHQNKNTYNKNYKFLSSSRCRQTSEQC
>d1dpya_ a.133.1.2 (A:) Snake phospholipase A2 {Indian krait (Bungarus caeruleus), different isoforms}
NLIQFKNMIQCAGTRIWTAYVAYGCYCGKGGSGTPVDELDRCCYTHDHCYNEAEKIPGCNPN
IKTYSYTCTQPNLTCTDSADTCAQFLCECDRTAAICFASAPYNSNNIMLSSSTSCQ
>d1fe5a_ a.133.1.2 (A:) Snake phospholipase A2 {Indian krait (Bungarus caeruleus), different isoforms}
NLIQFKNMIQCAGTRPWTAYVNYGCYCGKGGSGTPVDELDRCCYTHDNCYNEAEKIPGCNPN
IKTYSYTCTEPNLTCTDTADTCARFLCNCDRTAAICFASAPYNSNNVMISSSTNCQ
>d1kvoa_ a.133.1.2 (A:) Phospholipase A2 {Human (Homo sapiens), synovial fluid}
NLVNFHRMIKLTTGKEAALSYGFYGCHCGVGGRGSPKDATDRCCVTHDCCYKRLEKRGCGTK
FLSYKFSNSGSRITCAKQDSCRSQLCECDKAAATCFARNKTTYNKKYQYYSNKHCRGSTPRC
>d1g4ia_ a.133.1.2 (A:) Phospholipase A2 {Cow (Bos taurus), pancreas}
ALWQFNGMIKCKIPSSEPLLDFNNYGCYCGLGGSGTPVDDLDRCCQTHDNCYKQAKKLDSCK
VLVDNPYTNNYSYSCSNNEITCSSENNACEAFICNCDRNAAICFSKVPYNKEHKNLDKKNC

>d1hn4a_ a.133.1.2 (A:) Phospholipase A2 {Pig (Sus scrofa), pancreas}
GISSRALWQFRSMIKCAIPGSHPLMDFNNYGCYCGLGGSGTPVDELDRCCETHDNCYRDAKN
LDSCKFLVDNPYTESYSYSCSNTEITCNSKNNACEAFICNCDRNAAICFSKAPYNKEHKNLD
TKKYC
>d5p2pa_ a.133.1.2 (A:) Phospholipase A2 {Pig (Sus scrofa), pancreas}
ALFQFRSMIKCAIPGSHPLMDFNNYGCYCGWGGSGTPVDELDRCCETHDNCYRDAKNLSGCY
PYTESYSYSCSNTEITCNSKNNACEAFICNCDRNAAICFSKAPYNKEHKNLDTKKYC
>d1buna_ a.133.1.2 (A:) beta2-bungarotoxin, phospholipase A2 chain {Many-banded krait (Bungarus multicinctus), elapid}
NLINFMEMIRYTIPCEKTWGEYADYGCYCGAGGSGRPIDALDRCCYVHDNCYGDAEKKHKCN
PKTQSYSYKLTKRTIICYGAAGTCARIVCDCDRTAALCFGNSEYIEGHKNIDTARFCQ
>d1goda_ a.133.1.2 (A:) Myotoxin II {Bothrops godmani}
SMYQLWKMILQETGKNAVPSYGLYGCNCGVGSRGKPKDATDRCCFVHKCCYKKLTDCSPKTD
SYSYSWKDKTIVCGDNNPCLQEMCECDKAVAICLRENLDTYNKNYKIYPKPLCKKADAC
>d1gmza_ a.133.1.2 (A:) Myotoxin II {Snake (Bothrops pirajai), piratoxin III}
DLWQFGKMILKETGKLPFPYYVTYGCYCGVGGRGGPKDATDRCCFVHDCCYGKLTSCKPKTD
RYSYSRKDGTIVCGENDPCRKEICECDKAAAVCFRENLDTYNKKYMSYLKSLCKKXADDC
>d1poc__ a.133.1.1 (-) Phospholipase A2 {European honeybee (Apis mellifera)}
IIYPGTLWCGHGNKSSGPNELGRFKHTDACCRTHDMCPDVMSAGESKHGLTNTASHTRLSCD
CDDKFYDCLKNSADTISSYFVGKMYFNLIDTKCYKLEHPVTGCGERTEGRCLHYTVDKSKPK
VYQWFDLRKY
>d1faza_ a.133.1.3 (A:) Prokaryotic phospholipase A2 {Streptomyces violaceoruber}
APADKPQVLASFTQTSASSQNAWLAANRNQSAWAAYEFDWSTDLCTQAPDNPFGFPFNTACA
RHDFGYRNYKAAGSFDANKSRIDSAFYEDMKRVCTGYTGEKNTACNSTAWTYYQAVKIFG
>d1bxm__ a.134.1.1 (-) beta-cryptogein {Phytophthora cryptogea}
RGTCTATQQTAAYHTLVSILSDASFNQCSTDSGYSMLTAKALPTTAQYKLMCASTACNTMIK
KIVTLNPPNCDLTVPTSGLVLNVYSYANGFSNKCSSL
>d1g8qa_ a.135.1.1 (A:) CD81 extracellular domain {Human (Homo sapiens)}
FVNKDQIAKDVKQFYDQALQQAVVDDDANNAKAVVKTFHETLDCCGSSTLTALTTSVLKNNL
CPSGSNIISNLFKEDCHQKIDDLFSGKH
>d1dvoa_ a.136.1.1 (A:) Repressor of bacterial conjugation FinO {Escherichia coli}
PPKWKVKKQKLAEKAAREAELTAKKAQARQALSIYLNLPTLDEAVNTLKPWWPGLFDGDTPR
LLACGIRDVLLEDVAQRNIPLSHKKLRRAMKAITRSESYLCAMKAGACRYDTEGYVTEHISQ
EEEVYAAERLDKIRRQNRIKAELQAVLD
>d1jjsa_ a.153.1.1 (A:) Nuclear receptor coactivator CBP/p300 ibid domain {Mouse (Mus musculus)}
ALQDLLRTLKSPSSPQQQQQVLNILKSNPQLMAAFIKQRTAKYVAN

>d1kbhb_ a.153.1.1 (B:) Nuclear receptor coactivator CBP/p300 ibid domain {Mouse (Mus musculus)}
PNRSISPSALQDLLRTLKSPSSPQQQQQVLNILKSNPQLMAAFIKQRTAKYVANQPGMQ
>d1kbha_ a.153.1.1 (A:) Nuclear receptor coactivator ACTR {Human (Homo sapiens)}
EGQSDERALLDQLHTLLSNTDATGLEEIDRALGIPELVNQGQALEPK
>d1ffky_ a.137.1.1 (Y:) Ribosomal protein L39e {Archaeon Haloarcula marismortui}
GKKSKATKKRKAKLDNQNSRVPAYVMLKTDREVQRNHKRRHWRRNDTDE
>d1jj21_ a.137.1.1 (1:) Ribosomal protein L39e {Archaeon Haloarcula marismortui}
GKKSKATKKRLAKLDNQNSRVPAWVMLKTDEVQRNHKRRHWRRNDTDE
>d1g72b_ a.137.2.1 (B:) Methanol dehydrogenase, light chain {Methylophilus methylotrophus, w3a1}
YDGQNCKEPGNCWENKPGYPEKIAGSKYDPKHDPVELNKQEESIKAMDARNAKRIAN
>d4aahb_ a.137.2.1 (B:) Methanol dehydrogenase, light chain {Methylophilus methylotrophus, w3a1}
YDGQNCKEPGNCWENKPGYPEKIAGSKYDPKHDPVELNKQEESIKAMDARNAKRIANAKSSG
NFVFDVK
>d1h4ib_ a.137.2.1 (B:) Methanol dehydrogenase, light chain {Methylobacterium extorquens}
YDGTKCKAAGNCWEPKPGFPEKIAGSKYDPKHDPKELNKQADSIKQMEERNKKRVENFKKTG
KFEYDVAKISA
>d1gg2g_ a.137.3.1 (G:) Transducin (heterotrimeric G protein), gamma chain {Cow (Bos taurus)}
SIAQARKLVEQLKMEANIDRIKVSKAAADLMAYCEAHAKEDPLLTPVPASENPF
>d1gotg_ a.137.3.1 (G:) Transducin (heterotrimeric G protein), gamma chain {Cow (Bos taurus)}
LTEKDKLKMEVDQLKKEVTLERMLVSKCCEEFRDYVEERSGEDPLVKGIPEDKNPFKE
>d1tbge_ a.137.3.1 (E:) Transducin (heterotrimeric G protein), gamma chain {Cow (Bos taurus)}
APVINIEDLTEKDKLKMEVDQLKKEVTLERMLVSKCCEEFRDYVEERSGEDPLVKGIPEDKN
PFKELK
>d1hfes_ a.137.4.1 (S:) Fe-only hydrogenase smaller subunit {Desulfovibrio desulfuricans}
VKQIKDYMLDRINGVYGADAKFPVRASQDNTQVKALYKSYLEKPLGHKSHDLLHTHWFDKSK
GVKELTTAGKLPNPRASEFEGPYPYE
>d1ef1c_ a.137.5.1 (C:) Moesin tail domain {Human (Homo sapiens)}
AEASADLRADAMAKDRSEEERTTEAEKNERVQKHLKALTSELANARDESKKTANDMIHAENM
RLGRDKYKTLRQIRQGNTKQRIDEFESM
>d2prgc_ a.137.6.1 (C:) Nuclear receptor coactivator Src-1 {Human (Homo sapiens)}
QTSHKLVQLLTTTAEQQLRHADIDTSCKDVLSCTGTSNSASANSSGGSCPSSHSSLTERHKI
LHRLLQEGSPSDIT

>d1dp5b_ a.137.7.1 (B:) Proteinase A inhibitor IA3 {Baker's yeast (Saccharomyces cerevisiae)}
NTDQQKVSEIFQSSKEKLQGDAKVVSDAFMM
>d1dpjb_ a.137.7.1 (B:) Proteinase A inhibitor IA3 {Baker's yeast (Saccharomyces cerevisiae)}
TDQQKVSEIFQSSKEKLQGDAKVVSDAFK
>d1e79i_ a.137.8.1 (I:) Epsilon subunit of mitochondrial F1F0-ATP synthase {Cow (Bos taurus)}
VAYWRQAGLSYIRYSQICAKAVRDALKTEFKANAMKTSGSTIKIVKV
>d1jjuc_ a.137.9.1 (C:) Quinohemoprotein amine dehydrogenase C chain {Paracoccus denitrificans}
MNALVGCTTSFDPGWEVDAFGAVSNLCQPMEADLYGCADPCWXPAQVADTLNTYPNWSAGAD
DVMQDWRKLQSVFPETK
>d1jmxg_ a.137.9.1 (G:) Quinohemoprotein amine dehydrogenase C chain {Pseudomonas putida}
AVAGCTATTDPGWEVDAFGGVSSLCQPMEADLYGCSDPCWXPAQVPDMMSTYQDWNAQASNS
AEDWRNLGTVFPKDK
>d1aqe__ a.138.1.1 (-) Cytochrome c3 {Desulfovibrio desulfuricans, different strains}
TFEIPESVTMSPKQFEGYTPKKGDVTFNHASHMDIACQQCHHTVPDTYTIESCMTEGCHDNI
KERTEISSVERTFHTTKDSEKSCVGCHRELKRQGPSDAPLACNSCHVQ
>d1i77a_ a.138.1.1 (A:) Cytochrome c3 {Desulfovibrio desulfuricans, different strains}
APAAPDKPLEFKGSQKTVMFPHAVHAKVECVTCHHQVDGKESFAKCGSSGCHDDLAGKQGEK
SLYYVVHTKKELKHTNCIGCHSKVVEGKPELKKDLTACAKSKCHP
>d2cy3__ a.138.1.1 (-) Cytochrome c3 {Desulfovibrio desulfuricans, different strains}
ADAPGDDYVISAPEGMKAKPKGDKPGALQKTVPFPHTKHATVECVQCHHTLEADGGAVKKCT
TSGCHDSLEFRDKANAKDIKLVENAFHTQCIDCHKALKKDKKPTGPTACGKCHTTN
>d3cyr__ a.138.1.1 (-) Cytochrome c3 {Desulfovibrio desulfuricans, different strains}
APAVPNKPVEVKGSQKTVMFPHAPHEKVECVTCHHLVDGKESYAKCGSSGCHDDLTAKKGEK
SLYYVVHAKGELKHTSCLACHSKVVAEKPELKKDLTGCAKSKCHP
>d2cdv__ a.138.1.1 (-) Cytochrome c3 {Desulfovibrio vulgaris}
APKAPADGLKMDKTKQPVVFNHSTHKAVKCGDCHHPVNGKENYQKCATAGCHDNMDKKDKSA
KGYYHAMHDKGTKFKSCVGCHLETAGADAAKKKELTGCKGSKCHS
>d2ctha_ a.138.1.1 (A:) Cytochrome c3 {Desulfovibrio vulgaris}
APKAPADGLKMEATKQPVVFNHSTHKSVKCGDCHHPVNGKEDYRKCGTAGCHDSMDKKDKSA
KGYYHVMHDKNTKFKSCVGCHVEVAGADAAKKKDLTGCKKSKCHE
>d1wad__ a.138.1.1 (-) Cytochrome c3 {Desulfovibrio gigas}
VDVPADGAKIDFIAGGEKNLTVVFNHSTHKDVKCDDCHHDPGDKQYAGCTTDGCHNILDKAD
KSVNSWYKVVHDAKGGAKPTCISCHKDKAGDDKELKKKLTGCKGSACHP
>d3caoa_ a.138.1.1 (A:) Cytochrome c3 {Desulfovibrio africanus}
EDMTHVPTDAFGKLERPAAVFNHDEHNEKAGIESCNACHHVWVNGVLAEDEDSVGTPCSDCH

ALEQDGDTPGLQDAYHQQCWGCHEKQAKGPVMCGECHVKN
>d1hh5a_ a.138.1.1 (A:) Cytochrome c7 (cytochrome c551.5) {Desulfuromonas acetoxidans}
ADVVTYENKKGNVTFDHKAHAEKLGCDACHEGTPAKIAIDKKSAHKDACKTCHKSNNGPTKC
GGCHIK
>d19hca_ a.138.1.1 (A:) Nine-haem cytochrome c {Desulfovibrio desulfuricans, ATCC 27774}
AALEPTDSGAPSAIVMFPVGEKPNPKGAAMKPVVFNHLIHEKKIADCETCHHTGDPVSCSTC
HTVEGKAEGDYITLDRAMHATDIAARAKGNTPTSCVSCHQSETKERRECAGCHAITTPKDDE
AWCATCHDITPSMTPSEMQKGIAGTLLPGDNEALAAETVLAEATVAPVSPMLAPYKVVIDAL
ADKYEPSDFTHRRHLTSLMESIKDDKLAQAFHDKPEILCATCHHRSPLSLTPPKCGSCHTKE
IDAADPGRPNLMAAYHLECMGCHKGMAVARPRDTDCTTCHKAAA
>d1duwa_ a.138.1.1 (A:) Nine-haem cytochrome c {Desulfovibrio desulfuricans, ATCC 29577}
EPTDSGAPSAIVMFPVSAKPNPKGAAMKPAVFNHLAHEKKIANCETCHHTGDPVACSTCHTT
EGKAEGNFVTLDRAMHATNIAKRAKGNTPVSCVSCHEQQTKERRECAGCHAIVTPKRDQAWC
ATCHNVTSSMTPEQMQQGIKGKLPPDQNEALAAETVLNHKPVQPLTAMQGPYKVSIDALADK
YEPSNFTHRRHMASLMERIKGDKLAEAFHNKPETLCATCHHRSPLSATPPKCGSCHTKEIDP
ANPNRPNLKAAYHLQCMGCHQGMNVGRPKNTDCTTCHKARP
>d1dxrc_ a.138.1.2 (C:) Photosynthetic reaction centre (cytochrome subunit) {Rhodopseudomonas viridis}
CFEPPPATTTQTGFRGLSMGEVLHPATVKAKKERDAQYPPALAAVKAEGPPVSQVYKNVKVL
GNLTEAEFLRTMTAITEWVSPQEGCTYCHDENNLASEAKYPYVVARRMLEMTRAINTNWTQH
VAQTGVTCYTCHRGTPLPPYVRYLEPTLPLNNRETPTHVERVETRSGYVVRLAKYTAYSALN
YDPFTMFLANDKRQVRVVPQTALPLVGVSRGKERRPLSDAYATFALMMSISDSLGTNCTFCH
NAQTFESWGKKSTPQRAIAWWGIRMVRDLNMNYLAPLNASLPASRLGRQGEAPQADCRTCHQ
GVTKPLFGASRLKDYPELGPIK
>d1eysc_ a.138.1.2 (C:) Photosynthetic reaction centre (cytochrome subunit) {Thermochromatium tepidum}
CEGPPPGTEQIGYRGVGMENYYVKRQRALSIQANQPVESLPAADSTGPKASEVYQSVQVLKD
LSVGEFTRTMVAVTTWVSPKEGCNYCHVPGNWASDDIYTKVVSRRMFELVRAANSDWKAHVA
ETGVTCYTCHRGNPVPKYAWVTDPGPKYPSGLKPTGQNYGSKTVAYASLPFDPLTPFLDQAN
EIRITGNAALAGSNPASLKQAEWTFGLMMNISDSLGVGCTSCHNTRAFNDWTQSTPKRTTAW
YAIRHVRDINQNYIWPLNDVLPASRKGPYGDPLRVSCMTCHQAVNKPLYGAQMAKDYPGLYK
>d1fgja_ a.138.1.3 (A:) Hydroxylamine oxidoreductase, HAO {Nitrosomonas europaea}
DISTVPDETYDALKLDRGKATPKETYEALVKRYKDPAHGAGKGTMGDYWEPIAISIYMDPNT
FYKPPVSPKEVAERKDCVECHSDETPVWVRAWKRSTHANLDKIRNLKSDDPLYYKKGKLEEV
ENNLRSMGKLGEKETLKEVGCIDCHVDVNKKDKADHTKDIRMPTADTCGTCHLREFAERESE
RDTMVWPNGQWPAGRPSHALDYTANIETTVWATMPQREVAEGCTMCHTNQNKCDNCHTRHEF
SAAESRKPEACATCHSGVDHNNWEAYTMSKHGKLAEMNRDKWNWEVRLKDAFSKGGQNAPTC
AACHMEYEGEYTHNITRKTRWANYPFVPGIAENITSDWSEARLDSWVLTCTQCHSERFARSY
LDLMDKGTLEGLAKYQEANAIVHKMYEDGTLTGQKTNRPNPPEPEKPGFGIFTQLFWSKGNN
PASLELKVLEMGENNLAKMHVGLAHVNPGGWTYTEGWGPMNRAYVEIQDEYTKMQELSALQA

RVN
>d1ft5a_ a.138.1.3 (A:) Cytochrome c554 {Nitrosomonas europaea}
ADAPFEGRKKCSSCHKAQAQSWKDTAHAKAMESLKPNVKKEAKQKAKLDPAKDYTQDKDCVG
CHVDGFGQKGGYTIESPKPMLTGVGCESCHGPGRNFRGDHRKSGQAFEKSGKKTPRKDLAKK
GQDFHFEERCSACHLNYEGSPWKGAKAPYTPFTPEVDAKYTFKFDEMVKEVKAMHEHYKLEG
VFEGEPKFKFHDEFQASAKPAKKGK
>d1ddca_ a.138.1.3 (A:) Dimeric di-heme split-soret cytochrome c {Desulfovibrio desulfuricans, ATCC 27774}
RFDQVGGAFGWKPHKLDPKECAQVAYDGYWYKGFGCGFGAFYSIVGLMGEKYGAPYNQFPFA
MLEANKGGISDWGTIYGALYGAAATFSLFWGRKEVHPMVNELFRWYEVTKLPIFNPGDAAQG
VKGDLPMSASDSVLCHISVSKWCYENKIEATSKQRSERAGRLTADAAFKAAEIINTKIDQGK
DFKSTFPMQASVSSCGECHMTKGNDANWAKGIMDCTPCHSGTAATQNKFVNH
>d1qdba_ a.138.1.3 (A:) Cytochrome c nitrite reductase {Sulfurospirillum deleyianum}
GIAGKEKSEEWAKYYPRQFDSWKKTKEYDSFTDMLAKDPALVIAWSGYAFSKDYNSPRGHYY
ALQDNVNSLRTGAPVDAKTGPLPTACWTCKSPDVPRLIEEDGELEYFTGKWAKYGSQIVNVI
GCANCHDDKTAELKVRVPHLNRGLQAAGLKTFEESTHQDKRTLVCAQCHVEYYFKKTEWKDA
KGADKTAMVVTLPWANGVGKDGNAGVEGMIKYYDEINFSDWTHNISKTPMLKAQHPGFEFWK
SGIHGQKGVSCADCHMPYTQEGSVKYSDHQVKENPLDSMDQSCMNCHRESESKLRGIVHQKY
ERKEFLNKVAFDNIGKAHLETGKAIEAGASDEELKEVRKLIRHGQFKADMAIAAHGNYFHAP
EETLRLLAAGSDDAQKARLLLVKILAKHGVMDYIAPDFDTKDKAQKLAKVDIAALAAEKMKF
KQTLEQEWKKEAKAKGRANPELYKDVDTINDGKSSWNKK
>d1fs7a_ a.138.1.3 (A:) Cytochrome c nitrite reductase {Wolinella succinogenes}
KTAHSQGIEGKAMSEEWARYYPRQFDSWKKTKESDNITDMLKEKPALVVAWAGYPFSKDYNA
PRGHYYALQDNINTLRTGAPVDGKTGPLPSACWTCKSPDVPRIIEQDGELEYFTGKWAKYGD
EIVNTIGCYNCHDDKSAELKSKVPYLDRGLSAAGFKTFAESTHQEKRSLVCAQCHVEYYFKK
TEWKDDKGVDKTAMVVTLPWSKGISTEQMEAYYDEINFADWTHGISKTPMLKAQHPDWELYK
TGIHGQKGVSCADCHMPYTQEGAVKYSDHKVGNPLDNMDKSCMNCHRESEQKLKDIVKQKFE
RKEFLQDIAFDNIGKAHLETGKAMELGATDAELKEIRTHIRHAQWRADMAIAGHGSFFHAPE
EVLRLLASGNEEAQKARIKLVKVLAKYGAIDYVAPDFETKEKAQKLAKVDMEAFIAEKLKFK
QTLEQEWKKQAIAKGRLNPESLKGVDEKSSYYDKTKK
>d1e39a1 a.138.1.3 (A:1-102) Flavocytochrome c3 (respiratory fumarate reductase), N-terminal domain {Shewanella frigidimarina}
ADNLAEFHVQNQECDSCHTPDGELSNDSLTYENTQCVSCHGTLAEVAETTKHEHYNAHASHF
PGEVACTSCHSAHEKSMVYCDSCHSFDFNMPYAKKWLRDE
>d1qo8a1 a.138.1.3 (A:2-102) Flavocytochrome c3 (respiratory fumarate reductase), N-terminal domain {Shewanella frigidimarina}
TPDMGSFHADMGSCQSCHAKPIKVTDSETHENAQCKSCHGEYAELANDKLQFDPHNSHLGDI
NCTSCHKGHEEPKFYCNECHSFDIKPMPFSDAKKKKSWD
>d1d4ca1 a.138.1.3 (A:1-102) Flavocytochrome c3 (respiratory fumarate reductase), N-terminal domain {Shewanella putrefaciens}
APEVLADFHGEMGGCDSCHVSDKGGVTNDNLTHENGQCVSCHGDLKELAAAAPKDKVSPHKS
HLIGEIACTSCHKGHEKSVAYCDACHSFGFDMPFGGKWER

>d1neu__ b.1.1.1 (-) Myelin membrane adhesion molecule P0 {Rat (Rattus norvegicus)}
IVVYTDREVYGAVGSQVTLHCSFWSSEWVSDDISFTWRYQPEGGRDAISIFHYAKGQPYIDE
VGTFKERIQWVGDPSWKDGSIVIHNLDYSDNGTFTCDVKNPPDIVGKTSQVTLYVFE
>d1eaja_ b.1.1.1 (A:) Coxsackie virus and adenovirus receptor (Car), domain 1 {Human (Homo sapiens)}
FARSLSITTPEEMIEKAKGETAYLPCKFTLSPEDQGPLDIEWLISPADNQKVDQVIILYSGD
KIYDDYYPDLKGRVHFTSNDLKSGDASINVTNLQLSDIGTYQCKVKKAPGVANKKIHLVVLV
>d1qfoa_ b.1.1.1 (A:) N-terminal domain of sialoadhesin {Mouse (Mus musculus)}
TWGVSSPKNVQGLSGSCLLIPCIFSYPADVPVSNGITAIWYYDYSGKRQVVIHSGDPKLVDK
RFRGRAELMGNMDHKVCNLLLKDLKPEDSGTYNFRFEISDSNRWLDVKGTTVTVTT
>d1akjd_ b.1.1.1 (D:) CD8 {Human (Homo sapiens)}
SQFRVSPLDRTWNLGETVELKCQVLLSNPTSGCSWLFQPRGAAASPTFLLYLSQNKPKAAEG
LDTQRFSGKRLGDTFVLTLSDFRRENEGYYFCSALSNSIMYFSHFVPVFLPA
>d1bqhg_ b.1.1.1 (G:) CD8 {Mouse (Mus musculus)}
KPQAPELRIFPKKMDAELGQKVDLVCEVLGSVSQGCSWLFQNSSSKLPQPTFVVYMASSHNK
ITWDEKLNSSKLFSAMRDTNNKYVLTLNKFSKENEGYYFCSVISNSVMYFSSVVPVLQKV
>d1cdy_1 b.1.1.1 (1-97) N-terminal domain of CD4 {Human (Homo sapiens)}
KKVVLGKKGDTVELTCTASQKKSIQFHWKNSNQIKILGNQGSFLTKSPSKLNDRADSRRSLW
DQGNFPLIIKNLKIEDSDTYICEVEDQKEEVQLLV
>d1wioa2 b.1.1.1 (A:179-291) N-terminal domain of CD4 {Human (Homo sapiens)}
FQKASSIVYKKEGEQVEFSFPLAFTVEKLTGSGELWWQAERASSSKSWITFDLKNKEVSVKR
VTQDPKLQMGKKLPLHLTLPQALPQYAGSGNLTLALEAKTGKLHQEVNLVV
>d1cid_1 b.1.1.1 (1-105) N-terminal domain of CD4 {Rat (Rattus rattus)}
TSITAYKSEGESAEFSFPLNLGEESLQGELRWKAEKAPSSQSWITFSLKNQKVSVQKSTSNP
KFQLSETLPLTLQIPQVSLQFAGSGNLTLTLDRGILYQEVNLV
>d1hnf_1 b.1.1.1 (4-104) CD2, first domain {Human (Homo sapiens)}
TNALETWGALGQDINLDIPSFQMSDDIDDIKWEKTSDKKKIAQFRKEKETFKEKDTYKLFKN
GTLKIKHLKTDDQDIYKVSIYDTKGKNVLEKIFDLKIQE
>d1hnga1 b.1.1.1 (A:2-99) CD2, first domain {Rat (Rattus norvegicus)}
DSGTVWGALGHGINLNIPNFQMTDDIDEVRWERGSTLVAEFKRKMKPFLKSGAFEILANGDL
KIKNLTRDDSGTYNVTVYSTNGTRILNKALDLRILE
>d1ccza1 b.1.1.1 (A:1-93) CD2-binding domain of CD58, N-terminal domain {Human (Homo sapiens)}
FSQQIYGVVYGNVTFHVPSNVPLKEVLWKKQKDKVAELENSEFRAFSSFKNRVYLDTVSGSL
TIYNLTSSDEDEYEMESPNITDTMKFFLYVL
>d1qa9b_ b.1.1.1 (B:) CD2-binding domain of CD58, N-terminal domain {Human (Homo sapiens)}
SSQQIYGVKYGNVTFHVPSNQPLKEVLWKKQKDKVAELENSEFRAFSSFKNRVYLDTKSGSL

TIYNLTSSDEDEYEMESPNITDSMKFFLYVGES
>d1dr9a1 b.1.1.1 (A:1-105) CD80, N-terminal domain {Human (Homo sapiens)}
VIHVTKEVKEVATLSCGHNVSVEELAQTRIYWQKEKKMVLTMMSGDMNIWPEYKNRTIFDIT
NNLSIVILALRPSDEGTYECVVLKYEKDAFKREHLAEVTLSVK
>d1i85a_ b.1.1.1 (A:) CD86 (b7-2), N-terminal domain {Human (Homo sapiens)}
MLKIQAYFNETADLPCQFANSQNQSLSELVVFWQDQENLVLNEVYLGKEKFDSVHSKYMGRT
SFDSDSWTLRLHNLQIKDKGLYQCIIHHKKPTGMIRIHQMNSELSVLA
>d1f97a1 b.1.1.1 (A:27-128) Junction adhesion molecule, JAM, N-terminal domain {Mouse (Mus musculus)}
KGSVYTAQSDVQVPENESIKLTCTYSGFSSPRVEWKFVQGSTTALVCYNSQITAPYADRVTF
SSSGITFSSVTRKDNGEYTCMVSEEGGQNYGEVSIHLTVL
>d1jmaa_ b.1.1.1 (A:) HSV glycoprotein D {Herpes simplex virus type 1}
KYALADASLKMADPNRFRGKDLPVLDQLTDPPGVRRVYHIQAGLPDPFQPPSLPITVYYAVL
ERACRSVLLNAPSEAPQIVRGASEDVRKQPYNLTIAWFRMGGNCAIPITVMEYTECSYNKSL
GACPIRTQPRWNYYDSFSAVSEDNLGFLMHAPAFETAGTYLRLVKINDWTEITQFILEHRAK
GSCKYALPLRIPPSACLSPQAYQQGVTVDSIGMLPRFIPENQRTVAVYSLKIAGWHGPKAPY
TSTLLPPELSE
>d1igta1 b.1.1.1 (A:1-108) Immunoglobulin (variable domains of L and H chains) {Intact IgG2a antibody Mab231 (mouse), kappa L chain}
DIVLTQSPSSLSASLGDTITITCHASQNINVWLSWYQQKPGNIPKLLIYKASNLHTGVPSRF
SGSGSGTGFTLTISSLQPEDIATYYCQQGQSYPLTFGGGTKLEIKR
>d1igtb1 b.1.1.1 (B:1-114) Immunoglobulin (variable domains of L and H chains) {Intact IgG2a antibody Mab231 (mouse), kappa L chain}
EVKLQESGGGLVQPGGSLKLSCATSGFTFSDYYMYWVRQTPEKRLEWVAYISNGGGSTYYPD
TVKGRFTISRDNAKNTLYLQMSRLKSEDTAMYYCARHGGYYAMDYWGQGTTVTVSSA
>d1igya1 b.1.1.1 (A:2-107) Immunoglobulin (variable domains of L and H chains) {Intact IgG1 antibody Mab61.1.3 (mouse), kappa L chain}
KCAHTVSKSMSMSVGERVTLTCKASENVVTYVSWYQQKPEQSPKLLIYGASNRYTGVPDRFT
GSGSATDFTLTISSVQAEDLADYHCGQGYSYPYTFGGGTKLEIK
>d1igyb1 b.1.1.1 (B:2-113) Immunoglobulin (variable domains of L and H chains) {Intact IgG1 antibody Mab61.1.3 (mouse), kappa L chain}
VKLQESGAELARPGASVKMSCKASGYTFTTYTIHWIKQRPGQGLEWIGYINPSSVYTNYNQR
FKDKATLTRDRSSNTANIHLSSLTSDDSAVYYCVREGEVPYWGQGTTVTVSS
>d1hzhh1 b.1.1.1 (H:1-113) Immunoglobulin (variable domains of L and H chains) {Intact IgG B12 antibody (human), kappa L chain}
QVQLVQSGAEVKKPGASVKVSCQASGYRFSNFVIHWVRQAPGQRFEWMGWINPYNGNKEFSA
KFQDRVTFTADTSANTAYMELRSLRSADTAVYYCARVGPYSWDDSPQDNYYMDVWGKGTTVI
VSS
>d1hzhl1 b.1.1.1 (L:1-107) Immunoglobulin (variable domains of L

and H chains) {Intact IgG B12 antibody (human), kappa L chain}
EIVLTQSPGTLSLSPGERATFSCRSSHSIRSRRVAWYQHKPGQAPRLVIHGVSNRASGISDR
FSGSGSGTDFTLTITRVEPEDFALYYCQVYGASSYTFGQGTKLERK
>d8faba1 b.1.1.1 (A:3-105) Immunoglobulin (variable domains of L and H chains) {Fab HIL (human), lambda L chain}
ELTQPPSVSVSPGQTARITCSANALPNQYAYWYQQKPGRAPVMVIYKDTQRPSGIPQRFSSS
TSGTTVTLTISGVQAEDEADYYCQAWDNSASIFGGGTKLTV
>d8fabb1 b.1.1.1 (B:1-121) Immunoglobulin (variable domains of L and H chains) {Fab HIL (human), lambda L chain}
AVKLVQAGGGVVQPGRSLRLSCIASGFTFSNYGMHWVRQAPGKGLEWVAVIWYNGSRTYYGD
SVKGRFTISRDNSKRTLYMQMNSLRTEDTAVYYCARDPDILTAFSFDYWGQGVLVTVSS
>d7fabh1 b.1.1.1 (H:1-116) Immunoglobulin (variable domains of L and H chains) {Fab NEW (human), lambda L chain}
AVQLEQSGPGLVRPSQTLSLTCTVSGTSFDDYYWTWVRQPPGRGLEWIGYVFYTGTTLLDPS
LRGRVTMLVNTSKNQFSLRLSSVTAADTAVYYCARNLIAGGIDVWGQGSLVTVS
>d7fabl1 b.1.1.1 (L:1-103) Immunoglobulin (variable domains of L and H chains) {Fab NEW (human), lambda L chain}
ASVLTQPPSVSGAPGQRVTISCTGSSSNIGAGHNVKWYQQLPGTAPKLLIFHNNARFSVSKS
GTSATLAITGLQAEDEADYYCQSYDRSLRVFGGGTKLTVLR
>d1bafh1 b.1.1.1 (H:1-115) Immunoglobulin (variable domains of L and H chains) {Fab ANO2 (mouse), kappa L chain}
DVQLQESGPGLVKPSQSQSLTCTVTGYSITSDYAWNWIRQFPGNKLEWMGYMSYSGSTRYNP
SLRSRISITRDTSKNQFFLQLKSVTTEDTATYFCARGWPLAYWGQGTQVSVSE
>d1bafl1 b.1.1.1 (L:1-108) Immunoglobulin (variable domains of L and H chains) {Fab ANO2 (mouse), kappa L chain}
QIVLTQSPAIMSASPGEKVTMTCSASSSVYYMYWYQQKPGSSPRLLIYDTSNLASGVPVRFS
GSGSGTSYSLTISRMEAEDAATYYCQQWSSYPPITFGVGTKLELKR
>d1a3rh1 b.1.1.1 (H:2-119) Immunoglobulin (variable domains of L and H chains) {Fab 8F5 (mouse), kappa L chain}
VQLQQSGAELVRPGASVKLSCTTSGFNIKDIYIHWVKQRPEQGLEWIGRLDPANGYTKYDPK
FQGKATITVDTSSNTAYLHLSSLTSEDTAVYYCDGYYSYYDMDYWGPGTSVTVSSAKTTAP
>d1a3rl1 b.1.1.1 (L:1-114) Immunoglobulin (variable domains of L and H chains) {Fab 8F5 (mouse), kappa L chain}
DIVMTQSPSSLTVTTGEKVTMTCKSSQSLLNSRTQKNYLTWYQQKPGQSPKLLIYWASTRES
GVPDRFTGSGSGTDFTLSISGVQAEDLAVYYCQNNYNYPLTFGAGTKLELKRADAAPT
>d1bbjh1 b.1.1.1 (H:1-115) Immunoglobulin (variable domains of L and H chains) {Fab B72.3 (mouse/human chimera), kappa L chain}
EVQLQQSDAELVKPGASVKISCKASGYTFTDHAIHWAKQKPEQGLEWIGYISPGNDDIKYNE
KFKGKATLTADKSSSTAYMQLNSLTSEDSAVYFCKRSYYGHWGQGTTLTVSSA
>d1bbjl1 b.1.1.1 (L:1-109) Immunoglobulin (variable domains of L and H chains) {Fab B72.3 (mouse/human chimera), kappa L chain}
DIQMTQSPASLSVSVGETVTITCRASENIYSNLAWYQQKQGKSPQLLVYAATNLADGVPSRF
SGSGSGTQYSLKINSLQSEDFGSYYCQHFWGTPYTFGGGTRLEIKRA
>d1hila1 b.1.1.1 (A:1-108) Immunoglobulin (variable domains of L

and H chains) {Fab 17/9 (mouse), kappa L chain}
DIVMTQSPSSLTVTAGEKVTMSCTSSQSLFNSGKQKNYLTWYQQKPGQPPKVLIYWASTRES
GVPDRFTGSGSGTDFTLTISSVQAEDLAVYYCQNDYSNPLTFGGGTKLELKR
>d1hilb1 b.1.1.1 (B:1-112) Immunoglobulin (variable domains of L and H chains) {Fab 17/9 (mouse), kappa L chain}
EVQLVESGGDLVKPGGSLKLSCAASGFSFSSYGMSWVRQTPDKRLEWVATISNGGGYTYYPD
SVKGRFTISRDNAKNTLYLQMSSLKSEDSAMYYCARRERYDENGFAYWGQGTLVTVS
>d1dbbh1 b.1.1.1 (H:1-112) Immunoglobulin (variable domains of L and H chains) {Fab DB3 (mouse), kappa L chain}
QIQLVQSGPELKKPGETVKISCKASGYAFTNYGVNWVKEAPGKELKWMGWINIYTGEPTYVD
DFKGRFAFSLETSASTAYLEINNLKNEDTATYFCTRGDYVNWYFDVWGAGTTVTVS
>d1dbbl1 b.1.1.1 (L:1-107) Immunoglobulin (variable domains of L and H chains) {Fab DB3 (mouse), kappa L chain}
DVVMTQIPLSLPVNLGDQASISCRSSQSLIHSNGNTYLHWYLQKPGQSPKLLMYKVSNRFYG
VPDRFSGSGSGTDFTLKISRVEAEDLGIYFCSQSSHVPPTFGGGTKLEIK
>d1dfbh1 b.1.1.1 (H:1-126) Immunoglobulin (variable domains of L and H chains) {Fab 3D6 (human), kappa L chain}
EVQLVESGGGLVQPGRSLRLSCAASGFTFNDYAMHWVRQAPGKGLEWVSGISWDSSSIGYAD
SVKGRFTISRDNAKNSLYLQMNSLRAEDMALYYCVKGRDYYDSGGYFTVAFDIWGQGTMVTV
SS
>d1dfbl1 b.1.1.1 (L:1-106) Immunoglobulin (variable domains of L and H chains) {Fab 3D6 (human), kappa L chain}
DIQMTQSPSTLSASVGDRVTITCRASQSISRWLAWYQQKPGKVPKLLIYKASSLESGVPSRF
SGSGSGTEFTLTISSLQPDDFATYYCQQYNSYSFGPGTKVDIKR
>d1igfh1 b.1.1.1 (H:1-113) Immunoglobulin (variable domains of L and H chains) {Fab B13I2 (mouse), kappa L chain}
EVQLVESGGDLVKPGGSLKLSCAASGFTFSRCAMSWVRQTPEKRLEWVAGISSGGSYTFYPD
TVKGRFIISRNNARNTLSLQMSSLRSEDTAIYYCTRYSSDPFYFDYWGQGTTLTVSS
>d1igfl1 b.1.1.1 (L:1-107) Immunoglobulin (variable domains of L and H chains) {Fab B13I2 (mouse), kappa L chain}
DVLMTQTPLSLPVSLGDQASISCRSNQTILLSDGDTYLEWYLQKPGQSPKLLIYKVSNRFSG
VPDRFSGSGSGTDFTLKISRVEAEDLGVYYCFQGSHVPPTFGGGTKLEIK
>d1igja1 b.1.1.1 (A:1-107) Immunoglobulin (variable domains of L and H chains) {Fab 26-10 (mouse), kappa L chain}
DVVMTQTPLSLPVSLGDQASISCRSSQSLVHSNGNTYLNWYLQKAGQSPKLLIYKVSNRFSG
VPDRFSGSGSGTDFTLKISRVEAEDLGIYFCSQTTHVPPTFGGGTKLEIK
>d1igjb1 b.1.1.1 (B:2-114) Immunoglobulin (variable domains of L and H chains) {Fab 26-10 (mouse), kappa L chain}
VQLQQSGPELVKPGASVRMSCKSSGYIFTDFYMNWVRQSHGKSLDYIGYISPYSGVTGYNQK
FKGKATLTVDKSSSTAYMELRSLTSEDSAVYYCAGSSGNKWAMDYWGHGASVTVSSA
>d1igmh_ b.1.1.1 (H:) Immunoglobulin (variable domains of L and H chains) {Fv POT (human) IgM, kappa L chain}
EVHLLESGGNLVQPGGSLRLSCAASGFTFNIFVMSWVRQAPGKGLEWVSGVFGSGGNTDYAD
AVKGRFTITRDNSKNTLYLQMNSLRAEDTAIYYCAKHRVSYVLTGFDSWGQGTLVTVSSGSA

SAPTL
>d1igml_ b.1.1.1 (L:) Immunoglobulin (variable domains of L and H chains) {Fv POT (human) IgM, kappa L chain}
DIQMTQSPSSLSASVGDRVTITCQASQDISNYLAWYQQKPGKAPELRIYDASNLETGVPSRF
SGSGSGTDFTFTISSLQPEDIATYYCQQYQNLPLTFGPGTKVDIKRTVAAPSV
>d1dqlh_ b.1.1.1 (H:) Immunoglobulin (variable domains of L and H chains) {Fv MEZ (human) IgM, kappa L chain}
EVQLVESGGGLVQPGGSLRLSCAASGFTFSSYAMHWVRQAPGKGLEWVAVISSDGGNKYYTD
SVKGRFTISRNDSKNTLYLQMNSLRTEDTAVFYCARGNPPYSSGWGGGDYWGQGTMVTVSS
>d1dqll_ b.1.1.1 (L:) Immunoglobulin (variable domains of L and H chains) {Fv MEZ (human) IgM, kappa L chain}
DIQMTQSPSSLSASVGDRVTITCRASQDIRNDLGWYQQKPGKAPKKLIYAASSLQSGVPSRF
SGSGSGTDFTLTISSLQPEDFATYYCLQQNSNWTFGQGTKVDIK
>d1dn0a1 b.1.1.1 (A:1-107) Immunoglobulin (variable domains of L and H chains) {Fab Kau cold agglutinin (human) IgM, kappa L chain}
EIVLTQSPATLSLSPGERATLSCGASQSVSSNYLAWYQQKPGQAPRLLIYDASSRATGIPDR
FSGSGSGTDFTLTISRLEPEDFAVYYCQQYGSSPLTFGGGTKVEI
>d1dn0b1 b.1.1.1 (B:1-120) Immunoglobulin (variable domains of L and H chains) {Fab Kau cold agglutinin (human) IgM, kappa L chain}
EVQLQQWGAGLLKPSETLSLTCAVYGGSFSDYYWSWIRQPPGKGLEWIGEINHSGSTNYNPS
LKSRVTISVDTSKNQFSLKLSSVTAADTAVYYCARPPHDTSGHYWNYWGQGTLVTVSS
>d1indh1 b.1.1.1 (H:1-114) Immunoglobulin (variable domains of L and H chains) {Fab Cha255 (mouse), lambda L chain}
EVTLVESGGDSVKPGGSLKLSCAASGFTLSGETMSWVRQTPEKRLEWVATTLSGGGFTFYSA
SVKGRFTISRDNAQNNLYLQLNSLRSEDTALYFCASHRFVHWGHGTLVTVSA
>d1faih1 b.1.1.1 (H:1-123) Immunoglobulin (variable domains of L and H chains) {Fab R19.9 (mouse), kappa L chain}
QVQLQQSGAELVRAGSSVKMSCKASGYTFTSYGVNWVKQRPGQGLEWIGYINPGKGYLSYNE
KFKGKTTLTVDRSSSTAYMQLRSLTSEDAAVYFCARSFYGGSDLAVYYFDSWGQGTTLTVS
>d1fail1 b.1.1.1 (L:1-108) Immunoglobulin (variable domains of L and H chains) {Fab R19.9 (mouse), kappa L chain}
DIQMTQTTSSLSASLGDRVTISCRASQDISNYLNWYQQKPDGTVKLLIYYTSRLHSGVPSRF
SGSGSGTDYSLTISNLEHEDIATYFCQQGSTLPRTFGGGTKLEIKR
>d2fb4h1 b.1.1.1 (H:1-119) Immunoglobulin (variable domains of L and H chains) {Fab KOL (human), lambda L chain}
EVQLVQSGGGVVQPGRSLRLSCSSSGFIFSSYAMYWVRQAPGKGLEWVAIIWDDGSDQHYAD
SVKGRFTISRNDSKNTLFLQMDSLRPEDTGVYFCARDGGHGFCSSASCFGPDYWGQGTPVTV
SSA
>d2fb4l1 b.1.1.1 (L:1-109) Immunoglobulin (variable domains of L and H chains) {Fab KOL (human), lambda L chain}
QSVLTQPPSASGTPGQRVTISCSGTSSNIGSSTVNWYQQLPGMAPKLLIYRDAMRPSGVPDR
FSGSKSGASASLAIGGLQSEDETDYYCAAWDVSLNAYVFGTGTKVTVLG
>d2fbjh1 b.1.1.1 (H:1-118) Immunoglobulin (variable domains of L and H chains) {Fab J539 (mouse), kappa L chain}

EVKLLESGGGLVQPGGSLKLSCAASGFDFSKYWMSWVRQAPGKGLEWIGEIHPDSGTINYTP
SLKDKFIISRDNAKNSLYLQMSKVRSEDTALYYCARLHYYGYNAYWGQGTLVTVSA
>d2fbjl1 b.1.1.1 (L:1-109) Immunoglobulin (variable domains of L and H chains) {Fab J539 (mouse), kappa L chain}
EIVLTQSPAITAASLGQKVTITCSASSSVSSLHWYQQKSGTSPKPWIYEISKLASGVPARFS
GSGSGTSYSLTINTMEAEDAAIYYCQQWTYPLITFGAGTKLELKRAD
>d1fgvh_ b.1.1.1 (H:) Immunoglobulin (variable domains of L and H chains) {Fab H52 (synthetic, humanised version), kappa L chain}
EVQLVESGGGLVQPGGSLRLSCATSGYTFTEYTMHWMRQAPGKGLEWVAGINPKNGGTSYAD
SVKGRFTISVDKSKNTLYLQMNSLRAEDTAVYYCARWRGLNYGFDVRYFDVWGQGTLVTVSS
>d1fgvl_ b.1.1.1 (L:) Immunoglobulin (variable domains of L and H chains) {Fab H52 (synthetic, humanised version), kappa L chain}
DIQMTQSPSSLSASVGDRVTITCRASQDINNYLNWYQQKPGKAPKLLIYYTSTLESGVPSRF
SGSGSGTDYTLTISSLQPEDFATYYCQQGNTLPPTFGAGTKVEIK
>d2fgwh1 b.1.1.1 (H:1-124) Immunoglobulin (variable domains of L and H chains) {Fab H52 (synthetic, humanised version), kappa L chain}
EVQLVESGGGLVQPGGSLRLSCATSGYTFTEYTMHWMRQAPGKGLEWVAGINPKNGGTSHNQ
RFMDRFTISVDKSTSTAYMQMNSLRAEDTAVYYCARWRGLNYGFDVRYFDVWGQGTLVTVSS
>d1mcph1 b.1.1.1 (H:1-121) Immunoglobulin (variable domains of L and H chains) {Fab MCPC603 (human), kappa L chain}
EVKLVESGGGLVQPGGSLRLSCATSGFTFSDFYMEWVRQPPGKRLEWIAASRNKGNKYTTEY
SASVKGRFIVSRDTSQSILYLQMNALRAEDTAIYYCARNYYGSTWYFDVWGAGTTVTVS
>d2imn__ b.1.1.1 (-) Immunoglobulin (variable domains of L and H chains) {Fab MCPC603 (human), kappa L chain}
DIVMTQSPSSLSVSAGERVTMSCKSSQSLLYKDGKNFLAWYQQKPGQPPKLLIYGASTRESG
VPDRFTGSGSGTDFTLTISSVQAEDLAVYYCQNDHSYPLTFGAGTKLELKR
>d1fvca_ b.1.1.1 (A:) Immunoglobulin (variable domains of L and H chains) {Fab 4D5 (synthetic, humanised version), kappa L chain}
DIQMTQSPSSLSASVGDRVTITCRASQDVNTAVAWYQQKPGKAPKLLIYSASFLYSGVPSRF
SGSRSGTDFTLTISSLQPEDFATYYCQQHYTTPPTFGQGTKVEIKRT
>d1fvcb_ b.1.1.1 (B:) Immunoglobulin (variable domains of L and H chains) {Fab 4D5 (synthetic, humanised version), kappa L chain}
EVQLVESGGGLVQPGGSLRLSCAASGFNIKDTYIHWVRQAPGKGLEWVARIYPTNGYTRYAD
SVKGRFTISADTSKNTAYLQMNSLRAEDTAVYYCSRWGGDGFYAMDYWGQGTLVTVSS
>d1ggbh1 b.1.1.1 (H:1-112) Immunoglobulin (variable domains of L and H chains) {Fab 50.1 (mouse), kappa L chain}
QVQLQESGPGILQPSQTLSLTCSFSGFSLSTYGMGVSWIRQPSGKGLEWLAHIFWDGDKRYN
PSLKSRLKISKDTSNNQVFLKITSVDTADTATYYCVQEGYIYWGQGTSVTVS
>d1ggbl1 b.1.1.1 (L:1-107) Immunoglobulin (variable domains of L and H chains) {Fab 50.1 (mouse), kappa L chain}
DIVLTQSPGSLAVSLGQRATISCRASESVDDDGNSFLHWYQQKPGQPPKLLIYRSSNLISGI
PDRFSGSGSRTDFTLTINPVEADDVATYYCQQSNEDPLTFGAGTKLEIK
>d1ai1h1 b.1.1.1 (H:1-112) Immunoglobulin (variable domains of L

and H chains) {Fab 59.1 (mouse), kappa L chain}
QVKLQESGPAVIKPSQSLSLTCIVSGFSITRTNYCWHWIRQAPGKGLEWMGRICYEGSIYYS
PSIKSRSTISRDTSLNKFFIQLISVTNEDTAMYYCSRENHMYETYFDVWGQGTTVTVS
>d1ai1l1 b.1.1.1 (L:1-108) Immunoglobulin (variable domains of L and H chains) {Fab 59.1 (mouse), kappa L chain}
DIVMTQSPASLVVSLGQRATISCRASESVDSYGKSFMHWYQQKPGQPPKVLIYIASNLESGV
PARFSGSGSRTDFTLTIDPVEADDAATYYCQQNNEDPPTFGAGTKLEMRR
>d1mamh1 b.1.1.1 (H:1-119) Immunoglobulin (variable domains of L and H chains) {Fab Yst9.1 (mouse), kappa L chain}
EVKLVESGGGLVQPGGSLRLSCATSGFTFTDYYMSWVRQPPGKALEWLGFIRNKADGYTTEY
SASVKGRFTISRDNSQSILYLQMNTLRAEDSATYYCTRDPYGPAAYWGQGTLVTVSA
>d1maml1 b.1.1.1 (L:1-108) Immunoglobulin (variable domains of L and H chains) {Fab Yst9.1 (mouse), kappa L chain}
DIQMTQTTSSLSASLGDRVTISCRASQDIYNYLNWYQQKPDGTVKLLIYYTSRLHSGVPSRF
SGSGSGTDYSLTISNLNQEDMATYICQQGNTLPFTFGSGTKLEIKR
>d1mfa_1 b.1.1.1 (1L-111L) Immunoglobulin (variable domains of L and H chains) {Fab SE155-4 (mouse), lambda L chain}
QIVVTQESALTTSPGETVTLTCRSSTGTVTSGNHANWVQEKPDHLFTGLIGDTNNRAPGVPA
RFSGSLIGDKAALTITGAQPEDEAIYFCALWSNNHWIFGGGTKLTVLGQ
>d1mfa_2 b.1.1.1 (251H-367H) Immunoglobulin (variable domains of L and H chains) {Fab SE155-4 (mouse), lambda L chain}
EVQVQQSGTVVARPGASVKMSCKASGYTFTNYWMHWIKQRPGQGLEWIGAIYPGNSATFYNH
KFRAKTKLTAVTSTTTAYMELSSLTSEDSAVYYCTRGGHGYYGDYWGQGASLTVS
>d1nbvh1 b.1.1.1 (H:1-122) Immunoglobulin (variable domains of L and H chains) {Fab BV04-01 (mouse), kappa L chain}
EVQPVETGGGLVQPKGSLKLSCAASGFSFNTNAMNWVRQAPGKGLEWVARIRSKSNNYATYY
ADSVKDRFTISRDDSQNMLYLQMNNLKTEDTAMYYCVRDQTGTAWFAYWGQGTLVTVSAA
>d1teth1 b.1.1.1 (H:1-112) Immunoglobulin (variable domains of L and H chains) {Fab TE33 (mouse), kappa L chain}
QIQLVQSGPELKTPGETVRISCKASGYTFTTYGMSWVKQTPGKGFKWMGWINTYSGVPTYAD
DFKGRFAFSLETSASTAYLQINNLKNEDTATYFCARRSWYFDVWGTGTTVTVS
>d1tetl1 b.1.1.1 (L:1-107) Immunoglobulin (variable domains of L and H chains) {Fab TE33 (mouse), kappa L chain}
DVLMTQTPLSLPVSLGDQASISCKSSQSIVHSSGNTYFEWYLQKPGQSPKLLIYKVSNRFSG
VPDRFSGSGSGTDFTLKISRVEAEDLGVYYCFQGSHIPFTFGSGTKLEIK
>d1flrh1 b.1.1.1 (H:1-118) Immunoglobulin (variable domains of L and H chains) {Fab 4-4-20 (mouse), kappa L chain}
EVKLDETGGGLVQPGRPMKLSCVASGFTFSDYWMNWVRQSPEKGLEWVAQIRNKPYNYETYY
SDSVKGRFTISRDDSKSSVYLQMNNLRVEDMGIYYCTGSYYGMDYWGQGTSVTVSS
>d4fabl1 b.1.1.1 (L:1-112) Immunoglobulin (variable domains of L and H chains) {Fab 4-4-20 (mouse), kappa L chain}
DVVMTQTPLSLPVSLGDQASISCRSSQSLVHSQGNTYLRWYLQKPGQSPKVLIYKVSNRFSG
VPDRFSGSGSGTDFTLKISRVEAEDLGVYFCSQSTHVPWTFGGGTKLEIK
>d1jfqh1 b.1.1.1 (H:302-421) Immunoglobulin (variable domains of

L and H chains) {Fab 36-71 (mouse), kappa L chain}
VQLQQSGVELVRAGSSVKMSCKASGYTFTSNGINWVKQRPGQGLEWIGYNNPGNGYITYNEK
FKGKTTLTVDKSSNTAYMQLRSLTSEDSAVYFCARSEYYGGSYKFDYWGQGTTLTVSS
>d1jfql1 b.1.1.1 (L:1-108) Immunoglobulin (variable domains of L and H chains) {Fab 36-71 (mouse), kappa L chain}
DIQMTQIPSSLSASLGDRVSISCRASQDINNFLNWYQQKPDGTIKLLIYFTSRSQSGVPSRF
SGSGSGTDYSLTISNLEQEDIATYFCQQGNALPRTFGGGTKLEIKR
>d1gigh1 b.1.1.1 (H:1-120) Immunoglobulin (variable domains of L and H chains) {Fab HC19 (mouse), lambda L chain}
QVQLKESGPGLVAPSQSLSITCTVSGFLLISNGVHWVRQPPGKGLEWLGVIWAGGNTNYNSA
LMSRVSISKDNSKSQVFLKMKSLQTDDTAMYYCARDFYDYDVFYYAMDYWGQGTSVTV
>d2cgrh1 b.1.1.1 (H:1-117) Immunoglobulin (variable domains of L and H chains) {Fab, anti-sweetener (mouse), kappa L chain}
RVQLLESGAELMKPGASVQISCKATGYTFSEYWIEWVKERPGHGLEWIGEILPGSGRTNYRE
KFKGKATFTADTSSNTAYMQLSSLTSEDSAVYYCTRGYSSMDYWGQGTSVTVSAA
>d2cgrl1 b.1.1.1 (L:1-112) Immunoglobulin (variable domains of L and H chains) {Fab, anti-sweetener (mouse), kappa L chain}
ELVMTQSPLSLPVSLGDQASISCRPSQSLVHSNGNTYLHWYLQKPGQSPKLLIYRVSNRFSG
VPDRFSGSGSGTAFTLKISRVEAEDLGVYFCSQGTHVPYTFGGGTKLELK
>d1figh1 b.1.1.1 (H:1-113) Immunoglobulin (variable domains of L and H chains) {Fab 1F7 (mouse), kappa L chain}
DVQLQQSGPELEKPGASVKISCKASGFSLPGHNINWIVQRNGKSLEWIGNIDPYYGGTNFNP
KFKGKATLTVDKSSSTLYMHLTSLQSEDSAVYYCARRRDGNYGFTYWGQGTLVTVSA
>d1figl1 b.1.1.1 (L:1-108) Immunoglobulin (variable domains of L and H chains) {Fab 1F7 (mouse), kappa L chain}
ENVLTQSPAIMSASPGEKVTMACRASSSVSSTYLHWYQQKSGASPKLLIYSTSNLASGVPAR
FSGSGSGTSYSLTISSVEAEDAATYYCQQYSGYPLTFGAGTKLELKR
>d1frgh1 b.1.1.1 (H:218-336) Immunoglobulin (variable domains of L and H chains) {Fab 26/9 (mouse), kappa L chain}
EVLLVESGGDLVKPGGFLKLSCAASGFTFSSFGMSWVRHTPDKRLEWVATISNGGGYTYYQD
SVKGRFTISRDNAKNTLFLEMTSLKSEDAGLYYCARRERYDEKGFAYWGRGTLVTVS
>d1frgl1 b.1.1.1 (L:1-113) Immunoglobulin (variable domains of L and H chains) {Fab 26/9 (mouse), kappa L chain}
DIVMTQSPSSLTVTAGEKVTMSCKSSQSLFNSGKRKNFLTWYHQKPGQPPKLLIYWASTRES
GVPDRFSGSGSGTDFTLTITSVQAEDLAIYYCQNDYSHPLTFGAGTKLELK
>d1a2ya_ b.1.1.1 (A:) Immunoglobulin (variable domains of L and H chains) {Fab D1.3 (mouse), kappa L chain}
DIVLTQSPASLSASVGETVTITCRASGNIHNYLAWYQQKQGKSPQLLVYYTTTLADGVPSRF
SGSGSGTQYSLKINSLQPEDFGSYYCQHFWSTPRTFGGGTKLEIK
>d1a2yb_ b.1.1.1 (B:) Immunoglobulin (variable domains of L and H chains) {Fab D1.3 (mouse), kappa L chain}
QVQLQESGPGLVAPSQSLSITCTVSGFSLTGYGVNWVRQPPGKGLEWLGMIWGDGNTDYNSA
LKSRLSISKDNSKSQVFLKMNSLHTDDTARYYCARERDYRLDYWGQGTTLTVSS
>d1a7ql_ b.1.1.1 (L:) Immunoglobulin (variable domains of L and

H chains) {Fab D1.3 (mouse), kappa L chain}
DIVLTQSPASLSASVGETVTITCRAGGNTHNYLAWYQQKQGKSPQLLVYYTTTLAAGVPSRF
SGSGSGTQYSLKINSLQPDDFGSYYCQHFWSTPRSFGGGTKLEI
>d1cica1 b.1.1.1 (A:1-107) Immunoglobulin (variable domains of L and H chains) {Anti-idiotope (D1.3) Fab E225, (mouse), kappa L chain}
DIVMTQSHKFMSTSVGDRVSITCKASQDVRIAVAWYQQKPGQSPKLLIYWASTRHTGVPDRF
TGSGSGTDFTLTISNVQSEDLADYFCQHCGSYPFTFGSGTKLEIK
>d1cicb1 b.1.1.1 (B:1-117) Immunoglobulin (variable domains of L and H chains) {Anti-idiotope (D1.3) Fab E225, (mouse), kappa L chain}
QVQLQQPGSELVRPGASVKLSCKASGYTFTNYWMHWVKQRPGQGLEWIGNIYPGSGDSNYDE
KFKSKATLTVDTSSSTAYMQLSGLTSEDSAVYYCARGLAFYFDHWGQGTTLTVSS
>d1jhlh_ b.1.1.1 (H:) Immunoglobulin (variable domains of L and H chains) {Fv D11.15 (mouse), kappa L chain}
QVQLQQSGAELVRPGASVKLSCKASGYTFISYWINWVKQRPGQGLEWIGNIYPSDSYTNYNQ
KFKDKATLTVDKSSSTAYMQLSSPTSEDSAVYYCTRDDNYGAMDYWGQGTTVTV
>d1jhll_ b.1.1.1 (L:) Immunoglobulin (variable domains of L and H chains) {Fv D11.15 (mouse), kappa L chain}
DIELTQSPSYLVASPGETITINCRASKSISKSLAWYQEKPGKTNNLLIYSGSTLQSGIPSRF
SGSGSGTDFTLTISSLEPEDFAMYICQQHNEYPWTFGGGTKLEIKR
>d1bqlh1 b.1.1.1 (H:2-116) Immunoglobulin (variable domains of L and H chains) {Fab HyHEL-5 (mouse), kappa L chain}
VQLQQSGAELMKPGASVKISCKASGYTFSDYWIEWVKQRPGHGLEWIGEILPGSGSTNYHER
FKGKATFTADTSSSTAYMQLNSLTSEDSGVYYCLHGNYDFDGWGQGTTLTVSS
>d1bqll1 b.1.1.1 (L:1-106) Immunoglobulin (variable domains of L and H chains) {Fab HyHEL-5 (mouse), kappa L chain}
DIVLTQSPAIMSASPGEKVTMTCSASSSVNYMYWYQQKSGTSPKRWIYDTSKLASGVPVRFS
GSGSGTSYSLTISSMETEDAATYYCQQWGRNPTFGGGTKLEIKR
>d1ic7h_ b.1.1.1 (H:) Immunoglobulin (variable domains of L and H chains) {Fab HyHEL-10 (mouse), kappa L chain}
DVQLQESGPSLVKPSQTLSLTCSVTGDSITSAYWSWIRKFPGNRLEYMGYVSYSGSTYYNPS
LKSRISITRDTSKNQYYLDLNSVTTEDTATYYCANWAGDYWGQGTLVTVSAA
>d2jelh1 b.1.1.1 (H:1-113) Immunoglobulin (variable domains of L and H chains) {Fab JE142 (mouse), kappa L chain}
QVQLAQSGPELVRPGVSVKISCKGSGYTFTTYAMHWVKQSHAKSLEWIGLISTYSGYTNYNQ
KFKGKATMTVDKSSSTAYMELARLTSEDSAIYYCARVMGEQYFDVWGAGTTVIVSS
>d1ncbh1 b.1.1.1 (H:1-113) Immunoglobulin (variable domains of L and H chains) {Fab NC41 (mouse), kappa L chain}
QIQLVQSGPELKKPGETVKISCKASGYTFTNYGMNWVKQAPGKGLEWMGWINTNTGEPTYGE
EFKGRFAFSLETSASTANLQINNLKNEDKATFFCARGEDNFGSLSDYWGQGTTLTVSS
>d1ncbl1 b.1.1.1 (L:1-108) Immunoglobulin (variable domains of L and H chains) {Fab NC41 (mouse), kappa L chain}
DIVMTQSPKFMSTSVGDRVTITCKASQDVSTAVVWYQQKPGQSPKLLIYWASTRHIGVPDRF

AGSGSGTDYTLTISSVQAEDLALYYCQQHYSPPWTFGGGTKLEIKR
>d1forh1 b.1.1.1 (H:1-120) Immunoglobulin (variable domains of L and H chains) {Fab 17-Ia (mouse), kappa L chain}
QGQLQQSGAELVRPGSSVKISCKASGYAFSSFWVNWVKQRPGQGLEWIGQIYPGDGDNKYNG
KFKGKATLTADKSSTTAYMQLYSLTSEDSAVYFCARSGNYPYAMDYWGQGTSVTVSSA
>d1forl1 b.1.1.1 (L:1-107) Immunoglobulin (variable domains of L and H chains) {Fab 17-Ia (mouse), kappa L chain}
QIVLTQSPAIMSAFPGEKVTITCSATSSVNYMHWFQQKPGTSPKLWIYSSSNLASGVPARFS
GSGSGTSYSLTISRMEAEDAATYYCQQRSSYPITFGSGTKLEIKR
>d1knoa1 b.1.1.1 (A:1-108) Immunoglobulin (variable domains of L and H chains) {Fab CNJ206 (mouse), kappa L chain}
QIQMTQSPSSLSASLGERVSLTCRASQEISGYLSWLQQKPDGTIKRLIYAASTLDSGVPKRF
SGSRSGSDYSLTISSLESEDFADYYCLQYASSPYTFGGGTKLEILR
>d1knob1 b.1.1.1 (B:1-119) Immunoglobulin (variable domains of L and H chains) {Fab CNJ206 (mouse), kappa L chain}
DVKLVESGGGLVQPGGSRKLSCAASGFTFSSFGMHWVRQAPEKGLEWVAYISSGSSTIYYAD
TVKGRFTISRDNPKNTLFLQMTSLRSEDTAMYYCARGDYYGSRGAYWGQGTLVTVSA
>d1eapb1 b.1.1.1 (B:1-124) Immunoglobulin (variable domains of L and H chains) {Fab 17E8 (mouse), kappa L chain}
EVQLQESGTELVKPGASVKISCKASGYISTDHAIHWVKQRPEQGLEWIGYISPGNGDIKYNE
KFKVKATLTADQSSSTAYMQLNSLTSEDSAVYFCKRSYYGSSYVDYWGQGTTLTVSS
>d1mrdh1 b.1.1.1 (H:2-115) Immunoglobulin (variable domains of L and H chains) {Fab Jel 103 (mouse), kappa L chain}
VQLQQSGAELVKPGASVKLSCKASGYTFTSYWMQWVKQRPGQGLEWIGEIDPSDSYTNYNQK
FKGKATLTVDTSSSTAYMQLSSLTSEDSAVYYCANLRGYFDYWGQGTTLTVSSAK
>d1fbih1 b.1.1.1 (H:1-121) Immunoglobulin (variable domains of L and H chains) {Fab F9.13.7 (mouse), kappa L chain}
QVQLQQPGAELVKPGASVKLSCKASGYTFTSYWMHWVKQGPGQGLEWIGEIDPSDSYPNYNE
KFKGKATLTVDKSSSTAYMQLSSLTSEDSAVYYCASLYYYGTSYGVLDYWGQGTSVTVS
>d1fbil1 b.1.1.1 (L:1-107) Immunoglobulin (variable domains of L and H chains) {Fab F9.13.7 (mouse), kappa L chain}
DIQMTQTTSSLSASLGDRVTISCRASQDISNYLNWYQKKPDGTVKLLIYYTSRLHSGVPSRF
SGSGSGTDYSLTIRNLEQEDIATYFCQQGYTLPYTFGGGTKLEIK
>d1rmfh1 b.1.1.1 (H:1-119) Immunoglobulin (variable domains of L and H chains) {Fab R6.5 (mouse), kappa L chain}
QVQLQQSGPELVRPGVSVKISCKGSGYTFIDYAIHWVKESHAKSLEWIGVISAYSGDTNYNQ
KFKGKATMTVDKSSNTAYLELARLTSEDSAIYYCARGGWLLLSFDYWGQGTTLTVSS
>d1rmfl1 b.1.1.1 (L:1-112) Immunoglobulin (variable domains of L and H chains) {Fab R6.5 (mouse), kappa L chain}
DVVMTQSPLSLPVSLGDQASISCRSSQSLVHSNGNNYLHWYLQKSGQAPKLLIYKVSNRFSG
VPDRFSGSGSGTDFTLKISRVEAEDLGVYFCSQSTHVPLTFGGGTKLEIK
>d1fpth1 b.1.1.1 (H:1-113) Immunoglobulin (variable domains of L and H chains) {Fab C3, neutralizing type 1 poliovirus, (mouse), kappa L chain}

QVQLQQSGAELVRPGTSVKVSCKASGYAFTNYLIQWIKQRPGQGLEWIGVINPGSGGTDYNA
NFKGKATLTADKSSSIVYMQLSSLTSDDSAVYFCARDFYDYDVGFDYWGQGTTLTVSS
>d1fptl1 b.1.1.1 (L:1-108) Immunoglobulin (variable domains of L and H chains) {Fab C3, neutralizing type 1 poliovirus, (mouse), kappa L chain}
DVVMTQTPLSLPVSLGDQASISCSSSQSLVHSNGKTYLHWYLQKPGQSPKLLIYKVSNRFSG
VPDRFSGSGSGTYFTLKISRVEAEDLGVYFCSQSTHVPYTFGGGTKLEIKR
>d1ikfh1 b.1.1.1 (H:1-126) Immunoglobulin (variable domains of L and H chains) {Fab, anti-cyclosporin A, (mouse), kappa L chain}
EVKLVESGGGLVQPGGSLKLSCATSGFTFSDYYMYWVRQNSEKRLEWVAFISNGGGSAFYAD
IVKGRFTISRDNAKNTLYLQMSRLKSEDTAMYYCTRHTLYDTLYGNYPVWFADWGQGTLVTV
SA
>d1ikfl1 b.1.1.1 (L:1-107) Immunoglobulin (variable domains of L and H chains) {Fab, anti-cyclosporin A, (mouse), kappa L chain}
DIQMTQTTSSLSASLGDRVTISCRASQDISTYLNWYQQKPDGTVKLLIFYTSRLRSGVPSRF
SGSGSGTDYSLTISNLEQEDIATYFCQQGSRIPPTFGGGTKLEIL
>d1lmka1 b.1.1.1 (A:2-127) Immunoglobulin (variable domains of L and H chains) {scFv dimer (L5MK16 diabody), based on: (mouse), kappa L chain}
VQLQQSGTELMKPGRSLKISCKTTGYIFSNYWIEWVKQRPGHGLEWIGKILPGGGSNTYNDK
FKGKATFTADTSSNIAYMQLSSLTSEDSAVYYCARGEDYYAYWYVLDYWGQGTTVTVSSGGG
GS
>d1lmka2 b.1.1.1 (A:201-312) Immunoglobulin (variable domains of L and H chains) {scFv dimer (L5MK16 diabody), based on: (mouse), kappa L chain}
DIELTQSPLSLPVSLGDQASISCRSSQSLVHSNGNTSLHWYLKKPGQSPKLLIYKVSTRFSG
VPDRFSGSGSGTDFTLKISRVEAEDLGVYFCSQSTHVPFTFGSGTKLELK
>d1nqba2 b.1.1.1 (A:121-233) Immunoglobulin (variable domains of L and H chains) {scFv trivalent antibody, based on: (mouse), kappa L chain}
DIELTQTPLSLPVSLGDQASISCRSSQSIVHSNGNTYLEWYLQKPGQSPKLLIYKVSNRFSG
VPDRFSGSGSGTDFTLKISRVEAEDLGVYYCFQGSHVPYTFGGGTKLEIKR
>d1igch1 b.1.1.1 (H:1-119) Immunoglobulin (variable domains of L and H chains) {Fab MoPC21 (mouse), kappa L chain}
DVQLVESGGGLVQPGGSRKLSCAASGFTFSSFGMHWVRQAPEKGLEWVAYISSGSSTLHYAD
TVKGRFTISRDNPKNTLFLQMTSLRSEDTGMYYCARWGNYPYYAMDYWGQGTSVTVS
>d1igcl1 b.1.1.1 (L:1-108) Immunoglobulin (variable domains of L and H chains) {Fab MoPC21 (mouse), kappa L chain}
NIVMTQSPKSMSMSVGERVTLTCKASENVVTYVSWYQQKPEQSPKLLIYGASNRYTGVPDRF
TGSGSATDFTLTISSVQAEDLADYHCGQGNSYPYTFGGGTKLEIKR
>d1ibgh1 b.1.1.1 (H:2-113) Immunoglobulin (variable domains of L and H chains) {Fab 40-50 (mouse), kappa L chain}
VHLVQSGPGLVAPSQSLSITCTVSGFSLTTYGVHWFRQPPGKGLEWLGLIWAGGNTDYNSAL
MSRLSINKDNSKSQVFLKMNSLQADDTAMYYCARFRFASYYDYAVDYWGQGTSVTVSS

>d1ibgl1 b.1.1.1 (L:2-107) Immunoglobulin (variable domains of L and H chains) {Fab 40-50 (mouse), kappa L chain}
IVLTQSPASLAVSLGQRATISCRASKSVSTSGYSHIHWYQQKPGQPPKLLIYLASILESGVP
ARFSGSGSGTDFTLNIHPVEEEDAATYYCQHSREYPLTFGAGTELELK
>d1mlbb1 b.1.1.1 (B:1-118) Immunoglobulin (variable domains of L and H chains) {Fab D44.1 (mouse), kappa L chain}
QVQLQESGAEVMKPGASVKISCKATGYTFSTYWIEWVKQRPGHGLEWIGEILPGSGSTYYNE
KFKGKATFTADTSSNTAYMQLSSLTSEDSAVYYCARGDGNYGYWGQGTTLTVSSAS
>d1a14h_ b.1.1.1 (H:) Immunoglobulin (variable domains of L and H chains) {Fab NC10 (mouse), kappa L chain}
QVQLQQSGAELVKPGASVRMSCKASGYTFTNYNMYWVKQSPGQGLEWIGIFYPGNGDTSYNQ
KFKDKATLTADKSSNTAYMQLSSLTSEDSAVYYCARSGGSYRYDGGFDYWGQGTTVTV
>d1a14l_ b.1.1.1 (L:) Immunoglobulin (variable domains of L and H chains) {Fab NC10 (mouse), kappa L chain}
DIELTQTTSSLSASLGDRVTISCRASQDISNYLNWYQQNPDGTVKLLIYYTSNLHSEVPSRF
SGSGSGTDYSLTISNLEQEDIATYFCQQDFTLPFTFGGGTAA
>d1nmbl_ b.1.1.1 (L:) Immunoglobulin (variable domains of L and H chains) {Fab NC10 (mouse), kappa L chain}
DIQMTQTTSSLSASLGDRVTISCRASQDISNYLNWYQQNPDGTVKLLIYYTSNLHSEVPSRF
SGSGSGTDYSLTISNLEQEDIATYFCQQDFTLPFTFGGGTKLEIRRA
>d1bm3h1 b.1.1.1 (H:1-125) Immunoglobulin (variable domains of L and H chains) {Anti-integrin Fab OPG2 (mouse), kappa L chain}
EVQLVQSGGGLVNPGRSLKLSCAASGFTFSSYGMSWVRQTPEKRLEWVAAISGGGTYIHYPD
SVKGRFTISRDNAKNNLYLQMSSLRSEDTALYYCTRHPFYRYDGGNYYAMDHWGQGTSVTVS
A
>d1nsnh1 b.1.1.1 (H:1-114) Immunoglobulin (variable domains of L and H chains) {Fab N10 (mouse), kappa L chain}
DVQLQESGPGLVKPSQSLSLTCTVTGYSITSDYAWNWIRQFPGNKLEWMGYITYSGTTSYNP
SLKSRISISRDTSKNQFFMQLNSVTTEDTGTFYCTRGNGDWGQGTTLTVSSA
>d1nsnl1 b.1.1.1 (L:1-107) Immunoglobulin (variable domains of L and H chains) {Fab N10 (mouse), kappa L chain}
DIVLTQSPSSLAVSLGQRATISCRASQSVSTSSFRYMHWYQQKPGQPPRLLIKYASNLESGV
PARFSGSGSGTDFTLNIHPVEEEDTATYYCQHSWEIPYTFGGGTKLEIK
>d1iaih1 b.1.1.1 (H:1-121) Immunoglobulin (variable domains of L and H chains) {Fab 730.1.4 (mouse), kappa L chain}
QIQLVQSGPELKKPGETVKISCKASGYTFTNYGMNWVKQAPGKGLKWMAWINTYTGEPTYAD
DFKGRFAFSLETSASTAYLQINNLKNEDTATYFCARDGYYENYYAMDYWGQGTSVTVSS
>d1iail1 b.1.1.1 (L:1-108) Immunoglobulin (variable domains of L and H chains) {Fab 730.1.4 (mouse), kappa L chain}
DIVMTQSHKFMSTSVGDRVSITCKASQDVSTAVAWYQQKPGQSPKLLIYSASYQYTGVPDRF
TGSGSRTDFTFTINSVQAEDLAVYYCHQHYSTPFTFGSGTKLEIKR
>d1iaii1 b.1.1.1 (I:1-121) Immunoglobulin (variable domains of L and H chains) {Fab 409.5.3 (mouse), kappa L chain}
EVKLQESGGGLVQPGGSMKLSCVASGFTFNNYWMSWVRQSPEKGLEWVAEIRLNSDNFATHY

AESVKGKFIISRDDSKSRLYLQMNSLRAEDTGIYYCVLRPLFYYAVDYWGQGTSVTVSS
>d1iaim1 b.1.1.1 (M:1-109) Immunoglobulin (variable domains of L and H chains) {Fab 409.5.3 (mouse), kappa L chain}
DIQLTQSPAFMAASPGEKVTITCSVSSSISSSNLHWYQQKSETSPKPWIYGTSNLASGVPVR
FSGSGSGTSYSLTISSMEAEDAATYYCQQWNSYPYTFGGGTKLEIKR
>d1plgh1 b.1.1.1 (H:1-117) Immunoglobulin (variable domains of L and H chains) {Polysialic acid-binding Fab (mouse), kappa L chain}
QIQLQQSGPELVRPGASVKISCKASGYTFTDYYIHWVKQRPGEGLEWIGWIYPGSGNTKYNE
KFKGKATLTVDTSSSTAYMQLSSLTSEDSAVYFCARGGKFAMDYWGQGTSVTVSS
>d1plgl1 b.1.1.1 (L:1-112) Immunoglobulin (variable domains of L and H chains) {Polysialic acid-binding Fab (mouse), kappa L chain}
DVVMTQTPLSLPVSLGDQASISCRSSQSLVHSNGNTYLYWYLQKPGQSPKPLIYRVSNRFSG
VPDRFSGSGSGTDFTLKISRVEAEDLGVYFCFQGTHVPYTFGGGTRLEIK
>d1aj7h1 b.1.1.1 (H:1-114) Immunoglobulin (variable domains of L and H chains) {Fab 48G7 (mouse/human), kappa L chain}
QVQLQQSGAELVKPGASVKLSCTASGFNIKDTYMHWVKQRPEQGLEWIGRIDPANGNTKYDP
KFQGKATITADTSSNTAYLQLSSLTSEDTAVYYCASYYGIYWGQGTTLTVSSA
>d1gafh1 b.1.1.1 (H:1-114) Immunoglobulin (variable domains of L and H chains) {Fab 48G7 (mouse/human), kappa L chain}
QVQLQQSGAELVKPGASVKLSCTASGFNIKDTYMHWVKQRPKQGLEWIGRIDPANVDTKYDP
KFQDKATITADTSSKTTYLQLSSLTSEDTAVYYCASYYGIYWGQGTTLTVSS
>d1gafl1 b.1.1.1 (L:1-109) Immunoglobulin (variable domains of L and H chains) {Fab 48G7 (mouse/human), kappa L chain}
DIQMTQSPSSLSASLGERVSLTCRASQEINGYLGWLQQKPDGTIKRLIYAASTLHSGVPKRF
SGSRSGSDYSLTISSLESEDFADYYCLQYASYPRTFGGGTKVEIKRT
>d1vgeh1 b.1.1.1 (H:1-122) Immunoglobulin (variable domains of L and H chains) {Fab TR1.9 (mouse/human), kappa L chain}
QVKLLEQSGAEVKKPGASVKVSCKASGYSFTSYGLHWVRQAPGQRLEWMGWISAGTGNTKYS
QKFRGRVTFTRDTSATTAYMGLSSLRPEDTAVYYCARDPYGGGKSEFDYWGQGTLVTVSS
>d1vgel1 b.1.1.1 (L:1-107) Immunoglobulin (variable domains of L and H chains) {Fab TR1.9 (mouse/human), kappa L chain}
ELVMTQSPSSLSASVGDRVNIACRASQGISSALAWYQQKPGKAPRLLIYDASNLESGVPSRF
SGSGSGTDFTLTISSLQPEDFAIYYCQQFNSYPLTFGGGTKVEIK
>d1yuhb1 b.1.1.1 (B:1-118) Immunoglobulin (variable domains of L and H chains) {Fab anti-nitrophenol (mouse), lambda L chain}
QVQFQQSGAELVKPGASVKLSCKASGYTFTSYLMHWIKQRPGRGLEWIGRIDPNNVVTKFNE
KFKSKATLTVDKPSSTAYMELSSLTSEDSAVYYCARYAYCRPMDYWGQGTTVTVSS
>d1ucbh1 b.1.1.1 (H:1-113) Immunoglobulin (variable domains of L and H chains) {Fab CBR96 (mouse/human), kappa L chain}
EVNLVESGGGLVQPGGSLKVSCVTSGFTFSDYYMYWVRQTPEKRLEWVAYISQGGDITDYPD
TVKGRFTISRDNAKNSLYLQMSRLKSEDTAMYYCARGLDDGAWFAYWGQGTLVTVSV
>d1ucbl1 b.1.1.1 (L:4-108) Immunoglobulin (variable domains of L and H chains) {Fab CBR96 (mouse/human), kappa L chain}
MTQIPVSLPVSLGDQASISCRSSQIIVHNNGNTYLEWYLQKPGQSPQLLIYKVSNRFSGVPD

RFSGSGSGTDFTLKISRVEAEDLGVYYCFQGSHVPFTFGSGTKLEIKR
>d1dvfc_ b.1.1.1 (C:) Immunoglobulin (variable domains of L and H chains) {Fv E5.2 (mouse), kappa L chain}
DIQLTQSPSSLSASLGDRVTISCRASQDISNYLNWYQQKPDGTVKLLIYYTSRLHSGVPSRF
SGSGSGTDYSLTISNLEQEDIATYFCQQGNTLPWTFGGGTKLEIK
>d1dvfd_ b.1.1.1 (D:) Immunoglobulin (variable domains of L and H chains) {Fv E5.2 (mouse), kappa L chain}
QVQLQQSGTELVKSGASVKLSCTASGFNIKDTHMNWVKQRPEQGLEWIGRIDPANGNIQYDP
KFRGKATITADTSSNTAYLQLSLTSEDTAVYYCATKVIYYQGRGAMDYWGQGTTLTVS
>d1ghfh1 b.1.1.1 (H:1-115) Immunoglobulin (variable domains of L and H chains) {Fab GH1002 (mouse), kappa L chain}
VQLQQSGPELKKPGETVKISCKLWYTFTDYGMNWVKQAPGKGLKWMGWIQTNTEEPTYGAEF
KGRFAFSLETSAFTAYKQINNLKNEDMATYFCARVEAGFDYWAQGTTLTVSS
>d1ghfl1 b.1.1.1 (L:1-107) Immunoglobulin (variable domains of L and H chains) {Fab GH1002 (mouse), kappa L chain}
DIQMTQTTSSLSASLGDRVTISCRESQDISNSLNWYQQKPDGTVKLLIYYTSRLHSGVPSRF
SGSGTGTDYSLTISNLEQEDFATYFCQQGNTLPYTFGGGTKLEIK
>d1nldh1 b.1.1.1 (H:1-112) Immunoglobulin (variable domains of L and H chains) {Fab 1583, against an epitope of gp41 of HIV-1, (mouse), kappa L chain}
QVKLQQSGPGLVQPSQSLSITCTVSGFSLTCYGVHWVRQSPGKGLEWLGVIWSGGDTDYNAA
FISRLSITKDNSKSQVFFKMNSLQPNDRAIYYCARRGGDFWGQGTTVTVS
>d1nldl1 b.1.1.1 (L:1-107) Immunoglobulin (variable domains of L and H chains) {Fab 1583, against an epitope of gp41 of HIV-1, (mouse), kappa L chain}
DVVMTQTPLTLSVTIGQPASISCKSSQSLLDSDGKTYLNWLLQRPGQSPKRLIYLVSKLDSG
VPDRFTGSGSGTDFTLKISRVEAEDLGVYYCWQGTHFPRTFGGGTKLEIK
>d1kelh1 b.1.1.1 (H:1-115) Immunoglobulin (variable domains of L and H chains) {Fab 28B4 (mouse), kappa L chain}
EVKLVESGGGLGQPGGSLRLSCATSGFTFTDYYFNWARQPPGKALEWLGFIRNKAKGYTTEY
SASVKGRFTISRDNSQGILYLQMNTLRAEDSATYYCARWGSYAMDYWGQGTSV
>d1kell1 b.1.1.1 (L:1-112) Immunoglobulin (variable domains of L and H chains) {Fab 28B4 (mouse), kappa L chain}
DVLMTQTPLSLPVSLGDQASISCRFSQSIVHSNGNTYLEWYLQKSGQSPKLLIYKVSNRFSG
VPDRFSGSGSGTDFTLKISRVEAEDLGVYYCFQGSHVPRTFGGGTKLEIK
>d1osph1 b.1.1.1 (H:1-120) Immunoglobulin (variable domains of L and H chains) {Fab 184.1 (mouse), kappa L chain}
EVQLQESGPSLVKPSQTLSLTCSVTGEPITSGFWDWIRKFPGNKLEFMGYIRYGGGTYYNPS
LKSPISITRDTSKNHYYLQLNSVVTEDTATYYCARSRDYYGSSGFAFWGEGTLVTVSA
>d1ospl1 b.1.1.1 (L:1-107) Immunoglobulin (variable domains of L and H chains) {Fab 184.1 (mouse), kappa L chain}
DIQMSQSSSSFSVSLGDRVTITCKASEDIYSRLAWYQQKPGNAPRLLISGATSLETWVPSRF
SGSDSGKDYTLSITSLQTEDVATYFCQQYWSPPPTFGGGTKLEIK
>d1fj1a1 b.1.1.1 (A:1-107) Immunoglobulin (variable domains of L

and H chains) {Fab LA-2 (mouse), kappa L chain}
DIQMTQSPSSLSATLGGKVTITCKASQDINKYIAWYQHKPGKGPRLLIHYTSTLQPGNPSRF
SGSGSGRDYSFSISNLEAEDIAIYYCLQYDNLQRTFGGGTKVEIK
>d1fj1b1 b.1.1.1 (B:1-114) Immunoglobulin (variable domains of L and H chains) {Fab LA-2 (mouse), kappa L chain}
QIQLVQSGPELKKPGETVKISCKASGYTFTDYSMYWVKQAPGKGLKRMGWINTETGEPTYAD
DFKGRFALSLDTSASTAYLHISNLKNEDTATYFCARGLDSWGQGTSVTVSSA
>d1cloh1 b.1.1.1 (H:1-113) Immunoglobulin (variable domains of L and H chains) {Fab A5B7 (mouse), kappa L chain}
EVKLVESGGGLVQPGGSLRLSCATSGFTFTDYYMNWVRQPPGKALEWLGFIGNKANGYTTEY
SASVKGRFTISRDKSQSILYLQMNTLRAEDSATYYCTRDRGLRFYFDYWGQGTTLTVSS
>d1clol1 b.1.1.1 (L:1-107) Immunoglobulin (variable domains of L and H chains) {Fab A5B7 (mouse), kappa L chain}
QTVLSQSPAILSASPGEKVTMTCRASSSVTYIHWYQQKPGSSPKSWIYATSNLASGVPARFS
GSGSGTSYSLTISRVEAEDAATYYCQHWSSKPPTFGGGTKLEIK
>d1ad0a1 b.1.1.1 (A:1-107) Immunoglobulin (variable domains of L and H chains) {Fab A5B7 (engineered human construct), kappa L chain}
QTVLTQSPSSLSVSVGDRVTITCRASSSVTYIHWYQQKPGLAPKSLIYATSNLASGVPSRFS
GSGSGTDYTFTISSLQPEDIATYYCQHWSSKPPTFGQGTKVEVKR
>d1ad0b1 b.1.1.1 (B:1-113) Immunoglobulin (variable domains of L and H chains) {Fab A5B7 (engineered human construct), kappa L chain}
EVQLLESGGGLVQPGGSLRLSCATSGFTFTDYYMNWVRQAPGKGLEWLGFIGNKANGYTTEY
SASVKGRFTISRDKSKSTLYLQMNTLQAEDSAIYYCTRDRGLRFYFDYWGQGTLVTVSS
>d1mimh1 b.1.1.1 (H:1-115) Immunoglobulin (variable domains of L and H chains) {Fab CHI621 (mouse), kappa L chain}
QLQQSGTVLARPGASVKMSCKASGYSFTRYWMHWIKQRPGQGLEWIGAIYPGNSDTSYNQKF
EGKAKLTAVTSASTAYMELSSLTHEDSAVYYCSRDYGYYFDFWGQGTTLTVSS
>d1miml1 b.1.1.1 (L:1-105) Immunoglobulin (variable domains of L and H chains) {Fab CHI621 (mouse), kappa L chain}
QIVSTQSPAIMSASPGEKVTMTCSASSSRSYMQWYQQKPGTSPKRWIYDTSKLASGVPARFS
GSGSGTSYSLTISSMEAEDAATYYCHQRSSYTFGGGTKLEIKR
>d1afvh1 b.1.1.1 (H:1-120) Immunoglobulin (variable domains of L and H chains) {Fab 25.3 (mouse), kappa L chain}
QVQLQQPGSVLVRPGASVKLSCKASGYTFTSSWIHWAKQRPGQGLEWIGEIHPNSGNTNYNE
KFKGKATLTVDTSSSTAYVDLSSLTSEDSAVYYCARWRYGSPYYFDYWGQGTTLTVSS
>d1afvl1 b.1.1.1 (L:1-112) Immunoglobulin (variable domains of L and H chains) {Fab 25.3 (mouse), kappa L chain}
DIVLTQSPASLAVSLGQRATISCRASESVDNYGISFMNWFQQKPGQPPKLLIYAASNLGSGV
PARFSGSGSGTDFSLNIHPMEEEDTAMYFCQQSKEVPLTFGAGTKVELKR
>d2mpah1 b.1.1.1 (H:1-121) Immunoglobulin (variable domains of L and H chains) {Bactericidal Fab MN12H2, (mouse), kappa L chain}
EVNLQQSGTVLARPGASVRMSCKASGYSFTSYWLHWIKQRPGQGLEWIGGIYPGNRDTRYTQ
RFKDKAKLTAVTSANTAYMELSSLTNEDSAVYYCSIIYFDYADFIMDYWGQGTTVTVSS
>d2mpal1 b.1.1.1 (L:1-112) Immunoglobulin (variable domains of L

and H chains) {Bactericidal Fab MN12H2, (mouse), kappa L chain}
DIVMTQTPLSLPVSLGDKASISCRSSQALVHSNGNTYLHWYLQKPGQSPKLLIYKVSNRFSG
VPDRFSGSGSGTDFTLKISRVEAEDLGVFFCSQSTHVPRTFGGGTKLEIK
>d1qkzh1 b.1.1.1 (H:1-113) Immunoglobulin (variable domains of L and H chains) {Fab MN14C11.6 (mouse), kappa L chain}
DVKLVESGGGLVKPGRSLKLSCAASGFTFSDYYMFWVRQTPEQRLEWVATISDGGAYTYYPD
SVKGRFTISRDNAKNNLYLQMNSLKSEDTGMYYCARDPLEYYGMDYWGQGTSVAVSS
>d1qkzl1 b.1.1.1 (L:1-107) Immunoglobulin (variable domains of L and H chains) {Fab MN14C11.6 (mouse), kappa L chain}
NIVMTQTPLSLPVSLGDQASISCRSSQSLVHSNGNTYLHWYLQKPGQSPKLLIYTVSNRFSG
VPDRFSGSGSGTDFTLKISRVEAEDLGVYFCSQSTHFPTFGGGTKLEIK
>d1pskh1 b.1.1.1 (H:1-114) Immunoglobulin (variable domains of L and H chains) {Fab against a ganglioside (mouse), kappa L chain}
EVQLQQSGPELVKPGASVKISCKTSGYTFTKYTMHWVKQSHGKSLEWIGDINPNNGGTNYNQ
KFKGTATLTVHKSSTTAYMELRSLTSEDSAVYYCTSKSFDYWGQGTTLTVSS
>d1pskl1 b.1.1.1 (L:1-106) Immunoglobulin (variable domains of L and H chains) {Fab against a ganglioside (mouse), kappa L chain}
QIVLTQSPAIMSASPGEKVTITCSASSSVSNIHWFQQKPGTFPKLWIYSTSTLASGVPGRFS
GSGSGTSYSLTISRMGAEDAATYYCQQRSGYPFTFGSGTKLEIK
>d1yejh1 b.1.1.1 (H:1-113) Immunoglobulin (variable domains of L and H chains) {Fab D2.3 (mouse), kappa L chain}
EMQLQQSGAELLRPGTSVKLSCKTSGYIFTSYWIHWVKQRSGQGLEWIARIYPGTGSTYYNE
KFKGKATLTADKSSSTAYMQLSTLKSEDSAVYFCTRWGFIPVREDYVMDYWGQGTLVTVSS
>d1yejl1 b.1.1.1 (L:1-107) Immunoglobulin (variable domains of L and H chains) {Fab D2.3 (mouse), kappa L chain}
DIVMTQSPLTLSVTIGQPASISCKSSQSLLYSNGKTYLNWLLQRPGQSPKRLIHLVSKLDSG
VPDRITGSGSGTDFTLKISRVEAADLGVYYCVQGTHFPYTFGGGTKLEIL
>d1yedb1 b.1.1.1 (B:1-113) Immunoglobulin (variable domains of L and H chains) {Fab D2.4 (mouse), kappa L chain}
AVKLQQSGPELVRPGTSVKLSCKTSGYIFTSYWIHWLKQSSGQGLEWIARIYPGTGGTYYNE
KFKGKATLTADKSSSTAYMQLSSLKSEDSAVYFCTRWGFTTVRENYYAMDYWGQGTLVTVSS
>d1yeeh1 b.1.1.1 (H:1-113) Immunoglobulin (variable domains of L and H chains) {Fab D2.5 (mouse), kappa L chain}
EVKLQESGAELVRPGASVKLSCKTSGYIFTSYWIHWVKQRAAAGLEWIARIYPGTGSSYYNV
KFKGKATLTADKSSSTAYMQLSSLKSDDSAVYFCVRWGFIPVREDYVLDYWGQGTLVTVSS
>d1cfvh_ b.1.1.1 (H:) Immunoglobulin (variable domains of L and H chains) {Fv 4155 (mouse), kappa L chain}
QVQLQESGGGLVNLGGSMTLSCVASGFTFNTYYMSWVRQTPEKTLELVAAINSDGEPIYYPD
TLKGRVTISRDNAKKTLYLQMSSLNFEDTALYYCARLNYAVYGMDYWGQGTTVTVSS
>d1cfvl_ b.1.1.1 (L:) Immunoglobulin (variable domains of L and H chains) {Fv 4155 (mouse), kappa L chain}
DIELTQSPPSLPVSLGDQVSISCRSSQSLVSNNRRNYLHWYLQKPGQSPKLVIYKVSNRFSG
VPDRFSGSGSGTDFTLKISRVAAEDLGLYFCSQSSHVPLTFGSGTKLEIKR
>d1hyxh1 b.1.1.1 (H:1-113) Immunoglobulin (variable domains of L

and H chains) {Fab 6D9 (mouse), kappa L chain}
EVKLLESGGGLVKPGGSLKLSCAASGFTFSNYAMSWVRQTPEKRLEWVVSISSGGSIYYLDS
VKGRFTVSRDNARNILYLQMTSLRSEDTAMYFCARVSHYDGSRDWYFDVWGAGTSVTVSS
>d1hyxl1 b.1.1.1 (L:1-107) Immunoglobulin (variable domains of L and H chains) {Fab 6D9 (mouse), kappa L chain}
ELVMTQTPLSLPVSLGDQASISCRSSQTIVHSNGDTYLDWFLQKPGQSPKLLIYKVSNRFSG
VPDRFSGSGSGTDFTLKISRVEAEDLGVYYCFQGSHVPPTFGGGTKLEIK
>d2hrph1 b.1.1.1 (H:1-113) Immunoglobulin (variable domains of L and H chains) {Fab F11.2.32 (mouse), kappa L chain}
DVQLVESGGGLVQPGGSRKLSCAASGFTFMRFGMHWVRQAPEKGLEWVAYISSGSSTIYYAD
TVKGRFTISRDNPKNTLFLQMTSLRSEDTALYYCARSGGIERYDGTYYVMDYWGQGTSVTVS
S
>d2hrpl1 b.1.1.1 (L:1-107) Immunoglobulin (variable domains of L and H chains) {Fab F11.2.32 (mouse), kappa L chain}
DTVLTQSPASLAVSLGQRATISCRASESVDYYGKSFMNWFQQKPGQPPKLLIYAASNQGSGV
PARFSGSGSGTDFSLHIHPMEEDDSAMYFCQQSKEVPWTFGGGTKLEIK
>d2ap2a_ b.1.1.1 (A:) Immunoglobulin (variable domains of L and H chains) {scFv C219, (mouse sequence-based), kappa L chain}
FVRDIVMTQSPSSLTVTAGEKVTMSCKSSQSLLNSGNQKNYLTWYQQKPGQPPKLLIYWAST
RESGVPDRFTGSGSGTDFTLTISSVQAEDLAVYYCQNDYSYPLTFGAGTKLEP
>d2ap2b_ b.1.1.1 (B:) Immunoglobulin (variable domains of L and H chains) {scFv C219, (mouse sequence-based), kappa L chain}
EVQLQQSGAELVRPGASVKLSCTASGFNIKDDFMHWVKQRPEQGLEWIGRIDPANDNTKYAP
KFQDKATIIADTSSNTAYLQLSSLTSEDTAVYYCARREVYSYYSPLDVWGAGTTVTVPSG
>d1nfde1 b.1.1.1 (E:2-107) Immunoglobulin (variable domains of L and H chains) {Fab H57 (hamster), lambda L chain}
YELIQPSSASVTVGETVKITCSGDQLPKNFAYWFQQKSDKNILLLIYMDNKRPSGIPERFSG
STSGTTATLTISGAQPEDEAAYYCLSSYGDNNDLVFGSGTQLTVLR
>d1nfdf1 b.1.1.1 (F:1-114) Immunoglobulin (variable domains of L and H chains) {Fab H57 (hamster), lambda L chain}
EVYLVESGGDLVQPGSSLKVSCAASGFTFSDFWMYWVRQAPGKGLEWVGRIKNIPNNYATEY
ADSVRGRFTISRDDSRNSIYLQMNRLRVDDTAIYYCTRAGRFDHFDYWGQGTMVTVSSA
>d2h1ph1 b.1.1.1 (H:301-420) Immunoglobulin (variable domains of L and H chains) {Fab 2H1 (mouse), kappa L chain}
DVKLVESGGGLVKLGGSLKLSCAASGFTFSSYFLSWVRQTPEKRLELVATINSNGDKTYHPD
TMKGRFTISRDNAKNTLYLQMSSLKSEDTALYYCARRDSSASLYFDYWGQGTTLTVSS
>d2h1pl1 b.1.1.1 (L:1-113) Immunoglobulin (variable domains of L and H chains) {Fab 2H1 (mouse), kappa L chain}
DVVMTQTPLSLPVSLGDPASISCRSSQSLVHSNGNTYLHWYLQKPGQSPKLLIYKVSNRFSG
VPDKFSGSGSGTDFTLKISRVEAEDQGVYFCSQSTHVPWTFGGGTKLEIKR
>d1aqkh1 b.1.1.1 (H:1-123) Immunoglobulin (variable domains of L and H chains) {Fab B7-15A2 (human), lambda L chain}
EVQLVESGGGVVQPGRSLRLSCAASGFTFNNYAIHWVRQAPGKGLEWVAFISYDGSKNYYAD
SVKGRFTISRDNSKNTLFLQMNSLRPEDTAIYYCARVLFQQLVLYAPFDIWGQGTMVTVSS

>d1aqkl1 b.1.1.1 (L:1-111) Immunoglobulin (variable domains of L and H chains) {Fab B7-15A2 (human), lambda L chain}
ENVLTQPPSVSGAPGQRVTISCTGSNSNIGAGFTVHWYQHLPGTAPKLLIFANTNRPSGVPD
RFSGSKSGTSASLAITGLQAEDEADYYCQSYDSSLSARFGGGTRLTVLG
>d1axsa1 b.1.1.1 (A:1-107) Immunoglobulin (variable domains of L and H chains) {Oxy-cope catalytic Fab az-28, chimeric (mouse V domains/human C1 domains)}
ELVLTQSPSSMYASLGERVTITCKASQDINSYLNWFQQKPGKSPKTLIYRTNRLVDGVPSRF
SGSGSGQDYSLTISSLEYEDMGIYYCLQYDEFPYTFGSGTKLEIK
>d1d5ih1 b.1.1.1 (H:1-113) Immunoglobulin (variable domains of L and H chains) {Oxy-cope catalytic Fab az-28, chimeric (mouse V domains/human C1 domains)}
QVQLQQSGAELMKPGASVKISCKATGYTFSSYWIEWVKQRPGHGLEWIGEILPGSGSTNYNE
KFKGKATFTADTSSNTAYMQLSSLTSEDSAVYYCARGHSYYFYDGDYWGQGTSVTVSS
>d1d5il1 b.1.1.1 (L:1-107) Immunoglobulin (variable domains of L and H chains) {Oxy-cope catalytic Fab az-28, chimeric (mouse V domains/human C1 domains)}
DIKMTQSPSSMYASLGERVTITCKASQDINSYLSWFQQKPGKSPKTLIYRANRLVDGVPSRF
SGSGSGQDYSLTISSLEYEDMGIYYCLQYDEFPYTFGSGTKLEIK
>d1ar1c_ b.1.1.1 (C:) Immunoglobulin (variable domains of L and H chains) {Fv against Paracoccus denitrificans cytochrome c oxidase (mouse), kappa L chain}
EVKLQESGGDLVQPGGSLKLSCAASGFTFSSYTMSWVRQTPEKRLEWVASINNGGGRTYYPD
TVKGRFTISRDNAKNTLYLQMSSLKSEDTAMYYCVRHEYYYAMDYWGQGTTVTVSS
>d1ar1d_ b.1.1.1 (D:) Immunoglobulin (variable domains of L and H chains) {Fv against Paracoccus denitrificans cytochrome c oxidase (mouse), kappa L chain}
DIELTQTPVSLSASVGETVTITCRASENIYSYLAWYQQKQGKSPQFLVYNAKTLGEGVPSRF
SGSGSGTQFSLKINSLLPEDFGSYYCQHHYGTPPLTFGGGTKLEIK
>d1ae6h1 b.1.1.1 (H:1-114) Immunoglobulin (variable domains of L and H chains) {Fab CTM01 (mouse), kappa L chain}
QIQLQQSGPELVKPGASVKISCKASGYTFTDYYINWMKQKPGQGLEWIGWIDPGSGNTKYNE
KFKGKATLTVDTSSSTAYMQLSSLTSEDTAVYFCAREKTTYYYAMDYWGQGTSVTVSAA
>d1ae6l1 b.1.1.1 (L:1-106A) Immunoglobulin (variable domains of L and H chains) {Fab CTM01 (mouse), kappa L chain}
DIVMTQAAPSVPVTPGESLSISCRSSKSLLHSNGDTFLYWFLQRPGQSPQLLIYRMSNLASG
VPDRFSGSGSGTAFTLRVSRVEAEDVGVYYCMQHLEYPFTFGAGTKLELK
>d1ad9a1 b.1.1.1 (A:1-107) Immunoglobulin (variable domains of L and H chains) {Fab CTM01 (human construct), kappa L chain}
DIQMTQSPSTLSASVGDRVTITCRSSKSLLHSNGDTFLYWFQQKPGKAPKLLMYRMSNLASG
VPSRFSGSGSGTEFTLTISSLQPDDFATYYCMQHLEYPFTFGQGTKVEVKR
>d1ad9b1 b.1.1.1 (B:1-113) Immunoglobulin (variable domains of L and H chains) {Fab CTM01 (human construct), kappa L chain}
EIQLVQSGAEVKKPGSSVKVSCKASGYTFTDYYINWMRQAPGQGLEWIGWIDPGSGNTKYNE

KFKGRATLTVDTSTNTAYMELSSLRSEDTAFYFCAREKTTYYYAMDYWGQGTLVTVSS
>d1fgnh1 b.1.1.1 (H:1-117) Immunoglobulin (variable domains of L and H chains) {Anti-human tissue factor Fab 5G9 (mouse), kappa L chain}
EIQLQQSGAELVRPGALVKLSCKASGFNIKDYYMHWVKQRPEQGLEWIGLIDPENGNTIYDP
KFQGKASITADTSSNTAYLQLSSLTSEDTAVYYCARDNSYYFDYWGQGTTLTVSS
>d1fgnl1 b.1.1.1 (L:1-108) Immunoglobulin (variable domains of L and H chains) {Anti-human tissue factor Fab 5G9 (mouse), kappa L chain}
DIKMTQSPSSMYASLGERVTITCKASQDIRKYLNWYQQKPWKSPKTLIYYATSLADGVPSRF
SGSGSGQDYSLTISSLESDDTATYYCLQHGESPYTFGGGTKLEINR
>d1jpth1 b.1.1.1 (H:1-117) Immunoglobulin (variable domains of L and H chains) {Anti-human tissue humanised factor Fab D3H44}
EVQLVESGGGLVQPGGSLRLSCAASGFNIKEYYMHWVRQAPGKGLEWVGLIDPEQGNTIYDP
KFQDRATISADNSKNTAYLQMNSLRAEDTAVYYCARDTAAYFDYWGQGTLVTVSS
>d1jptl1 b.1.1.1 (L:1-107) Immunoglobulin (variable domains of L and H chains) {Anti-human tissue humanised factor Fab D3H44}
DIQMTQSPSSLSASVGDRVTITCRASRDIKSYLNWYQQKPGKAPKVLIYYATSLAEGVPSRF
SGSGSGTDYTLTISSLQPEDFATYYCLQHGESPWTFGQGTKVEIK
>d1jrhh1 b.1.1.1 (H:1-113) Immunoglobulin (variable domains of L and H chains) {Fab A6 (mouse), kappa L chain}
AVKLQESGPGILKPSQTLSLTCSFSGFSLTTYGMGVGWIRQSSGKGLEWLAHIWWDDDKYYN
PSLKSRLTISKDTSRNQVFLKITSVATADTATYYCARRAPFYGNHAMDYWGQGTTVTVSS
>d1jrhl1 b.1.1.1 (L:1-107) Immunoglobulin (variable domains of L and H chains) {Fab A6 (mouse), kappa L chain}
SVEMTQSPSSFSVSLGDRVTITCKASEDIYNRLAWYQQKPGNAPRLLISGATSLETEVPSRF
SGSGSGKDYTLSITSLQTEDVATYYCQQYWSTWTFGGGTKLEIK
>d1gpoh1 b.1.1.1 (H:1-113) Immunoglobulin (variable domains of L and H chains) {Fab M41 (artificial design)}
EVKLQESGPSLVKPSQTLSLTCSVTGDSITSDFWSWIRQFPGNRLEYMGFVQYSGETAYNPS
LKSRISITRDTSKNQYYLDLNSVTTEDTAVYYCANWHGDYWGQGTTVTVSS
>d1gpol1 b.1.1.1 (L:1-112) Immunoglobulin (variable domains of L and H chains) {Fab M41 (artificial design)}
DIELTQSPATLSVTPGNSVSISCRASQSLVNEDGNTYLFWYQQKSHESPRLLIKYASQSISG
IPSRFSGSGSGTDFTLSINSVETEDLAVYFCQQITDWPFTFGGGTKLEIK
>d1kb5h1 b.1.1.1 (H:1-113) Immunoglobulin (variable domains of L and H chains) {Fab Desire-1 (mouse), kappa L chain}
EVQLQQSGPELEKPGASVKISCKASGYSFTGYNMNWVKQSNGKSLEWIGNIDPYYGGISYNQ
KFKGRATLTVDKSSSTAYMQLKSLTSEDSAVYYCARSRTDLYYFDYWGQGTTLTVSS
>d1kb5l1 b.1.1.1 (L:1-108) Immunoglobulin (variable domains of L and H chains) {Fab Desire-1 (mouse), kappa L chain}
DIQMTQSPASLSASVGETVTITCRASKNIYSYLAWYQQKQGKSPQLLVYNAKTLGEGVPSRF
SGSGSGTQFSLKINSLQPEDFGSYYCQHHYGTPYTFGGGTKLEIKR
>d1a4jb1 b.1.1.1 (B:1-119) Immunoglobulin (variable domains of L

and H chains) {Diels alder catalytic Fab (mouse), kappa L chain}
QVQLLESGPELKKPGETVKISCKASGYTFTNYGMNWVKQAPGKGLKWMGWINTYTGEPTYAD
DFKGRFAFSLETSASTAYLQINNLKNEDTATYFCVQAERLRRTFDYWGAGTTVTVSS
>d1a4ka1 b.1.1.1 (A:1-112) Immunoglobulin (variable domains of L and H chains) {Diels alder catalytic Fab (mouse), kappa L chain}
ELVMTQTPLSLPVSLGDQASISCRSSQSLLHSNGNTYLHWYLQKPGQSPKLLIYKVSNRFSG
VPDRFSGSGSGTDFTLKISRVEAEDLGVYFCSQVTHVPPTFGGGTKLEIKRTVAA
>d1c1eh1 b.1.1.1 (H:1-119) Immunoglobulin (variable domains of L and H chains) {Diels alder catalytic Fab 1E9 (mouse), kappa L chain}
QIQLVQSGPELKKPGETVKISCKASGYMFTNYGMNWVKQAPGKALKLMGWINPYTGESTFAD
DFKGRFAFFLETSATTAYLQINNLKNEDMATYFCARGTTIVRAMDYWGQGTSLTVSSAKTTP
P
>d1c1el1 b.1.1.1 (L:1-112) Immunoglobulin (variable domains of L and H chains) {Diels alder catalytic Fab 1E9 (mouse), kappa L chain}
ELVMTQTPLSLPVSLGDQASISCRSSQSLVHSNGNTYLHWYLQKPGQSPKFLIYKVSNRFSG
VPDRFGGSGSGTDFILKISRVEAEDLGVYFCFQSTHFFPTFGGGTKLEIKSADAA
>d1a3lh1 b.1.1.1 (H:1-113) Immunoglobulin (variable domains of L and H chains) {Diels alder catalytic Fab 13G5 (mouse), kappa L chain}
EVQLEESGPELVRPGTSVKISCKASGYTFTNYWLGWVKQRPGHGFEWIGDIYPGGVYTTNNE
KFRGKAILTADTSSSTAYMQLSSLTSEDSAVYFCARAGGYYTGGDYWGQGTSVTVSS
>d1a3ll1 b.1.1.1 (L:1-107) Immunoglobulin (variable domains of L and H chains) {Diels alder catalytic Fab 13G5 (mouse), kappa L chain}
DIVLTQAAFSNPVTLGASASISCRSSKSLLNSNGIIHMYWYLQKPGQSPQLLIYQMSKLASG
APDRFSGSGSGTDFTLRISRVEAEDVGVYYCAQNLELPYTFGGGTKLEIK
>d1ay1h1 b.1.1.1 (H:1-115) Immunoglobulin (variable domains of L and H chains) {Fab TP7 (mouse), kappa L chain}
EVQLQESGPGLVKPYQSLSLSCTVTGYSITSDYAWNWIRQFPGNKLEWMGYITYSGTTDYNP
SLKSRISITRDTSKNQFFLQLNSVTTEDTATYYCARYYYGYWYFDVWGQGTTLTVSS
>d1ay1l1 b.1.1.1 (L:1-107) Immunoglobulin (variable domains of L and H chains) {Fab TP7 (mouse), kappa L chain}
DIQMTQSPAIMSASPGEKVTMTCSASSSVSYMYWYQQKPGSSPRLLIYDSTNLASGVPVRFS
GSGSGTSYSLTISRMEAEDAATYYCQQWSTYPLTFGAGTKLELK
>d1dsfh_ b.1.1.1 (H:) Immunoglobulin (variable domains of L and H chains) {Anticancer Fv B1 (mouse)}
QLVESGGGLVKPGGSLKLSCAASGFIFSDNYMYWVRQTPEKCLEWVATISDGGTYIDYSDSV
KGRFTISRDNAKNNLYLQMSSLRSEDTGMYYCGRSPIYYDYAPFTYWGQGTLVTVSA
>d1dsfl_ b.1.1.1 (L:) Immunoglobulin (variable domains of L and H chains) {Anticancer Fv B1 (mouse)}
DVVMTQTPLSLPVSLGDQASISCRSSQNLVHSDGKTYLHWFLQKPGQSPTLLIYKVSNRFSG
VPDRFSGSGSGTDFILKISRVEAEDLGVYFCSQSTHVPLTFGCGTKLELK
>d1a6ta1 b.1.1.1 (A:1-107) Immunoglobulin (variable domains of L and H chains) {Fab Mab1-IA (mouse), kappa L chain}
QSVLSQSPAILSASPGEKVIMTCSPSSSVSYMQWYQQKPGSSPKPWIYSTSNLASGVPGRFS
GGGSGTSFSLTISGVEAEDAATYYCQQYSSHPLTFGGGTKLELK

>d1a6tb1 b.1.1.1 (B:1-113) Immunoglobulin (variable domains of L and H chains) {Fab Mab1-IA (mouse), kappa L chain}
EVQLQQSGPDLVKPGASVKISCKASGYSFSTYYMHWVKQSHGKSLEWIGRVDPDNGGTSFNQ
KFKGKAILTVDKSSSTAYMELGSLTSEDSAVYYCARRDDYYFDFWGQGTSLTVSS
>d1a6wh_ b.1.1.1 (H:) Immunoglobulin (variable domains of L and H chains) {Fv B1-8 (mouse), lambda L chain}
QVQLQQPGAELVKPGASVKLSCKASGYTFTSYWMHWVKQRPGRGLEWIGRIDPNSGGTKYNE
KFKSKATLTVDKPSSTAYMQLSSLTSEDSAVYYCARYDYYGSSYFDYWGQGTTVTVSS
>d1a6wl_ b.1.1.1 (L:) Immunoglobulin (variable domains of L and H chains) {Fv B1-8 (mouse), lambda L chain}
AVVTQESALTTSPGETVTLTCRSSTGAVTTSNYANWVQEKPDHLFTGLIGGTNNRAPGVPAR
FSGSLIGNKAALTITGAQTEDEAIYFCALWYSNHWVFGGGTKLTVLE
>d1g9mh1 b.1.1.1 (H:1-129) Immunoglobulin (variable domains of L and H chains) {HIV-1 neutralizing Fab 17B (human), kappa L chain}
QVQLLESGAEVKKPGSSVKVSCKASGDTFIRYSFTWVRQAPGQGLEWMGRIITILDVAHYAP
HLQGRVTITADKSTSTVYLELRNLRSDDTAVYFCAGVYEGEADEGEYRNNGFLKHWGQGTLV
TVTSA
>d1g9ml1 b.1.1.1 (L:1-109) Immunoglobulin (variable domains of L and H chains) {HIV-1 neutralizing Fab 17B (human), kappa L chain}
ELELTQSPATLSVSPGERATLSCRASESVSSDLAWYQQKPGQAPRLLIYGASTRATGVPARF
SGSGSGAEFTLTISSLQSEDFAVYYCQQYNNWPPRYTFGQGTRLEIK
>d12e8h1 b.1.1.1 (H:1-114) Immunoglobulin (variable domains of L and H chains) {Fab 2E8 (mouse), kappa L chain}
EVQLQQSGAEVVRSGASVKLSCTASGFNIKDYYIHWVKQRPEKGLEWIGWIDPEIGDTEYVP
KFQGKATMTADTSSNTAYLQLSSLTSEDTAVYYCNAGHDYDRGRFPYWGQGTLVTVSAA
>d12e8l1 b.1.1.1 (L:1-107) Immunoglobulin (variable domains of L and H chains) {Fab 2E8 (mouse), kappa L chain}
DIVMTQSQKFMSTSVGDRVSITCKASQNVGTAVAWYQQKPGQSPKLMIYSASNRYTGVPDRF
TGSGSGTDFTLTISNMQSEDLADYFCQQYSSYPLTFGAGTKLELK
>d1adqh1 b.1.1.1 (H:1-113) Immunoglobulin (variable domains of L and H chains) {IgM rheumatoid factor Fab (human), lambda L chain}
EVQLVESGGGLVQPGRSLRLSCVTSGFTFDDYAMHWVRQSPGKGLEWVSGISWNTGTIIYAD
SVKGRFIISRDNAKNSLYLQMNSLRVEDTALYYCAKTRSYVVAAEYYFHYWGQGILVTVSS
>d1adql1 b.1.1.1 (L:2-107) Immunoglobulin (variable domains of L and H chains) {IgM rheumatoid factor Fab (human), lambda L chain}
YVLTQPPSVSVAPGQTARITCGGNNIGSKSVHWYQQKPGQAPVLVVYDDSDRPPGIPERFSG
SNSGNTATLTISRVEAGDEADYYCQVWDSSSDHAVFGGGTKLTVLG
>d2hmic1 b.1.1.1 (C:1-107) Immunoglobulin (variable domains of L and H chains) {Fab 28 against HIV-1 RT (mouse), kappa L chain}
DIQMTQTTSSLSASLGDRVTISCSASQDISSYLNWYQQKPEGTVKLLIYYTSSLHSGVPSAF
SGSGSGTDYSLTISNLEPEDFATYYCQQYSKFPWTFGGGTKLEIK
>d2hmid1 b.1.1.1 (D:1-123) Immunoglobulin (variable domains of L and H chains) {Fab 28 against HIV-1 RT (mouse), kappa L chain}
QITLKESGPGIVQPSQPFRLTCTFSGFSLSTSGIGVTWIRQPSGKGLEWLATIWWDDDNRYN

PSLKSRLTVSKDTSNNQAFLNMMTVETADTAIYYCAQSAITSVTDSAMDHWGQGTSVTVSS
>d1bvka_ b.1.1.1 (A:) Immunoglobulin (variable domains of L and H chains) {Humanized anti-lysozyme Fv HuLys11 (mouse), kappa L chain}
DIQMTQSPSSLSASVGDRVTITCRASGNIHNYLAWYQQKPGKAPKLLIYYTTTLADGVPSRF
SGSGSGTDYTFTISSLQPEDIATYYCQHFWSTPRTFGQGTKVEIKR
>d1bvkb_ b.1.1.1 (B:) Immunoglobulin (variable domains of L and H chains) {Humanized anti-lysozyme Fv HuLys11 (mouse), kappa L chain}
QVQLQESGPGLVRPSQTLSLTCTVSGFSLTGYGVNWVRQPPGRGLEWIGMIWGDGNTDYNSA
LKSRVTMLKDTSKNQFSLRLSSVTAADTAVYYCARERDYRLDYWGQGSLVTVSS
>d1a0qh1 b.1.1.1 (H:2-114) Immunoglobulin (variable domains of L and H chains) {Fab 29G11 (mouse), kappa L chain}
VQLQESDAELVKPGASVKISCKASGYTFTDHVIHWVKQKPEQGLEWIGYISPGNGDIKYNEK
FKGKATLTADKSSSTAYMQLNSLTSEDSAVYLCKRGYYGRSNVDYWGQGTTLTVSSA
>d1a0ql1 b.1.1.1 (L:2-108) Immunoglobulin (variable domains of L and H chains) {Fab 29G11 (mouse), kappa L chain}
IELTQSPSSLSASLGGKVTITCKASQDIKKYIGWYQHKPGKQPRLLIHYTSTLLPGIPSRFR
GSGSGRDYSFSISNLEPEDIATYYCLQYYNLRTFGGGTKLEIKR
>d1fnsh1 b.1.1.1 (H:215-336) Immunoglobulin (variable domains of L and H chains) {Fab NMC-4 (mouse), kappa L chain}
QVQLKESGPGLVAPSQSLSITCTVSGFSLTDYGVDWVRQPPGKGLEWLGMIWGDGSTDYNSA
LKSRLSITKDNSKSQVFLKMNSLQTDDTARYYCVRDPADYGNYDYALDYWGQGTSVTVSS
>d1fnsl1 b.1.1.1 (L:1-107) Immunoglobulin (variable domains of L and H chains) {Fab NMC-4 (mouse), kappa L chain}
DIQMTQSPSSLSASLGDRVTISCSASQDINKYLNWYQQKPDGAVKLLIFYTSSLHSGVPSRF
SGSGSGTDYSLTISNLEPEDIATYYCQQYEKLPWTFGGGTKLEVK
>d1qfuh1 b.1.1.1 (H:1-113) Immunoglobulin (variable domains of L and H chains) {Influenza virus hemagglutinin-neutralizing Fab (mouse), kappa L chain}
QVQLQQPGAELVRPGASVKLSCKASGYTLTTYWMNWFKQRPDQGLEWIGRIDPYDSETHYNQ
KFKDKAILTVDRSSSTAYMQLSSLTSEDSAVYYCTRFLQITTIIYGMDYWGQGTSVTVSS
>d1eo8h1 b.1.1.1 (H:1-113) Immunoglobulin (variable domains of L and H chains) {Influenza virus hemagglutinin-neutralizing Fab BH151 (mouse), kappa L chain}
QVQLQQSGAELMKPGPSVKISCKATGYSFSTYFIEWIRQRPGHGLEWIGEILPGSDNTNFNE
KFKDRATFTADTPSNTAYMQLSSLTSEDSAVYYCARPTGRLWFSYWGQGTLVTVSA
>d1eo8l1 b.1.1.1 (L:1-106B) Immunoglobulin (variable domains of L and H chains) {Influenza virus hemagglutinin-neutralizing Fab BH151 (mouse), kappa L chain}
QIILTQSPAIMSASPGEKVTMTCSASSDISYMHWYQQKSDTSPKIWIYDTSKLASGVPARFS
GSGSGTSYSLTISTMEAEDAATYYCHQRSSYPTFGGGTKLEIK
>d35c8h1 b.1.1.1 (H:1-113) Immunoglobulin (variable domains of L and H chains) {Catalytic Fab 5C8 (mouse), kappa L chain}

EVQLQQSGAELVKPGASVKLSCTASGFNIKDTYMHWVKQKPEQGLEWIAQIDPANGNTKYDP
KFQGKATITADTSSNTAYLHLSSLTSEDSAVYYCAADPPYYGHGDYWGQGTTLTVSS
>d35c8l1 b.1.1.1 (L:1-107) Immunoglobulin (variable domains of L and H chains) {Catalytic Fab 5C8 (mouse), kappa L chain}
DIVLTQSPAIMSASLGERVTMTCTASSSVSSSNLHWYQQKPGSSPKLWIYSTSNLASGVPAR
FSGSGSGTSYSLTISSMEAEDAATYYCHQYHRSPYTFGGGTKLEIK
>d1a5fh1 b.1.1.1 (H:1-120) Immunoglobulin (variable domains of L and H chains) {Anti-E-selectin Fab (mouse), kappa L chain}
EVALQQSGAELVKPGASVKLSCAASGFTIKDAYMHWVKQKPEQGLEWIGRIDSGSSNTNYDP
TFKGKATITADDSSNTAYLQMSSLTSEDTAVYYCARVGLSYWYAMDYWGQGTSVTVSS
>d1axth1 b.1.1.1 (H:1-113) Immunoglobulin (variable domains of L and H chains) {Catalytic Fab 33F12 (mouse), kappa L chain}
EVKLEESGGGLVQPGGSMKLSCVVSGLTFSRFWMSWVRQSPEKGLEWVAEIRLKSDNYATHY
AESVKGKFTISRDDSKSRLYLQMNSLRTEDTGIYYCKIYFYSFSYWGQGTLVTVSA
>d1axtl1 b.1.1.1 (L:1-107) Immunoglobulin (variable domains of L and H chains) {Catalytic Fab 33F12 (mouse), kappa L chain}
ELVMTQTPLSLPVSLGDQASISCRSSQSLVHSYGNTFLNWYLQKSGQSPKLLIYKVSNRFSG
VPDRFSGSGSGTDFTLKISRVEAEDLGVYFCSQGTHVPYTFGGGTKLEIK
>d1b2wh1 b.1.1.1 (H:1-117) Immunoglobulin (variable domains of L and H chains) {Humanized and chimeric anti-gamma-interferon Fab}
EVQLVQSGGGVVQPGRSLKLSCLASGYIFTSSWINWVKQRPGRGLEWIGRIDPSDGEVHYNQ
DFKDRFTISRDKSKNTLYLQMNSLRPEDTAVYYCARGFLPWFADWGQGTLVTVSS
>d1b2wl1 b.1.1.1 (L:1-107) Immunoglobulin (variable domains of L and H chains) {Humanized and chimeric anti-gamma-interferon Fab}
DIQMTQSPSTLSASVGDRVTITCKASENVDTYVSWYQQKPGKAPKLLIYGASNRYTGVPSRF
SGSGSGTDFTLTISSLQPDDFATYYCGQSYNYPFTFGQGTKVEVK
>d1b4jh1 b.1.1.1 (H:1-117) Immunoglobulin (variable domains of L and H chains) {Humanized and chimeric anti-gamma-interferon Fab}
EVQLQQPGADLVMPGAPVKLSCLASGYIFTSSWINWVKQRPGRGLEWIGRIDPSDGEVHYNQ
DFKDKATLTVDKSSSTAYIQLNSLTSEDSAVYYCARGFLPWFADWGQGTLVTVSA
>d1b4jl1 b.1.1.1 (L:1-107) Immunoglobulin (variable domains of L and H chains) {Humanized and chimeric anti-gamma-interferon Fab}
NIVMTQSPKSMYVSIGERVTLSCKASENVDTYVSWYQQKPEQSPKLLIYGASNRYTGVPDRF
TGSGSATDFTLTISSVQAEDLADYHCGQSYNYPFTFGSGTKLEIK
>d1bfoa1 b.1.1.1 (A:1-107) Immunoglobulin (variable domains of L and H chains) {CAMPATH-1G igg2b monoclonal fab (rat), kappa L chain}
DIKMTQSPSFLSASVGDRVTLNCKASQNIDKYLNWYQQKLGESPKLLIYNTNNLQTGIPSRF
SGSGSGTDFTLTISSLQPEDVATYFCLQHISRPRTFGTGTKLELK
>d1bfob1 b.1.1.1 (B:1-121) Immunoglobulin (variable domains of L and H chains) {CAMPATH-1G igg2b monoclonal fab (rat), kappa L chain}
EVKLLESGGGLVQPGGSMRLSCAGSGFTFTDFYMNWIRQPAGKAPEWLGFIRDKAKGYTTEY
NPSVKGRFTISRDNTQNMLYLQMNTLRAEDTATYYCAREGHTAAPFDYWGQGVMVTVSS
>d1ce1h1 b.1.1.1 (H:1-121) Immunoglobulin (variable domains of L and H chains) {Therapeutic CAMPATH-1H humanized fab (rat), kappa

L chain}
QVQLQESGPGLVRPSQTLSLTCTVSGFTFTDFYMNWVRQPPGRGLEWIGFIRDKAKGYTTEY
NPSVKGRVTMLVDTSKNQFSLRLSSVTAADTAVYYCAREGHTAAPFDYWGQGSLVTVSS
>d1ce1l1 b.1.1.1 (L:1-107) Immunoglobulin (variable domains of L and H chains) {Therapeutic CAMPATH-1H humanized fab (rat), kappa L chain}
DIQMTQSPSSLSASVGDRVTITCKASQNIDKYLNWYQQKPGKAPKLLIYNTNNLQTGVPSRF
SGSGSGTDFTFTISSLQPEDIATYYCLQHISRPRTFGQGTKVEIK
>d1bj1h1 b.1.1.1 (H:1-123) Immunoglobulin (variable domains of L and H chains) {VEGF neutralizing Fab-12 (mouse), kappa L chain}
EVQLVESGGGLVQPGGSLRLSCAASGYTFTNYGMNWVRQAPGKGLEWVGWINTYTGEPTYAA
DFKRRFTFSLDTSKSTAYLQMNSLRAEDTAVYYCAKYPHYYGSSHWYFDVWGQGTLVTVSS
>d1bj1j1 b.1.1.1 (J:1-107) Immunoglobulin (variable domains of L and H chains) {VEGF neutralizing Fab-12 (mouse), kappa L chain}
DIQMTQSPSSLSASVGDRVTITCSASQDISNYLNWYQQKPGKAPKVLIYFTSSLHSGVPSRF
SGSGSGTDFTLTISSLQPEDFATYYCQQYSTVPWTFGQGTKVEIK
>d1blna1 b.1.1.1 (A:1-107) Immunoglobulin (variable domains of L and H chains) {Anti-p-glycoprotein Fab MRK-16 (mouse), kappa L chain}
DVLMTQTPVSLSVSLGDQASISCRSSQSIVHSTGNTYLEWYLQKPGQSPKLLIYKISNRFSG
VPDRFSGSGSGTDFTLKISRVEAEDLGVYYCFQASHAPRTFGGGTKLEIK
>d1blnb1 b.1.1.1 (B:1-113) Immunoglobulin (variable domains of L and H chains) {Anti-p-glycoprotein Fab MRK-16 (mouse), kappa L chain}
EVILVESGGGLVKPGGSLKLSCAASGFTFSSYTMSWVRQTPEKRLEWVATISSGGGNTYYPD
SVKGRFTISRDNAKNNLYLQMSSLRSEDTALYYCARYYRYEAWFASWGQGTLVTVSA
>d1boga1 b.1.1.1 (A:1-107) Immunoglobulin (variable domains of L and H chains) {Anti-p24 (HIV-1) Fab CB 4-1 (mouse), kappa L chain}
DIKMTQSPSSMYTSLGERVTITCKASQDINSFLTWFLQKPGKSPKTLIYRANRLMIGVPSRF
SGSGSGQTYSLTISSLEYEDMGIYYCLQYDDFPLTFGAGTKLDLK
>d1bogb1 b.1.1.1 (B:1-112) Immunoglobulin (variable domains of L and H chains) {Anti-p24 (HIV-1) Fab CB 4-1 (mouse), kappa L chain}
QDQLQQSGAELVRPGASVKLSCKALGYIFTDYEIHWVKQTPVHGLEWIGGIHPGSSGTAYNQ
KFKGKATLTADKSSTTAFMELSSLTSEDSAVYYCTRKDYWGQGTLVTVSA
>d1f58h1 b.1.1.1 (H:1-113) Immunoglobulin (variable domains of L and H chains) {Anti-gp120 (HIV-1) Fab 58.2, (mouse), kappa L chain}
DVQLQQSGPDLVKPSQSLSLTCTVTGYSITSGYSWHWIRQFPGNKLEWMGYIHYSAGTNYNP
SLKSRISITRDTSKNQFFLQLNSVTTEDTATYYCAREEAMPYGNQAYYYAMDCWGQGTTVTV
SS
>d1f58l1 b.1.1.1 (L:1-107) Immunoglobulin (variable domains of L and H chains) {Anti-gp120 (HIV-1) Fab 58.2, (mouse), kappa L chain}
DIVLTQSPASLAVSLGQRATISCKASQGVDFDGASFMNWYQQKPGQPPKLLIFAASTLESGI
PARFSGRGSGTDFTLNIHPVEEEDAATYYCQQSHEDPLTFGAGTKLELK
>d1wejh1 b.1.1.1 (H:1-112) Immunoglobulin (variable domains of L

and H chains) {Anti-cytochrome c Fab E8, (mouse), kappa L chain}
EVQLQQSGAELVKPGASVKLSCTASGFNIKDTYMHWVKQRPEKGLEWIGRIDPASGNTKYDP
KFQDKATITADTSSNTAYLQLSSLTSEDTAVYYCAGYDYGNFDYWGQGTT
>d1wejl1 b.1.1.1 (L:1-107) Immunoglobulin (variable domains of L and H chains) {Anti-cytochrome c Fab E8, (mouse), kappa L chain}
DIQMTQSPASLSASVGETVTITCRASGNIHNYLAWYQQKQGKSPQLLVYNAKTLADGVPSRF
SGSGSGTQYSLKINSLQPEDFGSYYCQHFWSTPWTFGGGTKLEIK
>d1sbsh1 b.1.1.1 (H:1-123) Immunoglobulin (variable domains of L and H chains) {Anti-HCG Fab 3A2, (mouse), kappa L chain}
EVNLEESGGGLVQPGGSMKLSCVASGFTFSNYWMNWVRQSPEKGLEWVADIRLKSNNYATLY
AESVKGRFTISRDDSKSSVYLQMNNLRAEDTGIYYCTRGAYYRYDYAMDYWGQGTSVTVSS
>d1sbsl1 b.1.1.1 (L:1-113) Immunoglobulin (variable domains of L and H chains) {Anti-HCG Fab 3A2, (mouse), kappa L chain}
DIVMSQSPSSLAVSVGEKVTMTCKSSQSLLYSSNQMNYLAWYQQKPGQSPKLLIYWASTRES
GVPDRFTGSGSGTDFTLTISSVEAEDLAVYYCQQYHSYPFTFGSGTKLEIK
>d1sm3h1 b.1.1.1 (H:1-113) Immunoglobulin (variable domains of L and H chains) {Tumor-specific Fab SM3, (mouse), lambda L chain}
QVQLQESGGGLVQPGGSMKLSCVASGFTFSNYWMNWVRQSPEKGLEWVAEIRLKSNNYATHY
AESVKGRFTISRDDSKSSVYLQMNNLRAEDTGIYYCTGVGQFAYWGQGTTVTVSS
>d2pcpa1 b.1.1.1 (A:1-107) Immunoglobulin (variable domains of L and H chains) {Fab 6B5, (mouse), kappa L chain}
DVLMTQTPLSLPVSLGDQASISCRSSQTIVHSNGNTYLEWYLQKPGQSPKLLIYKVTNRFSG
VPDRFSGSGSGTDFTLKISRVEAEDLGVYYCFQGTHAPYTFGGGTKLEIK
>d2pcpb1 b.1.1.1 (B:1-113) Immunoglobulin (variable domains of L and H chains) {Fab 6B5, (mouse), kappa L chain}
EVQLQQSGPELVKPGASVKMSCKASGYTFTDYYIHWNKQSHGKSLEWIGYIYPNNGGNGYNH
KFKGKATLTVDKSSSTAYMDVRTLTSEDSAVYYCGRSTWDDFDYWGQGTTLTVSS
>d3fcta1 b.1.1.1 (A:1-107) Immunoglobulin (variable domains of L and H chains) {Mature metal chelatase catalytic Fab, (human), kappa L chain}
ELVMTQTPKFMSTTVGDRVSITCKASQNVGTPVAWYQQKPGQSPKLLIYSASNRYTGVPDRF
TGSGSGTDFTLTISNMQSEDLADYFCQQYSSYPLTFGGGTKVEIK
>d3fctb1 b.1.1.1 (B:1-114) Immunoglobulin (variable domains of L and H chains) {Mature metal chelatase catalytic Fab, (human), kappa L chain}
QVQLLESGAELVKPGASVKLSCKASGYTFTSYWMHWVKQRPGRGLEWIGMIDPNSGGTKYNE
KFKSKATLTVDKPSNTAYMQLSSLTSEDSAVYYCTRRDMDYWGAGTTVTVSS
>d1cf8h1 b.1.1.1 (H:1-113) Immunoglobulin (variable domains of L and H chains) {Catalytic Fab 19A4 (mouse), kappa L chain}
DVQLQESGPGLVKPSQSLSLTCTVTGYSITSGYAWNWIRQFPGNKLEWMGYIRYSGDTRYNP
SLKSRISITRDTSKNQFFLQLNSVTTEDTATYYCAIGYGNSDYWGQGTLVTVSA
>d1cf8l1 b.1.1.1 (L:1-107) Immunoglobulin (variable domains of L and H chains) {Catalytic Fab 19A4 (mouse), kappa L chain}
DIVLTQSPTIMSVSPGEKVTLTCSASSSVSSNYVYWYQQKPGSSPKVWIYSTSNLASGVPAR

FSGSGSGTSYSLTISSMEAEDAASYFCLQWSSFPYTFGGGTKLELK
>d1c12a1 b.1.1.1 (A:1-107) Immunoglobulin (variable domains of L and H chains) {Fab directed agains the musk odorant traseolide, (mouse), kappa L chain}
DIELTQSPSSMSVSLGDTVSITCHASQGISSNIGWLQQKPGKSFKGLIYHGTNLEDGVPSRF
SGSGSGADYSLTISSLESEDFADYYCVQYVQFPFTFGSGTKLEIK
>d1c12b1 b.1.1.1 (B:301-413) Immunoglobulin (variable domains of L and H chains) {Fab directed agains the musk odorant traseolide, (mouse), kappa L chain}
QVQLQESGPGLVKPSQSLSLTCTVTGYSITSDYAWNWIRQFPGNKLEWMGYISYSGSTSYSP
SLKSRISLTRDTSKNQFFLQLNSVTTEDTATYYCVTSLTWLLRRKRSYWGQGTTVTVSS
>d1dlfh_ b.1.1.1 (H:) Immunoglobulin (variable domains of L and H chains) {Anti-dansyl Fv, (mouse), kappa L chain}
EVKLEESGGGLVQPGGSMKLSCATSGFTFSDAWMDWVRQSPEKGLEWVAEIRNKANNHATYY
AESVKGRFTISRDDSKRRVYLQMNTLRAEDTGIYYCTGIYYHYPWFAYWGQGTLVTVS
>d1dlfl_ b.1.1.1 (L:) Immunoglobulin (variable domains of L and H chains) {Anti-dansyl Fv, (mouse), kappa L chain}
DVVMTQTPLSLPVSLGNQASISCRSSQSLVHSNGNTYLHWYLQKPGQSPKLLIYKVSNRFSG
VPDRFSGSGSGTDFTLKISRVEAEDLGVYFCSQSTHVPFTFGSGTKLEIKR
>d43c9a_ b.1.1.1 (A:) Immunoglobulin (variable domains of L and H chains) {Sterolytic and amidolytic Fv 43c9, (mouse), kappa L chain}
DVVMTQTPSSLAMSVGQKVTMSCKSSQSLLNISNQKNYLAWYQQKPGQSPKLLVYFASTRES
GVPDRFIGSGSGTDFTLTISSVQAEDQADYFCQQHYRAPRTFGGGTKLEIK
>d43c9b_ b.1.1.1 (B:) Immunoglobulin (variable domains of L and H chains) {Sterolytic and amidolytic Fv 43c9, (mouse), kappa L chain}
GQVQLVESGPGLVAPSQSLSITCTVSGISLSRYNVHWVRQSPGKGLEWLGMIWGGGSIEYNP
ALKSRLSISKDNSKSQIFLKMNSLQTDDSAMYYCVSYGYGGDRFSYWGQGTLVTVS
>d1bz7a1 b.1.1.1 (A:1-107) Immunoglobulin (variable domains of L and H chains) {Fab R24, (mouse), kappa L chain}
DIQMTQITSSLSVSLGDRVIISCRASQDIGNFLNWYQQKPDGSLKLLIYYTSRLQSGVPSRF
SGWGSGTDYSLTISNLEEEDIATFFCQQGKTLPYTFGGGTKLEIK
>d1bz7b1 b.1.1.1 (B:1-122) Immunoglobulin (variable domains of L and H chains) {Fab R24, (mouse), kappa L chain}
DVQLVESGGGLVQPGGSRKLSCAASGFTFSNFGMHWVRQAPEKGLEWVAYISSGGSSINYAD
TVKGRFTISRDNPKNTLFLQMTSLRSEDTAIYYCTRGGTGTRSLYYFDYWGQGATLIVSS
>d1ct8a1 b.1.1.1 (A:1-107) Immunoglobulin (variable domains of L and H chains) {Catalytic Fab 7C8, (mouse), kappa L chain}
ELVMTQTPATLSVTPGDSVSLSCRASQSVSNKLHWYQQKSHESPRLLIKFASQSIPGIPSRF
SGSGSGSDFTLSINSVETEDFGIYFCHQTHGRPLTFGAGTKLELK
>d1ct8b1 b.1.1.1 (B:1-113) Immunoglobulin (variable domains of L and H chains) {Catalytic Fab 7C8, (mouse), kappa L chain}
QVKLLESGAVLVKPGASVKLSCKTSGFTFSSSYINWLKQKPGQSLEWIAWIYAGSGGTVYNQ

HFTDKARLTVDTSSSTAYMQFSSLTTEDSAIYYCARYRYDEGFAYWGQGTLVTVSA
>d1c5da1 b.1.1.1 (A:1-106) Immunoglobulin (variable domains of L and H chains) {Fab against the main immunogenic region of the human muscle acetylcholine receptor, (rat), kappa L chain}
DIQMTQSPPSLSASLGDKVTITCQASQDINKYIAWYQQKPGKAPRQLIRYTSILVLGTPSRF
SGSGSGRDFSFSISNVASEDIASYYCLQYGNLYTFGAGTKLEIK
>d1c5db1 b.1.1.1 (B:1-117) Immunoglobulin (variable domains of L and H chains) {Fab against the main immunogenic region of the human muscle acetylcholine receptor, (rat), kappa L chain}
EVKLLESGPGLVQPSQTLSLTCTVSGFPLTTNGVSWVRQPPGKGLEWIAAISSGGSPYYNSA
LKSRLSINRDTSKSQVFLKMNSLQTEDTAIYFCTREDGWNYFDYWGPGTMVTVSS
>d1f3rb2 b.1.1.1 (B:139-257) Immunoglobulin (variable domains of L and H chains) {scFv MAB198, (rat), kappa L chain}
DIKLTQSPSLLSASVGDRVTLSCKGSQNINNYLAWYQQKLGEAPKLLIYNTNSLQTGIPSRF
SGSGSGTDYTLTISSLQPEDVATYFCYQYNNGYTFGAGTKLELKAAEQKLISEEDLN
>d1dqqa1 b.1.1.1 (A:1-107) Immunoglobulin (variable domains of L and H chains) {Anti-lysozyme Fab HYHEL-63, (mouse), kappa L chain}
DIVLTQSPATLSVTPGDSVSLSCRASQSISNNLHWYQQKSHESPRLLIKYASQSISGIPSRF
SGSGSGTDFTLSINSVETEDFGMYFCQQSNSWPYTFGGGTKLEIK
>d1dqqb1 b.1.1.1 (B:1-113) Immunoglobulin (variable domains of L and H chains) {Anti-lysozyme Fab HYHEL-63, (mouse), kappa L chain}
EVQLQESGPSLVKPSQTLSLTCSVTGDSVTSDYWSWIRKFPGNKLEYMGYISYSGSTYYHPS
LKSRISITRDTSKNQYYLQLNSVTTEDTATYYCASWGGDVWGAGTTVTVSS
>d1ejoh1 b.1.1.1 (H:2501-2619) Immunoglobulin (variable domains of L and H chains) {Anti-FMDV Fab 4C4, (mouse), kappa L chain}
QMLVESGGDLVKPGGSLKLSCAASGFTFSSYTMSWVRQTPEKRLEWVATISSGGAYTYYPDS
VKGRFTISDDNAESTLYLQMSSLRSEDTAMYYCVRRAFDSDVGFASWGHRTLVTVSA
>d1ejol1 b.1.1.1 (L:2001-2111) Immunoglobulin (variable domains of L and H chains) {Anti-FMDV Fab 4C4, (mouse), kappa L chain}
DIVLTQSPASLAVSLGQRATISCRASESVDSYGNSFMHWYQQKPGQPPKLLIYRASNLESGI
PARFSGSGSRTDFTLTINPVEADDVATYYCQQSNEDPLTFGAGTKLELK
>d1cr9h1 b.1.1.1 (H:1-110) Immunoglobulin (variable domains of L and H chains) {Anti-prion Fab 3F4, (mouse), kappa L chain}
KVKLQQSGAELVRSGASVKLSCTASGFNIKDYYIQWVKQRPEQGLEWIGWIDPENGNSEYAP
RFQGKATMTADTLSNTAYLQLSSLTSEDTAVYYCNADLHDYWGQGTTLTVSS
>d1cr9l1 b.1.1.1 (L:1-106A) Immunoglobulin (variable domains of L and H chains) {Anti-prion Fab 3F4, (mouse), kappa L chain}
DVVMTQTPLSLSVTIGQPASISCKSSQSLLDSDGKTYLIWVFQRPGQSPKRLIFLVSKRDSG
VPDRFTGSGSGTDFTLKISRVEAEDVGVYYCWQGTHFPHTVGGGTKLEIA
>d1qfwh_ b.1.1.1 (H:) Immunoglobulin (variable domains of L and H chains) {Anti-[gonadotropin alpha subunit] Fv, kappa L chain}
QLQQSGAELVKPGASVKLSCKASDYTFTSYWMHWVKQRPGQGLEWIGEINPTNGRTYYNEKF
KSKATLTVAASASTAAMQASSLTSEDSAVYYCARRYGNSFDYWGQGTTVTVSS
>d1qfwl_ b.1.1.1 (L:) Immunoglobulin (variable domains of L and

H chains) {Anti-[gonadotropin alpha subunit] Fv, kappa L chain}
DIELTQSPDSLAVSLGQRATISCRASESVDSYGNSFMQWYQQKPGQPPKLLIYRASNLESGI
PARFSGTGSRTDFTLTINPVEADDVATYYCQQSDEYPYMYTFGGGTKLEIKR
>d1qfwi_ b.1.1.1 (I:) Immunoglobulin (variable domains of L and
H chains) {Anti-[gonadotropin beta subunit] Fv, kappa L chain}
QVQLQESGGHLVKPGGSLKLSCAASGFAFSSFDMSWIRQTPEKRLEWVASITNVGTYTYYPG
SVKGRFSISRDNARNTLNLQMSSLRSEDTALYFCARQGTAAQPYWYFDVWGAGTTVTVS
>d1qfwm_ b.1.1.1 (M:) Immunoglobulin (variable domains of L and
H chains) {Anti-[gonadotropin beta subunit] Fv, kappa L chain}
DIELTQSPKSMSMSVGERVTLSCKASETVDSFVSWYQQKPEQSPKLLIFGASNRFSGVPDRF
TGSGSATDFTLTISSVQAEDFADYHCGQTYNHPYTFGGGTKLEIKR
>d32c2a1 b.1.1.1 (A:1-110) Immunoglobulin (variable domains of L
and H chains) {Fab 32C2 against P450-arom, (mouse), kappa L chain}
DIVLTQSPASLAVSLGQRATISCRASKSVSTSGYGYMHWNQQKPGQPPRLLIYLVSNLESGV
PARFSGSGSGTDFTLNIHPVEEEDAATYYCQHIREPLTFGGGTKLEIK
>d32c2b1 b.1.1.1 (B:1-119) Immunoglobulin (variable domains of L
and H chains) {Fab 32C2 against P450-arom, (mouse), kappa L chain}
DVQLQESGPGLVKPSQSLSLTCTVTGYSISSDYAWNWIRQFPGNKLEWMGYISYSGSTSYNP
SLKSRISITRDTSKNQFFLQLSSVTTEDTATYYCARGYYGSSHSPVWGAGTTVTVSS
>d1dqdh1 b.1.1.1 (H:1-122) Immunoglobulin (variable domains of L
and H chains) {Fab HGR-2 F6, (mouse), kappa L chain}
EVQLQESGPSLVKPSQTLSLTCSVTGDSITSGYWNWIRKFPGNKLEYMGYISYSGSTYYNPS
LKSRLSITRDTSRNQYYLQLKSVTPEDTATYYCASPPGYYGSGPYAMDYWGQGTSVTVSS
>d1dqdl1 b.1.1.1 (L:1-106) Immunoglobulin (variable domains of L
and H chains) {Fab HGR-2 F6, (mouse), kappa L chain}
DIVLSQSPAIMSASPGEKVTITCSASSSVSYMHWFQQKPGTSPKLCIYTTSNLASGVPARFS
GSGSGTSYSLTISRMEAEDAATYYCQQRSTYPPTFGSGTKLEIK
>d1deea1 b.1.1.1 (A:1-107) Immunoglobulin (variable domains of L
and H chains) {Fab of human IgM RF 2A2}
DIQMTQSPSSLSASVGDRVTITCRTSQSISSYLNWYQQKPGKAPKLLIYAASSLQSGVPSRF
SGSGSGTDFTLTISSLQPEDFATYYCQQSYSAPRTFGQGTKVEIK
>d1deeb1 b.1.1.1 (B:501-621) Immunoglobulin (variable domains of
L and H chains) {Fab of human IgM RF 2A2}
QVQLVESGGGVVQPGKSLRLSCAASGFTFSGYGMHWVRQAPGKGLEWVALISYDESNKYYAD
SVKGRFTISRDNSKNTLYLQMNSLRAEDTAVYYCAKVKFYDPTAPNDYWGQGTLVTVSS
>d1qnzh_ b.1.1.1 (H:) Immunoglobulin (variable domains of L and
H chains) {Anti-HIV Fv 0.5B, (mouse), kappa L chain}
QVQLQQSGAELVKPGASVKMSCKASGYTFTTYPIEWMKQNHGKSLEWIGNFHPYSDDTNYNE
KFKGKAKLTVEKSSSTVYLEFSRLTSDDSAVYYCAIHYGSAYAMDYWGQGTSVTVSS
>d1f4xh1 b.1.1.1 (H:1-117) Immunoglobulin (variable domains of L
and H chains) {Anti-carbohydrate Fab S-20-4 (mouse), lambda L chain}
EVQLEESGGGLVTPGGSLRLSCAASGYVFSTYDMSWVRQTPEKRLEWVAFISSGGGRTSYPD
TVKGRFTISRDDAKNTLYLQMSSLQSEDTAMYYCTRHFYAVLDYWGRGTTLTVSS
>d1f4xl1 b.1.1.1 (L:1-110) Immunoglobulin (variable domains of L

and H chains) {Anti-carbohydrate Fab S-20-4 (mouse), lambda L chain}
QAVVTQESALTTSPGETVTLTCRSSTGTVTTSNYANWVQEKPDHLFTGLIGATNNRAAGVPV
RFSGSLIGGKAALTITGAQTEDEAIYFCALWYSGHWVFGGGTKLTVLG
>d1f11b1 b.1.1.1 (B:1-113) Immunoglobulin (variable domains of L and H chains) {Anti-Pres2 Fab F124, (mouse), kappa L chain}
EVQLQQSGPELVKPGASVKMSCKASGYTFTDYYMKWVKQSHGKSLEWIGDINPNNGGTGYNQ
KFKGKATLTVDKSSSTAYMQLNSLTSEDSAVYYCANDYGSTYGFAYWGQGTLVTVSA
>d1f3dh1 b.1.1.1 (H:1-121) Immunoglobulin (variable domains of L and H chains) {Catalytic Fab 4B2, (mouse), kappa L chain}
EIQLQQSGPELVKPGASVKVSCKASGYSFIDYNIHWVKQSHGKSLEWIGYIVPYSGGTTFNQ
KFKGKATLTVDKSSSTAFMHLNSLTFEDSAVYYCANDYDGVYWGQGTTLTVSS
>d1f3dj1 b.1.1.1 (J:1-107) Immunoglobulin (variable domains of L and H chains) {Catalytic Fab 4B2, (mouse), kappa L chain}
DVLMTQTPLSLPVSLGDQVSISCRSSQSIFHSDGKTYLEWHLQKPGQSPKLLIYKVSKRFSG
VPDRFSGSGSGTDFTLKISRVEAEDLGVYYCFQGSHVPYTFGGGTKLEIK
>d1fh5h1 b.1.1.1 (H:4-120) Immunoglobulin (variable domains of L and H chains) {Fab MAK33, (human), kappa L chain}
SGGGLVKPAGSLKLSCAASGFTFSSYYMYWVRQTPDKRLEWVATISDGGSYTYYPDSVKGRF
TISRDNAKNNLYLQMSSLKSEDTAMYYCARDAMDYWGQGTLVTVSA
>d1fskb1 b.1.1.1 (B:1-107) Immunoglobulin (variable domains of L and H chains) {Anti bet v1 Fab BV16, (mouse), kappa L chain}
NIVLTQSPKSMSVSVGERVTLSCKASENVDTYVFWFQQKPDQSPKLLLYGPSNRYTGVPDRF
TGSGSTTDFTLTISSVQAEDLADYHCGQSYSYPYTFGGGTKLEIK
>d1fskc1 b.1.1.1 (C:1-118) Immunoglobulin (variable domains of L and H chains) {Anti bet v1 Fab BV16, (mouse), kappa L chain}
QVQLQQPGTELVRPGASVILSCKASGYTFTSYWINWVKQRPGQGLEWVGNIFPSDSYTNYNQ
KFKDKATLTVDKSSSTAYMQVNSPTSEDSAVYYCTRGARDTWFAYWGQGTLVTVSV
>d1c5ch1 b.1.1.1 (H:1-113) Immunoglobulin (variable domains of L and H chains) {Decarboxylase catalytic Fab 21D8, chimeric (mouse V domains/human C1 domains)}
QVQLLEPGTELVKPGASVKLSCRASGYSFTSYWMHWVKQRPGQGLEWIGLIDPSNGRTNFND
KFKSRATLTVDTSSSTAYMQLSSLTSEDSAVYYCVRIAYWGQGTLVTVSS
>d1c5cl1 b.1.1.1 (L:1-107) Immunoglobulin (variable domains of L and H chains) {Decarboxylase catalytic Fab 21D8, chimeric (mouse V domains/human C1 domains)}
EIQLTQSPSSLSASLGERVSLTCRTSQEISGYLSWLQQKPDGTIKRLIYDATKLDSGAPKRF
SGSRSGSDYSLTISSLESEDFADYYCLQYASFPRTFGGGTKLEIK
>d1etza1 b.1.1.1 (A:1-110) Immunoglobulin (variable domains of L and H chains) {Anti-sweetener Fab NC10.14, (mouse), lamba L chain}
FAVVTQESALTTSPGETVTLTCRSSTGAVTTSNYAIWVQEKPDHLFSGLIGGTNNRVPGVPA
RFSGSLIGDKAALTVTGAQTEDEAIYFCALWYSNHWVFGGGTKLTVLG
>d1etzb1 b.1.1.1 (B:1-126) Immunoglobulin (variable domains of L and H chains) {Anti-sweetener Fab NC10.14, (mouse), lamba L chain}
QVTLKESGPGILQPSQTLSLTCSFSGFSLSTSGMGVGWIRQPSGEGLEWLADIWWNDKKYYN

PSLKSRLTVSKDTSSNQVFLKITSVDTSDTATYHCARRTFSYYYGSSFYYFDNWGQGTTLTV
SS
>d1emth1 b.1.1.1 (H:1-116) Immunoglobulin (variable domains of L and H chains) {Anti-C60 fullerene Fab, (mouse), kappa L chain}
QVHLQESGPELVRPGASVKISCKTSGYVFSSSWMNWVKQRPGQGLKWIGRIYPGNGNTNYNE
KFKGKATLTADKSSNTAYMQLSSLTSVDSAVYFCATSSAYWGQGTLLTVSA
>d1emtl1 b.1.1.1 (L:1-107) Immunoglobulin (variable domains of L and H chains) {Anti-C60 fullerene Fab, (mouse), kappa L chain}
DIQMTQTTSSLSASLGDRVTFSCSASQDISNYLNWYQQKPDGTIKLLIYYTSSLRSGVPSRF
SGSGSGTDYSLTINNLEPEDIATYFCQQYSRLPFTFGSGTKLEIK
>d1fl3a1 b.1.1.1 (A:2-116) Immunoglobulin (variable domains of L and H chains) {Blue fluorescent Fab 19G2, (mouse), kappa L chain}
AALLESGGGLVKPGGSLKLSCTASGITFSRYIMSWVRQIPEKRLEWVASISSGGITYYPDSV
AGRFTISRDNVRNILYLQMSSLRSEDTALYYCARGQGRPYWGQGTSVTVSA
>d1fl3b1 b.1.1.1 (B:2-113) Immunoglobulin (variable domains of L and H chains) {Blue fluorescent Fab 19G2, (mouse), kappa L chain}
AALTQSPVSNPVTLGTSASISCRSTKSLLHSNGITYLYWYLQKPGQSPQLLIYQMSNLASGV
PNRFSSSGSGTDFTLRINTVEAEDVGVYYCAQNLELPPTFGAGTKLELKR
>d1dzba1 b.1.1.1 (A:1-117) Immunoglobulin (variable domains of L and H chains) {Anti-lysozyme scFv 1F9, (mouse), kappa L chain}
QVKLQQSGAELVKPGASVKLSCTASGFNIKDTYMHWVKQRPEQGLEWIGRIDPANGNTKYDP
KFQGKATITADTSSNTAYLQLSSLTSEDTAVYYCARWDWYFDVWGQGTTVTVSSG
>d1dzba2 b.1.1.1 (A:201-307) Immunoglobulin (variable domains of L and H chains) {Anti-lysozyme scFv 1F9, (mouse), kappa L chain}
DIELTQSPSSMYTSLGERVTITCKASQDINSYLRWFQQKPGKSPKTLIYYATSLADGVPSRF
SGSGSGQDYSLTISSLESDDTTTYYCLQHGESPYTFGGGTKLEIK
>d1qoka1 b.1.1.1 (A:27-147) Immunoglobulin (variable domains of L and H chains) {Anti-carcinoembryonic scFv MFE-23, (mouse), kappa L chain}
QVKLQQSGAELVRSGTSVKLSCTASGFNIKDSYMHWLRQGPEQGLEWIGWIDPENGDTEYAP
KFQGKATFTTDTSSNTAYLQLSSLTSEDTAVYYCNEGTPTGPYYFDYWGQGTTVTVSSG
>d1qoka2 b.1.1.1 (A:162-267) Immunoglobulin (variable domains of L and H chains) {Anti-carcinoembryonic scFv MFE-23, (mouse), kappa L chain}
ENVLTQSPAIMSASPGEKVTITCSASSSVSYMHWFQQKPGTSPKLWIYSTSNLASGVPARFS
GSGSGTSYSLTISRMEAEDAATYYCQQRSSYPLTFGAGTKLELK
>d1e6oh1 b.1.1.1 (H:1-120) Immunoglobulin (variable domains of L and H chains) {Fab 13B5 against HIV-1 capsid protein p24, (mouse), kappa L chain}
EVQLQQSGAELARPGASVKMSCKASGYTFTSYTMHWVKQRPGQGLEWIGYINPSSGYSNYNQ
KFKDKATLTADKSSSTAYMQLSSLTSEDSAVYYCSRPVVRLGYNFDYWGQGSTLTVSS
>d1e6ol1 b.1.1.1 (L:1-105) Immunoglobulin (variable domains of L and H chains) {Fab 13B5 against HIV-1 capsid protein p24, (mouse), kappa L chain}

EIVLTQSPAITAASLGQKVTITCSASSSVSYMHWYQQKSGTSPKPWIYEISKLASGVPARFS
GSGSGTSYSLTISSMEAEDAAIYYCQQWNYPFTFGSGTKLEIK
>d1dl7h_ b.1.1.1 (H:) Immunoglobulin (variable domains of L and H chains) {Fv M3C65, (human), lambda L chain}
QVQLKESGPGLVAPSQSLSITCTVSGFSLTGYGVNWVRQPPGKGLEWLGMIWGDGSTDYNSA
LKSRLNISKDKSKSQVFLRMYSLQTDDTARYYCARDYGPYWGQGTLVTVS
>d1dl7l_ b.1.1.1 (L:) Immunoglobulin (variable domains of L and H chains) {Fv M3C65, (human), lambda L chain}
QAVVTQESALTTSPGETVTLTCRSSTGAVTTSNYANWVQEKPDHLFTGLIGGTKHRTPGAPA
RFSGSLIGDKAALTITGAQTEDEAIYFCALWYSNHWVFGGGTKLTVL
>d1egjh1 b.1.1.1 (H:1-113) Immunoglobulin (variable domains of L and H chains) {Fab against cytokyne receptor common beta chain domain 4, (mouse), kappa L chain}
EVQLQQSGPELVKPGTSVKMSCKASGYTFTDYYMKWVKHSHGKSLEWIGDINPSNGGTLYNQ
KFKGKATLTVDKSSSTASMQLSRLTSEDSAVYYCSRGDGIHGGFAYWGQGTTVTVSS
>d1egjl1 b.1.1.1 (L:1-107) Immunoglobulin (variable domains of L and H chains) {Fab against cytokyne receptor common beta chain domain 4, (mouse), kappa L chain}
NIVLTQSPASLAVSLGQRATISCRANESVYSYGDSFMHWYQQKPGQPPKLLIYLASNLASGV
PARFSGSGSRTDFTLTIDPVETDDAATYYCQQNNEDPWTFGGGTKLEIK
>d1ehlh1 b.1.1.1 (H:1-113) Immunoglobulin (variable domains of L and H chains) {Anti-photoproduct Fab 64M-2, (mouse), kappa L chain}
EVQLQQSGTVLARPGASVKMSCKASGYSFTSFWMHWVKQRPGQGLEWIGTIYPGNSDTSYNQ
KFKGKAKLTAVTSASTAYMEVSSLTNEDSAVYYCTRRSGYKYYALDYWGQGTSVTVSS
>d1fe8h1 b.1.1.1 (H:1-115) Immunoglobulin (variable domains of L and H chains) {Fab RU5, (mouse), kappa L chain}
DVKLVQSGPGLVAPSQSLSITCTVSGFSLTTYGVSWVRQPPGKGLEWLGVIWGDGNTTYHSA
LISRLSISKDNSRSQVFLKLNSLHTDDTATYYCAGNYYGMDYWGQGTSVTVSS
>d1fe8l1 b.1.1.1 (L:1-107) Immunoglobulin (variable domains of L and H chains) {Fab RU5, (mouse), kappa L chain}
DIAMTQTTSSLSASLGQKVTISCRASQDIGNYLNWYQQKPDGTVRLLIYYTSRLHSGVPSRF
SGSGSGTDYSLTISNLESEDIATYFCQNGGTNPWTFGGGTKLEVK
>d1ezvx_ b.1.1.1 (X:) Immunoglobulin (variable domains of L and H chains) {Fv against Rieske protein from the yeast cytochrome bc1 complex, (mouse), kappa L chain}
EVKLQESGAGLVQPSQSLSLTCSVTGYSITSGYYWNWIRLFPGNKLEWVGYISNVGDNNYNP
SLKDRLSITRDTSKNQFFLKLNSVTTEDTATYYCARSEYYSVTGYAMDYWGQGTTVTVSSAW
RHP
>d1ezvy_ b.1.1.1 (Y:) Immunoglobulin (variable domains of L and H chains) {Fv against Rieske protein from the yeast cytochrome bc1 complex, (mouse), kappa L chain}
DIELTQTPVSLAASLGDRVTISCRASQDINNFLNWYQQKPDGTIKLLIYYTSRLHAGVPSRF
SGSGSGTDYSLTISNLEPEDIATYFCQHHIKFPWTFGAGTKLEIK
>d1il1a1 b.1.1.1 (A:3-121) Immunoglobulin (variable domains of L

and H chains) {Anti-HIV Fab G3-519, (mouse), kappa L chain}
QLQQSGAELVRSGASVKLSCATSDFNIKDYYIHWVRQRPEQGLEWIGWLDPENGDTESAPKF
QGKATMTADTSSNTAYLQLSSLTSEASAVYYCNAISTTRDYYALDYWGQGTSVTVSS
>d1il1b1 b.1.1.1 (B:1-112) Immunoglobulin (variable domains of L and H chains) {Anti-HIV Fab G3-519, (mouse), kappa L chain}
DIVMSQSPSSLAVSVGEKVTMSCKSSQSLLYSRNQMNYLSWYQQKPGQSPKLLIYWASTRES
GVPDRFTGSGSGTDFTLTISSVKAEDLAVYYCQQYYHYRTFGGGTRLEIR
>d1f8th1 b.1.1.1 (H:1-113) Immunoglobulin (variable domains of L and H chains) {Anti-IL2 Fab LNKB-2, (mouse), kappa L chain}
GVQLQESGPGLVKPSQSLSLTCTVTGYSITSDYAWNWIRQFPGNKLEWMGYITYSGSTGYNP
SLKSRISITRDTSKNQFFLQLNSVTTEDTATYYCASYDDYTWFTYWGQGTLVTVSA
>d1f8tl1 b.1.1.1 (L:1-107) Immunoglobulin (variable domains of L and H chains) {Anti-IL2 Fab LNKB-2, (mouse), kappa L chain}
DVQMTQTPLTLSVTIGQPASISCESSQSLLYSNGKTYLNWLLQRPGQSPKRLIYLVSKLDSG
VPDRFTGSGSGTDFTLRISRVEAEDLGVYYCVQGTHFPRTFGGGTKLEIK
>d1e4wh1 b.1.1.1 (H:1-110) Immunoglobulin (variable domains of L and H chains) {Anti-TGFalpha Fab TAB2, (mouse), kappa L chain}
QVQLQQPGAELVKPGASVKLSCKASGFTFTNYWMHWVKQRPGQGLEWIGEILPSNGRTNYNE
KFKTKATLTVDKSSNTAYMQLSSLTSEDSAVYYCARSPSDYWGQGTTLTVSS
>d1e4wl1 b.1.1.1 (L:1-107) Immunoglobulin (variable domains of L and H chains) {Anti-TGFalpha Fab TAB2, (mouse), kappa L chain}
DIQMTQTPSSLSASLGDRVTISCRASQDISHYLNWFQQKPDGTVKLLIYYTSTLHSGVPSRF
SGSGSGTDYSLTISNLEEEDIAFYFCQQGGALPFTFGSGTKLAIK
>d1h8na1 b.1.1.1 (A:3-109) Immunoglobulin (variable domains of L and H chains) {Anti-ampicillin scFv, (mouse), kappa L chain}
KDIVLTQSHKFMSTSVGDRVSITCKASQDVGTAVAWYQQKPGQSPKLLIYWASTRHTGVPDR
FTGSGSGTDFTLTISNVQSEDLADYFCQQYSSYPLTFGAGTKLEL
>d1h8na2 b.1.1.1 (A:132-243) Immunoglobulin (variable domains of L and H chains) {Anti-ampicillin scFv, (mouse), kappa L chain}
QVQLQESGGELVRPGASVKLSCKASGYTFTSYWINWVKQRPGQGLEWIGNIYPSDSYTNYNQ
KFKDKATLTVDKSSSTAYMQLSSLTSEDSAVYFCARWGYWGQGTLVTVSA
>d1i7za1 b.1.1.1 (A:1-107) Immunoglobulin (variable domains of L and H chains) {Fab GNC92H2, (mouse/human chimera), kappa L chain}
DLVLTQSPASLAVSLGQRATISCRASKSVSTSGYNYMHWYQQKPGQPPKLLIYLASNLASGV
PARFSGSGSGTDFTLNIHPVEEEDAATYYCLYSREFPPWTFGGGTKLEIK
>d1i7zb1 b.1.1.1 (B:1-113) Immunoglobulin (variable domains of L and H chains) {Fab GNC92H2, (mouse/human chimera), kappa L chain}
QVQLQQSGPELKKPGETVKISCKTSGYSFTNYGMNWVKQAPGKGLKWMGWINTYTGEPTYAD
DFRGRFAFSLATSASTAYLQIINLKNEDTATYFCETYDSPLGDYWGQGTTVTVSS
>d1iqda1 b.1.1.1 (A:2-108) Immunoglobulin (variable domains of L and H chains) {Fab BO2C11 against the C2 domain of factor VIII, (human), kappa L chain}
IALTQSPGTLSLSPGERATLSCRASQSFSSSYLAWYQQKPGQAPRLLIYGASTRATGIPDRF
SGSGSGTDFTLTISRLEPEDFAVYYCQKYGTSAITFGQGTRLEIK

>d1iqdb1 b.1.1.1 (B:1-114) Immunoglobulin (variable domains of L and H chains) {Fab BO2C11 against the C2 domain of factor VIII, (human), kappa L chain}
QVQLVQSGAEVKKPGASVKVSCKVSGYTLTELPVHWVRQAPGKGLEWVGSFDPESGESIYAR
EFQGSVTMTADTSTNIAYMELSSLRSDDTAVYYCAVPDPDAFDIWGQGTMVTVSS
>d1fn4a1 b.1.1.1 (A:1-107) Immunoglobulin (variable domains of L and H chains) {Fab 198 against actylcholine receptor, (rat)}
DIKLTQSPSLLSASVGDRVTLSCKGSQNINNYLAWYQQKLGEAPKLLIYNTNSLQTGIPSRF
SGSGSGTDYTLTISSLQPEDVATYFCYQYNNGYTFGAGTKLELKR
>d1fn4b1 b.1.1.1 (B:1-106) Immunoglobulin (variable domains of L and H chains) {Fab 198 against actylcholine receptor, (rat)}
QVQLLESGPGLVRPSETLSLTCTVSGFSLTSFSVSWVRHPSGKGPEWMGRMWYDGYTAYNSA
LKSRLSISRDTSKNQVFLKMNSLQTDDTGTYYCTRDLYGGYPLGFWYFDFWGPG
>d1jglh1 b.1.1.1 (H:1-113) Immunoglobulin (variable domains of L and H chains) {Anti-estradiol Fab 57-2, (mouse), kappa L chain}
QIQLVQSGPELKKPGETVRISCKASDYSFMTSGMQWVQQMPGKGLKWIGWLNTQSGVPEYAE
DFKGRFAFSLETSATTAYLQINNLKNEDTATYFCATWGGNSAYWGQGTTLTVSS
>d1jp5a1 b.1.1.1 (A:1-112) Immunoglobulin (variable domains of L and H chains) {scFv 1695, (mouse), kappa L chain}
DILMTQTPLYLPVSLGDQASISCRSSQTIVHNNGNTYLEWYLQKPGQSPQLLIYKVSNRFSG
VPDRFSGSGSGTDFTLKISRVEAEDLGIYYCFQGSHFPPTFGGGTKLEIK
>d1jp5a2 b.1.1.1 (A:128-247) Immunoglobulin (variable domains of L and H chains) {scFv 1695, (mouse), kappa L chain}
EVQLQQSGPELKKPGETVKISCKATNYAFTDYSMHWVKQAPGGDLKYVGWINTETDEPTFAD
DFKGRFAFSLDTSTSTAFLQINNLKNEDTATYFCVRDRHDYGEIFTYWGQGTTVTVSS
>d1fl5b1 b.1.1.1 (B:1-113) Immunoglobulin (variable domains of L and H chains) {Sulfide oxidase catalytic Fab 28b4 germline precursor, (mouse/human?), kappa L chain}
QVQLVESGGGLVQPGGSLRLSCATSGFTFTDYYMSWVRQPPGKALEWLGFIRNKANGYTTEY
SASVKGRFTISRDNSQSILYLQMNTLRAEDSATYYCARDGSYAMDYWGQGTSVTVSS
>d1k4ca1 b.1.1.1 (A:1-118) Immunoglobulin (variable domains of L and H chains) {Fab against potassium channel KcsA, (mouse), kappa L chain}
QVQLQQPGAELVKPGASVKLSCKASGYTFTSDWIHWVKQRPGHGLEWIGEIIPSYGRANYNE
KIQKKATLTADKSSSTAFMQLSSLTSEDSAVYYCARERGDGYFAVWGAGTTVTVSS
>d1k4cb1 b.1.1.1 (B:1-107) Immunoglobulin (variable domains of L and H chains) {Fab against potassium channel KcsA, (mouse), kappa L chain}
DILLTQSPAILSVSPGERVSFSCRASQSIGTDIHWYQQRTNGSPRLLIKYASESISGIPSRF
SGSGSGTDFTLSINSVESEDIANYYCQQSNRWPFTFGSGTKLEIK
>d1jguh1 b.1.1.1 (H:1-113) Immunoglobulin (variable domains of L and H chains) {Catalytic Fab 1D4, (mouse), kappa L chain}
EVKLVESRGGLVKPGGSLQLSCAASGFTFSGYAMSWFRLTPEKRLEWVASIYNGFRIHYLDS
VKGRFTISSDYARNILYLQMSTLRSEDTAMYYCSRGDAYSRYFDVWGAGTTVTVSA

>d1jgul1 b.1.1.1 (L:1-107) Immunoglobulin (variable domains of L and H chains) {Catalytic Fab 1D4, (mouse), kappa L chain}
EVVMTQSPLSLPVSLGDQASISCRSSQSLVHSNGNTYLHWYLQKPGQSPKLLIYKVSNRFSG
VPDRFSGSGSGTDFTLKISRVEAEDLGVYFCSQSTHVPPLTFGAGTKLELK
>d1i8ma1 b.1.1.1 (A:1-107) Immunoglobulin (variable domains of L and H chains) {Anti ssDNA Fab, (mouse), kappa L chain}
ELQMTQSPASLSASVGETVTITCRASENIYSYLAWYQQKQGKSPQLLVYNAKTLAEGVPSRF
SGSGSGTQFSLKINSLQPEDFGSYYCQHHYGTPLTFGAGTKLELK
>d1i8mb1 b.1.1.1 (B:1-113) Immunoglobulin (variable domains of L and H chains) {Anti ssDNA Fab, (mouse), kappa L chain}
QVKLLESGPELVKPGASVKMSCKASGYTFTSYVMHWVKQKPGQGLEWIGYINPYNDGTKYNE
KFKGKATLTSDKSSSTAYMELSSLTSEDSAVYYCVRGGYRPYYAMDYWGQGTSVTVSS
>d1jv5b_ b.1.1.1 (B:) Immunoglobulin (variable domains of L and H chains) {Anti-blood group A Fv, (human), kappa L chain}
QVQLQQPGAELVKPGTSVKLSCKASGYNFTSYWINWVKLRPGQGLEWIGDIYPGSGITNYNE
KFKSKATLTVDTSSSTAYMQLSSLASEDSALYYCAGQYGNLWFAYWGQGTLVTVS
>d1iqwh1 b.1.1.1 (H:1-121) Immunoglobulin (variable domains of L and H chains) {Anti-human Fas Fab hfe7a, (mouse), kappa L chain}
QVQLQQPGAELVKPGASVKLSCKASGYTFTSYWMQWVKQRPGQGLEWIGEIDPSDSYTNYNQ
KFKGKATLTVDTSSSTAYMQLSSLTSEDSAVYYCARNRDYSNNWYFDVWGTGTTVTVSS
>d1iqwl1 b.1.1.1 (L:1-111) Immunoglobulin (variable domains of L and H chains) {Anti-human Fas Fab hfe7a, (mouse), kappa L chain}
DIVLTQSPASLAVSLGQRATISCKASQSVDYDGDSYMNWYQQKPGQPPKLLIYAASNLESGI
PARFSGSGSGTDFTLNIHPVEEEDAATYYCQQSNEDPRTFGGGTKLEIK
>d1jnha1 b.1.1.1 (A:1-108) Immunoglobulin (variable domains of L and H chains) {Anti-estradiol Fab 10G6D6, (mouse), lambda L chain}
QAVVTQESALTTSPGETVTLTCRSSSGAITTSHYANWIQEKPDHLFTGLISGTNNRAPGVPA
RFSGSLIGDKAALTITGAQTEDEAIYICALWFSNQFIFGSGTKVTV
>d1jnhb1 b.1.1.1 (B:1-117) Immunoglobulin (variable domains of L and H chains) {Anti-estradiol Fab 10G6D6, (mouse), lambda L chain}
EVQLQQSGAELARPGASVKLSCRTSGYSFTTYWMQWVRQRPGQGLEWIAAIYPGDDDARYTQ
KFKGKATLTADRSSSIVYLQLNSLTSEDSAVYSCSRGRSLYYTMDYWGQGTSVTV
>d1jnlh1 b.1.1.1 (H:1-114) Immunoglobulin (variable domains of L and H chains) {Anti-estradiol Fab 17E12E5, (mouse), kappa L chain}
EVQLQQSGAELVKPGASVRLSCSASGFNIKDTYMFWVKQRPEQGLDWIGRINPANGISKYDP
RFQGKATLTADTSSNTAYLQLDNLTSEDTAVYYCAIEKDLPWGQGTLVTVSV
>d1jnll1 b.1.1.1 (L:1-107) Immunoglobulin (variable domains of L and H chains) {Anti-estradiol Fab 17E12E5, (mouse), kappa L chain}
QIVMTQTPASLSASVGETVTITCRASGNIYNYLAWYQQKQGKSPQLLVYNAKTLVDGVPLRF
SGSGSGTQYSLKINSLQPEDFGNYYCHHFWNTPYTFGGGTKLEIK
>d1vhp__ b.1.1.1 (-) Immunoglobulin (variable domains of L and H chains) {VH-P8 domain (human), camelized monomer}
EVQLVESGGGLVQPGGSLRLSCAASGFTFSSYAMSWVRQAPGKEREIVSAVSGSGGSTYYAD
SVKGRFTISRDNSKNTLYLQMNSLRAEDTAVYYCARLKKYAFDYWGQGTLVTVSS

>d1jtpa_ b.1.1.1 (A:) Immunoglobulin (variable domains of L and H chains) {Camel (Camelus dromedarius), anti-lysozyme antibody}
DVQLQASGGGSVQAGGSLRLSCAASGYTIGPYCMGWFRQAPGKEREGVAAINMGGGITYYAD
SVKGRFTISQDNAKNTVYLLMNSLEPEDTAIYYCAADSTIYASYYECGHGLSTGGYGYDSWG
QGTQVTVSSRR
>d1bzqk_ b.1.1.1 (K:) Immunoglobulin (variable domains of L and H chains) {Camel (Camelus dromedarius), anti-RNase A antibody}
QVQLVESGGGLVQAGGSLRLSCAASGYAYTYIYMGWFRQAPGKEREGVAAMDSGGGGTLYAD
SVKGRFTISRDKGKNTVYLQMDSLKPEDTATYYCAAGGYELRDRTYGQWGQGTQVTVSSRGR
>d1f2xk_ b.1.1.1 (K:) Immunoglobulin (variable domains of L and H chains) {Camel (Camelus dromedarius), antibody cab-ca05}
QVQLVESGGGSVQAGGSLRLSCAASGYTVSTYCMGWFRQAPGKEREGVATILGGSTYYGDSV
KGRFTISQDNAKNTVYLQMNSLKPEDTAIYYCAGSTVASTGWCSRLRPYDYHYRGQGTQVTV
SS
>d1hcv__ b.1.1.1 (-) Immunoglobulin (variable domains of L and H chains) {Llama (Lama glama), anti-gonadotropin alpha subunit VH domain}
VQLQESGGGLVQAGGSLRLSCAASGRTGSTYDMGWFRQAPGKERESVAAINWDSARTYYASS
VRGRFTISRDNAKKTVYLQMNSLKPEDTAVYTCGAGEGGTWDSWGQGTQVTVSS
>d1qd0a_ b.1.1.1 (A:) Immunoglobulin (variable domains of L and H chains) {Llama (Lama glama), anti-RR6 VH domain}
QVQLQESGGGLVQAGGSLRLSCAASGRAASGHGHYGMGWFRQVPGKEREFVAAIRWSGKETW
YKDSVKGRFTISRDNAKTTVYLQMNSLKGEDTAVYYCAARPVRVADISLPVGFDYWGQGTQV
TVSS
>d1i3ua_ b.1.1.1 (A:) Immunoglobulin (variable domains of L and H chains) {Llama (Lama glama), the dye RR1-binding VHh domain}
VQLQESGGGLVQAGDSLKLSCEASGDSIGTYVIGWFRQAPGKERIYLATIGRNLVGPSDFYT
RYADSVKGRFAVSRDNAKNTVNLQMNSLKPEDTAVYYCAAKTTTWGGNDPNNWNYWGQGTQV
TV
>d1ivla_ b.1.1.1 (A:) Immunoglobulin (variable domains of L and H chains) {VL domain (kappa) of antibody M29B, dimer synthetic}
DIELTQSPATLSVTPGNSVSISCRASQSIGNRLFWYQQKSHESPRLLIKYASQSISGIPSRF
SGSGSGTDFTLSINSVETEDLAVYFCQQVSEWPFTFGGGTKLEIK
>d1bwwa_ b.1.1.1 (A:) Immunoglobulin (variable domains of L and H chains) {Bence-Jones VL (kappa) dimer REI (human)}
TPDIQMTQSPSSLSASVGDRVTITCQASQDIIKYLNWYQQKPGKAPKLLIYEASNLQAGVPS
RFSGSGSGTDYTFTISSLQPEDIATYYCQQYQSLPYTFGQGTKLQIT
>d2rhe__ b.1.1.1 (-) Immunoglobulin (variable domains of L and H chains) {Bence-Jones VL (lambda) dimer RHE (human)}
ESVLTQPPSASGTPGQRVTISCTGSATDIGSNSVIWYQQVPGKAPKLLIYYNDLLPSGVSDR
FSASKSGTSASLAISGLESEDEADYYCAAWNDSLDEPGFGGGTKLTVLGQPK
>d1bjma1 b.1.1.1 (A:1-111) Immunoglobulin (variable domains of L and H chains) {Bence-Jones lambda L chain dimer LOC (human)}
ESVLTQPPSASGTPGQRVTISCSGSSSNIGENSVTWYQHLSGTAPKLLIYEDNSRASGVSDR

FSASKSGTSASLAISGLQPEDETDYYCAAWDDSLDVAVFGTGTKVTVLG
>d1wtla_ b.1.1.1 (A:) Immunoglobulin (variable domains of L and H chains) {Bence-Jones VL (kappa) dimer WAT (human)}
DIQMTQSPSSLSASVGDRVTITCRASQDITNYVNWFQQRPGQAPKVLIYGASILETGVPSRF
SGSGSGTDFTFTISSLQPEDIATYYCQQYDTLPLTFGGGTKVDIKR
>d1b0wa_ b.1.1.1 (A:) Immunoglobulin (variable domains of L and H chains) {Bence-Jones VL (kappa) dimer BRE (human)}
DIQMTQSPSSLSASVGDRVTITCQASQDISDYLIWYQQKLGKAPNLLIYDASTLETGVPSRF
SGSGSGTEYTFTISSLQPEDIATYYCQQYDDLPYTFGQGTKVEIKR
>d1eeqa_ b.1.1.1 (A:) Immunoglobulin (variable domains of L and H chains) {Bence-Jones VL (kappa) domain LEN (human)}
DIVLTQSPDSLAVSLGERATINCKSSQSVLDSSNSKNYLAWYQQKPGQPPKLLIYWASTRES
GVPDRFSGSGSGTDFTLTISSLQAEDVAVYYCQQYYSHPYSFGQGTKLEIK
>d1lila1 b.1.1.1 (A:2-107) Immunoglobulin (variable domains of L and H chains) {Bence-Jones lambda L chain dimer CLE (human)}
YEVTQPPSLSVSPGQTARITCSGEKLGDAYVCWYQQRPGQSPVVVIYQDNRRPSGIPERFSG
SSSGNTATLTISGTQTLDEADYYCQVWDSNASVVFGGGTKLTVLG
>d1cd0a_ b.1.1.1 (A:) Immunoglobulin (variable domains of L and H chains) {Bence-Jones VL (lambda) dimer JTO (human)}
NFMLNQPHSVSESPGKTVTISCTRSSGNIDSNYVQWYQQRPGSAPITVIYEDNQRPSGVPDR
FAGSIDRSSNSASLTISGLKTEDEADYYCQSYDARNVVFGGGTRLTVLG
>d2cd0a_ b.1.1.1 (A:) Immunoglobulin (variable domains of L and H chains) {Bence-Jones VL (lambda) dimer WIL (human)}
NFLLTQPHSVSESPGKTVTISCTRSSGSIANNYVHWYQQRPGSSPTTVIFEDDHRPSGVPDR
FSGSVDTSSNSASLTISGLKTEDEADYYCQSYDHNNQVFGGGTKLTVLG
>d1b6da1 b.1.1.1 (A:1-107) Immunoglobulin (variable domains of L and H chains) {Bence-Jones kappa L chain DEL (human)}
DIQMTQSPSSLSASVGDRVTITCQASQDISSYLNWYQQKPGKAPKLLIHAASSLETGVPSRF
SGSGSGTDFSFTISSLQPEDLATYYCQQYDSLPLTFGGGTKVEIK
>d1ek3a_ b.1.1.1 (A:) Immunoglobulin (variable domains of L and H chains) {Kappa-4 VL REC (human)}
DIVMTQSPDSLAVSPGERATINCKSSQNLLDSSFDTNTLAWYQQKPGQPPKLLIYWASSRES
GVPDRFSGSGSGTDFTLTISSLQAEDVAVYYCQQYYSTPPTFGGGTKVEIKR
>d1mcoh1 b.1.1.1 (H:1-117) Immunoglobulin (variable domains of L and H chains) {Intact antibody (lambda) MCG (human)}
PLVLQESGPGLVKPSEALSLTCTVSGDSINTILYYWSWIRQPPGKGLEWIGYIYYSGSTYGN
PSLKSRVTISVNTSKNQFYSKLSSVTAADTAVYYCARVPLVVNPWGQGTLVTVSS
>d1dcla1 b.1.1.1 (A:1-111) Immunoglobulin (variable domains of L and H chains) {Lambda L chain dimer MCG (human)}
PSALTQPPSASGSLGQSVTISCTGTSSNVGGYNYVSWYQQHAGKAPKVIIYEVNKRPSGVPD
RFSGSKSGNTASLTVSGLQAEDEADYYCSSYEGSDNFVFGTGTKVTVLG
>d1mcww1 b.1.1.1 (W:1-111) Immunoglobulin (variable domains of L and H chains) {Heterologous L chain dimer MCG-WEIR hybrid (human)}
ESALTQPASVSGSPGQSITVSCAGHTSDVADSNSISWFQQHPDKAPKLLIYAVTFRPSGIPL

RFSGSKSGNTASLTISGLLPDDEADYFCMSYLSDASFVFGSGTKVTVLR
>d1ac6a_ b.1.1.1 (A:) T-cell antigen receptor {Mouse (Mus musculus), alpha-chain}
DSVTQTEGQVALSEEDFLTIHCNYSASGYPALFWYVQYPGEGPQFLFRASRDKEKGSSRGFE
ATYNKEATSFHLQKASVQESDSAVYYCALSGGNNKLTFGAGTKLTIKP
>d1b88a_ b.1.1.1 (A:) T-cell antigen receptor {Mouse (Mus musculus), alpha-chain}
MQQVRQSPQSLTVWEGETAILNCSYENSAFDYFPWYQQFPGEGPALLISILSVSNKKEDGRF
TIFFNKREKKLSLHIADSQPGDSATYFCAASASFGDNSKLIWGLGTSLVVNP
>d1d9ka_ b.1.1.1 (A:) T-cell antigen receptor {Mouse (Mus musculus), alpha-chain}
QVRQSPQSLTVWEGETTILNCSYEDSTFDYFPWYRQFPGKSPALLIAISLVSNKKEDGRFTI
FFNKREKKLSLHITDSQPGDSATYFCAATGSFNKLTFGAGTRLAVSPY
>d1fo0a_ b.1.1.1 (A:) T-cell antigen receptor {Mouse (Mus musculus), alpha-chain}
KVTQTQTSISVMEKTTVTMDCVYETQDSSYFLFWYKQTASGEIVFLIRQDSYKKENATVGHY
SLNFQKPKSSIGLIITATQIEDSAVYFCAMRGDYGGSGNKLIFGTGTLLSVKP
>d1h5ba_ b.1.1.1 (A:) T-cell antigen receptor {Mouse (Mus musculus), alpha-chain}
GDQVEQSPSALSLHEGTDSALRCNFTTTMRSVQWFRQNSRGSLISLFYLASGTKENGRLKSA
FDSERARYSTLHIRDAQLEDSGTYFCAAEASSGSWQLIFGSGTQLTVMPVT
>d1i9ea_ b.1.1.1 (A:) T-cell antigen receptor {Mouse (Mus musculus), alpha-chain}
QSVTQPDARVTVSEGASLQLRCKYSYSATPYLFWYVQYPRQGLQLLLKYYSGDPVVQGVNGF
EAEFSKSNSSFHLRKASVHWSDSAVYFCAVSGFASALTFGSGTKVIVLPYIQN
>d1kb5a_ b.1.1.1 (A:) T-cell antigen receptor {Mouse (Mus musculus), alpha-chain}
QQVRQSPQSLTVWEGETAILNCSYEDSTFNYFPWYQQFPGEGPALLISIRSVSDKKEDGRFT
IFFNKREKKLSLHITDSQPGDSATYFCAARYQGGRALIFGTGTTVSVSPGSAD
>d1nfda1 b.1.1.1 (A:1-117) T-cell antigen receptor {Mouse (Mus musculus), alpha-chain}
DSVTQTEGLVTVTEGLPVKLNCTYQTTYLTIAFFWYVQYLNEAPQVLLKSSTDNKRTEHQGF
HATLHKSSSSFHLQKSSAQLSDSALYYCALSEGGNYKYVFGAGTRLKVIAH
>d1bd2d1 b.1.1.1 (D:1-117) T-cell antigen receptor {Human (Homo sapiens), alpha-chain}
QQVKQNSPSLSVQEGRISILNCDYTNSMFDYFLWYKKYPAEGPTFLISISSIKDKNADGRFT
VFLNKSAKHLSLHIVPSQPGDSAVYFCAAMEGAQKLVFGQGTRLTINPN
>d1fytd1 b.1.1.1 (D:1-117) T-cell antigen receptor {Human (Homo sapiens), alpha-chain}
QSVTQLGSHVSVSEGALVLLRCNYSSSVPPYLFWYVQYPNQGLQLLLKYTSAATLVKGINGF
EAEFKKSETSFHLTKPSAHMSDAAEYFCAVSESPFGNEKLTFGTGTRLTIIPN
>d1qrnd1 b.1.1.1 (D:1-117) T-cell antigen receptor {Human (Homo sapiens), alpha-chain}
KEVEQNSGPLSVPEGAIASLNCTYSDRGSQSFFWYRQYSGKSPELIMSIYSNGDKEDGRFTA

QLNKASQYVSLLIRDSQPSDSATYLCAVTTDSWGKLQFGAGTQVVVTPD
>d1bec_1 b.1.1.1 (3-117) T-cell antigen receptor {Mouse (Mus musculus), beta-chain}
AVTQSPRNKVAVTGGKVTLSCQQTNNHNNMYWYRQDTGHGLRLIHYSYGAGSTEKGDIPDGY
KASRPSQEQFSLILELATPSQTSVYFCASGGGRGSYAEQFFGPGTRLTVLE
>d1fo0b_ b.1.1.1 (B:) T-cell antigen receptor {Mouse (Mus musculus), beta-chain}
VTLLEQNPRWRLVPRGQAVNLRCILKNSQYPWMSWYQQDLQKQLQWLFTLRSPGDKEVKSLP
GADYLATRVTDTELRLQVANMSQGRTLYCTCSADRVGNTLYFGEGSRLIV
>d1kb5b_ b.1.1.1 (B:) T-cell antigen receptor {Mouse (Mus musculus), beta-chain}
VTLLEQNPRWRLVPRGQAVNLRCILKNSQYPWMSWYQQDLQKQLQWLFTLRSPGDKEVKSLP
GADYLATRVTDTELRLQVANMSQGRTLYCTCSAAPDWGASAETLYFGSGTRLTVL
>d1nfdb1 b.1.1.1 (B:1-117) T-cell antigen receptor {Mouse (Mus musculus), beta-chain}
DSGVVQSPRHIIKEKGGRSVLTCIPISGHSNVVWYQQTLGKELKFLIQHYEKVERDKGFLPS
RFSVQQFDDYHSEMNMSALELEDSAMYFCASSLRWGDEQYFGPGTRLTVLE
>d1tcrb1 b.1.1.1 (B:1-117) T-cell antigen receptor {Mouse (Mus musculus), beta-chain}
EAAVTQSPRNKVAVTGGKVTLSCNQTNNHNNMYWYRQDTGHGLRLIHYSYGAGSTEKGDIPD
GYKASRPSQENFSLILELATPSQTSVYFCASGGGGTLYFGAGTRLSVLE
>d1bd2e1 b.1.1.1 (E:3-118) T-cell antigen receptor {Human (Homo sapiens), beta-chain}
GVTQTPKFQVLKTGQSMTLQCAQDMNHEYMSWYRQDPGMGLRLIHYSVGAGITDQGEVPNGY
NVSRSTTEDFPLRLLSAAPSQTSVYFCASSYPGGGFYEQYFGPGTRLTVTE
>d1fyte1 b.1.1.1 (E:3-118) T-cell antigen receptor {Human (Homo sapiens), beta-chain}
KVTQSSRYLVKRTGEKVFLECVQDMDHENMFWYRQDPGLGLRLIYFSYDVKMKEKGDIPEGY
SVSREKKERFSLILESASTNQTSMYLCASSSTGLPYGYTFGSGTRLTVVE
>d1hxma1 b.1.1.1 (A:1-120) T-cell antigen receptor {Human (Homo sapiens), gamma-chain}
AIELVPEHQTVPVSIGVPATLRCSMKGEAIGNYYINWYRKTQGNTMTFIYREKDIYGPGFKD
NFQGDIDIAKNLAVLKILAPSERDEGSYYCACDTLGMGGEYTDKLIFGKGTRVTVEPR
>d1hxmb1 b.1.1.1 (B:1-123) T-cell antigen receptor {Human (Homo sapiens), delta-chain}
AGHLEQPQISSTKTLSKTARLECVVSGITISATSVYWYRERPGEVIQFLVSISYDGTVRKES
GIPSGKFEVDRIPETSTSTLTIHNVEKQDIATYYCALWEAQQELGKKIKVFGPGTKLIITD
>d1tvda_ b.1.1.1 (A:) T-cell antigen receptor {Human (Homo sapiens), delta-chain}
DKVTQSSPDQTVASGSEVVLLCTYDTVYSNPDLFWYRIRPDYSFQFVFYGDDSRSEGADFTQ
GRFSVKHILTQKAFHLVISPVRTEDSATYYCAFTLPPPTDKLIFGKGTRVTVEP
>d1ah1__ b.1.1.1 (-) Immunoreceptor CTLA-4 (CD152), N-terminal fragment {Human (Homo sapiens)}
AMHVAQPAVVLASSRGIASFVCEYASPGKATEVRVTVLRQADSQVTEVCAATYMMGNELTFL

DDSICTGTSSGNQVNLTIQGLRAMDTGLYICKVELMYPPPYYLGIGNGTQIYVIDPEPCPDS
DQEPK
>d1i8lc_ b.1.1.1 (C:) Immunoreceptor CTLA-4 (CD152), N-terminal fragment {Human (Homo sapiens)}
MHVAQPAVVLASSRGIASFVCEYASPGKATEVRVTVLRQADSQVTEVCAATYMMGNELTFLD
DSICTGTSSGNQVNLTIQGLRAMDTGLYICKVELMYPPPYYLGIGNGAQIYVIDPE
>d1dqta_ b.1.1.1 (A:) Immunoreceptor CTLA-4 (CD152), N-terminal fragment {Mouse (Mus musculus)}
IQVTQPSVVLASSHGVASFPCEYSPSHNTDEVRVTVLRQTNDQMTEVCATTFTEKNTVGFLD
YPFCSGTFNESRVNLTIQGLRAVDTGLYLCKVELMYPPPYFVGMGNGTQIYVIDP
>d3frua1 b.1.1.2 (A:179-269) Fc (IgG) receptor, alpha-3 domain and beta subunit {Rat (Rattus norvegicus)}
KEPPSMRLKARPGNSGSSVLTCAAFSFYPPELKFRFLRNGLASGSGNCSTGPNGDGSFHAWS
LLEVKRGDEHHYQCQVEHEGLAQPLTVDL
>d3frub1 b.1.1.2 (B:) Fc (IgG) receptor, alpha-3 domain and beta subunit {Rat (Rattus norvegicus)}
IQKTPQIQVYSRHPPENGKPNFLNCYVSQFHPPQIEIELLKNGKKIPNIEMSDLSFSKDWSF
YILAHTEFTPTETDVYACRVKHVTLKEPKTVTWDRDM
>d1bmg__ b.1.1.2 (-) Class I MHC, beta2-microglobulin and alpha-3 domain {Cow (Bos taurus)}
IQRPPKIQVYSRHPPEDGKPNYLNCYVYGFHPPQIEIDLLKNGEKIKSEQSDLSFSKDWSFY
LLSHAEFTPNSKDQYSCRVKHVTLEQPRIVKWDRDL
>d1i4fa1 b.1.1.2 (A:182-275) Class I MHC, beta2-microglobulin and alpha-3 domain {Human (Homo sapiens), HLA-A2.1}
TDAPKTHMTHHAVSDHEATLRCWALSFYPAEITLTWQRDGEDQTQDTELVETRPAGDGTFQK
WAAVVVPSGQEQRYTCHVQHEGLPKPLTLRWE
>d1i4fb1 b.1.1.2 (B:) Class I MHC, beta2-microglobulin and alpha-3 domain {Human (Homo sapiens), HLA-A2.1}
MIQRTPKIQVYSRHPAENGKSNFLNCYVSGFHPSDIEVDLLKNGERIEKVEHSDLSFSKDWS
FYLLYYTEFTPTEKDEYACRVNHVTLSQPKIVKWDRDM
>d1agda1 b.1.1.2 (A:182-276) Class I MHC, beta2-microglobulin and alpha-3 domain {Human (Homo sapiens), HLA-B0801}
ADPPKTHVTHHPISDHEATLRCWALGFYPAEITLTWQRDGEDQTQDTELVETRPAGDRTFQK
WAAVVVPSGEEQRYTCHVQHEGLPKPLTLRWEP
>d1qqda1 b.1.1.2 (A:182-274) Class I MHC, beta2-microglobulin and alpha-3 domain {Human (Homo sapiens), HLA-CW4}
AEHPKTHVTHHPVSDHEATLRCWALGFYPAEITLTWQWDGEDQTQDTELVETRPAGDGTFQK
WAAVVVPSGEEQRYTCHVQHEGLPEPLTLRW
>d1mhea1 b.1.1.2 (A:182-274) Class I MHC, beta2-microglobulin and alpha-3 domain {Human (Homo sapiens), HLA-E}
LEPPKTHVTHHPISDHEATLRCWALGFYPAEITLTWQQDGEGHTQDTELVETRPAGDGTFQK
WAAVVVPSGEEQRYTCHVQHEGLPEPVTLRW
>d1de4a1 b.1.1.2 (A:182-275) Class I MHC, beta2-microglobulin and alpha-3 domain {Human (Homo sapiens), hemochromatosis protein Hfe}

QQVPPLVKVTHHVTSSVTTLRCRALNYYPQNITMKWLKDKQPMDAKEFEPKDVLPNGDGTYQ
GWITLAVPPGEEQRYTCQVEHPGLDQPLIVIW
>d1fzka1 b.1.1.2 (A:182-274) Class I MHC, beta2-microglobulin and alpha-3 domain {Mouse (Mus musculus), H-2KB}
TDSPKAHVTHHSRPEDKVTLRCWALGFYPADITLTWQLNGEELIQDMELVETRPAGDGTFQK
WASVVVPLGKEQYYTCHVYHQGLPEPLTLRW
>d1fzkb1 b.1.1.2 (B:) Class I MHC, beta2-microglobulin and alpha-3 domain {Mouse (Mus musculus), H-2KB}
IQKTPQIQVYSRHPPENGKPNILNCYVTQFHPPHIEIQMLKNGKKIPKVEMSDMSFSKDWSF
YILAHTEFTPTETDTYACRVKHDSMAEPKTVYWDRDM
>d1jpfa1 b.1.1.2 (A:182-276) Class I MHC, beta2-microglobulin and alpha-3 domain {Mouse (Mus musculus), H-2DB}
TDSPKAHVTHHPRSKGEVTLRCWALGFYPADITLTWQLNGEELTQDMELVETRPAGDGTFQK
WASVVVPLGKEQNYTCRVYHEGLPEPLTLRWEP
>d1mhca1 b.1.1.2 (A:182-276) Class I MHC, beta2-microglobulin and alpha-3 domain {Mouse (Mus musculus), H-2M3}
ADPPKAHVAHHPRPKGDVTLRCWALGFYPADITLTWQKDEEDLTQDMELVETRPSGDGTFQK
WAAVVVPSGEEQRYTCYVHHEGLTEPLALKWRS
>d1qo3a1 b.1.1.2 (A:182-275) Class I MHC, beta2-microglobulin and alpha-3 domain {Mouse (Mus musculus), H-2DD}
TDPPKAHVTHHRRPEGDVTLRCWALGFYPADITLTWQLNGEELTQEMELVETRPAGDGTFQK
WASVVVPLGKEQKYTCHVEHEGLPEPLTLRWG
>d1k8da1 b.1.1.2 (A:182-274) Class I MHC, beta2-microglobulin and alpha-3 domain {Mouse (Mus musculus), IB QA-2}
TDPPKAHVTHHPRSYGAVTLRCWALGFYPADITLTWQLNGEELTQDMELVETRPAGDGTFQK
WASVVVPLGKEQNYTCHVNHEGLPEPLTLRW
>d1ed3a1 b.1.1.2 (A:182-275) Class I MHC, beta2-microglobulin and alpha-3 domain {Rat (Rattus norvegicus), RT1-AA}
SDPPEAHVTLHPRPEGDVTLRCWALGFYPADITLTWQLNGEDLTQDMELVETRPAGDGTFQK
WASVVVPLGKEQNYTCRVEHEGLPKPLSQRWE
>d1zaga1 b.1.1.2 (A:184-277) Zinc-alpha-2-glycoprotein, ZAG {Human (Homo sapiens)}
QDPPSVVVTSHQAPGEKKKLKCLAYDFYPGKIDVHWTRAGEVQEPELRGDVLHNGNGTYQSW
VVVAVPPQDTAPYSCHVQHSSLAQPLVVPWEA
>d1hyrc1 b.1.1.2 (C:181-274) MHC I homolog {Human (Homo sapiens), Mic-a}
TVPPMVNVTRSEASEGNITVTCRASGFYPWNITLSWRQDGVSLSHDTQQWGDVLPDGNGTYQ
TWVATRICQGEEQRFTCYMEHSGNHSTHPVPS
>d1c16a1 b.1.1.2 (A:181-276) MHC I homolog {Mouse (Mus musculus), t22}
RSDPPKAHVTRHPRPEGDVTLRCWALGFYPADITLTWQLNGEELTQDMELVETRPAGDGTFQ
KWAAVVVPLGKEQSYTCHVYHEGLPEPLILRWGG
>d1exua1 b.1.1.2 (A:177-267) MHC-related Fc receptor {Human (Homo sapiens)}

KEPPSMRLKARPSSPGFSVLTCSAFSFYPPELQLRFLRNGLAAGTGQGDFGPNSDGSFHASS
SLTVKSGDEHHYCCIVQHAGLAQPLRVEL
>d1igtb3 b.1.1.2 (B:236-361) Immunoglobulin (constant domains of L and H chains) {Intact IgG2a antibody Mab231 (mouse), kappa L chain}
PCPPCKCPAPNLLGGPSVFIFPPKIKDVLMISLSPIVTCVVVDVSEDDPDVQISWFVNNVEV
HTAQTQTHREDYNSTLRVVSALPIQHQDWMSGKEFKCKVNNKDLPAPIERTISKPKG
>d1igtb4 b.1.1.2 (B:363-474) Immunoglobulin (constant domains of L and H chains) {Intact IgG2a antibody Mab231 (mouse), kappa L chain}
SVRAPQVYVLPPPEEEMTKKQVTLTCMVTDFMPEDIYVEWTNNGKTELNYKNTEPVLDSDGS
YFMYSKLRVEKKNWVERNSYSCSVVHEGLHNHHTTKSFSR
>d1igyb3 b.1.1.2 (B:236-361) Immunoglobulin (constant domains of L and H chains) {Intact IgG1 antibody Mab61.1.3 (mouse), kappa L chain}
GCKPCICTVPEVSSVFIFPPKPKDTLLITVTPKVTCVVVDISKDDPEVQFSWFVDNVEVHTA
QTQPREEQFNSTFRVVSALPIMHQDWLNGKEFKCRVNSAAFPAPIEKTISKTKG
>d1igyb4 b.1.1.2 (B:363-474) Immunoglobulin (constant domains of L and H chains) {Intact IgG1 antibody Mab61.1.3 (mouse), kappa L chain}
KPRAPQVYTIPPPKEQMAKDKVSLTCMITDFFPEDITVEWQSDGQAPENYKNTQPIMDTDGS
YFVYSKLNVQKSNWEAGNTFTCSVLHEGLHNHHTEKSLSH
>d1hzhh3 b.1.1.2 (H:236-359) Immunoglobulin (constant domains of L and H chains) {Intact IgG B12 antibody (human), kappa L chain}
THTCPPCPAPELLGGPSVFLFPPKPKDTLMISRTPEVTCVVVDVSHEDPEVKFNWYVDGVEV
HNAKTKPREEQYNSTYRVVSVLTVLHQDWLNGKEYKCKVSNKALPAPIEKTISKA
>d8faba2 b.1.1.2 (A:106-208) Immunoglobulin (constant domains of L and H chains) {Fab HIL (human), lambda L chain}
LGQPKAAPSVTLFPPSSEELQANKATLVCLISDFYPGAVTVAWKADSSPIKAGVETTTPSKQ
SNNKYAASSYLSLTPEQWKSHRSYSCQVTHEGSTVEKTVAP
>d1dn0b2 b.1.1.2 (B:121-225) Immunoglobulin (constant domains of L and H chains) {Fab Kau cold agglutinin (human) IgM, kappa L chain}
GSASAPTLFPLVSCENSPSDTSSVAVGCLAQDFLPDSITFSWKYKNNSDISSTRGFPSVLRG
GKYAATSQVLLPSKDVMAGTDEHVVCKVQHPNGNKEKNVPLPV
>d2fb4l2 b.1.1.2 (L:110-214) Immunoglobulin (constant domains of L and H chains) {Fab KOL (human), lambda L chain}
QPKANPTVTLFPPSSEELQANKATLVCLISDFYPGAVTVAWKADGSPVKAGVETTKPSKQSN
NKYAASSYLSLTPEQWKSHRSYSCQVTHEGSTVEKTVAPTECS
>d2ig2h2 b.1.1.2 (H:120-231) Immunoglobulin (constant domains of L and H chains) {Fab KOL (human), lambda L chain}
STKGPSVFPLAPSSKSTSGGTAALGCLVKDYFPQPVTVSWNSGALTSGVHTFPAVLQSSGLY
SLSSVVTVPSSSLGTQTYICNVNHKPSNTKVDKRVEPKSCDKTHTCPPCP
>d2fbjh2 b.1.1.2 (H:119-220) Immunoglobulin (constant domains of L and H chains) {Fab J539 (mouse), kappa L chain}
ESARNPTIYPLTLPPALSSDPVIIGCLIHDYFPSGTMNVTWGKSGKDITTVNFPPALASGGR
YTMSNQLTLPAVECPEGESVKCSVQHDSNPVQELDVNCSG

>d1mfbl2 b.1.1.2 (L:112-212) Immunoglobulin (constant domains of L and H chains) {Fab SE155-4 (mouse), lambda L chain}
PKSSPSVTLFPPSSEELETNKATLVCTITDFYPGVVTVDWKVDGTPVTQGMETTQPSKQSNN
KYMASSYLTLTARAWERHSSYSCQVTHEGHTVEKSLSRA
>d1teth2 b.1.1.2 (H:113-213) Immunoglobulin (constant domains of L and H chains) {Fab TE33 (mouse), kappa L chain}
SAKTTPPSVYPLAPGSMVTLGCLVKGYFPEPVTVTWNSGSLSSGVHTFPAVLQSDLYTLSSS
VTVPSSPRPSETVTCNVAHPASSTKVDKKIVPR
>d2jelh2 b.1.1.2 (H:114-226) Immunoglobulin (constant domains of L and H chains) {Fab JE142 (mouse), kappa L chain}
AATTPPSVYPLAPGSGGQGNSMVTLGCLVKGYFPEPVTVTWNSGSLSSGVHTFPAVLAADLY
TLSSSVTVPSSPRPSETVTCNVAHPASSTKVDKKIAPG
>d2jell2 b.1.1.2 (L:109-212) Immunoglobulin (constant domains of L and H chains) {Fab JE142 (mouse), kappa L chain}
ADAAPTVSIFPPSSEQLTSGGASVVCFLNNFYPKDINVKWKIGDGARQNGVLNSWTDQDSKD
STYSMSSTLTLTKDEYERHNSYTCEATHKTSDSPIVKSFNRN
>d1eapb2 b.1.1.2 (B:125-221) Immunoglobulin (constant domains of L and H chains) {Fab 17E8 (mouse), kappa L chain}
AKTTPPSVYPLAPGCGDTTGSSVTLGCLVKGYFPESVTVTWNSGGLSSSVHTFPALLQSGLY
TMSSSVTVPGGGWPSATVTCSVAHPASSTTVDKKL
>d1yuhb2 b.1.1.2 (B:119-218) Immunoglobulin (constant domains of L and H chains) {Fab anti-nitrophenol (mouse/human), lambda L chain}
AATTPPSVYPLAPGSAAQTNSMVTLGCLVKGYFPEPVTVTWNSGALSSGVHTFPAVLQSDLY
TLSSSVTVPASTWPSGTVTCNVAHPASSTAVDKKIVPR
>d1clzh2 b.1.1.2 (H:115-231) Immunoglobulin (constant domains of L and H chains) {Fab MBR96 (mouse), kappa L chain}
TTTAPSVYPLVPGCSDTSGSSVTLGCLVKGYFPEPVTVKWNYGALSSGVRTVSSVLQSGFYS
LSSLVTVPSSTWPSQTVICNVAHPASKTELIKRIEPR
>d1nldh2 b.1.1.2 (H:113-215) Immunoglobulin (constant domains of L and H chains) {Fab 1583, against an epitope of gp41 of HIV-1, (mouse), kappa L chain}
SASTTAPSVYPLAPVSGDQTNSSVTLGCLVKGYFPEPVTLTWNSGSLSSGVHTFPAVLQSDL
YTLSSSVTVTSSPWPSETITCNVAHPASSTKVDKKIEPRGC
>d1kelh2 b.1.1.2 (H:116-218) Immunoglobulin (constant domains of L and H chains) {Fab 28B4 (mouse), kappa L chain}
TVSSAKTTPPSVYPLAPGSAAQTNSMVTLGCLVKGYFPEPVTVTWNSGSLSSGVHTFPAVLQ
SDLYTLSSSVTVPSSPRPSETVTCNVAHPASSTKVDKKIVP
>d1osph2 b.1.1.2 (H:121-218) Immunoglobulin (constant domains of L and H chains) {Fab 184.1 (mouse), kappa L chain}
AKTTPPSVYPLAPGCGDTTGSSVTLGCLVKGYFPESVTVTWNSGSLSSSVHTFPALLQSGLY
TMSSSVTVPSSTWPSQTVTCSVAHPASSTTVDKKLE
>d1nfde2 b.1.1.2 (E:108-215) Immunoglobulin (constant domains of L and H chains) {Fab H57 (hamster), lambda L chain}
GPKSSPKVTVFPPSPEELRTNKATLVCLVNDFYPGSATVTWKANGATINDGVKTTKPSKQGQ

NYMTSSYLSLTADQWKSHNRVSCQVTHEGETVEKSLSPAECL
>d1nfdf2 b.1.1.2 (F:115-228) Immunoglobulin (constant domains of L and H chains) {Fab H57 (hamster), lambda L chain}
TTTAPSVYPLAPACDSTTSTTDTVTLGCLVKGYFPEPVTVSWNSGALTSGVHTFPSVLHSGL
YSLSSSVTVPSSTWPKQPITCNVAHPASSTKVDKKIEPR
>d1aqkl2 b.1.1.2 (L:112-216) Immunoglobulin (constant domains of L and H chains) {Fab B7-15A2 (human), lambda L chain}
QPKAAPSVTLFPPSSEELQANKATLVCLISDFYPGAVTVAWKADSSPVNAGVETTKPSKQSN
NKYAASSYLSLTPEQWKSHKSYSCQVTHEGSTVEKTVAPAECS
>d1a4kh2 b.1.1.2 (H:120-211) Immunoglobulin (constant domains of L and H chains) {Diels alder catalytic Fab (mouse), kappa L chain}
SVFPLAPSSKSTSGGTAALGCLVKDYFPEPVTVSWNSGALTSGVHTFPAVLQSSGLYSLSSV
VTVPSSSLGTQTYICNVNHKPSNTKVDKKV
>d1c1eh2 b.1.1.2 (H:120-228) Immunoglobulin (constant domains of L and H chains) {Diels alder catalytic Fab 1E9 (mouse), kappa L chain}
SVYPLAPGSAAQTNSMVTLGCLVKGYFPEPVTVTWNSGSLSSGVHTFPAVLQSDLYTLSSSV
TVPSSPRPSETVTCNVAHPASSTKVDKKIVPR
>d2hmic2 b.1.1.2 (C:108-214) Immunoglobulin (constant domains of L and H chains) {Fab 28 against HIV-1 RT (mouse), kappa L chain}
RADAAPTVSIFPPSSEQLTSGGASVVCFLNNFYPKDINVAWAIDGSAAANGVLNSWTDQDSK
DSTYSMSSTLTLTADEYEAANSYTCAATHKTSTSPIVKSFNANEC
>d1a5fh2 b.1.1.2 (H:121-217) Immunoglobulin (constant domains of L and H chains) {Anti-E-selectin Fab (mouse), kappa L chain}
AKTTPPSVYPLAPGSAAQTNSMVTLGCLVKGYFPEPVTVTWNSGSLSSGVHTFPAVLQSDLY
TLSSSVSVPTSTETVTCNVAHAPSSTKVDKKIVPR
>d1bfoa2 b.1.1.2 (A:108-214) Immunoglobulin (constant domains of L and H chains) {CAMPATH-1G igg2b monoclonal fab (rat), kappa L chain}
RANAAPTVSIFPPSTEQLATGGASVVCLMNKFYPRDISVKWKIDGTERNGVLNSVTDQDSAD
STYSMSSTLSLTKADYQSHNLYTCQVVHKTSSSPVVAKNFNRNEC
>d1bogb2 b.1.1.2 (B:113-213) Immunoglobulin (constant domains of L and H chains) {Anti-p24 (HIV-1) Fab CB 4-1 (mouse), kappa L chain}
AKTTAPSVYPLVPVCGGTTGSSVTLGCLVKGYFPEPVTLTWNSGSLSSGVHTFPALLQSGLY
TLSSSVTVTSNTWPSQTITCNVAHPASSTKVDKKIEPRV
>d1wejh2 b.1.1.2 (H:113-223) Immunoglobulin (constant domains of L and H chains) {Anti-cytochrome c Fab E8, (mouse), kappa L chain}
LTVSSAETTPPSVYPLAPGTAALKSSMVTLGCLVKGYFPEPVTVTWNSGSLSSGVHTFPAVL
QSDLYTLTSSVTVPSSTWPSQTVTCNVAHPASSTKVDKKIVPRNCGGDC
>d1sm3h2 b.1.1.2 (H:114-213) Immunoglobulin (constant domains of L and H chains) {Tumor-specific Fab SM3, (mouse), lambda L chain}
AKTTPPTVYPLAPGSNAASQSMVTLGCLVKGYFPEPVTVTWNSGSLASGVHTFPAVLQSDLY
TLSSSVTVPSSTWPSETVTCNVAHPASSTKVDAKIVPR
>d1c12b2 b.1.1.2 (B:414-513) Immunoglobulin (constant domains of

L and H chains) {Fab directed agains the musk odorant traseolide, (mouse), kappa L chain}
ASTKGPSVYPLAPGSKAAASMVTLGCLVKGYFPEPVTVTWNSGSLSSGVHTFPAVLQSDLYT
LSSSVTVPSSPRPSETVTCNVAHPASSTKVDKKIVPE
>d1r24b2 b.1.1.2 (B:123-217) Immunoglobulin (constant domains of L and H chains) {Fab R24, (mouse), kappa L chain}
ATTTAPSVYPLVPGCSDTSGSSVTLGCLVKGYFPGPVTVKWNYGALSSGVRTVSSVLQSGFY
SLSSLVTVPSSTWPSQTVICNVAHPASKTDLIK
>d1c5da2 b.1.1.2 (A:107-213) Immunoglobulin (constant domains of L and H chains) {Fab against the main immunogenic region of the human muscle acetylcholine receptor, (rat), kappa L chain}
RADAAPTVSIFPPSTEQLATGGASVVCLMNNFYPRDISVKWKIDGTERRDGVLDSVTDQDSK
DSTYSMSSTLSLTKADYESHNLYTCEVVHKTSSSPVVKSFNRNEC
>d1c5db2 b.1.1.2 (B:118-215) Immunoglobulin (constant domains of L and H chains) {Fab against the main immunogenic region of the human muscle acetylcholine receptor, (rat), kappa L chain}
AQTTAPSVYPLAPGCGDTTSSTVTLGCLVKGYFPEPVTVTWNSGALSSDVHTFPAVLQSGLY
TLTSSVTSSTWPSQTVTCNVAHPASSTKVDKKLERR
>d32c2b2 b.1.1.2 (B:120-218) Immunoglobulin (constant domains of L and H chains) {Fab 32C2 against P450-arom, (mouse), kappa L chain}
AKTTPPPVYPLVPGSLAQTNSMVTLGCLVKGYFPEPVTVTWNSGSLSSGVHTFPAVLQSDLY
TLSSSVTVPSSTWPSETVTCNVAHPASSTKVDKKIEP
>d1deeb2 b.1.1.2 (B:622-723) Immunoglobulin (constant domains of L and H chains) {Fab of human IgM RF 2A2}
GSASAPTLFPLVSCENSNPSSTVAVGCLAQDFLPDSITFSWKYKNNSDISSTRGFPSVLRGG
KYAATSQVLLPSKDVAQGTNEHVVCKVQHPNGNKEKDVPL
>d1f3dh2 b.1.1.2 (H:122-223) Immunoglobulin (constant domains of L and H chains) {Catalytic Fab 4B2, (mouse), kappa L chain}
AKTTPPSVYPLAPGSAAQTNSMVTLGCLVKGYFPEPVTVTWNSGSLSSGVHTFPAVLQSDLY
TLSSSVTVPSSTWPSETVTCNVAHPASSTKVDKKIVPRDC
>d1fh5h2 b.1.1.2 (H:121-215) Immunoglobulin (constant domains of L and H chains) {Fab MAK33, (human), kappa L chain}
AKTTPPSVYPLAVTLGCLVKGYFPEPVTVTWNSGSLSSGVHTFPAVLQSDLYTLSSSVTVTS
STWPSETVTCNVAHPASSTKVDKKIVPR
>d1c5ch2 b.1.1.2 (H:114-230) Immunoglobulin (constant domains of L and H chains) {Decarboxylase catalytic Fab 21D8, chimeric (mouse V domains/human C1 domains)}
ASTKGPSVFPLAPSSKSTSGGTAALGCLVKDYFPEPVTVSWNSGALTSGVHTFPAVLQSSGL
YSLSSVVTVPSSSLGTQTYICNVNHKPSNTKVDKKVEPKSC
>d1c5cl2 b.1.1.2 (L:108-214) Immunoglobulin (constant domains of L and H chains) {Decarboxylase catalytic Fab 21D8, chimeric (mouse V domains/human C1 domains)}
RTVAAPSVFIFPPSDEQLKSGTASVVCLLNNFYPREAKVQWKVDNALQSGNSQESVTEQDSK
DSTYSLSSTLTLSKADYEKHKVYACEVTHQGLSSPVTKSFNRGEC

>d1fe8h2 b.1.1.2 (H:116-216) Immunoglobulin (constant domains of L and H chains) {Fab RU5, (mouse), kappa L chain}
AETTAPSVYKLEPVSSVTLGCLVKGYFPEPVTLTWNSGSLSSGVHTFPAVLQSDLYTLSSSV
TVTSSTWPSQSITCNVAHPASSTKVDKKIEPRG
>d1iqdb2 b.1.1.2 (B:115-212) Immunoglobulin (constant domains of L and H chains) {Fab BO2C11 against the C2 domain of factor VIII, (human), kappa L chain}
ASTKGPSVFPLAPCSRSTSESTAALGCLVKDYFPEPVTVSWNSGALTSGVHTFPAVLQSSGL
YSLSSVVTVPSSSLGTATYTCNVDHKPSNTKVDKRV
>d1fn4b2 b.1.1.2 (B:107-208) Immunoglobulin (constant domains of L and H chains) {Fab 198 against actylcholine receptor, (rat)}
TMVTVSSVFPLAPGSAAQTNSMVTLGCLVKGYFPEPVTVTWNSGALSSGVHTFPAVLQSGLY
TLTSSVTVPSSTWSSQAVTCNVAHPASSTKVDKKIVPRDC
>d1jguh2 b.1.1.2 (H:114-212) Immunoglobulin (constant domains of L and H chains) {Catalytic Fab 1D4, (mouse), kappa L chain}
AKTTAPSVYPLAPVCGDTTGSSVTLGCLVKGYFPEPVTLTWNSGSLSSGVHTFPAVLQSDLY
TLSSSVTVTSSTWPSQSITCNVAHPASSTKVDKKIEP
>d1jgul2 b.1.1.2 (L:108-214) Immunoglobulin (constant domains of L and H chains) {Catalytic Fab 1D4, (mouse), kappa L chain}
RADAAPTVSIFPPSSEQLTSGGASVVCFLNNFYPKDINVKWKIDGSERQNGVLNSWTDQDSK
DSTYSMSSTLTLTKDEYERHNSYTCEATHKTSTSPIVKSFNRNEC
>d1mcoh2 b.1.1.2 (H:118-219) Immunoglobulin (constant domains of L and H chains) {Intact antibody (lambda) MCG (human)}
ASTKGPSVFPLAPSSKSTSGGTAALGCLVKDYFPQPVTVSWNSGALTSGVHTFPAVLQSSGL
YSLSSVVTVPSSSLGTQTYICNVNHKPSNTKVDKRVAPEL
>d1adqa1 b.1.1.2 (A:238-341) Immunoglobulin (constant domains of L and H chains) {Fc (human) IgG1 class}
PSVFLFPPKPKDTLMISRTPEVTCVVVDVSQEDPQVQFNWYVDGVQVHNAKTKPREQQFNST
YRVVSVLTVLHQNWLDGKEYKCKVSNKGLPSSIEKTISKAKG
>d1dn2a1 b.1.1.2 (A:237-341) Immunoglobulin (constant domains of L and H chains) {Fc (human) IgG1 class}
GPSVFLFPPKPKDTLMISRTPEVTCVVVDVSHENPEVKFNWYVDGVEVHNAKTKPREEQYNS
TYRVVSVLTVLHQDWLNGKEYKCKVSNKALPAPIEKTISKAKG
>d1dn2a2 b.1.1.2 (A:342-443) Immunoglobulin (constant domains of L and H chains) {Fc (human) IgG1 class}
QPREPQVYTLPPSREEMTKNQVSLTCLVKGFYPSDIAVEWESNGQPENNYKTTPPVLDSDGS
FFLYSKLTVDKSRWQQGNVFSCSVMHEALHNHYTQKSLSL
>d1fc2d1 b.1.1.2 (D:238-341) Immunoglobulin (constant domains of L and H chains) {Fc (human) IgG1 class}
PSVFLFPPKPKDTLMISRTPEVTCVVVDVSHEDPQVKFNWYVDGVQVHNAKTKPREQQYNST
YRVVSVLTVLHQNWLDGKEYKCKVSNKALPAPIEKTISKAKG
>d1fp5a1 b.1.1.2 (A:336-438) Immunoglobulin (constant domains of L and H chains) {Fc (human) IgE}
VSAYLSRPSPFDLFIRKSPTITCLVVDLAPSKGTVNLTWSRASGKPVNHSTRKEEKQRNGTL

TVTSTLPVGTRDWIEGETYQCRVTHPHLPRALMRSTTKTSG
>d1fp5a2 b.1.1.2 (A:439-543) Immunoglobulin (constant domains of L and H chains) {Fc (human) IgE}
PRAAPEVYAFATPEWPGSRDKRTLACLIQNFMPEDISVQWLHNEVQLPDARHSTTQPRKTKG
SGFFVFSRLEVTRAEWEQKDEFICRAVHEAASPSQTVQRAVSV
>d1i1ca1 b.1.1.2 (A:239-341) Immunoglobulin (constant domains of L and H chains) {Fc (rat) IgG}
SVFIFPPKTKDVLGGGLTPKVTCVVVDISQNDPEVRFSWFIDDVEVHTAQTHAPEKQSNSTL
RSVSELPIVERDWLNGKTFKCKVNSGAFPAPIEKSISKPEG
>d1i1ca2 b.1.1.2 (A:342-443) Immunoglobulin (constant domains of L and H chains) {Fc (rat) IgG}
TPRGPQVYTMAPPKEEMTQSQVSITCMVKGFYPPDIYTEWKMNGQPQENYKNTPPTMDTDGS
YFLYSKLNVKKETWQQGNTFTCSVLHEGLENEHTEKSLSH
>d1pfc__ b.1.1.2 (-) Immunoglobulin (constant domains of L and H chains) {Fc (guinea pig)}
RTISKAKGPPRIPEVYLLPPPRNELSKKKVSLTCMITGFYPADINVEWDSSEPSDYKNTPPV
FDTDGSFFLYSRLKVDTDAWNNGESFTCSVMHEALPNHVIQKSISRSPG
>d1cqka_ b.1.1.2 (A:) Immunoglobulin (constant domains of L and H chains) {Fc MAK33 (mouse)}
PAAPQVYTIPPPLEQMAKDLVSLTCMITDFFPEDITVEWQWNGQPAENYKNTQPIMDTDGSY
FVYSKLNVQKSNWEAGNTFTCSVLHEGLHNHHTEKSLSH
>d1g84a_ b.1.1.2 (A:) Immunoglobulin (constant domains of L and H chains) {C epsilon2 domain from IgE (human)}
SRDFTPPTVKILQSSSDGGGHFPPTIQLLCLVSGYTPGTINITWLEDGQVMDVDLSTASTTQ
EGELASTQSELTLSQKHWLSDRTYTCQVTYQGHTFEDSTKKSA
>d1tcra2 b.1.1.2 (A:118-213) T-cell antigen receptor {Mouse (Mus musculus), alpha-chain}
IQNPEPAVYALKDPRSQDSTLCLFTDFDSQINVPKTMESGTFITDATVLDMKAMDSKSNGAI
AWSNQTSFTCQDIFKETNATYPSSDVPC
>d1bd2d2 b.1.1.2 (D:118-203) T-cell antigen receptor {Human (Homo sapiens), alpha-chain}
IQNPDPAVYQLRDSKSSDKSVCLFTDFDSQTNVSQSKDSDVYITDKTVLDMRSMDFKSNSAV
AWSNKSDFACANAFNNSIIPEDTF
>d1bec_2 b.1.1.2 (118-246) T-cell antigen receptor {Mouse (Mus musculus), beta-chain}
DLRQVTPPKVSLFEPSKAEIANKQKATLVCLARGFFPDHVELSWWVNGKEVHSGVSTDPQAY
KESNYSYCLSSRLRVSATFWHNPRNHFRCQVQFHGLSEEDKWPEGSPKPVTQNISAEAWGRA
D
>d1bd2e2 b.1.1.2 (E:119-247) T-cell antigen receptor {Human (Homo sapiens), beta-chain}
DLKNVFPPEVAVFEPSEAEISHTQKATLVCLATGFYPDHVELSWWVNGKEVHSGVSTDPQPL
KEQPALNDSRYALSSRLRVSATFWQDPRNHFRCQVQFYGLSENDEWTQDRAKPVTQIVSAEA
WGRAD
>d1hxma2 b.1.1.2 (A:121-206) T-cell antigen receptor {Human (Homo

sapiens), gamma-chain}
SQPHTKPSVFVMKNGTNVACLVKEFYPKDIRINLVSSKKITEFDPAIVISPSGKYNAVKLGK
YEDSNSVTCSVQHDNKTVHSTDFE
>d1hxmb2 b.1.1.2 (B:124-230) T-cell antigen receptor {Human (Homo sapiens), delta-chain}
KQLDADVSPKPTIFLPSIAETKLQKAGTYLCLLEKFFPDVIKIHWEEKKSNTILGSQEGNTM
KTNDTYMKFSWLTVPEKSLDKEHRCIVRHENNKNGVDQEIIFPPI
>d1cd1a1 b.1.1.2 (A:186-279) CD1, beta2-microglobulin and alpha-3 domain {Mouse (Mus musculus)}
QEKPVAWLSSVPSSAHGHRQLVCHVSGFYPKPVWVMWMRGDQEQQGTHRGDFLPNADETWYL
QATLDVEAGEEAGLACRVKHSSLGGQDIILYW
>d1hdma1 b.1.1.2 (A:94-196) Class II MHC, C-terminal domains of alpha and beta chains {Human (Homo sapiens), HLA-DM}
SRGFPIAEVFTLKPLEFGKPNTLVCFVSNLFPPMLTVNWHDHSVPVEGFGPTFVSAVDGLSF
QAFSYLNFTPEPSDIFSCIVTHEPDRYTAIAYWVPRNALPS
>d1hdmb1 b.1.1.2 (B:88-185) Class II MHC, C-terminal domains of alpha and beta chains {Human (Homo sapiens), HLA-DM}
TRPPSVQVAKTTPFNTREPVMLACYVWGFYPAEVTITWRKNGKLVMHSSAHKTAQPNGDWTY
QTLSHLALTPSYGDTYTCVVEHIGAPEPILRDWTPG
>d1aqdb1 b.1.1.2 (B:93-190) Class II MHC, C-terminal domains of alpha and beta chains {Human (Homo sapiens), HLA-DR1}
RRVEPKVTVYPSKTQPLQHHNLLVCSVSGFYPGSIEVRWFRNGQEEKAGVVSTGLIQNGDWT
FQTLVMLETVPRSGEVYTCQVEHPSVTSPLTVEWRA
>d1fv1a1 b.1.1.2 (A:82-181) Class II MHC, C-terminal domains of alpha and beta chains {Human (Homo sapiens), HLA-DR2}
ITNVPPEVTVLTNSPVELREPNVLICFIDKFTPPVVNVTWLRNGKPVTTGVSETVFLPREDH
LFRKFHYLPFLPSTEDVYDCRVEHWGLDEPLLKHWEFD
>d1fv1b1 b.1.1.2 (B:93-190) Class II MHC, C-terminal domains of alpha and beta chains {Human (Homo sapiens), HLA-DR2}
RRVEPKVTVYPARTQTLQHHNLLVCSVNGFYPGSIEVRWFRNSQEEKAGVVSTGLIQNGDWT
FQTLVMLETVPRSGEVYTCQVEHPSVTSPLTVEWRA
>d1d5zb1 b.1.1.2 (B:93-190) Class II MHC, C-terminal domains of alpha and beta chains {Human (Homo sapiens), HLA-DR4}
RRVYPEVTVYPAKTQPLQHHNLLVCSVNGFYPGSIEVRWFRNGQEEKTGVVSTGLIQNGDWT
FQTLVMLETVPRSGEVYTCQVEHPSLTSPLTVEWRA
>d1jk8a1 b.1.1.2 (A:85-181) Class II MHC, C-terminal domains of alpha and beta chains {Human (Homo sapiens), HLA-DQ8}
EVPEVTVFSKSPVTLGQPNTLICLVDNIFPPVVNITWLSNGHSVTEGVSETSFLSKSDHSFF
KISYLTFLPSDDEIYDCKVEHWGLDEPLLKHWEPE
>d1jk8b1 b.1.1.2 (B:95-192) Class II MHC, C-terminal domains of alpha and beta chains {Human (Homo sapiens), HLA-DQ8}
VEPTVTISPSRTEALNHHNLLVCSVTDFYPAQIKVRWFRNDQEETTGVVSTPLIRNGDWTFQ
ILVMLEMTPQRGDVYTCHVEHPSLQNPIIVEWRAQS
>d1iaka1 b.1.1.2 (A:82-181) Class II MHC, C-terminal domains of

alpha and beta chains {Mouse (Mus musculus), I-AK}
ATNEAPQATVFPKSPVLLGQPNTLICFVDNIFPPVINITWLRNSKSVTDGVYETSFFVNRDY
SFHKLSYLTFIPSDDDIYDCKVEHWGLEEPVLKHWEPE
>d1iakb1 b.1.1.2 (B:93-190) Class II MHC, C-terminal domains of alpha and beta chains {Mouse (Mus musculus), I-AK}
RLEQPSVVISLSRTEALNHHNTLVCSVTDFYPAKIKVRWFRNGQEETVGVSSTQLIRNGDWT
FQVLVMLEMTPRRGEVYTCHVEHPSLTSPITVEWRA
>d1fnga1 b.1.1.2 (A:82-182) Class II MHC, C-terminal domains of alpha and beta chains {Mouse (Mus musculus), I-EK}
DANVAPEVTVLSRSPVNLGEPNILICFIDKFSPPVVNVTWLRNGRPVTEGVSETVFLPRDDH
LFRKFHYLTFLPSTDDFYDCEVDHWGLEEPLRKHWEFEE
>d1fngb1 b.1.1.2 (B:93-188) Class II MHC, C-terminal domains of alpha and beta chains {Mouse (Mus musculus), I-EK}
RRVEPTVTVYPTKTQPLEHHNLLVCSVSDFYPGNIEVRWFRNGKEEKTGIVSTGLVRNGDWT
FQTLVMLETVPQSGEVYTCQVEHPSLTDPVTVEW
>d2iada1 b.1.1.2 (A:83-186) Class II MHC, C-terminal domains of alpha and beta chains {Mouse (Mus musculus), I-AD}
TNEAPQATVFPKSPVLLGQPNTLICFVDNIFPPVINITWLRNSKSVTDGVYETSFLVNRDHS
FHKLSYLTFIPSDDDIYDCKVEHWGLEEPVLKHWEPEISSADLVPR
>d1es0a1 b.1.1.2 (A:83-180) Class II MHC, C-terminal domains of alpha and beta chains {Mouse (Mus musculus), I-A(G7)}
TNEAPQATVFPKSPVLLGQPNTLICFVDNIFPPVINITWLRNSKSVTDGVYETSFLVNRDHS
FHKLSYLTFIPSDDDIYDCKVEHWGLEEPVLKHWSS
>d1es0b1 b.1.1.2 (B:94-189) Class II MHC, C-terminal domains of alpha and beta chains {Mouse (Mus musculus), I-A(G7)}
RLEQPNVAISLSRTEALNHHNTLVCSVTDFYPAKIKVRWFRNGQEETVGVSSTQLIRNGDWT
FQVLVMLEMTPHQGEVYTCHVEHPSLKSPITVEWS
>d1k8ia1 b.1.1.2 (A:93-191) Class II MHC, C-terminal domains of alpha and beta chains {Mouse (Mus musculus), H2-DM}
VSRGLPVAEVFTLKPLEFGKPNTLVCFISNLFPPTLTVNWQLHSAPVEGASPTSISAVDGLT
FQAFSYLNFTPEPFDLYSCTVTHEIDRYTAIAYWVPQ
>d1k8ib1 b.1.1.2 (B:95-190) Class II MHC, C-terminal domains of alpha and beta chains {Mouse (Mus musculus), H2-DM}
APSVRVAQTTPFNTREPVMLACYVWGFYPADVTITWMKNGQLVPSHSNKEKTAQPNGDWTYQ
TVSYLALTPSYGDVYTCVVQHSGTSEPIRGDWTP
>d1vcaa1 b.1.1.3 (A:91-199) Second domain of vascular cell adhesion molecule-1 (VCAM-1) {Human (Homo sapiens)}
FPKDPEIHLSGPLEAGKPITVKCSVADVYPFDRLEIDLLKGDHLMKSQEFLEDADRKSLETK
SLEVTFTPVIEDIGKVLVCRAKLHIDEMDSVPTVRQAVKELQVYISP
>d1iam_1 b.1.1.3 (83-185) Second domain of intercellular cell adhesion molecule-1 (ICAM-1) {Human (Homo sapiens)}
YWTPERVELAPLPSWQPVGKQLTLRCQVEGGAPRAQLTVVLLRGEKELKREPAVGEPAEVTT
TVLVRRDHHGAQFSCRTELDLRPQGLELFENTSAPYQLQTF
>d1ic1a1 b.1.1.3 (A:83-190) Second domain of intercellular cell

adhesion molecule-1 (ICAM-1) {Human (Homo sapiens)}
YWTPERVELAPLPSWQPVGKNLTLRCQVEGGAPRANLTVVLLRGEKELKREPAVGEPAEVTT
TVLVRRDHHGANFSCRTELDLRPQGLELFENTSAPYQLQTFVLPAT
>d1zxq_1 b.1.1.3 (87-192) Second domain of intercellular cell adhesion molecule-2 (ICAM-2) {Human (Homo sapiens)}
PPRQVILTLQPTLVAVGKSFTIECRVPTVEPLDSLTLFLFRGNETLHYETFGKAAPAPQEAT
ATFNSTADREDGHRNFSCLAVLDLMSRGGNIFHKHSAPKMLEIY
>d1cdy_2 b.1.1.3 (98-178) CD4 {Human (Homo sapiens)}
FGLTANSDTHLLQGQSLTLTLESPPGSSPSVQCRSPRGKNIQGGKTLSVSQLELQDSGTWTC
TVLQNQKKVEFKIDIVVLA
>d1wioa4 b.1.1.3 (A:292-363) CD4 {Human (Homo sapiens)}
MRATQLQKNLTCEVWGPTSPKLMLSLKLENKEAKVSKREKAVWVLNPEAGMWQCLLSDSGQV
LLESNIKVLP
>d1cid_2 b.1.1.3 (106-177) CD4 {Rat (Rattus rattus)}
VMKVTQPDSNTLTCEVMGPTSPKMRLILKQENQEARVSRQEKVIQVQAPEAGVWQCLLSEGE
EVKMDSKIQV
>d1hnf_2 b.1.1.3 (105-182) CD2, second domain {Human (Homo sapiens)}
RVSKPKISWTCINTTLTCEVMNGTDPELNLYQDGKHLKLSQRVITHKWTTSLSAKFKCTAGN
KVSKESSVEPVSCPEK
>d1ccza2 b.1.1.3 (A:94-171) CD2-binding domain of CD58, second domain {Human (Homo sapiens)}
EMVSKPMIYWECSNATLTCEVLEGTDVELKLYQGKEHLRSLRQKTMSYQWTNLRAPFKCKAV
NRVSQESEMEVVNCPE
>d1dr9a2 b.1.1.3 (A:106-200) CD80, second domain {Human (Homo sapiens)}
ADFPTPSISDFEIPTSNIRRIICSTSGGFPEPHLSWLENGEELNAINTTVSQDPETELYAVS
SKLDFNMTTNHSFMCLIKYGHLRVNQTFNWNTA
>d1vcaa2 b.1.1.4 (A:1-90) N-terminal domain of vascular cell adhesion molecule-1 (VCAM-1) {Human (Homo sapiens)}
FKIETTPESRYLAQIGDSVSLTCSTTGCESPFFSWRTQIDSPLNGKVTNEGTTSTLTMNPVS
FGNEHSYLCTATCESRKLEKGIQVEIYS
>d1iam_2 b.1.1.4 (1-82) N-terminal domain of intracellular adhesion molecule-1, ICAM-1 {Human (Homo sapiens)}
QTSVSPSKVILPRGGSVLVTCSTSCDQPKLLGIETPLPKKELLLPGNNRKVYELSNVQEDSQ
PMCYSNCPDGQSTAKTFLTV
>d1zxq_2 b.1.1.4 (1-86) N-terminal domain of intracellular adhesion molecule-2, ICAM-2 {Human (Homo sapiens)}
KVFEVHVRPKKLAVEPKGSLEVNCSTTCNQPEVGGLETSLNKILLDEQAQWKHYLVSNISHD
TVLQCHFTCSGKQESMNSNVSVYQ
>d1epfa1 b.1.1.4 (A:1-97) Neural cell adhesion molecule (NCAM) {Rat (Rattus norvegicus)}
LQVDIVPSQGEISVGESKFFLCQVAGDAKDKDISWFSPNGEKLSPNQQRISVVWNDDDSSTL
TIYNANIDDAGIYKCVVTAEDGTQSEATVNVKIFQ

>d1epfa2 b.1.1.4 (A:98-189) Neural cell adhesion molecule (NCAM) {Rat (Rattus norvegicus)}
KLMFKNAPTPQEFKEGEDAVIVCDVVSSLPPTIIWKHKGRDVILKKDVRFIVLSNNYLQIRG
IKKTDEGTYRCEGRILARGEINFKDIQVIV
>d1ie5a_ b.1.1.4 (A:) Neural cell adhesion molecule (NCAM) {Chicken (Gallus gallus)}
GKDIQVIVNVPPSVRARQSTMNATANLSQSVTLACDADGFPEPTMTWTKDGEPIEQEDNEEK
YSFNYDGSELIIKKVDKSDEAEYICIAENKAGEQDATIHLKVFAK
>d1gsma1 b.1.1.4 (A:1-90) Mucosal addressin cell adhesion molecule-1 (MADCAM-1) {Human (Homo sapiens)}
VKPLQVEPPEPVVAVALGASRQLTCRLACADRGASVQWRGLDTSLGAVQSDTGRSVLTVRNA
SLSAAGTRVCVGSCGGRTFQHTVQLLVY
>d1gsma2 b.1.1.4 (A:91-206) Mucosal addressin cell adhesion molecule-1 (MADCAM-1) {Human (Homo sapiens)}
AFPNQLTVSPAALVPGDPEVACTAHKVTPVDPNALSFSLLVGGQELEGAQALGPEVQEEEEE
PQGDEDVLFRVTERWRLPPLGTPVPPALYCQATMRLPGLELSHRQAIPVLIEGR
>d1fhga_ b.1.1.4 (A:) Telokin {Turkey (Meleagris gallopavo)}
AEEKPHVKPYFTKTILDMEVVEGSAARFDCKVEGYPDPEVMWFKDDNPVKESRHFQIDYDEE
GNCSLTISEVCGDDDAKYTCKAVNSLGEATCTAELLVETM
>d1g1ca_ b.1.1.4 (A:) Titin {Human (Homo sapiens), different modules}
SMEAPKIFERIQSQTVGQGSDAHFRVRVVGKPDPECEWYKNGVKIERSDRIYWYWPEDNVCE
LVIRDVTGEDSASIMVKAINIAGETSSHAFLLVQAK
>d1nct__ b.1.1.4 (-) Titin {Human (Homo sapiens), different modules}
SKTTLAARILTKPRSMTVYEGESARFSCDTDGEPVPTVTWLRKGQVLSTSARHQVTTTKYKS
TFEISSVQASDEGNYSVVVENSEGKQEAEFTLTIQK
>d1koa_1 b.1.1.4 (6265-6361) Twitchin {Nematode (Caenorhabditis elegans)}
QPRFIVKPYGTEVGEGQSANFYCRVIASSPPVVTWHKDDRELKQSVKYMKRYNGNDYGLTIN
RVKGDDKGEYTVRAKNSYGTKEEIVFLNVTRHSEP
>d1wiu__ b.1.1.4 (-) Twitchin {Nematode (Caenorhabditis elegans)}
LKPKILTASRKIKIKAGFTHNLEVDFIGAPDPTATWTVGDSGAALAPELLVDAKSSTTSIFF
PSAKRADSGNYKLKVKNELGEDEAIFEVIVQ
>d1tiu__ b.1.1.4 (-) Twitchin {Human (Homo sapiens), Ig repeat 27}
LIEVEKPLYGVEVFVGETAHFEIELSEPDVHGQWKLKGQPLTASPDCEIIEDGKKHILILHN
CQLGMTGEVSFQAANAKSAANLKVKEL
>d1iray1 b.1.1.4 (Y:1-101) Type-1 interleukin-1 receptor {Human (Homo sapiens)}
DKCKEREEKIILVSSANEIDVRPCPLNPNEHKGTITWYKDDSKTPVSTEQASRIHQHKEKLW
FVPAKVEDSGHYYCVVRNSSYCLRIKISAKFVENEPNLC
>d1iray2 b.1.1.4 (Y:102-204) Type-1 interleukin-1 receptor {Human (Homo sapiens)}
YNAQAIFKQKLPVAGDGGLVCPYMEFFKNENNELPKLQWYKDCKPLLLDNIHFSGVKDRLIV

MNVAEKHRGNYTCHASYTYLGKQYPITRVIEFITLEENKPT
>d1iray3 b.1.1.4 (Y:205-311) Type-1 interleukin-1 receptor {Human (Homo sapiens)}
RPVIVSPANETMEVDLGSQIQLICNVTGQLSDIAYWKWNGSVIDEDDPVLGEDYYSVENPAN
KRRSTLITVLNISEIESRFYKHPFTCFAKNTHGIDAAYIQLIYPV
>d1cvsc1 b.1.1.4 (C:149-250) Fibroblast growth factor receptor, FGFR {Human (Homo sapiens), FGFR1}
MPVAPYWTSPEKMEKKLHAVPAAKTVKFKCPSSGTPQPTLRWLKNGKEFKPDHRIGGYKVRY
ATWSIIMDSVVPSDKGNYTCIVENEYGSINHTYQLDVVER
>d1cvsc2 b.1.1.4 (C:251-359) Fibroblast growth factor receptor, FGFR {Human (Homo sapiens), FGFR1}
SPHRPILQAGLPANKTVALGSNVEFMCKVYSDPQPHIQWLKHIEVNGSKIGPDNLPYVQILK
TAGVNTTDKEMEVLHLRNVSFEDAGEYTCLAGNSIGLSHHSAWLTVL
>d1ev2e1 b.1.1.4 (E:150-250) Fibroblast growth factor receptor, FGFR {Human (Homo sapiens), FGFR2}
NKRAPYWTNTEKMEKRLHAVPAANTVKFRCPAGGNPMPTMRWLKNGKEFKQEHRIGGYKVRN
QHWSLIMESVVPSDKGNYTCVVENEYGSINHTYHLDVVE
>d1ev2e2 b.1.1.4 (E:251-360) Fibroblast growth factor receptor, FGFR {Human (Homo sapiens), FGFR2}
RSPHRPILQAGLPANASTVVGGDVEFVCKVYSDAQPHIQWIKHVEKNGSKYGPDGLPYLKVL
KAAGVNTTDKEIEVLYIRNVTFEDAGEYTCLAGNSIGISFHSAWLTVL
>d1biha1 b.1.1.4 (A:5-98) Hemolin {Moth (Hyalophora cecropia)}
KYPVLKDQPAEVLFRENNPTVLECIIEGNDQGVKYSWKKDGKSYNWQEHNAALRKDEGSLVF
LRPQASDEGHYQCFAETPAGVASSRVISFRKT
>d1biha2 b.1.1.4 (A:99-209) Hemolin {Moth (Hyalophora cecropia)}
YLIASPAKTHEKTPIEGRPFQLDCVLPNAYPKPLITWKKRLSGADPNADVTDFDRRITAGPD
GNLYFTIVTKEDVSDIYKYVCTAKNAAVDEEVVLVEYEIKGVTKDNSGY
>d1biha3 b.1.1.4 (A:210-306) Hemolin {Moth (Hyalophora cecropia)}
KGEPVPQYVSKDMMAKAGDVTMIYCMYGSNPMGYPNYFKNGKDVNGNPEDRITRHNRTSGKR
LLFKTTLPEDEGVYTCEVDNGVGKPQKHSLKLTVV
>d1biha4 b.1.1.4 (A:307-395) Hemolin {Moth (Hyalophora cecropia)}
SAPKYEQKPEKVIVVKQGQDVTIPCKVTGLPAPNVVWSHNAKPLSGGRATVTDSGLVIKGVK
NGDKGYYGCRATNEHGDKYFETLVQVN
>d1cs6a1 b.1.1.4 (A:7-103) Axonin-1 {Ckicken (Gallus gallus)}
RSYGPVFEEQPAHTLFPEGSAEEKVTLTCRARANPPATYRWKMNGTELKMGPDSRYRLVAGD
LVISNPVKAKDAGSYQCVATNARGTVVSREASLRF
>d1cs6a2 b.1.1.4 (A:104-208) Axonin-1 {Ckicken (Gallus gallus)}
GFLQEFSAEERDPVKITEGWGVMFTCSPPPHYPALSYRWLLNEFPNFIPADGRRFVSQTTGN
LYIAKTEASDLGNYSCFATSHIDFITKSVFSKFSQLSLAAEDA
>d1cs6a3 b.1.1.4 (A:209-299) Axonin-1 {Ckicken (Gallus gallus)}
RQYAPSIKAKFPADTYALTGQMVTLECFAFGNPVPQIKWRKLDGSQTSKWLSSEPLLHIQNV
DFEDEGTYECEAENIKGRDTYQGRIIIHA
>d1cs6a4 b.1.1.4 (A:300-388) Axonin-1 {Ckicken (Gallus gallus)}
QPDWLDVITDTEADIGSDLRWSCVASGKPRPAVRWLRDGQPLASQNRIEVSGGELRFSKLVL

EDSGMYQCVAENKHGTVYASAELTVQA
>d1gl4b_ b.1.1.4 (B:) Perlecan Ig3 domain {Mouse (Mus musculus)}
PIMVTVEEQRSQSVRPGADVTFICTAKSKSPAYTLVWTRLHNGKLPSRAMDFNGILTIRNVQ
PSDAGTYVCTGSNMFAMDQGTATLHVQ
>d1f97a2 b.1.1.4 (A:129-238) Junction adhesion molecule, JAM, C-terminal domain {Mouse (Mus musculus)}
VPPSKPTISVPSSVTIGNRAVLTCSEHDGSPPSEYSWFKDGISMLTADAKKTRAFMNSSFTI
DPKSGDLIFDPVTAFDSGEYYCQAQNGYGTAMRSEAAHMDAVELNVGG
>d1fltx_ b.1.1.4 (X:) Second domain of the Flt-1 receptor {Human (Homo sapiens)}
GRPFVEMYSEIPEIIHMTEGRELVIPCRVTSPNITVTLKKFPLDTLIPDGKRIIWDSRKGFI
ISNATYKEIGLLTCEATVNGHLYKTNYLTHRQT
>d1he7a_ b.1.1.4 (A:) NGF binding domain of trkA receptor {Human (Homo sapiens)}
SHMPASVQLHTAVEMHHWCIPFSVDGQPAPSLRWLFNGSVLNETSFIFTEFLEPAANETVRH
GCLRLNQPTHVNNGNYTLLAANPFGQASASIMAAFMDNPFEFNPE
>d1wwwx_ b.1.1.4 (X:) NGF binding domain of trkA receptor {Human (Homo sapiens)}
VSFPASVQLHTAVEMHHWCIPFSVDGQPAPSLRWLFNGSVLNETSFIFTEFLEPAANETVRH
GCLRLNQPTHVNNGNYTLLAANPFGQASASIMAAFMDNP
>d1wwbx_ b.1.1.4 (X:) Ligand binding domain of trkB receptor {Human (Homo sapiens)}
VHFAPTITFLESPTSDHHWCIPFTVKGNPKPALQWFYNGAILNESKYICTKIHVTNHTEYHG
CLQLDNPTHMNNGDYTLIAKNEYGKDEKQISAHFMGWPGID
>d1wwca_ b.1.1.4 (A:) NT3 binding domain of trkC receptor {Human (Homo sapiens)}
TVYYPPRVVSLEEPELRLEHCIEFVVRGNPPPTLHWLHNGQPLRESKIIHVEYYQEGEISEG
CLLFNKPTHYNNGNYTLIAKNPLGTANQTINGHFLKEPFPVDE
>d1fcga1 b.1.1.4 (A:4-88) Fc gamma receptor ectodomain (CD32) {Human (Homo sapiens), IIa}
APPKAVLKLEPPWINVLQEDSVTLTCQGARSPESDSIQWFHNGNLIPTHTQPSYRFKANNND
SGEYTCQTGQTSLSDPVHLTVLF
>d1fcga2 b.1.1.4 (A:89-174) Fc gamma receptor ectodomain (CD32) {Human (Homo sapiens), IIa}
EWLVLQTPHLEFQEGETIMLRCHSWKDKPLVKVTFFQNGKSQKFSHLDPTFSIPQANHSHSG
DYHCTGNIGYTLFSSKPVTITVQV
>d2fcba1 b.1.1.4 (A:6-90) Fc gamma receptor ectodomain (CD32) {Human (Homo sapiens), IIb}
APPKAVLKLEPQWINVLQEDSVTLTCRGTHSPESDSIQWFHNGNLIPTHTQPSYRFKANNND
SGEYTCQTGQTSLSDPVHLTVLS
>d2fcba2 b.1.1.4 (A:91-178) Fc gamma receptor ectodomain (CD32) {Human (Homo sapiens), IIb}
EWLVLQTPHLEFQEGETIVLRCHSWKDKPLVKVTFFQNGKSKKFSRSDPNFSIPQANHSHSG
DYHCTGNIGYTLYSSKPVTITVQAPA

>d1fnla1 b.1.1.4 (A:3-86) Fc gamma receptor ectodomain (CD32) {Human (Homo sapiens), III}
EDLPKAVVFLEPQWYSVLEKDSVTLKCQGAYSPEDNSTQWFHNESLISSQASSYFIDAATVN
DSGEYRCQTNLSTLSDPVQLEV
>d1fnla2 b.1.1.4 (A:87-175) Fc gamma receptor ectodomain (CD32) {Human (Homo sapiens), III}
HIGWLLLQAPRWVFKEEDPIHLRCHSWKNTALHKVTYLQNGKDRKYFHHNSDFHIPKATLKD
SGSYFCRGLVGSKNVSSETVNITITQA
>d1f2qa1 b.1.1.4 (A:4-85) IgE high affinity receptor alpha subunit {Human (Homo sapiens)}
KPKVSLNPPWNRIFKGENVTLTCNGNNFFEVSSTKWFHNGSLSEETNSSLNIVNAKFEDSGE
YKCQHQQVNESEPVYLEVFS
>d1f2qa2 b.1.1.4 (A:86-174) IgE high affinity receptor alpha subunit {Human (Homo sapiens)}
DWLLLQASAEVVMEGQPLFLRCHGWRNWDVYKVIYYKDGEALKYWYENHNISITNATVEDSG
TYYCTGKVWQLDYESEPLNITVIKAPR
>d1efxd1 b.1.1.4 (D:4-103) Killer cell inhibitory receptor {Human (Homo sapiens), kir2dl3}
VHRKPSLLAHPGRLVKSEETVILQCWSDVRFEHFLLHREGKFKDTLHLIGEHHDGVSKANFS
IGPMMQDLAGTYRCYGSVTHSPYQLSAPSDPLDIVITG
>d1efxd2 b.1.1.4 (D:104-200) Killer cell inhibitory receptor {Human (Homo sapiens), kir2dl3}
LYEKPSLSAQPGPTVLAGESVTLSCSSRSSYDMYHLSREGEAHECRFSAGPKVNGTFQADFP
LGPATHGGTYRCFGSFRDSPYEWSNSSDPLLVSVI
>d1nkr_1 b.1.1.4 (6-101) Killer cell inhibitory receptor {Human (Homo sapiens), p58-cl42 kir}
RKPSLLAHPGPLVKSEETVILQCWSDVMFEHFLLHREGMFNDTLRLIGEHHDGVSKANFSIS
RMTQDLAGTYRCYGSVTHSPYQVSAPSDPLDIVI
>d1nkr_2 b.1.1.4 (102-200) Killer cell inhibitory receptor {Human (Homo sapiens), p58-cl42 kir}
IGLYEKPSLSAQPGPTVLAGENVTLSCSSRSSYDMYHLSREGEAHERRLPAGPKVNGTFQAD
FPLGPATHGGTYRCFGSFHDSPYEWSKSSDPLLVSVT
>d1g0xa1 b.1.1.4 (A:2-97) Ligand binding domain of lir-1 (ilt2) {Human (Homo sapiens)}
HLPKPTLWAEPGSVITQGSPVTLRCQGGQETQEYRLYREKKTAPWITRIPQELVKKGQFPIP
SITWEHAGRYRCYYGSDTAGRSESSDPLELVVTG
>d1g0xa2 b.1.1.4 (A:98-198) Ligand binding domain of lir-1 (ilt2) {Human (Homo sapiens)}
AYIKPTLSAQPSPVVNSGGNVTLQCDSQVAFDGFILCKEGEDEHPQCLNSQPHARGSSRAIF
SVGPVSPSRRWWYRCYAYDSNSPYEWSLPSDLLELLVLG
>d1f42a1 b.1.1.4 (A:1-87) The p40 domain of interleukin-12 (IL-12 beta chain), N-teminal domain {Human (Homo sapiens)}
IWELKKDVYVVELDWYPDAPGEMVVLTCDTPEEDGITWTLDQSSEVLGSGKTLTIQVKEFGD
AGQYTCHKGGEVLSHSLLLLHKKED

>d1jbja1 b.1.1.4 (A:101-186) CD3 gamma chain ectodomain fragment {Mouse (Mus musculus)}
KKDGSQTNKAKNLVQVDGSRGDGSVLLTCGLTDKTIKWLKDGSIISPLNATKNTWNLGNNAK
DPRGTYQCQGAKETSNPLQVYYRM
>d1jbja2 b.1.1.4 (A:1-100) CD3 epsilon chain ectodomain fragment {Mouse (Mus musculus)}
DDAENIEYKVSISGTSVELTCPLDSDENLKWEKNGQELPQKHDKHLVLQDFSEVEDSGYYVC
YTPASNKNTYLYLKARVGSADDAKKDAAKKDDAKKDDA
>d1k3ia1 b.1.1.5 (A:538-639) Galactose oxidase, C-terminal domain {Fungi (Fusarium spp)}
GNLATRPKITRTSTQSVKVGGRITISTDSSISKASLIRYGTATHTVNTDQRRIPLTLTNNGG
NSYSFQVPSDSGVALPGYWMLFVMNSAGVPSVASTIRVTQ
>d1qba_1 b.1.1.5 (781-885) Bacterial chitobiase, c-terminal domain {Serratia marcescens}
GETHFVDTQALEKDWLRFANILGQRELAKLDKGGVAYRLPVPGARVAGGKLEANIALPGLGI
EYSTDGGKQWQRYDAKAKPAVSGEVQVRSVSPDGKRYSRAEKV
>d1svb_1 b.1.1.5 (303-395) Envelope glycoprotein, domain III (C-terminal) {Tick-borne encephalitis virus}
TYTMCDKTKFTWKRAPTDSGHDTVVMEVTFSGTKPCRIPVRAVAHGSPDVNVAMLITPNPTI
ENNGGGFIEMQLPPGDNIIYVGELSHQWFQK
>d1cgt_1 b.1.1.5 (495-579) Cyclodextrin glycosyltransferase, domain E {Bacillus circulans, different strains}
ETTPTIGHVGPVMGKPGNVVTIDGRGFGSTKGTVYFGTTAVTGAAITSWEDTQIKVTIPSVA
AGNYAVKVAASGVNSNAYNNFTI
>d1kcla1 b.1.1.5 (A:496-581) Cyclodextrin glycosyltransferase, domain E {Bacillus circulans, different strains}
TATPTIGHVGPMMAKPGVTITIDGRGFGSSKGTVYFGTTAVSGADITSWEDTQIKVKIPAVA
GGNYNIKVANAAGTASNVYDNFEV
>d1cyg_1 b.1.1.5 (492-574) Cyclodextrin glycosyltransferase, domain E {Bacillus stearothermophilus}
ESTPIIGHVGPMMGQVGHQVTIDGEGFGTNTGTVKFGTTAANVVSWSNNQIVVAVPNVSPGK
YNITVQSSSGQTSAAYDNFEV
>d1qhoa1 b.1.1.5 (A:496-576) Cyclodextrin glycosyltransferase, domain E {Bacillus stearothermophilus, maltogenic alpha-amylase}
ASAPQIGSVAPNMGIPGNVVTIDGKGFGTTQGTVTFGGVTATVKSWTSNRIEVYVPNMAAGL
TDVKVTAGGVSSNLYSYNI
>d1pama1 b.1.1.5 (A:497-582) Cyclodextrin glycosyltransferase, domain E {Bacillus sp., strain 1011}
TTPIIGNVGPMMAKPGVTITIDGRGFGSGKGTVYFGTTAVTGADIVAWEDTQIQVKIPAVPG
GIYDIRVANAAGAASNIYDNFEVL
>d1ciu_1 b.1.1.5 (496-578) Cyclodextrin glycosyltransferase, domain E {Thermoanaerobacterium thermosulfurigenes, EM1}
SNSPLIGHVGPTMTKAGQTITIDGRGFGTTSGQVLFGSTAGTIVSWDDTEVKVKVPSVTPGK
YNISLKTSSGATSNTYNNINI

>d1smaa1 b.1.1.5 (A:1-123) Maltogenic amylase, N-terminal domain {Thermus sp.}
MRKEAIHHRSTDNFAYAYDSETLHLRLQTKKNDVDHVELLFGDPYEWHDGAWQFQTMPMRKT
GSDGLFDYWLAEVKPPYRRLRYGFVLRAGGEKLVYTEKGFYHEAPSDDTAYYFCFPFLHRV
>d1bvza1 b.1.1.5 (A:1-120) Maltogenic amylase, N-terminal domain {Thermoactinomyces vulgaris, TVAII}
MLLEAIFHEAKGSYAYPISETQLRVRLRAKKGDVVRCEVLYADRYASPEEELAHALAGKAGS
DERFDYFEALLECSTKRVKYVFLLTGPQGEAVYFGETGFSAERSKAGVFQYAYIHRSE
>d1eh9a1 b.1.1.5 (A:1-90) Glycosyltrehalose trehalohydrolase, N-terminal domain {Archaeon Sulfolobus solfataricus, km1}
TFAYKIDGNEVIFTLWAPYQKSVKLKVLEKGLYEMERDEKGYFTITLNNVKVRDRYKYVLDD
ASEIPDPASRYQPEGVHGPSQIIQESKE
>d1bf2_1 b.1.1.5 (1-162) Isoamylase, N-terminal domain {Pseudomonas amyloderamosa}
AINSMSLGASYDAQQANITFRVYSSQATRIVLYLYSAGYGVQESATYTLSPAGSGVWAVTVP
VSSIKAAGITGAVYYGYRAWGPNWPYASNWGKGSQAGFVSDVDANGDRFNPNKLLLDPYAQE
VSQDPLNPSNQNGNVFASGASYRTTDSGIYAPKGVVLV
>d1lla_3 b.1.1.5 (380-628) Hemocyanin, C-terminal domain {Horseshoe crab (Limulus polyphemus)}
PYDHDVLNFPDIQVQDVTLHARVDNVVHTFMREQELELKHGINPGNARSIKARYYHLDHEPF
SYAVNVQNNSASDKHATVRIFLAPKYDELGNEIKADELRRTAIELDKFKTDLHPGKNTVVRH
SLDSSVTLSHQPTFEDLLHGVGLNEHKSEYCSCGWPSHLLVPKGNIKGMEYHLFVMLTDWDK
DKVDGSESVACVDAVSYCGARDHKYPDKKPMGFPFDRPIHTEHISDFLTNNMFIKDIKIKFH
E
>d1hc2_3 b.1.1.5 (399-653) Hemocyanin, C-terminal domain {Spiny lobster (Panulirus interruptus)}
PPYTHDNLEFSGMVVNGVAIDGELITFFDEFQYSLINAVDSGENIEDVEINARVHRLNHNEF
TYKITMSNNNDGERLATFRIFLCPIEDNNGITLTLDEARWFCIELDKFFQKVPSGPETIERS
SKDSSVTVPDMPSFQSLKEQADNAVNGGHDLDLSAYERSCGIPDRMLLPKSKPEGMEFNLYV
AVTDGDKDTEGHNGGHDYGGTHAQCGVHGEAYPDNRPLGYPLERRIPDERVIDGVSNIKHVV
VKIVHHL
>d1js8a2 b.1.1.5 (A:2792-2892) C-terminal domain of octopus hemocyanin {Giant octopus (Octopus dofleini)}
EDRVFAGFLLRTIGQSADVNFDVCTKDGECTFGGTFCILGGEHEMFWAFDRLFKYDITTSLK
HLRLDAHDDFDIKVTIKGIDGHVLSNKYLSPPTVFLAPA
>d1clc_2 b.1.1.5 (35-134) CelD cellulase, N-terminal domain {Clostridium thermocellum}
IETKVSAAKITENYQFDSRIRLNSIGFIPNHSKKATIAANCSTFYVVKEDGTIVYTGTATSM
FDNDTKETVYIADFSSVNEEGTYYLAVPGVGKSVNFKI
>d1f1sa2 b.1.1.5 (A:171-248) Hyaluronate lyase precatalytic domain {Streptococcus agalactiae}
SEHPQPVTTQIEKSVNTALNKNYVFNKADYQYTLTNPSLGKIVGGILYPNATGSTTVKISDK
SGKIIKEVPLSVTAST
>d1edqa1 b.1.1.5 (A:24-132) Chitinase A, N-terminal domain

{Serratia marcescens}
AAPGKPTIAWGNTKFAIVEVDQAATAYNNLVKVKNAADVSVSWNLWNGDTGTTAKVLLNGKE
AWSGPSTGSSGTANFKVNKGGRYQMQVALCNADGCTASDATEIVVAD
>d1f13a1 b.1.1.5 (A:5-190) Transglutaminase N-terminal domain
{Human (Homo sapiens)}
RTAFGGRRAVPPNNSNAAEDDLPTVELQGVVPRGVNLQEFLNVTSVHLFKERWDTNKVDHHT
DKYENNKLIVRRGQSFYVQIDFSRPYDPRRDLFRVEYVIGRYPQENKGTYIPVPIVSELQSG
KWGAKIVMREDRSVRLSIQSSPKCIVGKFRMYVAVWTPYGVLRTSRNPETDTYILFNPWCED
>d1g0da1 b.1.1.5 (A:6-140) Transglutaminase N-terminal domain {Red
sea bream (Chrysophrys major)}
GLIVDVNGRSHENNLAHRTREIDRERLIVRRGQPFSITLQCSDSLPPKHHLELVLHLGKRDE
VVIKVQKEHGARDKWWFNQQGAQDEILLTLHSPANAVIGHYRLAVLVMSPDGHIVERADKIS
FHMLFNPWCRD
>d1eut_1 b.1.1.5 (403-505) Sialidase, "linker" domain
{Micromonospora viridifaciens}
GICAPFTIPDVALEPGQQVTVPVAVTNQSGIAVPKPSLQLDASPDWQVQGSVEPLMPGRQAK
GQVTITVPAGTTPGRYRVGATLRTSAGNASTTFTVTVGLLD
>d1ksr__ b.1.1.5 (-) F-actin cross-linking gelation factor
(ABP-120), one repeat (ROD 4) {Slime mold (Dictyostelium
discoideum), different domains}
ADPEKSYAEGPGLDGGECFQPSKFKIHAVDPDGVHRTDGGDGFVVTIEGPAPVDPVMVDNGD
GTYDVEFEPKEAGDYVINLTLDGDNVNGFPKTVTVKPA
>d1qfha1 b.1.1.5 (A:646-749) F-actin cross-linking gelation factor
(ABP-120), one repeat (ROD 4) {Slime mold (Dictyostelium
discoideum), different domains}
KPAPSAEHSYAEGEGLVKVFDNAPAEFTIFAVDTKGVARTDGGDPFEVAINGPDGLVVDAKV
TDNNDGTYGVVYDAPVEGNYNVNVTLRGNPIKNMPIDVKCIE
>d1qfha2 b.1.1.5 (A:750-857) F-actin cross-linking gelation factor
(ABP-120), one repeat (ROD 4) {Slime mold (Dictyostelium
discoideum), different domains}
GANGEDSSFGSFTFTVAAKNKKGEVKTYGGDKFEVSITGPAEEITLDAIDNQDGTYTAAYSL
VGNGRFSTGVKLNGKHIEGSPFKQVLGNPGKKNPEVKSFTTTRTAN
>d1ds6b_ b.1.1.5 (B:) Rho GDP-dissociation inhibitor 1, RhoGDI
{Human (Homo sapiens)}
GNYKPPPQKSLKELQEMDKDDESLIKYKKTLLGDGPVVTDPKAPNVVVTRLTLVCESAPGPI
TMDLTGDLEALKKETIVLKEGSEYRVKIHFKVNRDIVSGLKYVQHTYRTGVKVDKATFMVGS
YGPRPEEYEFLTPVEEAPKGMLARGTYHNKSFFTDDDKQDHLSWEWNLSIKKEWG
>d1fsoa_ b.1.1.5 (A:) Rho GDP-dissociation inhibitor 1, RhoGDI
{Human (Homo sapiens)}
MVPNVVVTGLTLVCSSAPGPLELDLTGDLESFKKQSFVLKEGVEYRIKISFRVNREIVSGMK
YIQHTYRAGVAIDATDYMVGSYGPRAEEYEFLTPVEEAPKGMLARGSYSIKSRFTDDDKTDH
LSWEWNFTIKKDWK
>d1hh4e_ b.1.1.5 (E:) Rho GDP-dissociation inhibitor 1, RhoGDI
{Human (Homo sapiens)}

HSVNYKPPAQKSIQEIQELDKDDESLRKYKEALLGRVAVSADPNVPNVVVTGLTLVCSSAPG
PLELDLTGDLESFKKQSFVLKEGVEYRIKISFRVNREIVSGMKYIQHTYRKGVKIDKTDYMV
GSYGPRAEEYEFLTPVEEAPKGMLARGSYSIKSRFTDDDKTDHLSWEWNLTIKKDW
>d1rhoa_ b.1.1.5 (A:) Rho GDP-dissociation inhibitor 1, RhoGDI
{Human (Homo sapiens)}
VAVSADPNVPNVVVTGLTLVCSSAPGPLELDLTGDLESFKKQSFVLKEGVEYRIKISFRVNR
EIVSGMKYIEHTYRKGVKIDKTDYMVGSYGPRAEEYEFLTPVEEAPKGMLARGSYSIKSRFT
DDDKTDHLSWEWNLTIKKDWK
>d1ajw__ b.1.1.5 (-) Rho GDP-dissociation inhibitor 1, RhoGDI {Cow
(Bos taurus)}
AVSADPNVPNVVVTRLTLVCSTAPGPLELDLTGDLESFKKQSFVLKEGVEYRIKISFRVNRE
IVSGMKYIQHTYRKGVKIDKTDYMVGSYGPRAEEYEFLTPMEEAPKGMLARGSYNIKSRFTD
DDRTDHLSWEWNLTIKKEWKD
>d1doab_ b.1.1.5 (B:) Rho GDP-dissociation inhibitor 1, RhoGDI {Cow
(Bos taurus)}
EPTAEQLAQIAAENEEDEHSVNYKPPAQKSIQEIQELDKDDESLRKYKEALLGRVAVSADPN
VPNVVVTRLTLVCSTAPGPLELDLTGDLESFKKQSFVLKEGVEYRIKISFRVNREIVSGMKY
IQHTYRKGVKIDKTDYMVGSYGPRAEEYEFLTPMEEAPKGMLARGSYNIKSRFTDDDRTDHL
SWEWNLTIKKEWKD
>d1ayra2 b.1.1.5 (A:183-368) Arrestin {Cow (Bos taurus), visual
arrestin}
DMGPQPRAEASWQFFMSDKPLRLAVSLSKEIYYHGEPIPVTVAVTNSTEKTVKKIKVLVEQV
TNVVLYSSDYYIKTVAAEEAQEKVPPNSSLTKTLTLVPLLANNRERRGIALDGKIKHEDTNL
ASSTIIKEGIDKTVMGILVSYQIKVKLTVSGLLGELTSSEVATEVPFRLMHPQPEDPDTAKA
>d1cf1a1 b.1.1.5 (A:10-182) Arrestin {Cow (Bos taurus), visual
arrestin}
HVIFKKISRDKSVTIYLGKRDYIDHVERVEPVDGVVLVDPELVKGKRVYVSLTCAFRYGQED
IDVMGLSFRRDLYFSQVQVFPPVGASGATTRLQESLIKKLGANTYPFLLTFPDYLPCSVMLQ
PAPQDVGKSCGVDFEIKAFATHSTDVEEDKIPKKSSVRLLIRKVQHAPR
>d1cf1a2 b.1.1.5 (A:183-393) Arrestin {Cow (Bos taurus), visual
arrestin}
DMGPQPRAEASWQFFMSDKPLRLAVSLSKEIYYHGEPIPVTVAVTNSTEKTVKKIKVLVEQV
TNVVLYSSDYYIKTVAAEEAQEKVPPNSSLTKTLTLVPLLANNRERRGIALDGKIKHEDTNL
ASSTIIKEGIDKTVMGILVSYQIKVKLTVSGLLGELTSSEVATEVPFRLMHPQPEDPDTAKE
SFQDENFVFEEFARQNLKDAGEYKE
>d1g4ma1 b.1.1.5 (A:5-175) Arrestin {Cow (Bos taurus),
beta-arrestin 1}
GTRVFKKASPNGKLTVYLGKRDFVDHIDLVEPVDGVVLVDPEYLKERRVYVTLTCAFRYGRE
DLDVLGLTFRKDLFVANVQSFPPAPEDKKPLTRLQERLIKKLGEHAYPFTFEIPPNLPCSVT
LQPGPEDTGKACGVDYEVKAFCAENLEEKIHKRNSVRLVIRKVQYAP
>d1g4ma2 b.1.1.5 (A:176-393) Arrestin {Cow (Bos taurus),
beta-arrestin 1}
ERPGPQPTAETTRQFLMSDKPLHLEASLDKEIYYHGEPISVNVHVTNNTNKTVKKIKISVRQ
YADICLFNTAQYKCPVAMEEADDTVAPSSTFCKVYTLTPFLANNREKRGLALDGKLKHEDTN

LASSTLLREGANREILGIIVSYKVKVKLVVSRGGLLGDLASSDVAVELPFTLMHPKPKEEPP
HREVPEHETPVDTNLIELDTNDDDIVFEDFAR
>d1a02n1 b.1.1.5 (N:577-678) Transcription factor NFATC,
C-terminal domain {Human (Homo sapiens)}
LPMVERQDTDSCLVYGGQQMILTGQNFTSESKVVFTEKTTDGQQIWEMEATVDKDKSQPNML
FVEIPEYRNKHIRTPVKVNFYVINGKRKRSQPQHFTYHPV
>d1imhc1 b.1.1.5 (C:368-468) Transcription factor TONEBP,
C-terminal domain {Human (Homo sapiens)}
VPEILKKSLHSCSVKGEEEVFLIGKNFLKGTKVIFQENVSDENSWKSEAEIDMELFHQNHLI
VKVPPYHDQHITLPVSVGIYVVTNAGRSHDVQPFTYTPD
>d1bfs__ b.1.1.5 (-) p50 subunit of NF-kappa B transcription factor,
C-terminal domain {Mouse (Mus musculus)}
ASNLKIVRMDRTAGCVTGGEEIYLLCDKVQKDDIQIRFYEEEENGGVWEGFGDFSPTDVHRQ
FAIVFKTPKYKDVNITKPASVFVQLRRKSDLETSEPKPFLYYPE
>d1a3qa1 b.1.1.5 (A:227-327) p52 subunit of NF-kappa B (NFKB),
C-terminal domain {Human (Homo sapiens)}
NLKISRMDKTAGSVRGGDEVYLLCDKVQKDDIEVRFYEDDENGWQAFGDFSPTDVHKQYAIV
FRTPPYHKMKIERPVTVFLQLKRKRGGDVSDSKQFTYYP
>d1bfta_ b.1.1.5 (A:) p65 subunit of NF-kappa B (NFKB), C-terminal
domain {Mouse (Mus musculus)}
TAELKICRVNRNSGSCLGGDEIFLLCDKVQKEDIEVYFTGPGWEARGSFSQADVHRQVAIVF
RTPPYADPSLQAPVRVSMQLRRPSDRELSEPMEFQYLPD
>d1ikna1 b.1.1.5 (A:192-303) p65 subunit of NF-kappa B (NFKB),
C-terminal domain {Mouse (Mus musculus)}
AELKICRVNRNSGSCLGGDEIFLLCDKVQKEDIEVYFTGPGWEARGSFSQADVHRQVAIVFR
TPPYADPSLQAPVRVSMQLRRPSDRELSEPMEFQYLPDTDDRHRIEEKRK
>d1nfia1 b.1.1.5 (A:190-314) p65 subunit of NF-kappa B (NFKB),
C-terminal domain {Human (Homo sapiens)}
NTAELKICRVNRNSGSCLGGDEIFLLCDKVQKEDIEVYFTGPGWEARGSFSQADVHRQVAIV
FRTPPYADPSLQAPVRVSMQLRRPSDRELSEPMEFQYLPDTDDRHRIEEKRKRTYETFKSIM
K
>d1ahm__ b.1.1.5 (-) Major mite allergen {House-dust mite
(Dermatophagoides farinae), Der f 2}
DQVDVKDCANNEIKKVMVDGCHGSDPCIIHRGKPFTLEALFDANQNTKTAKIEIKASLDGLE
IDVPGIDTNACHFVKCPLVKGQQYDIKYTWNVPKIAPKSENVVVTVKLIGDNGVLACAIATH
GKIRD
>d1a9v__ b.1.1.5 (-) Major mite allergen {House-dust mite
(Dermatophagoides pteronyssinus), Der p 2}
SQVDVKDCANHEIKKVLVPGCHGSEPCIIHRGKPFQLEAVFEANQNTKTAKIEIKASIDGLE
VDVPGIDPNACHYMKCPLVKGQQYDIKYTWNVPKIAPKSENVVVTVKVMGDDGVLACAIATH
AKIRD
>d1soxa1 b.1.1.5 (A:344-466) Sulfite oxidase, C-terminal domain
{Chicken (Gallus gallus)}
ELPVQSAVTQPRPGAAVPPGELTVKGYAWSGGGREVVRVDVSLDGGRTWKVARLMGDKAPPG

RAWAWALWELTVPVEAGTELEIVCKAVDSSYNVQPDSVAPIWNLRGVLSTAWHRVRVSVQD
>d1cvra1 b.1.1.5 (A:351-432) Gingipain R (RgpB), C-terminal domain {Porphyromonas gingivalis}
PTEMQVTAPANISASAQTFEVACDYNGAIATLSDDGDMVGTAIVKDGKAIIKLNESIADETN
LTLTVVGYNKVTVIKDVKVE
>d1h6ta1 b.1.1.6 (A:241-321) Internalin B {Listeria monocytogenes}
ECLNKPINHQSNLVVPNTVKNTDGSLVTPEIISDDGDYEKPNVKWHLPEFTNEVSFIFYQPV
TIGKAKARFHGRVTQPLKE
>d1h6ua1 b.1.1.6 (A:263-343) Internalin H {Listeria monocytogenes}
TITNQPVFYNNNLVVPNVVKGPSGAPIAPATISDNGTYASPNLTWNLTSFINNVSYTFNQSV
TFKNTTVPFSGTVTQPLTE
>d1ehxa_ b.1.1.6 (A:) Cellulosomal scaffoldin protein CipC, module x2.1 {Clostridium cellulolyticum}
MQDPTINPTSISAKAGSFADTKITLTPNGNTFNGISELQSSQYTKGTNEVTLLASYLNTLPE
NTTKTLTFDFGVGTKNPKLTITVLPKDIPGLE
>d1im3d_ b.1.1.6 (D:) Cytomegalovirus protein US2 {Human cytomegalovirus}
PWFQIEDNRCYIDNGKLFARGSIVGNMSRFVFDPKADYGGVGENLYVHADDVEFVPGESLKW
NVRNLDVMPIFETLALRLVLQGDVIWLRCVPEL
>d1jjua3 b.1.1.6 (A:274-351) Quinohemoprotein amine dehydrogenase A chain, domains 4 and 5 {Paracoccus denitrificans}
AAPQVLAVAPARLKIGEETQLRVAGTGLGSDLTLPEGVAGSVESAGNGVTVLKLTATGTPGP
VSLELGGQKVDLVAYD
>d1jjua4 b.1.1.6 (A:352-489) Quinohemoprotein amine dehydrogenase A chain, domains 4 and 5 {Paracoccus denitrificans}
RPDRISIVPDLTIARIGGNGGPIPKVPAQFEAMGWLNGPDGQPGTGDDIALGAFPASWATDN
FDEEAEKMQDAKYAGSIDDTGLFTPAEAGPNPERPMQTNNAGNLKVIATVDAEGEPLSAEAH
LYATVQRFVDAPIR
>d1jmxa3 b.1.1.6 (A:282-363) Quinohemoprotein amine dehydrogenase A chain, domains 4 and 5 {Pseudomonas putida}
GKARLLAVQPAFIKAGGESEITLVGSGLAGKPDLGAGVEVTEVLEQTPTLVRLKARAAADAK
PGQREVAVGTLKGVNLAVYD
>d1jmxa4 b.1.1.6 (A:364-494) Quinohemoprotein amine dehydrogenase A chain, domains 4 and 5 {Pseudomonas putida}
KVEEVKVVPAFSIARIGENGASVPKVQGRFEAEAWGKDANGQPLRIGYLPASWKVEPFNERA
VEDEDVKFAGKMQADGVFVPGGAGPNPERKMMTNNAGNLKVIATLADGGQTGEGHMIVTVQR
WNNPPLP
>d2hft_1 b.1.2.1 (1-106) Extracellular region of human tissue factor {Human (Homo sapiens)}
SGTTNTVAAYNLTWKSTNFKTILEWEPKPVNQVYTVQISTKSGDWKSKCFYTTDTECDLTDE
IVKDVKQTYLARVFSYPAGNVESTGSAGEPLYENSPEFTPYLET
>d2hft_2 b.1.2.1 (107-211) Extracellular region of human tissue factor {Human (Homo sapiens)}
NLGQPTIQSFEQVGTKVNVTVEDERTLVRRNNTFLSLRDVFGKDLIYTLYYWKSSSQEKGEF

RSGKKTAKTNTNEFLIDVDKGENYCFSVQAVIPSRTVNRKSTDSPVECMG
>g1dan.1 b.1.2.1 (T:,U:91-106) Extracellular region of human tissue factor {Human (Homo sapiens)}
TVAAYNLTWKSTNFKTILEWEPKPVNQVYTVQISTKSGDWKSKCFYTTDTECDLTDEIVKDV
KQTYLARVFSYPAXEPLYENSPEFTPYLET
>d1a21a1 b.1.2.1 (A:4-106) Extracellular region of human tissue factor {Rabbit (Oryctolagus cuniculus)}
TGRAYNLTWKSTNFKTILEWEPKSIDHVYTVQISTRLENWKSKCFLTAETECDLTDEVVKDV
GQTYMARVLSYPARNGNTTGFPEEPPFRNSPEFTPYLDTNL
>d1a21a2 b.1.2.1 (A:107-208) Extracellular region of human tissue factor {Rabbit (Oryctolagus cuniculus)}
GQPTIQSFEQVGTKLNVTVQDARTLVRRNGTFLSLRAVFGKDLNYTLYYWRASSTGKKTATT
NTNEFLIDVDKGENYCFSVQAVIPSRKRKQRSPESLTECT
>d1fna__ b.1.2.1 (-) Fibronectin, different Fn3 modules {Human (Homo sapiens)}
RDLEVVAATPTSLLISWDAPAVTVRYYRITYGETGGNSPVQEFTVPGSKSTATISGLKPGVD
YTITVYAVTGRGDSPASSKPISINYRTEI
>d1fnf_1 b.1.2.1 (1142-1235) Fibronectin, different Fn3 modules {Human (Homo sapiens)}
PLSPPTNLHLEANPDTGVLTVSWERSTTPDITGYRITTTPTNGQQGNSLEEVVHADQSSCTF
DNLSPGLEYNVSVYTVKDDKESVPISDTIIPA
>d1fnf_2 b.1.2.1 (1236-1326) Fibronectin, different Fn3 modules {Human (Homo sapiens)}
VPPPTDLRFTNIGPDTMRVTWAPPPSIDLTNFLVRYSPVKNEEDVAELSISPSDNAVVLTNL
LPGTEYVVSVSSVYEQHESTPLRGRQKTG
>d1fnf_3 b.1.2.1 (1327-1415) Fibronectin, different Fn3 modules {Human (Homo sapiens)}
LDSPTGIDFSDITANSFTVHWIAPRATITGYRIRHHPEHFSGRPREDRVPHSRNSITLTNLT
PGTEYVVSIVALNGREESPLLIGQQST
>d1fnha1 b.1.2.1 (A:3-92) Fibronectin, different Fn3 modules {Human (Homo sapiens)}
PAPTDLKFTQVTPTSLSAQWTPPNVQLTGYRVRVTPKEKTGPMKEINLAPDSSSVVVSGLMV
ATKYEVSVYALKDTLTSRPAQGVVTTLE
>d1fnha2 b.1.2.1 (A:93-182) Fibronectin, different Fn3 modules {Human (Homo sapiens)}
NVSPPRRARVTDATETTITISWRTKTETITGFQVDAVPANGQTPIQRTIKPDVRSYTITGLQ
PGTDYKIYLYTLNDNARSSPVVIDASTA
>d1fnha3 b.1.2.1 (A:183-271) Fibronectin, different Fn3 modules {Human (Homo sapiens)}
IDAPSNLRFLATTPNSLLVSWQPPRARITGYIIKYEKPGSPPREVVPRPRPGVTEATITGLE
PGTEYTIYVIALKNNQKSEPLIGRKKT
>d1j8ka_ b.1.2.1 (A:) Fibronectin, different Fn3 modules {Human (Homo sapiens)}
NIDRPKGLAFTDVDVDSIKIAWESPQGQVSRYRVTYSSPEDGIHELFPAPDGEEDTAELQGL

RPGSEYTVSVVALHDDMESQPLIGTQSTAIPA
>d2fnba_ b.1.2.1 (A:) Fibronectin, different Fn3 modules {Human (Homo sapiens)}
MRGSEVPQLTDLSFVDITDSSIGLRWTPLNSSTIIGYRITVVAAGEGIPIFEDFVDSSVGYY
TVTGLEPGIDYDISVITLINGGESAPTTLTQQT
>d2mfn_1 b.1.2.1 (1-92) Fibronectin, different Fn3 modules {Mouse (Mus musculus)}
GLDSPTGFDSSDITANSFTVHWVAPRAPITGYIIRHHAEHSVGRPRQDRVPPSRNSITLTNL
NPGTEYVVSIIAVNGREESPPLIGQQATVS
>d2mfn_2 b.1.2.1 (93-184) Fibronectin, different Fn3 modules {Mouse (Mus musculus)}
DIPRDLEVIASTPTSLLISWEPPAVSVRYYRITYGETGGNSPVQEFTVPGSKSTATINNIKP
GADYTITLYAVTGRGDSPASSKPVSINYKT
>d1qr4a1 b.1.2.1 (A:1-87) Tenascin {Chicken (Gallus gallus)}
DNPKDLEVSDPTETTLSLRWRRPVAKFDRYRLTYVSPSGKKNEMEIPVDSTSFILRGLDAGT
EYTISLVAEKGRHKSKPTTIKGSTV
>d1qr4a2 b.1.2.1 (A:88-175) Tenascin {Chicken (Gallus gallus)}
VGSPKGISFSDITENSATVSWTPPRSRVDSYRVSYVPITGGTPNVVTVDGSKTRTKLVKLVP
GVDYNVNIISVKGFEESEPISGILKT
>d1ten__ b.1.2.1 (-) Tenascin {Human (Homo sapiens)}
RLDAPSQIEVKDVTDTTALITWFKPLAEIDGIELTYGIKDVPGDRTTIDLTEDENQYSIGNL
KPDTEYEVSLISRRGDMSSNPAKETFTT
>d1cfb_1 b.1.2.1 (610-709) Neuroglian, two amino proximal Fn3 repeats {Drosophila melanogaster}
IVQDVPNAPKLTGITCQADKAEIHWEQQGDNRSPILHYTIQFNTSFTPASWDAAYEKVPNTD
SSFVVQMSPWANYTFRVIAFNKIGASPPSAHSDSCTTQ
>d1cfb_2 b.1.2.1 (710-814) Neuroglian, two amino proximal Fn3 repeats {Drosophila melanogaster}
PDVPFKNPDNVVGQGTEPNNLVISWTPMPEIEHNAPNFHYYVSWKRDIPAAAWENNNIFDWR
QNNIVIADQPTFVKYLIKVVAINDRGESNVAAEEVVGYSGEDR
>d1qg3a1 b.1.2.1 (A:1126-1217) Integin beta-4 subunit {Human (Homo sapiens)}
DLGAPQNPNAKAAGSRKIHFNWLPPSGKPMGYRVKYWIQGDSESEAHLLDSKVPSVELTNLY
PYCDYEMKVCAYGAQGEGPYSSLVSCRTHQ
>d1qg3a2 b.1.2.1 (A:1218-1320) Integin beta-4 subunit {Human (Homo sapiens)}
EVPSEPGRLAFNVVSSTVTQLSWAEPAETNGEITAYEVCYGLVNDDNRPIGPMKKVLVDNPK
NRMLLIENLRESQPYRYTVKARNGAGWGPEREAIINLATQP
>d1axib1 b.1.2.1 (B:32-130) Growth hormone receptor {Human (Homo sapiens)}
EPKFTKCRSPERETFSCHWTDEVHHGTKNEGPIQLFYTRRNTQEWTQEWKECPDYVSAGENS
CYFNSSFTSIAIPYCIKLTSNGGTVDEKCFSVDEIVQ
>d1axib2 b.1.2.1 (B:131-236) Growth hormone receptor {Human (Homo sapiens)}

PDPPIALNWTLLNVSLTGIHADIQVRWEAPRNADIQKGWMVLEYELQYKEVNETKWKMMDPI
LTTSVPVYSLKVDKEYEVRVRSKQRNSGNYGEFSEVLYVTLPQM
>d1eerb1 b.1.2.1 (B:8-116) Erythropoietin (EPO) receptor {Human (Homo sapiens)}
DPKFESKAALLAARGPEELLCFTERLEDLVCFWEEAASAGVGPGQYSFSYQLEDEPWKLCRL
HQAPTARGAVRFWCSLPTADTSSFVPLELRVTAASGAPRYHRVIHIN
>d1eerb2 b.1.2.1 (B:117-220) Erythropoietin (EPO) receptor {Human (Homo sapiens)}
EVVLLDAPVGLVARLADESGHVVLRWLPPPETPMTSHIRYEVDVSAGQGAGSVQRVEILEGR
TECVLSNLRGRTRYTFAVRARMAEPSFGGFWSEWSEPVSLLT
>d1bp3b1 b.1.2.1 (B:202-300) Prolactin receptor {Human (Homo sapiens)}
LPPGKPEIFKCRSPNKETFTCWWRPGTDGGLPTNYSLTYHREGETLMHECPDYITGGPNSCH
FGKQYTSMWRTYIMMVNATNQMGSSFSDELYVDVTYI
>d1bp3b2 b.1.2.1 (B:301-404) Prolactin receptor {Human (Homo sapiens)}
VQPDPPLELAVEVKQPEDRKPYLWIKWSPPTLIDLKTGWFTLLYEIRLKPEKAAEWEIHFAG
QQTEFKILSLHPGQKYLVQVRCKPDHGYWSAWSPATFIQIPS
>d1f6fb1 b.1.2.1 (B:5-100) Prolactin receptor {Rat (Rattus norvegicus)}
GKPEIHKCRSPDKETFTCWWNPGTDGGLPTNYSLTYSKEGEKTTYECPDYKTSGPNSCFFSK
QYTSIWKIYIITVNATNQMGSSSSDPLYVDVTYI
>d1f6fb2 b.1.2.1 (B:101-203) Prolactin receptor {Rat (Rattus norvegicus)}
VEPEPPRNLTLEVKQLKDKKTYLWVKWSPPTITDVKTGWFTMEYEIRLKPEEAEEWEIHFTG
HQTQFKVFDLYPGQKYLVQTRCKPDHGYWSRWSQESSVEMP
>d1iarb1 b.1.2.1 (B:1-96) Interleukin-4 receptor alpha chain {Human (Homo sapiens)}
FKVLQEPTCVSDYMSISTCEWKMNGPTNCSTELRLLYQLVFLLSEAHTCIPENNGGAGCVCH
LLMDDVVSADNYTLDLWAGQQLLWKGSFKPSEHV
>d1iarb2 b.1.2.1 (B:97-197) Interleukin-4 receptor alpha chain {Human (Homo sapiens)}
KPRAPGNLTVHTNVSDTLLLTWSNPYPPDNYLYNHLTYAVNIWSENDPADFRIYNVTYLEPS
LRIAASTLKSGISYRARVRAWAQAYNTTWSEWSPSTKWH
>d1egja_ b.1.2.1 (A:) Common beta-chain in the GM-CSF, IL-3 and IL-5 receptors {Human (Homo sapiens)}
IQMAPPSLNVTKDGDSYSLRWETMKMRYEHIDHTFEIQYRKDTATWKDSKTETLQNAHSMAL
PALEPSTRYWARVRVRTSRTGYNGIWSEWSEARSWDTES
>d1gh7a1 b.1.2.1 (A:1-103) Common beta-chain in the GM-CSF, IL-3 and IL-5 receptors {Human (Homo sapiens)}
EETIPLQTLRCYNDYTSHITCRWADTQDAQRLVNVTLIRRVNEDLLEPVSCDLSDDMPWSAC
PHPRCVPRRCVIPCQSFVVTDVDYFSFQPDRPLGTRLTVTL
>d1gh7a2 b.1.2.1 (A:104-217) Common beta-chain in the GM-CSF, IL-3 and IL-5 receptors {Human (Homo sapiens)}

TQHVQPPEPRDLQISTDQDHFLLTWSVALGSPQSHWLSPGDLEFEVVYKRLQDSWEDAAILL
SNTSQATLGPEHLMPSSTYVARVRTRLAPGSRLSGRPSKWSPEVCWDSQPGD
>d1gh7a3 b.1.2.1 (A:218-316) Common beta-chain in the GM-CSF, IL-3 and IL-5 receptors {Human (Homo sapiens)}
EAQPQNLECFFDGAAVLSCSWEVRKEVASSVSFGLFYKPSPDAGSAVLLREEECSPVLREGL
GSLHTRHHCQIPVPDPATHGQYIVSVQPRRAEKHIKS
>d1cd9b1 b.1.2.1 (B:1-107) Granulocyte colony-stimulating factor (GC-SF) receptor {Mouse (Mus musculus)}
AGYPPASPSNLSCLMHLTTNSLVCQWEPGPETHLPTSFILKSFRSRADCQYQGDTIPDCVAK
KRQNNCSIPRKNLLLYQYMAIWVQAENMLGSSESPKLCLDPMDVV
>d1cd9b2 b.1.2.1 (B:108-213) Granulocyte colony-stimulating factor (GC-SF) receptor {Mouse (Mus musculus)}
KLEPPMLQALDIGPDVVSHQPGCLWLSWKPWKPSEYMEQECELRYQPQLKGANWTLVFHLPS
SKDQFELCGLHQAPVYTLQMRCIRSSLPGFWSPWSPGLQLRPTM
>d1fyhb1 b.1.2.1 (B:12-109) Interferon-gamma receptor alpha chain {Human (Homo sapiens)}
VPTPTNVTIESYNMNPIVYWEYQIMPQVPVFTVEVKNYGVKNSEWIDACINISHHYCNISDH
VGDPSNSLWVRVKARVGQKESAYAKSEEFAVCRDGK
>d1fyhb2 b.1.2.1 (B:110-223) Interferon-gamma receptor alpha chain {Human (Homo sapiens)}
IGPPKLDIRKEEKQIMIDIFHPSVFVNGDEQEVDYDPETTCYIRVYNVYVRMNGSEIQYKIL
TQKEDDCDEIQCQLAIPVSSLNSQYCVSAEGVLHVWGVTTEKSKEVCITIFN
>d1bqua1 b.1.2.1 (A:5-99) Cytokyne receptor gp130 cytokine-binding domains {Human (Homo sapiens)}
GLPPEKPKNLSCIVNEGKKMRCEWDGGRETHLETNFTLKSEWATHKFADCKAKRDTPTSCTV
DYSTVYFVNIEVWVEAENALGKVTSDHINFDPV
>d1bqua2 b.1.2.1 (A:100-214) Cytokyne receptor gp130 cytokine-binding domains {Human (Homo sapiens)}
YKVKPNPPHNLSVINSEELSSILKLTWTNPSIKSVIILKYNIQYRTKDASTWSQIPPEDTAS
TRSSFTVQDLKPFTEYVFRIRCMKEDGKGYWSDWSEEASGITYEDRPSKEPSF
>d1i1ra1 b.1.2.1 (A:2-101) Cytokyne receptor gp130 cytokine-binding domains {Human (Homo sapiens)}
LLDPCGYISPESPVVQLHSNFTAVCVLKEKCMDYFHVNANYIVWKTNHFTIPKEQYTIINRT
ASSVTFTDIASLNIQLTCNILTFGQLEQNVYGITIISG
>d1j7vr1 b.1.2.1 (R:2-100) Interleukin-10 receptor 1, IL-10R1 {Human (Homo sapiens)}
GTELPSPPSVWFEAEFFHHILHWTPIPQQSESTCYEVALLRYGIESWNSISQCSQTLSYDLT
AVTLDLYHSNGYRARVRAVDGSRHSQWTVTNTRFSVD
>d1j7vr2 b.1.2.1 (R:101-206) Interleukin-10 receptor 1, IL-10R1 {Human (Homo sapiens)}
EVTLTVGSVNLEIHNGFILGKIQLPRPKMAPAQDTYESIFSHFREYEIAIRKVPGQFTFTHK
KVKHEQFSLLTSGEVGEFCVQVKPSVASRSNKGMWSKEECISLT
>d1bpv__ b.1.2.1 (-) Type I titin module {Human (Homo sapiens)}
SPIDPPGKPVPLNITRHTVTLKWAKPEYTGGFKITSYIVEKRDLPNGRWLKANFSNILENEF

TVSGLTEDAAYEFRVIAKNAAGAISPPSEPSDAITCRDDVEA
>d1f42a2 b.1.2.1 (A:88-211) The p40 domain of interleukin-12 (IL-12 beta chain), domains 2 and 3 {Human (Homo sapiens)}
GIWSTDILKDQKEPKNKTFLRCEAKNYSGRFTCWWLTTISTDLTFSVKSSRGSSDPQGVTCG
AATLSAERVRGDNKEYEYSVECQEDSACPAAEESLPIEVMVDAVHKLKYENYTSSFFIRDII
>d1f42a3 b.1.2.1 (A:212-306) The p40 domain of interleukin-12 (IL-12 beta chain), domains 2 and 3 {Human (Homo sapiens)}
KPDPPKNLQLKPLKNSRQVEVSWEYPDTWSTPHSYFSLTFCVQVQGKSKREKKDRVFTDKTS
ATVICRKNASISVRAQDRYYSSSWSEWASVPCS
>d1b4ra_ b.1.3.1 (A:) Polycystein-1, PKD-1 {Human (Homo sapiens)}
ATLVGPHGPLASGQLAAFHIAAPLPVTATRWDFGDGSAEVDAAGPAASHRYVLPGRYHVTAV
LALGAGSALLGTDVQVEA
>d1jz8a1 b.1.4.1 (A:220-333) beta-Galactosidase, domains 2 and 4 {Escherichia coli}
TQISDFHVATRFNDDFSRAVLEAEVQMCGELRDYLRVTVSLWQGETQVASGTAPFGGEIIDE
RGGYADRVTLRLNVENPKLWSAEIPNLYRAVVELHTADGTLIEAEACDVGFR
>d1jz8a2 b.1.4.1 (A:626-730) beta-Galactosidase, domains 2 and 4 {Escherichia coli}
FFQFRLSGQTIEVTSEYLFRHSDNELLHWMVALDGKPLASGEVPLDVAPQGKQLIELPELPQ
PESAGQLWLTVRVVQPNATAWSEAGHISAWQQWRLAENLSVTL
>d1bhga1 b.1.4.1 (A:226-328) beta-Glucuronidase {Human (Homo sapiens)}
TYIDDITVTTSVEQDSGLVNYQISVKGSNLFKLEVRLLDAENKVVANGTGTQGQLKVPGVSL
WWPYLMHERPAYLYSLEVQLTAQTSLGPVSDFYTLPVGIRT
>d1f13a2 b.1.5.1 (A:516-627) Transglutaminase, two C-terminal domains {Human (Homo sapiens)}
SNVDMDFEVENAVLGKDFKLSITFRNNSHNRYTITAYLSANITFYTGVPKAEFKKETFDVTL
EPLSFKKEAVLIQAGEYMGQLLEQASLHFFVTARINETRDVLAKQKSTVL
>d1f13a3 b.1.5.1 (A:628-728) Transglutaminase, two C-terminal domains {Human (Homo sapiens)}
TIPEIIIKVRGTQVVGSDMTVTVEFTNPLKETLRNVWVHLDGPGVTRPMKKMFREIRPNSTV
QWEEVCRPWVSGHRKLIASMSSDSLRHVYGELDVQIQRR
>d1g0da2 b.1.5.1 (A:472-583) Transglutaminase, two C-terminal domains {Red sea bream (Chrysophrys major)}
RLQLSIKHAQPVFGTDFDVIVEVKNEGGRDAHAQLTMLAMAVTYNSLRRGECQRKTISVTVP
AHKAHKEVMRLHYDDYVRCVSEHHLIRVKALLDAPGENGPIMTVANIPLS
>d1g0da3 b.1.5.1 (A:584-684) Transglutaminase, two C-terminal domains {Red sea bream (Chrysophrys major)}
TPELLVQVPGKAVVWEPLTAYVSFTNPLPVPLKGGVFTLEGAGLLSATQIHVNGAVAPSGKV
SVKLSFSPMRTGVRKLLVDFDSDRLKDVKGVTTVVVHKK
>d1ncia_ b.1.6.1 (A:) N-cadherin (neural) {Mouse (Mus musculus)}
GSDWVIPPINLPENSRGPFPQELVRIRSGRDKNLSLRYSVTGPGADQPPTGIFIINPISGQL
SVTKPLDRELIARFHLRAHAVDINGNQVENPIDIVINVID
>d1ncja2 b.1.6.1 (A:102-215) N-cadherin (neural) {Mouse (Mus

musculus)}
NDNRPEFLHQVWNGSVPEGSKPGTYVMTVTAIDADDPNALNGMLRYRIVSQAPSTPSPNMFT
INNETGDIITVAAGLDREKVQQYTLIIQATDMEGNPTYGLSNTATAVITVTD
>d1edha1 b.1.6.1 (A:3-101) E-cadherin (epithelial) {Mouse (Mus musculus)}
VIPPISCPENEKGEFPKNLVQIKSNRDKETKVFYSITGQGADKPPVGVFIIERETGWLKVTQ
PLDREAIAKYILYSHAVSSNGEAVEDPMEIVITVTDQ
>d1edha2 b.1.6.1 (A:102-213) E-cadherin (epithelial) {Mouse (Mus musculus)}
NDNRPEFTQEVFEGSVAEGAVPGTSVMKVSATDADDDVNTYNAAIAYTIVSQDPELPHKNMF
TVNRDTGVISVLTSGLDRESYPTYTLVVQAADLQGEGLSTTAKAVITVKD
>d2mcm__ b.1.7.1 (-) Macromycin {Streptomyces macromomyceticus}
APGVTVTPATGLSNGQTVTVSATGLTPGTVYHVGQCAVVEPGVIGCDATTSTDVTADAAGKI
TAQLKVHSSFQAVVGADGTPWGTVNCKVVSCSAGLGSDSGEGAAQAITFA
>d1noa__ b.1.7.1 (-) Neocarzinostatin {Streptomyces carzinostaticus}
AAPTATVTPSSGLSDGTVVKVAGAGLQAGTAYDVGQCAWVDTGVLACNPADFSSVTADANGS
ASTSLTVRRSFEGFLFDGTRWGTVDCTTAACQVGLSDAAGNGPEGVAISFN
>d1acx__ b.1.7.1 (-) Actinoxanthin {Actinomyces globisporus, number 1131}
APAFSVSPASGASDGQSVSVSVAAAGETYYIAQCAPVGGQDACNPATATSFTTDASGAASFS
FTVRKSYAGQTPSGTPVGSVDCATDACNLGAGNSGLNLGHVALTFG
>d1hzka_ b.1.7.1 (A:) Antitumor antibiotic C-1027 apoprotein {Streptomyces globisporus}
APAFSVSPASGLSDGQSVSVSVSGAAAGETYYIAQCAPVGGQDACNPATATSFTTDASGAAS
FSFVVRKSYTGSTPEGTPVGSVDCATAACNLGAGNSGLDLGHVALTFG
>d1akp__ b.1.7.1 (-) Kedarcidin (apo form) {Actimomycete, strain L585-6}
ASAAVSVSPATGLADGATVTVSASGFATSTSATALQCAILADGRGACNVAEFHDFSLSGGEG
TTSVVVRRSFTGYVMPDGPEVGAVDCDTAPGGCEIVVGGNTGEYGNAAISFG
>d1cbja_ b.1.8.1 (A:) Cu,Zn superoxide dismutase, SOD {Cow (Bos taurus)}
ATKAVCVLKGDGPVQGTIHFEAKGDTVVVTGSITGLTEGDHGFHVHQFGDNTQGCTSAGPHF
NPLSKKHGGPKDEERHVGDLGNVTADKNGVAIVDIVDPLISLSGEYSIIGRTMVVHEKPDDL
GRGGNEESTKTGNAGSRLACGVIGIAK
>d1azva_ b.1.8.1 (A:) Cu,Zn superoxide dismutase, SOD {Human (Homo sapiens)}
ATKAVCVLKGDGPVQGIINFEQKESNGPVKVWGSIKRLTEGLHGFHVHEFGDNTAGCTSAGP
HFNPLSRKHGGPKDEERHVGDLGNVTADKDGVADVSIEDSVISLSGDHCIIGRTLVVHEKAD
DLGKGGNEESTKTGNAGSRLACGVIGIAQ
>d1xsoa_ b.1.8.1 (A:) Cu,Zn superoxide dismutase, SOD {African clawed frog (Xenopus laevis)}
VKAVCVLAGSGDVKGVVHFEQQDEGAVSVEGKIEGLTDGLHGFHIHVFGDNTNGCMSAGSHF
NPENKNHGAPGDTDRHVGDLGNVTAEGGVAQFKITDSLISLKGPNSIIGRTAVVHEKADDLG

KGGNDESLKTGNAGGRLACGVIGYSP
>d1srda_ b.1.8.1 (A:) Cu,Zn superoxide dismutase, SOD {Spinach (Spinacia oleracea)}
ATKKAVAVLKGTSNVEGVVTLTQEDDGPTTVNVRISGLAPGKHGFHLHEFGDTTNGCMSTGP
HFNPDKKTHGAPEDEVRHAGDLGNIVANTDGVAEATIVDNQIPLTGPNSVVGRALVVHELED
DLGKGGHELSPTTGNAGGRLACGVVGLTPV
>d1jcv__ b.1.8.1 (-) Cu,Zn superoxide dismutase, SOD {Baker's yeast (Saccharomyces cerevisiae)}
VQAVAVLKGDAGVSGVVKFEQASESEPTTVSYEIAGNSPNAERGFHIHEFGDATNGCVSAGP
HFNPFKKTHGAPTDEVRHVGDMGNVKTDENGVAKGSFKDSLIKLIGPTSVVGRSVVIHAGQD
DLGKGDTEESLKTGNAGPRPACGVIGLTN
>d1yaia_ b.1.8.1 (A:) Cu,Zn superoxide dismutase, SOD {Photobacterium leiognathi}
QDLTVKMTDLQTGKPVGTIELSQNKYGVVFTPELADLTPGMHGFHIHQNGSCASSEKDGKVV
LGGAAGGHYDPEHTNKHGFPWTDDNHKGDLPALFVSANGLATNPVLAPRLTLKELKGHAIMI
HAGGDNHSDMPKALGGGGARVACGVIQ
>d1eso__ b.1.8.1 (-) Cu,Zn superoxide dismutase, SOD {Escherichia coli}
ASEKVEMNLVTSQGVGQSIGSVTITETDKGLEFSPDLKALPPGEHGFHIHAKGSCQPATKDG
KASAAESAGGHLDPQNTGKHEGPEGAGHLGDLPALVVNNDGKATDAVIAPRLKSLDEIKDKA
LMVHVGGDNMSDQPKPLGGGGERYACGVIK
>d1eqwa_ b.1.8.1 (A:) Cu,Zn superoxide dismutase, SOD {Salmonella typhimurium}
NTLTVKMNDALSSGTGENIGEITVSETPYGLLFTPHLNGLTPGIHGFHVHTNPSCMPGMKDG
KEVPALMAGGHLDPEKTGKHLGPYNDKGHLGDLPGLVVNADGTATYPLLAPRLKSLSELKGH
SLMIHKGGDNYSDKPAPLGGGGARFACGVIE
>d2apsa_ b.1.8.1 (A:) Cu,Zn superoxide dismutase, SOD {Actinobacillus pleuropneumoniae}
EKLVVQVQQLDPVKGNKDVGTVEITESAYGLVFTPHLHGLAQGLHGFHIHQNPSCEPKEKDG
KLVAGLGAGGHWDPKETKQHGYPWSDNAHLGDLPALFVEHDGSATNPVLAPRLKKLDEVKGH
SLMIHEGGDNHSDHPAPLGGGGPRMACGVIK
>d1ej8a_ b.1.8.1 (A:) Copper chaperone for superoxide dismutase, C-terminal domain {Baker's yeast (Saccharomyces cerevisiae)}
SSAVAILETFQKYTIDQKKDTAVRGLARIVQVGENKTLFDITVNGVPEAGNYHASIHEKGDV
SKGVESTGKVWHKFDEPIECFNESDLGKNLYSGKTFLSAPLPTWQLIGRSFVISKSLNHPEN
EPSSVKDYSFLGVIAR
>d1jk9b1 b.1.8.1 (B:74-245) Copper chaperone for superoxide dismutase, C-terminal domain {Baker's yeast (Saccharomyces cerevisiae)}
GKPNSSAVAILETFQKYTIDQKKDTAVRGLARIVQVGENKTLFDITVNGVPEAGNYHASIHE
KGDVSKGVESTGKVWHKFDEPIECFNESDLGKNLYSGKTFLSAPLPTWQLIGRSFVISKSLN
HPENEPSSVKDYSFLGVIARSAGVWENNKQVCACTGKTVWEERKDALA
>d1do5a_ b.1.8.1 (A:) Copper chaperone for superoxide dismutase, C-terminal domain {Human (Homo sapiens)}

QNLGAAVAILGGPGTVQGVVRFLQLTPERCLIEGTIDGLEPGLHGLHVHQYGDLTNNCNSCG
NHFNPDGASHGGPQDSDRHRGDLGNVRADADGRAIFRMEDEQLKVWDVIGRSLIIDEGEDDL
GRGGHPLSKITGNSGERLACGIIARSAGLF
>d1d7ca_ b.1.9.1 (A:) Cytochrome domain of cellobiose dehydrogenase {Fungus (Phanerochaete chrysosporium)}
ESASQFTDPTTGFQFTGITDPVHDVTYGFVFPPLATSGAQSTEFIGEVVAPIASKWIGIALG
GAMNNDLLLVAWANGNQIVSSTRWATGYVQPTAYTGTATLTTLPETTINSTHWKWVFRCQGC
TEWNNGGGIDVTSQGVLAWAFSNVAVDDPSDPQSTFSEHTDFGFFGIDYSTAHSANYQNYLN
GDSG
>d1i8aa_ b.1.9.2 (A:) Xylanase 10A {Thermotoga maritima}
MVATAKYGTPVIDGEIDEIWNTTEEIETKAVAMGSLDKNATAKVRVLWDENYLYVLAIVKDP
VLNKDNSNPWEQDSVEIFIDENNHKTGYYEDDDAQFRVNYMNEQTFGTGGSPARFKTAVKLI
EGGYIVEAAIKWKTIKPTPNTVIGFNIQVNDANEKGQRVGIISWSDPTNNSWRDPSKFGNLR
LIK
>d1qtsa1 b.1.10.1 (A:692-824) Alpa-adaptin AP2, N-terminal subdomain {Mouse (Mus musculus)}
GSPGIRLGSSEDNFARFVCKNNGVLFENQLLQIGLKSEFRQNLGRMFIFYGNKTSTQFLNFT
PTLICADDLQTNLNLQTKPVDPTVDGGAQVQQVVNIECISDFTEAPVLNIQFRYGGTFQNVS
VKLPITLNK
>d1e42a1 b.1.10.1 (A:705-824) Beta2-adaptin AP2, N-terminal subdomain {Human (Homo sapiens)}
GGYVAPKAVWLPAVKAKGLEISGTFTHRQGHIYMEMNFTNKALQHMTDFAIQFNKNSFGVIP
STPLAIHTPLMPNQSIDVSLPLNTLGPVMKMEPLNNLQVAVKNNIDVFYFSCLIPLNV
>d1jv2a1 b.1.15.1 (A:439-598) Thigh, calf-1 and calf-2 domains of integrin alpha {Human (Homo sapiens)}
PVITVNAGLEVYPSILNQDNKTCSLPGTALKVSCFNVRFCLKADGKGVLPRKLNFQVELLLD
KLKQKGAIRRALFLYSRSPSHSKNMTISRGGLMQCEELIAYLRDESEFRDKLTPITIFMEYR
LDYRTAADTTGLQPILNQFTPANISRQAHILLDCGE
>d1jv2a2 b.1.15.1 (A:599-737) Thigh, calf-1 and calf-2 domains of integrin alpha {Human (Homo sapiens)}
DNVCKPKLEVSVDSDQKKIYIGDDNPLTLIVKAQNQGEGAYEAELIVSIPLQADFIGVVRNN
EALARLSCAFKTENQTRQVVCDLGNPMKAGTQLLAGLRFSVHQQSEMDTSVKFDLQIQSSNL
FDKVSPVVSHKVDLA
>d1jv2a3 b.1.15.1 (A:738-956) Thigh, calf-1 and calf-2 domains of integrin alpha {Human (Homo sapiens)}
VLAAVEIRGVSSPDHVFLPIPNWEHKENPETEEDVGPVVQHIYELRNNGPSSFSKAMLHLQW
PYKYNNNTLLYILHYDIDGPMNCTSDMEINPLRIKISSLQTTEKNDTVAGQGERDHLITKRD
LALSEGDIHTLGCGVAQCLKIVCQVGRLDRGKSAILYVKSLLWTETFMNKENQNHSYSLKSS
ASFNVIEFPYKNLPIEDITNSTLVTTNVTWGIQ
>d1jv2b1 b.1.15.1 (B:55-106,B:355-434) Hybrid domain of integrin beta {Human (Homo sapiens)}
EFPVSEARVLEDRPLSDKGSGDSSQVTQVSPQRIALRLRPDDSKNFSIQVRQXVELEVRDLP
EELSLSFNATCLNNEVIPGLKSCMGLKIGDTVSFSIEAKVRGCPQEKEKSFTIKPVGFKDSL
IVQVTFDCD

>d1qpxa1 b.1.11.1 (A:1-124) Pilus chaperone PapD, N-domain {Escherichia coli}
AVSLDRTRAVFDGSEKSMTLDISNDNKQLPYLAQAWIENENQEKIITGPVIATPPVQRLDPG
AKSMVRLSTTPDISKLPQDRESLFYFNLREIPPRSEKANVVQIALCTKIKLFYRPAAIKTRP
>d1quna1 b.1.11.1 (A:1-121) Periplasmic chaperone FimC {Escherichia coli}
GVALGATRVIYPAGQKQVQLAVTNNDENSTYLIQSWVENADGVKDGRFIVTPPLFAMKGKKE
NTLRILDATNNQLPQDRESLFWMNVKAIPSMDKSKLTENTLQLAIISRIKLYYRPAKLA
>d1mspa_ b.1.11.2 (A:) Major sperm protein, alpha isoform (recombinant), ph 4.6 {Pig roundworm (Ascaris suum)}
SVPPGDINTQPSQKIVFNAPYDDKHTYHIKITNAGGRRIGWAIKTTNMRRLSVDPPCGVLDP
KEKVLMAVSCDTFNAATEDLNNDRITIEWTNTPDGAAKQFRREWFQGDGMVRRKNLPIEYNL
>d4kbpa1 b.1.12.1 (A:9-120) Purple acid phosphatase, N-terminal domain {Kidney bean (Phaseolus vulgaris)}
RDMPLDSDVFRVPPGYNAPQQVHITQGDLVGRAMIISWVTMDEPGSSAVRYWSEKNGRKRIA
KGKMSTYRFFNYSSGFIHHTTIRKLKYNTKYYYEVGLRNTTRRFSFITPP
>d1dqia_ b.1.13.1 (A:) Superoxide reductase (SOR) {Archaeon Pyrococcus furiosus}
MISETIRSGDWKGEKHVPVIEYEREGELVKVKVQVGKEIPHPNTTEHHIRYIELYFLPEGEN
FVYQVGRVEFTAHGESVNGPNTSDVYTEPIAYFVLKTKKKGKLYALSYCNIHGLWENEVTLE
>d1dfx_1 b.1.13.1 (37-125) Desulfoferrodoxin C-terminal domain {Desulfovibrio desulfuricans}
VEGSTDGAMEKHVPVIEKVDGGYLIKVGSVPHPMEEKHWIEWIELLADGRSYTKFLKPGDAP
EAFFAIDASKVTAREYCNLHGHWKAEN
>d1f00i1 b.1.14.1 (I:658-752) Intimin {Escherichia coli}
ASITEIKADKTTAVANGQDAITYTVKVMKGDKPVSNQEVTFTTTLGKLSNSTEKTDTNGYAK
VTLTSTTPGKSLVSARVSDVAVDVKAPEVEFFT
>d1f00i2 b.1.14.1 (I:753-841) Intimin {Escherichia coli}
TLTIDDGNIEIVGTGVKGKLPTVWLQYGQVNLKASGGNGKYTWRSANPAIASVDASSGQVTL
KEKGTTTISVISSDNQTATYTIATPNS
>d1cwva1 b.1.14.1 (A:503-596) Invasin {Yersinia pseudotuberculosis}
LTLTAAVIGDGAPANGKTAITVEFTVADFEGKPLAGQEVVITTNNGALPNKITEKTDANGVA
RIALTNTTDGVTVVTAEVEGQRQSVDTHFVKG
>d1cwva2 b.1.14.1 (A:597-692) Invasin {Yersinia pseudotuberculosis}
TIAADKSTLAAVPTSIIADGLMASTITLELKDTYGDPQAGANVAFDTTLGNMGVITDHNDGT
YSAPLTSTTLGVATVTVKVDGAAFSVPSVTVNFT
>d1cwva3 b.1.14.1 (A:693-795) Invasin {Yersinia pseudotuberculosis}
ADPIPDAGRSSFTVSTPDILADGTMSSTLSFVPVDKNGHFISGMQGLSFTQNGVPVSISPIT
EQPDSYTATVVGNSVGDVTITPQVDTLILSTLQKKISLFPV
>d1cwva4 b.1.14.1 (A:796-886) Invasin {Yersinia pseudotuberculosis}

PTLTGILVNGQNFATDKGFPKTIFKNATFQLQMDNDVANNTQYEWSSSFTPNVSVNDQGQVT
ITYQTYSEVAVTAKSKKFPSYSVSYRFYP
>d1f0la1 b.2.1.1 (A:381-535) Diphtheria toxin, C-terminal domain {Corynebacterium diphtheriae}
SPGHKTQPFLHDGYAVSWNTVEDSIIRTGFQGESGHDIKITAENTPLPIAGVLLPTIPGKLD
VNKSKTHISVNGRKIRMRCRAIDGDVTFCRPKSPVYVGNGVHANLHVAFHRSSSEKIHSNEI
SSDSIGVLGYQKTVDHTKVNSKLSLFFEIKS
>d1exh__ b.2.2.1 (-) Exo-1,4-beta-D-glycanase (cellulase, xylanase), cellulose-binding domain, CBD {Cellulomonas fimi}
ASSGPAGCQVLWGVNQWNTGFTANVTVKNTSSAPVDGWTLTFSFPSGQQVTQAWSSTVTQSG
SAVTVRNAPWNGSIPAGGTAQFGFNGSHTGTNAAPTAFSLNGTPCTVG
>d1e5ba_ b.2.2.1 (A:) Endo-1,4-beta xylanase D, xylan binding domain, XBD {Cellulomonas fimi}
TGCSVTATRAEEWSDGFNVTYSVSGSSAWTVNLALNGSQTIQASWNANVTGSGSTRTVTPNG
SGNTFGVTVMKNGSSTTPAATCAGS
>d1hejc_ b.2.2.1 (C:) Endo-1,4-beta xylanase D, xylan binding domain, XBD {Cellulomonas fimi}
TGSCSVSAVRGEEWADRFNVTYSVSGSSSWVVTLGLNGGQSVQSSWNAALTGSSGTVTARPN
GSGNSFGVTFYKNGSSATPGATCATG
>d1nbca_ b.2.2.2 (A:) Cellusomal scaffolding protein A, scafoldin {Clostridium thermocellum}
NLKVEFYNSNPSDTTNSINPQFKVTNTGSSAIDLSKLTLRYYYTVDGQKDQTFWCDHAAIIG
SNGSYNGITSNVKGTFVKMSSSTNNADTYLEISFTGGTLEPGAHVQIQGRFAKNDWSNYTQS
NDYSFKSASQFVEWDQVTAYLNGVLVWGKEP
>d1g43a_ b.2.2.2 (A:) Cellusomal scaffolding protein A, scafoldin {Clostridium cellulolyticum}
AGTGVVSVQFNNGSSPASSNSIYARFKVTNTSGSPINLADLKLRYYYTQDADKPLTFWCDHA
GYMSGSNYIDATSKVTGSFKAVSPAVTNADHYLEVALNSDAGSLPAGGSIEIQTRFARNDWS
NFDQSNDWSYTAAGSYMDWQKISAFVGGTLAYGSTP
>d1tf4a2 b.2.2.2 (A:461-605) Endo/exocellulase:cellobiose E-4, C-terminal domain {Thermomonospora fusca}
PEIFVEAQINTPGTTFTEIKAMIRNQSGWPARMLDKGTFRYWFTLDEGVDPADITVSSAYNQ
CATPEDVHHVSGDLYYVEIDCTGEKIFPGGQSEHRREVQFRIAGGPGWDPSNDWSFQGIGNE
LAPAPYIVLYDDGVPVWGTAP
>d1anu__ b.2.2.2 (-) Cohesin domain {Clostridium thermocellum, cellulosome, various modules}
VVVEIGKVTGSVGTTVEIPVYFRGVPSKGIANCDFVFRYDPNVLEIIGIDPGDIIVDPNPTK
SFDTAIYPDRKIIVFLFAEDSGTGAYAITKDGVFAKIRATVKSSAPGYITFDEVGGFADNDL
VEQKVSFIDGGVNV
>d1aoha_ b.2.2.2 (A:) Cohesin domain {Clostridium thermocellum, cellulosome, various modules}
AVRIKVDTVNAKPGDTVRIPVRFSGIPSKGIANCDFVYSYDPNVLEIIEIEPGELIVDPNPT
KSFDTAVYPDRKMIVFLFAEDSGTGAYAITEDGVFATIVAKVKSGAPNGLSVIKFVEVGGFA
NNDLVEQKTQFFDGGVNVG

>d1g1ka_ b.2.2.2 (A:) Cohesin domain {Clostridium thermocellum, cellulosome, various modules}
ASLKVTVGTANGKPGDTVTVPVTFADVAKMKNVGTCNFYLGYDASLLEVVSVDAGPIVKNAA
VNFSSSASNGTISFLFLDNTITDELITADGVFANIKFKLKSVTAKTTTPVTFKDGGAFGDGT
MSKIASVTKTNGSVTIDPG
>d1qba_2 b.2.2.3 (28-200) Bacterial chitobiase, n-terminal domain {Serratia marcescens}
DQQLVDQLSQLKLNVKMLDNRAGENGVDCAALGADWASCNRVLFTLSNDGQAIDGKDWVIYF
HSPRQTLRVDNDQFKIAHLTGDLYKLEPTAKFSGFPAGKAVEIPVVAEYWQLFRNDFLPRWY
ATSGDAKPKMLANTDTENLDQFVAPFTGDQWKRTKDDKNILMTPASRFV
>d1amx__ b.2.3.1 (-) Collagen-binding domain of adhesin {Staphylococcus aureus}
TSSVFYYKTGDMLPEDTTHVRWFLNINNEKSYVSKDITIKDQIQGGQQLDLSTLNINVTGTH
SNYYSGQSAITDFEKAFPGSKITVDNTKNTIDVTIPQGYGSYNSFSINYKTKITNEQQKEFV
NNSQAWYQEHGKEEVNGKSFNHTVHN
>d1qunb1 b.2.3.2 (B:1-158) Mannose-specific adhesin FimH {Escherichia coli}
FACKTANGTAIPIGGGSANVYVNLAPVVNVGQNLVVDLSTQIFCHNDYPETITDYVTLQRGS
AYGGVLSNFSGTVKYSGSSYPFPTTSETPRVVYNSRTDKPWPVALYLTPVSSAGGVAIKAGS
LIAVLILRQTNNYNSDDFQFVWNIYANNDVVVPT
>d1qunb2 b.2.3.2 (B:159-279) Mannose-specific adhesin FimH {Escherichia coli}
GGCDVSARDVTVTLPDYPGSVPIPLTVYCAKSQNLGYYLSGTTADAGNSIFTNTASFSPAQG
VGVQLTRNGTIIPANNTVSLGAVGTSAVSLGLTANYARTGGQVTAGNVQSIIGVTFVYQ
>d1pdkb_ b.2.3.2 (B:) PapK pilus subunit {Escherichia coli}
LLDRPCHVSGDSLNKHVVFKTRASRDFWYPPGRSPTESFVIRLENCHATAVGKIVTLTFKGT
EEAALPGHLKVTGVNAGRLGIALLDTDGSSLLKPGTSHNKGQGEKVTGNSLELPFGAYVVAT
PEALRTKSVVPGDYEATATFELTYR
>d1j8ra_ b.2.3.3 (A:) PapG adhesin receptor-binding domain {Escherichia coli}
WNNIVFYSLGDVNSYQGGNVVITQRPQFITSWRPGIATVTWNQCNGPEFADGFWAYYREYIA
WVVFPKKVMTQNGYPLFIEVHNKGSWSEENTGDNDSYFFLKGYKWDERAFDAGNLCQKPGEI
TRLTEKFDDIIFKVALPADLPLGDYSVKIPYTSGMQRHFASYLGARFKIPYNVAKTLPRENE
MLFLFKNIGG
>d1edya_ b.2.4.1 (A:) alpha-1-macroglobulin {Rat (Rattus norvegicus)}
EAPFTLKVNTLPLNFDKAEHHRKFQIHINVSYIGERPNSNMVIVDVKMVSGFIPVKPSVKKL
QDQSNIQRTEVNTNHVLIYIEKLTNQTMGFSFAVEQDIPVKNLKPAPVKVYDYYETDEFAIE
EYSAPFSSDS
>d1bv8a_ b.2.4.1 (A:) alpha-2-macroglobulin {Human (Homo sapiens)}
EEFPFALGVQTLPQTCDEPKAHTSFQISLSVSYTGSRSASNMAIVDVKMVSGFIPLKPTVKM
LERSNHVSRTEVSSNHVLIYLDKVSNQTLSLFFTVLQDVPVRDLKPAIVKVYDYYETDEFAI
AEYNAPCSKDLGNA
>d1ayoa_ b.2.4.1 (A:) alpha-2-macroglobulin {Cow (Bos taurus)}

EFPFALEVQTLPQTCDGPKAHTSFQISLSVSYIGSRPASNMAIVDVKMVSGFIPLKPTVKML
ERSNVSRTEVSNNHVLIYLDKVTNETLTLTFTVLQDIPVRDLKPAIVKVYDYYETDEFAVAE
YSAPCS
>d1ycsa_ b.2.5.1 (A:) p53 tumor suppressor, DNA-binding domain {Human (Homo sapiens)}
VPSQKTYQGSYGFRLGFLHSGTAKSVTCTYSPALNKMFCQLAKTCPVQLWVDSTPPPGTRVR
AMAIYKQSQHMTEVVRRCPHHERCSDSDGLAPPQHLIRVEGNLRVEYLDDRNTFRHSVVVPY
EPPEVGSDCTTIHYNYMCNSSCMGGMNRRPILTIITLEDSSGNLLGRNSFEVRVCACPGRDR
RTEEE
>d1hu8a_ b.2.5.1 (A:) p53 tumor suppressor, DNA-binding domain {Mouse (Mus musculus)}
TYQGNYGFHLGFLQSGTAKSVMCTYSPPLNKLFCQLAKTCPVQLWVSATPPAGSRVRAMAIY
KKSQHMTEVVRRCPHHERCSDGDGLAPPQHLIRVEGNLAPEYLEDRQTFRHSVVVPYEPPEA
GSEYTTIHYKYMCNSSCMGGMNRRPILTIITLEDSSGNLLGRDSFEVRVCACPGRDRRTEEE
>d1a02n2 b.2.5.1 (N:399-576) Transcription factor NFATC, DNA-binding domain {Human (Homo sapiens)}
WPLSSQSGSYELRIEVQPKPHHRAHYETEGSRGAVKAPTGGHPVVQLHGYMENKPLGLQIFI
GTADERILKPHAFYQVHRITGKTVTTTSYEKIVGNTKVLEIPLEPKNNMRATIDCAGILKLR
NADIELRKGETDIGRKNTRVRLVFRVHIPESSGRIVSLQTASNPIECSQRSAHE
>d1a66a_ b.2.5.1 (A:) Transcription factor NFATC, DNA-binding domain {Human (Homo sapiens)}
MKDWQLPSHSGPYELRIEVQPKSHHRARYETEGSRGAVKASAGGHPIVQLHGYLENEPLMLQ
LFIGTADDRLLRPHAFYQVHRITGKTVSTTSHEAILSNTKVLEIPLLPENSMRAVIDCAGIL
KLRNSDIELRKGETDIGRKNTRVRLVFRVHVPQPSGRTLSLQVASNPIECSQRS
>d1imhc2 b.2.5.1 (C:188-367) Transcription factor TONEBP, DNA-binding domain {Human (Homo sapiens)}
KKSPMLCGQYPVKSEGKELKIVVQPETQHRARYLTEGSRGSVKDRTQQGFPTVKLEGHNEPV
VLQVFVGNDSGRVKPHGFYQACRVTGRNTTPCKEVDIEGTTVIEVGLDPSNNMTLAVDCVGI
LKLRNADVEARIGIAGSKKKSTRARLVFRVNIMRKDGSTLTLQTPSSPILCTQPAG
>d1nfka2 b.2.5.1 (A:39-250) p50 subunit of NF-kappa B (NFKB), N-terminal domain {Mouse (Mus musculus)}
GPYLQILEQPKQRGFRFRYVCEGPSHGGLPGASSEKNKKSYPQVKICNYVGPAKVIVQLVTN
GKNIHLHAHSLVGKHCEDGVCTVTAGPKDMVVGFANLGILHVTKKKVFETLEARMTEACIRG
YNPGLLVHSDLAYLQAEGGGDRQLTDREKEIIRQAAVQQTKEMDLSVVRLMFTAFLPDSTGS
FTRRLEPVVSDAIYDSKAPNASNLKI
>d1a3qa2 b.2.5.1 (A:37-226) p52 subunit of NF-kappa B (NFKB), N-terminal domain {Human (Homo sapiens)}
GPYLVIVEQPKQRGFRFRYGCEGPSHGGLPGASSEKGRKTYPTVKICNYEGPAKIEVDLVTH
SDPPRAHAHSLVGKQCSELGICAVSVGPKDMTAQFNNLGVLHVTKKNMMGTMIQKLQRQRLR
SRPQGLTEAEQRELEQEAKELKKVMDLSIVRLRFSAFLRSLPLKPVISQPIHDSKSPGAS
>d1ikna2 b.2.5.1 (A:19-191) p65 subunit of NF-kappa B (NFKB), N-terminal domain {Mouse (Mus musculus)}
PYVEIIEQPKQRGMRFRYKCEGRSAGSIPGERSTDTTKTHPTIKINGYTGPGTVRISLVTKD
PPHRPHPHELVGKDCRDGYYEADLCPDRSIHSFQNLGIQCVKKRDLEQAISQRIQTNNNPFH

VPIEEQRGDYDLNAVRLCFQVTVRDPAGRPLLLTPVLSHPIFDNRAPNT
>d1nfia2 b.2.5.1 (A:20-189) p65 subunit of NF-kappa B (NFKB), N-terminal domain {Human (Homo sapiens)}
YVEIIEQPKQRGMRFRYKCEGRSAGSIPGERSTDTTKTHPTIKINGYTGPGTVRISLVTKDP
PHRPHPHELVGKDCRDGFYEAELCPDRCIHSFQNLGIQCVKKRDLEQAISQRIQTNNNPFQV
PIEEQRGDYDLNAVRLCFQVTVRDPSGRPLRLPPVLPHPIFDNRAP
>d1bvoa_ b.2.5.1 (A:) Dorsal homologue Gambif1 {African malaria mosquito (Anopheles gambiae)}
PYVEITEQPHPKALRFRYECEGRSAGSIPGVNTTAEQKTFPSIQVHGYRGRAVVVVSCVTKE
GPEHKPHPHNLVGKEGCKKGVCTVEINSTTMSYTFNNLGIQCVKKKDVEEALRLRQEIRVDP
FRTGFGHAKEPGSIDLNAVRLCFQVFLEGQQRGRFTEPLTPVVSDIIYDKK
>d1xbra_ b.2.5.1 (A:) T domain from Brachyury transcription factor {African clawed frog (Xenopus laevis)}
ELKVSLEERDLWTRFKELTNEMIVTKNGRRMFPVLKVSMSGLDPNAMYTVLLDFVAADNHRW
KYVNGEWVPGGKPEPQAPSCVYIHPDSPNFGAHWMKDPVSFSKVKLTNKMNGGGQIMLNSLH
KYEPRIHIVRVGGTQRMITSHSFPETQFIAVTAYQNEEITALKIKHNPFAKAFLDAKERN
>d1bf5a2 b.2.5.1 (A:317-568) STAT-1, DNA-binding domain {Human (Homo sapiens)}
FVVERQPCMPTHPQRPLVLKTGVQFTVKLRLLVKLQELNYNLKVKVLFDKDVNERNTVKGFR
KFNILGTHTKVMNMEESTNGSLAAEFRHLQLKEQKNAGTRTNEGPLIVTEELHSLSFETQLC
QPGLVIDLETTSLPVVVISNVSQLPSGWASILWYNMLVAEPRNLSFFLTPPCARWAQLSEVL
SWQFSSVTKRGLNVDQLNMLGEKLLGPNASPDGLIPWTRFCKENINDKNFPFWLWIESILEL
IKKH
>d1bg1a2 b.2.5.1 (A:322-575) STAT3b {Mouse (Mus musculus)}
VVERQPCMPMHPDRPLVIKTGVQFTTKVRLLVKFPELNYQLKIKVCIDKDSGDVAALRGSRK
FNILGTNTKVMNMEESNNGSLSAEFKHLTLREQRCGNGGRANCDASLIVTEELHLITFETEV
YHQGLKIDLETHSLPVVVISNICQMPNAWASILWYNMLTNNPKNVNFFTKPPIGTWDQVAEV
LSWQFSSTTKRGLSIEQLTTLAEKLLGPGVNYSGCQITWAKFCKENMAGKGFSFWVWLDNII
DLVKKY
>d1e50q_ b.2.5.1 (Q:) Acute myeloid leukemia 1 protein (AML1), RUNT domain {Human (Homo sapiens)}
LVRTDSPNFLCSVLPTHWRCNKTLPIAFKVVALGDVPDGTLVTVMAGNDENYSAELRNATAA
MKNQVARFNDLRFVGRSGRGKSFTLTITVFTNPPQVATYHRAIKITVD
>d1h9da_ b.2.5.1 (A:) Acute myeloid leukemia 1 protein (AML1), RUNT domain {Human (Homo sapiens)}
VLADHPGELVRTDSPNFLCSVLPTHWRCNKTLPIAFKVVALGDVPDGTLVTVMAGNDENYSA
ELRNATAAMKNQVARFNDLRFVGRSGRGKSFTLTITVFTNPPQVATYHRAIKITVDGPREPR
R
>d1hcz_1 b.2.6.1 (1-167,231-250) Cytochrome f, large domain {Turnip (Brassica rapa)}
YPIFAQQNYENPREATGRIVCANCHLASKPVDIEVPQAVLPDTVFEAVVKIPYDMQLKQVLA
NGKKGALNVGAVLILPEGFELAPPDRISPEMKEKIGNLSFQNYRPNKKNILVIGPVPGQKYS
EITFPILAPDPATNKDVHFLKYPIYVGGNRGRGQIYPDGSKSNXPNVGGFGQGDAEIVLQDP
LR

>d1e2wa1 b.2.6.1 (A:1-168,A:233-251) Cytochrome f, large domain {Chlamydomonas reinhardtii}
YPVFAQQNYANPREANGRIVCANCHLAQKAVEIEVPQAVLPDTVFEAVIELPYDKQVKQVLA
NGKKGDLNVGMVLILPEGFELAPPDRVPAEIKEKVGNLYYQPYSPEQKNILVVGPVPGKKYS
EMVVPILSPDPAKNKNVSYLKYPIYFGGNRGRGQVYPDGKKSNFXNVGGFGQAETEIVLQNP
AR
>d1ci3m1 b.2.6.1 (M:1-169,M:232-249) Cytochrome f, large domain {Phormidium laminosum}
YPFWAQQNYANPREATGRIVCANCHLAAKPAEIEVPQAVLPDSVFKAVVKIPYDHSVQQVQA
DGSKGPLNVGAVLMLPEGFTIAPEDRIPEEMKEEVGPSYLFQPYADDKQNIVLVGPLPGDEY
EEIVFPVLSPNPATNKSVAFGKYSIHLGANRGRGQIYPTGEKSNNXNVGGFGQKDTEIVLQS
PN
>d1i31a_ b.2.7.1 (A:) Second domain of Mu2 adaptin subunit (ap50) of ap2 adaptor {Rat (Rattus norvegicus)}
IGWRREGIKYRRNELFLDVLESVNLLMSPQGQVLSAHVSGRVVMKSYLSGMPECKFGMNDKI
VIEKQGKGTADETSKSGKQSIAIDDCTFHQCVRLSKFDSERSISFIPPDGEFELMRYRTTKD
IILPFRVIPLVREVGRTKLEVKVVIKSNFKPSLLAQKIEVRIPTPLNTSGVQVICMKGKAKY
KASENAIVWKIKRMAGMKESQISAEIELLPTNDKKKWARPPISMNFEVPFAPSGLKVRYLKV
FEPKLNYSDHDVIKWVRYIGRSGIYETRC
>d1cgt_2 b.3.1.1 (580-684) Cyclodextrin glycosyltransferase, C-terminal domain {Bacillus circulans, different strains}
LTGDQVTVRFVVNNASTTLGQNLYLTGNVAELGNWSTGSTAIGPAFNQVIHQYPTWYYDVSV
PAGKQLEFKFFKKNGSTITWESGSNHTFTTPASGTATVTVNWQ
>d1kcla2 b.3.1.1 (A:582-686) Cyclodextrin glycosyltransferase, C-terminal domain {Bacillus circulans, different strains}
LSGDQVSVRFVVNNATTALGQNVYLTGSVSELGNWDPAKAIGPMYNQVVYQYPNWYYDVSVP
AGKTIEFKFLKKQGSTVTWEGGSNHTFTAPSSGTATINVNWQP
>d1cyg_2 b.3.1.1 (575-680) Cyclodextrin glycosyltransferase, C-terminal domain {Bacillus stearothermophilus}
LTNDQVSVRFVVNNATTNLGQNIYIVGNVYELGNWDTSKAIGPMFNQVVYSYPTWYIDVSVP
EGKTIEFKFIKKDSQGNVTWESGSNHVYTTPTNTTGKIIVDWQN
>d1qhoa2 b.3.1.1 (A:577-686) Cyclodextrin glycosyltransferase, C-terminal domain {Bacillus stearothermophilus, maltogenic alpha-amylase}
LSGTQTSVVFTVKSAPPTNLGDKIYLTGNIPELGNWSTDTSGAVNNAQGPLLAPNYPDWFYV
FSVPAGKTIQFKFFIKRADGTIQWENGSNHVATTPTGATGNITVTWQN
>d1pama2 b.3.1.1 (A:583-686) Cyclodextrin glycosyltransferase, C-terminal domain {Bacillus sp., strain 1011}
TGDQVTVRFVINNATTALGQNVFLTGNVSELGNWDPNNAIGPMYNQVVYQYPTWYYDVSVPA
GQTIEFKFLKKQGSTVTWEGGANRTFTTPTSGTATVNVNWQP
>d1ciu_2 b.3.1.1 (579-683) Cyclodextrin glycosyltransferase, C-terminal domain {Thermoanaerobacterium thermosulfurigenes, EM1}
LTGNQICVRFVVNNASTVYGENVYLTGNVAELGNWDTSKAIGPMFNQVVYQYPTWYYDVSVP
AGTTIQFKFIKKNGNTITWEGGSNHTYTVPSSSTGTVIVNWQQ

>d1acz__ b.3.1.1 (-) Glucoamilase, granular starch-binding domain {Aspergillus niger}
CTTPTAVAVTFDLTATTTYGENIYLVGSISQLGDWETSDGIALSADKYTSSDPLWYVTVTLP
AGESFEYKFIRIESDDSVEWESDPNREYTVPQACGTSTATVTDTWR
>d1cqya_ b.3.1.1 (A:) beta-amylase {Bacillus cereus}
TPVMQTIVVKNVPTTIGDTVYITGNRAELGSWDTKQYPIQLYYDSHSNDWRGNVVLPAERNI
EFKAFIKSKDGTVKSWQTIQQSWNPVPLKTTSHTSSW
>d1h8la1 b.3.2.1 (A:305-383) Carboxypeptidase D, a regulatory domain {Crested duck (Lophonetta specularioides)}
GIWGFVLDATDGRGILNATISVADINHPVTTYKDGDYWRLLVQGTYKVTASARGYDPVTKTV
EVDSKGGVQVNFTLSRT
>d1vcbc_ b.3.3.1 (C:) VHL {Human (Homo sapiens)}
LRSVNSREPSQVIFCNRSPRVVLPVWLNFDGEPQPYPTLPPGTGRRIHSYRGHLWLFRDAGT
HDGLLVNQTELFVPSLNVDGQPIFANITLPVYTLKERCLQVVRSLVKPENYRRLDIVRSLYE
DLEDHPNVQKDLERLTQE
>d1f86a_ b.3.4.1 (A:) Transthyretin (synonym: prealbumin) {Human (Homo sapiens)}
CPLMVKVLDAVRGSPAINVAVHVFRKAADDTWEPFASGKTSESGELHGLTTEEEFVEGIYKV
EIDTKSYWKALGISPFHEHAEVVFTANDSGPRRYTIAALLSPYSYSTMAVVTN
>d1ttba_ b.3.4.1 (A:) Transthyretin (synonym: prealbumin) {Human (Homo sapiens)}
GPTGTGESKCPLMVKVLDAVRGSPAINVAVHVFRKAADDTWEPFASGKTSESGELHGLTTEE
EFVEGIYKVEIDTKSYWKALGISPFHEHAEVVFTANDSGPRRYTIATLLSPYSYSTTAVVTN
PKE
>d1gkea_ b.3.4.1 (A:) Transthyretin (synonym: prealbumin) {Rat (Rattus norvegicus)}
SKCPLMVKVLDAVRGSPAVDVAVKVFKKTADGSWEPFASGKTAESGELHGLTTDEKFTEGVY
RVELDTKSYWKALGISPFHEYAEVVFTANDSGHRHYTIAALLSPYSYSTTAVVSNPQN
>d1tfpa_ b.3.4.1 (A:) Transthyretin (synonym: prealbumin) {Chicken (Gallus gallus)}
CPLMVKVLDAVRGSPAANVAVKVFKKAADGTWQDFATGKTTEFGEIHELTTEEQFVEGVYRV
EFDTSSYWKGLGLSPFHEYADVVFTANDSGHRHYTIAALLSPFSYSTTAVVS
>d1d2oa1 b.3.5.1 (A:535-624) B repeat unit of collagen binding surface protein (cna) {Staphylococcus aureus}
ETTSSIGEKVWDDKDNQDGKRPEKVSVNLLANGEKVKTLDVTSETNWKYEFKDLPKYDEGKK
IEYTVTEDHVKDYTTDINGTTITNKYTP
>d1d2oa2 b.3.5.1 (A:625-721) B repeat unit of collagen binding surface protein (cna) {Staphylococcus aureus}
GETSATVTKNWDDNNNQDGKRPTEIKVELYQDGKATGKTAILNESNNWTHTWTGLDEKAKGQ
QVKYTVEELTKVKGYTTHVDNNDMGNLITTNKYTP
>d1dmha_ b.3.6.1 (A:) Catechol 1,2-dioxygenase {Acinetobacter calcoaceticus}
VKIFNTQDVQDFLRVASGLEQEGGNPRVKQIIHRVLSDLYKAIEDLNITSDEYWAGVAYLNQ
LGANQEAGLLSPGLGFDHYLDMRMDAEDAALGIENATPRTIEGPLYVAGAPESVGYARMDDG

SDPNGHTLILHGTIFDADGKPLPNAKVEIWHANTKGFYSHFDPTGEQQAFNMRRSIITDENG
QYRVRTILPAGYGCPPEGPTQQLLNQLGRHGNRPAHIHYFVSADGHRKLTTQINVAGDPYTY
DDFAYATREGLVVDAVEHTDPEAIKANDVEGPFAEMVFDLKLTRLVDGVDNQVVDRPRLAV
>d3pcca_ b.3.6.1 (A:) Protocatechuate-3,4-dioxygenase, alpha chain {Pseudomonas aeruginosa}
PIELLPETPSQTAGPYVHIGLALEAAGNPTRDQEIWNRLAKPDAPGEHILLLGQVYDGNGHL
VRDSFLEVWQADANGEYQDAYNLENAFNSFGRTATTFDAGEWTLHTVKPGVVNNAAGVPMAP
HINISLFARGINIHLHTRLYFDDEAQANAKCPVLNLIEQPQRRETLIAKRCEVDGKTAYRFD
IRIQGEGETVFFDF
>d1eo9a_ b.3.6.1 (A:) Protocatechuate-3,4-dioxygenase, alpha chain {Acinetobacter calcoaceticus, adp1}
ELKETPSQTGGPYVHIGLLPKQANIEVFEHNLDNNLVQDNTQGQRIRLEGQVFDGLSLPLRD
VLIEIWQADTNGVYPSQADTQGKQVDPNFLGWGRTGADFGTGFWSFNTIKPGAVPGRKGSTQ
APHISLIIFARGINIGLHTRVYFDDEAEANAKDPVLNSIEWATRRQTLVAKREERDGEVVYR
FDIRIQGENETVFFDI
>d3pccm_ b.3.6.1 (M:) Protocatechuate-3,4-dioxygenase, beta chain {Pseudomonas aeruginosa}
PAQDNSRFVIRDRNWHPKALTPDYKTSIARSPRQALVSIPQSISETTGPNFSHLGFGAHDHD
LLLNFNNGGLPIGERIIVAGRVVDQYGKPVPNTLVEMWQANAGGRYRHKNDRYLAPLDPNFG
GVGRCLTDSDGYYSFRTIKPGPYPWRNGPNDWRPAHIHFGISGPSIATKLITQLYFEGDPLI
PMCPIVKSIANPEAVQQLIAKLDMNNANPMDCLAYRFDIVLRGQRKTHFE
>d1eo9b_ b.3.6.1 (B:) Protocatechuate-3,4-dioxygenase, beta chain {Acinetobacter calcoaceticus, adp1}
IIWGAYAQRNTEDHPPAYAPGYKTSVLRSPKNALISIAETLSEVTAPHFSADKFGPKDNDLI
LNYAKDGLPIGERVIVHGYVRDQFGRPVKNALVEVWQANASGRYRHPNDQYIGAMDPNFGGC
GRMLTDDNGYYVFRTIKPGPYPWRNRINEWRPAHIHFSLIADGWAQRLISQFYFEGDTLIDS
CPILKTIPSEQQRRALIALEDKSNFIEADSRCYRFDITLRGRRATYFENDLT
>d1c3ga1 b.4.1.1 (A:180-259) Heat shock protein 40 Sis1 {Baker's yeast (Saccharomyces cerevisiae)}
ETVQVNLPVSLEDLFVGKKKSFKIGRKGPHGASEKTQIDIQLKPGWKAGTKITYKNQGDYNP
QTGRRKTLQFVIQEKSHP
>d1c3ga2 b.4.1.1 (A:260-349) Heat shock protein 40 Sis1 {Baker's yeast (Saccharomyces cerevisiae)}
NFKRDGDDLIYTLPLSFKESLLGFSKTIQTIDGRTLPLSRVQPVQPSQTSTYPGQGMPTPKN
PSQRGNLIVKYKVDYPISLNDAQKRAID
>d1hd8a1 b.105.1.1 (A:263-356) Penicillin-binding protein 5, C-terminal domain {Escherichia coli}
FETVNPLKVGKEFASEPVWFGDSDRASLGVDKDVYLTIPRGRMKDLKASYVLNSSELHAPLQ
KNQVVGTINFQLDGKTIEQRPLVVLQEIPEGN
>d1hoe__ b.5.1.1 (-) alpha-Amylase inhibitor tendamistat {Streptomyces tendae}
DTTVSEPAPSCVTLYQSWRYSQADNGCAETVTVKVVYEDDTEGLCYAVAPGQITTVGDGYIG
SHGHARYLARCL
>d1aac__ b.6.1.1 (-) Amicyanin {Paracoccus denitrificans}

DKATIPSESPFAAAEVADGAIVVDIAKMKYETPELHVKVGDTVTWINREAMPHNVHFVAGVL
GEAALKGPMMKKEQAYSLTFTEAGTYDYHCTPHPFMRGKVVVE
>d1id2a_ b.6.1.1 (A:) Amicyanin {Paracoccus versutus (Thiobacillus versutus)}
QDKITVTSEKPVAAADVPADAVVVGIEKMKYLTPEVTIKAGETVYWVNGEVMPHNVAFKKGI
VGEDAFRGEMMTKDQAYAITFNEAGSYDYFCTPHPFMRGKVIVE
>d1plc__ b.6.1.1 (-) Plastocyanin {Poplar (Populus nigra), variant italica}
IDVLLGADDGSLAFVPSEFSISPGEKIVFKNNAGFPHNIVFDEDSIPSGVDASKISMSEEDL
LNAKGETFEVALSNKGEYSFYCSPHQGAGMVGKVTVN
>d9pcy__ b.6.1.1 (-) Plastocyanin {French bean (Phaseolus vulgaris)}
LEVLLGSGDGSLVFVPSEFSVPSGEKIVFKNNAGFPHNVVFDEDEIPAGVDAVKISMPEEEL
LNAPGETYVVTLDTKGTYSFYCSPHQGAGMVGKVTVN
>d1pla__ b.6.1.1 (-) Plastocyanin {Parsley (Petroselinum crispum)}
AEVKLGSDDGGLVFSPSSFTVAAGEKITFKNNAGFPHNIVFDEDEVPAGVNAEKISQPEYLN
GAGETYEVTLTEKGTYKFYCEPHAGAGMKGEVTVN
>d1ag6__ b.6.1.1 (-) Plastocyanin {Spinach (Spinacia oleracea)}
VEVLLGGDDGSLAFLPGDFSVASGEEIVFKNNAGFPHNVVFDEDEIPSGVDAAKISMSEEDL
LNAPGETYKVTLTEKGTYKFYCSPHQGAGMVGKVTVN
>d1bypa_ b.6.1.1 (A:) Plastocyanin {White campion (Silene pratensis)}
AEVLLGSSDGGLAFVPSDLSIASGEKITFKNNAGFPHNDLFDKKEVPAGVDVTKISMPEEDL
LNAPGEEYSVTLTEKGTYKFYCAPHAGAGMVGKVTVN
>d1iuz__ b.6.1.1 (-) Plastocyanin {Sea lettuce (Ulva pertusa)}
AQIVKLGGDDGSLAFVPSKISVAAGEAIEFVNNAGFPHNIVFDEDAVPAGVDADAISYDDYL
NSKGETVVRKLSTPGVYGVYCEPHAGAGMKMTITVQ
>d2plt__ b.6.1.1 (-) Plastocyanin {Green alga (Chlamydomonas reinhardtii)}
DATVKLGADSGALEFVPKTLTIKSGETVNFVNNAGFPHNIVFDEDAIPSGVNADAISRDDYL
NAPGETYSVKLTAAGEYGYYCEPHQGAGMVGKIIVQ
>d7pcy__ b.6.1.1 (-) Plastocyanin {Green alga (Enteromorpha prolifera)}
AAIVKLGGDDGSLAFVPNNITVGAGESIEFINNAGFPHNIVFDEDAVPAGVDADAISAEDYL
NSKGQTVVRKLTTPGTYGVYCDPHSGAGMKMTITVQ
>d1kdj__ b.6.1.1 (-) Plastocyanin {Fern (Adiantum capillus-veneris)}
AKVEVGDEVGNFKFYPDSITVSAGEAVEFTLVGETGHNIVFDIPAGAPGTVASELKAASMDE
NDLLSEDEPSFKAKVSTPGTYTFYCTPHKSANMKGTLTVK
>d1nin__ b.6.1.1 (-) Plastocyanin {Anabaena variabilis}
ETYTVKLGSDKGLLVFEPAKLTIKPGDTVEFLNNKVPPHNVVFDAALNPAKSADLAKSLSHK
QLLMSPGQSTSTTFPADAPAGEYTFYCEPHRGAGMVGKITVAG
>d1bawa_ b.6.1.1 (A:) Plastocyanin {Cyanobacterium (Phormidium laminosum)}

ETFTVKMGADSGLLQFEPANVTVHPGDTVKWVNNKLPPHNILFDDKQVPGASKELADKLSHS
QLMFSPGESYEITFSSDFPAGTYTYYCAPHRGAGMVGKITVEG
>d1pcs__ b.6.1.1 (-) Plastocyanin {Cyanobacterium (Synechocystis sp.), pcc 6803}
ANATVKMGSDSGALVFEPSTVTIKAGEEVKWVNNKLSPHNIVFDADGVPADTAAKLSHKGLL
FAAGESFTSTFTEPGTYTYYCEPHRGAGMVGKVVVE
>d1bxva_ b.6.1.1 (A:) Plastocyanin {Cyanobacterium (Synechocystis sp.), pcc 7942}
QTVAIKMGADNGMLAFEPSTIEIQAGDTVQWVNNKLAPHNVVVEGQPELSHKDLAFSPGETF
EATFSEPGTYTYYCEPHRGAGMVGKIVVQ
>d2b3ia_ b.6.1.1 (A:) Plastocyanin {Photosynthetic prokaryote (Prochlorothrix hollandica)}
ASVQIKMGTDKYAPLYEPKALSISAGDTVEFVMNKVGPHNVIFDKVPAGESAPALSNTKLAI
APGSFYSVTLGTPGTYSFYCTPHRGAGMVGTITVE
>d1paz__ b.6.1.1 (-) Pseudoazurin {Alcaligenes faecalis, strain s-6}
ENIEVHMLNKGAEGAMVFEPAYIKANPGDTVTFIPVDKGHNVESIKDMIPEGAEKFKSKINE
NYVLTVTQPGAYLVKCTPHYAMGMIALIAVGDSPANLDQIVSAKKPKIVQERLEKVIA
>d1pmy__ b.6.1.1 (-) Pseudoazurin {Methylobacterium extorquens, strain am1}
DEVAVKMLNSGPGGMMVFDPALVRLKPGDSIKFLPTDKGHNVETIKGMAPDGADYVKTTVGQ
EAVVKFDKEGVYGFKCAPHYMMGMVALVVVGDKRDNLEAAKSVQHNKLTQKRLDPLFAQIQ
>d1bqk__ b.6.1.1 (-) Pseudoazurin {Achromobacter cycloclastes}
ADFEVHMLNKGKDGAMVFEPASLKVAPGDTVTFIPTDKGHNVETIKGMIPDGAEAFKSKINE
NYKVTFTAPGVYGVKCTPHYGMGMVGVVQVGDAPANLEAVKGAKNPKKAQERLDAALAALGN
>d1adwa_ b.6.1.1 (A:) Pseudoazurin {Thiosphaera pantotropha}
ATHEVHMLNKGESGAMVFEPAFVRAEPGDVINFVPTDKSHNVEAIKEILPEGVESFKSKINE
SYTLTVTEPGLYGVKCTPHFGMGMVGLVQVGDAPENLDAAKTAKMPKKARERMDAELAQVN
>d2cbp__ b.6.1.1 (-) Plantacyanin {Cucumber (Cucumis sativus)}
AVYVVGGSGGWTFNTESWPKGKRFRAGDILLFNYNPSMHNVVVVNQGGFSTCNTPAGAKVYT
SGRDQIKLPKGQSYFICNFPGHCQSGMKIAVNAL
>d1f56a_ b.6.1.1 (A:) Plantacyanin {Spinach (Spinacia oleracea)}
AVYNIGWSFNVNGARGKSFRAGDVLVFKYIKGQHNVVAVNGRGYASCSAPRGARTYSSGQDR
IKLTRGQNYFICSFPGHCGGGMKIAINAK
>d1azca_ b.6.1.1 (A:) Azurin {Alcaligenes denitrificans}
AQCEATIESNDAMQYNLKEMVVDKSCKQFTVHLKHVGKMAKVAMGHNWVLTKEADKQGVATD
GMNAGLAQDYVKAGDTRVIAHTKVIGGGESDSVTFDVSKLTPGEAYAYFCSFPGHWAMMKGT
LKLSN
>d1dyza_ b.6.1.1 (A:) Azurin {Alcaligenes xylosoxidans, NCIMB (11015), different isoforms}
AQCEATVESNDAMQYNVKEIVVDKSCKQFTMHLKHVGKMAKVAMGHNLVLTKDADKQAVATD
GMGAGLAQDYVKAGDTRVIAHTKVIGGGESDSVTFDVSKIAAGENYAYFCSFPGHWAMMKGT
LKLGS
>d1rkra_ b.6.1.1 (A:) Azurin {Alcaligenes xylosoxidans, NCIMB

(11015), different isoforms}
AECSVDIAGNDGMQFDKKEITVSKSCKQFTVNLKHPGKLAKNVMGHNWVLTKQADMQGAVND
GMAAGLDNNYVKKDDARVIAHTKVIGGGETDSVTFDVSKLAAGEDYAYFCSFPGHFALMKGV
LKLVD
>d1cc3a_ b.6.1.1 (A:) Azurin {Pseudomonas aeruginosa}
AECSVDIQGNDQMQFNTNAITVDKSCKQFTVNLSHPGNLPKNVMGHNWVLSTAADMQGVVTD
GMASGLDKDYLKPDDSRVIAHTKLIGSGEKDSVTFDVSKLKEGEQYMFFCSELCGINHALMK
GTLTLK
>d1jzga_ b.6.1.1 (A:) Azurin {Pseudomonas aeruginosa}
AECSVDIQGNDQMQFNTNAITVDKSCKQFTVNLSHPGNLPKNVMGHNWVLSTAADMQGVVTD
GMASGLDKDYLKPDDSRVIAHTKLIGSGEKDSVTFDVSKLKEGEQYMFFCTFPGHSALMKGT
LTLK
>d1joi__ b.6.1.1 (-) Azurin {Pseudomonas fluorescens}
AECKVTVDSTDQMSFNTKAIEIDKSCKTFTVELTHSGSLPKNVMGHNWVLSSAADMPGIASD
GMAAGIDKNYLKEGDTRVIAHTKIIGAGEKDSVTFDVSKLAAGTDYAFFCSFPGHISMMKGT
VTVK
>d1nwpa_ b.6.1.1 (A:) Azurin {Pseudomonas putida}
AECKVTVDSTDQMSFNTKDIAIDKSCKTFTVELTHSGSLPKNVMGHNLVISKEADMQPIATD
GLSAGIDKQYLKDGDARVIAHTKVIGAGEKDSVTFDVSKLAAGEKYGFFCSFPGHISMMKGT
VTLK
>d1cuoa_ b.6.1.1 (A:) Azurin {Methylomonas sp. j}
ASCETTVTSGDTMTYSTRSISVPASCAEFTVNFEHKGHMPKTGMGHNWVLAKSADVGDVAKE
GAHAGADNNFVTPGDKRVIAFTPIIGGGEKTSVKFKVSALSKDEAYTYFCSYPGHFSMMRGT
LKLEE
>d1qhqa_ b.6.1.1 (A:) Auracyanin {Chloroflexus aurantiacus}
ANAPGGSNVVNETPAQTVEVRAAPDALAFAQTSLSLPANTVVRLDFVNQNNLGVQHNWVLVN
GGDDVAAAVNTAAQNNADALFVPPPDTPNALAWTAMLNAGESGSVTFRTPAPGTYLYICTFP
GHYLAGMKGTLTVTP
>d1e30a_ b.6.1.1 (A:) Rusticyanin {Thiobacillus ferrooxidans}
LDTTWKEATLPQVKAMLEKDTGKVSGDTVTYSGKTVHVVAAAVLPGFPFPSFEVHDKKNPTL
EIPAGATVDVTFINTNKGFGHSFDITKKGPPYAVMPVIDPIVAGTGFSPVPKDGKFGYTNFT
WHPTAGTYYYVCQIPGHAATGQFGKIVVK
>d1jer__ b.6.1.1 (-) Stellacyanin {Cucumber (Cucumis sativus)}
MQSTVHIVGDNTGWSVPSSPNFYSQWAAGKTFRVGDSLQFNFPANAHNVHEMETKQSFDACN
FVNSDNDVERTSPVIERLDELGMHYFVCTVGTHCSNGQKLSINVVAAN
>d1ibya_ b.6.1.4 (A:) Red copper protein nitrosocyanin
{Nitrosomonas europaea}
EHNFNVVINAYDTTIPELNVEGVTVKNIRAFNVLNEPETLVVKKGDAVKVVVENKSPISEGF
SIDAFGVQEVIKAGETKTISFTADKAGAFTIWCQLHPKNIHLPGTLNVVE
>d1qnia1 b.6.1.4 (A:451-581) Nitrous oxide reductase, C-terminal
domain {Pseudomonas nautica}
KIYERNDPYFASCRAQAEKDGVTLESDNKVIRDGNKVRVYMTSVAPQYGMTDFKVKEGDEVT
VYITNLDMVEDVTHGFCMVNHGVSMEISPQQTASVTFTAGKPGVYWYYCNWFCHALHMEMVG
RMLVEAA

>d1fwxa1 b.6.1.4 (A:452-581) Nitrous oxide reductase, C-terminal domain {Paracoccus denitrificans}
SVWDRNDPMWAETRAQAEADGVDIDNWTEEVIRDGNKVRVYMSSVAPSFSIESFTVKEGDEV
TVIVTNLDEIDDLTHGFTMGNYGVAMEIGPQMTSSVTFVAANPGVYWYYCQWFCHALHMEMR
GRMLVEPK
>d1cyx__ b.6.1.2 (-) Quinol oxidase (CyoA) {Escherichia coli}
KPITIEVVSMDWKWFFIYPEQGIATVNEIAFPANTPVYFKVTSNSVMHSFFIPRLGSQIYAM
AGMQTRLHLIANEPGTYDGICAEICGPGHSGMKFKAIATPDRAAFDQWVAKAKQSPNTMSDM
AAFEKLAAPSEYNQVEYFSNVKPDLFADVINKFM
>d1fftb1 b.6.1.2 (B:118-283) Quinol oxidase (CyoA) {Escherichia coli}
KPLAHDEKPITIEVVSMDWKWFFIYPEQGIATVNEIAFPANTPVYFKVTSNSVMNSFFIPRL
GSQIYAMAGMQTRLHLIANEPGTYDGISASYSGPGFSGMKFKAIATPDRAAFDQWVAKAKQS
PNTMSDMAAFEKLAAPSEYNQVEYFSNVKPDLFADVINKFMA
>d1ocrb1 b.6.1.2 (B:91-227) Cytochrome c oxidase {Cow (Bos taurus)}
NNPSLTVKTMGHQWYWSYEYTDYEDLSFDSYMIPTSELKPGELRLLEVDNRVVLPMEMTIRM
LVSSEDVLHSWAVPSLGLKTDAIPGRLNQTTLMSSRPGLYYGQCSEICGSNHSFMPIVLELV
PLKYFEKWSASML
>d1ar1b1 b.6.1.2 (B:108-252) Cytochrome c oxidase {Paracoccus denitrificans}
NDPDLVIKAIGHQWYWSYEYPNDGVAFDALMLEKEALADAGYSEDEYLLATDNPVVVPVGKK
VLVQVTATDVIHAWTIPAFAVKQDAVPGRIAQLWFSVDQEGVYFGQCSELCGINHAYMPIVV
KAVSQEKYEAWLAGAKEEFAA
>d2cuaa_ b.6.1.2 (A:) Cytochrome c oxidase {Thermus thermophilus, ba3 type}
AGKLERVDPTTVRQEGPWADPAQAVVQTGPNQYTVYVLAFAFGYQPNPIEVPQGAEIVFKIT
SPDVIHGFHVEGTNINVEVLPGEVSTVRYTFKRPGEYRIICNQYCGLGHQNMFGTIVVKE
>d2cuab_ b.6.1.2 (B:) Cytochrome c oxidase {Thermus thermophilus, ba3 type}
AYTLATHTAGVIPAGKLERVDPTTVRQEGPWADPAQAVVQTGPNQYTVYVLAFAFGYQPNPI
EVPQGAEIVFKITSPDVIHGFHVEGTNINVEVLPGEVSTVRYTFKRPGEYRIICNQYCGLGH
QNMFGTIVVKE
>d1nif_1 b.6.1.3 (8-166) Nitrite reductase, NIR {Achromobacter cycloclastes}
DISTLPRVKVDLVKPPFVHAHDQVAKTGPRVVEFTMTIEEKKLVIDREGTEIHAMTFNGSVP
GPLMVVHENDYVELRLINPDTNTLLHNIDFHAATGALGGGALTQVNPGEETTLRFKATKPGV
FVYHCAPEGMVPWHVTSGMNGAIMVLPRDGLKDEK
>d1nif_2 b.6.1.3 (167-340) Nitrite reductase, NIR {Achromobacter cycloclastes}
GQPLTYDKIYYVGEQDFYVPKDEAGNYKKYETPGEAYEDAVKAMRTLTPTHIVFNGAVGALT
GDHALTAAVGERVLVVHSQANRDTRPHLIGGHGDYVWATGKFRNPPDLDQETWLIPGGTAGA
AFYTFRQPGVYAYVNHNLIEAFELGAAGHFKVTGEWNDDLMTSVVKPASM
>d1j9qa1 b.6.1.3 (A:4-166) Nitrite reductase, NIR {Alcaligenes faecalis, strain s-6}

ATAAEIAALPRQKVELVDPPFVHAHSQVAEGGPKVVEFTMVIEEKKIVIDDAGTEVHAMAFN
GTVPGPLMVVHQDDYLELTLINPETNTLMHNINFHAATGALGGGGLTEINPGEKTILRFKAT
KPGVFVYHCAPPGMVPWHVVSGMNGAIMVLPREGLHDGK
>d1j9qa2 b.6.1.3 (A:167-339) Nitrite reductase, NIR {Alcaligenes faecalis, strain s-6}
GKALTYDKIYYVGEQDFYVPRDENGKYKKYEAPGDAYEDTVKVMRTLTPTHVVFNGAVGALT
GDKAMTAAVGEKVLIVHSQANRDTRPHLIGGHGDYVWATGKFNTPPDVDQETWFIPGGAAGA
AFYTFQQPGIYAYVNHNLIEAFELGAAAHFKVTGEWNDDLMTSVLAPSG
>d1gs7a1 b.6.1.3 (A:1-159) Nitrite reductase, NIR {Alcaligenes xylosoxidans}
QDADKLPHTKVTLVAPPQVHPHEQATKSGPKVVEFTMTIEEKKMVIDDKGTTLQAMTFNGSM
PGPTLVVHEGDYVQLTLVNPATNAMPHNVDFHGATGALGGAKLTNVNPGEQATLRFKADRSG
TFVYHCAPEGMVPWHVVSGMSGTLMVLPRDGLKDP
>d1gs7a2 b.6.1.3 (A:160-336) Nitrite reductase, NIR {Alcaligenes xylosoxidans}
QGKPLHYDRAYTIGEFDLYIPKGPDGKYKDYATLAESYGDTVQVMRTLTPSHIVFNGKVGAL
TGANALTAKVGETVLLIHSQANRDTRPHLIGGFGDWVWETGKFANPPQRDLETWFIRGGSAG
AALYTFKQPGVYAYLNHNLIEAFELGAAGHIKVEGKWNDDLMKQIKAPAPIPR
>d1ndsa1 b.6.1.3 (A:11-166) Nitrite reductase, NIR {Alcaligenes xylosoxidans}
GLPRVAVDLVAPPLVHPHSQVAAGAPKVVQFRMSIEEKKMVADDDGTTAQAMTFNGSVPGPT
LVVHEGDYIELTLVNPATNSMPHNVDFHAATGALGGAGLTQVVPGQEAVLRFKADRSGTFVY
HCAPAGMVPWHVVSGMNGALMVLPRDGLRDAA
>d1ndsa2 b.6.1.3 (A:167-340) Nitrite reductase, NIR {Alcaligenes xylosoxidans}
GAALAYDRVYTIGESDLYVPKAADGNYSDYPALASAYADTVAVMRTLTPSHAVFNGAVGALT
GANALTAAVGESVLIIHSQANRDSRPHLIGGHGDWVWTTGKFANPPQLNMETWFIPGGSAAA
ALYTFKQPGTYAYLSHNLIEAMELGAAAQASVEGQWDDDLMTSVAAPGPA
>d1kbva1 b.6.1.3 (A:13-163) Nitrite reductase, NIR {Neisseria gonorrhoeae, AniA}
ELPVIDAVTTHAPEVPPAIDRDYPAKVRVKMETVEKTMKMDDGVEYRYWTFDGDVPGRMIRV
REGDTVEVEFSNNPSSTVPHNVDFHAATGQGGGAAATFTAPGRTSTFSFKALQPGLYIYHCA
VAPVGMHIANGMYGLILVEPKEGLPKV
>d1kbva2 b.6.1.3 (A:164-314) Nitrite reductase, NIR {Neisseria gonorrhoeae, AniA}
DKEFYIVQGDFYTKGKKGAQGLQPFDMDKAVAEQPEYVVFNGHVGALTGDNALKAKAGETVR
MYVGNGGPNLVSSFHVIGEIFDKVYVEGGKLINENVQSTIVPAGGSAIVEFKVDIPGNYTLV
DHSIFRAFNKGALGQLKVEGAENPEIM
>d1kv7a1 b.6.1.3 (A:31-170) multi-copper oxidase CueO {Escherichia coli}
RPTLPIPDLLTTDARNRIQLTIGAGQSTFGGKTATTWGYNGNLLGPAVKLQRGKAVTVDIYN
QLTEETTLHWHGLEVPGEVDGGPQGIIPPGGKRSVTLNVDQPAATCWFHPHQHGKTGRQVAM
GLAGLVVIEDDEILKL
>d1kv7a2 b.6.1.3 (A:171-335) multi-copper oxidase CueO

{Escherichia coli}
MLPKQWGIDDVPVIVQDKKFSADGQIDYQLDVMTAAVGWFGDTLLTNGAIYPQHAAPRGWLR
LRLLNGCNARSLNFATSDNRPLYVIASDGGLLPEPVKVSELPVLMGERFEVLVEVNDNKPFD
LVTLPVSQMGMAIAPFDKPHPVMRIQPIAISASGALPDTLS
>d1kv7a3 b.6.1.3 (A:336-516) multi-copper oxidase CueO
{Escherichia coli}
SLPALPSLEGLTVRKLQLSMDPMLDMMGMQMLMEKYGDQAMAGMDHSQMMGHMGHGNMNHMN
HGGKFDFHHANKINGQAFDMNKPMFAAAKGQYERWVISGVGDMMLHPFHIHGTQFRILSENG
KPPAAHRAGWKDTVKVEGNVSEVLVKFNHDAPKEHAYMAHCHLLEHEDTGMMLGFTV
>d1aoza1 b.6.1.3 (A:1-129) Ascorbate oxidase {Zucchini (Cucurbita
pepo medullosa)}
SQIRHYKWEVEYMFWAPNCNENIVMGINGQFPGPTIRANAGDSVVVELTNKLHTEGVVIHWH
GILQRGTPWADGTASISQCAINPGETFFYNFTVDNPGTFFYHGHLGMQRSAGLYGSLIVDPP
QGKKE
>d1aoza2 b.6.1.3 (A:130-338) Ascorbate oxidase {Zucchini
(Cucurbita pepo medullosa)}
PFHYDGEINLLLSDWWHQSIHKQEVGLSSKPIRWIGEPQTILLNGRGQFDCSIAAKYDSNLE
PCKLKGSESCAPYIFHVSPKKTYRIRIASTTALAALNFAIGNHQLLVVEADGNYVQPFYTSD
IDIYSGESYSVLITTDQNPSENYWVSVGTRARHPNTPPGLTLLNYLPNSVSKLPTSPPPQTP
AWDDFDRSKNFTYRITAAMGSPK
>d1aoza3 b.6.1.3 (A:339-552) Ascorbate oxidase {Zucchini
(Cucurbita pepo medullosa)}
PPVKFNRRIFLLNTQNVINGYVKWAINDVSLALPPTPYLGAMKYNLLHAFDQNPPPEVFPED
YDIDTPPTNEKTRIGNGVYQFKIGEVVDVILQNANMMKENLSETHPWHLHGHDFWVLGYGDG
KFSAEEESSLNLKNPPLRNTVVIFPYGWTAIRFVADNPGVWAFHCHIEPHLHMGMGVVFAEG
VEKVGRIPTKALACGGTAKSLINNPKNP
>d1hfua1 b.6.1.3 (A:1-131) Laccase {Inky cap fungus (Coprinus
cinereus)}
AIVNSVDTMTLTNANVSPDGFTRAGILVNGVHGPLIRGGKNDNFELNVVNDLDNPTMLRPTS
IHWHGLFQRGTNWADGADGVNQCPISPGHAFLYKFTPAGHAGTFWYHSHFGTQYCDGLRGPM
VIYDDND
>d1hfua2 b.6.1.3 (A:132-303) Laccase {Inky cap fungus (Coprinus
cinereus)}
PHAALYDEDDENTIITLADWYHIPAPSIQGAAQPDATLINGKGRYVGGPAAELSIVNVEQGK
KYRMRLISLSCDPNWQFSIDGHELTIIEVDGELTEPHTVDRLQIFTGQRYSFVLDANQPVDN
YWIRAQPNKGRNGLAGTFANGVNSAILRYAGAANADPTTSANPNPAQL
>d1hfua3 b.6.1.3 (A:304-503) Laccase {Inky cap fungus (Coprinus
cinereus)}
NEADLHALIDPAAPGIPTPGAADVNLRFQLGFSGGRFTINGTAYESPSVPTLLQIMSGAQSA
NDLLPAGSVYELPRNQVVELVVPAGVLGGPHPFHLHGHAFSVVRSAGSSTYNFVNPVKRDVV
SLGVTGDEVTIRFVTDNPGPWFFHCHIEFHLMNGLAIVFAEDMANTVDANNPPVEWAQLCEI
YDDLPPEATSIQTV
>d1kcw_1 b.6.1.3 (1-192) Ceruloplasmin {Human (Homo sapiens)}
KEKHYYIGIIETTWDYASDHGEKKLISVDTEHSNIYLQNGPDRIGRLYKKALYLQYTDETFR

TTIEKPVWLGFLGPIIKAETGDKVYVHLKNLASRPYTFHSHGITYYKEHEGAIYPDNTTDFQ
RADDKVYPGEQYTYMLLATEEQSPGEGDGNCVTRIYHSHIDAPKDIASGLIGPLIICKKDSL
DKEKEK
>d1kcw_2 b.6.1.3 (193-338) Ceruloplasmin {Human (Homo sapiens)}
HIDREFVVMFSVVDENFSWYLEDNIKTYCSEPEKVDKDNEDFQESNRMYSVNGYTFGSLPGL
SMCAEDRVKWYLFGMGNEVDVHAAFFHGQALTNKNYRIDTINLFPATLFDAYMVAQNPGEWM
LSCQNLNHLKAGLQAFFQVQEC
>d1kcw_3 b.6.1.3 (347-553) Ceruloplasmin {Human (Homo sapiens)}
IRGKHVRHYYIAAEEIIWNYAPSGIDIFTKENLTAPGSDSAVFFEQGTTRIGGSYKKLVYRE
YTDASFTNRKERGPEEEHLGILGPVIWAEVGDTIRVTFHNKGAYPLSIEPIGVRFNKNNEGT
YYSPNYNPQSRSVPPSASHVAPTETFTYEWTVPKEVGPTNADPVCLAKMYYSAVDPTKDIFT
GLIGPMKICKKGSLHANGRQK
>d1kcw_4 b.6.1.3 (554-705) Ceruloplasmin {Human (Homo sapiens)}
DVDKEFYLFPTVFDENESLLLEDNIRMFTTAPDQVDKEDEDFQESNKMHSMNGFMYGNQPGL
TMCKGDSVVWYLFSAGNEADVHGIYFSGNTYLWRGERRDTANLFPQTSLTLHMWPDTEGTFN
VECLTTDHYTGGMKQKYTVNQCRRQSED
>d1kcw_5 b.6.1.3 (706-884) Ceruloplasmin {Human (Homo sapiens)}
STFYLGERTYYIAAVEVEWDYSPQREWEKELHHLQEQNVSNAFLDKGEFYIGSKYKKVVYRQ
YTDSTFRVPVERKAEEEHLGILGPQLHADVGDKVKIIFKNMATRPYSIHAHGVQTESSTVTP
TLPGETLTYVWKIPERSGAGTEDSACIPWAYYSTVDQVKDLYSGLIGPLIVCRRP
>d1kcw_6 b.6.1.3 (892-1040) Ceruloplasmin {Human (Homo sapiens)}
RRKLEFALLFLVFDENESWYLDDNIKTYSDHPEKVNKDDEEFIESNKMHAINGRMFGNLQGL
TMHVGDEVNWYLMGMGNEIDLHTVHFHGHSFQYKHRGVYSSDVFDIFPGTYQTLEMFPRTPG
IWLLHCHVTDHIHAGMETTYTVLQN
>d1qasa2 b.7.1.1 (A:626-756) PI-specific phospholipase C isozyme D1 (PLC-D1), C-terminal domain {Rat (Rattus norvegicus)}
WRPERLRVRIISGQQLPKVNKNKNSIVDPKVIVEIHGVGRDTGSRQTAVITNNGFNPRWDME
FEFEVTVPDLALVRFMVEDYDSSSKNDFIGQSTIPWNSLKQGYRHVHLLSKNGDQHPSATLF
VKISIQD
>d1rlw__ b.7.1.1 (-) Domain from cytosolic phospholipase A2 {Human (Homo sapiens)}
SSHKFTVVVLRATKVTKGAFGDMLDTPDPYVELFISTTPDSRKRTRHFNNDINPVWNETFEF
ILDPNQENVLEITLMDANYVMDETLGTATFTVSSMKVGEKKEVPFIFNQVTEMVLEMSLEVA
SS
>d1d5ra1 b.7.1.1 (A:188-351) Pten tumor suppressor (Phoshphoinositide phosphatase), C-terminal domain {Human (Homo sapiens)}
YRPVALLFHKMMFETIPMFSGGTCNPQFVVCQLKVKIYSSNSGPTRREDKFMYFEFPQPLPV
CGDIKVEFFHKQNKMLKKDKMFHFWVNTFFIPGPEEVDNDKEYLVLTLTKNDLDKANKDKAN
RYFSPNFKVKLYFTKTV
>d1e8xa2 b.7.1.1 (A:357-522) Phoshoinositide 3-kinase (PI3K) {Pig (Sus scrofa)}
CDRKFRVKIRGIDIPVLPRTADLTVFVEANIQYGQQVLCQRRTSPKPFTEEVLWNVWLEFSI
KIKDLPKGALLNLQIYCGKAPALSGKTSAEMPSPESKGKAQLLYYVNLLLIDHRFLLRHGEY

VLHMWQLSGKGEDQGSFNADKLTSATNPDKENSMSISILLDN
>d1e8ya2 b.7.1.1 (A:357-522) Phoshoinositide 3-kinase (PI3K) {Human (Homo sapiens)}
CDRKFRVKIRGIDIPVLPRNTDLTVFVEANIQHGQQVLCQRRTSPKPFTEEVLWNVWLEFSI
KIKDLPKGALLNLQIYCGKAPALSSKASAESPSSESKGKVRLLYYVNLLLIDHRFLLRRGEY
VLHMWQISGKGEDQGSFNADKLTSATNPDKENSMSISILLDN
>d1bdya_ b.7.1.1 (A:) Domain from protein kinase C delta {Rat (Rattus norvegicus)}
MAPFLRISFNSYELGSLQAEDDASQPFCAVKMKEALTTDRGKTLVQKKPTMYPEWKSTFDAH
IYEGRVIQIVLMRAAEDPMSEVTVGVSVLAERCKKNNGKAEFWLDLQPQAKVLMCVQYFLE
>d1gmia_ b.7.1.1 (A:) Domain from protein kinase C epsilon {Rat (Rattus rattus)}
MVVFNGLLKIKICEAVSLKPTAWSLRDAVGPRPQTFLLDPYIALNVDDSRIGQTATKQKTNS
PAWHDEFVTDVCNGRKIELAVFHDAPIGYDDFVANCTIQFEELLQNGSRHFEDWIDLEPEGK
VYVIIDLSGSSG
>d1byna_ b.7.1.2 (A:) Synaptogamin I {Rat (Rattus norvegicus)}
EKLGKLQYSLDYDFQNNQLLVGIIQAAELPALDMGGTSDPYVKVFLLPDKKKKFETKVHRKT
LNPVFNEQFTFKVPYSELGGKTLVMAVYDFDRFSKHDIIGEFKVPMNTVDFGHVTEEWRDLQ
SAEK
>d1dqva1 b.7.1.2 (A:295-424) Synaptogamin I {Rat (Rattus norvegicus)}
GAPCGRISFALRYLYGSDQLVVRILQALDLPAKDSNGFSDPYVKIYLLPDRKKKFQTKVHRK
TLNPIFNETFQFSVPLAELAQRKLHFSVYDFDRFSRHDLIGQVVLDNLLELAEQPPDRPLWR
DILEGG
>d1dqva2 b.7.1.2 (A:425-569) Synaptogamin I {Rat (Rattus norvegicus)}
SEKADLGELNFSLCYLPTAGLLTVTIIKASNLKAMDLTGFSDPYVKASLISEGRRLKKRKTS
IKKNTLNPTYNEALVFDVAPESVENVGLSIAVVDYDCIGHNEVIGVCRVGPEAADPHGREHW
AEMLANPRKPVEHWHQLVEEK
>d1k5wa_ b.7.1.2 (A:) Synaptogamin I {Rat (Rattus norvegicus)}
KLGDICFSLRYVPTAGKLTVVILEAKNLKKMDVGGLSDPYVKIHLMQNGKRLKKKKTTIKKN
TLNPYYNESFSFEVPFEQIQKVQVVVTVLDYDKIGKNDAIGKVFVGYNSTGAELRHWSDMLA
NPRRPIAQWHTLQVEEEVDAMLAV
>d1rsy__ b.7.1.2 (-) Synaptogamin I {Rat (Rattus norvegicus)}
GGGILDSMVEKEEPKEEEKLGKLQYSLDYDFQNNQLLVGIIQAAELPALDMGGTSDPYVKVF
LLPDKKKKFETKVHRKTLNPVFNEQFTFKVPYSELGGKTLVMAVYDFDRFSKHDIIGEFKVP
MNTVDFGHVTEEWRDLQSA
>d1dsya_ b.7.1.2 (A:) C2 domain from protein kinase c (alpha) {Rat (Rattus norvegicus)}
TEKRGRIYLKAEVTDEKLHVTVRDAKNLIPMDPNGLSDPYVKLKLIPDPKNESKQKTKTIRS
TLNPQWNESFTFKLKPSDKDRRLSVEIWDWDRTTRNDFMGSLSFGVSELMKMPASGWYKLLN
QEEGEYYNVPIPE
>d1a25a_ b.7.1.2 (A:) C2 domain from protein kinase c (beta) {Rat (Rattus norvegicus)}

ERRGRIYIQAHIDREVLIVVVRDAKNLVPMDPNGLSDPYVKLKLIPDPKSESKQKTKTIKCS
LNPEWNETFRFQLKESDKDRRLSVEIWDWDLTSRNDFMGSLSFGISELQKAGVDGWFKLLSQ
EEGEYFNV
>d3rpba_ b.7.1.2 (A:) C2b-domain of rabphilin {Rat (Rattus norvegicus)}
RGKILVSLMYSTQQGGLIVGIIRCVHLAAMDANGYSDPFVKLWLKPDMGKKAKHKTQIKKKT
LNPEFNEEFFYDIKHSDLAKKSLDISVWDYDIGKSNDYIGGCQLGISAKGERLKHWYECLKN
KDKKIERWHQLQNENH
>d1qpxa2 b.7.2.1 (A:125-215) PapD {Escherichia coli}
NEVWQDQLILNKVSGGYRIENPTPYYVTVIGLGGSEKQAEEGEFETVMLSPRSEQTVKSANY
NTPYLSYINDYGGRPVLSFICNGSRCSVK
>d1quna2 b.7.2.1 (A:122-205) FimC {Escherichia coli}
LPPDQAAEKLRFRRSANSLTLINPTPYYLTVTELNAGTRVLENALVPPMGESAVKLPSDAGS
NITYRTINDYGALTPKMTGVME
>d1who__ b.7.3.1 (-) Pollen allergen PHL P 2 {Timothy grass (Phleum pratense)}
VPKVTFTVEKGSNEKHLAVLVKYEGDTMAEVELREHGSDEWVAMTKGEGGVWTFDSEEPLQG
PFNFRFLTEKGMKNVFDDVVPEKYTIGATYAP
>d1dcea2 b.7.4.1 (A:241-350) Rab geranylgeranyltransferase alpha-subunit, insert domain {Rat (Rattus norvegicus)}
PHDVLCCVHVSREEACLSVCFSRPLTVGSRMGTLLLMVDEAPLSVEWRTPDGRNRPSHVWLC
DLPAASLNDQLPQHTFRVIWTGSDSQKECVLLKDRPECWCRDSATDEQ
>d1czya1 b.8.1.1 (A:350-501) TNF receptor associated factor 2 (TRAF2) {Human (Homo sapiens)}
YDGVFIWKISDFPRKRQEAVAGRIPAIFSPAFYTSRYGYKMCLRIYLNGDGTGRGTHLSLFF
VVMKGPNDALLRWPFNQKVTLMLLDQNNREHVIDAFRPDVTSSSFQRPVNDMNIASGCPLFC
PVSKMEAKNSYVRDDAIFIKAIVDLTGL
>d1flka1 b.8.1.1 (A:350-504) TNF receptor associated factor 3 (TRAF3) {Human (Homo sapiens)}
YNGVLIWKIRDYKRRKQEAVMGKTLSLYSQPFYTGYFGYKMCARVYLNGDGMGKGTHLSLFF
VIMRGEYDALLPWPFKQKVTLMLMDQGSSRRHLGDAFKPDPNSSSFKKPTGEMNIASGCPVF
VAQTVLENGTYIKDDTIFIKVIVDTSDLPDP
>d1k2fa_ b.8.1.2 (A:) SIAH, seven in absentia homolog {Mouse (Mus musculus)}
SVLFPCKYASSGCEITLPHTEKAEHEELCEFRPYSCPCPGASCKWQGSLDAVMPHLMHQHKS
ITTLQGEDIVFLATDINLPGAVDWVMMQSCFGFHFMLVLEKQEKYDGHQQFFAIVQLIGTRK
QAENFAYRLELNGHRRRLTWEATPRSIHEGIATAIMNSDCLVFDTSIAQLFAENGNLGINVT
ISMC
>d2bn2a_ b.9.1.1 (A:) Neurophysin II {Cow (Bos taurus)}
AMSDLELRQCLPCGPGGKGRCFGPSICCGDELGCFVGTAEALRCQEENYLPSPCQSGQKPCG
SGGRCAAAGICCNDESCVTEPEC
>d1kvp__ b.10.1.1 (-) Bacteriophage capsid proteins {Bacteriophage phi-X174}
SNIQTGAERMPHDLSHLGFLAGQIGRLITISTTPVIAGDSFEMDAVGALRLSPLRRGLAIDS

TVDIFTFYVPHRHVYGEQWIKFMKDGVNATPLPTVNTTGYIDHAAFLGTINPDTNKIPKHLF
QGYLNIYNNYFKAPWMPDRTEANPNELNQDDARYGFRCCHLKNIWTAPLPPETELSRQMTTS
TGMAPVTTKFRDVPNLSGTPLIFRDNKGRTIKTGQLGIGPVDAGFLVAQNTAQAANGERAIP
SNLWADLSNATSIDIMGLQAAYANLHTDQERDYFMQRYRDVISSFGGKTSYDADNRPLLVMR
SNLWASGYDVDGTDQTSLGQFSGRVQQTYKHSVPRFFVPEHGTMFTLALVRFPPTATKEIQY
LNAKGALTYTDIAGDPVLYGNLPPREISMKDVFRSGDSSKKFKIAEGQWYRYAPSYVSPAYH
LLEGFPFIQEPPSGDLQERVLIRHHDYDQCFQSVQLLQWNSQVKFNVTVYRNLPTTRDSIMT
S
>d2bpa1_ b.10.1.1 (1:) Bacteriophage capsid proteins
{Bacteriophage phi-X174}
SNIQTGAERMPHDLSHLGFLAGQIGRLITISTTPVIAGDSFEMDAVGALRLSPLRRGLAIDS
TVDIFTFYVPHRHVYGEQWIKFMKDGVNATPLPTVNTTGYIDHAAFLGTINPDTNKIPKHLF
QGYLNIYNNYFKAPWMPDRTEANPNELNQDDARYGFRCCHLKNIWTAPLPPETELSRQMTTS
TTSIDIMGLQAAYANLHTDQERDYFMQRYRDVISSFGGKTSYDADNRPLLVMRSNLWASGYD
VDGTDQTSLGQFSGRVQQTYKHSVPRFFVPEHGTMFTLALVRFPPTATKEIQYLNAKGALTY
TDIAGDPVLYGNLPPREISMKDVFRSGDSSKKFKIAEGQWYRYAPSYVSPAYHLLEGFPFIQ
EPPSGDLQERVLIRHHDYDQCFQSVQLLQWNSQVKFNVTVYRNLPTTRDSIMTS
>d2bpa2_ b.10.1.1 (2:) Bacteriophage capsid proteins
{Bacteriophage phi-X174}
MFQTFISRHNSNFFSDKLVLTSVTPASSAPVLQTPKATSSTLYFDSLTVNAGNGGFLHCIQM
DTSVNAANQVVSVGADIAFDADPKFFACLVRFESSSVPTTLPTAYDVYPLNGRHDGGYYTVK
DCVTIDVLPRTPGNNVYVGFMVWSNFTATKCRGLVSLNQVIKEIICLQPLK
>d1gff1_ b.10.1.1 (1:) Bacteriophage capsid proteins
{Bacteriophage G4}
VPHDLSHLVFEAGKIGRLKTISWTPVVAGDSFECDMVGAIRLSPLRRGLAVDSRVDIFSFYI
PHRHIYGQQWINFMKDGVNASPLPPVTCSSGWDSAAYLGTIPSSTLKVPKFLHQGYLNIYNN
YFKPPWSDDLTYANPSNMPSEDYKWGVRVANLKSIWTAPLPPDTRTSENMTTGTSTIDIMGL
QAAYAKLHTEQERDYFMTRYRDIMKEFGGHTSYDGDNRPLLLMRSEFWASGYDVDGTDQSSL
GQFSGRVQQTFNHKVPRFYVPEHGVIMTLAVTRFPPTHEMEMHYLVGKENLTYTDIACDPAL
MANLPPREVSLKEFFHSSPDSAKFKIAEGQWYRTQPDRVAFPYNALDGFPFYSALPSTDLKD
RVLVNTNNYDEIFQSMQLAHWNMQTKFNINVYRHMPTTRDSIMTS
>d1gff2_ b.10.1.1 (2:) Bacteriophage capsid proteins
{Bacteriophage G4}
MFQKFISKHNAPINSTQLAATKTPAVAAPVLSVPNLSRSTILINATTTAVTTHSGLCHVVRI
DETNPTNHHALSIAGSLSNVPADMIAFAIRFEVADGVVPTAVPALYDVYPIETFNNGKAISF
KDAVTIDSHPRTVGNDVYAGIMLWSNAWTASTISGVLSVNQVNREATVLQPLK
>d1stma_ b.10.1.2 (A:) SPMV coat protein {Satellite panicum mosaic
virus}
AAATSLVYDTCYVTLTERATTSFQRQSFPTLKGMGDRAFQVVAFTIQGVSAAPLMYNARLYN
PGDTDSVHATGVQLMGTVPRTVRLTPRVGQNNWFFGNTEEAETILAIDGLVSTKGANAPSNT
VIVTGCFRLAPSELQSS
>d1a34a_ b.10.1.2 (A:) STMV coat protein {Satellite tobacco mosaic
virus}
TGDNSNVVTMIRAGSYPKVNPTPTWVRAIPFEVSVQSGIAFKVPVGSLFSANFRTDSFTSVT

VMSVRAWTQLTPPVNEYSFVRLKPLFKTGDSTEEFEGRASNINTRASVGYRIPTNLRQNTVA
ADNVCEVRSNCRQVALVISCCFN
>d2stv__ b.10.1.2 (-) STNV coat protein {Satellite tobacco necrosis virus}
TMRAVKRMINTHLEHKRFALINSGNTNATAGTVQNLSNGIIQGDDINQRSGDQVRIVSHKLH
VRGTAITVSQTFRFIWFRDNMNRGTTPTVLEVLNTANFMSQYNPITLQQKRFTILKDVTLNC
SLTGESIKDRIINLPGQLVNYNGATAVAASNGPGAIFMLQIGDSLVGLWDSSYEAVYTDA
>d1smva_ b.10.1.2 (A:) SMV coat potein {Sesbania mosaic virus}
GAITVLHCELTAEIGVTDSIVVSSELVMPYTVGTWLRGVADNWSKYSWLSVRYTYIPSCPSS
TAGSIHMGFQYDMADTVPVSVNKLSNLRGYVSGQVWSGSAGLCFINNSRCSDTSTAISTTLD
VSELGKKWYPYKTSADYATAVGVDVNIATDLVPARLVIALLDGSSSTAVAAGRIYDTYTIQM
IEPTASALNL
>d1smvc_ b.10.1.2 (C:) SMV coat potein {Sesbania mosaic virus}
QAGISMAPSAQGAMVRIRNPAVSSSRGAITVLHCELTAEIGVTDSIVVSSELVMPYTVGTWL
RGVADNWSKYSWLSVRYTYIPSCPSSTAGSIHMGFQYDMADTVPVSVNKLSNLRGYVSGQVW
SGSAGLCFINNSRCSDTSTAISTTLDVSELGKKWYPYKTSADYATAVGVDVNIATDLVPARL
VIALLDGSSSTAVAAGRIYDTYTIQMIEPTASALNL
>d1f2na_ b.10.1.2 (A:) RYMV capsid protein {Rice yellow mottle virus}
LSSNTWPLHSVEFLADFKRSSTSADATTYDCVPFNLPRVWSLARCYSMWKPTRWDVVYLPEV
SATVAGSIEMCFLYDYADTIPRYTGKMSRTAGFVTSSVWYGAEGCHLLSGGSARNAVVASMD
CSRVGWKRVTSSIPSSVDPNVVNTILPARLAVRSSIKPTVSDTPGKLYVIASMVLRDPVDPT
LNT
>d1f2nc_ b.10.1.2 (C:) RYMV capsid protein {Rice yellow mottle virus}
AEPQLQRAPVAQASRISGTVPGPLSSNTWPLHSVEFLADFKRSSTSADATTYDCVPFNLPRV
WSLARCYSMWKPTRWDVVYLPEVSATVAGSIEMCFLYDYADTIPRYTGKMSRTAGFVTSSVW
YGAEGCHLLSGGSARNAVVASMDCSRVGWKRVTSSIPSSVDPNVVNTILPARLAVRSSIKPT
VSDTPGKLYVIASMVLRDPVDPTLNT
>d1bmv1_ b.10.1.2 (1:) BPMV coat protein {Bean pod mottle virus}
SISQQTVWNQMATVRTPLNFDSSKQSFCQFSVDLLGGGISVDKTGDWITLVQNSPISNLLRV
AAWKKGCLMVKVVMSGNAAVKRSDWASLVQVFLTNSNSTEHFDACRWTKSEPHSWELIFPIE
VCGPNNGFEMWSSEWANQTSWHLSFLVDNPKQSTTFDVLLGISQNFEIAGNTLMPAFSVPQ
>d1bmv2_ b.10.1.2 (2:) BPMV coat protein {Bean pod mottle virus}
METNLFKLSLDDVETPKGSMLDLKISQSKIALPKNTVGGTILRSDLLANFLTEGNFRASVDL
QRTHRIKGMIKMVATVGIPENTGIALACAMNSSIRGRASSDIYTICSQDCELWNPACTKAMT
MSFNPNPCSDAWSLEFLKRTGFHCDIICVTGWTATPMQDVQVTIDWFISSQECVPRTYCVLN
PQNPFVLNRWMGKLTFPQGTSRSVKRMPLSIGGGAGAKSAILMNMPNAVLSMWRYFVGDLVF
EVSKMTSPYIKCTVSFFIAFGNLADDTINFEAFPHKLVQFGEIQEKVVLKFSQEEFLTAWST
QVRPATTLLADGCPYLYAMVHDSSVSTIPGDFVIGVKLTIIENMCAYGLNPGISGSRLLGTI
PQ
>d1a6ca1 b.10.1.2 (A:1-176) TRSV capsid protein {Tobacco ringspot virus}
AVTVVPDPTCCGTLSFKVPKDAKKGKHLGTFDIRQAIMDYGGLHSQEWCAKGIVNPTFTVRM

HAPRNAFAGLSIACTFDDYKRIDLPALGNECPPSEMFELPTKVFMLKDADVHEWQFNYGELT
GHGLCNWANVATQPTLYFFVASTNQVTMAADWQCIVTMHVDMGPVIDRFELN
>d1a6ca2 b.10.1.2 (A:177-348) TRSV capsid protein {Tobacco ringspot virus}
PTMTWPIQLGDTFAIDRYYEAKEIKLDGSTSMLSISYNFGGPVKHSKKHAISYSRAVMSRNL
GWSGTISGSVKSVSSLFCTASFVIFPWECEAPPTLRQVLWGPHQIMHGDGQFEIAIKTRLHS
AATTEEGFGRLGILPLSGPIAPDAHVGSYEFIVHINTWRPDSQVHPPM
>d1a6ca3 b.10.1.2 (A:349-513) TRSV capsid protein {Tobacco ringspot virus}
FSSSELYNWFTLTNLKPDANTGVVNFDIPGYIHDFASKDATVTLASNPLSWLVAATGWHYGE
VDLCISWSRSKQAQAQEGSVSITTNYRDWGAYWQGQARIYDLRRTEAEIPIFLGSYAGATPS
GALGKQNYVRISIVNAKDIVALRVCLRPKSIKFWGRSATLF
>d4sbva_ b.10.1.2 (A:) SBMV coat protein {Southern bean mosaic virus, cow pea strain}
SSMDVTILSHCELSTELAVTVTIVVTSELVMPFTVGTWLRGVAQNWSKYAWVAIRYTYLPSC
PTTTSGAIHMGFQYDMADTLPVSVNQLSNLKGYVTGPVWEGQSGLCFVNNTKCPDTSRAITI
ALDTNEVSEKRYPFKTATDYATAVGVNANIGNILVPARLVTAMEGGSSKTAVNTGRLYASYT
IRLIEPIAAALNL
>d4sbvc_ b.10.1.2 (C:) SBMV coat protein {Southern bean mosaic virus, cow pea strain}
QAGVSMAPIAQGTMVKLRPPMLRSSMDVTILSHCELSTELAVTVTIVVTSELVMPFTVGTWL
RGVAQNWSKYAWVAIRYTYLPSCPTTTSGAIHMGFQYDMADTLPVSVNQLSNLKGYVTGPVW
EGQSGLCFVNNTKCPDTSRAITIALDTNEVSEKRYPFKTATDYATAVGVNANIGNILVPARL
VTAMEGGSSKTAVNTGRLYASYTIRLIEPIAAALNL
>d2tbva_ b.10.1.2 (A:) TBSV coat protein {Tomato bushy stunt virus}
GGVTVTSHREYLTQVNNSSGFVVNGGIVGNSLQLNPSNGTLFSWLPALASNFDQYSFNSVVL
DYVPLCGTTEVGRVALYFDKDSQDPEPADRVELANFGVLKETAPWAEAMLRIPTDKVKRYCN
DSATVDQKLIDLGQLGIATYGGAGADAVGELFLARSVTLYFPQPTNTLLSSKRLDLTGSLAD
ATGPGYLVLTRTPTVLTHTFRATGTFNLSGGLRCLTSLTLGATGAVVINDILAIDNVGTASD
YFLNCTVSSLPATVTFTVSGVAAGILLVGRARANVVNLL
>d2tbvc_ b.10.1.2 (C:) TBSV coat protein {Tomato bushy stunt virus}
IITHVGGVGGSIMAPVAVSRQLVGSKPKFTGRTSGGVTVTSHREYLTQVNNSSGFVVNGGIV
GNSLQLNPSNGTLFSWLPALASNFDQYSFNSVVLDYVPLCGTTEVGRVALYFDKDSQDPEPA
DRVELANFGVLKETAPWAEAMLRIPTDKVKRYCNDSATVDQKLIDLGQLGIATYGGAGADAV
GELFLARSVTLYFPQPTNTLLSSKRLDLTGSLADATGPGYLVLTRTPTVLTHTFRATGTFNL
SGGLRCLTSLTLGATGAVVINDILAIDNVGTASDYFLNCTVSSLPATVTFTVSGVAAGILLV
GRARANVVNLL
>d1cwpa_ b.10.1.2 (A:) Cowpea chlorotic mottle virus {Host: cowpea (Vigna unguiculta), (L.)}
KAIKAWTGYSVSKWTASCAAAEAKVTSAITISLPNELSSERNKQLKVGRVLLWLGLLPSVSG
TVKSCVTETQTTAAASFQVALAVADNSKDVVAAMYPEAFKGITLEQLAADLTIYLYSSAALT
EGDVIVHLEVEHVRPTFDDSFTPVY
>d1c8na_ b.10.1.2 (A:) TNV coat protein {Tobacco necrosis virus}
NSTVVSNSELILNLTPIALAYTVQSLPLIATQPAWLGTIADNYSKWRWVSLRIIYSPKCPTT

TSGTVAMCLSYDRNDVAPGSRVQLSQTYKAINFPPYAGYDGAAILNTDVTPTSAIYVDVDVT
RFDKAWYSTIGTAAFAALTAFDQNQFCPCTVHIGSDGGPAVAVPPGDIFFKYVIELIEPINP
TMN
>d1c8nc_ b.10.1.2 (C:) TNV coat protein {Tobacco necrosis virus}
GVSRAGGFVTAPVIGAMVTRPTVPRFGMRGNSTVVSNSELILNLTPIALAYTVQSLPLIATQ
PAWLGTIADNYSKWRWVSLRIIYSPKCPTTTSGTVAMCLSYDRNDVAPGSRVQLSQTYKAIN
FPPYAGYDGAAILNTDVTPTSAIYVDVDVTRFDKAWYSTIGTAAFAALTAFDQNQFCPCTVH
IGSDGGPAVAVPPGDIFFKYVIELIEPINPTMN
>d1auya_ b.10.1.2 (A:) TYMV coat protein {Turnip yellow mosaic virus}
SPLTIKQPFQSEVLFAGTKDAEASLTIANIDSVSTLTTFYRHASLESLWVTIHPTLQAPTFP
TTVGVCWVPAQSPVTPAQITKTYGGQIFCIGGAIQTLSPLIVKCPLEMMQPRVKDSIQYLDS
PKLLISITAQPTAPPASTCIITVSGTLSMHSPLITDTST
>d1auyb_ b.10.1.2 (B:) TYMV coat protein {Turnip yellow mosaic virus}
MEIDKELAPQDRTVTVATVLPAVPGPSPLTIKQPFQSEVLFAGTKDAEASLTIANIDSVSTL
TTFYRHASLESLWVTIHPTLQAPTFPTTVGVCWVPAQSPVTPAQITKTYGGQIFCIGGAIQT
LSPLIVKCPLEMMQPRVKDSIQYLDSPKLLISITAQPTAPPASTCIITVSGTLSMHSPLITD
TST
>d1e57a_ b.10.1.2 (A:) PHMV coat protein {Physalis mottle virus}
SPAIVLPFQFEATTFGTAETAAQVSLQTADPITKLTAPYRHAQIVECKAILTPTDLAVSNPL
TVYLAWVPANSPATPTQILRVYGGQSFVLGGAISAAKTIEVPLNLDSVNRMLKDSVTYTDTP
KLLAYSRAPTNPSKIPTASIQISGRIRLSKPMLIAN
>d1e57b_ b.10.1.2 (B:) PHMV coat protein {Physalis mottle virus}
VVKVKQASIPAPGSILSQPNTEQSPAIVLPFQFEATTFGTAETAAQVSLQTADPITKLTAPY
RHAQIVECKAILTPTDLAVSNPLTVYLAWVPANSPATPTQILRVYGGQSFVLGGAISAAKTI
EVPLNLDSVNRMLKDSVTYTDTPKLLAYSRAPTNPSKIPTASIQISGRIRLSKPMLIAN
>d1ddla_ b.10.1.2 (A:) DYMV coat protein {Desmodium yellow mottle tymovirus}
MEQDKILAHQASLNTKPSLLPPPVGNPPPVISYPFQITLASLGTEDAADSVSIASNSVLATY
TALYRHAQLKHLKATIHPTYMAPKYPTSVALVWVPANSTATSTQVLDTYGGLHFCIGGSVNS
VKPIDVEANLTNLNPIIKASTTFTDTPKLLYYSKAQATAPTSPTCYLTIQGQIELSSPLLQA
SS
>d1f15a_ b.10.1.2 (A:) CMV coat protein {Cucumber mosaic virus, strain fny}
ERCRPGYTFTSITLKPPKIDRGSYYGKRLLLPDSVTEYDKKLVSRLQIRVNPLPKFDSTVWV
TVRKVPASSDLSVAAISAMFADGASPVLVYQYAASGVQANNKLLYDLSAMRADIGDMRKYAV
LVYSKDDALETDELVLHVDIEHQRIPTSGVLPV
>d1f15b_ b.10.1.2 (B:) CMV coat protein {Cucumber mosaic virus, strain fny}
DANFRVLSQQLSRLNKTLAAGRPTINHPTFVGSERCRPGYTFTSITLKPPKIDRGSYYGKRL
LLPDSVTEYDKKLVSRLQIRVNPLPKFDSTVWVTVRKVPASSDLSVAAISAMFADGASPVLV
YQYAASGVQANNKLLYDLSAMRADIGDMRKYAVLVYSKDDALETDELVLHVDIEHQRIPTSG
VLPV

>d2bbva_ b.10.1.3 (A:) Nodavirus capsid protein {Black beetle virus}
LTRLSQPGLAFLKCAFAPPDFNTDPGKGIPDRFEGKVVTRKDVLNQSINFTANRDTFILIAP
TPGVAYWVADVPAGTFPISTTTFNAVNFPGFNSMFGNAAASRSDQVSSFRYASMNVGIYPTS
NLMQFAGSITVWKCPVKLSNVQFPVATTPATSALVHTLVGLDGVLAVGPDNFSESFIKGVFS
QSVCNEPDFEFSDILEGIQTLPPANVTVATSGQPFNLAAGAEAVSGIVGWGNMDTIVIRVSA
PTGAVNSAILKTWACLEYRPNPNAMLYQFGHDSPPCDEVALQEYRTVARSLPVAVIAAQN
>d2bbvc_ b.10.1.3 (C:) Nodavirus capsid protein {Black beetle virus}
TQTAPVPQQNVPKQPRRRRNRARRNRRQGRAMNMGALTRLSQPGLAFLKCAFAPPDFNTDPG
KGIPDRFEGKVVTRKDVLNQSINFTANRDTFILIAPTPGVAYWVADVPAGTFPISTTTFNAV
NFPGFNSMFGNAAASRSDQVSSFRYASMNVGIYPTSNLMQFAGSITVWKCPVKLSNVQFPVA
TTPATSALVHTLVGLDGVLAVGPDNFSESFIKGVFSQSVCNEPDFEFSDILEGIQTLPPANV
TVATSGQPFNLAAGAEAVSGIVGWGNMDTIVIRVSAPTGAVNSAILKTWACLEYRPNPNAML
YQFGHDSPPCDEVALQEYRTVARSLPVAVIAAQN
>d1nova_ b.10.1.3 (A:) Nodavirus capsid protein {Nodamura virus}
NMLKMSAPGLDFLKCAFASPDFSTDPGKGIPDKFQGLVLPKKHCLTQSITFTPGKQTMLLVA
PIPGIACLKAEANVGASFSGVPLASVEFPGFDQLFGTSATDTAANVTAFRYASMAAGVYPTS
NLMQFAGSIQVYKIPLKQVLNSYSQTVATVPPTNLAQNTIAIDGLEALDALPNNNYSGSFIE
GCYSQSVCNEPEFEFHPIMEGYASVPPANVTNAQASMFTNLTFSGARYTGLGDMDAIAILVT
TPTGAVNTAVLKVWACVEYRPNPNSTLYEFARESPANDEYALAAYRKIARDIPIAVACKDN
>d1novc_ b.10.1.3 (C:) Nodavirus capsid protein {Nodamura virus}
RRRAAPRQQQRQQSNRASNQPRRRRARRTRRQQRMAATNNMLKMSAPGLDFLKCAFASPDFS
TDPGKGIPDKFQGLVLPKKHCLTQSITFTPGKQTMLLVAPIPGIACLKAEANVGASFSGVPL
ASVEFPGFDQLFGTSATDTAANVTAFRYASMAAGVYPTSNLMQFAGSIQVYKIPLKQVLNSY
SQTVATVPPTNLAQNTIAIDGLEALDALPNNNYSGSFIEGCYSQSVCNEPEFEFHPIMEGYA
SVPPANVTNAQASMFTNLTFSGARYTGLGDMDAIAILVTTPTGAVNTAVLKVWACVEYRPNP
NSTLYEFARESPANDEYALAAYRKIARDIPIAVACKDN
>g1f8v.1 b.10.1.3 (A:,D:) Nodavirus capsid protein {Pariacoto virus}
NRRNKARKVVSRSTALVPMAPASQRTGPAPRKPRKRNQALVRNPRLTDAGLAFLKCAFAAPD
FSVDPGKGIPDNFHGRTLAIKDCNTTSVVFTPNTDTYIVVAPVPGFAYFRAEVAVGAQPTTF
VGVPYPTYATNFGAGSQNGLPAVNNYSKFRYASMACGLYPTSNMMQFSGSVQVWRVDLNLSE
AVNPAVTAITPAPGVFANFVDKRINGLRGIRPLAPRDNYSGNFIDGAYTFAFDKSTDFEWCD
FVRSLEFSESNVLGAATAMKLLAPGGGTDTTLTGLGNVNTLVYKISTPTGAVNTAILRTWNC
IELQPYTDSALFQFSGVSPPFDPLALECYHNLKMRFPVAVSSRENXSKFWEGVLRVLNQISG
TLSVIPGPVGTISAGVHQLTGMYM
>g1f8v.2 b.10.1.3 (B:,E:) Nodavirus capsid protein {Pariacoto virus}
NPRLTDAGLAFLKCAFAAPDFSVDPGKGIPDNFHGRTLAIKDCNTTSVVFTPNTDTYIVVAP
VPGFAYFRAEVAVGAQPTTFVGVPYPTYATNFGAGSQNGLPAVNNYSKFRYASMACGLYPTS
NMMQFSGSVQVWRVDLNLSEAVNPAVTAITPAPGVFANFVDKRINGLRGIRPLAPRDNYSGN
FIDGAYTFAFDKSTDFEWCDFVRSLEFSESNVLGAATAMKLLAPGGGTDTTLTGLGNVNTLV
YKISTPTGAVNTAILRTWNCIELQPYTDSALFQFSGVSPPFDPLALECYHNLKMRFPVAVSS

RENXSKFWEGVLRVLNQISGTLSVIP
>d1dnv__ b.10.1.3 (-) Galleria mellonella densovirus capsid protein {Wax moth (Galleria mellonella), densovirus}
VYIIPRPFSNFGKKLSTYTKSHKFMIFGLANNVIGPTGTGTTAVNRLLTTCLAEIPWQKLPL
YMNQSEFDLLPPGSRVVECNVKVIFRTNRIAFETSSTVTKQATLNQISNVQTAIGLNKLGWG
INRAFTAFQSDQPMIPTATTAPKYEPVTGDTGYRGMIADYYGADSTNDTAFGNAGNYPHHQV
SSFTFLQNYYCMYQQTNQGTGGWPCLAEHLQQFDSKTVNNQCLIDVTYKPKMGLIKSPLNYK
IIGQPTVKGTISVGDNLVNMRGAVVTNPPEATQNVAESTHNLTRNFPADLFNIYSDIEKSQV
LHKGPWGHENPQIQPSVHIGIQAVPALTTGALLINSSPLNSWTDSMGYIDVMSSCTVMEAQP
THFPFSTEANTNPGNTIYRINLTPNSLTSAFNGLYGNGATLGN
>d1b35a_ b.10.1.3 (A:) Cricket paralysis virus (CRPV) {Host: australian black field cricket (Teleogryllus commodus)}
VMGEDQQIPRNEAQHGVHPISIDTHRISNNWSPQAMCIGEKVVSIRQLIKRFGIFGDANTLQ
ADGSSFVVAPFTVTSPTKTLTSTRNYTQFDYYYYLYAFWRGSMRIKMVAETQDGTGTPRKKT
NFTWFVRMFNSLQDSFNSLISTSSSAVTTTVLPSGTINMGPSTQVIDPTVEGLIEVEVPYYN
ISHITPAVTIDDGTPSMEDYLKGHSPPCLLTFSPRDSISATNHIITASFMRALGDDFSFMYL
LGVPPLVNVARA
>d1b35b_ b.10.1.3 (B:) Cricket paralysis virus (CRPV) {Host: australian black field cricket (Teleogryllus commodus)}
ENSHIENEDKRLTSEQKEIVHFVSEGVTPSTTALPDIVNLSTNYLDKNTREDRIHSIKDFLS
RPIIIATNLWSVSDPVEKQLYTANFPEVLISNAMYQDKLKGFVGLRATLVVKVQVNSQPFQQ
GRLMLQYIPYAQYMPNRVTLINETLQGRSGCPRTDLELSVGTEVEMRIPYVSPHLYYNLITG
QGSFGSIYVVVYSQLHDQVSGTGSIEYTVWAHLEDVDVQYPTGANIFTGNEAYIKGTSRYDA
AQKAHAA
>d1b35c_ b.10.1.3 (C:) Cricket paralysis virus (CRPV) {Host: australian black field cricket (Teleogryllus commodus)}
SKPTVQGKIGECKLRGQGRMANFDGMDMSHKMALSSTNEIETNEGLAGTSLDVMDLSRVLSI
PNYWDRFTWKTSDVINTVLWDNYVSPFKVKPYSATITDRFRCTHMGKVANAFTYWRGSMVYT
FKFVKTQYHSGRLRISFIPYYYNTTISTGTPDVSRTQKIVVDLRTSTAVSFTVPYIGSRPWL
YCIRPESSWLSKDNTDGALMYNCVSGIVRVEVLNQLVAAQNVFSEIDVICEVNGGPDLEFAG
PTCPRYVPYAGDFTLADTRKIEAERTQEYSNNED
>d1sida_ b.10.1.4 (A:) Murine polyomavirus coat protein vp1 {Murine polyoma virus, strain small-plaque 16}
KACPRPAPVPKLLIKGGMEVLDLVTGPDSVTEIEAFLNPRMGQPPTPESLTEGGQYYGWSRG
INLATSDTEDSPGNNTLPTWSMAKLQLPMLNEDLTCDTLQMWEAVSVKTEVVGSGSLLDVHG
FNKPTDTVNTKGISTPVEGSQYHVFAVGGEPLDLQGLVTDARTKYKEEGVVTIKTITKKDMV
NKDQVLNPISKAKLDKDGMYPVEIWHPDPAKNENTRYFGNYTGGTTTPPVLQFTNTLTTVLL
DENGVGPLCKGEGLYLSCVDIMGWRVTRNYDVHHWRGLPRYFKITLRKRWVKNPYPMASLIS
SLFNNMLPQVQGQPMEGENTQVEEVRVYDGTEPVPGDPDMTRYVDRFGKTKTVFPG
>d1vpsa_ b.10.1.4 (A:) Murine polyomavirus coat protein vp1 {Murine polyoma virus, strain small-plaque 16}
GGMEVLDLVTGPDSVTEIEAFLNPRMGQPPTPESLTEGGQYYGWSRGINLATSDTEDSPGNN
TLPTWSMAKLQLPMLNEDLTCDTLQMWEAVSVKTEVVGSGSLLDVHGFNKPTDTVNTKGIST
PVEGSQYHVFAVGGEPLDLQGLVTDARTKYKEEGVVTIKTITKKDMVNKDQVLNPISKAKLD

KDGMYPVEIWHPDPAKNENTRYFGNYTGGTTTPPVLQFTNTLTTVLLDENGVGPLCKGEGLY
LSCVDIMGWRVTRNYDVHHWRGLPRYFKITLRKRWVK
>d1fmd1_ b.10.1.4 (1:) Foot-and-mouth desease virus
{Foot-and-mouth disease virus, (strain bfs, 1860)}
TTTTGESADPVTTTVENYGGETQVQRRHHTDVAFVLDRFVKVTVSDNQHTLDVMQAHKDNIV
GALLRAATYYFSDLEIAVTHTGKLTWVPNGAPVSALNNTTNPTAYHKGPVTRLALPYTAPHR
VLATAYTGTTTYTASARGDLAHLTTTHAAHLPTSFNFGAVKAETITELLVRMKRAELYCPRP
ILPIQPTGDRHKQPLVAPAKQ
>d1fmd2_ b.10.1.4 (2:) Foot-and-mouth desease virus
{Foot-and-mouth disease virus, (strain bfs, 1860)}
DKKTEETTLLEDRILTTRNGHTTSTTQSSVGVTFGYATAEDSTSGPNTSALETRVHQAERFF
KMALFDWVPSQNFGHMHKVVLPHEPKGVYGGLVKSYAYMRNGWDVEVTAVGNQFNGGCLLVA
LVPEMGDISDREKYQLTLYPHQFINPRTNMTAHITVPYVGVNRYDQYKQHRPWTLVVMVVAP
LTTNTAGAQQIKVYANIAPTNVHVAGELPSKE
>d1fmd3_ b.10.1.4 (3:) Foot-and-mouth desease virus
{Foot-and-mouth disease virus, (strain bfs, 1860)}
GIFPVACSDGYGNMVTTDPKTADPAYGKVYNPPRTALPGRFTNYLDVAEACPTFLMFENVPY
VSTRTDGQRLLAKFDVSLAAKHMSNTYLAGLAQYYTQYTGTINLHFMFTGPTDAKARYMVAY
VPPGMDAPDNPEEAAHCIHAEWDTGLNSKFTFSIPYISAADYTYTASHEAETTCVQGWVCVY
QITHGKADADALVVSASAGKDFELRLPVDARQQ
>d1qqp1_ b.10.1.4 (1:) Foot-and-mouth desease virus
{Foot-and-mouth disease virus, (strain bfs, 1860)}
TTSAGESADPVTTTVENYGGETQIQRRQHTDVSFIMDRFVKVTPQNQINILDLMQVPSHTLV
GALLRASTYYFSDLEIAVKHEGDLTWVPNGAPEKALDNTTNPTAYHKAPLTRLALPYTAPHR
VLATVYNGECRYSRNAVPNLRGDLQVLAQKVARTLPTSFNYGAIKATRVTELLYRMKRAETY
CPRPLLAIHPTEARHKQKIVAPVK
>d1qqp2_ b.10.1.4 (2:) Foot-and-mouth desease virus
{Foot-and-mouth disease virus, (strain bfs, 1860)}
DKKTEETTLLEDRILTTRNGHTTSTTQSSVGVTYGYATAEDFVSGPNTSGLETRVVQAERFF
KTHLFDWVTSDSFGRCHLLELPTDHKGVYGSLTDSYAYMRNGWDVEVTAVGNQFNGGCLLVA
MVPELCSIQKRELYQLTLFPHQFINPRTNMTAHITVPFVGVNRYDQYKVHKPWTLVVMVVAP
LTVNTEGAPQIKVYANIAPTNVHVAGEFPSKE
>d1qqp3_ b.10.1.4 (3:) Foot-and-mouth desease virus
{Foot-and-mouth disease virus, (strain bfs, 1860)}
GIFPVACSDGYGGLVTTDPKTADPVYGKVFNPPRNQLPGRFTNLLDVAEACPTFLRFEGGVP
YVTTKTDSDRVLAQFDMSLAAKHMSNTFLAGLAQYYTQYSGTINLHFMFTGPTDAKARYMVA
YAPPGMEPPKTPEAAAHCIHAEWDTGLNSKFTFSIPYLSAADYTYTASDVAETTNVQGWVCL
FQITHGKADGDALVVLASAGKDFELRLPVDARAE
>d1c8da_ b.10.1.4 (A:) Parvovirus (panleukopenia virus) capsid
{Host: dog (Canis familiaris)}
GVGISTGTFNNQTEFKFLENGWVYITANSSRLVHLNMPESENYRRVVVNNMDKTAVNGNMAL
DDIHAEIVTPWSLVDANAWGVWFNPGDWQLIVNTMSELHLVSFEQEIFNVVLKTVSESATQP
PTKVYNNDLTASLMVALDSNNTMPFTPAAMRSETLGFYPWKPTIPTPWRYYFQWDRTLIPSH
TGTSGTPTNIYHGTDPDDVQFYTIENSVPVHLLRTGDEFATGTFFFDCKPCRLTHTWQTNRA

LGLPPFLNSLPQSEGDTNFGDIGVQQDKRRGVTQMGNTNYITEATIMRPAEVGYSAPYYSFE
ASTQGPFKTPIAAGRGGAQTDENQAADGNPRYAFGRQHGQKTTTTGETPERFTYIAHQDTGR
YPEGDWIQNINFNLPVTNDNVLLPTDPIGGKTGINYTNIFNTYGPLTALNNVPPVYPNGQIW
DKEFDTDLKPRLHVNAPFVCQNNCPGQLFVKVAPNLTNQYDPDASANMSRIVTYSDFWWKGK
LVFKAKLRASHTWNPIQQMSINVDNQFNYVPSNIGGMKIVYEKSQLAPRKLY
>d1k3va_ b.10.1.4 (A:) Parvovirus (panleukopenia virus) capsid {Host: pig (Sus scrofa)}
GVGVSTGTFNNQTEFQYLGEGLVRITAHASRLIHLNMPEHETYKRIHVLNSESGVAGQMVQD
DAHTQMVTPWSLIDANAWGVWFNPADWQLISNNMTEINLVSFEQEIFNVVLKTITESATSPP
TKIYNNDLTASLMVALDTNNTLPYTPAAPRSETLGFYPWLPTKPTQYRYYLSCIRNLNPPTY
TGQSQQITDSIQTGLHSDIMFYTIENAVPIHLLRTGDEFSTGIYHFDTKPLKLTHSWQTNRS
LGLPPKLLTEPTTEGDQHPGTLPAANTRKGYHQTINNSYTEATAIRPAQVGYNTPYMNFEYS
NGGPFLTPIVPTADTQYNDDEPNGAIRFTMDYQHGHLTTSSQELERYTFNPQSKCGRAPKQQ
FNQQAPLNLENTNNGTLLPSDPIGGKSNMHFMNTLNTYGPLTALNNTAPVFPNGQIWDKELD
TDLKPRLHVTAPFVCKNNPPGQLFVKIAPNLTDDFNADSPQQPRIITYSNFWWKGTLTFTAK
MRSSNMWNPIQQHTTTAENIGNYIPTNIGGIRMFPEYSQLIPRKLY
>d1mvma_ b.10.1.4 (A:) MVM coat protein {Murine minute virus, strain i}
GVGVSTGSYDNQTHYRFLGDGWVEITALATRLVHLNMPKSENYCRIRVHNTTDTSVKGNMAK
DDAHEQIWTPWSLVDANAWGVWLQPSDWQYICNTMSQLNLVSLDQEIFNVVLKTVTEQDSGG
QAIKIYNNDLTACMMVAVDSNNILPYTPAANSMETLGFYPWKPTIASPYRYYFCVDRDLSVT
YENQEGTIEHNVMGTPKGMNSQFFTIENTQQITLLRTGDEFATGTYYFDTNPVKLTHTWQTN
RQLGQPPLLSTFPEADTDAGTLTAQGSRHGATQMEVNWVSEAIRTRPAQVGFCQPHNDFEAS
RAGPFAAPKVPADVTQGMDREANGSVRYSYGKQHGENWAAHGPAPERYTWDETNFGSGRDTR
DGFIQSAPLVVPPPLNGILTNANPIGTKNDIHFSNVFNSYGPLTTFSHPSPVYPQGQIWDKE
LDLEHKPRLHITAPFVCKNNAPGQMLVRLGPNLTDQYDPNGATLSRIVTYGTFFWKGKLTMR
AKLRANTTWNPVYQVSVEDNGNSYMSVTKWLPTATGNMQSVPLITRPVARNTY
>d1hxs1_ b.10.1.4 (1:) Poliovirus {Poliovirus type 1, strain Mahoney}
GSSSTDNTVRETVGAATSRDALPNTEASGPTHSKEIPALTAVETGATNPLVPSDTVQTRHVV
QHRSRSESSIESFFARGACVTIMTVDNPASTTNKDKLFAVWKITYKDTVQLRRKLEFFTYSR
FDMELTFVVTANFTETNNGHALNQVYQIMYVPPGAPVPEKWDDYTWQTSSNPSIFYTYGTAP
ARISVPYVGISNAYSHFYDGFSKVPLKDQSAALGDSLYGAASLNDFGILAVRVVNDHNPTKV
TSKIRVYLKPKHIRVWCPRPPRAVAYYGPGVDYKDGTLTPLSTKDLTTY
>d1hxs2_ b.10.1.4 (2:) Poliovirus {Poliovirus type 1, strain Mahoney}
ACGYSDRVLQLTLGNSTITTQEAANSVVAYGRWPEYLRDSEANPVDQPTEPDVAACRFYTLD
TVSWTKESRGWWWKLPDALRDMGLFGQNMYYHYLGRSGYTVHVQCNASKFHQGALGVFAVPE
MCLAGDSNTTTMHTSYQNANPGEKGGTFTGTFTPDNNQTSPARRFCPVDYLLGNGTLLGNAF
VFPHQIINLRTNNCATLVLPYVNSLSIDSMVKHNNWGIAILPLAPLNFASESSPEIPITLTI
APMCCEFNGLRNITLPRLQ
>d1hxs3_ b.10.1.4 (3:) Poliovirus {Poliovirus type 1, strain Mahoney}
GLPVMNTPGSNQYLTADNFQSPCALPEFDVTPPIDIPGEVKNMMELAEIDTMIPFDLSATKK

NTMEMYRVRLSDKPHTDDPILCLSLSPASDPRLSHTMLGEILNYYTHWAGSLKFTFLFCGSM
MATGKLLVSYAPPGADPPKKRKEAMLGTHVIWDIGLQSSCTMVVPWISNTTYRQTIDDSFTE
GGYISVFYQTRIVVPLSTPREMDILGFVSACNDFSVRLLRDTTHIEQKA
>d1pov0_ b.10.1.4 (0:) Poliovirus {Poliovirus type 1, strain Mahoney}
GAQVSSQKVGAHENSNRAYGGSTINYTTINYYRDSASNAASKQDFSQDPSKFTEPIKDVLIK
TAPMLNSPNIEACGYSDRVLQLTLGNSTITTQEAANSVVAYGRWPEYLRDSEANPVDQPTEP
DVAACRFYTLDTVSWTKESRGWWWKLPDALRDMGLFGQNMYYHYLGRSGYTVHVQCNASKFH
QGALGVFAVPEMCLAGDSNTTTMHTSYQNANPGEKGGTFTGTFTPDNNQTSPARRFCPVDYL
LGNGTLLGNAFVFPHQIINLRTNNCATLVLPYVNSLSIDSMVKHNNWGIAILPLAPLNFASE
SSPEIPITLTIAPMCCEFNGLRNITLPRLQ
>d1pov1_ b.10.1.4 (1:) Poliovirus {Poliovirus type 1, strain Mahoney}
QHRSRSESSIESFFARGACVTIMTVDNPASTTNKDKLFAVWKITYKDTVQLRRKLEFFTYSR
FDMELTFVVTANFTETNNGHALNQVYQIMYVPPGAPVPEKWDDYTWQTSSNPSIFYTYGTAP
ARISVPYVGISNAYSHFYDGFSKVPLKDQSAALGDSLYGAASLNDFGILAVRVVNDHNPTKV
TSKIRVYLKPKHIRVWCPRPPRAVAYYGPGVDYKDGTLTPLSTKDLTTY
>d1eah1_ b.10.1.4 (1:) Poliovirus {Poliovirus type 2, strain Lansing}
ANNLPDTQSSGPAHSKETPALTAVETGATNPLVPSDTVQTRHVIQKRTRSESTVESFFARGA
CVAIIEVDNDAPTKRASKLFSVWKITYKDTVQLRRKLEFFTYSRFDMEFTFVVTSNYTDANN
GHALNQVYQIMYIPPGAPIPGKWNDYTWQTSSNPSVFYTYGAPPARISVPYVGIANAYSHFY
DGFAKVPLAGQASTEGDSLYGAASLNDFGSLAVRVVNDHNPTKLTSKIRVYMKPKHVRVWCP
RPPRAVPYYGPGVDYKDGLAPLPGKGLTTY
>d1eah2_ b.10.1.4 (2:) Poliovirus {Poliovirus type 2, strain Lansing}
SVRVMQLTLGNSTITTQEAANSVVAYGRWPEYIKDSEANPVDQPTEPDVAACRFYTLDTVTW
RKESRGWWWKLPDALKDMGLFGQNMFYHYLGRAGYTVHVQCNASKFHQGALGVFAVPEMCLA
GDSTTHMFTKYENANPGEKGGEFKGSFTLDTNATNPARNFCPVDYLFGSGVLAGNAFVYPHQ
IINLRTNNCATLVLPYVNSLSIDSMTKHNNWGIAILPLAPLDFATESSTEIPITLTIAPMCC
EFNGLRNITVPRTQ
>d1eah3_ b.10.1.4 (3:) Poliovirus {Poliovirus type 2, strain Lansing}
GLPVLNTPGSNQYLTADNYQSPCAIPEFDVTPPIDIPGEVRNMMELAEIDTMIPLNLTNQRK
NTMDMYRVELNDAAHSDTPILCLSLSPASDPRLAHTMLGEILNYYTHWAGSLKFTFLFCGSM
MATGKLLVSYAPPGAEAPKSRKEAMLGTHVIWDIGLQSSCTMVVPWISNTTYRQTINDSFTE
GGYISMFYQTRVVVPLSTPRKMDILGFVSACNDFSVRLLRDTTHISQEA
>d1pvc1_ b.10.1.4 (1:) Poliovirus {Poliovirus type 3, strain Sabin}
QDSLPDTKASGPAHSKEVPALTAVETGATNPLAPSDTVQTRHVVQRRSRSESTIESFFARGA
CVAIIEVDNEQPTTRAQKLFAMWRITYKDTVQLRRKLEFFTYSRFDMEFTFVVTANFTNANN
GHALNQVYQIMYIPPGAPTPKSWDDYTWQTSSNPSIFYTYGAAPARISVPYVGLANAYSHFY
DGFAKVPLKTDANDQIGDSLYSAMTVDDFGVLAVRVVNDHNPTKVTSKVRIYMKPKHVRVWC
PRPPRAVPYYGPGVDYRNNLDPLSEKGLTTY
>d1pvc2_ b.10.1.4 (2:) Poliovirus {Poliovirus type 3, strain Sabin}

ACGYSDRVLQLTLGNSTITTQEAANSVVAYGRWPEFIRDDEANPVDQPTEPDVATCRFYTLD
TVMWGKESKGWWWKLPDALRDMGLFGQNMYYHYLGRSGYTVHVQCNASKFHQGALGVFAIPE
YCLAGDSDKQRYTSYANANPGERGGKFYSQFNKDNAVTSPKREFCPVDYLLGCGVLLGNAFV
YPHQIINLRTNNSATIVLPYVNALAIDSMVKHNNWGIAILPLSPLDFAQDSSVEIPITVTIA
PMCSEFNGLRNVTAPKFQ
>d1pvc3_ b.10.1.4 (3:) Poliovirus {Poliovirus type 3, strain Sabin}
GLPVLNTPGSNQYLTSDNHQSPCAIPEFDVTPPIDIPGEVKNMMELAEIDTMIPLNLESTKR
NTMDMYRVTLSDSADLSQPILCLSLSPAFDPRLSHTMLGEVLNYYTHWAGSLKFTFLFCGSM
MATGKILVAYAPPGAQPPTSRKEAMLGTHVIWDLGLQSSCTMVVPWISNVTYRQTTQDSFTE
GGYISMFYQTRIVVPLSTPKSMSMLGFVSACNDFSVRLLRDTTHISQSA
>d4rhv1_ b.10.1.4 (1:) Rhinovirus coat protein {Human rhinovirus 14}
TVASISSGPKHTQKVPILTANETGATMPVLPSDSIETRTTYMHFNGSETDVECFLGRAACVH
VTEIQNKDATGIDNHREAKLFNDWKINLSSLVQLRKKLELFTYVRFDSEYTILATASQPDSA
NYSSNLVVQAMYVPPGAPNPKEWDDYTWQSASNPSVFFKVGDTSRFSVPYVGLASAYNCFYD
GYSHDDAETQYGITVLNHMGSMAFRIVNEHDEHKTLVKIRVYHRAKHVEAWIPRAPRALPYT
SIGRTNYPKNTEPVIKKRKGDIKSY
>d4rhv2_ b.10.1.4 (2:) Rhinovirus coat protein {Human rhinovirus 14}
GYSDRVQQITLGNSTITTQEAANAVVCYAEWPEYLPDVDASDVNKTSKPDTSVCRFYTLDSK
TWTTGSKGWCWKLPDALKDMGVFGQNMFFHSLGRSGYTVHVQCNATKFHSGCLLVVVIPEHQ
LASHEGGNVSVKYTFTHPGERGIDLSSANEVGGPVKDVLYNMNGTLLGNLLIFPHQFINLRT
NNTATIVIPYINSVPIDSMTRHNNVSLMVIPIAPLTVPTGATPSLPITVTIAPMCTEFSGIR
SKSIVPQ
>d4rhv3_ b.10.1.4 (3:) Rhinovirus coat protein {Human rhinovirus 14}
GLPTTTLPGSGQFLTTDDRQSPSALPNYEPTPRIHIPGKVHNLLEIIQVDTLIPMNNTHTKD
EVNSYLIPLNANRQNEQVFGTNLFIGDGVFKTTLLGEIVQYYTHWSGSLRFSLMYTGPALSS
AKLILAYTPPGARGPQDRREAMLGTHVVWDIGLQSTIVMTIPWTSGVQFRYTDPDTYTSAGF
LSCWYQTSLILPPETTGQVYLLSFISACPDFKLRLMKDTQTISQTVALTE
>d1aym1_ b.10.1.4 (1:) Rhinovirus coat protein {Human rhinovirus 16}
NPVERYVDEVLNEVLVVPNINQSHPTTSNAAPVLDAAETGHTNKIQPEDTIETRYVQSSQTL
DEMSVESFLGRSGCIHESVLDIVDNYNDQSFTKWNINLQEMAQIRRKFEMFTYARFDSEITM
VPSVAAKDGHIGHIVMQYMYVPPGAPIPTTRDDYAWQSGTNASVFWQHGQPFPRFSLPFLSI
ASAYYMFYDGYDGDTYKSRYGTVVTNDMGTLCSRIVTSEQLHKVKVVTRIYHKAKHTKAWCP
RPPRAVQYSHTHTTNYKLSSEVHNDVAIRPRTNLTTV
>d1aym2_ b.10.1.4 (2:) Rhinovirus coat protein {Human rhinovirus 16}
SDRIIQITRGDSTITSQDVANAVVGYGVWPHYLTPQDATAIDKPTQPDTSSNRFYTLDSKMW
NSTSKGWWWKLPDALKDMGIFGENMFYHFLGRSGYTVHVQCNASKFHQGTLLVVMIPEHQLA
TVNKGNVNAGYKYTHPGEAGREVGTQVENEKQPSDDNWLNFDGTLLGNLLIFPHQFINLRSN
NSATLIVPYVNAVPMDSMVRHNNWSLVIIPVCQLQSNNISNIVPITVSISPMCAEFSGARAK
TVVQ

>d1aym3_ b.10.1.4 (3:) Rhinovirus coat protein {Human rhinovirus 16}
GLPVYVTPGSGQFMTTDDMQSPCALPWYHPTKEIFIPGEVKNLIEMCQVDTLIPINSTQSNI
GNVSMYTVTLSPQTKLAEEIFAIKVDIASHPLATTLIGEIASYFTHWTGSLRFSFMFCGTAN
TTLKVLLAYTPPGIGKPRSRKEAMLGTHVVWDVGLQSTVSLVVPWISASQYRFTTPDTYSSA
GYITCWYQTNFVVPPNTPNTAEMLCFVSGCKDFCLRMARDTDLHKQTGPITQ
>d1r1a1_ b.10.1.4 (1:) Rhinovirus coat protein {Human rhinovirus 1A}
NYIDEVLNEVLVVPNIKESHHTTSNSAPLLDAAETGHTSNVQPEDAIETRYVITSQTRDEMS
IESFLGRSGCVHISRIKVDYTDYNGQDINFTKWKITLQEMAQIRRKFELFTYVRFDSEITLV
PCIAGRGDDIGHIVMQYMYVPPGAPIPSKRNDFSWQSGTNMSIFWQHGQPFPRFSIPFLSIA
SAYYMFYDGYDGDNTSSKYGSVVTNDMGTICSRIVTEKQKLSVVITTHIYHKAKHTKAWCPR
PPRAVPYTHSHVTNYMPETGDVTTAIVRRNTITTA
>d1r1a2_ b.10.1.4 (2:) Rhinovirus coat protein {Human rhinovirus 1A}
DRIMQITRGDSTISSDDVANAVVGYGVWPHYLTPQDATAINKPTQPDTSSNRFYTLESKHWN
GSSKGWWWKLPDALKDMGIFGENMYYHFLGRSGYTVHVQCNASKFHQGTLLVAMIPEHQLAS
AKHGSVTAGYKLTHPGEAGRDVSQERDASLRQPSDDSWLNFDGTLLGNLLIFPHQFINLRSN
NSATLIVPYVNAVPMDSMLRHNNWCLVIIPISPLRSETTSSNIVPITVSISPMCAEFSGARA
KNIKQ
>d1r1a3_ b.10.1.4 (3:) Rhinovirus coat protein {Human rhinovirus 1A}
GLPVYITPGSGQFMTTDDMQSPCALPWYHPTKEISIPGEVKNLIEMCQVDTLIPVNNVGNNV
GNVSMYTVQLGNQTGMAQKVFSIKVDITSTPLATTLIGEIASYYTHWTGSLRFSFMFCGTAN
TTLKLLLAYTPPGIDEPTTRKDAMLGTHVVWDVGLQSTISLVVPWVSASHFRLTADNKYSMA
GYITCWYQTNLVVPPSTPQTADMLCFVSACKDFCLRMARDTDLHIQSGPIEQ
>d1fpn1_ b.10.1.4 (1:) Rhinovirus coat protein {Human rhinovirus 2}
LVVPNINSSNPTTSNSAPALDAAETGHTSSVQPEDVIETRYVQTSQTRDEMSLESFLGRSGC
IHESKLEVTLANYNKENFTVWAINLQEMAQIRRKFELFTYTRFDSEITLVPCISALSQDIGH
ITMQYMYVPPGAPVPNSRDDYAWQSGTNASVFWQHGQAYPRFSLPFLSVASAYYMFYDGYDE
QDQNYGTANTNNMGSLCSRIVTEKHIHKVHIMTRIYHKAKHVKAWCPRPPRALEYTRAHRTN
FKIEDRSIQTAIVTRPIITTA
>d1fpn2_ b.10.1.4 (2:) Rhinovirus coat protein {Human rhinovirus 2}
RIIQITRGDSTITSQDVANAIVAYGVWPHYLSSKDASAIDKPSQPDTSSNRFYTLRSVTWSS
SSKGWWWKLPDALKDMGIFGENMFYHYLGRSGYTIHVQCNASKFHQGTLIVALIPEHQIASA
LHGNVNVGYNYTHPGETGREVKAETRLNPDLQPTEEYWLNFDGTLLGNITIFPHQFINLRSN
NSATIIAPYVNAVPMDSMRSHNNWSLVIIPICPLETSSAINTIPITISISPMCAEFSGARAK
RQ
>d1fpn3_ b.10.1.4 (3:) Rhinovirus coat protein {Human rhinovirus 2}
GLPVFITPGSGQFLTTDDFQSPCALPWYHPTKEISIPGEVKNLVEICQVDSLVPINNTDTYI
NSENMYSVVLQSSINAPDKIFSIRTDVASQPLATTLIGEISSYFTHWTGSLRFSFMFCGTAN

TTVKLLLAYTPPGIAEPTTRKDAMLGTHVIWDVGLQSTISMVVPWISASHYRNTSPGRSTSG
YITCWYQTRLVIPPQTPPTARLLCFVSGCKDFCLRMARDTNLHLQSGAIAQ
>d1rhi1_ b.10.1.4 (1:) Rhinovirus coat protein {Human rhinovirus 3}
QTLASVSSGPKHTQSVPALTANETGATLPTRPSDNVETRTTYMHFNGSETDVESFLGRAACV
HVTEIKNKNAAGLDNHRKEGLFNDWKINLSSLVQLRKKLELFTYVRFDSEYTILATASQPEA
SSYSSNLTVQAMYVPPGAPNPKEWDDYTWQSASNPSVFFKVGETSRFSVPFVGIASAYNCFY
DGYSHDDPDTPYGITVLNHMGSMAFRVVNEHDVHTTIVKIRVYHRAKHVEAWIPRAPRALPY
VSIGRTNYPRDSKTIVKKRTNIKTY
>d1rhi2_ b.10.1.4 (2:) Rhinovirus coat protein {Human rhinovirus 3}
GYSDRVQQITLGNSTITTQEARNAIVCYAEWPEYLSDNDASDVNKTSKPDISVCRFYTLDSK
TWKATSKGWCWKLPDALKDMGVFGQNMFYHSLGRTGYTIHVQCNATKFHSGCLLVVVIPEHQ
LASHEGGTVSVKYKYTHPGDRGIDLDTVEVAGGPTSDAIYNMDGTLLGNLLIFPHQFINMRT
NNTATIVVPYINSVPIDSMTRHNNVSLMVVPIAPLNAPTGSSPTLPVTVTIAPMCTEFTGIR
SRSIVPQ
>d1rhi3_ b.10.1.4 (3:) Rhinovirus coat protein {Human rhinovirus 3}
GLPTTTLPGSGQFLTTDDRQSPSALPSYEPTPRIHIPGKVRNLLEIIQVGTLIPMNNTGTND
NVTNYLIPLHADRQNEQIFGTKLYIGDGVFKTTLLGEIAQYYTHWSGSLRISLMYTGPALSS
AKIILAYTPPGTRGPEDKKEAMLGTHVVWDIGLQSTIVMTIPWTSGVQFRYTDPDTYTSAGY
LSCWYLTSLILPPQTSGQVYLLSFISACPDFKLRLMKDTQTISQTDALTE
>d1bev1_ b.10.1.4 (1:) Bovine enterovirus coat protein {Bovine enterovirus, VG-5-27}
QAAGALVAGTSTSTHSVATDSTPALQAAETGATSTARDESMIETRTIVPTHGIHETSVESFF
GRSSLVGMPLLATGTSITHWRIDFREFVQLRAKMSWFTYMRFDVEFTIIATSSTGQNVTTEQ
HTTYQVMYVPPGAPVPSNQDSFQWQSGCNPSVFADTDGPPAQFSVPFMSSANAYSTVYDGYA
RFMDTDPDRYGILPSNFLGFMYFRTLEDAAHQVRFRIYAKIKHTSCWIPRAPRQAPYKKRYN
LVFSGDSDRICSNRASLTSY
>d1bev2_ b.10.1.4 (2:) Bovine enterovirus coat protein {Bovine enterovirus, VG-5-27}
EACGYSDRVAQLTLGNSTITTQEAANICVAYGCWPAKLSDTDATSVDKPTEPGVSADRFYTL
RSKPWQADSKGWYWKLPDALNNTGMFGQNAQFHYLYRGGWAVHVQCNATKFHQGTLLVLAIP
EHQIATQEQPAFDRTMPGSEGGTFQEPFWLEDGTSLGNSLIYPHQWINLRTNNSATLILPYV
NAIPMDSAIRHSNWTLAIIPVAPLKYAAETTPLVPITVTIAPMETEYNGLRRAIASNQ
>d1bev3_ b.10.1.4 (3:) Bovine enterovirus coat protein {Bovine enterovirus, VG-5-27}
GLPTKPGPGSYQFMTTDEDCSPCILPDFQPTPEIFIPGKVNNLLEIAQVESILEANNREGVE
GVERYVIPVSVQDALDAQIYALRLELGGSGPLSSSLLGTLAKHYTQWSGSVEITCMFTGTFM
TTGKVLLAYTPPGGDMPRNREEAMLGTHVIWDFGLQSSITLVIPWISASHFRGVSNDDVLNY
QYYAAGHVTIWYQTNMVIPPGFPNTAGIIMMIAAQPNFSFRIQKDREDMTQTAILQ
>d2mev1_ b.10.1.4 (1:) Mengo virus {Host: monkey brain; middle size plaque variant}
GVENAEKGVTENTDATADFVAQPVYLPENQTKVAFFYDRSSPIGAFAVKSGSLESGFAPFSN

KACPNSVILTPGPQFDPAYDQLRPQRLTEIWGNGNEETSEVFPLKTKQDYSFCLFSPFVYYK
CDLEVTLSPHTSGAHGLLVRWCPTGTPTKPTTQVLHEVSSLSEGRTPQVYSAGPGTSNQISF
VVPYNSPLSVLPAVWYNGHKRFDNTGDLGIAPNSDFGTLFFAGTKPDIKFTVYLRYKNMRVF
CPRPTVFFPWPTSGDKIDMT
>d2mev2_ b.10.1.4 (2:) Mengo virus {Host: monkey brain; middle size plaque variant}
ENLSDRVSQDTAGNTVTNTQSTVGRLVGYGTVHDGEHPASCADTASEKILAVERYYTFKVND
WTSTQKPFEYIRIPLPHVLSGEDGGVFGATLRRHYLVKTGWRVQVQCNASQFHAGSLLVFMA
PEYPTLDVFAMDNRWSKDNLPNGTRTQTNRKGPFAMDHQNFWQWTLYPHQFLNLRTNTTVDL
EVPYVNIAPTSSWTQHASWTLVIAVVAPLTYSTGASTSLDITASIQPVRPVFNGLRHEVLSR
Q
>d2mev3_ b.10.1.4 (3:) Mengo virus {Host: monkey brain; middle size plaque variant}
SPIPVTIREHAGTWYSTLPDSTVPIYGKTPVAPANYMVGEYKDFLEIAQIPTFIGNKVPNAV
PYIEASNTAVKTQPLAVYQVTLSCSCLANTFLAALSRNFAQYRGSLVYTFVFTGTAMMKGKF
LIAYTPPGAGKPTSRDQAMQATYAIWDLGLNSSYSFTVPFISPTHFRMVGTDQANITNVDGW
VTVWQLTPLTYPPGCPTSAKILTMVSAGKDFSLKMPISPAPWSPQ
>d1cov1_ b.10.1.4 (1:) Coxsackievirus B3 {Host: human (Homo sapiens)}
RVADTVGTGPTNSEAIPALTAAETGHTSQVVPSDTMQTRHVKNYHSRSESTIENFLCRSACV
YFTEYENSGAKRYAEWVITPRQAAQLRRKLEFFTYVRFDLELTFVITSTQQPSTTQNQDAQI
LTHQIMYVPPGGPVPDKVDSYVWQTSTNPSVFWTEGNAPPRMSVPFLSIGNAYSNFYDGWSE
FSRNGVYGINTLNNMGTLYARHVNAGSTGPIKSTIRIYFKPKHVKAWIPRPPRLCQYEKAKN
VNFQPSGVTTTRQSITTMTNT
>d1cov2_ b.10.1.4 (2:) Coxsackievirus B3 {Host: human (Homo sapiens)}
GYSDRVRSITLGNSTITTQECANVVVGYGVWPDYLKDSEATAEDQPTQPDVATCRFYTLDSV
QWQKTSPGWWWKLPDALSNLGLFGQNMQYHYLGRTGYTIHVQCNASKFHQGCLLVVCVPEAE
MGCATLNNTPSSAELLGGDTAKEFADKPVASGSNKLVQRVVYNAGMGVGVGNLTIFPHQWIN
LRTNNSATIVMPYTNSVPMDNMFRHNNVTLMVIPFVPLDYCPGSTTYVPITVTIAPMCAEYN
GLRLAGHQ
>d1cov3_ b.10.1.4 (3:) Coxsackievirus B3 {Host: human (Homo sapiens)}
GLPTMNTPGSCQFLTSDDFQSPSAMPQYDVTPEMRIPGEVKNLMEIAEVDSVVPVQNVGEKV
NSMEAYQIPVRSNEGSGTQVFGFPLQPGYSSVFSRTLLGEILNYYTHWSGSIKLTFMFCGSA
MATGKFLLAYSPPGAGAPTKRVDAMLGTHVVWDVGLQSSCVLCIPWISQTHYRYVASDEYTA
GGFITCWYQTNIVVPADAQSSCYIMCFVSACNDFSVRLLKDTPFISQENFFQ
>d1d4m1_ b.10.1.4 (1:) Coxsackievirus A9 {Host: human (Homo sapiens)}
GDVEEAIERAVVHVADTMRSGPSNSASVPALTAVETGHTSQVTPSDTMQTRHVKNYHSRSES
TVENFLGRSACVYMEEYKTTDNDVNKKFVAWPINTKQMVQMRRKLEMFTYLRFDMEVTFVIT
SRQDPGTTLAQDMPVLTHQIMYVPPGGPIPAKVDDYAWQTSTNPSIFWTEGNAPARMSIPFI
SIGNAYSNFYDGWSNFDQRGSYGYNTLNNLGHIYVRHVSGSSPHPITSTIRVYFKPKHTRAW
VPRPPRLCQYKKAFSVDFTPTPITDTRKDINTVTTV

>d1d4m2_ b.10.1.4 (2:) Coxsackievirus A9 {Host: human (Homo sapiens)}
SDRVRSITLGNSTITTQECANVVVGYGRWPTYLRDDEATAEDQPTQPDVATCRFYTLDSIKW
EKGSVGWWWKFPEALSDMGLFGQNMQYHYLGRAGYTIHVQCNASKFHQGCLLVVCVPEAEMG
GAVVGQAFSATAMANGDKAYEFTSATQSDQTKVQTAIHNAGMGVGVGNLTIYPHQWINLRTN
NSATIVMPYINSVPMDNMFRHYNFTLMVIPFVKLDYADTASTYVPITVTVAPMCAEYNGLRL
AQAQ
>d1d4m3_ b.10.1.4 (3:) Coxsackievirus A9 {Host: human (Homo sapiens)}
GLPTMNTPGSTQFLTSDDFQSPCALPQFDVTPSMNIPGEVKNLMEIAEVDSVVPVNNVQDTT
DQMEMFRIPVTINAPLQQQVFGLRLQPGLDSVFKHTLLGEILNYYAHWSGSMKLTFVFCGSA
MATGKFLIAYSPPGANPPKTRKDAMLGTHIIWDIGLQSSCVLCVPWISQTHYRLVQQDEYTS
AGYVTCWYQTGMIVPPGTPNSSSIMCFASACNDFSVRMLRDTPFISQDNKLQ
>d1ev11_ b.10.1.4 (1:) Echovirus type 1 {Host: human (Homo sapiens)}
GDVQNAVEGAMVRVADTVQTSATNSERVPNLTAVETGHTSQAVPGDTMQTRHVINNHVRSES
TIENFLARSACVFYLEYKTGTKEDSNSFNNWVITTRRVAQLRRKLEMFTYLRFDMEITVVIT
SSQDQSTSQNQNAPVLTHQIMYVPPGGPIPVSVDDYSWQTSTNPSIFWTEGNAPARMSIPFI
SIGNAYSNFYDGWSHFSQAGVYGFTTLNNMGQLFFRHVNKPNPAAITSVARIYFKPKHVRAW
VPRPPRLCPYINSTNVNFEPKPVTEVRTNIITT
>d1ev12_ b.10.1.4 (2:) Echovirus type 1 {Host: human (Homo sapiens)}
GYSDRVRSITLGNSTITTQECANVVVGYGEWPEYLSDNEATAEDQPTQPDVATCRFYTLDSV
QWENGSPGWWWKFPDALRDMGLFGQNMYYHYLGRAGYTIHVQCNASKFHQGCILVVCVPEAE
MGSAQTSGVVNYEHISKGEIASRFTTTTTAEDHGVQAAVWNAGMGVGVGNLTIFPHQWINLR
TNNSATIVMPYVNSVPMDNMYRHHNFTLMIIPFVPLDFSAGASTYVPITVTVAPMCAEYNGL
RLAGHQ
>d1ev13_ b.10.1.4 (3:) Echovirus type 1 {Host: human (Homo sapiens)}
GLPTMNTPGSNQFLTSDDFQSPSAMPQFDVTPEMHIPGEVRNLMEIAEVDSVMPINNDSAAK
VSSMEAYRVELSTNTNAGTQVFGFQLNPGAESVMNRTLMGEILNYYAHWSGSIKITFVFCGS
AMTTGKFLLSYAPPGAGAPKTRKDAMLGTHVVWDVGLQSSCVLCIPWISQTHYRFVEKDPYT
NAGFVTCWYQTSVVSPASNQPKCYMMCMVSACNDFSVRMLRDTKFIEQTSFYQ
>d1tme1_ b.10.1.4 (1:) Theiler's murine encephalomyelitis virus {Theiler's murine encephalomyelitis virus, strain da}
GSDNAEKGKVSNDDASVDFVAEPVKLPENQTRVAFFYDRAVPIGMLRPGQNIESTFVYQEND
LRLNCLLLTPLPSFCPDSTSGPVKTKAPVQWRWVRSGGTTNFPLMTKQDYAFLCFSPFTYYK
CDLEVTVSALGTDTVASVLRWAPTGAPADVTDQLIGYTPSLGETRNPHMWLVGAGNTQISFV
VPYNSPLSVLPAAWFNGWSDFGNTKDFGVAPNADFGRLWIQGNTSASVRIRYKKMKVFCPRP
TLFFPWPV
>d1tme2_ b.10.1.4 (2:) Theiler's murine encephalomyelitis virus {Theiler's murine encephalomyelitis virus, strain da}
DRVASDKAGNSATNTQSTVGRLCGYGEAHHGEHPASCADTATDKVLAAERYYTIDLASWTTT
QEAFSHIRIPLPHVLAGEDGGVFGATLRRHYLCKTGWRVQVQCNASQFHAGSLLVFMAPEFY
TGKGTKTGDMEPTDPFTMDTTWRAPQGAPTGYRYDSRTGFFAMNHQNQWQTVYPHQILNLR
TNTTVDLEVPYVNIAPTSSWTQHANWTLVVAVFSPLQYASGSSSDVQITASIQPVNPVFNGL
RHETVIA

>d1tme3_ b.10.1.4 (3:) Theiler's murine encephalomyelitis virus {Theiler's murine encephalomyelitis virus, strain da}
SPIAVTVREHKGCFYSTNPDTTVPIYGKTISTPNDYMCGEFSDLLELCKLPTFLGNPNSNNK
RYPYFSATNSVPTTSLVDYQVALSCSCMCNSMLAAVARNFNQYRGSLNFLFVFTGAAMVKGK
FLIAYTPPGAGKPTTRDQAMQATYAIWDLGLNSSFVFTAPFISPTHYRQTSYTSATIASVDG
WVTVWQLTPLTYPSGTPVNSDILTLVSAGDDFTLRMPISPTKWVPQ
>d1tmf1_ b.10.1.4 (1:) Theiler's murine encephalomyelitis virus {Theiler's murine encephalomyelitis virus, strain da}
GVDNAEKGKVSNDDASVDFVAEPVKLPENQTRVAFFYDRAVPIGMLRPGQNMETTFNYQEND
YRLNCLLLTPLPSFCPDSSSGPQKTKAPVQWRWVRSGGVNGANFPLMTKQDYAFLCFSPFTF
YKCDLEVTVSALGTDTVASVLRWAPTGAPADVTDQLIGYTPSLGETRNPHMWLVGAGNSQVS
FVVPYNSPLSVLPAAWFNGWSDFGNTKDFGVAPNADFGRLWIQGNTSASVRIRYKKMKVFCP
RPTLFFPWPTPTTTKINADNPVPILELE
>d1tmf2_ b.10.1.4 (2:) Theiler's murine encephalomyelitis virus {Theiler's murine encephalomyelitis virus, strain da}
DQNTEEMENLSDRVASDKAGNSATNTQSTVGRLCGYGKSHHGEHPASCADTATDKVLAAERY
YTIDLASWTTSQEAFSHIRIPLPHVLAGEDGGVFGATLRRHYLCKTGWRVQVQCNASQFHAG
SLLVFMAPEFYTGKGTKTGTMEPSDPFTMDTEWRSPQGAPTGYRYDSRTGFFATNHQNQWQW
TVYPHQILNLRTNTTVDLEVPYVNVAPSSSWTQHANWTLVVAVLSPLQYATGSSPDVQITAS
LQPVNPVFNGLRHETVIAQ
>d1sva1_ b.10.1.4 (1:) Simian virus 40 (SV40) coat protein {Simian virus 40}
PKKPKEPVQVPKLVIKGGIEVLGVKTGVDSFTEVECFLNPQMGNPDEHQKGLSKSLAAEKQF
TDDSPDKEQLPCYSVARIPLPNINEDLTCGNILMWEAVTVKTEVIGVTAMLNLHSGTQKTHE
NGAGKPIQGSNFHFFAVGGEPLELQGVLANYRTKYPAQTVTPKNATVDSQQMNTDHKAVLDK
DNAYPVECWVPDPSKNENTRYFGTYTGGENVPPVLHITNTATTVLLDEQGVGPLCKADSLYV
SAVDICGLFTNTSGTQQWKGLPRYFKITLRKRSVKNPYPISFLLSDLINRRTQRVDGQPMIG
MSSQVEEVRVYEDTEELPGDPDMIRYIDEFGQTTTRMQ
>d1dzla_ b.10.1.4 (A:) L1 protein {Human papillomavirus type 16}
KVVSTDEYVARTNIYYHAGTSRLLAVGHPYFPIKKPNNNKILVPKVSGLQYRVFRIHLPDPN
KFGFPDTSFYNPDTQRLVWACVGVEVGRGQPLGVGISGHPLLNKLDDTENASAYAANAGVDN
RECISMDYKQTQLCLIGCKPPIGEHWGKGSPCTQVAVQPGDCPPLELINTVIQDGDMVDTGF
GAMDFTTLQANKSEVPLDICTSICKYPDYIKMVSEPYGDSLFFYLRREQMFVRHLFNRAGTV
GENVPDDLYIKGSGSTANLASSNYFPTPSGSMVTSDAQIFNKPYWLQRAQGHNNGICWGNQL
FVTVVDTTRSTNMSLCAAISTSETTYKNTNFKEYLRHGEEYDLQFIFQLCKITLTADVMTYI
HSMNSTILEDWNFGLQPPPGGTLEDTYRFVTSQAIACQKHTPPAPKEDPLKKYTFWEVNLKE
KFSADLDQFPLGRKFLLQLGL
>d1ihma_ b.10.1.4 (A:) Calcivirus capsid protein {Norwalk virus}
DPLAMDPVAGSSTAVATAGQVNPIDPWIINNFVQAPQGEFTISPNNTPGDVLFDLSLGPHLN
PFLLHLSQMYNGWVGNMRVRIMLAGNAFTAGKIIVSCIPPGFGSHNLTIAQATLFPHVIADV
RTLDPIEVPLEDVRNVLFHNNDRNQQTMRLVCMLYTPLRTGGGTGDSFVVAGRVMTCPSPDF
NFLFLVPPTVEQKTRPFTLPNLPLSSLSNSRAPLPISSMGISPDNVQSVQFQNGRCTLDGRL
VGTTPVSLSHVAKIRGTSNGTVINLTELDGTPFHPFEGPAPIGFPDLGGCDWHINMTQFGHS
SQTQYDVDTTPDTFVPHLGSIQANGIGSGNYVGVLSWISPPSHPSGSQVDLWKIPNYGSSIT

EATHLAPSVYPPGFGEVLVFFMSKMPGPGAYNLPCLLPQEYISHLASEQAPTVGEAALLHYV
DPDTGRNLGEFKAYPDGFLTCVPNGASSGPQQLPINGVFVFVSWVSRFYQLKPVGTAS
>d1amm_1 b.11.1.1 (1-85) gamma-Crystallin {Cow (Bos taurus),
isoform II (B)}
GKITFYEDRGFQGHCYECSSDCPNLQPYFSRCNSIRVDSGCWMLYERPNYQGHQYFLRRGDY
PDYQQWMGFNDSIRSCRLIPQHT
>d1amm_2 b.11.1.1 (86-174) gamma-Crystallin {Cow (Bos taurus),
isoform II (B)}
GTFRMRIYERDDFRGQMSEITDDCPSLQDRFHLTEVHSLNVLEGSWVLYEMPSYRGRQYLLR
PGEYRRYLDWGAMNAKVGSLRRVMDFY
>d1elpa1 b.11.1.1 (A:1-85) gamma-Crystallin {Cow (Bos taurus),
isoform IIIb (D)}
GKITFYEDRGFQGRHYECSSDHSNLQPYLGRCNSVRVDSGCWMIYEQPNYLGPQYFLRRGDY
PDYQQWMGLNDSIRSCRLIPHAG
>d1elpa2 b.11.1.1 (A:87-174) gamma-Crystallin {Cow (Bos taurus),
isoform IIIb (D)}
SHRLRLYEREDYRGQMIEITEDCSSLQDRFHFNEIHSLNVLEGSWVLYELPNYRGRQYLLRP
GEYRRYHDWGAMNAKVGSLRRVIDIY
>d1a7ha_ b.11.1.1 (A:) gamma-Crystallin {Cow (Bos taurus), isoform
S}
MYKIQIFEKGDFNGQMHETTEDCPSIMEQFHMREVHSCKVLEGAWIFYELPNYRGRQYLLDK
KEYRKPVDWGAASPAVQSFRRIVE
>d1ha4a_ b.11.1.1 (A:) gamma-Crystallin {Human (Homo sapiens)}
GQYKIQIFEKGDFSGQMYETTEDCPSIMEQFHMREIHSCKVLEGVWIFYELPNYRGRQYLLD
KKEYRKPIDWGAASPAVQSFRRIVE
>d1a45_1 b.11.1.1 (1-84) gamma-Crystallin {Cow (Bos taurus),
isoform F}
GKITFYEDRGFQGRHYECSSDHSNLQPYFSRCNSIRVDSGCWMLYEQPNFQGPQYFLRRGDY
PDYQQWMGLNDSIRSCRLIPHT
>d1a45_2 b.11.1.1 (86-174) gamma-Crystallin {Cow (Bos taurus),
isoform F}
GSHRLRIYEREDYRGQMVEITEDCSSLHDRFHFSEIHSFNVLEGWWVLYEMTNYRGRQYLLR
PGDYRRYHDWGATNARVGSLRRAVDFY
>d2bb2_1 b.11.1.1 (-2-85) beta-Crystallin {Cow (Bos taurus)}
LNPKIIIFEQENFQGHSHELNGPCPNLKETGVEKAGSVLVQAGPWVGYEQANCKGEQFVFEK
GEYPRWDSWTSSRRTDSLSSLRPIKVDS
>d2bb2_2 b.11.1.1 (86-175) beta-Crystallin {Cow (Bos taurus)}
QEHKITLYENPNFTGKKMEVIDDDVPSFHAHGYQEKVSSVRVQSGTWVGYQYPGYRGLQYLL
EKGDYKDSGDFGAPQPQVQSVRRIRDMQW
>d1a5da1 b.11.1.1 (A:1-84) beta-Crystallin {Rat (Rattus
norvegicus), isoform E}
GKITFYEDRGFQGRHYECSTDHSNLQPYFSRCNSVRVDSGCWMLYEQPNFTGCQYFLRRGDY
PDYQQWMGFSDSVRSCRLIPHS
>d1a5da2 b.11.1.1 (A:85-174) beta-Crystallin {Rat (Rattus

norvegicus), isoform E}
SSHRIRIYEREDYRGQMVEITDDCPHLQDRFHFSDFHSFHVMEGYWVLYEMPNYRGRQYLLR
PGEYRRYHDWGAMNARVGSLRRIMDFY
>d1bd7a_ b.11.1.1 (A:) beta-Crystallin {Rat (Rattus norvegicus), isoform E}
EHKIILYENPNFTGKKMEIVDDDVPSFHAHGYQEKVSSVRVQSGTWVGYQYPGYRGLQYLLE
KGDYKDNSDFGAPHPQVQSVRRIRDMQGNPKIIIFEQENFQGHSHELSGPCPNLKETGMEKA
GSVLVQAGPWVGYEQANCKGEQFVFEKGEYPRWDSWTSSRRTDSLSSLRPIK
>d1npsa_ b.11.1.1 (A:) Protein S {Myxococcus xanthus}
ANITVFYNEDFQGKQVDLPPGNYTRAQLAALGIENNTISSVKVPPGVKAILYQNDGFAGDQI
EVVANAEELGPLNNNVSSIRVISVPV
>d1prr_2 b.11.1.1 (91-173) Protein S {Myxococcus xanthus}
PRARFFYKEQFDGKEVDLPPGQYTQAELERYGIDNNTISSVKPQGLAVVLFKNDNFSGDTLP
VNSDAPTLGAMNNNTSSIRIS
>d1hdfa_ b.11.1.1 (A:) Spherulin 3a (S3a) {Slime mold (Physarum polycephalum)}
SVCKGVSGNPAKGEVFLYKHVNFQGDSWKVTGNVYDFRSVSGLNDVVSSVKVGPNTKAFIFK
DDRFNGNFIRLEESSQVTDLTTRNLNDAISSMIVATFE
>d1wkt__ b.11.1.2 (-) Yeast killer toxin {Williopsis mrakii}
GDGYLIMCKNCDPNTGSCDWKQNWNTCVGIGANVHWMVTGGSTDGKQGCATIWEGSGCVGRS
TTMCCPANTCCNINTGFYIRSYRRVE
>d1bhu__ b.11.1.3 (-) Streptomyces metalloproteinase inhibitor, SMPI {Streptomyces nigrescens}
APSCPAGSLCTYSGTGLSGARTVIPASDMEKAGTDGVKLPASARSFANGTHFTLRYGPARKV
TCVRFPCYQYATVGKVAPGAQLRSLPSPGATVTVGQDLGD
>d1f53a_ b.11.1.4 (A:) Killer toxin-like protein SKLP {Streptomyces sp.}
IDHVPCRGGENFLKIWSHSGGQQSVDCYANRGRIDFGGWWVDKISTGNNDLIYYDANGDSVR
VDRWHDITYPNRPPKVNSIEIL
>d1g6ea_ b.11.1.6 (A:) Antifungal protein AFP1 {Streptomyces tendae, tu901}
MINRTDCNENSYLEIHNNEGRDTLCFANAGTMPVAIYGVNWVESGNNVVTLQFQRNLSDPRL
ETITLQKWGSWNPGHIHEILSIRIY
>d1c01a_ b.11.1.5 (A:) Plant antimicrobial protein MIAMP1 {Macadamia nut (Macadamia integrifolia)}
SAFTVWSGPGCNNRAERYSKCGCSAIHQKGGYDFSYTGQTAALYNQAGCSGVAHTRFGSSAR
ACNPFGWKSIFIQC
>d1f8na2 b.12.1.1 (A:6-149) Plant lipoxigenase {Soybean (Glycine max), isozyme L1}
HKIKGTVVLMPKNELEVNPDGSAVDNLNAFLGRSVSLQLISATKADAHGKGKVGKDTFLEGI
NTSLPTLGAGESAFNIHFEWDGSMGIPGAFYIKNYMQVEFFLKSLTLEAISNQGTIRFVCNS
WVYNTKLYKSVRIFFANHTY
>d1ik3a2 b.12.1.1 (A:9-167) Plant lipoxigenase {Soybean (Glycine max), isozyme L3}

GHKIKGTVVLMRKNVLDVNSVTSVGGIIGQGLDLVGSTLDTLTAFLGRSVSLQLISATKADA
NGKGKLGKATFLEGIITSLPTLGAGQSAFKINFEWDDGSGIPGAFYIKNFMQTEFFLVSLTL
EDIPNHGSIHFVCNSWIYNAKLFKSDRIFFANQTY
>d1lox_2 b.12.1.1 (2-112) 15-Lipoxygenase {Rabbit (Oryctolagus cuniculus)}
GVYRVCVSTGASIYAGSKNKVELWLVGQHGEVELGSCLRPTRNKEEEFKVNVSKYLGSLLFV
RLRKKHFLKEDAWFCNWISVQALGAAEDKYWFPCYRWVVGDGVQSLPVG
>d1hpla1 b.12.1.2 (A:337-449) Pancreatic lipase, C-terminal domain {Horse (Equus caballus)}
RWRYRVDVTLSGKKVTGHVLVSLFGNKGNSRQYEIFQGTLKPDNTYSNEFDSDVEVGDLEKV
KFIWYNNVINLTLPKVGASKITVERNDGSVFNFCSEETVREDVLLTLTAC
>d1etha1 b.12.1.2 (A:337-448) Pancreatic lipase, C-terminal domain {Pig (Sus scrofa)}
ARWRYKVSVTLSGKKVTGHILVSLFGNEGNSRQYEIYKGTLQPDNTHSDEFDSDVEVGDLQK
VKFIWYNVINPTLPRVGASKITVERNDGKVYDFCSQETVREEVLLTLNPC
>d1gpl_1 b.12.1.2 (337-449) Pancreatic lipase, C-terminal domain {Guinea pig (Cavia porcellus)}
RWRYKVSVTLSGKKVTGHILVSLFGNKGNSKQYEIFKGTLKPDSTHSNEFDSDVDVGDLQMV
KFIWYNNVINPTLPRVGASKIIVETNVGKQFNFCSPETVREEVLLTLTPC
>d1rp1_1 b.12.1.2 (337-449) Pancreatic lipase, C-terminal domain {Dog (Canis familiaris)}
RWRYGVSITLSGKRATGQAKVALFGSKGNTHQFNIFKGILKPGSTHSNEFDAKLDVGTIEKV
KFLWNNNNPTFPKVGAAKITVQKGEEKTVHSFCSESTVREDVLLTLTPC
>d1bu8a1 b.12.1.2 (A:337-449) Pancreatic lipase, C-terminal domain {Rat (Rattus norvegicus)}
RWRYKVSVTLSGAKKLSGYILVALYGNNGNSKQYEIFKGSLKPEARHVRDIDVDINVGEIQK
VKFLWNNKVINLFRPTLGASQITVQSGVDGKEYNFCSSDTVREDVLQSLYPC
>d1ca1_2 b.12.1.3 (250-370) Alpha-toxin, C-terminal domain {Clostridium perfringens}
SVGKNVKELVAYISTSGEKDAGTDDYMYFGIKTKDGKTQEWEMDNPGNDFMTGSKDTYTFKL
KDENLKIDDIQNMWIRKRKYTAFPDAYKPENIKVIANGKVVVDKDINEWISGNSTYNIK
>d1k5ja_ b.13.3.1 (A:) Nucleoplasmin core {Xenopus laevis}
VSLIWGCELNEQNKTFEFKVEDDEEKCEHQLALRTVCLGDKAKDEFHIVEIVTQEEGAEKSV
PIATLKPSILPMATMVGIELTPPVTFRLKAGSGPLYISGQHVA
>d1pgs_1 b.13.1.1 (4-140) Peptide:N-glycosidase F, PNGase F {Flavobacterium meningosepticum}
DNTVNIKTFDKVKNAFGDGLSQSAEGTFTFPADVTTVKTIKMFIKNECPNKTCDEWDRYANV
YVKNKTTGEWYEIGRFITPYWVGTEKLPRGLEIDVTDFKSLLSGNTELKIYTETWLAKGREY
SVDFDIVYGTPDY
>d1pgs_2 b.13.1.1 (141-314) Peptide:N-glycosidase F, PNGase F {Flavobacterium meningosepticum}
KYSAVVPVIQYNKSSIDGVPYGKAHTLGLKKNIQLPTNTEKAYLRTTISGWGHAKPYDAGSR
GCAEWCFRTHTIAINNANTFQHQLGALGCSANPINNQSPGNWTPDRAGWCPGMAVPTRIDVL
NNSLTGSTFSYEYKFQSWTNNGTNGDAFYAISSFVIAKSNTPISAPVVTN

>d1phm_1 b.13.1.2 (45-198) Peptidylglycine alpha-hydroxylating monooxygenase, PHM {Rat (Rattus norvegicus)}
NECLGTIGPVTPLDASDFALDIRMPGVTPKESDTYFCMSMRLPVDEEAFVIDFKPRASMDTV
HHMLLFGCNMPSSTGSYWFCDEGTCTDKANILYAWARNAPPTRLPKGVGFRVGGETGSKYFV
LQVHYGDISAFRDNHKDCSGVSVHLTRVPQ
>d1phm_2 b.13.1.2 (199-354) Peptidylglycine alpha-hydroxylating monooxygenase, PHM {Rat (Rattus norvegicus)}
PLIAGMYLMMSVDTVIPPGEKVVNADISCQYKMYPMHVFAYRVHTHHLGKVVSGYRVRNGQW
TLIGRQNPQLPQAFYPVEHPVDVTFGDILAARCVFTGEGRTEATHIGGTSSDEMCNLYIMYY
MEAKYALSFMTCTKNVAPDMFRTIPAEANIPI
>d1hx6a1 b.13.2.1 (A:15-244) Coat protein p3 {Bacteriophage prd1}
LRNQQAMAANLQARQIVLQQSYPVIQQVETQTFDPANRSVFDVTPANVGIVKGFLVKVTAAI
TNNHATEAVALTDFGPANLVQRVIYYDPDNQRHTETSGWHLHFVNTAKQGAPFLSSMVTDSP
IKYGDVMNVIDAPATIAAGATGELTMYYWVPLAYSETDLTGAVLANVPQSKQRLKLEFANNN
TAFAAVGANPLEAIYQGAGAADCEFEEISYTVYQSYLDQLPVGQ
>d1hx6a2 b.13.2.1 (A:245-384) Coat protein p3 {Bacteriophage prd1}
NGYILPLIDLSTLYNLENSAQAGLTPNVDFVVQYANLYRYLSTIAVFDNGGSFNAGTDINYL
SQRTANFSDTRKLDPKTWAAQTRRRIATDFPKGVYYCDNRDKPIYTLQYGNVGFVVNPKTVN
QNARLLMGYEYFTSRT
>d1dhx_1 b.13.2.2 (44-650) Adenovirus hexon {Human adenovirus type 2}
FRNPTVAPTHDVTTDRSQRLTLRFIPVDREDTAYSYKARFTLAVGDNRVLDMASTYFDIRGV
LDRGPTFKPYSGTAYNALAPKGAPNSCEWEQTEDSGRAVAEDEEEEDEDEEEEEEEQNARDQ
ATKKTHVYAQAPLSGETITKSGLQIGSDNAETQAKPVYADPSYQPEPQIGESQWNEADANAA
GGRVLKKTTPMKPCYGSYARPTNPFGGQSVLVPDEKGVPLPKVDLQFFSNTTSLNDRQGNAT
KPKVVLYSEDVNMETPDTHLSYKPGKGDENSKAMLGQQSMPNRPNYIAFRDNFIGLMYYNST
GNMGVLAGQASQLNAVVDLQDRNTELSYQLLLDSIGDRTRYFSMWNQAVDSYDPDVRIIENH
GTEDELPNYCFPLGGIGVTDTYQAIKANGNGSGDNGDTTWTKDETFATRNEIGVGNNFAMEI
NLNANLWRNFLYSNIALYLPDKLKYNPTNVEISDNPNTYDYMNKRVVAPGLVDCYINLGARW
SLDYMDNVNPFNHHRNAGLRYRSMLLGNGRYVPFHIQVPQKFFAIKNLLLLPGSYTYEWNFR
KDVNMVLQSSLGNDLRVDGASIKFDSICLYATFFPMAHNTASTLEAMLR
>d1ruxa1 b.13.2.2 (A:5-636) Adenovirus hexon {Human adenovirus type 5}
MMPQWSYMHISGQDASEYLSPGLVQFARATETYFSLNNKFRNPTVAPTHDVTTDRSQRLTLR
FIPVDREDTAYSYKARFTLAVGDNRVLDMASTYFDIRGVLDRGPTFKPYSGTAYNALAPKGA
PNPCEWDEAATALEINLEEEDDDNEDEVDEQAEQQKTHVFGQAPYSGINITKEGIQIGVEGQ
TPKYADKTFQPEPQIGESQWYETEINHAAGRVLKKTTPMKPCYGSYAKPTNENGGQGILVKQ
QNGKLESQVEMQFFSTTEATAGNGDNLTPKVVLYSEDVDIETPDTHISYMPTIKEGNSRELM
GQQSMPNRPNYIAFRDNFIGLMYYNSTGNMGVLAGQASQLNAVVDLQDRNTELSYQLLLDSI
GDRTRYFSMWNQAVDSYDPDVRIIENHGTEDELPNYCFPLGGVINTETLTKVKPKTGQENGW
EKDATEFSDKNEIRVGNNFAMEINLNANLWRNFLYSNIALYLPDKLKYSPSNVKISDNPNTY
DYMNKRVVAPGLVDCYINLGARWSLDYMDNVNPFNHHRNAGLRYRSMLLGNGRYVPFHIQVP
QKFFAIKNLLLLPGSYTYEWNFRKDVNMVLQSSLGNDLRVDGASIKFDSICLYATFFPMAHN
TASTLEAMLRND

>d1ruxa2 b.13.2.2 (A:637-946) Adenovirus hexon {Human adenovirus type 5}
TNDQSFNDYLSAANMLYPIPANATNVPISIPSRNWAAFRGWAFTRLKTKETPSLGSGYDPYY
TYSGSIPYLDGTFYLNHTFKKVAITFDSSVSWPGNDRLLTPNEFEIKRSVDGEGYNVAQCNM
TKDWFLVQMLANYNIGYQGFYIPESYKDRMYSFFRNFQPMSRQVVDDTKYKDYQQVGILHQH
NNSGFVGYLAPTMREGQAYPANFPYPLIGKTAVDSITQKKFLCDRTLWRIPFSSNFMSMGAL
TDLGQNLLYANSAHALDMTFEVDPMDEPTLLYVLFEVFDVVRVHRPHRGVIETVYLRTPFSA
>d1kful2 b.14.1.1 (L:356-514) Calpain large subunit, middle domain (domain III) {Human (Homo sapiens)}
WKLTKMDGNWRRGSTAGGCRNYPNTFWMNPQYLIKLEEEDEDEEDGESGCTFLVGLIQKHRR
RQRKMGEDMHTIGFGIYEVPEELSGQTNIHLSKNFFLTNRARERSDTFINLREVLNRFKLPP
GEYILVPSTFEPNKDGDFCIRVFSEKKADYQAVDD
>d1df0a2 b.14.1.1 (A:356-514) Calpain large subunit, middle domain (domain III) {Rat (Rattus norvegicus)}
WKLTKMDGNWRRGSTAGGCRNYPNTFWMNPQYLIKLEEEDEDDEDGERGCTFLVGLIQKHRR
RQRKMGEDMHTIGFGIYEVPEELTGQTNIHLSKNFFLTTRARERSDTFINLREVLNRFKLPP
GEYVLVPSTFEPHKNGDFCIRVFSEKKADYQTVDD
>d1f35a_ b.94.1.1 (A:) Olfactory marker protein {Mouse (Mus musculus)}
AEDGPQKQQLEMPLVLDQDLTQQMRLRVESLKQRGEKKQDGEKLIRPAESVYRLDFIQQQKL
QFDHWNVVLDKPGKVTITGTSQNWTPDLTNLMTRQLLDPAAIFWRKEDSDAMDWNEADALEF
GERLSDLAKIRKVMYFLITFGEGVEPANLKASVVFNQL
>d1shsa_ b.15.1.1 (A:) Small heat shock protein {Archaeon Methanococcus jannaschii}
TGIQISGKGFMPISIIEGDQHIKVIAWLPGVNKEDIILNAVGDTLEIRAKRSPLMITESERI
IYSEIPEEEEIYRTIKLPATVKEENASAKFENGVLSVILPKAESSIKKGINIE
>d1gmea_ b.15.1.1 (A:) Small heat shock protein {Wheat (Triticum aestivum)}
SIVRRSNVFDPFADLWADPFDTFRSIVPAISGGGSETAAFANARMDWKETPEAHVFKADLPG
VKKEEVKVEVEDGNVLVVSGERTKEKEDKNDKWHRVERSSGKFVRRFRLLEDAKVEEVKAGL
ENGVLTVTVPKAEVKKPEVKAIQISG
>d1gmeb_ b.15.1.1 (B:) Small heat shock protein {Wheat (Triticum aestivum)}
NARMDWKETPEAHVFKADLPGVKKEEVKVEVEDGNVLVVSGERTKEKEDKNDKWHRVERSSG
KFVRRFRLLEDAKVEEVKAGLENGVLTVTVPKAEVKKPEVKAIQISG
>d1ejfa_ b.15.1.2 (A:) Co-chaperone p23 {Human (Homo sapiens)}
MQPASAKWYDRRDYVFIEFCVEDSKDVNVNFEKSKLTFSCLGGSDNFKHLNEIDLFHCIDPN
DSKHKRTDRSILCCLRKGESGQSWPRLTKERAKLNWLSVDFNNWKDWE
>d1ezsa_ b.16.1.1 (A:) Ecotin, trypsin inhibitor {Escherichia coli}
PYPQAEKGMKRQVIQLTPQEDESTLKVELLIGQTLEVDCNLHRLGGKLENKTLEGAAAAYYV
FDKVSSPVSTRMACPDGKKEKKFVTAYLGDAGMLRYNSKLPIVVYTPDNVDVKYRVWKAEEK
IDNAVVR
>d1slua_ b.16.1.1 (A:) Ecotin, trypsin inhibitor {Escherichia

coli}
IAPYPQAEKGMKRQVIQLTPQEDESTLKVELLIGQTLEVDCNLHRLGGKLENKTLEGWGYDY
YVFDKVSSPVSTMMHCPDGKKEKKFVTAYLGDAGMLRYNSKLPIVVYTPDNVDVKYRVWKAE
EKIDNAVVR
>g1fi8.1 b.16.1.1 (C:,D:) Ecotin, trypsin inhibitor {Escherichia coli}
PLEKIAPYPQAEKGMKRQVIQLTPQEDESTLKVELLIGQTLEVDCNLHRLGGKLENKTLEGW
GYDYYVFDKVSSPIEPDXKFVTAYLGDAGMLRYNSKLPIVVYTPDNVDVKYRVWKAEEKIDN
AVVR
>d1beha_ b.17.1.1 (A:) Phosphatidylethanolamine binding protein, PEBP {Human (Homo sapiens)}
VDLSKWSGPLSLQEVDEQPQHPLHVTYAGAAVDELGKVLTPTQVKNRPTSISWDGLDSGKLY
TLVLTDPDAPSRKDPKYREWHHFLVVNMKGNDISSGTVLSDYVGSGPPKGTGLHRYVWLVYE
QDRPLKCDEPILSNRSGDHRGKFKVASFRKKYELRAPVAGTCYQAEWDDYVPKLYEQLSG
>d1a44__ b.17.1.1 (-) Phosphatidylethanolamine binding protein, PEBP {Cow (Bos taurus)}
PVDLSKWSGPLSLQEVDERPQHPLQVKYGGAEVDELGKVLTPTQVKNRPTSITWDGLDPGKL
YTLVLTDPDAPSRKDPKYREWHHFLVVNMKGNNISSGTVLSDYVGSGPPKGTGLHRYVWLVY
EQEGPLKCDEPILSNRSGDHRGKFKVASFRKKYELGAPVAGTCYQAEWDDYVPKLYEQLSG
>d1qoua_ b.17.1.1 (A:) Centroradialis protein Cen {Garden snapdragon (Antirrhinum majus)}
GRVIGDVVDHFTSTVKMSVIYNSNNSIKHVYNGHELFPSAVTSTPRVEVHGGDMRSFFTLIM
TDPDVPGPSDPYLREHLHWIVTDIPGTTDSSFGKEVVSYEMPRPNIGIHRFVFLLFKQKKRG
QAMLSPPVVCRDGFNTRKFTQENELGLPVAAVFFNCQRET
>d1fjja_ b.17.1.2 (A:) Hypothetical protein YbhB {Escherichia coli}
AMKLISNDLRDGDKLPHRHVFNGMGYDGDNISPHLAWDDVPAGTKSFVVTCYDPDAPTGSGW
WHWVVVNLPADTRVLPQGFGSGLVAMPDGVLQTRTDFGKTGYDGAAPPKGETHRYIFTVHAL
DIERIDVDEGASGAMVGFNVHFHSLASASITAMFS
>d1fuxa_ b.17.1.2 (A:) Hypothetical protein YbcL {Escherichia coli}
EFQVTSNEIKTGEQLTTSHVFSGFGCEGGNTSPSLTWSGVPEGTKSFAVTVYDPDAPTGSGW
WHWTVVNIPATVTYLPVDAGRRDGTKLPTGAVQGRNDFGYAGFGGACPPKGDKPHHYQFKVW
ALKTEKIPVDSNSSGALVGYMLNANKIATAEITPVYEIKLE
>d1g13a_ b.95.1.1 (A:) Ganglioside M2 (gm2) activator {Human (Homo sapiens)}
SSFSWDNCDEGKDPAVIRSLTLEPDPIIVPGNVTLSVMGSTSVPLSSPLKVDLVLEKEVAGL
WIKIPCTDYIGSCTFEHFCDVLDMLIPTGEPCPEPLRTYGLPCHCPFKEGTYSLPKSEFVVP
DLELPSWLTTGNYRIESVLSSSGKRLGCIKIAASLKGI
>d1i9ba_ b.96.1.1 (A:) Acetylcholine binding protein (ACHBP) {Great pond snail (Lymnaea stagnalis)}
FDRADILYNIRQTSRPDVIPTQRDRPVAVSVSLKFINILEVNEITNEVDVVFWQQTTWSDRT
LAWNSSHSPDQVSVPISSLWVPDLAAYNAISKPEVLTPQLARVVSDGEVLYMPSIRQRFSCD
VSGVDTESGATCRIKIGSWTHHSREISVDPTTENSDDSEYFSQYSRFEILDVTQKKNSVTYS

CCPEAYEDVEVSLNFRKKG
>d1k3ia2 b.18.1.1 (A:-12-150) Galactose oxidase, N-terminal domain {Fungi (Fusarium spp)}
IPEGSLQFLSLRASAPIGSAISRNNWAVTCDSAQSGNECNKAIDGNKDTFWHTFYGANGDPK
PPHTYTIDMKTTQNVNGLSMLPRQDGNQNGWIGRHEVYLSSDGTNWGSPVASGSWFADSTTK
YSNFETRPARYVRLVAITEANGQPWTSIAEINVFQASS
>d1eut_2 b.18.1.1 (506-647) Sialidase, C-terminal domain {Micromonospora viridifaciens}
QARMSIADVDSEETAREDGRASNVIDGNPSTFWHTEWSRADAPGYPHRISLDLGGTHTISGL
QYTRRQNSANEQVADYEIYTSLNGTTWDGPVASGRFTTSLAPQRAVFPARDARYIRLVALSE
QTGHKYAAVAELEVEGQR
>d1czsa_ b.18.1.2 (A:) C2 domain of factor V {Human (Homo sapiens)}
GCSTPLGMENGKIENKQITASSFKKSWWGDYWEPFRARLNAQGRVNAWQAKANNNKQWLEID
LLKIKKITAIITQGCKSLSSEMYVKSYTIHYSEQGVEWKPYRLKSSMVDKIFEGNTNTKGHV
KNFFNPPIISRFIRVIPKTWNQSITLRLELFGCDIY
>d1d7pm_ b.18.1.2 (M:) C2 domain of factor VIII {Human (Homo sapiens)}
LNSCSMPLGMESKAISDAQITASSYFTNMFATWSPSKARLHLQGRSNAWRPQVNNPKEWLQV
DFQKTMKVTGVTTQGVKSLLTSMYVKEFLISSSQDGHQWTLFFQNGKVKVFQGNQDSFTPVV
NCLDPPLLTRYLRIHPQSWVHQIALRMEVLGCEAQ
>d1jhja_ b.18.1.9 (A:) APC10/DOC1 subunit of the anaphase-promoting complex {Human (Homo sapiens)}
ATPNKTPPGADPKQLERTGTVREIGSQAVWSLSSCKPGFGVDQLRDDNLETYWQSDGSQPHL
VNIQFRRKTTVKTLCIYADYKSDESYTPSKISVRVGNNFHNLQEIRQLELVEPSGWIHVPLT
DNHKKPTRTFMIQIAVLANHQNGRDTHMRQIKIYTPV
>d1dlc_1 b.18.1.3 (500-644) delta-Endotoxin, C-terminal domain {Bacillus thuringiensis tenebrionis, CRYIIIA (BT13)}
FFNMIDSKKITQLPLVKAYKLQSGASVVAGPRFTGGDIIQCTENGSAATIYVTPDVSYSQKY
RARIHYASTSQITFTLSLDGAPFNQYYFDKTINKGDTLTYNSFNLASFSTPFELSGNNLQIG
VTGLSAGDKVYIDKIEFIPVN
>d1ji6a1 b.18.1.3 (A:503-652) delta-Endotoxin, C-terminal domain {Bacillus thuringiensis, CRY3bb1}
FFNTIDAEKITQLPVVKAYALSSGASIIEGPGFTGGNLLFLKESSNSIAKFKVTLNSAALLQ
RYRVRIRYASTTNLRLFVQNSNNDFLVIYINKTMNKDDDLTYQTFDLATTNSNMGFSGDKNE
LIIGAESFVSNEKIYIDKIEFIPVQL
>d1ciy_1 b.18.1.3 (462-609) delta-Endotoxin, C-terminal domain {Bacillus thuringiensis, CRYIA (A)}
NNIIPSSQITQIPLTKSTNLGSGTSVVKGPGFTGGDILRRTSPGQISTLRVNITAPLSQRYR
VRIRYASTTNLQFHTSIDGRPINQGNFSATMSSGSNLQSGSFRTVGFTTPFNFSNGSSVFTL
SAHVFNSGNEVYIDRIEFVPAEVT
>d1i5pa1 b.18.1.3 (A:473-633) delta-Endotoxin, C-terminal domain {Bacillus thuringiensis subsp. kurstaki, CRY2AA}
NIYAANENGTMIHLAPEDYTGFTISPIHATQVNNQTRTFISEKFGNQGDSLRFEQSNTTARY
TLRGNGNSYNLYLRVSSIGNSTIRVTINGRVYTVSNVNTTTNNDGVNDNGARFSDINIGNIV

ASDNTNVTLDINVTLNSGTPFDLMNIMFVPTNLPPLY
>d1nuka_ b.18.1.4 (A:) Ligand-binding domain of the ephb2 receptor tyrosine kinase {Mouse (Mus musculus)}
EETLMDSTTATAELGWMVHPPSGWEEVSGYDENMNTIRTYQVCNVFESSQNNWLRTKFIRRR
GAHRIHVEMKFSVRDCSSIPSVPGSCKETFNLYYYEADFDLATKTFPNWMENPWVKVDTIAA
DESFSQVDLGGRVMKINTEVRSFGPVSRNGFYLAFQDYGGCMSLIAVRVFYR
>d1jz8a3 b.18.1.5 (A:13-219) beta-Galactosidase {Escherichia coli}
RRDWENPGVTQLNRLAAHPPFASWRNSEEARTDRPSQQLRSLNGEWRFAWFPAPEAVPESWL
ECDLPEADTVVVPSNWQMHGYDAPIYTNVTYPITVNPPFVPTENPTGCYSLTFNVDESWLQE
GQTRIIFDGVNSAFHLWCNGRWVGYGQDSRLPSEFDLSAFLRAGENRLAVMVLRWSDGSYLE
DQDMWRMSGIFRDVSLLHKPT
>d1bhga2 b.18.1.5 (A:22-225) beta-Glucuronidase {Human (Homo sapiens)}
GLQGGMLYPQESPSRECKELDGLWSFRADFSDNRRRGFEEQWYRRPLWESGPTVDMPVPSSF
NDISQDWRLRHFVGWVWYEREVILPERWTQDLRTRVVLRIGSAHSYAIVWVNGVDTLEHEGG
YLPFEADISNLVQVGPLPSRLRITIAINNTLTPTTLPPGTIQYLTDTSKYPKGYFVQNTYFD
FFNYAGLQRSVLLYTTPT
>d1cx1a_ b.18.1.6 (A:) Cellulose-binding domain {Cellulomonas fimi}
ASLDSEVELLPHTSFAESLGPWSLYGTSEPVFADGRMCVDLPGGQGNPWDAGLVYNGVPVGE
GESYVLSFTASATPDMPVRVLVGEGGGAYRTAFEQGSAPLTGEPATREYAFTSNLTFPPDGD
APGQVAFHLGKAGAYEFCISQVSLTTSAT
>d1ulo__ b.18.1.6 (-) Cellulose-binding domain {Cellulomonas fimi}
ASPIGEGTFDDGPEGWVAYGTDGPLDTSTGALCVAVPAGSAQYGVGVVLNGVAIEEGTTYTL
RYTATASTDVTVRALVGQNGAPYGTVLDTSPALTSEPRQVTETFTASATYPATPAADDPEGQ
IAFQLGGFSADAWTLCLDDVALDSEVEL
>d1gmma_ b.18.1.10 (A:) Carbohydrate binding module from xylanase U {Clostridium thermocellum}
FSKIESEEYNSLKSSTIQTIGTSDGGSGIGYIESGDYLVFNKINFGNGANSFKARVASGADT
PTNIQLRLGSPTGTLIGTLTVASTGGWNNYEEKSCSITNTTGQHDLYLVFSGPVNIDYFIFD
SN
>d1gnya_ b.18.1.11 (A:) Xylan-binding module from xylanase 10c {Pseudomonas cellulosa}
GNVVIEVDMANGWRGNASGSTSHSGITYSADGVTFAALGDGVGAVFDIARPTTLEDAVIAMV
VNVSAEFKASEANLQIFAQLKEDWSKGEWDCLAGSSELTADTDLTLTCTIDEDDDKFNQTAR
DVQVGIQAKGTPAGTITIKSVTITLAQEA
>d1j83a_ b.18.1.12 (A:) Endo-1,4-beta glucanase EngF {Clostridium cellulovorans}
QPTAPKDFSSGFWDFNDGTTQGFGVNPDSPITAINVENANNALKISNLNSKGSNDLSEGNFW
ANVRISADIWGQSINIYGDTKLTMDVIAPTPVNVSIAAIPQSSTHGWGNPTRAIRVWTNNFV
AQTDGTYKATLTISTNDSPNFNTIATDAADSVVTNMILFVGSNSDNISLDNIKFTK
>d1dyoa_ b.18.1.7 (A:) Xylan-binding domain {Clostridium thermocellum}

PDAGYYYHDTFEGSVGQWTARGPAEVLLSGRTAYKGSESLLVRNRTAAWNGAQRALNPRTFV
PGNTYCFSVVASFIEGASSTTFCMKLQYVDGSGTQRYDTIDMKTVGPNQWVHLYNPQYRIPS
DATDMYVYVETADDTINFYIDEAIGAVAGTVI
>d1xnaa_ b.18.1.8 (A:) N-terminal domain of xrcc1 {Human (Homo sapiens)}
MPEIRLRHVVSCSSQDSTHCAENLLKADTYRKWRAAKAGEKTISVVLQLEKEEQIHSVDIGN
DGSAFVEVLVGSSAGGAGEQDYEVLLVTSSFMSPSESRSGSNPNRVRMFGPDKLVRAAAEKR
WDRVKIVCSQPYSKDSPFGLSFVRFHS
>d1ju3a1 b.18.1.13 (A:352-574) Bacterial cocaine esterase C-terminal domain {Rhodococcus sp. mb1}
PLPDTAYTPFYLGGSGAANTSTGGGTLSTSISGTESADTYLYDPADPVPSLGGTLLFHNGDN
GPADQRPIHDRDDVLCYSTEVLTDPVEVTGTVSARLFVSSSAVDTDFTAKLVDVFPDGRAIA
LCDGIVRMRYRETLVNPTLIEAGEIYEVAIDMLATSNVFLPGHRIMVQVSSSNFPKYDRNSN
TGGVIAREQLEEMCTAVNRIHRGPEHPSHIVLPIIKR
>d1bvp12 b.19.1.1 (1:121-254) Virus capsid protein vp7 (BTV-10 vp7), central (top) domain {Bluetongue virus}
PARQPYGFFLETEETFQPGRWFMRAAQAVTAVVCGPDMIQVSLNAGARGDVQQIFQGRNDPM
MIYLVWRRIENFAMAQGNSQQTQAGVTVSVGGVDMRAGRIIAWDGQAALHVHNPTQQNAMVQ
IQVVFYISMD
>d1ahsa_ b.19.1.1 (A:) Virus capsid protein vp7 (BTV-10 vp7), central (top) domain {African horse sickness virus}
TGPYAGAVEVQQSGRYYVPQGRTRGGYINSNIAEVCMDAGAAGQVNALLAPRRGDAVMIYFV
WRPLRIFCDPQGASLESAPGTFVTVDGVNVAAGDVVAWNTIAPVNVGNPGARRSILQFEVLW
YT
>d1qhda2 b.19.1.1 (A:149-332) vp6, the major capsid protein of group A rotavirus {Bovine rotavirus}
GFTFHKPNIFPYSASFTLNRSQPAHDNLMGTMWLNAGSEIQVAGFDYSCAINAPANTQQFEH
IVQLRRVLTTATITLLPDAERFSFPRVITSADGATTWYFNPVILRPNNVEIEFLLNGQIINT
YQARFGTIIARNFDTIRLSFQLMRPPNMTPAVAALFPNAQPFEHHATVGLTLRIESAVCE
>d1jsda_ b.19.1.2 (A:) Hemagglutinin {Influenza A virus, different strains}
DKICIGYQSTNSTETVDTLTETNVPVTHAKELLHTSHNGMLCATNLGHPLILDTCTIEGLIY
GNPSCDLLLGGREWSYIVERPSAVNGMCYPGNVENLEELRSLFSSASSYQRIQIFPDTIWNV
SYSGTSSACSDSFYRSMRWLTQKNNAYPIQDAQYTNNRGKSILFMWGINHPPTDTVQTNLYT
RTDTTTSVTTEDINRTFKPVIGPRPLVNGLHGRIDYYWSVLKPGQTLRVRSNGNLIAPWYGH
ILSGESHGRILKTDLNSGNCVVQCQTERGGLNTTLPFHNVSKYAFGNCPKYVGVKSLKLAVG
LRNVPAR
>d1jsma_ b.19.1.2 (A:) Hemagglutinin {Influenza A virus, different strains}
DQICIGYHANNSTEQVDTIMEKNVTVTHAQDILEKTHNGKLCDLNGVKPLILRDCSVAGWLL
GNPMCDEFLNVPEWSYIVEKDNPVNGLCYPENFNDYEELKHLLSSTNHFEKIRIIPRSSWSN
HDASSGVSSACPYNGRSSFFRNVVWLIKKNNAYPTIKRSYNNTNQEDLLILWGIHHPNDAAE
QTKLYQNPTTYVSVGTSTLNQRSVPEIATRPKVNGQSGRMEFFWTILKPNDAINFESNGNFI
APEYAYKIVKKGGSAIMKSGLEYGNCNTKCQTPMGAINSSMPFHNIHPLTIGECPKYVKSGR

LVLATGLRNVP
>d2visc_ b.19.1.2 (C:) Hemagglutinin {Influenza A virus, different strains}
VQSSSTGKICNNPHRILDGIDCTLIDALLGDPHCDVFQNETWDLFVERSKAFSNCYPYDVPD
YASLRSLVASSGTLEFITEGFTWTGVIQNGGSNACKRGPGSGFFSRLNWLTKSGSTYPVLNV
TMPNNDNFDKLYIWGIHHPSTNQEQTSLYVQASGRVTVSTRRSQQTIIPNIGSRPWVRGLSS
RISIYWTIVKPGDVLVINSNGNLIAPRGYFKMRTGKSSIMRSDAPIDTCISECITPNGSIPN
DKPFQNVNKITYGACPKYV
>d2viua_ b.19.1.2 (A:) Hemagglutinin {Influenza A virus, different strains}
STATLCLGHHAVPNGTLVKTITDDQIEVTNATELVQSSSTGKICNNPHRILDGIDCTLIDAL
LGDPHCDVFQNETWDLFVERSKAFSNCYPYDVPDYASLRSLVASSGTLEFITEGFTWTGVIQ
NGGSNACKRGPGSGFFSRLNWLTKSGSTYPVLNVTMPNNDNFDKLYIWGIHHPSTNQEQTSL
YVQASGRVTVSTRRSQQTIIPNIGSRPWVRGLSSRISIYWTIVKPGDVLVINSNGNLIAPRG
YFKMRTGKSSIMRSDAPIDTCISECITPNGSIPNDKPFQNVNKITYGACPKYVKQNTLKLAT
GMRNVPEKQT
>d1flca1 b.19.1.3 (A:151-306) Hemagglutinin domain of haemagglutinin-esterase-fusion glycoprotein HEF1 {Influenza C virus}
CMSLVNALDKTIPLQVTAGTAGNCNNSFLKNPALYTQEVKPSENKCGKENLAFFTLPTQFGT
YECKLHLVASCYFIYDSKEVYNKRGCDNYFQVIYDSFGKVVGGLDNRVSPYTGNSGDTPTMQ
CDMLQLKPGRYSVRSSPRFLLMPERSYCFDMK
>d1aol__ b.20.1.1 (-) F-MuLV receptor-binding domain {Friend murine leukemia virus}
QVYNITWEVTNGDRETVWAISGNHPLWTWWPVLTPDLCMLALSGPPHWGLEYQAPYSSPPGP
PCCSGSSGSSAGCSRDCDEPLTSLTPRCNTAWNRLKLDQVTHKSSEGFYVCPGSHRPREAKS
CGGPDSFYCASWGCETTGRVYWKPSSSWDYITVDNNLTTSQAVQVCKDNKWCNPLAIQFTNA
GKQVTSWTTGHYWGLRLYVSGRDPGLTFGIRLRYQNLGPRVP
>d1knb__ b.21.1.1 (-) Adenovirus fiber protein head domain (knob domain) {Human adenovirus type 5}
NDKLTLWTTPAPSPNCRLNAEKDAKLTLVLTKCGSQILATVSVLAVKGSLAPISGTVQSAHL
IIRFDENGVLLNNSFLDPEYWNFRNGDLTEGTAYTNAVGFMPNLSAYPKSHGKTAKSNIVSQ
VYLNGDKTKPVTLTITLNGTQETGDTTPSAYSMSFSWDWSGHNYINEIFATSSYTFSYIAQE
>d1qhva_ b.21.1.1 (A:) Adenovirus fiber protein head domain (knob domain) {Human adenovirus type 2}
AITIGNKNDDKLTLWTTPDPSPNCRIHSDNDCKFTLVLTKCGSQVLATVAALAVSGDLSSMT
GTVASVSIFLRFDQNGVLMENSSLKKHYWNFRNGNSTNANPYTNAVGFMPNLLAYPKTQSQT
AKNNIVSQVYLHGDKTKPMILTITLNGTSESTETSEVSTYSMSFTWSWESGKYTTETFATNS
YTFSYIAQE
>d1h7za_ b.21.1.1 (A:) Adenovirus fiber protein head domain (knob domain) {Human adenovirus type 3}
KNNTLWTGPKPEANCIIEYGKQNPDSKLTLILVKNGGIVNGYVTLMGASDYVNTLFKNKNVS
INVELYFDATGHILPDSSSLKTDLELKYKQTADFSARGFMPSTTAYPFVLPNAGTHNENYIF
GQCYYKASDGALFPLEVTVMLNKRLPDSRTSYVMTFLWSLNAGLAPETTQATLITSPFTFSY

IREDD
>d1kaca_ b.21.1.1 (A:) Adenovirus fiber protein head domain (knob domain) {Human adenovirus type 12}
TPYDPLTLWTTPDPPPNCSLIQELDAKLTLCLTKNGSIVNGIVSLVGVKGNLLNIQSTTTTV
GVHLVFDEQGRLITSTPTALVPQASWGYRQGQSVSTNTVTNGLGFMPNVSAYPRPNASEAKS
QMVSLTYLQGDTSKPITMKVAFNGITSLNGYSLTFMWSGLSNYINQPFSTPSCSFSYITQE
>d1kkea1 b.21.1.2 (A:250-312) Reovirus attachment protein sigma 1 {Reovirus}
EQSYVASAVTPLRLNSSTKVLDMLIDSSTLEINSSGQLTVRSTSPNLRYPIADVSGGIGMSP
N
>d1aly__ b.22.1.1 (-) Extracellular domain of CD40 ligand {Human (Homo sapiens)}
GDQNPQIAAHVISEASSKTTSVLQWAEKGYYTMSNNLVTLENGKQLTVKRQGLYYIYAQVTF
CSNREASSQAPFIASLCLKSPGRFERILLRAANTHSSAKPCGQQSIHLGGVFELQPGASVFV
NVTDPSQVSHGTGFTSFGLLKL
>d1c28a_ b.22.1.1 (A:) 30 kd adipocyte complement-related protein {Mouse (Mus musculus)}
MYRSAFSVGLETRVTVPNVPIRFTKIFYNQQNHYDGSTGKFYCNIPGLYYFSYHITVYMKDV
KVSLFKKDKAVLFTYDQYQEKNVDQASGSVLLHLEVGDQVWLQVYGDGDHNGLYADNVNDST
FTGFLLYHDT
>d1tnra_ b.22.1.1 (A:) Tumor necrosis factor (TNF) {Human (Homo sapiens)}
KPAAHLIGDPSKQNSLLWRANTDRAFLQDGFSLSNNSLLVPTSGIYFVYSQVVFSGKAYSPK
ATSSPLYLAHEVQLFSSQYPFHVPLLSSQKMVYPGLQEPWLHSMYHGAAFQLTQGDQLSTHT
DGIPHLVLSPSTVFFGAFAL
>d4tsva_ b.22.1.1 (A:) Tumor necrosis factor (TNF) {Human (Homo sapiens)}
DKPVAHVVANPQAEGQLQWSNRRANALLANGVELRDNQLVVPIEGLFLIYSQVLFKGQGCPS
THVLLTHTISRIAVSYQTKVNLLSAIKSPCQRETPEGAEAKPWYEPIYLGGVFQLEKGDRLS
AEINRPDYLDFAESGQVYFGIIAL
>d2tnfa_ b.22.1.1 (A:) Tumor necrosis factor (TNF) {Mouse (Mus musculus)}
SDKPVAHVVANHQVEEQLEWLSQRANALLANGMDLKDNQLVVPADGLYLVYSQVLFKGQGCP
DYVLLTHTVSRFAISYQEKVNLLSAVKSPCPKDTPEGAELKPWYEPIYLGGVFQLEKGDQLS
AEVNLPKYLDFAESGQVYFGVIAL
>d1dg6a_ b.22.1.1 (A:) Apoptosis-2 ligand, apo2l/TRAIL {Human (Homo sapiens)}
QRVAAHITGTRGRSNTLSSPNSKNEKALGRKINSWESSRSGHSFLSNLHLRNGELVIHEKGF
YYIYSQTYFRFQEEIKENTKNDKQMVQYIYKYTSYPAPILLMKSARNSCWSKDAEYGLYSIY
QGGIFELKENDRIFVSVTNEHLIDMDHEASFFGAFLVG
>d1jtzx_ b.22.1.1 (X:) TRANCE/RANKL cytokine {Mouse (Mus musculus)}
QPFAHLTINAASIPSGSHKVTLSSWYHDRGWAKISNMTLSNGKLRVNQDGFYYLYANICFRH
HETSGSVPTDYLQLMVYVVKTSIKIPSSHNLMKGGSTKNWSGNSEFHFYSINVGGFFKLRAG

EEISIQVSNPSLLDPDQDATYFGAFKVQDID
>d1jh5a_ b.22.1.1 (A:) Soluble part of TALL-1, sTALL-1 {Human (Homo sapiens)}
VTQDCLQLIADSETPTIQKGSYTFVPWLLSFKRGSALEEKENKILVKETGYFFIYGQVLYTD
KTYAMGHLIQRKKVHVFGDELSLVTLFRCIQNMPETLPNNSCYSAGIAKLEEGDELQLAIPR
ENAQISLDGDVTFFGALKLL
>d1gr3a_ b.22.1.1 (A:) Collagen X NC1 trimerisation domain {Human (Homo sapiens)}
MPVSAFTVILSKAYPAIGTPIPFDKILYNRQQHYDPRTGIFTCQIPGIYYFSYHVHVKGTHV
WVGLYKNGTPVMYTYDEYTKGYLDQASGSAIIDLTENDQVWLQLPNAESNGLYSSEYVHSSF
SGFLVAPM
>d1sfp__ b.23.1.1 (-) Acidic seminal fluid protein (ASFP) {Cow (Bos taurus)}
LPRNTNCGGILKEESGVIATYYGPKTNCVWTIQMPPEYHVRVSIQYLQLNCNKESLEIIDGL
PGSPVLGKICEGSLMDYRSSGSIMTVKYIREPEHPASFYEVLYFQDPQA
>d1sppa_ b.23.1.1 (A:) Major seminal plasma glycoprotein PSP-I {Pig (Sus scrofa)}
LDYHACGGRLTDDYGTIFTYKGPKTECVWTLQVDPKYKLLVSIPTLNLTCGKEYVEVLEGAP
GSKSLGKFCEGLSILNRGSSGMTVKYKRDSGHPASPYEIIFLRDSQG
>d1sppb_ b.23.1.1 (B:) Major seminal plasma glycoprotein PSP-II {Pig (Sus scrofa)}
ARINGPDECGRVIKDTSGSISNTDRQKNLCTWTILMKPDQKVRMAIPYLNLACGKEYVEVFD
GLLSGPSYGKLCAGAAIVFLSTANTMTIKYNRISGNSSSPFLIYFYGSSP
>d1cb8a2 b.24.1.1 (A:600-700) Chondroitinase AC {Pedobacter heparinus (Flavobacterium heparinum)}
PKVLANTNQLQAVYHQQLDMVQAIFYTAGKLSVAGIEIETDKPCAVLIKHINGKQVIWAADP
LQKEKTAVLSIRDLKTGKTNRVKIDFPQQEFAGATVELK
>d1egua2 b.24.1.1 (A:815-893) Hyaluronate lyase {Streptococcus pneumoniae}
SSLIENNETLQSVYDAKQGVWGIVKYDDSVSTISNQFQVLKRGVYTIRKEGDEYKIAYYNPE
TQESAPDQEVFKKLEQH
>d1f1sa3 b.24.1.1 (A:920-984) Hyaluronate lyase {Streptococcus agalactiae}
SKQQVIYDKNSQTWAVIKHDNQESLINNQFKMNKAGLYLVQKVGNDYQNVYYQPQTMTKTDQ
LAI
>d1iaza_ b.97.1.1 (A:) Equinatoxin II (eqtII, tenebrosin C) {European sea anemone (Actinia equina)}
AGAVIDGASLSFDILKTVLEALGNVKRKIAVGVDNESGKTWTALNTYFRSGTSDIVLPHKVP
HGKALLYNGQKDRGPVATGAVGVLAYLMSDGNTLAVLFSVPYDYNWYSNWWNVRIYKGKRRA
DQRMYEELYYNLSPFRGDNGWHTRNLGYGLKSRGFMNSSGHAILEIHVSKA
>d1aun__ b.25.1.1 (-) Pathogenesis-related protein 5d {Common tobacco (Nicotiana tabacum)}
SGVFEVHNNCPYTVWAAATPVGGGRRLERGQSWWFWAPPGTKMARIWGRTNCNFDGAGRGWC
QTGDCGGVLECKGWGKPPNTLAEYALNQFSNLDFWDISVIDGFNIPMSFGPTKPGPGKCHGI

QCTANINGECPGSLRVPGGCNNPCTTFGGQQYCCTQGPCGPTELSRWFKQRCPDAYSYPQDD
PTSTFTCTSWTTDYKVMFCPYG
>d1du5a_ b.25.1.1 (A:) Zeamatin {Maize (Zea mays)}
AVFTVVNQCPFTVWAASVPVGGGRQLNRGESWRITAPAGTTAARIWARTGCKFDASGRGSCR
TGDCGGVLQCTGYGRAPNTLAEYALKQFNNLDFFDISLIDGFNVPMSFLPDGGSGCSRGPRC
AVDVNARCPAELRQDGVCNNACPVFKKDEYCCVGSAANDCHPTNYSRYFKGQCPDAYSYPKD
DATSTFTCPAGTNYKVVFCP
>d1thw__ b.25.1.1 (-) Thaumatin {Ketemfe (Thaumatococcus daniellii)}
ATFEIVNRCSYTVWAAASKGDAALDAGGRQLNSGESWTINVEPGTKGGKIWARTDCYFDDSG
SGICKTGDCGGLLRCKRFGRPPTTLAEFSLNQYGKDYIDISNIKGFNVPMDFSPTTRGCRGV
RCAADIVGQCPAKLKAPGGGCNDACTVFQTSEYCCTTGKCGPTEYSRFFKRLCPDAFSYVLD
KPTTVTCPGSSNYRVTFCPTA
>d1dd1a_ b.26.1.1 (A:) Smad4 tumor suppressor C-terminal domain {Human (Homo sapiens)}
NGHLQHHPPMPPHPGHYWPVHNELAFQPPISNHPAPEYWCSIAYFEMDVQVGETFKVPSSCP
IVTVDGYVDPSGGDRFCLGQLSNVHRTEAIERARLHIGKGVQLECKGEGDVWVRCLSDHAVF
VQSYYLDREAGRAPGDAVHKIYPSAYIKVFDLRQCHRQMQQQAATAQAAAAAQAAAVAGNIP
GPGSVGGIAPAISLSAAAGIGVDDLRRLCILRMSFVKGWGPDYPRQSIKETPCWIEIHLHRA
LQLLDEVLHTMPI
>d1ygs__ b.26.1.1 (-) Smad4 tumor suppressor C-terminal domain {Human (Homo sapiens)}
APEYWCSIAYFEMDVQVGETFKVPSSCPIVTVDGYVDPSGGDRFCLGQLSNVHRTEAIERAR
LHIGKGVQLECKGEGDVWVRCLSDHAVFVQSYYLDREAGRAPGDAVHKIYPSAYIKVFDLRQ
CHRQMQQQAATAQAAAAAQAAAVAGNIPGPGSVGGIAPAISLSAAAGIGVDDLRRLCILRMS
FVKGWGPDYPRQSIKETPCWIEIHLHRALQLLDEVLHTM
>d1khxa_ b.26.1.1 (A:) Smad2 MH2 domain {Human (Homo sapiens)}
PVTYSEPAFWCSIAYYELNQRVGETFHASQPSLTVDGFTDPSNSERFCLGLLSNVNRNATVE
MTRRHIGRGVRLYYIGGEVFAECLSDSAIFVQSPNCNQRYGWHPATVCKIPPGCNLKIFNNQ
EFAALLAQSVNQGFEAVYQLTRMCTIRMSFVKGWGAEYRRQTVTSTPCWIELHLNGPLQWLD
KVLTQMGSPSVRCSSMS
>d1khua_ b.26.1.1 (A:) Smad1 {Human (Homo sapiens)}
PKHWCSIVYYELNNRVGEAFHASSTSVLVDGFTDPSNNKNRFCLGLLSNVNRNSTIENTRRH
IGKGVHLYYVGGEVYAECLSDSSIFVQSRNCNYHHGFHPTTVCKIPSGCSLKIFNNQEFAQL
LAQSVNHGFETVYELTKMCTIRMSFVKGWGAEYHRQDVTSTPCWIEIHLHGPLQWLDKVLTQ
MGSPHNPISSVS
>d1dmza_ b.26.1.2 (A:) Phosphotyrosine binding domain of Rad53 {Baker's yeast (Saccharomyces cerevisiae)}
GNGRFLTLKPLPDSIIQESLEIQQGVNPFFIGRSEDCNCKIEDNRLSRVHCFIFKKRHAVGK
SMYESPAQGLDDIWYCHTGTNVSYLNNNRMIQGTKFLLQDGDEIKIIWDKNNKFVIGFKVEI
NDTTGLFNEGLGMLQEQRVVLKQTAEEKDLVKKL
>d1g6ga_ b.26.1.2 (A:) Phosphotyrosine binding domain of Rad53 {Baker's yeast (Saccharomyces cerevisiae)}
GENIVCRVICTTGQIPIRDLSADISQVLKEKRSIKKVWTFGRNPACDYHLGNISRLSNKHFQ

ILLGEDGNLLLNDISTNGTWLNGQKVEKNSNQLLSQGDEITVGVGVESDILSLVIFINDKFK
QCL
>d1k3ja_ b.26.1.2 (A:) Phosphotyrosine binding domain of Rad53
{Baker's yeast (Saccharomyces cerevisiae)}
ATQRFLIEKFSQEQIGENIVCRVICTTGQIPIRDLSADISQVLKEKRSIKKVWTFGRNPACD
YHLGNISRLSNKHFQILLGEDGNLLLNDISTNGTWLNGQKVEKNSNQLLSQGDEITVGVGVE
SDILSLVIFINDKFKQCLEQNKVDRIR
>d1qu5a_ b.26.1.2 (A:) Phosphotyrosine binding domain of Rad53
{Baker's yeast (Saccharomyces cerevisiae)}
EAETREQKLLHSNNTENVKSSKKKGNGRFLTLKPLPDSIIQESLEIQQGVNPFFIGRSEDCN
CKIEDNRLSRVHCFIFKKRHAVGKSMYESPAQGLDDIWYCHTGTNVSYLNNNRMIQGTKFLL
QDGDEIKIIWDKNNKFVIGFKVEINDTTGLFNEGLGMLQEQRVVLKQTAEEKDLVKKL
>d1cq3a_ b.27.1.1 (A:) Soluble secreted chemokine inhibitor, VCCI
{Cowpox virus}
SFSSSSSCTEEENKHHMGIDVIIKVTKQDQTPTNDKICQSVTEVTESEDESEEVVKGDPTTY
YTVVGGGLTMDFGFTKCPKISSISEYSDGNTVNARLSSVSPGQGKDSPAITREEALSMIKDC
EMSINIKCSEEEKDSNIKTHPVLGSNISHKKVSYEDIIGSTIVDTKCVKNLEISVRIGDMCK
ESSELEVKDGFKYVDGSASEDAADDTSLINSAKLIACV
>d1p35a_ b.28.1.1 (A:) Paculovirus p35 {Nuclear polyhedrosis virus
(Autographa californica), ACMNPV}
CVIFPVEIDVSQTIIRDCQVDKQTRELVYINKIMNTQLTKPVLMMFNISGPIRSVTRKNNNL
RDRIKSKVDEQFDQLERDYSDQMDGFHDSIKYFKDEHYSVSCQNGSVLKSKFAKILKSHDYT
DKKSIEAYEKYCLPKLVDERNDYYVAVCVLKPGFENGSNQVLSFEYNPIGNKVIVPFAHEIN
DTGLYEYDVVAYVDSVQFDGEQFEEFVQSLILPSSFKNSEKVLYYNEASKNKSMIYKALEFT
TESSWGKSEKYNWKIFCNGFIYDKKSKVLYVKLHNVTSALNKNVILNTIKA
>d1nls__ b.29.1.1 (-) Concanavalin A {Jack bean (Canavalia
ensiformis)}
ADTIVAVELDTYPNTDIGDPSYPHIGIDIKSVRSKKTAKWNMQNGKVGTAHIIYNSVDKRLS
AVVSYPNADSATVSYDVDLDNVLPEWVRVGLSASTGLYKETNTILSWSFTSKLKSNSTHETN
ALHFMFNQFSKDQKDLILQGDATTGTDGNLELTRVSSNGSPQGSSVGRALFYAPVHIWESSA
VVASFEATFTFLIKSPDSHPADGIAFFISNIDSSIPSGSTGRLLGLFPDAN
>d2cna__ b.29.1.1 (-) Concanavalin A {Jack bean (Canavalia
ensiformis)}
ADTIVAVELDTYPNTDIGDPSYPHIGIDIKSVRSKKTAKWNMQDGKVGTAHIIYNSVDKRLS
AVVSYPNADATSVSYDVDLNDVLPEWVRVGLSASTGLYKETNTILSWSFTSKLKSNSTHQTD
ALHFMFNQFSKDQKDLILQGDATTGTDGNLELTRVSSNGSPEGSSVGRALFYAPVHIWESSA
TVSAFEATFAFLIKSPDSHPADGIAFFISNIDSSIPSGSTGRLLGLFPDAN
>g2ltn.1 b.29.1.1 (A:,B:) Legume lectin {Garden pea (Pisum
sativum)}
TETTSFLITKFSPDQQNLIFQGDGYTTKEKLTLTKAVKNTVGRALYSSPIHIWDRETGNVAN
FVTSFTFVINAPNSYNVADGFTFFIAPVDTKPQTGGGYLGVFNSAEYDKTTQTVAVEFDTFY
NAAWDPSNRDRHIGIDVNSIKSVNTKSWKLQNGEEANVVIAFNAATNVLTVSLTYPNXVTSY
TLSDVVSLKDVVPEWVRIGFSATTGAEYAAHEVLSWSFHSELSG
>g1len.1 b.29.1.1 (A:,B:) Legume lectin {Common lentil (Lens

culinaris)}
TETTSFSITKFSPDQQNLIFQGDGYTTKGKLTLTKAVKSTVGRALYSTPIHIWDRDTGNVAN
FVTSFTFVIDAPSSYNVADGFTFFIAPVDTKPQTGGGYLGVFNSKEYDKTSQTVAVEFDTFY
NAAWDPSNKERHIGIDVNSIKSVNTKSWNLQNGERANVVIAFNAATNVLTVTLTYPNXVTSY
TLNEVVPLKDVVPEWVRIGFSATTGAEFAAQEVHSWSFNSQLG
>d1led__ b.29.1.1 (-) Legume lectin {West-central african legume (Griffonia simplicifolia)}
ENTVNFTYPDFWSYSLKNGTEITFLGDATRIPGALQLTKTDANGNPVRSSAGQASYSEPVFL
WDSTGKAASFYTSFTFLLKNYGAPTADGLAFFLAPVDSSVKDYGGFLGLFRHETAADPSKNQ
VVAVEFDTWINKDWNDPPYPHIGIDVNSIVSVATTRWENDDAYGSSIATAHITYDARSKILT
VLLSYEHGRDYILSHVVDLAKVLPQKVRIGFSAGVGYDEVTYILSWHFFSTLDGTNK
>d1hqla_ b.29.1.1 (A:) Legume lectin {Griffonia simplicifolia, lectin I-b4}
SVSFTFPNFWSDVEDSIIFQGDANTTAGTLQLCKTNQYGTPLQWSAGRALYSDPVQLWDNKT
ESVASFYTEFTFFLKITGNGPADGLAFFLAPPDSDVKDAGEYLGLFNKSTATQPSKNQVVAV
EFDTWTNPNFPEPSYRHIGINVNSIVSVATKRWEDSDIFSGKIATARISYDGSAEILTVVLS
YPDGSDYILSHSVDMRQNLPESVRVGISASTGNNQFLTVYILSWRFSSNL
>d1ax0__ b.29.1.1 (-) Legume lectin {Coral tree (Erythrina corallodendron)}
VETISFSFSEFEPGNDNLTLQGAALITQSGVLQLTKINQNGMPAWDSTGRTLYAKPVHIWDM
TTGTVASFETRFSFSIEQPYTRPLPADGLVFFMGPTKSKPAQGYGYLGIFNNSKQDNSYQTL
GVEFDTFSNPWDPPQVPHIGIDVNSIRSIKTQPFQLDNGQVANVVIKYDASSKILHAVLVYP
SSGAIYTIAEIVDVKQVLPEWVDVGLSGATGAQRDAAETHDVYSWSFQASLPE
>d1wbfa_ b.29.1.1 (A:) Legume lectin {Winged bean (Psophocarpus tetragonolobus), basic agglutinin}
KTISFNFNQFHQNEEQLKLQRDARISSNSVLELTKVVNGVPTWNSTGRALYAKPVQVWDSTT
GNVASFETRFSFSIRQPFPRPHPADGLVFFIAPPNTQTGEGGGYFGIYNPLSPYPFVAVEFD
TFRNTWDPQIPHIGIDVNSVISTKTVPFTLDNGGIANVVIKYDASTKILHVVLVFPSLGTIY
TIADIVDLKQVLPESVNVGFSAATGDPSGKQRNATETHDILSWSFSASLPG
>d1f9ka_ b.29.1.1 (A:) Legume lectin {Winged bean (Psophocarpus tetragonolobus), acidic lectin}
ETQSFNFDHFEENSKELNLQRQASIKSNGVLELTKLTKNGVPVWKSTGRALYAEPIKIWDST
TGNVASFETRFSFNITQPYAYPEPADGLTFFMVPPNSPQGEDGGNLGVFKPPEGDNAFAVEF
DTFQNTWDPQVPHIGIDVNSIVSSKTLHFQLENGGVANVVIKYDSPTKILNVVLAFHSVGTV
YTLSNIVDLKQEFPNSEWVNVGLSATTGYQKNAVETHEIISWSFTSSL
>g1loe.1 b.29.1.1 (A:,B:) Legume lectin {Lathyrus ochrus, isolectin I}
TETTSFSITKFGPDQQNLIFQGDGYTTKERLTLTKAVRNTVGRALYSSPIHIWDSKTGNVAN
FVTSFTFVIDAPNSYNVADGFTFFIAPVDTKPQTGGGYLGVFNSKDYDKTSQTVAVEFDTFY
NTAWDPSNGDRHIGIDVNSIKSINTKSWALQNGKEANVVIAFNAATNVLTVSLTYPXTSYTL
NEVVPLKEFVPEWVRIGFSATTGAEFAAHEVLSWYFHSELA
>d2pela_ b.29.1.1 (A:) Legume lectin {Peanut (Arachis hypogaea)}
AETVSFNFNSFSEGNPAINFQGDVTVLSNGNIQLTNLNKVNSVGRVLYAMPVRIWSSATGNV
ASFLTSFSFEMKDIKDYDPADGIIFFIAPEDTQIPAGSIGGGTLGVSDTKGAGHFVGVEFDT

YSNSEYNDPPTDHVGIDVNSVDSVKTVPWNSVSGAVVKVTVIYDSSTKTLSVAVTNDNGDIT
TIAQVVDLKAKLPERVKFGFSASGSLGGRQIHLIRSWSFTSTLITT
>d1g9fa_ b.29.1.1 (A:) Legume lectin {Soybean (Glycine max)}
AETVSFSWNKFVPKQPNMILQGDAIVTSSGKLQLNKVDENGTPKPSSLGRALYSTPIHIWDK
ETGSVASFAASFNFTFYAPDTKRLADGLAFFLAPIDTKPQTHAGYLGLFNENESGDQVVAVE
FDTFRNSWDPPNPHIGINVNSIRSIKTTSWDLANNKVAKVLITYDASTSLLVASLVYPSQRT
SNILSDVVDLKTSLPEWVRIGFSAATGLDIPGESHDVLSWSFASNLPHASSNIDPLDLTSFV
LHE
>g1qmo.1 b.29.1.1 (A:,E:) Legume lectin {Field bean (Dolichos lab lab), Fril}
AQSLSFSFTKFDPNQEDLIFQGHATSTNNVLQVTKLDSAGNPVSSSAGRVLYSAPLRLWEDS
AVLTSFDTIINFEISTPYTSRIADGLAFFIAPPDSVISYHGGFLGLFPNANXSNVVAVEFDT
YLNPDYGDPNYIHIGIDVNSIRSKVTAKWDWQNGKIATAHISYNSVSKRLSVTSYYAGSKPA
TLSYDIELHTVLPEWVRVGLSASTGQDKERNTVHSWSFTSSLWTN
>d1g7ya_ b.29.1.1 (A:) Legume lectin {Horse gram (Dolichos biflorus), different isoforms}
ADIQSFSFKNFNSSSFILQGDATVSSSKLRLTKVKGNGLPTLSSLGRAFYSSPIQIYDKSTG
AVASWATSFTANIFAPNKSSSADGIAFALVPVGSEPKSNSGFLGVFDSDVYDNSAQTVAVEF
DTFSNTDWDPTSRHIGIDVNSIKSIRTASWGLANGQNAEILITYNAATSLLVASLVHPSRRT
SYIVSERVDITNELPEYVSIGFSATTGLSEGYTETHDVLSWSFASKLPDDSTTEPLDIASYL
VRNVL
>d1lu1__ b.29.1.1 (-) Legume lectin {Horse gram (Dolichos biflorus), different isoforms}
ANIQSFSFKNFNSPSFILQGDATVSSGKLQLTKVKENGIPTPSSLGRAFYSSPIQIYDKSTG
AVASWATSFTVKISAPSKASFADGIAFALVPVGSEPRRNGGYLGVFDSDVYNNSAQTVAVEF
DTLSNSGWDPSMKHIGIDVNSIKSIATVSWDLANGENAEILITYNAATSLLVASLVHPSRRT
SYILSERVDITNELPEYVSVGFSATTGLSEGYIETHDVLSWSFASKLPDDSTAEPLDLASYL
VRNVL
>d1h9wa_ b.29.1.1 (A:) Legume lectin {Duke (Dioclea guianensis)}
ADTIVAVELDSYPNTDIGDPSYPHIGIDIKSIRSKSTARWNMQTGKVGTAHISYNSVAKRLS
AVVSYTGSSSTTVSYDVDLNNVLPEWVRVGLSATTGLYKETNTILSWSFTSKLKTNSIADAN
SLHFSFNQFSQNPKDLILQGDATTDSDGNLELTKVSSSGDPQGSSVGRALFYAPVHIWEKSA
VVASFDATFTFLIKSPDRDPADGITFFIANTDTSIPSGSGGRLLGLFPDAN
>d1fx5a_ b.29.1.1 (A:) Legume lectin {Furze (Ulex europaeus), UEA-I}
SDDLSFKFKNFSQNGKDLSFQGNASVIETGVLQLNKVGNNLPDETGGIARYIAPIHIWNCNT
GELASFITSFSFFMETSANPKAATDGLTFFLAPPDSPLRRAGGYFGLFNDTKCDSSYQTVAV
EFDTIGSPVNFWDPGFPHIGIDVNCVKSINAERWNKRYGLNNVANVEIIYEASSKTLTASLT
YPSDQTSISVTSIVDLKEILPEWVSVGFSGSTYIGRQATHEVLNWYFTSTFINT
>d1qnwa_ b.29.1.1 (A:) Legume lectin {Furze (Ulex europaeus), UEA-II}
SDDLSFNFDKFVPNQKNIIFQGDASVSTTGVLQVTKVSKPTTTSIGRALYAAPIQIWDSITG
KVASFATSFSFVVKADKSDGVDGLAFFLAPANSQIPSGSSAGMFGLFSSSDSKSSNQIIAVE
FDTYFGKAYNPWDPDFKHIGIDVNSIKSIKTVKWDWRNGEVADVVITYRAPTKSLTVCLSYP

SDGTSNIITASVDLKAILPEWVSVGFSGGVGNAAEFETHDVLSWYFTSNLE
>d1dbna_ b.29.1.1 (A:) Legume lectin {Maackia amurensis, leukoagglutinin}
SDELSFTINNFVPNEADLLFQGEASVSSTGVLQLTKVENGQPQKYSVGRALYAAPVRIWGNT
TGSVASFSTSFTFVVKAPNPDITSDGLAFYLAPPDSQIPSGSVSKYLGLFNNSNSDSSNQIV
AVEFDTYFAHSYDPWDPNYRHIGIDVNGIESIKTVQWDWINGGVAFATITYLAPNKTLIASL
VYPSNQTTFSVAASVDLKEILPEWVRVGFSAATGYPTEVETHDVLSWSFTSTL
>d1fnya_ b.29.1.1 (A:) Legume lectin {Black locust (Robinia pseudoacacia)}
TGSLSFSFPKFAPNQPYLINQGDALVTSTGVLQLTNVVNGVPSSKSLGRALYAAPFQIWDST
TGNVASFVTSFTFIIQAPNPATTADGLAFFLAPVDTQPLDLGGMLGIFKDGYFNKSNQIVAV
EFDTFSNGDWDPKGRHLGINVNSIESIKTVPWNWTNGEVANVFISYEASTKSLTASLVYPSL
ETSFIIDAIVDVKIVLPEWVRFGFSATTGIDKGYVQTNDVLSWSFESNLPG
>d1avba_ b.29.1.1 (A:) Phytohemagglutinin-L, PHA-L, also arcelin {Kidney bean (Phaseolus vulgaris)}
SNDASFNVETFNKTNLILQGDATVSSEGHLLLTNVKGNEEDSMGRAFYSAPIQINDRTIDNL
ASFSTNFTFRINAKNIENSAYGLAFALVPVGSRPKLKGRYLGLFNTTNYDRDAHTVAVVFDT
VSNRIEIDVNSIRPIATESCNFGHNNGEKAEVRITYDSPKNDLRVSLLYPSSEEKCHVSATV
PLEKEVEDWVSVGFSATSGSKKETTETHNVLSWSFSSNFI
>d1dhkb_ b.29.1.1 (B:) Phytohemagglutinin-L, PHA-L, also arcelin {Kidney bean (Phaseolus vulgaris)}
ATETSFIIDAFNKTNLILQGDATVSSNGNLQLSYNSYDSMSRAFYSAPIQIRDSTTGNVASF
DTNFTMNIRTHRQANSAVGLDFVLVPVQPESKGDTVTVEFDTFLSRISIDVNNNDIKSVPWD
VHDYDGQNAEVRITYNSSTKVFSVSLSNPSTGKSNNVSTTVELEKEVYDWVSVGFSATSGAY
QWSYETHDVLSWSFSSKF
>d1g8wa_ b.29.1.1 (A:) Phytohemagglutinin-L, PHA-L, also arcelin {Kidney bean (Phaseolus vulgaris)}
SNDIYFNFQRFNETNLILQRDASVSSSGQLRLTNLNGNGEPRVGSLGRAFYSAPIQIWDNTT
GTVASFATSFTFNIQVPNNAGPADGLAFALVPVGSQPKDKGGFLGLFDGSNSNFHTVAVEFD
TLYNKDWDPTERHIGIDVNSIRSIKTTRWDFVNGENAEVLITYDSSTNLLVASLVYPSQKTS
FIVSDTVDLKSVLPEWVSVGFSATTGINKGNVETNDVLSWSFASKLS
>d1ioaa_ b.29.1.1 (A:) Phytohemagglutinin-L, PHA-L, also arcelin {Kidney bean (Phaseolus vulgaris), G02771, arcelin-5a}
ATETSFNFPNFHTDDKLILQGNATISSKGQLQLTGVGSNELPRVDSLGRAFYSDPIQIKDSN
NVASFNTNFTFIIRAKNQSISAYGLAFALVPVNSPPQKKQEFLGIFNTNNPEPNARTVAVVF
NTFKNRIDFDKNFIKPYVNENCDFHKYNGEKTDVQITYDSSNNDLRVFLHFTVSQVKCSVSA
TVHLEKEVDEWVSVGFSPTSGLTEDTTETHDVLSWSFSSKFR
>d1gbg__ b.29.1.2 (-) Bacillus 1-3,1-4-beta-glucanase {Bacillus licheniformis}
QTGGSFYEPFNNYNTGLWQKADGYSNGNMFNCTWRANNVSMTSLGEMRLSLTSPSYNKFDCG
ENRSVQTYGYGLYEVNMKPAKNVGIVSSFFTYTGPTDGTPWDEIDIEFLGKDTTKVQFNYYT
NGVGNHEKIVNLGFDAANSYHTYAFDWQPNSIKWYVDGQLKHTATTQIPQTPGKIMMNLWNG
AGVDEWLGSYNGVTPLYAHYNWVRYTKR
>d2ayh__ b.29.1.2 (-) Bacillus 1-3,1-4-beta-glucanase {Hybrid

protein: residues 1-16 from Bacillus amyloliquefaciens and Bacillus macerans}
QTGGSFFEPFNSYNSGTWEKADGYSNGGVFNCTWRANNVNFTNDGKLKLGLTSSAYNKFDCA
EYRSTNIYGYGLYEVSMKPAKNTGIVSSFFTYTGPAHGTQWDEIDIEFLGKDTTKVQFNYYT
NGVGGHEKVISLGFDASKGFHTYAFDWQPGYIKWYVDGVLKHTATANIPSTPGKIMMNLWNG
TGVDDWLGSYNGANPLYAEYDWVKYTSN
>d1ajka_ b.29.1.2 (A:) Bacillus 1-3,1-4-beta-glucanase {Bacillus macerans}
NTGIVSSFFTYTGPAHGTQWDEIDIEFLGKDTTKVQFNYYTNGVGGHEKVISLGFDASKGFH
TYAFDWQPGYIKWYVDGVLKHTATANIPSTPGKIMMNLWNGTGVDDWLGSYNGANPLYAEYD
WVKYTSNQTGGSFFEPFNSYNSGTWEKADGYSNGGVFNCTWRANNVNFTNDGKLKLGLTSSA
YNKFDCAEYRSTNIYGYGLYEVSMKPAK
>d1ajoa_ b.29.1.2 (A:) Bacillus 1-3,1-4-beta-glucanase {Bacillus macerans}
GHEKVISLGFDASKGFHTYAFDWQPGYIKWYVDGVLKHTATANIPSTPGKIMMNLWNGTGVD
DWLGSYNGANPLYAEYDWVKYTSNQTGGSFFEPFNSYNSGTWEKADGYSNGGVFNCTWRANN
VNFTNDGKLKLGLTSSAYNKFDCAEYRSTNIYGYGLYEVSMKPAKNTGIVSSFFTYTGPAHG
TQWDEIDIEFLGKDTTKVQFNYYTNG
>d1cpm__ b.29.1.2 (-) Bacillus 1-3,1-4-beta-glucanase {Bacillus macerans}
FDCAEYRSTNIYGYGLYEVSMKPAKNTGIVSSFFTYTGPAHGTQWDEIDIEFLGKDTTKVQF
NYYTNGVGGHEKVISLGFDASKGFHTYAFDWQPGYIKWYVDGVLKHTATANIPSTPGKIMMN
LWNGTGVDDWLGSYNGANPLYAEYDWVKYTSNQTGGSFFEPFNSYNSGTWEKADGYSNGGVF
NCTWRANNVNFTNDGKLKLGLTSSAYNA
>d1cpn__ b.29.1.2 (-) Bacillus 1-3,1-4-beta-glucanase {Bacillus macerans}
FDCAEYRSTNIYGYGLYEVSMKPAKNTGIVSSFFTYTGPAHGTQWDEIDIEFLGKDTTKVQF
NYYTNGVGGHEKVISLGFDASKGFHTYAFDWQPGYIKWYVDGVLKHTATANIPSTPGKIMMN
LWNGTGVDDWLGSYNGANPLYAEYDWVKYTSNGSVFWEPKSYFNPSTWEKADGYSNGGVFNC
TWRANNVNFTNDGKLKLGLTSS
>d1dypa_ b.29.1.2 (A:) kappa-Carrageenase, catalytic {Pseudoalteromonas carrageenovora}
SMQPPIAKPGETWILQAKRSDEFNVKDATKWNFQTENYGVWSWKNENATVSKGKLKLTTKRE
SHQRTFWDGCNQQQVANYPLYYTSGVAKSRATGNYGYYEARIKGASTFPGVSPAFWMYSTID
RSLTKEGDVQYSEIDVVELTQKSAVRESDHDLHNIVVKNGKPTWMRPGSFPQTNHNGYHLPF
DPRNDFHTYGVNVTKDKITWYVDGEIVGEKDNLYWHRQMNLTLSQGLRAPHTQWKCNQFYPS
ANKSAEGFPTSMEVDYVRTWVKV
>d1slta_ b.29.1.3 (A:) S-lectin, different isoforms {Cow (Bos taurus)}
CGLVASNLNLKPGECLRVRGEVAADAKSFLLNLGKDDNNLCLHFNPRFNAHGDVNTIVCNSK
DAGAWGAEQRESAFPFQPGSVVEVCISFNQTDLTIKLPDGYEFKFPNRLNLEAINYLSAGGD
FKIKCVAFE
>d1bkza_ b.29.1.3 (A:) S-lectin, different isoforms {Human (Homo sapiens)}

SNVPHKSSLPEGIRPGTVLRIRGLVPPNASRFHVNLLCGEEQGSDAALHFNPRLDTSEVVFN
SKEQGSWGREERGPGVPFQRGQPFEVLIIASDDGFKAVVGDAQYHHFRHRLPLARVRLVEVG
GDVQLDSVRIF
>d1hlca_ b.29.1.3 (A:) S-lectin, different isoforms {Human (Homo sapiens)}
ELEVKNMDMKPGSTLKITGSIADGTDGFVINLGQGTDKLNLHFNPRFSESTIVCNSLDGSNW
GQEQREDHLCFSPGSEVKFTVTFESDKFKVKLPDGHELTFPNRLGHSHLSYLSVRGGFNMSS
FKLKE
>d1qmja_ b.29.1.3 (A:) S-lectin, different isoforms {Chicken (Gallus gallus)}
QGLVVTQLDVQPGECVKVKGKILSDAKGFSVNVGKDSSTLMLHFNPRFDCHGDVNTVVCNSK
EDGTWGEEDRKADFPFQQGDKVEICISFDAAEVKVKVPEVEFEFPNRLGMEKIQYLAVEGDF
KVKAIKFS
>d1a78a_ b.29.1.3 (A:) S-lectin, different isoforms {Toad (Bufo arenarum)}
ASAGVAVTNLNLKPGHCVEIKGSIPPDCKGFAVNLGEDASNFLLHFNARFDLHGDVNKIVCN
SKEADAWGSEQREEVFPFQQGAEVMVCFEYQTQKIIIKFSSGDQFSFPVRKVLPSIPFLSLE
GLAFKSITTE
>d1hdka_ b.29.1.3 (A:) Charcot-Leyden crystal (CLC) protein {Human (Homo sapiens)}
SLLPVPYTEAASLSTGSTVTIKGRPLVCFLNEPYLQVDFHTEMKEESDIVFHFQVCFGRRVV
MNSREYGAWKQQVESKNMPFQDGQEFELSISVLPDKYQVMVNGQSSYTFDHRIKPEAVKMVQ
VWRDISLTKFNVSYL
>d1a3k__ b.29.1.3 (-) Galectin-3 CRD {Human (Homo sapiens)}
LIVPYNLPLPGGVVPRMLITILGTVKPNANRIALDFQRGNDVAFHFNPRFNENNRRVIVCNT
KLDNNWGREERQSVFPFESGKPFKIQVLVEPDHFKVAVNDAHLLQYNHRVKKLNEISKLGIS
GDIDLTSASYTMI
>d1c1la_ b.29.1.3 (A:) Congerin I {Conger eel (Conger myriaster)}
GGLQVKNFDFTVGKFLTVGGFINNSPQRFSVNVGESMNSLSLHLDHRFNYGADQNTIVMNST
LKGDNGWETEQRSTNFTLSAGQYFEITLSYDINKFYIDILDGPNLEFPNRYSKEFLPFLSLA
GDARLTLVKLE
>d1d2sa_ b.29.1.4 (A:) Sex hormone-binding globulin {Human (Homo sapiens)}
PPAVHLSNGPGQEPIAVMTFDLTKITKTSSSFEVRTWDPEGVIFYGDTNPKDDWFMLGLRDG
RPEIQLHNHWAQLTVGAGPRLDDGRWHQVEVKMEGDSVLLEVDGEEVLRLRQVSGHPIMRIA
LGGLLFPASNLRLPLVPALDGCLRRDSWLDKQAEISASAPTSLRSC
>d1dyka1 b.29.1.4 (A:2744-2932) Laminin alpha2 chain {Mouse (Mus musculus)}
HGPCVAESEPALLTGSKQFGLSRNSHIAIAFDDTKVKNRLTIELEVRTEAESGLLFYMARIN
HADFATVQLRNGFPYFSYDLGSGDTSTMIPTKINDGQWHKIKIVRVKQEGILYVDDASSQTI
SPKKADILDVVGILYVGGLPINYTTRRIGPVTYSLDGCVRNLHMEQAPVDLDQPTSSFHVGT
CFA
>d1dyka2 b.29.1.4 (A:2933-3117) Laminin alpha2 chain {Mouse (Mus musculus)}

NAESGTYFDGTGFAKAVGGFKVGLDLLVEFEFRTTRPTGVLLGVSSQKMDGMGIEMIDEKLM
FHVDNGAGRFTAIYDAEIPGHMCNGQWHKVTAKKIKNRLELVVDGNQVDAQSPNSASTSADT
NDPVFVGGFPGGLNQFGLTTNIRFRGCIRSLKLTKGTGKPLEVNFAKALELRGVQPVSCPT
>d1c4ra_ b.29.1.4 (A:) Ligand-binding domain of neurexin 1beta {Rat (Rattus norvegicus)}
HAGTTYIFSKGGGQITYKWPPNDRPSTRADRLAIGFSTVQKEAVLVRVDSSSGLGDYLELHI
HQGKIGVKFNVGTDDIAIEESNAIINDGKYHVVRFTRSGGNATLQVDSWPVIERYPAGRQLT
IFNSQATIIIGGKEQGQPFQGQLSGLYYNGLKVLNMAAENDANIAIVGNVRLVGEV
>d1saca_ b.29.1.5 (A:) Serum amyloid P component (SAP) {Human (Homo sapiens)}
HTDLSGKVFVFPRESVTDHVNLITPLEKPLQNFTLCFRAYSDLSRAYSLFSYNTQGRDNELL
VYKERVGEYSLYIGRHKVTSKVIEKFPAPVHICVSWESSSGIAEFWINGTPLVKKGLRQGYF
VEAQPKIVLGQEQDSYGGKFDRSQSFVGEIGDLYMWDSVLPPENILSAYQGTPLPANILDWQ
ALNYEIRGYVIIKPLVWV
>d1b09a_ b.29.1.5 (A:) C-reactive protein (CRP) {Human (Homo sapiens)}
QTDMSRKAFVFPKESDTSYVSLKAPLTKPLKAFTVCLHFYTELSSTRGYSIFSYATKRQDNE
ILIFWSKDIGYSFTVGGSEILFEVPEVTVAPVHICTSWESASGIVEFWVDGKPRVRKSLKKG
YTVGAEASIILGQEQDSFGGNFEGSQSLVGDIGNVNMWDFVLSPDEINTIYLGGPFSPNVLN
WRALKYEVQGEVFTKPQLWP
>d1jhna1 b.29.1.12 (A:61-262) Calnexin {Dog (Canis familiaris)}
YKAPVPSGEVYFADSFDRGTLSGWILSKAKKDDTDDEIAKYDGKWEVDEMKETKLPGDKGLV
LMSRAKHHAISAKLNKPFLFDTKPLIVQYEVNFQNGIECGGAYVKLLSKTPELNLDQFHDKT
PYTIMFGPDKCGEDYKLHFIFRHKNPKTGVYEEKHAKRPDADLKTYFTDKKTHLYTLILNPD
NSFEILVDQSIVNSGN
>d1jhna2 b.29.1.12 (A:412-458) Calnexin {Dog (Canis familiaris)}
LEPFKMTPFSAIGLELWSMTSDIFFDNFIVCGDRRVVDDWANDGWGL
>d1a8d_1 b.29.1.6 (1-247) Tetanus neurotoxin {Clostridium tetani}
MKNLDCWVDNEEDIDVILKKSTILNLDINNDIISDISGFNSSVITYPDAQLVPGINGKAIHL
VNNESSEVIVHKAMDIEYNDMFNNFTVSFWLRVPKVSASHLEQYGTNEYSIISSMKKHSLSI
GSGWSVSLKGNNLIWTLKDSAGEVRQITFRDLPDKFNAYLANKWVFITITNDRLSSANLYIN
GVLMGSAEITGLGAIREDNNITLKLDRCNNNNQYVSIDKFRIFCKALNPKEIEKLYTSYLS
>d3btaa1 b.29.1.6 (A:872-1078) Botulinum neurotoxin {Clostridium botulinum, serotype A}
IINTSILNLRYESNHLIDLSRYASKINIGSKVNFDPIDKNQIQLFNLESSKIEVILKNAIVY
NSMYENFSTSFWIRIPKYFNSISLNNEYTIINCMENNSGWKVSLNYGEIIWTLQDTQEIKQR
VVFKYSQMINISDYINRWIFVTITNNRLNNSKIYINGRLIDQKPISNLGNIHASNNIMFKLD
GCRDTHRYIWIKYFNLFDKEL
>d1epwa1 b.29.1.6 (A:862-1079) Botulinum neurotoxin {Clostridium botulinum, serotype B}
NIILNLRYKDNNLIDLSGYGAKVEVYDGVELNDKNQFKLTSSANSKIRVTQNQNIIFNSVFL
DFSVSFWIRIPKYKNDGIQNYIHNEYTIINCMKNNSGWKISIRGNRIIWTLIDINGKTKSVF
FEYNIREDISEYINRWFFVTITNNLNNAKIYINGKLESNTDIKDIREVIANGEIIFKLDGDI
DRTQFIWMKYFSIFNTELSQSNIEERYKIQSY

>d1ikpa1 b.29.1.7 (A:2-251) Exotoxin A, N-terminal domain {Pseudomonas aeruginosa}
EEAFDLWNECAKACVLDLKDGVRSSRMSVDPAIADTNGQGVLHYSMVLEGGNDALKLAIDNA
LSITSDGLTIRLEGGVEPNKPVRYSYTRQARGSWSLNWLVPIGHEKPSNIKVFIHELNAGNQ
LSHMSPIYTIEMGDELLAKLARDATFFVRAHESNEMQPTLAISHAGVSVVMAQAQPRREKRW
SEWASGKVLCLLDQLDGVYNYLAQQRCNLDDTWEGKIYRVLAGNPAKHDLDIKPTVISHRLH
FP
>d1kit_1 b.29.1.8 (25-216) Vibrio cholerae sialidase, N-terminal and insertion domains {Vibrio cholerae}
ALFDYNATGDTEFDSPAKQGWMQDNTNNGSGVLTNADGMPAWLVQGIGGRAQWTYSLSTNQH
AQASSFGWRMTTEMKVLSGGMITNYYANGTQRVLPIISLDSSGNLVVEFEGQTGRTVLATGT
AATEYHKFELVFLPGSNPSASFYFDGKLIRDNIQPTASKQNMIVWGNGSSNTDGVAAYRDIK
FEIQGD
>d1kit_2 b.29.1.8 (347-543) Vibrio cholerae sialidase, N-terminal and insertion domains {Vibrio cholerae}
DVTDQVKERSFQIAGWGGSELYRRNTSLNSQQDWQSNAKIRIVDGAANQIQVADGSRKYVVT
LSIDESGGLVANLNGVSAPIILQSEHAKVHSFHDYELQYSALNHTTTLFVDGQQITTWAGEV
SQENNIQFGNADAQIDGRLHVQKIVLTQQGHNLVEFDAFYLAQQTPEVEKDLEKLGWTKIKT
GNTMSLYGNAS
>d2sli_1 b.29.1.9 (81-276) Leech intramolecular trans-sialidase, N-terminal domain {North american leech (Macrobdella decora)}
IPEGILMEKNNVDIAEGQGYSLDQEAGAKYVKAMTQGTIILSYKSTSENGIQSLFSVGNSTA
GNQDRHFHIYITNSGGIGIELRNTDGVFNYTLDRPASVRALYKGERVFNTVALKADAANKQC
RLFANGELLATLDKDAFKFISDITGVDNVTLGGTKRQGKIAYPFGGTIGDIKVYSNALSDEE
LIQATGVTTY
>d6cel__ b.29.1.10 (-) Cellobiohydrolase I (cellulase, Endoglucanase I, CBH1) {Trichoderma reesei, Cel7A}
ESACTLQSETHPPLTWQKCSSGGTCTQQTGSVVIDANWRWTHATNSSTNCYDGNTWSSTLCP
DNETCAKNCCLDGAAYASTYGVTTSGNSLSIDFVTQSAQKNVGARLYLMASDTTYQEFTLLG
NEFSFDVDVSQLPCGLNGALYFVSMDADGGVSKYPTNTAGAKYGTGYCDSQCPRDLKFINGQ
ANVEGWEPSSNNANTGIGGHGSCCSQMDIWEANSISEALTPHPCTTVGQEICEGDGCGGTYS
DNRYGGTCDPDGCDWNPYRLGNTSFYGPGSSFTLDTTKKLTVVTQFETSGAINRYYVQNGVT
FQQPNAELGSYSGNELNDDYCTAEEAEFGGSSFSDKGGLTQFKKATSGGMVLVMSLWDDYYA
NMLWLDSTYPTNETSSTPGAVRGSCSTSSGVPAQVESQSPNAKVTFSNIKFGPIGSTGNPSG
>d1eg1a_ b.29.1.10 (A:) Cellobiohydrolase I (cellulase, Endoglucanase I, CBH1) {Trichoderma reesei, Endoglucanase I}
EQPGTSTPEVHPKLTTYKCTKSGGCVAQDTSVVLDWNYRWMHDANYNSCTVNGGVNTTLCPD
EATCGKNCFIEGVDYAASGVTTSGSSLTMNQYMPSSSGGYSSVSPRLYLLDSDGEYVMLKLN
GQELSFDVDLSALPCGENGSLYLSQMDENGGANQYNTAGANYGSGYCDAQCPVQTWRNGTLN
TSHQGFCCNEMDILEGNSRANALTPHSCTATACDSAGCGFNPYGSGYKSYYGPGDTVDTSKT
FTIITQFNTDNGSPSGNLVSITRKYQQNGVDIPSAQPGGDTISSCPSASAYGGLATMGKALS
SGMVLVFSIWNDNSQYMNWLDSGNAGPCSSTEGNPSNILANNPNTHVVFSNIRWGDIGSTT
>d3ovwa_ b.29.1.10 (A:) Cellobiohydrolase I (cellulase, Endoglucanase I, CBH1) {Fusarium oxysporum}

ETPDKAKEQHPKLETYRCTKASGCKKQTNYIVADAGIHGIRQKNGAGCGDWGQKPNATACPD
EASCAKNCILSGMDSNAYKNAGITTSGNKLRLQQLINNQLVSPRVYLLEENKKKYEMLHLTG
TEFSFDVEMEKLPCGMNGALYLSEMPQDGGKSTSRNSKAGAYYGAGYCDAQCYVTPFINGVG
NIKGQGVCCNELDIWEANSRATHIAPHPCSKPGLYGCTGDECGSSGICDKAGCGWNHNRINV
TDFYGRGKQYKVDSTRKFTVTSQFVANKQGDLIELHRHYIQDNKVIESAVVNISGPPKINFI
NDKYCAATGANEYMRLGGTKQMGDAMSRGMVLAMSVWWSEGDFMAWLDQGVAGPCDATEGDP
KNIVKVQPNPEVTFSNIRIGEIGSTSSV
>d1dyma_ b.29.1.10 (A:) Cellobiohydrolase I (cellulase, Endoglucanase I, CBH1) {Humicola insolens, Cel7b}
EKPGETKEVHPQLTTFRCTKRGGCKPATNFIVLDSLSHPIHRAEGLGPGGCGDWGNPPPKDV
CPDVESCAKNCIMEGIPDYSQYGVTTNGTSLRLQHILPDGRVPSPRVYLLDKTKRRYEMLHL
TGFEFTFDVDATKLPCGMNSALYLSEMHPTGAKSKYNPGGAYYGTGYCDAQCFVTPFINGLG
NIEGKGSCCNAMDIWEANSRASHVAPHTCNKKGLYLCEGEECAFEGVCDKNGCGWNNYRVNV
TDYYGRGEEFKVNTLKPFTVVTQFLANRRGKLEKIHRFYVQDGKVIESFYTNKEGVPYTNMI
DDEFCEATGSRKYMELGATQGMGEALTRGMVLAMSIWWDQGGNMEWLDHGEAGPCAKGEGAP
SNIVQVEPFPEVTYTNLRWGEIGSTY
>d1gpia_ b.29.1.10 (A:) Cellobiohydrolase I (cellulase, Endoglucanase I, CBH1) {Phanerochaete chrysosporium, Cel7d}
EQAGTNTAENHPQLQSQQCTTSGGCKPLSTKVVLDSNWRWVHSTSGYTNCYTGNEWDTSLCP
DGKTCAANCALDGADYSGTYGITSTGTALTLKFVTGSNVGSRVYLMADDTHYQLLKLLNQEF
TFDVDMSNLPCGLNGALYLSAMDADGGMSKYPGNKAGAKYGTGYCDSQCPKDIKFINGEANV
GNWTETGSNTGTGSYGTCCSEMDIWEANNDAAAFTPHPCTTTGQTRCSGDDCARNTGLCDGD
GCDFNSFRMGDKTFLGKGMTVDTSKPFTVVTQFLTNDNTSTGTLSEIRRIYIQNGKVIQNSV
ANIPGVDPVNSITDNFCAQQKTAFGDTNWFAQKGGLKQMGEALGNGMVLALSIWDDHAANML
WLDSDYPTDKDPSAPGVARGTCATTSGVPSDVESQVPNSQVVFSNIKFGDIGSTFSGTS
>d1xnb__ b.29.1.11 (-) Xylanase II {Bacillus circulans}
ASTDYWQNWTDGGGIVNAVNGSGGNYSVNWSNTGNFVVGKGWTTGSPFRTINYNAGVWAPNG
NGYLTLYGWTRSPLIEYYVVDSWGTYRPTGTYKGTVKSDGGTYDIYTTTRYNAPSIDGDRTT
FTQYWSVRQSKRPTGSNATITFTNHVNAWKSHGMNLGSNWAYQVMATEGYQSSGSSNVTVW
>d1qh7a_ b.29.1.11 (A:) Xylanase II {Bacillus agaradhaerens}
EIVTDNSIGNHDGYDYEFWKDSGGSGTMILNHGGTFSAQWNNVNNILFRKGKKFNETQTHQQ
VGNMSINYGANFQPNGNAYLCVYGWTVDPLVEYYIVDSWGNWRPPGATPKGTITVDGGTYDI
YETLRVNQPSIKGIATFKQYWSVRRSKRTSGTISVSNHFRAWENLGMNMGKMYEVALTVEGY
QSSGSANVYSNTLRINGNPLS
>d1hixa_ b.29.1.11 (A:) Xylanase II {Streptomyces sp. s38, xyl1}
ITTNQTGTNNGYYYSFWTDGGGSVSMNLASGGSYGTSWTNCGNFVAGKGWANGARRTVNYSG
SFNPSGNAYLTLYGWTANPLVEYYIVDNWGTYRPTGTYKGTVTSDGGTYDVYQTTRVNAPSV
EGTKTFNQYWSVRQSKRTGGSITAGNHFDAWARYGMPLGSFNYYMIMATEGYQSSGSSSIS
>d1xnd__ b.29.1.11 (-) Xylanase II {Trichoderma harzianum}
QTIGPGTGYSNGYYYSYWNDGHAGVTYTNGGGGSFTVNWSNSGNFVAGKGWQPGTKNKVINF
SGSYNPNGNSYLSIYGWSRNPLIEYYIVENFGTYNPSTGATKLGEVTSDGSVYDIYRTQRVN
QPSIIGTATFYQYWSVRRNHRSSGSVNTANHFNAWASHGLTLGTMDYQIVAVEGYFSSGSAS
ITVS
>d1xyn__ b.29.1.11 (-) Xylanase II {Trichoderma reesei, xynI}

ASINYDQNYQTGGQVSYSPSNTGFSVNWNTQDDFVVGVGWTTGSSAPINFGGSFSVNSGTGL
LSVYGWSTNPLVEYYIMEDNHNYPAQGTVKGTVTSDGATYTIWENTRVNEPSIQGTATFNQY
ISVRNSPRTSGTVTVQNHFNAWASLGLHLGQMNYQVVAVEGWGGSGSASQSVSN
>d1enxa_ b.29.1.11 (A:) Xylanase II {Trichoderma reesei, xynII}
ETIQPGTGYNNGYFYSYWNDGHGGVTYTNGPGGQFSVNWSNSGNFVGGKGWQPGTKNKVINF
SGSYNPNGNSYLSVYGWSRNPLIEYYIVENFGTYNPSTGATKLGEVTSDGSVYDIYRTQRVN
QPSIIGTATFYQYWSVRRNHRSSGSVNTANHFNAWAQQGLTLGTMDYQIVAVEGYFSSGSAS
ITVS
>d1yna__ b.29.1.11 (-) Xylanase II {Thermomyces lanuginosus}
ETTPNSEGWHDGYYYSWWSDGGAQATYTNLEGGTYEISWGDGGNLVGGKGWNPGLNARAIHF
EGVYQPNGNSYLAVYGWTRNPLVEYYIVENFGTYDPSSGATDLGTVECDGSIYRLGKTTRVN
APSIDGTQTFDQYWSVRQDKRTSGTVQTGCHFDAWARAGLNVNGDHYYQIVATEGYFSSGYA
RITVADVG
>d1bk1__ b.29.1.11 (-) Xylanase II {Aspergillus kawachii}
AGINYVQNYNGNLGDFTYDESAGTFSMYWEDGVSSDFVVGLGWTTGSSNAITYSAEYSASGS
SSYLAVYGWVNYPQAEYYIVEDYGDYNPCSSATSLGTVYSDGSTYQVCTDTRTNEPSITGTS
TFTQYFSVRESTRTSGTVTVANHFNFWAQHGFGNSDFNYQVMAVEAWSGAGSASVTIS
>d1pvxa_ b.29.1.11 (A:) Xylanase II {Paecilomyces variotii
bainier}
GTTPNSEGWHDGYYYSWWSDGGGDSTYTNNSGGTYEITWGNGGNLVGGKGWNPGLNARAIHF
TGVYQPNGTSYLSVYGWTRNPLVEYYIVENFGSSNPSSGSTDLGTVSCDGSTYTLGQSTRYN
APSIDGTQTFNQYWSVRQDKRSSGTVQTGCHFDAWASAGLNVTGDHYYQIVATEGYFSSGYA
RITVADVG
>d1f5ja_ b.29.1.11 (A:) Xylanase II {Dictyoglomus thermophilum}
ALTSNASGTFDGYYYELWKDTGNTTMTVYTQGRFSCQWSNINNALFRTGKKYNQNWQSLGTI
RITYSATYNPNGNSYLCIYGWSTNPLVEFYIVESWGNWRPPGATSLGQVTIDGGTYDIYRTT
RVNQPSIVGTATFDQYWSVRTSKRTSGTVTVTDHFRAWANRGLNLGTIDQITLCVEGYQSSG
SANITQNTFSQSS
>d2nlra_ b.29.1.11 (A:) Endo-1,4-beta-glucanase (cellulase)
catalytic domain {Streptomyces lividans, CelB2}
DTTICEPFGTTTIQGRYVVQNNRWGSTAPQCVTATDTGFRVTQADGSAPTNGAPKSYPSVFN
GCHYTNCSPGTDLPVRLDTVSAAPSSISYGFVDGAVYNASYDIWLDPTARTDGVNQTEIMIW
FNRVGPIQPIGSPVGTASVGGRTWEVWSGGNGSNDVLSFVAPSAISGWSFDVMDFVRATVAR
GLAENDWYLTSVQAGFEPWQNGAGLAVNSFSSTVET
>d1h8va_ b.29.1.11 (A:) Endo-1,4-beta-glucanase (cellulase)
catalytic domain {Trichoderma reesei, Cel12A}
ETSCDQWATFTGNGYTVSNNLWGASAGSGFGCVTAVSLSGGASWHADWQWSGGQNNVKSYQN
SQIAIPQKRTVNSISSMPTTASWSYSGSNIRANVAYDLFTAANPNHVTYSGDYELMIWLGKY
GDIGPIGSSQGTVNVGGQSWTLYYGYNGAMQVYSFVAQTNTTNYSGDVKNFFNYLRDNKGYN
AAGQYVLSYQFGTEPFTGSGTLNVASWTASIN
>d1jz8a4 b.30.1.1 (A:731-1023) beta-Galactosidase, domain 5
{Escherichia coli}
PAASHAIPHLTTSEMDFCIELGNKRWQFNRQSGFLSQMWIGDKKQLLTPLRDQFTRAPLDND
IGVSEATRIDPNAWVERWKAAGHYQAEAALLQCTADTLADAVLITTAHAWQHQGKTLFISRK

TYRIDGSGQMAITVDVEVASDTPHPARIGLNCQLAQVAERVNWLGLGPQENYPDRLTAACFD
RWDLPLSDMYTPYVFPSENGLRCGTRELNYGPHQWRGDFQFNISRYSQQQLMETSHRHLLHA
EEGTWLNIDGFHMGIGGDDSWSPSVSAEFQLSAGRYHYQLVWCQK
>d1oaca1 b.30.2.1 (A:301-724) Copper amine oxidase, domain 3 (catalytic) {Escherichia coli}
PAVKPMQIIEPEGKNYTITGDMIHWRNWDFHLSMNSRVGPMISTVTYNDNGTKRKVMYEGSL
GGMIVPYGDPDIGWYFKAYLDSGDYGMGTLTSPIARGKDAPSNAVLLNETIADYTGVPMEIP
RAIAVFERYAGPEYKHQEMGQPNVSTERRELVVRWISTVGNADYIFDWIFHENGTIGIDAGA
TGIEAVKGVKAKTMHDETAKDDTRYGTLIDHNIVGTTHQHIYNFRLDLDVDGENNSLVAMDP
VVKPNTAGGPRTSTMQVNQYNIGNEQDAAQKFDPGTIRLLSNPNKENRMGNPVSYQIIPYAG
GTHPVAKGAQFAPDEWIYHRLSFMDKQLWVTRYHPGERFPEGKYPNRSTHDTGLGQYSKDNE
SLDNTDAVVWMTTGTTHVARAEEWPIMPTEWVHTLLKPWNFFDETPTLGALK
>d1ksia1 b.30.2.1 (A:207-647) Copper amine oxidase, domain 3 (catalytic) {Pea seedling (Pisum sativum)}
VSKQSPPFGPKQHSLTSHQPQGPGFQINGHSVSWANWKFHIGFDVRAGIVISLASIYDLEKH
KSRRVLYKGYISELFVPYQDPTEEFYFKTFFDSGEFGFGLSTVSLIPNRDCPPHAQFIDTYV
HSANGTPILLKNAICVFEQYGNIMWRHTENGIPNESIEESRTEVNLIVRTIVTVGNADNVID
WEFKASGSIKPSIALSGILEIKGTNIKHKDEIKEDLHGKLVSANSIGIYHDHFYIYYLDFDI
DGTHNSFEKTSLKTVRIKDGSSKRKSYWTTETQTAKTESDAKITIGLAPAELVVVNPNIKTA
VGNEVGYRLIPAIPAHPLLTEDDYPQIRGAFTNYNVWVTAYNRTEKWAGGLYVDHSRGDDTL
AVWTKQNREIVNKDIVMWHVVGIHHVPAQEDFPIMPLLSTSFELRPTNFFERNPVLKTLSPR
DVAWPGC
>d1av4_1 b.30.2.1 (212-628) Copper amine oxidase, domain 3 (catalytic) {Arthrobacter globiformis}
PLRTTQKPISITQPEGPSFTVTGGNHIEWEKWSLDVGFDVREGVVLHNIAFRDGDRLRPIIN
RASIAEMVVPYGDPSPIRSWQNYFDTGEYLVGQYANSLELGCDCLGDITYLSPVISDAFGNP
REIRNGICMHEEDWGILAKHSDLWSGINYTRRNRRMVISFFTTIGNADYGFYWYLYLDGTIE
FEAKATGVVFTSAFPEGGSDNISQLAPGLGAPFHQHIFSARLDMAIDGFTNRVEEEDVVRQT
MGPGNERGNAFSRKRTVLTRESEAVREADARTGRTWIISNPESKNRLNEPVGYKLHAHNQPT
LLADPGSSIARRAAFATKDLWVTRYADDERYPTGDFVNQHSGGAGLPSYIAQDRDIDGQDIV
VWHTFGLTHFPRVEDWPIMPVDTVGFKLRPEGFFDRSPVLDVPAN
>d1a2va1 b.30.2.1 (A:237-672) Copper amine oxidase, domain 3 (catalytic) {Yeast (Hansenula polymorpha)}
PEAPPINVTQPEGVSFKMTGNVMEWSNFKFHIGFNYREGIVLSDVSYNDHGNVRPIFHRISL
SEMIVPYGSPEFPHQRKHALDIGEYGAGYMTNPLSLGCDCKGVIHYLDAHFSDRAGDPITVK
NAVCIHEEDDGLLFKHSDFRDNFATSLVTRATKLVVSQIFTAANAEYCLYWVFMQDGAIRLD
IRLTGILNTYILGDDEEAGPWGTRVYPNVNAHNHQHLFSLRIDPRIDGDGNSAAACDAKSSP
YPLGSPENMYGNAFYSEKTTFKTVKDSLTNYESATGRSWDIFNPNKVNPYSGKPPSYKLVST
QCPPLLAKEGSLVAKRAPWASHSVNVVPYKDNRLYPSGDHVPQWSGDGVRGMREWIGDGSEN
IDNTDILFFHTFGITHFPAPEDFPLMPAEPITLMLRPRHFFTENPGLDIQPSYAMTTSEAKR
AV
>d1cb8a3 b.30.3.1 (A:336-599) Chondroitinase AC {Pedobacter heparinus (Flavobacterium heparinum)}
IEPYHHQFWNGDYVQHLRPAYSFNVRMVSKRTRRSESGNKENLLGRYLSDGATNIQLRGPEY

YNIMPVWEWDKIPGITSRDYLTDRPLTKLWGEQGSNDFAGGVSDGVYGASAYALDYDSLQAK
KAWFFFDKEIVCLGAGINSNAPENITTTLNQSWLNGPVISTAGKTGRGKITTFKAQGQFWLL
HDAIGYYFPEGANLSLSTQSQKGNWFHINNSHSKDEVSGDVFKLWINHGARPENAQYAYIVL
PGINKPEEIKKYNGTA
>d1egua3 b.30.3.1 (A:541-814) Hyaluronate lyase {Streptococcus pneumoniae}
TSYLSAFNKMDKTAMYNAEKGFGFGLSLFSSRTLNYEHMNKENKRGWYTSDGMFYLYNGDLS
HYSDGYWPTVNPYKMPGTTETDAKRADSDTGKVLPSAFVGTSKLDDANATATMDFTNWNQTL
TAHKSWFMLKDKIAFLGSNIQNTSTDTAATTIDQRKLESSNPYKVYVNDKEASLTEQEKDYP
ETQSVFLESSDSKKNIGYFFFKKSSISMSKALQKGAWKDINEGQSDKEVENEFLTISQAHKQ
NGDSYGYMLIPNVDRATFNQMIKELE
>d1f1sa4 b.30.3.1 (A:620-919) Hyaluronate lyase {Streptococcus agalactiae}
LKSNLSTFNSMDRLAYYNAKKDFGFALSLHSKRTLNYEGMNDENTRGWYTGDGMFYIYNSDQ
SHYSNHFWPTVNPYKMAGTTEKDAKREDTTKEFMSKHSKDAKEKTGQVTGTSDFVGSVKLND
HFALAAMDFTNWDRTLTAQKGWVILNDKIVFLGSNIKNTNGIGNVSTTIDQRKDDSKTPYTT
YVNGKTIDLKQASSQQFTDTKSVFLESKEPGRNIGYIFFKNSTIDIERKEQTGTWNSINRTS
KNTSIVSNPFITISQKHDNKGDSYGYMMVPNIDRTSFDKLANSKEVELLENS
>d1h54a2 b.30.4.1 (A:1-268) Lactobacillus maltose phosphorylase, N-terminal domain {Lactobacillus brevis}
MKRIFEVQPWNVITHTFDPKDKRLQESMTSLGNGYMGMRGDFEEGYSGDSLQGIYLGGVWYP
DKTRVGWWKNGYPKYFGKVVNAVNFIKLPIEINGEPVDLAKDKISDFTLDLDMHQGVLNRSF
VVERGAVRVALNFQRFLSVAQPELSVQKVTVKNLSDAEVDVTLKPSIDADVMNEEANYDERF
WDVLATDQQADRGSIVAKTTPNPFGTPRFTSGMEMRLVTDLKNVAITQPNEKEVTTAYTGKL
APQASAELEKRVIVVTSRDY
>d1hs6a2 b.98.1.1 (A:1-208) Leukotriene A4 hydrolase N-terminal domain {Human (Homo sapiens)}
PEIVDTCSLASPASVCRTKHLHLRCSVDFTRRTLTGTAALTVQSQEDNLRSLVLDTKDLTIE
KVVINGQEVKYALGERQSYKGSPMEISLPIALSKNQEIVIEISFETSPKSSALQWLTPEQTS
GKEHPYLFSQCQAIHCRAILPCQDTPSVKLTYTAEVSVPKELVALMSAIRDGETPDPEDPSR
KIYKFIQKVPIPCYLIALVVGA
>d1es6a_ b.31.1.1 (A:) EV matrix protein {Ebola virus}
GDTPSNPLRPIADDTIDHASHTPGSVSSAFILEAMVNVISGPKVLMKQIPIWLPLGVADQKT
YSFDSTTAAIMLASYTITHFGKATNPLVRVNRLGPGIPDHPLRLLRIGNQAFLQEFVLPPVQ
LPQYFTFDLTALKLITQPLPAATWTDDTPTGSNGALRPGISFHPKLRPILLPNKSGKKGNSA
DLTSPEKIQAIMTSLQDFKIVPIDPTKNIMGIEVPETLVLKLTGKKVTSKNGQPIIPVLLPK
YIGLDPVAPGDLTMVITQDCDTCHSPASLP
>d1qexa_ b.32.1.1 (A:) gp9 {Bacteriophage T4}
MFIQEPKKLIDTGEIGNASTGDILFDGGNKINSDFNAIYNAFGDQRKMAVANGTGADGQIIH
ATGYYQKHSITEYATPVKVGTRHDIDTSTVGVKVIIERGELGDCVEFINSNGSISVTNPLTI
QAIDSIKGVSGNLVVTSPYSKVTLRCISSDNSTSVWNYSIESMFGQKESPAEGTWNISTSGS
VDIPLFHRTEYNMAKLLVTCQSVDGRKIKTAEINILVDTVNSEVISSEYAVMRVGNETEEDE
IANIAFSIKENYVTATISSSTVGMRAAVKVIATQKIGVAQ
>d1rie__ b.33.1.1 (-) ISP subunit of the mitochondrial cytochrome

bc1-complex, watersoluble domain {Cow (Bos taurus)}
AMSKIEIKLSDIPEGKNMAFKWRGKPLFVRHRTKKEIDQEAAVEVSQLRDPQHDLERVKKPE
WVILIGVCTHLGCVPIANAGDFGGYYCPCHGSHYDASGRIRKGPAPLNLEVPSYEFTSDDMV
IVG
>d1ezve1 b.33.1.1 (E:87-215) ISP subunit of the mitochondrial cytochrome bc1-complex, watersoluble domain {Baker's yeast (Saccharomyces cerevisiae)}
DVLAMAKVEVNLAAIPLGKNVVVKWQGKPVFIRHRTPHEIQEANSVDMSALKDPQTDADRVK
DPQWLIMLGICTHLGCVPIGEAGDFGGWFCPCHGSHYDISGRIRKGPAPLNLEIPAYEFDGD
KVIVG
>d1rfs__ b.33.1.1 (-) ISP subunit from chloroplast cytochrome bf complex {Spinach (Spinacia oleracea)}
TIAKDALGNDVIAAEWLKTHAPGDRTLTQGLKGDPTYLVVESDKTLATFGINAVCTHLGCVV
PFNAAENKFICPCHGSQYNNQGRVVRGPAPLSLALAHCDVDDGKVVFVPWTETDFRTGEAPW
WSA
>d1g8kb_ b.33.1.1 (B:) Arsenite oxidase Rieske subunit {Alcaligenes faecalis}
RTTLAYPATAVSVAKNLAANEPVSFTYPDTSSPCVAVKLGAPVPGGVGPDDDIVAYSVLCTH
MGCPTSYDSSSKTFSCPCHFTEFDAEKAGQMICGEATADLPRVLLRYDAASDALTAVGVDGL
IYGRQANVI
>d1fqta_ b.33.1.1 (A:) Rieske-type ferredoxin associated with biphenyl dioxygenase {Burkholderia cepacia}
MKFTRVCDRRDVPEGEALKVESGGTSVAIFNVDGELFATQDRCTHGDWSLSDGGYLEGDVVE
CSLHMGKFCVRTGKVKSPPPCEALKIFPIRIEDNDVLVDFEAGYLAP
>d1eg9a1 b.33.1.2 (A:1-154) Naphthalene 1,2-dioxygenase alpha subunit, N-domain {Pseudomonas putida}
MNYNNKILVSESGLSQKHLIHGDEELFQHELKTIFARNWLFLTHDSLIPAPGDYVTAKMGID
EVIVSRQNDGSIRAFLNVCRHRGKTLVSVEAGNAKGFVCSYHGWGFGSNGELQSVPFEKDLY
GESLNKKCLGLKEVARVESFHGFIYGCFDQ
>d1bia_2 b.34.1.1 (271-317) Biotin repressor/biotin holoenzyme synthetase, C-terminal domain {Escherichia coli}
FINRPVKLIIGDKEIFGISRGIDKQGALLLEQDGIIKPWMGGEISLR
>d1byma_ b.34.1.2 (A:) Diphtheria toxin repressor (DtxR) {Corynebacterium diphtheriae}
NPIPGLDELGVGNSDAAAPGTRVIDAATSMPRKVRIVQINEIFQVETDQFTQLLDADIRVGS
EVEIVDRDGHITLSHNGKDVELLDDLAHTIRIEEL
>d1c0wa3 b.34.1.2 (A:165-223) Diphtheria toxin repressor (DtxR) {Corynebacterium diphtheriae}
IVQINEIFQVETDQFTQLLDADIRVGSEVEIVDRDGHITLSHNGKDVELLDDLAHTIRI
>d1g3sa3 b.34.1.2 (A:148-225) Diphtheria toxin repressor (DtxR) {Corynebacterium diphtheriae}
PGTRVIDAATSMPRKVRIVQINEIFQVETDQFTQLLDADIRVGSEVEIVDRDGHITLSHNGK
DVELLDDLAHTIRIEE
>d1fx7a3 b.34.1.2 (A:145-230) Iron-dependent regulator IdeR

{Mycobacterium tuberculosis}
GADDANLVRLTELPAGSPVAVVVRQLTEHVQGDIDLITRLKDAGVVPNARVTVETTPGGGVT
IVIPGHENVTLPHEMAHAVKVEKV
>d1igqa_ b.34.1.3 (A:) Transcriptional repressor protein KorB
{Escherichia coli}
KKAIVQVEHDERPARLILNRRPPAEGYAWLKYEDDGQEFEANLADVKLVALIEG
>d1igub_ b.34.1.3 (B:) Transcriptional repressor protein KorB
{Escherichia coli}
PDPDKLKKAIVQVEHDERPARLILNRRPPAEGYAWLKYEDDGQEFEANLADVKLVALIEG
>d1ckaa_ b.34.2.1 (A:) C-Crk, N-terminal SH3 domain {Mouse (Mus
musculus)}
AEYVRALFDFNGNDEEDLPFKKGDILRIRDKPEEQWWNAEDSEGKRGMIPVPYVEKY
>d1efna_ b.34.2.1 (A:) Fyn proto-oncogene tyrosine kinase, SH3
domain {Human (Homo sapiens)}
ALFVALYDYEAITEDDLSFHKGEKFQILNSSEGDWWEARSLTTGETGYIPSNYVAPV
>d1shfa_ b.34.2.1 (A:) Fyn proto-oncogene tyrosine kinase, SH3
domain {Human (Homo sapiens)}
VTLFVALYDYEARTEDDLSFHKGEKFQILNSSEGDWWEARSLTTGETGYIPSNYVAPVD
>d1neb__ b.34.2.1 (-) SH3 domain from nebulin {Human (Homo sapiens)}
TAGKIFRAMYDYMAADADEVSFKDGDAIINVQAIDEGWMYGTVQRTGRTGMLPANYVEAI
>d1bbza_ b.34.2.1 (A:) Abl tyrosine kinase, SH3 domain {Human (Homo
sapiens)}
NLFVALYDFVASGDNTLSITKGEKLRVLGYNHNGEWCEAQTKNGQGWVPSNYITPVNS
>d2abl_1 b.34.2.1 (75-139) Abl tyrosine kinase, SH3 domain {Human
(Homo sapiens)}
MGPSENDPNLFVALYDFVASGDNTLSITKGEKLRVLGYNHNGEWCEAQTKNGQGWVPSNYIT
PVN
>d1pht__ b.34.2.1 (-) Phosphatidylinositol 3-kinase (p85-alpha
subunit, pi3k), SH3 domain {Human (Homo sapiens)}
AEGYQYRALYDYKKEREEDIDLHLGDILTVNKGSLVALGFSDGQEARPEEIGWLNGYNETTG
ERGDFPGTYVEYIGRKKISPP
>d1g2ba_ b.34.2.1 (A:) alpha-Spectrin, SH3 domain {Chicken (Gallus
gallus)}
MDRQGFVPAAYVKKLDSGTGKELVLALYDYQEKSPREVTMKKGDILTLLNSTNKDWWKVEVN
>d1pwt__ b.34.2.1 (-) alpha-Spectrin, SH3 domain {Chicken (Gallus
gallus)}
MGTGKELVLALYDYQEKSPREVTMKKGDILTLLNSTNKDWWKVEVNDRQGFVPAAYVKKLD
>d1qkwa_ b.34.2.1 (A:) alpha-Spectrin, SH3 domain {Chicken (Gallus
gallus)}
KELVLALYDYQEKSPREVTMKKGDILTLLNSTNKDWWKVEVGDRQGFVPAAYVKKLD
>d1tuc__ b.34.2.1 (-) alpha-Spectrin, SH3 domain {Chicken (Gallus
gallus)}
MGPREVTMKKGDILTLLNSTNKDWWKVEVNDRQGFVPAAYVKKLDSGTGKELVLALYDYQE
>d1awj__ b.34.2.1 (-) IL-2 inducible T-cell (Itc) kinase {Mouse

(Mus musculus)}
KKPLPPTPEDNRRSFQEPEETLVIALYDYQTNDPQELALRCDEEYYLLDSSEIHWWRVQDKN
GHEGYAPSSYLVEKS
>d1bu1a_ b.34.2.1 (A:) Hemapoetic cell kinase Hck {Human (Homo sapiens)}
IIVVALYDYEAIHHEDLSFQKGDQMVVLEESGEWWKARSLATRKEGYIPSNYVARVD
>d1qcfa1 b.34.2.1 (A:80-145) Hemapoetic cell kinase Hck {Human (Homo sapiens)}
SGIRIIVVALYDYEAIHHEDLSFQKGDQMVVLEESGEWWKARSLATRKEGYIPSNYVARVDS
LET
>d1cska_ b.34.2.1 (A:) c-src tyrosine kinase {Human (Homo sapiens)}
GTECIAKYNFHGTAEQDLPFCKGDVLTIVAVTKDPNWYKAKNKVGREGIIPANYVQKR
>d1fmk_1 b.34.2.1 (82-145) c-src tyrosine kinase {Human (Homo sapiens)}
MVTTFVALYDYESRTETDLSFKKGERLQIVNNTEGDWWLAHSLSTGQTGYIPSNYVAPSDSI
QA
>d1nloc_ b.34.2.1 (C:) c-src tyrosine kinase {Chicken (Gallus gallus)}
TFVALYDYESRTETDLSFKKGERLQIVNNTEGDWWLAHSLTTGQTGYIPSNYVAPS
>d1awx__ b.34.2.1 (-) Bruton's tyrosine kinase {Human (Homo sapiens)}
GSMSTSELKKVVALYDYMPMNANDLQLRKGDEYFILEESNLPWWRARDKNGQEGYIPSNYVT
EAEDS
>d1qlya_ b.34.2.1 (A:) Bruton's tyrosine kinase {Human (Homo sapiens)}
LKKVVALYDYMPMNANDLQLRKGDEYFILEESNLPWWRARDKNGQEGYIPSNYVTEAE
>d1gl5a_ b.34.2.1 (A:) tyrosine kinase tec {Mouse (Mus musculus)}
GSEIVVAMYDFQATEAHDLRLERGQEYIILEKNDLHWWRARDKYGSEGYIPSNYVTGKKSNN
LDQYD
>d1gcqa_ b.34.2.1 (A:) Growth factor receptor-bound protein 2 (GRB2), N- and C-terminal domains {Human (Homo sapiens)}
STYVQALFDFDPQEDGELGFRRGDFIHVMDNSDPNWWKGACHGQTGMFPRNYVTPV
>d1gria1 b.34.2.1 (A:1-56) Growth factor receptor-bound protein 2 (GRB2), N- and C-terminal domains {Human (Homo sapiens)}
MEAIAKYDFKATADDELSFKRGDILKVLNEECDQNWYKAELNGKDGFIPKNYIEMK
>d1gria2 b.34.2.1 (A:157-217) Growth factor receptor-bound protein 2 (GRB2), N- and C-terminal domains {Human (Homo sapiens)}
QPTYVQALFDFDPQEDGELGFRRGDFIHVMDNSDPNWWKGACHGQTGMFPRNYVTPVNRNV
>d1gbra_ b.34.2.1 (A:) Growth factor receptor-bound protein 2 (GRB2), N- and C-terminal domains {Mouse (Mus musculus)}
GSRRASVGSMEAIAKYDFKATADDELSFKRGDILKVLNEECDQNWYKAELNGKDGFIPKNYI
EMKPHPEFIVTD
>d1sema_ b.34.2.1 (A:) Growth factor receptor-bound protein 2 (GRB2), N- and C-terminal domains {Caenorhabditis elegans, SEM-5}

ETKFVQALFDFNPQESGELAFKRGDVITLINKDDPNWWEGQLNNRRGIFPSNYVCPYN
>d2hsp__ b.34.2.1 (-) Phospholipase C, SH3 domain {Human (Homo sapiens)}
GSPTFKCAVKALFDYKAQREDELTFIKSAIIQNVEKQEGGWWRGDYGGKKQLWFPSNYVEEM
VNPEGIHRD
>d1h92a_ b.34.2.1 (A:) p56-lck tyrosine kinase, SH3 domain {Human (Homo sapiens)}
GSPLQDNLVIALHSYEPSHDGDLGFEKGEQLRILEQSGEWWKAQSLTTGQEGFIPFNFVAKA
N
>d1lcka1 b.34.2.1 (A:63-116) p56-lck tyrosine kinase, SH3 domain {Human (Homo sapiens)}
DNLVIALHSYEPSHDGDLGFEKGEQLRILEQSGEWWKAQSLTTGQEGFIPFNFV
>d1ycsb2 b.34.2.1 (B:457-519) 53BP2 {Human (Homo sapiens)}
IMNKGVIYALWDYEPQNDDELPMKEGDCMTIIHREDEDEIEWWWARLNDKEGYVPRNLLGLY
P
>d1bb9__ b.34.2.1 (-) Amphiphysin 2 {Rat (Rattus norvegicus)}
TTGRLDLPPGFMFKVQAQHDYTATDTDELQLKAGDVVLVIPFQNPEEQDEGWLMGVKESDWN
QHKELEKCRGVFPENFTERVQ
>d1i07a_ b.34.2.1 (A:) EPS8 SH3 domain {Mouse (Mus musculus)}
KKYAKSKYDFVARNSSELSVMKDDVLEILDDRRQWWKVRNASGDSGFVPNNILDIMRTP
>d1gcqc_ b.34.2.1 (C:) Vav N-terminal SH3 domain {Mouse (Mus musculus)}
GSHMPKMEVFQEYYGIPPPPGAFGPFLRLNPGDIVELTKAEAEHNWWEGRNTATNEVGWFPC
NRVHPYV
>d1k1za_ b.34.2.1 (A:) Vav N-terminal SH3 domain {Mouse (Mus musculus)}
RAQDKKRNELGLPKMEVFQEYYGIPPPPGAFGGFLRLNPGDIVELTKAEAEHNWWEGRNTAT
NEVGWFPCNRVHPYVH
>d1i1ja_ b.34.2.1 (A:) Melanoma inhibitory activity protein {Human (Homo sapiens)}
GPMPKLADRKLCADQECSHPISMAVALQDYMAPDCRFLTIHRGQVVYVFSKLKGRGRLFWGG
SVQGDYYGDLAARLGYFPSSIVREDQTLKPGKVDVKTDKWDFYC
>d1kjwa1 b.34.2.1 (A:430-525) Psd-95 {Rat (Rattus norvegicus)}
GFYIRALFDYDKTKDCGFLSQALSFRFGDVLHVIDAGDEEWWQARRVHSDSETDDIGFIPSK
RRVERREWSRLKAKDWGSSSGSQGREDSVLSYET
>d1br2a1 b.34.3.1 (A:34-79) Myosin S1 fragment, N-terminal domain {Chicken (Gallus gallus), pectoral muscle}
LVWVPSEKHGFEAASIKEEKGDEVTVELQENGKKVTLSKDDIQKMN
>d2mysa1 b.34.3.1 (A:34-79) Myosin S1 fragment, N-terminal domain {Chicken (Gallus gallus), pectoral muscle}
AKSSVFVVHPKQSFVKGTIQSKEGGKVTVKTEGGETLTVKEDQVFS
>d1b7ta1 b.34.3.1 (A:29-76) Myosin S1 fragment, N-terminal domain {Bay scallop (Aequipecten irradians)}
DGKKNCWVPDEKEGFASAEIQSSKGDEITVKIVADSSTRTVKKDDIQS

>d1jwya1 b.34.3.1 (A:36-79) Myosin S1 fragment, N-terminal domain {Slime mold (Dictyostelium discoideum)}
FKLTVSDKRYIWYNPDPKERDSYECGEIVSETSDSFTFKTVDGQ
>d1lvk_1 b.34.3.1 (34-79) Myosin S1 fragment, N-terminal domain {Slime mold (Dictyostelium discoideum)}
YIWYNPDPKERDSYECGEIVSETSDSFTFKTSDGQDRQVKKDDANQ
>d1g5va_ b.34.9.1 (A:) Survival motor neuron protein 1, smn {Human (Homo sapiens)}
QQWKVGDKCSAIWSEDGCIYPATIASIDFKRETCVVVYTGYGNREEQNLSDLLSPI
>d1khca_ b.34.9.2 (A:) DNA methyltransferase DNMT3B {Mouse (Mus musculus)}
TEYQDDKEFGIGDLVWGKIKGFSWWPAMVVSWKATSKRQAMPGMRWVQWFGDGKFSEISADK
LVALGLFSQHFNLATFNKLVSYRKAMYHTLEKARVRAGKTFSSSPGESLEDQLKPMLEWAHG
GFKPTGIEGLKPN
>d1vie__ b.34.4.1 (-) R67 dihydrofolate reductase {Escherichia coli, plasmid PLZ1}
PSNATFGMGDRVRKKSGAAWQGQIVGWYCTNLTPEGYAVESEAHPGSVQIYPVAALERIN
>d1psf__ b.34.4.2 (-) Photosystem I accessory protein E (PsaE) {Cyanobacterium (Synechococcus sp.), pcc 7002}
AIERGSKVKILRKESYWYGDVGTVASIDKSGIIYPVIVRFNKVNYNGFSGSAGGLNTNNFAE
HELEVVG
>d1qp2a_ b.34.4.2 (A:) Photosystem I accessory protein E (PsaE) {Cyanobacterium (Nostoc sp.), strain pcc8009}
MVQRGSKVRILRPESYWFQDVGTVASVDQSGIKYPVIVRFEKVNYSGINTNNFAEDELVEVE
APKAKPKK
>d1jb0e_ b.34.4.2 (E:) Photosystem I accessory protein E (PsaE) {Synechococcus elongatus}
VQRGSKVKILRPESYWYNEVGTVASVDQTPGVKYPVIVRFDKVNYTGYSGSASGVNTNNFAL
HEVQEVA
>d1dj7b_ b.34.4.3 (B:) Ferredoxin thioredoxin reductase (FTR), alpha (variable) chain {Synechocystis sp.}
MNVGDRVRVTSSVVVYHHPEHKKTAFDLQGMEGEVAAVLTEWQGRPISANLPVLVKFEQRFK
AHFRPDEVTLI
>d2ahjb_ b.34.4.4 (B:) Nitrile hydratase beta chain {Rhodococcus erythropolis}
MDGVHDLAGVQGFGKVPHTVNADIGPTFHAEWEHLPYSLMFAGVAELGAFSVDEVRYVVERM
EPRHYMMTPYYERYVIGVATLMVEKGILTQDELESLAGGPFPLSRPSESEGRPAPVETTTFE
VGQRVRVRDEYVPGHIRMPAYCRGRVGTISHRTTEKWPFPDAIGHGRNDAGEEPTYHVKFAA
EELFGSDTDGGSVVVDLFEGYLEPA
>d1jj2s_ b.34.5.1 (S:) Ribosomal proteins L24 (L24p) {Archaeon Haloarcula marismortui}
SKQPDKQRKSQRRAPLHERHKQVRATLSADLREEYGQRNVRVNAGDTVEVLRGDFAGEEGEV
INVDLDKAVIHVEDVTLEKTDGEEVPRPLDTSNVRVTDLDLEDEKREARLESEDDSA
>d1jj2p_ b.34.5.1 (P:) Ribosomal proteins L21e {Archaeon

Haloarcula marismortui}
PSSNGPLEGTRGKLKNKPRDRGTSPPQRAVEEFDDGEKVHLKIDPSVPNGRFHPRFDGQTGT
VEGKQGDAYKVDIVDGGKEKTIIVTAAHLRRQE
>d2eifa1 b.34.5.2 (A:1-73) N-terminal domain of eukaryotic initiation translation factor 5a {Archaeon Methanococcus jannaschii}
VIIMPGTKQVNVGSLKVGQYVMIDGVPCEIVDISVSKPGKHGGAKARVVGIGIFEKVKKEFV
APTSSKVEVPI
>d1bkb_1 b.34.5.2 (4-74) N-terminal domain of eukaryotic initiation translation factor 5a {Archaeon Pyrobaculum aerophilum}
KWVMSTKYVEAGELKEGSYVVIDGEPCRVVEIEKSKTGKHGSAKARIVAVGVFDGGKRTLSL
PVDAQVEVP
>d1rl2a1 b.34.5.3 (A:126-195) C-terminal domain of ribosomal protein L2 {Bacillus stearothermophilus}
GNALPLENIPVGTLVHNIELKPGRGGQLVRAAGTSAQVLGKEGKYVIVRLASGEVRMILGKC
RATVGEVG
>d1jj2a1 b.34.5.3 (A:91-237) C-terminal domain of ribosomal protein L2 {Archaeon Haloarcula marismortui}
GNTLPLAEIPEGVPVCNVESSPGDGGKFARASGVNAQLLTHDRNVAVVKLPSGEMKRLDPQC
RATIGVVGGGGRTDKPFVKAGNKHHKMKARGTKWPNVRGVAMNAVDHPFGGGGRQHPGKPKS
ISRNAPPGRKVGDIASKRTGRGG
>d3vub__ b.34.6.1 (-) CcdB {Escherichia coli}
MQFKVYTYKRESRYRLFVDVQSDIIDTPGRRMVIPLASARLLSDKVSRELYPVVHIGDESWR
MMTTDMASVPVSVIGEEVADLSHRENDIKNAINLMFWGI
>d1hyoa1 b.34.8.1 (A:1-118) Fumarylacetoacetate hydrolase, FAH, N-terminal domain {Mouse (Mus musculus)}
MSFIPVAEDSDFPIQNLPYGVFSTQSNPKPRIGVAIGDQILDLSVIKHLFTGPALSKHQHVF
DETTLNNFMGLGQAAWKEARASLQNLLSASQARLRDDKELRQRAFTSQASATMHLP
>d1ex4a1 b.34.7.1 (A:223-270) DNA-binding domain of retroviral integrase {Human immunodeficiency virus type 1}
FRVYYRDSRNSLWKGPAKLLWKGEGAVVIQDNSDIKVVPRRKAKIIRD
>d1ihva_ b.34.7.1 (A:) DNA-binding domain of retroviral integrase {Human immunodeficiency virus type 1}
MIQNFRVYYRDSRDPVWKGPAKLLWKGEGAVVIQDNSDIKVVPRRKAKIIRD
>d1c0ma1 b.34.7.1 (A:217-269) DNA-binding domain of retroviral integrase {Rous sarcoma virus (RSV, avian sarcoma virus)}
VLTEGPPVKIRIETGEWEKGWNVLVWGRGYAAVKNRDTDKVIWVPSRKVKPDI
>d1c6vx_ b.34.7.1 (X:) DNA-binding domain of retroviral integrase {Simian immunodeficiency virus}
KNSKFKNFRVYYREGRDQLWKGPGELLWKGEGAVLLKVGTDIKVVPRRKAKIIKD
>d1aono_ b.35.1.1 (O:) Chaperonin-10 (GroES) {Escherichia coli}
MNIRPLHDRVIVKRKEVETKSAGGIVLTGSAAAKSTRGEVLAVGNGRILENGEVKPLDVKVG
DIVIFNDGYGVKSEKIDNEEVLIMSESDILAIVEA
>d1jh2a_ b.35.1.1 (A:) Chaperonin-10 (GroES) {Mycobacterium

tuberculosis}
AKVNIKPLEDKILVQANEAETTTASGLVIPDTAKEKPQEGTVVAVGPGRWDEDGEKRIPLDV
AEGDTVIYSKYGGTEIKYNGEEYLILSARDVLAVVSK
>d1lepa_ b.35.1.1 (A:) Chaperonin-10 (GroES) {Mycobacterium leprae}
AKVKIKPLEDKILVQAGEAETMTPSGLVIPENAKEKPQEGTVVAVGPGRWDEDGAKRIPVDV
SEGDIVIYSKYGGTEIKYNGEEYLILSARDVLAVVSK
>d1g31a_ b.35.1.1 (A:) GP31 co-chaperonin {Bacteriophage T4}
QQLPIRAVGEYVILVSEPAQAGDEEVTESGLIIGKRVQGEVPELCVVHSVGPDVPEGFCEVG
DLTSLPVGQIRNVPHPFVALGLKQPKEIKQKFVTCHYKAIPCLYK
>d1heta1 b.35.1.2 (A:1-174,A:325-374) Alcohol dehydrogenase {Horse (Equus caballus)}
STAGKVIKCKAAVLWEEKKPFSIEEVEVAPPKAHEVRIKMVATGICRSDDHVVSGTLVTPLP
VIAGHEAAGIVESIGEGVTTVRPGDKVIPLFTPQCGKCRVCKHPEGNFCLKNDLSMPRGTMQ
DGTSRFTCRGKPIHHFLGTSTFSQYTVVDEISVAKIDAASPLEKVCLIGCXKDSVPKLVADF
MAKKFALDPLITHVLPFEKINEGFDLLRSGESIRTILTF
>d1d1ta1 b.35.1.2 (A:1-174,A:325-374) Alcohol dehydrogenase {Human (Homo sapiens), different isozymes}
GTAGKVIKCKAAVLWEQKQPFSIEEIEVAPPKTKEVRIKILATGICRTDDHVIKGTMVSKFP
VIVGHEATGIVESIGEGVTTVKPGDKVIPLFLPQCRECNACRNPDGNLCIRSDITGRGVLAD
GTTRFTCKGKPVHHFLNTSTFTEYTVVDESSVAKIDDAAPPEKVCLIGCXRDDVPKLVTEFL
AKKFDLDQLITHVLPFKKISEGFELLNSGQSIRTVLTF
>d1hsoa1 b.35.1.2 (A:1-174,A:325-374) Alcohol dehydrogenase {Human (Homo sapiens), different isozymes}
STAGKVIKCKAAVLWELKKPFSIEEVEVAPPKAHEVRIKMVAVGICGTDDHVVSGTMVTPLP
VILGHEAAGIVESVGEGVTTVKPGDKVIPLAIPQCGKCRICKNPESNYCLKNDVSNPQGTLQ
DGTSRFTCRRKPIHHFLGISTFSQYTVVDENAVAKIDAASPLEKVCLIGCXKECVPKLVADF
MAKKFSLDALITHVLPFEKINEGFDLLHSGKSIRTILMF
>d1hsza1 b.35.1.2 (A:1-174,A:325-374) Alcohol dehydrogenase {Human (Homo sapiens), different isozymes}
STAGKVIKCKAAVLWEVKKPFSIEDVEVAPPKAYEVRIKMVAVGICRTDDHVVSGNLVTPLP
VILGHEAAGIVESVGEGVTTVKPGDKVIPLFTPQCGKCRVCKNPESNYCLKNDLGNPRGTLQ
DGTRRFTCRGKPIHHFLGTSTFSQYTVVDENAVAKIDAASPLEKVCLIGCXKEGIPKLVADF
MAKKFSLDALITHVLPFEKINEGFDLLHSGKSIRTVLTF
>d1ht0a1 b.35.1.2 (A:1-174,A:325-374) Alcohol dehydrogenase {Human (Homo sapiens), different isozymes}
STAGKVIKCKAAVLWELKKPFSIEEVEVAPPKAHEVRIKMVAAGICRSDEHVVSGNLVTPLP
VILGHEAAGIVESVGEGVTTVKPGDKVIPLFTPQCGKCRICKNPESNYCLKNDLGNPRGTLQ
DGTRRFTCSGKPIHHFVGVSTFSQYTVVDENAVAKIDAASPLEKVCLIGCXKESVPKLVADF
MAKKFSLDALITNVLPFEKINEGFDLLRSGKSIRTVLTF
>d1teha1 b.35.1.2 (A:3-174,A:325-374) Alcohol dehydrogenase {Human (Homo sapiens), different isozymes}
ANEVIKCKAAVAWEAGKPLSIEEIEVAPPKAHEVRIKIIATAVCHTDAYTLSGADPEGCFPV
ILGHEGAGIVESVGEGVTKLKAGDTVIPLYIPQCGECKFCLNPKTNLCQKIRVTQGKGLMPD

GTSRFTCKGKTILHYMGTSTFSEYTVVADISVAKIDPLAPLDKVCLLGCXVESVPKLVSEYM
SKKIKVDEFVTHNLSFDEINKAFELMHSGKSIRTVVKI
>d1e3ia1 b.35.1.2 (A:1-174,A:325-376) Alcohol dehydrogenase {Mouse (Mus musculus), class II}
GTQGKVIKCKAAIAWKTGSPLCIEEIEVSPPKACEVRIQVIATCVCPTDINATDPKKKALFP
VVLGHECAGIVESVGPGVTNFKPGDKVIPFFAPQCKRCKLCLSPLTNLCGKLRNFKYPTIDQ
ELMEDRTSRFTCKGRSIYHFMGVSSFSQYTVVSEANLARVDDEANLERVCXKSVDSVPNLVS
DYKNKKFDLDLLVTHALPFESINDAIDLMKEGKSIRTILTF
>d1cdoa1 b.35.1.2 (A:1-175,A:325-374) Alcohol dehydrogenase {Cod (Gadus callarias)}
ATVGKVIKCKAAVAWEANKPLVIEEIEVDVPHANEIRIKIIATGVCHTDLYHLFEGKHKDGF
PVVLGHEGAGIVESVGPGVTEFQPGEKVIPLFISQCGECRFCQSPKTNQCVKGWANESPDVM
SPKETRFTCKGRKVLQFLGTSTFSQYTVVNQIAVAKIDPSAPLDTVCLLGCXKDGVPKMVKA
YLDKKVKLDEFITHRMPLESVNDAIDLMKHGKCIRTVLSL
>d1keva1 b.35.1.2 (A:1-150,A:315-351) Bacterial secondary alcohol dehydrogenase {Clostridium beijerinckii}
MKGFAMLGINKLGWIEKERPVAGSYDAIVRPLAVSPCTSDIHTVFEGALGDRKNMILGHEAV
GEVVEVGSEVKDFKPGDRVIVPCTTPDWRSLEVQAGFQQHSNGMLAGWKFSNFKDGVFGEYF
HVNDADMNLAILPKDMPLENAVMITDXDLSKLVTHVYHGFDHIEEALLLMKDKPKDLIKAVV
IL
>d1ykfa1 b.35.1.2 (A:1-150,A:315-352) Bacterial secondary alcohol dehydrogenase {Thermoanaerobacter brockii}
MKGFAMLSIGKVGWIEKEKPAPGPFDAIVRPLAVAPCTSDIHTVFEGAIGERHNMILGHEAV
GEVVEVGSEVKDFKPGDRVVVPAITPDWRTSEVQRGYHQHSGGMLAGWKFSNVKDGVFGEFF
HVNDADMNLAHLPKEIPLEAAVMIPDXDPSKLVTHVFRGFDNIEKAFMLMKDKPKDLIKPVV
ILA
>d1e3ja1 b.35.1.2 (A:4-150,A:314-351) Ketose reductase (sorbitol dehydrogenase) {Silverleaf whitefly (Bemisia argentifolii)}
DNLSAVLYKQNDLRLEQRPIPEPKEDEVLLQMAYVGICGSDVHYYEHGRIADFIVKDPMVIG
HEASGTVVKVGKNVKHLKKGDRVAVEPGVPCRRCQFCKEGKYNLCPDLTFCATPPDDGNLAR
YYVHAADFCHKLPDNVSLEEGALXNVKQLVTHSFKLEQTVDAFEAARKKADNTIKVMISCRQ
>d1qora1 b.35.1.2 (A:2-135,A:266-327) Quinone oxidoreductase {Escherichia coli}
ATRIEFHKHGGPEVLQAVEFTPADPAENEIQVENKAIGINFIDTYIRSGLYPPPSLPSGLGT
EAAGIVSKVGSGVKHIKAGDRVVYAQSALGAYSSVHNIIADKAAILPAAISFEQAAASFLKG
LTVYYLLRKTXLQGYITTREELTEASNELFSLIASGVIKVDVAEQQKYPLKDAQRAHEILES
RATQGSSLLIP
>d1auua_ b.35.2.1 (A:) SacY {Bacillus subtilis}
MKIKRILNHNAIVVKDQNEEKILLGAGIAFNKKKNDIVDPSKIEKTFIRKDTPDY
>d1pdr__ b.36.1.1 (-) Discs large protein homolog {Human (Homo sapiens)}
ITREPRKVVLHRGSTGLGFNIVGGEDGEGIFISFILAGGPADLSGELRKGDRIISVNSVDLR
AASHEQAAAALKNAGQAVTIVAQYRPEEYSRQHA
>d1kwaa_ b.36.1.1 (A:) Cask/Lin-2 {Human (Homo sapiens)}

RSRLVQFQKNTDEPMGITLKMNELNHCIVARIMHGGMIHRQGTLHVGDEIREINGISVANQT
VEQLQKMLREMRGSITFKIVPSYREF
>d1be9a_ b.36.1.1 (A:) Synaptic protein PSD-95 {Rat (Rattus norvegicus)}
FLGEEDIPREPRRIVIHRGSTGLGFNIIGGEDGEGIFISFILAGGPADLSGELRKGDQILSV
NGVDLRNASHEQAAIALKNAGQTVTIIAQYKPEEYSRFEANSRVNSSGRIVTN
>d1qlca_ b.36.1.1 (A:) Synaptic protein PSD-95 {Rat (Rattus norvegicus)}
AEKVMEIKLIKGPKGLGFSIAGGVGNQHIPGDNSIYVTKIIEGGAAHKDGRLQIGDKILAVN
SVGLEDVMHEDAVAALKNTYDVVYLKVAKPSNA
>d1qava_ b.36.1.1 (A:) Syntrophin {Mouse (Mus musculus)}
GSLQRRRVTVRKADAGGLGISIKGGRENKMPILISKIFKGLAADQTEALFVGDAILSVNGED
LSSATHDEAVQALKKTGKEVVLEVKYMK
>d1b8qa_ b.36.1.1 (A:) Neuronal nitric oxide synthase, NNOS {Rat (Rattus norvegicus)}
GSHMIEPNVISVRLFKRKVGGLGFLVKERVSKPPVIISDLIRGGAAEQSGLIQAGDIILAVN
DRPLVDLSYDSALEVLRGIASETHVVLILRGPEGFTTHLETTFTGDGTPKTIRVTQPLGPPT
KAV
>d1qaua_ b.36.1.1 (A:) Neuronal nitric oxide synthase, NNOS {Rat (Rattus norvegicus)}
NVISVRLFKRKVGGLGFLVKERVSKPPVIISDLIRGGAAEQSGLIQAGDIILAVNDRPLVDL
SYDSALEVLRGIASETHVVLILRGPEGFTTHLETTFTGDGTPKTIRVTQP
>d3pdza_ b.36.1.1 (A:) Phosphatase hPTP1e {Human (Homo sapiens)}
PKPGDIFEVELAKNDNSLGISVTGGVNTSVRHGGIYVKAVIPQGAAESDGRIHKGDRVLAVN
GVSLEGATHKQAVETLRNTGQVVHLLLEKGQSPT
>d1g9oa_ b.36.1.1 (A:) Na+/H+ exchanger regulatory factor, NHERF {Human (Homo sapiens)}
RMLPRLCCLEKGPNGYGFHLHGEKGKLGQYIRLVEPGSPAEKAGLLAGDRLVEVNGENVEKE
THQQVVSRIRAALNAVRLLVVDPETDEQL
>d1ihja_ b.36.1.1 (A:) Inad {Fruit fly (Drosophila melanogaster)}
GELIHMVTLDKTGKKSFGICIVRGEVKDSPNTKTTGIFIKGIVPDSPAHLCGRLKVGDRILS
LNGKDVRNSTEQAVIDLIKEADFKIELEIQTF
>d1fc6a3 b.36.1.3 (A:157-248) Photosystem II D1 C-terminal processing protease {Algae (Scenedesmus obliquus)}
AGSVTGVGLEITYDGGSGKDVVVLTPAPGGPAEKAGARAGDVIVTVDGTAVKGMSLYDVSDL
LQGEADSQVEVVLHAPGAPSNTRTLQLTRQ
>d1k32a1 b.36.1.3 (A:763-853) Tricorn protease {Archaeon Thermoplasma acidophilum}
GRIACDFKLDGDHYVVAKAYAGDYSNEGEKSPIFEYGIDPTGYLIEDIDGETVGAGSNIYRV
LSEKAGTSARIRLSGKGGDKRDLMIDILD
>d1i16__ b.36.1.2 (-) Interleukin 16 {Human (Homo sapiens)}
MPDLNSSTDSAASASAASDVSVESTAEATVCTVTLEKMSAGLGFSLEGGKGSLHGDKPLTIN
RIFKGAASEQSETVQPGDEILQLGGTAMQGLTRFEAWNIIKALPDGPVTIVIRRKSLQSKET
TAAGDS

>d1g3p_1 b.37.1.1 (1-65) N-terminal domains of the minor coat protein g3p {Bacteriophage M13}
AETVESCLAKSHTENSFTNVWKDDKTLDRYANYEGCLWNATGVVVCTGDETQCYGTWVPIGL
AIP
>d1g3p_2 b.37.1.1 (91-217) N-terminal domains of the minor coat protein g3p {Bacteriophage M13}
EYGDTPIPGYTYINPLDGTYPPGTEQNPANPNPSLEESQPLNTFMFQNNRFRNRQGALTVYT
GTVTQGTDPVKTYYQYTPVSSKAMYDAYWNGKFRDCAFHSGFNEDIFVCEYQGQSSDLPQPP
VNA
>d1fgp__ b.37.1.1 (-) N-terminal domains of the minor coat protein g3p {Bacteriophage fd}
ETVESCLAKPHTENSFTNVWKDDKTLDRYANYEGCLWNATGVVVCTGDETQCYGTWVPIGLA
IPENAAAH
>d1b34a_ b.38.1.1 (A:) D1 core SNRNP protein {Human (Homo sapiens)}
KLVRFLMKLSHETVTIELKNGTQVHGTITGVDVSMNTHLKAVKMTLKNREPVQLETLSIRGN
NIRYFILPDSLPLDTLLV
>d1b34b_ b.38.1.1 (B:) D2 core SNRNP protein {Human (Homo sapiens)}
TGPLSVLTQSVKNNTQVLINCRNNKKLLGRVKAFDRHCNMVLENVKEMWTEVPKSGKGKKKS
KPVNKDRYISKMFLRGDSVIVVLRNPLIAGK
>d1d3ba_ b.38.1.1 (A:) D3 core SNRNP protein {Human (Homo sapiens)}
GVPIKVLHEAEGHIVTCETNTGEVYRGKLIEAEDNMNCQMSNITVTYRDGRVAQLEQVYIRG
CKIRFLILPD
>d1d3bb_ b.38.1.1 (B:) B core SNRNP protein {Human (Homo sapiens)}
SKMLQHIDYRMRCILQDGRIFIGTFKAFDKHMNLILCDCDEFRKIKPKNSKQAEREEKRVLG
LVLLRGENLVSMTVEGPPP
>d1d3bl_ b.38.1.1 (L:) B core SNRNP protein {Human (Homo sapiens)}
TVGKSSKMLQHIDYRMRCILQDGRIFIGTFKAFDKHMNLILCDCDEFRKIKPKNSKQAEREE
KRVLGLVLLRGENLVSMTVEGPPPKDTG
>d1i81a_ b.38.1.1 (A:) Archaeal homoheptameric Sm protein {Archaeon Methanobacterium thermoautotrophicum}
RVNVQRPLDALGNSLNSPVIIKLKGDREFRGVLKSFDLHMNLVLNDAEELEDGEVTRRLGTV
LIRGDNIVYISP
>d1jria_ b.38.1.1 (A:) Archaeal homoheptameric Sm protein {Archaeon Methanobacterium thermoautotrophicum}
QRPLDALGNSLNSPVIIKLKGDREFRGVLKSFDLHMNLVLNDAEELEDGEVTRRLGTVLIRG
DNIVYISRGK
>d1i8fa_ b.38.1.1 (A:) Archaeal homoheptameric Sm protein {Archaeon Pyrobaculum aerophilum}
ATLGATLQDSIGKQVLVKLRDSHEIRGILRSFDQHVNLLLEDAEEIIDGNVYKRGTMVVRGE
NVLFISPVP
>d1i4k1_ b.38.1.1 (1:) Archaeal homoheptameric Sm protein {Archaeon Archaeoglobus fulgidus}
PPRPLDVLNRSLKSPVIVRLKGGREFRGTLDGYDIHMNLVLLDAEEIQNGEVVRKVGSVVIR
GDTVVFVSPA

>d1h5pa_ b.99.1.1 (A:) Nuclear autoantigen Sp100b {Human (Homo sapiens)}
MDENINFKQSELPVTCGEVKGTLYKERFKQGTSKKCIQSEDKKWFTPREFEIEGDRGASKNW
KLSIRCGGYTLKVLMENKFLPEPPSTRKKVTIK
>d1whi__ b.39.1.1 (-) Ribosomal protein L14 {Bacillus stearothermophilus}
MIQQESRLKVADNSGAREVLVIKVLGGSGRRYANIGDVVVATVKDATPGGVVKKGQVVKAVV
VRTKRGVRRPDGSYIRFDENACVIIRDDKSPRGTRIFGPVARELRDKDFMKIISLAPEVI
>d1jj2j_ b.39.1.1 (J:) Ribosomal protein L14 {Archaeon Haloarcula marismortui}
MEALGADVTQGLEKGSLITCADNTGARELKVISVHGYSGTKNRHPKAGLGDKITVSVTKGTP
EMRRQVLEAVVVRQRKPIRRPDGTRVKFEDNAAVIVDENEDPRGTELKGPIAREVAQRFGSV
ASAATMIV
>d1ez6a_ b.40.1.1 (A:) Staphylococcal nuclease {Staphylococcus aureus}
LHKEPATLIKAIDGDTVKLMYKGQPMVFRLLLVDIPETKHPKKGVEKYGPEAAAFTKKMVEN
AKKIEVEFDKGQRTDKYGRGLAYIYADGKMVNEALVRQGLAKVAYVYKGNNTHEQLLRKAEA
QAKKEKLNIWS
>d1joqa_ b.40.1.1 (A:) Staphylococcal nuclease {Staphylococcus aureus}
ATSTKKLHKEPATLIKAIDGDTVKLMYKGQPMTFRLLLVDTPETKHPKKGVEKYGPEASAFT
KKMVENAKKIEVEFDKGQRTDKYGRGLAYIYADGKMVNEALVRQGLAKVAYVYKPNNTHEQL
LRKSEAQAKKEKLNIWSEDNADSGQ
>d1sty__ b.40.1.1 (-) Staphylococcal nuclease {Staphylococcus aureus}
KLHKEPATLIKAIDGDTVKLMYKGQPMTFRLLLVDTPETKHPKKGVEKYGPEASAFTKKMVE
NAKKIEVEFDKGQRTDKYGRGLAYIYADGKMVNEALVRQGLAKVAYVYKPNNTHEQHLRGKS
EAQAKKEKLNIWS
>d2sob__ b.40.1.1 (-) Staphylococcal nuclease {Staphylococcus aureus}
ATSTKKLHKEPATLIKAIDGDTVKLMYKGQPMTFRLLLVDTPETKHPKKGVEKYGPEASAFT
KKMLENAKKIEVEFDKGQRTDKYGRVLAYIYADGKMVNEAL
>d1djrd_ b.40.2.1 (D:) Heat-labile toxin {Escherichia coli, type IB}
APQTITELCSEYRNTQIYTINDKILSYTESMAGKREMVIITFKSGETFQVEVPGSQHIDSQK
KAIERMKDTLRITYLTETKIDKLCVWNNKTPNSIAAISMKN
>d1ltrd_ b.40.2.1 (D:) Heat-labile toxin {Escherichia coli, type IB}
APQSITELCSEYHNTQIYTINDKILSYTESMAGKREMVIITFKSGATFQVEVPGSQHIDSQK
KAIERMKDTLRITYLTETKIDKLCVWNNKTPNSIAAISMEKLYAGA
>d1tiid_ b.40.2.1 (D:) Heat-labile toxin {Escherichia coli, type IIB}
GASQFFKDNCNRTTASLVEGVELTKYISDINNNTDGMYVVSSTGGVWRISRAKDYPDNVMTA
EMRKIAMAAVLSGMRVNMCASPASSPNVIWAIELEA

>d3chbd_ b.40.2.1 (D:) Cholera toxin {Vibrio cholerae}
TPQNITDLCAEYHNTQIHTLNDKIFSYTESLAGKREMAIITFKNGATFQVEVPGSQHIDSQK
KAIERMKDTLRIAYLTEAKVEKLCVWNNKTPRAIAAISMAN
>d1c4qa_ b.40.2.1 (A:) Verotoxin-1/shiga-toxin, B-pentamer
{Escherichia coli}
TPDCVTGKVEYTKYNDDDTFTVKVGDKELATNRANLQSLLLSAQITGMTVTIKTNACHNGGG
FSEVIFR
>d2bosa_ b.40.2.1 (A:) Verotoxin-1/shiga-toxin, B-pentamer
{Escherichia coli}
ADCAKGKIEFSKYNEDNTFTVKVSGREYWTNRWNLQPLLQSAQLTGMTVTIISNTCSSGSGF
AEVQFN
>d1prtb1 b.40.2.1 (B:90-199) Pertussis toxin S2/S3 subunits,
C-terminal domain {Bordetella pertussis}
TTRNTGQPATDHYYSNVTATRLLSSTNSRLCAVFVRSGQPVIGACTSPYDGKYWSMYSRLRK
MLYLIYVAGISVRVHVSKEEQYYDYEDATFETYALTGISICNPGSSLC
>d1prtc1 b.40.2.1 (C:90-199) Pertussis toxin S2/S3 subunits,
C-terminal domain {Bordetella pertussis}
TIYKTGQPAADHYYSKVTATRLLASTNSRLCAVFVRDGQSVIGACASPYEGRYRDMYDALRR
LLYMIYMSGLAVRVHVSKEEQYYDYEDATFQTYALTGISLCNPAASIC
>d1prtd_ b.40.2.1 (D:) Pertussis toxin S4 subunit {Bordetella
pertussis}
DVPYVLVKTNMVVTSVAMKPYEVTPTRMLVCGIAAKLGAAASSPDAHVPFCFGKDLKRPGSS
PMEVMLRAVFMQQRPLRMFLGPKQLTFEGKPALELIRMVECSGKQDCP
>d1prtf_ b.40.2.1 (F:) Pertussis toxin S5 subunit {Bordetella
pertussis}
LPTHLYKNFTVQELALKLKGKNQEFCLTAFMSGRSLVRACLSDAGHEHDTWFDTMLGFAISA
YALKSRIALTVEDSPYPGTPGDLLELQICPLNGYCE
>d1esfa1 b.40.2.2 (A:1-120) Staphylococcal enterotoxin A, SEA
{Staphylococcus aureus}
SEKSEEINEKDLRKKSELQGTALGNLKQIYYYNEKAKTENKESHDQFLQHTILFKGFFTDHS
WYNDLLVDFDSKDIVDKYKGKKVDLYGAYYGYQCAGGTPNKTACMYGGVTLHDNNRLT
>d1i4pa1 b.40.2.2 (A:1-120) Staphylococcal enterotoxin C2, SEC2
{Staphylococcus aureus}
ESQPDPTPDELHKSSEFTGTMGNMKYLYDDHYVSATKVMSVDKFLAHDLIYNISDKKLKNYD
KVKTELLNEDLAKKYKDEVVDVYGSNYYVNCYFSSKDNVGKVTGGKTCMYGGITKHEG
>d3tss_1 b.40.2.2 (5-93) Toxic shock syndrome toxin-1 (TSST-1)
{Staphylococcus aureus}
NIKDLLDWYSSGSDTFTNSEVLDNSLGSMRIKNTDGSISLIIFPSPYYSPAFTKGEKVDLNT
KRIKKSQHTSEGTWIHFQISGVTNTEK
>d1sebd1 b.40.2.2 (D:2-121) Staphylococcal enterotoxin B, SEB
{Staphylococcus aureus}
SQPDPKPDELHKSSKFTGLMENMKVLYDDNHVSAINVKSIDQFLYFDLIYSIKDTYDNVRVE
FKNKDLADKYKDKYVDVFGANYYYQCYFSKKKTCMYGGVTEH
>d3seb_1 b.40.2.2 (1-121) Staphylococcal enterotoxin B, SEB

{Staphylococcus aureus}
ESQPDPKPDELHKSSKFTGLMENMKVLYDDNHVSAINVKSIDQFLYFDLIYSIKDTKLGNYD
NVRVEFKNKDLADKYKDKYVDVFGANYYYQCYFSKKTNDINSHQTDKRKTCMYGGVTEH
>d1jckb1 b.40.2.2 (B:1-121) Staphylococcal enterotoxin C3, SEC3
{Staphylococcus aureus}
ESQPDPMPDDLHKSSEFTGTMGNMKYLYDDHYVSATKVKSVDKFLAHDLIYNINDKKLNNYD
KVKTELLNEDLANKYKDEVVDVYGSNYYVNCYFSSKDNVGKVTSGKTCMYGGITKHEGN
>d1enfa1 b.40.2.2 (A:2-101) Staphylococcal enterotoxin H, SEH
{Staphylococcus aureus}
DLHDKSELTDLALANAYGQYNHPFIKENIKSDEISGEKDLIFRNQGDSGNDLRVKFATADLA
QKFKNKNVDIYGASFYYKCEKISENISECLYGGTTLNS
>d1an8_1 b.40.2.2 (3-95) Streptococcal superantigen Spe-C
{Streptococcus pyogenes}
KKDISNVKSDLLYAYTITPYDYKDCRVNFSTTHTLNIDTQKYRGKDYYISSEMSYEASQKFK
RDDHVDVFGLFYILNSHTGEYIYGGITPAQN
>d1et9a1 b.40.2.2 (A:1-95) Streptococcal superantigen Spe-H
{Streptococcus pyogenes}
NSYNTTNRHNLESLYKHDSNLIEADSIKNSPDIVTSHMLKYSVKDKNLSVFFEKDWISQEFK
DKEVDIYALSAQEVCECPGKRYEAFGGITLTNS
>d1eu3a1 b.40.2.2 (A:2A-96) Streptococcal superantigen Smez-2
{Streptococcus pyogenes}
GLEVDNNSLLRNIYSTIVYEYSDIVIDFKTSHNLVTKKLDVRDARDFFINSEMDEYAANDFK
TGDKIAVFSVPFDWNYLSKGKVTAYTYGGITPYQK
>d1bxta1 b.40.2.2 (A:1-119) Streptococcal superantigen SSA
{Streptococcus pyogenes}
SSQPDPTPEQLNKSSQFTGVMGNLRCLYDNHFVEGTNVRSTGQLLQHDLIFPIKDLKLKNYD
SVKTEFNSKDLATKYKNKDVDIFGSNYYYNCYYSEGNSCKNAKKTCMYGGVTEHHRN
>d1fnua1 b.40.2.2 (A:1-107) Streptococcal pyrogenic exotoxin A1
{Streptococcus pyogenes}
QQDPDPSQLHRSSLVKNLQNIYFLYEGDPVTHENVKSVDQLLSHDLIYNVSGPNYDKLKTEL
KNQEMATLFKDKNVDIYGVEYYHLCYLCENAERSACIYGGVTNHE
>d1d2ba_ b.40.3.1 (A:) TIMP-1 {Human (Homo sapiens)}
CTCVPPHPQTAFCNSDLVIRAKFVGTPEVNQTTLYQRYEIKMTKMYKGFQALGDAADIRFVY
TPAMESVCGYFHRSHNRSEEFLIAGKLQDGLLHITTCSFVAPWNSLSLAQRRGFTKTYTVGC
EE
>d1ueab_ b.40.3.1 (B:) TIMP-1 {Human (Homo sapiens)}
CTCVPPHPQTAFCNSDLVIRAKFVGTPEVAQTTLYQRYEIKMTKMYKGFQALGDAADIRFVY
TPAMESVCGYFHRSHARSEEFLIAGKLQDGLLHITTCSFVAPWNSLSLAQRRGFTKTYTVGC
EECTVFPCLSIPCKLQSGTHCLWTDQLLQGSEKGFQSRHLACLPREPGLCTWQSLRS
>d1br9__ b.40.3.1 (-) TIMP-2 {Human (Homo sapiens)}
CSCSPVHPQQAFCNADVVIRAKAVSEKEVDSGNDIYGNPIKRIQYEIKQIKMFKGPEKDIEF
IYTAPSSAVCGVSLDVGGKKEYLIAGKAEGDGKMHITLCDFIVPWDTLSTTQKKSLNHRYQM
GCECKITRCPMIPCYISSPDECLWMDWVTEKNINGHQAKFFACIKRSDGSCAWYRGAA
>d2tmp__ b.40.3.1 (-) TIMP-2 {Human (Homo sapiens)}

CSCSPVHPQQAFCNADVVIRTKAVSEKEVDSGNDIYGNPIKRIQYEIKQIKMFKGPEKDIEF
IYTAPSSAVCGVSLDVGGKKEYLIAGKAEGDGKMHITLCDFIVPWDTLSTTQKKSLNHRYQM
GCE
>d1bqqt_ b.40.3.1 (T:) TIMP-2 {Cow (Bos taurus)}
CSCSPVHPQQAFCNADIVIRAKAVNKKEVDSGNDIYGNPIKRIQYEIKQIKMFKGPDQDIEF
IYTAPAAAVCGVSLDIGGKKEYLIAGKAEGNGNMHITLCDFIVPWDTLSATQKKSLNHRYQM
GCECKITRCPMIPCYISSPDECLWMDWVTEKNINGHQAKFFACIKRSDGSCAWYRGAAPP
>d1jb3a_ b.40.3.2 (A:) The laminin-binding domain of agrin {Chicken (Gallus gallus)}
ELQRREEEANVVLTGTVEEIMNVDPVHHTYSCKVRVWRYLKGKDIVTHEILLDGGNKVVIGG
FGDPLICDNQVSTGDTRIFFVNPAPQYMWPAHRNELMLNSSLMRITLRNLEEVEHCVEEHRK
LLA
>d1k28a1 b.40.8.1 (A:6-129) Tail-associated lysozyme gp5, N-terminal domain {Bacteriophage T4}
NNLNWFVGVVEDRMDPLKLGRVRVRVVGLHPPQRAQGDVMGIPTEKLPWMSVIQPITSAAMS
GIGGSVTGPVEGTRVYGHFLDKWKTNGIVLGTYGGIVREKPNRLEGFSDPTGQYPRRLGNDT
>d1eova1 b.40.4.1 (A:71-204) Aspartyl-tRNA synthetase (AspRS) {Baker's yeast (Saccharomyces cerevisiae)}
AKDNYGKLPLIQSRDSDRTGQKRVKFVDLDEAKDSDKEVLFRARVHNTRQQGATLAFLTLRQ
QASLIQGLVKANKEGTISKNMVKWAGSLNLESIVLVRGIVKKVDEPIKSATVQNLEIHITKI
YTISETPEAL
>d1b8aa1 b.40.4.1 (A:1-103) Aspartyl-tRNA synthetase (AspRS) {Archaeon Pyrococcus kodakaraensis}
MYRTHYSSEITEELNGQKVKVAGWVWEVKDLGGIKFLWIRDRDGIVQITAPKKKVDPELFKL
IPKLRSEDVVAVEGVVNFTPKAKLGFEILPEKIVVLNRAET
>d1c0aa1 b.40.4.1 (A:1-106) Aspartyl-tRNA synthetase (AspRS) {Escherichia coli}
MRTEYCGQLRLSHVGQQVTLCGWVNRRRDLGSLIFIDMRDREGIVQVFFDPDRADALKLASE
LRNEFCIQVTGTVRARDEKNINRDMATGEIEVLASSLTIINRAD
>d1g51a1 b.40.4.1 (A:1-104) Aspartyl-tRNA synthetase (AspRS) {Thermus thermophilus}
MRRTHYAGSLRETHVGEEVVLEGWVNRRRDLGGLIFLDLRDREGLVQLVAHPASPAYATAER
VRPEWVVRAKGLVRLRPEPNPRLATGRVEVELSALEVLAEAK
>d1bbua1 b.40.4.1 (A:11-154) Lysyl-tRNA synthetase (LysRS) {Escherichia coli, gene lysS}
VVDLNNELKTRREKLANLREQGIAFPNDFRRDHTSDQLHAEFDGKENEELEALNIEVAVAGR
MMTRRIMGKASFVTLQDVGGRIQLYVARDDLPEGVYNEQFKKWDLGDILGAKGKLFKTKTGE
LSIHCTELRLLTKALRPLPD
>d1krs__ b.40.4.1 (-) Lysyl-tRNA synthetase (LysRS) {Escherichia coli, gene lysS}
FRRDHTSDQLHAEFDGKENEELEALNIEVAVAGRMMTRRIMGKASFVTLQDVGGRIQLYVAR
DDLPEGVYNEQFKKWDLGDILGAKGKLFKTKTGELSIHCTELRLLTKA
>d1e1oa1 b.40.4.1 (A:11-153) Lysyl-tRNA synthetase (LysRS) {Escherichia coli, gene lysU}

AIDFNDELRNRREKLAALRQQGVAFPNDFRRDHTSDQLHEEFDAKDNQELESLNIEVSVAGR
MMTRRIMGKASFVTLQDVGGRIQLYVARDSLPEGVYNDQFKKWDLGDIIGARGTLFKTQTGE
LSIHCTELRLLTKALRPLP
>d1gm5a2 b.40.4.9 (A:106-285) RecG "wedge" domain {Thermotoga maritima}
CSGEEVDLSTDIQYAKGVGPNRKKKLKKLGIETLRDLLEFFPRDYEDRRKIFKLNDLLPGEK
VTTQGKIVSVETKKFQNMNILTAVLSDGLVHVPLKWFNQDYLQTYLKQLTGKEVFVTGTVKS
NAYTGQYEIHNAEVTPKEGEYVRRILPIYRLTSGISQKQMRKIFEENIPSLCCSLK
>d1cuk_3 b.40.4.2 (1-64) DNA helicase RuvA subunit, N-terminal domain {Escherichia coli}
MIGRLRGIIIEKQPPLVLIEVGGVGYEVHMPMTCFYELPEAGQEAIVFTHFVVREDAQLLYG
FN
>d1bvsa3 b.40.4.2 (A:1-63) DNA helicase RuvA subunit, N-terminal domain {Mycobacterium leprae}
MIFSVRGEVLEVALDHAVIEAAGIGYRVNATPSALATLNQGSQARLVTAMVVREDSMTLYGF
S
>d3ulla_ b.40.4.3 (A:) ssDNA-binding protein {Human (Homo sapiens), mitochondria}
LERSLNRVHLLGRVGQDPVLRQVEGKNPVTIFSLATNEMWRSGDSEVYQLGDVSQKTTWHRI
SVFRPGLRDVAYQYVKKGSRIYLEGKIDYGEYMDKNNVRRQATTIIADNIIFL
>d1kawa_ b.40.4.3 (A:) ssDNA-binding protein {Escherichia coli}
RGVNKVILVGNLGQDPEVRYMPNGGAVANITLATSESWRDKATGEMKEQTEWHRVVLFGKLA
EVASEYLRKGSQVYIEGQLRTRKWTDQSGQDRYTTEVVVNVGGTMQML
>d1qvca_ b.40.4.3 (A:) ssDNA-binding protein {Escherichia coli}
ASRGVNKVILVGNLGQDPEVRYMPNGGAVANITLATSESWRDKATGEMKEQTEWHRVVLFGK
LAEVASEYLRKGSQVYIEGQLRTRKWTDQSGQDRYTTEVVVNVGGTMQMLGGRQGGGAPAGG
NIGGGQPQGGWGQPQQPQGGN
>d1ewia_ b.40.4.3 (A:) Replication protein A 70 KDa subunit (RPA70) fragment {Human (Homo sapiens)}
MVGQLSEGAIAAIMQKGDTNIKPILQVINIRPITTGNSPPRYRLLMSDGLNTLSSFMLATQL
NPLVEEEQLSSNCVCQIHRFIVNTLKDGRRVVILMELEVLKSAEAVGVKIGN
>d1fgua1 b.40.4.3 (A:181-298) Replication protein A 70 KDa subunit (RPA70) fragment {Human (Homo sapiens)}
MSKVVPIASLTPYQSKWTICARVTNKSQIRTWSNSRGEGKLFSLELVDESGEIRATAFNEQV
DKFFPLIEVNKVYYFSKGTLKIANKQFTAVKNDYEMTFNNETSVMPCEDDHHLPTV
>d1fgua2 b.40.4.3 (A:299-426) Replication protein A 70 KDa subunit (RPA70) fragment {Human (Homo sapiens)}
QFDFTGIDDLENKSKDSLVDIIGICKSYEDATKITVRSNNREVAKRNIYLMDTSGKVVTATL
WGEDADKFDGSRQPVLAIKGARVSDFGGRSLSVLSSSTIIANPDIPEAYKLRGWFDAEGQAL
DGVS
>d1quqa_ b.40.4.3 (A:) Replication protein A 32 KDa subunit (RPA32) fragment {Human (Homo sapiens)}
HIVPCTISQLLSATLVDEVFRIGNVEISQVTIVGIIRHAEKAPTNIVYKIDDMTAAPMDVRQ
WVDTDDTSSENTVVPPETYVKVAGHLRSFQNKKSLVAFKIMPLEDMNEFTTHILEVINAHMV

LSK
>d1quqb_ b.40.4.3 (B:) Replication protein A 14 KDa (RPA14) subunit {Human (Homo sapiens)}
DMMDLPRSRINAGMLAQFIDKPVCFVGRLEKIHPTGKMFILSDGEGKNGTIELMEPLDEEIS
GIVEVVGRVTAKATILCTSYVQFKEDSHPFDLGLYNEAVKIIHDFPQFYPLG
>d1jb7a1 b.40.4.3 (A:36-204) Telomere end binding protein alpha subunit {Oxytricha nova}
YEYVELAKASLTSAQPQHFYAVVIDATFPYKTNQERYICSLKIVDPTLYLKQQKGAGDASDY
ATLVLYAKRFEDLPIIHRAGDIIRVHRATLRLYNGQRQFNANVFYSSSWALFSTDKRSVTQE
INNQDAVSDTTPFSFSSKHATIEKNEISILQNLRKWANQYFSSYS
>d1jb7a2 b.40.4.3 (A:205-328) Telomere end binding protein alpha subunit {Oxytricha nova}
VISSDMYTALNKAQAQKGDFDVVAKILQVHELDEYTNELKLKDASGQVFYTLSLKLKFPHVR
TGEVVRIRSATYDETSTQKKVLILSHYSNIITFIQSSKLAKELRAKIQDDHSVEVASLKKNV
>d1jb7a3 b.40.4.3 (A:329-495) Telomere end binding protein alpha subunit {Oxytricha nova}
SLNAVVLTEVDKKHAALPSTSLQDLFHHADSDKELQAQDTFRTQFYVTKIEPSDVKEWVKGY
DRKTKKSSSLKGASGKGDNIFQVQFLVKDASTQLNNNTYRVLLYTQDGLGANFFNVKADNLH
KNADARKKLEDSAELLTKFNSYVDAVVERRNGFYLIKDTKLIY
>d1k8ga2 b.40.4.3 (A:205-315) Telomere end binding protein alpha subunit {Oxytricha nova}
VISSDMYTALNKAQAQKGDFDVVAKILQVHELDEYTNELKLKDASGQVFYTLSLKLKFPHVR
TGEVVRIRSATYDETSTQKKVLILSHYSNIITFIQSSKLAKELRAKIQD
>d1jb7b_ b.40.4.3 (B:) Core domain of telomere end binding protein beta subunit {Oxytricha nova}
QQQSAFKQLYTELFNNEGDFSKVSSNLKKPLKCYVKESYPHFLVTDGYFFVAPYFTKEAVNE
FHAKFPNVNIVDLTDKVIVINNWSLELRRVNSAEVFTSYANLEARLIVHSFKPNLQERLNPT
RYPVNLFRDDEFKTTIQHFRHTALQAAINKTVKGDNLVDISKVADAAGKKGKVDAGIVKASA
SKGDEFSDFSFKEGNTATLKIADIFVQEKG
>d1jjcb3 b.40.4.4 (B:39-151) Domain B2 of PheRS-beta, PheT {Thermus thermophilus (Thermus aquaticus)}
FPIPRGVVFARVLEAHPIPGTRLKRLVLDAGRTVEVVSGAENARKGIGVALALPGTELPGLG
QKVGERVIQGVRSFGMALSPRELGVGEYGGGLLEFPEDALPPGTPLSEAWP
>d1fl0a_ b.40.4.4 (A:) EMAP II {Human (Homo sapiens)}
IDVSRLDLRIGCIITARKHPDADSLYVEEVDVGEIAPRTVVSGLVNHVPLEQMQNRMVILLC
NLKPAKMRGVLSQAMVMCASSPEKIEILAPPNGSVPGDRITFDAFPGEPDKELNPKKKIWEQ
IQPDLHTNDECVATYKGVPFEVKGKGVCRAQTMSNSGIKL
>d1gd7a_ b.40.4.4 (A:) CsaA {Thermus thermophilus}
MTPLEAFQILDLRVGRVLRAEPHEKARKPSYKLWVDLGPLGVKQSSAQITELYRPEDLVGRL
VVCAVNLGAKRVAGFLSEVLVLGVPDEAGRVVLLAPDREVPLGGKVF
>d1mjc__ b.40.4.5 (-) Major cold shock protein {Escherichia coli}
SGKMTGIVKWFNADKGFGFITPDDGSKDVFVHFSAIQNDGYKSLDEGQKVSFTIESGAKGPA
AGNVTSL
>d1csp__ b.40.4.5 (-) Major cold shock protein {Bacillus subtilis}

MLEGKVKWFNSEKGFGFIEVEGQDDVFVHFSAIQGEGFKTLEEGQAVSFEIVEGNRGPQAAN
VTKEA
>d1c9oa_ b.40.4.5 (A:) Major cold shock protein {Bacillus
caldolyticus}
MQRGKVKWFNNEKGYGFIEVEGGSDVFVHFTAIQGEGFKTLEEGQEVSFEIVQGNRGPQAAN
VVKL
>d1g6pa_ b.40.4.5 (A:) Major cold shock protein {Thermotoga
maritima}
MRGKVKWFDSKKGYGFITKDEGGDVFVHWSAIEMEGFKTLKEGQVVEFEIQEGKKGPQAAHV
KVVE
>d1h95a_ b.40.4.5 (A:) Y-box protein 1 cold shock domain (YB1-CSD)
{Human (Homo sapiens)}
MKKVIATKVLGTVKWFNVRNGYGFINRNDTKEDVFVHQTAIKKNNPRKYLRSVGDGETVEFD
VVEGEKGAEAANVTGPG
>d1sro__ b.40.4.5 (-) S1 RNA-binding domain of polyribonucleotide
phosphorylase, PNPase {Escherichia coli}
AEIEVGRVYTGKVTRIVDFGAFVAIGGGKEGLVHISQIADKRVEKVTDYLQMGQEVPVKVLE
VDRQGRIRLSIKEA
>d1e3pa2 b.40.4.5 (A:656-717) S1 RNA-binding domain of
polyribonucleotide phosphorylase, PNPase {Streptomyces
antibioticus}
GSVVKTTTFGAFVSLLPGKDGLLHISQIRKLAGGKRVENVEDVLGVGQKVQVEIAEIDSRGK
>d1hh2p1 b.40.4.5 (P:127-198) S1 domain of NusA {Thermotoga
maritima}
FEKYSELKGTVTTAEVIRVMGEWADIRIGKLETRLPKKEWIPGEEIKAGDLVKVYIIDVVKT
TKGPKILVSR
>d1k0ra1 b.40.4.5 (A:108-183) S1 domain of NusA {Mycobacterium
tuberculosis}
STREGEIVAGVIQRDSRANARGLVVVRIGTETKASEGVIPAAEQVPGESYEHGNRLRCYVVG
VTRGAREPLITLSR
>d1go3e_ b.40.4.5 (E:) RNA polymerase II subunit RBP4 (RpoE)
{Archaeon Methanococcus jannaschii}
MYKILEIADVVKVPPEEFGKDLKETVKKILMEKYEGRLDKDVGFVLSIVDVKDIGEGKVVHG
DGSAYHPVVFETLVYIPEMYELIEGEVVDVVEFGSFVRLGPLDGLIHVSQIMDDYVSYDPKR
EAIIGKETGKVLEIGDYVRARIVAISLKAERKRGSKIALTMRQPYLGKLEWIEEEKAKKQ
>d1ah9__ b.40.4.5 (-) Translational initiation factor 1, IF1
{Escherichia coli}
AKEDNIEMQGTVLETLPNTMFRVELENGHVVTAHISGKMRKNYIRILTGDKVTVELTPYDLS
KGRIVFRSR
>d1hr0w_ b.40.4.5 (W:) Translational initiation factor 1, IF1
{Escherichia coli}
AKEKDTIRTEGVVTEALPNATFRVKLDSGPEILAYISGKMRMHYIRILPGDRVVVEITPYDP
TRGRIVYRK
>d1jt8a_ b.40.4.5 (A:) Archaeal initiation factor-1a, aIF1a

{Archaeon Methanococcus jannaschii}
MAEQQQEQQIRVRIPRKEENEILGIIEQMLGASRVRVRCLDGKTRLGRIPGRLKNRIWVREG
DVVIVKPWEVQGDQKCDIIWRYTKTQVEWLKRKGYLDELL
>d1d7qa_ b.40.4.5 (A:) Translation initiation factor-1a, eIF1a
{Human (Homo sapiens)}
PKNKGKGGKNRRRGKNENESEKRELVFKEDGQEYAQVIKMLGNGRLEAMCFDGVKRLCHIRG
KLRKKVWINTSDIILVGLRDYQDNKADVILKYNADEARSLKAYGELPEHAKINETDTFGPGD
DDEIQFDDIGDDDEDIDDI
>d1a62_2 b.40.4.5 (48-125) Rho termination factor, RNA-binding
domain {Escherichia coli}
DIFGDGVLEILQDGFGFLRSADSSYLAGPDDIYVSPSQIRRFNLRTGDTISGKIRPPKEGER
YFALLKVNEVNFDKPE
>d2eifa2 b.40.4.5 (A:74-132) C-terminal domain of eukaryotic
initiation translation factor 5a {Archaeon Methanococcus
jannaschii}
IDRRKGQVLAIMGDMVQIMDLQTYETLELPIPEGIEGLEPGGEVEYIEAVGQYKITRVI
>d1bkb_2 b.40.4.5 (75-139) C-terminal domain of eukaryotic
initiation translation factor 5a {Archaeon Pyrobaculum aerophilum}
IIEKFTAQILSVSGDVIQLMDMRDYKTIEVPMKYVEEEAKGRLAPGAEVEVWQILDRYKIIR
VKG
>d1rl2a2 b.40.4.5 (A:60-125) N-terminal domain of ribosomal
protein L2 {Bacillus stearothermophilus}
QYRIIDFKRDKDGIPGRVATIEYDPNRSANIALINYADGEKRYIIAPKNLKVGMEIMSGPDA
DIKI
>d1jj2a2 b.40.4.5 (A:1-90) N-terminal domain of ribosomal protein
L2 {Archaeon Haloarcula marismortui}
GRRIQGQRRGRGTSTFRAPSHRYKADLEHRKVEDGDVIAGTVVDIEHDPARSAPVAAVEFED
GDRRLILAPEGVGVGDELQVGVDAEIAP
>d1fjgl_ b.40.4.5 (L:) Ribosomal protein S12 {Thermus
thermophilus}
PTINQLVRKGREKVRKKSKVPALKGAPFRRGVCTVVRTVTPKKPNSALRKVAKVRLTSGYEV
TAYIPGEGHNLQEHSVVLIRGGRVKDLPGVRYHIVRGVYDAAGVKDRKKSRSKYGTKKPKEA
A
>d1fjgq_ b.40.4.5 (Q:) Ribosomal protein S17 {Thermus
thermophilus}
PKKVLTGVVVSDKMQKTVTVLVERQFPHPLYGKVIKRSKKYLAHDPEEKYKLGDVVEIIESR
PISKRKRFRVLRLVESGRMDLVEKYLIRRQNYQSLSKRGGKA
>d1i94q_ b.40.4.5 (Q:) Ribosomal protein S17 {Thermus
thermophilus}
PKKVLTGVVVSDKMQKTVTVLVERQFPHPLYGKVIKRSKKYLAHDPEERYKVGDVVEIIEAR
PISKRKRFRVLRLVEEGRLDLVEKYLVRRQNYASLSKRGGKA
>d1rip__ b.40.4.5 (-) Ribosomal protein S17 {Bacillus
stearothermophilus}
QRKVYVGRVVSDKMDKTITVLVETYKKHPLYGKRVKYSKKYKAHDEHNEAKVGDIVKIMETR

PLSATKRFRLVEIVEKAVR
>d1ckma1 b.40.4.6 (A:239-327) RNA guanylyltransferase (mRNA capping enzyme) {Chlorella virus, PBCV-1}
THHTIDFIIMSEDGTIGIFDPNLRKNVPVGKLDGYYNKGSIVECGFADGTWKYIQGRSDKNQ
ANDRLTYEKTLLNIEENITIDELLDLF
>d1a0i_1 b.40.4.6 (241-349) ATP-dependent DNA ligase {Bacteriophage T7}
PENEADGIIQGLVWGTKGLANEGKVIGFEVLLESGRLVNATNISRALMDEFTETVKEATLSQ
WGFFSPYGIGDNDACTINPYDGWACQISYMEETPDGSLRHPSFVMFR
>d1fvia1 b.40.4.6 (A:190-293) ATP-dependent DNA ligase {Chlorella virus, PBCV-1}
FKDAEATIISMTALFKNTNTKTKDNFGYSKRSTHKSGKVEEDVMGSIEVDYDGVVFSIGTGF
DADQRRDFWQNKESYIGKMVKFKYFEMGSKDCPRFPVFIGIR
>d1dgsa2 b.40.4.6 (A:315-400) NAD+-dependent DNA ligase {Thermus filiformis}
AEEKETRLLDVVFQVGRTGRVTPVGVLEPVFIEGSEVSRVTLHNESYIEELDIRIGDWVLVH
KAGGVIPEVLRVLKERRTGKERPI
>d1gvp__ b.40.4.7 (-) Gene V protein {Filamentous bacteriophage (f1, M13)}
MIKVEIKPSQAQFTTRSGVSRQGKPYSLNEQLCYVDLGNEYPVLVKITLDEGQPAYAPGLYT
VHLSSFKVGQFGSLMIDRLRLVPAK
>d1pfsa_ b.40.4.7 (A:) Gene V protein {Pseudomonas bacteriophage pf3}
MNIQITFTDSVRQGTSAKGNPYTFQEGFLHLEDKPHPLQCQFFVESVIPAGSYQVPYRINVN
NGRPELAFDFKAMKRA
>d1gpc__ b.40.4.7 (-) Gene 32 protein (gp32) core {Bacteriophage T4}
GFSSEDKGEWKLKLDNAGNGQAVIRFLPSKNDEQAPFAILVNHGFKKNGKWYIETCSSTHGD
YDSCPVCQYISKNDLYNTDNKEYSLVKRKTSYWANILVVKDPAAPENEGKVFKYRFGKKIWD
KINAMIAVDVEMGETPVDVTCPWEGANFVLKVKQVSGFSNYDESKFLNQSAIPNIDDESFQK
ELFEQMVDLSEMTSKDKFKSFEELNTKFGQVM
>d1je5a_ b.40.4.7 (A:) gp2.5 {Bacteriophage T7}
MAKKIFTSALGTAEPYAYIAKPDYGNEERGFGNPRGVYKVDLTIPNKDPRCQRMVDEIVKCH
EEAYAAAVEEYEANPPAVARGKKPLKPYEGDMPFFDNGDGTTTFKFKCYASFQDKKTKETKH
INLVVVDSKGKKMEDVPIIGGGSKLKVKYSLVPYKWNTAVGASVKLQLESVMLVELATFGGG
EDDWADEVEEN
>d1i50h_ b.40.4.8 (H:) RNA polymerase subunit RBP8 {Baker's yeast (Saccharomyces cerevisiae)}
SNTLFDDIFQVSEVDPGRYNKVCRIEAASTTQDQCKLTLDINVELFPVAAQDSLTVTIASSL
NLEDTPANDSSATRSWRPPQAGDRSLADDYDYVMYGTAYKFEEVSKDLIAVYYSFGGLLMRL
EGNYRNLNNLKQENAYLLIRR
>d1e9ga_ b.40.5.1 (A:) Inorganic pyrophosphatase {Baker's yeast (Saccharomyces cerevisiae)}
TYTTRQIGAKNTLEYKVYIEKDGKPVSAFHDIPLYADKENNIFNMVVEIPRWTNAKLEITKE

ETLNPIIQDTKKGKLRFVRNCFPHHGYIHNYGAFPQTWEDPNVSHPETKAVGDNDPIDVLEI
GETIAYTGQVKQVKALGIMALLDEGETDWKVIAIDINDPLAPKLNDIEDVEKYFPGLLRATN
EWFRIYKIPDGKPENQFAFSGEAKNKKYALDIIKETHDSWKQLIAGKSSDSKGIDLTNVTLP
DTPTYSKAASDAIPPASLKADAPIDKSIDKWFFISG
>d1qeza_ b.40.5.1 (A:) Inorganic pyrophosphatase {Archaeon Sulfolobus acidocaldarius}
KLSPGKNAPDVVNVLVEIPQGSNIKYEYDDEEGVIKVDRVLYTSMNYPFNYGFIPGTLEEDG
DPLDVLVITNYQLYPGSVIEVRPIGILYMKDEEGEDAKIVAVPKDKTDPSFSNIKDINDLPQ
ATKNKIVHFFEHYKELEPGKYVKISGWGSATEAKNRIQLAIKRVSG
>d1i40a_ b.40.5.1 (A:) Inorganic pyrophosphatase {Escherichia coli}
SLLNVPAGKDLPEDIYVVIEIPANADPIKYEIDKESGALFVDRFMSTAMFYPCNYGYINHTL
SLDGDPVDVLVPTPYPLQPGSVTRCRPVGVLKMTDEAGEDAKLVAVPHSKLSKEYDHIKDVN
DLPELLKAQIAHFFEHYKDLEKGKWVKVEGWENAEAAKAEIVASFERAKNK
>d2prd__ b.40.5.1 (-) Inorganic pyrophosphatase {Thermus thermophilus}
ANLKSLPVGDKAPEVVHMVIEVPRGSGNKYEYDPDLGAIKLDRVLPGAQFYPGDYGFIPSTL
AEDGDPLDGLVLSTYPLLPGVVVEVRVVGLLLMEDEKGGDAKVIGVVAEDQRLDHIQDIGDV
PEGVKQEIQHFFETYKALEAKKGKWVKVTGWRDRKAALEEVRACIARYKG
>d1fr3a_ b.40.6.1 (A:) Molybdate/tungstate binding protein MOP {Sporomusa ovata}
MKISGRNKLEATVKEIVKGTVMAKIVMDYKGTELVAAITIDSVADLDLVPGDKVTALVKATE
MEVLK
>d1guta_ b.40.6.1 (A:) Molybdate/tungstate binding protein MOP {Clostridium pasteurianum, MOP II}
SISARNQLKGKVVGLKKGVVTAEVVLEIAGGNKITSIISLDSVEELGVKEGAELTAVVKSTD
VMILA
>d1h9ma1 b.40.6.2 (A:1-73) Cytoplasmic molybdate-binding protein ModG {Azotobacter vinelandii}
MKISARNVFKGTVSALKEGAVNAEVDILLGGGDKLAAVVTLESARSLQLAAGKEVVAVVKAP
WVLLMTDSSGY
>d1h9ma2 b.40.6.2 (A:74-141) Cytoplasmic molybdate-binding protein ModG {Azotobacter vinelandii}
RLSARNILTGTVKTIETGAVNAEVTLALQGGTEITSMVTKEAVAELGLKPGASASAVIKASN
VILGVP
>d1h9ra1 b.40.6.2 (A:123-199) C-terminal domain of molybdate-dependent transcriptional regulator ModE {Escherichia coli}
MQTSARNQWFGTITARDHDDVQQHVDVLLADGKTRLKVAITAQSGARLGLDEGKEVLILLKA
PWVGITQDEAVAQNA
>d1h9ra2 b.40.6.2 (A:200-261) C-terminal domain of molybdate-dependent transcriptional regulator ModE {Escherichia coli}
DNQLPGIISHIERGAEQCEVLMALPDGQTLCATVPVNEATSLQQGQNVTAYFNADSVIIATL

>d1g2913 b.40.6.3 (1:241-301) Maltose transport protein MalK, C-terminal domain {Archaeon Thermococcus litoralis}
GSPPMNFLDAIVTEDGFVDFGEFRLKLLPDQFEVLGELGYVGREVIFGIRPEDLYDAMFAQ
>d1g2914 b.40.6.3 (1:302-372) Maltose transport protein MalK, C-terminal domain {Archaeon Thermococcus litoralis}
VRVPGENLVRAVVEIVENLGSERIVRLRVGGVTFVGSFRSESRVREGVEVDVVFDMKKIHIF
DKTTGKAIF
>d1b3qa2 b.40.7.1 (A:540-671) Histidine kinase CheA, C-terminal domain {Thermotoga maritima}
TLAIICALLVKVNNLVYAIPIANIDTILSISKEDIQRVQDRDVIVIRGEVIPVYRLWEVLQI
EHKEELEEMEAVIVRVGNRKYGIVVDDLLGQDDIVIKSLGKVFSEVKEFSGAAILGDGSIAL
IINVSGIV
>d1k0sa_ b.40.7.1 (A:) Chemotaxis protein CheW {Thermotoga maritima}
MKTLADALKEFEVLSFEIDEQALAFDVDNIEMVIEKSDITPVPKSRHFVEGVINLRGRIIPV
VNLAKILGISFDEQKMKSIIVARTKDVEVGFLVDRVLGVLRITENQLDLTNVSDKFGKKSKG
LVKTDGRLIIYLDIDKIIEEITVKEGV
>d1dxrh1 b.41.1.1 (H:37-258) Photosynthetic reaction centre {Rhodopseudomonas viridis}
RREGYPLVEPLGLVKLAPEDGQVYELPYPKTFVLPHGGTVTVPRRRPETRELKLAQTDGFEG
APLQPTGNPLVDAVGPASYAERAEVVDATVDGKAKIVPLRVATDFSIAEGDVDPRGLPVVAA
DGVEAGTVTDLWVDRSEHYFRYLELSVAGSARTALIPLGFCDVKKDKIVVTSILSEQFANVP
RLQSRDQITLREEDKVSAYYAGGLLYATPERAESLL
>d1qovh1 b.41.1.1 (H:36-250) Photosynthetic reaction centre {Rhodobacter sphaeroides}
MREGYPLENEDGTPAANQGPFPLPKPKTFILPHGRGTLTVPGPESEDRPIALARTAVSEGFP
HAPTGDPMKDGVGPASWVARRDLPELDGHGHNKIKPMKAAAGFHVSAGKNPIGLPVRGCDLE
IAGKVVDIWVDIPEQMARFLEVELKDGSTRLLPMQMVKVQSNRVHVNALSSDLFAGIPTIKS
PTEVTLLEEDKICGYVAGGLMYAAPKRKS
>d1eysh1 b.41.1.1 (H:59-259) Photosynthetic reaction centre {Thermochromatium tepidum}
PDLPDPKTFVLPHNGGTVVAPRVEAPVAVNATPFSPAPGSPLVPNGDPMLSGFGPAASPDRP
KHCDLTFEGLPKIVPMRVAKEFSIAEGDPDPRGMTVVGLDGEVAGTVSDVWVDRSEPQIRYL
EVEVAANKKKVLLPIGFSRFDKKARKVKVDAIKAAHFANVPTLSNPDQVTLYEEDKVCAYYA
GGKLYATAERAGPLL
>d1bfg__ b.42.1.1 (-) Basic FGF (FGF2) {Human (Homo sapiens)}
DPKRLYCKNGGFFLRIHPDGRVDGVREKSDPHIKLQLQAEERGVVSIKGVSANRYLAMKEDG
RLLASKSVTDECFFFERLESNNYNTYRSRKYTSWYVALKRTGQYKLGSKTGPGQKAILFLPM
SA
>d1bla__ b.42.1.1 (-) Basic FGF (FGF2) {Human (Homo sapiens)}
MAEGEITTLPALPEDGGSGAFPPGHFKDPKRLYCKNGGFFLRIHPDGRVDGVREKSDPHIKL
QLQAEERGVVSIKGVSANRYLAMKEDGRLLASKSVTDECFFFERLESNNYNTYRSRKYTSWY
VALKRTGQYKLGSKTGPGQKAILFLPMSAKS
>d1bara_ b.42.1.1 (A:) Acidic FGF (FGF1) {Cow (Bos taurus)}

PKLLYCSNGGYFLRILPDGTVDGTKDRSDQHIQLQLAAESIGEVYIKSTETGQFLAMDTDGL
LYGSQTPNEECLFLERLEENGYNTYISKKHAEKHWFVGLKKNGRSKLGPRTHFGQKAILFLP
LPV
>d1jqza_ b.42.1.1 (A:) Acidic FGF (FGF1) {Human (Homo sapiens)}
HHHHFNLPPGNYKKPKLLYCSNGGHFLRILPDGTVDGTRDRSDQHIQLQLSAESVGEVYIKS
TETGQYLAMDTDGLLYGSQTPNEECLFLERLEENHYNTYISKKHAEKNWFVGLKKNGSCKRG
PRTHYGQKAILFLPLPV
>d2afga_ b.42.1.1 (A:) Acidic FGF (FGF1) {Human (Homo sapiens)}
KPKLLYCSNGGHFLRILPDGTVDGTRDRSDQHIQLQLSAESVGEVYIKSTETGQYLAMDTDG
LLYGSQTPNEECLFLERLEENHYNTYISKKHAEKNWFVGLKKNGSCKRGPRTHYGQKAILFL
PLPVS
>d1fmms_ b.42.1.1 (S:) Acidic FGF (FGF1) {Eastern newt (Notophthalmus viridescens)}
QKPKLLYCSNGGYFLRIFPDGKVDGTRDRSDPYIQLQFYAESVGEVYIKSLETGQYLAMDSD
GQLYASQSPSEECLFLERLEENNYNTYKSKVHADKDWFVGIKKNGKTKPGSRTHFGQKAILF
LPLPVSSD
>d1ijta_ b.42.1.1 (A:) Fibroblast growth factor 4 (FGF4) {Human (Homo sapiens)}
GIKRLRRLYCNVGIGFHLQALPDGRIGGAHADTRDSLLELSPVERGVVSIFGVASRFFVAMS
SKGKLYGSPFFTDECTFKEILLPNNYNAYESYKYPGMFIALGKNGKTKKGNRVSPTMKVTHF
LPRL
>d1qqka_ b.42.1.1 (A:) Keratinocyte growth factor, FGF7 {Rat (Rattus norvegicus)}
DIRVRRLFCRTQWYLRIDKRGKVKGTQEMRNSYNIMEIRTVAVGIVAIKGVESEYYLAMNKE
GKLYAKKECNEDCNFKELILENHYNTYASAKWTHSGGEMFVALNQKGLPVKGKKTKKEQKTA
HFLPMAIT
>d1qqla_ b.42.1.1 (A:) Keratinocyte growth factor, FGF7 {Rat (Rattus norvegicus)}
DIRVRRLFCRTQWYLRIDKRGKVKGTQEMRNSYNIMEIRTVAVGIVAIKGVESEYYLAMNKE
GKLYAKQTPNEECLFLERLEENHYNTYISKKHAEKNWFVGLKKNGSCKRGPRTHYGQKAILF
LPLPVSS
>d1ihka_ b.42.1.1 (A:) Fibroblast growth factor 9, FGF9 {Human (Homo sapiens)}
TDLDHLKGILRRRQLYCRTGFHLEIFPNGTIQGTRKDHSRFGILEFISIAVGLVSIRGVDSG
LYLGMNEKGELYGSEKLTQECVFREQFEENWYNTYSSNLYKHVDTGRRYYVALNKDGTPREG
TRTKRHQKFTHFLPRPVDPDKVPELYKDILSQS
>d1i1b__ b.42.1.2 (-) Interleukin-1beta {Human (Homo sapiens)}
VRSLNCTLRDSQQKSLVMSGPYELKALHLQGQDMEQQVVFSMSFVQGEESNDKIPVALGLKE
KNLYLSCVLKDDKPTLQLESVDPKNYPKKKMEKRFVFNKIEINNKLEFESAQFPNWYISTSQ
AENMPVFLGGTKGGQDITDFTMQFVSS
>d8i1b__ b.42.1.2 (-) Interleukin-1beta {Mouse (Mus musculus)}
QLHYRLRDEQQKSLVLSDPYELKALHLNGQNINQQVIFSMSFVQGEPSNDKIPVALGLKGKN
LYLSCVMKDGTPTLQLESVDPKQYPKKKMEKRFVFNKIEVKSKVEFESAEFPNWYISTSQAE
HKPVFLGNNSGQDIIDFTMESV

>d1ilr1_ b.42.1.2 (1:) Interleukin-1 receptor antagonist protein {Human (Homo sapiens)}
SKMQAFRIWDVNQKTFYLRNNQLVAGYLQGPNVNLEEKIDVVPIEPHALFLGIHGGKMCLSC
VKSGDETRLQLEAVNITDLSENRKQDKRFAFIRSDSGPTTSFESAACPGWFLCTAMEADQPV
SLTNMPDEGVMVTKFYFQEDE
>d2ila__ b.42.1.2 (-) Interleukin-1alpha {Human (Homo sapiens)}
NVKYNFMRIIKYEFILNDALNQSIIRANAQYLTAAALHNLDEAVKFDMGAYKSSKDDAKITV
ILRISKTQLYVTAQDEDQPVLLKEMPEIPKTITGSETNLLFFWETHGTKNYFTSVAHPNLFI
ATKQDYWVCLAGGPPSITDFQILE
>d2aaib1 b.42.2.1 (B:1-135) Plant cytotoxin B-chain (lectin) {Castor bean (Ricinus communis), Ricin}
ADVCMDPEPIVRIVGRNGLCVDVRDGRFHNGNAIQLWPCKSNTDANQLWTLKRDNTIRSNGK
CLTTYGYSPGVYVMIYDCNTAATDATRWQIWDNGTIINPRSSLVLAATSGNSGTTLTVQTNI
YAVSQGWLPTN
>d2aaib2 b.42.2.1 (B:136-262) Plant cytotoxin B-chain (lectin) {Castor bean (Ricinus communis), Ricin}
NTQPFVTTIVGLYGLCLQANSGQVWIEDCSSEKAEQQWALYADGSIRPQQNRDNCLTSDSNI
RETVVKILSCGPASSGQRWMFKNDGTILNLYSGLVLDVRASDPSLKQIILYPLHGDPNQIWL
PLF
>d1abrb1 b.42.2.1 (B:1-140) Plant cytotoxin B-chain (lectin) {Abrus precatorius}
IVEKSKICSSRYEPTVRIGGRDGMCVDVYDNGYHNGNRIIMWKCKDRLEENQLWTLKSDKTI
RSNGKCLTTYGYAPGSYVMIYDCTSAVAEATYWEIWDNGTIINPKSALVLSAESSSMGGTLT
VQTNEYLMRQGWRTGN
>d1abrb2 b.42.2.1 (B:141-267) Plant cytotoxin B-chain (lectin) {Abrus precatorius}
NTSPFVTSISGYSDLCMQAQGSNVWMADCDSNKKEQQWALYTDGSIRSVQNTNNCLTSKDHK
QGSTILLMGCSNGWASQRWVFKNDGSIYSLYDDMVMDVKGSDPSLKQIILWPYTGKPNQIWL
TLF
>d1ce7b1 b.42.2.1 (B:1-133) Plant cytotoxin B-chain (lectin) {European mistletoe (Viscum album)}
CSASEPTVRIVGRNGMNVDVRDDDFHDGNQIQLWPSKSNNDPNQLWTIKRDGTIRSNGSCLT
TYGYTAGVYVMIFDCATAVGEATVWQIWGNGTIINPRSNLVLAASSGIKGTTLTVQTLDYTL
GQGWLAGND
>d1ce7b2 b.42.2.1 (B:134-255) Plant cytotoxin B-chain (lectin) {European mistletoe (Viscum album)}
TAPREVTIYGFNDLCMESGGGSVTVETCSSGKADKWALYGDGSIRPEQNQAQCLTSGGDSVA
GVNIVSCSGAASGQRWVFTNEGAILNLKNGLAMDVANPGGGRIIIYPATGKPNQMWLPVF
>d1hwmb1 b.42.2.1 (B:3-135) Plant cytotoxin B-chain (lectin) {Sambucus ebulus, ebulin}
ETCAIPAPFTRRIVGRDGLCVDVRNGYDTDGTPIQLWPCGTQRNQQWTFYNDKTIRSMGKCM
TANGLNSGSYIMITDCSTAAEDATKWEVLIDGSIINPSSGLVMTAPSGASRTTLLLENNIHA
ASQGWTVSN
>d1hwmb2 b.42.2.1 (B:136-266) Plant cytotoxin B-chain (lectin)

{Sambucus ebulus, ebulin}
DVQPIATLIVGYNEMCLQANGENNNVWMEDCDVTSVQQQWALFDDRTIRVNNSRGLCVTSNG
YVSKDLIVIRKCQGLATQRWFFNSDGSVVNLKSTRVMDVKESDVSLQEVIIFPATGNPNQQW
RTQVPQI
>d1xyfa1 b.42.2.1 (A:313-436) Endo-1,4-beta-xylanase C-terminal domain {Streptomyces olivaceoviridis}
GQIKGVGSGRCLDVPNASTTDGTQVQLYDCHSATNQQWTYTDAGELRVYGDKCLDAAGTGNG
TKVQIYSCWGGDNQKWRLNSDGSIVGVQSGLCLDAVGGGTANGTLIQLYSCSNGSNQRWTRT
>d1dqga_ b.42.2.2 (A:) Mannose receptor {Mouse (Mus musculus)}
DARQFLIYNEDHKRCVDALSAISVQTATCNPEAESQKFRWVSDSQIMSVAFKLCLGVPSKTD
WASVTLYACDSKSEYQKWECKNDTLFGIKGTELYFNYGNRQEKNIKLYKGSGLWSRWKVYGT
TDDLCSRGYE
>d1jlxa1 b.42.3.1 (A:1-153) Agglutinin {Love-lies-bleeding (Amaranthus caudatus)}
AGLPVIMCLKSNNHQKYLRYQSDNIQQYGLLQFSADKILDPLAQFEVEPSKTYDGLVHIKSR
YTNKYLVRWSPNHYWITASANEPDENKSNWACTLFKPLYVEEGNMKKVRLLHVQLGHYTQNY
TVGGSFVSYLFAESSQIDTGSKDVFHVID
>d1jlxa2 b.42.3.1 (A:154-299) Agglutinin {Love-lies-bleeding (Amaranthus caudatus)}
WKSIFQFPKGYVTFKGNNGKYLGVITINQLPCLQFGYDNLNDPKVAHQMFVTSNGTICIKSN
YMNKFWRLSTDDWILVDGNDPRETNEAAALFRSDVHDFNVISLLNMQKTWFIKRFTSGKPGF
INCMNAATQNVDETAILEIIEL
>d1wba__ b.42.4.1 (-) Winged bean albumin 1 {Goa bean (Psophocarpus tetragonolobus)}
DDPVYDAEGNKLVNRGKYTIVSFSDGAGIDVVATGNENPEDPLSIVKSTRNIMYATSISSED
KTPPQPRNILENMRLKINFATDPHKGDVWSVVDFQPDGQQLKLAGRYPNQVKGAFTIQKGSN
TPRTYKLLFCPVGSPCKNIGISTDPEGKKRLVVSYQSDPLVVKFHRH
>d1tie__ b.42.4.1 (-) Erythrina cafra trypsin inhibitor {Erythrina caffra}
VLLDGNGEVVQNGGTYYLLPQVWAQGGGVQLAKTGEETCPLTVVQSPNELSDGKPIRIESRL
RSAFIPDDDKVRIGFAYAPKCAPSPWWTVVEDEQEGLSVKLSEDESTQFDYPFKFEQVSDQL
HSYKLLYCEGKHEKCASIGINRDQKGYRRLVVTEDYPLTVVLKKDE
>d1eyla_ b.42.4.1 (A:) chymotrypsin inhibitor WCI {Winged bean (Psophocarpus tetragonolobus)}
EFDDDLVDAEGNLVENGGTYYLLPHIWAHGGGIETAKTGNEPCPLTVVRSPNEVSKGEPIRI
SSQFLSLFIPRGSLVALGFANPPSCAASPWWTVVDSPQGPAVKLSQQKLPEKDILVFKFEKV
SHSNIHVYKLLYCQHDEEDVKCDQYIGIHRDRNGNRRLVVTEENPLELVLLKAKS
>d1avwb_ b.42.4.1 (B:) Soybean trypsin inhibitor {Soybean (Glycine max)}
DFVLDNEGNPLENGGTYYILSDITAFGGIRAAPTGNERCPLTVVQSRNELDKGIGTIISSPY
RIRFIAEGHPLSLKFDSFAVIMLCVGIPTEWSVVEDLPEGPAVKIGENKDAMDGWFRLERVS
EFNNYKLVFCPQDKCGDIGISIDHDDGTRRLVVSKNKPLVVQFQKLD
>d1avac_ b.42.4.1 (C:) Amylase/subtilisin inhibitor {Barley (Hordeum vulgare), seed}

ADPPPVHDTDGHELRADANYYVLSANRAHGGGLTMAPGHGRHCPLFVSQDPNGQHDGFPVRI
TPYGVAPSDKIIRLSTDVRISFRAYTTCLQSTEWHIDSELAAGRRHVITGPVKDPSPSGREN
AFRIEKYSGAEVHEYKLMSCGDWCQDLGVFRDLKGGAWFLGATEPYHVVVFKKAPPA
>d1a8d_2 b.42.4.2 (248-452) Tetanus neurotoxin {Clostridium tetani}
ITFLRDFWGNPLRYDTEYYLIPVASSSKDVQLKNITDYMYLTNAPSYTNGKLNIYYRRLYNG
LKFIIKRYTPNNEIDSFVKSGDFIKLYVSYNNNEHIVGYPKDGNAFNNLDRILRVGYNAPGI
PLYKKMEAVKLRDLKTYSVQLKLYDDKNASLGLVGTHNGQIGNDPNRDILIASNWYFNHLKD
KILGCDWYFVPTDEGWTND
>d3btaa2 b.42.4.2 (A:1079-1295) Botulinum neurotoxin {Clostridium botulinum, serotype A}
NEKEIKDLYDNQSNSGILKDFWGDYLQYDKPYYMLNLYDPNKYVDVNNVGIRGYMYLKGPRG
SVMTTNIYLNSSLYRGTKFIIKKYASGNKDNIVRNNDRVYINVVVKNKEYRLATNASQAGVE
KILSALEIPDVGNLSQVVVMKSKNDQGITNKCKMNLQDNNGNDIGFIGFHQFNNIAKLVASN
WYNRQIERSSRTLGCSWEFIPVDDGWGERPL
>d1epwa2 b.42.4.2 (A:1080-1290) Botulinum neurotoxin {Clostridium botulinum, serotype B}
SEYLKDFWGNPLMYNKEYYMFNAGNKNSYIKLKKDSPVGEILTRSKYNQNSKYINYRDLYIG
EKFIIRRKSNSQSINDDIVRKEDYIYLDFFNLNQEWRVYTYKYFKKEEEKLFLAPISDSDEF
YNTIQIKEYDEQPTYSCQLLFKKDEESTDEIGLIGIHRFYESGIVFEEYKDYFCISKWYLKE
VKRKPYNLKLGCNWQFIPKDEGWTE
>d1dfca1 b.42.5.1 (A:1008-1140) Fascin {Human (Homo sapiens)}
EAVQIQFGLINCGNKYLTAEAFGFKVNASASSLKKKQIWTLEQPPDEAGSAAVCLRSHLGRY
LAADKDGNVTCEREVPGPDCRFLIVAHDDGRWSLQSEAHRRYFGGTEDRLSCFAQTVSPAEK
WSVHIAMHP
>d1dfca2 b.42.5.1 (A:1141-1259) Fascin {Human (Homo sapiens)}
QVNIYSVTRKRYAHLSARPADEIAVDRDVPWGVDSLITLAFQDQRYSVQTADHRFLRHDGRL
VARPEPATGYTLEFRSGKVAFRDCEGRYLAPSGPSGTLKAGKATKVGKDELFALEQS
>d1dfca3 b.42.5.1 (A:1260-1382) Fascin {Human (Homo sapiens)}
CAQVVLQAANERNVSTRQGMDLSANQDEETDQETFQLEIDRDTKKCAFRTHTGKYWTLTATG
GVQSTASSKNASCYFDIEWRDRRITLRASNGKFVTSKKNGQLAASVETAGDSELFLMKLIN
>d1dfca4 b.42.5.1 (A:1383-1493) Fascin {Human (Homo sapiens)}
RPIIVFRGEHGFIGCRKVTGTLDANRSSYDVFQLEFNDGAYNIKDSTGKYWTVGSDSAVTSS
GDTPVDFFFEFCDYNKVAIKVGGRYLKGDHAGVLKASAETVDPASLWEY
>d1hcd__ b.42.5.2 (-) Histidine-rich actin-binding protein (hisactophilin) {Dictyostelium discoideum}
MGNRAFKSHHGHFLSAEGEAVKTHHGHHDHHTHFHVENHGGKVALKTHCGKYLSIGDHKQVY
LSHHLHGDHSLFHLEHHGGKVSIKGHHHHYISADHHGHVSTKEHHDHDTTFEEIII
>d1i8da1 b.43.4.3 (A:1-93) Riboflavin synthase {Escherichia coli}
MFTGIVQGTAKLVSIDEKPNFRTHVVELPDHMLDGLETGASVAHNGCCLTVTEINGNHVSFD
LMKETLRITNLGDLKVGDWVNVERAAKFSDE
>d1i8da2 b.43.4.3 (A:94-206) Riboflavin synthase {Escherichia coli}
IGGHLMSGHIMTTAEVAKILTSENNRQIWFKVQDSQLMKYILYKGFIGIDGISLTVGEVTPT

RFCVHLIPETLERTTLGKKKLGARVNIEIDPQTQAVVDTVERVLAARENAM
>d1fnc_1 b.43.4.2 (19-154) Ferredoxin reductase (flavodoxin reductase) N-terminal domain {Spinach (Spinacia oleracea)}
HSKKMEEGITVNKFKPKTPYVGRCLLNTKITGDDAPGETWHMVFSHEGEIPYREGQSVGVIP
DGEDKNGKPHKLRLYSIASSALGDFGDAKSVSLCVKRLIYTNDAGETIKGVCSNFLCDLKPG
AEVKLTGPVGKE
>d1qfza1 b.43.4.2 (A:1-153) Ferredoxin reductase (flavodoxin reductase) N-terminal domain {Garden pea (Pisum sativum)}
QVTTEAPAKVVKHSKKQDENIVVNKFKPKEPYVGRCLLNTKITGDDAPGETWHMVFSTEGEV
PYREGQSIGIVPDGIDKNGKPHKLRLYSIASSAIGDFGDSKTVSLCVKRLVYTNDAGEVVKG
VCSNFLCDLKPGSEVKITGPVGKEMLMPK
>d1fb3a1 b.43.4.2 (A:67-207) Ferredoxin reductase (flavodoxin reductase) N-terminal domain {Paprika (Capsicum annuum)}
ISKKQDEGVVVNKFRPKEPYIGRCLLNTKITGDDAPGETWHMVFSTEGEIPYREGQSIGVIA
DGVDANGKPHKLRLYSIASSALGDFGDSKTVSLCVKRLVYTNDKGEEVKGVCSNFLCDLKPG
ADVKITGPVGKEMLMPK
>d1gawa1 b.43.4.2 (A:11-156) Ferredoxin reductase (flavodoxin reductase) N-terminal domain {Maize (Zea mays), leaf isoform}
PATAKAKKESKKQEEGVVTNLYKPKEPYVGRCLLNTKITGDDAPGETWHMVFSTEGKIPYRE
GQSIGVIADGVDKNGKPHKVRLYSIASSAIGDFGDSKTVSLCVKRLIYTNDAGEIVKGVCSN
FLCDLQPGDNVQITGPVGKEML
>d1jb9a1 b.43.4.2 (A:6-162) Ferredoxin reductase (flavodoxin reductase) N-terminal domain {Maize (Zea mays), root isoform}
SRSKVSVAPLHLESAKEPPLNTYKPKEPFTATIVSVESLVGPKAPGETCHIVIDHGGNVPYW
EGQSYGVIPPGENPKKPGAPQNVRLYSIASTRYGDNFDGRTGSLCVRRAVYYDPETGKEDPS
KNGVCSNFLCNSKPGDKIQLTGPSGKIMLLPEE
>d1que_1 b.43.4.2 (1-141) Ferredoxin reductase (flavodoxin reductase) N-terminal domain {Cyanobacterium (Anabaena sp.), pcc 7119}
TQAKAKHADVPVNLYRPNAPFIGKVISNEPLVKEGGIGIVQHIKFDLTGGNLKYIEGQSIGI
IPPGVDKNGKPEKLRLYSIASTRHGDDVDDKTISLCVRQLEYKHPESGETVYGVCSTYLTHI
EPGSEVKITGPVGKEML
>d1fdr_1 b.43.4.2 (2-100) Ferredoxin reductase (flavodoxin reductase) N-terminal domain {Escherichia coli}
ADWVTGKVTKVQNWTDALFSLTVHAPVLPFTAGQFTKLGLEIDGERVQRAYSYVNSPDNPDL
EFYLVTVPDGKLSPRLAALKPGDEVQVVSEAAGFFVL
>d1a8p_1 b.43.4.2 (2-100) Ferredoxin reductase (flavodoxin reductase) N-terminal domain {Azotobacter vinelandii}
SNLNVERVLSVHHWNDTLFSFKTTRNPSLRFENGQFVMIGLEVDGRPLMRAYSIASPNYEEH
LEFFSIKVQNGPLTSRLQHLKEGDELMVSRKPTGTLV
>d1qfja1 b.43.4.2 (A:1-97) NAD(P)H:flavin oxidoreductase {Escherichia coli}
TTLSCKVTSVEAITDTVYRVRIVPDAAFSFRAGQYLMVVMDERDKRPFSMASTPDEKGFIEL
HIGASEINLYAKAVMDRILKDHQIVVDIPHGEAWL

>d2cnd_1 b.43.4.2 (11-124) Nitrate reductase core domain {Corn (Zea mays)}
GRIHCRLVAKKELSRDVRLFRFSLPSPDQVLGLPIGKHIFVCATIEGKLCMRAYTPTSMVDE
IGHFDLLVKVYFKNEHPKFPNGGLMTQYLDSLPVGSYIDVKGPLGHVEYTGR
>d1ndh_1 b.43.4.2 (3-125) cytochrome b5 reductase {Pig (Sus scrofa), liver}
PAITLENPDIKYPLRLIDKEVVNHDTRRFRFALPSPEHILGLPVGQHIYLSARIDGNLVIRP
YTPVSSDDDKGFVDLVIKVYFKDTHPKFPAGGKMSQYLESMKIGDTIEFRGPNGLLVYQGK
>d1i7pa1 b.43.4.2 (A:29-153) cytochrome b5 reductase {Rat (Rattus norvegicus)}
HHHMITLENPDIKYPLRLIDKEILSHDTRRFRFALPSPQHILGLPIGQHIYLSTRIDGNLVI
RPYTPVSSDDDKGFVDLVVKVYFKETHPKFPAGGKMSQYLENMNIGDTIEFRGPNGLLVYQG
K
>d2pia_1 b.43.4.2 (1-103) Phthalate dioxygenase reductase {Pseudomonas cepacia, db01}
TTPQEDGFLRLKIASKEKIARDIWSFELTDPQGAPLPPFEAGANLTVAVPNGSRRTYSLCND
SQERNRYVIAVKRDSNGRGGSISFIDDTSEGDAVEVSLPRN
>d1ep3b1 b.43.4.2 (B:2-102) Dihydroorotate dehydrogenase B, PyrK subunit {Lactococcus lactis, isozyme B}
SQLQEMMTVVSQREVAYNIFEMVLKGTLVDEMDLPGQFLHLAVPNGAMLLRRPISISSWDKR
AKTCTILYRIGDETTGTYKLSKLESGAKVDVMGPLGNGF
>d1cqxa2 b.43.4.2 (A:151-261) Flavohemoglobin, central domain {Alcaligenes eutrophus}
WKGWRTFVIREKRPESDVITSFILEPADGGPVVNFEPGQYTSVAIDVPALGLQQIRQYSLSD
MPNGRTYRISVKREGGGPQPPGYVSNLLHDHVNVGDQVKLAAPYGSFHI
>d1ja1a1 b.43.4.1 (A:240-518) NADPH-cytochrome p450 reductase {Rat (Rattus norvegicus)}
SSIRQYELVVHEDMDVAKVYTGEMGRLKSYENQKPPFDAKNPFLAAVTANRKLNQGTERHLM
HLELDISDSKIRYESGDHVAVYPANDSALVNQIGEILGADLDVIMSLNNLDEESNKKHPFPC
PTTYRTALTYYLDITNPPRTNVLYELAQYASEPSEQEHLHKMASSSGEGKELYLSWVVEARR
HILAILQDYPSLRPPIDHLCELLPRLQARYYAIASSSKVHPNSVHICAVAVEYEAKSGRVNK
GVATSWLRAKEPAGENGGRALVPMFVRKSQF
>d1ddga1 b.43.4.1 (A:226-446) Sulfite reductase flavoprotein {Escherichia coli}
IHTSPYSKDAPLVASLSVNQKITGRNSEKDVRHIEIDLGDSGLRYQPGDALGVWYQNDPALV
KELVELLWLKGDEPVTVEGKTLPLNEALQWHFELTVNTANIVENYATLTRSETLLPLVGDKA
KLQHYAATTPIVDMVRFSPAQLDAEALINLLRPLTPRLYSIASSQAEVENEVHVTVGVVRYD
VEGRARAGGASSFLADRVEEEGEVRVFIEHNDNFR
>d1f20a1 b.43.4.1 (A:963-1232) Neuronal nitric-oxide synthase FAD/NADP+ domain {Rat (Rattus norvegicus)}
SWKRNKFRLTYVAEAPDLTQGLSNVHKKRVSAARLLSRQNLQSPKSSRSTIFVRLHTNGNQE
LQYQPGDHLGVFPGNHEDLVNALIERLEDAPPANHVVKVEMLEERNTALGVISNWKDESRLP
PCTIFQAFKYYLDITTPPTPLQLQQFASLATNEKEKQRLLVLSKGLQEYEEWKWGKNPTMVE
VLEEFPSIQMPATLLLTQLSLLQPRYYSISSSPDMYPDEVHLTVAIVSYHTRDGEGPVHHGV

CSSWLNRIQADDVVPCFVRGAP
>d1fuia1 b.43.2.1 (A:356-591) L-fucose isomerase, C-terminal domain {Escherichia coli}
AQVFADVRTYWSPEAIERVTGHKLDGLAEHGIIHLINSGSAALDGSCKQRDSEGNPTMKPHW
EISQQEADACLAATEWCPAIHEYFRGGGYSSRFLTEGGVPFTMTRVNIIKGLGPVLQIAEGW
SVELPKDVHDILNKRTNSTWPTTWFAPRLTGKGPFTDVYSVMANWGANHGVLTIGHVGADFI
TLASMLRIPVCMHNVEETKVYRPSAWAAHGMDIEGQDYRACQNYGPLYKR
>d1efca1 b.43.3.1 (A:205-296) Elongation factor Tu (EF-Tu), domain 2 {Escherichia coli}
AIDKPFLLPIEDVFSISGRGTVVTGRVERGIIKVGEEVEIVGIKETQKSTCTGVEMFRKLLD
EGRAGENVGVLLRGIKREEIERGQVLAKPG
>d1exma1 b.43.3.1 (A:213-312) Elongation factor Tu (EF-Tu), domain 2 {Thermus thermophilus}
PVRDVDKPFLMPVEDVFTITGRGTVATGRIERGKVKVGDEVEIVGLAPETRKTVVTGVEMHR
KTLQEGIAGDNVGVLLRGVSREEVERGQVLAKPGSITP
>d1d2ea1 b.43.3.1 (A:251-348) Elongation factor Tu (EF-Tu), domain 2 {Cow (Bos taurus), mitochondrial}
TRDLEKPFLLPVESVYSIPGRGTVVTGTLERGILKKGDECEFLGHSKNIRTVVTGIEMFHKS
LDRAEAGDNLGALVRGLKREDLRRGLVMAKPGSIQP
>d1f60a1 b.43.3.1 (A:241-334) Elongation factor eEF-1alpha, domain 2 {Baker's yeast (Saccharomyces cerevisiae)}
DKPLRLPLQDVYKIGGIGTVPVGRVETGVIKPGMVVTFAPAGVTTEVKSVEMHHEQLEQGVP
GDNVGFNVKNVSVKEIRRGNVCGDAKNDPPKG
>d1jnya1 b.43.3.1 (A:228-322) Elongation factor eEF-1alpha, domain 2 {Archaeon Sulfolobus solfataricus}
PVDKPLRIPIQDVYSISGVGTVPVGRVESGVLKVGDKIVFMPAGKVGEVRSIETHHTKMDKA
EPGDNIGFNVRGVEKKDIKRGDVVGHPNNPPTV
>d1dar_1 b.43.3.1 (283-400) Elongation factor G (EF-G), domain II {Thermus thermophilus}
PLDIPPIKGTTPEGEVVEIHPDPNGPLAALAFKIMADPYVGRLTFIRVYSGTLTSGSYVYNT
TKGRKERVARLLRMHANHREEVEELKAGDLGAVVGLKETITGDTLVGEDAPRVILE
>d1g7sa1 b.43.3.1 (A:228-328) Initiation factor IF2/eIF5b, domains 2 and 4 {Archaeon Methanobacterium thermoautotrophicum}
EDSPARGTILEVKEETGLGMTIDAVIYDGILRKDDTIAMMTSKDVISTRIRSLLKPRPLEEM
RESRKKFQKVDEVVAAAGIKIVAPGIDDVMAGSPLRVVT
>d1g7sa2 b.43.3.1 (A:460-587) Initiation factor IF2/eIF5b, domains 2 and 4 {Archaeon Methanobacterium thermoautotrophicum}
IIKPASIRLIPKLVFRQSKPAIGGVEVLTGVIRQGYPLMNDDGETVGTVESMQDKGENLKSA
SRGQKVAMAIKDAVYGKTIHEGDTLYVDIPENHYHILKEQLSGDLTDEELDLMDKIAEIKRK
KNPD
>d1d1na_ b.43.3.1 (A:) Initiation factor IF2/eIF5b, domains 2 and 4 {Bacillus stearothermophilus}
YEEKVIGQAEVRQTFKVSKVGTIAGCYVTDGKITRDSKVRLIRQGIVVYEGEIDSLKRYKDD
VREVAQGYECGLTIKNFNDIKEGDVIEAYVMQEVARA

>d1jj2b_ b.43.3.2 (B:) Ribosomal protein L3 {Archaeon Haloarcula marismortui}
PQPSRPRKGSLGFGPRKRSTSETPRFNSWPSDDGQPGVQGFAGYKAGMTHVVLVNDEPNSPR
EGMEETVPVTVIETPPMRAVALRAYEDTPYGQRPLTEVWTDEFHSELDRTLDVPEDHDPDAA
EEQIRDAHEAGDLGDLRLITHTVPDAVPSVPKKKPDVMETRVGGGSVSDRLDHALDIVEDGG
EHAMNDIFRAGEYADVAGVTKGKGTQGPVKRWGVQKRKGKHARQGWRRRIGNLGPWNPSRVR
STVPQQGQTGYHQRTELNKRLIDIGEGDEPTVDGGFVNYGEVDGPYTLVKGSVPGPDKRLVR
FRPAVRPNDQPRLDPEVRYVSNESNQG
>d1efca2 b.44.1.1 (A:297-393) Elongation factor Tu (EF-Tu) {Escherichia coli}
TIKPHTKFESEVYILSKDEGGRHTPFFKGYRPQFYFRTTDVTGTIELPEGVEMVMPGDNIKM
VVTLIHPIAMDDGLRFAIREGGRTVGAGVVAKVLS
>d1exma2 b.44.1.1 (A:313-405) Elongation factor Tu (EF-Tu) {Thermus thermophilus}
HTKFEASVYVLKKEEGGRHTGFFSGYRPQFYFRTTDVTGVVQLPPGVEMVMPGDNVTFTVEL
IKPVALEEGLRFAIREGGRTVGAGVVTKILE
>d1d2ea2 b.44.1.1 (A:349-451) Elongation factor Tu (EF-Tu) {Cow (Bos taurus), mitochondrial}
HQKVEAQVYILTKEEGGRHKPFVSHFMPVMFSLTWDMACRIILPPGKELAMPGEDLKLTLIL
RQPMILEKGQRFTLRDGNRTIGTGLVTDTPAMTEEDKNIKW
>d1f60a2 b.44.1.1 (A:335-441) Elongation factor eEF-1alpha, C-terminal domain {Baker's yeast (Saccharomyces cerevisiae)}
CASFNATVIVLNHPGQISAGYSPVLDCHTAHIACRFDELLEKNDRRSGKKLEDHPKFLKSGD
AALVKFVPSKPMCVEAFSEYPPLGRFAVRDMRQTVAVGVIKSVDK
>d1jnya2 b.44.1.1 (A:323-429) Elongation factor eEF-1alpha, C-terminal domain {Archaeon Sulfolobus solfataricus}
ADEFTARIIVVWHPTALANGYTPVLHVHTASVACRVSELVSKLDPRTGQEAEKNPQFLKQGD
VAIVKFKPIKPLCVEKYNEFPPLGRFAMRDMGKTVGVGIIVDVKP
>d1flma_ b.45.1.1 (A:) FMN-binding protein {Desulfovibrio vulgaris, strain Miyazaki F}
MLPGTFFEVLKNEGVVAIATQGEDGPHLVNTWNSYLKVLDGNRIVVPVGGMHKTEANVARDE
RVLMTLGSRKVAGRNGPGTGFLIRGSAAFRTDGPEFEAIARFKWARAALVITVVSAEQTL
>d1ci0a_ b.45.1.1 (A:) Pyridoxine 5'-phoshate oxidase (PNP oxidase) {Baker's yeast (Saccharomyces cerevisiae)}
FTLNEKQLTDDPIDLFTKWFNEAKEDPRETLPEAITFSSAELPSGRVSSRILLFKELDHRGF
TIYSNWGTSRKAHDIATNPNAAIVFFWKDLQRQVRVEGITEHVNRETSERYFKTRPRGSKIG
AWASRQSDVIKNREELDELTQKNTERFKDAEDIPCPDYWGGLRIVPLEIEFWQGRPSRLHDR
FVYRRKTENDPWKVVRLAP
>d1dnla_ b.45.1.1 (A:) Pyridoxine 5'-phoshate oxidase (PNP oxidase) {Escherichia coli}
GGLRRRDLPADPLTLFERWLSQACEAKLADPTAMVVATVDEHGQPYQRIVLLKHYDEKGMVF
YTNLGSRKAHQIENNPRVSLLFPWHTLERQVMVIGKAERLSTLEVMKYFHSRPRDSQIGAWV
SKQSSRISARGILESKFLELKQKFQQGEVPLPSFWGGFRVSLEQIEFWQGGEHRLHDRFLYQ
RENDAWKIDRLAP

>d1ejea_ b.45.1.2 (A:) FMN-binding protein MTH152 {Archaeon Methanobacterium thermoautotrophicum}
GSQAAHMMSMDFEDFPVESAHRILTPRPTVMVTTVDEEGNINAAPFSFTMPVSIDPPVVAFA
SAPDHHTARNIESTHEFVINITPADIIERMWVTARDIPAGENELEAAGLAWTSSRRVKPPRI
VEAPGHLECELLRMFEVGDHNLITGSVVSASVRSGAVKEGLLDVESVKPVLHVGGNKFVVGD
HVRHVE
>d1i0ra_ b.45.1.2 (A:) Ferric reductase {Archaeon Archaeoglobus fulgidus}
MDVEAFYKISYGLYIVTSESNGRKCGQIANTVFQLTSKPVQIAVCLNKENDTHNAVKESGAF
GVSVLELETPMEFIGRFGFRKSSEFEKFDGVEYKTGKTGVPLVTQHAVAVIEAKVVKECDVG
THTLFVGEAVDAEVLKDAEVLTYADYHLMKKGKTPRT
>d1k28d1 b.106.1.1 (D:4-200) Baseplate structural protein gp27 {Bacteriophage T4}
LQRPGYPNLSVKLFDSYDAWSNNRFVELAATITTLTMRDSLYGRNEGMLQFYDSKNIHTKMD
GNEIIQISVANANDINNVKTRIYGCKHFSVSVDSKGDNIIAIELGTIHSIENLKFGRPFFPD
AGESIKEMLGVIYQDRTLLTPAINAINAYVPDIPWTSTFENYLSYVREVALAVGSDKFVFVW
QDIMGVNMMDY
>d1k28d2 b.106.1.1 (D:201-376) Baseplate structural protein gp27 {Bacteriophage T4}
DMMINQEPYPMIVGEPSLIGQFIQELKYPLAYDFVWLTKSNPHKRDPMKNATIYAHSFLDSS
IPMITTGKGENSIVVSRSGAYSEMTYRNGYEEAIRLQTMAQYDGYAKCSTIGNFNLTPGVKI
IFNDSKNQFKTEFYVDEVIHELSNNNSVTHLYMFTNATKLETIDPVKVKNEF
>d1fmta1 b.46.1.1 (A:207-314) Methionyl-tRNAfmet formyltransferase, C-terminal domain {Escherichia coli}
LSKEEARIDWSLSAAQLERCIRAFNPWPMSWLEIEGQPVKVWKASVIDTATNAAPGTILEAN
KQGIQVATGDGILNLLSLQPAGKKAMSAQDLLNSRREWFVPGNRLV
>d1ewna_ b.46.1.2 (A:) 3-methyladenine DNA glycosylase (AAG, ANPG, MPG) {Human (Homo sapiens)}
HLTRLGLEFFDQPAVPLARAFLGQVLVRRLPNGTELRGRIVETQAYLGPEDEAAHSRGGRQT
PRNRGMFMKPGTLYVYIIYGMYFCMNISSQGDGACVLLRALEPLEGLETMRQLRSTLRKGTA
SRVLKDRELCSGPSKLCQALAINKSFDQRDLAQDEAVWLERGPLEPSEPAVVAAARVGVGHA
GEWARKPLRFYVRGSPWVSVVDRVAEQD
>d1arb__ b.47.1.1 (-) Achromobacter protease {Achromobacter lyticus, strain m497-1}
GVSGSCNIDVVCPEGDGRRDIIRAVGAYSKSGTLACTGSLVNNTANDRKMYFLTAHHCGMGT
ASTAASIVVYWNYQNSTCRAPNTPASGANGDGSMSQTQSGSTVKATYATSDFTLLELNNAAN
PAFNLFWAGWDRRDQNYPGAIAIHHPNVAEKRISNSTSPTSFVAWGGGAGTTHLNVQWQPSG
GVTEPGSSGSPIYSPEKRVLGQLHGGPSSCSATGTNRSDQYGRVFTSWTGGGAAASRLSDWL
DPASTGAQFIDGLDS
>d1qq4a_ b.47.1.1 (A:) alpha-Lytic protease {Lysobacter enzymogenes, 495}
ANIVGGIEYSINNASLCSVGFSVTRGATKGFVTAGHCGTVNATARIGGAVVGTFAARVFPGN
DRAWVSLTSAQTLLPRVANGSSFVTVRGSTEAAVGAAVCHSGRTTGYQCGTITAKNVTANYA
EGAVRGLTQSNACMGRGDSGGSWITSAGQAQGVMSGGNVQSNGNNCGIPASQRSSLFERLQP

ILSQYGLSLVTG
>d2sga__ b.47.1.1 (-) Protease A {Streptomyces griseus, strain k1}
IAGGEAITTGGSRCSLGFNVSVNGVAHALTAGHCTNISASWSIGTRTGTSFPNNDYGIIRHS
NPAAADGRVYLYNGSYQDITTAGNAFVGQAVQRSGSTTGLRSGSVTGLNATVNYGSSGIVYG
MIQTNVCAQPGDSGGSLFAGSTALGLTSGGSGNCRTGGTTFYQPVTEALSAYGATVL
>d1hpga_ b.47.1.1 (A:) Glutamic acid-specific protease
{Streptomyces griseus}
VLGGGAIYGGGSRCSAAFNVTKGGARYFVTAGHCTNISANWSASSGGSVVGVREGTSFPTND
YGIVRYTDGSSPAGTVDLYNGSTQDISSAANAVVGQAIKKSGSTTKVTSGTVTAVNVTVNYG
DGPVYNMVRTTACSAGGDSGGAHFAGSVALGIHSGSSGCSGTAGSAIHQPVTEALSAYGVTV
Y
>d1sgt__ b.47.1.1 (-) Trypsin {Streptomyces griseus, strain k1}
VVGGTRAAQGEFPFMVRLSMGCGGALYAQDIVLTAAHCVSGSGNNTSITATGGVVDLQSGAA
VKVRSTKVLQAPGYNGTGKDWALIKLAQPINQPTLKIATTTAYNQGTFTVAGWGANREGGSQ
QRYLLKANVPFVSDAACRSAYGNELVANEEICAGYPDTGGVDTCQGDSGGPMFRKDNADEWI
QVGIVSWGYGCARPGYPGVYTEVSTFASAIASAARTL
>d2sfa__ b.47.1.1 (-) Serine proteinase {Streptomyces fradiae}
IAGGEAIYAAGGGRCSLGFNVRSSSGATYALTAGHCTEIASTWYTNSGQTSLLGTRAGTSFP
GNDYGLIRHSNASAADGRVYLYNGSYRDITGAGNAYVGQTVQRSGSTTGLHSGRVTGLNATV
NYGGGDIVSGLIQTNVCAEPGDSGGALFAGSTALGLTSGGSGNCRTGGTTFFQPVTEALSAY
GVSIL
>d1sgpe_ b.47.1.1 (E:) Protease B {Streptomyces griseus, strain
k1}
ISGGDAIYSSTGRCSLGFNVRSGSTYYFLTAGHCTDGATTWWANSARTTVLGTTSGSSFPNN
DYGIVRYTNTTIPKDGTVGGQDITSAANATVGMAVTRRGSTTGTHSGSVTALNATVNYGGGD
VVYGMIRTNVCAEPGDSGGPLYSGTRAIGLTSGGSGNCSSGGTTFFQPVTEALVAYGVSVY
>d1agja_ b.47.1.1 (A:) Epidermolytic (exfoliative) toxin A
{Staphylococcus aureus}
EVSAEEIKKHEEKWNKYYGVNAFNLPKELFSKVDEKDRQKYPYNTIGNVFVKGQTSATGVLI
GKNTVLTNRHIAKFANGDPSKVSFRPSINTDDNGNTETPYGEYEVKEILQEPFGAGVDLALI
RLKPDQNGVSLGDKISPAKIGTSNDLKDGDKLELIGYPFDHKVNQMHRSEIELTTLSRGLRY
YGFTVPGNSGSGIFNSNGELVGIHSSKVSHLDREHQINYGVGIGNYVKRIINEKNE
>d1qtfa_ b.47.1.1 (A:) Exfoliative toxin B {Staphylococcus aureus}
KEYSAEEIRKLKQKFEVPPTDKELYTHITDNARSPYNSVGTVFVKGSTLATGVLIGKNTIVT
NYHVAREAAKNPSNIIFTPAQNRDAEKNEFPTPYGKFEAEEIKESPYGQGLDLAIIKLKPNE
KGESAGDLIQPANIPDHIDIAKGDKYSLLGYPYNYSAYSLYQSQIEMFNDSQYFGYTEVGNS
GSGIFNLKGELIGIHSGKGGQHNLPIGVFFNRKISSLYSVDNTFGDTLGNDLKKRAKLDK
>d1ezxc_ b.47.1.2 (C:) Trypsin(ogen) {Cow (Bos taurus)}
CGGSLINSQWVVSAAHCYKSGIQVRLGEDNINVVEGNEQFISASKSIVHPSYNSNTLNNDIM
LIKLKSAASLNSRVASISLPTSCASAGTQCLISGWGNTKSSGTSYPDVLKCLKAPILSDSSC
KSAYPGQITSNMFCAGYLEGGKDSCQGDSGGPVVCSGKLQGIVSWGSGCAQKNKPGVYTKVC
NYVSWIKQTIASN
>d1hj9a_ b.47.1.2 (A:) Trypsin(ogen) {Cow (Bos taurus)}
IVGGYTCGANTVPYQVSLNSGYHFCGGSLINSQWVVSAAHCYKSGIQVRLGEDNINVVEGNE

QFISASKSIVHPSYNSNTLNNDIMLIKLKSAASLNSRVASISLPTSCASAGTQCLISGWGNT
KSSGTSYPDVLKCLKAPILSDSSCKSAYPGQITSNMFCAGYLEGGKDSCQGDSGGPVVCSGK
LQGIVSWGSGCAQKNKPGVYTKVCNYVSWIKQTIASN
>d1mcta_ b.47.1.2 (A:) Trypsin(ogen) {Pig (Sus scrofa)}
IVGGYTCAANSIPYQVSLNSGSHFCGGSLINSQWVVSAAHCYKSRIQVRLGEHNIDVLEGNE
QFINAAKIITHPNFNGNTLDNDIMLIKLSSPATLNSRVATVSLPRSCAAAGTECLISGWGNT
KSSGSSYPSLLQCLKAPVLSNSSCKSSYPGQITGNMICVGFLQGGKDSCQGDSGGPVVCNGQ
LQGIVSWGYGCAQKNKPGVYTKVCNYVNWIQQTIAAN
>d1f7za_ b.47.1.2 (A:) Trypsin(ogen) {Rat (Rattus norvegicus)}
IVGGYTCQENSVPYQVSLNSGYHFCGGSLINDQWVVSAAHCYKSRIQVRLGEHNINVLEGNE
QFVNAAKIIKHPNFDRKTLNNDIMLIKLSSPVKLNARVATVALPSSCAPAGTQCLISGWGNT
LSSGVNEPDLLQCLDAPLLPQADCEASYPGKITDNMVCVGFLEGGKDSCQGDSGGPVVCNGE
LQGIVSWGYGCALPDNPGVYTKVCNYVDWIQDTIAAN
>d1trna_ b.47.1.2 (A:) Trypsin(ogen) {Human (Homo sapiens)}
IVGGYNCEENSVPYQVSLNSGYHFCGGSLINEQWVVSAGHCYKSRIQVRLGEHNIEVLEGNE
QFINAAKIIRHPQYDRKTLNNDIMLIKLSSRAVINARVSTISLPTAPPATGTKCLISGWGNT
ASSGADYPDELQCLDAPVLSQAKCEASYPGKITSNMFCVGFLEGGKDSCQGDSGGPVVCNGQ
LQGVVSWGDGCAQKNKPGVYTKVYNYVKWIKNTIAANS
>d1h4wa_ b.47.1.2 (A:) Trypsin(ogen) {Human (Homo sapiens),
trypsin IV (brain isoform)}
IVGGYTCEENSLPYQVSLNSGSHFCGGSLISEQWVVSAAHCYKTRIQVRLGEHNIKVLEGNE
QFINAVKIIRHPKYNRDTLDNDIMLIKLSSPAVINARVSTISLPTAPPAAGTECLISGWGNT
LSFGADYPDELKCLDAPVLTQAECKASYPGKITNSMFCVGFLEGGKDSCQRDSGGPVVCNGQ
LQGVVSWGHGCAWKNRPGVYTKVYNYVDWIKDTIAANS
>d1a0ja_ b.47.1.2 (A:) Trypsin(ogen) {North atlantic salmon (Salmo
salar)}
IVGGYECRKNSASYQASLQSGYHFCGGSLISSTWVVSAAHCYKSRIQVRLGEHNIAVNEGTE
QFIDSVKVIMHPSYNSRNLDNDIMLIKLSKPASLNSYVSTVALPSSCASSGTRCLVSGWGNL
SGSSSNYPDTLRCLDLPILSSSSCNSAYPGQITSNMFCAGFMEGGKDSCQGDSGGPVVCNGQ
LQGVVSWGYGCAQRNKPGVYTKVCNYRSWISSTMSSN
>d1hj8a_ b.47.1.2 (A:) Trypsin(ogen) {North atlantic salmon (Salmo
salar)}
IVGGYECKAYSQPHQVSLNSGYHFCGGSLVNENWVVSAAHCYKSRVEVRLGEHNIKVTEGSE
QFISSSRVIRHPNYSSYNIDNDIMLIKLSKPATLNTYVQPVALPTSCAPAGTMCTVSGWGNT
MSSTADSNKLQCLNIPILSYSDCNNSYPGMITNAMFCAGYLEGGKDSCQGDSGGPVVCNGEL
QGVVSWGYGCAEPGNPGVYAKVCIFNDWLTSTMASY
>d1gdna_ b.47.1.2 (A:) Trypsin(ogen) {Mold (Fusarium oxysporum)}
IVGGTSASAGDFPFIVSISRNGGPWCGGSLLNANTVLTAAHCVSGYAQSGFQIRAGSLSRTS
GGITSSLSSVRVHPSYSGNNNDLAILKLSTSIPSGGNIGYARLAASGSDPVAGSSATVAGWG
ATSEGGSSTPVNLLKVTVPIVSRATCRAQYGTSAITNQMFCAGVSSGGKDSCQGDSGGPIVD
SSNTLIGAVSWGNGCARPNYSGVYASVGALRSFIDTYA
>d1pytd_ b.47.1.2 (D:) (alpha,gamma)-chymotrypsin(ogen) {Cow (Bos
taurus)}
CGAPIFQPNLSARVVGGEDAIPHSWPWQISLQYLRDNTWRHTCGGTLITPNHVLTAAHCISN

TLTYRVALGKNNLEVEDEAGSLYVGVDTIFVHEKWNSFLVRNDIALIKLAETVELGDTIQVA
CLPSEGSLLPQDYPCFVTGWGRLYTNGPIAAELQQGLQPVVDYATCSQRDWWGTTVKETMVC
AGGDGVISACNGDSGGPLNCQADGQWDVRGIVSFGSGLSCNTFKKPTVFTRVSAYIDWINQK
LQL
>g1gg6.1 b.47.1.2 (A:,B:,C:) (alpha,gamma)-chymotrypsin(ogen)
{Cow (Bos taurus)}
CGVPAIQPVLXIVNGEEAVPGSWPWQVSLQDKTGFHFCGGSLINENWVVTAAHCGVTTSDVV
VAGEFDQGSSSEKIQKLKIAKVFKNSKYNSLTINNDITLLKLSTAASFSQTVSAVCLPSASD
DFAAGTTCVTTGWGLTRYXANTPDRLQQASLPLLSNTNCKKYWGTKIKDAMICAGASGVSSC
MGDSGGPLVCKKNGAWTLVGIVSWGSSTCSTSTPGVYARVTALVNWVQQTLAAN
>d1eq9a_ b.47.1.2 (A:) (alpha,gamma)-chymotrypsin(ogen) {Red fire
ant (Solenopsis invicta)}
IVGGKDAPVGKYPYQVSLRLSGSHRCGASILDNNNVLTAAHCVDGLSNLNRLKVHVGTNYLS
ESGDVYDVEDAVVNKNYDDFLLRNDVALVHLTNPIKFNDLVQPIKLSTNDEDLESNPCTLTG
WGSTRLGGNTPNALQEIELIVHPQKQCERDQWRVIDSHICTLTKRGEGACHGDSGGPLVANG
AQIGIVSFGSPCALGEPDVYTRVSSFVSWINANLKK
>d1npma_ b.47.1.2 (A:) Neuropsin {Mouse (Mus musculus)}
ILEGRECIPHSQPWQAALFQGERLICGGVLVGDRWVLTAAHCKKQKYSVRLGDHSLQSRDQP
EQEIQVAQSIQHPCYNNSNPEDHSHDIMLIRLQNSANLGDKVKPVQLANLCPKVGQKCIISG
WGTVTSPQENFPNTLNCAEVKIYSQNKCERAYPGKITEGMVCAGSSNGADTCQGDSGGPLVC
DGMLQGITSWGSDPCGKPEKPGVYTKICRYTTWIKKTMD
>d1azza_ b.47.1.2 (A:) Crab collagenase {Atlantic sand fiddler crab
(Uca pugilator)}
IVGGVEAVPNSWPHQAALFIDDMYFCGGSLISPEWILTAAHCMDGAGFVDVVLGAHNIREDE
ATQVTIQSTDFTVHENYNSFVISNDIAVIRLPVPVTLTAAIATVGLPSTDVGVGTVVTPTGW
GLPSDSALGISDVLRQVDVPIMSNADCDAVYGIVTDGNICIDSTGGKGTCNGDSGGPLNYNG
LTYGITSFGAAAGCEAGYPDAFTRVTYFLDWIQTQTGITP
>d2hlca_ b.47.1.2 (A:) HL collagenase {Common cattle grub
(Hypoderma lineatum)}
IINGYEAYTGLFPYQAGLDITLQDQRRVWCGGSLIDNKWILTAAHCVHDAVSVVVYLGSAVQ
YEGEAVVNSERIISHSMFNPDTYLNDVALIKIPHVEYTDNIQPIRLPSGEELNNKFENIWAT
VSGWGQSNTDTVILQYTYNLVIDNDRCAQEYPPGIIVESTICGDTSDGKSPCFGDSGGPFVL
SDKNLLIGVVSFVSGAGCESGKPVGFSRVTSYMDWIQQNTGIKF
>g1h8d.1 b.47.1.2 (L:,H:) Thrombin {Human (Homo sapiens)}
EADCGLRPLFEKKSLEDKTERELLESYISXIVEGSDAEIGMSPWQVMLFRKSPQELLCGASL
ISDRWVLTAAHCLLYPPWDKNFTENDLLVRIGKHSRTRYERNIEKISMLEKIYIHPRYNWRE
NLDRDIALMKLKKPVAFSDYIHPVCLPDRETAASLLQAGYKGRVTGWGNLKETGQPSVLQVV
NLPIVERPVCKDSTRIRITDNMFCAGYKPDEGKRGDACEGDSGGPFVMKSPFNNRWYQMGIV
SWGEGCDRDGKYGFYTHVFRLKKWIQKVIDQFGCSSVLIVVC
>g1jou.1 b.47.1.2 (A:,B:) Thrombin {Human (Homo sapiens)}
SEYQTFFNPRTFGSGEADCGLRPLFEKKSLEDKTERELLESYIDGXIVEGSDAEIGMSPWQV
MLFRKSPQELLCGASLISDRWVLTAAHCLLYPPWDKNFTENDLLVRIGKHSRTRYERNIEKI
SMLEKIYIHPRYNWRENLDRDIALMKLKKPVAFSDYIHPVCLPDRETAASLLQAGYKGRVTG
WGNLKETWTANVGKGQPSVLQVVNLPIVERPVCKDSTRIRITDNMFCAGYKPDEGKRGDACE

GDAGGPFVMKSPFNNRWYQMGIVSWGEGCDRDGKYGFYTHVFRLKKWIQKVIDQFGE
>g1vr1.1 b.47.1.2 (L:,H:) Thrombin {Human (Homo sapiens)}
ADCGLRPLFEKKSLEDKTERELLESYIXIVEGSDAEIGMSPWQVMLFAKHRRSPGERFLCGA
SLISDRWVLTAAHCLLYPPWDKNFTENDLLVRIGKHSRTRYERNIEKISMLEKIYIHPRYNW
RENLDRDIALMKLKKPVAFSDYIHPVCLPDRETAASLLQAGYKGRVTGWGNLKETWTANVGK
GQPSVLQVVNLPIVERPVCKDSTRIRITDNMFCAYYKPDEGKRGDACEGDSGGPFVMKSPFN
NRWYQMGIVSWGEGCDRDGKYGFYTHVFRLKKWIQKVID
>g2hnt.1 b.47.1.2 (L:,C:,E:,F:) Thrombin {Human (Homo sapiens)}
ADCGLRPLFEKKSLEDKTERELLESYIDXIVEGSDAEIGMSPWQVMLFRKSPQELLCGASLI
SDRWVLTAAHCLLYPPWDKNFTENDLLVRIGKHSXEKISMLEKIYIHPRYNWRENLDRDIAL
MKLKKPVAFSDYIHPVCLPDRETAASLLQAGYKGRVTGWGXPSVLQVVNLPIVERPVCKDST
RIRITDNMFCAGYKPDEGKRGDACEGDSGGPFVMKSPFNNRWYQMGIVSWGEGCDRDGKYGF
YTHVFRLKKWIQKVIDQ
>g1etr.1 b.47.1.2 (L:,H:) Thrombin {Cow (Bos taurus)}
TFGAGEADCGLRPLFEKKQVQDQTEKELFESYIEGRXIVEGQDAEVGLSPWQVMLFRKSPQE
LLCGASLISDRWVLTAAHCLLYPPWDKNFTVDDLLVRIGKHSRTRYERKVEKISMLDKIYIH
PRYNWKENLDRDIALLKLKRPIELSDYIHPVCLPDKQTAAKLLHAGFKGRVTGWGNRRETWT
TSVAEVQPSVLQVVNLPLVERPVCKASTRIRITDNMFCAGYKPGEGKRGDACEGDSGGPFVM
KSPYNNRWYQMGIVSWGEGCDRDGKYGFYTHVFRLKKWIQKVIDRLGS
>d1fona_ b.47.1.2 (A:) Procarboxypeptidase A-S6 subunit III
(zymogen E) {Cow (Bos taurus)}
SWSWQVSLQYEKDGAFHHTCGGSLIAPDWVVTAGHCISTSRTYQVVLGEYDRSVLEGSEQVI
PINAGDLFVHPLWNSNCVACGNDIALVKLSRSAQLGDKVQLANLPPAGDILPNEAPCYISGW
GRLYTGGPLPDKLQQALLPTVDYEHCSQWDWWGITVKKTMVCAGGDTRSGCNGDSGGPLNCP
AADGSWQVHGVTSFVSAFGCNTIKKPTVFTRVSAFIDWIDETIASN
>d1pytc_ b.47.1.2 (C:) Procarboxypeptidase A-S6 subunit III
(zymogen E) {Cow (Bos taurus)}
SRPSSRVVNGEDAVPYSWSWQVSLQYEKDGAFHHTCGGSLIAPDWVVTAGHCISTSRTYQVV
LGEYDRSVLQGSEQVIPINAGDLFVHPLWNSNCVACGNDIALVKLSRSAQLGDKVQLANLPP
AGDILPNEAPCYISGWGRLYTGGPLPDKLQEALLPVVDYEHCSQYDWWGITVKKTMVCAGGD
TRSGCDGDSGGPLNCPAADGSWQVHGVTSFVSAFGCNTIKKPTVFTRVSAFIDWINETIASN
>d1ppfe_ b.47.1.2 (E:) Elastase {Human (Homo sapiens)}
IVGGRRARPHAWPFMVSLQLRGGHFCGATLIAPNFVMSAAHCVANVNVRAVRVVLGAHNLSR
REPTRQVFAVQRIFENGYDPVNLLNDIVILQLNGSATINANVQVAQLPAQGRRLGNGVQCLA
MGWGLLGRNRGIASVLQELNVTVVTSLCRRSNVCTLVRGRQAGVCFGDSGSPLVCNGLIHGI
ASFVRGGCASGLYPDAFAPVAQFVNWIDSIIQ
>d1brup_ b.47.1.2 (P:) Elastase {Pig (Sus scrofa)}
VVGGEDARPNSWPWQVSLQYDSSGQWRHTCGGTLVDQSWVLTAAHCISSSRTYRVVLGRHSL
STNEPGSLAVKVSKLVVHQDWNSNQLSNGNDIALLKLASPVSLTDKIQLGCLPAAGTILPNN
YVCYVTGWGRLQTNGASPDILQQGQLLVVDYATCSKPGWWGSTVKTNMICAGGDGIISSCNG
DSGGPLNCQGANGQWQVHGIVSFGSSLGCNYYHKPSVFTRVSNYIDWINSVIANN
>d1qnja_ b.47.1.2 (A:) Elastase {Pig (Sus scrofa)}
VVGGTEAQRNSWPSQISLQYRSGSSWAHTCGGTLIRQNWVMTAAHCVDRELTFRVVVGEHNL
NQNDGTEQYVGVQKIVVHPYWNTDDVAAGYDIALLRLAQSVTLNSYVQLGVLPRAGTILANN

SPCYITGWGLTRTNGQLAQTLQQAYLPTVDYAICSSSSYWGSTVKNSMVCAGGDGVRSGCQG
DSGGPLHCLVNGQYAVHGVTSFVSRLGCNVTRKPTVFTRVSAYISWINNVIASN
>d1elt__ b.47.1.2 (-) Elastase {Salmon (Salmo salar)}
VVGGRVAQPNSWPWQISLQYKSGSSYYHTCGGSLIRQGWVMTAAHCVDSARTWRVVLGEHNL
NTNEGKEQIMTVNSVFIHSGWNSDDVAGGYDIALLRLNTQASLNSAVQLAALPPSNQILPNN
NPCYITGWGKTSTGGPLSDSLKQAWLPSVDHATCSSSGWWGSTVKTTMVCAGGGANSGCNGD
SGGPLNCQVNGSYYVHGVTSFVSSSGCNASKKPTVFTRVSAYISWMNGIM
>d1ekbb_ b.47.1.2 (B:) Enteropeptidase (enterokinase light chain) {Cow (Bos taurus)}
IVGGSDSREGAWPWVVALYFDDQQVCGASLVSRDWLVSAAHCVYGRNMEPSKWKAVLGLHMA
SNLTSPQIETRLIDQIVINPHYNKRRKNNDIAMMHLEMKVNYTDYIQPICLPEENQVFPPGR
ICSIAGWGALIYQGSTADVLQEADVPLLSNEKCQQQMPEYNITENMVCAGYEAGGVDSCQGD
SGGPLMCQENNRWLLAGVTSFGYQCALPNRPGVYARVPRFTEWIQSFLH
>d1a7s__ b.47.1.2 (-) Heparin binding protein, HBP {Human (Homo sapiens)}
IVGGRKARPRQFPFLASIQNQGRHFCGGALIHARFVMTAASCFPGVSTVVLGAYDLRRRERQ
SRQTFSISSMSENGYDPQQNLNDLMLLQLDREANLTSSVTILPLPLQNATVEAGTRCQVAGW
GSQRSGGRLSRFPRFVNVTVTPEDQCRPNNVCTGVLTRRGGICNGDGGTPLVCEGLAHGVAS
FSLGPCGRGPDFFTRVALFRDWIDGVLNNPGPGPA
>d1a0la_ b.47.1.2 (A:) beta-Tryptase {Human (Homo sapiens)}
IVGGQEAPRSKWPWQVSLRVHGPYWMHFCGGSLIHPQWVLTAAHCVGPDVKDLAALRVQLRE
QHLYYQDQLLPVSRIIVHPQFYTAQIGADIALLELEEPVKVSSHVHTVTLPPASETFPPGMP
CWVTGWGDVDNDERLPPPFPLKQVKVPIMENHICDAKYHLGAYTGDDVRIVRDDMLCAGNTR
RDSCQGDSGGPLVCKVNGTWLQAGVVSWGEGCAQPNRPGIYTRVTYYLDWIHHYVPKK
>d1cgha_ b.47.1.2 (A:) Cathepsin G {Human (Homo sapiens)}
IIGGRESRPHSRPYMAYLQIQSPAGQSRCGGFLVREDFVLTAAHCWGSNINVTLGAHNIQRR
ENTQQHITARRAIRHPQYNQRTIQNDIMLLQLSRRVRRNRNVNPVALPRAQEGLRPGTLCTV
AGWGRVSMRRGTDTLREVQLRVQRDRQCLRIFGSYDPRRQICVGDRRERKAAFKGDSGGPLL
CNNVAHGIVSYGKSSGVPPEVFTRVSSFLPWIRTTMRS
>d1danh_ b.47.1.2 (H:) Coagulation factor VIIa {Human (Homo sapiens)}
IVGGKVCPKGECPWQVLLLVNGAQLCGGTLINTIWVVSAAHCFDKIKNWRNLIAVLGEHDLS
EHDGDEQSRRVAQVIIPSTYVPGTTNHDIALLRLHQPVVLTDHVVPLCLPERTFSERTLAFV
RFSLVSGWGQLLDRGATALELMVLNVPRLMTQDCLQQSRKVGDSPNITEYMFCAGYSDGSKD
SCKGDSGGPHATHYRGTWYLTGIVSWGQGCATVGHFGVYTRVSQYIEWLQKLMRSEPRPGVL
LRAPFP
>d3rp2a_ b.47.1.2 (A:) Chymase (Proteinase II) {Rat (Rattus rattus)}
IIGGVESIPHSRPYMAHLDIVTEKGLRVICGGFLISRQFVLTAAHCKGREITVILGAHDVRK
RESTQQKIKVEKQIIHESYNSVPNLHDIMLLKLEKKVELTPAVNVVPLPSPSDFIHPGAMCW
AAGWGKTGVRDPTSYTLREVELRIMDEKACVDYRYYEYKFQVCVGSPTTLRAAFMGDSGGPL
LCAGVAHGIVSYGHPDAKPPAIFTRVSTYVPWINAVIN
>d1klt__ b.47.1.2 (-) Chymase (Proteinase II) {Human (Homo sapiens)}

IIGGTESKPHSRPYMAYLEIVTSNGPSKFCGGFLIRRNFVLTAAHCAGRSITVTLGAHNITE
EEDTWQKLEVIKQFRHPKYNTSTLHHDIMLLKLKEKASLTLAVGTLPFPSQFNFVPPGRMCR
VAGWGRTGVLKPGSDTLQEVKLRLMDPQACSHFRDFDHNLQLCVGNPRKTKSAFKGDSGGPL
LCAGVAQGIVSYGRSDAKPPAVFTRISHYRPWINQILQAN
>g2pka.1 b.47.1.2 (A:,B:) Kallikrein A {Pig (Sus scrofa)}
IIGGRECEKNSHPWQVAIYHYSSFQCGGVLVNPKWVLTAAHCKNDNYEVWLGRHNLFENENT
AQFFGVTADFPHPGFNLSXADGKDYSHDLMLLRLQSPAKITDAVKVLELPTQEPELGSTCEA
SGWGSIEPGPDDFEFPDEIQCVQLTLLQNTFCADAHPDKVTESMLCAGYLPGGKDTCMGDSG
GPLICNGMWQGITSWGHTPCGSANKPSIYTKLIFYLDWIDDTITENP
>d1ton__ b.47.1.2 (-) Tonin {Rat (Rattus rattus)}
IVGGYKCEKNSQPWQVAVINEYLCGGVLIDPSWVITAAHCYSNNYQVLLGRNNLFKDEPFAQ
RRLVRQSFRHPDYIPLIVTNDTEQPVHDHSNDLMLLHLSEPADITGGVKVIDLPTKEPKVGS
TCLASGWGSTNPSEMVVSHDLQCVNIHLLSNEKCIETYKDNVTDVMLCAGEMEGGKDTCAGD
SGGPLICDGVLQGITSGGATPCAKPKTPAIYAKLIKFTSWIKKVMKENP
>d1sgfa_ b.47.1.2 (A:) 7S NGF protease subunits {Mouse (Mus musculus)}
NSQPWHVAVYRFNKYQCGGVLLDRNWVLTAAHCYNDKYQVWLGKNNFLEDEPSDQHRLVSKA
IPHPDFNMSLLNEHTPQPEDDYSNDLMLLRLSKPADITDVVKPITLPTEEPKLGSTCLASGW
GSTTPIKYPDDLQCVNLKLLPNEDCDKAHEMKVTDAMLCAGEMDGGSYTCEHDSGGPLICDG
ILQGITSWGPEPCGEPTEPSVYTKLIKFSSWIRETMANNP
>d1sgfg_ b.47.1.2 (G:) 7S NGF protease subunits {Mouse (Mus musculus)}
IVGGFKCEKNSQPWHVAVYRYTQYLCGGVLLDPNWVLTAAHCYDDNYKVWLGKNNLFKDEPS
AQHRFVSKAIPHPGFNMSLMRFLEYDYSNDLMLLRLSKPADITDTVKPITLPTEEPKLGSTC
LASGWGSITPTKFQFTDDLYCVNLKLLPNEDCAKAHIEKVTDAMLCAGEMDGGKDTCKGDSG
GPLICDGVLQGITSWGHTPCGEPDMPGVYTKLNKFTSWIKDTMAKNP
>d1dlea_ b.47.1.2 (A:) Factor B {Human (Homo sapiens)}
ADPDESQSLSLCGMVWEHRKGTDYHKQPWQAKISVIRPSKGHESCMGAVVSEYFVLTAAHCF
TVDDKEHSIKVSVGGEKRDLEIEVVLFHPNYNINGKKEAGIPEFYDYDVALIKLKNKLKYGQ
TIRPICLPCTEGTTRALRLPPTTTCQQQKEELLPAQDIKALFVSEEEKKLTRKEVYIKNGDK
KGSCERDAQYAPGYDKVKDISEVVTPRFLCTGGVSPYADPNTCRGDSGGPLIVHKRSRFIQV
GVISWGVVDVCKNQKRQKQVPAHARDFHINLFQVLPWLKEKLQDEDLGFL
>d1bio__ b.47.1.2 (-) Factor D {Human (Homo sapiens)}
ILGGREAEAHARPYMASVQLNGAHLCGGVLVAEQWVLSAAHCLEDAADGKVQVLLGAHSLSQ
PEPSKRLYDVLRAVPHPDSQPDTIDHDLLLLQLSEKATLGPAVRPLPWQRVDRDVAPGTLCD
VAGWGIVNHAGRRPDSLQHVLLPVLDRATCNRRTHHDGAITERLMCAESNRRDSCKGDSGGP
LVCGGVLEGVVTSGSRVCGNRKKPGIYTRVASYAAWIDSVLA
>g1rtf.1 b.47.1.2 (A:,B:) Two-chain tissue plasminogen activator (TC)-T-PA {Human (Homo sapiens)}
TCGLRQYSXIKGGLFADIASHPWQAAIFAKHRRSPGERFLCGGILISSCWILSAAHCFQERF
PPHHLTVILGRTYRVVPGEEEQKFEVEKYIVHKEFDDDTYDNDIALLQLKSDSSRCAQESSV
VRTVCLPPADLQLPDWTECELSGYGKHEALSPFYSERLKEAHVRLYPSSRCTSQHLLNRTVT
DNMLCAGDTRSGGPQANLHDACQGDSGGPLVCLNDGRMTLVGIISWGLGCGQKDVPGVYTKV
TNYLDWIRDNMRP

>d1a5ia_ b.47.1.2 (A:) Single chain tissue plasminogen activator {Vampire bat (Desmodus rotundus)}
TCGLRKYKEPQLHSTGGLFTDITSHPWQAAIFAQNRRSSGERFLCGGILISSCWVLTAAHCF
QESYLPDQLKVVLGRTYRVKPGEEEQTFKVKKYIVHKEFDDDTYNNDIALLQLKSDSPQCAQ
ESDSVRAICLPEANLQLPDWTECELSGYGKHKSSSPFYSEQLKEGHVRLYPSSRCAPKFLFN
KTVTNNMLCAGDTRSGEIYPNVHDACQGDSGGPLVCMNDNHMTLLGIISWGVGCGEKDVPGV
YTKVTNYLGWIRDNMHL
>d1bqya_ b.47.1.2 (A:) Plasminogen activator from snake venom, TSV-PA {Chinese green tree viper (Trimeresurus stejnejeri)}
VFGGDECNINEHRSLVVLFNSNGFLCGGTLINQDWVVTAAHCDSNNFQLLFGVHSKKILNED
EQTRDPKEKFFCPNRKKDDEVDKDIMLIKLDSSVSNSEHIAPLSLPSSPPSVGSVCRIMGWG
KTIPTKEIYPDVPHCANINILDHAVCRTAYSWRQVANTTLCAGILQGGRDTCHFDSGGPLIC
NGIFQGIVSWGGHPCGQPGEPGVYTKVFDYLDWIKSIIAGNKDATCPP
>d1ao5a_ b.47.1.2 (A:) Kallikrein-13 {Mouse (Mus musculus)}
VVGGFNCEKNSQPWQVAVYYQKEHICGGVLLDRNWVLTAAHCYVDQYEVWLGKNKLFQEEPS
AQHRLVSKSFPHPGFNMSLLMLQTIPPGADFSDDLMLLRLSKPADITDVVKPIALPTKEPKP
GSKCLASGWGSITPTRWQKPDDLQCVFITLLPNENCAKVYLQKVTDVMLCAGEMGGGKDTCR
DDSGGPLICDGILQGTTSYGPVPCGKPGVPAIYTNLIKFNSWIKDTMMKNA
>d1pfxc_ b.47.1.2 (C:) Coagulation factor IXa, protease domain {Pig (Sus scrofa)}
IVGGENAKPGQFPWQVLLNGKIDAFCGGSIINEKWVVTAAHCIEPGVKITVVAGEYNTEETE
PTEQRRNVIRAIPHHSYNATVNKYSHDIALLELDEPLTLNSYVTPICIADKEYTNIFLKFGS
GYVSGWGRVFNRGRSATILQYLKVPLVDRATCLRSTKFTIYSNMFCAGFHEGGKDSCQGDSG
GPHVTEVEGTSFLTGIISWGEECAVKGKYGIYTKVSRYVNWIKEKTKLT
>d1rfna_ b.47.1.2 (A:) Coagulation factor IXa, protease domain {Human (Homo sapiens)}
VVGGEDAKPGQFPWQVVLNGKVDAFCGGSIVNEKWIVTAAHCVETGVKITVVAGEHNIEETE
HTEQKRNVIRIIPHHNYNAAINKYNHDIALLELDEPLVLNSYVTPICIADKEYTNIFLKFGS
GYVSGWGRVFHKGRSALVLQYLRVPLVDRATCLRSTKFTIYNNMFCAGFHEGGRDSCQGDSG
GPHVTEVEGTSFLTGIISWGEECAMKGKYGIYTKVSRYVNWIKEKTKLT
>d1fjsa_ b.47.1.2 (A:) Coagulation factor Xa (Chrismas factor), protease domain {Human (Homo sapiens)}
IVGGQECKDGECPWQALLINEENEGFCGGTILSEFYILTAAHCLYQAKRFKVRVGDRNTEQE
EGGEAVHEVEVVIKHNRFTKETYDFDIAVLRLKTPITFRMNVAPACLPERDWAESTLMTQKT
GIVSGFGRTHEKGRQSTRLKMLEVPYVDRNSCKLSSSFIITQNMFCAGYDTKQEDACQGDSG
GPHVTRFKDTYFVTGIVSWGEGCARKGKYGIYTKVTAFLKWIDRSMKT
>d1kigh_ b.47.1.2 (H:) Coagulation factor Xa (Chrismas factor), protease domain {Cow (Bos taurus)}
IVGGRDCAEGECPWQALLVNEENEGFCGGTILNEFYVLTAAHCLHQAKRFTVRVGDRNTEQE
EGNEMAHEVEMTVKHSRFVKETYDFDIAVLRLKTPIRFRRNVAPACLPEKDWAEATLMTQKT
GIVSGFGRTHEKGRLSSTLKMLEVPYVDRSTCKLSSSFTITPNMFCAGYDTQPEDACQGDSG
GPHVTRFKDTYFVTGIVSWGEGCARKGKFGVYTKVSNFLKWIDKIMKARAGAAGS
>d1fxya_ b.47.1.2 (A:) Coagulation factor Xa-trypsin chimera {Synthetic, based on Homo sapiens sequence}

IVGGYNCKDGEVPWQALLINEENEGFCGGTILSEFYILTAAHCLYQAKRFKVRVGDRNTEQE
EGGEAVHEVEVVIKHNRFTKETYDFDIAVLRLKTPITFRMNVAPASLPTAPPATGTKCLISG
WGNTASSGADYPDELQCLDAPVLSQAKCEASYPGKITSNMFCVGFLEGGKDSCQGDSGGPVV
CNGQLQGVVSWGDGCAQKNKPGVYTKVYNYVKWIKNTIAANS
>d1elva1 b.47.1.2 (A:410-668) Complement C1S protease, catalytic domain {Human (Homo sapiens)}
CGVPREPFEEKQRIIGGSDADIKNFPWQVFFDNPWAGGALINEYWVLTAAHVVEGNREPTMY
VGSTSVQTSRLAKSKMLTPEHVFIHPGWKLLAVPEGRTNFDNDIALVRLKDPVKMGPTVSPI
CLPGTSSDYNLMDGDLGLISGWGRTEKRDRAVRLKAARLPVAPLRKCKEVKVEKPTADAEAY
VFTPNMICAGGEKGMDSCKGDSGGAFAVQDPNDKTKFYAAGLVSWGPQCGTYGLYTRVKNYV
DWIMKTMQENS
>d1autc_ b.47.1.2 (C:) Activated protein c (autoprothrombin IIa) {Human (Homo sapiens)}
LIDGKMTRRGDSPWQVVLLDSKKKLACGAVLIHPSWVLTAAHCMDESKKLLVRLGEYDLRRW
EKWELDLDIKEVFVHPNYSKSTTDNDIALLHLAQPATLSQTIVPICLPDSGLAERELNQAGQ
ETLVTGWGYHSSREKEAKRNRTFVLNFIKIPVVPHNECSEVMSNMVSENMLCAGILGDRQDA
CEGDSGGPMVASFHGTWFLVGLVSWGEGCGLLHNYGVYTKVSRYLDWIHGHIRD
>d1fuja_ b.47.1.2 (A:) Myeloblastin, PR3 {Human (Homo sapiens)}
IVGGHEAQPHSRPYMASLQMRGNPGSHFCGGTLIHPSFVLTAAHCLRDIPQRLVNVVLGAHN
VRTQEPTQQHFSVAQVFLNNYDAENKLNDILLIQLSSPANLSASVATVQLPQQDQPVPHGTQ
CLAMGWGRVGAHDPPAQVLQELNVTVVTFFCRPHNICTFVPRRKAGICFGDSGGPLICDGII
QGIDSFVIWGCATRLFPDFFTRVALYVDWIRSTLR
>g1c5y.1 b.47.1.2 (A:,B:) Urokinase-type plasminogen activator (LMW U-PA), catalytic domain {Human (Homo sapiens)}
LKFQCGQKTXIIGGEFTTIENQPWFAAIYRRHRGGSVTYVCGGSLMSPCWVISATHCFIDYP
KKEDYIVYLGRSRLNSNTQGEMKFEVENLILHKDYSADTLAHHNDIALLKIRSKEGRCAQPS
RTIQTICLPSMYNDPQFGTSCEITGFGKEASTDYLYPEQLKMTVVKLISHRECQQPHYYGSE
VTTKMLCAADPQWKTDSCQGDSGGPLVCSLQGRMTLTGIVSWGRGCALKDKPGVYTRVSHFL
PWIRSHTKEE
>d1ddja_ b.47.1.2 (A:) Plasmin(ogen), catalytic domain {Human (Homo sapiens)}
SFDCGKPQVEPKKCPGRVVGGCVAHPHSWPWQVSLRTRFGMHFCGGTLISPEWVLTAAHCLE
KSPRPSSYKVILGAHQEVNLEPHVQEIEVSRLFLEPTRKDIALLKLSSPAVITDKVIPACLP
SPNYVVADRTECFITGWGETQGTFGAGLLKEAQLPVIENKVCNRYEFLNGRVQSTELCAGHL
AGGTDSCQGDAGGPLVCFEKDKYILQGVTSWGLGCARPNKPGVYVRVSRFVTWIEGVMRNN
>d1fi8a_ b.47.1.2 (A:) Granzyme B {Rat (Rattus norvegicus)}
IIGGHEAKPHSRPYMAYLQIMDEYSGSKKCGGFLIREDFVLTAAHCSGSKIQVTLGAHNIKE
QEKMQQIIPVVKIIPHPAYNSKTISNDIMLLKLKSKAKRSSAVKPLNLPRRNVKVKPGDVCY
VAGWGKLGPMGKYSDTLQEVELTVQEDQKCESYLKNYFDKANEICAGDPKIKRASFRGDSGG
PLVCKKVAAGIVSYGQNDGSTPRAFTKVSTFLSWIKKTMKK
>d1iaua_ b.47.1.2 (A:) Granzyme B {Human (Homo sapiens)}
IIGGHEAKPHSRPYMAYLMIWDQKSLKRCGGFLIRDDFVLTAAHCWGSSINVTLGAHNIKEQ
EPTQQFIPVKRPIPHPAYNPKNFSNDIMLLQLERKAKRTRAVQPLRLPSNKAQVKPGQTCSV
AGWGQTAPLGKHSHTLQEVKMTVQEDRKCESDLRHYYDSTIELCVGDPEIKKTSFKGDSGGP

LVCNKVAQGIVSYGRNNGMPPRACTKVSSFVHWIKKTMKR
>d1eufa_ b.47.1.2 (A:) Duodenase {Cow (Bos taurus)}
IIGGHEAKPHSRPYMAFLLFKTSGKSHICGGFLVREDFVLTAAHCLGSSINVTLGAHNIMER
ERTQQVIPVRRPIPHPDYNDETLANDIMLLKLTRKADITDKVSPINLPRSLAEVKPGMMCSV
AGWGRLGVNMPSTDKLQEVDLEVQSEEKCIARFKNYIPFTQICAGDPSKRKNSFSGDSGGPL
VCNGVAQGIVSYGRNDGTTPDVYTRISSFLSWIHSTMR
>g1fiw.1 b.47.1.2 (L:,A:) Beta-acrosin {Sheep (Ovis aries)}
TTCDGPCGVRFRQNXIIGGQDAAHGAWPWMVSLQIFTYHNNRRYHVCGGSLLNSQWLLTAAH
CFRIKKKVTDWRLIFGAKEVEWGTNKPVKPPLQERYVEKIIIHEKYSASSEANDIALMKITP
PVTCGHFIGPGCLPQFRAGPPRVPQTCWVAGWGFLQENARRTSPMLQEARVDLIDLGLCNST
RWYNGRIRSTNVCAGYPEGKIDTCQGDSGGPLMCKDSAENSYVVVGITSWGVGCARAKRPGV
YTSTWSYLNWIASKIGSTAVHMIQLPT
>g1fiz.1 b.47.1.2 (L:,A:) Beta-acrosin {Pig (Sus scrofa)}
ATCDGPCGLRFRQXVVGGMSAEPGAWPWMVSLQIFMYHNNRRYHTCGGILLNSHWVLTAAHC
FKNKKKVTDWRLIFGANEVVWGSNKPVKPPLQERFVEEIIIHEKYVSGLEINDIALIKITPP
VPCGPFIGPGCLPQFKAGPPRAPQTCWVTGWGYLKEKGPRTSPTLQEARVALIDLELCNSTR
WYNGRIRSTNVCAGYPRGKIDTCQGDSGGPLMCRDRAENTFVVVGITSWGVGCARAKRPGVY
TSTWPYLNWIASKIGSNALQMVQLGTPPR
>d1eaxa_ b.47.1.2 (A:) Matriptase MTSP1 {Human (Homo sapiens)}
VVGGTDADEGEWPWQVSLHALGQGHICGASLISPNWLVSAAHCYIDDRGFRYSDPTQWTAFL
GLHDQSQRSAPGVQERRLKRIISHPFFNDFTFDYDIALLELEKPAEYSSMVRPICLPDASHV
FPAGKAIWVTGWGHTQYGGTGALILQKGEIRVINQTTCENLLPQQITPRMMCVGFLSGGVDS
CQGDSGGPLSSVEADGRIFQAGVVSWGDGCAQRNKPGVYTRLPLFRDWIKENTGV
>d1svpa_ b.47.1.3 (A:) Viral capsid protein {Sindbis virus}
ALKLEADRLFDVKNEDGDVIGHALAMEGKVMKPLHVKGTIDHPVLSKLKFTKSSAYDMEFAQ
LPVNMRSEAFTYTSEHPEGFYNWHHGAVQYSGGRFTIPRGVGGRGDAGRPIMDNSGRVVAIV
LGGADEGTRTALSVVTWNSKGKTIKTTPEGTEEWSA
>d1vcpa_ b.47.1.3 (A:) Viral capsid protein {Semliki forest virus}
CIFEVKHEGKVTGYACLVGDKVMKPAHVKGVIDNADLAKLAFKKSSKYDLECAQIPVHMRSD
ASKYTHEKPEGHYNWHHGAVQYSGGRFTIPTGAGKPGDSGRPIFDNKGRVVAIVLGGANEGS
RTALSVVTWNKDMVTRVTPEGSEEW
>d1a1qa_ b.47.1.3 (A:) NS3 protease {Human hepatitis C virus (HCV), different isolates}
PITAYSQQTRGLLGCIITSLTGRDKNQVEGEVQVVSTATQSFLATCVNGVCWTVYHGAGSKT
LAGPKGPITQMYTNVDQDLVGWQAPPGARSLTPCTCGSSDLYLVTRHADVIPVRRRGDSRGS
LLSPRPVSYLKGSSGGPLLCPSGHAVGIFRAAVCTRGVAKAVDFVPVESMETTMRSPVF
>d1cu1a1 b.47.1.3 (A:705-720,A:3-186) NS3 protease {Human hepatitis C virus (HCV), different isolates}
GSVVIVGRIILSGSGSXITAYSQQTRGLLGCIITSLTGRDKNQVEGEVQVVSTATQSFLATC
VNGVCWTVYHGAGSKTLAGPKGPITQMYTNVDQDLVGWQAPPGARSLTPCTCGSSDLYLVTR
HADVIPVRRRGDSRGSLLSPRPVSYLKGSSGGPLLCPSGHAVGIFRAAVCTRGVAKAVDFVP
VESMETTMRSPVFTD
>d1dxwa_ b.47.1.3 (A:) NS3 protease {Human hepatitis C virus (HCV), different isolates}

TGRDKNQVEGEVQVVSTATQSFLATCVNGVCWTVYHGAGSKTLAGPKGPITQMYTNVDQDLV
GWQAPPGARSLTPCTCGSSDLYLVTRHADVIPVRRRGDSRGSLLSPRPVSYLKGSSGGPLLC
PSGHAVGIFRAAVCTRGVAKAVDFVPVESMETTMRASKKKK
>g1a1r.1 b.47.1.3 (A:,C:) NS3 protease {Human hepatitis C virus (HCV), different isolates}
VEGEVQIVSTATQTFLATCINGVCWTVYHGAGTRTIASPKGPVIQMYTNVDQDLVGWPAPQG
SRSLTPCTCGSSDLYLVTRHADVIPVRRRGDSRGSLLSPRPISYLKGSSGGPLLCPTGHAVG
LFRAAVCTRGVAKAVDFIPVENLETTMRXGSVVIVGRIVLSGKPA
>g1a1r.2 b.47.1.3 (B:,D:) NS3 protease {Human hepatitis C virus (HCV), different isolates}
PITAYAQQTRGLLGCIITSLTGRDKNQVEGEVQIVSTATQTFLATCINGVCWTVYHGAGTRT
IASPKGPVIQMYTNVDQDLVGWPAPQGSRSLTPCTCGSSDLYLVTRHADVIPVRRRGDSRGS
LLSPRPISYLKGSSGGPLLCPTGHAVGLFRAAVCTRGVAKAVDFIPVENLETTMRXKGSVVI
VGRIVLSGKPAIIPK
>g1dy9.1 b.47.1.3 (A:,C:) NS3 protease {Human hepatitis C virus (HCV), different isolates}
APITAYSQQTRGLLGCIITSLTGRDKNQVDGEVQVLSTATQSFLATCVNGVCWTVYHGAGSK
TLAGPKGPITQMYTNVDQDLVGWPAPPGARSMTPCTCGSSDLYLVTRHADVIPVRRRGDSRG
SLLSPRPVSYLKGSSGGPLLCPSGHVVGIFRAAVCTRGVAKAVDFIPVESMXGSVVIVGRII
LS
>g1ns3.1 b.47.1.3 (A:,C:) NS3 protease {Human hepatitis C virus (HCV), different isolates}
ITAYSQQTRGLLGCIITSLTGRDKNQVEGEVQVVSTATQSFLATCVNGVCWTVYHGAGSKTL
AGPKGPITQMYTNVDQDLVGWQAPPGARSLTPCTCGSSDLYLVTRHADVIPVRRRGDSRGSL
LSPRPVSYLKGSSGGPLLCPSGHAVGIFRAAVCTRGVAKAVDFVPVESMETTMRXKGSVVIV
GRIILS
>d1befa_ b.47.1.3 (A:) NS3 protease {Dengue virus serotype 2}
WDVPSPPPVGKAELEDGAYRIKQKGILGYSQIGAGVYKEGTFHTMWHVTRGAVLMHKGKRIE
PSWADVKKDLVSCGGGWKLEGEWKEGEEVQVLALEPGKNPRAVQTKPGLFKTNAGTIGAVSL
DFSPGTSGSPIIDKKGKVVGIYGNGVVTRSGAYVSAIAQTEKSIEDNPEIEDD
>d1cqqa_ b.47.1.4 (A:) 3C cysteine protease (picornain 3C) {Human rhinovirus type 2}
GPEEEFGMSLIKHNSCVITTENGKFTGLGVYDRFVVVPTHADPGKEIQVDGITTKVIDSYDL
YNKNGIKLEITVLKLDRNEKFRDIRRYIPNNEDDYPNCNLALLANQPEPTIINVGDVVSYGN
ILLSGNQTARMLKYSYPTKSGYCGGVLYKIGQVLGIHVGGNGRDGFSAMLLRSYFT
>d1hava_ b.47.1.4 (A:) 3C cysteine protease (picornain 3C) {Human hepatitis A virus}
STLEIAGLVRKNLVQFGVGEKNGSVRWVMNALGVKDDWLLVPSHAYKFEKDYEMMEFYFNRG
GTYYSISAGNVVIQSLDVGFQDVVLMKVPTIPKFRDITQHFIKKGDVPRALNRLATLVTTVN
GTPMLISEGPLKMEEKATYVHKKNDGTTVDLTVDQAWRGKGEGLPGMCGGALVSSNQSIQNA
ILGIHVAGGNSILVAKLVTQEMFQNIDKKI
>d2hrva_ b.47.1.4 (A:) 2A cysteine proteinase {Human rhinovirus 2}
GPSDMYVHVGNLIYRNLHLFNSEMHESILVSYSSDLIIYRTNTVGDDYIPSCDCTQATYYCK

HKNRYFPITVTSHDWYEIQESEYYPKHIQYNLLIGEGPCEPGDCGGKLLCKHGVIGIVTAGG
DNHVAFIDLRHFHCA
>d1bco_1 b.48.1.1 (481-560) mu transposase, C-terminal domain {Bacteriophage mu}
TEEQKRMLLLPAEAVNVSRKGEFTLKVGGSLKGAKNVYYNMALMNAGVKKVVVRFDPQQLHS
TVYCYTLDGRFICEAECL
>d1e79a2 b.49.1.1 (A:19-94) N-terminal domain of alpha and beta subunits of F1 ATP synthase {Cow (Bos taurus)}
ADTSVDLEETGRVLSIGDGIARVHGLRNVQAEEMVEFSSGLKGMSLNLEPDNVGVVVFGNDK
LIKEGDIVKRTGAI
>d1e79d2 b.49.1.1 (D:9-81) N-terminal domain of alpha and beta subunits of F1 ATP synthase {Cow (Bos taurus)}
TTGRIVAVIGAVVDVQFDEGLPPILNALEVQGRETRLVLEVAQHLGESTVRTIAMDGTEGLV
RGQKVLDSGAP
>d1maba2 b.49.1.1 (A:10-94) N-terminal domain of alpha and beta subunits of F1 ATP synthase {Rat (Rattus norvegicus)}
SSILEERILGADTSVDLEETGRVLSIGDGIARVHGLRNVQAEEMVEFSSGLKGMSLNLEPDN
VGVVVFGNDKLIKEGDIVKRTGAI
>d1mabb2 b.49.1.1 (B:1-81) N-terminal domain of alpha and beta subunits of F1 ATP synthase {Rat (Rattus norvegicus)}
SAAPKAGTATGQIVAVIGAVVDVQFDEGLPPILNALEVQGRESRLVLEVAQHLGESTVRTIA
MDGTEGLVRGQKVLDSGAP
>d1skyb2 b.49.1.1 (B:21-95) N-terminal domain of alpha and beta subunits of F1 ATP synthase {Bacillus sp., strain ps3}
SQIQVSDVGTVIQVGDGIARAHGLDNVMSGEAVEFANAVMGMALNLEENNVGIVILGPYTGI
KEGDEVRRTGRIM
>d1skye2 b.49.1.1 (E:1-82) N-terminal domain of alpha and beta subunits of F1 ATP synthase {Bacillus sp., strain ps3}
MTRGRVIQVMGPVVDVKFENGHLPAIYNALKIQHKARNENEVDIDLTLEVALHLGDDTVRTI
AMASTDGLIRGMEVIDTGAP
>d1fx0a2 b.49.1.1 (A:25-96) N-terminal domain of alpha and beta subunits of F1 ATP synthase {Spinach (Spinacia oleracea), chloroplast}
KVVNTGTVLQVGDGIARIHGLDEVMAGELVEFEEGTIGIALNLESNNVGVVLMGDGLMIQEG
SSVKATGRIA
>d1fx0b2 b.49.1.1 (B:19-97) N-terminal domain of alpha and beta subunits of F1 ATP synthase {Spinach (Spinacia oleracea), chloroplast}
NLGRIAQIIGPVLNVAFPPGKMPNIYNALIVKGRDTAGQPMNVTCEVQQLLGNNRVRAVAMS
ATDGLTRGMEVIDTGAP
>d1bd0a1 b.49.2.1 (A:2-11,A:245-382) Alanine racemase {Bacillus stearothermophilus}
NDFHRDTWAEXFSLHSRLVHVKKLQPGEKVSYGATYTAQTEEWIGTIPIGYADGWLRRLQHF
HVLVDGQKAPIVGRICMDQCMIRLPGPLPVGTKVTLIGRQGDEVISIDDVARHLETINYEVP

CTISYRVPRIFFRHKRIMEVRNAIG
>d1d7ka1 b.49.2.1 (A:7-43,A:284-427) Eukaryotic ornithine decarboxylase {Human (Homo sapiens)}
EEFDCHFLDEGFTAKDILDQKINEVSSSDDKDAFYVAXFTLAVNIIAKKIVLKEQTGSDDED
ESSEQTFMYYVNDGVYGSFNCILYDHAHVKPLLQKRPKPDERYYSSSIWGPTCDGLDRIVER
CDLPEMHVGDWMLFENMGAYTVAAASTFNGFQRPTIYYVMSGPAWQLMQQFQNPDFPP
>d7odca1 b.49.2.1 (A:2-43,A:284-418) Eukaryotic ornithine decarboxylase {Mouse (Mus musculus)}
SSFTKDEFDCHILDEGFTAKDILDQKINEVSSSDDKDAFYVAXFTLAVNIIAKKTVWKEQPG
SDDEDESNEQTFMYYVNDGVYGSFNCILYDHAHVKALLQKRPKPDEKYYSSSIWGPTCDGLD
RIVERCNLPEMHVGDWMLFENMGAYTVAAASTFNGFQRPNIYYVMSRPMWQLMK
>d1f3ta1 b.49.2.1 (A:14-43,A:284-422) Eukaryotic ornithine decarboxylase {Trypanosoma brucei}
RFLEGFNTRDALCKKISMNTCDEGDPFFVAXFTLAVNVIAKKVTPGVQTDVGAHAESNAQSF
MYYVNDGVYGSFNCILYDHAVVRPLPQREPIPNEKLYPSSVWGPTCDGLDQIVERYYLPEMQ
VGEWLLFEDMGAYTVVGTSSFNGFQSPTIYYVVSGLPDHVVRELKS
>d2toda1 b.49.2.1 (A:37-43,A:284-410) Eukaryotic ornithine decarboxylase {Trypanosoma brucei}
GDPFFVAXFTLAVNVIAKKVTPGVQTDVGAHAESNAQSFMYYVNDGVYGSFNCILYDHAVVR
PLPQREPIPNEKLYPSSVWGPTCDGLDQIVERYYLPEMQVGEWLLFEDMGAYTVVGTSSFNG
FQSPTIYYVVS
>d1b6la_ b.50.1.1 (A:) Human immunodeficiency virus type 1 protease {Human immunodeficiency virus type 1}
PQITLWKRPLVTIRIGGQLKEALLDTGADDTVIEEMNLPGKWKPKMIGGIGGFIKVRQYDQI
PVEIXGHKAIGTVLVGPTPVNIIGRNLLTQIGXTLNF
>d1bdqa_ b.50.1.1 (A:) Human immunodeficiency virus type 1 protease {Human immunodeficiency virus type 1}
PQITLWQRPLVTIKIGGQLKEALLDTGADDSIVAGIELPGRWKPKMVGGIGGFIKVRQYDQI
LIEICGHKAIGTVLVGPTPINIIGRNLLTQIGCTLNF
>d1c6ya_ b.50.1.1 (A:) Human immunodeficiency virus type 1 protease {Human immunodeficiency virus type 1}
PQITLWQRPVVTIKIGGQLMEALIDTGADDTVLEEMDLPGRWKPKIIGGIGGFVKVRQYDQI
PIEICGHKVIGTVLVGPTPTNIIGRNLLTQIGCTLNF
>d1dazc_ b.50.1.1 (C:) Human immunodeficiency virus type 1 protease {Human immunodeficiency virus type 1}
PQITLWKRPLVTIKIGGQLKEALLDTGADDTVIEEMSLPGRWKPIMIGGIGGFIKVRQYDQI
IIEIAGHKAIGTVLVGPTPVNIIGRNLLTQIGATLNF
>d1difa_ b.50.1.1 (A:) Human immunodeficiency virus type 1 protease {Human immunodeficiency virus type 1}
PQITLWQRPLVTIKIGGQLKEALLDTGADDTVLEEMSLPGRWKPKMIGGIGGFIKVRQYDQI
LIEICGHKAIGTVLVGPTPVNIIGRNLLTQIGCTLNF
>d1hvc__ b.50.1.1 (-) Human immunodeficiency virus type 1 protease {Human immunodeficiency virus type 1}
PQITLWQRPLVTIRIGGQLKEALLDTGADDTVLEEMNLPGKWKPKMIGGIGGFIKVRQYDQI

LIEICGHKAIGTVLVGPTPVNIIGRNLLTQIGCTLNFGGSSGPQITLWQRPLVTIKIGGQLK
EALLDTGADDTVLEEMSLPGRWKPKMIGGIGGFIKVRQYDQILIEICGHKAIGTVLVGPTPV
NIIGRNLLTQIGCTLNF
>d1idaa_ b.50.1.1 (A:) Human immunodeficiency virus type 2 (HIV-2) protease {Human immunodeficiency virus type 2}
PQFSLWKRPVVTAYIEGQPVEVLLDTGADDSIVAGIELGNNYSPKIVGGIGGFINTKEYKNV
EIEVLNKKVRATIMTGDTPINIFGRNILTALGMSLNL
>d1az5__ b.50.1.1 (-) Simian immunodeficiency virus (SIV) protease {Simian immunodeficiency virus, different strains}
PQFHLWKRPVVTAHIEGQPVEVLLDTGADDSIVTGIELGPHYTPKIVGGIGGFINTKEYKNV
EVEVLGKRIKGTIMTGDTPINIFGRNLLTALGMSLNF
>d1k6va_ b.50.1.1 (A:) Simian immunodeficiency virus (SIV) protease {Simian immunodeficiency virus, different strains}
PQITLWKRPLVTIRIGGQLKEALLDTGADDTVLEEMNLPGRWKPKMIGGIGGFIKVRQYDQI
PIEICGHKAIGTVLVGPTPTNVIGRNLLTQIGCTLNF
>d1siva_ b.50.1.1 (A:) Simian immunodeficiency virus (SIV) protease {Simian immunodeficiency virus, different strains}
PQFSLWRRPVVTAHIEGQPVEVLLDTGADDSIVTGIELGPHYTPKIVGGIGGFINTKEYKNV
KIEVLGKRIKGTIMTGDTPINIFGRNLLTALGMSLNL
>d4fiv__ b.50.1.1 (-) Feline immunodeficiency virus (FIV) protease {Feline immunodeficiency virus}
VGTTTTLEKRPEILIFVNGYPIKFLLDTGADITILNRRDFQVKNSIENGRQNMIGVGGGKRG
TNYINVHLEIRDENYKTQCIFGNVCVLEDNSLIQPLLGRDNMIKFNIRLVM
>d1baia_ b.50.1.1 (A:) Rous sarcoma virus protease {Rous sarcoma virus, strain pr-C}
LAMTMEHKDRPLVRVILTNTGSHPVKQRSVYITALLDTGADDTVISEEDWPTDWPVMEAANP
QIHGIGGGIPVRKSRDMIELGVINRDGSLERPLLLFPLVAMTPVNILGRDCLQGLGLRLTNL
>d2rspa_ b.50.1.1 (A:) Rous sarcoma virus protease {Rous sarcoma virus, strain pr-C}
LAMTMEHKDRPLVRVILTNTGSHPVKQRSVYITALLDSGADITIISEEDWPTDWPVMEAANP
QIHGIGGGIPMRKSRDMIELGVINRDGSLERPLLLFPAVAMVRGSILGRDCLQGLGLRLTNL
>d1fmb__ b.50.1.1 (-) EIAV protease {Equine infectious anemia virus}
VTYNLEKRPTTIVLINDTPLNVLLDTGADTSVLTTAHYNRLKYRGRKYQGTGIGGVGGNVET
FSTPVTIKKKGRHIKTRMLVADIPVTILGRDILQDLGAKLVL
>d2er7e_ b.50.1.2 (E:) Endothiapepsin {Chestnut blight fungus (Endothia parasitica)}
STGSATTTPIDSLDDAYITPVQIGTPAQTLNLDFDTGSSDLWVFSSETTASEVDGQTIYTPS
KSTTAKLLSGATWSISYGDGSSSSGDVYTDTVSVGGLTVTGQAVESAKKVSSSFTEDSTIDG
LLGLAFSTLNTVSPTQQKTFFDNAKASLDSPVFTADLGYHAPGTYNFGFIDTTAYTGSITYT
AVSTKQGFWEWTSTGYAVGSGTFKSTSIDGIADTGTTLLYLPATVVSAYWAQVSGAKSSSSV
GGYVFPCSATLPSFTFGVGSARIVIPGDYIDFGPISTGSSSCFGGIQSSAGIGINIFGDVAL
KAAFVVFNGATTPTLGFASK
>d1bxoa_ b.50.1.2 (A:) Acid protease {Fungus (Penicillium

janthinellum), penicillopepsin}
AASGVATNTPTANDEEYITPVTIGGTTLNLNFDTGSADLWVFSTELPASQQSGHSVYNPSAT
GKELSGYTWSISYGDGSSASGNVFTDSVTVGGVTAHGQAVQAAQQISAQFQQDTNNDGLLGL
AFSSINTVQPQSQTTFFDTVKSSLAQPLFAVALKHQQPGVYDFGFIDSSKYTGSLTYTGVDN
SQGFWSFNVDSYTAGSQSGDGFSGIADTGTTLLLLDDSVVSQYYSQVSGAQQDSNAGGYVFD
CSTNLPDFSVSISGYTATVPGSLINYGPSGDGSTCLGGIQSNSGIGFSIFGDIFLKSQYVVF
DSDGPQLGFAPQA
>d1ibqa_ b.50.1.2 (A:) Acid protease {Fungus (Aspergillus
phoenicis), aspergillopepsin}
SKGSAVTTPQNNDEEYLTPVTVGKSTLHLDFDTGSADLWVFSDELPSSEQTGHDLYTPSSSA
TKLSGYSWDISYGDGSSASGDVYRDTVTVGGVTTNKQAVEAASKISSEFVQDTANDGLLGLA
FSSINTVQPKAQTTFFDTVKSQLDSPLFAVQLKHDAPGVYDFGYIDDSKYTGSITYTDADSS
QGYWGFSTDGYSIGDGSSSSSGFSAIADTGTTLILLDDEIVSAYYEQVSGAQESYEAGGYVF
SCSTDLPDFTVVIGDYKAVVPGKYINYAPVSTGSSTCYGGIQSNSGLGLSILGDVFLKSQYV
VFNSEGPKLGFAAQA
>d2apr__ b.50.1.2 (-) Acid protease {Bread mold (Rhizopus
chinensis)}
AGVGTVPMTDYGNDIEYYGQVTIGTPGKKFNLDFDTGSSDLWIASTLCTNCGSGQTKYDPNQ
SSTYQADGRTWSISYGDGSSASGILAKDNVNLGGLLIKGQTIELAKREAASFASGPNDGLLG
LGFDTITTVRGVKTPMDNLISQGLISRPIFGVYLGKAKNGGGGEYIFGGYDSTKFKGSLTTV
PIDNSRGWWGITVDRATVGTSTVASSFDGILDTGTTLLILPNNIAASVARAYGASDNGDGTY
TISCDTSAFKPLVFSINGASFQVSPDSLVFEEFQGQCIAGFGYGNWGFAIIGDTFLKNNYVV
FNQGVPEVQIAPVAE
>d2asi__ b.50.1.2 (-) Acid protease {Rhizomucor miehei}
GSVDTPGYYDFDLEEYAIPVSIGTPGQDFLLLFDTGSSDTWVPHKGCTKSEGCVGSRFFDPS
ASSTFKATNYNLNITYGTGANGLYFEDSIAIGDITVTKQILAYVDNVRGPTAEQSPNADIFL
DGLFGAAYPDNTAMEAEYGSTYNTVHVNLYKQGLISSPLFSVYMNTNSGTGEVVFGGVNNTL
LGGDIAYTDVMSRYGGYYFWDAPVTGITVDGSAAVRFSRPQAFTIDTGTNFFIMPSSAASKI
VKAALPDATETQQGWVVPCASYQNSKSTISIVMQKSGSSSDTIEISVPVSKMLLPVDQSNET
CMFIILPDGGNQYIVGNLFLRFFVNVYDFGNNRIGFAPLASAYENE
>d1eaga_ b.50.1.2 (A:) Acid protease {Yeast (Candida albicans)}
QAVPVTLHNEQVTYAADITVGSNNQKLNVIVDTGSSDLWVPDVNVDCQVTYSDQTADFCKQK
GTYDPSGSSASQDLNTPFKIGYGDGSSSQGTLYKDTVGFGGVSIKNQVLADVDSTSIDQGIL
GVGYKTNEAGGSYDNVPVTLKKQGVIAKNAYSLYLNSPDAATGQIIFGGVDNAKYSGSLIAL
PVTSDRELRISLGSVEVSGKTINTDNVDVLLDSGTTITYLQQDLADQIIKAFNGKLTQDSNG
NSFYEVDCNLSGDVVFNFSKNAKISVPASEFAASLQGDDGQPYDKCQLLFDVNDANILGDNF
LRSAYIVYDLDDNEISLAQVKYTSASSISALT
>d1j71a_ b.50.1.2 (A:) Acid protease {Yeast (Candida tropicalis)}
SDVPTTLINEGPSYAADIVVGSNQQKQTVVIDTGSSDLWVVDTDAECQVTYSGQTNNFCKQE
GTFDPSSSSSAQNLNQDFSIEYGDLTSSQGSFYKDTVGFGGISIKNQQFADVTTTSVDQGIM
GIGFTADEAGYNLYDNVPVTLKKQGIINKNAYSLYLNSEDASTGKIIFGGVDNAKYTGTLTA
LPVTSSVELRVHLGSINFDGTSVSTNADVVLDSGTTITYFSQSTADKFARIVGATWDSRNEI
YRLPSCDLSGDAVFNFDQGVKITVPLSELILKDSDSSICYFGISRNDANILGDNFLRRAYIV
YDLDDKTISLAQVKYTSSSDISAL

>d1dpja_ b.50.1.2 (A:) Acid protease {Baker's yeast (Saccharomyces cerevisiae), proteinase A}
GGHDVPLTNYLNAQYYTDITLGTPPQNFKVILDTGSSNLWVPSNECGSLACFLHSKYDHEAS
SSYKANGTEFAIQYGTGSLEGYISQDTLSIGDLTIPKQDFAEATSEPGLTFAFGKFDGILGL
GYDTISVDKVVPPFYNAIQQDLLDEKRFAFYLGDTSKDTENGGEATFGGIDESKFKGDITWL
PVRRKAYWEVKFEGIGLGDEYAELESHGAAIDTGTSLITLPSGLAEMINAEIGAKKGWTGQY
TLDCNTRDNLPDLIFNFNGYNFTIGPYDYTLEVSGSCISAITPMDFPEPVGPLAIVGDAFLR
KYYSIYDLGNNAVGLAKAI
>g1b5f.1 b.50.1.2 (A:,B:) Plant acid proteinase, phytepsin {Cynara cardunculus}
GSAVVALTNDRDTSYFGEIGIGTPPQKFTVIFDTGSSVLWVPSSKCINSKACRAHSMYESSD
SSTYKENGTFGAIIYGTGSITGFFSQDSVTIGDLVVKEQDFIEATDEADNVFLHRLFDGILG
LSFQTISVPVWYNMLNQGLVKERRFSFWLNRNVDEEEGGELVFGGLDPNHFRGDHTYVPVTY
QYYWQFGIGDVLIGDKSTGFCAPGCQAFADSGTSLLSGPTAIVTQINHAIGANXEELQVDCN
TLSSMPNVSFTIGGKKFGLTPEQYILKVGKGEATQCISGFTAMDATLLGPLWILGDVFMRPY
HTVFDYGNLLVGFAEAA
>d1qdma2 b.50.1.2 (A:2-247,A:248-338) Plant acid proteinase, phytepsin {Barley (Hordeum vulgare)}
EEEGDIVALKNYMNAQYFGEIGVGTPPQKFTVIFDTGSSNLWVPSAKCYFSIACYLHSRYKA
GASSTYKKNGKPAAIQYGTGSIAGYFSEDSVTVGDLVVKDQEFIEATKEPGITFLVAKFDGI
LGLGFKEISVGKAVPVWYKMIEQGLVSDPVFSFWLNRHVDEGEGGEIIFGGMDPKHYVGEHT
YVPVTQKGYWQFDMGDVLVGGKSTGFCAGGCAAIADSGTSLLAGPTAIITEINEKIGAAGXS
PMGESAVDCGSLGSMPDIEFTIGGKKFALKPEEYILKVGEGAAAQCISGFTAMDIPPPRGPL
WILGDVFMGPYHTVFDYGKLRIGFAKAA
>d3psg__ b.50.1.2 (-) Pepsin(ogen) {Pig (Sus scrofa)}
LVKVPLVRKKSLRQNLIKDGKLKDFLKTHKHNPASKYFPEAAALIGDEPLENYLDTEYFGTI
GIGTPAQDFTVIFDTGSSNLWVPSVYCSSLACSDHNQFNPDDSSTFEATSQELSITYGTGSM
TGILGYDTVQVGGISDTNQIFGLSETEPGSFLYYAPFDGILGLAYPSISASGATPVFDNLWD
QGLVSQDLFSVYLSSNDDSGSVVLLGGIDSSYYTGSLNWVPVSVEGYWQITLDSITMDGETI
ACSGGCQAIVDTGTSLLTGPTSAIANIQSDIGASENSDGEMVISCSSIDSLPDIVFTIDGVQ
YPLSPSAYILQDDDSCTSGFEGMDVPTSSGELWILGDVFIRQYYTVFDRANNKVGLAPVA
>d4pep__ b.50.1.2 (-) Pepsin(ogen) {Pig (Sus scrofa)}
IGDEPLENYLDTEYFGTIGIGTPAQDFTVIFDTGSSNLWVPSVYCSSLACSDHNQFNPDDSS
TFEATSQELSITYGTGSMTGILGYDTVQVGGISDTNQIFGLSETEPGSFLYYAPFDGILGLA
YPSISASGATPVFDNLWDQGLVSQDLFSVYLSSNDDSGSVVLLGGIDSSYYTGSLNWVPVSV
EGYWQITLDSITMDGETIACSGGCQAIVDTGTSLLTGPTSAIANIQSDIGASENSDGEMVIS
CSSIDSLPDIVFTIDGVQYPLSPSAYILQDDDSCTSGFEGMDVPTSSGELWILGDVFIRQYY
TVFDRANNKVGLAPVA
>d1psoe_ b.50.1.2 (E:) Pepsin(ogen) {Human (Homo sapiens), 3A}
VDEQPLENYLDMEYFGTIGIGTPAQDFTVVFDTGSSNLWVPSVYCSSLACTNHNRFNPEDSS
TYQSTSETVSITYGTGSMTGILGYDTVQVGGISDTNQIFGLSETEPGSFLYYAPFDGILGLA
YPSISSSGATPVFDNIWNQGLVSQDLFSVYLSADDQSGSVVIFGGIDSSYYTGSLNWVPVTV
EGYWQITVDSITMNGEAIACAEGCQAIVDTGTSLLTGPTSPIANIQSDIGASENSDGDMVVS
CSAISSLPDIVFTINGVQYPVPPSAYILQSEGSCISGFQGMNLPTESGELWILGDVFIRQYF

TVFDRANNQVGLAPVA
>g1htr.1 b.50.1.2 (P:,B:) Pepsin(ogen) {Human (Homo sapiens), progastricsin (pepsinogen C)}
AVVKVPLKKFKSIRETMKEKGLLGEFLRTHKYDPAWKYRFGDLXSVTYEPMAYMDAAYFGEI
SIGTPPQNFLVLFDTGSSNLWVPSVYCQSQACTSHSRFNPSESSTYSTNGQTFSLQYGSGSL
TGFFGYDTLTVQSIQVPNQEFGLSENEPGTNFVYAQFDGIMGLAYPALSVDEATTAMQGMVQ
EGALTSPVFSVYLSNQQGSSGGAVVFGGVDSSLYTGQIYWAPVTQELYWQIGIEEFLIGGQA
SGWCSEGCQAIVDTGTSLLTVPQQYMSALLQATGAQEDEYGQFLVNCNSIQNLPSLTFIING
VEFPLPPSSYILSNNGYCTVGVEPTYLSSQNGQPLWILGDVFLRSYYSVYDLGNNRVGFATA
A
>d1am5__ b.50.1.2 (-) Pepsin(ogen) {Atlantic cod (Gadus morhua)}
RVTEQMKNEADTEYYGVISIGTPPESFKVIFDTGSSNLWVSSSHCSAQACSNHNKFKPRQSS
TYVETGKTVDLTYGTGGMRGILGQDTVSVGGGSDPNQELGESQTEPGPFQAAAPFDGILGLA
YPSIAAAGAVPVFDNMGSQSLVEKDLFSFYLSGGGANGSEVMLGGVDNSHYTGSIHWIPVTA
EKYWQVALDGITVNGQTAACEGCQAIVDTGTSKIVAPVSALANIMKDIGASENQGEMMGNCA
SVQSLPDITFTINGVKQPLPPSAYIEGDQAFCTSGLGSSGVPSNTSELWIFGDVFLRNYYTI
YDRTNNKVGFAPAA
>d1mpp__ b.50.1.2 (-) Pepsin {Mucor pusillus}
GSVDTPGLYDFDLEEYAIPVSIGTPGQDFYLLFDTGSSDTWVPHKGCDNSEGCVGKRFFDPS
SSSTFKETDYNLNITYGTGGANGIYFRDSITVGGATVKQQTLAYVDNVSGPTAEQSPDSELF
LDGIFGAAYPDNTAMEAEYGDTYNTVHVNLYKQGLISSPVFSVYMNTNDGGGQVVFGGVNNT
LLGGDIQYTDVLKSRGGYFFWDAPVTGVKIDGSDAVSFDGAQAFTIDTGTNFFIAPSSFAEK
VVKAALPDATESQQGYTVPCSKYQDSKTTFSLVLQKSGSSSDTIDVSVPISKMLLPVDKSGE
TCMFIVLPDGGNQFIVGNLFLRFFVNVYDFGKNRIGFAPLASGYEND
>g1lyb.1 b.50.1.2 (A:,B:) Cathepsin D {Human (Homo sapiens)}
GPIPEVLKNYMDAQYYGEIGIGTPPQCFTVVFDTGSSNLWVPSIHCKLLDIACWIHHKYNSD
KSSTYVKNGTSFDIHYGSGSLSGYLSQDTVSVPCQXGGVKVERQVFGEATKQPGITFIAAKF
DGILGMAYPRISVNNVLPVFDNLMQQKLVDQNIFSFYLSRDPDAQPGGELMLGGTDSKYYKG
SLSYLNVTRKAYWQVHLDQVEVASGLTLCKEGCEAIVDTGTSLMVGPVDEVRELQKAIGAVP
LIQGEYMIPCEKVSTLPAITLKLGGKGYKLSPEDYTLKVSQAGKTLCLSGFMGMDIPPPSGP
LWILGDVFIGRYYTVFDRDNNRVGFAEAA
>d3cms__ b.50.1.2 (-) Chymosin (synonim: renin) {Cow (Bos taurus)}
GEVASVPLTNYLDSQYFGKIYLGTPPQEFTVLFDTGSSDFWVPSIYCKSNACKNHQRFDPRK
SSTFQNLGKPLSIHYGTGSMQGILGYDTVTVSNIVDIQQTVGLSTQEPGDFFTYAEFDGILG
MAYPSLASEYSIPVFDNMMNRHLVAQDLFSVYMDRNGQESMLTLGAIDPSYYTGSLHWVPVT
VQQYWQFTVDSVTISGVVVACEGGCQAILDTGTSKLVGPSSDILNIQQAIGATQNQYGEFDI
DCDNLSYMPTVVFEINGKMYPLTPSAYTSQDQGFCTSGFQSENHSQKWILGDVFIREYYSVF
DRANNLVGLAKAI
>d1hrna_ b.50.1.2 (A:) Chymosin (synonim: renin) {Human (Homo sapiens)}
GNTTSSVILTNYMDTQYYGEIGIGTPPQTFKVVFDTGSSNVWVPSSKCSRLYTACVYHKLFD
ASDSSSYKHNGTELTLRYSTGTVSGFLSQDIITVGGITVTQMFGEVTEMPALPFMLAEFDGV
VGMGFIEQAIGRVTPIFDNIISQGVLKEDVFSFYYNRDSENSQSLGGQIVLGGSDPQHYEGN
FHYINLIKTGVWQIQMKGVSVGSSTLLCEDGCLALVDTGASYISGSTSSIEKLMEALGAKKR

LFDYVVKCNEGPTLPDISFHLGGKEYTLTSADYVFQESYSSKKLCTLAIHAMDIPPPTGPTW
ALGATFIRKFYTEFDRRNNRIGFALAR
>d1smra_ b.50.1.2 (A:) Chymosin (synonim: renin) {Mouse (Mus musculus)}
TDLISPVVLTNYLNSQYYGEIGIGTPPQTFKVIFDTGSANLWVPSTKCSRLYLACGIHSLYE
SSDSSSYMENGDDFTIHYGSGRVKGFLSQDSVTVGGITVTQTFGEVTQLPLIPFMLAQFDGV
LGMGFPAQAVGGVTPVFDHILSQGVLKEKVFSVYYNRGPHLLGGEVVLGGSDPQHYQGDFHY
VSLSKTDSWQITMKGVSVGSSTLLCEEGCEVVVDTGSSFISAPTSSLKLIMQALGAKEKRLH
EYVVSCSQVPTLPDISFNLGGRAYTLSSTDYVLQYPNRRDKLCTVALHAMDIPPPTGPVWVL
GATFIRKFYTEFDRHNNRIGFALAR
>d1fkna_ b.50.1.2 (A:) beta-secretase (memapsin) {Human (Homo sapiens)}
RRGSFVEMVDNLRGKSGQGYYVEMTVGSPPQTLNILVDTGSSNFAVGAAPHPFLHRYYQRQL
SSTYRDLRKGVYVPYTQGKWEGELGTDLVSIPHGPNVTVRANIAAITESDKFFINGSNWEGI
LGLAYAEIARPDDSLEPFFDSLVKQTHVPNLFSLQLCGAGFPLNQSEVLASVGGSMIIGGID
HSLYTGSLWYTPIRREWYYEVIIVRVEINGQDLKMDCKEYNYDKSIVDSGTTNLRLPKKVFE
AAVKSIKAASSTEKFPDGFWLGEQLVCWQAGTTPWNIFPVISLYLMGEVTNQSFRITILPQQ
YLRPVEDVATSQDDCYKFAISQSSTGTVMGAVIMEGFYVVFDRARKRIGFAVSACHVHDEFR
TAAVEGPFVTLDMEDCGYN
>d1pfza_ b.50.1.2 (A:) Plasmepsin ii (a hemoglobin-degrading enzyme) {Plasmodium falciparum}
HLTIGFKVENAHDRILKTIKTHKLKNYIKESVNFLNSGLTKTNYLGSSNDNIELVDFQNIMF
YGDAEVGDNQQPFTFILDTGSANLWVPSVKCTTAGCLTKHLYDSSKSRTYEKDGTKVEMNYV
SGTVSGFFSKDLVTVGNLSLPYKFIEVIDTNGFEPTYTASTFDGILGLGWKDLSIGSVDPIV
VELKNQNKIENALFTFYLPVHDKHTGFLTIGGIEERFYEGPLTYEKLNHDLYWQITLDAHVG
NIMLEKANCIVDSGTSAITVPTDFLNKMLQNLDVIKVPFLPFYVTLCNNSKLPTFEFTSENG
KYTLEPEYYLQHIEDVGPGLCMLNIIGLDFPVPTFILGDPFMRKYFTVFDYDNHSVGIALAK
KNL
>d1smea_ b.50.1.2 (A:) Plasmepsin ii (a hemoglobin-degrading enzyme) {Plasmodium falciparum}
SSNDNIELVDFQNIMFYGDAEVGDNQQPFTFILDTGSANLWVPSVKCTTAGCLTKHLYDSSK
SRTYEKDGTKVEMNYVSGTVSGFFSKDLVTVGNLSLPYKFIEVIDTNGFEPTYTASTFDGIL
GLGWKDLSIGSVDPIVVELKNQNKIENALFTFYLPVHDKHTGFLTIGGIEERFYEGPLTYEK
LNHDLYWQITLDAHVGNIMLEKANCIVDSGTSAITVPTDFLNKMLQNLDVIKVPFLPFYVTL
CNNSKLPTFEFTSENGKYTLEPEYYLQHIEDVGPGLCMLNIIGLDFPVPTFILGDPFMRKYF
TVFDYDNHSVGIALAKKNL
>d1qs8a_ b.50.1.2 (A:) Plasmepsin ii (a hemoglobin-degrading enzyme) {Plasmodium vivax}
SENDVIELDDVANIMFYGEGEVGDNHQKFMLIFDTGSANLWVPSKKCNSSGCSIKNLYDSSK
SKSYEKDGTKVDITYGSGTVKGFFSKDLVTLGHLSMPYKFIEVIDTDDLEPIYSSVEFDGIL
GLGWKDLSIGSIDPIVVELKNQNKIDNALFTFYLPVHDVHAGYLTIGGIEEKFYEGNITYEK
LNHDLYWQIDLDVHFGKQTMEKANVIVDSGTTTITAPSEFLNKFFANLNVIKVPFLPFYVTT
CDNKEMPTLEFKSANNTYTLEPEYYMNPILEVDDTLCMITMLPVDIDSNTFILGDPFMRKYF
TVFDYDKESVGFAIAKN

>d1ile_2 b.51.1.1 (198-386) Isoleucyl-tRNA synthetase (IleRS) {Thermus thermophilus}
KEIQDPSVYVRFPLKEPKKLGLEKASLLIWTTTPWTLPGNVAAAVHPEYTYAAFQVGDEALI
LEEGLGRKLLGEGTQVLKTFPGKALEGLPYTPPYPQALEKGYFVVLADYVSQEDGTGIVHQA
PAFGAEDLETARVYGLPLLKTVDEEGKLLVEPFKGLYFREANRAILRDLRGRGLLFKEESYL
HSY
>d1ffya2 b.51.1.1 (A:201-394) Isoleucyl-tRNA synthetase (IleRS) {Staphylococcus aureus}
HDKRSASIYVAFNVKDDKGVVDADAKFIIWTTTPWTIPSNVAITVHPELKYGQYNVNGEKYI
IAEALSDAVAEALDWDKASIKLEKEYTGKELEWVVAQHPFLDRESLVINGDHVTTDAGTGCV
HTAPGHGEDDYIVGQQYELPVISPIDDKGVFTEEGGQFEGMFYDKANKAVTDLLTEKGALLK
LDFITHSY
>d1gaxa2 b.51.1.1 (A:190-342) Valyl-tRNA synthetase (ValRS) {Thermus thermophilus}
TEPTPGKLYTLRYEVEGGGFIEIATVRPETVFADQAIAVHPEDERYRHLLGKRARIPLTEVW
IPILADPAVEKDFGTGALKVTPAHDPLDYEIGERHGLKPVSVINLEGRMEGERVPEALRGLD
RFEARRKAVELFREAGHLVKEEDYTIALA
>d1gmua1 b.107.1.1 (A:1-70) Urease metallochaperone UreE, N-terminal domain {Klebsiella aerogenes}
MLYLTQRLEIPAAATASVTLPIDVRVKSRVKVTLNDGRDAGLLLPRGLLLRGGDVLSNEEGT
EFVQVIAA
>d1eara1 b.107.1.1 (A:1-74) Urease metallochaperone UreE, N-terminal domain {Bacillus pasteurii}
MVITKIVGHIDDLSHQIKKVDWLEVEWEDLNKRILRKETENGTDIAIKLENSGTLRYGDVLY
ESDDTLIAIRTK
>d2eng__ b.52.1.1 (-) Endoglucanase V {Humicola insolens}
ADGRSTRYWDCCKPSCGWAKKAPVNQPVFSCNANFQRITDFDAKSGCEPGGVAYSCADQTPW
AVNDDFALGFAATSIAGSNEAGWCCACYELTFTSGPVAGKKMVVQSTSTGGDLGSNHFDLNI
PGGGVGIFDGCTPQFGGLPGQRYGGISSRNECDRFPDALKPGCYWRFDWFKNADNPSFSFRQ
VQCPAELVARTGCRRNDDGNFPAV
>d1bw3__ b.52.1.2 (-) Barwin {Barley (Hordeum vulgare)}
EQANDVRATYHYYRPAQNNWDLGAPAVSAYCATWDASKPLSWRSKYGWTAFCGPAGPRGQAA
CGKCLRVTNPATGAQITARIVDQCANGGLDLDWDTVFTKIDTNGIGYQQGHLNVNYQFVDCR
D
>g1aw8.1 b.52.2.1 (A:,B:) Pyruvoyl dependent aspartate decarboxylase, ADC {Escherichia coli}
MIRTMLQGKLHRVKVTHADLHYEGXSCAIDQDFLDAAGILENEAIDIWNVTNGKRFSTYAIA
AERGSRIISVNGAAAHCASVGDIVIIASFVTMPDEEARTWRPNVAYFEGDNEMK
>d1eu1a1 b.52.2.2 (A:626-780) Dimethylsulfoxide reductase (DMSO reductase) {Rhodobacter sphaeroides}
ERLGGAGAKYPLHVVASHPKSRLHSQLNGTSLRDLYAVAGHEPCLINPADAAARGIADGDVL
RVFNDRGQILVGAKVSDAVMPGAIQIYEGGWYDPLDPSEEGTLDKYGDVNVLSLDVGTSKLA
QGNCGQTILADVEKYAGAPVTVTVFDTPKGA
>d1dmr_1 b.52.2.2 (626-781) Dimethylsulfoxide reductase (DMSO

reductase) {Rhodobacter capsulatus}
ERLDGPGAKYPLHIAASHPFNRLHSQLNGTVLREGYAVQGHEPCLMHPDDAAARGIADGDVV
RVHNDRGQILTGVKVTDAVMKGVIQIYEGGWYDPSDVTEPGTLDKYGDVNVLSADIGTSKLA
QGNCGQTVLAEVEKYTGPAVTLTGFVAPKAAE
>d1aa6_1 b.52.2.2 (565-715) Formate dehydrogenase H {Escherichia coli}
PIDKLTDEYPMVLSTVREVGHYSCRSMTGNCAALAALADEPGYAQINTEDAKRLGIEDEALV
WVHSRKGKIITRAQVSDRPNKGAIYMTYQWWIGACNELVTENLSPITKTPEYKYCAVRVEPI
ADQRAAEQYVIDEYNKLKTRLREAALA
>d1tmo_1 b.52.2.2 (632-798) Trimethylamine N-oxide reductase {Shewanella massilia}
ERSHGGPGSDKHPIWLQSCHPDKRLHSQMCESREYRETYAVNGREPVYISPVDAKARGIKDG
DIVRVFNDRGQLLAGAVVSDNFPKGIVRIHEGAWYGPVGKDGSTEGGAEVGALCSYGDPNTL
TLDIGTSKLAQACSAYTCLVEFEKYQGKVPKVSSFDGPIEVEI
>d1g8ka1 b.52.2.2 (A:683-825) Arsenite oxidase large subunit {Alcaligenes faecalis}
LPATVQQQKDKYRFWLNNGRNNEVWQTAYHDQYNSLMQERYPMAYIEMNPDDCKQLDVTGGD
IVEVYNDFGSTFAMVYPVAEIKRGQTFMLFGYVNGIQGDVTTDWTDRDIIPYYKGTWGDIRK
VGSMSEFKRTVSFKSRRFG
>d2napa1 b.52.2.2 (A:601-723) Dissimilatory nitrate reductase (NAP) {Desulfovibrio desulfuricans}
AAEEPDAEYPLYLTSMRVIDHWHTATMTGKVPELQKANPIAFVEINEEDAARTGIKHGDSVI
VETRRDAMELPARVSDVCRPGLIAVPFFDPKKLVNKLFLDATDPVSREPEYKICAARVRKA
>d1qcsa1 b.52.2.3 (A:0-85) N-terminal domain of NSF-N, NSF-Nn {Hamster (Cricetulus griseus)}
NMAGRSMQAARCPTDELSLSNCAVVSEKDYQSGQHVIVRTSPNHKYIFTLRTHPSVVPGSVA
FSLPQRKWAGLSIGQEIEVALYSF
>d1cr5a1 b.52.2.3 (A:26-107) N-terminal domain of NSF-N, NSF-Nn {Baker's yeast (Saccharomyces cerevisiae), sec18p}
TRHLKVSNCPNNSYALANVAAVSPNDFPNNIYIIIDNLFVFTTRHSNDIPPGTIGFNGNQRT
WGGWSLNQDVQAKAFDLFKY
>d1cz4a1 b.52.2.3 (A:1-91) N-terminal domain of VAT-N, VAT-Nn {Archaeon Thermoplasma acidophilum}
MESNNGIILRVAEANSTDPGMSRVRLDESSRRLLDAEIGDVVEIEKVRKTVGRVYRARPEDE
NKGIVRIDSVMRNNCGASIGDKVKVRKVR
>d1e32a1 b.52.2.3 (A:21-106) Membrane fusion atpase p97 N-terminal domain , P97-Nn {Mouse (Mus musculus)}
NRPNRLIVDEAINEDNSVVSLSQPKMDELQLFRGDTVLLKGKKRREAVCIVLSDDTCSDEKI
RMNRVVRNNLRVRLGDVISIQPCP
>d1dfup_ b.53.1.1 (P:) Ribosomal protein L25 {Escherichia coli}
MFTINAEVRKEQGKGASRRLRAANKFPAIIYGGKEAPLAIELDHDKVMNMQAKAEFYSEVLT
IVVDGKEIKVKAQDVQRHPYKPKLQHIDFVRA
>d1feua_ b.53.1.1 (A:) Ribosomal protein TL5 (general stress protein CTC) {Thermus thermophilus}

MEYRLKAYYREGEKPSALRRAGKLPGLMYNRHLNRKVYVDLVEFDKVFRQASIHHVIVLELP
DGQSLPTLVRQVNLDKRRRRPEHVDFFVLSDEPVEMYVPLRFVGTPAGVRAGGVLQEIHRDI
LVKVSPRNIPEFIEVDVSGLEIGDSLHASDLKLPPGVELAVSPEETIAAVVPPEDVEKLAE
>d1gtra1 b.53.1.2 (A:339-547) Gln-tRNA synthetase (GlnRS), C-terminal (anticodon-binding) domain {Escherichia coli}
APRAMAVIDPVKLVIENYQGEGEMVTMPNHPNKPEMGSRQVPFSGEIWIDRADFREEANKQY
KRLVLGKEVRLRNAYVIKAERVEKDAEGNITTIFCTYDADTLSKDPADGRKVKGVIHWVSAA
HALPVEIRLYDRLFSVPNPGAADDFLSVINPESLVIKQGFAEPSLKDAVAGKAFQFEREGYF
CLDSRHSTAEKPVFNRTVGLRDT
>d1h9db_ b.54.1.1 (B:) Core binding factor beta, CBF {Human (Homo sapiens)}
PRVVPDQRSKFENEEFFRKLSRECEIKYTGFRDRPHEERQARFQNACRDGRSEIAFVATGTN
LSLQFFPASWQGEQRQTPSREYVDLEREAGKVYLKAPMILNGVCVIWKGWIDLQRLDGMGCL
EFDEERAQQE
>d1mai__ b.55.1.1 (-) Phospholipase C delta-1 {Rat (Rattus norvegicus)}
GLQDDPDLQALLKGSQLLKVKSSSWRRERFYKLQEDCKTIWQESRKVMRSPESQLFSIEDIQ
EVRMGHRTEGLEKFARDIPEDRCFSIVFKDQRNTLDLIAPSPADAQHWVQGLRKIIH
>d1btn__ b.55.1.1 (-) beta-spectrin {Mouse (Mus musculus), brain}
MEGFLNRKHEWEAHNKKASSRSWHNVYCVINNQEMGFYKDAKSAASGIPYHSEVPVSLKEAI
CEVALDYKKKKHVFKLRLSDGNEYLFQAKDDEEMNTWIQAISSA
>d1dro__ b.55.1.1 (-) beta-spectrin {Fruit fly (Drosophila melanogaster)}
GSGTGAGEGHEGYVTRKHEWDSTTKKASNRSWDKVYMAAKAGRISFYKDQKGYKSNPELTFR
GEPSYDLQNAAIEIASDYTKKKHVLRVKLANGALFLLQAHDDTEMSQWVTSLKAQSDSTA
>d1dyna_ b.55.1.1 (A:) Dynamin {Human (Homo sapiens)}
ILVIRKGWLTINNIGIMKGGSKEYWFVLTAENLSWYKDDEEKEKKYMLSVDNLKLRDVEKGF
MSSKHIFALFNTEQRNVYKDYRQLELACETQEEVDSWKASFLRAGVYPERV
>d1btka_ b.55.1.1 (A:) Bruton's tyrosine kinase {Human (Homo sapiens)}
AAVILESIFLKRSQQKKKTSPLNFKKCLFLLTVHKLSYYEYDFERGRRGSKKGSIDVEKITC
VETVVPEKNPPPERQIPRRGEESSEMEQISIIERFPYPFQVVYDEGPLYVFSPTEELRKRWI
HQLKNVIRYNSDLVQKYHPCFWIDGQYLCCSQTAKNAMGCQILEN
>d1pls__ b.55.1.1 (-) Pleckstrin, N-terminal domain {Human (Homo sapiens)}
MEPKRIREGYLVKKGSVFNTWKPMWVVLLEDGIEFYKKKSDNSPKGMIPLKGSTLTSPCQDF
GKRMFVFKITTTKQQDHFFQAAFLEERDAWVRDINKAIKCIEGLEHHHHHH
>d1dbha2 b.55.1.1 (A:418-550) Son of sevenless-1 (sos-1) {Human (Homo sapiens)}
AIKKMNEIQKNIDGWEGKDIGQCCNEFIMEGTLTRVGAKHERHIFLFDGLMICCKSNHGQPR
LPGASNAEYRLKEKFFMRKVQINDKDDTNEYKHAFEIILKDENSVIFSAKSAEEKNNWMAAL
ISLQYRSTL
>d1foea2 b.55.1.1 (A:1240-1401) GEF of TIAM1 (T-Lymphoma invasion and metastasis indusing protein 1) {Mouse (Mus musculus)}

EFGAVFDQLIAEQTGEKKEVADLSMGDLLLHTSVIWLNPPASLGKWKKEPELAAFVFKTAVV
LVYKDGSKQKKKLVGSHRLSIYEEWDPFRFRHMIPTEALQVRALPSADAEANAVCEIVHVKS
ESEGRPERVFHLCCSSPESRKDFLKSVHSILRDKHRRQ
>d1bak__ b.55.1.1 (-) G-protein coupled receptor kinase 2
(beta-adrenergic receptor kinase 1) {Human (Homo sapiens)}
GSHMGKDCIMHGYMSKMGNPFLTQWQRRYFYLFPNRLEWRGEGEAPQSLLTMEEIQSVEETQ
IKERKCLLLKIRGGKQFILQCDSDPELVQWKKELRDAYREAQQLVQRVPKMKNKPRS
>d1faoa_ b.55.1.1 (A:) Dual adaptor of phosphotyrosine and
3-phosphoinositides DAPP1/PHISH {Human (Homo sapiens)}
PSLGTKEGYLTKQGGLVKTWKTRWFTLHRNELKYFKDQMSPEPIRILDLTECSAVQFDYSQE
RVNCFCLVFPFRTFYLCAKTGVEADEWIKILRWKLSQI
>d1fgya_ b.55.1.1 (A:) Grp1 {Mouse (Mus musculus)}
TFFNPDREGWLLKLGGRVKTWKRRWFILTDNCLYYFEYTTDKEPRGIIPLENLSIREVLDPR
KPNCFELYNPSHKGQVIKACKTEADGRVVEGNHVVYRISAPSPEEKEEWMKSIKASISRDPF
YDM
>d1fhoa_ b.55.1.1 (A:) UNC-89 {Nematode (Caenorhabditis elegans)}
MGDTGKLGRIIRHDAFQVWEGDEPPKLRYVFLFRNKIMFTEQDASTSPPSYTHYSSIRLDKY
NIRQHTTDEDTIVLQPQEPGLPSFRIKPKDFETSEYVRKAWLRDIAEEQEKYAAERD
>d1aqca_ b.55.1.2 (A:) X11 {Human (Homo sapiens)}
MEDLIDGIIFAANYLGSTQLLSDKTPSKNVRMMQAQEAVSRIKMAQKLAKSRKKAPEGESQP
MTEVDLFILTQRIKVLNADTQETMMDHPLRTISYIADIGNIVVLMARRRIPRSNSQENVEAS
HPSQDGKRQYKMICHVFESEDAQLIAQSIGQAFSVAYQEFLR
>d1qqga1 b.55.1.2 (A:12-114) Insulin receptor substrate 1, IRS-1
{Human (Homo sapiens)}
DVRKVGYLRKPKSMHKRFFVLRAASEAGGPARLEYYENEKKWRHKSSAPKRSIPLESCFNIN
KRADSKNKHLVALYTRDEHFAIAADSEAEQDSWYQALLQLH
>d1qqga2 b.55.1.2 (A:159-262) Insulin receptor substrate 1, IRS-1
{Human (Homo sapiens)}
AFKEVWQVILKPKGLGQTKNLIGIYRLCLTSKTISFVKLNSEAAAVVLQLMNIRRCGHSENF
FFIEVGRSAVTGPGEFWMQVDDSVVAQNMHETILEAMRAMSD
>d1shca_ b.55.1.2 (A:) Shc adaptor protein {Human (Homo sapiens)}
GSHMGQLGGEEWTRHGSFVNKPTRGWLHPNDKVMGPGVSYLVRYMGCVEVLQSMRALDFNTR
TQVTREAISLVCEAVPGAKGATRRRKPCSRPLSSILGRSNLKFAGMPITLTVSTSSLNLMAA
DCKQIIANHHMQSISFASGGDPDTAEYVAYVAKDPVNQRACHILECPEGLAQDVISTIGQAF
ELRFKQYLR
>d1ddma_ b.55.1.2 (A:) Numb {Fruit fly (Drosophila melanogaster)}
HQWQADEEAVRSATCSFSVKYLGCVEVFESRGMQVCEEALKVLRQSRRRPVRGLLHVSGDGL
RVVDDETKGLIVDQTIEKVSFCAPDRNHERGFSYICRDGTTRRWMCHGFLACKDSGERLSHA
VGCAFAVCLER
>d1rrpb_ b.55.1.3 (B:) Nuclear pore complex protein Nup358 {Human
(Homo sapiens)}
HFEPVVPLPDKIEVKTGEEDEEEFFCNRAKLFRFDVESKEWKERGIGNVKILRHKTSGKIRL
LMRREQVLKICANHYISPDMKLTPNAGSDRSFVWHALDYADELPKPEQLAIRFKTPEEAALF
KCKFEEAQSI

>d1k5db_ b.55.1.3 (B:) Ran-binding protein 1, Ranbp1 {Human (Homo sapiens)}
NHDPQFEPIVSLPEQEIKTLEEDEEELFKMRAKLFRFASENDLPEWKERGTGDVKLLKHKEK
GAIRLLMRRDKTLKICANHYITPMMELKPNAGSDRAWVWNTHADFADECPKPELLAIRFLNA
ENAQKFKTKFEECRKEIEEREK
>d1evha_ b.55.1.4 (A:) Enabled {Mouse (Mus musculus)}
SEQSICQARAAVMVYDDANKKWVPAGGSTGFSRVHIYHHTGNNTFRVVGRKIQDHQVVINCA
IPKGLKYNQATQTFHQWRDARQVYGLNFGSKEDANVFASAMMHALEVLN
>d1qc6a_ b.55.1.4 (A:) Ena/vasp-like protein {Mouse (Mus musculus)}
MSEQSICQARASVMVYDDTSKKWVPIKPGQQGFSRINIYHNTASSTFRVVGVKLQDQQVVIN
YSIVKGLKYNQATPTFHQWRDARQVYGLNFASKEEATTFSNAMLFALNIMN
>d1egxa_ b.55.1.4 (A:) Vasodilator-stimulated phosphoprotein (VASP) {Human (Homo sapiens)}
MSETVICSSRATVMLYDDGNKRWLPAGTGPQAFSRVQIYHNPTANSFRVVGRKMQPDQQVVI
NCAIVRGVKYNQATPNFHQWRDARQVWGLNFGSKEDAAQFAAGMASALEALEG
>d1ddwa_ b.55.1.4 (A:) Homer {Rat (Rattus norvegicus)}
MGEQPIFSTRAHVFQIDPNTKKNWVPTSKHAVTVSYFYDSTRNVYRIISLDGSKAIINSTIT
PNMTFTKTSQKFGQWADSRANTVYGLGFSSEHHLSKFAEKFQEFKEAAR
>d1i7aa_ b.55.1.4 (A:) Homer {Mouse (Mus musculus), 2b/vesl 2}
EQPIFTTRAHVFQIDPSTKKNWVPASKQAVTVSYFYDVTRNSYRIISVDGAKVIINSTITPN
MTFTKTSQKFGQWADSRANTVFGLGFSSELQLTKFAEKFQEVREAAR
>d1e5wa2 b.55.1.5 (A:199-346) Moesin {Human (Homo sapiens)}
EMYGVNYFSIKNKKGSELWLGVDALGLNIYEQNDRLTPKIGFPWSEIRNISFNDKKFVIKPI
DKKAPDFVFYAPRLRINKRILALCMGNHELYMRRRKPDTIEVQQMKAQAREEKHQKQMERAM
LENEKKKREMAEKEKEKIEREKEE
>d1ef1a2 b.55.1.5 (A:199-297) Moesin {Human (Homo sapiens)}
EMYGVNYFSIKNKKGSELWLGVDALGLNIYEQNDRLTPKIGFPWSEIRNISFNDKKFVIKPI
DKKAPDFVFYAPRLRINKRILALCMGNHELYMRRRKP
>d1gc7a2 b.55.1.5 (A:199-297) Radixin {Mouse (Mus musculus)}
EMYGVNYFEIKNKKGTELWLGVDALGLNIYEHDDKLTPKIGFPWSEIRNISFNDKKFVIKPI
DKKAPDFVFYAPRLRINKRILALCMGNHELYMRRRKP
>d1gg3a2 b.55.1.5 (A:188-279) Erythroid membrane protein 4.1R {Human (Homo sapiens)}
GVDLHKAKDLEGVDIILGVCSSGLLVYKDKLRINRFPWPKVLKISYKRSSFFIKIRPGEQEQ
YESTIGFKLPSYRAAKKLWKVCVEHHTFFR
>d1h4ra2 b.55.1.5 (A:215-313) Merlin {Human (Homo sapiens)}
EMYGVNYFAIRNKKGTELLLGVDALGLHIYDPENRLTPKISFPWNEIRNISYSDKEFTIKPL
DKKIDVFKFNSSKLRVNKLILQLCIGNHDLFMRRRKA
>d1ytfc1 b.56.1.1 (C:) Transcription factor IIA (TFIIA), N-terminal domain {Baker's yeast (Saccharomyces cerevisiae)}
ENLMLCLYDKVTRTKARWKCSLKDGVVTINRNDYTFQKAQVEAEWV
>d1ytfd2 b.56.1.1 (D:55-119) Transcription factor IIA (TFIIA), N-terminal domain {Baker's yeast (Saccharomyces cerevisiae)}

NTQSKLTVKGNLDTYGFCDDVWTFIVKNCQVTVEDSHRDASQNGSGDSQSVISVDKLRIVAC
NSK
>d1iega_ b.57.1.1 (A:) Human cytomegalovirus protease {Human cytomegalovirus}
QAVAPVYVGGFLARYDQSPDEAELLLPRDVVEHWLHAQGQGQPSLSVALPLNINHDDTAVVG
HVAAMQSVRDGLFCLGCVTSPRFLEIVRRASEKSELVSRGPVSPLQPDKVVEFLSGSYAGLS
LASRRCDDVEQATSLSGSETTPFKAVALCSVGRRRGTLAVYGRDPEWVTQRFPDLTAADRDG
LRAQWQRCGSTAVDASGDPFRSDSYGLLGNSVDALYIRERLPKLRYDKQLVGVTERESYVKA
>d1jq6a_ b.57.1.1 (A:) Human cytomegalovirus protease {Human cytomegalovirus}
VAPVYVGGFLARYDQSPDEAELLLPRDVVEHWLHAQGQGQPSLSVALPLNINHDDTAVVGHV
AAMQSVRDGLFCLGCVTSPRFLEIVRRASEKSELVSRGPVSPLQPDKVVEFLSGSYAGLSLS
SRRCDDVEQATSLSGSETTPFKHVALCSVGRRRGTLAVYGRDPEWVTQRFPDLTAADRDGLR
AQWQRCGSTAVDASGDPFRSDSYGLLGNYVDALY
>d1at3a_ b.57.1.1 (A:) HSV-2 protease {Herpes simplex virus type 2}
RAVPIYVAGFLALYDSGDPGELALDPDTVRAALPPENPLPINVDHRARCEVGRVLAVVNDPR
GPFFVGLIACVQLERVLETAASAAIFERRGPALSREERLLYLITNYLPSVSLSTKRRGDEVP
PDRTLFAHVALCAIGRRLGTIVTYDTSLDAAIAPFRHLDPATREGVRREAAEAELALAGRTW
APGVEALTHTLLSTAVNNMMLRDRWSLVAERRRQAGIAGHTYLQA
>d1fl1a_ b.57.1.1 (A:) KSHV protease {Kaposi's sarcoma-associated herpes virus}
AQGLYVGGFVDVVSCPKLEQELYLDPDQVTDYLPVTEPLPITIEHLPETEVGWTLGLFQVSH
GIFCTGAITSPAFLELASRLADTSHVARAPVKNLPKEPLLEILHTWLPGLSLSSIHPRELSQ
TPSGPVFQHVSLCALGRRRGTVAVYGHDAEWVVSRFSSVSKSERAHILQHVSSCRLEDLSTP
NFVSPLETLMAKAIDAGFIRDRLDLLKTDRGVASILSPVYLKA
>d1vzv__ b.57.1.1 (-) VZV protease {Varicella-Zoster virus}
EALYVAGYLALYSKDEGELNITPEIVRSALPPTSKIPINIDHRKDCVVGEVIAIIEDIRGPF
FLGIVRCPQLHAVLFEAAHSNFFGNRDSVLSPLERALYLVTNYLPSVSLSSKRLFTHVALCV
VGRRVGTVVNYDCTPESSIEPFRVLSMESKARLLSLVKDYAGLNKVWKVSEDKLAKVLLSTA
VNNMLLRDRWDVVAKRRREAGIMGH
>d1a49a1 b.58.1.1 (A:116-217) Pyruvate kinase (PK) {Rabbit (Oryctolagus cuniculus)}
PEIRTGLIKGSGTAEVELKKGATLKITLDNAYMEKCDENILWLDYKNICKVVDVGSKVYVDD
GLISLQVKQKGPDFLVTEVENGGFLGSKKGVNLPGAAVDL
>d1pkm_1 b.58.1.1 (116-217) Pyruvate kinase (PK) {Cat (Felis domestica)}
PEIRTGLIKGSGTAEVELKKGATLKITLDNAYMEKCDENVLWLDYKNICKVVEVGSKVYVDD
GLISLLVKEKGADFLVTEVENGGSLGSKKGVNLPGAAVDL
>d1pkla1 b.58.1.1 (A:88-186) Pyruvate kinase (PK) {Leishmania mexicana}
EIRTGQFVGGDAVMERGATCYVTTDPAFADKGTKDKFYIDYQNLSKVVRPGNYIYIDDGILI
LQVQSHEDEQTLECTVTNSHTISDRRGVNLPGCDVDL
>d1a3wa1 b.58.1.1 (A:88-188) Pyruvate kinase (PK) {Baker's yeast

(Saccharomyces cerevisiae)}
PEIRTGTTTNDVDYPIPPNHEMIFTTDDKYAKACDDKIMYVDYKNITKVISAGRIIYVDDGV
LSFQVLEVVDDKTLKVKALNAGKICSHKGVNLPGTDVDL
>d1e0ta1 b.58.1.1 (A:70-167) Pyruvate kinase (PK) {Escherichia coli}
PEIRTMKLEGGNDVSLKAGQTFTFTTDKSVIGNSEMVAVTYEGFTTDLSVGNTVLVDDGLIG
MEVTAIEGNKVICKVLNNGDLGENKGVNLPGVSIAL
>d1g8fa1 b.58.1.2 (A:2-168) ATP sulfurylase N-terminal domain {Baker's yeast (Saccharomyces cerevisiae)}
PAPHGGILQDLIARDALKKNELLSEAQSSDILVWNLTPRQLCDIELILNGGFSPLTGFLNEN
DYSSVVTDSRLADGTLWTIPITLDVDEAFANQIKPDTRIALFQDDEIPIAILTVQDVYKPNK
TIEAERVFRGDPEHPAISYLFNVAGDYYVGGSLEAIQLPQHYD
>d1i2da1 b.58.1.2 (A:2-170) ATP sulfurylase N-terminal domain {Fungus (Penicillium chrysogenum)}
ANAPHGGVLKDLLARDAPRQAELAAEAESLPAVTLTERQLCDLELIMNGGFSPLEGFMNQAD
YDRVCEDNRLADGNVFSMPITLDASQEVIDEKKLQAGSRITLRDFRDDRNLAILTIDDIYRP
DKTKEAKLVFGGDPEHPAIVYLNNTVKEFYIGGKIEAVNKLNHYD
>d1jhda1 b.58.1.2 (A:1-173) ATP sulfurylase N-terminal domain {unnamed symbiont of Riftia pachyptila}
MIKPVGSDELKPLFVYDPEEHHKLSHEAESLPSVVISSQAAGNAVMMGAGYFSPLQGFMNVA
DAMGAAEKMTLSDGSFFPVPVLCLLENTDAIGDAKRIALRDPNVEGNPVLAVMDIEAIEEVS
DEQMAVMTDKVYRTTDMDHIGVKTFNSQGRVAVSGPIQVLNFSYFQADF
>d1ik9a1 b.59.1.1 (A:1-117) XRCC4, N-terminal domain {Human (Homo sapiens)}
MERKISRIHLVSEPSITHFLQVSWEKTLESGFVITLTDGHSAWTGTVSESEISQEADDMAME
KGKYVGELRKALLSGAGPADVYTFNFSKESAYFFFEKNLKDVSFRLGSFNLEKVE
>d1hbq__ b.60.1.1 (-) Retinol binding protein {Cow (Bos taurus)}
ERDCRVSSFRVKENFDKARFAGTWYAMAKKDPEGLFLQDNIVAEFSVDENGQMSATAKGRVR
LLNNWDVCADMVGTFTDTEDPAKFKMKYWGVASFLQKGNDDHWIIDTDYETFAVQYSCRLLN
LDGTCADSYSFVFARDPSGFSPEVQKIVRQRQEELCLARQYRLIPHNGYCNGK
>d1aqb__ b.60.1.1 (-) Retinol binding protein {Pig (Sus scrofa domestica)}
ERDCRVSSFRVKENFDKARFSGTWYAMAKKDPEGLFLQDNIVAEFSVDENGHMSATAKGRVR
LLNNWDVCADMVGTFTDTEDPAKFKMKYWGVASFLQKGNDDHWIIDTDYDTYAVQYSCRLQN
LDGTCADSYSFVFARDPHGFSPEVQKIVRQRQEELCLARQYRIITHNGYCD
>d1rbp__ b.60.1.1 (-) Retinol binding protein {Human (Homo sapiens)}
ERDCRVSSFRVKENFDKARFSGTWYAMAKKDPEGLFLQDNIVAEFSVDETGQMSATAKGRVR
LLNNWDVCADMVGTFTDTEDPAKFKMKYWGVASFLQKGNDDHWIVDTDYDTYAVQYSCRLLN
LDGTCADSYSFVFSRDPNGLPPEAQKIVRQRQEELCLARQYRLIVHNGYCD
>d1iiua_ b.60.1.1 (A:) Retinol binding protein {Chicken (Gallus gallus), plasma isoform}
MDCRVSSFKVKENFDKNRYSGTWYAMAKKDPEGLFLQDNVVAQFTVDENGQMSATAKGRVRL
FNNWDVCADMIGSFTDTEDPAKFKMKYWGVASFLQKGNDDHWVVDTDYDTYALHYSCRELNE

DGTCADSYSFVFSRDPKGLPPEAQKIVRQRQIDLCLDRKYRVIVHNGFCS
>d1hn2a_ b.60.1.1 (A:) Odorant-binding protein {Cow (Bos taurus)}
AQEEEAEQNLSELSGPWRTVYIGSTNPEKIQENGPFRTYFRELVFDDEKGTVDFYFSVKRDG
KWKNVHVKATKQDDGTYVADYEGQNVFKIVSLSRTHLVAHNINVDKHGQTTELTELFVKLNV
EDEDLEKFWKLTEDKGIDKKNVVNFLENENHPHPE
>d1dzka_ b.60.1.1 (A:) Odorant-binding protein {Pig (Sus scrofa)}
FELSGKWITSYIGSSDLEKIGENAPFQVFMRSIEFDDKESKVYLNFFSKENGICEEFSLIGT
KQEGNTYDVNYAGNNKFVVSYASETALIISNINVDEEGDKTIMTGLLGKGTDIEDQDLEKFK
EVTRENGIPEENIVNIIERDDCPA
>d1bj7__ b.60.1.1 (-) Lipocalin allergen {Cow (Bos taurus), bos d 2}
IDPSKIPGEWRIIYAAADNKDKIVEGGPLRNYYRRIECINDCESLSITFYLKDQGTCLLLTE
VAKRQEGYVYVLEFYGTNTLEVIHVSENMLVTYVENYDGERITKMTEGLAKGTSFTPEELEK
YQQLNSERGVPNENIENLIKTDNCPP
>d1ew3a_ b.60.1.1 (A:) Lipocalin allergen {Horse (Equus caballus), equ c 1}
VAIRNFDISKISGEWYSIFLASDVKEKIEENGSMRVFVDVIRALDNSSLYAEYQTKVNGECT
EFPMVFDKTEEDGVYSLNYDGYNVFRISEFENDEHIILYLVNFDKDRPFQLFEFYAREPDVS
PEIKEEFVKIVQKRGIVKENIIDLTKIDRCFQLRG
>d1e5pa_ b.60.1.1 (A:) Aphrodisin, a sex pheromone {Golden hamster (Mesocricetus auratus)}
FAELQGKWYTIVIAADNLEKIEEGGPLRFYFRHIDCYKNCSEMEITFYVITNNQCSKTTVIG
YLKGNGTYETQFEGNNIFQPLYITSDKIFFTNKNMDRAGQETNMIVVAGKGNALTPEENEIL
VQFAHEKKIPVENILNILATDTCPE
>d1beba_ b.60.1.1 (A:) beta-Lactoglobulin {Cow (Bos taurus)}
QTMKGLDIQKVAGTWYSLAMAASDISLLDAQSAPLRVYVEELKPTPEGDLEILLQKWENGEC
AQKKIIAEKTKIPAVFKIDALNENKVLVLDTDYKKYLLFCMENSAEPEQSLVCQCLVRTPEV
DDEALEKFDKALKALPMHIRLSFNPTQLEEQC
>d1exsa_ b.60.1.1 (A:) beta-Lactoglobulin {Pig (Sus scrofa)}
VEVTPIMTELDTQKVAGTWHTVAMAVSDVSLLDAKSSPLKAYVEGLKPTPEGDLEILLQKRE
NDKCAQEVLLAKKTDIPAVFKINALDENQLFLLDTDYDSHLLLCMENSASPEHSLVCQSLAR
TLEVDDQIREKFEDALKTLSVPMRILPAQLEEQCRV
>d1epba_ b.60.1.1 (A:) Retinoic acid-binding protein {Rat (Rattus norvegicus), albino}
VKDFDISKFLGFWYEIAFASKMGTPGLAHKEEKMGAMVVELKENLLALTTTYYSEDHCVLEK
VTATEGDGPAKFQVTRLSGKKEVVVEATDYLTYAIIDITSLVAGAVHRTMKLYSRSLDDNGE
ALYNFRKITSDHGFSETDLYILKHDLTCVKVLQSAA
>d1jv4a_ b.60.1.1 (A:) Major urinary protein/alpha-2u-globulin {Mouse (Mus musculus)}
EEASSTGRNFNVEKINGEWHTIILASDKREKIEDNGNFRLFLEQIHVLEKSLVLKFHTVRDE
ECSELSMVADKTEKAGEYSVTYDGFNTFTIPKTDYDNFLMAHLINEKDGETFQLMGLYGREP
DLSSDIKERFAQLCEEHGILRENIIDLSNANRC
>d2a2ua_ b.60.1.1 (A:) Major urinary protein/alpha-2u-globulin {Rat (Rattus norvegicus)}

EEASSTRGNLDVAKLNGDWFSIVVASNKREKIEENGSMRVFMQHIDVLENSLGFKFRIKENG
ECRELYLVAYKTPEDGEYFVEYDGGNTFTILKTDYDRYVMFHLINFKNGETFQLMVLYGRTK
DLSSDIKEKFAKLCEAHGITRDNIIDLTKTDRCL
>d1qqsa_ b.60.1.1 (A:) Neutrophil gelatinase-associated lipocalin (NGAL) {Human (Homo sapiens)}
TSDLIPAPPLSKVPLQQNFQDNQFQGKWYVVGLAGNAILREDKDPQKMYATIYEEKEDASYN
VTSVLFRKKKCDYAIRTFVPGCQPGEFTLGNIKSYPGLTSYLVRVVSTNYNQHAMVFFKKVS
QNREYFKITLYGRTKELTSELKNNFIRFSKSLGLPENHIVFPVPIDQCID
>d1bbpa_ b.60.1.1 (A:) Bilin-binding protein {Cabbage butterfly (Pieris brassicae)}
NVYHDGACPEVKPVDNFDWSNYHGKWWEVAKYPNSVEKYGKCGWAEYTPEGKSVKVSNYHVI
HGKEYFIEGTAYPVGDSKIGKIYHKLTYGGVTKENVFNVLSTDNKNYIIGYYCKYDEDKKGH
QDFVWVLSRSKVLTGEAKTAVENYLIGSPVVDSQKLVYSDFSEAACKVN
>d1i4ua_ b.60.1.1 (A:) Alpha-crustacyanin {European lobster (Homarus gammarus)}
DKIPDFVVPGKCASVDRNKLWAEQTPNRNSYAGVWYQFALTNNPYQLIEKCVRNEYSFDGKQ
FVIESTGIAYDGNLLKRNGKLYPNPFGEPHLSIDYENSFAAPLVILETDYSNYACLYSCIDY
NFGYHSDFSFIFSRSANLADQYVKKCEAAFKNINVDTTRFVKTVQGSSCPYDTQKTL
>d1qfta_ b.60.1.1 (A:) Histamine binding protein {Brown ear tick (Rhipicephalus appendiculatus)}
NQPDWADEAANGAHQDAWKSLKADVENVYYMVKATYKNDPVWGNDFTCVGVMANDVNEDEKS
IQAEFLFMNNADTNMQFATEKVTAVKMYGYNRENAFRYETEDGQVFTDVIAYSDDNCDVIYV
PGTDGNEEGYELWTTDYDNIPANCLNKFNEYAVGRETRDVFTSACLEIAAA
>d1np1a_ b.60.1.1 (A:) Nitrophorin 1 {Rhodnius prolixus}
KCTKNALAQTGFNKDKYFNGDVWYVTDYLDLEPDDVPKRYCAALAAGTASGKLKEALYHYDP
KTQDTFYDVSELQEESPGKYTANFKKVEKNGNVKVDVTSGNYYTFTVMYADDSSALIHTCLH
KGNKDLGDLYAVLNRNKDTNAGDKVKGAVTAASLKFSDFISTKDNKCEYDNVSLKSLLTK
>d1euoa_ b.60.1.1 (A:) Nitrophorin 2 (prolixin-s) {Rhodnius prolixus}
MDCSTNISPKQGLDKAKYFSGKWYVTHFLDKDPQVTDQYCSSFTPRESDGTVKEALYHYNAN
KKTSFYNIGEGKLESSGLQYTAKYKTVDKKKAVLKEADEKNSYTLTVLEADDSSALVHICLR
EGSKDLGDLYTVLTHQKDAEPSAKVKSAVTQAGLQLSQFVGTKDLGCQYDDQFTSL
>d1koia_ b.60.1.1 (A:) Nitrophorin 4 {Rhodnius prolixus}
ACTKNAIAQTGFNKDKYFNGDVWYVTDYLDLEPDDVPKRYCAALAAGTASGKLKEALYHYDP
KTQDTFYDVSELQVESLGKYTANFKKVDKNGNVKVAVTAGNYYTFTVMYADDSSALIHTCLH
KGNKDLGDLYAVLNRNKDAAAGDKVKSAVSAATLEFSKFISTKENNCAYDNDSLKSLLTK
>d1hms__ b.60.1.2 (-) Muscle fatty acid binding protein (m-fabp) {Human (Homo sapiens)}
VDAFLGTWKLVDSKNFDDYMKSLGVGFATRQVASMTKPTTIIEKNGDILTLKTHSTFKNTEI
SFKLGVEFDETTADDRKVKSIVTLDGGKLVHLQKWDGQETTLVRELIDGKLILTLTHGTAVC
TRTYEKE
>d1bwya_ b.60.1.2 (A:) Muscle fatty acid binding protein (m-fabp) {Cow (Bos taurus)}
VDAFVGTWKLVDSKNFDDYMKSLGVGFATRQVGNMTKPTTIIEVNGDTVIIKTQSTFKNTEI

SFKLGVEFDETTADDRKVKSIVTLDGGKLVHVQKWNGQETSLVREMVDGKLILTLTHGTAVC
TRTYEKQA
>d1a57__ b.60.1.2 (-) Intestinal fatty acid binding protein {Rat (Rattus norvegicus)}
AFDGTWKVDRNENYSGAHDNLKLTITQEGNKFTVKESSNFRNIDVVFELGVDFAYSLADGTE
LTGTWTMEGNKLVGKFKRVDNGKELIAVREISGNELIQTYTYEGVEAKRIFKKE
>d1ifc__ b.60.1.2 (-) Intestinal fatty acid binding protein {Rat (Rattus norvegicus)}
AFDGTWKVDRNENYEKFMEKMGINVVKRKLGAHDNLKLTITQEGNKFTVKESSNFRNIDVVF
ELGVDFAYSLADGTELTGTWTMEGNKLVGKFKRVDNGKELIAVREISGNELIQTYTYEGVEA
KRIFKKE
>d3ifba_ b.60.1.2 (A:) Intestinal fatty acid binding protein {Human (Homo sapiens)}
AFDSTWKVDRSENYDKFMEKMGVNIVKRKLAAHDNLKLTITQEGNKFTVKESSAFRNIEVVF
ELGVTFNYNLADGTELRGTWSLEGNKLIGKFKRTDNGNELNTVREIIGDELVQTYVYEGVEA
KRIFKKD
>d1fdqa_ b.60.1.2 (A:) Brain fatty acid binding protein {Human (Homo sapiens)}
VEAFCATWKLTNSQNFDEYMKALGVGFATRQVGNVTKPTVIISQEGDKVVIRTLSTFKNTEI
SFQLGEEFDETTADDRNCKSVVSLDGDKLVHIQKWDGKETNFVREIKDGKMVMTLTFGDVVA
VRHYEKA
>d1b56__ b.60.1.2 (-) Epidermal fatty acid binding protein {Human (Homo sapiens)}
TVQQLEGRWRLVDSKGFDEYMKELGVGIALRKMGAMAKPDCIITCDGKNLTIKTESTLKTTQ
FSCTLGEKFEETTADGRKTQTVCNFTDGALVQHQEWDGKESTITRKLKDGKLVVECVMNNVT
CTRIYEKVE
>d1lid__ b.60.1.2 (-) Adipocyte lipid-binding protein, ALBP {Mouse (Mus musculus)}
CDAFVGTWKLVSSENFDDYMKEVGVGFATRKVAGMAKPNMIISVNGDLVTIRSESTFKNTEI
SFKLGVEFDEITADDRKVKSIITLDGGALVQVQKWDGKSTTIKRKRDGDKLVVECVMKGVTS
TRVYERA
>d1mdc__ b.60.1.2 (-) Fatty acid-binding protein {Tobacco hornworm (Manduca sexta)}
SYLGKVYSLVKQENFDGFLKSAGLSDDKIQALVSDKPTQKMEANGDSYSNTSTGGGGAKTVS
FKSGVEFDDVIGAGDSVKSMYTVDGNVVTHVVKGDAGVATFKKEYNGDDLVVTITSSNWDGV
ARRYYKA
>d1ftpa_ b.60.1.2 (A:) Fatty acid-binding protein {Desert locust (Schistocerca gregaria)}
VKEFAGIKYKLDSQTNFEEYMKAIGVGAIERKAGLALSPVIELEILDGDKFKLTSKTAIKNT
EFTFKLGEEFDEETLDGRKVKSTITQDGPNKLVHEQKGDHPTIIIREFSKEQCVITIKLGDL
VATRIYKAQ
>d1cbs__ b.60.1.2 (-) Cellular retinoic-acid-binding protein (CRABP) {Human (Homo sapiens), CRABP-II}
PNFSGNWKIIRSENFEELLKVLGVNVMLRKIAVAAASKPAVEIKQEGDTFYIKTSTTVRTTE

INFKVGEEFEEQTVDGRPCKSLVKWESENKMVCEQKLLKGEGPKTSWTRELTNDGELILTMT
ADDVVCTRVYVRE
>d1cbia_ b.60.1.2 (A:) Cellular retinoic-acid-binding protein (CRABP) {Cow and mouse (Bos taurus) and (Mus musculus), CRABP-I, identical sequences}
PNFAGTWKMRSSENFDELLKALGVNAMLRKVAVAAASKPHVEIRQDGDQFYIKTSTTVRTTE
INFKVGEGFEEETVDGRKCRSLPTWENENKIHCTQTLLEGDGPKTYWTRELANDELILTFGA
DDVVCTRIYVRE
>d1crb__ b.60.1.2 (-) Cellular retinol-binding protein II (CRBP) {Rat (Rattus norvegicus)}
PVDFNGYWKMLSNENFEEYLRALDVNVALRKIANLLKPDKEIVQDGDHMIIRTLSTFRNYIM
DFQVGKEFEEDLTGIDDRKCMTTVSWDGDKLQCVQKGEKEGRGWTQWIEGDELHLEMRAEGV
TCKQVFKKVH
>d1opaa_ b.60.1.2 (A:) Cellular retinol-binding protein II (CRBP) {Rat (Rattus norvegicus)}
TKDQNGTWEMESNENFEGYMKALDIDFATRKIAVRLTQTKIIVQDGDNFKTKTNSTFRNYDL
DFTVGVEFDEHTKGLDGRNVKTLVTWEGNTLVCVQKGEKENRGWKQWVEGDKLYLELTCGDQ
VCRQVFKKK
>d1ggla_ b.60.1.2 (A:) Cellular retinol-binding protein III {Human (Homo sapiens)}
PPNLTGYYRFVSQKNMEDYLQALNISLAVRKIALLLKPDKEIEHQGNHMTVRTLSTFRNYTV
QFDVGVEFEEDLRSVDGRKCQTIVTWEEEHLVCVQKGEVPNRGWRHWLEGEMLYLELTARDA
VCEQVFRKVH
>d1lfo__ b.60.1.2 (-) Liver fatty acid binding protein {Rat (Rattus norvegicus)}
MNFSGKYQVQSQENFEPFMKAMGLPEDLIQKGKDIKGVSEIVHEGKKVKLTITYGSKVIHNE
FTLGEECELETMTGEKVKAVVKMEGDNKMVTTFKGIKSVTEFNGDTITNTMTLGDIVYKRVS
KRI
>d1pmpa_ b.60.1.2 (A:) P2 myelin protein {Cow (Bos taurus), caudal spinal root myelin}
SNKFLGTWKLVSSENFDEYMKALGVGLATRKLGNLAKPRVIISKKGDIITIRTESPFKNTEI
SFKLGQEFEETTADNRKTKSTVTLARGSLNQVQKWNGNETTIKRKLVDGKMVVECKMKDVVC
TRIYEKV
>d1eal__ b.60.1.2 (-) Ileal lipid binding protein {Pig (Sus scrofa)}
AFTGKYEIESEKNYDEFMKRLALPSDAIDKARNLKIISEVKQDGQNFTWSQQYPGGHSITNT
FTIGKECDIETIGGKKFKATVQMEGGKVVVNSPNYHHTAEIVDGKLVEVSTVGGVSYERVSK
KLA
>d1avgi_ b.60.1.3 (I:) Thrombin inhibitor {Triatomine bug (Triatoma pallidipennis)}
AEGDDCSIEKAMGDFKPEEFFNGTWYLAHGPGVTSPAVCQKFTTSGSKGFTQIVEIGYNKFE
SNVKFQCNQVDNKNGEQYSFKCKSSDNTEFEADFTFISVSYDNFALVCRSITFTSQPKEDRY
LVFERTKSDTDPDAKEIC
>d1swga_ b.61.1.1 (A:) Streptavidin {Streptomyces avidinii}
SRYVLTGRYDSAPATDGSGTALGWTVAWKNNYRNAHSATTWSGQYVGGAEARINTQWLLTSG

TTEANAWKSTLVGHDTFTKVKPSAASGGGSAEAGITGTWYNQLGSTFIVTAGADGALTGTYE
SA
>d1swua_ b.61.1.1 (A:) Streptavidin {Streptomyces avidinii}
GITGTWYNQLGSTFIVTAGADGALTGTFESAVGNAESRYVLTGRYDSAPATDGSGTALGWTV
AWKNNYRNAHSATTWSGQYVGGAEARINTQWLLTSGTTEANAWKSTLVGHDTFTKVKP
>d1ij8a_ b.61.1.1 (A:) Avidin {Chicken (Gallus gallus)}
KCSLTGKWTNDLGSNMTIGAVNSRGEFTGTYTTAVTATSNEIKESPLHGTENTINKRTQPTF
GFTVNWKFSESTTVFTGQCFIDRNGKEVLKTMWLLRSSVNDIGDDWKATRVGINIFTRL
>d1smpi_ b.61.2.1 (I:) Metalloprotease inhibitor {Erwinia
chrysanthemi}
SSLRLPSAAELSGQWVLSGAEQHCDIRLNTDVLDGTTWKLAGDTACLQKLLPEAPVGWRPTP
DGLTLTQADGSAVAFFSRNRDRYEHKLVDGSVRTLKKK
>d1jiwi_ b.61.2.1 (I:) Metalloprotease inhibitor {Pseudomonas
aeruginosa, aprin}
SSLILLSASDLAGQWTLQQDEAPAICHLELRDSEVAEASGYDLGGDTACLTRWLPSEPRAWR
PTPAGIALLERGGLTLMLLGRQGEGDYRVQKGDGGQLVLRRAT
>d1ei5a1 b.61.3.1 (A:336-417) D-aminopeptidase, middle and
C-terminal domains {Ochrobactrum anthropi}
EVSRVEADSAWFGSWLDDETGLVLSLEDAGHGRMKARFGTSPEMMDVVSANEARSAVTTIRR
DGETIELVRASENLRLSMKR
>d1ei5a2 b.61.3.1 (A:418-520) D-aminopeptidase, middle and
C-terminal domains {Ochrobactrum anthropi}
VKGEAKHDIIGRYHSDELDADLLLVSEGGAIYGAFEGFLGKSDMYPLYSVGSDVWLLPVQRS
MDAPSPGEWKLVFRRDDKGEITGLSVGCWLARGVEYRRVQP
>d1jjua5 b.61.4.1 (A:166-273) Quinohemoprotein amine
dehydrogenase A chain, domain 3 {Paracoccus denitrificans}
PDAYADDASGAYVLAGRQPGRGDYTGRLVLKKAGEDYEVTMTLDFADGSRSFSGTGRILGAG
EWRATLSDGTVTIRQIFALQDGRFSGRWHDADSDVIGGRLAAVKAD
>d1jmxa5 b.61.4.1 (A:163-281) Quinohemoprotein amine
dehydrogenase A chain, domain 3 {Pseudomonas putida}
ESAAWAEWQKARPKADALPGQWAFSGHMLAKGDVRGVMSVTPDQGDTFKVEVKGAYADGTPF
NGSGSAILYNGYEWRGNVKVGDANLRQVFAALDGEMKGRMFEAEHDERGLDFTAVKE
>d2cpl__ b.62.1.1 (-) Cyclophilin (eukaryotic) {Human (Homo
sapiens), variant A}
VNPTVFFDIAVDGEPLGRVSFELFADKVPKTAENFRALSTGEKGFGYKGSCFHRIIPGFMCQ
GGDFTRHNGTGGKSIYGEKFEDENFILKHTGPGILSMANAGPNTNGSQFFICTAKTEWLDGK
HVVFGKVKEGMNIVEAMERFGSRNGKTSKKITIADCGQLE
>d1cyna_ b.62.1.1 (A:) Cyclophilin (eukaryotic) {Human (Homo
sapiens), variant B}
GPKVTVKVYFDLRIGDEDVGRVIFGLFGKTVPKTVDNFVALATGEKGFGYKNSKFHRVIKDF
MIQGGDFTRGDGTGGKSIYGERFPDENFKLKHYGPGWVSMANAGKDTNGSQFFITTVKTAWL
DGKHVVFGKVLEGMEVVRKVESTKTDSRDKPLKDVIIADCGKIEVEKPFAIAKE
>d1qoia_ b.62.1.1 (A:) Cyclophilin (eukaryotic) {Human (Homo
sapiens), U4/U6 snRNP-specific cyclophilin snucyp-20}

NSSPVNPVVFFDVSIGGQEVGRMKIELFADVVPKTAENFRQFCTGEFRKDGVPIGYKGSTFH
RVIKDFMIQGGDFVNGDGTGVASIYRGPFADENFKLRHSAPGLLSMANSGPSTNGCQFFITC
SKCDWLDGKHVVFGKIIDGLLVMRKIENVPTGPNNKPKLPVVISQCGEM
>d2rmca_ b.62.1.1 (A:) Cyclophilin (eukaryotic) {Mouse (Mus musculus), variant C}
KRGPSVTDKVFFDVRIGDKDVGRIVIGLFGNVVPKTVENFVALATGEKGYGYKGSIFHRVIK
DFMIQGGDFTARDGTGGMSIYGETFPDENFKLKHYGIGWVSMANAGPDTNGSQFFITLTKPT
WLDGKHVVFGKVLDGMTVVHSIELQATDGHDRPLTDCTIVNSGKIDVKTPFVVEVPDW
>d1a33__ b.62.1.1 (-) Cyclophilin (eukaryotic) {Nematode (Brugia malayi)}
KDRRRVFLDVTIDGNLAGRIVMELYNDIAPRTCNNFLMLCTGMAGTGKISGKPLHYKGSTFH
RVIKNFMIQGGDFTKGDGTGGESIYGGMFDDEEFVMKHDEPFVVSMANKGPNTNGSQFFITT
TPAPHLNNIHVVFGKVVSGQEVVTKIEYLKTNSKNRPLADVVILNCGELV
>d1dywa_ b.62.1.1 (A:) Cyclophilin (eukaryotic) {Caenorhabditis elegans, isoform 3}
MSRSKVFFDITIGGKASGRIVMELYDDVVPKTAGNFRALCTGENGIGKSGKPLHFKGSKFHR
IIPNFMIQGGDFTRGNGTGGESIYGEKFPDENFKEKHTGPGVLSMANAGPNTNGSQFFLCTV
KTEWLDGKHVVFGRVVEGLDVVKAVESNGSQSGKPVKDCMIADCGQLK
>d1qnga_ b.62.1.1 (A:) Cyclophilin (eukaryotic) {Plasmodium falciparum}
SKRSKVFFDISIDNSNAGRIIFELFSDITPRTCENFRALCTGEKIGSRGKNLHYKNSIFHRI
IPQFMCQGGDITNGNGSGGESIYGRSFTDENFNMKHDQPGLLSMANAGPNTNSSQFFITLVP
CPWLDGKHVVFGKVIEGMNVVREMEKEGAKSGYVKRSVVITDCGEL
>d1ihga2 b.62.1.1 (A:2-196) Cyclophilin 40 isomerase domain {Cow (Bos taurus)}
SHPSPQAKPSNPSNPRVFFDVDIGGERVGRIVLELFADIVPKTAENFRALCTGEKGIGPTTG
KPLHFKGCPFHRIIKKFMIQGGDFSNQNGTGGESIYGEKFEDENFHYKHDKEGLLSMANAGS
NTNGSQFFITTVPTPHLDGKHVVFGQVIKGMGVAKILENVEVKGEKPAKLCVIAECGELKEG
DDWGIFPKD
>d1clh__ b.62.1.1 (-) Bacterial cyclophilin {Escherichia coli}
AKGDPHVLLTTSAGNIELELDKQKAPVSVQNFVDYVNSGFYNNTTFHRVIPGFMIQGGGFTE
QMQQKKPNPPIKNEADNGLRNTRGTIAMARTADKDSATSQFFINVADNAFLDHGQRDFGYAV
FGKVVKGMDVADKISQVPTHDVGPYQNVPSKPVVILSAKVLP
>d1lopa_ b.62.1.1 (A:) Bacterial cyclophilin {Escherichia coli}
MVTFHTNHGDIVIKTFDDKAPETVKNFLDYCREGFYNNTIFHRVINGFMIQGGGFEPGMKQK
ATKEPIKNEANNGLKNTRGTLAMARTQAPHSATAQFFINVVDNDFLNFSGESLQGWGYCVFA
EVVDGMDEVDKIKGVATGRSGMHQDVPKEDVIIESVTVSE
>d1jsg__ b.63.1.1 (-) p14-TCL1 {Human (Homo sapiens)}
CPTLGEAVTDHPDRLWAWEKFVYLDEKQHAWLPLTIEIKDRLQLRVLLRREDVVLGRPMTPT
QIGPSLLPIMWQLYPDGRYRSSDSSFWRLVYHIKIDGVEDMLLELLPDD
>d1jnpa_ b.63.1.1 (A:) p14-TCL1 {Mouse (Mus musculus)}
RAETPAHPNRLWIWEKHVYLDEFRRSWLPVVIKSNEKFQVILRQEDVTLGEAMSPSQLVPYE
LPLMWQLYPKDRYRSADSMYWQILYHIKFRDVEDMLLEL
>d1a1x__ b.63.1.1 (-) p13-MTCP1 {Human (Homo sapiens)}

AGEDVGAPPDHLWVHQEGIYRDEYQRTWVAVVEEETSFLRARVQQIQVPLGDAARPSHLLTS
QLPLMWQLYPEERYMDNNSRLWQIQHHLMVRGVQELLLKLLPDD
>d1ijaa_ b.100.1.1 (A:) Sortase {Staphylococcus aureus}
MQAKPQIPKDKSKVAGYIEIPDADIKEPVYPGPATPEQLNRGVSFAEENESLDDQNISIAGH
TFIDRPNYQFTNLKAAKKGSMVYFKVGNETRKYKMTSIRDVKPTDVGVLDEQKGKDKQLTLI
TCDDYNEKTGVWEKRKIFVATEVK
>d1c39a_ b.64.1.1 (A:) Cation-dependent mannose 6-phosphate
receptor, extracytoplasmic domain {Cow (Bos taurus)}
EKTCDLVGEKGKESEKELALLKRLTPLFQKSFESTVGQSPDMYSYVFRVCREAGQHSSGAGL
VQIQKSNGKETVVGRFNETQIFQGSNWIMLIYKGGDEYDNHCGREQRRAVVMISCNRHTLAD
NFNPVSEERGKVQDCFYLFEMDSSLACS
>d1e6fa_ b.64.1.1 (A:) Cation-independent mannose-6-phosphate
receptor (MIR-receptor) {Human (Homo sapiens)}
DDCQVTNPSTGHLFDLSSLSGRAGFTAAYSEKGLVYMSICGENENCPPGVGACFGQTRISVG
KANKRLRYVDQVLQLVYKDGSPCPSKSGLSYKSVISFVCRPEAGPTNRPMLISLDKQTCTLF
FSWHTPLACE
>d1f3ua_ b.65.1.1 (A:) TFIIF beta subunit, Rap30 {Human (Homo
sapiens)}
AERGELDLTGAKQNTGVWLVKVPKYLSQQWAKASGRGEVGKLRIAKTQGRTEVSFTLNEDLA
NIHDIGGKPASVSAPREHPFVLQSVGGQTLTVFTESSSDKLSLEGIVVQRAECRPA
>d1f3ub_ b.65.1.1 (B:) TFIIF alpha subunit, Rap74 {Human (Homo
sapiens)}
GPSSQNVTEYVVRVPKNTTKKYNIMAFNAADKVNFATWNQARLERDLSNKKIYQEEEMPESG
AGSEFNRKLREEARRKKYGIVLKEFRPEDQPWLLRVNGKSGRKFKGIKKGGVTENTSYYIFT
QCPDGAFEAFPVHNWYNFTPLARHR
>d1f3ud_ b.65.1.1 (D:) TFIIF alpha subunit, Rap74 {Human (Homo
sapiens)}
SSQNVTEYVVRVPKNTTKKYNIMAFNAADKVNFATWNQARLERDLSNKKIYQEEEMPESGAG
SEFNRKLREEARRKKYGIVLKEFRPEDQPWLLRVNGKSGRKFKGIKKGGVTENTSYYIFTQC
PDGAFEAFPVHNWYNFTPLARHRTLTAEEAEEEWERRN
>d1hxn__ b.66.1.1 (-) Hemopexin {Rabbit (Oryctolagus cuniculus)}
ESTRCDPDLVLSAMVSDNHGATYVFSGSHYWRLDTNRDGWHSWPIAHQWPQGPSTVDAAFSW
EDKLYLIQDTKVYVFLTKGGYTLVNGYPKRLEKELGSPPVISLEAVDAAFVCPGSSRLHIMA
GRRLWWLDLKSGAQATWTELPWPHEKVDGALCMEKPLGPNSCSTSGPNLYLIHGPNLYCYRH
VDKLNAAKNLPQPQRVSRLLGCTH
>d1qhua1 b.66.1.1 (A:24-215) Hemopexin {Rabbit (Oryctolagus
cuniculus)}
IEQCSDGWSFDATTLDDNGTMLFFKDEFVWKSHRGIRELISERWKNFIGPVDAAFRHGHTSV
YLIKGDKVWVYTSEKNEKVYPKSLQDEFPGIPFPLDAAVECHRGECQDEGILFFQGNRKWFW
DLTTGTKKERSWPAVGNCTSALRWLGRYYCFQGNQFLRFNPVSGEVPPGYPLDVRDYFLSCP
GRGHRS
>d1gen__ b.66.1.1 (-) Gelatinase A (MMP-2), C-terminal domain
{Human (Homo sapiens)}
LGPVTPEICKQDIVFDGIAQIRGEIFFFKDRFIWRTVTPRDKPMGPLLVATFWPELPEKIDA

VYEAPQEEKAVFFAGNEYWIYSASTLERGYPKPLTSLGLPPDVQRVDAAFNWSKNKKTYIFA
GDKFWRYNEVKKKMDPGFPKLIADAWNAIPDNLDAVVDLQGGGHSYFFKGAYYLKLENQSLK
SVKFGSIKSDWLGC
>d1fbl_1 b.66.1.1 (272-466) Collagenase, C-terminal domain {Pig (Sus scrofa)}
PQTPQVCDSKLTFDAITTLRGELMFFKDRFYMRTNSFYPEVELNFISVFWPQVPNGLQAAYE
IADRDEVRFFKGNKYWAVRGQDVLYGYPKDIHRSFGFPSTVKNIDAAVFEEDTGKTYFFVAH
ECWRYDEYKQSMDTGYPKMIAEEFPGIGNKVDAVFQKDGFLYFFHGTRQYQFDFKTKRILTL
QKANSWFNC
>d1pex__ b.66.1.1 (-) Collagenase-3 (MMP-13), C-terminal domain {Human (Homo sapiens)}
TPDKCDPSLSLDAITSLRGETMIFKDRFFWRLHPQQVDAELFLTKSFWPELPNRIDAAYEHP
SHDLIFIFRGRKFWALNGYDILEGYPKKISELGLPKEVKKISAAVHFEDTGKTLLFSGNQVW
RYDDTNHIMDKDYPRLIEEDFPGIGDKVDAVYEKNGYIYFFNGPIQFEYSIWSNRIVRVMPA
NSILWC
>d1tl2a_ b.67.1.1 (A:) Tachylectin-2 {Japanese horseshoe crab (Tachypleus tridentatus)}
GGESMLRGVYQDKFYQGTYPQNKNDNWLARATLIGKGGWSNFKFLFLSPGGELYGVLNDKIY
KGTPPTHDNDNWMGRAKKIGNGGWNQFQFLFFDPNGYLYAVSKDKLYKASPPQSDTDNWIAR
ATEVGSGGWSGFKFLFFHPNGYLYAVHGQQFYKALPPVSNQDNWLARATKIGQGGWDTFKFL
FFSSVGTLFGVQGGKFYEDYPPSYAYDNWLARAKLIGNGGWDDFRFLFF
>d3sil__ b.68.1.1 (-) Salmonella sialidase {Salmonella typhimurium, strain lt2}
EKSVVFKAEGEHFTDQKGNTIVGSGSGGTTKYFRIPAMCTTSKGTIVVFADARHNTASDQSF
IDTAAARSTDGGKTWNKKIAIYNDRVNSKLSRVMDPTCIVANIQGRETILVMVGKWNNNDKT
WGAYRDKAPDTDWDLVLYKSTDDGVTFSKVETNIHDIVTKNGTISAMLGGVGSGLQLNDGKL
VFPVQMVRTKNITTVLNTSFIYSTDGITWSLPSGYCEGFGSENNIIEFNASLVNNIRNSGLR
RSFETKDFGKTWTEFPPMDKKVDNRNHGVQGSTITIPSGNKLVAAHSSAQNKNNDYTRSDIS
LYAHNLYSGEVKLIDDFYPKVGNASGAGYSCLSYRKNVDKETLYVVYEANGSIEFQDLSRHL
PVIKSYN
>d1f8ea_ b.68.1.1 (A:) Influenza neuraminidase {Influenza A virus, different strains}
RDFNNLTKGLCTINSWHIYGKDNAVRIGEDSDVLVTREPYVSCDPDECRFYALSQGTTIRGK
HSNGTIHDRSQYRALISWPLSSPPTVYNSRVECIGWSSTSCHDGKTRMSICISGPNNNASAV
IWYNRRPVTEINTWARNILRTQESECVCHNGVCPVVFTDGSATGPAETRIYYFKEGKILKWE
PLAGTAKHIEECSCYGERAEITCTCRDNWQGSNRPVIRIDPVAMTHTSQYICSPVLTDNPRP
NDPTVGKCNDPYPGNNNNGVKGFSYLDGVNTWLGRTISIASRSGYEMLKVPNALTDDKSKPT
QGQTIVLNTDWSGYSGSFMDYWAEGECYRACFYVELIRGRPKEDKVWWTSNSIVSMCSSTEF
LGQWDWPDGAKIEYFL
>d2bat__ b.68.1.1 (-) Influenza neuraminidase {Influenza A virus, different strains}
VEYRNWSKPQCQITGFAPFSKDNSIRLSAGGDIWVTREPYVSCDPVKCYQFALGQGTTLDNK
HSNDTVHDRIPHRTLLMNELGVPFHLGTRQVCIAWSSSSCHDGKAWLHVCITGDDKNATASF
IYDGRLVDSIGSWSQNILRTQESECVCINGTCTVVMTDGSASGRADTRILFIEEGKIVHISP

LAGSAQHVEECSCYPRYPGVRCICRDNWKGSNRPVVDINMEDYSIDSSYVCSGLVGDTPRND
DRSSNSNCRNPNNERGTQGVKGWAFDNGNDLWMGRTISKDLRSGYETFKVIGGWSTPNSKSQ
INRQVIVDSDNRSGYSGIFSVEGKSCINRCFYVELIRGRKQETRVWWTSNSIVVFCGTSGTY
GTGSWPDGANINFMPI
>d1inv__ b.68.1.1 (-) Influenza neuraminidase {Influenza B virus, different strains}
EPEWTYPRLSCQGSTFQKALLISPHRFGEIKGNSAPLIIREPFVACGPKECRHFALTHYAAQ
PGGYYNGTRKDRNKLRHLVSVKLGKIPTVENSIFHMAAWSGSACHDGREWTYIGVDGPDNDA
LVKIKYGEAYTDTYHSYAHNILRTQESACNCIGGDCYLMITDGSASGISKCRFLKIREGRII
KEILPTGRVEHTEECTCGFASNKTIECACRDNSYTAKRPFVKLNVETDTAEIRLMCTKTYLD
TPRPDDGSIAGPCESNGDKWLGGIKGGFVHQRMASKIGRWYSRTMSKTNRMGMELYVRYDGD
PWTDSDALTLSGVMVSIEEPGWYSFGFEIKDKKCDVPCIGIEMVHDGGKDTWHSAATAIYCL
MGSGQLLWDTVTGVDMAL
>d1nsca_ b.68.1.1 (A:) Influenza neuraminidase {Influenza B virus, different strains}
EPEWTYPRLSCQGSTFQKALLISPHRFGEARGNSAPLIIREPFIACGPKECKHFALTHYAAQ
PGGYYNGTREDRNKLRHLISVKLGKIPTVENSIFHMAAWSGSACHDGREWTYIGVDGPDSNA
LIKIKYGEAYTDTYHSYANNILRTQESACNCIGGDCYLMITDGSASGISKCRFLKIREGRII
KEIFPTGRVEHTEECTCGFASNKTIECACRDNSYTAKRPFVKLNVETDTAEIRLMCTETYLD
TPRPDDGSITGPCESNGDKGRGGIKGGFVHQRMASKIGRWYSRTMSKTERMGMELYVRYDGD
PWTDSDALAHSGVMVSMKEPGWYSFGFEIKDKKCDVPCIGIEMVHDGGKKTWHSAATAIYCL
MGSGQLLWDTVTGVDMAL
>d1e8ua_ b.68.1.1 (A:) Paramyxovirus hemagglutinin-neuraminidase head domain {Newcastle disease virus}
GAPIHDPDFIGGIGKELIVDNASDVTSFYPSAFQEHLNFIPAPTTGSGCTRIPSFDMSATHY
CYTHNVILSGCRDHSHSHQYLALGVLRTTATGRIFFSTLRSISLDDTQNRKSCSVSATPLGC
DMLCSKVTETEEEDYNSAVPTLMAHGRLGFDGQYHEKDLDVTTLFEDWVANYPGVGGGSFID
GRVWFSVYGGLKPNSPSDTVQEGKYVIYKRYNDTCPDEQDYQIRMAKSSYKPGRFGGKRIQQ
AILSIKVSTSLGEDPVLTVPPNTVTLMGAEGRILTVGTSHFLYQRGSSYFSPALLYPMTVSN
KTATLHSPYTFNAFTRPGSIPCQASARCPNSCVTGVYTDPYPLIFYRNHTLRGVFGTMLDSE
QARLNPASAVFDSTSRSRITRVSSSSTKAAYTTSTCFKVVKTNKTYCLSIAEISNTLFGEFR
IVPLLVEILKND
>d1eur__ b.68.1.1 (-) Micromonospora sialidase, N-terminal domain {Micromonospora viridifaciens}
GEPLYTEQDLAVNGREGFPNYRIPALTVTPDGDLLASYDGRPTGIDAPGPNSILQRRSTDGG
RTWGEQQVVSAGQTTAPIKGFSDPSYLVDRETGTIFNFHVYSQRQGFAGSRPGTDPADPNVL
HANVATSTDGGLTWSHRTITADITPDPGWRSRFAASGEGIQLRYGPHAGRLIQQYTIINAAG
AFQAVSVYSDDHGRTWRAGEAVGVGMDENKTVELSDGRVLLNSRDSARSGYRKVAVSTDGGH
SYGPVTIDRDLPDPTNNASIIRAFPDAPAGSARAKVLLFSNAASQTSRSQGTIRMSCDDGQT
WPVSKVFQPGSMSYSTLTALPDGTYGLLYEPGTGIRYANFNLAWLGGICAP
>d2sli_2 b.68.1.1 (277-759) Leech intramolecular trans-sialidase, C-terminal domain {North american leech (Macrobdella decora)}
GENIFYAGDVTESNYFRIPSLLTLSTGTVISAADARYGGTHDSKSKINIAFAKSTDGGNTWS
EPTLPLKFDDYIAKNIDWPRDSVGKNVQIQGSASYIDPVLLEDKLTKRIFLFADLMPAGIGS

SNASVGSGFKEVNGKKYLKLRWHKDAGRAYDYTIREKGVIYNDATNQPTEFRVDGEYNLYQH
DTNLTCKQYDYNFSGNNLIESKTDVDVNMNIFYKNSVFKAFPTNYLAMRYSDDEGASWSDLD
IVSSFKPEVSKFLVVGPGIGKQISTGENAGRLLVPLYSKSSAELGFMYSDDHGDNWTYVEAD
NLTGGATAEAQIVEMPDGSLKTYLRTGSNCIAEVTSIDGGETWSDRVPLQGISTTSYGTQLS
VINYSQPIDGKPAIILSSPNATNGRKNGKIWIGLVNDTGNTGIDKYSVEWKYSYAVDTPQMG
YSYSCLAELPDGQVGLLYEKYDSWSRNELHLKDILKFEKYSISELTGQA
>d1kit_3 b.68.1.1 (217-346,544-781) Vibrio cholerae sialidase {Vibrio cholerae}
VIFRGPDRIPSIVASSVTPGVVTAFAEKRVGGGDPGALSNTNDIITRTSRDGGITWDTELNL
TEQINVSDEFDFSDPRPIYDPSSNTVLVSYARWPTDAAQNGDRIKPWMPNGIFYSVYDVASG
NWQAPIXVNPGPGHGITLTRQQNISGSQNGRLIYPAIVLDRFFLNVMSIYSDDGGSNWQTGS
TLPIPFRWKSSSILETLEPSEADMVELQNGDLLLTARLDFNQIVNGVNYSPRQQFLSKDGGI
TWSLLEANNANVFSNISTGTVDASITRFEQSDGSHFLLFTNPQGNPAGTNGRQNLGLWFSFD
EGVTWKGPIQLVNGASAYSDIYQLDSENAIVIVETDNSNMRILRMPITLLKQKLTLSQN
>d1crua_ b.68.2.1 (A:) Soluble quinoprotein glucose dehydrogenase {Acinetobacter calcoaceticus}
DVPLTPSQFAKAKSENFDKKVILSNLNKPHALLWGPDNQIWLTERATGKILRVNPESGSVKT
VFQVPEIVNDADGQNGLLGFAFHPDFKNNPYIYISGTFKNPKSTDKELPNQTIIRRYTYNKS
TDTLEKPVDLLAGLPSSKDHQSGRLVIGPDQKIYYTIGDQGRNQLAYLFLPNQAQHTPTQQE
LNGKDYHTYMGKVLRLNLDGSIPKDNPSFNGVVSHIYTLGHRNPQGLAFTPNGKLLQSEQGP
NSDDEINLIVKGGNYGWPNVAGYKDDSGYAYANYSAAANKSIKDLAQNGVKVAAGVPVTKES
EWTGKNFVPPLKTLYTVQDTYNYNDPTCGEMTYICWPTVAPSSAYVYKGGKKAITGWENTLL
VPSLKRGVIFRIKLDPTYSTTYDDAVPMFKSNNRYRDVIASPDGNVLYVLTDTAGNVQKDDG
SVTNTLENPGSLIKFT
>d1h6la_ b.68.3.1 (A:) Thermostable phytase (3-phytase) {Bacillus amyloliquefaciens}
KLSDPYHFTVNAAAETEPVDTAGDAADDPAIWLDPKNPQNSKLITTNKKSGLAVYSLEGKML
HSYHTGKLNNVDIRYDFPLNGKKVDIAAASNRSEGKNTIEIYAIDGKNGTLQSITDPNRPIA
SAIDEVYGFSLYHSQKTGKYYAMVTGKEGEFEQYELNADKNGYISGKKVRAFKMNSQTEGMA
ADDEYGSLYIAEEDEAIWKFSAEPDGGSNGTVIDRADGRHLTPDIEGLTIYYAADGKGYLLA
SSQGNSSYAIYERQGQNKYVADFQITDGPETDGTSDTDGIDVLGFGLGPEYPFGLFVAQNGE
NIDHGQKANQNFKMVPWERIADKIGFHPQVNKQVDPRKMTDRS
>d1crza1 b.68.4.1 (A:141-409) TolB, C-terminal domain {Escherichia coli}
AFRTRIAYVVQTNGGQFPYELRVSDYDGYNQFVVHRSPQPLMSPAWSPDGSKLAYVTFESGR
SALVIQTLANGAVRQVASFPRHNGAPAFSPDGSKLAFALSKTGSLNLYVMDLASGQIRQVTD
GRSNNTEPTWFPDSQNLAFTSDQAGRPQVYKVNINGGAPQRITWEGSQNQDADVSSDGKFMV
MVSSNGGQQHIAKQDLATGGVQVLSSTFLDETPSLAPNGTMVIYSSSQGMGSVLNLVSTDGR
FKARLPATDGQVKFPAWSPYL
>d1ijqa1 b.68.5.1 (A:377-642) Low density lipoprotein (LDL) reseptor YWTD domain {Human (Homo sapiens)}
IAYLFFTNRHEVRKMTLDRSEYTSLIPNLRNVVALDTEVASNRIYWSDLSQRMICSTQLDRA
HGVSSYDTVISRDIQAPDGLAVDWIHSNIYWTDSVLGTVSVADTKGVKRKTLFRENGSKPRA
IVVDPVHGFMYWTDWGTPAKIKKGGLNGVDIYSLVTENIQWPNGITLDLLSGRLYWVDSKLH

SISSIDVNGGNRKTILEDEKRLAHPFSLAVFEDKVFWTDIINEAIFSANRLTGSDVNLLAEN
LLSPEDMVLFHNLTQPRG
>d1e1aa_ b.68.6.1 (A:) Diisopropylfluorophosphatase
(phosphotriesterase, DFP) {Squid (Loligo vulgaris)}
IPVIEPLFTKVTEDIPGAEGPVFDKNGDFYIVAPEVEVNGKPAGEILRIDLKTGKKTVICKP
EVNGYGGIPAGCQCDRDANQLFVADMRLGLLVVQTDGTFEEIAKKDSEGRRMQGCNDCAFDY
EGNLWITAPAGEVAPADYTRSMQEKFGSIYCFTTDGQMIQVDTAFQFPNGIAVRHMNDGRPY
QLIVAETPTKKLWSYDIKGPAKIENKKVWGHIPGTHEGGADGMDFDEDNNLLVANWGSSHIE
VFGPDGGQPKMRIRCPFEKPSNLHFKPQTKTIFVTEHENNAVWKFEWQRNGKKQYCETLKFG
IF
>d1k32a2 b.68.7.1 (A:39-319) Tricorn protease N-terminal domain
{Archaeon Thermoplasma acidophilum}
MPNLLLNPDIHGDRIIFVCCDDLWEHDLKSGSTRKIVSNLGVINNARFFPDGRKIAIRVMRG
SSLNTADLYFYNGENGEIKRITYFSGKSTGRRMFTDVAGFDPDGNLIISTDAMQPFSSMTCL
YRVENDGINFVPLNLGPATHILFADGRRVIGRNTFELPHWKGYRGGTRGKIWIEVNSGAFKK
IVDMSTHVSSPVIVGHRIYFITDIDGFGQIYSTDLDGKDLRKHTSFTDYYPRHLNTDGRRIL
FSKGGSIYIFNPDTEKIEKIEIGDLESPEDRII
>d1k3ia3 b.69.1.1 (A:151-537) Galactose oxidase, central domain
{Fungi (Fusarium spp)}
YTAPQPGLGRWGPTIDLPIVPAAAAIEPTSGRVLMWSSYRNDAFGGSPGGITLTSSWDPSTG
IVSDRTVTVTKHDMFCPGISMDGNGQIVVTGGNDAKKTSLYDSSSDSWIPGPDMQVARGYQS
SATMSDGRVFTIGGSWSGGVFEKNGEVYSPSSKTWTSLPNAKVNPMLTADKQGLYRSDNHAW
LFGWKKGSVFQAGPSTAMNWYYTSGSGDVKSAGKRQSNRGVAPDAMCGNAVMYDAVKGKILT
FGGSPDYQDSDATTNAHIITLGEPGTSPNTVFASNGLYFARTFHTSVVLPDGSTFITGGQRR
GIPFEDSTPVFTPEIYVPEQDTFYKQNPNSIVRVYHSISLLLPDGRVFNGGGGLCGDCTTNH
FDAQIFTPNYLYNSN
>d1mdah_ b.69.2.1 (H:) Methylamine dehydrogenase, H-chain
{Paracoccus denitrificans}
EKSKVAGSAAAASAAAASDGSSCDHGPGAISRRSHITLPAYFAGTTENWVSCAGCGVTLGHS
LGAFLSLAVAGHSGSDFALASTSFARSAKGKRTDYVEVFDPVTFLPIADIELPDAPRFSVGP
RVHIIGNCASSACLLFFLFGSSAAAGLSVPGASDDQLTKSASCFHIHPGAAATHYLGSCPAS
LAASDLAAAPAAAGIVGAQCTGAQNCSSQAAQANYPGMLVWAVASSILQGDIPAAGATMKAA
IDGNESGRKADNFRSAGFQMVAKLKNTDGIMILTVEHSRSCLAAAENTSSVTASVGQTSGPI
SNGHDSDAIIAAQDGASDNYANSAGTEVLDIYDAASDQDQSSVELDKGPESLSVQNEA
>d2bbkh_ b.69.2.1 (H:) Methylamine dehydrogenase, H-chain
{Paracoccus denitrificans}
DEPRILEAPAPDARRVYVNDPAHFAAVTQQFVIDGEAGRVIGMIDGGFLPNPVVADDGSFIA
HASTVFSRIARGERTDYVEVFDPVTLLPTADIELPDAPRFLVGTYPWMTSLTPDGKTLLFYQ
FSPAPAVGVVDLEGKAFKRMLDVPDCYHIFPTAPDTFFMHCRDGSLAKVAFGTEGTPEITHT
EVFHPEDEFLINHPAYSQKAGRLVWPTYTGKIHQIDLSSGDAKFLPAVEALTEAERADGWRP
GGWQQVAYHRALDRIYLLVDQRDEWRHKTASRFVVVLDAKTGERLAKFEMGHEIDSINVSQD
EKPLLYALSTGDKTLYIHDAESGEELRSVNQLGHGPQVITTADMG
>d2madh_ b.69.2.1 (H:) Methylamine dehydrogenase, H-chain {Gram
negative methylotrophic bacteria (Thiobacillus versutus)}

SSASAAAAAAAAAALAAGAADGPTNDEAPGADGRRSYINLPAHHSAIIQQWVLDAGSGSILGH
VNGGFLPNPVAAHSGSEFALASTSFSRIAKGKRTDYVEVFDPVTFLPIADIELPDAPRFDVG
PYSWMNANTPNNADLLFFQFAAGPAVGLVVQGGSSDDQLLSSPTCYHIHPGAPSTFYLLCAQ
GGLAKTDHAGGAAGAGLVGAMLTAAQNLLTQPAQANKSGRIVWPVYSGKILQADISAAGATN
KAPIDALSGGRKADTWRPGGWQQVAYLKSSDGIYLLTSEQSAWKLHAAAKEVTSVTGLVGQT
SSQISLGHDVDAISVAQDGGPDLYALSAGTEVLHIYDAGAGDQDQSTVELGSGPQVLSVMNE
A
>d1jjub_ b.69.2.2 (B:) Quinohemoprotein amine dehydrogenase B chain {Paracoccus denitrificans}
RDYILAPARPDKLVVIDTEKMAVDKVITIADAGPTPMVPMVAPGGRIAYATVNKSESLVKID
LVTGETLGRIDLSTPEERVKSLFGAALSPDGKTLAIYESPVRLELTHFEVQPTRVALYDAET
LSRRKAFEAPRQITMLAWARDGSKLYGLGRDLHVMDPEAGTLVEDKPIQSWEAETYAQPDVL
AVWNQHESSGVMATPFYTARKDIDPADPTAYRTGLLTMDLETGEMAMREVRIMDVFYFSTAV
NPAKTRAFGAYNVLESFDLEKNASIKRVPLPHSYYSVNVSTDGSTVWLGGALGDLAAYDAET
LEKKGQVDLPGNASMSLASVRLFTRDE
>d1jmxb_ b.69.2.2 (B:) Quinohemoprotein amine dehydrogenase B chain {Pseudomonas putida}
GPALKAGHEYMIVTNYPNNLHVVDVASDTVYKSCVMPDKFGPGTAMMAPDNRTAYVLNNHYG
DIYGIDLDTCKNTFHANLSSVPGEVGRSMYSFAISPDGKEVYATVNPTQRLNDHYVVKPPRL
EVFSTADGLEAKPVRTFPMPRQVYLMRAADDGSLYVAGPDIYKMDVKTGKYTVALPLRNWNR
KGYSAPDVLYFWPHQSPRHEFSMLYTIARFKDDKQDPATADLLYGYLSVDLKTGKTHTQEFA
DLTELYFTGLRSPKDPNQIYGVLNRLAKYDLKQRKLIKAANLDHTYYCVAFDKKGDKLYLGG
TFNDLAVFNPDTLEKVKNIKLPGGDMSTTTPQVFIR
>d1qnia2 b.69.3.1 (A:10-450) Nitrous oxide reductase, N-terminal domain {Pseudomonas nautica}
AHVAPGELDEYYGFWSGGHQGEVRVLGVPSMRELMRIPVFNVDSATGWGITNESKEILGGDQ
QYLNGDCHHPHISMTDGRYDGKYLFINDKANTRVARIRLDIMKTDKITHIPNVQAIHGLRLQ
KVPKTNYVFCNAEFVIPQPNDGTDFSLDNSYTMFTAIDAETMDVAWQVIVDGNLDNTDADYT
GKYATSTCYNSERAVDLAGTMRNDRDWVVVFNVERIAAAVKAGNFKTIGDSKVPVVDGRGES
EFTRYIPVPKNPHGLNTSPDGKYFIANGKLSPTVSVIAIDKLDDLFEDKIELRDTIVAEPEL
GLGPLHTTFDGRGNAYTTLFIDSQVCKWNIADAIKHYNGDRVNYIRQKLDVQYQPGHNHASL
TESRDADGKWLVVLSKFSKDRFLPVGPLHPENDQLIDISGEEMKLVHDGPTYAEPHDCILVR
RDQIKTK
>d1fwxa2 b.69.3.1 (A:8-451) Nitrous oxide reductase, N-terminal domain {Paracoccus denitrificans}
ADGSVAPGQLDDYYGFWSSGQSGEMRILGIPSMRELMRVPVFNRCSATGWGQTNESVRIHER
TMSERTKKFLAANGKRIHDNGDLHHVHMSFTEGKYDGRFLFMNDKANTRVARVRCDVMKCDA
ILEIPNAKGIHGLRPQKWPRSNYVFCNGEDETPLVNDGTNMEDVANYVNVFTAVDADKWEVA
WQVLVSGNLDNCDADYEGKWAFSTSYNSEKGMTLPEMTAAEMDHIVVFNIAEIEKAIAAGDY
QELNGVKVVDGRKEASSLFTRYIPIANNPHGCNMAPDKKHLCVAGKLSPTVTVLDVTRFDAV
FYENADPRSAVVAEPELGLGPLHTAFDGRGNAYTSLFLDSQVVKWNIEDAIRAYAGEKVDPI
KDKLDVHYQPGHLKTVMGETLDATNDWLVCLSKFSKDRFLNVGPLKPENDQLIDISGDKMVL
VHDGPTFAEPHDAIAVHPSILSDIK
>d1tbga_ b.69.4.1 (A:) beta1-subunit of the signal-transducing G

protein heterotrimer {Cow (Bos taurus)}
MSELDQLRQEAEQLKNQIRDARKACADATLSQITNNIDPVGRIQMRTRRTLRGHLAKIYAMH
WGTDSRLLVSASQDGKLIIWDSYTTNKVHAIPLRSSWVMTCAYAPSGNYVACGGLDNICSIY
NLKTREGNVRVSRELAGHTGYLSCCRFLDDNQIVTSSGDTTCALWDIETGQQTTTFTGHTGD
VMSLSLAPDTRLFVSGACDASAKLWDVREGMCRQTFTGHESDINAICFFPNGNAFATGSDDA
TCRLFDLRADQELMTYSHDNIICGITSVSFSKSGRLLLAGYDDFNCNVWDALKADRAGVLAG
HDNRVSCLGVTDDGMAVATGSWDSFLKIWN
>d1erja_ b.69.4.1 (A:) Tup1, C-therminal domain {Baker's yeast (Saccharomyces cerevisiae)}
HYLVPYNQRANHSKPIPPFLLDLDSQSVPDALKKQTNDYYILYNPALPREIDVELHKSLDHT
SVVCCVKFSNDGEYLATGCNKTTQVYRVSDGSLVARLSDDSAANKDPENLNTSSSPSSDLYI
RSVCFSPDGKFLATGAEDRLIRIWDIENRKIVMILQGHEQDIYSLDYFPSGDKLVSGSGDRT
VRIWDLRTGQCSLTLSIEDGVTTVAVSPGDGKYIAAGSLDRAVRVWDSETGFLVERLDSENE
SGTGHKDSVYSVVFTRDGQSVVSGSLDRSVKLWNLQNANNKSDSKTPNSGTCEVTYIGHKDF
VLSVATTQNDEYILSGSKDRGVLFWDKKSGNPLLMLQGHRNSVISVAVANGSSLGPEYNVFA
TGSGDCKARIWKYKKI
>d1k8kc_ b.69.4.1 (C:) Arp2/3 complex 41 kDa subunit ARPC1 {Cow (Bos taurus)}
AYHSFLVEPISCHAWNKDRTQIAICPNNHEVHIYEKSGNKWVQVHELKEHNGQVTGVDWAPD
SNRIVTCGTDRNAYVWTLKGRTWKPTLVILRINRAARCVRWAPNEKKFAVGSGSRVISICYF
EQENDWWVCKHIKKPIRSTVLSLDWHPNSVLLAAGSCDFKCRIFSAYIKEVEERPAPTPWGS
KMPFGELMFESSSSCGWVHGVCFSANGSRVAWVSHDSTVCLADADKKMAVATLASETLPLLA
VTFITESSLVAAGHDCFPVLFTYDSAAGKLSFGGRLDVPKQSSQRGLTARERFQNLDKKASS
EGSAAAGAGLDSLHKNSVSQISVLSGGKAKCSQFCTTGMDGGMSIWDVRSLESALKDLKIV
>d1a12a_ b.69.5.1 (A:) Regulator of chromosome condensation RCC1 {Human (Homo sapiens)}
KKVKVSHRSHSTEPGLVLTLGQGDVGQLGLGENVMERKKPALVSIPEDVVQAEAGGMHTVCL
SKSGQVYSFGCNDEGALGRDTSVEGSEMVPGKVELQEKVVQVSAGDSHTAALTDDGRVFLWG
SFRDNNGVIGLLEPMKKSMVPVQVQLDVPVVKVASGNDHLVMLTADGDLYTLGCGEQGQLGR
VPELFANRGGRQGLERLLVPKCVMLKSRGSRGHVRFQDAFCGAYFTFAISHEGHVYGFGLSN
YHQLGTPGTESCFIPQNLTSFKNSTKSWVGFSGGQHHTVCMDSEGKAYSLGRAEYGRLGLGE
GAEEKSIPTLISRLPAVSSVACGASVGYAVTKDGRVFAWGMGTNYQLGTGQDEDAWSPVEMM
GKQLENRVVLSVSSGGQHTVLLVKDKEQS
>d1jtdb_ b.69.5.2 (B:) of beta-lactamase inhibitor protein-II, BLIP-II {Streptomyces exfoliatus}
VAATSVVAWGGNNDWGEATVPAEAQSGVDAIAGGYFHGLALKGGKVLGWGANLNGQLTMPAA
TQSGVDAIAAGNYHSLALKDGEVIAWGGNEDGQTTVPAEARSGVDAIAAGAWASYALKDGKV
IAWGDDSDGQTTVPAEAQSGVTALDGGVYTALAVKNGGVIAWGDNYFGQTTVPAEAQSGVDD
VAGGIFHSLALKDGKVIAWGDNRYKQTTVPTEALSGVSAIASGEWYSLALKNGKVIAWGSSR
TAPSSVQSGVSSIEAGPNAAYALKG
>d1c9la2 b.69.6.1 (A:3-330) Clathrin heavy-chain terminal domain {Rat (Rattus norvegicus)}
QILPIRFQEHLQLQNLGINPANIGFSTLTMESDKFICIREKVGEQAQVVIIDMNDPSNPIRR
PISADSAIMNPASKVIALKAGKTLQIFNIEMKSKMKAHTMTDDVTFWKWISLNTVALVTDNA

VYHWSMEGESQPVKMFDRHSSLAGCQIINYRTDAKQKWLLLTGISAQQNRVVGAMQLYSVDR
KVSQPIEGHAASFAQFKMEGNAEESTLFCFAVRGQAGGKLHIIEVGTPPTGNQPFPKKAVDV
FFPPEAQNDFPVAMQISEKHDVVFLITKYGYIHLYDLETGTCIYMNRISGETIFVTAPHEAT
AGIIGVNRKGQVLSVCVE
>d1jv2a4 b.69.8.1 (A:1-438) Integrin alpha N-terminal domain
{Human (Homo sapiens)}
FNLDVDSPAEYSGPEGSYFGFAVDFFVPSASSRMFLLVGAPKANTTQPGIVEGGQVLKCDWS
STRRCQPIEFDATGNRDYAKDDPLEFKSHQWFGASVRSKQDKILACAPLYHWRTEMKQEREP
VGTCFLQDGTKTVEYAPCRSQDIDADGQGFCQGGFSIDFTKADRVLLGGPGSFYWQGQLISD
QVAEIVSKYDPNVYSIKYNNQLATRTAQAIFDDSYLGYSVAVGDFNGDGIDDFVSGVPRAAR
TLGMVYIYDGKNMSSLYNFTGEQMAAYFGFSVAATDINGDDYADVFIGAPLFMDRGSDGKLQ
EVGQVSVSLQRASGDFQTTKLNGFEVFARFGSAIAPLGDLDQDGFNDIAIAAPYGGEDKKGI
VYIFNGRSTGLNAVPSQILEGQWAARSMPPSFGYSMKGATDIDKNGYPDLIVGAFGVDRAIL
YRAR
>d1qfma1 b.69.7.1 (A:1-430) Prolyl oligopeptidase, N-terminal
domain {Pig (Sus scrofa)}
MLSFQYPDVYRDETAIQDYHGHKVCDPYAWLEDPDSEQTKAFVEAQNKITVPFLEQCPIRGL
YKERMTELYDYPKYSCHFKKGKRYFYFYNTGLQNQRVLYVQDSLEGEARVFLDPNILSDDGT
VALRGYAFSEDGEYFAYGLSASGSDWVTIKFMKVDGAKELPDVLERVKFSCMAWTHDGKGMF
YNAYPQQDGKSDGTETSTNLHQKLYYHVLGTDQSEDILCAEFPDEPKWMGGAELSDDGRYVL
LSIREGCDPVNRLWYCDLQQESNGITGILKWVKLIDNFEGEYDYVTNEGTVFTFKTNRHSPN
YRLINIDFTDPEESKWKVLVPEHEKDVLEWVACVRSNFLVLCYLHDVKNTLQLHDLATGALL
KIFPLEVGSVVGYSGQKKDTEIFYQFTSFLSPGIIYHCDLTKEELEPRVFREVTVKGI
>d1k32a3 b.69.9.1 (A:320-679) Tricorn protease N-terminal domain
{Archaeon Thermoplasma acidophilum}
SIPSKFAEDFSPLDGDLIAFVSRGQAFIQDVSGTYVLKVPEPLRIRYVRRGGDTKVAFIHGT
REGDFLGIYDYRTGKAEKFEENLGNVFAMGVDRNGKFAVVANDRFEIMTVDLETGKPTVIER
SREAMITDFTISDNSRFIAYGFPLKHGETDGYVMQAIHVYDMEGRKIFAATTENSHDYAPAF
DADSKNLYYLSYRSLDPSPDRVVLNFSFEVVSKPFVIPLIPGSPNPTKLVPRSMTSEAGEYD
LNDMYKRSSPINVDPGDYRMIIPLESSILIYSVPVHGEFAAYYQGAPEKGVLLKYDVKTRKV
TEVKNNLTDLRLSADRKTVMVRKDDGKIYTFPLEKPEDERTVETDKRPLV
>d1g72a_ b.70.1.1 (A:) Methanol dehydrogenase, heavy chain
{Methylophilus methylotrophus, w3a1}
DADLDKQVNTAGAWPIATGGYYSQHNSPLAQINKSNVKNVKAAWSFSTGVLNGHEGAPLVIG
DMMYVHSAFPNNTYALNLNDPGKIVWQHKPKQDASTKAVMCCDVVDRGLAYGAGQIVKKQAN
GHLLALDAKTGKINWEVEVCDPKVGSTLTQAPFVAKDTVLMGCSGAELGVRGAVNAFDLKTG
ELKWRAFATGSDDSVRLAKDFNSANPHYGQFGLGTKTWEGDAWKIGGGTNWGWYAYDPKLNL
FYYGSGNPAPWNETMRPGDNKWTMTIWGRDLDTGMAKWGYQKTPHDEWDFAGVNQMVLTDQP
VNGKMTPLLSHIDRNGILYTLNRENGNLIVAEKVDPAVNVFKKVDLKTGTPVRDPEFATRMD
HKGTNICPSAMGFHNQGVDSYDPESRTLYAGLNHICMDWEPFMLPYRAGQFFVGATLAMYPG
PNGPTKKEMGQIRAFDLTTGKAKWTKWEKFAAWGGTLYTKGGLVWYATLDGYLKALDNKDGK
ELWNFKMPSGGIGSPMTYSFKGKQYIGSMYGVGGWPGVGLVFDLTDPSAGLGAVGAFRELQN
HTQMGGGLMVFSL
>d1h4ia_ b.70.1.1 (A:) Methanol dehydrogenase, heavy chain

{Methylobacterium extorquens}
NDKLVELSKSDDNWVMPGKNYDSNNFSDLKQINKGNVKQLRPAWTFSTGLLNGHEGAPLVVD
GKMYIHTSFPNNTFALGLDDPGTILWQDKPKQNPAARAVACCDLVNRGLAYWPGDGKTPALI
LKTQLDGNVAALNAETGETVWKVENSDIKVGSTLTIAPYVVKDKVIIGSSGAELGVRGYLTA
YDVKTGEQVWRAYATGPDKDLLLASDFNIKNPHYGQKGLGTGTWEGDAWKIGGGTNWGWYAY
DPGTNLIYFGTGNPAPWNETMRPGDNKWTMTIFGRDADTGEAKFGYQKTPHDEWDYAGVNVM
MLSEQKDKDGKARKLLTHPDRNGIVYTLDRTDGALVSANKLDDTVNVFKSVDLKTGQPVRDP
EYGTRMDHLAKDICPSAMGYHNQGHDSYDPKRELFFMGINHICMDWEPFMLPYRAGQFFVGA
TLNMYPGPKGDRQNYEGLGQIKAYNAITGDYKWEKMERFAVWGGTMATAGDLVFYGTLDGYL
KARDSDTGDLLWKFKIPSGAIGYPMTYTHKGTQYVAIYYGVGGWPGVGLVFDLADPTAGLGA
VGAFKKLANYTQMGGGVVVFSLDGKGPYDDPNVGEWK
>d1flga_ b.70.1.1 (A:) Ethanol dehydrogenase {Pseudomonas
aeruginosa}
KDVTWEDIANDDKTTGDVLQYGMGTHAQRWSPLKQVNADNVFKLTPAWSYSFGDEKQRGQES
QAIVSDGVIYVTASYSRLFALDAKTGKRLWTYNHRLPDDIRPCCDVVNRGAAIYGDKVFFGT
LDASVVALNKNTGKVVWKKKFADHGAGYTMTGAPTIVKDGKTGKVLLIHGSSGDEFGVVGRL
FARDPDTGEEIWMRPFVEGHMGRLNGKDSTVTGDVKAPSWPDDRNSPTGKVESWSHGGGAPW
QSASFDAETNTIIVGAGNPGPWNTWARTAKGGNPHDYDSLYTSGQVGVDPSSGEVKWFYQHT
PNDAWDFSGNNELVLFDYKAKDGKIVKATAHADRNGFFYVVDRSNGKLQNAFPFVDNITWAS
HIDLKTGRPVEREGQRPPLPEPGQKHGKAVEVSPPFLGGKNWNPMAYSQDTGLFYVPANHWK
EDYWTEEVSYTKGSAYLGMGFRIKRMYDDHVGSLRAMDPVSGKVVWEHKEHLPLWAGVLATA
GNLVFTGTGDGYFKAFDAKSGKELWKFQTGSGIVSPPITWEQDGEQYLGVTVGYGGAVPLWG
GDMADLTRPVAQGGSFWVFKLPSW
>d1kb0a2 b.70.1.1 (A:1-573) Quinoprotein alcohol dehydrogenase,
N-terminal domain {Comamonas testosteroni}
TGPAAQAAAAVQRVDGDFIRANAARTPDWPTIGVDYAETRYSRLDQINAANVKDLGLAWSYN
LESTRGVEATPVVVDGIMYVSASWSVVHAIDTRTGNRIWTYDPQIDRSTGFKGCCDVVNRGV
ALWKGKVYVGAWDGRLIALDAATGKEVWHQNTFEGQKGSLTITGAPRVFKGKVIIGNGGAEY
GVRGYITAYDAETGERKWRWFSVPGDPSKPFEDESMKRAARTWDPSGKWWEAGGGGTMWDSM
TFDAELNTMYVGTGNGSPWSHKVRSPKGGDNLYLASIVALDPDTGKYKWHYQETPGDNWDYT
STQPMILADIKIAGKPRKVILHAPKNGFFFVLDRTNGKFISAKNFVPVNWASGYDKHGKPIG
IAAARDGSKPQDAVPGPYGAHNWHPMSFNPQTGLVYLPAQNVPVNLMDDKKWEFNQAGPGKP
QSGTGWNTAKFFNAEPPKSKPFGRLLAWDPVAQKAAWSVEHVSPWNGGTLTTAGNVVFQGTA
DGRLVAYHAATGEKLWEAPTGTGVVAAPSTYMVDGRQYVSVAVGWGGVYGLAARATERQGPG
TVYTFVVGGKARMPE
>d1nira2 b.70.2.1 (A:118-543) C-terminal (heme d1) domain of
cytochrome cd1-nitrite reductase {Pseudomonas aeruginosa}
EWGMPEMRESWKVLVKPEDRPKKQLNDLDLPNLFSVTLRDAGQIALVDGDSKKIVKVIDTGY
AVHISRMSASGRYLLVIGRDARIDMIDLWAKEPTKVAEIKIGIEARSVESSKFKGYEDRYTI
AGAYWPPQFAIMDGETLEPKQIVSTRGMTVDTQTYHPEPRVAAIIASHEHPEFIVNVKETGK
VLLVNYKDIDNLTVTSIGAAPFLHDGGWDSSHRYFMTAANNSNKVAVIDSKDRRLSALVDVG
KTPHPGRGANFVHPKYGPVWSTSHLGDGSISLIGTDPKNHPQYAWKKVAELQGQGGGSLFIK
THPKSSHLYVDTTFNPDARISQSVAVFDLKNLDAKYQVLPIAEWADLGEGAKRVVQPEYNKR
GDEVWFSVWNGKNDSSALVVVDDKTLKLKAVVKDPRLITPTGKFNVYNTQHDVY

>d1qksa2 b.70.2.1 (A:136-567) C-terminal (heme d1) domain of cytochrome cd1-nitrite reductase {Paracoccus denitrificans}
EFGMKEMRESWKVHVAPEDRPTQQMNDWDLENLFSVTLRDAGQIALIDGSTYEIKTVLDTGY
AVHISRLSASGRYLFVIGRDGKVNMIDLWMKEPTTVAEIKIGSEARSIETSKMEGWEDKYAI
AGAYWPPQYVIMDGETLEPKKIQSTRGMTYDEQEYHPEPRVAAILASHYRPEFIVNVKETGK
ILLVDYTDLNNLKTTEISAERFLHDGGLDGSHRYFITAANARNKLVVIDTKEGKLVAIEDTG
GQTPHPGRGANFVHPTFGPVWATSHMGDDSVALIGTDPEGHPDNAWKILDSFPALGGGSLFI
KTHPNSQYLYVDATLNPEAEISGSVAVFDIKAMTGDGSDPEFKTLPIAEWAGITEGQPRVVQ
GEFNKDGTEVWFSVWNGKDQESALVVVDDKTLELKHVIKDERLVTPTGKFNVYNTMTDTY
>d1e43a1 b.71.1.1 (A:394-483) Bacterial alpha-Amylase {Bacillus licheniformis}
YAYGAQHDYFDHHDIVGWTREGDSSVANSGLAALITDGPGGAKRMYVGRQNAGETWHDITGN
RSEPVVINSEGWGEFHVNGGSVSIYVQR
>d1g94a1 b.71.1.1 (A:355-448) Bacterial alpha-Amylase {Pseudoalteromonas haloplanctis (Alteromonas haloplanctis)}
NWAVTNWWDNTNNQISFGRGSSGHMAINKEDSTLTATVQTDMASGQYCNVLKGELSADAKSC
SGEVITVNSDGTINLNIGAWDAMAIHKNAKLN
>d1bag_1 b.71.1.1 (348-425) Bacterial alpha-Amylase {Bacillus subtilis}
QPEELSNPNGNNQIFMNQRGSHGVVLANAGSSSVSINTATKLPDGRYDNKAGAGSFQVNDGK
LTGTINARSVAVLYPD
>d1hvxa1 b.71.1.1 (A:394-483) Bacterial alpha-Amylase {Bacillus stearothermophilus}
YAYGTQHDYLDHSDIIGWTREGVTEKPGSGLAALITDGPGGSKWMYVGKQHAGKVFYDLTGN
RSDTVTINSDGWGEFKVNGGSVSVWVPR
>d1gjwa1 b.71.1.1 (A:573-636) Maltosyltransferase {Thermotoga maritima}
GKFENLTTKDLVMYSYEKNGQKIVIAANVGKEPKEITGGRVWNGKWSDEEKVVLKPLEFALV
VQ
>d1cgt_3 b.71.1.1 (407-494) Cyclodextrin glycosyltransferase {Bacillus circulans, different strains}
GSTQQRWINNDVYVYERKFGKSVAVVAVNRNLSTSASITGLSTSLPTGSYTDVLGGVLNGNN
ITSTNGSINNFTLAAGATAVWQYTTA
>d1kcla3 b.71.1.1 (A:407-495) Cyclodextrin glycosyltransferase {Bacillus circulans, different strains}
GSTQERWINNDVLIYERKFGSNVAVVAVNRNLNAPASISGLVTSLPQGSYNDVLGGLLNGNT
LSVGSGGAASNFTLAAGGTAVWQYTAA
>d1cyg_3 b.71.1.1 (403-491) Cyclodextrin glycosyltransferase {Bacillus stearothermophilus}
GDTEQRWINGDVYVYERQFGKDVVLVAVNRSSSSNYSITGLFTALPAGTYTDQLGGLLDGNT
IQVGSNGSVNAFDLGPGEVGVWAYSAT
>d1qhoa3 b.71.1.1 (A:408-495) Cyclodextrin glycosyltransferase {Bacillus stearothermophilus, maltogenic alpha-amylase}
GTTTQRWINNDVYIYERKFFNDVVLVAINRNTQSSYSISGLQTALPNGSYADYLSGLLGGNG

ISVSNGSVASFTLAPGAVSVWQYSTS
>d1pama3 b.71.1.1 (A:407-496) Cyclodextrin glycosyltransferase {Bacillus sp., strain 1011}
GSTHERWINNDVIIYERKFGNNVAVVAINRNMNTPASITGLVTSLPRGSYNDVLGGILNGNT
LTVGAGGAASNFTLAPGGTAVWQYTTDA
>d1ciu_3 b.71.1.1 (407-495) Cyclodextrin glycosyltransferase {Thermoanaerobacterium thermosulfurigenes, EM1}
GTTQQRWINNDVYIYERKFGNNVALVAINRNLSTSYNITGLYTALPAGTYTDVLGGLLNGNS
ISVASDGSVTPFTLSAGEVAVWQYVSS
>d1hx0a1 b.71.1.1 (A:404-496) Animal alpha-amylase {Pig (Sus scrofa)}
QPFANWWDNGSNQVAFGRGNRGFIVFNNDDWQLSSTLQTGLPGGTYCDVISGDKVGNSCTGI
KVYVSSDGTAQFSISNSAEDPFIAIHAESKL
>d1smd_1 b.71.1.1 (404-496) Animal alpha-amylase {Human (Homo sapiens)}
QPFTNWYDNGSNQVAFGRGNRGFIVFNNDDWTFSLTLQTGLPAGTYCDVISGDKINGNCTGI
KIYVSDDGKAHFSISNSAEDPFIAIHAESKL
>d1jae_1 b.71.1.1 (379-471) Animal alpha-amylase {Yellow mealworm (Tenebrio molitor), larva}
GTQVENWWSNDDNQIAFSRGSQGFVAFTNGGDLNQNLNTGLPAGTYCDVISGELSGGSCTGK
SVTVGDNGSADISLGSAEDDGVLAIHVNAKL
>d2aaa_1 b.71.1.1 (382-476) Fungal alpha-amylase {Aspergillus niger, acid amylase}
YANDAFYTDSNTIAMAKGTSGSQVITVLSNKGSSGSSYTLTLSGSGYTSGTKLIEAYTCTSV
TVDSSGDIPVPMASGLPRVLLPASVVDSSSLCG
>d2taaa1 b.71.1.1 (A:382-478) Fungal alpha-amylase {Aspergillus oryzae, Taka-amylase}
YKNPYIKDDTTIAMRKGTDGSQIVTILSNKGASGDSYTLSLSGASYTAGQQLTEVIGCTTVT
VGSDGNVPVPMAGGLPRVLYPTEKLAGSKICSDSS
>d7taa_1 b.71.1.1 (382-476) Fungal alpha-amylase {Aspergillus oryzae, Taka-amylase}
YKNWPIYKDDTTIAMRKGTDGSQIVTILSNKGASGDSYTLSLSGAGYTAGQQLTEVIGCTTV
TVGSDGNVPVPMAGGLPRVLYPTEKLAGSKICS
>d1smaa2 b.71.1.1 (A:506-588) Maltogenic amylase {Thermus sp.}
GDVAFLTADDEVNHLVYAKTDGNETVMIIINRSNEAAEIPMPIDARGKWLVNLLTGERFAAE
AETLCVSLPPYGFVLYAVESW
>d1bvza2 b.71.1.1 (A:503-585) Maltogenic amylase {Thermoactinomyces vulgaris, TVAII}
GNVRSWHADKQANLYAFVRTVQDQHVGVVLNNRGEKQTVLLQVPESGGKTWLDCLTGEEVHG
KQGQLKLTLRPYQGMILWNGR
>d1eh9a2 b.71.1.1 (A:491-557) Glycosyltrehalose trehalohydrolase {Archaeon Sulfolobus solfataricus, km1}
CDRRVNVVNGENWLIIKGREYFSLYVFSKSSIEVKYSGTLLLSSNNSFPQHIEEGKYEFDKG
FALYK

>d1bf2_2 b.71.1.1 (638-750) Isoamylase {Pseudomonas amyloderamosa}
YSGSQLTWYQPSGAVADSNYWNNTSNYAIAYAINGPSLGDSNSIYVAYNGWSSSVTFTLPAP
PSGTQWYRVTDTCDWNDGASTFVAPGSETLIGGAGTTYGQCGQSLLLLISK
>d1gcya1 b.71.1.1 (A:358-418) G4-amylase (1,4-alpha-D-glucan maltotetrahydrolase) {Pseudomonas stutzeri}
RADSAISFHSGYSGLVATVSGSQQTLVVALNSDLGNPGQVASGSFSEAVNASNGQVRVWRS
>d1avaa1 b.71.1.1 (A:347-403) Plant alpha-amylase {Barley (Hordeum vulgare), seeds, AMY2 isozyme}
HNESKLQIIEADADLYLAEIDGKVIVKLGPRYDVGNLIPGGFKVAAHGNDYAVWEKI
>d1uok_1 b.71.1.1 (480-558) Oligo-1,6-glucosidase {Bacillus cereus}
GSYDLILENNPSIFAYVRTYGVEKLLVIANFTAEECIFELPEDISYSEVELLIHNYDVENGP
IENITLRPYEAMVFKLK
>d1g5aa1 b.71.1.1 (A:555-628) Amylosucrase {Neisseria polysaccharea}
RLVTFNTNNKHIIGYIRNNALLAFGNFSEYPQTVTAHTLQAMPFKAHDLIGGKTVSLNQDLT
LQPYQVMWLEIA
>d1f8ab1 b.72.1.1 (B:1-42) Mitotic rotamase PIN1 {Human (Homo sapiens)}
GSHGMADEEKLPPGWEKRMSRSSGRVYYFNHITNASQWERPS
>d1i8hb_ b.72.1.1 (B:) Mitotic rotamase PIN1 {Human (Homo sapiens)}
KLPPGWEKRMSRSSGRVYYFNHITNASQWERPSGNSSSG
>d1pina1 b.72.1.1 (A:6-39) Mitotic rotamase PIN1 {Human (Homo sapiens)}
KLPPGWEKRMSRSSGRVYYFNHITNASQWERPSG
>d1e0la_ b.72.1.1 (A:) Formin binding protein FBP28 domain {Domestic mouse (Mus musculus)}
GATAVSEWTEYKTADGKTYYYNNRTLESTWEKPQELK
>d1eg3a3 b.72.1.1 (A:47-84) Dystrophin {Human (Homo sapiens)}
PASQHFLSTSVQGPWERAISPNKVPYYINHETQTTCWD
>d1i5hw_ b.72.1.1 (W:) Ubiquitin ligase NEDD4 WWIII domain {Rat (Rattus norvegicus)}
GSPVDSNDLGPLPPGWEERTHTDGRVFFINHNIKKTQWEDPRMQNVAITG
>d1e0na_ b.72.1.1 (A:) Hypothetical protein Yjq8 (Set2p) {Baker's yeast (Saccharomyces cerevisiae)}
PGWEIIHENGRPLYYNAEQKTKLHYPP
>d1jmqa_ b.72.1.1 (A:) Yap65 ww domain {Human (Homo sapiens)}
FEIPDDVPLPAGWEMAKTSSGQRYFKNHIDQTTTWQDPRKAMLSQM
>d1k9ra_ b.72.1.1 (A:) Yap65 ww domain {Human (Homo sapiens)}
FEIPDDVPLPAGWEMAKTSSGQRYFLNHIDQTTTWQDPRK
>d1aiw__ b.72.2.1 (-) Cellulose-binding domain of endoglucanase Z {Erwinia chrysanthemi}
MGDCANANVYPNWVSKDWAGGQPTHNEAGQSIVYKGNLYTANWYTASVPGSDSSWTQVGSCN

>d1ed7a_ b.72.2.1 (A:) Chitin-binding domain of chitinase A1 {Bacillus circulans}
AWQVNTAYTAGQLVTYNGKTYKCLQPHTSLAGWEPSNVPALWQLQ
>d1goia1 b.72.2.1 (A:447-498) Chitinase B, C-terminal domain {Serratia marcescens}
NLPIMTAPAYVPGTTYAQGALVSYQGYVWQTKWGYITSAPGSDSAWLKVGRV
>d1dkga1 b.73.1.1 (A:139-197) Head domain of nucleotide exchange factor GrpE {Escherichia coli}
VEVIAETNVPLDPNVHQAIAMVESDDVAPGNVLGIMQKGYTLNGRTIRAAMVTVAKAKA
>d1e44b_ b.101.1.1 (B:) Ribonuclease domain of colicin E3 {Escherichia coli}
GFKDYGHDYHPAPKTENIKGLGDLKPGIPKTPKQNGGGKRKRWTGDKGRKIYEWDSQHGELE
GYRASDGQHLGSFDPKTGNQLKGPDPKRNIKKYL
>d1hcb__ b.74.1.1 (-) Carbonic anhydrase {Human (Homo sapiens), erythrocytes, isozyme I}
PDWGYDDKNGPEQWSKLYPIANGNNQSPVDIKTSETKHDTSLKPISVSYNPATAKEIINVGH
SFHVNFEDNDNRSVLKGGPFSDSYRLFQFHFHWGSTNEHGSEHTVDGVKYSAELHVAHWNSA
KYSSLAEAASKADGLAVIGVLMKVGEANPKLQKVLDALQAIKTKGKRAPFTNFDPSTLLPSS
LDFWTYPGSLTHPPLYESVTWIICKESISVSSEQLAQFRSLLSNVEGDNAVPMQHNNRPTQP
LKGRTVRASF
>d2cba__ b.74.1.1 (-) Carbonic anhydrase {Human (Homo sapiens), erythrocytes, isozyme II}
HHWGYGKHNGPEHWHKDFPIAKGERQSPVDIDTHTAKYDPSLKPLSVSYDQATSLRILNNGH
AFNVEFDDSQDKAVLKGGPLDGTYRLIQFHFHWGSLDGQGSEHTVDKKKYAAELHLVHWNTK
YGDFGKAVQQPDGLAVLGIFLKVGSAKPGLQKVVDVLDSIKTKGKSADFTNFDPRGLLPESL
DYWTYPGSLTTPPLLECVTWIVLKEPISVSSEQVLKFRKLNFNGEGEPEELMVDNWRPAQPL
KNRQIKASFK
>d1flja_ b.74.1.1 (A:) Carbonic anhydrase {Rat (Rattus norvegicus), isozyme III}
AKEWGYASHNGPEHWHELYPIAKGDNQSPIELHTKDIRHDPSLQPWSVSYDPGSAKTILNNG
KTCRVVFDDTFDRSMLRGGPLSGPYRLRQFHLHWGSSDDHGSEHTVDGVKYAAELHLVHWNP
KYNTFGEALKQPDGIAVVGIFLKIGREKGEFQILLDALDKIKTKGKEAPFNHFDPSCLFPAC
RDYWTYHGSFTTPPCEECIVWLLLKEPMTVSSDQMAKLRSLFASAENEPPVPLVGNWRPPQP
IKGRVVRASFK
>d1znca_ b.74.1.1 (A:) Carbonic anhydrase {Human (Homo sapiens), isozyme IV}
WCYEVQAESSNYPCLVPVKWGGNCQKDRQSPINIVTTKAKVDKKLGRFFFSGYDKKQTWTVQ
NNGHSVMMLLENKASISGGGLPAPYQAKQLHLHWSDLPYKGSEHSLDGEHFAMEMHIVHEKE
KGTSRNVKEAQDPEDEIAVLAFLVEAGTQVNEGFQPLVEALSNIPKPEMSTTMAESSLLDLL
PKEEKLRHYFRYLGSLTTPTCDEKVVWTVFREPIQLHREQILAFSQKLYYDKEQTVSMKDNV
RPLQQLGQRTVIKS
>d2znc__ b.74.1.1 (-) Carbonic anhydrase {Mouse (Mus musculus), isozyme IV}
WCYEIQTEDPRSSCLGPEKWPGACKENQQSPINIVTARTKVNPRLTPFILVGYDQKQQWPIK

NNQHTVEMTLGGGACIIGGDLPARYEAVQLHLHWSNGNDNGSEHSIDGRHFAMEMHIVHKKL
TSSKEDSKDKFAVLAFMIEVGDKVNKGFQPLVEALPSISKPHSTSTVRESSLQDMLPPSTKM
YTYFRYNGSLTTPNCDETVIWTVYKQPIKIHKNQFLEFSKNLYYDEDQKLNMKDNVRPLQPL
GKRQVFKSHA
>d1dmxa_ b.74.1.1 (A:) Carbonic anhydrase {Mouse (Mus musculus), liver, isozyme V}
GTRQSPINIQWKDSVYDPQLAPLRVSYDAASCRYLWNTGYFFQVEFDDSCEDSGISGGPLGN
HYRLKQFHFHWGATDEWGSEHAVDGHTYPAELHLVHWNSTKYENYKKASVGENGLAVIGVFL
KLGAHHQALQKLVDVLPEVRHKDTQVAMGPFDPSCLMPACRDYWTYPGSLTTPPLAESVTWI
VQKTPVEVSPSQLSMFRTLLFSGRGEEEDVMVNNYRPLQPLRDRKLRSSFR
>d1jd0a_ b.74.1.1 (A:) Carbonic anhydrase {Human (Homo sapiens), isozyme XII}
KWTYFGPDGENSWSKKYPSCGGLLQSPIDLHSDILQYDASLTPLEFQGYNLSANKQFLLTNN
GHSVKLNLPSDMHIQGLQSRYSATQLHLHWGNPNDPHGSEHTVSGQHFAAELHIVHYNSDLY
PDASTASNKSEGLAVLAVLIEMGSFNPSYDKIFSHLQHVKYKGQEAFVPGFNIEELLPERTA
EYYRYRGSLTTPPCNPTVLWTVFRNPVQISQEQLLALETALYCTHMDDPSPREMINNFRQVQ
KFDERLVYTSFS
>d1kopa_ b.74.1.1 (A:) Carbonic anhydrase {Neisseria gonorrhoeae}
HTHWGYTGHDSPESWGNLSEEFRLCSTGKNQSPVNITETVSGKLPAIKVNYKPSMVDVENNG
HTIQVNYPEGGNTLTVNGRTYTLKQFHFHVPSENQIKGRTFPMEAHFVHLDENKQPLVLAVL
YEAGKTNGRLSSIWNVMPMTAGKVKLNQPFDASTLLPKRLKYYRFAGSLTTPPCTEGVSWLV
LKTYDHIDQAQAEKFTRAVGSENNRPVQPLNARVVIE
>d1koqa_ b.74.1.1 (A:) Carbonic anhydrase {Neisseria gonorrhoeae}
THWGYTGHDSPESWGNLSEEFRLCSTGKNQSPVNITETVSGKLPAIKVNYKPSMVDVENNGH
TIQVNYPEGGNTLTVNGRTYTLKQFHFLTVNGRTYTLKQFHFHVPSENQIKGRTFPMEAHFV
HLDENKQPLVLAVLYEAGKTNGRLSLAVLYEAGKTNGRLSSIWNVMPMTAGKVKLNQPFDAS
TLLPKRLKYYRFAGSLTTPPCTEGVSWLVLKTYDHIDQAQAEKFTRAVGSENNRPVQPLNAR
VVIE
>d4bcl__ b.75.1.1 (-) Bacteriochlorophyll A protein {Prosthecochloris aestuarii, strain 2k}
TTTAHSDYEIILEGGSSSWGQVKGRAKVNVPAAIPLLPTDCNIRIDAKPLDAQKGVVRFTTK
IESVVDSVKNTLNVEVDIANETKDRRIAVGEGSLSVGDFSHSFSFEGSVVNMYYYRSDAVRR
NIPNPIYMQGRQFHDILMKVPLDNNDLVDTWEGFQQSISGGGANFGDWIREFWFIGPAFAAI
NEGGQRISPIVVNSSNVEGGEKGPVGVTRWKFSHAGSGVVDSISRWTELFPVEQLNKPASIE
GGFRSDSQGIEVKVDGNLPGVSRDAGGGLRRILNHPLIPLVHHGMVGKFNDFTVDTQLKIVL
PKGYKIRYAAPQFRSQNLEEYRWSGGAYARWVEHVCKGGTGQFEVLYAQ
>d1ksaa_ b.75.1.1 (A:) Bacteriochlorophyll A protein {Green sulfur bacterium (Chlorobium tepidum)}
TTAHSDYEIVLEGGSSSWGKVKARAKVNAPPASPLLPADCDVKLNVKPLDPAKGFVRISAVF
ESIVDSTKNKLTIEADIANETKERRISVGEGMVSVGDFSHTFSFEGSVVNLFYYRSDAVRRN
VPNPIYMQGRQFHDILMKVPLDNNDLIDTWEGTVKAIGSTGAFNDWIRDFWFIGPAFTALNE
GGQRISRIEVNGLNTESGPKGPVGVSRWRFSHGGSGMVDSISRWAELFPSDKLNRPAQVEAG
FRSDSQGIEVKVDGEFPGVSVDAGGGLRRILNHPLIPLVHHGMVGKFNNFNVDAQLKVVLPK
GYKIRYAAPQYRSQNLEEYRWSGGAYARWVEHVCKGGVGQFEILYAQ

>d1ospo_ b.76.1.1 (O:) Outer surface protein A {Lyme disease spirochete (Borrelia burgdorferi)}
SLDEKNSVSVDLPGEMKVLVSKEKNKDGKYDLIATVDKLELKGTSDKNNGSGVLEGVKADKC
KVKLTISDDLGQTTLEVFKEDGKTLVSKKVTSKDKSSTEEKFNEKGEVSEKIITRADGTRLE
YTGIKSDGSGKAKEVLKGYVLEGTLTAEKTTLVVKEGTVTLSKNISKSGEVSVELNDTDSSA
ATKKTAAWNSGTSTLTITVNSKKTKDLVFTKENTITVQQYDSNGTKLEGSAVEITKLDEIKN
ALK
>d1vmoa_ b.77.1.1 (A:) Vitelline membrane outer protein-I (VMO-I) {Hen (Gallus gallus)}
RTREYTSVITVPNGGHWGKWGIRQFCHSGYANGFALKVEPSQFGRDDTALNGIRLRCLDGSV
IESLVGKWGTWTSFLVCPTGYLVSFSLRSEKSQGGGDDTAANNIQFRCSDEAVLVGDGLSWG
RFGPWSKRCKICGLQTKVESPQGLRDDTALNNVRFFCCK
>d1dlc_2 b.77.2.1 (290-499) delta-Endotoxin (insectocide), middle domain {Bacillus thuringiensis tenebrionis, CRYIIIA (BT13)}
LYPKEVKTELTRDVLTDPIVGVNNLRGYGTTFSNIENYIRKPHLFDYLHRIQFHTRFQPGYY
GNDSFNYWSGNYVSTRPSIGSNDIITSPFYGNKSSEPVQNLEFNGEKVYRAVANTNLAVWPS
AVYSGVTKVEFSQYNDQTDEASTQTYDSKRNVGAVSWDSIDQLPPETTDEPLEKGYSHQLNY
VMCFLMQGSRGTIPVLTWTHKSVD
>d1ji6a2 b.77.2.1 (A:291-502) delta-Endotoxin (insectocide), middle domain {Bacillus thuringiensis, CRY3bb1}
LYSKGVKTELTRDIFTDPIFSLNTLQEYGPTFLSIENSIRKPHLFDYLQGIEFHTRLQPGYF
GKDSFNYWSGNYVETRPSIGSSKTITSPFYGDKSTEPVQKLSFDGQKVYRTIANTDVAAWPN
GKVYLGVTKVDFSQYDDQKNETSTQTYDSKRNNGHVSAQDSIDQLPPETTDEPLEKAYSHQL
NYAECFLMQDRRGTIPFFTWTHRSVD
>d1ciy_2 b.77.2.1 (256-461) delta-Endotoxin (insectocide), middle domain {Bacillus thuringiensis, CRYIA (A)}
PIRTVSQLTREIYTNPVLENFDGSFRGMAQRIEQNIRQPHLMDILNSITIYTDVHRGFNYWS
GHQITASPVGFSGPEFAFPLFGNAGNAAPPVLVSLTGLGIFRTLSSPLYRRIILGSGPNNQE
LFVLDGTEFSFASLTTNLPSTIYRQRGTVDSLDVIPPQDNSVPPRAGFSHRLSHVTMLSQAA
GAVYTLRAPTFSWQHRSAEF
>d1i5pa2 b.77.2.1 (A:264-472) delta-Endotoxin (insectocide), middle domain {Bacillus thuringiensis subsp. kurstaki, CRY2AA}
YQSLMVSSGANLYASGSGPQQTQSFTAQNWPFLYSLFQVNSNYILSGISGTRLSITFPNIGG
LPGSTTTHSLNSARVNYSGGVSSGLIGATNLNHNFNCSTVLPPLSTPFVRSWLDSGTDRAGV
ATSTNWQTESFQTTLSLRCGAFSARGNSNYFPDYFIRNISGVPLVIRNEDLTRPLHYNQIRN
IESPSGTPGGARAYLVSVHNRKN
>g1jac.5 b.77.3.1 (B:,A:) Jacalin {Jackfruit (Artocarpus integrifolia)}
SGKSQTVIVGSWGAKXGKAFDDGAFTGIREINLSYNKETAIGDFQVVYDLNGSPYVGQNHKS
FITGFTPVKISLDFPSEYIMEVSGYTGNVSGYVVVRSLTFKTNKKTYGPYGVTSGTPFNLPI
ENGLIVGFKGSIGYWLDYFSMYLSL
>g1jot.2 b.77.3.1 (B:,A:) Lectin MPA {Osage orange (Maclura pomifera)}
RNGKSQSIIVGPWGDRXGVTFDDGAYTGIREINFEYNSETAIGGLRVTYDLNGMPFVAEDHK

SFITGFKPVKISLEFPSEYIVEVSGYVGKVEGYTVIRSLTFKTNKQTYGPYGVTNGTPFSLP
IENGLIVGFKGSIGYWLDYFSIYLSL
>d1c3ma_ b.77.3.1 (A:) Heltuba lectin {Jerusalem artishoke
(Helianthus tuberosus)}
ASDIAVQAGPWGGNGGKRWLQTAHGGKITSIIIKGGTCIFSIQFVYKDKDNIEYHSGKFGVL
GDKAETITFAEDEDITAISGTFGAYYHMTVVTSLTFQTNKKVYGPFGTVASSSFSLPLTKGK
FAGFFGNSGDVLDSIGGVVVP
>d1jpc__ b.78.1.1 (-) Lectin (agglutinin) {Snowdrop (Galanthus
nivalis)}
DNILYSGETLSTGEFLNYGSFVFIMQEDCNLVLYDVDKPIWATNTGGLSRSCFLSMQTDGNL
VVYNPSNKPIWASNTGGQNGNYVCILQKDRNVVIYGTDRWATGTHT
>d1bwua_ b.78.1.1 (A:) Lectin (agglutinin) {Garlic (Allium
sativum)}
RNILRNDEGLYGGQSLDVNPYHFIMQEDCNLVLYDHSTSVWASNTGILGKKGCRAVLQSDGN
FVVYDAEGRSLWASHSVRGNGNYVLVLQEDGNVVIYRSDIWSTN
>d1bwud_ b.78.1.1 (D:) Lectin (agglutinin) {Garlic (Allium
sativum)}
RNILTNDEGLYGGQSLDVNPYHLIMQEDCNLVLYDHSTAVWSSNTDIPGKKGCKAVLQSDGN
FVVYDAEGASLWASHSVRGNGNYVLVLQEDGNVVIYRSDIWSTNTYR
>d1kj1a_ b.78.1.1 (A:) Lectin (agglutinin) {Garlic (Allium
sativum)}
RNLLTNGEGLYAGQSLDVEPYHFIMQEDCNLVLYDHSTSVWASNTGILGKKGCKAVLQSDGN
FVVYDAEGRSLWASHSVRGNGNYVLVLQEDGNVVIYGSDIWSTGTYK
>d1kj1d_ b.78.1.1 (D:) Lectin (agglutinin) {Garlic (Allium
sativum)}
RNILMNDEGLYAGQSLDVEPYHLIMQEDCNLVLYDHSTAVWTTNTDIPGKKGCKAVLQSDGN
FVVYDAEGRSLWASHSVRGNGNYVLVLQEDGNVVIYGSDIWSTNTYK
>d1npla_ b.78.1.1 (A:) Lectin (agglutinin) {Daffodil (Narcissus
pseudonarcissus)}
DNILYSGETLSPGEFLNNGRYVFIMQEDCNLVLYDVDKPIWATNTGGLDRRCHLSMQSDGNL
VVYSPRNNPIWASNTGGENGNYVCVLQKDRNVVIYGTARWATGTNIH
>d1b2pa_ b.78.1.1 (A:) Lectin (agglutinin) {Bluebell (Scilla
campanulata)}
NNIIFSKQPDDNHPQILHATESLEILFGTHVYRFIMQTDCNLVLYDNNNPIWATNTGGLGNG
CRAVLQPDGVLVVITNENVTVWQSPVAGKAGHYVLVLQPDRNVVIYGDALWATQTVR
>d1dlpa1 b.78.1.1 (A:1-115) Fetuin-binding protein Scafet
precursor {Bluebell (Scilla campanulata)}
NNILFGLSHEGSHPQTLHAAQSLELSSFRFTMQSDCNLVLFDSDVRVWASNTAGATGCRAVL
QSDGLLVILTAQNTIRWSSGTKGSIGNYVLVLQPDRTVTIYGPGLWDSGTSNK
>d1dlpa2 b.78.1.1 (A:116-235) Fetuin-binding protein Scafet
precursor {Bluebell (Scilla campanulata)}
GSVVVANNGNSILYSTQGNDNHPQTLHATQSLQLSPYRLSMETDCNLVLFDRDDRVWSTNTA
GKGTGCRAVLQPNGRMDVLTNQNIAVWTSGNSRSAGRYVFVLQPDRNLAIYGGALWTT
>d1kapp1 b.79.1.1 (P:247-470) Metalloprotease, C-terminal domain

{Pseudomonas aeruginosa, alkaline protease}
GANLTTRTGDTVYGFNSNTERDFYSATSSSSKLVFSVWDAGGNDTLDFSGFSQNQKINLNEK
ALSDVGGLKGNVSIAAGVTVENAIGGSGSDLLIGNDVANVLKGGAGNDILYGGLGADQLWGG
AGADTFVYGDIAESSAAAPDTLRDFVSGQDKIDLSGLDAFVNGGLVLQYVDAFAGKAGQAIL
SYDAASKAGSLAIDFSGDAHADFAINLIGQATQADIVV
>d1sat_1 b.79.1.1 (247-471) Metalloprotease, C-terminal domain {Serratia marcescens}
GANLSTRTGDTVYGFNSNTGRDFLSTTSNSQKVIFAAWDAGGNDTFDFSGYTANQRINLNEK
SFSDVGGLKGNVSIAAGVTIENAIGGSGNDVIVGNAANNVLKGGAGNDVLFGGGGADELWGG
AGKDIFVFSAASDSAPGASDWIRDFQKGIDKIDLSFFDKEANSSSFIHFVDHFSGTAGEALL
SYNASSNVTDLSVNIGGHAAPDFLVKIVGQVDVATDFIV
>d1air__ b.80.1.1 (-) Pectate lyase {Erwinia chrysanthemi, type C}
ATDTGGYAATAGGNVTGAVSKTATSMQDIVNIIDAARLDANGKKVKGGAYPLVITYTGNEDS
LINAAAANICGQWSKDPRGVEIKEFTKGITIIGANGSSANFGIWIKKSSDVVVQNMRIGYLP
GGAKDGDMIRVDDSPNVWVDHNELFAANHECDGTPDNDTTFESAVDIKGASNTVTVSYNYIH
GVKKVGLDGSSSSDTGRNITYHHNYYNDVNARLPLQRGGLVHAYNNLYTNITGSGLNVRQNG
QALIENNWFEKAINPVTSRYDGKNFGTWVLKGNNITKPADFSTYSITWTADTKPYVNADSWT
STGTFPTVAYNYSPVSAQCVKDKLPGYAGVGKNLATLTSTAC
>d1pcl__ b.80.1.1 (-) Pectate lyase {Erwinia chrysanthemi, type E}
AVETDAATTGWATQNGGTTGGAKAAKAVEVKNISDFKKALNGTDSSAKIIKVTGPIDISGGK
AYTSFDDQKARSQISIPSNTTIIGVGSNGKFTNGSLVIKGVKNVILRNLYIETPVDVAPHYE
SGDGWNAEWDAAVIDNSTNVWVDHVTISDGSFTDDKYTTKDGEKYVQHDGALDIKKGSDYVT
ISYSRFELHDKTILIGHSDSNGSQDSGKLRVTFHNNVFDRVTERAPRVRFGSIHAYNNVYLG
DVKHSVYPYLYSFGLGTSGSILSESNSFTLSNLKSIDGKNPECSIVKQFNSKVFSDKGSLVN
GSTTTKLDTCGLTAYKPTLPYKYSAQTMTSSLATSINNNAGYGKL
>d1bn8a_ b.80.1.1 (A:) Pectate lyase {Bacillus subtilis}
ADLGHQTLGSNDGWGAYSTGTTGGSKASSSNVYTVSNRNQLVSALGKETNTTPKIIYIKGTI
DMNVDDNLKPLGLNDYKDPEYDLDKYLKAYDPSTWGKKEPSGTQEEARARSQKNQKARVMVD
IPANTTIVGSGTNAKVVGGNFQIKSDNVIIRNIEFQDAYDYFPQWDPTDGSSGNWNSQYDNI
TINGGTHIWIDHCTFNDGSRPDSTSPKYYGRKYQHHDGQTDASNGANYITMSYNYYHDHDKS
SIFGSSDSKTSDDGKLKITLHHNRYKNIVQRAPRVRFGQVHVYNNYYEGSTSSSSYPFSYAW
GIGKSSKIYAQNNVIDVPGLSAAKTISVFSGGTALYDSGTLLNGTQINASAANGLSSSVGWT
PSLHGSIDASANVKSNVINQAGAGKLN
>d1ee6a_ b.80.1.1 (A:) Pectate lyase {Bacillus sp., strain ksmp15}
APTVVHETIRVPAGQTFDGKGQTYVANPNTLGDGSQAENQKPIFRLEAGASLKNVVIGAPAA
DGVHCYGDCTITNVIWEDVGEDALTLKSSGTVNISGGAAYKAYDKVFQINAAGTINIRNFRA
DDIGKLVRQNGGTTYKVVMNVENCNISRVKDAILRTDSSTSTGRIVNTRYSNVPTLFKGFKS
GNTTASGNTQY
>d1idk__ b.80.1.2 (-) Pectin lyase {Aspergillus niger, type A}
VGVSGSAEGFAKGVTGGGSATPVYPDTIDELVSYLGDDEARVIVLTKTFDFTDSEGTTTGTG
CAPWGTASACQVAIDQDDWCENYEPDAPSVSVEYYNAGTLGITVTSNKSLIGEGSSGAIKGK
GLRIVSGAENIIIQNIAVTDINPKYVWGGDAITLDDCDLVWIDHVTTARIGRQHYVLGTSAD

NRVSLTNNYIDGVSDYSATCDGYHYWAIYLDGDADLVTMKGNYIYHTSGRSPKVQDNTLLHA
VNNYWYDISGHAFEIGEGGYVLAEGNVFQNVDTVLETYEGEAFTVPSSTAGEVCSTYLGRDC
VINGFGSSGTFSEDSTSFLSDFEGKNIASASAYTSVASRVVANAGQGNL
>d1qcxa_ b.80.1.2 (A:) Pectin lyase {Aspergillus niger, type B}
AGVVGAAEGFAHGVTGGGSASPVYPTTTDELVSYLGDNEPRVIILDQTFDFTGTEGTETTTG
CAPWGTASQCQVAINLHSWCDNYQASAPKVSVTYDKAGILPITVNSNKSIVGQGTKGVIKGK
GLRVVSGAKNVIIQNIAVTDINPKYVWGGDAITVDDSDLVWIDHVTTARIGRQHIVLGTSAD
NRVTISYSLIDGRSDYSATCNGHHYWGVYLDGSNDMVTLKGNYFYNLSGRMPKVQGNTLLHA
VNNLFHNFDGHAFEIGTGGYVLAEGNVFQDVNVVVETPISGQLFSSPDANTNQQCASVFGRS
CQLNAFGNSGSMSGSDTSIISKFAGKTIAAAHPPGAIAQWTMKNAGQGK
>d1rmg__ b.80.1.3 (-) Rhamnogalacturonase A {Aspergillus aculeatus}
QLSGSVGPLTSASTKGATKTCNILSYGAVADNSTDVGPAITSAWAACKSGGLVYIPSGNYAL
NTWVTLTGGSATAIQLDGIIYRTGTASGNMIAVTDTTDFELFSSTSKGAVQGFGYVYHAEGT
YGARILRLTDVTHFSVHDIILVDAPAFHFTMDTCSDGEVYNMAIRGGNEGGLDGIDVWGSNI
WVHDVEVTNKDECVTVKSPANNILVESIYCNWSGGCAMGSLGADTDVTDIVYRNVYTWSSNQ
MYMIKSNGGSGTVSNVLLENFIGHGNAYSLDIDGYWSSMTAVAGDGVQLNNITVKNWKGTEA
NGATRPPIRVVCSDTAPCTDLTLEDIAIWTESGSSELYLCRSAYGSGYCLKDSSSHTSYTTT
STVTAAPSGYSATTMAADLATAFGLTASIPIPTIPTSFYPGLTPYSALAG
>d1bhe__ b.80.1.3 (-) Polygalacturonase {Erwinia carotovora, subsp. carotovora}
SDSRTVSEPKTPSSCTTLKADSSTATSTIQKALNNCDQGKAVRLSAGSTSVFLSGPLSLPSG
VSLLIDKGVTLRAVNNAKSFENAPSSCGVVDKNGKGCDAFITAVSTTNSGIYGPGTIDGQGG
VKLQDKKVSWWELAADAKVKKLKQNTPRLIQINKSKNFTLYNVSLINSPNFHVVFSDGDGFT
AWKTTIKTPSTARNTDGIDPMSSKNITIAYSNIATGDDNVAIKAYKGRAETRNISILHNDFG
TGHGMSIGSETMGVYNVTVDDLKMNGTTNGLRIKSDKSAAGVVNGVRYSNVVMKNVAKPIVI
DTVYEKKEGSNVPDWSDITFKDVTSETKGVVVLNGENAKKPIEVTMKNVKLTSDSTWQIKNV
NVKK
>d1ia5a_ b.80.1.3 (A:) Polygalacturonase {Fungus (Aspergillus aculeatus)}
ATTCTFSGSNGASSASKSKTSCSTIVLSNVAVPSGTTLDLTKLNDGTHVIFSGETTFGYKEW
SGPLISVSGSDLTITGASGHSINGDGSRWWDGEGGNGGKTKPKFFAAHSLTNSVISGLKIVN
SPVQVFSVAGSDYLTLKDITIDNSDGDDNGGHNTDAFDIGTSTYVTISGATVYNQDDCVAVN
SGENIYFSGGYCSGGHGLSIGSVGGRSDNTVKNVTFVDSTIINSDNGVRIKTNIDTTGSVSD
VTYKDITLTSIAKYGIVVQQNYGDTSSTPTTGVPITDFVLDNVHGSVVSSGTNILISCGSGS
CSDWTWTDVSVSGGKTSSKCTNVPSGASC
>d1czfa_ b.80.1.3 (A:) Polygalacturonase {Fungus (Aspergillus niger), endo-polygalacturonase II}
DSCTFTTAAAAKAGKAKCSTITLNNIEVPAGTTLDLTGLTSGTKVIFEGTTTFQYEEWAGPL
ISMSGEHITVTGASGHLINCDGARWWDGKGTSGKKKPKFFYAHGLDSSSITGLNIKNTPLMA
FSVQANDITFTDVTINNADGDTQGGHNTDAFDVGNSVGVNIIKPWVHNQDDCLAVNSGENIW
FTGGTCIGGHGLSIGSVGDRSNNVVKNVTIEHSTVSNSENAVRIKTISGATGSVSEITYSNI
VMSGISDYGVVIQQDYEDGKPTGKPTNGVTIQDVKLESVTGSVDSGATEIYLLCGSGSCSDW
TWDDVKVTGGKKSTACKNFPSVASC

>d1hg8a_ b.80.1.3 (A:) Polygalacturonase {Fusarium moniliforme}
DPCSVTEYSGLATAVSSCKNIVLNGFQVPTGKQLDLSSLQNDSTVTFKGTTTFATTADNDFN
PIVISGSNITITGASGHVIDGNGQAYWDGKGSNSNSNQKPDHFIVVQKTTGNSKITNLNIQN
WPVHCFDITGSSQLTISGLILDNRAGDKPNAKSGSLPAAHNTDGFDISSSDHVTLDNNHVYN
QDDCVAVTSGTNIVVSNMYCSGGHGLSIGSVGGKSDNVVDGVQFLSSQVVNSQNGCRIKSNS
GATGTINNVTYQNIALTNISTYGVDVQQDYLNGGPTGKPTNGVKISNIKFIKVTGTVASSAQ
DWFILCGDGSCSGFTFSGNAITGGGKTSSCNYPTNTCPS
>d1dbga_ b.80.1.4 (A:) Chondroitinase B {Flavobacterium heparinum}
QVVASNETLYQVVKEVKPGGLVQIADGTYKDVQLIVSNSGKSGLPITIKALNPGKVFFTGDA
KVELRGEHLILEGIWFKDGNRAIQAWKSHGPGLVAIYGSYNRITACVFDCFDEANSAYITTS
LTEDGKVPQHCRIDHCSFTDKITFDQVINLNNTARAIKDGSVGGPGMYHRVDHCFFSNPQKP
GNAGGGIRIGYYRNDIGRCLVDSNLFMRQDSEAEIITSKSQENVYYGNTYLNCQGTMNFRHG
DHQVAINNFYIGNDQRFGYGGMFVWGSRHVIACNYFELSETIKSRGNAALYLNPGAMASEHA
LAFDMLIANNAFINVNGYAIHFNPLDERRKEYCAANRLKFETPHQLMLKGNLFFKDKPYVYP
FFKDDYFIAGKNSWTGNVALGVEKGIPVNISANRSAYKPVKIKDIQPIEGIALDLNALISKG
ITGKPLSWDEVRPYWLKEMPGTYALTARLSADRAAKFKAVIKRNKEH
>d1h80a_ b.80.1.8 (A:) iota-carrageenase {Alteromonas sp., atcc 43554}
VSPKTYKDADFYVAPTQQDVNYDLVDDFGANGNDTSDDSNALQRAINAISRKPNGGTLLIPN
GTYHFLGIQMKSNVHIRVESDVIIKPTWNGDGKNHRLFEVGVNNIVRNFSFQGLGNGFLVDF
KDSRDKNLAVFKLGDVRNYKISNFTIDDNKTIFASILVDVTERNGRLHWSRNGIIERIKQNN
ALFGYGLIQTYGADNILFRNLHSEGGIALRMETDNLLMKNYKQGGIRNIFADNIRCSKGLAA
VMFGPHFMKNGDVQVTNVSSVSCGSAVRSDSGFVELFSPTDEVHTRQSWKQAVESKLGRGCA
QTPYARGNGGTRWAARVTQKDACLDKAKLEYGIEPGSFGTVKVFDVTARFGYNADLKQDQLD
YFSTSNPMCKRVCLPTKEQWSKQGQIYIGPSLAAVIDTTPETSKYDYDVKTFNVKRINFPVN
SHKTIDTNTESSRVCNYYGMSECSSSRWER
>d1qjva_ b.80.1.5 (A:) Pectin methylesterase PemA {Erwinia chrysanthemi}
ATTYNAVVSKSSSDGKTFKTIADAIASAPAGSTPFVILIKNGVYNERLTITRNNLHLKGESR
NGAVIAAATAAGTLKSDGSKWGTAGSSTITISAKDFSAQSLTIRNDFDFPANQAKSDSDSSK
IKDTQAVALYVTKSGDRAYFKDVSLVGYQDTLYVSGGRSFFSDCRISGTVDFIFGDGTALFN
NCDLVSRYRADVKSGNVSGYLTAPSTNINQKYGLVITNSRVIRESDSVPAKSYGLGRPWHPT
TTFSDGRYADPNAIGQTVFLNTSMDNHIYGWDKMSGKDKNGNTIWFNPEDSRFFEYKSYGAG
AAVSKDRRQLTDAQAAEYTQSKVLGDWTPTLP
>d1qq1a_ b.80.1.6 (A:) P22 tailspike protein {Salmonella phage P22}
YSIEADKKFKYSVKLSDYPTLQDAASAAVDGLLIDRDYNFYGGETVDFGGKVLTIECKAKFI
GDGNLIFTKLGKGSRIAGVFMESTTTPWVIKPWTDDNQWLTDAAAVVATLKQSKTDGYQPTV
SDYVKFPGIETLLPPNAKGQNITSTLEIRECIGVEVHRASGLMAGFLFRGCHFCKMVDANNP
SGGKDGIITFENLSGDWGKGNYVIGGRTSYGSVSSAQFLRNNGGFERDGGVIGFTSYRAGGS
GVKTWQGTVGSTTSRNYNLQFRDSVVIYPVWDGFDLGADTDMNPELDRPGDYPITQYPLHQL
PLNHLIDNLLVRGALGVGFGMDGKGMYVSNITVEDCAGSGAYLLTHESVFTNIAIIDTNTKD
FQANQIYISGACRVNGLRLIGIRSTDGQSLTIDAPNSTVSGITGMVDPSRINVANLAEEGLG
NIRANSFGYDSAAIKLRIHKLSKTLDSGALYSHINGGAGSGSAYTQLTAISGSTPDAVSLKV
NHKDCRGAEIPFVPDIASDDFIKDSSCFLPYWENNSTSLKALVKKPNGELVRLTLATL

>d1daba_ b.80.1.7 (A:) Virulence factor P.69 pertactin {Bordetella pertussis}
DWNNQSIVKTGERQHGIHIQGSDPGGVRTASGTTIKVSGRQAQGILLENPAAELQFRNGSVT
SSGQLSDDGIRRFLGTVTVKAGKLVADHATLANVGDTWDDDGIALYVAGEQAQASIADSTLQ
GAGGVQIERGANVTVQRSAIVDGGLHIGALQSLQPEDLPPSRVVLRDTNVTAVPASGAPAAV
SVLGASELTLDGGHITGGRAAGVAAMQGAVVHLQRATIRRGDALAGGAVPGGAVPGGAVPGG
FGPGGFGPVLDGWYGVDVSGSSVELAQSIVEAPELGAAIRVGRGARVTVPGGSLSAPHGNVI
ETGGARRFAPQAAPLSITLQAGAHAQGKALLYRVLPEPVKLTLTGGADAQGDIVATELPSIP
GTSIGPLDVALASQARWTGATRAVDSLSIDNATWVMTDNSNVGALRLASDGSVDFQQPAEAG
RFKVLTVNTLAGSGLFRMNVFADLGLSDKLVVMQDASGQHRLWVRNSGSEPASANTLLLVQT
PLGSAATFTLANKDGKVDIGTYRYRLAANGNGQWSLVGAKAPP
>d1ezga_ b.80.2.1 (A:) Insect cysteine-rich antifreeze protein {Yellow mealworm (Tenebrio molitor)}
QCTGGADCTSCTGACTGCGNCPNAVTCTNSQHCVKANTCTGSTDCNTAQTCTNSKDCFEANT
CTDSTNCYKATACTNSSGCP
>d1hf2a1 b.80.3.1 (A:100-206) Cell-division inhibitor MinC, C-terminal domain {Thermotoga maritima}
TGKVIKRNIRSGQTVVHSGDVIVFGNVNKGAEILAGGSVVVFGKAQGNIRAGLNEGGQAVVA
ALDLQTSLIQIAGFITHSKGEENVPSIAHVKGNRIVIEPFDKVSF
>d1ea0a1 b.80.4.1 (A:1203-1472) Alpha subunit of glutamate synthase, C-terminal domain {Azospirillum brasilense}
GRNEVPDTLDARIVADARPLFEEGEKMQLAYNARNTQRAIGTRLSSMVTRKFGMFGLQPGHI
TIRLRGTAGQSLGAFAVQGIKLEVMGDANDYVGKGLSGGTIVVRPTTSSPLETNKNTIIGNT
VLYGATAGKLFAAGQAGERFAVRNSGATVVVEGCGSNGCEYMTGGTAVILGRVGDNFAAGMT
GGMAYVYDLDDSLPLYINDESVIFQRIEVGHYESQLKHLIEEHVTETQSRFAAEILNDWARE
VTKFWQVVPKEMLNRLEVPVHL
>d1kq5a_ b.80.5.1 (A:) C-terminal domain of adenylylcyclase associated protein {Baker's yeast (Saccharomyces cerevisiae)}
MPPRKELVGNKWFIENYENETESLVIDANKDESIFIGKCSQVLVQIKGKVNAISLSETESCS
VVLDSSISGMDVIKSNKFGIQVNHSLPQISIDKSDGGNIYLSKESLNTEIYTSCSTAINVNL
PIGEDDDYVEFPISEQMKHSFADGKFKSAVFE
>d1lxa__ b.81.1.1 (-) UDP N-acetylglucosamine acyltransferase {Escherichia coli, gene lpxA}
MIDKSAFVHPTAIVEEGASIGANAHIGPFCIVGPHVEIGEGTVLKSHVVVNGHTKIGRDNEI
YQFASIGEVNQDLKYAGEPTRVEIGDRNRIRESVTIHRGTVQGGGLTKVGSDNLLMINAHIA
HDCTVGNRCILANNATLAGHVSVDDFAIIGGMTAVHQFCIIGAHVMVGGCSGVAQDVPPYVI
AQGNHATPFGVNIEGLKRRGFSREAITAIRNAYKLIYRSGKTLDEVKPEIAELAETYPEVKA
FTDFFARSTRGLIR
>d3tdt__ b.81.1.2 (-) Tetrahydrodipicolinate-N-succinlytransferase, THDP-succinlytransferase, DapD {Mycobacterium bovis}
MQQLQNVIESAFERRADITPANVDTVTREAVNQVIGLLDSGALRVAEKIDGQWVTHQWLKKA
VLLSFRINDNKVMDGAETRYYDKVPMKFADYDEARFQKEGFRVVPPATVRQGAFIARNTVLM
PSYVNIGAYVDEGTMVDTWATVGSCAQIGKNVHLSGGVGIGGVLEPLQANPTIIEDNCFIGA

RSEVVEGVIVEEGSVISMGVYLGQSTRIYDRETGEIHYGRVPAGSVVVSGNLPSKDGSYSLY
CAVIVKKVDAKTRGKVGINELLRTID
>d1xat__ b.81.1.3 (-) Xenobiotic acetyltransferase {Pseudomonas aeruginosa}
NYFESPFRGKLLSEQVSNPNIRVGRYSYYSGYYHGHSFDDCARYLMPDRDDVDKLVIGSFCS
IGSGAAFIMAGNQGHRAEWASTFPFHFMHEEPAFAGAVNGYQPAGDTLIGHEVWIGTEAMFM
PGVRVGHGAIIGSRALVTGDVEPYAIVGGNPARTIRKRFSDGDIQNLLEMAWWDWPLADIEA
AMPLLCTGDIPALYQHWKQRQA
>d1kk6a_ b.81.1.3 (A:) Xenobiotic acetyltransferase {Enterococcus faecium, VAT(D)}
MGPNPMKMYPIEGNKSVQFIKPILEKLENVEVGEYSYYDSKNGETFDKQILYHYPILNDKLK
IGKFCSIGPGVTIIMNGANHRMDGSTYPFNLFGNGWEKHMPKLDQLPIKGDTIIGNDVWIGK
DVVIMPGVKIGDGAIVAANSVVVKDIAPYMLAGGNPANEIKQRFDQDTINQLLDIKWWNWPI
DIINENIDKILDNSIIREVIW
>d1fxja1 b.81.1.4 (A:252-329) N-acetylglucosamine 1-phosphate uridyltransferase GlmU, C-terminal domain {Escherichia coli}
VMLRDPARFDLRGTLTHGRDVEIDTNVIIEGNVTLGHRVKIGTGCVIKNSVIGDDCEISPYT
VVEDANLAAACTIGPF
>d1hv9a1 b.81.1.4 (A:252-452) N-acetylglucosamine 1-phosphate uridyltransferase GlmU, C-terminal domain {Escherichia coli}
VMLRDPARFDLRGTLTHGRDVEIDTNVIIEGNVTLGHRVKIGTGCVIKNSVIGDDCEISPYT
VVEDANLAAACTIGPFARLRPGAELLEGAHVGNFVEMKKARLGKGSKAGHLTYLGDAEIGDN
VNIGAGTITCNYDGANKFKTIIGDDVFVGSDTQLVAPVTVGKGATIAAGTTVTRNVGENALA
ISRVPQTQKEGWRRP
>d1hm9a1 b.81.1.4 (A:252-459) N-acetylglucosamine 1-phosphate uridyltransferase GlmU, C-terminal domain {Streptococcus pneumoniae}
VSFVNPEATYIDIDVEIAPEVQIEANVILKGQTKIGAETVLTNGTYVVDSTIGAGAVITNSM
IEESSVADGVTVGPYAHIRPNSSLGAQVHIGNFVEVKGSSIGENTKAGHLTYIGNCEVGSNV
NFGAGTITVNYDGKNKYKTVIGDNVFVGSNSTIIAPVELGDNSLVGAGSTITKDVPADAIAI
GRGRQINKDEYATRLPHHPKNQ
>d1qrea_ b.81.1.5 (A:) Carbonic anhydrase {Archaeon Methanosarcina thermophila}
TVDEFSNIRENPVTPWNPEPSAPVIDPTAYIDPQASVIGEVTIGANVMVSPMASIRSDEGMP
IFVGDRSNVQDGVVLHALETINEEGEPIEDNIVEVDGKEYAVYIGNNVSLAHQSQVHGPAAV
GDDTFIGMQAFVFKSKVGNNCVLEPRSAAIGVTIPDGRYIPAGMVVTSQAEADKLPEVTDDY
AYSHTNEAVVYVNVHLAEGYKETS
>d1ewwa_ b.81.2.1 (A:) An insect antifreeze protein {Spruce budworm (Choristoneura fumiferana)}
DGSCTNTNSQLSANSKCEKSTLTNCYVDKSEVYGTTCTGSRFDGVTITTSTSTGSRISGPGC
KISTCIITGGVPAPSAACKISGCTFSAN
>d1dzra_ b.82.1.1 (A:) dTDP-4-dehydrorhamnose 3,5-epimerase RmlC {Salmonella typhimurium}
MMIVIKTAIPDVLILEPKVFGDERGFFFESYNQQTFEELIGRKVTFVQDNHSKSKKNVLRGL

HFQRGENAQGKLVRCAVGEVFDVAVDIRKESPTFGQWVGVNLSAENKRQLWIPEGFAHGFVT
LSEYAEFLYKATNYYSPSSEGSILWNDEAIGIEWPFSQLPELSAKDAAAPLLDQALLTE
>d1ep0a_ b.82.1.1 (A:) dTDP-4-dehydrorhamnose 3,5-epimerase RmlC {Archaeon Methanobacterium thermoautotrophicum}
EFRFIKTSLDGAIIIEPEVYTDERGYFMETFNEAIFQENGLEVRFVQDNESMSVRGVLRGLH
FQREKPQGKLVRVIRGEIFDVAVDLRKNSDTYGEWTGVRLSDENRREFFIPEGFAHGFLALS
DECIVNYKCTELYHPEYDSGIPWDDPDIGIDWPLEMVDDLIISEKDRNWKPLRENPVYL
>d1fi2a_ b.82.1.2 (A:) Germin {Barley (Hordeum vulgare)}
TDPDPLQDFCVADLDGKAVSVNGHTCKPMSEAGDDFLFSSKLTKAGNTSTPNGSAVTELDVA
EWPGTNTLGVSMNRVDFAPGGTNPPHIHPRATEIGMVMKGELLVGILGSLDSGNKLYSRVVR
AGETFVIPRGLMHFQFNVGKTEAYMVVSFNSQNPGIVFVPLTLFGSDPPIPTPVLTKALRVE
AGVVELLKSKFAGGS
>d2phla1 b.82.1.2 (A:11-210) Seed storage 7S protein {French bean (Phaseolus vulgaris), phaseolin}
DNPFYFNSDNSWNTLFKNQYGHIRVLQRFDQQSKRLQNLEDYRLVEFRSKPETLLLPQQADA
ELLLVVRSGSAILVLVKPDDRREYFFLTSDNPIFSDHQKIPAGTIFYLVNPDPKEDLRIIQL
AMPVNNPQIHEFFLSSTEAQQSYLQEFSKHILEASFNSKFEEINRVLFEEEGQQEGVIVNID
SEQIKELSKHAKSS
>d2phla2 b.82.1.2 (A:220-381) Seed storage 7S protein {French bean (Phaseolus vulgaris), phaseolin}
NTIGNEFGNLTERTDNSLNVLISSIEMEEGALFVPHYYSKAIVILVVNEGEAHVELVGPKGN
KETLEYESYRAELSKDDVFVIPAAYPVAIKATSNVNFTGFGINANNNNRNLLAGKTDNVISS
IGRALDGKDVLGLTFSGSGDEVMKLINKQSGSYFVDAH
>d1dgwa_ b.82.1.2 (A:) Seed storage 7S protein {Jack bean (Canavalia ensiformis), canavalin/vinculin}
NNPYLFRSNKFLTLFKNQHGSLRLLQRFNEDTEKLENLRDYRVLEYCSKPNTLLLPHHSDSD
LLVLVLEGQAILVLVNPDGRDTYKLDQGDAIKIQAGTPFYLINPDNNQNLRILKFAITFRRP
GTVEDFFLSSTKRLPSYLSAFSKNFLEASYDSPYDEIEQTLLQEEQEGVIVKMP
>g1dgr.3 b.82.1.2 (M:,N:) Seed storage 7S protein {Jack bean (Canavalia ensiformis), canavalin/vinculin}
QLRRYAATLSEGDIIVIPSSFPVALKAASDLNMVGIGVNAENNERNFLAGHKENVIRQIPRQ
VSDLTFPGSGEEVEELLENQKESYFVDGQPXDKPFNLRSRDPIYSNNYGKLYEITPEKNSQL
RDLDILLNCLQMNEGALFVPHYNSRATVILVANEGRAEVELVGLE
>g1dgw.1 b.82.1.2 (X:,Y:) Seed storage 7S protein {Jack bean (Canavalia ensiformis), canavalin/vinculin}
DKPFNLRSRDPIYSNNYGKLYEITPEKNSQLRDLDILLNCLQMNEGALFVPHYNSRATVILV
ANEGRAEVELVGLEXQLRRYAATLSEGDIIVIPSSFPVALKAASDLNMVGIGVNAENNERNF
LAGHKENVIRQIPRQVSDLTFPGSGEEVEELLENQKESYFVDGQP
>d1fxza1 b.82.1.2 (A:10-248) Seed storage 7S protein {Soybean (Glycine max), proglycinin}
NECQIQKLNALKPDNRIESEGGLIETWNPNNKPFQCAGVALSRCTLNRNALRRPSYTNGPQE
IYIQQGKGIFGMIYPGCPSTFEEPQQPQQRGQSSRPQDRHQKIYNFREGDLIAVPTGVAWWM
YNNEDTPVVAVSIIDTNSLENQLDQMPRRFYLAGNQEQEFLKYQQEQGGHQSQKGKHQQEEE
NEGGSILSGFTLEFLEHAFSVDKQIAKNLQGENEGEDKGAIVTVKGGLSVIKP

>d1fxza2 b.82.1.2 (A:297-470) Seed storage 7S protein {Soybean (Glycine max), proglycinin}
ICTMRLRHNIGQTSSPDIYNPQAGSVTTATSLDFPALSWLRLSAEFGSLRKNAMFVPHYNLN
ANSIIYALNGRALIQVVNCNGERVFDGELQEGRVLIVPQNFVVAARSQSDNFEYVSFKTNDT
PMIGTLAGANSLLNALPEEVIQHTFNLKSQQARQIKNNNPFKFLVPPQES
>d1pmi__ b.82.1.3 (-) Phosphomannose isomerase {Yeast (Candida albicans)}
SSEKLFRIQCGYQNYDWGKIGSSSAVAQFVHNSDPSITIDETKPYAELWMGTHPSVPSKAID
LNNQTLRDLVTAKPQEYLGESIITKFGSSKELPFLFKVLSIEKVLSIQAHPDKKLGAQLHAA
DPKNYPDDNHKPEMAIAVTDFEGFCGFKPLDQLAKTLATVPELNEIIGQELVDEFISGIKLP
AEVGSQDDVNNRKLLQKVFGKLMNTDDDVIKQQTAKLLERTDREPQVFKDIDSRLPELIQRL
NKQFPNDIGLFCGCLLLNHVGLNKGEAMFLQAKDPHAYISGDIIECMAASDNVVRAGFTPKF
KDVKNLVEMLTYSYESVEKQKMPLQEFPRSKGDAVKSVLYDPPIAEFSVLQTIFDKSKGGKQ
VIEGLNGPSIVIATNGKGTIQITGDDSTKQKIDTGYVFFVAPGSSIELTADSANQDQDFTTY
RAFVEA
>d1eyba_ b.82.1.4 (A:) Homogentisate dioxygenase {Human (Homo sapiens)}
AELKYISGFGNECSSEDPRCPGSLPEGQNNPQVCPYNLYAEQLSGSAFTCPRSTNKRSWLYR
ILPSVSHKPFESIDEGHVTHNWDEVDPDPNQLRWKPFEIPKASQKKVDFVSGLHTLCGAGDI
KSNNGLAIHIFLCNTSMENRCFYNSDGDFLIVPQKGNLLIYTEFGKMLVQPNEICVIQRGMR
FSIDVFEETRGYILEVYGVHFELPDLGPIGANGLANPRDFLIPIAWYEDRQVPGGYTVINKY
QGKLFAAKQDVSPFNVVAWHGNYTPYKYNLKNFMVINSVAFDHADPSIFTVLTAKSVRPGVA
IADFVIFPPRWGVADKTFRPPYYHRNCMSEFMGLIRGHYEAKQGGFLPGGGSLHSTMTPHGP
DADCFEKASKVKLAPERIADGTMAFMFESSLSLAVTKWGLKASRCLDENYHKCWEPLKSHFT
PNSRN
>d1qjea_ b.82.2.1 (A:) Isopenicillin N synthase {Emericella nidulans}
SKANVPKIDVSPLFGDDQAAKMRVAQQIDAASRDTGFFYAVNHGINVQRLSQKTKEFHMSIT
PEEKWDLAIRAYNKEHQDQVRAGYYLSIPGKKAVESFCYLNPNFTPDHPRIQAKTPTHEVNV
WPDETKHPGFQDFAEQYYWDVFGLSSALLKGYALALGKEENFFARHFKPDDTLASVVLIRYP
YLDPYPEAAIKTAADGTKLSFEWHEDVSLITVLYQSNVQNLQVETAAGYQDIEADDTGYLIN
CGSYMAHLTNNYYKAPIHRVKWVNAERQSLPFFVNLGYDSVIDPFDPREPNGKSDREPLSYG
DYLQNGLVSLINKNGQT
>d1dcs__ b.82.2.1 (-) Deacetoxycephalosporin C synthase {Streptomyces clavuligerus}
MDTTVPTFSLAELQQGLHQDEFRRCLRDKGLFYLTDCGLTDTELKSAKDLVIDFFEHGSEAE
KRAVTSPVPTMRRGFTGLESESTAQITNTGSYSDYSMCYSMGTADNLFPSGDFERIWTQYFD
RQYTASRAVAREVLRATGTEPDGGVEAFLDCEPLLRFRYFPQVPEHRSAEEQPLRMAPHYDL
SMVTLIQQTPCANGFVSLQAEVGGAFTDLPYRPDAVLVFCGAIATLVTGGQVKAPRHHVAAP
RRDQIAGSSRTSSVFFLRPNADFTFSVPLARECGFDVSLDGETATFQDWIGGNYVNIRRTSK
A
>d1gp6a_ b.82.2.1 (A:) Anthocyanidin synthase {Mouse-ear cress (Arabidopsis thaliana)}
VAVERVESLAKSGIISIPKEYIRPKEELESINDVFLEEKKEDGPQVPTIDLKNIESDDEKIR

ENCIEELKKASLDWGVMHLINHGIPADLMERVKKAGEEFFSLSVEEKEKYANDQATGKIQGY
GSKLANNASGQLEWEDYFFHLAYPEEKRDLSIWPKTPSDYIEATSEYAKCLRLLATKVFKAL
SVGLGLEPDRLEKEVGGLEELLLQMKINYYPKCPQPELALGVEAHTDVSALTFILHNMVPGL
QLFYEGKWVTAKCVPDSIVMHIGDTLEILSNGKYKSILHRGLVNKEKVRISWAVFCEPPKDK
IVLKPLPEMVSVESPAKFPPRTFAQHIEHKLFGKEQEEL
>d1ds1a_ b.82.2.2 (A:) Clavaminate synthase {Streptomyces clavuligerus}
TSVDCTAYGPELRALAARLPRTPRADLYAFLDAAHTAAASLPGALATALDTFNAEGSEDGHL
LLRGLPVEADADLPTTPSSTPAPEDRSLLTMEAMLGLVGRRLGLHTGYRELRSGTVYHDVYP
SPGAHHLSSETSETLLEFHTEMAYHRLQPNYVMLACSRADHERTAATLVASVRKALPLLDER
TRARLLDRRMPCCVDVAFRGGVDDPGAIAQVKPLYGDADDPFLGYDRELLAPEDPADKEAVA
ALSKALDEVTEAVYLEPGDLLIVDNFRTTHARTPFSPRWDGKDRWLHRVYIRTDRNGQLSGG
ERAGDVVAFTPRG
>d1jr7a_ b.82.2.3 (A:) Gab protein (hypothetical protein YgaT) {Escherichia coli}
GQDYSGFTLTPSAQSPRLLELTFTEQTTKQFLEQVAEWPVQALEYKSFLRFRVAKILDDLCA
NQLQPLLLKTLLNRAEGALLINAVGVDDVKQADEMVKLATAVAHLIGRSNFDAMSGQYYARF
VVKNVDNSDSYLRQPHRVMELHNDGTYVEEITDYVLMMKIDEQNMQGGNSLLLHLDDWEHLD
NYFRHPLARRPMRFAAPPSKNVSKDVFHPVFDVDQQGRPVMRYIDQFVQPKDFEEGVWLSEL
SDAIETSKGILSVPVPVGKFLLINNLFWLHGRDRFTPHPDLRRELMRQRGYFAYASNHYQTH
Q
>d1e5sa_ b.82.2.4 (A:) Type II Proline 3-hydroxylase (proline oxidase) {Streptomyces sp.}
MRSHILGKIELDQTRLAPDLAYLAAVPTVEEEYDEFSNGFWKHVPLWNASGDSEDRLYRDLK
DAAAQPTAHVEHVPYLKEIVTTVFDGTHLQMARSRNLKNAIVIPHRDFVELDREVDRYFRTF
MVLEDSPLAFHSNEDTVIHMRPGEIWFLDAATVHSAVNFSEISRQSLCVDFAFDGPFDEKEI
FADATLYAPGSTPDLPERRPFTAEHRRRILSLGQVIERENFRDILFLLSKVHYKYDVHPSET
YDWLIEISKQAGDEKMVVKAEQIRDFAVEARALSERFSLTSW
>d1ft9a2 b.82.3.1 (A:2-133) CO-sensing protein CooA, N-terminal domain {Rhodospirillum rubrum}
PPRFNIANVLLSPDGETFFRGFRSKIHAKGSLVCTGEGDENGVFVVVDGRLRVYLVGEEREI
SLFYLTSGDMFCMHSGCLVEATERTEVRFADIRTFEQKLQTCPSMAWGLIAILGRALTSCMR
TIEDLMFH
>d1hw5a2 b.82.3.2 (A:1-137) Catabolite gene activator protein, N-terminal domain {Escherichia coli}
VLGKPQTDPTLEWFLSHCHIHKYPSKSTLIHQGEKAETLYYIVKGSVAVLIKDEEGKEMILS
YLNQGDFIGELGLFEEGQERSAWVRAKTACEVAEISYKKFRQLIQVNPDILMRLSAQMARRL
QVLAEKVGNLAFL
>d1rgs_1 b.82.3.2 (113-244) Regulatory subunit of Protein kinase A {Cow (Bos taurus)}
RKVIPKDYKTMAALAKAIEKNVLFSHLDDNERSDIFDAMFPVSFIAGETVIQQGDEGDNFYV
IDQGEMDVYVNNEWATSVGEGGSFGELALIYGTPRAATVKAKTNVKLWGIDRDSYRRILMGS
TLRKRKMY
>d1rgs_2 b.82.3.2 (245-376) Regulatory subunit of Protein kinase

A {Cow (Bos taurus)}
EEFLSKVSILESLDKWERLTVADALEPVQFEDGQKIVVQGEPGDEFFIILEGSAAVLQRRSE
NEEFVEVGRLGPSDYFGEIALLMNRPRAATVVARGPLKCVKLDRPRFERVLGPCSDILKRNI
QQYNSFVS
>d1cx4a1 b.82.3.2 (A:130-265) Regulatory subunit of Protein kinase A {Rat (Rattus norvegicus)}
RIIHPKTDDQRNRLQEACKDILLFKNLDPEQMSQVLDAMFEKLVKEGEHVIDQGDDGDNFYV
IDRGTFDIYVKCDGVGRCVGNYDNRGSFGELALMYNTPRAATITATSPGALWGLDRVTFRRI
IVKNNAKKRKMY
>d1cx4a2 b.82.3.2 (A:266-412) Regulatory subunit of Protein kinase A {Rat (Rattus norvegicus)}
ESFIESLPFLKSLEVSERLKVVDVIGTKVYNDGEQIIAQGDSADSFFIVESGEVRITMKRKG
KSDIEENGAVEIARCLRGQYFGELALVTNKPRAASAHAIGTVKCLAMDVQAFERLLGPCMEI
MKRNIATYEEQLVALFGTNMDIV
>d2arca_ b.82.4.1 (A:) Regulatory protein AraC {Escherichia coli}
DPLLPGYSFNAHLVAGLTPIEANGYLDFFIDRPLGMKGYILNLTIRGQGVVKNQGREFVCRP
GDILLFPPGEIHHYGRHPEAREWYHQWVYFRPRAYWHEWLNWPSIFANTGFFRPDEAHQPHF
SDLFGQIINAGQGEGRYSELLAINLLEQLLLRRMEAI
>d1ig3a1 b.82.6.1 (A:179-263) Thiamin pyrophosphokinase, substrate-binding domain {Mouse (Mus musculus)}
DSLIYLLQPGKHRLHVDTGMEGSWCGLIPVGQPCNQVTTTGLKWNLTNDVLGFGTLVSTSNT
YDGSGLVTVETDHPLLWTMAIKS
>d1ig0a1 b.82.6.1 (A:224-319) Thiamin pyrophosphokinase, substrate-binding domain {Baker's yeast (Saccharomyces cerevisiae)}
TDLIFLIKKNGTLIEYDPQFRNTCIGNCGLLPIGEATLVKETRGLKWDVKNWPTSVVTGRVS
SSNRFVGDNCCFIDTKDDIILNVEIFVDKLIDFL
>d1wapa_ b.82.5.1 (A:) Trp RNA-binding attenuation protein (TRAP) {Bacillus subtilis}
DFVVIKAVEDGVNVIGLTRGTDTKFHHSEKLDKGEVIIAQFTEHTSAIKVRGEALIQTAYGE
MKSEKK
>d1c9sa_ b.82.5.1 (A:) Trp RNA-binding attenuation protein (TRAP) {Bacillus stearothermophilus}
SDFVVIKALEDGVNVIGLTRGADTRFHHSEKLDKGEVLIAQFTEHTSAIKVRGKAYIQTRHG
VIESEGK
>g1h6w.1 b.108.1.1 (A:,B:) Heat- and protease-stable fragment of the short fibre {Bacteriophage T4}
TGATLNGRGSTTSMRGVVKLTTTAGSQSGGDASSALAWNADVIHQRGGQTINGTLRINNTLT
IASGGANITGTVNMTGGYIQGKRVVTQNEIDRTIPVGAIMMWAADSLPSDAWRFCHGGTVSA
SDCPLYASRIGTRYGGSSSNPGLPDMRXSLNYIIKVKE
>d1k28a2 b.108.1.2 (A:362-584) Tail-associated lysozyme gp5, C-terminal domain {Bacteriophage T4}
DPADPPIPNDSRILFKEPVSSYKGEYPYVHTMETESGHIQEFDDTPGQERYRLVHPTGTYEE
VSPSGRRTRKTVDNLYDITNADGNFLVAGDKKTNVGGSEIYYNMDNRLHQIDGSNTIFVRGD

ETKTVEGNGTILVKGNVTIIVEGNADITVKGDATTLVEGNQTNTVNGNLSWKVAGTVDWDVG
GDWTEKMASMSSISSGQYTIDGSRIDIGSVDHHHHHH
>d1qiua2 b.83.1.1 (A:319-395) Adenovirus {Human adenovirus type 2}
VSIKKSSGLNFDNTAIAINAGKGLEFDTNTSESPDINPIKTKIGSGIDYNENGAMITKLGAG
LSFDNSGAITIGNKN
>d1kkea2 b.83.1.2 (A:313-455) Reovirus attachment protein sigma 1 {Reovirus}
YRFRQSMWIGIVSYSGSGLNWRVQVNSDIFIVDDYIHICLPAFDGFSIADGGDLSLNFVTGL
LPPLLTGDTEPAFHNDVVTYGAQTVAIGLSSGGAPQYMSKNLWVEQWQDGVLRLRVEGGGSI
THSNSKWPAMTVSYPRSFT
>d1h8ga_ b.109.1.1 (A:) Choline binding domain of autolysin C-LytA {Streptococcus pneumoniae}
TDGNWYWFDNSGEMATGWKKIADKWYYFNEEGAMKTGWVKYKDTWYYLDAKEGAMVSNAFIQ
SADGTGWYYLKPDGTLADRPEFTVEPDGLITVK
>d1hcxa_ b.109.1.1 (A:) Choline binding domain of autolysin C-LytA {Streptococcus pneumoniae}
GSYPKDKFEKINGTWYYFDSSGYMLADRWRKHTDGNWYWFDNSGEMATGWKKIADKWYYFNE
EGAMKTGWVKYKDTWYYLDAKEGAMVSNAFIQSADGTGWYYLKPDGTLADRPEFTVEPDGLI
TVK
>d1bdo__ b.84.1.1 (-) Biotinyl domain of acetyl-CoA carboxylase {Escherichia coli}
EISGHIVRSPMVGTFYRTPSPDAKAFIEVGQKVNVGDTLCIVEAMKMMNQIEADKSGTVKAI
LVESGQPVEFDEPLVVIE
>d1dd2a_ b.84.1.1 (A:) Biotin carboxyl carrier domain of transcarboxylase (TC 1.3S) {Propionibacterium freudenreichii, subsp. shermanii}
AGAGKAGEGEIPAPLAGTVSKILVKEGDTVKAGQTVLVLEAMKMETEINAPTDGKVEKVLVK
ERDAVQGGQGLIKIG
>d1htp__ b.84.1.1 (-) Protein H of glycine cleavage system {Pea (Pisum sativum)}
SNVLDGLKYAPSHEWVKHEGSVATIGITDHAQDHLGEVVFVELPEPGVSVTKGKGFGAVESV
KATSDVNSPISGEVIEVNTGLTGKPGLINSSPYEDGWMIKIKPTSPDELESLLGAKEYTKFC
EEEDAAH
>d1lac__ b.84.1.1 (-) Ipoyl domain of dihydrolipoamide acetyltransferase {Bacillus stearothermophilus}
AFEFKLPDIGEGIHEGEIVKWFVKPGDEVNEDDVLCEVQNDKAVVEIPSPVKGKVLEILVPE
GTVATVGQTLITLDAPGY
>d1iyu__ b.84.1.1 (-) Ipoyl domain of dihydrolipoamide acetyltransferase {Azotobacter vinelandii}
SEIIRVPDIGGDGEVIELLVKTGDLIEVEQGLVVLESAKASMEVPSPKAGVVKSVSVKLGDK
LKEGDAIIELEPAAGAR
>d1qjoa_ b.84.1.1 (A:) Ipoyl domain of dihydrolipoamide acetyltransferase {Escherichia coli}

MVKEVNVPDIGGDEVEVTEVMVKVGDKVAAEQSLITVEGDKASMEVPAPFAGVVKELKVNVG
DKVKTGSLIMIFEVEGAA
>d1gjxa_ b.84.1.1 (A:) Ipoyl domain of dihydrolipoamide acetyltransferase {Neisseria meningitidis}
ALVELKVPDIGGHENVDIIAVEVNVGDTIAVDDTLITLETDKATMDVPAEVAGVVKEVKVKV
GDKISEGGLIVVVEAEGTA
>d1fyc__ b.84.1.1 (-) Ipoyl domain of dihydrolipoamide acetyltransferase {Human (Homo sapiens)}
GSNMSYPPHMQVLLPALSPTMTMGTVQRWEKKVGEKLSEGDLLAEIETDKATIGFEVQEEGY
LAKILVPEGTRDVPLGTPLCIIVEKEADISAFADYRPTEVTDLK
>d1ghk__ b.84.1.1 (-) Lipoyl domain of the 2-oxoglutarate dehydrogenase complex {Azotobacter vinelandii}
AIDIKAPTFPESIADGTVATWHKKPGEAVKRDELIVDIETDKVVMEVLAEADGVIAEIVKNE
GDTVLSGELLGKLTEGG
>d1pmr__ b.84.1.1 (-) Lipoyl domain of the 2-oxoglutarate dehydrogenase complex {Escherichia coli}
SSVDILVPDLPESVADATVATWHKKPGDAVVRDEVLVEIETDKVVLEVPASADGILDAVLED
EGTTVTSRQILGRLREGN
>d1k8ma_ b.84.1.1 (A:) Lipoyl domain of the mitochondrial branched-chain alpha-ketoacid dehydrogenase {Human (Homo sapiens)}
MGQVVQFKLSDIGEGIREVTVKEWYVKEGDTVSQFDSICEVQSDKASVTITSRYDGVIKKLY
YNLDDIAYVGKPLVDIETEALKDLE
>d1dv1a1 b.84.2.1 (A:331-446) Biotin carboxylase subunit of acetyl-CoA carboxylase, C-terminal domain {Escherichia coli}
RGHAVECRINAEDPNTFLPSPGKITRFHAPGGFGVRWESHIYAGYTVPPYYDSMIGKLICYG
ENRDVAIARMKNALQELIIDGIKTNVDLQIRIMNDENFQHGGTNIHYLEKKLGL
>d1gsoa1 b.84.2.1 (A:328-426) Glycinamide ribonucleotide synthetase (GAR-syn). {Escherichia coli}
ERASLGVVMAAGGYPGDYRTGDVIHGLPLEEVAGGKVFHAGTKLADDEQVVTNGGRVLCVTA
LGHTVAEAQKRAYALMTDIHWDDCFCRKDIGWRAIER
>d1b6ra1 b.84.2.1 (A:277-355) N5-carboxyaminoimidazole ribonucleotide synthetase, AIRC, PurK {Escherichia coli}
NNPSVMINLIGSDVNYDWLKLPLVHLHWYDKEVRPGRKVGHLNLTDSDTSRLTATLEALIPL
LPPEYASGVIWAQSKFG
>d1eyza1 b.84.2.1 (A:319-392) Glycinamide ribonucleotide transformylase PurT {Escherichia coli}
GPAASAVILPQLTSQNVTFDNVQNAVGADLQIRLFGKPEIDGSRRLGVALATAESVVDAIER
AKHAAGQVKVQG
>d1hcz_2 b.84.2.2 (168-230) Cytochrome f, small domain {Turnip (Brassica rapa)}
NTVYNATAGGIISKILRKEKGGYEITIVDASNERQVIDIIPRGLELLVSEGESIKLDQPLTS
N
>d1e2wa2 b.84.2.2 (A:169-232) Cytochrome f, small domain

{Chlamydomonas reinhardtii}
TIYNASAAGKIVAITALSEKKGGFEVSIEKANGEVVVDKIPAGPDLIVKEGQTVQADQPLTN
NP
>d1ci3m2 b.84.2.2 (M:170-231) Cytochrome f, small domain
{Phormidium laminosum}
AVYNASAAGVITAIAKADDGSAEVKIRTEDGTTIVDKIPAGPELIVSEGEEVAAGAALTNNP
>d1gpr__ b.84.3.1 (-) Glucose permease IIa domain, IIa-glc
{Bacillus subtilis}
EPLQNEIGEEVFVSPITGEIHPITDVPDQVFSGKMMGDGFAILPSEGIVVSPVRGKILNVFP
TKHAIGLQSDGGREILIHFGIDTVSLKGEGFTSFVSEGDRVEPGQKLLEVDLDAVKPNVPSL
MTPIVFTNLAEGETVSIKASGSVNREQEDIVKIE
>d2gpr__ b.84.3.1 (-) Glucose permease IIa domain, IIa-glc
{Mycoplasma capricolum}
MWFFNKNLKVLAPCDGTIITLDEVEDEVFKERMLGDGFAINPKSNDFHAPVSGKLVTAFPTK
HAFGIQTKSGVEILLHIGLDTVSLDGNGFESFVTQDQEVNAGDKLVTVDLKSVAKKVPSIKS
PIIFTNNGGKTLEIVKMGEVKQGDVVAILK
>d1glaf_ b.84.3.1 (F:) Glucose-specific factor III (glsIII)
{Escherichia coli}
GLFDKLKSLVSDDKKDTGTIEIIAPLSGEIVNIEDVPDVVFAEKIVGDGIAIKPTGNKMVAP
VDGTIGKIFETNHAFSIESDSGVELFVHFGIDTVELKGEGFKRIAEEGQRVKVGDTVIEFDL
PLLEEKAKSTLTPVVISNMDEIKELIKLSGSVTVGETPVIRIKK
>d2f3ga_ b.84.3.1 (A:) Glucose-specific factor III (glsIII)
{Escherichia coli}
TIEIIAPLSGEIVNIEDVPDVVFAEKIVGDGIAIKPTGNKMVAPVDGTIGKIFETNHAFSIE
SDSGVELFVHFGIDTVELKGEGFKRIAEEGQRVKVGDTVIEFDLPLLEEKAKSTLTPVVISN
MDEIKELIKLSGSVTVGETPVIRIKK
>d1hg7a_ b.85.1.1 (A:) Type III antifreeze protein {Ocean pout
(Macrozoarces americanus), different isoforms}
MNQASVVANQLIPINTALTLVMMRSEVVTPVGIPAEDIPRLVSMQVNRAVPLGTTLMPDMVK
GYAA
>d1ops__ b.85.1.1 (-) Type III antifreeze protein {Ocean pout
(Macrozoarces americanus), different isoforms}
SQSVVATQLIPMNTALTPAMMEGKVTNPIGIPFAEMSQLVGKQVNTPVAKGQTLMPNMVKTY
AA
>d1c8aa2 b.85.1.1 (A:69-134) Type III antifreeze protein
{Antarctic eel pout (Austrolycichthys brachycephalus) and
(Lycodichthys dearborni)}
SPGLKSVVANQLIPINTALTLVMMKAEEVSPKGIPSEEISKLVGMQVNRAVYLDQTLMPDMV
KNYE
>d3rdn__ b.85.1.1 (-) Type III antifreeze protein {Antarctic eel
pout (Austrolycichthys brachycephalus) and (Lycodichthys
dearborni)}
NKASVVANQLIPINTALTLIMMKAEVVTPMGIPAEEIPNLVGMQVNRAVPLGTTLMPDMVKN
YEDGTTSPGLK

>d1c5ea_ b.85.2.1 (A:) Head decoration protein D (gpD, major capsid protein D) {Bacteriophage lambda}
SDPAHTATAPGGLSAKAPAMTPLMLDTSSRKLVAWDGTTDGAAVGILAVAADQTSTTLTFYK
SGTFRYEDVLWPEAASDETKKRTAFAGTAISIV
>d1ejrb_ b.85.3.1 (B:) Urease, beta-subunit {Klebsiella aerogenes}
MIPGEYHVKPGQIALNTGRATCRVVVENHGDRPIQVGSHYHFAEVNPALKFDRQQAAGYRLN
IPAGTAVRFEPGQKREVELVAFAGHRAVFGFRGEVMGPL
>d4ubpb_ b.85.3.1 (B:) Urease, beta-subunit {Bacillus pasteurii}
NYIVPGEYRVAEGEIEINAGREKTTIRVSNTGDRPIQVGSHIHFVEVNKELLFDRAEGIGRR
LNIPSGTAARFEPGEEMEVELTELGGNREVFGISDLTNGSVDNKELILQRAKELGYKGVE
>d1e9ya1 b.85.3.1 (A:106-238) Urease, beta-subunit {Helicobacter pylori}
LVPGELFLKNEDITINEGKKAVSVKVKNVGDRPVQIGSHFHFFEVNRCLDFDREKTFGKRLD
IAAGTAVRFEPGEEKSVELIDIGGNRRIFGFNALVDRQADNESKKIALHRAKERGFHGAKSD
DNYVKTIKE
>d1g8la1 b.85.6.1 (A:327-409) Molybdenum cofactor biosynthesis protein MoeA, C-terminal domain {Escherichia coli}
LPARQRVRTASRLKKTPGRLDFQRGVLQRNADGELEVTTTGHQGSHIFSSFSLGNCFIVLER
DRGNVEVGEWVEVEPFNALFG
>d1euwa_ b.85.4.1 (A:) Deoxyuridine 5'-triphosphate nucleotidohydrolase (dUTPase) {Escherichia coli}
MMKKIDVKILDPRVGKEFPLPTYATSGSAGLDLRACLNDAVELAPGDTTLVPTGLAIHIADP
SLAAMMLPRSGLGHKHGIVLGNLVGLIDSDYQGQLMISVWNRGQDSFTIQPGERIAQMIFVP
VVQAEFNLVEDF
>d1f7da_ b.85.4.1 (A:) Deoxyuridine 5'-triphosphate nucleotidohydrolase (dUTPase) {Feline immunodeficiency virus}
MIIEGDGILDKRSEDAGYDLLAAKEIHLLPGEVKVIPTGVKLMLPKGYWGLIIGKSSIGSKG
LDVLGGVIDEGYRGEIGVIMINVSRKSITLMERQKIAQLIILPCKHEVLEQGKVVM
>d1f7ra_ b.85.4.1 (A:) Deoxyuridine 5'-triphosphate nucleotidohydrolase (dUTPase) {Feline immunodeficiency virus}
MIIEGDGILDKRSEDAGYDLLAAKEIHLLPGEVKVIPTGVKLMLPKGYWGLIIGKSSIGSKG
LDVLGGVIDEGYRGEIGVIMINVSRKSITLMERQKIAQLIILPCKHEVLEQGKVVMDSERGD
NGYGSTGVF
>d1dun__ b.85.4.1 (-) Deoxyuridine 5'-triphosphate nucleotidohydrolase (dUTPase) {Equine infectious anemia virus}
MLAYQGTQIKEKRDEDAGFDLCVPYDIMIPVSDTKIIPTDVKIQVPPNSFGWVTGKSSMAKQ
GLLINGGIIDEGYTGEIQVICTNIGKSNIKLIEGQKFAQLIILQHHSNSRQPWDENKI
>d1tul__ b.85.5.1 (-) ACMNPV telokin-like protein {Baculovirus (Autographa californica), nuclear polyhedrosis virus}
GTPDIIVNAQINSEDENVLDFIIEDEYYLKKRGVGAHIIKVASSPQLRLLYKNAYSTVSCGN
YGVLCNLVQNGEYDLNAIMFNCAEIKLNKGQMLFQTKIWR
>d1at0__ b.86.1.1 (-) Hedgehog {Fruit fly (Drosophila melanogaster)}
CFTPESTALLESGVRKPLGELSIGDRVLSMTANGQAVYSEVILFMDRNLEQMQNFVQLHTDG

GAVLTVTPAHLVSVWQPESQKLTFVFADRIEEKNQVLVRDVETGELRPQRVVKVGSVRSKGV
VAPLTREGTIVVNSVAASCYA
>d1dfaa1 b.86.1.2 (A:1-180,A:416-454) PI-Scei intein {Baker's yeast (Saccharomyces cerevisiae)}
CFAKGTNVLMADGSIECIENIEVGNKVMGKDGRPREVIKLPRGRETMYSVVQKSQHRAHKSD
SSREVPELLKFTCNATHELVVRTPRSVRRLSRTIKGVEYFEVITFEMGQKKAPDGRIVELVK
EVSKSYPISEGPERANELVESYRKASNKAYFEWTIEARDLSLLGSHVRKATYQTYAXCRGFY
FELQELKEDDYYGITLSDDSDHQFLLANQVVVHN
>d1dq3a1 b.86.1.2 (A:1-128,A:415-454) PI-Pfui intein {Archaeon Pyrococcus furiosus}
CIDGKAKIIFENEGEEHLTTMEEMYERYKHLGEFYDEEYNRWGIDVSNVPIYVKSFDPESKR
VVKGKVNVIWKYELGKDVTKYEIITNKGTKILTSPWHPFFVLTPDFKIVEKRADELKEGDIL
IGGMXGLEVVRHITTTNEPRTFYDLTVENYQNYLAGENGMIFVHN
>d1am2__ b.86.1.2 (-) GyrA intein {Mycobacterium xenopi}
ASITGDALVALPEGESVRIADIVPGARPNSDNAIDLKVLDRHGNPVLADRLFHSGEHPVYAV
RTVEGLRVTGTANHPLLCLVDVAGVPTLLWKLIDEIKPGDYAVIQRSAFSVDCAGFARGKPE
FAPTTYTVGVPGLVRFLEAHHRDPDAKAIADELTDGRFYYAKVASVTDAGVQPVYSLRVDTA
DHAFITNGFVSHN
>d1umua_ b.87.1.1 (A:) UmuD' {Escherichia coli}
DYVEQRIDLNQLLIQHPSATYFVKASGDSMIDGGISDGDLLIVDSAITASHGDIVIAAVDGE
FTVKKLQLRPTVQLIPMNSAYSPITISSEDTLDVFGVVIHVVK
>d1jhfa2 b.87.1.1 (A:73-198) LexA C-terminal domain {Escherichia coli}
EEGLPLVGRVAADEPLLAQQHIEGHYQVDPSLFKPNADFLLRVSGMSMKDIGIMDGDLLAVH
KTQDVRNGQVVVARIDDEVTVKRLKKQGNKVELLPENSEFKPIVVDLRQQSFTIEGLAVGVI
RN
>d1f39a_ b.87.1.1 (A:) lambda repressor C-terminal domain {Bacteriophage lambda virus}
ASASAFWLEVEGNSMTAPTGSKPSFPDGMLILVDPEQAVEPGDFCIARLGGDEFTFKKLIRD
SGQVFLQPLNPQYPMIPCNESCSVVGKVIASQWPEETFG
>d1b12a_ b.87.1.2 (A:) Type 1 signal peptidase {Escherichia coli}
RSFIYEPFQIPSGSMMPTLLIGDFILVEKFAYGIKDPIYQKTLIETGHPKRGDIVVFKYPED
PKLDYIKRAVGLPGDKVTYDPVSKELTIQPGCSSGQACENALPVTYSNVEPSDFVQTFSRRN
GGEATSGFFEVPKNETKENGIRLSERKETLGDVTHRILTVPIAQDQVGMYYQQPGQQLATWI
VPPGQYFMMGDNRDNSADSRYWGFVPEANLVGRATAIWMSFDKQEGEWPTGLRLSRIGGIH
>d1jcha2 b.110.1.1 (A:84-315) Colicin E3 translocation domain {Escherichia coli}
VAAPVAFGFPALSTPGAGGLAVSISAGALSAAIADIMAALKGPFKFGLWGVALYGVLPSQIA
KDDPNMMSKIVTSLPADDITESPVSSLPLDKATVNVNVRVVDDVKDERQNISVVSGVPMSVP
VVDAKPTERPGVFTASIPGAPVLNISVNNSTPAVQTLSPGVTNNTDKDVRPAFGTQGGNTRD
AVIRFPKDSGHNAVYVSVSDVLSPDQVKQRQDEENRRQQEWDATHP
>d1hxra_ b.88.1.1 (A:) RabGEF Mss4 {Rat (Rattus norvegicus)}
ELVSAEGRNRKAVLCQRCGSRVLQPGTALFSRRQLFLPSMRKKPDLVDGSNPDGDVLEEHWL
VNDMFIFENVGFTKDVGNVKFLVCADCEIGPIGWHCLDDKNSFYVALERVSHE

>d1fwqa_ b.88.1.1 (A:) RabGEF Mss4 {Human (Homo sapiens)}
ELVSAEGRNRKAVLCQRCGSRVLQPGTALFSRRQLFLPSMRKKPALSDGSNPDGDLLQEHWL
VEDMFIFENVGFTKDVGNIKFLVCADCEIGPIGWHCLDDKNSFYVALERVSHE
>d1h6qa_ b.88.1.2 (A:) Translationally controlled
tumor-associated protein tctp, p23fyp {Fission yeast
(Schizosaccharomyces pombe)}
MLLYKDVISGDELVSDAYDLKEVDDIVYEADCQMVTVKQGGDVDIGANPSAEDAEENAEEGT
ETVNNLVYSFRLSPTSFDKKSYMSYIKGYMKAIKARLQESNPERVPVFEKNAIGFVKKILAN
FKDYDFYIGESMDPDAMVVLMNYREDGITPYMIFFKDGLVSEKF
>d3ezma_ b.89.1.1 (A:) Cyanovirin-N {Cyanobacterium (Nostoc
ellipsosporum)}
LGKFSQTCYNSAIQGSVLTSTCERTNGGYNTSSIDLNSVIENVDGSLKWQPSNFIETCRNTQ
LAGSSELAAECKTRAQQFVSTKINLDDHIANIDGTLKYE
>d1fjra_ b.102.1.1 (A:) Methuselah ectodomain {Fruit fly
(Drosophila melanogaster)}
DILECDYFDTVDISAAQKLQNGSYLFEGLLVPAILTGEYDFRILPDDSKQKVARHIRGCVCK
LKPCVRFCCPHDHIMDNGVCYDNMSDEELAELDPFLNVTLDDGSVSRRHFKNELIVQWDLPM
PCDGMFYLDNREEQDKYTLFENGTFFRHFDRVTLRKREYCLQHLTFADGNATSIRIAPHNCL
IV
>d1lkta_ b.90.1.1 (A:) Head-binding domain of phage P22 tailspike
protein {Salmonella bacteriophage P22}
ANVVVSNPRPIFTESRSFKAVANGKIYIGQIDTDPVNPANQIPVYIENEDGSHVQITQPLII
NAAGKIVYNGQLVKIVTVQGHSMAIYDANGSQVDYIANVLKY
>d1qqha_ b.91.1.1 (A:) E2 regulatory, transactivation domain
{Human papillomavirus type 18}
KSKAHKAIELQMALQGLAQSAYKTEDWTLQDTCEELWNTEPTHCFKKGGQTVQVYFDGNKDN
CMTYVAWDSVYYMTDAGTWDKTATCVSHRGLYYVKEGYNTFYIEFKSECEKYGNTGTWEVHF
GNNVIDCNDSMCSTSDDTVS
>d1dtoa_ b.91.1.1 (A:) E2 regulatory, transactivation domain
{Human papillomavirus type 16}
HMETLCQRLNVCQDKILTHYENDSTDLRDHIDYWKHMRLECAIYYKAREMGFKHINHQVVPT
LAVSKNKALQAIELQLTLETIYNSQYSNEKWTLQDVSLEVYLTAPTGCIKKHGYTVEVQFDG
DICNTMHYTNWTHIYICEEASVTVVEGQVDYYGLYYVHEGIRTYFVQFKDDAEKYSKNKVWE
VHAGGQVILCPTSVFS
>d1g8la2 b.103.1.1 (A:7-177) Molybdenum cofactor biosynthesis
protein MoeA, N-terminal and linker domains {Escherichia coli}
LMSLDTALNEMLSRVTPLTAQETLPLVQCFGRILASDVVSPLDVPGFDNSAMDGYAVRLADI
ASGQPLPVAGKSFAGQPYHGEWPAGTCIRIMTGAPVPEGCEAVVMQEQTEQMDNGVRFTAEV
RSGQNIRRRGEDISAGAVVFPAGTRLTTAELPVIASLGIAEVPVIRK
>d1k6wa1 b.92.1.2 (A:4-55,A:376-426) Cytosine deaminase
{Escherichia coli}
ALQTIINARLPGEEGLWQIHLQDGKISAIDAQSGVMPITENSLDAEQGLVIPXLIILPAENG
FDALRRQVPVRYSVRGGKVIASTQPAQTTVYLEQPEAIDYKR
>d1ejrc1 b.92.1.1 (C:1002-1129,C:1423-1475) alpha-Subunit of

urease {Klebsiella aerogenes}
SNISRQAYADMFGPTVGDKVRLADTELWIEVEDDLTTYGEEVKFGGGKVIRDGMGQGQMLAA
DCVDLVLTNALIVDHWGIVKADIGVKDGRIFAIGKAGNPDIQPNVTIPIGAATEVIAAEGKI
VTAGXSIEVGKLADLVVWSPAFFGVKPATVIKGGMIAIAPMGDINASIPTPQPVHYRP
>d4ubpc1 b.92.1.1 (C:1-131,C:435-483) alpha-Subunit of urease {Bacillus pasteurii}
MKINRQQYAESYGPTVGDEVRLADTDLWIEVEKDYTTYGDEVNFGGGKVLREGMGENGTYTR
TENVLDLLLTNALILDYTGIYKADIGVKDGYIVGIGKGGNPDIMDGVTPNMIVGTATEVIAA
EGKIVTAXLVLWEPKFFGVKADRVIKGGIIAYAQIGDPSASIPTPQPVMGRRMYGTV
>d1e9yb1 b.92.1.1 (B:1-131,B:432-480) alpha-Subunit of urease {Helicobacter pylori}
MKKISRKEYVSMYGPTTGDKVRLGDTDLIAEVEHDYTIYGEELKFGGGKTLREGMSQSNNPS
KEELDLIITNALIVDYTGIYKADIGIKDGKIAGIGKGGNKDMQDGVKNNLSVGPATEALAGE
GLIVTAGXADLVLWSPAFFGVKPNMIIKGGFIALSQMGDANASIPTPQPVYYREMFA
>d1aqt_2 b.93.1.1 (2-86) Epsilon subunit of F1F0-ATP synthase N-terminal domain {Escherichia coli}
STYHLDVVSAEQQMFSGLVEKIQVTGSEGELGIYPGHAPLLTAIKPGMIRIVKQHGHEEFIY
LSGGILEVQPGNVTVLADTAIRG
>d1e79h2 b.93.1.1 (H:15-100) Epsilon subunit of F1F0-ATP synthase N-terminal domain {Cow (Bos taurus)}
QMSFTFASPTQVFFNSANVRQVDVPTQTGAFGILAAHVPTLQVLRPGLVVVHAEDGTTSKYF
VSSGSVTVNADSSVQLLAEEAVTL
>d1hhna_ b.104.1.1 (A:) Calreticulin {Rat (Rattus norvegicus)}
SKKIKDPDAAKPEDWDERAKIDDPTDSKPEDWDKPEHIPDPDAKKPEDWDEEMDGEWEPPVI
QNPEYKGEWKPRQIDNPDYKGTWIHPEIDNPEYSPDANI
>d1jhna3 b.104.1.1 (A:270-411) Calnexin {Dog (Canis familiaris)}
PVNPSREIEDPEDQKPEDWDERPKIPDPDAVKPDDWNEDAPAKIPDEEATKPDGWLDDEPEY
VPDPDAEKPEDWDEDMDGEWEAPQIANPKCESAPGCGVWQRPMIDNPNYKGKWKPPMIDNPN
YQGIWKPRKIPNPDFFED
>d1tph1_ c.1.1.1 (1:) Triosephosphate isomerase {Chicken (Gallus gallus)}
RKFFVGGNWKMNGDKKSLGELIHTLNGAKLSADTEVVCGAPSIYLDFARQKLDAKIGVAAQN
CYKVPKGAFTGEISPAMIKDIGAAWVILGHSERRHVFGESDELIGQKVAHALAEGLGVIACI
GEKLDEREAGITEKVVFEQTKAIADNVKDWSKVVLAYEPVWAIGTGKTATPQQAQEVHEKLR
GWLKTHVSDAVAQSTRIIYGGSVTGGNCKELASQHDVDGFLVGGASLKPEFVDIINAKH
>d1htia_ c.1.1.1 (A:) Triosephosphate isomerase {Human (Homo sapiens)}
APSRKFFVGGNWKMNGRKQSLGELIGTLNAAKVPADTEVVCAPPTAYIDFARQKLDPKIAVA
AQNCYKVTNGAFTGEISPGMIKDCGATWVVLGHSERRHVFGESDELIGQKVAHALAEGLGVI
ACIGEKLDEREAGITEKVVFEQTKVIADNVKDWSKVVLAYEPVWAIGTGKTATPQQAQEVHE
KLRGWLKSNVSDAVAQSTRIIYGGSVTGATCKELASQPDVDGFLVGGASLKPEFVDIINAKQ
>d1i45a_ c.1.1.1 (A:) Triosephosphate isomerase {Baker's yeast (Saccharomyces cerevisiae)}
ARTFFVGGNFKLNGSKQSIKEIVERLNTASIPENVEVVICPPATYLDYSVSLVKKPQVTVGA

QNAYLKASGAFTGENSVDQIKDVGAKYVILGHSERRSYFHEDDKFIADKTKFALGQGVGVIL
CIGETLEEKKAGKTLDVVERQLNAVLEEVKDFTNVVVAYEPVWAIGTGLAATPEDAQDIHAS
IRKFLASKLGDKAASELRILYGGSANGSNAVTFKDKADVDGFLVGGASLKPEFVDIINSRN
>d1ttj__ c.1.1.1 (-) Triosephosphate isomerase {Trypanosoma brucei}
SKPQPIAAANWKCNGSQQSLSELIDLFNSTSINHDVQCVVASTSSHLAMTKERLSHPKFVIA
AQNAGNADALASLKDFGVNWIVLGHSERRAYYGETNEIVADKVAAAVASGFMVIACIGETLQ
ERESGRTAVVVLTQIAAIAKKLKKADWAKVVIAYEPVWAIGTGKVATPQQAQEAHALIRSWV
SSKIGADVAGELRILYGGSVNGKNARTLYQQRDVNGFLVGGASLKPEFVDIIKATQ
>d5tima_ c.1.1.1 (A:) Triosephosphate isomerase {Trypanosoma brucei}
SKPQPIAAANWKCNGSQQSLSELIDLFNSTSINHDVQCVVASTFVHLAMTKERLSHPKFVIA
AQNAIAKSGAFTGEVSLPILKDFGVNWIVLGHSERRAYYGETNEIVADKVAAAVASGFMVIA
CIGETLQERESGRTAVVVLTQIAAIAKKLKKADWAKVVIAYEPVWAIGTGKVATPQQAQEAH
ALIRSWVSSKIGADVAGELRILYGGSVNGKNARTLYQQRDVNGFLVGGASLKPEFVDIIKAT
Q
>d1tcda_ c.1.1.1 (A:) Triosephosphate isomerase {Trypanosoma cruzi}
KPQPIAAANWKCNGSESLLVPLIETLNAATFDHDVQCVVAPTFLHIPMTKARLTNPKFQIAA
QNAITRSGAFTGEVSLQILKDYGISWVVLGHSERRLYYGETNEIVAEKVAQACAAGFHVIVC
VGETNEEREAGRTAAVVLTQLAAVAQKLSKEAWSRVVIAYEPVWAIGTGKVATPQQAQEVHE
LLRRWVRSKLGTDIAAQLRILYGGSVTAKNARTLYQMRDINGFLVGGASLKPEFVEIIEATK
>d1ydva_ c.1.1.1 (A:) Triosephosphate isomerase {Plasmodium falciparum}
RKYFVAANWKCNGTLESIKSLTNSFNNLDFDPSKLDVVVFPVSVHYDHTRKLLQSKFSTGIQ
NVSKFGNGSYTGEVSAEIAKDLNIEYVIIGHFERRKYFHETDEDVREKLQASLKNNLKAVVC
FGESLEQREQNKTIEVITKQVKAFVDLIDNFDNVILVYEPLWAIGTGKTATPEQAQLVHKEI
RKIVKDTCGEKQANQIRILYGGSVNTENCSSLIQQEDIDGFLVGNASLKESFVDIIKSAM
>d1amk__ c.1.1.1 (-) Triosephosphate isomerase {Leishmania mexicana}
SAKPQPIAAANWKCNGTTASIEKLVQVFNEHTISHDVQCVVAPTFVHIPLVQAKLRNPKYVI
SAENAIAKSGAFTGEVSMPILKDIGVHWVILGHSERRTYYGETDEIVAQKVSEACKQGFMVI
ACIGETLQQREANQTAKVVLSQTSAIAAKLTKDAWNQVVLAYEPVWAIGTGKVATPEQAQEV
HLLLRKWVSENIGTDVAAKLRILYGGSVNAANAATLYAKPDINGFLVGGASLKPEFRDIIDA
TR
>d1trea_ c.1.1.1 (A:) Triosephosphate isomerase {Escherichia coli}
MRHPLVMGNWKLNGSRHMVHELVSNLRKELAGVAGCAVAIAPPEMYIDMAKREAEGSHIMLG
AQNVNLNLSGAFTGETSAAMLKDIGAQYIIIGHSERRTYHKESDELIAKKFAVLKEQGLTPV
LCIGETEAENEAGKTEEVCARQIDAVLKTQGAAAFEGAVIAYEPVWAIGTGKSATPAQAQAV
HKFIRDHIAKVDANIAEQVIIQYGGSVNASNAAELFAQPDIDGALVGGASLKADAFAVIVKA
AEAAKQA
>d2btma_ c.1.1.1 (A:) Triosephosphate isomerase {Bacillus stearothermophilus}
RKPIIAGNWKMNGTLAEAVQFVEDVKGHVPPADEVISVVCAPFLFLDRLVQAADGTDLKIGA

QTMHFADQGAYTGEVSPVMLKDLGVTYVILGHSERRQMFAETDETVNKKVLAAFTRGLIPII
CCGESLEEREAGQTNAVVASQVEKALAGLTPEQVKQAVIAYEPIWAIGTGKSSTPEDANSVC
GHIRSVVSRLFGPEAAEAIRIQYGGSVKPDNIRDFLAQQQIDGALVGGASLEPASFLQLVEA
GRH
>d1aw1a_ c.1.1.1 (A:) Triosephosphate isomerase {Vibrio marinus}
RHPVVMGNWKLNGSKEMVVDLLNGLNAELEGVTGVDVAVAPPALFVDLAERTLTEAGSAIIL
GAQNTDLNNSGAFTGDMSPAMLKEFGATHIIIGHSERREYHAESDEFVAKKFAFLKENGLTP
VLCIGESDAQNEAGETMAVCARQLDAVINTQGVEALEGAIIAYEPIWAIGTGKAATAEDAQR
IHAQIRAHIAEKSEAVAKNVVIQYGGSVKPENAAAYFAQPDIDGALVGGAALDAKSFAAIAK
AAAEAKA
>d1b9ba_ c.1.1.1 (A:) Triosephosphate isomerase {Thermotoga
maritima}
TRKLILAGNWKMHKTISEAKKFVSLLVNELHDVKEFEIVVCPPFTALSEVGEILSGRNIKLG
AQNVFYEDQGAFTGEISPLMLQEIGVEYVIVGHSERRRIFKEDDEFINRKVKAVLEKGMTPI
LCVGETLEEREKGLTFCVVEKQVREGFYGLDKEEAKRVVIAYEPVWAIGTGRVATPQQAQEV
HAFIRKLLSEMYDEETAGSIRILYGGSIKPDNFLGLIVQKDIDGGLVGGASLKESFIELARI
MRGV
>d1hg3a_ c.1.1.1 (A:) Triosephosphate isomerase {Archaeon
Pyrococcus woesei}
AKLKEPIIAINFKTYIEATGKRALEIAKAAEKVYKETGVTIVVAPQLVDLRMIAESVEIPVF
AQHIDPIKPGSHTGHVLPEAVKEAGAVGTLLNHSENRMILADLEAAIRRAEEVGLMTMVCSN
NPAVSAAVAALNPDYVAVEPPELIGTGIPVSKAKPEVITNTVELVKKVNPEVKVLCGAGIST
GEDVKKAIELGTVGVLLASGVTKAKDPEKAIWDLVSGI
>d1qo2a_ c.1.2.1 (A:) Phosphoribosylformimino-5-aminoimidazole
carboxamide ribotite isomerase HisA {Thermotoga maritima}
MLVVPAIDLFRGKVARMIKGRKENTIFYEKDPVELVEKLIEEGFTLIHVVDLSNAIENSGEN
LPVLEKLSEFAEHIQIGGGIRSLDYAEKLRKLGYRRQIVSSKVLEDPSFLKSLREIDVEPVF
SLDTRGGRVAFKGWLAEEEIDPVSLLKRLKEYGLEEIVHTEIEKDGTLQEHDFSLTKKIAIE
AEVKVLAAGGISSENSLKTAQKVHTETNGLLKGVIVGRAFLEGILTVEVMKRYAR
>d1thfd_ c.1.2.1 (D:) Cyclase subunit (or domain) of
imidazoleglycerolphosphate synthase HisF {Thermotoga maritima}
MLAKRIIACLDVKDGRVVKGSNFENLRDSGDPVELGKFYSEIGIDELVFLDITASVEKRKTM
LELVEKVAEQIDIPFTVGGGIHDFETASELILRGADKVSINTAAVENPSLITQIAQTFGSQA
VVVAIDAKRVDGEFMVFTYSGKKNTGILLRDWVVEVEKRGAGEILLTSIDRDGTKSGYDTEM
IRFVRPLTTLPIIASGGAGKMEHFLEAFLAGADAALAASVFHFREIDVRELKEYLKKHGVNV
RLEGL
>d1jvna1 c.1.2.1 (A:230-552) Cyclase subunit (or domain) of
imidazoleglycerolphosphate synthase HisF {Baker's yeast
(Saccharomyces cerevisiae), His7}
DYSNYGLTRRIIACLDVRTNDQGDLVVTKGDQYDVREKSDGKGVRNLGKPVQLAQKYYQQGA
DEVTFLNITSFRDCPLKDTPMLEVLKQAAKTVFVPLTVGGGIKDIVDVDGTKIPALEVASLY
FRSGADKVSIGTDAVYAAEKYYELGNRGDGTSPIETISKAYGAQAVVISVDPKRVYVNSQAD
TKNKVFETEYPGPNGEKYCWYQCTIKGGRESRDLGVWELTRACEALGAGEILLNCIDKDGSN
SGYDLELIEHVKDAVKIPVIASSGAGVPEHFEEAFLKTRADACLGAGMFHRGEFTVNDVKEY

LLEHGLKVRMDEE
>d1h5ya_ c.1.2.1 (A:) Cyclase subunit (or domain) of imidazoleglycerolphosphate synthase HisF {Archaeon Pyrobaculum aerophilum}
HMALRIIPCLDIDGGAKVVVKGVNFQGIREVGDPVEMAVRYEEEGADEIAILDITAAPEGRA
TFIDSVKRVAEAVSIPVLVGGGVRSLEDATTLFRAGADKVSVNTAAVRNPQLVALLAREFGS
QSTVVAIDAKWNGEYYEVYVKGGREATGLDAVKWAKEVEELGAGEILLTSIDRDGTGLGYDV
ELIRRVADSVRIPVIASGGAGRVEHFYEAAAAGADAVLAASLFHFRVLSIAQVKRYLKERGV
EVRI
>d1rpxa_ c.1.2.2 (A:) D-ribulose-5-phosphate 3-epimerase {Potato (Solanum tuberosum)}
SRVDKFSKSDIIVSPSILSANFSKLGEQVKAIEQAGCDWIHVDVMDGRFVPNITIGPLVVDS
LRPITDLPLDVHLMIVEPDQRVPDFIKAGADIVSVHCEQSSTIHLHRTINQIKSLGAKAGVV
LNPGTPLTAIEYVLDAVDLVLIMSVNPGFGGQSFIESQVKKISDLRKICAERGLNPWIEVDG
GVGPKNAYKVIEAGANALVAGSAVFGAPDYAEAIKGIKTSKRPE
>d1dbta_ c.1.2.3 (A:) Orotidine 5'-monophosphate decarboxylase (OMP decarboxylase) {Bacillus subtilis}
MKNNLPIIALDFASAEETLAFLAPFQQEPLFVKVGMELFYQEGPSIVKQLKERNCELFLDLK
LHDIPTTVNKAMKRLASLGVDLVNVHAAGGKKMMQAALEGLEEGTPAGKKRPSLIAVTQLTS
TSEQIMKDELLIEKSLIDTVVHYSKQAEESGLDGVVCSVHEAKAIYQAVSPSFLTVTPGIRM
SEDAANDQVRVATPAIAREKGSSAIVVGRSITKAEDPVKAYKAVRLEWEGI
>d1eixa_ c.1.2.3 (A:) Orotidine 5'-monophosphate decarboxylase (OMP decarboxylase) {Escherichia coli}
VTNSPVVVALDYHNRDDALAFVDKIDPRDCRLKVGKEMFTLFGPQFVRELQQRGFDIFLDLK
FHDIPNTAAHAVAAAADLGVWMVNVHASGGARMMTAAREALVPFGKDAPLLIAVTVLTSMEA
SDLVDLGMTLSPADYAERLAALTQKCGLDGVVCSAQEAVRFKQVFGQEFKLVTPGIRPQGSE
AGDQRRIMTPEQALSAGVDYMVIGRPVTQSVDPAQTLKAINASLQ
>d1dvja_ c.1.2.3 (A:) Orotidine 5'-monophosphate decarboxylase (OMP decarboxylase) {Archaeon Methanobacterium thermoautotrophicum}
MDVMNRLILAMDLMNRDDALRVTGEVREYIDTVKIGYPLVLSEGMDIIAEFRKRFGCRIIAD
FKVADIPETNEKICRATFKAGADAIIVHGFPGADSVRACLNVAEEMGREVFLLTEMSHPGAE
MFIQGAADEIARMGVDLGVKNYVGPSTRPERLSRLREIIGQDSFLISPGVGAQGGDPGETLR
FADAIIVGRSIYLADNPAAAAAGIIESIKDLLIPEDPAANKARKEAELAAATA
>d1dvjb_ c.1.2.3 (B:) Orotidine 5'-monophosphate decarboxylase (OMP decarboxylase) {Archaeon Methanobacterium thermoautotrophicum}
RLILAMDLMNRDDALRVTGEVREYIDTVKIGYPLVLSEGMDIIAEFRKRFGCRIIADFKVAD
IPETNEKICRATFKAGADAIIVHGFPGADSVRACLNVAEEMGREVFLLTEMSHPGAEMFIQG
AADEIARMGVDLGVKNYVGPSTRPERLSRLREIIGQDSFLISPGVGAQGGDPGETLRFADAI
IVGRSIYLADNPAAAAAGIIESIKD
>d1dqwa_ c.1.2.3 (A:) Orotidine 5'-monophosphate decarboxylase (OMP decarboxylase) {Baker's yeast (Saccharomyces cerevisiae)}
MHKATYKERAATHPSPVAAKLFNIMHEKQTNLCASLDVRTTKELLELVEALGPKICLLKTHV

DILTDFSMEGTVKPLKALSAKYNFLLFEDRKFADIGNTVKLQYSAGVYRIAEWADITNAHGV
VGPGIVSGLKQAAEEVTKEPRGLLMLAELSCKGSLSTGEYTKGTVDIAKSDKDFVIGFIAQR
DMGGRDEGYDWLIMTPGVGLDDKGDALGQQYRTVDDVVSTGSDIIIVGRGLFAKGRDAKVEG
ERYRKAGWEAYLRRCGQQD
>d1pii_2 c.1.2.4 (255-452) N-(5'phosphoribosyl)antranilate isomerase, PRAI {Escherichia coli}
GENKVCGLTRGQDAKAAYDAGAIYGGLIFVATSPRCVNVEQAQEVMAAAPLQYVGVFRNHDI
ADVVDKAKVLSLAAVQLHGNEEQLYIDTLREALPAHVAIWKALSVGETLPAREFQHVDKYVL
DNGQGGSGQRFDWSLLNGQSLGNVLLAGGLGADNCVEAAQTGCAGLDFNSAVESQPGIKDAR
LLASVFQTLRAY
>d1nsj__ c.1.2.4 (-) N-(5'phosphoribosyl)antranilate isomerase, PRAI {Thermotoga maritima}
MVRVKICGITNLEDALFSVESGADAVGFVFYPKSKRYISPEDARRISVELPPFVFRVGVFVN
EEPEKILDVASYVQLNAVQLHGEEPIELCRKIAERILVIKAVGVSNERDMERALNYREFPIL
LDTKTPEYGGSGKTFDWSLILPYRDRFRYLVLSGGLNPENVRSAIDVVRPFAVDVSSGVEAF
PGKKDHDSIKMFIKNAKGL
>d1pii_1 c.1.2.4 (1-254) Indole-3-glycerophosphate synthase, IPGS {Escherichia coli}
MQTVLAKIVADKAIWVEARKQQQPLASFQNEVQPSTRHFYDALQGARTAFILECKKASPSKG
VIRDDFDPARIAAIYKHYASAISVLTDEKYFQGSFNFLPIVSQIAPQPILCKDFIIDPYQIY
LARYYQADACLLMLSVLDDDQYRQLAAVAHSLEMGVLTEVSNEEEQERAIALGAKVVGINNR
DLRDLSIDLNRTRELAPKLGHNVTVISESGINTYAQVRELSHFANGFLIGSALMAHDDLHAA
VRRVLL
>d1a53__ c.1.2.4 (-) Indole-3-glycerophosphate synthase, IPGS {Archaeon Sulfolobus solfataricus}
PRYLKGWLKDVVQLSLRRPSFRASRQRPIISLNERILEFNKRNITAIIAEYKRKSPSGLDVE
RDPIEYSKFMERYAVGLSILTEEKYFNGSYETLRKIASSVSIPILMKDFIVKESQIDDAYNL
GADTVLLIVKILTERELESLLEYARSYGMEPLIEINDENDLDIALRIGARFIGINSRDLETL
EINKENQRKLISMIPSNVVKVAESGISERNEIEELRKLGVNAFLIGSSLMRNPEKIKEFIL
>d1qopa_ c.1.2.4 (A:) Trp synthase alpha-subunit {Salmonella typhimurium}
MERYENLFAQLNDRREGAFVPFVTLGDPGIEQSLKIIDTLIDAGADALELGVPFSDPLADGP
TIQNANLRAFAAGVTPAQCFEMLAIIREKHPTIPIGLLMYANLVFNNGIDAFYARCEQVGVD
SVLVADVPVEESAPFRQAALRHNIAPIFICPPNADDDLLRQVASYGRGYTYLLSRSGVTGAE
NRGALPLHHLIEKLKEYHAAPALQGFGISSPEQVSAAVRAGAAGAISGSAIVKIIEKNLASP
KQMLAELRSFVSAMKAASR
>d1geqa_ c.1.2.4 (A:) Trp synthase alpha-subunit {Archaeon Pyrococcus furiosus}
MFKDGSLIPYLTAGDPDKQSTLNFLLALDEYAGAIELGIPFSDPIADGKTIQESHYRALKNG
FKLREAFWIVKEFRRHSSTPIVLMTYYNPIYRAGVRNFLAEAKASGVDGILVVDLPVFHAKE
FTEIAREEGIKTVFLAAPNTPDERLKVIDDMTTGFVYLVSLYGTTGAREEIPKTAYDLLRRA
KRICRNKVAVGFGVSKREHVVSLLKEGANGVVVGSALVKIIGEKGREATEFLKKKVEELLGI
>d2tpsa_ c.1.3.1 (A:) Thiamin phosphate synthase {Bacillus subtilis}

HGIRMTRISREMMKELLSVYFIMGSNNTKADPVTVVQKALKGGATLYQFREKGGDALTGEAR
IKFAEKAQAACREAGVPFIVNDDVELALNLKADGIHIGQEDANAKEVRAAIGDMILGVSAHT
MSEVKQAEEDGADYVGLGPIYPTETKKDTRAVQGVSLIEAVRRQGISIPIVGIGGITIDNAA
PVIQAGADGVSMISAISQAEDPESAARKFREEIQTYKTGR
>d1ho1a_ c.1.24.1 (A:) Pyridoxine 5'-phosphate synthase {Escherichia coli}
AELLLGVNIDHIATLRNARGTAYPDPVQAAFIAEQAGADGITVHLREDRRHITDRDVRILRQ
TLDTRMNLEMAVTEEMLAIAVETKPHFCCLVPEKRQEVTTEGGLDVAGQRDKMRDACKRLAD
AGIQVSLFIDADEEQIKAAAEVGAPFIEIHTGCYADAKTDAEQAQELARIAKAATFAASLGL
KVNAGHGLTYHNVKAIAAIPEMHELNIGHAIIGRAVMTGLKDAVAEMKRLMLEARG
>d2dora_ c.1.4.1 (A:) Dihydroorotate dehydrogenase {Lactococcus lactis, isozyme A}
MLNTTFANAKFANPFMNASGVHCMTIEDLEELKASQAGAYITKSSTLEKREGNPLPRYVDLE
LGSINSMGLPNLGFDYYLDYVLKNQKENAQEGPIFFSIAGMSAAENIAMLKKIQESDFSGIT
ELNLSCPNVPGKPQLAYDFEATEKLLKEVFTFFTKPLGVKLPPYFDLVHFDIMAEILNQFPL
TYVNSVNSIGNGLFIDPEAESVVIKPKDGFGGIGGAYIKPTALANVRAFYTRLKPEIQIIGT
GGIETGQDAFEHLLCGATMLQIGTALHKEGPAIFDRIIKELEEIMNQKGYQSIADFHGKLKS
L
>d1ep3a_ c.1.4.1 (A:) Dihydroorotate dehydrogenase {Lactococcus lactis, isozyme B}
MTENNRLSVKLPGLDLKNPIIPASGCFGFGEEYAKYYDLNKLGSIMVKATTLHPRFGNPTPR
VAETASGMLNAIGLQNPGLEVIMTEKLPWLNENFPELPIIANVAGSEEADYVAVCAKIGDAA
NVKAIELNISCPNVKHGGQAFGTDPEVAAALVKACKAVSKVPLYVKLSPNVTDIVPIAKAVE
AAGADGLTMINTLMGVRFDLKTRQPILANITGGLSGPAIKPVALKLIHQVAQDVDIPIIGMG
GVANAQDVLEMYMAGASAVAVGTANFADPFVCPKIIDKLPELMDQYRIESLESLIQEVKEGK
K
>d1d3ga_ c.1.4.1 (A:) Dihydroorotate dehydrogenase {Human (Homo sapiens)}
MATGDERFYAEHLMPTLQGLLDPESAHRLAVRFTSLGLLPRARFQDSDMLEVRVLGHKFRNP
VGIAAGFDKHGEAVDGLYKMGFGFVEIGSVTPKPQEGNPRPRVFRLPEDQAVINRYGFNSHG
LSVVEHRLRARQQKQAKLTEDGLPLGVNLGKNKTSVDAAEDYAEGVRVLGPLADYLVVNVSS
PNTAGLRSLQGKAELRRLLTKVLQERDGLRRVHRPAVLVKIAPDLTSQDKEDIASVVKELGI
DGLIVTNTTVSRPAGLQGALRSETGGLSGKPLRDLSTQTIREMYALTQGRVPIIGVGGVSSG
QDALEKIRAGASLVQLYTALTFWGPPVVGKVKRELEALLKEQGFGGVTDAIGADHRR
>d1oyb__ c.1.4.1 (-) Old yellow enzyme (OYE) {Brewer's yeast (Saccharomyces carlsbergensis)}
SFVKDFKPQALGDTNLFKPIKIGNNELLHRAVIPPLTRMRALHPGNIPNRDWAVEYYTQRAQ
RPGTMIITEGAFISPQAGGYDNAPGVWSEEQMVEWTKIFNAIHEKKSFVWVQLWVLGWAAFP
DNLARDGLRYDSASDNVFMDAEQEAKAKKANNPQHSLTKDEIKQYIKEYVQAAKNSIAAGAD
GVEIHSANGYLLNQFLDPHSNTRTDEYGGSIENRARFTLEVVDALVEAIGHEKVGLRLSPYG
VFNSMSGGAETGIVAQYAYVAGELEKRAKAGKRLAFVHLVEPRVTNPFLTEGEGEYEGGSND
FVYSIWKGPVIRAGNFALHPEVVREEVKDKRTLIGYGRFFISNPDLVDRLEKGLPLNKYDRD
TFYQMSAHGYIDYPTYEEALKLGWDKK
>d1icpa_ c.1.4.1 (A:) 12-oxophytodienoate reductase 1 (OYE homolog)

{Tomato (Lycopersicon esculentum)}
QVDKIPLMSPCKMGKFELCHRVVLAPLTRQRSYGYIPQPHAILHYSQRSTNGGLLIGEATVI
SETGIGYKDVPGIWTKEQVEAWKPIVDAVHAKGGIFFCQIWHVGRVSNKDFQPNGEDPISCT
DRGLTPQIMSNGIDIAHFTRPRRLTTDEIPQIVNEFRVAARNAIEAGFDGVEIHGAHGYLID
QFMKDQVNDRSDKYGGSLENRCRFALEIVEAVANEIGSDRVGIRISPFAHYNEAGDTNPTAL
GLYMVESLNKYDLAYCHVVEPRMKTAWEKIECTESLVPMRKAYKGTFIVAGGYDREDGNRAL
IEDRADLVAYGRLFISNPDLPKRFELNAPLNKYNRDTFYTSDPIVGYTDYPFLE
>d1gox__ c.1.4.1 (-) Glycolate oxidase {Spinach (Spinacia oleracea)}
MEITNVNEYEAIAKQKLPKMVYDYYASGAEDQWTLAENRNAFSRILFRPRILIDVTNIDMTT
TILGFKISMPIMIAPTAMQKMAHPEGEYATARAASAAGTIMTLSSWATSSVEEVASTGPGIR
FFQLYVYKDRNVVAQLVRRAERAGFKAIALTVDTPRLGRREADIKNRFVLPPFLTLKNFEGI
DLGKMDKANDSGLSSYVAGQIDRSLSWKDVAWLQTITSLPILVKGVITAEDARLAVQHGAAG
IIVSNHGARQLDYVPATIMALEEVVKAAQGRIPVFLDGGVRRGTDVFKALALGAAGVFIGRP
VVFSLAAEGEAGVKKVLQMMRDEFELTMALSGCRSLKEISRSHIAADWD
>d1huva_ c.1.4.1 (A:) Membrane-associated (S)-mandelate dehydrogenase {Pseudomonas putida}
NLFNVEDYRKLAQKRLPKMVYDYLEGGAEDEYGVKHNRDVFQQWRFKPKRLVDVSRRSLQAE
VLGKRQSMPLLIGPTGLNGALWPKGDLALARAATKAGIPFVLSTASNMSIEDLARQCDGDLW
FQLYVIHREIAQGMVLKALHTGYTTLVLTTDVAVNGYRERDLHNRFKIPPFLTLKNFEGIDL
GKMDKANLEMQAALMSRQMDASFNWEALRWLRDLWPHKLLVKGLLSAEDADRCIAEGADGVI
LSNHGGRQLDCAISPMEVLAQSVAKTGKPVLIDSGFRRGSDIVKALALGAEAVLLGRATLYG
LAARGETGVDEVLTLLKADIDRTLAQIGCPDITSLSPDYLQNE
>d1h61a_ c.1.4.1 (A:) Pentaerythritol tetranirate reductase {Enterobacter cloacae}
SAEKLFTPLKVGAVTAPNRVFMAPLTRLRSIEPGDIPTPLMGEYYRQRASAGLIISEATQIS
AQAKGYAGAPGLHSPEQIAAWKKITAGVHAEDGRIAVQLWHTGRISHSSIQPGGQAPVSASA
LNANTRTSLRDENGNAIRVDTTTPRALELDEIPGIVNDFRQAVANAREAGFDLVELHSAHGY
LLHQFLSPSSNQRTDQYGGSVENRARLVLEVVDAVCNEWSADRIGIRVSPIGTFQNVDNGPN
EEADALYLIEELAKRGIAYLHMSETDLAGGKPYSEAFRQKVRERFHGVIIGAGAYTAEKAED
LIGKGLIDAVAFGRDYIANPDLVARLQKKAELNPQRPESFYGGGAEGYTDYPSL
>d1djna1 c.1.4.1 (A:1-340) Trimethylamine dehydrogenase, N-terminal domain {Methylophilus methylotrophus, w3a1}
ARDPKHDILFEPIQIGPKTLRNRFYQVPHCIGAGSDKPGFQSAHRSVKAEGGWAALNTEYCS
INPESDDTHRLSARIWDEGDVRNLKAMTDEVHKYGALAGVELWYGGAHAPNMESRATPRGPS
QYASEFETLSYCKEMDLSDIAQVQQFYVDAAKRSRDAGFDIVYVYGAHSYLPLQFLNPYYNK
RTDKYGGSLENRARFWLETLEKVKHAVGSDCAIATRFGVDTVYGPGQIEAEVDGQKFVEMAD
SLVDMWDITIGDIAEWGEDAGPSRFYQQGHTIPWVKLVKQVSKKPVLGVGRYTDPEKMIEIV
TKGYADIIGCARPSIADPFLPQKVEQGRYD
>d1ltda1 c.1.4.1 (A:98-511) Flavocytochrome b2, C-terminal domain {Baker's yeast (Saccharomyces cerevisiae)}
APGETKEDIARKEQLKSLLPPLDNIINLYDFEYLASQTLTKQAWAYYSSGANDEVTHRENHN
AYHRIFFKPKILVDVRKVDISTDMLGSHVDVPFYVSATALCKLGNPLEGEKDVARGCGQGVT
KVPQMISTLASCSPEEIIEAAPSDKQIQWYQLYVNSDRKITDDLVKNVEKLGVKALFVTVDA

PSLGQREKDMKLKFSNTKAGPKAMKKTNVEESQGASRALSKFIDPSLTWKDIEELKKKTKLP
IVIKGVQRTEDVIKAAEIGVSGVVLSNHGGRQLDFSRAPIEVLAETMPILEQRNLKDKLEVF
VDGGVRRGTDVLKALCLGAKGVGLGRPFLYANSCYGRNGVEKAIEILRDEIEMSMRLLGVTS
IAELKPDLLDLSTLKARTVGVPNDVLYNEVYEGPTLTEFEDA
>d1h7wa2 c.1.4.1 (A:533-844) Dihydropyrimidine dehydrogenase, domain 4 {Pig (Sus scrofa)}
ISVEMAGLKFINPFGLASAAPTTSSSMIRRAFEAGWGFALTKTFSLDKDIVTNVSPRIVRGT
TSGPMYGPGQSSFLNIELISEKTAAYWCQSVTELKADFPDNIVIASIMCSYNKNDWMELSRK
AEASGADALELNLSCPHGMGERGMGLACGQDPELVRNICRWVRQAVQIPFFAKLTPNVTDIV
SIARAAKEGGADGVTATNTVSGLMGLKADGTPWPAVGAGKRTTYGGVSGTAIRPIALRAVTT
IARALPGFPILATGGIDSAESGLQFLHSGASVLQVCSAVQNQDFTVIQDYCTGLKALLYLKS
IE
>d1ea0a2 c.1.4.1 (A:423-1193) Alpha subunit of glutamate synthase, central and FMN domains {Azospirillum brasilense}
TTHLDELVKTASLKGEPSDMDKAELRRRQQAFGLTMEDMELILHPMVEDGKEAIGSMGDDSP
IAVLSDKYRGLHHFFRQNFSQVTNPPIDSLRERRVMSLKTRLGNLGNILDEDETQTRLLQLE
SPVLTTAEFRAMRDYMGDTAAEIDATFPVDGGPEALRDALRRIRQETEDAVRGGATHVILTD
EAMGPARAAIPAILATGAVHTHLIRSNLRTFTSLNVRTAEGLDTHYFAVLIGVGATTVNAYL
AQEAIAERHRRGLFGSMPLEKGMANYKKAIDDGLLKIMSKMGISVISSYRGGGNFEAIGLSR
ALVAEHFPAMVSRISGIGLNGIQKKVLEQHATAYNEEVVALPVGGFYRFRKSGDRHGWEGGV
IHTLQQAVTNDSYTTFKKYSEQVNKRPPMQLRDLLELRSTKAPVPVDEVESITAIRKRFITP
GMSMGALSPEAHGTLNVAMNRIGAKSDSGEGGEDPARFRPDKNGDNWNSAIKQVASGRFGVT
AEYLNQCRELEIKVAQGAKPGEGGQLPGFKVTEMIARLRHSTPGVMLISPPPHHDIYSIEDL
AQLIYDLKQINPDAKVTVKLVSRSGIGTIAAGVAKANADIILISGNSGGTGASPQTSIKFAG
LPWEMGLSEVHQVLTLNRLRHRVRLRTDGGLKTGRDIVIAAMLGAEEFGIGTASLIAMGCIM
VRQCHSNTCPVGVCVQDDKLRQKFVGTPEKVVNLFTFLAEEVREILAGLGFRSLNEVIGRTD
LLHQVSRGAEHLDDLDLNPRLAQVDPG
>d1eepa_ c.1.5.1 (A:) Inosine monophosphate dehydrogenase (IMPDH) {Lyme disease spirochete (Borrelia burgdorferi)}
NKITKEALTFDDVSLIPRKSSVLPSEVSLKTQLTKNISLNIPFLSSAMDTVTESQMAIAIAK
EGGIGIIHKNMSIEAQRKEIEKVKTYKFQKTINTNGDTNEQKPEIFTAKQHLEKSDAYKNAE
HKEDFPNACKDLNNKLRVGAAVSIDIDTIERVEELVKAHVDILVIDSAHGHSTRIIELIKKI
KTKYPNLDLIAGNIVTKEAALDLISVGADCLKVGIGPGSICTTRIVAGVGVPQITAICDVYE
ACNNTNICIIADGGIRFSGDVVKAIAAGADSVMIGNLFAGTKESPSEEIIYNGKKFKSYVGM
GSISAMKRGSKSRYFQLENNEPKKLVPEGIEGMVPYSGKLKDILTQLKGGLMSGMGYLGAAT
ISDLKINSKFVKISHS
>d1zfja1 c.1.5.1 (A:2-94,A:221-492) Inosine monophosphate dehydrogenase (IMPDH) {Streptococcus pyogenes}
SNWDTKFLKKGYTFDDVLLIPAESHVLPNEVDLKTKLADNLTLNIPIITAAMDTVTGSKMAI
AIARAGGLGVIHKNMSITEQAEEVRKVKRSEXGRLLVAAAVGVTSDTFERAEALFEAGADAI
VIDTAHGHSAGVLRKIAEIRAHFPNRTLIAGNIATAEGARALYDAGVDVVKVGIGPGSICTT
RVVAGVGVPQVTAIYDAAAVAREYGKTIIADGGIKYSGDIVKALAAGGNAVMLGSMFAGTDE
APGETEIYQGRKYKTYRGMGSIAAMKKGSSDRYFQGSVNEANKLVPEGIEGRVAYKGAASDI
VFQMLGGIRSGMGYVGAGDIQELHENAQFVEMSGAGLIESHPHDVQITNEAPNYSV

>d1ak5_1 c.1.5.1 (2-101,222-483) Inosine monophosphate dehydrogenase (IMPDH) {Tritrichomonas foetus}
AKYYNEPCHTFNEYLLIPGLSTVDCIPSNVNLSTPLVKFQKGQQSEINLKIPLVSAIMQSVS
GEKMAIALAREGGISFIFGSQSIESQAAMVHAVKNFKAXHNELVDSQKRYLVGAGINTRDFR
ERVPALVEAGADVLCIDSSDGFSEWQKITIGWIREKYGDKVKVGAGNIVDGEGFRYLADAGA
DFIKIGIGGGSICITREQKGIGRGQATAVIDVVAERNKYFEETGIYIPVCSDGGIVYDYHMT
LALAMGADFIMLGRYFARFEESPTRKVTINGSVMKEYWGEGSSRARNWQRYDLGGKQKLSFE
EGVDSYVPYAGKLKDNVEASLNKVKSTMCNCGALTIPQLQSKAKITLVSSVSI
>d1jr1a1 c.1.5.1 (A:17-112,A:233-514) Inosine monophosphate dehydrogenase (IMPDH) {Chinese hamster (Cricetulus griseus)}
GLTAQQLFNCGDGLTYNDFLILPGYIDFTADQVDLTSALTKKITLKTPLVSSPMDTVTEAGM
AIAMALTGGIGFIHHNCTPEFQANEVRKVKKYEQXYPLASKDAKKQLLCGAAIGTHEDDKYR
LDLLALAGVDVVVLDSSQGNSIFQINMIKYMKEKYPNLQVIGGNVVTAAQAKNLIDAGVDAL
RVGMGCGSICITQEVLACGRPQATAVYKVSEYARRFGVPVIADGGIQNVGHIAKALALGAST
VMMGSLLAATTEAPGEYFFSDGIRLKKYRGMGSLDAMDKHLSSQNRYFSEADKIKVAQGVSG
AVQDKGSIHKFVPYLIAGIQHSCQDIGAKSLTQVRAMMYSGELKFEKRTSSAQVEGGVHSLH
SYEKRLF
>d1bd0a2 c.1.6.1 (A:12-244) Alanine racemase {Bacillus stearothermophilus}
VDLDAIYDNVENLRRLLPDDTHIMAVVKANAYGHGDVQVARTALEAGASRLAVAFLDEALAL
REKGIEAPILVLGASRPADAALAAQQRIALTVFRSDWLEEASALYSGPFPIHFHLKMDTGMG
RLGVKDEEETKRIVALIERHPHFVLEGLYTHFATADEVNTDYFSYQYTRFLHMLEWLPSRPP
LVHCANSAASLRFPDRTFNMVRFGIAMYGLAPSPGIKPLLPYPLKEA
>d1d7ka2 c.1.6.1 (A:44-283) Eukaryotic ornithine decarboxylase {Human (Homo sapiens)}
DLGDILKKHLRWLKALPRVTPFYAVKCNDSKAIVKTLAATGTGFDCASKTEIQLVQSLGVPP
ERIIYANPCKQVSQIKYAANNGVQMMTFDSEVELMKVARAHPKAKLVLRIATDDSKAVCRLS
VKFGATLRTSRLLLERAKELNIDVVGVSFHVGSGCTDPETFVQAISDARCVFDMGAEVGFSM
YLLDIGGGFPGSEDVKLKFEEITGVINPALDKYFPSDSGVRIIAEPGRYYVASA
>d7odca2 c.1.6.1 (A:44-283) Eukaryotic ornithine decarboxylase {Mouse (Mus musculus)}
DLGDILKKHLRWLKALPRVTPFYAVKCNDSRAIVSTLAAIGTGFDCASKTEIQLVQGLGVPA
ERVIYANPCKQVSQIKYAASNGVQMMTFDSEIELMKVARAHPKAKLVLRIATDDSKAVCRLS
VKFGATLKTSRLLLERAKELNIDVIGVSFHVGSGCTDPDTFVQAVSDARCVFDMATEVGFSM
HLLDIGGGFPGSEDTKLKFEEITSVINPALDKYFPSDSGVRIIAEPGRYYVASA
>d2toda2 c.1.6.1 (A:44-283) Eukaryotic ornithine decarboxylase {Trypanosoma brucei}
DLGDIVRKHETWKKCLPRVTPFYAVACNDDWRVLGTLAALGTGFDCASNTEIQRVRGIGVPP
EKIIYANPCKQISHIRYARDSGVDVMTFDCVDELEKVAKTHPKAKMVLRISTDDSLARCRLS
VKFGAKVEDCRFILEQAKKLNIDVTGVSFHVGSGSTDASTFAQAISDSRFVFDMGTELGFNM
HILDIGGGFPGTRDAPLKFEEIAGVINNALEKHFPPDLKLTIVAEPGRYYVASA
>d1ct5a_ c.1.6.2 (A:) "Hypothetical" protein ybl036c {Baker's yeast (Saccharomyces cerevisiae)}
TGITYDEDRKTQLIAQYESVREVVNAEAKNVHVNENASKILLLVVSKLKPASDIQILYDHGV

REFGENYVQELIEKAKLLPDDIKWHFIGGLQTNKCKDLAKVPNLYSVETIDSLKKAKKLNES
RAKFQPDCNPILCNVQINTSHEDQKSGLNNEAEIFEVIDFFLSEECKYIKLNGLMTIGSWNV
SHEDSKENRDFATLVEWKKKIDAKFGTSLKLSMGMSADFREAIRQGTAEVRIGTDIFG
>d1frb__ c.1.7.1 (-) FR-1 (fibroblast growth factor-induced)
protein {Mouse (Mus musculus)}
ATFVELSTKAKMPIVGLGTWKSPPNQVKEAVKAAIDAGYRHIDCAYAYCNENEVGEAIQEKI
KEKAVQREDLFIVSKLWPTCFEKKLLKEAFQKTLTDLKLDYLDLYLIHWPQGLQPGKELFPK
DDQGRILTSKTTFLEAWEGMEELVDQGLVKALGVSNFNHFQIERLLNKPGLKHKPVTNQVEC
HPYLTQEKLIQYCHSKGISVTAYSPLGSPDRPSAKPEDPSLLEDPKIKEIAAKHEKTSAQVL
IRFHIQRNVVVIPKSVTPSRIQENIQVFDFQLSDEEMATILSFNRNWRACLLPETVNMEEYP
YDAEY
>d1exba_ c.1.7.1 (A:) Voltage-dependent K+ channel beta subunit
{Rat (Rattus norvegicus)}
LQFYRNLGKSGLRVSCLGLGTWVTFGGQITDEMAEHLMTLAYDNGINLFDTAEVYAAGKAEV
VLGNIIKKKGWRRSSLVITTKIFWGGKAETERGLSRKHIIEGLKASLERLQLEYVDVVFANR
PDPNTPMEETVRAMTHVINQGMAMYWGTSRWSSMEIMEAYSVARQFNLIPPICEQAEYHMFQ
REKVEVQLPELFHKIGVGAMTWSPLACGIVSGKYDSGIPPYSRASLKGYQWLKDKILSEEGR
RQQAKLKELQAIAERLGCTLPQLAIAWCLRNEGVSSVLLGASNAEQLMENIGAIQVLPKLSS
SIVHEIDSILGNKPYS
>d1ads__ c.1.7.1 (-) Aldose reductase (aldehyde reductase) {Human
(Homo sapiens)}
ASRLLLNNGAKMPILGLGTWKSPPGQVTEAVKVAIDVGYRHIDCAHVYQNENEVGVAIQEKL
REQVVKREELFIVSKLWCTYHEKGLVKGACQKTLSDLKLDYLDLYLIHWPTGFKPGKEFFPL
DESGNVVPSDTNILDTWAAMEELVDEGLVKAIGISNFNHLQVEMILNKPGLKYKPAVNQIEC
HPYLTQEKLIQYCQSKGIVVTAYSPLGSPDRPWAKPEDPSLLEDPRIKAIAAKHNKTTAQVL
IRFPMQRNLVVIPKSVTPERIAENFKVFDFELSSQDMTTLLSYNRNWRVCALLSCTSHKDYP
FHEEF
>d2alr__ c.1.7.1 (-) Aldose reductase (aldehyde reductase) {Human
(Homo sapiens)}
AASCVLLHTGQKMPLIGLGTWKSEPGQVKAAVKYALSVGYRHIDCAAIYGNEPEIGEALKED
VGPGKAVPREELFVTSKLWNTKHHPEDVEPALRKTLADLQLEYLDLYLMHWPYAFERGDNPF
PKNADGTICYDSTHYKETWKALEALVAKGLVQALGLSNFNSRQIDDILSVASVRPAVLQVEC
HPYLAQNELIAHCQARGLEVTAYSPLGSSDRAWRDPDEPVLLEEPVVLALAEKYGRSPAQIL
LRWQVQRKVICIPKSITPSRILQNIKVFDFTFSPEEMKQLNALNKNWRYIVPMLTVDGKRVP
RDAGHPLYPFNDPY
>d1ah4__ c.1.7.1 (-) Aldose reductase (aldehyde reductase) {Pig
(Sus scrofa)}
ASHLVLYTGAKMPILGLGTWKSPPGKVTEAVKVAIDLGYRHIDCAHVYQNENEVGLGLQEKL
QGQVVKREDLFIVSKLWCTDHEKNLVKGACQTTLRDLKLDYLDLYLIHWPTGFKPGKDPFPL
DGDGNVVPDESDFVETWEAMEELVDEGLVKAIGVSNFNHLQVEKILNKPGLKYKPAVNQIEV
HPYLTQEKLIEYCKSKGIVVTAYSPLGSPDRPWAKPEDPSLLEDPRIKAIAAKYNKTTAQVL
IRFPMQRNLIVIPKSVTPERIAENFQVFDFELSPEDMNTLLSYNRNWRVCALMSCASHKDYP
FHEEY
>d1hqta_ c.1.7.1 (A:) Aldose reductase (aldehyde reductase) {Pig

(Sus scrofa)}
AASCVLLHTGQKMPLIGLGTWKSEPGQVKAAIKYALTVGYRHIDCAAIFGNELEIGEALQET
VGPGKAVPREELFVTSKLWNTKHHPEDVEPALRKTLADLQLEYLDLYLMHWPYAFERGDNPF
PKNADGTIRYDATHYKDTWKALEALVAKGLVRALGLSNFSSRQIDDVLSVASVRPAVLQVEC
HPYLAQNELIAHCQARGLEVTAYSPLGSSDRAWRDPNEPVLLEEPVVQALAEKYNRSPAQIL
LRWQVQRKVICIPKSVTPSRIPQNIQVFDFTFSPEEMKQLDALNKNLRFIVPMLTVDGKRVP
RDAGHPLYPFNDPY
>d1afsa_ c.1.7.1 (A:) 3-alpha-hydroxysteroid dehydrogenase {Rat (Rattus norvegicus)}
MDSISLRVALNDGNFIPVLGFGTTVPEKVAKDEVIKATKIAIDNGFRHFDSAYLYEVEEEVG
QAIRSKIEDGTVKREDIFYTSKLWSTFHRPELVRTCLEKTLKSTQLDYVDLYIIHFPMALQP
GDIFFPRDEHGKLLFETVDICDTWEAMEKCKDAGLAKSIGVSNFNCRQLERILNKPGLKYKP
VCNQVECHLYLNQSKMLDYCKSKDIILVSYCTLGSSRDKTWVDQKSPVLLDDPVLCAIAKKY
KQTPALVALRYQLQRGVVPLIRSFNAKRIKELTQVFEFQLASEDMKALDGLNRNFRYNNAKY
FDDHPNHPF
>d1ihia_ c.1.7.1 (A:) 3-alpha-hydroxysteroid dehydrogenase {Human (Homo sapiens), type III}
SKYQCVKLNDGHFMPVLGFGTYAPAEVPKSKALEAVKLAIEAGFHHIDSAHVYNNEEQVGLA
IRSKIADGSVKREDIFYTSKLWSNSHRPELVRPALERSLKNLQLDYVDLYLIHFPVSVKPGE
EVIPKDENGKILFDTVDLCATWEAMEKCKDAGLAKSIGVSNFNHRLLEMILNKPGLKYKPVC
NQVECHPYFNQRKLLDFCKSKDIVLVAYSALGSHREEPWVDPNSPVLLEDPVLCALAKKHKR
TPALIALRYQLQRGVVVLAKSYNEQRIRQNVQVFEFQLTSEEMKAIDGLNRNVRYLTLDIFA
GPPNYPFSDE
>d1c9wa_ c.1.7.1 (A:) CHO reductase {Chinese hampster (Cricetulus griseus)}
STFVELSTKAKMPIVGLGTWQSPPGQVKEAVKVAIDAGYRHIDCAYAYYNEHEVGEAIQEKI
KEKAVRREDLFIVSKLWPTCFERKLLKEAFQKTLTDLKLDYLDLYLIHWPQGLQPGKELFPK
DDQGNVLTSKITFLDAWEVMEELVDEGLVKALGVSNFNHFQIERILNKPGLKHKPVTNQVEC
HPYLTQEKLIEYCHSKGITVTAYSPLGSPNRPWAKPEDPSLLEDPKIKEIAAKHKKTSAQVL
IRFHIQRNVVVIPKSVTPARIHENFQVFDFQLSDQEMATILGFNRNWRACLLPETVNMEEYP
YDAEY
>d1hw6a_ c.1.7.1 (A:) 2,5-diketo-D-gluconic acid reductase A {Corynebacterium sp.}
TVPSIVLNDGNSIPQLGYGVFKVPPADTQRAVEEALEVGYRHIDTAAIYGNEEGVGAAIAAS
GIARDDLFITTKLWNDRHDGDEPAAAIAESLAKLALDQVDLYLVHWPTPAADNYVHAWEKMI
ELRAAGLTRSIGVSNHLVPHLERIVAATGVVPAVNQIELHPAYQQREITDWAAAHDVKIESW
GPLGQGKYDLFGAEPVTAAAAAHGKTPAQAVLRWHLQKGFVVFPKSVRRERLEENLDVFDFD
LTDTEIAAIDAMDP
>d1bli_2 c.1.8.1 (3-393) Bacterial alpha-amylase {Bacillus licheniformis}
LNGTLMQYFEWYMPNDGQHWKRLQNDSAYLAEHGITAVWIPPAYKGTSQADVGYGAYDLYDL
GEFHQKGTVRTKYGTKGELQSAIKSLHSRDINVYGDVVINHKGGADATEDVTAVEVDPADRN
RVISGEHLIKAWTHFHFPGRGSTYSDFKWHWYHFDGTDWDESRKLNRIYKFQGKAWDWEVSN
EFGNYDYLMYADIDYDHPDVAAEIKRWGTWYANELQLDGFRLDAVKHIKFSFLRDWVNHVRE

KTGKEMFTVAEYWSYDLGALENYLNKTNFNHSVFDVPLHYQFHAASTQGGGYDMRKLLNGTV
VSKHPLKSVTFVDNHDTQPGQSLESTVQTWFKPLAYAFILTRESGYPQVFYGDMYGTKGDSQ
REIPALKHKIEPILKARKQ
>d1e43a2 c.1.8.1 (A:1-393) Bacterial alpha-amylase {Bacillus amyloliquefaciens/Bacillus licheniformis chimera}
VNGTLMQYFEWYTPNDGQHWKRLQNDAEHLSDIGITAVWIPPAYKGLSQSDNGYGPYDLYDL
GEFQQKGTVRTKYGTKSELQDAIGSLHSRNVQVYGDVVLNHKAGADATEDVTAVEVNPANRN
QETSEEYQIKAWTDFRFPGRGNTYSDFKWHWYHFDGADWDESRKISRIFKFRGEGKAWDWEV
SSENGNYDYLMYADVDYDHPDVVAETKKWGIWYANELSLDGFRIDAAKHIKFSFLRDWVQAV
RQATGKEMFTVAEYWQNNAGKLENYLNKTSFNQSVFDVPLHFNLQAASSQGGGYDMRKLLNG
TVVSKHPLKSVTFVDNHDTQPGQSLESTVQTWFKPLAYAFILTRESGYPQVFYGDMYGTKGD
SQREIPALKHKIEPILKARKQ
>d1g94a2 c.1.8.1 (A:1-354) Bacterial alpha-amylase {Pseudoalteromonas haloplanctis (Alteromonas haloplanctis)}
TPTTFVHLFEWNWQDVAQECEQYLGPKGYAAVQVSPPNEHITGSQWWTRYQPVSYELQSRGG
NRAQFIDMVNRCSAAGVDIYVDTLINHMAAGSGTGTAGNSFGNKSFPIYSPQDFHESCTINN
SDYGNDRYRVQNCELVGLADLDTASNYVQNTIAAYINDLQAIGVKGFRFDASKHVAASDIQS
LMAKVNGSPVVFQEVIDQGGEAVGASEYLSTGLVTEFKYSTELGNTFRNGSLAWLSNFGEGW
GFMPSSSAVVFVDNHDNQRGHGGAGNVITFEDGRLYDLANVFMLAYPYGYPKVMSSYDFHGD
TDAGGPNVPVHNNGNLECFASNWKCEHRWSYIAGGVDFRNNTAD
>d1bag_2 c.1.8.1 (1-347) Bacterial alpha-amylase {Bacillus subtilis}
LTAPSIKSGTILHAWNWSFNTLKHNMKDIHDAGYTAIQTSPINQVKEGNQGDKSMSNWYWLY
QPTSYQIGNRYLGTEQEFKEMCAAAEEYGIKVIVDAVINHTTFDYAAISNEVKSIPNWTHGN
TQIKNWSDRWDVTQNSLLGLYDWNTQNTQVQSYLKRFLERALNDGADGFRFDAAKHIELPDD
GSYGSQFWPNITNTSAEFQYGQILQDSASRDAAYANYMDVTASNYGHSIRSALKNRNLGVSN
ISHYASDVSADKLVTWVESHDTYANDDEESTWMSDDDIRLGWAVIASRSGSTPLFFSRPEGG
GNGVRFPGKSQIGDRGSALFEDQAITAVNRFHNVMAG
>d1hvxa2 c.1.8.1 (A:1-393) Bacterial alpha-amylase {Bacillus stearothermophilus}
AAPFNGTMMQYFEWYLPDDGTLWTKVANEANNLSSLGITALWLPPAYKGTSRSDVGYGVYDL
YDLGEFNQKGAVRTKYGTKAQYLQAIQAAHAAGMQVYADVVFDHKGGADGTEWVDAVEVNPS
DRNQEISGTYQIQAWTKFDFPGRGNTYSSFKWRWYHFDGVDWDESRKLSRIYKFRGIGKAWD
WEVDTENGNYDYLMYADLDMDHPEVVTELKSWGKWYVNTTNIDGFRLDAVKHIKFSFFPDWL
SYVRSQTGKPLFTVGEYWSYDINKLHNYIMKTNGTMSLFDAPLHNKFYTASKSGGTFDMRTL
MTNTLMKDQPTLAVTFVDNHDTEPGQALQSWVDPWFKPLAYAFILTRQEGYPCVFYGDYYGI
PQYNIPSLKSKIDPLLIARRD
>d1gjwa2 c.1.8.1 (A:1-572) Maltosyltransferase {Thermotoga maritima}
MLLREINRYCKEKATGKRIYAVPKLWIPGFFKKFDEKSGRCFVDPYELGAEITDWILNQSRE
WDYSQPLSFLKGEKTPDWIKRSVVYGSLPRTTAAYNHKGSGYYEENDVLGFREAGTFFKMML
LLPFVKSLGADAIYLLPVSRMSDLFKKGDAPSPYSVKNPMELDERYHDPLLEPFKVDEEFKA
FVEACHILGIRVILDFIPRTAARDSDLIREHPDWFYWIKVEELADYTPPRAEELPFKVPDED
ELEIIYNKENVKRHLKKFTLPPNLIDPQKWEKIKREEGNILELIVKEFGIITPPGFSDLIND

PQPTWDDVTFLRLYLDHPEASKRFLDPNQPPYVLYDVIKASKFPGKEPNRELWEYLAGVIPH
YQKKYGIDGARLDMGHALPKELLDLIIKNVKEYDPAFVMIAEELDMEKDKASKEAGYDVILG
SSWYFAGRVEEIGKLPDIAEELVLPFLASVETPDTPRIATRKYASKMKKLAPFVTYFLPNSI
PYVNTGQEIGEKQPMNLGLDTDPNLRKVLSPTDEFFGKLAFFDHYVLHWDSPDRGVLNFIKK
LIKVRHEFLDFVLN
>d1cgt_4 c.1.8.1 (1-406) Cyclodextrin glycosyltransferase
{Bacillus circulans, different strains}
DPDTAVTNKQSFSTDVIYQVFTDRFLDGNPSNNPTGAAYDATCSNLKLYCGGDWQGLINKIN
DNYFSDLGVTALWISQPVENIFATINYSGVTNTAYHGYWARDFKKTNPYFGTMADFQNLITT
AHAKGIKIVIDFAPNHTSPAMETDTSFAENGRLYDNGTLVGGYTNDTNGYFHHNGGSDFSSL
ENGIYKNLYDLADFNHNNATIDKYFKDAIKLWLDMGVDGIRVDAVKHMPLGWQKSWMSSIYA
HKPVFTFGEWFLGSAASDADNTDFANKSGMSLLDFRFNSAVRNVFRDNTSNMYALDSMINST
ATDYNQVNDQVTFIDNHDMDRFKTSAVNNRRLEQALAFTLTSRGVPAIYYGTEQYLTGNGDP
DNRAKMPSFSKSTTAFNVISKLAPLRKSNPAIAY
>d1kcla4 c.1.8.1 (A:1-406) Cyclodextrin glycosyltransferase
{Bacillus circulans, different strains}
APDTSVSNKQNFSTDVIYQIFTDRFSDGNPANNPTGAAFDGTCTNLRLYCGGDWQGIINKIN
DGYLTGMGVTAIWISQPVENIYSIINYSGVNNTAYHGYWARDFKKTNPAYGTIADFQNLIAA
AHAKNIKVIIDFAPNHTSPASSDQPSFAENGRLYDNGTLLGGYTNDTQNLFHHNLGTDFSTT
ENGIYKNLYDLADLNHNNSTVDVYLKDAIKMWLDLGIDGIRMDAVKHMPFGWQKSFMAAVNN
YKPVFTFGEWFLGVNEVSPENHKFANESGMSLLDFRFAQKVRQVFRDNTDNMYGLKAMLEGS
AADYAQVDDQVTFIDNHDMERFHASNANRRKLEQALAFTLTSRGVPAIYYGTEQYMSGGTDP
DNRARIPSFSTSTTAYQVIQKLAPLRKCNPAIAY
>d1cyg_4 c.1.8.1 (1-402) Cyclodextrin glycosyltransferase
{Bacillus stearothermophilus}
AGNLNKVNFTSDVVYQIVVDRFVDGNTSNNPSGALFSSGCTNLRKYCGGDWQGIINKINDGY
LTDMGVTAIWISQPVENVFSVMNDASGSASYHGYWARDFKKPNPFFGTLSDFQRLVDAAHAK
GIKVIIDFAPNHTSPASETNPSYMENGRLYDNGTLLGGYTNDANMYFHHNGGTTFSSLEDGI
YRNLFDLADLNHQNPVIDRYLKDAVKMWIDMGIDGIRMDAVKHMPFGWQKSLMDEIDNYRPV
FTFGEWFLSENEVDANNHYFANESGMSLLDFRFGQKLRQVLRNNSDNWYGFNQMIQDTASAY
DEVLDQVTFIDNHDMDRFMIDGGDPRKVDMALAVLLTSRGVPNIYYGTEQYMTGNGDPNNRK
MMSSFNKNTRAYQVIQKLSSLRRNNPALAY
>d1qhoa4 c.1.8.1 (A:1-407) Cyclodextrin glycosyltransferase
{Bacillus stearothermophilus, maltogenic alpha-amylase}
SSSASVKGDVIYQIIIDRFYDGDTTNNNPAKSYGLYDPTKSKWKMYWGGDLEGVRQKLPYLK
QLGVTTIWLSPVLDNLDTLAGTDNTGYHGYWTRDFKQIEEHFGNWTTFDTLVNDAHQNGIKV
IVDFVPNHSTPFKANDSTFAEGGALYNNGTYMGNYFDDATKGYFHHNGDISNWDDRYEAQWK
NFTDPAGFSLADLSQENGTIAQYLTDAAVQLVAHGADGLRIDAVKHFNSGFSKSLADKLYQK
KDIFLVGEWYGDDPGTANHLEKVRYANNSGVNVLDFDLNTVIRNVFGTFTQTMYDLNNMVNQ
TGNEYKYKENLITFIDNHDMSRFLSVNSNKANLHQALAFILTSRGTPSIYYGTEQYMAGGND
PYNRGMMPAFDTTTTAFKEVSTLAGLRRNNAAIQY
>d1pama4 c.1.8.1 (A:1-406) Cyclodextrin glycosyltransferase
{Alkalophilic bacillus sp., strain 1011}
APDTSVSNKQNFSTDVIYQIFTDRFSDGNPANNPTGAAFDGSCTNLRLYCGGDWQGIINKIN

DGYLTGMGITAIWISQPVENIYSVINYSGVNNTAYHGYWARDFKKTNPAYGTMQDFKNLIDT
AHAHNIKVIIDFAPNHTSPASSDDPSFAENGRLYDNGNLLGGYTNDTQNLFHHYGGTDFSTI
ENGIYKNLYDLADLNHNNSSVDVYLKDAIKMWLDLGVDGIRVDAVKHMPFGWQKSFMATINN
YKPVFTFGEWFLGVNEISPEYHQFANESGMSLLDFRFAQKARQVFRDNTDNMYGLKAMLEGS
EVDYAQVNDQVTFIDNHDMERFHTSNGDRRKLEQALAFTLTSRGVPAIYYGSEQYMSGGNDP
DNRARLPSFSTTTTAYQVIQKLAPLRKSNPAIAY
>d1ciu_4 c.1.8.1 (1-406) Cyclodextrin glycosyltransferase {Thermoanaerobacterium thermosulfurigenes, EM1}
ASDTAVSNVVNYSTDVIYQIVTDRFVDGNTSNNPTGDLYDPTHTSLKKYFGGDWQGIINKIN
DGYLTGMGVTAIWISQPVENIYAVLPDSTFGGSTSYHGYWARDFKRTNPYFGSFTDFQNLIN
TAHAHNIKVIIDFAPNHTSPASETDPTYAENGRLYDNGTLLGGYTNDTNGYFHHYGGTDFSS
YEDGIYRNLFDLADLNQQNSTIDSYLKSAIKVWLDMGIDGIRLDAVKHMPFGWQKNFMDSIL
SYRPVFTFGEWFLGTNEIDVNNTYFANESGMSLLDFRFSQKVRQVFRDNTDTMYGLDSMIQS
TASDYNFINDMVTFIDNHDMDRFYNGGSTRPVEQALAFTLTSRGVPAIYYGTEQYMTGNGDP
YNRAMMTSFNTSTTAYNVIKKLAPLRKSNPAIAY
>d1hx0a2 c.1.8.1 (A:1-403) Animal alpha-amylase {Pig (Sus scrofa)}
EYAPQTQSGRTSIVHLFEWRWVDIALECERYLGPKGFGGVQVSPPNENIVVTNPSRPWWERY
QPVSYKLCTRSGNENEFRDMVTRCNNVGVRIYVDAVINHMCGSGAAAGTGTTCGSYCNPGNR
EFPAVPYSAWDFNDGKCKTASGGIESYNDPYQVRDCQLVGLLDLALEKDYVRSMIADYLNKL
IDIGVAGFRIDASKHMWPGDIKAVLDKLHNLNTNWFPAGSRPFIFQEVIDLGGEAIKSSEYF
GNGRVTEFKYGAKLGTVVRKWSGEKMSYLKNWGEGWGFMPSDRALVFVDNHDNQRGHGAGGS
SILTFWDARLYKIAVGFMLAHPYGFTRVMSSYRWARNFVNGEDVNDWIGPPNNNGVIKEVTI
NADTTCGNDWVCEHRWREIRNMVWFRNVVDG
>d1smd_2 c.1.8.1 (1-403) Animal alpha-amylase {Human (Homo sapiens)}
EYSSNTQQGRTSIVHLFEWRWVDIALECERYLAPKGFGGVQVSPPNENVAIHNPFRPWWERY
QPVSYKLCTRSGNEDEFRNMVTRCNNVGVRIYVDAVINHMCGNAVSAGTSSTCGSYFNPGSR
DFPAVPYSGWDFNDGKCKTGSGDIENYNDATQVRDCRLSGLLDLALGKDYVRSKIAEYMNHL
IDIGVAGFRIDASKHMWPGDIKAILDKLHNLNSNWFPEGSKPFIYQEVIDLGGEPIKSSDYF
GNGRVTEFKYGAKLGTVIRKWNGEKMSYLKNWGEGWGFMPSDRALVFVDNHDNQRGHGAGGA
SILTFWDARLYKMAVGFMLAHPYGFTRVMSSYRWPRYFENGKDVNDWVGPPNDNGVTKEVTI
NPDTTCGNDWVCEHRWRQIRNMVNFRNVVDG
>d1jae_2 c.1.8.1 (1-378) Animal alpha-amylase {Yellow mealworm (Tenebrio molitor), larva}
EKDANFASGRNSIVHLFEWKWNDIADECERFLQPQGFGGVQISPPNEYLVADGRPWWERYQP
VSYIINTRSGDESAFTDMTRRCNDAGVRIYVDAVINHMTGMNGVGTSGSSADHDGMNYPAVP
YGSGDFHSPCEVNNYQDADNVRNCELVGLRDLNQGSDYVRGVLIDYMNHMIDLGVAGFRVDA
AKHMSPGDLSVIFSGLKNLNTDYGFADGARPFIYQEVIDLGGEAISKNEYTGFGCVLEFQFG
VSLGNAFQGGNQLKNLANWGPEWGLLEGLDAVVFVDNHDNQRTGGSQILTYKNPKPYKMAIA
FMLAHPYGTTRIMSSFDFTDNDQGPPQDGSGNLISPGINDDNTCSNGYVCEHRWRQVYGMVG
FRNAVE
>d2aaa_2 c.1.8.1 (1-381) Fungal alpha-amylases {Aspergillus niger, acid amylase}
LSAASWRTQSIYFLLTDRFGRTDNSTTATCNTGNEIYCGGSWQGIIDHLDYIEGMGFTAIWI

SPITEQLPQDTADGEAYHGYWQQKIYDVNSNFGTADNLKSLSDALHARGMYLMVDVVPDHMG
YAGNGNDVDYSVFDPFDSSSYFHPYCLITDWDNLTMVEDCWEGDTIVSLPDLDTTETAVRTI
WYDWVADLVSNYSVDGLRIDSVLEVQPDFFPGYNKASGVYCVGEIDNGNPASDCPYQKVLDG
VLNYPIYWQLLYAFESSSGSISNLYNMIKSVASDCSDPTLLGNFIENHDNPRFAKYTSDYSQ
AKNVLSYIFLSDGIPIVYAGEEQHYAGGKVPYNREATWLSGYDTSAELYTWIATTNAIRKLA
IAADSAYIT
>d7taa_2 c.1.8.1 (1-381) Fungal alpha-amylases {Aspergillus oryzae, Taka-amylase}
ATPADWRSQSIYFLLTDRFARTDGSTTATCNTADQKYCGGTWQGIIDKLDYIQGMGFTAIWI
TPVTAQLPQTTAYGDAYHGYWQQDIYSLNENYGTADDLKALSSALHERGMYLMVDVVANHMG
YDGAGSSVDYSVFKPFSSQDYFHPFCFIQNYEDQTQVEDCWLGDNTVSLPDLDTTKDVVKNE
WYDWVGSLVSNYSIDGLRIDTVKHVQKDFWPGYNKAAGVYCIGEVLDGDPAYTCPYQNVMDG
VLNYPIYYPLLNAFKSTSGSMDDLYNMINTVKSDCPDSTLLGTFVENHDNPRFASYTNDIAL
AKNVAAFIILNDGIPIIYAGQEQHYAGGNDPANREATWLSGYPTDSELYKLIASANAIRNYA
ISKDTGFVT
>d1smaa3 c.1.8.1 (A:124-505) Maltogenic amylase, central domain {Thermus sp.}
DLFQAPDWVKDTVWYQIFPERFANGNPAISPKGARPWGSEDPTPTSFFGGDLQGIIDHLDYL
ADLGITGIYLTPIFRAPSNHKYDTADYFEIDPHFGDKETLKTLVKRCHEKGIRVMLDAVFNH
CGYEFAPFQDVLKNGAASRYKDWFHIREFPLQTEPRPNYDTFAFVPHMPKLNTAHPEVKRYL
LDVATYWIREFDIDGWRLDVANEIDHQFWREFRQAVKALKPDVYILGEIWHDAMPWLRGDQF
DAVMNYPLADAALRFFAKEDMSASEFADRLMHVLHSYPKQVNEAAFNLLGSHDTPRLLTVCG
GDVRKVKLLFLFQLTFTGSPCIYYGDEIGMTGGNDPECRKCMVWDPEKQNKELYEHVKQLIA
LRKQYRALRR
>d1bvza3 c.1.8.1 (A:121-502) Maltogenic amylase, central domain {Thermoactinomyces vulgaris, TVAII}
VFTTPEWAKEAVIYQIFPERFANGDPSNDPPGTEQWAKDARPRHDSFYGGDLKGVIDRLPYL
EELGVTALYFTPIFASPSHHKYDTADYLAIDPQFGDLPTFRRLVDEAHRRGIKIILDAVFNH
AGDQFFAFRDVLQKGEQSRYKDWFFIEDFPVSKTSRTNYETFAVQVPAMPKLRTENPEVKEY
LFDVARFWMEQGIDGWRLDVANEVDHAFWREFRRLVKSLNPDALIVGEIWHDASGWLMGDQF
DSVMNYLFRESVIRFFATGEIHAERFDAELTRARMLYPEQAAQGLWNLLDSHDTERFLTSCG
GNEAKFRLAVLFQMTYLGTPLIYYGDEIGMAGATDPDCRRPMIWEEKEQNRGLFEFYKELIR
LRHRLASLTR
>d1eh9a3 c.1.8.1 (A:91-490) Glycosyltrehalose trehalohydrolase, central domain {Archaeon Sulfolobus solfataricus, km1}
FNNETFLKKEDLIIYEIHVGTFTPEGTFEGVIRKLDYLKDLGITAIEIMPIAQFPGKRDWGY
DGVYLYAVQNSYGGPEGFRKLVDEAHKKGLGVILDVVYNHVGPEGNYMVKLGPYFSQKYKTP
WGLTFNFDDAESDEVRKFILENVEYWIKEYNVDGFRLDAVHAIIDTSPKHILEEIADVVHKY
NRIVIAESDLNDPRVVNPKEKCGYNIDAQWVDDFHHSIHAYLTGERQGYYTDFGNLDDIVKS
YKDVFVYDGKYSNFRRKTHGEPVGELDGCNFVVYIQNHDQVGNRGKGERIIKLVDRESYKIA
AALYLLSPYIPMIFMGEEYGEENPFYFFSDFSDSKLIQGVREGRKKENGQDTDPQDESTFNA
SKLSWKIDEEIFSFYKILIKMRKELSIA
>d1bf2_3 c.1.8.1 (163-637) Isoamylase, central domain {Pseudomonas amyloderamosa}

PSTQSTGTKPTRAQKDDVIYEVHVRGFTEQDTSIPAQYRGTYYGAGLKASYLASLGVTAVEF
LPVQETQNDANDVVPNSDANQNYWGYMTENYFSPDRRYAYNKAAGGPTAEFQAMVQAFHNAG
IKVYMDVVYNHTAEGGTWTSSDPTTATIYSWRGLDNATYYELTSGNQYFYDNTGIGANFNTY
NTVAQNLIVDSLAYWANTMGVDGFRFDLASVLGNSCLNGAYTASAPNCPNGGYNFDAADSNV
AINRILREFTVRPAAGGSGLDLFAEPWAIGGNSYQLGGFPQGWSEWNGLFRDSLRQAQNELG
SMTIYVTQDANDFSGSSNLFQSSGRSPWNSINFIDVHDGMTLKDVYSCNGANNSQAWPYGPS
DGGTSTNYSWDQGMSAGTGAAVDQRRAARTGMAFEMLSAGTPLMQGGDEYLRTLQCNNNAYN
LDSSANWLTYSWTTDQSNFYTFAQRLIAFRKAHPALRPSSW
>d1gcya2 c.1.8.1 (A:1-357) G4-amylase (1,4-alpha-D-glucan maltotetrahydrolase) {Pseudomonas stutzeri}
DQAGKSPNAVRYHGGDEIILQGFHWNVVREAPNDWYNILRQQAATIAADGFSAIWMPVPWRD
FSSWSDGSKSGGGEGYFWHDFNKNGRYGSDAQLRQAASALGGAGVKVLYDVVPNHMNRGYPD
KEINLPAGQGFWRNDCADPGNYPNDCDDGDRFIGGDADLNTGHPQVYGMFRDEFTNLRSQYG
AGGFRFDFVRGYAPERVNSWMTDSADNSFCVGELWKGPSEYPNWDWRNTASWQQIIKDWSDR
AKCPVFDFALKERMQNGSIADWKHGLNGNPDPRWREVAVTFVDNHDTGYSPGQNGGQHHWAL
QDGLIRQAYAYILTSPGTPVVYWDHMYDWGYGDFIRQLIQVRRAAGV
>d1avaa2 c.1.8.1 (A:1-346) Plant alpha-amylase {Barley (Hordeum vulgare), seeds, AMY2 isozyme}
QVLFQGFNWESWKHNGGWYNFLMGKVDDIAAAGITHVWLPPASQSVAEQGYMPGRLYDLDAS
KYGNKAQLKSLIGALHGKGVKAIADIVINHRTAEHKDGRGIYCIFEGGTPDARLDWGPHMIC
RDDRPYADGTGNPDTGADFGAAPDIDHLNLRVQKELVEWLNWLKADIGFDGWRFDFAKGYSA
DVAKIYIDRSEPSFAVAEIWTSLAYGGDGKPNLNQDQHRQELVNWVDKVGGKGPATTFDFTT
KGILNVAVEGELWRLRGTDGKAPGMIGWWPAKAVTFVDNHDTGSTQHMWPFPSDRVMQGYAY
ILTHPGTPCIFYDHFFDWGLKEEIDRLVSVRTRHGI
>d1uok_2 c.1.8.1 (1-479) Oligo-1,6, glucosidase {Bacillus cereus}
MEKQWWKESVVYQIYPRSFMDSNGDGIGDLRGIISKLDYLKELGIDVIWLSPVYESPNDDNG
YDISDYCKIMNEFGTMEDWDELLHEMHERNMKLMMDLVVNHTSDEHNWFIESRKSKDNKYRD
YYIWRPGKEGKEPNNWGAAFSGSAWQYDEMTDEYYLHLFSKKQPDLNWDNEKVRQDVYEMMK
FWLEKGIDGFRMDVINFISKEEGLPTVETEEEGYVSGHKHFMNGPNIHKYLHEMNEEVLSHY
DIMTVGEMPGVTTEEAKLYTGEERKELQMVFQFEHMDLDSGEGGKWDVKPCSLLTLKENLTK
WQKALEHTGWNSLYWNNHDQPRVVSRFGNDGMYRIESAKMLATVLHMMKGTPYIYQGEEIGM
TNVRFESIDEYRDIETLNMYKEKVMERGEDIEKVMQSIYIKGRDNARTPMQWDDQNHAGFTT
GEPWITVNPNYKEINVKQAIQNKDSIFYYYKKLIELRKNNEIVVY
>d1g5aa2 c.1.8.1 (A:1-554) Amylosucrase {Neisseria polysaccharea}
SPNSQYLKTRILDIYTPEQRAGIEKSEDWRQFSRRMDTHFPKLMNELDSVYGNNEALLPMLE
MLLAQAWQSYSQRNSSLKDIDIARENNPDWILSNKQVGGVCYVDLFAGDLKGLKDKIPYFQE
LGLTYLHLMPLFKCPEGKSDGGYAVSSYRDVNPALGTIGDLREVIAALHEAGISAVVDFIFN
HTSNEHEWAQRCAAGDPLFDNFYYIFPDRRMPDQYDRTLREIFPDQHPGGFSQLEDGRWVWT
TFNSFQWDLNYSNPWVFRAMAGEMLFLANLGVDILRMDAVAFIWKQMGTSCENLPQAHALIR
AFNAVMRIAAPAVFFKSEAIVHPDQVVQYIGQDECQIGYNPLQMALLWNTLATREVNLLHQA
LTYRHNLPEHTAWVNYVRSHDDIGWTFADEDAAYLGISGYDHRQFLNRFFVNRFDGSFARGV
PFQYNPSTGDCRVSGTAAALVGLAQDDPHAVDRIKLLYSIALSTGGLPLIYLGDEVGTLNDD
DWSQDSNKSDDSRWAHRPRYNEALYAQRNDPSTAAGQIYQDLRHMIAVRQSNPRFDGG
>d1eswa_ c.1.8.1 (A:) Amylomaltase MalQ {Thermus aquaticus}

MELPRAFGLLLHPTSLPGPYGVGVLGREARDFLRFLKEAGGRYWQVLPLGPTGYGDSPYQSF
SAFAGNPYLIDLRPLAERGYVRLEDPGFPQGRVDYGLLYAWKWPALKEAFRGFKEKASPEER
EAFAAFREREAWWLEDYALFMALKGAHGGLPWNRWPLPLRKREEKALREAKSALAEEVAFHA
FTQWLFFRQWGALKAEAEALGIRIIGDMPIFVAEDSAEVWAHPEWFHLDEEGRPTVVAGVPP
DYFSETGQRWGNPLYRWDVLEREGFSFWIRRLEKALELFHLVRIDHFRGFEAYWEIPASCPT
AVEGRWVKAPGEKLFQKIQEVFGEVPVLAEDLGVITPEVEALRDRFGLPGMKVLQFAFDDGM
ENPFLPHNYPAHGRVVVYTGTHDNDTTLGWYRTATPHEKAFMARYLADWGITFREEEEVPWA
LMHLGMKSVARLAVYPVQDVLALGSEARMNYPGRPSGNWAWRLLPGELSPEHGARLRAMAEA
TERL
>d1byb__ c.1.8.2 (-) beta-Amylase {Soybean (Glycine max)}
SNMLLNYVPVYVMLPLGVVNVDNVFEDPDGLKEQLLQLRAAGVDGVMVDVWWGIIELKGPKQ
YDWRAYRSLFQLVQECGLTLQAIMSFHQCGGNVGDIVNIPIPQWVLDIGESNHDIFYTNRSG
TRNKEYLTVGVDNEPIFHGRTAIEIYSDYMKSFRENMSDFLESGLIIDIEVGLGPAGELRYP
SYPQSQGWEFPRIGEFQCYDKYLKADFKAAVARAGHPEWELPDDAGKYNDVPESTGFFKSNG
TYVTEKGKFFLTWYSNKLLNHGDQILDEANKAFLGCKVKLAIKVSGIHWWYKVENHAAELTA
GYYNLNDRDGYRPIARMLSRHHAILNFTCLEMRDSEQPSDAKSGPQELVQQVLSGGWREDIR
VAGENALPRYDATAYNQIILNAKPQGVNNNGPPKLSMFGVTYLRLSDDLLQKSNFNIFKKFV
LKMHADQDYCANPQKYNHAITPLKPSAPKIPIEVLLEATKPTLPFPWLPETDMKVDG
>d1b1ya_ c.1.8.2 (A:) beta-Amylase {Barley (Hordeum vulgare)}
MKGNYVQVYVMLPLDAVSVNNRFEKGDELRAQLRKLVEAGVDGVMVDVWWGLVEGKGPKAYD
WSAYKQLFELVQKAGLKLQAIMSFHQCGGNVGDAVNIPIPQWVRDVGTRDPDIFYTDGHGTR
NIEYLTLGVDNQPLFHGRSAVQMYADYMTSFRENMKDFLDAGVIVDIEVGLGPAGELRYPSY
PQSHGWSFPGIGEFICYDKYLQADFKAAAAAVGHPEWEFPNDAGQYNDTPERTQFFRDNGTY
LSEKGRFFLAWYSNNLIKHGDRILDEANKVFLGYKVQLAIKIAGVHWWYKVPSHAAELTAGY
YNLHDRDGYRTIARMLKRHRASINFTCAEMRDSEQPPDAMSAPEELVQQVLSAGWREGLNVS
CENALPRYDPTAYNTILRNARPHGINQSGPPEHKLFGFTYLRLSNQLVEGQNYVNFKTFVDR
MHANLPRDPYVDPMAPLPRSGPEISIEMILQAAQPKIQPFPFQEHTDLPVGPTGGMGGQAEG
PTCG
>d1fa2a_ c.1.8.2 (A:) beta-Amylase {Sweet potato (Ipomoea batatas)}
APIPGVMPIGNYVSLYVMLPLGVVNADNVFPDKEKVEDELKQVKAGGCDGVMVDVWWGIIEA
KGPKQYDWSAYRELFQLVKKCGLKIQAIMSFHQCGGNVGDAVFIPIPQWILQIGDKNPDIFY
TNRAGNRNQEYLSLGVDNQRLFQGRTALEMYRDFMESFRDNMADFLKAGDIVDIEVGCGAAG
ELRYPSYPETQGWVFPGIGEFQCYDKYMVADWKEAVKQAGNADWEMPGKGAGTYNDTPDKTE
FFRPNGTYKTDMGKFFLTWYSNKLIIHGDQVLEEANKVFVGLRVNIAAKVSGIHWWYNHVSH
AAELTAGFYNVAGRDGYRPIARMLARHHATLNFTCLEMRDSEQPAEAKSAPQELVQQVLSSG
WKEYIDVAGENALPRYDATAYNQMLLKLRPNGVNLNGPPKLKMSGLTYLRLSDDLLQTDNFE
LFKKFVKKMHADLDPSPNAISPAVLERSNSAITIDELMEATKGSRPFPWYDVTDMPVDGSNP
FD
>d1b9za2 c.1.8.2 (A:1-417) Bacterial beta-amylase, catalytic domain {Bacillus cereus}
AVNGKGMNPDYKAYLMAPLKKIPEVTNWETFENDLRWAKQNGFYAITVDFWWGDMEKNGDQQ
FDFSYAQRFAQSVKNAGMKMIPIISTHQCGGNVGDDCNVPIPSWVWNQKSDDSLYFKSETGT
VNKETLNPLASDVIRKEYGELYTAFAAAMKPYKDVIAKIYLSGGPAGELRYPSYTTSDGTGY

PSRGKFQAYTEFAKSKFRLWVLNKYGSLNEVNKAWGTKLISELAILPPSDGEQFLMNGYLSM
YGKDYLEWYQGILENHTKLIGELAHNAFDTTFQVPIGAKIAGVHWQYNNPTIPHGAEKPAGY
NDYSHLLDAFKSAKLDVTFTCLEMTDKGSYPEYSMPKTLVQNIATLANEKGIVLNGENALSI
GNEEEYKRVAEMAFNYNFAGFTLLRYQDVMYNNSLMGKFKDLLGV
>d1xyza_ c.1.8.3 (A:) Xylanase {Clostridium thermocellum, XynZ}
NALRDYAEARGIKIGTCVNYPFYNNSDPTYNSILQREFSMVVCENEMKFDALQPRQNVFDFS
KGDQLLAFAERNGMQMRGHTLIWHNQNPSWLTNGNWNRDSLLAVMKNHITTVMTHYKGKIVE
WDVANECMDDSGNGLRSSIWRNVIGQDYLDYAFRYAREADPDALLFYNDYNIEDLGPKSNAV
FNMIKSMKERGVPIDGVGFQCHFINGMSPEYLASIDQNIKRYAEIGVIVSFTEIDIRIPQSE
NPATAFQVQANNYKELMKICLANPNCNTFVMWGFTDKYTWIPGTFPGYGNPLIYDSNYNPKP
AYNAIKEALM
>d1hiza_ c.1.8.3 (A:) Xylanase {Bacillus stearothermophilus, Xt6}
SYAKKPHISALNAPQLDQRYKNEFTIGAAVEPYQLQNEKDVQMLKRHFNSIVAENVMKPISI
QPEEGKFNFEQADRIVKFAKANGMDIRFHTLVWHSQVPQWFFLDKEGKPMVNETDPVKREQN
KQLLLKRLETHIKTIVERYKDDIKYWDVVNEVVGDDGKLRNSPWYQIAGIDYIKVAFQAARK
YGGDNIKLYMNDYNTEVEPKRTALYNLVKQLKEEGVPIDGIGHQSHIQIGWPSEAEIEKTIN
MFAALGLDNQITELDVSMYGWPPRAYPTYDAIPKQKFLDQAARYDRLFKLYEKLSDKISNVT
FWGIADNHTWLDSRADVYYDANGNVVVDPNAPYAKVEKGKGKDAPFVFGPDYKVKPAYWAII
DHK
>d1bg4__ c.1.8.3 (-) Xylanase {Penicillium simplicissimum}
EASVSIDAKFKAHGKKYLGTIGDQYTLTKNTKNPAIIKADFGQLTPENSMKWDATEPNRGQF
TFSGSDYLVNFAQSNGKLIRGHTLVWHSQLPGWVSSITDKNTLISVLKNHITTVMTRYKGKI
YAWDVLNEIFNEDGSLRNSVFYNVIGEDYVRIAFETARSVDPNAKLYINDYNLDSAGYSKVN
GMVSHVKKWLAAGIPIDGIGSQTHLGAGAGSAVAGALNALASAGTKEIAITELDIAGASSTD
YVNVVNACLNQAKCVGITVWGVADPDSWRSSSSPLLFDGNYNPKAAYNAIANAL
>d1edg__ c.1.8.3 (-) Endoglucanase CelA {Clostridium
cellulolyticum}
MYDASLIPNLQIPQKNIPNNDGMNFVKGLRLGWNLGNTFDAFNGTNITNELDYETSWSGIKT
TKQMIDAIKQKGFNTVRIPVSWHPHVSGSDYKISDVWMNRVQEVVNYCIDNKMYVILNTHHD
VDKVKGYFPSSQYMASSKKYITSVWAQIAARFANYDEHLIFEGMNEPRLVGHANEWWPELTN
SDVVDSINCINQLNQDFVNTVRATGGKNASRYLMCPGYVASPDGATNDYFRMPNDISGNNNK
IIVSVHAYCPWNFAGLAMADGGTNAWNINDSKDQSEVTWFMDNIYNKYTSRGIPVIIGECGA
VDKNNLKTRVEYMSYYVAQAKARGILCILWDNNNFSGTGELFGFFDRRSCQFKFPEIIDGMV
KYAFGLIN
>d1ceo__ c.1.8.3 (-) Endoglucanase CelC {Clostridium thermocellum}
MVSFKAGINLGGWISQYQVFSKEHFDTFITEKDIETIAEAGFDHVRLPFDYPIIESDDNVGE
YKEDGLSYIDRCLEWCKKYNLGLVLDMHHAPGYRFQDFKTSTLFEDPNQQKRFVDIWRFLAK
RYINEREHIAFELLNQVVEPDSTRWNKLMLECIKAIREIDSTMWLYIGGNNYNSPDELKNLA
DIDDDYIVYNFHFYNPFFFTHQKAHWSESAMAYNRTVKYPGQYEGIEEFVKNNPKYSFMMEL
NNLKLNKELLRKDLKPAIEFREKKKCKLYCGEFGVIAIADLESRIKWHEDYISLLEEYDIGG
AVWNYKKMDFEIYNEDRKPVSQELVNILAR
>d1cz1a_ c.1.8.3 (A:) Exo-beta-(1,3)-glucanase {Yeast (Candida
albicans)}
AWDYDNNVIRGVNLGGWFVLEPYMTPSLFEPFQNGNDQSGVPVDEYHWTQTLGKEAALRILQ

KHWSTWITEQDFKQISNLGLNFVRIPIGYWAFQLLDNDPYVQGQVQYLEKALGWARKNNIRV
WIDLHGAPGSQNGFDNSGLRDSYNFQNGDNTQVTLNVLNTIFKKYGGNEYSDVVIGIELLNE
PLGPVLNMDKLKQFFLDGYNSLRQTGSVTPVIIHDAFQVFGYWNNFLTVAEGQWNVVVDHHH
YQVFSGGELSRNINDHISVACNWGWDAKKESHWNVAGEWSAALTDCAKWLNGVNRGARYEGA
YDNAPYIGSCQPLLDISQWSDEHKTDTRRYIEAQLDAFEYTGGWVFWSWKTENAPEWSFQTL
TYNGLFPQPVTDRQFPNQCGFH
>d1ecea_ c.1.8.3 (A:) Endocellulase E1 {Acidothermus cellulolyticus}
AGGGYWHTSGREILDANNVPVRIAGINWFGFETCNYVVHGLWSRDYRSMLDQIKSLGYNTIR
LPYSDDILKPGTMPNSINFYQMNQDLQGLTSLQVMDKIVAYAGQIGLRIILDRHRPDCSGQS
ALWYTSSVSEATWISDLQALAQRYKGNPTVVGFDLHNEPHDPACWGCGDPSIDWRLAAERAG
NAVLSVNPNLLIFVEGVQSYNGDSYWWGGNLQGAGQYPVVLNVPNRLVYSAHDYATSVYPQT
WFSDPTFPNNMPGIWNKNWGYLFNQNIAPVWLGEFGTTLQSTTDQTWLKTLVQYLRPTAQYG
ADSFQWTFWSWNPDSGDTGGILKDDWQTVDTVKDGYLAPIKSSIFDPV
>d7a3ha_ c.1.8.3 (A:) Endoglucanase Cel5a {Bacillus agaradhaerens}
SVVEEHGQLSISNGELVNERGEQVQLKGMSSHGLQWYGQFVNYESMKWLRDDWGINVFRAAM
YTSSGGYIDDPSVKEKVKEAVEAAIDLDIYVIIDWHILSDNDPNIYKEEAKDFFDEMSELYG
DYPNVIYEIANEPNGSDVTWGNQIKPYAEEVIPIIRNNDPNNIIIVGTGTWSQDVHHAADNQ
LADPNVMYAFHFYAGTHGQNLRDQVDYALDQGAAIFVSEWGTSAATGDGGVFLDEAQVWIDF
MDERNLSWANWSLTHKDESSAALMPGANPTGGWTEAELSPSGTFVREKIRES
>d1egza_ c.1.8.3 (A:) Endoglucanase Cel5a {Erwinia chrysanthemi}
SVEPLSVNGNKIYAGEKAKSFAGNSLFWSNNGWGGEKFYTADTVASLKKDWKSSIVRAAMGV
QESGGYLQDPAGNKAKVERVVDAAIANDMYAIIGWHSHSAENNRSEAIRFFQEMARKYGNKP
NVIYEIYNEPLQVSWSNTIKPYAEAVISAIRAIDPDNLIIVGTPSWSQNVDEASRDPINAKN
IAYTLHFYAGTHGESLRNKARQALNNGIALFVTEWGTVNADGNGGVNQTETDAWVTFMRDNN
ISNANWALNDKNEGASTYYPDSKNLTESGKKVKSIIQSWPYKA
>d1g0ca_ c.1.8.3 (A:) Alkaline cellulase K catalytic domain {Bacillus sp.}
PAGMQAVKSPSEAGALQLVELNGQLTLAGEDGTPVQLRGMSTHGLQWFGEIVNENAFVALSN
DWGSNMIRLAMYIGENGYATNPEVKDLVYEGIELAFEHDMYVIVDWHVHAPGDPRADVYSGA
YDFFEEIADHYKDHPKNHYIIWELANEPSPNNNGGPGLTNDEKGWEAVKEYAEPIVEMLREK
GDNMILVGNPNWSQRPDLSADNPIDAENIMYSVHFYTGSHGASHIGYPEGTPSSERSNVMAN
VRYALDNGVAVFATEWGTSQANGDGGPYFDEADVWLNFLNKHNISWANWSLTNKNEISGAFT
PFELGRTDATDLDPGANQVWAPEELSLSGEYVRARIKGIEYTPIDRTK
>d1bqca_ c.1.8.3 (A:) Beta-mannanase {Thermomonospora fusca}
ATGLHVKNGRLYEANGQEFIIRGVSHPHNWYPQHTQAFADIKSHGANTVRVVLSNGVRWSKN
GPSDVANVISLCKQNRLICMLEVHDTTGYGEQSGASTLDQAVDYWIELKSVLQGEEDYVLIN
IGNEPYGNDSATVAAWATDTSAAIQRLRAAGFEHTLVVDAPNWGQDWTNTMRNNADQVYASD
PTGNTVFSIHMYGVYSQASTITSYLEHFVNAGLPLIIGEFGHDHSDGNPDEDTIMAEAERLK
LGYIGWSWSGNGGGVEYLDMVYNFDGDNLSPWGERIFYGPNGIASTAKEAVIFG
>d1qnra_ c.1.8.3 (A:) Beta-mannanase {Trichoderma reesei}
ASSFVTISGTQFNIDGKVGYFAGTNCYWCSFLTNHADVDSTFSHISSSGLKVVRVWGFNDVN
TQPSPGQIWFQKLSATGSTINTGADGLQTLDYVVQSAEQHNLKLIIPFVNNWSDYGGINAYV
NAFGGNATTWYTNTAAQTQYRKYVQAVVSRYANSTAIFAWELGNEPRCNGCSTDVIVQWATS

VSQYVKSLDSNHLVTLGDEGLGLSTGDGAYPYTYGEGTDFAKNVQIKSLDFGTFHLYPDSWG
TNYTWGNGWIQTHAAACLAAGKPCVFEEYGAQQNPCTNEAPWQTTSLTTRGMGGDMFWQWGD
TFANGAQSNSDPYTVWYNSSNWQCLVKNHVDAIN
>d1j9ya_ c.1.8.3 (A:) Mannanase 26A {Pseudomonas fluorescens, subsp. cellulosa}
PVTVKLVDSQATMETRSLFAFMQEQRRHSIMFGHQHETTQGLTITRTDGTQSDTFNAVGDFA
AVYGWDTLSIVAPKAEGDIVAQVKKAYARGGIITVSSHFDNPKTDTQKGVWPVGTSWDQTPA
VVDSLPGGAYNPVLNGYLDQVAEWANNLKDEQGRLIPVIFRLYHENTGSWFWWGDKQSTPEQ
YKQLFRYSVEYLRDVKGVRNFLYAYSPNNFWDVTEANYLERYPGDEWVDVLGFDTYGPVADN
ADWFRNVVANAALVARMAEARGKIPVISEIGIRAPDIEAGLYDNQWYRKLISGLKADPDARE
IAFLLVWRNAPQGVPGPNGTQVPHYWVPANRPENINNGTLEDFQAFYADEFTAFNRDIEQVY
QRPT
>d1ghsa_ c.1.8.3 (A:) Plant beta-glucanases {Barley (Hordeum vulgare), 1,3-beta-glucanase}
IGVCYGVIGNNLPSRSDVVQLYRSKGINGMRIYFADGQALSALRNSGIGLILDIGNDQLANI
AASTSNAASWVQNNVRPYYPAVNIKYIAAGNEVQGGATQSILPAMRNLNAALSAAGLGAIKV
STSIRFDEVANSFPPSAGVFKNAYMTDVARLLASTGAPLLANVYPYFAYRDNPGSISLNYAT
FQPGTTVRDQNNGLTYTSLFDAMVDAVYAALEKAGAPAVKVVVSESGWPSAGGFAASAGNAR
TYNQGLINHVGGGTPKKREALETYIFAMFNENQKTGDATERSFGLFNPDKSPAYNIQF
>d1aq0a_ c.1.8.3 (A:) Plant beta-glucanases {Barley (Hordeum vulgare), 1,3-1,4-beta-glucanase}
IGVCYGMSANNLPAASTVVSMFKSNGIKSMRLYAPNQAALQAVGGTGINVVVGAPNDVLSNL
AASPAAAASWVKSNIQAYPKVSFRYVCVGNEVAGGATRNLVPAMKNVHGALVAAGLGHIKVT
TSVSQAILGVFSPPSAGSFTGEAAAFMGPVVQFLARTNAPLMANIYPYLAWAYNPSAMDMGY
ALFNASGTVVRDGAYGYQNLFDTTVDAFYTAMGKHGGSSVKLVVSESGWPSGGGTAATPANA
RFYNQHLINHVGRGTPRHPGAIETYIFAMFNENQKDSGVEQNWGLFYPNMQHVYPINF
>d1jz8a5 c.1.8.3 (A:334-625) beta-Galactosidase, domain 3 {Escherichia coli}
EVRIENGLLLLNGKPLLIRGVNRHEHHPLHGQVMDEQTMVQDILLMKQNNFNAVRCSHYPNH
PLWYTLCDRYGLYVVDEANIETHGMVPMNRLTDDPRWLPAMSERVTRMVQRDRNHPSVIIWS
LGNESGHGANHDALYRWIKSVDPSRPVQYEGGGADTTATDIICPMYARVDEDQPFPAVPKWS
IKKWLSLPGETRPLILCQYAHAMGNSLGGFAKYWQAFRQYPRLQGGFVWDWVDQSLIKYDEN
GNPWSAYGGDFGDTPNDRQFCMNGLVFADRTPHPALTEAKHQQQ
>d1bhga3 c.1.8.3 (A:329-632) beta-Glucuronidase, domain 3 {Human (Homo sapiens)}
VAVTKSQFLINGKPFYFHGVNKHEDADIRGKGFDWPLLVKDFNLLRWLGANAFRTSHYPYAE
EVMQMCDRYGIVVIDECPGVGLALPQFFNNVSLHHHMQVMEEVVRRDKNHPAVVMWSVANEP
ASHLESAGYYLKMVIAHTKSLDPSRPVTFVSNSNYAADKGAPYVDVICLNSYYSWYHDYGHL
ELIQLQLATQFENWYKKYQKPIIQSEYGAETIAGFHQDPPLMFTEEYQKSLLEQYHLGLDQK
RRKYVVGELIWNFADFMTEQSPTRVLGNKKGIFTRQRQPKSAAFLLRERYWKIANE
>d1e0wa_ c.1.8.3 (A:) Xylanase A, catalytic core {Streptomyces lividans}
AESTLGAAAAQSGRYFGTAIASGRLSDSTYTSIAGREFNMVTAENEMKIDATEPQRGQFNFS
SADRVYNWAVQNGKQVRGHTLAWHSQQPGWMQSLSGSALRQAMIDHINGVMAHYKGKIVQWD

VVNEAFADGSSGARRDSNLQRSGNDWIEVAFRTARAADPSAKLCYNDYNVENWTWAKTQAMY
NMVRDFKQRGVPIDCVGFQSHFNSGSPYNSNFRTTLQNFAALGVDVAITELDIQGAPASTYA
NVTNDCLAVSRCLGITVWGVRDSDSWRSEQTPLLFNNDGSKKAAYTAVLDALNG
>d1clxa_ c.1.8.3 (A:) Xylanase A, catalytic core {Pseudomonas fluorescens}
GLASLADFPIGVAVAASGGNADIFTSSARQNIVRAEFNQITAENIMKMSYMYSGSNFSFTNS
DRLVSWAAQNGQTVHGHALVWHPSYQLPNWASDSNANFRQDFARHIDTVAAHFAGQVKSWDV
VNEALFDSADDPDGRGSANGYRQSVFYRQFGGPEYIDEAFRRARAADPTAELYYNDFNTEEN
GAKTTALVNLVQRLLNNGVPIDGVGFQMHVMNDYPSIANIRQAMQKIVALSPTLKIKITELD
VRLNNPYDGNSSNDYTNRNDCAVSCAGLDRQKARYKEIVQAYLEVVPPGRRGGITVWGIADP
DSWLYTHQNLPDWPLLFNDNLQPKPAYQGVVEALS
>d1fxma_ c.1.8.3 (A:) Xylanase A, catalytic core {Thermoascus aurantiacus}
QAAQSVDQLIKARGKVYFGVATDQNRLTTGKNAAIIQADFGQVTPENSMKWDATEPSQGNFN
FAGADYLVNWAQQNGKLIRGHTLVWHSQLPSWVSSITDKNTLTNVMKNHITTLMTRYKGKIR
AWDVVNEAFNEDGSLRQTVFLNVIGEDYIPIAFQTARAADPNAKLYINDYNLDSASYPKTQA
IVNRVKQWRAAGVPIDGIGSQTHLSAGQGAGVLQALPLLASAGTPEVAITELDVAGASPTDY
VNVVNACLNVQSCVGITVWGVADPDSWRASTTPLLFDGNFNPKPAYNAIVQDLQ
>d1tux__ c.1.8.3 (-) Xylanase A, catalytic core {Thermoascus aurantiacus}
AAAQSVDQLIDARGKVYFGVATDQNRLTTGKNAAIIQADFGQVTPENSMKWDATEPSQGNFN
FAGADYLVNWAQQNGKLIRGHTLVWHSQLPSWVVSITDKNTLTNVMKNHITTIMTRYIGKIR
AWDVVNEAFNEDGSLRQTVFNNVIGEDYIPIAFRTARAADPNAKLYINDYNLDSASKPKTSA
IVKRVKKWRAAGVPIDGIGSQTHLSAGQGASIDAALPNLASAGTPEVAITELDIAGATSTDY
VDVVNACLDVDSCIGITVWGVADPDSWRASTTPLLFDGNFNPKPAYNAIVQLL
>d1xyfa2 c.1.8.3 (A:1-303) Xylanase A, catalytic core {Streptomyces olivaceoviridis}
AESTLGAAAAQSGRYFGTAIASGKLGDSAYTTIASREFNMVTAENEMKIDATEPQRGQFNFS
AGDRVYNWAVQNGKQVRGHTLAWHSQQPGWMQSLSGSTLRQAMIDHINGVMGHYKGKIAQWD
VVNEAFSDDGSGGRRDSNLQRTGNDWIEVAFRTARAADPAAKLCYNDYNIENWTWAKTQGVY
NMVRDFKQRGVPIDCVGFQSHFNSGSPYNSNFRTTLQNFAALGVDVAITELDIQGASSSTYA
AVTNDCLAVSRCLGITVWGVRDTDSWRSGDTPLLFNGDGSKKAAYTAVLNALNGG
>d1fh9a_ c.1.8.3 (A:) Xylanase A, catalytic core {Cellulomonas fimi}
ATTLKEAADGAGRDFGFALDPNRLSEAQYKAIADSEFNLVVAENAMKWDATEPSQNSFSFGA
GDRVASYAADTGKELYGHTLVWHSQLPDWAKNLNGSAFESAMVNHVTKVADHFEGKVASWDV
VNEAFADGGGRRQDSAFQQKLGNGYIETAFRAARAADPTAKLCINDYNVEGINAKSNSLYDL
VKDFKARGVPLDCVGFQSHLIVGQVPGDFRQNLQRFADLGVDVRITELDIRMRTPSDATKLA
TQAADYKKVVQACMQVTRCQGVTVWGITDKYSWVPDVFPGEGAALVWDASYAKKPAYAAVME
AF
>d1e4mm_ c.1.8.4 (M:) Plant beta-glucosidase (myrosinase) {White mustard (Sinapis alba)}
EITCQENLPFTCGNTDALNSSSFSSDFIFGVASSAYQIEGTIGRGLNIWDGFTHRYPNKSGP
DHGNGDTTCDSFSYWQKDIDVLDELNATGYRFSIAWSRIIPRGKRSRGVNEKGIDYYHGLIS

GLIKKGITPFVTLFHWDLPQTLQDEYEGFLDPQIIDDFKDYADLCFEEFGDSVKYWLTINQL
YSVPTRGYGSALDAPGRCSPTVDPSCYAGNSSTEPYIVAHHQLLAHAKVVDLYRKNYTHQGG
KIGPTMITRWFLPYNDTDRHSIAATERMKEFFLGWFMGPLTNGTYPQIMIDTVGERLPSFSP
EESNLVKGSYDFLGLNYYFTQYAQPSPNPVNSTNHTAMMDAGAKLTYINASGHYIGPLFEKD
KADSTDNIYYYPKGIYSVMDYFKNKYYNPLIYVTENGISTPGDENRNQSMLDYTRIDYLCSH
LCFLNKVIKEKDVNVKGYLAWALGDNYEFNKGFTVRFGLSYIDWNNVTDRDLKKSGQWYQSF
ISP
>d1cbg__ c.1.8.4 (-) Plant beta-glucosidase (myrosinase) {Creeping white clover (Trifolium repens)}
FKPLPISFDDFSDLNRSCFAPGFVFGTASSAFQYEGAAFEDGKGPSIWDTFTHKYPEKIKDR
TNGDVAIDEYHRYKEDIGIMKDMNLDAYRFSISWPRVLPKGKLSGGVNREGINYYNNLINEV
LANGMQPYVTLFHWDVPQALEDEYRGFLGRNIVDDFRDYAELCFKEFGDRVKHWITLNEPWG
VSMNAYAYGTFAPGRCSDWLKLNCTGGDSGREPYLAAHYQLLAHAAAARLYKTKYQASQNGI
IGITLVSHWFEPASKEKADVDAAKRGLDFMLGWFMHPLTKGRYPESMRYLVRKRLPKFSTEE
SKELTGSFDFLGLNYYSSYYAAKAPRIPNARPAIQTDSLINATFEHNGKPLGPMAASSWLCI
YPQGIRKLLLYVKNHYNNPVIYITENGRNEFNDPTLSLQESLLDTPRIDYYYRHLYYVLTAI
GDGVNVKGYFAWSLFDNMEWDSGYTVRFGLVFVDFKNNLKRHPKLSAHWFKSFLKK
>d1e55a_ c.1.8.4 (A:) Plant beta-glucosidase (myrosinase) {Maize (Zea mays), zmglu1}
VQMLSPSEIPQRDWFPSDFTFGAATSAYQIEGAWNEDGKGESNWDHFCHNHPERILDGSNSD
IGANSYHMYKTDVRLLKEMGMDAYRFSISWPRILPKGTKEGGINPDGIKYYRNLINLLLENG
IEPYVTIFHWDVPQALEEKYGGFLDKSHKSIVEDYTYFAKVCFDNFGDKVKNWLTFNDPQTF
TSFSYGTGVFAPGRCSPGLDCAYPTGNSLVEPYTAGHNILLAHAEAVDLYNKHYKRDDTRIG
LAFDVMGRVPYGTSFLDKQAEERSWDINLGWFLEPVVRGDYPFSMRSLARERLPFFKDEQKE
KLAGSYNMLGLNYYTSRFSKNIDISPNYSPVLNTDDAYASQEVNGPDGKPIGPPMGNPWIYM
YPEGLKDLLMIMKNKYGNPPIYITENGIGDVDTKETPLPMEAALNDYKRLDYIQRHIATLKE
SIDLGSNVQGYFAWSLLDNFEWFAGFTERYGIVYVDRNNNCTRYMKESAKWLKEFNTA
>d1pbga_ c.1.8.4 (A:) 6-phospho-beta-D-galactosidase, PGAL {Lactococcus lactis}
MTKTLPKDFIFGGATAAYQAEGATHTDGKGPVAWDKYLEDNYWYTAEPASDFYHKYPVDLEL
AEEYGVNGIRISIAWSRIFPTGYGEVNEKGVEFYHKLFAECHKRHVEPFVTLHHFDTPEALH
SNGDFLNRENIEHFIDYAAFCFEEFPEVNYWTTFNEIGPIGDGQYLVGKFPPGIKYDLAKVF
QSHHNMMVSHARAVKLYKDKGYKGEIGVVHALPTKYPYDPENPADVRAAELEDIIHNKFILD
ATYLGHYSDKTMEGVNHILAENGGELDLRDEDFQALDAAKDLNDFLGINYYMSDWMQAFDGE
TEIIHNGKGEKGSSKYQIKGVGRRVAPDYVPRTDWDWIIYPEGLYDQIMRVKNDYPNYKKIY
ITENGLGYKDEFVDNTVYDDGRIDYVKQHLEVLSDAIADGANVKGYFIWSLMDVFSWSNGYE
KRYGLFYVDFDTQERYPKKSAHWYKKLAETQVIE
>d1e4ia_ c.1.8.4 (A:) Beta-glucosidase A {Bacillus polymyxa}
TIFQFPQDFMWGTATAAYQIEGAYQEDGRGLSIWDTFAHTPGKVFNGDNGNVACDSYHRYEE
DIRLMKELGIRTYRFSVSWPRIFPNGDGEVNQKGLDYYHRVVDLLNDNGIEPFCTLYHWDLP
QALQDAGGWGNRRTIQAFVQFAETMFREFHGKIQHWLTFNEPWCIAFLSNMLGVHAPGLTNL
QTAIDVGHHLLVAHGLSVRRFRELGTSGQIGIAPNVSWAVPYSTSEEDKAACARTISLHSDW
FLQPIYQGSYPQFLVDWFAEQGATVPIQDGDMDIIGEPIDMIGINYYSMSVNRFNPEAGFLQ
SEEINMGLPVTDIGWPVESRGLYEVLHYLQKYGNIDIYITENGACINDEVVNGKVQDDRRIS

YMQQHLVQVHRTIHDGLHVKGYMAWSLLDNFEWAEGYNMRFGMIHVDFRTQVRTPKQSYYWY
RNVVSNNWLETRR
>d1qoxa_ c.1.8.4 (A:) Beta-glucosidase A {Bacillus circulans, subsp. alkalophilus}
SIHMFPSDFKWGVATAAYQIEGAYNEDGRGMSIWDTFAHTPGKVKNGDNGNVACDSYHRVEE
DVQLLKDLGVKVYRFSISWPRVLPQGTGEVNRAGLDYYHRLVDELLANGIEPFCTLYHWDLP
QALQDQGGWGSRITIDAFAEYAELMFKELGGKIKQWITFNEPWCMAFLSNYLGVHAPGNKDL
QLAIDVSHHLLVAHGRAVTLFRELGISGEIGIAPNTSWAVPYRRTKEDMEACLRVNGWSGDW
YLDPIYFGEYPKFMLDWYENLGYKPPIVDGDMELIHQPIDFIGINYYTSSMNRYNPGEAGGM
LSSEAISMGAPKTDIGWEIYAEGLYDLLRYTADKYGNPTLYITENGACYNDGLSLDGRIHDQ
RRIDYLAMHLIQASRAIEDGINLKGYMEWSLMDNFEWAEGYGMRFGLVHVDYDTLVRTPKDS
FYWYKGVISRGWLDL
>d1gowa_ c.1.8.4 (A:) beta-Glycosidase {Archaeon Sulfolobus solfataricus}
MYSFPNSFRFGWSQAGFQSEMGTPGSEDPNTDWYKWVHDPENMAAGLVSGDLPENGPGYWGN
YKTFHDNAQKMGLKIARLNSEWSRQFPNPLPRPQNFDESKQDVTEVEINENELKRLDEYANK
DALNHYREIFKDLKSRGLYFIQNMYHWPLPLWLHDPIRVRRGDFTGPSGWLSTRTVYEFARF
SAYTAWKFDDLVDEYSTMNEPNVVGGLGYVGVKSGFPPGYLSFELSRRAMYNIIQAHARAYD
GIKSVSKKPVGIIYANSSFQPLTDKDMEAVEMAENDNRWWFFDAIIRGEITRGNEKIVRDDL
KGRLDWIGVNYYTRTVVKRTEKGYVSLGGYGHGCERNSVSLAGLPTSDFGWEFFPEGLYDVL
TKYWNRYHLYMYVTENGIADDADYQRPYYLVSHVYQVHRAINSGADVRGYLHWSLADNYEWA
SGFSMRFGLLKVDYNTKRLYWRPSALVYREIATNGAITDEIEHLNSVPPVKPLRH
>d1qvba_ c.1.8.4 (A:) beta-Glycosidase {Archaeon Thermosphaera aggregans}
MKFPKDFMIGYSSSPFQFEAGIPGSEDPNSDWWVWVHDPENTAAGLVSGDFPENGPGYWNLN
QNDHDLAEKLGVNTIRVGVEWSRIFPKPTFNVKVPVERDENGSIVHVDVDDKAVERLDELAN
KEAVNHYVEMYKDWVERGRKLILNLYHWPLPLWLHNPIMVRRMGPDRAPSGWLNEESVVEFA
KYAAYIAWKMGELPVMWSTMNEPNVVYEQGYMFVKGGFPPGYLSLEAADKARRNMIQAHARA
YDNIKRFSKKPVGLIYAFQWFELLEGPAEVFDKFKSSKLYYFTDIVSKGSSIINVEYRRDLA
NRLDWLGVNYYSRLVYKIVDDKPIILHGYGFLCTPGGISPAENPCSDFGWEVYPEGLYLLLK
ELYNRYGVDLIVTENGVSDSRDALRPAYLVSHVYSVWKAANEGIPVKGYLHWSLTDNYEWAQ
GFRQKFGLVMVDFKTKKRYLRPSALVFREIATHNGIPDELQHLTLIQ
>d2hvm__ c.1.8.5 (-) Hevamine A (chitinase/lysozyme) {Para rubber tree (Hevea brasiliensis)}
GGIAIYWGQNGNEGTLTQTCSTRKYSYVNIAFLNKFGNGQTPQINLAGHCNPAAGGCTIVSN
GIRSCQIQGIKVMLSLGGGIGSYTLASQADAKNVADYLWNNFLGGKSSSRPLGDAVLDGIDF
DIEHGSTLYWDDLARYLSAYSKQGKKVYLTAAPQCPFPDRYLGTALNTGLFDYVWVQFYNNP
PCQYSSGNINNIINSWNRWTTSINAGKIFLGLPAAPEAAGSGYVPPDVLISRILPEIKKSPK
YGGVMLWSKFYDDKNGYSSSILDSV
>d1nar__ c.1.8.5 (-) Seed storage protein {Vicia narbonensis, Narbonin}
PKPIFREYIGVKPNSTTLHDFPTEIINTETLEFHYILGFAIESYYESGKGTGTFEESWDVEL
FGPEKVKNLKRRHPEVKVVISIGGRGVNTPFDPAEENVWVSNAKESLKLIIQKYSDDSGNLI
DGIDIHYEHIRSDEPFATLMGQLITELKKDDDLNINVVSIAPSENNSSHYQKLYNAKKDYIN

WVDYQFSNQQKPVSTDDAFVEIFKSLEKDYHPHKVLPGFSTDPLDTKHNKITRDIFIGGCTR
LVQTFSLPGVFFWNANDSVIPKRDGDKPFIVELTLQQLLAA
>d1cnv__ c.1.8.5 (-) Seed storage protein {Jack bean (Canavalia ensiformis), Concanavalin B}
DISSTEIAVYWGQREDGLLRDTCKTNNYKIVFISFLDKFGCEIRKPELELEGVCGPSVGNPC
SFLESQIKECQRMGVKVFLALGGPKGTYSACSADYAKDLAEYLHTYFLSERREGPLGKVALD
GIHFDIQKPVDELNWDNLLEELYQIKDVYQSTFLLSAAPGCLSPDEYLDNAIQTRHFDYIFV
RFYNDRSCQYSTGNIQRIRNAWLSWTKSVYPRDKNLFLELPASQATAPGGGYIPPSALIGQV
LPYLPDLQTRYAGIALWNRQADKETGYSTNIIRYL
>d2ebn__ c.1.8.5 (-) Endo-beta-N-acetylglucosaminidase {Flavobacterium meningosepticum, endoglycosidase F1}
TTKANIKLFSFTEVNDTNPLNNLNFTLKNSGKPLVDMVVLFSANINYDAANDKVFVSNNPNV
QHLLTNRAKYLKPLQDKGIKVILSILGNHDRSGIANLSTARAKAFAQELKNTCDLYNLDGVF
FDDEYSAYQTPPPSGFVTPSNNAAARLAYETKQAMPNKLVTVYVYSRTSSFPTAVDGVNAGS
YVDYAIHDYGGSYDLATNYPGLAKSGMVMSSQEFNQGRYATAQALRNIVTKGYGGHMIFAMD
PNRSNFTSGQLPALKLIAKELYGDELVYSNTPYSKDW
>d1eoka_ c.1.8.5 (A:) Endo-beta-N-acetylglucosaminidase {Flavobacterium meningosepticum, endoglycosidase F3}
NGVCIAYYITDGRNPTFKLKDIPDKVDMVILFGLKYWSLQDTTKLPGGTGMMGSFKSYKDLD
TQIRSLQSRGIKVLQNIDDDVSWQSSKPGGFASAAAYGDAIKSIVIDKWKLDGISLDIEHSG
AKPNPIPTFPGYAATGYNGWYSGSMAATPAFLNVISELTKYFGTTAPNNKQLQIASGIDVYA
WNKIMENFRNNFNYIQLQSYGANVSRTQLMMNYATGTNKIPASKMVFGAYAEGGTNQANDVE
VAKWTPTQGAKGGMMIYTYNSNVSYANAVRDAVK
>d1edt__ c.1.8.5 (-) Endo-beta-N-acetylglucosaminidase {Streptomyces plicatus, endoglycosidase H}
KQGPTSVAYVEVNNNSMLNVGKYTLADGGGNAFDVAVIFAANINYDTGTKTAYLHFNENVQR
VLDNAVTQIRPLQQQGIKVLLSVLGNHQGAGFANFPSQQAASAFAKQLSDAVAKYGLDGVDF
DDEYAEYGNNGTAQPNDSSFVHLVTALRANMPDKIISLYNIGPAASRLSYGGVDVSDKFDYA
WNPYYGTWQVPGIALPKAQLSPAAVEIGRTSRSTVADLARRTVDEGYGVYLTYNLDGGDRTA
DVSAFTRELYGSEAVRT
>d1edqa2 c.1.8.5 (A:133-443,A:517-563) Chitinase A, catalytic domain {Serratia marcescens}
TDGSHLAPLKEPLLEKNKPYKQNSGKVVGSYFVEWGVYGRNFTVDKIPAQNLTHLLYGFIPI
CGGNGINDSLKEIEGSFQALQRSCQGREDFKVSIHDPFAALQKAQKGVTAWDDPYKGNFGQL
MALKQAHPDLKILPSIGGWTLSDPFFFMGDKVKRDRFVGSVKEFLQTWKFFDGVDIDWEFPG
GKGANPNLGSPQDGETYVLLMKELRAMLDQLSVETGRKYELTSAISAGKDKIDKVAYNVAQN
SMDHIFLMSYDFYGAFDLKNLGHQTALNAPAWKPDTAYTTVNGVNALLAQGVKPGKIVVGTA
MXDARSVQAKGKYVLDKQLGGLFSWEIDADNGDILNSMNASLGNSAGVQ
>d1goia2 c.1.8.5 (A:3-291,A:380-446) Chitinase B, catalytic domain {Serratia marcescens}
TRKAVIGYYFIPTNQINNYTETDTSVVPFPVSNITPAKAKQLTHINFSFLDINSNLECAWDP
ATNDAKARDVVNRLTALKAHNPSLRIMFSIGGWYYSNDLGVSHANYVNAVKTPASRAKFAQS
CVRIMKDYGFDGVNIDWEYPQAAEVDGFIAALQEIRTLLNQQTITDGRQALPYQLTIAGAGG
AFFLSRYYSKLAQIVAPLDYINLMTYDLAGPWEKVTNHQAALFGDAAGPTFYNALREANLGW

SWEELTRAFPSPFSLTVDAAVQQHLMMEGVPSAKIVMGVPFXDDAESFKYKAKYIKQQQLGG
VMFWHLGQDNRNGDLLAALDRYFNAADYDDSQLDMGTGLRYTGVGPG
>d1d2ka1 c.1.8.5 (A:36-292,A:355-427) Chitinase 1 {Fungus (Coccidioides immitis)}
GGFRSVVYFVNWAIYGRGHNPQDLKADQFTHILYAFANIRPSGEVYLSDTWADTDKHYPGDK
WDEPGNNVYGCIKQMYLLKKNNRNLKTLLSIGGWTYSPNFKTPASTEEGRKKFADTSLKLMK
DLGFDGIDIDWEYPEDEKQANDFVLLLKACREALDAYSAKHPNGKKFLLTIASPAGPQNYNK
LKLAEMDKYLDFWNLMAYDFSGSWDKVSGHMSNVFPSTTKPESTPFSSDKAVKDYIKAGVPA
NKIVLGMPLXDTVKIAGKKAEYITKNGMGGGMWWESSSDKTGNESLVGTVVNGLGGTGKLEQ
RENELSYPESVYDNLKNGMPS
>d1e9la1 c.1.8.5 (A:22-266,A:337-393) Chitinase-like lectin ym1, saccharide binding domain {Mouse (Mus musculus)}
YQLMCYYTSWAKDRPIEGSFKPGNIDPCLCTHLIYAFAGMQNNEITYTHEQDLRDYEALNGL
KDKNTELKTLLAIGGWKFGPAPFSAMVSTPQNRQIFIQSVIRFLRQYNFDGLNLDWQYPGSR
GSPPKDKHLFSVLVKEMRKAFEEESVEKDIPRLLLTSTGAGIIDVIKSGYKIPELSQSLDYI
QVMTYDLHDPKDGYTGENSPLYKSPYDIGKSADLNVDSIISYWKDHGAASEKLIVGFPAXDN
VRSFKLKAQWLKDNNLGGAVVWPLDMDDFSGSFCHQRHFPLTSTLKGDLNIHSAS
>d1jfxa_ c.1.8.8 (A:) Streptomyces lysozyme {Streptomyces coelicolor, "mueller" dsm3030}
DTSGVQGIDVSHWQGSINWSSVKSAGMSFAYIKATEGTNYKDDRFSANYTNAYNAGIIRGAY
HFARPNASSGTAQADYFASNGGGWSRDNRTLPGVLDIEHNPSGAMCYGLSTTQMRTWINDFH
ARYKARTTRDVVIYTTASWWNTCTGSWNGMAAKSPFWVAHWGVSAPTVPSGFPTWTFWQYSA
TGRVGGVSGDVDRNKFNGSAARLLALANNTA
>d1qba_3 c.1.8.6 (338-780) Bacterial chitobiase (beta-N-acetylhexosaminidase) {Serratia marcescens}
FPYRGIFLDVARNFHKKDAVLRLLDQMAAYKLNKFHFHLSDDEGWRIEIPGLPELTEVGGQR
CHDLSETTCLLPQYGQGPDVYGGFFSRQDYIDIIKYAQARQIEVIPEIDMPAHARAAVVSME
ARYKKLHAAGKEQEANEFRLVDPTDTSNTTSVQFFNRQSYLNPCLDSSQRFVDKVIGEIAQM
HKEAGQPIKTWHFGGDEAKNIRLGAGYTDKAKPEPGKGIIDQGNEDKPWAKSQVCQTMIKEG
KVADMEHLPSYFGQEVSKLVKAHGIDRMQAWQDGLKDAESSKAFATSRVGVNFWDTLYWGGF
DSVNDWANKGYEVVVSNPDYVYMDFPYEVNPDERGYYWGTRFSDERKVFSFAPDNMPQNAET
SVDRDGNHFNAKSDKPWPGAYGLSAQLWSETQRTDPQMEYMIFPRALSVAERSWHRAGWEQD
YRAGREYKG
>d1jaka1 c.1.8.6 (A:151-506) beta-N-acetylhexosaminidase {Streptomyces plicatus}
YAWRSAMLDVSRHFFGVDEVKRYIDRVARYKYNKLHLHLSDDQGWRIAIDSWPRLATYGGST
EVGGGPGGYYTKAEYKEIVRYAASRHLEVVPEIDMPGHTNAALASYAELNCDGVAPPLYTGT
KVGFSSLCVDKDVTYDFVDDVIGELAALTPGRYLHIGGDEAHSTPKADFVAFMKRVQPIVAK
YGKTVVGWHQLAGAEPVEGALVQYWGLDRTGDAEKAEVAEAARNGTGLILSPADRTYLDMKY
TKDTPLGLSWAGYVEVQRSYDWDPAGYLPGAPADAVRGVEAPLWTETLSDPDQLDYMAFPRL
PGVAELGWSPASTHDWDTYKVRLAAQAPYWEAAGIDFYRSPQVPWT
>d1iexa1 c.1.8.7 (A:1-388) Beta-D-glucan exohydrolase, N-terminal domain {Barley (Hordeum vulgare)}
DYVLYKDATKPVEDRVADLLGRMTLAEKIGQMTQIERLVATPDVLRDNFIGSLLSGGGSVPR

KGATAKEWQDMVDGFQKACMSTRLGIPMIYGIDAVHGQNNVYGATIFPHNVGLGATRDPYLV
KRIGEATALEVRATGIQYAFAPCIAVCRDPRWGRCYESYSEDRRIVQSMTELIPGLQGDVPK
DFTSGMPFVAGKNKVAACAKHFVGDGGTVDGINENNTIINREGLMNIHMPAYKNAMDKGVST
VMISYSSWNGVKMHANQDLVTGYLKDTLKFKGFVISDWEGIDRITTPAGSDYSYSVKASILA
GLDMIMVPNKYQQFISILTGHVNGGVIPMSRIDDAVTRILRVKFTMGLFENPYADPAMAEQL
GKQEHRDLAREAARKS
>d1fcqa_ c.1.8.9 (A:) Bee venom hyaluronidase {Honeybee (Apis mellifera)}
EFNVYWNVPTFMCHKYGLRFEEVSEKYGILQNWMDKFRGEEIAILYDPGMFPALLKDPNGNV
VARNGGVPQLGNLTKHLQVFRDHLINQIPDKSFPGVGVIDFESWRPIFRQNWASLQPYKKLS
VEVVRREHPFWDDQRVEQEAKRRFEKYGQLFMEETLKAAKRMRPAANWGYYAYPYCYNLTPN
QPSAQCEATTMQENDKMSWLFESEDVLLPSVYLRWNLTSGERVGLVGGRVKEALRIARQMTT
SRKKVLPYYWYKYQDRRDTDLSRADLEATLRKITDLGADGFIIWGSSDDINTKAKCLQFREY
LNNELGPAVKR
>d1a4ma_ c.1.9.1 (A:) Adenosine deaminase (ADA) {Mouse (Mus musculus)}
TPAFNKPKVELHVHLDGAIKPETILYFGKKRGIALPADTVEELRNIIGMDKPLSLPGFLAKF
DYYMPVIAGCREAIKRIAYEFVEMKAKEGVVYVEVRYSPHLLANSKVDPMPWNQTEGDVTPD
DVVDLVNQGLQEGEQAFGIKVRSILCCMRHQPSWSLEVLELCKKYNQKTVVAMDLAGDETIE
GSSLFPGHVEAYEGAVKNGIHRTVHAGEVGSPEVVREAVDILKTERVGHGYHTIEDEALYNR
LLKENMHFEVCPWSSYLTGAWDPKTTHAVVRFKNDKANYSLNTDDPLIFKSTLDTDYQMTKK
DMGFTEEEFKRLNINAAKSSFLPEEEKKELLERLYREYQ
>d1j79a_ c.1.9.4 (A:) Dihydroorotase {Escherichia coli}
SQVLKIRRPDDWHLHLRDGDMLKTVVPYTSEIYGRAIVMPNLAPPVTTVEAAVAYRQRILDA
VPAPHDFTPLMTCYLTDSLDPNELERGFNEGVFTAAKLYPANATTNSSHGVTSVDAIMPVLE
RMEKIGMPLLVHGEVTHADIDIFDREARFIESVMEPLRQRLTALKVVFEHITTKDAADYVRD
GNERLAATITPQHLMFNRNHMLVGGVRPHLYCLPILKRNIHQQALRELVASGFQRVFLGTDS
APHARHRKESSCGCAGCFNAPTALGSYATVFEEMNALQHFEAFCSVNGPQFYGLPVNDTFIE
LVREEQQVAESIALTDDTLVPFLAGETVRWSVK
>d1k6wa2 c.1.9.5 (A:56-375) Cytosine deaminase catalytic domain {Escherichia coli}
PFVEPHIHLDTTQTAGQPNWNQSGTLFEGIERWAERKALLTHDDVKQRAWQTLKWQIANGIQ
HVRTHVDVSDATLTALKAMLEVKQEVAPWIDLQIVAFPQEGILSYPNGEALLEEALRLGADV
VGAIPHFEFTREYGVESLHKTFALAQKYDRLIDVHCDEIDDEQSRFVETVAALAHHEGMGAR
VTASHTTAMHSYNGAYTSRLFRLLKMSGINFVANPLVNIHLQGRFDTYPKRRGITRVKEMLE
SGINVCFGHDDVFDPWYPLGTANMLQVLHMGLHVCQLMGYGQINDGLNLITHHSARTLNLQD
YGIAAGNSAN
>d1ejrc2 c.1.9.2 (C:1130-1422,C:1476-1567) alpha-subunit of urease, catalytic domain {Klebsiella aerogenes}
GIDTHIHWICPQQAEEALVSGVTTMVGGGTGPAAGTHATTCTPGPWYISRMLQAADSLPVNI
GLLGKGNVSQPDALREQVAAGVIGLKIHEAWGATPAAIDCALTVADEMDIQVALHSDTLNES
GFVEDTLAAIGGRTIHTFHTEGAGGGHAPDIITACAHPNILPSSTNPTLPYTLNTIDEHLDM
LMVCHHLDPDIAEDVAFAESRIRRETIAAEDVLHDLGAFSLTSSDSQAMGRVGEVILRTWQV
AHRMKVQRGALAEETGDNDNFRVKRYIAKYTINPALTHGIAHEVGXMFGALGSARHHCRLTF

LSQAAAANGVAERLNLRSAIAVVKGCRTVQKADMVHNSLQPNITVDAQTYEVRVDGELITSE
PADVLPMAQRYFLF
>d4ubpc2 c.1.9.2 (C:132-434,C:484-570) alpha-subunit of urease, catalytic domain {Bacillus pasteurii}
GGIDTHVHFINPDQVDVALANGITTLFGGGTGPAEGSKATTVTPGPWNIEKMLKSTEGLPIN
VGILGKGHGSSIAPIMEQIDAGAAGLKIHEDWGATPASIDRSLTVADEADVQVAIHSDTLNE
AGFLEDTLRAINGRVIHSFHVEGAGGGHAPDIMAMAGHPNVLPSSTNPTRPFTVNTIDEHLD
MLMVCHHLKQNIPEDVAFADSRIRPETIAAEDILHDLGIISMMSTDALAMGRAGEMVLRTWQ
TADKMKKQRGPLAEEKNGSDNFRLKRYVSKYTINPAIAQGIAHEVGSIEEGKFADXGDLIHD
TNITFMSKSSIQQGVPAKLGLKRRIGTVKNCRNIGKKDMKWNDVTTDIDINPETYEVKVDGE
VLTCEPVKELPMAQRYFLF
>d1e9yb2 c.1.9.2 (B:132-431,B:481-569) alpha-subunit of urease, catalytic domain {Helicobacter pylori}
GIDTHIHFISPQQIPTAFASGVTTMIGGGTGPADGTNATTITPGRRNLKWMLRAAEEYSMNL
GFLAKGNASNDASLADQIEAGAIGFKIHEDWGTTPSAINHALDVADKYDVQVAIHTDTLNEA
GCVEDTMAAIAGRTMHTFHTEGAGGGHAPDIIKVAGEHNILPASTNPTIPFTVNTEAEHMDM
LMVCHHLDKSIKEDVQFADSRIRPQTIAAEDTLHDMGAFSITSSDSQAMGRVGEVITRTWQT
ADKNKKEFGRLKEEKGDNDNFRIKRYLSKYTINPAIAHGISEYVGSVEVGKVXHHGKAKYDA
NITFVSQAAYDKGIKEELGLERQVLPVKNCRNVTKKDMQFNNTTAHIEVNPETYHVFVDGKE
VTSKPANKVSLAQLFSIF
>d1i0da_ c.1.9.3 (A:) Phosphotriesterase {Pseudomonas diminuta}
DRINTVRGPITISEAGFTLTHEHICGSSAGFLRAWPEFFGSRKALAEKAVRGLRRARAAGVR
TIVDVSTFDIGRDVSLLAEVSRAADVHIVAATGLWFDPPLSMRLRSVEELTQFFLREIQYGI
EDTGIRAGIIKVATTGKATPFQELVLKAAARASLATGVPVTTHTAASQRDGEQQAAIFESEG
LSPSRVCIGHSDDTDDLSYLTALAARGYLIGLDHIPHSAIGLEDNASASALLGIRSWQTRAL
LIKALIDQGYMKQILVSNDWLFGFSSYVTNIMDVMDRVNPDGMAFIPLRVIPFLREKGVPQE
TLAGITVTNPARFLSPTLRAS
>d1bf6a_ c.1.9.3 (A:) Phosphotriesterase homology protein {Escherichia coli}
SFDPTGYTLAHEHLHIDLSGFKNNVDCRLDQYAFICQEMNDLMTRGVRNVIEMTNRYMGRNA
QFMLDVMRETGINVVACTGYYQDAFFPEHVATRSVQELAQEMVDEIEQGIDGTELKAGIIAE
IGTSEGKITPLEEKVFIAAALAHNQTGRPISTHTSFSTMGLEQLALLQAHGVDLSRVTVGHC
DLKDNLDNILKMIDLGAYVQFDTIGKNSYYPDEKRIAMLHALRDRGLLNRVMLSMDITRRSH
LKANGGYGYDYLLTTFIPQLRQSGFSQADVDVMLRENPSQFFQ
>d1jcla_ c.1.10.1 (A:) Deoxyribose-phosphate aldolase DeoC {Escherichia coli}
HMTDLKASSLRALKLMDLTTLNDDDTDEKVIALCHQAKTPVGNTAAICIYPRFIPIARKTLK
EQGTPEIRIATVTNFPHGNDDIDIALAETRAAIAYGADEVDVVFPYRALMAGNEQVGFDLVK
ACKEACAAANVLLKVIIETGELKDEALIRKASEISIKAGADFIKTSTGKVAVNATPESARIM
MEVIRDMGVEKTVGFKPAGGVRTAEDAQKYLAIADELFGADWADARHYRFGASSLLASLLKA
LGHG
>d1nal1_ c.1.10.1 (1:) N-acetylneuraminate lyase {Escherichia coli}
NLRGVMAALLTPFDQQQALDKASLRRLVQFNIQQGIDGLYVGGSTGEAFVQSLSEREQVLEI

VAEEGKGKIKLIAHVGCVTTAESQQLAASAKRYGFDAVSAVTPFYYPFSFEEHCDHYRAIID
SADGLPMVVYNIPALSGVKLTLDQINTLVTLPGVGALKQTSGDLYQMEQIRREHPDLVLYNG
YDEIFASGLLAGADGGIGSTYNIMGWRYQGIVKALKEGDIQTAQKLQTECNKVIDLLIKTGV
FRGLKTVLHYMDVVSVPLCRKPFGPVDEKYQPELKALAQQLMQ
>d1f74a_ c.1.10.1 (A:) N-acetylneuraminate lyase {Haemophilus influenzae}
MRDLKGIFSALLVSFNEDGTINEKGLRQIIRHNIDKMKVDGLYVGGSTGENFMLSTEEKKEI
FRIAKDEAKDQIALIAQVGSVNLKEAVELGKYATELGYDCLSAVTPFYYKFSFPEIKHYYDT
IIAETGSNMIVYSIPFLTGVNMGIEQFGELYKNPKVLGVKFTAGDFYLLERLKKAYPNHLIW
AGFDEMMLPAASLGVDGAIGSTFNVNGVRARQIFELTKAGKLKEALEIQHVTNDLIEGILAN
GLYLTIKELLKLEGVDAGYCREPMTSKATAEQVAKAKDLKAKFLS
>d1dhpa_ c.1.10.1 (A:) Dihydrodipicolinate synthase {Escherichia coli}
MFTGSIVAIVTPMDEKGNVCRASLKKLIDYHVASGTSAIVSVGTTGESATLNHDEHADVVMM
TLDLADGRIPVIAGTGANATAEAISLTQRFNDSGIVGCLTVTPYYNRPSQEGLYQHFKAIAE
HTDLPQILYNVPSRTGCDLLPETVGRLAKVKNIIGIKEATGNLTRVNQIKELVSDDFVLLSG
DDASALDFMQLGGHGVISVTANVAARDMAQMCKLAAEGHFAEARVINQRLMPLHNKLFVEPN
PIPVKWACKELGLVATDTLRLPMTPITDSGRETVRAALKHAGLL
>d1qo5b_ c.1.10.1 (B:) Fructose-1,6-bisphosphate aldolase {Human (Homo sapiens), liver isozyme}
AHRFPALTQEQKKELSEIAQSIVANGKGILAADESVGTMGNRLQRIKVENTEENRRQFREIL
FSVDSSINQSIGGVILFHETLYQKDSQGKLFRNILKEKGIVVGIKLDQGGAPLAGTNKETTI
QGLDGLSERCAQYKKDGVDFGKWRAVLRIADQCPSSLAIQENANALARYASICQQNGLVPIV
EPEVIPDGDHDLEHCQYVTEKVLAAVYKALNDHHVYLEGTLLKPNMVTAGHACTKKYTPEQV
AMATVTALHRTVPAAVPGICFLSGGMSEEDATLNLNAINLCPLPKPWKLSFSYGRALQASAL
AAWGGKAANKEATQEAFMKRAMANCQAAKGQYVHTGSS
>d1adoa_ c.1.10.1 (A:) Fructose-1,6-bisphosphate aldolase {Rabbit (Oryctolagus cuniculus), muscle isozyme}
PHSHPALTPEQKKELSDIAHRIVAPGKGILAADESTGSIAKRLQSIGTENTEENRRFYRQLL
LTADDRVNPCIGGVILFHETLYQKADDGRPFPQVIKSKGGVVGIKVDKGVVPLAGTNGETTT
QGLDGLSERCAQYKKDGADFAKWRCVLKIGEHTPSALAIMENANVLARYASICQQNGIVPIV
EPEILPDGDHDLKRCQYVTEKVLAAVYKALSDHHIYLEGTLLKPNMVTPGHACTQKYSHEEI
AMATVTALRRTVPPAVTGVTFLSGGQSEEEASINLNAINKCPLLKPWALTFSYGRALQASAL
KAWGGKKENLKAAQEEYVKRALANSLACQGKYTSSGQAGAAASESLFISNHAY
>d1fdja_ c.1.10.1 (A:) Fructose-1,6-bisphosphate aldolase {Rabbit (Oryctolagus cuniculus), liver isozyme}
AHRFPALTPEQKKELSDIAQRIVANGKGILAADESVGTMGNRLQRIKVENSEENRRQFREIL
FTVDNSINQSIGGVILFHETLYQKDSQGKLFRNILKEKGIVVGIKLDQGGAPLAGTNKETTI
QGLDGLSERCAQYKKDGVDFGKWRAVLRIADQCPSSLAIQENANTLARYASICQQNGLVPIV
EPEVIPDGDHDLEHCQYVTEKVLAAVYKALNDHHVYLEGTLLKPNMVTAGHACTKKYTPEQV
AMATVTALHRTVPAAVPGICFLSGGMSEEDATLNLNAINLCPLPKPWKLSFSYGRALQASAL
AAWGGKAENKKATQEAFMKRAVVNCQAAKGQYVHTGSSGAASTQSLFTASYTY
>d1fbaa_ c.1.10.1 (A:) Fructose-1,6-bisphosphate aldolase {Drosophila melanogaster}

TTYFNYPSKELQDELREIAQKIVAPGKGILAADESGPTMGKRLQDIGVENTEDNRRAYRQLL
FSTDPKLAENISGVILFHETLYQKADDGTPFAEILKKKGIILGIKVDKGVVPLFGSEDEVTT
QGLDDLAARCAQYKKDGCDFAKWRCVLKIGKNTPSYQSILENANVLARYASICQSQRIVPIV
EPEVLPDGDHDLDRAQKVTETVLAAVYKALSDHHVYLEGTLLKPNMVTAGQSAKKNTPEEIA
LATVQALRRTVPAAVTGVTFLSGGQSEEEATVNLSAINNVPLIRPWALTFSYGRALQASVLR
AWAGKKENIAAGQNELLKRAKANGDAAQGKYVAGSAGAGSGSLFVANHAY
>d1a5ca_ c.1.10.1 (A:) Fructose-1,6-bisphosphate aldolase
{Plasmodium falciparum}
LPADVAEELATTAQKLVQAGKGILAADESTQTIKKRFDNIKLENTIENRASYRDLLFGTKGL
GKFISGAILFEETLFQKNEAGVPMVNLLHNENIIPGIKVDKGLVNIPCTDEEKSTQGLDGLA
ERCKEYYKAGARFAKWRTVLVIDTAKGKPTDLSIHETAWGLARYASICQQNRLVPIVEPEIL
ADGPHSIEVCAVVTQKVLSCVFKALQENGVLLEGALLKPNMVTAGYECTAKTTTQDVGFLTV
RTLRRTVPPALPGVVFLSGGQSEEEASVNLNSINALGPHPWALTFSYGRALQASVLNTWQGK
KENVAKAREVLLQRAEANSLATYGKYKGGAGG
>d1epxa_ c.1.10.1 (A:) Fructose-1,6-bisphosphate aldolase
{Trypanosome (Leishmania mexicana)}
MSRVTVLQSQLPAYNRLKTPYESELIATVKKLTTPGKGLLAADESIGSCTKRFQPIGLSNTE
EHRRQYRALMLEAEGFEQYISGVILHDETVGQKASNGQTFPEYLTARGVVPGIKTDMGLCPL
LEGAEGEQMTEGLDGYVKRASAYYKKGCRFCKWRNVYKIQNGTVSESAVRFNAETLARYAIL
SQMSGLVPIVEPEVMIDGKHDIDTCQRVSEHVWREVVAALQRHGVIWEGCLLKPNMVVPGAE
SGKTAAPEQVAHYTVMTLARTMPAMLPGVMFLSGGLSEVQASEYLNAINNSPLPRPYFLSFS
YARALQSSALKAWGGKESGLAAGRRAFLHRARMNSMAQLGKYKRSDD
>d1f2ja_ c.1.10.1 (A:) Fructose-1,6-bisphosphate aldolase
{Trypanosome (Trypanosoma brucei)}
SKRVEVLLTQLPAYNRLKTPYEAELIETAKKMTAPGKGLLAADESTGSCSKRFAGIGLSNTA
EHRRQYRALMLECEGFEQYISGVILHDETVYQKAKTGETFPQYLRRRGVVPGIKTDCGLEPL
VEGAKGEQMTAGLDGYIKRAKKYYAMGCRFCKWRNVYKIQNGTVSEAVVRFNAETLARYAIL
SQLCGLVPIVEPEVMIDGTHDIETCQRVSQHVWSEVVSALHRHGVVWEGCLLKPNMVVPGAE
SGLKGHAEQVAEYTVKTLARVIPPALPGVTFLSGGLSEVMASEYLNAMNNCPLPRPWKLTFS
YARALQSSAIKRWGGKESGVEAGRRAFMHRAKMNSLAQLGKYNRADD
>d1euaa_ c.1.10.1 (A:) KDPG aldolase {Escherichia coli}
MKNWKTSAESILTTGPVVPVIVVKKLEHAVPMAKALVAGGVRVLEVTLRTECAVDAIRAIAK
EVPEAIVGAGTVLNPQQLAEVTEAGAQFAISPGLTEPLLKAATEGTIPLIPGISTVSELMLG
MDYGLKEFKFFPAEANGGVKALQAIAGPFSQVRFCPTGGISPANYRDYLALKSVLCIGGSWL
VPADALEAGDYDRITKLAREAVEGAKL
>d1qfea_ c.1.10.1 (A:) Type I 3-dehydroquinate dehydratase
{Salmonella typhi}
MKTVTVKNLIIGEGMPKIIVSLMGRDINSVKAEALAYREATFDILEWRVDHFMDIASTQSVL
TAARVIRDAMPDIPLLFTFRSAKEGGEQTITTQHYLTLNRAAIDSGLVDMIDLELFTGDADV
KATVDYAHAHNVYVVMSNHDFHQTPSAEEMVSRLRKMQALGADIPKIAVMPQSKHDVLTLLT
ATLEMQQHYADRPVITMSMAKEGVISRLAGEVFGSAATFGAVKQASAPGQIAVNDLRSVLMI
LHNA
>d1i2oa_ c.1.10.1 (A:) Transaldolase {Escherichia coli}
TDKLTSLRQYTTVVADTGDIAAMKLYQPQDATTNPSLILNAAQIPEYRKLIDDAVAWAKQQS

NDRAQQIVDATDKLAVNIGLEILKLVPGRISTAVDARLSYDTEASIAKAKRLIKLYNDAGIS
NDRILIKLASTWQGIRAAEQLEKEGINCNLTLLFSFAQARACAEAGVFLISPFVGRILDWYK
ANTDKKEYAPAEDPGVVSVSEIYQYYKEHGYETVVMGASFRNIGEILELAGCDRLTIAPALL
KELAESEGAIERKLSYTGEVKARPARITESEFLWQHNQDPMAVDKLAEGIRKFAIDQEKLEK
MIGDLL
>d1f05a_ c.1.10.1 (A:) Transaldolase {Human (Homo sapiens)}
MESALDQLKQFTTVVADTGDFHAIDEYKPQDATTNPSLILAAAQMPAYQELVEEAIAYGRKL
GGSQEDQIKNAIDKLFVLFGAEILKKIPGRVSTEVDARLSFDKDAMVARARRLIELYKEAGI
SKDRILIKLSSTWEGIQAGKELEEQHGIHCNMTLLFSFAQAVACAEAGVTLISPFVGRILDW
HVANTDKKSYEPLEDPGVKSVTKIYNYYKKFSYKTIVMGASFRNTGEIKALAGCDFLTISPK
LLGELLQDNAKLVPVLSAKAAQASDLEKIHLDEKSFRWLHNEDQMAVEKLSDGIRKFAADAV
KLERMLTERMFN
>d1dosa_ c.1.10.2 (A:) Fructose-bisphosphate aldolase
{Escherichia coli}
SKIFDFVKPGVITGDDVQKVFQVAKENNFALPAVNCVGTDSINAVLETAAKVKAPVIVQFSN
GGASFIAGKGVKSDVPQGAAILGAISGAHHVHQMAEHYGVPVILHTDHCAKKLLPWIDGLLD
AGEKHFAATGKPLFSSHMIDLSEESLQENIEICSKYLERMSKIGMTLEIELGCTGGEEDGVD
NSHMDASALYTQPEDVDYAYTELSKISPRFTIAASFGNVHGVYKAGNVVLTPTILRDSQEYV
SKKHNLPHNSLNFVFHGGSGSTAQEIKDSVSYGVVKMNIDTDTQWATWEGVLNYYKANEAYL
QGQLGNPKGEDQPNKKYYDPRVWLRAGQTSMIARLEKAFQELNAIDVL
>d1h7na_ c.1.10.3 (A:) 5-aminolaevulinate dehydratase, ALAD
(porphobilinogen synthase) {Baker's yeast (Saccharomyces
cerevisiae)}
MHTAEFLETEPTEISSVLAGGYNHPLLRQWQSERQLTKNMLIFPLFISDNPDDFTEIDSLPN
INRIGVNRLKDYLKPLVAKGLRSVILFGVPLIPGTKDPVGTAADDPAGPVIQGIKFIREYFP
ELYIICDVCLCEYTSHGHCGVLYDDGTINRERSVSRLAAVAVNYAKAGAHCVAPSDMIDGRI
RDIKRGLINANLAHKTFVLSYAAKFSGNLYGPFRDAACSAPSNGDRKCYQLPPAGRGLARRA
LERDMSEGADGIIVKPSTFYLDIMRDASEICKDLPICAYHVSGEYAMLHAAAEKGVVDLKTI
AFESHQGFLRAGARLIITYLAPEFLDWLDE
>d1e51a_ c.1.10.3 (A:) 5-aminolaevulinate dehydratase, ALAD
(porphobilinogen synthase) {Human (Homo sapiens)}
MQPQSVLHSGYFHPLLRAWQTATTTLNASNLIYPIFVTDVPDDIQPITSLPGVARYGVKRLE
EMLRPLVEEGLRCVLIFGVPSRVPKDERGSAADSEESPAIEAIHLLRKTFPNLLVACDVCLC
PYTSHGHCGLLSENGAFRAEESRQRLAEVALAYAKAGCQVVAPSDMMDGRVEAIKEALMAHG
LGNRVSVMSYSAKFASCFYGPFRDAAKSSPAFGDRRCYQLPPGARGLALRAVDRDVREGADM
LMVKPGMPYLDIVREVKDKHPDLPLAVYHVSGEFAMLWHGAQAGAFDLKAAVLEAMTAFRRA
GADIIITYYTPQLLQWLK
>d1b4ka_ c.1.10.3 (A:) 5-aminolaevulinate dehydratase, ALAD
(porphobilinogen synthase) {Pseudomonas aeruginosa}
YPYTRLRRNRRDDFSRRLVRENVLTVDDLILPVFVLDGVNQRESIPSMPGVERLSIDQLLIE
AEEWVALGIPALALFPVTPVEKKSLDAAEAYNPEGIAQRATRALRERFPELGIITDVALDPF
TTHGQDGILDDDGYVLNDVSIDVLVRQALSHAEAGAQVVAPSDMMDGRIGAIREALESAGHT
NVRIMAYSAKYASAYYGPFRDAVGSASNLGKGNKATYQMDPANSDEALHEVAADLAEGADMV
MVKPGMPYLDIVRRVKDEFRAPTFVYQVSGEYAMHMGAIQNGWLAESVILESLTAFKRAGAD

GILTYFAKQAAEQLRR
>d1b4ea_ c.1.10.3 (A:) 5-aminolaevulinate dehydratase, ALAD (porphobilinogen synthase) {Escherichia coli}
TDLSQRPRRLRKSPALRAMFEETTLSLNDLVLPIFVEEEIDDYKAVEAMPGVMRIPEKHLAR
EIERIANAGIRSVMTFGISHHTDETGSDAWREDGLVARMSRICKQTVPEMIVMSDTCFCEYT
SHGHCGVLKEHGVDNDATLENLGKQAVVAAAAGADFIAPSAAMDGQVQAIRQALDAAGFKDT
AIMSYSTKFASSFYGPFREAAGSALKGDRKSYQMNPMNRREAIRESLLDEAQGADCLMVKPA
GAYLDIVRELRERTELPIGAYQVSGEYAMIKFAALAGAIDEEKVVLESLGSIKRAGADLIFS
YFALDLAEKKILR
>d1gg1a_ c.1.10.4 (A:)
3-deoxy-D-arabino-heptulosonate-7-phosphate synthase (DAHP synthase, AroG) {Escherichia coli}
DLRIKEIKELLPPVALLEKFPATENAANTVAHARKAIHKILKGNDDRLLVVIGPCSIHDPVA
AKEYATRLLALREELKDELEIVMRVYFEKPRTTVGWKGLINDPHMDNSFQINDGLRIARKLL
LDINDSGLPAAGEFLDMITPQYLADLMSWGAIGARTTESQVHRELASGLSCPVGFKNGTDGT
IKVAIDAINAAGAPHCFLSVTKWGHSAIVNTSGNGDCHIILRGGKEPNYSAKHVAEVKEGLN
KAGLPAQVMIDFSHANSSKQFKKQMDVCADVCQQIAGGEKAIIGVMVESHLVEGNQSLESGE
PLAYGKSITDACIGWEDTDALLRQLANAVKARR
>d1d9ea_ c.1.10.4 (A:) 3-deoxy-D-manno-octulosonate 8-phosphate synthase (KDO8P synthase) {Escherichia coli}
MKQKVVSIGDINVANDLPFVLFGGMNVLESRDLAMRICEHYVTVTQKLGIPYVFKASFDKAN
RSSIHSYRGPGLEEGMKIFQELKQTFGVKIITDVHEPSQAQPVADVVDVIQLPAFLARQTDL
VEAMAKTGAVINVKKPQFVSPGQMGNIVDKFKEGGNEKVILCDRGANFGYDNLVVDMLGFSI
MKKVSGNSPVIFDVTHALQCRDPFGAASGGRRAQVAELARAGMAVGLAGLFIEAHPDPEHAK
CDGPSALPLAKLEPFLKQMKAIDDLVKGFEELDTSK
>d1jcxa_ c.1.10.4 (A:) 3-deoxy-D-manno-octulosonate 8-phosphate synthase (KDO8P synthase) {Aquifex aeolicus}
EKFLVIAGPCAIESEELLLKVGEEIKRLSEKFKEVEFVFKSSFDKANRSSIHSFRGHGLEYG
VKALRKVKEEFGLKITTDIHESWQAEPVAEVADIIQIPAFLCRQTDLLLAAAKTGRAVNVKK
GQFLAPWDTKNVVEKLKFGGAKEIYLTERGTTFGYNNLVVDFRSLPIMKQWAKVIYDATHSV
QLPGGLGDKSGGMREFIFPLIRAAVAVGCDGVFMETHPEPEKALSDASTQLPLSQLEGIIEA
ILEIREVASKYYETI
>d1onea1 c.1.11.1 (A:142-436) Enolase {Baker's yeast (Saccharomyces cerevisiae)}
SPYVLPVPFLNVLNGGSHAGGALALQEFMIAPTGAKTFAEALRIGSEVYHNLKSLTKKRYGA
SAGNVGDEGGVAPNIQTAEEALDLIVDAIKAAGHDGKVKIGLDCASSEFFKDGKYDLDFKNP
NSDKSKWLTGPQLADLYHSLMKRYPIVSIEDPFAEDDWEAWSHFFKTAGIQIVADDLTVTNP
KRIATAIEKKAADALLLKVNQIGTLSESIKAAQDSFAAGWGVMVSHRSGETEDTFIADLVVG
LRTGQIKTGAPARSERLAKLNQLLRIEEELGDNAVFAGENFHHGDKL
>d1pdz_1 c.1.11.1 (140-433) Enolase {Lobster (Homarus vulgaris)}
DEVILPVPAFNVINGGSHAGNKLAMQEFMILPTGATSFTEAMRMGTEVYHHLKAVIKARFGL
DATAVGDEGGFAPNILNNKDALDLIQEAIKKAGYTGKIEIGMDVAASEFYKQNNIYDLDFKT
ANNDGSQKISGDQLRDMYMEFCKDFPIVSIEDPFDQDDWETWSKMTSGTTIQIVGDDLTVTN
PKRITTAVEKKACKCLLLKVNQIGSVTESIDAHLLAKKNGWGTMVSHRSGETEDCFIADLVV

GLCTGQIKTGAPCRSERLAKYNQILRIEEELGSGAKFAGKNFRAPS
>d1e9ia1 c.1.11.1 (A:140-430) Enolase {Escherichia coli}
PGKYSMPVPMMNIINGGEHADNNVDIQEFMIQPVGAKTVKEAIRMGSEVFHHLAKVLKAKGM
NTAVGDEGGYAPNLGSNAEALAVIAEAVKAAGYELGKDITLAMDCAASEFYKDGKYVLAGEG
NKAFTSEEFTHFLEELTKQYPIVSIEDGLDESDWDGFAYQTKVLGDKIQLVGDDLFVTNTKI
LKEGIEKGIANSILIKFNQIGSLTETLAAIKMAKDAGYTAVISHRSGETEDATIADLAVGTA
AGQIKTGSMSRSDRVAKYNQLIRIEEALGEKAPYNGRKEIKGQ
>d1bqg_1 c.1.11.2 (144-422) D-glucarate dehydratase {Pseudomonas putida}
EGQQRDAVEMLGYLFYVGDRNKTDLGYRSEHEADNEWFRLRNKEALTPESVVALAEAAYDRY
GFKDFKLKGGVLRGEDEIAAVTALSERFPDARITLDPNGAWSLKEAVALCRDQHHVLAYAED
PCGAENGYSGREVMAEFRRSTGLRTATNMIATDWRQMGHAIQLQSVDIPLADPHFWTMQGSV
RVAQMCNEWGLTWGSHSNNHFDISLAMFTHVAAAAPGNITAIDTHWIWQDGQRLTKEPLQIK
GGLVEVPKKPGLGVELDWDALMKAHEVYKSM
>d1ec7a1 c.1.11.2 (A:138-446) D-glucarate dehydratase {Escherichia coli}
DGQQRSEVEMLGYLFFVGNRKATPLPYQSQPDDSCDWYRLRHEEAMTPDAVVRLAEAAYEKY
GFNDFKLKGGVLAGEEEAESIVALAQRFPQARITLDPNGAWSLNEAIKIGKYLKGSLAYAED
PCGAEQGFSGREVMAEFRRATGLPTATNMIATDWRQMGHTLSLQSVDIPLADPHFWTMQGSV
RVAQMCHEFGLTWGSHSNNHFDISLAMFTHVAAAAPGKITAIDTHWIWQEGNQRLTKEPFEI
KGGLVQVPEKPGLGVEIDMDQVMKAHELYQKHGLGARDDAMGMQYLIPGWTFDNKRPCMVR
>d1fhua1 c.1.11.2 (A:100-320) O-succinylbenzoate synthase {Escherichia coli}
QAANYRAAPLCNGDPDDLILKLADMPGEKVAKVKVGLYEAVRDGMVVNLLLEAIPDLHLRLD
ANRAWTPLKGQQFAKYVNPDYRDRIAFLEEPCKTRDDSRAFARETGIAIAWDESLREPDFAF
VAEEGVRAVVIKPTLTGSLEKVREQVQAAHALGLTAVISSSIESSLGLTQLARIAAWLTPDT
IPGLDTLDLMQAQQVRRWPGSTLPVVEVDALERLL
>d1muca1 c.1.11.2 (A:131-372) Muconate-lactonizing enzyme {Pseudomonas putida}
RVRDSLEVAWTLASGDTARDIAEARHMLEIRRHRVFKLKIGANPVEQDLKHVVTIKRELGDS
ASVRVDVNQYWDESQAIRACQVLGDNGIDLIEQPISRINRGGQVRLNQRTPAPIMADESIES
VEDAFSLAADGAASIFALKIAKNGGPRAVLRTAQIAEAAGIGLYGGTMLEGSIGTLASAHAF
LTLRQLTWGTELFGPLLLTEEIVNEPPQYRDFQLHIPRTPGLGLTLDEQRLARFAR
>d2mnr_1 c.1.11.2 (133-359) Mandelate racemase {Pseudomonas putida}
PVQAYDSHSLDGVKLATERAVTAAELGFRAVKTKIGYPALDQDLAVVRSIRQAVGDDFGIMV
DYNQSLDVPAAIKRSQALQQEGVTWIEEPTLQHDYEGHQRIQSKLNVPVQMGENWLGPEEMF
KALSIGACRLAMPDAMKIGGVTGWIRASALAQQFGIPMSSHLFQEISAHLLAATPTAHWLER
LDLAGSVIEPTLTFEGGNAVIPDLPGVGIIWREKEIGKYLV
>d2chr_1 c.1.11.2 (127-370) Chlormuconate cycloisomerase {Alcaligenes eutrophus}
PLRSAIPIAWTLASGDTKRDLDSAVEMIERRRHNRFKVKLGFRSPQDDLIHMEALSNSLGSK
AYLRVDVNQAWDEQVASVYIPELEALGVELIEQPVGRENTQALRRLSDNNRVAIMADESLST
LASAFDLARDRSVDVFSLKLCNMGGVSATQKIAAVAEASGIASYGGTMLDSTIGTSVALQLY

STVPSLPFGCELIGPFVLADTLSHEPLEIRDYELQVPTGVGHGMTLDEDKVRQYARVS
>d1jpdx1 c.1.11.2 (X:114-321) L-Ala-D/L-Glu epimerase {Escherichia coli}
TLPETVITAQTVVIGTPDQMANSASTLWQAGAKLLKVKLDNHLISERMVAIRTAVPDATLIV
DANESWRAEGLAARCQLLADLGVAMLEQPLPAQDDAALENFIHPLPICADESCHTRSNLKAL
KGRYEMVNIKLDKTGGLTEALALATEARAQGFSLMLGCMLCTSRAISAALPLVPQVSFADLD
GPTWLAVDVEPALQFTTGELHL
>d1jpma1 c.1.11.2 (A:126-359) L-Ala-D/L-Glu epimerase {Bacillus subtilis}
YRDTLETDYTVSVNSPEEMAADAENYLKQGFQTLKIKVGKDDIATDIARIQEIRKRVGSAVK
LRLDANQGWRPKEAVTAIRKMEDAGLGIELVEQPVHKDDLAGLKKVTDATDTPIMADESVFT
PRQAFEVLQTRSADLINIKLMKAGGISGAEKINAMAEACGVECMVGSMIETKLGITAAAHFA
ASKRNITRFDFDAPLMLKTDVFNGGITYSGSTISMPGKPGLGIIGAAL
>d1kcza1 c.1.11.2 (A:161-413) beta-Methylaspartase {Clostridium tetanomorphum}
GAEINAVPVFAQSGDDRYDNVDKMIIKEADVLPHALINNVEEKLGLKGEKLLEYVKWLRDRI
IKLRVREDYAPIFHIDVYGTIGAAFDVDIKAMADYIQTLAEAAKPFHLRIEGPMDVEDRQKQ
MEAMRDLRAELDGRGVDAELVADEWCNTVEDVKFFTDNKAGHMVQIKTPDLGGVNNIADAIM
YCKANGMGAYCGGTCNETNRSAEVTTNIGMACGARQVLAKPGMGVDEGMMIVKNEMNRVLAL
VGRRK
>d1kkoa1 c.1.11.2 (A:161-411) beta-Methylaspartase {Citrobacter amalonaticus}
PCVPEAIPLFGQSGDDRYIAVDKMILKGVDVLPHALINNVEEKLGFKGEKLREYVRWLSDRI
LSLRSSPRYHPTLHIDVYGTIGLIFDMDPVRCAEYIASLEKEAQGLPLYIEGPVDAGNKPDQ
IRMLTAITKELTRLGSGVKIVADEWCNTYQDIVDFTDAGSCHMVQIKTPDLGGIHNIVDAVL
YCNKHGMEAYQGGTCNETEISARTCVHVALAARPMRMLIKPGMGFDEGLNIVFNEMNRTIAL
LQT
>d1a49a2 c.1.12.1 (A:12-115,A:218-395) Pyruvate kinase, N-terminal domain {Rabbit (Oryctolagus cuniculus)}
IQTQQLHAAMADTFLEHMCRLDIDSAPITARNTGIICTIGPASRSVETLKEMIKSGMNVARM
NFSHGTHEYHAETIKNVRTATESFASDPILYRPVAVALDTKGXPAVSEKDIQDLKFGVEQDV
DMVFASFIRKAADVHEVRKILGEKGKNIKIISKIENHEGVRRFDEILEASDGIMVARGDLGI
EIPAEKVFLAQKMIIGRCNRAGKPVICATQMLESMIKKPRPTRAEGSDVANAVLDGADCIML
SGETAKGDYPLEAVRMQHLIAREAEAAMFHRKLFE
>d1pkla2 c.1.12.1 (A:1-87,A:187-357) Pyruvate kinase, N-terminal domain {Leishmania mexicana}
SQLAHNLTLSIFDPVANYRAARIICTIGPSTQSVEALKGLIQSGMSVARMNFSHGSHEYHQT
TINNVRQAAAELGVNIAIALDTKGPXPAVSAKDRVDLQFGVEQGVDMIFASFIRSAEQVGDV
RKALGPKGRDIMIICKIENHQGVQNIDSIIEESDGIMVARGDLGVEIPAEKVVVAQKILISK
CNVAGKPVICATQMLESMTYNPRPTRAEVSDVANAVFNGADCVMLSGETAKGKYPNEVVQYM
ARICLEAQSAL
>d1a3wa2 c.1.12.1 (A:2-87,A:189-366) Pyruvate kinase, N-terminal domain {Baker's yeast (Saccharomyces cerevisiae)}
SRLERLTSLNVVAGSDLRRTSIIGTIGPKTNNPETLVALRKAGLNIVRMNFSHGSYEYHKSV

IDNARKSEELYPGRPLAIALDTKGXPALSEKDKEDLRFGVKNGVHMVFASFIRTANDVLTIR
EVLGEQGKDVKIIVKIENQQGVNNFDEILKVTDGVMVARGDLGIEIPAPEVLAVQKKLIAKS
NLAGKPVICATQMLESMTYNPRPTRAEVSDVGNAILDGADCVMLSGETAKGNYPINAVTTMA
ETAVIAEQAIAYLPNYD
>d1e0ta2 c.1.12.1 (A:1-69,A:168-344) Pyruvate kinase, N-terminal domain {Escherichia coli}
MKKTKIVCTIGPKTESEEMLAKMLDAGMNVMRLNFSHGDYAEHGQRIQNLRNVMSKTGKTAA
ILLDTKGXPALAEKDKQDLIFGCEQGVDFVAASFIRKRSDVIEIREHLKAHGGENIHIISKI
ENQEGLNNFDEILEASDGIMVARGDLGVEIPVEEVIFAQKMMIEKCIRARKVVITATMMLDS
MIKNPRPTDAEAGDVANAILDGTDAVMLSGESAKGKYPLEAVSIMATICERTDRVMNSRLE
>d1kbla1 c.1.12.2 (A:510-873) Pyruvate phosphate dikinase, C-terminal domain {Clostridium symbiosum}
IETQEASVSGSFERIMVWADKFRTLKVRTNADTPEDTLNAVKLGAEGIGLCRTEHMFFEADR
IMKIRKMILSDSVEAREEALNELIPFQKGDFKAMYKALEGRPMTVRYLDPPLHEFVPHTEEE
QAELAKNMGLTLAEVKAKVDELHEFNPMMGHRGCRLAVTYPEIAKMQTRAVMEAAIEVKEET
GIDIVPEIMIPLVGEKKELKFVKDVVVEVAEQVKKEKGSDMQYHIGTMIEIPRAALTADAIA
EEAEFFSFGTNDLTQMTFGFSRDDAGKFLDSYYKAKIYESDPFARLDQTGVGQLVEMAVKKG
RQTRPGLKCGICGEHGGDPSSVEFCHKVGLNYVSCSPFRVPIARLAAAQAALNN
>d1fiy__ c.1.12.3 (-) Phosphoenolpyruvate carboxylase {Escherichia coli}
QYSALRSNVSMLGKVLGETIKDALGEHILERVETIRKLSKSSRAGNDANRQELLTTLQNLSN
DELLPVARAFSQFLNLANTAEQYHSISPKGEAASNPEVIARTLRKLKNQPELSEDTIKKAVE
SLSLELVLTAHPTEITRRTLIHKMVEVNACLKQLDNKDIADYEHNQLMRRLRQLIAQSWHTD
EIRKLRPSPVDEAKWGFAVVENSLWQGVPNYLRELNEQLEENLGYKLPVEFVPVRFTSWMGG
DRDGNPNVTADITRHVLLLSRWKATDLFLKDIQVLVSELSMVEATPELLALVGEEGAAEPYR
YLMKNLRSRLMATQAWLEARLKGEELPKPEGLLTQNEELWEPLYACYQSLQACGMGIIANGD
LLDTLRRVKCFGVPLVRIDIRQESTRHTEALGELTRYLGIGDYESWSEADKQAFLIRELNSK
RPLLPRNWQPSAETREVLDTCQVIAEAPQGSIAAYVISMAKTPSDVLAVHLLLKEAGIGFAM
PVAPLFETLDDLNNANDVMTQLLNIDWYRGLIQGKQMVMIGYSDSAKDAGVMAASWAQYQAQ
DALIKTCEKAGIELTLFHGRGGSIGRGGAPAHAALLSQPPGSLKGGLRVTEQGEMIRFKYGL
PEITVSSLSLYTGAILEANLLPPPEPKESWRRIMDELSVISCDVYRGYVRENKDFVPYFRSA
TPEQELGKLPLGSRPAKRRPTGGVESLRAIPWIFAWTQNRLMLPAWLGAGTALQKVVEDGKQ
SELEAMCRDWPFFSTRLGMLEMVFAKADLWLAEYYDQRLVDKALWPLGKELRNLQEEDIKVV
LAIANDSHLMADLPWIAESIQLRNIYTDPLNVLQAELLHRSRQAEKEGQEPDPRVEQALMVT
IAGIAAGMRNTG
>d1pyma_ c.1.12.4 (A:) Phosphoenolpyruvate mutase {Blue mussel (Mytilus edulis)}
VKKTTQLKQMLNSKDLEFIMEAHNGLSARIVQEAGFKGIWGSGLSVSAQLGVRDSNEASWTQ
VVEVLEFMSDASDVPILLDADTGYGNFNNARRLVRKLEDRGVAGACLEDKLFPKTNSLHDGR
AQPLADIEEFALKIKACKDSQTDPDFCIVARVEAFIAGWGLDEALKRAEAYRNAGADAILMH
SKKADPSDIEAFMKAWNNQGPVVIVPTKYYKTPTDHFRDMGVSMVIWANHNLRASVSAIQQT
TKQIYDDQSLVNVEDKIVSVKEIFRLQRDDELVQAEDKYLPKN
>d1dxea_ c.1.12.5 (A:) 2-dehydro-3-deoxy-galactarate aldolase {Escherichia coli}

DVFPNKFKAALAAKQVQIGCWSALSNPISTEVLGLAGFDWLVLDGEHAPNDISTFIPQLMAL
KGSASAPVVRVPTNEPVIIKRLLDIGFYNFLIPFVETKEEAELAVASTRYPPEGIRGVSVSH
RANMFGTVADYFAQSNKNITILVQIESQQGVDNVDAIAATEGVDGIFVGPSDLAAALGHLGN
ASHPDVQKAIQHIFNRASAHGKPSGILAPVEADARRYLEWGATFVAVGSDLGVFRSATQKLA
DTFKK
>d1dqua_ c.1.12.6 (A:) Isocitrate lyase {Aspergillus nidulans}
SYIEEEDQRYWDEVAAVKNWWKDSRWRYTKRPFTAEQIVAKRGNLKIEYPSNVQAKKLWGIL
ERNFKNKEASFTYGCLDPTMVTQMAKYLDTVYVSGWQSSSTASSTDEPSPDLADYPMNTVPN
KVNHLWMAQLFHDRKQREERMTTPKDQRHKVANVDYLRPIIADADTGHGGLTAVMKLTKLFV
ERGAAGIHIEDQAPGTKKCGHMAGKVLVPISEHINRLVAIRAQADIMGTDLLAIARTDSEAA
TLITSTIDHRDHPFIIGSTNPDIQPLNDLMVMAEQAGKNGAELQAIEDEWLAKAGLKLFNDA
VVDAINNSPLPNKKAAIEKYLTQSKGKSNLEARAIAKEIAGTDIYFDWEAPRTREGYYRYQG
GTQCAINRAVAYAPFADLIWMESKLPDYKQAKEFADGVHAVWPEQKLAYNLSPSFNWKKAMP
RDEQETYIKRLGALGYAWQFITLAGLHTTALISDTFAKAYAKQGMRAYGELVQEPEMANGVD
VVTHQKWSGANYVDNMLKMITGG
>d1f8ma_ c.1.12.6 (A:) Isocitrate lyase {Mycobacterium tuberculosis}
ASVVGTPKSAEQIQQEWDTNPRWKDVTRTYSAEDVVALQGSVVEEHTLARRGAEVLWEQLHD
LEWVNALGALTGNMAVQQVRAGLKAIYLSGWQVAGDANLSGHTYPDQSLYPANSVPQVVRRI
NNALQRADQIAKIEGDTSVENWLAPIVADGEAGFGGALNVYELQKALIAAGVAGSHWEDQLA
SEKKCGHLGGKVLIPTQQHIRTLTSARLAADVADVPTVVIARTDAEAATLITSDVDERDQPF
ITGERTREGFYRTKNGIEPCIARAKAYAPFADLIWMETGTPDLEAARQFSEAVKAEYPDQML
AYNCSPSFNWKKHLDDATIAKFQKELAAMGFKFQFITLAGFHALNYSMFDLAYGYAQNQMSA
YVELQEREFAAEERGYTATKHQREVGAGYFDRIATTVDPNSSTTALTGSTEEGQF
>d1igwa_ c.1.12.6 (A:) Isocitrate lyase {Escherichia coli}
KTRTQQIEELQKEWTQPRWEGITRPYSAEDVVKLRGSVNPECTLAQLGAAKMWRLLHGESKK
GYINSLGALTGGQALQQAKAGIEAVYLSGWQVAADANLAASMYPDQSLYPANSVPAVVERIN
NTFRRADQIQWSAGIEPGDPRYVDYFLPIVADAEAGFGGVLNAFELMKAMIEAGAAAVHFED
QLASVKKCGHMGGKVLVPTQEAIQKLVAARLCADVTGVPTLLVARTDADAADLITSDCDPYD
SEFITGERTSEGFFRTHAGIEQAISRGLAYAPYADLVWCETSTPDLELARRFAQAIHAKYPG
KLLAYNCSPSFNWQKNLDDKTIASFQQQLSDMGYKFQFITLAGIHSMWFNMFDLANAYAQGE
GMKHYVEKVQQPEFAAAKDGYTFVSHQQEVGTGYFDKVTTIIQG
>d1d8ca_ c.1.13.1 (A:) Malate synthase G {Escherichia coli}
QTITQSRLRIDANFKRFVDEEVLPGTGLDAAAFWRNFDEIVHDLAPENRQLLAERDRIQAAL
DEWHRSNPGPVKDKAAYKSFLRELGYLVPQPERVTVETTGIDSEITSQAGPQLVVPAMNARY
ALNAANARWGSLYDALYGSDIIPQEGAMVSGYDPQRGEQVIAWVRRFLDESLPLENGSYQDV
VAFKVVDKQLRIQLKNGKETTLRTPAQFVGYRGDAAAPTCILLKNNGLHIELQIDANGRIGK
DDPAHINDVIVEAAISTILDCEDSVAAVDAEDKILLYRNLLGLMQGTLQEKMEKNGRQIVRK
LNDDRHYTAADGSEISLHGRSLLFIRNVGHLMTIPVIWDSEGNEIPEGILDGVMTGAIALYD
LKVQKNSRTGSVYIVKPKMHGPQEVAFANKLFTRIETMLGMAPNTLKMGIMDEERRTSLNLR
SCIAQARNRVAFINTGFLDRTGDEMHSVMEAGPMLRKNQMKSTPWIKAYERNNVLSGLFCGL
RGKAQIGKGMWAMPDLMADMYSQKGDQLRAGANTAWVPSPTAATLHALHYHQTNVQSVQANI
AQTEFNAEFEPLLDDLLTIPVAENANWSAQEIQQELDNNVQGILGYVVRWVEQGIGCSKVPD
IHNVALMEDRATLRISSQHIANWLRHGILTKEQVQASLENMAKVVDQQNAGDPAYRPMAGNF

ANSCAFKAASDLIFLGVKQPNGYTEPLLHAWRLREKES
>d3rubl1 c.1.14.1 (L:148-467) Ribulose 1,5-bisphosphate carboxylase-oxygenase {Tobacco (Nicotiana tabacum), variant turkish samsun}
FQGPPHGIQVERDKLNKYGRPLLGCTIKPKLGLSAKNYGRAVYECLRGGLDFTKDDENVNSQ
PFMRWRDRFLFCAEALYKAQAETGEIKGHYLNATAGTCEEMIKRAVFARELGVPIVMHDYLT
GGFTANTSLAHYCRDNGLLLHIHRAMHAVIDRQKNHGIHFRVLAKALRMSGGDHIHSGTVVG
KLEGERDITLGFVDLLRDDFVEQDRSRGIYFTQDWVSLPGVLPVASGGIHVWHMPALTEIFG
DDSVLQFGGMTLGHPWGNAPGAVANRVALEACVKARNEGRDLAQEGNEIIREACKWSPELAA
ACEVWKEIVF
>d1bura1 c.1.14.1 (A:148-475) Ribulose 1,5-bisphosphate carboxylase-oxygenase {Spinach (Spinacia oleracea)}
FQGPPHGIQVERDKLNKYGRPLLGCTIKPKLGLSAKNYGRAVYECLRGGLDFTKDDENVNSQ
PFMRWRDRFLFCAEALYKAQAETGEIKGHYLNATAGTCEDMMKRAVFARELGVPIVMHDYLT
GGFTANTTLSHYCRDNGLLLHIHRAMHAVIDRQKNHGMHFRVLAKALRLSGGDHIHSGTVVG
KLEGERDITLGFVDLLRDDYTEKDRSRGIYFTQSWVSTPGVLPVASGGIHVWHMPALTEIFG
DDSVLQFGGGTLGHPWGNAPGAVANRVALEACVQARNEGRDLAREGNTIIREATKWSPELAA
ACEVWKEIKFEFPAMDTV
>d1bwva1 c.1.14.1 (A:150-478) Ribulose 1,5-bisphosphate carboxylase-oxygenase {Galdieria partita}
GPATGVILERERLDKFGRPLLGCTTKPKLGLSGKNYGRVVYEALKGGLDFVKDDENINSQPF
MRWRERYLFTMEAVNKASAATGEVKGHYLNVTAATMEEMYARANFAKELGSVIIMIDLVIGY
TAIQTMAKWARDNDMILHLHRAGNSTYSRQKNHGMNFRVICKWMRMAGVDHIHAGTVVGKLE
GDPIITRGFYKTLLLPKLERNLQEGLFFDMEWASLRKVMPVASGGIHAGQMHQLIHYLGEDV
VLQFGGGTIGHPDGIQAGATANRVALEAMILARNENRDYLTEGPEILREAAKTCGALRTALD
LWKDITFNYTSTDTSDFV
>d1gk8a1 c.1.14.1 (A:150-475) Ribulose 1,5-bisphosphate carboxylase-oxygenase {Chlamydomonas reinhardtii}
GPPHGIQVERDKLNKYGRGLLGCTIKPKLGLSAKNYGRAVYECLRGGLDFTKDDENVNSQPF
MRWRDRFLFVAEAIYKAQAETGEVKGHYLNATAGTCEEMMKRAVCAKELGVPIIMHDYLTGG
FTANTSLAIYCRDNGLLLHIHRAMHAVIDRQRNHGIHFRVLAKALRMSGGDHLHSGTVVGKL
EGEREVTLGFVDLMRDDYVEKDRSRGIYFTQDWCSMPGVMPVASGGIHVWHMPALVEIFGDD
ACLQFGGGTLGHPWGNAPGAAANRVALEACTQARNEGRDLAREGGDVIRSACKWSPELAAAC
EVWKEIKFEFDTIDKL
>d1bxna1 c.1.14.1 (A:151-467) Ribulose 1,5-bisphosphate carboxylase-oxygenase {Alcaligenes eutrophus}
FAGPSTGIIVERERLDKFGRPLLGATTKPKLGLSGRNYGRVVYEGLKGGLDFMKDDENINSQ
PFMHWRDRFLFVMDAVNKASAATGEVKGSYLNVTAGTMEEMYRRAEFAKSLGSVIIMVDLIV
GWTCIQSMSNWCRQNDMILHLHRAGHGTYTRQKNHGVSFRVIAKWLRLAGVDHMHTGTAVGK
LEGDPLTVQGYYNVCRDAYTQTDLTRGLFFDQDWASLRKVMPVASGGIHAGQMHQLIHLFGD
DVVLQFGGGTIGHPQGIQAGATANRVALEAMVLARNEGRDILNEGPEILRDAARWCGPLRAA
LDTWGDI
>d1rbla1 c.1.14.1 (A:148-475) Ribulose 1,5-bisphosphate carboxylase-oxygenase {Synechococcus sp., strain pcc 6301}

FQGPPHGIQVERDLLNKYGRPMLGCTIKPKLGLSAKNYGRAVYECLRGGLDFTKDDENINSQ
PFQRWRDRFLFVADAIHKSQAETGEIKGHYLNVTAPTCEEMMKRAEFAKELGMPIIMHDFLT
AGFTANTTLAKWCRDNGVLLHIHRAMHAVIDRQRNHGIHFRVLAKCLRLSGGDHLHSGTVVG
KLEGDKASTLGFVDLMREDHIEADRSRGVFFTQDWASMPGVLPVASGGIHVWHMPALVEIFG
DDSVLQFGGGTLGHPWGNAPGATANRVALEACVQARNEGRDLYREGGDILREAGKWSPELAA
ALDLWKEIKFEFETMDKL
>d5ruba1 c.1.14.1 (A:138-457) Ribulose 1,5-bisphosphate
carboxylase-oxygenase {Rhodospirillum rubrum}
GPSVNISALWKVLGRPEVDGGLVVGTIIKPKLGLRPKPFAEACHAFWLGGDFIKNDEPQGNQ
PFAPLRDTIALVADAMRRAQDETGEAKLFSANITADDPFEIIARGEYVLETFGENASHVALL
VDGYVAGAAAITTARRRFPDNFLHYHRAGHGAVTSPQSKRGYTAFVHCKMARLQGASGIHTG
TMGFGKMEGESSDRAIAYMLTQDEAQGPFYRQSWGGMKACTPIISGGMNALRMPGFFENLGN
ANVILTAGGGAFGHIDGPVAGARSLRQAWQAWRDGVPVLDYAREHKELARAFESFPGDADQI
YPGWRKALGV
>d1geha1 c.1.14.1 (A:137-443) Ribulose 1,5-bisphosphate
carboxylase-oxygenase {Archaeon Thermococcus kodakaraensis}
DGPAFGIEGVRKMLEIKDRPIYGVVPKPKVGYSPEEFEKLAYDLLSNGADYMKDDENLTSPW
YNRFEERAEIMAKIIDKVENETGEKKTWFANITADLLEMEQRLEVLADLGLKHAMVDVVITG
WGALRYIRDLAADYGLAIHGHRAMHAAFTRNPYHGISMFVLAKLYRLIGIDQLHVGTAGAGK
LEGGKWDVIQNARILRESHYKPDENDVFHLEQKFYSIKAAFPTSSGGLHPGNIQPVIEALGT
DIVLQLGGGTLGHPDGPAAGARAVRQAIDAIMQGIPLDEYAKTHKELARALEKWGHVTP
>d1qtwa_ c.1.15.1 (A:) Endonuclease IV {Escherichia coli}
MKYIGAHVSAAGGLANAAIRAAEIDATAFALFTKNQRQWRAAPLTTQTIDEFKAACEKYHYT
SAQILPHDSYLINLGHPVTEALEKSRDAFIDEMQRCEQLGLSLLNFHPGSHLMQISEEDCLA
RIAESINIALDKTQGVTAVIENTAGQGSNLGFKFEHLAAIIDGVEDKSRVGVCIDTCHAFAA
GYDLRTPAECEKTFADFARTVGFKYLRGMHLNDAKSTFGSRVDRHHSLGEGNIGHDAFRWIM
QDDRFDGIPLILETINPDIWAEEIAWLKAQQTEKAVA
>d1d8wa_ c.1.15.2 (A:) L-rhamnose isomerase {Escherichia coli}
TQLEQAWELAKQRFAAVGIDVEEALRQLDRLPVSMHCWQGDDVSGFENPEGSLTGGIQATGN
YPGKARNASELRADLEQAMRLIPGPKRLNLHAIYLESDTPVSRDQIKPEHFKNWVEWAKANQ
LGLDFNPSCFSHPLSADGFTLSHADDSIRQFWIDHCKASRRVSAYFGEQLGTPSVMNIWIPD
GMKDITVDRLAPRQRLLAALDEVISEKLNPAHHIDAVESKLFGIGAESYTVGSNEFYMGYAT
SRQTALCLDAGHFHPTEVISDKISAAMLYVPQLLLHVSRPVRWDSDHVVLLDDETQAIASEI
VRHDLFDRVHIGLDFFDASINRIAAWVIGTRNMKKALLRALLEPTAELRKLEAPGDYTARLA
LLEEQKSLPWQAVWEMYCQRHDTPAGSEWLESVRAYEKEILSRR
>d1dxia_ c.1.15.3 (A:) D-xylose isomerase {Streptomyces murinus}
MSFQPTPEDRFTFGLWTVGWQGRDPFGDATRPALDPVETVQRLAELGAYGVTFHDDDLIPFG
SSDTERESHIKRFRQALDATGMTVPMATTNLFTHPVFKDGGFTANDRDVRRYALRKTIGNID
LAAELGAKTYVAWGGREGAESGGAKDVRDALDRMKEAFDLLGEYVTAQGYDLRFAIEPKPNE
PRGDILLPTVGHALAFIERLERPELYGVNPEVGHEQMAGLNFPHGIAQALWAGKLFHIDLNG
QSGIKYDQDLRFGAGDLRAAFWLVDLLETAGYEGPRHFDFKPPRTEDFDGVWASAAGCMRNY
LILKDRAAAFRADPEVQEALRAARLDQLAQPTAADGLDALLADRAAFEDFDVDAAAARGMAF
EHLDQLAMDHLLGARG
>d2gyia_ c.1.15.3 (A:) D-xylose isomerase {Streptomyces

olivochromogenes}
YQPTPEDRFTFGLWTVGWQGRDPFGDATRPALDPVETVQRLAELGAHGVTFHDDDLIPFGSS
DTERESHIKRFRQALDATGMTVPMATTNLFTHPVFKDGGFTANDRDVRRYALRKTIRNIDLA
VELGAKTYVAWGGREGAESGAAKDVRVALDRMKEAFDLLGEYVTSQGYDTRFAIEPKPNEPR
GDILLPTVGHALAFIERLERPELYGVNPEVGHEQMAGLNFPHGIAQALWAGKLFHIDLNGQS
GIKYDQDLRFGAGDLRAAFWLVDLLESAGYEGPRHFDFKPPRTEDIDGVWASAAGCMRNYLI
LKERAAAFRADPEVQEALRASRLDELAQPTAADGVQELLADRTAFEDFDVDAAAARGMAFER
LDQLAMDHLLGAR
>d1xis__ c.1.15.3 (-) D-xylose isomerase {Streptomyces
rubiginosus}
NYQPTPEDRFTFGLWTVGWQGRDPFGDATRRALDPVESVRRLAELGAHGVTFHDDDLIPFGS
SDSEREEHVKRFRQALDDTGMKVPMATTNLFTHPVFKDGGFTANDRDVRRYALRKTIRNIDL
AVELGAETYVAWGGREGAESGGAKDVRDALDRMKEAFDLLGEYVTSQGYDIRFAIEPKPNEP
RGDILLPTVGHALAFIERLERPELYGVNPEVGHEQMAGLNFPHGIAQALWAGKLFHIDLNGQ
NGIKYDQDLRFGAGDLRAAFWLVDLLESAGYSGPRHFDFKPPRTEDFDGVWASAAGCMRNYL
ILKERAAAFRADPEVQEALRASRLDELARPTAADGLQALLDDRSAFEEFDVDAAAARGMAFE
RLDQLAMDHLLGARG
>d1qt1a_ c.1.15.3 (A:) D-xylose isomerase {Streptomyces
diastaticus, M1033}
SYQPTPEDKFTFGLWTVGWQGRDPFGDATRGALDPAESVRRLAELGAHGVTFHDDDLIPFGA
TDSERAEHIKRFRQGLDETGMKVPMATTNLFTHPVFKDGGFTANDRDVRRYALRKTIRNIDL
AVELGAQTYVAWGGREGAESGAAKDVRVALDRMKEAFDLLGEYVTSQGYDTPFAIEPKPNEP
RGDILLPTIGHALAFIDGLERPELYGVNPEVGHEQMAGLNFPHGIAQALWAGKLFHIDLNGQ
SGIKYDQDLRFGPGDLRAAFWLVDLLESAGYEGPRHFDFKPPRTEDFDGVWASAAGCMRNYL
ILKERAAAFRADPEVQEALRAARLDELAQPTAGDGLQALLPDRSAFEDFDPDAAAARGMAFE
RLDQLAMDHLLGARG
>d4xiaa_ c.1.15.3 (A:) D-xylose isomerase {Arthrobacter, strain
b3728}
VQPTPADHFTFGLWTVGWTGADPFGVATRANLDPVEAVHKLAELGAYGITFHDNDLIPFDAT
AAEREKILGDFNQALADTGLKVPMVTTNLFSHPVFKDGGFTSNDRSIRRFALAKVLHNIDLA
AEMGAETFVMWGGREGSEYDGSKDLAAALDRMREGVDTAAGYIKDKGYNLRIALEPKPNEPR
GDIFLPTVGHGLAFIEQLEHGDIVGLNPETGHEQMAGLNFTHGIAQALWAEKLFHIDLNGQR
GIKYDQDLVFGHGDLTSAFFTVDLLENGFPNGGPKYTGPRHFDYKPSRTDGYDGVWDSAKAN
MSMYLLLKERALAFRADPEVQEAMKTSGVFELGETTLNAGESAADLMNDSASFAGFDAEAAA
ERNFAFIRLNQLAIEHLLGSR
>d1xima_ c.1.15.3 (A:) D-xylose isomerase {Actinoplanes
missouriensis}
VQATREDKFSFGLWTVGWQARDAFGDATRTALDPVEAVHKLAEIGAYGITFHDDDLVPFGSD
AQTRDGIIAGFKKALDETGLIVPMVTTNLFTHPVFKDGGFTSNDRSVRRYAIRKVLRQMDLG
AELGAKTLVLWGGREGAEYDSAKDVSAALDRYREALNLLAQYSEDRGYGLRFAIEPKPNEPR
GDILLPTAGHAIAFVQELERPELFGINPETGHEQMSNLNFTQGIAQALWHKKLFHIDLNGQH
GPKFDQDLVFGHGDLLNAFSLVDLLENGPDGAPAYDGPRHFDYKPSRTEDYDGVWESAKANI
RMYLLLKERAKAFRADPEVQEALAASKVAELKTPTLNPGEGYAELLADRSAFEDYDADAVGA
KGFGFVKLNQLAIEHLLGAR

>d1a0ca_ c.1.15.3 (A:) D-xylose isomerase {Clostridium thermosulfurogenes, also known as Thermoanaerobacter thermosulfurigenes}
NKYFENVSKIKYEGPKSNNPYSFKFYNPEEVIDGKTMEEHLRFSIAYWHTFTADGTDQFGKA
TMQRPWNHYTDPMDIAKARVEAAFEFFDKINAPYFCFHDRDIAPEGDTLRETNKNLDTIVAM
IKDYLKTSKTKVLWGTANLFSNPRFVHGASTSCNADVFAYSAAQVKKALEITKELGGENYVF
WGGREGYETLLNTDMEFELDNFARFLHMAVDYAKEIGFEGQFLIEPKPKEPTKHQYDFDVAN
VLAFLRKYDLDKYFKVNIEANHATLAFHDFQHELRYARINGVLGSIDANTGDMLLGWDTDQF
PTDIRMTTLAMYEVIKMGGFDKGGLNFDAKVRRASFEPEDLFLGHIAGMDAFAKGFKVAYKL
VKDRVFDKFIEERYASYKDGIGADIVSGKADFRSLEKYALERSQIVNKSGRQELLESILNQY
LFA
>d1a0da_ c.1.15.3 (A:) D-xylose isomerase {Bacillus stearothermophilus}
PYFDNISTIAYEGPASKNPLAFKFYNPEEKVGDKTMEEHLRFSVAYWHTFTGDGSDPFGAGN
MIRPWNKYSGMDLAKARVEAAFEFFEKLNIPFFCFHDVDIAPEGETLKETYKNLDIIVDMIE
EYMKTSKTKLLWNTANLFTHPRFVHGAATSCNADVFAYAAAKVKKGLEIAKRLGAENYVFWG
GREGYETLLNTDMKLELDNLARFLHMAVDYAKEIGFDGQFLIEPKPKEPTKHQYDFDVATAL
AFLQTYGLKDYFKFNIEANHATLAGHTFEHELRVARIHGMLGSVDANQGDMLLGWDTDEFPT
DLYSTTLAMYEILKNGGLGRGGLNFDAKVRRGSFEPEDLFYAHIAGMDSFAVGLKVAHRLIE
DRVFDEFIEERYKSYTEGIGREIVEGTADFHKLEAHALQLGEIQNQSGRQERLKTLLNQYLL
EVC
>d1a0ea_ c.1.15.3 (A:) D-xylose isomerase {Thermotoga neapolitana}
AEFFPEIPKVQFEGKESTNPLAFKFYDPEEIIDGKPLKDHLKFSVAFWHTFVNEGRDPFGDP
TADRPWNRYTDPMDKAFARVDALFEFCEKLNIEYFCFHDRDIAPEGKTLRETNKILDKVVER
IKERMKDSNVKLLWGTANLFSHPRYMHGAATTCSADVFAYAAAQVKKALEITKELGGEGYVF
WGGREGYETLLNTDLGFELENLARFLRMAVDYAKRIGFTGQFLIEPKPKEPTKHQYDFDVAT
AYAFLKSHGLDEYFKFNIEANHATLAGHTFQHELRMARILGKLGSIDANQGDLLLGWDTDQF
PTNVYDTTLAMYEVIKAGGFTKGGLNFDAKVRRASYKVEDLFIGHIAGMDTFALGFKVAYKL
VKDGVLDKFIEEKYRSFREGIGRDIVEGKVDFEKLEEYIIDKETIELPSGKQEYLESLINSY
IVKTILELR
>d1bxca_ c.1.15.3 (A:) D-xylose isomerase {Thermus aquaticus, subsp. Caldophilus}
MYEPKPEHRFTFGLWTVGNVGRDPFGDAVRERLDPVYVGHKLAELGVHGVNLHDEDLIPRGT
PPQERDQIVRRFKRALDETGLKVPMVTGNLFSDPGFKDGGFTSRDPWVRAYAFRKSLETMDL
GAELGAEIYVVWPGREGAEVEATGKARKVWDWVREPLNFMAAYAEDQGYGYRFALEPKPNEP
RGDIYFATVGSMLALIHTLERPERFGLNPEFAHETMAGLNFVHAVAQALDAGKLLHIDLNGQ
RMNRFDQDLRFGSENLKAAFLLVDLLESSGYQGPRHFDAHALRTEDEEGVWAFARGCMRTYL
ILKERAEAFREDPEVKELLAAYYQEDPAALPLMDPYSHEKAEALKRAELPLEAKRHRGYALE
RLDQLAVEYLLGVRG
>d1bxba_ c.1.15.3 (A:) D-xylose isomerase {Thermus aquaticus, subsp. Thermophilus}
MYEPKPEHRFTFGLWTVGNVGRDPFGDAVRERLDPVYVVHKLAELGAYGVNLHDEDLIPRGT
PPQERDQIVRRFKKALDETGLKVPMVTANLFSDPAFKDGAFTSPDPWVRAYALRKSLETMDL
GAELGAEIYVVWPGREGAEVEATGKARKVWDWVREALNFMAAYAEDQGYGYRFALEPKPNEP

RGDIYFATVGSMLAFIHTLDRPERFGLNPEFAHETMAGLNFVHAVAQALDAGKLFHIDLNDQ
RMSRFDQDLRFGSENLKAAFFLVDLLESSGYQGPRHFDAHALRTEDEEGVWAFARGCMRTYL
ILKERAEAFREDPEVKELLAAYYQEDPAALALLGPYSREKAEALKRAELPLEAKRRRGYALE
RLDQLAVEYLLGVRG
>d1luca_ c.1.16.1 (A:) Bacterial luciferase (alkanal monooxygenase)
{Vibrio harveyi}
MKFGNFLLTYQPPELSQTEVMKRLVNLGKASEGCGFDTVWLLEHHFTEFGLLGNPYVAAAHL
LGATETLNVGTAAIVLPTAHPVRQAEDVNLLDQMSKGRFRFGICRGLYDKDFRVFGTDMDNS
RALMDCWYDLMKEGFNEGYIAADNEHIKFPKIQLNPSAYTQGGAPVYVVAESASTTEWAAER
GLPMILSWIINTHEKKAQLDLYNEVATEHGYDVTKIDHCLSYITSVDHDSNRAKDICRNFLG
HWYDSYVNATKIFDDSDQTKGYDFNKGQWRDFVLKGHKDTNRRIDYSYEINPVGTPEECIAI
IQQDIDATGIDNICCGFEANGSEEEIIASMKLFQSDVMPYLKEKQ
>d1lucb_ c.1.16.1 (B:) Bacterial luciferase (alkanal monooxygenase)
{Vibrio harveyi}
MKFGLFFLNFMNSKRSSDQVIEEMLDTAHYVDQLKFDTLAVYENHFSNNGVVGAPLTVAGFL
LGMTKNAKVASLNHVITTHHPVRVAEEACLLDQMSEGRFAFGFSDCEKSADMRFFNRPTDSQ
FQLFSECHKIINDAFTTGYCHPNNDFYSFPKISVNPHAFTEGGPAQFVNATSKEVVEWAAKL
GLPLVFRWDDSNAQRKEYAGLYHEVAQAHGVDVSQVRHKLTLLVNQNVDGEAARAEARVYLE
EFVRESYSNTDFEQKMGELLSENAIGTYEESTQAARVAIECCGAADLLMSFESMEDKAQQRA
VIDVVNANIV
>d1nfp__ c.1.16.2 (-) Non-fluorescent flavoprotein (luxF, FP390)
{Photobacterium leiognathi}
MTKWNYGVFFLNFYHVGQQEPSLTMSNALETLRIIDEDTSIYDVVAFSEHHIDKSYNDETKL
APFVSLGKQIHVLATSPETVVKAAKYGMPLLFKWDDSQQKRIELLNHYQAAAAKFNVDIANV
RHRLMLFVNVNDNPTQAKAELSIYLEDYLSYTQAETSIDEIINSNAAGNFDTCLHHVAEMAQ
GLNNKVDFLFCFESMKDQENKKSLMINFDKRVINYRKEHNLN
>d1fvpa_ c.1.16.2 (A:) Non-fluorescent flavoprotein (luxF, FP390)
{Photobacterium phosphoreum}
MNKWNYGVFFVNFYNKGQQEPSKTMNNALETLRIIDEDTSIYDVINIDDHYLVKKDSEDKKL
APFITLGEKLYVLATSENTVDIAAKYALPLVFKWDDINEERLKLLSFYNASASKYNKNIDLV
RHQLMLHVNVNEAETVAKEELKLYIENYVACTQPSNFNGSIDSIIQSNVTGSYKDCLSYVAN
LAGKFDNTVDFLLCFESMQDQNKKKSVMIDLNNQVIKFRQDNNLI
>d1ezwa_ c.1.16.3 (A:) Coenzyme F420 dependent
tetrahydromethanopterin reductase {Archaeon Methanopyrus
kandleri}
AEVSFGIELLPDDKPTKIAHLIKVAEDNGFEYAWICDHYNNYSYMGVLTLAAVITSKIKLGP
GITNPYTRHPLITASNIATLDWISGGRAIIGMGPGDKATFDKMGLPFPCKIPIWNPEAEDEV
GPATAIREVKEVIYQYLEGGPVEYEGKYVKTGTADVKARSIQGSDIPFYMGAQGPIMLKTAG
EIANGVLVNASNPKDFEVAVPKIEEGAKEAGRSLDEIDVAAYTCFSIDKDEDKAIEATKIVV
AFIVMGSPDVVLERHGIDTEKAEQIAEAIGKGDFGTAIGLVDEDMIEAFSIAGDPDTVVDKI
EELLKAGVTQVVVGSPIGPDKEKAIELVGQEVIPHFK
>d1f07a_ c.1.16.3 (A:) Coenzyme F420 dependent
tetrahydromethanopterin reductase {Archaeon Methanobacterium
thermoautotrophicum}

MKFGIEFVPNEPIEKIVKLVKLAEDVGFEYAWITDHYNNKNVYETLALIAEGTETIKLGPGV
TNPYVRSPAITASAIATLDELSNGRATLGIGPGDKATFDALGIEWVKPVSTIRDAIAMMRTL
LAGEKTESGAQLMGVKAVQEKIPIYMGAQGPMMLKTAGEISDGALINASNPKDFEAAVPLIK
EGAEAAGKSIADIDVAAYTCCSIDEDAAAAANAAKIVVAFIAAGSPPPVFERHGLPADTGKK
FGELLGKGDFGGAIGAVDDALMEAFSVVGTPDEFIPKIEALGEMGVTQYVAGSPIGPDKEKS
IKLLGEVIASF
>d1qapa1 c.1.17.1 (A:130-296) Quinolinic acid phosphoribosyltransferase, C-terminal domain {Salmonella typhimurium}
VASEVRRYVGLLAGTQTQLLDTRKTLPGLRTALKYAVLCGGGANHRLGLTDAFLIKENHIIA
SGSVRQAVEKAFWLHPDVPVEVEVENLDELDDALKAGADIIMLDNFNTDQMREAVKRVNGQA
RLEVSGNVTAETLREFAETGVDFISVGALTKHVRALDLSMRFC
>d1qpoa1 c.1.17.1 (A:117-285) Quinolinic acid phosphoribosyltransferase, C-terminal domain {Mycobacterium tuberculosis}
IATATAAWVDAVRGTKAKIRDTRKTLPGLRALQKYAVRTGGGVNHRLGLGDAALIKDNHVAA
AGSVVDALRAVRNAAPDLPCEVEVDSLEQLDAVLPEKPELILLDNFAVWQTQTAVQRRDSRA
PTVMLESSGGLSLQTAATYAETGVDYLAVGALTHSVRVLDIGLDM
>d1qasa3 c.1.18.1 (A:299-625) Phospholipase C isozyme D1 (PLC-D1) {Rat (Rattus norvegicus)}
DQPLSHYLVSSSHNTYLLEDQLTGPSSTEAYIRALCKGCRCLELDCWDGPNQEPIIYHGYTF
TSKILFCDVLRAIRDYAFKASPYPVILSLENHCSLEQQRVMARHLRAILGPILLDQPLDGVT
TSLPSPEQLKGKILLKGKKLGGLLPAGGENGSEATDVSDEVEAAEMEDEAVRSQVQHKPKED
KLKLVPELSDMIIYCKSVHFGGFSSPGTSGQAFYEMASFSESRALRLLQESGNGFVRHNVSC
LSRIYPAGWRTDSSNYSPVEMWNGGCQIVALNFQTPGPEMDVYLGCFQDNGGCGYVLKPAFL
RDPNTTFNSRALTQGPW
>d2ptd__ c.1.18.2 (-) Phosphatidylinositol-specific phospholipase C {Bacillus cereus}
ASSVNELENWSKWMQPIPDSIPLARISIPGTHDSGTFKLQNPIKQVWGMTQEYDFRYQMDHG
ARIFDIRGRLTDDNTIVLHHGPLYLYVTLHEFINEAKQFLKDNPSETIIMSLKKEYEDMKGA
EDSFSSTFEKKYFVDPIFLKTEGNIKLGDARGKIVLLKRYSGSNEPGGYNNFYWPDNETFTT
TVNQNANVTVQEKYKVSYDEKVKSIKDTMDETMNNSEDLNHLYINFTSLSSGGTAWNSPYYY
ASYINPEIANYIKQKNPARVGWVIQDYINEKWSPLLYQEVIRANKSLI
>d2plc__ c.1.18.2 (-) Phosphatidylinositol-specific phospholipase C {Listeria monocytogenes}
VTTKQWMSALPDTTNLAALSIPGTHDTMSYNGDITWTLTKPLAQTQTMSLYQQLEAGIRYID
IRAKDNLNIYHGPIFLNASLSGVLETITQFLKKNPKETIIMRLKDEQNSNDSFDYRIQPLIN
IYKDYFYTTPRTDTSNKIPTLKDVRGKILLLSENHTKKPLVINSRKFGMQFGAPNQVIQDDY
NGPSVKTKFKEIVQTAYQASKADNKLFLNHISATSLTFTPRQYAAALNNKVEQFVLNLTSEK
VRGLGILIMDFPEKQTIKNIIKNNKF
>d7reqa1 c.1.19.1 (A:4-560) Methylmalonyl-CoA mutase, alpha and beta subunits {Propionibacterium freudenreichii, subsp. shermanii}
LPRFDSVDLGNAPVPADAARRFEELAAKAGTGEAWETAEQIPVGTLFNEDVYKDMDWLDTYA

GIPPFVHGPYATMYAFRPWTIRQYAGFSTAKESNAFYRRNLAAGQKGLSVAFDLPTHRGYDS
DNPRVAGDVGMAGVAIDSIYDMRELFAGIPLDQMSVSMTMNGAVLPILALYVVTAEEQGVKP
EQLAGTIQNDILKEFMVRNTYIYPPQPSMRIISEIFAYTSANMPKWNSISISGYHMQEAGAT
ADIEMAYTLADGVDYIRAGESVGLNVDQFAPRLSFFWGIGMNFFMEVAKLRAARMLWAKLVH
QFGPKNPKSMSLRTHSQTSGWSLTAQDVYNNVVRTCIEAMAATQGHTQSLHTNSLDEAIALP
TDFSARIARNTQLFLQQESGTTRVIDPWSGSAYVEELTWDLARKAWGHIQEVEKVGGMAKAI
EKGIPKMRIEEAAARTQARIDSGRQPLIGVNKYRLEHEPPLDVLKVDNSTVLAEQKAKLVKL
RAERDPEKVKAALDKITWAAGNPDDKDPDRNLLKLCIDAGRAMATVGEMSDALEKVFGRYT
>d7reqb1 c.1.19.1 (B:16-475) Methylmalonyl-CoA mutase, alpha and beta subunits {Propionibacterium freudenreichii, subsp. shermanii}
LTPTTLSLAGDFPKATEEQWEREVEKVLNRGRPPEKQLTFAECLKRLTVHTVDGIDIVPMYR
PKDAPKKLGYPGVAPFTRGTTVRNGDMDAWDVRALHEDPDEKFTRKAILEGLERGVTSLLLR
VDPDAIAPEHLDEVLSDVLLEMTKVEVFSRYDQGAAAEALVSVYERSDKPAKDLALNLGLDP
IGFAALQGTEPDLTVLGDWVRRLAKFSPDSRAVTIDANIYHNAGAGDVAELAWALATGAEYV
RALVEQGFTATEAFDTINFRVTATHDQFLTIARLRALREAWARIGEVFGVDEDKRGARQNAI
TSWRELTREDPYVNILRGSIATFSASVGGAESITTLPFTQALGLPEDDFPLRIARNTGIVLA
EEVNIGRVNDPAGGSYYVESLTRSLADAAWKEFQEVEKLGGMSKAVMTEHVTKVLDACNAER
AKRLANRKQPITAVSEFPMIGARSIE
>d1ccwb_ c.1.19.2 (B:) Glutamate mutase, large subunit {Clostridium cochlearium}
MELKNKKWTDEEFHKQREEVLQQWPTGKEVDLQEAVDYLKKIPAEKNFAEKLVLAKKKGITM
AQPRAGVALLDEHIELLRYLQDEGGADFLPSTIDAYTRQNRYDECENGIKESEKAGRSLLNG
FPGVNFGVKGCRKVLEAVNLPLQARHGTPDSRLLAEIIHAGGWTSNEGGGISYNVPYAKNVT
IEKSLLDWQYCDRLVGFYEEQGVHINREPFGPLTGTLVPPSMSNAVGITEALLAAEQGVKNI
TVGYGECGNMIQDIAALRCLEEQTNEYLKAYGYNDVFVTTVFHQWMGGFPQDESKAFGVIVT
ATTIAALAGATKVIVKTPHEAIGIPTKEANAAGIKATKMALNMLEGQRMPMSKELETEMAVI
KAETKCILDKMFELGKGDLAIGTVKAFETGVMDIPFGPSKYNAGKMMPVRDNLGCVRYLEFG
NVPFTEEIKNYNRERLQERAKFEGRDVSFQMVIDDIFAVGKGRLIGRPE
>d1eexa_ c.1.19.3 (A:) Diol dehydratase, alpha subunit {Klebsiella oxytoca}
MRSKRFEALAKRPVNQDGFVKEWIEEGFIAMESPNDPKPSIKIVNGAVTELDGKPVSDFDLI
DHFIARYGINLNRAEEVMAMDSVKLANMLCDPNVKRSEIVPLTTAMTPAKIVEVVSHMNVVE
MMMAMQKMRARRTPSQQAHVTNVKDNPVQIAADAAEGAWRGFDEQETTVAVARYAPFNAIAL
LVGSQVGRPGVLTQCSLEEATELKLGMLGHTCYAETISVYGTEPVFTDGDDTPWSKGFLASS
YASRGLKMRFTSGSGSEVQMGYAEGKSMLYLEARCIYITKAAGVQGLQNGSVSCIGVPSAVP
SGIRAVLAENLICSSLDLECASSNDQTFTHSDMRRTARLLMQFLPGTDFISSGYSAVPNYDN
MFAGSNEDAEDFDDYNVIQRDLKVDGGLRPVREEDVIAIRNKAARALQAVFAGMGLPPITDE
EVEAATYAHGSKDMPERNIVEDIKFAQEIINKNRNGLEVVKALAQGGFTDVAQDMLNIQKAK
LTGDYLHTSAIIVGDGQVLSAVNDVNDYAGPATGYRLQGERWEEIKNIPGALDPN
>d1f3ea_ c.1.20.1 (A:) tRNA-guanine transglycosylase {Zymomonas mobilis}
RPRFSFSIAAREGKARTGTIEMKRGVIRTPAFMPVGTAATVKALKPETVRATGADIILGNTY
HLMLRPGAERIAKLGGLHSFMGWDRPILTDSGGYQVMSLSSLTKQSEEGVTFKSHLDGSRHM

LSPERSIEIQHLLGSDIVMAFDECTPYPATPSRAASSMERSMRWAKRSRDAFDSRKEQAENA
ALFGIQQGSVFENLRQQSADALAEIGFDGYAVGGLAVGEGQDEMFRVLDFSVPMLPDDKPHY
LMGVGKPDDIVGAVERGIDMFDCVLPTRSGRNGQAFTWDGPINIRNARFSEDLKPLDSECHC
AVCQKWSRAYIHHLIRAGEILGAMLMTEHNIAFYQQLMQKIRDSISEGRFSQFAQDFRARYF
>d1aj2__ c.1.21.1 (-) Dihydropteroate synthetase {Escherichia coli}
MKLFAQGTSLDLSHPHVMGILNVTPDSFSDGGTHNSLIDAVKHANLMINAGATIIDVGGEST
RPGAAEVSVEEELQRVIPVVEAIAQRFEVWISVDTSKPEVIRESAKVGAHIINDIRSLSEPG
ALEAAAETGLPVCLMHMQGNPKTMQEAPKYDDVFAEVNRYFIEQIARCEQAGIAKEKLLLDP
GFGFGKNLSHNYSLLARLAEFHHFNLPLLVGMSRKSMIGQLLNVGPSERLSGSLACAVIAAM
QGAHIIRVHDVKETVEAMRVVEATLSAKENKRYE
>d1ad1a_ c.1.21.1 (A:) Dihydropteroate synthetase {Staphylococcus aureus}
TKTKIMGILNVTPDSFSDGGKFNNVESAVTRVKAMMDEGADIIDVGGVSTRPGHEMITVEEE
LNRVLPVVEAIVGFDVKISVDTFRSEVAEACLKLGVDIINDQWAGLYDHRMFQVVAKYDAEI
VLMHNGNGNRDEPVVEEMLTSLLAQAHQAKIAGIPSNKIWLDPGIGFAKTRNEEAEVMARLD
ELVATEYPVLLATSRKRFTKEMMGYDTTPVERDEVTAATTAYGIMKGVRAVRVHNVELNAKL
AKGIDFLKENENARHN
>d1eyea_ c.1.21.1 (A:) Dihydropteroate synthetase {Mycobacterium tuberculosis}
PVQVMGVLNVTDDSFSDGGCYLDLDDAVKHGLAMAAAGAGIVDVGGESSRPGATRVDPAVET
SRVIPVVKELAAQGITVSIDTMRADVARAALQNGAQMVNDVSGGRADPAMGPLLAEADVPWV
LMHWRAVSADTPHVPVRYGNVVAEVRADLLASVADAVAAGVDPARLVLDPGLGFAKTAQHNW
AILHALPELVATGIPVLVGASRKRFLGALLAGPDGVMRPTDGRDTATAVISALAALHGAWGV
RVHDVRASVDAIKVVEAWMGAE
>d1f6ya_ c.1.21.2 (A:) Methyltetrahydrofolate: corrinoid/iron-sulfur protein methyltransferase MetR {Moorella thermoacetica}
MLIIGERINGMFGDIKRAIQERDPAPVQEWARRQEEGGARALDLNVGPAVQDKVSAMEWLVE
VTQEVSNLTLCLDSTNIKAIEAGLKKCKNRAMINSTNAEREKVEKLFPLAVEHGAALIGLTM
NKTGIPKDSDTRLAFAMELVAAADEFGLPMEDLYIDPLILPANVAQDHAPEVLKTLQQIKML
ADPAPKTVLGLSNVSQNCQNRPLINRTFLAMAMACGLMSAIADACDEALIETAATAEILLNQ
TVYCDSFVKMFKTR
>d1uroa_ c.1.22.1 (A:) Uroporphyrinogen decarboxylase, UROD {Human (Homo sapiens)}
GFPELKNDTFLRAAWGEETDYTPVWCMRQAGRYLPEFRETRAAQDFFSTCRSPEACCELTLQ
PLRRFPLDAAIIFSDILVVPQALGMEVTMVPGKGPSFPEPLREEQDLERLRDPEVVASELGY
VFQAITLTRQRLAGRVPLIGFAGAPWTLMTYMVEGGGSSTMAQAKRWLYQRPQASHQLLRIL
TDALVPYLVGQVVAGAQALQLFESHAGHLGPQLFNKFALPYIRDVAKQVKARLREAGLAPVP
MIIFAKDGHFALEELAQAGYEVVGLDWTVAPKKARECVGKTVTLQGNLDPCALYASEEEIGQ
LVKQMLDDFGPHRYIANLGHGLYPDMDPEHVGAFVDAVHKHSRLLRQ
>d1j93a_ c.1.22.1 (A:) Uroporphyrinogen decarboxylase, UROD {Tobacco (Nicotiana tabacum), UROD-III}
TQPLLLDAVRGKEVERPPVWLMRQAGRYMKSYQLLCEKYPLFRDRSENVDLVVEISLQPWKV

FRPDGVILFSDILTPLSGMNIPFDIIKGKGPVIFDPLRTAADVEKVREFIPEKSVPYVGEAL
TILRKEVNNQAAVLGFVGAPFTLASYVVEGGSSKNFTKIKRLAFAEPKVLHALLQKFATSMA
KYIRYQADSGAQAVQIFDSWATELSPVDFEEFSLPYLKQIVDSVKLTHPNLPLILYASGSGG
LLERLPLTGVDVVSLDWTVDMADGRRRLGPNVAIQGNVDPGVLFGSKEFITNRINDTVKKAG
KGKHILNLGHGIKVGTPEENFAHFFEIAKGLRY
>d1b5ta_ c.1.23.1 (A:) Methylenetetrahydrofolate reductase {Escherichia coli}
GQINVSFEFFPPRTSEMEQTLWNSIDRLSSLKPKFVSVTYGANSGERDRTHSIIKGIKDRTG
LEAAPHLTCIDATPDELRTIARDYWNNGIRHIVALRGDLPPGSGKPEMYASDLVTLLKEVAD
FDISVAAYPEVHPEAKSAQADLLNLKRKVDAGANRAITQFFFDVESYLRFRDRCVSAGIDVE
IIPGILPVSNFKQAKKFADMTNVRIPAWMAQMFDGLDDDAETRKLVGANIAMDMVKILSREG
VKDFHFYTLNRAEMSYAICHTLGVRPA
>d1heta2 c.2.1.1 (A:175-324) Alcohol dehydrogenase {Horse (Equus caballus)}
GFSTGYGSAVKVAKVTQGSTCAVFGLGGVGLSVIMGCKAAGAARIIGVDINKDKFAKAKEVG
ATECVNPQDYKKPIQEVLTEMSNGGVDFSFEVIGRLDTMVTALSCCQEAYGVSVIVGVPPDS
QNLSMNPMLLLSGRTWKGAIFGGFKS
>d1d1ta2 c.2.1.1 (A:175-324) Alcohol dehydrogenase {Human (Homo sapiens), different isozymes}
GFSTGYGAAVKTGKVKPGSTCVVFGLGGVGLSVIMGCKSAGASRIIGIDLNKDKFEKAMAVG
ATECISPKDSTKPISEVLSEMTGNNVGYTFEVIGHLETMIDALASCHMNYGTSVVVGVPPSA
KMLTYDPMLLFTGRTWKGCVFGGLKS
>d1ht0a2 c.2.1.1 (A:175-324) Alcohol dehydrogenase {Human (Homo sapiens), different isozymes}
GFSTGYGSAVKVAKVTPGSTCAVFGLGGVGLSVVMGCKAAGAARIIAVDINKDKFAKAKELG
ATECINPQDYKKPIQEVLKEMTDGGVDFSFEVIGQLDTMMASLLCCHEACGTSVIVGVPPDS
QNLSINPMLLLTGRTWKGAIFGGFKS
>d1teha2 c.2.1.1 (A:175-324) Alcohol dehydrogenase {Human (Homo sapiens), different isozymes}
GISTGYGAAVNTAKLEPGSVCAVFGLGGVGLAVIMGCKVAGASRIIGVDINKDKFARAKEFG
ATECINPQDFSKPIQEVLIEMTDGGVDYSFECIGNVKVMRAALEACHKGWGVSVVVGVAASG
EEIATRPFQLVTGRTWKGTAFGGWKS
>d1e3ia2 c.2.1.1 (A:175-324) Alcohol dehydrogenase {Mouse (Mus musculus), class II}
LIGCGFSSGYGAAINTAKVTPGSTCAVFGLGCVGLSAIIGCKIAGASRIIAIDINGEKFPKA
KALGATDCLNPRELDKPVQDVITELTAGGVDYSLDCAGTAQTLKAAVDCTVLGWGSCTVVGA
KVDEMTIPTVDVILGRSINGTFFGGW
>d1cdoa2 c.2.1.1 (A:176-324) Alcohol dehydrogenase {Cod (Gadus callarias)}
GVSTGFGAAVNTAKVEPGSTCAVFGLGAVGLAAVMGCHSAGAKRIIAVDLNPDKFEKAKVFG
ATDFVNPNDHSEPISQVLSKMTNGGVDFSLECVGNVGVMRNALESCLKGWGVSVLVGWTDLH
DVATRPIQLIAGRTWKGSMFGGFKG
>d1keva2 c.2.1.1 (A:151-314) Bacterial secondary alcohol dehydrogenase {Clostridium beijerinckii}

MMTTGFHGAELADIQMGSSVVVIGIGAVGLMGIAGAKLRGAGRIIGVGSRPICVEAAKFYGA
TDILNYKNGHIVDQVMKLTNGKGVDRVIMAGGGSETLSQAVSMVKPGGIISNINYHGSGDAL
LIPRVEWGCGMAHKTIKGGLCPGGRLRAEMLRDMVVYNRV
>d1ykfa2 c.2.1.1 (A:151-314) Bacterial secondary alcohol dehydrogenase {Thermoanaerobacter brockii}
MMTTGFHGAELADIELGATVAVLGIGPVGLMAVAGAKLRGAGRIIAVGSRPVCVDAAKYYGA
TDIVNYKDGPIESQIMNLTEGKGVDAAIIAGGNADIMATAVKIVKPGGTIANVNYFGEGEVL
PVPRLEWGCGMAHKTIKGGLCPGGRLRMERLIDLVFYKRV
>d1e3ja2 c.2.1.1 (A:151-313) Ketose reductase (sorbitol dehydrogenase) {Silverleaf whitefly (Bemisia argentifolii)}
LEPLSVGVHACRRAGVQLGTTVLVIGAGPIGLVSVLAAKAYGAFVVCTARSPRRLEVAKNCG
ADVTLVVDPAKEEESSIIERIRSAIGDLPNVTIDCSGNEKCITIGINITRTGGTLMLVGMGS
QMVTVPLVNACAREIDIKSVFRYCNDYPIALEMVASGRC
>d1qora2 c.2.1.1 (A:136-265) Quinone oxidoreductase {Escherichia coli}
YEIKPDEQFLFHAAAGGVGLIACQWAKALGAKLIGTVGTAQKAQSALKAGAWQVINYREEDL
VERLKEITGGKKVRVVYDSVGRDTWERSLDCLQRRGLMVSFGNSSGAVTGVNLGILNQKGSL
YVTRPS
>d1udc__ c.2.1.2 (-) Uridine diphosphogalactose-4-epimerase (UDP-galactose 4-epimerase) {Escherichia coli}
MRVLVTGGSGYIGSHTCVQLLQNGHDVIILDNLCNSKRSVLPVIERLGGKHPTFVEGDIRNE
ALMTEILHDHAIDTVIHFAGLKAVGESVQKPLEYYDNNVNGTLRLISAMRAANVKNFIFSSS
ATVYGDQPKIPYVESFPTGTPQSPYGKSKLMVEQILTDLQKAQPDWSIALLRYFNPVGAHPS
GDMGEDPQGIPNNLMPYIAQVAVGRRDSLAIFGNDYPTEDGTGVRDYIHVMDLADGHVVAME
KLANKPGVHIYNLGAGVGNSVLDVVNAFSKACGKPVNYHFAPRREGDLPAYWADASKADREL
NWRVTRTLDEMAQDTWHWQSRHPQGYPD
>d1ek6a_ c.2.1.2 (A:) Uridine diphosphogalactose-4-epimerase (UDP-galactose 4-epimerase) {Human (Homo sapiens)}
MAEKVLVTGGAGYIGSHTVLELLEAGYLPVVIDNFHNAFRGGGSLPESLRRVQELTGRSVEF
EEMDILDQGALQRLFKKYSFMAVIHFAGLKAVGESVQKPLDYYRVNLTGTIQLLEIMKAHGV
KNLVFSSSATVYGNPQYLPLDEAHPTGGCTNPYGKSKFFIEEMIRDLCQADKTWNAVLLRYF
NPTGAHASGCIGEDPQGIPNNLMPYVSQVAIGRREALNVFGNDYDTEDGTGVRDYIHVVDLA
KGHIAALRKLKEQCGCRIYNLGTGTGYSVLQMVQAMEKASGKKIPYKVVARREGDVAACYAN
PSLAQEELGWTAALGLDRMCEDLWRWQKQNPSGFGT
>d1bxka_ c.2.1.2 (A:) dTDP-glucose 4,6-dehydratase (RmlB) {Escherichia coli}
MRKILITGGAGFIGSALVRYIINETSDAVVVVDKLTYAGNLMSLAPVAQSERFAFEKVDICD
RAELARVFTEHQPDCVMHLAAESHVDRSIDGPAAFIETNIVGTYTLLEAARAYWNALTEDKK
SAFRFHHISTDEVYGDLHSTDDFFTETTPYAPSSPYSASKASSDHLVRAWLRTYGLPTLITN
CSNNYGPYHFPEKLIPLMILNALAGKSLPVYGNGQQIRDWLYVEDHARALYCVATTGKVGET
YNIGGHNERKNLDVVETICELLEELAPNKPHGVAHYRDLITFVADRPGHDLRYAIDASKIAR
ELGCVPQETFESGMRKTVQWYLANESWWKQVQDGSYQGER
>d1kepa_ c.2.1.2 (A:) dTDP-glucose 4,6-dehydratase (RmlB) {Streptococcus suis, serotype 2}

QFKNIIVTGGAGFIGSNFVHYVYNNHPDVHVTVLDKLTYAGNKANLEAILGDRVELVVGDIA
DAELVDKLAAKADAIVHYAAESHNDNSLNDPSPFIHTNFIGTYTLLEAARKYDIRFHHVSTD
EVYGDLPLREDLPGHGEGPGEKFTAETNYNPSSPYSSTKAASDLIVKAWVRSFGVKATISNC
SNNYGPYQHIEKFIPRQITNILAGIKPKLYGEGKNVRDWIHTNDHSTGVWAILTKGRMGETY
LIGADGEKNNKEVLELILEKMGQPKDAYDHVTDRAGHDLRYAIDASKLRDELGWTPQFTDFS
EGLEETIQWYTDNQDWWKAEKEAVEANYAKTQEVIK
>d1kewa_ c.2.1.2 (A:) dTDP-glucose 4,6-dehydratase (RmlB)
{Streptococcus suis, serotype 2}
MKILITGGAGFIGSAVVRHIIKNTQDTVVNIDKLTYAGNLESLSDISESNRYNFEHADICDS
AEITRIFEQYQPDAVMHLAAESHVDRSITGPAAFIETNIVGTYALLEVARKYWSALGEDKKN
NFRFHHISTDEVYGDLPHPDEVENSVTLPLFTETTAYAPSSPYSASKASSDHLVRAWRRTYG
LPTIVTNCSNNYGPYHFPEKLIPLVILNALEGKPLPIYGKGDQIRDWLYVEDHARALHMVVT
EGKAGETYNIGGHNEKKNLDVVFTICDLLDEIVPKATSYREQITYVADRPGHDRRYAIDAGK
ISRELGWKPLETFESGIRKTVEWYLANTQWVNNVKSGAYQSWIEQNYEGRQ
>d1e6ua_ c.2.1.2 (A:) GDP-4-keto-6-deoxy-d-mannose
epimerase/reductase (GDP-fucose synthetase) {Escherichia coli}
AKQRVFIAGHRGMVGSAIRRQLEQRGDVELVLRTRDELNLLDSRAVHDFFASERIDQVYLAA
AKVGGIVANNTYPADFIYQNMMIESNIIHAAHQNDVNKLLFLGSSCIYPKLAKQPMAESELL
QGTLEPTNEPYAIAKIAGIKLCESYNRQYGRDYRSVMPTNLYGPHDNFHPSNSHVIPALLRR
FHEATAQKAPDVVVWGSGTPMREFLHVDDMAAASIHVMELAHEVWLENTQPMLSHINVGTGV
DCTIRELAQTIAKVVGYKGRVVFDASKPDGTPRKLLDVTRLHQLGWYHEISLEAGLASTYQW
FLENQ
>d1db3a_ c.2.1.2 (A:) GDP-mannose 4,6-dehydratase {Escherichia
coli}
SKVALITGVTGQDGSYLAEFLLEKGYEVHGIKRRASSFNTERVDHIYQDPHTCNPKFHLHYG
DLSDTSNLTRILREVQPDEVYNLGAMSHVAVSFESPEYTADVDAMGTLRLLEAIRFLGLEKK
TRFYQASTSELYGLVQEIPQKETTPFYPRSPYAVAKLYAYWITVNYRESYGMYACNGILFNH
ESPRRGETFVTRKITRAIANIAQGLESCLYLGNMDSLRDWGHAKDYVKMQWMMLQQEQPEDF
VIATGVQYSVRQFVEMAAAQLGIKLRFEGTGVEEKGIVVSVTGHDAPGVKPGDVIIAVDPRY
FRPAEVETLLGDPTKAHEKLGWKPEITLREMVSEMVANDLEAAKKHS
>d1eq2a_ c.2.1.2 (A:) ADP-L-glycero-D-mannoheptose 6-epimerase
{Escherichia coli}
MIIVTGGAGFIGSNIVKALNDKGITDILVVDNLKDGTKFVNLVDLNIADYMDKEDFLIQIMA
GEEFGDVEAIFHEGACSSTTEWDGKYMMDNNYQYSKELLHYCLEREIPFLYASSAATYGGRT
SDFIESREYEKPLNVYGYSKFLFDEYVRQILPEANSQIVGFRYFNVYGPREGHKGSMASVAF
HLNTQLNNGESPKLFEGSENFKRDFVYVGDVADVNLWFLENGVSGIFNLGTGRAESFQAVAD
ATLAYHKKGQIEYIPFPDKLKGRYQAFTQADLTNLRAAGYDKPFKTVAEGVTEYMAWLN
>d1qrra_ c.2.1.2 (A:) Sulfolipid biosynthesis protein SQD1 {Thale
cress (Arabidopsis thaliana)}
KRVMVIGGDGYCGWATALHLSKKNYEVCIVDNLVRRLFDHQLGLESLTPIASIHDRISRWKA
LTGKSIELYVGDICDFEFLAESFKSFEPDSVVHFGEQRSAPYSMIDRSRAVYTQHNNVIGTL
NVLFAIKEFGEECHLVKLGTMGEYGTPNIDIEEGYITITHNGRTDTLPYPKQASSFYHLSKV
HDSHNIAFTCKAWGIRATDLNQGVVYGVKTDETEMHEELRNRLDYDAVFGTALNRFCVQAAV
GHPLTVYGKGGQTRGYLDIRDTVQCVEIAIANPAKAGEFRVFNQFTEQFSVNELASLVTKAG

SKLGLDVKKMTVPNPRVEAEEHYYNAKHTKLMELGLEPHYLSDSLLDSLLNFAVQFKDRVDT
KQIMPSVSWKKIGVKTKS
>d1k6xa_ c.2.1.2 (A:) Negative transcriptional regulator NmrA
{Aspergillus nidulans}
QQKKTIAVVNATGRQAASLIRVAAAVGHHVRAQVHSLKGLIAEELQAIPNVTLFQGPLLNNV
PLMDTLFEGAHLAFINTTSQAGDEIAIGKDLADAAKRAGTIQHYIYSSMPDHSLYGPWPAVP
MWAPKFTVENYVRQLGLPSTFVYAGIYNNNFTSLPYPLFQMELMPDGTFEWHAPFDPDIPLP
WLDAEHDVGPALLQIFKDGPQKWNGHRIALTFETLSPVQVCAAFSRALNRRVTYVQVPKVEI
KVNIPVGYREQLEAIEVVFGEHKAPYFPLPEFSRPAAGSPKGLGPANGKGAGAGMMQGPGGV
ISQRVTDEARKLWSGWRDMEEYAREVFPIEEEANGLDWML
>d1cyda_ c.2.1.2 (A:) Carbonyl reductase {Mouse (Mus musculus)}
LNFSGLRALVTGAGKGIGRDTVKALHASGAKVVAVTRTNSDLVSLAKECPGIEPVCVDLGDW
DATEKALGGIGPVDLLVNNAALVIMQPFLEVTKEAFDRSFSVNLRSVFQVSQMVARDMINRG
VPGSIVNVSSMVAHVTFPNLITYSSTKGAMTMLTKAMAMELGPHKIRVNSVNPTVVLTDMGK
KVSADPEFARKLKERHPLRKFAEVEDVVNSILFLLSDRSASTSGGGILVDAGYLAS
>d1oaa__ c.2.1.2 (-) Sepiapterin reductase {Mouse (Mus musculus)}
ADGLGCAVCVLTGASRGFGRALAPQLARLLSPGSVMLVSARSESMLRQLKEELGAQQPDLKV
VLAAADLGTEAGVQRLLSAVRELPRPEGLQRLLLINNAATLGDVSKGFLNVNDLAEVNNYWA
LNLTSMLCLTSGTLNAFQDSPGLSKTVVNISSLCALQPYKGWGLYCAGKAARDMLYQVLAAE
EPSVRVLSYAPGPLDNDMQQLARETSKDPELRSKLQKLKSDGALVDCGTSAQKLLGLLQKDT
FQSGAHVDFYD
>d1hdr__ c.2.1.2 (-) Dihydropteridin reductase (pteridine
reductase) {Human (Homo sapiens)}
EARRVLVYGGRGALGSRCVQAFRARNWWVASVDVVENEEASASIIVKMTDSFTEQADQVTAE
VGKLLGEEKVDAILCVAGGWAGGNAKSKSLFKNCDLMWKQSIWTSTISSHLATKHLKEGGLL
TLAGAKAALDGTPGMIGYGMAKGAVHQLCQSLAGKNSGMPPGAAAIAVLPVTLDTPMNRKSM
PEADFSSWTPLEFLVETFHDWITGKNRPSSGSLIQVVTTEGRTELTPAYF
>d1e7wa_ c.2.1.2 (A:) Dihydropteridin reductase (pteridine
reductase) {Leishmania major}
TVPVALVTGAAKRLGRSIAEGLHAEGYAVCLHYHRSAAEANALSATLNARRPNSAITVQADL
SNVATAPVSGADGSAPVTLFTRCAELVAACYTHWGRCDVLVNNASSFYPTPLLRNDEDGHEP
CVGDREAMETATADLFGSNAIAPYFLIKAFAHRVAGTPAKHRGTNYSIINMVDAMTNQPLLG
YTIYTMAKGALEGLTRSAALELAPLQIRVNGVGPGLSVLVDDMPPAVWEGHRSKVPLYQRDS
SAAEVSDVVIFLCSSKAKYITGTCVKVDGGYSLTRA
>d1fds__ c.2.1.2 (-) Human estrogenic 17beta-hydroxysteroid
dehydrogenase {Human (Homo sapiens)}
ARTVVLITGCSSGIGLHLAVRLASDPSQSFKVYATLRDLKTQGRLWEAARALACPPGSLETL
QLDVRDSKSVAAARERVTEGRVDVLVCNAGLGLLGPLEALGEDAVASVLDVNVVGTVRMLQA
FLPDMKRRGSGRVLVTGSVGGLMGLPFNDVYCASKFALEGLCESLAVLLLPFGVHLSLIECG
PVHTAFMEKVLGSPEEVLDRTDIHTFHRFYQYLAHSKQVFREAAQNPEEVAEVFLTALRAPK
PTLRYFTTERFLPLLRMRLDDPSGSNYVTAMHREVFGDV
>d1fmca_ c.2.1.2 (A:) 7-alpha-hydroxysteroid dehydrogenase
{Escherichia coli}
MFNSDNLRLDGKCAIITGAGAGIGKEIAITFATAGASVVVSDINADAANHVVDEIQQLGGQA

FACRCDITSEQELSALADFAISKLGKVDILVNNAGGGGPKPFDMPMADFRRAYELNVFSFFH
LSQLVAPEMEKNGGGVILTITSMAAENKNINMTSYASSKAAASHLVRNMAFDLGEKNIRVNG
IAPGAILTDALKSVITPEIEQKMLQHTPIRRLGQPQDIANAALFLCSPAASWVSGQILTVSG
GGVQELN
>d1hdca_ c.2.1.2 (A:) 3-alpha,20-beta-hydroxysteroid
dehydrogenase {Streptomyces hydrogenans}
NDLSGKTVIITGGARGLGAEAARQAVAAGARVVLADVLDEEGAATARELGDAARYQHLDVTI
EEDWQRVVAYAREEFGSVDGLVNNAGISTGMFLETESVERFRKVVEINLTGVFIGMKTVIPA
MKDAGGGSIVNISSAAGLMGLALTSSYGASKWGVRGLSKLAAVELGTDRIRVNSVHPGMTYT
PMTAETGIRQGEGNYPNTPMGRVGEPGEIAGAVVKLLSDTSSYVTGAELAVDGGWTTGPTVK
YVMGQ
>d1fjha_ c.2.1.2 (A:) 3-alpha-hydroxysteroid dehydrogenase
{Comamonas testosteroni}
MSIIVISGCATGIGAATRKVLEAAGHQIVGIDIRDAEVIADLSTAEGRKQAIADVLAKCSKG
MDGLVLCAGLGPQTKVLGNVVSVNYFGATELMDAFLPALKKGHQPAAVVISSVASAHLAFDK
NPLALALEAGEEAKARAIVEHAGEQGGNLAYAGSKNALTVAVRKRAAAWGEAGVRLNTIAPG
ATETPLLQAGLQDPRYGESIAKFVPPMGRRAEPSEMASVIAFLMSPAASYVHGAQIVIDGGI
DAVMRPTQF
>d1bdb__ c.2.1.2 (-)
Cis-biphenyl-2,3-dihydrodiol-2,3-dehydrogenase {Pseudomonas sp.,
lb400}
MKLKGEAVLITGGASGLGRALVDRFVAEGAKVAVLDKSAERLAELETDHGDNVLGIVGDVRS
LEDQKQAASRCVARFGKIDTLIPNAGIWDYSTALVDLPEESLDAAFDEVFHINVKGYIHAVK
ACLPALVASRGNVIFTISNAGFYPNGGGPLYTAAKHAIVGLVRELAFELAPYVRVNGVGSGG
INSDLRGPSSLGMGSKAISTVPLADMLKSVLPIGRMPEVEEYTGAYVFFATRGDAAPATGAL
LNYDGGLGVRGFFSGAGGNDLLEQLNIH
>d1b16a_ c.2.1.2 (A:) Drosophila alcohol dehydrogenase {Fruit fly
(Drosophila lebanonensis)}
MDLTNKNVIFVAALGGIGLDTSRELVKRNLKNFVILDRVENPTALAELKAINPKVNITFHTY
DVTVPVAESKKLLKKIFDQLKTVDILINGAGILDDHQIERTIAINFTGLVNTTTAILDFWDK
RKGGPGGIIANICSVTGFNAIHQVPVYSASKAAVVSFTNSLAKLAPITGVTAYSINPGITRT
PLVHTFNSWLDVEPRVAELLLSHPTQTSEQCGQNFVKAIEANKNGAIWKLDLGTLEAIEWTK
HWDSHI
>d1gcoa_ c.2.1.2 (A:) Glucose dehydrogenase {Bacillus megaterium}
MYKDLEGKVVVITGSSTGLGKSMAIRFATEKAKVVVNYRSKEDEANSVLEEIKKVGGEAIAV
KGDVTVESDVINLVQSAIKEFGKLDVMINNAGLENPVSSHEMSLSDWNKVIDTNLTGAFLGS
REAIKYFVENDIKGTVINMSSVHEKIPWPLFVHYAASKGGMKLMTETLALEYAPKGIRVNNI
GPGAINTPINAEKFADPEQRADVESMIPMGYIGEPEEIAAVAAWLASSEASYVTGITLFADG
GMTQYPSFQAGRG
>d1gega_ c.2.1.2 (A:) meso-2,3-butanediol dehydrogenase
{Klebsiella pneumoniae}
KKVALVTGAGQGIGKAIALRLVKDGFAVAIADYNDATAKAVASEINQAGGHAVAVKVDVSDR
DQVFAAVEQARKTLGGFDVIVNNAGVAPSTPIESITPEIVDKVYNINVKGVIWGIQAAVEAF
KKEGHGGKIINACSQAGHVGNPELAVYSSSKFAVRGLTQTAARDLAPLGITVNGYCPGIVKT

PMWAEIDRQVSEAAGKPLGYGTAEFAKRITLGRLSEPEDVAACVSYLASPDSDYMTGQSLLI
DGGMVFN
>d1h5qa_ c.2.1.2 (A:) Mannitol dehydrogenase {Mushroom (Agaricus bisporus)}
PGFTISFVNKTIIVTGGNRGIGLAFTRAVAAAGANVAVIYRSAADAVEVTEKVGKEFGVKTK
AYQCDVSNTDIVTKTIQQIDADLGPISGLIANAGVSVVKPATELTHEDFAFVYDVNVFGVFN
TCRAVAKLWLQKQQKGSIVVTSSMSSQIINQSSLNGSLTQVFYNSSKAACSNLVKGLAAEWA
SAGIRVNALSPGYVNTDQTAHMDKKIRDHQASNIPLNRFAQPEEMTGQAILLLSDHATYMTG
GEYFIDGGQLIW
>d1edoa_ c.2.1.2 (A:) beta-keto acyl carrier protein reductase {Oil seed rape (Brassica napus)}
SPVVVVTGASRGIGKAIALSLGKAGCKVLVNYARSAKAAEEVSKQIEAYGGQAITFGGDVSK
EADVEAMMKTAIDAWGTIDVVVNNAGITRDTLLIRMKKSQWDEVIDLNLTGVFLCTQAATKI
MMKKRKGRIINIASVVGLIGNIGQANYAAAKAGVIGFSKTAAREGASRNINVNVVCPGFIAS
DMTAKLGEDMEKKILGTIPLGRTGQPENVAGLVEFLALSPAASYITGQAFTIDGGIAI
>d1i01a_ c.2.1.2 (A:) beta-keto acyl carrier protein reductase {Escherichia coli}
MNFEGKIALVTGASRGIGRAIAETLAARGAKVIGTATSENGAQAISDYLGANGKGLMLNVTD
PASIESVLEKIRAEFGEVDILVNNAGITRDNLLMRMKDEEWNDIIETNLSSVFRLSKAVMRA
MMKKRHGRIITIGSVVGTMGNGGQANYAAAKAGLIGFSKSLAREVASRGITVNVVAPGFIET
DMTRALSDDQRAGILAQVPAGRLGGAQEIANAVAFLASDEAAYITGETLHVNGGM
>d1eno__ c.2.1.2 (-) Enoyl-ACP reductase {Oil seed rape (Brassica napus)}
LPIDLRGKRAFIAGIADDNGYGWAVAKSLAAAGAEILVGTWVPALNIFETSLRRGKFDQSRV
LPDGSLMEIKKVYPLDAVFDNPEDVPEDVKANKRYAGSSNWTVQEAAECVRQDFGSIDILVH
SLANGPEVSKPLLETSRKGYLAAISASSYSFVSLLSHFLPIMNPGGASISLTYIASERIIPG
YGGGMSSAKAALESDTRVLAFEAGRKQNIRVNTISAGPLGSRAAKAIGFIDTMIEYSYNNAP
IQKTLTADEVGNAAAFLVSPLASAITGATIYVDNGLNSMGVALDSPVFK
>d1eny__ c.2.1.2 (-) Enoyl-ACP reductase {Mycobacterium tuberculosis, TB, gene InhA}
AGLLDGKRILVSGIITDSSIAFHIARVAQEQGAQLVLTGFDRLRLIQRITDRLPAKAPLLEL
DVQNEEHLASLAGRVTEAIGAGNKLDGVVHSIGFMPQTGMGINPFFDAPYADVSKGIHISAY
SYASMAKALLPIMNPGGSIVGMDFDPSRAMPAYNWMTVAKSALESVNRFVAREAGKYGVRSN
LVAAGPIRTLAMSAIVGGALGEEAGAQIQLLEEGWDQRAPIGWNMKDATPVAKTVCALLSDW
LPATTGDIIYADGGAHTQLL
>d1qg6a_ c.2.1.2 (A:) Enoyl-ACP reductase {Escherichia coli}
GFLSGKRILVTGVASKLSIAYGIAQAMHREGAELAFTYQNDKLKGRVEEFAAQLGSDIVLQC
DVAEDASIDTMFAELGKVWPKFDGFVHSIGFAPGDQLDGDYVNAVTREGFKIAHDISSYSFV
AMAKACRSMLNPGSALLTLSYLGAERAIPNYNVMGLAKASLEANVRYMANAMGPEGVRVNAI
SAGPIRTLAASGIKDFRKMLAHCEAVTPIRRTVTIEDVGNSAAFLCSDLSAGISGEVVHVDG
GFSIAAMNE
>d1ae1a_ c.2.1.2 (A:) Tropinone reductase {Jimsonweed (Datura stramonium), I}
RWSLKGTTALVTGGSKGIGYAIVEELAGLGARVYTCSRNEKELDECLEIWREKGLNVEGSVC

DLLSRTERDKLMQTVAHVFDGKLNILVNNAGVVIHKEAKDFTEKDYNIIMGTNFEAAYHLSQ
IAYPLLKASQNGNVIFLSSIAGFSALPSVSLYSASKGAINQMTKSLACEWAKDNIRVNSVAP
GVILTPLVETAIKKNPHQKEEIDNFIVKTPMGRAGKPQEVSALIAFLCFPAASYITGQIIWA
DGGFTANGGF
>d2ae2a_ c.2.1.2 (A:) Tropinone reductase {Jimsonweed (Datura stramonium), II}
AGRWNLEGCTALVTGGSRGIGYGIVEELASLGASVYTCSRNQKELNDCLTQWRSKGFKVEAS
VCDLSSRSERQELMNTVANHFHGKLNILVNNAGIVIYKEAKDYTVEDYSLIMSINFEAAYHL
SVLAHPFLKASERGNVVFISSVSGALAVPYEAVYGATKGAMDQLTRCLAFEWAKDNIRVNGV
GPGVIATSLVEMTIQDPEQKENLNKLIDRCALRRMGEPKELAAMVAFLCFPAASYVTGQIIY
VDGGLMANCGF
>d1g0oa_ c.2.1.2 (A:) 1,3,8-trihydroxynaphtalene reductase (THNR, naphtol reductase) {Rice blast fungus (Magnaporthe grisea)}
KYDAIPGPLGPQSASLEGKVALVTGAGRGIGREMAMELGRRGCKVIVNYANSTESAEEVVAA
IKKNGSDAACVKANVGVVEDIVRMFEEAVKIFGKLDIVCSNSGVVSFGHVKDVTPEEFDRVF
TINTRGQFFVAREAYKHLEIGGRLILMGSITGQAKAVPKHAVYSGSKGAIETFARCMAIDMA
DKKITVNVVAPGGIKTDMYHAVCREYIPNGENLSNEEVDEYAAVQWSPLRRVGLPIDIARVV
CFLASNDGGWVTGKVIGIDGGACM
>d1ja9a_ c.2.1.2 (A:) 1,3,6,8-tetrahydroxynaphthalene reductase {Rice blast fungus (Magnaporthe grisea)}
SKPLAGKVALTTGAGRGIGRGIAIELGRRGASVVVNYGSSSKAAEEVVAELKKLGAQGVAIQ
ADISKPSEVVALFDKAVSHFGGLDFVMSNSGMEVWCDELEVTQELFDKVFNLNTRGQFFVAQ
QGLKHCRRGGRIILTSSIAAVMTGIPNHALYAGSKAAVEGFCRAFAVDCGAKGVTVNCIAPG
GVKTDMFDENSWHYAPGGYKGMPQEKIDEGLANMNPLKRIGYPADIGRAVSALCQEESEWIN
GQVIKLTGGGI
>d1hdoa_ c.2.1.2 (A:) Biliverdin IX beta reductase {Human (Homo sapiens)}
MAVKKIAIFGATGQTGLTTLAQAVQAGYEVTVLVRDSSRLPSEGPRPAHVVVGDVLQAADVD
KTVAGQDAVIVLLGTRNDLSPTTVMSEGARNIVAAMKAHGVDKVVACTSAFLLWDPTKVPPR
LQAVTDDHIRMHKVLRESGLKYVAVMPPHIGDQPLTGAYTVTLDGRGPSRVISKHDLGHFML
RCLTTDEYDGHSTYPSHQY
>d1e6wa_ c.2.1.2 (A:) 3-hydroxyacyl-CoA dehydrogenase {Rat (Rattus norvegicus)}
SVKGLVAVITGGASGLGLSTAKRLVGQGATAVLLDVPNSEGETEAKKLGGNCIFAPANVTSE
KEVQAALTLAKEKFGRIDVAVNCAGIAVAIKTYHEKKNQVHTLEDFQRVINVNLIGTFNVIR
LVAGVMGQNEPDQGGQRGVIINTASVAAFEGQVGQAAYSASKGGIVGMTLPIARDLAPIGIR
VVTIAPGLFATPLLTTLPDKVRNFLASQVPFPSRLGDPAEYAHLVQMVIENPFLNGEVIRLD
GAIRMQP
>d1hu4a_ c.2.1.2 (A:) Carbonyl reductase/20beta-hydroxysteroid dehydrogenase {Pig (Sus scrofa)}
SSNTRVALVTGANKGIGFAIVRDLCRQFAGDVVLTARDVARGQAAVKQLQAEGLSPRFHQLD
IIDLQSIRALCDFLRKEYGGLDVLVNNAAIAFQLDNPTPFHIQAELTMKTNFMGTRNVCTEL
LPLIKPQGRVVNVSSTEGVRALNECSPELQQKFKSETITEEELVGLMNKFVEDTKNGVHRKE
GWSDSTYGVTKIGVSVLSRIYARKLREQRAGDKILLNACCPGWVRTDMGGPKAPKSPEVGAE

TPVYLALLPSDAEGPHGQFVTDKKVVEWGVPPESYPWVNA
>d1gado1 c.2.1.3 (O:0-148,O:313-330) Glyceraldehyde-3-phosphate dehydrogenase (GAPDH) {Escherichia coli}
TIKVGINGFGRIGRIVFRAAQKRSDIEIVAINDLLDADYMAYMLKYDSTHGRFDGTVEVKDG
HLIVNGKKIRVTAERDPANLKWDEVGVDVVAEATGLFLTDETARKHITAGAKKVVMTGPSKD
NTPMFVKGANFDKYAGQDIVSNASXNETGYSNKVLDLIAHISK
>d1gd1o1 c.2.1.3 (O:0-148,O:313-333) Glyceraldehyde-3-phosphate dehydrogenase (GAPDH) {Bacillus stearothermophilus, nca 1503}
AVKVGINGFGRIGRNVFRAALKNPDIEVVAVNDLTDANTLAHLLKYDSVHGRLDAEVSVNGN
NLVVNGKEIIVKAERDPENLAWGEIGVDIVVESTGRFTKREDAAKHLEAGAKKVIISAPAKN
EDITIVMGVNQDKYDPKAHHVISNASXNETGYSHRVVDLAAYIASKGL
>d1cero1 c.2.1.3 (O:1-148,O:313-333) Glyceraldehyde-3-phosphate dehydrogenase (GAPDH) {Thermus aquaticus}
MKVGINGFGRIGRQVFRILHSRGVEVALINDLTDNKTLAHLLKYDSIYHRFPGEVAYDDQYL
YVDGKAIRATAVKDPKEIPWAEAGVGVVIESTGVFTDADKAKAHLEGGAKKVIITAPAKGED
ITIVMGVNHEAYDPSRHHIISNASXNEWGYANRVADLVELVLRKGV
>d1hdgo1 c.2.1.3 (O:1-148,O:313-331) Glyceraldehyde-3-phosphate dehydrogenase (GAPDH) {Thermotoga maritima}
ARVAINGFGRIGRLVYRIIYERKNPDIEVVAINDLTDTKTLAHLLKYDSVHKKFPGKVEYTE
NSLIVDGKEIKVFAEPDPSKLPWKDLGVDFVIESTGVFRNREKAELHLQAGAKKVIITAPAK
GEDITVVIGCNEDQLKPEHTIISCASXNEYGYSNRVVDTLELLLKM
>d1b7go1 c.2.1.3 (O:1-138,O:301-340) Glyceraldehyde-3-phosphate dehydrogenase (GAPDH) {Archaeon Sulfolobus solfataricus}
MVNVAVNGYGTIGKRVADAIIKQPDMKLVGVAKTSPNYEAFIAHRRGIRIYVPQQSIKKFEE
SGIPVAGTVEDLIKTSDIVVDTTPNGVGAQYKPIYLQLQRNAIFQGGEKAEVADISFSALCN
YNEALGKKYIRVVSXESIVVPENIDAIRASMKLMSAEDSMRITNESLGILKGYLI
>d1cf2o1 c.2.1.3 (O:1-138,O:304-336) Glyceraldehyde-3-phosphate dehydrogenase (GAPDH) {Archaeon Methanothermus fervidus}
MKAVAINGYGTVGKRVADAIAQQDDMKVIGVSKTRPDFEARMALKKGYDLYVAIPERVKLFE
KAGIEVAGTVDDMLDEADIVIDCTPEGIGAKNLKMYKEKGIKAIFQGGEKHEDIGLSFNSLS
NYEESYGKDYTRVVXIVPENVDAVRAILEMEEDKYKSINKTNKAMNIL
>d1ggaa1 c.2.1.3 (A:1-164,A:334-358) Glyceraldehyde-3-phosphate dehydrogenase (GAPDH) {Trypanosoma brucei brucei, glycosome}
TIKVGINGFGRIGRMVFQALCDDGLLGNEIDVVAVVDMNTDARYFAYQMKYDSVHGKFKHSV
STTKSKPSVAKDDTLVVNGHRILCVKAQRNPADLPWGKLGVEYVIESTGLFTVKSAAEGHLR
GGARKVVISAPASGGAKTFVMGVNHNNYNPREQHVVSNASXNEWGYSHRVVDLVRHMAARDR
AAKL
>d1i32a1 c.2.1.3 (A:1-165,A:335-358) Glyceraldehyde-3-phosphate dehydrogenase (GAPDH) {Leishmania mexicana}
APIKVGINGFGRIGRMVFQAICDQGLIGTEIDVVAVVDMSTNAEYFAYQMKHDTVHGRPKYT
VEAVKSSPSVETADVLVVNGHRIKCVKAQRNPADLPWGKLGVDYVIESTGLFTDKLKAEGHI
KGGAKKVVISAPASGGAKTIVMGVNQHEYSPASHHVVSNASXNEWAYSHRVVDLVRYMAAKD
AASS
>d1dssg1 c.2.1.3 (G:1-148,G:313-334) Glyceraldehyde-3-phosphate

dehydrogenase (GAPDH) {Lobster (Palinurus versicolor)}
SKIGINGFGRIGRLVLRAALEMGAQVVAVNDPFIALEYMVYMFKYDSTHGMFKGEVKAEDGA
LVVDGKKITVFNEMKPENIPWSKAGAEYIVESTGVFTTIEKASAHFKGGAKKVIISAPSADA
PMFVCGVNLEKYSKDMKVVSNASXNEFGYSQRVIDLIKHMQKVDSA
>d3gpdg1 c.2.1.3 (G:1-150,G:315-334) Glyceraldehyde-3-phosphate dehydrogenase (GAPDH) {Human (Homo sapiens)}
GKVKVGVDGFGRIGRLVTRAAFNSGKVDIVAINDPFIDLHYMVYMFQYDSTHGKFHGTVKAE
DGKLVIDGKAITIFQERDPENIKWGDAGTAYVVESTGVFTTMEKAGAHLKGGAKRIVISAPS
ADAPMFVMGVNHFKYANSLKIISNASXNEFGYSERVVDLMAHMASKE
>d1jn0a1 c.2.1.3 (A:0-148,A:313-333) Glyceraldehyde-3-phosphate dehydrogenase (GAPDH) {Spinach (Spinacia oleracea)}
KLKVAINGFGRIGRNFLRCWHGKDSPLDVVVINDTGGVKQASHLLKYDSILGTFDADVKTAG
DSAISVGKVIKVVSDRNPVNLPWGDMGIDLVIEGTGVFVDRDGAGKHLQAGAKKVLITAPGK
GDIPTYVVGVNEEGYTHADTIISNASXNEWGYSQRVVDLADIVANKWQ
>d1gl3a1 c.2.1.3 (A:1-133,A:355-367) Aspartate beta-semialdehyde dehydrogenase {Escherichia coli}
MKNVGFIGWRGMVGSVLMQRMVEERDFDAIRPVFFSTSQLGQAAPSFGGTTGTLQDAFDLEA
LKALDIIVTCQGGDYTNEIYPKLRESGWQGYWIDAASSLRMKDDAIIILDPVNQDVITDGLN
NGIRTFVGGXAAEPLRRMLRQLA
>d1ebfa1 c.2.1.3 (A:2-150,A:341-359) Homoserine dehydrogenase {Baker's yeast (Saccharomyces cerevisiae)}
STKVVNVAVIGAGVVGSAFLDQLLAMKSTITYNLVLLAEAERSLISKDFSPLNVGSDWKAAL
AASTTKTLPLDDLIAHLKTSPKPVILVDNTSSAYIAGFYTKFVENGISIATPNKKAFSSDLA
TWKALFSNKPTNGFVYHEATVGAGLXAAVTAAGVLGDVIKIAQRL
>d1e5qa1 c.2.1.3 (A:2-124,A:392-450) Saccharopine reductase {Rice blast fungus (Magnaporthe grisea)}
ATKSVLMLGSGFVTRPTLDVLTDSGIKVTVACRTLESAKKLSAGVQHSTPISLDVNDDAALD
AEVAKHDLVISLIPYTFHATVIKSAIRQKKHVVTTSYVSPAMMELDQAAKDAGITVMNEIGX
YSAMAKLVGVPCAVAVKFVLDGTISDRGVLAPMNSKINDPLMKELKEKYGIECKEKVVA
>d1f06a1 c.2.1.3 (A:1-118,A:269-320) Diaminopimelic acid dehydrogenase (DAPDH) {Corynebacterium glutamicum}
MTNIRVAIVGYGNLGRSVEKLIAKQPDMDLVGIFSRRATLDTKTPVFDVADVDKHADDVDVL
FLCMGSATDIPEQAPKFAQFACTVDTYDNHRDIPRHRQVMNEAATAAGNVALVSTGXRNPDF
TASSQIAFGRAAHRMKQQGQSGAFTVLEVAPYLLSPENLDDLIARDV
>d1dih_1 c.2.1.3 (2-130,241-273) Dihydrodipicolinate reductase {Escherichia coli}
HDANIRVAIAGAGGRMGRQLIQAALALEGVQLGAALEREGSSLLGSDAGELAGAGKTGVTVQ
SSLDAVKDDFDVFIDFTRPEGTLNHLAFCRQHGKGMVIGTTGFDEAGKQAIRDAAADIAIVF
AANFSXMTFANGAVRSALWLSGKESGLFDMRDVLDLNNL
>d1k5ha2 c.2.1.3 (A:1-125,A:275-300) 1-deoxy-D-xylulose-5-phosphate reductoisomerase {Escherichia coli}
MKQLTILGSTGSIGCSTLDVVRHNPEHFRVVALVAGKNVTRMVEQCLEFSPRYAVMDDEASA
KLLKTMLQQQGSRTEVLSGQQAACDMAALEDVDQVMAAIVGAAGLLPTLAAIRAGKTILLAN

KXDMRTPIAHTMAWPNRVNSGVKPLDFC
>d1gcua1 c.2.1.3 (A:1-128,A:247-292) Biliverdin reductase {Rat (Rattus norvegicus)}
MDAEPKRKFGVVVVGVGRAGSVRLRDLKDPRSAAFLNLIGFVSRRELGSLDEVRQISLEDAL
RSQEIDVAYICSESSSHEDYIRQFLQAGKHVLVEYPMTLSFAAAQELWELAAQKGRVLHEEH
VELLXKNIFLKDQDIFVQKLLDQVSAEDLAAEKKRIMHCLGLASDIQKLCH
>d1evja1 c.2.1.3 (A:30-160,A:323-381) Glucose-fructose oxidoreductase, N-terminal domain {Zymomonas mobilis}
RRFGYAIVGLGKYALNQILPGFAGCQHSRIEALVDGNAEKAKIVAAEYGVDPRKIYDYSNFD
KIAKDPKIDAVYIILPNSLHAEFAIRAFKAGKHVMCEKPMATSVADCQRMIDAAKAANKKLM
IGYRCHYXNQFSAQLDHLAEAVINNKPVRSPGEEGMQDVRLIQAIYEAARTGRPVNTDWGYV
RQGGY
>d1h6da1 c.2.1.3 (A:51-212,A:375-433) Glucose-fructose oxidoreductase, N-terminal domain {Zymomonas mobilis}
QAATLPAGASQVPTTPAGRPMPYAIRPMPEDRRFGYAIVGLGKYALNQILPGFAGCQHSRIE
ALVSGNAEKAKIVAAEYGVDPRKIYDYSNFDKIAKDPKIDAVYIILPNSLHAEFAIRAFKAG
KHVMCEKPMATSVADCQRMIDAAKAANKKLMIGYRCHYXNQFSAQLDHLAEAVINNKPVRSP
GEEGMQDVRLIQAIYEAARTGRPVNTDWGYVRQGGY
>d1dpga1 c.2.1.3 (A:1-181,A:413-426) Glucose 6-phosphate dehydrogenase, N-terminal domain {Leuconostoc mesenteroides}
VSEIKTLVTFFGGTGDLAKRKLYPSVFNLYKKGYLQKHFAIVGTARQALNDDEFKQLVRDCI
KDFTDDQAQAEAFIEHFSYRAHDVTDAASYAVLKEAIEEAADKFDIDGNRIFYMSVAPRFFG
TIAKYLKSEGLLADTGYNRLMIEKPFGTSYDTAAELQNDLENAFDDNQLFRIDHYLGXEPYE
RMIHDTMNGD
>d1qkia1 c.2.1.3 (A:12-199,A:435-449) Glucose 6-phosphate dehydrogenase, N-terminal domain {Human (Homo sapiens)}
VCGILREELFQGDAFHQSDTHIFIIMGASGDLAKKKIYPTIWWLFRDGLLPENTFIVGYARS
RLTVADIRKQSEPFFKATPEEKLKLEDFFARNSYVAGQYDDAASYQRLNSHMNALHLGSQAN
RLFYLALPPTVYEAVTKNIHESCMSQIGWNRIIVEKPFGRDLQSSDRLSNHISSLFREDQIY
RIXDAYERLILDVFCGSQ
>d2naca1 c.2.1.4 (A:148-335) Formate dehydrogenase {Pseudomonas sp., strain 101}
ISVAEHVVMMILSLVRNYLPSHEWARKGGWNIADCVSHAYDLEAMHVGTVAAGRIGLAVLRR
LAPFDVHLHYTDRHRLPESVEKELNLTWHATREDMYPVCDVVTLNCPLHPETEHMINDETLK
LFKRGAYIVNTARGKLCDRDAVARALESGRLAGYAGDVWFPQPAPKDHPWRTMPYNGMTPHI
SG
>d1qp8a1 c.2.1.4 (A:83-263) Putative formate dehydrogenase {Archaeon Pyrobaculum aerophilum}
ADAVAEFALALLLAPYKRIIQYGEKMKRGDYGRDVEIPLIQGEKVAVLGLGEIGTRVGKILA
ALGAQVRGFSRTPKEGPWRFTNSLEEALREARAAVCALPLNKHTRGLVKYQHLALMAEDAVF
VNVGRAEVLDRDGVLRILKERPQFIFASDVWWGRNDFAKDAEFFSLPNVVATPWVAG
>d1dxy_1 c.2.1.4 (101-299) D-2-hydroxyisocaproate dehydrogenase {Lactobacillus casei}
SPAAIAEFALTDTLYLLRNMGKVQAQLQAGDYEKAGTFIGKELGQQTVGVMGTGHIGQVAIK

LFKGFGAKVIAYDPYPMKGDHPDFDYVSLEDLFKQSDVIDLHVPGIEQNTHIINEAAFNLMK
PGAIVINTARPNLIDTQAMLSNLKSGKLAGVGIDTYEYETEDLLNLAKHGSFKDPLWDELLG
MPNVVLSPHIAYY
>d1gdha1 c.2.1.4 (A:101-291) D-glycerate dehydrogenase
{Hyphomicrobium methylovorum}
VTVATAEIAMLLLLGSARRAGEGEKMIRTRSWPGWEPLELVGEKLDNKTLGIYGFGSIGQAL
AKRAQGFDMDIDYFDTHRASSSDEASYQATFHDSLDSLLSVSQFFSLNAPSTPETRYFFNKA
TIKSLPQGAIVVNTARGDLVDNELVVAALEAGRLAYAGFDVFAGEPNINEGYYDLPNTFLFP
HIGSA
>d1psda1 c.2.1.4 (A:108-295) Phosphoglycerate dehydrogenase
{Escherichia coli}
NTRSVAELVIGELLLLLRGVPEANAKAHRGVWNKLAAGSFEARGKKLGIIGYGHIGTQLGIL
AESLGMYVYFYDIENKLPLGNATQVQHLSDLLNMSDVVSLHVPENPSTKNMMGAKEISLMKP
GSLLINASRGTVVDIPALCDALASKHLAGAAIDVFPTEPATNSDPFTSPLCEFDNVLLTPHI
GG
>d2dlda1 c.2.1.4 (A:104-300) D-lactate dehydrogenase
{Lactobacillus helveticus}
PNAIAEHAAIQAARVLRQDKRMDEKMAKRDLRWAPTIGREVRDQVVGVVGTGHIGQVFMRIM
EGFGAKVIAYDIFKNPELEKKGYYVDSLDDLYKQADVISLHVPDVPANVHMINDKSIAEMKD
GVVIVNCSRGRLVDTDAVIRGLDSGKIFGFVMDTYEDEVGVFNKDWEGKEFPDKRLADLIDR
PNVLVTPHTAF
>d1pjca1 c.2.1.4 (A:136-303) L-alanine dehydrogenase {Phormidium
lapideum}
AGRLSVQFGARFLERQQGGRGVLLGGVPGVKPGKVVILGGGVVGTEAAKMAVGLGAQVQIFD
INVERLSYLETLFGSRVELLYSNSAEIETAVAEADLLIGAVLVPGRRAPILVPASLVEQMRT
GSVIVDVAVDQGGCVETLHPTSHTQPTYEVFGVVHYGVPNMPGA
>d1f8ga1 c.2.1.4 (A:144-326) Nicotinamide nucleotide
transhydrogenase dI component {Rhodospirillum rubrum}
AGYRAVIDGAYEFARAFPMMMTAAGTVPPARVLVFGVGVAGLQAIATAKRLGAVVMATDVRA
ATKEQVESLGGKFITVDDEAMKTAETAGGYAKEMGEEFRKKQAEAVLKELVKTDIAITTALI
PGKPAPVLITEEMVTKMKPGSVIIDLAVEAGGNCPLSEPGKIVVKHGVKIVGHTNVPSR
>d1b3ra1 c.2.1.4 (A:190-352) S-adenosylhomocystein hydrolase {Rat
(Rattus norvegicus)}
NLYGCRESLIDGIKRATDVMIAGKVAVVAGYGDVGKGCAQALRGFGARVIITEIDPINALQA
AMEGYEVTTMDEACKEGNIFVTTTGCVDIILGRHFEQMKDDAIVCNIGHFDVEIDVKWLNEN
AVEKVNIKPQVDRYLLKNGHRIILLAEGRLVNLGCAMGH
>d1gpja2 c.2.1.10 (A:144-302) Glutamyl tRNA-reductase middle
domain {Archaeon Methanopyrus kandleri}
SEGAVSIGSAAVELAERELGSLHDKTVLVVGAGEMGKTVAKSLVDRGVRAVLVANRTYERAV
ELARDLGGEAVRFDELVDHLARSDVVVSATAAPHPVIHVDDVREALRKRDRRSPILIIDIAN
PRDVEEGVENIEDVEVRTIDDLRVIARENLERRRK
>d1mlda1 c.2.1.5 (A:1-144) Malate dehydrogenase {Pig (Sus scrofa)}
AKVAVLGASGGIGQPLSLLLKNSPLVSRLTLYDIAHTPGVAADLSHIETRATVKGYLGPEQL
PDCLKGCDVVVIPAGVPRKPGMTRDDLFNTNATIVATLTAACAQHCPDAMICIISNPVNSTI

PITAEVFKKHGVYNPNKIFG
>d5mdha1 c.2.1.5 (A:1-154) Malate dehydrogenase {Pig (Sus scrofa)}
SEPIRVLVTGAAGQIAYSLLYSIGNGSVFGKDQPIILVLLDITPMMGVLDGVLMELQDCALP
LLKDVIATDKEEIAFKDLDVAILVGSMPRRDGMERKDLLKANVKIFKCQGAALDKYAKKSVK
VIVVGNPANTNCLTASKSAPSIPKENFSCL
>d7mdha1 c.2.1.5 (A:23-197) Malate dehydrogenase {Sorghum (Sorghum vulgare), chloroplast}
DCFGVFCTTYDLKAEDKTKSWKKLVNIAVSGAAGMISNHLLFKLASGEVFGQDQPIALKLLG
SERSFQALEGVAMELEDSLYPLLREVSIGIDPYEVFEDVDWALLIGAKPRGPGMERAALLDI
NGQIFADQGKALNAVASKNVKVLVVGNPCNTNALICLKNAPDIPAKNFHAL
>d1civa1 c.2.1.5 (A:12-193) Malate dehydrogenase {Flaveria bidentis, chloroplast}
LPAKQKPECFGVFCLTYDLKAEEETKSWKKIINVAVSGAAGMISNHLLFKLASGEVFGPDQP
ISLKLLGSERSFAALEGVAMELEDSLYPLLRQVSIGIDPYEIFQDAEWALLIGAKPRGPGME
RADLLDINGQIFAEQGKALNAVASPNVKVMVVGNPCNTNALICLKNAPNIPPKNFHAL
>d2cmd_1 c.2.1.5 (1-145) Malate dehydrogenase {Escherichia coli}
MKVAVLGAAGGIGQALALLLKTQLPSGSELSLYDIAPVTPGVAVDLSHIPTAVKIKGFSGED
ATPALEGADVVLISAGVRRKPGMDRSDLFNVNAGIVKNLVQQVAKTCPKACIGIITNPVNTT
VAIAAEVLKKAGVYDKNKLFG
>d1bdma1 c.2.1.5 (A:0-154) Malate dehydrogenase {Thermus flavus}
MKAPVRVAVTGAAGQIGYSLLFRIAAGEMLGKDQPVILQLLEIPQAMKALEGVVMELEDCAF
PLLAGLEATDDPDVAFKDADYALLVGAAPRKAGMERRDLLQVNGKIFTEQGRALAEVAKKDV
KVLVVGNPANTNALIAYKNAPGLNPRNFTAM
>d2hlpa1 c.2.1.5 (A:22-162) Malate dehydrogenase {Archaeon Haloarcula marismortui}
TKVSVVGAAGTVGAAAGYNIALRDIADEVVFVDIPDKEDDTVGQAADTNHGIAYDSNTRVRQ
GGYEDTAGSDVVVITAGIPRQPGQTRIDLAGDNAPIMEDIQSSLDEHNDDYISLTTSNPVDL
LNRHLYEAGDRSREQVIG
>d1b8pa1 c.2.1.5 (A:3-158) Malate dehydrogenase {Aquaspirillum arcticum}
KTPMRVAVTGAAGQICYSLLFRIANGDMLGKDQPVILQLLEIPNEKAQKALQGVMMEIDDCA
FPLLAGMTAHADPMTAFKDADVALLVGARPRGPGMERKDLLEANAQIFTVQGKAIDAVASRN
IKVLVVGNPANTNAYIAMKSAPSLPAKNFTAM
>d1guya1 c.2.1.5 (A:1-143) Malate dehydrogenase {Chloroflexus aurantiacus}
MRKKISIIGAGFVGSTTAHWLAAKELGDIVLLDIVEGVPQGKALDLYEASPIEGFDVRVTGT
NNYADTANSDVIVVTSGAPRKPGMSREDLIKVNADITRACISQAAPLSPNAVIIMVNNPLDA
MTYLAAEVSGFPKERVIGQ
>d1gv0a1 c.2.1.5 (A:1-142) Malate dehydrogenase {Chlorobium tepidum}
MKITVIGAGNVGATTAFRLAEKQLARELVLLDVVEGIPQGKALDMYESGPVGLFDTKVTGSN
DYADTANSDIVVITAGLPRKPGMTREDLLSMNAGIVREVTGRIMEHSKNPIIVVVSNPLDIM
THVAWQKSGLPKERVIGM
>d1guza1 c.2.1.5 (A:1-142) Malate dehydrogenase {Chlorobium

vibrioforme}
MKITVIGAGNVGATTAFRLAEKQLARELVLLDVVEGIPQGKALDMYESGPVGLFDTKVTGSN
DYADTANSDIVIITAGLPRKPGMTREDLLMKNAGIVKEVTDNIMKHSKNPIIIVVSNPLDIM
THVAWVRSGLPKERVIGM
>d1hyha1 c.2.1.5 (A:21-166) L-2-hydroxyisocapronate dehydrogenase, L-HICDH {Lactobacillus confusus}
ARKIGIIGLGNVGAAVAHGLIAQGVADDYVFIDANEAKVKADQIDFQDAMANLEAHGNIVIN
DWAALADADVVISTLGNIKLQQDNPTGDRFAELKFTSSMVQSVGTNLKESGFHGVLVVISNP
VDVITALFQHVTGFPAHKVIGT
>d5ldh_1 c.2.1.5 (1-162) Lactate dehydrogenase {Pig (Sus scrofa)}
ATLKEKLIAPVAQQETTIPDNKITVVGVGQVGMACAISILGKSLTDELALVDVLEDKLKGEM
MDLQHGSLFLQTPKIVANKDYSVTANSKIVVVTAGVRQQEGESRLNLVQRNVNVFKFIIPQI
VKYSPNCIIIVVSNPVDILTYVAWKLSGLPKHRVIG
>d9ldta1 c.2.1.5 (A:1-162) Lactate dehydrogenase {Pig (Sus scrofa)}
ATLKDQLIHNLLKEEHVPHNKITVVGVGAVGMACAISILMKELADEIALVDVMEDKLKGEMM
DLQHGSLFLRTPKIVSGKDYNVTANSRLVVITAGARQQEGESRLNLVQRNVNIFKFIIPNIV
KYSPNCKLLVVSNPVDILTYVAWKISGFPKNRVIG
>d1i0za1 c.2.1.5 (A:1-160) Lactate dehydrogenase {Human (Homo sapiens), heart isoform (H chain)}
ATLKEKLIAPVAEEEATVPNNKITVVGVGQVGMACAISILGKSLADELALVDVLEDKLKGEM
MDLQHGSLFLQTPKIVADKDYSVTANSKIVVVTAGVRQQEGESRLNLVQRNVNVFKFIIPQI
VKYSPDCIIIVVSNPVDILTYVTWKLSGLPKHRVIG
>d1i10a1 c.2.1.5 (A:1-159) Lactate dehydrogenase {Human (Homo sapiens), muscle isoform (M chain)}
ATLKDQLIYNLLKEEQTPQNKITVVGVGAVGMACAISILMKDLADELALVDVIEDKLKGEMM
DLQHGSLFLRTPKIVSGKDYNVTANSKLVIITAGARQQEGESRLNLVQRNVNIFKFIIPNVV
KYSPNCKLLIVSNPVDILTYVAWKISGFPKNRVIG
>d2ldx_1 c.2.1.5 (1-159) Lactate dehydrogenase {Mouse (Mus musculus)}
STVKEQLIQNLVPEDKLSRCKITVVGVGDVGMACAISILLKGLADELALVDADTDKLRGEAL
DLQHGSLFLSTPKIVFGKDYNVSANSKLVIITAGARMVSGQTRLDLLQRNVAIMKAIVPGVI
QNSPDCKIIVVTNPVDILTYVVWKISGFPVGRVIG
>d1ldm_1 c.2.1.5 (1-160) Lactate dehydrogenase {Dogfish (Squalus acanthias)}
ATLKDKLIGHLATSQEPRSYNKITVVGVGAVGMACAISILMKDLADEVALVDVMEDKLKGEM
MDLQHGSLFLHTAKIVSGKDYSVSAGSKLVVITAGARQQEGESRLNLVQRNVNIFKFIIPNI
VKHSPDCIILVVSNPVDVLTYVAWKLSGLPMHRIIG
>d1ceqa1 c.2.1.5 (A:19-163) Lactate dehydrogenase {Malaria parasite (Plasmodium falciparum)}
PKAKIVLVGSGMIGGVMATLIVQKNLGDVVLFDIVKNMPHGKALDTSHTNVMAYSNCKVSGS
NTYDDLAGSDVVIVTAGFTKAPGKSDKEWNRDDLLPLNNKIMIEIGGHIKKNCPNAFIIVVT
NPVDVMVQLLHQHSGVPKNKIIGL
>d1ldna1 c.2.1.5 (A:15-162) Lactate dehydrogenase {Bacillus

stearothermophilus}
MKNNGGARVVVIGAGFVGASYVFALMNQGIADEIVLIDANESKAIGDAMDFNHGKVFAPKPV
DIWHGDYDDCRDADLVVICAGANQKPGETRLDLVDKNIAIFRSIVESVMASGFQGLFLVATN
PVDILTYATWKFSGLPHERVIGSG
>d1llc_1 c.2.1.5 (13-164) Lactate dehydrogenase {Lactobacillus casei}
ASITDKDHQKVILVGDGAVGSSYAFAMVLQGIAQEIGIVDIFKDKTKGDAIDLSNALPFTSP
KKIYSAEYSDAKDADLVVITAGAPKQPGETRLDLVNKNLKILKSIVDPIVDSGFNLIFLVAA
NPVDILTYATWKLSGFPKNRVVGSG
>d1ez4a1 c.2.1.5 (A:16-162) Lactate dehydrogenase {Lactobacillus pentosus}
SMPNHQKVVLVGDGAVGSSYAFAMAQQGIAEEFVIVDVVKDRTKGDALDLEDAQAFTAPKKI
YSGEYSDCKDADLVVITAGAPQKPGESRLDLVNKNLNILSSIVKPVVDSGFDGIFLVAANPV
DILTYATWKFSGFPKERVIGSG
>d1llda1 c.2.1.5 (A:7-149) Lactate dehydrogenase {Bifidobacterium longum, strain am101-2}
PTKLAVIGAGAVGSTLAFAAAQRGIAREIVLEDIAKERVEAEVLDMQHGSSFYPTVSIDGSD
DPEICRDADMVVITAGPRQKPGQSRLELVGATVNILKAIMPNLVKVAPNAIYMLITNPVDIA
THVAQKLTGLPENQIFGSG
>d1a5z_1 c.2.1.5 (22-163) Lactate dehydrogenase {Thermotoga maritima}
MKIGIVGLGRVGSSTAFALLMKGFAREMVLIDVDKKRAEGDALDLIHGTPFTRRANIYAGDY
ADLKGSDVVIVAAGVPQKPGETRLQLLGRNARVMKEIARNVSKYAPDSIVIVVTNPVDVLTY
FFLKESGMDPRKVFGS
>d1hyea1 c.2.1.5 (A:1-145) MJ0490, lactate/malate dehydrogenase {Archaeon Methanococcus jannaschii}
MKVTIIGASGRVGSATALLLAKEPFMKDLVLIGREHSINKLEGLREDIYDALAGTRSDANIY
VESDENLRIIDESDVVIITSGVPRKEGMSRMDLAKTNAKIVGKYAKKIAEICDTKIFVITNP
VDVMTYKALVDSKFERNQVFG
>d1qmga2 c.2.1.6 (A:82-307) Acetohydroxy acid isomeroreductase, ketoacid reductoisomerase (KARI) {Spinach (Spinacia oleracea)}
SATTFDFDSSVFKKEKVTLSGHDEYIVRGGRNLFPLLPDAFKGIKQIGVIGWGSQAPAQAQN
LKDSLTEAKSDVVVKIGLRKGSNSFAEARAAGFSEENGTLGDMWETISGSDLVLLLISDSAQ
ADNYEKVFSHMKPNSILGLSHGFLLGHLQSLGQDFPKNISVIAVCPKGMGPSVRRLYVQGKE
VNGAGINSSFAVHQDVDGRATDVALGWSIALGSPFTFATT
>d2pgd_2 c.2.1.6 (1-176) 6-phosphogluconate dehydrogenase {Sheep (Ovis orientalis aries)}
AQADIALIGLAVMGQNLILNMNDHGFVVCAFNRTVSKVDDFLANEAKGTKVLGAHSLEEMVS
KLKKPRRIILLVKAGQAVDNFIEKLVPLLDIGDIIIDGGNSEYRDTMRRCRDLKDKGILFVG
SGVSGGEDGARYGPSLMPGGNKEAWPHIKAIFQGIAAKVGTGEPCCDWVGDD
>d1pgja2 c.2.1.6 (A:1-178) 6-phosphogluconate dehydrogenase {Trypanosoma brucei}
SMDVGVVGLGVMGANLALNIAEKGFKVAVFNRTYSKSEEFMKANASAPFAGNLKAFETMEAF
AASLKKPRKALILVQAGAATDSTIEQLKKVFEKGDILVDTGNAHFKDQGRRAQQLEAAGLRF

LGMGISGGEEGARKGPAFFPGGTLSVWEEIRPIVEAAAAKADDGRPCVTMNGSG
>d1f0ya2 c.2.1.6 (A:12-203) Short chain L-3-hydroxyacyl CoA dehydrogenase {Human (Homo sapiens)}
KIIVKHVTVIGGGLMGAGIAQVAAATGHTVVLVDQTEDILAKSKKGIEESLRKVAKKKFAEN
PKAGDEFVEKTLSTIATSTDAASVVHSTDLVVEAIVENLKVKNELFKRLDKFAAEHTIFASN
TSSLQITSIANATTRQDRFAGLHFFNPVPVMKLVEVIKTPMTSQKTFESLVDFSKALGKHPV
SCKDTP
>d1dlja2 c.2.1.6 (A:1-196) UDP-glucose dehydrogenase (UDPGDH) {Streptococcus pyogenes}
MKIAVAGSGYVGLSLGVLLSLQNEVTIVDILPSKVDKINNGLSPIQDEYIEYYLKSKQLSIK
ATLDSKAAYKEAELVIIATPTNYNSRINYFDTQHVETVIKEVLSVNSHATLIIKSTIPIGFI
TEMRQKFQTDRIIFSPEFLRESKALYDNLYPSRIIVSCEENDSPKVKADAEKFALLLKSAAK
KNNVPVLIMG
>d1bg6_2 c.2.1.6 (4-187) N-(1-D-carboxylethyl)-L-norvaline dehydrogenase {Arthrobacter, strain 1c}
SKTYAVLGLGNGGHAFAAYLALKGQSVLAWDIDAQRIKEIQDRGAIIAEGPGLAGTAHPDLL
TSDIGLAVKDADVILIVVPAIHHASIAANIASYISEGQLIILNPGATGGALEFRKILRENGA
PEVTIGETSSMLFTCRSERPGQVTVNAIKGAMDFACLPAAKAGWALEQIGSVLPQYVAVE
>d1evya2 c.2.1.6 (A:9-188) Glycerol-3- phosphate dehydrogenase {Trypanosome (Leishmania mexicana)}
KDELLYLNKAVVFGSGAFGTALAMVLSKKCREVCVWHMNEEEVRLVNEKRENVLFLKGVQLA
SNITFTSDVEKAYNGAEIILFVIPTQFLRGFFEKSGGNLIAYAKEKQVPVLVCTKGIERSTL
KFPAEIIGEFLPSPLLSVLAGPSFAIEVATGVFTCVSIASADINVARRLQRIMSTG
>d1ks9a2 c.2.1.6 (A:1-167) Ketopantoate reductase PanE {Escherichia coli}
MKITVLGCGALGQLWLTALCKQGHEVQGWLRVPQPYCSVNLVETDGSIFNESLTANDPDFLA
TSDLLLVTLKAWQVSDAVKSLASTLPVTTPILLIHNGMGTIEELQNIQQPLLMGTTTHAARR
DGNVIIHVANGITHIGPARQQDGDYSYLADILQTVLPDVAWHN
>d1jaya_ c.2.1.6 (A:) Coenzyme F420H2:NADP+ oxidoreductase (FNO) {Archaeon Archaeoglobus fulgidus}
MRVALLGGTGNLGKGLALRLATLGHEIVVGSRREEKAEAKAAEYRRIAGDASITGMKNEDAA
EACDIAVLTIPWEHAIDTARDLKNILREKIVVSPLVPVSRGAKGFTYSSERSAAEIVAEVLE
SEKVVSALHTIPAARFANLDEKFDWDVPVCGDDDESKKVVMSLISEIDGLRPLDAGPLSNSR
LVESLTPLILNIMRFNGMGELGIKFL
>d1bgva1 c.2.1.7 (A:195-449) Glutamate dehydrogenase {Clostridium symbiosum}
KARSFGGSLVRPEATGYGSVYYVEAVMKHENDTLVGKTVALAGFGNVAWGAAKKLAELGAKA
VTLSGPDGYIYDPEGITTEEKINYMLEMRASGRNKVQDYADKFGVQFFPGEKPWGQKVDIIM
PCATQNDVDLEQAKKIVANNVKYYIEVANMPTTNEALRFLMQQPNMVVAPSKAVNAGGVLVS
GFEMSQNSERLSWTAEEVDSKLHQVMTDIHDGSAAAAERYGLGYNLVAGANIVGFQKIADAM
MAQGIAW
>d1gtma1 c.2.1.7 (A:181-419) Glutamate dehydrogenase {Archaeon Pyrococcus furiosus}
GGSLGRIEATARGASYTIREAAKVLGWDTLKGKTIAIQGYGNAGYYLAKIMSEDFGMKVVAV

SDSKGGIYNPDGLNADEVLKWKNEHGSVKDFPGATNITNEELLELEVDVLAPAAIEEVITKK
NADNIKAKIVAEVANGPVTPEADEILFEKGILQIPDFLCNAGGVTVSYFEWVQNITGYYWTI
EEVRERLDKKMTKAFYDVYNIAKEKNIHMRDAAYVVAVQRVYQAMLDRGWVKH
>d1euza1 c.2.1.7 (A:181-419) Glutamate dehydrogenase {Archaeon Thermococcus profundus}
IGGSLGRGTATAQGAIFTIREAAKALGIDLKGKKIAVQGYGNAGYYTAKLAKEQLGMTVVAV
SDSRGGIYNPDGLDPDEVLKWKREHGSVKDFPGATNITNEELLELEVDVLAPAAIEEVITEK
NADNIKAKIVAEVANGPVTPEADDILREKGILQIPDFLCNAGGVTVSYFEWVQNINGYYWTE
EEVREKLDKKMTKAFWEVYNTHKDKNIHMRDAAYVVAVSRVYQAMKDRGWVKK
>d1bvua1 c.2.1.7 (A:181-418) Glutamate dehydrogenase {Archaeon Thermococcus litoralis}
GGIVARMDATARGASYTVREAAKALGMDLKGKTIAIQGYGNAGYYMAKIMSEEYGMKVVAVS
DTKGGIYNPDGLNADEVLAWKKKTGSVKDFPGATNITNEELLELEVDVLAPSAIEEVITKKN
ADNIKAKIVAELANGPTTPEADEILYEKGILIIPDFLCNAGGVTVSYFEWVQNITGDYWTVE
ETRAKLDKKMTKAFWDVYNTHKEKNINMRDAAYVVAVSRVYQAMKDRGWIKK
>d1b26a1 c.2.1.7 (A:179-412) Glutamate dehydrogenase {Thermotoga maritima}
GGSKGREEATGRGVKVCAGLAMDVLGIDPKKATVAVQGFGNVGQFAALLISQELGSKVVAVS
DSRGGIYNPEGFDVEELIRYKKEHGTVVTYPKGERITNEELLELDVDILVPAALEGAIHAGN
AERIKAKAVVEGANGPTTPEADEILSRRGILVVPDILANAGGVTVSYFEWVQDLQSFFWDLD
QVRNALEKMMKGAFNDVMKVKEKYNVDMRTAAYILAIDRVAYATKKRG
>d1hwxa1 c.2.1.7 (A:209-501) Glutamate dehydrogenase {Cow (Bos taurus)}
HGRISATGRGVFHGIENFIENASYMSILGMTPGFGDKTFAVQGFGNVGLHSMRYLHRFGAKC
VAVGESDGSIWNPDGIDPKELEDFKLQHGTILGFPKAKIYEGSILEVDCDILIPAASEKQLT
KSNAPRVKAKIIAEGANGPTTPQADKIFLERNIMVIPDLYLNAGGVTVSYFQILKNLNHVSY
GRLTFKYERDSNYHLLMSVQESLERKFGKHGGTIPIVPTAEFQDRISGASEKDIVHSGLAYT
MERSARQIMRTAMKYNLGLDLRTAAYVNAIEKVFRVYNEAGVTFT
>d1leha1 c.2.1.7 (A:135-364) Leucine dehydrogenase {Bacillus sphaericus}
GISPAFGSSGNPSPVTAYGVYRGMKAAAKEAFGSDSLEGLAVSVQGLGNVAKALCKKLNTEG
AKLVVTDVNKAAVSAAVAEEGADAVAPNAIYGVTCDIFAPCALGAVLNDFTIPQLKAKVIAG
SADNQLKDPRHGKYLHELGIVYAPDYVINAGGVINVADELYGYNRTRAMKRVDGIYDSIEKI
FAISKRDGVPSYVAADRMAEERIAKVAKARSQFLQDQRNILNGR
>d1c1da1 c.2.1.7 (A:149-349) Phenylalanine dehydrogenase {Rhodococcus sp., M4}
SAFTTAVGVFEAMKATVAHRGLGSLDGLTVLVQGLGAVGGSLASLAAEAGAQLLVADTDTER
VAHAVALGHTAVALEDVLSTPCDVFAPCAMGGVITTEVARTLDCSVVAGAANNVIADEAASD
ILHARGILYAPDFVANAGGAIHLVGREVLGWSESVVHERAVAIGDTLNQVFEISDNDGVTPD
EAARTLAGRRAREAS
>d1a4ia1 c.2.1.7 (A:127-296) Methylenetetrahydrofolate dehydrogenase/cyclohydrolase {Human (Homo sapiens)}
LTSINAGRLARGDLNDCFIPCTPKGCLELIKETGVPIAGRHAVVVGRSKIVGAPMHDLLLWN
NATVTTCHSKTAHLDEEVNKGDILVVATGQPEMVKGEWIKPGAIVIDCGINYVPDDKKPNGR

KVVGDVAYDEAKERASFITPVPGGVGPMTVAMLMQSTVESAKRFLE
>d1b0aa1 c.2.1.7 (A:123-288) Methylenetetrahydrofolate dehydrogenase/cyclohydrolase {Escherichia coli}
FHPYNVGRLCQRAPRLRPCTPRGIVTLLERYNIDTFGLNAVVIGASNIVGRPMSMELLLAGC
TTTVTHRFTKNLRHHVENADLLIVAVGKPGFIPGDWIKEGAIVIDVGINRLENGKVVGDVVF
EDAAKRASYITPVPGGVGPMTVATLIENTLQACVEYHDPQDE
>d1edza1 c.2.1.7 (A:149-319) Methylenetetrahydrofolate dehydrogenase/cyclohydrolase {Baker's yeast (Saccharomyces cerevisiae)}
PCTPLAIVKILEFLKIYNNLLPEGNRLYGKKCIVINRSEIVGRPLAALLANDGATVYSVDVN
NIQKFTRGESLKLNKHHVEDLGEYSEDLLKKCSLDSDVVITGVPSENYKFPTEYIKEGAVCI
NFACTKNFSDDVKEKASLYVPMTGKVTIAMLLRNMLRLVRNVELSKE
>d1do8a1 c.2.1.7 (A:280-573) Mitochondrial NAD(P)-dependenent malic enzyme {Human (Homo sapiens)}
IQGTAAVALAGLLAAQKVISKPISEHKILFLGAGEAALGIANLIVMSMVENGLSEQEAQKKI
WMFDKYGLLVKGRKAKIDSYQEPFTHSAPESIPDTFEDAVNILKPSTIIGVAGAGRLFTPDV
IRAMASINERPVIFALSNPTAQAECTAEEAYTLTEGRCLFASGSPFGPVKLTDGRVFTPGQG
NNVYIFPGVALAVILCNTRHISDSVFLEAAKALTSQLTDEELAQGRLYPPLANIQEVSINIA
IKVTEYLYANKMAFRYPEPEDKAKYVKERTWRSEYDSLLPDVYEWP
>d1id1a_ c.2.1.9 (A:) Rck domain from putative potassium channel Kch {Escherichia coli}
HRKDHFIVCGHSILAINTILQLNQRGQNVTVISNLPEDDIKQLEQRLGDNADVIPGDSNDSS
VLKKAGIDRCRAILALSDNDADNAFVVLSAKDMSSDVKTVLAVSDSKNLNKIKMVHPDIILS
PQLFGSEILARVLNGEEINNDMLVSMLLN
>d1jkja1 c.2.1.8 (A:1-121) Succinyl-CoA synthetase, alpha-chain, N-terminal (CoA-binding) domain {Escherichia coli}
SILIDKNTKVICQGFTGSQGTFHSEQAIAYGTKMVGGVTPGKGGTTHLGLPVFNTVREAVAA
TGATASVIYVPAPFCKDSILEAIDAGIKLIITITEGIPTLDMLTVKVKLDEAGVRMIGP
>d1euca1 c.2.1.8 (A:1-130) Succinyl-CoA synthetase, alpha-chain, N-terminal (CoA-binding) domain {Pig (Sus scrofa)}
CSYTASRKHLYVDKNTKVICQGFTGKQGTFHSQQALEYGTNLVGGTTPGKGGKTHLGLPVFN
TVKEAKEQTGATASVIYVPPPFAAAAINEAIDAEVPLVVCITEGIPQQDMVRVKHRLLRQGK
TRLIGP
>d1djna2 c.3.1.1 (A:490-645) Trimethylamine dehydrogenase, C-terminal domain {Methylophilus methylotrophus, w3a1}
RWNTDGTNCLTHDPIPGADASLPDQLTPEQVMDGKKKIGKRVVILNADTYFMAPSLAEKLAT
AGHEVTIVSGVHLANYMHFTLEYPNMMRRLHELHVEELGDHFCSRIEPGRMEIYNIWGDGSK
RTYRGPGVSPRDANTSHRWIEFDSLVLVTGRH
>d1cjca1 c.3.1.1 (A:107-331) Adrenodoxin reductase of mitochondrial p450 systems {Cow (Bos taurus)}
HQALDIPGEELPGVFSARAFVGWYNGLPENRELAPDLSCDTAVILGQGNVALDVARILLTPP
DHLEKTDITEAALGALRQSRVKTVWIVGRRGPLQVAFTIKELREMIQLPGTRPMLDPADFLG
LQDRIKEAARPRKRLMELLLRTATEKPGVEEAARRASASRAWGLRFFRSPQQVLPSPDGRRA
AGIRLAVTRLEGIGEATRAVPTGDVEDLPCGLVLSSIGY

>d1h7wa3 c.3.1.1 (A:288-440) Dihydropyrimidine dehydrogenase, domain 3 {Pig (Sus scrofa)}
PKTDDIFQGLTQDQGFYTSKDFLPLVAKSSKAGMCACHSPLPSIRGAVIVLGAGDTAFDCAT
SALRCGARRVFLVFRKGFVNIRAVPEEVELAKEEKCEFLPFLSPRKVIVKGGRIVAVQFVRT
EQDETGKWNEDEDQIVHLKADVVISAFGS
>d1coy_1 c.3.1.2 (4-318,451-506) Cholesterol oxidase {Brevibacterium sterolicum}
RTLADGDRVPALVIGSGYGGAVAALRLTQAGIPTQIVEMGRSWDTPGSDGKIFCGMLNPDKR
SMWLADKTDQPVSNFMGFGINKSIDRYVGVLDSERFSGIKVYQGRGVGGGSLVNGGMAVTPK
RNYFEEILPSVDSNEMYNKYFPRANTGLGVNNIDQAWFESTEWYKFARTGRKTAQRSGFTTA
FVPNVYDFEYMKKEAAGQVTKSGLGGEVIYGNNAGKKSLDKTYLAQAAATGKLTITTLHRVT
KVAPATGSGYSVTMEQIDEQGNVVATKVVTADRVFFAAGSVGTSKLLVSMKAQGHLPNLSSQ
VGEGWXGVLLNKATDNFGRLPEYPGLYVVDGSLVPGNVGVNPFVTITALAERNMDKIISSDI
>d1ijha1 c.3.1.2 (A:9-318,A:451-506) Cholesterol oxidase {Streptomyces sp.}
GYVPAVVIGTGYGAAVSALRLGEAGVQTLMLEMGQLWNQPGPDGNIFCGMLNPDKRSSWFKN
RTEAPLGSFLWLDVVNRNIDPYAGVLDRVNYDQMSVYVGRGVGGGSLVNGGMAVEPKRSYFE
EILPRVDSSEMYDRYFPRANSMLRVNHIDTKWFEDTEWYKFARVSREQAGKAGLGTVFVPNV
YDFGYMQREAAGEVPKSALATEVIYGNNHGKQSLDKTYLAAALGTGKVTIQTLHQVKTIRQT
KDGGYALTVEQKDTDGKLLATKEISCRYLFLGAGSLGSTELLVRARDTGTLPNLNSEVGAGW
XGCVLGKATDDYGRVAGYKNLYVTDGSLIPGSVGVLPFVTITALAERNVERIIKQDV
>d1k0ia1 c.3.1.2 (A:1-173,A:276-394) p-Hydroxybenzoate hydroxylase, PHBH {Pseudomonas aeruginosa}
MKTQVAIIGAGPSGLLLGQLLHKAGIDNVILERQTPDYVLGRIRAGVLEQGMVDLLREAGVD
RRMARDGLVHEGVEIAFAGQRRRIDLKRLSGGKTVTVYGQTEVTRDLMEAREACGATTVYQA
AEVRLHDLQGERPYVTFERDGERLRLDCDYIAGCDGFHGISRQSIPAERXMQHGRLFLAGDA
AHIVPPTGAKGLNLAASDVSTLYRLLLKAYREGRGELLERYSAICLRRIWKAERFSWWMTSV
LHRFPDTDAFSQRIQQTELEYYLGSEAGLATIAENYVGLPYEEIE
>d1el8a1 c.3.1.2 (A:1-217,A:322-385) Sarcosine oxidase {Bacillus sp., strain b0618}
STHFDVIVVGAGSMGMAAGYQLAKQGVKTLLVDAFDPPHTNGSHHGDTRIIRHAYGEGREYV
PLALRSQELWYELEKETHHKIFTKTGVLVFGPKGESAFVAETMEAAKEHSLTVDLLEGDEIN
KRWPGITVPENYNAIFEPNSGVLFSENCIRAYRELAEARGAKVLTHTRVEDFDISPDSVKIE
TANGSYTADKLIVSMGAWNSKLLSKLNLDIPXDEHFIIDLHPEHSNVVIAAGFSGHGFKFSS
GVGEVLSQLALTGKTEHDISIFSINRPALKESLQ
>d1foha5 c.3.1.2 (A:1-240,A:342-461) Phenol hydroxylase {Soil-living yeast (Trichosporon cutaneum)}
TKYSESYCDVLIVGAGPAGLMAARVLSEYVRQKPDLKVRIIDKRSTKVYNGQADGLQCRTLE
SLKNLGLADKILSEANDMSTIALYNPDENGHIRRTDRIPDTLPGISRYHQVVLHQGRIERHI
LDSIAEISDTRIKVERPLIPEKMEIDSSKAEDPEAYPVTMTLRYMSDHESTPLQFGHKTENS
LFHSNLQTQEEEDANYRLPEGKEAGEIETVHCKYVIGCDGGHSWVRRTLGFEMIXVTEKFSK
DERVFIAGDACHTHSPKAGQGMNTSMMDTYNLGWKLGLVLTGRAKRDILKTYEEERHAFAQA
LIDFDHQFSRLFSGRPAKDVADEMGVSMDVFKEAFVKGNEFASGTAINYDE
>d1cf3a1 c.3.1.2 (A:3-324,A:521-583) Glucose oxidase {Aspergillus

niger}
GIEASLLTDPKDVSGRTVDYIIAGGGLTGLTTAARLTENPNISVLVIESGSYESDRGPIIED
LNAYGDIFGSSVDHAYETVELATNNQTALIRSGNGLGGSTLVNGGTWTRPHKAQVDSWETVF
GNEGWNWDNVAAYSLQAERARAPNAKQIAAGHYFNASCHGVNGTVHAGPRDTGDDYSPIVKA
LMSAVEDRGVPTKKDFGCGDPHGVSMFPNTLHEDQVRSDAAREWLLPNYQRPNLQVLTGQYV
GKVLLSQNGTTPRAVGVEFGTHKGNTHNVYAKHEVLLAAGSAVSPTILEYSGIGMKSILEPL
GIDTVVDLPVGLXCSMMPKEMGGVVDNAARVYGVQGLRVIDGSIPPTQMSSHVMTVFYAMAL
KISDAILEDYASMQ
>d1gpea1 c.3.1.2 (A:1-328,A:525-587) Glucose oxidase {Penicillium amagasakiense}
YLPAQQIDVQSSLLSDPSKVAGKTYDYIIAGGGLTGLTVAAKLTENPKIKVLVIEKGFYESN
DGAIIEDPNAYGQIFGTTVDQNYLTVPLINNRTNNIKAGKGLGGSTLINGDSWTRPDKVQID
SWEKVFGMEGWNWDNMFEYMKKAEAARTPTAAQLAAGHSFNATCHGTNGTVQSGARDNGQPW
SPIMKALMNTVSALGVPVQQDFLCGHPRGVSMIMNNLDENQVRVDAARAWLLPNYQRSNLEI
LTGQMVGKVLFKQTASGPQAVGVNFGTNKAVNFDVFAKHEVLLAAGSAISPLILEYSGIGLK
SVLDQANVTQLLDLPVGIXCSMMSRELGGVVDATAKVYGTQGLRVIDGSIPPTQVSSHVMTI
FYGMALKVADAILDDYAKSA
>d1b5qa1 c.3.1.2 (A:5-293,A:406-463) Polyamine oxidase {Maize (Zea mays)}
PRVIVVGAGMSGISAAKRLSEAGITDLLILEATDHIGGRMHKTNFAGINVELGANWVEGVNG
GKMNPIWPIVNSTLKLRNFRSDFDYLAQNVYKEDGGVYDEDYVQKRIELADSVEEMGEKLSA
TLHASGRDDMSILAMQRLNEHQPNGPATPVDMVVDYYKFDYEFAEPPRVTSLQNTVPLATFS
DFGDDVYFVADQRGYEAVVYYLAGQYLKTDDKSGKIVDPRLQLNKVVREIKYSPGGVTVKTE
DNSVYSADYVMVSASLGVLQSDLIQFKPKLPTWKVRAIYQFXWPVGVNRYEYDQLRAPVGRV
YFTGEHTSEHYNGYVHGAYLSGIDSAEILINCAQKKMC
>d1f8ra1 c.3.1.2 (A:4-319,A:433-486) L-amino acid oxidase {Malayan pit viper (Calloselasma rhodostoma)}
RNPLAECFQENDYEEFLEIARNGLKATSNPKHVVIVGAGMAGLSAAYVLAGAGHQVTVLEAS
ERPGGRVRTYRNEEAGWYANLGPMRLPEKHRIVREYIRKFDLRLNEFSQENDNAWYFIKNIR
KKVGEVKKDPGLLKYPVKPSEAGKSAGQLYEESLGKVVEELKRTNCSYILNKYDTYSTKEYL
IKEGDLSPGAVDMIGDLLNEDSGYYVSFIESLKHDDIFAYEKRFDEIVDGMDKLPTAMYRDI
QDKVHFNAQVIKIQQNDQKVTVVYETLSKETPSVTADYVIVCTTSRAVRLIKFNPPLLPKKA
HALRSVXFTPYQFQHFSDPLTASQGRIYFAGEYTAQAHGWIDSTIKSGLRAARDVNLASEN
>d1gosa1 c.3.1.2 (A:4-289,A:402-500) Monoamine oxidase B {Human (Homo sapiens)}
KCDVVVVGGGISGMAAAKLLHDSGLNVVVLEARDRVGGRTYTLRNQKVKYVDLGGSYVGPTQ
NRILRLAKELGLETYKVNEVERLIHHVKGKSYPFRGPFPPVWNPITYLDHNNFWRTMDDMGR
EIPSDAPWKAPLAEEWDNMTMKELLDKLCWTESAKQLATLFVNLCVTAETHEVSALWFLWYV
KQCGGTTRIISTTNGGQERKFVGGSGQVSERIMDLLGDRVKLERPVIYIDQTRENVLVETLN
HEMYEAKYVISAIPPTLGMKIHFNPPLPMMRNQMITRVXFPPGILTQYGRVLRQPVDRIYFA
GTETATHWSGYMEGAVEAGERAAREILHAMGKIPEDEIWQSEPESVDVPAQPITTTFLERHL
PSVPGLLRLIGLTT
>d1d5ta1 c.3.1.3 (A:-2-291,A:389-431) Guanine nucleotide dissosiation inhibitor, GDI {Cow (Bos taurus)}

HHMDEEYDVIVLGTGLTECILSGIMSVNGKKVLHMDRNPYYGGESSSITPLEELYKRFQLLE
GPPETMGRGRDWNVDLIPKFLMANGQLVKMLLYTEVTRYLDFKVVEGSFVYKGGKIYKVPST
ETEALASNLMGMFEKRRFRKFLVFVANFDENDPKTFEGVDPQNTSMRDVYRKFDLGQDVIDF
TGHALALYRTDDYLDQPCLETINRIKLYSESLARYGKSPYLYPLYGLGELPQGFARLSAIYG
GTYMLNKPVDDIIMENGKVVGVKSEGEVARCKQLICDPSYVPDRVXPIDDGSESQVFCSCSY
DATTHFETTCNDIKDIYKRMAGSAFDF
>d1chua2 c.3.1.4 (A:2-237,A:354-422) L-aspartate oxidase
{Escherichia coli}
NTLPEHSCDVLIIGSGAAGLSLALRLADQHQVIVLSKGPVTEGSTFYAQGGIAAVFDETDSI
DSHVEDTLIAGAGICDRHAVEFVASNARSCVQWLIDQGVLFDTHIQPNGEESYHLTREGGHS
HRRILHAADATGREVETTLVSKALNHPNIRVLERTNAVDLIVSDKIGLPGTRRVVGAWVWNR
NKETVETCHAKAVVLATGGASKVYQYTTNPDISSGDGIAMAWRAGCRVANXCGGVMVDDHGR
TDVEGLYAIGEVSYTGLHGANRMASNSLLECLVYGWSAAEDITRRMPYAHDISTLPPW
>d1fuma2 c.3.1.4 (A:1-225,A:358-442) Fumarate reductase
flavoprotein subunit {Escherichia coli}
QTFQADLAIVGAGGAGLRAAIAAAQANPNAKIALISKVYPMRSHTVAAEGGSAAVAQDHDSF
EYHFHDTVAGGDWLCEQDVVDYFVHHCPTEMTQLELWGCPWSRRPDGSVNVRRFGGMKIERT
WFAADKTGFHMLHTLFQTSLQFPQIQRFDEHFVLDILVDDGHVRGLVAMNMMEGTLVQIRAN
AVVMATGGAGRVYRYNTNGGIVTGDGMGMALSHGVPLRDXMGGIETDQNCETRIKGLFAVGE
CSSVGLHGANRLGSNSLAELVVFGRLAGEQATERAATAGNGNEAAIEAQAAGVEQRLKDLVN
Q
>d1qlaa2 c.3.1.4 (A:1-250,A:372-457) Fumarate reductase
flavoprotein subunit {Wolinella succinogenes}
MKVQYCDSLVIGGGLAGLRAAVATQQKGLSTIVLSLIPVKRSHSAAAQGGMQASLGNSKMSD
GDNEDLHFMDTVKGSDWGCDQKVARMFVNTAPKAIRELAAWGVPWTRIHKGDRMAIINAQKT
TITEEDFRHGLIHSRDFGGTKKWRTCYTADATGHTMLFAVANECLKLGVSIQDRKEAIALIH
QDGKCYGAVVRDLVTGDIIAYVAKGTLIATGGYGRIYKNTTNAVVCEGTGTAIALETGIAQL
GNXMGGIRTDYRGEAKLKGLFSAGEAACWDMHGFNRLGGNSVSEAVVAGMIVGEYFAEHCAN
TQVDLETKTLEKFVKGQEAYMKSLVES
>d1e39a2 c.3.1.4 (A:103-359,A:506-568) Flavocytochrome c3
(respiratory fumarate reductase) {Shewanella frigidimarina}
PTIAELAKDKSERQAALASAPHDTVDVVVVGSGGAGFSAAISATDSGAKVILIEKEPVIGGN
AKLAAGGMNAAWTDQQKAKKITDSPELMFEDTMKGGQNINDPALVKVLSSHSKDSVDWMTAM
GADLTDVGMMGGASVNRAHRPTGGAGVGAHVVQVLYDNAVKRNIDLRMNTRGIEVLKDDKGT
VKGILVKGMYKGYYWVKADAVILATGGFAKNNERVAKLDPSLKGFISTNQPGAVGDGLDVAE
NAGGALKDMXTMGGVMIDTKAEVMNAKKQVIPGLYGAGEVTGGVHGANRLGGNAISDIITFG
RLAGEEAAKYS
>d1qo8a2 c.3.1.4 (A:103-359,A:506-565) Flavocytochrome c3
(respiratory fumarate reductase) {Shewanella frigidimarina}
DGWDQDKIQKAIAAGPSETTQVLVVGAGSAGFNASLAAKKAGANVILVDKAPFSGGNSMISA
GGMNAVGTKQQTAHGVEDKVEWFIEDAMKGGRQQNDIKLVTILAEQSADGVQWLESLGANLD
DLKRSGGARVDRTHRPHGGKSSGPEIIDTLRKAAKEQGIDTRLNSRVVKLVVNDDHSVVGAV
VHGKHTGYYMIGAKSVVLATGGYGMNKEMIAYYRPTMKDMTSSNNITATGDGVLMAKEIGAS
MTDIDWVQAXAINTTASVLDLQSKPIDGLFAAGEVTGGVHGYNRLGGNAIADTVVFGRIAGD

NAAKHALD
>d1d4ca2 c.3.1.4 (A:103-359,A:506-570) Flavocytochrome c3 (respiratory fumarate reductase) {Shewanella putrefaciens}
KFVPVDADKAAQDKAIAAGVKETTDVVIIGSGGAGLAAAVSARDAGAKVILLEKEPIPGGNT
KLAAGGMNAAETKPQAKLGIEDKKQIMIDDTMKGGRNINDPELVKVLANNSSDSIDWLTSMG
ADMTDVGRMGGASVNRSHRPTGGAGVGAHVAQVLWDNAVKRGTDIRLNSRVVRILEDASGKV
TGVLVKGEYTGYYVIKADAVVIAAGGFAKNNERVSKYDPKLKGFKATNHPGATGDGLDVALQ
AGAATRDLEXMGGLVIDTKAEVKSEKTGKPITGLYAAGEVTGGVHGANRLGGNAISDIVTYG
RIAGASAAKFAKD
>d1jnra2 c.3.1.4 (A:2-256,A:402-502) Adenylylsulfate reductase A subunit {Archaeon Archaeoglobus fulgidus}
VYYPKKYELYKADEVPTEVVETDILIIGGGFSGCGAAYEAAYWAKLGGLKVTLVEKAAVERS
GAVAQGLSAINTYIDLTGRSERQNTLEDYVRYVTLDMMGLAREDLVADYARHVDGTVHLFEK
WGLPIWKTPDGKYVREGQWQIMIHGESYKPIIAEAAKMAVGEENIYERVFIFELLKDNNDPN
AVAGAVGFSVREPKFYVFKAKAVILATGGATLLFRPRSTGEAAGRTWYAIFDTGSGYYMGLK
AGAMLTQXAGFWVCGPEDLMPEEYAKLFPLKYNRMTTVKGLFAIGDCAGANPHKFSSGSFTE
GRIAAKAAVRFILEQKPNPEIDDAVVEELKKKAYAPMERFMQYKDLS
>d3grs_1 c.3.1.5 (18-165,291-363) Glutathione reductase {Human (Homo sapiens)}
VASYDYLVIGGGSGGLASARRAAELGARAAVVESHKLGGTCVNVGCVPKKVMWNTAVHSEFM
HDHADYGFPSCEGKFNWRVIKEKRDAYVSRLNAIYQNNLTKSHIEIIRGHAAFTSDPKPTIE
VSGKKYTAPHILIATGGMPSTPHEXRVPNTKDLSLNKLGIQTDDKGHIIVDEFQNTNVKGIY
AVGDVCGKALLTPVAIAAGRKLAHRLFEYKEDSKLD
>d3grs_2 c.3.1.5 (166-290) Glutathione reductase {Human (Homo sapiens)}
SQIPGASLGITSDGFFQLEELPGRSVIVGAGYIAVEMAGILSALGSKTSLMIRHDKVLRSFD
SMISTNCTEELENAGVEVLKFSQVKEVKKTLSGLEVSMVTAVPGRLPVMTMIPDVDCLLWAI
G
>d1gera2 c.3.1.5 (A:147-262) Glutathione reductase {Escherichia coli}
DIPGVEYGIDSDGFFALPALPERVAVVGAGYIAVELAGVINGLGAKTHLFVRKHAPLRSFDP
MISETLVEVMNAEGPQLHTNAIPKAVVKNTDGSLTLELEDGRSETVDCLIWAIG
>d1gesa1 c.3.1.5 (A:3-146,A:263-335) Glutathione reductase {Escherichia coli}
KHYDYIAIGGGSGGIASINRAAMYGQKCALIEAKELGGTCVNVGCVPKKVMWHAAQIREAIH
MYGPDYGFDTTINKFNWETLIASRTAYIDRIHTSYENVLGKNNVDVIKGFARFVDAKTLEVN
GETITADHILIATGGRPSHPXREPANDNINLEAAGVKTNEKGYIVVDKYQNTNIEGIYAVGD
NTGAVELTPVAVAAGRRLSERLFNNKPDEHLD
>d1gesa2 c.3.1.5 (A:147-262) Glutathione reductase {Escherichia coli}
DIPGVEYGIDSDGFFALPALPERVAVVGAGYIGVELGGVINGLGAKTHLFEMFDAPLPSFDP
MISETLVEVMNAEGPQLHTNAIPKAVVKNTDGSLTLELEDGRSETVDCLIWAIG
>d1feca1 c.3.1.5 (A:1-169,A:287-357) Trypanothione reductase {Crithidia fasciculata}

SRAYDLVVIGAGSGGLEAGWNAASLHKKRVAVIDLQKHHGPPHYAALGGTCVNVGCVPKKLM
VTGANYMDTIRESAGFGWELDRESVRPNWKALIAAKNKAVSGINDSYEGMFADTEGLTFHQG
FGALQDNHTVLVRESADPNSAVLETLDTEYILLATGSWPQHLGIEXVPRSQTLQLEKAGVEV
AKNGAIKVDAYSKTNVDNIYAIGDVTDRVMLTPVAINEGAAFVDTVFANKPRATD
>d1feca2 c.3.1.5 (A:170-286) Trypanothione reductase {Crithidia fasciculata}
GDDLCITSNEAFYLDEAPKRALCVGGGYISIEFAGIFNAYKARGGQVDLAYRGDMILRGFDS
ELRKQLTEQLRANGINVRTHENPAKVTKNADGTRHVVFESGAEADYDVVMLAIGR
>d1aoga1 c.3.1.5 (A:3-169,A:287-357) Trypanothione reductase {Trypanosoma cruzi}
SKIFDLVVIGAGSGGLEAAWNAATLYKKRVAVIDVQMVHGPPFFSALGGTCVNVGCVPKKLM
VTGAQYMEHLRESAGFGWEFDRTTLRAEWKNLIAVKDEAVLNINKSYDEMFRDTEGLEFFLG
WGSLESKNVVNVRESADPASAVKERLETEHILLASGSWPHMPNXGRSPRTKDLQLQNAGVMI
KNGGVQVDEYSRTNVSNIYAIGDVTNRVMLTPVAINEAAALVDTVFGTTPRKT
>d1aoga2 c.3.1.5 (A:170-286) Trypanothione reductase {Trypanosoma cruzi}
IPGIEHCISSNEAFYLPEPPRRVLTVGGGFISVEFAGIFNAYKPKDGQVTLCYRGEMILRGF
DHTLREELTKQLTANGIQILTKENPAKVELNADGSKSVTFESGKKMDFDLVMMAI
>d1h6va1 c.3.1.5 (A:10-170,A:293-366) Mammalian thioredoxin reductase {Rat (Rattus norvegicus)}
SYDFDLIIIGGGSGGLAAAKEAAKFDKKVMVLDFVTPTPLGTNWGLGGTCVNVGCIPKKLMH
QAALLGQALKDSRNYGWKLEDTVKHDWEKMTESVQNHIGSLNWGYRVALREKKVVYENAYGK
FIGPHKIMATNNKGKEKVYSAERFLIATGERPRYLGIXRDSCTRTIGLETVGVKINEKTGKI
PVTDEEQTNVPYIYAIGDILEGKLELTPVAIQAGRLLAQRLYGGSTVKCD
>d1h6va2 c.3.1.5 (A:171-292) Mammalian thioredoxin reductase {Rat (Rattus norvegicus)}
PGDKEYCISSDDLFSLPYCPGKTLVVGASYVALECAGFLAGIGLDVTVMVRSILLRGFDQDM
ANKIGEHMEEHGIKFIRQFVPTKIEQIEAGTPGRLKVTAKSTNSEETIEDEFNTVLLAVG
>d1trb_1 c.3.1.5 (1-118,245-316) Thioredoxin reductase {Escherichia coli}
GTTKHSKLLILGSGPAGYTAAVYAARANLQPVLITGMEKGGQLTTTTEVENWPGDPNDLTGP
LLMERMHEHATKFETEIIFDHINKVDLQNRPFRLNGDNGEYTCDALIIATGASARYXHSPNT
AIFEGQLELENGYIKVQSGIHGNATQTSIPGVFAAGDVMDHIYRQAITSAGTGCMAALDAER
YLDGL
>d1trb_2 c.3.1.5 (119-244) Thioredoxin reductase {Escherichia coli}
LGLPSEEAFKGRGVSACATSDGFFYRNQKVAVIGGGNTAVEEALYLSNIASEVHLIHRRDGF
RAEKILIKRLMDKVENGNIILHTNRTLEEVTGDQMGVTGVRLRDTQNSDNIESLDVAGLFVA
IG
>d1vdc_1 c.3.1.5 (1-117,244-316) Thioredoxin reductase {Mouse-ear cress (Arabidopsis thaliana)}
LETHNTRLCIVGSGPAAHTAAIYAARAELKPLLFEGWMANDIAPGGQLTTTTDVENFPGFPE
GILGVELTDKFRKQSERFGTTIFTETVTKVDFSSKPFKLFTDSKAILADAVILAIGAVAKXG
HEPATKFLDGGVELDSDGYVVTKPGTTQTSVPGVFAAGDVQDKKYRQAITAAGTGCMAALDA

EHYLQEI
>d1vdc_2 c.3.1.5 (118-243) Thioredoxin reductase {Mouse-ear cress (Arabidopsis thaliana)}
RLSFVGSGEVLGGFWNRGISACAVCDGAAPIFRNKPLAVIGGGDSAMEEANFLTKYGSKVYI
IHRRDAFRASKIMQQRALSNPKIDVIWNSSVVEAYGDGERDVLGGLKVKNVVTGDVSDLKVS
GLFFAI
>d1hyua1 c.3.1.5 (A:199-325,A:452-521) Alkyl hydroperoxide reductase subunit F (AhpF), C-terminal domains {Salmonella typhimurium}
AEKRAAEALNKRDAYDVLIVGSGPAGAAAAVYSARKGIRTGLMGERFGGQVLDTVDIENYIS
VPKTEGQKLAGALKAHVSDYDVDVIDSQSASKLVPAATEGGLHQIETASGAVLKARSIIIAT
GAKXLPNTHWLEGALERNRMGEIIIDAKCETSVKGVFAAGDCTTVPYKQIIIATGEGAKASL
SAFDYLIRTKIA
>d1fl2a1 c.3.1.5 (A:212-325,A:452-521) Alkyl hydroperoxide reductase subunit F (AhpF), C-terminal domains {Escherichia coli}
AYDVLIVGSGPAGAAAAIYSARKGIRTGLMGERFGGQILDTVDIENYISVPKTEGQKLAGAL
KVHVDEYDVDVIDSQSASKLIPAAVEGGLHQIETASGAVLKARSIIVATGAKXLPNTNWLEG
AVERNRMGEIIIDAKCETNVKGVFAAGDCTTVPYKQIIIATGEGAKASLSAFDYLIRTKTA
>d1fl2a2 c.3.1.5 (A:326-451) Alkyl hydroperoxide reductase subunit F (AhpF), C-terminal domains {Escherichia coli}
WRNMNVPGEDQYRTKGVTYCPHCDGPLFKGKRVAVIGGGNSGVEAAIDLAGIVEHVTLLEFA
PEMKADQVLQDKLRSLKNVDIILNAQTTEVKGDGSKVVGLEYRDRVSGDIHNIELAGIFVQI
GL
>d1nhp_1 c.3.1.5 (1-119,243-321) NADH peroxidase {Enterococcus faecalis}
MKVIVLGSSHGGYEAVEELLNLHPDAEIQWYEKGDFISFLSAGMQLYLEGKVKDVNSVRYMT
GEKMESRGVNVFSNTEITAIQPKEHQVTVKDLVSGEERVENYDKLIISPGAVPFELDXGVRP
NTAWLKGTLELHPNGLIKTDEYMRTSEPDVFAVGDATLIKYNPADTEVNIALATNARKQGRF
AVKNLEEPVKPFP
>d1nhp_2 c.3.1.5 (120-242) NADH peroxidase {Enterococcus faecalis}
IPGKDLDNIYLMRGRQWAIKLKQKTVDPEVNNVVVIGSGYIGIEAAEAFAKAGKKVTVIDIL
DRPLGVYLDKEFTDVLTEEMEANNITIATGETVERYEGDGRVQKVVTDKNAYDADLVVVAV
>d1d7ya1 c.3.1.5 (A:5-115,A:237-308) NADH-dependent ferredoxin reductase, BphA4 {Pseudomonas sp., KKS102}
ALKAPVVVLGAGLASVSFVAELRQAGYQGLITVVGDEAERPYDRPPLSKDFMAHGDAEKIRL
DCKRAPEVEWLLGVTAQSFDPQAHTVALSDGRTLPYGTLVLATGAAPRAXVLANDALARAAG
LACDDGIFVDAYGRTTCPDVYALGDVTRQRNPLSGRFERIETWSNAQNQGIAVARHLVDP
>d1d7ya2 c.3.1.5 (A:116-236) NADH-dependent ferredoxin reductase, BphA4 {Pseudomonas sp., KKS102}
LPTLQGATMPVHTLRTLEDARRIQAGLRPQSRLLIVGGGVIGLELAATARTAGVHVSLVETQ
PRLMSRAAPATLADFVARYHAAQGVDLRFERSVTGSVDGVVLLDDGTRIAADMVVVGIG
>d1lvl_1 c.3.1.5 (1-150,266-335) Dihydrolipoamide dehydrogenase {Pseudomonas putida}
QQTIQTTLLIIGGGPGGYVAAIRAGQLGIPTVLVEGQALGGTCLNIGCIPSKALIHVAEQFH

QASRFTEPSPLGISVASPRLDIGQSVAWKDGIVDRLTTGVAALLKKHGVKVVHGWAKVLDGK
QVEVDGQRIQCEHLLLATGSSSVELPXRRPRTKGFNLECLDLKMNGAAIAIDERCQTSMHNV
WAIGDVAGEPMLAHRAMAQGEMVAEIIAGKARRFE
>d1lvl_2 c.3.1.5 (151-265) Dihydrolipoamide dehydrogenase
{Pseudomonas putida}
MLPLGGPVISSTEALAPKALPQHLVVVGGGYIGLELGIAYRKLGAQVSVVEARERILPTYDS
ELTAPVAESLKKLGIALHLGHSVEGYENGCLLANDGKGGQLRLEADRVLVAVG
>d1lpfa1 c.3.1.5 (A:1-158,A:278-348) Dihydrolipoamide
dehydrogenase {Pseudomonas fluorescens}
SQKFDVVVIGAGPGGYVAAIRAAQLGLKTACIEKYIGKEGKVALGGTCLNVGCIPSKALLDS
SYKYHEAKEAFKVHGIEAKGVTIDVPAMVARKANIVKNLTGGIATLFKANGVTSFEGHGKLL
ANKQVEVTGLDGKTQVLEAENVIIASGSRPVEIPXRRPVTTDLLAADSGVTLDERGFIYVDD
HCKTSVPGVFAIGDVVRGAMLAHKASEEGVMVAERIAGHKAQMN
>d1lpfa2 c.3.1.5 (A:159-277) Dihydrolipoamide dehydrogenase
{Pseudomonas fluorescens}
PAPLSDDIIVDSTGALEFQAVPKKLGVIGAGVIGLELGSVWARLGAEVTVLEALDKFLPAAD
EQIAKEALKVLTKQGLNIRLGARVTASEVKKKQVTVTFTDANGEQKETFDKLIVAVG
>d3lada1 c.3.1.5 (A:1-158,A:278-348) Dihydrolipoamide
dehydrogenase {Azotobacter vinelandii}
SQKFDVIVIGAGPGGYVAAIKSAQLGLKTALIEKYKGKEGKTALGGTCLNVGCIPSKALLDS
SYKFHEAHESFKLHGISTGEVAIDVPTMIARKDQIVRNLTGGVASLIKANGVTLFEGHGKLL
AGKKVEVTAADGSSQVLDTENVILASGSKPVEIPXRRPVTTDLLAADSGVTLDERGFIYVDD
YCATSVPGVYAIGDVVRGAMLAHKASEEGVVVAERIAGHKAQMN
>d3lada2 c.3.1.5 (A:159-277) Dihydrolipoamide dehydrogenase
{Azotobacter vinelandii}
PAPVDQDVIVDSTGALDFQNVPGKLGVIGAGVIGLELGSVWARLGAEVTVLEAMDKFLPAVD
EQVAKEAQKILTKQGLKILLGARVTGTEVKNKQVTVKFVDAEGEKSQAFDKLIVAVG
>d1ebda1 c.3.1.5 (A:7-154,A:272-346) Dihydrolipoamide
dehydrogenase {Bacillus stearothermophilus}
AIETETLVVGAGPGGYVAAIRAAQLGQKVTIVEKGNLGGVCLNVGCIPSKALISASHRYEQA
KHSEEMGIKAENVTIDFAKVQEWKASVVKKLTGGVEGLLKGNKVEIVKGEAYFVDANTVRVV
NGDSAQTYTFKNAIIATGSRPIELXVGRRPNTDELGLEQIGIKMTNRGLIEVDQQCRTSVPN
IFAIGDIVPGPALAHKASYEGKVAAEAIAGHPSAVDYV
>d1ebda2 c.3.1.5 (A:155-271) Dihydrolipoamide dehydrogenase
{Bacillus stearothermophilus}
PNFKFSNRILDSTGALNLGEVPKSLVVIGGGYIGIELGTAYANFGTKVTILEGAGEILSGFE
KQMAAIIKKRLKKKGVEVVTNALAKGAEEREDGVTVTYEANGETKTIDADYVLVT
>d1ojt_1 c.3.1.5 (117-275,401-470) Dihydrolipoamide dehydrogenase
{Neisseria meningitidis}
GSADAEYDVVVLGGGPGGYSAAFAAADEGLKVAIVERYKTLGGVCLNVGCIPSKALLHNAAV
IDEVRHLAANGIKYPEPELDIDMLRAYKDGVVSRLTGGLAGMAKSRKVDVIQGDGQFLDPHH
LEVSLTAGDAYEQAAPTGEKKIVAFKNCIIAAGSRXAPNGKLISAEKAGVAVTDRGFIEVDK
QMRTNVPHIYAIGDIVGQPMLAHKAVHEGHVAAENCAGHKAYFD
>d1ojt_2 c.3.1.5 (276-400) Dihydrolipoamide dehydrogenase

{Neisseria meningitidis}
VTKLPFIPEDPRIIDSSGALALKEVPGKLLIIGGGIIGLEMGTVYSTLGSRLDVVEMMDGLM
QGADRDLVKVWQKQNEYRFDNIMVNTKTVAVEPKEDGVYVTFEGANAPKEPQRYDAVLVAAG
R
>d1jeha1 c.3.1.5 (A:1-160,A:283-355) Dihydrolipoamide
dehydrogenase {Baker's yeast (Saccharomyces cerevisiae)}
TINKSHDVVIIGGGPAGYVAAIKAAQLGFNTACVEKRGKLGGTCLNVGCIPSKALLNNSHLF
HQMHTEAQKRGIDVNGDIKINVANFQKAKDDAVKQLTGGIELLFKKNKVTYYKGNGSFEDET
KIRVTPVDGLEGTVKEDHILDVKNIIVATGSEVTPFXVGRRPYIAGLGAEKIGLEVDKRGRL
VIDDQFNSKFPHIKVVGDVTFGPMLAHKAEEEGIAAVEMLKTGHGHVN
>d1jeha2 c.3.1.5 (A:161-282) Dihydrolipoamide dehydrogenase
{Baker's yeast (Saccharomyces cerevisiae)}
PGIEIDEEKIVSSTGALSLKEIPKRLTIIGGGIIGLEMGSVYSRLGSKVTVVEFQPQIGASM
DGEVAKATQKFLKKQGLDFKLSTKVISAKRNDDKNVVEIVVEDTKTNKQENLEAEVLLVA
>d1dxla1 c.3.1.5 (A:4-152,A:276-347) Dihydrolipoamide
dehydrogenase {Garden pea (Pisum sativum)}
SDENDVVIIGGGPGGYVAAIKAAQLGFKTTCIEKRGALGGTCLNVGCIPSKALLHSSHMYHE
AKHSFANHGVKVSNVEIDLAAMMGQKDKAVSNLTRGIEGLFKKNKVTYVKGYGKFVSPSEIS
VDTIEGENTVVKGKHIIIATGSDVKXGRTPFTSGLNLDKIGVETDKLGRILVNERFSTNVSG
VYAIGDVIPGPMLAHKAEEDGVACVEYLAGKVGHVD
>d1dxla2 c.3.1.5 (A:153-275) Dihydrolipoamide dehydrogenase
{Garden pea (Pisum sativum)}
SLPGVTIDEKKIVSSTGALALSEIPKKLVVIGAGYIGLEMGSVWGRIGSEVTVVEFASEIVP
TMDAEIRKQFQRSLEKQGMKFKLKTKVVGVDTSGDGVKLTVEPSAGGEQTIIEADVVLVSA
>d1fcda1 c.3.1.5 (A:1-114,A:256-327) Flavocytochrome c sulfide
dehydrogenase, FCSD, flavin-binding subunit {Purple phototrophic
bacterium (Chromatium vinosum)}
AGRKVVVVGGGTGGATAAKYIKLADPSIEVTLIEPNTDYYTCYLSNEVIGGDRKLESIKHGY
DGLRAHGIQVVHDSATGIDPDKKLVKTAGGAEFGYDRCVVAPGIELIYDKIEXQRAGKIAQI
AGLTNDAGWCPVDIKTFESSIHKGIHVIGDASIANPMPKSGYSANSQGKVAAAAVVVLLKGE
E
>d1fcda2 c.3.1.5 (A:115-255) Flavocytochrome c sulfide
dehydrogenase, FCSD, flavin-binding subunit {Purple phototrophic
bacterium (Chromatium vinosum)}
GYSEEAAAKLPHAWKAGEQTAILRKQLEDMADGGTVVIAPPAAPFRCPPGPYERASQVAYYL
KAHKPMSKVIILDSSQTFSKQSQFSKGWERLYGFGTENAMIEWHPGPDSAVVKVDGGEMMVE
TAFGDEFKADVINLIPP
>d1djna3 c.4.1.1 (A:341-489,A:646-729) Trimethylamine
dehydrogenase, middle domain {Methylophilus methylotrophus, w3a1}
DIRVCIGCNVCISRWEIGGPPMICTQNATAGEEYRRGWHPEKFRQTKNKDSVLIVGAGPSGS
EAARVLMESGYTVHLTDTAEKIGGHLNQVAALPGLGEWSYHRDYRETQITKLLKKNKESQLA
LGQKPMTADDVLQYGADKVIIATGAXSECTLWNELKARESEWAENDIKGIYLIGDAEAPRLI
ADATFTGHRVAREIEEANPQIAIPYKRETIAWGTPHMPGGNFKIEYKV
>d1cjca2 c.4.1.1 (A:6-106,A:332-460) Adrenodoxin reductase of

mitochondrial p450 systems {Cow (Bos taurus)}
TPQICVVGSGPAGFYTAQHLLKHHSRAHVDIYEKQLVPFGLVRFGVAPDHPEVKNVINTFTQ
TARSDRCAFYGNVEVGRDVTVQELQDAYHAVVLSYGAEDXKSRPIDPSVPFDPKLGVVPNME
GRVVDVPGLYCSGWVKRGPTGVITTTMTDSFLTGQILLQDLKAGHLPSGPRPGSAFIKALLD
SRGVWPVSFSDWEKLDAEEVSRGQASGKPREKLLDPQEMLRLLGH
>d1h7wa4 c.4.1.1 (A:184-287,A:441-532) Dihydropyrimidine dehydrogenase, domain 2 {Pig (Sus scrofa)}
EAYSAKIALLGAGPASISCASFLARLGYSDITIFEKQEYVGGLSTSEIPQFRLPYDVVNFEI
ELMKDLGVKIICGKSLSENEITLNTLKEEGYKAAFIGIGLPEXVLRDPKVKEALSPIKFNRW
DLPEVDPETMQTSEPWVFAGGDIVGMANTTVESVNDGKQASWYIHKYIQAQYGASVSAKPEL
PLFYTPVDLVD
>d1an9a1 c.4.1.2 (A:1-194,A:288-340) D-amino acid oxidase, N-terminal domain {Pig (Sus scrofa)}
MRVVVIGAGVIGLSTALCIHERYHSVLQPLDVKVYADRFTPFTTTDVAAGLWQPYTSEPSNP
QEANWNQQTFNYLLSHIGSPNAANMGLTPVSGYNLFREAVPDPYWKDMVLGFRKLTPRELDM
FPDYRYGWFNTSLILEGRKYLQWLTERLTERGVKFFLRKVESFEEVARGGADVIINCTGVWA
GVLQPDPLXQVRLEREQLRFGSSNTEVIHNYGHGGYGLTIHWGCALEVAKLFGKVLEERNLL
>d1c0pa1 c.4.1.2 (A:999-1193,A:1289-1361) D-amino acid oxidase, N-terminal domain {Yeast (Rhodotorula gracilis)}
LMMHSQKRVVVLGSGVIGLSSALILARKGYSVHILARDLPEDVSSQTFASPWAGANWTPFMT
LTDGPRQAKWEESTFKKWVELVPTGHAMWLKGTRRFAQNEDGLLGHWYKDITPNYRPLPSSE
CPPGAIGVTYDTLSVHAPKYCQYLARELQKLGATFERRTVTSLEQAFDGADLVVNATGLGAK
SIAGIDDQAXRGGPRVEAERIVLPLDRTKSPLSLGRGSARAAKEKEVTLVHAYGFSSAGYQQ
SWGAAEDVAQLVDEAFQRYHG
>d1i8ta1 c.4.1.3 (A:1-244,A:314-367) UDP-galactopyranose mutase, N-terminal domain {Escherichia coli}
MYDYIIVGSGLFGAVCANELKKLNKKVLVIEKRNHIGGNAYTEDCEGIQIHKYGAHIFHTND
KYIWDYVNDLVEFNRFTNSPLAIYKDKLFNLPFNMNTFHQMWGVKDPQEAQNIINAQKKKYG
DKVPENLEEQAISLVGEDLYQALIKGYTEKQWGRSAKELPAFIIKRIPVRFTFDNNYFSDRY
QGIPVGGYTKLIEKMLEGVDVKLGIDFLKDKDSLASKAHRIIYTGPIDQYFDYRFGALXNDN
KNMELFKKYRELASREDKVIFGGRLAEYKYYDMHQVISAALYQVKNIMSTD
>d2uaga1 c.5.1.1 (A:1-93) UDP-N-acetylmuramoyl-L-alanine:D-glutamate ligase MurD, N-terminal domain {Escherichia coli}
ADYQGKNVVIIGLGLTGLSCVDFFLARGVTPRVMDTRMTPPGLDKLPEAVERHTGSLNDEWL
MAADLIVASPGIALAHPSLSAAADAGIEIVG
>d1tml__ c.6.1.1 (-) Cellulase E2 {Thermomonospora fusca, strain yx}
NDSPFYVNPNMSSAEWVRNNPNDPRTPVIRDRIASVPQGTWFAHHNPGQITGQVDALMSAAQ
AAGKIPILVVYNAPGRDCGNHSSGGAPSHSAYRSWIDEFAAGLKNRPAYIIVEPDLISLMSS
CMQHVQQEVLETMAYAGKALKAGSSQARIYFDAGHSAWHSPAQMASWLQQADISNSAHGIAT
NTSNYRWTADEVAYAKAVLSAIGNPSLRAVIDTSRNGNGPAGNEWCDPSGRAIGTPSTTNTG
DPMIDAFLWIKLPGEADGCIAGAGQFVPQAAYEMAIAA
>d1qjwa_ c.6.1.1 (A:) Cellobiohydrolase II (Cel6) {Trichoderma

reesei, Cel6a}
ATYSGNPFVGVTPWANAYYASEVSSLAIPSLTGAMATAAAAVAKVPSFMWLDTLDKTPLMEQ
TLADIRTANKNGGNYAGQFVVFDLPDRDCAALASNGEYSIADGGVAKYKNYIDTIRQIVVEY
SDIRTLLVIEPDSLANLVTNLGTPKCANAQSAYLECINYAVTQLNLPNVAMYLDAGHAGWLG
WPANQDPAAQLFANVYKNASSPRALRGLATNVANYNGWNITSPPSYTQGNAVYNEKLYIHAI
GPLLANHGWSNAFFITDQGRSGKQPTGQQQWGDWCNVIGTGFGIRPSANTGDSLLDSFVWVK
PGGECDGTSDSSAPRFDSHCALPDALQPAPQAGAWFQAYFVQLLTNANPSFL
>d2bvwa_ c.6.1.1 (A:) Cellobiohydrolase II (Cel6) {Humicola
insolens, Cel6a}
NGNPFEGVQLWANNYYRSEVHTLAIPQITDPALRAAASAVAEVPSFQWLDRNVTVDTLLVQT
LSEIREANQAGANPQYAAQIVVYDLPDRDCAAAASNGEWAIANNGVNNYKAYINRIREILIS
FSDVRTILVIEPDSLANMVTNMNVPKCSGAASTYRELTIYALKQLDLPHVAMYMDAGHAGWL
GWPANIQPAAELFAKIYEDAGKPRAVRGLATNVANYNAWSVSSPPPYTSPNPNYDEKHYIEA
FRPLLEARGFPAQFIVDQGRSGKQPTGQKEWGHWCNAIGTGFGMRPTANTGHQYVDAFVWVK
PGGECDGTSDTTAARYDYHCGLEDALKPAPEAGQWFNEYFIQLLRNANPPF
>d1dysa_ c.6.1.1 (A:) Cellobiohydrolase II (Cel6) {Humicola
insolens, Cel6b}
GNPFSGRTLLVNSDYSSKLDQTRQAFLSRGDQTNAAKVKYVQEKVGTFYWISNIFLLRDIDV
AIQNARAAKARGENPIVGLVLYNLPDRDCSAGESSGELKLSQNGLNRYKNEYVNPFAQKLKA
ASDVQFAVILEPDAIGNMVTGTSAFCRNARGPQQEAIGYAISQLQASHIHLYLDVANGGWLG
WADKLEPTAQEVATILQKAGNNAKIRGFSSNVSNYNPYSTSNPPPYTSGSPSPDESRYATNI
ANAMRQRGLPTQFIIDQSRVALSGARSEWGQWCNVNPAGFGQPFTTNTNNPNVDAIVWVKPG
GESDGQCGMGGAPAAGMWFDAYAQMLTQNAHDEIA
>d1cm5a_ c.7.1.1 (A:) Pyruvate formate-lyase, PFL {Escherichia
coli}
SELNEKLATAWEGFTKGDWQNEVNVRDFIQKNYTPYEGDESFLAGATEATTTLWDKVMEGVK
LENRTHAPVDFDTAVASTITSHDAGYINKQLEKIVGLQTEAPLKRALIPFGGIKMIEGSCKA
YNRELDPMIKKIFTEYRKTHNQGVFDVYTPDILRCRKSGVLTGLPDAYGRGRIIGDYRRVAL
YGIDYLMKDKLAQFTSLQADLENGVNLEQTIRLREEIAEQHRALGQMKEMAAKYGYDISGPA
TNAQEAIQWTYFGYLAAVKSQNGAAMSFGRTSTFLDVYIERDLKAGKITEQEAQEMVDHLVM
KLRMVRFLRTPEYDELFSGDPIWATESIGGMGLDGRTLVTKNSFRFLNTLYTMGPSPEPNMT
ILWSEKLPLNFKKFAAKVSIDTSSLQYENDDLMRPDFNNDDYAIAAAVSPMIVGKQMQFFGA
RANLAKTMLYAINGGVDEKLKMQVGPKSEPIKGDVLNYDEVMERMDHFMDWLAKQYITALNI
IHYMHDKYSYEASLMALHDRDVIRTMACGIAGLSVAADSLSAIKYAKVKPIRDEDGLAIDFE
IEGEYPQFGNNDPRVDDLAVDLVERFMKKIQKLHTYRDAIPTQSVLTITSNVVYGKKTGNTP
DGRRAGAPFGPGANPMHGRDQKGAVASLTSVAKLPFAYAKDGISYTFSIVPNALGKDDEVRK
TNLAGLMDGYFHHEASIEGGQHLNVNVMNREMLLDAMENPEKYPQLTIRVSGYAVRFNSLTK
EQQQDVITRTFTQSM
>d1qhma_ c.7.1.1 (A:) Pyruvate formate-lyase, PFL {Escherichia
coli}
NEKLATAWEGFTKGDWQNEVNVRDFIQKNYTPYEGDESFLAGATEATTTLWDKVMEGVKLEN
RTHAPVDFDTAVASTITSHDAGYINKQLEKIVGLQTEAPLKRALIPFGGIKMIEGSCKAYNR
ELDPMIKKIFTEYRKTHNQGVFDVYTPDILRCRKSGVLTGLPDAYGRGRIIGDYRRVALYGI
DYLMKDKLAQFTSLQADLENGVNLEQTIRLREEIAEQHRALGQMKEMAAKYGYDISGPATNA

QEAIQWTYFGYLAAVKSQNGAAMSFGRTSTFLDVYIERDLKAGKITEQEAQEMVDHLVMKLR
MVRFLRTPEYDELFSGDPIWATESIGGMGLDGRTLVTKNSFRFLNTLYTMGPSPEPNMTILW
SEKLPLNFKKFAAKVSIDTSSLQYENDDLMRPDFNNDDYAIACCVSPMIVGKQMQFFGARAN
LAKTMLYAINGGVDEKLKMQVGPKSEPIKGDVLNYDEVMERMDHFMDWLAKQYITALNIIHY
MHDKYSYEASLMALHDRDVIRTMACGIAGLSVAADSLSAIKYAKVKPIRDEDGLAIDFEIEG
EYPQFGNNDPRVDDLAVDLVERFMKKIQKLHTYRDAIPTQSVLTITSNVVYGKK
>d1rlr_2 c.7.1.2 (222-748) R1 subunit of ribonucleotide reductase, C-terminal domain {Escherichia coli}
FSSCVLIECGDSLDSINATSSAIVKYVSQRAGIGINAGRIRALGSPIRGGEAFHTGCIPFYK
HFQTAVKSCSQGGVRGGAATLFYPMWHLEVESLLVLKNNRGVEGNRVRHMDYGVQINKLMYT
RLLKGEDITLFSPSDVPGLYDAFFADQEEFERLYTKYEKDDSIRKQRVKAVELFSLMMQERA
STGRIYIQNVDHCNTHSPFDPAIAPVRQSNLCLEIALPTKPLNDVNDENGEIALCTLSAFNL
GAINNLDELDELAILAVRALDALLDYQDYPIPAAKRGAMGRRTLGIGVINFAYYLAKHGKRY
SDGSANNLTHKTFEAIQYYLLKASNELAKEQGACPWFNETTYAKGILPIDTYKKDLDTIANE
PLHYDWEALRESIKTHGLRNSTLSALMPSETSSQISNATNGIEPPRGYVSIKASKDGILRQV
VPDYEHLHDAYELLWEMPGNDGYLQLVGIMQKFIDQSISANTNYDPSRFPSGKVPMQQLLKD
LLTAYKFGVKTLYYQNTRDDIDDLSNFQL
>d1b8ba_ c.7.1.3 (A:) Class III anaerobic ribonucleotide triphosphate reductase NRDD subunit {Bacteriophage T4}
SRVFPTQRDLMAGIVSKHIAKNMVPSFIMKAHESGIIHVHDIDYSPALPFTNCCLVDLKGML
ENGFKLGNAQIETPKSIGVATAIMAQITAQVASHQYGGTTFANVDKVLSPYVKRTYAKHIED
AEKWQIADALNYAQSKTEKDVYDAFQAYEYEVNTLFSSNGQTPFVTLTFGTGTDWTERMIQK
AILKNRIKGLGRDGITPIFPKLVMFVEEGVNLYKDDPNYDIKQLALECASKRMYPDIISAKN
NKAITGSSVPVSPMGCRSFLSVWKDSTGNEILDGRNNLGVVTLNLPRIALDSYIGTQFNEQK
FVELFNERMDLCFEALMCRISSLKGVKATVAPILYQEGAFGVRLKPDDDIIELFKNGRSSVS
LGYIGIHELNILVGRDIGREILTKMNAHLKQWTERTGFAFSLYSTPAENLCYRFCKLDTEKY
GSVKDVTDKGWYTNSFHVSVEENITPFEKISREAPYHFIATGGHISYVELPDMKNNLKGLEA
VWDYAAQHLDYFGVNMPVDKCFTCGSTHEMTPTENGFVCSICGETDPKKMNTIRRTCAYLGN
PNERG
>d1kbla2 c.8.1.1 (A:377-509) Pyruvate phosphate dikinase, central domain {Clostridium symbiosum}
LHPTFNPAALKAGEVIGSALPASPGAAAGKVYFTADEAKAAHEKGERVILVRLETSPEDIEG
MHAAEGILTVRGGMTSHAAVVARGMGTCCVSGCGEIKINEEAKTFELGGHTFAEGDYISLDG
STGKIYKGD
>d1zyma2 c.8.1.2 (A:3-21,A:145-249) N-terminal domain of enzyme I of the PEP:sugar phosphotransferase system {Escherichia coli}
SGILASPGIAFGKALLLKEXKIIDLSAIQDEVILVAADLTPSETAQLNLKKVLGFITDAGGR
TSHTSIMARSLELPAIVGTGSVTSQVKNDDYLILDAVNNQVYVNPTNEVIDKMRAVQEQVAS
E
>d1aco_1 c.8.2.1 (529-754) Aconitase, C-terminal domain {Cow (Bos taurus)}
VDVSPTSQRLQLLEPFDKWDGKDLEDLQILIKVKGKCTTDHISAAGPWLKFRGHLDNISNNL
LIGAINSENRKANSVRNAVTQEFGPVPDTARYYKQHGIRWVVIGDENYGEGSSREHSALEPR
FLGGRAIITKSFARIHETNLKKQGLLPLTFADPADYNKIHPVDKLTIQGLKDFAPGKPLTCI

IKHPNGTQETILLNHTFNETQIEWFRAGSALNRMKELQQK
>d1a9xb1 c.8.3.1 (B:1502-1652) Carbamoyl phosphate synthetase, small subunit N-terminal domain {Escherichia coli}
IKSALLVLEDGTQFHGRAIGATGSAVGEVVFNTSMTGYQEILTDPSYSRQIVTLTYPHIGNV
GTNDADEESSQVHAQGLVIRDLPLIASNFRNTEDLSSYLKRHNIVAIADIDTRKLTRLLREK
GAQNGCIIAGDNPDAALALEKARAFPG
>d1de4c2 c.8.4.1 (C:190-382) Transferrin receptor ectodomain, apical domain {Human (Homo sapiens)}
IQVKDSAQNSVIIVDKNGRLVYLVENPGGYVAYSKAATVTGKLVHANFGTKKDFEDLYTPVN
GSIVIVRAGKITFAEKVANAESLNAIGVLIYMDQTKFPIVNAELSFFGHAHLGTGDPYTPGF
PSFNHTQFPPSRSSGLPNIPVQTISRAAAEKLFGNMEGDCPSDWKTDSTCRMVTSESKNVKL
TVSNVLK
>d1dk7a_ c.8.5.1 (A:) GroEL {Escherichia coli}
EGMQFDRGYLSPYFINKPETGAVELESPFILLADKKISNIREMLPVLEAVAKAGKPLLIIAE
DVEGEALATLVVNTMRGIVKVAAVKAPGFGDRRKAMLQDIATLTGGTVISEEIGMELEKATL
EDLGQAKRVVINKDTTTIIDGV
>d1kid__ c.8.5.1 (-) GroEL {Escherichia coli}
GLVPRGSEGMQFDRGYLSPYFINKPETGAVELESPFILLADKKISNIREMLPVLEAVAKAGK
PLLIIAEDVEGEALATLVVNTMRGIVKVAAVKAPGFGDRRKAMLQDIATLTGGTVISEEIGM
ELEKATLEDLGQAKRVVINKDTTTIIDGVGEEAAIQGRVAQIRQQIEEATSDYDREKLQERV
AKLAGGV
>d1oela2 c.8.5.1 (A:191-366) GroEL {Escherichia coli}
EGMQFDRGYLSPYFINKPETGAVELESPFILLADKKISNIREMLPVLEAVAKAGKPLLIIAE
DVEGEALATAVVNTIRGIVKVAAVKAPGFGDRRKAMLQDIATLTGGTVISEEIGMELEKATL
EDLGQAKRVVINKDTTTIIDGVGEEAAIQGRVAQIRQQIEEATSDYDREKLQ
>d1ioka2 c.8.5.1 (A:191-366) GroEL {Paracoccus denitrificans}
EGMQFDRGYLSPYFVTNADKMIAELEDAYILLHEKKLSSLQPMVPLLESVIQSQKPLLIVAE
DVEGEALATLVVNKLRGGLKIAAVKAPGFGDRRKAMLQDIAILTGGQVISEDLGMKLENVTI
DMLGRAKKVSINKDNTTIVDGAGEKAEIEARVSQIRQQIEETTSDYDREKLQ
>d1srva_ c.8.5.1 (A:) GroEL {Thermus thermophilus}
GYQFDKGYISPYFVTNPETMEAVLEDAFILIVEKKVSNVRELLPILEQVAQTGKPLLIIAED
VEGEALATLVVNKLRGTLSVAAVKAPGFGDRRKEMLKDIAAVTGGTVISEELGFKLENATLS
MLGRAERVRITKDETTIVGGK
>d1a6db2 c.8.5.2 (B:216-367) Thermosome {Archaeon Thermoplasma acidophilum}
GIIVDKEKVHPGMPDVVKDAKIALLDAPLEIKKPEFDTNLRIEDPSMIQKFLAQEENMLREM
VDKIKSVGANVVITQKGIDDMAQHYLSRAGIYAVRRVKKSDMDKLAKATGASIVSTIDEISS
SDLGTAERVEQVKVGEDYMTFVTGCKNP
>d1ass__ c.8.5.2 (-) Thermosome {Archaeon Thermoplasma acidophilum}
MSGIVIDKEKVHSKMPDVVKNAKIALIDSALEIKKTEIEAKVQISDPSKIQDFLNQETNTFK
QMVEKIKKSGANVVLCQKGIDDVAQHYLAKEGIYAVRRVKKSDMEKLAKATGAKIVTDLDDL
TPSVLGEAETVEERKIGDDRMTFVMGCK
>d1ay7b_ c.9.1.1 (B:) Barstar (barnase inhibitor) {Bacillus

amyloliquefaciens}
KKAVINGEQIRSISDLHQTLKKELALPEYYGENLDALWDCLTGWVEYPLVLEWRQFEQSKQL
TENGAESVLQVFREAKAEGCDITIILS
>d1jj2x_ c.9.2.1 (X:) Ribosomal protein L32e {Archaeon Haloarcula marismortui}
TELQARGLTEKTPDLSDEDARLLTQRHRVGKPQFNRQDHHKKKRVSTSWRKPRGQLSKQRRG
IKGKGDTVEAGFRSPTAVRGKHPSGFEEVRVHNVDDLEGVDGDTEAVRIASKVGARKRERIE
EEAEDAGIRVLNPTYVEV
>d1e8ca1 c.98.1.1 (A:3-87) UDP-N-acetylmuramyl tripeptide synthetase MurE {Escherichia coli}
RNLRDLLAPWVPDAPSRALREMTLDSRVAAAGDLFVAVVGHQADGRRYIPQAIAQGVAAIIA
EAKDEATDGEIREMHGVPVIYLS
>d1gg4a3 c.98.1.1 (A:1-81) UDP-murNac-tripeptide D-alanyl-D-alanine-adding enzyme MurF {Escherichia coli}
MISVTLSQLTDILNGELQGADITLDAVTTDTRKLTPGCLFVALKGERFDAHDFADQAKAGGA
GALLVSRPLDIDLPQLIVK
>d2bnh__ c.10.1.1 (-) Ribonuclease inhibitor {Pig (Sus scrofa)}
MNLDIHCEQLSDARWTELLPLLQQYEVVRLDDCGLTEEHCKDIGSALRANPSLTELCLRTNE
LGDAGVHLVLQGLQSPTCKIQKLSLQNCSLTEAGCGVLPSTLRSLPTLRELHLSDNPLGDAG
LRLLCEGLLDPQCHLEKLQLEYCRLTAASCEPLASVLRATRALKELTVSNNDIGEAGARVLG
QGLADSACQLETLRLENCGLTPANCKDLCGIVASQASLRELDLGSNGLGDAGIAELCPGLLS
PASRLKTLWLWECDITASGCRDLCRVLQAKETLKELSLAGNKLGDEGARLLCESLLQPGCQL
ESLWVKSCSLTAACCQHVSLMLTQNKHLLELQLSSNKLGDSGIQELCQALSQPGTTLRVLCL
GDCEVTNSGCSSLASLLLANRSLRELDLSNNCVGDPGVLQLLGSLEQPGCALEQLVLYDTYW
TEEVEDRLQALEGSKPGLRVIS
>d1a4ya_ c.10.1.1 (A:) Ribonuclease inhibitor {Human (Homo sapiens)}
SLDIQSLDIQCEELSDARWAELLPLLQQCQVVRLDDCGLTEARCKDISSALRVNPALAELNL
RSNELGDVGVHCVLQGLQTPSCKIQKLSLQNCCLTGAGCGVLSSTLRTLPTLQELHLSDNLL
GDAGLQLLCEGLLDPQCRLEKLQLEYCSLSAASCEPLASVLRAKPDFKELTVSNNDINEAGV
RVLCQGLKDSPCQLEALKLESCGVTSDNCRDLCGIVASKASLRELALGSNKLGDVGMAELCP
GLLHPSSRLRTLWIWECGITAKGCGDLCRVLRAKESLKELSLAGNELGDEGARLLCETLLEP
GCQLESLWVKSCSFTAACCSHFSSVLAQNRFLLELQISNNRLEDAGVRELCQGLGQPGSVLR
VLWLADCDVSDSSCSSLAATLLANHSLRELDLSNNCLGDAGILQLVESVRQPGCLLEQLVLY
DIYWSEEMEDRLQALEKDKPSLRVIS
>d1yrga_ c.10.1.2 (A:) Rna1p (RanGAP1), N-terminal domain {Fission yeast (Schizosaccharomyces pombe)}
ARFSIEGKSLKLDAITTEDEKSVFAVLLEDDSVKEIVLSGNTIGTEAARWLSENIASKKDLE
IAEFSDIFTGRVKDEIPEALRLLLQALLKCPKLHTVRLSDNAFGPTAQEPLIDFLSKHTPLE
HLYLHNNGLGPQAGAKIARALQELAVNKKAKNAPPLRSIICGRNRLENGSMKEWAKTFQSHR
LLHTVKMVQNGIRPEGIEHLLLEGLAYCQELKVLDLQDNTFTHLGSSALAIALKSWPNLREL
GLNDCLLSARGAAAVVDAFSKLENIGLQTLRLQYNEIELDAVRTLKTVIDEKMPDLLFLELN
GNRFSEEDDVVDEIREVFSTRGRGELDELDDME
>d1fqva2 c.10.1.3 (A:146-431) Cyclin A/CDK2-associated p19, Skp2

{Human (Homo sapiens)}
ESLWQTLDLTGKNLHPDVTGRLLSQGVIAFRCPRSFMDQPLAEHFSPFRVQHMDLSNSVIEV
STLHGILSQCSKLQNLSLEGLRLSDPIVNTLAKNSNLVRLNLSGCSGFSEFALQTLLSSCSR
LDELNLSWCFDFTEKHVQVAVAHVSETITQLNLSGYRKNLQKSDLSTLVRRCPNLVHLDLSD
SVMLKNDCFQEFFQLNYLQHLSLSRCYDIIPETLLELGEIPTLKTLQVFGIVPDGTLQLLKE
ALPHLQINCSHFTTIARPTIGNKKNQEIWGIKCRLTLQ
>d1fs2a2 c.10.1.3 (A:146-401) Cyclin A/CDK2-associated p19, Skp2
{Human (Homo sapiens)}
ESLWQTLDEFRVQHMDLSNSVIEVSTLHGILSQCSKLQNLSLEGLRLSDPIVNTLAKNSNLV
RLNLSGCSGFSEFALQTLLSSCSRLDELNLSWCFDFTEKHVQVAVAHVSETITQLNLSGYRK
NLQKSDLSTLVRRCPNLVHLDLSDSVMLKNDCFQEFFQLNYLQHLSLSRCYDIIPETLLELG
EIPTLKTLQVFGIVPDGTLQLLKEALPHLQIN
>d1h6ta2 c.10.2.1 (A:31-240) Internalin B {Listeria monocytogenes}
GPLGSETITVPTPIKQIFSDDAFAETIKDNLKKKSVTDAVTQNELNSIDQIIANNSDIKSVQ
GIQYLPNVTKLFLNGNKLTDIKPLANLKNLGWLFLDENKVKDLSSLKDLKKLKSLSLEHNGI
SDINGLVHLPQLESLYLGNNKITDITVLSRLTKLDTLSLEDNQISDIVPLAGLTKLQNLYLS
KNHISDLRALAGLKNLDVLELFSQ
>d1h6ua2 c.10.2.1 (A:36-262) Internalin H {Listeria monocytogenes}
GSITQPTAINVIFPDPALANAIKIAAGKSNVTDTVTQADLDGITTLSAFGTGVTTIEGVQYL
NNLIGLELKDNQITDLAPLKNLTKITELELSGNPLKNVSAIAGLQSIKTLDLTSTQITDVTP
LAGLSNLQVLYLDLNQITNISPLAGLTNLQYLSIGNAQVSDLTPLANLSKLTTLKADDNKIS
DISPLASLPNLIEVHLKNNQISDVSPLANTSNLFIVTLTNQ
>d1jl5a_ c.10.2.6 (A:) Leucine rich effector protein YopM {Yersinia
pestis}
KSKTEYYNAWSEWERNAPPGNGEQREMAVSRLRDCLDRQAHELELNNLGLSSLPELPPHLES
LVASCNSLTELPELPQSLKSLLVDNNNLKALSDLPPLLEYLGVSNNQLEKLPELQNSSFLKI
IDVDNNSLKKLPDLPPSLEFIAAGNNQLEELPELQNLPFLTAIYADNNSLKKLPDLPLSLES
IVAGNNILEELPELQNLPFLTTIYADNNLLKTLPDLPPSLEALNVRDNYLTDLPELPQSLTF
LDVSENIFSGLSELPPNLYYLNASSNEIRSLCDLPPSLEELNVSNNKLIELPALPPRLERLI
ASFNHLAEVPELPQNLKQLHVEYNPLREFPDIPESVEDLRMNS
>d1dcea3 c.10.2.2 (A:444-567) Rab geranylgeranyltransferase
alpha-subunit, C-terminal domain {Rat (Rattus norvegicus)}
RVLHLAHKDLTVLCHLEQLLLVTHLDLSHNRLRALPPALAALRCLEVLQASDNALENVDGVA
NLPRLQELLLCNNRLQQSAAIQPLVSCPRLVLLNLQGNSLCQEEGIQERLAEMLPSVSSILT
>d1koha1 c.10.2.3 (A:201-362) mRNA export factor tap {Human (Homo
sapiens)}
LNELKPEQVEQLKLIMSKRYDGSQQALDLKGLRSDPDLVAQNIDVVLNRRSSMAATLRIIEE
NIPELLSLNLSNNRLYRLDDMSSIVQKAPNLKILNLSGNELKSERELDKIKGLKLEELWLDG
NSLSDTFRDQSTYISAIRERFPKLLRLDGHELPPPIAF
>d1a9na_ c.10.2.4 (A:) Splicesomal U2A' protein {Human (Homo
sapiens)}
VKLTAELIEQAAQYTNAVRDRELDLRGYKIPVIENLGATLDQFDAIDFSDNEIRKLDGFPLL
RRLKTLLVNNNRICRIGEGLDQALPDLTELILTNNSLVELGDLDPLASLKSLTYLCILRNPV
TNKKHYRLYVIYKVPQVRVLDFQKVKLKERQEAEKMFK

>d1igra1 c.10.2.5 (A:1-149) L1 and L2 domains of the type 1 insulin-like growth factor receptor {Human (Homo sapiens)}
EICGPGIDIRNDYQQLKRLENCTVIEGYLHILLISKAEDYRSYRFPKLTVITEYLLLFRVAG
LESLGDLFPNLTVIRGWKLFYNYALVIFEMTNLKDIGLYNLRNITRGAIRIEKNADLCYLST
VDWSLILDAVSNNYIVGNKPPKECG
>d1igra2 c.10.2.5 (A:300-478) L1 and L2 domains of the type 1 insulin-like growth factor receptor {Human (Homo sapiens)}
KVCEEEKKTKTIDSVTSAQMLQGCTIFKGNLLINIRRGNNIASELENFMGLIEVVTGYVKIR
HSHALVSLSFLKNLRLILGEEQLEGNYSFYVLDNQNLQQLWDWDHRNLTIKAGKMYFAFNPK
LCVSEIYRMEEVTGTKGRQSKGDINTRNNGERASCESDVDDDDKEQKLISEEDLN
>d1ds9a_ c.11.1.1 (A:) Outer arm dynein light chain 1 {Green algae (Chlamydomonas reinhardtii)}
MAKATTIKDAIRIFEERKSVVATEAEKVELHGMIPPIEKMDATLSTLKACKHLALSTNNIEK
ISSLSGMENLRILSLGRNLIKKIENLDAVADTLEELWISYNQIASLSGIEKLVNLRVLYMSN
NKITNWGEIDKLAALDKLEDLLLAGNPLYNDYKENNATSEYRIEVVKRLPNLKKLDGMPVDV
DEREQANVARGG
>d1jj2k_ c.12.1.1 (K:) Ribosomal protein L15 (L15p) {Archaeon Haloarcula marismortui}
TSKKKRQRGSRTHGGGSHKNRRGAGHRGGRGDAGRDKHEFHNHEPLGKSGFKRPQKVQEEAA
TIDVREIDENVTLLAADDVAEVEDGGFRVDVRDVVEEADDADYVKVLGAGQVRHELTLIADD
FSEGAREKVEGAGGSVELTDLGEERQ
>d1jj2n_ c.12.1.1 (N:) Ribosomal protein L18e {Archaeon Haloarcula marismortui}
SKTNPRLSSLIADLKSAARSSGGAVWGDVAERLEKPRRTHAEVNLGRIERYAQEDETVVVPG
KVLGSGVLQKDVTVAAVDFSGTAETKIDQVGEAVSLEQAIENNPEGSHVRVIR
>d1aua_2 c.13.1.1 (97-299) C-terminal domain of phosphatidylinositol transfer protein sec14p {Baker's yeast (Saccharomyces cerevisiae)}
YDEKPLIAKFYPQYYHKTDKDGRPVYFEELGAVNLHEMNKVTSEERMLKNLVWEYESVVQYR
LPACSRAAGHLVETSCTIMDLKGISISSAYSVMSYVREASYISQNYYPERMGKFYIINAPFG
FSTAFRLFKPFLDPVTVSKIFILGSSYQKELLKQIPAENLPVKFGGKSEVDESKGGLYLSDI
GPWRDPKYIGPEGEAPE
>d1h4xa_ c.13.2.1 (A:) Anti-sigma factor antagonist SpoIIaa {Bacillus sphaericus}
AFQLEMVTRETVVIRLFGELDHHAVEQIRAKISTAIFQGAVTTIIWNFERLSFMDSSGVGLV
LGRMRELEAVAGRTILLNPSPTMRKVFQFSGLGPWMMDATEEEAIDRVR
>d1auz__ c.13.2.1 (-) Anti-sigma factor antagonist SpoIIaa {Bacillus subtilis}
SLGIDMNVKESVLCIRLTGELDHHTAETLKQKVTQSLEKDDIRHIVLNLEDLSFMDSSGLGV
ILGRYKQIKQIGGEMVVCAISPAVKRLFDMSGLFKIIRFEQSEQQALLTLGVAS
>d1tyfa_ c.14.1.1 (A:) Clp protease, ClpP subunit {Escherichia coli}
SRGERSFDIYSRLLKERVIFLTGQVEDHMANLIVAQMLFLEAENPEKDIYLYINSPGGVITA
GMSIYDTMQFIKPDVSTICMGQAASMGAFLLTAGAKGKRFCLPNSRVMIHQPLGGYQGQATD

IEIHAREILKVKGRMNELMALHTGQSLEQIERDTERDRFLSAPEAVEYGLVDSILTHRN
>d1fc6a4 c.14.1.2 (A:78-156,A:249-463) Photosystem II D1
C-terminal processing protease {Algae (Scenedesmus obliquus)}
VTSEQLLFLEAWRAVDRAYVDKSFNGQSWFKLRETYLKKEPMDRRAQTYDAIRKMLAVLDDP
FTRFLEPSRLAALRRGTXKVTINPVTFTTCSNVAAAALPPGAAKQQLGYVRLATFNSNTTAA
AQQAFTELSKQGVAGLVLDIRNNGGGLFPAGVNVARMLVDRGDLVLIADSQGIRDIYSADGN
SIDSATPLVVLVNRGTASASEVLAGALKDSKRGLIAGERTFGKGLIQTVVDLSDGSGVAVTV
ARYQTPAGVDINKIGVSPDVQLDPEVLPTDLEGVCRVLGSDAAPRLF
>d1k32a4 c.14.1.2 (A:680-762,A:854-1061) Tricorn protease
{Archaeon Thermoplasma acidophilum}
SSIHEEFLQMYDEAWKLARDNYWNEAVAKEISERIYEKYRNLVPLCKTRYDLSNVIVEMQGE
YRTSHSYEMGGTFTDKDPFRSXDDRFIRYRSWVEANRRYVHERSKGTIGYIHIPDMGMMGLN
EFYRLFINESSYQGLIVDVRFNGGGFVSQLIIEKLMNKRIGYDNPRRGTLSPYPTNSVRGKI
IAITNEYAGSDGDIFSFSFKKLGLGKLIGTRTWGGVVGITPKRRLIDGTVLTQPEFAFWFRD
AGFGVENYGVDPDVEIEYAPHDYLSGKDPQIDYAIDALIEELRN
>d1j7xa_ c.14.1.2 (A:) Interphotoreceptor retinoid-binding
protein IRBP {African clawed frog (Xenopus laevis)}
DPSVTHVLHQLCDILANNYAFSERIPTLLQHLPNLDYSTVISEEDIAAKLNYELQSLTEDPR
LVLKSKTDTLVMPGDSIQAENIPEDEAMLQALVNTVFKVSILPGNIGYLRFDQFADVSVIAK
LAPFIVNTVWEPITITENLIIDLRYNVGGSSTAVPLLLSYFLDPETKIHLFTLHNRQQNSTD
EVYSHPKVLGKPYGSKKGVYVLTSHQTATAAEEFAYLMQSLSRATIIGEITSGNLMHSKVFP
FGDTQLSVTVPIINFIDSNGDYWLGGGVVPDAIVLADEALDKAKEIIAFHPPLA
>d1nzya_ c.14.1.3 (A:) 4-Chlorobenzoyl-CoA dehalogenase
{Pseudomonas sp., strain CBS-3}
MYEAIGHRVEDGVAEITIKLPRHRNALSVKAMQEVTDALNRAEEDDSVGAVMITGAEDAFCA
GFYLREIPLDKGVAGVRDHFRIAALWWHQMIHKIIRVKRPVLAAINGVAAGGGLGISLASDM
AICADSAKFVCAWHTIGIGNDTATSYSLARIVGMRRAMELMLTNRTLYPEEAKDWGLVSRVY
PKDEFREVAWKVARELAAAPTHLQVMAKERFHAGWMQPVEECTEFEIQNVIASVTHPHFMPC
LTRFLDGHRADRPQVELPAGV
>d1ey3a_ c.14.1.3 (A:) Enoyl-CoA hydratase (crotonase) {Rat
(Rattus norvegicus)}
FQYIITEKKGKNSSVGLIQLNRPKALNALCNGLIEELNQALETFEEDPAVGAIVLTGGEKAF
AAGADIKEMQNRTFQDCYSGKFLSHWDHITRIKKPVIAAVNGYALGGGCELAMMCDIIYAGE
KAQFGQPEILLGTIPGAGGTQRLTRAVGKSLAMEMVLTGDRISAQDAKQAGLVSKIFPVETL
VEEAIQCAEKIANNSKIIVAMAKESVNAAFEMTLTEGNKLEKKLFYSTFATDDRREGMSAFV
EKRKANFKDH
>d1dcia_ c.14.1.3 (A:) Dienoyl-CoA isomerase
(delta3-delta2-enoyl-CoA isomerase) {Rat (Rattus norvegicus)}
AYESIQVTSAQKHVLHVQLNRPEKRNAMNRAFWRELVECFQKISKDSDCRAVVVSGAGKMFT
SGIDLMDMASDILQPPGDDVARIAWYLRDLISRYQKTFTVIEKCPKPVIAAIHGGCIGGGVD
LISACDIRYCTQDAFFQVKEVDVGLAADVGTLQRLPKVIGNRSLVNELTFTARKMMADEALD
SGLVSRVFPDKDVMLNAAFALAADISSKSPVAVQGSKINLIYSRDHSVDESLDYMATWNMSM
LQTQDIIKSVQAAMEKKDSKSITFSKL
>d1hnua_ c.14.1.3 (A:) Dienoyl-CoA isomerase

(delta3-delta2-enoyl-CoA isomerase) {Baker's yeast (Saccharomyces cerevisiae)}
NEKISYRIEGPFFIIHLINPDNLNALEGEDYIYLGELLELADRNRDVYFTIIQSSGRFFSSG
ADFKGIAKAQGDDTNKYPSETSKWVSNFVARNVYVTDAFIKHSKVLICCLNGPAIGLSAALV
ALCDIVYSINDKVYLLYPFANLGLITEGGTTVSLPLKFGTNTTYECLMFNKPFKYDIMCENG
FISKNFNMPSSNAEAFNAKVLEELREKVKGLYLPSCLGMKKLLKSNHIDAFNKANSVEVNES
LKYWVDGEPLKRFRQ
>d1hzda_ c.14.1.3 (A:) AUH protein {Human (Homo sapiens)}
EDELRVRHLEEENRGIVVLGINRAYGKNSLSKNLIKMLSKAVDALKSDKKVRTIIIRSEVPG
IFCAGADLKERAKMSSSEVGPFVSKIRAVINDIANLPVPTIAAIDGLALGGGLELALACDIR
VAASSAKMGLVETKLAIIPGGGGTQRLPRAIGMSLAKELIFSARVLDGKEAKAVGLISHVLE
QNQEGDAAYRKALDLAREFLPQGPVAMRVAKLAINQGMEVDLVTGLAIEEACYAQTIPTKDR
LEGLLAFKEKRPPRYKGE
>d1ef8a_ c.14.1.3 (A:) Methylmalonyl CoA decarboxylase {Escherichia coli}
MSYQYVNVVTINKVAVIEFNYGRKLNALSKVFIDDLMQALSDLNRPEIRCIILRAPSGSKVF
SAGHDIHELPSGGRDPLSYDDPLRQITRMIQKFPKPIISMVEGSVWGGAFEMIMSSDLIIAA
STSTFSMTPVNLGVPYNLVGIHNLTRDAGFHIVKELIFTASPITAQRALAVGILNHVVEVEE
LEDFTLQMAHHISEKAPLAIAVIKEELRVLGEAHTMNSDEFERIQGMRRAVYDSEDYQEGMN
AFLEKRKPNFVGH
>d1jnxx1 c.15.1.3 (X:1649-1757) Breast cancer associated protein, BRCA1 {Human (Homo sapiens)}
RMSMVVSGLTPEEFMLVYKFARKHHITLTNLITEETTHVVMKTDAEFVCERTLKYFLGIAGG
KWVVSYFWVTQSIKERKMLNEHDFEVRGDVVNGRNHQGPKRARESQD
>d1jnxx2 c.15.1.3 (X:1758-1859) Breast cancer associated protein, BRCA1 {Human (Homo sapiens)}
RKIFRGLEICCYGPFTNMPTDQLEWMVQLCGASVVKELSSFTLGTGVHPIVVVQPDAWTEDN
GFHAIGQMCEAPVVTREWVLDSVALYQCQELDTYLIPQIP
>d1cdza_ c.15.1.1 (A:) DNA-repair protein XRCC1 {Human (Homo sapiens)}
ELPDFFQGKHFFLYGEFPGDERRKLIRYVTAFNGELEDYMSDRVQFVITAQEWDPSFEEALM
DNPSLAFVRPRWIYSCNEKQKLLPHQLYGVVPQA
>d1in1a_ c.15.1.2 (A:) DNA ligase III alpha {Human (Homo sapiens)}
GSADETLCQTKVLLDIFTGVRLYLPPSTPDFSRLRRYFVAFDGDLVQEFDMTSATHVLGSRD
KNPAAQQVSPEWIWACIRKRRLVAPC
>d1dgtb3 c.15.1.2 (B:2582-2660) NAD+-dependent DNA ligase, domain 4 {Thermus filiformis}
EEVSDLLSGLTFVLTGELSRPREEVKALLGRLGAKVTDSVSRKTSYLVVGENPGSKLEKARA
LGVAVLTEEEFWRFLKE
>d1rvv1_ c.16.1.1 (1:) Lumazine synthase {Bacillus subtilis}
MNIIQGNLVGTGLKIGIVVGRFNDFITSKLLSGAEDALLRHGVDTNDIDVAWVPGAFEIPFA
AKKMAETKKYDAIITLGTVIRGATTHYDYVCNEAAKGIAQAANTTGVPVIFGIVTTENIEQA
IERAGTKAGNKGVDCAVSAIEMANLNRSFE
>d1di0a_ c.16.1.1 (A:) Lumazine synthase {Brucella abortus}

TSFKIAFIQARWHADIVDEARKSFVAELAAKTGGSVEVEIFDVPGAYEIPLHAKTLARTGRY
AAIVGAAFVIDGGIYDHDFVATAVINGMMQVQLETEVPVLSVVLTPHHFHESKEHHDFFHAH
FKVKGVEAAHAALQIVSERSRIAA
>d1hqka_ c.16.1.1 (A:) Lumazine synthase {Aquifex aeolicus}
MQIYEGKLTAEGLRFGIVASRFNHALVDRLVEGAIDCIVRHGGREEDITLVRVPGSWEIPVA
AGELARKEDIDAVIAIGVLIRGATPHFDYIASEVSKGLANLSLELRKPITFGVITADTLEQA
IERAGTKHGNKGWEAALSAIEMANLFKSLR
>d1c41a_ c.16.1.1 (A:) Lumazine synthase {Rice blast fungus (Magnaporthe grisea)}
GPTPQQHDGSALRIGIVHARWNETIIEPLLAGTKAKLLACGVKESNIVVQSVPGSWELPIAV
QRLYSASQLQTPSSGPSLSAGDLLGSSTTDLTALPTTTASSTGPFDALIAIGVLIKGETMHF
EYIADSVSHGLMRVQLDTGVPVIFGVLTVLTDDQAKARAGVIEGSHNHGEDWGLAAVEMGVR
RRDWAAGKT
>d1c2ya_ c.16.1.1 (A:) Lumazine synthase {Spinach (Spinacia oleracea)}
MNELEGYVTKAQSFRFAIVVARFNEFVTRRLMEGALDTFKKYSVNEDIDVVWVPGAYELGVT
AQALGKSGKYHAIVCLGAVVKGDTSHYDAVVNSASSGVLSAGLNSGVPCVFGVLTCDNMDQA
INRAGGKAGNKGAESALTAIEMASLFEHHLK
>d1ejba_ c.16.1.1 (A:) Lumazine synthase {Baker's yeast (Saccharomyces cerevisiae)}
AVKGLGKPDQVYDGSKIRVGIIHARWNRVIIDALVKGAIERMASLGVEENNIIIETVPGSYE
LPWGTKRFVDRQAKLGKPLDVVIPIGVLIKGSTMHFEYISDSTTHALMNLQEKVDMPVIFGL
LTCMTEEQALARAGIDEAHSMHNHGEDWGAAAVEMAVKFGKNAF
>d1cp3a_ c.17.1.1 (A:) Apopain (caspase-3, cpp32) {Human (Homo sapiens)}
NSYKMDYPEMGLCIIINNKNFHKSTGMTSRSGTDVDAANLRETFRNLKYEVRNKNDLTREEI
VELMRDVSKEDHSKRSSFVCVLLSHGEEGIIFGTNGPVDLKKITNFFRGDRCRSLTGKPKLF
IIQACRGTELDCGIETDSGVDDDMACHKIPVDADFLYAYSTAPGYYSWRNSKDGSWFIQSLC
AMLKQYADKLEFMHILTRVNRKVATEFESFSFDATFHAKKQIPCIVSMLTKELYFYH
>g1ibc.1 c.17.1.1 (A:,B:) Interleukin-1beta converting enzyme (a cysteine protease) {Human (Homo sapiens)}
GNVKLCSLEEAQRIWKQKSAEIYPIMDKSSRTRLALIICNEEFDSIPRRTGAEVDITGMTML
LQNLGYSVDVKKNLTASDMTTELEAFAHRPEHKTSDSTFLVFMSHGIREGICGKKHSEQVPD
ILQLNAIFNMLNTKNCPSLKDKPKVIIIQACRGDSPGVVWFKDXAIKKAHIEKDFIAFCSST
PDNVSWRHPTMGSVFIGRLIEHMQEYACSCDVEEIFRKVRFSFEQPAGRAQMPTTERVTLTR
CFYLFPGH
>d1f1ja_ c.17.1.1 (A:) Caspase-7 {Human (Homo sapiens)}
YQYNMNFEKLGKCIIINNKNFDKVTGMGVRNGTDKDAEALFKCFRSLGFDVIVYNDCSCAKM
QDLLKKASEEDHTNAACFACILLSHGEENVIYGKDGVTPIKDLTAHFRGDRSKTLLEKPKLF
FIQACRGTELDDGIQADSGPINDTDANPRYKIPVEADFLFAYSTVPGYYSWRSPGRGSWFVQ
ALCSILEEHGKDLEIMQILTRVNDRVARHFESQSDDPHFHEKKQIPCVVSMLTKELYFS
>g1qtn.1 c.17.1.1 (A:,B:) Caspase-8 {Human (Homo sapiens)}
DKVYQMKSKPRGYCLIINNHNFAKAREKVPKLHSIRDRNGTHLDAGALTTTFEELHFEIKPH
DDCTVEQIYEILKIYQLMDHSNMDCFICCILSHGDKGIIYGTDGQEAPIYELTSQFTGLKCP

SLAGKPKVFFIQACQGDNYQKGIPVETDXTRYIPDEADFLLGMATVNNCVSYRNPAEGTWYI
QSLCQSLRERCPRGDDILTILTEVNYEVSNKDDKKNMGKQMPQPTFTLRKKLVFPSD
>d1jxqa_ c.17.1.1 (A:) Caspase-9 {Human (Homo sapiens)}
MGALESLRGNADLAYILSMEPCGHCLIINNVNFCRESGLRTRTGSNIDCEKLRRRFSSLHFM
VEVKGDLTAKKMVLALLELARQDHGALDCCVVVILSHGCQASHLQFPGAVYGTDGCPVSVEK
IVNIFNGTSCPSLGGKPKLFFIQACGGEQKDHGFEVASTSPEDESPGSNPEPDATPFQEGLR
TFDQLDAISSLPTPSDIFVSYSTFPGFVSWRDPKSGSWYVETLDDIFEQWAHSEDLQSLLLR
VANAVSVKGIYKQMPGCFNFLRKKLFFKTS
>d1cvra2 c.17.1.2 (A:1-350) Gingipain R (RgpB), N-terminal domain {Porphyromonas gingivalis}
YTPVEEKENGRMIVIVAKKYEGDIKDFVDWKNQRGLRTEVKVAEDIASPVTANAIQQFVKQE
YEKEGNDLTYVLLVGDHKDIPAKITPGIKSDQVYGQIVGNDHYNEVFIGRFSCESKEDLKTQ
IDRTIHYERNITTEDKWLGQALCIASAEGGPSADNGESDIQHENVIANLLTQYGYTKIIKCY
DPGVTPKNIIDAFNGGISLVNYTGHGSETAWGTSHFGTTHVKQLTNSNQLPFIFDVACVNGD
FLFSMPCFAEALMRAQKDGKPTGTVAIIASTIDQYWAPPMRGQDEMNEILCEKHPNNIKRTF
GGVTMNGMFAMVEKYKKDGENMLDTWTVFGDPSLLVRTLV
>d1akz__ c.18.1.1 (-) Uracil-DNA glycosylase {Human (Homo sapiens)}
MEFFGESWKKHLSGEFGKPYFIKLMGFVAEERKHYTVYPPPHQVFTWTQMCDIKDVKVVILG
QDPYHGPNQAHGLCFSVQRPVPPPPSLENIYKELSTDIEDFVHPGHGDLSGWAKQGVLLLNA
VLTVRAHQANSHKERGWEQFTDAVVSWLNQNSNGLVFLLWGSYAQKKGSAIDRKRHHVLQTA
HPSPLSVYRGFFGCRHFSKTNELLQKSGKKPIDWKEL
>d1laue_ c.18.1.1 (E:) Uracil-DNA glycosylase {Herpes simplex virus type 1}
LDWTTFRRVFLIDDAWRPLMEPELANPLTAHLLAEYNRRCQTEEVLPPREDVFSWTRYCTPD
EVRVVIIGQDPYHHPGQAHGLAFSVRANVPPPPSLRNVLAAVKNCYPEARMSGHGCLEKWAR
DGVLLLNTTLTVKRGAAASHSRIGWDRFVGGVIRRLAARRPGLVFMLWGTHAQNAIRPDPRV
HCVLKFSHPSPLSKVPFGTCQHFLVANRYLETRSISPIDWSV
>d3euga_ c.18.1.1 (A:) Uracil-DNA glycosylase {Escherichia coli}
LTWHDVLAEEKQQPHFLNTLQTVASERQSGVTIYPPQKDVFNAFRFTELGDVKVVILGQDPY
HGPGQAHGLAFSVRPGIAIPPSLLNMYKELENTIPGFTRPNHGYLESWARQGVLLLNTVLTV
RAGQAHSHASLGWETFTDKVISLINQHREGVVFLLWGSHAQKKGAIIDKQRHHVLKAPHPSP
LSAHRGFFGCNHFVLANQWLEQHGETPIDWMPVLPAESE
>d1muga_ c.18.1.2 (A:) G:T/U mismatch-specific DNA glycosylase {Escherichia coli}
MVEDILAPGLRVVFCGINPGLSSAGTGFPFAHPANRFWKVIYQAGFTDRQLKPQEAQHLLDY
RCGVTKLVDRPTVQANEVSKQELHAGGRKLIEKIEDYQPQALAILGKQAYEQGFSQRGAQWG
KQTLTIGSTQIWVLPNPSGLSRVSLEKLVEAYRELDQALVV
>d1mla_1 c.19.1.1 (3-127,198-307) Catalytic domain of malonyl-CoA ACP transacylase {Escherichia coli}
QFAFVFPGQGSQTVGMLADMAASYPIVEETFAEASAALGYDLWALTQQGPAEELNKTWQTQP
ALLTASVALYRVWQQQGGKAPAMMAGHSLGEYSALVCAGVIDFADAVRLVEMRGKFMQEAVP
EXVPSHCALMKPAADKLAVELAKITFNAPTVPVVNNVDVKCETNGDAIRDALVRQLYNPVQW
TKSVEYMAAQGVEHLYEVGPGKVLTGLTKRIVDTLTASALNEPSAMAAAL

>d1g7sa3 c.20.1.1 (A:329-459) Initiation factor IF2/eIF5b, domain 3 {Archaeon Methanobacterium thermoautotrophicum}
DPEKVREEILSEIEDIKIDTDEAGVVVKADTLGSLEAVVKILRDMYVPIKVADIGDVSRRDV
VNAGIALQEDRVYGAIIAFNVKVIPSAAQELKNSDIKLFQGNVIYRLMEEYEEWVRGIEEEK
KKKWMEA
>d1jj2i_ c.21.1.1 (I:) Ribosomal protein L13 {Archaeon Haloarcula marismortui}
AEFDADVIVDARDCIMGRVASQVAEQALDGETVAVVNAERAVITGREEQIVEKYEKRVDIGN
DNGYFYPKRPDGIFKRTIRGMLPHKKQRGREAFESVRVYLGNPYDEDGEVLDGTSLDRLSNI
KFVTLGEISETLGANKTW
>d1dmga_ c.22.1.1 (A:) Ribosomal protein L4 {Thermotoga maritima}
AQVDLLNVKGEKVGTLEISDFVFNIDPNYDVMWRYVDMQLSNRRAGTASTKTRGEVSGGGRK
PWPQKHTGRARHGSIRSPIWRHGGVVHGPKPRDWSKKLNKKMKKLALRSALSVKYRENKLLV
LDDLKLERPKTKSLKEILQNLQLSDKKTLIVLPWKEEGYMNVKLSGRNLPDVKVIIADNPNN
SKNGEKAVRIDGLNVFDMLKYDYLVLTRDMVSKIEEVLG
>d1jj2c_ c.22.1.1 (C:) Ribosomal protein L4 {Archaeon Haloarcula marismortui}
MQATIYDLDGNTDGEVDLPDVFETPVRSDLIGKAVRAAQANRKQDYGSDEYAGLRTPAESFG
SGRGQAHVPKLDGRARRVPQAVKGRSAHPPKTEKDRSLDLNDKERQLAVRSALAATADADLV
ADRGHEFDRDEVPVVVSDDFEDLVKTQEVVSLLEALDVHADIDRADETKIKAGQGSARGRKY
RRPASILFVTSDEPSTAARNLAGADVATASEVNTEDLAPGGAPGRLTVFTESALAEVAER
>d1hey__ c.23.1.1 (-) CheY protein {Escherichia coli}
DKELKFLVVGNGGTGKSTVRNLLKELGFNNVEDAEDGVDALNKLQAGGYGFVISDWNMPNMD
GLELLKTIRADGAMSALPVLMVTAEAKKENIIAAAQAGASGYVVKPFTAATLEEKLNKIFEK
LGM
>d1jbea_ c.23.1.1 (A:) CheY protein {Escherichia coli}
ADKELKFLVVDDFSTMRRIVRNLLKELGFNNVEEAEDGVDALNKLQAGGYGFVISDWNMPNM
DGLELLKTIRADGAMSALPVLMVTAEAKKENIIAAAQAGASGYVVKPFTAATLEEKLNKIFE
KLGM
>d1tmy__ c.23.1.1 (-) CheY protein {Thermotoga maritima}
GKRVLIVDDAAFMRMMLKDIITKAGYEVAGEATNGREAVEKYKELKPDIVTMDITMPEMNGI
DAIKEIMKIDPNAKIIVCSAMGQQAMVIEAIKAGAKDFIVKPFQPSRVVEALNKVS
>d1a04a2 c.23.1.1 (A:5-142) Nitrate/nitrite response regulator (NARL), receiver domain {Escherichia coli}
EPATILLIDDHPMLRTGVKQLISMAPDITVVGEASNGEQGIELAESLDPDLILLDLNMPGMN
GLETLDKLREKSLSGRIVVFSVSNHEEDVVTALKRGADGYLLKDMEPEDLLKALHQAAAGEM
VLSEALTPVLAASL
>d1ntr__ c.23.1.1 (-) NTRC receiver domain {Salmonella typhimurium}
MQRGIVWVVDDDSSIRWVLERALAGAGLTCTTFENGNEVLAALASKTPDVLLSDIRMPGMDG
LALLKQIKQRHPMLPVIIMTAHSDLDAAVSAYQQGAFDYLPKPFDIDEAVALVERAISHYQE
>d1dbwa_ c.23.1.1 (A:) Transcriptional regulatory protein FixJ, receiver domain {Rhizobium meliloti}
MQDYTVHIVDDEEPVRKSLAFMLTMNGFAVKMHQSAEAFLAFAPDVRNGVLVTDLRMPDMSG

VELLRNLGDLKINIPSIVITGHGDVPMAVEAMKAGAVDFIEKPFEDTVIIEAIERASEHLV
>d1qkka_ c.23.1.1 (A:) Transcriptional regulatory protein DctD, receiver domain {Sinorhizobium meliloti}
PSVFLIDDDRDLRKAMQQTLELAGFTVSSFASATEALAGLSADFAGIVISDIRMPGMDGLAL
FRKILALDPDLPMILVTGHGDIPMAVQAIQDGAYDFIAKPFAADRLVQSARRAEEKRRLVME
NRSLRRAAEAASEGL
>d1dz3a_ c.23.1.1 (A:) Sporulation response regulator Spo0A {Bacillus stearothermophilus}
SIKVCIADDNRELVSLLDEYISSQPDMEVIGTAYNGQDCLQMLEEKRPDILLLDIIMPHLDG
LAVLERIRAGFEHQPNVIMLTAFGQEDVTKKAVELGASYFILKPFDMENLAHHIRQVYGKT
>d1nat__ c.23.1.1 (-) Sporulation response regulator Spo0F {Bacillus subtilis}
NEKILIVDDQYGIRILLNEVFNKEGYQTFQAANGLQALDIVTKERPDLVLLDMKIPGMDGIE
ILKRMKVIDENIRVIIMTAYGELDMIQESKELGALTHFAKPFDIDEIRDAVKKYLPL
>d1a2oa1 c.23.1.1 (A:1-140) Methylesterase CheB, N-terminal domain {Salmonella typhimurium}
MSKIRVLSVDDSALMRQIMTEIINSHSDMEMVATAPDPLVARDLIKKFNPDVLTLDVEMPRM
DGLDFLEKLMRLRPMPVVMVSSLTGKGSEVTLRALELGAIDFVTKPQLGIREGMLAYSEMIA
EKVRTAARARIAAHKP
>d1kgsa2 c.23.1.1 (A:2-123) PhoB receiver domain {Thermotoga maritima}
NVRVLVVEDERDLADLITEALKKEMFTVDVCYDGEEGMYMALNEPFDVVILDIMLPVHDGWE
ILKSMRESGVNTPVLMLTALSDVEYRVKGLNMGADDYLPKPFDLRELIARVRALIRRKSE
>d1b00a_ c.23.1.1 (A:) PhoB receiver domain {Escherichia coli}
ARRILVVEDEAPIREMVCFVLEQNGFQPVEAEDYDSAVNQLNEPWPDLILLDWMLPGGSGIQ
FIKHLKRESMTRDIPVVMLTARGEEEDRVRGLETGADDYITKPFSPKELVARIKAVMRRI
>d1dcfa_ c.23.1.2 (A:) Receiver domain of the ethylene receptor {Thale cress (Arabidopsis thaliana)}
HMSNFTGLKVLVMDENGVSRMVTKGLLVHLGCEVTTVSSNEECLRVVSHEHKVVFMDVCMPG
VENYQIALRIHEKFTKQRHQRPLLVALSGNTDKSTKEKCMSFGLDGVLLKPVSLDNIRDVLS
DLLEPRVLYE
>d1qo0d_ c.23.1.3 (D:) Negative regulator of the amidase operon AmiR {Pseudomonas aeruginosa}
SANSLLGSLRELQVLVLNPPGEVSDALVLQLIRIGCSVRQCWPPPEAFDVPVDVVFTSIFQN
RHHDEIAALLAAGTPRTTLVALVEYESPAVLSQIIELECHGVITQPLDAHRVLPVLVSARRI
SEEMAKLKQKTEQLQDRIAGQARINQAKVLLMQRHGWDEREAHQHLSREAMKRREPILKIAQ
ELL
>d1fyva_ c.23.2.1 (A:) Toll-like receptor 1, TLR1 {Human (Homo sapiens)}
NIPLEELQRNLQFHAFISYSGHDSFWVKNELLPNLEKEGMQICLHERNFVPGKSIVENIITC
IEKSYKSIFVLSPNFVQSEWCHYELYFAHHNLFHEGSNSLILILLEPIPQYSIPSSYHKLKS
LMARRTYLEWPKEKSKRGLFWANLRAAINIKLTEQAK
>d1fywa_ c.23.2.1 (A:) Toll-like receptor 2, TLR2 {Human (Homo sapiens)}

SRNICYDAFVSYSERDAYWVENLMVQELENFNPPFKLCLHKRDFIPGKWIIDNIIDSIEKSH
KTVFVLSENFVKSEWCKYELDFSHFRLFDENNDAAILILLEPIEKKAIPQRFCKLRKIMNTK
TYLEWPMDEAQREGFWVNLRAAIKS
>d1eiwa_ c.23.3.1 (A:) Hypothetical protein MTH538 {Archaeon Methanobacterium thermoautotrophicum}
VTAEIRLYITEGEVEDYRVFLERLEQSGLEWRPATPEDADAVIVLAGLWGTRRDEILGAVDL
ARKSSKPIITVRPYGLENVPPELEAVSSEVVGWNPHCIRDALEDALDVI
>d1jkja2 c.23.4.1 (A:122-287) Succinyl-CoA synthetase, alpha-chain, C-terminal domain {Escherichia coli}
NCPGVITPGECKIGIQPGHIHKPGKVGIVSRSGTLTYEAVKQTTDYGFGQSTCVGIGGDPIP
GSNFIDILEMFEKDPQTEAIVMIGEIGGSAEEEAAAYIKEHVTKPVVGYIAGVTAPKGKRMG
HAGAIIAGGKGTADEKFAALEAAGVKTVRSLADIGEALKTVL
>d1euca2 c.23.4.1 (A:131-306) Succinyl-CoA synthetase, alpha-chain, C-terminal domain {Pig (Sus scrofa)}
NCPGVINPGECKIGIMPGHIHKKGRIGIVSRSGTLTYEAVHQTTQVGLGQSLCVGIGGDPFN
GTDFTDCLEIFLNDPATEGIILIGEIGGNAEENAAEFLKQHNSGPKSKPVVSFIAGLTAPPG
RRMGHAGAIIAGGKGGAKEKITALQSAGVVVSMSPAQLGTTIYKEFEKRKML
>d1jkjb1 c.23.4.1 (B:239-388) Succinyl-CoA synthetase, beta-chain, C-terminal domain {Escherichia coli}
DPREAQAAQWELNYVALDGNIGCMVNGAGLAMGTMDIVKLHGGEPANFLDVGGGATKERVTE
AFKIILSDDKVKAVLVNIFGGIVRCDLIADGIIGAVAEVGVNVPVVVRLEGNNAELGAKKLA
DSGLNIIAAKGLTDAAQQVVAAVEGK
>d1eucb1 c.23.4.1 (B:246-393) Succinyl-CoA synthetase, beta-chain, C-terminal domain {Pig (Sus scrofa)}
EPIENEAAKYDLKYIGLDGNIACFVNGAGLAMATCDIIFLNGGKPANFLDLGGGVKESQVYQ
AFKLLTADPKVEAILVNIFGGIVNCAIIANGITKACRELELKVPLVVRLEGTNVHEAQNILT
NSGLPITSAVDLEDAAKKAVASVT
>d2fcr__ c.23.5.1 (-) Flavodoxin {Chondrus crispus}
KIGIFFSTSTGNTTEVADFIGKTLGAKADAPIDVDDVTDPQALKDYDLLFLGAPTWNTGADT
ERSGTSWDEFLYDKLPEVDMKDLPVAIFGLGDAEGYPDNFCDAIEEIHDCFAKQGAKPVGFS
NPDDYDYEESKSVRDGKFLGLPLDMVNDQIPMEKRVAGWVEAVVSETGV
>d1f4pa_ c.23.5.1 (A:) Flavodoxin {Desulfovibrio vulgaris}
PKALIVYGSTTGNTEYTAETIARELADAGYEVDSRDAASVEAGGLFEGFDLVLLGCSTWGDD
SIELQDDFIPLFDSLEETGAQGRKVACFGCGDSSWEYFCGAVDAIEEKLKNLGAEIVQDGLR
IDGDPRAARDDIVGWAHDVRGAI
>d1rcf__ c.23.5.1 (-) Flavodoxin {Anabaena, pcc 7119 and 7120}
SKKIGLFYGTQTGKTESVAEIIRDEFGNDVVTLHDVSQAEVTDLNDYQYLIIGCPTWNIGEL
QSDWEGLYSELDDVDFNGKLVAYFGTGDQIGYADNFQDAIGILEEKISQRGGKTVGYWSTDG
YDFNDSKALRNGKFVGLALDEDNQSDLTDDRIKSWVAQLKSEFGL
>d1ag9a_ c.23.5.1 (A:) Flavodoxin {Escherichia coli}
AITGIFFGSDTGNTENIAKMIQKQLGKDVADVHDIAKSSKEDLEAYDILLLGIPTWYYGEAQ
CDWDDFFPTLEEIDFNGKLVALFGCGDQEDYAEYFCDALGTIRDIIEPRGATIVGHWPTAGY
HFEASKGLADDDHFVGLAIDEDRQPELTAERVEKWVKQISEELHLDEILNA
>d1czna_ c.23.5.1 (A:) Flavodoxin {Anacystis nidulans and

Synechococcus, pcc 7942}
AKIGLFYGTQTGVTQTIAESIQQEFGGESIVDLNDIANADASDLNAYDYLIIGCPTWNVGEL
QSDWEGIYDDLDSVNFQGKKVAYFGAGDQVGYSDNFQDAMGILEEKISSLGSQTVGYWPIEG
YDFNESKAVRNNQFVGLAIDEDNQPDLTKNRIKTWVSQLKSEFGL
>d5nul__ c.23.5.1 (-) Flavodoxin {Clostridium beijerinckii}
MKIVYWSGTGNTEKMAELIAKGIIESGKDVNTINVSDVNIDELLNEDILILGCSAMTDEVLE
ESEFEPFIEEISTKISGKKVALFGSYGWGDGKWMRDFEERMNGYGCVVVETPLIVQNEPDEA
EQDCIEFGKKIANI
>d1fuea_ c.23.5.1 (A:) Flavodoxin {Helicobacter pylori}
GKIGIFFGTDSGNAEAIAEKISKAIGNAEVVDVAKASKEQFNGFTKVILVAPTAGAGDLQTD
WEDFLGTLEASDFANKTIGLVGLGDQDTYSETFAEGIFHIYEKAKAGKVVGQTSTDGYHFAA
SKAVEGGKFVGLVIDEDNQDDLTDERIAKWVEQVRGSFA
>d1bvyf_ c.23.5.1 (F:) FMN-binding domain of the cytochrome
P450bm-3 {Bacillus megaterium}
NTPLLVLYGSNMGTAEGTARDLADIAMSKGFAPQVATLDSHAGNLPREGAVLIVTASYNGHP
PDNAKQFVDWLDQASADEVKGVRYSVFGCGDKNWATTYQKVPAFIDETLAAKGAENIADRGE
ADASDDFEGTYEEWREHMWSDVAAYFNL
>d1e5da1 c.23.5.1 (A:251-402) Rubredoxin oxygen:oxidoreductase
(ROO), C-terminal domain {Desulfovibrio gigas}
PTNKVVIFYDSMWHSTEKMARVLAESFRDEGCTVKLMWCKACHHSQIMSEISDAGAVIVGSP
THNNGILPYVAGTLQYIKGLRPQNKIGGAFGSFGWSGESTKVLAEWLTGMGFDMPATPVKVK
NVPTHADYEQLKTMAQTIARALKAKLAA
>d1ja1a2 c.23.5.2 (A:63-239) NADPH-cytochrome p450 reductase,
N-terminal domain {Rat (Rattus norvegicus)}
PVKESSFVEKMKKTGRNIIVFYGSQTGTAEEFANRLSKDAHRYGMRGMSADPEEYDLADLSS
LPEIDKSLVVFCMATYGEGDPTDNAQDFYDWLQETDVDLTGVKFAVFGLGNKTYEHFNAMGK
YVDQRLEQLGAQRIFELGLGDDDGNLEEDFITWREQFWPAVCEFFGVEATGEE
>d1b1ca_ c.23.5.2 (A:) NADPH-cytochrome p450 reductase, N-terminal
domain {Human (Homo sapiens)}
SSFVEKMKKTGRNIIVFYGSQTGTAEEFANRLSKDAHRYGMRGMSADPEEYDLADLSSLPEI
DNALVVFCMATYGEGDPTDNAQDFYDWLQETDVDLSGVKFAVFGLGNKTYEHFNAMGKYVDK
RLEQLGAQRIFELGLGDDDGNLEEDFITWREQFWPAVCEHFGV
>d1dxqa_ c.23.5.3 (A:) NAD(P)H:quinone reductase {Mouse (Mus
musculus)}
AARRALIVLAHSEKTSFNYAMKEAAVEALKKRGWEVLESDLYAMNFNPIISRNDITGELKDS
KNFQYPSESSLAYKEGRLSPDIVAEHKKLEAADLVIFQFPLQWFGVPAILKGWFERVLVAGF
AYTYAAMYDNGPFQNKKTLLSITTGGSGSMYSLQGVHGDMNVILWPIQSGILRFCGFQVLEP
QLVYSIGHTPPDARMQILEGWKKRLETVWEETPLYFAPSSLFDLNFQAGFLLMKEVQEEQKK
NKFGLSVGHHLGKSIPADNQIKARK
>d1qrda_ c.23.5.3 (A:) NAD(P)H:quinone reductase {Rat (Rattus
rattus)}
AVRRALIVLAHAERTSFNYAMKEAAVEALKKKGWEVVESDLYAMNFNPLISRNDITGEPKDS
ENFQYPVESSLAYKEGRLSPDIVAEQKKLEAADLVIFQFPLYWFGVPAILKGWFERVLVAGF
AYTYATMYDKGPFQNKKTLLSITTGGSGSMYSLQGVHGDMNVILWPIQSGILRFCGFQVLEP

QLVYSIGHTPPDARVQVLEGWKKRLETVWEESPLYFAPSSLFDLNFQAGFLLKKEVQEEQKK
NKFGLSVGHHLGKSIPADNQIKARK
>d1d4aa_ c.23.5.3 (A:) NAD(P)H:quinone reductase {Human (Homo sapiens)}
VGRRALIVLAHSERTSFNYAMKEAAAAALKKKGWEVVESDLYAMNFNPIISRKDITGKLKDP
ANFQYPAESVLAYKEGHLSPDIVAEQKKLEAADLVIFQFPLQWFGVPAILKGWFERVFIGEF
AYTYAAMYDKGPFRSKKAVLSITTGGSGSMYSLQGIHGDMNVILWPIQSGILHFCGFQVLEP
QLTYSIGHTPADARIQILEGWKKRLENIWDETPLYFAPSSLFDLNFQAGFLMKKEVQDEEKN
KKFGLSVGHHLGKSIPTDNQIKARK
>d1qr2a_ c.23.5.3 (A:) Quinone reductase type 2 (menadione reductase) {Human (Homo sapiens)}
AGKKVLIVYAHQEPKSFNGSLKNVAVDELSRQGCTVTVSDLYAMNFEPRATDKDITGTLSNP
EVFNYGVETHEAYKQRSLASDITDEQKKVREADLVIFQFPLYWFSVPAILKGWMDRVLCQGF
AFDIPGFYDSGLLQGKLALLSVTTGGTAEMYTKTGVNGDSRYFLWPLQHGTLHFCGFKVLAP
QISFAPEIASEEERKGMVAAWSQRLQTIWKEEPIPCTAHWHFGQ
>d1bmta2 c.23.6.1 (A:741-896) Methionine synthase, C-terminal domain {Escherichia coli}
EQGKTNGKMVIATVKGDVHDIGKNIVGVVLQCNNYEIVDLGVMVPAEKILRTAKEVNADLIG
LSGLITPSLDEMVNVAKEMERQGFTIPLLIGGATTSKAHTAVKIEQNYSGPTVYVQNASRTV
GVVAALLSDTQRDDFVARTRKEYETVRIQHGR
>d1fmfa_ c.23.6.1 (A:) Glutamate mutase, small subunit {Clostridium tetanomorphum}
MEKKTIVLGVIGSDCHAVGNKILDHSFTNAGFNVVNIGVLSSQEDFINAAIETKADLICVSS
LYGQGEIDCKGLREKCDEAGLKGIKLFVGGNIVVGKQNWPDVEQRFKAMGFDRVYPPGTSPE
TTIADMKEVLGVE
>d1ccwa_ c.23.6.1 (A:) Glutamate mutase, small subunit {Clostridium cochlearium}
MEKKTIVLGVIGSDCHAVGNKILDHAFTNAGFNVVNIGVLSPQELFIKAAIETKADAILVSS
LYGQGEIDCKGLRQKCDEAGLEGILLYVGGNIVVGKQHWPDVEKRFKDMGYDRVYAPGTPPE
VGIADLKKDLNIE
>d7reqa2 c.23.6.1 (A:561-728) Methylmalonyl-CoA mutase C-terminal domains of alpha and beta subunits {Propionibacterium freudenreichii, subsp. shermanii}
AQIRTISGVYSKEVKNTPEVEEARELVEEFEQAEGRRPRILLAKMGQDGHDRGQKVIATAYA
DLGFDVDVGPLFQTPEETARQAVEADVHVVGVSSLAGGHLTLVPALRKELDKLGRPDILITV
GGVIPEQDFDELRKDGAVEIYTPGTVIPESAISLVKKLRASLDA
>d7reqb2 c.23.6.1 (B:476-638) Methylmalonyl-CoA mutase C-terminal domains of alpha and beta subunits {Propionibacterium freudenreichii, subsp. shermanii}
TKPFPAAPARKGLAWHRDSEVFEQLMDRSTSVSERPKVFLACLGTRRDFGGREGFSSPVWHI
AGIDTPQVEGGTTAEIVEAFKKSGAQVADLCSSAKVYAQQGLEVAKALKAAGAKALYLSGAF
KEFGDDAAEAEKLIDGRLFMGMDVVDTLSSTLDILGVAK
>d1c4ka1 c.23.7.1 (A:1-107) Ornithine decarboxylase N-terminal "wing" domain {Lactobacillus sp., strain 30a}

SSSLKIASTQEARQYFDTDRVVVDAVGSDFTDVGAVIAMDYETDVIDAADATKFGIPVFAVT
KDAQAISADELKKIFHIIDLENKFDATVNAREIETAVNNYEDSIL
>d1qcza_ c.23.8.1 (A:) N5-carboxyaminoimidazole ribonucleotide (N5-CAIR) mutase PurE {Escherichia coli}
PARVAIVMGSKSDWATMQFAAEIFEILNVPHHVEVVSAHRTPDKLFSFAESAEENGYQVIIA
GAGGAAHLPGMIAAKTLVPVLGVPVQSAALSGVDSLYSIVQMPRGIPVGTLAIGKAGAANAA
LLAAQILATHDKELHQRLNDWRKAQTDEVLENPDPRGAA
>d1cex__ c.23.9.1 (-) Cutinase {Fungus (Fusarium solani), subsp. pisi}
RTTRDDLINGNSASCADVIFIYARGSTETGNLGTLGPSIASNLESAFGKDGVWIQGVGGAYR
ATLGDNALPRGTSSAAIREMLGLFQQANTKCPDATLIAGGYSQGAALAAASIEDLDSAIRDK
IAGTVLFGYTKNLQNRGRIPNYPADRTKVFCNTGDLVCTGSLIVAAPHLAYGPDARGPAPEF
LIEKVRAVRGS
>d1g66a_ c.23.9.1 (A:) Acetylxylan esterase {Penicillium purpurogenum}
SCPAIHVFGARETTASPGYGSSSTVVNGVLSAYPGSTAEAINYPACGGQSSCGGASYSSSVA
QGIAAVASAVNSFNSQCPSTKIVLVGYSQGGEIMDVALCGGGDPNQGYTNTAVQLSSSAVNM
VKAAIFMGDPMFRAGLSYEVGTCAAGGFDQRPAGFSCPSAAKIKSYCDASDPYCCNGSNAAT
HQGYGSEYGSQALAFVKSKLG
>d1qoza_ c.23.9.1 (A:) Acetylxylan esterase {Trichoderma reesei}
ECPAIHVFGARETTVSQGYGSSATVVNLVIQAHPGTTSEAIVYPACGGQASCGGISYANSVV
NGTNAAAAAINNFHNSCPDTQLVLVGYSQGAQIFDNALCGGGDPGEGITNTAVPLTAGAVSA
VKAAIFMGDPRNIHGLPYNVGTCTTQGFDARPAGFVCPSASKIKSYCDAADPYCCTGNDPNV
HQGYGQEYGQQALAFINSQLS
>d1esc__ c.23.10.1 (-) Esterase {Streptomyces scabies}
DPVPTVFFGDSYTANFGIAPVTNQDSERGWCFQAKENYPAVATRSLADKGITLDVQADVSCG
GALIHHFWEKQELPFGAGELPPQQDALKQDTQLTVGSLGGNTLGFNRILKQCSDELRKPSLL
PGDPVDGDEPAAKCGEFFGTGDGKQWLDDQFERVGAELEELLDRIGYFAPDAKRVLVGYPRL
VPEDTTKCLTAAPGQTQLPFADIPQDALPVLDQIQKRLNDAMKKAAADGGADFVDLYAGTGA
NTACDGADRGIGGLLEDSQLELLGTKIPWYAHPNDKGRDIQAKQVADKIEEILN
>d1flca2 c.23.10.2 (A:1-150,A:307-427) Esterase domain of haemagglutinin-esterase-fusion glycoprotein HEF1 {Influenza C virus}
EKIKICLQKQVNSSFSLHNGFGGNLYATEEKRMFELVKPKAGASVLNQSTWIGFGDSRTDKS
NSAFPRSADVSAKTADKFRFLSGGSLMLSMFGPPGKVDYLYQGCGKHKVFYEGVNWSPHAAI
NCYRKNWTDIKLNFQKNIYELASQSHXEKGPVTAVQSIWGKGRESDYAVDQACLSTPGCMLI
QKQKPYIGEADDHHGDQEMRELLSGLDYEARCISQSGWVNETSPFTEKYLLPPKFGRCPLAA
KEESIPKIPDGLLIPTSGTDTTVT
>d1es9a_ c.23.10.3 (A:) Platelet-activating factor acetylhydrolase {Cow (Bos taurus)}
ENPASKPTPVQDVQGDGKWMSLHHRFVADSKDKEPEVVFIGDSLVQLMHQCEIWRELFSPLH
ALNFGIGGDSTQHVLWRLENGELEHIRPKIVVVWVGTNNHGHTAEQVTGGIKAIVQLVNERQ
PQARVVVLGLLPRGQHPNPLREKNRRVNELVRAALAGHPRAHFLDADPGFVHSDGTISHHDM
YDYLHLSRLGYTPVCRALHSLLLRLL

>d1fxwf_ c.23.10.3 (F:) Platelet-activating factor acetylhydrolase {Cow (Bos taurus)}
SNPAAIPHAAEDIQGDDRWMSQHNRFVLDCKDKEPDVLFVGDSMVQLMQQYEIWRELFSPLH
ALNFGIGGDTTRHVLWRLKNGELENIKPKVIVVWVGTNNHENTAEEVAGGIEAIVQLINTRQ
PQAKIIVLGLLPRGEKPNPLRQKNAKVNQLLKVSLPKLANVQLLDTDGGFVHSDGAISCHDM
FDFLHLTGGGYAKICKPLHELIMQLL
>d1k7ca_ c.23.10.4 (A:) Rhamnogalacturonan acetylesterase {Fungus (Aspergillus aculeatus)}
TTVYLAGDSTMAKNGGGSGTNGWGEYLASYLSATVVNDAVAGRSARSYTREGRFENIADVVT
AGDYVIVEFGHNDGGSLSTDNGRTDCSGTGAEVCYSVYDGVNETILTFPAYLENAAKLFTAK
GAKVILSSQTPNNPWETGTFVNSPTRFVEYAELAAEVAGVEYVDHWSYVDSIYETLGNATVN
SYFPIDHTHTSPAGAEVVAEAFLKAVVCTGTSLKSVLTTTSFEGTCL
>d1iexa2 c.23.11.1 (A:389-603) Beta-D-glucan exohydrolase, C-terminal domain {Barley (Hordeum vulgare)}
LVLLKNGKTSTDAPLLPLPKKAPKILVAGSHADNLGYQCGGWTIEWQGDTGRTTVGTTILEA
VKAAVDPSTVVVFAENPDAEFVKSGGFSYAIVAVGEHPYTETKGDNLNLTIPEPGLSTVQAV
CGGVRCATVLISGRPVVVQPLLAASDALVAAWLPGSEGQGVTDALFGDFGFTGRLPRTWFKS
VDQLPMNVGDAHYDPLFRLGYGLTTNATK
>d2naca2 c.23.12.1 (A:1-147,A:336-374) Formate dehydrogenase {Pseudomonas sp., strain 101}
AKVLCVLYDDPVDGYPKTYARDDLPKIDHYPGGQTLPTPKAIDFTPGQLLGSVSGELGLRKY
LESNGHTLVVTSDKDGPDSVFERELVDADVVISQPFWPAYLTPERIAKAKNLKLALTAGIGS
DHVDLQSAIDRNVTVAEVTYCNSXTTLTAQARYAAGTREILECFFEGRPIRDEYLIVQGGAL
A
>d1qp8a2 c.23.12.1 (A:1-82,A:264-302) Putative formate dehydrogenase {Archaeon Pyrobaculum aerophilum}
MELYVNFELPPEAEEELRKYFKIVRGGDLGNVEAALVSRITAEELAKMPRLKFIQVVTAGLD
HLPWESIPPHVTVAGNAGSNXGYGNERVWRQMVMEAVRNLITYATGGRPRNIAKREDYIG
>d1dxy_2 c.23.12.1 (1-100,300-330) D-2-hydroxyisocaproate dehydrogenase {Lactobacillus casei}
MKIIAYGARVDEIQYFKQWAKDTGNTLEYHTEFLDENTVEWAKGFDGINSLQTTPYAAGVFE
KMHAYGIKFLTIRNVGTDNIDMTAMKQYGIRLSNVPAYXTETAVHNMVYFSLQHLVDFLTKG
ETSTEVTG
>d1gdha2 c.23.12.1 (A:2-100,A:292-321) D-glycerate dehydrogenase {Hyphomicrobium methylovorum}
KKKILITWPLPEAAMARARESYDVIAHGDDPKITIDEMIETAKSVDALLITLNEKCRKEVID
RIPENIKCISTYSIGFDHIDLDACKARGIKVGNAPHGXATQAREDMAHQANDLIDALFGGAD
MSYALA
>d1psda2 c.23.12.1 (A:7-107,A:296-326) Phosphoglycerate dehydrogenase {Escherichia coli}
EKDKIKFLLVEGVHQKALESLRAAGYTNIEFHKGALDDEQLKESIRDAHFIGLRSRTHLTED
VINAAEKLVAIGCFCIGTNQVDLDAAAKRGIPVFNAPFSXSTQEAQENIGLEVAGKLIKYSD
NGSTLSAVN
>d2dlda2 c.23.12.1 (A:1-103,A:301-337) D-lactate dehydrogenase

{Lactobacillus helveticus}
MTKVFAYAIRKDEEPFLNEWKEAHKDIDVDYTDKLLTPETAKLAKGADGVVVYQQLDYTADT
LQALADAGVTKMSLRNVGVDNIDMDKAKELGFQITNVPVYSXYTTHAVRNMVVKAFNNNLKL
INGEKPDSPVALNKNKF
>d1pjca2 c.23.12.2 (A:1-135,A:304-361) L-alanine dehydrogenase
{Phormidium lapideum}
MEIGVPKEIKNQEFRVGLSPSSVRTLVEAGHTVFIETQAGIGAGFADQDYVQAGAQVVPSAK
DAWSREMVVKVKEPLPAEYDLMQKDQLLFTYLHLAAARELTEQLMRVGLTAIAYETVELPNR
SLPLLTPMSIIXVPWTATQALNNSTLPYVVKLANQGLKALETDDALAKGLNVQAHRLVHPAV
QQVFPDLA
>d1f8ga2 c.23.12.2 (A:1-143,A:327-384) Nicotinamide nucleotide
transhydrogenase dI component {Rhodospirillum rubrum}
MKIAIPKERRPGEDRVAISPEVVKKLVGLGFEVIVEQGAGVGASITDDALTAAGATIASTAA
QALSQADVVWKVQRPMTAEEGTDEVALIKEGAVLMCHLGALTNRPVVEALTKRKITAYAMEL
MPRISRAQSMDILSSQSNLXVAADASPLFAKNLLNFLTPHVDKDTKTLVMKLEDETVSGTCV
TRDGAIVHPALTGQGA
>d1a7aa2 c.23.12.3 (A:2-189,A:353-432) S-adenosylhomocystein
hydrolase {Human (Homo sapiens)}
SDKLPYKVADIGLAAWGRKALDIAENEMPGLMRMRERYSASKPLKGARIAGCLHMTVETAVL
IETLVTLGAEVQWSSCNIFSTQNHAAAAIAKAGIPVYAWKGETDEEYLWCIEQTLYFKDGPL
NMILDDGGDLTNLIHTKYPQLLPGIRGISEETTTGVHNLYKMMANGILKVPAINVNDSVTKS
KFXHPSFVMSNSFTNQVMAQIELWTHPDKYPVGVHFLPKKLDEAVAEAHLGKLNVKLTKLTE
KQAQYLGMSCDGPFKPDHYRY
>d1b3ra2 c.23.12.3 (A:4-189,A:353-431) S-adenosylhomocystein
hydrolase {Rat (Rattus norvegicus)}
LPYKVADIGLAAWGRKALDIAENEMPGLMRMREMYSASKPLKGARIAGCLHMTVETAVLIET
LVALGAEVRWSSCNIFSTQDHAAAAIAKAGIPVFAWKGETDEEYLWCIEQTLHFKDGPLNMI
LDDGGDLTNLIHTKHPQLLSGIRGISEETTTGVHNLYKMMANGILKVPAINVNDSVTKSKFD
XPSFVMSNSFTNQVMAQIELWTHPDKYPVGVHFLPKKLDEAVAEAHLGKLNVKLTKLTEKQA
QYLGMPINGPFKPDHYRY
>d2dhqa_ c.23.13.1 (A:) Type II 3-dehydroquinate dehydratase
{Mycobacterium tuberculosis}
LIVNVINGPNLGRLGRREPAVYGGTTHDELVALIEREAAELGLKAVVRQSDSEAQLLDWIHQ
AADAAEPVILNAGGLTHTSVALRDACAELSAPLIEVHISNVHAREEFRRHSYLSPIATGVIV
GLGIQGYLLALRYLAEH
>d1d0ia_ c.23.13.1 (A:) Type II 3-dehydroquinate dehydratase
{Streptomyces coelicolor}
PRSLANAPIMILNGPNLNLLGQRQPEIYGSDTLADVEALCVKAAAAHGGTVDFRQSNHEGEL
VDWIHEARLNHCGIVINPAAYSHTSVAILDALNTCDGLPVVEVHISNIHQREPFRHHSYVSQ
RADGVVAGCGVQGYVFGVERIAALAG
>d1f8ya_ c.23.14.1 (A:) Nucleoside 2-deoxyribosyltransferase
{Lactobacillus leichmannii}
PKKTIYFGAGWFTDRQNKAYKEAMEALKENPTIDLENSYVPLDNQYKGIRVDEHPEYLHDKV
WATATYNNDLNGIKTNDIMLGVYIPDEEDVGLGMELGYALSQGKYVLLVIPDEDYGKPINLM

SWGVSDNVIKMSQLKDFNFNKPRFDFYEGAVY
>d1fjgb_ c.23.15.1 (B:) Ribosomal protein S2 {Thermus thermophilus}
VKELLEAGVHFGHERKRWNPKFARYIYAERNGIHIIDLQKTMEELERTFRFIEDLAMRGGTI
LFVGTKKQAQDIVRMEAERAGMPYVNQRWLGGMLTNFKTISQRVHRLEELEALFASPEIEER
PKKEQVRLKHELERLQKYLSGFRLLKRLPDAIFVVDPTKEAIAVREARKLFIPVIALADTDS
DPDLVDYIIPGNDDAIRSIQLILSRAVDLIIQARGGVVEPSPSYALVQEAE
>d1f2va_ c.23.17.1 (A:) Precorrin-8x methylmutase {Pseudomonas denitrificans}
PEYDYIRDGNAIYERSFAIIRAEADLSRFSEEEADLAVRMVHACGSVEATRQFVFSPDFVSS
ARAALKAGAPILCDAEMVAHGVTRARLPAGNEVICTLRDPRTPALAAEIGNTRSAAALKLWS
ERLAGSVVAIGNAPTALFFLLEMLRDGAPKPAAILGMPVGFVGAAESKDALAENSYGVPFAI
VRGRLGGSAMTAAALNSLARPGL
>d1gpma2 c.23.16.1 (A:3-207) GMP synthetase {Escherichia coli}
ENIHKHRILILDFGSQYTQLVARRVRELGVYCELWAWDVTEAQIRDFNPSGIILSGGPESTT
EENSPRAPQYVFEAGVPVFGVCYGMQTMAMQLGGHVEASNEREFGYAQVEVVNDSALVRGIE
DALTADGKPLLDVWMSHGDKVTAIPSDFITVASTESCPFAIMANEEKRFYGVQFHPEVTHTR
QGMRMLERFVRDICQCEAL
>d1a9xb2 c.23.16.1 (B:1653-1880) Carbamoyl phosphate synthetase, small subunit C-terminal domain {Escherichia coli}
LNGMDLAKEVTTAEAYSWTQGSWTLTGGLPQAKKEDELPFHVVAYDFGAKRNILRMLVDRGC
RLTIVPAQTSAEDVLKMNPDGIFLSNGPGDPAPCDYAITAIQKFLETDIPVFGICLGHQLLA
LASGAKTVKMKFGHHGGNHPVKDVEKNVVMITAQNHGFAVDEATLPANLRVTHKSLFDGTLQ
GIHRTDKPAFSFQGNPEASPGPHDAAPLFDHFIELIEQYRKT
>d1qdlb_ c.23.16.1 (B:) Anthranilate synthase GAT subunit, TrpG {Archaeon Sulfolobus solfataricus}
MDLTLIIDNYDSFVYNIAQIVGELGSYPIVIRNDEISIKGIERIDPDRLIISPGPGTPEKRE
DIGVSLDVIKYLGKRTPILGVCLGHQAIGYAFGAKIRRARKVFHGKISNIILVNNSPLSLYY
GIAKEFKATRYHSLVVDEVHRPLIVDAISAEDNEIMAIHHEEYPIYGVQFHPESVGTSLGYK
ILYNFLNRV
>d1i1qb_ c.23.16.1 (B:) Anthranilate synthase GAT subunit, TrpG {Salmonella typhimurium}
ADILLLDNIDSFTWNLADQLRTNGHNVVIYRNHIPAQTLIDRLATMKNPVLMLSPGPGVPSE
AGCMPELLTRLRGKLPIIGICLGHQAIVEAYGGYVGQAGEILHGKATSIEHDGQAMFAGLAN
PLPVARYHSLVGSNVPAGLTINAHFNGMVMAVRHDADRVCGFQFHPESILTTQGARLLEQTL
AWAQQK
>d1i7qb_ c.23.16.1 (B:) Anthranilate synthase GAT subunit, TrpG {Serratia marcescens}
ADILLLDNVDSFTYNLVDQLRASGHQVVIYRNQIGAEVIIERLQHMEQPVLMLSPGPGTPSE
AGCMPELLQRLRGQLPIIGICLGHQAIVEAYGGQVGQAGEILHGKASAIAHDGEGMFAGMAN
PLPVARYHSLVGSNIPADLTVNARFGEMVMAVRDDRRRVCGFQFHPESILTTHGARLLEQTL
AWALAK
>d1k9vf_ c.23.16.1 (F:) GAT subunit (or domain) of imidazoleglycerolphosphate synthase HisF {Thermotoga maritima}

MRIGIISVGPGNIMNLYRGVKRASENFEDVSIELVESPRNDLYDLLFIPGVGHFGEGMRRLR
ENDLIDFVRKHVEDERYVVGVCLGMQLLFEESEEAPGVKGLSLIEGNVVKLRSRRLPHMGWN
EVIFKDTFPNGYYYFVHTYRAVCEEEHVLGTTEYDGEIFPSAVRKGRILGFQFHPEKSSKIG
RKLLEKVIECSLSR
>d1jvna2 c.23.16.1 (A:-3-229) GAT subunit (or domain) of imidazoleglycerolphosphate synthase HisF {Baker's yeast (Saccharomyces cerevisiae), His7}
GSHMPVVHVIDVESGNLQSLTNAIEHLGYEVQLVKSPKDFNISGTSRLILPGVGNYGHFVDN
LFNRGFEKPIREYIESGKPIMGICVGLQALFAGSVESPKSTGLNYIDFKLSRFDDSEKPVPE
IGWNSCIPSENLFFGLDPYKRYYFVHSFAAILNSEKKKNLENDGWKIAKAKYGSEEFIAAVN
KNNIFATQFHPEKSGKAGLNVIENFLKQQSPPIPNYSAEEKELLMN
>d1g2ia_ c.23.16.2 (A:) Intracellular protease {Archaeon Pyrococcus horikoshii}
MKVLFLTANEFEDVELIYPYHRLKEEGHEVYIASFERGTITGKHGYSVKVDLTFDKVNPEEF
DALVLPGGRAPERVRLNEKAVSIARKMFSEGKPVASICHGPQILISAGVLRGRKGTSYPGIK
DDMINAGVEWVDAEVVVDGNWVSSRVPADLYAWMREFVKLLK
>d1cf9a1 c.23.16.3 (A:598-753) Catalase, C-terminal domain {Escherichia coli, HPII}
VKGRVVAILLNDEVRSADLLAILKALKAKGVHAKLLYSRMGEVTADDGTVLPIAATFAGAPS
LTVDAVIVPCGNIADIADNGDANYYLMEAYKHLKPIALAGDARKFKATIKIADQGEEGIVEA
DSADGSFMDELLTLMAAHRVWSRIPKIDKIPA
>d1fyea_ c.23.16.4 (A:) Aspartyl dipeptidase PepE {Salmonella typhimurium}
MELLLLSNSTLPGKAWLEHALPLIANQLNGRRSAVFIPFAGVTQTWDEYTDKTAEVLAPLGV
NVTGIHRVADPLAAIEKAEIIIVGGGNTFQLLKESRERGLLAPMADRVKRGALYIGWSAGAN
LACPTIRTTNDMPIVDPNGFDALDLFPLQINPHFTNALPEGHKGETREQRIRELLVVAPELT
VIGLPEGNWIQVSNGQAVLGGPNTTWVFKAGEEAVALEAGHRF
>d1a9xa2 c.24.1.1 (A:936-1073) Carbamoyl phosphate synthetase, large subunit allosteric, C-terminal domain {Escherichia coli}
NSTMKKHGRALLSVREGDKERVVDLAAKLLKQGFELDATHGTAIVLGEAGINPRLVNKVHEG
RPHIQDRIKNGEYTYIINTTSGRRAIEDSRVIRRSALQYKVHYDTTLNGGFATAMALNADAT
EKVISVQEMHAQIK
>d1b93a_ c.24.1.2 (A:) Methylglyoxal synthase, MgsA {Escherichia coli}
MELTTRTLPARKHIALVAHDHCKQMLMSWVERHQPLLEQHVLYATGTTGNLISRATGMNVNA
MLSGPMGGDQQVGALISEGKIDVLIFFWDPLNAVPHDPDVKALLRLATVWNIPVATNVATAD
FIIQSPHFNDAVDILIPDYQRYLA
>d1g8ma1 c.24.1.3 (A:4-200) IMP cyclohydrolase domain of bifunctional purine biosynthesis enzyme ATIC {Chicken (Gallus gallus)}
RQQLALLSVSEKAGLVEFARSLNALGLGLIASGGTATALRDAGLPVRDVSDLTGFPEMLGGR
VKTLHPAVHAGILARNIPEDNADMNKQDFSLVRVVVCNLYPFVKTVSSPGVTVPEAVEKIDI
GGVALLRAAAKNHARVTVVCDPADYSSVAKEMAASKDKDTSVETRRHLALKAFTHTAQYDAA
ISDYFRKEYSK

>d1fnc_2 c.25.1.1 (155-314) Ferredoxin reductase (flavodoxin reductase) {Spinach (Spinacia oleracea)}
MLMPKDPNATIIMLGTGTGIAPFRSFLWKMFFEKHDDYKFNGLAWLFLGVPTSSSLLYKEEF
EKMKEKAPDNFRLDFAVSREQTNEKGEKMYIQTRMAQYAVELWEMLKKDNTYVYMCGLKGME
KGIDDIMVSLAAAEGIDWIEYKRQLKKAEQWNVEVY
>d1qfza2 c.25.1.1 (A:154-308) Ferredoxin reductase (flavodoxin reductase) {Garden pea (Pisum sativum)}
DPNATVIMLGTGTGIAPFRSFLWKMFFEKHEDYQFNGLAWLFLGVPTSSSLLYKEEFEKMKE
KAPENFRLDFAVSREQVNDKGEKMYIQTRMAQYAEELWELLKKDNTFVYMCGLKGMEKGIDD
IMVSLAAKDGIDWIEYKRTLKKAEQWNVEVS
>d1fb3a2 c.25.1.1 (A:208-362) Ferredoxin reductase (flavodoxin reductase) {Paprika (Capsicum annuum)}
DPNATVIMLGTGTGIAPFRSFLWKMFFEKHDDYKFNGLAWLFLGVPTSSSLLYKEEFEKMKE
KAPENFRLDFAVSREQTNEKGEKMYIQTRMAQYAEELWTLLKKDNTFVYMCGLKGMEQGIDD
IMSSLAAKEGIDWADYKKQLKKAEQWNVEVY
>d1gawa2 c.25.1.1 (A:157-314) Ferredoxin reductase (flavodoxin reductase) {Maize (Zea mays), leaf isoform}
MPKDPNATIIMLATGTGIAPFRSFLWKMFFEKHDDYKFNGLGWLFLGVPTSSSLLYKEEFGK
MKERAPENFRVDYAVSREQTNAAGERMYIQTRMAEYKEELWELLKKDNTYVYMCGLKGMEKG
IDDIMVSLAEKDGIDWFDYKKQLKRGDQWNVEVY
>d1jb9a2 c.25.1.1 (A:163-316) Ferredoxin reductase (flavodoxin reductase) {Maize (Zea mays), root isoform}
DPNATHIMIATGTGVAPFRGYLRRMFMEDVPNYRFGGLAWLFLGVANSDSLLYDEEFTSYLK
QYPDNFRYDKALSREQKNRSGGKMYVQDKIEEYSDEIFKLLDGGAHIYFCGLKGMMPGIQDT
LKKVAERRGESWDQKLAQLKKNKQWHVEVY
>d1que_2 c.25.1.1 (142-303) Ferredoxin reductase (flavodoxin reductase) {Cyanobacterium (Anabaena sp.), pcc 7119}
LPDDPEANVIMLATGTGIAPMRTYLWRMFKDAERAANPEYQFKGFSWLVFGVPTTPNILYKE
ELEEIQQKYPDNFRLTYAISREQKNPQGGRMYIQDRVAEHADQLWQLIKNQKTHTYICGLRG
MEEGIDAALSAAAAKEGVTWSDYQKDLKKAGRWHVETY
>d1fdr_2 c.25.1.1 (101-248) Ferredoxin reductase (flavodoxin reductase) {Escherichia coli}
DEVPHCETLWMLATGTAIGPYLSILRLGKDLDRFKNLVLVHAARYAADLSYLPLMQELEKRY
EGKLRIQTVVSRETAAGSLTGRIPALIESGELESTIGLPMNKETSHVMLCGNPQMVRDTQQL
LKETRQMTKHLRRRPGHMTAEHYW
>d1a8p_2 c.25.1.1 (101-258) Ferredoxin reductase (flavodoxin reductase) {Azotobacter vinelandii}
TSDLLPGKHLYMLSTGTGLAPFMSLIQDPEVYERFEKVVLIHGVRQVNELAYQQFITEHLPQ
SEYFGEAVKEKLIYYPTVTRESFHNQGRLTDLMRSGKLFEDIGLPPINPQDDRAMICGSPSM
LDESCEVLDGFGLKISPRMGEPGDYLIERAFVEK
>d1qfja2 c.25.1.1 (A:98-232) NAD(P)H:flavin oxidoreductase {Escherichia coli}
RDDEERPMILIAGGTGFSYARSILLTALARNPNRDITIYWGGREEQHLYDLCELEALSLKHP
GLQVVPVVEQPEAGWRGRTGTVLTAVLQDHGTLAEHDIYIAGRFEMAKIARDLFCSERNARE

DRLFGDAFAFI
>d2cnd_2 c.25.1.1 (125-270) Nitrate reductase {Corn (Zea mays)}
GSFVINGKQRNARRLAMICGGSGITPMYQIIQAVLRDQPEDHTEMHLVYANRTEDDILLRDE
LDRWAAEYPDRLKVWYVIDQVKRPEEGWKYSVGFVTEAVLREHVPEGGDDTLALACGPPPMI
QFAISPNLEKMKYDMANSFVVF
>d1ndh_2 c.25.1.1 (126-272) cytochrome b5 reductase {Pig (Sus scrofa), liver}
GKFAIRPDKKSSPVIKTVKSVGMIAGGTGITPMLQVIRAIMKDPDDHTVCHLLFANQTEKDI
LLRPELEELRNEHSARFKLWYTVDRAPEAWDYSQGFVNEEMIRDHLPPPEEEPLVLMCGPPP
MIQYACLPNLERVGHPKERCFAF
>d1i7pa2 c.25.1.1 (A:154-300) cytochrome b5 reductase {Rat (Rattus norvegicus)}
GKFAIRADKKSNPVVRTVKSVGMIAGGTGITPMLQVIRAVLKDPNDHTVCYLLFANQSEKDI
LLRPELEELRNEHSSRFKLWYTVDKAPDAWDYSQGFVNEEMIRDHLPPPGEETLILMCGPPP
MIQFACLPNLERVGHPKERCFTF
>d2pia_2 c.25.1.2 (104-223) Phthalate dioxygenase reductase {Pseudomonas cepacia, db01}
EFPLDKRAKSFILVAGGIGITPMLSMARQLRAEGLRSFRLYYLTRDPEGTAFFDELTSDEWR
SDVKIHHDHGDPTKAFDFWSVFEKSKPAQHVYCCGPQALMDTVRDMTGHWPSGTVHFE
>d1ep3b2 c.25.1.3 (B:103-262) Dihydroorotate dehydrogenase B, PyrK subunit {Lactococcus lactis, isozyme B}
PVAEVTSTDKILIIGGGIGVPPLYELAKQLEKTGCQMTILLGFASENVKILENEFSNLKNVT
LKIATDDGSYGTKGHVGMLMNEIDFEVDALYTCGAPAMLKAVAKKYDQLERLYISMESRMAC
GIGACYACVEHDKEDESHALKVCEDGPVFLGKQLSL
>d1ja1a3 c.25.1.4 (A:519-678) NADPH-cytochrome p450 reductase {Rat (Rattus norvegicus)}
RLPFKSTTPVIMVGPGTGIAPFMGFIQERAWLREQGKEVGETLLYYGCRRSDEDYLYREELA
RFHKDGALTQLNVAFSREQAHKVYVQHLLKRDREHLWKLIHEGGAHIYVAGDARNMAKDVQN
TFYDIVAEFGPMEHTQAVDYVKKLMTKGRYSLNVWS
>d1ddga2 c.25.1.4 (A:447-599) Sulfite reductase flavoprotein {Escherichia coli}
LPANPETPVIMIGPGTGIAPFRAFMQQRAADEAPGKNWLFFGNPHFTEDFLYQVEWQRYVKE
GVLTRIDLAWSRDQKEKVYVQDKLREQGAELWRWINDGAHIYVCGDANRMAKDVEQALLEVI
AEFGGMDTEAADEFLSELRVERRYQRDVY
>d1f20a2 c.25.1.4 (A:1233-1397) Neuronal nitric-oxide synthase FAD/NADP+ domain {Rat (Rattus norvegicus)}
SFHLPRNPQVPCILVGPGTGIAPFRSFWQQRQFDIQHKGMNPCPMVLVFGCRQSKIDHIYRE
ETLQAKNKGVFRELYTAYSREPDRPKKYVQDVLQEQLAESVYRALKEQGGHIYVCGDVTMAA
DVLKAIQRIMTQQGKLSEEDAGVFISRLRDDNRYHEDIFGV
>d1cqxa3 c.25.1.5 (A:262-403) Flavohemoglobin, C-terminal domain {Alcaligenes eutrophus}
DVDAKTPIVLISGGVGLTPMVSMLKVALQAPPRQVVFVHGARNSAVHAMRDRLREAAKTYEN
LDLFVFYDQPLPEDVQGRDYDYPGLVDVKQIEKSILLPDADYYICGPIPFMRMQHDALKNLG
IHEARIHYEVFGPDLFAE

>d4ts1a_ c.26.1.1 (A:) Tyrosyl-tRNA synthetase (TyrRS) {Bacillus stearothermophilus, nca 1503}
MDLLAELQWRGLVNQTTDEDGLRKLLNEERVTLYCGFDPTADSLHIGHLATILTMRRFQQAG
HRPIALVGGATGLIGDPSGKKSERTLNAKETVEAWSARIKEQLGRFLDFEADGNPAKIKNNY
DWIGPLDVITFLRDVGKHFSVNYMMAKESVQSRIETGISFTEFSYMMLQAYDFLRLYETEGC
RLQIGGSDQWGNITAGLELIRKTKGEARAFGLTIPLVTKADGTKFGKTESGTIWLDKEKTSP
YEFYQFWINTDDRDVIRYLKYFTFLSKEEIEALEQELREAPEKRAAQKTLAEEVTKLVHGEE
ALRQAIRYA
>d1jila_ c.26.1.1 (A:) Tyrosyl-tRNA synthetase (TyrRS) {Staphylococcus aureus}
TNVLIEDLKWRGLIYQQTDEQGIEDLLNKEQVTLYCGADPTADSLHIGHLLPFLTLRRFQEH
GHRPIVLIGGGTGMIGDPSGKSEERVLQTEEQVDKNIEGISKQMHNIFEFGTDHGAVLVNNR
DWLGQISLISFLRDYGKHVGVNYMLGKDSIQSRLEHGISYTEFTYTILQAIDFGHLNRELNC
KIQVGGSDQWGNITSGIELMRRMYGQTDAYGLTIPLVTKSDGKKFGKSESGAVWLDAEKTSP
YEFYQFWINQSDEDVIKFLKYFTFLGKEEIDRLEQSKNEAPHLREAQKTLAEEVTKFIHGED
ALNDAIRISQALF
>d1i6la_ c.26.1.1 (A:) Tryptophanyl-tRNA synthetase (TrpRS) {Bacillus stearothermophilus}
MKTIFSGIQPSGVITIGNYIGALRQFVELQHEYNCYFCIVDQHAITVWQDPHELRQNIRRLA
ALYLAVGIDPTQATLFIQSEVPAHAQAAWMLQCIVYIGELERMTQFKEKSAGKEAVSAGLLT
YPPLMAADILLYNTDIVPVGEDQKQHIELTRDLAERFNKRYGELFTIPEARIPKVGARIMSL
VDPTKKMSKSDPNPKAYITLLDDAKTIEKKIKSAVTDSEGTIRYDKEAKPGISNLLNIYSTL
SGQSIEELERQYEGKGYGVFKADLAQVVIETLRPIQERYHHWMESEELDRVLDEGAEKANRV
ASEMVRKMEQAMGLGR
>d1gtra2 c.26.1.1 (A:8-338) Glutaminyl-tRNA synthetase (GlnRS) {Escherichia coli}
TNFIRQIIDEDLASGKHTTVHTRFPPEPNGYLHIGHAKSICLNFGIAQDYKGQCNLRFDDTN
PVKEDIEYVESIKNDVEWLGFHWSGNVRYSSDYFDQLHAYAIELINKGLAYVDELTPEQIRE
YRGTLTQPGKNSPYRDRSVEENLALFEKMRAGGFEEGKACLRAKIDMASPFIVMRDPVLYRI
KFAEHHQTGNKWCIYPMYDFTHCISDALEGITHSLCTLEFQDNRRLYDWVLDNITIPVHPRQ
YEFSRLNLEYTVMSKRKLNLLVTDKHVEGWDDPRMPTISGLRRRGYTAASIREFCKRIGVTK
QDNTIEMASLESCIREDLNEN
>d1gln_2 c.26.1.1 (1-305) Glutamyl-tRNA synthetase (GluRS) {Thermus thermophilus}
MVVTRIAPSPTGDPHVGTAYIALFNYAWARRNGGRFIVRIEDTDRARYVPGAEERILAALKW
LGLSYDEGPDVAAPTGPYRQSERLPLYQKYAEELLKRGWAYRAFETPEELEQIRKEKGGYDG
RARNIPPEEAEERARRGEPHVIRLKVPRPGTTEVKDELRGVVVYDNQEIPDVVLLKSDGYPT
YHLANVVDDHLMGVTDVIRAEEWLVSTPIHVLLYRAFGWEAPRFYHMPLLRNPDKTKISKRK
SHTSLDWYKAEGFLPEALRNYLCLMGFSMPDGREIFTLEEFIQAFTWERVSLGGPVF
>d1a8h_2 c.26.1.1 (1-348) Methionyl-tRNA synthetase (MetRS) {Thermus thermophilus}
MEKVFYVTTPIYYVNAEPHLGHAYTTVVADFLARWHRLDGYRTFFLTGTDEHGETVYRAAQA
AGEDPKAFVDRVSGRFKRAWDLLGIAYDDFIRTTEERHKKVVQLVLKKVYEAGDIYYGEYEG
LYCVSCERFYTEKELVEGLCPIHGRPVERRKEGNYFFRMEKYRPWLQEYIQENPDLIRPEGY

RNEVLAMLAEPIGDLSISRPKSRVPWGIPLPWDENHVTYVWFDALLNYVSALDYPEGEAYRT
FWPHAWHLIGKDILKPHAVFWPTMLKAAGIPMYRHLNVGGFLLGPDGRKMSKTLGNVVDPFA
LLEKYGRDALRYYLLREIPYGQDTPVSEEALRTRYEAD
>d1f4la2 c.26.1.1 (A:4-140,A:176-388) Methionyl-tRNA synthetase (MetRS) {Escherichia coli}
AKKILVTCALPYANGSIHLGHMLEHIQADVWVRYQRMRGHEVNFICADDAHGTPIMLKAQQL
GITPEQMIGEMSQEHQTDFAGFNISYDNYHSTHSEENRQLSELIYSRLKENGFIKNRTISQL
YDPEKGMFLPDRFXVVSGATPVMRDSEHFFFDLPSFSEMLQAWTRSGALQEQVANKMQEWFE
SGLQQWDISRDAPYFGFEIPNAPGKYFYVWLDAPIGYMGSFKNLCDKRGDSVSFDEYWKKDS
TAELYHFIGKDIVYFHSLFWPAMLEGSNFRKPSNLFVHGYVTVNGAKMSKSRGTFIKASTWL
NHFDADSLRYYYTAKLSSRIDDIDLNLEDFVQRVNADIVNK
>d1ile_3 c.26.1.1 (1-197,387-641) Isoleucyl-tRNA synthetase (IleRS) {Thermus thermophilus}
MFKEVGEPNFPKLEEEVLAFWKREKIFQKSVENRKGGPRYTVYEGPPTANGLPHVGHAQARS
YKDLFPRYKTMRGYYAPRRAGWDTHGLPVELEVEKKLGLKSKREIEAYGIERFNQACRESVF
TYEKEWEAFTERIAYWVDLEDAYATLEPTYIESIWWSLKNLFDRGLLYRDHKVVPYCPRCGT
PLSSHEVALGYXPHCWRCSTPLMYYATESWFIKNTLFKDELIRNNQEIHWVPPHIKEGRYGE
WLKNLVDWALSRNRYWGTPLPIWVCQACGKEEAIGSFQELKARATKPLPEPFDPHRPYVDQV
ELACACGGTMRRVPYVIDVWYDSGAMPFASLHYPFEHEEVFRESFPADFIAEGIDQTRGWFN
SLHQLGVMLFGSIAFKNVICHGLILDEKGQKMSKSKGNVVDPWDIIRKFGADALRWYIYVSA
PPEADRRFGPNLVRETVRD
>d1ffya3 c.26.1.1 (A:1-200,A:395-644) Isoleucyl-tRNA synthetase (IleRS) {Staphylococcus aureus}
MDYEKTLLMPKTDFPMRGGLPNKEPQIQEKWDAEDQYHKALEKNKGNETFILHDGPPYANGN
LHMGHALNKILKDFIVRYKTMQGFYAPYVPGWDTHGLPIEQALTKKGVDRKKMSTAEFREKC
KEFALEQIELQKKDFRRLGVRGDFNDPYITLKPEYEAAQIRIFGEMADKGLIYKGKKPVYWS
PSSESSLAEAEIEYXPHDWRTKKPVIFRATPQWFASISKVRQDILDAIENTNFKVNWGKTRI
YNMVRDRGEWVISRQRVWGVPLPVFYAENGEIIMTKETVNHVADLFAEHGSNIWFEREAKDL
LPEGFTHPGSPNGTFTKETDIMDVWFDSGSSHRGVLETRPELSFPADMYLEGSDQYRGWFNS
SITTSVATRGVSPYKFLLSHGFVMDGEGKKMSKSLGNVIVPDQVVKQKGADIARLWVSSTDY
LADVRISDEILKQTSDD
>d1gaxa3 c.26.1.1 (A:1-189,A:343-578) Valyl-tRNA synthetase (ValRS) {Thermus thermophilus}
MDLPKAYDPKSVEPKWAEKWAKNPFVANPKSGKPPFVIFMPPPNVTGSLHMGHALDNSLQDA
LIRYKRMRGFEAVWLPGTDHAGIATQVVVERLLLKEGKTRHDLGREKFLERVWQWKEESGGT
ILKQLKRLGASADWSREAFTMDEKRSRAVRYAFSRYYHEGLAYRAPRLVNWCPRCETTLSDL
EVEXTCSRCGTPIEYAIFPQWWLRMRPLAEEVLKGLRRGDIAFVPERWKKVNMDWLENVKDW
NISRQLWWGHQIPAWYCEDCQAVNVPRPERYLEDPTSCEACGSPRLKRDEDVFDTWFSSALW
PLSTLGWPEETEDLKAFYPGDVLVTGYDILFLWVSRMEVSGYHFMGERPFKTVLLHGLVLDE
KGQKMSKSKGNVIDPLEMVERYGADALRFALIYLATGGQDIRLDLRWLEMARNF
>d1f7ua2 c.26.1.1 (A:136-483) Arginyl-tRNA synthetase (ArgRS) {Baker's yeast (Saccharomyces cerevisiae)}
SCKLVENKKVIIEFSSPNIAKPFHAGHLRSTIIGGFLANLYEKLGWEVIRMNYLGDWGKQFG
LLAVGFERYGNEEALVKDPIHHLFDVYVRINKDIEEEGDSIPLEQSTNGKAREYFKRMEDGD

EEALKIWKRFREFSIEKYIDTYARLNIKYDVYSGESQVSKESMLKAIDLFKEKGLTHEDKGA
VLIDLTKFNKKLGKAIVQKSDGTTLYLTRDVGAAMDRYEKYHFDKMIYVIASQQDLHAAQFF
EILKQMGFEWAKDLQHVNFGMVQGMSTRKGTVVFLDNILEETKEKMHEVMKKNENKYAQIEH
PEEVADLVGISAVMIQDMQGKRINNYEFKWERMLSFEG
>d1iq0a2 c.26.1.1 (A:97-466) Arginyl-tRNA synthetase (ArgRS) {Thermus thermophilus}
PFPRRPGVVLVEHTSVNPNKELHVGHLRNIALGDAIARILAYAGREVLVLNYIDDTGRQAAE
TLFALRHYGLTWDGKEKYDHFAGRAYVRLHQDPEYERLQPAIEEVLHALERGELREEVNRIL
LAQMATMHALNARYDLLVWESDIVRAGLLQKALALLEQSPHVFRPREGKYAGALVMDASPVI
PGLEDPFFVLLRSNGTATYYAKDIAFQFWKMGILEGLRFRPYENPYYPGLRTSAPEGEAYTP
KAEETINVVDVRQSHPQALVRAALALAGYPALAEKAHHLAYETVLLEGRQMSGRKGLAVSVD
EVLEEATRRARAIVEEKNPDHPDKEEAARMVALGAIRFSMVKTEPKKQIDFRYQEALSFE
>d1coza_ c.26.1.2 (A:) CTP:glycerol-3-phosphate cytidylyltransferase {Bacillus subtilis}
MKKVITYGTFDLLHWGHIKLLERAKQLGDYLVVAISTDEFNLQKQKKAYHSYEHRKLILETI
RYVDEVIPEKNWEQKKQDIIDHNIDVFVMGDDWEGKFDFLKDQCEVVYLPRTEGISTTKIKE
EI
>d1qjca_ c.26.1.3 (A:) Phosphopantetheine adenylyltransferase {Escherichia coli}
KRAIYPGTFDPITNGHIDIVTRATQMFDHVILAIAASPSKKPMFTLEERVALAQQATAHLGN
VEVVGFSDLMANFARNQHATVLIRGLRAVADFEYEMQLAHMNRHLMPELESVFLMPSKEWSF
ISSSLVKEVARHQGDVTHFLPENVHQALMAKLA
>d1f9aa_ c.26.1.3 (A:) Nicotinamide mononucleotide (NMN) adenylyltransferase {Archaeon Methanococcus jannaschii}
LRGFIIGRFQPFHKGHLEVIKKIAEEVDEIIIGIGSAQKSHTLENPFTAGERILMITQSLKD
YDLTYYPIPIKDIEFNSIWVSYVESLTPPFDIVYSGNPLVRVLFEERGYEVKRPEMFNRKEY
SGTEIRRRMLNGEKWEHLVPKAVVDVIKEIKGVERLRKLA
>d1ej2a_ c.26.1.3 (A:) Nicotinamide mononucleotide (NMN) adenylyltransferase {Archaeon Methanobacterium thermoautotrophicum}
MRGLLVGRMQPFHRGHLQVIKSILEEVDELIICIGSAQLSHSIRDPFTAGERVMMLTKALSE
NGIPASRYYIIPVQDIECNALWVGHIKMLTPPFDRVYSGNPLVQRLFSEDGYEVTAPPLFYR
DRYSGTEVRRRMLDDGDWRSLLPESVVEVIDEINGVERIKHLA
>d1ihoa_ c.26.1.4 (A:) Pantothenate synthetase (Pantoate-beta-alanine ligase, PanC) {Escherichia coli}
MLIIETLPLLRQQIRRLRMEGKRVALVPTMGNLHDGHMKLVDEAKARADVVVVSIFVNPMQF
DRPEDLARYPRTLQEDCEKLNKRKVDLVFAPSVKEIYPNGTETHTYVDVPGLSTMLEGASRP
GHFRGVSTIVSKLFNLVQPDIACFGEKDFQQLALIRKMVADMGFDIEIVGVPIMRAKDGLAL
SSRNGYLTAEQRKIAPGLYKVLSSIADKLQAGERDLDEIITIAGQELNEKGFRADDIQIRDA
DTLLEVSETSKRAVILVAAWLGDARLIDNKMVEL
>d1g8fa2 c.26.1.5 (A:169-389) ATP sulfurylase central domain {Baker's yeast (Saccharomyces cerevisiae)}
YPGLRKTPAQLRLEFQSRQWDRVVAFQTRNPMHRAHRELTVRAAREANAKVLIHPVVGLTKP
GDIDHHTRVRVYQEIIKRYPNGIAFLSLLPLAMRMSGDREAVWHAIIRKNYGASHFIVGRDH

AGPGKNSKGVDFYGPYDAQELVESYKHELDIEVVPFRMVTYLPDEDRYAPIDQIDTTKTRTL
NISGTELRRRLRVGGEIPEWFSYPEVVKILRESNP
>d1i2da2 c.26.1.5 (A:171-390) ATP sulfurylase central domain
{Fungus (Penicillium chrysogenum)}
YVALRYTPAELRVHFDKLGWSRVVAFQTRNPMHRAHRELTVRAARSRQANVLIHPVVGLTKP
GDIDHFTRVRAYQALLPRYPNGMAVLGLLGLAMRMGGPREAIWHAIIRKNHGATHFIVGRDH
AGPGSNSKGEDFYGPYDAQHAVEKYKDELGIEVVEFQMVTYLPDTDEYRPVDQVPAGVKTLN
ISGTELRRRLRSGAHIPEWFSYPEVVKILRESNP
>d1jhda2 c.26.1.5 (A:174-396) ATP sulfurylase central domain
{unnamed symbiont of Riftia pachyptila}
PDTFRTAVEIRNEIKEHGWSKVVAFQTRNPMHRAHEELCRMAMESLDADGVVVHMLLGKLKK
GDIPAPVRDAAIRTMAEVYFPPNTVMVTGYGFDMLYAGPREAVLHAYFRQNMGATHFIIGRD
HAGVGDYYGAFDAQTIFDDEVPEGAMEIEIFRADHTAYSKKLNKIVMMRDVPDHTKEDFVLL
SGTKVREMLGQGIAPPPEFSRPEVAKILMDYYQSINS
>d1gpma1 c.26.2.1 (A:208-404) GMP synthetase, central domain
{Escherichia coli}
WTPAKIIDDAVARIREQVGDDKVILGLSGGVDSSVTAMLLHRAIGKNLTCVFVDNGLLRLNE
AEQVLDMFGDHFGLNIVHVPAEDRFLSALAGENDPEAKRKIIGRVFVEVFDEEALKLEDVKW
LAQGTIYPDVIESAASATGKAHVIKSHHNVGGLPKEMKMGLVEPLKELFKDEVRKIGLELGL
PYDMLYRHPFP
>d1ih8a_ c.26.2.1 (A:) NH3-dependent NAD+-synthetase {Bacillus
subtilis}
SMQEKIMRELHVKPSIDPKQEIEDRVNFLKQYVKKTGAKGFVLGISGGQDSTLAGRLAQLAV
ESIREEGGDAQFIAVRLPHGTQQDEDDAQLALKFIKPDKSWKFDIKSTVSAFSDQYQQETGD
QLTDFNKGNVKARTRMIAQYAIGGQEGLLVLGTDHAAEAVTGFFTKYGDGGADLLPLTGLTK
RQGRTLLKELGAPERLYLKEPTADLLDEKPQQSDETELGISYDEIDDYLEGKEVSAKVSEAL
EKRYSMTEHKRQVPASMFDDWWK
>d1ct9a1 c.26.2.1 (A:193-516) Asparagine synthetase B, C-terminal
domain {Escherichia coli}
RDWFDYDAVKDNVTDKNELRQALEDSVKSHLMSDVPYGVLLSGGLDSSIISAITKKYAARRV
EDQERSEAWWPQLHSFAVGLPGSPDLKAAQEVANHLGTVHHEIHFTVQEGLDAIRDVIYHIE
TYDVTTIRASTPMYLMSRKIKAMGIKMVLSGEGSDEVFGGYLYFHKAPNAKELHEETVRKLL
ALHMYDCARANKAMSAWGVEARVPFLDKKFLDVAMRINPQDKMCGNGKMEKHILRECFEAYL
PASVAWRQKEQFSDGVGYSWIDTLKEVAAQQVSDQQLETARFRFPYNTPTSKEAYLYREIFE
ELFPLPSAAECVPG
>d1jgta1 c.26.2.1 (A:210-508) beta-Lactam synthetase
{Streptomyces clavuligerus}
PGLSRRILPEGEAVAAVRAALEKAVAQRVTPGDTPLVVLSGGIDSSGVAACAHRAAGELDTV
SMGTDTSNEFREARAVVDHLRTRHREITIPTTELLAQLPYAVWASESVDPDIIEYLLPLTAL
YRALDGPERRILTGYGADIPLGGMHREDRLPALDTVLAHDMATFDGLNEMSPVLSTLAGHWT
THPYWDREVLDLLVSLEAGLKRRHGRDKWVLRAAMADALPAETVNRPKLGVHEGSGTTSSFS
RLLLDHGVAEDRVHEAKRQVVRELFDLTVGGGRHPSEVDTDDVVRSVADRT
>d1k92a1 c.26.2.1 (A:1-188) Argininosuccinate synthetase,
N-terminal domain {Escherichia coli}

TTILKHLPVGQRIGIAFSGGLDTSAALLWMRQKGAVPYAYTANLGQPDEEDYDAIPRRAMEY
GAENARLIDCRKQLVAEGIAAIQCGAFHNTTGGLTYFNTTPLGRAVTGTMLVAAMKEDGVNI
WGDGSTYKGNDIERFYRYGLLTNAELQIYKPWLDTDFIDELGGRHEMSEFMIACGFDYKMSV
EK
>d1sur__ c.26.2.2 (-) Phosphoadenylyl sulphate (PAPS) reductase {Escherichia coli}
SKLDLNALNELPKVDRILALAETNAELEKLDAEGRVAWALDNLPGEYVLSSSFGIQAAVSLH
LVNQIRPDIPVILTDTGYLFPETYRFIDELTDKLKLNLKVYRATESAAWQEARYGKLWEQGV
EGIEKYNDINKVEPMNRALKELNAQTWFAGLRREQSGSRANLPVLAIQRGVFKVLPIIDWDN
RTIYQYLQKHGLKYHPLWDEGYLSVGDTH
>d1dlja3 c.26.3.1 (A:295-402) UDP-glucose dehydrogenase (UDPGDH), C-terminal (UDP-binding) domain {Streptococcus pyogenes}
AKQIINVLKEQESPVKVVGVYRLIMKSNSDNFRESAIKDVIDILKSKDIKIIIYEPMLNKLE
SEDQSVLVNDLENFKKQANIIVTNRYDNELQDVKNKVYSRDIFGRD
>d2tpt_2 c.27.1.1 (71-335) Thymidine phosphorylase {Escherichia coli}
DWKSLHLNGPIVDKHSTGGVGDVTSLMLGPMVAACGGYIPMISGRGLGHTGGTLDKLESIPG
FDIFPDDNRFREIIKDVGVAIIGQTSSLAPADKRFYATRDITATVDSIPLITASILAKKLAE
GLDALVMDVKVGSGAFMPTYELSEALAEAIVGVANGAGVRTTALLTDMNQVLASSAGNAVEV
REAVQFLTGEYRNPRLFDVTMALCVEMLISGKLAKDDAEARAKLQAVLDNGKAAEVFGRMVA
AQKGPTDFVENYAKYLP
>d1brwa2 c.27.1.1 (A:71-330) Pyrimidine nucleoside phosphorylase {Bacillus stearothermophilus}
LSSIRGVKVDKHSTGGVGDTTTLVLGPLVASVGVPVAKMSGRGLGHTGGTIDKLESVPGFHV
EISKDEFIRLVNENGIAIIGQTGDLTPADKKLYALRDVTATVNSIPLIASSIMSKKIAAGAD
AIVLDVKTGAGAFMKKLDEARRLARVMVDIGKRVGRRTMAVISDMSQPLGYAVGNALEVKEA
IETLKGNGPHDLTELCLTLGSHMVYLAEKAPSLDEARRLLEEAIRSGAAIAAFKTFLAAQGG
DASVVDDLDKLP
>d1dnpa2 c.28.1.1 (A:1-200) N-terminal domain of DNA photolyase {Escherichia coli}
TTHLVWFRQDLRLHDNLALAAACRNSSARVLALYIATPRQWATHNMSPRQAELINAQLNGLQ
IALAEKGIPLLFREVDDFVASVEIVKQVCAENSVTHLFYNYQYEVNERARDVEVERALRNVV
CEGFDDSVILPPGAVMTGNHEMYKVFTPFKNAWLKRLREGMPECVAAPKVRSSGSIEPSPSI
TLNYPRQSFDTAHF
>d1iqra2 c.28.1.1 (A:2-171) N-terminal domain of DNA photolyase {Thermus thermophilus}
GPLLVWHRGDLRLHDHPALLEALARGPVVGLVVLDPNNLKTTPRRRAWFLENVRALREAYRA
RGGALWVLEGLPWEKVPEAARRLKAKAVYALTSHTPYGRYRDGRVREALPVPLHLLPAPHLL
PPDLPRAYRVYTPFSRLYRGAAPPLPPPEALPKGPEEGEIPREDPG
>d1qnf_2 c.28.1.1 (1-204) N-terminal domain of DNA photolyase {Anacystis nidulans}
MAAPILFWHRRDLRLSDNIGLAAARAQSAQLIGLFCLDPQILQSADMAPARVAYLQGCLQEL
QQRYQQAGSRLLLLQGDPQHLIPQLAQQLQAEAVYWNQDIEPYGRDRDGQVAAALKTAGIRA
VQLWDQLLHSPDQILSGSGNPYSVYGPFWKNWQAQPKPTPVATPTELVDLSPEQLTAIAPLL

LSELPTLKQLGFDWDGGF
>d1efva1 c.29.1.1 (A:20-207) Electron transfer flavoprotein, ETFP {Human (Homo sapiens)}
QSTLVIAEHANDSLAPITLNTITAATRLGGEVSCLVAGTKCDKVAQDLCKVAGIAKVLVAQH
DVYKGLLPEELTPLILATQKQFNYTHICAGASAFGKNLLPRVAAKLEVAPISDIIAIKSPDT
FVRTIYAGNALCTVKCDEKVKVFSVRGTSFDAAATSGGSASSEKASSTSPVEISEWLDQKLT
KS
>d1efvb1 c.29.1.1 (B:) Electron transfer flavoprotein, ETFP {Human (Homo sapiens)}
LRVLVAVKRVIDYAVKIRVKPDRTGVVTDGVKHSMNPFCEIAVEEAVRLKEKKLVKEVIAVS
CGPAQCQETIRTALAMGADRGIHVEVPPAEAERLGPLQVARVLAKLAEKEKVDLVLLGKQAI
DDDCNQTGQMTAGFLDWPQGTFASQVTLEGDKLKVEREIDGGLETLRLKLPAVVTADLRLNE
PRYATLPNIMKAKKKKIEVIKPGDLGVDLTSKLSVISVEDPPQRTAGVKVETTEDLVAKLKE
IGRI
>d1efpa1 c.29.1.1 (A:2-184) Electron transfer flavoprotein, ETFP {Paracoccus denitrificans}
AVLLLGEVTNGALNRDATAKAVAAVKALGDVTVLCAGASAKAAAEEAAKIAGVAKVLVAEDA
LYGHRLAEPTAALIVGLAGDYSHIAAPATTDAKNVMPRVAALLDVMVLSDVSAILDADTFER
PIYAGNAIQVVKSKDAKKVFTIRTASFDAAGEGGTAPVTETAAAADPGLSSWVADEVAE
>d1efpb1 c.29.1.1 (B:) Electron transfer flavoprotein, ETFP {Paracoccus denitrificans}
MKVLVPVKRLIDYNVKARVKSDGSGVDLANVKMSMNPFDEIAVEEAIRLKEKGQAEEIIAVS
IGVKQAAETLRTALAMGADRAILVVAADDVQQDIEPLAVAKILAAVARAEGTELIIAGKQAI
DNDMNATGQMLAAILGWAQATFASKVEIEGAKAKVTREVDGGLQTIAVSLPAVVTADLRLNE
PRYASLPNIMKAKKKPLDEKTAADYGVDVAPRLEVVSVREPEGRKAGIKVGSVDELVGKL
>d1mjha_ c.29.1.2 (A:) "Hypothetical" protein MJ0577 {Archaeon Methanococcus jannaschii}
VMYKKILYPTDFSETAEIALKHVKAFKTLKAEEVILLHVIDEREIKKRDIFSLLLGVAGLNK
SVEEFENELKNKLTEEAKNKMENIKKELEDVGFKVKDIIVVGIPHEEIVKIAEDEGVDIIIM
GSHGKTNLKEILLGSVTENVIKKSNKPVLVVKRKNS
>d1jmva_ c.29.1.2 (A:) Universal stress protein A, UspA {Haemophilus influenzae}
MYKHILVAVDLSEESPILLKKAVGIAKRHDAKLSIIHVDVNFSDLYTGLIDVNMSSMQDRIS
TETQKALLDLAESVDYPISEKLSGSGDLGQVLSDAIEQYDVDLLVTGHHQDFWSKLMSSTRQ
VMNTIKIDMLVVPLRD
>d1dv1a2 c.30.1.1 (A:1-114) Biotin carboxylase (BC) subunit of acetyl-CoA carboxylase {Escherichia coli}
MLDKIVIANRGEIALRILRACKELGIKTVAVHSSADRDLKHVLLADETVCIGPAPSVKSYLN
IPAIISAAEITGAVAIHPGYGFLSENANFAEQVERSGFIFIGPKAETIRLMG
>d1gsoa2 c.30.1.1 (A:-2-103) Glycinamide ribonucleotide synthetase (GAR-syn). {Escherichia coli}
EFMKVLVIGNGGREHALAWKAAQSPLVETVFVAPGNAGTALEPALQNVAIGVTDIPALLDFA
QNEKIDLTIVGPEAPLVKGVVDTFRAAGLKIFGPTAGAAQLEG
>d1b6ra2 c.30.1.1 (A:1-78) N5-carboxyaminoimidazole

ribonucleotide synthetase, AIRC, PurK {Escherichia coli}
MKQVCVLGNGQLGRMLRQAGEPLGIAVWPVGLDAEPAAVPFQQSVITAEIERWPETALTRQL
ARHPAFVNRDVFPIIA
>d1eyza2 c.30.1.1 (A:2-112) Glycinamide ribonucleotide transformylase PurT {Escherichia coli}
TLLGTALRPAATRVMLLGSGELGKEVAIECQRLGVEVIAVDRYADAPAMHVAHRSHVINMLD
GDALRRVVELEKPHYIVPEIEAIATDMLIQLEEEGLNVVPCARATKLTM
>d1a9xa3 c.30.1.1 (A:1-127) Carbamoyl phosphate synthetase (CPS), large subunit {Escherichia coli}
MPKRTDIKSILILGAGPIVIGQACEFDYSGAQACKALREEGYRVINVNSNPATIMTDPEMAD
ATYIEPIHWEVVRKIIEKERPDAVLPTMGGQTALNCALELERQGVLEEFGVTMIGATADAID
KAE
>d1a9xa4 c.30.1.1 (A:556-676) Carbamoyl phosphate synthetase (CPS), large subunit {Escherichia coli}
STDREKIMVLGGGPNRIGQGIEFDYCCVHASLALREDGYETIMVNCNPETVSTDYDTSDRLY
FEPVTLEDVLEIVRIEKPKGVIVQYGGQTPLKLARALEAAGVPVIGTSPDAIDRAEDRE
>d1iow_1 c.30.1.2 (1-96) D-Ala-D-Ala ligase {Escherichia coli, gene ddlB}
MTDKIAVLLGGTSAEREVSLNSGAAVLAGLREGGIDAYPVDPKEVDVTQLKSMGFQKVFIAL
HGRGGEDGTLQGMLELMGLPYTGSGVMASALSMD
>d1ehia1 c.30.1.2 (A:3-134) D-alanine:D-lactate ligase, VanA {Leuconostoc mesenteroides, Ddl2}
KKRVALIFGGNSSEHDVSKRSAQNFYNAIEATGKYEIIVFAIAQNGFFLDTESSKKILALED
EQPIVDAFMKTVDASDPLARIHALKSAGDFDIFFPVVHGNLGEDGTLQGLFKLLDKPYVGAP
LRGHAVSF
>d1e4ea1 c.30.1.2 (A:2-131) D-alanine:D-lactate ligase, VanA {Enterococcus faecium}
NRIKVAILFGGCSEEHDVSVKSAIEIAANINKEKYEPLYIGITKSGVWKMCEKPCAEWENEN
CYSAVLSPDKKMHGLLVKKNHEYEINHVDVAFSALHGKSGEDGSIQGLFELSGIPFVGCDIQ
SSAICM
>d1gsa_1 c.30.1.3 (1-122) Prokaryotic glutathione synthetase, N-terminal domain {Escherichia coli}
MIKLGIVMDPIANINIKKDSSFAMLLEAQRRGYELHYMEMGDLYLINGEARAHTRTLNVKQN
YEEWFSFVGEQDLPLADLDVILMRKDPPFDTEFIYATYILERAEEKGTLIVNKPQSLRDC
>d2hgsa1 c.30.1.4 (A:202-303) Eukaryotic glutathione synthetase {Human (Homo sapiens)}
PNALVLLIAQEKERNIFDQRAIENELLARNIHVIRRTFEDISEKGSLDQDRRLFVDGQEIAV
VYFRDGYMPRQYSLQNWEARLLLERSHAAKCPDIATQLAG
>d1auva1 c.30.1.5 (A:112-213) Synapsin Ia domain {Cow (Bos taurus)}
AARVLLVIDEPHTDWAKYFKGKKIHGEIDIKVEQAEFSDLNLVAHANGGFSVDMEVLRNGVK
VVRSLKPDFVLIRQHAFSMARNGDYRSLVIGLQYAGIPSI
>d1dhs__ c.31.1.1 (-) Deoxyhypusine synthase, DHS {Human (Homo sapiens)}
APAGALAAVLKHSSTLPPESTQVRGYDFNRGVNYRALLEAFGTTGFQATNFGRAVQQVNAMI

EKKLEPLTSCTIFLGYTSNLISSGIRETIRYLVQHNMVDVLVTTAGGVEEDLIKCLAPTYLG
EFSLRGKELRENGINRIGNLLVPNENYCKFEDWLMPILDQMVMEQNTEGVKWTPSKMIARLG
KEINNPESVYYWAQKNHIPVFSPALTDGSLGDMIFFHSYKNPGLVLDIVEDLRLINTQAIFA
KCTGMIILGGGVVKHHIANANLMRNGADYAVYINTAQEFDGSDSGARPDEAVSWGKIRVDAQ
PVKVYADASLVFPLLVAETFAQKMDAFMHEKNED
>d1efva2 c.31.1.2 (A:208-331) C-terminal domain of the electron transfer flavoprotein alpha subunit {Human (Homo sapiens)}
DRPELTGAKVVVSGGRGLKSGENFKLLYDLADQLHAAVGASRAAVDAGFVPNDMQVGQTGKI
VAPELYIAVGISGAIQHLAGMKDSKTIVAINKDPEAPIFQVADYGIVADLFKVVPEMTEILK
>d1efpa2 c.31.1.2 (A:185-308) C-terminal domain of the electron transfer flavoprotein alpha subunit {Paracoccus denitrificans}
SDRPELTSARRVVSGGRGLGSKESFAIIEELADKLGAAVGASRAAVDSGYAPNDWQVGQTGK
VVAPELYVAVGISGAIQHLAGMKDSKVIVAINKDEEAPIFQIADYGLVGDLFSVVPELTGKL
>d1poxa1 c.31.1.3 (A:183-365) Pyruvate oxidase {Lactobacillus plantarum}
YASANNYQTPLLPEPDVQAVTRLTQTLLAAERPLIYYGIGARKAGKELEQLSKTLKIPLMST
YPAKGIVADRYPAYLGSANRVAQKPANEALAQADVVLFVGNNYPFAEVSKAFKNTRYFLQID
IDPAKLGKRHKTDIAVLADAQKTLAAILAQVSERESTPWWQANLANVKNWRAYLASLED
>d1pvda1 c.31.1.3 (A:182-360) Pyruvate decarboxylase {Baker's yeast (Saccharomyces cerevisiae)}
QTPIDMSLKPNDAESEKEVIDTILALVKDAKNPVILADACCSRHDVKAETKKLIDLTQFPAF
VTPMGKGSISEQHPRYGGVYVGTLSKPEVKEAVESADLILSVGALLSDKTKNIVEFHSDHMK
IRNATFPGVQMKFVLQKLLTNIADAAKGYKPVAVPARTPANAAVP
>d1qpba1 c.31.1.3 (A:182-360) Pyruvate decarboxylase {Baker's yeast (Saccharomyces cerevisiae)}
QTPIDMSLKPNDAESEKEVIDTILVLIKDAKNPVILADACCSRHDVKAETKKLIDLTQFPAF
VTPMGKGSIDEQHPRYGGVYVGTLSKPEVKEAVESADLILSVGALLSDFNTGSFSYSYKTKN
IVEFHSDHMKIRNATFPGVQMKFVLQKLLTAIADAAKGYKPVAVPARTPANAAVP
>d1zpda1 c.31.1.3 (A:188-362) Pyruvate decarboxylase {Zymomonas mobilis}
EASDEASLNAAVDETLKFIANRDKVAVLVGSKLRAAGAEEAAVKFTDALGGAVATMAAAKSF
FPEENALYIGTSWGEVSYPGVEKTMKEADAVIALAPVFNDYSTTGWTDIPDPKKLVLAEPRS
VVVNGIRFPSVHLKDYLTRLAQKVSKKTGSLDFFKSLNAGELKKAAPADPS
>d1bfd_1 c.31.1.3 (182-341) Benzoylformate decarboxylase {Pseudomonas putida}
SVRLNDQDLDILVKALNSASNPAIVLGPDVDAANANADCVMLAERLKAPVWVAPSAPRCPFP
TRHPCFRGLMPAGIAAISQLLEGHDVVLVIGAPVFRYHQYDPGQYLKPGTRLISVTCDPLEA
ARAPMGDAIVADIGAMASALANLVEESSRQLPTAAP
>d1jsca1 c.31.1.3 (A:280-460) Acetohydroxyacid synthase catalytic subunit {Baker's yeast (Saccharomyces cerevisiae)}
AQDEFVMQSINKAADLINLAKKPVLYVGAGILNHADGPRLLKELSDRAQIPVTTTLQGLGSF
DQEDPKSLDMLGMHGCATANLAVQNADLIIAVGARFDDRVTGNISKFAPEARRAAAEGRGGI
IHFEVSPKNINKVVQTQIAVEGDATTNLGKMMSKIFPVKERSEWFAQINKWKKEYPY
>d1d4oa_ c.31.1.4 (A:) Transhydrogenase domain III (dIII) {Cow (Bos

taurus)}
GTHTEINLDNAIDMIREANSIIITPGYGLCAAKAQYPIADLVKMLSEQGKKVRFGIHPVAGR
MPGQLNVLLAEAGVPYDIVLEMDEINHDFPDTDLVLVIGANDTVNSAAQEDPNSIIAGMPVL
EVWKSKQVIVMKRSLGVGYAAVDNPIFYKPNTAMLLGDAKKTCDALQAKVRES
>d1hzzc_ c.31.1.4 (C:) Transhydrogenase domain III (dIII) {Rhodospirillum rubrum}
SVKAGSAEDAAFIMKNASKVIIVPGYGMAVAQAQHALREMADVLKKEGVEVSYAIHPVAGRM
PGHMNVLLAEANVPYDEVFELEEINSSFQTADVAFVIGANDVTNPAAKTDPSSPIYGMPILD
VEKAGTVLFIKRSMASGYAGVENELFFRNNTMMLFGDAKKMTEQIVQAMN
>d1icia_ c.31.1.5 (A:) AF1676 sir2 homolog {Archaeon Archaeoglobus fulgidus}
GSHHHHHHGSHMDEKLLKTIAESKYLVALTGAGVSAESGIPTFRGKDGLWNRYRPEELANPQ
AFAKDPEKVWKWYAWRMEKVFNAQPNKAHQAFAELERLGVLKCLITQNVDDLHERAGSRNVI
HLHGSLRVVRCTSCNNSFEVESAPKIPPLPKCDKCGSLLRPGVVWFGEMLPPDVLDRAMREV
ERADVIIVAGTSAVVQPAASLPLIVKQRGGAIIEINPDETPLTPIADYSLRGKAGEVMDELV
RHVRKALS
>d1j8fa_ c.31.1.5 (A:) Sirt2 histone deacetylase {Human (Homo sapiens)}
GEADMDFLRNLFSQTLSLGSQKERLLDELTLEGVARYMQSERCRRVICLVGAGISTSAGIPD
FRSPSTGLYDNLEKYHLPYPEAIFEISYFKKHPEPFFALAKELYPGQFKPTICHYFMRLLKD
KGLLLRCYTQNIDTLERIAGLEQEDLVEAHGTFYTSHCVSASCRHEYPLSWMKEKIFSEVTP
KCEDCQSLVKPDIVFFGESLPARFFSCMQSDFLKVDLLLVMGTSLQVQPFASLISKAPLSTP
RLLINKEKAGQSDPFLGMIMGLGGGMDFDSKKAYRDVAWLGECDQGCLALAELLGWKKELED
LVRREHASIDAQS
>d1fsz_1 c.32.1.1 (23-231) Cell-division protein FtsZ {Archaeon Methanococcus jannaschii}
SPEDKELLEYLQQTKAKITVVGCGGAGNNTITRLKMEGIEGAKTVAINTDAQQLIRTKADKK
ILIGKKLTRGLGAGGNPKIGEEAAKESAEEIKAAIQDSDMVFITCGLGGGTGTGSAPVVAEI
SKKIGALTVAVVTLPFVMEGKVRMKNAMEGLERLKQHTDTLVVIPNEKLFEIVPNMPLKLAF
KVADEVLINAVKGLVELITKDGL
>d1tuba1 c.32.1.1 (A:1-245) Tubulin alpha-subunit {Pig (Sus scrofa)}
MRECISIHVGQAGVQIGNACWELYCLEHGIQPDGQMPSDKTIGGGDDSFNTFFSETGAGKHV
PRAVFVDLEPTVIDEVRTGTYRQLFHPEQLITGKEDAANNYARGHYTIGKEIIDLVLDRIRK
LADQCTGLQGFSVFHSFGGGTGSGFTSLLMERLSVDYGKKSKLEFSIYPAPQVSTAVVEPYN
SILTTHTTLEHSDCAFMVDNEAIYDICRRNLDIERPTYTNLNRLIGQIVSSITASLRFD
>d1tubb1 c.32.1.1 (B:1-245) Tubulin beta-subunit {Pig (Sus scrofa)}
MREIVHIQAGQCGNQIGAKFWEVISDEHGIDPTGSYHGDSDLQLERINVYYNEAAGNKYVPR
AILVDLEPGTMDSVRSGPFGQIFRPDNFVFGQSGAGNNWAKGHYTEGAELVDSVLDVVRKES
ESCDCLQGFQLTHSLGGGTGSGMGTLLISKIREEYPDRIMNTFSVVPSPKVSDTVVEPYNAT
LSVHQLVENTDETYCIDNEALYDICFRTLKLTTPTYGDLNHLVSATMSGVTTCLRFP
>d1nbaa_ c.33.1.1 (A:) N-carbamoylsarcosine amidohydrolase {Arthrobacter sp.}

TFNDIEARLAAVLEEAFEAGTSIYNERGFKRRIGYGNRPAVIHIDLANAWTQPGHPFSCPGM
ETIIPNVQRINEAARAKGVPVFYTTNVYRNRDASSGTNDMGLWYSKIPTETLPADSYWAQID
DRIAPADGEVVIEKNRASAFPGTNLELFLTSNRIDTLIVTGATAAGCVRHTVEDAIAKGFRP
IIPRETIGDRVPGVVQWNLYDIDNKFGDVESTDSVVQYLDALPQFEDTVPKTLSDPQPEVEA
PADPV
>d1im5a_ c.33.1.2 (A:) Pyrazinamidase/nicotinamidase {Archaeon Pyrococcus horikoshii}
PEEALIVVDMQRDFMPGGALPVPEGDKIIPKVNEYIRKFKEKGALIVATRDWHPENHISFRE
RGGPWPRHCVQNTPGAEFVVDLPEDAVIISKATEPDKEAYSGFEGTDLAKILRGNGVKRVYI
CGVATEYCVRATALDALKHGFEVYLLRDAVKGIKPEDEERALEEMKSRGIKIVQF
>d1yaca_ c.33.1.2 (A:) YcaC {Escherichia coli}
TKPYVRLDKNDAAVLLVDHQAGLLSLVRDIEPDKFKNNVLALGDLAKYFNLPTILTTSAETG
PNGPLVPELKAQFPDAPYIARPGNINAWDNEDFVKAVKATGKKQLIIAGVVTEVCVAFPALS
AIEEGFDVFVVTDASGTFNEITRHSAWDRMSQAGAQLMTWFGVACELHRDWRNDIAGLATLF
SNHIPDYRNLMTSYDTLT
>d1hi9a_ c.99.1.1 (A:) Zn-dependent D-aminopeptidase DppA {Bacillus subtilis}
MKLYMSVDMEGISGLPDDTFVDSGKRNYERGRLIMTEEANYCIAEAFNSGCTEVLVNDSHSK
MNNLMVEKLHPEADLISGDVKPFSMVEGLDDTFRGALFLGYHARASTPGVMSHSMIFGVRHF
YINDRPVGELGLNAYVAGYYDVPVLMVAGDDRAAKEAEELIPNVTTAAVKQTISRSAVKCLS
PAKRGRLLTEKTAFALQNKDKVKPLTPPDRPVLSIEFANYGQAEWANLMPGTEIKTGTTTVQ
FQAKDMLEAYQAMLVMTELAMRTSFC
>d1e20a_ c.34.1.1 (A:) Halotolerance protein Hal3 {Mouse-ear cress (Arabidopsis thaliana)}
RKPRVLLAASGSVAAIKFGNLCHCFTEWAEVRAVVTKSSLHFLDKLSLPQEVTLYTDEDEWS
SWNKIGDPVLHIELRRWADVLVIAPLSANTLGKIAGGLCDNLLTCIIRAWDYTKPLFVAPAM
NTLMWNNPFTERHLLSLDELGITLIPPIKKRLACGDYGNGAMAEPSLIYSTVRLFWESQAH
>d1g5qa_ c.34.1.1 (A:) Epidermin modifying enzyme (peptidyl-cysteine decarboxylase) EpiD {Staphylococcus epidermidis}
MYGKLLICATASINVININHYIVELKQHFDEVNILFSPSSKNFINTDVLKLFCDNLYDEIKD
PLLNNINIVENHEYILVLPASANTINKIANGICDNLLTTVCLTGYQKLFIFPNMNIRMWGNP
FLQKNIDLLKNNDVKVYSPDMNKSFEISSGRYKNNITMPNIENVLNFVLN
>d1k1ea_ c.108.1.5 (A:) Probable phosphatase YrbI {Haemophilus influenzae, HI1679}
KLENIKFVITDVDGVLTDGQLHYDANGEAIKSFHVRDGLGIKMLMDADIQVAVLSGRDSPIL
RRRIADLGIKLFFLGKLEKETACFDLMKQAGVTAEQTAYIGDDSVDLPAFAACGTSFAVADA
PIYVKNAVDHVLSTHGGKGAFREMSDMILQAQGKSSVFDTAQGFLKSVKSMGQ
>d1zrn__ c.108.1.1 (-) L-2-Haloacid dehalogenase, HAD {Pseudomonas sp., strain YL}
YIKGIAFDLYGTLFDVHSVVGRCDEAFPGRGREISALWRQKQLEYTWLRSLMNRYVNFQQAT
EDALRFTCRHLGLDLDARTRSTLCDAYLRLAPFSEVPDSLRELKRRGLKLAILSNGSPQSID
AVVSHAGLRDGFDHLLSVDPVQVYKPDNRVYELAEQALGLDRSAILFVASNAWDATGARYFG
FPTCWINRTGNVFEEMGQTPDWEVTSLRAVVELF

>d1qq5a_ c.108.1.1 (A:) L-2-Haloacid dehalogenase, HAD {Xanthobacter autotrophicus}
MIKAVVFDAYGTLFDVQSVADATERAYPGRGEYITQVWRQKQLEYSWLRALMGRYADFWSVT
REALAYTLGTLGLEPDESFLADMAQAYNRLTPYPDAAQCLAELAPLKRAILSNGAPDMLQAL
VANAGLTDSFDAVISVDAKRVFKPHPDSYALVEEVLGVTPAEVLFVSSNGFDVGGAKNFGFS
VARVARLSQEALARELVSGTIAPLTMFKALRMREETYAEAPDFVVPALGDLPRLVRGMA
>d1ek1a1 c.108.1.2 (A:4-225) Epoxide hydrolase, N-terminal domain {Mouse (Mus musculus)}
RVAAFDLDGVLALPSIAGAFRRSEEALALPRDFLLGAYQTEFPEGPTEQLMKGKITFSQWVP
LMDESYRKSSKACGANLPENFSISQIFSQAMAARSINRPMLQAAIALKKKGFTTCIVTNNWL
DDGDKRDSLAQMMCELSQHFDFLIESCQVGMIKPEPQIYNFLLDTLKAKPNEVVFLDDFGSN
LKPARDMGMVTILVHNTASALRELEKVTGTQFPEAP
>d1feza_ c.108.1.3 (A:) Phosphonoacetaldehyde hydrolase {Bacillus cereus}
KIEAVIFDWAGTTVDYGCFAPLEVFMEIFHKRGVAITAEEARKPMGLLKIDHVRALTEMPRI
ASEWNRVFRQLPTEADIQEMYEEFEEILFAILPRYASPINAVKEVIASLRERGIKIGSTTGY
TREMMDIVAKEAALQGYKPDFLVTPDDVPAGRPYPWMCYKNAMELGVYPMNHMIKVGDTVSD
MKEGRNAGMWTVGVILGSSELGLTEEEVENMDSVELREKIEVVRNRFVENGAHFTIETMQEL
ESVMEHIE
>d1j97a_ c.108.1.4 (A:) Phosphoserine phosphatase {Archaeon Methanococcus jannaschii}
EKKKKLILFDFDSTLVNNETIDEIAREAGVEEEVKKITKEAMEGKLNFEQSLRKRVSLLKDL
PIEKVEKAIKRITPTEGAEETIKELKNRGYVVAVVSGGFDIAVNKIKEKLGLDYAFANRLIV
KDGKLTGDVEGEVLKENAKGEILEKIAKIEGINLEDTVAVGDGANDISMFKKAGLKIAFCAK
PILKEKADICIEKRDLREILKYIK
>d1fs5a_ c.35.1.1 (A:) Glucosamine 6-phosphate deaminase/isomerase {Escherichia coli}
MRLIPLTTAEQVGKWAARHIVNRINAFKPTADRPFVLGLPTGGTPMTTYKALVEMHKAGQVS
FKHVVTFNMDEYVGLPKEHPESYYSFMHRNFFDHVDIPAENINLLNGNAPDIDAECRQYEEK
IRSYGKIHLFMGGVGNDGHIAFNEPASSLASRTRIKTLTHDTRVANSRFFDNDVNQVPKYAL
TVGVGTLLDAEEVMILVLGSQKALALQAAVEGCVNHMWTISCLQLHPKAIMVCDEPSTMELK
VKTLRYFNELEAENIKGL
>d1d9ta_ c.35.1.1 (A:) Glucosamine 6-phosphate deaminase/isomerase {Human (Homo sapiens)}
MKLIILEHYSQASEWAAKYIRNRIIQFNPGPEKYFTLGLPTGSTPLGCYKKLIEYYKNGDLS
FKYVKTFNMDEYVGLPRDHPESYHSFMWNNFFKHIDIHPENTHILDGNAVDLQAECDAFEEK
IKAAGGIELFVGGIGPDGHIAFNEPGSSLVSRTRVKTLAMDTILANARFFDGELTKVPTMAL
TVGVGTVMDAREVMILITGAHKAFALYKAIEEGVNHMWTVSAFQQHPRTVFVCDEDATLELK
VKTVKYFKGLMLVHNKLVDPLYSIKEKETEKSQ
>d1ig3a2 c.100.1.1 (A:10-178) Thiamin pyrophosphokinase, catalytic domain {Mouse (Mus musculus)}
HSSGLVPRGSHMEHAFTPLEPLLPTGNLKYCLVVLNQPLDARFRHLWKKALLRACADGGANH
LYDLTEGERESFLPEFVSGDFDSIRPEVKEYYTKKGCDLISTPDQDHTDFTKCLQVLQRKIE
EKELQVDVIVTLGGLGGRFDQIMASVNTLFQATHITPVPIIIIQK

>d1ig0a2 c.100.1.1 (A:3-223) Thiamin pyrophosphokinase, catalytic domain {Baker's yeast (Saccharomyces cerevisiae)}
EECIENPERIKIGTDLINIRNKMNLKELIHPNEDENSTLLILNQKIDIPRPLFYKIWKLHDL
KVCADGAANRLYDYLDDDETLRIKYLPNYIIGDLDSLSEKVYKYYRKNKVTIIKQTTQYSTD
FTKCVNLISLHFNSPEFRSLISNKDNLQSNHGIELEKGIHTLYNTMTESLVFSKVTPISLLA
LGGIGGRFDQTVHSITQLYTLSENASYFKLCYMTP
>d1f75a_ c.101.1.1 (A:) Undecaprenyl diphosphate synthase {Micrococcus luteus}
NINAAQIPKHIAIIMDGNGRWAKQKKMPRIKGHYEGMQTVRKITRYASDLGVKYLTLYAFST
ENWSRPKDEVNYLMKLPGDFLNTFLPELIEKNVKVETIGFIDDLPDHTKKAVLEAKEKTKHN
TGLTLVFALNYGGRKEIISAVQLIAERYKSGEISLDEISETHFNEYLFTANMPDPELLIRTS
GEERLSNFLIWQCSYSEFVFIDEFWPDFNEESLAQCISIYQNR
>d1jp3a_ c.101.1.1 (A:) Undecaprenyl diphosphate synthase {Escherichia coli}
LPAHGCRHVAIIMDGNGRWAKKQGKIRAFGHKAGAKSVRRAVSFAANNGIEALTLYAFSSEN
WNRPAQEVSALMELFVWALDSEVKSLHRHNVRLRIIGDTSRFNSRLQERIRKSEALTAGNTG
LTLNIAANYGGRWDIVQGVRQLAEKVQQGNLQPDQIDEEMLNQHVCMHELAPVDLVIRTGGE
HRISNFLLWQIAYAELYFTDVLWPDFDEQDFEGALNAFANRE
>d1pvda2 c.36.1.1 (A:2-181) Pyruvate decarboxylase {Baker's yeast (Saccharomyces cerevisiae)}
SEITLGKYLFERLKQVNVNTVFGLPGDFNLSLLDKIYEVEGMRWAGNANELNAAYAADGYAR
IKGMSCIITTFGVGELSALNGIAGSYAEHVGVLHVVGVPSISHHTLGNGDFTVFHRMSANIS
ETTAMITDIATAPAEIDRCIRTTYVTQRPVYLGLPANLVDLNVPAKLL
>d1pvda3 c.36.1.1 (A:361-556) Pyruvate decarboxylase {Baker's yeast (Saccharomyces cerevisiae)}
ASTPLKQEWMWNQLGNFLQEGDVVIAETGTSAFGINQTTFPNNTYGISQVLWGSIGFTTGAT
LGAAFAAEEIDPKKRVILFIGDGSLQLTVQEISTMIRWGLKPYLFVLNNDGYTIEKLIHGPK
AQYNEIQGWDHLSLLPTFGAKDYETHRVATTGEWDKLTQDKSFNDNSKIRMIEIMLPVFDAP
QNLVKQAKLT
>d1zpda2 c.36.1.1 (A:2-187) Pyruvate decarboxylase {Zymomonas mobilis}
SYTVGTYLAERLVQIGLKHHFAVAGDYNLVLLDNLLLNKNMEQVYCCNELNCGFSAEGYARA
KGAAAAVVTYSVGALSAFDAIGGAYAENLPVILISGAPNNNDHAAGHVLHHALGKTDYHYQL
EMAKNITAAAEAIYTPEEAPAKIDHVIKTALREKKPVYLEIACNIASMPCAAPGPASALFND
>d1zpda3 c.36.1.1 (A:363-566) Pyruvate decarboxylase {Zymomonas mobilis}
APLVNAEIARQVEALLTPNTTVIAETGDSWFNAQRMKLPNGARVEYEMQWGHIGWSVPAAFG
YAVGAPERRNILMVGDGSFQLTAQEVAQMVRLKLPVIIFLINNYGYTIEVMIHDGPYNNIKN
WDYAGLMEVFNGNGGYDSGAAKGLKAKTGGELAEAIKVALANTDGPTLIECFIGREDCTEEL
VKWGKRVAAANSRKPVNK
>d1poxa2 c.36.1.1 (A:9-182) Pyruvate oxidase {Lactobacillus plantarum}
TNILAGAAVIKVLEAWGVDHLYGIPGGSINSIMDALSAERDRIHYIQVRHEEVGAMAAAADA
KLTGKIGVCFGSAGPGGTHLMNGLYDAREDHVPVLALIGQFGTTGMNMDTFQEMNENPIYAD

VADYNVTAVNAATLPHVIDEAIRRAYAHQGVAVVQIPVDLPWQQISAEDW
>d1poxa3 c.36.1.1 (A:366-593) Pyruvate oxidase {Lactobacillus plantarum}
KQEGPLQAYQVLRAVNKIAEPDAIYSIDVGDINLNANRHLKLTPSNRHITSNLFATMGVGIP
GAIAAKLNYPERQVFNLAGDGGASMTMQDLVTQVQYHLPVINVVFTNCQYGFIKDEQEDTNQ
NDFIGVEFNDIDFSKIADGVHMQAFRVNKIEQLPDVFEQAKAIAQHEPVLIDAVITGDRPLP
AEKLRLDSAMSSAADIEAFKQRYEAQDLQPLSTYLKQFGLDD
>d1bfd_2 c.36.1.1 (2-181) Benzoylformate decarboxylase {Pseudomonas putida}
ASVHGTTYELLRRQGIDTVFGNPGSNELPFLKDFPEDFRYILALQEACVVGIADGYAQASRK
PAFINLHSAAGTGNAMGALSNAWNSHSPLIVTAGQQTRAMIGVEALLTNVDAANLPRPLVKW
SYEPASAAEVPHAMSRAIHMASMAPQGPVYLSVPYDDWDKDADPQSHHLFDRHVSS
>d1bfd_3 c.36.1.1 (342-524) Benzoylformate decarboxylase {Pseudomonas putida}
EPAKVDQDAGRLHPETVFDTLNDMAPENAIYLNESTSTTAQMWQRLNMRNPGSYYFCAAGGL
GFALPAAIGVQLAEPERQVIAVIGDGSANYSISALWTAAQYNIPTIFVIMNNGTYGALRWFA
GVLEAENVPGLDVPGIDFRALAKGYGVQALKADNLEQLKGSLQEALSAKGPVLIEVSTV
>d1jsca2 c.36.1.1 (A:83-270) Acetohydroxyacid synthase catalytic subunit {Baker's yeast (Saccharomyces cerevisiae)}
PDMDTSFVGLTGGQIFNEMMSRQNVDTVFGYPGGAILPVYDAIHNSDKFNFVLPKHEQGAGH
MAEGYARASGKPGVVLVTSGPGATNVVTPMADAFADGIPMVVFTGQVPTSAIGTDAFQEADV
VGISRSCTKWNVMVKSVEELPLRINEAFEIATSGRPGPVLVDLPKDVTAAILRNPIPTKTTL
PS
>d1jsca3 c.36.1.1 (A:461-648) Acetohydroxyacid synthase catalytic subunit {Baker's yeast (Saccharomyces cerevisiae)}
AYMEETPGSKIKPQTVIKKLSKVANDTGRHVIVTTGVGQHQMWAAQHWTWRNPHTFITSGGL
GTMGYGLPAAIGAQVAKPESLVIDIDGDASFNMTLTELSSAVQAGTPVKILILNNEEQGMVT
QWQSLFYEHRYSHTHQLNPDFIKLAEAMGLKGLRVKKQEELDAKLKEFVSTKGPVLLEVEVD
KK
>d1gpua1 c.36.1.2 (A:3-337) Transketolase, TK {Baker's yeast (Saccharomyces cerevisiae)}
QFTDIDKLAVSTIRILAVDTVSKANSGHPGAPLGMAPAAHVLWSQMRMNPTNPDWINRDRFV
LSNGHAVALLYSMLHLTGYDLSIEDLKQFRQLGSRTPGHPEFELPGVEVTTGPLGQGISNAV
GMAMAQANLAATYNKPGFTLSDNYTYVFLGDGCLQEGISSEASSLAGHLKLGNLIAIYDDNK
ITIDGATSISFDEDVAKRYEAYGWEVLYVENGNEDLAGIAKAIAQAKLSKDKPTLIKMTTTI
GYGSLHAGSHSVHGAPLKADDVKQLKSKFGFNPDKSFVVPQEVYDHYQKTILKPGVEANNKW
NKLFSEYQKKFPELGAELARRLSGQ
>d1gpua2 c.36.1.2 (A:338-534) Transketolase, TK {Baker's yeast (Saccharomyces cerevisiae)}
LPANWESKLPTYTAKDSAVATRKLSETVLEDVYNQLPELIGGSADLTPSNLTRWKEALDFQP
PSSGSGNYSGRYIRYGIREHAMGAIMNGISAFGANYKPYGGTFLNFVSYAAGAVRLSALSGH
PVIWVATHDSIGVGEDGPTHQPIETLAHFRSLPNIQVWRPADGNEVSAAYKNSLESKHTPSI
IALSRQNLPQL
>d1dtwa1 c.36.1.3 (A:) Branched-chain alpha-keto acid

dehydrogenase {Human (Homo sapiens)}
PQFPGASAEFIDKLEFIQPNVISGIPIYRVMDRQGQIINPSEDPHLPKEKVLKLYKSMTLLN
TMDRILYESQRQGRISFYMTNYGEEGTHVGSAAALDNTDLVFGQYREAGVLMYRDYPLELFM
AQCYGNISDLGKGRQMPVHYGCKERHFVTISSPLATQIPQAVGAAYAAKRANANRVVICYFG
EGAASEGDAHAGFNFAATLECPIIFFCRNNGYAISTPTSEQYRGDGIAARGPGYGIMSIRVD
GNDVFAVYNATKEARRRAVAENQPFLIEAMTYRIGHHSTSDDSSAYRSVDEVNYWDKQDHPI
SRLRHYLLSQGWWDEEQEKAWRKQSRRKVMEAFEQAERKPKPNPNLLFSDVYQEMPAQLRKQ
QESLARHLQTYGEHYPLDHFDK
>d1dtwb1 c.36.1.3 (B:17-204) Branched-chain alpha-keto acid
dehydrogenase {Human (Homo sapiens)}
QTQKMNLFQSVTSALDNSLAKDPTAVIFGEDVAFGGVFRCTVGLRDKYGKDRVFNTPLCEQG
IVGFGIGIAVTGATAIAEIQFADYIFPAFDQIVNEAAKYRYRSGDLFNCGSLTIRSPWGCVG
HGALYHSQSPEAFFAHCPGIKVVIPRSPFQAKGLLLSCIEDKNPCIFFEPKILYRAAAEEVP
IE
>d1qs0a1 c.36.1.3 (A:) 2-oxoisovalerate dehydrogenase, E1B
{Pseudomonas putida}
NEYAPLRLHVPEPTGRPGCQTDFSYLRLNDAGQARKPPVDVDAADTADLSYSLVRVLDEQGD
AQGPWAEDIDPQILRQGMRAMLKTRIFDSRMVVAQRQKKMSFYMQSLGEEAIGSGQALALNR
TDMCFPTYRQQSILMARDVSLVEMICQLLSNERDPLKGRQLPIMYSVREAGFFTISGNLATQ
FVQAVGWAMASAIKGDTKIASAWIGDGATAESDFHTALTFAHVYRAPVILNVVNNQWAISTF
QAIAGGESTTFAGRGVGCGIASLRVDGNDFVAVYAASRWAAERARRGLGPSLIEWVTYRAGP
HSTSDDPSKYRPADDWSHFPLGDPIARLKQHLIKIGHWSEEEHQATTAEFEAAVIAAQKEAE
QYGTLANGHIPSAASMFEDVYKEMPDHLRRQRQEL
>d1qs0b1 c.36.1.3 (B:2-205) 2-oxoisovalerate dehydrogenase, E1B
{Pseudomonas putida}
ATTTMTMIQALRSAMDVMLERDDNVVVYGQDVGYFGGVFRCTEGLQTKYGKSRVFDAPISES
GIVGTAVGMGAYGLRPVVEIQFADYFYPASDQIVSEMARLRYRSAGEFIAPLTLRMPCGGGI
YGGQTHSQSPEAMFTQVCGLRTVMPSNPYDAKGLLIASIECDDPVIFLEPKRLYNGPFDGHH
DRPVTPWSKHPHSAVPDG
>d1ik6a1 c.36.1.3 (A:1-191) E1-beta subunit of pyruvate
dehydrogenase {Archaeon Pyrobaculum aerophilum}
VAGVVMMANMAKAINMALHEEMERDERVVVLGEDVGKKGGVFLVTEGLYERFGPERVIDTPL
NEGGILGFAMGMAMAGLKPVAEIQFVDFIWLGADELLNHIAKLRYRSGGNYKAPLVVRTPVG
SGTRGGLYHSNSPEAIFVHTPGLVVVMPSTPYNAKGLLKAAIRGDDPVVFLEPKILYRAPRE
EVPEG
>d1keka1 c.36.1.4 (A:2-258) Pyruvate-ferredoxin oxidoreductase,
PFOR, domains I and VI {Desulfovibrio africanus}
GKKMMTTDGNTATAHVAYAMSEVAAIYPITPSSTMGEEADDWAAQGRKNIFGQTLTIREMQS
EAGAAGAVHGALAAGALTTTFTASQGLLLMIPNMYKISGELLPGVFHVTARAIAAHALSIFG
DHQDIYAARQTGFAMLASSSVQEAHDMALVAHLAAIESNVPFMHFFDGFRTSHEIQKIEVLD
YADMASLVNQKALAEFRAKSMNPEHPHVRGTAQNPDIYFQGREAANPYYLKVPGIVAEYMQK
VASLTGRSY
>d1keka2 c.36.1.4 (A:786-1232) Pyruvate-ferredoxin oxidoreductase,
PFOR, domains I and VI {Desulfovibrio africanus}

VKSEVLPRDSLKGSQFQEPLMEFSGACSGCGETPYVRVITQLFGERMFIANATGCSSIWGAS
APSMPYKTNRLGQGPAWGNSLFEDAAEYGFGMNMSMFARRTHLADLAAKALESDASGDVKEA
LQGWLAGKNDPIKSKEYGDKLKKLLAGQKDGLLGQIAAMSDLYTKKSVWIFGGDGWAYDIGY
GGLDHVLASGEDVNVFVMDTEVYSNTGGQSSKATPTGAVAKFAAAGKRTGKKDLARMVMTYG
YVYVATVSMGYSKQQFLKVLKEAESFPGPSLVIAYATCINQGLRKGMGKSQDVMNTAVKSGY
WPLFRYDPRLAAQGKNPFQLDSKAPDGSVEEFLMAQNRFAVLDRSFPEDAKRLRAQVAHELD
VRFKELEHMAATNIFESFAPAGGKADGSVDFGEGAEFCTRDDTPMMARPDSGEACDQNRAGT
SEQQGDLSKRTKK

>d1gky__ c.37.1.1 (-) Guanylate kinase {Baker's yeast (Saccharomyces cerevisiae)}
SRPIVISGPSGTGKSTLLKKLFAEYPDSFGFSVSSTTRTPRAGEVNGKDYNFVSVDEFKSMI
KNNEFIEWAQFSGNYYGSTVASVKQVSKSGKTCILDIDMQGVKSVKAIPELNARFLFIAPPS
VEDLKKRLEGRGTETEESINKRLSAAQAELAYAETGAHDKVIVNDDLDKAYKELKDFIFAEK

>d1kgda_ c.37.1.1 (A:) Guanylate kinase-like domain of Cask {Human (Homo sapiens)}
HMRKTLVLLGAHGVGRRHIKNTLITKHPDRFAYPIPHTTRPPKKDEENGKNYYFVSHDQMMQ
DISNNEYLEYGSHEDAMYGTKLETIRKIHEQGLIAILDVEPQALKVLRTAEFAPFVVFIAAP
TITPGLNEDESLQRLQKESDILQRTYAHYFDLTIINNEIDETIRHLEEAVELVC

>d1kjwa2 c.37.1.1 (A:526-724) Guanylate kinase-like domain of Psd-95 {Rat (Rattus norvegicus)}
VTQMEVHYARPIIILGPTKDRANDDLLSEFPDKFGSCVPHTTRPKREYEIDGRDYHFVSSRE
KMEKDIQAHKFIEAGQYNSHLYGTSVQSVREVAEQGKHCILDVSANAVRRLQAAHLHPIAIF
IRPRSLENVLEINKRITEEQARKAFDRATKLEQEFTECFSAIVEGDSFEEIYHKVKRVIEDL
SGPYIWVPARERL

>d1ukz__ c.37.1.1 (-) Uridylate kinase {Baker's yeast (Saccharomyces cerevisiae)}
PAFSPDQVSVIFVLGGPGAGKGTQCEKLVKDYSFVHLSAGDLLRAEQGRAGSQYGELIKNCI
KEGQIVPQEITLALLRNAISDNVKANKHKFLIDGFPRKMDQAISFERDIVESKFILFFDCPE
DIMLERLLERGKTSGRSDDNIESIKKRFNTFKETSMPVIEYFETKSKVVRVRCDRSVEDVYK
DVQDAIRDSL

>d1deka_ c.37.1.1 (A:) Deoxynucleoside monophosphate kinase {Bacteriophage T4}
MKLIFLSGVKRSGKDTTADFIMSNYSAVKYQLAGPIKDALAYAWGVFAANTDYPCLTRKEFE
GIDYDRETNLNLTKLEVITIMEQAFCYLNGKSPIKGVFVFDDEGKESVNFVAFNKITDVINN
IEDQWSVRRLMQALGTDLIVNNFDRMYWVKLFALDYLDKFNSGYDYYIVPDTRQDHEMDAAR
AMGATVIHVVRPGQKSNDTHITEAGLPIRDGDLVITNDGSLEELFSKIKNTLKVL

>d1j90a_ c.37.1.1 (A:) Deoxyribonucleoside kinase {Fruit fly (Drosophila melanogaster)}
TQPFTVLIEGNIGSGKTTYLNHFEKYKNDICLLTEPVEKWRNVNGVNLLELMYKDPKKWAMP
FQSYVTLTMLQSHTAPTNKKLKIMERSIFSARYCFVENMRRNGSLEQGMYNTLEEWYKFIEE
SIHVQADLIIYLRTSPEVAYERIRQRARSEESCVPLKYLQELHELHEDWLIHQRRPQSCKVL
VLDADLNLE

>d1jaga_ c.37.1.1 (A:) Deoxyguanosine kinase {Human (Homo sapiens)}

GPRRLSIEGNIAVGKSTFVKLLTKTYPEWHVATEPVATWQNIQAAGNQKACTAQSLGNLLDM
MYREPARWSYTFQTFSFLSRLKVQLEPFPEKLLQARKPVQIFERSVYSDRYIFAKNLFENGS
LSDIEWHIYQDWHSFLLWEFASRITLHGFIYLQASPQVCLKRLYQRAREEEKGIELAYLEQL
HGQHEAWLIHKTTKLHFEALMNIPVLVLDVNDDFSEEVTKQEDLMREVNTFVKNL
>d1ckea_ c.37.1.1 (A:) CMP kinase {Escherichia coli}
AIAPVITIDGPSGAGKGTLCKAMAEALQWHLLDSGAIYRVLALAALHHHVDVASEDALVPLA
SHLDVRFVSTNGNLEVILEGEDVSGEIRTQEVANAASQVAAFPRVREALLRRQRAFRELPGL
IADGRDMGTVVFPDAPVKIFLDASSEERAHRRMLQLQVKGFSVNFERLLAEIKERDDRDRNR
AVAPLVPAADALVLDSTTLSIEQVIEKALQYARQKLALA
>d1qf9a_ c.37.1.1 (A:) UMP/CMP kinase {Dictyostelium discoideum}
MEKSKPNVVFVLGGPGSGKGTQCANIVRDFGWVHLSAGDLLRQEQQSGSKDGEMIATMIKNG
EIVPSIVTVKLLKNAIDANQGKNFLVDGFPRNEENNNSWEENMKDFVDTKFVLFFDCPEEVM
TQRLLKRGESSGRSDDNIESIKKRFNTFNVQTKLVIDHYNKFDKVKIIPANRDVNEVYNDVE
NLFKSMGF
>d1e2ka_ c.37.1.1 (A:) Thymidine kinase {Herpes simplex virus type 1, different strains}
MPTLLRVYIDGPHGMGKTTTTQLLVALGSRDDIVYVPEPMTYWRVLGASETIANIYTTQHRL
DQGEISAGDAAVVMTSAQITMGMPYAVTDAVLAPHIGGEAGSSHAPPPALTLIFDRHPIAAL
LCYPAARYLMGSMTPQAVLAFVALIPPTLPGTNIVLGALPEDRHIDRLAKRQRPGERLDLAM
LAAIRRVYGLLANTVRYLQCGGSWREDWGQLSGTAVPPQGAEPQSNAGPRPHIGDTLFTLFR
APELLAPNGDLYNVFAWALDVLAKRLRSMHVFILDYDQSPAGCRDALLQLTSGMVQTHVTTP
GSIPTICDLARTFAREMGE
>d3adk__ c.37.1.1 (-) Adenylate kinase {Pig (Sus scrofa)}
MEEKLKKSKIIFVVGGPGSGKGTQCEKIVQKYGYTHLSTGDLLRAEVSSGSARGKMLSEIME
KGQLVPLETVLDMLRDAMVAKVDTSKGFLIDGYPREVKQGEEFERKIGQPTLLLYVDAGPET
MTKRLLKRGETSGRVDDNEETIKKRLETYYKATEPVIAFYEKRGIVRKVNAEGSVDDVFSQV
CTHLDTLK
>d1nksa_ c.37.1.1 (A:) Adenylate kinase {Archaeon Sulfolobus acidocaldarius}
MKIGIVTGIPGVGKSTVLAKVKEILDNQGINNKIINYGDFMLATALKLGYAKDRDEMRKLSV
EKQKKLQIDAAKGIAEEARAGGEGYLFIDTHAVIRTPSGYLPGLPSYVITEINPSVIFLLEA
DPKIILSRQKRDTTRNRNDYSDESVILETINFARYAATASAVLAGSTVKVIVNVEGDPSIAA
NEIIRSMK
>d2ak3a1 c.37.1.1 (A:0-124,A:162-225) Adenylate kinase {Cow (Bos taurus), mitochondrial izozyme-3}
GASARLLRAAIMGAPGSGKGTVSSRITKHFELKHLSSGDLLRDNMLRGTEIGVLAKTFIDQG
KLIPDDVMTRLVLHELKNLTQYNWLLDGFPRTLPQAEALDRAYQIDTVINLNVPFEVIKQRL
TXDRPETVVKRLKAYEAQTEPVLEYYRKKGVLETFSGTETNKIWPHVYAFLQTKLPQRSQET
SVTP
>d1ak2_1 c.37.1.1 (14-146,177-233) Adenylate kinase {Cow (Bos taurus), mitochondrial izozyme-2}
PKGVRAVLLGPPGAGKGTQAPKLAKNFCVCHLATGDMLRAMVASGSELGKKLKATMDAGKLV
SDEMVLELIEKNLETPPCKNGFLLDGFPRTVRQAEMLDDLMEKRKEKLDSVIEFSIPDSLLI
RRITGRLIHXSDDNKKALKIRLEAYHTQTTPLVEYYSKRGIHSAIDASQTPDVVFASILAAF

SKATS
>d1aky_1 c.37.1.1 (3-130,169-220) Adenylate kinase {Baker's yeast (Saccharomyces cerevisiae)}
ESIRMVLIGPPGAGKGTQAPNLQERFHAAHLATGDMLRSQIAKGTQLGLEAKKIMDQGGLVS
DDIMVNMIKDELTNNPACKNGFILDGFPRTIPQAEKLDQMLKEQGTPLEKAIELKVDDELLV
ARITXNADALKKRLAAYHAQTEPIVDFYKKTGIWAGVDASQPPATVWADILNKLGKN
>d1e4ya1 c.37.1.1 (A:1-121,A:157-214) Adenylate kinase {Escherichia coli}
MRIILLGALVAGKGTQAQFIMEKYGIPQISTGDMLRAAVKSGSELGKQAKDIMDAGKLVTDE
LVIALVKERIAQEDCRNGFLLDGFPRTIPQADAMKEAGINVDYVLEFDVPDELIVDRIVXKD
DQEETVRKRLVEYHQMTAPLIGYYSKEAEAGNTKYAKVDGTKPVAEVRADLEKILG
>d1zaka1 c.37.1.1 (A:3-127,A:159-222) Adenylate kinase {Maize (Zea mays)}
ADPLKVMISGAPASGKGTQCELIKTKYQLAHISAGDLLRAEIAAGSENGKRAKEFMEKGQLV
PDEIVVNMVKERLRQPDAQENGWLLDGYPRSYSQAMALETLEIRPDTFILLDVPDELLVERV
VXFDDTEEKVKLRLETYYQNIESLLSTYENIIVKVQGDATVDAVFAKIDELLGSILEKKNEM
VSST
>d1zin_1 c.37.1.1 (1-125,161-217) Adenylate kinase {Bacillus stearothermophilus}
MNLVLMGLPGAGKGTQAEKIVAAYGIPHISTGDMFRAAMKEGTPLGLQAKQYMDRGDLVPDE
VTIGIVRERLSKDDCQNGFLLDGFPRTVAQAEALETMLADIGRKLDYVIHIDVRQDVLMERL
TXADDNEATVANRLEVNMKQMKPLVDFYEQKGYLRNINGEQDMEKVFADIRELLGGLAR
>d1tmka_ c.37.1.1 (A:) Thymidylate kinase {Baker's yeast (Saccharomyces cerevisiae)}
GRGKLILIEGLDRTGKTTQCNILYKKLQPNCKLLKFPERSTRIGGLINEYLTDDSFQLSDQA
IHLLFSANRWEIVDKIKKDLLEGKNIVMDRYVYSGVAYSAAKGTNGMDLDWCLQPDVGLLKP
DLTLFLSTQDVDNNAEKSGFGDERYETVKFQEKVKQTFMKLLDKEIRKGDESITIVDVTNKG
IQEVEALIWQIVEPVLSTHIDHDKFSFF
>d1e9ea_ c.37.1.1 (A:) Thymidylate kinase {Human (Homo sapiens)}
ARRGALIVLEGVDRAGKSTQSRKLVEALCAAGHRAELLRFPERSTEIGKLLSSYLQKKSDVE
DHSVHLLFSANRWEQVPLIKEKLSQGVTLVVDRYAFSGVAYTGAKENFSLDWCKQPDVGLPK
PDLVLFLQLQLADAAKRGAFGHERYENGAFQERALRCFHQLMKDTTLNWKMVDASKSIEAVH
EDIRVLSEDAIATATEKPLKELWK
>d4tmka_ c.37.1.1 (A:) Thymidylate kinase {Escherichia coli}
RSKYIVIEGLEGAGKTTARNVVVETLEQLGIRDMVFTREPGGTQLAEKLRSLLLDIKSVGDE
VITDKAEVLMFYAARVQLVETVIKPALANGTWVIGDRHDLSTQAYQGGGRGIDQHMLATLRD
AVLGDFRPDLTLYLDVTPEVGLKRARARGELDRIEQESFDFFNRTRARYLELAAQDKSIHTI
DATQPLEAVMDAIRTTVTHWVKEL
>d1g3ua_ c.37.1.1 (A:) Thymidylate kinase {Mycobacterium tuberculosis}
MLIAIEGVDGAGKRTLVEKLSGAFRAAGRSVATLAFPRYGQSVAADIAAEALHGEHGDLASS
VYAMATLFALDRAGAVHTIQGLCRGYDVVILDRYVASNAAYSAARLHENAAGKAAAWVQRIE
FARLGLPKPDWQVLLAVSAELAGERSRGRAQRDPGRARDNYERDAELQQRTGAVYAELAAQG
WGGRWLVVGADVDPGRLAATLA

>d1e6ca_ c.37.1.2 (A:) Shikimate kinase {Erwinia chrysanthemi}
MTEPIFMVGARGCGMTTVGRELARALGYEFVDTDIFMQHTSGMTVADVVAAEGWPGFRRRES
EALQAVATPNRVVATGGGMVLLEQNRQFMRAHGTVVYLFAPAEELALRLQASLQAHQRPTLT
GRPIAEEMEAVLREREALYQDVAHYVVDATQPPAAIVCELMQTMRL
>d1qhxa_ c.37.1.3 (A:) Chloramphenicol phosphotransferase
{Streptomyces venezuelae}
MTTRMIILNGGSSAGKSGIVRCLQSVLPEPWLAFGVDSLIEAMPLKMQSAEGGIEFDADGGV
SIGPEFRALEGAWAEGVVAMARAGARIIIDDVFLGGAAAQERWRSFVGDLDVLWVGVRCDGA
VAEGRETARGDRVAGMAAKQAYVVHEGVEYDVEVDTTHKESIECAWAIAAHVVP
>d1d6ja_ c.37.1.4 (A:) Adenosine-5'phosphosulfate kinase (APS
kinase) {Penicillium chrysogenum}
HASALTRSERTELRNQRGLTIWLTGLSASGKSTLAVELEHQLVRDRRVHAYRLDGDNIRFGL
NKDLGFSEADRNENIRRIAEVAKLFADSNSIAITSFISPYRKDRDTARQLHEVATPGEETGL
PFVEVYVDVPVEVAEQRDPKGLYKKAREGVIKEFTGISAPYEAPANPEVHVKNYELPVQDAV
KQIIDYLDTKGYLPAKK
>d1g8fa3 c.37.1.15 (A:390-511) ATP sulfurylase C-terminal domain
{Baker's yeast (Saccharomyces cerevisiae)}
PRPKQGFSIVLGNSLTVSREQLSIALLSTFLQFGGGRYYKIFEHNNKTELLSLIQDFIGSGS
GLIIPDQWEDDKDSVVGKQNVYLLDTSSSADIQLESADEPISHIVQKVVLFLEDNGFFVF
>d1i2da3 c.37.1.15 (A:391-573) ATP sulfurylase C-terminal domain
{Fungus (Penicillium chrysogenum)}
PRATQGFTIFLTGYMNSGKDAIARALQVTLNQQGGRSVSLLLGDTVRHELSSELGFTREDRH
TNIQRIAFVATELTRAGAAVIAAPIAPYEESRKFARDAVSQAGSFFLVHVATPLEHCEQSDK
RGIYAAARRGEIKGFTGVDDPYETPEKADLVVDFSKQSVRSIVHEIILVLESQGFLERQ
>d1aqua_ c.37.1.5 (A:) Estrogen sulfotransferase {Mouse (Mus
musculus)}
EYYEVFGEFRGVLMDKRFTKYWEDVEMFLARPDDLVIATYPKSGTTWISEVVYMIYKEGDVE
KCKEDAIFNRIPYLECRNEDLINGIKQLKEKESPRIVKTHLPPKLLPASFWEKNCKMIYLCR
NAKDVAVSYYYFLLMITSYPNPKSFSEFVEKFMQGQVPYGSWYDHVKAWWEKSKNSRVLFMF
YEDMKEDIRREVVKLIEFLERKPSAELVDRIIQHTSFQEMKNNPSTNYTMMPEEMMNQKVSP
FMRKGIIGDWKNHFPEALRERFDEHYKQQMKDCTVKFRME
>d1efha_ c.37.1.5 (A:) Hydroxysteroid sulfotransferase {Human
(Homo sapiens)}
DFLWFEGIAFPTMGFRSETLRKVRDEFVIRDEDVIILTYPKSGTNWLAEILCLMHSKGDAKW
IQSVPIWERSPWVESEIGYTALSETESPRLFSSHLPIQLFPKSFFSSKAKVIYLMRNPRDVL
VSGYFFWKNMKFIKKPKSWEEYFEWFCQGTVLYGSWFDHIHGWMPMREEKNFLLLSYEELKQ
DTGRTIEKICQFLGKTLEPEELNLILKNSSFQSMKENKMSNYSLLSVDYVVDKAQLLRKGVS
GDWKNHFTVAQAEDFDKLFQEKMADLPRKLAAALE
>d1cjma_ c.37.1.5 (A:) Aryl sulfotransferase sult1a3 {Human (Homo
sapiens)}
SRPPLEYVKGVPLIKYFAEALGPLQSFQARPDDLLINTYPKSGTTWVSQILDMIYQGGDLEK
CNRAPIYVRVPFLEVNDPGEPSGLETLKDTPPPRLIKSHLPLALLPQTLLDQKVKVVYVARN
PKDVAVSYYHFHRMEKAHPEPGTWDSFLEKFMAGEVSYGSWYQHVQEWWELSRTHPVLYLFY
EDMKENPKREIQKILEFVGRSLPEETMDFMVQHTSFKEMKKNPMTNYTTVPQELMDHSISPF

MRKGMAGDWKTTFTVAQNERFDADYAEKMAGCSLSFRS
>d1nsta_ c.37.1.5 (A:) Heparan sulfate
N-deacetylase/N-sulfotransferase domain {Human (Homo sapiens)}
DPLWQDPCEDKRHKDIWSKEKTCDRFPKLLIIGPQKTGTTALYLFLGMHPDLSSNYPSSETF
EEIQFFNGHNYHKGIDWYMEFFPIPSNTTSDFYFEKSANYFDSEVAPRRAAALLPKAKVLTI
LINPADRAYSWYQHQRAHDDPVALKYTFHEVITAGSDASSKLRALQNRCLVPGWYATHIERW
LSAYHANQILVLDGKLLRTEPAKVMDMVQKFLGVTNTIDYHKTLAFDPKKGFWCQLLEGGKT
KCLGKSKGRKYPEMDLDSRAFLKDYYRDHNIELSKLLYKMGQTLPTWLREDLQ
>d1fmja_ c.37.1.5 (A:) Retinol dehydratase {Fall armyworm
(Spodoptera frugiperda)}
PFPYEFRELNPEEDKLVKANLGAFPTTYVKLGPKGYMVYRPYLKDAANIYNMPLRPTDVFVA
SYQRSGTTMTQELVWLIENDLNFEAAKTYMSLRYIYLDGFMIYDPEKQEEYNDILPNPENLD
MERYLGLLEYSSRPGSSLLAAVPPTEKRFVKTHLPLSLMPPNMLDTVKMVYLARDPRDVAVS
SFHHARLLYLLNKQSNFKDFWEMFHRGLYTLTPYFEHVKEAWAKRHDPNMLFLFYEDYLKDL
PGCIARIADFLGKKLSEEQIQRLCEHLNFEKFKNNGAVNMEDYREIGILADGEHFIRKGKAG
CWRDYFDEEMTKQAEKWIKDNLKDTDLRYPNM
>d1a7j__ c.37.1.6 (-) Phosphoribulokinase {Rhodobacter
sphaeroides}
SKKHPIISVTGSSGAGTSTVKHTFDQIFRREGVKAVSIEGDAFHRFNRADMKAELDRRYAAG
DATFSHFSYEANELKELERVFREYGETGQGRTRTYVHDDAEAARTGVAPGNFTDWRDFDSDS
HLLFYEGLHGAVVNSEVNIAGLADLKIGVVPVINLEWIQKIHRDRATRGYTTEAVTDVILRR
MHAYVHCIVPQFSQTDINFQRVPVVDTSNPFIARWIPTADESVVVIRFRNPRGIDFPYLTSM
IHGSWMSRANSIVVPGNKLDLAMQLILTPLIDRVVRESKV
>d1esma_ c.37.1.6 (A:) Pantothenate kinase PanK {Escherichia coli}
QTLMTPYLQFDRNQWAALRDSVPMTLSEDEIARLKGINEDLSLEEVAEIYLPLSRLLNFYIS
SNLRRQAVLEQFLGTNGQRIPYIISIAGSVAVGKSTTARVLQALLSRWPEHRRVELITTDGF
LHPNQVLKERGLMKKKGFPESYDMHRLVKFVSDLKSGVPNVTAPVYSHLIYDVIPDGDKTVV
QPDILILEGLNVLQSGMDYPHDPHHVFVSDFVDFSIYVDAPEDLLQTWYINRFLKFREGAFT
DPDSYFHNYAKLTKEEAIKTAMTLWKEINWLNLKQNILPTRERASLILTKSANHAVEEVRLR
K
>d1bif_1 c.37.1.7 (37-249)
6-phosphofructo-2-kinase/fructose-2,6-bisphosphatase, kinase
domain {Rat (Rattus norvegicus)}
CPTLIVMVGLPARGKTYISKKLTRYLNFIGVPTREFNVGQYRRDMVKTYKSFEFFLPDNEEG
LKIRKQCALAALNDVRKFLSEEGGHVAVFDATNTTRERRAMIFNFGEQNGYKTFFVESICVD
PEVIAANIVQVKLGSPDYVNRDSDEATEDFMRRIECYENSYESLDEEQDRDLSYIKIMDVGQ
SYVVNRVADHIQSRIVYYLMNIHVTPR
>d1ctqa_ c.37.1.8 (A:) cH-p21 Ras protein {Human (Homo sapiens)}
MTEYKLVVVGAGGVGKSALTIQLIQNHFVDEYDPTIEDSYRKQVVIDGETCLLDILDTAGQE
EYSAMRDQYMRTGEGFLCVFAINNTKSFEDIHQYREQIKRVKDSDDVPMVLVGNKCDLAART
VESRQAQDLARSYGIPYIETSAKTRQGVEDAFYTLVREIRQH
>d1ds6a_ c.37.1.8 (A:) Rac {Human (Homo sapiens)}
MQAIKCVVVGDGAVGKTCLLISYTTNAFPGEYIPTVFDNYSANVMVDSKPVNLGLWDTAGQE
DYDRLRPLSYPQTDVFLICFSLVSPASYENVRAKWFPEVRHHCPSTPIILVGTKLDLRDDKD

TIEKLKEKKLAPITYPQGLALAKEIDSVKYLECSALTQRGLKTVFDEAIRAVLCPQP
>d1mh1__ c.37.1.8 (-) Rac {Human (Homo sapiens)}
GSPQAIKCVVVGDGAVGKTCLLISYTTNAFPGEYIPTVFDNYSANVMVDGKPVNLGLWDTAG
QEDYDRLRPLSYPQTDVSLICFSLVSPASFENVRAKWYPEVRHHCPNTPIILVGTKLDLRDD
KDTIEKLKEKKLTPITYPQGLAMAKEIGAVKYLECSALTQRGLKTVFDEAIRAVLCPPP
>d1c1ya_ c.37.1.8 (A:) Rap1A {Human (Homo sapiens)}
MREYKLVVLGSGGVGKSALTVQFVQGIFVEKYDPTIEDSYRKQVEVDCQQCMLEILDTAGTE
QFTAMRDLYMKNGQGFALVYSITAQSTFNDLQDLREQILRVKDTEDVPMILVGNKCDLEDER
VVGKEQGQNLARQWCNCAFLESSAKSKINVNEIFYDLVRQINR
>d1kao__ c.37.1.8 (-) Rap2a {Human (Homo sapiens)}
MREYKVVVLGSGGVGKSALTVQFVTGTFIEKYDPTIEDFYRKEIEVDSSPSVLEILDTAGTE
QFASMRDLYIKNGQGFILVYSLVNQQSFQDIKPMRDQIIRVKRYEKVPVILVGNKVDLESER
EVSSSEGRALAEEWGCPFMETSAKSKTMVDELFAEIVRQMNYA
>d3raba_ c.37.1.8 (A:) Rab3a {Rat (Rattus norvegicus)}
NFDYMFKILIIGNSSVGKTSFLFRYADDSFTPAFVSTVGIDFKVKTIYRNDKRIKLQIWDTA
GQERYRTITTAYYRGAMGFILMYDITNEESFNAVQDWSTQIKTYSWDNAQVLLVGNKCDMED
ERVVSSERGRQLADHLGFEFFEASAKDNINVKQTFERLVDVICEK
>d1huqa_ c.37.1.8 (A:) Rab5c {Mouse (Mus musculus)}
ICQFKLVLLGESAVGKSSLVLRFVKGQFHEYQESTIGAAFLTQTVCLDDTTVKFEIWDTAGQ
ERYHSLAPMYYRGAQAAIVVYDITNTDTFARAKNWVKELQRQASPNIVIALAGNKADLASKR
AVEFQEAQAYADDNSLLFMETSAKTAMNVNEIFMAIAKKL
>d1d5ca_ c.37.1.8 (A:) Rab6 {Malaria parasite (Plasmodium
falciparum)}
KYKLVFLGEQAVGKTSIITRFMYDTFDNNYQSTIGIDFLSKTLYLDEGPVRLQLWDTAGQER
FRSLIPSYIRDSAAAIVVYDITNRQSFENTTKWIQDILNERGKDVIIALVGNKTDLGDLRKV
TYEEGMQKAQEYNTMFHETSAKAGHNIKVLFKKTASKL
>d1g16a_ c.37.1.8 (A:) Rab-related protein Sec4 {Baker's yeast
(Saccharomyces cerevisiae)}
SIMKILLIGDSGVGKSCLLVRFVEDKFNPSFITTIGIDFKIKTVDINGKKVKLQIWDTAGQE
RFRTITTAYYRGAMGIILVYDITDERTFTNIKQWFKTVNEHANDEAQLLLVGNKSDMETRVV
TADQGEALAKELGIPFIESSAKNDDNVNEIFFTLAKLIQEKI
>d1byua_ c.37.1.8 (A:) Ran {Dog (Canis familiaris)}
EPQVQFKLVLVGDGGTGKTTFVKRHLTGEFEKKYVPTLGVEVHPLVFHTNRGPIKFNVWDTA
GQEKFGGLRDGYYIQAQCAIIMFDVTSRVTYKNVPNWHRDLVRVCENIPIVLCGNKVDIKDR
KVKAKSIVFHRKKNLQYYDISAKSNYNFEKPFLWLARKLIGDPNLEFVAMPALAPPEVVMDP
ALAAQYEHDLEVAQTT
>d1i2ma_ c.37.1.8 (A:) Ran {Human (Homo sapiens)}
QVQFKLVLVGDGGTGKTTFVKRHLTGEFEKKYVATLGVEVHPLVFHTNRGPIKFNVWDTAGQ
EKFGGLRDGYYIQAQCAIIMFDVTSRVTYKNVPNWHRDLVRVCENIPIVLCGNKVDIKDRKV
KAKSIVFHRKKNLQYYDISAKSNYNFEKPFLWLARKLIGDPNLEFV
>d1tx4b_ c.37.1.8 (B:) RhoA {Human (Homo sapiens)}
AIRKKLVIVGDGACGKTCLLIVNSKDQFPEVYVPTVFENYVADIEVDGAQVELALWDTAGQE
DYDRLRPLSYPDTDVILMCFSIDSPDSLENIPEKWTPEVKHFCPNVPIILVGNKKDLRNDEH
TRRELAKMKQEPVKPEEGRDMANRIGAFGYMECSAKTKDGVREVFEMATRAAL

>d1hura_ c.37.1.8 (A:) ADP-ribosylation factor {Human (Homo sapiens), ARF1}
GNIFANLFKGLFGKKEMRILMVGLDAAGKTTILYKLKLGEIVTTIPTIGFNVETVEYKNISF
TVWDVGGQDKIRPLWRHYFQNTQGLIFVVDSNDRERVNEAREELMRMLAEDELRDAVLLVFA
NKQDLPNAMNAAEITDKLGLHSLRHRNWYIQATCATSGDGLYEGLDWLSNQLRNQK
>d1e0sa_ c.37.1.8 (A:) ADP-ribosylation factor {Human (Homo sapiens), ARF6}
GKVLSKIFGNKEMRILMLGLDAAGKTTILYKLKLGQSVTTIPTVGFNVETVTYKNVKFNVWD
VGGQDKIRPLWRHYYTGTQGLIFVVDCADRDRIDEARQELHRIINDREMRDAIILIFANKQD
LPDAMKPHEIQEKLGLTRIRDRNWYVQPSCATSGDGLYEGLTWLTSNYK
>d1fzqa_ c.37.1.8 (A:) ADP-ribosylation factor {Mouse (Mus musculus), ARL3}
GLLSILRKLKSAPDQEVRILLLGLDNAGKTTLLKQLASEDISHITPTQGFNIKSVQSQGFKL
NVWDIGGQRKIRPYWRSYFENTDILIYVIDSADRKRFEETGQELTELLEEEKLSCVPVLIFA
NKQDLLTAAPASEIAEGLNLHTIRDRVWQIQSCSALTGEGVQDGMNWVCKNV
>d1f6ba_ c.37.1.8 (A:) SAR1 {Chinese hamster (Cricetulus griseus)}
SSVLQFLGLYKKTGKLVFLGLDNAGKTTLLHMLKDDRLGQHVPTLHPTSEELTIAGMTFTTF
DLGGHIQARRVWKNYLPAINGIVFLVDCADHERLLESKEELDSLMTDETIANVPILILGNKI
DRPEAISEERLREMFGLYGQTTGKGSVSLKELNARPLEVFMCSVLKRQGYGEGFRWMAQYID
>d2ngra_ c.37.1.8 (A:) CDC42 {Human (Homo sapiens)}
MQTIKCVVVGDGAVGKTCLLISYTTNKFPSEYVPTVFDNYAVTVMIGGEPYTLGLFDTAGQE
DYDRLRPLSYPQTDVFLVCFSVVSPSSFENVKEKWVPEITHHCPKTPFLLVGTQIDLRDDPS
TIEKLAKNKQKPITPETAEKLARDLKAVKYVECSALTQKGLKNVFDEAILAALEPPEPKKSR
RCVLL
>d1ek0a_ c.37.1.8 (A:) Ypt51 {Baker's yeast (Saccharomyces cerevisiae)}
VTSIKLVLLGEAAVGKSSIVLRFVSNDFAENKEPTIGAAFLTQRVTINEHTVKFEIWDTAGQ
ERFASLAPMYYRNAQAALVVYDVTKPQSFIKARHWVKELHEQASKDIIIALVGNKIDMLQEG
GERKVAREEGEKLAEEKGLLFFETSAKTGENVNDVFLGIGEKIPLK
>d1h65a_ c.37.1.8 (A:) Chloroplast protein translocon GTPase Toc34 {Garden pea (Pisum sativum)}
VREWSGINTFAPATQTKLLELLGNLKQEDVNSLTILVMGKGGVGKSSTVNSIIGERVVSISP
FQSEGPRPVMVSRSRAGFTLNIIDTPGLIEGGYINDMALNIIKSFLLDKTIDVLLYVDRLDA
YRVDNLDKLVAKAITDSFGKGIWNKAIVALTHAQFSPPDGLPYDEFFSKRSEALLQVVRSGA
SLKKDAQASDIPVVLIENSGRCNKNDSDEKVLPNGIAWIPHLVQTITEVALNKSESIFVDKN
LIDKLAAAD
>d1azsc2 c.37.1.8 (C:36-66,C:202-393) Transducin (alpha subunit) {Cow (Bos taurus)}
VYRATHRLLLLGAGESGKSTIVKQMRILHVNXVLTSGIFETKFQVDKVNFHMFDVGGQRDER
RKWIQCFNDVTAIIFVVASSSYNMVIREDNQTNRLQEALNLFKSIWNNRWLRTISVILFLNK
QDLLAEKVLAGKSKIEDYFPEFARYTTPEDATPEPGEDPRVTRAKYFIRDEFLRISTASGDG
RHYCYPHFTCAVDTENIRRVFNDCRDIIQRMHLRQYEL
>d1tada2 c.37.1.8 (A:27-56,A:178-342) Transducin (alpha subunit) {Cow (Bos taurus)}

ARTVKLLLLGAGESGKSTIVKQMKIIHQDGXTGIIETQFSFKDLNFRMFDVGGQRSERKKWI
HCFEGVTCIIFIAALSAYDMVLVEDDEVNRMHESLHLFNSICNHRYFATTSIVLFLNKKDVF
SEKIKKAHLSICFPDYNGPNTYEDAGNYIKVQFLELNMRRDVKEIYSHMTCATDTQNVKFVF
DAVTDIIIKE
>d1bof_2 c.37.1.8 (10-60,182-354) Transducin (alpha subunit) {Rat (Rattus norvegicus)}
KAAVERSKMIDRNLREDGEKAAREVKLLLLGAGESGKSTIVKQMKIIHEAGXTGIVETHFTF
KDLHFKMFDVGGQRSERKKWIHCFEGVTAIIFCVALSDYDLVLAEDEEMNRMHESMKLFDSI
CNNKWFTDTSIILFLNKKDLFEEKIKKSPLTICYPEYAGSNTYEEAAAYIQCQFEDLNKRKD
TKEIYTHFTCATDTKNVQFVFDAVTDVIIKNNLKDCGLF
>d1cipa2 c.37.1.8 (A:32-60,A:182-347) Transducin (alpha subunit) {Rat (Rattus norvegicus)}
REVKLLLLGAGESGKSTIVKQMKIIHEAGXTGIVETHFTFKDLHFKMFDVGGQRSERKKWIH
CFEGVTAIIFCVALSDYDLVLAEDEEMNRMHESMKLFDSICNNKWFTDTSIILFLNKKDLFE
EKIKKSPLTICYPEYAGSNTYEEAAAYIQCQFEDLNKRKDTKEIYTHFTCATDTKNVQFVFD
AVTDVIIKNN
>d1fqja2 c.37.1.8 (A:28-60,A:182-344) Transducin (alpha subunit) {Rat (Rattus norvegicus)}
RTVKLLLLGAGESGKSTIVKQMKIIHQDGYSLEXETQFSFKDLNFRMFDVGGQRSERKKWIH
CFEGVTAIIFCVALSDYDLVLAEDEEMNRMHESMKLFDSICNNKWFTDTSIILFLNKKDLFE
EKIKKSPLTICYPEYAGSNTYEEAGNYIKVQFLELNMRRDVKEIYSHMTCATDTQNVKFVFD
AVTDIIIKENL
>d1gota2 c.37.1.8 (A:6-60,A:182-343) Transducin (alpha subunit) {Rat (Rattus norvegicus)}
SAEEKHSRELEKKLKEDAEKDARTVKLLLLGAGESGKSTIVKQMKIIHQDGYSLEXETQFSF
KDLNFRMFDVGGQRSERKKWIHCFEGVTAIIFCVALSDYDLVLAEDEEMNRMHESMKLFDSI
CNNKWFTDTSIILFLNKKDLFEEKIKKSPLTICYPEYAGSNTYEEAGNYIKVQFLELNMRRD
VKEIYSHMTCATDTQNVKFVFDAVTDIIIKEN
>d1efca3 c.37.1.8 (A:8-204) Elongation factor Tu (EF-Tu), N-terminal (G) domain {Escherichia coli}
TKPHVNVGTIGHVDHGKTTLTAAITTVLAKTYGGAARAFDQIDNAPEEKARGITINTSHVEY
DTPTRHYAHVDCPGHADYVKNMITGAAQMDGAILVVAATDGPMPQTREHILLGRQVGVPYII
VFLNKCDMVDDEELLELVEMEVRELLSQYDFPGDDTPIVRGSALKALEGDAEWEAKILELAG
FLDSYIPEPER
>d1efm_1 c.37.1.8 (12-190) Elongation factor Tu (EF-Tu), N-terminal (G) domain {Escherichia coli}
VNVGTIGHVDHGKTTLTAAITTVLAKTYGGAARXXXXXXXXXXXXXXGITINTSHVEYDTPT
RHYAHVDCPGHADYVKNMITGAAQMDGAILVVAATDGPMPQTREHILLGRQVGVPYIIVFLN
KCDMVDDEELLELVEMEVRELLSQYDFPGDDTPIVRGSALKALEGDAEWEAKILE
>d1etu_1 c.37.1.8 (5-200) Elongation factor Tu (EF-Tu), N-terminal (G) domain {Escherichia coli}
FERTKPHVNVGTIGHVDHGKTTLTAAITTVLAKTYGGAAXXXXXXXXXXXXXXXXGITINTSH
VEYDTPTRHYAHVDCPGHADYVKNMITGAAQMDGAILVVAATDGPMPQTREHILLGRQVGVP
YIIVFLNKCDMVDDEELLELVEMEVRELLSQYDFPGDDTPIVRGSALKALEGDAEWEAKILE

LAGFLDSYIP
>d1exma3 c.37.1.8 (A:3-212) Elongation factor Tu (EF-Tu), N-terminal (G) domain {Thermus thermophilus}
GEFIRTKPHVNVGTIGHVDHGKTTLTAALTFVTAAENPNVEVKDYGDIDKAPEERARGITIN
TAHVEYETAKRHYSHVDCPGHADYIKNMITGAAQMDGAILVVSAADGPMPQTREHILLARQV
GVPYIVVFMNKVDMVDDPELLDLVEMEVRDLLNQYEFPGDEVPVIRGSALLALEQMHRNPKT
RRGENEWVDKIWELLDAIDEYIPT
>d1d2ea3 c.37.1.8 (A:55-250) Elongation factor Tu (EF-Tu), N-terminal (G) domain {Cow (Bos taurus), mitochondrial}
KPHVNVGTIGHVDHGKTTLTAAITKILAEGGGAKFKKYEEIDNAPEERARGITINAAHVEYS
TAARHYAHTDCPGHADYVKNMITGTAPLDGCILVVAANDGPMPQTREHLLLARQIGVEHVVV
YVNKADAVQDSEMVELVELEIRELLTEFGYKGEETPIIVGSALCALEQRDPELGLKSVQKLL
DAVDTYIPVP
>d1f60a3 c.37.1.8 (A:2-240) Elongation factor eEF-1alpha, N-terminal (G) domain {Baker's yeast (Saccharomyces cerevisiae)}
GKEKSHINVVVIGHVDSGKSTTTGHLIYKCGGIDKRTIEKFEKEAAELGKGSFKYAWVLDKL
KAERERGITIDIALWKFETPKYQVTVIDAPGHRDFIKNMITGTSQADCAILIIAGGVGEFEA
GISKDGQTREHALLAFTLGVRQLIVAVNKMDSVKWDESRFQEIVKETSNFIKKVGYNPKTVP
FVPISGWNGDNMIEATTNAPWYKGWEKETKAGVVKGKTLLEAIDAIEQPSRPT
>d1jnya3 c.37.1.8 (A:4-227) Elongation factor eEF-1alpha, N-terminal (G) domain {Archaeon Sulfolobus solfataricus}
KPHLNLIVIGHVDHGKSTLVGRLLMDRGFIDEKTVKEAEEAAKKLGKESEKFAFLLDRLKEE
RERGVTINLTFMRFETKKYFFTIIDAPGHRDFVKNMITGASQADAAILVVSAKKGEYEAGMS
VEGQTREHIILAKTMGLDQLIVAVNKMDLTEPPYDEKRYKEIVDQVSKFMRSYGFNTNKVRF
VPVVAPSGDNITHKSENMKWYNGPTLEEYLDQLELPPK
>d1dar_2 c.37.1.8 (1-282) Elongation factor G (EF-G), N-terminal (G) domain {Thermus thermophilus}
MAVKVEYDLKRLRNIGIAAHIDAGKTTTTERILYYTGRIHKIGEVHEGAATMDFMEQERERG
ITITAAVTTCFWKDHRINIIDTPGHVDFTIEVERSMRVLDGAIVVFDSSQGVEPQSETVWRQ
AEKYKVPRIAFANKMDKTGADLWLVIRTMQERLGARPVVMQLPIGREDTFSGIIDVLRMKAY
TYGNDLGTDIREIPIPEEYLDQAREYHEKLVEVAADFDENIMLKYLEGEEPTEEELVAAIRK
GTIDLKITPVFLGSALKNKGVQLLLDAVVDYLPS
>d1g7sa4 c.37.1.8 (A:1-227) Initiation factor IF2/eIF5b, N-terminal (G) domain {Archaeon Methanobacterium thermoautotrophicum}
MKIRSPIVSVLGHVDHGKTTLLDHIRGSAVASREAGGITQHIGATEIPMDVIEGICGDFLKK
FSIRETLPGLFFIDTPGHEAFTTLRKRGGALADLAILIVDINEGFKPQTQEALNILRMYRTP
FVVAANKIDRIHGWRVHEGRPFMETFSKQDIQVQQKLDTKVYELVGKLHEEGFESERFDRVT
DFASQVSIIPISAITGEGIPELLTMLMGLAQQYLREQLKIE
>d1egaa1 c.37.1.8 (A:4-182) GTPase Era, N-terminal domain {Escherichia coli}
DKSYCGFIAIVGRPNVGKSTLLNKLLGQKISITSRKAQTTRHRIVGIHTEGAYQAIYVDTPG
LHMEEKRAINRLMNKAASSSIGDVELVIFVVEGTRWTPDDEMVLNKLREGKAPVILAVNKVD
NVQEKADLLPHLQFLASQMNFLDIVPISAETGLNVDTIAAIVRKHLPEATHHFPE

>d1f5na2 c.37.1.8 (A:7-283) Interferon-induced guanylate-binding protein 1 (GBP1), N-terminal domain {Human (Homo sapiens)}
MTGPMCLIENTNGRLMANPEALKILSAITQPMVVVAIVGLYRTGKSYLMNKLAGKKKGFSLG
STVQSHTKGIWMWCVPHPKKPGHILVLLDTEGLGDVEKGDNQNDSWIFALAVLLSSTFVYNS
IGTINQQAMDQLYYVTELTHRIRSKSSPDENENEVEDSADFVSFFPDFVWTLRDFSLDLEAD
GQPLTPDEYLTYSLKLKKGTSQKDETFNLPRLCIRKFFPKKKCFVFDRPVHRRKLAQLEKLQ
DEELDPEFVQQVADFCSYIFSNSKTKTLS
>d1jwyb_ c.37.1.8 (B:) Dynamin G domain {Slime mold (Dictyostelium discoideum)}
DQLIPVINKLQDVFNTLGSDPLDLPQIVVVGSQSSGKSSVLENIVGRDFLPRGSGIVTRRPL
ILQLTHLPIADDGSQTQEWGEFLHKPNDMFYDFSEIREEIIRDTDRMTGKNKGISAQPINLK
IYSPHVVNLTLVDLPGITKVPVGDQPTDIEQQIRRMVMAYIKKQNAIIVAVTPANTDLANSD
ALQLAKEVDPEGKRTIGVITKLDLMDKGTDAMEVLTGRVIPLTLGFIGVINRSQEDIIAKKS
IRESLKSEILYFKNHPIYKSIANRSGTAYLSKTLNKLLMFHIRDTLPDLKVKVSKMLS
>d1br2a2 c.37.1.9 (A:80-789) Myosin S1, motor domain {Chicken (Gallus gallus), pectoral muscle}
PPKFSKVEDMAELTCLNEASVLHNLRERYFSGLIYTYSGLFCVVINPYKQLPIYSEKIIDMY
KGKKRHEMPPHIYAIADTAYRSMLQDREDQSILCTGESGAGKTENTKKVIQYLAVVASSHKG
KKDTSITQGPSFSYGELEKQLLQANPILEAFGNAKTVKNDNSSRFGKFIRINFDVTGYIVGA
NIETYLLEKSRAIRQAKDERTFHIFYYLIAGASEQMRNDLLLEGFNNYTFLSNGHVPIPAQQ
DDEMFQETLEAMTIMGFTEEEQTSILRVVSSVLQLGNIVFKKERNTDQASMPDNTAAQKVCH
LMGINVTDFTRSILTPRIKVGRDVVQKAQTKEQADFAIEALAKAKFERLFRWILTRVNKALD
KTKRQGASFLGILDIAGFEIFEINSFEQLCINYTNEKLQQLFNHTMFILEQEEYQREGIEWN
FIDFGLDLQPCIELIERPTNPPGVLALLDEECWFPKATDTSFVEKLIQEQGNHAKFQKSKQL
KDKTEFCILHYAGKVTYNASAWLTKNMDPLNDNVTSLLNQSSDKFVADLWKDVDRIVGLDQM
AKMTESSLPSASKTKKGMFRTVGQLYKEQLTKLMTTLRNTNPNFVRCIIPNHEKRAGKLDAH
LVLEQLRCNGVLEGIRICRQGFPNRIVFQEFRQRYEILAANAIPKGFMDGKQACILMIKALE
LDPNLYRIGQSKIFFRTGVLAHLEEERD
>d2mysa2 c.37.1.9 (A:4-33,A:80-843) Myosin S1, motor domain {Chicken (Gallus gallus), pectoral muscle}
DAEMAAFGEAAPYLRKSEKERIEAQNKPFDXMNPPKYDKIEDMAMMTHLHEPAVLYNLKERY
AAWMIYTYSGLFCVTVNPYKWLPVYNPKVVLAYRGKKRQEAPPHIFSISDNAYQFMLTDREN
QSILITGESGAGKTVNTKRVIQYFATIAASGEKKKEEQSGKMQGTLEDQIISANPLLEAFGN
AKTVRNDNSSRFGKFIRIHFGATGKLASADIETYLLEKSRVTFQLPAERSYHIFYQIMSNKK
PELIDMLLITTNPYDYHYVSEGEITVPSIDDQEELMATDSAIDILGFSADEKTAIYKLTGAV
MHYGNLKFKQKQREEQAEPDGTEVADKAAYLMGLNSAELLKALCYPRVGVGNEAVTKGETVS
EVHNSVGALAKAVYEKMFLWMVIRINQQLDTKQPRQYFIGVLDIAGFEIFDFNSFEQLCINF
TNEKLQQFFNHHMFVLEQEEYKKEGIEWEFIDFGMDLAACIELIEKPMGIFSILEEECMFPK
ATDTSFKNKLYDEHLGKSNNFQKPKPAKGKAEAHFSLVHYAGTVDYNISGWLEKNKDPLNET
VIGLYQKSSVKTLALLFATYGGEAEGGGGKKGGKKKGSSFQTVSALFRENLNKLMANLRSTH
PHFVRCIIPNETKTPGAMEHELVLHQLRCNGVLEGIRICRKGFPSRVLYADFKQRYRVLNAS
AIPEGQFMDSKKASEKLLGGGDVDHTQYAFGHTKVFFKAGLLGLLEEMRDDKLAEIITATQA
RCRGFLMRVEYRAMVERRESIFCIQYNVRSFMNVKHWPWMKLFFKIKPLLK
>d1b7ta4 c.37.1.9 (A:5-28,A:77-835) Myosin S1, motor domain {Bay

scallop (Aequipecten irradians)}
FSDPDFQYLAVDRKKLMKEQTAAFXMNPPKFEKLEDMANMTYLNEASVLYNLRSRYTSGLIY
TYSGLFCIAVNPYRRLPIYTDSVIAKYRGKRKTEIPPHLFSVADNAYQNMVTDRENQSCLIT
GESGAGKTENTKKVIMYLAKVACAVKKKDEEASDKKEGSLEDQIIQANPVLEAYGNAKTTRN
NNSSRFGKFIRIHFGPTGKIAGADIETYLLEKSRVTYQQSAERNYHIFYQICSNAIPELNDV
MLVTPDSGLYSFINQGCLTVDNIDDVEEFKLCDEAFDILGFTKEEKQSMFKCTASILHMGEM
KFKQRPREEQAESDGTAEAEKVAFLCGINAGDLLKALLKPKVKVGTEMVTKGQNMNQVVNSV
GALAKSLYDRMFNWLVRRVNKTLDTKAKRNYYIGVLDIAGFEIFDFNSFEQLCINYTNERLQ
QFFNHHMFILEQEEYKKEGIAWEFIDFGMDLQMCIDLIEKPMGILSILEEECMFPKADDKSF
QDKLYQNHMGKNRMFTKPGKPTRPNQGPAHFELHHYAGNVPYSITGWLEKNKDPINENVVAL
LGASKEPLVAELFKAPEEPAGGGKKKKGKSSAFQTISAVHRESLNKLMKNLYSTHPHFVRCI
IPNELKQPGLVDAELVLHQLQCNGVLEGIRICRKGFPSRLIYSEFKQRYSILAPNAIPQGFV
DGKTVSEKILAGLQMDPAEYRLGTTKVFFKAGVLGNLEEMRDERLSKIISMFQAHIRGYLIR
KAYKKLQDQRIGLSVIQRNIRKWLVLRNWQWWKLYSKVKP
>d1lvk_2 c.37.1.9 (2-33,80-759) Myosin S1, motor domain {Slime mold (Dictyostelium discoideum)}
NPIHDRTSDYHKYLKVKQGDSDLFKLTVSDKRXRNPIKFDGVEDMSELSYLNEPAVFHNLRV
RYNQDLIYTYSGLFLVAVNPFKRIPIYTQEMVDIFKGRRRNEVAPHIFAISDVAYRSMLDDR
QNQSLLITGESGAGKTENTKKVIQYLASVAGRNQANGSGVLEQQILQANPILEAFGNAKTTR
NNNSSRFGKFIEIQFNNAGFISGASIQSYLLEKSRVVFQSTSERNYHIFYQLLAGATAEEKK
ALHLAGPESFNYLNQSGCVDIKGVSDEDEFKITRQAMDIVGFSQEEQMSIFKIIAGILHLGN
IKFEKGAGEGAVLKDKTALNAASTVFGVNPSVLEKALMEPRILAGRDLVAQHLNVEKSSSSR
DALVKALYGRLFLWLVKKINNVLCSERKAYFIGVLDISGFEIFKVNSFEQLCINYTNEKLQQ
FFNHHMFKVEQEKYLKEKINWTFIDFGLDSQATIDLIDGRQPPGILALLDEQSVFPNATDNT
LITKLHSHFSKKNAKYEEPRFSKTEFGVTHYAGQVMYEIQDWLEKNKDPLQQDLELCFKDSS
DNVVTKLFNDPNIASRAKKGANFLTVAAQYKEQLASLMATLETTNPHFVRCIIPNNKQLPAK
LEDKVVLDQLRCNGVLEGIRITRKGFPNRIIYADFVKRYYDLAPNVPRDAEDSQKATDAVLK
HLNIDPEQFRFGITKIFFRAGQLARIEEARE
>d1mnd_2 c.37.1.9 (2-33,80-690) Myosin S1, motor domain {Slime mold (Dictyostelium discoideum)}
NPIHDRTSDYHKYLKVKQGDSDLFKLTVSDKRXRNPIKFDGVEDMSELSYLNEPAVFHNLRV
RYNQDLIYTYSGLFLVAVNPFKRIPIYTQEMVDIFKGRRRNEVAPHIFAISDVAYRSMLDDR
QNQSLLITGESGAGKTENTKKVIQYLASVAGRNQANGSGVLEQQILQANPILEAFGNAKTTR
NNNSSRFGKFIEIQFNNAGFISGASIQSYLLEKSRVVFQSETERNYHIFYQLLAGATAEEKK
ALHLAGPESFNYLNQSGCVDIKGVSDEDEFKITRQAMDIVGFSQEEQMSIFKIIAGILHLGN
IKFEKGAGEGAVLKDKTALNAASTVFGVNPSVLEKALMEPRILAGRDLVAQHLNVEKSSSSR
DALVKALYGRLFLWLVKKINNVLCSERAAYFIGVLDISGFEIFKVNSFEQLCINYTNEKLQQ
FFNHHMFKVEQEEYLKEKINWTFIDFGLDSQATIDLIDGRQPPGILALLDEQSVFPNATDNT
LITKLHSHFSKKNAKYEEPRFSKTEFGVTHYAGQVMYEIQDWLEKNKDPLQQDLELCFKDSS
DNVVTKLFNDPNIASRAKKGANFITVAAQYKEQLASLMATLETTNPHFVRCIIPNNKQLPAK
LEDKVVLDQLRCNGVLEGIRITRK
>d1bg2__ c.37.1.9 (-) Kinesin {Human (Homo sapiens)}
DLAECNIKVMCRFRPLNESEVNRGDKYIAKFQGEDTVVIASKPYAFDRVFQSSTSQEQVYND
CAKKIVKDVLEGYNGTIFAYGQTSSGKTHTMEGKLHDPEGMGIIPRIVQDIFNYIYSMDENL

EFHIKVSYFEIYLDKIRDLLDVSKTNLSVHEDKNRVPYVKGCTERFVCSPDEVMDTIDEGKS
NRHVAVTNMNEHSSRSHSIFLINVKQENTQTEQKLSGKLYLVDLAGSEKVSKTGAEGAVLDE
AKNINKSLSALGNVISALAEGSTYVPYRDSKMTRILQDSLGGNCRTTIVICCSPSSYNESET
KSTLLFGQRAKTI
>g2kin.1 c.37.1.9 (A:,B:) Kinesin {Rat (Rattus norvegicus)}
ADPAECSIKVMCRFRPLNEAEILRGDKFIPKFKGEETVVIGQGKPYVFDRVLPPNTTQEQVY
NACAKQIVKDVLEGYNGTIFAYGQTSSGKTHTMEGKLHDPQLMGIIPRIAHDIFDHIYSMDE
NLEFHIKVSYFEIYLDKIRDLLDVSKTNLAVHEDKNRVPYVKGCTERFVSSPEEVMDVIDEG
KANRHVAVTNMNEHSSRSHSIFLINIKQENVETEKKLSGKLYLVDLAGSEKVXAKNINKSLS
ALGNVISALAEGTKTHVPYRDSKMTRILQDSLDGNCRTTIVICCSPSVFNEAETKSTLMFGQ
RAKTIKNTVSVNLELTAEEWKKKYEKEKE
>d1i6ia_ c.37.1.9 (A:) Kinesin {Mouse (Mus musculus), kif1a}
GASVKVAVRVRPFNSREMSRDSKCIIQMSGSTTTIVNPKQPKETPKSFSFDYSYWSHTSPED
INYASQKQVYRDIGEEMLQHAFEGYNVCIFAYGQTGAGKSYTMMGKQEKDQQGIIPQLCEDL
FSRINDTTNDNMSYSVEVSYMEIYCERVRDLLNPKNKGNLRVREHPLLGPYVEDLSKLAVTS
YNDIQDLMDSGNKARTVAATNMNETSSRSHAVFNIIFTQKRHDAETNITTEKVSKISLVDLA
GSERADSTGAKGTRLKEGANINKSLTTLGKVISALAEMDSGPNKNKKKKKTDFIPYRDSVLT
WLLRENLGGNSRTAMVAALSPADINYDETLSTLRYADRAKQIRNTVSVNHHHHH
>d1ii6a_ c.37.1.9 (A:) Kinesin {Human (Homo sapiens), mitotic
kinesin eg5}
GKNIQVVVRCRPFNLAERKASAHSIVECDPVRKEVSVRTGGLADKSSRKTYTFDMVFGASTK
QIDVYRSVVCPILDEVIMGYNCTIFAYGQTGTGKTFTMEGERSPNEEYTWEEDPLAGIIPRT
LHQIFEKLTDNGTEFSVKVSLLEIYNEELFDLLNPSSDVSERLQMFDDPRNKRGVIIKGLEE
ITVHNKDEVYQILEKGAAKRTTAATLMNAYSSRSHSVFSVTIHMKETTIDGEELVKIGKLNL
VDLAGSENIGRSGAVDKRAREAGNINQSLLTLGRVITALVERTPHVPYRESKLTRILQDSLG
GRTRTSIIATISPASLNLEETLSTLEYAHRAKNILNKPEV
>d1goja_ c.37.1.9 (A:) Kinesin {Neurospora crassa}
SSSANSIKVVARFRPQNRVEIESGGQPIVTFQGPDTCTVDSKEAQGSFTFDRVFDMSCKQSD
IFDFSIKPTVDDILNGYNGTVFAYGQTGAGKSYTMMGTSIDDPDGRGVIPRIVEQIFTSILS
SAANIEYTVRVSYMEIYMERIRDLLAPQNDNLPVHEEKNRGVYVKGLLEIYVSSVQEVYEVM
RRGGNARAVAATNMNQESSRSHSIFVITITQKNVETGSAKSGQLFLVDLAGSEKVGKTGASG
QTLEEAKKINKSLSALGMVINALTDGKSSHVPYRDSKLTRILQESLGGNSRTTLIINCSPSS
YNDAETLSTLRFGMRAKSIKNKAKVNAELSPAELKQMLAKAKTQ
>d2ncda_ c.37.1.9 (A:) Kinesin motor Ncd (non-claret disjunctional)
{Fruit fly (Drosophila melanogaster)}
LRQRTEELLRCNEQQAAELETCKEQLFQSNMERKELHNTVMDLRGNIRVFCRIRPPLESEEN
RMCCTWTYHDESTVELQSIDAQAKSKMGQQIFSFDQVFHPLSSQSDIFEMVSPLIQSALDGY
NICIFAYGQTGSGKTYTMDGVPESVGVIPRTVDLLFDSIRGYRNLGWEYEIKATFLEIYNEV
LYDLLSNEQKDMEIRMAKNNKNDIYVSNITEETVLDPNHLRHLMHTAKMNRATASTAGNERS
SRSHAVTKLELIGRHAEKQEISVGSINLVDLAGSESPKTSTRMTETKNINRSLSELTNVILA
LLQKQDHIPYRNSKLTHLLMPSLGGNSKTLMFINVSPFQDCFQESVKSLRFAASVNSC
>d1f9va_ c.37.1.9 (A:) Kinesin motor Ncd (non-claret disjunctional)
{Baker's yeast (Saccharomyces cerevisiae), Kar}
GNIRVYCRIRPALKNLENSDTSLINVNEFDDNSGVQSMEVTKIQNTAQVHEFKFDKIFDQQD

TNVDVFKEVGQLVQSSLDGYNVCIFAYGQTGSGKTFTMLNPGDGIIPSTISHIFNWINKLKT
KGWDYKVNCEFIEIYNENIVDLLRSDNNNKEDTSIGLKHEIRHDQETKTTTITNVTSCKLES
EEMVEIILKKANKLRSTASTASNEHSSASHSIFIIHLSGSNAKTGAHSYGTLNLVDLAGSER
INVSQVVGDRLRETQNINKSLSCLGDVIHALGQPDSTKRHIPFRNSKLTYLLQYSLTGDSKT
LMFVNISPSSSHINETLNSLRFASKVNSTRLV
>d1byi__ c.37.1.10 (-) Dethiobiotin synthetase {Escherichia coli}
SKRYFVTGTDTEVGKTVASCALLQAAKAAGYRTAGYKPVASGSEKTPEGLRNSDALALQRNS
SLQLDYATVNPYTFAEPTSPHIISAQEGRPIESLVMSAGLRALEQQADWVLVEGAGGWFTPL
SDTFTFADWVTQEQLPVILVVGVKLGCINHAMLTAQVIQHAGLTLAGWVANDVTPPGKRHAE
YMTTLTRMIPAPLLGEIPWLAENPENAATGKYINLALL
>d1qf5a_ c.37.1.10 (A:) Adenylosuccinate synthetase, PurA
{Escherichia coli}
GNNVVVLGTQWGDEGKGKIVDLLTERAKYVVRYQGGHNAGHTLVINGEKTVLHLIPSGILRE
NVTSIIGNGVVLSPAALMKEMKELEDRGIPVRERLLLSEACPLILDYHVALDNAREKARGAK
AIGTTGRGIGPAYEDKVARRGLRVGDLFDKETFAEKLKEVMEYHNFQLVNYYKAEAVDYQKV
LDDTMAVADILTSMVVDVSDLLDQARQRGDFVMFEGAQGTLLDIDHGTYPYVTSSNTTAGGV
ATGSGLGPRYVDYVLGILKAYSTRVGAGPFPTELFDETGEFLCKQGNEFGATTGRRRRTGWL
DTVAVRRAVQLNSLSGFCLTKLDVLDGLKEVKLCVAYRMPDGREVTTTPLAADDWKGVEPIY
ETMPGWSESTFGVKDRSGLPQAALNYIKRIEELTGVPIDIISTGPDRTETMILRDPFDA
>d1dj2a_ c.37.1.10 (A:) Adenylosuccinate synthetase, PurA
{Mouse-ear cress (Arabidopsis thaliana)}
IGSLSQVSGVLGCQWGDEGKGKLVDILAQHFDIVARCQGGANAGHTIYNSEGKKFALHLVPS
GILNEDTTCVIGNGVVVHLPGLFKEIDGLESNGVSCKGRILVSDRAHLLFDFHQEVDGLRES
ELAKSFIGTTKRGIGPAYSSKVIRNGIRVGDLRHMDTLPQKLDLLLSDAAARFQGFKYTPEM
LREEVEAYKRYADRLEPYITDTVHFINDSISQKKKVLVEGGQATMLDIDFGTYPFVTSSSPS
AGGICTGLGIAPSVVGDLIGVVKAYTTRVGSGPFPTENLGTGGDLLRLAGQEFGTTTGRPRR
CGWLDIVALKFSCQINGFASLNLTKLDVLSDLNEIQLGVAYKRSDGTPVKSFPGDLRLLEEL
HVEYEVLPGWKSDISSVRNYSDLPKAAQQYVERIEELVGVPIHYIGIGPGRDALIYK
>d1dj3a_ c.37.1.10 (A:) Adenylosuccinate synthetase, PurA {Bread
wheat (Triticum aestivum)}
ADRVSSLSNVSGVLGSQWGDEGKGKLVDVLAPRFDIVARCQGGANAGHTIYNSEGKKFALHL
VPSGILHEGTLCVVGNGAVIHVPGFFGEIDGLQSNGVSCDGRILVSDRAHLLFDLHQTVDGL
REAELANSFIGTTKRGIGPCYSSKVTRNGLRVCDLRHMDTFGDKLDVLFEDAAARFEGFKYS
KGMLKEEVERYKKFAERLEPFIADTVHVLNESIRQKKKILVEGGQATMLDIDFGTYPFVTSS
SPSAGGICTGLGIAPRVIGDLIGVVKAYTTRVGSGPFPTELLGEEGDVLRKAGMEFGTTTGR
PRRCGWLDIVALKYCCDINGFSSLNLTKLDVLSGLPEIKLGVSYNQMDGEKLQSFPGDLDTL
EQVQVNYEVLPGWDSDISSVRSYSELPQAARRYVERIEELAGVPVHYIGVGPGRDALIYK
>d1j4ba_ c.37.1.10 (A:) Adenylosuccinate synthetase, PurA {Mouse
(Mus musculus)}
AAATGSRVTVVLGAQWGDEGKGKVVDLLATDADIVSRCQGGNNAGHTVVVDGKEYDFHLLPS
GIINTKAVSFIGNGVVIHLPGLFEEAEKNEKKGLKDWEKRLIISDRAHLVFDFHQAVDGLQE
VQRQAQEGKNIGTTKKGIGPTYSSKAARTGLRICDLLSDFDEFSARFKNLAHQHQSMFPTLE
IDVEGQLKRLKGFAERIRPMVRDGVYFMYEALHGPPKKVLVEGANAALLDIDFGTYPFVTSS
NCTVGGVCTGLGIPPQNIGDVYGVVKAYTTRVGIGAFPTEQINEIGDLLQNRGHEWGVTTGR

KRRCGWLDLMILRYAHMVNGFTALALTKLDILDVLSEIKVGISYKLNGKRIPYFPANQEILQ
KVEVEYETLPGWKADTTGARKWEDLPPQAQSYVRFVENHMGVAVKWVGVGKSRESMIQLF
>d1eg7a_ c.37.1.10 (A:) Formyltetrahydrofolate synthetase
{Moorella thermoacetica}
DIEIAQAAKMKPVMELARGLGIQEDEVELYGKYKAKISLDVYRRLKDKPDGKLILVTAITPT
PAGEGKTTTSVGLTDALARLGKRVMVCLREPSLGPSFGIKGGAAGGGYAQVVPMEDINLHFT
GDIHAVTYAHNLLAAMVDNHLQQGNVLNIDPRTITWRRVIDLNERALRNIVIGLGGKANGVP
RETGFDISVASEVMACLCLASDLMDLKERFSRKVVGYTYDGKPVTAGDLEAQGSMALLMKDA
IKPNLVQTLENTPAFIHGGPFANIAHGCNSIIATKTALKLADYVVTEAGFGADLGAEKFYDV
KCRYAGFKPDATVIVATVRALKMHGGVPKSDLATENLEALREGFANLEKHIENIGKFGVPAV
VAINAFPTDTEAELNLLYELCAKAGAEVALSWAKGGEGGLELARKVLQTLESRPSNFHVLYN
LDLSIKDKIAKIATEIYGADGVNYTAEADKAIQRYESLGYGNLPVVMAKTQYSFSDDMTKLG
RPRNFTITVREVRLSAGGRLIVPITGAIMTMPGLPKRPAACNIDIDADGVITG
>d1fp6a_ c.37.1.10 (A:) Nitrogenase iron protein {Azotobacter
vinelandii}
AMRQCAIYGKGGIGKSTTTQNLVAALAEMGKKVMIVGCDPKADSTRLILHSKAQNTIMEMAA
EAGTVEDLELEDVLKAGYGGVKCVESGGPEPGVGCAGRGVITAINFLEEEGAYEDDLDFVFY
DVLGDVVCGGFAMPIRENKAQEIYIVCSGEMMAMYAANNISKGIVKYANSGSVRLGGLICNS
RNTDREDELIIALANKLGTQMIHFVPRDNVVQRAEIRRMTVIEYDPKAKQADEYRALARKVV
DNKLLVIPNPITMDELEELLMEFGIMEVEDESIVGKTAEEV
>d1cp2a_ c.37.1.10 (A:) Nitrogenase iron protein {Clostridium
pasteurianum}
MRQVAIYGKGGIGKSTTTQNLTSGLHAMGKTIMVVGCDPKADSTRLLLGGLAQKSVLDTLRE
EGEDVELDSILKEGYGGIRCVESGGPEPGVGCAGRGIITSINMLEQLGAYTDDLDYVFYDVL
GDVVCGGFAMPIREGKAQEIYIVASGEMMALYAANNISKGIQKYAKSGGVRLGGIICNSRKV
ANEYELLDAFAKELGSQLIHFVPRSPMVTKAEINKQTVIEYDPTCEQAEEYRELARKVDANE
LFVIPKPMTQERLEEILMQYG
>d1hyqa_ c.37.1.10 (A:) Cell division regulator MinD {Archaeon
Archaeoglobus fulgidus}
VRTITVASGKGGTGKTTITANLGVALAQLGHDVTIVDADITMANLELILGMEGLPVTLQNVL
AGEARIDEAIYVGPGGVKVVPAGVSLEGLRKANPEKLEDVLTQIMESTDILLLDAPAGLERS
AVIAIAAAQELLLVVNPEISSITDGLKTKIVAERLGTKVLGVVVNRITTLGIEMAKNEIEAI
LEAKVIGLIPEDPEVRRAAAYGKPVVLRSPNSPAARAIVELANYIA
>d1g3qa_ c.37.1.10 (A:) Cell division regulator MinD {Archaeon
Pyrococcus furiosus}
MGRIISIVSGKGGTGKTTVTANLSVALGDRGRKVLAVDGDLTMANLSLVLGVDDPDVTLHDV
LAGEANVEDAIYMTQFDNVYVLPGAVDWEHVLKADPRKLPEVIKSLKDKFDFILIDCPAGLQ
LDAMSAMLSGEEALLVTNPEISCLTDTMKVGIVLKKAGLAILGFVLNRYGRSDRDIPPEAAE
DVMEVPLLAVIPEDPAIREGTLEGIPAVKYKPESKGAKAFVKLAEEIEKLA
>d1iona_ c.37.1.10 (A:) Cell division regulator MinD {Archaeon
Pyrococcus horikoshii}
MTRIISIVSGKGGTGKTTVTANLSVALGEMGRKVLAVDGDLTMANLSLVLGVDDVNITLHDV
LAGDAKLEDAIYMTQFENVYILPGAVDWEHVIKADPRKLPEVIKSLKGKYDFILIDCPAGLQ
LRAMSAMLSGEEAILVTNPEISCLTDTMKVGMVLKKAGLAILGFILNRYGRSERDIPPEAAQ

DVMDVPLLAVIPEDPVIREGTLEGIPAVKYKPESKGAQAFIKLAEEVDKLAGIKAKI
>d1jpna2 c.37.1.10 (A:89-296) GTPase domain of the signal sequence recognition protein Ffh {Thermus aquaticus}
EARLPVLKDRNLWFLVGLQGSGKTTTAAKLALYYKGKGRRPLLVAADTQRPAAREQLRLLGE
KVGVPVLEVMDGESPESIRRRVEEKARLEARDLILVDTAGRLQIDEPLMGELARLKEVLGPD
EVLLVLDAMTGQEALSVARAFDEKVGVTGLVLTKLDGDARGGAALSARHVTGKPIYFAGVSE
KPEGLEPFYPERLAGRILGMGD
>d1j8mf2 c.37.1.10 (F:87-297) GTPase domain of the signal sequence recognition protein Ffh {Archaeon Acidianus ambivalens}
DKEPKVIPDKIPYVIMLVGVQGTGKTTTAGKLAYFYKKKGFKVGLVGADVYRPAALEQLQQL
GQQIGVPVYGEPGEKDVVGIAKRGVEKFLSEKMEIIIVDTAGRHGYGEEAALLEEMKNIYEA
IKPDEVTLVIDASIGQKAYDLASKFNQASKIGTIIITKMDGTAKGGGALSAVAATGATIKFI
GTGEKIDELEVFNPRRFVARLHHHH
>d1fts_2 c.37.1.10 (285-495) GTPase domain of the signal recognition particle receptor FtsY {Escherichia coli}
PLNVEGKAPFVILMVGVNGVGKTTTIGKLARQFEQQGKSVMLAAGDTFRAAAVEQLQVWGQR
NNIPVIAQHTGADSASVIFDAIQAAKARNIDVLIADTAGRLQNKSHLMEELKKIVRVMKKLD
VEAPHEVMLTIDASTGQNAVSQAKLFHEAVGLTGITLTKLDGTAKGGVIFSVADQFGIPIRY
IGVGERIEDLRPFKADDFIEALFAR
>d1ihua1 c.37.1.10 (A:1-296) Arsenite-translocating ATPase ArsA {Escherichia coli}
MQFLQNIPPYLFFTGKGGVGKTSISCATAIRLAEQGKRVLLVSTDPASNVGQVFSQTIGNTI
QAIASVPGLSALEIDPQAAAQQYRARIVDPIKGVLPDDVVSSINEQLSGACTTEIAAFDEFT
GLLTDASLLTRFDHIIFDTAPTGHTIRLLQLPGAWSSFIDSNPEGASCLGPMAGLEKQREQY
AYAVEALSDPKRTRLVLVARLQKSTLQEVARTHLELAAIGLKNQYLVINGVLPKTEAANDTL
AAAIWEREQEALANLPADLAGLPTDTLFLQPVNMVGVSALSRLLSTQP
>d1ihua2 c.37.1.10 (A:308-586) Arsenite-translocating ATPase ArsA {Escherichia coli}
QRPDIPSLSALVDDIARNEHGLIMLMGKGGVGKTTMAAAIAVRLADMGFDVHLTTSDPAAHL
SMTLNGSLNNLQVSRIDPHEETERYRQHVLETKGKELDEAGKRLLEEDLRSPCTEEIAVFQA
FSRVIREAGKRFVVMDTAPTGHTLLLLDATGAYHREIAKKMGEKGHFTTPMMLLQDPERTKV
LLVTLPETTPVLEAANLQADLERAGIHPWGWIINNSLSIADTRSPLLRMRAQQELPQIESVK
RQHASRVALVPVLASEPTGIDKLKQLAGHHH
>d2reb_1 c.37.1.11 (3-268) RecA protein, ATPase-domain {Escherichia coli}
DENKQKALAAALGQIEKQFGKGSIMRLGEDRSMDVETISTGSLSLDIALGAGGLPMGRIVEI
YGPESSGKTTLTLQVIAAAQREGKTCAFIDAEHALDPIYARKLGVDIDNLLCSQPDTGEQAL
EICDALARSGAVDVIVVDSVAALTPKAEIEGEIGDSHMGLAARMMSQAMRKLAGNLKQSNTL
LIFINQIRMKIGVMFGNPETTTGGNALKFYASVRLDIRRIGAVKEGENVVGSETRVKVVKNK
IAAPFKQAEFQILYGEGI
>d1g19a1 c.37.1.11 (A:1-269) RecA protein, ATPase-domain {Mycobacterium tuberculosis}
MTQTPDREKALELAVAQIEKSYGKGSVMRLGDEARQPISVIPTGSIALDVALGIGGLPRGRV
IEIYGPESSGKTTVALHAVANAQAAGGVAAFIDAEHALDPDYAKKLGVDTDSLLVSQPDTGE

QALEIADMLIRSGALDIVVIDSVAALVPRAELEGEMGDSHVGLQARLMSQALRKMTGALNNS
GTTAIFINQLRDKIGVMFGSPETTTGGKALKFYASVRMDVRRVETLKDGTNAVGNRTRVKVV
KNKCLAPFKQAEFDILYGKGI
>d1cr1a_ c.37.1.11 (A:) Gene 4 protein (g4p, DNA primase), helicase domain {Bacteriophage T7}
MRERIREHLSSEESVGLLFSGCTGINDKTLGARGGEVIMVTSGSGMGKSTFVRQQALQWGTA
MGKKVGLAMLEESVEETAEDLIGLHNRVRLRQSDSLKREIIENGKFDQWFDELFGNDTFHLY
DSFAEAETDRLLAKLAYMRSGLGCDVIILDHISIVVSASGESDERKMIDNLMTKLKGFAKST
GVVLVVICHLKNPDKGKAHEEGRPVSITDLRGSGALRQLSDTIIALERNQQGDMPNLVLVRI
LKCRFTGDTGIAGYMEYNKETGWLEPSSY
>d1g8ya_ c.37.1.11 (A:) Hexameric replicative helicase repA {Escherichia coli, plasmid rsf1010}
ATHKPINILEAFAAAPPPLDYVLPNMVAGTVGALVSPGGAGKSMLALQLAAQIAGGPDLLEV
GELPTGPVIYLPAEDPPTAIHHRLHALGAHLSAEERQAVADGLLIQPLIGSLPNIMAPEWFD
GLKRAAEGRRLMVLDTLRRFHIEEENASGPMAQVIGRMEAIAADTGCSIVFLHHASKGAAMM
GAGDQQQASRGSSVLVDNIRWQSYLSSMTSAEAEEWGVDDDQRRFFVRFGVSKANYGAPFAD
RWFRRHDGGVLKPA
>d1e9ra_ c.37.1.11 (A:) Bacterial conjugative coupling protein TrwB {Escherichia coli}
VGQGEFGGAPFKRFLRGTRIVSGGKLKRMTREKAKQVTVAGVPMPRDAEPRHLLVNGATGTG
KSVLLRELAYTGLLRGDRMVIVDPNGDMLSKFGRDKDIILNPYDQRTKGWSFFNEIRNDYDW
QRYALSVVPRGKTDEAEEWASYGRLLLRETAKKLALIGTPSMRELFHWTTIATFDDLRGFLE
GTLAESLFAGSNEASKALTSARFVLSDKLPEHVTMPDGDFSIRSWLEDPNGGNLFITWREDM
GPALRPLISAWVDVVCTSILSLPEEPKRRLWLFIDELASLEKLASLADALTKGRKAGLRVVA
GLQSTSQLDDVYGVKEAQTLRASFRSLVVLGGSRTDPKTNEDMSLSLGEHEVERDRYSKNTG
KHHSTGRALERVRERVVMPAEIANLPDLTAYVGFAGNRPIAKVPLEIKQFANRQPAFVEGT
>d1e79a3 c.37.1.11 (A:95-379) Central domain of alpha and beta subunits of F1 ATP synthase {Cow (Bos taurus)}
VDVPVGEELLGRVVDALGNAIDGKGPIGSKARRRVGLKAPGIIPRISVREPMQTGIKAVDSL
VPIGRGQRELIIGDRQTGKTSIAIDTIINQKRFNDGTDEKKKLYCIYVAIGQKRSTVAQLVK
RLTDADAMKYTIVVSATASDAAPLQYLAPYSGCSMGEYFRDNGKHALIIYDDLSKQAVAYRQ
MSLLLRRPPGREAYPGDVFYLHSRLLERAAKMNDAFGGGSLTALPVIETQAGDVSAYIPTNV
ISITDGQIFLETELFYKGIRPAINVGLSVSRVGSAAQ
>d1e79d3 c.37.1.11 (D:82-357) Central domain of alpha and beta subunits of F1 ATP synthase {Cow (Bos taurus)}
IRIPVGPETLGRIMNVIGEPIDERGPIKTKQFAAIHAEAPEFVEMSVEQEILVTGIKVVDLL
APYAKGGKIGLFGGAGVGKTVLIMELINNVAKAHGGYSVFAGVGERTREGNDLYHEMIESGV
INLKDATSKVALVYGQMNEPPGARARVALTGLTVAEYFRDQEGQDVLLFIDNIFRFTQAGSE
VSALLGRIPSAVGYQPTLATDMGTMQERITTTKKGSITSVQAIYVPADDLTDPAPATTFAHL
DATTVLSRAIAELGIYPAVDPLDSTSRI
>d1skyb3 c.37.1.11 (B:96-371) Central domain of alpha and beta subunits of F1 ATP synthase {Bacillus sp., strain ps3}
EVPVGETLIGRVVNPLGQPVDGLGPVETTETRPIESRAPGVMDRRSVHEPLQTGIKAIDALV
PIGRGQRELIIGDRQTGKTSVAIDTIINQKDQNMICIYVAIGQKESTVATVVETLAKHGAPD

YTIVVTASASQPAPLLFLAPYAGVAMGEYFMIMGKHVLVVIDDLSKQAAAYRQLSLLLRRPP
GREAYPGDIFYLHSRLLERAAKLSDAKGGGSLTALPFVETQAGDISAYIPTNVISITDGQIF
LQSDLFFSGVRPAINAGLSVSRVGGAAQ
>d1skye3 c.37.1.11 (E:83-356) Central domain of alpha and beta subunits of F1 ATP synthase {Bacillus sp., strain ps3}
ISVPVGQVTLGRVFNVLGEPIDLEGDIPADARRDPIHRPAPKFEELATEVEILETGIKVVDL
LAPYIKGGKIGLFGGAGVGKTVLIQELIHNIAQEHGGISVFAGVGERTREGNDLYHEMKDSG
VISKTAMVFGQMNEPPGARMRVALTGLTMAEYFRDEQGQDGLLFIDNIFRFTQAGSEVSALL
GRMPSAIGYQPTLATEMGQLQERITSTAKGSITSIQAIYVPADDYTDPAPATTFSHLDATTN
LERKLAEMGIYPAVDPLVSTSRALAP
>d1fx0a3 c.37.1.11 (A:97-372) Central domain of alpha and beta subunits of F1 ATP synthase {Spinach (Spinacia oleracea), chloroplast}
QIPVSEAYLGRVINALAKPIDGRGEITASESRLIESPAPGIMSRRSVYEPLQTGLIAIDAMI
PVGRGQRELIIGDRQTGKTAVATDTILNQQGQNVICVYVAIGQKASSVAQVVTNFQERGAME
YTIVVAETADSPATLQYLAPYTGAALAEYFMYRERHTLIIYDDLSKQAQAYRQMSLLLRRPP
GREAYPGDVFYLHSRLLERAAKLSSLLGEGSMTALPIVETQAGDVSAYIPTNVISITDGQIF
LSADLFNAGIRPAINVGISVSRVGSAAQ
>d1fx0b3 c.37.1.11 (B:98-377) Central domain of alpha and beta subunits of F1 ATP synthase {Spinach (Spinacia oleracea), chloroplast}
LSVPVGGPTLGRIFNVLGEPVDNLRPVDTRTTSPIHRSAPAFTQLDTKLSIFETGIKVVNLL
APYRRGGKIGLFGGAGVGKTVLIMELINNIAKAHGGVSVFGGVGERTREGNDLYMEMKESGV
INEQNIAESKVALVYGQMNEPPGARMRVGLTALTMAEYFRDVNEQDVLLFIDNIFRFVQAGS
EVSALLGRMPSAVGYQPTLSTEMGSLQERITSTKEGSITSIQAVYVPADDLTDPAPATTFAH
LDATTVLSRGLAAKGIYPAVDPLDSTSTMLQP
>d1cbua_ c.37.1.11 (A:) Adenosylcobinamide kinase/adenosylcobinamide phosphate guanylyltransferase CobU {Salmonella typhimurium}
MILVTGGARSGKSRHAEALIGDAPQVLYIATSQILDDEMAARIQHHKDGRPAHWRTAECWRH
LDTLITADLAPDDAILLECITTMVTNLLFALGGENDPEQWDYAAMERAIDDEIQILIAACQR
CPAKVVLVTNEVGMGIVPENRLARHFRDIAGRVNQRLAAAADEVWLVVSGIGVKIK
>d1g5ta_ c.37.1.11 (A:) ATP:corrinoid adenosyltransferase CobA {Salmonella typhimurium}
ERGIIIVFTGNGKGKTTAAFGTAARAVGHGKNVGVVQFIKGTWPNGERNLLEPHGVEFQVMA
TGFTWETQNREADTAACMAVWQHGKRMLADPLLDMVVLDELTYMVAYDYLPLEEVISALNAR
PGHQTVIITGRGCHRDILDLADTVSELRPVKHA
>d1g64b_ c.37.1.11 (B:) ATP:corrinoid adenosyltransferase CobA {Salmonella typhimurium}
QQRQQKVKDRVDARVAQAQEERGIIIVFTGNGKGKTTAAFGTAARAVGHGKNVGVVQFIKGT
WPNGERNLLEPHGVEFQVMATGFTWETQNREADTAACMAVWQHGKRMLADPLLDMVVLDELT
YMVAYDYLPLEEVISALNARPGHQTVIITGRGCHRDILDLADTVSELRPVKHAFDAGVKAQM
GIDY
>d1b0ua_ c.37.1.12 (A:) ATP-binding subunit of the histidine

permease {Salmonella typhimurium}
NKLHVIDLHKRYGGHEVLKGVSLQARAGDVISIIGSSGSGKSTFLRCINFLEKPSEGAIIVN
GQNINLVRDKDGQLKVADKNQLRLLRTRLTMVFQHFNLWSHMTVLENVMEAPIQVLGLSKHD
ARERALKYLAKVGIDERAQGKYPVHLSGGQQQRVSIARALAMEPDVLLFDEPTSALDPELVG
EVLRIMQQLAEEGKTMVVVTHEMGFARHVSSHVIFLHQGKIEEEGDPEQVFGNPQSPRLQQF
LKGSLKKLEH
>d1g6ha_ c.37.1.12 (A:) MJ1267 {Archaeon Methanococcus jannaschii}
TMEILRTENIVKYFGEFKALDGVSISVNKGDVTLIIGPNGSGKSTLINVITGFLKADEGRVY
FENKDITNKEPAELYHYGIVRTFQTPQPLKEMTVLENLLIGEICPGESPLNSLFYKKWIPKE
EEMVEKAFKILEFLKLSHLYDRKAGELSGGQMKLVEIGRALMTNPKMIVMDEPIAGVAPGLA
HDIFNHVLELKAKGITFLIIEHRLDIVLNYIDHLYVMFNGQIIAEGRGEEEIKNVLSDPKVV
EIYIGE
>d1f3oa_ c.37.1.12 (A:) MJ0796 {Archaeon Methanococcus jannaschii}
MIKLKNVTKTYKMGEEIIYALKNVNLNIKEGEFVSIMGPSGSGKSTMLNIIGCLDKPTEGEV
YIDNIKTNDLDDDELTKIRRDKIGFVFQQFNLIPLLTALENVELPLIFKYRGAMSGEERRKR
ALECLKMAELEERFANHKPNQLSGGQQQRVAIARALANNPPIILADEPTGALDSKTGEKIMQ
LLKKLNEEDGKTVVVVTHDINVARFGERIIYLKDGEVEREEKLRGF
>d1jj7a_ c.37.1.12 (A:) Peptide transporter Tap1, C-terminal ABC
domain {Human (Homo sapiens)}
GLLTPLHLEGLVQFQDVSFAYPNRPDVLVLQGLTFTLRPGEVTALVGPNGSGKSTVAALLQN
LYQPTGGQLLLDGKPLPQYEHRYLHRQVAAVGQEPQVFGRSLQENIAYGLTQKPTMEEITAA
AVKSGAHSFISGLPQGYDTEVDEAGSQLSGGQRQAVALARALIRKPCVLILDDATSALDANS
QLQVEQLLYESPERYSRSVLLITQHLSLVEQADHILFLEGGAIREGGTHQQLMEKKGCYWAM
VQA
>d1g2912 c.37.1.12 (1:1-240) Maltose transport protein MalK,
N-terminal domain {Archaeon Thermococcus litoralis}
MAGVRLVDVWKVFGEVTAVREMSLEVKDGEFMILLGPSGCGKTTTLRMIAGLEEPSRGQIYI
GDKLVADPEKGIFVPPKDRDIAMVFQSYALYPHMTVYDNIAFPLKLRKVPRQEIDQRVREVA
ELLGLTELLNRKPRELSGGQRQRVALGRAIVRKPQVFLMDEPLSNLDAKLRVRMRAELKKLQ
RQLGVTTIYVTHDQVEAMTMGDRIAVMNRGVLQQVGSPDEVYDKPANTFVAGFI
>g1f2t.1 c.37.1.12 (A:,B:) Rad50 {Archaeon Pyrococcus furiosus}
MKLERVTVKNFRSHSDTVVEFKEGINLIIGQNGSGKSSLLDAILVGLYWPLRIKDIKKDEFT
KVGARDTYIDLIFEKDGTKYRITRRFLKGYSSGEIHAMKRLVGNEWKHVTEPSSKAISAFME
KLIPYNIFLNAIYIRQGQIDAILESXAREAALSKIGELASEIFAEFTEGKYSEVVVRAEENK
VRLFVVWEGKERPLTFLSGGERIALGLAFRLAMSLYLAGEISLLILDEPTPYLDEERRRKLI
TIMERYLKKIPQVILVSHDEELKDAADHVIRISLENGSSKVEVVS
>g1ii8.1 c.37.1.12 (A:,B:) Rad50 {Archaeon Pyrococcus furiosus}
MKLERVTVKNFRSHSDTVVEFKEGINLIIGQNGSGKSSLLDAILVGLYWPLRIKDIKKDEFT
KVGARDTYIDLIFEKDGTKYRITRRFLKGYSSGEIHAMKRLVGNEWKHVTEPSSKAISAFME
KLIPYNIFLNAIYIRQGQIDAILESDEAREKVVREVLNLDKFETAYKKLSELKKTINNRIKE
YRDILARTEXRERVKKEIKDLEKAKDFTEELIEKVKKYKALAREAALSKIGELASEIFAEFT
EGKYSEVVVRAEENKVRLFVVWEGKERPLTFLSGGERIALGLAFRLAMSLYLAGEISLLILD
EPTPYLDEERRRKLITIMERYLKKIPQVILVSHDEELKDAADHVIRISLENGSSKVEVVS
>d1e69a_ c.37.1.12 (A:) Smc head domain {Thermotoga maritima}

MRLKKLYLKGFKSFGRPSLIGFSDRVTAIVGPNGSGKSNIIDAIKWVFGEQSKKELRASEKF
DMIFAGSENLPPAGSAYVELVFEENGEEITVARELKRTGENTYYLNGSPVRLKDIRDRFAGT
GLGVDFYSIVGQGQIDRIVNASPEELRLESSKHPTSLVPRGSYQRVNESFNRFISLLFFGGE
GRLNIVSEAKSILDAGFEISIRKPGRRDQKLSLLSGGEKALVGLALLFALMEIKPSPFYVLD
EVDSPLDDYNAERFKRLLKENSKHTQFIVITHNKIVMEAADLLHGVTMVNGVSAIVPVEV
>d1qhla_ c.37.1.12 (A:) Cell division protein MukB {Escherichia coli}
RGKFRSLTLINWNGFFARTFDLDELVTTLSGGNGAGKSTTMAAFVTALIPDLTLLHFRNTTE
AGATSGSRDKGLHGKLKAGVCYSMLDTINSRHQRVVVGVRLQQVAGRDRKVDIKPFAIQGLP
MSVQPTQLVTETLNERQARVLPLNELKDKLEAMEGVQFKQFNSITDYHSLMFDLGIIARRLR
SASDRSKFYRLIEASLYGGISSAITRSLRDYLLPEN
>d1ewqa2 c.37.1.12 (A:542-765) DNA repair protein MutS, the C-terminal domain {Thermus aquaticus}
YVRPRFGDRLQIRAGRHPVVERRTEFVPNDLEMAHELVLITGPNMAGKSTFLRQTALIALLA
QVGSFVPAEEAHLPLFDGIYTRIGASDDLAGGKSTFMVEMEEVALILKEATENSLVLLDEVG
RGTSSLDGVAIATAVAEALHERRAYTLFATHYFELTALGLPRLKNLHVAAREEAGGLVFYHQ
VLPGPASKSYGVEVAAMAGLPKEVVARARALLQAMAAR
>d1e3ma2 c.37.1.12 (A:567-800) DNA repair protein MutS, the C-terminal domain {Escherichia coli}
YTCPTFIDKPGIRITEGRHPVVEQVLNEPFIANPLNLSPQRRMLIITGPNMGGKSTYMRQTA
LIALMAYIGSYVPAQKVEIGPIDRIFTRVGAADDLASGRSTFMVEMTETANILHNATEYSLV
LMDEIGRGTSTYDGLSLAWACAENLANKIKALTLFATHYFELTQLPEKMEGVANVHLDALEH
GDTIAFMHSVQDGAASKSYGLAVAALAGVPKEVIKRARQKLRELESIS
>d1pjr_1 c.37.1.13 (1-318) DEXX box DNA helicase {Bacillus stearothermophilus, PcrA}
MNFLSEQLLAHLNKEQQEAVRTTEGPLLIMAGAGSGKTRVLTHRIAYLMAEKHVAPWNILAI
TFTNKAAREMRERVQSLLGGAAEDVWISTFHSMCVRILRRDIDRIGINRNFSILDPTDQLSV
MKTILKEKNIDPKKFEPRTILGTISAAKNELLPPEQFAKRASTYYEKVVSDVYQEYQQRLLR
NHSLDFDDLIMTTIQLFDRVPDVLHYYQYKFQYIHIDEYQDTNRAQYTLVKKLAERFQNICA
VGDADQSIYRWRGADIQNILSFERDYPNAKVILLEQNYRSTKRILQAANEVIEHNVNRKPKR
IWTENPEG
>d1pjr_2 c.37.1.13 (319-651) DEXX box DNA helicase {Bacillus stearothermophilus, PcrA}
KPILYYEAMNEADEAQFVAGRIREAVERGERRYRDFAVLYRTNAQSRVMEEMLLKANIPYQI
VGGLKFYDRKEIKDILAYLRVIANPDDDLSLLRIINVPKRGIGASTIDKLVRYAADHELSLF
EALGELEMIGLGAKAAGALAAFRSQLEQWTQLQEYVSVTELVEEVLDKSGYREMLKAERTIE
AQSRLENLDEFLSVTKHFENVSDDKSLIAFLTDLALISDLDELDGTEQAAEGDAVMLMTLHA
AKGLEFPVVFLIGMEEGIFPHNRSLEDDDEMEEERRLAYVGITRAEEELVLTSAQMRTLFGN
IQMDPPSRFLNEIPAHLLETASR
>g1qhh.1 c.37.1.13 (A:,B:,C:,D:) DEXX box DNA helicase {Bacillus stearothermophilus, PcrA}
MNFLSEQLLAHLNKEQQEAVRTTEGPLLIMAGAGSGKTRVLTHRIAYLMAEKHVAPWNILAI
TFTNKAAREMRERVQSLLGGAAEDVWISTFHSMCVRILRRDIDRIGINRNFSILDPTDQLSV
MKTILKEKNIDPKKFEPRTILGTISAAKNELLPPEQFAKRXYYEKVVSDVYQEYQQRLLRNH

SLDFDDLIMTTIQLFDRVPDVLHYYQYKFQYIHIDEYQDTNRAQYTLVKKLAERFQNICAVG
DADQSIYRWRGADIQNILSFERDYPNAKVILLEQNYRSTKRILQAANEVIEHNVNRKPKRIW
TENPEGKPILYYEAMNEADEAQFVAGRIREAVERGERRYRDFAVLYRTNAQSRVMEEMLLKA
NIPYQIVGGLKFYDRKEIKDILAYLRVIANPDDDLSLLRIINVPKRGIGASTIDXLFEALGE
LEMIGLGAKAAGALAAFRSQLEQWTQLQEYVSVTELVEEVLDKSGYREMLKAERTIEAQSRL
ENLDEFLSVTKHFENVSDDKSLIAFLTDLALISXGDAVMLMTLHAAKGLEFPVVFLIGMEEG
IFPHNRSLEDDDEMEEERRLAYVGITRAEEELVLTSAQMRTLFGNIQMDPPSRFLNEIPAHL
LETASR
>d1uaaa1 c.37.1.13 (A:2-307) DEXX box DNA helicase {Escherichia coli, RepD}
RLNPGQQQAVEFVTGPCLVLAGAGSGKTRVITNKIAHLIRGCGYQARHIAAVTFTNKAAREM
KERVGQTLGRKEARGLMISTFHTLGLDIIKREYAALGMKANFSLFDDTDQLALLKELTEGLI
EDDKVLLQQLISTISNWKNDLKTPSQAAASAIGERDRIFAHCYGLYDAHLKACNVLDFDDLI
LLPTLLLQANEEVRKRWQNKIRYLLVDEYQDTNTSQYELVKLLVGSRARFTVVGDDDQSIYS
WRGARPQNLVLLSQDFPALKVIKLEQNYRSSGRILKAANILIANNPHVFEKRLFSELG
>d1uaaa2 c.37.1.13 (A:308-640) DEXX box DNA helicase {Escherichia coli, RepD}
YGAELKVLSANNEEHEAERVTGELIAHHFVNKTQYKDYAILYRGNHQSRVFEKFLMQNRIPY
KISGGTSFFSRPEIKDLLAYLRVLTNPDDDSAFLRIVNTPKREIGPATLKKLGEWAMTRNKS
MFTASFDMGLSQTLSGRGYEALTRFTHWLAEIQRLAEREPIAAVRDLIHGMDYESWLYETSP
SPKAAEMRMKNVNQLFSWMTEMLEGSELDEPMTLTQVVTRFTLRDMMERGESEEELDQVQLM
TLHASKGLEFPYVYMVGMEEGFLPHQSSIDEDNIDEERRLAYVGITRAQKELTFTLCKERRQ
YGELVRPEPSRFLLELPQDDLIW
>d1hv8a1 c.37.1.13 (A:3-210) Putative DEAD box RNA helicase {Archaeon Methanococcus jannaschii}
VEYMNFNELNLSDNILNAIRNKGFEKPTDIQMKVIPLFLNDEYNIVAQARTGSGKTASFAIP
LIELVNENNGIEAIILTPTRELAIQVADEIESLKGNKNLKIAKIYGGKAIYPQIKALKNANI
VVGTPGRILDHINRGTLNLKNVKYFILDEADEMLNMGFIKDVEKILNACNKDKRILLFSATM
PREILNLAKKYMGDYSFIKAKI
>d1hv8a2 c.37.1.13 (A:211-365) Putative DEAD box RNA helicase {Archaeon Methanococcus jannaschii}
NANIEQSYVEVNENERFEALCRLLKNKEFYGLVFCKTKRDTKELASMLRDIGFKAGAIHGDL
SQSQREKVIRLFKQKKIRILIATDVMSRGIDVNDLNCVINYHLPQNPESYMHRIGRTGRAGK
KGKAISIINRREYKKLRYIERAMKLKIKKLK
>d1gm5a3 c.37.1.13 (A:286-549) RecG helicase domain {Thermotoga maritima}
ETLPERILEKRKLLGVKDAYYGMHFPKTFYHLEKARERLAYEELFVLQLAFQKIRKEREKHG
GIPKKIEGKLAEEFIKSLPFKLTNAQKRAHQEIRNDMISEKPMNRLLQGDVGSGKTVVAQLA
ILDNYEAGFQTAFMVPTSILAIQHYRRTVESFSKFNIHVALLIGATTPSEKEKIKSGLRNGQ
IDVVIGTHALIQEDVHFKNLGLVIIDEQHRFGVKQREALMNKGKMVDTLVMSATPIPRSMAL
AFYGDLDVTVIDEMPP
>d1gm5a4 c.37.1.13 (A:550-755) RecG helicase domain {Thermotoga maritima}
GRKEVQTMLVPMDRVNEVYEFVRQEVMRGGQAFIVYPLIEESDKLNVKSAVEMYEYLSKEVF

PEFKLGLMHGRLSQEEKDRVMLEFAEGRYDILVSTTVIEVGIDVPRANVMVIENPERFGLAQ
LHQLRGRVGRGGQEAYCFLVVGDVGEEAMERLRFFTLNTDGFKIAEYDLKTRGPGEFFGVKQ
HGLSGFKVADLYRDLKLLEW
>d1fuka_ c.37.1.13 (A:) Initiation factor 4a {Baker's yeast (Saccharomyces cerevisiae)}
IKQFYVNVEEEEYKYECLTDLYDSISVTQAVIFCNTRRKVEELTTKLRNDKFTVSAIYSDLP
QQERDTIMKEFRSGSSRILISTDLLARGIDVQQVSLVINYDLPANKENYIHRIGRGGRFGRK
GVAINFVTNEDVGAMRELEKFYSTQIEELPSDIATLLN
>d1qdea_ c.37.1.13 (A:) Initiation factor 4a {Baker's yeast (Saccharomyces cerevisiae)}
IQTNYDKVVYKFDDMELDENLLRGVFGYGFEEPSAIQQRAIMPIIEGHDVLAQAQSGTGKTG
TFSIAALQRIDTSVKAPQALMLAPTRELALQIQKVVMALAFHMDIKVHACIGGTSFVEDAEG
LRDAQIVVGTPGRVFDNIQRRRFRTDKIKMFILDEADEMLSSGFKEQIYQIFTLLPPTTQVV
LLSATMPNDVLEVTTKFMRNPVRILV
>d1c4oa1 c.37.1.13 (A:2-409) Nucleotide excision repair enzyme UvrB {Thermus thermophilus}
TFRYRGPSPKGDQPKAIAGLVEALRDGERFVTLLGATGTGKTVTMAKVIEALGRPALVLAPN
KILAAQLAAEFRELFPENAVEYFISYYDYYQPEAYVPGKDLYIEKDASINPEIERLRHSTTR
SLLTRRDVIVVASVSAIYGLGDPREYRARNLVVERGKPYPREVLLERLLELGYQRNDIDLSP
GRFRAKGEVLEIFPAYETEPIRVELFGDEVERISQVHPVTGERLRELPGFVLFPATHYLSPE
GLEEILKEIEKELWERVRYFEERGEVLYAQRLKERTLYDLEMLRVMGTCPGVENYARYFTGK
APGEPPYTLLDYFPEDFLVFLDESHVTVPQLQGMYRGDYARKKTLVDYGFRLPSALDNRPLR
FEEFLERVSQVVFVSATPGPFELAHSGRVVEQIIRP
>d1c4oa2 c.37.1.13 (A:410-583) Nucleotide excision repair enzyme UvrB {Thermus thermophilus}
TGLLDPLVRVKPTENQILDLMEGIRERAARGERTLVTVLTVRMAEELTSFLVEHGIRARYLH
HELDAFKRQALIRDLRLGHYDCLVGINLLREGLDIPEVSLVAILDADKEGFLRSERSLIQTI
GRAARNARGEVWLYADRVSEAMQRAIEETNRRRALQEAYNLEHGITPETV
>d1d9xa1 c.37.1.13 (A:2-414) Nucleotide excision repair enzyme UvrB {Bacillus caldotenax}
EGRFQLVAPYEPQGDQPQAIAKLVDGLRRGVKHQTLLGATGTGKTFTISNVIAQVNKPTLVI
AHNKTLAGQLYSELKEFFPHNAVEYFVSYYDYYQPEAYVPQTDTYIEKDAKINDEIDKLRHS
ATSALFERRDVIIVASVSCIYGLGSPEEYRELVVSLRVGMEIERNALLRRLVDIQYDRNDID
FRGTFRVRGDVVEIFPASRDEHCIRVEFFGDEIERIRAEVDALTGKVLGEREHVAIFPASHF
VTREEKMRLAIQNIEQELEERLAELRAQGKLLEAQRLEQRTRYDLEMMREMGFCSGIENYSR
HLALRPPGSTPYTLLDYFPDDFLIIVDESHVTLPQLRGMYNGDRARKQVLVDHGFRLPSALD
NRPLTFEEFEQKINQIIYVSATPGPYELEHSPGVVEQIIRP
>d1d9xa2 c.37.1.13 (A:415-595) Nucleotide excision repair enzyme UvrB {Bacillus caldotenax}
TGLLDPTIDVRPTKGQIDDLIGEIRERVERNERTLVTTLTKKMAEDLTDYLKEAGIKVAYLH
SEIKTLERIEIIRDLRLGKYDVLVGINLLREGLDIPEVSLVAILDADKEGFLRSERSLIQTI
GRAARNANGHVIMYADTITKSMEIAIQETKRRRAIQEEYNRKHGIVPRTVKKEIRDV
>d1jr3a2 c.37.1.13 (A:3-242) gamma subunit of DNA polymerase III, N-domain {Escherichia coli}

YQVLARKWRPQTFADVVGQEHVLTALANGLSLGRIHHAYLFSGTRGVGKTSIARLLAKGLNC
ETGITATPCGVCDNCREIEQGRFVDLIEIDAASRTKVEDTRDLLDNVQYAPARGRFKVYLID
EVHMLSRHSFNALLKTLEEPPEHVKFLLATTDPQKLPVTILSRCLQFHLKALDVEQIRHQLE
HILNEEHIAHEPRALQLLARAAEGSLRDALSLTDQAIASGDGQVSTQAVSAMLG
>d1a5t_2 c.37.1.13 (1-207) delta prime subunit of DNA polymerase III, N-domain {Escherichia coli}
MRWYPWLRPDFEKLVASYQAGRGHHALLIQALPGMGDDALIYALSRYLLCQQPQGHKSCGHC
RGCQLMQAGTHPDYYTLAPEKGKNTLGVDAVREVTEKLNEHARLGGAKVVWVTDAALLTDAA
ANALLKTLEEPPAETWFFLATREPERLLATLRSRCRLHYLAPPPEQYAVTWLSREVTMSQDA
LLAALRLSAGSPGAALALFQG
>d1jqlb_ c.37.1.13 (B:) delta subunit of DNA polymerase III, N-domain {Escherichia coli}
MIRLYPEQLRAQLNEGLRAAYLLLGNDPLLLQESQDAVRQVAAAQGFEEHHTFSIDPNTDWN
AIFSLCQAMSLFASRQTLLLLLPENGPNAAINEQLLTLTGLLHDDLLLIVRGNKLSKAQENA
AWFTALANRSVQVTCQ
>d1jr3d2 c.37.1.13 (D:1-211) delta subunit of DNA polymerase III, N-domain {Escherichia coli}
MIRLYPEQLRAQLNEGLRAAYLLLGNDPLLLQESQDAVRQVAAAQGFEEHHTFSIDPNTDWN
AIFSLCQAMSLFASRQTLLLLLPENGPNAAINEQLLTLTGLLHDDLLLIVRGNKLSKAQENA
AWFTALANRSVQVTCQTPEQAQLPRWVAARAKQLNLELDDAANQVLCYCYEGNLLALAQALE
RLSLLWPDGKLTLPRVEQAVNDAAH
>d1iqpa2 c.37.1.13 (A:2-232) Replication factor C {Archaeon Pyrococcus furiosus}
SEEIREVKVLEKPWVEKYRPQRLDDIVGQEHIVKRLKHYVKTGSMPHLLFAGPPGVGKTTAA
LALARELFGENWRHNFLELNASDERGINVIREKVKEFARTKPIGGASFKIIFLDEADALTQD
AQQALRRTMEMFSSNVRFILSCNYSSKIIEPIQSRCAIFRFRPLRDEDIAKRLRYIAENEGL
ELTEEGLQAILYIAEGDMRRAINILQAAAALDKKITDENVFMVAS
>d1hqca2 c.37.1.13 (A:5-242) Holliday junction helicase RuvB {Thermus thermophilus}
ALRPKTLDEYIGQERLKQKLRVYLEAAKARKEPLEHLLLFGPPGLGKTTLAHVIAHELGVNL
RVTSGPAIEKPGDLAAILANSLEEGDILFIDEIHRLSRQAEEHLYPAMEDFVMDIVIGQGPA
ARTIRLELPRFTLIGATTRPGLITAPLLSRFGIVEHLEYYTPEELAQGVMRDARLLGVRITE
EAALEIGRRSRGTMRVAKRLFRRVRDFAQVAGEEVITRERALEALAALGLDE
>d1in4a2 c.37.1.13 (A:17-254) Holliday junction helicase RuvB {Thermotoga maritima}
QFLRPKSLDEFIGQENVKKKLSLALEAAKMRGEVLDHVLLAGPPGLGKTTLAHIIASELQTN
IHVTSGPVLVKQGDMAAILTSLERGDVLFIDEIHRLNKAVEELLYSAIEDFQIDIMIGKGPS
AKSIRIDIQPFTLVGATTRSGLLSSPLRSRFGIILELDFYTVKELKEIIKRAASLMDVEIED
AAAEMIAKRSRGTPRIAIRLTKRVRDMLTVVKADRINTDIVLKTMEVLNIDD
>d1fnna2 c.37.1.13 (A:1-276) CDC6, N-domain {Archaeon Pyrobaculum aerophilum}
AIVVDDSVFSPSYVPKRLPHREQQLQQLDILLGNWLRNPGHHYPRATLLGRPGTGKTVTLRK
LWELYKDKTTARFVYINGFIYRNFTAIIGEIARSLNIPFPRRGLSRDEFLALLVEHLRERDL
YMFLVLDDAFNLAPDILSTFIRLGQEADKLGAFRIALVIVGHNDAVLNNLDPSTRGIMGKYV

IRFSPYTKDQIFDILLDRAKAGLAEGSYSEDILQMIADITGAQTPLDTNRGDARLAIDILYR
SAYAAQQNGRKHIAPEDVRKSSKEVLFG
>d1d2na_ c.37.1.13 (A:) Hexamerization domain of
N-ethylmalemide-sensitive fusion (NSF) protein {Chinese hamster
(Cricetulus griseus)}
EDYASYIMNGIIKWGDPVTRVLDDGELLVQQTKNSDRTPLVSVLLEGPPHSGKTALAAKIAE
ESNFPFIKICSPDKMIGFSETAKCQAMKKIFDDAYKSQLSCVVVDDIERLLDYVPIGPRFSN
LVLQALLVLLKKAPPQGRKLLIIGTTSRKDVLQEMEMLNAFSTTIHVPNIATGEQLLEALEL
LGNFKDKERTTIAQQVKGKKVWIGIKKLLMLIEMSLQMDPEYRVRKFLALLREEGASPLD
>d1e32a2 c.37.1.13 (A:201-458) Membrane fusion atpase p97, D1
domain {Mouse (Mus musculus)}
VGYDDVGGCRKQLAQIKEMVELPLRHPALFKAIGVKPPRGILLYGPPGTGKTLIARAVANET
GAFFFLINGPEIMSKLAGESESNLRKAFEEAEKNAPAIIFIDELDAIAPKREKTHGEVERRI
VSQLLTLMDGLKQRAHVIVMAATNRPNSIDPALRRFGRFDREVDIGIPDATGRLEILQIHTK
NMKLADDVDLEQVANETHGHVGADLAALCSEAALQAIRKKMDLIDLEDETIDAEVMNSLAVT
MDDFRWALSQ
>d1g6oa_ c.37.1.13 (A:) Hexameric traffic ATPase, HP0525
{Helicobacter pylori}
LSAEDKKFLEVERALKEAALNPLRHATEELFGDFLKMENITEICYNGNKVVWVLKNNGEWQP
FDVRDRKAFSLSRLMHFARCCASFKKKTIDNYENPILSSNLANGERVQIVLSPVTVNDETIS
ISIRIPSKTTYPHSFFEEQGFYNLLDNKEQAISAIKDGIAIGKNVIVCGGTGSGKTTYIKSI
MEFIPKEERIISIEDTEEIVFKHHKNYTQLFFGGNITSADCLKSCLRMRPDRIILGELRSSE
AYDFYNVLCSGHKGTLTTLHAGSSEEAFIRLANMSSSNSAARNIKFESLIEGFKDLIDMIVH
INHHKQCDEFYIK
>d1ht1e_ c.37.1.13 (E:) HslU {Escherichia coli}
HSEMTPREIVSELDKHIIGQDNAKRSVAIALRNRWRRMQLNEELRHEVTPKNILMIGPTGVG
KTEIARRLAKLANAPFIKVEATKFTEVGYVGKEVDSIIRDLTDAAVKMVRVQAIEKNRYRAE
ELAEERILDVLIPPAKNNWGQTEQQQEPSAARQAFRKKLREGQLDDKEIEIDLAAAPMGVEI
MAPPGMEEMTSQLQSMFQNLGGQKQKARKLKIKDAMKLLIEEEAAKLVNPEELKQDAIDAVE
QHGIVFIDEIDKICKRGESSGPDVSREGVQRDLLPLVEGCTVSTKHGMVKTDHILFIASGAF
QIAKPSDLIPELQGRLPIRVELQALTTSDFERILTEPNASITVQYKALMATEGVNIEFTDSG
IKRIAEAAWQVNESTENIGARRLHTVLERLMEEISYDASDLSGQNITIDADYVSKHLDALVA
DEDLSRFIL
>d1g41a_ c.37.1.13 (A:) HslU {Haemophilus influenzae}
SEMTPREIVSELDQHIIGQADAKRAVAIALRNRWRRMQLQEPLRHEVTPKNILMIGPTGVGK
TEIARRLAKLANAPFIKVEATKFTEVGYVGKEVDSIIRDLTDSAMKLVRQQEIAKNRARAED
VAEERILDALLPPAKNQWGEVENHDSHSSTRQAFRKKLREGQLDDKEIEIDVSAGVSMGVEI
MAPPGMEEMTNQLQSLFQNLGSDKTKKRKMKIKDALKALIDDEAAKLINPEELKQKAIDAVE
QNGIVFIDEIDKICKKGEYSGADVSREGVQRDLLPLVEGSTVSTKHGMVKTDHILFIASGAF
QVARPSDLIPELQGRLPIRVELTALSAADFERILTEPHASLTEQYKALMATEGVNIAFTTDA
VKKIAEAAFRVNEKTENIGARRLHTVMERLMDKISFSASDMNGQTVNIDAAYVADALGEVVE
NEDLSRFIL
>d1g8pa_ c.37.1.13 (A:) ATPase subunit of magnesium chelatase, BchI
{Rhodobacter capsulatus}

RPVFPFSAIVGQEDMKLALLLTAVDPGIGGVLVFGDRGTGKSTAVRALAALLPEIEAVEGCP
VSSPNVEMIPDWATVLSTNVIRKPTPVVDLPLGVSEDRVVGALDIERAISKGEKAFEPGLLA
RANRGYLYIDECNLLEDHIVDLLLDVAQSGENVVERDGLSIRHPARFVLVGSGNPEEGDLRP
QLLDRFGLSVEVLSPRDVETRVEVIRRRDTYDADPKAFLEEWRPKDMDIRNQILEARERLPK
VEAPNTALYDCAALCIALGSDGLRGELTLLRSARALAALEGATAVGRDHLKRVATMALSHRL
RRDPLDEAGSTARVARTVEETLP
>d1a1va1 c.37.1.14 (A:190-325) HCV helicase domain {Human hepatitis C virus (HCV), different isolates}
PPAVPQSFQVAHLHAPTGSGKSTKVPAAYAAQGYKVLVLNPSVAATLGFGAYMSKAHGVDPN
IRTGVRTITTGSPITYSTYGKFLADGGCSGGAYDIIICDECHSTDATSILGIGTVLDQAETA
GARLVVLATATP
>d1a1va2 c.37.1.14 (A:326-624) HCV helicase domain {Human hepatitis C virus (HCV), different isolates}
PGSVTVPHPNIEEVALSTTGEIPFYGKAIPLEVIKGGRHLIFCHSKKKCDELAAKLVALGIN
AVAYYRGLDVSVIPTSGDVVVVATDALMTGFTGDFDSVIDCNTCVTQTVDFSLDPTFTIETT
TLPQDAVSRTQRRGRTGRGKPGIYRFVAPGERPSGMFDSSVLCECYDAGCAWYELTPAETTV
RLRAYMNTPGLPVCQDHLEFWEGVFTGLTHIDAHFLSQTKQSGENFPYLVAYQATVCARAQA
PPPSWDQMWKCLIRLKPTLHGPTPLLYRLGAVQNEVTLTHPITKYIMTCMS
>d1cu1a3 c.37.1.14 (A:326-631) HCV helicase domain {Human hepatitis C virus (HCV), different isolates}
PGSVTVPHPNIEEVALSNTGEIPFYGKAIPIEAIRGGRHLIFCHSKKKCDELAAKLSGLGIN
AVAYYRGLDVSVIPTIGDVVVVATDALMTGYTGDFDSVIDCNTCVTQTVDFSLDPTFTIETT
TVPQDAVSRSQRRGRTGRGRRGIYRFVTPGERPSGMFDSSVLCECYDAGCAWYELTPAETSV
RLRAYLNTPGLPVCQDHLEFWESVFTGLTHIDAHFLSQTKQAGDNFPYLVAYQATVCARAQA
PPPSWDQMWKCLIRLKPTLHGPTPLLYRLGAVQNEVTLTHPITKYIMACMSADLEVVT
>d1gkub1 c.37.1.16 (B:1-250) Helicase-like "domain" of reverse gyrase {Archaeon Archaeoglobus fulgidus}
AAAAAAAAAAAAAAAAAAAAAAAAAAAAAAAASLCLFPEDFLLKEFVEFFRKCVGEPRAIQK
MWAKRILRKESFAATAPTGVGKTSFGLAMSLFLALKGKRCYVIFPTSLLVIQAAETIRKYAE
KAGVGTENLIGYYHGRIPKREKENFMQNLRNFKIVITTTQFLSKHYRELGHFDFIFVDDVDA
ILKASKNVDKLLHLLGFHYDLKTKSWVGEARGCLMVSTATAKKGKKAELFRQLLNFDIGSSR
IT
>d1gkub2 c.37.1.16 (B:251-498) Helicase-like "domain" of reverse gyrase {Archaeon Archaeoglobus fulgidus}
VRNVEDVAVNDESISTLSSILEKLGTGGIIYARTGEEAEEIYESLKNKFRIGIVTATKKGDY
EKFVEGEIDHLIGTAHYYGTLVRGLDLPERIRFAVFVGCPSFRVTIEDIDSLSPQMVKLLAY
LYRNVDEIERLLPAVERHIDEVREILKKVMGKERPQAKDVVVREGEVIFPDLRTYIQGSGRT
SRLFAGGLTKGASFLLEDDSELLSAFIERAKLYDIEFKSIDEVDFEKLSRELDESRDRYRRR
>d1gl9b1 c.37.1.16 (B:2-250) Helicase-like "domain" of reverse gyrase {Archaeon Archaeoglobus fulgidus}
IPVVYSNLCPVCGGDLESKEIEKHVCFRKKRSLCLFPEDFLLKEFVEFFRKCVGEPRAIQKM
WAKRILRKESFAATAPTGVGKTSFGLAMSLFLALKGKRCYVIFPTSLLVIQAAETIRKYAEK
AGVGTENLIGYYHGRIPKREKENFMQNLRNFKIVITTTQFLSKHYRELGHFDFIFVDDVDAI
LKASKNVDKLLHLLGFHYDLKTKSWVGEARGCLMVSTATAKKGKKAELFRQLLNFDIGSSRI

T
>d1ble__ c.38.1.1 (-) Fructose permease, subunit IIb {Bacillus subtilis}
MNIVLARIDDRFIHGQILTRWIKVHAADRIIVVSDDIAQDEMRKTLILSVAPSNVKASAVSV
SKMAKAFHSPRYEGVTAMLLFENPSDIVSLIEAGVPIKTVNVGGMRFENHRRQITKSVSVTE
QDIKAFETLSDKGVKLELRQLPSDASEDFVQILRNVT
>d1d0va_ c.39.1.1 (A:) Nicotinate mononucleotide:5,6-dimethylbenzimidazole phosphoribosyltransferase (CobT) {Salmonella typhimurium}
LHALLRDIPAPDAEAMARTQQHIDGLLKPPGSLGRLETLAVQLAGMPGLNGTPQVGEKAVLV
MCADHGVWDEGVAVSPKIVTAIQAANMTRGTTGVCVLAAQAGAKVHVIDVGIDAEPIPGVVN
MRVARGCGNIAVGPAMSRLQAEALLLEVSRYACDLAQRGVTLFGVGELGMANTTPAAAMVSV
FTGSDAKEVVGIGANLPPSRIDNKVDVVRRAIAINQPNPRDGIDVLSKVGGFDLVGMTGVML
GAARCGLPVLLDGFLSYSAALAACQIAPAVRPYLIPSHFSAEKGARIALAHLSMEPYLHMAM
RLGEGSGAALAMPIVEAACAMFHNMGELAASNIVLP
>d1chd__ c.40.1.1 (-) Methylesterase CheB, C-terminal domain {Salmonella typhimurium}
LLSSEKLIAIGASTGGTEAIRHVLQPLPLSSPAVIITQHMPPGFTRSFAERLNKLCQISVKE
AEDGERVLPGHAYIAPGDKHMELARSGANYQIKIHDGPPVNRHRPSVDVLFHSVAKHAGRNA
VGVILTGMGNDGAAGMLAMYQAGAWTIAQNEASCVVFGMPREAINMGGVSEVVDLSQVSQQM
LAKISAGQAIRI
>d1csee_ c.41.1.1 (E:) Subtilisin {Bacillus subtilis, carlsberg}
AQTVPYGIPLIKADKVQAQGFKGANVKVAVLDTGIQASHPDLNVVGGASFVAGEAYNTDGNG
HGTHVAGTVAALDNTTGVLGVAPSVSLYAVKVLNSSGSGSYSGIVSGIEWATTNGMDVINMS
LGGASGSTAMKQAVDNAYARGVVVVAAAGNSGNSGSTNTIGYPAKYDSVIAVGAVDSNSNRA
SFSSVGAELEVMAPGAGVYSTYPTNTYATLNGTSMASPHVAGAAALILSKHPNLSASQVRNR
LSSTATYLGSSFYYGKGLINVEAAAQ
>d1bh6a_ c.41.1.1 (A:) Subtilisin {Bacillus licheniformis}
AQTVPYGIPLIKADKVQAQGYKGANVKVGIIDTGIASSHTDLKVVGGASFVSGESYNTDGNG
HGTHVAGTVAALDNTTGVLGVAPNVSLYAIKVLNSSGSGSYSAIVSGIEWATQNGLDVINMS
LGGPSGSTALKQAVDKAYASGIVVVAAAGNSGNSGSQNTIGYPAKYDSVIAVGAVDSNKNRA
SFSSVGSELEVMAPGVSVYSTYPSNTYTSLNGTSMASPHVAGAAALILSKYPTLSASQVRNR
LSSTATNLGDSFYYGKGLINVEAAAQ
>d1gci__ c.41.1.1 (-) Subtilisin {Bacillus lentus}
AQSVPWGISRVQAPAAHNRGLTGSGVKVAVLDTGISTHPDLNIRGGASFVPGEPSTQDGNGH
GTHVAGTIAALNNSIGVLGVAPSAELYAVKVLGASGSGSVSSIAQGLEWAGNNGMHVANLSL
GSPSPSATLEQAVNSATSRGVLVVAASGNSGAGSISYPARYANAMAVGATDQNNNRASFSQY
GAGLDIVAPGVNVQSTYPGSTYASLNGTSMATPHVAGAAALVKQKNPSWSNVQIRNHLKNTA
TSLGSTNLYGSGLVNAEAATR
>d1s01__ c.41.1.1 (-) Subtilisin {Bacillus amyloliquefaciens, Novo/BPN'}
AQSVPYGVSQIKAPALHSQGYTGSNVKVAVIDSGIDSSHPDLKVAGGASFVPSETPNFQDDN
SHGTHVAGTVAALDNSIGVLGVAPSSALYAVKVLGDAGSGQYSWIINGIEWAIANNMDVINM
SLGGPSGSAALKAAVDKAVASGVVVVAAAGNEGSTGSSSTVGYPGKYPSVIAVGAVDASNQR

ASFSSVGPELDVMAPGVSICSTLPGNKYGAKSGTSMASPHVAGAAALILSKHPNWTNTQVRS
SLQNTTTKLGDSFYYGKGLINVQAAAQ
>d1sue__ c.41.1.1 (-) Subtilisin {Bacillus amyloliquefaciens, Novo/BPN'}
AKCVSYGVSQIKAPALHSQGYTGSNVKVAVIDSGIDSSHPDLNVAGGASFVPSETNPFQDNN
SHGTHVAGTVLAVAPSASLYAVKVLGADGSGQYSWIINGIEWAIANNMDVINMSLGGPSGSA
ALKAAVDKAVASGVVVVAAAGNEGTSGSSSTVGYPGKYPSVIAVGAVDSSNQRASFSSVGPE
LDVMAPGVSICSTLPGNKYGAKSGTSMASPHVAGAAALILSKHPNWTNTQVRSSLENTTTKL
GDSFYYGKGLINVEAAAQ
>d1sup__ c.41.1.1 (-) Subtilisin {Bacillus amyloliquefaciens, Novo/BPN'}
AQSVPYGVSQIKAPALHSQGYTGSNVKVAVIDSGIDSSHPDLKVAGGASMVPSETNPFQDNN
SHGTHVAGTVAALNNSIGVLGVAPSASLYAVKVLGADGSGQYSWIINGIEWAIANNMDVINM
SLGGPSGSAALKAAVDKAVASGVVVVAAAGNEGTSGSSSTVGYPGKYPSVIAVGAVDSSNQR
ASFSSVGPELDVMAPGVSIQSTLPGNKYGAYNGTSMASPHVAGAAALILSKHPNWTNTQVRS
SLENTTTKLGDSFYYGKGLINVQAAAQ
>d1meea_ c.41.1.1 (A:) Messentericopeptidase {Bacillus mesentericus}
AQSVPYGISQIKAPALHSQGYTGSNVKVAVIDSGIDSSHPDLNVRGGASFVPSETNPYQDGS
SHGTHVAGTIAALNNSIGVLGVAPSASLYAVKVLDSTGSGQYSWIINGIEWAISNNMDVINM
SLGGPTGSTALKTVVDKAVSSGIVVAAAAGNEGSSGSTSTVGYPAKYPSTIAVGAVNSANQR
ASFSSAGSELDVMAPGVSIQSTLPGGTYGAYNGTSMATPHVAGAAALILSKHPTWTNAQVRD
RLESTATYLGSSFYYGKGLINVQAAAQ
>d1dbia_ c.41.1.1 (A:) Thermostable serine protease {Bacillus sp., AK.1}
WTPNDTYYQGYQYGPQNTYTDYAWDVTKGSSGQEIAVIDTGVDYTHPDLDGKVIKGYDFVDN
DYDPMDLNNHGTHVAGIAAAETNNATGIAGMAPNTRILAVRALDRNGSGTLSDIADAIIYAA
DSGAEVINLSLGCDCHTTTLENAVNYAWNKGSVVVAAAGNNGSSTTFEPASYENVIAVGAVD
QYDRLASFSNYGTWVDVVAPGVDIVSTITGNRYAYMSGTSMASPHVAGLAALLASQGRNNIE
IRQAIEQTADKISGTGTYFKYGRINSYNAVTY
>d1thm__ c.41.1.1 (-) Thermitase {Thermoactinomyces vulgaris}
YTPNDPYFSSRQYGPQKIQAPQAWDIAEGSGAKIAIVDTGVQSNHPDLAGKVVGGWDFVDND
STPQNGNGHGTHCAGIAAAVTNNSTGIAGTAPKASILAVRVLDNSGSGTWTAVANGITYAAD
QGAKVISLSLGGTVGNSGLQQAVNYAWNKGSVVVAAAGNAGNTAPNYPAYYSNAIAVASTDQ
NDNKSSFSTYGSWVDVAAPGSSIYSTYPTSTYASLSGTSMATPHVAGVAGLLASQGRSASNI
RAAIENTADKISGTGTYWAKGRVNAYKAVQY
>d1ic6a_ c.41.1.1 (A:) Proteinase K {Fungus (Tritirachium album), strain limber}
AAQTNAPWGLARISSTSPGTSTYYYDESAGQGSCVYVIDTGIEASHPEFEGRAQMVKTYYYS
SRDGNGHGTHCAGTVGSRTYGVAKKTQLFGVKVLDDNGSGQYSTIIAGMDFVASDKNNRNCP
KGVVASLSLGGGYSSSVNSAAARLQSSGVMVAVAAGNNNADARNYSPASEPSVCTVGASDRY
DRRSSFSNYGSVLDIFGPGTDILSTWIGGSTRSISGTSMATPHVAGLAAYLMTLGKTTAASA
CRYIADTANKGDLSNIPFGTVNLLAYNNYQA
>d1ga6a_ c.41.1.2 (A:) Serine-carboxyl proteinase PSCP

{Pseudomonas sp.}
AGTAKGHNPTEFPTIYDASSAPTAANTTVGIITIGGVSQTLQDLQQFTSANGLASVNTQTIQ
TGSSNGDYSDDQQGQGEWDLDSQSIVGSAGGAVQQLLFYMADQSASGNTGLTQAFNQAVSDN
VAKVINVSLGWCEADANADGTLQAEDRIFATAAAQGQTFSVSSGDEGVYECNNRGYPDGSTY
SVSWPASSPNVIAVGGTTLYTTSAGAYSNETVWNEGLDSNGKLWATGGGYSVYESKPSWQSV
VSGTPGRRLLPDISFDAAQGTGALIYNYGQLQQIGGTSLASPIFVGLWARLQSANSNSLGFP
AASFYSAISSTPSLVHDVKSGNNGYGGYGYNAGTGWDYPTGWGSLDIAKLSAYIRSNGF
>d1d3va_ c.42.1.1 (A:) Arginase {Rat (Rattus norvegicus)}
KPIEIIGAPFSKGQPRGGVEKGPAALRKAGLVEKLKETEYNVRDHGDLAFVDVPNDSPFQIV
KNPRSVGKANEQLAAVVAETQKNGTISVVLGGDHSMAIGSISGHARVHPDLCVIWVDAHTDI
NTPLTTSSGNLHGQPVAFLLKELKGKFPDVPGFSWVTPCISAKDIVYIGLRDVDPGEHYIIK
TLGIKYFSMTEVDKLGIGKVMEETFSYLLGRKKRPIHLSFDVDGLDPVFTPATGTPVVGGLS
YREGLYITEEIYKTGLLSGLDIMEVNPTLGKTPEEVTRTVNTAVALTLSCFGTKREGNHK
>d2ceva_ c.42.1.1 (A:) Arginase {Bacillus caldovelox}
KPISIIGVPMDLGQTRRGVDMGPSAMRYAGVIERLERLHYDIEDLGDIPIGKAERLHEQGDS
RLRNLKAVAEANEKLAAAVDQVVQRGRFPLVLGGDHSIAIGTLAGVAKHYERLGVIWYDAHG
DVNTAETSPSGNIHGMPLAASLGFGHPALTQIGGYSPKIKPEHVVLIGVRSLDEGEKKFIRE
KGIKIYTMHEVDRLGMTRVMEETIAYLKERTDGVHLSLDLDGLDPSDAPGVGTPVIGGLTYR
ESHLAMEMLAEAQIITSAEFVEVNPILDERNKTASVAVALMGSLFGEKLM
>d1c3pa_ c.42.1.2 (A:) HDAC homologue {Aquifex aeolicus}
KKVKLIGTLDYGKYRYPKNHPLKIPRVSLLLRFKDAMNLIDEKELIKSRPATKEELLLFHTE
DYINTLMEAERCQCVPKGAREKYNIGGYENPVSYAMFTGSSLATGSTVQAIEEFLKGNVAFN
PAGGMHHAFKSRANGFCYINNPAVGIEYLRKKGFKRILYIDLDAHHCDGVQEAFYDTDQVFV
LSLHQSPEYAFPFEKGFLEEIGEGKGKGYNLNIPLPKGLNDNEFLFALEKSLEIVKEVFEPE
VYLLQLGTDPLLEDYLSKFNLSNVAFLKAFNIVREVFGEGVYLGGGGYHPYALARAWTLIWC
ELSGREVPEKLNNKAKELLKSIDFEEFDDEVDRSYMLETLKDPWRGGEVRKEVKDTLEKAKA
>d1hf2a2 c.102.1.1 (A:1-99) Cell-division inhibitor MinC, N-terminal domain {Thermotoga maritima}
MVDFKMTKEGLVLLIKDYQNLEEVLNAISARITQMGGFFAKGDRISLMIENHNKHSQDIPRI
VSHLRNLGLEVSQILVGSTVEGKENDLKVQSRTTVES
>d1jkea_ c.110.1.1 (A:) D-Tyr tRNAtyr deacylase {Escherichia coli}
MIALIQRVTRASVTVEGEVTGEIGAGLLVLLGVEKDDDEQKANRLCERVLGYRIFSDAEGKM
NLNVQQAGGSVLVVSQFTLAADTERGMRPSFSKGASPDRAEALYDYFVERCRQQEMNTQTGR
FAADMQVSLVNDGPVTFWLQV
>d1nocb_ c.43.1.1 (B:) Chloramphenicol acetyltransferase {Escherichia coli}
ITGYTTVDISQWHRKEHFEAFQSVAQCTYNQTVQLDITAFLKTVKKNKHKFYPAFIHILARL
MNAHPEFRMAMKDGELVIWDSVHPCYTVFHEQTETFSSLWSEYHDDFRQFLHIYSQDVACYG
ENLAYFPKGFIENMFFVSANPWVSFTSFDLNVANMDNFFAPVFTMGKYYTQGDKVLMPLAIQ
VHHAVCDGFHVGRMLNELQQYCDEWQG
>d3cla__ c.43.1.1 (-) Chloramphenicol acetyltransferase {Escherichia coli}
MNYTKFDVKNWVRREHFEFYRHRLPCGFSLTSKIDITTLKKSLDDSAYKFYPVMIYLIAQAV
NQFDELRMAIKDDELIVWDSVDPQFTVFHQETETFSALSCPYSSDIDQFMVNYLSVMERYKS

DTKLFPQGVTPENHLNISALPWVNFDSFNLNVANFTDYFAPIITMAKYQQEGDRLLLPLSVQ
VHHAVCDGFHVARFINRLQELCNSKLK
>d1eaf__ c.43.1.1 (-) Dihydrolipoamide acetyltransferase
{Azotobacter vinelandii}
IPPIPPVDFAKYGEIEEVPMTRLMQIGATNLHRSWLNVPHVTQFESADITELEAFRVAQKAV
AKKAGVKLTVLPLLLKACAYLLKELPDFNSSLAPSGQALIRKKYVHIGFAVDTPDGLLVPVI
RNVDQKSLLQLAAEAAELAEKARSKKLGADAMQGACFTISSLGHIGGTAFTPIVNAPEVAIL
GVSKASMQPVWDGKAFQPRLMLPLSLSYDHRVINGAAAARFTKRLGDLLADIRAILL
>d1b5sa_ c.43.1.1 (A:) Dihydrolipoamide acetyltransferase
{Bacillus stearothermophilus}
AAAKPATTEGEFPETREKMSGIRRAIAKAMVHSKHTAPHVTLMDEADVTKLVAHRKKFKAIA
AEKGIKLTFLPYVVKALVSALREYPVLNTSIDDETEEIIQKHYYNIGIAADTDRGLLVPVIK
HADRKPIFALAQEINELAEKARDGKLTPGEMKGASCTITNIGSAGGQWFTPVINHPEVAILG
IGRIAEKPIVRDGEIVAAPMLALSLSFDHRMIDGATAQKALNHIKRLLSDPELLLM
>d1e2o__ c.43.1.1 (-) Dihydrolipoamide succinyltransferase
{Escherichia coli}
ARSEKRVPMTRLRKRVAERLLEAKNSTAMLTTFNEVNMKPIMDLRKQYGEAFEKRHGIRLGF
MSFYVKAVVEALKRYPEVNASIDGDDVVYHNYFDVSMAVSTPRGLVTPVLRDVDTLGMADIE
KKIKELAVKGRDGKLTVEDLTGGNFTITNGGVFGSLMSTPIINPPQSAILGMHAIKDRPMAV
NGQVEILPMMYLALSYDHRLIDGRESVGFLVTIKELLEDPTRLLLDV
>d1phr__ c.44.1.1 (-) Tyrosine phosphatase {Cow (Bos taurus)}
VTKSVLFVCLGNICRSPIAEAVFRKLVTDQNISDNWVIDSGAVSDWNVGRSPDPRAVSCLRN
HGINTAHKARQVTKEDFVTFDYILCMDESNLRDLNRKSNQVKNCRAKIELLGSYDPQKQLII
EDPYYGNDADFETVYQQCVRCCRAFLEKVR
>d5pnt__ c.44.1.1 (-) Tyrosine phosphatase {Human (Homo sapiens)}
AEQATKSVLFVCLGNICRSPIAEAVFRKLVTDQNISENWRVDSAATSGYEIGNPPDYRGQSC
MKRHGIPMSHVARQITKEDFATFDYILCMDESNLRDLNRKSNQVKTCKAKIELLGSYDPQKQ
LIIEDPYYGNDSDFETVYQQCVRCCRAFLEKAH
>d1d1qa_ c.44.1.1 (A:) Tyrosine phosphatase {Baker's yeast
(Saccharomyces cerevisiae)}
IEKPKISVAFIALGNFCRSPMAEAIFKHEVEKANLENRFNKIDSFGTSNYHVGESPDHRTVS
ICKQHGVKINHKGKQIKTKHFDEYDYIIGMDESNINNLKKIQPEGSKAKVCLFGDWNTNDGT
VQTIIEDPWYGDIQDFEYNFKQITYFSKQFLKKEL
>d1jf8a_ c.44.1.1 (A:) Arsenate reductase ArsC {Staphylococcus
aureus}
DKKTIYFISTGNSARSQMAEGWGKEILGEGWNVYSAGIETHGVNPKAIEAMKEVDIDISNHT
SDLIDNDILKQSDLVVTLCSDADNNCPILPPNVKKEHWGFDDPAGKEWSEFQRVRDEIKLAI
EKFKLR
>d1jl3a_ c.44.1.1 (A:) Arsenate reductase ArsC {Bacillus subtilis}
NKIIYFLCTGNSCRSQMAEGWAKQYLGDEWKVYSAGIEAHGLNPNAVKAMKEVGIDISNQTS
DIIDSDILNNADLVVTLCGDAADKCPMTPPHVKREHWGFDDPARAQGTEEEKWAFFQRVRDE
IGNRLKEFAETGK
>d1iiba_ c.44.2.1 (A:) Enzyme IIB-cellobiose {Escherichia coli}
KKHIYLFSSAGMSTSLLVSKMRAQAEKYEVPVIIEAFPETLAGEKGQNADVVLLGPQIAYML

PEIQRLLPNKPVEVIDSLLYGKVDGLGVLKAAVAAIKKAAA
>d1vhra_ c.45.1.1 (A:) VH1-related dual-specificity phosphatase, VHR {Human (Homo sapiens)}
SVQDLNDLLSDGSGCYSLPSQPCNEVTPRIYVGNASVAQDIPKLQKLGITHVLNAAEGRSFM
HVNTNANFYKDSGITYLGIKANDTQEFNLSAYFERAADFIDQALAQKNGRVLVHCREGYSRS
PTLVIAYLMMRQKMDVKSALSIVRQNREIGPNDGFLAQLCQLNDRLAKEGKLKP
>d1mkp__ c.45.1.1 (-) Mapk phosphatase Pyst1 (mkp3) {Human (Homo sapiens)}
ASFPVEILPFLYLGCAKDSTNLDVLEEFGIKYILNVTPNLPNLFENAGEFKYKQIPISDHWS
QNLSQFFPEAISFIDEARGKNCGVLVHSLAGISRSVTVTVAYLMQKLNLSMNDAYDIVKMKK
SNISPNFNFMGQLLDFERTL
>d1d5ra2 c.45.1.1 (A:14-187) Phoshphoinositide phosphatase Pten (Pten tumor suppressor), N-terminal domain {Human (Homo sapiens)}
RRYQEDGFDLDLTYIYPNIIAMGFPAERLEGVYRNNIDDVVRFLDSKHKNHYKIYNLCAERH
YDTAKFNCRVAQYPFEDHNPPQLELIKPFCEDLDQWLSEDDNHVAAIHCKAGKGRTGVMICA
YLLHRGKFLKAQEALDFYGEVRTRDKKGVTIPSQRRYVYYYSYLLKNHLD
>d1i9sa_ c.45.1.1 (A:) mRNA capping enzyme, triphosphatase domain {Mouse (Mus musculus)}
KIPPRWLNCPRRGQPVAGRFLPLKTMLGPRYDSQVAEENRFHPSMLSNYLKSLKVKMSLLVD
LTNTSRFYDRNDIEKEGIKYIKLQCKGHGECPTTENTETFIRLCERFNERSPPELIGVHCTH
GFNRTGFLICAFLVEKMDWSIEAAVATFAQARPPGIYKGDYLKELFRRYGDIEEAPPPPVLP
DWCFEDED
>d1fpza_ c.45.1.1 (A:) Kinase associated phosphatase (kap) {Human (Homo sapiens)}
TPIHISWLSLSRVNCSQFLGLCALPGCKFKDVRRNVQKDTEELKSCGIQDIFVFCTRGELSK
YRVPNLLDLYQQCGIITHHHPIADGGTPDIASCCEIMEELTTCLKNYRKTLIHSYGGLGRSC
LVAACLLLYLSDTISPEQAIDSLRDLRGSGAIQTIKQYNYLHEFRDKLAAHL
>d1eeoa_ c.45.1.2 (A:) Tyrosine phosphatase {Human (Homo sapiens), 1B}
EMEKEFEQIDKSGSWAAIYQDIRHEASDFPCRVAKLPKNKNRNRYRDVSPFDHSRIKLHQED
NDYINASLIKMEEAQRSYILTQGPLPNTCGHFWEMVWEQKSRGVVMLNRVMEKGSLKCAQYW
PQKEEKEMIFEDTNLKLTLISEDIKSYYTVRQLELENLTTQETREILHFHYTTWPDFGVPES
PASFLNFLFKVRESGSLSPEHGPVVVHSSAGIGRSGTFCLADTCLLLMDKRKDPSSVDIKKV
LLEMRKFRMGLIQTADQLRFSYLAVIEGAKFIMGDSSVQDQWKELSHED
>d1rpma_ c.45.1.2 (A:) Tyrosine phosphatase {Human (Homo sapiens), mu}
AIRVADLLQHITQMKCAEGYGFKEEYESFFEGQSAPWDSAKKDENRMKNRYGNIIAYDHSRV
RLQTIEGDTNSDYINGNYIDGYHRPNHYIATQGPMQETIYDFWRMVWHENTASIIMVTNLVE
VGRVKCCKYWPDDTEIYKDIKVTLIETELLAEYVIRTFAVEKRGVHEIREIRQFHFTGWPDH
GVPYHATGLLGFVRQVKSKSPPSAGPLVVHCSAGAGRTGCFIVIDIMLDMAEREGVVDIYNC
VRELRSRRVNMVQTEEQYVFIHDAILEACL
>d1yfoa_ c.45.1.2 (A:) Tyrosine phosphatase {Mouse (Mus musculus)}
KYPPLPVDKLEEEINRRMADDNKLFREEFNALPACPIQATCEAASKEENKEKNRYVNILPYD
HSRVHLTPVEGVPDSDYINASFINGYQEKNKFIAAQGPKEETVNDFWRMIWEQNTATIVMVT

NLKERKECKCAQYWPDQGCWTYGNVRVSVEDVTVLVDYTVRKFCIQQVGDVTNRKPQRLITQ
FHFTSWPDFGVPFTPIGMLKFLKKVKACNPQYAGAIVVHCSAGVGRTGTFVVIDAMLDMMHS
ERKVDVYGFVSRIRAQRCQMVQTDMQYVFIYQALLEHYLY
>d2shpa1 c.45.1.2 (A:219-525) Tyrosine phosphatase {Human (Homo sapiens), shp-2}
TRINAAEIESRVRELSKLAETTDKVKQGFWEEFETLQQQECKLLYSRKEGQRQENKNKNRYK
NILPFDHTRVVLHDGDPNEPVSDYINANIIMPEFETKCNNSKPKKSYIATQGCLQNTVNDFW
RMVFQENSRVIVMTTKEVERGKSKCVKYWPDEYALKEYGVMRVRNVKESAAHDYTLRELKLS
KVGQGNTERTVWQYHFRTWPDHGVPSDPGGVLDFLEEVHHKQESIMDAGPVVVHCSAGIGRT
GTFIVIDILIDIIREKGVDCDIDVPKTIQMVRSQRSGMVQTEAQYRSIYMAVQHYIETL
>d1fpra_ c.45.1.2 (A:) Tyrosine phosphatase {Human (Homo sapiens), shp-1}
GFWEEFESLQKQEVKNLHQRLEGQRPENKGKNRYKNILPFDHSRVILQGRDSNIPGSDYINA
NYIKNQLLGPDENAKTYIASQGCLEATVNDFWQMAWQENSRVIVMTTREVEKGRNKCVPYWP
EVGMQRAYGPYSVTNCGEHDTTEYKLRTLQVSPLDNGDLIREIWHYQYLSWPDHGVPSEPGG
VLSFLDQINQRQESLPHAGPIIVHSSAGIGRTGTIIVIDMLMENISTKGLDCDIDIQKTIQM
VRAQRSGMVQTEAQYKFIYVAIAQFIETTKKKLEVL
>d1jlna_ c.45.1.2 (A:) Tyrosine phosphatase {Mouse (Mus musculus), ptp-sl/br7}
GSPREKVAMEYLQSASRVLTRSQLRDVVASSHLLQSEFMEIPMNFVDPKEIDIPRHGTKNRY
KTILPNPLSRVCLRPKNITDSLSTYINANYIRGYSGKEKAFIATQGPMINTVNDFWQMVWQE
DSPVIVMITKLKEKNEKCVLYWPEKRGIYGKVEVLVTGVTECDNYTIRNLVLKQGSHTQHVK
HYWYTSWPDHKTPDSAQPLLQLMLDVEEDRLASEGRGPVVVHCSAGIGRTGCFIATSIGCQQ
LKEEGVVDALSIVCQLRVDRGGMVQTSEQYEFVHHALCLFESRLSPETV
>d1ypta_ c.45.1.2 (A:) Tyrosine phosphatase {Yersinia enterocolitica}
PEARAELSSRLTTLRNTLAPATNDPRYLQACGGEKLNRFRDIQCRRQTAVRADLNANYIQVG
NTRTIACQYPLQSQLESHFRMLAENRTPVLAVLASSSEIANQRFGMPDYFRQSGTYGSITVE
SKMTQQVGLGDGIMADMYTLTIREAGQKTISVPVVHVGNWPDQTAVSSEVTKALASLVDQTA
ETKRNMYESKGSSAVADDSKLRPVIHCRAGVGRTAQLIGAMCMNDSRNSQLSVEDMVSQMRV
QRNGIMVQKDEQLDVLIKLAEGQGRPLLNS
>d1g4us2 c.45.1.2 (S:297-539) SptP tyrosine phosphatase, catalytic domain {Salmonella typhimurium}
PQTMSGPTLGLARFAVSSIPINQQTQVKLSDGMPVPVNTLTFDGKPVALAGSYPKNTPDALE
AHMKMLLEKECSCLVVLTSEDQMQAKQLPPYFRGSYTFGEVHTNSQKVSSASQGEAIDQYNM
QLSCGEKRYTIPVLHVKNWPDHQPLPSTDQLEYLADRVKNSNQNGAPGRSSSDKHLPMIHCL
GGVGRTGTMAAALVLKDNPHSNLEQVRADFRDSRNNRMLEDASQFVQLKAMQAQLLM
>d1lara1 c.45.1.2 (A:1307-1623) RPTP Lar {Human (Homo sapiens)}
MITDLADNIERLKANDGLKFSQEYESIDPGQQFTWENSNLEVNKPKNRYANVIAYDHSRVIL
TSIDGVPGSDYINANYIDGYRKQNAYIATQGPLPETMGDFWRMVWEQRTATVVMMTRLEEKS
RVKCDQYWPARGTETCGLIQVTLLDTVELATYTVRTFALHKSGSSEKRELRQFQFMAWPDHG
VPEYPTPILAFLRRVKACNPLDAGPMVVHCSAGVGRTGCFIVIDAMLERMKHEKTVDIYGHV
TCMRSQRNYMVQTEDQYVFIHEALLEAATCGHTEVPARNLYAHIQKLGQVPPGESVTAMELE
FKLLASS

>d1lara2 c.45.1.2 (A:1628-1876) RPTP Lar {Human (Homo sapiens)}
SRFISANLPCNKFKNRLVNIMPYELTRVCLQPIRGVEGSDYINASFLDGYRQQKAYIATQGP
LAESTEDFWRMLWEHNSTIIVMLTKLREMGREKCHQYWPAERSARYQYFVVDPMAEYNMPQY
ILREFKVTDARDGQSRTIRQFQFTDWPEQGVPKTGEGFIDFIGQVHKTKEQFGQDGPITVHC
SAGVGRTGVFITLSIVLERMRYEGVVDMFQTVKTLRTQRPAMVQTEDQYQLCYRAALEYLGS
F
>d1larb1 c.45.1.2 (B:1340-1623) RPTP Lar {Human (Homo sapiens)}
TWENSNLEVNKPKNRYANVIAYDHSRVILTSIDGVPGSDYINANYIDGYRKQNAYIATQGPL
PETMGDFWRMVWEQRTATVVMMTRLEEKSRVKCDQYWPARGTETCGLIQVTLLDTVELATYT
VRTFALHKSGSSEKRELRQFQFMAWPDHGVPEYPTPILAFLRRVKACNPLDAGPMVVHCSAG
VGRTGCFIVIDAMLERMKHEKTVDIYGHVTCMRSQRNYMVQTEDQYVFIHEALLEAATCGHT
EVPARNLYAHIQKLGQVPPGESVTAMELEFKLLASS
>d1c25__ c.46.1.1 (-) CDC25a {Human (Homo sapiens)}
MLIGDFSKGYLFHTVAGKHQDLKYISPEIMASVLNGKFANLIKEFVIIDCRYPYEYEGGHIK
GAVNLHMEEEVEDFLLKKPIVPTDGKRVIVVFHCEFSSERGPRMCRYVRERDRLGNEYPKLH
YPELYVLKGGYKEFFMKCQSYCEPPSYRPMHHEDFKE
>d1qb0a_ c.46.1.1 (A:) CDC25b {Human (Homo sapiens)}
DHRELIGDYSKAFLLQTVDGKHQDLKYISPETMVALLTGKFSNIVDKFVIVDCRYPYEYEGG
HIKTAVNLPLERDAESFLLKSPIAPCSLDKRVILIFHCEFSSERGPRMCRFIRERDRAVNDY
PSLYYPEMYILKGGYKEFFPQHPNFCEPQDYRPMNHEAFKDELKTFRLKTRSWA
>d1hzma_ c.46.1.1 (A:) Erk2 binding domain of Mapk phosphatase mkp-3 {Human (Homo sapiens)}
MIDTLRPVPFASEMAISKTVAWLNEQLELGNERLLLMDCRPQELYESSHIESAINVAIPGIM
LRRLQKGNLPVRALFTRGEDRDRFTRRCGTDTVVLYDESSSDWNENTGGESLLGLLLKKLKD
EGCRAFYLEGGFSKFQAEFSLHCETNLDGS
>d1gmxa_ c.46.1.3 (A:) Sulfurtransferase GlpE {Escherichia coli}
MDQFECINVADAHQKLQEKEAVLVDIRDPQSFAMGHAVQAFHLTNDTLGAFMRDNDFDTPVM
VMCYHGNSSKGAAQYLLQQGYDVVYSIDGGFEAWQRQFPAEVAYGA
>d1rhs_1 c.46.1.2 (1-149) Rhodanese {Cow (Bos taurus)}
VHQVLYRALVSTKWLAESVRAGKVGPGLRVLDASWYSPGTREARKEYLERHVPGASFFDIEE
CRDKASPYEVMLPSEAGFADYVGSLGISNDTHVVVYDGDDLGSFYAPRVWWMFRVFGHRTVS
VLNGGFRNWLKEGHPVTSEPSRPEP
>d1rhs_2 c.46.1.2 (150-293) Rhodanese {Cow (Bos taurus)}
AIFKATLNRSLLKTYEQVLENLESKRFQLVDSRAQGRYLGTQPEPDAVGLDSGHIRGSVNMP
FMNFLTEDGFEKSPEELRAMFEAKKVDLTKPLIATCRKGVTACHIALAAYLCGKPDVAIYDG
SWFEWFHRAPPETWVSQGKG
>d1e0ca1 c.46.1.2 (A:1-135) Sulfurtransferase {Azotobacter vinelandii}
MDDFASLPLVIEPADLQARLSAPELILVDLTSAARYAEGHIPGARFVDPKRTQLGQPPAPGL
QPPREQLESLFGELGHRPEAVYVVYDDEGGGWAGRFIWLLDVIGQQRYHYLNGGLTAWLAED
RPLSRELPAPA
>d1e0ca2 c.46.1.2 (A:136-271) Sulfurtransferase {Azotobacter vinelandii}
GGPVALSLHDEPTASRDYLLGRLGAADLAIWDARSPQEYRGEKVLAAKGGHIPGAVNFEWTA

AMDPSRALRIRTDIAGRLEELGITPDKEIVTHCQTHHRSGLTYLIAKALGYPRVKGYAGSWG
EWGNHPDTPVEL
>d2trxa_ c.47.1.1 (A:) Thioredoxin {Escherichia coli}
SDKIIHLTDDSFDTDVLKADGAILVDFWAEWCGPCKMIAPILDEIADEYQGKLTVAKLNIDQ
NPGTAPKYGIRGIPTLLLFKNGEVAATKVGALSKGQLKEFLDANLA
>d1thx__ c.47.1.1 (-) Thioredoxin {Anabaena sp., pcc 7120}
SKGVITITDAEFESEVLKAEQPVLVYFWASWCGPCQLMSPLINLAANTYSDRLKVVKLEIDP
NPTTVKKYKVEGVPALRLVKGEQILDSTEGVISKDKLLSFLDTHLN
>d1dbya_ c.47.1.1 (A:) Thioredoxin {Chlamydomonas reinhardtii}
MEAGAVNDDTFKNVVLESSVPVLVDFWAPWCGPCRIIAPVVDEIAGEYKDKLKCVKLNTDES
PNVASEYGIRSIPTIMVFKGGKKCETIIGAVPKATIVQTVEKYLN
>d1ep7a_ c.47.1.1 (A:) Thioredoxin {Chlamydomonas reinhardtii}
GGSVIVIDSKAAWDAQLAKGKEEHKPIVVDFTATWCGPCKMIAPLFETLSNDYAGKVIFLKV
DVDAVAAVAEAAGITAMPTFHVYKDGVKADDLVGASQDKLKALVAKHAAA
>d1quwa_ c.47.1.1 (A:) Thioredoxin {Bacillus acidocaldarius}
ATMTLTDANFQQAIQGDKPVLVDFWAAWCGPCRMMAPVLEEFAEAHADKVTVAKLNVDENPE
TTSQFGIMSIPTLILFKGGRPVKQLIGYQPKEQLEAQLADVLQ
>d1f9ma_ c.47.1.1 (A:) Thioredoxin {Spinach (Spinacia oleracea), thioredoxin F}
MEAIVGKVTEVNKDTFWPIVKAAGDKPVVLDMFTQWCGPCKAMAPKYEKLAEEYLDVIFLKL
DCNQENKTLAKELGIRVVPTFKILKENSVVGEVTGAKYDKLLEAIQAARS
>d1fb6a_ c.47.1.1 (A:) Thioredoxin {Spinach (Spinacia oleracea), thioredoxin M}
VQDVNDSSWKEFVLESEVPVMVDFWAPWCGPCKLIAPVIDELAKEYSGKIAVYKLNTDEAPG
IATQYNIRSIPTVLFFKNGERKESIIGAVPKSTLTDSIEKYL
>d1erv__ c.47.1.1 (-) Thioredoxin {Human (Homo sapiens)}
MVKQIESKTAFQEALDAAGDKLVVVDFSATWCGPCKMIKPFFHSLSEKYSNVIFLEVDVDDC
QDVASECEVKSMPTFQFFKKGQKVGEFSGANKEKLEATINELV
>d1aba__ c.47.1.1 (-) Glutaredoxin (Thioltransferase) {Bacteriophage T4}
MFKVYGYDSNIHKCGPCDNAKRLLTVKKQPFEFINIMPEKGVFDDEKIAELLTKLGRDTQIG
LTMPQVFAPDGSHIGGFDQLREYFK
>d1qfna_ c.47.1.1 (A:) Glutaredoxin (Thioltransferase) {Escherichia coli}
MQTVIFGRSGCPYSVRAKDLAEKLSNERDDFQYQYVDIRAEGITKEDLQQKAGKPVETVPQI
FVDQQHIGGYTDFAAWVKENLDA
>d3grx__ c.47.1.1 (-) Glutaredoxin (Thioltransferase) {Escherichia coli, GRX3}
ANVEIYTKETCPYSHRAKALLSSKGVSFQELPIDGNAAKREEMIKRSGRTTVPQIFIDAQHI
GGYDDLYALDARGGLDPLLK
>d1kte__ c.47.1.1 (-) Glutaredoxin (Thioltransferase) {Pig (Sus scrofa)}
AQAFVNSKIQPGKVVVFIKPTCPFCRKTQELLSQLPFKEGLLEFVDITATSDTNEIQDYLQQ
LTGARTVPRVFIGKECIGGCTDLESMHKRGELLTRLQQVGAVK

>d1jhb__ c.47.1.1 (-) Glutaredoxin (Thioltransferase) {Human (Homo sapiens)}
AQEFVNCKIQPGKVVVFIKPTCPYCRRAQEILSQLPIKQGLLEFVDITATNHTNEIQDYLQQ
LTGARTVPRVFIGKDCIGGCSDLVSLQQSGELLTRLKQIGALQ
>d1h75a_ c.47.1.1 (A:) Glutaredoxin-like NRDH-redoxin {Escherichia coli}
MRITIYTRNDCVQCHATKRAMENRGFDFEMINVDRVPEAAEALRAQGFRQLPVVIAGDLSWS
GFRPDMINRLHPAP
>d1fo5a_ c.47.1.1 (A:) MJ0307, thioredoxin/glutaredoxin-like protein {Archaeon Methanococcus jannaschii}
MSKVKIELFTSPMCPHCPAAKRVVEEVANEMPDAVEVEYINVMENPQKAMEYGIMAVPTIVI
NGDVEFIGAPTKEALVEAIKKRL
>d1iloa_ c.47.1.1 (A:) MTH985, a thioredoxin {Archaeon Methanobacterium thermoautotrophicum}
MMKIQIYGTGCANCQMLEKNAREAVKELGIDAEFEKIKEMDQILEAGLTALPGLAVDGELKI
MGRVASKEEIKKILS
>d1gh2a_ c.47.1.1 (A:) Thioredoxin-like protein, N-terminal domain {Human (Homo sapiens)}
VGVKPVGSDPDFQPELSGAGSRLAVVKFTMRGCGPCLRIAPAFSSMSNKYPQAVFLEVDVHQ
CQGTAATNNISATPTFQFFRNKVRIDQYQGADAVGLEEKIKQHLE
>d1bjx__ c.47.1.2 (-) Protein disulfide isomerase, PDI {Human (Homo sapiens)}
AATTLPDGAAAESLVESSEVAVIGFFKDVESDSAKQFLQAAEAIDDIPFGITSNSDVFSKYQ
LDKDGVVLFKKFDEGRNNFEGEVTKENLLDFIKHNQLPLVIEFTEQTA
>d1mek__ c.47.1.2 (-) Protein disulfide isomerase, PDI {Human (Homo sapiens)}
DAPEEEDHVLVLRKSNFAEALAAHKYLLVEFYAPWCGHCKALAPEYAKAAGKLKAEGSEIRL
AKVDATEESDLAQQYGVRGYPTIKFFRNGDTASPKEYTAGREADDIVNWLKKRTGPAA
>d1a8l_1 c.47.1.2 (1-119) Protein disulfide isomerase, PDI {Archaeon Pyrococcus furiosus}
MGLISDADKKVIKEEFFSKMVNPVKLIVFVRKDHCQYCDQLKQLVQELSELTDKLSYEIVDF
DTPEGKELAKRYRIDRAPATTITQDGKDFGVRYFGLPAGHEFAAFLEDIVDVSREET
>d1a8l_2 c.47.1.2 (120-226) Protein disulfide isomerase, PDI {Archaeon Pyrococcus furiosus}
NLMDETKQAIRNIDQDVRILVFVTPTCPYCPLAVRMAHKFAIENTKAGKGKILGDMVEAIEY
PEWADQYNVMAVPKIVIQVNGEDRVEFEGAYPEKMFLEKLLSALS
>d1hyua3 c.47.1.2 (A:1-102) Alkyl hydroperoxide reductase subunit F (AhpF), N-terminal domain {Salmonella typhimurium}
MLDTNMKTQLRAYLEKLTKPVELIATLDDSAKSAEIKELLAEIAELSDKVTFKEDNTLPVRK
PSFLITNPGSQQGPRFAGSPLGHEFTSLVLALLWTGGHPS
>d1hyua4 c.47.1.2 (A:103-198) Alkyl hydroperoxide reductase subunit F (AhpF), N-terminal domain {Salmonella typhimurium}
KEAQSLLEQIRDIDGDFEFETYYSLSCHNCPDVVQALNLMAVLNPRIKHTAIDGGTFQNEIT
ERNVMGVPAVFVNGKEFGQGRMTLTEIVAKVDTG

>d1a8y_1 c.47.1.3 (3-126) Calsequestrin {Rabbit (Oryctolagus cuniculus)}
GLDFPEYDGVDRVINVNAKNYKNVFKKYEVLALLYHEPPEDDKASQRQFEMEELILELAAQV
LEDKGVGFGLVDSEKDAAVAKKLGLTEEDSIYVFKEDEVIEYDGEFSADTLVEFLLDVLEDP
>d1a8y_2 c.47.1.3 (127-228) Calsequestrin {Rabbit (Oryctolagus cuniculus)}
VELIEGERELQAFENIEDEIKLIGYFKNKDSEHYKAFKEAAEEFHPYIPFFATFDSKVAKKL
TLKLNEIDFYEAFMEEPVTIPDKPNSEEEIVNFVEEHRRS
>d1a8y_3 c.47.1.3 (229-347) Calsequestrin {Rabbit (Oryctolagus cuniculus)}
TLRKLKPESMYETWEDDMDGIHIVAFAEEADPDGYEFLEILKSVAQDNTDNPDLSIIWIDPD
DFPLLVPYWEKTFDIDLSAPQIGVVNVTDADSVWMEMDDEEDLPSAEELEDWLEDVL
>d1fvka2 c.47.1.4 (A:1-64,A:129-188) Disulphide-bond formation facilitator (DSBA) {Escherichia coli}
AQYEDGKQYTTLEKPVAGAPQVLEFFSFFCPHCYQFEEVLHISDNVKKKLPEGVKMTKYHVN
FMXFVVKSLVAQQEKAAADVQLRGVPAMFVNGKYQLNPQGMDTSNMDVFVQQYADTVKYLSE
K
>d1bed_2 c.47.1.4 (1-62,127-181) Disulphide-bond formation facilitator (DSBA) {Vibrio cholerae}
AQFKEGEHYQVLKTPASSSPVVSEFFSFYCPHCNTFEPIIAQLKQQLPEGAKFQKNHVSFMG
XFAVDSMVRRFDKQFQDSGLTGVPAVVVNNRYLVQGQSVKSLDEYFDLVNYLLTLK
>d1aqwa2 c.47.1.5 (A:1-76) Glutathione S-transferase {Human (Homo sapiens), class pi}
PPYTVVYFPVRGRCAALRMLLADQGQSWKEEVVTVETWQEGSLKASCLYGQLPKFQDGDLTL
YQSNTILRHLGRTL
>d2gsra2 c.47.1.5 (A:1-76) Glutathione S-transferase {Pig (Sus scrofa), class pi}
PPYTITYFPVRGRCEAMRMLLADQDQSWKEEVVTMETWPPLKPSCLFRQLPKFQDGDLTLYQ
SNAILRHLGRSFGL
>d1glqa2 c.47.1.5 (A:1-78) Glutathione S-transferase {Mouse (Mus musculus), class pi}
PPYTIVYFPVRGRCEAMRMLLADQGQSWKEEVVTIDTWMQGLLKPTCLYGQLPKFEDGDLTL
YQSNAILRHLGRSLGL
>d1gtua2 c.47.1.5 (A:1-84) Glutathione S-transferase {Human (Homo sapiens), class mu}
PMILGYWDIRGLAHAIRLLLEYTDSSYEEKKYTMGDAPDYDRSQWLNEKFKLGLDFPNLPYL
IDGAHKITQSNAILCYIARKHN
>d1hna_2 c.47.1.5 (1-84) Glutathione S-transferase {Human (Homo sapiens), class mu}
PMTLGYWNIRGLAHSIRLLLEYTDSSYEEKKYTMGDAPDYDRSQWLNEKFKLGLDFPNLPYL
IDGTHKITQSNAILRYIARKHN
>d3gtub2 c.47.1.5 (B:1-84) Glutathione S-transferase {Human (Homo sapiens), class mu}
SCESSMVLGYWDIRGLAHAIRLLLEFTDTSYEEKRYTCGEAPDYDRSQWLDVKFKLDLDFPN

LPYLLDGKNKITQSNAILRYIA
>d2gsta2 c.47.1.5 (A:1-84) Glutathione S-transferase {Rat (Rattus norvegicus), class mu}
PMILGYWNVRGLTHPIRLLLEYTDSSYEEKRYAMGDAPDYDRSQWLNEKFKLGLDFPNLPYL
IDGSRKITQSNAIMRYLARKHH
>d1gsua2 c.47.1.5 (A:1-84) Glutathione S-transferase {Chicken (Gallus gallus), class mu}
VVTLGYWDIRGLAHAIRLLLEYTETPYQERRYKAGPAPDFDPSDWTNEKEKLGLDFPNLPYL
IDGDVKLTQSNAILRYIARKHN
>d1gsea2 c.47.1.5 (A:2-80) Glutathione S-transferase {Human (Homo sapiens), class alpha (a1-1)}
AEKPKLHYFNARGKMESTRWLLAAAGVEFEEKFIKSAEDLDKLRNDGYLMFQQVPMVEIDGM
KLVQTRAILNYIASKYN
>d1gula2 c.47.1.5 (A:4-80) Glutathione S-transferase {Human (Homo sapiens), class alpha (a1-1)}
RPKLHYPNGRGRMESVRWVLAAAGVEFDEEFLETKEQLYKLQDGNHLLFQQVPMVEIDGMKL
VQTRSILHYIADKHN
>d1f3aa2 c.47.1.5 (A:1-79) Glutathione S-transferase {Mouse (Mus musculus), class alpha (a1-1)}
AGKPVLHYFNARGRMECIRWLLAAAGVEFEEKFIQSPEDLEKLKKDGNLMFDQVPMVEIDGM
KLAQTRAILNYIATKYD
>d1b48a2 c.47.1.5 (A:2-79) Glutathione S-transferase {Mouse (Mus musculus), class alpha (a1-4)}
AAKPKLYYFNGRGRMESIRWLLAAAGVEFEEEFLETREQYEKMQKDGHLLFGQVPLVEIDGM
MLTQTRAILSYLAAKY
>d1ljra2 c.47.1.5 (A:1-79) Glutathione S-transferase {Human (Homo sapiens), class theta}
MGLELFLDLVSQPSRAVYIFAKKNGIPLELRTVDLVKGQHKSKEFLQINSLGKLPTLKDGDF
ILTESSAILIYLSCKYQ
>d1pd212 c.47.1.5 (1:1-75) Glutathione S-transferase {Rat (Rattus norvegicus), class sigma}
MPNYKLLYFNMRGRAEIIRYIFAYLDIKYEDHRIEQADWPKIKPTLPFGKIPVLEVEGLTLH
QSLAIARYLTKNT
>d2gsq_2 c.47.1.5 (1-75) Glutathione S-transferase {Squid (Ommastrephes sloani pacificus), class sigma}
PKYTLHYFPLMGRAELCRFVLAAHGEEFTDRVVEMADWPNLKATMYSNAMPVLDIDGTKMSQ
SMCIARHLAREFG
>d1eema2 c.47.1.5 (A:5-102) Glutathione S-transferase {Human (Homo sapiens), class omega}
SARSLGKGSAPPGPVPEGSIRIYSMRFCPFAERTRLVLKAKGIRHEVININLKNKPEWFFKK
NPFGLVPVLENSQGQLIYESAITCEYLDEAYPGKKL
>d1fw1a2 c.47.1.5 (A:5-87) Glutathione S-transferase {Human (Homo sapiens), class zeta}
KPILYSYFRSSCSWRVRIALALKGIDYKTVPINLIKDGGQQFSKDFQALNPMKQVPTLKIDG

ITIHQSLAIIEYLEETRPTPR
>d1duga2 c.47.1.5 (A:1-80) Glutathione S-transferase {Schistosoma japonicum}
SPILGYWKIKGLVQPTRLLLEYLEEKYEEHLYERDEGDKWRNKKFELGLEFPNLPYYIDGDV
KLTQSMAIIRYIADKHNM
>d1fhe_2 c.47.1.5 (1-80) Glutathione S-transferase {Fasciola hepatica}
PAKLGYWKLRGLAQPVRLFLEYLGEEYEEHLYGRDDREKWMSEKFNMGLDLPNLPYYIDDKC
KLTQSVAIMRYIADKHGM
>d2fhea2 c.47.1.5 (A:1-80) Glutathione S-transferase {Fasciola hepatica}
PAKLGYWKIRGLQQPVRLLLEYLGEKYEEQIYERDDGEKWFSKKFELGLDLPNLPYYIDDKC
KLTQSLAILRYIADKHGM
>d1gnwa2 c.47.1.5 (A:2-85) Glutathione S-transferase {Mouse-ear cress (Arabidopsis thaliana)}
GIKVFGHPASIATRRVLIALHEKNLDFELVHVELKDGEHKKEPFLSRNPFGQVPAFEDGDLK
LFESRAITQYIAHRYENQGTNL
>d1axda2 c.47.1.5 (A:1-80) Glutathione S-transferase {Maize (Zea mays), type I}
APMKLYGAVMSWNLTRCATALEEAGSDYEIVPINFATAEHKSPEHLVRNPFGQVPALQDGDL
YLFESRAICKYAARKNKP
>d1aw9_2 c.47.1.5 (2-82) Glutathione S-transferase {Maize (Zea mays), type III}
APLKLYGMPLSPNVVRVATVLNEKGLDFEIVPVDLTTGAHKQPDFLALNPFGQIPALVDGDE
VLFESRAINRYIASKYASE
>d1e6ba2 c.47.1.5 (A:8-87) Glutathione S-transferase {Mouse-ear cress (Arabidopsis thaliana), class zeta}
KLKLYSYWRSSCAHRVRIALALKGLDYEYIPVNLLKGDQFDSDFKKINPMGTVPALVDGDVV
INDSFAIIMYLDEKYPEP
>d1a0fa2 c.47.1.5 (A:1-80) Glutathione S-transferase {Escherichia coli}
MKLFYKPGACSLASHITLRESGKDFTLVSVDLMKKRLENGDDYFAVNPKGQVPALLLDDGTL
LTEGVAIMQYLADSVPDR
>d1pmt_2 c.47.1.5 (1-80) Glutathione S-transferase {Proteus mirabilis}
MKLYYTPGSCSLSPHIVLRETGLDFSIERIDLRTKKTESGKDFLAINPKGQVPVLQLDNGDI
LTEGVAIVQYLADLKPDR
>d1f2ea2 c.47.1.5 (A:1-80) Glutathione S-transferase {Sphingomonas paucimobilis}
MKLFISPGACSLAPHIALRETGADFEAVKVDLAVRKTEAGEDFLTVNPSGKVPALTLDSGET
LTENPAILLYIADQNPAS
>d1g7oa2 c.47.1.5 (A:1-75) Glutaredoxin 2 {Escherichia coli}
MKLYIYDHCPYCLKARMIFGLKNIPVELHVLLNDDAETPTRMVGQKQVPILQKDDSRYMPES
MDIVHYVDKLDGK

>d1k0da2 c.47.1.5 (A:109-200) Yeast prion protein ure2p, nitrogen regulation fragment {Baker's yeast (Saccharomyces cerevisiae)}
QPLEGYTLFSHRSAPNGFKVAIVLSELGFHYNTIFLDFNLGEHRAPEFVSVNPNARVPALID
HGMDNLSIWESGAILLHLVNKYYKETGNPL
>d1k0dd2 c.47.1.5 (D:99-200) Yeast prion protein ure2p, nitrogen regulation fragment {Baker's yeast (Saccharomyces cerevisiae)}
YSRITKFFQEQPLEGYTLFSHRSAPNGFKVAIVLSELGFHYNTIFLDFNLGEHRAPEFVSVN
PNARVPALIDHGMDNLSIWESGAILLHLVNKYYKETGNPL
>d1k0ma2 c.47.1.5 (A:6-91) Chloride intracellular channel 1 (clic1) {Human (Homo sapiens)}
PQVELFVKAGSDGAKIGNCPFSQRLFMVLWLKGVTFNVTTVDTKRRTETVQKLCPGGELPFL
LYGTEVHTDTNKIEEFLEAVLCPP
>d2trcp_ c.47.1.6 (P:) Phosducin {Rat (Rattus norvegicus)}
EGQATHTGPKGVINDWRKFKLESEDGDSIPPSKKEILRQMSSPQSRDDKDSKERMSRKMSIQ
EYELIHQDKEDEGCLRKYRRQCMQDMHQKLSFGPRYGFVYELETGEQFLETIEKEQKVTTIV
VNIYEDGVRGCDALNSSLECLAAEYPMVKFCKIRASNTGAGDRFSSDVLPTLLVYKGGELIS
NFISVAEQFAEDFFAADVESFLNEYGLLPER
>d1a0rp_ c.47.1.6 (P:) Phosducin {Cow (Bos taurus)}
FEGQASHTGPKGVINDWRKFKLESEDSDSVAHSKKEILRQMSSPQSRDDKDSKERFSRKMSV
QEYELIHKDKEDENCLRKYRRQCMQDMHQKLSFGPRYGFVYELESGEQFLETIEKEQKITTI
VVHIYEDGIKGCDALNSSLICLAAEYPMVKFCKIKASNTGAGDRFSSDVLPTLLVYKGGELL
SNFISVTEQLAEEFFTGDVESFLNEYGLLPEK
>d1g7ea_ c.47.1.7 (A:) Endoplasmic reticulum protein ERP29, N-domain {Rat (Rattus norvegicus)}
LHTKGALPLDTVTFYKVIPKSKFVLVKFDTQYPYGEKQDEFKRLAENSASSDDLLVAEVGIS
DYGDKLNMELSEKYKLDKESYPVFYLFRDGDFENPVPYSGAVKVGAIQRWLKGQGVYLGM
>d1qgva_ c.47.1.8 (A:) spliceosomal protein U5-15Kd {Human (Homo sapiens)}
SYMLPHLHNGWQVDQAILSEEDRVVVIRFGHDWDPTCMKMDEVLYSIAEKVKNFAVIYLVDI
TEVPDFNKMYELYDPCTVMFFFRNKHIMIDLGTGNNNKINWAMEDKQEMVDIIETVYRGARK
GRGLVVSPKDYST
>d1eeja1 c.47.1.9 (A:61-216) Disulfide bond isomerase, DsbC, C-terminal domain {Escherichia coli}
NVTNKMLLKQLNALEKEMIVYKAPQEKHVITVFTDITCGYCHKLHEQMADYNALGITVRYLA
FPRQGLDSDAEKEMKAIWCAKDKNKAFDDVMAGKSVAPASCDVDIADHYALGVQLGVSGTPA
VVLSNGTLVPGYQPPKEMKEFLDEHQKMTSGK
>d1gp1a_ c.47.1.10 (A:) Glutathione peroxidase {Cow (Bos taurus)}
RTVYAFSARPLAGGEPFNLSSLRGKVLLIENVASLXGTTVRDYTQMNDLQRRLGPRGLVVLG
FPCNQFGHQENAKNEEILNCLKYVRPGGGFEPNFMLFEKCEVNGEKAHPLFAFLREVLPTPS
DDATALMTDPKFITWSPVCRNDVSWNFEKFLVGPDGVPVRRYSRRFLTIDIEPDIETLLSQ
>d1qk8a_ c.47.1.10 (A:) Tryparedoxin I {Crithidia fasciculata}
GLDKYLPGIEKLRRGDGEVEVKSLAGKLVFFYFSASWCPPCRGFTPQLIEFYDKFHESKNFE
VVFCTWDEEEDGFAGYFAKMPWLAVPFAQSEAVQKLSKHFNVESIPTLIGVDADSGDVVTTR
ARATLVKDPEGEQFPWKDA

>d1i5ga_ c.47.1.10 (A:) Tryparedoxin II {Crithidia fasciculata}
SGLKKFFPYSTNVLKGAAADIALPSLAGKTVFFYFSASWCPPSRAFTPQLIDFYKAHAEKKN
FEVMLISWDESAEDFKDYYAKMPWLALPFEDRKGMEFLTTGFDVKSIPTLVGVEADSGNIIT
TQARTMVVKDPEAKDFPWPN
>d1e2ya_ c.47.1.10 (A:) Tryparedoxin peroxidase (thioredoxin peroxidase homologue) {Crithidia fasciculata}
GAAKLNHPAPEFDDMALMPNGTFKKVSLSSYKGKYVVLFFYPMDFTFVCPTEIIQFSDDAKR
FAEINTEVISCSCDSEYSHLQWTSVDRKKGGLGPMAIPMLADKTKAIARAYGVLDEDSGVAY
RGVFIIDPNGKLRQIIINDMPIGRNVEEVIRLVEALQFVEEHG
>d1qq2a_ c.47.1.10 (A:) Thioredoxin peroxidase 2 (thioredoxin peroxidase B, 2-cys peroxiredoxin) {Norway rat (Rattus norvegicus)}
SGNAKIGHPAPSFKATAVMPDGQFKDISLSDYKGKYVVFFFYPLDFTFVCPTEIIAFSDRAE
EFKKLNCQVIGASVDSHFSHLAWINTPKKQGGLGPMNIPLVSDPKRTIAQDYGVLKADEGIS
FRGLFIIDDKGILRQITINDLPVGRSVDEILRLVQAFQFTDKHGEVCPA
>d1qmva_ c.47.1.10 (A:) Thioredoxin peroxidase 2 (thioredoxin peroxidase B, 2-cys peroxiredoxin) {Human (Homo sapiens)}
SGNARIGKPAPDFKATAVVDGAFKEVKLSDYKGKYVVLFFYPLDFTFVAPTEIIAFSNRAED
FRKLGCEVLGVSVDSQFTHLAWINTPRKEGGLGPLNIPLLADVTRRLSEDYGVLKTDEGIAY
RGLFIIDGKGVLRQITVNDLPVGRSVDEALRLVQAFQYTDEHGEVCPAGWKPGSDTIKPNVD
DSKEYFSKHN
>d1hd2a_ c.47.1.10 (A:) Peroxiredoxin 5 {Human (Homo sapiens)}
APIKVGDAIPAVEVFEGEPGNKVNLAELFKGKKGVLFGVPGAFTPGCSKTHLPGFVEQAEAL
KAKGVQVVACLSVNDAFVTGEWGRAHKAEGKVRLLADPTGAFGKETDLLLDDSLVSIFGNRR
LKRFSMVVQDGIVKALNVEPDGTGLTCSLAPNIISQL
>d1prxa_ c.47.1.10 (A:) HorF6 peroxidase {Human (Homo sapiens)}
LLLGDVAPNFEANTTVGRIRFHDFLGDSWGILFSHPRDFTPVCTTELGRAAKLAPEFAKRNV
KLIALSIDSVEDHLAWSKDINAYNSEEPTEKLPFPIIDDRNRELAILLGMLDPAEKDEKGMP
VTARVVFVFGPDKKLKLSILYPATTGRNFDEILRVVISLQLTAEKRVATPVDWKDGDSVMVL
PTIPEEEAKKLFPKGVFTKELPSGKKYLRYTPQP
>d1kyga_ c.47.1.10 (A:) Alkyl hydroperoxide reductase AhpC {Salmonella typhimurium}
SLINTKIKPFKNQAFKNGEFIEVTEKDTEGRWSVFFFYPADFTFVCPTELGDVADHYEELQK
LGVDVYSVSTDTHFTHKAWHSSSETIAKIKYAMIGDPTGALTRNFDNMREDEGLADRATFVV
DPQGIIQAIEVTAEGIGRDASDLLRKIKAAQYVAAHPGEVCP
>d1jfua_ c.47.1.10 (A:) Membrane-anchored thioredoxin-like protein TlpA, soluble domain {Bradyrhizobium japonicum}
TGDPACRAAVATAQKIAPLAHGEVAALTMASAPLKLPDLAFEDADGKPKKLSDFRGKTLLVN
LWATWCVPCRKEMPALDELQGKLSGPNFEVVAINIDTRDPEKPKTFLKEANLTRLGYFNDQK
AKVFQDLKAIGRALGMPTSVLVDPQGCEIATIAGPAEWASEDALKLIRAATG
>d1foha3 c.47.1.10 (A:462-662) Phenol hydroxylase, C-terminal domain {Soil-living yeast (Trichosporon cutaneum)}
NLVTDKKSSKQELAKNCVVGTRFKSQPVVRHSEGLWMHFGDRLVTDGRFRIIVFAGKATDAT
QMSRIKKFSAYLDSENSVISLYTPKVSDRNSRIDVITIHSCHRDDIEMHDFPAPALHPKWQY

DFIYADCDSWHHPHPKSYQAWGVDETKGAVVVVRPDGYTSLVTDLEGTAEIDRYFSGILVEP
KEKSGAQTEADWTKS
>d1f37a_ c.47.1.11 (A:) Thioredoxin-like 2Fe-2S ferredoxin
{Aquifex aeolicus}
AEFKHVFVCVQDRPPGHPQGSCAQRGSREVFQAFMEKIQTDPQLFMTTVITPTGCMNACMMG
PVVVVYPDGVWYGQVKPEDVDEIVEKHLKGGEPVERLVISKGKPPGM
>d1j9ba_ c.47.1.12 (A:) Arsenate reductase ArsC {Escherichia coli}
NITIYHNPACGTSRNTLEMIRNSGTEPTIILYLENPPSRDELVKLIADMGISVRALLRKNVE
PYEQLGLAEDKFTDDQLIDFMLQHPILINRPIVVTPLGTRLCRPSEVVLDILQDAQKGAFTK
EDGEKVVDEAGKRL
>d1qmha1 c.47.2.1 (A:185-279) RNA 3'-terminal phosphate cyclase,
RPTC, insert domain {Escherichia coli}
ERGNIVQMRGEVLLAGVPRHVAEREIATLAGSFSLHEQNIHNLPRDQGPGNTVSLEVESENI
TERFFVVGEKRVSAEVVAAQLVKEVKRYLASTA
>d1gpua3 c.48.1.1 (A:535-680) Transketolase {Baker's yeast
(Saccharomyces cerevisiae)}
EGSSIESASKGGYVLQDVANPDIILVATGSEVSLSVEAAKTLAAKNIKARVVSLPDFFTFDK
QPLEYRLSVLPDNVPIMSVEVLATTCWGKYAHQSFGIDRFGASGKAPEVFKFFGFTPEGVAE
RAQKTIAFYKGDKLISPLKKAF
>d1dtwb2 c.48.1.2 (B:205-342) Branched-chain alpha-keto acid
dehydrogenase {Human (Homo sapiens)}
PYNIPLSQAEVIQEGSDVTLVAWGTQVHVIREVASMAKEKLGVSCEVIDLRTIIPWDVDTIC
KSVIKTGRLLISHEAPLTGGFASEISSTVQEECFLNLEAPISRVCGYDTPFPHIFEPFYIPD
KWKCYDALRKMINY
>d1qs0b2 c.48.1.2 (B:206-339) 2-oxoisovalerate dehydrogenase E1b
{Pseudomonas putida}
YYTVPLDKAAITRPGNDVSVLTYGTTVYVAQVAAEESGVDAEVIDLRSLWPLDLDTIVESVK
KTGRCVVVHEATRTCGFGAELVSLVQEHCFHHLEAPIERVTGWDTPYPHAQEWAYFPGPSRV
GAALKKVMEV
>d1ik6a2 c.48.1.2 (A:192-326) E1-beta subunit of pyruvate
dehydrogenase {Archaeon Pyrobaculum aerophilum}
DYVVEIGKARVAREGDDVTLVTYGAVVHKALEAAERVKASVEVVDLQTLNPLDFDTVLKSVS
KTGRLIIAHDSPKTGGLGAEVRALVAEKALDRLTAPVIRLAGPDVPQSPIAADAAYAPTVER
IIKAIEYVMRY
>d1keka3 c.48.1.3 (A:259-415) Pyruvate-ferredoxin oxidoreductase,
PFOR, domain II {Desulfovibrio africanus}
KLFDYVGAPDAERVIVSMGSSCETIEEVINHLAAKGEKIGLIKVRLYRPFVSEAFFAALPAS
AKVITVLDRTKEPGAPGDPLYLDVCSAFVERGEAMPKILAGRYGLGSKEFSPAMVKSVYDNM
SGAKKNHFTVGIEDDVTGTSLPVDNAFADTTPK
>d1a49a3 c.49.1.1 (A:396-530) Pyruvate kinase, C-terminal domain
{Rabbit (Oryctolagus cuniculus)}
ELARSSSHSTDLMEAMAMGSVEASYKCLAAALIVLTESGRSAHQVARYRPRAPIIAVTRNHQ
TARQAHLYRGIFPVVCKDPVQEAWAEDVDLRVNLAMNVGKARGFFKKGDVVIVLTGWRPGSG
FTNTMRVVPVP

>d1pkla3 c.49.1.1 (A:358-498) Pyruvate kinase, C-terminal domain {Leishmania mexicana}
NEYVFFNSIKKLQHIPMSADEAVCSSAVNSVYETKAKAMVVLSNTGRSARLVAKYRPNCPIV
CVTTRLQTCRQLNITQGVESVFFDADKLGHDEGKEHRVAAGVEFAKSKGYVQTGDYCVVIHA
DHKVKGYANQTRILLVE
>d1a3wa3 c.49.1.1 (A:367-500) Pyruvate kinase, C-terminal domain {Baker's yeast (Saccharomyces cerevisiae)}
DMRNCTPKPTSTTETVAASAVAAVFEQKAKAIIVLSTSGTTPRLVSKYRPNCPIILVTRCPR
AARFSHLYRGVFPFVFEKEPVSDWTDDVEARINFGIEKAKEFGILKKGDTYVSIQGFKAGAG
HSNTLQVSTV
>d1e0ta3 c.49.1.1 (A:354-470) Pyruvate kinase, C-terminal domain {Escherichia coli}
ITEAVCRGAVETAEKLDAPLIVVATQGGKSARAVRKYFPDATILALTTNEKTAHQLVLSKGV
VPQLVKEITSTDDFYRLGKELALQSGLAHKGDVVVMVSGALVPSGTTNTASVHVL
>d1e79g_ c.49.2.1 (G:) ATP syntase (F1-ATPase), gamma subunit {Cow (Bos taurus)}
ATLKDITRRLKSIKNIQKITKSMKMVAAAKYARAERELKPARVYGVGSLALYEKADIKTPED
KKKHLIIGVSSDRGLCGAIHSSVAKQMKSEAANLAAAGKEVKIIGVGDKIRSILHRTHSDQF
LVTFKEVGRRPPTFGDASVIALELLNSGYEFDEGSIIFNRFRSVISYKTEEKPIFSLDTISS
AESMSIYDDIDADVLRNYQEYSLANIIYYSLKESTTSEQSARMTAMDNASKNASEMIDKLTL
TFNRTRQAVITKELIEIISGAAAL
>d1mabg_ c.49.2.1 (G:) ATP syntase (F1-ATPase), gamma subunit {Rat (Rattus norvegicus)}
RDITRRLKSIKNIQKITKSMKMVAAAKYARAERELKPARVYGTGSLALYEKAEIKGPEDKKK
HLIIGVSSDRGLCGAIHSSVAKQMKNDMAALTAAGKEVMIVGIGEKIKSILYRTHSDQFLVS
FKDVGRKPPTFGDASVIALELLNSGYEFDEGSIIFNQFKSVISYKTEEKPIFSFSTVVAAEN
MSIYDDIDADVLQNYQEYNLANIIYYSLKESTTSEQSARMTAMDNASKNASDMIDKLTLTFN
RTRQAVITKELIEIISGAAALD
>d1fs0g_ c.49.2.1 (G:) ATP syntase (F1-ATPase), gamma subunit {Escherichia coli}
KITKAMEMVAASKMRKSQDRMAASRPYAETMRKVIGHLAHGNLEYKHPYLEDRDVKRVGYLV
VSTDRGLCGGLNINLFKKLLAEMKTWTDKGVQCDLAMIGSKGVSFFNSVGGNVVAQVTGMGD
NPSLSELIGPVKVMLQAYDEGRLDKLYIVSNKFINTMSQVPTISQLLPLPASDDDDLKHKSW
DYLYEPDPKALLDTLLRRYVESQVYQGVVENLASEQAARMVAMK
>d1lam_1 c.50.1.1 (1-159) Leucine aminopeptidase, N-terminal domain {Cow (Bos taurus)}
TKGLVLGIYSKEKEEDEPQFTSAGENFNKLVSGKLREILNISGPPLKAGKTRTFYGLHEDFP
SVVVVGLGKKTAGIDEQENWHEGKENIRAAVAAGCRQIQDLEIPSVEVDPCGDAQAAAEGAV
LGLYEYDDLKQKRKVVVSAKLHGSEDQEAWQRGVL
>d1kmma1 c.51.1.1 (A:326-424) Histidyl-tRNA synthetase (HisRS), C-terminal domain {Escherichia coli}
DPVVDIYLVASGADTQSAAMALAERLRDELPGVKLMTNHGGGNFKKQFARADKWGARVAVVL
GESEVANGTAVVKDLRSGEQTAVAQDSVAAHLRTLLG
>d1qe0a1 c.51.1.1 (A:326-420) Histidyl-tRNA synthetase (HisRS),

C-terminal domain {Staphylococcus aureus}
IEENLDLFIVTMGDQADRYAVKLLNHLRHNGIKADKDYLQRKIKGQMKQADRLGAKFTIVIG
DQELENNKIDVKNMTTGESETIELDALVEYFKK
>d1h4vb1 c.51.1.1 (B:326-421) Histidyl-tRNA synthetase (HisRS), C-terminal domain {Thermus thermophilus}
EKGPDLYLIPLTEEAVAEAFYLAEALRPRLRAEYALAPRKPAKGLEEALKRGAAFAGFLGED
ELRAGEVTLKRLATGEQVRLSREEVPGYLLQALG
>d1atia1 c.51.1.1 (A:395-505) Glycyl-tRNA synthetase (GlyRS), C-terminal domain {Thermus thermophilus}
QLAPIKVAVIPLVKNRPEITEYAKRLKARLLALGLGRVLYEDTGNIGKAYRRHDEVGTPFAV
TVDYDTIGQSKDGTTRLKDTVTVRDRDTMEQIRLHVDELEGFLRERLRW
>d1qf6a1 c.51.1.1 (A:533-642) Threonyl-tRNA synthetase (ThrRS), C-terminal domain {Escherichia coli}
FPTWLAPVQVVIMNITDSQSEYVNELTQKLSNAGIRVKADLRNEKIGFKIREHTLRRVPYML
VCGDKEVESGKVAVRTRRGKDLGSMDVNEVIEKLQQEIRSRSLKQLEE
>d1hc7a1 c.51.1.1 (A:277-403) C-terminal domain of ProRS {Thermus thermophilus}
RGLVLPPRLAPIQVVIVPIYKDESRERVLEAAQGLRQALLAQGLRVHLDDRDQHTPGYKFHE
WELKGVPFRVELGPKDLEGGQAVLASRLGGKETLPLAALPEALPGKLDAFHEELYRRALAFR
EDH
>d1g5ha1 c.51.1.1 (A:343-469) The aaRS-like accessory subunit of mitochondrial polymerase gamma, C-terminal domain {Mouse (Mus musculus)}
RKVLKLHPCLAPIKVALDVGKGPTVELRQVCQGLLNELLENGISVWPGYSETVHSSLEQLHS
KYDEMSVLFSVLVTETTLENGLIQLRSRDTTMKEMMHISKLRDFLVKYLASASNVAAALDHH
HHH
>d1crza2 c.51.2.1 (A:7-140) TolB, N-terminal domain {Escherichia coli}
DSGVDSGRPIGVVPFQWAGPGAAPEDIGGIVAADLRNSGKFNPLDRARLPQQPGSAQEVQPA
AWSALGIDAVVVGQVTPNPDGSYNVAYQLVDTGGAPGTVLAQNSYKVNKQWLRYAGHTASDE
VFEKLTGIKG
>d1eexb_ c.51.3.1 (B:) Diol dehydratase, beta subunit {Klebsiella oxytoca}
GFLTEVGEARQGTQQDEVIIAVGPAFGLAQTVNIVGIPHKSILREVIAGIEEEGIKARVIRC
FKSSDVAFVAVEGNRLSGSGISIGIQSKGTTVIHQQGLPPLSNLELFPQAPLLTLETYRQIG
KNAARYAKRESPQPVPTLNDQMARPKYQAKSAILHIKETKYVVTGKNPQELRVA
>d1b78a_ c.51.4.1 (A:) XTP pyrophosphatase {Archaeon Methanococcus jannaschii}
KIYFATGNPNKIKEANIILKDLKDVEIEQIKISYPEIQGTLEEVAEFGAKWVYNILKKPVIV
EDSGFFVEALNGFPGTYSKFVQETIGNEGILKLLEGKDNRNAYFKTVIGYCDENGVRLFKGI
VKGRVSEEIRSKGYGFAYDSIFIPEEEERTFAEMTTEEKSQISHRKKAFEEFKKFLLDRI
>d1ex2a_ c.51.4.2 (A:) Maf protein {Bacillus subtilis}
MTKPLILASQSPRRKELLDLLQLPYSIIVSEVEEKLNRNFSPEENVQWLAKQKAKAVADLHP
HAIVIGADTMVCLDGECLGKPQDQEEAASMLRRLSGRSHSVITAVSIQAENHSETFYDKTEV

AFWSLSEEEIWTYIETKEPMDKAGAYGIQGRGALFVKKIDGDYYSVMGLPISKTMRALRHF
>d1ihna_ c.103.1.1 (A:) Hypothetical protein MT938 (MTH938) {Archaeon Methanobacterium thermoautotrophicum}
SHMFSDCRFGSVTYRGREYRSDIVVHVDGSVTPRRKEISRRKYGTSHVMAEEELEELLEEKP
ESIIIGSGVHGALETGFRSDATVLPTCEAIKRYNEERSAGRRVAAIIHVTC
>d1ckqa_ c.52.1.1 (A:) Restriction endonuclease EcoRI {Escherichia coli}
SQGVIGIFGDYAKAHDLAVGEVSKLVKKALSNEYPQLSFRYRDSIKKTEINEALKKIDPDLG
GTLFVSNSSIKPDGGIVEVKDDYGEWRVVLVAEAKHQGKDIINIRNGLLVGKRGDQDLMAAG
NAIERSHKNISEIANFMLSESHFPYVLFLEGSNFLTENISITRPDGRVVNLEYNSGILNRLD
RLTAANYGMPINSNLCINKFVNHKDKSIMLQAASIYTQGDGREWDSKIMFEIMFDISTTSLR
VLGRDLFEQLTSK
>d1az3a_ c.52.1.2 (A:) Restriction endonuclease EcoRV {Escherichia coli}
SLRSDLINALYDENQKYDVCGIISAEGKIYPLGSDTKVLSTIFELFSRPIINKIAEKHGYIV
EEPKQQNHYPDFTLYKPSEPNKKIAIDIKTTYTNKENEKIKFTLGGYTSFIRNNTKNIVYPF
DQYIAHWIIGYVYTRVATRKSSLKTYNINELNEIPKPYKGVKVFLQDKWVIAGDLAGSGNTT
NIGSIHAHYKDFVEGKGIFDSEDEFLDYWR
>d1eona_ c.52.1.2 (A:) Restriction endonuclease EcoRV {Escherichia coli}
SLRSDLINALYDENQKYDVCGIISAEGKIYPLGSDTKVLSTIFELFSRPIINKIAEKHGYIV
EEPKQQNHYPDFTLYKPSEPNKKIAIDIKTTYTNKENEKIKFTLGGYTSFIRNNTKNIVYPF
DQYIAHWIIGYVYTRVATRKSSLKTYNINELNEIPKPYKGVKVFLQDKWVIAGDLAGSGNTT
NIGSIHAHYKDFVEGKGIFDSEDEFLDYWRNYERTSQLRNDKYNNISEYRNWIYRGRK
>d1bam__ c.52.1.3 (-) Restriction endonuclease BamHI {Bacillus amyloliquefaciens}
MEVEKEFITDEAKELLSKDKLIQQAYNEVKTSICSPIWPATSKTFTINNTEKNCNGVVPIKE
LCYTLLEDTYNWYREKPLDILKLEKKKGGPIDVYKEFIENSELKRVGMEFETGNISSAHRSM
NKLLLGLKHGEIDLAIILMPIKQLAYYLTDRVTNFEELEPYFELTEGQPFIFIGFNAEAYNS
NVPLIPKGSDGMSKRSIKKWKDKVENK
>d1dmua_ c.52.1.4 (A:) Restriction endonuclease BglI {Bacillus subtilis}
MYNLHREKIFMSYNQNKQYLEDNPEIQEKIELYGLNLLNEVISDNEEEIRADYNEANFLHPF
WMNYPPLDRGKMPKGDQIPWIEVGEKAVGSKLTRLVSQREDITVREIGLPTGPDERYLLTSP
TIYSLTNGFTDSIMMFVDIKSVGPRDSDYDLVLSPNQVSGNGDWAQLEGGIQNNQQTIQGPR
SSQIFLPTIPPLYILSDGTIAPVVHLFIKPIYAMRSLTKGDTGQSLYKIKLASVPNGLGLFC
NPGYAFDSAYKFLFRPGKDDRTKSLLQKRVRVDLRVLDKIGPRVMTIDMDK
>d1dfma_ c.52.1.5 (A:) Restriction endonuclease BglII {Bacillus subtilis}
MKIDITDYNHADEILNPQLWKEIEETLLKMPLHVKASDQASKVGSLIFDPVGTNQYIKDELV
PKHWKNNIPIPKRFDFLGTDIDFGKRDTLVEVQFSNYPFLLNNTVRSELFHKSNMDIDEEGM
KVAIIITKGHMFPASNSSLYYEQAQNQLNSLAEYNVFDVPIRLVGLIEDFETDIDIVSTTYA
DKRYSRTITKRDTVKGKVIDTNTPNTRRRKRGTIVTY
>d3pvia_ c.52.1.6 (A:) Restriction endonuclease PvuII {Proteus

vulgaris}
SHPDLNKLLELWPHIQEYQDLALKHGINDIFQGNGGKLLQVLLITGLTVLPGREGNDAVDNA
GQEYELKSINIDLTKGFSTHHHMNPVIIAKYRQVPWIFAIYRGIAIEAIYRLEPKDLEFYYD
KWERKWYSDGHKDINNPKIPVKYVMEHGTKIY
>d1cfr__ c.52.1.7 (-) Restriction endonuclease Cfr10I {Citrobacter freundii}
MDIISKSGEGNKYTINSAIAFVAYASHIDINTTEFSKVLSGLRDFINDEAIRLGGKISDGSF
NKCNGDWYEWLIGIRAIEFFLESETNFIVVKMPNATSFDVMSIYKSCLSEFIYDLRSKLSLN
NVNLITSNPDFSIIDIRGRREELKSMLKDISFSNISLSTISEIDNLYKNFIDYAELEHIKSF
LSVKTTFRPDRRLQLAHEGSLMKALYTHLQTRTWTINPTGIRYYAAATSIGNADVIGLKTVA
THSITDVKSLPQSAVDEIFKINSVLDVDSCLSHIL
>d1knva_ c.52.1.7 (A:) Restriction endonuclease Bse634I {Bacillus stearothermophilus}
NLTNSNCVEEYKENGKTKIRIKPFNALIELYHHQTPTGSIKENLDKLENYVKDVVKAKGLAI
PTSGAFSNTRGTWFEVMIAIQSWNYRVKRELNDYLIIKMPNVKTFDFRKIFDNETREKLHQL
EKSLLTHKQQVRLITSNPDLLIIRQKDLIKSEYNLPINKLTHENIDVALTLFKDIEGKCKWD
SLVAGVGLKTSLRPDRRLQLVHEGNILKSLFAHLKMRYWNPKAEFKYYGASSEPVSKADDDA
LQTAATHTIVNVNSTPERAVDDIFSLTSFEDIDKMLDQIIKK
>d1d02a_ c.52.1.8 (A:) Restriction endonuclease MunI {Eubacteria (Mycoplasma unidentified)}
LSGRLNWQALAGLKASGAEQNLYNVFNAVFEGTKYVLYEKPKHLKNLYAQVVLPDDVIKEIF
NPLIDLSTTQWGVSPAFAIENTETHKILFGEIKRQDGWVEGKDPSAGRGNAHERSCKLFTPG
LLKAYRTIGGINDEEILPFWVVFEGDITRDPKRVREITFWYDHYQDNYFMWRPNESGEKLVQ
HFNEKLKKYLD
>d1ev7a_ c.52.1.9 (A:) Restriction endonuclease NaeI {Nocardia aerocolonigenes}
EPDDDLERVRATLYSLDPDGDRTAGVLRDTLDQLYDGQRTGRWNFDQLHKTEKTHMGTLVEI
NLHREFQFGDGFETDYEIAGVQVDCKFSMSQGAWMLPPESIGHICLVIWASDQQCAWTAGLV
KVIPQFLGTANRDLKRRLTPEGRAQVVKLWPDHGKLQENLLLHIPGDVRDQIFSAKSSRGNQ
HGQARVNELFRRVHGRLIGRAVIATVAQQDDFMKRVRGSGGARSILRPEGIIILGHQDNDPK
VANDLGLPVPRKGQVVAARVVPADEGDQRQTAEIQGRRWAVAVPGDPIVEAPVV
>d1fiua_ c.52.1.10 (A:) Restriction endonuclease NgoIV {Neisseria gonorrhoeae}
MQPLFTQERRIFHKKLLDGNILATNNRGVVSNADGSNTRSFNIAKGIADLLHSETVSERLPG
QTSGNAFEAICSEFVQSAFEKLQHIRPGDWNVKQVGSRNRLEIARYQQYAHLTALAKAAEEN
PELAAALGSDYTITPDIIVTRNLIADAEINRNEFLVDENIATYASLRAGNGNMPLLHASISC
KWTIRSDRAQNARSEGLNLVRNRKGRLPHIVVVTAEPTPSRISSIALGTGEIDCVYHFALYE
LEQILQSLNYEDALDLFYIMVNGKRLKDISDLPLDLAV
>d1dc1a_ c.52.1.11 (A:) Restriction endonuclease BsobI {Bacillus stearothermophilus}
KPFENHLKSVDDLKTTYEEYRAGFIAFALEKNKRSTPYIERARALKVAASVAKTPKDLLYLE
DIQDALLYASGISDKAKKFLTEDDKKESINNLIENFLEPAGEEFIDELIFRYLLFQGDSLGG
TMRNIAGALAQQKLTRAIISALDIANIPYKWLDSRDKKYTNWMDKPEDDYELETFAKGISWT
INGKHRTLMYNITVSLVKKNVDICLFNCEPEIYTPQKVHQQPEKYLLLGELKGGIDPAGADE

HWKTANTALTRIRNKFSEKGLSPKTIFIGAAIEHSMAEEIWDQLQSGSLTNSANLTKTEQVG
SLCRWIINI
>d1kc6a_ c.52.1.19 (A:) Restriction endonuclease HincII
{Haemophilus influenzae}
SFIKPIYQDINSILIGQKVKRPKSGTLSGHAAGEPFEKLVYKFLKENLSDLTFKQYEYLNDL
FMKNPAIIGHEARYKLFNSPTLLFLLSRGKAATENWSIENLFEEKQNDTADILLVKDQFYEL
LDVKTRNISKSAQAPNIISAYKLAQTCAKMIDNKEFDLFDINYLEVDWELNGEDLVCVSTSF
AELFKSEPSELYINWAAAMQIQFHVRDLDQGFNGTREEWAKSYLKHFVTQAEQRAISMIDKF
VKPFKKYIL
>d2foka4 c.52.1.12 (A:387-579) Restriction endonuclease FokI,
C-terminal (catalytic) domain {Flavobacterium okeanokoites}
KSELEEKKSELRHKLKYVPHEYIELIEIARNSTQDRILEMKVMEFFMKVYGYRGKHLGGSRK
PDGAIYTVGSPIDYGVIVDTKAYSGGYNLPIGQADEMQRYVEENQTRNKHINPNEWWKVYPS
SVTEFKFLFVSGHFKGNYKAQLTRLNHITNCNGAVLSVEELLIGGEMIKAGTLTLEEVRRKF
NNGEINF
>d1avqa_ c.52.1.13 (A:) lambda exonuclease {Bacteriophage lambda}
SHMTPDIILQRTGIDVRAVEQGDDAWHKLRLGVITASEVHNVIAKPRSGKKWPDMKMSYFHT
LLAEVCTGVAPEVNAKALAWGKQYENDARTLFEFTSGVNVTESPIIYRDESMRTACSPDGLC
SDGNGLELKCPFTSRDFMKFRLGGFEAIKSAYMAQVQYSMWVTRKNAWYFANYDPRMKREGL
HYVVIERDEKYMASFDEIVPEFIEKMDEALAEIGFVFGEQWR
>d1azo__ c.52.1.14 (-) DNA mismatch repair protein MutH from
{Escherichia coli}
PRPLLSPPETEEQLLAQAQQLSGYTLGELAALVGLVTPENLKRDKGWIGVLLEIWLGASAGS
KPEQDFAALGVELKTIPVDSLGRPLETTFVCVAPLTGNSGVTWETSHVRHKLKRVLWIPVEG
EASIPLAQRRVGSPLLWSPNEEEDRQLREDWEELMDMIVLGQVERITARHGEYLQIRPKAAN
AKALTEAIGARGERILTLPRGFYLKKNFTSALLARHFLIQ
>d1cw0a_ c.52.1.15 (A:) Very short patch repair (VSR) endonuclease
{Escherichia coli}
ADVHDKATRSKNMRAIATRDTAIEKRLASLLTGQGLAFRVQDASLPGRPDFVVDEYRCVIFT
HGCFWHHHHCYLFKVPATRTEFWLEKIGKNVERDRRDISRLQELGWRVLIVWECALRGREKL
TDEALTERLEEWICGEGASAQIDTQGIHLLA
>d1vsra_ c.52.1.15 (A:) Very short patch repair (VSR) endonuclease
{Escherichia coli}
AIEKRLASLLTGQGLAFRVQDASLPGRPDFVVDEYRCVIFTHGCFWHHHHCYLFKVPATRTE
FWLEKIGKNVERDRRDISRLQELGWRVLIVWECALRGREKLTDEALTERLEEWICGEGASAQ
IDTQGIHLLA
>d1f1za2 c.52.1.16 (A:8-168) TnsA endonuclease, N-terminal domain
{Escherichia coli}
FSEVQIARRIKEGRGQGHGKDYIPWLTVQEVPSSGRSHRIYSHKTGRVHHLLSDLELAVFLS
LEWESSVLDIREQFPLLPSDTRQIAIDSGIKHPVIRGVDQVMSTDFLVDCKDGPFEQFAIQV
KPAAALQDERTLEKLELERRYWQQKQIPWFIFTDKEI
>d1fzra_ c.52.1.17 (A:) Endonuclease I (Holliday junction
resolvase) {Bacteriophage T7}
SGLEDKVSKQLESKGIKFEYEEWKVPYVIPASNHTYTPDFLLPNGIFVKTKGLWESDDRKKH

LLIREQHPELDIRIVFSSSRTKLYKGSPTSYGEFCEKHGIKFADKLIPAEWIKEPKKEVPFD
RLKRK
>d1gefa_ c.52.1.18 (A:) Archaeal Holliday junction resolvase Hjc {Archaeon Pyrococcus furiosus}
MYRKGAQAERELIKLLEKHGFAVVRSAGSKKVDLVAGNGKKYLCIEVKVTKKDHLYVGKRDM
GRLIEFSRRFGGIPVLAVKFLNVGWRFIEVSPKIEKFVFTPSSGVSLEVLLGIQKTLE
>d1hh1a_ c.52.1.18 (A:) Archaeal Holliday junction resolvase Hjc {Archaeon Sulfolobus solfataricus}
SAVERNIVSRLRDKGFAVVRAPASGSKRKDPIPDIIALKNGVIILIEMKSRKDIEGKIYVRR
EQAEGIIEFARKSGGSLFLGVKKPGVLKFIPFEKLRRTETGNYVADSEIEGLDLEDLVRLVE
AKISRTLD
>d1a79a1 c.52.2.1 (A:83-179) tRNA splicing endonuclease, C-terminal domain {Archaeon Methanococcus jannaschii}
ERLCLKYLVYKDLRTRGYIVKTGLKYGADFRLYERGANIDKEHSVYLVKVFPEDSSFLLSEL
TGFVRVAHSVRKKLLIAIVDADGDIVYYNMTYVKP
>d1dzfa1 c.52.3.1 (A:5-143) Eukaryotic RPB5 N-terminal domain {Baker's yeast (Saccharomyces cerevisiae)}
NERNISRLWRAFRTVKEMVKDRGYFITQEEVELPLEDFKAKYCDSMGRPQRKMMSFQANPTE
ESISKFPDMGSLWVEFCDEPSVGVKTMKTFVIHIQEKNFQTGIFVYQNNITPSAMKLVPSIP
PATIETFNEAALVVN
>d1gdta2 c.53.1.1 (A:1-140) gamma,delta resolvase, catalytic domain {Escherichia coli}
MRLFGYARVSTSQQSLDIQVRALKDAGVKANRIFTDKASGSSSDRKGLDLLRMKVEEGDVIL
VKKLDRLGRDTADMIQLIKEFDAQGVSIRFIDDGISTDGEMGKMVVTILSAVAQAERQRILE
RTNEGRQEAMAKGVVF
>d1hx7a_ c.53.1.1 (A:) gamma,delta resolvase, catalytic domain {Escherichia coli}
MRLFGYARVSTSQQSLDIQVRALKDAGVKANRIFTDKASGSSSDRKGLDLLRMKVEEGDVIL
VKKLDRLGRDTADMIQLIKEFDAQGVSIRFIDDGISTDGEMGK
>d2rsla_ c.53.1.1 (A:) gamma,delta resolvase, catalytic domain {Escherichia coli}
MRLFGYARVSTSQQSLDIQVRALKDAGVKANRIFTDKASGSSSDRKGLDLLRMKVEEGDVIL
VKKLDRLGRDTADMIQLIKEFDAQGVSIRFIDDGISTDGEMGKMVVTILSAVAQAERQRI
>d1tfr_2 c.53.1.2 (12-180) T4 RNase H {Bacteriophage T4}
KEGICLIDFSQIALSTALVNFPDKEKINLSMVRHLILNSIKFNVKKAKTLGYTKIVLCIDNA
KSGYWRRDFAYYYKKNRGKAREESTWDWEGYFESSHKVIDELKAYMPYIVMDIDKYEADDHI
AVLVKKFSLEGHKILIISSDGDFTQLHKYPNVKQWSPMHKKWVKI
>d1bgxt2 c.53.1.2 (T:1-173) 5' to 3' exonuclease domain of DNA polymerase <i>Taq</i> {Thermus aquaticus}
MRGMLPLFEPKGRVLLVDGHHLAYRTFHALKGLTTSRGEPVQAVYGFAKSLLKALKEDGDAV
IVVFDAKAPSFRHEAYGGYKAGRAPTPEDFPRQLALIKELVDLLGLARLEVPGYEADDVLAS
LAKKAEKEGYEVRILTADKDLYQLLSDRIHVLHPEGYLITPAWLWEKYG
>d1xo1a2 c.53.1.2 (A:19-185) T5 5'-exonuclease {Bacteriophage T5}
RRNLMIVDGTNLGFRFKHNNSKKPFASSYVSTIQSLAKSYSARTTIVLGDKGKSVFRLEHLP

EYAGNRDEKYAQRTEEEKALDEQFFEYLKDAFELCKTTFPTFTIRGVEADDMAAYIVKLIGH
LYDHVWLISTDGDWDTLLTDKVSRFSFTTRREYHLRDMYEHHN
>d1a77_2 c.53.1.2 (2-208) Flap endonuclease-1 {Archaeon
Methanococcus jannaschii}
GVQFGDFIPKNIISFEDLKGKKVAIDGMNALYQFLTSIRLRDGSPLRNRKGEITSAYNGVFY
KTIHLLENDITPIWVFDGEPPKLKEKTRKVRREMKEKAELKMKEAIKKEDFEEAAKYAKRVS
YLTPKMVENCKYLLSLMGIPYVEAPSEGEAQASYMAKKGDVWAVVSQDYDALLYGAPRVVRN
LTTTKEMPELIELNEVLEDLR
>d1b43a2 c.53.1.2 (A:1-219) Fen-1 nuclease {Archaeon Pyrococcus
furiosus}
GVPIGEIIPRKEIELENLYGKKIAIDALNAIYQFLSTIRQKDGTPLMDSKGRITSHLSGLFY
RTINLMEAGIKPVYVFDGEPPEFKKKELEKRREAREEAEEKWREALEKGEIEEARKYAQRAT
RVNEMLIEDAKKLLELMGIPIVQAPSEGEAQAAYMAAKGSVYASASQDYDSLLFGAPRLVRN
LTITGKRKLPGKNVYVEIKPELIILEEVLKELK
>d1ekja_ c.53.2.1 (A:) beta-carbonic anhydrase {Pea (Pisum
sativum)}
EASERIKTGFLHFKKEKYDKNPALYGELAKGQSPPFMVFACSDSRVCPSHVLDFQPGEAFVV
RNVANLVPPYDQAKYAGTGAAIEYAVLHLKVSNIVVIGHSACGGIKGLLSFPFDGTYSTDFI
EEWVKIGLPAKAKVKAQHGDAPFAELCTHCEKEAVNASLGNLLTYPFVREGLVNKTLALKGG
YYDFVKGSFELWGLEFGLSSTFSV
>d1g5ca_ c.53.2.1 (A:) beta-carbonic anhydrase {Archaeon
Methanobacterium thermoautotrophicum}
IIKDILRENQDFRFRDLSDLKHSPKLCIITCMDSRLIDLLERALGIGRGDAKVIKNAGNIVD
DGVIRSAAVAIYALGDNEIIIVGHTDCGMARLDEDLIVSRMRELGVEEEVIENFSIDVLNPV
GDEEENVIEGVKRLKSSPLIPESIGVHGLIIDINTGRLKPLYLDE
>d1i6pa_ c.53.2.1 (A:) beta-carbonic anhydrase {Escherichia coli}
KDIDTLISNNALWSKMLVEEDPGFFEKLAQAQKPRFLWIGCSDSRVPAERLTGLEPGELFVH
RNVANLVIHTDLNCLSVVQYAVDVLEVEHIIICGHYGCGGVQAAVENPELGLINNWLLHIRD
IWFKHSSLLGEMPQERRLDTLCELNVMEQVYNLGHSTIMQSAWKRGQKVTIHGWAYGIHDGL
LRDLDVTATNRETLEQRYRHGISNLKLK
>d1ddza1 c.53.2.1 (A:84-325) beta-carbonic anhydrase {Red alga
(Porphyridium purpureum)}
VMSDLEKKFIELEAKLVAQPAGQAMPGKSNIFANNEAWRQEMLKQDPEFFNRLANGQSPEYL
WIGCADSRVPANQLLDLPAGEVFVHRNIANQCIHSDISFLSVLQYAVQYLKVKHILVCGHYG
CGGAKAALGDSRLGLIDNWLRHIRDVRRMNAKYLDKCKDGDEELNRLIELNVLEQVHNVCAT
SIVQDAWDAGQELTVQGVVYGVGDGKLRDLGVVVNSSDDISKFYRTKSDSGALKAG
>d1ddza2 c.53.2.1 (A:326-564) beta-carbonic anhydrase {Red alga
(Porphyridium purpureum)}
NPNAPLVQVTKGGESELDSTMEKLTAELVQQTPGKLKEGANRVFVNNENWRQKMLKQDPQFF
SNLAHTQTPEILWIGCADSRVPANQIINLPAGEVFVHRNIANQCIHSDMSFLSVLQYAVQYL
KVKRVVVCGHYACGGCAAALGDSRLGLIDNWLRHIRDVRRHNQAELSRITDPKDSLNRLIEI
NVLEQMHNVCATSIVQDAWDAGQELEVQGVVYGVGDGKLRDMGVVAKANDDIG
>d1pdo__ c.54.1.1 (-) IIA domain of mannose transporter, IIA-Man
{Escherichia coli}

TIAIVIGTHGWAAEQLLKTAEMLLGEQENVGWIDFVPGENAETLIEKYNAQLAKLDTTKGVL
FLVDTWGGSPFNAASRIVVDKEHYEVIAGVNIPMLVETLMARDDDPSFDELVALAVETGREG
VKALK
>d1bupa1 c.55.1.1 (A:4-188) Heat shock protein 70kDa, ATPase fragment {Cow (Bos taurus)}
GPAVGIDLGSTYSCVGVFQHGKVEIIANDQGNRTTPSYVAFTDTERLIGDAAKNQVAMNPTN
TVFDAKRLIGRRFDDAVVQSDMKHWPFMVVNDAGRPKVQVEYKGETKSFYPEEVSSMVLTKM
KEIAEAYLGKTVTNAVVTVPAYFNDSQRQATKDAGTIAGLNVLRIINEPTAAAIAYGLDKK
>d1bupa2 c.55.1.1 (A:189-381) Heat shock protein 70kDa, ATPase fragment {Cow (Bos taurus)}
VGAERNVLIFDLGGGTFDVSILTIEDGIFEVKSTAGDTHLGGEDFDNRMVNHFIAEFKRKHK
KDISENKRAVRRLRTACERAKRTLSSSTQASIEIDSLYEGIDFYTSITRARFEELNADLFRG
TLDPVEKALRDAKLDKSQIHDIVLVGGSTRIPKIQKLLQDFFNGKELNKSINPDEAVAYGAA
VQAAILS
>d1hjoa1 c.55.1.1 (A:3-188) Heat shock protein 70kDa, ATPase fragment {Human (Homo sapiens)}
KAAAIGIDLGTTYSCVGVFQHGKVEIIANDQGNRTTPSYVAFTDTERLIGDAAKNQVALNPQ
NTVFDAKRLIGRKFGDPVVQSDMKHWPFQVINDGDKPKVQVSYKGETKAFYPEEISSMVLTK
MKEIAEAYLGYPVTNAVITVPAYFNDSQRQATKDAGVIAGLNVLRIINEPTAAAIAYGLDRT
>d1hjoa2 c.55.1.1 (A:189-382) Heat shock protein 70kDa, ATPase fragment {Human (Homo sapiens)}
GKGERNVLIFDLGGGTFDVSILTIDDGIFEVKATAGDTHLGGEDFDNRLVNHFVEEFKRKHK
KDISQNKRAVRRLRTACERAKRTLSSSTQASLEIDSLFEGIDFYTSITRARFEELCSDLFRS
TLEPVEKALRDAKLDKAQIHDLVLVGGSTRIPKVQKLLQDFFNGRDLNKSINPDEAVAYGAA
VQAAILMG
>d1dkgd1 c.55.1.1 (D:3-185) Heat shock protein 70kDa, ATPase fragment {Escherichia coli, gene dnaK}
KIIGIDLGTTNSCVAIMDGTTPRVLENAEGDRTTPSIIAYTQDGETLVGQPAKRQAVTNPQN
TLFAIKRLIGRRFQDEEVQRDVSIMPFKIIAADNGDAWVEVKGQKMAPPQISAEVLKKMKKT
AEDYLGEPVTEAVITVPAYFNDAQRQATKDAGRIAGLEVKRIINEPTAAALAYGLDKGT
>d1dkgd2 c.55.1.1 (D:186-383) Heat shock protein 70kDa, ATPase fragment {Escherichia coli, gene dnaK}
GNRTIAVYDLGGGTFDISIIEIDEVDGEKTFEVLATNGDTHLGGEDFDSRLINYLVEEFKKD
QGIDLRNDPLAMQRLKEAAEKAKIELSSAQQTDVNLPYITADATGPKHMNIKVTRAKLESLV
EDLVNRSIELLKVALQDAGLSVSDIDDVILVGGQTRMPMVQKKVAEFFGKEPRKDVNPDEAV
AIGAAVQGGVLT
>d1j6za1 c.55.1.1 (A:4-146) Actin {Rabbit (Oryctolagus cuniculus)}
ETTALVCDNGSGLVKAGFAGDDAPRAVFPSIVGRPRHQGVMVGMGQKDSYVGDEAQSKRGIL
TLKYPIEHGIITNWDDMEKIWHHTFYNELRVAPEEHPTLLTEAPLNPKANREKMTQIMFETF
NVPAMYVAIQAVLSLYASG
>d1j6za2 c.55.1.1 (A:147-372) Actin {Rabbit (Oryctolagus cuniculus)}
RTTGIVLDSGDGVTHNVPIYEGYALPHAIMRLDLAGRDLTDYLMKILTERGYSFVTTAEREI
VRDIKEKLCYVALDFENEMATAASSSSLEKSYELPDGQVITIGNERFRCPETLFQPSFIGME

SAGIHETTYNSIMKCDIDIRKDLYANNVMSGGTTMYPGIADRMQKEITALAPSTMKIKIIAP
PERKYSVWIGGSILASLSTFQQMWITKQEYDEAGPSIVHR
>d1d4xa1 c.55.1.1 (A:4-146) Actin {Nematode (Caenorhabditis elegans)}
EVAALVVDNGSGMCKAGFAGDDAPRAVFPSIVGRPRHQGVMVGMGQKDSYVGDEAQSKRGIL
TLKYPIEHGIVTNWDDMEKIWHHTFYNELRVAPEEHPVLLTEAPLNPKANREKMTQIMFETF
NTPAMYVAIQAVLSLYASG
>d1d4xa2 c.55.1.1 (A:147-375) Actin {Nematode (Caenorhabditis elegans)}
RTTGVVLDSGDGVTHTVPIYEGYALPHAILRLDLAGRDLTDYLMKILTERGYSFTTTAEREI
VRDIKEKLCYVALDFEQEMATAASSSSLEKSYELPDGQVITVGNERFRCPEAMFQPSFLGME
SAGIHETSYNSIMKCDIDIRKDLYANTVLSGGTTMYPGIADRMQKEITALAPSTMKIKIIAP
PERKYSVWIGGSILASLSTFQQMWISKQEYDESGPSIVHRKCF
>d1c0fa1 c.55.1.1 (A:1-146) Actin {Slime mold (Dictyostelium discoideum)}
DGEDVQALVIDNGSGMCKAGFAGDDAPRAVFPSIVGRPRHTGKDSYVGDEAQSKRGILTLKY
PIEHGIVTNWDDMEKIWHHTFYNELRVAPEEHPVLLTEAPLNPKANREKMTQIMFETFNTPA
MYVAIQAVLSLYASG
>d1dgaa2 c.55.1.1 (A:147-375) Actin {Slime mold (Dictyostelium discoideum)}
RTTGIVMDSGDGVSHTVPIYEGYALPHAILRLDLAGRDLTDYMMKILTERGYSFTTTAEREI
VRDIKEKLAYVALDFEQEMATAASSSALEKSYELPDGQVITIGNERFRCPEALFQPSFLGME
SAGIHETTYNSIMKCDVDIRKDLYGNVVLSGGTTMFPGIADRMNKELTALAPSTMKIKIIAP
PERKYSVWIGGSILASLSTFQQMWISKEEYDESGPSIVHRKCF
>d1yaga1 c.55.1.1 (A:4-146) Actin {Baker's yeast (Saccharomyces cerevisiae)}
EVAALVIDNGSGMCKAGFAGDDAPRAVFPSIVGRPRHQGIMVGMGQKDSYVGDEAQSKRGIL
TLRYPIEHGIVTNWDDMEKIWHHTFYNELRVAPEEHPVLLTEAPMNPKSNREKMTQIMFETF
NVPAFYVSIQAVLSLYSSG
>d1yaga2 c.55.1.1 (A:147-375) Actin {Baker's yeast (Saccharomyces cerevisiae)}
RTTGIVLDSGDGVTHVVPIYAGFSLPHAILRIDLAGRDLTDYLMKILSERGYSFSTTAEREI
VRDIKEKLCYVALDFEQEMQTAAQSSSIEKSYELPDGQVITIGNERFRAPEALFHPSVLGLE
SAGIDQTTYNSIMKCDVDVRKELYGNIVMSGGTTMFPGIAERMQKEITALAPSSMKVKIIAP
PERKYSVWIGGSILASLTTFQQMWISKQEYDESGPSIVHHKCF
>d1jcfa1 c.55.1.1 (A:1-140) Prokaryotic actin homolog MreB {Thermotoga maritima}
MLRKDIGIDLGTANTLVFLRGKGIVVNEPSVIAIDSTTGEILKVGLEAKNMIGKTPATIKAI
RPMRDGVIADYTVALVMLRYFINKAKGGMNLFKPRVVIGVPIGITDVERRAILDAGLEAGAS
KVFLIEEPMAAAIGSN
>d1jcfa2 c.55.1.1 (A:141-336) Prokaryotic actin homolog MreB {Thermotoga maritima}
LNVEEPSGNMVVDIGGGTTEVAVISLGSIVTWESIRIAGDEMDEAIVQYVRETYRVAIGERT
AERVKIEIGNVFPSKENDELETTVSGIDLSTGLPRKLTLKGGEVREALRSVVVAIVESVRTT

LEKTPPELVSDIIERGIFLTGGGSLLRGLDTLLQKETGISVIRSEEPLTAVAKGAGMVLDKV
NILKKLQGAG
>d1k8ka1 c.55.1.1 (A:3-160) Actin-related protein 3, Arp3 {Cow (Bos taurus)}
GRLPACVVDCGTGYTKLGYAGNTEPQFIIPSCIAIKESAKVGDQAQRRVMKGVDDLDFFIGD
EAIEKPTYATKWPIRHGIVEDWDLMERFMEQVIFKYLRAEPEDHYFLLTEPPLNTPENREYT
AEIMFESFNVPGLYIAVQAVLALAASWTSRQVGE
>d1k8ka2 c.55.1.1 (A:161-418) Actin-related protein 3, Arp3 {Cow (Bos taurus)}
RTLTGTVIDSGDGVTHVIPVAEGYVIGSCIKHIPIAGRDITYFIQQLLRDREVGIPPEQSLE
TAKAVKERYSYVCPDLVKEFNKYDTDGSKWIKQYTGINAISKKEFSIDVGYERFLGPEIFFH
PEFANPDFTQPISEVVDEVIQNCPIDVRRPLYKNIVLSGGSTMFRDFGRRLQRDLKRTVDAR
LKLSEELSGGRLKPKPIDVQVITHHMQRYAVWFGGSMLASTPEFYQVCHTKKDYEEIGPSIC
RHNPVFGVMS
>d1k8kb1 c.55.1.1 (B:154-343) Actin-related protein 2, Arp2 {Cow (Bos taurus)}
GVVVDSGDGVTHICPVYEGFSLPHLTRRLDIAGRDITRYLIKLLLLRGYAFNHSADFETVRM
IKEKLCYVGYNIEQEQKLALETTVLVESYTLPDGRIIKVGGERFEAPEALFQPHLINVEGVG
VAELLFNTIQAADIDTRSEFYKHIVLSGGSTMYPGLPSRLERELKQLYLERVLKGDVEKLSK
FKIR
>d1e4ft1 c.55.1.1 (T:7-199) Cell division protein FtsA {Thermotoga maritima}
TVFYTSIDIGSRYIKGLVLGKRDQEWEALAFSSVKSRGLDEGEIKDAIAFKESVNTLLKELE
EQLQKSLRSDFVISFSSVSFEREDTVIERDFGEEKRSITLDILSEMQSEALEKLKENGKTPL
HIFSKRYLLDDERIVFNPLDMKASKIAIEYTSIVVPLKVYEMFYNFLQDTVKSPFQLKSSLV
STAEGVL
>d1e4ft2 c.55.1.1 (T:200-390) Cell division protein FtsA {Thermotoga maritima}
TTPEKDRGVVVVNLGYNFTGLIAYKNGVPIKISYVPVGMKHVIKDVSAVLDTSFEESERLII
THGNAVYNDLKEEEIQYRGLDGNTIKTTTAKKLSVIIHARLREIMSKSKKFFREVEAKIVEE
GEIGIPGGVVLTGGGAKIPRINELATEVFKSPVRTGCYANSDRPSIINADEVANDPSFAAAF
GNVFA
>d1huxa_ c.55.1.5 (A:) Hydroxyglutaryl-CoA dehydratase component A {Acidaminococcus fermentans}
SIYTLGIDVGSTASKCIILKDGKEIVAKSLVAVGTGTSGPARSISEVLENAHMKKEDMAFTL
ATGYGRNSLEGIADKQMSELSCHAMGASFIWPNVHTVIDIGGQDVKVIHVENGTMTNFQMND
KCAAGTGRFLDVMANILEVKVSDLAELGAKSTKRVAISSTCTVFAESEVISQLSKGTDKIDI
IAGIHRSVASRVIGLANRVGIVKDVVMTGGVAQNYGVRGALEEGLGVEIKTSPLAQYNGALG
AALYAYKKAAK
>d1g99a1 c.55.1.2 (A:1-156) Acetate kinase {Archaeon Methanosarcina thermophila}
MKVLVINAGSSSLKYQLIDMTNESALAVGLCERIGIDNSIITQKKFDGKKLEKLTDLPTHKD
ALEEVVKALTDDEFGVIKDMGEINAVGHRVVHGGEKFTTSALYDEGVEKAIKDCFELAPLHN
PPNMMGISACAEIMPGTPMVIVFDTAFHQTMP

>d1g99a2 c.55.1.2 (A:157-398) Acetate kinase {Archaeon Methanosarcina thermophila}
PYAYMYALPYDLYEKHGVRKYGFHGTSHKYVAERAALMLGKPAEETKIITCHLGNGSSITAV
EGGKSVETSMGFTPLEGLAMGTRCGSIDPAIVPFLMEKEGLTTREIDTLMNKKSGVLGVSGL
SNDFRDLDEAASKGNRKAELALEIFAYKVKKFIGEYSAVLNGADAVVFTAGIGENSASIRKR
ILTGLDGIGIKIDDEKNKIRGQEIDISTPDAKVRVFVIPTNEELAIARETKEIVET
>d1ig8a1 c.55.1.3 (A:18-224) Hexokinase {Baker's yeast (Saccharomyces cerevisiae), pII}
DVPKELMQQIENFEKIFTVPTETLQAVTKHFISELEKGLSKKGGNIPMIPGWVMDFPTGKES
GDFLAIDLGGTNLRVVLVKLGGDRTFDTTQSKYRLPDAMRTTQNPDELWEFIADSLKAFIDE
QFPQGISEPIPLGFTFSFPASQNKINEGILQRWTKGFDIPNIENHDVVPMLQKQITKRNIPI
EVVALINDTTGTLVASYYTDP
>d1ig8a2 c.55.1.3 (A:225-486) Hexokinase {Baker's yeast (Saccharomyces cerevisiae), pII}
ETKMGVIFGTGVNGAYYDVCSDIEKLQGKLSDDIPPSAPMAINCEYGSFDNEHVVLPRTKYD
ITIDEESPRPGQQTFEKMSSGYYLGEILRLALMDMYKQGFIFKNQDLSKFDKPFVMDTSYPA
RIEEDPFENLEDTDDLFQNEFGINTTVQERKLIRRLSELIGARAARLSVCGIAAICQKRGYK
TGHIAADGSVYNRYPGFKEKAANALKDIYGWTQTSLDDYPIKIVPAEDGSGAGAAVIAALAQ
KRIAEGKSVGIIGA
>d1bdg_1 c.55.1.3 (13-222) Hexokinase {Blood fluke (Schistosoma mansoni)}
FSDQQLFEKVVEILKPFDLSVVDYEEICDRMGESMRLGLQKSTNEKSSIKMFPSYVTKTPNG
TETGNFLALDLGGTNYRVLSVTLEGKGKSPRIQERTYCIPAEKMSGSGTELFKYIAETLADF
LENNGMKDKKFDLGFTFSFPCVQKGLTHATLVRWTKGFSADGVEGHNVAELLQTELDKRELN
VKCVAVVNDTVGTLASCALEDP
>d1bdg_2 c.55.1.3 (223-460) Hexokinase {Blood fluke (Schistosoma mansoni)}
KCAVGLIVGTGTNVAYIEDSSKVELMDGVKEPEVVINTEWGAFGEKGELDCWRTQFDKSMDI
DSLHPGKQLYEKMVSGMYLGELVRHIIVYLVEQKILFRGDLPERLKVRNSLLTRYLTDVERD
PAHLLYNTHYMLTDDLHVPVVEPIDNRIVRYACEMVVKRAAYLAGAGIACILRRINRSEVTV
GVDGSLYKFHPKFCERMTDMVDKLKPKNTRFCLRLSEDGSGKGAAAIAASC
>d1czan1 c.55.1.3 (N:16-222) Mammalian type I hexokinase {Human (Homo sapiens)}
DDQVKKIDKYLYAMRLSDETLIDIMTRFRKEMKNGLSRDFNPTATVKMLPTFVRSIPDGSEK
GDFIALDLGGSSFRILRVQVNHEKNQNVHMESEVYDTPENIVHGSGSQLFDHVAECLGDFME
KRKIKDKKLPVGFTFSFPCQQSKIDEAILITWTKRFKASGVEGADVVKLLNKAIKKRGDYDA
NIVAVVNDTVGTMMTCGYDDQ
>d1czan2 c.55.1.3 (N:223-465) Mammalian type I hexokinase {Human (Homo sapiens)}
HCEVGLIIGTGTNACYMEELRHIDLVEGDEGRMCINTEWGAFGDDGSLEDIRTEFDRAIDAY
SLNPGKQLFEKMVSGMYLGELVRLILVKMAKEGLLFEGRITPELLTRGKFNTSDVSAIEKNK
EGLHNAKEILTRLGVEPSDDDCVSVQHVCTIVSFRSANLVAATLGAILNRLRDNKGTPRLRT
TVGVDGSLYKTHPQYSRRFHKTLRRLVPDSDVRFLLSESGSGKGAAMVTAVAYRLAE
>d1czan3 c.55.1.3 (N:466-670) Mammalian type I hexokinase {Human

(Homo sapiens)}
QHRQIEETLAHFHLTKDMLLEVKKRMRAEMELGLRKQTHNNAVVKMLPSFVRRTPDGTENGD
FLALDLGGTNFRVLLVKIRSGKKRTVEMHNKIYAIPIEIMQGTGEELFDHIVSCISDFLDYM
GIKGPRMPLGFTFSFPCQQTSLDAGILITWTKGFKATDCVGHDVVTLLRDAIKRREEFDLDV
VAVVNDTVGTMMTCAYEEP
>d1czan4 c.55.1.3 (N:671-913) Mammalian type I hexokinase {Human
(Homo sapiens)}
TCEVGLIVGTGSNACYMEEMKNVEMVEGDQGQMCINMEWGAFGDNGCLDDIRTHYDRLVDEY
SLNAGKQRYEKMISGMYLGEIVRNILIDFTKKGFLFRGQISETLKTRGIFETKFLSQIESDR
LALLQVRAILQQLGLNSTCDDSILVKTVCGVVSRRAAQLCGAGMAAVVDKIRENRGLDRLNV
TVGVDGTLYKLHPHFSRIMHQTVKELSPKCNVSFLLSEDGSGKGAALITAVGVRLRT
>d1bg3a1 c.55.1.3 (A:1-222) Mammalian type I hexokinase {Rat
(Rattus norvegicus)}
MIAAQLLAYYFTELKDDQVKKIDKYLYAMRLSDEILIDILTRFKKEMKNGLSRDYNPTASVK
MLPTFVRSIPDGSEKGDFIALDLGGSSFRILRVQVNHEKNQNVSMESEIYDTPENIVHGSGT
QLFDHVADCLGDFMEKKKIKDKKLPVGFTFSFPCRQSKIDEAVLITWTKRFKASGVEGADVV
KLLNKAIKKRGDYDANIVAVVNDTVGTMMTCGYDDQ
>d1bg3a2 c.55.1.3 (A:223-465) Mammalian type I hexokinase {Rat
(Rattus norvegicus)}
QCEVGLIIGTGTNACYMEELRHIDLVEGDEGRMCINTEWGAFGDDGSLEDIRTEFDRELDRG
SLNPGKQLFEKMVSGMYMGELVRLILVKMAKEGLLFEGRITPELLTRGKFNTSDVSAIEKDK
EGIQNAKEILTRLGVEPSDVDCVSVQHICTIVSFRSANLVAATLGAILNRLRDNKGTPRLRT
TVGVDGSLYKMHPQYSRRFHKTLRRLVPDSDVRFLLSESGTGKGAAMVTAVAYRLAE
>d1bg3a3 c.55.1.3 (A:466-670) Mammalian type I hexokinase {Rat
(Rattus norvegicus)}
QHIRQIEETLAHFRLSKQTLMEVKKRLRTEMEMGLRKETNSKATVKMLPSFVRSIPDGTEHG
DFLALDLGGTNFRVLLVKIRSGKKRTVEMHNKIYSIPLEIMQGTGDELFDHIVSCISDFLDY
MGIKGPRMPLGFTFSFPCHQTNLDCGILISWTKGFKATDCEGHDVASLLRDAVKRREEFDLD
VVAVVNDTVGTMMTCAYEEP
>d1bg3a4 c.55.1.3 (A:671-911) Mammalian type I hexokinase {Rat
(Rattus norvegicus)}
TCEIGLIVGTGTNACYMEEMKNVEMVEGNQGQMCINMEWGAFGDNGCLDDIRTDFDKVVDEY
SLNSGKQRFEKMISGMYLGEIVRNILIDFTKKGFLFRGQISEPLKTRGIFETKFLSQIESDR
LALLQVRAILQQLGLNSTCDDSILVKTVCGVVSKRAAQLCGAGMAAVVEKIRENRGLDHLNV
TVGVDGTLYKLHPHFSRIMHQTVKELSPKCTVSFLLSEDGSGKGAALITAVGVRL
>d1bu6o1 c.55.1.4 (O:3-253) Glycerol kinase {Escherichia coli}
KKYIVALDQGTTSSRAVVMDHDANIISVSQREFEQIYPKPGWVEHDPMEIWATQSSTLVEVL
TKADISSDQIAAIGITNQRETTIVWEKETGKPIYNAIVWQCRRTAEICEHLKRDGLEDYIRS
NTGLVIDPYFSGTKVKWILDHVEGSRERARRGELLFGTVDTWLIWKMTQGRVHVTDYTNASR
TMLFNIHTLDWDDKMLEVLDIPREMLPEVRRSSEVYGQTNIGGKGGTRIPISGIAGDQQAAL
FGQ
>d1bu6o2 c.55.1.4 (O:254-499) Glycerol kinase {Escherichia coli}
LCVKEGMAKNTYGTGCFMLMNTGEKAVKSENGLLTTIACGPTGEVNYALEGAVFMAGASIQW
LRDEMKLINDAYDSEYFATKVQNTNGVYVVPAFTGLGAPYWDPYARGAIFGLTRGVNANHII

RATLESIAYQTRDVLEAMQADSGIRLHALRVDGGAVANNFLMQFQSDILGTRVERPEVREVT
ALGAAYLAGLAVGFWQNLDELQEKAVIEREFRPGIETTERNYRYAGWKKAVKRAMAWEEH
>d1chma1 c.55.2.1 (A:2-156) Creatinase {Pseudomonas putida}
QMPKTLRIRNGDKVRSTFSAQEYANRQARLRAHLAAENIDAAIFTSYHNINYYSDFLYCSFG
RPYALVVTEDDVISISANIDGGQPWRRTVGTDNIVYTDWQRDNYFAAIQQALPKARRIGIEH
DHLNLQNRDKLAARYPDAELVDVAAACMRMR
>d1az9_1 c.55.2.1 (1-176) Aminopeptidase P {Escherichia coli}
SEISRQEFQRRRQALVEQMQPGSAALIFAAPEVTRSADSEYPYRQNSDFWYFTGFNEPEAVL
VLIKSDDTHNHSVLFNRVRDLTAEIWFGRRLGQDAAPEKLGVDRALAFSEINQQLYQLLNGL
DVVYHAQGEYAYADVIVNSALEKLRKGSRQNLTAPATMIDWRPVVHEMRLFK
>d1jl1a_ c.55.3.1 (A:) RNase H (RNase HI) {Escherichia coli}
KQVEIFTAGSALGNPGPGGYGAILRYRGREKTFSAGYTRTTNNRMELMAAIVALEALKEHAE
VILSTDSQYVRQGITQWIHNWKKRGWKTADKKPVKNVDLWQRLDAALGQHQIKWEWVKGHAG
HPENERADELARAAAMNPTLEDTGYQVE
>d1ril__ c.55.3.1 (-) RNase H (RNase HI) {Thermus thermophilus}
RKRVALFTDGACLGNPGPGGWAALLRFHAHEKLLSGGEACTTNNRMELKAAIEGLKALKEPC
EVDLYTDSHYLKKAFTEGWLEGWRKRGWRTAEGKPVKNRDLWEALLLAMAPHRVRFHFVKGH
TGHPENERVDREARRQAQSQAKT
>d1jl2a_ c.55.3.1 (A:) RNase H (RNase HI) {Chimeric (Escherichia coli/Thermus thermophilus)}
KQVEIFTDGSALGNPGPGGYGAILRYRGREKTFSAGYTRTTNNRMELKAAIEGLKALKEPAE
VDLYTDSHYLKKAFTEGWLEGWRKRGWRTAEGKPVKNRDLWEALLLAMAPHRVRFHFVKGHA
GHPENERADELARAAAMNPTLEDTGY
>d1ekea_ c.55.3.1 (A:) Class II ribonuclease H (RNase HII) {Archaeon Methanococcus jannaschii}
MIIIGIDEAGRGPVLGPMVVCAFAIEKEREEELKKLGVKDSKELTKNKRAYLKKLLENLGYV
EKRILEAEEINQLMNSINLNDIEINAFSKVAKNLIEKLNIRDDEIEIYIDACSTNTKKFEDS
FKDKIEDIIKERNLNIKIIAEHKADAKYPVVSAASIIAKAERDEIIDYYKKIYGDIGSGYPS
DPKTIKFLEDYFKKHKKLPDIARTHWKTCKRILDKSKQT
>d1i39a_ c.55.3.1 (A:) Class II ribonuclease H (RNase HII) {Archaeon Archaeoglobus fulgidus}
MKAGIDEAGKGCVIGPLVVAGVACSDEDRLRKLGVKDSKKLSQGRREELAEEIRKICRTEVL
KVSPENLDERMAAKTINEILKECYAEIILRLKPEIAYVDSPDVIPERLSRELEEITGLRVVA
EHKADEKYPLVAAASIIAKVEREREIERLKEKFGDFGSGYASDPRTREVLKEWIASGRIPSC
VRMRWKTVSNLRQK
>d1io2a_ c.55.3.1 (A:) Class II ribonuclease H (RNase HII) {Archaeon Thermococcus kodakaraensis}
MKIAGIDEAGRGPVIGPMVIAAVVVDENSLPKLEELKVRDSKKLTPKRREKLFNEILGVLDD
YVILELPPDVIGSREGTLNEFEVENFAKALNSLKVKPDVIYADAADVDEERFARELGERLNF
EAEVVAKHKADDIFPVVSAASILAKVTRDRAVEKLKEEYGEIGSGYPSDPRTRAFLENYYRE
HGEFPPIVRKGWKTLKKIAEKVESEKK
>d1c9ra1 c.55.3.1 (A:430-558) HIV RNase H (Domain of reverse transcriptase) {Human immunodeficiency virus type 1}
EKEPIVGAETFYVDGAANAATKLGKAGYVTNKGRQKVVPLTNTTNQKTELQAIYLALQDSGL

EVNIVTDSQYALGIIQAQPDKSESELVNQIIEALIKKEAVYLAWVPAHAGIGGNAAVDALVS
AGIAA
>d1hrha1 c.55.3.1 (A:432-556) HIV RNase H (Domain of reverse transcriptase) {Human immunodeficiency virus type 1}
EPIVGAETFYVDGAANRETKLGKAGYVTNKGRQKVVPLTNTTNQKTELQAIYLALQDSGLEV
NIVTDSQYALGIIQAQPDKSESELVNQIIEQLIKKEKVYLAWVPAHKGIGGNEQVDKLVSAG
I
>d1jlaa1 c.55.3.1 (A:430-553) HIV RNase H (Domain of reverse transcriptase) {Human immunodeficiency virus type 1}
EKEPIVGAETFYVDGAANRETKLGKAGYVTNRGRQKVVTLTDTTNQKTELQAIYLALQDSGL
EVNIVTDSQYALGIIQAQPDQSESELVNQIIEQLIKKEKVYLAWVPAHKGIGGNEQVDKLVS
>d1vrta1 c.55.3.1 (A:430-539) HIV RNase H (Domain of reverse transcriptase) {Human immunodeficiency virus type 1}
EKEPIVGAETFYVDGAANRETKLGKAGYVTNRGRQKVVTLTDTTNQKTELQAIYLALQDSGL
EVNIVTDSQYALGIIQAQPDQSESELVNQIIEQLIKKEKVYLAWVPAH
>d1asu__ c.55.3.2 (-) Retroviral integrase, catalytic domain {Rous sarcoma virus (RSV, avian sarcoma virus)}
PLREPRGLGPLQIWQTDFTLEPRMAPRSWLAVTVDTASSAIVVTQHGRVTSVAAQHHWATAI
AVLGRPKAIKTDNGSCFTSKSTREWLARWGIAHTTGIPGNSQGQAMVERANRLLKDKIRVLA
EGDGFMKRIPTSKQGELLAKAMYALNHFERGENTKTNL
>d1c0ma2 c.55.3.2 (A:49-216) Retroviral integrase, catalytic domain {Rous sarcoma virus (RSV, avian sarcoma virus)}
GVNPRGLGPLQIWQTDFTLEPRMAPRSWLAVTVDTASSAIVVTQHGRVTSVAVQHHWATAIA
VLGRPKAIKTDNGSCFTSKSTREWLARWGIAHTTGIPGNSQGQAMVERANRLLKDRIRVLAE
GDGFMKRIPTSKQGELLAKAMYALNHKERGENTKTPIQKHWRPT
>d1cxqa_ c.55.3.2 (A:) Retroviral integrase, catalytic domain {Rous sarcoma virus (RSV, avian sarcoma virus)}
GRGLGPLQIWQTDFTLEPRMAPRSWLAVTVDTASSAIVVTQHGRVTSVAAQHHWATAIAVLG
RPKAIKTDNGSCFTSKSTREWLARWGIAHTTGIPGNSQGQAMVERANRLLKDKIRVLAEGDG
FMKRIPTSKQGELLAKAMYALNH
>d1exqa_ c.55.3.2 (A:) Retroviral integrase, catalytic domain {Human immunodeficiency virus type 1}
SSPGIWQLDCTHLEGKVILVAVHVASGYIEAEVIPAETGQETAYFLLKLAGRWPVKTIHTDN
GSNFTGATVRAACDWAGIKQEDGIPYNPQSQGVVESMNKELKKIIGQVRDQAEHLKTAVQMA
VFIHNKKRKGGIGGYSAGERIVDIIATDIQ
>d1hyva_ c.55.3.2 (A:) Retroviral integrase, catalytic domain {Human immunodeficiency virus type 1}
SPGIWQLDCTHLEGKVILVAVHVASGYIEAEVIPAETGQETAYFLLKLAGRWPVKTVHTDNG
SNFTSTTVKAACWWAGIKQEFGIPYNPQSQGVIESMNKELKKIIGQVRDQAEHLKTAVQMAV
FIHNKKRKGGIGGYSAGERIVDIIATDIQT
>d1c6va_ c.55.3.2 (A:) Retroviral integrase, catalytic domain {Simian immunodeficiency virus}
NSDLGTWQMDCTHLEGKIVIVAVHVASGFIEAEVIPQETGRQTALFLLKLAGRWPITHLHTD
NGANFASQEVKMVAWWAGIEHTFGVPYNPQSQGVVEAMNHHLKNQIDRIREQANSVETIVLM

AVHCMNHKRRGGIGDMTPAERLINMITTEQEIQFQ
>d1bco_2 c.55.3.3 (258-480) mu transposase, core domain {Bacteriophage mu}
EHLDAMQWINGDGYLHNVFVRWFNGDVIRPKTWFWQDVKTRKILGWRCDVSENIDSIRLSFM
DVVTRYGIPEDFHITIDNTRGAANKWLTGGAPNRYRFKVKEDDPKGLFLLMGAKMHWTSVVA
GKGWGQAKPVERAFGVGGLEEYVDKHPALAGAYTGPNPQAKPDNYGDRAVDAELFLKTLAEG
VAMFNARTGRETEMCGGKLSFDDVFEREYARTIVRKP
>d1b7ea_ c.55.3.4 (A:) Transposase inhibitor (Tn5 transposase) {Escherichia coli}
SAEAIRKAGAMQTVKLAQEFPELLAIEDTTSLSYRHQVAEELGKLGSIQDKSRGWWVHSVLL
LEATTFRTVGLLHQEWWMRPDDPADADEKESGKWLAAAATSRLRMGSMMSNVIAVCDREADI
HAYLQDKLAHNERFVVRSKHPRKDVESGLYLYDHLKNQPELGGYQISIPQKGVVDKRGKRKN
RPARKASLSLRSGRITLKQGNITLNAVLAEEINPPKGETPLKWLLLTSEPVESLAQALRVID
IYTHRWRIEEFHKAWKTGAGAERQRMEEPDNLERMVSILSFVAVRLLQLRESFTLPQALRAQ
GLLKEAEHVESQSAETVLTPDECQLLGYLDKGKRKRKEKGSLQWAYMAIARLGGFMDSKRTG
IASWGALWEGWEALQSKLDGFLAAKDLMAQ
>d1f3ia_ c.55.3.4 (A:) Transposase inhibitor (Tn5 transposase) {Escherichia coli}
ALHRAADWAKSVFSSAALGDPRRTARLVNVAAQLAKYSGKSITISSEGSKAAQEGAYRFIRN
PNVSAEAIRKAGAMQTVKLAQEFPELLAIEDTTSLSYRHQVAEELGKLGSIQDKSRGWWVHS
VLLLEATTFRTVGLLHQEWWMRPDDPADADEKESGKWLAAAATSRLRMGSMMSNVIAVCDRE
ADIHAYLQDKLAHNERFVVRSKHPRKDVESGLYLYDHLKNQPELGGYQISIPQKGVVDKRGK
RKNRPARKASLSLRSGRITLKQGNITLNAVLAEEINPPKGETPLKWLLLTSEPVESLAQALR
VIDIYTHRWRIEEFHKAWKTGAGAERQRMEEPDNLERMVSILSFVAVRLLQLRESFTLPQAL
RAQGLLKEAEHVESQSAETVLTPDECQLLGYLDKGKRKRKEKAGSLQWAYMAIARLGGFMDS
KRTGIASWGALWEGWEALQSKLDGFLAAKDLMAQGIKIG
>d1kfsa1 c.55.3.5 (A:324-518) Exonuclease domain of prokaryotic DNA polymerase {Escherichia coli}
MISYDNYVTILDEETLKAWIAKLEKAPVFAFDTETDSLDNISANLVGLSFAIEPGVAAYIPV
AHDYLDAPDQISRERALELLKPLLEDEKALKVGQNLKYDRGILANYGIELRGIAFDTMLESY
ILNSVAGRHDMDSLAERWLKHKTITFEEIAGKGKNQLTFNQIALEEAGRYAAEDADVTLQLH
LKMWPDLQK
>d1qtma1 c.55.3.5 (A:293-422) Exonuclease domain of prokaryotic DNA polymerase {Thermus aquaticus}
ALEEAPWPPPEGAFVGFVLSRKEPMWADLLALAAARGGRVHRAPEPYKALRDLKEARGLLAK
DLSVLALREGLGLPPGDDPMLLAYLLDPSNTTPEGVARRYGGEWTEEAGERAALSERLFANL
WGRLEG
>d1xwl_1 c.55.3.5 (297-468) Exonuclease domain of prokaryotic DNA polymerase {Bacillus stearothermophilus, newly identified strain as yet unnamed}
AKMAFTLADRVTEEMLADKAALVVEVVEENYHDAPIVGIAVVNEHGRFFLRPETALADPQFV
AWLGDETKKKSMFDSKRAAVALKWKGIELCGVSFDLLLAAYLLDPAQGVDDVAAAAKMKQYE
AVRPDEAVYGKGAKRAVPDEPVLAEHLVRKAAAIWELERPFLDELRRN
>d1t7pa1 c.55.3.5 (A:1-210) Exonuclease domain of T7 DNA polymerase

{Bacteriophage T7}
MIVSDIEANALLESVTKFHCGVIYDYSTAEYVSYRPSDFGAYLDALEAEVARGGLIVFHNGH
KYDVPALTKLAKLQLNREFHLPRENCIDTLVLSRLIHSNLKDTDMGLLRSGKLPGALEAWGY
RLGEMKGEYKDDFKRMLEEQGEEYVDGMEWWNFNEEMMDYNVQDVVVTKALLEKLLSDKHYF
PPEIDFTDVGYTTFWSES
>d1noya_ c.55.3.5 (A:) Exonuclease domain of family B (archaeal and phage) DNA polymerases {Bacteriophage T4}
DEFYISIETVGNNIVERYIDENGKERTREVEYLPTMFRHCKEESKYKDIYGKNCAPQKFPSM
KDARDWMKRMEDIGLEALGMNDFKLAYISDTYGSEIVYDRKFVRVANCDIEVTGDKFPDPMK
AEYEIDAITHYDSIDDRFYVFDLLNSMYGSVSKWDAKLAAKLDCEGGDEVPQEILDRVIYMP
FDNERDMLMEYINLWEQKRPAIFTGWNIEGFDVPYIMNRVKMILGERSMKRFSPIGRVKSKL
LQNMYGSKEIYSIDGVSILDYLDLYKKFAFTNLPSFSLESVAQHETKKGKLPYDGPINKLRE
TNHQRYISYNIIDVESVQAIDKIRGFIDLVLSMSYYAKMPFSGVMSPIKTWDAIIFNSLKGE
>d1ih7a1 c.55.3.5 (A:1-375) Exonuclease domain of family B (archaeal and phage) DNA polymerases {Bacteriophage RB69}
MKEFYLTVEQIGDSIFERYIDSNGRERTREVEYKPSLFAHCPESQATKYFDIYGKPCTRKLF
ANMRDASQWIKRMEDIGLEALGMDDFKLAYLSDTYNYEIKYDHTKIRVANFDIEVTSPDGFP
EPSQAKHPIDAITHYDSIDDRFYVFDLLNSPYGNVEEWSIEIAAKLQEQGGDEVPSEIIDKI
IYMPFDNEKELLMEYLNFWQQKTPVILTGWNVESFDIPYVYNRIKNIFGESTAKRLSPHRKT
RVKVIENMYGSREIITLFGISVLDYIDLYKKFSFTNQPSYSLDYISEFELNVGKLKYDGPIS
KLRESNHQRYISYNIIDVYRVLQIDAKRQFINLSLDMGYYAKIQIQSVFSPIKTWDAIIFNS
LKE
>d1tgoa1 c.55.3.5 (A:1-347) Exonuclease domain of family B (archaeal and phage) DNA polymerases {Archaeon Thermococcus gorgonarius}
MILDTDYITEDGKPVIRIFKKENGEFKIDYDRNFEPYIYALLKDDSAIEDVKKITAERHGTT
VRVVRAEKVKKKFLGRPIEVWKLYFTHPQDVPAIRDKIKEHPAVVDIYEYDIPFAKRYLIDK
GLIPMEGDEELKMLAFDIETLYHEGEEFAEGPILMISYADEEGARVITWKNIDLPYVDVVST
EKEMIKRFLKVVKEKDPDVLITYNGDNFDFAYLKKRSEKLGVKFILGREGSEPKIQRMGDRF
AVEVKGRIHFDLYPVIRRTINLPTYTLEAVYEAIFGQPKEKVYAEEIAQAWETGEGLERVAR
YSMEDAKVTYELGKEFFPMEAQLSRLVGQSLWDVSRS
>d1qhta1 c.55.3.5 (A:1-347) Exonuclease domain of family B (archaeal and phage) DNA polymerases {Archaeon Thermococcus sp., 9on-7}
MILDTDYITENGKPVIRVFKKENGEFKIEYDRTFEPYFYALLKDDSAIEDVKKVTAKRHGTV
VKVKRAEKVQKKFLGRPIEVWKLYFNHPQDVPAIRDRIRAHPAVVDIYEYDIPFAKRYLIDK
GLIPMEGDEELTMLAFAIATLYHEGEEFGTGPILMISYADGSEARVITWKKIDLPYVDVVST
EKEMIKRFLRVVREKDPDVLITYNGDNFDFAYLKKRCEELGIKFTLGRDGSEPKIQRMGDRF
AVEVKGRIHFDLYPVIRRTINLPTYTLEAVYEAVFGKPKEKVYAEEIAQAWESGEGLERVAR
YSMEDAKVTYELGREFFPMEAQLSRLIGQSLWDVSRS
>d1d5aa1 c.55.3.5 (A:1-347) Exonuclease domain of family B (archaeal and phage) DNA polymerases {Archaeon Desulfurococcus tok}
MILDADYITEDGKPVIRVFKKEKGEFKIDYDRDFEPYIYALLKDDSAIEDIKKITAERHGTT

VRVTRAERVKKKFLGRPVEVWKLYFTHPQDVPAIRDKIREHPAVVDIYEYDIPFAKRYLIDR
GLIPMEGDEELRMLAFDIETLAHAGAAAGAGPILMISYADEEGARVITWKNIDLPYVESVST
EKEMIKRFLKVIQEKDPDVLITYNGDNFDFAYLKKRSEMLGVKFILGRDGSEPKIQRMGDRF
AVEVKGRIHFDLYPVIRRTINLPTYTLETVYEPVFGQPAEKVYAEEIAEAWASGEGLERVAR
YSMEDAKATYELGKEFFPMEAQLSRLVGQSLWDVSRS
>d1gcxa1 c.55.3.5 (A:1-347) Exonuclease domain of family B (archaeal and phage) DNA polymerases {Archaeon Pyrococcus kodakaraensis}
MILDTDYITEDGKPVIRIFKKENGEFKIEYDRTFEPYFYALLKDDSAIEEVKKITAERHGTV
VTVKRVEKVQKKFLGRPVEVWKLYFTHPQDVPAIRDKIREHPAVIDIYEYDIPFAKRYLIDK
GLVPMEGDEELKMLAFDIETLYHEGEEFAEGPILMISYADEEGARVITWKNVDLPYVDVVST
EREMIKRFLRVVKEKDPDVLITYNGDNFDFAYLKKRCEKLGINFALGRDGSEPKIQRMGDRF
AVEVKGRIHFDLYPVIRRTINLPTYTLEAVYEAVFGQPKEKVYAEEITTAWETGENLERVAR
YSMEDAKVTYELGKEFLPMEAQLSRLIGQSLWDVSRS
>d1fxxa_ c.55.3.5 (A:) Exonuclease I {Escherichia coli}
QSTFLFHDYETFGTHPALDRPAQFAAIRTDSEFNVIGEPEVFYCKPADDYLPQPGAVLITGI
TPQEARAKGENEAAFAARIHSLFTVPKTCILGYNNVRFDDEVTRNIFYRNFYDPYAWSWQHD
NSRWDLLDVMRACYALRPEGINWPENDDGLPSFRLEHLTKANGIEHSNAHDAMADVYATIAM
AKLVKTRQPRLFDYLFTHRNKHKLMALIDVPQMKPLVHVSGMFGAWRGNTSWVAPLAWHPEN
RNAVIMVDLAGDISPLLELDSDTLRERLYTAKTDLGDNAAVPVKLVHINKCPVLAQANTLRP
EDADRLGINRQHCLDNLKILRENPQVREKVVAIFAEAEPFTPSDNVDAQLYNGFFSDADRAA
MKIVLETEPRNLPALDITFVDKRIEKLLFNYRARNFPGTLDYAEQQRWLEHRRQVFTPEFLQ
GYADELQMLVQQYADDKEKVALLKALWQYADEIVEH
>d1hjra_ c.55.3.6 (A:) RuvC resolvase {Escherichia coli}
AIILGIDPGSRVTGYGVIRQVGRQLSYLGSGCIRTKVDDLPSRLKLIYAGVTEIITQFQPDY
FAIEQVFMAKNADSALKLGQARGVAIVAAVNQELPVFEYAARQVKQTVVGIGSAEKSQVQHM
VRTLLKLPANPQADAADALAIAITHCHVSQNAMQ
>d1kcfa2 c.55.3.7 (A:39-256) Mitochondrial resolvase ydc2 catalytic domain {Fission yeast (Schizosaccharomyces pombe)}
TSRVLGIDLGIKNFSYCFASQNEDSKVIIHNWSVENLTEKNGLDIQWTEDFQPSSMADLSIQ
LFNTLHEKFNPHVILMERQRYRSGIATIPEWTLRVNMLESMLYALHYAEKRNSIEQKIQYPF
LLSLSPKSTYSYWASVLNTKASFSKKKSRVQMVKELIDGQKILFENEEALYKWNNGSRVEFK
KDDMADSALIASGWMRWQAQLKHYRNFCKQFL
>d1jj2m_ c.55.4.1 (M:) Ribosomal protein L18 (L18p) {Archaeon Haloarcula marismortui}
ATGPRYKVPMRRRREARTDYHQRLRLLKSGKPRLVARKSNKHVRAQLVTLGPNGDDTLASAH
SSDLAEYGWEAPTGNMPSAYLTGLLAGLRAQEAGVEEAVLDIGLNSPTPGSKVFAIQEGAID
AGLDIPHNDDVLADWQRTRGAHIAEYDEQLEEPLYSGDFDAADLPEHFDELRETLLDGDIEL
>d1fjgk_ c.55.4.1 (K:) Ribosomal protein S11 {Thermus thermophilus}
KRQVASGRAYIHASYNNTIVTITDPDGNPITWSSGGVIGYKGSRKGTPYAAQLAALDAAKKA
MAYGMQSVDVIVRGTGAGREQAIRALQASGLQVKSIVDDTPVPHNGCRPKKKFRKAS
>d1dt9a1 c.55.4.2 (A:143-276) Middle domain of eukaryotic peptide chain release factor subunit 1, ERF1 {Human (Homo sapiens)}

DSKFGFIVIDGSGALFGTLQGNTREVLHKFTVDLPKKHGRGGQSALRFARLRMEKRHNYVRK
VAETAVQLFISGDKVNVAGLVLAGSADFKTELSQSDMFDQRLQSKVLKLVDISYGGENGFNQ
AIELSTEVLS
>d1eo1a_ c.55.5.1 (A:) Hypothetical protein MTH1175 {Archaeon Methanobacterium thermoautotrophicum}
MKIAIASSGTDLGSEVSRFFGRAPYFMIVEMKKGNIESSEVIENPSASASGGAGIRTAQIIA
NNGVKAVIASSPGPNAFEVLNELGIKIYRATGTSVEENLKLFTEGNLEEIRSPGSGRGRRRR
>d1ewqa3 c.55.6.1 (A:121-266) DNA repair protein MutS, domain II {Thermus aquaticus}
LLQESLLPREANYLAAIATGDGWGLAFLDVSTGEFKGTVLKSKSALYDELFRHRPAEVLLAP
ELLENGAFLDEFRKRFPVMLSEAPFEPEGEGPLALRRARGALLAYAQRTQGGALSLQPFRFY
DPGAFMRLPEATLRALEVFEPL
>d1e3ma3 c.55.6.1 (A:117-269) DNA repair protein MutS, domain II {Escherichia coli}
GTISDEALLQERQDNLLAAIWQDSKGFGYATLDISSGRFRLSEPADRETMAAELQRTNPAEL
LYAEDFAEMSLIEGRRGLRRRPLWEFEIDTARQQLNLQFGTRDLVGFGVENAPRGLCAAGCL
LQYAKDTQRTTLPHIRSITMEREQDSIIM
>d1sfe_2 c.55.7.1 (12-92) Ada DNA repair protein {Escherichia coli}
LAVRYALADCELGRCLVAESERGICAILLGDDDATLISELQQMFPAADNAPADLMFQQHVRE
VIASLNQRDTPLTLPLDIR
>d1qnta2 c.55.7.1 (A:6-91) O6-alkylguanine-DNA alkyltransferase {Human (Homo sapiens)}
EMKRTTLDSPLGKLELSGCEQGLHEIKLLGKGTSAADAVEVPAPAAVLGGPEPLMQCTAWLN
AYFHQPEAIEEFPVPALHHPVFQQ
>d1mgta2 c.55.7.1 (A:1-88) O6-alkylguanine-DNA alkyltransferase {Archaeon Pyrococcus kodakaraensis}
MLSVEKFRVGERVVWIGVIFSGRVQGIAFAFDRGTLMKRIHDLAEHLGKRGVSISLDVQPSD
YPEKVFKVLIGELDNASFLRELSFEG
>d1cfza_ c.56.1.1 (A:) Hydrogenase maturating endopeptidase HybD {Escherichia coli}
MRILVLGVGNILLTDEAIGVRIVEALEQRYILPDYVEILDGGTAGMELLGDMANRDHLIIAD
AIVSKKNAPGTMMILRDEEVPALFTNKISPHQLGLADVLSALRFTGEFPKKLTLVGVIPESL
EPHIGLTPTVEAMIEPALEQVLAALRESGVEAIPRSDS
>d1c8ba_ c.56.1.2 (A:) Germination protease {Bacillus megaterium}
MEKELDLSQYSVRTDLAVEAKDIALENQPKPNNQSEIKGVIVKEKEEQGVKISMVEITEEGA
EAIGKKKGRYVTLESVGIREQDTEKQEEAMEEVFAKELNFFIKSLNIPDDASCLVVGLGNLS
VTPDALGPKAVDNLLITRHLFELQPESVQDGFRPVSAIVPGVMGMTGIETSDIIFGVVKKVN
PDFIIAIDALAARSIERVNATIQISDSGIHPGSGVGNKRKEISYETLGIPVIAIGIPTVVDA
VSITSDTIDFILKHFGREMKEQGKPSKSLLPSGMTFGEKKKLTEDDLPNEEQRQTYLGMIGT
LPDEEKRRLIHEVLAPLGHNLMVTPKEVDMFIEDMANVVAGGLNAALHHEVDQENFGAYTH
>d1ulb__ c.56.2.1 (-) Purine nucleoside phosphorylase, PNP {Human (Homo sapiens)}
MENGYTYEDYKNTAEWLLSHTKHRPQVAIICGSGLGGLTDKLTQAQIFDYSEIPNFPRSTVP
GHAGRLVFGFLNGRACVMMQGRFHMYEGYPLWKVTFPVRVFHLLGVDTLVVTNAAGGLNPKF

EVGDIMLIRDHINLPGFSGQNPLRGPNDERFGDRFPAMSDAYDRTMRQRALSTWKQMGEQRE
LQEGTYVMVAGPSFETVAECRVLQKLGADAVGMSTVPEVIVARHCGLRVFGFSLITNKVIMD
YESLEKANHEEVLAAGKQAAQKLEQFVSILMASIPLPDKAS
>d1b8oa_ c.56.2.1 (A:) Purine nucleoside phosphorylase, PNP {Cow (Bos taurus)}
NGYTYEDYQDTAKWLLSHTEQRPQVAVICGSGLGGLVNKLTQAQTFDYSEIPNFPESTVPGH
AGRLVFGILNGRACVMMQGRFHMYEGYPFWKVTFPVRVFRLLGVETLVVTNAAGGLNPNFEV
GDIMLIRDHINLPGFSGENPLRGPNEERFGVRFPAMSDAYDRDMRQKAHSTWKQMGEQRELQ
EGTYVMLGGPNFETVAECRLLRNLGADAVGMSTVPEVIVARHCGLRVFGFSLITNKVIMDYE
SQGKANHEEVLEAGKQAAQKLEQFVSLLMASI
>d1k9sa_ c.56.2.1 (A:) Purine nucleoside phosphorylase, PNP {Escherichia coli}
ATPHINAEMGDFADVVLMPGDPLRAKYIAETFLEDAREVNNVRGMLGFTGTYKGRKISVMGH
GMGIPSCSIYTKELITDFGVKKIIRVGSCGAVLPHVKLRDVVIGMGACTDSKVNRIRFKDHD
FAAIADFDMVRNAVDAAKALGIDARVGNLFSADLFYSPDGEMFDVMEKYGILGVEMEAAGIY
GVAAEFGAKALTICTVSDHIRTHEQTTAAERQTTFNDMIKIALESVLLGDK
>d1qe5a_ c.56.2.1 (A:) Purine nucleoside phosphorylase, PNP {Cellulomonas sp.}
PPLDDPATDPFLVARAAADHIAQATGVEGHDMALVLGSGWGGAAELLGEVVAEVPTHEIPGF
SSVTRSIRVERADGSVRHALVLGSRTHLYEGKGVRAVVHGVRTAAATGAETLILTNGCGGLN
QEWGAGTPVLLSDHINLTARSPLEGPTFVDLTDVYSPRLRELAHRVDPTLPEGVYAQFPGPH
YETPAEVRMAGILGADLVGMSTTLEAIAARHCGLEVLGVSLVTNLAAGISPTPLSHAEVIEA
GQAAGPRISALLADIAKR
>d1g2oa_ c.56.2.1 (A:) Purine nucleoside phosphorylase, PNP {Mycobacterium tuberculosis}
DPDELARRAAQVIADRTGIGEHDVAVVLGSGWLPAVAALGSPTTVLPQAELPGFVPPTAAGH
AGELLSVPIGAHRVLVLAGRIHAYEGHDLRYVVHPVRAARAAGAQIMVLTNAAGGLRADLQV
GQPVLISDHLNLTARSPLVGGEFVDLTDAYSPRLRELARQSDPQLAEGVYAGLPGPHYETPA
EIRMLQTLGADLVGMSTVHETIAARAAGAEVLGVSLVTNLAAGITGEPLSHAEVLAAGAASA
TRMGALLADVIARF
>d1k3fa_ c.56.2.1 (A:) Uridine phosphorylase {Escherichia coli}
MSKSDVFHLGLTKNDLQGATLAIVPGDPDRVEKIAALMDKPVKLASHREFTTWRAELDGKPV
IVCSTGIGGPSTSIAVEELAQLGIRTFLRIGTTGAIQPHINVGDVLVTTASVRLDGASLHFA
PLEFPAVADFECTTALVEAAKSIGATTHVGVTASSDTFYPGQERYDTYSGRVVRHFKGSMEE
WQAMGVMNYEMESATLLTMCASQGLRAGMVAGVIVNRTQQEIPNAETMKQTESHAVKIVVEA
ARRLL
>d1cb0a_ c.56.2.1 (A:) 5'-deoxy-5'-methylthioadenosine phosphorylase {Human (Homo sapiens)}
AVKIGIIGGTGLDDPEILEGRTEKYVDTPFGKPSDALILGKIKNVDCVLLARHGRQHTIMPS
KVNYQANIWALKEEGCTHVIVTTACGSLREEIQPGDIVIIDQFIDRTTMRPQSFYDGSHSCA
RGVCHIPMAEPFCPKTREVLIETAKKLGLRCHSKGTMVTIEGPRFSSRAESFMFRTWGADVI
NMTTVPEVVLAKEAGICYASIAMATDYDCWKEHEEAVSVDRVLKTLKENANKAKSLLLTTIP
QIGSTEWSETLHNLKNMAQFSVLLP
>d1je0a_ c.56.2.1 (A:) 5'-deoxy-5'-methylthioadenosine

phosphorylase {Archaeon Sulfolobus solfataricus}
PVHILAKKGEVAERVLVVGDPGRARLLSTLLQNPKLTNENRGFLVYTGKYNGETVSIATHGI
GGPSIAIVLEELAMLGANVFIRYGTTGALVPYINLGEYIIVTGASYNQGGLFYQYLRDNACV
ASTPDFELTNKLVTSFSKRNLKYYVGNVFSSDAFYAEDEEFVKKWSSRGNIAVEMECATLFT
LSKVKGWKSATVLVVSDNLAKGGIWITKEELEKSVMDGAKAVLDTLTS
>d2pth__ c.56.3.1 (-) Peptidyl-tRNA hydrolase {Escherichia coli}
TIKLIVGLANPGAEYAATRHNAGAWFVDLLAERLRAPLREEAKFFGYTSRVTLGGEDVRLLV
PTTFMNLSGKAVAAMASFFRINPDEILVAHDELDLPPGVAKFKLGGGHGGHNGLKDIISKLG
NNPNFHRLRIGIGHPGDKNKVVGFVLGKPPVSEQKLIDEAIDEAARCTEMWFTDGLTKATNR
LHAFKAQ
>d1a2za_ c.56.4.1 (A:) Pyrrolidone carboxyl peptidase
(pyroglutamate aminopeptidase) {Archaeon Thermococcus litoralis}
MKKVLITGFEPFGGDSKNPTEQIAKYFDRKQIGNAMVYGRVLPVSVKRATIELKRYLEEIKP
EIVINLGLAPTYSNITVERIAVNIIDARIPDNDGYQPIDEKIEEDAPLAYMATLPVRAITKT
LRDNGIPATISYSAGTYLCNYVMFKTLHFSKIEGYPLKAGFIHVPYTPDQVVNKFFLLGKNT
PSMCLEAEIKAIELAVKVSLDYLEKDRDDIKIPL
>d1auga_ c.56.4.1 (A:) Pyrrolidone carboxyl peptidase
(pyroglutamate aminopeptidase) {Bacillus amyloliquefaciens}
MEKKVLLTGFDPFGGETVNPSWEAVKRLNGAAEGPASIVSEQVPTVFYKSLAVLREAIKKHQ
PDIIICVGQAGGRMQITPERVAINLNEARIPDNEGNQPVGEDISQGGPAAYWTGLPIKRIVE
EIKKEGIPAAVSYTAGTFVCNHLFYGLMDEISRHHPHIRGGFIHIPYIPEQTLQKSAPSLSL
DHITKALKIAAVTAAVHEDDIETG
>d1iofa_ c.56.4.1 (A:) Pyrrolidone carboxyl peptidase
(pyroglutamate aminopeptidase) {Archaeon Pyrococcus furiosus}
MKVLVTGFEPFGGEKINPTERIAKDLDGIKIGDAQVFGRVLPVVFGKAKEVLEKTLEEIKPD
IAIHVGLAPGRSAISIERIAVNAIDARIPDNEGKKIEDEPIVPGAPTAYFSTLPIKKIMKKL
HERGIPAYISNSAGLYLCNYVMYLSLHHSATKGYPKMSGFIHVPYIPEQIIDKIGKGQVPPS
MCYEMELEAVKVAIEVALEELL
>d2ctc__ c.56.5.1 (-) Carboxypeptidase A {Cow (Bos taurus)}
ARSTNTFNYATYHTLDEIYDFMDLLVAEHPQLVSKLQIGRSYEGRPIYVLKFSTGGSNRPAI
WIDLGIHSREWITQATGVWFAKKFTEDYGQDPSFTAILDSMDIFLEIVTNPDGFAFTHSQNR
LWRKTRSVTSSSLCVGVDANRNWDAGFGKAGASSSPCSETYHGKYANSEVEVKSIVDFVKDH
GNFKAFLSIHSYSQLLLYPYGYTTQSIPDKTELNQVAKSAVEALKSLYGTSYKYGSIITTIY
QASGGSIDWSYNQGIKYSFTFELRDTGRYGFLLPASQIIPTAQETWLGVLTIMEHTLNN
>d1pca_1 c.56.5.1 (1-308) Carboxypeptidase A {Pig (Sus scrofa)}
ARTTSTFNYATYHTLEEIYDFMDILVAEHPALVSKLQIGRSYEGRPIYVLKFSTGGSNRPAI
WIDSGIHSREWITQASGVWFAKKITENYGQNSSFTAILDSMDIFLEIVTNPNGFAFTHSDNR
LWRKTRSKASGSLCVGSDSNRNWDAGFGGAGASSSPCAETYHGKYPNSEVEVKSITDFVKNN
GNIKAFISIHSYSQLLLYPYGYKTQSPADKSELNQIAKSAVAALKSLYGTSYKYGSIITVIY
QASGGVIDWTYNQGIKYSFSFELRDTGRRGFLLPASQIIPTAQETWLALLTIMEHTLNNS
>d1dtda_ c.56.5.1 (A:) Carboxypeptidase A {Human (Homo sapiens)}
FNFGAYHTLEEISQEMDNLVAEHPGLVSKVNIGSSFENRPMNVLKFSTGGDKPAIWLDAGIH
AREWVTQATALWTANKIVSDYGKDPSITSILDALDIFLLPVTNPDGYVFSQTKNRMWRKTRS
KVSAGSLCVGVDPNRNWDAGFGGPGASSNPCSDSYHGPSANSEVEVKSIVDFIKSHGKVKAF

IILHSYSQLLMFPYGYKCTKLDDFDELSEVAQKAAQSLSRLHGTKYKVGPICSVIYQASGGS
IDWSYDYGIKYSFAFELRDTGRYGFLLPARQILPTAEETWLGLKAIMEHVRDHPY
>d1nsa_1 c.56.5.1 (4-308) Carboxypeptidase B {Pig (Sus scrofa)}
TTGHSYEKYNNWETIEAWTEQVTSKNPDLISRSAIGTTFDGDNIYLLKVGKPGSNKPAIFMD
CGFHAREWISQAFCQWFVRDAVRTYGYEAHMTEFLDNLDFYVLPVLNIDGYIYTWTKNRMWR
KTRSTNAGSSCTGTDPNRNFNAGWCTVGASVNPCNETYCGSAAESEKETKALADFIRNNLSS
IKAYLTIHSYSQMILYPYSYDYKLPENDAELNSLAKGAVKELASLYGTSYSYGPGSTTIYPA
AGGSDDWAYNQGIKYSFTFELRDKGRFGFVLPESQIQATCQETMLAVKYVTNYTLEHL
>d1cpb__ c.56.5.1 (-) Carboxypeptidase B {Cow (Bos taurus)}
TTGHSYEKYNNWETIEAWTEQVASENPDLISRSAIGTTFLGNTIYLLKVGKPGSNKPAVFMD
CGFHAREWISPAFCQWFVREXXXXXXXEIHMTEFLDKLDFYVLPVVNIDGYIYTWTTNRMWR
KTRSTRAGSSCTGTDLNRNFDAGWCSIGASNNPCSETYCGSAAESEKESKAVADFIRNHLSS
IKAYLTIHSYSQMMLYPYSYDYKLPKNNVELNTLAKGAVKKLASLHGTTYSYGPGATTIYPA
SGGSDDWAYDQGIKYSFTFELRDKGRYGFVLPESQIQPTCEETMLAIKYVTSYVLEHL
>d1h8la2 c.56.5.1 (A:4-304) Carboxypeptidase D, catalytic domain {Crested duck (Lophonetta specularioides)}
QAVQPVDFRHHHFSDMEIFLRRYANEYPSITRLYSVGKSVELRELYVMEISDNPGIHEAGEP
EFKYIGNMHGNEVVGRELLLNLIEYLCKNFGTDPEVTDLVQSTRIHIMPSMNPDGYEKSQEG
DRGGTVGRNNSNNYDLNRNFPDQFFQVTDPPQPETLAVMSWLKTYPFVLSANLHGGSLVVNY
PFDDDEQGIAIYSKSPDDAVFQQLALSYSKENKKMYQGSPCKDLYPTEYFPHGITNGAQWYN
VPGGMQDWNYLNTNCFEVTIELGCVKYPKAEELPKYWEQNRRSLLQFIKQVHR
>d1obr__ c.56.5.2 (-) Carboxypeptidase T {Thermoactinomyces vulgaris}
DFPSYDSGYHNYNEMVNKINTVASNYPNIVKKFSIGKSYEGRELWAVKISDNVGTDENEPEV
LYTALHHAREHLTVEMALYTLDLFTQNYNLDSRITNLVNNREIYIVFNINPDGGEYDISSGS
YKSWRKNRQPNSGSSYVGTDLNRNYGYKWGCCGGSSGSPSSETYRGRSAFSAPETAAMRDFI
NSRVVGGKQQIKTLITFHTYSELILYPYGYTYTDVPSDMTQDDFNVFKTMANTMAQTNGYTP
QQASDLYITDGDMTDWAYGQHKIFAFTFEMYPTSYNPGFYPPDEVIGRETSRNKEAVLYVAE
KADCPYSVIGKSC
>d1lam_2 c.56.5.3 (160-484) Leucine aminopeptidase, C-terminal domain {Cow (Bos taurus)}
FASGQNLARRLMETPANEMTPTKFAEIVEENLKSASIKTDVFIRPKSWIEEQEMGSFLSVAK
GSEEPPVFLEIHYKGSPNASEPPLVFVGKGITFDSGGISIKAAANMDLMRADMGGAATICSA
IVSAAKLDLPINIVGLAPLCENMPSGKANKPGDVVRARNGKTIQVDNTDAEGRLILADALCY
AHTFNPKVIINAATLTGAMDIALGSGATGVFTNSSWLWNKLFEASIETGDRVWRMPLFEHYT
RQVIDCQLADVNNIGKYRSAGACTAAAFLKEFVTHPKWAHLDIAGVMTNKDEVPYLRKGMAG
RPTRTLIEFLFRFSQ
>d1amp__ c.56.5.4 (-) Aminopeptidase {Aeromonas proteolytica}
MPPITQQATVTAWLPQVDASQITGTISSLESFTNRFYTTTSGAQASDWIASEWQALSASLPN
ASVKQVSHSGYNQKSVVMTITGSEAPDEWIVIGGHLDSTIGSHTNEQSVAPGADDDASGIAA
VTEVIRVLSENNFQPKRSIAFMAYAAEEVGLRGSQDLANQYKSEGKNVVSALQLDMTNYKGS
AQDVVFITDYTDSNFTQYLTQLMDEYLPSLTYGFDTCGYACSDHASWHNAGYPAAMPFESKF
NDYNPRIHTTQDTLANSDPTGSHAKKFTQLGLAYAIEMGSATG
>d1qq9a_ c.56.5.4 (A:) Aminopeptidase {Streptomyces griseus}

APDIPLANVKAHLTQLSTIAANNGGNRAHGRPGYKASVDYVKAKLDAAGYTTTLQQFTSGGA
TGYNLIANWPGGDPNKVLMAGAHLDSVSSGAGINDNGSGSAAVLETALAVSRAGYQPDKHLR
FAWWGAEELGLIGSKFYVNNLPSADRSKLAGYLNFDMIGSPNPGYFVYDDDPVIEKTFKNYF
AGLNVPTEIETEGDGRSDHAPFKNVGVPVGGLFTGAGYTKSAAQAQKWGGTAGQAFDRCYHS
SCDSLSNINDTALDRNSDAAAHAIWTLSS
>d1cg2a1 c.56.5.4 (A:26-213,A:327-414) Carboxypeptidase G2, catalytic domain {Pseudomonas sp., strain rs-16}
QKRDNVLFQAATDEQPAVIKTLEKLVNIETGTGDAEGIAAAGNFLEAELKNLGFTVTRSKSA
GLVVGDNIVGKIKGRGGKNLLLMSHMDTVYLKGILAKAPFRVEGDKAYGPGIADDKGGNAVI
LHTLKLLKEYGVRDYGTITVLFNTDEEKGSFGSRDLIQEEAKLADYVLSFEPTSAGDEKLSL
GTXFNAGEGGKKLVDKAVAYYKEAGGTLGVEERTGGGTDAAYAALSGKPVIESLGLPGFGYH
SDKAEYVDISAIPRRLYMAARLIMDLGAG
>d1de4c3 c.56.5.5 (C:122-189,C:383-608) Transferrin receptor ectodomain, protease-like domain {Human (Homo sapiens)}
LYWDDLKRKLSEKLDSTDFTSTIKLLNENSYVPREAGSQKDENLALYVENQFREFKLSKVWR
DQHFVKXEIKILNIFGVIKGFVEPDHYVVVGAQRDAWGPGAAKSGVGTALLLKLAQMFSDMV
LKDGFQPSRSIIFASWSAGDFGSVGATEWLEGYLSSLHLKAFTYINLDKAVLGTSNFKVSAS
PLLYTLIEKTMQNVKHPVTGQFLYQDSNWASKVEKLTLDNAAFPFLAYSGIPAVSFCFCEDT
DYPYLGTTMDTYKELIERIPELNKVARAAAEVAGQFVIKLTHDVELN
>d1boub_ c.56.6.1 (B:) LigB subunit of an aromatic-ring-opening dioxygenase LigAB {Pseudomonas paucimobilis}
ARVTTGITSSHIPALGAAIQTGTSDNDYWGPVFKGYQPIRDWIKQPGNMPDVVILVYNDHAS
AFDMNIIPTFAIGCAETFKPADEGWGPRPVPDVKGHPDLAWHIAQSLILDEFDMTIMNQMDV
DHGCTVPLSMIFGEPEEWPCKVIPFPVNVVTYPPPSGKRCFALGDSIRAAVESFPEDLNVHV
WGTGGMSHQLQGPRAGLINKEFDLNFIDKLISDPEELSKMPHIQYLRESGSEGVELVMWLIM
RGALPEKVRDLYTFYHIPASNTALGAMILQPEETAGTPLEPRKVMSGHSL
>d1di6a_ c.57.1.1 (A:) MogA {Escherichia coli}
ATLRIGLVSISDRASSGVYQDKGIPALEEWLTSALTTPFELETRLIPDEQAIIEQTLCELVD
EMSCHLVLTTGGTGPARRDVTPDATLAVADREMPGFGEQMRQISLHFVPTAILSRQVGVIRK
QALILNLPGQPKSIKETLEGVKDAEGNVVVHGIFASVPYCIQLLEGPYVETAPEVVAAFRPK
SARR
>d1jlja_ c.57.1.1 (A:) Gephyrin N-terminal domain {Human (Homo sapiens)}
HQIRVGVLTVSDSCFRNLAEDRSGINLKDLVQDPSLLGGTISAYKIVPDEIEEIKETLIDWC
DEKELNLILTTGGTGFAPRDVTPEATKEVIEREAPGMALAMLMGSLNVTPLGMLSRPVCGIR
GKTLIINLPGSKKGSQECFQFILPALPHAIDLLRDAIVKVKEVHD
>d1eava_ c.57.1.1 (A:) Plant CNX1 G domain {Mouse-ear cress (Arabidopsis thaliana)}
GPEYKVAILTVSDTVSAGAGPDRSGPRAVSVVDSSSEKLGGAKVVATAVVPDEVERIKDILQ
KWSDVDEMDLILTLGGTGFTPRDVTPEATKKVIERETPGLLFVMMQESLKITPFAMLSRSAA
GIRGSTLIINMPGNPNAVAECMEALLPALKHALKQI
>d1g8la3 c.57.1.2 (A:178-326) MoeA, central domain {Escherichia coli}
VRVALFSTGDELQLPGQPLGDGQIYDTNRLAVHLMLEQLGCEVINLGIIRDDPHALRAAFIE

ADSQADVVISSGGVSVGEADYTKTILEELGEIAFWKLAIKPGKPFAFGKLSNSWFCGLPGNP
VSATLTFYQLVQPLLAKLSGNTASG
>d1bgva2 c.58.1.1 (A:1-194) Glutamate dehydrogenase {Clostridium symbiosum}
SKYVDRVIAEVEKKYADEPEFVQTVEEVLSSLGPVVDAHPEYEEVALLERMVIPERVIEFRV
PWEDDNGKVHVNTGYRVQFNGAIGPYKGGLRFAPSVNLSIMKFLGFEQAFKDSLTTLPMGGA
KGGSDFDPNGKSDREVMRFCQAFMTELYRHIGPDIDVPAGDLGVGAREIGYMYGQYRKIVGG
FYNGVLTG
>d1gtma2 c.58.1.1 (A:3-180) Glutamate dehydrogenase {Archaeon Pyrococcus furiosus}
ADPYEIVIKQLERAAQYMEISEEALEFLKRPQRIVEVTIPVEMDDGSVKVFTGFRVQHNWAR
GPTKGGIRWHPEETLSTVKALAAWMTWKTAVMDLPYGGGKGGIIVDPKKLSDREKERLARGY
IRAIYDVISPYEDIPAPDVYTNPQIMAWMMDEYETISRRKTPAFGIITGKPLSI
>d1euza2 c.58.1.1 (A:4-180) Glutamate dehydrogenase {Archaeon Thermococcus profundus}
IDPFEMAVKQLERAAQYMDISEEALEWLKKPMRIVEVSVPIEMDDGSVKVFTGFRVQHNWAR
GPTKGGIRWHPAETLSTVKALATWMTWKVAVVDLPYGGGKGGIIVNPKELSEREQERLARAY
IRAVYDVIGPWTDIPAPDVYTNPKIMGWMMDEYETIMRRKGPAFGVITGKPLS
>d1bvua2 c.58.1.1 (A:3-180) Glutamate dehydrogenase {Archaeon Thermococcus litoralis}
QDPFEIAVKQLERAAQYMDISEEALEFLKRPQRIVEVSIPVEMDDGSVKVFTGFRVQYNWAR
GPTKGGIRWHPEETLSTVKALAAWMTWKTAVMDLPYGGGKGGVICNPKEMSDREKERLARGY
VRAIYDVISPYTDIPAPDVYTNPQIMAWMMDEYETISRRKDPSFGVITGKPPSV
>d1b26a2 c.58.1.1 (A:4-178) Glutamate dehydrogenase {Thermotoga maritima}
SLYEMAVEQFNRAASLMDLESDLAEVLRRPKRVLIVEFPVRMDDGHVEVFTGYRVQHNVARG
PAKGGIRYHPDVTLDEVKALAFWMTWKTAVMNLPFGGGKGGVRVDPKKLSRNELERLSRRFF
SEIQVIIGPYNDIPAPDVNTNADVIAWYMDTYSMNVGHTVLGIVTGKPVEL
>d1hwxa2 c.58.1.1 (A:1-208) Glutamate dehydrogenase {Cow (Bos taurus)}
ADREDDPNFFKMVEGFFDRGASIVEDKLVEDLKTRQTQEQKRNRVRGILRIIKPCNHVLSLS
FPIRRDDGSWEVIEGYRAQHSHQRTPCKGGIRYSTDVSVDEVKALASLMTYKCAVVDVPFGG
AKAGVKINPKNYTDEDLEKITRRFTMELAKKGFIGPGVDVPAPNMSTGEREMSWIADTYAST
IGHYDINAHACVTGKPISQGGI
>d1leha2 c.58.1.1 (A:1-134) Leucine dehydrogenase {Bacillus sphaericus}
MEIFKYMEKYDYEQLVFCQDEASGLKAVIAIHDTTLGPALGGARMWTYNAEEEAIEDALRLA
RGMTYKNAAAGLNLGGGKTVIIGDPFADKNEDMFRALGRFIQGLNGRYITAEDVGTTVDDMD
LIHQETDYVT
>d1c1da2 c.58.1.1 (A:1-148) Phenylalanine dehydrogenase {Rhodococcus sp., M4}
SIDSALNWDGEMTVTRFDAMTGAHFVIRLDSTQLGPAAGGTRAAQYSNLADALTDAGKLAGA
MTLKMAVSNLPMGGGKSVIALPAPRHSIDPSTWARILRIHAENIDKLSGNYWTGPDVNTNSA
DMDTLNDTTEFVFGRSLERGGAGS

>d1a4ia2 c.58.1.2 (A:2-126) Tetrahydrofolate dehydrogenase/cyclohydrolase {Human (Homo sapiens)}
APAEILNGKEISAQIRARLKNQVTQLKEQVPGFTPRLAILQVGNRDDSNLYINVKLKAAEEI
GIKATHIKLPRTTTESEVMKYITSLNEDSTVHGFLVQLPLDSENSINTEEVINAIAPEKDVD
G
>d1b0aa2 c.58.1.2 (A:2-122) Tetrahydrofolate dehydrogenase/cyclohydrolase {Escherichia coli}
AAKIIDGKTIAQQVRSEVAQKVQARIAAGLRAPGLAVVLVGSNPASQIYVASKRKACEEVGF
VSRSYDLPETTSEAELLELIDTLNADNTIDGILVQLPLPAGIDNVKVLERIHPDKDVDG
>d1edza2 c.58.1.2 (A:3-148) Tetrahydrofolate dehydrogenase/cyclohydrolase {Baker's yeast (Saccharomyces cerevisiae)}
KPGRTILASKVAETFNTEIINNVEEYKKTHNGQGPLLVGFLANNDPAAKMYATWTQKTSESM
GFRYDLRVIEDKDFLEEAIIQANGDDSVNGIMVYFPVFGNAQDQYLQQVVCKEKDVEGLNHV
YYQNLYHNVRYLDKENRLKSIL
>d1do8a2 c.58.1.3 (A:21-279) Mitochondrial NAD(P)-dependenent malic enzyme {Human (Homo sapiens)}
IKEKGKPLMLNPRTNKGMAFTLQERQMLGLQGLLPPKIETQDIQALRFHRNLKKMTSPLEKY
IYIMGIQERNEKLFYRILQDDIESLMPIVYTPTVGLACSQYGHIFRRPKGLFISISDRGHVR
SIVDNWPENHVKAVVVTDGERILGLGDLGVYGMGIPVGKLCLYTACAGIRPDRCLPVCIDVG
TDNIALLKDPFYMGLYQKRDRTQQYDDLIDEFMKAITDRYGRNTLIQFEDFGNHNAFRFLRK
YREKYCTFNDD
>d2uaga2 c.59.1.1 (A:298-437) UDP-N-acetylmuramoyl-L-alanine:D-glutamate ligase MurD {Escherichia coli}
GLPHRFEVVLEHNGVRWINDSKATNVGSTEAALNGLHVDGTLHLLLGGDGKSADFSPLARYL
NGDNVRLYCFGRDGAQLAALRPEVAEQTETMEQAMRLLAPRVQPGDMVLLSPACASLDQFKN
FEQRGNEFARLAKELG
>d1e8ca2 c.59.1.1 (A:338-497) UDP-N-acetylmuramyl tripeptide synthetase MurE {Escherichia coli}
VCGRMEVFTAPGKPTVVVDYAHTPDALEKALQAARLHCAGKLWCVFGCGGDRDKGKRPLMGA
IAEEFADVAVVTDDNPRTEEPRAIINDILAGMLDAGHAKVMEGRAEAVTCAVMQAKENDVVL
VAGKGHEDYQIVGNQRLDYSDRVTVARLLGVIARSH
>d1gg4a1 c.59.1.1 (A:313-447) UDP-murNac-tripeptide D-alanyl-D-alanine-adding enzyme MurF {Escherichia coli}
VPGRLFPIQLAENQLLLDDSYNANVGSMTAAVQVLAEMPGYRVLVVGDMAELGAESEACHVQ
VGEAAKAAGIDRVLSVGKQSHAISTASGVGEHFADKTALITRLKLLIAEQQVITILVKGSRS
AAMEEVVRALQ
>d1jbwa1 c.59.1.2 (A:297-425) Folylpolyglutamate synthetase, C-terminal domain {Lactobacillus casei}
WPARLEKISDTPLIVIDGAHNPDGINGLITALKQLFSQPITVIAGILADKDYAAMADRLTAA
FSTVYLVPVPGTPRALPEAGYEALHEGRLKDSWQEALAASLNDVPDQPIVITGSLYLASAVR
QTLLG
>d1qhfa_ c.60.1.1 (A:) Phosphoglycerate mutase {Baker's yeast

(Saccharomyces cerevisiae)}
PKLVLVRHGQSEWNEKNLFTGWVDVKLSAKGQQEAARAGELLKEKKVYPDVLYTSKLSRAIQ
TANIALEKADRLWIPVNRSWRLNERHYGDLQGKDKAETLKKFGEEKFNTYRRSFDVPPPPID
ASSPFSQKGDERYKYVDPNVLPETESLALVIDRLLPYWQDVIAKDLLSGKTVMIAAHGNSLR
GLVKHLEGISDADIAKLNIPTGIPLVFELDENLKPSKPSYYLDPEAAAAGAAAV
>d3pgm__ c.60.1.1 (-) Phosphoglycerate mutase {Baker's yeast
(Saccharomyces cerevisiae)}
PKLVLVRHGQSEWNEKNLFTGWVDVKLSAKGQQEAARAGELLKEKGVNVLVDYTSKLSRAIQ
TANIALEKADRLWIPVNRSWRLNERHYGDLQGKDKAQTLKKFGEEKFNTYRRSFDVPPPPID
ASSPFSQKGDERYKYVDPNVLPETESLALVIDRLLPYWQDVIAKLVGKTSMIAAHGNSLRGL
VKHLEGISDADIAKLNIPPGTILVFELDENLKPSKPSYYLDPEA
>d1fzta_ c.60.1.1 (A:) Phosphoglycerate mutase {Yeast
(Schizosaccharomyces pombe)}
MTTEAAPNLLVLTRHGESEWNKLNLFTGWKDPALSETGIKEAKLGGERLKSRGYKFDIAFTS
ALQRAQKTCQIILEEVGEPNLETIKSEKLNERYYGDLQGLNKDDARKKWGAEQVQIWRRSYD
IAPPNGESLKDTAERVLPYYKSTIVPHILKGEKVLIAAHGNSLRALIMDLEGLTGDQIVKRE
LATGVPIVYHLDKDGKYVSKELIDN
>d1e58a_ c.60.1.1 (A:) Phosphoglycerate mutase {Escherichia coli}
AVTKLVLVRHGESQWNKENRFTGWYDVDLSEKGVSEAKAAGKLLKEEGYSFDFAYTSVLKRA
IHTLWNVLDELDQAWLPVEKSWKLNERHYGALQGLNKAETAEKYGDEQVKQWRRGFAVTPPE
LTKDDERYPGHDPRYAKLSEKELPLTESLALTIDRVIPYWNETILPRMKSGERVIIAAHGNS
LRALVKYLDNMSEEEILELNIPTGVPLVYEFDENFKPLKRYYLGNADEIAAKAAAVANQGK
>d1ebba_ c.60.1.1 (A:) Broad specificity phosphatase YhfR
{Bacillus stearothermophilus}
ATTLYLTRHGETKWNVERRMQGWQDSPLTEKGRQDAMRLGKRLEAVELAAIYTSTSGRALET
AEIVRGGRLIPIYQDERLREIHLGDWEGKTHDEIRQMDPIAFDHFWQAPHLYAPQRGERFCD
VQQRALEAVQSIVDRHEGETVLIVTHGVVLKTLMAAFKDTPLDHLWSPPYMYGTSVTIIEVD
GGTFHVAVEGDVSHIE
>d1rpa__ c.60.1.2 (-) Acid phosphatase {Rat (Rattus norvegicus)}
KELKFVTLVFRHGDRGPIETFPNDPIKESSWPQGFGQLTKWGMGQHYELGSYIRRRYGRFLN
NSYKHDQVYIRSTDVDRTLMSAMTNLAALFPPEGNSIWNPRLLWQPIPVHTVSLSEDRLLYL
PFRDCPRFQELKSETLKSEEFLKRLQPYKSFIDTLPSLSGFEDQDLFEIWSRLYDPLYCESV
HNFTLPTWATEDAMTKLKELSELSLLSLYGIHKQKEKSRLQGGVLVNEILKNMKLATQPQKA
RKLIMYSAHDTTVSGLQMALDVYNGLLPPYASCHIMELYQDNGGHFVEMYYRNETQNEPYPL
TLPGCTHSCPLEKFAELLDPVIPQDWATECMG
>d2hpaa_ c.60.1.2 (A:) Acid phosphatase {Human (Homo sapiens)}
KELKFVTLVFRHGDRSPIDTFPTDPIKESSWPQGFGQLTQLGMEQHYELGEYIRKRYRKFLN
ESYKHEQVYIRSTDVDRTLMSAMTNLAALFPPEGVSIWNPILLWQPIPVHTVPLSEDQLLYL
PFRNCPRFQELESETLKSEEFQKRLHPYKDFIATLGKLSGLHGQDLFGIWSKVYDPLYCESV
HNFTLPSWATEDTMTKLRELSELSLLSLYGIHKQKEKSRLQGGVLVNEILNHMKRATQIPSY
KKLIMYSAHDTTVSGLQMALDVYNGLLPPYASCHLTELYFEKGEYFVEMYYRNETQHEPYPL
MLPGCSPSCPLERFAELVGPVIPQDWSTECMT
>d1ihp__ c.60.1.3 (-) Phytase
(myo-inositol-hexakisphosphate-3-phosphohydrolase) {Aspergillus

ficuum}
SCDTVDQGYQCFSETSHLWGQYAPFFSLANESVISPEVPAGCRVTFAQVLSRHGARYPTDSK
GKKYSALIEEIQQNATTFDGKYAFLKTYNYSLGADDLTPFGEQELVNSGIKFYQRYESLTRN
IVPFIRSSGSSRVIASGKKFIEGFQSTKLKDPRAQPGQSSPKIDVVISEASSSNNTLDPGTC
TVFEDSELADTVEANFTATFVPSIRQRLENDLSGVTLTDTEVTYLMDMCSFDTISTSTVDTK
LSPFCDLFTHDEWINYDYLQSLKKYYGHGAGNPLGPTQGVGYANELIARLTHSPVHDDTSSN
HTLDSSPATFPLNSTLYADFSHDNGIISILFALGLYNGTKPLSTTTVENITQTDGFSSAWTV
PFASRLYVEMMQCQAEQEPLVRVLVNDRVVPLHGCPVDALGRCTRDSFVRGLSFARSGGDWA
ECFA
>d1qfxa_ c.60.1.3 (A:) Phytase
(myo-inositol-hexakisphosphate-3-phosphohydrolase) {Aspergillus
niger}
KQFSQEFRDGYSILKHYGGNGPYSERVSYGIARDPPTSCEVDQVIMVKRHGERYPSPSAGKD
IEEALAKVYSINTTEYKGDLAFLNDWTYYVPNECYYNAETTSGPYAGLLDAYNHGNDYKARY
GHLWNGETVVPFFSSGYGRVIETARKFGEGFFGYNYSTNAALNIISESEVMGADSLTPTCDT
DNDQTTCDNLTYQLPQFKVAAARLNSQNPGMNLTASDVYNLMVMASFELNARPFSNWINAFT
QDEWVSFGYVEDLNYYYCAGPGDKNMAAVGAVYANASLTLLNQGPKEAGSLFFNFAHDTNIT
PILAALGVLIPNEDLPLDRVAFGNPYSIGNIVPMGGHLTIERLSCQATALSDEGTYVRLVLN
EAVLPFNDCTSGPGYSCPLANYTSILNKNLPDYTTTCNVSASYPQYLSFWWNYNTTTELNYR
SSPIACQEGDAMD
>d1dkla_ c.60.1.3 (A:) Phytase
(myo-inositol-hexakisphosphate-3-phosphohydrolase) {Escherichia
coli}
SEPELKLESVVIVSRHGVRAPTKATQLMQDVTPDAWPTWPVKLGWLTPRGGELIAYLGHYQR
QRLVADGLLAKKGCPQSGQVAIIADVDERTRKTGEAFAAGLAPDCAITVHTQADTSSPDPLF
NPLKTGVCQLDNANVTDAILSRAGGSIADFTGHRQTAFRELERVLNFPQSNLCLKREKQDES
CSLTQALPSELKVSADNVSLTGAVSLASMLTEIFLLQQAQGMPEPGWGRITDSHQWNTLLSL
HNAQFYLLQRTPEVARSRATPLLDLIKTALTPHPPQKQAYGVTLPTSVLFIAGHDTNLANLG
GALELNWTLPGQPDNTPPGGELVFERWRRLSDNSQWIQVSLVFQTLQQMRDKTPLSLNTPPG
EVKLTLAGCEERNAQGMCSLAGFTQIVNEARIPACSL
>d1bif_2 c.60.1.4 (250-468)
6-phosphofructo-2-kinase/fructose-2,6-bisphosphatase,
phosphatase domain {Rat (Rattus norvegicus)}
SIYLCRHGESELNLKGRIGGDPGLSPRGREFSKHLAQFISDQNIKDLKVFTSQMKRTIQTAE
ALSVPYEQFKVLNEIDAGVCEEMTYEEIQDHYPLEFALRDQDKYRYRYPKGESYEDLVQRLE
PVIMELERQENVLVICHQAVMRCLLAYFLDKAAEELPYLKCPLHTVLKLTPVAYGCKVESIF
LNVAAVNTHRDRPQNVDISRPSEEALVTVPAHQ
>d1fbta_ c.60.1.4 (A:)
6-phosphofructo-2-kinase/fructose-2,6-bisphosphatase,
phosphatase domain {Rat (Rattus norvegicus)}
RSIYLCRHGESELNLRGRIGGDSGLSARGKQYAYALANFIRSQGISSLKVWTSHMKRTIQTA
EALGVPYEQWKALNEIDAGVCEEMTYEEIQEHYPEEFALRDQDKYRYRYPKGESYEDLVQRL
EPVIMELERQENVLVICHQAVMRCLLAYFLDKSSDELPYLKCPLHTVLKLTPVAYGCRVESI
YLNV

>d1nula_ c.61.1.1 (A:) Xantine-guanine PRTase (XPRTase) {Escherichia coli}
EKYIVTWDMLQIHARKLASRLMPSEQWKGIIAVSRGGLVPGALLARELGIRHVDTVCISSYD
HDNQRELKVLKRAEGDGEGFIVIDDLVDTGGTAVAIREMYPKAHFVTIFAKPAGRPLVDDYV
VDIPQDTWIEQPWDMGVVFVPPISGR
>d1hgxa_ c.61.1.1 (A:) Hypoxantine-guanine-xanthine PRTase {Tritrichomonas foetus}
MDDLERVLYNQDDIQKRIRELAAELTEFYEDKNPVMICVLTGAVFFYTDLLKHLDFQLEPDY
IICSSYSGTKSTGNLTISKDLKTNIEGRHVLVVEDIIDTGLTMYQLLNNLQMRKPASLKVCT
LCDKDIGKKAYDVPIDYCGFVVENRYIIGYGFDFHNKYRNLPVIGILKE
>d1fsga_ c.61.1.1 (A:) Hypoxantine-guanine-xanthine PRTase {Toxoplasma gondii}
GSHMASKPIEDYGKGKGRIEPMYIPDNTFYNADDFLVPPHCKPYIDKILLPGGLVKDRVEKL
AYDIHRTYFGEELHIICILKGSRGFFNLLIDYLATIQKYSGRESSVPPFFEHYVRLKSYQND
NSTGQLTVLSDDLSIFRDKHVLIVEDIVDTGFTLTEFGERLKAVGPKSMRIATLVEKRTDRS
NSLKGDFVGFSIEDVWIVGCCYDFNEMFRDFDHVAVLSDAARKKFEK
>d1gph11 c.61.1.1 (1:235-465) Glutamine PRPP amidotransferase, C-terminal domain {Bacillus subtilis}
ICSMEYIYFSRPDSNIDGINVHSARKNLGKMLAQESAVEADVVTGVPDSSISAAIGYAEATG
IPYELGLIKNRYVGRTFIQPSQALREQGVRMKLSAVRGVVEGKRVVMVDDSIVRGTTSRRIV
TMLREAGATEVHVKISSPPIAHPCFYGIDTSTHEELIASSHSVDEIRQEIGADTLSFLSVEG
LLKGIGRKYDDSNCGQCLACFTGKYPTEIYQDTVLPHVKEAVLTK
>d1ecfa1 c.61.1.1 (A:250-492) Glutamine PRPP amidotransferase, C-terminal domain {Escherichia coli}
NPCLFEYVYFARPDSFIDKISVYSARVNMGTKLGEKIAREWEDLDIDVVIPIPETSCDIALE
IARILGKPYRQGFVKNRYVGRTFIMPGQQLRRKSVRRKLNANRAEFRDKNVLLVDDSIVRGT
TSEQIIEMAREAGAKKVYLASAAPEIRFPNVYGIDMPSATELIAHGREVDEIRQIIGADGLI
FQDLNDLIDAVRAENPDIQQFECSVFNGVYVTKDVDQGYLDFLDTLRNDDAKAVQRQ
>d1dqna_ c.61.1.1 (A:) Guanine PRTase {Giardia lamblia}
MICSVTGKPVKDVLSTFFKDRNDVLESEVKKFHLLATFEECKALAADTARRMNEYYKDVAEP
VTLVALLTGAYLYASLLTVHLTFPYTLHFVKVSSYKGTRQESVVFDEEDLKQLKEKREVVLI
DEYVDSGHTIFSIQEQIKHAKICSCFVKDVDAIKKHSALADTKMFYGYTPMPKGSWLIGFGL
DDNGLRRGWAHLFDINLSESEVTEFRRRLTEHIKGLNINGVNRY
>d1bzya_ c.61.1.1 (A:) Hypoxantine-guanine PRTase (HGPRTase) {Human (Homo sapiens)}
SPGVVISDDEPGYDLDLFCIPNHYAEDLERVFIPHGLIMDRTERLARDVMKEMGGHHIVALC
VLKGGYKFFADLLDYIKALNRNSDRSIPMTVDFIRLKSYCNDQSTGDIKVIGGDDLSTLTGK
NVLIVEDIIDTGKTMQTLLSLVRQYNPKMVKVASLLVKRTPRSVGYKPDFVGFEIPDKFVVG
YALDYNEYFRDLNHVCVISETGKAKYKA
>d1cjba_ c.61.1.1 (A:) Hypoxantine-guanine PRTase (HGPRTase) {Plasmodium falciparum}
PIPNNPGAGENAFDPVFVNDDDGYDLDSFMIPAHYKKYLTKVLVPNGVIKNRIEKLAYDIKK
VYNNEEFHILCLLKGSRGFFTALLKHLSRIHNYSAVETSKPLFGEHYVRVKSYCNDQSTGTL
EIVSEDLSCLKGKHVLIVEDIIDTGKTLVKFCEYLKKFEIKTVAIACLFIKRTPLWNGFKAD

FVGFSIPDHFVVGYSLDYNEIFRDLDHCCLVNDEGKKKYKAT
>d1tc1a_ c.61.1.1 (A:) Hypoxanthine PRTase {Trypanosoma cruzi}
YEFAEKILFTEEEIRTRIKEVAKRIADDYKGKGLRPYVNPLVLISVLKGSFMFTADLCRALC
DFNVPVRMEFICVSSYGEGLTSSGQVRMLLDTRHSIEGHHVLIVEDIVDTALTLNYLYHMYF
TRRPASLKTVVLLDKREGRRVPFSADYVVANIPNAFVIGYGLDYDDTYRELRDIVVLRPE
>d1qb7a_ c.61.1.1 (A:) Adenine PRTase {Leishmania donovani}
PFKEVSPNSFLLDDSHALSQLLKKSYRWYSPVFSPRNVPRFADVSSITESPETLKAIRDFLV
QRYRAMSPAPTHILGFDARGFLFGPMIAVELEIPFVLMRKADKNAGLLIRSEPYEKEYKEAA
PEVMTIRYGSIGKGSRVVLIDDVLATGGTALSGLQLVEASDAVVVEMVSILSIPFLKAAEKI
HSTANSRYKDIKFISLLSDDALTEENCGDSKNYTGPRVLSCGDVLAEHPH
>d1g2qa_ c.61.1.1 (A:) Adenine PRTase {Baker's yeast
(Saccharomyces cerevisiae)}
MPIASYAQELKLALHQYPNFPSEGILFEDFLPIFRNPGLFQKLIDAFKLHLEEAFPEVKIDY
IVGLESRGFLFGPTLALALGVGFVPVRKAGKLPGECFKATYEKEYGSDLFEIQKNAIPAGSN
VIIVDDIIATGGSAAAAGELVEQLEANLLEYNFVMELDFLKGRSKLNAPVFTLL
>d1oroa_ c.61.1.1 (A:) Orotate PRTase {Escherichia coli}
MKPYQRQFIEFALSKQVLKFGEFTLKSGRKSPYFFNAGLFNTGRDLALLGRFYAEALVDSGI
EFDLLFGPAYKGIPIATTTAVALAEHHDLDLPYCFNRKEAKDHGEGGNLVGSALQGRVMLVD
DVITAGTAIRESMEIIQANGATLAGVLISLDRQERGRGEISAIQEVERDYNCKVISIITLKD
LIAYLEEKPEMAEHLAAVKAYREEFGV
>d1a3c__ c.61.1.1 (-) Uracil PRTase {Bacillus subtilis}
QKAVILDEQAIRRALTRIAHEMIERNKGMNNCILVGIKTRGIYLAKRLAERIEQIEGNPVTV
GEIDITLYRDDLSKKTSNDEPLVKGADIPVDITDQKVILVDDVLYTGRTVRAGMDALVDVGR
PSSIQLAVLVDRGHRELPIRADYIGKNIPTSKSEKVMVQLDEVDQNDLVAIYEN
>d1bd3a_ c.61.1.1 (A:) Uracil PRTase {Toxoplasma gondii}
QEESILQDIITRFPNVVLMKQTAQLRAMMTIIRDKETPKEEFVFYADRLIRLLIEEALNELP
FQKKEVTTPLDVSYHGVSFYSKICGVSIVRAGESMESGLRAVCRGVRIGKILIQRDETTAEP
KLIYEKLPADIRERWVMLLDPMCATAGSVCKAIEVLLRLGVKEERIIFVNILAAPQGIERVF
KEYPKVRMVTAAVDICLNSRYYIVPGIGDFGDRYFGTM
>d1dkra1 c.61.1.2 (A:8-166) Phosphoribosylpyrophosphate
synthetase {Bacillus subtilis}
NLKIFSLNSNPELAKEIADIVGVQLGKCSVTRFSDGEVQINIEESIRGCDCYIIQSTSDPVN
EHIMELLIMVDALKRASAKTINIVIPYYGYARQDRKARSREPITAKLFANLLETAGATRVIA
LDLHAPQIQGFFDIPIDHLMGVPILGEYFEGKNLE
>d1dkra2 c.61.1.2 (A:167-316) Phosphoribosylpyrophosphate
synthetase {Bacillus subtilis}
DIVIVSPDHGGVTRARKLADRLKAPIAIIDKRRPRPNVAEVMNIVGNIEGKTAILIDDIIDT
AGTITLAANALVENGAKEVYACCTHPVLSGPAVERINNSTIKELVVTNSIKLPEEKKIERFK
QLSVGPLLAEAIIRVHEQQSVSYLFS
>d1lfaa_ c.62.1.1 (A:) Integrin CD11a/CD18 (Leukocyte function
associated antigen-1, LFA-1) {Human (Homo sapiens)}
GNVDLVFLFDGSMSLQPDEFQKILDFMKDVMKKLSNTSYQFAAVQFSTSYKTEFDFSDYVKR
KDPDALLKHVKHMLLLTNTFGAINYVATEVFREELGARPDATKVLIIITDGEATDSGNIDAA
KDIIRYIIGIGKHFQTKESQETLHKFASKPASEFVKILDTFEKLKDLFTELQKKIYVIE

>d1atza_ c.62.1.1 (A:) von Willebrand factor A3 domain {Human (Homo sapiens)}
QPLDVILLLDGSSSFPASYFDEMKSFAKAFISKANIGPRLTQVSVLQYGSITTIDVPWNVVP
EKAHLLSLVDVMQREGGPSQIGDALGFAVRYLTSEMHGARPGASKAVVILVTDVSVDSVDAA
ADAARSNRVTVFPIGIGDRYDAAQLRILAGPAGDSNVVKLQRIEDLPTMVTLGNSFLHKL
>d1fnsa_ c.62.1.1 (A:) von Willebrand factor A1 domain {Human (Homo sapiens)}
MYCSRLLDLVFLLDGSSRLSEAEFEVLKAFVVDMMERLRVSQKWVRVAVVEYHDGSHAYIGL
KDRKRPSELRRIASQVKYAGSQVASTSEVLKYTLFQIFSKIDRPEASRIALLLMASQEPQRM
SRNFVRYVQGLKKKKVIVIPVGIGPHANLKQIRLIEKQAPENKAFVLSSVDELEQQRDEIVS
YLCDLAPEAP
>d1ido__ c.62.1.1 (-) Integrin CR3 (CD11b/CD18, Mac-1), alpha subunit {Human (Homo sapiens)}
DSDIAFLIDGSGSIIPHDFRRMKEFVSTVMEQLKKSKTLFSLMQYSEEFRIHFTFKEFQNNP
NPRSLVKPITQLLGRTHTATGIRKVVRELFNITNGARKNAFKILVVITDGEKFGDPLGYEDV
IPEADREGVIRYVIGVGDAFRSEKSRQELNTIASKPPRDHVFQVNNFEALKTIQNQLREK
>d1qc5a_ c.62.1.1 (A:) Integrin alpha1-beta1 {Human (Homo sapiens)}
STQLDIVIVLDGSNSIYPWDSVTAFLNDLLERMDIGPKQTQVGIVQYGENVTHEFNLNKYSS
TEEVLVAAKKIVQRGGRQTMTALGTDTARKEAFTEARGARRGVKKVMVIVTDGESHDNHRLK
KVIQDCEDENIQRFSIAILGSYNRGNLSTEKFVEEIKSIASEPTEKHFFNVSDEIALVTIVK
TLGERI
>d1ck4a_ c.62.1.1 (A:) Integrin alpha1-beta1 {Rat (Rattus norvegicus)}
TQLDIVIVLDGSNSIYPWESVIAFLNDLLKRMDIGPKQTQVGIVQYGENVTHEFNLNKYSST
EEVLVAANKIGRQGGLQTMTALGIDTARKEAFTEARGARRGVKKVMVIVTDGESHDNYRLKQ
VIQDCEDENIQRFSIAILGHYNRGNLSTEKFVEEIKSIASEPTEKHFFNVSDELALVTIVKA
LGERIFA
>d1aoxa_ c.62.1.1 (A:) Integrin alpha2-beta1 {Human (Homo sapiens)}
SCPSLIDVVVVCDESNSIYPWDAVKNFLEKFVQGLDIGPTKTQVGLIQYANNPRVVFNLNTY
KTKEEMIVATSQTSQYGGDLTNTFGAIQYARKYAYSAASGGRRSATKVMVVVTDGESHDGSM
LKAVIDQCNHDNILRFGIAVLGYLNRNALDTKNLIKEIKAIASIPTERYFFNVSDEAALLEK
AGTLGEQIFSIEGGT
>d1jv2b2 c.62.1.1 (B:107-354) Integrin beta A domain {Human (Homo sapiens)}
VEDYPVDIYYLMDLSYSMKDDLWSIQNLGTKLATQMRKLTSNLRIGFGAFVDKPVSPYMYIS
PPEALENPCYDMKTTCLPMFGYKHVLTLTDQVTRFNEEVKKQSVSRNRDAPEGGFDAIMQAT
VCDEKIGWRNDASHLLVFTTDAKTHIALDGRLAGIVQPNDGQCHVGSDNHYSASTTMDYPSL
GLMTEKLSQKNINLIFAVTENVVNLYQNYSELIPGTTVGVLSMDSSNVLQLIVDAYGKIRSK
>d1poia_ c.63.1.1 (A:) Glutaconate-CoA transferase alpha {Acidaminococcus fermentans}
SKVMTLKDAIAKYVHSGDHIALGGFTTDRKPYAAVFEILRQGITDLTGLGGAAGGDWDMLIG
NGRVKAYINCYTANSGVTNVSRRFRKWFEAGKLTMEDYSQDVIYMMWHAAALGLPFLPVTLM

QGSGLTDEWGISKEVRKTLDKVPDDKFKYIDNPFKPGEKVVAVPVPQVDVAIIHAQQASPDG
TVRIWGGKFQDVDIAEAAKYTIVTCEEIISDEEIRRDPTKNDIPGMCVDAVVLAPYGAHPSQ
CYGLYDYDNPFLKVYDKVSKTQEDFDAFCKEWVFDLKDHDEYLNKLGATRLINLKVVPGLGY
HIDMTKE
>d1poib_ c.63.1.1 (B:) Glutaconate-CoA transferase beta
{Acidaminococcus fermentans}
DYTNYTNKEMQAVTIAKQIKNGQVVTVGTGLPLIGASVAKRVYAPDCHIIVESGLMDCSPVE
VPRSVGDLRFMAHCGCIWPNVRFVGFEINEYLHKANRLIAFIGGAQIDPYGNVNSTSIGDYH
HPKTRFTGSGGANGIATYSNTIIMMQHEKRRFMNKIDYVTSPGWIDGPGGRERLGLPGDVGP
QLVVTDKGILKFDEKTKRMYLAAYYPTSSPEDVLENTGFDLDVSKAVELEAPDPAVIKLIRE
EIDPGQAFIQVP
>d1keka4 c.64.1.1 (A:416-668) Pyruvate-ferredoxin oxidoreductase,
PFOR, domain III {Desulfovibrio africanus}
GTIQCQFWGLGADGTVGANKQAIKIIGDNTDLFAQGYFSYDSKKSGGITISHLRFGEKPIQS
TYLVNRADYVACHNPAYVGIYDILEGIKDGGTFVLNSPWSSLEDMDKHLPSGIKRTIANKKL
KFYNIDAVKIATDVGLGGRINMIMQTAFFKLAGVLPFEKAVDLLKKSIHKAYGKKGEKIVKM
NTDAVDQAVTSLQEFKYPDSWKDAPAETKAEPMTNEFFKNVVKPILTQQGDKLPVSAFEADG
RFPLG
>d1jkxa_ c.65.1.1 (A:) Glycinamide ribonucleotide transformylase,
GART {Escherichia coli}
MNIVVLISGNGSNLQAIIDACKTNKIKGTVRAVFSNKADAFGLERARQAGIATHTLIASAFD
SREAYDRELIHEIDMYAPDVVVLAGFMRILSPAFVSHYAGRLLNIHPSLLPKYPGLHTHRQA
LENGDEEHGTSVHFVTDELDGGPVILQAKVPVFAGDSEDDITARVQTQEHAIYPLVISWFAD
GRLKMHENAAWLDGQRLPPQGYA
>d1fmta2 c.65.1.1 (A:1-206) Methionyl-tRNAfmet formyltransferase
{Escherichia coli}
SESLRIIFAGTPDFAARHLDALLSSGHNVVGVFTQPDRPAGRGKKLMPSPVKVLAEEKGLPV
FQPVSLRPQENQQLVAELQADVMVVVAYGLILPKAVLEMPRLGCINVHGSLLPRWRGAAPIQ
RSLWAGDAETGVTIMQMDVGLDTGDMLYKLSCPITAEDTSGTLYDKLAELGPQGLITTLKQL
ADGTAKPEVQDETLVTYAEK
>d1vid__ c.66.1.1 (-) Catechol O-methyltransferase, COMT {Rat
(Rattus norvegicus)}
TKEQRILRYVQQNAKPGDPQSVLEAIDTYCTQKEWAMNVGDAKGQIMDAVIREYSPSLVLEL
GAYCGYSAVRMARLLQPGARLLTMEMNPDYAAITQQMLNFAGLQDKVTILNGASQDLIPQLK
KKYDVDTLDMVFLDHWKDRYLPDTLLLEKCGLLRKGTVLLADNVIVPGTPDFLAYVRGSSSF
ECTHYSSYLEYMKVVDGLEKAIYQGPS
>d1fp1d2 c.66.1.12 (D:129-372) Chalcone O-methyltransferase
{Alfalfa (Medicago sativa)}
RGYLASFTTFLCYPALLQVWMNFKEAVVDEDIDLFKNVHGVTKYEFMGKDKKMNQIFNKSMV
DVCATEMKRMLEIYTGFEGISTLVDVGGGSGRNLELIISKYPLIKGINFDLPQVIENAPPLS
GIEHVGGDMFASVPQGDAMILKAVCHNWSDEKCIEFLSNCHKALSPNGKVIIVEFILPEEPN
TSEESKLVSTLDNLMFITVGGRERTEKQYEKLSKLSGFSKFQVACRAFNSLGVMEFYK
>d1fp2a2 c.66.1.12 (A:109-352) Isoflavone O-methyltransferase
{Alfalfa (Medicago sativa)}

LCLAPMVECVLDPTLSGSYHELKKWIYEEDLTLFGVTLGSGFWDFLDKNPEYNTSFNDAMAS
DSKLINLALRDCDFVFDGLESIVDVGGGTGTTAKIICETFPKLKCIVFDRPQVVENLSGSNN
LTYVGGDMFTSIPNADAVLLKYILHNWTDKDCLRILKKCKEAVTNDGKRGKVTIIDMVIDKK
KDENQVTQIKLLMDVNMACLNGKERNEEEWKKLFIEAGFQHYKISPLTGFLSLIEIYP
>d1ej0a_ c.66.1.2 (A:) RNA methyltransferase FtsJ {Escherichia coli}
GLRSRAWFKLDEIQQSDKLFKPGMTVVDLGAAPGGWSQYVVTQIGGKGRIIACDLLPMDPIV
GVDFLQGDFRDELVMKALLERVGDSKVQVVMSDMAPNMSGTPAVDIPRAMYLVELALEMCRD
VLAPGGSFVVKVFQGEGFDEYLREIRSLFTKVKVRKPDSSRARSREVYIVATGRKP
>d1fbna_ c.66.1.3 (A:) Fibrillarin homologue {Archaeon Methanococcus jannaschii}
MEDIKIKEIFENIYEVDLGDGLKRIATKSIVKGKKVYDEKIIKIGDEEYRIWNPNKSKLAAA
IIKGLKVMPIKRDSKILYLGASAGTTPSHVADIADKGIVYAIEYAPRIMRELLDACAERENI
IPILGDANKPQEYANIVEKVDVIYEDVAQPNQAEILIKNAKWFLKKGGYGMIAIKARSIDVT
KDPKEIFKEQKEILEAGGFKIVDEVDIEPFEKDHVMFVGIWEGK
>d1dusa_ c.66.1.4 (A:) Hypothetical protein MJ0882 {Archaeon Methanococcus jannaschii}
FSEKPTTKSDVKIVEDILRGKKLKFKTDSGVFSYGKVDKGTKILVENVVVDKDDDILDLGCG
YGVIGIALADEVKSTTMADINRRAIKLAKENIKLNNLDNYDIRVVHSDLYENVKDRKYNKII
TNPPIRAGKEVLHRIIEEGKELLKDNGEIWVVIQTKQGAKSLAKYMKDVFGNVETVTIKGGY
RVLKSKKL
>d1im8a_ c.66.1.14 (A:) Hypothetical protein HI0319 (YecO) {Haemophilus influenzae}
FIFDENVAEVFPDMIQRSVPGYSNIITAIGMLAERFVTADSNVYDLGCSRGAATLSARRNIN
QPNVKIIGIDNSQPMVERCRQHIAAYHSEIPVEILCNDIRHVEIKNASMVILNFTLQFLPPE
DRIALLTKIYEGLNPNGVLVLSEKFRFEDTKINHLLIDLHHQFKRANGYSELEVSQKRTALE
NVMRTDSIETHKVRLKNVGFSQVELWFQCFNFGSMIAVK
>d1d2ha_ c.66.1.5 (A:) Glycine <i>N</i>-methyltransferase {Rat (Rattus norvegicus)}
TAEYKAWLLGLLRQHGCHRVLDVACGTGVDSIMLVEEGFSVTSVDASDKMLKYALKERWNRR
KEPAFDKWVIEEANWLTLDKDVPAGDGFDAVICLGNSFAHLPDSKGDQSEHRLALKNIASMV
RPGGLLVIDHKNYDYILSTGCAPPGKNIYYKSDLTKDITTSVLTVNNKAHMVTLDYTVQVPG
AGRDGAPGFSKFRLSYYPHCLASFTELVQEAFGGRCQHSVLGDFKPYRPGQAYVPCYFIHVL
KKTG
>d1xvaa_ c.66.1.5 (A:) Glycine <i>N</i>-methyltransferase {Rat (Rattus norvegicus)}
VDSVYRTRSLGVAAEGIPDQYADGEAARVWQLYIGDTRSRTAEYKAWLLGLLRQHGCHRVLD
VACGTGVDSIMLVEEGFSVTSVDASDKMLKYALKERWNRRKEPAFDKWVIEEANWLTLDKDV
PAGDGFDAVICLGNSFAHLPDSKGDQSEHRLALKNIASMVRPGGLLVIDHRNYDYILSTGCA
PPGKNIYYKSDLTKDITTSVLTVNNKAHMVTLDYTVQVPGAGRDGAPGFSKFRLSYYPHCLA
SFTELVQEAFGGRCQHSVLGDFKPYRPGQAYVPCYFIHVLKKTG
>d1hnna_ c.66.1.15 (A:) Phenylethanolamine N-methyltransferase, PNMTase {Human (Homo sapiens)}
AVASAYQRFEPRAYLRNNYAPPRGDLCNPNGVGPWKLRCLAQTFATGEVSGRTLIDIGSGPT

VYQLLSACSHFEDITMTDFLEVNRQELGRWLQEEPGAFNWSMYSQHACLIEGKGECWQDKER
QLRARVKRVLPIDVHQPQPLGAGSPAPLPADALVSAFCLEAVSPDLASFQRALDHITTLLRP
GGHLLLIGALEESWYLAGEARLTVVPVSEEEVREALVRSGYKVRDLRTYIMPAHLQTGVDDV
KGVFFAWAQKVGL
>d1khha_ c.66.1.16 (A:) Guanidinoacetate methyltransferase {Rat (Rattus norvegicus)}
RWETPYMHSLAAAAASRGGRVLEVGFGMAIAASRVQQAPIKEHWIIECNDGVFQRLQNWALK
QPHKVVPLKGLWEEVAPTLPDGHFDGILYDTYPLSEETWHTHQFNFIKTHAFRLLKPGGILT
YCNLTSWGELMKSKYTDITAMFEETQVPALLEAGFQRENICTEVMALVPPADCRYYAFPQMI
TPLVTKH
>d1g6q1_ c.66.1.6 (1:) Arginine methyltransferase, HMT1 {Baker's yeast (Saccharomyces cerevisiae)}
DYYFDSYDHYGIHEEMLQDTVRTLSYRNAIIQNKDLFKDKIVLDVGCGTGILSMFAAKHGAK
HVIGVDMSSIIEMAKELVELNGFSDKITLLRGKLEDVHLPFPKVDIIISEWMGYFLLYESMM
DTVLYARDHYLVEGGLIFPDKCSIHLAGLEDSQYKDEKLNYWQDVYGFDYSPFVPLVLHEPI
VDTVERNNVNTTSDKLIEFDLNTVKISDLAFKSNFKLTAKRQDMINGIVTWFDIVFPAPKGK
RPVEFSTGPHAPYTHWKQTIFYFPDDLDAETGDTIEGELVCSPNEKNNRDLNIKISYKFESN
GIDGNSRSRKNEGSYLMH
>d1f3la_ c.66.1.6 (A:) Arginine methyltransferase, HMT1 {Rat (Rattus norvegicus)}
DLQEDEDGVYFSSYGHYGIHEEMLKDKVRTESYRDFIYQNPHIFKDKVVLDVGCGTGILSMF
AAKAGAKKVIAVDQSEILYQAMDIIRLNKLEDTIVLIKGKIEEVSLPVEKVDVIISEWMGYF
LLFESMLDSVLYAKSKYLAKGGSVYPDICTISLVAVSDVSKHADRIAFWDDVYGFNMSCMKK
AVIPEAVVEVVDHKTLISDPCDIKHIDCHTTSISDLEFSSDFTLRTTKTAMCTAVAGYFDIY
FEKNCHNRVVFSTGPQSTKTHWKQTIFLLEKPFPVKAGEALKGKITVHKNKKDPRSLIVTLT
LNSSTQTYSLQ
>d1dl5a1 c.66.1.7 (A:1-213) Protein-L-isoaspartyl O-methyltransferase {Thermotoga maritima}
MREKLFWILKKYGVSDHIAKAFLEIPREEFLTKSYPLSYVYEDIVLVSYDDGEEYSTSSQPS
LMALFMEWVGLDKGMRVLEIGGGTGYNAAVMSRVVGEKGLVVSVEYSRKICEIAKRNVERLG
IENVIFVCGDGYYGVPEFSPYDVIFVTVGVDEVPETWFTQLKEGGRVIVPINLKLSRRQPAF
LFKKKDPYLVGNYKLETRFITAGGNLG
>d1jg1a_ c.66.1.7 (A:) Protein-L-isoaspartyl O-methyltransferase {Archaeon Pyrococcus furiosus}
EKELYEKWMRTVEMLKAEGIIRSKEVERAFLKYPRYLSVEDKYKKYAHIDEPLPIPAGQTVS
APHMVAIMLEIANLKPGMNILEVGTGSGWNAALISEIVKTDVYTIERIPELVEFAKRNLERA
GVKNVHVILGDGSKGFPPKAPYDVIIVTAGAPKIPEPLIEQLKIGGKLIIPVGSYHLWQELL
EVRKTKDGIKIKNHGGVAFVPLIGEYGWK
>d1kr5a_ c.66.1.7 (A:) Protein-L-isoaspartyl O-methyltransferase {Human (Homo sapiens)}
ASHSELIHNLRKNGIIKTDKVFEVMLATDRSHYAKCNPYMDSPQSIGFQATISAPHMHAYAL
ELLFDQLHEGAKALDVGSGSGILTACFARMVGCTGKVIGIDHIKELVDDSVNNVRKDDPTLL
SSGRVQLVVGDGRMGYAEEAPYDAIHVGAAAPVVPQALIDQLKPGGRLILPVGPAGGNQMLE
QYDKLQDGSIKMKPLMGVIYVPLTDKEKQWSR

>d1i9ga_ c.66.1.13 (A:) Probable methyltransferase Rv2118c {Mycobacterium tuberculosis}
TGPFSIGERVQLTDAKGRRYTMSLTPGAEFHTHRGSIAHDAVIGLEQGSVVKSSNGALFLVL
RPLLVDYVMSMPRGPQVIYPKDAAQIVHEGDIFPGARVLEAGAGSGALTLSLLRAVGPAGQV
ISYEQRADHAEHARRNVSGCYGQPPDNWRLVVSDLADSELPDGSVDRAVLDMLAPWEVLDAV
SRLLVAGGVLMVYVATVTQLSRIVEALRAKQCWTEPRAWETLQRGWNVVGLAVRPQHSMRGH
TAFLVATRRLAPGAVA
>d1af7_2 c.66.1.8 (92-284) Chemotaxis receptor methyltransferase CheR, C-terminal domain {Salmonella typhimurium}
NLTAFFREAHHFPILAEHARRRHGEYRVWSAAASTGEEPYSIAITLADALGMAPGRWKVFAS
DIDTEVLEKARSGIYRLSELKTLSPQQLQRYFMRGTGPHEGLVRVRQELANYVEFSSVNLLE
KQYNVPGPFDAIFCRNVMIYFDKTTQEDILRRFVPLLKPDGLLFAGHSENFSNLVREFSLRG
QTVYALS
>d3mag__ c.66.1.9 (-) Polymerase regulatory subunit VP39 {Vaccinia virus}
MDVVSLDKPFMYFEEIDNELDYEPESANEVAKKLPYQGQLKLLLGELFFLSKLQRHGILDGA
TVVYIGSAPGTHIRYLRDHFYNLGVIIKWMLIDGRHHDPILNGLRDVTLVTRFVDEEYLRSI
KKQLHPSKIILISDVRSPKRGGNESTADLLSNYALQNVMISILNPVASSLKWRCPFPDQWIK
DFYIPHGNKMLQPFAPSYSAEMRLLSIYTGENMRLTRVTKSDAVNYEKKMYYLNKIVRNKVV
VNFDYPNQEYDYFHMYFMLRTVYCNKTFPTTKAKVLFLQQSIFRFLNIP
>d1yub__ c.66.1.9 (-) rRNA methyltransferase {Streptococcus pneumoniae, Ermam}
MNKNIKYSQNFLTSEKVLNQIIKQLNLKETDTVYEIGTGKGHLTTKLAKISKQVTSIELDSH
LFNLSSEKLKLNTRVTLIHQDILQFQFPNKQRYKIVGNIPYHLSTQIIKKVVFESRASDIYL
IVEEGFYKRTLDIHRTLGLLLHTQVSIQQLLKLPAECFHPKPKVNSVLIKLTRHTTDVPDKY
WKLYTYFVSKWVNREYRQLFTKNQFHQAMKHAKVNNLSTITYEQVLSIFNSYLLFNGRK
>d1qama_ c.66.1.9 (A:) rRNA methyltransferase {Bacillus subtilis, Ermc'}
QNFITSKHNIDKIMTNIRLNEHDNIFEIGSGKGHFTLELVQRCNFVTAIEIDHKLCKTTENK
LVDHDNFQVLNKDILQFKFPKNQSYKIFGNIPYNISTDIIRKIVFDSIADEIYLIVEYGFAK
RLLNTKRSLALFLMAEVDISILSMVPREYFHPKPKVNSSLIRLNRKKSRISHKDKQKYNYFV
MKWVNKEYKKIFTKNQFNNSLKHAGIDDLNNISFEQFLSLFNSYKLFNK
>d1i4wa_ c.66.1.9 (A:) Transcription factor sc-mtTFB {Baker's yeast (Saccharomyces cerevisiae)}
PIPGIKDISKLKFFYGFKYLWNPTVYNKIFDKLDLTKTYKHPEELKVLDLYPGVGIQSAIFY
NKYCPRQYSLLEKRSSLYKFLNAKFEGSPLQILKRDPYDWSTYSNLIDEERIFVPEVQSSDH
INDKFLTVANVTGEGSEGLIMQWLSCIGNKNWLYRFGKVKMLLWMPSTTARKLLARPGMHSR
SKCSVVREAFTDTKLIAISDANELKGFDSQCIEEWDPILFSAAEIWPTKGKPIALVEMDPID
FDFDVDNWDYVTRHLMILKRTPLNTVMDSLGHGGQQYFNSRITDKDLLKKCPIDLTNDEFIY
LTKLFMEWPFKP
>d6mhta_ c.66.1.10 (A:) DNA methylase HhaI, coenzyme-binding domain {Haemophilus haemolyticus}
MIEIKDKQLTGLRFIDLFAGLGGFRLALESCGAECVYSNEWDKYAQEVYEMNFGEKPEGDIT
QVNEKTIPDHDILCAGFPCQAFSISGKQKGFEDSRGTLFFDIARIVREKKPKVVFMENVKNF

ASHDNGNTLEVVKNTMNELDYSFHAKVLNALDYGIPQKRERIYMICFRNDLNIQNFQFPKPF
ELNTFVKDLLLPDSEVEHLVIDRKDLVMTNQEIEQTTPKTVRLGIVGKGGQGERIYSTRGIA
ITLSAYGGGIFAKTGGYLVNGKTRKLHPRECARVMGYPDSYKVHPSTSQAYKQFGNSVVINV
LQYIAYNIGSSLNFKPY
>d1g38a_ c.66.1.10 (A:) DNA methylase TaqI, coenzyme-binding domain {Thermus aquaticus}
VETPPEVVDFMVSLAEAPRGGRVLEPACAHGPFLRAFREAHGTGYRFVGVEIDPKALDLPPW
AEGILADFLLWEPGEAFDLILGNPPYGIVGEASKYPIHVFKAVKDLYKKAFSTWKGKYNLYG
AFLEKAVRLLKPGGVLVFVVPATWLVLEDFALLREFLAREGKTSVYYLGEVFPQKKVSAVVI
RFQKSGKGLSLWDTQESESGFTPILWAEYPHWEGEIIRFETEETRKLEISGMPLGDLFHIRF
AARSPEFKKHPAVRKEPGPGLVPVLTGRNLKPGWVDYEKNHSGLWMPKERAKELRDFYATPH
LVVAHTKGTRVVAAWDERAYPWREEFHLLPKEGVRLDPSSLVQWLNSEAMQKHVRTLYRDFV
PHLTLRMLERLPVRREYGFHT
>d1dcta_ c.66.1.10 (A:) DNA methylase HaeIII, coenzyme-binding domain {Haemophilus aegyptius}
MNLISLFSGAGGLDLGFQKAGFRIICANEYDKSIWKTYESNHSAKLIKGDISKISSDEFPKC
DGIIGGPPCQSWSEGGSLRGIDDPRGKLFYEYIRILKQKKPIFFLAENVKGMMAQRHNKAVQ
EFIQEFDNAGYDVHIILLNANDYGVAQDRKRVFYIGFRKELNINYLPPIPHLIKPTFKDVIW
DLKDNPIPALDKNKTNGNKCIYPNHEYFIGSYSTIFMSRNRVRQWNEPAFTVQASGRQCQLH
PQAPVMLKVSKNLNKFVEGKEHLYRRLTVRECARVQGFPDDFIFHYESLNDGYKMIGNAVPV
NLAYEIAKTIKSAL
>d2dpma_ c.66.1.10 (A:) DpnM DNA adenine methyltransferase {Streptococcus pneumoniae}
TLQPFTKWTGGKRQLLPVIRELIPKTYNRYFEPFVGGGALFFDLAPKDAVINDFNAELINCY
QQIKDNPQELIEILKVHQEYNSKEYYLDLRSADRDERIDMMSEVQRAARILYMLRVNFNGLY
RVNSKNQFNVPYGRYKNPKIVDEELISAISVYINNNQLEIKVGDFEKAIVDVRTGDFVYFDP
PYIPLSETSAFTSYTHEGFSFADQVRLRDAFKRLSDTGAYVMLSNSSSALVEELYKDFNIHY
VEATRTNGAKSSSRGKISEIIVTNYEK
>d1g55a_ c.66.1.10 (A:) DNMT2 {Human (Homo sapiens)}
EPLRVLELYSGVGGMHHALRESCIPAQVVAAIDVNTVANEVYKYNFPHTQLLAKTIEGITLE
EFDRLSFDMILMSPPCQPFTRIGRQGDMTDSRTNSFLHILDILPRLQKLPKYILLENVKGFE
VSSTRDLLIQTIENCGFQYQEFLLSPTSLGIPNSRLRYFLIAKLQSEPLPFQAPGQVLMEFP
KIEIHRKNQQDSDLSVKMLKDFLEDDTDVNQYLLPPKSLLRYALLLDIVQPTCRRSVCFTKG
YGSYIEGTGSVLQTAEDVQVENIYKSLTNLSQEEQITKLLILKLRYFTPKEIANLLGFPPEF
GFPEKITVKQRYRLLGNSLNVHVVAKLIKILYE
>d1booa_ c.66.1.11 (A:) m.PvuII N4 cytosine-specific DNA methyltransferase {Proteus vulgaris}
NFGKKPAYTTSNGSMYIGDSLELLESFPEESISLVMTSPPFALQRKKEYGNLEQHEYVDWFL
SFAKVVNKKLKPDGSFVVDFGGAYMKGVPARSIYNFRVLIRMIDEVGFFLAEDFYWFNPSKL
PSPIEWVNKRKIRVKDAVNTVWWFSKTEWPKSDITKVLAPYSDRMKKLIEDPDKFYTPKTRP
SGHDIGKSFSKDNGGSIPPNLLQISNSESNGQYLANCKLMGIKAHPARFPAKLPEFFIRMLT
EPDDLVVDIFGGSNTTGLVAERESRKWISFEMKPEYVAASAFRFLDNNISEEKITDIYNRIL
NGESLDLNSI
>d1eg2a_ c.66.1.11 (A:) m.RsrI N6 adenosine-specific DNA

methyltransferase {Rhodobacter sphaeroides}
GTTRHVYDVCDCLDTLAKLPDDSVQLIICDPPYNIMLADWDDHMDYIGWAKRWLAEAERVLS
PTGSIAIFGGLQYQGEAGSGDLISIISHMRQNSKMLLANLIIWNYPNGMSAQRFFANRHEEI
AWFAKTKKYFFDLDAVREPYDEETKAAYMKDKRLNPESVEKGRNPTNVWRMSRLNGNSLERV
GHPTQKPAAVIERLVRALSHPGSTVLDFFAGSGVTARVAIQEGRNSICTDAAPVFKEYYQKQ
LTFLQDDGLIDKARSYEIVEGAANFGAALQR
>d1inla_ c.66.1.17 (A:) Spermidine synthase {Thermotoga maritima}
RTLKELERELQPRQHLWYFEYYTGNNVGLFMKMNRVIYSGQSDIQRIDIFENPDLGVVFALD
GITMTTEKDEFMYHEMLAHVPMFLHPNPKKVLIIGGGDGGTLREVLKHDSVEKAILCEVDGL
VIEAARKYLKQTSCGFDDPRAEIVIANGAEYVRKFKNEFDVIIIDSTDPTAGQGGHLFTEEF
YQACYDALKEDGVFSAETEDPFYDIGWFKLAYRRISKVFPITRVYLGFMTTYPSGMWSYTFA
SKGIDPIKDFDPEKVRKFNKELKYYNEEVHVASFALPNFVKKELGLM
>d1kpga_ c.66.1.18 (A:) Mycolic acid cyclopropane synthase CmaA1
{Mycobacterium tuberculosis}
DELKPHFANVQAHYDLSDDFFRLFLDPTQTYSCAYFERDDMTLQEAQIAKIDLALGKLGLQP
GMTLLDVGCGWGATMMRAVEKYDVNVVGLTLSKNQANHVQQLVANSENLRSKRVLLAGWEQF
DEPVDRIVSIGAFEHFGHERYDAFFSLAHRLLPADGVMLLHTITGLHPKEIHERGLPMSFTF
ARFLKFIVTEIFPGGRLPSIPMVQECASANGFTVTRVQSLQPHYAKTLDLWSAALQANKGQA
IALQSEEVYERYMKYLTGCAEMFRIGYIDVNQFTCQK
>d1kpia_ c.66.1.18 (A:) Mycolic acid cyclopropane synthase CmaA1
{Mycobacterium tuberculosis}
QLKPPVEAVRSHYDKSNEFFKLWLDPSMTYSCAYFERPDMTLEEAQYAKRKLALDKLNLEPG
MTLLDIGCGWGSTMRHAVAEYDVNVIGLTLSENQYAHDKAMFDEVDSPRRKEVRIQGWEEFD
EPVDRIVSLGAFEHFADGAGDAGFERYDTFFKKFYNLTPDDGRMLLHTITIPDKEEAQELGL
TSPMSLLRFIKFILTEIFPGGRLPRISQVDYYSSNAGWKVERYHRIGANYVPTLNAWADALQ
AHKDEAIALKGQETCDIYMHYLRGCSDLFRDKYTDVCQFTLVK
>d7aata_ c.67.1.1 (A:) Aspartate aminotransferase, AAT {Chicken
(Gallus gallus), mitochondria}
SSWWSHVEMGPPDPILGVTEAFKRDTNSKKMNLGVGAYRDDNGKPYVLNCVRKAEAMIAAKK
MDKEYLPIAGLADFTRASAELALGENSEAFKSGRYVTVQGISGTGSLRVGANFLQRFFKFSR
DVYLPKPSWGNHTPIFRDAGLQLQAYRYYDPKTCSLDFTGAMEDISKIPEKSIILLHACAHN
PTGVDPRQEQWKELASVVKKRNLLAYFDMAYQGFASGDINRDAWALRHFIEQGIDVVLSQSY
AKNMGLYGERAGAFTVICRDAEEAKRVESQLKILIRPMYSNPPMNGARIASLILNTPELRKE
WLVEVKGMADRIISMRTQLVSNLKKEGSSHNWQHITDQIGMFCFTGLKPEQVERLTKEFSIY
MTKDGRISVAGVASSNVGYLAHAIHQVTK
>d2csta_ c.67.1.1 (A:) Aspartate aminotransferase, AAT {Chicken
(Gallus gallus), cytosolic form}
AASIFAAVPRAPPVAVFKLTADFREDGDSRKVNLGVGAYRTDEGQPWVLPVVRKVEQLIAGD
GSLNHEYLPILGLPEFRANASRIALGDDSPAIAQKRVGSVQGLGGTGALRIGAEFLRRWYNG
NNNTATPVYVSSPTWENHNSVFMDAGFKDIRTYRYWDAAKRGLDLQGLLDDMEKAPEFSIFI
LHACAHNPTGTDPTPDEWKQIAAVMKRRCLFPFFDSAYQGFASGSLDKDAWAVRYFVSEGFE
LFCAQSFSKNFGLYNERVGNLSVVGKDEDNVQRVLSQMEKIVRTTWSNPPSQGARIVATTLT
SPQLFAEWKDNVKTMADRVLLMRSELRSRLESLGTPGTWNHITDQIGMFSFTGLNPKQVEYM
IKEKHIYLMASGRINMCGLTTKNLDYVAKSIHEAVTKIQ

>d1ajsa_ c.67.1.1 (A:) Aspartate aminotransferase, AAT {Pig (Sus scrofa), cytosolic form}
APPSVFAEVPQAQPVLVFKLIADFREDPDPRKVNLGVGAYRTDDCQPWVLPVVRKVEQRIAN
NSSLNHEYLPILGLAEFRTCASRLALGDDSPALQEKRVGGVQSLGGTGALRIGAEFLARWYN
GTNNKDTPVYVSSPTWENHNGVFTTAGFKDIRSYRYWDTEKRGLDLQGFLSDLENAPEFSIF
VLHACAHNPTGTDPTPEQWKQIASVMKRRFLFPFFDSAYQGFASGNLEKDAWAIRYFVSEGF
ELFCAQSFSKNFGLYNERVGNLTVVAKEPDSILRVLSQMQKIVRVTWSNPPAQGARIVARTL
SDPELFHEWTGNVKTMADRILSMRSELRARLEALKTPGTWNHITDQIGMFSFTGLNPKQVEY
LINQKHIYLLPSGRINMCGLTTKNLDYVATSIHEAVTKIQ
>d1yaaa_ c.67.1.1 (A:) Aspartate aminotransferase, AAT {Baker's yeast (Saccharomyces cerevisiae), cytosolic form}
SATLFNNIELLPPDALFGIKQRYGQDQRATKVDLGIGAYRDDNGKPWVLPSVKAAEKLIHND
SSYNHEYLGITGLPSLTSNAAKIIFGTQSDALQEDRVISVQSLSGTGALHISAKFFSKFFPD
KLVYLSKPTWANHMAIFENQGLKTATYPYWANETKSLDLNGFLNAIQKAPEGSIFVLHSCAH
NPTGLDPTSEQWVQIVDAIASKNHIALFDTAYQGFATGDLDKDAYAVRLGVEKLSTVSPVFV
CQSFAKNAGMYGERVGCFHLALTKQAQNKTIKPAVTSQLAKIIRSEVSNPPAYGAKIVAKLL
ETPELTEQWHKDMVTMSSRITKMRHALRDHLVKLGTPGNWDHIVNQCGMFSFTGLTPQMVKR
LEETHAVYLVASGRASIAGLNQGNVEYVAKAIDEVVRFYA
>d1qisa_ c.67.1.1 (A:) Aspartate aminotransferase, AAT {Escherichia coli}
MFENITAAPADPILGLADLFRADERPGKINLGIGVYKDETGKTPVLTSVKKAEQYLLENETT
KNYLGIDGIPEFGRCTQELLFGKGSALINDKRARTAQTPGGTGALRVAADFLAKNTSVKRVW
VSNPSWPNHKSVFNSAGLEVREYAYYDAENHTLDFDALINSLNEAQAGDVVLFHGFCHNPTG
IDPTLEQWQTLAQLSVEKGWLPLFDFAYQGFARGLEEDAEGLRAFAAMHKELIVASSYSKNF
GLYNERVGACTLVAADSETVDRAFSQMKAAIRANYSNPPAHGASVVATILSNDALRAIWEQE
LTDMRQRIQRMRQLFVNTLQEKGANRDFSFIIKQNGMFSFSGLTKEQVLRLREEFGVYAVAS
GRVNVAGMTPDNMAPLCEAIVAVL
>d1bjwa_ c.67.1.1 (A:) Aspartate aminotransferase, AAT {Thermus thermophilus}
MRGLSRRVQAMKPSATVAVNAKALELRRQGVDLVALTAGEPDFDTPEHVKEAARRALAQGKT
KYAPPAGIPELREALAEKFRRENGLSVTPEETIVTVGGKQALFNLFQAILDPGDEVIVLSPY
WVSYPEMVRFAGGVVVEVETLPEEGFVPDPERVRRAITPRTKALVVNSPNNPTGAVYPKEVL
EALARLAVEHDFYLVSDEIYEHLLYEGEHFSPGRVAPEHTLTVNGAAKAFAMTGWRIGYACG
PKEVIKAMASVSSQSTTSPDTIAQWATLEALTNQEASRAFVEMAREAYRRRRDLLLEGLTAL
GLKAVRPSGAFYVLMDTSPIAPDEVRAAERLLEAGVAVVPGTDFAAFGHVRLSYATSEENLR
KALERFARVL
>d2ay1a_ c.67.1.1 (A:) Aromatic aminoacid aminotransferase, AroAT {Paracoccus denitrificans}
MLGNLKPQAPDKILALMGEFRADPRQGKIDLGVGVYKDATGHTPIMRAVHAAEQRMLETETT
KTYAGLSGEPEFQKAMGELILGDGLKSETTATLATVGGTGALRQALELARMANPDLRVFVSD
PTWPNHVSIMNFMGLPVQTYRYFDAETRGVDFEGMKADLAAAKKGDMVLLHGCCHNPTGANL
TLDQWAEIASILEKTGALPLIDLAYQGFGDGLEEDAAGTRLIASRIPEVLIAASCSKNFGIY
RERTGCLLALCADAATRELAQGAMAFLNRQTYSFPPFHGAKIVSTVLTTPELRADWMAELEA
VRSGMLRLREQLAGELRDLSGSDRFGFVAEHRGMFSRLGATPEQVKRIKEEFGIYMVGDSRI

NIAGLNDNTIPILARAIIEVGV
>d3tata_ c.67.1.1 (A:) Aromatic aminoacid aminotransferase, AroAT {Escherichia coli}
MFQKVDAYAGDPILTLMERFKEDPRSDKVNLSIGLYYNEDGIIPQLQAVAEAEARLNAQPHG
ASLYLPMEGLNCYRHAIAPLLFGADHPVLKQQRVATIQTLGGSGALKVGADFLKRYFPESGV
WVSDPTWENHVAIFAGAGFEVSTYPWYDEATNGVRFNDLLATLKTLPARSIVLLHPCCHNPT
GADLTNDQWDAVIEILKARELIPFLDIAYQGFGAGMEEDAYAIRAIASAGLPALVSNSFSKI
FSLYGERVGGLSVMCEDAEAAGRVLGQLKATVRRNYSSPPNFGAQVVAAVLNDEALKASWLA
EVEEMRTRILAMRQELVKVLSTEMPERNFDYLLNQRGMFSYTGLSAAQVDRLREEFGVYLIA
SGRMCVAGLNTANVQRVAKAFAAVM
>d1gdea_ c.67.1.1 (A:) Aromatic aminoacid aminotransferase, AroAT {Archaeon Pyrococcus horikoshii}
ALSDRLELVSASEIRKLFDIAAGMKDVISLGIGEPDFDTPQHIKEYAKEALDKGLTHYGPNI
GLLELREAIAEKLKKQNGIEADPKTEIMVLLGANQAFLMGLSAFLKDGEEVLIPTPAFVSYA
PAVILAGGKPVEVPTYEEDEFRLNVDELKKYVTDKTRALIINSPCNPTGAVLTKKDLEEIAD
FVVEHDLIVISDEVYEHFIYDDARHYSIASLDGMFERTITVNGFSKTFAMTGWRLGFVAAPS
WIIERMVKFQMYNATCPVTFIQYAAAKALKDERSWKAVEEMRKEYDRRRKLVWKRLNEMGLP
TVKPKGAFYIFPRIRDTGLTSKKFSELMLKEARVAVVPGSAFGKAGEGYVRISYATAYEKLE
EAMDRMERVLKERKLV
>d1bw0a_ c.67.1.1 (A:) Tyrosine aminotransferase (TAT) {Trypanosoma cruzi}
WDVSMSNHAGLVFNPIRTVSDNAKPSPSPKPIIKLSVGDPTLDKNLLTSAAQIKKLKEAIDS
QECNGYFPTVGSPEAREAVATWWRNSFVHKEELKSTIVKDNVVLCSGGSHGILMAITAICDA
GDYALVPQPGFPHYETVCKAYGIGMHFYNCRPENDWEADLDEIRRLKDDKTKLLIVTNPSNP
CGSNFSRKHVEDIVRLAEELRLPLFSDEIYAGMVFKGKDPNATFTSVADFETTVPRVILGGT
AKNLVVPGWRLGWLLYVDPHGNGPSFLEGLKRVGMLVCGPCTVVQAALGEALLNTPQEHLDQ
IVAKIEESAMYLYNHIGECIGLAPTMPRGAMYLMSRIDLEKYRDIKTDVEFFEKLLEEENVQ
VLPGTIFHAPGFTRLTTTRPVEVYREAVERIKAFCQRHAA
>d1fg7a_ c.67.1.1 (A:) Histidinol-phosphate aminotransferase {Escherichia coli}
TVTITDLARENVRNLTPYQSARRLGGNGDVWLNANEYPTAVEFQLTQQTLNRYPECQPKAVI
ENYAQYAGVKPEQVLVSRGADEGIELLIRAFCEPGKDAILYCPPTYGMYSVSAETIGVECRT
VPTLDNWQLDLQGISDKLDGVKVVYVCSPNNPTGQLINPQDFRTLLELTRGKAIVVADEAYI
EFCPQASLAGWLAEYPHLAILRTLSKAFALAGLRCGFTLANEEVINLLMKVIAPYPLSTPVA
DIAAQALSPQGIVAMRERVAQIIAEREYLIAALKEIPCVEQVFDSETNYILARFKASSAVFK
SLWDQGIILRDQNKQPSLSGCLRITVGTREESQRVIDALRAEQV
>d1kusa_ c.67.1.1 (A:) L-threonine-O-3-phosphate decarboxylase CobD {Salmonella enterica}
HGGNIREPATVLGISPDQLLDFSANINPLGMPVSVKRALIDNLDCIERYPDADYFHLHQALA
RHHQVPASWILAGNGETESIFTVASGLKPRRAMIVTPGFAEYGRALAQSGCEIRRWSLREAD
GWQLTDAILEALTPDLDCLFLCTPNNPTGLLPERPLLQAIADRCKSLNINLILDEAFIDFIP
HETGFIPALKDNPHIWVLRSLTKFYAIPGLRLGYLVNSDDAAMARMRRQQMPWSVNALAALA
GEVALQDSAWQQATWHWLREEGARFYQALCQLPLLTVYPGRANYLLLRCEREDIDLQRRLLT
QRILIRSCANYPGLDSRYYRVAIRSAAQNERLLAALRNVLTGIAP

>d1jg8a_ c.67.1.1 (A:) Low-specificity threonine aldolase {Thermatoga maritima}
MIDLRSDTVTKPTEEMRKAMAQAEVGDDVYGEDPTINELERLAAETFGKEAALFVPSGTMGN
QVSIMAHTQRGDEVILEADSHIFWYEVGAMAVLSGVMPHPVPGKNGAMDPDDVRKAIRPRNI
HFPRTSLIAIENTHNRSGGRVVPLENIKEICTIAKEHGINVHIDGARIFNASIASGVPVKEY
AGYADSVMFCLSKGLCAPVGSVVVGDRDFIERARKARKMLGGGMRQAGVLAAAGIIALTKMV
DRLKEDHENARFLALKLKEIGYSVNPEDVKTNMVILRTDNLKVNAHGFIEALRNSGVLANAV
SDTEIRLVTHKDVSRNDIEEALNIFEKLFRKFS
>d1tpla_ c.67.1.2 (A:) Tyrosine phenol-lyase {Citrobacter intermedius}
MNYPAEPFRIKSVETVSMIPRDERLKKMQEAGYNTFLLNSKDIYIDLLTDSGTNAMSDKQWA
GMMMGDEAYAGSENFYHLERTVQELFGFKHIVPTHQGRGAENLLSQLAIKPGQYVAGNMYKN
GAVFVDIVRDEAHDAGLNIAFKGDIDLKKLQKLIDEKGAENIAYICLAVTVNLAGGQPVSMA
NMRAVRELTAAHGIKVFYDATRCVENAYFIKEQEQGFENKSIAEIVHEMFSYADGCTMSGKK
DCLVNIGGFLCMNDDEMFSSAKELVVVYEGMPSYGGLAGRDMEAMAIGLREAMQYEYIEHRV
KQVRYLGDKLKAAGVPIVEPVGGHAVFLDARRFCEHLTQDEFPAQSLAASIYVETGVRSMER
GIKLETVRLTIPRRVYTYAHMDVVADGIIKLYQHKEDIRGLKFIYFFTARFDYI
>d1ax4a_ c.67.1.2 (A:) Tryptophan indol-lyase (tryptophanase) {Proteus vulgaris}
AKRIVEPFRIKMVEKIRVPSREEREAALKEAGYNPFLLPSSAVYIDLLTDSGTNAMSDHQWA
AMITGDEAYAGSRNYYDLKDKAKELFNYDYIIPAHQGRGAENILFPVLLKYKQKEGKAKNPV
FISNFHFDTTAAHVELNGCKAINIVTEKAFDSETYDDWKGDFDIKKLKENIAQHGADNIVAI
VSTVTCNSAGGQPVSMSNLKEVYEIAKQHGIFVVMDSARFCENAYFIKARDPKYKNATIKEV
IFDMYKYADALTMSAKKDPLLNIGGLVAIRDNEEIFTLARQRCVPMEGFVTYGGLAGRDMAA
MVQGLEEGTEEEYLHYRIGQVKYLGDRLREAGIPIQYPTGGHAVFVDCKKLVPQIPGDQFPA
QAVINALYLESGVRAVEIGSFLLGRDPATGEQKHADMEFMRLTIARRVYTNDHMDYIADALI
GLKEKFATLKGLEFEYEPPVLRHFTARLKPI
>d1js3a_ c.67.1.6 (A:) DOPA decarboxylase {Pig (Sus scrofa)}
MNASDFRRRGKEMVDYMADYLEGIEGRQVYPDVQPGYLRPLIPATAPQEPDTFEDILQDVEK
IIMPGVTHWHSPYFFAYFPTASSYPAMLADMLCGAIGCIGFSWAASPACTELETVMMDWLGK
MLQLPEAFLAGEAGEGGGVIQGSASEATLVALLAARTKVVRRLQAASPGLTQGAVLEKLVAY
ASDQAHSSVERAGLIGGVKLKAIPSDGKFAMRASALQEALERDKAAGLIPFFVVATLGTTSC
CSFDNLLEVGPICHEEDIWLHVDAAYAGSAFICPEFRHLLNGVEFADSFNFNPHKWLLVNFD
CSAMWVKRRTDLTGAFKLDPVYLKHSHQGSGLITDYRHWQLPLGRRFRSLKMWFVFRMYGVK
GLQAYIRKHVQLSHEFEAFVLQDPRFEVCAEVTLGLVCFRLKGSDGLNEALLERINSARKIH
LVPCRLRGQFVLRFAICSRKVESGHVRLAWEHIRGLAAELLA
>d1cl1a_ c.67.1.3 (A:) Cystathionine beta-lyase, CBL {Escherichia coli}
KLDTQLVNAGRSKKYTLGAVNSVIQRASSLVFDSVEAKKHATRNRANGELFYGRRGTLTHFS
LQQAMCELEGGAGCVLFPCGAAAVANSILAFIEQGDHVLMTNTAYEPSQDFCSKILSKLGVT
TSWFDPLIGADIVKHLQPNTKIVFLESPGSITMEVHDVPAIVAAVRSVVPDAIIMIDNTWAA
GVLFKALDFGIDVSIQAATKYLVGHSDAMIGTAVCNARCWEQLRENAYLMGQMVDADTAYIT
SRGLRTLGVRLRQHHESSLKVAEWLAEHPQVARVNHPALPGSKGHEFWKRDFTGSSGLFSFV
LKKKLNNEELANYLDNFSLFSMAYSWGGYESLILANQPEHIAAIRPQGEIDFSGTLIRLHIG

LEDVDDLIADLDAGFARIV
>d1ibja_ c.67.1.3 (A:) Cystathionine beta-lyase, CBL {Thale cress (Arabidopsis thaliana)}
ASVSTLLVNLDNKFDPFDAMSTPLYQTATFKQPSAIENGPYDYTRSGNPTRDALESLLAKLD
KADRAFCFTSGMAALSAVTHLIKNGEEIVAGDDVYGGSDRLLSQVVPRSGVVVKRVNTTKLD
EVAAAIGPQTKLVWLESPTNPRQQISDIRKISEMAHAQGALVLVDNSIMSPVLSRPLELGAD
IVMHSATKFIAGHSDVMAGVLAVKGEKLAKEVYFLQNSEGSGLAPFDCWLCLRGIKTMALRI
EKQQENARKIAMYLSSHPRVKKVYYAGLPDHPGHHLHFSQAKGAGSVFSFITGSVALSKHLV
ETTKYFSIAVSFGSVKSLISMPCFMSHASIPAEVREARGLTEDLVRISAGIEDVDDLISDLD
IAFKTFPL
>d1cs1a_ c.67.1.3 (A:) Cystathionine gamma-synthase, CGS {Escherichia coli}
RKQATIAVRSGLNDDEQYGCVVPPIHLSSTYNFTGFNEPRAHDYSRRGNPTRDVVQRALAEL
EGGAGAVLTNTGMSAIHLVTTVFLKPGDLLVAPHDCYGGSYRLFDSLAKRGCYRVLFVDQGD
EQALRAALAEKPKLVLVESPSNPLLRVVDIAKICHLAREVGAVSVVDNTFLSPALQNPLALG
ADLVLHSCTKYLNGHSDVVAGVVIAKDPDVVTELAWWANNIGVTGGAFDSYLLLRGLRTLVP
RMELAQRNAQAIVKYLQTQPLVKKLYHPSLPENQGHEIAARQQKGFGAMLSFELDGDEQTLR
RFLGGLSLFTLAESLGGVESLISHAATMTHAGMAPEARAAAGISETLLRISTGIEDGEDLIA
DLENGFRAANKG
>d1qgna_ c.67.1.3 (A:) Cystathionine gamma-synthase, CGS {Common tobacco (Nicotiana tabacum)}
MKYASFLNSDGSVAIHAGERLGRGIVTDAITTPVVNTSAYFFNKTSELIDFKEKRRASFEYG
RYGNPTTVVLEEKISALEGAESTLLMASGMCASTVMLLALVPAGGHIVTTTDCYRKTRIFIE
TILPKMGITATVIDPADVGALELALNQKKVNLFFTESPTNPFLRCVDIELVSKLCHEKGALV
CIDGTFATPLNQKALALGADLVLHSATKFLGGHNDVLAGCISGPLKLVSEIRNLHHILGGAL
NPNAAYLIIRGMKTLHLRVQQQNSTALRMAEILEAHPKVRHVYYPGLQSHPEHHIAKKQMTG
FGGAVSFEVDGDLLTTAKFVDALKIPYIAPSFGGCESIVDQPAIMSYWDLSQSDRAKYGIMD
NLVRFSFGVEDFDDLKADILQALDSI
>d1e5ea_ c.67.1.3 (A:) Methionine gamma-lyase, MGL {Trichomonas vaginalis}
ERMTPATACIHANPQKDQFGAAIPPIYQTSTFVFDNCQQGGNRFAGQESGYIYTRLGNPTVS
NLEGKIAFLEKTEACVATSSGMGAIAATVLTILKAGDHLISDECLYGCTHALFEHALTKFGI
QVDFINTAIPGEVKKHMKPNTKIVYFETPANPTLKIIDMERVCKDAHSQEGVLVIADNTFCS
PMITNPVDFGVDVVVHSATKYINGHTDVVAGLICGKADLLQQIRMVGIKDITGSVISPHDAW
LITRGLSTLNIRMKAESENAMKVAEYLKSHPAVEKVYYPGFEDHEGHDIAKKQMRMYGSMIT
FILKSGFEGAKKLLDNLKLITLAVSLGGCESLIQHPASMTHAVVPKEEREAAGITDGMIRLS
VGIEDADELIADFKQGLDALLR
>d1d2fa_ c.67.1.3 (A:) Modulator in mal gene expression, MalY {Escherichia coli}
LLPFTISDMDFATAPCIIEALNQRLMHGVFGYSRWKNDEFLAAIAHWFSTQHYTAIDSQTVV
YGPSVIYMVSELIRQWSETGEGVVIHTPAYDAFYKAIEGNQRTVMPVALEKQADGWFCDMGK
LEAVLAKPECKIMLLCSPQNPTGKVWTCDELEIMADLCERHGVRVISDEIHMDMVWGEQPHI
PWSNVARGDWALLTSGSKSFNIPALTGAYGIIENSSSRDAYLSALKGRDGLSSPSVLALTAH
IAAYQQGAPWLDALRIYLKDNLTYIADKMNAAFPELNWQIPQSTYLAWLDLRPLNIDDNALQ

KALIEQEKVAIMPGYTYGEEGRGFVRLNAGCPRSKLEKGVAGLINAIRAVR
>d1c7na_ c.67.1.3 (A:) Cystalysin {Treponema denticola}
MIYDFTTKISRKNLGSLKWDLMYSQNPEVGNEVVPLSVADMEFKNPPELIEGLKKYLDETVL
GYTGPTEEYKKTVKKWMKDRHQWDIQTDWIINTAGVVPAVFNAVREFTKPGDGVIIITPVYY
PFFMAIKNQERKIIECELLEKDGYYTIDFQKLEKLSKDKNNKALLFCSPHNPVGRVWKKDEL
QKIKDIVLKSDLMLWSDEIHFDLIMPGYEHTVFQSIDEQLADKTITFTAPSKTFNIAGMGMS
NIIIKNPDIRERFTKSRDATSGMPFTTLGYKACEICYKECGKWLDGCIKVIDKNQRIVKDFF
EVNHPEIKAPLIEGTYLQWIDFRALKMDHKAMEEFMIHKAQIFFDEGYIFGDGGIGFERINL
AAPSSVIQESLERLNKALKDLK
>d1eg5a_ c.67.1.3 (A:) NifS-like protein/selenocysteine lyase
{Thermotoga maritima}
MRVYFDNNATTRVDDRVLEEMIVFYREKYGNPNSAHGMGIEANLHMEKAREKVAKVLGVSPS
EIFFTSCATESINWILKTVAETFEKRKRTIITTPIEHKAVLETMKYLSMKGFKVKYVPVDSR
GVVKLEELEKLVDEDTFLVSIMAANNEVGTIQPVEDVTRIVKKKNKETLVHVDAVQTIGKIP
FSLEKLEVDYASFSAHKFHGPKGVGITYIRKGVPIRPLIHGGGQERGLRSGTQNVPGIVGAA
RAMEIAVEELSEAAKHMEKLRSKLVSGLMNLGAHIITPLEISLPNTLSVSFPNIRGSTLQNL
LSGYGIYVSTSSACTSKDERLRHVLDAMGVDRRIAQGAIRISLCKYNTEEEVDYFLKKIEEI
LSFL
>d1jf9a_ c.67.1.3 (A:) NifS-like protein/selenocysteine lyase
{Escherichia coli}
IFSVDKVRADFPVLSREVNGLPLAYLDSAASAQKPSQVIDAEAEFYRHGYAAVHRGIHTLSA
QATEKMENVRKRASLFINARSAEELVFVRGTTEGINLVANSWGNSNVRAGDNIIISQMEHHA
NIVPWQMLCARVGAELRVIPLNPDGTLQLETLPTLFDEKTRLLAITHVSNVLGTENPLAEMI
TLAHQHGAKVLVDGAQAVMHHPVDVQALDCDFYVFSGHKLYGPTGIGILYVKEALLQEMPPW
EGGGSMIATVSLSEGTTWTKAPWRFEAGTPNTGGIIGLGAALEYVSALGLNNIAEYEQNLMH
YALSQLESVPDLTLYGPQNRLGVIAFNLGKHHAYDVGSFLDNYGIAVRTGHHCAMPLMAYYN
VPAMCRASLAMYNTHEEVDRLVTGLQRIHRLLG
>d1elua_ c.67.1.3 (A:) Cystine C-S lyase {Synechocystis sp.}
QFPGLANKTYFNFGGQGILPTVALEAITAMYGYLQENGPFSIAANQHIQQLIAQLRQALAET
FNVDPNTITITDNVTTGCDIVLWGLDWHQGDEILLTDCEHPGIIAIVQAIAARFGITYRFFP
VAATLNQGDAAAVLANHLGPKTRLVILSHLLWNTGQVLPLAEIMAVCRRHQGNYPVRVLVDG
AQSAGSLPLDFSRLEVDYYAFTGHKWFAGPAGVGGLYIHGDCLGEINPTYVGWRSITYGAKG
EPTGWAEGGKRFEVATSAYPQYAGLLAALQLHQRQGTAEERYQAICQRSEFLWRGLNQLPHV
HCLATSAPQAGLVSFTVDSPLGHRAIVQKLEEQRIYLRTIADPDCIRACCHYITDEEEINHL
LARLADFGP
>d2dkb__ c.67.1.4 (-) Dialkylglycine decarboxylase {Pseudomonas
cepacia}
LNDDATFWRNARHHLVRYGGTFEPMIIERAKGSFVYDADGRAILDFTSGQMSAVLGHCHPEI
VSVIGEYAGKLDHLFSEMLSRPVVDLATRLANITPPGLDRALLLSTGAESNEAAIRMAKLVT
GKYEIVGFAQSWHGMTGAAASATYSAGRKGVGPAAVGSFAIPAPFTYRPRFERNGAYDYLAE
LDYAFDLIDRQSSGNLAAFIAEPILSSGGIIELPDGYMAALKRKCEARGMLLILDEAQTGVG
RTGTMFACQRDGVTPDILTLSKTLGAGLPLAAIVTSAAIEERAHELGYLFYTTHVSDPLPAA
VGLRVLDVVQRDGLVARANVMGDRLRRGLLDLMERFDCIGDVRGRGLLLGVEIVKDRRTKEP
ADGLGAKITRECMNLGLSMNIVQLPGMGGVFRIAPPLTVSEDEIDLGLSLLGQAIERAL

>d2gsaa_ c.67.1.4 (A:) Glutamate-1-semialdehyde aminomutase (aminotransferase) {Synechococcus sp., strain GR6}
FKTIKSDEIFAAAQKLMPGGVSSPVRAFKSVGGQPIVFDRVKDAYAWDVDGNRYIDYVGTWG
PAICGHAHPEVIEALKVAMEKGTSFGAPCALENVLAEMVNDAVPSIEMVRFVNSGTEACMAV
LRLMRAYTGRDKIIKFEGCYHGHADMFLVKAGSGVATLGLPSSPGVPKKTTANTLTTPYNDL
EAVKALFAENPGEIAGVILEPIVGNSGFIVPDAGFLEGLREITLEHDALLVFDEVMTGFRIA
YGGVQEKFGVTPDLTTLGKIIGGGLPVGAYGGKREIMQLVAPAGPMYQAGTLSGNPLAMTAG
IKTLELLRQPGTYEYLDQITKRLSDGLLAIAQETGHAACGGQVSGMFGFFFTEGPVHNYEDA
KKSDLQKFSRFHRGMLEQGIYLAPSQFEAGFTSLAHTEEDIDATLAAARTVMSAL
>d2oata_ c.67.1.4 (A:) Ornithine aminotransferase {Human (Homo sapiens)}
GPPTSDDIFEREYKYGAHNYHPLPVALERGKGIYLWDVEGRKYFDFLSSYSAVNQGHCHPKI
VNALKSQVDKLTLTSRAFYNNVLGEYEEYITKLFNYHKVLPMNTGVEAGETACKLARKWGYT
VKGIQKYKAKIVFAAGNFWGRTLSAISSSTDPTSYDGFGPFMPGFDIIPYNDLPALERALQD
PNVAAFMVEPIQGEAGVVVPDPGYLMGVRELCTRHQVLFIADEIQTGLARTGRWLAVDYENV
RPDIVLLGKALSGGLYPVSAVLCDDDIMLTIKPGEHGSTYGGNPLGCRVAIAALEVLEEENL
AENADKLGIILRNELMKLPSDVVTAVRGKGLLNAIVIKETKDWDAWKVCLRLRDNGLLAKPT
HGDIIRFAPPLVIKEDELRESIEIINKTILSF
>d1gtxa_ c.67.1.4 (A:) 4-aminobutyrate aminotransferase, GABA-aminotransferase {Pig (Sus scrofa)}
FDYDGPLMKTEVPGPRSRELMKQLNIIQNAEAVHFFCNYEESRGNYLVDVDGNRMLDLYSQI
SSIPIGYSHPALVKLVQQPQNVSTFINRPALGILPPENFVEKLRESLLSVAPKGMSQLITMA
CGSCSNENAFKTIFMWYRSKERGQSAFSKEELETCMINQAPGCPDYSILSFMGAFHGRTMGC
LATTHSKAIHKIDIPSFDWPIAPFPRLKYPLEEFVKENQQEEARCLEEVEDLIVKYRKKKKT
VAGIIVEPIQSEGGDNHASDDFFRKLRDISRKHGCAFLVDEVQTGGGSTGKFWAHEHWGLDD
AADVMTFSKKMMTGGFFHKEEFRPNAPYRIFNTWLGDPSKNLLLAEVINIIKREDLLSNAAH
AGKVLLTGLLDLQARYPQFISRVRGRGTFCSFDTPDESIRNKLISIARNKGVMLGGCGDKSI
RFRPTLVFRDHHAHLFLNIFSDILADFK
>d1bt4a_ c.67.1.4 (A:) Phosphoserine aminotransferase, PSAT {Bacillus circulans, subsp. alkalophilus}
SERAYNFNAGPAALPLEVLERAQAEFVDYQHTGMSIMEMSHRGAVYEAVHNEAQARLLALLG
NPTGYKVLFIQGGASTQFAMIPMNFLKEGQTANYVMTGSWASKALKEAKLIGDTHVAASSEA
SNYMTLPKLQEIQLQDNAAYLHLTSNETIEGAQFKAFPDTGSVPLIGDMSSDILSRPFDLNQ
FGLVYAGAQKNLGPSGVTVVIVREDLVAESPKHLPTMLRYDTYVKNNSLYNTPPSFGIYMVN
EVLKWIEERGGLEGVQQANRKKASLIYDAIDQSGGFYRGCVDVDSRSDMNITFRLASEELEK
EFVKASEQEGFVGLKGHRSVGGLRASIYNAVPYESCEALVQFMEHFKRSRG
>d1bjna_ c.67.1.4 (A:) Phosphoserine aminotransferase, PSAT {Escherichia coli}
QIFNFSSGPAMLPAEVLKQAQQELRDWNGLGTSVMEVSHRGKEFIQVAEEAEKDFRDLLNVP
SNYKVLFCHGGGRGQFAAVPLNILGDKTTADYVDAGYWAASAIKEAKKYCTPNVFDAKVTVD
GLRAVKPMREWQLSDNAAYMHYCPNETIDGIAIDETPDFGADVVVAADFSSTILSRPIDVSR
YGVIYAGAQKNIGPAGLTIVIVREDLLGKANIACPSILDYSILNDNGSMFNTPPTFAWYLSG
LVFKWLKANGGVAEMDKINQQKAELLYGVIDNSDFYRNDVAKRNRSRMNVPFQLADSALDKL
FLEESFAAGLHALKGHRVVGGMRASIYNAMPLEGVKALTDFMVEFERRHG

>d1cj0a_ c.67.1.4 (A:) Serine hydroxymethyltransferase {Rabbit (Oryctolagus cuniculus)}
WSSHEQMLAQPLKDSDAEVYDIIKKESNRQRVGLELIASENFASRAVLEALGSCLNNKYSEG
YPGQRYYGGTEHIDELETLCQKRALQAYGLDPQCWGVNVQPYSGSPANFAVYTALVEPHGRI
MGLDLPDGGHLTHGFMTDKKKISATSIFFESMAYKVNPDTGYIDYDRLEENARLFHPKLIIA
GTSCYSRNLDYGRLRKIADENGAYLMADMAHISGLVVAGVVPSPFEHCHVVTTTTHKTLRGC
RAGMIFYRRGVRSVDPKTGKEILYNLESLINSAVFPGLQGGPHNHAIAGVAVALKQAMTPEF
KEYQRQVVANCRALSAALVELGYKIVTGGSDNHLILVDLRSKGTDGGRAEKVLEACSIACNK
NTCPGDKSALRPSGLRLGTPALTSRGLLEKDFQKVAHFIHRGIELTVQIQDDTGPRATLKEF
KEKLAGDEKHQRAVRALRQEVESFAALFPLPGLPGF
>d1ejia_ c.67.1.4 (A:) Serine hydroxymethyltransferase {Mouse (Mus musculus)}
MADRDATLWASHEKMLSQPLKDSDAEVYSIIKKESNRQRVGLELIASENFASRAVLEALGSS
LNNKYSEGYPGQRYYGGTEFIDELEMLCQKRALQAYHLDPQCWGVNVQPYSGSPANFAVYTA
LVEPHGRIMGLDLPDGGHLTHGFMTDKKKISATSIFFESMPYKVYPETGYINYDQLEENASL
FHPKLIIAGTSCYSRNLDYARLRKIADDNGAYLMADMAHISGLVAAGVVPSPFEHCHVVTTT
THKTLRGCRAGMIFYRKGVRSVDPKTGKETYYELESLINSAVFPGLQGGPHNHAIAGVAVAL
KQAMTTEFKIYQLQVLANCRALSDALTELGYKIVTGGSDNHLILMDLRSKGTDGGRAEKVLE
ACSIACNKNTCPGDKSALRPSGLRLGTPALTSRGLLEEDFQKVAHFIHRGIELTLQIQSHMA
TKATLKEFKEKLAGDEKIQSAVATLREEVENFASNFSLPGLPDF
>d1bj4a_ c.67.1.4 (A:) Serine hydroxymethyltransferase {Human (Homo sapiens)}
DADLWSSHDAMLAQPLKDSDVEVYNIIKKESNRQRVGLELIASENFASRAVLEALGSCLNNK
YSEGYPGQRYYGGTEFIDELETLCQKRALQAYKLDPQCWGVNVQPYSGSPANFAVYTALVEP
HGRIMGLDLPDGGHLTHGFMTDKKKISATSIFFESMPYKVNPDTGYINYDQLEENARLFHPK
LIIAGTSCYSRNLEYARLRKIADENGAYLMADMAHISGLVAAGVVPSPFEHCHVVTTTTHKT
LRGCRAGMIFYRKGVKSVDPATGKEILYNLESLINSAVFPGLQGGPHNHAIAGVAVALKQAM
TLEFKVYQHQVVANCRALSEALTELGYKIVTGGSDNHLILVDLRSKGTDGGRAEKVLEACSI
ACNKNTCPGDRSALRPSGLRLGTPALTSRGLLEKDFQKVAHFIHRGIELTLQIQSDTGVAAT
LKEFKERLAGDKYQAAVQALREEVESFASLFPLPGL
>d1dfoa_ c.67.1.4 (A:) Serine hydroxymethyltransferase {Escherichia coli}
LKREMNIADYDAELWQAMEQEKVRQEEHIELIASENYTSPRVMQAQGSQLTNKYAEGYPGKR
YYGGCEYVDIVEQLAIDRAKELFGADYANVQPHSGSQANFAVYTALLEPGDTVLGMNLAHGG
HLTHGSPVNFSGKLYNIVPYGIDATGHIDYADLEKQAKEHKPKMIIGGFSAYSGVVDWAKMR
EIADSIGAYLFVDMAHVAGLVAAGVYPNPVPHAHVVTTTTHKTLAGPRGGLILAKGGSEELY
KKLNSAVFPGGQGGPLMHVIAGKAVALKEAMEPEFKTYQQQVAKNAKAMVEVFLERGYKVVS
GGTDNHLFLVDLVDKNLTGKEADAALGRANITVNKNSVPNDPKSPFVTSGIRVGTPAITRRG
FKEAEAKELAGWMCDVLDSINDEAVIERIKGKVLDICARYPVYA
>d1b9ha_ c.67.1.4 (A:) 3-amino-5-hydroxybenzoic acid synthase (AHBA synthase) {Amycolatopsis mediterranei}
KAPEFPAWPQYDDAERNGLVRALEQGQWWRMGGDEVNSFEREFAAHHGAAHALAVTNGTHAL
ELALQVMGVGPGTEVIVPAFTFISSSQAAQRLGAVTVPVDVDAATYNLDPEAVAAAVTPRTK
VIMPVHMAGLMADMDALAKISADTGVPLLQDAAHAHGARWQGKRVGELDSIATFSFQNGKLM

TAGEGGAVVFPDGETEKYETAFLRHSCGRPRDDRRYFHKIAGSNMRLNEFSASVLRAQLARL
DEQIAVRDERWTLLSRLLGAIDGVVPQGGDVRADRNSHYMAMFRIPGLTEERRNALVDRLVE
AGLPAFAAFRAIYRTDAFWELGAPDESVDAIARRCPNTDAISSDCVWLHHRVLLAGEPELHA
TAEIIADAVARA
>d1fc4a_ c.67.1.4 (A:) 2-amino-3-ketobutyrate CoA ligase
{Escherichia coli}
GSHMRGEFYQQLTNDLETARAEGLFKEERIITSAQQADITVADGSHVINFCANNYLGLANHP
DLIAAAKAGMDSHGFGMASVRFICGTQDSHKELEQKLAAFLGMEDAILYSSCFDANGGLFET
LLGAEDAIISDALNHASIIDGVRLCKAKRYRYANNDMQELEARLKEAREAGARHVLIATDGV
FSMDGVIANLKGVCDLADKYDALVMVDDSHAVGFVGENGRGSHEYCDVMGRVDIITGTLGKA
LGGASGGYTAARKEVVEWLRQRSRPYLFSNSLAPAIVAASIKVLEMVEAGSELRDRLWANAR
QFREQMSAAGFTLAGADHAIIPVMLGDAVVAQKFARELQKEGIYVTGFFYPVVPKGQARIRT
QMSAAHTPEQITRAVEAFTRIGKQLGVIA
>d1bs0a_ c.67.1.4 (A:) PLP-dependent acyl-CoA synthase
(8-amino-7-oxonanoate synthase, AONS) {Escherichia coli}
SWQEKINAALDARRAADALRRRYPVAQGAGRWLVADDRQYLNFSSNDYLGLSHHPQIIRAWQ
QGAEQFGIGSGGSGHVSGYSVVHQALEEELAEWLGYSRALLFISGFAANQAVIAAMMAKEDR
IAADRLSHASLLEAASLSPSQLRRFAHNDVTHLARLLASPCPGQQMVVTEGVFSMDGDSAPL
AEIQQVTQQHNGWLMVDDAHGTGVIGEQGRGSCWLQKVKPELLVVTFGKGFGVSGAAVLCSS
TVADYLLQFARHLIYSTSMPPAQAQALRASLAVIRSDEGDARREKLAALITRFRAGVQDLPF
TLADSCSAIQPLIVGDNSRALQLAEKLRQQGCWVTAIRPPTVPAGTARLRLTLTAAHEMQDI
DRLLEVLHGNG
>d1qj5a_ c.67.1.4 (A:) Adenosylmethionine-8-amino-7-oxononanoate
aminotransferase, BioA {Escherichia coli}
MTTDDLAFDQRHILHPYTSMTSPLPVYPVVSAEGCELILSDGRRLVDGMSSWWAAIHGYNHP
QLNAAMKSQIDAMSHVMFGGITHAPAIELCRKLVAMTPQPLECVFLADSGSVAVEVAMKMAL
QYWQAKGEARQRFLTFRNGYHGDTFGAMSVCDPDNSMHSLWKGYLPENLFAPAPQSRMDGEW
DERDMVGFARLMAAHRHEIAAVIIEPIVQGAGGMRMYHPEWLKRIRKICDREGILLIADEIA
TGFGRTGKLFACEHAEIAPDILCLGKALTGGTMTLSATLTTREVAETISNGEAGCFMHGPTF
MGNPLACAAANASLAILESGDWQQQVADIEVQLREQLAPARDAEMVADVRVLGAIGVVETTH
PVNMAALQKFFVEQGVWIRPFGKLIYLMPPYIILPQQLQRLTAAVNRAVQDETFFCQ
>d1b8ga_ c.67.1.4 (A:) 1-aminocyclopropane-1-carboxylate synthase
(ACC synthase) {Apple (Malus domestica)}
MLSRNATFNSHGQDSSYFLGWQEYEKNPYHEVHNTNGIIQMGLAENQLCFDLLESWLAKNPE
AAAFKKNGESIFAELALFQDYHGLPAFKKAMVDFMAEIRGNKVTFDPNHLVLTAGATSANET
FIFCLADPGEAVLIPTPYYPGFDRDLKWRTGVEIVPIHCTSSNGFQITETALEEAYQEAEKR
NLRVKGVLVTNPSNPLGTTMTRNELYLLLSFVEDKGIHLISDEIYSGTAFSSPSFISVMEVL
KDRNCDENSEVWQRVHVVYSLSKDLGLPGFRVGAIYSNDDMVVAAATKMSSFGLVSSQTQHL
LSAMLSDKKLTKNYIAENHKRLKQRQKKLVSGLQKSGISCLNGNAGLFCWVDMRHLLRSNTF
EAEMELWKKIVYEVHLNISPGSSCHCTEPGWFRVCFANLPERTLDLAMQRLKAFVG
>d1iaya_ c.67.1.4 (A:) 1-aminocyclopropane-1-carboxylate synthase
(ACC synthase) {Tomato (Lycopersicon esculentum)}
ILSKLATNEEHGENSPYFDGWKAYDSDPFHPLKNPNGVIQMGLAENQLCLDLIEDWIKRNPK
GSICSEGIKSFKAIANFQDYHGLPEFRKAIAKFMEKTRGGRVRFDPERVVMAGGATGANETI

IFCLADPGDAFLVPSPYYPAFNRDLRWRTGVQLIPIHCESSNNFKITSKAVKEAYENAQKSN
IKVKGLILTNPSNPLGTTLDKDTLKSVLSFTNQHNIHLVCDEIYAATVFDTPQFVSIAEILD
EQEMTYCNKDLVHIVYSLSKDMGLPGFRVGIIYSFNDDVVNCARKMSSFGLVSTQTQYFLAA
MLSDEKFVDNFLRESAMRLGKRHKHFTNGLEVVGIKCLKNNAGLFCWMDLRPLLRESTFDSE
MSLWRVIINDVKLNVSPGSSFECQEPGWFRVCFANMDDGTVDIALARIRRFVGVEK
>d1c4ka2 c.67.1.5 (A:108-569) Ornithine decarboxylase major domain {Lactobacillus sp., strain 30a}
PPFFKSLKEYVSRYLIQFDCPGHQGGQYYRKHPAGREFYDFFGETVFRADLCNADVALGDLL
IHEGPAVAAEKHAARVYNADKTYFVLGGSSNANNTVTSALVSNGDLVLFDRNNHKSVYNSAL
AMAGGRPVYLQTNRNPYGFIGGIYDSDFDEKKIRELAAKVDPERAKWKRPFRLAVIQLGTYD
GTIYNAHEVVKRIGHLCDYIEFDSAWVGYEQFIPMMRNSSPLLIDDLGPEDPGIIVVQSVHK
QQAGFSQTSQIHKKDSHIKGQLRYCDHKHFNNSFNLFMSTSPFYPMYAALDVNAAMQEGEAG
RKLWHDLLITTIEARKKLIKAGSMFRPFVPPVVNGKKWEDGDTEDMANNIDYWRFEKGAKWH
AYEGYGDNQYYVDPNKFMLTTPGINPETGDYEDFGVPATIVANYLRDHGIIPEKSDLNSILF
LMTPAETPAKMNNLITQLLQLQRLIEED
>d1qg8a_ c.68.1.1 (A:) Spore coat polysaccharide biosynthesis protein SpsA {Bacillus subtilis}
PKVSVIMTSYNKSDYVAKSISSILSQTFSDFELFIMDDNSNEETLNVIRPFLNDNRVRFYQS
DISGVKERTEKTRYAALINQAIEMAEGEYITYATDDNIYMPDRLLKMVRELDTHPEKAVIYS
ASKTYHLNENRDIVKETVRPAAQVTWNAPCAIDHCSVMHRYSVLEKVKEKFGSYWDESPAFY
RIGDARFFWRVNHFYPFYPLDEELDLNYITDQSIHFQLFELEKNEFVRNLPPQRNCRELRES
LKKLGMG
>d1j8wb_ c.68.1.2 (B:) beta 1,4 galactosyltransferase (b4GalT1) {Cow (Bos taurus)}
TACPEESPLLVGPMLIEFNIPVDLKLVEQQNPKVKLGGRYTPMDCISPHKVAIIIPFRNRQE
HLKYWLYYLHPILQRQQLDYGIYVINQAGESMFNRAKLLNVGFKEALKDYDYNCFVFSDVDL
IPMNDHNTYRCFSQPRHISVAMDKFGFSLPYVQYFGGVSALSKQQFLSINGFPNNYWGWGGE
DDDIYNRLAFRGMSVSRPNAVIGKCRMIRHSRDKKNEPNPQRFDRIAHTKETMLSDGLNSLT
YMVLEVQRYPLYTKITVDIGTPS
>d1ezia_ c.68.1.3 (A:) CMP acylneuraminate synthetase {Neisseria meningitidis}
MEKQNIAVILARQNSKGLPLKNLRKMNGISLLGHTINAAISSKCFDRIIVSTDGGLIAEEAK
NFGVEVVLRPAELASDTASSISGVIHALETIGSNSGTVTLLQPTSPLRTGAHIREAFSLFDE
KIKGSVVSACPMEHHPLKTLLQINNGEYAPMRHLSDLEQPRQQLPQAFRPNGAIYINDTASL
IANNCFFIAPTKLYIMSHQDSIDIDTELDLQQAENILN
>d1ga8a_ c.68.1.4 (A:) Galactosyltransferase LgtC {Neisseria meningitidis}
MDIVFAADDNYAAYLCVAAKSVEAAHPDTEIRFHVLDAGISEANRAAVAANLRGGGGNIRFI
DVNPEDFAGFPLNIRHISITTYARLKLGEYIADCDKVLYLDIDVLVRDSLTPLWDTDLGDNW
LGASIDLFVERQEGYKQKIGMADGEYYFNAGVLLINLKKWRRHDIFKMSSEWVEQYKDVMQY
QDQDILNGLFKGGVCYANSRFNFMPTNYAFMANWFASRHTDPLYRDRTNTVMPVAVSHYCGP
AKPWHRDCTAWGAERFTELAGSLTTVPEEWRGKL
>d1g93a_ c.68.1.9 (A:) alpha-1,3-galactosyltransferase catalytic domain {Cow (Bos taurus)}

KLKLSDWFNPFKRPEVVTMTKWKAPVVWEGTYNRAVLDNYYAKQKITVGLTVFAVGRYIEHY
LEEFLTSANKHFMVGHPVIFYIMVDDVSRMPLIELGPLRSFKVFKIKPEKRWQDISMMRMKT
IGEHIVAHIQHEVDFLFCMDVDQVFQDKFGVETLGESVAQLQAWWYKADPNDFTYERRKESA
AYIPFGEGDFYYHAAIFGGTPTQVLNITQECFKGILKDKKNDIEAQWHDESHLNKYFLLNKP
TKILSPEYCWDYHIGLPADIKLVKMSWQT
>d1hv9a2 c.68.1.5 (A:4-251) N-acetylglucosamine 1-phosphate uridyltransferase GlmU, N-terminal domain {Escherichia coli}
NAMSVVILAAGKGTRMYSDLPKVLHTLAGKAMVQHVIDAANELGAAHVHLVYGHGGDLLKQA
LKDDNLNWVLQAEQLGTGHAMQQAAPFFADDEDILMLYGDVPLISVETLQRLRDAKPQGGIG
LLTVKLDDPTGYGRITRENGKVTGIVEHKDATDEQRQIQEINTGILIANGADMKRWLAKLTN
NNAQGEYYITDIIALAYQEGREIVAVHPQRLSEVEGVNNRLQLSRLERVYQSEQAEKLLLAG
>d1hm9a2 c.68.1.5 (A:2-251) N-acetylglucosamine 1-phosphate uridyltransferase GlmU, N-terminal domain {Streptococcus pneumoniae}
SNFAIILAAGKGTRMKSDLPKVLHKVAGISMLEHVFRSVGAIQPEKTVTVVGHKAELVEEVL
AGQTEFVTQSEQLGTGHAVMMTEPILEGLSGHTLVIAGDTPLITGESLKNLIDFHINHKNVA
TILTAETDNPFGYGRIVRNDNAEVLRIVEQKDATDFEKQIKEINTGTYVFDNERLFEALKNI
NTNNAQGEYYITDVIGIFRETGEKVGAYTLKDFDESLGVNDRVALATAESVMRRRINHKHMV
NG
>d1fxoa_ c.68.1.6 (A:) glucose-1-phosphate thymidylyltransferase RmlA {Pseudomonas aeruginosa}
KRKGIILAGGSGTRLHPATLAISKQLLPVYDKPMIYYPLSTLMLAGIREILIISTPQDTPRF
QQLLGDGSNWGLDLQYAVQPSPDGLAQAFLIGESFIGNDLSALVLGDNLYYGHDFHELLGSA
SQRQTGASVFAYHVLDPERYGVVEFDQGGKAISLEEKPLEPKSNYAVTGLYFYDQQVVDIAR
DLKPSPRGELEITDVNRAYLERGQLSVEIMGRGYAWLDTGTHDSLLEAGQFIATLENRQGLK
VACPEEIAYRQKWIDAAQLEKLAAPLAKNGYGQYLKRLLTETVY
>d1iina_ c.68.1.6 (A:) glucose-1-phosphate thymidylyltransferase RmlA {Salmonella enterica}
MKTRKGIILAGGSGTRLYPVTMAVSQQLLPIYDKPMIYYPLSTLMLAGIRDILIISTPQDTP
RFQQLLGDGSQWGLNLQYKVQPSPDGLAQAFIIGEEFIGHDDCALVLGDNIFYGHDLPKLME
AAVNKESGATVFAYHVNDPERYGVVEFDQKGTAVSLEEKPLQPKSNYAVTGLYFYDNSVVEM
AKNLKPSARGELEITDINRIYMEQGRLSVAMMGRGYAWLDTGTHQSLIEASNFIATIEERQG
LKVSCPEEIAFRKNFINAQQVIELAGPLSKNDYGKYLLKMV
>d1h5ra_ c.68.1.6 (A:) glucose-1-phosphate thymidylyltransferase RmlA {Escherichia coli}
KMRKGIILAGGSGTRLYPVTMAVSKQLLPIYDKPMIYYPLSTLMLAGIRDILIISTPQDTPR
FQQLLGDGSQWGLNLQYKVQPSPDGLAQAFIIGEEFIGGDDCALVLGDNIFYGHDLPKLMEA
AVNKESGATVFAYHVNDPERYGVVEFDKNGTAISLEEKPLEPKSNYAVTGLYFYDNDVVQMA
KNLKPSARGELEITDINRIYLEQGRLSVAMMGRGYAWLDTGTHQSLIEASNFIATIEERQGL
KVSCPEEIAFRKGFIDVEQVRKLAVPLIKNNYGQYLYKMTKD
>d1fgga_ c.68.1.7 (A:) 1,3-Glucuronyltransferase I (glcAT-I) {Human (Homo sapiens)}
MTIYVVTPTYARLVQKAELVRLSQTLSLVPRLHWLLVEDAEGPTPLVSGLLAASGLLFTHLV
VLTPKAQRLREGEPGWVHPRGVEQRNKALDWLRGRGGAVGGEKDPPPPGTQGVVYFADDDNT

YSRELFEEMRWTRGVSVWPVGLVGGLRFEGPQVQDGRVVGFHTAWEPSRPFPVDMAGFAVAL
PLLLDKPNAQFDSTAPRGHLESSLLSHLVDPKDLEPRAANCTRVLVWHTRTEKPKMKQEEQL
QRQGRGSDPAIEV
>d1fo8a_ c.68.1.10 (A:) N-acetylglucosaminyltransferase I {Rabbit (Oryctolagus cuniculus)}
LAVIPILVIACDRSTVRRCLDKLLHYRPSAELFPIIVSQDCGHEETAQVIASYGSAVTHIRQ
PDLSNIAVQPDHRKFQGYYKIARHYRWALGQIFHNFNYPAAVVVEDDLEVAPDFFEYFQATY
PLLKADPSLWCVSAWNDNGKEQMVDSSKPELLYRTDFFPGLGWLLLAELWAELEPKWPKAFW
DDWMRRPEQRKGRACVRPEISRTMTFGRKGVSHGQFFDQHLKFIKLNQQFVPFTQLDLSYLQ
QEAYDRDFLARVYGAPQLQVEKVRTNDRKELGEVRVQYTGRDSFKAFAKALGVMDDLKSGVP
RAGYRGIVTFLFRGRRVHLAPPQTWDGYDPSWT
>d1e5ka_ c.68.1.8 (A:) Molybdenum cofactor biosynthesis protein MobA {Escherichia coli}
MTTITGVVLAGGKARRMGGVDKGLLELNGKPLWQHVADALMTQLSHVVVNANRHQEIYQASG
LKVIEDSLADYPGPLAGMLSVMQQEAGEWFLFCPCDTPYIPPDLAARLNHQRKDAPVVWVHD
GERDHPTIALVNRAIEPLLLEYLQAGERRVMVFMRLAGGHAVDFSDHKDAFVNVNTPEELAR
WQ
>d1i52a_ c.68.1.13 (A:) 4-diphosphocytidyl-2-c-methylerythritol (CDP-me) synthase (YgbP) {Escherichia coli}
HLDVCAVVPAAGFGRRMQTECPKQYLSIGNQTILEHSVHALLAHPRVKRVVIAISPGDSRFA
QLPLANHPQITVVDGGDERADSVLAGLKAAGDAQWVLVHDAARPCLHQDDLARLLALSETSR
TGGILAAPVRDTMKRAEPGKNAIAHTVDRNGLWHALTPQFFPRELLHDCLTRALNEGATITD
EASALEYCGFHPQLVEGRADNIKVTRPEDLALAEFYLTR
>d1h7ea_ c.68.1.13 (A:) CMP:2-keto-3-deoxy-manno-octonic acid (CMP-KDO)synthetase, KdsB {Escherichia coli}
SKAVIVIPARYGSSRLPGKPLLDIVGKPMIQHVYERALQVAGVAEVWVATDDPRVEQAVQAF
GGKAIMTRNDHESGTDRLVEVMHKVEADIYINLQGDEPMIRPRDVETLLQGMRDDPALPVAT
LCHAISAAEAAEPSTVKVVVNTRQDALYFSRSPIPYPRNAEKARYLKHVGIYAYRRDVLQNY
SQLPESMPEQAESLEQLRLMNAGINIRTFEVAATGPGVDTPACLEKVRALMAQELAENA
>d1jyka_ c.68.1.13 (A:) CTP:phosphocholine cytidylytransferase LicC {Streptococcus pneumoniae}
EIRVKAIILAAGLGTRLRPLTENTPKALVQVNQKPLIEYQIEFLKEKGINDIIIIVGYLKEQ
FDYLKEKYGVRLVFNDKYADYNNFYSLYLVKEELANSYVIDADNYLFKNMFRNDLTRSTYFS
VYREDCTNEWFLVYGDDYKVQDIIVDSKAGRILSGVSFWDAPTAEKIVSFIDKAYVSGEFVD
LYWDNMVKDNIKELDVYVEELEGNSIYEIDSVQDYRKLEEILK
>d1jw9b_ c.111.1.1 (B:) Molybdenum cofactor biosynthesis protein MoeB {Escherichia coli}
AELSDQEMLRYNRQIILRGFDFDGQEALKDSRVLIVGLGGLGCAASQYLASAGVGNLTLLDF
DTVSLSNLQRQTLHSDATVGQPKVESARDALTRINPHIAITPVNALLDDAELAALIAEHDLV
LDCTDNVAVRNQLNAGCFAAKVPLVSGAAIRMEGQITVFTYQDGEPCYRCLSRLFGENALTC
VEAGVMAPLIGVIGSLQAMEAIKMLAGYGKPASGKIVMYDAMTCQFREMKLMRNPGCEVCG
>d1ea5a_ c.69.1.1 (A:) Acetylcholinesterase {Electric ray (Torpedo californica)}
SELLVNTKSGKVMGTRVPVLSSHISAFLGIPFAEPPVGNMRFRRPEPKKPWSGVWNASTYPN

NCQQYVDEQFPGFSGSEMWNPNREMSEDCLYLNIWVPSPRPKSTTVMVWIYGGGFYSGSSTL
DVYNGKYLAYTEEVVLVSLSYRVGAFGFLALHGSQEAPGNVGLLDQRMALQWVHDNIQFFGG
DPKTVTIFGESAGGASVGMHILSPGSRDLFRRAILQSGSPNCPWASVSVAEGRRRAVELGRN
LNCNLNSDEELIHCLREKKPQELIDVEWNVLPFDSIFRFSFVPVIDGEFFPTSLESMLNSGN
FKKTQILLGVNKDEGSFFLLYGAPGFSKDSESKISREDFMSGVKLSVPHANDLGLDAVTLQY
TDWMDDNNGIKNRDGLDDIVGDHNVICPLMHFVNKYTKFGNGTYLYFFNHRASNLVWPEWMG
VIHGYEIEFVFGLPLVKELNYTAEEEALSRRIMHYWATFAKTGNPNEPHSQESKWPLFTTKE
QKFIDLNTEPMKVHQRLRVQMCVFWNQFLPKLLNAT
>d1maaa_ c.69.1.1 (A:) Acetylcholinesterase {Mouse (Mus musculus)}
EDPQLLVRVRGGQLRGIRLKAPGGPVSAFLGIPFAEPPVGSRRFMPPEPKRPWSGVLDATTF
QNVCYQYVDTLYPGFEGTEMWNPNRELSEDCLYLNVWTPYPRPASPTPVLIWIYGGGFYSGA
ASLDVYDGRFLAQVEGAVLVSMNYRVGTFGFLALPGSREAPGNVGLLDQRLALQWVQENIAA
FGGDPMSVTLFGESAGAASVGMHILSLPSRSLFHRAVLQSGTPNGPWATVSAGEARRRATLL
ARLVGCPPGGAGGNDTELIACLRTRPAQDLVDHEWHVLPQESIFRFSFVPVVDGDFLSDTPE
ALINTGDFQDLQVLVGVVKDEGSYFLVYGVPGFSKDNESLISRAQFLAGVRIGVPQASDLAA
EAVVLHYTDWLHPEDPTHLRDAMSAVVGDHNVVCPVAQLAGRLAAQGARVYAYIFEHRASTL
TWPLWMGVPHGYEIEFIFGLPLDPSLNYTTEERIFAQRLMKYWTNFARTGDPNDPRDRKSPQ
WPPYTTAAQQYVSLNLKPLEVRRGLRAQTCAFWNRFLPKLLSAT
>d1f8ua_ c.69.1.1 (A:) Acetylcholinesterase {Human (Homo sapiens)}
DAELLVTVRGGRLRGIRLKTPGGPVSAFLGIPFAEPPMGPRRFLPPEPKQPWSGVVDATTFQ
SVCYQYVDTLYPGFEGTEMWNPNRELSEDCLYLNVWTPYPRPTSPTPVLVWIYGGGFYSGAS
SLDVYDGRFLVQAERTVLVSMNYRVGAFGFLALPGSREAPGNVGLLDQRLALQWVQENVAAF
GGDPTSVTLFGQSAGAASVGMHLLSPPSRGLFHRAVLQSGAPNGPWATVGMGEARRRATQLA
HLVGCPPGGTGGNDTELVACLRTRPAQVLVNHEWHVLPQESVFRFSFVPVVDGDFLSDTPEA
LINAGDFHGLQVLVGVVKDEGSYFLVYGAPGFSKDNESLISRAEFLAGVRVGVPQVSDLAAE
AVVLHYTDWLHPEDPARLREALSDVVGDHNVVCPVAQLAGRLAAQGARVYAYVFEHRASTLS
WPLWMGVPHGYEIEFIFGIPLDPSRNYTAEEKIFAQRLMRYWANFARTGDPNEPRDPKAPQW
PPYTAGAQQYVSLDLRPLEVRRGLRAQACAFWNRFLPKLLSAT
>d1dx4a_ c.69.1.1 (A:) Acetylcholinesterase {Fruit fly (Drosophila melanogaster)}
DRLVVQTSSGPVRGRSVTVQGREVHVYTGIPYAKPPVEDLRFRKPVPAEPWHGVLDATGLSA
TCVQERYEYFPGFSGEEIWNPNTNVSEDCLYINVWAPAKARLRHGRGANGGEHPNGKQADTD
HLIHNGNPQNTTNGLPILIWIYGGGFMTGSATLDIYNADIMAAVGNVIVASFQYRVGAFGFL
HLAPEMPSEFAEEAPGNVGLWDQALAIRWLKDNAHAFGGNPEWMTLFGESAGSSSVNAQLMS
PVTRGLVKRGMMQSGTMNAPWSHMTSEKAVEIGKALINDCNCNASMLKTNPAHVMSCMRSVD
AKTISVQQWNSYSGILSFPSAPTIDGAFLPADPMTLMKTADLKDYDILMGNVRDEGTYFLLY
DFIDYFDKDDATALPRDKYLEIMNNIFGKATQAEREAIIFQYTSWEGNPGYQNQQQIGRAVG
DHFFTCPTNEYAQALAERGASVHYYYFTHRTSTSLWGEWMGVLHGDEIEYFFGQPLNNSLQY
RPVERELGKRMLSAVIEFAKTGNPAQDGEEWPNFSKEDPVYYIFSTDDKIEKLARGPLAARC
SFWNDYLPKVRSW
>d2bce__ c.69.1.1 (-) Bile-salt activated lipase (cholesterol esterase) {Cow (Bos taurus)}
AKLGSVYTEGGFVEGVNKKLSLFGDSVDIFKGIPFAAAPKALEKPERHPGWQGTLKAKSFKK
RCLQATLTQDSTYGNEDCLYLNIWVPQGRKEVSHDLPVMIWIYGGAFLMGASQGANFLSNYL

YDGEEIATRGNVIVVTFNYRVGPLGFLSTGDSNLPGNYGLWDQHMAIAWVKRNIEAFGGDPD
NITLFGESAGGASVSLQTLSPYNKGLIKRAISQSGVGLCPWAIQQDPLFWAKRIAEKVGCPV
DDTSKMAGCLKITDPRALTLAYKLPLGSTEYPKLHYLSFVPVIDGDFIPDDPVNLYANAADV
DYIAGTNDMDGHLFVGMDVPAINSNKQDVTEEDFYKLVSGLTVTKGLRGANATYEVYTEPWA
QDSSQETRKKTMVDLETDILFLIPTKIAVAQHKSHAKSANTYTYLFSQPSRMPIYPKWMGAD
HADDLQYVFGKPFATPLGYRAQDRTVSKAMIAYWTNFARTGDPNTGHSTVPANWDPYTLEDD
NYLEINKQMDSNSMKLHLRTNYLQFWTQTYQALPTVTSAGASLLPPEDNSQASPVPPADNSG
APTEPSAGDSEVAQMPVVIGF
>d1f6wa_ c.69.1.1 (A:) Bile-salt activated lipase (cholesterol esterase) {Human (Homo sapiens)}
AKLGAVYTEGGFVEGVNKKLGLLGDSVDIFKGIPFAAPTKALENPQPHPGWQGTLKAKNFKK
RCLQATITQDSTYGDEDCLYLNIWVPQGRKQVSRDLPVMIWIYGGAFLMGSGHGANFLNNYL
YDGEEIATRGNVIVVTFNYRVGPLGFLSTGDANLPGNYGLRDQHMAIAWVKRNIAAFGGDPD
NITLFGESAGGASVSLQTLSPYNKGLIRRAISQSGVALSPWVIQKNPLFWAKKVAEKVGCPV
GDAARMAQCLKVTDPRALTLAYKVPLAGLEYPMLHYVGFVPVIDGDFIPDDPINLYANAADI
DYIAGTNNMDGHIFASIDMPAINKGNKKVTEEDFYKLVSEFTITKGLRGAKTTFDVYTESWA
QDPSQENKKKTVVDFETDVLFLVPTEIALAQHRANAKSAKTYAYLFSHPSRMPVYPKWVGAD
HADDIQYVFGKPFATPTGYRPQDRTVSKAMIAYWTNFAKTGDPNMGDSAVPTHWEPYTTENS
GYLEITKKMGSSSMKRSLRTNFLRYWTLTYLALPTVT
>d1qe3a_ c.69.1.1 (A:) Thermophylic para-nitrobenzyl esterase (PNB esterase) {Bacillus subtilis}
THQIVTTQYGKVKGTTENGVHKWKGIPYAKPPVGQWRFKAPEPPEVWEDVLDATAYGPICPQ
PSDLLSLSYTELPRQSEDCLYVNVFAPDTPSQNLPVMVWIHGGAFYLGAGSEPLYDGSKLAA
QGEVIVVTLNYRLGPFGFLHLSSFDEAYSDNLGLLDQAAALKWVRENISAFGGDPDNVTVFG
ESAGGMSIAALLAMPAAKGLFQKAIMESGASRTMTKEQAASTAAAFLQVLGINESQLDRLHT
VAAEDLLKAADQLRIAEKENIFQLFFQPALDPKTLPEEPEKSIAEGAASGIPLLIGTTRDEG
YLFFTPDSDVHSQETLDAALEYLLGKPLAEKAADLYPRSLESQIHMMTDLLFWRPAVAYASA
QSHYAPVWMYRFDWHPEKPPYNKAFHALELPFVFGNLDGLERMAKAEITDEVKQLSHTIQSA
WITFAKTGNPSTEAVNWPAYHEETRETVILDSEITIENDPESEKRQKLF
>d1jkma_ c.69.1.2 (A:) Carboxylesterase {Bacillus subtilis, brefeldin A esterase}
PGRLGDESSGPRTDPRFSPAMVEALATFGLDAVAAAPPVSASDDLPTVLAAVGASHDGFQAV
YDSIALDLPTDRDDVETSTETILGVDGNEITLHVFRPAGVEGVLPGLVYTHGGGMTILTTDN
RVHRRWCTDLAAAGSVVVMVDFRNAWTAEGHHPFPSGVEDCLAAVLWVDEHRESLGLSGVVV
QGESGGGNLAIATTLLAKRRGRLDAIDGVYASIPYISGGYAWDHERRLTELPSLVENDGYFI
ENGGMALLVRAYDPTGEHAEDPIAWPYFASEDELRGLPPFVVAVNELDPLRDEGIAFARRLA
RAGVDVAARVNIGLVHGADVIFRHWLPAALESTVRDVAGFAADRARLR
>d1evqa_ c.69.1.2 (A:) Carboxylesterase {Alicyclobacillus acidocaldarius}
LDPVIQQVLDQLNRMPAPDYKHLSAQQFRSQQSLFPPVKKEPVAEVREFDMDLPGRTLKVRM
YRPEGVEPPYPALVYYHGGGWVVGDLETHDPVCRVLAKDGRAVVFSVDYRLAPEHKFPAAVE
DAYDALQWIAERAADFHLDPARIAVGGDSAGGNLAAVTSILAKERGGPALAFQLLIYPSTGY
DPAHPPASIEENAEGYLLTGGMMLWFRDQYLNSLEELTHPWFSPVLYPDLSGLPPAYIATAQ
YDPLRDVGKLYAEALNKAGVKVEIENFEDLIHGFAQFYSLSPGATKALVRIAEKLRDALA

>d1jjia_ c.69.1.2 (A:) Carboxylesterase {Archaeon Archaeoglobus fulgidus}
MLDMPIDPVYYQLAEYFDSLPKFDQFSSAREYREAINRIYEERNRQLSQHERVERVEDRTIK
GRNGDIRVRVYQQKPDSPVLVYYHGGGFVICSIESHDALCRRIARLSNSTVVSVDYRLAPEH
KFPAAVYDCYDATKWVAENAEELRIDPSKIFVGGDSAGGNLAAAVSIMARDSGEDFIKHQIL
IYPVVNFVAPTPSLLEFGEGLWILDQKIMSWFSEQYFSREEDKFNPLASVIFADLENLPPAL
IITAEYDPLRDEGEVFGQMLRRAGVEASIVRYRGVLHGFINYYPVLKAARDAINQIAALLVF
D
>d1jjfa_ c.69.1.2 (A:) Feruloyl esterase domain of the cellulosomal xylanase z {Clostridium thermocellum}
SLPTMPPSGYDQVRNGVPRGQVVNISYFSTATNSTRPARVYLPPGYSKDKKYSVLYLLHGIG
GSENDWFEGGGRANVIADNLIAEGKIKPLIIVTPNTNAAGPGIADGYENFTKDLLNSLIPYI
ESNYSVYTDREHRAIAGLSMGGGQSFNIGLTNLDKFAYIGPISAAPNTYPNERLFPDGGKAA
REKLKLLFIACGTNDSLIGFGQRVHEYCVANNINHVYWLIQGGGHDFNVWKPGLWNFLQMAD
EAGLTRD
>d1gkla_ c.69.1.2 (A:) Feruloyl esterase domain of the cellulosomal xylanase y {Clostridium thermocellum}
SFKYESAVQYRPAPDSYLNPCPQAGRIVKETYTGINGTKSLNVYLPYGYDPNKKYNIFYLMH
GGGENENTIFSNDVKLQNILDHAIMNGELEPLIVVTPTFNGGNCTAQNFYQEFRQNVIPFVE
SKYSTYAESTTPQGIAASRMHRGFGGFAMGGLTTWYVMVNCLDYVAYFMPLSGDYWYGNSPQ
DKANSIAEAINRSGLSKREYFVFAATGSEDIAYANMNPQIEAMKALPHFDYTSDFSKGNFYF
LVAPGATHWWGYVRHYIYDALPYFFHELEHHHHHH
>d1f0na_ c.69.1.3 (A:) Antigen 85b {Mycobacterium tuberculosis}
SRPGLPVEYLQVPSPSMGRDIKVQFQSGGNNSPAVYLLDGLRAQDDYNGWDINTPAFEWYYQ
SGLSIVMPVGGQSSFYSDWYSPACGKAGCQTYKWETFLTSELPQWLSANRAVKPTGSAAIGL
SMAGSSAMILAAYHPQQFIYAGSLSALLDPSQGMGPSLIGLAMGDAGGYKAADMWGPSSDPA
WERNDPTQQIPKLVANNTRLWVYCGNGTPNELGGANIPAEFLENFVRSSNLKFQDAYNAAGG
HNAVFNFPPNGTHSWEYWGAQLNAMKGDLQSSLGAG
>d1dqza_ c.69.1.3 (A:) Antigen 85c {Mycobacterium tuberculosis}
RPGLPVEYLQVPSASMGRDIKVQFQGGGPHAVYLLDGLRAQDDYNGWDINTPAFEEYYQSGL
SVIMPVGGQSSFYTDWYQPSQSNGQNYTYKWETFLTREMPAWLQANKGVSPTGNAAVGLSMS
GGSALILAAYYPQQFPYAASLSGFLNPSESWWPTLIGLAMNDSGGYNANSMWGPSSDPAWKR
NDPMVQIPRLVANNTRIWVYCGNGTPSDLGGDNIPAKFLEGLTLRTNQTFRDTYAADGGRNG
VFNFPPNGTHSWPYWNEQLVAMKADIQHVLNG
>d1ju3a2 c.69.1.21 (A:5-351) Bacterial cocaine esterase N-terminal domain {Rhodococcus sp. mb1}
NYSVASNVMVPMRDGVRLAVDLYRPDADGPVPVLLVRNPYDKFDVFAWSTQSTNWLEFVRDG
YAVVIQDTRGLFASEGEFVPHVDDEADAEDTLSWILEQAWCDGNVGMFGVSYLGVTQWQAAV
SGVGGLKAIAPSMASADLYRAPWYGPGGALSVEALLGWSALIGTGLITSRSDARPEDAADFV
QLAAILNDVAGAASVTPLAEQPLLGRLIPWVIDQVVDHPDNDESWQSISLFERLGGLATPAL
ITAGWYDGFVGESLRTFVAVKDNADARLVVGPWSHSNLTGRNADRKFGIAATYPIQEATTMH
KAFFDRHLRGETDALAGVPKVRLFVMGIDEWRDETDW
>d1qfma2 c.69.1.4 (A:431-710) Prolyl oligopeptidase, C-terminal domain {Pig (Sus scrofa)}

DASDYQTVQIFYPSKDGTKIPMFIVHKKGIKLDGSHPAFLYGYGGFNISITPNYSVSRLIFV
RHMGGVLAVANIRGGGEYGETWHKGGILANKQNCFDDFQCAAEYLIKEGYTSPKRLTINGGS
NGGLLVATCANQRPDLFGCVIAQVGVMDMLKFHKYTIGHAWTTDYGCSDSKQHFEWLIKYSP
LHNVKLPEADDIQYPSMLLLTADHDDRVVPLHSLKFIATLQYIVGRSRKQNNPLLIHVDTKA
GHGAGKPTAKVIEEVSDMFAFIARCLNIDWIP
>g1wht.1 c.69.1.5 (A:,B:) Serine carboxypeptidase II {Wheat (Triticum vulgaris)}
GHAADRIARLPGQPAVDFDMYSGYITVDEGAGRSLFYLLQEAPEDAQPAPLVLWLNGGPGCS
SVAYGASEELGAFRVKPRGAGLVLNEYRWNKVANVLFLDSPAGVGFSYTNTSSDIYTSGDNR
TAHDSYAFLAKWFERFPHYKYRDFYIAGESYAGHYVPELSQLVHRSKNPVINLKGFMVGNGL
IDDYHDYVGTFEFWWNHGIVSDDTYRRLKEACLHDSFIHPSPACDAATDVATAEQGNIDMYS
LYTPVCNIXSYDPCTERYSTAYYNRRDVQMALHANVTGAMNYTWATCSDTINTHWHDAPRSM
LPIYRELIAAGLRIWVFSGDTDAVVPLTATRYSIGALGLPTTTSWYPWYDDQEVGGWSQVYK
GLTLVSVRGAGHEVPLHRPRQALVLFQYFLQGKPMPGQ
>d1cpy__ c.69.1.5 (-) Serine carboxypeptidase II {Baker's yeast (Saccharomyces cerevisiae)}
KIKDPKILGIDPNVTQYTGYLDVEDEDKHFFFWTFESRNDPAKDPVILWLNGGPGCSSLTGL
FFALGPSSIGPDLKPIGNPYSWNSNATVIFLDQPVNVGFSYSGSSGVSNTVAAGKDVYNFLE
LFFDQFPEYVNKGQDFHIAGASYAGHYIPVFASEILSHKDRNFNLTSVLIGNGLTDPLTQYN
YYEPMACGEGGEPSVLPSEECSAMEDSLERCLGLIESCYDSQSVWSCVPATIYCNNAQLAPY
QRTGRNVYDIRKDCEGGNLCYPTLQDIDDYLNQDYVKEAVGAEVDHYESCNFDINRNFLFAG
DWMKPYHTAVTDLLNQDLPILVYAGDKDFICNWLGNKAWTDVLPWKYDEEFASQKVRNWTAS
ITDEVAGEVKSYKHFTYLRVFNGGHMVPFDVPENALSMVNEWIHGGFSL
>d1ac5__ c.69.1.5 (-) Serine carboxypeptidase II {Baker's yeast (Saccharomyces cerevisiae), kex1(delta)p}
LPSSEEYKVAYELLPGLSEVPDPSNIPQMHAGHIPLRSEDADEQDSSDLEYFFWKFTNNDSN
GNVDRPLIIWLNGGPGCSSMDGALVESGPFRVNSDGKLYLNEGSWISKGDLLFIDQPTGTGF
SVEQNKDEGKIDKNKFDEDLEDVTKHFMDFLENYFKIFPEDLTRKIILSGESYAGQYIPFFA
NAILNHNKFSKIDGDTYDLKALLIGNGWIDPNTQSLSYLPFAMEKKLIDESNPNFKHLTNAH
ENCQNLINSASTDEAAHFSYQECENILNLLLSYTRESSQKGTADCLNMYNFNLKDSYPSCGM
NWPKDISFVSKFFSTPGVIDSLHLDSDKIDHWKECTNSVGTKLSNPISKPSIHLLPGLLESG
IEIVLFNGDKDLICNNKGVLDTIDNLKWGGIKGFSDDAVSFDWIHKSKSTDDSEEFSGYVKY
DRNLTFVSVYNASHMVPFDKSLVSRGIVDIYSNDVMIIDNNGKNVMITT
>d1ivya_ c.69.1.5 (A:) Human 'protective protein', HPP {Human (Homo sapiens)}
APDQDEIQRLPGLAKQPSFRQYSGYLKSSGSKHLHYWFVESQKDPENSPVVLWLNGGPGCSS
LDGLLTEHGPFLVQPDGVTLEYNPYSWNLIANVLYLESPAGVGFSYSDDKFYATNDTEVAQS
NFEALQDFFRLFPEYKNNKLFLTGESYAGIYIPTLAVLVMQDPSMNLQGLAVGNGLSSYEQN
DNSLVYFAYYHGLLGNRLWSSLQTHCCSQNKCNFYDNKDLECVTNLQEVARIVGNSGLNIYN
LYAPCAGGVPSHFRYEKDTVVVQDLGNIFTRLPLKRMWHQALLRSGDKVRMDPPCTNTTAAS
TYLNNPYVRKALNIPEQLPQWDMCNFLVNLQYRRLYRSMNSQYLKLLSSQKYQILLYNGDVD
MACNFMGDEWFVDSLNQKMEVQRRPWLVKYGDSGEQIAGFVKEFSHIAFLTIKGAGHMVPTD
KPLAAFTMFSRFLNKQPY
>d1hlga_ c.69.1.6 (A:) Gastric lipase {Human (Homo sapiens)}

SPEVTMNISQMITYWGYPNEEYEVVTEDGYILEVNRIPYGKKNSGNTGQRPVVFLQHGLLAS
ATNWISNLPNNSLAFILADAGYDVWLGNSRGNTWARRNLYYSPDSVEFWAFSFDEMAKYDLP
ATIDFIVKKTGQKQLHYVGHSQGTTIGFIAFSTNPSLAKRIKTFYALAPVATVKYTKSLINK
LRFVPQSLFKFIFGDKIFYPHNFFDQFLATEVCSREMLNLLCSNALFIICGFDSKNFNTSRL
DVYLSHNPAGTSVQNMFHWTQAVKSGKFQAYDWGSPVQNRMHYDQSQPPYYNVTAMNVPIAV
WNGGKDLLADPQDVGLLLPKLPNLIYHKEIPFYNHLDFIWAMDAPQEVYNDIVSMISEDKK
>d1azwa_ c.69.1.7 (A:) Proline iminopeptidase {Xanthomonas campestris, pv. citri}
MRTLYPEITPYQQGSLKVDDRHTLYFEQCGNPHGKPVVMLHGGPGGGCNDKMRRFHDPAKYR
IVLFDQRGSGRSTPHADLVDNTTWDLVADIERLRTHLGVDRWQVFGGSWGSTLALAYAQTHP
QQVTELVLRGIFLLRRFELEWFYQEGASRLFPDAWEHYLNAIPPVERADLMSAFHRRLTSDD
EATRLAAAKAWSVWEGATSFLHVDEDFVTGHEDAHFALAFARIENHYFVNGGFFEVEDQLLR
DAHRIADIPGVIVHGRYDVVCPLQSAWDLHKAWPKAQLQISPASGHSAFEPENVDALVRATD
GFA
>d1qtra_ c.69.1.7 (A:) Proline iminopeptidase {Serratia marcescens}
LRGLYPPLAAYDSGWLDTGDGHRIYWELSGNPNGKPAVFIHGGPGGGISPHHRQLFDPERYK
VLLFDQRGCGRSRPHASLDNNTTWHLVADIERLREMAGVEQWLVFGGSWGSTLALAYAQTHP
ERVSEMVLRGIFTLRKQRLHWYYQDGASRFFPEKWERVLSILSDDERKDVIAAYRQRLTSAD
PQVQLEAAKLWSVWEGETVTLLPSRESASFGEDDFALAFARIENHYFTHLGFLESDDQLLRN
VPLIRHIPAVIVHGRYDMACQVQNAWDLAKAWPEAELHIVEGAGHSYDEPGILHQLMIATDR
FAGK
>d1b6g__ c.69.1.8 (-) Haloalkane dehalogenase {Xanthobacter autotrophicus}
MVNAIRTPDQRFSNLDQYPFSPNYLDDLPGYPGLRAHYLDEGNSDAEDVFLCLHGEPTWSYL
YRKMIPVFAESGARVIAPDFFGFGKSDKPVDEEDYTFEFHRNFLLALIERLDLRNITLVVQD
WGGFLGLTLPMADPSRFKRLIIMNACLMTDPVTQPAFSAFVTQPADGFTAWKYDLVTPSDLR
LDQFMKRWAPTLTEAEASAYAAPFPDTSYQAGVRKFPKMVAQRDQACIDISTEAISFWQNDW
NGQTFMAIGMKDKLLGPDVMYPMKALINGCPEPLEIADAGHFVQEFGEQVAREALKHFAETE
>d1bn7a_ c.69.1.8 (A:) Haloalkane dehalogenase {Rhodococcus sp.}
IGTGFPFDPHYVEVLGERMHYVDVGPRDGTPVLFLHGNPTSSYLWRNIIPHVAPSHRCIAPD
LIGMGKSDKPDLDYFFDDHVRYLDAFIEALGLEEVVLVIHDWGSALGFHWAKRNPERVKGIA
CMEFIRPIPTWDEWPEFARETFQAFRTADVGRELIIDQNAFIEGVLPKCVVRPLTEVEMDHY
REPFLKPVDREPLWRFPNEIPIAGEPANIVALVEAYMNWLHQSPVPKLLFWGTPGVLIPPAE
AARLAESLPNCKTVDIGPGLHYLQEDNPDLIGSEIARWLPGLA
>d1cv2a_ c.69.1.8 (A:) Haloalkane dehalogenase {Sphingomonas paucimobilis, UT26, LinB}
GAKPFGEKKFIEIKGRRMAYIDEGTGDPILFQHGNPTSSYLWRNIMPHCAGLGRLIACDLIG
MGDSDKLDPSGPERYAYAEHRDYLDALWEALDLGDRVVLVVHDWGSALGFDWARRHRERVQG
IAYMEAIAMPIEWADFPEQDRDLFQAFRSQAGEELVLQDNVFVEQVLPGLILRPLSEAEMAA
YREPFLAAGEARRPTLSWPRQIPIAGTPADVVAIARDYAGWLSESPIPKLFINAEPGALTTG
RMRDFCRTWPNQTEITVAGAHFIQEDSPDEIGAAIAAFVRRLRPA
>d1din__ c.69.1.9 (-) Dienelactone hydrolase {Pseudomonas sp., B13}

MLTEGISIQSYDGHTFGALVGSPAKAPAPVIVIAQEIFGVNAFMRETVSWLVDQGYAAVCPD
LYARQAPGTALDPQDERQREQAYKLWQAFDMEAGVGDLEAAIRYARHQPYSNGKVGLVGYAL
GGALAFLVAAKGYVDRAVGYYGVGLEKQLNKVPEVKHPALFHMGGQDHFVPAPSRQLITEGF
GANPLLQVHWYEEAGHSFARTSSSGYVASAAALANERTLDFLAPLQS
>d1c4xa_ c.69.1.10 (A:)
2-hydroxy-6-oxo-6-phenylhexa-2,4-dienoate hydrolase (BPHD)
{Rhodococcus sp., strain rha1}
TVEIIEKRFPSGTLASHALVAGDPQSPAVVLLHGAGPGAHAASNWRPIIPDLAENFFVVAPD
LIGFGQSEYPETYPGHIMSWVGMRVEQILGLMNHFGIEKSHIVGNSMGGAVTLQLVVEAPER
FDKVALMGSVGAPMNARPPELARLLAFYADPRLTPYRELIHSFVYDPENFPGMEEIVKSRFE
VANDPEVRRIQEVMFESMKAGMESLVIPPATLGRLPHDVLVFHGRQDRIVPLDTSLYLTKHL
KHAELVVLDRCGHWAQLERWDAMGPMLMEHFRA
>d1ek1a2 c.69.1.11 (A:226-544) Mammalian epoxide hydrolase,
C-terminal domain {Mouse (Mus musculus)}
LPVPCNPNDVSHGYVTVKPGIRLHFVEMGSGPALCLCHGFPESWFSWRYQIPALAQAGFRVL
AIDMKGYGDSSSPPEIEEYAMELLCKEMVTFLDKLGIPQAVFIGHDWAGVMVWNMALFYPER
VRAVASLNTPFMPPDPDVSPMKVIRSIPVFNYQLYFQEPGVAEAELEKNMSRTFKSFFRASD
ETGFIAVHKATEIGGILVNTPEDPNLSKITTEEEIEFYIQQFKKTGFRGPLNWYRNTERNWK
WSCKGLGRKILVPALMVTAEKDIVLRPEMSKNMEKWIPFLKRGHIEDCGHWTQIEKPTEVNQ
ILIKWLQTE
>d1ehya_ c.69.1.11 (A:) Bacterial epoxide hydrolase {Agrobacterium
radiobacter}
AIRRPEDFKHYEVQLPDVKIHYVREGAGPTLLLLHGWPGFWWEWSKVIGPLAEHYDVIVPDL
RGFGDSEKPDLNDLSKYSLDKAADDQAALLDALGIEKAYVVGHDFAAIVLHKFIRKYSDRVI
KAAIFDPIQPDFGPVYFGLGHVHESWYSQFHQLDMAVEVVGSSREVCKKYFKHFFDHWSYRD
ELLTEEELEVHVDNCMKPDNIHGGFNYYRANIRPDAALWTDLDHTMSDLPVTMIWGLGDTCV
PYAPLIEFVPKYYSNYTMETIEDCGHFLMVEKPEIAIDRIKTAFR
>d1qo7a_ c.69.1.11 (A:) Bacterial epoxide hydrolase {Aspergillus
niger}
KAFAKFPSSASISPNPFTVSIPDEQLDDLKTLVRLSKIAPPTYESLQADGRFGITSEWLTTM
REKWLSEFDWRPFEARLNSFPQFTTEIEGLTIHFAALFSEREDAVPIALLHGWPGSFVEFYP
ILQLFREEYTPETLPFHLVVPSLPGYTFSSGPPLDKDFGLMDNARVVDQLMKDLGFGSGYII
QGGDIGSFVGRLLGVGFDACKAVHLNLCAMRAPPEGPSIESLSAAEKEGIARMEKFMTDGLA
YAMEHSTRPSTIGHVLSSSPIALLAWIGEKYLQWVDKPLPSETILEMVSLYWLTESFPRAIH
TYRETTPTASAPNGATMLQKELYIHKPFGFSFFPKDLCPVPRSWIATTGNLVFFRDHAEGGH
FAALERPRELKTDLTAFVEQVW
>d1brt__ c.69.1.12 (-) Bromoperoxidase A2 {Streptomyces
aureofaciens}
PFITVGQENSTSIDLYYEDHGTGQPVVLIHGFPLSGHSWERQSAALLDAGYRVITYDRRGFG
QSSQPTTGYDYDTFAADLNTVLETLDLQDAVLVGFSTGTGEVARYVSSYGTARIAKVAFLAS
LEPFLLKTDDNPDGAAPQEFFDGIVAAVKADRYAFYTGFFNDFYNLDENLGTRISEEAVRNS
WNTAASGGFFAAAAAPTTWYTDFRADIPRIDVPALILHGTGDRTLPIENTARVFHKALPSAE
YVEVEGAPHGLLWTHAEEVNTALLAFLAK
>d1a8q__ c.69.1.12 (-) Bromoperoxidase A1 {Streptomyces

aureofaciens}
PICTTRDGVEIFYKDWGQGRPVVFIHGWPLNGDAWQDQLKAVVDAGYRGIAHDRRGHGHSTP
VWDGYDFDTFADDLNDLLTDLDLRDVTLVAHSMGGGELARYVGRHGTGRLRSAVLLSAIPPV
MIKSDKNPDGVPDEVFDALKNGVLTERSQFWKDTAEGFFSANRPGNKVTQGNKDAFWYMAMA
QTIEGGVRCVDAFGYTDFTEDLKKFDIPTLVVHGDDDQVVPIDATGRKSAQIIPNAELKVYE
GSSHGIAMVPGDKEKFNRDLLEFLNK
>d1a88a_ c.69.1.12 (A:) Chloroperoxidase L {Streptomyces lividans}
GTVTTSDGTNIFYKDWGPRDGLPVVFHHGWPLSADDWDNQMLFFLSHGYRVIAHDRRGHGRS
DQPSTGHDMDTYAADVAALTEALDLRGAVHIGHSTGGGEVARYVARAEPGRVAKAVLVSAVP
PVMVKSDTNPDGLPLEVFDEFRAALAANRAQFYIDVPSGPFYGFNREGATVSQGLIDHWWLQ
GMMGAANAHYECIAAFSETDFTDDLKRIDVPVLVAHGTDDQVVPYADAAPKSAELLANATLK
SYEGLPHGMLSTHPEVLNPDLLAFVKS
>d1a8s__ c.69.1.12 (-) Chloroperoxidase F {Pseudomonas fluorescens}
TTFTTRDGTQIYYKDWGSGQPIVFSHGWPLNADSWESQMIFLAAQGYRVIAHDRRGHGRSSQ
PWSGNDMDTYADDLAQLIEHLDLRDAVLFGFSTGGGEVARYIGRHGTARVAKAGLISAVPPL
MLKTEANPGGLPMEVFDGIRQASLADRSQLYKDLASGPFFGFNQPGAKSSAGMVDWFWLQGM
AAGHKNAYDCIKAFSETDFTEDLKKIDVPTLVVHGDADQVVPIEASGIASAALVKGSTLKIY
SGAPHGLTDTHKDQLNADLLAFIKG
>d1thta_ c.69.1.13 (A:) Myristoyl-ACP-specific thioesterase {Vibrio harveyi}
QCKTIAHVLRVNNGQELHVWETPPKENVPFKNNTILIASGFARRMDHFAGLAEYLSTNGFHV
FRYDSLHHVGLSSGSIDEFTMTTGKNSLCTVYHWLQTKGTQNIGLIAASLSARVAYEVISDL
ELSFLITAVGVVNLRDTLEKALGFDYLSLPIDELPNDLDFEGHKLGSEVFVRDCFEHHWDTL
DSTLDKVANTSVPLIAFTANNDDWVKQEEVYDMLAHIRTGHCKLYSLLGSSHDLGENLVVLR
NFYQSVTKAAIAMDGGSLEIDVDFIEPDFEQLTIATVNERRLKAEIENRTPEMA
>d1ei9a_ c.69.1.13 (A:) Palmitoyl protein thioesterase 1 {Cow (Bos taurus)}
DPPAPLPLVIWHGMGDSCCNPLSMGAIKKMVEKKIPGIHVLSLEIGKTLREDVENSFFLNVN
SQVTTVCQILAKDPKLQQGYNAMGFSQGGQFLRAVAQRCPSPPMVNLISVGGQHQGVFGLPR
CPGESSHICDFIRKTLNAGAYNKAIQERLVQAEYWHDPIREDIYRNHSIFLADINQERGVNE
SYKKNLMALKKFVMVKFLNDTIVDPVDSEWFGFYRSGQAKETIPLQESTLYTQDRLGLKAMD
KAGQLVFLALEGDHLQLSEEWFYAHIIPFLE
>d1auoa_ c.69.1.14 (A:) Carboxylesterase {Pseudomonas fluorescens}
MTEPLILQPAKPADACVIWLHGLGADRYDFMPVAEALQESLLTTRFVLPQAPTRPVTINGGY
EMPSWYDIKAMSPARSISLEELEVSAKMVTDLIEAQKRTGIDASRIFLAGFSQGGAVVFHTA
FINWQGPLGGVIALSTYAPTFGDELELSASQQRIPALCLHGQYDDVVQNAMGRSAFEHLKSR
GVTVTWQEYPMGHEVLPQEIHDIGAWLAARLG
>d1fj2a_ c.69.1.14 (A:) Acyl protein thioesterase 1 {Human (Homo sapiens)}
MDPEFMSTPLPAIVPAARKATAAVIFLHGLGDTGHGWAEAFAGIRSSHIKYICPHAPVRPVT
LNMNVAMPSWFDIIGLSPDSQEDESGIKQAAENIKALIDQEVKNGIPSNRIILGGFSQGGAL
SLYTALTTQQKLAGVTALSCWLPLRASFPQGPIGGANRDISILQCHGDCDPLVPLMFGSLTV

EKLKTLVNPANVTFKTYEGMMHSSCQQEMMDVKQFIDKLLPPI
>d1qlwa_ c.69.1.15 (A:) A novel bacterial esterase {Alcaligenes sp.}
VPKTPAGPLTLSGQGSFFVGGRDVTSETLSLSPKYDAHGTVTVDQMYVRYQIPQRAKRYPIT
LIHGCCLTGMTWETTPDGRMGWDEYFLRKGYSTYVIDQSGRGRSATDISAINAVKLGKAPAS
SLPDLFAAGHEAAWAIFRFGPRYPDAFKDTQFPVQAQAELWQQMVPDWLGSMPTPNPTVANL
SKLAIKLDGTVLLSHSQSGIYPFQTAAMNPKGITAIVSVEPGECPKPEDVKPLTSIPVLVVF
GDHIEEFPRWAPRLKACHAFIDALNAAGGKGQLMSLPALGVHGNSHMMMQDRNNLQVADLIL
DWIGRNTA
>d1jfra_ c.69.1.16 (A:) Lipase {Streptomyces exfoliatus}
NPYERGPAPTNASIEASRGPYATSQTSVSSLVASGFGGGTIYYPTSTADGTFGAVVISPGFT
AYQSSIAWLGPRLASQGFVVFTIDTNTTLDQPDSRGRQLLSALDYLTQRSSVRTRVDATRLG
VMGHSMGGGGSLEAAKSRTSLKAAIPLTGWNTDKTWPELRTPTLVVGADGDTVAPVATHSKP
FYESLPGSLDKAYLELRGASHFTPNTSDTTIAKYSISWLKRFIDSDTRYEQFLCPIPRPSLT
IAEYRGTCPHTS
>d1tca__ c.69.1.17 (-) Triacylglycerol lipase {Yeast (Candida antarctica), form b}
LPSGSDPAFSQPKSVLDAGLTCQGASPSSVSKPILLVPGTGTTGPQSFDSNWIPLSTQLGYT
PCWISPPPFMLNDTQVNTEYMVNAITALYAGSGNNKLPVLTWSQGGLVAQWGLTFFPSIRSK
VDRLMAFAPDYKGTVLAGPLDALAVSAPSVWQQTTGSALTTALRNAGGLTQIVPTTNLYSAT
DEIVQPQVSNSPLDSSYLFNGKNVQAQAVCGPLFVIDHAGSLTSQFSYVVGRSALRSTTGQA
RSADYGITDCNPLPANDLTPEQKVAAAALLAPAAAAIVAGPKQNCEPDLMPYARPFAVGKRT
CSGIVTP
>d3tgl__ c.69.1.17 (-) Triacylglycerol lipase {Rhizomucor miehei}
GIRAATSQEINELTYYTTLSANSYCRTVIPGATWDCIHCDATEDLKIIKTWSTLIYDTNAMV
ARGDSEKTIYIVFRGSSSIRNWIADLTFVPVSYPPVSGTKVHKGFLDSYGEVQNELVATVLD
QFKQYPSYKVAVTGHSLGGATVLLCALDLYQREEGLSSSNLFLYTQGQPRVGDPAFANYVVS
TGIPYRRTVNERDIVPHLPPAAFGFLHAGEEYWITDNSPETVQVCTSDLETSDCSNSIVPFT
SVLDHLSYFGINTGLCT
>d1tia__ c.69.1.17 (-) Triacylglycerol lipase {Penicillium camembertii}
DVSTSELDQFEFWVQYAAASYYEADYTAQVGDKLSCSKGNCPEVEATGATVSYDFSDSTITD
TAGYIAVDHTNSAVVLAFRGSYSVRNWVADATFVHTNPGLCDGCLAELGFWSSWKLVRDDII
KELKEVVAQNPNYELVVVGHSLGAAVATLAATDLRGKGYPSAKLYAYASPRVGNAALAKYIT
AQGNNFRFTHTNDPVPKLPLLSMGYVHVSPEYWITSPNNATVSTSDIKVIDGDVSFDGNTGT
GLPLLTDFEAHIWYFVQVDAGKG
>d1tib__ c.69.1.17 (-) Triacylglycerol lipase {Thermomyces (Humicola) lanuginosa}
EVSQDLFNQFNLFAQYSAAAYCGKNNDAPAGTNITCTGNACPEVEKADATFLYSFEDSGVGD
VTGFLALDNTNKLIVLSFRGSRSIENWIGNLNFDLKEINDICSGCRGHDGFTSSWRSVADTL
RQKVEDAVREHPDYRVVFTGHSLGGALATVAGADLRGNGYDIDVFSYGAPRVGNRAFAEFLT
VQTGGTLYRITHTNDIVPRLPPREFGYSHSSPEYWIKSGTLVPVTRNDIVKIEGIDATGGNN
QPNIPDIPAHLWYFGLIGTCL
>d1lgya_ c.69.1.17 (A:) Triacylglycerol lipase {Rhizopus niveus}

KVVAATTAQIQEFTKYAGIAATAYCRSVVPGNKWDCVQCQKWVPDGKIITTFTSLLSDTNGY
VLRSDKQKTIYLVFRGTNSFRSAITDIVFNFSDYKPVKGAKVHAGFLSSYEQVVNDYFPVVQ
EQLTAHPTYKVIVTGHSLGGAQALLAGMDLYQREPRLSPKNLSIFTVGGPRVGNPTFAYYVE
STGIPFQRTVHKRDIVPHVPPQSFGFLHPGVESWIKSGTSNVQICTSEIETKDCSNSIVPFT
SILDHLSYFDINEGSCL
>d1thg__ c.69.1.17 (-) Type-B carboxylesterase/lipase {Fungus (Geotrichum candidum), ATCC 34614}
EAPTAVLNGNEVISGVLEGKVDTFKGIPFADPPLNDLRFKHPQPFTGSYQGLKANDFSPACM
QLDPGNSLTLLDKALGLAKVIPEEFRGPLYDMAKGTVSMNEDCLYLNVFRPAGTKPDAKLPV
MVWIYGGAFVYGSSAAYPGNSYVKESINMGQPVVFVSINYRTGPFGFLGGDAITAEGNTNAG
LHDQRKGLEWVSDNIANFGGDPDKVMIFGESAGAMSVAHQLIAYGGDNTYNGKKLFHSAILQ
SGGPLPYHDSSSVGPDISYNRFAQYAGCDTSASANDTLECLRSKSSSVLHDAQNSYDLKDLF
GLLPQFLGFGPRPDGNIIPDAAYELFRSGRYAKVPYISGNQEDEGTAFAPVALNATTTPHVK
KWLQYIFYDASEASIDRVLSLYPQTLSVGSPFRTGILNALTPQFKRVAAILSDMLFQSPRRV
MLSATKDVNRWTYLSTHLHNLVPFLGTFHGNELIFQFNVNIGPANSYLRYFISFANHHDPNV
GTNLLQWDQYTDEGKEMLEIHMTDNVMRTDDYRIEGISNFETDVNLYG
>d1lpp__ c.69.1.17 (-) Type-B carboxylesterase/lipase {Fungus (Candida rugosa), formerly Cylindracea}
APTATLANGDTITGLNAIINEAFLGIPFAEPPVGNLRFKDPVPYSGSLDGQKFTSYGPSCMQ
QNPEGTYEENLPKAALDLVMQSKVFEAVSPSSEDCLTINVVRPPGTKAGANLPVMLWIFGGG
FEVGGTSTFPPAQMITKSIAMGKPIIHVSVNYRVSSWGFLAGDEIKAEGSANAGLKDQRLGM
QWVADNIAAFGGDPTKVTIFGESAGSMSVMCHILWNDGDNTYKGKPLFRAGIMQSGAMVPSD
AVDGIYGNEIFDLLASNAGCGSASDKLACLRGVSSDTLEDATNNTPGFLAYSSLRLSYLPRP
DGVNITDDMYALVREGKYANIPVIIGDQNDEGTFFGTSSLNVTTDAQAREYFKQSFVHASDA
EIDTLMTAYPGDITQGSPFDTGILNALTPQFKRISAVLGDLGFTLARRYFLNHYTGGTKYSF
LSKQLSGLPVLGTFHSNDIVFQDYLLGSGSLIYNNAFIAFATDLDPNTAGLLVKWPEYTSSS
QSGNNLMMINALGLYTGKDNFRTAGYDALFSNPPSFFV
>d1clea_ c.69.1.17 (A:) Type-B carboxylesterase/lipase {Candida cylindracea, cholesterol esterase}
APTAKLANGDTITGLNAIINEAFLGIPFAEPPVGNLRFKDPVPYSGSLNGQKFTSYGPSCMQ
QNPEGTFEENLGKTALDLVMQSKVFQAVLPQSEDCLTINVVRPPGTKAGANLPVMLWIFGGG
FEIGSPTIFPPAQMVTKSVLMGKPIIHVAVNYRVASWGFLAGDDIKAEGSGNAGLKDQRLGM
QWVADNIAGFGGDPSKVTIFGESAGSMSVLCHLIWNDGDNTYKGKPLFRAGIMQSGAMVPSD
PVDGTYGNEIYDLFVSSAGCGSASDKLACLRSASSDTLLDATNNTPGFLAYSSLRLSYLPRP
DGKNITDDMYKLVRDGKYASVPVIIGDQNDEGTVFGLSSLNVTTNAQARAYFKQSFIHASDA
EIDTLMAAYPQDITQGSPFDTGIFNAITPQFKRISAVLGDLAFIHARRYFLNHFQGGTKYSF
LSKQLSGLPIMGTFHANDIVWQDYLLGSGSVIYNNAFIAFATDLDPNTAGLLVNWPKYTSSS
QSGNNLMMINALGLYTGKDNFRTAGYDALMTNPSSFFV
>d1i6wa_ c.69.1.18 (A:) Lipase A {Bacillus subtilis}
HNPVVMVHGIGGASFNFAGIKSYLVSQGWSRDKLYAVDFWDKTGTNYNNGPVLSRFVQKVLD
ETGAKKVDIVAHSMGGANTLYYIKNLDGGNKVANVVTLGGANRLTTGKALPGTDPNQKILYT
SIYSSADMIVMNYLSRLDGARNVQIHGVGHIGLLYSSQVNSLIKEGLNGGGQNTN
>d4lipd_ c.69.1.18 (D:) Lipase {Burkholderia cepacia (formerly Pseudomonas cepacia)}

DNYAATRYPIILVHGLTGTDKYAGVLEYWYGIQEDLQQRGATVYVANLSGFQSDDGPNGRGE
QLLAYVKTVLAATGATKVNLVGHSQGGLTSRYVAAVAPDLVASVTTIGTPHRGSEFADFVQG
VLAYDPTGLSSTVIAAFVNVFGILTSSSNNTNQDALAALKTLTTAQAATYNQNYPSAGLGAP
GSCQTGAPTETVGGNTHLLYSWAGTAIQPTISVFGVTGATDTSTIPLVDPANALDPSTLALF
GTGTVMVNRGSGQNDGVVSKCSALYGQVLSTSYKWNHLDEINQLLGVRGANAEDPVAVIRTH
ANRLKLAGV
>d1ex9a_ c.69.1.18 (A:) Lipase {Pseudomonas aeruginosa}
STYTQTKYPIVLAHGMLGFDNILGVDYWFGIPSALRRDGAQVYVTEVSQLDTSEVRGEQLLQ
QVEEIVALSGQPKVNLIGHSHGGPTIRYVAAVRPDLIASATSVGAPHKGSDTADFLRQIPPG
SAGEAVLSGLVNSLGALISFLSSGSTGTQNSLGSLESLNSEGAARFNAKYPQGIPTSACGEG
AYKVNGVSYYSWSGSSPLTNFLDPSDAFLGASSLTFKNGTANDGLVGTCSSHLGMVIRDNYR
MNHLDEVNQVFGLTSLFETSPVSVYRQHANRLKNASL
>d1cvl__ c.69.1.18 (-) Lipase {Chromobacterium viscosum}
ADTYAATRYPVILVHGLAGTDKFANVVDYWYGIQSDLQSHGAKVYVANLSGFQSDDGPNGRG
EQLLAYVKQVLAATGATKVNLIGHSQGGLTSRYVAAVAPQLVASVTTIGTPHRGSEFADFVQ
DVLKTDPTGLSSTVIAAFVNVFGTLVSSSHNTDQDALAALRTLTTAQTATYNRNFPSAGLGA
PGSCQTGAATETVGGSQHLLYSWGGTAIQPTSTVLGVTGATDTSTGTLDVANVTDPSTLALL
ATGAVMINRASGQNDGLVSRCSSLFGQVISTSYHWNHLDEINQLLGVRGANAEDPVAVIRTH
VNRLKLQGV
>d1hpla2 c.69.1.19 (A:1-336) Pancreatic lipase, N-terminal domain {Horse (Equus caballus)}
NEVCYERLGCFSDDSPWAGIVERPLKILPWSPEKVNTRFLLYTNENPDNFQEIVADPSTIQS
SNFNTGRKTRFIIHGFIDKGEESWLSTMCQNMFKVESVNCICVDWKSGSRTAYSQASQNVRI
VGAEVAYLVGVLQSSFDYSPSNVHIIGHSLGSHAAGEAGRRTNGAVGRITGLDPAEPCFQGT
PELVRLDPSDAQFVDVIHTDIAPFIPNLGFGMSQTAGHLDFFPNGGKEMPGCQKNVLSQIVD
IDGIWQGTRDFAACNHLRSYKYYTDSILNPDGFAGFSCASYSDFTANKCFPCSSEGCPQMGH
YADRFPGRTKGVGQLFYLNTGDASNFA
>d1etha2 c.69.1.19 (A:1-336) Pancreatic lipase, N-terminal domain {Pig (Sus scrofa)}
SEVCFPRLGCFSDDAPWAGIVQRPLKILPWSPKDVDTRFLLYTNQNQNNYQELVADPSTITN
SNFRMDRKTRFIIHGFIDKGEEDWLSNICKNLFKVESVNCICVDWKGGSRTGYTQASQNIRI
VGAEVAYFVEVLKSSLGYSPSNVHVIGHSLGSHAAGEAGRRTNGTIERITGLDPAEPCFQGT
PELVRLDPSDAKFVDVIHTDAAPIIPNLGFGMSQTVGHLDFFPNGGKQMPGCQKNILSQIVD
IDGIWEGTRDFVACNHLRSYKYYADSILNPDGFAGFPCDSYNVFTANKCFPCPSEGCPQMGH
YADRFPGKTNGVSQVFYLNTGDASNF
>d1lpbb2 c.69.1.19 (B:1-336) Pancreatic lipase, N-terminal domain {Human (Homo sapiens)}
KEVCYERLGCFSDDSPWSGITERPLHILPWSPKDVNTRFLLYTNENPNNFQEVAADSSSISG
SNFKTNRKTRFIIHGFIDKGEENWLANVCKNLFKVESVNCICVDWKGGSRTGYTQASQNIRI
VGAEVAYFVEFLQSAFGYSPSNVHVIGHSLGAHAAGEAGRRTNGTIGRITGLDPAEPCFQGT
PELVRLDPSDAKFVDVIHTDGAPIVPNLGFGMSQVVGHLDFFPNGGVEMPGCKKNILSQIVD
IDGIWEGTRDFAACNHLRSYKYYTDSIVNPDGFAGFPCASYNVFTANKCFPCPSGGCPQMGH
YADRYPGKTNDVGQKFYLDTGDASNFA
>d1gpl_2 c.69.1.19 (1-336) Pancreatic lipase, N-terminal domain

{Guinea pig (Cavia porcellus)}
AEVCYSHLGCFSDEKPWAGTSQRPIKSLPSDPKKINTRFLLYTNENQNSYQLITATDIATIK
ASNFNLNRKTRFIIHGFTDSGENSWLSDMCKNMFQVEKVNCICVDWKGGSKAQYSQASQNIR
VVGAEVAYLVQVLSTSLNYAPENVHIIGHSLGAHTAGEAGKRLNGLVGRITGLDPAEPYFQD
TPEEVRLDPSDAKFVDVIHTDISPILPSLGFGMSQKVGHMDFFPNGGKDMPGCKTGISCNHH
RSIEYYHSSILNPEGFLGYPCASYDEFQESGCFPCPAKGCPKMGHFADQYPGKTNAVEQTFF
LNTGASDNFT
>d1rp1_2 c.69.1.19 (1-336) Pancreatic lipase, N-terminal domain
{Dog (Canis familiaris)}
KEVCYEQIGCFSDAEPWAGTAIRPLKVLPWSPERIGTRFLLYTNKNPNNFQTLLPSDPSTIG
ASNFQTDKKTRFIIHGFIDKGEENWLLDMCKNMFKVEEVNCICVDWKKGSQTSYTQAANNVR
VVGAQVAQMLSMLSANYSYSPSQVQLIGHSLGAHVAGEAGSRTPGLGRITGLDPVEASFQGT
PEEVRLDPTDADFVDVIHTDAAPLIPFLGFGTSQQMGHLDFFPNGGEEMPGCKKNALSQIVD
LDGIWEGTRDFVACNHLRSYKYYSESILNPDGFASYPCASYRAFESNKCFPCPDQGCPQMGH
YADKFAQKYFLNTGDSSNFA
>d1bu8a2 c.69.1.19 (A:1-336) Pancreatic lipase, N-terminal domain
{Rat (Rattus norvegicus)}
KEVCYGHLGCFSNDKPWAGMLQRPLKIFPWSPEDIDTRFLLYTNENPNNYQKISATEPDTIK
FSNFQLDRKTRFIVHGFIDKGEDGWLLDMCKKMFQVEKVNCICVDWRRGSRTEYTQASYNTR
VVGAEIAFLVQVLSTEMGYSPENVHLIGHSLGAHVVGEAGRRLEGHVGRITGLDPAEPCFQG
LPEEVRLDPSDAMFVDVIHTDSAPIIPYLGFGMSQKVGHLDFFPNGGKEMPGCQKNILSTIV
DINGIWEGTQNFVACNHLRSYKYYASSILNPDGFLGYPCSSYEKFQQNDCFPCPEEGCPKMG
HYADQFEGKTATVEQTVYLNTGDSGNFT
>d1qj4a_ c.69.1.20 (A:) Hydroxynitrile lyase {Rubber tree (Hevea
brasiliensis)}
AFAHFVLIHTICHGAWIWHKLKPLLEALGHKVTALDLAASGVDPRQIEEIGSFDEYSEPLLT
FLEALPPGEKVILVGESCGGLNIAIAADKYCEKIAAAVFHNSVLPDTEHCPSYVVDKLMEVF
PDWKDTTYFTYTKDGKEITGLKLGFTLLRENLYTLCGPEEYELAKMLTRKGSLFQNILAKRP
FFTKEGYGSIKKIYVWTDQDEIFLPEFQLWQIENYKPDKVYKVEGGDHKLQLTKTKEIAEIL
QEVADTYN
>d1e89a_ c.69.1.20 (A:) Hydroxynitrile lyase {Cassava (Manihot
esculenta)}
PISKMVTAHFVLIHTICHGAWIWHKLKPALERAGHKVTALDMAASGIDPRQIEQINSFDEYS
EPLLTFLEKLPQGEKVIIVGEACAGLNIAIAADRYVDKIAAGVFHNSLLPDTVHSPSYTVEK
LLESFPDWRDTEYFTFTNITGETITTMKLGFVLLRENLFTKCTDGEYELAKMVMRKGSLFQN
VLAQRPKFTEKGYGSIKKVYIWTDQDKIFLPDFQRWQIANYKPDKVYQVQGGDHKLQLTKTE
EVAHILQEVADAYA
>d1keza_ c.69.1.22 (A:) Erythromycin polyketide synthase
{Saccharopolyspora erythraea}
SSALRDGYRQAGVSGRVRSYLDLLAGLSDFREHFDGSDGFSLDLVDMADGPGEVTVICCAGT
AAISGPHEFTRLAGALRGIAPVRAVPQPGYEEGEPLPSSMAAVAAVQADAVIRTQGDKPFVV
AGHSAGALMAYALATELLDRGHPPRGVVLIDVYPPGHQDAMNAWLEELTATLFDRETVRMDD
TRLTALGAYDRLTGQWRPRETGLPTLLVSAGEPMGPWPDDSWKPTWPFEHDTVAVPGDHFTM
VQEHADAIARHIDAWLGGG

>d2masa_ c.70.1.1 (A:) Inosine-uridine nucleoside N-ribohydrolase, IU-NH {Crithidia fasciculata}
AKKIILDCDPGLDDAVAILLAHGNPEIELLAITTVVGNQTLAKVTRNAQLVADIAGITGVPI
AAGCDKPLVRKIMTAGHIHGESGMGTVAYPAEFKNKVDERHAVNLIIDLVMSHEPKTITLVP
TGGLTNIAMAARLEPRIVDRVKEVVLMGGGYHEGNATSVAEFNIIIDPEAAHIVFNESWQVT
MVGLDLTHQALATPPILQRVKEVDTNPARFMLEIMDYYTKIYQSNRYMAAAAVHDPCAVAYV
IDPSVMTTERVPVDIELTGKLTLGMTVADFRNPRPEHCHTQVAVKLDFEKFWGLVLDALERI
GDP
>d1ezra_ c.70.1.1 (A:) Nucleoside hydrolase {Leishmania major}
PRKIILDCDPGIDDAVAIFLAHGNPEIELLAITTVVGNQSLEKVTQNARLVADVAGIVGVPV
AAGCTKPLVRGVRNASHIHGETGMGNVSYPPEFKTKLDGRHAVQLIIDLIMSHEPKTITLVP
TGGLTNIAMAVRLEPRIVDRVKEVVLMGGGYHTGNASPVAEFNVFIDPEAAHIVFNESWNVT
MVGLDLTHLALATPAVQKRVREVGTKPAAFMLQILDFYTKVYEKEHDTYGKVHDPCAVAYVI
DPTVMTTERVPVDIELNGALTTGMTVADFRYPRPKNCRTQVAVKLDFDKFWCLVIDALERIG
DP
>d1hoza_ c.70.1.1 (A:) Inosine-adenosine-guanosine preferring nucleoside hydrolase {Trypanosoma vivax}
GSAKNVVLDHDGNLDDFVAMVLLASNTEKVRLIGALCTDADCFVENGFNVTGKIMCLMHNNM
NLPLFPIGKSAATAVNPFPKEWRCLAKNMDDMPILNIPENVELWDKIKAENEKYEGQQLLAD
LVMNSEEKVTICVTGPLSNVAWCIDKYGEKFTSKVEECVIMGGAVDVRGNVFLPSTDGTAEW
NIYWDPASAKTVFGCPGLRRIMFSLDSTNTVPVRSPYVQRFGEQTNFLLSILVGTMWAMCTH
CELLRDGDGYYAWDALTAAYVVDQKVANVDPVPIDVVVDKQPNEGATVRTDAENYPLTFVAR
NPEAEFFLDMLLRSARAC
>d1ra9__ c.71.1.1 (-) Dihydrofolate reductase, prokaryotic type {Escherichia coli}
MISLIAALAVDRVIGMENAMPWNLPADLAWFKRNTLDKPVIMGRHTWESIGRPLPGRKNIIL
SSQPGTDDRVTWVKSVDEAIAACGDVPEIMVIGGGRVYEQFLPKAQKLYLTHIDAEVEGDTH
FPDYEPDDWESVFSEFHDADAQNSHSYCFEILERR
>d3dfr__ c.71.1.1 (-) Dihydrofolate reductase, prokaryotic type {Lactobacillus casei}
TAFLWAQNRNGLIGKDGHLPWHLPDDLHYFRAQTVGKIMVVGRRTYESFPKRPLPERTNVVL
THQEDYQAQGAVVVHDVAAVFAYAKQHLDQELVIAGGAQIFTAFKDDVDTLLVTRLAGSFEG
DTKMIPLNWDDFTKVSSRTVEDTNPALTHTYEVWQKKA
>d1df7a_ c.71.1.1 (A:) Dihydrofolate reductase, prokaryotic type {Mycobacterium tuberculosis}
MVGLIWAQATSGVIGRGGDIPWRLPEDQAHFREITMGHTIVMGRRTWDSLPAKVRPLPGRRN
VVLSRQADFMASGAEVVGSLEEALTSPETWVIGGGQVYALALPYATRCEVTEVDIGLPREAG
DALAPVLDETWRGETGEWRFSRSGLRYRLYSYHRS
>d1d1ga_ c.71.1.1 (A:) Dihydrofolate reductase, prokaryotic type {Thermotoga maritima}
AKVIFVLAMDVSGKIASSVESWSSFEDRKNFRKITTEIGNVVMGRITFEEIGRPLPERLNVV
LTRRPKTSNNPSLVFFNGSPADVVKFLEGKGYERVAVIGGKTVFTEFLREKLVDELFVTVEP
YVFGKGIPFFDEFEGYFPLKLLEMRRLNERGTLFLKYSVE
>d1vdra_ c.71.1.1 (A:) Dihydrofolate reductase, prokaryotic type

{Haloferax volcanii}
ELVSVAALAENRVIGRDGELPWPSIPADKKQYRSRIADDPVVLGRTTFESMRDDLPGSAQIV
MSRSERSFSVDTAHRAASVEEAVDIAASLDAETAYVIGGAAIYALFQPHLDRMVLSRVPGEY
EGDTYYPEWDAAEWELDAETDHEGFTLQEWVRS
>d8dfr__ c.71.1.1 (-) Dihydrofolate reductases, eukaryotic type
{Chicken (Gallus gallus)}
VRSLNSIVAVCQNMGIGKDGNLPWPPLRNEYKYFQRMTSTSHVEGKQNAVIMGKKTWFSIPE
KNRPLKDRINIVLSRELKEAPKGAHYLSKSLDDALALLDSPELKSKVDMVWIVGGTAVYKAA
MEKPINHRLFVTRILHEFESDTFFPEIDYKDFKLLTEYPGVPADIQEEDGIQYKFEVYQKSV
>d1hfq__ c.71.1.1 (-) Dihydrofolate reductases, eukaryotic type
{Human (Homo sapiens)}
VGSLNCIVAVSQNMGIGKNGDLPWPPLRNESRYFQRMTTTSSVEGKQNLVIMGKKTWFSIPE
KNRPLKGRINLVLSRELKEPPQGAHFLSRSLDDALKLTEQPELANKVDMVWIVGGSSVYKEA
MNHPGHLKLFVTRIMQDFESDTFFPEIDLEKYKLLPEYPGVLSDVQEEKGIKYKFEVYEKND
>d1dyr__ c.71.1.1 (-) Dihydrofolate reductases, eukaryotic type
{Fungus (Pneumocystis carinii)}
NQQKSLTLIVALTTSYGIGRSNSLPWKLKKEISYFKRVTSFVPTFDSFESMNVVLMGRKTWE
SIPLQFRPLKGRINVVITRNESLDLGNGIHSAKSLDHALELLYRTYGSESSVQINRIFVIGG
AQLYKAAMDHPKLDRIMATIIYKDIHCDVFFPLKFRDKEWSSVWKKEKHSDLESWVGTKVPH
GKINEDGFDYEFEMWTRDL
>d1aoea_ c.71.1.1 (A:) Dihydrofolate reductases, eukaryotic type
{Yeast (Candida albicans)}
MLKPNVAIIVAALKPALGIGYKGKMPWRLRKEIRYFKDVTTRTTKPNTRNAVIMGRKTWESI
PQKFRPLPDRLNIILSRSYENEIIDDNIIHASSIESSLNLVSDVERVFIIGGAEIYNELINN
SLVSHLLITEIEHPSPESIEMDTFLKFPLESWTKQPKSELQKFVGDTVLEDDIKEGDFTYNY
TLWTRK
>d1ekqa_ c.72.1.2 (A:) Hydroxyethylthiazole kinase (THZ kinase,
ThiK) {Bacillus subtilis}
MDAQSAAKCLTAVRRHSPLVHSITNNVVTNFTANGLLALGASPVMAYAKEEVADMAKIAGAL
VLNIGTLSKESVEAMIIAGKSANEHGVPVILDPVGAGATPFRTESARDIIREVRLAAIRGNA
AEIAHTVGVTDWLIKGVDAGEGGGDIIRLAQQAAQKLNTVIAITGEVDVIADTSHVYTLHNG
HKLLTKVTGAGCLLTSVVGAFCAVEENPLFAAIAAISSYGVAAQLAAQQTADKGPGSFQIEL
LNKLSTVTEQDVQEWATIERV
>d1jxha_ c.72.1.2 (A:)
4-amino-5-hydroxymethyl-2-methylpyrimidine phosphate kinase
(HMP-phosphate kinase, ThiD) {Salmonella typhimurium}
MQRINALTIAGTDPSGGAGIQADLKTFSALGAYGCSVITALVAENTCGVQSVYRIEPDFVAA
QLDSVFSDVRIDTTKIGMLAETDIVEAVAERLQRHHVRNVVLDTVMLAKSGDPLLSPSAIET
LRVRLLPQVSLITPNLPEAAALLDAPHARTEQEMLAQGRALLAMGCEAVLMKGGHLEDAQSP
DWLFTREGEQRFSAPRVNTKNTHGTGCTLSAALAALRPRHRSWGETVNEAKAWLSAALAQAD
TLEVGKGIGPVHHFHAWW
>d1rkd__ c.72.1.1 (-) Ribokinase {Escherichia coli}
AGSLVVLGSINADHILNLQSFPTPGETVTGNHYQVAFGGKGANQAVAAGRSGANIAFIACTG
DDSIGESVRQQLATDNIDITPVSVIKGESTGVALIFVNGEGENVIGIHAGANAALSPALVEA

QRERIANASALLMQLESPLESVMAAAKIAHQNKTIVALNPAPARELPDELLALVDIITPNET
EAEKLTGIRVENDEDAAKAAQVLHEKGIRTVLITLGSRGVWASVNGEGQRVPGFRVQAVDTI
AAGDTFNGALITALLEEKPLPEAIRFAHAAAAIAVTRKGAQPSVPWREEIDAFLDRQR
>d1bx4a_ c.72.1.1 (A:) Adenosine kinase {Human (Homo sapiens)}
VRENILFGMGNPLLDISAVVDKDFLDKYSLKPNDQILAEDKHKELFDELVKKFKVEYHAGGS
TQNSIKVAQWMIQQPHKAATFFGCIGIDKFGEILKRKAAEAHVDAHYYEQNEQPTGTCAACI
TGDNRSLIANLAAANCYKKEKHLDLEKNWMLVEKARVCYIAGFFLTVSPESVLKVAHHASEN
NRIFTLNLSAPFISQFYKESLMKVMPYVDILFGNETEAATFAREQGFETKDIKEIAKKTQAL
PKMNSKRQRIVIFTQGRDDTIMATESEVTAFAVLDQDQKEIIDTNGAGDAFVGGFLSQLVSD
KPLTECIRAGHYAASIIIRRTGCTFPEKPDFH
>d1dgya_ c.72.1.1 (A:) Adenosine kinase {Toxoplasma gondii}
GPMRVFAIGNPILDLVAEVPSSFLDEFFLKRGDATLATPEQMRIYSTLDQFNPTSLPGGSAL
NSVRVVQKLLRKPGSAGYMGAIGDDPRGQVLKELCDKEGLATRFMVAPGQSTGTCAVLINEK
ERTLCTHLGACGSFRIPENWTTFASGALIFYATAYTLTATPKNALEVAGYAHGIPNAIFTLN
LSAPFCVELYKDAMQSLLLHTNILFGNEEEFAHLAKVHNLVAAEKVALSVANKEHAVEVCTG
ALRLLTAGQNTGATKLVVMTRGHNPVIAAEQTADGTVVVHEVGVPVVAAEKIVDTNGAGDAF
VGGFLYGLSQGKTVKQCIMCGNACAQDVIQHVGFSLSF
>d1gc5a_ c.72.1.3 (A:) ADP-dependent glucokinase {Archaeon
Thermococcus litoralis}
MKESLKDRIRLWKRLYVNAFENALNAIPNVKGVLLAYNTNIDAIKYLDADDLEKRVTEKGKE
KVFEIIENPPEKISSIEELLGGILRSIKLGKAMEWFVESEEVRRYLREWGWDELRIGGQAGI
MANLLGGVYRIPTIVHVPQNPKLQAELFVDGPIYVPVFEGNKLKLVHPKDAIAEEEELIHYI
YEFPRGFQVFDVQAPRENRFIANADDYNARVYMRREFREGFEEITRNVELAIISGLQVLKEY
YPDGTTYKDVLDRVESHLNILNRYNVKSHFEFAYTANRRVREALVELLPKFTSVGLNEVELA
SIMEIIGDEELAKEVLEGHIFSVIDAMNVLMDETGIERIHFHTYGYYLALTQYRGEEVRDAL
LFASLAAAAKAMKGNLERIEQIRDALSVPTNERAIVLEEELEKEFTEFENGLIDMVDRQLAF
VPTKIVASPKSTVGIGDTISSSAFVSEFGMRKR
>d2uaga3 c.72.2.1 (A:94-297)
UDP-N-acetylmuramoyl-L-alanine:D-glutamate ligase MurD
{Escherichia coli}
DIELFCREAQAPIVAITGSNGKSTVTTLVGEMAKAAGVNVGVGGNIGLPALMLLDDECELYV
LELSSFQLETTSSLQAVAATILNVTEDHMDRYPFGLQQYRAAKLRIYENAKVCVVNADDALT
MPIRGADERCVSFGVNMGDYHLNHQQGETWLRVKGEKVLNVKEMKLSGQHNYTNALAALALA
DAAGLPRASSLKALTTFT
>d1e8ca3 c.72.2.1 (A:88-337) UDP-N-acetylmuramyl tripeptide
synthetase MurE {Escherichia coli}
QLNERLSALAGRFYHEPSDNLRLVGVTGTNGKTTTTQLLAQWSQLLGEISAVMGTVGNGLLG
KVIPTENTTGSAVDVQHELAGLVDQGATFCAMEVSSHGLVQHRVAALKFAASVFTNLSRDHL
DYHGDMEHYEAAKWLLYSEHHCGQAIINADDEVGRRWLAKLPDAVAVSMEDHINPNCHGRWL
KATEVNYHDSGATIRFSSSWGDGEIESHLMGAFNVSNLLLALATLLALGYPLADLLKTAARL
QP
>d1gg4a4 c.72.2.1 (A:82-312) UDP-murNac-tripeptide
D-alanyl-D-alanine-adding enzyme MurF {Escherichia coli}
DTRLAFGELAAWVRQQVPARVVALTGSSGKTSVKEMTAAILSQCGNTLYTAGNLNNDIGVPM

TLLRLTPEYDYAVIELGANHQGEIAWTVSLTRPEAALVNNLAAAHLEGFGSLAGVAKAKGEI
FSGLPENGIAIMNADNNDWLNWQSVIGSRKVWRFSPNAANSDFTATNIHVTSHGTEFTLQTP
TGSVDVLLPLPGRHNIANALAAAALSMSVGATLDAIKAGLANLKA
>d1jbwa2 c.72.2.2 (A:1-296) Folylpolyglutamate synthetase {Lactobacillus casei}
MNYTETVAYIHSFPRLAKTGDHRRILTLLHALGNPQQQGRYIHVTGTNGKGSAANAIAHVLE
ASGLTVGLYTSPFIMRFNERIMIDHEPIPDAALVNAVAFVRAALERLQQQQADFNVTEFEFI
TALAYWYFRQRQVDVAVIEVGIGGDTDSTNVITPVVSVLTEVALDHQKLLGHTITAIAKHKA
GIIKRGIPVVTGNLVPDAAAVVAAKVATTGSQWLRFDRDFSVPKAKLHGWGQRFTYEDQDGR
ISDLEVPLVGDYQQRNMAIAIQTAKVYAKQTEWPLTPQNIRQGLAASH
>d1jzta_ c.104.1.1 (A:) Hypothetical protein YNL200c (YNU0_YEAST) {Baker's yeast (Saccharomyces cerevisiae)}
LKVVSSKLAAEIDKELMGPQIGFTLQQLMELAGFSVAQAVCRQFPLRGKTETEKGKHVFVIA
GPGNNGGDGLVCARHLKLFGYNPVVFYPKRSERTEFYKQLVHQLNFFKVPVLSQDEGNWLEY
LKPEKTLCIVDAIFGFSFKPPMREPFKGIVEELCKVQNIIPIVSVDVPTGWDVDKGPISQPS
INPAVLVSLTVPKPCSSHIRENQTTHYVGGRFIPRDFANKFGFEPFGYESTDQILKL
>d1b7ba_ c.73.1.1 (A:) Carbamate kinase {Enterococcus faecium}
GKKMVVALGGNAILSNDASAHAQQQALVQTSAYLVHLIKQGHRLIVSHGNGPQVGNLLLQQQ
AADSEKNPAMPLDTCVAMTQGSIGYWLSNALNQELNKAGIKKQVATVLTQVVVDPADEAFKN
PTKPIGPFLTEAEAKEAMQAGAIFKEDAGRGWRKVVPSPKPIDIHEAETINTLIKNDIITIS
CGGGGIPVVGQELKGVEAVIDKDFASEKLAELVDADALVILTGVDYVCINYGKPDEKQLTNV
TVAELEEYKQAGHFAPGSMLPKIEAAIQFVESQPNKQAIITSLENLGSMSGDEIVGTVV
>d1e19a_ c.73.1.1 (A:) Carbamate kinase {Archaeon Pyrococcus furiosus}
GKRVVIALGGNALQQRGQKGSYEEMMDNVRKTARQIAEIIARGYEVVITHGNGPQVGSLLLH
MDAGQATYGIPAQPMDVAGAMSQGWIGYMIQQALKNELRKRGMEKKVVTIITQTIVDKNDPA
FQNPTKPVGPFYDEETAKRLAREKGWIVKEDSGRGWRRVVPSPDPKGHVEAETIKKLVERGV
IVIASGGGGVPVILEDGEIKGVEAVIDKDLAGEKLAEEVNADIFMILTDVNGAALYYGTEKE
QWLREVKVEELRKYYEEGHFKAGSMGPKVLAAIRFIEWGGERAIIAHLEKAVEALEGKTGTQ
VLP
>d1eqja1 c.105.1.1 (A:77-310)
2,3-Bisphosphoglycerate-independent phosphoglycerate mutase, substrate-binding domain {Bacillus stearothermophilus}
QSLTRINIAIREGEFDRNETFLAAMNHVKQHGTSLHLFGLLSDGGVHSHIHHLYALLRLAAK
EGVKRVYIHGFLDGRDVGPQTAPQYIKELQEKIKEYGVGEIATLSGRYYSMDRDKRWDRVEK
AYRAMVYGEGPTYRDPLECIEDSYKHGIYDEFVLPSVIVREDGRPVATIQDNDAIIFYNFRP
DRAIQISNTFTNEDFREFDRGPKHPKHLFFVCLTHFSETVAGYVAFKP
>d1eqja2 c.76.1.3 (A:3-76,A:311-510)
2,3-Bisphosphoglycerate-independent phosphoglycerate mutase, catalytic domain {Bacillus stearothermophilus}
KKPVALIILDGFALRDETYGNAVAQANKPNFDRYWNEYPHTTLKACGEAVGLPEGQMGNSEV
GHLNIGAGRIVYXTNLDNTIGEVLSQHGLRQLRIAETEKYPHVTFFMSGGREEEFPGEDRIL
INSPKVPTYDLKPEMSAYEVTDALLKEIEADKYDAIILNYANPDMVGHSGKLEPTIKAVEAV
DECLGKVVDAILAKGGIAIITADHGNADEVLTPDGKPQTAHTTNPVPVIVTKKGIKLRDGGI

LGDLAPTMLDLLGLPQPKEMTGKSLIV
>d1ed8a_ c.76.1.1 (A:) Alkaline phosphatase {Escherichia coli}
TPEMPVLENRAAQGDITAPGGARRLTGDQTAALRDSLSDKPAKNIILLIGDGMGDSEITAAR
NYAEGAGGFFKGIDALPLTGQYTHYALNKKTGKPDYVTDSAASATAWSTGVKTYNGALGVDI
HEKDHPTILEMAKAAGLATGNVSTAELQDATPAALVAHVTSRKCYGPSATSEKCPGNALEKG
GKGSITEQLLNARADVTLGGGAKTFAETATAGEWQGKTLREQAQARGYQLVSDAASLNSVTE
ANQQKPLLGLFADGNMPVRWLGPKATYHGNIDKPAVTCTPNPQRNDSVPTLAQMTDKAIELL
SKNEKGFFLQVEGASIDKQDHAANPCGQIGETVDLDEAVQRALEFAKKEGNTLVIVTADHAH
ASQIVAPDTKAPGLTQALNTKDGAVMVMSYGNSEEDSQEHTGSQLRIAAYGPHAANVVGLTD
QTDLFYTMKAALGLK
>d1ew2a_ c.76.1.1 (A:) Alkaline phosphatase {Human (Homo sapiens)}
IIPVEEENPDFWNREAAEALGAAKKLQPAQTAAKNLIIFLGDGMGVSTVTAARILKGQKKDK
LGPEIPLAMDRFPYVALSKTYNVDKHVPDSGATATAYLCGVKGNFQTIGLSAAARFNQCNTT
RGNEVISVMNRAKKAGKSVGVVTTTRVQHASPAGTYAHTVNRNWYSDADVPASARQEGCQDI
ATQLISNMDIDVILGGGRKYMFRMGTPDPEYPDDYSQGGTRLDGKNLVQEWLAKRQGARYVW
NRTELMQASLDPSVTHLMGLFEPGDMKYEIHRDSTLDPSLMEMTEAALRLLSRNPRGFFLFV
EGGRIDHGHHESRAYRALTETIMFDDAIERAGQLTSEEDTLSLVTADHSHVFSFGGYPLRGS
SIFGLAPGKARDRKAYTVLLYGNGPGYVLKDGARPDVTESESGSPEYRQQSAVPLDEETHAG
EDVAVFARGPQAHLVHGVQEQTFIAHVMAFAACLEPYTACDLAPP
>d1auk__ c.76.1.2 (-) Arylsulfatase A {Human (Homo sapiens)}
RPPNIVLIFADDLGYGDLGCYGHPSSTTPNLDQLAAGGLRFTDFYVPVSLCTPSRAALLTGR
LPVRMGMYPGVLVPSSRGGLPLEEVTVAEVLAARGYLTGMAGKWHLGVGPEGAFLPPHQGFH
RFLGIPYSHDQGPCQNLTCFPPATPCDGGCDQGLVPIPLLANLSVEAQPPWLPGLEARYMAF
AHDLMADAQRQDRPFFLYYASHHTHYPQFSGQSFAERSGRGPFGDSLMELDAAVGTLMTAIG
DLGLLEETLVIFTADNGPETMRMSRGGCSGLLRCGKGTTYEGGVREPALAFWPGHIAPGVTH
ELASSLDLLPTLAALAGAPLPNVTLDGFDLSPLLLGTGKSPRQSLFFYPSYPDEVRGVFAVR
TGKYKAHFFTQGSAHSDTTADPACHASSSLTAHEPPLLYDLSKDPGENYNLLGGVAGATPEV
LQALKQLQLLKAQLDAAVTFGPSQVARGEDPALQICCHPGCTPRPACCHCP
>d1fsu__ c.76.1.2 (-) Arylsulfatase B (4-sulfatase) {Human (Homo sapiens)}
SRPPHLVFLLADDLGWNDVGFHGSRIRTPHLDALAAGGVLLDNYYTQPLXTPSRSQLLTGRY
QIRTGLQHQIIWPCQPSCVPLDEKLLPQLLKEAGYTTHMVGKWHLGMYRKECLPTRRGFDTY
FGYLLGSEDYYSHERCTLIDALNVTRCALDFRDGEEVATGYKNMYSTNIFTKRAIALITNHP
PEKPLFLYLALQSVHEPLQVPEEYLKPYDFIQDKNRHHYAGMVSLMDEAVGNVTAALKSSGL
WNNTVFIFSTDNGGQTLAGGNNWPLRGRKWSLWEGGVRGVGFVASPLLKQKGVKNRELIHIS
DWLPTLVKLARGHTNGTKPLDGFDVWKTISEGSPSPRIELLHNIDPNFVDSSPCPRNSMAPA
KDDSSLPEYSAFNTSVHAAIRHGNWKLLTGYPGCGYWFPPPSQYNVSEIPSSDPPTKTLWLF
DIDRDPEERHDLSREYPHIVTKLLSRLQFYHKHSVPVYFPAQDPRCDPKATGVWGPWM
>d1hdha_ c.76.1.2 (A:) Arylsulfatase B (4-sulfatase) {Pseudomonas aeruginosa}
KRPNFLVIVADDLGFSDIGAFGGEIATPNLDALAIAGLRLTDFHTASTCSPTRSMLLTGTDH
HIAGIGTMAEALTPELEGKPGYEGHLNERVVALPELLREAGYQTLMAGKWHLGLKPEQTPHA
RGFERSFSLLPGAANHYGFEPPYDESTPRILKGTPALYVEDERYLDTLPEGFYSSDAFGDKL
LQYLKERDQSRPFFAYLPFSAPHWPLQAPREIVEKYRGRYDAGPEALRQERLARLKELGLVE

ADVEAHPVLALTREWEALEDEERAKSARAMEVYAAMVERMDWNIGRVVDYLRRQGELDNTFV
LFMSDNGAEGALLEAFPKFGPDLLGFLDRHYDNSLENIGRANSYVWYGPRWAQAATAPSRLY
KAFTTQGGIRVPALVRYPRLSRQGAISHAFATVMDVTPTLLDLAGVRHPGKRWRGREIAEPR
GRSWLGWLSGETEAAHDENTVTGWELFGMRAIRQGDWKAVYLPAPVGPATWQLYDLARDPGE
IHDLADSQPGKLAELIEHWKRYVSETGVV
>d1k30a_ c.112.1.1 (A:) Glycerol-3-phosphate (1)-acyltransferase
{Cushaw squash (Cucurbita moschata)}
SHSRKFLDVRSEEELLSCIKKETEAGKLPPNVAAGMEELYQNYRNAVIESGNPKADEIVLSN
MTVALDRILLDVEDPFVFSSHHKAIREPFDYYIFGQNYIRPLIDFGNSFVGNLSLFKDIEEK
LQQGHNVVLISNHQTEADPAIISLLLEKTNPYIAENTIFVAGDRVLADPLCKPFSIGRNLIC
VYSKKHMFDIPELTETKRKANTRSLKEMALLLRGGSQLIWIAPSGGRDRPDPSTGEWYPAPF
DASSVDNMRRLIQHSDVPGHLFPLALLCHDIMPPPSQVEIEIGEKRVIAFNGAGLSVAPEIS
FEEIAATHKNPEEVREAYSKALFDSVAMQYNVLKTAISGKQGLGASTADVSLSQPW
>d1e4bp_ c.74.1.1 (P:) L-fuculose-1-phosphate aldolase
{Escherichia coli}
MERNKLARQIIDTCLEMTRLGLNQGTAGQVSVRYQDGMLITPTGIPYEKLTESHIVFIDGNG
KHEEGKLPSSEWRFHMAAYQSRPDANAVVHNHAVHCTAVSILNRSIPAIHYMIAAAGGNSIP
CAPYATFGTRELSEHVALALKNRKATLLQHHGLIACEVNLEKALWLAHEVEVLAQLYLTTLA
ITDPVPVLSDEEIAVVLEKF
>d1jdia_ c.74.1.1 (A:) L-ribulose-5-phosphate 4-epimerase
{Escherichia coli}
MLEDLKRQVLEANLALPKHNLVTLTWGNVSAVDRERGVFVIKPSGVDYSIMTADDMVVVSIE
TGEVVEGAKKPSSDTPTHRLLYQAFPSIGGIVHTHSRHATIWAQAGQSIPATGTTHADYFYG
TIPCTRKMTDAEINGEYEWETGNVIVETFEKQGIDAAQMPGVLVHSHGPFAWGKNAEDAVHN
AIVLEEVAYMGIFCRQLAPQLPDMQQTLLNKHYLRKH
>d1j9la_ c.106.1.1 (A:) SurE homolog TM1662 (TM107 ?) {Thermotoga
maritima}
MRILVTNDDGIQSKGIIVLAELLSEEHEVFVVAPDKERSATGHSITIHVPLWMKKVFISERV
VAYSTTGTPADCVKLAYNVVMDKRVDLIVSGVNRGPNMGMDILHSGTVSGAMEGAMMNIPSI
AISSANYESPDFEGAARFLIDFLKEFDFSLLDPFTMLNINVPAGEIKGWRFTRQSRRRWNDY
FEERVSPFGEKYYWMMGEVIEDDDRDDVDYKAVREGYVSITPIHPFLTNEQCLKKLREVYD
>d1cjya2 c.75.1.1 (A:142-721) Cytosolic phospholipase A2 catalytic
domain {Human (Homo sapiens)}
PDLRFSMALCDQEKTFRQQRKEHIRESMKKLLGPKNSEGLHSARDVPVVAILGSGGGFRAMV
GFSGVMKALYESGILDCATYVAGLSGSTWYMSTLYSHPDFPEKGPEEINEELMKNVSHNPLL
LLTPQKVKRYVESLWKKKSSGQPVTFTDIFGMLIGETLIHNRMNTTLSSLKEKVNTAQCPLP
LFTCLHVKPDVSELMFADWVEFSPYEIGMAKYGTFMAPDLFGSKFFMGTVVKKYEENPLHFL
MGVWGSAFSILFNRVLGVSGSQSRGSTMEEELENITTKHIVSNDSSDSDDESHEPKGTENED
AGSDYQSDNQASWIHRMIMALVSDSALFNTREGRAGKVHNFMLGLNLNTSYPLSPLSDFATQ
DSFDDDELDAAVADPDEFERIYEPLDVKSKKIHVVDSGLTFNLPYPLILRPQRGVDLIISFD
FSARPSDSSPPFKELLLAEKWAKMNKLPFPKIDPYVFDREGLKECYVFKPKNPDMEKDCPTI
IHFVLANINFRKYKAPGVPRETEEEKEIADFDIFDDPESPFSTFNFQYPNQAFKRLHDLMHF
NTLNNIDVIKEAMVESIEYRRQ
>d1xaa__ c.77.1.1 (-) 3-isopropylmalate dehydrogenase, IPMDH

{Thermus thermophilus}
MKVAVLPGDGIGPEVTEAALKVLRALDEAEGLGLAYEVFPFGGAAIDAFGEPFPEPTRKGVE
EAEAVLLGSVGGPKWDGLPRKIRPETGLLSLRKSQDLFANLRPAKVFPGLERLSPLKEEIAR
GVDVLIVRELTGGIYFGEPRGMSEAEAWNTERYSKPEVERVARVAFEAARKRRKHVVSVDKA
NVLEVGEFWRKTVEEVGRGYPDVALEHQYVDAMAMHLVRSPARFDVVVTGNIFGDILSDLAS
VLPGSLGLLPSASLGRGTPVFEPVHGSAPDIAGKGIANPTAAILSAAMMLEHAFGLVELARK
VEDAVAKALLETPPPDLGGSAGTEAFTATVLRHLA
>d1xad__ c.77.1.1 (-) 3-isopropylmalate dehydrogenase, IPMDH
{Chimera (Thermus thermophilus) and (Bacillus subtilis)}
MKVAVLPGDGIGPEVTEAALKVLRALDEAEGLGLAYEVFPFGGAAIDAFGEPFPEPTRKGVE
EAEAVLLGSVGGPKWDQNPRELRPEKGLLSIRKQLDLFANLRPVKVFESLSDASPLKKEYID
NVDFVIVRELTGGIYFGEPRGMSEAEAWNTERYSKPEVERVARVAFEAARKRRKHVVSVDKA
NVLEVGEFWRKTVEEVGRGYPDVALEHQYVDAMAMHLVRSPARFDVVVTGNIFGDILSDLAS
VLPGSLGLLPSASLGRGTPVFEPVHGSAPDIAGKGIANPTAAILSAAMMLEHAFGLVELARK
VEDAVAKALLETPPPDLGGSAGTEAFTATVLRHLA
>d2ayqa_ c.77.1.1 (A:) 3-isopropylmalate dehydrogenase, IPMDH
{Bacillus coagulans}
MKMKLAVLPGDGIGPEVMDAAIRVLKTVLDNDGHEAVFENALIGGAAIDEAGTPLPEETLDI
CRRSDAILLGAVGGPKWDHNPASLRPEKGLLGLRKEMGLFANLRPVKAYATLLNASPLKRER
VENVDLVIVRELTGGLYFGRPSERRGPGENEVVDTLAYTREEIERIIEKAFQLAQIRRKKLA
SVDKANVLESSRMWREIAEETAKKYPDVELSHMLVDSTSMQLIANPGQFDVIVTENMFGDIL
SDEASVITGSLGMLPSASLRSDRFGMYEPVHGSAPDIAGQGKANPLGTVLSAALMLRYSFGL
EKEAAAIEKAVDDVLQDGYCTGDLQVANGKVVSTIELTDRLIEKLN
>d1a05a_ c.77.1.1 (A:) 3-isopropylmalate dehydrogenase, IPMDH
{Thiobacillus ferrooxidans}
MKKIAIFAGDGIGPEIVAAARQVLDAVDQAAHLGLRCTEGLVGGAALDASDDPLPAASLQLA
MAADAVILGAVGGPRWDAYPPAKRPEQGLLRLRKGLDLYANLRPAQIFPQLLDASPLRPELV
RDVDILVVRELTGDIYFGQPRGLEVIDGKRRGFNTMVYDEDEIRRIAHVAFRAAQGRRKQLC
SVDKANVLETTRLWREVVTEVARDYPDVRLSHMYVDNAAMQLIRAPAQFDVLLTGNMFGDIL
SDEASQLTGSIGMLPSASLGEGRAMYEPIHGSAPDIAGQDKANPLATILSVAMMLRHSLNAE
PWAQRVEAAVQRVLDQGLRTADIAAPGTPVIGTKAMGAAVVNALNLK
>d1cnza_ c.77.1.1 (A:) 3-isopropylmalate dehydrogenase, IPMDH
{Salmonella typhimurium}
MSKNYHIAVLPGDGIGPEVMAQALKVMDAVRSRFDMRITTSHYDVGGIAIDNHGHPLPKATV
EGCEQADAILFGSVGGPKWENLPPESQPERGALLPLRKHFKLFSNLRPAKLYQGLEAFCPLR
ADIAANGFDILCVRELTGGIYFGQPKGREGSGQYEKAFDTEVYHRFEIERIARIAFESARKR
RRKVTSIDKANVLQSSILWREIVNDVAKTYPDVELAHMYIDNATMQLIKDPSQFDVLLCSNL
FGDILSDECAMITGSMGMLPSASLNEQGFGLYEPAGGSAPDIAGKNIANPIAQILSLALLLR
YSLDANDAATAIEQAINRALEEGVRTGDLARGAAAVSTDEMGDIIARYVAEGV
>d1cm7a_ c.77.1.1 (A:) 3-isopropylmalate dehydrogenase, IPMDH
{Escherichia coli}
MSKNYHIAVLPGDGIGPEVMTQALKVLDAVRNRFAMRITTSHYDVGGAAIDNHGQPLPPATV
EGCEQADAVLFGSVGGPKWEHLPPDQQPERGALLPLRKHFKLFSNLRPAKLYQGLEAFCPLR
ADIAANGFDILCVRELTGGIYFGQPKGREGSGQYEKAFDTEVYHRFEIERIARIAFESARKR

RHKVTSIDKANVLQSSILWREIVNEIATEYPDVELAHMYIDNATMQLIKDPSQFDVLLCSNL
FGDILSDECAMITGSMGMLPSASLNEQGFGLYEPAGGSAPDIAGKNIANPIAQILSLALLLR
YSLDADDAACAIERAINRALEEGIRTGDLARGAAAVSTDEMGDIIARYVAEGV
>d1iso__ c.77.1.1 (-) Isocitrate dehydrogenase, ICDH {Escherichia coli}
SKVVVPAQGKKITLQNGKLNVPENPIIPYIEGDGIGVDVTPAMLKVVDAAVEKAYKGERKIS
WMEIYTGEKSTQVYGQDVWLPAETLDLIREYRVAIKGPLTTPVGGGIRSLNVALRQELDLYI
CLRPVRYYQGTPSPVKHPELTDMVIFRENSEDIYAGIEWKADSADAEKVIKFLREEMGVKKI
RFPEHCGIGIKPMSEEGTKRLVRAAIEYAIANDRDSVTLVHKGNIMKFTEGAFKDWGYQLAR
EEFGGELIDGGPWLKVKNPNTGKEIVIKDVIADAFLQQILLRPAEYDVIACMNLNGDYISDA
LAAQVGGIGIAPGANIGDEYALFEATHGTAPDIAGQDKANPGSIILSAEMMLRHMGWTEAAD
LIVKGMEGAINAKTVTKDFESLMDGAKLLKCSEFGDAIIENM
>d1hqsa_ c.77.1.1 (A:) Isocitrate dehydrogenase, ICDH {Bacillus subtilis}
MAQGEKITVSNGVLNVPNNPIIPFIEGDGTGPDIWNAASKVLEAAVEKAYKGEKKITWKEVY
AGEKAYNKTGEWLPAETLDVIREYFIAIKGPLTTPVGGGIRSLNVALRQELDLFVCLRPVRY
FTGVPSPVKRPEDTDMVIFRENTEDIYAGIEYAKGSEEVQKLISFLQNELNVNKIRFPETSG
IGIKPVSEEGTSRLVRAAIDYAIEHGRKSVTLVHKGNIMKFTEGAFKNWGYELAEKEYGDKV
FTWAQYDRIAEEQGKDAANKAQSEAEAAGKIIIKDSIADIFLQQILTRPNEFDVVATMNLNG
DYISDALAAQVGGIGIAPGANINYETGHAIFEATHGTAPKYAGLDKVNPSSVILSGVLLLEH
LGWNEAADLVIKSMEKTIASKVVTYDFARLMDGATEVKCSEFGEELIKNMD
>d1ekxa1 c.78.1.1 (A:1-150) Aspartate carbamoyltransferase catalytic subunit {Escherichia coli}
ANPLYQKHIISINDLSRDDLNLVLATAAKLKANPQPELLKHKVIASCFFEASTRTRLSFETS
MHRLGASVVGFSDSANTSLGKKGETLADTISVISTYVDAIVMRHPQEGAARLATEFSGNVPV
LNAGDGSNQHPTQTLLDLFTIQETQG
>d1ekxa2 c.78.1.1 (A:151-310) Aspartate carbamoyltransferase catalytic subunit {Escherichia coli}
RLDNLHVAMVGDLKYGRTVHSLTQALAKFDGNRFYFIAPDALAMPQYILDMLDEKGIAWSLH
SSIEEVMAEVDILYMTRVQKERLDPSEYANVKAQFVLRASDLHNAKANMKVLHPLPRVDEIA
TDVDKTPHAWYFQQAGNGIFARQALLALVLNRDLVL
>d2atca2 c.78.1.1 (A:151-305) Aspartate carbamoyltransferase catalytic subunit {Escherichia coli}
RLNNLHVAMVGDLKYGRTVHSLTQALAKFDGNRFYFIAPDALAMPEYILDMLDEKGIAWSLH
SSIEEVMTRVQKERLDPSEYABVKAQFLVRANSLGGLHNAKMNAKVLHPLPRVDEIATDVDK
TPHAWYFQQAGNGIFARQALLALVLNRDLVL
>d2at2a1 c.78.1.1 (A:1-144) Aspartate carbamoyltransferase catalytic subunit {Bacillus subtilis}
MKHLTTMSELSTEEIKDLLQTAQELKSGKTDNQLTGKFAANLFFEPSTRTRFSFEVAEKKLG
MNVLNLDGTSTSVQKGETLYDTIRTLESIGVDVCVIRHSEDEYYEELVSQVNIPILNAGDGC
GQHPTQSLLDLMTIYEEFNT
>d2at2a2 c.78.1.1 (A:145-295) Aspartate carbamoyltransferase catalytic subunit {Bacillus subtilis}
FKGLTVSIHGDIKHSRVARSNAEVLTRLGARVLFSGPSEWQDEENTFGTYVSMDEAVESSDV

VMLLRIQNERHQSAVSQEGYLNKYGLTVERAERMKRHAIIMHPAPVNRGVEIDDSLVESEKS
RIFKQMKNGVFIRMAVIQCALQTNVKR
>d1dxha1 c.78.1.1 (A:1-150) Ornithine transcarbamoylase
{Pseudomonas aeruginosa}
AFNMHNRNLLSLMHHSTRELRYLLDLSRDLKRAKYTGTEQQHLKRKNIALIFEKTSTRTRCA
FEVAAYDQGANVTYIDPNSSQIGHKESMKDTARVLGRMYDAIEYRGFKQEIVEELAKFAGVP
VFNGLTDEYHPTQMLADVLTMREHSD
>d1dxha2 c.78.1.1 (A:151-335) Ornithine transcarbamoylase
{Pseudomonas aeruginosa}
KPLHDISYAYLGDARNNMGNSLLLIGAKLGMDVRIAAPKALWPHDEFVAQCKKFAEESGAKL
TLTEDPKEAVKGVDFVHTDVWVSMGEPVEAWGERIKELLPYQVNMEIMKATGNPRAKFMHCL
PAFHNSETKVGKQIAEQYPNLANGIEVTEDVFESPYNIAFEQAENRMHTIKAILVSTLADI
>d1duvg1 c.78.1.1 (G:1-150) Ornithine transcarbamoylase
{Escherichia coli}
SGFYHKHFLKLLDFTPAELNSLLQLAAKLKADKKSGKEEAKLTGKNIALIFEKDSTRTRCSF
EVAAYDQGARVTYLGPSGSQIGHKESIKDTARVLGRMYDGIQYRGYGQEIVETLAEYASVPV
WNGLTNEFHPTQLLADLLTMQEHLPG
>d1duvg2 c.78.1.1 (G:151-333) Ornithine transcarbamoylase
{Escherichia coli}
KAFNEMTLVYAGDARNNMGNSMLEAAALTGLDLRLVAPQACWPEAALVTECRALAQQNGGNI
TLTEDVAKGVEGADFIYTDVWVSMGEAKEKWAERIALLREYQVNSKMMQLTGNPEVKFLHCL
PAFHDDQTTLGKKMAEEFGLHGGMEVTDEVFESAASIVFDQAENRMHTIKAVMVATLSK
>d1a1s_1 c.78.1.1 (1-150) Ornithine transcarbamoylase {Archaeon
Pyrococcus furiosus}
VVSLAGRDLLCLQDYTAEEIWTILETAKMFKIWQKIGKPHRLLEGKTLAMIFQKPSTRTRVS
FEVAMAHLGGHALYLNAQDLQLRRGETIADTARVLSRYVDAIMARVYDHKDVEDLAKYATVP
VINGLSDFSHPCQALADYMTIWEKKG
>d1a1s_2 c.78.1.1 (151-313) Ornithine transcarbamoylase {Archaeon
Pyrococcus furiosus}
TIKGVKVVYVGDGNNVAHSLMIAGTKLGADVVVATPEGYEPDEKVIKWAEQNAAESGGSFEL
LHDPVKAVKDADVIYTDVWASMGQEAEAEERRKIFRPFQVNKDLVKHAKPDYMFMHCLPAHR
GEEVTDDVIDSPNSVVWDQAENRLHAQKAVLALVMGGIK
>d1otha1 c.78.1.1 (A:34-184) Ornithine transcarbamoylase {Human
(Homo sapiens)}
KVQLKGRDLLTLKNFTGEEIKYMLWLSADLKFRIKQKGEYLPLLQGKSLGMIFEKRSTRTRL
STETGFALLGGHPCFLTTQDIHLGVNESLTDTARVLSSMADAVLARVYKQSDLDTLAKEASI
PIINGLSDLYHPIQILADYLTLQEHYS
>d1otha2 c.78.1.1 (A:185-354) Ornithine transcarbamoylase {Human
(Homo sapiens)}
SLKGLTLSWIGDGNNILHSIMMSAAKFGMHLQAATPKGYEPDASVTKLAEQYAKENGTKLLL
TNDPLEAAHGGNVLITDTWISMGREEEKKKRLQAFQGYQVTMKTAKVAASDWTFLHCLPRKP
EEVDDEVFYSPRSLVFPEAENRKWTIMAVMVSLLTDYSPQLQKPKF
>d1b74a1 c.78.2.1 (A:1-105) Glutamate racemase {Aquifex
pyrophilus}

MKIGIFDSGVGGLTVLKAIRNRYRKVDIVYLGDTARVPYGIRSKDTIIRYSLECAGFLKDKG
VDIIVVACNTASAYALERLKKEINVPVFGVIEPGVKEALKKSR
>d1b74a2 c.78.2.1 (A:106-252) Glutamate racemase {Aquifex
pyrophilus}
NKKIGVIGTPATVKSGAYQRKLEEGGADVFAKACPLFAPLAEEGLLEGEITRKVVEHYLKEF
KGKIDTLILGCTHYPLLKKEIKKFLGDAEVVDSSEALSLSLHNFIKDDGSSSLELFFTDLSP
NLQFLIKLILGRDYPVKLAEGVF
>d1qopb_ c.79.1.1 (B:) Tryptophan synthase, beta-subunit
{Salmonella typhimurium}
TTLLNPYFGEFGGMYVPQILMPALNQLEEAFVSAQKDPEFQAQFADLLKNYAGRPTALTKCQ
NITAGTRTTLYLKREDLLHGGAHKTNQVLGQALLAKRMGKSEIIAETGAGQHGVASALASAL
LGLKCRIYMGAKDVERQSPNVFRMRLMGAEVIPVHSGSATLKDACNEALRDWSGSYETAHYM
LGTAAGPHPYPTIVREFQRMIGEETKAQILDKEGRLPDAVIACVGGGSNAIGMFADFINDTS
VGLIGVEPGGHGIETGEHGAPLKHGRVGIYFGMKAPMMQTADGQIEESYSISAGLDFPSVGP
QHAYLNSIGRADYVSITDDEALEAFKTLCRHEGIIPALESSHALAHALKMMREQPEKEQLLV
VNLSGRGDKDIFTVHDIL
>d1fcja_ c.79.1.1 (A:) O-acetylserine sulfhydrylase (Cystein
synthase) {Salmonella typhimurium}
SKIYEDNSLTIGHTPLVRLNRIGNGRILAKVESRNPSFSVKCRIGANMIWDAEKRGVLKPGV
ELVEPTNGNTGIALAYVAAARGYKLTLTMPETMSIERRKLLKALGANLVLTEGAKGMKGAIQ
KAEEIVASDPQKYLLLQQFSNPANPEIHEKTTGPEIWEDTDGQVDVFISGVGTGGTLTGVTR
YIKGTKGKTDLITVAVEPTDSPVIAQALAGEEIKPGPHKIQGIGAGFIPGNLDLKLIDKVVG
ITNEEAISTARRLMEEEGILAGISSGAAVAAALKLQEDESFTNKNIVVILPSSG
>d1tdj_1 c.79.1.1 (5-335) Allosteric threonine deaminase
N-terminal domain {Escherichia coli}
QPLSGAPEGAEYLRAVLRAPVYEAAQVTPLQKMEKLSSRLDNVILVKREDRQPVHSFKLRGA
YAMMAGLTEEQKAHGVITASAGNHAQGVAFSSARLGVKALIVMPTATADIKVDAVRGFGGEV
LLHGANFDEAKAKAIELSQQQGFTWVPPFDHPMVIAGQGTLALELLQQDAHLDRVFVPVGGG
GLAAGVAVLIKQLMPQIKVIAVEAEDSACLKAALDAGHPVDLPRVGLFAEGVAVKRIGDETF
RLCQEYLDDIITVDSDAICAAMKDLFEDVRAVAEPSGALALAGMKKYIALHNIRGERLAHIL
SGANVNFHGLRYVSERCELGE
>d1e5xa_ c.79.1.1 (A:) Threonine synthase {Mouse-ear cress
(Arabidopsis thaliana)}
IETAVKPPHRTEDNIRDEARRNRSNAVNPFSAKYVPFNAAPGSTESYSLDEIVYRSRSGGLL
DVEHDMEALKRFDGAYWRDLFDSRVGKSTWPYGSGVWSKKEWVLPEIDDDDIVSAFEGNSNL
FWAERFGKQFLGMNDLWVKHCGISHTGSFKDLGMTVLVSQVNRLRKMKRPVVGVGCASTGDT
SAALSAYCASAGIPSIVFLPANKISMAQLVQPIANGAFVLSIDTDFDGCMKLIREITAELPI
YLANSLNSLRLEGQKTAAIEILQQFDWQVPDWVIVPGGNLGNIYAFYKGFKMCQELGLVDRI
PRMVCAQAANANPLYLHYKSGWKDFKPMTASTTFASAIQIGDPVSIDRAVYALKKCNGIVEE
ATEEELMDAMAQADSTGMFICPHTGVALTALFKLRNQGVIAPTDRTVVVSTAHGLKFTQSKI
DYHSNAIPDMACRFSNPPVDVKADFGAVMDVLKSYLGSNTLTS
>d1f2da_ c.79.1.1 (A:) 1-aminocyclopropane-1-carboxylate
deaminase {Yeast (Hansenula saturnus)}
AGVAKFAKYPLTFGPSPISNLNRLSQHLGSKVNVYAKREDCNSGLAFGGNKLRKLEYIVPDI

VEGDYTHLVSIGGRQSNQTRMVAALAAKLGKKCVLIQEDWVPIPEAEKDVYNRVGNIELSRI
MGADVRVIEDGFDIGMRKSFANALQELEDAGHKPYPIPAGCSEHKYGGLGFVGFADEVINQE
VELGIKFDKIVVCCVTGSTTAGILAGMAQYGRQDDVIAIDASFTSEKTKEQTLRIANNTAKL
IGVEHEFKDFTLDTRFAYPCYGVPNEGTIEAIRTCAEQEGVLTDPVYEGKSMQGLIALIKED
YFKPGANVLYVHLGGAPALSAYSSFFPTKTA
>d1jbqa_ c.79.1.1 (A:) Cystathionine beta-synthase {Human (Homo sapiens)}
WIRPDAPSRCTWQLGRPASESPHHHTAPAKSPKILPDILKKIGDTPMVRINKIGKKFGLKCE
LLAKCEFFNAGGSVKDRISLRMIEDAERDGTLKPGDTIIEPTSGNTGIGLALAAAVRGYRCI
IVMPEKMSSEKVDVLRALGAEIVRTPTNARFDSPESHVGVAWRLKNEIPNSHILDQYRNASN
PLAHYDTTADEILQQCDGKLDMLVASVGTGGTITGIARKLKEKCPGCRIIGVDPEGSILAEP
EELNQTEQTTYEVEGIGYDFIPTVLDRTVVDKWFKSNDEEAFTFARMLIAQEGLLCGGSAGS
TVAVAVKAAQELQEGQRCVVILPDSVRNYMTKFLSDRWMLQKGFL
>d1jeoa_ c.80.1.3 (A:) Probable 3-hexulose-6-phosphate isomerase MJ1247 {Archaeon Methanococcus jannaschii}
LEELDIVSNNILILKKFYTNDEWKNKLDSLIDRIIKAKKIFIFGVGRSGYIGRCFAMRLMHL
GFKSYFVGETTTPSYEKDDLLILISGSGRTESVLTVAKKAKNINNNIIAIVCECGNVVEFAD
LTIPLEVKKSKYLPMGTTFEETALIFLDLVIAEIMKRLNLDESEIIKRHCNLL
>d1moq__ c.80.1.1 (-) "Isomerase domain" of glucosamine 6-phosphate synthase (GLMS) {Escherichia coli}
GDKGIYRHYMQKEIYEQPNAIKNTLTGRISHGQVDLSELGPNADELLSKVEHIQILACGTSY
NSGMVSRYWFESLAGIPCDVEIASEFRYRKSAVRRNSLMITLSQSGETADTLAGLRLSKELG
YLGSLAICNVPGSSLVRESDLALMTNAGTEIGVASTKAFTTQLTVLLMLVAKLSRLKGLDAS
IEHDIVHGLQALPSRIEQMLSQDKRIEALAEDFSDKHHALFLGRGDQYPIALEGALKLKEIS
YIHAEAYAAGELKHGPLALIDADMPVIVVAPNNELLEKLKSNIEEVRARGGQLYVFADQDAG
FVSSDNMHIIEMPHVEEVIAPIFYTVPLQLLAYHVALIKGTDVDQPRNLAKSVTVE
>d1g98a_ c.80.1.2 (A:) Phosphoglucose isomerase, PGI {Rabbit (Oryctolagus cuniculus)}
AALTRNPQFQKLQQWHREHGSELNLRHLFDTDKERFNHFSLTLNTNHGHILLDYSKNLVTEE
VMHMLLDLAKSRGVEAARESMFNGEKINSTEDRAVLHVALRNRSNTPIVVDGKDVMPEVNKV
LDKMKAFCQRVRSGDWKGYTGKTITDVINIGIGGSDLGPLMVTEALKPYSSGGPRVWFVSNI
DGTHIAKTLACLNPESSLFIIASKTFTTQETITNAKTAKDWFLLSAKDPSTVAKHFVALSTN
TAKVKEFGIDPQNMFEFWDWVGGRYSLWSAIGLSIALHVGFDNFEQLLSGAHWMDQHFRTTP
LEKNAPVLLAMLGIWYINCFGCETQAVLPYDQYLHRFAAYFQQGDMESNGKYITKSGARVDH
QTGPIVWGEPGTNGQHAFYQLIHQGTKMIPCDFLIPVQTQHPIRKGLHHKILLANFLAQTEA
LMKGKSTEEARKELQAAGKSPEDLMKLLPHKVFEGNRPTNSIVFTKLTPFILGALIAMYEHK
IFVQGVVWDINSFDQWGVELGKQLAKKIEPELDGSSPVTSHDSSTNGLINFIKQQREAK
>d1iata_ c.80.1.2 (A:) Phosphoglucose isomerase, PGI {Human (Homo sapiens)}
AALTRDPQFQKLQQWYREHRSELNLRRLFDANKDRFNHFSLTLNTNHGHILVDYSKNLVTED
VMRMLVDLAKSRGVEAARERMFNGEKINYTEGRAVLHVALRNRSNTPILVDGKDVMPEVNKV
LDKMKSFCQRVRSGDWKGYTGKTITDVINIGIGGSDLGPLMVTEALKPYSSGGPRVWYVSNI
DGTHIAKTLAQLNPESSLFIIASKTFTTQETITNAETAKEWFLQAAKDPSAVAKHFVALSTN
TTKVKEFGIDPQNMFEFWDWVGGRYSLWSAIGLSIALHVGFDNFEQLLSGAHWMDQHFRTTP

LEKNAPVLLALLGIWYINCFGCETHAMLPYDQYLHRFAAYFQQGDMESNGKYITKSGTRVDH
QTGPIVWGEPGTNGQHAFYQLIHQGTKMIPCDFLIPVQTQHPIRKGLHHKILLANFLAQTEA
LMRGKSTEEARKELQAAGKSPEDLERLLPHKVFEGNRPTNSIVFTKLTPFMLGALVAMYEHK
IFVQGIIWDINSFDQWGVELGKQLAKKIEPELDGSAQVTSHDASTNGLINFIKQQREARV
>d1c7qa_ c.80.1.2 (A:) Phosphoglucose isomerase, PGI {Bacillus stearothermophilus}
AISFDYSNALPFMQENELDYLSEFVKAAHHMLHERKGPGSDFLGWVDWPIRYDKNEFSRIKQ
AAERIRNHSDALVVIGIGGSYLGARAAIEALSHTFHNQMNDTTQIYFAGQNISSTYISHLLD
VLEGKDLSINVISKSGTTTEPAIAFRIFRDYMEKKYGKEEARKRIYVTTDRTKGALKKLADQ
EGYETFVIPDNIGGRYSVLTAVGLLPIAVAGLNIDRMMEGAASAYHKYNNPDLLTNESYQYA
AVRNILYRKGKAIELLVNYEPSLHYVSEWWKQLFGESEGKDQKGLFPASVDFTTDLHSMGQY
VQEGRRNLIETVLHVKKPQIELTIQEDPENIDGLNFLAGKTLDEVNKKAFQGTLLAHVDGGV
PNLIVELDEMNEYTFGEMVYFFEKACGISGHLLGVNPFDQPGVEAYKKNMFALLGKPGFEDE
KAALMKRL
>d1eu1a2 c.81.1.1 (A:4-625) Dimethylsulfoxide reductase (DMSO reductase) {Rhodobacter sphaeroides}
ANGEVMSGCHWGVFKARVENGRAVAFEPWDKDPAPSHQLPGVLDSIYSPTRIKYPMVRREFL
EKGVNADRSTRGNGDFVRVTWDEALDLVARELKRVQESYGPTGTFGGSYGWKSPGRLHNCQV
LMRRALNLAGGFVNSSGDYSTAAAQIIMPHVMGTLEVYEQQTAWPVVVENTDLMVFWAADPM
KTNEIGWVIPDHGAYAGMKALKEKGTRVIAINPVRTETADYFGADVVSPRPQTDVALMLGMA
HTLYSEDLHDKDFLENCTTGFDLFAAYLTGESDGTPKTAEWAAEICGLPAEQIRELARSFVA
GRTMLAAGWSIQRMHHGEQAHWMLVTLASMIGQIGLPGGGFGLSYHYSNGGSPTSDGPALGG
ISDGGKAVEGAAWLSESGATSIPCARVVDMLLNPGGEFQFNGATATYPDVKLAYWAGGNPFA
HHQDRNRMLKAWEKLETFIVQDFQWTATARHADIVLPATTSYERNDIESVGDYSNRAILAMK
KVVDPLYEARSDYDIFAALAERLGKGAEFTEGRDEMGWISSFYEAAVKQAEFKNVAMPSFED
FWSEGIVEFPITEGANFVRYADFREDPLFNPLGTPSGLIEIYSKNIEKMGYDDCPAHPTWME
PA
>d1dmr_2 c.81.1.1 (3-625) Dimethylsulfoxide reductase (DMSO reductase) {Rhodobacter capsulatus}
LANGTVMSGSHWGVFTATVENGRATAFTPWEKDPHPSPMLAGVLDSIYSPTRIKYPMVRREF
LEKGVNADRSTRGNGDFVRVSWDQALDLVAAEVKRVEETYGPEGVFGGSYGWKSPGRLHNCT
TLLRRMLTLAGGYVNGAGDYSTGAAQVIMPHVVGTLEVYEQQTAWPVLAENTEVMVFWAADP
IKTSQIGWVIPEHGAYPGLEALKAKGTKVIVIDPVRTKTVEFFGAEHITPKPQTDVAIMLGM
AHTLVAEDLYDKDFIANYTSGFDKFLPYLDGETDSTPKTAEWAEGISGVPAETIKELARLFE
SKRTMLAAGWSMQRMHHGEQAHWMLVTLASMLGQIGLPGGGFGLSYHYSGGGTPSTSGPALA
GITDGGAATKGPEWLAASGASVIPVARVVDMLENPGAEFDFNGTRSKFPDVKMAYWVGGNPF
VHHQDRNRMVKAWEKLETFVVHDFQWTPTARHADIVLPATTSYERNDIETIGDYSNTGILAM
KKIVEPLYEARSDYDIFAAVAERLGKGAEFTEGKDEMGWIKSFYDDAAKQGKAAGVQMPAFD
AFWAEGIVEFPVTDGADFVRYASFREDPLLNPLGTPTGLIEIYSKNIEKMGYDDCPAHPTWM
EPL
>d1aa6_2 c.81.1.1 (1-564) Formate dehydrogenase H {Escherichia coli}
MKKVVTVCPYCASGCKINLVVDNGKIVRAEAAQGKTNQGTLCLKGYYGWDFINDTQILTPRL
KTPMIRRQRGGKLEPVSWDEALNYVAERLSAIKEKYGPDAIQTTGSSRGTGNETNYVMQKFA

RAVIGTNNVDCCARVCHGPSVAGLHQSVGNGAMSNAINEIDNTDLVFVFGYNPADSHPIVAN
HVINAKRNGAKIIVCDPRKIETARIADMHIALKNGSNIALLNAMGHVIIEENLYDKAFVASR
TEGFEEYRKIVEGYTPESVEDITGVSASEIRQAARMYAQAKSAAILWGMGVTQFYQGVETVR
SLTSLAMLTGNLGKPHAGVNPVRGQNNVQGACDMGALPDTYPGYQYVKDPANREKFAKAWGV
ESLPAHTGYRISELPHRAAHGEVRAAYIMGEDPLQTDAELSAVRKAFEDLELVIVQDIFMTK
TASAADVILPSTSWGEHEGVFTAADRGFQRFFKAVEPKWDLKTDWQIISEIATRMGYPMHYN
NTQEIWDELRHLCPDFYGATYEKMGELGFIQWPCRDTSDADQGTSYLFKEKFDTPNGLAQFF
TCDWVA
>d1tmo_2 c.81.1.1 (5-631) Trimethylamine N-oxide reductase
{Shewanella massilia}
NEDEWLTTGSHFGAFKMKRKNGVIAEVKPFDLDKYPTDMINGIRGMVYNPSRVRYPMVRLDF
LLKGHKSNTHQRGDFRFVRVTWDKALTLFKHSLDEVQTQYGPSGLHAGQTGWRATGQLHSST
SHMQRAVGMHGNYVKKIGDYSTGAGQTILPYVLGSTEVYAQGTSWPLILEHSDTIVLWSNDP
YKNLQVGWNAETHESFAYLAQLKEKVKQGKIRVISIDPVVTKTQAYLGCEQLYVNPQTDVTL
MLAIAHEMISKKLYDDKFIQGYSLGFEEFVPYVMGTKDGVAKTPEWAAPICGVEAHVIRDLA
KTLVKGRTQFMMGWCIQRQQHGEQPYWMAAVLATMIGQIGLPGGGISYGHHYSSIGVPSSGA
AAPGAFPRNLDENQKPLFDSSDFKGASSTIPVARWIDAILEPGKTIDANGSKVVYPDIKMMI
FSGNNPWNHHQDRNRMKQAFHKLECVVTVDVNWTATCRFSDIVLPACTTYERNDIDVYGAYA
NRGILAMQKMVEPLFDSLSDFEIFTRFAAVLGKEKEYTRNMGEMEWLETLYNECKAANAGKF
EMPDFATFWKQGYVHFGDGEVWTRHADFRNDPEINPLGTPSGLIEIFSRKIDQFGYDDCKGH
PTWMEKT
>d1g8ka2 c.81.1.1 (A:4-682) Arsenite oxidase large subunit
{Alcaligenes faecalis}
NDRITLPPANAQRTNMTCHFCIVGCGYHVYKWPELEEGGRAPEQNALGLDFRKQLPPLAVTL
TPAMTNVVTEHDGARYDIMVVPDKACVVNSGLSSTRGGKMASYMYTPTGDGKERLSAPRLYA
ADEWVDTTWDHAMALYAGLIKKTLDKDGPQGVFFSCFDHGGAGGGFENTWGTGKLMFSAIQT
PMVRIHNRPAYNSECHATREMGIGELNNAYEDAQLADVIWSIGNNPYESQTNYFLNHWLPNL
QGATTSKKKERFPNENFPQARIIFVDPRETPSVAIARHVAGNDRVLHLAIEPGTDTALFNGL
FTYVVEQGWIDKPFIEAHTKGFDDAVKTNRLSLDECSNITGVPVDMLKRAAEWSYKPKASGQ
APRTMHAYEKGIIWGNDNYVIQSALLDLVIATHNVGRRGTGCVRMGGHQEGYTRPPYPGDKK
IYIDQELIKGKGRIMTWWGCNNFQTSNNAQALREAILQRSAIVKQAMQKARGATTEEMVDVI
YEATQNGGLFVTSINLYPTKLAEAAHLMLPAAHPGEMNLTSMNGERRIRLSEKFMDPPGTAM
ADCLIAARIANALRDMYQKDGKAEMAAQFEGFDWKTEEDAFNDGFRRAGQPGAPAIDSQGGS
TGHLVTYDRLRKSGNNGVQLPVVSWDESKGLVGTEMLYTEGKFDTDDGKAHFKPAPWNG
>d2napa2 c.81.1.1 (A:4-600) Dissimilatory nitrate reductase (NAP)
{Desulfovibrio desulfuricans}
RPEKWVKGVCRYCGTGCGVLVGVKDGKAVAIQGNPNNHNAGLLCLKGSLLIPVLNSKERVTQ
PLVRRHKGGKLEPVSWDEALDLMASRFRSSIDMYGPNSVAWYGSGQCLTEESYVANKIFKGG
FGTNNVDGNPRLCMASAVGGYVTSFGKDEPMGTYADIDQATCFFIIGSNTSEAHPVLFRRIA
RRKQVEPGVKIIVADPRRTNTSRIADMHVAFRPGTDLAFMHSMAWVIINEELDNPRFWQRYV
NFMDAEGKPSDFEGYKAFLENYRPEKVAEICRVPVEQIYGAARAFAESAATMSLWCMGINQR
VQGVFANNLIHNLHLITGQICRPGATSFSLTGQPNACGGVRDGGALSHLLPAGRAIPNAKHR
AEMEKLWGLPEGRIAPEPGYHTVALFEALGRGDVKCMIICETNPAHTLPNLNKVHKAMSHPE
SFIVCIEAFPDAVTLEYADLVLPPAFWCERDGVYGCGERRYSLTEKAVDPPGQCRPTVNTLV

EFARRAGVDPQLVNFRNAEDVWNEWRMVSKGTTYDFWGMTRERLRKESGLIWPCPSEDHPGT
SLRYVRGQDPCVPADHPDRFFFYGKPDGRAVIWMRPAKG
>d1ad3a_ c.82.1.1 (A:) Aldehyde reductase (dehydrogenase), ALDH
{Rat (Rattus norvegicus)}
SISDTVKRAREAFNSGKTRSLQFRIQQLEALQRMINENLKSISGALASDLGKNEWTSYYEEV
AHVLEELDTTIKELPDWAEDEPVAKTRQTQQDDLYIHSEPLGVVLVIGAWNYPFNLTIQPMV
GAVAAGNAVILKPSEVSGHMADLLATLIPQYMDQNLYLVVKGGVPETTELLKERFDHIMYTG
STAVGKIVMAAAAKHLTPVTLELGGKSPCYVDKDCDLDVACRRIAWGKFMNSGQTCVAPDYI
LCDPSIQNQIVEKLKKSLKDFYGEDAKQSRDYGRIINDRHFQRVKGLIDNQKVAHGGTWDQS
SRYIAPTILVDVDPQSPVMQEEIFGPVMPIVCVRSLEEAIQFINQREKPLALYVFSNNEKVI
KKMIAETSSGGVTANDVIVHITVPTLPFGGVGNSGMGAYHGKKSFETFSHRRSCLVKSLLNE
EAHKARYPPSPA
>d1bi9a_ c.82.1.1 (A:) Aldehyde reductase (dehydrogenase), ALDH
{Rat (Rattus norvegicus), retinal type II}
MASLQLLPSPTPNLEIKYTKIFINNEWQNSESGRVFPVCNPATGEQVCEVQEADKVDIDKAV
QAARLAFSLGSVWRRMDASERGRLLDKLADLVERDRATLATMESLNGGKPFLQAFYIDLQGV
IKTLRYYAGWADKIHGMTIPVDGDYFTFTRHEPIGVCGQIIPWNFPLLMFTWKIAPALCCGN
TVVIKPAEQTPLSALYMGALIKEAGFPPGVVNILPGYGPTAGAAIASHIGIDKIAFTGSTEV
GKLIQEAAGRSNLKRVTLELGGKSPNIIFADADLDYAVEQAHQGVFFNQGQCCTAGSRIFVE
ESIYEEFVKRSVERAKRRIVGSPFDPTTEQGPQIDKKQYNKILELIQSGVAEGAKLECGGKG
LGRKGFFIEPTVFSNVTDDMRIAKEEIFGPVQEILRFKTMDEVIERANNSDFGLVAAVFTND
INKALMVSSAMQAGTVWINCYNALNAQSPFGGFKMSGNGREMGEFGLREYSEVKTVTVKIPQ
KNS
>d1ag8a_ c.82.1.1 (A:) Aldehyde reductase (dehydrogenase), ALDH
{Cow (Bos taurus), mitochondrial}
VPTPNQQPEVLYNQIFINNEWHDAVSKKTFPTVNPSTGDVICHVAEGDKADVDRAVKAARAA
FQLGSPWRRMDASERGRLLNRLADLIERDRTYLAALETLDNGKPYIISYLVDLDMVLKCLRY
YAGWADKYHGKTIPIDGDYFSYTRHEPVGVCGQIIPWNFPLLMQAWKLGPALATGNVVVMKV
AEQTPLTALYVANLIKEAGFPPGVVNVIPGFGPTAGAAIASHEDVDKVAFTGSTEVGHLIQV
AAGKSNLKRVTLEIGGKSPNIIMSDADMDWAVEQAHFALFFNQGQCCCAGSRTFVQEDIYAE
FVERSVARAKSRVVGNPFDSRTEQGPQVDETQFKKVLGYIKSGKEEGLKLLCGGGAAADRGY
FIQPTVFGDLQDGMTIAKEEIFGPVMQILKFKSMEEVVGRANNSKYGLAAAVFTKDLDKANY
LSQALQAGTVWVNCYDVFGAQSPFGGYKLSGSGRELGEYGLQAYTEVKTVTVRVPQKNS
>d1cw3a_ c.82.1.1 (A:) Aldehyde reductase (dehydrogenase), ALDH
{Human (Homo sapiens), mitochondrial}
AVPAPNQQPEVFCNQIFINNEWHDAVSRKTFPTVNPSTGEVICQVAEGDKEDVDKAVKAARA
AFQLGSPWRRMDASHRGRLLNRLADLIERDRTYLAALETLDNGKPYVISYLVDLDMVLKCLR
YYAGWADKYHGKTIPIDGDFFSYTRHEPVGVCGQIIPWNFPLLMQAWKLGPALATGNVVVMK
VAEQTPLTALYVANLIKEAGFPPGVVNIVPGFGPTAGAAIASHEDVDKVAFTGSTEIGRVIQ
VAAGSSNLKRVTLELGGKSPNIIMSDADMDWAVEQAHFALFFNQGQCCCAGSRTFVQEDIYD
EFVERSVARAKSRVVGNPFDSKTEQGPQVDETQFKKILGYINTGKQEGAKLLCGGGIAADRG
YFIQPTVFGDVQDGMTIAKEEIFGPVMQILKFKTIEEVVGRANNSTYGLAAAVFTKDLDKAN
YLSQALQAGTVWVNCYDVFGAQSPFGGYKMSGSGRELGEYGLQAYTEVKTVTVKVPQKNS
>d1bxsa_ c.82.1.1 (A:) Aldehyde reductase (dehydrogenase), ALDH

{Sheep (Ovis aries)}
DVPAPLTNLQFKYTKIFINNEWHSSVSGKKFPVFNPATEEKLCEVEEGDKEDVDKAVKAARQ
AFQIGSPWRTMDASERGRLLNKLADLIERDRLLLATMEAMNGGKLFSNAYLMDLGGCIKTLR
YCAGWADKIQGRTIPMDGNFFTYTRSEPVGVCGQIIPWNFPLLMFLWKIGPALSCGNTVVVK
PAEQTPLTALHMGSLIKEAGFPPGVVNIVPGYGPTAGAAISSHMDVDKVAFTGSTEVGKLIK
EAAGKSNLKRVSLELGGKSPCIVFADADLDNAVEFAHQGVFYHQGQCCIAASRLFVEESIYD
EFVRRSVERAKKYVLGNPLTPGVSQGPQIDKEQYEKILDLIESGKKEGAKLECGGGPWGNKG
YFIQPTVFSDVTDDMRIAKEEIFGPVQQIMKFKSLDDVIKRANNTFYGLSAGIFTNDIDKAI
TVSSALQSGTVWVNCYSVVSAQCPFGGFKMSGNGRELGEYGFHEYTEVKTVTIKISQKNS
>d1a4sa_ c.82.1.1 (A:) Aldehyde reductase (dehydrogenase), ALDH
{Baltic cod (Gadus callarias)}
AQLVDSMPSASTGSVVVTDDLNYWGGRRIKSKDGATTEPVFEPATGRVLCQMVPCGAEEVDQ
AVQSAQAAYLKWSKMAGIERSRVMLEAARIIRERRDNIAKLEVINNGKTITEAEYDIDAAWQ
CIEYYAGLAPTLSGQHIQLPGGAFAYTRREPLGVCAGILAWNYPFMIAAWKCAPALACGNAV
VFKPSPMTPVTGVILAEIFHEAGVPVGLVNVVQGGAETGSLLCHHPNVAKVSFTGSVPTGKK
VMEMSAKTVKHVTLELGGKSPLLIFKDCELENAVRGALMANFLTQGQVCTNGTRVFVQREIM
PQFLEEVVKRTKAIVVGDPLLTETRMGGLISKPQLDKVLGFVAQAKKEGARVLCGGEPLTPS
DPKLKNGYFMSPCVLDNCRDDMTCVKEEIFGPVMSVLPFDTEEEVLQRANNTTFGLASGVFT
RDISRAHRVAANLEAGTCYINTYSISPVEVPFGGYKMSGFGRENGQATVDYYSQLKTVIVEM
GDVDSLF
>d1euha_ c.82.1.1 (A:) Aldehyde reductase (dehydrogenase), ALDH
{Streptococcus mutans}
TKQYKNYVNGEWKLSENEIKIYEPASGAELGSVPAMSTEEVDYVYASAKKAQPAWRALSYIE
RAAYLHKVADILMRDKEKIGAILSKEVAKGYKSAVSEVVRTAEIINYAAEEGLRMEGEVLEG
GSFEAASKKKIAVVRREPVGLVLAISPFNYPVNLAGSKIAPALIAGNVIAFKPPTQGSISGL
LLAEAFAEAGLPAGVFNTITGRGSEIGDYIVEHQAVNFINFTGSTGIGERIGKMAGMRPIML
ELGGKDSAIVLEDADLELTAKNIIAGAFGYSGQRCTAVKRVLVMESVADELVEKIREKVLAL
TIGNPEDDADITPLIDTKSADYVEGLINDANDKGATALTEIKREGNLICPILFDKVTTDMRL
AWEEPFGPVLPIIRVTSVEEAIEISNKSEYGLQASIFTNDFPRAFGIAEQLEVGTVHINNKT
QRGTDNFPFLGAKKSGAGIQGVKYSIEAMTTVKSVVFDIK
>d1ez0a_ c.82.1.1 (A:) Aldehyde reductase (dehydrogenase), ALDH
{Vibrio harveyi}
TDNVFYATNAFTGEALPLAFPVHTEVEVNQAATAAAKVARDFRRLNNSKRASLLRTIASELE
ARSDDIIARAHLETALPEVRLTGEIARTANQLRLFADVVNSGSYHQAILDTPNPTRAPLPKP
DIRRQQIALGPVAVFGASNFPLAFSAAGGDTASALAAGCPVIVKGHTAHPGTSQIVAECIEQ
ALKQEQLPQAIFTLLQGNQRALGQALVSHPEIKAVGFTGSVGGGRALFNLAHERPEPIPFYG
ELGAINPTFIFPSAMRAKADLADQFVASMTMGCGQFCTKPGVVFALNTPETQAFIETAQSLI
RQQSPSTLLTPGIRDSYQSQVVSRGSDDGIDVTFSQAESPCVASALFVTSSENWRKHPAWEE
EIFGPQSLIVVCENVADMLSLSEMLAGSLTATIHATEEDYPQVSQLIPRLEEIAGRLVFNGW
PTGVEVGYAMVHGGPYPASTHSASTSVGAEAIHRWLRPVAYQALPESLLPDSLKAENPLEIA
RAVDGKAA
>d1k75a_ c.82.1.2 (A:) L-histidinol dehydrogenase HisD
{Escherichia coli}
NTIIDWNSCTAEQQRQLLMRPAISASESITRTVNDILDNVKARGDEALREYSAKFDKTTVTA

LKVSAEEIAAASERLSDELKQAMAVAVKNIETFHTAQKLPPVDVETQPGVRCQQVTRPVASV
GLYIPGGSAPLFSTVLMLATPASIAGCKKVVLCSPPPIADEILYAAQLCGVQDVFNVGGAQA
IAALAFGTESVPKVDKIFGPGNAFVTEAKRQVSQRLDGAAIDMPAGPSEVLVIADSGATPDF
VASDLLSQAEHGPDSQVILLTPAADMARRVAEAVERQLAELPRAETARQALNASRLIVTKDL
AQCVEISNQYGPEHLIIQTRNARELVDSITSAGSVFLGDWSPESAGDYASGTNHVLPTYGYT
ATCSSLGLADFQKRMTVQELSKEGFSALASTIETLAAAERLTAHKNAVTLRVNALKEQA
>d1aco_2 c.83.1.1 (2-528) Aconitase, first 3 domains {Cow (Bos taurus)}
RAKVAMSHFEPHEYIRYDLLEKNIDIVRKRLNRPLTLSEKIVYGHLDDPANQEIERGKTYLR
LRPDRVAMQDATAQMAMLQFISSGLPKVAVPSTIHCDHLIEAQLGGEKDLRRAKDINQEVYN
FLATAGAKYGVGFWRPGSGIIHQIILENYAYPGVLLIGTDSHTPNGGGLGGICIGVGGADAV
DVMAGIPWELKCPKVIGVKLTGSLSGWTSPKDVILKVAGILTVKGGTGAIVEYHGPGVDSIS
CTGMATICNMGAEIGATTSVFPYNHRMKKYLSKTGRADIANLADEFKDHLVPDSGCHYDQLI
EINLSELKPHINGPFTPDLAHPVAEVGSVAEKEGWPLDIRVGLIGSCTNSSYEDMGRSAAVA
KQALAHGLKCKSQFTITPGSEQIRATIERDGYAQVLRDVGGIVLANACGPCIGQWDRKDIKK
GEKNTIVTSYNRNFTGRNDANPETHAFVTSPEIVTALAIAGTLKFNPETDFLTGKDGKKFKL
EAPDADELPRAEFDPGQDTYQHPPKDSSGQR
>d3pmga1 c.84.1.1 (A:1-190) Phosphoglucomutase {Rabbit (Oryctolagus cuniculus)}
VKIVTVKTKAYPDQKPGTSGLRKRVKVFQSSTNYAENFIQSIISTVEPAQRQEATLVVGGDG
RFYMKEAIQLIVRIAAANGIGRLVIGQNGILSTPAVSCIIRKIKAIGGIILTASHNPGGPNG
DFGIKFNISNGGPAPEAITDKIFQISKTIEEYAICPDLKVDLGVLGKQQFDLENKFKPFTVE
IVDS
>d3pmga2 c.84.1.1 (A:191-303) Phosphoglucomutase {Rabbit (Oryctolagus cuniculus)}
VEAYATMLRNIFDFNALKELLSGPNRLKIRIDAMHGVVGPYVKKILCEELGAPANSAVNCVP
LEDFGGHHPDPNLTYAADLVETMKSGEHDFGAAFDGDGDRNMILGKHGFFV
>d3pmga3 c.84.1.1 (A:304-420) Phosphoglucomutase {Rabbit (Oryctolagus cuniculus)}
NPSDSVAVIAANIFSIPYFQQTGVRGFARSMPTSGALDRVANATKIALYETPTGWKFFGNLM
DASKLSLCGEESFGTGSDHIREKDGLWAVLAWLSILATRKQSVEDILKDHWHKFG
>d1kfia1 c.84.1.1 (A:3-205) Exocytosis-sensitive phosphoprotein, pp63/parafusin {Paramecium tetraurelia}
QVIPAPRVQVTQPYAGQKPGTSGLRKKVSEATQPNYLENFVQSIFNTLRKDELKPKNVLFVG
GDGRYFNRQAIFSIIRLAYANDISEVHVGQAGLMSTPASSHYIRKVNEEVGNCIGGIILTAS
HNPGGKEHGDFGIKFNVRTGAPAPEDFTDQIYTHTTKIKEYLTVDYEFEKHINLDQIGVYKF
EGTRLEKSHFEVKVVDT
>d1kfia2 c.84.1.1 (A:206-323) Exocytosis-sensitive phosphoprotein, pp63/parafusin {Paramecium tetraurelia}
VQDYTQLMQKLFDFDLLKGLFSNKDFSFRFDGMHGVAGPYAKHIFGTLLGCSKESLLNCDPS
EDFGGGHPDPNLTYAHDLVELLDIHKKKDVGTVPQFGAACDGDADRNMILGRQFFV
>d1kfia3 c.84.1.1 (A:324-443) Exocytosis-sensitive phosphoprotein, pp63/parafusin {Paramecium tetraurelia}
TPSDSLAVIAANANLIFKNGLLGAARSMPTSGALDKVAAKNGIKLFETPTGWKFFGNLMDAG

LINLCGEESFGTGSNHIREKDGIWAVLAWLTILAHKNKNTDHFVTVEEIVTQYWQQFG
>d1k2yx1 c.84.1.1 (X:5-154)
Phosphomannomutase/phosphoglucomutase {Pseudomonas aeruginosa}
KAPTLPASIFRAYDIRGVVGDTLTAETAYWIGRAIGSESLARGEPCVAVGRDGRLSGPELVK
QLIQGLVDCGCQVSDVGMVPTPVLYYAANVLEGKSGVMLTGAHNPPDYNGFKIVVAGETLAN
EQIQALRERIEKNDLASGVGSVEQVD
>d1k2yx2 c.84.1.1 (X:155-258)
Phosphomannomutase/phosphoglucomutase {Pseudomonas aeruginosa}
ILPRYFKQIRDDIAMAKPMKVVVDCGNGVAGVIAPQLIEALGCSVIPLYCEVDGNFPNHHPD
PGKPENLKDLIAKVKAENADLGLAFDGDGDRVGVVTNTGTII
>d1k2yx3 c.84.1.1 (X:259-367)
Phosphomannomutase/phosphoglucomutase {Pseudomonas aeruginosa}
YPDRLLMLFAKDVVSRNPGADIIFDVKCTRRLIALISGYGGRPVMWKTGHSLIKKKMKETGA
LLAGEMSGHVFFKERWFGFDDGIYSAARLLEILSQDQRDSEHVFSAF
>d1k2yx4 c.84.1.1 (X:368-463)
Phosphomannomutase/phosphoglucomutase {Pseudomonas aeruginosa}
PSDISTPEINITVTEDSKFAIIEALQRDAQWGEGNITTLDGVRVDYPKGWGLVRASNTTPVL
VLRFEADTEEELERIKTVFRNQLKAVDSSLPVPF
>d1fuia2 c.85.1.1 (A:1-355) L-fucose isomerase, N-terminal and second domains {Escherichia coli}
MKKISLPKIGIRPVIDGRRMGVRESLEEQTMNMAKATAALLTEKLRHACGAAVECVISDTCI
AGMAEAAACEEKFSSQNVGLTITVTPCWCYGSETIDMDPTRPKAIWGFNGTERPGAVYLAAA
LAAHSQKGIPAFSIYGHDVQDADDTSIPADVEEKLLRFARAGLAVASMKGKSYLSLGGVSMG
IAGSIVDHNFFESWLGMKVQAVDMTELRRRIDQKIYDEAELEMALAWADKNFRYGEDENNKQ
YQRNAEQSRAVLRESLLMAMCIRDMMQGNSKLADIGRVEESLGYNAIAAGFQGQRHWTDQYP
NGDTAEAILNSSFDWNGVREPFVVATENDSLNGVAMLMGHQLTGT
>d1fw8a_ c.86.1.1 (A:) Phosphoglycerate kinase {Baker's yeast (Saccharomyces cerevisiae)}
SKYSLAPVAKELQSLLGKDVTFLNDCVGPEVEAAVKASAPGSVILLENLRYHIEEEGSRKVD
GQKVKASKEDVQKFRHELSSLADVYINDAFGTAHRAHSSMVGFDLPQRAAGFLLEKELKYFG
KALENPTRPFLAILGGAKVADKIQLIDNLLDKVDSIIIGGGMAFTFKKVLENTEIGDSIFDK
AGAEIVPKLMEKAKAKGVEVVLPVDFIIADAFSADANTKTVTDKEGIPAGWQGLDNGPESRK
LFAATVAKAKTIVWNGPPGVFEFEKFAAGTKALLDEVVKSSAAGNTVIIGGGDTATVAKKYG
VTDKISHVSTGGGASLELLEGKELPGVAFLSEKKSLSSKLSVQDLDLKDKRVFIRVDFNVPL
DGKKITSNQRIVAALPTIKYVLEHHPRYVVLASHLGRPNGERN
>d1qpg__ c.86.1.1 (-) Phosphoglycerate kinase {Baker's yeast (Saccharomyces cerevisiae)}
SLSSKLSVQDLDLKDKRVFIRVDFNVPLDGKKITSNQRIVAALPTIKYVLEHHPRYVVLASH
LGQPNGERNEKYSLAPVAKELQSLLGKDVTFLNDCVGPEVEAAVKASAPGSVILLENLRYHI
EEEGSRKVDGQKVKASKEDVQKFRHELSSLADVYINDAFGTAHRAHSSMVGFDLPQRAAGFL
LEKELKYFGKALENPTRPFLAILGGAKVADKIQLIDNLLDKVDSIIIGGGMAFTFKKVLENT
EIGDSIFDKAGAEIVPKLMEKAKAKGVEVVLPVDFIIADAFSADANTKTVTDKEGIPAGWQG
LDNGPESRKLFAATVAKAKTIVWNGPPGVFEFEKFAAGTKALLDEVVKSSAAGNTVIIGGGD
TATVAKKYGVTDKISHVSTGGGASLELLEGKELPGVAFLSEKK

>d1php__ c.86.1.1 (-) Phosphoglycerate kinase {Bacillus stearothermophilus}
MNKKTIRDVDVRGKRVFCRVDFNVPMEQGAITDDTRIRAALPTIRYLIEHGAKVILASHLGR
PKGKVVEELRLDAVAKRLGELLERPVAKTNEAVGDEVKAAVDRLNEGDVLLLENVRFYPGEE
KNDPELAKAFAELADLYVNDAFGAAHRAHASTEGIAHYLPAVAGFLMEKELEVLGKALSNPD
RPFTAIIGGAKVKDKIGVIDNLLEKVDNLIIGGGLAYTFVKALGHDVGKSLLEEDKIELAKS
FMEKAKEKGVRFYMPVDVVVADRFANDANTKVVPIDAIPADWSALDIGPKTRELYRDVIRES
KLVVWNGPMGVFEMDAFAHGTKAIAEALAEALDTYSVIGGGDSAAAVEKFGLADKMDHISTG
GGASLEFMEGKQLPGVVALEDK
>d1vpe__ c.86.1.1 (-) Phosphoglycerate kinase {Thermotoga maritima}
EKMTIRDVDLKGKRVIMRVDFNVPVKDGVVQDDTRIRAALPTIKYALEQGAKVILLSHLGRP
KGEPSPEFSLAPVAKRLSELLGKEVKFVPAVVGDEVKKAVEELKEGEVLLLENTRFHPGETK
NDPELAKFWASLADIHVNDAFGTAHRAHASNVGIAQFIPSVAGFLMEKEIKFLSKVTYNPEK
PYVVVLGGAKVSDKIGVITNLMEKADRILIGGAMMFTFLKALGKEVGSSRVEEDKIDLAKEL
VEKAKEKGVEIVLPVDAVIAQKIEPGVEKKVVRIDDGIPEGWMGLDIGPETIELFKQKLSDA
KTVVWNGPMGVFEIDDFAEGTKQVALAIAALTEKGAITVVGGGDSAAAVNKFGLEDKFSHVS
TGGGASLEFLEGKELPGIASMRIKKA
>d16pk__ c.86.1.1 (-) Phosphoglycerate kinase {Trypanosoma brucei}
EKKSINECDLKGKKVLIRVDFNVPVKNGKITNDYRIRSALPTLKKVLTEGGSCVLMSHLGRP
KGIPMAQAGKIRSTGGVPGFQQKATLKPVAKRLSELLLRPVTFAPDCLNAADVVSKMSPGDV
VLLENVRFYKEEGSKKAKDREAMAKILASYGDVYISDAFGTAHRDSATMTGIPKILGNGAAG
YLMEKEISYFAKVLGNPPRPLVAIVGGAKVSDKIQLLDNMLQRIDYLLIGGAMAYTFLKAQG
YSIGKSKCEESKLEFARSLLKKAEDRKVQVILPIDHVCHTEFKAVDSPLITEDQNIPEGHMA
LDIGPKTIEKYVQTIGKCKSAIWNGPMGVFEMVPYSKGTFAIAKAMGRGTHEHGLMSIIGGG
DSASAAELSGEAKRMSHVSTGGGASLELLEGKTLPGVTVLDDK
>d1hdia_ c.86.1.1 (A:) Phosphoglycerate kinase {Pig (Sus scrofa)}
NKLTLDKLNVKGKRVVMRVDFNVPMAAAQITNNARIKAAVPSIKFCLDDGAKSVVLMSHLGR
PDGSPMPDKYSLQPVAAELKSALGKAVLFLKDCVGPAVEKACADPAAGSVILLENLRFHVEE
EGKGKDASGNKAAGEPAKIKAFRASLSALGDVYVNDAFGTAHRAHSSMVGVNLPKKAGAFLM
KKELNYFAAAAESPERPFLAILGGAKVADKIQLINNMLDKVNEMIIGGGMAFTFLKVLNNME
IGTSLFDEAGKKIVKNLMSKAAANGVKITLPVDFVTADKFDEQAKIGQATVASGIPAGWMGL
DCGPKSSAKYSEAVARAKQIVWNGPVGVFEWEAFAQGTKALMDEVVKATSRGCITIIGGGDT
ATCCAKWNTEDNVSHVSTGGGASLELLEGKVLPGVDALSNV
>d1jixa_ c.87.1.1 (A:) beta-Glucosyltransferase (DNA-modifying) {Bacteriophage T4}
MKIAIINMGNNVINFKTVPSSETIYLFKVISEMGLNVDIISLKNGVYTKSFDEVDVNDYDRL
IVVNSSINFFGGKPNLAILSAQKFMAKYKSKIYYLFTDIRLPFSQSWPNVKNRPWAYLYTEE
ELLIKSPIKVISQGINLDIAKAAHKKVDNVIEFEYFPIEQYKIHMNDFQLSKPTKKTLDVIY
GGSFRSGQRESKMVEFLFDTGLNIEFFGNAREKQFKNPKYPWTKAPVFTGKIPMNMVSEKNS
QAIAALIIGDKNYNDNFITLRVWETMASDAVMLIDEEFDTKHRIINDARFYVNNRAELIDRV
NELKHSDVLRKEMLSIQHDILNKTRAKKAEWQDAFKKAIDL
>d1f0ka_ c.87.1.2 (A:) Peptidoglycan biosynthesys glycosyltransferase MurG {Escherichia coli}

KRLMVMAGGTGGHVFPGLAVAHHLMAQGWQVRWLGTADRMEADLVPKHGIEIDFIRISGLRG
KGIKALIAAPLRIFNAWRQARAIMKAYKPDVVLGMGGYVSGPGGLAAWSLGIPVVLHEQNGI
AGLTNKWLAKIATKVMQAFPGAFPNAEVVGNPVRTDVLALPLPQQRLAGREGPVRVLVVGGS
QGARILNQTMPQVAAKLGDSVTIWHQSGKGSQQSVEQAYAEAGQPQHKVTEFIDDMAAAYAW
ADVVVCRSGALTVSEIAAAGLPALFVPFQHKDRQQYWNALPLEKAGAAKIIEQPQLSVDAVA
NTLAGWSRETLLTMAERARAASIPDATERVANEVSRVARAL
>d1f6da_ c.87.1.3 (A:) UDP-N-acetylglucosamine 2-epimerase
{Escherichia coli}
MKVLTVFGTRPEAIKMAPLVHALAKDPFFEAKVCVTAQHREMLDQVLKLFSIVPDYDLNIMQ
PGQGLTEITCRILEGLKPILAEFKPDVVLVHGDTTTTLATSLAAFYQRIPVGHVEAGLRTGD
LYSPWPEEANRTLTGHLAMYHFSPTETSRQNLLRENVADSRIFITGNTVIDALLWVRDQVMS
SDKLRSELAANYPFIDPDKKMILVTGHRRESFGRGFEEICHALADIATTHQDIQIVYPVHLN
PNVREPVNRILGHVKNVILIDPQEYLPFVWLMNHAWLILTDSGGIQEEAPSLGKPVLVMRDT
TERPEAVTAGTVRLVGTDKQRIVEEVTRLLKDENEYQAMSRAHNPYGDGQACSRILEALKNN
RISL
>d1iira_ c.87.1.5 (A:) UDP-glucosyltransferase GtfB
{Amycolatopsis orientalis}
MRVLLATCGSRGDTEPLVALAVRVRDLGADVRMCAPPDCAERLAEVGVPHVPVGPSARAPIQ
RAKPLTAEDVRRFTTEAIATQFDEIPAAAEGCAAVVTTGLLAAAIGVRSVAEKLGIPYFYAF
HCPSYVPSPYYPPPPLGEPSTQDTIDIPAQWERNNQSAYQRYGGLLNSHRDAIGLPPVEDIF
TFGYTDHPWVAADPVLAPLQPTDLDAVQTGAWILPDERPLSPELAAFLDAGPPPVYLGFGSL
GAPADAVRVAIDAIRAHGRRVILSRGWADLVLPDDGADCFAIGEVNHQVLFGRVAAVIHHGG
AGTTHVAARAGAPQILLPQMADQPYYAGRVAELGVGVAHDGPIPTFDSLSAALATALTPETH
ARATAVAGTIRTDGAAVAARLLLDAVSRE
>d1em6a_ c.87.1.4 (A:) Glycogen phosphorylase {Human (Homo
sapiens)}
ENVAELKKSFNRHLHFTLVKDRNVATTRDYYFALAHTVRDHLVGRWIRTQQHYYDKCPKRVY
YLSLEFYMGRTLQNTMINLGLQNACDEAIYQLGLDIEELEEIEEDAGLGNGGLGRLAACFLD
SMATLGLAAYGYGIRYEYGIFNQKIRDGWQVEEADDWLRYGNPWEKSRPEFMLPVHFYGKVE
HTNTGTKWIDTQVVLALPYDTPVPGYMNNTVNTMRLWSARAPNDFNLRDFNVGDYIQAVLDR
NLAENISRVLYPNDNFFEGKELRLKQEYFVVAATLQDIIRRFKASKFGSTRGAGTVFDAFPD
QVAIQLNDTHPALAIPELMRIFVDIEKLPWSKAWELTQKTFAYTNHTVLPEALERWPVDLVE
KLLPRHLEIIYEINQKHLDRIVALFPKDVDRLRRMSLIEEEGSKRINMAHLCIVGSHAVNGV
AKIHSDIVKTKVFKDFSELEPDKFQNKTNGITPRRWLLLCNPGLAELIAEKIGEDYVKDLSQ
LTKLHSFLGDDVFLRELAKVKQENKLKFSQFLETEYKVKINPSSMFDVQVKRIHEYKRQLLN
CLHVITMYNRIKKDPKKLFVPRTVIIGGKAAPGYHMAKMIIKLITSVADVVNNDPMVGSKLK
VIFLENYRVSLAEKVIPATDLSEQISTAGTEASGTGNMKFMLNGALTIGTMDGANVEMAEEA
GEENLFIFGMRIDDVAALDKKGYEAKEYYEALPELKLVIDQIDNGFFSPKQPDLFKDIINML
FYHDRFKVFADYEAYVKCQDKVSQLYMNPKAWNTMVLKNIAASGKFSSDRTIKEYAQNIWNV
EPS
>d1a8i__ c.87.1.4 (-) Glycogen phosphorylase {Rabbit (Oryctolagus
cuniculus)}
QEKRKQISVRGLAGVENVTELKKNFNRHLHFTLVKDRNVATPRDYYFALAHTVRDHLVGRWI
RTQQHYYEKDPKRIYYLSLEFYMGRTLQNTMVNLALENACDEATYQLGLDMEELEEIEEDAG

LGNGGLGRLAACFLDSMATLGLAAYGYGIRYEFGIFNQKICGGWQMEEADDWLRYGNPWEKA
RPEFTLPVHFYGRVEHTSQGAKWVDTQVVLAMPYDTPVPGYRNNVVNTMRLWSAKAPNDFNL
KDFNVGGYIQAVLDRNLAENISRVLYPNDNFFEGKELRLKQEYFVVAATLQDIIRRFKSSKF
GCRDPVRTNFDAFPDKVAIQLNDTHPSLAIPELMRVLVDLERLDWDKAWEVTVKTCAYTNHT
VIPEALERWPVHLLETLLPRHLQIIYEINQRFLNRVAAAFPGDVDRLRRMSLVEEGAVKRIN
MAHLCIAGSHAVNGVARIHSEILKKTIFKDFYELEPHKFQNKTNGITPRRWLVLCNPGLAEI
IAERIGEEYISDLDQLRKLLSYVDDEAFIRDVAKVKQENKLKFAAYLEREYKVHINPNSLFD
VQVKRIHEYKRQLLNCLHVITLYNRIKKEPNKFVVPRTVMIGGKAAPGYHMAKMIIKLITAI
GDVVNHDPVVGDRLRVIFLENYRVSLAEKVIPAADLSEQISTAGTEASGTGNMKFMLNGALT
IGTMDGANVEMAEEAGEENFFIFGMRVEDVDRLDQRGYNAQEYYDRIPELRQIIEQLSSGFF
SPKQPDLFKDIVNMLMHHDRFKVFADYEEYVKCQERVSALYKNPREWTRMVIRNIATSGKFS
SDRTIAQYAREIWGVEPSRQRLP
>d1ygpa_ c.87.1.4 (A:) Glycogen phosphorylase {Baker's yeast (Saccharomyces cerevisiae)}
TRRLTGFLPQEIKSIDTMIPLLSRALWNKHQVKKFNKAEDFQDRFIDHVETTLARSLYNCDD
MVAYEAASMSIRDNLVIDWNKTQQKFTTRDPKRVYYLSLEFLMGRALDNALINMKIEDPEDP
AASKGKPREMIKGALDELGFKLEDVLDQEPDAGLGNGGLGRLAACFVDSMATEGIPAWGYGL
RYEYGIFAQKIIDGYQVETPDYWLNSGNPWEIERNEVQIPVTFYGYVDRPEGGKTTLSASQW
IGGERVLAVAYDFPVPGFKTSNVNNLRLWQARPTTEFDLNKFNNGDYKNSVAQQQRAESITA
VLYPNDNFAQGKELRLKQQYFWCAASLHDILRRFKKSKRPWTEFPDQVAIQLNDTHPTLAIV
ELQRVLVDLEKLDWHEAWDIVTKTFAYTNHTVMQEALEKWPRRLFGHLLPRHLEIIYDINWF
FLEDVAKKFPKDVDLLSRISIIEENSPERQIRMAFLAIVGSHKVNGVVELHSELIKTTIFKD
FIKFYGPSKFVNVTNGITPRRWLKQANPSLAKLISETLNDPTEEYLLDMAKLTQLEKYVEDK
EFLKKWNQVKLNNKIRLVDLIKKENDGVDIINREYLDDTLFDMQVKRIHEYKRQQLNVFGII
YRYLAMKNMLKNGASIEEVARKYPRKVSIFGGKSAPGYYMAKLIIKLINCVADIVNNDESIE
HLLKVVFVADYNVSKAEIIIPASDLSEHISTAGTEASGTSNMKFVMNGGLIIGTVDGANVEI
TREIGEDNVFLFGNLSENVEELRYNHQYHPQDLPSSLDSVLSYIESGQFSPENPNEFKPLVD
SIKYHGDYYLVSDDFESYLATHELVDQEFHNQRSEWLKKSVLSLANVGFFSSDRCIEEYSDT
IWNVEPVT
>d1qm5a_ c.87.1.4 (A:) Maltodextrin phosphorylase (MALP) {Escherichia coli}
SQPIFNDKQFQEALSRQWQRYGLNSAAEMTPRQWWLAVSEALAEMLRAQPFAKPVANQRHVN
YISMEFLIGRLTGNNLLNLGWYQDVQDSLKAYDINLTDLLEEEIDPALGNGGLGRLAACFLD
SMATVGQSATGYGLNYQYGLFRQSFVDGKQVEAPDDWHRSNYPWFRHNEALDVQVGIGGKVT
KDGRWEPEFTITGQAWDLPVVGYRNGVAQPLRLWQATHAHPFDLTKFNDGDFLRAEQQGINA
EKLTKVLYPNDNHTAGKKLRLMQQYFQCACSVADILRRHHLAGRKLHELADYEVIQLNDTHP
TIAIPELLRVLIDEHQMSWDDAWAITSKTFAYTNHTLMPEALERWDVKLVKGLLPRHMQIIN
EINTRFKTLVEKTWPGDEKVWAKLAVVHDKQVHMANLCVVGGFAVNGVAALHSDLVVKDLFP
EYHQLWPNKFHNVTNGITPRRWIKQCNPALAALLDKSLQKEWANDLDQLINLEKFADDAKFR
DQYREIKQANKVRLAEFVKVRTGIEINPQAIFDIQIKRLHEYKRQHLNLLHILALYKEIREN
PQADRVPRVFLFGAKAAPGYYLAKNIIFAINKVADVINNDPLVGDKLKVVFLPDYCVSAAEK
LIPAADISEQISTAGKEASGTGNMKLALNGALTVGTLDGANVEIAEKVGEENIFIFGHTVEQ
VKAILAKGYDPVKWRKKDKVLDAVLKELESGKYSDGDKHAFDQMLHSIGKQGGDPYLVMADF
AAYVEAQKQVDVLYRDQEAWTRAAILNTARCGMFSSDRSIRDYQARIWQAKR

>d4ecaa_ c.88.1.1 (A:) Asparaginase type II {Escherichia coli}
LPNITILATGGTIAGGGDSATKSNYTVGKVGVENLVNAVPQLKDIANVKGEQVVNIGSQDMN
DNVWLTLAKKINTDCDKTDGFVITHGVDTMEETAYFLDLTVKCDKPVVMVGAMRPSTSMSAD
GPFNLYNAVVTAADKASANRGVLVVMNDTVLDGRDVTKTNTTDVATFKSVNYGPLGYIHNGK
IDYQRTPARKHTSDTPFDVSKLNELPKVGIVYNYANASDLPAKALVDAGYDGIVSAGVGNGN
LYKSVFDTLATAAKTGTAVVRSSRVPTGATTQDAEVDDAKYGFVASGTLNPQKARVLLQLAL
TQTKDPQQIQQIFNQY
>d1wsaa_ c.88.1.1 (A:) Asparaginase type II {Wolinella succinogenes}
KPQVTILATGGTIAGSGESSVKSSYSAGAVTVDKLLAAVPAINDLATIKGEQISSIGSQEMT
GKVWLKLAKRVNELLAQKETEAVIITHGTDTMEETAFFLNLTVKSQKPVVLVGAMRPGSSMS
ADGPMNLYNAVNVAINKASTNKGVVIVMNDEIHAAREATKLNTTAVNAFASPNTGKIGTVYY
GKVEYFTQSVRPHTLASEFDISKIEELPRVDILYAHPDDTDVLVNAALQAGAKGIIHAGMGN
GNPFPLTQNALEKAAKSGVVVARSSRVGSGSTTQEAEVDDKKLGFVATESLNPQKARVLLML
ALTKTSDREAIQKIFSTY
>d1jsra_ c.88.1.1 (A:) Asparaginase type II {Erwinia chrysanthemi}
LPNIVILATGGTIAGSAATGTQTTGYKAGALGVDTLINAVPEVKKLANVKGEQFSNMASENM
TGDVVLKLSQRVNELLARDDVDGVVITHGTDTVEESAYFLHLTVKSDKPVVFVAAMRPATAI
SADGPMNLLEAVRVAGDKQSRGRGVMVVINDRIGSARYITKTNASTLDTFRANEEGYLGVII
GNRIYYQNRIDKLHTTRSVFDVRGLTSLPKVDILYGYQDDPEYLYDAAIQHGVKGIVYAGMG
AGSVSVRGIAGMRKALEKGVVVMRSTRTGNGIVPPDEELPGLVSDSLNPAHARILLMLALTR
TSDPKVIQEYFHTY
>d1agx__ c.88.1.1 (-) Glutaminase-asparaginase {Acinetobacter glutaminasificans}
KNNVVIVATGGTIAGAGASSTNSATYSAAKVPVDALIKAVPQVNDLANITGIQALQVASESI
TDKELLSLARQVNDLVKKPSVNGVVITHGTDTMEETAFFLNLVVHTDKPIVLVGSMRPSTAL
SADGPLNLYSAVALASSNEAKNKGVMVLMNDSIFAARDVTKGINIHTHAFVSQWGALGTLVE
GKPYWFRSSVKKHTNNSEFNIEKIQGDALPGVQIVYGSDNMMPDAYQAFAKAGVKAIIHAGT
GNGSMANYLVPEVRKLHDEQGLQIVRSSRVAQGFVLRNAEQPDDKYGWIAAHDLNPQKARLL
MALALTKTNDAKEIQNMFWNY
>d4pgaa_ c.88.1.1 (A:) Glutaminase-asparaginase {Pseudomonas sp., 7A}
KLANVVILATGGTIAGAGASAANSATYQAAKVGVDKLIAGVPELADLANVRGEQVMQIASES
ITNDDLLKLGKRVAELADSNDVDGIVITHGTDTLEETAYFLNLVQKTDKPIVVVGSMRPGTA
MSADGMLNLYNAVAVASNKDSRGKGVLVTMNDEIQSGRDVSKSINIKTEAFKSAWGPLGMVV
EGKSYWFRLPAKRHTVNSEFDIKQISSLPQVDIAYSYGNVTDTAYKALAQNGAKALIHAGTG
NGSVSSRVVPALQQLRKNGTQIIRSSHVNQGGFVLRNAEQPDDKNDWVVAHDLNPEKARILA
MVAMTKTQDSKELQRIFWEY
>d1pfka_ c.89.1.1 (A:) Phosphofructokinase {Escherichia coli}
MIKKIGVLTSGGDAPGMNAAIRGVVRSALTEGLEVMGIYDGYLGLYEDRMVQLDRYSVSDMI
NRGGTFLGSARFPEFRDENIRAVAIENLKKRGIDALVVIGGDGSYMGAMRLTEMGFPCIGLP
GTIDNDIKGTDYTIGFFTALSTVVEAIDRLRDTSSSHQRISVVEVMGRYCGDLTLAAAIAGG
CEFVVVPEVEFSREDLVNEIKAGIAKGKKHAIVAITEHMCDVDELAHFIEKETGRETRATVL
GHIQRGGSPVPYDRILASRMGAYAIDLLLAGYGGRCVGIQNEQLVHHDIIDAIENMKRPFKG

DWLDCAKKLY
>d3pfk__ c.89.1.1 (-) Phosphofructokinase {Bacillus stearothermophilus}
MKRIGVLTSGGDSPGMNAAIRSVVRKAIYHGVEVYGVYHGYAGLIAGNIKKLEVGDVGDIIH
RGGTILYTARCPEFKTEEGQKKGIEQLKKHGIQGLVVIGGDGSYQGAKKLTEHGFPCVGVPG
TIDNDIPGTDFTIGFDTALNTVIDAIDKIRDTATSHERTYVIEVMGRHAGDIALWSGLAGGA
ETILIPEADYDMNDVIARLKRGHERGKKHSIIIVAEGVGSGVDFGRQIQEATGFETRVTVLG
HVQRGGSPTAFDRVLASRLGARAVELLLEGKGGRCVGIQNNQLVDHDIAEALANKHTIDQRM
YALSKELSI
>d1cbf__ c.90.1.1 (-) Cobalt precorrin-4 methyltransferase CbiF {Bacillus megaterium}
GLVPRGSHMKLYIIGAGPGDPDLITVKGLKLLQQADVVLYADSLVSQDLIAKSKPGAEVLKT
AGMHLEEMVGTMLDRMREGKMVVRVHTGDPAMYGAIMEQMVLLKREGVDIEIVPGVTSVFAA
AAAAEAELTIPDLTQTVILTRAEGRTPVPEFEKLTDLAKHKCTIALFLSSTLTKKVMKEFIN
AGWSEDTPVVVVYKATWPDEKIVRTTVKDLDDAMRTNGIRKQAMILAGWALDP
>d1i74a_ c.107.1.1 (A:) Manganese-dependent inorganic pyrophosphatase (family II) {Streptococcus mutans}
SKILVFGHQNPDSDAIGSSMAYAYLKRQLGVDAQAVALGNPNEETAFVLDYFGIQAPPVVKS
AQAEGAKQVILTDHNEFQQSIADIREVEVVEVVDHHRVANFETANPLYMRLEPVGSASSIVY
RLYKENGVAIPKEIAGVMLSGLISDTLLLKSPTTHASDPAVAEDLAKIAGVDLQEYGLAMLK
AGTNLASKTAAQLVDIDAKTFELNGSQVRVAQVNTVDINEVLERQNEIEEAIKASQAANGYS
DFVLMITDILNSNSEILALGNNTDKVEAAFNFTLKNNHAFLAGAVSRKKQVVPQLTESFNG
>d1k20a_ c.107.1.1 (A:) Manganese-dependent inorganic pyrophosphatase (family II) {Streptococcus gordonii}
SKILVFGHQNPDSDAIGSSYAFAYLAREAYGLDTEAVALGEPNEETAFVLDYFGVAAPRVIT
SAKAEGAEQVILTDHNEFQQSVADIAEVEVYGVVDHHRVANFETANPLYMRLEPVGSASSIV
YRMFKEHSVAVSKEIAGLMLSGLISDTLLLKSPTTHPTDKAIAPELAELAGVNLEEYGLAML
KAGTNLASKSAEELIDIDAKTFELNGNNVRVAQVNTVDIAEVLERQAEIEAAIEKAIADNGY
SDFVLMITDIINSNSEILAIGSNMDKVEAAFNFVLENNHAFLAGAVSRKKQVVPQLTESFNA
>d1k23a_ c.107.1.1 (A:) Manganese-dependent inorganic pyrophosphatase (family II) {Bacillus subtilis}
MEKILIFGHQNPDTDTICSAIAYADLKNKLGFNAEPVRLGQVNGETQYALDYFKQESPRLVE
TAANEVNGVILVDHNERQQSIKDIEEVQVLEVIDHHRIANFETAEPLYYRAEPVGCTATILN
KMYKENNVKIEKEIAGLMLSAIISDSLLFKSPTCTDQDVAAAKELAEIAGVDAEEYGLNMLK
AGADLSKKTVEELISLDAKEFTLGSKKVEIAQVNTVDIEDVKKRQAELEAVISKVVAEKNLD
LFLLVITDILENDSLALAIGNEAAKVEKAFNVTLENNTALLKGVVSRKKQVVPVLTDAM
>d1ayl_1 c.91.1.1 (228-540) Phosphoenolpyruvate (PEP) carboxykinase (ATP-oxaloacetate carboxy-liase) {Escherichia coli}
IASMHCSANVGEKGDVAVFFGLSGTGKTTLSTDPKRRLIGDDEHGWDDDGVFNFEGGCYAKT
IKLSKEAEPEIYNAIRRDALLENVTVREDGTIDFDDGSKTENTRVSYPIYHIDNIVKPVSKA
GHATKVIFLTADAFGVLPPVSRLTADQTQYHFLSGFTAKLAGTERGITEPTPTFSACFGAAF
LSLHPTQYAEVLVKRMQAAGAQAYLVNTGWNGTGKRISIKDTRAIIDAILNGSLDNAETFTL
PMFNLAIPTELPGVDTKILDPRNTYASPEQWQEKAETLAKLFIDNFDKYTDTPAGAALVAAG
PKL

>d1ii2a1 c.91.1.1 (A:201-523) Phosphoenolpyruvate (PEP) carboxykinase (ATP-oxaloacetate carboxy-liase) {Trypanosoma cruzi}
HLCMHASANVGKQGDVTVFFGLSGTGKTTLSADPHRNLIGDDEHVWTDRGVFNIEGGCYAKA
IGLNPKTEKDIYDAVRFGAVAENCVLDKRTGEIDFYDESICKNTRVAYPLSHIEGALSKAIA
GHPKNVIFLTNDAFGVMPPVARLTSAQAMFWFVMGYTANVPGVEAGGTRTARPIFSSCFGGP
FLVRHATFYGEQLAEKMQKHNSRVWLLNTGYAGGRADRGAKRMPLRVTRAIIDAIHDGTLDR
TEYEEYPGWGLHIPKYVAKVPEHLLNPRKAWKDVRQFNETSKELVAMFQESFSARFAAKASQ
EMKSAVPRYVEFA
>d1khba1 c.91.1.1 (A:260-622) Cytosolic phosphoenolpyruvate carboxykinase (GTP-hydrolysing) {Human (Homo sapiens)}
WLAEHMLVLGITNPEGEKKYLAAAFPSACGKTNLAMMNPSLPGWKVECVGDDIAWMKFDAQG
HLRAINPENGFFGVAPGTSVKTNPNAIKTIQKNTIFTNVAETSDGGVYWEGIDEPLASGVTI
TSWKNKEWSSEDGEPCAHPNSRFCTPASQCPIIDAAWESPEGVPIEGIIFGGRRPAGVPLVY
EALSWQHGVFVGAAMRSEATAAAEHKGKIIMHDPFAMRPFFGYNFGKYLAHWLSMAQHPAAK
LPKIFHVNWFRKDKEGKFLWPGFGENSRVLEWMFNRIDGKASTKLTPIGYIPKEDALNLKGL
GHINMMELFSISKEFWDKEVEDIEKYLVDQVNADLPCEIEREILALKQRISQM
>d1jb1a_ c.91.1.2 (A:) HPr kinase HprK C-terminal domain {Lactobacillus casei}
ERRSMHGVLVDIYGLGVLITGDSGVGKSETALELVQRGHRLIADDRVDVYQQDEQTIVGAAP
PILSHLLEIRGLGIIDVMNLFGAGAVREDTTISLIVHLENWTPDKTFDRLGSGEQTQLIFDV
PVPKITVPVKVGRNLAIIIEVAAMNFRAKSMGYDATKTFEKNLNHLIEH
>d1ayl_2 c.109.1.1 (1-227) Phosphoenolpyruvate (PEP) carboxykinase (ATP-oxaloacetate carboxy-liase) {Escherichia coli}
MRVNNGLTPQELEAYGISDVHDIVYNPSYDLLYQEELDPSLTGYERGVLTNLGAVAVDTGIF
TGRSPKDKYIVRDDTTRDTFWWADKGKGKNDNKPLSPETWQHLKGLVTRQLSGKRLFVVDAF
CGANPDTRLSVRFITEVAWQAHFVKNMFIRPSDEELAGFKPDFIVMNGAKCTNPQWKEQGLN
SENFVAFNLTERMQLIGGTWYGGEMKKGMFSMMNYLLPLKG
>d1ii2a2 c.109.1.1 (A:2-200) Phosphoenolpyruvate (PEP) carboxykinase (ATP-oxaloacetate carboxy-liase) {Trypanosoma cruzi}
PPTIHRNLLSPELVQWALKIEKDSRLTARGALAVMSYAKTGRSPLDKRIVDTDDVRENVDWG
KVNMKLSEESFARVRKIAKEFLDTREHLFVVDCFAGHDERYRLKVRVFTTRPYHALFMRDML
IVPTPEELATFGEPDYVIYNAGECKADPSIPGLTSTTCVALNFKTREQVILGTEYAGEMKKG
ILTVMFELMPQMN
>d1khba2 c.109.1.1 (A:10-259) Cytosolic phosphoenolpyruvate carboxykinase (GTP-hydrolysing) {Human (Homo sapiens)}
NLSAKVVQGSLDSLPQAVREFLENNAELCQPDHIHICDGSEEENGRLLGQMEEEGILRRLKK
YDNCWLALTDPRDVARIESKTVIVTQEQRDTVPIPKTGLSQLGRWMSEEDFEKAFNARFPGC
MKGRTMYVIPFSMGPLGSPLSKIGIELTDSPYVVASMRIMTRMGTPVLEALGDGEFVKCLHS
VGCPLPLQKPLVNNWPCNPELTLIAHLPDRREIISFGSGYGGNSLLGKKCFALRMASRLAKE
EG
>d1doza_ c.92.1.1 (A:) Ferrochelatase {Bacillus subtilis}
SRKKMGLLVMAYGTPYKEEDIERYYTHIRRGRKPEPEMLQDLKDRYEAIGGISPLAQITEQQ

AHNLEQHLNEIQDEITFKAYIGLKHIEPFIEDAVAEMHKDGITEAVSIVLAPHFSTFSVQSY
NKRAKEEAEKLGGLTITSVESWYDEPKFVTYWVDRVKETYASMPEDERENAMLIVSAHSLPE
KIKEFGDPYPDQLHESAKLIAEGAGVSEYAVGWQSEGNTPDPWLGPDVQDLTRDLFEQKGYQ
AFVYVPVGFVADHLEVLYDNDYECKVVTDDIGASYYRPEMPNAKPEFIDALATVVLKKLGR
>d1hrka_ c.92.1.1 (A:) Ferrochelatase {Human (Homo sapiens)}
RKPKTGILMLNMGGPETLGDVHDFLLRLFLDRDLMTLPIQNKLAPFIAKRLTPKIQEQYRRI
GGGSPIKIWTSKQGEGMVKLLDELSPNTAPHKYYIGFRYVHPLTEEAIEEMERDGLERAIAF
TQYPQYSCSTTGSSLNAIYRYYNQVGRKPTMKWSTIDRWPTHHLLIQCFADHILKELDHFPL
EKRSEVVILFSAHSLPMSVVNRGDPYPQEVSATVQKVMERLEYCNPYRLVWQSKVGPMPWLG
PQTDESIKGLCERGRKNILLVPIAFTSDHIETLYELDIEYSQVLAKECGVENIRRAESLNGN
PLFSKALADLVHSHIQSNELCSKQLTLSCPLCVNPVCRETKSFFTSQQL
>d1qgoa_ c.92.1.2 (A:) Cobalt chelatase CbiK {Salmonella typhimurium}
KKALLVVSFGTSYHDTCEKNIVACERDLAASCPDRDLFRAFTSGMIIRKLRQRDGIDIDTPL
QALQKLAAQGYQDVAIQSLHIINGDEYEKIVREVQLLRPLFTRLTLGVPLLSSHNDYVQLMQ
ALRQQMPSLRQTEKVVFMGHGASHHAFAAYACLDHMMTAQRFPARVGAVESYPEVDILIDSL
RDEGVTGVHLMPLMLVAGDHAINDMASDDGDSWKMRFNAAGIPATPWLSGLGENPAIRAMFV
AHLHQALNM
>d1efdn_ c.92.2.1 (N:) Periplasmic ferric siderophore binding protein FhuD {Escherichia coli}
GIDPNRIVALEWLPVELLLALGIVPYGVADTINYRLWVSEPPLPDSVIDVGLRTEPNLELLT
EMKPSFMVWSAGYGPSPEMLARIAPGRGFNFSDGKQPLAMARKSLTEMADLLNLQSAAETHL
AQYEDFIRSMKPRFVKRGARPLLLTTLIDPRHMLVFGPNSLFQEILDEYGIPNAWQGETNFW
GSTAVSIDRLAAYKDVDVLCFDHDNSKDMDALMATPLWQAMPFVRAGRFQRVPAVWFYGATL
SAMHFVRVLDNAIG
>d1toaa_ c.92.2.2 (A:) Periplasmic zinc binding protein TroA {Treponema pallidum}
GKPLVVTTIGMIADAVKNIAQGDVHLKGLMGPGVDPHLYTATAGDVEWLGNADLILYNGLHL
ETKMGEVFSKLRGSRLVVAVSETIPVSQRLSLEEAEFDPHVWFDVKLWSYSVKAVYESLCKL
LPGKTREFTQRYQAYQQQLDKLDAYVRRKAQSLPAERRVLVTAHDAFGYFSRAYGFEVKGLQ
GVSTASEASAHDMQELAAFIAQRKLPAIFIESSIPHKNVEALRDAVQARGHVVQIGGELFSD
AMGDAGTSEGTYVGMVTHNIDTIVAALAR
>d1psza_ c.92.2.2 (A:) Pneumococcal surface antigen PssA {Pneumococcus (Streptococcus pneumoniae)}
KKDTTSGQKLKVVATNSIIADITKNIAGDKIDLHSIVPIGQDPHEYEPLPEDVKKTSEADLI
FYNGINLETGGNAWFTKLVENAKKTENKDYFAVSDGVDVIYLEGQNEKGKEDPHAWLNLENG
IIFAKNIAKQLSAKDPNNKEFYEKNLKEYTDKLDKLDKESKDKFNKIPAEKKLIVTSEGAFK
YFSKAYGVPSAYIWEINTEEEGTPEQIKTLVEKLRQTKVPSLFVESSVDDRPMKTVSQDTNI
PIYAQIFTDSIAEQGKEGDSYYSMMKYNLDKIAEGLAK
>d1mioa_ c.92.2.3 (A:) Nitrogenase iron-molybdenum protein, alpha and beta chains {Clostridium pasteurianum}
SENLKDEILEKYIPKTKKTRSGHIVIKTEETPNPEIVANTRTVPGIITARGCAYAGCKGVVM
GPIKDMVHITHGPIGCSFYTWGGRRFKSKPENGTGLNFNEYVFSTDMQESDIVFGGVNKLKD
AIHEAYEMFHPAAIGVYATCPVGLIGDDILAVAATASKEIGIPVHAFSCEGYKGVSQSAGHH

IANNTVMTDIIGKGNKEQKKYSINVLGEYNIGGDAWEMDRVLEKIGYHVNATLTGDATYEKV
QNADKADLNLVQCHRSINYIAEMMETKYGIPWIKCNFIGVDGIVETLRDMAKCFDDPELTKR
TEEVIAEEIAAIQDDLDYFKEKLQGKTACLYVGGSRSHTYMNMLKSFGVDSLVAGFEFAHRD
DYEGREVIPTIKIDADSKNIPEITVTPDEQKYRVVIPEDKVEELKKAGVPLSSYGGMMKEMH
DGTILIDDMNHHDMEVVLEKLKPDMFFAGIKEKFVIQKGGVLSKQLHSYDYNGPYAGFRGVV
NFGHELVNGIYTPAWKMITPPWKKASSES
>d1miob_ c.92.2.3 (B:) Nitrogenase iron-molybdenum protein, alpha and beta chains {Clostridium pasteurianum}
LDATPKEIVERKALRINPAKTCQPVGAMYAALGIHNCLPHSHGSQGCCSYHRTVLSRHFKEP
AMASTSSFTEGASVFGGGSNIKTAVKNIFSLYNPDIIAVHTTCLSETLGDDLPTYISQMEDA
GSIPEGKLVIHTNTPSYVGSHVTGFANMVQGIVNYLSENTGAKNGKINVIPGFVGPADMREI
KRLFEAMDIPYIMFPDTSGVLDGPTTGEYKMYPEGGTKIEDLKDTGNSDLTLSLGSYASDLG
AKTLEKKCKVPFKTLRTPIGVSATDEFIMALSEATGKEVPASIEEERGQLIDLMIDAQQYLQ
GKKVALLGDPDEIIALSKFIIELGAIPKYVVTGTPGMKFQKEIDAMLAEAGIEGSKVKVEGD
FFDVHQWIKNEGVDLLISNTYGKFIAREENIPFVRFGFPIMDRYGHYYNPKVGYKGAIRLVE
EITNVILDKIERECTEEDFEVVR
>d2mina_ c.92.2.3 (A:) Nitrogenase iron-molybdenum protein, alpha and beta chains {Azotobacter vinelandii}
SREEVESLIQEVLEVYPEKARKDRNKHLAVNDPAVTQSKKCIISNKKSQPGLMTIRGCAYAG
SKGVVWGPIKDMIHISHGPVGCGQYSRAGRRNYYIGTTGVNAFVTMNFTSDFQEKDIVFGGD
KKLAKLIDEVETLFPLNKGISVQSECPIGLIGDDIESVSKVKGAELSKTIVPVRCEGFRGVS
QSLGHHIANDAVRDWVLGKRDEDTTFASTPYDVAIIGDYNIGGDAWSSRILLEEMGLRCVAQ
WSGDGSISEIELTPKVKLNLVHCYRSMNYISRHMEEKYGIPWMEYNFFGPTKTIESLRAIAA
KFDESIQKKCEEVIAKYKPEWEAVVAKYRPRLEGKRVMLYIGGLRPRHVIGAYEDLGMEVVG
TGYEFAHNDDYDRTMKEMGDSTLLYDDVTGYEFEEFVKRIKPDLIGSGIKEKFIFQKMGIPF
REMHSWDYSGPYHGFDGFAIFARDMDMTLNNPCWKKLQAPWE
>d2minb_ c.92.2.3 (B:) Nitrogenase iron-molybdenum protein, alpha and beta chains {Azotobacter vinelandii}
SQQVDKIKASYPLFLDQDYKDMLAKKRDGFEEKYPQDKIDEVFQWTTTKEYQELNFQREALT
VNPAKACQPLGAVLCALGFEKTMPYVHGSQGCVAYFRSYFNRHFREPVSCVSDSMTEDAAVF
GGQQNMKDGLQNCKATYKPDMIAVSTTCMAEVIGDDLNAFINNSKKEGFIPDEFPVPFAHTP
SFVGSHVTGWDNMFEGIARYFTLKSMDDKVVGSNKKINIVPGFETYLGNFRVIKRMLSEMGV
GYSLLSDPEEVLDTPADGQFRMYAGGTTQEEMKDAPNALNTVLLQPWHLEKTKKFVEGTWKH
EVPKLNIPMGLDWTDEFLMKVSEISGQPIPASLTKERGRLVDMMTDSHTWLHGKRFALWGDP
DFVMGLVKFLLELGCEPVHILCHNGNKRWKKAVDAILAASPYGKNATVYIGKDLWHLRSLVF
TDKPDFMIGNSYGKFIQRDTLHKGKEFEVPLIRIGFPIFDRHHLHRSTTLGYEGAMQILTTL
VNSILERLDEETRGMQATDYNHDLVR
>d1qgua_ c.92.2.3 (A:) Nitrogenase iron-molybdenum protein, alpha and beta chains {Klebsiella pneumoniae}
TNATGERNLALIQEVLEVFPETARKERRKHMMVSDPKMKSVGKCIISNRKSQPGVMTVRGCA
YAGSKGVVFGPIKDMAHISHGPVGCGQYSRAGRRNYYTGVSGVDSFGTLNFTSDFQERDIVF
GGDKKLSKLIEEMELLFPLTKGITIQSECPVGLIGDDISAVANASSKALDKPVIPVRCEGFR
GVSQSLGHHIANDVVRDWILNNREGQPFETTPYDVAIIGDYNIGGDAWASRILLEEMGLRVV
AQWSGDGTLVEMENTPFVKLNLVHCYRSMNYIARHMEEKHQIPWMEYNFFGPTKIAESLRKI

ADQFDDTIRANAEAVIARYEGQMAAIIAKYRPRLEGRKVLLYMGGLRPRHVIGAYEDLGMEI
IAAGYEFAHNDDYDRTLPDLKEGTLLFDDASSYELEAFVKALKPDLIGSGIKEKYIFQKMGV
PFRQMHSWDYSGPYHGYDGFAIFARDMDMTLNNPAWNELTAPWL
>d1qgub_ c.92.2.3 (B:) Nitrogenase iron-molybdenum protein, alpha and beta chains {Klebsiella pneumoniae}
SQTIDKINSCYPLFEQDEYQELFRNKRQLEEAHDAQRVQEVFAWTTTAEYEALNFRREALTV
DPAKACQPLGAVLCSLGFANTLPYVHGSQGCVAYFRTYFNRHFKEPIACVSDSMTEDAAVFG
GNNNMNLGLQNASALYKPEIIAVSTTCMAEVIGDDLQAFIANAKKDGFVDSSIAVPHAHTPS
FIGSHVTGWDNMFEGFAKTFTADYQGQPGKLPKLNLVTGFETYLGNFRVLKRMMEQMAVPCS
LLSDPSEVLDTPADGHYRMYSGGTTQQEMKEAPDAIDTLLLQPWQLLKSKKVVQEMWNQPAT
EVAIPLGLAATDELLMTVSQLSGKPIADALTLERGRLVDMMLDSHTWLHGKKFGLYGDPDFV
MGLTRFLLELGCEPTVILSHNANKRWQKAMNKMLDASPYGRDSEVFINCDLWHFRSLMFTRQ
PDFMIGNSYGKFIQRDTLAKGKAFEVPLIRLGFPLFDRHHLHRQTTWGYEGAMNIVTTLVNA
VLEKLDSDTSQLGKTDYSFDLVR
>d1jr2a_ c.113.1.1 (A:) Uroporphyrinogen III synthase (U3S, HemD) {Human (Homo sapiens)}
MKVLLLKDAKEDDCGQDPYIRELGLYGLEATLIPVLSFEFLSLPSFSEKLSHPEDYGGLIFT
SPRAVEAAELCLEQNNKTEVWERSLKEKWNAKSVYVVGNATASLVSKIGLDTEGETCGNAEK
LAEYICSRESSALPLLFPCGNLKREILPKALKDKGIAMESITVYQTVAHPGIQGNLNSYYSQ
QGVPASITFFSPSGLTYSLKHIQELSGDNIDQIKFAAIGPTTARALAAQGLPVSCTAESPTP
QALATGIRKALQ
>d2dri__ c.93.1.1 (-) D-ribose-binding protein {Escherichia coli, strain k-12}
KDTIALVVSTLNNPFFVSLKDGAQKEADKLGYNLVVLDSQNNPAKELANVQDLTVRGTKILL
INPTDSDAVGNAVKMANQANIPVITLDRQATKGEVVSHIASDNVLGGKIAGDYIAKKAGEGA
KVIELQGIAGTSAARERGEGFQQAVAAHKFNVLASQPADFDRIKGLNVMQNLLTAHPDVQAV
FAQNDEMALGALRALQTAGKSDVMVVGFDGTPDGEKAVNDGKLAATIAQLPDQIGAKGVETA
DKVLKGEKVQAKYPVDLKLVVKQ
>d8abp__ c.93.1.1 (-) L-arabinose-binding protein {Escherichia coli}
NLKLGFLVKQPEEPWFQTEWKFADKAGKDLGFEVIKIAVPDGEKTLNAIDSLAASGAKGFVI
CTPDPKLGSAIVAKARGYDMKVIAVDDQFVNAKGKPMDTVPLVMLAATKIGERQGQELYKEM
QKRGWDVKESAVMAITANELDTARRRTTGSMDALKAAGFPEKQIYQVPTKSNDIPGAFDAAN
SMLVQHPEVKHWLIVGMNDSTVLGGVRATEGQGFKAADIIGIGINGVDAVSELSKAQATGFY
GSLLPSPDVHGYKSSEMLYNWVAKDVEPPKFTEVTDVVLITRDNFKEELEKKGLGGK
>d1rpja_ c.93.1.1 (A:) D-allose-binding protein {Escherichia coli}
AAEYAVVLKTLSNPFWVDMKKGIEDEAKTLGVSVDIFASPSEGDFQSQLQLFEDLSNKNYKG
IAFAPLSSVNLVMPVARAWKKGIYLVNLDEKIDMDNLKKAGGNVEAFVTTDNVAVGAKGASF
IIDKLGAEGGEVAIIEGKAGNASGEARRNGATEAFKKASQIKLVASQPADWDRIKALDVATN
VLQRNPNIKAIYCANDTMAMGVAQAVANAGKTGKVLVVGTDGIPEARKMVEAGQMTATVAQN
PADIGATGLKLMVDAEKSGKVIPLDKAPEFKLVDSILVTQ
>d2gbp__ c.93.1.1 (-) Galactose/glucose-binding protein {Escherichia coli}
ADTRIGVTIYKYDDNFMSVVRKAIEQDAKAAPDVQLLMNDSQNDQSKQNDQIDVLLAKGVKA

LAINLVDPAAAGTVIEKARGQNVPVVFFNKEPSRKALDSYDKAYYVGTDSKESGIIQGDLIA
KHWAANQGWDLNKDGQIQFVLLKGEPGHPDAEARTTYVIKELNDKGIKTEQLQLDTAMWDTA
QAKDKMDAWLSGPNANKIEVVIANNDAMAMGAVEALKAHNKSSIPVFGVDALPEALALVKSG
ALAGTVLNDANNQAKATFDLAKNLADGKGAADGTNWKIDNKVVRVPYVGVDKDNLAEFSKK
>d1gca__ c.93.1.1 (-) Galactose/glucose-binding protein
{Salmonella typhimurium, strain lt2}
ADTRIGVTIYKYDDNFMSVVRKAIEKDGKSAPDVQLLMNDSQNDQSKQNDQIDVLLAKGVKA
LAINLVDPAAAGTVIEKARGQNVPVVFFNKEPSRKALDSYDKAYYVGTDSKESGVIQGDLIA
KHWQANQGWDLNKDGKIQYVLLKGEPGHPDAEARTTYVVKELNDKGIQTEQLALDTAMWDTA
QAKDKMDAWLSGPNANKIEVVIANNDAMAMGAVEALKAHNKSSIPVFGVDALPEALALVKSG
AMAGTVLNDANNQAKATFDLAKNLAEGKGAADGTSWKIENKIVRVPYVGVDKDNLSEFTQK
>d1pea__ c.93.1.1 (-) Amide receptor/negative regulator of the
amidase operon (AmiC) {Pseudomonas aeruginosa}
PLIGLLFSETGVTADIERSQRYGALLAVEQLNREGGVGGRPIETLSQDPGGDPDRYRLCAED
FIRNRGVRFLVGCYMSHTRKAVMPVVERADALLCYPTPYEGFEYSPNIVYGGPAPNQNSAPL
AAYLIRHYGERVVFIGSDYIYPRESNHVMRHLYRQHGGTVLEEIYIPLYPSDDDLQRAVERI
YQARADVVFSTVVGTGTAELYRAIARRYGDGRRPPIASLTTSEAEVAKMESDVAEGQVVVAP
YFSSIDTPASRAFVQACHGFFPENATITAWAEAAYWQTLLLGRAAQAAGNWRVEDVQRHLYD
IDIDAPQGPVRVERQNNHSRLSSRIAEIDARGVFQVRWQSPEPIRPDPYVVVHNLDDW
>d1jx6a_ c.93.1.1 (A:) Quorum-sensing signal (autoinducer-2)
binding protein LuxP {Vibrio harveyi}
GYWGYQEFLDEFPEQRNLTNALSEAVRAQPVPLSKPTQRPIKISVVYPGQQVSDYWVRNIAS
FEKRLYKLNINYQLNQVFTRPNADIKQQSLSLMEALKSKSDYLIFTLDTTRHRKFVEHVLDS
TNTKLILQNITTPVREWDKHQPFLYVGFDHAEGSRELATEFGKFFPKHTYYSVLYFSEGYIS
DVRGDTFIHQVNRDNNFELQSAYYTKATKQSGYDAAKASLAKHPDVDFIYACSTDVALGAVD
ALAELGREDIMINGWGGGSAELDAIQKGDLDITVMRMNDDTGIAMAEAIKWDLEDKPVPTVY
SGDFEIVTKADSPERIEALKKRAFRYSD
>d1dbqa_ c.93.1.1 (A:) Purine repressor (PurR), C-terminal domain
{Escherichia coli}
KSIGLLATSSEAAYFAEIIEAVEKNCFQKGYTLILGNAWNNLEKQRAYLSMMAQKRVDGLLV
MCSEYPEPLLAMLEEYRHIPMVVMDWGEAKADFTDAVIDNAFEGGYMAGRYLIERGHREIGV
IPGPLERNTGAGRLAGFMKAMEEAMIKVPESWIVQGDFEPESGYRAMQQILSQPHRPTAVFC
GGDIMAMGALCAADEMGLRVPQDVSLIGYDNVRNARYFTPALTTIHQPKDSLGETAFNMLLD
RIVNKREEPQSIEVHPRLIERRSVADGPFRDYRR
>d1jyea_ c.93.1.1 (A:) Lac-repressor (lacR) core (C-terminal
domain) {Escherichia coli}
LLIGVATSSLALHAPSQIVAAILSRADQLGASVVVSMVERSGVEACKTAVHNLLAQRVSGLI
INYPLDDQDAIAVEAACTNVPALFLDVSDQTPINSIIFSHEDGTRLGVEHLVALGHQQIALL
AGPLSSVSARLRLAGWHKYLTRNQIQPIAEREGDWSAMSGFQQTMQMLNEGIVPTAMLVAND
QMALGAMRAITESGLRVGADISVVGYDDTEDSSCYIPPLTTIKQDFRLLGQTSVDRLLQLSQ
GQAVKGNQLLPVSLVKRKTTLAP
>d1tlfa_ c.93.1.1 (A:) Lac-repressor (lacR) core (C-terminal
domain) {Escherichia coli}
SLLIGVATSSLALHAPSQIVAAIKSRADQLGASVVVSMVERSGVEACKAAVHNLLAQRVSGL

IINYPLDDQDAIAVEAACTNVPALFLDVSDQTPINSIIFSHEDGTRLGVEHLVALGHQQIAL
LAGPLSSVSARLRLAGWHKYLTRNQIQPIAEREGDWSAMSGFQQTMQMLNEGIVPTAMLVAN
DQMALGAMRAITESGLRVGADISVVGYDDTEDSSCYIPPLTTIKQDFRLLGQTSVDRLLQLS
QGQAVKGNQLLPVSLVKRKTTLAPNTQTASPRALADSLMQLARQVSRL
>d1byka_ c.93.1.1 (A:) Trehalose repressor, C-terminal domain
{Escherichia coli}
SDKVVAIIVTRLDSLSENLAVQTMLPAFYEQGYDPIMMESQFSPQLVAEHLGVLKRRNIDGV
VLFGFTGITEEMLAHWQSSLVLLARDAKGFASVCYDDEGAIKILMQRLYDQGHRNISYLGVP
HSDVTTGKRRHEAYLAFCKAHKLHPVAALPGLAMKQGYENVAKVITPETTALLCATDTLALG
ASKYLQEQRIDTLQLASVGNTPLMKFLHPEIVTVDPGYAEAGRQAACQLIAQVTGRSEPQQI
IIPATLS
>d2liv__ c.93.1.1 (-) Leucine-,isoleucine-,valine-binding (LIV)
protein {Escherichia coli}
EDIKVAVVGAMSGPVAQYGDQEFTGAEQAVADINAKGGIKGNKLQIAKYDDACDPKQAVAVA
NKVVNDGIKYVIGHLCSSSTQPASDIYEDEGILMITPAATAPELTARGYQLILRTTGLDSDQ
GPTAAKYILEKVKPQRIAIVHDKQQYGEGLARAVQDGLKKGNANVVFFDGITAGEKDFSTLV
ARLKKENIDFVYYGGYHPEMGQILRQARAAGLKTQFMGPEGVANVSLSNIAGESAEGLLVTK
PKNYDQVPANKPIVDAIKAKKQDPSGAFVWTTYAALQSLQAGLNQSDDPAEIAKYLKANSVD
TVMGPLTWDEKGDLKGFEFGVFDWHANGTATDAK
>d2lbp__ c.93.1.1 (-) Leucine-binding protein {Escherichia coli}
DDIKVAVVGAMSGPIAQWGIMEFNGAEQAIKDINAKGGIKGDKLVGVEYDDACDPKQAVAVA
NKIVNDGIKYVIGHLCSSSTQPASDIYEDEGILMISPGATAPELTQRGYQHIMRTAGLDSSQ
GPTAAKYILETVKPQRIAIIHDKQQYGEGLARSVQDGLKAANANVVFFDGITAGEKDFSALI
ARLKKENIDFVYYGGYYPEMGQMLRQARSVGLKTQFMGPEGVGNASLSNIAGDAAEGMLVTM
PKRYDQDPANQGIVDALKADKKDPSGPYVWITYAAVQSLATALERTGSDEPLALVKDLKANG
ANTVIGPLNWDEKGDLKGFDFGVFQWHADGSSTKAK
>d1dp4a_ c.93.1.1 (A:) Hormone binding domain of the atrial
natriuretic peptide receptor {Rat (Rattus norvegicus)}
SDLTVAVVLPLTNTSYPWSWARVGPAVELALARVKARPDLLPGWTVRMVLGSSENAAGVCSD
TAAPLAAVDLKWEHSPAVFLGPGCVYSAAPVGRFTAHWRVPLLTAGAPALGIGVKDEYALTT
RTGPSHVKLGDFVTALHRRLGWEHQALVLYADRLGDDRPCFFIVEGLYMRVRERLNITVNHQ
EFVEGDPDHYPKLLRAVRRKGRVIYICSSPDAFRNLMLLALNAGLTGEDYVFFHLDVFGQSL
KSAQGLVPQKPWERGDGQDRSARQAFQAAKIITYKEPDNPEYLEFLKQLKLLADKKFNFTVE
DGLKNIIPASFHDGLLLYVQAVTETLAQGGTVTDGENITQRMWNRSFQGVTGYLKIDRNGDR
DTDFSLWDMDPETGAFRVVLNYNGTSQELMAVSEHKLYWPLGYPPPDVPKCGF
>d1jdpa_ c.93.1.1 (A:) Hormone binding domain of the atrial
natriuretic peptide receptor {Human (Homo sapiens)}
EALPPQKIEVLVLLPQDDSYLFSLTRVRPAIEYALRSVEGNGTGRRLLPPGTRFQVAYEDSD
CGNRALFSLVDRVAAARGAKPDLILGPVCEYAAAPVARLASHWDLPMLSAGALAAGFQHKDS
EYSHLTRVAPAYAKMGEMMLALFRHHHWSRAALVYSDDKLERNCYFTLEGVHEVFQEEGLHT
SIYSFDETKDLDLEDIVRNIQASERVVIMCASSDTIRSIMLVAHRHGMTSGDYAFFNIELFN
SSSYGDGSWKRGDKHDFEAKQAYSSLQTVTLLRTVKPEFEKFSMEVKSSVEKQGLNMEDYVN
MFVEGFHDAILLYVLALHEVLRAGYSKKDGGKIIQQTWNRTFEGIAGQVSIDANGDRYGDFS
VIAMTDVEAGTQEVIGDYFGKEGRFEMRP

>d1ewka_ c.93.1.1 (A:) Metabotropic glutamate receptor subtype 1 {Rat (Rattus norvegicus)}
RSVARMDGDVIIGALFSVHHQPPAEKVPERKCGEIREQYGIQRVEAMFHTLDKINADPVLLP
NITLGSEIRDSCWHSSVALEQSIEFIRDSLISIRDEKDGLNRCLPDGQTLPPGRTKKPIAGV
IGPGSSSVAIQVQNLLQLFDIPQIAYSATSIDLSDKTLYKYFLRVVPSDTLQARAMLDIVKR
YNWTYVSAVHTEGNYGESGMDAFKELAAQEGLCIAHSDKIYSNAGEKSFDRLLRKLRERLPK
ARVVVCFCEGMTVRGLLSAMRRLGVVGEFSLIGSDGWADRDEVIEGYEVEANGGITIKLQSP
EVRSFDDYFLKLRLDTNTRNPWFPEFWQHRFQCRLPGHLLENPNFKKVCTGNESLEENYVQD
SKMGFVINAIYAMAHGLQNMHHALCPGHVGLCDAMKPIDGRKLLDFLIKSSFVGVSGEEVWF
DEKGDAPGRYDIMNLQYTEANRYDYVHVGTWHEGVLNIDDYKI
>d1jeta_ c.94.1.1 (A:) Oligo-peptide binding protein (OPPA) {Salmonella typhimurium}
ADVPAGVQLADKQTLVRNNGSEVQSLDPHKIEGVPESNVSRDLFEGLLISDVEGHPSPGVAE
KWENKDFKVWTFHLRENAKWSDGTPVTAHDFVYSWQRLADPNTASPYASYLQYGHIANIDDI
IAGKKPATDLGVKALDDHTFEVTLSEPVPYFYKLLVHPSVSPVPKSAVEKFGDKWTQPANIV
TNGAYKLKNWVVNERIVLERNPQYWDNAKTVINQVTYLPISSEVTDVNRYRSGEIDMTYNNM
PIELFQKLKKEIPNEVRVDPYLCTYYYEINNQKAPFNDVRVRTALKLALDRDIIVNKVKNQG
DLPAYSYTPPYTDGAKLVEPEWFKWSQQKRNEEAKKLLAEAGFTADKPLTFDLLYNTSDLHK
KLAIAVASIWKKNLGVNVNLENQEWKTFLDTRHQGTFDVARAGWCADYNEPTSFLNTMLSDS
SNNTAHYKSPAFDKLIADTLKVADDTQRSELYAKAEQQLDKDSAIVPVYYYVNARLVKPWVG
GYTGKDPLDNIYVKNLYIIKH
>d1pda_1 c.94.1.1 (3-219) Porphobilinogen deaminase (hydroxymethylbilane synthase), N-terminal domain {Escherichia coli}
DNVLRIATRQSPLALWQAHYVKDKLMASHPGLVVELVPMVTRGDVILDTPLAKVGGKGLFVK
ELEVALLENRADIAVHSMKDVPVEFPQGLGLVTICEREDPRDAFVSNNYDSLDALPAGSIVG
TSSLRRQCQLAERRPDLIIRSLRGNVGTRLSKLDNGEYDAIILAVAGLKRLGLESRIRAALP
PEISLPAVGQGAVGIECRLDDSRTRELLAAL
>d1lst__ c.94.1.1 (-) Lysine-,arginine-,ornithine-binding (LAO) protein {Salmonella typhimurium}
ALPQTVRIGTDTTYAPFSSKDAKGEFIGFDIDLGNEMCKRMQVKCTWVASDFDALIPSLKAK
KIDAIISSLSITDKRQQEIAFSDKLYAADSRLIAAKGSPIQPTLESLKGKHVGVLQGSTQEA
YANDNWRTKGVDVVAYANQDLIYSDLTAGRLDAALQDEVAASEGFLKQPAGKEYAFAGPSVK
DKKYFGDGTGVGLRKDDTELKAAFDKALTELRQDGTYDKMAKKYFDFNVYGDK
>d1sbp__ c.94.1.1 (-) Sulphate-binding protein {Salmonella typhimurium}
KDIQLLNVSYDPTRELYEQYNKAFSAHWKQETGDNVVIDQSHGGSGKQATSVINGIEADTVT
LALAYDVNAIAERGRIDKNWIKRLPDDSAPYTSTIVFLVRKGNPKQIHDWNDLIKPGVSVIT
PNPKSSGGARWNYLAAWGYALHHNNNDQAKAEDFVKALFKNVEVLDSGARGSTNTFVERGIG
DVLIAWENEALLATNELGKDKFEIVTPSESILAEPTVSVVDKVVEKKDTKAVAEAYLKYLYS
PEGQEIAAKNFYRPRDADVAKKYDDAFPKLKLFTIDEVFGGWAKAQKDHFADGGTFDQISK
>d1ixh__ c.94.1.1 (-) Phosphate-binding protein {Escherichia coli}
EASLTGAGATFPAPVYAKWADTYQKETGNKVNYQGIGSSGGVKQIIANTVDFGASDAPLSDE
KLAQEGLFQFPTVIGGVVLAVNIPGLKSGELVLDGKTLGDIYLGKIKKWDDEAIAKLNPGLK

LPSQNIAVVRRADGSGTSFVFTSYLAKVNEEWKNNVGTGSTVKWPIGLGGKGNDGIAAFVQR
LPGAIGYVEYAYAKQNNLAYTKLISADGKPVSPTEENFANAAKGADWSKTFAQDLTNQKGED
AWPITSTTFILIHKDQKKPEQGTEVLKFFDWAYKTGAKQANDLDYASLPDSVVEQVRAAWKT
NIKDSSGKPLY
>d3mbp__ c.94.1.1 (-) D-maltodextrin-binding protein, MBP
{Escherichia coli}
KIEEGKLVIWINGDKGYNGLAEVGKKFEKDTGIKVTVEHPDKLEEKFPQVAATGDGPDIIFW
AHDRFGGYAQSGLLAEITPDKAFQDKLYPFTWDAVRYNGKLIAYPIAVEALSLIYNKDLLPN
PPKTWEEIPALDKELKAKGKSALMFNLQEPYFTWPLIAADGGYAFKYENGKYDIKDVGVDNA
GAKAGLTFLVDLIKNKHMNADTDYSIAEAAFNKGETAMTINGPWAWSNIDTSKVNYGVTVLP
TFKGQPSKPFVGVLSAGINAASPNKELAKEFLENYLLTDEGLEAVNKDKPLGAVALKSYEEE
LAKDPRIAATMENAQKGEIMPNIPQMSAFWYAVRTAVINAASGRQTVDEALKDAQTRITK
>d1elja_ c.94.1.1 (A:) D-maltodextrin-binding protein, MBP
{Archaeon Pyrococcus furiosus}
MKIEEGKVVIWHAMQPNELEVFQSLAEEYMALCPEVEIVFEQKPNLEDALKAAIPTGQGPDL
FIWAHDWIGKFAEAGLLEPIDEYVTEDLLNEFAPMAQDAMQYKGHYYALPFAAETVAIIYNK
EMVSEPPKTFDEMKAIMEKYYDPANEKYGIAWPINAYFISAIAQAFGGYYFDDKTEQPGLDK
PETIEGFKFFFTEIWPYMAPTGDYNTQQSIFLEGRAPMMVNGPWSINDVKKAGINFGVVPLP
PIIKDGKEYWPRPYGGVKLIYFAAGIKNKDAAWKFAKWLTTSEESIKTLALELGYIPVLTKV
LDDPEIKNDPVIYGFGQAVQHAYLMPKSPKMSAVWGGVDGAINEILQDPQNADIEGILKKYQ
QEILNNMQ
>d1eu8a_ c.94.1.1 (A:) D-maltodextrin-binding protein, MBP
{Archaeon Thermococcus litoralis}
IEEGKIVFAVGGAPNEIEYWKGVIAEFEKKYPGVTVELKRQATDTEQRRLDLVNALRGKSSD
PDVFLMDVAWLGQFIASGWLEPLDDYVQKDNYDLSVFFQSVINLADKQGGKLYALPVYIDAG
LLYYRKDLLEKYGYSKPPETWQELVEMAQKIQSGERETNPNFWGFVWQGKQYEGLVCDFVEY
VYSNGGSLGEFKDGKWVPTLNKPENVEALQFMVDLIHKYKISPPNTYTEMTEEPVRLMFQQG
NAAFERNWPYAWGLHNADDSPVKGKVGVAPLPHFPGHKSAATLGGWHIGISKYSDNKALAWE
FVKFVESYSVQKGFAMNLGWNPGRVDVYDDPAVVSKSPHLKELRAVFENAVPRPIVPYYPQL
SEIIQKYVNSALAGKISPQEALDKAQKEAEELVKQ
>d3thia_ c.94.1.1 (A:) Thiaminase I {Paenibacillus
thiaminolyticus}
ITLKVAIYPYVPDPARFQAAVLDQWQRQEPGVKLEFTDWDSYSADPPDDLDVFVLDSIFLSH
FVDAGYLLPFGSQDIDQAEDVLPFALQGAKRNGEVYGLPQILCTNLLFYRKGDLKIGQVDNI
YELYKKIGTSHSEQIPPPQNKGLLINMAGGTTKASMYLEALIDVTGQYTEYDLLPPLDPLND
KVIRGLRLLINMAGEKPSQYVPEDGDAYVRASWFAQGSGRAFIGYSESMMRMGDYAEQVRFK
PISSSAGQDIPLFYSDVVSVNSKTAHPELAKKLANVMASADTVEQALRPQADGQYPQYLLPA
RHQVYEALMQDYPIYSELAQIVNKPSNRVFRLGPEVRTWLKDAKQVLPEALG
>d1mrp__ c.94.1.1 (-) Ferric-binding protein {Haemophilus
influenzae}
DITVYNGQHKEAATAVAKAFEQETGIKVTLNSGKSEQLAGQLKEEGDKTPADVFYTEQTATF
ADLSEAGLLAPISEQTIQQTAQKGVPLAPKKDWIALSGRSRVVVYDHTKLSEKDMEKSVLDY
ATPKWKGKIGYVSTSGAFLEQVVALSKMKGDKVALNWLKGLKENGKLYAKNSVALQAVENGE
VPAALINNYYWYNLAKEKGVENLKSRLYFVRHQDPGALVSYSGAAVLKASKNQAEAQKFVDF

LASKKGQEALVAARAEYPLRADVVSPFNLEPYEKLEAPVVSATTAQDKEHAIKLIEEAGLK
>d1d9ya_ c.94.1.1 (A:) Ferric-binding protein {Neisseria gonorrhoeae}
DITVYNGQHKEAAQAVADAFTRATGIKVKLNCAKGDQLAGQIKEEGSRSPADVFYSEQIPAL
ATLSAANLLEPLPASTINETRGKGVPVAAKKDWVALSGRSRVVVYDTRKLSEKDLEKSVLNY
ATPKWKNRIGYVPTSGAFLEQIVAIVKLKGEAAALKWLKGLKEYGKPYAKNSVALQAVENGE
IDAALINNYYWHAFAREKGVQNVHTRLNFVRHRDPGALVTYSGAAVLKSSQNKDEAKKFVAF
LAGKEGQRALTAVRAEYPLNPHVVSTFNLEPIAKLEAPQVSATTVSEKEHATRLLEQAGMK
>d1dpe__ c.94.1.1 (-) Dipeptide-binding protein {Escherichia coli}
KTLVYCSEGSPEGFNPQLFISGTTYDASSVPLYNRLVEFKIGTTEVIPGLAEKWEVSEDGKT
YTFHLRKGVKWHDNKEFKPTRELNADDVVFSFDRQKNAQNPYHKVSGGSYEYFEGMGLPELI
SEVKKVDDNTVQFVLTRPEAPFLADLAMDFASILSKEYADAMMKAGTPEKLDLNPIGTGPFQ
LQQYQKDSRIRYKAFDGYWGTKPQIDTLVFSITPDASVRYAKLQKNECQVMPYPNPADIARM
KQDKSINLMEMPGLNVGYLSYNVQKKPLDDVKVRQALTYAVNKDAIIKAVYQGAGVSAKNLI
PPTMWGYNDDVQDYTYDPEKAKALLKEAGLEKGFSIDLWAMPVQRPYNPNARRMAEMIQADW
AKVGVQAKIVTYEWGEYLKRAKDGEHQTVMMGWTGDNGDPDNFFATEFSCAASEQGSNYSKW
CYKPFEDLIQPARATDDHNKRVELYKQAQVVMHDQAPALIIAHSTVFEPVRKEVKGYVVDPL
GKHHFENVSIE
>d1hsla_ c.94.1.1 (A:) Histidine-binding protein {Escherichia coli}
AIPQKIRIGTDPTYAPFESKNAQGELVGFDIDLAKELCKRINTQCTFVENPLDALIPSLKAK
KIDAIMSSLSITEKRQQEIAFTDKLYAADSRLVVAKNSDIQPTVASLKGKRVGVLQGTTQET
FGNEHWAPKGIEIVSYQGQDNIYSDLTAGRIDAAFQDEVAASEGFLKQPVGKDYKFGGPAVK
DEKLFGVGTGMGLRKEDNELREALNKAFAEMRADGTYEKLAKKYFDFDVYGG
>d1pot__ c.94.1.1 (-) Spermidine/putrescine-binding protein PotD {Escherichia coli}
NNTLYFYNWTEYVPPGLLEQFTKETGIKVIYSTYESNETMYAKLKTYKDGAYDLVVPSTYYV
DKMRKEGMIQKIDKSKLTNFSNLDPDMLNKPFDPNNDYSIPYIWGATAIGVNGDAVDPKSVT
SWADLWKPEYKGSLLLTDDAREVFQMALRKLGYSGNTTDPKEIEAAYNELKKLMPNVAAFNS
DNPANPYMEGEVNLGMIWNGSAFVARQAGTPIDVVWPKEGGIFWMDSLAIPANAKNKEGALK
LINFLLRPDVAKQVAETIGYPTPNLAARKLLSPEVANDKTLYPDAETIKNGEWQNDVGAASS
IYEEYYQKLKAG
>d1a99a_ c.94.1.1 (A:) Putrescine receptor (PotF) {Escherichia coli}
QKTLHIYNWSDYIAPDTVANFEKETGIKVVYDVFDSNEVLEGKLMAGSTGFDLVVPSASFLE
RQLTAGVFQPLDKSKLPEWKNLDPELLKLVAKHDPDNKFAMPYMWATTGIGYNVDKVKAVLG
ENAPVDSWDLILKPENLEKLKSCGVSFLDAPEEVFATVLNYLGKDPNSTKADDYTGPATDLL
LKLRPNIRYFHSSQYINDLANGDICVAIGWAGDVWQASNRAKEAKNGVNVSFSIPKEGAMAF
FDVFAMPADAKNKDEAYQFLNYLLRPDVVAHISDHVFYANANKAATPLVSAEVRENPGIYPP
ADVRAKLFTLKVQDPKIDRVRTRAWTKVKSG
>d1wdna_ c.94.1.1 (A:) Glutamine-binding protein {Escherichia coli}
KLVVATDTAFVPFEFKQGDLYVGFDVDLWAAIAKELKLDYELKPMDFSGIIPALQTKNVDLA
LAGITITDERKKAIDFSDGYYKSGLLVMVKANNNDVKSVKDLDGKVVAVKSGTGSVDYAKAN

IKTKDLRQFPNIDNAYMELGTNRADAVLHDTPNILYFIKTAGNGQFKAVGDSLEAQQYGIAF
PKGSDELRDKVNGALKTLRENGTYNEIYKKWFGTEPK
>d1ftka_ c.94.1.1 (A:) Glutamate receptor ligand binding core {Rat (Rattus norvegicus), GluR2}
KTVVVTTILESPYVMMKKNHEMLEGNERYEGYCVDLAAEIAKHCGFKYKLTIVGDGKYGARD
ADTKIWNGMVGELVYGKADIAIAPLTITLVREEVIDFSKPFMSLGISIMIKKPGTDGNPIES
AEDLSKQTEIAYGTLDSGSTKEFFRRSKIAVFDKMWTYMRSAEPSVFVRTTAEGVARVRKSK
GKYAYLLESTMNEYIEQRKPCDTMKVGGNLDSKGYGIATPKGSSLGNAVNLAVLKLNEQGLL
DKLKNKWWYDKGEC
>d1ii5a_ c.94.1.1 (A:) Glutamate receptor ligand binding core {Synechocystis sp., GluR0}
GSAMALKVGVVGNPPFVFYGEGKNAAFTGISLDVWRAVAESQKWNSEYVRQNSISAGITAVA
EGELDILIGPISVTPERAAIEGITFTQPYFSSGIGLLIPGTATPLFRSVGDLKNKEVAVVRD
TTAVDWANFYQADVRETNNLTAAITLLQKKQVEAVMFDRPALIYYTRQNPNLNLEVTEIRVS
LEPYGFVLKENSPLQKTINVEMLNLLYSRVIAEFTERWLG
>d1amf__ c.94.1.1 (-) Molybdate-binding protein, ModA {Escherichia coli}
GKITVFAAASLTNAMQDIATQFKKEKGVDVVSSFASSSTLARQIEAGAPADLFISADQKWMD
YAVDKKAIDTATRQTLLGNSLVVVAPKASVQKDFTIDSKTNWTSLLNGGRLAVGDPEHVPAG
IYAKEALQKLGAWDTLSPKLAPAEDVRGALALVERNEAPLGIVYGSDAVASKGVKVVATFPE
DSHKKVEYPVAVVEGHNNATVKAFYDYLKGPQAAEIFKRYGFTIK
>d1atg__ c.94.1.1 (-) Molybdate-binding protein, ModA {Azotobacter vinelandii}
ELKVVTATNFLGTLEQLAGQFAKQTGHAVVISSGSSGPVYAQIVNGAPYNVFFSADEKSPEK
LDNQGFALPGSRFTYAIGKLVLWSAKPGLVDNQGKVLAGNGWRHIAISNPQIAPYGLAGTQV
LTHLGLLDKLTAQERIVEANSVGQAHSQTASGAADLGFVALAQIIQAAAKIPGSHWFPPANY
YEPIVQQAVITKSTAEKANAEQFMSWMKGPKAVAIIKAAGYVLPQ
>d1al3__ c.94.1.1 (-) Cofactor-binding fragment of CysB {Klebsiella aerogenes}
TWPDKGSLYVATTHTQARYALPGVIKGFIERYPRVSLHMHQGSPTQIAEAVSKGNADFAIAT
EALHLYDDLVMLPCYHWNRSIVVTPEHPLATKGSVSIEELAQYPLVTYTFGFTGRSELDTAF
NRAGLTPRIVFTATDADVIKTYVRLGLGVGVIASMAVDPVSDPDLVKLDANGIFSHSTTKIG
FRRSTFLRSYMYDFIQRFAPHLTRDVVDTAVALRSNEDIEAMFKDIKLPEK
>d1i6aa_ c.94.1.1 (A:) Hydrogen peroxide-inducible genes activator OxyR, regulatory domain {Escherichia coli}
ETMSGPLHIGLIPTVGPYLLPHIIPMLHQTFPKLEMYLHEAQTHQLLAQLDSGKLDAVILAL
VKESEAFIEVPLFDEPMLLAIYEDHPWANREAVPMADLAGEKLLMLEDGHCLRDQAMGFCFE
AGADEDTHFRATSLETLRNMVAAGSGITLLPALAVPPERKRDGVVYLPAIKPEPRRTIGLVY
RPGSPLRSRYEQLAEAIRARMDGHFD
>d1kwha_ c.94.1.1 (A:) Alginate-binding periplasmic protein AlgQ2 {Sphingomonas sp.}
KEATWVTDKPLTLKIHMHFRDKWVWDENWPVAKESFRLTNVKLQSVANKAATNSQEQFNLMM
ASGDLPDVVGGDNLKDKFIQYGQEGAFVPLNKLIDQYAPHIKAFFKSHPEVERAIKAPDGNI
YFIPYVPDGVVARGYFIREDWLKKLNLKPPQNIDELYTVLKAFKEKDPNGNGKADEVPFIDR

HPDEVFRLVNFWGARSSGSDNYMDFYIDNGRVKHPWAETAFRDGMKHVAQWYKEGLIDKEIF
TRKAKAREQMFGGNLGGFTHDWFASTMTFNEGLAKTVPGFKLIPIAPPTNSKGQRWEEDSRQ
KVRPDGWAITVKNKNPVETIKFFDFYFSRPGRDISNFGVPGVTYDIKNGKAVFKDSVLKSPQ
PVNNQLYDMGAQIPIGFWQDYDYERQWTTPEAQAGIDMYVKGKYVMPGFEGVNMTREERAIY
DKYWADVRTYMYEMGQAWVMGTKDVDKTWDEYQRQLKLRGLYQVLQMMQQAYDRQYKN
>d1cb6a2 c.94.1.2 (A:1335-1691) Lactoferrin {Human (Homo sapiens)}
EEEVAARRARVVWCAVGEQELRKCNQWSGLSEGSVTCSSASTTEDCIALVLKGEADAMSLDG
GYVYTAGKCGLVPVLAENYKSQQSSDPDPNCVDRPVEGYLAVAVVRRSDTSLTWNSVKGKKS
CHTAVDRTAGWNIPMGLLFNQTGSCKFDEYFSQSCAPGSDPRSNLCALCIGDEQGENKCVPN
SNERYYGYTGAFRCLAENAGDVAFVKDVTVLQNTDGNNNEAWAKDLKLADFALLCLDGKRKP
VTEARSCHLAMAPNHAVVSRMDKVERLKQVLLHQQAKFGRNGSDCPDKFCLFQSETKNLLFN
DNTECLARLHGKTTYEKYLGPQYVAGITNLKKCSTSPLLEACEFLRK
>d1lct__ c.94.1.2 (-) Lactoferrin {Human (Homo sapiens)}
RSVQWCAVSNPEATKCFQWQRNMRKVRGPPVSCIKRDSPIQCIQAIAENRADAVTLDGGFIY
EAGLAPYKLRPVAAEVYGTERQPRTHYYAVAVVKKGGSFQLNELQGLKSCHTGLRRTAGWNV
PIGTLRPFLNWTGPPEPIEAAVARFFSASCVPGADKGQFPNLCRLCAGTGENKCAFSSQEPY
FSYSGAFKCLRDGAGDVAFIRESTVFEDLSDEAERDEYELLCPDNTRKPVDKFKDCHLARVP
SHAVVARSVNGKEDAIWNLLRQAQEKFGKDKSPKFQLFGSPSGQKDLLFKDSAIGFSRVPPR
IDSGLYLGSGYFTA
>d1lgbc_ c.94.1.2 (C:) Lactoferrin {Human (Homo sapiens)}
HYYAVAVVKKGGSFQLNELQGLKSCHTGLRRTAGWNVPIGTLRPFLNWTGPPEPIEAAVARF
FSASCVPGADKGQFPNLCRLCAGTGENKCAFSSQEPYFSYSGAFKCLKDGAGDVAFIRESTV
FEDLSDEAERDEYELLCPDNTRKPVDKFKDCHLAR
>d1ce2a1 c.94.1.2 (A:1-333) Lactoferrin {Domestic water buffalo (Bubalus arnee bubalis)}
APRKNVRWCTISQPEWLKCHRWQWRMKKLGAPSITCVRRASVLECIRAITEKKADAVTLDGG
MVFEAGRDPYKLRPVAAEIYGTKESPQTHYYAVAVVKKGSNFQLDQLQGRNSCHTGLGRSAG
WNIPMGILRPYLSWTESLEPLQGAVAKFFSASCVPCVDRQAYPNLCQLCKGEGENQCACSPR
EPYFGYSGAFKCLQDGAGDVAFVKETTVFENLPEKADRDQYELLCLNNTRAPVDAFKECHLA
QVPSHAVVARSVDGKEDLIWKLLSKAQEKFGKNKSGSFQLFGSPPGQRDLLFKDSALGFLRI
PSKVDSALYLGSRYLTALKNLRE
>d1ce2a2 c.94.1.2 (A:334-689) Lactoferrin {Domestic water buffalo (Bubalus arnee bubalis)}
TAEEVQARRARVVWCAVGPEEQKKCQQWSQQSGQIVTCATASTTDDCIALVLKGEADALSLD
GGYIYTAGKCGLVPVLAENRKSSKHSSLDCVLRPTEGYLAVAVVKKANEGLTWNSLKGKKSC
HTAVDRTAGWNIPMGLIANQTGSCAFDEFFSQSCAPGADPKSRLCALCAGDDQGLDKCVPNS
KEKYYGYTGAFRCLAEDVGDVAFVKNDTVWENTNGESTADWAKNLNREDFRLLCLDGTRKPV
TEAQSCHLAVAPNHAVVSLSERAAHVEQVLLHQQALFGENGKNCPDKFCLFKSETKNLLFND
NTECLAKLGGRPTYEEYLGTEYVTAIANLKKCSTSPLLEACAFLTR
>d1b1xa1 c.94.1.2 (A:1-333) Lactoferrin {Horse (Equus caballus)}
APRKSVRWCTISPAEAAKCAKFQRNMKKVRGPSVSCIRKTSSFECIQAIAANKADAVTLDGG
LVYEAGLHPYKLRPVAAEVYQTRGKPQTRYYAVAVVKKGSGFQLNQLQGVKSCHTGLGRSAG
WNIPIGTLRPYLNWTGPPEPLQKAVANFFSASCVPCADGKQYPNLCRLCAGTEADKCACSSQ
EPYFGYSGAFKCLENGAGDVAFVKDSTVFENLPDEAERDKYELLCPDNTRKPVDAFKECHLA

RVPSHAVVARSVDGREDLIWKLLHRAQEEFGRNKSSAFQLFGSTPGEQDLLFKDSALGFVRI
PSQIDSGLYLGANYLTATQNLRE
>d1b1xa2 c.94.1.2 (A:334-689) Lactoferrin {Horse (Equus caballus)}
TAAEVAARRERVVWCAVGPEEERKCKQWSDVSNRKVACASASTTEECIALVLKGEADALNLD
GGFIYVAGKCGLVPVLAENQKSQNSNAPDCVHRPPEGYLAVAVVRKSDADLTWNSLSGKKSC
HTGVGRTAAWNIPMGLLFNQTGSCKFDKFFSQSCAPGADPQSSLCALCVGNNENENKCMPNS
EERYYGYTGAFRCLAEKAGDVAFVKDVTVLQNTDGKNSEPWAKDLKQEDFELLCLDGTRKPV
AEAESCHLARAPNHAVVSQSDRAQHLKKVLFLQQDQFGGNGPDCPGKFCLFKSETKNLLFND
NTECLAELQGKTTYEQYLGSEYVTSITNLRRCSSSPLLEACAFLRA
>d1dtza1 c.94.1.2 (A:1-333) Lactoferrin {Arabian camel (Camelus dromedarius)}
ASKKSVRWCTTSPAESKKCAQWQRRMKKVRGPSVTCVKKTSRFECIQAISTEKADAVTLDGG
LVYDAGLDPYKLRPIAAEVYGTENQPQTHYYAVAIAKKGTNFQLNQLQGLKSCHTGLGRSAG
WNIPMGLLRPFLDWTGPPEPLQKAVAKFFSASCVPCVDGKEYPNLCQLCAGTGENKCACSSQ
EPYFGYSGAFKCLQDGAGDVAFVKDSTVFESLPAKADRDQYELLCPNNTRKPVDAFQECHLA
RVPSHAVVARSVNGKEDLIWKLLVKAQEKFGRGKPSAFQLFGSPAGQKDLLFKDSALGLLRI
PKKIDSGLYLGSNYITAIRGLRE
>d1dtza2 c.94.1.2 (A:334-689) Lactoferrin {Arabian camel (Camelus dromedarius)}
TAAEVELRRAQVVWCAVGSDEQLKCQEWSRQSNQSVVCATASTTEDCIALVLKGEADALSLD
GGYIYIAGKCGLVPVLAESQQSPESSGLDCVHRPVKGYLAVAVVRKANDKITWNSLRGKKSC
HTAVDRTAGWNIPMGPLFKDTDSCRFDEFFSQSCAPGSDPRSKLCALCAGNEEGQLKCVPNS
SERLYGYTGAFRCLAENVGDVAFVKDVTVLDNTDGKGTEQWAKDLKLGDFELLCLNGTRKPV
TEAESCHLPVAPNHAVVSRIDKVAHLRQVLLRQQAHFGRNGEDCPGKFCLFQSKTKNLLFND
NTECLAKLQGKTTYDEYLGPQYVTAIAKLRRCSTSPLLEACAFLMR
>d1dot_1 c.94.1.2 (1-334) Ovotransferrin {Duck (Anas platyrhynchos)}
APPKTTVRWCTISSAEEKKCNSLKDHMQQERVTLSCVQKATYLDCIKAISNNEADAISLDGG
QVFEAGLAPYKLKPIAAEVYERSGGSTTSYYAVAVVKKGTDFMIKDLRGKTSCHTGLGRSAG
WNIPIGTLIHREDIEWEGIESGISEQAVAKFFSASCVPGATIEQKLCRQCKGDAKTKCLRNG
PYSGYSGAFQCLKDGKGDVAFVKHTTVQENAPEEKDEYELLCLDGSRQPVDSYKTCNWARVA
AHAVVARDDSKIDDIWSFLGMQAYSLGVDTTSDFHLFGPPGKKDPVLKDLLFKDSAIMLKRV
PELMDSQLYLGFEYYSAIQSLRKD
>d1dot_2 c.94.1.2 (335-686) Ovotransferrin {Duck (Anas platyrhynchos)}
QLTVGPRENKIQWCAVGKDEKSKCDRWSVVSNGEVECTILDDNKDCIVKITKGEADAISLDG
GFVYTAGVCGLVPVVGESYEDETQCSKDEEQPAYYFAVAVVKKSSAITWNNLQGKKSCHTAV
GRTAGWNIPMGLIHNKTGSCDFDDYFSEGCAPGSPPNSRLCKLCQGSGENLLEKCVASSHEK
YYGYTGALRCLVEQGDVAFIKHSTVGENVSGSNKDDWAKGLTRDDFELLCTNGKRAKTMDYK
TCHLAKVPTHAVVARPEKANKIRELLEGQEKLFGLHGTEKERFMMFQSQTKDLLFKALTKCL
VKLRQGITYKEFLGDEYYASVASLNTCNPSDLLQVCTFLEDK
>d1gv8a_ c.94.1.2 (A:) Ovotransferrin {Duck (Anas platyrhynchos)}
SYYAVAVVKKGTDFMIKDLRGKTSCHTGLGRSAGWNIPIGTLIHRGDIEWEGIESGSVEQAV
AKFFSASCVPGATTEQKLCRQCKGDAKTKCLRNAPYSGYSGAFQCLKDGKGDVAFVKHTTVQ

ENAPEEKDEYELLCLDGTRQPVDSYKTCNWARVAA
>d1ieja_ c.94.1.2 (A:) Ovotransferrin {Chicken (Gallus gallus)}
KSVIRWCTISSPEEKKCNNLRDLTQQERISLTCVQKATYLDCIKAIANNEADAITLDGGQVF
EAGLAPYKLKPIAAEVYEHTEGSTTSYYAVAVVKKGTEFTVNDLQGKTSCHTGLGRSAGWNI
PIGTLLHRGAIEWEGIESGSVEQAVAKFFSASCVPGATIEQKLCRQCKGDPKTKCARNAPYS
GYSGAFHCLKDGKGDVAFVKHTTVNENAPDQKDEYELLCLDGSRQPVDNYKTCNWARVAAHA
VVARDDNKVEDIWSFLSKAQSDFGVDTKSDFHLFGPPGKKDPVLKDLLFKDSAIMLKRVPSL
MDSQLYLGFEYYSAIQSMR
>d1iq7a_ c.94.1.2 (A:) Ovotransferrin {Chicken (Gallus gallus)}
RIQWCAVGKDEKSKCDRWSVVSNGDVECTVVDETKDCIIKIMKGEADAVALDGGLVYTAGVC
GLVPVMAERYDDESQCSKTDERPASYFAVAVARKDSNVNWNNLKGKKSCHTAVGRTAGWVIP
MGLIHNRTGTCNFDEYFSEGCAPGSPPNSRLCQLCQGSGGIPPEKCVASSHEKYFGYTGALR
CLVEKGDVAFIQHSTVEENTGGKNKADWAKNLQMDDFELLCTDGRRANVMDYRECNLAEVPT
HAVVVRPEKANKIRDLLERQEKRFGVNGSEKSKFMMFESQNKDLLFKDLTKCLFKVREGTTY
KEFLGDKFYTVISSLKTCNPSDILQMCSFLEGK
>d1jnfa1 c.94.1.2 (A:3-334) Transferrin {Rabbit (Oryctolagus cuniculus)}
EKTVRWCAVNDHEASKCANFRDSMKKVLPEDGPRIICVKKASYLDCIKAIAAHEADAVTLDA
GLVHEAGLTPNNLKPVVAEFYGSKENPKTFYYAVALVKKGSNFQLNELQGKKSCHTGLGRSA
GWNIPIGLLLCDLPEPRKPLEKAVASFFSGSCVPCADGADFPQLCQLCPGCGCSSVQPYFGY
SGAFKCLKDGLGDVAFVKQETIFENLPSKDERDQYELLCLDNTRKPVDEYEQCHLARVPSHA
VVARSVDGKEDLIWELLNQAQEHFGKDKSGDFQLFSSPHGKNLLFKDSAYGFFKVPPRMDAN
LYLGYEYVTAVRNLREGICPDP
>d1jnfa2 c.94.1.2 (A:335-676) Transferrin {Rabbit (Oryctolagus cuniculus)}
LQDECKAVKWCALGHHERLKCDEWSVTSGGLIECESAETPEDCIAKIMNGEADAMSLDGGYV
YIAGQCGLVPVLAENYESTDCKKAPEEGYLSVAVVKKSNPDINWNNLEGKKSCHTAVDRTAG
WNIPMGLLYNRINHCRFDEFFRQGCAPGSQKNSSLCELCVGPSVCAPNNREGYYGYTGAFRC
LVEKGDVAFVKSQTVLQNTGGRNSEPWAKDLKEEDFELLCLDGTRKPVSEAHNCHLAKAPNH
AVVSRKDKAACVKQKLLDLQVEFGNTVADCSSKFCMFHSKTKDLLFRDDTKCLVDLRGKNTY
EKYLGADYIKAVSNLRKCSTSRLLEACTFHKH
>d1tfd__ c.94.1.2 (-) Transferrin {Rabbit (Oryctolagus cuniculus)}
VRWCAVNDHEASKCANFRDSMKKVLPEDGPRIICVKKASYLDCIKAIAAHEADAVTLDAGLV
HEAGLTPNNLKPVVAEFYGSKENPKTFYYAVALVKKGSNFQLNELQGKKSCHTGLGRSAGWN
IPIGLLYCDLPEPRKPLEKAVASFFSGSCVPCADGADFPQLCQLCPGCGCSSSQPYFGYSGA
FKCLKDGLGDVAFVKQETIFENLPSKDERDQYELLCLDNTRKPVDEYEQCHLARVPSHAVVA
RSVDGKEDLIWELLNQAQEHFGKDKSGDFQLFSSPHGKNLLFKDSAYGFFK
>d1a8e__ c.94.1.2 (-) Transferrin {Human (Homo sapiens)}
DKTVRWCAVSEHEATKCQSFRDHMKSVIPSDGPSVACVKKASYLDCIRAIAANEADAVTLDA
GLVYDAYLAPNNLKPVVAEFYGSKEDPQTFYYAVAVVKKDSGFQMNQLRGKKSCHTGLGRSA
GWNIPIGLLYCDLPEPRKPLEKAVANFFSGSCAPCADGTDFPQLCQLCPGCGCSTLNQYFGY
SGAFKCLKDGAGDVAFVKHSTIFENLANKADRDQYELLCLDNTRKPVDEYKDCHLAQVPSHT
VVARSMGGKEDLIWELLNQAQEHFGKDKSKEFQLFSSPHGKDLLFKDSAHGFLKVPPRMDAK
MYLGYEYVTAIRNLREGTC

>d1h76a1 c.94.1.2 (A:3-333) Transferrin {Pig (Sus scrofa)}
QKTVRWCTISNQEANKCSSFRENMSKAVKNGPLVSCVKKSSYLDCIKAIRDKEADAVTLDAG
LVFEAGLAPYNLKPVVAEFYGQKDNPQTHYYAVAVVKKGSNFQWNQLQGKRSCHTGLGRSAG
WIIPMGLLYDQLPEPRKPIEKAVASFFSSSCVPCADPVNFPKLCQQCAGKGAEKCACSNHEP
YFGYAGAFNCLKEDAGDVAFVKHSTVLENLPDKADRDQYELLCRDNTRRPVDDYENCYLAQV
PSHAVVARSVDGQEDSIWELLNQAQEHFGRDKSPDFQLFSSSHGKDLLFKDSANGFLKIPSK
MDSSLYLGYQYVTALRNLREE
>d1h76a2 c.94.1.2 (A:342-687) Transferrin {Pig (Sus scrofa)}
ECKKVRWCAIGHEETQKCDAWSINSGGKIECVSAENTEDCIAKIVKGEADAMSLDGGYIYIA
GKCGLVPVLAENYKTEGENCVNTPEKGYLAVAVVKKSSGPDLNWNNLKGKKSCHTAVDRTAG
WNIPMGLLYNKINSCKFDQFFGEGCAPGSQRNSSLCALCIGSERAPGRECLANNHERYYGYT
GAFRCLVEKGDVAFVKDQVVQQNTDGKNKDDWAKDLKQMDFELLCQNGAREPVDNAENCHLA
RAPNHAVVARDDKVTCVAEELLKQQAQFGRHVTDCSSSFCMFKSNTKDLLFRDDTQCLARVG
KTTYESYLGADYITAVANLRKCSTSKLLEACTFHSA
>d1afwa1 c.95.1.1 (A:25-293) Thiolase {Baker's yeast
(Saccharomyces cerevisiae)}
KNSLLEKRPEDVVIVAANRSAIGKGFKGAFKDVNTDYLLYNFLNEFIGRFPEPLRADLNLIE
EVACGNVLNVGAGATEHRAACLASGIPYSTPFVALNRQCSSGLTAVNDIANKIKVGQIDIGL
ALGVESMTNNYKNVNPLGMISSEELQKNREAKKCLIPMGITNENVAANFKISRKDQDEFAAN
SYQKAYKAKNEGLFEDEILPIKLPDGSICQSDEGPRPNVTAESLSSIRPAFIKDRGTTTAGN
ASQVSDGVAGVLLARRSVANQ
>d1afwa2 c.95.1.1 (A:294-417) Thiolase {Baker's yeast
(Saccharomyces cerevisiae)}
LNLPVLGRYIDFQTVGVPPEIMGVGPAYAIPKVLEATGLQVQDIDIFEINEAFAAQALYCIH
KLGIDLNKVNPRGGAIALGHPLGCTGARQVATILRELKKDQIGVVSMCIGTGMGAAAIFIKE
>d1qfla1 c.95.1.1 (A:4-268) Biosynthetic thiolase {Zoogloea
ramigera}
SIVIASAARTAVGSFNGAFANTPAHELGATVISAVLERAGVAAGEVNEVILGQVLPAGEGQN
PARQAAMKAGVPQEATAWGMNQLCGSGLRAVALGMQQIATGDASIIVAGGMESMSMAPHCAH
LRGGVKMGDFKMIDTMIKDGLTDAFYGYHMGTTAENVAKQWQLSRDEQDAFAVASQNKAEAA
QKDGRFKDEIVPFIVKGRKGDITVDADEYIRHGATLDSMAKLRPAFDKEGTVTAGNASGLND
GAAAALLMSEAEASRRG
>d1qfla2 c.95.1.1 (A:269-392) Biosynthetic thiolase {Zoogloea
ramigera}
IQPLGRIVSWATVGVDPKVMGTGPIPASRKALERAGWKIGDLDLVEANEAFAAQACAVNKDL
GWDPSIVNVNGGAIAIGHPIGASGARILNTLLFEMKRRGARKGLATLCIGGGMGVAMCIESL
>d1ek4a1 c.95.1.1 (A:1-253) Beta-ketoacyl-ACP synthase I
{Escherichia coli}
MKRVVITGLGIVSSIGNNQQEVLASLREGRSGITFSQELKDSGMRSHVWGNVKLDTTGLIDR
KVVRFMSDASIYAFLSMEQAIADAGLSPEAYQNNPRVGLIAGSGGGSPRFQVFGADAMRGPR
GLKAVGPYVVTKAMASGVSACLATPFKIHGVNYSISSASATSAHCIGNAVEQIQLGKQDIVF
AGGGEELCWEMACEFDAMGALSTKYNDTPEKASRTYDAHRDGFVIAGGGGMVVVEELEHALA
RGAHI
>d1ek4a2 c.95.1.1 (A:254-406) Beta-ketoacyl-ACP synthase I

{Escherichia coli}
YAEIVGYGATSDGADMVAPSGEGAVRCMKMAMHGVDTPIDYLNSHGTSTPVGDVKELAAIRE
VFGDKSPAISATKAMTGHSLGAAGVQEAIYSLLMLEHGFIAPSINIEELDEQAAGLNIVTET
TDRELTTVMSNSFGFGGTNATLVMRKLKD
>d1kas_1 c.95.1.1 (2-251) Beta-ketoacyl-ACP synthase II
{Escherichia coli}
KRRVVVTGLGMLSPVGNTVESTWKALLAGQSGISLIDHFDTSAYATKFAGLVKDFNCEDIIS
RKEQRKMDAFIQYGIVAGVQAMQDSGLEITEENATRIGAAIGSGIGGLGLIEENHTSLMNGG
PRKISPFFVPSTIVNMVAGHLTIMYGLRGPSISIATACTSGVHNIGHAARIIAYGDADVMVA
GGAEKASTPLGVGGFGAARALSTRNDNPQAASRPWDKERDGFVLGDGAGMLVLEEYEHAKKR
GA
>d1kas_2 c.95.1.1 (252-412) Beta-ketoacyl-ACP synthase II
{Escherichia coli}
KIYAELVGFGMSSDAYHMTSPPENGAGAALAMANALRDAGIEASQIGYVNAHGTSTPAGDKA
EAQAVKTIFGEAASRVLVSSTKSMTGHLLGAAGAVESIYSILALRDQAVPPTINLDNPDEGC
DLDFVPHEARQVSGMEYTLCNSFGFGGTNGSLIFKKI
>d1e5ma1 c.95.1.1 (A:6-255) Beta-ketoacyl-ACP synthase II
{Synechocystis sp.}
KKRVVVTGLGAITPIGNTLQDYWQGLMEGRNGIGPITRFDASDQACRFGGEVKDFDATQFLD
RKEAKRMDRFCHFAVCASQQAINDAKLVINELNADEIGVLIGTGIGGLKVLEDQQTILLDKG
PSRCSPFMIPMMIANMASGLTAINLGAKGPNNCTVTACAAGSNAIGDAFRLVQNGYAKAMIC
GGTEAAITPLSYAGFASARALSFRNDDPLHASRPFDKDRDGFVMGEGSGILILEELESALAR
GA
>d1e5ma2 c.95.1.1 (A:256-416) Beta-ketoacyl-ACP synthase II
{Synechocystis sp.}
KIYGEMVGYAMTCDAYHITAPVPDGRGATRAIAWALKDSGLKPEMVSYINAHGTSTPANDVT
ETRAIKQALGNHAYNIAVSSTKSMTGHLLGGSGGIEAVATVMAIAEDKVPPTINLENPDPEC
DLDYVPGQSRALIVDVALSNSFGFGGHNVTLAFKKYQ
>d1hnja1 c.95.1.1 (A:1-174) Ketoacyl-ACP synthase III (FabH)
{Escherichia coli}
MYTKIIGTGSYLPEQVRTNADLEKMVDTSDEWIVTRTGIRERHIAAPNETVSTMGFEAATRA
IEMAGIEKDQIGLIVVATTSATHAFPSAACQIQSMLGIKGCPAFDVAAACAGFTYALSVADQ
YVKSGAVKYALVVGSDVLARTCDPTDRGTIIIFGDGAGAAVLAASEEPGI
>d1hnja2 c.95.1.1 (A:175-317) Ketoacyl-ACP synthase III (FabH)
{Escherichia coli}
ISTHLHADGSYGELLTLPNADRVNPENSIHLTMAGNEVFKVAVTELAHIVDETLAANNLDRS
QLDWLVPHQANLRIISATAKKLGMSMDNVVVTLDRHGNTSAASVPCALDEAVRDGRIKPGQL
VLLEAFGGGFTWGSALVRF
>d1hzpa1 c.95.1.1 (A:-10-174) Ketoacyl-ACP synthase III (FabH)
{Mycobacterium tuberculosis}
MTEIATTSGARSVGLLSVGAYRPERVVTNDEICQHIDSSDEWIYTRTGIKTRRFAADDESAA
SMATEACRRALSNAGLSAADIDGVIVTTNTHFLQTPPAAPMVAASLGAKGILGFDLSAGCAG
FGYALGAAADMIRGGGAATMLVVGTEKLSPTIDMYDRGNCFIFADGAAAVVVGETPFQGI
>d1hzpa2 c.95.1.1 (A:175-317) Ketoacyl-ACP synthase III (FabH)

{Mycobacterium tuberculosis}
GPTVAGSDGEQADAIRQDIDWITFAQNPSGPRPFVRLEGPAVFRWAAFKMGDVGRRAMDAAG
VRPDQIDVFVPHQANSRINELLVKNLQLRPDAVVANDIEHTGNTSAASIPLAMAELLTTGAA
KPGDLALLIGYGAGLSYAAQVVRM
>d1bi5a1 c.95.1.2 (A:1-235) Chalcone synthase {Alfalfa (Medicago sativa)}
MVSVSEIRKAQRAEGPATILAIGTANPANCVEQSTYPDFYFKITNSEHKTELKEKFQRMCDK
SMIKRRYMYLTEEILKENPNVCEYMAPSLDARQDMVVVEVPRLGKEAAVKAIKEWGQPKSKI
THLIVCTTSGVDMPGADYQLTKLLGLRPYVKRYMMYQQGAFAGGTVLRLAKDLAENNKGARV
LVVCSEVTAVTFRGPSDTHLDSLVGQALFGDGAAALIVGSDPVPEIEKP
>d1bi5a2 c.95.1.2 (A:236-389) Chalcone synthase {Alfalfa (Medicago sativa)}
IFEMVWTAQTIAPDSEGAIDGHLREAGLTFHLLKDVPGIVSKNITKALVEAFEPLGISDYNS
IFWIAHPGGPAILDQVEQKLALKPEKMNATREVLSEYGNMSSACVLFILDEMRKKSTQNGLK
TTGEGLEWGVLFGFGPGLTIETVVLRSVAI
>d1ee0a1 c.95.1.2 (A:20-235) Pyrone synthase (PyS, chalcone synthase 2) {Gerbera hybrida}
GLATILAIGTATPPNCVAQADYADYYFRVTKSEHMVDLKEKFKRICEKTAIKKRYLALTEDY
LQENPTMCEFMAPSLNARQDLVVTGVPMLGKEAAVKAIDEWGLPKSKITHLIFCTTAGVDMP
GADYQLVKLLGLSPSVKRYMLYQQGAAAGGTVLRLAKDLAENNKGSRVLIVCSEITAILFHG
PNENHLDSLVAQALFGDGAAALIVGSGPHL
>d1ee0a2 c.95.1.2 (A:236-395) Pyrone synthase (PyS, chalcone synthase 2) {Gerbera hybrida}
AVERPIFEIVSTDQTILPDTEKAMKLHLREGGLTFQLHRDVPLMVAKNIENAAEKALSPLGI
TDWNSVFWMVHPGGRAILDQVERKLNLKEDKLRASRHVLSEYGNLISACVLFIIDEVRKRSM
AEGKSTTGEGLDCGVLFGFGPGMTVETVVLRSVRVT
>d1feha1 c.96.1.1 (A:210-574) Fe-only hydrogenase, catalytic domain {Clostridium pasteurianum}
HMDRVKNALNAPEKHVIVAMAPSVRASIGELFNMGFGVDVTGKIYTALRQLGFDKIFDINFG
ADMTIMEEATELVQRIENNGPFPMFTSCCPGWVRQAENYYPELLNNLSSAKSPQQIFGTASK
TYYPSISGLDPKNVFTVTVMPCTSKKFEADRPQMEKDGLRDIDAVITTRELAKMIKDAKIPF
AKLEDSEADPAMGEYSGAGAIFGATGGVMEAALRSAKDFAENAELEDIEYKQVRGLNGIKEA
EVEINNNKYNVAVINGASNLFKFMKSGMINEKQYHFIEVMACHGGCVNGGGQPHVNPKDLEK
VDIKKVRASVLYNQDEHLSKRKSHENTALVKMYQNYFGKPGEGRAHEILHFKYKK
>d1hfel1 c.96.1.1 (L:87-398) Fe-only hydrogenase larger subunit, C-domain {Desulfovibrio desulfuricans}
WVPEVEKKLKDGKVKCIAMPAPAVRYALGDAFGMPVGSVTTGKMLAALQKLGFAHCWDTEFT
ADVTIWEEGSEFVERLTKKSDMPLPQFTSCCPGWQKYAETYYPELLPHFSTCKSPIGMNGAL
AKTYGAERMKYDPKQVYTVSIMPCIAKKYEGLRPELKSSGMRDIDATLTTRELAYMIKKAGI
DFAKLPDGKRDSLMGESTGGATIFGVTGGVMEAALRFAYEAVTGKKPDSWDFKAVRGLDGIK
EATVNVGGTDVKVAVVHGAKRFKQVCDDVKAGKSPYHFIEYMACPGGCVCGGGQPVMPGVLE
AM
>d1aln_1 c.97.1.1 (1-150) Cytidine deaminase {Escherichia coli}
MHPRFQTAFAQLADNLQSALEPILADKYFPALLTGEQVSSLKSATGLDEDALAFALLPLAAA

CARTPLSNFNVGAIARGVSGTWYFGANMEFIGATMQQTVHAEQSAISHAWLSGEKALAAITV
NYTPCGHCRQFMNELNSGLDLRIHLP
>d1aln_2 c.97.1.1 (151-294) Cytidine deaminase {Escherichia coli}
GREAHALRDYLPDAFGPKDLEIKTLLMDEQDHGYALTGDALSQAAIAAANRSHMPYSKSPSG
VALECKDGRIFSGSYAENAAFNPTLPPLQGALILLNLKGYDYPDIQRAVLAEKADAPLIQWD
ATSATLKALGCHSIDRVLLA
>d1g8ma2 c.97.2.1 (A:201-593) AICAR transformylase domain of bifunctional purine biosynthesis enzyme ATIC {Chicken (Gallus gallus)}
GVSQLPLRYGMNPHQSPAQLYTTRPKLPLTVVNGSPGFINLCDALNAWQLVKELKQALGIPA
AASFKHVSPAGAAVGIPLSEEEAQVCMVHDLHKTLTPLASAYARSRGADRMSSFGDFIALSD
ICDVPTAKIISREVSDGVVAPGYEEEALKILSKKKNGGYCVLQMDPNYEPDDNEIRTLYGLQ
LMQKRNNAVIDRSLFKNIVTKNKTLPESAVRDLIVASIAVKYTQSNSVCYAKDGQVIGIGAG
QQSRIHCTRLAGDKANSWWLRHHPRVLSMKFKAGVKRAEVSNAIDQYVTGTIGEDEDLVKWQ
AMFEEVPAQLTEAEKKQWIAKLTAVSLSSDAFFPFRDNVDRAKRIGVQFIVAPSGSAADEVV
IEACNELGITLIHTNLRLFHH
>d1rgea_ d.1.1.1 (A:) RNase Sa {Streptomyces aureofaciens}
DVSGTVCLSALPPEATDTLNLIASDGPFPYSQDGVVFQNRESVLPTQSYGYYHEYTVITPGA
RTRGTRRIITGEATQEDYYTGDHYATFSLIDQTC
>d1fus__ d.1.1.1 (-) RNase F1 {Fusarium moniliforme}
ESATTCGSTNYSASQVRAAANAACQYYQNDDTAGSSTYPHTYNNYEGFDFPVDGPYQEFPIK
SGGVYTGGSPGADRVVINTNCEYAGAITHTGASGNNFVGCSGTN
>d1i0va_ d.1.1.1 (A:) RNase T1 {Aspergillus oryzae}
ACDYTCGSNCYSSSDVSTAQAAGYKLHEDGETVGSNSYPHKYNNYEGFDFSVSSPYYEWPIL
SSGDVYSGGSPGADRVVFNENNQLAGVITHTGASGNNFVECT
>d1rtu__ d.1.1.1 (-) RNase U2 {Ustilago sphaerogena}
CDIPQSTNCGGNVYSNDDINTAIQGALDDVANGDRPDNYPHQYYXEASEDITLCCGSGPWSE
FPLVYNGPYYSSRDNYVSPGPDRVIYQTNTGEFCATVTHTGAASYDGFTQCS
>d1a2pa_ d.1.1.1 (A:) Barnase/Binase {Bacillus amyloliquefaciens}
VINTFDGVADYLQTYHKLPDNYITKSEAQALGWVASKGNLADVAPGKSIGGDIFSNREGKLP
GKSGRTWREADINYTSGFRNSDRILYSSDWLIYKTTDHYQTFTKIR
>d1goua_ d.1.1.1 (A:) Barnase/Binase {Bacillus intermedius}
AVINTFDGVADYLIRYKRLPNDYITKSQASALGWVASKGDLAEVAPGKSIGGDVFSNREGRL
PSAGSRTWREADINYVSGFRNADRLVYSSDWLIYKTTDHYATFTRIR
>d2rbia_ d.1.1.1 (A:) Barnase/Binase {Bacillus intermedius}
VINTFDGVADYLIRYKRLPDNYITKSQASALGWVASKGNLAEVAPGKSIGGDVFSNREGRLP
SASGRTWREADINYVSGFRNADRLVYSSDWLIYKTTDNYATFTRIR
>d1rds__ d.1.1.1 (-) RNase Ms {Molsin (Aspergillus saitoi)}
ESCEYTCGSTCYWSSDVSAAKAKGYSLYESGDTIDDYPHEYHDYEGFDFPVSGTYYEYPIMS
DYDVYTGGSPGADRVIFNGDDELAGVITHTGASGDDFVACSSS
>d0rst__ d.1.1.1 (-) RNase St {Streptomyces erythreus}
QAPCGDTSGFEEVRLADLPPEATDTYELIEKGGPYPYPEDGTVFENREGILPDCAEGYYHEY
TVKTPSGDDRGARRFVVGDGGEYFYTEDHYESFRLTIVN
>d1aqza_ d.1.1.1 (A:) Ribotoxin {Fungus (Aspergillus restrictus),

restrictocin}
ATWTCINQQLNPKTNKWEDKRLLYSQAKAESNSHHAPLSDGKTGSSYPHWFTNGYDGNGKLI
KGRTPIKFGKADCDRPPKHSQNGMGKDDHYLLEFPTFPDGHDYKFDSKKPKENPGPARVIYT
YPNKVFCGIVAHQRGNQGDLRLCSH
>d1de3a_ d.1.1.1 (A:) Ribotoxin {Fungus (Aspergillus giganteus), alpha-Sarcin}
AVTWTCLNDQKNPKTNKYETKRLLYNQNKAESNSHHAPLSDGKTGSSYPHWFTNGYDGDGKL
PKGRTPIKFGKSDCDRPPKHSKDGNGKTDHYLLEFPTFPDGHDYKFDSKKPKENPGPARVIY
TYPNKVFCGIIAHTKENQGELKLCSH
>d1cnsa_ d.2.1.1 (A:) Plant class II chitinase {Barley (Hordeum vulgare)}
SVSSIVSRAQFDRMLLHRNDGACQAKGFYTYDAFVAAAAAFSGFGTTGSADVQKREVAAFLA
QTSHETTGGWATAPDGAFAWGYCFKQERGASSDYCTPSAQWPCAPGKRYYGRGPIQLSHNYN
YGPAGRAIGVDLLANPDLVATDATVSFKTAMWFWMTAQPPKPSSHAVIVGQWSPSGADRAAG
RVPGFGVITNIINGGIECGHGQDSRVADRIGFYKRYCDILGVGYGNNLDCYSQRPFA
>d1dxja_ d.2.1.1 (A:) Plant class II chitinase {Jack bean (Canavalia ensiformis)}
DVGSVIDASLFDQLLKHRNDPACEGKGFYSYNAFVTAARSFGGFGTTGDTNTRKREVAAFLA
QTSHETTGGAAGSPDGPYAWGYCFVTERDKSNKYCDPGTPCPAGKSYYGRGPIQLTHNYNYA
QAGRALGVDLINNPDLVARDAVISFKTAIWFWMTPQGNKPSCHDVITNRWTPSAADVAANRT
PGFGVITNIINGGIECGRGPSPASGDRIGFYKRYCDVLHLSYGPNLNCRDQRPFGG
>d1lsg_1 d.2.1.2 (1-144) Lysozyme {Chicken (Gallus gallus)}
MKVFGRCELAAAMKRHGLDNYRGYSLGNWVCAAKFESNFNTQATNRNTDGSTDYGILQINSR
WWCNDGRTPGSRNLCNIPCSALLSSDITASVNCAKKIVSDGNGMNAWVAWRNRCKGTDVQAW
IRGCRLQQHHLGGAKQAGDV
>d3lzt__ d.2.1.2 (-) Lysozyme {Chicken (Gallus gallus)}
KVFGRCELAAAMKRHGLDNYRGYSLGNWVCAAKFESNFNTQATNRNTDGSTDYGILQINSRW
WCNDGRTPGSRNLCNIPCSALLSSDITASVNCAKKIVSDGNGMNAWVAWRNRCKGTDVQAWI
RGCRL
>d1jse__ d.2.1.2 (-) Lysozyme {Turkey (Meleagris gallopavo)}
KVYGRCELAAAMKRLGLDNYRGYSLGNWVCAAKFESNFNTHATNRNTDGSTDYGILQINSRW
WCNDGRTPGSKNLCNIPCSALLSSDITASVNCAKKIASGGNGMNAWVAWRNRCKGTDVHAWI
RGCRL
>d1hhl__ d.2.1.2 (-) Lysozyme {Guinea fowl (Numida meleagris)}
KVFGRCELAAAMKRHGLDNYRGYSLGNWVCAAKFESNFNSQATNRNTDGSTDYGVLQINSRW
WCNDGRTPGSRNLCNIPCSALQSSDITATANCAKKIVSDGDGMNAWVAWRKHCKGTDVRVWI
KGCRL
>d1ghla_ d.2.1.2 (A:) Lysozyme {Pheasant (Phasianus colchicus)}
GKVYGRCELAAAMKRMGLDNYRGYSLGNWVCAAKFESNFNTGATNRNTDGSTDYGILQINSR
WWCNDGRTPGSKNLCHIPCSALLSSDITASVNCAKKIVSDGNGMNAWVAWRKHCKGTDVNVW
IRGCRL
>d1jsf__ d.2.1.2 (-) Lysozyme {Human (Homo sapiens)}
KVFERCELARTLKRLGMDGYRGISLANWMCLAKWESGYNTRATNYNAGDRSTDYGIFQINSR
YWCNDGKTPGAVNACHLSCSALLQDNIADAVACAKRVVRDPQGIRAWVAWRNRCQNRDVRQY

VQGCGV
>d2eql__ d.2.1.2 (-) Lysozyme {Horse (Equus caballus), milk}
KVFSKCELAHKLKAQEMDGFGGYSLANWVCMAEYESNFNTRAFNGKNANGSSDYGLFQLNNK
WWCKDNKRSSSNACNIMCSKLLDENIDDDISCAKRVVRDPKGMSAWKAWVKHCKDKDLSEYL
ASCNL
>d1qqya_ d.2.1.2 (A:) Lysozyme {Dog (Canis familiaris), milk}
MKIFSKCELARKLKSMGMDGFHGYSLANWVCMAEYESNFNTQAFNGRNSNGSSDYGIFQLNS
KWWCKSNSHSSANACNIMCSKFLDDNIDDDIACAKRVVKDPNGMSAWVAWVKHCKGKDLSKY
LASCNL
>d1jug__ d.2.1.2 (-) Lysozyme {Australian echidna (Tachyglossus aculeatus)}
KILKKQELCKNLVAQGMNGYQHITLPNWVCTAFHESSYNTRATNHNTDGSTDYGILQINSRY
WCHDGKTPGSKNACNISCSKLLDDDITDDLKCAKKIAGEAKGLTPWVAWKSKCRGHDLSKFK
C
>d1lmq__ d.2.1.2 (-) Lysozyme {Rainbow trout (Oncorhynchus mykiss)}
KVYDRCELARALKASGMDGYAGNSLPNWVCLSKWESSYNTQATNRNTDGSTDYGIFQINSRY
WCDDGRTPGAKNVCGIRCSQLLTDDLTVAIRCAKRVVLDPNGIGAWVAWRLHCQNQDLRSYV
AGCGV
>d1gd6a_ d.2.1.2 (A:) Lysozyme {Silkworm (Bombyx mori)}
KTFTRCGLVHELRKHGFEENLMRNWVCLVEHESSRDTSKTNTNRNGSKDYGLFQINDRYWCS
KGASPGKDCNVKCSDLLTDDITKAAKCAKKIYKRHRFDAWYGWKNHCQGSLPDISSC
>d1iiza_ d.2.1.2 (A:) Lysozyme {Tasar silkworm (Antheraea mylitta)}
KRFTRCGLVNELRKQGFDENLMRDWVCLVENESARYTDKIANVNKNGSRDYGLFQINDKYWC
SKGSTPGKDCNVTCSQLLTDDITVASTCAKKIYKRTKFDAWSGWDNHCNHSNPDISSC
>d1alc__ d.2.1.2 (-) alpha-Lactalbumin {Baboon (Papio cynocephalus)}
KQFTKCELSQNLYDIDGYGRIALPELICTMFHTSGYDTQAIVENDESTEYGLFQISNALWCK
SSQSPQSRNICDITCDKFLDDDITDDIMCAKKILDIKGIDYWIAHKALCTEKLEQWLCEK
>d1b9oa_ d.2.1.2 (A:) alpha-Lactalbumin {Human (Homo sapiens)}
KQFTKCELSQLLKDIDGYGGIALPELICTMFHTSGYDTQAIVENDESTEYGLFQISNKLWCK
SSQVPQSRNICDISCDKFLDDDITDDIMCAKKILDIKGIDYWLAHKALCTEKLEQWLCEKL
>d1hfx__ d.2.1.2 (-) alpha-Lactalbumin {Guinea pig (Cavia porcellus)}
KQLTKCALSHELNDLAGYRDITLPEWLCIIFHISGYDTQAIVKNSDHKEYGLFQINDKDFCE
SSTTVQSRNICDISCDKLLDDDLTDDIMCVKKILDIKGIDYWLAHKPLCSDKLEQWYCEAQ
>d1fkqa_ d.2.1.2 (A:) alpha-Lactalbumin {Goat (Capra hircus)}
MEQLTKCEVFQKLKDLKDYGGVSLPEWVCVAFHTSGYDTQAIVQNNDSTEYGLFQINNKIWC
KDDQNPHSRNICNISCDKFLDDDLTDDIVCAKKILDKVGINYWLAHKALCSEKLDQWLCEKL
>d1f6ra_ d.2.1.2 (A:) alpha-Lactalbumin {Cow (Bos taurus)}
EQLTKCEVFRELKDLKGYGGVSLPEWVCTTFHTSGYDTQAIVQNNDSTEYGLFQINNKIWCK
DDQNPHSSNICNISCDKFLDDDLTDDIMCVKKILDKVGINYWLAHKALCSEKLDQWLCEKL
>d1j8wa_ d.2.1.2 (A:) alpha-Lactalbumin {Mouse (Mus musculus)}

TELTKCKVSHAIKDMDGYQGISLLEWTCVLFHTSGYDSQAVVNDNGSTEYGLFQISERFWCK
SSEFPESENICGISCDKLLDDELDDDIVCAKKIVAIKGIDYWKAYKPMCSEKLEQWRCEKP
>d169la_ d.2.1.3 (A:) Phage T4 lysozyme {Bacteriophage T4}
MNIFEMLRIDEGLRLKIYKDTEGYYTIGIGHLLTKSPSLNAAKSELDKAIGRNCNGVITKDE
AEKLFNQDVDAAVRGILRNAKLKPVYDSLDAVRRCALINMVFQMGETGVAGFTNSLRMLQQK
RWDAAAAALAAAAWAAATPNRAKRVITTFRTGTWDAYK
>d174la_ d.2.1.3 (A:) Phage T4 lysozyme {Bacteriophage T4}
MNIFEMLRIDEGLRLKIYKDTEGYYTIGIGHLLAAADLAAAKAALAAAIGRNTNGVITKDE
AEKLFNQDVDAAVRGILRNAKLKPVYDSLDAVRRAALINMVFQMGETGVAGFTNSLRMLQQK
RWDEAAVNLAKSRWYNQTPNRAKRVITTFRTGTWDAYKNL
>d176la_ d.2.1.3 (A:) Phage T4 lysozyme {Bacteriophage T4}
MNIFEMLRIDEGLRLKIYKDTEGYYTIGIGHTLKVDGNSNAAKSELDKAIGRNTNGVITKDE
AEKLFNQDVDAAVRGILRNAKLKPVYDSLDAVRRAALINMVFQMGETGVAGFTNSLRMLQQK
RWDEAAVNLAKSRWYNQTPNRAKRVITTFRTGTWDAYKNL
>d189l__ d.2.1.3 (-) Phage T4 lysozyme {Bacteriophage T4}
MNLFEMLRIDEGLRLKIYKDTEGYYTIGIGHLLTKSPDLNVAKSELDKAIGRNCNGVITKDE
AEKLFNQDVDAAVRGILRNPKLKPVYDSLDAVRRCALINMVFQMGETGVAGFTDSLRMLQQK
RWDEAAANLAKSRWYNQTPDRAKRVITTFRTGTWDAYKNL
>d191l__ d.2.1.3 (-) Phage T4 lysozyme {Bacteriophage T4}
MNIFEMLRIDEGLRLKIYKDTEGYYTIGIGHLLTKSPSLNAAKSELDKAIGRACAGAITKDE
AEKLFNQDVDAAVRGILRNAKLKPVYDSLDAVRRCALINMVFQMGETGVAGFTNSLRMLQQK
RWDAAAAALAKSRWYNQTPNRAKRVITTFRTGTWDAYK
>d192l__ d.2.1.3 (-) Phage T4 lysozyme {Bacteriophage T4}
MNIFEMLRIDEGLRLKIYKDTEGYYTIGIGHLLTKSPSLAAAKAALAAAIGRNTNGVITKDE
AEKLFNQDVDAAVRGILRNAKLKPVYDSLDAVRRAALINMVFQMGETGVAGFTNSLRMLQQK
RWAAAAAALAKSRWYNQTPNRAKRVITTFRTGTWDAYK
>d217l__ d.2.1.3 (-) Phage T4 lysozyme {Bacteriophage T4}
MNIFEMLRIDEGLRLKIYKDTEGYYTIGIGHLLTKSPSLNAAKEELDKAIGRNTNGVITKDE
AEKLFNQDVDAAVRGILRNAKLKPVYDSLDAVRRAALINMVFQMGETGVAGFTNSLRMLQQK
RWDEAAVNLAKSRWYNQTPNRAKRVITTFRTGTWDAYK
>d1k28a3 d.2.1.3 (A:130-345) Tail-associated lysozyme gp5,
catalytic domain {Bacteriophage T4}
NVLNQGGEVGYDSSSNVIQDSNLDTAINPDDRPLSEIPTDDNPNMSMAEMLRRDEGLRLKVY
WDTEGYPTIGIGHLIMKQPVRDMAQINKVLSKQVGREITGNPGSITMEEATTLFERDLADMQ
RDIKSHSKVGPVWQAVNRSRQMALENMAFQMGVGGVAKFNTMLTAMLAGDWEKAYKAGRDSL
WYQQTKGRASRVTMIILTGNLESYGVEVKT
>d1am7a_ d.2.1.4 (A:) Lambda lysozyme {Bacteriophage lambda}
MVEINNQRKAFLDMLAWSEGTDNGRQKTRNHGYDVIVGGELFTDYSDHPRKLVTLNPKLKST
GAGRYQLLSRWWDAYRKQLGLKDFSPKSQDAVALQQIKERGALPMIDRGDIRQAIDRCSNIW
ASLPGAGYGQFEHKADSLIAKFKEAGGTVR
>d153l__ d.2.1.5 (-) Lysozyme {Goose (Anser anser anser)}
RTDCYGNVNRIDTTGASCKTAKPEGLSYCGVSASKKIAERDLQAMDRYKTIIKKVGEKLCVE
PAVIAGIISRESHAGKVLKNGWGDRGNGFGLMQVDKRSHKPQGTWNGEVHITQGTTILINFI
KTIQKKFPSWTKDQQLKGGISAYNAGAGNVRSYARMDIGTTHDDYANDVVARAQYYKQHGY

>d1qsaa2 d.2.1.6 (A:451-618) 70 kDa soluble lytic transglycosylase, SLT70 {Escherichia coli}
LAYNDLFKRYTSGKEIPQSYAMAIARQESAWNPKVKSPVGASGLMQIMPGTATHTVKMFSIP
GYSSPGQLLDPETNINIGTSYLQYVYQQFGNNRIFSSAAYNAGPGRVRTWLGNSAGRIDAVA
FVESIPFSETRGYVKNVLAYDAYYRYFMGDKPTLMSATEWGRRY
>d1qusa_ d.2.1.6 (A:) 36 kDa soluble lytic transglycosylase, SLT35 {Escherichia coli}
MVEPQHNVMQMGGDFANNPNAQQFIDKMVNKHGFDRQQLQEILSQAKRLDSVLRLMDNQAPT
TSVKPPSGPNGAWLRYRKKFITPDNVQNGVVFWNQYEDALNRAWQVYGVPPEIIVGIIGVET
RWGRVMGKTRILDALATLSFNYPRRAEYFSGELETFLLMARDEQDDPLNLKGSFAGAMGYGQ
FMPSSYKQYAVDFSGDGHINLWDPVDAIGSVANYFKAHGWVKGDQVAVMANGQAPGLPNGFK
TKYSISQLAAAGLTPQQPLGNHQQASLLRLDVGTGYQYWYGLPNFYTITRYNHSTHYAMAVW
QLGQAVALARVQ
>d1chka_ d.2.1.7 (A:) Endochitosanase {Streptomyces sp., strain N174}
AGAGLDDPHKKEIAMELVSSAENSSLDWKAQYKYIEDIGDGRGYTGGIIGFCSGTGDMLELV
QHYTDLEPGNILAKYLPALKKVNGSASHSGLGTPFTKDWATAAKDTVFQQAQNDERDRVYFD
PAVSQAKADGLRALGQFAYYDAIVMHGPGNDPTSFGGIRKTAMKKARTPAQGGDETTYLNGF
LDARKAAMLTEAAHDDTSRVDTEQRVFLKAGNLDLNPPLKWKTYGDPYVINS
>d1qgia_ d.2.1.7 (A:) Endochitosanase {Bacillus circulans}
ASPDDNFSPETLQFLRNNTGLDGEQWNNIMKLINKPEQDDLNWIKYYGYCEDIEDERGYTIG
LFGATTGGSRDTHPDGPDLFKAYDAAKGASNPSADGALKRLGINGKMKGSILEIKDSEKVFC
GKIKKLQNDAAWRKAMWETFYNVYIRYSVEQARQRGFTSAVTIGSFVDTALNQGATGGSDTL
QGLLARSGSSSNEKTFMKNFHAKRTLVVDTNKYNKPPNGKNRVKQWDTLVDMGKMNLKNVDS
EIAQVTDWEMK
>d1aec__ d.3.1.1 (-) Actinidin {Chinese gooseberry or kiwifruit (Actinidia chinensis)}
LPSYVDWRSAGAVVDIKSQGECGGCWAFSAIATVEGINKIVTGVLISLSEQELIDCGRTQNT
RGCNGGYITDGFQFIINNGGINTEENYPYTAQDGECNVDLQNEKYVTIDTYENVPYNNEWAL
QTAVTYQPVSVALDAAGDAFKQYSSGIFTGPCGTAIDHAVTIVGYGTEGGIDYWIVKNSWDT
TWGEEGYMRILRNVGGAGTCGIATMPSYPVKY
>d2act__ d.3.1.1 (-) Actinidin {Chinese gooseberry or kiwifruit (Actinidia chinensis)}
LPSYVDWRSAGAVVDIKSQGECGGCWAFSAIATVEGINKITSGSLISLSEQELIDCGRTQNT
RGCDGGYITDGFQFIINDGGINTEENYPYTAQDGDCDVALQDQKYVTIDTYENVPYNNEWAL
QTAVTYQPVSVALDAAGDAFKQYASGIFTGPCGTAVDHAIVIVGYGTEGGVDYWIVKNSWDT
TWGEEGYMRILRNVGGAGTCGIATMPSYPVKY
>d1ppn__ d.3.1.1 (-) Papain {Papaya (Carica papaya)}
IPEYVDWRQKGAVTPVKNQGSCGSCWAFSAVVTIEGIIKIRTGNLNEYSEQELLDCDRRSYG
CNGGYPWSALQLVAQYGIHYRNTYPYEGVQRYCRSREKGPYAAKTDGVRQVQPYNEGALLYS
IANQPVSVVLEAAGKDFQLYRGGIFVGPCGNKVDHAVAAVGYGPNYILIKNSWGTGWGENGY
IRIKRGTGNSYGVCGLYTSSFYPVKN
>d1pcia_ d.3.1.1 (A:) Caricain (protease omega) {Papaya (Carica papaya)}

LTSTERLIQLFNSWMLNHNKFYENVDEKLYRFEIFKDNLNYIDETNKKNNSYWLGLNEFADL
SNDEFNEKYVGSLIDATIEQSYDEEFINEDIVNLPENVDWRKKGAVTPVRHQGSCGSCWAFS
AVATVEGINKIRTGKLVELSEQELVDCERRSHGCKGGYPPYALEYVAKNGIHLRSKYPYKAK
QGTCRAKQVGGPIVKTSGVGRVQPNNEGNLLNAIAKQPVSVVVESKGRPFQLYKGGIFEGPC
GTKVDGAVTAVGYGKSGGKGYILIKNSWGTAWGEKGYIRIKRAPGNSPGVCGLYKSSYYPTK
N
>d1ppo__ d.3.1.1 (-) Caricain (protease omega) {Papaya (Carica papaya)}
LPENVDWRKKGAVTPVRHQGSCGSCWAFSAVATVEGINKIRTGKLVELSEQELVDCERRSHG
CKGGYPPYALEYVAKNGIHLRSKYPYKAKQGTCRAKQVGGPIVKTSGVGRVQPNNEGNLLNA
IAKQPVSVVVESKGRPFQLYKGGIFEGPCGTKVDHAVTAVGYGKSGGKGYILIKNSWGTAWG
EKGYIRIKRAPGNSPGVCGLYKSSYYPTKN
>d1yal__ d.3.1.1 (-) Chymopapain {Papaya (Carica papaya)}
YPQSIDWRAKGAVTPVKNQGACGSCWAFSTIATVEGINKIVTGNLLELSEQELVDCDKHSYG
CKGGYQTTSLQYVANNGVHTSKVYPYQAKQYKCRATDKPGPKVKITGYKRVPSNCETSFLGA
LANQPLSVLVEAGGKPFQLYKSGVFDGPCGTKLDHAVTAVGYGTSDGKNYIIIKNSWGPNWG
EKGYMRLKRQSGNSQGTCGVYKSSYYPFKGFA
>d1gece_ d.3.1.1 (E:) Glycyl endopeptidase {Papaya (Carica papaya)}
LPESVDWRAKGAVTPVKHQGYCESCWAFSTVATVEGINKIKTGNLVELSEQELVDCDLQSYG
CNRGYQSTSLQYVAQNGIHLRAKYPYIAKQQTCRANQVGGPKVKTNGVGRVQSNNEGSLLNA
IAHQPVSVVVESAGRDFQNYKGGIFEGSCGTKVDHAVTAVGYGKSGGKGYILIKNSWGPGWG
ENGYIRIRRASGNSPGVCGVYRSSYYPIKN
>d1cqda_ d.3.1.1 (A:) Proline-specific cysteine protease {Ginger rhizome (Zingiber officinale)}
LPDSIDWRENGAVVPVKNQGGCGSCWAFSTVAAVEGINQIVTGDLISLSEQQLVDCTTANHG
CRGGWMNPAFQFIVNNGGINSEETYPYRGQDGICNSTVNAPVVSIDSYENVPSHNEQSLQKA
VANQPVSVTMDAAGRDFQLYRSGIFTGSCNISANHALTVVGYGTENDKDFWIVKNSWGKNWG
ESGYIRAERNIENPDGKCGITRFASYPVKK
>d3gcb__ d.3.1.1 (-) Bleomycin hydrolase {Baker's yeast (Saccharomyces cerevisiae), Gal6}
AFQGAMASSIDISKINSWNKEFQSDLTHQLATTVLKNYNADDALLNKTRLQKQDNRVFNTVV
STDSTPVTNQKSSGRAWLFAATNQLRLNVLSELNLKEFELSQAYLFFYDKLEKANYFLDQIV
SSADQDIDSRLVQYLLAAPTEDGGQYSMFLNLVKKYGLIPKDLYGDLPYSTTASRKWNSLLT
TKLREFAETLRTALKERSADDSIIVTLREQMQREIFRLMSLFMDIPPVQPNEQFTWEYVDKD
KKIHTIKSTPLEFASKYAKLDPSTPVSLINDPRHPYGKLIKIDRLGNVLGGDAVIYLNVDNE
TLSKLVVKRLQNNKAVFFGSHTPKFMDKKTGVMDIELWNYPAIGYNLPQQKASRIRYHESLM
THAMLITGCHVDETSKLPLRYRVENSWGKDSGKDGLYVMTQKYFEEYCFQIVVDINELPKEL
ASKFTSGKEEPIVLPIWDPMGALA
>d2cb5a_ d.3.1.1 (A:) Bleomycin hydrolase {Human (Homo sapiens)}
SSSGLNSEKVAALIQKLNSDPQFVLAQNVGTTHDLLDICLKRATVQRAQHVFQHAVPQEGKP
ITNQKSSGRSWIFSCLNVMRLPFMKKLNIEEFEFSQSYLFFWDKVERCYFFLSAFVDTAQRK
EPEDGRLVQFLLMNPANDGGQWDMLVNIVEKYGVIPKKCFPESYTTEATRRMNDILNHKMRE
FCIRLRNLVHSGATKGEISATQDVMMEEIFRVVCICLGNPPETFTWEYRDKDKNYEKIGPIT

PLEFYREHVKPLFNMEDKICLVNDPRPQHKHNKLYTVEYLSNMVGGRKTLYNNQPIDFLKKM
VAASIKDGEAVWFGCDVGKHFNSKLGLSDMNLYDHELVFGVSLKNMNKAERLTFGESLMTHA
MTFTAVSEKDDQDGAFTKWRVENSWGEDHGHKGYLCMTDEWFSEYVYEVVVDRKHVPEEVLA
VLEQEPIILPAWDPMGALA
>d1f2aa_ d.3.1.1 (A:) Cruzain {Trypanosoma cruzi}
APAAVDWRARGAVTAVKDQGQCGSCWAFSAIGNVECQWFLAGHPLTNLSEQMLVSCDKTDSG
CSGGLMNNAFEWIVQENNGAVYTEDSYPYASGEGISPPCTTSGHTVGATITGHVELPQDEAQ
IAAWLAVNGPVAVAVDASSWMTYTGGVMTSCVSEQLDHGVLLVGYNDSAAVPYWIIKNSWTT
QWGEEGYIRIAKGSNQCLVKEEASSAVVG
>d3pbh__ d.3.1.1 (-) (Pro)cathepsin B {Human (Homo sapiens)}
MRSRPSFHPLSDELVNYVNKRNTTWQAGHNFYNVDMSYLKRLCGTFLGGPKPPQRVMFTEDL
KLPASFDAREQWPQCPTIKEIRDQGSCGSCWAFGAVEAISDRICIHTNAHVSVEVSAEDLLT
CCGSMCGDGCNGGYPAEAWNFWTRKGLVSGGLYESHVGCRPYSIPPCEHHVNGSRPPCTGEG
DTPKCSKICEPGYSPTYKQDKHYGYNSYSVSNSEKDIMAEIYKNGPVEGAFSVYSDFLLYKS
GVYQHVTGEMMGGHAIRILGWGVENGTPYWLVANSWNTDWGDNGFFKILRGQDHCGIESEVV
AGIPRTD
>g1huc.1 d.3.1.1 (A:,B:) (Pro)cathepsin B {Human (Homo sapiens)}
LPASFDAREQWPQCPTIKEIRDQGSCGSCWAFGAVEAISDRICIHTNXVSVEVSAEDLLTCC
GSMCGDGCNGGYPAEAWNFWTRKGLVSGGLYESHVGCRPYSIPPCEHHVNGSRPPCTGEGDT
PKCSKICEPGYSPTYKQDKHYGYNSYSVSNSEKDIMAEIYKNGPVEGAFSVYSDFLLYKSGV
YQHVTGEMMGGHAIRILGWGVENGTPYWLVANSWNTDWGDNGFFKILRGQDHCGIESEVVAG
IPRTD
>d1mira_ d.3.1.1 (A:) (Pro)cathepsin B {Rat (Rattus norvegicus)}
SDDMINYINKQNTTWQAGRNFYNVDISYLKKLCGTVLGGPKLPERVGFSEDINLPESFDARE
QWSNCPTIAQIRDQGSCGSSWAFGAVEAMSDRICIHTNGRVNVEVSAEDLLTCCGIQCGDGC
NGGYPSGAWNFWTRKGLVSGGVYNSHIGCLPYTIPPCEHHVNGARPPCTGEGDTPKCNKMCE
AGYSTSYKEDKHYGYTSYSVSDSEKEIMAEIYKNGPVEGAFTVFSDFLTYKSGVYKHEAGDV
MGGHAIRILGWGIENGVPYWLVANSWNADWGDNGFFKILRGENHCGIESEIVAGIPRTQQYW
GRF
>d1thea_ d.3.1.1 (A:) (Pro)cathepsin B {Rat (Rattus norvegicus)}
LPESFDAREQWSNCPTIAQIRDQGSCGSCWAFGAVEAMSDRICIHTNGRVNVEVSAEDLLTC
CGIQCGDGCNGGYPSGAWNFWTRKGLVSGGVYNSHIGCLPYTIPPCEHHVNGARPPCTGEGD
TPKCNKMCEAGYSTSYKEDKHYGYTSYSVSDSEKEIMAEIYKNGPVEGAFTVFSDFLTYKSG
VYKHEAGDVMGGHAIRILGWGIENGVPYWLVANSWNADWGDNGFFKILRGENHCGIESEIVA
GIPRT
>d1qdqa_ d.3.1.1 (A:) (Pro)cathepsin B {Cow (Bos taurus)}
LPESFDAREQWPNCPTIKEIRDQGSCGSCWAFGAVEAISDRICIHSNGRVNVEVSAEDMLTC
CGGECGDGCNGGEPSGAWNFWTKKGLVSGGLYNSHVGCRPYSIPPCEHHVNGSRPPCTGEGD
TPKCSKTCEPGYSPSYKEDKHFGCSSYSVANNEKEIMAEIYKNGPVEGAFSVYSDFLLYKSG
VYQHVSGEIMGGHAIRILGWGVENGTPYWLVANSWNTDWGDNGFFKILRGQDHCGIESEIVA
GMPCT
>d1cs8a_ d.3.1.1 (A:) (Pro)cathepsin L {Human (Homo sapiens)}
SLTFDHSLEAQWTKWKAMHNRLYGMNEEGWRRAVWEKNMKMIELHNQEYREGKHSFTMAMNA
FGDMTSEEFRQVMNGFQNRKPRKGKVFQEPLFYEAPRSVDWREKGYVTPVKNQGQCGSCWAF

SATGALEGQMFRKTGRLISLSEQNLVDCSGPQGNEGCNGGLMDYAFQYVQDNGGLDSEESYP
YEATEESCKYNPKYSVANDAGFVDIPKQEKALMKAVATVGPISVAIDAGHESFLFYKEGIYF
EPDCSSEDMDHGVLVVGYGFESTESDNNKYWLVKNSWGEEWGMGGYVKMAKDRRNHCGIASA
ASYPTV
>g1icf.1 d.3.1.1 (A:,B:) (Pro)cathepsin L {Human (Homo sapiens)}
APRSVDWREKGYVTPVKNQGQCGSCWAFSATGALEGQMFRKTGRLISLSEQNLVDCSGPQGN
EGCNGGLMDYAFQYVQDNGGLDSEESYPYEATEESCKYNPKYSVANDTGFVDIPKQEKALMK
AVATVGPISVAIDAGHESFLFYKEGIYFEPDCSSEDMDHGVLVVGYGFESTXNNKYWLVKNS
WGEEWGMGGYVKMAKDRRNHCGIASAASYPTV
>d1mema_ d.3.1.1 (A:) (Pro)cathepsin K {Human (Homo sapiens)}
APDSVDYRKKGYVTPVKNQGQCGSCWAFSSVGALEGQLKKKTGKLLNLSPQNLVDCVSENDG
CGGGYMTNAFQYVQKNRGIDSEDAYPYVGQEESCMYNPTGKAAKCRGYREIPEGNEKALKRA
VARVGPVSVAIDASLTSFQFYSKGVYYDESCNSDNLNHAVLAVGYGIQKGNKHWIIKNSWGE
NWGNKGYILMARNKNNACGIANLASFPKM
>d7pcka_ d.3.1.1 (A:) (Pro)cathepsin K {Human (Homo sapiens)}
LYPEEILDTHWELWKKTHRKQYNNKVDEISRRLIWEKNLKYISIHNLEASLGVHTYELAMNH
LGDMTSEEVVQKMTGLKVPLSHSRSNDTLYIPEWEGRAPDSVDYRKKGYVTPVKNQGQCGSC
WAFSSVGALEGQLKKKTGKLLNLSPQNLVDCVSENDGCGGGYMTNAFQYVQKNRGIDSEDAY
PYVGQEESCMYNPTGKAAKCRGYREIPEGNEKALKRAVARVGPVSVAIDASLTSFQFYSKGV
YYDESCNSDNLNHAVLAVGYGIQKGNKHWIIKNSWGENWGNKGYILMARNKNNACGIANLAS
FPKM
>d8pcha_ d.3.1.1 (A:) (Pro)cathepsin K {Pig (Sus scrofa)}
YPPSMDWRKKGNFVSPVKNQGSCGSCWTFSTTGALESAVAIATGKMLSLAEQQLVDCAQNFN
NHGCQGGLPSQAFEYIRYNKGIMGEDTYPYKGQDDHCKFQPDKAIAFVKDVANITMNDEEAM
VEAVALYNPVSFAFEVTNDFLMYRKGIYSSTSCHKTPDKVNHAVLAVGYGEENGIPYWIVKN
SWGPQWGMNGYFLIERGKNMCGLAACASYPIPLV
>d1fh0a_ d.3.1.1 (A:) (Pro)cathepsin V {Human (Homo sapiens)}
LPKSVDWRKKGYVTPVKNQKQCGSCWAFSATGALEGQMFRKTGKLVSLSEQNLVDCSRPQGN
QGCNGGFMARAFQYVKENGGLDSEESYPYVAVDEICKYRPENSVAQDTGFTVVAPGKEKALM
KAVATVGPISVAMDAGHSSFQFYKSGIYFEPDCSSKNLDHGVLVVGYGFEGANSDNSKYWLV
KNSWGPEWGSNGYVKIAKDKNNHCGIATAASYPNV
>d1deua_ d.3.1.1 (A:) (Pro)cathepsin X {Human (Homo sapiens)}
RGQTCYRPLRGDGLAPLGRTTYPRPHEYLSPADLPKSWDWRNVDGVNYASITRNQHIPQYCG
SCWAHASTSAMADRINIKRKGAWPSTLLSVQNVIDCGNAGSCEGGNDLSVWDYAHQHGIPDE
TCNNYQAKDQECDKFNQCGTCNEFKECHAIRNYTLWRVGDYGSLSGREKMMAEIYANGPISC
GIMATERLANYTGGIYAEYQDTTYINHVVSVAGWGISDGTEYWIVRNSWGEPWGERGWLRIV
TSTYKDGKGARYNLAIEEHCTFGDPIV
>d1ef7a_ d.3.1.1 (A:) (Pro)cathepsin X {Human (Homo sapiens)}
LPKSWDWRNVDGVNYASITRNQHIPQYCGSCWAHASTSAMADRINIKRKGAWPSTLLSVQNV
IDCGNAGSCEGGNDLSVWDYAHQHGIPDETCNNYQAKDQECDKFNQCGTCNEFKECHAIRNY
TLWRVGDYGSLSGREKMMAEIYANGPISCGIMATERLANYTGGIYAEYQDTTYINHVVSVAG
WGISDGTEYWIVRNSWGEPWGERGWLRIVTSTYKDGKGARYNLAIEEHCTFGDPIV
>d1cv8__ d.3.1.1 (-) Staphopain {Staphylococcus aureus}
NEQYVNKLENFKIRETQGNNGWCAGYTMSALLNATYNTNKYHAEAVMRFLHPNLQGQQFQFT

GLTPREMIYFGQTQGRSPQLLNRMTTYNEVDNLTKNNKGIAILGSRVESRNGMHAGHAMAVV
GNAKLNNGQEVIIIWNPWDNGFMTQDAKNNVIPVSNGDHYQWYSSIYGY
>d1dkia_ d.3.1.1 (A:) Streptococcal pyrogenic exotoxin B {Streptococcus pyogenes}
LDKVNLGGELSGSNMYVYNISTGGFVIVSGDKRSPEILGYSTSGSFDVNGKENIASFMESYV
EQIKENKKLDSTYAGTAEIKQPVVKSLLDSKGIHYNQGNPYNLLTPVIEKVKPGEQSFVGQH
AATGSVATATAQIMKYHNYPNKGLKDYTYTLSSNNPYFNHPKNLFAAISTRQYNWNNILPTY
SGRESNVQKMAISELMADVGISVDMDYGPSSGSAGSSRVQRALKENFGYNQSVHQINRGDFS
KQDWEAQIDKELSQNQPVYYQGVGKVGGHAFVIDGADGRNFYHVNWGWGGVSDGFFRLDALN
PSALGTGGGAGGFNGYQSAVVGIKP
>d1qmya_ d.3.1.2 (A:) FMDV leader protease {Foot-and-mouth disease virus}
MELTLYNGEKKTFYSRPNNHDNAWLNAILQLFRYVEEPFFDWVYSSPENLTLEAIKQLEDLT
GLELHEGGPPALVIWNIKHLLHTGIGTASRPSEVCVVDGTDMSLADFHAGIFLKGQEHAVFA
CVTSNGWYAIDDEDFYPWTPDPSDVLVFVPYD
>d1qola_ d.3.1.2 (A:) FMDV leader protease {Foot-and-mouth disease virus}
MELTLYNGEKKTFYSRPNNHDNAWLNAILQLFRYVEEPFFDWVYSSPENLTLEAIKQLEDLT
GLELHEGGPPALVIWNIKHLLHTGIGTASRPSEVCVVDGTDMCLADFHAGIFLKGQEHAVFA
CVTSNGWYAIDDEDFYPWTPDPSDVLVFVPYDQEPLNGEWKAKVQRKLK
>d1kful3 d.3.1.3 (L:2-355) Calpain large subunit, catalytic domain (domain II) {Human (Homo sapiens)}
AGIAAKLAKDREAAEGLGSHERAIKYLNQDYEALRNECLEAGTLFQDPSFPAIPSALGFKEL
GPYSSKTRGMRWKRPTEICADPQFIIGGATRTDICQGALGDCWLLAAIASLTLNEEILARVV
PLNQSFQENYAGIFHFQFWQYGEWVEVVVDDRLPTKDGELLFVHSAEGSEFWSALLEKAYAK
INGCYEALSGGATTEGFEDFTGGIAEWYELKKPPPNLFKIIQKALQKGSLLGCSIDITSAAD
SEAITFQKLVKGHAYSVTGAEEVESNGSLQKLIRIRNPWGEVEWTGRWNDNCPSWNTIDPEE
RERLTRRHEDGEFWMSFSDFLRHYSRLEICNLTPDTLTSDTYKK
>d1df0a3 d.3.1.3 (A:2-355) Calpain large subunit, catalytic domain (domain II) {Rat (Rattus norvegicus)}
AGIAMKLAKDREAAEGLGSHERAIKYLNQDYETLRNECLEAGALFQDPSFPALPSSLGFKEL
GPYSSKTRGIEWKRPTEICADPQFIIGGATRTDICQGALGDSWLLAAIASLTLNEEILARVV
PLDQSFQENYAGIFHFQFWQYGEWVEVVVDDRLPTKDGELLFVHSAEGSEFWSALLEKAYAK
INGCYEALSGGATTEGFEDFTGGIAEWYELRKPPPNLFKIIQKALEKGSLLGCSIDITSAAD
SEAVTYQKLVKGHAYSVTGAEEVESSGSLQKLIRIRNPWGQVEWTGKWNDNCPSWNTVDPEV
RANLTERQEDGEFWMSFSDFLRHYSRLEICNLTPDTLTCDSYKK
>d1f13a4 d.3.1.4 (A:191-515) Transglutaminase catalytic domain {Human (Homo sapiens)}
DAVYLDNEKEREEYVLNDIGVIFYGEVNDIKTRSWSYGQFEDGILDTCLYVMDRAQMDLSGR
GNPIKVSRVGSAMVNAKDDEGVLVGSWDNIYAYGVPPSAWTGSVDILLEYRSSENPVRYGQC
WVFAGVFNTFLRCLGIPARIVTNYFSAHDNDANLQMDIFLEEDGNVNSKLTKDSVWNYHCWN
EAWMTRPDLPVGFGGWQAVDSTPQENSDGMYRCGPASVQAIKHGHVCFQFDAPFVFAEVNSD
LIYITAKKDGTHVVENVDATHIGKLIVTKQIGGDGMMDITDTYKFQEGQEEERLALETALMY
GAKKPLNTEGVMKSR

>d1g0da4 d.3.1.4 (A:141-461) Transglutaminase catalytic domain {Red sea bream (Chrysophrys major)}
DMVYLPDESKLQEYVMNEDGVIYMGTWDYIRSIPWNYGQFEDYVMDICFEVLDNSPAALKNS
EMDIEHRSDPVYVGRTITAMVNSNGDRGVLTGRWEEPYTDGVAPYRWTGSVPILQQWSKAGV
RPVKYGQCWVFAAVACTVLRCLGIPTRPITNFASAHDVDGNLSVDFLLNERLESLDSRQRSD
SSWNFHCWVESWMSREDLPEGNDGWQVLDPTPQELSDGEFCCGPCPVAAIKEGNLGVKYDAP
FVFAEVNADTIYWIVQKDGQRRKITEDHASVGKNISTKSVYGNHREDVTLHYKYPEGSQKER
EVYKKAGRRVT
>d1e2ta_ d.3.1.5 (A:) Arylamine N-acetyltransferase {Salmonella typhimurium}
HMTSFLHAYFTRLHCQPLGVPTVEALRTLHLAHNCAIPFENLDVLLPREIQLDETALEEKLL
YARRGGYCFELNGLFERALRDIGFNVRSLLGRVILSHPASLPPRTHRLLLVDVEDEQWIADV
GFGGQTLTAPLRLQAEIAQQTPHGEYRLMQEGSTWILQFRHHEHWQSMYCFDLGVQQQSDHV
MGNFWSAHWPQSHFRHHLLMCRHLPDGGKLTLTNFHFTRYHQGHAVEQVNVPDVPSLYQLLQ
QQFGLGVNDVKHGFTEAELAAVMAAF
>d1uch__ d.3.1.6 (-) Ubiquitin C-terminal hydrolase UCH-l3 {Human (Homo sapiens)}
RWLPLEANPEVTNQFLKQLGLHPNWQFVDVYGMDPELLSMVPRPVCAVLLLFPITEKYEVFR
TEEEEKIKSQGQDVTSSVYFMKQTISNACGTIGLIHAIANNKDKMHFESGSTLKKFLEESVS
MSPEERARYLENYDAIRVTHETSAHEGQTEAPSIDEKVDLHFIALVHVDGHLYELDGRKPFP
INHGETSDETLLEDAIEVCKKFMERDPDELRFNAIALSAA
>d1cmxa_ d.3.1.6 (A:) Ubiquitin C-terminal hydrolase UCH-l3 {Synthetic, based on Saccharomyces cerevisiae sequence}
RAVVPIESNPEVFTNFAHKLGLKNEWAYFDIYSLTEPELLAFLPRPVKAIVLLFPINEDRKS
STSQQITSSYDVIWFKQSVKNACGLYAILHSLSNNQSLLEPGSDLDNFLKSQSDTSSSKNRF
DDVTTDQFVLNVIKENVQTFSTGQSEAPEATADTNLHYITYVEENGGIFELDGRNLSGPLYL
GKSDPTATDLIEQELVRVRVASYMENANEEDVLNFAMLGLGPN
>d1avpa_ d.3.1.7 (A:) Human adenovirus 2 proteinase {Mastadenovirus H2}
MGSSEQELKAIVKDLGCGPYFLGTYDKRFPGFVSPHKLACAIVNTAGRETGGVHWMAFAWNP
RSKTCYLFEPFGFSDQRLKQVYQFEYESLLRRSAIASSPDRCITLEKSTQSVQGPNSAACGL
FCCMFLHAFANWPQTPMDHNPTMNLITGVPNSMLNSPQVQPTLRRNQEQLYSFLERHSPYFR
SHSAQIRSATSFCHLKNM
>d1euva_ d.3.1.7 (A:) Ulp1 protease C-terminal domain {Baker's yeast (Saccharomyces cerevisiae)}
GSLVPELNEKDDDQVQKALASRENTQLMNRDNIEITVRDFKTLAPRRWLNDTIIEFFMKYIE
KSTPNTVAFNSFFYTNLSERGYQGVRRWMKRKKTQIDKLDKIFTPINLNQSHWALGIIDLKK
KTIGYVDSLSNGPNAMSFAILTDLQKYVMEESKHTIGEDFDLIHLDCPQQPNGYDCGIYVCM
NTLYGSADAPLDFDYKDAIRMRRFIAHLILTDALK
>d7ceib_ d.4.1.1 (B:) DNase domain of colicin E7 {Escherichia coli}
RNKPGKATGKGKPVNNKWLNNAGKDLGSPVPDRIANKLRDKEFKSFDDFRKKFWEEVSKDPE
LSKQFSRNNNDRMKVGKAPKTRTQDVSGKRTSFELHHEKPISQNGGVYDMDNISVVTPKRHI
DIH
>d1emvb_ d.4.1.1 (B:) DNase domain of colicin E9 {Escherichia coli}

MESKRNKPGKATGKGKPVGDKWLDDAGKDSGAPIPDRIADKLRDKEFKSFDDFRKAVWEEVS
KDPELSKNLNPSNKSSVSKGYSPFTPKNQQVGGRKVYELHHDKPISQGGEVYDMDNIRVTTP
KRHIDIH
>d1ql0a_ d.4.1.2 (A:) Sm endonuclease {Serratia marcescens}
SIDNCAVGCPTGGSSKVSIVRHAYTLNNNSTTKFANWVAYHITKDTPASGKTRNWKTDPALN
PADTLAPADYTGANAALKVDRGHQAPLASLAGVSDWESLNYLSNITPQKSDLNQGAWARLED
QERKLIDRADISSVYTVTGPLYERDMGKLPGTQKAHTIPSAYWKVIFINNSPAVNHYAAFLF
DQNTPKGADFCQFRVTVDEIEKRTGLIIWAGLPDDVQASLKSKPGVLPELMGCKN
>d1a73a_ d.4.1.3 (A:) Intron-encoded homing endonuclease I-PpoI
{Slime mold (Physarum polycephalum)}
ALTNAQILAVIDSWEETVGQFPVITHHVPLGGGLQGTLHCYEIPLAAPYGVGFAKNGPTRWQ
YKRTINQVVHRWGSHTVPFLLEPDNINGKTCTASHLCHNTRCHNPLHLCWESLDDNKGRNWC
PGPNGGCVHAVVCLRQGPLYGPGATVAGPQQRGSHFVV
>d1e7la2 d.4.1.5 (A:1-103) Recombination endonuclease VII,
C-terminal and dimerization domains {Bacteriophage T4}
MLLTGKLYKEEKQKFYDAQNGKCLICQRELNPDVQANHLDHDHELNGPKAGKVRGLLCNLCD
AAEGQMKHKFNRSGLKGQGVDYLEWLENLLTYLKSDYTQNN
>d1dy5a_ d.5.1.1 (A:) Ribonuclease A (also ribonuclease B, S) {Cow
(Bos taurus)}
KETAAAKFERQHMDSSTSAASSSNYCNQMMKSRNLTKDRCKPVNTFVHESLADVQAVCSQKN
VACKNGQTNCYQSYSTMSITDCRETGSSKYPNCAYKTTQANKHIIVACEGNPYVPVHFDASV
>d1h8xa_ d.5.1.1 (A:) Ribonuclease A (also ribonuclease B, S) {Cow
(Bos taurus)}
KESAAAKFERQHMDSGNSPSSSSTYCNQMMRRRNMTQGRCKPVNTFVHEPLVDVQNVCFQEK
VTCKNGQGNCYKSNSSMHITDCRLTNGSRYPNCAYRTSQKERHIIVACEGSPYVPVHFDASV
E
>d1rbd__ d.5.1.1 (-) Ribonuclease A (also ribonuclease B, S) {Cow
(Bos taurus)}
SSSNYCNQMMKSRNLTKDRCKPVNTFVHESLADVQAVCSQKNVACKNGQTNCYQSYSTMSIT
DCRETGSSKYPNCAYKTTQANKHIIVACEGNPYVPVHFDASV
>g1d5d.1 d.5.1.1 (A:,B:) Ribonuclease A (also ribonuclease B, S)
{Cow (Bos taurus)}
KETAAAKMERQHLDSXNYCNQMMKSRNLTKDRCKPVNTFVHESLADVQAVCSQKNVACKNGQ
TNCYQSYSTMSITDCRETGSSKYPNCAYKTTQANKHIIVACEGNPYVPVHFDASV
>d1e21a_ d.5.1.1 (A:) Ribonuclease A (also ribonuclease B, S) {Human
(Homo sapiens), des1-7}
AFQRQHMDSDSSPSSSSTYCNQMMRRRNMTQGRCKPVNTFVHEPLVDVQNVCFQEKVTCKNG
QGNCYKSNSSMHITDCRLTNGSRYPNCAYRTSPKERHIIVACEGSPYVPVHFDASVE
>d1rraa_ d.5.1.1 (A:) Ribonuclease A (also ribonuclease B, S) {Rat
(Rattus norvegicus)}
AESSADKFKRQHMDTEGPSKSSPTYCNQMMKRQGMTKGSCKPVNTFVHEPLEDVQAICSQGQ
VTCKNGRNNCHKSSSTLRITDCRLKGSSKYPNCDYTTTDSQKHIIIACDGNPYVPVHFDASV
>d1onc__ d.5.1.1 (-) P-30 protein {Frog (Rana pipiens)}
EDWLTFQKKHITNTRDVDCDNIMSTNLFHCKDKNTFIYSRPEPVKAICKGIIASKNVLTTSE

FYLSDCNVTSRPCKYKLKKSTNKFCVTCENQAPVHFVGVGSC
>d1bc4__ d.5.1.1 (-) Cytotoxic ribonuclease {Bullfrog (Rana catesbeiana)}
ENWATFQQKHIINTPIINCNTIMDNNIYIVGGQCKRVNTFIISSATTVKAICTGVINMNVLS
TTRFQLNTCTRTSITPRPCPYSSRTETNYICVKCENQYPVHFAGIGRCP
>d11bga_ d.5.1.1 (A:) Seminal ribonucleasease {Cow (Bos taurus)}
KESAAAKFERQHMDSGNSPSSSSNYCNLMMCCRKMTQGKCKPVNTFVHESLADVKAVCSQKK
VTCKNGQTNCYQSKSTMRITDCRETGSSKYPNCAYKTTQVEKHIIVACGGKPSVPVHFDASV
>d1b6va_ d.5.1.1 (A:) Hybrid between ribonuclease A and seminal ribonuclease {Cow (Bos taurus)}
KETAAAKFERQHMDSSTSAASSSNYCNQMMKSRNLTKDRCKPVNTFVHESLADVKAVCSQKK
VTCKNGQTNCYQSKSTMRITDCRETGSSKYPNCAYKTTQANKHIIVACGGKPYVPVHFDASV
>d1dyta_ d.5.1.1 (A:) Eosinophil cationic protein (ECP), ribonuclease 3 {Human (Homo sapiens)}
RPPQFTRAQWFAIQHISLNPPRCTIAMRAINNYRWRCKNQNTFLRTTFANVVNVCGNQSIRC
PHNRTLNNCHRSRFRVPLLHCDLINPGAQNISNCRYADRPGRRFYVVACDNRDPRDSPRYPV
VPVHLDTTI
>d1hi2a_ d.5.1.1 (A:) Eosinophil-derived neurotoxin (EDN) {Human (Homo sapiens)}
MKPPQFTWAQWFETQHINMTSQQCTNAMQVINNYQRRCKNQNTFLLTTFANVVNVCGNPNMT
CPSNKTRKNCHHSGSQVPLIHCNLTTPSPQNISNCRYAQTPANMFYIVACDNRDQRRDPPQY
PVVPVHLDRII
>d1rnfa_ d.5.1.1 (A:) Ribonuclease 4 {Human (Homo sapiens)}
MQDGMYQRFLRQHVHPEETGGSDRYCNLMMQRRKMTLYHCKRFNTFIHEDIWNIRSICSTTN
IQCKNGKMNCHEGVVKVTDCRDTGSSRAPNCRYRAIASTRRVVIACEGNPQVPVHFDG
>d1b1ia_ d.5.1.1 (A:) Angiogenin {Human (Homo sapiens)}
EDNSRYTHFLTQHYDAKPQGRDDRYCESIMRRRGLTSPCKDINTFIHGNKRSIKAICENKNG
NPHRENLRISKSSFQVTTCKLHGGSPWPPCQYRATAGFRNVVVACENGLPVHLDQSIFRRP
>d1agi__ d.5.1.1 (-) Angiogenin {Cow (Bos taurus)}
AQDDYRYIHFLTQHYDAKPKGRNDEYCFNMMKNRRLTRPCKDRNTFIHGNKNDIKAICEDRN
GQPYRGDLRISKSEFQITICKHKGGSSRPPCRYGATEDSRVIVVGCENGLPVHFDESFITPR
H
>d1ag2__ d.6.1.1 (-) Prion protein domain {Mouse (Mus musculus)}
GLGGYMLGSAMSRPMIHFGNDWEDRYYRENMYRYPNQVYYRPVDQYSNQNNFVHDCVNITIK
QHTVTTTTKGENFTETDVKMMERVVEQMCVTQYQKESQAYY
>d1b10a_ d.6.1.1 (A:) Prion protein domain {Golden hamster (Mesocricetus auratus)}
LGGYMLGSAMSRPMMHFGNDWEDRYYRENMNRYPNQVYYRPVDQYNNQNNFVHDCVNITIKQ
HTVTTTTKGENFTETDIKIMERVVEQMCTTQYQKESQAYYDG
>d1fo7a_ d.6.1.1 (A:) Prion protein domain {Human (Homo sapiens)}
LGGYMLGSAMSRPIIHFGSDYEDRYYRENMHRYPNQVYYRPMDEYSNQNNFVHDCVNITIKQ
HTVTTTTKGENFTKTDVKMMERVVEQMCITQYERESQAYYQRGSS
>d1i4ma_ d.6.1.1 (A:) Prion protein domain {Human (Homo sapiens)}
GAVVGGLGGYMLGSAMSRPIIHFGSDYEDRYYRENMHRYPNQVYYRPMDEYSNQNNFVHDCV

NITIKQHTVTTTTKGENFTETDVKMMERVVEQMCITQYERESQAYY
>d1dwya_ d.6.1.1 (A:) Prion protein domain {Cow (Bos taurus)}
GLGGYMLGSAMSRPLIHFGSDYEDRYYRENMHRYPNQVYYRPVDQYSNQNNFVHDCVNITVK
EHTVTTTTKGENFTETDIKMMERVVEQMCITQYQRESQAYYQ
>d1i17a_ d.6.1.1 (A:) Prion-like protein Doppel {Mouse (Mus musculus)}
RVAENRPGAFIKQGRKLDIDFGAEGNRYYAANYWQFPDGIYYEGCSEANVTKEMLVTSCVNA
TQAANQAEFSREKQDSKLHQRVLWRLIKEICSAKHCDFWLERGAA
>d1e01a_ d.7.1.1 (A:) Membrane-bound lytic murein transclycosylase D, MltD {Escherichia coli}
DSITYRVRKGDSLSSIAKRHGVNIKDVMRWNSDTANLQPGDKLTLFVK
>d1hywa_ d.186.1.1 (A:) Head-to-tail joining protein W, gpW {Bacteriophage lambda}
MTRQEELAAARAALHDLMTGKRVATVQKDGRRVEFTATSVSDLKKYIAELEVQTGMTQ
>d1ejra_ d.8.1.1 (A:) Urease, gamma-subunit {Klebsiella aerogenes}
MELTPREKDKLLLFTAALVAERRLARGLKLNYPESVALISAFIMEGARDGKSVASLMEEGRH
VLTREQVMEGVPEMIPDIQVEATFPDGSKLVTVHNPII
>d4ubpa_ d.8.1.1 (A:) Urease, gamma-subunit {Bacillus pasteurii}
MHLNPAEKEKLQIFLASELLLRRKARGLKLNYPEAVAIITSFIMEGARDGKTVAMLMEEGKH
VLTRDDVMEGVPEMIDDIQAEATFPDGTKLVTVHNPIS
>d1e9ya2 d.8.1.1 (A:1-105) Urease, gamma-subunit {Helicobacter pylori}
MKLTPKELDKLMLHYAGELAKKRKEKGIKLNYVEAVALISAHIMEEARAGKKTAAELMQEGR
TLLKPDDVMDGVASMIHEVGIEAMFPDGTKLVTVHTPIEANGK
>d1qe6b_ d.9.1.1 (B:) Interleukin-8, IL-8 {Human (Homo sapiens)}
AKECRCQCIKTYSKPFHPKFIKELRVIESGPCCANTEIIVKLSDGRELCLDPKENWVQRVVE
KFLKRAENS
>d3il8__ d.9.1.1 (-) Interleukin-8, IL-8 {Human (Homo sapiens)}
LRCQCIKTYSKPFHPKFIKELRVIESGPHCANTEIIVKLSDGRELCLDPKENWVQRVVEKFL
KRAENS
>d1plfa_ d.9.1.1 (A:) Platelet factor 4, PF4 {Cow (Bos taurus)}
LQCVCLKTTSGINPRHISSLEVIGAGLHCPSPQLIATLKTGRKICLDQQNPLYKKIIKRLLK
S
>d1pfma_ d.9.1.1 (A:) Platelet factor 4, PF4 {Human (Homo sapiens)}
MSAKELRCQCVKTTSQVRPRHITSLEVIKAGPHCPTAQLIATLKNGRKICLDLQAPLYKKII
KKLLES
>d1rhpa_ d.9.1.1 (A:) Platelet factor 4, PF4 {Human (Homo sapiens)}
DLQCLCVKTTSQVRPRHITSLEVIKAGPHCPTAQLIATLKNGRKICLDLQAPLYKKIIKKLL
ES
>d1mgsa_ d.9.1.1 (A:) Melanoma growth stimulating activity (MGSA) {Human (Homo sapiens)}
ASVATELRCQCLQTLQGIHPKNIQSVNVKSPGPHCAQTEVIATLKNGRKACLNPASPIVKKI
IEKMLNSDKSN
>d1roda_ d.9.1.1 (A:) IL-8/MGSA chimeric protein CIL-8M {Human

(Homo sapiens)}
SAKELRCQCIKTYSKPFHPKFIKELRVIESGPHCANTEIIVKLSDGRELCLDPASPIVKKII
EKMLNSDKSN
>d1huna_ d.9.1.1 (A:) Macrophage inflammatory protein, MIP {Human
(Homo sapiens), 1-beta}
APMGSDPPTACCFSYTARKLPRNFVVDYYETSSLCSQPAVVFQTKRSKQVCADPSESWVQEY
VYDLELN
>d1b50a_ d.9.1.1 (A:) Macrophage inflammatory protein, MIP {Human
(Homo sapiens), 1-alpha}
SLAADTPTACCFSYTSRQIPQNFIAAYFETSSQCSKPGVIFLTKRSRQVCADPSEEWVQKYV
SDLELSA
>d1ha6a_ d.9.1.1 (A:) Macrophage inflammatory protein, MIP {Mouse
(Mus musculus), ccl20/mip-3a}
ASNYDCCLSYIQTPLPSRAIVGFTRQMADEACDINAIIFHTKKRKSVCADPKQNWVKRAVNL
LSLRVKKM
>d1cm9a_ d.9.1.1 (A:) Macrophage inflammatory protein, MIP
{Kaposi's sarcoma herpes virus, VMIP-II}
SWHRPDKCCLGYQKRPLPQVLLSSWYPTSQLCSKPGVIFLTKRGRQVCADKSKDWVKKLMQQ
LPVTAR
>d1b3aa_ d.9.1.1 (A:) RANTES (regulated upon activation, normal
T-cell expressed and secreted) {Human (Homo sapiens)}
PYSSDTTPCCFAYIARPLPRAHIKEYFYTSGKCSNPAVVFVTRKNRQVCANPEKKWVREYIN
SLEMS
>d1doka_ d.9.1.1 (A:) Monocyte chemoattractant protein-1 (MCP-1,
MCAF) {Human (Homo sapiens)}
MQPDAINAPVTCCYNFTNRKISVQRLASYRRITSSKCPKEAVIFKTIVAKEICADPKQKWVQ
DSMDHLDKQT
>d1esra_ d.9.1.1 (A:) Monocyte chemoattractant protein-2 (MCP-2)
{Human (Homo sapiens)}
EPDSVSIPITCCFNVINRKIPIQRLESYTRITNIQCPKEAVIFKTQRGKEVCADPKERWVRD
SMKHLDQIFQNLKP
>d1el0a_ d.9.1.1 (A:) CC chemokine I-309 {Human (Homo sapiens)}
SKSMQVPFSRCCFSFAEQEIPLRAILCYRNTSSICSNEGLIFKLKRGKEACALDTVGWVQRH
RKMLRHCPSKRK
>d1eot__ d.9.1.1 (-) Eotaxin {Human (Homo sapiens)}
GPASVPTTCCFNLANRKIPLQRLESYRRITSGKCPQKAVIFKTKLAKDICADPKKKWVQDSM
KYLDQKSPTPKP
>d1eiha_ d.9.1.1 (A:) Eotaxin-2 {Human (Homo sapiens)}
VVIPSPCCMFFVSKRIPENRVVSYQLSSRSTCLKAGVIFTTKKGQQSCGDPKQEWVQRYMKN
LDAKQKKASPR
>d1j9oa_ d.9.1.1 (A:) Lymphotactin {Human (Homo sapiens)}
VGSEVSDKRTCVSLTTQRLPVSRIKTYTITEGSLRAVIFITKRGLKVCADPQATWVRDVVRS
MDRKSNTRNNMIQTKPTGTQQSTNTAVTLTG
>d1bo0__ d.9.1.1 (-) Monocyte chemoattractant protein-3 (MCP-3)

{Human (Homo sapiens)}
QPVGINTSTTCCYRFINKKIPKQRLESYRRTTSSHCPREAVIFKTKLDKEICADPTQKWVQD
FMKHLDKKTQTPKL
>d1b2ta_ d.9.1.1 (A:) Chemokine domain of fractalkine {Human (Homo sapiens)}
MQHHGVTKCNITCSKMTSKIPVALLIHYQQNQASCGKRAIILETRQHRLFCADPKEQWVKDA
MQHLDRQAAALTRNG
>d1f2la_ d.9.1.1 (A:) Chemokine domain of fractalkine {Human (Homo sapiens)}
VTKCNITCSKMTSKIPVALLIHYQQNQASCGKRAIILETRQHRLFCADPKEQWVKDAMQHLD
RQ
>d1tvxa_ d.9.1.1 (A:) Neutrophil-activating peptide-2 (NAP-2) {Human (Homo sapiens)}
LRCLCIKTTSGIHPKNIQSLEVIGKGTHCNQVEVIATLKDGRKICLDPDAPRIKKIVQKKLA
GD
>d1tvxb_ d.9.1.1 (B:) Neutrophil-activating peptide-2 (NAP-2) {Human (Homo sapiens)}
DSDLYAELRCLCIKTTSGIHPKNIQSLEVIGKGTHCNQVEVIATLKDGRKICLDPDAPRIKK
IVQKKLAGD
>d1a15a_ d.9.1.1 (A:) Stromal cell-derived factor-1 (SDF-1) {Human (Homo sapiens)}
KPVSLSYRCPCRFFESHVARANVKHLKILNTPACALQIVARLKNNNRQVCIDPKLKWIQEYL
EKALN
>d1a15b_ d.9.1.1 (B:) Stromal cell-derived factor-1 (SDF-1) {Human (Homo sapiens)}
RCPCRFFESHVARANVKHLKILNTPACALQIVARLKNNNRQVCIDPKLKWIQEYLEK
>d1qg7a_ d.9.1.1 (A:) Stromal cell-derived factor-1 (SDF-1) {Human (Homo sapiens)}
SYRCPCRFFESHVARANVKHLKILNTPNCALQIVARLKNNNRQVCIDPKLKWIQEYLEKALN
>d1mi2a_ d.9.1.1 (A:) Macrophage inflammatory protein-2 {Mouse (Mus musculus)}
AVVASELRCQCLKTLPRVDFKNIQSLSVTPPGPHCAQTEVIATLKGGQKVCLDPEAPLVQKI
IQKILNKGKAN
>d2hcc__ d.9.1.1 (-) Chemokine hcc-2 (macrophage inflammatory protein-5) {Human (Homo sapiens)}
HFAADCCTSYISQSIPCSLMKSYFETSSECSKPGVIFLTKKGRQVCAKPSGPGVQDCMKKLK
PYSI
>d1qnka_ d.9.1.1 (A:) Gro beta {Human (Homo sapiens)}
TELRCQCLQTLQGIHLKNIQSVKVKSPGPHCAQTEVIATLKNGQKACLNPASPMVKKIIEKM
LKNGKSN
>d1g91a_ d.9.1.1 (A:) Myeloid progenitor inhibitory factor-1 (MPIF-1) {Human (Homo sapiens)}
MDRFHATSADCCISYTPRSIPCSLLESYFETNSECSKPGVIFLTKKGRRFCANPSDKQVQVC
MRMLKLDTRIKTRKN

>d1bf4a_ d.9.2.1 (A:) DNA-binding protein {Archaeon Sulfolobus solfataricus, Sso7d}
ATVKFKYKGEEKEVDISKIKKVWRVGKMISFTYDEGGGKTGRGAVSEKDAPKELLQMLEKQK
K
>d1azpa_ d.9.2.1 (A:) DNA-binding protein {Archaeon Sulfolobus acidocaldarius, Sac7d}
MVKVKFKYKGEEKEVDTSKIKKVWRVGKMVSFTYDDNGKTGRGAVSEKDAPKELLDMLARAE
REKK
>d1ap0__ d.9.2.2 (-) Modifier protein 1 (M31, HP1 beta) {Mouse (Mus musculus)}
HMVEEVLEEEEEEYVVEKVLDRRVVKGKVEYLLKWKGFSDEDNTWEPEENLDCPDLIAEFLQ
SQKTAHETDKS
>d1dz1a_ d.9.2.2 (A:) Modifier protein 1 (M31, HP1 beta) {Mouse (Mus musculus)}
HMKEESEKPRGFARGLEPERIIGATDSSGELMFLMKWKNSDEADLVPAKEANVKCPQVVISF
YEERLTWH
>d1e0ba_ d.9.2.2 (A:) HP1 homologue SWI6 {Fission yeast (Schizosaccharomyces pombe)}
QVENYDSWEDLVSSIDTIERKDDGTLEIYLTWKNGAISHHPSTITNKKCPQKMLQFYESHL
>d1g6za_ d.9.2.2 (A:) Histone methyltransferase clr4 chromo domain {Fission yeast (Schizosaccharomyces pombe)}
ISSPKQEEYEVERIVDEKLDRNGAVKLYRIRWLNYSSRSDTWEPPENLSGCSAVLAEWKRRK
RRLKGSNS
>d1bb8__ d.10.1.1 (-) DNA-binding domain from tn916 integrase {Enterococcus faecalis}
EKRRDNRGRILKTGESQRKDGRYLYKYIDSFGEPQFVYSWKLVATDRVPAGKRDCISLREKI
AELQKDIHD
>d1gcca_ d.10.1.2 (A:) GCC-box binding domain {Mouse-ear cress (Arabidopsis thaliana)}
KHYRGVRQRPWGKFAAEIRDPAKNGARVWLGTFETAEDAALAYDRAAFRMRGSRALLNFPLR
V
>d1qk9a_ d.10.1.3 (A:) Methyl-CpG-binding protein 2, MECP2 {Human (Homo sapiens)}
ASASPKQRRSIIRDRGPMYDDPTLPEGWTRKLKQRKSGRSAGKYDVYLINPQGKAFRSKVEL
IAYFEKVGDTSLDPNDFDFTVTGRGSGSGC
>d1d9na_ d.10.1.3 (A:) Methylation-dependent transcriptional repressor MBD1/PCM1 {Human (Homo sapiens)}
MAEDWLDCPALGPGWKRREVFRKSGATCGRSDTYYQSPTGDRIRSKVELTRYLGPACDLTLF
DFKQGILCYPAPK
>d1k25a1 d.11.1.1 (A:632-692) Penicillin-binding protein 2x (pbp-2x), c-terminal domain {Streptococcus pneumoniae}
TESSYAMPSIKDISPGELAEALRRNIVQPIVVGTGTKIKETSVEEGTNLAPNQQVLLLSDK
>d1k25a2 d.11.1.1 (A:693-750) Penicillin-binding protein 2x (pbp-2x), c-terminal domain {Streptococcus pneumoniae}

VEEIPDMYGWKKETAETFAKWLDIELEFEGSGSVVQKQDVRTNTAIKNIKKIKLTLGD
>d1qmea1 d.11.1.1 (A:632-692) Penicillin-binding protein 2x (pbp-2x), c-terminal domain {Streptococcus pneumoniae}
QQSPYPMPSVKDISPGDLAEELRRNLVQPIVVGTGTKIKNSSAEEGKNLAPNQQVLILSDK
>d1qmea2 d.11.1.1 (A:693-750) Penicillin-binding protein 2x (pbp-2x), c-terminal domain {Streptococcus pneumoniae}
AEEVPDMYGWTKETAETLAKWLNIELEFQGSGSTVQKQDVRANTAIKDIKKITLTLGD
>d1jj2r_ d.12.1.1 (R:) Ribosomal protein L23 {Archaeon Haloarcula marismortui}
SWDVIKHPHVTEKAMNDMDFQNKLQFAVDDRASKGEVADAVEEQYDVTVEQVNTQNTMDGEK
KAVVRLSEDDDAQEVASRI
>d1ffki_ d.12.1.2 (I:) Ribosomal protein L15e {Archaeon Haloarcula marismortui}
MKSMYAYIREAWKRPYEGYVGELMWHRLQKWRREPAVVRIPRPTRLDRARALGYKAKKGIIV
VRVRIRRGGRRATRPNKGRKSKKMMVNRRPRKKNLQWIAEERANRKYPNMEVLNSYWVGEDG
RYKWFEVILVDRDHPAIKSDPQLSWVSRTRGRVYRGLTSAGRKARGLRRKGRGAEKVRPSLR
ANFRKKRR
>d1jj2l_ d.12.1.2 (L:) Ribosomal protein L15e {Archaeon Haloarcula marismortui}
ARSAYSYIREAWKRPKEGQIAELMWHRMQEWRNEPAVVRIERPTRLDRARSLGYKAKQGIIV
VRVAIRKGSSRRTRFNKGRRSKRMMVNRITRKKNIQRIAEERANRKFPNLRVLNSYSVGEDG
RHKWHEVILIDPDHPAIKSDDQLSWISRTRHRLRTFRGLTSAGRRCRGLRGQGKGSEKVRPS
LRVNGAKA
>d4rhn__ d.13.1.1 (-) Histidine triad nucleotide-binding protein (HINT) {Rabbit (Oryctolagus cuniculus)}
RPGGDTIFGKIIRKEIPAKIIFEDDQCLAFHDISPQAPTHFLVIPKKHISQISAAEDADESL
LGHLMIVGKKCAADLGLKKGYRMVVNEGSDGGQSVYHVHLHVLGGRQMNWPPG
>d1fit__ d.13.1.1 (-) FHIT (fragile histidine triad protein) {Human (Homo sapiens)}
SFRFGQHLIKPSVVFLKTELSFALVNRKPVVPGHVLVCPLRPVERFHDLRPDEVADLFQTTQ
RVGTVVEKHFHGTSLTFSMQDGPEAGQTVKHVHVHVLPRKAGDFHRNDSIYEELQKHDKEDF
PASWRSEEEMAAEAAALRVYFQ
>d1kpf__ d.13.1.1 (-) Protein kinase C inhibitor-1, PKCI-1 {Human (Homo sapiens)}
DTIFGKIIRKEIPAKIIFEDDRCLAFHDISPQAPTHFLVIPKKHISQISVAEDDDESLLGHL
MIVGKKCAADLGLNKGYRMVVNEGSDGGQSVYHVHLHVLGGRQMHWPPG
>d1emsa1 d.13.1.1 (A:281-440) NIT-FHIT fusion protein, C-terminal domain {Nematode (Caenorhabditis elegans)}
RSDLYTLHINEKSSETGGLKFARFNIPADHIFYSTPHSFVFVNLKPVTDGHVLVSPKRVVPR
LTDLTDAETADLFIVAKKVQAMLEKHHNVTSTTICVQDGKDAGQTVPHVHIHILPRRAGDFG
DNEIYQKLASHDKEPERKPRSNEQMAEEAVVYRNLM
>d1guqa1 d.13.1.2 (A:2-177) Galactose-1-phosphate uridylyltransferase {Escherichia coli}
TQFNPVDHPHRRYNPLTGQWILVSPHRAKRPWQGAQETPAKQVLPAHDPDCFLCAGNVRVTG

DKNPDYTGTYVFTNDFAALMSDTPDAPESHDPLMRCQSARGTSRVICFSPDHSKTLPELSVA
ALTEIVKTWQEQTAELGKTYPWVQVFENKGAAMGCSNPHPGGQIWANSFLPN
>d1guqa2 d.13.1.2 (A:178-348) Galactose-1-phosphate uridylyltransferase {Escherichia coli}
EAEREDRLQKEYFAEQKSPMLVDYVQRELADGSRTVVETEHWLAVVPYWAAWPFETLLLPKA
HVLRITDLTDAQRSDLALALKKLTSRYDNLFQCSFPYSMGWHGAPFNGEENQHWQLHAHFYP
PLLRSATVRKFMVGYEMLAETQRDLTAEQAAERLRAVSDIHFRESGV
>d1jyaa_ d.198.1.1 (A:) YopE chaperone SycE {Yersinia pestis}
YSFEQAITQLFQQLSLSIPDTIEPVIGVKVGEFACHITEHPVGQILMFTLPSLDNNDEKETL
LSHNIFSQDILKPILSWDEVGGHPVLWNRQPLNSLDNNSLYTQLEMLVQGAERLQ
>d1k6za_ d.198.1.1 (A:) YopE chaperone SycE {Yersinia pestis}
SFEQAITQLFQQLSLSIPDTIEPVIGVKVGEFACHITEHPVGQILMFTLPSLDNNDEKETLL
SHNIFSQDILKPILSWDEVGGHPVLWNRQPLNSLDNNSLYTQLEMLVQGAERLQTSSLISPP
RSFSHH
>d1jyoa_ d.198.1.1 (A:) Virulence effector SptP secretion chaperone SicP {Salmonella typhimurium}
LQAHQDIIANIGEKLGLPLTFDDNNQCLLLLDSDIFTSIEAKDDIWLLNGMIIPLSPVCGDS
IWRQIMVINGELAANNEGTLAYIDAAETLLLIHAITDLTNTYHIISQLESFVNQQEALKNIL
QEYAKV
>d1k3ea_ d.198.1.1 (A:) Secretion chaperone CesT {Escherichia coli}
MSSRSELLLEKFAEKIGIGSISFNENRLCSFAIDEIYYISLSDANDEYMMIYGVCGKFPTDN
SNFALEILNANLWFAENGGPYLCYEAGAQSLLLALRFPLDDATPEKLENEIEVVVKSMENLY
LVLHNQGITLENEHMKIEEISS
>d1k3sa_ d.198.1.1 (A:) Secretion chaperone SigE {Salmonella enterica}
MESLLNRLYDALGLDAPEDEPLLIIDDGIQVYFNESDHTLEMCCPFMPLPDDILTLQHFLRL
NYTSAVTIGADADNTALVALYRLPQTSTEEEALTGFELFISNVKQLKEHYA
>d1k8kf_ d.198.2.1 (F:) ARPC4 (20 kDa subunit) {Cow (Bos taurus)}
TATLRPYLSAVRATLQAALCLENFSSQVVERHNKPEVEVRSSKELLLQPVTISRNEKEKVLI
EGSINSVRVSIAVKQADEIEKILCHKFMRFMMMRAENFFILRRKPVEGYDISFLITNFHTEQ
MYKHKLVDFVIHFMEEIDKEISEMKLSVNARARIVAEEFLKNF
>d1k8kd1 d.198.2.1 (D:1-120) ARPC2 (34 kDa subunit) {Cow (Bos taurus)}
MILLEVNNRIIEETLALKFENAAAGNKPEAVEVTFADFDGVLYHISNPNGDKTKVMVSISLK
FYKELQAHGADELLKRVYGSYLVNPESGYNVSLLYDLENLPASKDSIVHQAGMLKRNC
>d1k8kd2 d.198.2.1 (D:121-284) ARPC2 (34 kDa subunit) {Cow (Bos taurus)}
FASVFEKYFQFQEEGKEGENRAVIHYRDDETMYVESKKDRVTVVFSTVFKDDDDVVIGKVFM
QEFKEGRRASHTAPQVLFSHREPPLELKDTDAAVGDNIGYITFVLFPRHTNASARDNTINLI
HTFRDYLHYHIKCSKAYIHTRMRAKTSDFLKVLNRARPDA
>d1kafa_ d.199.1.1 (A:) DNA-binding C-terminal domain of the transcription factor MotA {Bacteriophage T4}
MEITSDMEEDKDLMLKLLDKNGFVLKKVEIYRSNYLAILEKRTNGIRNFEINNNGNMRIFGY

KMMEHHIQKFTDIGMSCKIAKNGNVYLDIKRSAENIEAVITVASEL
>d1dar_3 d.14.1.1 (476-599) Elongation factor G (EF-G), domain IV {Thermus thermophilus}
VGKPQVAYRETITKPVDVEGKFIRQTGGRGQYGHVKIKVEPLPRGSGFEFVNAIVGGVIPKE
YIPAVQKGIEEAMQSGPLIGFPVVDIKVTLYDGSYHEVDSSEMAFKIAGSMAIKEAVQKGDP
>d1pkp_1 d.14.1.1 (78-148) Ribosomal protein S5, C-terminal domain {Bacillus stearothermophilus}
GTTIPHEVIGHFGAGEIILKPASEGTGVIAGGPARAVLELAGISDILSKSIGSNTPINMVRA
TFDGLKQLK
>d1fjge1 d.14.1.1 (E:74-154) Ribosomal protein S5, C-terminal domain {Thermus thermophilus}
GTIPHEIEVEFGASKIVLKPAAPGTGVIAGAVPRAILELAGVTDILTKELGSRNPINIAYAT
MEALRQLRTKADVERLRKG
>d1fjgi_ d.14.1.1 (I:) Ribosomal protein S9 {Thermus thermophilus}
EQYYGTGRRKEAVARVFLRPGNGKVTVNGQDFNEYFQGLVRAVAALEPLRAVDALGRFDAYI
TVRGGGKSGQIDAIKLGIARALVQYNPDYRAKLKPLGFLTRDARVVERKKYGKHKARRAPQY
SKR
>d1a6f__ d.14.1.2 (-) RNase P protein {Bacillus subtilis}
AHLKKRNRLKKNEDFQKVFKHGTSVANRQFVLYTLDQPENDELRVGLSVSKKIGNAVMRNRI
KRLIRQAFLEEKERLKEKDYIIIARKPASQLTYEETKKSLQHLFRKSSLYK
>d1d6ta_ d.14.1.2 (A:) RNase P protein {Staphylococcus aureus}
MLLEKAYRIKKNADFQRIYKKGHSVANRQFVVYTCNNKEIDHFRLGISVSKKLGNAVLRNKI
KRAIRENFKVHKSHILAKDIIVIARQPAKDMTTLQIQNSLEHVLKIAKVFNKKIK
>d1b63a1 d.14.1.3 (A:217-331) DNA mismatch repair protein MutL {Escherichia coli}
GTAFLEQALAIEWQHGDLTLRGWVADPNHTTPALAEIQYCYVNGRMMRDRLINHAIRQACED
KLGADQQPAFVLYLEIDPHQVDVNVHPAKHEVRFHQSRLVHDFIYQGVLSVLQ
>d1h7sa1 d.14.1.3 (A:232-365) DNA mismatch repair protein PMS2 {Human (Homo sapiens)}
GQKQLQSLIPFVQLPPSDSVCEEYGLSCSDALHNLFYISGFISQCTHGVGRSSTDRQFFFIN
RRPCDPAKVCRLVNEVYHMYNRHQYPFVVLNISVDSECVDINVTPDKRQILLQEEKLLLAVL
KTSLIGMFDS
>d1ei1a1 d.14.1.3 (A:221-392) DNA gyrase B {Escherichia coli}
GIKAFVEYLNKNKTPIHPNIFYFSTEKDGIGVEVALQWNDGFQENIYCFTNNIPQRDGGTHL
AGFRAAMTRTLNAYMDKEGYSKKAKVSATGDDAREGLIAVVSVKVPDPKFSSQTKDKLVSSE
VKSAVEQQMNELLAEYLLENPTDAKIVVGKIIDAARAREAARRAREMT
>d1e3ha2 d.14.1.4 (A:3-151) Polynucleotide phosphorylase/guanosine pentaphosphate synthase (PNPase/GPSI), domains 1 and 4 {Streptomyces antibioticus}
NETHYAEAVIDNGAFGTRTIRFETGRLARQAAGSAVAYLDDDTMVLSATTASKNPKDQLDFF
PLTVDVEERMYAAGKIPGSFFRREGRPSEDAILTCRLIDRPLRPSFKKGLRNEIQVVATIMA
LNPDHLYDVVAINAASASTQLAGLP
>d1e3ha3 d.14.1.4 (A:346-482) Polynucleotide phosphorylase/guanosine pentaphosphate synthase (PNPase/GPSI),

domains 1 and 4 {Streptomyces antibioticus}
TDIRTLAAEVEAIPRVHGSALFERGETQILGVTTLNMLRMEQQLDTLSPVTRKRYMHNYNFP
PYSVGETGRVGSPKRREIGHGALAERAIVPVLPTREEFPYAIRQVSEALGSNGSTSMGSVCA
STMSLLNAGVPLK
>d1h72c1 d.14.1.5 (C:5-167) Homoserine kinase {Archaeon Methanococcus jannaschii}
MKVRVKAPCTSANLGVGFDVFGLCLKEPYDVIEVEAIDDKEIIIEVDDKNIPTDPDKNVAGI
VAKKMIDDFNIGKGVKITIKKGVKAGSGLGSSAASSAGTAYAINELFKLNLDKLKLVDYASY
GELASSGAKHADNVAPAIFGGFTMVTNYEPLEVLHIPID
>d1fi4a1 d.14.1.5 (A:3-190) Mevalonate 5-diphosphate decarboxylase {Baker's yeast (Saccharomyces cerevisiae)}
VYTASVTAPVNIATLKYWGKRDTKLNLPTNSSISVTLSQDDLRTLTSAATAPEFERDTLWLN
GEPHSIDNERTQNCLRDLRQLRKEMESKDASLPTLSQWKLHIVSENNFPTAAGLASSAAGFA
ALVSAIAKLYQLPQSTSEISRIARKGSGSACRSLFGGYVAWEMGKAEDGHDSMAVQIADSSD
WP
>d1c3ta_ d.15.1.1 (A:) Ubiquitin {Human (Homo sapiens)}
MQLFVKTLTGKTLTVELEPSDTVENLKAKIQDKEGIPPDQQRLIFAGKQLEDGRTLSDYNLQ
KESTIHLVLRLRGG
>d1gjza_ d.15.1.1 (A:) Ubiquitin {Human (Homo sapiens)}
GSMQIFVKTLTGKTITLEVEPSDTIENVKAKIQDKEGIPPDQQRLIFAGKQLE
>d1ubi__ d.15.1.1 (-) Ubiquitin {Human (Homo sapiens)}
MQIFVKTLTGKTITLEVEPSDTIENVKAKIQDKEGIPPDQQRLIFAGKQLEDGRTLSDYNIQ
KESTLHLVLRLRGG
>d1ud7a_ d.15.1.1 (A:) Ubiquitin {Human (Homo sapiens)}
MQVFLKTLTGKTVTIEVEPSDTVENFKAKIQDKEGIPPDQQRLIFAGKQLEDGRTLSDYNIQ
KESTIHLVLRLRGG
>d1a5r__ d.15.1.1 (-) SUMO-1 (smt3 homologue) {Human (Homo sapiens)}
GSMSDQEAKPSTEDLGDKKEGEYIKLKVIGQDSSEIHFKVKMTTHLKKLKESYCQRQGVPMN
SLRFLFEGQRIADNHTPKELGMEEEDVIEVYQEQTGGHSTV
>d1euvb_ d.15.1.1 (B:) SUMO-1 (smt3 homologue) {Baker's yeast (Saccharomyces cerevisiae)}
PETHINLKVSDGSSEIFFKIKKTTPLRRLMEAFAKRQGKEMDSLRFLYDGIRIQADQTPEDL
DMEDNDIIEAHREQIGG
>d1ndda_ d.15.1.1 (A:) Nedd8 {Human (Homo sapiens)}
MLIKVKTLTGKEIEIDIEPTDKVERIKERVEEKEGIPPQQQRLIYSGKQMNDEKTAADYKIL
GGSVLHLVLALR
>d1vcba_ d.15.1.1 (A:) Elongin B {Human (Homo sapiens)}
MDVFLMIRRHKTTIFTDAKESSTVFELKRIVEGILKRPPDEQRLYKDDQLLDDGKTLGECGF
TSQTARPQAPATVGLAFRADDTFEALCIEPFSSPPE
>d1bt0a_ d.15.1.1 (A:) Rub1 {Mouse-ear cress (Arabidopsis thaliana)}
MLIKVKTLTGKEIEIDIEPTDTIDRIKERVEEKEGIPPVQQRLIYAGKQLADDKTAKDYNIE
GGSVLHLVLAL

>d1h8ca_ d.15.1.2 (A:) Fas-assosiated factor 1, Faf1 {Human (Homo sapiens)}
NAEPVSKLRIRTPSGEFLERRFLASNKLQIVFDFVASKGFPWDEYKLLSTFPRRDVTQLDPN
KSLLEVKLFPQETLFLEAKE
>d1i42a_ d.15.1.2 (A:) p47 {Rat (Rattus norvegicus)}
KASSSILINEAEPTTNIQIRLADGGRLVQKFNHSHRISDIRLFIVDARPAMAATSFVLMTTF
PNKELADENQTLKEANLLNAVIVQRLT
>d1eo6a_ d.15.1.3 (A:) Golgi-associated ATPase enhancer of 16 kD, Gate-16 {Cow (Bos taurus)}
MKWMFKEDHSLEHRCVESAKIRAKYPDRVPVIVEKVSGSQIVDIDKRKYLVPSDITVAQFMW
IIRKRIQLPSEKAIFLFVDKTVPQSSLTMGQLYEKEKDEDGFLYVAYSGENTFG
>d1gnua_ d.15.1.3 (A:) GABA(A) receptor associated protein GABARAP {Human (Homo sapiens)}
MKFVYKEEHPFEKRRSEGEKIRKKYPDRVPVIVEKAPKARIGDLDKKKYLVPSDLTVGQFYF
LIRKRIHLRAEDALFFFVNNVIPPTSATMGQLYQEHHEEDFFLYIAYSDESVYGL
>d1ef1a3 d.15.1.4 (A:4-87) Moesin {Human (Homo sapiens)}
TISVRVTTMDAELEFAIQPNTTGKQLFDQVVKTIGLREVWFFGLQYQDTKGFSTWLKLNKKV
TAQDVRKESPLLFKFRAKFYPE
>d1gc7a3 d.15.1.4 (A:1-87) Radixin {Mouse (Mus musculus)}
MPKPINVRVTTMDAELEFAIQPNTTGKQLFDQVVKTVGLREVWFFGLQYVDSKGYSTWLKLN
KKVTQQDVKKENPLQFKFRAKFFPE
>d1gg3a3 d.15.1.4 (A:1-81) Erythroid membrane protein 4.1R {Human (Homo sapiens)}
MHCKVSLLDDTVYECVVEKHAKGQDLLKRVCEHLNLLEEDYFGLAIWDNATSKTWLDSAKEI
KKQVRGVPWNFTFNVKFYP
>d1h4ra3 d.15.1.4 (A:20-103) Merlin {Human (Homo sapiens)}
KTFTVRIVTMDAEMEFNCEMKWKGKDLFDLVCRTLGLRETWFFGLQYTIKDTVAWLKMDKKV
LDHDVSKEEPVTFHFLAKFYPE
>d1c1yb_ d.15.1.5 (B:) c-Raf1 RBD {Human (Homo sapiens)}
SNTIRVFLPNKQRTVVNVRNGMSLHDCLMKALKVRGLQPECCAVFRLLHEHKGKKARLDWNT
DAASLIGEELQVDFL
>d1lfda_ d.15.1.5 (A:) Ral guanosine-nucleotide exchange factor, RalGDS {Rat (Rattus norvegicus)}
GDCCIIRVSLDVDNGNMYKSILVTSQDKAPTVIRKAMDKHNLDEDEPEDYELLQIISEDHKL
KIPENANVFYAMNSAANYDFILKKR
>d1raxa_ d.15.1.5 (A:) Ral guanosine-nucleotide exchange factor, RalGDS {Human (Homo sapiens)}
QQVGDCCIIRVSLDVDNGNMYKSILVTSQDKAPAVIRKAMDKHNLEEEEPEDYELLQILSDD
RKLKIPENANVFYAMNSTANYDFVLKKRTFT
>d1rlf__ d.15.1.5 (-) RalGDS-like factor, Rlf {Mouse (Mus musculus)}
GSSDCRIIRVQMELGEDGSVYKSILVTSQDKAPSVISRVLKKNNRDSAVASEFELVQLLPGD
RELTIPHSANVFYAMDGASHDFLLRQRR
>d1ef5a_ d.15.1.5 (A:) Rgl {Mouse (Mus musculus)}

EDTCIIRISVEDNNGNMYKSIMLTSQDKTPAVIQRAMSKHNLESDPAEEYELVQVISEDKEL
VIPDSANVFYAMNSQVNFDFILRKKN
>d1e8xa3 d.15.1.5 (A:142-321) Phoshoinositide 3-kinase (PI3K) {Pig (Sus scrofa)}
AASEETLAFQRQLNALIGYDVTDVSNVHDDELEFTRRRLVTPRMAEVAGRDPKLYAMHPWVT
SKPLPEYLLKKITNNCVFIVIHRSTTSQTIKVSADDTPGTILQSFFTKMAKKKSLMDIPESQ
NERDFVLRVCGRDEYLVGETPIKNFQWVRQCLKNGEEIHLVLDTPPDPALDEVRKE
>d1e8ya3 d.15.1.5 (A:143-322) Phoshoinositide 3-kinase (PI3K) {Human (Homo sapiens)}
MSEESQAFQRQLTALIGYDVTDVSNVHDDELEFTRRGLVTPRMAEVASRDPKLYAMHPWVTS
KPLPEYLWKKIANNCIFIVIHRSTTSQTIKVSPDDTPGAILQSFFTKMAKKKSLMDIPESQS
EQDFVLRVCGRDEYLVGETPIKNFQWVRHCLKNGEEIHVVLDTPPDPALDEVRKEE
>d1i35a_ d.15.1.5 (A:) Protein kinase byr2 {Yest (Schizosaccharomyces pombe)}
CILRFIACNGQTRAVQSRGDYQKTLAIALKKFSLEDASKFIVCVSQSSRIKLITEEEFKQIC
FNSSSPERDRLIIVPKEKPCPSFEDLRRSWEIE
>d1d4ba_ d.15.2.1 (A:) Cell death-inducing effector B (CIDE-B), N-terminal domain {Human (Homo sapiens)}
MEYLSALNPSDLLRSVSNISSEFGRRVWTSAPPPQRPFRVCDHKRTIRKGLTAATRQELLAK
ALETLLLNGVLTLVLEEDGTAVDSEDFFQLLEDDTCLMVLQSGQSWSPTRSGVLHHHHHH
>d1c9fa_ d.15.2.1 (A:) Caspase-activated DNase (CAD), DFF40, N-terminal domain {Mouse (Mus musculus)}
MCAVLRQPKCVKLRALHSACKFGVAARSCQELLRKGCVRFQLPMPGSRLCLYEDGTEVTDDC
FPGLPNDAELLLLTAGETWHGYVSD
>d1ibxa_ d.15.2.1 (A:) Caspase-activated DNase (CAD), DFF40, N-terminal domain {Human (Homo sapiens)}
MLQKPKSVKLRALRSPRKFGVAGRSCQEVLRKGCLRFQLPERGSRLCLYEDGTELTEDYFPS
VPDNAELVLLTLGQAWQGH
>d1f2ri_ d.15.2.1 (I:) Inhibitor of caspase-activated DNase (ICAD), DFF45, N-terminal domain {Mouse (Mus musculus)}
MELSRGASAPDPDDVRPLKPCLLRRNHSRDQHGVAASSLEELRSKACELLAIDKSLTPITLV
LAEDGTIVDDDDYFLCLPSNTKFVALACNEKWTYNDSD
>d1ibxb_ d.15.2.1 (B:) Inhibitor of caspase-activated DNase (ICAD), DFF45, N-terminal domain {Human (Homo sapiens)}
SGEIRTLKPCLLRRNYSREQHGVAASCLEDLRSKACDILAIDKSLTPVTLVLAEDGTIVDDD
DYFLCLPSNTKFVALASNEKWAYNNSD
>d1ip9a_ d.15.2.2 (A:) Bud emergence mediator Bemp1 {Baker's yeast (Saccharomyces cerevisiae)}
GAMGSSTSGLKTTKIKFYYKDDIFALMLKGDTTYKELRSKIAPRIDTDNFKLQTKLFDGSGE
EIKTDSQVSNIIQAKLKISVHDI
>d1fm0d_ d.15.3.1 (D:) Molybdopterin synthase subunit MoaD {Escherichia coli}
MIKVLFFAQVRELVGTDATEVAADFPTVEALRQHMAAQSDRWALALEDGKLLAAVNQTLVSF
DHPLTDGDEVAFFPPVTGG

>d1f0za_ d.15.3.2 (A:) Thiamin biosynthesis sulfur carrier protein ThiS {Escherichia coli}
MQILFNDQAMQCAAGQTVHELLEQLDQRQAGAALAINQQIVPREQWAQHIVQDGDQILLFQV
IAGG
>d1jsba_ d.15.3.2 (A:) Hypothetical protein MTH1743 {Archaeon Methanobacterium thermoautotrophicum}
MVIGMKFTVITDDGKKILESGAPRRIKDVLGELEIPIETVVVKKNGQIVIDEEEIFDGDIIE
VIRVIYGG
>d1czpa_ d.15.4.1 (A:) 2Fe-2S ferredoxin {Cyanobacterium (Anabaena sp.), pcc 7119 and 7120}
ATFKVTLINEAEGTKHEIEVPDDEYILDAAEEQGYDLPFSCRAGACSTCAGKLVSGTVDQSD
QSFLDDDQIEAGYVLTCVAYPTSDVVIQTHKEEDLY
>d1frd__ d.15.4.1 (-) 2Fe-2S ferredoxin {Cyanobacterium (Anabaena sp.), pcc 7119 and 7120}
ASYQVRLINKKQDIDTTIEIDEETTILDGAEENGIELPFSCHSGSCSSCVGKVVEGEVDQSD
QIFLDDEQMGKGFALLCVTYPRSNCTIKTHQEPYLA
>d4fxc__ d.15.4.1 (-) 2Fe-2S ferredoxin {Spirulina platensis}
ATYKVTLINEAEGINETIDCDDDTYILDAAEEAGLDLPYSCRAGACSTCAGTITSGTIDQSD
QSFLDDDQIEAGYVLTCVAYPTSDCTIKTHQEEGLY
>d1fxia_ d.15.4.1 (A:) 2Fe-2S ferredoxin {Cyanobacterium (Aphanothece sacrum)}
ASYKVTLKTPDGDNVITVPDDEYILDVAEEEGLDLPYSCRAGACSTCAGKLVSGPAPDEDQS
FLDDDQIQAGYILTCVAYPTGDCVIETHKEEALY
>d1dox__ d.15.4.1 (-) 2Fe-2S ferredoxin {Synechocystis sp., pcc 6803}
ASYTVKLITPDGESSIECSDDTYILDAAEEAGLDLPYSCRAGACSTCAGKITAGSVDQSDQS
FLDDDQIEAGYVLTCVAYPTSDCTIETHKEEDLY
>d2cjn__ d.15.4.1 (-) 2Fe-2S ferredoxin {Synechococcus elongatus}
ATYKVTLVRPDGSETTIDVPEDEYILDVAEEQGLDLPFSCRAGACSTCAGKLLEGEVDQSDQ
SFLDDDQIEKGFVLTCVAYPRSDCKILTNQEEELY
>d1awd__ d.15.4.1 (-) 2Fe-2S ferredoxin {Chlorella fusca}
YKVTLKTPSGEETIECPEDTYILDAAEEAGLDLPYSCRAGACSSCAGKVESGEVDQSDQSFL
DDAQMGKGFVLTCVAYPTSDVTILTHQEAALY
>d1frra_ d.15.4.1 (A:) 2Fe-2S ferredoxin {Equisetum arvense}
AYKTVLKTPSGEFTLDVPEGTTILDAAEEAGYDLPFSCRAGACSSCLGKVVSGSVDESEGSF
LDDGQMEEGFVLTCIAIPESDLVIETHKEEELF
>d1doi__ d.15.4.1 (-) 2Fe-2S ferredoxin {Archaeon Haloarcula marismortui}
PTVEYLNYEVVDDNGWDMYDDDVFGEASDMDLDDEDYGSLEVNEGEYILEAAEAQGYDWPFS
CRAGACANCAAIVLEGDIDMDMQQILSDEEVEDKNVRLTCIGSPDADEVKIVYNAKHLDYLQ
NRVI
>d1e0za_ d.15.4.1 (A:) 2Fe-2S ferredoxin {Archaeon Halobacterium halobium}
PTVEYLNYETLDDQGWDMDDDDLFEKAADAGLDGEDYGTMEVAEGEYILEAAEAQGYDWPFS

CRAGACANCASIVKEGEIDMDMQQILSDEEVEEKDVRLTCIGSPAADEVKIVYNAKHLDYLQ
NRVI
>d1pfd__ d.15.4.1 (-) 2Fe-2S ferredoxin {Parsley (Petroselinum crispum)}
ATYNVKLITPDGEVEFKCDDDVYVLDQAEEEGIDIPYSCRAGSCSSCAGKVVSGSIDQSDQS
FLDDEQMDAGYVLTCHAYPTSDVVIETHKEEEIV
>d1a70__ d.15.4.1 (-) 2Fe-2S ferredoxin {Spinach (Spinacia oleracea)}
AAYKVTLVTPTGNVEFQCPDDVYILDAAEEEGIDLPYSCRAGSCSSCAGKLKTGSLNQDDQS
FLDDDQIDEGWVLTCAAYPVSDVTIETHKKEELTA
>d1gaqb_ d.15.4.1 (B:) 2Fe-2S ferredoxin {Maize (Zea mays)}
ATYNVKLITPEGEVELQVPDDVYILDQAEEDGIDLPYSCRAGSCSSCAGKVVSGSVDQSDQS
YLDDGQIADGWVLTCHAYPTSDVVIETHKEEELTGA
>d1e9ma_ d.15.4.1 (A:) Ferredoxin VI {Rhodobacter capsulatus}
AKIIFIEHNGTRHEVEAKPGLTVMEAARDNGVPGIDADCGGACACSTCHAYVDPAWVDKLPK
ALPTETDMIDFAYEPNPATSRLTCQIKVTSLLDGLVVHLPEKQI
>d1put__ d.15.4.1 (-) Putidaredoxin {Pseudomonas putida}
SKVVYVSHDGTRRQLDVADGVSLMQAAVSNGIYDIVGDCGGSASCATCHVYVNEAFTDKVPA
ANEREIGMLECVTAELKPNSRLCCQIIMTPELDGIVVDVPDRQW
>d1b9ra_ d.15.4.1 (A:) Terpredoxin {Pseudomonas sp.}
PRVVFIDEQSGEYAVDAQDGQSLMEVATQNGVPGIVAECGGSCVCATCRIEIEDAWVEIVGE
ANPDENDLLQSTGEPMTAGTRLSCQVFIDPSMDGLIVRVPLPA
>d1ayfa_ d.15.4.1 (A:) Adrenodoxin {Cow (Bos taurus)}
KITVHFINRDGETLTTKGKIGDSLLDVVVQNNLDIDGFGACEGTLACSTCHLIFEQHIFEKL
EAITDEENDMLDLAYGLTDRSRLGCQICLTKAMDNMTVRVP
>d1feha2 d.15.4.2 (A:1-126) Fe-only hydrogenase, N-terminal domain {Clostridium pasteurianum}
MKTIIINGVQFNTDEDTTILKFARDNNIDISALCFLNNCNNDINKCEICTVEVEGTGLVTAC
DTLIEDGMIINTNSDAVNEKIKSRISQLLDIHEFKCGPCNRRENCEFLKLVIKYKARASKPF
LP
>d1hlra2 d.15.4.2 (A:1-80) Aldehyde oxidoreductase, N-terminal domain {Desulfovibrio gigas}
MIQKVITVNGIEQNLFVDAEALLSDVLRQQLGLTGVKVGCEQGQCGACSVILDGKVVRACVT
KMKRVADGAQITTIEGVG
>d1dgja2 d.15.4.2 (A:1-80) Aldehyde oxidoreductase, N-terminal domain {Desulfovibrio desulfuricans}
METKTLIVNGMARRLLVSPNDLLVDVLRSQLQLTSVKVGCGKGQCGACTVILDGKVVRACII
KMSRVAENASVTTLEGIG
>d1fo4a2 d.15.4.2 (A:3-92) Xanthine oxidase, N-terminal domain {Cow (Bos taurus)}
ADELVFFVNGKKVVEKNADPETTLLAYLRRKLGLRGTKLGCGEGGCGACTVMLSKYDRLQDK
IIHFSANACLAPICTLHHVAVTTVEGIG
>d1jroa2 d.15.4.2 (A:1-84) Xanthine dehydrogenase chain A, N-terminal domain {Rhodobacter capsulatus}

MEIAFLLNGETRRVRIEDPTQSLLELLRAEGLTGTKEGCNEGDCGACTVMIRDAAGSRAVNA
CLMMLPQIAGKALRTIEGIAAP
>d1qj2a2 d.15.4.2 (A:3-81) Carbone monoxide (CO) dehydrogenase iron-sulfur protein, N-domain {Pseudomonas carboxydovorans}
KAHIELTINGHPVEALVEPRTLLIHFIREQQNLTGAHIGCDTSHCGACTVDLDGMSVKSCTM
FAVQANGASITTIEGMA
>d1ffva2 d.15.4.2 (A:3-81) Carbone monoxide (CO) dehydrogenase iron-sulfur protein, N-domain {Hydrogenophaga pseudoflava}
KKIITVNVNGKAQEKAVEPRTLLIHFLREELNLTGAHIGCETSHCGACTVDIDGRSVKSCTH
LAVQCDGSEVLTVEGLA
>d2pia_3 d.15.4.2 (224-321) Phthalate dioxygenase reductase, C-terminal domain {Pseudomonas cepacia, db01}
SFGATNTNARENTPFTVRLSRSGTSFEIPANRSILEVLRDANVRVPSSCESGTCGSCKTALC
SGEADHRDMVLRDDEKGTQIMVCVSRAKSAELVLDL
>d1fumb2 d.15.4.2 (B:1-105) Fumarate reductase iron-sulfur protein, N-terminal domain {Escherichia coli}
AEMKNLKIEVVRYNPEVDTAPHSAFYEVPYDATTSLLDALGYIKDNLAPDLSYRWSCRMAIC
GSCGMMVNNVPKLACKTFLRDYTDGMKVEALANFPIERDLVVD
>d1qlab2 d.15.4.2 (B:1-106) Fumarate reductase iron-sulfur protein, N-terminal domain {Wolinella succinogenes}
MGRMLTIRVFKYDPQSAVSKPHFQEYKIEEAPSMTIFIVLNMIRETYDPDLNFDFVCRAGIC
GSCGMMINGRPSLACRTLTKDFEDGVITLLPLPAFKLIKDLSVD
>d1jq4a_ d.15.4.2 (A:) Methane monooxygenase reductase N-terminal domain {Methylococcus capsulatus}
MQRVHTITAVTEDGESLRFECRSDEDVITAALRQNIFLMSSCREGGCATCKALCSEGDYDLK
GCSVQALPPEEEEEGLVLLCRTYPKTDLEIELPYTH
>d1ssn__ d.15.5.1 (-) Staphylokinase {Staphylococcus aureus}
SSSFDKGKYKKGDDASYFEPTGPYLMVNVTGVDSKGNELLSPHYVEFPIKPGTTLTKEKIEY
YVEWALDATAYKEFRVVELDPSAKIEVTYYDKNKKKEETKSFPITEKGFVVPDLSEHIKNPG
FNLITKVVIEKK
>d2sak__ d.15.5.1 (-) Staphylokinase {Staphylococcus aureus}
SYFEPTGPYLMVNVTGVDSKGNELLSPHYVEFPIKPGTTLTKEKIEYYVEWALDATAYKEFR
VVELDPSAKIEVTYYDKNKKKEETKSFPITEKGFVVPDLSEHIKNPGFNLITKVVIEKK
>d1bmlc1 d.15.5.1 (C:12-148) Streptokinase {Streptococcus equisimilis}
SVNNSQLVVSVAGTVEGTNQDISLKFFEIDLTSRPAHGGKTEQGLSPKSKPFATDSGAMPHK
LEKADLLKAIQEQLIANVHSNDDYFEVIDFASDATITDRNGKVYFADKDGSVTLPTQPVQEF
LLSGHVRVRPYKE
>d1bmlc3 d.15.5.1 (C:285-372) Streptokinase {Streptococcus equisimilis}
DPFDRSHLKLFTIKYVDVNTNELLKSEQLLTASERNLDFRDLYDPRDKAKLLYNNLDAFGIM
DYTLTGKVEDNHDDTNRIITVYMGKR
>d1c4pc_ d.15.5.1 (C:) Streptokinase {Streptococcus equisimilis}
KPIQNQAKSVDVEYTVQFTPLNPDDDFRPGLKDTKLLKTLAIGDTITSQELLAQAQSILNKT

HPGYTIYERDSSIVTHDNDIFRTILPMDQEFTYHVKNREQAYEINKKSGLNEEINNTDLISE
KYYVLKKG
>d1qqra_ d.15.5.1 (A:) Streptokinase {Streptococcus equisimilis}
IQNQAKSVDVEYTVQFTPLNPDDDFRPGLKLTKLLKTLAIGDTITSQELLAQAQSILNKNHP
GYTIYERDSSIVTHDNDIFRTILPMDQEFTYRVKNREQAYRINKKSGLNEEINNTDLISEKY
YVLKKGEKPYDPFD
>d1esfa2 d.15.6.1 (A:121-233) Staphylococcal enterotoxin A, SEA
{Staphylococcus aureus}
EEKKVPINLWLDGKQNTVPLETVKTNKKNVTVQELDLQARRYLQEKYNLYNSDVFDGKVQRG
LIVFHTSTEPSVNYDLFGAQGQYSNTLLRIYRDNKTINSENMHIDIYLYTS
>d1i4pa2 d.15.6.1 (A:121-239) Staphylococcal enterotoxin C2, SEC2
{Staphylococcus aureus}
NHFDNGNLQNVLIRVYENKRNTISFEVQTDKKSVTAQELDIKARNFLINKKNLYEFNSSPYE
TGYIKFIENNGNTFWYDMMPAPGDKFDQSKYLMMYNDNKTVDSKSVKIEVHLTTKNG
>d3tss_2 d.15.6.1 (94-194) Toxic shock syndrome toxin-1 (TSST-1)
{Staphylococcus aureus}
LPTPIELPLKVKVHGKDSPLKYWPKFDKKQLAISTLDFKIRHQLTQTHGLYRSSDKTGGYWK
ITMNDGSTYQSDLSKKFEYNTEKPPINIDEIKTIEAEIN
>d1sebd2 d.15.6.1 (D:127-235) Staphylococcal enterotoxin B, SEB
{Staphylococcus aureus}
DKYRSITVRVFEDGKNLLSFDVQTNKKKVTAQELDYLTRHYLVKNKKLYETGYIKFIENENS
FWYDMMPAPGDKFDQSKYLMMYNDNKMVDSKDVKIEVYLT
>d3seb_2 d.15.6.1 (122-238) Staphylococcal enterotoxin B, SEB
{Staphylococcus aureus}
NGNQLDKYRSITVRVFEDGKNLLSFDVQTNKKKVTAQELDYLTRHYLVKNKKLYEFNNSPYE
TGYIKFIENENSFWYDMMPAPGDKFDQSKYLMMYNDNKMVDSKDVKIEVYLTTKK
>d1enfa2 d.15.6.1 (A:102-213) Staphylococcal enterotoxin H, SEH
{Staphylococcus aureus}
EKLAQERVIGANVWVDGIQKETELIRTNKKNVTLQELDIKIRKILSDKYKIYYKDSEISKGL
IEFDMKTPRDYSFDIYDLKGENDYEIDKIYEDNKTLKSDDISHIDVNLYT
>d1an8_2 d.15.6.1 (96-208) Streptococcal superantigen Spe-C
{Streptococcus pyogenes}
NKVNHKLLGNLFISGESQQNLNNKIILEKDIVTFQEIDFKIRKYLMDNYKIYDATSPYVSGR
IEIGTKDGKHEQIDLFDSPNEGTRSDIFAKYKDNRIINMKNFSHFDIYLEK
>d1et9a2 d.15.6.1 (A:96-204) Streptococcal superantigen Spe-H
{Streptococcus pyogenes}
EKKEIKVPVNVWDKSKQQPPMFITVNKPKVTAQEVDIKVRKLLIKKYDIYNNREQKYSKGTV
TLDLNSGKDIVFDLYYFGNGDFNSMLKIYSNNERIDSTQFHVDVSIS
>d1eu3a2 d.15.6.1 (A:97-209) Streptococcal superantigen Smez-2
{Streptococcus pyogenes}
TSIPKNIPVNLWINGKQISVPYNEISTNKTTVTAQEIDLKVRKFLIAQHQLYSSGSSYKSGR
LVFHTNDNSDKYSFDLFYVGYRDKESIFKVYKDNKSFNIDKIGHLDIEIDS
>d1bxta2 d.15.6.1 (A:120-234) Streptococcal superantigen SSA
{Streptococcus pyogenes}

QIEGKFPNITVKVYEDNENILSFDITTNKKQVTVQELDCKTRKILVSRKNLYEFNNSPYETG
YIKFIESSGDSFWYDMMPAPGAIFDQSKYLMLYNDNKTVSSSAIAIEVHLTKK
>d1fnua2 d.15.6.1 (A:108-221) Streptococcal pyrogenic exotoxin A1 {Streptococcus pyogenes}
GNHLEIPKKIVVKVSIDGIQSLSFDIETNKKMVTAQELDYKVRKYTIDNKQLYTNGPSKYET
GYIKFIPKNKESFWFDFFPEPEFTQSKYLMIYKDNETLDNKTSQIEVYLTTK
>d1fcla_ d.15.7.1 (A:) Immunoglobulin-binding protein G, different constituent domains {Streptococcus sp., group G}
TTFKLIINGKTLKGETTTEAVDAATAEKVLKQYINDNGIDGEWTYDDATKTWTVTE
>d1fd6a_ d.15.7.1 (A:) Immunoglobulin-binding protein G, different constituent domains {Streptococcus sp., group G}
MTTFKLIINGKTLKGETTTEAVDAATAEKVFKQYANDNGIDGEWTYDDATKTFTVTE
>d1gb4__ d.15.7.1 (-) Immunoglobulin-binding protein G, different constituent domains {Streptococcus sp., group G}
MTTFKLIINGKTLKGEITIEAVDAAEAEKIFKQYANDNGIDGEWTYDDATKTFTVTE
>d1pgb__ d.15.7.1 (-) Immunoglobulin-binding protein G, different constituent domains {Streptococcus sp., group G}
MTYKLILNGKTLKGETTTEAVDAATAEKVFKQYANDNGVDGEWTYDDATKTFTVTE
>d1pgx__ d.15.7.1 (-) Immunoglobulin-binding protein G, different constituent domains {Streptococcus sp., group G}
ELTPAVTTYKLVINGKTLKGETTTKAVDAETAEKAFKQYANDNGVDGVWTYDDATKTFTVTE
MVTEVPVA
>d1qkza_ d.15.7.1 (A:) Immunoglobulin-binding protein G, different constituent domains {Streptococcus sp., group G}
VTTYKLVINGKTLKGETTTKAVDAATAEKVFKQYANDNGVDGEWTYDDATKTFTVTEK
>d2igd__ d.15.7.1 (-) Immunoglobulin-binding protein G, different constituent domains {Streptococcus sp., group G}
MTPAVTTYKLVINGKTLKGETTTKAVDAETAEKAFKQYANDNGVDGVWTYDDATKTFTVTE
>d2igg__ d.15.7.1 (-) Immunoglobulin-binding protein G, different constituent domains {Streptococcus sp., group G}
LTPAVTTYKLVINGKTLKGETTTEAVDAATAEKVFKQYANDNGVDGEWTYDDATKTFTVTEK
PE
>d1heze_ d.15.7.1 (E:) Immunoglobulin light chain-binding domain of protein L {Peptostreptococcus magnus}
EVTIKVNLIFADGKIQTAEFKGTFEEATAEAYRYADLLAKVNGEYTADLEDGGNHMNIKFA
>d1hz6a_ d.15.7.1 (A:) Immunoglobulin light chain-binding domain of protein L {Peptostreptococcus magnus}
HHAMEEVTIKANLIFANGSTQTAEFKGTFEKATSEAYAYADTLKKDNGEWTVDVADKGYTLN
IKFAG
>d1jmla_ d.15.7.1 (A:) Immunoglobulin light chain-binding domain of protein L {Peptostreptococcus magnus}
MHHHHHHGMEEVTIKANLIFANGSTQTAEFKGTFEKATSEAYAYADTLKKDNGEWTVDVVPK
AYTLNIKFAG
>d1k52a_ d.15.7.1 (A:) Immunoglobulin light chain-binding domain

of protein L {Peptostreptococcus magnus}
MHHHHHHAMEEVTIKANLIFANGSTQTAEFKGTFEKATSEAYAYADTLKKDNGEWTVDVADG
GYTLNIKFAG
>d1kh0a_ d.15.7.1 (A:) Immunoglobulin light chain-binding domain of protein L {Peptostreptococcus magnus}
EEVTIKANLIFANGSTQTAEFKGTKEKALSEVLAYADTLKKDNGEWTIDKRVTNGVIILNIK
FAG
>d2ptl__ d.15.7.1 (-) Immunoglobulin light chain-binding domain of protein L {Peptostreptococcus magnus}
ENKEETPETPETDSEEEVTIKANLIFANGSTQTAEFKGTFEKATSEAYAYADTLKKDNGEYT
VDVADKGYTLNIKFAG
>d1tif__ d.15.8.1 (-) Translation initiation factor IF3, N-terminal domain {Bacillus stearothermophilus}
KDFIINEQIRAREVRLIDQNGDQLGIKSKQEALEIAARRNLDLVLVAPNAKPPVCRIMDYGK
FRFEQQKKEKEARK
>d1f52a1 d.15.9.1 (A:1-100) Glutamine synthetase, N-terminal domain {Salmonella typhimurium}
SAEHVLTMLNEHEVKFVDLRFTDTKGKEQHVTIPAHQVNAEFFEEGKMFDGSSIGGWKGINE
SDMVLMPDASTAVIDPFFADSTLIIRCDILEPGTLQGY
>d1coy_2 d.16.1.1 (319-450) Cholesterol oxidase {Brevibacterium sterolicum}
GNNGNIMVGRANHMWDATGSKQATIPTMGIDNWADPTAPIFAEIAPLPAGLETYVSLYLAIT
KNPERARFQFNSGTGKVDLTWAQSQNQKGIDMAKKVFDKINQKEGTIYRTDLFGVYYKTWGD
DFTYHPLG
>d1ijha2 d.16.1.1 (A:319-450) Cholesterol oxidase {Streptomyces sp.}
GPNGNIMTARANHMWNPTGAHQSSIPALGIDAWDNSDSSVFAEIAPMPAGLETWVSLYLAIT
KNPQRGTFVYDAATDRAKLNWTRDQNAPAVNAAKALFDRINKANGTIYRYDLFGTQLKAFAD
DFCYHPLG
>d1k0ia2 d.16.1.2 (A:174-275) p-Hydroxybenzoate hydroxylase (PHBH) {Pseudomonas aeruginosa}
LKVFERVYPFGWLGLLADTPPVSHELIYANHPRGFALCSQRSATRSQYYVQVPLSEKVEDWS
DERFWTELKARLPSEVAEKLVTGPSLEKSIAPLRSFVVEP
>d1foha4 d.16.1.2 (A:241-341) Phenol hydroxylase {Soil-living yeast (Trichosporon cutaneum)}
GEQTDYIWGVLDAVPASNFPDIRSRCAIHSAESGSIMIIPRENNLVRFYVQLQARAEKGGRV
DRTKFTPEVVIANAKKIFHPYTFDVQQLDWFTAYHIGQR
>d1an9a2 d.16.1.3 (A:195-287) D-amino acid oxidase {Pig (Sus scrofa)}
LQPGRGQIIKVDAPWLKNFIITHDLERGIYNSPYIIPGLQAVTLGGTFQVGNWNEINNIQDH
NTIWEGCCRLEPTLKDAKIVGEYTGFRPVRP
>d1c0pa2 d.16.1.3 (A:1194-1288) D-amino acid oxidase {Yeast (Rhodotorula gracilis)}
AEPIRGQTVLVKSPCKRCTMDSSDPASPAYIIPRPGGEVICGGTYGVGDWDLSVNPETVQRI

LKHCLRLDPTISSDGTIEGIEVLRHNVGLRPAR
>d1el8a2 d.16.1.3 (A:218-321) Sarcosine oxidase {Bacillus sp., strain b0618}
LQPYRQVVGFFESDESKYSNDIDFPGFMVEVPNGIYYGFPSFGGCGLKLGYHTFGQKIDPDT
INREFGVYPEDESNLRAFLEEYMPGANGELKRGAVCMYTKTL
>d1i8ta2 d.16.1.7 (A:245-313) UDP-galactopyranose mutases {Escherichia coli}
EYRSLKFETERHEFPNFQGNAVINFTDANVPYTRIIEHKHFDYVETKHTVVTKEYPLEWKVG
DEPYYPV
>d1cf3a2 d.16.1.4 (A:325-520) Glucose oxidase {Aspergillus niger}
NLQDQTTATVRSRITSAGAGQGQAAWFATFNETFGDYSEKAHELLNTKLEQWAEEAVARGGF
HNTTALLIQYENYRDWIVNHNVAYSELFLDTAGVASFDVWDLLPFTRGYVHILDKDPYLHHF
AYDPQYFLNELDLLGQAAATQLARNISNSGAMQTYFAGETIPGDNLAYDADLSAWTEYIPYH
FRPNYHGVGT
>d1gpea2 d.16.1.4 (A:329-524) Glucose oxidase {Penicillium amagasakiense}
NMQDQTTTTVSSRASSAGAGQGQAVFFANFTETFGDYAPQARDLLNTKLDQWAEETVARGGF
HNVTALKVQYENYRNWLLDEDVAFAELFMDTEGKINFDLWDLIPFTRGSVHILSSDPYLWQF
ANDPKFFLNEFDLLGQAAASKLARDLTSQGAMKEYFAGETLPGYNLVQNATLSQWSDYVLQN
FRPNWHAVSS
>d1b5qa2 d.16.1.5 (A:294-405) Polyamine oxidase {Maize (Zea mays)}
DMAVYTKIFLKFPRKFWPEGKGREFFLYASSRRGYYGVWQEFEKQYPDANVLLVTVTDEESR
RIEQQSDEQTKAEIMQVLRKMFPGKDVPDATDILVPRWWSDRFYKGTFSN
>d1f8ra2 d.16.1.5 (A:320-432) L-amino acid oxidase {Malayan pit viper (Calloselasma rhodostoma)}
HYRSGTKIFLTCTTKFWEDDGIHGGKSTTDLPSRFIYYPNHNFTNGVGVIIAYGIGDDANFF
QALDFKDCADIVFNDLSLIHQLPKKDIQSFCYPSVIQKWSLDKYAMGGITT
>d1gosa2 d.16.1.5 (A:290-401) Monoamine oxidase B {Human (Homo sapiens)}
PLGSVIKCIVYYKEPFWRKKDYCGTMIIDGEEAPVAYTLDDTKPEGNYAAIMGFILAHKARK
LARLTKEERLKKLCELYAKVLGSLEALEPVHYEEKNWCEEQYSGGCYTTY
>d1d5ta2 d.16.1.6 (A:292-388) Guanine nucleotide dissosiation inhibitor, GDI {Cow (Bos taurus)}
RKAGQVIRIICILSHPIKNTNDANSCQIIIPQNQVNRKSDIYVCMISYAHNVAAQGKYIAIA
STTVETTDPEKEVEPALGLLEPIDQKFVAISDLYE
>d1mola_ d.17.1.1 (A:) Monellin, B & A chains together {Serendipity berry (Dioscoreophyllum cumminsii)}
GEWEIIDIGPFTQNLGKFAVDEENKIGQYGRLTFNKVIRPCMKKTIYENEREIKGYEYQLYV
YASDKLFRADISEDYKTRGRKLLRFNGPVPPP
>d1eqka_ d.17.1.2 (A:) Phytocystatin {Japanese rice (Oryza sativa), subsp. japonica, oryzacystatin-I}
MSSDGGPVLGGVEPVGNENDLHLVDLARFAVTEHNKKANSLLEFEKLVSVKQQVVAGTLYYF
TIEVKEGDAKKLYEAKVWEKPWMDFKELQEFKPVDASANA
>d1cewi_ d.17.1.2 (I:) Cystatin {Chicken (Gallus gallus)}

GAPVPVDENDEGLQRALQFAMAEYNRASNDKYSSRVVRVISAKRQLVSGIKYILQVEIGRTT
CPKSSGDLQSCEFHDEPEMAKYTTCTFVVYSIPWLNQIKLLESKCQ
>d1dvd__ d.17.1.2 (-) Cystatin A (stefin A) {Human (Homo sapiens)}
MIPGGLSEAKPATPEIQEIVDKVKPQLEEKTNETYGKLEAVQYKTQVVAGTNYYIKVRAGDN
KYMHLKVFKSLPGQNEDLVLTGYQVDKNKDDELTGF
>d1stfi_ d.17.1.2 (I:) Cystatin B (stefin B) {Human (Homo sapiens)}
MMSGAPSATQPATAETQHIADQVRSQLEEKYNKKFPVFKAVSFKSQVVAGTNYFIKVHVGDE
DFVHLRVFQSLPHENKPLTLSNYQTNKAKHDELTYF
>d1g96a_ d.17.1.2 (A:) Cystatin C {Human (Homo sapiens)}
VGGPMDASVEEEGVRRALDFAVGEYNKASNDMYHSRALQVVRARKQIVAGVNYFLDVELGRT
TCTKTQPNLDNCPFHDQPHLKRKAFCSFQIYAVPWQGTMTLSKSTCQDA
>d1oaca2 d.17.2.1 (A:91-185) Copper amine oxidase, domains 1 and
2 {Escherichia coli}
KRPHPLNALTADEIKQAVEIVKASADFKPNTRFTEISLLPPDKEAVWAFALENKPVDQPRKA
DVIMLDGKHIIEAVVDLQNNKLLSWQPIKDAHG
>d1oaca3 d.17.2.1 (A:186-300) Copper amine oxidase, domains 1 and
2 {Escherichia coli}
MVLLDDFASVQNIINNSEEFAAAVKKRGITDAKKVITTPLTVGYFDGKDGLKQDARLLKVIS
YLDVGDGNYWAHPIENLVAVVDLEQKKIVKIEEGPVVPVPMTARPFDGRDRVA
>d1ksia2 d.17.2.1 (A:6-98) Copper amine oxidase, domains 1 and 2
{Pea seedling (Pisum sativum)}
VQHPLDPLTKEEFLAVQTIVQNKYPISNNRLAFHYIGLDDPEKDHVLRYETHPTLVSIPRKI
FVVAIINSQTHEILINLRIRSIVSDNIHNGY
>d1ksia3 d.17.2.1 (A:99-206) Copper amine oxidase, domains 1 and
2 {Pea seedling (Pisum sativum)}
GFPILSVDEQSLAIKLPLKYPPFIDSVKKRGLNLSEIVCSSFTMGWFGEEKNVRTVRLDCFM
KESTVNIYVRPITGITIVADLDLMKIVEYHDRDIEAVPTAENTEYQ
>d1av4_2 d.17.2.1 (9-96) Copper amine oxidase, domains 1 and 2
{Arthrobacter globiformis}
ASPFRLASAGEISEVQGILRTAGLLGPEKRIAYLGVLDPARGAGSEAEDRRFRVFIHDVSGA
RPQEVTVSVTNGTVISAVELDTAATG
>d1av4_3 d.17.2.1 (97-211) Copper amine oxidase, domains 1 and 2
{Arthrobacter globiformis}
ELPVLEEEFEVVEQLLATDERWLKALAARNLDVSKVRVAPLSAGVFEYAEERGRRILRGLAF
VQDFPEDSAWAHPVDGLVAYVDVVSKEVTRVIDTGVFPVPAEHGNYTDPELTG
>d1a2va2 d.17.2.1 (A:18-115) Copper amine oxidase, domains 1 and
2 {Yeast (Hansenula polymorpha)}
PARPAHPLDPLSTAEIKAATNTVKSYFAGKKISFNTVTLREPARKAYIQWKEQGGPLPPRLA
YYVILEAGKPGVKEGLVDLASLSVIETRALETVQPI
>d1a2va3 d.17.2.1 (A:116-236) Copper amine oxidase, domains 1 and
2 {Yeast (Hansenula polymorpha)}
LTVEDLCSTEEVIRNDPAVIEQCVLSGIPANEMHKVYCDPWTIGYDERWGTGKRLQQALVYY
RSDEDDSQYSHPLDFCPIVDTEEKKVIFIDIPNRRRKVSKHKHANFYPKHMIEKVGAMR
>d1eeja2 d.17.3.1 (A:1-60) Disulfide bond isomerase, DsbC,

N-terminal domain {Escherichia coli}
DDAAIQQTLAKMGIKSSDIQPAPVAGMKTVLTNSGVLYITDDGKHIIQGPMYDVSGTAPV
>d3stda_ d.17.4.1 (A:) Scytalone dehydratase {Fungus (Magnaporthe grisea)}
GEITFSDYLGLMTCVYEWADSYDSKDWDRLRKVIAPTLRIDYRSFLDKLWEAMPAEEFVGMV
SSKQVLGDPTLRTQHFIGGTRWEKVSEDEVIGYHQLRVPHQRYKDTTMKEVTMKGHAHSANL
HWYKKIDGVWKFAGLKPDIRWGEFDFDRIFEDGRETFG
>d1ouna_ d.17.4.2 (A:) Nuclear transport factor-2 (NTF2) {Rat (Rattus norvegicus)}
GDKPIWEQIGSSFIQHYYQLFDNDRTQLGAIYIDASCLTWEGQQFQGKAAIVEKLSSLPFQK
IQHSITAQDHQPTPDSCIISMVVGQLKADEDPIMGFHQMFLLKNINDAWVCTNDMFRLALHN
F
>d1jkga_ d.17.4.2 (A:) NTF2-related export protein 1 (p15) {Human (Homo sapiens)}
ASVDFKTYVDQACRAAEEFVNVYYTTMDKRRRLLSRLYMGTATLVWNGNAVSGQESLSEFFE
MLPSSEFQISVVDCQPVHDEATPSQTTVLVVICGSVKFEGNKQRDFNQNFILTAQASPSNTV
WKIASDCFRFQDWAS
>d1jkgb_ d.17.4.2 (B:) NTF2-like domain of Tip associating protein, TAP {Human (Homo sapiens)}
APPCKGSYFGTENLKSLVLHFLQQYYAIYDSGDRQGLLDAYHDGACCSLSIPFIPQNPARSS
LAEYFKDSRNVKKLKDPTLRFRLLKHTRLNVVAFLNELPKTQHDVNSFVVDISAQTSTLLCF
SVNGVFKEVDGKSRDSLRAFTRTFIAVPASNSGLCIVNDELFVRNASSEEIQRAFAMPAPTP
>d1qjga_ d.17.4.3 (A:) Delta-5-3-ketosteroid isomerase, steroid delta-isomerase, KSI {Comamonas testosteroni and Pseudomonas testosteroni}
MNTPEHMTAVVQRYVAALNAGDLDGIVALFADDATVENPVGSEPRSGTAAIREFYANSLKLP
LAVELTQEVRAVANEAAFAFIVSFEYQGRKTVVAPIDHFRFNGAGKVVSMRALFGEKNIHAG
A
>d1ea2a_ d.17.4.3 (A:) Delta-5-3-ketosteroid isomerase, steroid delta-isomerase, KSI {Pseudomonas putida}
NLPTAQEVQGLMARFIELVDVGDIEAIVQMYADDATVEDPFGQPPIHGREQIAAFYRQGLGG
GKVRACLTGPVRASHNGCGAMPFRVEMVWNGQPCALDVIDVMRFDEHGRIQTMQAYWSEVNL
SV
>d1eg9b_ d.17.4.4 (B:) Naphthalene 1,2-dioxygenase beta subunit {Pseudomonas putida}
MINIQEDKLVSAHDAEEILRFFNCHDSALQQEATTLLTQEAHLLDIQAYRAWLEHCVGSEVQ
YQVISRELRAASERRYKLNEAMNVYNENFQQLKVRVEHQLDPQNWGNSPKLRFTRFITNVQA
AMDVNDKELLHIRSNVILHRARRGNQVDVFYAAREDKWKRGEGGVRKLVQRFVDYPERILQT
HNLMVFL
>d1euic_ d.17.5.1 (C:) Uracil-DNA glycosylase inhibitor protein {Bacteriophage pbs2}
QLVIQESILMLPEEVEEVIGNKPESDILVHTAYDESTDENVMLLTSDAPEYKPWALVIQDSN
GENKIKML
>d1ugia_ d.17.5.1 (A:) Uracil-DNA glycosylase inhibitor protein

{Bacteriophage pbs2}
TNLSDIIEKETGKQLVIQESILMLPEEVEEVIGNKPESDILVHTAYDESTDENVMLLTSDAP
EYKPWALVIQDSNGENKIKML
>d1pcfa_ d.18.1.1 (A:) Transcriptional coactivator PC4 C-terminal domain {Human (Homo sapiens)}
AMFQIGKMRYVSVRDFKGKVLIDIREYWMDPEGEMKPGRKGISLNPEQWSQLKEQISDIDDA
VRKL
>d1jb0d_ d.187.1.1 (D:) Photosystem I subunit PsaD {Synechococcus elongatus}
TTLTGQPPLYGGSTGGLLSAADTEEKYAITWTSPKEQVFEMPTAGAAVMREGENLVYFARKE
QCLALAAQQLRPRKINDYKIYRIFPDGETVLIHPKDGVFPEKVNKGREAVNSVPRSIGQNPN
PSQLKFTGKKPYDP
>d3frua2 d.19.1.1 (A:1-178) Fc (IgG) receptor, alpha-1 and alpha-2 domains {Rat (Rattus norvegicus)}
AEPRLPLMYHLAAVSDLSTGLPSFWATGWLGAQQYLTYNNLRQEADPCGAWIWENQVSWYWE
KETTDLKSKEQLFLEAIRTLENQINGTFTLQGLLGCELAPDNSSLPTAVFALNGEEFMRFNP
RTGNWSGEWPETDIVGNLWMKQPEAARKESEFLLTSCPERLLGHLERGRQNLEW
>d1cd1a2 d.19.1.1 (A:7-185) CD1, alpha-1 and alpha-2 domains {Mouse (Mus musculus)}
NYTFRCLQMSSFANRSWSRTDSVVWLGDLQTHRWSNDSATISFTKPWSQGKLSNQQWEKLQH
MFQVYRVSFTRDIQELVKMMSPKEDYPIEIQLSAGCEMYPGNASESFLHVAFQGKYVVRFWG
TSWQTVPGAPSWLDLPIKVLNADQGTSATVQMLLNDTCPLFVRGLLEAGKSDLEK
>d1hdma2 d.19.1.1 (A:13-93) MHC class II, N-terminal domains of alpha and beta chains {Human (Homo sapiens), HLA-DM}
LQNHTFLHTVYCQDGSPSVGLSEAYDEDQLFFFDFSQNTRVPRLPEFADWAQEQGDAILFDK
EFCEWMIQQIPKLDGKIPV
>d1hdmb2 d.19.1.1 (B:3-87) MHC class II, N-terminal domains of alpha and beta chains {Human (Homo sapiens), HLA-DM}
FVAHVESTCLLDDAGTPKDFTYCISFNKDLLTCWDPEENKMAPCNSLANVLSQHLNQKDTLM
QRLNGLQNCATHTQPFWGSLTNR
>d1aqdb2 d.19.1.1 (B:4-92) MHC class II, N-terminal domains of alpha and beta chains {Human (Homo sapiens), HLA-DR1}
RPRFLWQLKFECHFFNGTERVRLLERCIYNQEESVRFDSDVGEYRAVTELGRPDAEYWNSQK
DLLEQRRAAVDTYCRHNYGVGESFTVQ
>d1bx2b2 d.19.1.1 (B:3-92) MHC class II, N-terminal domains of alpha and beta chains {Human (Homo sapiens), HLA-DR2}
TRPRFLWQPKRECHFFNGTERVRFLDRYFYNQEESVRFDSDVGEFRAVTELGRPDAEYWNSQ
KDILEQARAAVDTYCRHNYGVVESFTVQ
>d1fv1a2 d.19.1.1 (A:3-81) MHC class II, N-terminal domains of alpha and beta chains {Human (Homo sapiens), HLA-DR2}
EEHVIIQAEFYLNPDQSGEFMFDFDGDEIFHVDMAKKETVWRLEEFGRFASFEAQGALANIA
VDKANLEIMTKRSNYTP
>d1fv1b2 d.19.1.1 (B:1-92) MHC class II, N-terminal domains of alpha and beta chains {Human (Homo sapiens), HLA-DR2}

GDTRPRFLQQDKYECHFFNGTERVRFLHRDIYNQEEDLRFDSDVGEYRAVTELGRPDAEYWN
SQKDFLEDRRAAVDTYCRHNYGVGESFTVQ
>d1a6ab2 d.19.1.1 (B:5-92) MHC class II, N-terminal domains of alpha and beta chains {Human (Homo sapiens), HLA-DR3}
PRFLEYSTSECHFFNGTERVRYLDRYFHNQEENVRFDSDVGEFRAVTELGRPDAEYWNSQKD
LLEQKRGRVDNYCRHNYGVVESFTVQ
>d1d5zb2 d.19.1.1 (B:1-92) MHC class II, N-terminal domains of alpha and beta chains {Human (Homo sapiens), HLA-DR4}
GDTRPRFLEQVKHECHFFNGTERVRFLDRYFYHQEEYVRFDSDVGEYRAVTELGRPDAEYWN
SQKDLLEQKRAAVDTYCRHNYGVGESFTVQ
>d1jk8a2 d.19.1.1 (A:2-84) MHC class II, N-terminal domains of alpha and beta chains {Human (Homo sapiens), HLA-DQ8}
VADHVASYGVNLYQSYGPSGQYSHEFDGDEEFYVDLERKETVWQLPLFRRFRRFDPQFALTN
IAVLKHNLNIVIKRSNSTAATN
>d1jk8b2 d.19.1.1 (B:3-94) MHC class II, N-terminal domains of alpha and beta chains {Human (Homo sapiens), HLA-DQ8}
SPEDFVYQFKGMCYFTNGTERVRLVTRYIYNREEYARFDSDVGVYRAVTPLGPPAAEYWNSQ
KEVLERTRAELDTVCRHNYQLELRTTLQRR
>d1iaka2 d.19.1.1 (A:1-81) MHC class II, N-terminal domains of alpha and beta chains {Mouse (Mus musculus), I-AK}
IEADHVGSYGITVYQSPGDIGQYTFEFDGDELFYVDLDKKETVWMLPEFAQLRRFEPQGGLQ
NIATGKHNLEILTKRSNSTP
>d1iakb2 d.19.1.1 (B:5-92) MHC class II, N-terminal domains of alpha and beta chains {Mouse (Mus musculus), I-AK}
GSFVHQFQPFCYFTNGTQRIRLVIRYIYNREEYVRFDSDVGEYRAVTELGRPDAEYWNKQYL
ERTRAELDTVCRHNYEKTETPTSLR
>d1fnga2 d.19.1.1 (A:1-81) MHC class II, N-terminal domains of alpha and beta chains {Mouse (Mus musculus), I-EK}
IKEEHTIIQAEFYLLPDKRGEFMFDFDGDEIFHVDIEKSETIWRLEEFAKFASFEAQGALAN
IAVDKANLDVMKERSNNTP
>d1fngb2 d.19.1.1 (B:4-92) MHC class II, N-terminal domains of alpha and beta chains {Mouse (Mus musculus), I-EK}
RPWFLEYCKSECHFYNGTQRVRLLVRYFYNLEENLRFDSDVGEFRAVTELGRPDAENWNSQP
EFLEQKRAEVDTVCRHNYEIFDNFLVP
>d1i3rb2 d.19.1.1 (B:1-120) MHC class II, N-terminal domains of alpha and beta chains {Mouse (Mus musculus), I-EK}
GKKVITAFNEGLKGGGGSLVGGGSGGGGSRPWFLEYCKSECHFYNGTQRVRLLVRYFYNLEE
NLRFDSDVGEFRAVTELGRPDAENWNSQPEFLEQKRAEVDTVCRHNYEIFDNFLVPRR
>d2iadb2 d.19.1.1 (B:5-93) MHC class II, N-terminal domains of alpha and beta chains {Mouse (Mus musculus), I-AD}
RHFVVQFKGECYYTNGTQRIRLVTRYIYNREEYVRYDSDVGEYRAVTELGRPDAEYWNSQPE
ILERTRAEVDTACRHNYEGPETSTSLR
>d1es0a2 d.19.1.1 (A:1B-82) MHC class II, N-terminal domains of alpha and beta chains {Mouse (Mus musculus), I-A(G7)}

DIEADHVGFYGTTVYQSPGDIGQYTHEFDGDELFYVDLDKKKTVWRLPEFGQLILFEPQGGL
QNIAAEKHNLGILTKRSNFTPA
>d1es0b2 d.19.1.1 (B:5-93) MHC class II, N-terminal domains of alpha and beta chains {Mouse (Mus musculus), I-A(G7)}
RHFVHQFKGECYFTNGTQRIRLVTRYIYNREEYLRFDSDVGEYRAVTELGRHSAEYYNKQYL
ERTRAELDTACRHNYEETEVPTSLR
>d1k8ia2 d.19.1.1 (A:11-92) MHC class II, N-terminal domains of alpha and beta chains {Mouse (Mus musculus), H2-DM}
QNHTFRHTLFCQDGIPNIGLSETYDEDELFSFDFSQNTRVPRLPDFAEWAQGQGDASAIAFD
KSFCEMLMREVSPKLEGQIP
>d1k8ib2 d.19.1.1 (B:1-94) MHC class II, N-terminal domains of alpha and beta chains {Mouse (Mus musculus), H2-DM}
GFVAHVESTCVLNDAGTPQDFTYCVSFNKDLLACWDPDVGKIVPCEFGVLSRLAEIISNILN
EQESLIHRLQNGLQDCATHTQPFWDVLTHRTR
>d1i4fa2 d.19.1.1 (A:1-181) MHC class I, alpha-1 and alpha-2 domains {Human (Homo sapiens), HLA-A2.1}
GSHSMRYFFTSVSRPGRGEPRFIAVGYVDDTQFVRFDSDAASQRMEPRAPWIEQEGPEYWDG
ETRKVKAHSQTHRVDLGTLRGYYNQSEAGSHTVQRMYGCDVGSDWRFLRGYHQYAYDGKDYI
ALKEDLRSWTAADMAAQTTKHKWEAAHVAEQLRAYLEGTCVEWLRRYLENGKETLQR
>d1hsaa2 d.19.1.1 (A:1-181) MHC class I, alpha-1 and alpha-2 domains {Human (Homo sapiens), HLA-B2705}
GSHSMRYFHTSVSRPGRGEPRFITVGYVDDTLFVRFDSDAASPREEPRAPWIEQEGPEYWDR
ETQICKAKAQTDREDLRTLLRYYNQSEAGSHTLQNMYGCDVGPDGRLLRGYHQDAYDGKDYI
ALNEDLSSWTAADTAAQITQRKWEAARVAEQLRAYLEGECVEWLRRYLENGKETLQR
>d1hsba2 d.19.1.1 (A:1-181) MHC class I, alpha-1 and alpha-2 domains {Human (Homo sapiens), HLA-AW68}
GSHSMRYFYTSVSRPGRGEPRFIAVGYVDDTQFVRFDSDAASQRMEPRAPWIEQEGPEYWDR
NTRNVKAQSQTDRVDLGTLRGYYNQSEAGSHTIQMMYGCDVGSDGRFLRGYRQDAYDGKDYI
ALKEDLRSWTAADMAAQTTKHKWEAAHVAEQWRAYLEGTCVEWLRRYLENGKETLQR
>d1agda2 d.19.1.1 (A:1-181) MHC class I, alpha-1 and alpha-2 domains {Human (Homo sapiens), HLA-B0801}
GSHSMRYFDTAMSRPGRGEPRFISVGYVDDTQFVRFDSDAASPREEPRAPWIEQEGPEYWDR
NTQIFKTNTQTDRESLRNLRGYYNQSEAGSHTLQSMYGCDVGPDGRLLRGHNQYAYDGKDYI
ALNEDLRSWTAADTAAQITQRKWEAARVAEQDRAYLEGTCVEWLRRYLENGKDTLER
>d1a1na2 d.19.1.1 (A:1-181) MHC class I, alpha-1 and alpha-2 domains {Human (Homo sapiens), HLA-B*3501}
GSHSMRYFYTAMSRPGRGEPRFIAVGYVDDTQFVRFDSDAASPRTEPRPPWIEQEGPEYWDR
NTQIFKTNTQTYRESLRNLRGYYNQSEAGSHIIQRMYGCDLGPDGRLLRGHDQSAYDGKDYI
ALNEDLSSWTAADTAAQITQRKWEAARVAEQLRAYLEGLCVEWLRRYLENGKETLQR
>d1e27a2 d.19.1.1 (A:1-181) MHC class I, alpha-1 and alpha-2 domains {Human (Homo sapiens), HLA-B*5101}
GSHSMRYFYTAMSRPGRGEPRFIAVGYVDDTQFVRFDSDAASPRTEPRAPWIEQEGPEYWDR
NTQIFKTNTQTYRENLRIALRYYNQSEAGSHTWQTMYGCDVGPDGRLLRGHNQYAYDGKDYI
ALNEDLSSWTAADTAAQITQRKWEAAREAEQLRAYLEGLCVEWLRRHLENGKETLQR

>d1efxa2 d.19.1.1 (A:1-181) MHC class I, alpha-1 and alpha-2 domains {Human (Homo sapiens), HLA-CW3}
GSHSMRYFYTAVSRPGRGEPHFIAVGYVDDTQFVRFDSDAASPRGEPRAPWVEQEGPEYWDR
ETQKYKRQAQTDRVSLRNLRGYYNQSEAGSHIIQRMYGCDVGPDGRLLRGYDQYAYDGKDYI
ALNEDLRSWTAADTAAQITQRKWEAAREAEQLRAYLEGLCVEWLRRYLKNGKETLQR
>d1qqda2 d.19.1.1 (A:2-181) MHC class I, alpha-1 and alpha-2 domains {Human (Homo sapiens), HLA-CW4}
SHSMRYFSTSVSWPGRGEPRFIAVGYVDDTQFVRFDSDAASPRGEPREPWVEQEGPEYWDRE
TQKYKRQAQADRVNLRKLRGYYNQSEDGSHTLQRMFGCDLGPDGRLLRGYNQFAYDGKDYIA
LNEDLRSWTAADTAAQITQRKWEAAREAEQRRAYLEGTCVEWLRRYLENGKETLQR
>d1mhea2 d.19.1.1 (A:2-181) MHC class I, alpha-1 and alpha-2 domains {Human (Homo sapiens), HLA-E}
SHSLKYFHTSVSRPGRGEPRFISVGYVDDTQFVRFDNDAASPRMVPRAPWMEQEGSEYWDRE
TRSARDTAQIFRVNLRTLRGYYNQSEAGSHTLQWMHGCELGPDRRFLRGYEQFAYDGKDYLT
LNEDLRSWTAVDTAAQISEQKSNDASEAEHQRAYLEDTCVEWLHKYLEKGKETLLH
>d1de4a2 d.19.1.1 (A:4-181) MHC class I, alpha-1 and alpha-2 domains {Human (Homo sapiens), hemochromatosis protein Hfe}
RSHSLHYLFMGASEQDLGLSLFEALGYVDDQLFVFYDHESRRVEPRTPWVSSRISSQMWLQL
SQSLKGWDHMFTVDFWTIMENHNHSKESHTLQVILGCEMQEDNSTEGYWKYGYDGQDHLEFC
PDTLDWRAAEPRAWPTKLEWERHKIRARQNRAYLERDCPAQLQQLLELGRGVLD
>d1fzka2 d.19.1.1 (A:1-181) MHC class I, alpha-1 and alpha-2 domains {Mouse (Mus musculus), H-2KB}
GPHSLRYFVTAVSRPGLGEPRYMEVGYVDDTEFVRFDSDAENPRYEPRARWMEQEGPEYWER
ETQKAKGNEQSFRVDLRTLLGYYNQSKGGSHTIQVISGCEVGSDGRLLRGYQQYAYDGCDYI
ALNEDLKTWTAADMAALITKHKWEQAGAAEYYRAYLEGTCVEWLRRYLKNGNATLLR
>d1jpfa2 d.19.1.1 (A:2-181) MHC class I, alpha-1 and alpha-2 domains {Mouse (Mus musculus), H-2DB}
PHSMRYFETAVSRPGLEEPRYISVGYVDNKEFVRFDSDAENPRYEPRAPWMEQEGPEYWERE
TQKAKGQEQWFRVSLRNLLGYYNQSAGGSHTLQQMSGCDLGSDWRLLRGYLQFAYEGRDYIA
LNEDLKTWTAADMAAQITRRKWEQSGAAEHYKAYLEGECVEWLHRYLKNGNATLLR
>d1mhca2 d.19.1.1 (A:1-181) MHC class I, alpha-1 and alpha-2 domains {Mouse (Mus musculus), H-2M3}
GSHSLRYFHTAVSRPGRGEPQYISVGYVDDVQFQRCDSIEEIPRMEPRAPWMEKERPEYWKE
LKLKVKNIAQSARANLRTLLRYYNQSEGGSHILQWMVSCEVGPDMRLLGAHYQAAYDGSDYI
TLNEDLSSWTAVDMVSQITKSRLESAGTAEYFRAYVEGECLELLHRFLRNGKEILQR
>d1ld9a2 d.19.1.1 (A:1-181) MHC class I, alpha-1 and alpha-2 domains {Mouse (Mus musculus), H-2LD}
GPHSMRYFETAVSRPGLGEPRYISVGYVDNKEFVRFDSDAENPRYEPQAPWMEQEGPEYWER
ITQIAKGQEQWFRVNLRTLLGYYNQSAGGTHTLQWMYGCDVGSDGRLLRGYEQFAYDGCDYI
ALNEDLKTWTAADMAAQITRRKWEQAGAAEYYRAYLEGECVEWLHRYLKNGNATLLR
>d1qo3a2 d.19.1.1 (A:2-181) MHC class I, alpha-1 and alpha-2 domains {Mouse (Mus musculus), H-2DD}
SHSLRYFVTAVSRPGFGEPRYMEVGYVDNTEFVRFDSDAENPRYEPRARWIEQEGPEYWERE
TRRAKGNEQSFRVDLRTALRYYNQSAGGSHTLQWMAGCDVESDGRLLRGYWQFAYDGCDYIA

LNEDLKTWTAADMAAQITRRKWEQAGAAERDRAYLEGECVEWLRRYLKNGNATLLR
>d1k8da2 d.19.1.1 (A:1-181) MHC class I, alpha-1 and alpha-2 domains {Mouse (Mus musculus), IB QA-2}
GQHSLQYFHTAVSRPGLGEPWFISVGYVDDTQFVRFDSDAENPRMEPRARWMEQEGPEYWER
ETQIAKGHEQSFRGSLRTAQSYYNQSKGGSHTLQWMYGCDMGSDGRLLRGYLQFAYEGRDYI
ALNEDLKTWTAVDMAAQITRRKWEQAGIAEKDQAYLEGTCMQSLRRYLELGKETLLR
>d1ed3a2 d.19.1.1 (A:1-181) MHC class I, alpha-1 and alpha-2 domains {Rat (Rattus norvegicus), RT1-AA}
GSHSLRYFYTAVSRPGLGEPRFIAVGYVDDTEFVRFDSDAENPRMEPRARWMEREGPEYWEQ
QTRIAKEWEQIYRVDLRTLRGYYNQSEGGSHTIQEMYGCDVGSDGSLLRGYRQDAYDGRDYI
ALNEDLKTWTAADFAAQITRNKWERARYAERLRAYLEGTCVEWLSRYLELGKETLLR
>d1zaga2 d.19.1.1 (A:5-183) Zinc-alpha-2-glycoprotein, ZAG {Human (Homo sapiens)}
DGRYSLTYIYTGLSKHVEDVPAFQALGSLNDLQFFRYNSKDRKSQPMGLWRQVEGMEDWKQD
SQLQKAREDIFMETLKDIVEYYNDSNGSHVLQGRFGCEIENNRSSGAFWKYYYDGKDYIEFN
KEIPAWVPFDPAAQITKQKWEAEPVYVQRAKAYLEEECPATLRKYLKYSKNILDR
>d1hyrc2 d.19.1.1 (C:0-180) MHC I homolog {Human (Homo sapiens), Mic-a}
MEPHSLRYNLTVLSWDGSVQSGFLTEVHLDGQPFLRCDRQKCRAKPQGQWAEDVLGNKTWDR
ETRDLTGNGKDLRMTLAHIKDQKEGLHSLQEIRVCEIHEDNSTRSSQHFYYDGELFLSQNLE
TKEWTMPQSSRAQTLAMNVRNFLKEDAMKTKTHYHAMHADCLQELRRYLKSGVVLRR
>d1c16a2 d.19.1.1 (A:1-180) MHC I homolog {Mouse (Mus musculus), t22}
GSHSLRYFYTAVSRPGLGEPWFIIVGYVDDMQVLRFSSKEETPRMAPWLEQEEADNWEQQTR
IVTIQGQLSERNLMTLVHFYNKSMDDSHTLQWLQGCDVEPDRHLCLWYNQLAYDSEDLPTLN
ENPSSCTVGNSTVPHISQDLKSHCSDLLQKYLEKGKERLL
>d1exua2 d.19.1.1 (A:4-176) Class I MHC-related Fc receptor {Human (Homo sapiens)}
HLSLLYHLTAVSSPAPGTPAFWVSGWLGPQQYLSYNSLRGEAEPCGAWVWENQVSWYWEKET
TDLRIKEKLFLEAFKALGGKGPYTLQGLLGCELGPDNTSVPTAKFALNGEEFMNFDLKQGTW
GGDWPEALAISQRWQQQDKAANKELTFLLFSCPHRLREHLERGRGNLEW
>d1kcgc_ d.19.1.1 (C:) Class I MHC-related molecule Ulbp3 {Human (Homo sapiens)}
DAHSLWYNFTIIHLPRHGQQWCEVQSQVDQKNFLSYDCGSDKVLSMGHLEEQLYATDAWGKQ
LEMLREVGQRLRLELADTELEDFTPSGPLTLQVRMSCECEADGYIRGSWQFSFDGRKFLLFD
SNNRKWTVVHAGARRMKEKWEKDSGLTTFFKMVSMRDCKSWLRDFLMHRKKRLE
>d1jfma_ d.19.1.1 (A:) NK cell ligand RAE-1 beta {Mouse (Mus musculus)}
DAHSLRCNLTIKDPTPADPLWYEAKCFVGEILILHLSNINKTMTSGDPGETANATEVKKCLT
QPLKNLCQKLRNKVSNTKVDTHKTNGYPHLQVTMIYPQSQGRTPSATWEFNISDSYFFTFYT
ENMSWRSANDESGVIMNKWKDDGEFVKQLKFLIHECSQKMDEFLKQSKEK
>d2aak__ d.20.1.1 (-) Ubiquitin conjugating enzyme {Arabidopsis thaliana}
MSTPARKRLMRDFKRLQQDPPAGISGAPQDNNIMLWNAVIFGPDDTPWDGGTFKLSLQFSED

YPNKPPTVRFVSRMFHPNIYADGSICLDILQNQWSPIYDVAAILTSIQSLLCDPNPNSPANS
EAARMYSESKREYNRRVRDVVEQSWT
>d1fzya_ d.20.1.1 (A:) Ubiquitin conjugating enzyme {Baker's yeast (Saccharomyces cerevisiae), ubc1}
SRAKRIMKEIQAVKDDPAAHITLEFVSESDIHHLKGTFLGPPGTPYEGGKFVVDIEVPMEYP
FKPPKMQFDTKVYHPNISSVTGAICLDILKNAWSPVITLKSALISLQALLQSPEPNDPQDAE
VAQHYLRDRESFNKTAALWTRLYAS
>d1ayza_ d.20.1.1 (A:) Ubiquitin conjugating enzyme {Baker's yeast (Saccharomyces cerevisiae), ubc2 (RAD6)}
STPARRRLMRDFKRMKEDAPPGVSASPLPDNVMVWNAMIIGPADTPYEDGTFRLLLEFDEEY
PNKPPHVKFLSEMFHPNVYANGEICLDILQNRWTPTYDVASILTSIQSLFNDPNPASPANVE
AATLFKDHKSQYVKRVKETVEKSWEDDMD
>d1qcqa_ d.20.1.1 (A:) Ubiquitin conjugating enzyme {Baker's yeast (Saccharomyces cerevisiae), ubc4}
MSSSKRIAKELSDLERDPPTSCSAGPVGDDLYHWQASIMGPADSPYAGGVFFLSIHFPTDYP
FKPPKISFTTKIYHPNINANGNICLDILKDQWSPALTLSKVLLSICSLLTDANPDDPLVPEI
AHIYKTDRPKYEATAREWTKKYAV
>d2ucz__ d.20.1.1 (-) Ubiquitin conjugating enzyme {Baker's yeast (Saccharomyces cerevisiae), ubc7}
SKTAQKRLLKELQQLIKDSPPGIVAGPKSENNIFIWDCLIQGPPDTPYADGVFNAKLEFPKD
YPLSPPKLTFTPSILHPNIYPNGEVCISILHSPGDDPNMYELAEERWSPVQSVEKILLSVMS
MLSEPNIESGANIDACILWRDNRPEFERQVKLSILKSLGF
>d1jata_ d.20.1.1 (A:) Ubiquitin conjugating enzyme {Baker's yeast (Saccharomyces cerevisiae), ubc13}
AASLPKRIIKETEKLVSDPVPGITAEPHDDNLRYFQVTIEGPEQSPYEDGIFELELYLPDDY
PMEAPKVRFLTKIYHPNIDRLGRICLDVLKTNWSPALQIRTVLLSIQALLASPNPNDPLAND
VAEDWIKNEQGAKAKAREWTKLYAKKKP
>d1j7db_ d.20.1.1 (B:) Ubiquitin conjugating enzyme {Human (Homo sapiens), ubc13}
AGLPRRIIKETQRLLAEPVPGIKAEPDESNARYFHVVIAGPQDSPFEGGTFKLELFLPEEYP
MAAPKVRFMTKIYHPNVDKLGRICLDILKDKWSPALQIRTVLLSIQALLSAPNPDDPLANDV
AEQWKTNEAQAIETARAWTRLYAMN
>d1jatb_ d.20.1.1 (B:) Ubiquitin conjugating enzyme {Baker's yeast (Saccharomyces cerevisiae), mms2}
SKVPRNFRLLEELEKGEKGFGPESCSYGLADSDDITMTKWNGTILGPPHSNHENRIYSLSID
CGPNYPDSPPKVTFISKINLPCVNPTTGEVQTDFHTLRDWKRAYTMETLLLDLRKEMATPAN
KKLRQPKEGETF
>d1j7da_ d.20.1.1 (A:) Ubiquitin conjugating enzyme {Human (Homo sapiens), mms2}
GVKVPRNFRLLEELEEGQKGVGDGTVSWGLEDDEDMTLTRWTGMIIGPPRTNYENRIYSLKV
ECGPKYPEAPPSVRFVTKINMNGINNSSGMVDARSIPVLAKWQNSYSIKVVLQELRRLMMSK
ENMKLPQPPEGQTYNN
>d1c4zd_ d.20.1.1 (D:) Ubiquitin conjugating enzyme {Human (Homo sapiens), ubch7}

SRRLMKELEEIRKCGMKNFRNIQVDEANLLTWQGLIVPDNPPYDKGAFRIEINFPAEYPFKP
PKITFKTKIYHPNIDEKGQVCLPVISAENWKPATKTDQVIQSLIALVNDPQPEHPLRADLAE
EYSKDRKKFCKNAEEFTKKY
>d1u9aa_ d.20.1.1 (A:) Ubiquitin conjugating enzyme {Human (Homo sapiens), ubc9}
LNMSGIALSRLAQERKAWRKDHPFGFVAVPTKNPDGTMNLMNWECAIPGKKGTPWEGGLFKL
RMLFKDDYPSSPPKCKFEPPLFHPNVYPSGTVCLSILEEDKDWRPAITIKQILLGIQELLNE
PNIQDPAQAEAYTIYCQNRVEYEKRVRAQAKKFAPS
>d1i7ka_ d.20.1.1 (A:) Ubiquitin conjugating enzyme {Human (Homo sapiens), ubch10}
PVGKRLQQELMTLMMSGDKGISAFPESDNLFKWVGTIHGAAGTVYEDLRYKLSLEFPSGYPY
NAPTVKFLTPCYHPNVDTQGNISLDILKEKWSALYDVRTILLSIQSLLGEPNIDSPLNTHAA
ELWKNPTAFKKYLQETYSKQVT
>d2e2c__ d.20.1.1 (-) Ubiquitin conjugating enzyme {Clam (Spisula solidissima), E-2C}
MTTSKERHSVSKRLQQELRTLLMSGDPGITAFPDGDNLFKWVATLDGPKDTVYESLKYKLTL
EFPSDYPYKPPVVKFTTPCWHPNVDQSGNICLDILKENWTASYDVRTILLSLQSLLGEPNNA
SPLNAQAADMWSNQTEYKKVLHEKYKTAQSDK
>d1bwza1 d.21.1.1 (A:1-130) Diaminopimelate epimerase {Haemophilus influenzae}
MQFSKMHGLGNDFVVVDGVTQNVFFTPETIRRLANRHCGIGFDQLLIVEAPYDPELDFHYRI
FNADGSEVSQCGNGARCFARFVTLKGLTNKKDISVSTQKGNMVLTVKDMNQIRVNMGEPIWE
PAKIPF
>d1bwza2 d.21.1.1 (A:131-274) Diaminopimelate epimerase {Haemophilus influenzae}
TANKFEKNYILRTDIQTVLCGAVSMGNPHCVVQVDDIQTANVEQLGPLLESHERFPERVNAG
FMQIINKEHIKLRVYERGAGETQACGSGACAAVAVGIMQGLLNNNVQVDLPGGSLMIEWNGV
GHPLYMTGEATHIYDGFITL
>d1h6ra_ d.22.1.1 (A:) Green fluorescent protein, GFP {Jellyfish (Aequorea victoria)}
SKGEELFTGVVPILVELDGDVNGHKFSVSGEGEGDATYGKLTLKFIVTTGKLPVPWPTLVTT
FAYGLQCFARYPDHMKRHDFFKSAMPEGYVQERTIFFKDDGNYKTRAEVKFEGDTLVNRIEL
KGIDFKEDGNILGHKLEYNYNSHCVYIVADKQKNGIKVNFKIRHNIEDGSVQLADHYQQNTP
IGDGPVLLPDNHYLCYQSALSKDPNEKRDHMVLLEFVTAAGITH
>d2emd__ d.22.1.1 (-) Green fluorescent protein, GFP {Jellyfish (Aequorea victoria)}
ELFTGVVPILVELDGDVNGHKFSVSGEGEGDATYGKLTLKFICTTGKLPVPWPTLVTTLSYG
VQCFSRYPDHMKRHDFFKSAMPEGYVQERTIFFKDDGNYKTRAEVKFEGDTLVNRIELKGID
FKEDGNILGHKLEYNYNSHNVYIMADKQKNGIKVNFKIRHNIEDGSVQLADHYQQNTPIGDG
PVLLPDNHYLSTQSALSKDPNEKRDHMVLLEFVTAAGIT
>d1ggxa_ d.22.1.1 (A:) Red fluorescent protein (fp583 or dsred(clontech)) {Coral (Discosoma sp.)}
VIKEFMRFKVRMEGTVNGHEFEIEGEGEGRPYEGHNTVKLKVTKGGPLPFAWDILSPQFQYG
SKVYVKHPADIPDYKKLSFPEGFKWERVMNFEDGGVVTVTQDSSLQDGCFIYKVKFIGVNFP

SDGPVMQKKTMGWEASTERLYPRDGVLKGEIHKALKLKDGGHYLVEFKSIYMAKKPVQLPGY
YYVDSKLDITSHNEDYTIVEQYERTEGRHHLFL
>d1gl4a1 d.22.1.2 (A:399-631) Domain G2 of nidogen-1 {Mouse (Mus musculus)}
GSPQRVNGKVKGRIFVGSSQVPVVFENTDLHSYVVMNHGRSYTAISTIPETVGYSLLPLAPI
GGIIGWMFAVEQDGFKNGFSITGGEFTRQAEVTFLGHPGKLVLKQQFSGIDEHGHLTISTEL
EGRVPQIPYGASVHIEPYTELYHYSSSVITSSSTREYTVMEPDQDGAAPSHTHIYQWRQTIT
FQECAHDDARPALPSTQQLSVDSVFVLYNKEERILRYALSNSIGPVR
>d1c8za_ d.23.1.1 (A:) Transcriptional factor tubby, C-terminal domain {Mouse (Mus musculus)}
GSVDIEVQDLEEFALRPAPQGITIKCRITRDKKGMDRGMFPTYFLHLDREDGKKVFLLAGRK
RKKSKTSNYLISVDPTDLSRGGDSYIGKLRSNLMGTKFTVYDNGVNPQKASSSTLESGTLRQ
ELAAVCYETNVLGFKGPRKMSVIVPGMNMVHERVCIRPRNEHETLLARWQNKNTESIIELQN
KTPVWNDDTQSYVLNFHGRVTQASVKNFQIIHGNDPDYIVMQFGRVAEDVFTMDYNYPLCAL
QAFAIALSSFDSKLACE
>d2pil__ d.24.1.1 (-) Pilin {Gc (Neisseria gonorrhoeae)}
FTLIELMIVIAIVGILAAVALPAYQDYTARAQVSEAILLAEGQKSAVTEYYLNHGKWPENNT
SAGVASPPSDIKGKYVKEVEVKNGVVTATMLSSGVNNEIKGKKLSLWARRENGSVKWFCGQP
VTRTDDDTVADAKDGKEIDTKHLPSTCRDNFDAK
>d1hpwa_ d.24.1.1 (A:) Pilin {Pseudomonas aeruginosa}
ALEGTEFARAQLSEAMTLASGLKTKVSDIFSQDGSCPANTAATAGIEKDTDINGKYVAKVTT
GGTAAASGGCTIVATMKASDVATPLRGKTLTLTLGNADKGSYTWACTSNADNKYLPKTCQTA
TTTTP
>d1dzoa_ d.24.1.1 (A:) Pilin {Pseudomonas aeruginosa, type IV pilin, pak pilin}
GTEFARSEGASALASVNPLKTTVEEALSRGWSVKSGTGTEDATKKEVPLGVAADANKLGTIA
LKPDPADGTADITLTFTMGGAGPKNKGKIITLTRTAADGLWKCTSDQDEQFIPKGCSR
>d1p32a_ d.25.1.1 (A:) Acidic mitochondrial matrix protein p32 {Human (Homo sapiens)}
MHTDGDKAFVDFLSDEIKEERKIQKHKTLPKMSGGWELELNGTEAKLVRKVAGEKITVTFNI
NNSIPPTFDGEEEPSQGQKVEEQEPELTSTPNFVVEVIKNDDGKKALVLDCHYPEDEVGQED
EAESDIFSIREVSFQSTGESEWKDTNYTLNTDSLDWALYDHLMDFLADRGVDNTFADELVEL
STALEHQEYITFLEDLKSFVKSQ
>d1bkf__ d.26.1.1 (-) FK-506 binding protein (FKBP12), an immunophilin {Human (Homo sapiens)}
GVQVETISPGDGRTFPKRGQTCVVHYTGMLEDGKKFDSSRDKNKPFKFMLGKQEVIRGWEEG
VAQMSVGQRAKLTISPDYAYGATGVPGIIPPHATLVFDVELLKLE
>d1c9ha_ d.26.1.1 (A:) Calcineurin (FKBP12.6) {Human (Homo sapiens)}
GVEIETISPGDGRTFPKKGQTCVVHYTGMLQNGKKFDSSRDRNKPFKFRIGKQEVIKGFEEG
AAQMSLGQRAKLTCTPDVAYGATGHPGVIPPNATLIFDVELLNLE
>d1yat__ d.26.1.1 (-) Calcineurin (FKBP12.6) {Baker's yeast (Saccharomyces cerevisiae)}
SEVIEGNVKIDRISPGDGATFPKTGDLVTIHYTGTLENGQKFDSSVDRGSPFQCNIGVGQVI

KGWDVGIPKLSVGEKARLTIPGPYAYGPRGFPGLIPPNSTLVFDVELLKVN
>d1pbk__ d.26.1.1 (-) FKBP25 {Human (Homo sapiens)}
PKYTKSVLKKGDKTNFPKKGDVVHCWYTGTLQDGTVFDTNIQTSAKKKKNAKPLSFKVGVGK
VIRGWDEALLTMSKGEKARLEIEPEWAYGKKGQPDAKIPPNAKLTFEVELVDID
>d1rot__ d.26.1.1 (-) FKBP59-I, N-terminal domain {Rabbit (Oryctolagus cuniculus)}
GVDISPKQDEGVLKVIKREGTGTETPMIGDRVFVHYTGWLLDGTKFDSSLDRKDKFSFDLGK
GEVIKAWDIAVATMKVGELCRITCKPEYAYGSAGSPPKIPPNATLVFEVELFEFKG
>d1pina2 d.26.1.1 (A:45-163) Mitotic rotamase PIN1, domain 2 {Human (Homo sapiens)}
GKNGQGEPARVRCSHLLVKHSQSRRPSSWRQEKITRTKEEALELINGYIQKIKSGEEDFESL
ASQFSDCSSAKARGDLGAFSRGQMQKPFEDASFALRTGEMSGPVFTDSGIHIILRTE
>d1eq3a_ d.26.1.1 (A:) Parvulin {Human (Homo sapiens), hpar14}
NAVKVRHILCEKHGKIMEAMEKLKSGMRFNEVAAQYSEDKARQGGDLGWMTRGSMVGPFQEA
AFALPVSGMDKPVFTDPPVKTKFGYHIIMVEGRK
>d1fd9a_ d.26.1.1 (A:) Macrophage infectivity potentiator protein (MIP) {Legionella pneumophila}
TDKDKLSYSIGADLGKNFKNQGIDVNPEAMAKGMQDAMSGAQLALTEQQMKDVLNKFQKDLM
AKRTAEFNKKADENKVKGEAFLTENKNKPGVVVLPSGLQYKVINSGNGVKPGKSDTVTVEYT
GRLIDGTVFDSTEKTGKPATFQVSQVIPGWTEALQLMPAGSTWEIYVPSGLAYGPRSVGGPI
GPNETLIFKIHLISVKKS
>d1grj_2 d.26.1.2 (80-158) GreA transcript cleavage factor, C-terminal domain {Escherichia coli}
MPNNGRVIFGATVTVLNLDSDEEQTYRIVGDDEADFKQNLISVNSPIARGLIGKEEDDVVVI
KTPGGEVEFEVIKVEYL
>d3eipa_ d.26.2.1 (A:) Colicin E3 immunity protein {Escherichia coli}
GLKLDLTWFDKSTEDFKGEEYSKDFGDDGSVMESLGVPFKDNVNNGCFDVIAEWVPLLQPYF
NHQIDISDNEYFVSFDYRDGDW
>d1edqa3 d.26.3.1 (A:444-516) Chitinase A {Serratia marcescens}
YGRGWTGVNGYQNNIPFTGTATGPVKGTWENGIVDYRQIAGQFMSGEWQYTYDATAEAPYVF
KPSTGDLITFD
>d1goia3 d.26.3.1 (A:292-379) Chitinase B {Serratia marcescens}
YGRAFKGVSGGNGGQYSSHSTPGEDPYPSTDYWLVGCEECVRDKDPRIASYRQLEQMLQGNY
GYQRLWNDKTKTPYLYHAQNGLFVTY
>d1d2ka2 d.26.3.1 (A:293-354) Chitinase 1 {Fungus (Coccidioides immitis)}
YGRAFASTDGIGTSFNGVGGGSWENGVWDYKDMPQQGAQVTELEDIAASYSYDKNKRYLISY
>d1e9la2 d.26.3.1 (A:267-336) Chitinase-like lectin ym1 {Mouse (Mus musculus)}
YGHTFILSDPSKTGIGAPTISTGPPGKYTDESGLLAYYEVCTFLNEGATEVWDAPQEVPYAY
QGNEWVGY
>d1fjgp_ d.27.1.1 (P:) Ribosomal protein S16 {Thermus thermophilus}

MVKIRLARFGSKHNPHYRIVVTDARRKRDGKYIEKIGYYDPRKTTPDWLKVDVERARYWLSV
GAQPTDTARRLLRQAGVFRQE
>d1fjgs_ d.28.1.1 (S:) Ribosomal protein S19 {Thermus thermophilus}
PRSLKKGVFVDDHLLEKVLELNAKGEKRLIKTWSRRSTIVPEMVGHTIAVYNGKQHVPVYIT
ENMVGHKLGEFAPTRTYRGHGK
>d1qkha_ d.28.1.1 (A:) Ribosomal protein S19 {Thermus thermophilus}
GVFVDDHLLEKVLELNAKGEKRLIKTWSRRSTIVPEMVGHTIAVYNGKQHVPVYITENMVGH
KLGEFAPTRTY
>d1jj2w_ d.29.1.1 (W:) Ribosomal protein L31e {Archaeon Haloarcula marismortui}
ERVVTIPLRDARAEPNHKRADKAMILIREHLAKHFSVDEDAVRLDPSINEAAWARGRANTPS
KIRVRAARFEEEGEAIVEAE
>d1b33n_ d.30.1.1 (N:) Allophycocyanin linker chain (domain) {Mastigocladus laminosus}
GRLFKITACVPSQTRIRTQRELQNTYFTKLVPYENWFREQQRIQKMGGKIVKVELATGKQGI
NTGLA
>d1qcsa2 d.31.1.1 (A:86-201) C-terminal domain of NSF-N, NSF-Nc {Hamster (Cricetulus griseus)}
DKAKQCIGTMTIEIDFLQKKNIDSNPYDTDKMAAEFIQQFNNQAFSVGQQLVFSFNDKLFGL
LVKDIEAMDPSILKGEPASGKRQKIEVGLVVGNSQVAFEKAENSSLNLIGKAKT
>d1cr5a2 d.31.1.1 (A:108-210) C-terminal domain of NSF-N, NSF-Nc {Baker's yeast (Saccharomyces cerevisiae), sec18p}
SGKQSYLGSIDIDISFRARGKAVSTVFDQDELAKQFVRCYESQIFSPTQYLIMEFQGHFFDL
KIRNVQAIDLGDIEPTSAVATGIETKGILTKQTQINFFKGR
>d1cz4a2 d.31.1.1 (A:92-185) C-terminal domain of VAT-N, VAT-Nc {Archaeon Thermoplasma acidophilum}
TEIAKKVTLAPIIRKDQRLKFGEGIEEYVQRALIRRPMLEQDNISVPGLTLAGQTGLLFKVV
KTLPSKVPVEIGEETKIEIREEPASEVLEEGG
>d1e32a3 d.31.1.1 (A:107-200) Membrane fusion atpase p97 domain 2, P97-Nc {Mouse (Mus musculus)}
DVKYGKRIHVLPIDDTVEGITGNLFEVYLKPYFLEAYRPIRKGDIFLVRGGMRAVEFKVVET
DPSPYCIVAPDTVIHCEGEPIKREDEEESLNE
>d1qipa_ d.32.1.1 (A:) Glyoxalase I (lactoylglutathione lyase) {Human (Homo sapiens)}
GGLTDEAALSCCSDADPSTKDFLLQQTMLRVKDPKKSLDFYTRVLGMTLIQKCDFPIMKFSL
YFLAYEDKNDIPKEKDEKIAWALSRKATLELTHNWGTEDDETQSYHNGNSDPRGFGHIGIAV
PDVYSACKRFEELGVKFVKKPDDGKMKGLAFIQDPDGYWIEILNPNKMATLM
>d1f9za_ d.32.1.1 (A:) Glyoxalase I (lactoylglutathione lyase) {Escherichia coli}
MRLLHTMLRVGDLQRSIDFYTKVLGMKLLRTSENPEYKYSLAFVGYGPETEEAVIELTYNWG
VDKYELGTAYGHIALSVDNAAEACEKIRQNGGNVTREAGPVKGGTTVIAFVEDPDGYKIELI
EEKDAGRGLGN

>d1qtoa_ d.32.1.2 (A:) Bleomycin resistance protein, BRP {Streptomyces verticillus}
MVKFLGAVPVLTAVDVPANVSFWVDTLGFEKDFGDRDFAGVRRGDIRLHISRTEHQIVADNT
SAWIEVTDPDALHEEWARAVSTDYADTSGPAMTPVGESPAGREFAVRDPAGNCVHFTAGE
>d1byla_ d.32.1.2 (A:) Bleomycin resistance protein, BRP {Streptoalloteichus hindustanus}
FMAKLTSAVPVLTARDVAGAVEFWTDRLGFSRDFVEDDFAGVVRDDVTLFISAVQDQVVPDN
TLAWVWVRGLDELYAEWSEVVSTNFRDASGPAMTEIGEQPWGREFALRDPAGNCVHFVAE
>d1ecsa_ d.32.1.2 (A:) Bleomycin resistance protein, BRP {Klebsiella pneumoniae}
TDQATPNLPSRDFDSTAAFYERLGFGIVFRDAGWMILQRGDLMLEFFAHPGLDPLASWFSCC
LRLDDLAEFYRQCKSVGIQETSSGYPRIHAPELQGWGGTMAALVDPDGTLLRLIQNEL
>d1jc4a_ d.32.1.4 (A:) Methylmalonyl-CoA epimerase {Propionibacterium shermanii}
NEDLFICIDHVAYACPDADEASKYYQETFGWHELHREENPEQGVVEIMMAPAAKLTEHMTQV
QVMAPLNDESTVAKWLAKHNGRAGLHHMAWRVDDIDAVSATLRERGVQLLYDEPKLGTGGNR
INFMHPKSGKGVLIELTQYPK
>d1gdga1 d.32.1.3 (A:1-132) 2,3-Dihydroxybiphenyl dioxygenase (DHBD, BPHC enzyme) {Pseudomonas sp.}
SIERLGYLGFAVKDVPAWDHFLTKSVGLMAAGSAGDAALYRADQRAWRIAVQPGELDDLAYA
GLEVDDAAALERMADKLRQAGVAFTRGDEALMQQRKVMGLLCLQDPFGLPLEIYYGPAEIFH
EPFLPSAP
>d1gdga2 d.32.1.3 (A:133-288) 2,3-Dihydroxybiphenyl dioxygenase (DHBD, BPHC enzyme) {Pseudomonas sp.}
VSGFVTGDQGIGHFVRCVPDTAKAMAFYTEVLGFVLSDIIDIQMGPETSVPAHFLHCNGRHH
TIALAAFPIPKRIHHFMLQANTIDDVGYAFDRLDAAGRITSLLGRHTNDQTLSFYADTPSPM
IEVEFGWGPRTVDSSWTVARHSRTAMWGHKSV
>d1han_1 d.32.1.3 (2-132) 2,3-Dihydroxybiphenyl dioxygenase (DHBD, BPHC enzyme) {Burkholderia cepacia (formerly Pseudomonas cepacia)}
SIRSLGYMGFAVSDVAAWRSFLTQKLGLMEAGTTDNGDLFRIDSRAWRIAVQQGEVDDLAFA
GYEVADAAGLAQMADKLKQAGIAVTTGDASLARRRGVTGLITFADPFGLPLEIYYGASEVFE
KPFLPGA
>d1han_2 d.32.1.3 (133-289) 2,3-Dihydroxybiphenyl dioxygenase (DHBD, BPHC enzyme) {Burkholderia cepacia (formerly Pseudomonas cepacia)}
AVSGFLTGEQGLGHFVRCVPDSDKALAFYTDVLGFQLSDVIDMKMGPDVTVPAYFLHCNERH
HTLAIAAFPLPKRIHHFMLEVASLDDVGFAFDRVDADGLITSTLGRHTNDHMVSFYASTPSG
VEVEYGWSARTVDRSWVVVRHDSPSMWGHKSVR
>d1mpya1 d.32.1.3 (A:1-145) Catechol 2,3-dioxygenase (metapyrocatechase) {Pseudomonas putida, mt2}
MNKGVMRPGHVQLRVLDMSKALEHYVELLGLIEMDRDDQGRVYLKAWTEVDKFSLVLREADE
PGMDFMGFKVVDEDALRQLERDLMAYGCAVEQLPAGELNSCGRRVRFQAPSGHHFELYADKE
YTGKWGLNDVNPEAWPRDLKG
>d1mpya2 d.32.1.3 (A:146-307) Catechol 2,3-dioxygenase

(metapyrocatechase) {Pseudomonas putida, mt2}
MAAVRFDHALMYGDELPATYDLFTKVLGFYLAEQVLDENGTRVAQFLSLSTKAHDVAFIHHP
EKGRLHHVSFHLETWEDLLRAADLISMTDTSIDIGPTRHGLTHGKTIYFFDPSGNRNEVFCG
GDYNYPDHKPVTWTTDQLGKAIFYHDRILNERFMTVLT
>d1cjxa1 d.32.1.3 (A:4-153) 4-hydroxyphenylpyruvate dioxygenase, HppD {Pseudomonas fluorescens}
YENPMGLMGFEFIEFASPTPGTLEPIFEIMGFTKVATHRSKNVHLYRQGEINLILNNEPNSI
ASYFAAEHGPSVCGMAFRVKDSQKAYNRALELGAQPIHIDTGPMELNLPAIKGIGGAPLYLI
DRFGEGSSIYDIDFVYLEGVERNPVG
>d1cjxa2 d.32.1.3 (A:154-356) 4-hydroxyphenylpyruvate dioxygenase, HppD {Pseudomonas fluorescens}
AGLKVIDHLTHNVYRGRMVYWANFYEKLFNFREARYFDIKGEYTGLTSKAMSAPDGMIRIPL
NEESSKGAGQIEEFLMQFNGEGIQHVAFLTDDLVKTWDALKKIGMRFMTAPPDTYYEMLEGR
LPDHGEPVDQLQARGILLDGSSVEGDKRLLLQIFSETLMGPVFFEFIQRKGDDGFGEGNFKA
LFESIERDQVRRGVLAT
>d1fx3a_ d.33.1.1 (A:) Bacterial protein-export protein SecB {Haemophilus influenzae}
QPVLQIQRIYVKDVSFEAPNLPHIFQQEWKPKLGFDLSTETTQVGDDLYEVVLNISVETTLE
DSGDVAFICEVKQAGVFTISGLEDVQMAHCLTSQCPNMLFPYARELVSNLVNRGTFPALNLS
PVNFDALFVEYMNRQQAEN
>d1bm8__ d.34.1.1 (-) DNA-binding domain of Mlu1-box binding protein MBP1 {Baker's yeast (Saccharomyces cerevisiae)}
QIYSARYSGVDVYEFIHSTGSIMKRKKDDWVNATHILKAANFAKAKRTRILEKEVLKETHEK
VQGGFGKYQGTWVPLNIAKQLAEKFSVYDQLKPLFDF
>d1dk0a_ d.35.1.1 (A:) Heme-binding protein A (HasA) {Serratia marcescens}
AFSVNYDSSFGGYSIHDYLGQWASTFGDVNHTNGNVTDANSGGFYGGSLSGSQYAISSTANQ
VTAFVAGGNLTYTLFNEPAHTLYGQLDSLSFGDGLSGGDTSPYSIQVPDVSFGGLNLSSLQA
QGHDGVVHQVVYGLMSGDTGALETALNGILDDYGLSVNSTFDQVAAATA
>d1eyqa_ d.36.1.1 (A:) Chalcone isomerase {Alfalfa (Medicago sativa)}
SITAITVENLEYPAVVTSPVTGKSYFLGGAGERGLTIEGNFIKFTAIGVYLEDIAVASLAAK
WKGKSSEELLETLDFYRDIISGPFEKLIRGSKIRELSGPEYSRKVMENCVAHLKSVGTYGDA
EAEAMQKFAEAFKPVNFPPGASVFYRQSPDGILGLSFSPDTSIPEKEAALIENKAVSSAVLE
TMIGEHAVSPDLKRCLAARLPALLNE
>d1b3ob2 d.37.1.1 (B:112-159) Type II inosine monophosphate dehydrogenase {Human (Homo sapiens)}
QGFITDPVVLSPKDRVRDVFEAKARHGFCGIPITDTGRMGSRLVGIIS
>d1jr1a2 d.37.1.1 (A:113-155) Type II inosine monophosphate dehydrogenase {Chinese hamster (Cricetulus griseus)}
GFITDPVVLSPKDRVRDVFEAKARHGFCGIPITDTGRMGSRLV
>d1jr1a3 d.37.1.1 (A:178-232) Type II inosine monophosphate dehydrogenase {Chinese hamster (Cricetulus griseus)}
IMTKREDLVVAPAGITLKEANEILQRSKKGKLPIVNENDELVAIIARTDLKKNRD

>d1zfja2 d.37.1.1 (A:95-158) Type II inosine monophosphate dehydrogenase {Streptococcus pyogenes}
NGVIIDPFFLTPEHKVSEAEELMQRYRISGVPIVETLANRKLVGIITNRDMRFISDYNAPIS
EH
>d1zfja3 d.37.1.1 (A:159-220) Type II inosine monophosphate dehydrogenase {Streptococcus pyogenes}
MTSEHLVTAAVGTDLETAERILHEHRIEKLPLVDNSGRLSGLITIKDIEKVIEFPHAAKDEF
>d1bvqa_ d.38.1.1 (A:) 4-hydroxybenzoyl-CoA thioesterase {Pseudomonas sp., CBS-3}
ARSITMQQRIEFGDCDPAGIVWYPNYHRWLDAASRNYFIKCGLPPWRQTVVERGIVGTPIVS
CNASFVCTASYDDVLTIETCIKEWRRKSFVQRHSVSRTTPGGDVQLVMRADEIRVFAMNDGE
RLRAIEVPADYIELC
>d1mkaa_ d.38.1.2 (A:) beta-Hydroxydecanol thiol ester dehydrase {Escherichia coli}
VDKRESYTKEDLLASGRGELFGAKGPQLPAPNMLMMDRVVKMTETGGNFDKGYVEAELDINP
DLWFFGCHFIGDPVMPGCLGLDAMWQLVGFYLGWLGGEGKGRALGVGEVKFTGQVLPTAKKV
TYRIHFKRIVNRRLIMGLADGEVLVDGRLIYTASDLKVGLFQDTSAF
>d1c8ua1 d.38.1.3 (A:2-115) Thioesterase II (TesB) {Escherichia coli}
SQALKNLLTLLNLEKIEEGLFRGQSEDLGLRQVFGGQVVGQALYAAKETVPEERLVHSFHSY
FLRPGDSKKPIIYDVETLRDGNSFSARRVAAIQNGKPIFYMTASFQAPEAGF
>d1c8ua2 d.38.1.3 (A:116-286) Thioesterase II (TesB) {Escherichia coli}
EHQKTMPSAPAPDGLPSETQIAQSLAHLLPPVLKDKFICDRPLEVRPVEFHNPLKGHVAEPH
RQVWIRANGSVPDDLRVHQYLLGYASDLNFLPVALQPHGIGFLEPGIQIATIDHSMWFHRPF
NLNEWLLYSVESTSASSARGFVRGEFYTQDGVLVASTVQEGVMRNHN
>d1cmia_ d.39.1.1 (A:) Dynein light chain 8 (DLC8) {Human (Homo sapiens)}
KAVIKNADMSEEMQQDSVECATQALEKYNIEKDIAAHIKKEFDKKYNPTWHCIVGRNFGSYV
THETKHFIYFYLGQVAILLFKSG
>d1csei_ d.40.1.1 (I:) Eglin C {Leech (Hirudo medicinalis)}
KSFPEVVGKTVDQAREYFTLHYPQYNVYFLPEGSPVTLDLRYNRVRVFYNPGTNVVNHVPHV
G
>d1egl__ d.40.1.1 (-) Eglin C {Leech (Hirudo medicinalis)}
TEFGSELKSFPEVVGKTVDQAREYFTLHYPQYDVYFLPEGSPVTLDLRYNRVRVFYNPGTNV
VNHVPHVG
>d1ypci_ d.40.1.1 (I:) Chymotrypsin inhibitor CI-2 {Barley (Hordeum vulgare)}
MKTEWPELVGKSVAAAKKVILQDKPEAQIIVLPVGTIVTMEYRIDRVRLFVDKLDNIAQVPR
VG
>d2snii_ d.40.1.1 (I:) Chymotrypsin inhibitor CI-2 {Barley (Hordeum vulgare)}
LKTEWPELVGKSVEEAKKVILQDKPEAQIIVLPVGTIVTMEYRIDRVRLFVDKLDNIAEVPR
VG

>g1cq4.1 d.40.1.1 (A:,B:) Chymotrypsin inhibitor CI-2 {Barley (Hordeum vulgare)}
KTEWPELVGKSVEEAKKVILQDKPEAQIIVLPVGTIVXYRIDRVRLFVDKLDNIAQVPRVG
>d1tin__ d.40.1.1 (-) Trypsin inhibitor V {Pumpkin (Cucurbita maxima)}
SSCPGKSSWPHLVGVGGSVAKAIIERQNPNVKAVILEEGTPVTKDFRCNRVRIWVNKRGLVV
SPPRIG
>d1dwma_ d.40.1.1 (A:) Trypsin inhibitor LUTI {Flax (Linum usitatissimum)}
SRRCPGKNAWPELVGKSGNMAAATVERENRNVHAIVLKEGSAMTKDFRCDRVWVIVNDHGVV
TSVPHIT
>d1jv2b3 d.200.1.1 (B:606-690) Integrin beta tail domain {Human (Homo sapiens)}
DACTFKKECVECKKFDREPYMTENTCNRYCRDEIESVKELKDTGKDAVNCTYKNEDDCVVRF
QYYEDSSGKSILYVVEEPECPKG
>d1hlra3 d.41.1.1 (A:194-310) Aldehyde oxidoreductase, domain 3 {Desulfovibrio gigas}
DYGADLGLKMPAGTLHLAMVQAKVSHANIKGIDTSEALTMPGVHSVITHKDVKGKNRITGLI
TFPTNKGDGWDRPILCDEKVFQYGDCIALVCADSEANARAAAEKVKVDLEELPAY
>d1dgja3 d.41.1.1 (A:194-310) Aldehyde oxidoreductase, domain 3 {Desulfovibrio desulfuricans}
EFGADAALRMPENTLHLALAQAKVSHALIKGIDTSEAEKMPGVYKVLTHKDVKGKNRITGLI
TFPTNKGDGWERPILNDSKIFQYGDALAIVCADSEANARAAAEKVKFDLELLPEY
>d1fiqc1 d.41.1.1 (C:571-694) Xanthine oxidase, domain 5 (?) {Cow (Bos taurus)}
DTVGRPLPHLAAAMQASGEAVYCDDIPRYENELFLRLVTSTRAHAKIKSIDVSEAQKVPGFV
CFLSADDIPGSNETGLFNDETVFAKDTVTCVGHIIGAVVADTPEHAERAAHVVKVTYEDLPA
>d1fo4a3 d.41.1.1 (A:537-694) Xanthine oxidase, domain 5 (?) {Cow (Bos taurus)}
KLDPTYTSATLLFQKHPPANIQLFQEVPNGQSKEDTVGRPLPHLAAAMQASGEAVYCDDIPR
YENELFLRLVTSTRAHAKIKSIDVSEAQKVPGFVCFLSADDIPGSNETGLFNDETVFAKDTV
TCVGHIIGAVVADTPEHAERAAHVVKVTYEDLPA
>d1jrob1 d.41.1.1 (B:2-123) Xanthine dehydrogenase chain B, N-terminal domain {Rhodobacter capsulatus}
SVGKPLPHDSARAHVTGQARYLDDLPCPANTLHLAFGLSTEASAAITGLDLEPVRESPGVIA
VFTAADLPHDNDASPAPSPEPVLATGEVHFVGQPIFLVAATSHRAARIAARKARITYAPR
>d1qj2b1 d.41.1.1 (B:10-146) Carbon monoxide (CO) dehydrogenase molybdoprotein, N-domain {Pseudomonas carboxydovorans}
TSAERAEKLQGMGCKRKRVEDIRFTQGKGNYVDDVKLPGMLFGDFVRSSHAHARIKSIDTSK
AKALPGVFAVLTAADLKPLNLHYMPTLAGDVQAVLADEKVLFQNQEVAFVVAKDRYVAADAI
ELVEVDYEPLPVL
>d1ffvb1 d.41.1.1 (B:7-146) Carbon monoxide (CO) dehydrogenase molybdoprotein, N-domain {Hydrogenophaga pseudoflava}
DAEARELALAGMGASRLRKEDARFIQGKGNYVDDIKMPGMLHMDIVRAPIAHGRIKKIHKDA

ALAMPGVHAVLTAEDLKPLKLHWMPTLAGDVAAVLADEKVHFQMQEVAIVIADDRYIAADAV
EAVKVEYDELPVVIDP
>d1qapa2 d.41.2.1 (A:8-129) Quinolinic acid phosphoribosyltransferase, N-terminal domain {Salmonella typhimurium}
DDRRDALLERINLDIPAAVAQALREDLGGEVDAGNDITAQLLPADTQAHATVITREDGVFCG
KRWVEEVFIQLAGDDVRLTWHVDDGDAIHANQTVFELQGPARVLLTGERTALNFVQTLSG
>d1qpoa2 d.41.2.1 (A:2-116) Quinolinic acid phosphoribosyltransferase, N-terminal domain {Mycobacterium tuberculosis}
GLSDWELAAARAAIARGLDEDLRYGPDVTTLATVPASATTTASLVTREAGVVAGLDVALLTL
NEVLGTNGYRVLDRVEDGARVPPGEALMTLEAQTRGLLTAERTMLNLVGHLSG
>d2tpt_3 d.41.3.1 (336-440) Thymidine phosphorylase {Escherichia coli}
TAMLTKAVYADTEGFVSEMDTRALGMAVVAMGGGRRQASDTIDYSVGFTDMARLGDQVDGQR
PLAVIHAKDENNWQEAAKAVKAAIKLADKAPESTPTVYRRISE
>d1brwa3 d.41.3.1 (A:331-433) Pyrimidine nucleoside phosphorylase {Bacillus stearothermophilus}
KAAYTSTVTAAADGYVAEMAADDIGTAAMWLGAGRAKKEDVIDLAVGIVLHKKIGDRVQKGE
ALATIHSNRPDVLDVKEKIEAAIRLSPQPVARPPLIYETIV
>d1ffkf_ d.41.4.1 (F:) Ribosomal protein L10e {Archaeon Haloarcula marismortui}
KPGAHFRNSIKPAYTRREYISGIPGKGIAQFKMGNNGAGPTYPAQVENVVEKPVQIRHNALE
AARNAANRFVQNSGAAANYKFRIRKFPFHVIREQDGDGMRAPFGKSVGTAARSHGANHDFIA
WVNPDPAVEFAWRRAYMKVTPTVNIDSSPAGNA
>d1jj2h_ d.41.4.1 (H:) Ribosomal protein L10e {Archaeon Haloarcula marismortui}
KPGAMYRNSSKPAYTRREYISGIPGKKIAQFDMGNNGAGPTYPAQVELVVEKPVQIRHNALE
AARVAANRYVQNSGAAANYKFRIRKFPFHVIRENKAAAAAAAAAADGMRAPFGKPVGTAAR
VHGANHIFIAWVNPDPNVEEAWRRAKMKVTPTINIDSSPAGNA
>d1fm0e_ d.41.5.1 (E:) Molybdopterin synthase subunit MoaE {Escherichia coli}
AETKIVVGPQPFSVGEEYPWLAERDEDGAVVTFTGKVRNHNLGDSVNALTLEHYPGMTEKAL
AEIVDEARNRWPLGRVTVIHRIGELWPGDEIVFVGVTSAHRSSAFEAGQFIMDYLKTRAPFW
KREATPEGDRWVEARESDQQAAKRW
>d1buoa_ d.42.1.1 (A:) Promyelocytic leukemia zinc finger (PLZF) protein BTB domain {Human (Homo sapiens)}
MGMIQLQNPSHPTGLLCKANQMRLAGTLCDVVIMVDSQEFHAHRTVLACTSKMFEILFHRNS
QHYTLDFLSPKTFQQILEYAYTATLQAKAEDLDDLLYAAEILEIEYLEEQCLKMLETIQ
>d1vcbb_ d.42.1.1 (B:) Elongin C {Human (Homo sapiens)}
MYVKLISSDGHEFIVKREHALTSGTIKAMLSGPGQFAENETNEVNFREIPSHVLSKVCMYFT
YKVRYTNSSTEIPEFPIAPEIALELLMAANFLDC
>d1hv2a_ d.42.1.1 (A:) Elongin C {Baker's yeast (Saccharomyces cerevisiae)}

MSQDFVTLVSKDDKEYEISRSAAMISPTLKAMIEGPFRESKGRIELKQFDSHILEKAVEYLN
YNLKYSGVSEDDDEIPEFEIPTEMSLELLLAADYLSI
>d1a68__ d.42.1.2 (-) Shaker potassium channel {California sea hare (Aplysia californica)}
ERVVINVSGLRFETQLKTLNQFPDTLLGNPQKRNRYYDPLRNEYFFDRNRPSFDAILYFYQS
GGRLRRPVNVPLDVFSEEIKFYELG
>d1t1da_ d.42.1.2 (A:) Shaker potassium channel {California sea hare (Aplysia californica)}
ERVVINVSGLRFETQLKTLNQFPDTLLGNPQKRNRYYDPLRNEYFFDRNRPSFDAILYFYQS
GGRLRRPVNVPLDVFSEEIKFYELGENAFERYREDEGF
>d3kvt__ d.42.1.2 (-) akv3.1 voltage-gated potassium channel {California sea hare (Aplysia californica)}
ENRVIINVGGIRHETYKATLKKIPATRLSRLTEGMLNYDPVLNEYFFDRHPGVFAQIINYYR
SGKLHYPTDVCGPLFEEELEFWGLDSNQVEPCCWMTYTAHR
>d1exbe_ d.42.1.2 (E:) KV1.1 {Rat (Rattus norvegicus)}
CERVVINISGLRFETQLKTLAQFPNTLLGNPKKRMRYFDPLRNEYFFDRNRPSFDAILYYYQ
SGGRLRRPVNVPLDMFSEEIKFYELGEEA
>d1dsxa_ d.42.1.2 (A:) Kv1.2 {Rat (Rattus norvegicus)}
ERVVINISGLRFEVQLKTLAQFPETLLGDPKKRMRYFDPLRNEYFFDRNRPSFDAILYYYQS
GGRLRRPVNVPLDIFSEEIRFYELG
>d1qdva_ d.42.1.2 (A:) Kv1.2 {Rat (Rattus norvegicus)}
ERVVINISGLRFETQLKTLAQFPETLLGDPKKRMRYFDPLRNEYFFDRNRPSFDAILYYYQS
GGRLRRPVNVPLDIFSEEIRFYELGEEAMEMFREDEG
>d1fs1b2 d.42.1.2 (B:2-68) Cyclin A/CDK2-associated p45, Skp1 {Human (Homo sapiens)}
PSIKLQSSDGEIFEVDVEIAKQSVTIKTMLEDLGMDPVPLPNVNAAILKKVIQWCTHHKDD
>d1efub2 d.43.1.1 (B:140-282) Elongation factor Ts (EF-Ts), dimerisation domain {Escherichia coli}
DVLGSYQHGARIGVLVAAKGADEELVKHIAMHVAASKPEFIKPEDVSAEVVEKEYQVQLDIA
MQSGKPKEIAEKMVEGRMKKFTGEVSLTGQPFVMEPSKTVGQLLKEHNAEVTGFIRFEVGEG
IEKVETDFAAEVAAMSKQS
>d1efub4 d.43.1.1 (B:55-139) Elongation factor Ts (EF-Ts), dimerisation domain {Escherichia coli}
VAADGVIKTKIDGNYGIILEVNCQTDFVAKDAGFQAFADKVLDAAVAGKITDVEVLKAQFEE
ERVALVAKIGENINIRRVAALEG
>d1tfe__ d.43.1.1 (-) Elongation factor Ts (EF-Ts), dimerisation domain {Thermus thermophilus}
AREGIIGHYIHHNQRVGVLVELNCETDFVARNELFQNLAKDLAMHIAMMNPRYVSAEEIPAE
ELEKERQIYIQAALNEGKPQQIAEKIAEGRLKKYLEEVVLLEQPFVKDDKVKVKELIQQAIA
KIGENIVVRRFCRFELGA
>d1i0ha2 d.44.1.1 (A:91-205) Mn superoxide dismutase (MnSOD) {Escherichia coli}
GTTLQGDLKAAIERDFGSVDNFKAEFEKAAASRFGSGWAWLVLKGDKLAVVSTANQDSPLMG
EAISGASGFPIMGLDVWEHAYFLKFQNRRPDYIKEFWNVVNWDEAAARFAAKK

>d1mnga2 d.44.1.1 (A:93-203) Mn superoxide dismutase (MnSOD) {Thermus thermophilus}
GGAKEPVGELKKAIDEQFGGFQALKEKLTQAAMGRFGSGWAWLVKDPFGKLHVLSTPNQDNP
VMEGFTPIVGIDVWEHAYYLKYQNRRADYLQAIWNVLNWDVAEEFFKKA
>d1ap6a2 d.44.1.1 (A:84-198) Mn superoxide dismutase (MnSOD) {Human (Homo sapiens)}
NGGGEPKGELLEAIKRDFGSFDKFKEKLTAASVGVQGSGWGWLGFNKERGHLQIAACPNQDP
LQGTTGLIPLLGIDVWEHAYYLQYKNVRPDYLKAIWNVINWENVTERYMACKK
>d1kkca2 d.44.1.1 (A:98-213) Mn superoxide dismutase (MnSOD) {Aspergillus fumigatus}
EKSGGGKIDQAPVLKAAIEQRWGSFDKFKDAFNTTLLGIQGSGWGWLVTDGPKGKLDITTTH
DQDPVTGAAPVFGVDMWEHAYYLQYLNDKASYAKGIWNVINWAEAENRYIAGDK
>d1dt0a2 d.44.1.1 (A:84-197) Fe superoxide dismutase (FeSOD) {Pseudomonas ovalis}
AGGQPTGALADAINAAFGSFDKFKEEFTKTSVGTFGSGWGWLVKKADGSLALASTIGAGCPL
TIGDTPLLTCDVWEHAYYIDYRNLRPKYVEAFWNLVNWAFVAEQFEGKTYKV
>d3sdpa2 d.44.1.1 (A:84-190) Fe superoxide dismutase (FeSOD) {Pseudomonas ovalis}
DAGGQPTGALADAINAAFGSFDKFKEEFTKTSVGTFGSGWAWLVKADGSLALCSTIGAGAPL
TSGDTPLLTCDVWEHAYYIDYRNLRPKYVEAFWNLVNWAFVAEEG
>d1isaa2 d.44.1.1 (A:83-192) Fe superoxide dismutase (FeSOD) {Escherichia coli}
NAGGEPTGKVAEAIAASFGSFADFKAQFTDAAIKNFGSGWTWLVKNSDGKLAIVSTSNAGTP
LTTDATPLLTVDVWEHAYYIDYRNARPGYLEHFWALVNWEFVAKNLAA
>d1idsa2 d.44.1.1 (A:86-199) Fe superoxide dismutase (FeSOD) {Mycobacterium tuberculosis}
NGGDKPTGELAAAIADAFGSFDKFRAQFHAAATTVQGSGWAALGWDTLGNKLLIFQVYDHQT
NFPLGIVPLLLLDMWEHAFYLQYKNVKVDFAKAFWNVVNWADVQSRYAAATS
>d1coja2 d.44.1.1 (A:91-212) Fe superoxide dismutase (FeSOD) {Aquifex pyrophilus}
GGKGEPSEALKKKIEEDIGGLDACTNELKAAAMAFRGWAILGLDIFSGRLVVNGLDAHNVYN
LTGLIPLIVIDTYEHAYYVDYKNKRPPYIDAFFKNINWDVVNERFEKAMKAYEALKDFIK
>d1sssa2 d.44.1.1 (A:93-208) Fe superoxide dismutase (FeSOD) {Archaeon Sulfolobus solfataricus}
PSGKGGGKPGGALADLINKQYGSFDRFKQVFTETANSLPGTGWAVLYYDTESGNLQIMTFEN
HFQNHIAEIPIILILDEFEHAYYLQYKNKRADYVNAWWNVVNWDAAEKKLQKYL
>d1b06a2 d.44.1.1 (A:93-210) Fe superoxide dismutase (FeSOD) {Archaeon Sulfolobus acidocaldarius}
PAGKGGGKPGGALADLIDKQYGSFDRFKQVFSESANSLPGSGWTVLYYDNESGNLQIMTVEN
HFMNHIAELPVILIVDEFEHAYYLQYKNKRGDYLNAWWNVVNWDDAEKRLQKYLNK
>d1bsma2 d.44.1.1 (A:87-201) Cambialistic superoxide dismutase {Propionibacterium shermanii}
SAPERPTDELGAAIDEFFGSFDNMKAQFTAAATGIQGSGWASLVWDPLGKRINTLQFYDHQN
NLPAGSIPLLQLDMWEHAFYLQYKNVKGDYVKSWWNVVNWDDVALRFSEARVA

>d1qnna2 d.44.1.1 (A:85-191) Cambialistic superoxide dismutase {Porphyromonas gingivalis}
KGGAPKGKLGEAIDKQFGSFEKFKEEFNTAGTTLFGSGWVWLASDANGKLSIEKEPNAGNPV
RKGLNPLLGFDVWEHAYYLTYQNRRADHLKDLWSIVDWDIVESRY
>d1ctf__ d.45.1.1 (-) Ribosomal protein L7/12, C-terminal domain {Escherichia coli}
EFDVILKAAGANKVAVIKAVRGATGLGLKEAKDLVESAPAALKEGVSKDDAEALKKALEEAG
AEVEVK
>d1dd3a2 d.45.1.1 (A:58-128) Ribosomal protein L7/12, C-terminal domain {Thermotoga maritima}
EFDVVLKSFGQNKIQVIKVVREITGLGLKEAKDLVEKAGSPDAVIKSGVSKEEAEEIKKKLE
EAGAEVELK
>d1ekta_ d.46.1.1 (A:) Transcription-state regulator AbrB, the N-terminal DNA recognition domain {Bacillus subtilis}
MKSTGIVRKVDELGRVVIPIELRRTLGIAEKDALEIYVDDEKIILKKYKPNMT
>d1mmsa2 d.47.1.1 (A:8-70) Ribosomal protein L11, N-terminal domain {Thermotoga maritima}
QIKLQLPAGKATPAPPVGPALGQHGVNIMEFCKRFNAETADKAGMILPVVITVYEDKSFTFI
I
>d2reb_2 d.48.1.1 (269-328) RecA protein, C-terminal domain {Escherichia coli}
NFYGELVDLGVKEKLIEKAGAWYSYKGEKIGQGKANATAWLKDNPETAKEIEKKVRELLL
>d1g19a2 d.48.1.1 (A:270-329) RecA protein, C-terminal domain {Mycobacterium tuberculosis}
SREGSLIDMGVDQGLIRKSGAWFTYEGEQLGQGKENARNFLVENADVADEIEKKIKEKLG
>d1e8oa_ d.49.1.1 (A:) Signal recognition particle alu RNA binding heterodimer, SRP9/14 {Human (Homo sapiens)}
PQYQTWEEFSRAAEKLYLADPMKARVVLKYRHSDGNLCVKVTDDLVCLVYKTDQAQDVKKIE
KFHSQLMRLMVA
>d1e8ob_ d.49.1.1 (B:) Signal recognition particle alu RNA binding heterodimer, SRP9/14 {Human (Homo sapiens)}
VLLESEQFLTELTRLFQKCRTSGSVYITLKKYDGRTKPIPKKGTVEGFEPADNKCLLRATDG
KKKISTVVSSKEVNKFQMAYSNLLRANMDGLK
>d1914__ d.49.1.1 (-) Signal recognition particle alu RNA binding heterodimer, SRP9/14 {Mouse (Mus musculus)}
MVLLESEQFLTELTRLFQKCRSSGSVFITLKKYDGRTKPIPRKSSVEGLEPAENKCLLRATD
GKRKISTVVSSKEVNKFQMAYSNLLRANMDGLKKRDKKNKSKKSKPAQGGEQKLISEEDDSA
GSPMPQFQTWEEFSRAAEKLYLADPMKVRVVLKYRHVDGNLCIKVTDDLVCLVYRTDQAQDV
KKIEKFHSQLMRLMVAKESRNV
>d1di2a_ d.50.1.1 (A:) Double-stranded RNA-binding protein A, second dsRBD {Xenopus laevis}
MPVGSLQELAVQKGWRLPEYTVAQESGPPHKREFTITCRVETFVETGSGTSKQVAKRVAAEK
LLTKFKT
>d1ekza_ d.50.1.1 (A:) Staufen, domain III {Fruit fly (Drosophila

melanogaster)}
MDEGDKKSPISQVHEIGIKRNMTVHFKVLREEGPAHMKNFITACIVGSIVTEGEGNGKKVSK
KRAAEKMLVELQKL
>d1stu__ d.50.1.1 (-) Staufen, domain III {Fruit fly (Drosophila
melanogaster)}
PISQVHEIGIKRNMTVHFKVLREEGPAHMKNFITACIVGSIVTEGEGNGKKVSKKRAAEKML
VELQKL
>d1qu6a1 d.50.1.1 (A:1-90) dsRNA-dependent protein kinase pkr
{Human (Homo sapiens)}
GSHMEMAGDLSAGFFMEELNTYRQKQGVVLKYQELPNSGPPHDRRFTFQVIIDGREFPEGEG
RSKKEAKNAAAKLAVEILNKEKKAVSPL
>d1qu6a2 d.50.1.1 (A:91-179) dsRNA-dependent protein kinase pkr
{Human (Homo sapiens)}
LLTTTNSSEGLSMGNYIGLINRIAQKKRLTVNYEQCASGVHGPEGFHYKCKMGQKEYSIGTG
STKQEAKQLAAKLAYLQILSEETGSGC
>d1pkp_2 d.50.1.2 (4-77) Ribosomal S5 protein, N-terminal domain
{Bacillus stearothermophilus}
INPNKLELEERVVAVNRVAKVVKGGRRLRFSALVVVGDKNGHVGFGTGKAQEVPEAIRKAIE
DAKKNLIEVPIV
>d1fjge2 d.50.1.2 (E:5-73) Ribosomal S5 protein, N-terminal domain
{Thermus thermophilus}
DFEEKMILIRRTARMQAGGRRFRFGALVVVGDRQGRVGLGFGKAPEVPLAVQKAGYYARRNM
VEVPLQN
>d1ah5_2 d.50.2.1 (220-313) Porphobilinogen deaminase
(hydroxymethylbilane synthase), C-terminal domain {Escherichia
coli}
NHHETALRVTAERAMNTRLEGGCQVPIGSYAELIDGEIWLRALVGAPDGSQIIRGERRGAPQ
DAEQMGISLAEELLNNGAREILAEVYNGDAPA
>d1pda_2 d.50.2.1 (220-307) Porphobilinogen deaminase
(hydroxymethylbilane synthase), C-terminal domain {Escherichia
coli}
NHHETALRVTAERAMNTRLEGACQVPIGSYAELIDGEIWLRGLVGAPDGSQIIRGERRGAPQ
DAEQMGISLAEELLNNGAREILAEVY
>d1dq3a2 d.50.3.1 (A:336-414) PI-Pfui intein middle domain
{Archaeon Pyrococcus furiosus}
GNFGLPLNFNAFKEWASEYGVEFKTNGSQTIAIINDERISLGQWHTRNRVSKAVLVKMLRKL
YEATKDEEVKRMLHLIE
>d1jida_ d.201.1.1 (A:) SRP19 {Human (Homo sapiens)}
AARSPADQDRFICIYPAYLNNKKTIAEGRRIPISKAVENPTATEIQDVCSAVGLNVFLEKNK
MYSREWNRDVQYRGRVRVQLKQEDGSLCLVQFPSRKSVMLYAAEMIPKLKTR
>d1dt4a_ d.51.1.1 (A:) Neuro-oncological ventral antigen 1, nova-1,
KH3 {Human (Homo sapiens)}
MKDVVEIAVPENLVGAILGKGGKTLVEYQELTGCRIQISKKGEFLPGTRNRKVTITGTPAAT
QAAQYLITQRI

>d1dtja_ d.51.1.1 (A:) Neuro-oncological ventral antigen 2, nova-2, KH3 {Human (Homo sapiens)}
MKELVEMAVPENLVGAILGKGGKTLVEYQELTGARIQISKKGEFLPGTRNRRVTITGSPAAT
QAAQYLISQRVT
>d1ec6a_ d.51.1.1 (A:) Neuro-oncological ventral antigen 2, nova-2, KH3 {Human (Homo sapiens)}
MKELVEIAVPENLVGAILGKGGKTLVEYQELTGARIQISKKGEFLPGTRNRRVTITGSPAAT
QAAQYLISQRVTYEQGVRASNPQKV
>d1vig__ d.51.1.1 (-) Vigilin, KH6 {Human (Homo sapiens)}
INRMDYVEINIDHKFHRHLIGKSGANINRIKDQYKVSVRIPPDSEKSNLIRIEGDPQGVQQA
KRELLELAS
>d2fmr__ d.51.1.1 (-) Fragile X protein, KH1 {Human (Homo sapiens)}
ASRFHEQFIVREDLMGLAIGTHGANIQQARKVPGVTAIDLDEDTCTFHIYGEDQDAVKKARS
FLE
>d1khma_ d.51.1.1 (A:) HnRNP K, KH3 {Human (Homo sapiens)}
GSPNSYGDLGGPIITTQVTIPKDLARSIIGKGGQRIKQIRHESGASIKIDEPLEGSEDRIIT
ITGTQDQIQNAQYLLQNSVKQYSGKFF
>d1k1ga_ d.51.1.1 (A:) RNA splicing factor 1 {Human (Homo sapiens)}
TRVSDKVMIPQDEYPEINFVGLLIGPRGNTLKNIEKECNAKIMIRGKGSVKEGKVGRKDGQM
LPGEDEPLHALVTANTMENVKKAVEQIRNILKQGIETPEDQNDLRKMQLRELARLNGTLR
>d2proc1 d.52.1.1 (C:18-85) Alpha-lytic protease prodomain {Lysobacter enzymogenes}
IFPTQLPQYLQTEKLARTQAAAIEREFGAQFAGSWIERNEDGSFKLVAATSGARKSSTLGGV
EVRNVR
>d3proc1 d.52.1.1 (C:6-85) Alpha-lytic protease prodomain {Lysobacter enzymogenes}
PQLKFAMQRDLGIFPTQLPQYLQTEKLARTQAAAIEREFGAQFAGSWIERNEDGSFKLVAAT
SGARKSSTLGGVEVRNVR
>d3proc2 d.52.1.1 (C:86-163) Alpha-lytic protease prodomain {Lysobacter enzymogenes}
YSLKQLQSAMEQLDAGANARVKGVSKPLDGVQSWYVDPRSNAVVVKVDDGATDAGVDFVALS
GADSAQVRIESSPGKL
>d1gpma3 d.52.2.1 (A:405-525) GMP synthetase, C-terminal, dimerisation domain {Escherichia coli}
GPGLGVRVLGEVKKEYCDLLRRADAIFIEELRKADLYDKVSQAFTVFLPVRSVGVMGDGRKY
DWVVSLRAVETIDFMTAHWAHLPYDFLGRVSNRIINEVNGISRVVYDISGKPPATIEWE
>d1fjgc1 d.52.3.1 (C:2-106) Ribosomal protein S3 N-terminal domain {Thermus thermophilus}
GNKIHPIGFRLGITRDWESRWYAGKKQYRHLLLEDQRIRGLLEKELYSAGLARVDIERAADN
VAVTVHVAKPGVVIGRGGERIRVLREELAKLTGKNVALNVQEV
>d1egaa2 d.52.3.1 (A:183-295) GTPase Era C-terminal domain {Escherichia coli}
DYITDRSQRFMASEIIREKLMRFLGAELPYSVTVEIERFVSNERGGYDINGLILVEREGQKK
MVIGNKGAKIKTIGIEARKDMQEMFEAPVHLELWVKVKSGWADDERALRSL

>d1hh2p2 d.52.3.1 (P:199-276) Transcription factor NusA, C-terminal domains {Thermotoga maritima}
RVPEFVIGLMKLEIPEVENGIVEIKAIAREPGVRTKVAVASNDPNVDPIGACIGEGGSRIAA
ILKELKGEKLDVLKWS
>d1hh2p3 d.52.3.1 (P:277-344) Transcription factor NusA, C-terminal domains {Thermotoga maritima}
DDPKQLIANALAPATVIEVEILDKENKAARVLVPPTQLSLAIGKGGQNARLAAKLTGWKIDI
KPIMNL
>d1k0ra2 d.52.3.1 (A:184-262) Transcription factor NusA, C-terminal domains {Mycobacterium tuberculosis}
THPNLVRKLFSLEVPEIADGSVEIVAVAREAGHRSKIAVRSNVAGLNAKGACIGPMGQRVRN
VMSELSGEKIDIIDYDD
>d1k0ra3 d.52.3.1 (A:263-329) Transcription factor NusA, C-terminal domains {Mycobacterium tuberculosis}
DPARFVANALSPAKVVSVSVIDQTARAARVVVPDFQLSLAIGKEGQNARLAARLTGWRIDIR
GDAPP
>d1e3ha4 d.52.3.1 (A:579-632) Polynucleotide phosphorylase/guanosine pentaphosphate synthase (PNPase/GPSI), domain 6 {Streptomyces antibioticus}
APRIITVKIPVDKIGEVIGPKRQMINQIQEDTGAEITIEDDGTIYIGAADGPAA
>d1fjgc2 d.53.1.1 (C:107-207) Ribosomal protein S3 C-terminal domain {Thermus thermophilus}
QNPNLSAPLVAQRVAEQIERRFAVRRAIKQAVQRVMESGAKGAKVIVSGRIGGAEQARTEWA
AQGRVPLHTLRANIDYGFALARTTYGVLGVKAYIFLGEV
>d1hh2p4 d.202.1.1 (P:1-126) Transcription factor NusA, N-terminal domain {Thermotoga maritima}
MNIGLLEALDQLEEEKGISKEEVIPILEKALVSAYRKNFGNSKNVEVVIDRNTGNIKVYQLL
EVVEEVEDPATQISLEEAKKIDPLAEVGSIVKKELNVKNFGRIAAQTAKQVLIQRIRELEKE
KQ
>d1k0ra4 d.202.1.1 (A:-4-99) Transcription factor NusA, N-terminal domain {Mycobacterium tuberculosis}
VSRRHMNIDMAALHAIEVDRGISVNELLETIKSALLTAYRHTQGHQTDARIEIDRKTGVVRV
IARETDEAGNLISEWDDTPEGFGRIAATTARQVMLQRFRDAE
>d1onea2 d.54.1.1 (A:1-141) Enolase {Baker's yeast (Saccharomyces cerevisiae)}
AVSKVYARSVYDSRGNPTVEVELTTEKGVFRSIVPSGASTGVHEALEMRDGDKSKWMGKGVL
HAVKNVNDVIAPAFVKANIDVKDQKAVDDFLISLDGTANKSKLGANAILGVSLAASRAAAAE
KNVPLYKHLADLSKSKT
>d1pdz_2 d.54.1.1 (1-139) Enolase {Lobster (Homarus vulgaris)}
SITKVFARTIFDSRGNPTVEVDLYTSKGLFRAAVPSGASTGVHEALEMRDGDKSKYHGKSVF
NAVKNVNDVIVPEIIKSGLKVTQQKECDEFMCKLDGTENKSSLGANAILGVSLAICKAGAAE
LGIPLYRHIANLANY
>d1e9ia2 d.54.1.1 (A:1-139) Enolase {Escherichia coli}
SKIVKIIGREIIDSRGNPTVEAEVHLEGGFVGMAAAPSGASTGSREALELRDGDKSRFLGKG

VTKAVAAVNGPIAQALIGKDAKDQAGIDKIMIDLDGTENKSKFGANAILAVSLANAKAAAAA
KGMPLYEHIAELNGT
>d1bqg_2 d.54.1.1 (12-143) D-glucarate dehydratase {Pseudomonas putida}
GAPVITDLKVVPVAGHDSMLLNLSGAHGPLFTRNILILTDSSGHVGVGEVPGGEGIRKTLED
ARHLLINQSIGNYQSLLNKVRNAFADRDVGGRGLQTFDLRIAVHAVTAVESALLDLLGQHLQ
VPVAALLG
>d1ec7a2 d.54.1.1 (A:5-137) D-glucarate dehydratase {Escherichia coli}
FTTPVVTEMQVIPVAGHDSMLMNLSGAHAPFFTRNIVIIKDNSGHTGVGEIPGGEKIRKTLE
DAIPLVVGKTLGEYKNVLTLVRNTFADRDAGGRGLQTFDLRTTIHVVTGIEAAMLDLLGQHL
GVNVASLLG
>d1fhua2 d.54.1.1 (A:1-99) O-succinylbenzoate synthase {Escherichia coli}
MRSAQVYRWQIPMDAGVVLRDRRLKTRDGLYVCLREGEREGWGEISPLPGFSQETWEEAQSV
LLAWVNNWLAGDCELPQMPSVAFGVSCALAELTDTLP
>d1muca2 d.54.1.1 (A:4-130) Muconate-lactonizing enzyme (cis muconate cycloisomerase) {Pseudomonas putida}
ALIERIDAIIVDLPTIRPHKLAMHTMQQQTLVVLRVRCSDGVEGIGEATTIGGLAYGYESPE
GIKANIDAHLAPALIGLAADNINAAMLKLDKLAKGNTFAKSGIESALLDAQGKRLGLPVSEL
LGG
>d2mnr_2 d.54.1.1 (3-132) Mandelate racemase {Pseudomonas putida}
EVLITGLRTRAVNVPLAYPVHTAVGTVGTAPLVLIDLATSAGVVGHSYLFAYTPVALKSLKQ
LLDDMAAMIVNEPLAPVSLEAMLAKRFCLAGYTGLIRMAAAGIDMAAWDALGKVHETPLVKL
LGANAR
>d2chr_2 d.54.1.1 (1-126) Chlormuconate cycloisomerase {Alcaligenes eutrophus}
MKIDAIEAVIVDVPTKRPIQMSITTVHQQSYVIVRVYSEGLVGVGEGGSVGGPVWSAECAET
IKIIVERYLAPHLLGTDAFNVSGALQTMARAVTGNASAKAAVEMALLDLKARALGVSIAELL
GG
>d1jpdx2 d.54.1.1 (X:-2-113) L-Ala-D/L-Glu epimerase {Escherichia coli}
GSHMRTVKVFEEAWPLHTPFVIARGSRSEARVVVVELEEEGIKGTGECTPYPRYGESDASVM
AQIMSVVPQLEKGLTREELQKILPAGAARNALDCALWDLAARRQQQSLADLIGI
>d1jpma2 d.54.1.1 (A:1-125) L-Ala-D/L-Glu epimerase {Bacillus subtilis}
MKIIRIETSRIAVPLTKPFKTALRTVYTAESVIVRITYDSGAVGWGEAPPTLVITGDSMDSI
ESAIHHVLKPALLGKSLAGYEAILHDIQHLLTGNMSAKAAVEMALYDGWAQMCGLPLYQMLG
G
>d1kcza2 d.54.1.1 (A:1-160) beta-Methylaspartase {Clostridium tetanomorphum}
MKIVDVLCTPGLTGFYFDDQRAIKKGAGHDGFTYTGSTVTEGFTQVRQKGESISVLLVLEDG
QVAHGDCAAVQYSGAGGRDPLFLAKDFIPVIEKEIAPKLIGREITNFKPMAEEFDKMTVNGN
RLHTAIRYGITQAILDAVAKTRKVTMAEVIRDEYNP

>d1kkoa2 d.54.1.1 (A:1-160) beta-Methylaspartase {Citrobacter amalonaticus}
MKIKQALFTAGYSSFYFDDQQAIKNGAGHDGFIYTGDPVTPGFTSVRQAGECVSVQLILENG
AVAVGDCAAVQYSGAGGRDPLFLAEHFIPFLNDHIKPLLEGRDVDAFLPNARFFDKLRIDGN
LLHTAVRYGLSQALLDATALASGRLKTEVVCDEWQL
>d1bxea_ d.55.1.1 (A:) Ribosomal protein L22 {Thermus aquaticus, subsp. Thermus thermophilus}
MEAKAIARYVRISPRKVRLVVDLIRGKSLEEARNILRYTNKRGAYFVAKVLESAAANAVNNH
DMLEDRLYVKAAYVDEGPALKRVLPRARGRADIIKKRTSHITVILGEK
>d1jj2q_ d.55.1.1 (Q:) Ribosomal protein L22 {Archaeon Haloarcula marismortui}
GISYSVEADPDTTAKAMLRERQMSFKHSKAIAREIKGKTAGEAVDYLEAVIEGDQPVPFKQH
NSGVGHKSKVDGWDAGRYPEKASKAFLDLLENAVGNADHQGFDGEAMTIKHVAAHKVGEQQG
RKPRAMGRASAWNSPQVDVELILEEP
>d1gd8a_ d.188.1.1 (A:) Prokaryotic ribosomal protein L17 {Thermus thermophilus}
SSHRLALYRNQAKSLLTHGRITTTVPKAKELRGFVDHLIHLAKRGDLHARRLVLRDLQDVKL
VRKLFDEIAPRYRDRQGGYTRVLKLAERRRGDGAPLALVELVE
>d1oela3 d.56.1.1 (A:137-190,A:367-409) GroEL {Escherichia coli}
PCSDSKAIAQVGTISANSDETVGKLIAEAMDKVGKEGVITVEDGTGLQDELDVVXERVAKLA
GGVAVIKVGAATEVEMKEKKARVEDALHATRAAVEE
>d1ioka3 d.56.1.1 (A:137-190,A:367-409) GroEL {Paracoccus denitrificans}
PVNDSSEVAQVGTISANGESFIGQQIAEAMQRVGNEGVITVEENKGMETEVEVVXERVAKLA
GGVAVIRVGGMTEIEVKERKDRVDDALNATRAAVQE
>d1a6da3 d.56.1.2 (A:146-214,A:368-403) Thermosome {Archaeon Thermoplasma acidophilum}
TDDATLRKIALTALSGKNTGLSNDFLADLVVKAVNAVAEVRDGKTIVDTANIKVDKKNGGSV
NDTQFISXAVSILIRGGTDHVVSEVERALNDAIRVVAITKEDGK
>d1a6db3 d.56.1.2 (B:145-215,B:368-403) Thermosome {Archaeon Thermoplasma acidophilum}
GADEKALLLKMAQTSLNSKSASVAKDKLAEISYEAVKSVAELRDGKYYVDFDNIQVVKKQGG
AIDDTQLINXKAVSILVRGETEHVVDEMERSITDSLHVVASALEDG
>d1h6ha_ d.189.1.1 (A:) p40phox NADPH oxidase {Human (Homo sapiens)}
AVAQQLRAESDFEQLPDDVAISANIADIEEKRGFTSHFVFVIEVKTKGGSKYLIYRRYRQFH
ALQSKLEERFGPDSKSSALACTLPTLPAKVYVGVKQEIAEMRIPALNAYMKSLLSLPVWVLM
DEDVRIFFYQSPYDSEQVP
>d1gd5a_ d.189.1.1 (A:) p47phox NADPH oxidase {Human (Homo sapiens)}
GSMGDTFIRHIALLGFEKRFVPSQHYVYMFLVKWQDLSEKVVYRRFTEIYEFHKTLKEMFPI
EAGAINPENRIIPHLPAPKWFDGQRAAENRQGTLTEYCSTLMSLPTKISRCPHLLDFFKVRP
DDLKLP
>d1ji8a_ d.203.1.1 (A:) DsrC, the gamma subunit of dissimilatory

sulfite reductase {Archaeon Pyrobaculum aerophilum}
MPVKCPGEYQVDGKKVILDEDCFMQNPEDWDEKVAEWLARELEGIQKMTEEHWKLVKYLREY
WETFGTCPPIKMVTKETGFSLEKIYQLFPSGPAHGACKVAGAPKPTGCV
>d1ghha_ d.57.1.1 (A:) DNA damage-inducible protein DinI
{Escherichia coli}
MRIEVTIAKTSPLPAGAIDALAGELSRRIQYAFPDNEGHVSVRYAAANNLSVIGATKEDKQR
ISEILQETWESADDWFVSE
>d1fxd__ d.58.1.1 (-) Ferredoxin II {Desulfovibrio gigas}
PIEVNDDCMACEACVEICPDVFEMNEEGDKAVVINPDSDLDCVEEAIDSCPAEAIVRS
>d1dura_ d.58.1.1 (A:) Ferredoxin II {Peptostreptococcus
asaccharolyticus}
AYVINDSCIACGACKPECPVNCIQEGSIYAIDADSCIDCGSCASVCPVGAPNPED
>d1fca__ d.58.1.1 (-) Ferredoxin II {Clostridium acidi-urici}
AYVINEACISCGACEPECPVDAISQGGSRYVIDADTCIDCGACAGVCPVDAPVQA
>d2fdn__ d.58.1.1 (-) Ferredoxin II {Clostridium acidi-urici}
AYVINEACISCGACEPECPVNAISSGDDRYVIDADTCIDCGACAGVCPVDAPVQA
>d1clf__ d.58.1.1 (-) Ferredoxin II {Clostridium pasteurianum}
AYKIADSCVSCGACASECPVNAISQGDSIFVIDADTCIDCGNCANVCPVGAPVQE
>d1blu__ d.58.1.1 (-) Ferredoxin II {Chromatium vinosum}
ALMITDECINCDVCEPECPNGAISQGDETYVIEPSLCTECVGHYETSQCVEVCPVDCIIKDP
SHEETEDELRAKYERITG
>d7fd1a_ d.58.1.2 (A:) Ferredoxin {Azotobacter vinelandii}
AFVVTDNCIKCKYTDCVEVCPVDCFYEGPNFLVIHPDECIDCALCEPECPAQAIFSEDEVPE
DMQEFIQLNAELAEVWPNITEKKDPLPDAEDWDGVKGKLQHLER
>d1bc6__ d.58.1.2 (-) Ferredoxin {Bacillus schlegelii}
AYVITEPCIGTKDASCVEVCPVDCIHEGEDQYYIDPDVCIDCGACEAVCPVSAIYHEDFVPE
EWKSYIQKNRDFFKK
>d1h98a_ d.58.1.2 (A:) Ferredoxin {Thermus thermophilus}
PHVICEPCIGVKDQSCVEVCPVECIYDGGDQFYIHPEECIDCGACVPACPVNAIYPEEDVPE
QWKSYIEKNRKLAGL
>d1xer__ d.58.1.3 (-) Ferredoxin {Archaeon Sulfolobus sp.}
GIDPNYRTNRQVVGEHSGHKVYGPVEPPKVLGIHGTIVGVDFDLCIADGSCINACPVNVFQW
YDTPGHPASEKKADPVNEQACIFCMACVNVCPVAAIDVKPP
>d1vjw__ d.58.1.4 (-) Ferredoxin {Thermotoga maritima}
MKVRVDADACIGCGVCENLCPDVFQLGDDGKAKVLQPETDLPCAKDAADSCPTGAISVE
>d1fxra_ d.58.1.4 (A:) Ferredoxin I {Sulfate-reducing bacteria
(Desulfovibrio africanus)}
ARKFYVDQDECIACESCVEIAPGAFAMDPEIEKAYVKDVEGASQEEVEEAMDTCPVQCIHWE
DE
>d1iqza_ d.58.1.4 (A:) Ferredoxin {Bacillus thermoproteolyticus}
PKYTIVDKETCIACGACGAAAPDIYDYDEDGIAYVTLDDNQGIVEVPDILIDDMMDAFEGCP
TDSIKVADEPFDGDPNKFE
>d1jb0c_ d.58.1.4 (C:) Photosystem I iron-sulfur protein PsaC
{Synechococcus elongatus}

AHTVKIYDTCIGCTQCVRACPTDVLEMVPWDGCKAGQIASSPRTEDCVGCKRCETACPTDFL
SIRVYLGAETTRSMGLAY
>d1feha3 d.58.1.5 (A:127-209) Fe-only hydrogenase, second domain {Clostridium pasteurianum}
KDKTEYVDERSKSLTVDRTKCLLCGRCVNACGKNTETYAMKFLNKNGKTIIGAEDEKCFDDT
NCLLCGQCIIACPVAALSEKS
>d1hfel2 d.58.1.5 (L:2-86) Fe-only hydrogenase larger subunit, N-domain {Desulfovibrio desulfuricans}
SRTVMERIEYEMHTPDPKADPDKLHFVQIDEAKCIGCDTCSQYCPTAAIFGEMGEPHSIPHI
EACINCGQCLTHCPENAIYEAQS
>d1keka5 d.58.1.5 (A:669-785) Pyruvate-ferredoxin oxidoreductase, PFOR, domain V {Desulfovibrio africanus}
TSQFEKRGVAINVPQWVPENCIQCNQCAFVCPHSAILPVLAKEEELVGAPANFTALEAKGKE
LKGYKFRIQINTLDCMGCGNCADICPPKEKALVMQPLDTQRDAQVPNLEYAARIP
>d1h7wa5 d.58.1.5 (A:845-1017) Dihydropyrimidine dehydrogenase, C-terminal domain {Pig (Sus scrofa)}
ELQGWDGQSPGTESHQKGKPVPRIAELMGKKLPNFGPYLEQRKKIIAEEKMRLKEQNAAFPP
LERKPFIPKKPIPAIKDVIGKALQYLGTFGELSNIEQVVAVIDEEMCINCGKCYMTCNDSGY
QAIQFDPETHLPTVTDTCTGCTLCLSVCPIIDCIRMVSRTTPYEPKRGL
>d1jnrb_ d.58.1.5 (B:) Adenylylsulfate reductase B subunit {Archaeon Archaeoglobus fulgidus}
PSFVNPEKCDGCKALERTACEYICPNDLMTLDKEKMKAYNREPDMCWECYSCVKMCPQGAID
VRGYVDYSPLGGACVPMRGTSDIMWTVKYRNGKVLRFKFAIRTTPWGSIQPFEGFPEPTEEA
LKSELLAGEPEIIGTSEFPQVKKKA
>d1d09b1 d.58.2.1 (B:1-100) Aspartate carbamoyltransferase {Escherichia coli}
MTHDNKLQVEAIKRGTVIDHIPAQIGFKLLSLFKLTETDQRITIGLNLPSGEMGRKDLIKIE
NTFLSEDQVDQLALYAPQATVNRIDNYEVVGKSRPSLP
>d2atcb1 d.58.2.1 (B:1-100) Aspartate carbamoyltransferase {Escherichia coli}
MTHNDKLQVAEIKRGTVINHIPAEIGFKLLSLFKLTETQDRITIGLNLPSGEMGRKDLIKIE
NTFLSEDEVDELALYAPQATVNRINDYEVVGKSRPSLP
>d1pca_2 d.58.3.1 (4A-99A) Procarboxypeptidase A {Pig (Sus scrofa)}
KEDFVGHQVLRISVDDEAQVQKVKELEDLEHLQLDFWRGPARPGFPIDVRVPFPSIQAVKVF
LEAHGIRYTIMIEDVQLLLDEEQEQMFASQGR
>d1pyta_ d.58.3.1 (A:) Procarboxypeptidase A {Cow (Bos taurus)}
KEDFVGHQVLRITAADEAEVQTVKELEDLEHLQLDFWRGPGQPGSPIDVRVPFPSLQAVKVF
LEAHGIRYRIMIEDVQSLLDEEQEQMFASQSR
>d1aye_2 d.58.3.1 (4A-99A) Procarboxypeptidase A {Human (Homo sapiens)}
LETFVGDQVLEIVPSNEEQIKNLLQLEAQEHLQLDFWKSPTTPGETAHVRVPFVNVQAVKVF
LESQGIAYSIMIEDVQVLLDKENEEMLFNRRR
>d1nsa_2 d.58.3.1 (7A-95A) Procarboxypeptidase B {Pig (Sus

scrofa)}
FEGEKVFRVNVEDENDISELHELASTRQIDFWKPDSVTQIKPHSTVDFRVKAEDILAVEDFL
EQNELQYEVLINNLRSVLEAQFDSVSR
>d1pba__ d.58.3.1 (-) Procarboxypeptidase B {Pig (Sus scrofa)}
HHSGEHFEGEKVFRVNVEDENDISELHELASTRQIDFWKPDSVTQIKPHSTVDFRVKAEDIL
AVEDFLEQNELQYEVLINN
>d1spbp_ d.58.3.2 (P:) Subtilisin prosegment {Bacillus amyloliquefaciens}
EKKYIVGFKQTMSTMSAAKKKDVISEKGGKVQKQFKYVDAASATLNEKAVKELKKDPSVAYV
EEDHVAHAY
>d1scjb_ d.58.3.2 (B:) Subtilisin prosegment {Bacillus subtilis}
EKKYIVGFKQTMSAMSSAKKKDVISQKGGKVEKQFKYVNAAAATLDEKAVKELKKDPSVAYV
EEDHIAHEY
>d1itpa_ d.58.3.2 (A:) Proteinase A inhibitor 1, POIA1 {Oyster mushroom (Pleurotus ostreatus)}
GSAGKFIVIFKNDVSEDKIRETKDEVIAEGGTITNEYNMPGMKGFAGELTPQSLTKFQGLQG
DLIDSIEEDGIVTTQ
>d1mli__ d.58.4.1 (-) Muconalactone isomerase {Pseudomonas putida}
MLFHVKMTVKLPVDMDPAKATQLKADEKELAQRLQREGTWRHLWRIAGHYANYSVFDVPSVE
ALHDTLMQLPLFPYMDIEVDGLCRHPSSIHSDDR
>d2pii__ d.58.5.1 (-) PII (product of glnB) {Escherichia coli}
MKKIDAIIKPFKLDDVREALAEVGITGMTVTEVKGFGRQKGHTELYRGAEYMVDFLPKVKIE
IVVPDDIVDTCVDTIIRTAQTGKIGDGKIFVFDVARVIRIRTGEEDDAAI
>d1gnka_ d.58.5.1 (A:) PII-homolog GlnK {Escherichia coli}
MKLVTVIIKPFKLEDVREALSSIGIQGLTVTEVKGFGRQKGHAELYRGAEYSVNFLPKVKID
VAIADDQLDEVIDIVSKAAYTGKIGDGKIFVAELQRVIRIRTGEADEAAL
>d1nuea_ d.58.6.1 (A:) Nucleoside diphosphate kinases {Human (Homo sapiens)}
ANLERTFIAIKPDGVQRGLVGEIIKRFEQKGFRLVAMKFLRASEEHLKQHYIDLKDRPFFPG
LVKYMNSGPVVAMVWEGLNVVKTGRVMLGETNPADSKPGTIRGDFCIQVGRNIIHGSDSVKS
AEKEISLWFKPEELVDYKSCAHDWVYE
>d1ehwa_ d.58.6.1 (A:) Nucleoside diphosphate kinases {Human (Homo sapiens), NDK4}
HMGTRERTLVAVKPDGVQRRLVGDVIQRFERRGFTLVGMKMLQAPESVLAEHYQDLRRKPFY
PALIRYMSSGPVVAMVWEGYNVVRASRAMIGHTDSAEAAPGTIRGDFSVHISRNVIHASDSV
EGAQREIQLWFQSSELVSW
>d1be4a_ d.58.6.1 (A:) Nucleoside diphosphate kinases {Cow (Bos taurus)}
ANSERTFIAIKPDGVQRGLMGEIIKRFEQKGFRLVAMKFMRASEDLLKEHYIDLKDRPFFAG
LVKYMHSGPVVAMVWEGLNVVKTGRVMLGETNPADSKPGTIRGDFCIQVGRNIIHGSDSVES
AEKEIALWFRPEELVNYKSCAQNWIYE
>d1hlwa_ d.58.6.1 (A:) Nucleoside diphosphate kinases {Dictyostelium discoideum}
VNKERTFLAVKPDGVARGLVGEIIARYEKKGFVLVGLKQLVPTKDLAESHYAEHKERPFFGG

LVSFITSGPVVAMVFEGKGVVASARLMIGVTNPLASAPGSIRGDFGVDVGRNIIAGSDSVES
ANREIALWFKPEELLTEVKPNPNLYE
>d1nsqa_ d.58.6.1 (A:) Nucleoside diphosphate kinases {Drosophila melanogaster}
AANKERTFIMVKPDGVQRGLVGKIIERFEQKGFKLVALKFTWASKELLEKHYADLSARPFFP
GLVNYMNSGPVVPMVWEGLNVVKTGRQMLGATNPADSLPGTIRGDFCIQVGRNIIHGSDAVE
SAEKEIALWFNEKELVTWTPAAKDWIYE
>d1nhkl_ d.58.6.1 (L:) Nucleoside diphosphate kinases {Myxococcus xanthus}
AIERTLSIIKPDGLEKGVIGKIISRFEEKGLKPVAIRLQHLSQAQAEGFYAVHKARPFFKDL
VQFMISGPVVLMVLEGENAVLANRDIMGATNPAQAAEGTIRKDFATSIDKNTVHGSDSLENA
KIEIAYFFRETEIHSYPYQ
>d1ha1_1 d.58.7.1 (8-92) Nuclear ribonucleoprotein A1, RNP A1, UP1 {Human (Homo sapiens)}
EPEQLRKLFIGGLSFETTDESLRSHFEQWGTLTDCVVMRDPNTKRSRGFGFVTYATVEEVDA
AMNARPHKVDGRVVEPKRAVSRE
>d1ha1_2 d.58.7.1 (99-180) Nuclear ribonucleoprotein A1, RNP A1, UP1 {Human (Homo sapiens)}
AHLTVKKIFVGGIKEDTEEHHLRDYFEQYGKIEVIEIMTDRGSGKKRGFAFVTFDDHDSVDK
IVIQKYHTVNGHNCEVRKAL
>d2up1a2 d.58.7.1 (A:99-190) Nuclear ribonucleoprotein A1, RNP A1, UP1 {Human (Homo sapiens)}
GAHLTVKKIFVGGIKEDTEEHHLRDYFEQYGKIEVIEIMTDRGSGKKRGFAFVTFDDHDSVD
KIVIQKYHTVNGHNCEVRKALSKQEMASAS
>d1fht__ d.58.7.1 (-) Splicesomal U1A protein {Human (Homo sapiens)}
AVPETRPNHTIYINNLNEKIKKDELKKSLYAIFSQFGQILDILVSRSLKMRGQAFVIFKEVS
SATNALRSMQGFPFYDKPMRIQYAKTDSDIIAKMKGTFVERDRKREKRKPKSQE
>d1nrca_ d.58.7.1 (A:) Splicesomal U1A protein {Human (Homo sapiens)}
TRPNHTIYINNLNEKIKKDELKKSLYAIFSQFGQILDILVSRSLKMRGQAFVIFKEVSSATN
ALRSMQGFPFYDKPMRICYAKTD
>d1urna_ d.58.7.1 (A:) Splicesomal U1A protein {Human (Homo sapiens)}
AVPETRPNHTIYINNLNEKIKKDELKKSLHAIFSRFGQILDILVSRSLKMRGQAFVIFKEVS
SATNALRSMQGFPFYDKPMRIQYAKTDSDIIAKM
>d2u1a__ d.58.7.1 (-) Splicesomal U1A protein {Human (Homo sapiens)}
MAPAQPLSENPPNHILFLTNLPEETNELMLSMLFNQFPGFKEVRLVPGRHDIAFVEFDNEVQ
AGAARDALQGFKITQNNAMKISFAKK
>d1a9nb_ d.58.7.1 (B:) Splicing factor U2B'' {Human (Homo sapiens)}
IRPNHTIYINNMNDKIKKEELKRSLYALFSQFGHVVDIVALKTMKMRGQAFVIFKELGSSTN
ALRQLQGFPFYGKPMRIQYAKTDSDIISKMRG
>d1u2fa_ d.58.7.1 (A:) Splicing factor U2AF 65 KDa subunit {Human

(Homo sapiens)}
ARRLYVGNIPFGITEEAMMDFFNAQMRLGGLTQAPGNPVLAVQINQDKNFAFLEFRSVDETT
QAMAFDGIIFQGQSLKIRRPHDYQPLPG
>d2u2fa_ d.58.7.1 (A:) Splicing factor U2AF 65 KDa subunit {Human
(Homo sapiens)}
AHKLFIGGLPNYLNDDQVKELLTSFGPLKAFNLVKDSATGLSKGYAFCEYVDINVTDQAIAG
LNGMQLGDKKLLVQRASVGAKNA
>d1b7fa1 d.58.7.1 (A:123-204) Sex-lethal protein {Drosophila
melanogaster}
SNTNLIVNYLPQDMTDRELYALFRAIGPINTCRIMRDYKTGYSYGYAFVDFTSEMDSQRAIK
VLNGITVRNKRLKVSYARPG
>d1b7fa2 d.58.7.1 (A:205-289) Sex-lethal protein {Drosophila
melanogaster}
GESIKDTNLYVTNLPRTITDDQLDTIFGKYGSIVQKNILRDKLTGRPRGVAFVRYNKREEAQ
EAISALNNVIPEGGSQPLSVRLA
>d1sxl__ d.58.7.1 (-) Sex-lethal protein {Drosophila melanogaster}
MSYARPGGESIKDTNLYVTNLPRTITDDQLDTIFGKYGSIVQKNILRDKLTGRPRGVAFVRY
NKREEAQEAISALNNVIPEGGSQPLSVRLAEEHGK
>d1d8za_ d.58.7.1 (A:) Hu antigen C (Huc) {Mouse (Mus musculus)}
MDSKTNLIVNYLPQNMTQDEFKSLFGSIGDIESCKLVRDKITGQSLGYGFVNYSDPNDADKA
INTLNGLKLQTKTIKVSYARPSSASIR
>d1d9aa_ d.58.7.1 (A:) Hu antigen C (Huc) {Mouse (Mus musculus)}
DANLYVSGLPKTMSQKEMEQLFSQYGRIITSRILLDQATGVSRGVGFIRFDKRIEAEEAIKG
LNGQKPLGAAEPITVKFANNPSQ
>d1fxla1 d.58.7.1 (A:37-118) Hu antigen D (Hud) {Human (Homo
sapiens)}
SKTNLIVNYLPQNMTQEEFRSLFGSIGEIESCKLVRDKITGQSLGYGFVNYIDPKDAEKAIN
TLNGLRLQTKTIKVSYARPS
>d1fxla2 d.58.7.1 (A:119-203) Hu antigen D (Hud) {Human (Homo
sapiens)}
SASIRDANLYVSGLPKTMTQKELEQLFSQYGRIITSRILVDQVTGVSRGVGFIRFDKRIEAE
EAIKGLNGQKPSGATEPITVKFA
>d1hd1a_ d.58.7.1 (A:) Heterogeneous nuclear ribonucleoprotein d0
{Human (Homo sapiens)}
KMFIGGLSWDTTKKDLKDYFSKFGEVVDCTLKLDPITGRSRGFGFVLFKESESVDKVMDQKE
HKLNGKVIDPKRA
>d2msta_ d.58.7.1 (A:) Neural RNA-binding protein Musashi-1 {Mouse
(Mus musculus)}
KIFVGGLSVNTTVEDVKHYFEQFGKVDDAMLMFDKTTNRHRGFGFVTFESEDIVEKVCEIHF
HEINNKMVECKKA
>d1cvja1 d.58.7.1 (A:11-90) Poly(A)-binding protein {Human (Homo
sapiens)}
ASLYVGDLHPDVTEAMLYEKFSPAGPILSIRVCRDMITRRSLGYAYVNFQQPADAERALDTM
NFDVIKGKPVRIMWSQRD

>d1cvja2 d.58.7.1 (A:91-179) Poly(A)-binding protein {Human (Homo sapiens)}
PSLRKSGVGNIFIKNLDKSIDNKALYDTFSAFGNILSCKVVCDENGSKGYGFVHFETQEAAE
RAIEKMNGMLLNDRKVFVGRFKSRKER
>d1qm9a1 d.58.7.1 (A:1-110) Polypyrimidine tract-binding protein {Human (Homo sapiens)}
MGNSVLLVSNLNPERVTPQSLFILFGVYGDVQRVKILFNKKENALVQMADGNQAQLAMSHLN
GHKLHGKPIRITLSKHQNVQLPREGQEDQGLTKDYGNSPLHRFKKPGS
>d1qm9a2 d.58.7.1 (A:111-198) Polypyrimidine tract-binding protein {Human (Homo sapiens)}
KNFQNIFPPSATLHLSNIPPSVSEEDLKVLFSSNGGVVKGFKFFQKDRKMALIQMGSVEEAV
QALIDLHNHDLGENHHLRVSFSKSTI
>d1fj7a_ d.58.7.1 (A:) Nucleolin {Golden hamster (Mesocricetus auratus)}
GSHMLEDPVEGSESTTPFNLFIGNLNPNKSVAELKVAISELFAKNDLAVVDVRTGTNRKFGY
VDFESAEDLEKALELTGLKVFGNEIKLEKPKGRDGTRGC
>d1fjca_ d.58.7.1 (A:) Nucleolin {Golden hamster (Mesocricetus auratus)}
SHMLEDPCTSKKVRAARTLLAKNLSFNITEDELKEVFEDALEIRLVSQDGKSKGIAYIEFKS
EADAEKNLEEKQGAEIDGRSVSLYYTGEKGGTRG
>d1fjeb1 d.58.7.1 (B:1-91) Nucleolin {Golden hamster (Mesocricetus auratus)}
GSHMVEGSESTTPFNLFIGNLNPNKSVAELKVAISELFAKNDLAVVDVRTGTNRKFGYVDFE
SAEDLEKALELTGLKVFGNEIKLEKPKGR
>d1fjeb2 d.58.7.1 (B:92-175) Nucleolin {Golden hamster (Mesocricetus auratus)}
DSKKVRAARTLLAKNLSFNITEDELKEVFEDALEIRLVSQDGKSKGIAYIEFKSEADAEKNL
EEKQGAEIDGRSVSLYYTGEKG
>d1h6kx_ d.58.7.1 (X:) CBP20, 20KDa nuclear cap-binding protein {Human (Homo sapiens)}
KSCTLYVGNLSFYTTEEQIYELFSKSGDIKKIIMGLDKMTACGFCFVEYYSRADAENAMRYI
NGTRLDDRIIRTDWDAG
>d1fo1a2 d.58.7.2 (A:123-191) mRNA export factor tap {Human (Homo sapiens)}
TIPYGRKYDKAWLLSMIQSKCSVPFTPIEFHYENTRAQFFVEDASTASALKAVNYKILDREN
RRISIII
>d1ft8a2 d.58.7.2 (A:118-199) mRNA export factor tap {Human (Homo sapiens)}
NWFKITIPYGRKYDKAWLLSMIQSKCSVPFTPIEFHYENTRAQFFVEDASTASALKAVNYKI
LDRENRRISIIINSSAPPHT
>d1ft8e1 d.58.7.2 (E:) mRNA export factor tap {Human (Homo sapiens)}
WFKITIPYGRKYDKAWLLSMIQSKCSVPFTPIEFHYENTRAQFFVEDASTASALKAV
>d1koha2 d.58.7.2 (A:105-200) mRNA export factor tap {Human (Homo sapiens)}

RGGAGTSQDGTSKNWFKITIPYGRKYDKAWLLSMIQSKSSVPFTPIEFHYENTRAQFFVEDA
STASALKAVNYKILDRENRRISIIINSSAPPHTI
>d1jmta_ d.58.7.3 (A:) U2AF35 (35 KDa subunit) {Human (Homo sapiens)}
SQTIALLNIYRNPQNSSQSADGLRSAVSDVEMQEHYDEFFEEVFTEMEEKYGEVEEMNVCDN
LGDHLVGNVYVKFRREEDAEKAVIDLNNRWFNGQPIHAELSP
>d1dbda_ d.58.8.1 (A:) Papillomavirus-1 E2 protein {Bovine papillomavirus type 1}
RRTTNDGFHLLKAGGSCFALISGTANQVKCYRFRVKKNHRHRYENCTTTWFTVADNGAERQG
QAQILITFGSPSQRQDFLKHVPLPPGMNISGFTASLDF
>d2bopa_ d.58.8.1 (A:) Papillomavirus-1 E2 protein {Bovine papillomavirus type 1}
SCFALISGTANQVKCYRFRVKKNHRHRYENCTTTWFTVADNGAERQGQAQILITFGSPSQRQ
DFLKHVPLPPGMNISGFTASLDF
>d1a7ge_ d.58.8.1 (E:) Papillomavirus-1 E2 protein {Human papillomavirus type 31}
ATTPIIHLKGDANILKCLRYRLSKYKQLYEQVSSTWHWTCTDGKHKNAIVTLTYISTSQRDD
FLNTVVIPNTVSVSTGYMTI
>d1by9__ d.58.8.1 (-) Papillomavirus-1 E2 protein {Human papillomavirus type 16}
TTPIVHLKGDANTLKCLRYRFKKHCTLYTAVSSTWHWTGHNVKHKSAIVTLTYDSEWQRDQF
LSQVKIPKTITVSTGFMS
>d1f9fa_ d.58.8.1 (A:) Papillomavirus-1 E2 protein {Human papillomavirus type 18}
HMTPIIHLKGDRNSLKCLRYRLRKHSDHYRDISSTWHWTGAGNEKTGILTVTYHSETQRTKF
LNTVAIPDSVQILVGYMTM
>d1b3ta_ d.58.8.1 (A:) Epstein barr virus nuclear antigen-1 (ebna1) {Epstein-Barr virus}
KGGWFGKHRGQGGSNPKFENIAEGLRALLARSHVERTTDEGTWVAGVFVYGGSKTSLYNLRR
GTALAIPQCRLTPLSRLPFGMAPGPGPQPGPLRESIVCYFMVFLQTHIFAEVLKDAIKDLVM
TKPAPTCNIRVTVCSFDDGVDLP
>d1vhib_ d.58.8.1 (B:) Epstein barr virus nuclear antigen-1 (ebna1) {Epstein-Barr virus}
PKFENIAEGLRALLARSHVERTTDEGTWVAGVFVYGGSKTSLYNLRRGTALAIPQCRLTPLS
RLPFGMAPGPGPQPGPLRESIVCYFMVFLQTHIFAEVLKDAIKDLVMTKPAPTCNIRVTVCS
FDDGVDLP
>d3rubl2 d.58.9.1 (L:22-147) Ribulose 1,5-bisphosphate carboxylase-oxygenase {Tobacco (Nicotiana tabacum), variant turkish samsun}
LTYYTPEYQTKDTDILAAFRVTPQPGVPPEEAGAAVAAESSTGTWTTVWTDGLTSLDRYKGR
CYRIERVVGEKDQYIAYVAYPLDLFEEGSVTNMFTSIVGNVFGFKALRALRLEDLRIPPAYV
KT
>d1bura2 d.58.9.1 (A:12-147) Ribulose 1,5-bisphosphate carboxylase-oxygenase {Spinach (Spinacia oleracea)}

EFKAGVKDYKLTYYTPEYETLDTDILAAFRVSPQPGVPPEEAGAAVAAESSTGTWTTVWTDG
LTNLDRYKGRCYHIEPVAGEENQYICYVAYPLDLFEEGSVTNMFTSIVGNVFGFKALRALRL
EDLRIPVAYVKT
>d1bwva2 d.58.9.1 (A:7-149) Ribulose 1,5-bisphosphate carboxylase-oxygenase {Galdieria partita}
RIKNSRYESGVIPYAKMGYWNPDYQVKDTDVLALFRVTPQPGVDPIEAAAAVAGESSTATWT
VVWTDLLTAADLYRAKAYKVDQVPNNPEQYFAYIAYELDLFEEGSIANLTASIIGNVFGFKA
VKALRLEDMRLPLAYLKTFQ
>d1gk8a2 d.58.9.1 (A:7-149) Ribulose 1,5-bisphosphate carboxylase-oxygenase {Chlamydomonas reinhardtii}
TKAGAGFKAGVKDYRLTYYTPDYVVRDTDILAAFRMTPQPGVPPEECGAAVAAESSTGTWTT
VWTDGLTSLDRYKGRCYDIEPVPGEDNQYIAYVAYPIDLFEEGSVTNMFTSIVGNVFGFKAL
RALRLEDLRIPPAYVKTFV
>d1bxna2 d.58.9.1 (A:22-150) Ribulose 1,5-bisphosphate carboxylase-oxygenase {Alcaligenes eutrophus}
YKMGYWDGDYVPKDTDLLALFRITPQDGVDPVEAAAAVAGESSTATWTVVWTDRLTACDMYR
AKAYRVDPVPNNPEQFFCYVAYDLSLFEEGSIANLTASIIGNVFSFKPIKAARLEDMRFPVA
YVKT
>d1rbla2 d.58.9.1 (A:9-147) Ribulose 1,5-bisphosphate carboxylase-oxygenase {Synechococcus sp., strain pcc 6301}
SAAGYKAGVKDYKLTYYTPDYTPKDTDLLAAFRFSPQPGVPADEAGAAIAAESSTGTWTTVW
TDLLTDMDRYKGKCYHIEPVAGEENSYFAFIAYPLDLFEEGSVTNILTSIVGNVFGFKAIRS
LRLEDIRFPVALVKT
>d5ruba2 d.58.9.1 (A:2-137) Ribulose 1,5-bisphosphate carboxylase-oxygenase {Rhodospirillum rubrum}
DQSSRYVNLALKEEDLIAGGEHVLCAYIMKPKAGYGYVATAAHFAAESSTGTNVEVCTTDDF
TRGVDALVYEVDEARELTKIAYPVALFDRNITDGKAMIASFLTLTMGNNQGMGDVEYAKMHD
FYVPEAYRALFD
>d1geha2 d.58.9.1 (A:12-136) Ribulose 1,5-bisphosphate carboxylase-oxygenase {Archaeon Thermococcus kodakaraensis}
YVDKGYEPSKKRDIIAVFRVTPAEGYTIEQAAGAVAAESSTGTWTTLYPWYEQERWADLSAK
AYDFHDMGDGSWIVRIAYPFHAFEEANLPGLLASIAGNIFGMKRVKGLRLEDLYFPEKLIRE
F
>d2acy__ d.58.10.1 (-) Acylphosphatase {Cow (Bos taurus)}
AEGDTLISVDYEIFGKVQGVFFRKYTQAEGKKLGLVGWVQNTDQGTVQGQLQGPASKVRHMQ
EWLETKGSPKSHIDRASFHNEKVIVKLDYTDFQIVK
>d1aps__ d.58.10.1 (-) Acylphosphatase {Horse (Equus caballus)}
STARPLKSVDYEVFGRVQGVCFRMYAEDEARKIGVVGWVKNTSKGTVTGQVQGPEEKVNSMK
SWLSKVGSPSSRIDRTNFSNEKTISKLEYSNFSVRY
>d1i1ga2 d.58.37.1 (A:62-141) LprA {Archaeon Pyrococcus furiosus}
YSLVTITGVDTKPEKLFEVAEKLKEYDFVKELYLSSGDHMIMAVIWAKDGEDLAEIISNKIG
KIEGVTKVCPAIILEKLK
>d1dar_4 d.58.11.1 (600-689) Elongation factor G (EF-G), domains III and V {Thermus thermophilus}

VILEPIMRVEVTTPEEYMGDVIGDLNARRGQILGMEPRGNAQVIRAFVPLAEMFGYATDLRS
KTQGRGSFVMFFDHYQEVPKQVQEKLIK
>d1fnma4 d.58.11.1 (A:404-482) Elongation factor G (EF-G), domains III and V {Thermus thermophilus}
VPEPVIDVAIEPKTKADQEKLSQALARLAEEDPTFRVSTHPETGQTIISGMGELHLEIIVDR
LKREFKVDANVGKPQVA
>d1b64__ d.58.12.1 (-) Guanine nucleotide exchange factor (GEF) domain from elongation factor-1 beta {Human (Homo sapiens)}
MLVAKSSILLDVKPWDDETDMAKLEECVRSIQADGLVWGSSKLVPVGYGIKKLQIQCVVEDD
KVGTDMLEEQITAFEDYVQSMDVAAFNKI
>d1f60b_ d.58.12.1 (B:) Guanine nucleotide exchange factor (GEF) domain from elongation factor-1 beta {Baker's yeast (Saccharomyces cerevisiae)}
PAAKSIVTLDVKPWDDETNLEEMVANVKAIEMEGLTWGAHQFIPIGFGIKKLQINCVVEDDK
VSLDDLQQSIEEDEDHVQSTDIAAMQKL
>d1gh8a_ d.58.12.1 (A:) aEF-1beta {Archaeon Methanobacterium thermoautotrophicum}
MGDVVATIKVMPESPDVDLEALKKEIQERIPEGTELHKIDEEPIAFGLVALNVMVVVGDAEG
GTEAAEESLSGIEGVSNIEVTDVRRLM
>d1b7yb4 d.58.13.1 (B:682-775) Phenylalanyl-tRNA synthetase {Thermus thermophilus (Thermus aquaticus)}
LAFQDPSRHPAAFRDLAVVVPAPTPYGEVEALVREAAGPYLESLALFDLYQGPPLPEGHKSL
AFHLRFRHPKRTLRDEEVEEAVSRVAEALRAR
>d1jjcb4 d.58.13.1 (B:682-785) Phenylalanyl-tRNA synthetase {Thermus thermophilus (Thermus aquaticus)}
LAFQDPSRHPAAFRDLAVVVPAPTPYGEVEALVREAAGPYLESLALFDLYQGPPLPEGHKSL
AFHLRFRHPKRTLRDEEVEEAVSRVAEALRARGFGLRGLDTP
>d1loua_ d.58.14.1 (A:) Ribosomal protein S6 {Thermus thermophilus}
MRRYEVNIVLNPNLDQSQLALEKEIIQRAAENYGARVEKVEELGLRRLAYPIAKDPQGYFLW
YQVEMPEDRVNDLARELRIRDNVRRVMVVKSQEPF
>d1qjha_ d.58.14.1 (A:) Ribosomal protein S6 {Thermus thermophilus}
MRRYEVNIVLNPNLDQSQLALEKEIIQRALENYGARVEKVAILGLMVLAYPIAKDPQGYFLW
YQVEMPEDRVNDLARELRIRDNVRRVMVVKS
>d1fjgj_ d.58.15.1 (J:) Ribosomal protein S10 {Thermus thermophilus}
KIRIKLRGFDHKTLDASAQKIVEAARRSGAQVSGPIPLPTRVRRFTVIRGPFKHKDSREHFE
LRTHNRLVDIINPNRKTIEQLMTLDLPTGVEIEIKT
>d1fa0a1 d.58.16.1 (A:352-523) Poly(A) polymerase, C-terminal domain {Baker's yeast (Saccharomyces cerevisiae)}
NDFFFRYKFYLEITAYTRGSDEQHLKWSGLVESKVRLLVMKLEVLAGIKIAHPFTKPFESSY
CCPTEDDYEMIQDKYGSHKTETALNALKLVTDENKEEESIKDAPKAYLSTMYIGLDFNIENK
KEKVDIHIPCTEFVNLCRSFNEDYGDHKVFNLALRFVKGYDLPDEVFD

>d1f5aa1 d.58.16.1 (A:365-498) Poly(A) polymerase, C-terminal domain {Cow (Bos taurus)}
PNFFQKYKHYIVLLASAPTEKQRLEWVGLVESKIRILVGSLEKNEFITLAHVNPQSFPAPKE
NPDKEEFRTMWVIGLVFKKTENSENLSVDLTYDIQSFTDTVYRQAINSKMFEVDMKIAAMHV
KRKQLHQLLP
>d1afj__ d.58.17.1 (-) Mercuric ion binding protein MerP {Shigella flexneri}
ATQTVTLAVPGMTCAACPITVKKALSKVEGVSKVDVGFEKREAVVTFDDTKASVQKLTKATA
DAGYPSSVKQ
>d1fvqa_ d.58.17.1 (A:) Copper transporter domain ccc2a {Baker's yeast (Saccharomyces cerevisiae)}
AREVILAVHGMTCSACTNTINTQLRALKGVTKCDISLVTNECQVTYDNEVTADSIKEIIEDC
GFDCEILRDS
>d1aw0__ d.58.17.1 (-) Menkes copper-transporting ATPase {Human (Homo sapiens)}
LTQETVINIDGMTCNSCVQSIEGVISKKPGVKSIRVSLANSNGTVEYDPLLTSPETLRGAIE
DMGFDATLSD
>d1cc8a_ d.58.17.1 (A:) ATX1 metallochaperone protein (ATOX1) {Baker's yeast (Saccharomyces cerevisiae)}
AEIKHYQFNVVMTCSGCSGAVNKVLTKLEPDVSKIDISLEKQLVDVYTTLPYDFILEKIKKT
GKEVRSGKQL
>d1fe0a_ d.58.17.1 (A:) ATX1 metallochaperone protein (ATOX1) {Human (Homo sapiens), HAH1}
PKHEFSVDMTCGGCAEAVSRVLNKLGGVKYDIDLPNKKVCIESEHSMDTLLATLKKTGKTVS
YLGL
>d1cpza_ d.58.17.1 (A:) Copper chaperone {Enterococcus hirae}
AQEFSVKGMSCNHCVARIEEAVGRISGVKKVKVQLKKEKAVVKFDEANVQATEICQAINELG
YQAEVI
>d1k0va_ d.58.17.1 (A:) Copper chaperone {Bacillus subtilis, CopZ}
MEQKTLQVEGMSCQHCVKAVETSVGELDGVSAVHVNLEAGKVDVSFDADKVSVKDIADAIED
QGYDVAKIEGR
>d1qupa2 d.58.17.1 (A:2-73) Copper chaperone for superoxide dismutase, N-terminal domain {Baker's yeast (Saccharomyces cerevisiae)}
TTNDTYEATYAIPMHCENCVNDIKACLKNVPGINSLNFDIEQQIMSVESSVAPSTIINTLRN
CGKDAIIRGA
>d1gmua2 d.58.38.1 (A:71-138) Urease metallochaperone UreE, C-terminal domain {Klebsiella aerogenes}
DEEVSVVRCDDPFMLAKACYALGNRHVPLQIMPGELRYHHDHVLDDMLRQFGLTVTFGQLPF
EPEAGA
>d1eara2 d.58.38.1 (A:75-142) Urease metallochaperone UreE, C-terminal domain {Bacillus pasteurii}
LEKVYVIKPQTMQEMGKMAFEIGNRHTMCIIEDDEILVRYDKTLEKLIDEVGVSYEQSERRF
KEPFKY

>d1psda3 d.58.18.1 (A:327-410) Phosphoglycerate dehydrogenase, regulatory (C-terminal) domain {Escherichia coli}
FPEVSLPLHGGRRLMHIHENRPGVLTALNKIFAEQGVNIAAQYLQTSAQMGYVVIDIEADED
VAEKALQAMKAIPGTIRARLLY
>d1tdj_2 d.58.18.2 (336-423) Allosteric threonine deaminase C-terminal domain {Escherichia coli}
QREALLAVTIPEEKGSFLKFCQLLGGRSVTEFNYRFADAKNACIFVGVRLSRGLEERKEILQ
MLNDGGYSVVDLSDDEMAKLHVRYMV
>d1tdj_3 d.58.18.2 (424-514) Allosteric threonine deaminase C-terminal domain {Escherichia coli}
GGRPSHPLQERLYSFEFPESPGALLRFLNTLGTYWNISLFHYRSHGTDYGRVLAAFELGDHE
PDFETRLNELGYDCHDETNNPAFRFFLAG
>d1phza1 d.58.18.3 (A:19-115) Phenylalanine hydroxylase N-terminal domain {Rat (Rattus norvegicus)}
GQETSYIEDNSNQNGAISLIFSLKEEVGALAKVLRLFEENDINLTHIESRPSRLNKDEYEFF
TYLDKRTKPVLGSIIKSLRNDIGATVHELSRDKEK
>d1cg2a2 d.58.19.1 (A:214-326) Carboxypeptidase G2, dimerisation domain {Pseudomonas sp., strain rs-16}
SGIAYVQVNITGKASHAGAAPELGVNALVEASDLVLRTMNIDDKAKNLRFNWTIAKAGNVSN
IIPASATLNADVRYARNEDFDAAMKTLEERAQQKKLPEADVKVIVTRGRPA
>d1dqaa1 d.58.20.1 (A:587-703) NAD-binding domain of HMG-CoA reductase {Human (Homo sapiens)}
GMTRGPVVRLPRACDSAEVKAWLETSEGFAVIKEAFDSTSRFARLQKLHTSIAGRNLYIRFQ
SRSGDAMGMNMISKGTEKALSKLHEYFPEMQILAVSGNYCTDKKPAAINWIEGRG
>d1qaxa1 d.58.20.1 (A:111-220) NAD-binding domain of HMG-CoA reductase {Pseudomonas mevalonii}
LMHAQVQIVGIQDPLNARLSLLRRKDEIIELANRKDQLLNSLGGGCRDIEVHTFADTPRGPM
LVAHLIVDVRDAMGANTVNTMAEAVAPLMEAITGGQVRLRILSNLADL
>d1ekra_ d.58.21.1 (A:) Molybdenum cofactor biosynthesis protein C, MoaC {Escherichia coli}
GEAHMVDVSAKAETVREARAEAFVTMRSETLAMIIDGRHHKGDVFATARIAGIQAAKRTWDL
IPLCHPLMLSKVEVNLQAEPEHNRVRIETLCRLTGKTGVEMEALTAASVAALTIYDMCKAVQ
KDMVIGPVRLLAKSGGKSGDFK
>d1f3va_ d.58.22.1 (A:) TRADD, N-terminal domain {Human (Homo sapiens)}
HEEWVGSAYLFVESSLDKVVLSDAYAHPQQKVAVYRALQAALAESGGSPDVLQMLKIHRSDP
QLIVQLRFCGRQPCGRFLRAYREGALRAALQRSLAAALAQHSVPLQLELRAGAERLDALLAD
EERCLSCILAQQPDRLRDEELAELEDALRNLKCG
>d1mla_2 d.58.23.1 (128-197) Probable ACP-binding domain of malonyl-CoA ACP transacylase {Escherichia coli}
GTGAMAAIIGLDDASIAKACEEAAEGQVVSPVNFNSPGQVVIAGHKEAVERAGAACKAAGAK
RALPLPVS
>d1ffgb_ d.58.24.1 (B:) CheY-binding domain of CheA {Escherichia coli}

PRRIILSRLKAGEVDLLEEELGHLTTLTDVVKGADSLSAILPGDIAEDDITAVLCFVIEADQ
ITFETV
>d1kp6a_ d.58.25.1 (A:) Killer toxin KP6 alpha-subunit {Smut fungus (Ustilago maydis)}
NNAFCAGFGLSCKWECWCTAHGTGNELRYATAAGCGDHLSKSYYDARAGHCLFSDDLRNQFY
SHCSSLNNNMSCRSLSK
>d1h72c2 d.58.26.1 (C:168-300) Homoserine kinase, C-terminal domain {Archaeon Methanococcus jannaschii}
FKLDILIAIPNISINTKEAREILPKAVGLKDLVNNVGKACGMVYALYNKDKSLFGRYMMSDK
VIEPVRGKLIPNYFKIKEEVKDKVYGITISGSGPSIIAFPKEEFIDEVENILRDYYENTIRT
EVGKGVEVV
>d1fi4a2 d.58.26.2 (A:191-393) Mevalonate 5-diphosphate decarboxylase {Baker's yeast (Saccharomyces cerevisiae)}
QMKACVLVVSDIKKDVSSTQGMQLTVATSELFKERIEHVVPKRFEVMRKAIVEKDFATFAKE
TMMDSNSFHATCLDSFPPIFYMNDTSKRIISWCHTINQFYGETIVAYTFDAGPNAVLYYLAE
NESKLFAFIYKLFGSVPGWDKKFTTEQLEAFNHQFESSNFTARELDLELQKDVARVILTQVG
SGPQETNESLIDAKTGL
>d1regx_ d.58.27.1 (X:) Translational regulator protein regA {Bacteriophage T4}
MIEITLKKPEDFLKVKETLTRMGIANNKDKVLYQSCHILQKKGLYYIVHFKEMLRMDGRQVE
MTEEDEVRRDSIAWLLEDWGLIEIVPGQRTFMKDLTNNFRVISFKQKHEWKLVPKYTIGN
>d1fvga_ d.58.28.1 (A:) Peptide methionine sulfoxide reductase {Cow (Bos taurus)}
KIVSPQEALPGRKEPLVVAAKHHVNGNRTVEPFPEGTQMAVFGMGCFWGAERKFWTLKGVYS
TQVGFAGGYTPNPTYKEVCSGKTGHAEVVRVVFQPEHISFEELLKVFWENHDPTQGMRQGND
HGSQYRSAIYPTSAEHVGAALKSKEDYQKVLSEHGFGLITTDIREGQTFYYAEDYHQQYLSK
DPDGYC
>d1ff3a_ d.58.28.1 (A:) Peptide methionine sulfoxide reductase {Escherichia coli}
SLFDKKHLVSPADALPGRNTPMPVATLHAVNGHSMTNVPDGMEIAIFAMGCFWGVERLFWQL
PGVYSTAAGYTGGYTPNPTYREVCSGDTGHAEAVRIVYDPSVISYEQLLQVFWENHDPAQGM
RQGNDHGTQYRSAIYPLTPEQDAAARASLERFQAAMLAADDDRHITTEIANATPFYYAEDDH
QQYLHKNPYGYCGIGGIGVCLPPEA
>d1ff3c_ d.58.28.1 (C:) Peptide methionine sulfoxide reductase {Escherichia coli}
LVSPADALPGRNTPMPVATLHAVNGHSMTNVPDGMEIAIFAMGCFWGVERLFWQLPGVYSTA
AGYTGGYTPNPTYREVCSGDTGHAEAVRIVYDPSVISYEQLLQVFWENHDPAQGMRQGNDHG
TQYRSAIYPLTPEQDAAARASLERFQAAMLAADDDRHITTEIANATPFYYAEDDHQQYLHK
>d1azsa_ d.58.29.1 (A:) Adenylyl cyclase VC1, domain C1a {Dog (Canis familiaris)}
DMMFHKIYIQKHDNVSILFADIEGFTSLASQCTAQELVMTLNELFARFDKLAAENHCLRIKI
LGDCYYCVSGLPEARADHAHCCVEMGMDMIEAISLVREMTGVNVNMRVGIHSGRVHCGVLGL
RKWQFDVWSNDVTLANHMEAGGKAGRIHITKATLSYLNGDYEVEPGCGGERNAYLKEHSIET
FLIL

>d1azsb_ d.58.29.1 (B:) Adenylyl cyclase IIC1, domain C2a {Rat (Rattus norvegicus)}
HQSYDCVCVMFASIPDFKEFYTESDVNKEGLECLRLLNEIIADFDDLLSKPKFSGVEKIKTI
GSTYMAATGLSAIPSQEHAQEPERQYMHIGTMVEFAYALVGKLDAINKHSFNDFKLRVGINH
GPVIAGVIGAQKPQYDIWGNTVNVASRMDSTGVLDKIQVTEETSLILQTLGYTCTCRGIINV
KGKGDLKTYFVNT
>d1fx2a_ d.58.29.1 (A:) Receptor-type monomeric adenylyl cyclase {Trypanosome (Trypanosoma brucei), different isoform}
NNNRAPKEPTDPVTLIFTDIESSTALWAAHPDLMPDAVAAHHRMVRSLIGRYKCYEVKTVGD
SFMIASKSPFAAVQLAQELQLCFLHHDWGTNALDDSYREFEEQRAEGECEYTPPTAHMDPEV
YSRLWNGLRVRVGIHTGLCDIRHDEVTKGYDYYGRTPNMAARTESVANGGQVLMTHAAYMSL
SAEDRKQIDVTALGDVALRGVSDPVKMYQLNTVPSRNFAALRLDREYFD
>d1fx4a_ d.58.29.1 (A:) Receptor-type monomeric adenylyl cyclase {Trypanosome (Trypanosoma brucei), different isoform}
DNDSAPKEPTGPVTLIFTDIESSTALWAAHPDLMPDAVATHHRLIRSLITRYECYEVKTVGD
SFMIASKSPFAAVQLAQELQLCFLRLDWETNAVDESYREFEEQRAEGECEYTPPTASLDPEV
YSRLWNGLRVRVGIHTGLCDIRYDEVTKGYDYYGRTSNMAARTESVANGGQVLMTHAAYMSL
SGEDRNQLDVTTLGATVLRGVPEPVRMYQLNAVPGRNFAALRLDR
>d1eqoa_ d.58.30.1 (A:) 6-hydroxymethyl-7,8-dihydropterin pyrophosphokinase, HPPK {Escherichia coli}
TVAYIAIGSNLASPLEQVNAALKALGDIPESHILTVSSFYRTPPLGPQDQPDYLNAAVALET
SLAPEELLNHTQRIELQQGRVRKAERWGPRTLDLDIMLFGNEVINTERLTVPHYDMKNRGFM
LWPLFEIAPELVFPDGEMLRQILHTRAFDKLNKW
>d1cbka_ d.58.30.1 (A:) 6-hydroxymethyl-7,8-dihydropterin pyrophosphokinase, HPPK {Haemophilus influenzae}
MITAYIALGSNLNTPVEQLHAALKAISQLSNTHLVTTSSFYKSKPLGPQDQPDYVNAVAKIE
TELSPLKLLDELQRIENEQGRVRLRRWGERTLDLDILLYGNEIIQNERLTIPHYDMHNREFV
IVPLFEIASDLVLPNSQIITELVKQFADHKMIKLNP
>d1gpja3 d.58.39.1 (A:1-143) Glutamyl tRNA-reductase catalytic, N-terminal domain {Archaeon Methanopyrus kandleri}
MEDLVSVGITHKEAEVEELEKARFESDEAVRDIVESFGLSGSVLLQTSNRVEVYASGARDRA
EELGDLIHDDAWVKRGSEAVRHLFRVASGLESMMVGEQEILRQVKKAYDRAARLGTLDEALK
IVFRRAINLGKRAREETRI
>d1hbnc_ d.58.31.1 (C:) Methyl-coenzyme M reductase gamma chain {Archaeon Methanobacterium thermoautotrophicum}
AQYYPGTTKVAQNRRNFCNPEYELEKLREISDEDVVKILGHRAPGEEYPSVHPPLEEMDEPE
DAIREMVEPIDGAKAGDRVRYIQFTDSMYFAPAQPYVRSRAYLCRYRGADAGTLSGRQIIET
RERDLEKISKELLETEFFDPARSGVRGKSVHGHSLRLDEDGMMFDMLRRQIYNKDTGRVEMV
KNQIGDELDEPVDLGEPLDEETLMEKTTIYRVDGEAYRDDVEAVEIMQRIHVLRSQGGFNL
>d1e6vc_ d.58.31.1 (C:) Methyl-coenzyme M reductase gamma chain {Archaeon Methanopyrus kandleri}
FYYPGETDVAENRRKYMNPNYELKKLREIPDEDIVRLMGHREPGEEYPSVHPPLEEMEEPEC
PIRELVEPTEGAKAGDRIRYIQFTDSVYFAPIHPYIRARMYMWRYRGVDTGSLSGRQIIEVR
ERDLEKIAKELLETEIFDPARSGVRGATVHGHALRLDENGLMLHALRRYRLNEETGEVEYVK

DQVGIELDEPIPVGAPADEDDLKERTTIYRIDGTPYREDEELLQVVQRIHELRTLAGYRPEE
>d1e6yc_ d.58.31.1 (C:) Methyl-coenzyme M reductase gamma chain {Archaeon Methanosarcina barkeri}
AYERQYYPGATSVAANRRKHMSGKLEKLREISDEDLTAVLGHRAPGSDYPSTHPPLAEMGEP
ACSTRENVAATPGAAAGDRVRYIQFADSMYNAPATPYFRSYFAAINFRGVDPGTLSGRQIVE
ARERDMEQCAKVQMETEITDHALAGVRGATVHGHSVRLQEDGVMFDMLDRRRLENGTIIMDK
DQVAIPLDRKVDLGKPMSSEEAAKRTTIYRVDNVAFRDDAEVVEWVHRIFDQRTKFGFQPK
>d1hbna2 d.58.31.2 (A:2-269) Alpha chain {Archaeon Methanobacterium thermoautotrophicum}
ADKLFINALKKKFEESPEEKKTTFYTLGGWKQSERKTEFVNAGKEVAAKRGIPQYNPDIGTP
LGQRVLMPYQVSTTDTYVEGDDLHFVNNAAMQQMWDDIRRTVIVGLNHAHAVIEKRLGKEVT
PETITHYLETVNHAMPGAAVVQEHMVETHPALVADSYVKVFTGNDEIADEIDPAFVIDINKQ
FPEDQAETLKAEVGDGIWQVVRIPTIVSRTCDGATTSRWSAMQIGMSMISAYKQAAGEAATG
DFAYAAKHAEVIHMGTYLPV
>d1e6va2 d.58.31.2 (A:8-272) Alpha chain {Archaeon Methanopyrus kandleri}
LFMKALKEKFEESPEEKYTKFYIFGGWKQSERKKEFKEWADKIVEERGVPHYNPDIGVPLGQ
RKLMSYQVSGTDVFVEGDDLTFVNNAAMQQMWDDIRRTVIVGMDTAHRVLERRLGKEVTPET
INEYMETLNHALPGGAVVQEHMVEIHPGLTWDCYAKIITGDLELADEIDDKFLIDIEKLFPE
EQAEQLIKAIGNRTYQVCRMPTIVGHVCDGATMYRWAAMQIAMSFICAYKIAAGEAAVSDFA
FASKHAEVINMGEMLPA
>d1e6ya2 d.58.31.2 (A:1002-1283) Alpha chain {Archaeon Methanosarcina barkeri}
AADIFSKFKKDMEVKFAQEFGSNKQTGGDITDKTAKFLRLGPEQDPRKVEMIKAGKEIAEKR
GIAFYNPMMHSGAPLGQRAITPYTISGTDIVCEPDDLHYVNNAAMQQMWDDIRRTCIVGLDM
AHETLEKRLGKEVTPETINHYLEVLNHAMPGAAVVQEMMVETHPALVDDCYVKVFTGDDALA
DEIDKQFLIDINKEFSEEQAAQIKASIGKTSWQAIHIPTIVSRTTDGAQTSRWAAMQIGMSF
ISAYAMCAGEAAVADLSFAAKHAALVSMGEMLPA
>d1hbnb2 d.58.31.2 (B:2-188) Beta chain {Archaeon Methanobacterium thermoautotrophicum}
AKFEDKVDLYDDRGNLVEEQVPLEALSPLRNPAIKSIVQGIKRTVAVNLEGIENALKTAKVG
GPACKIMGRELDLDIVGNAESIAAAAKEMIQVTEDDDTNVELLGGGKRALVQVPSARFDVAA
EYSAAPLVTATAFVQAIINEFDVSMYDANMVKAAVLGRYPQSVEYMGANIATMLDIPQKLEG
P
>d1e6vb2 d.58.31.2 (B:7-189) Beta chain {Archaeon Methanopyrus kandleri}
DTVDLYDDRGNCVAEEVPIEVLSPMRNEAIQSIVNDIKRTVAVDLEGIENALQNATVGGKGM
KIPGREMDVDIVDNAEAIADEIEKMIRVYQDDDTNVEPMYDGKRLLVQLPSERVKVMADPYS
GTLQAGMAVVHAIIDVCEVDMWDANMVKAAVFGRYPQTIDYFGGNVASMLDVPMKQEGV
>d1e6yb2 d.58.31.2 (B:2002-2185) Beta chain {Archaeon Methanosarcina barkeri}
SDTVDIYDDRGKLLESNVDIMSLAPTRNAAIQSIIMDTKRSVAVNLAGIQGALASGKMGGKG
RQILGRGLNYDIVGNADAIAENVKKLVQVDEGDDTNVIKVKGGKSLLIQSPKSRIIAGADFM
SATTVGAAAVTQTIMDMFGTDPYDAPIVKSAVWGSYPQTMDLMGGQVQGILSIPQNNEGL

>d1e8ga1 d.58.32.1 (A:274-560) Vanillyl-alcohol oxidase {Fungus (Penicillium simplicissimum)}
GGYQSYLITLPKDGDLKQAVDIIRPLRLGMALQNVPTIRHILLDAAVLGDKRSYSSRTEPLS
DEELDKIAKQLNLGRWNFYGALYGPEPIRRVLWETIKDAFSAIPGVKFYFPEDTPENSVLRV
RDKTMQGIPTYDELKWIDWLPNGAHLFFSPIAKVSGEDAMMQYAVTKKRCQEAGLDFIGTFT
VGMREMHHIVCIVFNKKDLIQKRKVQWLMRTLIDDCAANGWGEYRTHLAFMDQIMETYNWNN
SSFLRFNEVLKNAVDPNGIIAPGKSGVWPSQYSHVTWKL
>d1diqa1 d.58.32.1 (A:243-521) Flavoprotein subunit of p-cresol methylhydroxylase {Pseudomonas putida}
PVFKPFEVIFEDEADIVEIVDALRPLRMSNTIPNSVVIASTLWEAGSAHLTRAQYTTEPGHT
PDSVIKQMQKDTGMGAWNLYAALYGTQEQVDVNWKIVTDVFKKLGKGRIVTQEEAGDTQPFK
YRAQLMSGVPNLQEFGLYNWRGGGGSMWFAPVSEARGSECKKQAAMAKRVLHKYGLDYVAEF
IVAPRDMHHVIDVLYDRTNPEETKRADACFNELLDEFEKEGYAVYRVNTRFQDRVAQSYGPV
KRKLEHAIKRAVDPNNILAPGRSGIDLNNDF
>d1f0xa1 d.58.32.2 (A:274-567) D-lactate dehydrogenase {Escherichia coli}
KNQQVFYIGTNQPEVLTEIRRHILANFENLPVAGEYMHRDIYDIAEKYGKDTFLMIDKLGTD
KMPFFFNLKGRTDAMLEKVKFFRPHFTDRAMQKFGHLFPSHLPPRMKNWRDKYEHHLLLKMA
GDGVGEAKSWLVDYFKQAEGDFFVCTPEEGSKAFLHRFAAAGAAIRYQAVHSDEVEDILALD
IALRRNDTEWYEHLPPEIDSQLVHKLYYGHFMCYVFHQDYIVKKGVDVHALKEQMLELLQQR
GAQYPAEHNVGHLYKAPETLQKFYRENDPTNSMNPGIGKTSKRKNW
>d1i19a1 d.58.32.3 (A:274-613) Cholesterol oxidase {Brevibacterium sterolicum}
FRQRCQSYTDIPWRELFAPKGADGRTFEKFVAESGGAEAIWYPFTEKPWMKVWTVSPTKPDS
SNEVGSLGSAGSLVGKPPQAREVSGPYNYIFSDNLPEPITDMIGAINAGNPGIAPLFGPAMY
EITKLGLAATNANDIWGWSKDVQFYIKATTLRLTEGGGAVVTSRANIATVINDFTEWFHERI
EFYRAKGEFPLNGPVEIRCCGLDQAADVKVPSVGPPTISATRPRPDHPDWDVAIWLNVLGVP
GTPGMFEFYREMEQWMRSHYNNDDATFRPEWSKGWAFGPDPYTDNDIVTNKMRATYIEGVPT
TENWDTARARYNQIDPHRVFTNGFMDKLLP
>d1ftra1 d.58.33.1 (A:1-148) Formylmethanofuran:tetrahydromethanopterin formyltransferase {Archaeon Methanopyrus kandleri}
MEINGVEIEDTFAEAFEAKMARVLITAASHKWAMIAVKEATGFGTSVIMCPAEAGIDCGYVP
PEETPDGRPGVTIMIGHNDEDELKEQLLDRIGQCVMTAPTASAFDAMPEAEKEDEDRVGYKL
SFFGDGYQEEDELDGRKVWKIPVV
>d1ftra2 d.58.33.1 (A:149-296) Formylmethanofuran:tetrahydromethanopterin formyltransferase {Archaeon Methanopyrus kandleri}
EGEFIVEDSFGITTGVAGGNFYIMAESQPAGLQAAEAAVDAIKGVEGAYAPFPGGIVASASK
VGSKQYDFLPASTNDAYCPTVEDNELPEGVKCVYEIVINGLNEEAVKEAMRVGIEAACQQPG
VVKISAGNFGGKLGQYEIHLHDLF
>d1qd1a1 d.58.34.1 (A:2-180) Formiminotransferase domain of formiminotransferase-cyclodeaminase. {Pig (Sus scrofa)}
SQLVECVPNFSEGKNQEVIDAISRAVAQTPGCVLLDVDSGPSTNRTVYTFVGRPEDVVEGAL

NAARAAYQLIDMSRHHGEHPRMGALDVCPFIPVRGVTMDECVRCAQAFGQRLAEELGVPVYL
YGEAARTAGRQSLPALRAGEYEALPEKLKQAEWAPDFGPSAFVPSWGATVAGARK
>d1qd1a2 d.58.34.1 (A:181-326) Formiminotransferase domain of formiminotransferase-cyclodeaminase. {Pig (Sus scrofa)}
FLLAFNINLLSTREQAHRIALDLREQGRGKDQPGRLKKVQAIGWYLDEKNLAQVSTNLLDFE
VTGLHTVFEETCREAQELSLPVVGSQLVGLVPLKALLDAAAFYCEKENLFLLQDEHRIRLVV
NRLGLDSLAPFKPKERIIEYLV
>d1dj0a1 d.58.35.1 (A:7-114) Pseudouridine synthase I {Escherichia coli}
PPVYKIALGIEYDGSKYYGWQRQNEVRSVQEKLEKALSQVANEPITVFCAGRTDAGVHGTGQ
VVHFETTALRKDAAWTLGVNANLPGDIAVRWVKTVPDDFHARFSAT
>d1dj0a2 d.58.35.1 (A:115-270) Pseudouridine synthase I {Escherichia coli}
ARRYRYIIYNHRLRPAVLSKGVTHFYEPLDAERMHRAAQCLLGENDFTSFRAVQCQSRTPWR
NVMHINVTRHGPYVVVDIKANAFVHHMVRNIVGSLMEVGAHNQPESWIAELLAAKDRTLAAA
TAKAEGLYLVAVDYPDRYDLPKPPMGPLFLAD
>d1k8wa1 d.58.35.2 (A:9-73) Pseudouridine synthase II TruB {Escherichia coli}
MDINGVLLLDKPQGMSSNDALQKVKRIYNANRAGHTGALDPLATGMLPICLGEATKFSQYLL
DSD
>d1k8wa2 d.58.35.2 (A:74-312) Pseudouridine synthase II TruB {Escherichia coli}
KRYRVIARLGQRTDTSDADGQIVEERPVTFSAEQLAAALDTFRGDIEQIPSMYSALKYQGKK
LYEYARQGIEVPREARPITVYELLFIRHEGNELELEIHCSKGTYIRTIIDDLGEKLGCGAHV
IYLRRLAVSKYPVERMVTLEHLRELVEQAEQQDIPAAELLDPLLMPMDSPASDYPVVNLPLT
SSVYFKNGNPVRTSGAPLEGLVRVTEGENGKFIGMGEIDDEGRVAPRRLVVEY
>d1aop_1 d.58.36.1 (81-145) Sulfite reductase, domains 1 and 3 {Escherichia coli}
LLRCRLPGGVITTKQWQAIDKFAGENTIYGSIRLTNRQTFQFHGILKKNVKPVHQMLHSVGL
DAL
>d1aop_2 d.58.36.1 (346-425) Sulfite reductase, domains 1 and 3 {Escherichia coli}
IGWVKGIDDNWHLTLFIENGRILDYPARPLKTGLLEIAKIHKGDFRITANQNLIIAGVPESE
KAKIEKIAKESGLMNAVT
>d1bxya_ d.59.1.1 (A:) Prokaryotic ribosomal protein L30 {Thermus thermophilus}
MPRLKVKLVKSPIGYPKDQKAALKALGLRRLQQERVLEDTPAIRGNVEKVAHLVRVEVVE
>d1jj2v_ d.59.1.1 (V:) Archaeal L30 (L30a) {Archaeon Haloarcula marismortui}
MHALVQLRGEVNMHTDIQDTLEMLNIHHVNHCTLVPETDAYRGMVAKVNDFVAFGEPSQETL
ETVLATRAEPLEGDADVDDEWVAEHTDYDDISGLAFALLSEETTLREQGLSPTLRLHPPRGG
HDGVKHPVKEGGQLGKHDTEGIDDLLEAMR
>d1fw9a_ d.190.1.1 (A:) Chorismate lyase {Escherichia coli}
SHPALTQLRALRYSKEIPALDPQLLDWLLLEDSMTKRFEQQGKTVSVTMIREGFVEQNEIPE

ELPLLPKESRYWLREILLSADGEPWLAGRTVVPVSTLSGPELALQKLGKTPLGRYLFTSSTL
TRDFIEIGRDAGLWGRRSRLRLSGKPLLLTELFLPASPLY
>d1bowa_ d.60.1.1 (A:) Multidrug-binding domain of transcription activator BmrR {Bacillus subtilis}
RLGEVFVLDEEEIRIIQTEAEGIGPENVLNASYSKLKKFIESADGFTNNSYGATFSFQPYTS
IDEMTYRHIFTPVLTNKQISSITPDMEITTIPKGRYACIAYNFSPEHYFLNLQKLIKYIADR
QLTVVSDVYELIIPIHYSPKKQEEYRVEMKIRIL
>d1d5ya3 d.60.1.2 (A:122-294) Rob transcription factor, C-terminal domain {Escherichia coli}
EFTMPEHKFVTLEDTPLIGVTQSYSCSLEQISDFRHEMRYQFWHDFLGNAPTIPPVLYGLNE
TRPSQDKDDEQEVFYTTALAQDQADGYVLTGHPVMLQGGEYVMFTYEGLGTGVQEFILTVYG
TCMPMLNLTRRKGQDIERYYPAEDAKAGDRPINLRCELLIPIRRKLAAA
>d1jh6a_ d.61.1.1 (A:) tRNA splicing product Appr>p cyclic nucleotide phosphodiesterase {Thale cress (Arabidopsis thaliana)}
MEEVKKDVYSVWALPDEESEPRFKKLMEALRSEFTGPRFVPHVTVAVSAYLTADEAKKMFES
ACDGLKAYTATVDRVSTGTFFFQCVFLLLQTTPEVMEAGEHCKNHFNCSTTTPYMPHLSLLY
AELTEEEKKNAQEKAYTLDSSLDGLSFRLNRLALCKTDTEDKTLETWETVAVCNLNP
>d1f32a_ d.62.1.1 (A:) Pepsin inhibitor-3 {Pig roundworm (Ascaris suum)}
FLFSMSTGPFICTVKDNQVFVANLPWTMLEGDDIQVGKEFAARVEDCTNVKHDMAPTCTKPP
PFCGPQDMKMFNFVGCSVLGNKLFIDQKYVRDLTAKDHAEVQTFREKIAAFEEQQENQPPSS
GMPHGAVPAGGLSPPPPPSFCTV
>d1d8ia_ d.63.1.1 (A:) mRNA triphosphatase CET1 {Baker's yeast (Saccharomyces cerevisiae)}
HMYRNVPIWAQKWKPTIKALQSINVKDLKIDPSFLNIIPDDDLTKSVQDWVYATIYSIAPEL
RSFIELEMKFGVIIDAKGPDRVNPPVSSQCVFTELDAHLTPNIDASLFKELSKYIRGISEVT
ENTGKFSIIESQTRDSVYRVGLSTQRPRFLRMSTDIKTGRVGQFIEKRHVAQLLLYSPKDSY
DVKISLNLELPVPDNDPPEKYKSQSPISERTKDRVSYIHNDSCTRIDITKVENHNQNSKSRQ
SETTHEVELEINTPALLNAFDNITNDSKEYASLIRTFLNNGTIIRRKLSSLSY
>d2if1__ d.64.1.1 (-) Eukaryotic translation initiation factor eIF-1 (SUI1) {Human (Homo sapiens)}
MRGSHHHHHHTDPMSAIQNLHSFDPFADASKGDDLLPAGTEDYIHIRIQQRNGRKTLTTVQG
IADDYDKKKLVKAFKKKFACNGTVIEHPEYGEVIQLQGDQRKNICQFLVEIGLAKDDQLKVH
GF
>d1d1ra_ d.64.1.1 (A:) YciH {Escherichia coli}
KGDGVVRIQRQTSGRKGKGVCLITGVDLDDAELTKLAAELKKKCGCGGAVKDGVIEIQGDKR
DLLKSLLEAKGMKVKLAGGLE
>d1lbu_2 d.65.1.1 (84-213) Zn2+ DD-carboxypeptidase, C-terminal, catalytic domain {Streptomyces albus G}
VNFTYAELNRCNSDWSGGKVSAATARANALVTMWKLQAMRHAMGDKPITVNGGFRSVTCNSN
VGGASNSRHMYGHAADLGAGSQGFCALAQAARNHGFTEILGPGYPGHNDHTHVAGGDGRFWS
APSCGI
>d1vhh__ d.65.1.2 (-) Sonic hedgehog {Mouse (Mus musculus)}
KLTPLAYKQFIPNVAEKTLGASGRYEGKITRNSERFKELTPNYNPDIIFKDEENTGADRLMT

QRCKDKLNALAISVMNQWPGVKLRVTEGWDEDGHHSEESLHYEGRAVDITTSDRDRSKYGML
ARLAVEAGFDWVYYESKAHIHCSVKAENSVAAK
>d1qf6a2 d.66.1.1 (A:2-62) Threonyl-tRNA synthetase (ThrRS), N-terminal 'additional' domain {Escherichia coli}
PVITLPDGSQRHYDHAVSPMDVALDIGPGLAKACIAGRVNGELVDACDLIENDAQLSIITA
>d1fjgd_ d.66.1.2 (D:) Ribosomal protein S4 {Thermus thermophilus}
GRYIGPVCRLCRREGVKLYLKGERCYSPKCAMERRPYPPGQHGQKRARRPSDYAVRLREKQK
LRRIYGISERQFRNLFEEASKKKGVTGSVFLGLLESRLDNVVYRLGFAVSRRQARQLVRHGH
ITVNGRRVDLPSYRVRPGDEIAVAEKSRNLELIRQNLEAMKGRKVGPWLSLDVEGMKGKFLR
LPDREDLALPVQENLVIEFYSR
>d1c06a_ d.66.1.2 (A:) Ribosomal protein S4 {Bacillus stearothermophilus}
MKLSEYGLQLQEKQKLRHMYGVNERQFRKTFEEAGKMPGKHGENFMILLESRLDNLVYRLGL
ARTRRQARQLVTHGHILVDGSRVNIPSYRVKPGQTIAVREKSRNLQVIKEALEANNYIPDYL
SFDPEKMEGTYTRLPERSELPAEINEALIVEFYSR
>d1dm9a_ d.66.1.3 (A:) Heat shock protein 15 kD {Escherichia coli}
PAVEVRLDKWLWAARFYKTRALAREMIEGGKVHYNGQRSKPSKIVELNATLTLRQGNDERTV
IVKAITEQRRPASEAALLYEETAESVEKREKMALARKLNALT
>d1qf6a3 d.67.1.1 (A:63-241) Threonyl-tRNA synthetase (ThrRS), second 'additional' domain {Escherichia coli}
KDEEGLEIIRHSCAHLLGHAIKQLWPHTKMAIGPVIDNGFYYDVDLDRTLTQEDVEALEKRM
HELAEKNYDVIKKKVSWHEARETFANRGESYKVSILDENIAHDDKPGLYFHEEYVDMCRGPH
VPNMRFCHHFKLMKTAGAYWRGDSNNKMLQRIYGTAWADKKALNAYLQRLEEAAK
>d1f7ua3 d.67.2.1 (A:2-135) Arginyl-tRNA synthetase (ArgRS), N-terminal 'additional' domain {Baker's yeast (Saccharomyces cerevisiae)}
ASTANMISQLKKLSIAEPAVAKDSHPDVNIVDLMRNYISQELSKISGVDSSLIFPALEWTNT
MERGDLLIPIPRLRIKGANPKDLAVQWAEKFPCGDFLEKVEANGPFIQFFFNPQFLAKLVIP
DILTRKEDYG
>d1iq0a3 d.67.2.1 (A:1-96) Arginyl-tRNA synthetase (ArgRS), N-terminal 'additional' domain {Thermus thermophilus}
MLRRALEEAIAQALKEMGVPVRLKVARAPKDKPGDYGVPLFALAKELRKPPQAIAQELKDRL
PLPEFVEEAVPVGGYLNFRLRTEALLREALRPKA
>d1dd5a_ d.67.3.1 (A:) Ribosome recycling factor, RRF {Thermotoga maritima}
VNPFIKEAKEKMKRTLEKIEDELRKMRTGKPSPAILEEIKVDYYGVPTPVNQLATISISEER
TLVIKPWDKSVLSLIEKAINASDLGLNPINDGNVIRLVFPSPTTEQREKWVKKAKEIVEEGK
IAIRNIRREILKKIKEDQKEGLIPEDDAKRLENEIQKLTDEFIEKLDEVFEIKKEEIMEF
>d1eh1a_ d.67.3.1 (A:) Ribosome recycling factor, RRF {Thermus thermophilus}
MTLKELYAETRSHMQKSLEVLEHNLAGLRTGRANPALLLHLKVEYYGAHVPLNQIATVTAPD
PRTLVVQSWDQNALKAIEKAIRDSDLGLNPSNKGDALYINIPPLTEERRKDLVRAVRQYAEE
GRVAIRNIRREALDKLKKLAKELHLSEDETKRAEAEIQKITDEFIAKADQLAEKKEQEILG
>d1ek8a_ d.67.3.1 (A:) Ribosome recycling factor, RRF {Escherichia

coli}
MISDIRKDAEVRMDKCVEAFKTQISKIRTGRASPSLLDGIVVEYYGTPTPLRQLASVTVEDS
RTLKINVFDRSMSPAVEKAIMASDLGLNPNSAGSDIRVPLPPLTEERRKDLTKIVRGEAEQA
RVAVRNVRRDANDKVKALLKDKEISEDDDRRSQDDVQKLTDAAIKKIEAALADKEAELMQF
>d1ge9a_ d.67.3.1 (A:) Ribosome recycling factor, RRF {Aquifex aeolicus}
MIKELEDIFKEAEKDMKKAVEYYKNEIAGLRTSRASTALVEEIKVEYYGSKVPIKQLGTISV
PEHNQIVIQVWDQNAVPAIEKAIREELNLNPTVQGNVIRVTLPPLTEERRRELVRLLHKITE
EARVRVRNVRREAKEMIEELEGISEDEKKRALERLQKLTDKYIDEINKLMEAKEKEIMSV
>d1j98a_ d.185.1.2 (A:) Autoinducer-2 production protein LuxS {Bacillus subtilis}
VESFELDHNAVVAPYVRHCGVHKVGTDGVVNKFDIRFCQPNKQAMKPDTIHTLEHLLAFTIR
SHAEKYDHFDIIDISPMGCQTGYYLVVSGETTSAEIVDLLEDTMKEAVEITEIPAANEKQCG
QAKLHDLEGAKRLMRFWLSQDKEELLKVFG
>d1inna_ d.185.1.2 (A:) Autoinducer-2 production protein LuxS {Deinococcus radiodurans}
NVESFDLDHTKVKAPYVRLAGVKTTPKGDQISKYDLRFLQPNQGAIDPAAIHTLEHLLAGYM
RDHLEGVVDVSPMGCRTGMYMAVIGEPDEQGVMKAFEAALKDTAGHDQPIPGVSELECGNYR
DHDLAAARQHARDVLDQGLKVQETILL
>d1j6wa_ d.185.1.2 (A:) Autoinducer-2 production protein LuxS {Haemophilus influenzae}
LLDSFKVDHTKMNAPAVRIAKTMLTPKGDNITVFDLRFCIPNKEILSPKGIHTLEHLFAGFM
RDHLNGDSIEIIDISPMGCRTGFYMSLIGTPNEQKVSEAWLASMQDVLGVQDQASIPELNIY
QCGSYTEHSLEDAHEIAKNVIARGIGVNKNEDLSLDN
>d1j6xa_ d.185.1.2 (A:) Autoinducer-2 production protein LuxS {Helicobacter pylori}
MKMNVESFNLDHTKVKAPYVRIADRKKGVNGDLIVKYDVRFKQPNRDHMDMPSLHSLEHLVA
EIIRNHANYVVDWSPMGCQTGFYLTVLNHDNYTEILEVLEKTMQDVLKAKEVPASNEKQCGW
AANHTLEGAQNLARAFLDKRAEWSEVG
>d1hr6b1 d.185.1.1 (B:24-245) Mitochondrial processing peptidase (MPP) beta chain {Baker's yeast (Saccharomyces cerevisiae)}
PGTRTSKLPNGLTIATEYIPNTSSATVGIFVDAGSRAENVKNNGTAHFLEHLAFKGTQNRPQ
QGIELEIENIGSHLNAYTSRENTVYYAKSLQEDIPKAVDILSDILTKSVLDNSAIERERDVI
IRESEEVDKMYDEVVFDHLHEITYKDQPLGRTILGPIKNIKSITRTDLKDYITKNYKGDRMV
LAGAGAVDHEKLVQYAQKYFGHVPKSESPVPLGSPR
>d1hr6b2 d.185.1.1 (B:246-462) Mitochondrial processing peptidase (MPP) beta chain {Baker's yeast (Saccharomyces cerevisiae)}
GPLPVFCRGERFIKENTLPTTHIAIALEGVSWSAPDYFVALATQAIVGNWDRAIGTGTNSPS
PLAVAASQNGSLANSYMSFSTSYADSGLWGMYIVTDSNEHNVRLIVNEILKEWKRIKSGKIS
DAEVNRAKAQLKAALLLSLDGSTAIVEDIGRQVVTTGKRLSPEEVFEQVDKITKDDIIMWAN
YRLQNKPVSMVALGNTSTVPNVSYIEEKLNQ
>d1hr6a1 d.185.1.1 (A:14-233) Mitochondrial processing peptidase (MPP) alpha chain {Baker's yeast (Saccharomyces cerevisiae)}
ARTDNFKLSSLANGLKVATSNTPGHFSALGLYIDAGSRFEGRNLKGCTHILDRLAFKSTEHV

EGRAMAETLELLGGNYQCTSSRENLMYQASVFNQDVGKMLQLMSETVRFPKITEQELQEQKL
SAEYEIDEVWMKPELVLPELLHTAAYSGETLGSPLICPRGLIPSISKYYLLDYRNKFYTPEN
TVAAFVGVPHEKALELTGKYLGDWQSTHPPITKK
>d1hr6a2 d.185.1.1 (A:234-470) Mitochondrial processing peptidase (MPP) alpha chain {Baker's yeast (Saccharomyces cerevisiae)}
VAQYTGGESCIPPAPVFGNLPELFHIQIGFEGLPIDHPDIYALATLQTLLGGGGSFSAGGPG
KGMYSRLYTHVLNQYYFVENCVAFNHSYSDSGIFGISLSCIPQAAPQAVEVIAQQMYNTFAN
KDLRLTEDEVSRAKNQLKSSLLMNLESKLVELEDMGRQVLMHGRKIPVNEMISKIEDLKPDD
ISRVAEMIFTGNVNNAGNGKGRATVVMQGDRGSFGDVENVLKAYGLGNSSS
>d1be3a1 d.185.1.1 (A:1-233) Cytochrome bc1 core subunit 1 {Cow (Bos taurus)}
TATYAQALQSVPETQVSQLDNGLRVASEQSSQPTCTVGVWIDAGSRYESEKNNGAGYFVEHL
AFKGTKNRPGNALEKEVESMGAHLNAYSTREHTAYYIKALSKDLPKAVELLADIVQNCSLED
SQIEKERDVILQELQENDTSMRDVVFNYLHATAFQGTPLAQSVEGPSENVRKLSRADLTEYL
SRHYKAPRMVLAAAGGLEHRQLLDLAQKHFSGLSGTYDEDAVPTLSP
>d1be3a2 d.185.1.1 (A:234-446) Cytochrome bc1 core subunit 1 {Cow (Bos taurus)}
CRFTGSQICHREDGLPLAHVAIAVEGPGWAHPDNVALQVANAIIGHYDCTYGGGAHLSSPLA
SIAATNKLCQSFQTFNICYADTGLLGAHFVCDHMSIDDMMFVLQGQWMRLCTSATESEVLRG
KNLLRNALVSHLDGTTPVCEDIGRSLLTYGRRIPLAEWESRIAEVDARVVREVCSKYFYDQC
PAVAGFGPIEQLPDYNRIRSGMFWLRF
>d1bcca1 d.185.1.1 (A:4-232) Cytochrome bc1 core subunit 1 {Chicken (Gallus gallus)}
YAQALQSVPETQVSQLDNGVRVASEQSSQPTCTVGVWIDAGSRYESEKNNGAGYFLEHLAFK
GTKNRPQNALEKEVESMGAHLNAYSSREHTAYYIKALSKDVPKAVELLADIVQNCSLEDSQI
EKERDVIVRELQENDTSMREVVFNYLHATAFQGTGLAQSVEGPSENIRKLSRADLTEYLSTH
YTAPRMVLAAAGGVEHQQLLELAQKHFGGVPFTYDDDAVPTLS
>d1bcca2 d.185.1.1 (A:233-445) Cytochrome bc1 core subunit 1 {Chicken (Gallus gallus)}
KCRFTGSQIRHREDGLPLAHVAIAVEGPGWAHPDLVALQVANAIIGHYDRTYGGGLHSSSPL
ASIAVTNKLCQSFQTFSICYSETGLFGFYFVCDRMSIDDMMFVLQGQWMRLCTSISESEVLR
GKNFLRNALVSHLDGTTPVCEDIGRELLTYGRRIPLEEWEERLAEVDARMVREVCSKYIYDQ
CPAVAGPGPIEQLPDYNRIRSGMFWLR
>d1ezva1 d.185.1.1 (A:27-239) Cytochrome bc1 core subunit 1 {Baker's yeast (Saccharomyces cerevisiae)}
AEVTQLSNGIVVATEHNPAHTASVGVVFGSGAANENPYNNGVSNLWKNIFLSKENSAVAAKE
GLALSSNISRDFQSYIVSSLPGSTDKSLDFLNQSFIQQKANLLSSSNFEATKKSVLKQVQDF
EDNDHPNRVLEHLHSTAFQNTPLSLPTRGTLESLENLVVADLESFANNHFLNSNAVVVGTGN
IKHEDLVNSIESKNLSLQTGTKPVLKK
>d1ezva2 d.185.1.1 (A:240-456) Cytochrome bc1 core subunit 1 {Baker's yeast (Saccharomyces cerevisiae)}
KAAFLGSEVRLRDDTLPKAWISLAVEGEPVNSPNYFVAKLAAQIFGSYNAFEPASRLQGIKL
LDNIQEYQLCDNFNHFSLSYKDSGLWGFSTATRNVTMIDDLIHFTLKQWNRLTISVTDTEVE
RAKSLLKLQLGQLYESGNPVNDANLLGAEVLIKGSKLSLGEAFKKIDAITVKDVKAWAGKRL

WDQDIAIAGTGQIEGLLDYMRIRSDMSMMRW
>d1be3b1 d.185.1.1 (B:21-235) Cytochrome bc1 core subunit 2 {Cow (Bos taurus)}
PQDLEFTRLPNGLVIASLENYAPASRIGLFIKAGSRYENSNNLGTSHLLRLASSLTTKGASS
FKITRGIEAVGGKLSVTSTRENMAYTVECLRDDVDILMEFLLNVTTAPEFRRWEVAALQPQL
RIDKAVALQNPQAHVIENLHAAAYRNALANSLYCPDYRIGKVTPVELHDYVQNHFTSARMAL
IGLGVSHPVLKQVAEQFLNIRGGLGLSGA
>d1be3b2 d.185.1.1 (B:236-439) Cytochrome bc1 core subunit 2 {Cow (Bos taurus)}
KAKYHGGEIREQNGDSLVHAALVAESAAIGSAEANAFSVLQHVLGAGPHVKRGSNATSSLYQ
AVAKGVHQPFDVSAFNASYSDSGLFGFYTISQAASAGDVIKAAYNQVKTIAQGNLSNPDVQA
AKNKLKAGYLMSVESSEGFLDEVGSQALAAGSYTPPSTVLQQIDAVADADVINAAKKFVSGR
KSMAASGNLGHTPFIDEL
>d1bccb1 d.185.1.1 (B:18-235) Cytochrome bc1 core subunit 2 {Chicken (Gallus gallus)}
PPHPQDLEITKLPNGLVIASLENYSPGSTIGVFIKAGSRYENSSNLGTSHLLRLASSLTTKG
ASSFKITRGIEAVGGKLSVESTRENMAYTVECLRDDVEILMEFLLNVTTAPEFRPWEVADLQ
PQLKIDKAVAFQNPQTHVIENLHAAAYRNALADSLYCPDYRIGKVTSVELHDFVQNHFTSAR
MALVGLGVSHPVLKNVAEQLLNIRGGLGLSGA
>d1bccb2 d.185.1.1 (B:236-439) Cytochrome bc1 core subunit 2 {Chicken (Gallus gallus)}
KAKYRGGEIREQNGDSLVHAAIVAESAAIGGAEANAFSVLQHVLGANPHVKRGLNATSSLYQ
AVAKGVHQPFDVSAFNASYSDSGLFGFYTISQAAYAGQVIKAAYNQVKTIAQGNVSNENVQA
AKNKLKAKYLMSVESSEGFLEEVGSQALAAGSYNPPSTVLQQIDAVADADVIKAAKKFVSRQ
KSMAASGNLGHTPFVDEL
>d1ezvb1 d.185.1.1 (B:17-218) Cytochrome bc1 core subunit 2 {Baker's yeast (Saccharomyces cerevisiae)}
LTVSARDAPTKISTLAVKVHGGSRYATKDGVAHLLNRFNFQNTNTRSALKLVRESELLGGTF
KSTLDREYITLKATFLKDDLPYYVNALADVLYKTAFKPHELTESVLPAARYDYAVAEQCPVK
SAEDQLYAITFRKGLGNPLLYDGVERVSLQDIKDFADKVYTKENLEVSGENVVEADLKRFVD
ESLLSTLPAGKSLVSK
>d1ezvb2 d.185.1.1 (B:219-368) Cytochrome bc1 core subunit 2 {Baker's yeast (Saccharomyces cerevisiae)}
SEPKFFLGEENRVRFIGDSVAAIGIPVNKASLAQYEVLANYLTSALSELSGLISSAKLDKFT
DGGLFTLFVRDQDSAVVSSNIKKIVADLKKGKDLSPAINYTKLKNAVQNESVSSPIELNFDA
VKDFKLGKFNYVAVGDVSNLPYLDEL
>d1tig__ d.68.1.1 (-) Translation initiation factor IF3, C-terminal domain {Bacillus stearothermophilus}
INVKEVRLSPTIEEHDFNTKLRNARKFLEKGDKVKATIRFKGRAITHKEIGQRVLDRLSEAC
ADIAVVETAPKMDGRNMFLVLAPKND
>d2ifea_ d.68.1.1 (A:) Translation initiation factor IF3, C-terminal domain {Escherichia coli}
VIQVKEIKFRPGTDEGDYQVKLRSLIRFLEEGDKAKITLRFRGREMAHQQIGMEVLNRVKDD
LQELAVVESFPTKIEGRQMIMVLAPKKKQ

>d1i96v_ d.68.1.1 (V:) Translation initiation factor IF3, C-terminal domain {Thermus thermophilus}
EVKSIKFRVKIDEHDYQTKLGHIKRFLQEGHKVKVTIMFRGREVAHPELGERILNRVTEDLK
DLAVVEMKPEMLGRDMNMLLAPVK
>d1dcja_ d.68.3.1 (A:) SirA {Escherichia coli}
MTDLFSSPDHTLDALGLRCPEPVMMVRKTVRNMQPGETLLIIADDPATTRDIPGFCTFMEHE
LVAKETDGLPYRYLIRKGG
>d1jdqa_ d.68.3.2 (A:) Hypothetical protein TM0983 {Thermotoga maritima}
GSSHHHHHHSSGLVPRGSHMAKYQVTKTLDVRGEVCPVPDVETKRALQNMKPGEILEVWIDY
PMSKERIPETVKKLGHEVLEIEEVGPSEWKIYIKVK
>d1qmha2 d.68.2.1 (A:5-184,A:280-338) RNA 3'-terminal phosphate cyclase, RPTC {Escherichia coli}
MIALDGAQGEGGGQILRSALSLSMITGQPFTITSIRAGRAKPGLLRQHLTAVKAATEICGAT
VEGAELGSQRLLFRPGTVRGGDYRFAIGSAGSCTLVLQTVLPALWFADGPSRVEVSGGTDNP
SAPPADFIRRVLEPLLAKIGIHQQTTLLRHGFYPAGGGVVATEVSPVASFNTLQLGXAVGEY
LADQLVLPMALAGAGEFTVAHPSCHLLTNIAVVERFLPVRFSLIETDGVTRVSI
>d1uae__ d.68.2.2 (-) UDP-N-acetylglucosamine enolpyruvyl transferase (EPT, MurA, MurZ) {Escherichia coli}
MDKFRVQGPTKLQGEVTISGAKNAALPILFAALLAEEPVEIQNVPKLKDVDTSMKLLSQLGA
KVERNGSVHIDARDVNVFCAPYDLVKTMRASIWALGPLVARFGQGQVSLPGGCTIGARPVDL
HISGLEQLGATIKLEEGYVKASVDGRLKGAHIVMDKVSVGATVTIMCAATLAEGTTIIENAA
REPEIVDTANFLITLGAKISGQGTDRIVIEGVERLGGGVYRVLPDRIETGTFLVAAAISRGK
IICRNAQPDTLDAVLAKLRDAGADIEVGEDWISLDMHGKRPKAVNVRTAPHPAFPTDMQAQF
TLLNLVAEGTGFITETVFENRFMHVPELSRMGAHAEIESNTVICHGVEKLSGAQVMATDLRA
SASLVLAGCIAEGTTVVDRIYHIDRGYERIEDKLRALGANIERVKG
>d1ejda_ d.68.2.2 (A:) UDP-N-acetylglucosamine enolpyruvyl transferase (EPT, MurA, MurZ) {Enterobacter cloacae}
MDKFRVQGPTRLQGEVTISGAKNAALPILFAALLAEEPVEIQNVPKLKDIDTTMKLLTQLGT
KVERXGSVWIDASNVNNFSAPYDLVKTMRASIWALGPLVARFGQGQVSLPGGCAIGARPVDL
HIFGLEKLGAEIKLEEGYVKASVNGRLKGAHIVMDKVSVGATVTIMSAATLAEGTTIIENAA
REPEIVDTANFLVALGAKISGQGTDRITIEGVERLGGGVYRVLPDRIETGTFLVAAAISGGK
IVCRNAQPDTLDAVLAKLREAGADIETGEDWISLDMHGKRPKAVTVRTAPHPAFPTDMQAQF
TLLNLVAEGTGVITETIFENRFMHVPELIRMGAHAEIESNTVICHGVEKLSGAQVMATDLRA
SASLVLAGCIAEGTTVVDRIYHIDRGYERIEDKLRALGANIERVKGE
>d1g6sa_ d.68.2.2 (A:) 5-enol-pyruvyl shikimate-3-phosphate(EPSP) synthase {Escherichia coli}
MESLTLQPIARVDGTINLPGSKSVSNRALLLAALAHGKTVLTNLLDSDDVRHMLNALTALGV
SYTLSADRTRCEIIGNGGPLHAEGALELFLGNAGTAMRPLAAALCLGSNDIVLTGEPRMKER
PIGHLVDALRLGGAKITYLEQENYPPLRLQGGFTGGNVDVDGSVSSQFLTALLMTAPLAPED
TVIRIKGDLVSKPYIDITLNLMKTFGVEIENQHYQQFVVKGGQSYQSPGTYLVEGDASSASY
FLAAAAIKGGTVKVTGIGRNSMQGDIRFADVLEKMGATICWGDDYISCTRGELNAIDMDMNH
IPDAAMTIATAALFAKGTTTLRNIYNWRVKETDRLFAMATELRKVGAEVEEGHDYIRITPPE
KLNFAEIATYNDHRMAMCFSLVALSDTPVTILDPKCTAKTFPDYFEQLARISQAA

>d1imua_ d.204.1.1 (A:) Ribosome binding protein Y (HI0257, YfiA homologue) {Haemophilus influenzae}
MTLNITSKQMDITPAIREHLEERLAKLGKWQTQLISPHFVLNKVPNGFSVEASIGTPLGNLL
ASATSDDMYKAINEVEEKLERQLNKLQHKSESRRADERLKDSFEN
>d1tola2 d.69.1.1 (A:125-216) C-terminal domain of TolA {Escherichia coli}
SGADINNYAGQIKSAIESKFYDASSYAGKTCTLRIKLAPDGMLLDIKPEGGDPALCQAALAA
AKLAKIPKPPSQAVYEVFKNAPLDFKPAAA
>d1kpta_ d.70.1.1 (A:) Virally encoded KP4 toxin {Ustilago maydis, P4 strain}
LGINCRGSSQCGLSGGNLMVRIRDQACGNQGQTWCPGERRAKVCGTGNSISAYVQSTNNCIS
GTEACRHLTNLVNHGCRVCGSDPLYAGNDVSRGQLTVNYVNSC
>g1kve.1 d.70.1.2 (A:,B:) SMK toxin {Halotolerant yeast (Pichia farinosa)}
WSLRWRMQKSTTIAAIAGCSGAATFGGLAGGIVGCIAAGILAILQGFEVNWHNGGGGDRSNP
VXGEATTIWGVGADEAIDKGTPSKNDLQNMSADLAKNGFKGHQGVACSTVKDGNKDVYMIKF
SLAGGSNDPGGSPCSDD
>d1ev0a_ d.71.1.1 (A:) Cell division protein MinE topological specificity domain {Escherichia coli}
RSDAEPHYLPQLRKDILEVICKYVQIDPEMVTVQLEQKDGDISILELNVTLPEAEELK
>d1dw9a2 d.72.1.1 (A:87-156) Cyanase C-terminal domain {Escherichia coli}
RIPTDPTMYRFYEMLQVYGTTLKALVHEKFGDGIISAINFKLDVKKVADPEGGERAVITLDG
KYLPTKPF
>d3rubs_ d.73.1.1 (S:) Ribulose 1,5-bisphosphate carboxylase-oxygenase {Tobacco (Nicotiana tabacum), variant turkish samsun}
MQVWPPINKKKYETLSYLPDLSQEQLLSEVEYLLKNGWVPCLEFETEHGFVYRENNKSPGYY
DGRYWTMWKLPMFGCTDATQVLAEVEEAKKAYPQAWIRIIGFDNVRQVQCISFIAYKPEGY
>d1burs_ d.73.1.1 (S:) Ribulose 1,5-bisphosphate carboxylase-oxygenase {Spinach (Spinacia oleracea)}
MQVWPILGMKKYETLSYLPPLTTEQLLAEVNYLLVNNWIPCLEFEVKDGFVYREHLKSPGYY
DGRYWTMWKLPMFGCTDPAQVLNELEECKKAYPDAFIRIIGFDNKRQVQCISFIAYKPAGY
>d8ruci_ d.73.1.1 (I:) Ribulose 1,5-bisphosphate carboxylase-oxygenase {Spinach (Spinacia oleracea)}
MQVWPILNLKKYETLSYLPPLTTDQLARQVDYLLNNKWVPCLEFETDHGFVYREHHNSPGYY
DGRYWTMWKLPMFGCTDPAQVLNELEECKKEYPNAFIRIIGFDSNREVQCISFIAYKPAGY
>d1bwvs_ d.73.1.1 (S:) Ribulose 1,5-bisphosphate carboxylase-oxygenase {Galdieria partita}
VRITQGTFSFLPDLTDEQIKKQIDYMISKKLAIGIEYTNDIHPRNAYWEIWGLPLFDVTDPA
AVLFEINACRKARSNFYIKVVGFSSVRGIESTIISFIVNRPKHEPGFNLMRQEDKSRSIKYT
IHSYESYKPEDERY
>d1gk8i_ d.73.1.1 (I:) Ribulose 1,5-bisphosphate carboxylase-oxygenase {Chlamydomonas reinhardtii}

MVWTPVNNKMFETFSYLPPLTDEQIAAQVDYIVANGWIPCLEFAEADKAYVSNESAIRFGSV
SCLYYDNRYWTMWKLPMFGCRDPMQVLREIVACTKAFPDAYVRLVAFDNQKQVQIMGFLVQR
P
>d1bxni_ d.73.1.1 (I:) Ribulose 1,5-bisphosphate
carboxylase-oxygenase {Alcaligenes eutrophus}
MRITQGTFSFLPELTDEQITKQLEYCLNQGWAVGLEYTDDPHPRNTYWEMFGLPMFDLRDAA
GILMEINNARNTFPNHYIRVTAFDSTHTVESVVMSFIVNRPADEPGFRLVRQEEPGRTLRYS
IESYA
>d1rblm_ d.73.1.1 (M:) Ribulose 1,5-bisphosphate
carboxylase-oxygenase {Synechococcus sp., strain pcc 6301}
SMKTLPKERRFETFSYLPPLSDRQIAAQIEYMIEQGFHPLIEFNEHSNPEEFYWTMWKLPLF
ACAAPQQVLDEVRECRSEYGDCYIRVAGFDNIKECQTSSFIVHRPGR
>d1dcpa_ d.74.1.1 (A:) Pterin-4a-carbinolamine dehydratase
(PCD)/dimerization cofactor of HNF1 (DCoH) {Rat (Rattus
norvegicus)}
HRLSAEERDQLLPNLRAVGWNELEGRDAIFKQFHFKDFNRAFGFMTRVALQAEKLDHHPEWF
NVYNKVHITLSTHECAGLSERDINLASFIEQVAVSMT
>d1xxaa_ d.74.2.1 (A:) C-terminal domain of arginine repressor
{Escherichia coli}
LKNLVLDIDYNDAVVVIHTSPGAAQLIARLLDSLGKAEGILGTIAGDDTIFTTPANGFTVKD
LYEAILELF
>d1b4ba_ d.74.2.1 (A:) C-terminal domain of arginine repressor
{Bacillus stearothermophilus}
ALVDVFIKLDGTGNLLVLRTLPGNAHAIGVLLDNLDWDEIVGTICGDDTCLIICRTPKDAKK
VSNQLLSML
>d1f9na2 d.74.2.1 (A:79-149) C-terminal domain of arginine
repressor {Bacillus subtilis}
ALMDAFVKIDSASHMIVLKTMPGNAQAIGALMDNLDWDEMMGTICGDDTILIICRTPEDTEG
VKNRLLELL
>d1i50k_ d.74.3.2 (K:) RPB11 {Baker's yeast (Saccharomyces
cerevisiae)}
MNAPDRFELFLLGEGESKLKIDPDTKAPNAVVITFEKEDHTLGNLIRAELLNDRKVLFAAYK
VEHPFFARFKLRIQTTEGYDPKDALKNACNSIINKLGALKTNFETEWNLQTL
>d1bdfa1 d.74.3.1 (A:2-52,A:179-232) RNA polymerase alpha
{Escherichia coli}
QGSVTEFLKPRLVDIEQVSSTHAKVTLEPLERGFGHTLGNALRAILLSSMPXPVERIAYNVE
AARVEQRTDLDKLVIEMETNGTIDPEEAIRRAATILAEQLEAFV
>d1i6va1 d.74.3.1 (A:6-49,A:173-229) RNA polymerase alpha {Thermus
aquaticus}
LKAPVFTATTQGDHYGEFVLEPLERGFGVTLGNPLRRILLSSIPXPVRRVAFQVEDTRLGQR
TDLDKLTLRIWTDGSVTPLEALNQAVAILKEHLNYFANPE
>d1i50c1 d.74.3.1 (C:3-41,C:173-268) RPB3 {Baker's yeast
(Saccharomyces cerevisiae)}
EEGPQVKIREASKDNVDFILSNVDLAMANSLRRVMIAEIXAAAIEFEYDPWNKLKHTDYWYE

QDSAKEWPQSKNCEYEDPPNEGDPFDYKAQADTFYMNVESVGSIPVDQVVVRGIDTLQKKVA
SILLALTQMDQD
>d1c0aa2 d.74.4.1 (A:288-420) Prokaryotic AspRS, insert domain
{Escherichia coli}
NPMELTDVADLLKSVEFAVFAGPANDPKGRVAALRVPGGASLTRKQIDEYGNFVKIYGAKGL
AYIKVNERAKGLEGINSPVAKFLNAEIIEDILDRTAAQDGDMIFFGADNKKIVADAMGALRL
KVGKDLGLT
>d1g51a2 d.74.4.1 (A:295-414) Prokaryotic AspRS, insert domain
{Thermus thermophilus}
FGLELKEVGPLFRQSGFRVFQEAESVKALALPKALSRKEVAELEEVAKRHKAQGLAWARVEE
GGFSGGVAKFLEPVREALLQATEARPGDTLLFVAGPRKVAATALGAVRLRAADLLGLK
>d1a79a2 d.75.1.1 (A:9-82) tRNA splicing endonuclease EdnA,
N-terminal domain {Archaeon Methanococcus jannaschii}
KITGLLDGDRVIVFDKNGISKLSARHYGNVEGNFLSLSLVEALYLINLGWLEVKYKDNKPLS
FEELYEYARNVE
>d1ewqa4 d.75.2.1 (A:1-120) DNA repair protein MutS, domain I
{Thermus aquaticus}
MEGMLKGEGPGPLPPLLQQYVELRDQYPDYLLLFQVGDFYECFGEDAERLARALGLVLTHKT
SKDFTTPMAGIPLRAFEAYAERLLKMGFRLAVADQVEPAEEAEGLVRREVTQLLTPGT
>d1e3ma4 d.75.2.1 (A:2-116) DNA repair protein MutS, domain I
{Escherichia coli}
SAIENFDAHTPMMQQYLRLKAQHPEILLFYRMGDFYELFYDDAKRASQLLDISLTKRGASAG
EPIPMAGIPYHAVENYLAKLVNQGESVAICEQIGDPATSKGPVERKVVRIVTP
>d1e3mb4 d.75.2.1 (B:14-116) DNA repair protein MutS, domain I
{Escherichia coli}
MQQYLRLKAQHPEILLFYRMGDFYELFYDDAKRASQLLDISLTKRGASAGEPIPMAGIPYHA
VENYLAKLVNQGESVAICEQIGDPATSKGPVERKVVRIVTP
>d1gyfa_ d.76.1.1 (A:) GYF domain from cd2bp2 protein {Human (Homo
sapiens)}
DVMWEYKWENTGDAELYGPFTSAQMQTWVSEGYFPDGVYCRKLDPPGGQFYNSKRIDFDLYT
>d1jg5a_ d.205.1.1 (A:) GTP cyclohydrolase I feedback regulatory
protein, GFRP {Rat (Rattus norvegicus)}
PYLLISTQIRMEVGPTMVGDEHSDPELMQQLGASKRRVLGNNFYEYYVNDPPRIVLDKLECR
GFRVLSMTGVGQTLVWCLHKE
>d1iq4a_ d.77.1.1 (A:) Ribosomal protein L5 {Bacillus
stearothermophilus}
MNRLKEKYLNEVVPALMSKFNYKSIMQVPKIEKIVINMGVGDAVQNPKALDSAVEELTLIAG
QRPVVTRAKKSIAGFRLRQGMPIGAKVTLRGERMYEFLDKLISVSLPRARDFRGVSKKSFDG
RGNYTLGIKEQLIFPEIDYDKVNKVRGMDIVIVTTANTDEEARELLALLGMPFQK
>d1jj2d_ d.77.1.1 (D:) Ribosomal protein L5 {Archaeon Haloarcula
marismortui}
FHEMREPRIEKVVVHMGIGHGGRDLANAEDILGEITGQMPVRTKAKRTVGEFDIREGDPIGA
KVTLRDEMAEEFLQTALPLAELATSQFDDTGNFSFGVEEHTEFPSQEYDPSIGIYGLDVTVN
LVRPGYRVAKRDKASRSIPTKHRLNPADAVAFIESTYDVEV

>d1eika_ d.78.1.1 (A:) RNA polymerase subunit RBP5 (RNA polymerase subunit H) {Archaeon Methanobacterium thermoautotrophicum}
MKREILKHQLVPEHVILNESEAKRVLKELDAHPEQLPKIKTTDPVAKAIGAKRGDIVKIIRK
SPTAEEFVTYRLVQD
>d1hmja_ d.78.1.1 (A:) RNA polymerase subunit RBP5 (RNA polymerase subunit H) {Archaeon Methanococcus jannaschii}
PKHEIVPKEEVEEILKRYNIKIQQLPKIYEDDPVIQEIGAKEGDVVRVIRKSPTAGVSIAYR
LVIKRI
>d1dzfa2 d.78.1.1 (A:144-215) Eukaryotic RPB5 C-terminal domain {Baker's yeast (Saccharomyces cerevisiae)}
ITHHELVPKHIRLSSDEKRELLKRYRLKESQLPRIQRADPVALYLGLKRGEVVKIIRKSETS
GRYASYRICM
>d1qkla_ d.78.1.2 (A:) RPB6 {Human (Homo sapiens)}
MSDNEDNFDGDDFDDVEEDEGLDDLENAEEEGQENVEILPSGERPQANQKRITTPYMTKYER
ARVLGTRALQIAMCAPVMVELEGETDPLLIAMKELKARKIPIIIRRYLPDGSYEDWGVDELI
ITD
>d1i50f_ d.78.1.2 (F:) RPB6 {Baker's yeast (Saccharomyces cerevisiae)}
KAIPKDQRATTPYMTKYERARILGTRALQISMNAPVFVDLEGETDPLRIAMKELAEKKIPLV
IRRYLPDGSFEDWSVEELIVDL
>d1qu9a_ d.79.1.1 (A:) Conserved 'hypothetical' protein YjgF {Escherichia coli}
SKTIATENAPAAIGPYVQGVDLGNMIITSGQIPVNPKTGEVPADVAAQARQSLDNVKAIVEA
AGLKVGDIVKTTVFVKDLNDFATVNATYEAFFTEHNATFPARSCVEVARLPKDVKIEIEAIA
VRR
>d1qd9a_ d.79.1.1 (A:) Purine regulatory protein YabJ {Bacillus subtilis}
TKAVHTKHAPAAIGPYSQGIIVNNMFYSSGQIPLTPSGEMVNGDIKEQTHQVFSNLKAVLEE
AGASFETVVKATVFIADMEQFAEVNEVYGQYFDTHKPARSCVEVARLPKDALVEIEVIALVK
>d1jd1a_ d.79.1.1 (A:) Highdosage growth inhibitor YER057cp (YEO7_YEAST) {Baker's yeast (Saccharomyces cerevisiae)}
TTLTPVICESAPAAAASYSHAMKVNNLIFLSGQIPVTPDNKLVEGSIADKAEQVIQNIKNVL
EASNSSLDRVVKVNIFLADINHFAEFNSVYAKYFNTHKPARSCVAVAALPLGVDMEMEAIAA
ER
>d1dbfa_ d.79.1.2 (A:) Chorismate mutase {Bacillus subtilis}
MMIRGIRGATTVERDTEEEILQKTKQLLEKIIEENHTKPEDVVQMLLSATPDLHAVFPAKAV
RELSGWQYVPVTCMQEMDVTGGLKKCIRVMMTVQTDVPQDQIRHVYLEKAVVLRPDLSLTKN
TEL
>d1fnja_ d.79.1.2 (A:) Chorismate mutase {Bacillus subtilis}
MIRGIRGATTVERDTEEEILQKTKQLLEKIIEENHTKPEDVVQMLLSATPDLHAVFPAKAVR
ELSGWQYVPVTCMQEMDVTGGLKKSIKVMMTVQTDVPQDQIRHVYLEKAVVLR
>d1jy8a_ d.79.5.1 (A:) 2C-methyl-D-erythritol 2,4-cyclodiphosphate synthase IspF {Escherichia coli}
MRIGHGFDVHAFGGEGPIIIGGVRIPYEKGLLAHSDGDVALHALTDALLGAAALGDIGKLFP

DTDPAFKGADSRELLREAWRRIQAKGYTLGNVDVTIIAQAPKMLPHIPQMRVFIAEDLGCHM
DDVNVKATTTEKLGFTGRGEGIACEAVALLI
>d1fsz_2 d.79.2.1 (232-356) Cell-division protein FtsZ {Archaeon Methanococcus jannaschii}
INVDFADVKAVMNNGGLAMIGIGESDSEKRAKEAVSMALNSPLLDVDIDGATGALIHVMGPE
DLTLEEAREVVATVSSRLDPNATIIWGATIDENLENTVRVLLVITGVQSRIEFTDTGLKRKK
L
>d1tuba2 d.79.2.1 (A:246-440) Tubulin alpha-subunit {Pig (Sus scrofa)}
GALNVDLTEFQTNLVPYPRGHFPLATYAPVISAEKAYHEQLSVAEITNACFEPANQMVKCDP
RHGKYMACCLLYRGDVVPKDVNAAIATIKTKRTIQFVDWCPTGFKVGINYEPPTVVPGGDLA
KVQRAVCMLSNTTAIAEAWARLDHKFDLMYAKRAFVHWYVGEGMEEGEFSEAREDMAALEKD
YEEVGVDSV
>d1tubb2 d.79.2.1 (B:246-437) Tubulin beta-subunit {Pig (Sus scrofa)}
GQLNADLRKLAVNMVPFPRLHFFMPGFAPLTSRGSQQYRALTVPELTQQMFDAKNMMAACDP
RHGRYLTVAAVFRGRMSMKEVDEQMLNVQNKNSSYFVEWIPNNVKTAVCDIPPRGLKMSATF
IGNSTAIQELFKRISEQFTAMFRRKAFLHWYTGEGMDEMEFTEAESNMNDLVSEYQQYQD
>d1ck9a_ d.79.3.1 (A:) Eukaryotic ribosomal protein L30 (L30e) {Baker's yeast (Saccharomyces cerevisiae)}
APVKSQESINQKLALVIKSGKYTLGYKSTVKSLRQGKSKLIIIAANTPVLRKSELEYYAMLS
KTKVYYFQGGNNELGTAVGKLFRVGVVSILEAGDSDILTTLA
>d1jj2f_ d.79.3.1 (F:) Ribosomal protein L7ae {Archaeon Haloarcula marismortui}
PVYVDFDVPADLEDDALEALEVARDTGAVKKGTNETTKSIERGSAELVFVAEDVQPEEIVMH
IPELADEKGVPFIFVEQQDDLGHAAGLEVGSAAAAVTDAGAAATVLEEIADKVEELR
>d1e7ka_ d.79.3.1 (A:) Spliceosomal 15.5kd protein {Human (Homo sapiens)}
ADVNPKAYPLADAHLTKKLLDLVQQSCNYKQLRKGANEATKTLNRGISEFIVMAADAEPLEI
ILHLPLLCEDKNVPYVFVRSKQALGRACGVSRPVIACSVTIKEGSQLKQQIQSIQQSIERLL
V
>d1dt9a2 d.79.3.2 (A:277-422) C-terminal domain of eukaryotic peptide chain release factor subunit 1, ERF1 {Human (Homo sapiens)}
NVKFIQEKKLIGRYFDEISQDTGKYCFGVEDTLKALEMGAVEILIVYENLDIMRYVLHCQGT
EEEKILYLTPEQEKDKSHFTDKETGQEHELIESMPLLEWFANNYKKFGATLEIVTDKSQEGS
QFVKGFGGIGGILRYRVDFQGM
>d1clia1 d.79.4.1 (A:5-170) Aminoimidazole ribonucleotide synthetase (PurM) N-terminal domain {Escherichia coli}
TSLSYKDAGVDIDAGNALVGRIKGVVKKTRRPEVMGGLGGFGALCALPQKYREPVLVSGTDG
VGTKLRLAMDLKRHDTIGIDLVAMCVNDLVVQGAEPLFFLDYYATGKLDVDTASAVISGIAE
GCLQSGCSLVGGETAEMPGMYHGEDYDVAGFCVGVVEKSEII
>d1clib1 d.79.4.1 (B:1021-1170) Aminoimidazole ribonucleotide synthetase (PurM) N-terminal domain {Escherichia coli}
ALVGRIKGVVKKTRRPEVMGGLGGFGALCALPQKYREPVLVSGTDGVGTKLRLAMDLKRHDT

IGIDLVAMCVNDLVVQGAEPLFFLDYYATGKLDVDTASAVISGIAEGCLQSGCSLVGGETAE
MPGMYHGEDYDVAGFCVGVVEKSEII
>d1otfa_ d.80.1.1 (A:) 4-oxalocrotonate tautomerase {Pseudomonas sp., DmpI}
PIAQLYIIEGRTDEQKETLIRQVSEAMANSLDAPLERVRVLITEMPKNHFGIGGEPASK
>d1bjpa_ d.80.1.1 (A:) 4-oxalocrotonate tautomerase {Pseudomonas putida, XylH}
PIAQIHILEGRSDEQKETLIREVSEAISRSLDAPLTSVRVIITEMAKGHFGIGGELASKVRR
>d1otga_ d.80.1.2 (A:) 5-carboxymethyl-2-hydroxymuconate isomerase (CHMI) {Escherichia coli}
PHFIVECSDNIREEADLPGLFAKVNPTLAATGIFPLAGIRSRVHWVDTWQMADGQHDYAFVH
MTLKIGAGRSLESRQQAGEMLFELIKTHFAALMESRLLALSFEIEELHPTLNFKQNNVHALF
K
>d1gd0a_ d.80.1.3 (A:) Microphage migration inhibition factor (MIF) {Human (Homo sapiens)}
PMFIVNTNVPRASVPDGFLSELTQQLAQATGKPPQYIAVHVVPDQLMAFGGSSEPCALCSLH
SIGKIGGAQNRSYSKLLCGLLAERLRISPDRVYINYYDMNAANVGWNNSTFALEHH
>d1fim__ d.80.1.3 (-) Microphage migration inhibition factor (MIF) {Rat (Rattus norvegicus)}
PAFIVNTNVPRASVPEGFLSELTQQLAQATGKPAQYIAVHVVPDQLMTFSGTSDPCALCSLH
SIGKIGGAQNRNYSKLLCGLLSDRLHISPDRVYINYYDANA
>d1mfia_ d.80.1.3 (A:) Microphage migration inhibition factor (MIF) {Mouse (Mus musculus)}
PMFIVNTNVPRASVPEGFLSELTQQLAQATGKPAQYIAVHVVPDQLMTFSGTNDPCALCSLH
SIGKIGGAQNRNYSKLLCGLLSDRLHISPDRVYINYYDMNAANVGWNGSTFA
>d1hfoa_ d.80.1.3 (A:) Microphage migration inhibition factor (MIF) {Trichina (Trichinella spiralis)}
PIFTLNTNIKATDVPSDFLSSTSALVGNILSKPGSYVAVHINTDQQLSFGGSTNPAAFGTLM
SIGGIEPSRNRDHSAKLFDHLNTKLGIPKNRMYIHFVNLNGDDVGWNGTTF
>d1dpta_ d.80.1.3 (A:) D-dopachrome tautomerase {Human (Homo sapiens)}
PFLELDTNLPANRVPAGLEKRLCAAAASILGKPADRVNVTVRPGLAMALSGSTEPCAQLSIS
SIGVVGTAEDNRSHSAHFFEFLTKELALGQDRILIRFFPLESWQIGKIGTVMTFL
>d1gado2 d.81.1.1 (O:149-312) Glyceraldehyde-3-phosphate dehydrogenase (GAPDH) {Escherichia coli}
CTTNCLAPLAKVINDNFGIIEGLMTTVHATTATQKTVDGPSHKDWRGGRGASQNIIPSSTGA
AKAVGKVLPELNGKLTGMAFRVPTPNVSVVDLTVRLEKAATYEQIKAAVKAAAEGEMKGVLG
YTEDDVVSTDFNGEVCTSVFDAKAGIALNDNFVKLVSWYD
>d1gd1o2 d.81.1.1 (O:149-312) Glyceraldehyde-3-phosphate dehydrogenase (GAPDH) {Bacillus stearothermophilus, nca 1503}
CTTNCLAPFAKVLHEQFGIVRGMMTTVHSYTNDQRILDLPHKDLRRARAAAESIIPTTTGAA
KAVALVLPELKGKLNGMAMRVPTPNVSVVDLVAELEKEVTVEEVNAALKAAAEGELKGILAY
SEEPLVSRDYNGSTVSSTIDALSTMVIDGKMVKVVSWYD
>d1cero2 d.81.1.1 (O:149-312) Glyceraldehyde-3-phosphate

dehydrogenase (GAPDH) {Thermus aquaticus}
CTTNSLAPVMKVLEEAFGVEKALMTTVHSYTNDQRLLDLPHKDLRRARAAAINIIPTTTGAA
KATALVLPSLKGRFDGMALRVPTATGSISDITALLKREVTAEEVNAALKAAAEGPLKGILAY
TEDEIVLQDIVMDPHSSIVDAKLTKALGNMVKVFAWYD
>d1hdgo2 d.81.1.1 (O:149-312) Glyceraldehyde-3-phosphate
dehydrogenase (GAPDH) {Thermotoga maritima}
CTTNSIAPIVKVLHEKFGIVSGMLTTVHSYTNDQRVLDLPHKDLRRARAAAVNIIPTTTGAA
KAVALVVPEVKGKLDGMAIRVPTPDGSITDLTVLVEKETTVEEVNAVMKEATEGRLKGIIGY
NDEPIVSSDIIGTTFSGIFDATITNVIGGKLVKVASWYD
>d1b7go2 d.81.1.1 (O:139-300) Glyceraldehyde-3-phosphate
dehydrogenase (GAPDH) {Archaeon Sulfolobus solfataricus}
CNTTALLRTICTVNKVSKVEKVRATIVRRAADQKEVKKGPINSLVPDPATVPSHHAKDVNSV
IRNLDIATMAVIAPTTLMHMHFINITLKDKVEKKDILSVLENTPRIVLISSKYDAEATAELV
EVARDLKRDRNDIPEVMIFSDSIYVKDDEVMLMYAVHQ
>d1cf2o2 d.81.1.1 (O:139-303) Glyceraldehyde-3-phosphate
dehydrogenase (GAPDH) {Archaeon Methanothermus fervidus}
SCNTTGLCRTLKPLHDSFGIKKVRAVIVRRGADPAQVSKGPINAIIPNPPKLPSHHGPDVKT
VLDINIDTMAVIVPTTLMHQHNVMVEVEETPTVDDIIDVFEDTPRVILISAEDGLTSTAEIM
EYAKELGRSRNDLFEIPVWRESITVVDNEIYYMQAVHQESD
>d1ggaa2 d.81.1.1 (A:165-333) Glyceraldehyde-3-phosphate
dehydrogenase (GAPDH) {Trypanosoma brucei brucei, glycosome}
CTTNCLAPLVHVLVKEGFGISTGLMTTVHSYTATQKTVDGVSVKDWRGGRAAALNIIPSTTG
AAKAVGMVIPSTQGKLTGMAFRVPTADVSVVDLTFIATRDTSIKEIDAALKRASKTYMKNIL
GYTDEELVSADFISDSRSSIYDSKATLQNNLPNERRFFKIVSWYD
>d1i32a2 d.81.1.1 (A:166-334) Glyceraldehyde-3-phosphate
dehydrogenase (GAPDH) {Leishmania mexicana}
CTTNCLAPIVHVLTKENFGIETGLMTTIHSYTATQKTVDGVSLKDWRGGRAAAVNIIPSTTG
AAKAVGMVIPSTKGKLTGMSFRVPTPDVSVVDLTFRATRDTSIQEIDKAIKKAAQTYMKGIL
GFTDEELVSADFINDNRSSVYDSKATLQNNLPGEKRFFKVVSWYD
>d1dssg2 d.81.1.1 (G:149-312) Glyceraldehyde-3-phosphate
dehydrogenase (GAPDH) {Lobster (Palinurus versicolor)}
CTTNCLAPVAKVLHENFEIVEGLMTTVHAVTATQKTVDGPSAKDWRGGRGAAQNIIPSSTGA
AKAVGKVIPELDGKLTGMAFRVPTPNVSVVDLTVRLGKECSYDDIKAAMKAASEGPLQGVLG
YTEDDVVSCDFTGDNRSSIFDAKAGIQLSKTFVKVVSWYD
>d3gpdg2 d.81.1.1 (G:151-314) Glyceraldehyde-3-phosphate
dehydrogenase (GAPDH) {Human (Homo sapiens)}
CTTNCLAPLAKVIHDHFGIVEGLMTTVHAITATQKTVDSPSGKLWRGGRGAAQNLIPASTGA
AKAVGKVIPELDGKLTGMAFRVPTANVSVLDLTCRLEKPAKYDDIKKVVKEASEGPLKGILG
YTEDEVVSDDFNGSNHSSIFDAGAGIELNDTFVKLVSWYD
>d1jn0a2 d.81.1.1 (A:149-312) Glyceraldehyde-3-phosphate
dehydrogenase (GAPDH) {Spinach (Spinacia oleracea)}
CTTNCLAPFVKVLDQKFGIIKGTMTTTHSYTGDQRLLDASHRDLRRARAACLNIVPTSTGAA
KAVALVLPQLKGKLNGIALRVPTPNVSVVDLVVQVSKKTFAEEVNAAFRESADQELKGILSV
CDEPLVSIDFRCTDVSSTIDSSLTMVMGDDMVKVIAWYD

>d1gl3a2 d.81.1.1 (A:134-354) Aspartate beta-semialdehyde dehydrogenase {Escherichia coli}
NCTVSLMLMSLGGLFANDLVDWVSVATYQAASGGGARHMRELLTQMGHLYGHVADELATPSS
AILDIERKVTTLTRSGELPVDNFGVPLAGSLIPWIDKQLDNGQSREEWKGQAETNKILNTSS
VIPVDGLCVRVGALRCHSQAFTIKLKKDVSIPTVEELLAAHNPWAKVVPNDREITMRELTPA
AVTGTLTTPVGRLRKLNMGPEFLSAFTVGDQLLWG
>d1ebfa2 d.81.1.2 (A:151-340) Homoserine dehydrogenase {Baker's yeast (Saccharomyces cerevisiae)}
PIISFLREIIQTGDEVEKIEGIFSGTLSYIFNEFSTSQANDVKFSDVVKVAKKLGYTEPDPR
DDLNGLDVARKVTIVGRISGVEVESPTSFPVQSLIPKPLESVKSADEFLEKLSDYDKDLTQL
KKEAATENKVLRFIGKVDVATKSVSVGIEKYDYSHPFASLKGSDNVISIKTKRYTNPVVIQG
AGAG
>d1e5qa2 d.81.1.2 (A:125-391) Saccharopine reductase {Rice blast fungus (Magnaporthe grisea)}
LDPGIDHLYAIKTIEEVHAAGGKIKTFLSYCGGLPAPESSDNPLGYKFSWSSRGVLLALRNA
ASFYKDGKVTNVAGPELMATAKPYFIYPGFAFVAYPNRDSTPYKERYQIPEADNIVRGTLRY
QGFPQFIKVLVDIGFLSDEEQPFLKEAIPWKEATQKIVKASSASEQDIVSTIVSNATFESTE
EQKRIVAGLKWLGIFSDKKITPRGNALDTLCATLEEKMQFEEGERDLVMLQHKFEIENKDGS
RETRTSSLCEYGAPIGSGG
>d1f06a2 d.81.1.3 (A:119-268) Diaminopimelic acid dehydrogenase (DAPDH) {Corynebacterium glutamicum}
WDPGMFSINRVYAAAVLAEHQQHTFWGPGLSQGHSDALRRIPGVQKAVQYTLPSEDALEKAR
RGEAGDLTGKQTHKRQCFVVADAADHERIENDIRTMPDYFVGYEVEVNFIDEATFDSEHTGM
PHGGHVITTGDTGGFNHTVEYILKLD
>d1dih_2 d.81.1.3 (131-240) Dihydrodipicolinate reductase {Escherichia coli}
VGVNVMLKLLEKAAKVMGDYTDIEIIEAHHRHKVDAPSGTALAMGEAIAHALDKDLKDCAVY
SREGHTGERVPGTIGFATVRAGDIVGEHTAMFADIGERLEITHKASSR
>d1k5ha3 d.81.1.3 (A:126-274) 1-deoxy-D-xylulose-5-phosphate reductoisomerase {Escherichia coli}
ESLVTCGRLFMDAVKQSKAQLLPVDSEHNAIFQSLPQPIQHNLGYADLEQNGVVSILLTGSG
GPFRETPLRDLATMTPDQACRHPNWSMGRKISVDSATMMNKGLEYIEARWLFNASASQMEVL
IHPQSVIHSMVRYQDGSVLAQLGEP
>d1gcua2 d.81.1.4 (A:129-246) Biliverdin reductase {Rat (Rattus norvegicus)}
MEEFEFLRREVLGKELLKGSLRFTASPLEEERFGFPAFSGISRLTWLVSLFGELSLISATLE
ERKEDQYMKMTVQLETQNKGLLSWIEEKGPGLKRNRYVNFQFTSGSLEEVPSVGVN
>d1h6da2 d.81.1.5 (A:213-374) Glucose-fructose oxidoreductase {Zymomonas mobilis}
DPMNRAAVKLIRENQLGKLGMVTTDNSDVMDQNDPAQQWRLRRELAGGGSLMDIGIYGLNGT
RYLLGEEPIEVRAYTYSDPNDERFVEVEDRIIWQMRFRSGALSHGASSYSTTTTSRFSVQGD
KAVLLMDPATGYYQNLISVQTPGHANQSMMPQFIMPAN
>d1dpga2 d.81.1.5 (A:182-412,A:427-485) Glucose 6-phosphate dehydrogenase {Leuconostoc mesenteroides}

KEMVQNIAALRFGNPIFDAAWNKDYIKNVQVTLSEVLGVEERAGYYDTAGALLDMIQNHTMQ
IVGWLAMEKPESFTDKDIRAAKNAAFNALKIYDEAEVNKYFVRAQYGAGDSADFKPYLEELD
VPADSKNNTFIAGELQFDLPRWEGVPFYVRSGKRLAAKQTRVDIVFKAGTFNFGSEQEAQEA
VLSIIIDPKGAIELKLNAKSVEDAFNTRTIDLGWTVSDEDKKNTPXGSNFADWNGVSIAWKF
VDAISAVYTADKAPLETYKSGSMGPEASDKLLAANGDAWVFKG
>d1qkia2 d.81.1.5 (A:200-434,A:450-511) Glucose 6-phosphate dehydrogenase {Human (Homo sapiens)}
DHYLGKEMVQNLMVLRFANRIFGPIWNRDNIACVILTFKEPFGTEGRGGYFDEFGIIRDVMQ
NHLLQMLCLVAMEKPASTNSDDVRDEKVKVLKCISEVQANNVVLGQYVGNPDGEGEATKGYL
DDPTVPRGSTTATFAAVVLYVENERWDGVPFILRCGKALNERKAEVRLQFHDVAGDIFHQQC
KRNELVIRVQPNEAVYTKMMTKKPGMFFNPEESELDLTYGNRYKNVKLPXMHFVRSDELLEA
WRIFTPLLHQIELEKPKPIPYIYGSRGPTEADELMKRVGFQYEGTYKWVN
>d1oaca4 d.82.1.1 (A:5-90) Copper amine oxidase, domain N {Escherichia coli}
AHMVPMDKTLKEFGADVQWDDYAQLFTLIKDGAYVKVKPGAQTAIVNGQPLALQVPVVMKDN
KAWVSDTFINDVFQSGLDQTFQVE
>d1ekga_ d.82.2.1 (A:) C-terminal domain of frataxin {Human (Homo sapiens)}
LDETTYERLAEETLDSLAEFFEDLADKPYTFEDYDVSFGSGVLTVKLGGDLGTYVINKQTPN
KQIWLSSPSSGPKRYDWTGKNWVYSHDGVSLHELLAAELTKALKTKLDLSSLAYSGK
>d1ew4a_ d.82.2.1 (A:) CyaY {Escherichia coli}
MNDSEFHRLADQLWLTIEERLDDWDGDSDIDCEINGGVLTITFENGSKIIINRQEPLHQVWL
ATKQGGYHFDLKGDEWICDRSGETFWDLLEQAATQQAGETVSFR
>d1ewfa1 d.83.1.1 (A:1-217) Bactericidal permeability-increasing protein, BPI {Human (Homo sapiens)}
VNPGVVVRISQKGLDYASQQGTAALQKELKRIKIPDYSDSFKIKHLGKGHYSFYSMDIREFQ
LPSSQISMVPNVGLKFSISNANIKISGKWKAQKRFLKMSGNFDLSIEGMSISADLKLGSNPT
SGKPTITCSSCSSHINSVHVHISKSKVGWLIQLFHKKIESALRNKMNSQVCEKVTNSVSSEL
QPYFQTLPVMTKIDSVAGINYGLVAPPATTA
>d1ewfa2 d.83.1.1 (A:218-456) Bactericidal permeability-increasing protein, BPI {Human (Homo sapiens)}
ETLDVQMKGEFYSENHHNPPPFAPPVMEFPAAHDRMVYLGLSDYFFNTAGLVYQEAGVLKMT
LRDDMIPKESKFRLTTKFFGTFLPEVAKKFPNMKIQIHVSASTPPHLSVQPTGLTFYPAVDV
QAFAVLPNSALASLFLIGMHTTGSMEVSAESNRLVGELKLDRLLLELKHSNIGPFPVELLQD
IMNYIVPILVLPRVNEKLQKGFPLPTPARVQLYNVVLQPHQNFLLFGADVVYK
>d1ihra_ d.191.1.1 (A:) Dimeric C-terminal domain of membrane protein TonB {Escherichia coli}
ARAQALRIEGQVKVKFDVTPDGRVDNVQILSAKPANMFEREVKNAMRRWRYEPGKPGSGIVV
NILFKINGTTE
>d2sici_ d.84.1.1 (I:) Subtilisin inhibitor {Streptomyces albogriseolus, s-3253}
YAPSALVLTVGKGVSATTAAPERAVTLTCAPGPSGTHPAAGSACADLAAVGGDLNALTRGED
VMCPMVYDPVLLTVDGVWQGKRVSYERVFSNECEMNAHGSSVFAF
>d1e6ta_ d.85.1.1 (A:) MS2 virus coat protein {Bacteriophage MS2}

ASNFTQFVLVDNGGTGDVTVAPSNFANGVAEWISSNSRSQAYKVTCSVRQSSAQNRKYTIKV
EVPKVATQTVGGVELPVAAWRSYLNMELTIPIFATNSDCELIVKAMQGLLKDGNPIPSAIAA
NSGIY
>d1unaa_ d.85.1.1 (A:) GA coat protein {Bacteriophage GA}
ATLHSFVLVDNGGTGNVTVVPVSNANGVAEWLSNNSRSQAYRVTASYRASGADKRKYTIKLE
VPKIVTQVVNGVELPVSAWKAYASIDLTIPIFAATDDVTVISKSLTGLFKVGNPIAEAISSQ
SGFYA
>d1frsa_ d.85.1.1 (A:) fr coat protein {Bacteriophage FR}
ASNFEEFVLVDNGGTGDVKVAPSNFANGVAEWISSNSRSQAYKVTCSVRQSSANNRKYTVKV
EVPKVATQVQGGVELPVAAWRSYMNMELTIPVFATNDDCALIVKALQGTFKTGNPIATAIAA
NSGIY
>d1qbea_ d.85.1.1 (A:) Qbeta coat protein {Bacteriophage Qbeta}
AKLETVTLGNIGKDGKQTLVLNPRGVNPTNGVASLSQAGAVPALEKRVTVSVSQPSRNRKNY
KVQVKIQNPTACTANGSCDPSVTRQAYADVTFSFTQYSTDEERAFVRTELAALLASPLLIDA
IDQLNPAY
>d1dwna_ d.85.1.1 (A:) PP7 coat protein {Bacteriophage PP7}
SKTIVLSVGEATRTLTEIQSTADRQIFEEKVGPLVGRLRLTASLRQNGAKTAYRVNLKLDQA
DVVDCSTSVCGELPKVRYTQVWSHDVTIVANSTEASRKSLYDLTKSLVATSQVEDLVVNLVP
LGR
>d1ej1a_ d.86.1.1 (A:) Translation initiation factor eIF4e {Mouse
(Mus musculus)}
KHPLQNRWALWFFKNDKSKTWQANLRLISKFDTVEDFWALYNHIQLSSNLMPGCDYSLFKDG
IEPMWEDEKNKRGGRWLITLNKQQRRSDLDRFWLETLLCLIGESFDDYSDDVCGAVVNVRAK
GDKIAIWTTECENRDAVTHIGRVYKERLGLPPKIVIGYQSHADTATKSGSTTKNRFVV
>d1ap8__ d.86.1.1 (-) Translation initiation factor eIF4e {Baker's
yeast (Saccharomyces cerevisiae)}
MSVEEVSKKFEENVSVDDTTATPKTVLSDSAHFDVKHPLNTKWTLWYTKPAVDKSESWSDLL
RPVTSFQTVEEFWAIIQNIPEPHELPLKSDYHVFRNDVRPEWEDEANAKGGKWSFQLRGKGA
DIDELWLRTLLAVIGETIDEDDSQINGVVLSIRKGGNKFALWTKSEDKEPLLRIGGKFKQVL
KLTDDGHLEFFPHSSANGRHPQPSITL
>d3grs_3 d.87.1.1 (364-478) Glutathione reductase {Human (Homo
sapiens)}
YNNIPTVVFSHPPIGTVGLTEDEAIHKYGIENVKTYSTSFTPMYHAVTKRKTKCVMKMVCAN
KEEKVVGIHMQGLGCDEMLQGFAVAVKMGATKADFDNTVAIHPTSSEELVTLR
>d1gesa3 d.87.1.1 (A:336-450) Glutathione reductase {Escherichia
coli}
YSNIPTVVFSHPPIGTVGLTEPQAREQYGDDQVKVYKSSFTAMYTAVTTHRQPCRMKLVCVG
SEEKIVGIHGIGFGMDEMLQGFAVALKMGATKKDFDNTVAIHPTAAEEFVTMR
>d1feca3 d.87.1.1 (A:358-485) Trypanothione reductase {Crithidia
fasciculata}
HTKVACAVFSIPPMGVCGYVEEDAAKKYDQVAVYESSFTPLMHNISGSTYKKFMVRIVTNHA
DGEVLGVHMLGDSSPEIIQSVAICLKMGAKISDFYNTIGVHPTSAEELCSMRTPAYFYEKGK
RVEK
>d1aoga3 d.87.1.1 (A:358-487) Trypanothione reductase

{Trypanosoma cruzi}
DHTRVASAVFSIPPIGTCGLIEEVASKRYEVVAVYLSSFTPLMHKVSGSKYKTFVAKIITNH
SDGTVLGVHLLGDNAPEIIQGIGICLKLNAKISDFYNTIGVHPTSAEELCSMRTPSYYYVKG
EKMEKP
>d1h6va3 d.87.1.1 (A:367-499) Mammalian thioredoxin reductase {Rat (Rattus norvegicus)}
YDNVPTTVFTPLEYGCCGLSEEKAVEKFGEENIEVYHSFFWPLEWTVPSRDNNKCYAKVICN
LKDNERVVGFHVLGPNAGEVTQGFAAALKCGLTKQQLDSTIGIHPVCAEIFTTLSVTKRSGG
DILQSGCCG
>d1nhp_3 d.87.1.1 (322-447) NADH peroxidase {Enterococcus faecalis}
GVQGSSGLAVFDYKFASTGINEVMAQKLGKETKAVTVVEDYLMDFNPDKQKAWFKLVYDPET
TQILGAQLMSKADLTANINAISLAIQAKMTIEDLAYADFFFQPAFDKPWNIINTAALEAVKQ
ER
>d1d7ya3 d.87.1.1 (A:309-405) NADH-dependent ferredoxin reductase, BphA4 {Pseudomonas sp., KKS102}
TAPGYAELPWYWSDQGALRIQVAGLASGDEEIVRGEVSLDAPKFTLIELQKGRIVGATCVNN
ARDFAPLRRLLAVGAKPDRAALADPATDLRKLAAA
>d1lvl_3 d.87.1.1 (336-458) Dihydrolipoamide dehydrogenase {Pseudomonas putida}
PAAIAAVCFTDPEVVVVGKTPEQASQQGLDCIVAQFPFAANGRAMSLESKSGFVRVVARRDN
HLILGWQAVGVAVSELSTAFAQSLEMGACLEDVAGTIHAHPTLGEAVQEAALRALGHALHI
>d1lpfa3 d.87.1.1 (A:349-472) Dihydrolipoamide dehydrogenase {Pseudomonas fluorescens}
YDLIPSVIYTHPEIAWVGKTEQTLKAEGVEVNVGTFPFAASGRAMAANDTTGLVKVIADAKT
DRVLGVHVIGPSAAELVQQGAIGMEFGTSAEDLGMMVFSHPTLSEALHEAALAVNGHAIHIA
>d3lada3 d.87.1.1 (A:349-472) Dihydrolipoamide dehydrogenase {Azotobacter vinelandii}
YDLIPAVIYTHPEIAGVGKTEQALKAEGVAINVGVFPFAASGRAMAANDTAGFVKVIADAKT
DRVLGVHVIGPSAAELVQQGAIAMEFGTSAEDLGMMVFAHPALSEALHEAALAVSGHAIHVA
>d1ebda3 d.87.1.1 (A:347-461) Dihydrolipoamide dehydrogenase {Bacillus stearothermophilus}
AIPAVVFSDPECASVGYFEQQAKDEGIDVIAAKFPFAANGRALALNDTDGFLKLVVRKEDGV
IIGAQIIGPNASDMIAELGLAIEAGMTAEDIALTIHAHPTLGEIAMEAAEVAL
>d1ojt_3 d.87.1.1 (471-598) Dihydrolipoamide dehydrogenase {Neisseria meningitidis}
ARVIPGVAYTSPEVAWVGETELSAKASARKITKANFPWAASGRAIANGCDKPFTKLIFDAET
GRIIGGGIVGPNGGDMIGEVCLAIEMGCDAADIGKTIHPHPTLGESIGMAAEVALGTCTDLP
PQKK
>d1jeha3 d.87.1.1 (A:356-478) Dihydrolipoamide dehydrogenase {Baker's yeast (Saccharomyces cerevisiae)}
YNNIPSVMYSHPEVAWVGKTEEQLKEAGIDYKIGKFPFAANSRAKTNQDTEGFVKILIDSKT
ERILGAHIIGPNAGEMIAEAGLALEYGASAEDVARVCHAHPTLSEAFKEANMAAYDKAIHC
>d1dxla3 d.87.1.1 (A:348-470) Dihydrolipoamide dehydrogenase

{Garden pea (Pisum sativum)}
YDKVPGVVYTNPEVASVGKTEEQVKETGVEYRVGKFPFMANSRAKAIDNAEGLVKIIAEKET
DKILGVHIMAPNAGELIHEAAIALQYDASSEDIARVCHAHPTMSEAIKEAAMATYDKPIHI
>d1fcda3 d.87.1.1 (A:328-401) Flavocytochrome c sulfide dehydrogenase, FCSD, flavin-binding subunit {Purple phototrophic bacterium (Chromatium vinosum)}
PGTPSYLNTCYSILAPAYGISVAAIYRPNADGSAIESVPDSGGVTPVDAPDWVLEREVQYAY
SWYNNIVHDTFG
>d1qj2c1 d.87.2.1 (C:178-285) Carbon monoxide (CO) dehydrogenase flavoprotein, C-terminal domain {Pseudomonas carboxydovorans}
GHGYAYEKLKRKIGDYATAAAAVVLTMSGGKCVTASIGLTNVANTPLWAEEAGKVLVGTALD
KPALDKAVALAEAITAPASDGRGPAEYRTKMAGVMLRRAVERAKAR
>d1ffvc1 d.87.2.1 (C:178-287) Carbon monoxide (CO) dehydrogenase flavoprotein, C-terminal domain {Hydrogenophaga pseudoflava}
GTGWAYEKLKRKTGDWATAGCAVVMRKSGNTVSHIRIALTNVAPTALRAEAAEAALLGKAFT
KEAVQAAADAAIAICEPAEDLRGDADYKTAMAGQMVKRALNAAWARCA
>d1fo4a4 d.87.2.1 (A:415-531) Xanthine oxidase, domain 4 (?) {Cow (Bos taurus)}
DEFFSAFKQASRREDDIAKVTCGMRVLFQPGSMQVKELALCYGGMADRTISALKTTQKQLSK
FWNEKLLQDVCAGLAEELSLSPDAPGGMIEFRRTLTLSFFFKFYLTVLKKLGKDS
>d1jroa3 d.87.2.1 (A:346-462) Xanthine dehydrogenase chain A, domain 4 {Rhodobacter capsulatus}
PGLRCYKLSKRFDQDISAVCGCLNLTLKGSKIETARIAFGGMAGVPKRAAAFEAALIGQDFR
EDTIAAALPLLAQDFTPLSDMRASAAYRMNAAQAMALRYVRELSGEAVAVLEVMP
>d1srsa_ d.88.1.1 (A:) Serum response factor (SRF) core {Human (Homo sapiens)}
TRGRVKIKMEFIDNKLRRYTTFSKRKTGIMKKAYELSTLTGTQVLLLVASETGHVYTFATRK
LQPMITSETGKALIQTCLNSPD
>d1mnma_ d.88.1.1 (A:) MCM1 transcriptional regulator {Baker's yeast (Saccharomyces cerevisiae)}
QKERRKIEIKFIENKTRRHVTFSKRKHGIMKKAFELSVLTGTQVLLLVVSETGLVYTFSTPK
FEPIVTQQEGRNLIQACLNAPDD
>d1egwa_ d.88.1.1 (A:) Mef2a core {Human (Homo sapiens)}
GRKKIQITRIMDERNRQVTFTKRKFGLMKKAYELSVLCDCEIALIIFNSSNKLFQYASTDMD
KVLLKYTEY
>d1tbd__ d.89.1.1 (-) The origin DNA-binding domain of SV40 T-antigen {Simian virus 40}
GSKVEDPKDFPSELLSFLSHAVFSNRTLACFAIYTTKEKAALLYKKIMEKYSVTFISRHNSY
NHNILFFLTPHRHRVSAINNYAQKLCTFSFLICKGVNKEYLMYSALTRDPFSVIEESLPGGL
KEHDFNPESS
>d1f08a_ d.89.1.2 (A:) Replication initiation protein E1 {Bovine papillomavirus}
GSRATVFKLGLFKSLFLCSFHDITRLFKNDKTTNQQWVLAVFGLAEVFFEASFELLKKQCSF
LQMQKRSHEGGTCAVYLICFNTAKSRETVRNLMANMLNVREECLMLQPPKIRGLSAALFWFK

SSLSPATLKHGALPEWIRAQTTLN
>d1nox__ d.90.1.1 (-) NADH oxidase {Thermus thermophilus, HB8}
PVLDAKTAALKRRSIRRYRKDPVPEGLLREILEAALRAPSAWNLQPWRIVVVRDPATKRALR
EAAFGQAHVEEAPVVLVLYADLEDALAHLDEVIHPGVQGERREAQKQAIQRAFAAMGQEARK
AWASGQSYILLGYLLLLLEAYGLGSVPMLGFDPERVRAILGLPSRAAIPALVALGYPAEEGY
PSHRLPLERVVLWR
>d1bkja_ d.90.1.1 (A:) Flavin reductase P (NADPH:FMN oxidoreductase) {Vibrio harveyi}
NNTIETILAHRSIRKFTAVPITDEQRQTIIQAGLAASSSSMLQVVSIVRVTDSEKRNELAQF
AGNQAYVESAAEFLVFCIDYQRHATINPDVQADFTELTLIGAVDSGIMAQNCLLAAESMGLG
GVYIGGLRNSAAQVDELLGLPENSAVLFGMCLGHPDQNPEVKPRLPAHVVVHENQYQELNLD
DIQSYDQTMQAYYASRTSNQKLSTWSQEVTGKLAGESRPHILPYLNSKGLAKR
>d1vfra_ d.90.1.1 (A:) Flavin reductase P (NADPH:FMN oxidoreductase) {Vibrio fischeri}
THPIIHDLENRYTSKKYDPSKKVSQEDLAVLLEALRLSASSINSQPWKFIVIESDAAKQRMH
DSFANMHQFNQPHIKACSHVILFANKLSYTRDDYDVVLSKAVADKRITEEQKEAAFASFKFV
ELNCDENGEHKAWTKPQAYLALGNALHTLARLNIDSTTMEGIDPELLSEIFADELKGYECHV
ALAIGYHHPSEDYNASLPKSRKAFEDVITIL
>d1kqba_ d.90.1.1 (A:) Nitroreductase {Enterobacter cloacae}
DIISVALKRHSTKAFDASKKLTAEEAEKIKTLLQYSPSSTNSQPWHFIVASTEEGKARVAKS
AAGTYVFNERKMLDASHVVVFCAKTAMDDAWLERVVDQEEADGRFNTPEAKAANHKGRTYFA
DMHRVDLKDDDQWMAKQVYLNVGNFLLGVGAMGLDAVPIEGFDAAILDEEFGLKEKGFTSLV
VVPVGHHSVEDFNATLPKSRLPLSTIVTEC
>d1icra_ d.90.1.1 (A:) Nitroreductase {Escherichia coli, minor form}
DIISVALKRHSTKAFDASKKLTPEQAEQIKTLLQYSPSSTNSQPWHFIVASTEEGKARVAKS
AAGNYVFNERKMLDASHVVVFCAKTAMDDVWLKLVVDQEDADGRFATPEAKAANDKGRKFFA
DMHRKDLHDDAEWMAKQVYLNVGNFLLGVAALGLDAVPIEGFDAAILDAEFGLKEKGYTSLV
VVPVGHHSVEDFNATLPKSRLPQNITLTEV
>d1f5va_ d.90.1.1 (A:) Nitroreductase {Escherichia coli, oxygen-insensitive form}
MTPTIELICGHRSIRHFTDEPISEAQREAIINSARATSSSSFLQCSSIIRITDKALREELVT
LTGGQKHVAQAAEFWVFCADFNRHLQICPDAQLGLAEQLLLGVVDTAMMAQNALIAAESLGL
GGVYIGGLRNNIEAVTKLLKLPQHVLPLFGLCLGWPADNPDLKPRLPASILVHENSYQPLDK
GALAQYDEQLAEYYLTRGSNNRRDTWSDHIRRTIIKESRPFILDYLHKQGWATR
>d1dt9a3 d.91.1.1 (A:5-142) N-terminal domain of eukaryotic peptide chain release factor subunit 1, ERF1 {Human (Homo sapiens)}
PSAADRNVEIWKIKKLIKSLEAARGNGTSMISLIIPPKDQISRVAKMLADEFGTASNIKSRV
NRLSVLGAITSVQQRLKLYNKVPPNGLVVYCGTIVTEEGKEKKVNIDFEPFKPINTSLYLCD
NKFHTEALTALLSD
>d1c7ka_ d.92.1.1 (A:) Zinc protease {Streptomyces caespitosus}
TVTVTYDPSNAPSFQQEIANAAQIWNSSVRNVQLRAGGNADFSYYEGNDSRGSYAQTDGHGR
GYIFLDYQQNQQYDSTRVTAHETGHVLGLPDHYQGPCSELMSGGGPGPSCTNPYPNAQERSR
VNALWANG

>d1g12a_ d.92.1.12 (A:) Fungal zinc peptidase {Grifola frondosa}
TYNGCSSSEQSALAAAASAAQSYVAESLSYLQTHTAATPRYTTWFGSYISSRHSTVLQHYTD
MNSNDFSSYSFDCTCTAAGTFAYVYPNRFGTVYLCGAFWKAPTTGTDSQAGTLVHESSHFTR
NGGTKDYAYGQAAAKSLATMDPDKAVMNADNHEYFSENNPAQS
>d1eb6a_ d.92.1.12 (A:) Fungal zinc peptidase {Aspergillus oryzae, deuterolysin}
TEVTDCKGDAESSLTTALSNAAKLANQAAEAAESGDESKFEEYFKTTDQQTRTTVAERLRAV
AKEAGSTSGGSTTYHCNDPYGYCEPNVLAYTLPSKNEIANCDIYYSELPPLAQKCHAQDQAT
TTLHEFTHAPGVYQPGTEDLGYGYDAATQLSAQDALNNADSYALYANAIELKC
>d1ezm__ d.92.1.2 (-) Elastase {Pseudomonas aeruginosa}
AEAGGPGGNQKIGKYTYGSDYGPLIVNDRCEMDDGNVITVDMNSSTDDSKTTPFRFACPTNT
YKQVNGAYSPLNDAHFFGGVVFKLYRDWFGTSPLTHKLYMKVHYGRSVENAYWDGTAMLFGD
GATMFYPLVSLDVAAHEVSHGFTEQNSGLIYRGQSGGMNEAFSDMAGEAAEFYMRGKNDFLI
GYDIKKGSGALRYMDQPSRDGRSIDNASQYYNGIDVHHSSGVYNRAFYLLANSPGWDTRKAF
EVFVDANRYYWTATSNYNSGACGVIRSAQNRNYSAADVTRAFSTVGVTCP
>d8tlne_ d.92.1.2 (E:) Thermolysin {Bacillus thermoproteolyticus}
ITGTSTVGVGRGVLGDQKNINTTYSTYYYLQDNTRGDGIFTYDAKYRTTLPGSLWADADNQF
FASYDAPAVDAHYYAGVTYDYYKNVHNRLSYDGNNAAIRSSVHYSQGYNNAFWNGSEMVYGD
GDGQTFIPLSGGIDVVAHELTHAVTDYTAGLIYQNESGAINEAISDIFGTLVEFYANKNPDW
EIGEDVYTPGISGDSLRSMSDPAKYGDPDHYSKRYTGTQDNGGVHINSGIINKAAYLISQGG
THYGVSVVGIGRDKLGKIFYRALTQYLTPTSNFSQLRAAAVQSATDLYGSTSQEVASVKQAF
DAVGVK
>d1npc__ d.92.1.2 (-) Neutral protease {Bacillus cereus, strain dsm 3101}
VTGTNKVGTGKGVLGDTKSLNTTLSGSSYYLQDNTRGATIFTYDAKNRSTLPGTLWADADNV
FNAAYDAAAVDAHYYAGKTYDYYKATFNRNSINDAGAPLKSTVHYGSNYNNAFWNGSQMVYG
DGDGVTFTSLSGGIDVIGHELTHAVTENSSNLIYQNESGALNEAISDIFGTLVEFYDNRNPD
WEIGEDIYTPGKAGDALRSMSDPTKYGDPDHYSKRYTGSSDNGGVHTNSGIINKQAYLLANG
GTHYGVTVTGIGKDKLGAIYYRANTQYFTQSTTFSQARAGAVQAAADLYGANSAEVAAVKQS
FSAVGVN
>d1bqba_ d.92.1.2 (A:) Aureolysin {Staphylococcus aureus}
AAATGTGKGVLGDTKDININSIDGGFSLEDLTHQGKLSAYNFNDQTGQATLITNEDENFVKD
DQRAGVDANYYAKQTYDYYKNTFGRESYDNHGSPIVSLTHVNHYGGQDNRNNAAWIGDKMIY
GDGDGRTFTNLSGANDVVAHEITHGVTQQTANLEYKDQSGALNESFSDVFGYFVDDEDFLMG
EDVYTPGKEGDALRSMSNPEQFGQPSHMKDYVYTEKDNGGVHTNSGIPNKAAYNVIQAIGKS
KSEQIYYRALTEYLTSNSNFKDLKDALYQAAKDLYEQQTAEQVYEAWNEVGVE
>d1hs6a3 d.92.1.13 (A:209-460) Leukotriene A4 hydrolase catalytic domain {Human (Homo sapiens)}
LESRQIGPRTLVWSEKEQVEKSAYEFSETESMLKIAEDLGGPYVWGQYDLLVLPPSFPYGGM
ENPCLTFVTPTLLAGDKSLSNVIAHEISHSWTGNLVTNKTWDHFWLNEGHTVYLERHICGRL
FGEKFRHFNALGGWGELQNSVKTFGETHPFTKLVVDLTDIDPDVAYSSVPYEKGFALLFYLE
QLLGGPEIFLGFLKAYVEKFSYKSITTDDWKDFLYSYFKDKVDVLNQVDWNAWLYSPGLPPI
KPNY
>d1dmta_ d.92.1.4 (A:) Neutral endopeptidase (neprilysin) {Human

(Homo sapiens)}
GICKSSDCIKSAARLIQNMDATTEPCTDFFKYACGGWLKRNVIPETSSRYGNFDILRDELEV
VLKDVLQEPKTEDIVAVQKAKALYRSCINESAIDSRGGEPLLKLLPDIYGWPVATENWEQKY
GASWTAEKAIAQLNSKYGKKVLINLFVGTDDKNSVNHVIHIDQPRLGLPSRDYYECTGIYKE
ACTAYVDFMISVARLIRQEERLPIDENQLALEMNKVMELEKEIANATAKPEDRNDPMLLYNK
MTLAQIQNNFSLEINGKPFSWLNFTNEIMSTVNISITNEEDVVVYAPEYLTKLKPILTKYSA
RDLQNLMSWRFIMDLVSSLSRTYKESRNAFRKALYGTTSETATWRRCANYVNGNMENAVGRL
YVEAAFAGESKHVVEDLIAQIREVFIQTLDDLTWMDAETKKRAEEKALAIKERIGYPDDIVS
NDNKLNNEYLELNYKEDEYFENIIQNLKFSQSKQLKKLREKVDKDEWISGAAVVNAFYSSGR
NQIVFPAGILQPPFFSAQQSNSLNYGGIGMVIGHEITHGFDDNGRNFNKDGDLVDWWTQQSA
SNFKEQSQCMVYQYGNFSWDLAGGQHLNGINTLGENIADNGGLGQAYRAYQNYIKKNGEEKL
LPGLDLNHKQLFFLNFAQVWCGTYRPEYAVNSIKTDVHSPGNFRIIGTLQNSAEFSEAFHCR
KNSYMNPEKKCRVW
>d1i1ip_ d.92.1.5 (P:) Neurolysin (endopeptidase 24.16) {Rat
(Rattus norvegicus)}
MSSYTAAGRNVLRWDLSPEQIKTRTEQLIAQTKQVYDTVGTIALKEVTYENCLQVLADIEVT
YIVERTMLDFPQHVSSDREVRAASTEADKKLSRFDIEMSMREDVFQRIVHLQETCDLEKIKP
EARRYLEKSIKMGKRNGLHLSEHIRNEIKSMKKRMSELCIDFNKNLNEDDTSLVFSKAELGA
LPDDFIDSLEKTDEDKYKVTLKYPHYFPVMKKCCVPETRRKMEMAFHTRCKQENTAILQQLL
PLRAQVAKLLGYNTHADFVLELNTAKSTSRVAAFLDDLSQKLKPLGEAEREFILSLKKKECE
ERGFEYDGKINAWDLHYYMTQTEELKYSVDQESLKEYFPIEVVTEGLLSIYQELLGLSFEQV
PDAHVWNKSVSLYTVKDKATGEVLGQFYLDLYPREGKYNHAACFGLQPGCLLPDGSRMMSVA
ALVVNFSQPVAGRPSLLRHDEVRTYFHEFGHVMHQICAQTDFARFSGTNVETDFVEVPSQML
ENWVWDVDSLRKLSKHYKDGHPITDELLEKLVASRLVNTGLLTLRQIVLSKVDQSLHTNATL
DAASEYAKYCTEILGVAATPGTNMPATFGHLAGGYDGQYYGYLWSEVFSMDMFHSCFKKEGI
MNPEVGMKYRNLILKPGGSLDGMDMLQNFLQREPNQKAFLMSRGL
>d1j7na1 d.92.1.14 (A:27-263) Anthrax toxin lethal factor, N- and
C-terminal domains {Bacillus anthracis}
ERNKTQEEHLKEIMKHIVKIEVKGEEAVKKEAAEKLLEKVPSDVLEMYKAIGGKIYIVDGDI
TKHISLEALSEDKKKIKDIYGKDALLHEHYVYAKEGYEPVLVIQSSEDYVENTEKALNVYYE
IGKILSRDILSKINQPYQKFLDVLNTIKNASDSDGQDLLFTNQLKEHPTDFSVEFLEQNSNE
VQEVFAKAFAYYIEPQHRDVLQLYAPEAFNYMDKFNEQEINLSLEELKDQR
>d1j7na2 d.92.1.14 (A:551-773) Anthrax toxin lethal factor, N- and
C-terminal domains {Bacillus anthracis}
PKSKIDTKIQEAQLNINQEWNKALGLPKYTKLITFNVHNRYASNIVESAYLILNEWKNNIQS
DLIKKVTNYLVDGNGRFVFTDITLPNIAEQYTHQDEIYEQVHSKGLYVPESRSILLHGPSKG
VELRNDSEGFIHEFGHAVDDYAGYLLDKNQSDLVTNSKKFIDIFKEEGSNLTSYGRTNEAEF
FAEAFRLMHSTDHAERLKVQKNAPKTFQFINDQIKFI
>d1lml__ d.92.1.3 (-) Leishmanolysin {Leishmania major}
VVRDVNWGALRIAVSTEDLTDPAYHCARVGQHVKDHAGAIVTCTAEDILTNEKRDILVKHLI
PQAVQLHTERLKVQQVQGKWKVTDMVGDICGDFKVPQAHITEGFSNTDFVMYVASVPSEEGV
LAWATTCQTFSDGHPAVGVINIPAANIASRYDQLVTRVVTHEMAHALGFSGPFFEDARIVAN
VPNVRGKNFDVPVINSSTAVAKAREQYGCDTLEYLEVEDQGGAGSAGSHIKMRNAQDELMAP
AAAAGYYTALTMAIFQDLGFYQADFSKAEVMPWGQNAGCAFLTNKCMEQSVTQWPAMFCNES

EDAIRCPTSRLSLGACGVTRHPGLPPYWQYFTDPSLAGVSAFMDYCPVVVPYSDGSCTQRAS
EAHASLLPFNVFSDAARCIDGAFRPKATDGIVKSYAGLCANVQCDTATRTYSVQVHGSNDYT
NCTPGLRVELSTVSNAFEGGGYITCPPYVEVCQGNVQAAKD
>d1kapp2 d.92.1.6 (P:1-246) Metallo protease, catalytic (N-terminal) domain {Pseudomonas aeruginosa, alkaline protease}
GRSDAYTQVDNFLHAYARGGDELVNGHPSYTVDQAAEQILREQASWQKAPGDSVLTLSYSFL
TKPNDFFNTPWKYVSDIYSLGKFSAFSAQQQAQAKLSLQSWSDVTNIHFVDAGQGDQGDLTF
GNFSSSVGGAAFAFLPDVPDALKGQSWYLINSSYSANVNPANGNYGRQTLTHEIGHTLGLSH
PGDYNAGEGDPTYADATYAEDTRAYSVMSYWEEQNTGQDFKGAYSSAPLLDDIAAIQKLY
>d1sat_2 d.92.1.6 (4-246) Metallo protease, catalytic (N-terminal) domain {Serratia marcescens}
TGYDAVDDLLHYHERGNGIQINGKDSFSNEQAGLFITRENQTWNGYKVFGQPVKLTFSFPDY
KFSSTNVAGDTGLSKFSAEQQQQAKLSLQSWADVANITFTEVAAGQKANITFGNYSQDRPGH
YDYGTQAYAFLPNTIWQGQDLGGQTWYNVNQSNVKHPATEDYGRQTFTHEIGHALGLSHPGD
YNAGEGDPTYADVTYAEDTRQFSLMSYWSETNTGGDNGGHYAAAPLLDDIAAIQHLY
>d3btaa3 d.92.1.7 (A:1-546) Botulinum neurotoxin {Clostridium botulinum, serotype A}
PFVNKQFNYKDPVNGVDIAYIKIPNVGQMQPVKAFKIHNKIWVIPERDTFTNPEEGDLNPPP
EAKQVPVSYYDSTYLSTDNEKDNYLKGVTKLFERIYSTDLGRMLLTSIVRGIPFWGGSTIDT
ELKVIDTNCINVIQPDGSYRSEELNLVIIGPSADIIQFECKSFGHEVLNLTRNGYGSTQYIR
FSPDFTFGFEESLEVDTNPLLGAGKFATDPAVTLAHELIHAGHRLYGIAINPNRVFKVNTNA
YYEMSGLEVSFEELRTFGGHDAKFIDSLQENEFRLYYYNKFKDIASTLNKAKSIVGTTASLQ
YMKNVFKEKYLLSEDTSGKFSVDKLKFDKLYKMLTEIYTEDNFVKFFKVLNRKTYLNFDKAV
FKINIVPKVNYTIYDGFNLRNTNLAANFNGQNTEINNMNFTKLKNFTGLFEFYKLLCVRGII
TSKTKSLDKGYNKALNDLCIKVNNWDLFFSPSEDNFTNDLNKGEEITSDTNIEAAEENISLD
LIQQYYLTFNFDNEPENISIENLSSDIIGQLELMPNIERFPNGKKYELDK
>d1epwa3 d.92.1.7 (A:1-533) Botulinum neurotoxin {Clostridium botulinum, serotype B}
PVTINNFNYNDPIDNNNIIMMEPPFARGTGRYYKAFKITDRIWIIPERYTFGYKPEDFNKSS
GIFNRDVCEYYDPDYLNTNDKKNIFLQTMIKLFNRIKSKPLGEKLLEMIINGIPYLGDRRVP
LEEFNTNIASVTVNKLISNPGEVERKKGIFANLIIFGPGPVLNENETIDIGIQNHFASREGF
GGIMQMKFCPEYVSVFNNVQENKGASIFNRRGYFSDPALILMHELIHVLHGLYGIKVDDLPI
VPNEKKFFMQSTDAIQAEELYTFGGQDPSIITPSTDKSIYDKVLQNFRGIVDRLNKVLVCIS
DPNININIYKNKFKDKYKFVEDSEGKYSIDVESFDKLYKSLMFGFTETNIAENYKIKTRASY
FSDSLPPVKIKNLLDNEIYTIEEGFNISDKDMEKEYRGQNKAINKQAYEEISKEHLAVYKIQ
MCKSVKAPGICIDVDNEDLFFIADKNSFSDDLSKNERIEYNTQSNYIENDFPINELILDTDL
ISKIELPSENTESLTDFNVDVPVYEKQPAIKKIFTDE
>d1f83a_ d.92.1.7 (A:) Botulinum neurotoxin {Clostridium botulinum, serotype B}
PVTINNFNYNDPIDNNNIIMMEPPFARGTGRYYKAFKITDRIWIIPERYTFGYKPEDFNKSS
GIFNRDVCEYYDPDYLNTNDKKNIFLQTMIKLFNRIKSKPLGEKLLEMIINGIPYLGDRRVP
LEEFNTNIASVTVNKLISNPGEVERKKGIFANLIIFGPGPVLNENETIDIGIQNHFASREGF
GGIMQMKFCPEYVSVFNNVQENKGASIFNRRGYFSDPALILMHELIHVLHGLYGIKVDDLPI
VPNEKKFFMQSTDAIQAEELYTFGGQDPSIITPSTDKSIYDKVLQNFRGIVDRLNKVLVCIS

DPNININIYKNKFKDKYKFVEDSEGKYSIDVESFDKLYKSLMFGFTETNIAENYKIKTRASY
FSDSLPPVKIKNLLDNEIYTIEEGFNISDKDMEKEYRGQNKAINKQAYEEISK
>d1ast__ d.92.1.8 (-) Astacin {European fresh water crayfish (Astacus astacus)}
AAILGDEYLWSGGVIPYTFAGVSGADQSAILSGMQELEEKTCIRFVPRTTESDYVEIFTSGS
GCWSYVGRISGAQQVSLQANGCVYHGTIIHELMHAIGFYHEHTRMDRDNYVTINYQNVDPSM
TSNFDIDTYSRYVGEDYQYYSIMHYGKYSFSIQWGVLETIVPLQNGIDLTDPYDKAHMLQTD
ANQINNLYTNECSL
>d4aig__ d.92.1.9 (-) Snake venom metalloprotease {Eastern diamondback rattlesnake (Crotalus adamanteus), adamalysin II}
NLPQRYIELVVVADRRVFMKYNSDLNIIRTRVHEIVNIINEFYRSLNIRVSLTDLEIWSGQD
FITIQSSSSNTLNSFGEWRERVLLTRKRHDNAQLLTAINFEGKIIGKAYTSSMCNPRSSVGI
VKDHSPINLLVAVTMAHELGHNLGMEHDGKDCLRGASLCIMRPGLTPGRSYEFSDDSMGYYQ
KFLNQYKPQCILNKP
>d1atla_ d.92.1.9 (A:) Snake venom metalloprotease {Western diamonback rattlesnake (Crotalus atrox), atrolysin C}
LPQRYIELVVVADHRVFMKYNSDLNTIRTRVHEIVNFINGFYRSLNIHVSLTDLEIWSNEDQ
INIQSASSDTLNAFAEWRETDLLNRKSHDNAQLLTAIELDEETLGLAPLGTMCDPKLSIGIV
QDHSPINLLMGVTMAHELGHNLGMEHDGKDCLRGASLCIMRPGLTKGRSYEFSDDSMHYYER
FLKQYKPQCILNKP
>d1buda_ d.92.1.9 (A:) Snake venom metalloprotease {Five-pace snake (Agkistrodon acutus), acutolysin A}
FQRYMEIVIVVDHSMVKKYNGDSDSIKAWVYEMINTITESYSYLKIDISLSGLEIWSGKDLI
DVEASAGNTLKSFGEWRAKDLIHRISHDNAQLLTATDFDGATIGLAYVASMCNPKRSVGVIQ
DHSSVNRLVAITLAHEMAHNLGVSHDEGSCSCGGKSCIMSPSISDETIKYFSDCSYIQCRDY
ISKENPPCILN
>d1quaa_ d.92.1.9 (A:) Snake venom metalloprotease {Chinese five-pace snake (Agkistrodon acutus), acutolysin C}
PAPQTSIELFLIVDHSMYAKYNSNSSKITTTLKARVNIMNAIYSSLNLVITLSGIEMWSAAD
LITVQSSSRNTLKLFASWRETDLLKRTSNDNAQLLTATNFNGNTVGLAYLKTMCNSKYSVGL
IQDHSAIPLLMAVTMAHELGHNLGMNHDGAGCSCATCIMAPVLSSGPAKSFSDCSKHDYQSF
LTIHKPQCLLN
>d1bkca_ d.92.1.10 (A:) TNF-alpha converting enzyme, TACE, catalytic domain {Human (Homo sapiens)}
DPMKNTCKLLVVADHRFYRYMGRGEESTTTNYLIELIDRVDDIYRNTAWDNAGFKGYGIQIE
QIRILKSPQEVKPGEKHYNMAKSYPNEEKDAWDVKMLLEQFSFDIAEEASKVCLAHLFTYQD
FDMGTLGLAYVGSPRANSHGGVCPKAYYSPVGKKNIYLNSGLTSTKNYGKTILTKEADLVTT
HELGHNFGAEHDPDGLAECAPNEDQGGKYVMYPIAVSGDHENNKMFSQCSKQSIYKTIESKA
QECFQER
>d1cgla_ d.92.1.11 (A:) Fibroblast collagenase (MMP-1) {Human (Homo sapiens)}
VLTEGNPRWEQTHLRYRIENYTPDLPRADVDHAIEKAFQLWSDVTPLTFTKVSEGQADIMIS
FVRGDHRDNSPFDGPGGNLAHAFDPGPGIGGDAHFDEDERWTNNFREYNLHRVAAHELGHSL
GLSHSTDIGALMYPSYTFSGDVQLAQDDIDGIQAIYGRSQNPVQ

>d1hfc__ d.92.1.11 (-) Fibroblast collagenase (MMP-1) {Human (Homo sapiens)}
PRWEQTHLTYRIENYTPDLPRADVDHAIEKAFQLWSNVTPLTFTKVSEGQADIMISFVRGDH
RDNSPFDGPGGNLAHAFQPGPGIGGDAHFDEDERWTNNFREYNLHRVAAHELGHSLGLSHST
DIGALMYPSYTFSGDVQLAQDDIDGIQAIYGRS
>d1fbl_2 d.92.1.11 (100-271) Fibroblast collagenase (MMP-1) {Pig (Sus scrofa)}
FVLTPGNPRWENTHLTYRIENYTPDLSREDVDRAIEKAFQLWSNVSPLTFTKVSEGQADIMI
SFVRGDHRDNSPFDGPGGNLAHAFQPGPGIGGDAHFDEDERWTKNFRDYNLYRVAAHELGHS
LGLSHSTDIGALMYPNYIYTGDVQLSQDDIDGIQAIYGPSENPVQPSG
>d1hova_ d.92.1.11 (A:) MMP-2 {Human (Homo sapiens)}
MYNFFPRKPKWDKNQITYRIIGYTPDLDPETVDDAFARAFQVWSDVTPLRFSRIHDGEADIM
INFGRWEHGDGYPFDGKDGLLAHAFAPGTGVGGDSHFDDDELWTNTSANYSLFLVAAHEFGH
AMGLEHSQDPGALMAPIYTYTKNFRLSQDDIKGIQELYG
>d1i76a_ d.92.1.11 (A:) Neutrophil collagenase (MMP-8) {Human (Homo sapiens)}
MLTPGNPKWERTNLTYRIRNYTPQLSEAEVERAIKDAFELWSVASPLIFTRISQGEADINIA
FYQRDHGDNSPFDGPNGILAHAFQPGQGIGGDAHFDAEETWTNTSANYNLFLVAAHEFGHSL
GLAHSSDPGALMYPNYAFRETSNYSLPQDDIDGIQAIYG
>d1qiba_ d.92.1.11 (A:) Gelatinase A {Human (Homo sapiens)}
RKPKWDKNQITYRIIGYTPDLDPETVDDAFARAFQVWSDVTPLRFSRIHDGEADIMINFGRW
EHGDGYPFDGKDGLLAHAFAPGTGVGGDSHFDDDELWSLGKGVGYSLFLVAAHEFGHAMGLE
HSQDPGALMAPIYTYTKNFRLSQDDIKGIQELYGASP
>d1hy7a_ d.92.1.11 (A:) Stromelysin-1 (MMP-3) {Human (Homo sapiens), fibroblast}
FRTFPGIPKWRKTHLTYRIVNYTPDLPKDAVDSAVEKALKVWEEVTPLTFSRLYEGEADIMI
SFAVREHGDFYPFDGPGNVLAHAYAPGPGINGDAHFDDDEQWTKDTTGTNLFLVAAHEIGHS
LGLFHSANTEALMYPLYHSLTDLTRFRLSQDDINGIQSLYGPPP
>d1mmq__ d.92.1.11 (-) Matrilysin (MMP-9) {Human (Homo sapiens)}
YSLFPNSPKWTSKVVTYRIVSYTRDLPHITVDRLVSKALNMWGKEIPLHFRKVVWGTADIMI
GFARGAHGDSYPFDGPGNTLAHAFAPGTGLGGDAHFDEDERWTDGSSLGINFLYAATHELGH
SLGMGHSSDPNAVMYPTYGNGDPQNFKLSQDDIKGIQKLYGK
>d1hv5a_ d.92.1.11 (A:) Stromelysin-3 (MMP-11) {Mouse (Mus musculus)}
MFVLSGGRWEKTDLTYRILRFPWQLVREQVRQTVAEALQVWSEVTPLTFTEVHEGRADIMID
FARYWHGDNLPFDGPGGILAHAFFPKTHREGDVHFDYDETWTIGDNQGTDLLQVAAHEFGHV
LGLQHTTAAKALMSPFYTFRYPLSLSPDDRRGIQHLYG
>d1jk3a_ d.92.1.11 (A:) Macrophage elastase (MMP-12) {Human (Homo sapiens)}
GPVWRKHYITYRINNYTPDMNREDVDYAIRKAFQVWSNVTPLKFSKINTGMADILVVFARGA
HGDFHAFDGKGGILAHAFGPGSGIGGDAHFDEDEFWTTHSGGTNLFLTAVHAIGHSLGLGHS
SDPKAVMFPTYKYVDINTFRLSADDIRGIQSLYG
>d830ca_ d.92.1.11 (A:) Collagenase-3 (MMP-13) {Human (Homo sapiens)}

YNVFPRTLKWSKMNLTYRIVNYTPDMTHSEVEKAFKKAFKVWSDVTPLNFTRLHDGIADIMI
SFGIKEHGDFYPFDGPSGLLAHAFPPGPNYGGDAHFDDDETWTSSSKGYNLFLVAAHEFGHS
LGLDHSKDPGALMFPIYTYTGKSHFMLPDDDVQGIQSLYGPGDE
>d1cxva_ d.92.1.11 (A:) Collagenase-3 (MMP-13) {Mouse (Mus musculus)}
YNVFPRTLKWSQTNLTYRIVNYTPDMSHSEVEKAFRKAFKVWSDVTPLNFTRIYDGTADIMI
SFGTKEHGDFYPFDGPSGLLAHAFPPGPNYGGDAHFDDDETWTSSSKGYNLFIVAAHELGHS
LGLDHSKDPGALMFPIYTYTGKSHFMLPDDDVQGIQFLYG
>d1bqqm_ d.92.1.11 (M:) Membrane-type matrix metalloproteinase (CDMT1-MMP) {Human (Homo sapiens)}
IQGLKWQHNEITFCIQNYTPKVGEYATYEAIRKAFRVWESATPLRFREVPYAYIREGHEKQA
DIMIFFAEGFHGDSTPFDGEGGFLAHAYFPGPNIGGDTHFDSAEPWTVRNEDLNGNDIFLVA
VHELGHALGLEHSSDPSAIMAPFYQWMDTENFVLPDDDRRGIQQLYGGES
>d1qba_4 d.92.2.1 (201-337) Bacterial chitobiase, Domain 2 {Serratia marcescens}
SNADLQTLPAGALRGKIVPTPMQVKVHAQDADLRKGVALDLSTLVKPAADVVSQRFALLGVP
VQTNGYPIKTDIQPGKFKGAMAVSGAYELKIGKKEAQVIGFDQAGVFYGLQSILSLVPSDGS
GKIATLDASDAPR
>d1jaka2 d.92.2.1 (A:8-150) beta-N-acetylhexosaminidase, N-terminal domain {Streptomyces plicatus}
DRKAPVRPTPLDRVIPAPASVDPGGAPYRITRGTHIRVDDSREARRVGDYLADLLRPATGYR
LPVTAHGHGGIRLRLAGGPYGDEGYRLDSGPAGVTITARKAAGLFHGVQTLRQLLPPAVEKD
SAQPGPWLVAGGTIEDTPR
>d1cwdl_ d.93.1.1 (L:) p56-lck tyrosine kinase {Human (Homo sapiens)}
GSWFFKNLSRKDAERQLLAPGNTHGSFLIRESESTAGSFSLSVRDFDQNQGEVVKHYKIRNL
DNGGFYISPRITFPGLHELVRHYTNASDGLCTRLSR
>d1lkka_ d.93.1.1 (A:) p56-lck tyrosine kinase {Human (Homo sapiens)}
LEPEPWFFKNLSRKDAERQLLAPGNTHGSFLIRESESTAGSFSLSVRDFDQNQGEVVKHYKI
RNLDNGGFYISPRITFPGLHELVRHYTNASDGLCTRLSRPCQT
>d1bkl__ d.93.1.1 (-) v-src tyrosine kinase {Rous sarcoma virus, Schmidt-ruppin strain a}
EEWYFGKITRRESESLLLNPENPRGTFLVRESETTKGAYCLSVSDFDNAKGLNVKHYKIRKL
DSGGFYITSRTQFSSLQQLVAYYSKHADGLCHRLTNVCPTSKEFIVTD
>d1shaa_ d.93.1.1 (A:) v-src tyrosine kinase {Rous sarcoma virus, Schmidt-ruppin strain a}
AEEWYFGKITRRESERLLLNPENPRGTFLVRESETTKGAYCLSVSDFDNAKGLNVKHYKIRK
LDSGGFYITSRTQFSSLQQLVAYYSKHADGLCHRLTNVCPT
>d1a09a_ d.93.1.1 (A:) c-src tyrosine kinase {Human (Homo sapiens)}
DSIQAEEWYFGKITRRESERLLLNAENPRGTFLVRESETTKGAYCLSVSDFDNAKGLNVKHY
KIRKLDSGGFYITSRTQFNSLQQLVAYYSKHADGLCHRLTTVCP
>d1g83a2 d.93.1.1 (A:142-245) Tyrosine kinase Fyn {Human (Homo sapiens)}

DSIQAEEWYFGKLGRKDAERQLLSFGNPRGTFLIRESETTKGAYSLSIRDWDDMKGDHVKHY
KIRKLDNGGYYITTRAQFETLQQLVQHYSERAAGLSSRLVVP
>d1ayaa_ d.93.1.1 (A:) Tyrosine phosphatase Syp {Mouse (Mus musculus)}
MRRWFHPNITGVEAENLLLTRGVDGSFLARPSKSNPGDFTLSVRRNGAVTHIKIQNTGDYYD
LYGGEKFATLAELVQYYMEHHGQLKEKNGDVIELKYPLN
>d1fhs__ d.93.1.1 (-) Growth factor receptor-bound protein 2 (GRB2) {Human (Homo sapiens)}
GIEMKPHPWFFGKIPRAKAEEMLSKQRHDGAFLIRESESAPGDFSLSVKFGNDVQHFKVLRD
GAGKYFLWVVKFNSLNELVDYHRSTSVSRNQQIFLRDIEQVPQQPTYVQA
>d1zfpe_ d.93.1.1 (E:) Growth factor receptor-bound protein 2 (GRB2) {Human (Homo sapiens)}
KPHPWFFGKIPRAKAEEMLSKQRHDGAFLIRESESAPGDFSLSVKFGNDVQHFKVLRDGAGK
YFLWVVKFNSLNELVDYHRSTSVSRNQQIFLRDIEQ
>d1qcfa2 d.93.1.1 (A:146-248) Hemopoetic cell kinase Hck {Human (Homo sapiens)}
EEWFFKGISRKDAERQLLAPGNMLGSFMIRDSETTKGSYSLSVRDYDPRQGDTVKHYKIRTL
DNGGFYISPRSTFSTLQELVDHYKKGNDGLCQKLSVPCMSS
>d1mil__ d.93.1.1 (-) Shc adaptor protein {Human (Homo sapiens)}
GSQLRGEPWFHGKLSRREAEALLQLNGDFLVRESTTTPGQYVLTGLQSGQPKHLLLVDPEGV
VRTKDHRFESVSHLISYHMDNHLPIISAGSELCLQQPVERKL
>d1qada_ d.93.1.1 (A:) Phosphatidylinositol 3-kinase, p85-alpha subunit {Cow (Bos taurus)}
EDLPHHDEKTWNVGSSNRNKAENLLRGKRDGTFLVRESSKQGCYACSVVVDGEVKHCVINKT
ATGYGFAEPYNLYSSLKELVLHYQHTSLVQHNDSLNVTLAYPVYA
>d1pica_ d.93.1.1 (A:) Phosphatidylinositol 3-kinase, p85-alpha subunit {Human (Homo sapiens)}
GSPIPHHDEKTWNVGSSNRNKAENLLRGKRDGTFLVRESSKQGCYACSVVVDGEVKHCVINK
TATGYGFAEPYNLYSSLKELVLHYQHTSLVQHNDSLNVTLAYPVYAQQRR
>d1fu6a_ d.93.1.1 (A:) Phosphatidylinositol 3-kinase, p85-alpha subunit {Rat (Rattus norvegicus)}
GMNNNMSLQDAEWYWGDISREEVNEKLRDTADGTFLVRDASTKMHGDYTLTLRKGGNNKSIK
IFHRDGKYGFSDPLTFNSVVELINHYRNESLAQYNPKLDVKLLYPVSKY
>d1ab2__ d.93.1.1 (-) Proto-oncogen tyrosine kinase {Human (Homo sapiens)}
GSGNSLEKHSWYHGPVSRNAAEYLLSSGINGSFLVRESESSPGQRSISLRYEGRVYHYRINT
ASDGKLYVSSESRFNTLAELVHHHSTVADGLITTLHYPAPKRGIHRD
>d1a81a1 d.93.1.1 (A:9-137) Syk tyrosine kinase {Human (Homo sapiens)}
SANHLPFFFGNITREEAEDYLVQGGMSDGLYLLRQSRNYLGGFALSVAHGRKAHHYTIEREL
NGTYAIAGGRTHASPADLCHYHSQESDGLVCLLKKPFNRPQGVQPKTGPFEDLKENLIREYV
KQTWN
>d1a81a2 d.93.1.1 (A:138-262) Syk tyrosine kinase {Human (Homo sapiens)}

LQGQALEQAIISQKPQLEKLIATTAHEKMPWFHGKISREESEQIVLIGSKTNGKFLIRARDN
NGSYALCLLHEGKVLHYRIDKDKTGKLSIPEGKKFDTLWQLVEHYSYKADGLLRVLTVPCQK
I
>d1a81e1 d.93.1.1 (E:9-117) Syk tyrosine kinase {Human (Homo sapiens)}
SANHLPFFFGNITREEAEDYLVQGGMSDGLYLLRQSRNYLGGFALSVAHGRKAHHYTIEREL
NGTYAIAGGRTHASPADLCHYHSQESDGLVCLLKKPFNRPQGVQPKT
>d1a81e2 d.93.1.1 (E:152-262) Syk tyrosine kinase {Human (Homo sapiens)}
PQLEKLIATTAHEKMPWFHGKISREESEQIVLIGSKTNGKFLIRARDNNGSYALCLLHEGKV
LHYRIDKDKTGKLSIPEGKKFDTLWQLVEHYSYKADGLLRVLTVPCQKI
>d1csya_ d.93.1.1 (A:) Syk tyrosine kinase {Human (Homo sapiens)}
GSRRASVGSHEKMPWFHGKISREESEQIVLIGSKTNGKFLIRARDNNGSYALCLLHEGKVLH
YRIDKDKTGKLSIPEGKKFDTLWQLVEHYSYKADGLLRVLTVPCQKIGTQ
>d2plda_ d.93.1.1 (A:) Phospholipase C-gamma-1 {Cow (Bos taurus)}
GSPGIHESKEWYHASLTRAQAEHMLMRVPRDGAFLVRKRNEPNSYAISFRAEGKIKHCRVQQ
EGQTVMLGNSEFDSLVDLISYYEKHPLYRKMKLRYPINEENSS
>d1blk__ d.93.1.1 (-) P55 Blk protein tyrosine kinase {Mouse (Mus musculus)}
GSVAPVETLEVEKWFFRTISRKDAERQLLAPMNKAGSFLIRESESNKGAFSLSVKDITTQGE
VVKHYKIRSLDNGGYYISPRITFPTLQALVQHYSKKGDGLCQKLTLPCVNLA
>d2abl_2 d.93.1.1 (140-237) Abl tyrosine kinase {Human (Homo sapiens)}
SLEKHSWYHGPVSRNAAEYLLSSGINGSFLVRESESSPGQRSISLRYEGRVYHYRINTASDG
KLYVSSESRFNTLAELVHHHSTVADGLITTLHYPAP
>d1jwoa_ d.93.1.1 (A:) Csk homologous kinase Chk {Human (Homo sapiens)}
LSLMPWFHGKISGQEAVQQLQPPEDGLFLVRESARHPGDYVLCVSFGRDVIHYRVLHRDGHL
TIDEAVFFCNLMDMVEHYSKDKGAICTKLVRPKRK
>d1bf5a3 d.93.1.1 (A:569-710) STAT-1 {Human (Homo sapiens)}
LLPLWNDGCIMGFISKERERALLKDQQPGTFLLRFSESSREGAITFTWVERSQNGGEPDFHA
VEPYTKKELSAVTFPDIIRNYKVMAAENIPENPLKYLYPNIDKDHAFGKYYSRGYIKTELIS
VS
>d1bg1a3 d.93.1.1 (A:576-716) STAT3b {Mouse (Mus musculus)}
ILALWNEGYIMGFISKERERAILSTKPPGTFLLRFSESSKEGGVTFTWVEKDISGSTQIQSV
EPYTKQQLNNMSFAEIIMGYKIMDATNILVSPLVYLYPDIPKEEAFGKYCRPESQEHPEADP
GSAAPYLKTKFICVTPF
>d2cbla3 d.93.1.1 (A:264-351) Cbl {Human (Homo sapiens)}
THPGYMAFLTYDEVKARLQKFIHKPGSYIFRLSCTRLGQWAIGYVTADGNILQTIPHNKPLF
QALIDGFREGFYLFPDGRNQNPDLTG
>d2shpa2 d.93.1.1 (A:2-110) Tyrosine phoshatase shp-2 {Human (Homo sapiens)}
KSRRWFHPNITGVEAENLLLTRGVDGSFLARPSKSNPGDLTLSVRRNGAVTHIKIQNTGDYY
DLYGGEKFATLAELVQYYMEHHGQLKEKNGDVIELKYPLNCADPTSE

>d2shpa3 d.93.1.1 (A:111-218) Tyrosine phoshatase shp-2 {Human (Homo sapiens)}
RWFHGHLSGKEAEKLLTEKGKHGSFLVRESQSHPGDFVLSVRTGDDKGESNDGKSKVTHVMI
RCQELKYDVGGGERFDSLTDLVEHYKKNPMVETLGTVLQLKQPLNT
>d1d4ta_ d.93.1.1 (A:) The Xlp protein Sap {Human (Homo sapiens)}
MDAVAVYHGKISRETGEKLLLATGLDGSYLLRDSESVPGVYCLCVLYHGYIYTYRVSQTETG
SWSAETAPGVHKRYFRKIKNLISAFQKPDQGIVIPLQYPVEK
>d1spha_ d.94.1.1 (A:) Histidine-containing phosphocarrier protein (HPr) {Bacillus subtilis}
AQKTFKVTADSGIHARPATVLVQTASKYDADVNLEYNGKTVNLKDIMGVMSLGIAKGAEITI
SASGADENDALNALEETMKSEGLGE
>d1ptf__ d.94.1.1 (-) Histidine-containing phosphocarrier protein (HPr) {Enterococcus faecalis}
MEKKEFHIVAETGIHARPATLLVQTASKFNSDINLEYKGKSVNLKSIMGVMSLGVGQGSDVT
ITVDGADEAEGMAAIVETLQKEGLA
>d1opd__ d.94.1.1 (-) Histidine-containing phosphocarrier protein (HPr) {Escherichia coli}
MFEQEVTITAPNGLHTRPAAQFVKEAKGFTSEITVTSNGKSASAKDLFKLQTLGLTQGTVVT
ISAEGEDEQKAVEHLVKLMAELE
>d1pch__ d.94.1.1 (-) Histidine-containing phosphocarrier protein (HPr) {Mycoplasma capricolum}
AKFSAIITDKVGLHARPASVLAKEASKFSSNITIIANEKQGNLKSIMNVMAMAIKTGTEITI
QADGNDADQAIQAIKQTMIDTALIQG
>d1zer__ d.94.1.1 (-) Histidine-containing phosphocarrier protein (HPr) {Staphylococcus aureus}
MEQNSYVIIDETGIHARPATMLVQTASKFDSDIQLEYNGKKVNLKSIMGVMSLGVGKDAEIT
IYADGSDESDAIQAISDVLSKEGLT
>d1qr5a_ d.94.1.1 (A:) Histidine-containing phosphocarrier protein (HPr) {Staphylococcus carnosus}
MEQQSYTIIDETGIHARPATMLVQTASKFDSDIQLEYNGKKVNLKSIMGVMSLGVGKDAEIT
IYADGSDEADAIQAITDVLSKEGLTE
>d1k1ca_ d.94.1.1 (A:) Crh, catabolite repression HPr-like protein {Bacillus subtilis}
VQQKVEVRLKTGLQARPAALFVQEANRFTSDVFLEKDGKKVNAKSIMGLMSLAVSTGTEVTL
IAQGEDEQEALEKLAAYVQEEV
>d1jrma_ d.206.1.1 (A:) Hypothetical protein MTH637 {Archaeon Methanobacterium thermoautotrophicum}
VITMDCLREVGDDLLVNIEVSPASGKFGIPSYNEWRKRIEVKIHSPPQKGKANREIIKEFSE
TFGRDVEIVSGQKSRQKTIRIQGMGRDLFLKLVSEKFGLEIP
>d1iba__ d.95.1.1 (-) Glucose permease domain IIB {Escherichia coli}
MAPALVAAFGGKENITNLDACITRLRVSVADVSKVDQAGLKKLGAAGVVVAGSGVQAIFGTK
SDNLKTEMDEYIRNFG
>d1af5__ d.95.2.1 (-) DNA endonuclease I-CreI {Chlamydomonas

reinhardtii}
KYNKEFLLYLAGFVDGDGSIIAQIKPNQSYKFKHQLSLTFQVTQKTQRRWFLGKLVDEIGVG
YVRDRGSVSDYILSEIKPLHNFLTQLQPFLKLKQKQANLVLKIIEQLPLEVCTWVDQIAALN
DS
>d1g9za_ d.95.2.1 (A:) DNA endonuclease I-CreI {Chlamydomonas reinhardtii}
NTKYNKEFLLYLAGFVDGDGSIIAQIKPNQSYKFKHQLSLTFQVTQKTQRRWFLDKLVDEIG
VGYVRDRGSVSDYILSEIKPLHNFLTQLQPFLKLKQKQANLVLKIIEQLPSAKESPDKFLEV
CTWVDQIAALNDSKTRKTTSETVRAVLD
>d1b24a1 d.95.2.1 (A:7-99) I-dmoI {Archaeon Desulfurococcus mobilis}
VSGISAYLLGLIIGDGGLYKLKYKGNRSEYRVVITQKSENLIKQHIAPLMQFLIDELNVKSK
IQIVKGDTRYELRVSSKKLYYYFANMLERIR
>d1b24a2 d.95.2.1 (A:100-179) I-dmoI {Archaeon Desulfurococcus mobilis}
LFNMREQIAFIKGLYVAEGDKTLKRLRIWNKNKALLEIVSRWLNNLGVRNTIHLDDHRHGVY
VLNISLRDRIKFVHTILS
>d1dfaa2 d.95.2.2 (A:181-298) PI-SceI {Baker's yeast (Saccharomyces cerevisiae)}
PILYENDHFFDYMQKSKFHLTIEGPKVLAYLLGLWIGDGLSDRATFSVDSRDTSLMERVTEY
AEKLNLCAEYKDRKEPQVAKTVNLYSKVVRGNGIRNNLNTENPLWDAIVGLGFLKD
>d1dfaa3 d.95.2.2 (A:299-415) PI-SceI {Baker's yeast (Saccharomyces cerevisiae)}
GVKNIPSFLSTDNIGTRETFLAGLIDSDGYVTDEHGIKATIKTIHTSVRDGLVSLARSLGLV
VSVNAEPAKVDMNGTKHKISYAIYMSGGDVLLNVLSKCAGSKKFRPAPAAAFARE
>d1dq3a3 d.95.2.2 (A:129-226) PI-Pfui intein {Archaeon Pyrococcus furiosus}
PDGEDYKFIFDYWLAGFIAGDGCFDKYHSHVKGHEYIYDRLRIYDYRIETFEIINDYLEKTF
GRKYSIQKDRNIYYIDIKARNITSHYLKLLEGIDNG
>d1dq3a4 d.95.2.2 (A:227-335) PI-Pfui intein {Archaeon Pyrococcus furiosus}
IPPQILKEGKNAVLSFIAGLFDAEGHVSNKPGIELGMVNKRLIEDVTHYLNALGIKARIREK
LRKDGIDYVLHVEEYSSLLRFYELIGKNLQNEEKREKLEKVLSNHKG
>d1a8ra_ d.96.1.1 (A:) GTP cyclohydrolase I {Escherichia coli}
PSLSKEAALVHEALVARGLETPLRPPVHEMDNETRKSLIAGHMTEIMQLLNLDLADDSLMET
PHRIAKMYVDEIFSGLDYANFPKITLIENKMKVDEMVTVRDITLTSTCESHFVTIDGKATVA
YIPKDSVIGLSKINRIVQFFAQRPQVQERLTQQILIALQTLLGTNNVAVSIDAVHYCVKARG
IRDATSATTTTSLGGLFKSSQNTRHEFLRAVRHHN
>d1is8a_ d.96.1.1 (A:) GTP cyclohydrolase I {Rat (Rattus norvegicus)}
RPRSEEDNELNLPNLAAAYSSILRSLGEDPQRQGLLKTPWRAATAMQFFTKGYQETISDVLN
DAIFDEDHDEMVIVKDIDMFSMCEHHLVPFVGRVHIGYLPNKQVLGLSKLARIVEIYSRRLQ
VQERLTKQIAVAITEALQPAGVGVVIEATHMCMVMRGVQKMNSKTVTSTMLGVFREDPKTRE
EFLTLIRS

>d1b66a_ d.96.1.2 (A:) 6-pyruvoyl tetrahydropterin synthase {Rat (Rattus norvegicus)}
LRRRARLSRLVSFSASHRLHSPSLSAEENLKVFGKCNNPNGHGHNYKVVVTIHGEIDPVTGM
VMNLTDLKEYMEEAIMKPLDHKNLDLDVPYFADVVSTTENVAVYIWENLQRLLPVGALYKVK
VYETDNNIVVYKGE
>d1dhn__ d.96.1.3 (-) 7,8-dihidroneopterin aldolase {Staphylococcus aureus}
MQDTIFLKGMRFYGYHGALSAENEIGQIFKVDVTLKVDLSEAGRTDNVIDTVHYGEVFEEVK
SIMEGKAVNLLEHLAERIANRINSQYNRVMETKVRITKENPPIPGHYDGVGIEIVRENK
>d1b9la_ d.96.1.3 (A:) 7,8-dihydroneopterin triphosphate epimerase {Escherichia coli}
AQPAAIIRIKNLRLRTFIGIKEEEINNRQDIVINVTIHYPADKARTSEDINDALNYRTVTKN
IIQHVENNRFSLLEKLTQDVLDIAREHHWVTYAEVEIDKLHALRYADSVSMTLSWQR
>d1uox_1 d.96.1.4 (1-136) Urate oxidase (uricase) {Aspergillus flavus}
SAVKAARYGKDNVRVYKVHKDEKTGVQTVYEMTVCVLLEGEIETSYTKADNSVIVATDSIKN
TIYITAKQNPVTPPELFGSILGTHFIEKYNHIHAAHVNIVCHRWTRMDIDGKPHPHSFIRDS
EEKRNVQVDVVE
>d1uox_2 d.96.1.4 (137-295) Urate oxidase (uricase) {Aspergillus flavus}
GKGIDIKSSLSGLTVLKSTNSQFWGFLRDEYTTLKETWDRILSTDVDATWQWKNFSGLQEVR
SHVPKFDATWATAREVTLKTFAEDNSASVQATMYKMAEQILARQQLIETVEYSLPNKHYFEI
DLSWHKGLQNTGKNAEVFAPQSDPNGLIKCTVGRS
>d1puc__ d.97.1.1 (-) suc1 {Fission yeast (Schizosaccharomyces pombe)}
SKSGVPRLLTASERERLEPFIDQIHYSPRYADDEYEYRHVMLPKAMLKAIPTDYFNPETGTL
RILQEEEWRGLGITQSLGWEMYEVHVPEPHILLFKREKD
>d1qb3a_ d.97.1.1 (A:) cks1 {Baker's yeast (Saccharomyces cerevisiae)}
HAFQGRKLTDQERARVLEFQDSIHYSPRYSDDNYEYRHVMLPKAMLKVIPSDYFNSEVGTLR
ILTEDEWRGLGITQSLGWEHYECHAPEPHILLFKRPLNYEAELRAATAAAQ
>d1cksa_ d.97.1.1 (A:) CksHs2 {Human (Homo sapiens)}
AHKQIYYSDKYFDEHYEYRHVMLPRELSKQVPKTHLMSEEEWRRLGVQQSLGWVHYMIHEPE
PHILLFRRPLPK
>d1buhb_ d.97.1.1 (B:) CksHs1 {Human (Homo sapiens)}
QIYYSDKYDDEEFEYRHVMLPKDIAKLVPKTHLMSESEWRNLGVQQSQGWVHYMIHEPEPHI
LLFRRPLP
>d1jtgb_ d.98.1.1 (B:) beta-lactamase-inhibitor protein, BLIP {Streptomyces clavuligerus}
AGVMTGAKFTQIQFGMTRQQVLDIAGAENCETGGSFGDSIHCRGHAAGDYYAYATFGFTSAA
ADAKVDSKSQEKLLAPSAPTLTLAKFNQVTVGMTRAQVLATVGQGSCTTWSEYYPAYPSTAG
VTLSLSCFDVDGYSSTGFYRGSAHLWFTDGVLQGKRQWDLV
>d1div_1 d.99.1.1 (56-149) Ribosomal protein L9 C-domain {Bacillus stearothermophilus}

RQAAEELANAKKLKEQLEKLTVTIPAKAGEGGRLFGSITSKQIAESLQAQHGLKLDKRKIEL
ADAIRALGYTNVPVKLHPEVTATLKVHVTEQK
>d1div_2 d.100.1.1 (1-55) Ribosomal protein L9 N-domain {Bacillus stearothermophilus}
MKVIFLKDVKGKGKKGEIKNVADGYANNFLFKQGLAIEATPANLKALEAQKQKEQ
>d1qhka_ d.100.1.2 (A:) N-terminal domain of RNase HI {Baker's yeast (Saccharomyces cerevisiae)}
GNFYAVRKGRETGIYNTWNECKNQVDGYGGAIYKKFNSYEQAKSFLG
>d1e3ha5 d.101.1.1 (A:152-262) Polynucleotide phosphorylase/guanosine pentaphosphate synthase (PNPase/GPSI), domains 2 and 5 {Streptomyces antibioticus}
FSGPIGGVRVALIRGQWVAFPTHTELEDAVFDMVVAGRVLEDGDVAIMMVEAEATEKTIQLV
KDGAEAPTEEVVAAGLDAAKPFIKVLCKAQADLAAKAAKPTGEFPVFLD
>d1e3ha6 d.101.1.1 (A:483-578) Polynucleotide phosphorylase/guanosine pentaphosphate synthase (PNPase/GPSI), domains 2 and 5 {Streptomyces antibioticus}
APVAGIAMGLISQEINGETHYVALTDILGAEDAFGDMDFKVAGTKEFVTALQLDTKLDGIPA
SVLAAALKQARDARLHILDVMMEAIDTPDEMSPN
>d1efnb_ d.102.1.1 (B:) Regulatory factor Nef {Human immunodeficiency virus type 1}
RPQVPLRPMTYKAAVDLSHFLKEKGGLEGLIHSQRRQDILDLWIYHTQGYFPDWQNYTPGPG
VRYPLTFGWCYKLVPVEPDKVEEANKGENTSLLHPVSLHGMDDPEREVLEWRFDSRLAFHHV
ARELHPEYF
>d2nef__ d.102.1.1 (-) Regulatory factor Nef {Human immunodeficiency virus type 1}
AWLEAQEEEEVGFPVTPQVPLRPMTYKAAVDLSHFLKEKGGLEGLIHSQRRQDILDLWIYHT
QGYFPDWQNYTPGPGIRYPLTFGWCYKLVPVEPEKLEEANKDDPEREVLEWRFDSRLAFHHM
ARELHPEYFKNA
>d1cby__ d.103.1.1 (-) Mosquitocidal delta-endotoxin CytB {Bacillus thuringiensis, strain Kyushuensis}
CSAPIIRKPFKHIVLTVPSSDLDNFNTVFYVQPQYINQALHLANAFQGAIDPLNLNFNFEKA
LQIANGIPNSAIVKTLNQSVIQQTVEISVMVEQLKKIIQEVLGLVINSTSFWNSVEATIKGT
FTNLDTQIDEAWIFWHSLSAHNTSYYYNILFSIQNEDTGAVMAVLPLAFEVSVDVEKQKVLF
FTIKDSARYEVKMKALTLVQALHSSNAPIVDIFNVNNYNLY
>d1seta2 d.104.1.1 (A:111-421) Seryl-tRNA synthetase (SerRS) {Thermus thermophilus, strain hb27}
VGGEEANREIKRVGGPPEFSFPPLDHVALMEKNGWWEPRISQVSGSRSYALKGDLALYELAL
LRFAMDFMARRGFLPMTLPSYAREKAFLGTGHFPAYRDQVWAIAETDLYLTGTAEVVLNALH
SGEILPYEALPLRYAGYAPAFRSEAGSFGKDVRGLMRVHQFHKVEQYVLTEASLEASDRAFQ
ELLENAEEILRLLELPYRLVEVATGDMGPGKWRQVDIEVYLPSEGRYRETHSCSALLDWQAR
RANLRYRDPEGRVRYAYTLNNTALATPRILAMLLENHQLQDGRVRVPQALIPYMGKEVLEPC
G
>d1e1oa2 d.104.1.1 (A:161-502) Lysyl-tRNA synthetase (LysRS) {Escherichia coli, gene lysU}

DQEVRYRQRYLDLIANDKSRQTFVVRSKILAAIRQFMVARGFMEVETPMMQVIPGGASARPF
ITHHNALDLDMYLRIAPELYLKRLVVGGFERVFEINRNFRNEGISVRHNPEFTMMELYMAYA
DYHDLIELTESLFRTLAQEVLGTTKVTYGEHVFDFGKPFEKLTMREAIKKYRPETDMADLDN
FDAAKALAESIGITVEKSWGLGRIVTEIFDEVAEAHLIQPTFITEYPAEVSPLARRNDVNPE
ITDRFEFFIGGREIGNGFSELNDAEDQAERFQEQVNAKAAGDDEAMFYDEDYVTALEYGLPP
TAGLGIGIDRMIMLFTNSHTIRDVILFPAMRP
>d1bbua2 d.104.1.1 (A:161-502) Lysyl-tRNA synthetase (LysRS)
{Escherichia coli, gene lysS}
DQEARYRQRYLDLISNDESRNTFKVRSQILSGIRQFMVNRGFMEVETPMMQVIPGGAAARPF
ITHHNALDLDMYLRIAPELYLKRLVVGGFERVFEINRNFRNEGISVRHNPEFTMMELYMAYA
DYKDLIELTESLFRTLAQDILGKTEVTYGDVTLDFGKPFEKLTMREAIKKYRPETDMADLDN
FDSAKAIAESIGIHVEKSWGLGRIVTEIFEEVAEAHLIQPTFITEYPAEVSPLARRNDVNPE
ITDRFEFFIGGREIGNGFSELNDAEDQAQRFLDQVAAKDAGDDEAMFYDEDYVTALEHGLPP
TAGLGIGIDRMVMLFTNSHTIRDVILFPAMRP
>d1kmma2 d.104.1.1 (A:4-325) Histidyl-tRNA synthetase (HisRS)
{Escherichia coli}
NIQAIRGMNDYLPGETAIWQRIEGTLKNVLGSYGYSEIRLPIVEQTPLFKRAIGEVTDVVEK
EMYTFEDRNGDSLTLRPEGTAGCVRAGIEHGLLYNQEQRLWYIGPMFRHERPQKGRYRQFHQ
LGCEVFGLQGPDIDAELIMLTARWWRALGISEHVTLELNSIGSLEARANYRDALVAFLEQHK
EKLDEDCKRRMYTNPLRVLDSKNPEVQALLNDAPALGDYLDEESREHFAGLCKLLESAGIAY
TVNQRLVRGLDYYNRTVFEWVTNSLGSQGTVCAGGRYDGLVEQLGGRATPAVGFAMGLERLV
LLVQAVNPEFKA
>d1qe0a2 d.104.1.1 (A:1-325) Histidyl-tRNA synthetase (HisRS)
{Staphylococcus aureus}
MIKIPRGTQDILPEDSKKWRYIENQLDELMTFYNYKEIRTPIFESTDLFARGVGDSTDVVQK
EMYTFKDKGDRSITLRPEGTAAVVRSYIEHKMQGNPNQPIKLYYNGPMFRYERKQKGRYRQF
NQFGVEAIGAENPSVDAEVLAMVMHIYQSFGLKHLKLVINSVGDMASRKEYNEALVKHFEPV
IHEFCSDCQSRLHTDPMRILDCKVDRDKEAIKTAPRITDFLNEESKAYYEQVKAYLDDLGIP
YTEDPNLVRGLDYYTHTAFELMMDNPNYDGAITTLCGGGRYNGLLELLDGPSETGIGFALSI
ERLLLALEEEGIELD
>d1h4vb2 d.104.1.1 (B:2-325) Histidyl-tRNA synthetase (HisRS)
{Thermus thermophilus}
TARAVRGTKDLFGKELRMHQRIVATARKVLEAAGALELVTPIFEETQVFEKGVGAATDIVRK
EMFTFQDRGGRSLTLRPEGTAAMVRAYLEHGMKVWPQPVRLWMAGPMFRAERPQKGRYRQFH
QVNYEALGSENPILDAEAVVLLYECLKELGLRRLKVKLSSVGDPEDRARYNAYLREVLSPHR
EALSEDSKERLEENPMRILDSKSERDQALLKELGVRPMLDFLGEEARAHLKEVERHLERLSV
PYELEPALVRGLDYYVRTAFEVHHEEIGAQSALGGGGRYDGLSELLGGPRVPGVGFAFGVER
VALALEAEGFGLPE
>d1atia2 d.104.1.1 (A:1-394) Glycyl-tRNA synthetase (GlyRS)
{Thermus thermophilus}
AASSLDELVALCKRRGFIFQSSEIYGGLQGVYDYGPLGVELKNNLKQAWWRRNVYERDDMEG
LDASVLTHRLVLHYSGHEATFADPMVDNRITKKRYRLDHLLKEQPEEVLKRLYRAMEVEEEN
LHALVQAMMQAPERAGGAMTAAGVLDPASGEPGDWTPPRYFNMMFQDLRGPRGGRGLLAYLR
PETAQGIFVNFKNVLDATSRKLGFGIAQIGKAFRNEITPRNFIFRVREFEQMEIEYFVRPGE

DEYWHRYWVEERLKWWQEMGLSRENLVPYQQPPESSAHYAKATVDILYRFPHGSLELEGIAQ
RTDFDLGSHTKDQEALGITARVLRNEHSTQRLAYRDPETGKWFVPYVIEPSAGVDRGVLALL
AEAFTREELPNGEERIVLKLKP
>d1b76a2 d.104.1.1 (A:1-394) Glycyl-tRNA synthetase (GlyRS)
{Thermus thermophilus}
AASSLDELVALCKRRGFIFQSSEIYGGLQGVYDYGPLGVELKNNLKQAWWRRNVYERDDMEG
LDASVLTHRLVLHYSGHEATFADPMVDNAKARYWTPPRYFNMMFQDLRGPRGGRGLLAYLRP
ETAQGIFVNFKNVLDATSRKLGFGIAQIGKAFRNEITPRNFIFRVREFEQMEIEYFVRPGED
EYWHRYWVEERLKWWQEMGLSRENLVPYQQPPESSAHYAKATVDILYRFPHGSLELEGIAQR
TDFDLGSHTKDQEALGITARVLRNEHSTQRLAYRDPETGKWFVPYVIEPSAGVDRGVLALLA
EAFTREELPNGEERIVLKLKP
>d1qf6a4 d.104.1.1 (A:242-532) Threonyl-tRNA synthetase (ThrRS)
{Escherichia coli}
RDHRKIGKQLDLYHMQEEAPGMVFWHNDGWTIFRELEVFVRSKLKEYQYQEVKGPFMMDRVL
WEKTGHWDNYKDAMFTTSSENREYCIKPMNCPGHVQIFNQGLKSYRDLPLRMAEFGSCHRNE
PSGSLHGLMRVRGFTQDDAHIFCTEEQIRDEVNGCIRLVYDMYSTFGFEKIVVKLSTRPEKR
IGSDEMWDRAEADLAVALEENNIPFEYQLGEGAFYGPKIEFTLYDCLDRAWQCGTVQLDFSL
PSRLSASYVGEDNERKVPVMIHRAILGSMERFIGILTEEFAGF
>d1eova2 d.104.1.1 (A:205-557) Aspartyl-tRNA synthetase (AspRS)
{Baker's yeast (Saccharomyces cerevisiae)}
PILLEDASRSEAEAEAAGLPVVNLDTRLDYRVIDLRTVTNQAIFRIQAGVCELFREYLATKK
FTEVHTPKLLGAPSEGGSSVFEVTYFKGKAYLAQSPQFNKQQLIVADFERVYEIGPVFRAEN
SNTHRHMTEFTGLDMEMAFEEHYHEVLDTLSELFVFIFSELPKRFAHEIELVRKQYPVEEFK
LPKDGKMVRLTYKEGIEMLRAAGKEIGDFEDLSTENEKFLGKLVRDKYDTDFYILDKFPLEI
RPFYTMPDPANPKYSNSYDFFMRGEEILSGAQRIHDHALLQERMKAHGLSPEDPGLKDYCDG
FSYGCPPHAGGGIGLERVVMFYLDLKNIRRASLFPRDPKRLRP
>d1b8aa2 d.104.1.1 (A:104-438) Aspartyl-tRNA synthetase (AspRS)
{Archaeon Pyrococcus kodakaraensis}
PLPLDPTGKVKAELDTRLNNRFMDLRRPEVMAIFKIRSSVFKAVRDFFHENGFIEIHTPKII
ATATEGGTELFPMKYFEEDAFLAESPQLYKEIMMASGLDRVYEIAPIFRAEEHNTTRHLNEA
WSIDSEMAFIEDEEEVMSFLERLVAHAINYVREHNAKELDILNFELEEPKLPFPRVSYDKAL
EILGDLGKEIPWGEDIDTEGERLLGKYMMENENAPLYFLYQYPSEAKPFYIMKYDNKPEICR
AFDLEYRGVEISSGGQREHRHDILVEQIKEKGLNPESFEFYLKAFRYGMPPHGGFGLGAERL
IKQMLDLPNIREVILFPRDRRRLTP
>d1c0aa3 d.104.1.1 (A:107-287,A:421-585) Aspartyl-tRNA synthetase
(AspRS) {Escherichia coli}
VLPLDSNHVNTEEARLKYRYLDLRRPEMAQRLKTRAKITSLVRRFMDDHGFLDIETPMLTKA
TPEGARDYLVPSRVHKGKFYALPQSPQLFKQLLMMSGFDRYYQIVKCFRDEDLRADRQPEFT
QIDVETSFMTAPQVREVMEALVRHLWLEVKGVDLGDFPVMTFAEAERRYGSDKPDLRXDESK
WAPLWVIDFPMFEDDGEGGLTAMHHPFTSPKDMTAAELKAAPENAVANAYDMVINGYEVGGG
SVRIHNGDMQQTVFGILGINEEEQREKFGFLLDALKYGTPPHAGLAFGLDRLTMLLTGTDNI
RDVIAFPKTTAAACLMTEAPSFANPTALAELSIQVVK
>d1g51a3 d.104.1.1 (A:105-294,A:415-580) Aspartyl-tRNA synthetase
(AspRS) {Thermus thermophilus}

TPPFPVDAGWRGEEEKEASEELRLKYRYLDLRRRRMQENLRLRHRVIKAIWDFLDREGFVQV
ETPFLTKSTPEGARDFLVPYRHEPGLFYALPQSPQLFKQMLMVAGLDRYFQIARCFRDEDLR
ADRQPDFTQLDLEMSFVEVEDVLELNERLMAHVFREALGVELPLPFPRLSYEEAMERYGSDK
PDLRXREGFRFLWVVDFPLLEWDEEEEAWTYMHHPFTSPHPEDLPLLEKDPGRVRALAYDLV
LNGVEVGGGSIRIHDPRLQARVFRLLGIGEEEQREKFGFFLEALEYGAPPHGGIAWGLDRLL
ALMTGSPSIREVIAFPKNKEGKDPLTGAPSPVPEEQLRELGLMVVRP
>d1jjca_ d.104.1.1 (A:) Phenyl-tRNA synthetase (PheRS) alpha subunit, PheS {Thermus thermophilus and (Thermus aquaticus)}
RVDVSLPGASLFSGGLHPITLMERELVEIFRALGYQAVEGPEVESEFFNFDALNIPEHHPAR
DMWDTFWLTGEGFRLEGPLGEEVEGRLLLRTHTSPMQVRYMVAHTPPFRIVVPGRVFRFEQT
DATHEAVFHQLEGLVVGEGIAMAHLKGAIYELAQALFGPDSKVRFQPVYFPFVEPGAQFAVW
WPEGGKWLELGGAGMVHPKVFQAVDAYRERLGLPPAYRGVTGFAFGLGVERLAMLRYGIPDI
RYFFGGRLKFLEQFKGVL
>d1jjcb5 d.104.1.1 (B:475-681) Phenyl-tRNA synthetase (PheRS) beta subunit, PheT, central domain {Thermus thermophilus (Thermus aquaticus)}
ALPAFFPAPDNRGVEAPYRKEQRLREVLSGLGFQEVYTYSFMDPEDARRFRLDPPRLLLLNP
LAPEKAALRTHLFPGLVRVLKENLDLDRPERALLFEVGRVFREREETHLAGLLFGEGVGLPW
AKERLSGYFLLKGYLEALFARLGLAFRVEAQAFPFLHPGVSGRVLVEGEEVGFLGALHPEIA
QELELPPVHLFELRLPLPDKP
>d1hc7a2 d.104.1.1 (A:5-276) Prolyl-tRNA synthetase (ProRS) {Thermus thermophilus}
KGLTPQSQDFSEWYLEVIQKAELADYGPVRGTIVVRPYGYAIWENIQQVLDRMFKETGHQNA
YFPLFIPMSFLRKEAEHVEGFSPELAVVTHAGGEELEEPLAVRPTSETVIGYMWSKWIRSWR
DLPQLLNQWGNVVRWEMRTRPFLRTSEFLWQEGHTAHATREEAEEEVRRMLSIYARLAREYA
AIPVIEGLKTEKEKFAGAVYTTTIEALMKDGKALQAGTSHYLGENFARAFDIKFQDRDLQVK
YVHTTSWGLSWRFIGAIIMTHGDD
>d1g5ha2 d.104.1.1 (A:41-330) The aaRS-like accessory subunit of mitochondrial polymerase gamma, N-terminal domain {Mouse (Mus musculus)}
EALVDLCRRRHFLSGTPQQLSTAALLSGCHARFGPLGVELRKNLASQWWSSMVVFREQVFAV
DSLHQEPGSSQPRDSAFRLVSPESIREILQDREPSKEQLVAFLENLLKTSGKLRATLLHGAL
EHYVNCLDLVNRKLPFGLAQIGVCFHPVSNSNQTPSSVTRVGEKTEASLVWFTPTRTSSQWL
DFWLRHRLLWWRKFAMSPSNFSSADCQDELGRKGSKLYYSFPWGKEPIETLWNLGDQELLHT
YPGNVSTIQGRDGRKNVVPCVLSVSGDVDLGTLAYLYDSFQL
>d12asa_ d.104.1.1 (A:) Asparagine synthetase {Escherichia coli}
AYIAKQRQISFVKSHFSRQLEERLGLIEVQAPILSRVGDGTQDNLSGAEKAVQVKVKALPDA
QFEVVHSLAKWKRQTLGQHDFSAGEGLYTHMKALRPDEDRLSPLHSVYVDQWDWERVMGDGE
RQFSTLKSTVEAIWAGIKATEAAVSEEFGLAPFLPDQIHFVHSQELLSRYPDLDAKGRERAI
AKDLGAVFLVGIGGKLSDGHRHDVRAPDYDDWSTPSELGHAGLNGDILVWNPVLEDAFELSS
MGIRVDADTLKHQLALTGDEDRLELEWHQALLRGEMPQTIGGGIGQSRLTMLLLQLPHIGQV
QAGVWPAAVRESVPSLL
>d1bia_3 d.104.1.2 (64-270) Biotin repressor/biotin holoenzyme synthetase, catalytic (central) domain {Escherichia coli}

IQLLNAKQILGQLDGGSVAVLPVIDSTNQYLLDRIGELKSGDACIAEYQQAGRGRRGRKWFS
PFGANLYLSMFWRLEQGPAAAIGLSLVIGIVMAEVLRKLGADKVRVKWPNDLYLQDRKLAGI
LVELTGKTGDAAQIVIGAGINMAMRRVEESVVNQGWITLQEAGINLDRNTLAAMLIRELRAA
LELFEQEGLAPYLSRWEKLDN
>d1qtsa2 d.105.1.1 (A:825-938) Alpa-adaptin AP2, C-terminal subdomain {Mouse (Mus musculus)}
FFQPTEMASQDFFQRWKQLSNPQQEVQNIFKAKHPMDTEITKAKIIGFGSALLEEVDPNPAN
FVGAGIIHTKTTQIGCLLRLEPNLQAQMYRLTLRTSKDTVSQRLCELLSEQF
>d1e42a2 d.105.1.1 (A:825-937) Beta2-adaptin AP2, C-terminal subdomain {Human (Homo sapiens)}
LFVEDGKMERQVFLATWKDIPNENELQFQIKECHLNADTVSSKLQNNNVYTIAKRNVEGQDM
LYQSLKLTNGIWILAELRIQPGNPNYTLSLKCRAPEVSQYIYQVYDSILKN
>d1c44a_ d.106.1.1 (A:) Sterol carrier protein 2 (SCP2) {Rabbit (Oryctolagus cuniculus)}
SSAGDGFKANLVFKEIEKKLEEEGEQFVKKIGGIFAFKVKDGPGGKEATWVVDVKNGKGSVL
PNSDKKADCTITMADSDLLALMTGKMNPQSAFFQGKLKITGNMGLAMKLQNLQLQPGKAKL
>d1ikta_ d.106.1.1 (A:) SCP2-like domain of MFE-2 {Human (Homo sapiens)}
LQSTFVFEEIGRRLKDIGPEVVKKVNAVFEWHITKGGNIGAKWTIDLKSGSGKVYQGPAKGA
ADTTIILSDEDFMEVVLGKLDPQKAFFSGRLKARGNIMLSQKLQMILKDYAKL
>d1eq6a_ d.107.1.1 (A:) Ran-binding protein mog1p {Baker's yeast (Saccharomyces cerevisiae)}
SMNNKEVELYGGAITTVVPPGFIDASTLREVPDTQEVYVNSRRDEEEFEDGLATNESIIVDL
LETVDKSDLKEAWQFHVEDLTELNGTTKWEALQEDTVQQGTKFTGLVMEVANKWGKPDLAQT
VVIGVALIRLTQFDTDVVISINVPLTKEEASQASNKELPARCHAVYQLLQEMVRKFHVVDTS
LFA
>d1b87a_ d.108.1.1 (A:) Aminoglycoside 6'-N-acetyltransferase {Enterococcus faecium}
MIISEFDRNNPVLKDQLSDLLRLTWPEEYGDSSAEEVEEMMNPERIAVAAVDQDELVGFIGA
IPQYGITGWELHPLVVESSRRKNQIGTRLVNYLEKEVASRGGITIYLGTDDLDHGTTLSQTD
LYEHTFDKVASIQNLREHPYEFYEKLGYKIVGVLPNANGWDKPDIWMAKTIIPRPDS
>d1bo4a_ d.108.1.1 (A:) Aminoglycoside 3-N-acetyltransferase {Serratia marcescens}
GIIRTCRLGPDQVKSMRAALDLFGREFGDVATYSQHQPDSDYLGNLLRSKTFIALAAFDQEA
VVGALAAYVLPKFEQPRSEIYIYDLAVSGEHRRQGIATALINLLKHEANALGAYVIYVQADY
GDDPAVALYTKLG
>d1cm0a_ d.108.1.1 (A:) Histone acetyltransferase domain of P300/CBP associating factor, PCAF {Human (Homo sapiens)}
KVIEFHVVGNSLNQKPNKKILMWLVGLQNVFSHQLPRMPKEYITRLVFDPKHKTLALIKDGR
VIGGICFRMFPSQGFTEIVFCAVTSNEQVKGYGTHLMNHLKEYHIKHDILNFLTYADEYAIG
YFKKQGFSKEIKIPKTKYVGYIKDYEGATLMGCELNPRI
>d1ygha_ d.108.1.1 (A:) Catalytic domain of GCN5 histone acetyltransferase {Baker's yeast (Saccharomyces cerevisiae)}
KIEFRVVNNDNTKENMMVLTGLKNIFQKQLPKMPKEYIARLVYDRSHLSMAVIRKPLTVVGG

ITYRPFDKREFAEIVFCAISSTEQVRGYGAHLMNHLKDYVRNTSNIKYFLTYADNYAIGYFK
KQGFTKEITLDKSIWMGYIKDYEGGTLMQCSMLPRIRYLD
>d1qsta_ d.108.1.1 (A:) Catalytic domain of GCN5 histone acetyltransferase {Tetrahymena thermophila}
LDFDILTNDGTHRNMKLLIDLKNIFSRQLPKMPKEYIVKLVFDRHHESMVILKNKQKVIGGI
CFRQYKPQRFAEVAFLAVTANEQVRGYGTRLMNKFKDHMQKQNIEYLLTYADNFAIGYFKKQ
GFTKEHRMPQEKWKGYIKDYDGGTLMECYIHPYVDY
>d1qsma_ d.108.1.1 (A:) Histone acetyltransferase HPA2 {Baker's yeast (Saccharomyces cerevisiae)}
DNITVRFVTENDKEGWQRLWKSYQDFYEVSFPDDLDDFNFGRFLDPNIKMWAAVAVESSSEK
IIGMINFFNHMTTWDFKDKIYINDLYVDENSRVKGAGGKLIQFVYDEADKLGTPSVYWCTDE
SNHRAQLLYVKVGYKAPKILYKRKGY
>d1bob__ d.108.1.1 (-) Histone acetyltransferase HAT1 {Baker's yeast (Saccharomyces cerevisiae)}
FKPETWTSSANEALRVSIVGENAVQFSPLFTYPIYGDSEKIYGYKDLIIHLAFDSVTFKPYV
NVKYSAKLGDDNIVDVEKKLLSFLPKDDVIVRDEAKWVDCFAEERKTHNLSDVFEKVSEYSL
NGEEFVVYKSSLVDDFARRMHRRVQIFSLLFIEAANYIDETDPSWQIYWLLNKKTKELIGFV
TTYKYWHYLGAKSFDEDIDKKFRAKISQFLIFPPYQNKGHGSCLYEAIIQSWLEDKSITEIT
VEDPNEAFDDLRDRNDIQRLRKLGYDAVFQKHSDLSDEFLESSRKSLKLEERQFNRLVEMLL
LLNNS
>d1fy7a_ d.108.1.1 (A:) Histone acetyltransferase ESA1 {Baker's yeast (Saccharomyces cerevisiae)}
ARVRNLNRIIMGKYEIEPWYFSPYPIELTDEDFIYIDDFTLQYFGSKKQYERYRKKCTLRHP
PGNEIYRDDYVSFFEIDGRKQRTWCRNLCLLSKLFLDHKTLYYDVDPFLFYCMTRRDELGHH
LVGYFSKEKESADGYNVACILTLPQYQRMGYGKLLIEFSYELSKKENKVGSPEKPLSDLGLL
SYRAYWSDTLITLLVEHQKEITIDEISSMTSMTTTDILHTAKTLNILRYYKGQHIIFLNEDI
LDRYNRLKAKKRRTIDPNRLIWKPP
>d1cjwa_ d.108.1.1 (A:) Serotonin N-acetyltranferase {Sheep (Ovis aries)}
HTLPANEFRCLTPEDAAGVFEIEREAFISVSGNCPLNLDEVQHFLTLCPELSLGWFVEGRLV
AFIIGSLWDEERLTQESLALHRPRGHSAHLHALAVHRSFRQQGKGSVLLWRYLHHVGAQPAV
RRAVLMCEDALVPFYQRFGFHPAGPCAIVVGSLTFTEMHCSL
>d1i12a_ d.108.1.1 (A:) Glucosamine-phoshate N-acetyltransferase GNA1 {Baker's yeast (Saccharomyces cerevisiae)}
LPDGFYIRRMEEGDLEQVTETLKVLTTVGTITPESFCKLIKYWNEATVWNDNEDKKIMQYNP
MVIVDKRTETVAATGNIIIERKIIHELGLCGHIEDIAVNSKYQGQGLGKLLIDQLVTIGFDY
GCYKIILDCDEKNVKFYEKCGFSNAGVEMQIRK
>d1iica1 d.108.1.2 (A:34-218) N-myristoyl transferase, NMT {Baker's yeast (Saccharomyces cerevisiae)}
AMKDHKFWRTQPVKDFDEKVVEEGPIDKPKTPEDISDKPLPLLSSFEWCSIDVDNKKQLEDV
FVLLNENYVEDRDAGFRFNYTKEFFNWALKSPGWKKDWHIGVRVKETQKLVAFISAIPVTLG
VRGKQVPSVEINFLCVHKQLRSKRLTPVLIKEITRRVNKCDIWHALYTAGIVLPAPVSTCR
>d1iica2 d.108.1.2 (A:219-455) N-myristoyl transferase, NMT {Baker's yeast (Saccharomyces cerevisiae)}

YTHRPLNWKKLYEVDFTGLPDGHTEEDMIAENALPAKTKTAGLRKLKKEDIDQVFELFKRYQ
SRFELIQIFTKEEFEHNFIGEESLPLDKQVIFSYVVEQPDGKITDFFSFYSLPFTILNNTKY
KDLGIGYLYYYATDADFQFKDRFDPKATKALKTRLCELIYDACILAKNANMDVFNALTSQDN
TLFLDDLKFGPGDGFLNFYLFNYRAKPITGGLNPDNSNDIKRRSNVGVVML
>d1nmta1 d.108.1.2 (A:60-224) N-myristoyl transferase, NMT {Yeast (Candida albicans)}
EGPIDKLKTPEDVPNDPLPLISDFEWSTLDIDDNLQLDELYKLLYDNYVEDIDATFRFKYSH
EFFQWALKPPGWRKDWHVGVRVKSTGKLVAFIAATPVTFKLNKSNKVIDSVEINFLCIHKKL
RNKRLAPVLIKEITRRVNKQNIWQALYTGGSILPTPLTTCR
>d1nmta2 d.108.1.2 (A:225-451) N-myristoyl transferase, NMT {Yeast (Candida albicans)}
YQHRPINWSKLHDVGFSHLPPNQTKSSMVASYTLPNNPKLKGLRPMTGKDVSTVLSLLYKYQ
ERFDIVQLFTEEEFKHWMLGHDENSDSNVVKSYVVEDENGIITDYFSYYLLPFTVLDNAQHD
ELGIAYLFYYASDSFEKPNYKKRLNELITDALITSKKFGVDVFNCLTCQDNTYFLKDCKFGS
GDGFLNYYLFNYRTFPMDGGIDKKTKEVVEDQTSGIGVVLL
>d2vik__ d.109.1.1 (-) Villin, domain 1 (res. 1-126) {Chicken (Gallus gallus)}
VELSKKVTGKLDKTTPGIQIWRIENMEMVPVPTKSYGNFYEGDCYVLLSTRKTGSGFSYNIH
YWLGKNSSQDEQGAAAIYTTQMDEYLGSVAVQHREVQGHESETFRAYFKQGLIYKQGGVASG
MK
>d1svy__ d.109.1.1 (-) Severin, domain 2 {Dictyostelium discoideum}
EYKPRLLHISGDKNAKVAEVPLATSSLNSGDCFLLDAGLTIYQFNGSKSSPQEKNKAAEVAR
AIDAERKGLPKVEVFCETDSDIPAEFWKLLGGKGAIAAKH
>d1d0na2 d.109.1.1 (A:153-262) Gelsolin {Horse (Equus caballus)}
VPNEVVVQRLLQVKGRRVVRATEVPVSWESFNNGDCFILDLGNNIYQWCGSKSNRFERLKAT
QVSKGIRDNERSGRAQVSVFEEGAEPEAMLQVLGPKPTLPEATEDTVK
>d1d0na3 d.109.1.1 (A:263-383) Gelsolin {Horse (Equus caballus)}
EDAANRKLAKLYKVSNGAGPMVVSLVADENPFAQGALRSEDCFILDHGKDGKIFVWKGKQAN
MEERKAALKTASDFISKMDYPKQTQVSVLPEGGETPLFRQFFKNWRDPDQTEGLGLAYL
>d1d0na4 d.109.1.1 (A:384-532) Gelsolin {Horse (Equus caballus)}
SSHIAHVERVPFDAATLHTSTAMAAQHGMDDDGTGQKQIWRVEGSNKVPVDPATYGQFYGGD
SYIILYNYRHGSRQGQIIYNWQGAQSTQDEVAASAILTAQLDEELGGTPVQSRVVQGKEPAH
LMSLFGGKPMIVYKGGTSREGGQTA
>d1d0na5 d.109.1.1 (A:533-628) Gelsolin {Horse (Equus caballus)}
PASTRLFQVRASSSGATRAVEIIPKAGALNSNDAFVLKTPSAAYLWVGAGASEAEKTGAQEL
LRVLRAQPVQVAEGSEPDSFWEALGGKATYRTSP
>d1d0na6 d.109.1.1 (A:629-755) Gelsolin {Horse (Equus caballus)}
RLKDKKMDAHPPRLFACSNKIGRFVIEEVPGEFMQEDLATDDVMLLDTWDQVFVWVGKDSQD
EEKTEALTSAKRYIDTDPAHRDRRTPITVVKQGFEPPSFVGWFLGWDDSYWSVDPLDRALAE
LAA
>d1d4xg_ d.109.1.1 (G:) Gelsolin {Human (Homo sapiens)}
VEHPEFLKAGKEPGLQIWRVEKFDLVPVPTNLYGDFFTGDAYVILKTVQLRNGNLQYDLHYW
LGNECSQDESGAAAIFTVQLDDYLNGRAVQHREVQGFESATFLGYFKSGLKYKKGGVASGFK

>d1db0b1 d.109.1.1 (B:412-532) Gelsolin {Human (Homo sapiens)}
MDDDGTGQKQIWRIEGSNKVPVDPATYGQFYGGDSYIILYNYRHGGRQGQIIYNWQGAQSTQ
DEVAASAILTAQLDEELGGTPVQSRVVQGKEPAHLMSLFGGKPMIIYKGGTSREGGQTA
>d1db0b3 d.109.1.1 (B:629-742) Gelsolin {Human (Homo sapiens)}
RLKDKKMDAHPPRLFACSNKIGRFVIEEVPGELMQEDLATDDVMLLDTWDQVFVWVGKDSQD
EEKTEALTSAKRYIETDPANRDRRTPITVVKQGFEPPSFVGWFLGWDDSYWS
>d1kcqa_ d.109.1.1 (A:) Gelsolin {Human (Homo sapiens)}
VVQRLFQVKGRRVVRATEVPVSWESFNNGDCFILDLGNNIHQWCGSNSNRYERLKATQVSKG
IRDNERSGRARVHVSEEGTEPEAMLQVLGPKPALPAGTEDTA
>d1f7sa_ d.109.1.2 (A:) Cofilin (actin depolymerizing factor, ADF)
{Plant (Arabidopsis thaliana), ADF1}
ASGMAVHDDCKLRFLELKAKRTHRFIVYKIEEKQKQVVVEKVGQPIQTYEEFAACLPADECR
YAIYDFDFVTAENCQKSKIFFIAWCPDIAKVRSKMIYASSKDRFKRELDGIQVELQATDPTE
>d1cfya_ d.109.1.2 (A:) Cofilin (actin depolymerizing factor, ADF)
{Baker's yeast (Saccharomyces cerevisiae)}
VAVADESLTAFNDLKLGKKYKFILFGLNDAKTEIVVKETSTDPSYDAFLEKLPENDCLYAIY
DFEYEINGNEGKRSKIVFFTWSPDTAPVRSKMVYASSKDALRRALNGVSTDVQGTDFSEVSY
DSVLERVSR
>d1cnua_ d.109.1.2 (A:) Cofilin (actin depolymerizing factor, ADF)
{Amoeba (Acanthamoeba castellanii), actophorin}
GIAVSDDCVQKFNELKLGHQHRYVTFKMNASNTEVVVEHVGGPNATYEDFKSQLPERDCRYA
IFDYEFQVDGGQRNKITFILWAPDSAPIKSKMMYTSTKDSIKKKLVGIQVEVQATDAAEISE
DAVSERAKKD
>d1hqz1_ d.109.1.2 (1:) Cofilin-like domain of actin-binding
protein abp1p {Baker's yeast (Saccharomyces cerevisiae)}
LEPIDYTTHSREIDAEYLKIVRGSDPDTTWLIISPNAKKEYEPESTGSSFHDFLQLFDETKV
QYGLARVSPPGSDVEKIIIIGWCPDSAPLKTRASFAANFAAVANNLFKGYHVQVTARDEDDL
DENELLMKISNAAGA
>d1ak7__ d.109.1.2 (-) Destrin {Human and pig (Homo sapiens) and
(Sus scrofa)}
TMITPSSGNSASGVQVADEVCRIFYDMKVRKCSTPEEIKKRKKAVIFCLSADKKCIIVEEGK
EILVGDVGVTITDPFKHFVGMLPEKDCRYALYDASFETKESRKEELMFFLWAPELAPLKSKM
IYASSKDAIKKKFQGIKHECQANGPEDLNRACIAEKLGGSLIVAFEGCPV
>d1pne__ d.110.1.1 (-) Profilin (actin-binding protein) {Cow (Bos
taurus)}
AGWNAYIDNLMADGTCQDAAIVGYKDSPSVWAAVPGKTFVNITPAEVGILVGKDRSSFFVNG
LTLGGQKCSVIRDSLLQDGEFTMDLRTKSTGGAPTFNITVTMTAKTLVLLMGKEGVHGGMIN
KKCYEMASHLRRSQY
>d1fil__ d.110.1.1 (-) Profilin (actin-binding protein) {Human
(Homo sapiens), isoform I}
AGWNAYIDNLMADGTCQDAAIVGYKDSPSVWAAVPGKTFVNITPAEVGVLVGKDRSSFYVNG
LTLGGQKCSVIRDSLLQDGEFSMDLRTKSTGGAPTFNVTVTKTDKTLVLLMGKEGVHGGLIN
KKCYEMASHLRRSQY
>d1d1ja_ d.110.1.1 (A:) Profilin (actin-binding protein) {Human

(Homo sapiens), isoform II}
AGWQSYVDNLMCDGCCQEAAIVGYCDAKYVWAATAGGVFQSITPIEIDMIVGKDREGFFTNG
LTLGAKKCSVIRDSLYVDGDCTMDIRTKSQGGEPTYNVAVGRAGRALVIVMGKEGVHGGTLN
KKAYELALYLRRSD
>d1acf__ d.110.1.1 (-) Profilin (actin-binding protein)
{Acanthamoeba castellanii}
SWQTYVDTNLVGTGAVTQAAILGLDGNTWATSAGFAVTPAQGTTLAGAFNNADAIRAGGFDL
AGVHYVTLRADDRSIYGKKGSSGVITVKTSKAILVGVYNEKIQPGTAANVVEKLADYLIGQG
F
>d1f2ka_ d.110.1.1 (A:) Profilin (actin-binding protein)
{Acanthamoeba castellanii}
SWQTYVDTNLVGTGAVTQAAIIGHDGNTWATSAGFAVSPANGAALANAFKDATAIRSNGFEL
AGTRYVTIRADDRSVYGKKGSAGVITVKTSKAILIGVYNEKIQPGTAANVVEKLADYLIGQG
F
>d1ypra_ d.110.1.1 (A:) Profilin (actin-binding protein) {Baker's
yeast (Saccharomyces cerevisiae)}
SWQAYTDNLIGTGKVDKAVIYSRAGDAVWATSGGLSLQPNEIGEIVQGFDNPAGLQSNGLHI
QGQKFMLLRADDRSIYGRHDAEGVVCVRTKQTVIIAHYPPTVQAGEATKIVEQLADYLIGVQ
Y
>d1cqa__ d.110.1.1 (-) Profilin (actin-binding protein) {Birch
(Betula verrucosa)}
SWQTYVDEHLMCDIDGQGEELAASAIVGHDGSVWAQSSSFPQFKPQEITGIMKDFEEPGHLA
PTGLHLGGIKYMVIQGEAGAVIRGKKGSGGITIKKTGQALVFGIYEEPVTPGQCNMVVERLG
DYLIDQGL
>d3nul__ d.110.1.1 (-) Profilin (actin-binding protein) {Mouse-ear
cress (Arabidopsis thaliana)}
SWQSYVDDHLMCDVEGNHLTAAAILGQDGSVWAQSAKFPQLKPQEIDGIKKDFEEPGFLAPT
GLFLGGEKYMVIQGEQGAVIRGKKGPGGVTIKKTNQALVFGFYDEPMTGGQCNLVVERLGDY
LIESEL
>d1g5ua_ d.110.1.1 (A:) Profilin (actin-binding protein) {Para
rubber tree (Hevea brasiliensis), hevb8}
SWQTYVDDHLMCDIDGHRLTAAAIIGHDGSVWAQSSSFPQFKSDEVAAVMKDFDEPGSLAPT
GLHLGGTKYMVIQGEPGAVIRGKKGSGGITVKRTGQALIIGIYDEPLTPGQCNMIVERLGDY
LLDQGL
>d1f5ma_ d.110.2.1 (A:) Hypothetical protein ykl069wp {Baker's
yeast (Saccharomyces cerevisiae)}
STGFHHADHVNYSSNLNKEEILEQLLLSYEGLSDGQVNWVCNLSNASSLIWHAYKSLAVDIN
WAGFYVTQASEENTLILGPFQGKVACQMIQFGKGVCGTAASTKETQIVPDVNKYPGHIACDG
ETKSEIVVPIISNDGKTLGVIDIDCLDYEGFDHVDKEFLEKLAKLINKSCVF
>d3pyp__ d.110.3.1 (-) Photoactive yellow protein, PYP
{Ectothiorhodospira halophila}
MEHVAFGSEDIENTLAKMDDGQLDGLAFGAIQLDGDGNILQYNAAEGDITGRDPKQVIGKNF
FKDVAPCTDSPEFYGKFKEGVASGNLNTMFEYTFDYQMTPTKVKVHMKKALSGDSYWVFVKR
V

>d1ew0a_ d.110.3.2 (A:) Histidine kinase FixL heme domain {Rhizobium meliloti}
GSHMLETEDVVRARDAHLRSILDTVPDATVVSATDGTIVSFNAAAVRQFGYAEEEVIGQNLR
ILMPEPYRHEHDGYLQRYMATGEKRIIGIDRVVSGQRKDGSTFPMKLAVGEMRSGGERFFTG
FIRDLT
>d1dp6a_ d.110.3.2 (A:) Histidine kinase FixL heme domain {Bradyrhizobium japonicum}
DAMIVIDGHGIIQLFSTAAERLFGWSELEAIGQNVNILMPEPDRSRHDSYISRYRTTSDPHI
IGIGRIVTGKRRDGTTFPMHLSIGEMQSGGEPYFTGFVRDLTEHQQTQARLQEL
>d1bywa_ d.110.3.3 (A:) Erg potassium channel, N-terminal domain {Human (Homo sapiens)}
SRKFIIANARVENCAVIYCNDGFCELCGYSRAEVMQRPCTCDFLHGPCTQRRAAAQIAQALL
GAEERKVEIAFYRKDGSCFLCLVDVVPVKNEDGAVIMFILNFEVVMEK
>d1g28a_ d.110.3.4 (A:) Photoreceptor phy3 flavin-binding domain, lov2 {Maidenhair fern (Adiantum capillus-veneris)}
KSFVITDPRLPDNPIIFASDRFLELTEYTREEVLGNNCRFLQGRGTDRKAVQLIRDAVKEQR
DVTVQVLNYTKGGRAFWNLFHLQVMRDENGDVQYFIGVQQEM
>d1ifqa_ d.110.4.1 (A:) Sec22b {Mouse (Mus musculus)}
SVLLTMIARVADGLPLAASMQEDEQSGRDLQQYQSQAKQLFRKLNEQSPTRCTLEAGAMTFH
YIIEQGVCYLVLCEAAFPKKLAFAYLEDLHSEFDEQHGKKVPTVSRPYSFIEFDTFIQKTKK
LYI
>d1h8ma_ d.110.4.1 (A:) Synaptobrevin homolog 1 ykt6 {Baker's yeast (Saccharomyces cerevisiae)}
MRIYYIGVFRSGGEKALELSEVKDLSQFGFFERSSVGQFMTFFAETVASRTGAGERQSIEEG
NYIGHVYARSEGICGVLITDKQYPVRPAYTLLNKILDEYLVAHPKEEWADVTETNDALKMKQ
LDTYISKYQDPSQADA
>d1cfe__ d.111.1.1 (-) Pathogenesis-related protein 1 (PR1) {Tomato (Lycopersicon esculentum), P14a}
QNSPQDYLAVHNDARAQVGVGPMSWDANLASRAQNYANSRAGDCNLIHSGAGENLAKGGGDF
TGRAAVQLWVSERPSYNYATNQCVGGKKCRHYTQVVWRNSVRLGCGRARCNNGWWFISCNYD
PVGNWIGQRPY
>d1qnxa_ d.111.1.1 (A:) Insect allergen 5 (AG5) {Yellow jacket (Vespula vulgaris), Ves v 5}
AEAEFNNYCKIKCLKGGVHTACKYGSLKPNCGNKVVVSYGLTKQEKQDILKEHNDFRQKIAR
GLETRGNPGPQPPAKNMKNLVWNDELAYVAQVWANQCQYGHDTCRDVAKYQVGQNVALTGST
AAKYDDPVKLVKMWEDEVKDYNPKKKFSGNDFLKTGHYTQMVWANTKEVGCGSIKYIQEKWH
KHYLVCNYGPSGNFKNEELYQTK
>d1a6ja_ d.112.1.1 (A:) Nitrogen regulatory bacterial protein IIa-ntr {Escherichia coli}
LQLSSVLNRECTRSRVHCQSKKRALEIISELAAKQLSLPPQVVFEAILTREKMGSTGIGNGI
AIPHGKLEEDTLRAVGVFVQLETPIAFDAIDNQPVDLLFALLVPADQTKTHLHTLSLVAKRL
ADKTICRRLRAAQSDEELYQIITDTE
>d1a3aa_ d.112.1.1 (A:) Phosphotransferase IIa-mannitol {Escherichia coli}

LFKLGAENIFLGRKAATKEEAIRFAGEQLVKGGYVEPEYVQAMLDREKLTPTYLGESIAVPH
GTVEAKDRVLKTGVVFCQYPEGVRFGEEEDDIARLVIGIAARNNEHIQVITSLTNALDDESV
IERLAHTTSVDEVLELLAGRK
>d1hynp_ d.112.1.2 (P:) Erythrocite membrane Band 3 {Human (Homo sapiens)}
KVYVELQELVMDEKNQELRWMEAARWVQLEENLGENGAWGRPHLSHLTFWSLLELRRVFTKG
TVLLDLQETSLAGVANQLLDRFIFEDQIRPQDREELLRALLLKHSHAGELEALGGVKPAVLT
RSGDPSQPLLPQHSSLETQLFCEQGDGGTEGHSPSGILEKIPPDSEATLVLVGRADFLEQPV
LGFVRLQEAAELEAVELPVPIRFLFVLLGPEAPHIDYTQLGRAAATLMSERVFRIDAYMAQS
RGELLHSLEGFLDCSLVLPPTDAPSEQALLSLVPVQRELLRRRYQ
>d1mut__ d.113.1.1 (-) Nucleoside triphosphate pyrophosphorylase (MutT) {Escherichia coli}
MKKLQIAVGIIRNENNEIFITRRAADAHMANKLEFPGGKIEMGETPEQAVVRELQEEVGITP
QHFSLFEKLEYEFPDRHITLWFWLVERWEGEPWGKEGQPGEWMSLVGLNADDFPPANEPVIA
KLKRL
>d1g0sa_ d.113.1.1 (A:) ADP-ribose pyrophosphatase {Escherichia coli}
MLKPDNLPVTFGKNDVEIIARETLYRGFFSLDLYRFRHRLFNGQMSHEVRREIFERGHAAVL
LPFDPVRDEVVLIEQIRIAAYDTSETPWLLEMVAGMIEEGESVEDVARREAIEEAGLIVKRT
KPVLSFLASPGGTSERSSIMVGEVDATTASGIHGLADENEDIRVHVVSREQAYQWVEEGKID
NAASVIALQWLQLHHQALKNEWA
>d1jkna_ d.113.1.1 (A:) Diadenosine tetraphosphate hydrolase {Narrow-leaved blue lupine (Lupinus angustifolius)}
GPLGSMDSPPEGYRRNVGICLMNNDKKIFAASRLDIPDAWQMPQGGIDEGEDPRNAAIRELR
EETGVTSAEVIAEVPYWLTYDFPPKVREKLNIQWGSDWKGQAQKWFLFKFTGQDQEINLLGD
GSEKPEFGEWSWVTPEQLIDLTVEFKKPVYKEVLSVFAPHL
>d1hx3a_ d.113.1.2 (A:) Isopentenyl diphosphate isomerase {Escherichia coli}
EHVILLNAQGVPTGTLEKYAAHTADTRLHLAFSSWLFNAKGQLLVTRRALSKKAWPGVWTNS
VAGHPQLGESNEDAVIRRCRYELGVEITPPESIYPDFRYRATDPSGIVENEVCPVFAARTTS
ALQINDDEVMDYQWCDLADVLHGIDATPWAFSPWMVMQATNREARKRLSAFT
>d1hzta_ d.113.1.2 (A:) Isopentenyl diphosphate isomerase {Escherichia coli}
LHLAFSSWLFNAKGQLLVTRRALSKKAWPGVWTNSVCGHPQLGESNEDAVIRRCRYELGVEI
TPPESIYPDFRYRATDPSGIVENEVCPVFAARTTSALQINDDEVMDYQWCDLADVLHGIDAT
PWAFSPWMVMQATNREARKRLSAFTQLKL
>d1ush_1 d.114.1.1 (363-550) 5'-nucleotidase (syn. UDP-sugar hydrolase), C-terminal domain {Escherichia coli}
KIGETNGRLEGDRDKVRFVQTNMGRLILAAQMDRTGADFAVMSGGGIRDSIEAGDISYKNVL
KVQPFGNVVVYADMTGKEVIDYLTAVAQMKPDSGAYPQFANVSFVAKDGKLNDLKIKGEPVD
PAKTYRMATLNFNATGGDGYPRLDNKPGYVNTGFIDAEVLKAYIQKSSPLDVSVYEPKGEVS
WQ
>d1hrua_ d.115.1.1 (A:) Hypothetical protein YrdC {Escherichia coli}

NNLQRDAIAAAIDVLNEERVIAYPTEAVFGVGCDPDSETAVMRLLELKQRPVDKGLILIAAN
YEQLKPYIDDTMLTDVQRETIFSRWPGPVTFVFPAPATTPRWLTGRFDSLAVRVTDHPLVVA
LCQAYGKPLVSTSANLSGLPPCRTVDEVRAQFGAAFPVVPGETGGRLNPSEIRDALTGELFR
>d1g57a_ d.115.1.2 (A:) 3,4-dihydroxy-2-butanone 4-phosphate synthase, DHBP synthase, RibB {Escherichia coli}
LLSSFGTPFERVENALAALREGRGVMVLDDEDRENEGDMIFPAETMTVEQMALTIRHGSGIV
CLCITEDRRKQLDLPMMVENNTSAYGTGFTVTIEAAEGVTTGVSAADRITTVRAAIADGAKP
SDLNRPGHVFPLRAQAGGVLTRGGHTEATIDLMTLAGFKPAGVLCELTNDDGTMARAPECIE
FANKHNMALVTIEDLVAYRQAHE
>d1dbxa_ d.116.1.1 (A:) Hypothetical protein HI1434 (YbaK homologue) {Haemophilus influenzae}
TPAIDLLKKQKIPFILHTYDHDPNNQHFGDEAAEKLGIDPNRSFKTLLVAENGDQKKLACFV
LATANMLNLKKAAKSIGVKKVEMADKDAAQKSTGYLVGGISPLGQKKRVKTVINSTALEFET
IYVSGGKRGLSVEIAPQDLAKVLGAEFTDIVDE
>d1qqqa_ d.117.1.1 (A:) Thymidylate synthase {Escherichia coli}
MKQYLELMQKVLDEGTQKNDRTGTGTLSIFGHQMRFNLQDGFPLVTTKRCHLRSIIHELLWF
LQGDTNIAYLHENNVTIWDEWADENGDLGPVYGKQWRAWPTPDGRHIDQITTVLNQLKNDPD
SRRIIVSAWNVGELDKMALAPCHAFFQFYVADGKLSCQLYQRSCDVFLGLPFNIASYALLVH
MMAQQCDLEVGDFVWTGGDTHLYSNHMDQTHLQLSREPRPLPKLIIKRKPESIFDYRFEDFE
IEGYDSHPGIKAPVAI
>d1tsy__ d.117.1.1 (-) Thymidylate synthase {Lactobacillus casei}
MLEQPYLDLAKKVLDEGHFKPDRTHTGTYSIFGHQMRFDLSKGFPLLTTKKVPFGLIKSELL
WFLHGDTNIRFLLQHRNHIWDEWAFEKWVKSDEYHGPDMTDFGHRSQKDPEFAAVYHEEMAK
FDDRVLHDDAFAAKYGDLGLVYGSQWRAWHTSKGDTIDQLGDVIEQIKTHPYSRKLIVSAWN
PEDVPTMALPPCHTLYQFYVNDGKLSLQLYQRSADIFLGVPFNIASYALLTHLVAHECGLEV
GEFIHTFGDAHLYVNHLDQIKEQLSRTPRPAPTLQLNPDKHDIFDFDMKDIKLLNYDPYPAI
KAPVAV
>d1bkpa_ d.117.1.1 (A:) Thymidylate synthase {Bacillus subtilis}
TQFDKQYNSIIKDIINNGISDEEFDVRTKWDSDGTPAHTLSVISKQMRFDNSEVPILTTKKV
AWKTAIKELLWIWQLKSNDVNDLNMMGVHIWDQWKQEDGTIGHAYGFQLGKKNRSLNGEKVD
QVDYLLHQLKNNPSSRRHITMLWNPDELDAMALTPCVYETQWYVKHGKLHLEVRARSNDMAL
GNPFNVFQYNVLQRMIAQVTGYELGEYIFNIGDCHVYTRHIDNLKIQMEREQFEAPELWINP
EVKDFYDFTIDDFKLINYKHGDKLLFEVAV
>d1tis__ d.117.1.1 (-) Thymidylate synthase {Bacteriophage T4}
MKQYQDLIKDIFENGYETDDRTGTGTIALFGSKLRWDLTKGFPAVTTKKLAWKACIAELIWF
LSGSTNVNDLRLIQHDSLIQGKTVWDENYENQAKDLGYHSGELGPIYGKQWRDFGGVDQIIE
VIDRIKKLPNDRRQIVSAWNPAELKYMALPPCHMFYQFNVRNGYLDLQWYQRSVDVFLGLPF
NIASYATLVHIVAKMCNLIPGDLIFSGGNTHIYMNHVEQCKEILRREPKELCELVISGLPYK
FRYLSTKEQLKYVLKLRPKDFVLNNYVSHPPIKGKMAV
>d1f28a_ d.117.1.1 (A:) Thymidylate synthase {Pneumocystis carinii}
NAEEQQYLNLVQYIINHGEDRPDRTGTGTLSVFAPSPLKFSLRNKTFPLLTTKRVFIRGVIE
ELLWFIRGETDSLKLREKNIHIWDANGSREYLDSIGLTKRQEGDLGPIYGFQWRHFGAEYID
CKTNYIGQGVDQLANIIQKIRTSPYDRRLILSAWNPADLEKMALPPCHMFCQFYVHIPSNNH

RPELSCQLYQRSCDMGLGVPFNIASYALLTCMIAHVCDLDPGDFIHVMGDCHIYKDHIEALQ
QQLTRSPRPFPTLSLNRSITDIEDFTLDDFNIQNYHPYETIKMKMSI
>d2tsra_ d.117.1.1 (A:) Thymidylate synthase {Rat (Rattus norvegicus)}
QHGELQYLRQVEHIMRCGFKKEDRTGTGTLSVFGMQARYSLRDEFPLLTTKRVFWKGVLEEL
LWFIKGSTNAKELSSKGVRIWDANGSRDFLDSLGFSARQEGDLGPVYGFQWRHFGADYKDMD
SDYSGQGVDQLQKVIDTIKTNPDDRRIIMCAWNPKDLPLMALPPCHALCQFYVVNGELSCQL
YQRSGDMGLGVPFNIASYALLTYMIAHITGLQPGDFVHTLGDAHIYLNHIEPLKIQLQREPR
PFPKLRILRKVETIDDFKVEDFQIEGYNPHPTI
>d1hvya_ d.117.1.1 (A:) Thymidylate synthase {Human (Homo sapiens)}
PPHGELQYLGQIQHILRCGVRKDDRTGTGTLSVFGMQARYSLRDEFPLLTTKRVFWKGVLEE
LLWFIKGSTNAKELSSKGVKIWDANGSRDFLDSLGFSTREEGDLGPVYGFQWRHFGAEYRDM
ESDYSGQGVDQLQRVIDTIKTNPDDRRIIMCAWNPRDLPLMALPPCHALCQFYVVNSELSCQ
LYQRSGDMGLGVPFNIASYALLTYMIAHITGLKPGDFIHTLGDAHIYLNHIEPLKIQLQREP
RPFPKLRILRKVEKIDDFKAEDFQIEGYNPHPTIKMEMAV
>d1b5ea_ d.117.1.1 (A:) dCMP hydroxymethylase {Bacteriophage T4}
MISDSMTVEEIRLHLGLALKEKDFVVDKTGVKTIEIIGASFVADEPFIFGALNDEYIQRELE
WYKSKSLFVKDIPGETPKIWQQVASSKGEINSNYGWAIWSEDNYAQYDMCLAELGQNPDSRR
GIMIYTRPSMQFDYNKDGMSDFMCTNTVQYLIRDKKINAVVNMRSNDVVFGFRNDYAWQKYV
LDKLVSDLNAGDSTRQYKAGSIIWNVGSLHVYSRHFYLVDHWWKTGETHISKKDY
>d1kq4a_ d.207.1.1 (A:) Thy1 homologue {Thermotoga maritima, TM0449}
HMKIDILDKGFVELVDVMGNDLSAVRAARVSFDMGLKDEERDRHLIEYLMKHGHETPFEHIV
FTFHVKAPIFVARQWFRHRIASYNELSGRYSKLSYEFYIPSPERLEGYKTTIPPERVTEKIS
EIVDKAYRTYLELIESGVPREVARIVLPLNLYTRFFWTVNARSLMNFLNLRADSHAQWEIQQ
YALAIARIFKEKCPWTFEAFLKYAYKGDIL
>d1lba__ d.118.1.1 (-) Bacteriophage T7 lysozyme (Zn amidase) {Bacteriophage T7}
AKQRESTDAIFVHCSATKPSQNVGVREIRQWHKEQGWLDVGYHFIIKRDGTVEAGRDEMAVG
SHAKGYNHNSIGVCLVGGIDDKGKFDANFTPAQMQSLRSLLVTLLAKYEGAVLRAHHEVAPK
ACPSFDLKRWWEKNELVTSDRG
>d1cyo__ d.120.1.1 (-) Cytochrome b5 {Cow (Bos taurus)}
SKAVKYYTLEEIQKHNNSKSTWLILHYKVYDLTKFLEEHPGGEEVLREQAGGDATENFEDVG
HSTDARELSKTFIIGELHPDDRSKIT
>d1f03a_ d.120.1.1 (A:) Cytochrome b5 {Cow (Bos taurus)}
AVKYYTLEEIQKHNNSKSTWLILHYKVYDLTKFLEEHPGGEAVLRAQAGGDATANFEAVGHS
TDARELSKTFIIGELHPDDR
>d1aqa__ d.120.1.1 (-) Cytochrome b5 {Rat (Rattus norvegicus)}
KYYTLEEIQKHKDSKSTWVILHHKVYDLTKFLEEHPGGEEVLREQAGGDATENFEDVGHSTD
ARELSKTYIIGELHPDDRSKIA
>d1axx__ d.120.1.1 (-) Cytochrome b5 {Rat (Rattus norvegicus)}
DKDVKYYTLEEIQKHKDSKSTWVILHHKVYDLTKFLEEHPGGEEVLREQAGGDATENFEDVG
HSTDARELSKTYIIGELHPDDRSKIAKPSETL

>d1euea_ d.120.1.1 (A:) Cytochrome b5 {Rat (Rattus norvegicus)}
DPAVTYYRLEEVAKRNTAEETWMVIHGRVYDITRFLSEHPGGEEILLEQAGADATESFEDIG
HSPDAREMLKQYYIGDVHPNDLKP
>d1icca_ d.120.1.1 (A:) Cytochrome b5 {Rat (Rattus norvegicus)}
DPAVTYYRLEEVAKRNTSEETWMVIHGRVYDLTRFLSEHPGGEEVLREQAGADATESFEDVG
HSPDAREMLKQYYIGDVHPNDLKPK
>d1do9a_ d.120.1.1 (A:) Cytochrome b5 {Rabbit (Oryctolagus cuniculus)}
DKDVKYYTLEEIKKHNHSKSTWLILHHKVYDLTKFLEEHPGGEEVLREQAGGDATENFEDVG
HSTDARELSKTFIIGELHPDDRSKLSKPMETL
>d1cxya_ d.120.1.1 (A:) Cytochrome b558 {Ectothiorhodospira vacuolata}
TLPVFTLEQVAEHHSPDDCWMAIHGKVYDLTPYVPNHPGPAGMMLVWCGQESTEAWETKSYG
EPHSSLAARLLQRYLIGTL
>d1ltda2 d.120.1.1 (A:10-97) Flavocytochrome b2, N-terminal domain {Baker's yeast (Saccharomyces cerevisiae)}
KISPAEVAKHNKPDDCWVVINGYVYDLTRFLPNHPGGQDVIKFNAGKDVTAIFEPLHAPNVI
DKYIAPEKKLGPLQGSMPPELVCPPY
>d1soxa2 d.120.1.1 (A:3-93) Sulfite oxidase, N-terminal domain {Chicken (Gallus gallus)}
SYPEYTREEVGRHRSPEERVWVTHGTDVFDVTDFVELHPGGPDKILLAAGGALEPFWALYAV
HGEPHVLELLQQYKVGELSPDEAPAAPDA
>d1vcc__ d.121.1.1 (-) Vaccinia DNA topoisomerase I, 9 kDa N-terminal fragment {Vaccinia virus, strain WR}
MRALFYKDGKLFTDNNFLNPVSDDNPAYEVLQHVKIPTHLTDVVVYEQTWEEALTRLIFVGS
DSKGRRQYFYGKMHV
>d1amw__ d.122.1.1 (-) HSP90 {Baker's yeast (Saccharomyces cerevisiae)}
ASETFEFQAEITQLMSLIINTVYSNKEIFLRELISNASDALDKIRYKSLSDPKQLETEPDLF
IRITPKPEQKVLEIRDSGIGMTKAELINNLGTIAKSGTKAFMEALSAGADVSMIGQFGVGFY
SLFLVADRVQVISKSNDDEQYIWESNAGGSFTVTLDEVNERIGRGTILRLFLKDDQLEYLEE
KRIKEVIKRHSEFVAYPIQLVVTKEVE
>d1byqa_ d.122.1.1 (A:) HSP90 {Human (Homo sapiens)}
PMEEEEVETFAFQAEIAQLMSLIINTFYSNKEIFLRELISNSSDALDKIRYETLTDPSKLDS
GKELHINLIPNKQDRTLTIVDTGIGMTKADLINNLGTIAKSGTKAFMEALQAGADISMIGQF
GVGFYSAYLVAEKVTVITKHNDDEQYAWESSAGGSFTVRTDTGEPMGRGTKVILHLKEDQTE
YLEERRIKEIVKKHSQFIGYPITLFVE
>d1ei1a2 d.122.1.2 (A:2-220) DNA gyrase B {Escherichia coli}
SNSSDSSSIKVLKGLDAVRKRPGMYIGDTDDGTGLHHMVFEVVDNAIDEALAGHCKEIIVTI
HADNSVSVQDDGRGIPTGIHPEEGVSAAEVIMTVLHAGGKFDDNSYKVSGGLHGVGVSVVNA
LSQKLELVIQREGKIHRQIYEHGVPQAPLAVTGETEKTGTMVRFWPSLETFTNVTEFEYEIL
AKRLRELSFLDSGVSIRLRDKRDGKEDHFHYEG
>d1b63a2 d.122.1.2 (A:-2-216) DNA mismatch repair protein MutL {Escherichia coli}

SHMPIQVLPPQLANQIAAGEVVERPASVVKELVENSLDAGATRIDIDIERGGAKLIRIRDNG
CGIKKDELALALARHATSKIASLDDLEAIISLGFRGEALASISSVSRLTLTSRTAEQQEAWQ
AYAEGRDMNVTVKPAAHPVGTTLEVLDLFYNTPARRKFLRTEKTEFNHIDEIIRRIALARFD
VTINLSHNGKIVRQYRAVPEGGQKERRLGAIC
>d1bkna2 d.122.1.2 (A:20-216) DNA mismatch repair protein MutL {Escherichia coli}
VERPASVVKELVENSLDAGATRIDIDIERGGAKLIRIRDNGCGIKKDELALALARHATSKIA
SLDDLEAIISLGFRGEALASISSVSRLTLTSRTAEQQEAWQAYAEGRDMNVTVKPAAHPVGT
TLEVLDLFYNTPARRKFLRTEKTEFNHIDEIIRRIALARFDVTINLSHNGKIVRQYRAVPEG
GQKERRLGAIC
>d1h7sa2 d.122.1.2 (A:29-231) DNA mismatch repair protein PMS2 {Human (Homo sapiens)}
GQVVLSLSTAVKELVENSLDAGATNIDLKLKDYGVDLIEVSDNGCGVEEENFEGLTLKHHTS
KIQEFADLTQVETFGFRGEALSSLCALSDVTISTCHASAKVGTRLMFDHNGKIIQKTPYPRP
RGTTVSVQQLFSTLPVRHKEFQRNIKKEYAKMVQVLHAYCIISAGIRVSCTNQLGQGKRQPV
VCTGGSPSIKENIGSVF
>d1bxda_ d.122.1.3 (A:) Histidine kinase domain of the osmosensor EnvZ {Escherichia coli}
TGQEMPMEMADLNAVLGEVIAAESGYEREIETALYPGSIEVKMHPLSIKRAVANMVVNAARY
GNGWIKVSSGTEPNRAWFQVEDDGPGIAPEQRKHLFQPFVRGDSARTISGTGLGLAIVQRIV
DNHNGMLELGTSERGGLSIRAWLPVPVTRAQGTTKEG
>d1i58a_ d.122.1.3 (A:) Histidine kinase CheA {Thermotoga maritima}
GSHMVPISFVFNRFPRMVRDLAKKMNKEVNFIMRGEDTELDRTFVEEIGEPLLHLLRNAIDH
GIEPKEERIAKGKPPIGTLILSARHEGNNVVIEVEDDGRGIDKEKIIRKAIEKGLIDESKAA
TLSDQEILNFLFVPGFSTKEKVSEVSGRGVGMDVVKNVVESLNGSISIESEKDKGTKVTIRL
PLT
>d1id0a_ d.122.1.3 (A:) Histidine kinase PhoQ domain {Escherichia coli}
RELHPVAPLLDNLTSALNKVYQRKGVNISLDISPEISFVGEQNDFVEVMGNVLDNACKYCLE
FVEISARQTDEHLYIVVEDDGPGIPLSKREVIFDRGQRVDTLRPGQGVGLAVAREITEQYEG
KIVAGESMLGGARMEVIFGRQH
>d1gkza2 d.122.1.4 (A:186-378) Branched-chain alpha-ketoacid dehydrogenase kinase (BCK) {Rat (Rattus norvegicus)}
DFVGIICTRLSPKKIIEKWVDFARRLCEHKYGNAPRVRINGHVAARFPFIPMPLDYILPELL
KNAMRATMESHLDTPYNVPDVVITIANNDVDLIIRISDRGGGIAHKDLDRVMDYHFTTAEAS
TQDPRISPLFGHLDMHSGGQSGPMHGFGFGLPTSRAYAEYLGGSLQLQSLQGIGTDVYLRLR
HIDGREE
>d1jm6a2 d.122.1.4 (A:1177-1366) Pyruvate dehydrogenase kinase {Rat (Rattus norvegicus), isozyme 2}
PKHIGSIDPNCSVSDVVKDAYDMAKLLCDKYYMASPDLEIQEVNATNATQPIHMVYVPSHLY
HMLFELFKNAMRATVESHESSLTLPPIKIMVALGEEDLSIKMSDRGGGVPLRKIERLFSYMY
STAPTPQPGTGGTPLAGFGYGLPISRLYAKYFQGDLQLFSMEGFGTDAVIYLKALSTDSVER
LPVY

>d1ixma_ d.123.1.1 (A:) Sporulation responce regulatory protein Spo0B {Bacillus subtilis}
SDTALTNELIHLLGHSRHDWMNKLQLIKGNLSLQKYDRVFEMIEEMVIDAKHESKLSNLKTP
HLAFDFLTFNWKTHYMTLEYEVLGEIKDLSAYDQKLAKLMRKLFHLFDQAVSRESENHLTVS
LQTDHPDRQLILYLDFHGAFADPSAFDDIRQNGYEDVDIMRFEITSHECLIEIGL
>d1bola_ d.124.1.1 (A:) Ribonuclease Rh {Rhizopus niveus}
SSCSSTALSCSNSANSDTCCSPEYGLVVLNMQWAPGYGPDNAFTLHGLWPDKCSGAYAPSGG
CDSNRASSSIASVIKSKDSSLYNSMLTYWPSNQGNNNVFWSHEWSKHGTCVSTYDPDCYDNY
EEGEDIVDYFQKAMDLRSQYNVYKAFSSNGITPGGTYTATEMQSAIESYFGAKAKIDCSSGT
LSDVALYFYVRGRDTYVITDALSTGSCSGDVEYPTK
>d1bk7a_ d.124.1.1 (A:) Ribonuclease MC1 {Bitter gourd (Momordica charantia)}
FDSFWFVQQWPPAVCSFQKSGSCPGSGLRTFTIHGLWPQQSGTSLTNCPGSPFDITKISHLQ
SQLNTLWPNVLRANNQQFWSHEWTKHGTCSESTFNQAAYFKLAVDMRNNYDIIGALRPHAAG
PNGRTKSRQAIKGFLKAKFGKFPGLRCRTDPQTKVSYLVQVVACFAQDGSTLIDCTRDTCGA
NFIF
>d1dixa_ d.124.1.1 (A:) RNase LE {Tomatoes (Lycopersicon esculentum)}
ASGSKDFDFFYFVQQWPGSYCDTKQSCCYPTTGKPAADFGIHGLWPNNNDGTYPSNCDPNSP
YDQSQISDLISSMQQNWPTLACPSGSGSTFWSHEWEKHGTCAESVLTNQHAYFKKALDLKNQ
IDLLSILQGADIHPDGESYDLVNIRNAIKSAIGYTPWIQCNVDQSGNSQLYQVYICVDGSGS
SLIECPIFPGGKCGTSIEFPTF
>d1iqqa_ d.124.1.1 (A:) S3-RNase {Japanese pear (Pyrus pyrifolia)}
YDYFQFTQQYQLAVCNSNRTLCKDPPDKLFTVHGLWPSNMVGPDPSKCPIKNIRKREKLLEH
QLEIIWPNVFDRTKNNLFWDKEWMKHGSCGYPTIDNENHYFETVIKMYISKKQNVSRILSKA
KIEPDGKKRALLDIENAIRNGADNKKPKLKCQKKGTTTELVEITLCSDKSGEHFIDCPHPFE
PISPHYCPTNNIKY
>d1g2ra_ d.192.1.1 (A:) Hypothetical cytosolic protein SP0554 {Streptococcus pneumoniae}
RKIPLRKSVVSNEVIDKRDLLRIVKNKEGQVFIDPTGKANGRGAYIKLDNAEALEAKKKKVF
NRSFSMEVEESFYDELIAYVDHKVKRRELGLE
>d1c4ka3 d.125.1.1 (A:570-730) Ornithine decarboxylase C-terminal domain {Lactobacillus sp., strain 30a}
APLKQVLPSIYAANEERYNGYTIRELCQELHDFYKNNNTFTYQKRLFLREFFPEQGMLPYEA
RQEFIRNHNKLVPLNKIEGEIALEGALPYPPGVFCVAPGEKWSETAVKYFTILQDGINNFPG
FAPEIQGVYFKQEGDKVVAYGEVYDAEVAKNDDRYNN
>d1g61a_ d.126.1.1 (A:) Ribosome anti-association factor eIF6 (aIF6) {Archaeon Methanococcus jannaschii}
MIIRKYFSGIPTIGVLALTTEEITLLPIFLDKDDVNEVSEVLETKCLQTNIGGSSLVGSLSV
ANKYGLLLPKIVEDEELDRIKNFLKENNLDLNVEIIKSKNTALGNLILTNDKGALISPELKD
FKKDIEDSLNVEVEIGTIAELPTVGSNAVVTNKGCLTHPLVEDDELEFLKSLFKVEYIGKGT
ANKGTTSVGACIIANSKGAVVGGDTTGPELLIIEDALGL
>d1g62a_ d.126.1.1 (A:) Ribosome anti-association factor eIF6 (aIF6) {Baker's yeast (Saccharomyces cerevisiae)}

MATRTQFENSNEIGVFSKLTNTYCLVAVGGSENFYSAFEAELGDAIPIVHTTIAGTRIIGRM
TAGNRRGLLVPTQTTDQELQHLRNSLPDSVKIQRVEERLSALGNVICCNDYVALVHPDIDRE
TEELISDVLGVEVFRQTISGNILVGSYCSLSNQGGLVHPQTSVQDQEELSSLLQVPLVAGTV
NRGSSVVGAGMVVNDYLAVTGLDTTAPELSVIESIFRL
>d1jdw__ d.126.1.2 (-) L-arginine: glycine amidinotransferase {Human (Homo sapiens)}
CPVSSYNEWDPLEEVIVGRAENACVPPFTIEVKANTYEKYWPFYQKQGGHYFPKDHLKKAVA
EIEEMCNILKTEGVTVRRPDPIDWSLKYKTPDFESTGLYSAMPRDILIVVGNEIIEAPMAWR
SRFFEYRAYRSIIKDYFHRGAKWTTAPKPTMADELYNQDYPIHSVEDRHKLAAQGKFVTTEF
EPCFDAADFIRAGRDIFAQRSQVTNYLGIEWMRRHLAPDYRVHIISFKDPNPMHIDATFNII
GPGIVLSNPDRPCHQIDLFKKAGWTIITPPTPIIPDDHPLWMSSKWLSMNVLMLDEKRVMVD
ANEVPIQKMFEKLGITTIKVNIRNANSLGGGFHCWTCDVRRRGTLQSYLD
>d1bwda_ d.126.1.2 (A:) L-arginine: inosamine-phosphate amidinotransferase {Streptomyces griseus}
RSLVSVHNEWDPLEEVIVGTAVGARVPTADRSVFAVEYAGDYESQEQIPSGAYPDRVLKETE
EELHVLAAELTKLGVTVRRPGPRDHSALIKTPDWETDGFHDYCPRDGLLSVGQTIIETPMAL
RSRFLESLAYKDLLLEYFASGSRWLSAPKPRLTDDSYAPQAPAGERLTDEEPVFDAANVLRF
GTDLLYLVSDSGNELGAKWLQSAVGDTYTVHPCRKLYASTHVDSTIVPLRPGLVLTNPSRVN
DENMPDFLRSWENITCPELVDIGFTGDKPHCSVWIGMNLLVVRPDLAVVDRRQTALIRLLEK
HGMNVLPLQLTHSRTLGGGFHCATLDVRRTGALETYQF
>d1h70a_ d.126.1.3 (A:) Dimethylarginine dimethylaminohydrolase DDAH {Pseudomonas aeruginosa}
FMFKHIIARTPARSLVDGLTSSHLGKPDYAKALEQHNAYIRALQTCDVDITLLPPDERFPDS
VFVEDPVLCTSRCAIITRPGAESRRGETEIIEETVQRFYPGKVERIEAPGTVEAGDIMMVGD
HFYIGESARTNAEGARQMIAILEKHGLSGSVVRLEKVLHLKTGLAYLEHNNLLAAGEFVSKP
EFQDFNIIEIPEEESYAANCIWVNERVIMPAGYPRTREKIARLGYRVIEVDTSEYRKIDGGV
SSMSLRF
>d1chma2 d.127.1.1 (A:157-402) Creatinase, catalytic (C-terminal) domain {Pseudomonas putida}
MIKSAEEHVMIRHGARIADIGGAAVVEALGDQVPEYEVALHATQAMVRAIADTFEDVELMDT
WTWFQSGINTDGAHNPVTTRKVNKGDILSLNCFPMIAGYYTALERTLFLDHCSDDHLRLWQV
NVEVHEAGLKLIKPGARCSDIARELNEIFLKHDVLQYRTFGYGHSFGTLSHYYGREAGLELR
EDIDTVLEPGMVVSMEPMIMLPEGLPGAGGYREHDILIVNENGAENITKFPYGPEKNIIR
>d1c22a_ d.127.1.1 (A:) Methionine aminopeptidase {Escherichia coli}
AISIKTPEDIEKMRVAGRLAAEVLEMIEPYVKPGVSTGELDRICNDYIVNEQHAVSACLGYH
GYPKSVCISINEVVCHGIPDDAKLLKDGDIVNIDVTVIKDGFHGDTSKMFIVGKPTIMGERL
CRITQESLYLALRMVKPGINLREIGAAIQKFVEAEGFSVVREYCGHGIGQGFHEEPQVLHYD
SRETNVVLKPGMTFTIEPMVNAGKKEIRTMKDGWTVKTKDRSLSAQYEHTIVVTDNGCEILT
LRKDDTIPAIISHD
>d1xgsa2 d.127.1.1 (A:1-194,A:272-295) Methionine aminopeptidase {Archaeon Pyrococcus furiosus}
MDTEKLMKAGEIAKKVREKAIKLARPGMLLLELAESIEKMIMELGGKPAFPVNLSINEIAAH
YTPYKGDTTVLKEGDYLKIDVGVHIDGFIADTAVTVRVGMEEDELMEAAKEALNAAISVARA

GVEIKELGKAIENEIRKRGFKPIVNLSGHKIERYKLHAGISIPNIYRPHDNYVLKEGDVFAI
EPFATIGAXRNGIVAQFEHTIIVEKDSVIVTTE
>d1b6a_2 d.127.1.1 (110-374,449-478) Methionine aminopeptidase {Human (Homo sapiens)}
KVQTDPPSVPICDLYPNGVFPKGQECEYPPTQDGRTAAWRTTSEEKKALDQASEEIWNDFRE
AAEAHRQVRKYVMSWIKPGMTMIEICEKLEDCSRKLIKENGLNAGLAFPTGCSLNNCAAHYT
PNAGDTTVLQYDDICKIDFGTHISGRIIDCAFTVTFNPKYDTLLKAVKDATNTGIKCAGIDV
RLCDVGEAIQEVMESYEVEIDGKTYQVKPIRNLNGHSIGQYRIHAGKTVPIVKGGEATRMEE
GEVYAIETFGSTGKGVVXDIKGSYTAQFEHTILLRPTCKEVVSRGDDY
>d1az9_2 d.127.1.1 (177-440) Aminopeptidase P, C-terminal domain {Escherichia coli}
SPEEIAVLRRAGEITAMAHTRAMEKCRPGMFEYHLEGEIHHEFNRHGARYPSYNTIVGSGEN
GCILHYTENECEMRDGDLVLIDAGCEYKGYAGDITRTFPVNGKFTQAQREIYDIVLESLETS
LRLYRPGTSILEVTGEVVRIMVSGLVKLGILKGDVDELIAQNAHRPFFMHGLSHWLGLDVHD
VGVYGQDRSRILEPGMVLTVEPGLYIAPDAEVPEQYRGIGIRIEDDIVITETGNENLTASVV
KKPEEIEALMVAARKQ
>d1f52a2 d.128.1.1 (A:101-468) Glutamine synthetase, C-terminal domain {Salmonella typhimurium}
DRDPRSIAKRAEDYLRATGIADTVLFGPEPEFFLFDDIRFGASISGSHVAIDDIEGAWNSST
KYEGGNKGHRPGVKGGYFPVPPVDSAQDIRSEMCLVMEQMGLVVEAHHHEVATAGQNEVATR
FNTMTKKADEIQIYKYVVHNVAHRFGKTATFMPKPMFGDNGSGMHCHMSLAKNGTNLFSGDK
YAGLSEQALYYIGGVIKHAKAINALANPTTNSYKRLVPGYEAPVMLAYSARNRSASIRIPVV
ASPKARRIEVRFPDPAANPYLCFAALLMAGLDGIKNKIHPGEPMDKNLYDLPPEEAKEIPQV
AGSLEEALNALDLDREFLKAGGVFTDEAIDAYIALRREEDDRVRMTPHPVEFELYYSV
>d1crka2 d.128.1.2 (A:99-380) Creatine kinase, C-terminal domain {Chicken (Gallus gallus), mitochondria}
TMKHHTDLDASKITHGQFDERYVLSSRVRTGRSIRGLSLPPACSRAERREVENVVVTALAGL
KGDLSGKYYSLTNMSERDQQQLIDDHFLFDKPVSPLLTCAGMARDWPDARGIWHNNDKTFLV
WINEEDHTRVISMEKGGNMKRVFERFCRGLKEVERLIKERGWEFMWNERLGYVLTCPSNLGT
GLRAGVHVKLPRLSKDPRFPKILENLRLQKRGTGGVDTAAVADVYDISNLDRMGRSEVELVQ
IVIDGVNYLVDCEKKLEKGQDIKVPPPLPQFGRK
>d1qh4a2 d.128.1.2 (A:103-381) Creatine kinase, C-terminal domain {Chicken (Gallus gallus), brain-type}
TDEHKTDLNADNLQGGDDLDPNYVLSSRVRTGRSIRGFCLPPHCSRGERRAIEKLSVEALGS
LGGDLKGKYYALRNMTDAEQQQLIDDHFLFDKPVSPLLLASGMARDWPDARGIWHNDNKTFL
VWINEEDHLRVISMQKGGNMKEVFTRFCTGLTQIETLFKSKNYEFMWNPHLGYILTCPSNLG
TGLRAGVHIKLPNLGKHEKFGEVLKRLRLQKRGTGGVDTAAVGGVFDVSNADRLGFSEVELV
QMVVDGVKLLIEMEKRLEKGQSIDDLMPAQK
>d1qk1a2 d.128.1.2 (A:103-379) Creatine kinase, C-terminal domain {Human (Homo sapiens), mitochondria}
TTDLDASKIRSGYFDERYVLSSRVRTGRSIRGLSLPPACTRAERREVERVVVDALSGLKGDL
AGRYYRLSEMTEAEQQQLIDDHFLFDKPVSPLLTAAGMARDWPDARGIWHNNEKSFLIWVNE
EDHTRVISMEKGGNMKRVFERFCRGLKEVERLIQERGWEFMWNERLGYILTCPSNLGTGLRA
GVHIKLPLLSKDSRFPKILENLRLQKRGTGGVDTAATGGVFDISNLDRLGKSEVELVQLVID

GVNYLIDCERRLERGQDIRIPTPVIHTKH
>d2crka2 d.128.1.2 (A:103-381) Creatine kinase, C-terminal domain {Rabbit (Oryctolagus cuniculus)}
TDKHKTDLNHENLKGGDDLDPHYVLSSRVRTGRSIKGYTLPPHCSRGERRAVEKLSVEALNS
LTGEFKGKYYPLKSMTEQEQQQLIDDHFLFDKPVSPLLLASGMARDWPDARGIWHNDNKSFL
VWVNEEDHLRVISMEKGGNMKEVFRRFCVGLQKIEEIFKKAGHPFMWNEHLGYVLTCPSNLG
TGLRGGVHVKLAHLSKHPKFEEILTRLRLQKRGTSVFDTAAVGSVFDISNADRLGSSEVEQV
QLVVDGVKLMVEMEKKLEKGQSIDDMIPAQK
>d1g0wa2 d.128.1.2 (A:103-381) Creatine kinase, C-terminal domain {Cow (Bos taurus), retinal isoform}
TDEHKTDLNPDNLQGGDDLDPNYVLSSRVRTGRSIRGFCLPPHCSRGERRAIEKLAVEALSS
LDGDLAGRYYALKSMTEAEQQQLIDDHFLFDKPVSPLLLASGMARDWPDARGIWHNDNKTFL
VWINEEDHLRVISMQKGGNMKEVFTRFCNGLTQIETLFKSKNYEFMWNPHLGYILTCPSNLG
TGLRAGVHIKLPHLGKHEKFSEVLKRLRLQKRGTGGVDTAAVGGVFDVSNADRLGFSEVELV
QMVVDGVKLLIEMEQRLEQGQAIDDLMPAQK
>d1bg0_2 d.128.1.2 (96-357) Arginine kinase {Horseshoe crab (Limulus polyphemus)}
TDKHPPKQWGDINTLVGLDPAGQFIISTRVRCGRSLQGYPFNPCLTAEQYKEMEEKVSSTLS
SMEDELKGTYYPLTGMSKATQQQLIDDHFLFKEGDRFLQTANACRYWPTGRGIFHNDAKTFL
VWVNEEDHLRIISMQKGGDLKTVYKRLVTAVDNIESKLPFSHDDRFGFLTFCPTNLGTTMRA
SVHIQLPKLAKDRKVLEDIASKFNLQVRGTRGEHTESEGGVYDISNKRRLGLTEYQAVREMQ
DGILEMIKMEKAAA
>d1cdwa1 d.129.1.1 (A:155-252) TATA-box binding protein (TBP), C-terminal domain {Human (Homo sapiens)}
SGIVPQLQNIVSTVNLGCKLDLKTIALRARNAEYNPKRFAAVIMRIREPRTTALIFSSGKMV
CTGAKSEENSRLAARKYARVVQKLGFPAKFLDFKIQ
>d1cdwa2 d.129.1.1 (A:253-333) TATA-box binding protein (TBP), C-terminal domain {Human (Homo sapiens)}
NMVGSCDVKFPIRLEGLVLTHQQFSSYEPELFPGLIYRMIKPRIVLLIFVSGKVVLTGAKVR
AEIYEAFENIYPILKGFRK
>d1qnaa1 d.129.1.1 (A:17-115) TATA-box binding protein (TBP), C-terminal domain {Arabidopsis thaliana}
HPSGIVPTLQNIVSTVNLDCKLDLKAIALQARNAEYNPKRFAAVIMRIREPKTTALIFASGK
MVCTGAKSEDFSKMAARKYARIVQKLGFPAKFKDFKI
>d1qnaa2 d.129.1.1 (A:116-198) TATA-box binding protein (TBP), C-terminal domain {Arabidopsis thaliana}
QNIVGSCDVKFPIRLEGLAYSHAAFSSYEPELFPGLIYRMKVPKIVLLIFVSGKIVITGAKM
RDETYKAFENIYPVLSEFRKI
>d1ytba1 d.129.1.1 (A:61-155) TATA-box binding protein (TBP), C-terminal domain {Baker's yeast (Saccharomyces cerevisiae)}
SGIVPTLQNIVATVTLGCRLDLKTVALHARNAEYNPKRFAAVIMRIREPKTTALIFASGKMV
VTGAKSEDDSKLASRKYARIIQKIGFAAKFTDF
>d1ytba2 d.129.1.1 (A:156-240) TATA-box binding protein (TBP), C-terminal domain {Baker's yeast (Saccharomyces cerevisiae)}

KIQNIVGSCDVKFPIRLEGLAFSHGTFSSYEPELFPGLIYRMVKPKIVLLIFVSGKIVLTGA
KQREEIYQAFEAIYPVLSEFRKM
>d1aisa1 d.129.1.1 (A:1-92) TATA-box binding protein (TBP), C-terminal domain {Archaeon Pyrococcus woesei}
MVDMSKVKLRIENIVASVDLFAQLDLEKVLDLCPNSKYNPEEFPGIICHLDDPKVALLIFSS
GKLVVTGAKSVQDIERAVAKLAQKLKSIGV
>d1aisa2 d.129.1.1 (A:93-181) TATA-box binding protein (TBP), C-terminal domain {Archaeon Pyrococcus woesei}
KFKRAPQIDVQNMVFSGDIGREFNLDVVALTLPNCEYEPEQFPGVIYRVKEPKSVILLFSSG
KIVCSGAKSEADAWEAVRKLLRELDKY
>d1mpga2 d.129.1.2 (A:1-99) 3-Methyladenine DNA glycosylase II (gene alkA or aidA) {Escherichia coli}
MYTLNWQPPYDWSWMLGFLAARAVSSVETVADSYYARSLAVGEYRGVVTAIPDIARHTLHIN
LSAGLEPVAAECLAKMSRLFDLQCNPQIVNGALGRLG
>d1ko9a2 d.129.1.2 (A:12-135) 8-oxoguanine glycosylase {Human (Homo sapiens)}
GHRTLASTPALWASIPCPRSELRLDLVLPSGQSFRWREQSPAHWSGVLADQVWTLTQTEEQL
HCTVYRGDKSQASRPTPDELEAVRKYFQLDVTLAQLYHHWGSVDSHFQEVAQKFQGVRLLRQ
>d1f46a_ d.129.4.1 (A:) Cell-division protein ZipA, C-terminal domain {Escherichia coli}
RKEAVIIMNVAAHHGSELNGELLLNSIQQAGFIFGDMNIYHRHLSPDGSGPALFSLANMVKP
GTFDPEMKDFTTPGVTIFMQVPSYGDELQLFKLMLQSAQHIADEVGGVVLDDQRRMMTPQKL
REYQDIIREVKDANA
>d3pmga4 d.129.2.1 (A:421-561) Phosphoglucomutase {Rabbit (Oryctolagus cuniculus)}
RNFFTRYDYEEVEAEGATKMMKDLEALMFDRSFVGKQFSANDKVYTVEKADNFEYHDPVDGS
VSKNQGLRLIFADGSRIIFRLSGTGSAGATIRLYIDSYEKDNAKINQDPQVMLAPLISIALK
VSQLQERTGRTAPTVIT
>d1kfia4 d.129.2.1 (A:444-572) Exocytosis-sensitive phosphoprotein, pp63/parafusin {Paramecium tetraurelia}
RNYYSRYDYEQVDSAGANKMMEHLKTKFQYFEQLKQGNKADIYDYVDPVDQSVSKNQGVRFV
FGDGSRIIFRLSGTGSVGATIRIYFEQFEQQQIQHETATALANIIKLGLEISDIAQFTGRNE
PTVIT
>d1bv1__ d.129.3.1 (-) Major tree pollen allergen {White birch (Betula verrucosa), Bet v 1}
GVFNYETETTSVIPAARLFKAFILDGDNLFPKVAPQAISSVENIEGNGGPGTIKKISFPEGL
PFKYVKDRVDEVDHTNFKYNYSVIEGGPIGDTLEKISNEIKIVATPDGGSILKISNKYHTKG
DHEVKAEQVKASKEMGETLLRAVESYLLAHSDAYN
>d1e09a_ d.129.3.1 (A:) Major tree pollen allergen {Sweet cherry (Prunus avium), pru av 1}
GVFTYESEFTSEIPPPRLFKAFVLDADNLVPKIAPQAIKHSEILEGDGGPGTIKKITFGEGS
QYGYVKHKIDSIDKENYSYSYTLIEGDALGDTLEKISYETKLVASPSGGSIIKSTSHYHTKG
NVEIKEEHVKAGKEKASNLFKLIETYLKGHPDAYN
>d1em2a_ d.129.3.2 (A:) Lipid transport domain of Mln64 {Human (Homo

sapiens)}
SFSAQEREYIRQGKEATAVVDQILAQEENWKFEKNNEYGDTVYTIEVPFHGKTFILKTFLPC
PAELVYQEVILQPERMVLWNKTVTACQILQRVEDNTLISYDVSAGAAGGVVSPRDFVNVRRI
ERRRDRYLSSGIATSHSAKPPTHKYVRGENGPGGMIVLKSASNPRVCTFVWILNTDLKGRLP
RYLIHQSLAATMFEFAFHLRQRISELGA
>d1fvza_ d.129.3.4 (A:) Phoshatidylinositol transfer protein, PITP {Rat (Rattus norvegicus)}
VLLKEYRVILPVSVDEYQVGQLYSVAEASKNETGGGEGVEVLVNEPYEKDDGEKGQYTHKIY
HLQSKVPTFVRMLAPEGALNIHEKAWNAYPYCRTVITNEYMKEDFLIKIETWHKPDLGTQEN
VHKLEPEAWKHVEVIYIDIADRSQVLSKDYKAEEDPAKFKSIKTGRGPLGPNWKQELVNQKD
CPYMCAYKLVTVKFKWWGLQNKVENFIHKQEKRLFTNFHRQLFCWLDKWVDLTMDDIRRMEE
ETKRQLDEMRQKDPVKGMTAD
>d1eg9a2 d.129.3.3 (A:155-447) Naphthalene 1,2-dioxygenase alpha subunit, C-domain {Pseudomonas putida}
EAPPLMDYLGDAAWYLEPMFKHSGGLELVGPPGKVVIKANWKAPAENFVGDAYHVGWTHASS
LRSGESIFSSLAGNAALPPEGAGLQMTSKYGSGMGVLWDGYSGVHSADLVPELMAFGGAKQE
RLNKEIGDVRARIYRSHLNCTVFPNNSMLTCSGVFKVWNPIDANTTEVWTYAIVEKDMPEDL
KRRLADSVQRTFGPAGFWESDDNDNMETASQNGKKYQSRDSDLLSNLGFGEDVYGDAVYPGV
VGKSAIGETSYRGFYRAYQAHVSSSNWAEFEHASSTWHTELTKTT
>d1mxa_1 d.130.1.1 (1-102) S-adenosylmethionine synthetase {Escherichia coli}
AKHLFTSESVSEGHPDKIADQISDAVLDAILEQDPKARVACETYVKTGMVLVGGEITTSAWV
DIEEITRNTVREIGYVHSDMGFDANSCAVLSAIGKQSPDI
>d1mxa_2 d.130.1.1 (108-231) S-adenosylmethionine synthetase {Escherichia coli}
RADPLEQGAGDQGLMFGYATNETDVLMPAPITYAHRLVQRQAEVRKNGTLPWLRPDAKSQVT
FQYDDGKIVGIDAVVLSTQHSEEIDQKSLQEAVMEEIIKPILPAEWLTSATKFFINPTGRFV
>d1mxa_3 d.130.1.1 (232-383) S-adenosylmethionine synthetase {Escherichia coli}
IGGPMGDCGLTGRKIIVDTYGGMARHGGGAFSGKDPSKVDRSAAYAARYVAKNIVAAGLADR
CEIQVSYAIGVAEPTSIMVETFGTEKVPSEQLTLLVREFFDLRPYGLIQMLDLLHPIYKETA
AYGHFGREHFPWEKTDKAQLLRDAAGLK
>d1qm4a1 d.130.1.1 (A:17-116) S-adenosylmethionine synthetase {Rat (Rattus norvegicus)}
GAFMFTSESVGEGHPDKICDQISDAVLDAHLKQDPNAKVACETVCKTGMVLLCGEITSMAMI
DYQRVVRDTIKHIGYDDSAKGFDFKTCNVLVALEQQSP
>d1qm4a2 d.130.1.1 (A:129-252) S-adenosylmethionine synthetase {Rat (Rattus norvegicus)}
EDVGAGDQGLMFGYATDETEECMPLTIVLAHKLNTRMADLRRSGVLPWLRPDSKTQVTVQYV
QDNGAVIPVRVHTIVISVQHNEDITLEAMREALKEQVIKAVVPAKYLDEDTIYHLQPSGRFV
>d1qm4a3 d.130.1.1 (A:253-396) S-adenosylmethionine synthetase {Rat (Rattus norvegicus)}
IGGPQGDAGVTGRKIIVDTYGGWGAHGGGAFSGKDYTKVDRSAAYAARWVAKSLVKAGLCRR
VLVQVSYAIGVAEPLSISIFTYGTSKKTERELLEVVNKNFDLRPGVIVRDLDLKKPIYQKTA

CYGHFGRSEFPWEVPKKLVF
>d2pola1 d.131.1.1 (A:1-122) DNA polymerase III, beta subunit {Escherichia coli}
MKFTVEREHLLKPLQQVSGPLGGRPTLPILGNLLLQVADGTLSLTGTDLEMEMVARVALVQP
HEPGATTVPARKFFDICRGLPEGAEIAVQLEGERMLVRSGRSRFSLSTLPAADFPNLDDW
>d2pola2 d.131.1.1 (A:123-244) DNA polymerase III, beta subunit {Escherichia coli}
QSEVEFTLPQATMKRLIEATQFSMAHQDVRYYLNGMLFETEGEELRTVATDGHRLAVCSMPI
GQSLPSHSVIVPRKGVIELMRMLDGGDNPLRVQIGSNNIRAHVGDFIFTSKLVDGRFPDY
>d2pola3 d.131.1.1 (A:245-366) DNA polymerase III, beta subunit {Escherichia coli}
RRVLPKNPDKHLEAGCDLLKQAFARAAILSNEKFRGVRLYVSENQLKITANNPEQEEAEEIL
DVTYSGAEMEIGFNVSYVLDVLNALKCENVRMMLTDSVSSVQIEDAASQSAAYVVMPMRL
>d1b77a1 d.131.1.2 (A:1-110) gp45 sliding clamp {Bacteriophage RB69}
MKLSKDTIAILKNFASINSGILLSQGKFIMTRAVNGTTYAEANISDEIDFDVALYDLNSFLS
ILSLVSDDAEISMHTDGNIKIADTRSTVYWPAADKSTIVFPNKPIQFP
>d1b77a2 d.131.1.2 (A:111-228) gp45 sliding clamp {Bacteriophage RB69}
VASVITEIKAEDLQQLLRVSRGLQIDTIAITNKDGKIVINGYNKVEDSGLTRPKYSLTLTDY
DGSNNFNFVINMANMKIQPGNYKVMLWGAGDKVAAKFESSQVSYVIAMEADSTHDF
>d1czda1 d.131.1.2 (A:1001-1110) gp45 sliding clamp {Bacteriophage T4}
MKLSKDTTALLKNFATINSGIMLKSGQFIMTRAVNGTTYAEANISDVIDFDVAIYDLNGFLG
ILSLVNDDAEISQSEDGNIKIADARSTIFWPAADPSTVVAPNKPIPFP
>d1czda2 d.131.1.2 (A:1111-1228) gp45 sliding clamp {Bacteriophage T4}
VASAVTEIKAEDLQQLLRVSRGLQIDTIAITVKEGKIVINGFNKVEDSALTRVKYSLTLGDY
DGENTFNFIINMANMKMQPGNYKLLLWAKGKQGAAKFEGEHANYVVALEADSTHDF
>d1dmla1 d.131.1.2 (A:29-169) UL42 {Human herpes virus type 1}
APCQVVLQGAELNGILQAFAPLRTSLLDSLLVMGDRGILIHNTIFGEQVFLPLEHSQFSRYR
WRGPTAAFLSLVDQKRSLLSVFRANQYPDLRRVELAITGQAPFRTLVQRIWTTTSDGEAVEL
ASETLMKRELTSFVVLV
>d1dmla2 d.131.1.2 (A:170-319) UL42 {Human herpes virus type 1}
PQGTPDVQLRLTRPQLTKVLNATGADSATPTTFELGVNGKFSVFTTSTCVTFAAREEGVSSS
TSTQVQILSNALTKAGQAAANAKTVYGENTHRTFSVVVDDCSMRAVLRRLQVGGGTLKFFLT
TPVPSLCVTATGPNAVSAVFLLKPQK
>d1plq_1 d.131.1.2 (1-126) Prolifirating cell nuclear antigen (PCNA) {Baker's yeast (Saccharomyces cerevisiae)}
MLEAKFEEASLFKRIIDGFKDCVQLVNFQCKEDGIIAQAVDDSRVLLVSLEIGVEAFQEYRC
DHPVTLGMDLTSLSKILRCGNNTDTLTLIADNTPDSIILLFEDTKKDRIAEYSLKLMDIDAD
FL
>d1plq_2 d.131.1.2 (127-258) Prolifirating cell nuclear antigen (PCNA) {Baker's yeast (Saccharomyces cerevisiae)}

KIEELQYDSTLSLPSSEFSKIVRDLSQLSDSINIMITKETIKFVADGDIGSGSVIIKPFVDM
EHPETSIKLEMDQPVDLTFGAKYLLDIIKGSSLSDRVGIRLSSEAPALFQFDLKSGFLQFFL
APKFNDEE
>d1axca1 d.131.1.2 (A:1-126) Prolifirating cell nuclear antigen (PCNA) {Human (Homo sapiens)}
MFEARLVQGSILKKVLEALKDLINEACWDISSSGVNLQSMDSSHVSLVQLTLRSEGFDTYRC
DRNLAMGVNLTSMSKILKCAGNEDIITLRAEDNADTLALVFEAPNQEKVSDYEMKLMDLDVE
QL
>d1axca2 d.131.1.2 (A:127-255) Prolifirating cell nuclear antigen (PCNA) {Human (Homo sapiens)}
GIPEQEYSCVVKMPSGEFARICRDLSHIGDAVVISCAKDGVKFSASGELGNGNIKLSQTSNV
DKEEEAVTIEMNEPVQLTFALRYLNFFTKATPLSSTVTLSMSADVPLVVEYKIADMGHLKYY
LAPKI
>d1ge8a1 d.131.1.2 (A:2-117) Prolifirating cell nuclear antigen (PCNA) {Archaeon Pyrococcus furiosus}
PFEIVFEGAKEFAQLIDTASKLIDEAAFKVTEDGISMRAMDPSRVVLIDLNLPSSIFSKYEV
VEPETIGVNLDHLKKILKRGKAKDTLILKKGEENFLEITIQGTATRTFRVPLID
>d1ge8a2 d.131.1.2 (A:126-247) Prolifirating cell nuclear antigen (PCNA) {Archaeon Pyrococcus furiosus}
PELPFTAKVVVLGEVLKDAVKDASLVSDSIKFIARENEFIMKAEGETQEVEIKLTLEDEGLL
DIEVQEETKSAYGVSYLSDMVKGLGKADEVTIKFGNEMPMQMEYYIRDEGRLTFLLAPRV
>d1hlra4 d.133.1.1 (A:311-907) Aldehyde oxidoreductase {Desulfovibrio gigas}
MSGPAAAAEDAIEIHPGTPNVYFEQPIVKGEDTGPIFASADVTVEGDFYVGRQPHMPIEPDV
AFAYMGDDGKCYIHSKSIGVHLHLYMIAPGVGLEPDQLVLVANPMGGTFGYKFSPTSEALVA
VAAMATGRPVHLRYNYQQQQQYTGKRSPWEMNVKFAAKKDGTLLAMESDWLVDHGPYSEFGD
LLTLRGAQFIGAGYNIPNIRGLGRTVATNHVWGSAFRGYGAPQSMFASECLMDMLAEKLGMD
PLELRYKNAYRPGDTNPTGQEPEVFSLPDMIDQLRPKYQAALEKAQKESTATHKKGVGISIG
VYGSGLDGPDASEAWAELNADGTITVHTAWEDHGQGADIGCVGTAHEALRPMGVAPEKIKFT
WPNTATTPNSGPSGGSREQVMTGNAIRVACENLLKACEKPGGGYYTYDELKAADKPTKITGN
WTASGATHCDAVTGLGKPFVVYMYGVFMAEVTVDVATGQTTVDGMTLMADLGSLCNQLATDG
QIYGGLAQGIGLALSEDFEDIKKHATLVGAGFPFIKQIPDKLDIVYVNHPRPDGPFGASGVG
ELPLTSPHAAIINAIKSATGVRIYRLPAYPEKVLEALKA
>d1dgja4 d.133.1.1 (A:311-906) Aldehyde oxidoreductase {Desulfovibrio desulfuricans}
MSAPEAMAPDAIEIHPGTPNVYYDQLEEKGEDTVPFFNDPANVVAEGSYYTQRQPHLPIEPD
VGYGYINEQGQVVIHSKSVAIHLHALMIAPGLGLEFPKDLVLVQNTTGGTFGYKFSPTMEAL
VGVAVMATGRPCHLRYNYEQQQNYTGKRSPFWTTMRYAADRQGKILAMETDWSVDHGPYSEF
GDLLTLRGAQYIGAGYGIANIRGTGRTVATNHCWGAAFRGYGAPESEFPSEVLMDELAEKLG
MDPFELRALNCYREGDTTSSGQIPEVMSLPEMFDKMRPYYEESKKRVKERSTAEIKRGVGVA
LGVYGAGLDGPDTSEAWVELNDDGSVTLGNSWEDHGQGADAGSLGTAHEALRPLGITPENIH
LVMNDTSKTPNSGPAGGSRSQVVTGNAIRVACEMLIEGMRKPGGGFFTPAEMKAEGRPMRYD
GKWTAPAKDCDAKGQGSPFACYMYGLFLTEVAVEVATGKATVEKMVCVADIGKICNKLVVDG
QIYGGLAQGVGLALSEDYEDLKKHSTMGGAGIPSIKMIPDDIEIVYVETPRKDGPFGASGVG

EMPLTAPHAAIINGIYNACGARVRHLPARPEKVLEAMP
>d1fo4a5 d.133.1.1 (A:695-1332) Xanthine oxidase, C-terminal domain {Cow (Bos taurus)}
IITIEDAIKNNSFYGSELKIEKGDLKKGFSEADNVVSGELYIGGQDHFYLETHCTIAIPKGE
EGEMELFVSTQNAMKTQSFVAKMLGVPVNRILVRVKRMGGGFGGKETRSTLVSVAVALAAYK
TGHPVRCMLDRNEDMLITGGRHPFLARYKVGFMKTGTIVALEVDHYSNAGNSRDLSHSIMER
ALFHMDNCYKIPNIRGTGRLCKTNLSSNTAFRGFGGPQALFIAENWMSEVAVTCGLPAEEVR
WKNMYKEGDLTHFNQRLEGFSVPRCWDECLKSSQYYARKSEVDKFNKENCWKKRGLCIIPTK
FGISFTVPFLNQAGALIHVYTDGSVLVSHGGTEMGQGLHTKMVQVASKALKIPISKIYISET
STNTVPNSSPTAASVSTDIYGQAVYEACQTILKRLEPFKKKNPDGSWEDWVMAAYQDRVSLS
TTGFYRTPNLGYSFETNSGNAFHYFTYGVACSEVEIDCLTGDHKNLRTDIVMDVGSSLNPAI
DIGQVEGAFVQGLGLFTLEELHYSPEGSLHTRGPSTYKIPAFGSIPTEFRVSLLRDCPNKKA
IYASKAVGEPPLFLGASVFFAIKDAIRAARAQHTNNNTKELFRLDSPATPEKIRNACVDKFT
TLCVTGAPGNCKPWSLRV
>d1jrob2 d.133.1.1 (B:124-777) Xanthine dehydrogenase chain B, C-terminal domain {Rhodobacter capsulatus}
PAILTLDQALAADSRFEGGPVIWARGDVETALAGAAHLAEGCFEIGGQEHFYLEGQAALALP
AEGGVVIHCSSQHPSEIQHKVAHALGLAFHDVRVEMRRMGGGFGGKESQGNHLAIACAVAAR
ATGRPCKMRYDRDDDMVITGKRHDFRIRYRIGADASGKLLGADFVHLARCGWSADLSLPVCD
RAMLHADGSYFVPALRIESHRLRTNTQSNTAFRGFGGPQGALGMERAIEHLARGMGRDPAEL
RALNFYDPPERGGLSAPPSPPEPIATKKTQTTHYGQEVADCVLGELVTRLQKSANFTTRRAE
IAAWNSTNRTLARGIALSPVKFGISFTLTHLNQAGALVQIYTDGSVALNHGGTEMGQGLHAK
MVQVAAAVLGIDPVQVRITATDTSKVPNTSATAASSGADMNGMAVKDACETLRGRLAGFVAA
REGCAARDVIFDAGQVQASGKSWRFAEIVAAAYMARISLSATGFYATPKLSWDRLRGQGRPF
LYFAYGAAITEVVIDRLTGENRILRTDILHDAGASLNPALDIGQIEGAYVQGAGWLTTEELV
WDHCGRLMTHAPSTYKIPAFSDRPRIFNVALWDQPNREETIFRSKAVGEPPFLLGISAFLAL
HDACAACGPHWPDLQAPATPEAVLAAVRRAEGRA
>d1qj2b2 d.133.1.1 (B:147-809) Carbon monoxide (CO) dehydrogenase molybdoprotein {Pseudomonas carboxydovorans}
VDPFKAMEPDAPLLREDIKDKMTGAHGARKHHNHIFRWEIGDKEGTDATFAKAEVVSKDMFT
YHRVHPSPLETCQCVASMDKIKGELTLWGTFQAPHVIRTVVSLISGLPEHKIHVIAPDIGGG
FGNKVGAYSGYVCAVVASIVLGVPVKWVEDRMENLSTTSFARDYHMTTELAATKDGKILAMR
CHVLADHGAFDACADPSKWPAGFMNICTGSYDMPVAHLAVDGVYTNKASGGVAYRXSFRVTE
AVYAIERAIETLAQRLEMDSADLRIKNFIQPEQFPYMAPLGWEYDSGNYPLAMKKAMDTVGY
HQLRAEQKAKQEAFKRGETREIMGIGISFFTEIVGAGPSKNCDILGVSMFDSAEIRIHPTGS
VIARMGTKSQGQGHETTYAQIIATELGIPADDIMIEEGNTDTAPYGLGTYGSRSTPTAGAAT
AVAARKIKAKAQMIAAHMLEVHEGDLEWDVDRFRVKGLPEKFKTMKELAWASYNSPPPNLEP
GLEAVNYYDPPNMTYPFGAYFCIMDIDIDTGVAKTRRFYALDDCGTRINPMIIEGQVHGGLT
EAFAVAMGQEIRYDEQGNVLGASFMDFFLPTAVETPKWETDYTVTPSPHHPIGAKGVGESPH
VGGVPCFSNAVNDAYAFLNAGHIQMPHDAWRLWKVGEQLGLHV
>d1ffvb2 d.133.1.1 (B:147-803) Carbon monoxide (CO) dehydrogenase molybdoprotein {Hydrogenophaga pseudoflava}
IDALKPDAPVLREDLAGKTSGAHGPREHHNHIFTWGAGDKAATDAVFANAPVTVSQHMYYPR
VHPCPLETCGCVASFDPIKGDLTTYITSQAPHVVRTVVSMLSGIPESKVRIVSPDIGGGFGN

KVGIYPGYVCAIVASIVLGRPVKWVEDRVENISTTAFARDYHMDGELAATPDGKILGLRVNV
VADHGAFDACADPTKFPAGLFHICSGSYDIPRAHCSVKGVYTNKAPGGVAYRCSFRVTEAVY
LIERMVDVLAQKLNMDKAEIRAKNFIRKEQFPYTTQFGFEYDSGDYHTALKKVLDAVDYPAL
RAEQAARRADPNSPTLMGIGLVTFTEVVGAGPSKMCDILGVGMFDSCEIRIHPTGSAIARMG
TITQGQGHQTTYAQIIATELGIPSEVIQVEEGDTSTAPYGLGTYGSRSTPVAGAAIALAARK
IHAKARKIAAHMLEVNENDLDWEVDRFKVKGDDSKFKTMADIAWQAYHQPPAGLEPGLEAVH
YYDPPNFTYPFGIYLCVVDIDRATGETKVRRFYALDDCGTRINPMIIEGQIHGGLTEGYAVA
MGQQMPFDAQGNLLGNTLMDYFLPTAVETPHWETDHTVTPSPHHPIGAKGVAESPHVGSIPT
FTAAVVDAFAHVGVTHLDMPHTSYRVWKSLKEHNLAL
>d1aop_3 d.134.1.1 (149-345) Sulfite reductase hemoprotein (SiRHP), domains 2 and 4 {Escherichia coli}
NDMNRNVLCTSNPYESQLHAEAYEWAKKISEHLLPRTRAYAEIWLDQEKVATTDEEPILGQT
YLPRKFKTTVVIPPQNDIDLHANDMNFVAIAENGKLVGFNLLVGGGLSIEHGNKKTYARTAS
EFGYLPLEHTLAVAEAVVTTQRDWGNRTDRKNAKTKYTLERVGVETFKAEVERRAGIKFEPI
RPYEFTGRGDR
>d1aop_4 d.134.1.1 (426-570) Sulfite reductase hemoprotein (SiRHP), domains 2 and 4 {Escherichia coli}
PQRENSMACVSFPTCPLAMAEAERFLPSFIDNIDNLMAKHGVSDEHIVMRVTGCPNGCGRAM
LAEVGLVGKAPGRYNLHLGGNRIGTRIPRMYKENITEPEILASLDELIGRWAKEREAGEGFG
DFTVRAGIIRPVLDPARDLWD
>d1klqa_ d.135.1.1 (A:) The spindle assembly checkpoint protein mad2 {Human (Homo sapiens)}
GSITLRGSAEIVAEFFSFGINSILYQRGIYPSETFTRVQKYGLTLLVTTDLELIKYLNNVVE
QLKDWLYKCSVQKLVVVISNIESGEVLERWQFDIECDKTAKDDSAPREKSQKAIQDEIRSVI
RQITATVTFLPLLEVSCSFDLLIYTDKDLVVPEKWEESGPQFITNSEEVRLRSFTTTIHKVN
SMVAYKIPVND
>d1byra_ d.136.1.1 (A:) Nuclease Nuc {Salmonella typhimurium}
EPSVQVGYSPEGSARVLVLSAIDSAKTSIRMMAYSFTAPDIMKALVAAKKRGVDVKIVIDER
GNTGRASIAAMNYIANSGIPLRTDSNFPIQHDKVIIVDNVTVETGSFNFTKAAETKNSENAV
VIWNMPKLAESFLEHWQDRWNQGRDYRS
>d1f0ia1 d.136.1.2 (A:6-263) Phospholipase D {Streptomyces sp.}
AATPHLDAVEQTLRQVSPGLEGDVWERTSGNKLDGSAADPSDWLLQTPGCWGDDKCADRVGT
KRLLAKMTENIGNATRTVDISTLAPFPNGAFQDAIVAGLKESAAKGNSLKVRILVGAAPVYH
MNGIPSKYRDKLTAKLGKAAENITLNVASMTTSKTAFSWNHSKILVVDGQSALTGGINSWKD
DYLDTTHPVSDVDLALTGPAAGSAGRYLDTLWTWTCKNKSNIASVWFAASGNAGCMPTMHKD
TNPKASPATG
>d1f0ia2 d.136.1.2 (A:264-514) Phospholipase D {Streptomyces sp.}
NVPVIAVGGLGVGIKDVDPKSTFRPDLPTASDTKCVVGLHDNTNADRDYDTVNPEESALRAL
VASAKGHIEISQQDLNATCPPLPRYDIRLYDALAAKMAAGVKVRIVVSDPANRGAVGSGGYS
QIKSLSEISDTLRNRLANITGGQQAAKTAMCSNLQLATFRSSPNGKWADGHPYAQHHKLVSV
DSSTFYIGSKNLYPSWLQDFGYIVESPEAAKQLDAKLLDPQWKYSQETATVDYARGICGA
>d1jy1a1 d.136.1.3 (A:145-350) Tyrosyl-DNA phosphodiesterase TDP1 {Human (Homo sapiens)}
LEDPGEGQDIWDMLDKGNPFQFYLTRVSGVKPKYNSGALHIKDILSPLFGTLVSSAQFNYCF

DVDWLVKQYPPEFRKKPILLVHGDKREAKAHLHAQAKPYENISLCQAKLDIAFGTHHTKMML
LLYEEGLRVVIHTSNLIHADWHQKTQGIWLSPLYPRIADGTHKSGESPTHFKANLISYLTAY
NAPSLKEWIDVIHKHDLSET
>d1jy1a2 d.136.1.3 (A:351-608) Tyrosyl-DNA phosphodiesterase TDP1 {Human (Homo sapiens)}
NVYLIGSTPGRFQGSQKDNWGHFRLKKLLKDHASSMPNAESWPVVGQFSSVGSLGADESKWL
CSEFKESMLTLGKESKTPGKSSVPLYLIYPSVENVRTSLEGYPAGGSLPYSIQTAEKQNWLH
SYFHKWSAETSGRSNAMPHIKTYMRPSPDFSKIAWFLVTSANLSKAAWGALEKNGTQLMIRS
YELGVLFLPSALGLDSFKVKQKFFAGSQEPMATFPVPYDLPPELYGSKDRPWIWNIPYVKAP
DTHGNMWVPS
>d1ckv__ d.137.1.1 (-) Soluble methane monooxygenase regulatory protein B {Escherichia coli}
MSVNSNAYDAGIMGLKGKDFADQFFADENQVVHESDTVVLVLKKSDEINTFIEEILLTDYKK
NVNPTVNVEDRAGYWWIKANGKIEVDCDEISELLGRQFNVYDFLVDVSSTIGRAYTLGNKFT
ITSELMGLDRKLEDYHA
>d2moba_ d.137.1.1 (A:) Soluble methane monooxygenase regulatory protein B {Methylosinus trichosporium}
SNAVVLVLMKSDEIDAIIEDIVLKGGKAKNPSIVVEDKAGFWWIKADGAIEIDAAEAGELLG
KPFSVYDLLINVSSTVGRAYTLGTKFTITSEL
>d1g10a_ d.137.1.1 (A:) Toluene-4-monooxygenase catalytic effector protein {Pseudomonas mendocina}
STLADQALHNNNVGPIIRAGDLVEPVIETAEIDNPGKEITVEDRRAYVRIAAEGELILTRKT
LEEQLGRPFNMQELEINLASFAGQIQADEDQIRFYFDKTM
>d1hqi__ d.137.1.1 (-) Phenol hydroxylase P2 protein {Pseudomonas sp., CF600}
MSSLVYIAFQDNDNARYVVEAIIQDNPHAVVQHHPAMIRIEAEKRLEIRRETVEENLGRAWD
VQEMLVDVITIGGNVDEDDDRFVLEWKN
>d1jjcb6 d.138.1.1 (B:191-399) B3/B4 domain of PheRS, PheT {Thermus thermophilus (Thermus aquaticus)}
LKAEALPLPFALKVEDPEGAPHFTLGYAFGLRVAPSPLWMQRALFAAGMRPINNVVDVTNYV
MLERAQPMHAFDLRFVGEGIAVRRAREGERLKTLDGVERTLHPEDLVIAGWRGEESFPLGLA
GVMGGAESEVREDTEAIALEVACFDPVSIRKTARRHGLRTEASHRFERGVDPLGQVPAQRRA
LSLLQALAGARVAEALLEAGSPK
>d1clia2 d.139.1.1 (A:171-345) Aminoimidazole ribonucleotide synthetase (PurM) C-terminal domain {Escherichia coli}
DGSKVSDGDVLIALGSSGPHSNGYSLVRKILEVSGCDPQTTELDGKPLADHLLAPTRIYVKS
VLELIEKVDVHAIAHLTGGGFWENIPRVLPDNTQAVIDESSWQWPEVFNWLQTAGNVEHHEM
YRTFNCGVGMIIALPAPEVDKALALLNANGENAWKIGIIKASDSEQRVVIE
>d1hw7a_ d.193.1.1 (A:) Heat shock protein 33, Hsp33 {Escherichia coli}
HDQLHRYLFENFAVRGELVTVSETLQQILENHDYPQPVKNVLAELLVATSLLTATLKFDGDI
TVQLQGDGPMNLAVINGNNNQQMRGVARVQGEIPENADLKTLVGNGYVVITITPSEGERYQG
VVGLEGDTLAACLEDYFMRSEQLPTRLFIRTGDVDGKPAAGGMLLQVMPAQNAQQDDFDHLA
TLTETIKTEELLTLPANEVLWRLYHEEEVTVYDPQDVEFKCTC

>d1jw3a_ d.208.1.1 (A:) Hypothetical protein MTH1598 {Archaeon Methanobacterium thermoautotrophicum}
MKGFEFFDVTADAGFWAYGHDLEEVFENAALAMFEVMTDTSLVEAAEERRVEITSEDRVSLL
YDWLDELLFIHDTEFILFSKFKVKIDEKDDGLHLTGTAMGEEIKEGHERRDEVKAVTFHMME
ILDEDGLIKARVILDL
>d1dl5a2 d.197.1.1 (A:214-317) Protein-L-isoaspartyl O-methyltransferase, C-terminal domain {Thermotoga maritima}
NLLERNRKLLREFPFNREILLVRSHIFVELVDLLTRRLTEIDGTFYYAGPNGVVEFLDDRMR
IYGDAPEIENLLTQWESCGYRSFEYLMLHVGYNAFSHISCSI
>d1seia_ d.140.1.1 (A:) Ribosomal protein S8 {Bacillus stearothermophilus}
VMTDPIADMLTAIRNANMVRHEKLEVPASKIKREIAEILKREGFIRDYEYIEDNKQGILRIF
LKYGPNERVITGLKRISKPGLRVYVKAHEVPRVLNGLGIAILSTSQGVLTDKEARQKGTGGE
IIAYVI
>d1an7a_ d.140.1.1 (A:) Ribosomal protein S8 {Thermus thermophilus}
TDPIADMLTRIRNATRVYKESTDVPASRFKEEILRILAREGFIKGYERVDVDGKPYLRVYLK
YGPRRQGPDPRPEQVIHHIRRISKPGRRVYVGVKEIPRVRRGLGIAILSTSKGVLTDREARK
LGVGGELICEVW
>d1i94h_ d.140.1.1 (H:) Ribosomal protein S8 {Thermus thermophilus}
MLTDPIADMLTRIRNATRVYKESTEVPASRFKEEILKILAREGFIKGYERVEVDGKPYLRIH
LKYGPRRQGPDPRPEQVIKHIRRISRPGRRVYVGVKEIPRVRRGLGIAILSTPKGVLTDREA
RKLGVGGELICEVW
>d1i6ua_ d.140.1.1 (A:) Ribosomal protein S8 {Archaeon Methanococcus jannaschii}
SLMDPLANALNHISNCERVGKKVVYIKPASKLIGRVLKVMQDNGYIGEFEFIEDGRAGIFKV
ELIGKINKCGAIKPRFPVKKFGYEKFEKRYLPARDFGILIVSTTQGVMSHEEAKKRGLGGRL
LAYVY
>d1rl6a1 d.141.1.1 (A:7-81) Ribosomal protein L6 {Bacillus stearothermophilus}
PIEIPAGVTVTVNGNTVTVKGPKGELTRTFHPDMTITVEGNVITVTRPSDEKHHRALHGTTR
SLLANMVEGVSKG
>d1rl6a2 d.141.1.1 (A:82-170) Ribosomal protein L6 {Bacillus stearothermophilus}
YEKALELVGVGYRASKQGKKLVLSVGYSHPVEIEPEEGLEIEVPSQTKIIVKGADKQRVGEL
AANIRAVRPPEPYKGKGIRYEGELVRL
>d1jj2e1 d.141.1.1 (E:1-79) Ribosomal protein L6 {Archaeon Haloarcula marismortui}
PRVELEIPEDVDAEQDHLDITVEGDNGSVTRRLWYPDIDVSVDGDTVVIESDEDNAKTMSTI
GTFQSHIENMFHGVTEG
>d1jj2e2 d.141.1.1 (E:80-172) Ribosomal protein L6 {Archaeon Haloarcula marismortui}
WEYGMEVFYSHFPMQVNVEGDEVVIENFLGEKAPRRTTIHGDTDVEIDGEELTVSGPDIEAV

GQTAADIEQLTRINDKDVRVFQDGVYITRKP
>d1gsa_2 d.142.1.1 (123-314) Glutathione synthetase {Escherichia coli}
NEKLFTAWFSDLTPETLVTRNKAQLKAFWEKHSDIILKPLDGMGGASIFRVKEGDPNLGVIA
ETLTEHGTRYCMAQNYLPAIKDGDKRVLVVDGEPVPYCLARIPQGGETRGNLAAGGRGEPRP
LTESDWKIARQIGPTLKEKGLIFVGLDIIGDRLTEINVTSPTCIREIEAEFPVSITGMLMDA
IEARLQ
>d1iow_2 d.142.1.1 (97-306) D-ala-D-ala ligase {Escherichia coli, gene ddlB}
KLRSKLLWQGAGLPVAPWVALTRAEFEKGLSDKQLAEISALGLPVIVKPSREGSSVGMSKVV
AENALQDALRLAFQHDEEVLIEKWLSGPEFTVAILGEEILPSIRIQPSGTFYDYEAKFLSDE
TQYFCPAGLEASQEANLQALVLKAWTTLGCKGWGRIDVMLDSDGQFYLLEANTSPGMTSHSL
VPMAARQAGMSFSQLVVRILELAD
>d1ehia2 d.142.1.1 (A:135-362) D-alanine:D-lactate ligase, VanA {Leuconostoc mesenteroides, Ddl2}
DKALTKELLTVNGIRNTKYIVVDPESANNWSWDKIVAELGNIVFVKAANQGSSVGISRVTNA
EEYTEALSDSFQYDYKVLIEEAVNGARELEVGVIGNDQPLVSEIGAHTVPNQGSGDGWYDYN
NKFVDNSAVHFQIPAQLSPEVTKEVKQMALDAYKVLNLRGEARMDFLLDENNVPYLGEPNTL
PGFTNMSLFKRLWDYSDINNAKLVDMLIDYGFEDFAQNKKLS
>d1e4ea2 d.142.1.1 (A:132-342) D-alanine:D-lactate ligase, VanA {Enterococcus faecium}
DKSLTYIVAKNAGIATPAFWVINKDDRPVAATFTYPVFVKPARSGSSFGVKKVNSADELDYA
IESARQYDSKILIEQAVSGCEVGCAVLGNSAALVVGEVDQIRLQYGIFRIHQEVEPEKGSEN
AVITVPADLSAEERGRIQETVKKIYKTLGCRGLARVDMFLQDNGRIVLNEVNTLPGFTSYSR
YPRMMAAAGISLPELIDRLIVLALK
>d1dv1a3 d.142.1.2 (A:115-330) Biotin carboxylase subunit of acetyl-CoA carboxylase {Escherichia coli}
DKVSAIAAMKKAGVPCVPGSDGPLGDDMDKNRAIAKRIGYPVIIKASGGGGGRGMRVVRGDA
ELAQSISMTRAEAKAAFSNDMVYMEKYLENPRHVEIQVLADGQGNAIYLAERDCSMQRRHQK
VVEEAPAPGITPELRRYIGERCAKACVDIGYRGAGTFEFLFENGEFYFIEMNTRIQVEHPVT
EMITGVDLIKEQLRIAAGQPLSIKQEEVHV
>d1gsoa3 d.142.1.2 (A:104-327) Glycinamide ribonucleotide synthetase (GAR-syn) {Escherichia coli}
SKAFTKDFLARHKIPTAEYQNFTEVEPALAYLREKGAPIVIKADGLAAGKGVIVAMTLEEAE
AAVHDMLAGNAFGDAGHRIVIEEFLDGEEASFIVMVDGEHVLPMATSQDHKRVGDKDTGPNT
GGMGAYSPAPVVTDDVHQRTMERIIWPTVKGMAAEGNTYTGFLYAGLMIDKQGNPKVIEFNC
RFGDLETQPIMLRMKSDLVELCLAACESKLDEKTSEWD
>d1b6ra3 d.142.1.2 (A:79-276) N5-carboxyaminoimidazole ribonucleotide synthetase, AIRC, PurK {Escherichia coli}
DRLTQKQLFDKLHLPTAPWQLLAERSEWPAVFDRLGELAIVKRRTGGYDGRGQWRLRANETE
QLPAECYGECIVEQGINFSGEVSLVGARGFDGSTVFYPLTHNLHQDGILRTSVAFPQANAQQ
QARAEEMLSAIMQELGYVGVMAMECFVTPQGLLINELAPRVHNSGHWTQNGASISQFELHLR
AITDLPLPQPVV
>d1eyza3 d.142.1.2 (A:113-318) Glycinamide ribonucleotide

transformylase PurT {Escherichia coli}
NREGIRRLAAEELQLPTSTYRFADSESLFREAVADIGYPCIVKPVMSSSGKGQTFIRSAEQL
AQAWKYAQQGGRAGAGRVIVEGVVKFDFEITLLTVSAVDGVHFCAPVGHRQEDGDYRESWQP
QQMSPLALERAQEIARKVVLALGGYGLFGVELFVCGDEVIFSEVSPRPHDTGMVTLISQDLS
EFALHVRAFLGLPVGGIRQY
>d1a9xa5 d.142.1.2 (A:128-402) Carbamoyl phosphate synthetase (CPS), large subunit {Escherichia coli}
DRRRFDVAMKKIGLETARSGIAHTMEEALAVAADVGFPCIIRPSFTMGGSGGGIAYNREEFE
EICARGLDLSPTKELLIDESLIGWKEYEMEVVRDKNDNCIIVCSIENFDAMGIHTGDSITVA
PAQTLTDKEYQIMRNASMAVLREIGVETGGSNVQFAVNPKNGRLIVIEMNPRVSRSSALASK
ATGFPIAKVAAKLAVGYTLDELMNDITGGRTPASFEPSIDYVVTKIPRFNFEKFAGANDRLT
TQMKSVGEVMAIGRTQQESLQKALRGL
>d1a9xa6 d.142.1.2 (A:677-935) Carbamoyl phosphate synthetase (CPS), large subunit {Escherichia coli}
RFQHAVERLKLKQPANATVTAIEMAVEKAKEIGYPLVVRAAMEIVYDEADLRRYFQTAVLLD
HFLDDAVEVDVDAICDGEMVLIGGIMEHIEQAGVHSGDSACSLPAYTLSQEIQDVMRQQVQK
LAFELQVRGLMNVQFAVKNNEVYLIEVNPRAARTVPFVSKATGVPLAKVAARVMAGKSLAEQ
GVTKEVIPPYYSVKEVVLPFNKFPGVDPLLGPEMRSTGEVMGVGRTFAEAFAKAQLGS
>d1auva2 d.142.1.3 (A:214-417) Synapsin Ia, C-terminal domain {Cow (Bos taurus)}
NSLHSVYNFCDKPWVFAQMVRLHKKLGTEEFPLINQTFYPNHKEMLSSTTYPVVVKMGHAHS
GMGKVKVDNQHDFQDIASVVALTKTYATTEPFIDAKYDVRIQKIGQNYKAYMRTSVSGNWKT
NTGSAMLEQIAMSDRYKLWVDTCSEIFGGLDICAVEALHGKDGRDHIIEVVGSSMPLIGDHQ
DEDKQLIVELVVNKMAQA
>d1jkjb2 d.142.1.4 (B:1-238) Succinyl-CoA synthetase, beta-chain, N-terminal domain {Escherichia coli}
MNLHEYQAKQLFARYGLPAPVGYACTTPREAEEAASKIGAGPWVVKCQVHAGGRGKAGGVKV
VNSKEDIRAFAENWLGKRLVTYQTDANGQPVNQILVEAATDIAKELYLGAVVDRSSRRVVFM
ASTEGGVEIEKVAEETPHLIHKVALDPLTGPMPYQGRELAFKLGLEGKLVQQFTKIFMGLAT
IFLERDLALIEINPLVITKQGDLICLDGKLGADGNALFRQPDLREMRDQSQE
>d1eucb2 d.142.1.4 (B:0-245) Succinyl-CoA synthetase, beta-chain, N-terminal domain {Pig (Sus scrofa)}
MVNLQEYQSKKLMSDNGVKVQRFFVADTANEALEAAKRLNAKEIVLKAQILAGGRGKGVFSS
GLKGGVHLTKDPEVVGQLAKQMIGYNLATKQTPKEGVKVNKVMVAEALDISRETYLAILMDR
SCNGPVLVGSPQGGVDIEEVAASNPELIFKEQIDIIEGIKDSQAQRMAENLGFLGPLQNQAA
DQIKKLYNLFLKIDATQVEVNPFGETPEGQVVCFDAKINFDDNAEFRQKDIFAMDDKSEN
>d1kbla3 d.142.1.5 (A:2-376) Pyruvate phosphate dikinase, N-terminal domain {Clostridium symbiosum}
AKWVYKFEEGNASMRNLLGGKGCNLAEMTILGMPIPQGFTVTTEACTEYYNSGKQITQEIQD
QIFEAITWLEELNGKKFGDTEDPLLVSVRSGARASMPGMMDTILNLGLNDVAVEGFAKKTGN
PRFAYDSYRRFIQMYSDVVMEVPKSHFEKIIDAMKEEKGVHFDTDLTADDLKELAEKFKAVY
KEAMNGEEFPQEPKDQLMGAVKAVFRSWDNPRAIVYRRMNDIPGDWGTAVNVQTMVFGNKGE
TSGTGVAFTRNPSTGEKGIYGEYLINAQGEDVVAGVRTPQPITQLENDMPDCYKQFMDLAMK
LEKHFRDMQDMEFTIEEGKLYFLQTRNGKRTAPAALQIACDLVDEGMITEEEAVVRIEAKSL

DQL
>d2hgsa2 d.142.1.6 (A:304-474) Eukaryotic glutathione synthetase {Human (Homo sapiens)}
TKKVQQELSRPGMLEMLLPGQPEAVARLRATFAGLYSLDVGEEGDQAIAEALAAPSRFVLKP
QREGGGNNLYGEEMVQALKQLKDSEERASYILMEKIEPEPFENCLLRPGSPARVVQCISELG
IFGVYVRQEKTLVMNKHVGHLLRTKAIEHADGGVAAGVAVLDNPYPV
>d2hgsa3 d.142.1.6 (A:3-201) Eukaryotic glutathione synthetase {Human (Homo sapiens)}
TNWGSLLQDKQQLEELARQAVDRALAEGVLLRTSQEPTSSEVVSYAPFTLFPSLVPSALLEQ
AYAVQMDFNLLVDAVSQNAAFLEQTLSSTIKQDDFTARLFDIHKQVLKEGIAQTVFLGLNRS
DYMFQRSADGSPALKQIEINTISASFGGLASRTPAVHRHVLSVLSKTKEAGKILSNNPSKGL
ALGIAKAWELYGS
>d1a0i_2 d.142.2.1 (2-240) ATP-dependent DNA ligase, N-terminal domain {Bacteriophage T7}
VNIKTNPFKAVSFVESAIKKALDNAGYLIAEIKYDGVRGNICVDNTANSYWLSRVSKTIPAL
EHLNGFDVRWKRLLNDDRCFYKDGFMLDGELMVKGVDFNTGSGLLRTKWTDTKNQEFHEELF
VEPIRKKDKVPFKLHTGHLHIKLYAILPLHIVESGEDCDVMTLLMQEHVKNMLPLLQEYFPE
IEWQAAESYEVYDMVELQQLYEQKRAEGHEGLIVKDPMCIYKRGKKSGWWKMK
>d1fvia2 d.142.2.1 (A:2-189) ATP-dependent DNA ligase, N-terminal domain {Chlorella virus, PBCV-1}
AITKPLLAATLENIEDVQFPCLATPKIAGIRSVKQTQMLSRTFKPIRNSVMNRLLTELLPEG
SDGEISIEGATFQDTTSAVMTGHKMYNAKFSYYWFDYVTDDPLKKYIDRVEDMKNYITVHPH
ILEHAQVKIIPLIPVEINNITELLQYERDVLSKGFEGVMIRKPDGKYKFGRSTLKEGILLKM
KQ
>d1b04a_ d.142.2.2 (A:) Adenylation domain of NAD+-dependent DNA ligase {Bacillus stearothermophilus}
DRQQAERRAAELRELLNRYGYEYYVLDRPSVPDAEYDRLMQELIAIEEQYPELKTSDSPTQR
IGGPPLEAFRKVAHRVPMMSLANAFGEGDLRDFDRRVRQEVGEAAYVCELAIDGLAVSVRYE
DGYFVQGATRGDGTTGEDITENLKTIRSLPLRLKEPVSLEARGEAFMPKASFLRLNEERKAR
GEELFANPRNAAAGSLRQLDPKVAASRQLDLFVYGLADAEALGIASHSEALDYLQALGFKVN
PERRRCANIDEVIAFVSEWHDKRPQLPYEIDGIVIKVDSFAQQRALGATAKSPRWAIAYKFP
AE
>d1dgsa3 d.142.2.2 (A:1-314) Adenylation domain of NAD+-dependent DNA ligase {Thermus filiformis}
MTREEARRRINELRDLIRYHNYRYYVLADPEISDAEYDRLLRELKELEERFPEFKSPDSPTE
QVGARPLEPTFRPVRHPTRMYSLDNAFTYEEVLAFEERLEREAEAPSLYTVEHKVDGLSVLY
YEEGVWSTGSGDGEVGEEVTQNLLTIPTIPRRLKGVPDRLEVRGEVYMPIEAFLRLNEELEE
RGEKVFKNPRNAAAGSLRQKDPRVTAKRGLRATFYALGLGLGLEESGLKSQYELLLWLKEKG
FPVEHCYEKALGAEGVEEVYRRGLAQRHALPFEADGVVLKLDDLTLWGELGYTARAPRFALA
YKFP
>d1ckma2 d.142.2.3 (A:11-238) RNA guanylyltransferase (mRNA capping enzyme), N-terminal domain {Chlorella virus, PBCV-1}
NITTERAVLTLNGLQIKLHKVVGESRDDIVAKMKDLAMDDHKFPRLPGPNPVSIERKDFEKL
KQNKYVVSEKTDGIRFMMFFTRVFGFKVCTIIDRAMTVYLLPFKNIPRVLFQGSIFDGELCV

DIVEKKFAFVLFDAVVVSGVTVSQMDLASRFFAMKRSLKEFKNVPEDPAILRYKEWIPLEHP
TIIKDHLKKANAIYHTDGLIIMSVDEPVIYGRNFNLFKLKPG
>d1a48__ d.143.1.1 (-) SAICAR synthase {Baker's yeast (Saccharomyces cerevisiae)}
SITKTELDGILPLVARGKVRDIYEVDAGTLLFVATDRISAYDVIMENSIPEKGILLTKLSEF
WFKFLSNDVRNHLVDIAPGKTIFDYLPAKLSEPKYKTQLEDRSLLVHKHKLIPLEVIVRGYI
TGSAWKEYVKTGTVHGLKQPQGLKESQEFPEPIFTPSTKAEQGEHDENISPAQAAELVGEDL
SRRVAELAVKLYSKCKDYAKEKGIIIADTKFEFGIDEKTNEIILVDEVLTPDSSRFWNGASY
KVGESQDSYDKQFLRDWLTANKLNGVNGVKMPQDIVDRTRAKYIEAYETLTGSKWSH
>d1bo1a_ d.143.1.2 (A:) Phosphatidylinositol phosphate kinase IIbeta, PIPK IIbeta {Human (Homo sapiens)}
KLFRASEPILSVLMWGVNHTINELSNVPVPVMLMPDDFKAYSKIKVDNHLFNKENLPSRFKF
KEYCPMVFRNLRERFGIDDQDYQNSVTRSAPINSDSQGRCGTRFLTTYDRRFVIKTVSSEDV
AEMHNILKKYHQFIVECHGNTLLPQFLGMYRLTVDGVETYMVVTRNVFSHRLTVHRKYDLKG
STVAREASDKEKAKDLPTFKDNDFLNEGQKLHVGEESKKNFLEKLKRDVEFLAQLKIMDYSL
LVGIHDVDRAEQEEMEVEERAEDEECENDGVGGNLLCSYGTPPDSPGNLLSFPRFFGPGEFD
PSVDVYAMKSHESSPKKEVYFMAIIDILTPYDTKKKAAHAAKTVKHGAGAEISTVNPEQYSK
RFNEFMSNILT
>d1blxa_ d.144.1.1 (A:) Cyclin-dependent PK (CDK, different isozymes) {Human (Homo sapiens)}
GLCRADQQYECVAEIGEGAYGKVFKARDLKNGGRFVALKRVRVQTGEEGMPLSTIREVAVLR
HLETFEHPNVVRLFDVCTVSRTDRETKLTLVFEHVDQDLTTYLDKVPEPGVPTETIKDMMFQ
LLRGLDFLHSHRVVHRDLKPQNILVTSSGQIKLADFGLARIYSFQMALTSVVVTLWYRAPEV
LLQSSYATPVDLWSVGCIFAEMFRRKPLFRGSSDVDQLGKILDVIGLPGEEDWPRDVALPRQ
AFHSKSAQPIEKFVTDIDELGKDLLLKCLTFNPAKRISAYSALSHPYFQDLERCKEN
>d1jvpp_ d.144.1.1 (P:) Cyclin-dependent PK (CDK, different isozymes) {Human (Homo sapiens)}
MENFQKVEKIGEGTYGVVYKARNKLTGEVVALKKIRLDTETEGVPSTAIREISLLKELNHPN
IVKLLDVIHTENKLYLVFEFLHQDLKKFMDASALTGIPLPLIKSYLFQLLQGLAFCHSHRVL
HRDLKPQNLLINTEGAIKLADFGLARAFGVPVRTYTHEVVTLWYRAPEILLGCKYYSTAVDI
WSLGCIFAEMVTRRALFPGDSEIDQLFRIFRTLGTPDEVVWPGVTSMPDYKPSFPKWARQDF
SKVVPPLDEDGRSLLSQMLHYDPNKRISAKAALAHPFFQDVTKPVPHLRL
>d1apme_ d.144.1.1 (E:) cAMP-dependent PK, catalytic subunit {Mouse (Mus musculus)}
SEQESVKEFLAKAKEDFLKKWETPSQNTAQLDQFDRIKTLGTGSFGRVMLVKHKESGNHYAM
KILDKQKVVKLKQIEHTLNEKRILQAVNFPFLVKLEFSFKDNSNLYMVMEYVAGGEMFSHLR
RIGRFAEPHARFYAAQIVLTFEYLHSLDLIYRDLKPENLLIDQQGYIQVTDFGFAKRVKGRT
WTLCGTPEYLAPEIILSKGYNKAVDWWALGVLIYEMAAGYPPFFADQPIQIYEKIVSGKVRF
PSHFSSDLKDLLRNLLQVDLTKRFGNLKNGVNDIKNHKWFATTDWIAIYQRKVEAPFIPKFK
GPGDTSNFDDYEEEEIRVSINEKCGKEFTEF
>d1fota_ d.144.1.1 (A:) cAMP-dependent PK, catalytic subunit {Baker's yeast (Saccharomyces cerevisiae)}
YSLQDFQILRTLGTGSFGRVHLIRSRHNGRYYAMKVLKKEIVVRLKQVEHTNDERLMLSIVT
HPFIIRMWGTFQDAQQIFMIMDYIEGGELFSLLRKSQRFPNPVAKFYAAEVCLALEYLHSKD

IIYRDLKPENILLDKNGHIKITDFGFAKYVPDVTYTLCGTPDYIAPEVVSTKPYNKSIDWWS
FGILIYEMLAGYTPFYDSNTMKTYEKILNAELRFPPFFNEDVKDLLSRLITRDLSQRLGNLQ
NGTEDVKNHPWFKEVVWEKLLSRNIETPYEPPIQQGQGDTSQFDKYPEEDINYGVQGEDPYA
DLFRDF
>d1a06__ d.144.1.1 (-) Calmodulin-dependent protein kinase {Rat (Rattus norvegicus)}
WKQAEDIRDIYDFRDVLGTGAFSEVILAEDKRTQKLVAIKCIAKKALEGKEGSMENEIAVLH
KIKHPNIVALDDIYESGGHLYLIMQLVSGGELFDRIVEKGFYTERDASRLIFQVLDAVKYLH
DLGIVHRDLKPENLLYYSLDEDSKIMISDFGLSKMEDPGSVLSTACGTPGYVAPEVLAQKPY
SKAVDCWSIGVIAYILLCGYPPFYDENDAKLFEQILKAEYEFDSPYWDDISDSAKDFIRHLM
EKDPEKRFTCEQALQHPWIAGDTALDKNIHQSVSEQIKKNFAKSKWKQAFNATAVVRHM
>d1ia8a_ d.144.1.1 (A:) Cell cycle checkpoint kinase chk1 {Human (Homo sapiens)}
AVPFVEDWDLVQTLGEGAYGEVQLAVNRVTEEAVAVKIVDMKRAVDCPENIKKEICINKMLN
HENVVKFYGHRREGNIQYLFLEYCSGGELFDRIEPDIGMPEPDAQRFFHQLMAGVVYLHGIG
ITHRDIKPENLLLDERDNLKISDFGLATVFRYNNRERLLNKMCGTLPYVAPELLKRREFHAE
PVDVWSCGIVLTAMLAGELPWDQPSDSCQEYSDWKEKKTYLNPWKKIDSAPLALLHKILVEN
PSARITIPDIKKDRWYNKPLKKGAKRP
>d1phk__ d.144.1.1 (-) gamma-subunit of glycogen phosphorylase kinase (Phk) {Rabbit (Oryctolagus cuniculus)}
FYENYEPKEILGRGVSSVVRRCIHKPTCKEYAVKIIDVTGGGSFSAEEVQELREATLKEVDI
LRKVSGHPNIIQLKDTYETNTFFFLVFDLMKKGELFDYLTEKVTLSEKETRKIMRALLEVIC
ALHKLNIVHRDLKPENILLDDDMNIKLTDFGFSCQLDPGEKLREVCGTPSYLAPEIIECSMN
DNHPGYGKEVDMWSTGVIMYTLLAGSPPFWHRKQMLMLRMIMSGNYQFGSPEWDDYSDTVKD
LVSRFLVVQPQKRYTAEEALAHPFFQQYV
>d1h8fa_ d.144.1.1 (A:) Glycogen synthase kinase-3 beta (Gsk3b) {Human (Homo sapiens)}
SKVTTVVATPGQGPDRPQEVSYTDTKVIGNGSFGVVYQAKLCDSGELVAIKKVLQGKAFKNR
ELQIMRKLDHCNIVRLRYFFYSSGEKKDEVYLNLVLDYVPETVYRVARHYSRAKQTLPVIYV
KLYMYQLFRSLAYIHSFGICHRDIKPQNLLLDPDTAVLKLCDFGSAKQLVRGEPNVSYICSR
YYRAPELIFGATDYTSSIDVWSAGCVLAELLLGQPIFPGDSGVDQLVEIIKVLGTPTREQIR
EMNPNYTEFAFPQIKAHPWTKVFRPRTPPEAIALCSRLLEYTPTARLTPLEACAHSFFDELR
DPNVKLPNGRDTPALFNFTTQELSSNPPLATILIPPHARIQA
>d1tkia_ d.144.1.1 (A:) Titin, kinase domain {Human (Homo sapiens)}
KELYEKYMIAEDLGRGEFGIVHRCVETSSKKTYMAKFVKVKGTDQVLVKKEISILNIARHRN
ILHLHESFESMEELVMIFEFISGLDIFERINTSAFELNEREIVSYVHQVCEALQFLHSHNIG
HFDIRPENIIYQTRRSSTIKIIEFGQARQLKPGDNFRLLFTAPEYYAPEVHQHDVVSTATDM
WSLGTLVYVLLSGINPFLAETNQQIIENIMNAEYTFDEEAFKEISIEAMDFVDRLLVKERKS
RMTASEALQHPWLKQKIERVSTKVIRTLKHRRYYHTLIKKDLNMVVSAARISCGGAIRSQKG
VSVAKVKVASI
>d1koba_ d.144.1.1 (A:) Twitchin, kinase domain {California sea hare (Aplysia californica), twk43}
INDYDKFYEDIWKKYVPQPVEVKQGSVYDYYDILEELGSGAFGVVHRCVEKATGRVFVAKFI
NTPYPLDKYTVKNEISIMNQLHHPKLINLHDAFEDKYEMVLILEFLSGGELFDRIAAEDYKM

SEAEVINYMRQACEGLKHMHEHSIVHLDIKPENIMCETKKASSVKIIDFGLATKLNPDEIVK
VTTATAEFAAPEIVDREPVGFYTDMWAIGVLGYVLLSGLSPFAGEDDLETLQNVKRCDWEFD
EDAFSSVSPEAKDFIKNLLQKEPRKRLTVHDALEHPWLKGDHSNLTSRIPSSRYNKIRQKIK
EKYADWPAPQPAIGRIANFSSLRKHRPQEYQIYDSYFDRKEAV
>d1koa_2 d.144.1.1 (5915-6264) Twitchin, kinase domain
{Caenorhabditis elegans, pjk4}
YDNYVFDIWKQYYPQPVEIKHDHVLDHYDIHEELGTGAFGVVHRVTERATGNNFAAKFVMTP
HESDKETVRKEIQTMSVLRHPTLVNLHDAFEDDNEMVMIYEFMSGGELFEKVADEHNKMSED
EAVEYMRQVCKGLCHMHENNYVHLDLKPENIMFTTKRSNELKLIDFGLTAHLDPKQSVKVTT
GTAEFAAPEVAEGKPVGYYTDMWSVGVLSYILLSGLSPFGGENDDETLRNVKSCDWNMDDSA
FSGISEDGKDFIRKLLLADPNTRMTIHQALEHPWLTPGNAPGRDSQIPSSRYTKIRDSIKTK
YDAWPEPLPPLGRISNYSSLRKHRPQEYSIRDAFWDRSEA
>d1p38__ d.144.1.1 (-) MAP kinase p38 {Mouse (Mus musculus)}
ERPTFYRQELNKTIWEVPERYQNLSPVGSGAYGSVCAAFDTKTGHRVAVKKLSRPFQSIIHA
KRTYRELRLLKHMKHENVIGLLDVFTPARSLEEFNDVYLVTHLMGADLNNIVKCQKLTDDHV
QFLIYQILRGLKYIHSADIIHRDLKPSNLAVNEDCELKILDFGLARHTDDEMTGYVATRWYR
APEIMLNWMHYNQTVDIWSVGCIMAELLTGRTLFPGTDHIDQLKLILRLVGTPGAELLKKIS
SESARNYIQSLAQMPKMNFANVFIGANPLAVDLLEKMLVLDSDKRITAAQALAHAYFAQYHD
PDDEPVADPYDQSFESRDLLIDEWKSLTYDEVISFVPPPLD
>d1cm8a_ d.144.1.1 (A:) MAP kinase p38-gamma {Human (Homo sapiens)}
RSGFYRQEVTKTAWEVRAVYRDLQPVGSGAYGAVCSAVDGRTGAKVAIKKLYRPFQSELFAK
RAYRELRLLKHMRHENVIGLLDVFTPDETLDDFTDFYLVMPFMGTDLGKLMKHEKLGEDRIQ
FLVYQMLKGLRYIHAAGIIHRDLKPGNLAVNEDCELKILDFGLARQADSEMTGYVVTRWYRA
PEVILNWMRYTQTVDIWSVGCIMAEMITGKTLFKGSDHLDQLKEIMKVTGTPPAEFVQRLQS
DEAKNYMKGLPELEKKDFASILTNASPLAVNLLEKMLVLDAEQRVTAGEALAHPYFESLHDT
EDEPQVQKYDDSFDDVDRTLDEWKRVTYKEVLSFKP
>d1pme__ d.144.1.1 (-) MAP kinase Erk2 {Human (Homo sapiens)}
GQVFDVGPRYTNLSYIGEGAYGMVCSAYDNVNKVRVAIKKISPFEHQTYCQRTLREIKILLR
FRHENIIGINDIIRAPTIEQMKDVYLVTHLMGADLYKLLKTQHLSNDHICYFLYQILRGLKY
IHSANVLHRDLKPSNLLLNTTCDLKICDFGLARVADPDHDHTGFLTEYVATRWYRAPEIMLN
SKGYTKSIDIWSVGCILAEMLSNRPIFPGKHYLDQLNHILGILGSPSQEDLNCIINLKARNY
LLSLPHKNKVPWNRLFPNADSKALDLLDKMLTFNPHKRIEVEQALAHPYLEQYYDPSDEPIA
EAPFKFDMELDDLPKEKLKELIFEETARFQPGYRS
>d1jnk__ d.144.1.1 (-) c-jun N-terminal kinase (jnk3s) {Human (Homo
sapiens)}
DNQFYSVEVGDSTFTVLKRYQNLKPIGSGAQGIVCAAYDAVLDRNVAIKKLSRPFQNQTHAK
RAYRELVLMKCVNHKNIISLLNVFTPQKTLEEFQDVYLVMELMDANLCQVIQMELDHERMSY
LLYQMLCGIKHLHSAGIIHRDLKPSNIVVKSDCTLKILDFGLARTAGTSFMMTPYVVTRYYR
APEVILGMGYKENVDIWSVGCIMGEMVRHKILFPGRDYIDQWNKVIEQLGTPCPEFMKKLQP
TVRNYVENRPKYAGLTFPKLFPDSLFPADSEHNKLKASQARDLLSKMLVIDPAKRISVDDAL
QHPYINVWYDPAEVEAPPPQIYDKQLDEREHTIEEWKELIYKEVMN
>d1ckia_ d.144.1.1 (A:) Casein kinase-1, CK1 {Rat (Rattus
norvegicus)}
MELRVGNRYRLGRKIGSGSFGDIYLGTDIAAGEEVAIKLECVKTKHPQLHIESKIYKMMQGG

VGIPTIRWCGAEGDYNVMVMELLGPSLEDLFNFCSRKFSLKTVLLLADQMISRIEYIHSKNF
IHRDVKPDNFLMGLGKKGNLVYIIDFGLAKKYRDARTHQHIPYRENKNLTGTARYASINTHL
GIEQSRRDDLESLGYVLMYFNLGSLPWQGLKAATKRQKYERISEKKMSTPIEVLCKGYPSEF
ATYLNFCRSLRFDDKPDYSYLRQLFRNLFHRQGFSYDYVFDWNMLKFGASR
>d1csn__ d.144.1.1 (-) Casein kinase-1, CK1 {Fission yeast (Schizosaccharomyces pombe)}
NVVGVHYKVGRRIGEGSFGVIFEGTNLLNNQQVAIKFEPRRSDAPQLRDEYRTYKLLAGCTG
IPNVYYFGQEGLHNVLVIDLLGPSLEDLLDLCGRKFSVKTVAMAAKQMLARVQSIHEKSLVY
RDIKPDNFLIGRPNSKNANMIYVVDFGMVKFYRDPVTKQHIPYREKKNLSGTARYMSINTHL
GREQSRRDDLEALGHVFMYFLRGSLPWQGLKAATNKQKYERIGEKKQSTPLRELCAGFPEEF
YKYMHYARNLAFDATPDYDYLQGLFSKVLERLNTTEDENFDWNLL
>d1a6o__ d.144.1.1 (-) Protein kinase CK2, alpha subunit {Maize (Zea mays)}
SKARVYADVNVLRPKEYWDYEALTVQWGEQDDYEVVRKVGRGKYSEVFEGINVNNNEKCIIK
ILKPVKKKKIKREIKILQNLCGGPNIVKLLDIVRDQHSKTPSLIFEYVNNTDFKVLYPTLTD
YDIRYYIYELLKALDYCHSQGIMHRDVKPHNVMIDHELRKLRLIDWGLAEFYHPGKEYNVRV
ASRYFKGPELLVDLQDYDYSLDMWSLGCMFAGMIFRKEPFFYGHDNHDQLVKIAKVLGTDGL
NVYLNKYRIELDPQLEALVGRHSRKPWLKFMNADNQHLVSPEAIDFLDKLLRYDHQERLTAL
EAMTHPYFQQVRAAENS
>d1b6cb_ d.144.1.1 (B:) Type I TGF-beta receptor R4 {Human (Homo sapiens)}
TTLKDLIYDMTTSGSGSGLPLLVQRTIARTIVLQESIGKGRFGEVWRGKWRGEEVAVKIFSS
REERSWFREAEIYQTVMLRHENILGFIAADNKDNGTWTQLWLVSDYHEHGSLFDYLNRYTVT
VEGMIKLALSTASGLAHLHMEIVGTQGKPAIAHRDLKSKNILVKKNGTCCIADLGLAVRHDS
ATDTIDIAPNHRVGTKRYMAPEVLDDSINMKHFESFKRADIYAMGLVFWEIARRCSIGGIHE
DYQLPYYDLVPSDPSVEEMRKVVCEQKLRPNIPNRWQSCEALRVMAKIMRECWYANGAARLT
ALRIKKTLSQLSQQEG
>d1f3mc_ d.144.1.1 (C:) pak1 {Human (Homo sapiens)}
SDEEILEKLRSIVSVGDPKKKYTRFEKIGQGASGTVYTAMDVATGQEVAIRQMNLQQQPKKE
LIINEILVMRENKNPNIVNYLDSYLVGDELWVVMEYLAGGSLTDVVTETCMDEGQIAAVCRE
CLQALEFLHSNQVIHRDIKSDNILLGMDGSVKLTDFGFCAQITPEQSKRSTMVGTPYWMAPE
VVTRKAYGPKVDIWSLGIMAIEMIEGEPPYLNENPLRALYLIATNGTPELQNPEKLSAIFRD
FLNRCLDMDVEKRGSAKELLQHQFLKIAKPLSSLTPLIAAAKEATK
>d1howa_ d.144.1.1 (A:) Sky1p {Baker's yeast (Saccharomyces cerevisiae)}
FHPAFKGEPYKDARYILVRKLGWGHFSTVWLAKDMVNNTHVAMKIVRGDKVYTEAAEDEIKL
LQRVNDADNTKEDSMGANHILKLLDHFNHKGPNGVHVVMVFEVLGENLLALIKKYEHRGIPL
IYVKQISKQLLLGLDYMHRRCGIIHTDIKPENVLMEIVDSPENLIQIKIADLGNACWYDEHY
TNSIQTREYRSPEVLLGAPWGCGADIWSTACLIFELITGDFLFEPDEGHSYTKDDDHIAQII
ELLGELPSYLLRNGKYTRTFFNSRGLLRNISKLKFWPLEDVLTEKYKFSKDEAKEISDFLSP
MLQLDPRKRADAGGLVNHPWLKDTLGMEEIRVPDRELYGSGSDIPGWFEEVR
>d1qcfa3 d.144.1.2 (A:249-531) Haemopoetic cell kinase Hck {Human (Homo sapiens)}
KPQKPWEKDAWEIPRESLKLEKKLGAGQFGEVWMATYNKHTKVAVKTMKPGSMSVEAFLAEA

NVMKTLQHDKLVKLHAVVTKEPIYIITEFMAKGSLLDFLKSDEGSKQPLPKLIDFSAQIAEG
MAFIEQRNYIHRDLRAANILVSASLVCKIADFGLARVIEDNEYTAREGAKFPIKWTAPEAIN
FGSFTIKSDVWSFGILLMEIVTYGRIPYPGMSNPEVIRALERGYRMPRPENCPEELYNIMMR
CWKNRPEERPTFEYIQSVLDDFYTATESQYEEIP
>d1qpca_ d.144.1.2 (A:) Lymphocyte kinase (lck) {Human (Homo sapiens)}
KPWWEDEWEVPRETLKLVERLGAGQFGEVWMGYYNGHTKVAVKSLKQGSMSPDAFLAEANLM
KQLQHQRLVRLYAVVTQEPIYIITEYMENGSLVDFLKTPSGIKLTINKLLDMAAQIAEGMAF
IEERNYIHRDLRAANILVSDTLSCKIADFGLARLIEDNEYTAREGAKFPIKWTAPEAINYGT
FTIKSDVWSFGILLTEIVTHGRIPYPGMTNPEVIQNLERGYRMVRPDNCPEELYQLMRLCWK
ERPEDRPTFDYLRSVLEDFFTATE
>d1fmk_3 d.144.1.2 (249-533) c-src tyrosine kinase {Human (Homo sapiens)}
KPQTQGLAKDAWEIPRESLRLEVKLGQGCFGEVWMGTWNGTTRVAIKTLKPGTMSPEAFLQE
AQVMKKLRHEKLVQLYAVVSEEPIYIVTEYMSKGSLLDFLKGETGKYLRLPQLVDMAAQIAS
GMAYVERMNYVHRDLRAANILVGENLVCKVADFGLARLIEDNEYTARQGAKFPIKWTAPEAA
LYGRFTIKSDVWSFGILLTELTTKGRVPYPGMVNREVLDQVERGYRMPCPPECPESLHDLMC
QCWRKEPEERPTFEYLQAFLEDYFTSTEPQYQPGENL
>d1fgka_ d.144.1.2 (A:) Fibroblast growth factor receptor 1 {Human (Homo sapiens)}
ELPEDPRWELPRDRLVLGKPLGEGAFGQVVLAEAIGLDKDKPNRVTKVAVKMLKSDATEKDL
SDLISEMEMMKMIGKHKNIINLLGACTQDGPLYVIVEYASKGNLREYLQARRPPGLEYSYNP
SHNPEEQLSSKDLVSCAYQVARGMEYLASKKCIHRDLAARNVLVTEDNVMKIADFGLARDIH
HIDYYKKTTNGRLPVKWMAPEALFDRIYTHQSDVWSFGVLLWEIFTLGGSPYPGVPVEELFK
LLKEGHRMDKPSNCTNELYMMMRDCWHAVPSQRPTFKQLVEDLDRIVALTS
>d1vr2a_ d.144.1.2 (A:) Vascular endothelial growth factor receptor 2 (kdr) {Human (Homo sapiens)}
LPYDASKWEFPRDRLKLGKPLGRGAFGQVIEADAFGIDKTATCRTVAVKMLKEGATHSEHRA
LMSELKILIHIGHHLNVVNLLGACTKPGGPLMVIVEFCKFGNLSTYLRSKRNEFVPYKVAPE
DLYKDFLTLEHLICYSFQVAKGMEFLASRKCIHRDLAARNILLSEKNVVKICDFGLARDIYK
DPDYVRKGDARLPLKWMAPETIFDRVYTIQSDVWSFGVLLWEIFSLGASPYPGVKIDEEFCR
RLKEGTRMRAPDYTTPEMYQTMLDCWHGEPSQRPTFSELVEHLGNLLQANA
>d1ir3a_ d.144.1.2 (A:) Insulin receptor {Human (Homo sapiens)}
SSVFVPDEWEVSREKITLLRELGQGSFGMVYEGNARDIIKGEAETRVAVKTVNESASLRERI
EFLNEASVMKGFTCHHVVRLLGVVSKGQPTLVVMELMAHGDLKSYLRSLRPEAENNPGRPPP
TLQEMIQMAAEIADGMAYLNAKKFVHRDLAARNCMVAHDFTVKIGDFGMTRDIYETDYYRKG
GKGLLPVRWMAPESLKDGVFTTSSDMWSFGVVLWEITSLAEQPYQGLSNEQVLKFVMDGGYL
DQPDNCPERVTDLMRMCWQFNPKMRPTFLEIVNLLKDDLHPSFPEVSFFHSEENK
>d1k3aa_ d.144.1.2 (A:) Insulin-like growth factor 1 receptor {Human (Homo sapiens)}
VPDEWEVAREKITMSRELGQGSFGMVYEGVAKGVVKDEPETRVAIKTVNEAASMRERIEFLN
EASVMKEFNCHHVVRLLGVVSQGQPTLVIMELMTRGDLKSYLRSLRPEMENNPVLAPPSLSK
MIQMAGEIADGMAYLNANKFVHRDLAARNCMVAEDFTVKIGDFGMTRDIYETDYYRKGGKGL
LPVRWMSPESLKDGVFTTYSDVWSFGVVLWEIATLAEQPYQGLSNEQVLRFVMEGGLLDKPD

NCPDMLLELMRMCWQYNPKMRPSFLEIISSIKEEMEPGFREVSFYYSEENK
>d1byga_ d.144.1.2 (A:) C-terminal src kinase (csk) {Human (Homo sapiens)}
GWALNMKELKLLQTIGKGEFGDVMLGDYRGNKVAVKCIKNDATAQAFLAEASVMTQLRHSNL
VQLLGVIVEEKGGLYIVTEYMAKGSLVDYLRSRGRSVLGGDCLLKFSLDVCEAMEYLEGNNF
VHRDLAARNVLVSEDNVAKVSDFGLTKEASSTQDTGKLPVKWTAPEALREKKFSTKSDVWSF
GILLWEIYSFGRVPYPRIPLKDVVPRVEKGYKMDAPDGCPPAVYEVMKNCWHLDAAMRPSFL
QLREQLEHIKTHEL
>d1iepa_ d.144.1.2 (A:) Abelsone tyrosine kinase (abl) {Mouse (Mus musculus)}
MDPSSPNYDKWEMERTDITMKHKLGGGQYGEVYEGVWKKYSLTVAVKTLKEDTMEVEEFLKE
AAVMKEIKHPNLVQLLGVCTREPPFYIITEFMTYGNLLDYLRECNRQEVSAVVLLYMATQIS
SAMEYLEKKNFIHRDLAARNCLVGENHLVKVADFGLSRLMTGDTYTAHAGAKFPIKWTAPES
LAYNKFSIKSDVWAFGVLLWEIATYGMSPYPGIDLSQVYELLEKDYRMERPEGCPEKVYELM
RACWQWNPSDRPSFAEIHQAFETMFQ
>d1jpaa_ d.144.1.2 (A:) ephb2 receptor tyrosine kinase {Mouse (Mus musculus)}
KIFIDPFTFEDPNEAVREFAKEIDISCVKIEQVIGAGEFGEVCSGHLKLPGKREIFVAIKTL
KSGYTEKQRRDFLSEASIMGQFDHPNVIHLEGVVTKSTPVMIITEFMENGSLDSFLRQNDGQ
FTVIQLVGMLRGIAAGMKYLADMNYVHRDLAARNILVNSNLVCKVSDFGLSRFLEDDTSDPT
YTSALGGKIPIRWTAPEAIQYRKFTSASDVWSYGIVMWEVMSYGERPYWDMTNQDVINAIEQ
DYRLPPPMDCPSALHQLMLDCWQKDRNHRPKFGQIVNTLDKMIRNPNSLKA
>d1fvra_ d.144.1.2 (A:) Tie2 kinase {Human (Homo sapiens)}
PTIYPVLDWNDIKFQDVIGEGNFGQVLKARIKKDGLRMDAAIKRMKEYASKDDHRDFAGELE
VLCKLGHHPNIINLLGACEHRGYLYLAIEYAPHGNLLDFLRKSRVLETDPAFAIANSTASTL
SSQQLLHFAADVARGMDYLSQKQFIHRDLAARNILVGENYVAKIADFGLSRGQEVYVKKTMG
RLPVRWMAIESLNYSVYTTNSDVWSYGVLLWEIVSLGGTPYCGMTCAELYEKLPQGYRLEKP
LNCDDEVYDLMRQCWREKPYERPSFAQILVSLNRMLEERKTYVNTTLYEKFTYAGIDCSAE
>d1cjaa_ d.144.1.3 (A:) Actin-fragmin kinase, catalytic domain {Slime mold (Physarum polycephalum)}
AGALWEIEKELFTKLPAPSSAINSHLQPAKPKVPQKKPSKWDPPAEFKVDLSTAVSYNDIGD
INWKNLQQFKGIERSEKGTEGLFFVETESGVFIVKRSTNIESETFCSLLCMRLGLHAPKVRV
VSSNSEEGTNMLECLAAIDKSFRVITTLANQANILLMELVRGITLNKLTTTSAPEVLTKSTM
QQLGSLMALDVIVNNSDRLPIAWTNEGNLDNIMLSERGATVVPIDSKIIPLDASHPHGERVR
ELLRTLIAHPGHESSQFHSIRDIITLYTGYDVGTEGSISMQEGFLATVRECASFDLDAFERE
LLSWQESLQKCHNLSISPQAIPFILRMLRIFH
>d1ia9a_ d.144.1.5 (A:) Trp Ca-channel kinase domain {Mouse (Mus musculus)}
YYYSAVERNNLMRLSQSIPFVPVPPRGEPVTVYRLEESSPSILNNSMSSWSQLGLCAKIEFL
SKEEMGGGLRRAVKVLCTWSEHDILKSGHLYIIKSFLPEVINTWSSIYKEDTVLHLCLREIQ
QQRAAQKLTFAFNQMKPKSIPYSPRFLEVFLLYCHSAGQWFAVEECMTGEFRKYNNNNGDEI
IPTNTLEEIMLAFSHWTYEYTRGELLVLDLQGVGENLTDPSVIKAEEKRSCDMVFGPANLGE
DAIKNFRAKHHCNSCCRKLKLPDLKRNDYT
>d1e8xa4 d.144.1.4 (A:726-1092) Phoshoinositide 3-kinase (PI3K),

catalytic domain {Pig (Sus scrofa)}
TAMLHDFTQQVQVIDMLQKVTIDIKSLSAEKYDVSSQVISQLKQKLENLQNLNLPQSFRVPY
DPGLKAGALVIEKCKVMASKKKPLWLEFKCADPTALSNETIGIIFKHGDDLRQDMLILQILR
IMESIWETESLDLCLLPYGCISTGDKIGMIEIVKDATTIAKIQQSTVGNTGAFKDEVLSHWL
KEKCPIEEKFQAAVERFVYSCAGYCVATFVLGIGDRHNDNIMISETGNLFHIDFGHILGNYK
SFLGINKERVPFVLTPDFLFVMGTSGKKTSLHFQKFQDVCVKAYLALRHHTNLLIILFSMML
MTGMPQLTSKEDIEYIRDALTVGKSEEDAKKYFLDQIEVCRDKGWTVQFNWFLHLVL
>d1j7la_ d.144.1.6 (A:) Type IIIa 3',5"-aminoglycoside
phosphotransferase {Enterococcus faecalis}
AKMRISPELKKLIEKYRCVKDTEGMSPAKVYKLVGENENLYLKMTDSRYKGTTYDVEREKDM
MLWLEGKLPVPKVLHFERHDGWSNLLMSEADGVLCSEEYEDEQSPEKIIELYAECIRLFHSI
DISDCPYTNSLDSRLAELDYLLNNDLADVDCENWEEDTPFKDPRELYDFLKTEKPEEELVFS
HGDLGDSNIFVKDGKVSGFIDLGRSGRADKWYDIAFCVRSIREDIGEEQYVELFFDLLGIKP
DWEKIKYYILLDELF
>d1e8ga2 d.145.1.1 (A:6-273) Vanillyl-alcohol oxidase {Fungus
(Penicillium simplicissimum)}
EFRPLTLPPKLSLSDFNEFIQDIIRIVGSENVEVISSKDQIVDGSYMKPTHTHDPTHVMDQD
YFLASAIVAPRNVADVQSIVGLANKFSFPLWPISIGRNSGYGGAAPRVSGSVVLDMGKNMNR
VLEVNVEGAYCVVEPGVTYHDLHNYLEANNLRDKLWLDVPDLGGGSVLGNAVERGVGYTPYG
DHWMMHSGMEVVLANGELLRTGMGALPDPKRPETMGLKPEDQPWSKIAHLFPYGFGPYIDGL
FSQSNMGIVTKIGIWLMPNP
>d1diqa2 d.145.1.1 (A:7-242) Flavoprotein subunit of p-cresol
methylhydroxylase {Pseudomonas putida}
AVLPKGVTQGEFNKAVQKFRALLGDDNVLVESDQLVPYNKIMMPVENAAHAPSAAVTATTVE
QVQGVVKICNEHKIPIWTISTGRNFGYGSAAPVQRGQVILDLKKMNKIIKIDPEMCYALVEP
GVTFGQMYDYIQENNLPVMLSFSAPSAIAGPVGNTMDRGVGYTPYGEHFMMQCGMEVVLANG
DVYRTGMGGVPGSNTWQIFKWGYGPTLDGMFTQANYGICTKMGFWLMPKP
>d1f0xa2 d.145.1.1 (A:9-273) D-lactate dehydrogenase {Escherichia
coli}
NKAFLNELARLVGSSHLLTDPAKTARYRKGFRSGQGDALAVVFPGSLLELWRVLKACVTADK
IILMQAANTGLTEGSTPNGNDYDRDVVIISTLRLDKLHVLGKGEQVLAYPGTTLYSLEKALK
PLGREPHSVIGSSCIGASVIGGICNNSGGSLVQRGPAYTEMSLFARINEDGKLTLVNHLGID
LGETPEQILSKLDDDRIKDDDVRHDGRHAHDYDYVHRVRDIEADTPARYNADPDRLFESSGC
AGKLAVFAVRLDTFEAE
>d1i19a2 d.145.1.1 (A:57-273) Cholesterol oxidase {Brevibacterium
sterolicum}
VAPLPTPPNFPNDIALFQQAYQNWSKEIMLDATWVCSPKTPQDVVRLANWAHEHDYKIRPRG
AMHGWTPLTVEKGANVEKVILADTMTHLNGITVNTGGPVATVTAGAGASIEAIVTELQKHDL
GWANLPAPGVLSIGGALAVNAHGAALPAVGQTTLPGHTYGSLSNLVTELTAVVWNGTTYALE
TYQRNDPRITPLLTNLGRCFLTSVTMQAGPN
>d1uxy_1 d.145.1.2 (3-200) Uridine
diphospho-N-Acetylenolpyruvylglucosamine reductase (MurB),
N-terminal domain {Escherichia coli}
HSLKPWNTFGIDHNAQHIVCAEDEQQLLNAWQYATAEGQPVLILGEGSNVLFLEDYRGTVII

NRIKGIEIHDEPDAWYLHVGAGENWHRLVKYTLQEGMPGLENLALIPGCVGSSPIQNIGAYG
VELQRVCAYVDSVELATGKQVRLTAKECRFGYRDSIFKHEYQDRFAIVAVGLRLPKEWQPVL
TYGDLTRLDPTT
>d1hska1 d.145.1.2 (A:15-208) Uridine
diphospho-N-Acetylenolpyruvylglucosamine reductase (MurB),
N-terminal domain {Staphylococcus aureus}
NKDIYQALQQLIPNEKIKVDEPLKRYTYTKTGGNADFYITPTKNEEVQAVVKYAYQNEIPVT
YLGNGSNIIIREGGIRGIVISLLSLDHIEVSDDAIIAGSGAAIIDVSRVARDYALTGLEFAC
GIPGSIGGAVYMNAGAYGGEVKDCIDYALCVNEQGSLIKLTTKELELDYRNSIIQKEHLVVL
EAAFTLAP
>d1qj2c2 d.145.1.3 (C:1-177) Carbon monoxide (CO) dehydrogenase
flavoprotein, N-terminal domain {Pseudomonas carboxydovorans}
MIPGSFDYHRPKSIADAVALLTKLGEDARPLAGGHSLIPIMKTRLATPEHLVDLRDIGDLVG
IREEGTDVVIGAMTTQHALIGSDFLAAKLPIIRETSLLIADPQIRYMGTIGGNAANGDPGND
MPALMQCLGAAYELTGPEGARIVAARDYYQGAYFTAIEPGELLTAIRIPVPPT
>d1ffvc2 d.145.1.3 (C:1-177) Carbon monoxide (CO) dehydrogenase
flavoprotein, N-terminal domain {Hydrogenophaga pseudoflava}
MIPPRFEYHAPKSVGEAVALLGQLGSDAKLLAGGHSLLPMMKLRFAQPEHLIDINRIPELRG
IREEGSTVVIGAMTVENDLISSPIVQARLPLLAEAAKLIADPQVRNRGTIGGDIAHGDPGND
HPALSIAVEAHFVLEGPNGRRTVPADGFFLGTYMTLLEENEVMVEIRVPAFAQ
>d1fiqb2 d.145.1.3 (B:224-414) Xanthine oxidase, domain 3 (?) {Cow
(Bos taurus)}
PKQLRFEGERVTWIQASTLKELLDLKAQHPEAKLVVGNTEIGIEMKFKNQLFPMIICPAWIP
ELNAVEHGPEGISFGAACALSSVEKTLLEAVAKLPTQKTEVFRGVLEQLRWFAGKQVKSVAS
LGGNIITASPISDLNPVFMASGTKLTIVSRGTRRTVPMDHTFFPSYRKTLLGPEEILLSIEI
PYSRE
>d1fo4a6 d.145.1.3 (A:192-414) Xanthine oxidase, domain 3 (?) {Cow
(Bos taurus)}
SPSLFNPEEFMPLDPTQEPIFPPELLRLKDVPPKQLRFEGERVTWIQASTLKELLDLKAQHP
EAKLVVGNTEIGIEMKFKNQLFPMIICPAWIPELNAVEHGPEGISFGAACALSSVEKTLLEA
VAKLPTQKTEVFRGVLEQLRWFAGKQVKSVASLGGNIITASPISDLNPVFMASGTKLTIVSR
GTRRTVPMDHTFFPSYRKTLLGPEEILLSIEIPYSRE
>d1jroa4 d.145.1.3 (A:179-345) Xanthine dehydrogenase chain A,
domain 3 {Rhodobacter capsulatus}
PAFLPETSDALADWYLAHPEATLIAGGTDVSLWVTKALRDLPEVAFLSHCKDLAQIRETPDG
YGIGAGVTIAALRAFAEGPHPALAGLLRRFASEQVRQVATIGGNIANGSPIGDGPPALIAMG
ASLTLRRGQERRRMPLEDFFLEYRKQDRRPGEFVESVTLPKSA
>d1uxy_2 d.146.1.1 (201-342) Uridine
diphospho-N-Acetylenolpyruvylglucosamine reductase, MurB,
C-terminal domain {Escherichia coli}
VTPQQVFNAVCHMRTTKLPDPKVNGNAGAFFKNPVVSAETAKALLSQFPTAPNYPQADGSVK
LAAGWLIDQCQLKGMQIGGAAVHRQQALVLINEDNAKSEDVVQLAHHVRQKVGEKFNVWLEP
EVRFIGASGEVSAVETIS
>d1hska2 d.146.1.1 (A:209-317) Uridine

diphospho-N-Acetylenolpyruvylglucosamine reductase, MurB, C-terminal domain {Staphylococcus aureus}
GKMTEIQAKMDDLTERRESKQPLEYPSCGSVFQRPPGHFAGKLIQDSNLQGHRIGGVEVSTK
HAGFMVNVDNGTATDYENLIHYVQKTVKEKFGIELNREVRIIGEHPK
>d1qlma_ d.147.1.1 (A:) Methenyltetrahydromethanopterin cyclohydrolase {Archaeon Methanopyrus kandleri}
MVSVNENALPLVERMIERAELLNVEVQELENGTTVIDCGVEAAGGFEAGLLFSEVCMGGLAT
VELTEFEHDGLCLPAVQVTTDHPAVSTLAAQKAGWQVQVGDYFAMGSGPARALALKPKETYE
EIDYEDDADVAILCLESSELPDEDVAEHVADECGVDPENLYLLVAPTASIVGSVQVSARVVE
TGLYKLLEVLEYDVTRVKYATGTAPIAPVADDDGEAMGRTNDCILYGGTVYLYVEGDDELPE
VVEELPSEASEDYGKPFMKIFEEADYDFYKIDPGVFAPARVVVNDLSTGKTYTAGEINVDVL
KESFSL
>d1c4za_ d.148.1.1 (A:) Ubiquitin-protein ligase E3a, Hect catalytic domain (E6ap) {Human (Homo sapiens)}
NPYLRLKVRRDHIIDDALVRLEMIAMENPADLKKQLYVEFEGEQGVDEGGVSKEFFQLVVEE
IFNPDIGMFTYDESTKLFWFNPSSFETEGQFTLIGIVLGLAIYNNCILDVHFPMVVYRKLMG
KKGTFRDLGDSHPVLYQSLKDLLEYEGNVEDDMMITFQISQTDLFGNPMMYDLKENGDKIPI
TNENRKEFVNLYSDYILNKSVEKQFKAFRRGFHMVTNESPLKYLFRPEEIELLICGSRNLDF
QALEETTEYDGGYTRDSVLIREFWEIVHSFTDEQKRLFLQFTTGTDRAPVGGLGKLKMIIAK
NGPDTERLPTSHTCFNVLLLPEYSSKEKLKERLLKAITYA
>d2ahja_ d.149.1.1 (A:) Nitrile hydratase alpha chain {Rhodococcus erythropolis}
IDHTTENAAPAQAPVSDRAWALFRALDGKGLVPDGYVEGWKKTFEEDFSPRRGAELVARAWT
DPEFRQLLLTDGTAAVAQYGYLGPQGEYIVAVEDTPTLKNVIVCSLASCTAWPILGLPPTWY
KSFEYRARVVREPRKVLSEMGTEIASDIEIRVYDTTAETRYMVLPQRPAGTEGWSQEQLQEI
VTKDCLIGVAIPQV
>d1f7la_ d.150.1.2 (A:) Holo-(acyl carrier protein) synthase ACPS {Bacillus subtilis}
GIYGIGLDITELKRIASMAGRQKRFAERILTRSELDQYYELSEKRKNEFLAGRFAAKEAFSK
AFGTGIGRQLSFQDIEIRKDQNGKPYIICTKLSPAAVHVSITHTKEYAAAQVVIER
>d1ftha_ d.150.1.2 (A:) Holo-(acyl carrier protein) synthase ACPS {Streptococcus pneumoniae}
MIVGHGIDIEELASIESAVTRHEGFAKRVLTALEMERFTSLKGRRQIEYLAGRWSAKEAFSK
AMGTGISKLGFQDLEVLNNERGAPYFSQAPFSGKIWLSISHTDQFVTASVILEEN
>d1qr0a1 d.150.1.1 (A:1-101) 4'-Phosphopantetheinyl transferase SFP {Bacillus subtilis}
MKIYGIYMDRPLSQEENERFMTFISPEKREKCRRFYHKEDAHRTLLGDVLVRSVISRQYQLD
KSDIRFSTQEYGKPCIPDLPDAHFNISHSGRWVIGAFDS
>d1qr0a2 d.150.1.1 (A:102-228) 4'-Phosphopantetheinyl transferase SFP {Bacillus subtilis}
QPIGIDIEKTKPISLEIAKRFFSKTEYSDLLAKDKDEQTDYFYHLWSMKESFIKQEGKGLSL
PLDSFSVRLHQDGQVSIELPDSHSPCYIKTYEVDPGYKMAVCAAHPDFPEDITMVSYEELLR
AAA
>d1ako__ d.151.1.1 (-) DNA-repair enzyme exonuclease III

{Escherichia coli}
MKFVSFNINGLRARPHQLEAIVEKHQPDVIGLQETKVHDDMFPLEEVAKLGYNVFYHGQKGH
YGVALLTKETPIAVRRGFPGDDEEAQRRIIMAEIPSLLGNVTVINGYFPQGESRDHPIKFPA
KAQFYQNLQNYLETELKRDNPVLIMGDMNISPTDLDIGIGEENRKRWLRTGKCSFLPEEREW
MDRLMSWGLVDTFRHANPQTADRFSWFDYRSKGFDDNRGLRIDLLLASQPLAECCVETGIDY
EIRSMEKPSDHAPVWATFRR
>d1hd7a_ d.151.1.1 (A:) DNA repair endonuclease Hap1 {Human (Homo sapiens)}
LYEDPPDQKTSPSGKPATLKICSWNVDGLRAWIKKKGLDWVKEEAPDILCLQETKCSENKLP
AELQELPGLSHQYWSAPSDKEGYSGVGLLSRQCPLKVSYGIGDEEHDQEGRVIVAEFDSFVL
VTAYVPNAGRGLVRLEYRQRWDEAFRKFLKGLASRKPLVLCGDLNVAHEEIDLRNPKGNKKN
AGFTPQERQGFGELLQAVPLADSFRHLYPNTPYAYTFWTYMMNARSKNVGWRLDYFLLSHSL
LPALCDSKIRSKALGSDHCPITLYLAL
>d2dnja_ d.151.1.1 (A:) Deoxyribonuclease I {Cow (Bos taurus)}
LKIAAFNIRTFGETKMSNATLASYIVRIVRRYDIVLIQEVRDSHLVAVGKLLDYLNQDDPNT
YHYVVSEPLGRNSYKERYLFLFRPNKVSVLDTYQYDDGCESCGNDSFSREPAVVKFSSHSTK
VKEFAIVALHSAPSDAVAEINSLYDVYLDVQQKWHLNDVMLMGDFNADCSYVTSSQWSSIRL
RTSSTFQWLIPDSADTTATSTNCAYDRIVVAGSLLQSSVVPGSAAPFDFQAAYGLSNEMALA
ISDHYPVEVTLT
>d1i9za_ d.151.1.2 (A:) Synaptojanin, IPP5C domain {Yeast (Schizosaccharomyces pombe)}
YDPIHEYVNHELRKRENEFSEHKNVKIFVASYNLNGCSATTKLENWLFPENTPLADIYVVGF
QEIVQLTPQQVISADPAKRREWESCVKRLLNGKCTSGPGYVQLRSGQLVGTALMIFCKESCL
PSIKNVEGTVKKTGLGGVSGNKGAVAIRFDYEDTGLCFITSHLAAGYTNYDERDHDYRTIAS
GLRFRRGRSIFNHDYVVWFGDFNYRISLTYEEVVPCIAQGKLSYLFEYDQLNKQMLTGKVFP
FFSELPITFPPTYKFDIGTDIYDTSDKHRVPAWTDRILYRGELVPHSYQSVPLYYSDHRPIY
ATYEANIVKVDREKKKILFEELYNQRKQEVRDASQ
>d1aora2 d.152.1.1 (A:1-210) Aldehyde ferredoxin oxidoreductase {Archaeon Pyrococcus furiosus}
MYGNWGRFIRVNLSTGDIKVEEYDEELAKKWLGSRGLAIYLLLKEMDPTVDPLSPENKLIIA
AGPLTGTSAPTGGRYNVVTKSPLTGFITMANSGGYFGAELKFAGYDAIVVEGKAEKPVYIYI
KDEHIEIRDASHIWGKKVSETEATIRKEVGSEKVKIASIGPAGENLVKFAAIMNDGHRAAGR
GGVGAVMGSKNLKAIAVEGSKTVP
>d1b25a2 d.152.1.1 (A:1-210) Formaldehyde ferredoxin oxidoreductase {Archaeon Pyrococcus furiosus}
MYGWWGRILRVNLTTGEVKVQEYPEEVAKKFIGGRGLAAWILWNEARGVEPLSPENKLIFAA
GPFNGLPTPSGGKLVVAAKSPLTGGYGDGNLGTMASVHLRRAGYDALVVEGKAKKPVYIYIE
DDNVSILSAEGLWGKTTFETERELKEIHGKNVGVLTIGPAGENLVKYAVVISQEGRAAGRPG
MGAVMGSKKLKAVVIRGTKEIPVA
>d1gdoa_ d.153.1.1 (A:) Glucosamine 6-phosphate synthase, N-terminal domain {Escherichia coli}
CGIVGAIAQRDVAEILLEGLRRLEYRGYDSAGLAVVDAEGHMTRLRRLGKVQMLAQAAEEHP
LHGGTGIAHTRWATHGEPSEVNAHPHVSEHIVVVHNGIIENHEPLREELKARGYTFVSETDT
EVIAHLVNWELKQGGTLREAVLRAIPQLRGAYGTVIMDSRHPDTLLAARSGSPLVIGLGMGE

NFIASDQLALLPVTRRFIFLEEGDIAEITRRSVNIFDKTGAEVKRQDIESNL
>d1gph12 d.153.1.1 (1:1-234) Glutamine PRPP amidotransferase, N-terminal domain {Bacillus subtilis}
CGVFGIWGHEEAPQITYYGLHSLQHRGQEGAGIVATDGEKLTAHKGQGLITEVFQNGELSKV
KGKGAIGHVRYATAGGGGYENVQPLLFRSQNNGSLALAHNGNLVNATQLKQQLENQGSIFQT
SSDTEVLAHLIKRSGHFTLKDQIKNSLSMLKGAYAFLIMTETEMIVALDPNGLRPLSIGMMG
DAYVVASETCAFDVVGATYLREVEPGEMLIINDEGMKSERFSMNINRS
>d1ecfa2 d.153.1.1 (A:1-249) Glutamine PRPP amidotransferase, N-terminal domain {Escherichia coli}
CGIVGIAGVMPVNQSIYDALTVLQHRGQDAAGIITIDANNCFRLRKANGLVSDVFEARHMQR
LQGNMGIGHVRYPTAGSSSASEAQPFYVNSPYGITLAHNGNLTNAHELRKKLFEEKRRHINT
TSDSEILLNIFASELDNFRHYPLEADNIFAAIAATNRLIRGAYACVAMIIGHGMVAFRDPNG
IRPLVLGKRDIDENRTEYMVASESVALDTLGFDFLRDVAPGEAIYITEEGQLFTRQCADNPV
S
>d1ct9a2 d.153.1.1 (A:1-192) Asparagine synthetase B, N-terminal domain {Escherichia coli}
ASIFGVFDIKTDAVELRKKALELSRLMRHRGPDWSGIYASDNAILAHERLSIVDVNAGAQPL
YNQQKTHVLAVNGEIYNHQALRAEYGDRYQFQTGSDCEVILALYQEKGPEFLDDLQGMFAFA
LYDSEKDAYLIGRDHLGIIPLYMGYDEHGQLYVASEMKALVPVCRTIKEFPAGSYLWSQDGE
IRSYYH
>d1jgta2 d.153.1.1 (A:4-209) beta-Lactam synthetase {Streptomyces clavuligerus}
PVLPAAFGFLASARTGGGRAPGPVFATRGSHTDIDTPQGERSLAATLVHAPSVAPDRAVARS
LTGAPTTAVLAGEIYNRDELLSVLPAGPAPEGDAELVLRLLERYDLHAFRLVNGRFATVVRT
GDRVLLATDHAGSVPLYTCVAPGEVRASTEAKALAAHRDPKGFPLADARRVAGLTGVYQVPA
GAVMDIDLGSGTAVTHRTWT
>d1ea0a3 d.153.1.1 (A:1-422) Alpha subunit of glutamate synthase, N-terminal domain {Azospirillum brasilense}
CGVGFIAAIDGKPRRSVVEKGIEALKAVWHRGAVDADGKTGDGAGIHVAVPQKFFKDHVKVI
GHRAPDNKLAVGQVFLPRISLDAQEACRCIVETEILAFGYYIYGWRQVPINVDIIGEKANAT
RPEIEQIIVGNNKGVSDEQFELDLYIIRRRIEKAVKGEQINDFYICSLSARSIIYKGMFLAE
QLTTFYPDLLDERFESDFAIYHQRYSTNTFPTWPLAQPFRMLAHNGEINTVKGNVNWMKAHE
TRMEHPAFGTHMQDLKPVIGVGLSDSGSLDTVFEVMVRAGRTAPMVKMMLVPQALTSSQTTP
DNHKALIQYCNSVMEPWDGPAALAMTDGRWVVGGMDRNGLRPMRYTITTDGLIIGGSETGMV
KIDETQVIEKGRLGPGEMIAVDLQSGKLYRDRELKDHLATLKPWDKWVQN
>g1gk9.1 d.153.1.2 (A:,B:) Penicillin acylase {Escherichia coli}
QSSSEIKIVRDEYGMPHIYANDTWHLFYGYGYVVAQDRLFQMEMARRSTQGTVAEVLGKDFV
KFDKDIRRNYWPDAIRAQIAALSPEDMSILQGYADGMNAWIDKVNTNPETLLPKQFNTFGFT
PKRWEPFDVAMIFVGTMANRFSDSTSEIDNLALLTALKDKYGVSQGMAVFNQLKWLVNPSAP
TTIAVQESNYPLKFNQQNSQTAXSNMWVIGKSKAQDAKAIMVNGPQFGWYAPAYTYGIGLHG
AGYDVTGNTPFAYPGLVFGHNGVISWGSTAGFGDDVDIFAERLSAEKPGYYLHNGKWVKMLS
REETITVKNGQAETFTVWRTVHGNILQTDQTTQTAYAKSRAWDGKEVASLLAWTHQMKAKNW
QEWTQQAAKQALTINWYYADVNGNIGYVHTGAYPDRQSGHDPRLPVPGTGKWDWKGLLPFEM
NPKVYNPQSGYIANWNNSPQKDYPASDLFAFLWGGADRVTEIDRLLEQKPRLTADQAWDVIR

QTSRQDLNLRLFLPTLQAATSGLTQSDPRRQLVETLTRWDGINLLNDDGKTWQQPGSAILNV
WLTSMLKRTVVAAVPMPFDKWYSASGYETTQDGPTGSLNISVGAKILYEAVQGDKSPIPQAV
DLFAGKPQQEVVLAALEDTWETLSKRYGNNVSNWKTPAMALTFRANNFFGVPQAAAEETRHQ
AEYQNRGTENDMIVFSPTTSDRPVLAWDVVAPGQSGFIAPDGTVDKHYEDQLKMYENFGRKS
LWLTKQDVEAHKESQEVLHVQR
>g1cp9.1 d.153.1.2 (A:,B:) Penicillin acylase {Providencia
rettgeri}
ESTQIKIERDNYGVPHIYANDTYSLFYGYGYAVAQDRLFQMEMAKRSTQGTVSEVFGKDYIS
FDKEIRNNYWPDSIHKQINQLPSQEQDILRGYADGMNAWIKQINTKPDDLMPKQFIDYDFLP
SQWTSFDVAMIMVGTLANRFSDMNSEIDNLALLTALKDKYGEQLGVEFFNQINWLNNPNAPT
TISSEEFTYSDXSNVWLVGKTKASGAKAILLNGPQFGWFNPAYTYGIGLHGAGFNIVGNTPF
AYPAILFGHNGHVSWGSTAGFGDGVDIFAEQVSPEDPNSYLHQGQWKKMLSRQETLNVKGEQ
PITFEIYRTVHGNVVKRDKTTHTAYSKARAWDGKELTSLMAWVKQGQAQNWQQWLDQAQNQA
LTINWYYADKDGNIGYVHTGHYPDRQINHDPRLPVSGTGEWDWKGIQPFANNPKVYNPKSGY
IANWNNSPAKNYPASDLFAFLWGSADRVKEIDNRIEAYDKLTADDMWAILQQTSRVDLNHRL
FTPFLTQATQGLPSNDNSVKLVSMLQQWDGINQLSSDGKHYIHPGSAILDIWLKEMLKATLG
QTVPAPFDKWYLASGYETTQEGPTGSLNISTGAKLLYESLLEDKSPISQSIDLFSGQPQNDV
IRKTLNTTYQKMIEKYGDNPANWQTPATALTFRENNFFGIPQALPQENFHQNEYHNRGTEND
LIVFTEEGVSAWDVVAPGQSGFISPQGKPSPHYQDQLSLYQQFGKKPLWLNSEDVAPYIEST
ETLIIER
>g1fm2.1 d.153.1.2 (A:,B:) Cephalosporin acylase {Brevundimonas
diminuta}
QAPIAAYKPRSNEILWDGYGVPHIYGVDAPSAFYGYGWAQARSHGDNILRLYGEARGKGAEY
WGPDYEQTTVWLLTNGVPERAQQWYAQQSPDFRANLDAFAAGINAYAQQNPDDISPEVRQVL
PVSGADVVAHAHRLMNFLYVASPGRTLGXSNSWAVAPGKTANGNALLLQNPHLSWTTDYFTY
YEAHLVTPDFEIYGATQIGLPVIRFAFNQRMGITNTVNGMVGATNYRLTLQDGGYLYDGQVR
PFERRQASYRLRQADGSTVDKPLEIRSSVHGPVFERADGTAVAVRVAGLDRPGMLEQYFDMI
TAHSFDDYEAAMARMQVPTFNIVYADREGTINYSFNGVAPKRAEGDIAFWQGNVPGDSSRYL
WTETHPLDDLPRVTNPPGGFVQNSNDPPWTPTWPVTYCPANHPSYLAPQTPHSLRAQQSVRL
MSENDDLTLERFMALQFSHRAVMADRTLPDLIPAALIDPDPEVQAAARLLAAWDRDFTSDSR
AALLFEEWARLFAGQNFAGQAAFATPWSLDKPVSTPYGVRDPKAAVDQLRTAIANTKRKYGA
IDRPFGDASRMILNDVNVPGAAGYGNLGSFRVFTWSDPDENGIRTPVHGETWVAMIEFSTPV
RAYGLMSYGNSRQPGTTHYSDQIERVSRADFRELLLRREQVEAAVQERTPFNF
>d2pvaa_ d.153.1.3 (A:) Penicillin V acylase {Bacillus sphaericus}
CSSLSIRTTDDKSLFARTMDFTMEPDSKVIIVPRNYGIRLLEKENVVINNSYAFVGMGSTDI
TSPVLYDGVNEKTYADEPKKGTGLMGAMLYYATFATYADEPKKGTTGINPVYVISQVLGNCV
TVDDVIEKLTSYTLLNEANIILGFAPPLHYTFTDASGESIVIEPDKTGITIHRKTIGVMTNS
PGYEWHQTNLRAYIGVTPNPPQDIMMGDLDLTPFGQGAGGLGLPGDFTPSARFLRVAYWKKY
TEKAKNETEGVTNLFHILSSVNIPKGVVLTNEGKTDYTIYTSAMCAQSKNYYFKLYDNSRIS
AVSLMAENLNSQDLITFEWDRKQDIKQLNQVN
>d1pma1_ d.153.1.4 (1:) Proteasome beta subunit (catalytic)
{Archaeon Thermoplasma acidophilum}
TTTVGITLKDAVIMATERRVTMENFIMHKNGKKLFQIDTYTGMTIAGLVGDAQVLVRYMKAE
LELYRLQRRVNMPIEAVATLLSNMLNQVKYMPYMVQLLVGGIDTAPHVFSIDAAGGSVEDIY

ASTGSGSPFVYGVLESQYSEKMTVDEGVDLVIRAISAAKQRDSASGGMIDVAVITRKDGYVQ
LPTDQIESRIRKLGLIL
>d1ryp1_ d.153.1.4 (1:) Proteasome beta subunit (catalytic)
{Baker's yeast (Saccharomyces cerevisiae)}
QFNPYGDNGGTILGIAGEDFAVLAGDTRNITDYSINSRYEPKVFDCGDNIVMSANGFAADGD
ALVKRFKNSVKWYHFDHNDKKLSINSAARNIQHLLYGKRFFPYYVHTIIAGLDEDGKGAVYS
FDPVGSYEREQCRAGGAAASLIMPFLDNQVNFKNQYEPGTNGKVKKPLKYLSVEEVIKLVRD
SFTSATERHIQVGDGLEILIVTKDGVRKEFYELKRD
>d1ryp2_ d.153.1.4 (2:) Proteasome beta subunit (catalytic)
{Baker's yeast (Saccharomyces cerevisiae)}
TQQPIVTGTSVISMKYDNGVIIAADNLGSYGSLLRFNGVERLIPVGDNTVVGISGDISDMQH
IERLLKDLVTENAYDNPLADAEEALEPSYIFEYLATVMYQRRSKMNPLWNAIIVAGVQSNGD
QFLRYVNLLGVTYSSPTLATGFGAHMANPLLRKVVDRESDIPKTTVQVAEEAIVNAMRVLYY
RDARSSRNFSLAIIDKNTGLTFKKNLQVENMKWDFAKDIKGYGTQKI
>d1ryph_ d.153.1.4 (H:) Proteasome beta subunit (catalytic)
{Baker's yeast (Saccharomyces cerevisiae)}
LKKGEVSLGASIMAVTFKDGVILGADSRTTTGAYIANRVTDKLTRVHDKIWCCRSGSAADTQ
AIADIVQYHLELYTSQYGTPSTETAASVFKELCYENKDNLTAGIIVAGYDDKNKGEVYTIPL
GGSVHKLPYAIAGSGSTFIYGYCDKNFRENMSKEETVDFIKHSLSQAIKWDGSSGGVIRMVV
LTAAGVERLIFYPDEYEQL
>d1rypi_ d.153.1.4 (I:) Proteasome beta subunit (catalytic)
{Baker's yeast (Saccharomyces cerevisiae)}
TTIVGVKFNNGVVIAADTRSTQGPIVADKNCAKLHRISPKIWCAGAGTAADTEAVTQLIGSN
IELHSLYTSREPRVVSALQMLKQHLFKYQGHIGAYLIVAGVDPTGSHLFSIHAHGSTDVGYY
LSLGSGSLAAMAVLESHWKQDLTKEEAIKLASDAIQAGIWNDLGSGSNVDVCVMEIGKDAEY
LRNYLTPNVREEKQKSYKFPRGTTAVLKESIVNICD
>d1rypj_ d.153.1.4 (J:) Proteasome beta subunit (catalytic)
{Baker's yeast (Saccharomyces cerevisiae)}
SDPSSINGGIVVAMTGKDCVAIACDLRLGSQSLGVSNKFEKIFHYGHVFLGITGLATDVTTL
NEMFRYKTNLYKLKEERAIEPETFTQLVSSSLYERRFGPYFVGPVVAGINSKSGKPFIAGFD
LIGCIDEAKDFIVSGTASDQLFGMCESLYEPNLEPEDLFETISQALLNAADRDALSGWGAVV
YIIKKDEVVKRYLKMRQD
>d1rypk_ d.153.1.4 (K:) Proteasome beta subunit (catalytic)
{Baker's yeast (Saccharomyces cerevisiae)}
MDIILGIRVQDSVILASSKAVTRGISVLKDSDDKTRQLSPHTLMSFAGEAGDTVQFAEYIQA
NIQLYSIREDYELSPQAVSSFVRQELAKSIRSRRPYQVNVLIGGYDKKKNKPELYQIDYLGT
KVELPYGAHGYSGFYTFSLLDHHYRPDMTTEEGLDLLKLCVQELEKRMPMDFKGVIVKIVDK
DGIRQVDDFQAQ
>d1rypl_ d.153.1.4 (L:) Proteasome beta subunit (catalytic)
{Baker's yeast (Saccharomyces cerevisiae)}
TTTLAFRFQGGIIVAVDSRATAGNWVASQTVKRVIEINPFLLGTMAGGAADCQFWETWLGSQ
CRLHELREKERISVAAASKILSNLVYQYKGAGLSMGTMICGYTRKEGPTIYYVDSDGTRLKG
DIFCVGSGQTFAYGVLDSNYKWDLSVEDALYLGKRSILAAAHRDAYSGGSVNLYHVTEDGWI
YHGNHDVGELFWKVKEEEGSFNNVIG

>d1pmaa_ d.153.1.4 (A:) Proteasome alpha subunit (non-catalytic) {Archaeon Thermoplasma acidophilum}
TVFSPDGRLFQVEYAREAVKKGSTALGMKFANGVLLISDKKVRSRLIEQNSIEKIQLIDDYV
AAVTSGLVADARVLVDFARISAQQEKVTYGSLVNIENLVKRVADQMQQYTQYGGVRPYGVSL
IFAGIDQIGPRLFDCDPAGTINEYKATAIGSGKDAVVSFLEREYKENLPEKEAVTLGIKALK
SSLEEGEELKAPEIASITVGNKYRIYDQEEVKKFL
>d1rypa_ d.153.1.4 (A:) Proteasome alpha subunit (non-catalytic) {Baker's yeast (Saccharomyces cerevisiae)}
AGYDRHITIFSPEGRLYQVEYAFKATNQTNINSLAVRGKDCTVVISQKKVPDKLLDPTTVSY
IFCISRTIGMVVNGPIPDARNAALRAKAEAAEFRYKYGYDMPCDVLAKRMANLSQIYTQRAY
MRPLGVILTFVSVDEELGPSIYKTDPAGYYVGYKATATGPKQQEITTNLENHFKKSKIDHIN
EESWEKVVEFAITHMIDALGTEFSKNDLEVGVATKDKFFTLSAENIEERLVAIAEQD
>d1rypb_ d.153.1.4 (B:) Proteasome alpha subunit (non-catalytic) {Baker's yeast (Saccharomyces cerevisiae)}
MTDRYSFSLTTFSPSGKLGQIDYALTAVKQGVTSLGIKATNGVVIATEKKSSSPLAMSETLS
KVSLLTPDIGAVYSGMGPDYRVLVDKSRKVAHTSYKRIYGEYPPTKLLVSEVAKIMQEATQS
GGVRPFGVSLLIAGHDEFNGFSLYQVDPSGSYFPWKATAIGKGSVAAKTFLEKRWNDELELE
DAIHIALLTLKESVEGEFNGDTIELAIIGDENPDLLGYTGIPTDKGPRFRKLTSQEINDRLE
AL
>d1rypc_ d.153.1.4 (C:) Proteasome alpha subunit (non-catalytic) {Baker's yeast (Saccharomyces cerevisiae)}
GSRRYDSRTTIFSPEGRLYQVEYALESISHAGTAIGIMASDGIVLAAERKVTSTLLEQDTST
EKLYKLNDKIAVAVAGLTADAEILINTARIHAQNYLKTYNEDIPVEILVRRLSDIKQGYTQH
GGLRPFGVSFIYAGYDDRYGYQLYTSNPSGNYTGWKAISVGANTSAAQTLLQMDYKDDMKVD
DAIELALKTLSKTTDSSALTYDRLEFATIRKGANDGEVYQKIFKPQEIKDILVKTGIT
>d1rypd_ d.153.1.4 (D:) Proteasome alpha subunit (non-catalytic) {Baker's yeast (Saccharomyces cerevisiae)}
GYDRALSIFSPDGHIFQVEYALEAVKRGTCAVGVKGKNCVVLGCERRSTLKLQDTRITPSKV
SKIDSHVVLSFSGLNADSRILIEKARVEAQSHRLTLEDPVTVEYLTRYVAGVQQRYTQSGGV
RPFGVSTLIAGFDPRDDEPKLYQTEPSGIYSSWSAQTIGRNSKTVREFLEKNYDRKEPPATV
EECVKLTVRSLLEVVQTGAKNIEITVVKPDSDIVALSSEEINQYVTQIEQEKQEQ
>d1rype_ d.153.1.4 (E:) Proteasome alpha subunit (non-catalytic) {Baker's yeast (Saccharomyces cerevisiae)}
DRGVSTFSPEGRLFQVEYSLEAIKLGSTAIGIATKEGVVLGVEKRATSPLLESDSIEKIVEI
DRHIGCAMSGLTADARSMIEHARTAAVTHNLYYDEDINVESLTQSVCDLALRFGEGASGEER
LMSRPFGVALLIAGHDADDGYQLFHAEPSGTFYRYNAKAIGSGSEGAQAELLNEWHSSLTLK
EAELLVLKILKQVMEEKLDENNAQLSCITKQDGFKIYDNEKTAELIKELKEKEAAE
>d1rypf_ d.153.1.4 (F:) Proteasome alpha subunit (non-catalytic) {Baker's yeast (Saccharomyces cerevisiae)}
FRNNYDGDTVTFSPTGRLFQVEYALEAIKQGSVTVGLRSNTHAVLVALKRNADELSSYQKKI
IKCDEHMGLSLAGLAPDARVLSNYLRQQCNYSSLVFNRKLAVERAGHLLCDKAQKNTQSYGG
RPYGVGLLIIGYDKSGAHLLEFQPSGNVTELYGTAIGARSQGAKTYLERTLDTFIKIDGNPD
ELIKAGVEAISQSLRDESLTVDNLSIAIVGKDTPFTIYDGEAVAKYI
>d1rypg_ d.153.1.4 (G:) Proteasome alpha subunit (non-catalytic)

{Baker's yeast (Saccharomyces cerevisiae)}
GTGYDLSNSVFSPDGRNFQVEYAVKAVENGTTSIGIKCNDGVVFAVEKLITSKLLVPQKNVK
IQVVDRHIGCVYSGLIPDGRHLVNRGREEAASFKKLYKTPIPIPAFADRLGQYVQAHTLYNS
VRPFGVSTIFGGVDKNGAHLYMLEPSGSYWGYKGAATGKGRQSAKAELEKLVDHHPEGLSAR
EAVKQAAKIIYLAHEDNKEKDFELEISWCSLSETNGLHKFVKGDLLQEAIDFAQKEIN
>d1ht1a_ d.153.1.4 (A:) HslV (ClpQ) protease {Escherichia coli}
TTIVSVRRNGHVVIAGDGQATLGNTVMKGNVKKVRRLYNDKVIAGFAGGTADAFTLFELFER
KLEMHQGHLVKAAVELAKDWRTDRMLRKLEALLAVADETASLIITGNGDVVQPENDLIAIGS
GGPYAQAAARALLENTELSAREIAEKALDIAGDICIYTNHFHTIEELSYK
>d1jjwa_ d.153.1.4 (A:) HslV (ClpQ) protease {Haemophilus influenzae}
TTIVSVRRNGQVVVGGDGQVSLGNTVMKGNARKVRRLYNGKVLAGFAGGTADAFTLFELFER
KLEMHQGHLLKSAVELAKDWRTDRALRKLEAMLIVADEKESLIITGIGDVVQPEEDQILAIG
SGGNYALSAARALVENTELSAHEIVEKSLRIAGDICVFTNTNFTIEELP
>g1apy.1 d.153.1.5 (A:,B:) Glycosylasparaginase (aspartylglucosaminidase, AGA) {Human (Homo sapiens)}
SPLPLVVNTWPFKNATEAAWRALASGGSALDAVESGCAMCEREQCDGSVGFGGSPDELGETT
LDAMIMDGTTMDVGAVGDLRRIKNAIGVARKVLEHTTHTLLVGESATTFAQSMGFINEDLST
SASQALHSDWLARNCQPNYWRNVIPDPSKYCGPYKPPXTIGMVVIHKTGHIAAGTSTNGIKF
KIHGRVGDSPIPGAGAYADDTAGAAAATGNGDILMRFLPSYQAVEYMRRGEDPTIACQKVIS
RIQKHFPEFFGAVICANVTGSYGAACNKLSTFTQFSFMVYNSEKNQPTEEKVDCI
>g2gac.1 d.153.1.5 (A:,B:) Glycosylasparaginase (aspartylglucosaminidase, AGA) {Flavobacterium meningosepticum}
NKPIVLSTWNFGLHANVEAWKVLSKGGKALDAVEKGVRLVEDDPTERSVGYGGRPDRDGRVT
LDACIMDENYNIGSVACMEHIKNPISVARAVMEKTPHVMLVGDGALEFALSQGFKKENLLTA
ESEKEWKEWLKTXCIGMIALDAQGNLSGACTTSGMAYKMHGRVGDSPIIGAGLFVDNEIGAA
TATGHGEEVIRTVGTHLVVELMNQGRTPQQACKEAVERIVKIVNRRGKNLKDIQVGFIALNK
KGEYGAYCIQDGFNFAVHDQKGNRLETP
>d1b65a_ d.154.1.1 (A:) L-aminopeptidase D-Ala-esterase/amidase {Ochrobactrum anthropi}
KPRARDLGLPFTGVTGPYNAITDVDGVGVGFQTIIENEPRPGRKRPARSGVTAILPHMQSET
PVPVYAGVHRFNGNGEMTGTHWIEDGGYFLGPVVITNTHGIGMAHHATVRWMVDRYASTYQT
DDFLWIMPVVAETYDGALNDINGFPVTEADVRKALDNVASGPVQEGNCGGGTGMITYGFKGG
TGTASRVVEFGGRSFTIGALVQANHGQRDWLTIAGVPVGQHMRDGTPQSQLQERGSIIVVLA
TDLPLMPHQLKRLARRASIGIGRNGTPGGNNSGDIFIAFSTANQRPMQHRSAPFLDVEMVND
EPLDTVYLAAVDSVEEAVVNAMIAAEDMGGTPFDRLLVQAIDHERLRAVLRQYGRLA
>g1pya.1 d.155.1.1 (A:,B:) Histidine decarboxylase {Lactobacillus sp., strain 30a}
SELDAKLNKLGVDRIAISPYKQWTRGYMEPGNIGNGYVTGLKVDAGVRDKSDDDVLDGIVSY
DRAETKNAYIGQINMTTASXFTGVQGRVIGYDILRSPEVDKAKPLFTETQWDGSELPIYDAK
PLQDALVEYFGTEQDRRHYPAPGSFIVCANKGVTAERPKNDADMKPGQGYGVWSAIAISFAK
DPTKDSSMFVEDAGVWETPNEDELLEYLEGRRKAMAKSIAECGQDAHASFESSWIGFAYTMM
EPGQIGNAITVAPYVSLPIDSIPGGSILTPDKDMEIMENLTMPEWLEKMGYKSLSANNALKY
>d1jl0a_ d.156.1.1 (A:) S-adenosylmethionine decarboxylase {Human

(Homo sapiens)}
HFFEGTEKLLEVWFSRQQPDANQGSGDLRTIPRSEWDILLKDVQCSIISVTKTDKQEAYVLS
ESSMFVSKRRFILKTCGTTLLLKALVPLLKLARDYSGFDSIQSFFYSRKNFMKPSHQGYPHR
NFQEEIEFLNAIFPNGAYCMGRMNSDCWYLYTLDFPESRVISQPDQTLEILMSELDPAVMD
QFYMKDGVTAKDVTRESGIRDLIPGSVIDATMFNPCGYSMNGMKSDGTYWTIAITPEPEFSY
VSFETNLSQTSYDDLIRKVVEVFKPGKFVTTLFVNQSSKCRTVLASPQKIEGFKRLDCQSAM
FNDYNFVFTSFAKKQ
>d2bc2a_ d.157.1.1 (A:) Zn metallo-beta-lactamase {Bacillus cereus}
TVIKNETGTISISQLNKNVWVHTELGSFNGEAVPSNGLVLNTSKGLVLVDSSWDDKLTKELI
EMVEKKFQKRVTDVIITHAHADRIGGIKTLKERGIKAHSTALTAELAKKNGYEEPLGDLQTV
TNLKFGNMKVETFYPGKGHTEDNIVVWLPQYNILVGGALVKSTSAKDLGNVADAYVNEWSTS
IENVLKRYRNINAVVPGHGEVGDKGLLLHTLDLLK
>d1a7ta_ d.157.1.1 (A:) Zn metallo-beta-lactamase {Bacteroides fragilis}
SVKISDDISITQLSDKVYTYVSLAEIEGWGMVPSNGMIVINNHQAALLDTPINDAQTEMLVN
WVTDSLHAKVTTFIPNHWHGDCIGGLGYLQRKGVQSYANQMTIDLAKEKGLPVPEHGFTDSL
TVSLDGMPLQCYYLGGGHATDNIVVWLPTENILFGGCMLKDNQTTSIGNISDADVTAWPKTL
DKVKAKFPSARYVVPGHGNYGGTELIEHTKQIVNQYIESTS
>d1smla_ d.157.1.1 (A:) Zn metallo-beta-lactamase {Xanthomonas maltophilia}
EVPLPQLRAYTVDASWLQPMAPLQIADHTWQIGTEDLTALLVQTPDGAVLLDGGMPQMASHL
LDNMKARGVTPRDLRLILLSHAHADHAGPVAELKRRTGAKVAANAESAVLLARGGSDDLHFG
DGITYPPANADRIVMDGEVITVGGIVFTAHFMAGHTPGSTAWTWTDTRNGKPVRIAYADSLS
APGYQLQGNPRYPHLIEDYRRSFATVRALPCDVLLTPHPGASNWDYAAGARAGAKALTCKAY
ADAAEQKFDGQLAKETAG
>d1jjea_ d.157.1.1 (A:) Zn metallo-beta-lactamase {Pseudomonas aeruginosa, IMP-1}
SLPDLKIEKLDEGVYVHTSFEEVNGWGVVPKHGLVVLVNAEAYLIDTPFTAKDTEKLVTWFV
ERGYKIKGSISSHFHSDSTGGIEWLNSRSIPTYASELTNELLKKDGKVQATNSFSGVNYWLV
KNKIEVFYPGPGHTPDNVVVWLPERKILFGGCFIKPYGLGNLGDANIEAWPKSAKLLKSKYG
KAKLVVPSHSEVGDASLLKLTLEQAVKGLNESKK
>d1qh5a_ d.157.1.2 (A:) Glyoxalase II (hydroxyacylglutathione hydrolase) {Human (Homo sapiens)}
MKVEVLPALTDNYMYLVIDDETKEAAIVDPVQPQKVVDAARKHGVKLTTVLTTHHHWDHAGG
NEKLVKLESGLKVYGGDDRIGALTHKITHLSTLQVGSLNVKCLATPCHTSGHICYFVSKPGG
SEPPAVFTGDTLFVAGCGKFYEGTADEMCKALLEVLGRLPPDTRVYCGHEYTINNLKFARHV
EPGNAAIREKLAWAKEKYSIGEPTVPSTLAEEFTYNPFMRVREKTVQQHAGETDPVTTMRAV
RREKDQFKMPRD
>d1e5da2 d.157.1.3 (A:2-250) Rubredoxin oxygen:oxidoreductase (ROO), N-terminal domain {Desulfovibrio gigas}
QATKIIDGFHLVGAIDWNSRDFHGYTLSPMGTTYNAYLVEDEKTTLFDTVKAEYKGELLCGI
ASVIDPKKIDYLVIQHLELDHAGALPALIEACQPEKIFTSSLGQKAMESHFHYKDWPVQVVK
HGETLSLGKRTVTFYETRMLHWPDSMVSWFADEKVLISNDIFGQNIAASERFSDQIPVHTLE

RAMREYYANIVNPYAPQTLKAIETLVGAGVAPEFICPDHGVIFRGADQCTFAVQKYVEYAEQ
K
>d1a6q__ d.158.1.1 (-) Protein serine/threonine phosphatase 2C {Human (Homo sapiens)}
GAFLDKPKMEKHNAQGQGNGLRYGLSSMQGWRVEMEDAHTAVIGLPSGLESWSFFAVYDGHA
GSQVAKYCCEHLLDHITNNQDFKGSAGAPSVENVKNGIRTGFLEIDEHMRVMSEKKHGADRS
GSTAVGVLISPQHTYFINCGDSRGLLCRNRKVHFFTQDHKPSNPLEKERIQNAGGSVMIQRV
NGSLAVSRALGDFDYKCVHGKGPTEQLVSPEPEVHDIERSEEDDQFIILACDGIWDVMGNEE
LCDFVRSRLEVTDDLEKVCNEVVDTCLYKGSRDNMSVILICFPNAPKVSPEAVKKEAELDKY
LECRVEEIIKKQGEGVPDLVHVMRTLASENIPSLPPGGELASKRNVIEAVYNRLNPY
>d4kbpa2 d.159.1.1 (A:121-432) Plant purple acid phosphatase, catalytic domain {Kidney bean (Phaseolus vulgaris)}
QTGLDVPYTFGLIGDLGQSFDSNTTLSHYELSPKKGQTVLFVGDLSYADRYPNHDNVRWDTW
GRFTERSVAYQPWIWTAGNHEIEFAPEINETEPFKPFSYRYHVPYEASQSTSPFWYSIKRAS
AHIIVLSSYSAYGRGTPQYTWLKKELRKVKRSETPWLIVLMHSPLYNSYNHHFMEGEAMRTK
FEAWFVKYKVDVVFAGHVHAYERSERVSNIAYKITDGLCTPVKDQSAPVYITIGDAGNYGVI
DSNMIQPQPEYSAFREASFGHGMFDIKNRTHAHFSWNRNQDGVAVEADSVWFFNRHWYPVDD
ST
>d1qhwa_ d.159.1.1 (A:) Mammalian purple acid phosphatase {Rat (Rattus norvegicus)}
STLRFVAVGDWGGVPNAPFHTAREMANAKEIARTVQIMGADFIMSLGDNFYFTGVHDANDKR
FQETFEDVFSDRALRNIPWYVLAGNHDHLGNVSAQIAYSKISKRWNFPSPYYRLRFKVPRSN
ITVAIFMLDTVMLCGNSDDFVSQQPEMPRDLGVARTQLSWLKKQLAAAKEDYVLVAGHYPIW
SIAEHGPTRCLVKNLRPLLAAYGVTAYLCGHDHNLQYLQDENGVGYVLSGAGNFMDPSVRHQ
RKVPNGYLRFHYGSEDSLGGFTYVEIGSKEMSITYVEASGKSLFKTSLPRRP
>d1utea_ d.159.1.1 (A:) Mammalian purple acid phosphatase {Pig (Sus scrofa)}
PTPILRFVAVGDWGGVPNAPFHTAREMANAKAIATTVKTLGADFILSLGDNFYFTGVHDAKD
KRFQETFEDVFSDPSLRNVPWHVLAGNHDHLGNVSAQIAYSKISKRWNFPSPYYRLRFKIPR
SNVSVAIFMLDTVTLCGNSDDFVSQQPERPRNLALARTQLAWIKKQLAAAKEDYVLVAGHYP
VWSIAEHGPTHCLVKQLLPLLTTHKVTAYLCGHDHNLQYLQDENGLGFVLSGAGNFMDPSKK
HLRKVPNGYLRFHFGAENSLGGFAYVEITPKEMSVTYIEASGKSLFKTKLPRRA
>d1ii7a_ d.159.1.4 (A:) Mre11 {Archaeon Pyrococcus furiosus}
MKFAHLADIHLGYEQFHKPQREEEFAEAFKNALEIAVQENVDFILIAGDLFHSSRPSPGTLK
KAIALLQIPKEHSIPVFAIEGNHDRTQRGPSVLNLLEDFGLVYVIGMRKEKVENEYLTSERL
GNGEYLVKGVYKDLEIHGMKYMSSAWFEANKEILKRLFRPTDNAILMLHQGVREVSEARGED
YFEIGLGDLPEGYLYYALGHIHKRYETSYSGSPVVYPGSLERWDFGDYEVRYEWDGIKFKER
YGVNKGFYIVEDFKPRFVEIKVRPFIDVKIKGSEEEIRKAIKRLIPLIPKNAYVRLNIGWRK
PFDLTEIKELLNVEYLKIDTWRI
>d1ush_2 d.159.1.2 (26-362) 5'-nucleotidase (syn. UDP-sugar hydrolase), N-terminal domain {Escherichia coli}
YEQDKTYKITVLHTNDHHGHFWRNEYGEYGLAAQKTLVDGIRKEVAAEGGSVLLLSGGDINT
GVPESDLQDAEPDFRGMNLVGYDAMAIGNHEFDNPLTVLRQQEKWAKFPLLSANIYQKSTGE
RLFKPWALFKRQDLKIAVIGLTTDDTAKIGNPEYFTDIEFRKPADEAKLVIQELQQTEKPDI

IIAATHMGHYDNGEHGSNAPGDVEMARALPAGSLAMIVGGHSQDPVCMAAENKKQVDYVPGT
PCKPDQQNGIWIVQAHEWGKYVGRADFEFRNGEMKMVNYQLIPVNLKKKVTWEDGKSERVLY
TPEIAENQQMISLLSPFQNKGKAQLEV
>d1jk7a_ d.159.1.3 (A:) Protein phosphatase-1 (PP-1) {Human (Homo sapiens)}
KLNIDSIIQRLLEVRGSKPGKNVQLQENEIRGLCLKSREIFLSQPILLELEAPLKICGDIHG
QYYDLLRLFEYGGFPPESNYLFLGDYVDRGKQSLETICLLLAYKIKYPENFFLLRGNHECAS
INRIYGFYDECKRRYNIKLWKTFTDCFNCLPIAAIVDEKIFCCHGGLSPDLQSMEQIRRIMR
PTDVPDQGLLCDLLWSDPDKDVLGWGENDRGVSFTFGAEVVAKFLHKHDLDLICRAHQVVED
GYEFFAKRQLVTLFSAPNYCGEFDNAGAMMSVDETLMCSFQILKPA
>d1tcoa_ d.159.1.3 (A:) Protein phosphatase-2B (PP-2B, calcineurin A subunit) {Cow (Bos taurus)}
VPFPPSHRLTAKEVFDNDGKPRVDILKAHLMKEGRLEETVALRIITEGASILRQEKNLLDID
APVTVCGDIHGQFFDLMKLFEVGGSPANTRYLFLGDYVDRGYFSIECVLYLWALKILYPKTL
FLLRGNHECRHLTEYFTFKQECKIKYSERVYDACMDAFDCLPLAALMNQQFLCVHGGLSPEI
NTLDDIRKLDRFKEPPAYGPMCDILWSDPLEDFGNEKTQEHFTHNTVRGCSYFYSYPAVCEF
LQHNNLLSILRAHEAQDAGYRMYRKSQTTGFPSLITIFSAPNYLDVYNNKAAVLKYENNVMN
IRQFNCSPHPYWLPNFMDVFTWSLPFVGEKVTEMLVNVLNIC
>d1auia_ d.159.1.3 (A:) Protein phosphatase-2B (PP-2B, calcineurin A subunit) {Human (Homo sapiens)}
TDRVVKAVPFPPSHRLTAKEVFDNDGKPRVDILKAHLMKEGRLEESVALRIITEGASILRQE
KNLLDIDAPVTVCGDIHGQFFDLMKLFEVGGSPANTRYLFLGDYVDRGYFSIECVLYLWALK
ILYPKTLFLLRGNHECRHLTEYFTFKQECKIKYSERVYDACMDAFDCLPLAALMNQQFLCVH
GGLSPEINTLDDIRKLDRFKEPPAYGPMCDILWSDPLEDFGNEKTQEHFTHNTVRGCSYFYS
YPAVCEFLQHNNLLSILRAHEAQDAGYRMYRKSQTTGFPSLITIFSAPNYLDVYNNKAAVLK
YENNVMNIRQFNCSPHPYWLPNFMDVFTWSLPFVGEKVTEMLVNVLNICSDDELGSEEDGFD
GATAAARKEVIRNKIRAIGKMARVFSVLREESESVLTLKGLTPTGMLPSGVLSGGKQTLQSA
TVEAIEADEAIKGFSPQHKITSFEEAKGLDRINERMPPR
>d1g5ba_ d.159.1.3 (A:) lambda ser/thr protein phosphatase {Bacteriophage lambda}
MRYYEKIDGSKYRNIWVVGDLHGCYTNLMNKLDTIGFDNKKDLLISVGDLVDRGAENVECLE
LITFPWFRAVRGNHEQMMIDGLSERGNVNHWLLNGGGWFFNLDYDKEILAKALAHKADELPL
IIELVSKDKKYVICHADYPFDEYEFGKPVDHQQVIWNRERISNSQNGIVKEIKGADTFIFGH
TPAVKPLKFANQMYIDTGAVFCGNLTLIQVQGA
>d1emsa2 d.160.1.1 (A:10-280) NIT-FHIT fusion protein, N-terminal domain {Nematode (Caenorhabditis elegans)}
MATGRHFIAVCQMTSDNDLEKNFQAAKNMIERAGEKKCEMVFLPECFDFIGLNKNEQIDLAM
ATDCEYMEKYRELARKHNIWLSLGGLHHKDPSDAAHPWNTHLIIDSDGVTRAEYNKLHLFDL
EIPGKVRLMESEFSKAGTEMIPPVDTPIGRLGLSICYDVRFPELSLWNRKRGAQLLSFPSAF
TLNTGLAHWETLLRARAIENQCYVVAAAQTGAHNPKRQSYGHSMVVDPWGAVVAQCSERVDM
CFAEIDLSYVDTLREMQPVFSHR
>d1f89a_ d.160.1.1 (A:) hypothetical protein yl85 {Baker's yeast (Saccharomyces cerevisiae)}
SASKILSQKIKVALVQLSGSSPDKMANLQRAATFIERAMKEQPDTKLVVLPECFNSPYSTDQ

FRKYSEVINPKEPSTSVQFLSNLANKFKIILVGGTIPELDPKTDKIYNTSIIFNEDGKLIDK
HRKVHLFDVDIPNGISFHESETLSPGEKSTTIDTKYGKFGVGICYDMRFPELAMLSARKGAF
AMIYPSAFNTVTGPLHWHLLARSRAVDNQVYVMLCSPARNLQSSYHAYGHSIVVDPRGKIVA
EAGEGEEIIYAELDPEVIESFRQAVPLTKQRRF
>d1erza_ d.160.1.2 (A:) N-carbamoyl-D-aminoacid amidohydrolase
{Agrobacterium sp.}
TRQMILAVGQQGPIARAETREQVVVRLLDMLTKAASRGANFIVFPELALTTFFPRWHFTDEA
ELDSFYETEMPGPVVRPLFEKAAELGIGFNLGYAELVVEGGVKRRFNTSILVDKSGKIVGKY
RKIHLPGHKEYEAYRPFQHLEKRYFEPGDLGFPVYDVDAAKMGMFICNDRRWPEAWRVMGLR
GAEIICGGYNTPTHNPPVPQHDHLTSFHHLLSMQAGSYQNGAWSAAAGKVGMEENCMLLGHS
CIVAPTGEIVALTTTLEDEVITAAVDLDRCRELREHIFNFKQHRQPQHYGLIAEL
>d1hq0a_ d.194.1.1 (A:) Type 1 cytotoxic necrotizing factor,
catalytic domain {Escherichia coli}
SIESTSKSNFQKLSRGNIDVLKGRGSISSTRQRAIYPYFEAANADEQQPLFFYIKKDRFDNH
GYDQYFYDNTVGPNGIPTLNTYTGEIPSDSSSLGSTYWKKYNLTNETSIIRVSNSARGANGI
KIALEEVQEGKPVIITSGNLSGCTTIVARKEGYIYKVHTGTTKSLAGFTSTTGVKKAVEVLE
LLTKEPIPRVEGIMSNDFLVDYLSENFEDSLITYSSSEKKPDSQITIIRDNVSVFPYFLDNI
PEHGFGTSATVLVRVDGNVVVRSLSESYSLNADASEISVLKVFSKKF
>d1qdla_ d.161.1.1 (A:) Anthranilate synthase
aminodeoxyisochorismate synthase/lyase subunit, TrpE {Archaeon
Sulfolobus solfataricus}
AMEVHPISEFASPFEVFKCIERDFKVAGLLESIGGPQYKARYSVIAWSTNGYLKIHDDPVNI
LNGYLKDLKLADIPGLFKGGMIGYISYDAVRFWEKIRDLKPAAEDWPYAEFFTPDNIIIYDH
NEGKVYVNADLSSVGGCGDIGEFKVSFYDESLNKNSYERIVSESLEYIRSGYIFQVVLSRFY
RYIFSGDPLRIYYNLRRINPSPYMFYLKFDEKYLIGSSPELLFRVQDNIVETYPIAGTRPRG
ADQEEDLKLELELMNSEKDKAEHLMLVDLARNDLGKVCVPGTVKVPELMYVEKYSHVQHIVS
KVIGTLKKKYNALNVLSATFPAGTVSGAPKPMAMNIIETLEEYKRGPYAGAVGFISADGNAE
FAIAIRTAFLNKELLRIHAGAGIVYDSNPESEYFETEHKLKALKTAIGVR
>d1i1qa_ d.161.1.1 (A:) Anthranilate synthase
aminodeoxyisochorismate synthase/lyase subunit, TrpE {Salmonella
typhimurium}
KPTLELLTCDAAYRENPTALFHQVCGDRPATLLLESADIDSKDDLKSLLLVDSALRITALGD
TVTIQALSDNGASLLPLLDTALPAGVENDVLPAGRVLRFPPVSPLLDENARLCSLSVFDAFR
LLQGVVNIPTQEREAMFFGGLFAYDLVAGFEALPHLEAGNNCPDYCFYLAETLMVIDHQKKS
TRIQASLFTASDREKQRLNARLAYLSQQLTQPAPPLPVTPVPDMRCECNQSDDAFGAVVRQL
QKAIRAGEIFQVVPSRRFSLPCPSPLAAYYVLKKSNPSPYMFFMQDNDFTLFGASPESSLKY
DAASRQIEIYPIAGTRPRGRRADGTLDRDLDSRIELDMRTDHKELSEHLMLVDLARNDLARI
CTPGSRYVADLTKVDRYSYVMHLVSRVVGELRHDLDALHAYRACMNMGTLSGAPKVRAMQLI
ADAEGQRRGSYGGAVGYFTAHGDLDTCIVIRSALVENGIATVQAGAGIVLDSVPQSEADETR
NKARAVLRAIATAHHA
>d1i7qa_ d.161.1.1 (A:) Anthranilate synthase
aminodeoxyisochorismate synthase/lyase subunit, TrpE {Serratia
marcescens}
TKPQLTLLKVQASYRGDPTTLFHQLCGARPATLLLESAEINDKQNLQSLLVIDSALRITALG

HTVSVQALTANGPALLPLLDEALPPEVRNQARPNGRELTFPAIDAVQDEDARLRSLSVFDAL
RTILTLVDSPADEREAVMLGGLFAYDLVAGFENLPALRQDQRCPDFCFYLAETLLVLDHQRG
SARLQASVFSEQASEAQRLQHRLEQLQAELQQPPQPIPHQKLENMQLSCNQSDEEYGAVVSE
LQEAIRQGEIFQVVPSRRFSLPCPAPLGPYQTLKDNNPSPYMFFMQDDDFTLFGASPESALK
YDAGNRQIEIYPIAGTRPRGRRADGSLDLDLDSRIELEMRTDHKELAEHLMLVDLARNDLAR
ICQAGSRYVADLTKVDRYSFVMHLVSRVVGTLRADLDVLHAYQACMNMGTLSGAPKVRAMQL
IAALRSTRRGSYGGRVGYFTAVRNLDTCIVIRSAYVEDGHRTVQAGAGVVQDSIPEREADET
RNKARAVLRAIATAHHAKEVF
>d1k0ga_ d.161.1.1 (A:) P-aminobenzoate synthase component I
{Escherichia coli}
MKTLSPAVITLLWRQDAAEFYFSRLSHLPWAMLLHSGYADHPYSRFDIVVAEPICTLTTFGK
ETVVSESEKRTTTTDDPLQVLQQVLDRADIRPTHNEDLPFQGGALGLFGYDLGRRFESLPEI
AEQDIVLPDMAVGIYDWALIVDHQRHTVSLLSHNDVNARRAWLESQQFSPQEDFTLTSDWQS
NMTREQYGEKFRQVQEYLHSGDCYQVNLAQRFHATYSGDEWQAFLQLNQANRAPFSAFLRLE
QGAILSLSPERFILCDNSEIQTRPIKGTLPRLPDPQEDSKQAVKLANSAKDRAENLMIVDLM
RNDIGRVAVAGSVKVPELFVVEPFPAVHHLVSTITAQLPEQLHASDLLRAAFPGGSITGAPK
VRAMEIIDELEPQRRNAWCGSIGYLSFCGNMDTSITIRTLTAINGQIFCSAGGGIVADSQEE
AEYQETFDKVNRILKQLEK
>d1mlda2 d.162.1.1 (A:145-313) Malate dehydrogenase {Pig (Sus
scrofa)}
VTTLDIVRANAFVAELKGLDPARVSVPVIGGHAGKTIIPLISQCTPKVDFPQDQLSTLTGRI
QEAGTEVVKAKAGAGSATLSMAYAGARFVFSLVDAMNGKEGVVECSFVKSQETDCPYFSTPL
LLGKKGIEKNLGIGKISPFEEKMIAEAIPELKASIKKGEEFVKNM
>d5mdha2 d.162.1.1 (A:155-333) Malate dehydrogenase {Pig (Sus
scrofa)}
TRLDHNRAKAQIALKLGVTSDDVKNVIIWGNHSSTQYPDVNHAKVKLQAKEVGVYEAVKDDS
WLKGEFITTVQQRGAAVIKARKLSSAMSAAKAICDHVRDIWFGTPEGEFVSMGIISDGNSYG
VPDDLLYSFPVTIKDKTWKIVEGLPINDFSREKMDLTAKELAEEKETAFEFLSSA
>d7mdha2 d.162.1.1 (A:198-385) Malate dehydrogenase {Sorghum
(Sorghum vulgare), chloroplast}
TRLDENRAKCQLALKAGVFYDKVSNVTIWGNHSTTQVPDFLNAKIDGRPVKEVIKRTKWLEE
EFTITVQKRGGALIQKWGRSSAASTAVSIADAIKSLVTPTPEGDWFSTGVYTTGNPYGIAED
IVFSMPCRSKGDGDYELATDVSNDDFLWERIKKSEAELLAEKKCVAHLTGEGNAYCDVPEDT
ML
>d1civa2 d.162.1.1 (A:194-385) Malate dehydrogenase {Flaveria
bidentis, chloroplast}
TRLDENRAKCQLALKAGVFYDKVSNVTIWGNHSTTQVPDFLNAKIHGIPVTEVIRDRKWLED
EFTNMVQTRGGVLIKKWGRSSAASTAVSIVDAIRSLVTPTPEGDWFSTGVYTNGNPYGIAED
IVFSMPCRSKGDGDYEFVKDVIFDDYLSKKIKKSEDELLAEKKCVAHLTGEGIAVCDLPEDT
MLPGEM
>d2cmd_2 d.162.1.1 (146-312) Malate dehydrogenase {Escherichia
coli}
VTTLDIIRSNTFVAELKGKQPGEVEVPVIGGHSGVTILPLLSQVPGVSFTEQEVADLTKRIQ
NAGTEVVEAKAGGGSATLSMGQAAARFGLSLVRALQGEQGVVECAYVEGDGQYARFFSQPLL

LGKNGVEERKSIGTLSAFEQNALEGMLDTLKKDIALGQEFVNK
>d1bdma2 d.162.1.1 (A:155-332) Malate dehydrogenase {Thermus flavus}
TRLDHNRAKAQLAKKTGTGVDRIRRMTVWGNHSSIMFPDLFHAEVDGRPALELVDMEWYEKV
FIPTVAQRGAAIIQARGASSAASAANAAIEHIRDWALGTPEGDWVSMAVPSQGEYGIPEGIV
YSFPVTAKDGAYRVVEGLEINEFARKRMEITAQELLDEMEQVKALGLI
>d2hlpa2 d.162.1.1 (A:163-330) Malate dehydrogenase {Archaeon Haloarcula marismortui}
FGGRLDSARFRYVLSEEFDAPVQNVEGTILGEHGDAQVPVFSKVRVDGTDPEFSGDEKEQLL
GDLQESAMDVIERKGATEWGPARGVAHMVEAILHDTGRVLPASVKLEGEFGHEDTAFGVPVR
LGSNGVEEIVEWDLDDYEQDLMADAAEKLSDQYDKIS
>d1b8pa2 d.162.1.1 (A:159-329) Malate dehydrogenase {Aquaspirillum arcticum}
LRLDHNRALSQIAAKTGKPVSSIEKLFVWGNHSPTMYADYRYAQIDGASVKDMINDDAWNRD
TFLPTVGKRGAAIIDARGVSSAASAANAAIDHIHDWVLGTAGKWTTMGIPSDGSYGIPEGVI
FGFPVTTENGEYKIVQGLSIDAFSQERINVTLNELLEEQNGVQHLLG
>d1guya2 d.162.1.1 (A:144-306) Malate dehydrogenase {Chloroflexus aurantiacus}
AGVLDAARYRTFIAMEAGVSVEDVQAMLMGGHGDEMVPLPRFSTISGIPVSEFIAPDRLAQI
VERTRKGGGEIVNLLKTGSAYYAPAAATAQMVEAVLKDKKRVMPVAAYLTGQYGLNDIYFGV
PVILGAGGVEKILELPLNEEEMALLNASAKAVRATLDTL
>d1gv0a2 d.162.1.1 (A:143-305) Malate dehydrogenase {Chlorobium tepidum}
AGVLDSARFRSFIAMELGVSMQDVTACVLGGHGDAMVPVVKYTTVAGIPVADLISAERIAEL
VERTRTGGAEIVNHLKQGSAFYSPATSVVEMVESIVLDRKRVLTCAVSLDGQYGIDGTFVGV
PVKLGKNGVEHIYEIKLDQSDLDLLQKSAKIVDENCKML
>d1guza2 d.162.1.1 (A:143-305) Malate dehydrogenase {Chlorobium vibrioforme}
AGVLDAARFRSFIAMELGVSMQDINACVLGGHGDAMVPVVKYTTVAGIPISDLLPAETIDKL
VERTRNGGAEIVEHLKQGSAFYAPASSVVEMVESIVLDRKRVLPCAVGLEGQYGIDKTFVGV
PVKLGRNGVEQIYEINLDQADLDLLQKSAKIVDENCKML
>d1hyha2 d.162.1.1 (A:167-329) L-2-hydroxyisocapronate dehydrogenase, L-HICDH {Lactobacillus confusus}
GTLLDTARMQRAVGEAFDLDPRSVSGYNLGEHGNSQFVAWSTVRVMGQPIVTLADAGDIDLA
AIEEEARKGGFTVLNGKGYTSYGVATSAIRIAKAVMADAHAELVVSNRRDDMGMYLSYPAII
GRDGVLAETTLDLTTDEQEKLLQSRDYIQQRFDEIVDTL
>d9ldta2 d.162.1.1 (A:163-331) Lactate dehydrogenase {Pig (Sus scrofa)}
SGCNLDSARFRYLMGERLGVHPLSCHGWILGEHGDSSVPVWSGVNVAGVSLKNLHPELGTDA
DKEHWKAVHKEVVDSAYEVIKLKGYTSWAIGLSVADLAESIMKNLRRVHPISTMIKGLYGIK
ENVFLSVPCILGQNGISDVVKVTLTPEEEAHLKKSADTLWGIQKELQF
>d1i0za2 d.162.1.1 (A:161-332) Lactate dehydrogenase {Human (Homo sapiens), heart isoform (H chain)}
SGCNLDSARFRYLMAEKLGIHPSSCHGWILGEHGDSSVAVWSGVNVAGVSLQELNPEMGTDN

DSENWKEVHKMVVESAYEVIKLKGYTNWAIGLSVADLIESMLKNLSRIHPVSTMVKGMYGIE
NEVFLSLPCILNARGLTSVINQKLKDDEVAQLKKSADTLWDIQKDLKD
>d1i10a2 d.162.1.1 (A:160-331) Lactate dehydrogenase {Human (Homo sapiens), muscle isoform (M chain)}
SGCNLDSARFRYLMGERLGVHPLSCHGWVLGEHGDSSVPVWSGMNVAGVSLKTLHPDLGTDK
DKEQWKEVHKQVVESAYEVIKLKGYTSWAIGLSVADLAESIMKNLRRVHPVSTMIKGLYGIK
DDVFLSVPCILGQNGISDLVKVTLTSEEEARLKKSADTLWGIQKELQF
>d2ldx_2 d.162.1.1 (160-331) Lactate dehydrogenase {Mouse (Mus musculus)}
SGCNLDSARFRYLIGEKLGVNPTSCHGWVLGEHGDSSVPIWSGVNVAGVTLKSLNPAIGTDK
NKQHWKNVHKQVVEGGYEVLDMKGYTSWAIGLSVTDLARSILKNLKRVHPVTTLVKGFHGIK
EEVFLSIPCVLGESGITDFVKVNMTAEEEGLLKKSADTLWNMQKNLEL
>d1ldm_2 d.162.1.1 (161-329) Lactate dehydrogenase {Dogfish (Squalus acanthias)}
SGCNLDSARFRYLMGERLGVHSCSCHGWVIGEHGDSVPSVWSGMNVASIKLHPLDGTNKDKQ
DWKKLHKDVVDSAYEVIKLKGYTSWAIGLSVADLAETIMKNLCRVHPVSTMVKDFYGIKDNV
FLSLPCVLNDHGISNIVKMKLKPNEEQQLQKSATTLWDIQKDLKF
>d1ceqa2 d.162.1.1 (A:164-329) Lactate dehydrogenase {Malaria parasite (Plasmodium falciparum)}
GGVLDTSRLKYYISQKLNVCPRDVNAHIVGAHGNKMVLLKRYITVGGIPLQEFINNKLISDA
ELEAIFDRTVNTALEIVNLHASPYVAPAAAIIEMAESYLKDLKKVLICSTLLEGQYGHSDIF
GGTPVVLGANGVEQVIELQLNSEEKAKFDEAIAETKRMKALA
>d1ldna2 d.162.1.1 (A:163-330) Lactate dehydrogenase {Bacillus stearothermophilus}
TILDTARFRFLLGEYFSVAPQNVHAYIIGEHGDTELPVWSQAYIGVMPIRKLVESKGEEAQK
DLERIFVNVRDAAYQIIEKKGATYYGIAMGLARVTRAILHNENAILTVSAYLDGLYGERDVY
IGVPAVINRNGIREVIEIELNDDEKNRFHHSAATLKSVLARAFT
>d1llc_2 d.162.1.1 (165-334) Lactate dehydrogenase {Lactobacillus casei}
TSLDTARFRQSIAEMVNVDARSVHAYIMGEHGDTEFPVWSHANIGGVTIAEWVKAHPEIKED
KLVKMFEDVRDAAYEIIKLKGATFYGIATALARISKAILNDENAVLPLSVYMDGQYGINDLY
IGTPAVINRNGIQNILEIPLTDHEEESMQKSASQLKKVLTDAFAKNDI
>d1ez4a2 d.162.1.1 (A:163-334) Lactate dehydrogenase {Lactobacillus pentosus}
TSLDSSRLRVALGKQFNVDPRSVDAYIMGEHGDSEFAAYSTATIGTRPVRDVAKEQGVSDDD
LAKLEDGVRNKAYDIINLKGATFYGIGTALMRISKAILRDENAVLPVGAYMDGQYGLNDIYI
GTPAIIGGTGLKQIIESPLSADELKKMQDSAATLKKVLNDGLAELEN
>d1llda2 d.162.1.1 (A:150-319) Lactate dehydrogenase {Bifidobacterium longum, strain am101-2}
TNLDSARLRFLIAQQTGVNVKNVHAYIAGEHGDSEVPLWESATIGGVPMSDWTPLPGHDPLD
ADKREEIHQEVKNAAYKIINGKGATNYAIGMSGVDIIEAVLHDTNRILPVSSMLKDFHGISD
ICMSVPTLLNRQGVNNTINTPVSDKELAALKRSAETLKETAAQFGF
>d1a5z_2 d.162.1.1 (164-333) Lactate dehydrogenase {Thermotoga maritima}

GTVLDTARLRTLIAQHCGFSPRSVHVYVIGEHGDSEVPVWSGAMIGGIPLQNMCQVCQKCDS
KILENFAEKTKRAAYEIIERKGATHYAIALAVADIVESIFFDEKRVLTLSVYLEDYLGVKDL
CISVPVTLGKHGVERILELNLNEEELEAFRKSASILKNAINEITAEEN
>d1hyea2 d.162.1.1 (A:146-313) MJ0490, lactate/malate dehydrogenase {Archaeon Methanococcus jannaschii}
LGTHLDSLRFKVAIAKFFGVHIDEVRTRIIGEHGDSMVPLLSATSIGGIPIQKFERFKELPI
DEIIEDVKTKGEQIIRLKGGSEFGPAAAILNVVRCIVNNEKRLLTLSAYVDGEFDGIRDVCI
GVPVKIGRDGIEEVVSIELDKDEIIAFRKSAEIIKKYCEEVKNL
>d1aiha_ d.163.1.1 (A:) Integrase {Bacteriophage HP1}
ETELAFLYERDIYRLLAECDNSRNPDLGLIVRICLATGARWSEAETLTQSQVMPYKITFTNT
KSKKNRTVPISDELFDMLPKKRGRLFNDAYESFENAVLRAEIELPKGQLTHVLRHTFASHFM
MNGGNILVLKEILGHSTIEMTMRYAHFAPSHLESAVKFNPLSNPAQ
>d1ae9a_ d.163.1.1 (A:) Integrase (Int) {Bacteriophage lambda}
RSRLTADEYLKIYQAAESSPCWLRLAMELAVVTGQRVGDLCEMKWSDIVDGYLYVEQSKTGV
KIAIPTALHIDALGISMKETLDKCKEILGGETIIASTRREPLSSGTVSRYFMRARKASGLSF
EGDPPTFHELRSLSARLYEKQISDKFAQHLLGHKSDTMASQFRDDRGREWDKIEI
>d1f44a2 d.163.1.1 (A:130-343) Cre recombinase {Bacteriophage P1}
RAKQALAFERTDFDQVRSLMENSDRCQDIRNLAFLGIAYNTLLRIAEIARIRVKDISRTDGG
RMLIHIGRTKTLVSTAGVEKALSLGVTKLVERWISVSGVADDPNNYLFCRVRKNGVAAPSAT
SQLSTRALEGIFEATHRLIYGAKDDSGQRYLAWSGHSARVGAARDMARAGVSIPEIMQAGGW
TNVNIVMNFIRNLDSETGAMVRLLEDGD
>d5crxb2 d.163.1.1 (B:130-314) Cre recombinase {Bacteriophage P1}
RAKQALAFERTDFDQVRSLMENSDRCQDIRNLAFLGIAYNTLLRIAEIARIRVKDISRTDGG
RMLIHIGRTKTLVSTAGVEKALSLGVTKLVERWISVSGVADDPNNYLFCRVRKNGVAAPSAT
SQLSTRALEGIFEATHRLIYGAKDDSGQRYLAWSGHSARVGAARDMARAGVSIPEIMQAGG
>d1a0p_2 d.163.1.1 (111-292) Recombinase XerD {Escherichia coli}
KDLSEAQVERLLQAPLIDQPLELRDKAMLEVLYATGLRVSELVGLTMSDISLRQGVVRVIGK
GNKERLVPLGEEAVYWLETYLEHGRPWLLNGVSIDVLFPSQRAQQMTRQTFWHRIKHYAVLA
GIDSEKLSPHVLRHAFATHLLNHGADLRVVQMLLGHSDLSTTQIYTHVATERLRQLHQ
>d1floa2 d.163.1.1 (A:135-423) Flp recombinase {Baker's yeast (Saccharomyces cerevisiae)}
KGNSHSKKMLKALLSEGESIWEITEKILNSFEYTSRFTKTKTLYQFLFLATFINCGRFSDIK
NVDPKSFKLVQNKYLGVIIQCLVTETKTSVSRHIYFFSARGRIDPLVYLDEFLRNSEPVLKR
VNRTGNSSSNKQEYQLLKDNLVRSYNKALKKNAPYSIFAIKNGPKSHIGRHLMTSFLSMKGL
TELTNVVGNWSDKRASAVARTTYTHQITAIPDHYFALVSRYYAYDPISKEMIALKDETNPIE
EWQHIEQLKGSAEGSIRYPAWNGIISQEVLDYLSSYINRRI
>d1a31a1 d.163.1.2 (A:431-626,A:720-765) Eukaryotic DNA topoisomerase I, catalytic core {Human (Homo sapiens)}
PSSRIKGEKDWQKYETARRLKKCVDKIRNQYREDWKSKEMKVRQRAVALYFIDKLALRAGNE
KEEGETADTVGCCSLRVEHINLHPELDGQEYVVEFDFLGKDSIRYYNKVPVEKRVFKNLQLF
MENKQPEDDLFDRLNTGILNKHLQDLMEGLTAKVFRTYNASITLQQQLKELTAPDENIPAKI
LSYNRANRAVXKLNYLDPRITVAWCKKWGVPIEKIYNKTQREKFAWAIDMADEDYEF
>d1a41__ d.163.1.2 (-) Eukaryotic DNA topoisomerase I, catalytic core {Vaccinia virus}

NAKRDRIFVRVYNVMKRINCFINKNIKKSSTDSNYQLAVFMLMETMFFIRFGKMKYLKENET
VGLLTLKNKHIEISPDEIVIKFVGKDKVSHEFVVHKSNRLYKPLLKLTDDSSPEEFLFNKLS
ERKVYECIKQFGIRIKDLRTYGVNYTFLYNFWTNVKSISPLPSPKKLIALTIKQTAEVVGHT
PSISKRAYMATTILEMVKDKNFLDVVSKTTFDEFLSIVVDHVKS
>d1mhda_ d.164.1.1 (A:) SMAD MH1 domain {Human (Homo sapiens)}
PIVKRLLGWKKGEQNGQEEKWCEKAVKSLVKKLKKTGQLDELEKAITTQNVNTKCITIPRSL
DGRLQVSHRKGLPHVIYCRLWRWPDLHSHHELRAMELCEFAFNMKKDEVCVNPYHYQRVET
>d1hufa_ d.195.1.1 (A:) YopH tyrosine phosphatase N-terminal domain {Yersinia pestis}
LSLSDLHRQVSRLVQQESGDCTGKLRGNVAANKETTFQGLTIASGARESEKVFAQTVLSHVA
NVVLTQEDTAKLLQSTVKHNLNNYDLRSVGNGNSVLVSLRSDQMTLQDAKVLLEAALRQES
>d1mrj__ d.165.1.1 (-) alpha-Trichosanthin {Mongolian snake gourd (Trichosanthes kirilowii maxim)}
DVSFRLSGATSSSYGVFISNLRKALPNERKLYDIPLLRSSLPGSQRYALIHLTNYADETISV
AIDVTNVYIMGYRAGDTSYFFNEASATEAAKYVFKDAMRKVTLPYSGNYERLQTAAGKIREN
IPLGLPALDSAITTLFYYNANSAASALMVLIQSTSEAARYKFIEQQIGKRVDKTFLPSLAII
SLENSWSALSKQIQIASTNNGQFESPVVLINAQNQRVTITNVDAGVVTSNIALLLNRNNMA
>d1bryy_ d.165.1.1 (Y:) Bryodin {Red briony (Bryonia dioica)}
DVSFRLSGATTTSYGVFIKNLREALPYERKVYNIPLLRSSISGSGRYTLLHLTNYADETISV
AVDVTNVYIMGYLAGDVSYFFNEASATEAAKFVFKDAKKKVTLPYSGNYERLQTAAGKIREN
IPLGLPALDSAITTLYYYTASSAASALLVLIQSTAESARYKFIEQQIGKRVDKTFLPSLATI
SLENNWSALSKQIQIASTNNGQFESPVVLIDGNNQRVSITNASARVVTSNIALLLNRNNIA
>d1mrg__ d.165.1.1 (-) alpha-Momorcharin (momordin) {Bitter gourd (Momordica charantia)}
DVSFRLSGADPRSYGMFIKDLRNALPFREKVYNIPLLLPSVSGAGRYLLMHLFNRDGKTITV
AVDVTNIYIMGYLADTTSYFFNEPAAELASQYVFRDARRKITLPYSGDYERLQIAAGKPREK
IPIGLPALDSAISTLLHYDSTAAAGALLVLIQTTAEAARFKYIEQQIQERAYRDEVPSLATI
SLENSWSGLSKQIQLAQGNNGIFRTPIVLVDNKGNRVQITNVTSKVVTSNIQLLLNTRNI
>d1cf5a_ d.165.1.1 (A:) Beta-momorcharin {Bitter gourd (Momordica charantia)}
DVNFDLSTATAKTYTKFIEDFRATLPFSHKVYDIPLLYSTISDSRRFILLNLTSYAYETISV
AIDVTNVYVVAYRTRDVSYFFKESPPEAYNILFKGTRKITLPYTGNYENLQTAAHKIRENID
LGLPALSSAITTLFYYNAQSAPSALLVLIQTTAEAARFKYIERHVAKYVATNFKPNLAIISL
ENQWSALSKQIFLAQNQGGKFRNPVDLIKPTGQRFQVTNVDSDVVKGNIKLLLNSRASTADE
N
>d1ce7a_ d.165.1.1 (A:) Mistletoe lectin I A-chain {European mistletoe (Viscum album)}
YERGDLDVTAQTTGAGYFSFITLLRDYVSSGSFSNAIPLLSQSGGGGEAGRFVLVELTNSGG
DGITVAIDVTNLYVVAYQAGSQSYFLSGPGGRHGFTGTTRSSLPFNGSYPDLEQYGGQRKQI
PLGIDQLIQSVTALKFPGSTRTGARSILILIQMISEAARFNPILWRARQYINSGASFLPDVY
MLELETSWGQQSTQVQHSTDGVFNNPIALADPGGGVTLTNVRDVIASLAIMLFVC
>d1abra_ d.165.1.1 (A:) Abrin A-chain {Abrus precatorius}
EDRPIKFSTEGATSQSYKQFIEALRERLRGGLIHDIPVLPDPTTLQERNRYITVELSNSDTE
SIEVGIDVTNAYVVAYRAGTQSYFLRDAPSSASDYLFTGTDQHSLPFYGTYGDLERWAHQSR

QQIPLGLQALTHGISFFRSGGNDNEEKARTLIVIIQMVAEAARFRYISNRVRVSIQTGTAFQ
PDAAMISLENNWDNLSRGVQESVQDTFPNQVTLTNIRNEPVIVDSLSHPTVAVLALMLFVCN
PPN
>d1apa__ d.165.1.1 (-) Pokeweed antiviral protein alpha {Pokeweed (Phytolacca americana)}
INTITFDVGNATINKYATFMKSIHNQAKDPTLKCYGIPMLPNTNLTPKYLLVTLQDSSLKTI
TLMLKRNNLYVMGYADTYNGKCRYHIFKDISNTTERNDVMTTLCPNPSSRVGKNINYDSSYP
ALEKKVGRPRSQVQLGIQILNSGIGKIYGVDSFTEKTEAEFLLVAIQMVSEAARFKYIENQV
KTNFNRAFYPNAKVLNLEESWGKISTAIHNAKNGALTSPLELKNANGSKWIVLRVDDIEPDV
GLLKYVNGTCQAT
>d1d6aa_ d.165.1.1 (A:) Pokeweed antiviral protein alpha {Pokeweed (Phytolacca americana)}
VNTIIYNVGSTTISKYATFLNDLRNEAKDPSLKCYGIPMLPNTNTNPKYVLVELQGSNKKTI
TLMLRRNNLYVMGYSDPFETNKCRYHIFNDISGTERQDVETTLCPNANSRVSKNINFDSRYP
TLESKAGVKSRSQVQLGIQILDSNIGKISGVMSFTEKTEAEFLLVAIQMVSEAARFKYIENQ
VKTNFNRAFNPNPKVLNLQETWGKISTAIHDAKNGVLPKPLELVDASGAKWIVLRVDEIKPD
VALLNYVGGSCQTT
>d1qi7a_ d.165.1.1 (A:) Saporin So6 {Common soapwort (Saponaria officinalis)}
VTSITLDLVNPTAGQYSSFVDKIRNNVKDPNLKYGGTDIAVIGPPSKEKFLRINFQSSRGTV
SLGLKRDNLYVVAYLAMDNTNVNRAYYFKSEITSAELTALFPEATTANQKALEYTEDYQSIE
KNAQITQGDKSRKELGLGIDLLLTFMEAVNKKARVVKNEARFLLIAIQMTAEVARFRYIQNL
VTKNFPNKFDSDNKVIQFEVSWRKISTAIYGDAKNGVFNKDYDFGFGKVRQVKDLQMGLLMY
LGKPK
>d1ift__ d.165.1.1 (-) Ricin A-chain {Castor bean (Ricinus communis)}
YPIINFTTAGATVQSYTNFIRAVRGRLTTGADVRHEIPVLPNRVGLPINQRFILVELSNHAE
LSVTLALDVTNAYVVGYRAGNSAYFFHPDNQEDAEAITHLFTDVQNRYTFAFGGNYDRLEQL
AGNLRENIELGNGPLEEAISALYYYSTGGTQLPTLARSFIICIQMISEAARFQYIEGEMRTR
IRYNRRSAPDPSVITLENSWGRLSTAIQESNQGAFASPIQLQRRNGSKFSVYDVSILIPIIA
LMVYRCAPPP
>d1hwma_ d.165.1.1 (A:) Ebulin A-chain {Sambucus ebulus}
IDYPSVSFNLAGAKSTTYRDFLKNLRDRVATGTYEVNGLPVLRRESEVQVKNRFVLVRLTNY
NGDTVTSAVDVTNLYLVAFSANGNSYFFKDATELQKSNLFLGTTQHTLSFTGNYDNLETAAG
TRRESIELGPNPLDGAITSLWYDGGVARSLLVLIQMVPEAARFRYIEQEVRRSLQQLTSFTP
NALMLSMENNWSSMSLEVQLSGDNVSPFSGTVQLQNYDHTPRLVDNFEELYKITGIAILLFR
CVA
>d1dm0a_ d.165.1.2 (A:) Shiga toxin, A-chain {Shigella dysenteriae}
KEFTLDFSTAKTYVDSLNVIRSAIGTPLQTISSGGTSLLMIDSGTGDNLFAVDVRGIDPEEG
RFNNLRLIVERNNLYVTGFVNRTNNVFYRFADFSHVTFPGTTAVTLSGDSSYTTLQRVAGIS
RTGMQINRHSLTTSYLDLMSHSGTSLTQSVARAMLRFVTVTAEALRFRQIQRGFRTTLDDLS
GRSYVMTAEDVDLTLNWGRLSSVLPDYHGQDSVRVGRISFGSINAILGSVALILNCHHHASR
VARMASDEFPSMCPADGRVRGITHNKILWDSSTLGAILM

>g1lts.1 d.166.1.1 (A:,C:) Heat-labile toxin, A-chain {Escherichia coli, type IB}
RLYRADSRPPDEIKRSGGLMPRGHNEYFDRGTQMNINLYDHARGTQTGFVRYDDGYVSTSLS
LRSAHLAGQSILSGYSTYYIYVIATAPNMFNVNDVLGVYSPHPYEQEVSALGGIPYSQIYGW
YRVNFGVIDERLHRNREYRDRYYRNLNIAPAEDGYRLAGFPPDHQAWREEPWIHHAPQGCGX
GDTCNEETQNLSTIYLREYQSKVKRQIFSDYQSEVDIYNRI
>g1tii.1 d.166.1.1 (A:,C:) Heat-labile toxin, A-chain {Escherichia coli, type IIB}
NDYFRADSRTPDEVRRSGGLIPRGQDEAYERGTPININLYDHARGTATGNTRYNDGYVSTTT
TLRQAHLLGQNMLGGYNEYYIYVVAAAPNLFDVNGVLGRYSPYPSENEYAALGGIPLSQIIG
WYRVSFGAIEGGMHRNRDYRRDLFRGLSAAPNEDGYRIAGFPDGFPAWEEVPWREFAPNSCL
PXTTCASLTNKLSQHDLADFKKYIKRKFTLMTLLSINN
>d1f0la2 d.166.1.1 (A:1-187) Diphtheria toxin, N-terminal domain {Corynebacterium diphtheriae}
GADDVVDSSKSFVMENFSSYHGTKPGYVDSIQKGIQKPKSGTQGNYDDDWKGFYSTDNKYDA
AGYSVDNENPLSGKAGGVVKVTYPGLTKVLALKVDNAETIKKELGLSLTEPLMEQVGTEEFI
KRFGDGASRVVLSLPFAEGSSSVEYINNWEQAKALSVELEINFETRGKRGQDAMYEYMAQAC
A
>d1ikpa2 d.166.1.1 (A:395-606) Exotoxin A, C-terminal domain {Pseudomonas aeruginosa}
PTGAEFLGDGGDVSFSTRGTQNWTVERLLQAHRQLEERGYVFVGYHGTFLEAAQSIVFGGVR
ARSQDLDAIWRGFYIAGDPALAYGYAQDQEPDARGRIRNGALLRVYVPRSSLPGFYRTSLTL
AAPEAAGEVERLIGHPLPLRLDAITGPEEEGGRLETILGWPLAERTVVIPSAIPTDPRNVGG
DLDPSSIPDKEQAISALPDYASQPGK
>d1prta_ d.166.1.1 (A:) Pertussis toxin, S1 subunit {Bordetella pertussis}
DPPATVYRYDSRPPEDVFQNGFTAWGNNDNVLEHLTGRSCQVGSSNSAFVSTSSSRRYTEVY
LEHRMQEAVEAERAGRGTGHFIGYIYEVRADNNFYGAASSYFEYVDTYGDNAGRILAGALAT
YQSEYLAHRRIPPENIRRVTRVYHNGITGETTTTEYSNARYVSQQTRANPNPYTSRRSVASI
VGTLVRMAPVVGACMARQAESSEAMAAWSERAGEAMVLVYYESIAYSF
>g1xtc.1 d.166.1.1 (A:,C:) Cholera toxin {Vibrio cholerae}
NDDKLYRADSRPPDEIKQSGGLMPRGQSEYFDRGTQMNINLYDHARGTQTGFVRHDDGYVST
SISLRSAHLVGQTILSGHSTYYLYVLATAPNMFNVNDVLGAYSPHPDEQEVSALGGIPYSQI
YGWYRVHFGVLDEQLHRNRGYRDRYYSNLDIAPAADGYGLAGFPPEHRAWREEPWIHHAPPG
CGNAPRXSNTCDEKTQSLGVKFLDEYQSKVKRQIFSGYQSDIDTHNRIKDEL
>d1qs1a1 d.166.1.1 (A:60-264) Vegetative insecticidal protein 2 (VIP2) {Bacillus cereus}
TDKVEDFKEDKEKAKEWGKEKEKEKEWKLTATEKGKMNNFLDNKNDIKTNYKEITFSMAGSFED
EIKDLKEIDKMFDKTNLSNSIITYKNVEPTTIGFNKSLTEGNTINSDAMAQFKEQFLDRDIK
FDSYLDTHLTAQQVSSKERVILKVTVPSGKGSTTPTKAGVILNNSEYKMLIDNGYMVHVDKV
SKVVKKGVECLQIEGTLKK
>d1qs1a2 d.166.1.1 (A:265-461) Vegetative insecticidal protein 2 (VIP2) {Bacillus cereus}
SLDFKNDINAEAHSWGMKNYEEWAKDLTDSQREALDGYARQDYKEINNYLRNQGGSGNEKLD

AQIKNISDALGKKPIPENITVYRWCGMPEFGYQISDPLPSLKDFEEQFLNTIKEDKGYMSTS
LSSERLAAFGSRKIILRLQVPKGSTGAYLSAIGGFASEKEILLDKDSKYHIDKVTEVIIKGV
KRYVVDATLLT
>d1g24a_ d.166.1.1 (A:) Exoenzyme c3 {Clostridium botulinum}
AYSNTYQEFTNIDQAKAWGNAQYKKYGLSKSEKEAIVSYTKSASEINGKLRQNKGVINGFPS
NLIKQVELLDKSFNKMKTPENIMLFRGDDPAYLGTEFQNTLLNSNGTINKTAFEKAKAKFLN
KDRLEYGYISTSLMNVSQFAGRPIITKFKVAKGSKAGYIDPISAFAGQLEMLLPRHSTYHID
DMRLSSDGKQIIITATMMGTAINPK
>d1j7na3 d.166.1.1 (A:264-550) Anthrax toxin lethal factor, middle domain {Bacillus anthracis}
MLSRYEKWEKIKQHYQHWSDSLSEEGRGLLKKLQIPIEPKKDDIIHSLSQEEKELLKRIQID
SSDFLSTEEKEFLKKLQIDIRDSLSEEEKELLNRIQVDSSNPLSEKEKEFLKKLKLDIQPYD
INQRLQDTGGLIDSPSINLDVRKQYKRDIQNIDALLHQSIGSTLYNKIYLYENMNINNLTAT
LGADLVDSTDNTKINRGIFNEFKKNFKYSISSNYMIVDINERPALDNERLKWRIQLSPDTRA
GYLENGKLILQRNIGLEIKDVQIIKQSEKEYIRIDAKVV
>d1a26_2 d.166.1.2 (797-1012) Poly(ADP-ribose) polymerase, C-terminal domain {Chicken (Gallus gallus)}
LRTDIKVVDKDSEEAKIIKQYVKNTHAATHNAYDLKVVEIFRIEREGESQRYKPFKQLHNRQ
LLWHGSRTTNFAGILSQGLRIAPPEAPVTGYMFGKGIYFADMVSKSANYCHTSQADPIGLIL
LGEVALGNMYELKNASHITKLPKGKHSVKGLGKTAPDPTATTTLDGVEVPLGNGISTGINDT
CLLYNEYIVYDVAQVNLKYLLKLKFNYKTS
>d1g2aa_ d.167.1.1 (A:) Peptide deformylase {Escherichia coli}
SVLQVLHIPDERLRKVAKPVEEVNAEIQRIVDDMFETMYAEEGIGLAATQVDIHQRIIVIDV
SENRDERLVLINPELLEKSGETGIEEGCLSIPEQRALVPRAEKVKIRALDRDGKPFELEADG
LLAICIQHEMDHLVGKLFMDYLSPLKQQRIRQKVEKLDRL
>d2def__ d.167.1.1 (-) Peptide deformylase {Escherichia coli}
VLQVLHIPDERLRKVAKPVEEVNAEIQRIVDDMFETMYAEEGIGLAATQVDIHQRIIVIDVS
ENRDERLVLINPELLEKSGETGIEEGCLSIPEQRALVPRAEKVKIRALDRDGKPFELEADGL
LAICIQHEMDHLVGKLFMDYLS
>d1jbia_ d.209.1.1 (A:) Cochlin {Human (Homo sapiens)}
TAPIAITCFTRGLDIRKEKADVLCPGGCPLEEFSVYGNIVYASVSSICGAAVHRGVISNSGG
PVRVYSLPGRENYSSVDANGIQSQMLSRWSASFTVTLE
>d1chua3 d.168.1.1 (A:238-353) L-aspartate oxidase {Escherichia coli}
LEFNQFHPTALYHPQARNFLLTEALRGEGAYLKRPDGTRFMPDFDERGELAPRDIVARAIDH
EMKRLGADCMFLDISHKPADFIRQHFPMIYEKLLGLGIDLTQEPVPIVPAAHYT
>d1fuma3 d.168.1.1 (A:226-357) Fumarate reductase flavoprotein subunit {Escherichia coli}
MEFVQYHPTGLPGSGILMTEGCRGEGGILVNKNGYRYLQDYGMGPETPLGEPKNKYMELGPR
DKVSQAFWHEWRKGNTISTPRGDVVYLDLRHLGEKKLHERLPFICELAKAYVGVDPVKEPIP
VRPTAHYT
>d1qlaa3 d.168.1.1 (A:251-371) Fumarate reductase flavoprotein subunit {Wolinella succinogenes}
MEAVQFHPTPLFPSGILLTEGCRGDGGILRDVDGHRFMPDYEPEKKELASRDVVSRRMIEHI

RKGKGVQSPYGQHLWLDISILGRKHIETNLRDVQEICEYFAGIDPAEKWAPVLPMQHYS
>d1e39a3 d.168.1.1 (A:360-505) Flavocytochrome c3 (respiratory fumarate reductase) {Shewanella frigidimarina}
QYIQAAPTLSVKGGVMVTEAVRGNGAILVNREGKRFVNEITTRDKASAAILAQTGKSAYLIF
DDSVRKSLSKIDKYIGLGVAPTADSLVKLGKMEGIDGKALTETVARYNSLVSSGKDTDFERP
NLPRALNEGNYYAIEVTPGVHH
>d1qo8a3 d.168.1.1 (A:360-505) Flavocytochrome c3 (respiratory fumarate reductase) {Shewanella frigidimarina}
HPTVGKDSRILISETVRGVGAVMVNKDGNRFISELTTRDKASDAILKQPGQFAWIIFDNQLY
KKAKMVRGYDHLEMLYKGDTVEQLAKSTGMKVADLAKTVSDYNGYVASGKDTAFGRADMPLN
MTQSPYYAVKVAPGIHHTMGGV
>d1d4ca3 d.168.1.1 (A:360-505) Flavocytochrome c3 (respiratory fumarate reductase) {Shewanella putrefaciens}
YIQAHPTYSPAGGVMITEAVRGNGAIVVNREGNRFMNEITTRDKASAAILQQKGESAYLVFD
DSIRKSLKAIEGYVHLNIVKEGKTIEELAKQIDVPAAELAKTVTAYNGFVKSGKDAQFERPD
LPRELVVAPFYALEIAPAVHHT
>d1jnra3 d.168.1.1 (A:257-401) Adenylylsulfate reductase A subunit {Archaeon Archaeoglobus fulgidus}
FEHRFIPFRFKDGYGPVGAWFLFFKCKAKNAYGEEYIKTRAAELEKYKPYGAAQPIPTPLRN
HQVMLEIMDGNQPIYMHTEEALAELAGGDKKKLKHIYEEAFEDFLDMTVSQALLWACQNIDP
QEQPSEAAPAEPYIMGSHSGE
>d1lit__ d.169.1.1 (-) Lithostathine, inhibitor of stone formation {Human (Homo sapiens)}
CPEGTNAYRSYCYYFNEDRETWVDADLYCQNMNSGNLVSVLTQAEGAFVASLIKESGTDDFN
VWIGLHDPKKNRRWHWSSGSLVSYKSWGIGAPSSVNPGYCVSLTSSTGFQKWKDVPCEDKFS
FVCKFKN
>d1qdda_ d.169.1.1 (A:) Lithostathine, inhibitor of stone formation {Human (Homo sapiens)}
QEAQTELPQARISCPEGTNAYRSYCYYFNEDRETWVDADLYCQNMNSGNLVSVLTQAEGAFV
ASLIKESGTDDFNVWIGLHDPKKNRAWHWSSGSLVSYKSWGIGAPSSVNPGYCVSLTSSTGF
QKWKDVPCEDKFSFVCKFKN
>d1b6e__ d.169.1.1 (-) CD94 {Human (Homo sapiens)}
CSCQEKWVGYRCNCYFISSEQKTWNESRHLCASQKSSLLQLQNTDELDFMSSSQQFYWIGLS
YSEEHTAWLWENGSALSQYLFPSFETFNTKNCIAYNPNGNALDESCEDKNRYICKQQLI
>d1e87a_ d.169.1.1 (A:) CD69 {Human (Homo sapiens)}
SSCSEDWVGYQRKCYFISTVKRSWTSAQNACSEHGATLAVIDSEKDMNFLKRYAGREEHWVG
LKKEPGHPWKWSNGKEFNNWFNVTGSDKCVFLKNTEVSSMECEKNLYWICNKPYK
>d1hq8a_ d.169.1.1 (A:) NK cell-activating receptor nkg2d {Mouse (Mus musculus)}
GYCGPCPNNWICHRNNCYQFFNEEKTWNQSQASCLSQNSSLLKIYSKEEQDFLKLVKSYHWM
GLVQIPANGSWQWEDGSSLSYNQLTLVEIPKGSCAVYGSSFKAYTEDCANLNTYICMKRAV
>d1hyra_ d.169.1.1 (A:) NK cell-activating receptor nkg2d {Human (Homo sapiens)}
ESYCGPCPKNWICYKNNCYQFFDESKNWYESQASCMSQNASLLKVYSKEDQDLLKLVKSYHW

MGLVHIPTNGSWQWEDGSILSPNLLTIIEMQKGDCALYASSFKGYIENCSTPNTYICMQRTV
>d1egia_ d.169.1.1 (A:) Macrophage mannose receptor, CRD4 {Human (Homo sapiens)}
CPEDWGASSRTSLCFKLYAKGKHEKKTWFESRDFCRALGGDLASINNKEEQQTIWRLITASG
SYHKLFWLGLTYGSPSEGFTWSDGSPVSYENWAYGEPNNYQNVEYCGELKGDPTMSWNDINC
EHLNNWICQIQ
>d1bj3a_ d.169.1.1 (A:) Snake coagglutinin {Habu snake (Trimeresurus flavoviridis)}
DCPSGWSSYEGHCYKPFKLYKTWDDAERFCTEQAKGGHLVSIESAGEADFVAQLVTENIQNT
KSYVWIGLRVQGKEKQCSSEWSDGSSVSYENWIEAESKTCLGLEKETGFRKWVNIYCGQQNP
FVCEA
>d1ixxa_ d.169.1.1 (A:) Snake coagglutinin {Habu snake (Trimeresurus flavoviridis)}
DCLSGWSSYEGHCYKAFEKYKTWEDAERVCTEQAKGAHLVSIESSGEADFVAQLVTQNMKRL
DFYIWIGLRVQGKVKQCNSEWSDGSSVSYENWIEAESKTCLGLEKETDFRKWVNIYCGQQNP
FVCEA
>d1ixxb_ d.169.1.1 (B:) Snake coagglutinin {Habu snake (Trimeresurus flavoviridis)}
DCPSDWSSYEGHCYKPFSEPKNWADAENFCTQQHAGGHLVSFQSSEEADFVVKLAFQTFGHS
IFWMGLSNVWNQCNWQWSNAAMLRYKAWAEESYCVYFKSTNNKWRSRACRMMAQFVCEFQA
>d1c3aa_ d.169.1.1 (A:) Snake coagglutinin {Habu snake (Trimeresurus flavoviridis), flavocetin-A}
DFDCIPGWSAYDRYCYQAFSKPKNWEDAESFCEEGVKTSHLVSIESSGEGDFVAQLVAEKIK
TSFQYVWIGLRIQNKEQQCRSEWSDASSVNYENLVKQFSKKCYALKKGTELRTWFNVYCGTE
NPEVCKYTPEC
>d1c3ab_ d.169.1.1 (B:) Snake coagglutinin {Habu snake (Trimeresurus flavoviridis), flavocetin-A}
GFCCPLGWSSYDEHCYQVFQQKMNWEDAEKFCTQQHKGSHLVSFHSSEEVDFVTSKTFPILK
YDFVWIGLSNVWNECTKEWSDGTKLDYKAWSGGSDCIVSKTTDNQWLSMDCSSKYYVVCKFQ
A
>d1fvua_ d.169.1.1 (A:) Snake coagglutinin {Snake (Bothrops jararaca), botrocetin}
DCPSGWSSYEGNCYKFFQQKMNWADAERFCSEQAKGGHLVSIKIYSKEKDFVGDLVTKNIQS
SDLYAWIGLRVENKEKQCSSEWSDGSSVSYENVVERTVKKCFALEKDLGFVLWINLYCAQKN
PFVCKSPPP
>d1fvub_ d.169.1.1 (B:) Snake coagglutinin {Snake (Bothrops jararaca), botrocetin}
DCPPDWSSYEGHCYRFFKEWMHWDDAEEFCTEQQTGAHLVSFQSKEEADFVRSLTSEMLKGD
VVWIGLSDVWNKCRFEWTDGMEFDYDDYYLIAEYECVASKPTNNKWWIIPCTRFKNFVCEFQ
A
>d1ioda_ d.169.1.1 (A:) Snake coagglutinin {Sharp-nosed viper (Deinagkistrodon acutus)}
DCSSGWSSYEGHCYKVFKQSKTWADAESFCTKQVNGGHLVSIESSGEADFVGQLIAQKIKSA
KIHVWIGLRAQNKEKQCSIEWSDGSSISYENWIEEESKKCLGVHIETGFHKWENFYCEQQDP

FVCEA
>d1iodb_ d.169.1.1 (B:) Snake coagglutinin {Sharp-nosed viper (Deinagkistrodon acutus)}
DCPSDWSSYEGHCYKPFNEPKNWADAENFCTQQHTGSHLVSFQSTEEADFVVKLAFQTFDYG
IFWMGLSKIWNQCNWQWSNAAMLKYTDWAEESYCVYFKSTNNKWRSITCRMIANFVCEFQA
>d1jwia_ d.169.1.1 (A:) Snake coagglutinin {Puff adder (Bitis arientans), bitiscetin}
CLPDWSSYKGHCYKVFKKVGTWEDAEKFCVENSGHLASIDSKEEADFVTKLASQTLTKFVYD
AWIGLRDESKTQQCSPQWTDGSSVVYENVDEPTKCFGLDVHTEYRTWTDLPCGEKNPFICKS
>d1jwib_ d.169.1.1 (B:) Snake coagglutinin {Puff adder (Bitis arientans), bitiscetin}
GCLPDWSSYKGHCYKVFKVEKTWADAEKFCKELVNGGHLMSVNSREEGEFISKLALEKMRIV
LVWIGLSHFWRICPLRWTDGARLDYRALSDEPICFVAESFHNKWIQWTCNRKKSFVCKYRV
>d2afpa_ d.169.1.1 (A:) Type II antifreeze protein {Sea raven (Hemitripterus americanus)}
QRAGPNCPAGWQPLGDRCIYYETTAMTWALAETNCMKLGGHLASIHSQEEHSFIQTLNAGVV
WIGGSACLQAGAWTWSDGTPMNFRSWCSTKPDDVLAACCMQMTAAADQCWDDLPCPASHKSV
CAMTF
>d1h8ua_ d.169.1.1 (A:) Eosinophil major basic protein {Human (Homo sapiens)}
RYLLVRSLQTFSQAWFTCRRCYRGNLVSIHNFNINYRIQCSVSALNQGQVWIGGRITGSGRC
RRFQWVDGSRWNFAYWAAHQPWSRGGHCVALCTRGGYWRRAHCLRRLPFICSY
>d1qo3c_ d.169.1.1 (C:) NK cell receptor ly49a {Mouse (Mus musculus)}
STVLDSLQHTGRGDKVYWFCYGMKCYYFVMDRKTWSGCKQTCQSSSLSLLKIDDEDELKFLQ
LVVPSDSCWVGLSYDNKKKDWAWIDNRPSKLALNTRKYNIRDGGCMLLSKTRLDNGNCDQVF
ICICGKRLD
>d1qo3d_ d.169.1.1 (D:) NK cell receptor ly49a {Mouse (Mus musculus)}
DKVYWFCYGMKCYYFVMDRKTWSGCKQTCQSSSLSLLKIDDEDELKFLQLVVPSDSCWVGLS
YDNKKKDWAWIDNRPSKLALNTRKYNIRDGGCMLLSKTRLDNGNCDQVFICICGKRLDK
>d1dv8a_ d.169.1.1 (A:) H1 subunit of the asialoglycoprotein receptor {Human (Homo sapiens)}
CPVNWVEHERSCYWFSRSGKAWADADNYCRLEDAHLVVVTSWEEQKFVQHHIGPVNTWMGLH
DQNGPWKWVDGTDYETGFKNWRPEQPDDWYGHGLGGGEDCAHFTDDGRWNDDVCQRPYRWVC
ETEL
>d1k9ia_ d.169.1.1 (A:) DC-SIGN (dendritic cell-specific ICAM-3 grabbing nonintegrin) {Human (Homo sapiens)}
PCPWEWTFFQGNCYFMSNSQRNWHDSITACKEVGAQLVVIKSAEEQNFLQLQSSRSNRFTWM
GLSDLNQEGTWQWVDGSPLLPSFKQYWNRGEPNNVGEEDCAEFSGNGWNDDKCNLAKFWICK
KSAA
>d1k9ja_ d.169.1.1 (A:) DC-SIGNR (DC-SIGN related receptor) {Human (Homo sapiens)}
CRHCPKDWTFFQGNCYFMSNSQRNWHDSVTACQEVRAQLVVIKTAEEQNFLQLQTSRSNRFS

WMGLSDLNQEGTWQWVDGSPLSPSFQRYWNSGEPNNSGNEDCAEFSGSGWNDNRCDVDNYWI
CKKPAA
>d1g1sa1 d.169.1.1 (A:1-118) E-selectin {Human (Homo sapiens)}
WTYHYSTKAYSWNISRKYCQNRYTDLVAIQNKNEIDYLNKVLPYYSSYYWIGIRKNNKTWTW
VGTKKALTNEAENWADNEPNNKRNNEDCVEIYIKSPSAPGKWNDEHCLKKKHALCY
>d1g1ta1 d.169.1.1 (A:1-118) E-selectin {Human (Homo sapiens)}
WSYNTSTEAMTYDEASAYCQQRYTHLVAIQNKEEIEYLNSILSYSPSYYWIGIRKVNNVWVW
VGTQKPLTEEAKNWAPGEPNNRQKDEDCVEIYIKREKDVGMWNDERCSKKKLALCY
>d1hup_1 d.169.1.1 (112-228) Mannose-binding protein A, lectin
domain {Human (Homo sapiens)}
KQVGNKFFLTNGEIMTFEKVKALCVKFQASVATPRNAAENGAIQNLIKEEAFLGITDEKTEG
QFVDLTGNRLTYTNWNEGEPNNAGSDEDCVLLLKNGQWNDVPCSTSHLAVCEFPI
>d1fifa1 d.169.1.1 (A:105-226) Mannose-binding protein A, lectin
domain {Rat (Rattus norvegicus)}
KKSGKKFFVTNHERMPFSKVKALCSELRGTVAIPRNAEENKAIQEVAKTSAFLGITDEVTEG
QFMYVTGGRLTYSNWKKDQPDDWYGHGLGGGEDCVHIVDNGLWNDDSCQRPYTAVCEFPA
>d1rdl1_ d.169.1.1 (1:) Mannose-binding protein A, lectin domain
{Rat (Rattus norvegicus)}
KYFMSSVRRMPLNRAKALCSELQGTVATPRNAEENRAIQNVAKDVAFLGITDQRTENVFEDL
TGNRVRYTNWNEGEPNNVGSGENCVVLLTNGKWNDVPCSDSFLVVCEFS
>d2msba_ d.169.1.1 (A:) Mannose-binding protein A, lectin domain
{Rat (Rattus norvegicus)}
KKFFVTNHERMPFSKVKALCSELRGTVAIPRNAEENKAIQEVAKTSAFLGITDEVTEGQFMY
VTGGRLTYSNWKKDEPNDHGSGEDCVTIVDNGLWNDISCQASHTAVCEFP
>d1b08a1 d.169.1.1 (A:235-355) Surfactant protein, lectin domain
{Human (Homo sapiens), SP-D}
PNGQSVGEKIFKTAGFVKPFTEAQLLCTQAGGQLASPRSAAENAALQQLVVAKNEAAFLSMT
DSKTEGKFTYPTGESLVYSNWAPGEPNDDGGSEDCVEIFTNGKWNDRACGEKRLVVCEF
>d1byfa_ d.169.1.1 (A:) Lectin TC14 {Tunicate (Polyandrocarpa
misakiensis)}
DYEILFSDETMNYADAGTYCQSRGMALVSSAMRDSTMVKAILAFTEVKGHDYWVGADNLQDG
AYNFLWNDGVSLPTDSDLWSPNEPSNPQSWQLCVQIWSKYNLLDDVGCGGARRVICEKELD
>d1tn3__ d.169.1.1 (-) Tetranectin {Human (Homo sapiens)}
ALQTVCLKGTKVHMKCFLAFTQTKTFHEASEDCISRGGTLSTPQTGSENDALYEYLRQSVGN
EAEIWLGLNDMAAEGTWVDMTGARIAYKNWETEITAQPDGGKTENCAVLSGAANGKWFDKRC
RDQLPYICQFGIV
>d1prtb2 d.169.1.2 (B:4-89) Pertussis toxin, S2/S3 subunits,
N-terminal domain {Bordetella pertussis}
GIVIPPQEQITQHGSPYGRCANKTRALTVAELRGSGDLQEYLRHVTRGWSIFALYDGTYLGG
EYGGVIKDGTPGGAFDLKTTFCIM
>d1prtc2 d.169.1.2 (C:4-89) Pertussis toxin, S2/S3 subunits,
N-terminal domain {Bordetella pertussis}
GIVIPPKALFTQQGGAYGRCPNGTRALTVAELRGNAELQTYLRQITPGWSIYGLYDGTYLGQ
AYGGIIKDAPPGAGFIYRETFCIT

>d1prea1 d.169.1.2 (A:2-84) Proaerolysin, N-terminal domain {Aeromonas hydrophila}
EPVYPDQLRLFSLGQGVCGDKYRPVNREEAQSVKSNIVGMMGQWQISGLANGWVIMGPGYNG
EIKPGTASNTWCYPTNPVTGE
>d1f00i3 d.169.1.3 (I:842-939) Intimin {Escherichia coli}
LIVPNMSKRVTYNDAVNTCKNFGGKLPSSQNELENVFKAWGAANKYEYYKSSQTIISWVQQT
AQDAKSGVASTYDLVKQNPLNNIKASESNAYATCVK
>d1cwva5 d.169.1.3 (A:887-986) Invasin {Yersinia pseudotuberculosis}
NRWIYDGGRSLVSSLEASRQCQGSDMSAVLESSRATNGTRAPDGTLWGEWGSLTAYSSDWQS
GEYWVKKTSTDFETMNMDTGALQPGPAYLAFPLCALSI
>d1tsg__ d.169.1.4 (-) TSG-6, Link module {Human (Homo sapiens)}
GVYHREARSGKYKLTYAEAKAVCEFEGGHLATYKQLEAARKIGFHVCAAGWMAKGRVGYPIV
KPGPNCGFGKTGIIDYGIRLNRSERWDAYCYNPHAK
>d1bnla_ d.169.1.5 (A:) Endostatin {Human (Homo sapiens)}
HSHRDFQPVLHLVALNAPLSGGMRGIRGADFQCFQQARAVGLAGTFRAFLSSRLQDLYSIVR
RADRAAVPIVNLKDELLFPSWEALFSGSEGPLKPGARIFSFDGKDVLRHPTWPQKSVWHGSD
PNGRRLTESYCETWRTEAPSATGQASSLLGGRLLGQSAASCHHAYIVLCIENSF
>d1koe__ d.169.1.5 (-) Endostatin {Mouse (Mus musculus)}
QPVLHLVALNTPLSGGMRGIRGADFQCFQQARAVGLSGTFRAFLSSRLQDLYSIVRRADRGS
VPIVNLKDEVLSPSWDSLFSGSQGQLQPGARIFSFDGRDVLRHPAWPQKSVWHGSDPSGRRL
MESYCETWRTETTGATGQASSLLSGRLLEQKAASCHNSYIVLCIENSF
>d1dy2a_ d.169.1.5 (A:) Endostatin domain of collagen alpha1(xv) {Mouse (Mus musculus)}
RPVLHLVALNTPVAGDIRADFQCFQQARAAGLLSTFRAFLSSHLQDLSTVVRKAERFGLPIV
NLKGQVLFNNWDSIFSGDGGQFNTHIPIYSFDGRDVMTDPSWPQKVVWHGSNPHGVRLVDKY
CEAWRTTDMAVTGFASPLSTGKILDQKAYSCANRLIVLCIENSF
>d1by2__ d.170.1.1 (-) M2BP {Human (Homo sapiens)}
AVNDGDMRLADGGATNQGRVEIFYRGQWGTVCDNLWDLTDASVVCRALGFENATQALGRAAF
GQGSGPIMLDEVQCTGTEASLADCKSLGWLKSNCRHERDAGVVCTNETRSTHTL
>d1mwpa_ d.170.2.1 (A:) N-terminal domain of the amyloid precursor protein {Human (Homo sapiens)}
LLAEPQIAMFCGRLNMHMNVQNGKWDSDPSGTKTCIDTKEGILQYCQEVYPELQITNVVEAN
QPVTIQNWCKRGRKQCKTHPHFVIPYRCLVGEFV
>d1fid__ d.171.1.1 (-) Fibrinogen C-terminal domains {Human (Homo sapiens), gamma}
QIHDITGKDCQDIANKGAKQSGLYFIKPLKANQQFLVYCEIDGSGNGWTVFQKRLDGSVDFK
KNWIQYKEGFGHLSPTGTTEFWLGNEKIHLISTQSAIPYALRVELEDWNGRTSTADYAMFKV
GPEADKYRLTYAYFAGGDAGDAFDGFDFGDDPSDKFFTSHNGMQFSTWDNDNDKFEGNCAEQ
DGSGWWMNKCHAGHLNGVYYQGGTYSKASTPNGYDNGIIWATWKTRWYSMKKTTMKIIPFNR
LTIGEGQQHHL
>d1fzcb1 d.171.1.1 (B:200-458) Fibrinogen C-terminal domains {Human (Homo sapiens), beta}
SCNIPVVSGKECEEIIRKGGETSEMYLIQPDSSVKPYRVYCDMNTENGGWTVIQNRQDGSVD

FGRKWDPYKQGFGNVATNTDGKNYCGLPGEYWLGNDKISQLTRMGPTELLIEMEDWKGDKVK
AHYGGFTVQNEANKYQISVNKYRGTAGNALMDGASQLMGENRTMTIHNGMFFSTYDRDNDGW
LTSDPRKQCSKEDGGGWWYNRCHAANPNGRYYWGGQYTWDMAKHGTDDGVVWMNWKGSWYSM
RKMSMKIRPFF
>d1fzda_ d.171.1.1 (A:) Fibrinogen C-terminal domains {Human (Homo sapiens), fibrinogen-420, alpha-E}
GGWLLIQQRMDGSLNFNRTWQDYKRGFGSLNDEGEGEFWLGNDYLHLLTQRGSVLRVELEDW
AGNEAYAEYHFRVGSEAEGYALQVSSYEGTAGDALIEGSVEEGAEYTSHNNMQFSTFDRDAD
QWEENCAEVYGGGWWYNNCQAANLNGIYYPGGSYDPRNNSPYEIENGVVWVSFRGADYSLRA
VRMKIRPLVTQ
>d1jfec1 d.171.1.1 (C:142-393) Fibrinogen C-terminal domains {Chicken (Gallus gallus), gamma}
TAEIQETTGRDCQDIANKGARKSGLYFIKPQKAKQSFLVYCEIDTYGNGWTVLQRRLDGSED
FRRNWVQYKEGFGHLSPDDTTEFWLGNEKIHLITTQSTLPYALRIELEDWSGKKGTADYAVF
KVGTEEDKYRLTYAYFIGGEAGDAFDGFNFGDDPSDKSYTYHNGMRFSTFDNDNDNFEGNCA
EQDGSGWWMNRCHAGHLNGPYYIGGVYSRDTGTNSYDNGIIWATWRDRWYSMKKTTMKIIPF
NRLS
>d1jfeb1 d.171.1.1 (B:200-464) Fibrinogen C-terminal domains {Chicken (Gallus gallus), beta}
SPCVASCNIPVVSGRECEDIYRKGGETSEMYIIQPDPFTTPYRVYCDMETDNGGWTLIQNRQ
DGSVNFGRAWDEYKRGFGNIAKSGGKKYCDTPGEYWLGNDKISQLTKIGPTKVLIEMEDWNG
DKVSALYGGFTIHNEGNKYQLSVSNYKGNAGNALMEGASQLYGENRTMTIHNGMYFSTYDRD
NDGWLTTDPRKQCSKEDGGGWWYNRCHAANPNGRYYWGGTYSWDMAKHGTDDGIVWMNWKGS
WYSMKKMSMKIKPYFPD
>d1jc9a_ d.171.1.1 (A:) Tachylectin 5a {Japanese horseshoe crab (Tachypleus tridentatus)}
DPTDCADILLNGYRSSGGYRIWPKSWMTVGTLNVYCDMETDGGGWTVIQRRGNYGNPSDYFY
KPWKNYKLGFGNIEKDFWLGNDRIFALTNQRNYMIRFDLKDKENDTRYAIYQDFWIENEDYL
YCLHIGNYSGDAGNSFGRHNGHNFSTIDKDHDTHETHCAQTYKGGWWYDRCHESNLNGLYLN
GEHNSYADGIEWRAWKGYHYSLPQVEMKIRPVEF
>d1g9mg_ d.172.1.1 (G:) gp120 core {Human immunodeficiency virus type 1}
EVVLVNVTENFNMWKNDMVEQMHEDIISLWDQSLKPCVKLTPLCVGAGSCNTSVITQACPKV
SFEPIPIHYCAPAGFAILKCNNKTFNGTGPCTNVSTVQCTHGIRPVVSTQLLLNGSLAEEEV
VIRSVNFTDNAKTIIVQLNTSVEINCTGAGHCNISRAKWNNTLKQIASKLREQFGNNKTIIF
KQSSGGDPEIVTHSFNCGGEFFYCNSTQLFNSTWFNSTWSTEGSNNTEGSDTITLPCRIKQI
INMWQKVGKAMYAPPISGQIRCSSNITGLLLTRDGGNSNNESEIFRPGGGDMRDNWRSELYK
YKVVKIE
>d1g9ng_ d.172.1.1 (G:) gp120 core {Human immunodeficiency virus type 1}
LENVTENFNMWKNNMVEQMHEDIISLWDQSLKPCVKLTPLCVGAGSCNTSVITQACPKVSFE
PIPIHYCAPAGFAILKCNDKKFNGTGPCTNVSTVQCTHGIRPVVSTQLLLNGSLAEEEIVIR
SENFTNNAKTIIVQLNESVVINCTGAGHCNLSKTQWENTLEQIAIKLKEQFGNNKTIIFNPS
SGGDPEIVTHSFNCGGEFFYCNSTQLFTWNDTRKLNNTGRNITLPCRIKQIINMWQEVGKAM

YAPPIRGQIRCSSNITGLLLTRDGGKDTNGTEIFRPGGGDMRDNWRSELYKYKVVKIE
>d1msk__ d.173.1.1 (-) Methionine synthase (activation domain) {Escherichia coli}
TPPVTLEAARDNDFAFDWQAYTPPVAHRLGVQEVEASIETLRNYIDWTPFFMTWSLAGKYPR
ILEDEVVGVEAQRLFKDANDMLDKLSAEKTLNPRGVVGLFPANRVGDDIEIYRDETRTHVIN
VSHHLRQQTEKTGFANYCLADFVAPKLSGKADYIGAFAVTGGLEEDALADAFEAQHDDYNKI
MVKALADRLAEAFAEYLHERVRKVYWGYAPNENLSNEELIRENYQGIRPAPGYPACPEHTEK
ATIWELLEVEKHTGMKLTESFAMWPGASVSGWYFSHPDSKYYAVAQIQRDQVEDYARRKGMS
VTEVERWLAPNLGYDAD
>d1jwka_ d.174.1.1 (A:) Nitric oxide (NO) synthase oxygenase domain {Mouse (Mus musculus)}
QYVRIKNWGSGEILHDTLHHKATSCDFTCKSKSCLGSIMNPKSLTRGPRDKPTPLEELLPHA
IEFINQYYGSFKEAKIEEHLARLEAVTKEIETTGTYQLTLDELIFATKMAWRNAPRCIGRIQ
WSNLQVFDARNCSTAQEMFQHICRHILYATNNGNIRSAITVFPQRSDGKHDFRLWNSQLIRY
AGYQMPDGTIRGDAATLEFTQLCIDLGWKPRYGRFDVLPLVLQADGQDPEVFEIPPDLVLEV
TMEHPKYEWFQELGLKWYALPAVANMLLEVGGLEFPACPFNGWYMGTEIGVRDFCDTQRYNI
LEEVGRRMGLETHTLASLWKDRAVTEINVAVLHSFQKQNVTIMDHHTASESFMKHMQNEYRA
RGGCPADWIALVPPVSGSITPVFHQEMLNYVLSPFYYYQIEPWKTHIWQN
>d1nos__ d.174.1.1 (-) Nitric oxide (NO) synthase oxygenase domain {Mouse (Mus musculus)}
NPKSLTRGPRDKPTPLEELLPHAIEFINQYYGSFKEAKIEEHLARLEAVTKEIETTGTYQLT
LDELIFATKMAWRNAPRCIGRIQWSNLQVFDARNCSTAQEMFQHICRHILYATNNGNIRSAI
TVFPQRSDGKHDFRLWNSQLIRYAGYQMPDGTIRGDAATLEFTQLCIDLGWKPRYGRFDVLP
LVLQADGQDPEVFEIPPDLVLEVTMEHPKYEWFQELGLKWYALPAVANMLLEVGGLEFPACP
FNGWYMGTEIGVRDFCDTQRYNILEEVGRRMGLETHTLASLWKDRAVTEINVAVLHSFQKQN
VTIMDHHTASESFMKHMQNEYRARGGCPADWIWLVPPVSGSITPVFHQEMLNYVLSPFYYYQ
IEPWKTHIWQNEHHHH
>d3nosa_ d.174.1.1 (A:) Nitric oxide (NO) synthase oxygenase domain {Human (Homo sapiens)}
KFPRVKNWEVGSITYDTLSAQAQQDGPCTPRRCLGSLVFPRKLQGRPSPGPPAPEQLLSQAR
DFINQYYSSIKRSGSQAHEQRLQEVEAEVAATGTYQLRESELVFGAKQAWRNAPRCVGRIQW
GKLQVFDARDCRSAQEMFTYICNHIKYATNRGNLRSAITVFPQRCPGRGDFRIWNSQLVRYA
GYRQQDGSVRGDPANVEITELCIQHGWTPGNGRFDVLPLLLQAPDEPPELFLLPPELVLEVP
LEHPTLEWFAALGLRWYALPAVSNMLLEIGGLEFPAAPFSGWYMSTEIGTRNLCDPHRYNIL
EDVAVCMDLDTRTTSSLWKDKAAVEINVAVLHSYQLAKVTIVDHHAATASFMKHLENEQKAR
GGCPADWAWIVPPISGSLTPVFHQEMVNYFLSPAFRYQPDPW
>d4nosa_ d.174.1.1 (A:) Nitric oxide (NO) synthase oxygenase domain {Human (Homo sapiens)}
RHVRIKNWGSGMTFQDTLHHKAKGILTCRSKSCLGSIMTPKSLTRGPRDKPTPPDELLPQAI
EFVNQYYGSFKEAKIEEHLARVEAVTKEIETTGTYQLTGDELIFATKQAWRNAPRCIGRIQW
SNLQVFDARSCSTAREMFEHICRHVRYSTNNGNIRSAITVFPQRSDGKHDFRVWNAQLIRYA
GYQMPDGSIRGDPANVEFTQLCIDLGWKPKYGRFDVVPLVLQANGRDPELFEIPPDLVLEVA
MEHPKYEWFRELELKWYALPAVANMLLEVGGLEFPGCPFNGWYMGTEIGVRDFCDVQRYNIL
EEVGRRMGLETHKLASLWKDQAVVEINIAVIHSFQKQNVTIMDHHSAAESFMKYMQNEYRSR

GGCPADWIWLVPPMSGSITPVFHQEMLNYVLSPFYYYQVEAWKTHVWQD
>d1d0ca_ d.174.1.1 (A:) Nitric oxide (NO) synthase oxygenase domain {Cow (Bos taurus)}
GPKFPRVKNWELGSITYDTLCAQSQQDGPCTPRRCLGSLVLPRKLQTRPSPGPPPAEQLLSQ
ARDFINQYYSSIKRSGSQAHEERLQEVEAEVASTGTYHLRESELVFGAKQAWRNAPRCVGRI
QWGKLQVFDARDCSSAQEMFTYICNHIKYATNRGNLRSAITVFPQRAPGRGDFRIWNSQLVR
YAGYRQQDGSVRGDPANVEITELCIQHGWTPGNGRFDVLPLLLQAPDEAPELFVLPPELVLE
VPLEHPTLEWFAALGLRWYALPAVSNMLLEIGGLEFSAAPFSGWYMSTEIGTRNLCDPHRYN
ILEDVAVCMDLDTRTTSSLWKDKAAVEINLAVLHSFQLAKVTIVDHHAATVSFMKHLDNEQK
ARGGCPADWAWIVPPISGSLTPVFHQEMVNYILSPAFRYQPDPW
>d1k25a3 d.175.1.1 (A:67-263) Penicillin-binding protein 2x (pbp-2x), N-terminal domain {Streptococcus pneumoniae}
QITRTVPAKRGTIYDRNGVPIAEDATSYNVYAVIDKKYKSATGKILYVEDAQFNKVAEVFHK
YLDMEESYVREQLSQPNLKQVSFGSKGNGITYANMMAIKKELETAEVKGIDFTTSPNRSYPN
GQFASSFIGLAQLHENEDGSKSLLGTSGMESSLNSILAGTDGIITYEKDRVGNIVPGTELVS
QQTVDGKDVYT
>d1qmea3 d.175.1.1 (A:71-263) Penicillin-binding protein 2x (pbp-2x), N-terminal domain {Streptococcus pneumoniae}
TVPAKRGTIYDRNGVPIAEDATSYNVYAVIDENYKSATGKILYVEKTQFNKVAEVFHKYLDM
EESYVREQLSQPNLKQVSFGAKGNGITYANMMSIKKELEAAEVKGIDFTTSPNRSYPNGQFA
SSFIGLAQLHENEDGSKSLLGTSGMESSLNSILAGTDGIITYEKDRLGNIVPGTEQVSQRTM
DGKDVYT
>d1soxa3 d.176.1.1 (A:94-343) Sulfite oxidase, middle catalytic domain {Chicken (Gallus gallus)}
QDPFAGDPPRHPGLRVNSQKPFNAEPPAELLAERFLTPNELFFTRNHLPVPAVEPSSYRLRV
DGPGGGTLSLSLAELRSRFPKHEVTATLQCAGNRRSEMSRVRPVKGLPWDIGAISTARWGGA
RLRDVLLHAGFPEELQGEWHVCFEGLDADPGGAPYGASIPYGRALSPAADVLLAYEMNGTEL
PRDHGFPVRVVVPGVVGARSVKWLRRVAVSPDESPSHWQQNDYKGFSPCVDWDTVDYRTAPA
IQ
>d1hyoa2 d.177.1.1 (A:119-416) Fumarylacetoacetate hydrolase, FAH, C-terminal domain {Mouse (Mus musculus)}
ATIGDYTDFYSSRQHATNVGIMFRGKENALLPNWLHLPVGYHGRASSIVVSGTPIRRPMGQM
RPDNSKPPVYGACRLLDMELEMAFFVGPGNRFGEPIPISKAHEHIFGMVLMNDWSARDIQQW
EYVPLGPFLGKSFGTTISPWVVPMDALMPFVVPNPKQDPKPLPYLCHSQPYTFDINLSVSLK
GEGMSQAATICRSNFKHMYWTMLQQLTHHSVNGCNLRPGDLLASGTISGSDPESFGSMLELS
WKGTKAIDVGQGQTRTFLLDGDEVIITGHCQGDGYRVGFGQCAGKVLPAL
>d1i7oa1 d.177.1.1 (A:1-213) 4-hydroxyphenylacetate degradation bifunctional isomerase/decarboxylase HpcE {Escherichia coli}
MKGTIFAVALNHRSQLDAWQEAFQQSPYKAPPKTAVWFIKPRNTVIGCGEPIPFPQGEKVLS
GATVALIVGKTATKVREEDAAEYIAGYALANDVSLPEESFYRPAIKAKCRDGFCPIGETVAL
SNVDNLTIYTEINGRPADHWNTADLQRNAAQLLSALSEFATLNPGDAILLGTPQARVEIQPG
DRVRVLAEGFPPLENPVVDEREVTTRK
>d1i7oa2 d.177.1.1 (A:214-429) 4-hydroxyphenylacetate degradation bifunctional isomerase/decarboxylase HpcE {Escherichia coli}

SFPTLPHPHGTLFALGLNYADHASELEFKPPEEPLVFLKAPNTLTGDNQTSVRPNNIEYMHY
EAELVVVIGKQARNVSEADAMDYVAGYTVCNDYAIRDYLENYYRPNLRVKSRDGLTPMLSTI
VPKEAIPDPHNLTLRTFVNGELRQQGTTADLIFSVPFLIAYLSEFMTLNPGDMIATGTPKGL
SDVVPGDEVVVEVEGVGRLVNRIVSEETAK
>d1toh__ d.178.1.1 (-) Tyrosine hydroxylase {Rat (Rattus norvegicus)}
KVPWFPRKVSELDKCHHLVTKFDPDLDLDHPGFSDQVYRQRRKLIAEIAFQYKHGEPIPHVE
YTAEEIATWKEVYVTLKGLYATHACREHLEGFQLLERYCGYREDSIPQLEDVSRFLKERTGF
QLRPVAGLLSARDFLASLAFRVFQCTQYIRHASSPMHSPEPDCCHELLGHVPMLADRTFAQF
SQDIGLASLGASDEEIEKLSTVYWFTVEFGLCKQNGELKAYGAGLLSSYGELLHSLSEEPEV
RAFDPDTAAVQPYQDQTYQPVYFVSESFNDAKDKLRNYASRIQRPFSVKFDPYTLAIDVLDS
PHTIQRSLEGVQDELHTLAHALSAIS
>d3pah__ d.178.1.1 (-) Phenylalanine hydroxylase {Human (Homo sapiens)}
TVPWFPRTIQELDRFANQILSYGAELDADHPGFKDPVYRARRKQFADIAYNYRHGQPIPRVE
YMEEEKKTWGTVFKTLKSLYKTHACYEYNHIFPLLEKYCGFHEDNIPQLEDVSQFLQTCTGF
RLRPVAGLLSSRDFLGGLAFRVFHCTQYIRHGSKPMYTPEPDICHELLGHVPLFSDRSFAQF
SQEIGLASLGAPDEYIEKLATIYWFTVEFGLCKQGDSIKAYGAGLLSSFGELQYCLSEKPKL
LPLELEKTAIQNYTVTEFQPLYYVAESFNDAKEKVRNFAATIPRPFSVRYDPYTQRIEVL
>d1phza2 d.178.1.1 (A:116-427) Phenylalanine hydroxylase {Rat (Rattus norvegicus)}
NTVPWFPRTIQELDRFANQILSYGAELDADHPGFKDPVYRARRKQFADIAYNYRHGQPIPRV
EYTEEEKQTWGTVFRTLKALYKTHACYEHNHIFPLLEKYCGFREDNIPQLEDVSQFLQTCTG
FRLRPVAGLLSSRDFLGGLAFRVFHCTQYIRHGSKPMYTPEPDICHELLGHVPLFSDRSFAQ
FSQEIGLASLGAPDEYIEKLATIYWFTVEFGLCKEGDSIKAYGAGLLSSFGELQYCLSDKPK
LLPLELEKTACQEYSVTEFQPLYYVAESFSDAKEKVRTFAATIPRPFSVRYDPYTQRVEVLD
NT
>d1dqaa4 d.179.1.1 (A:462-586,A:704-870) Substrate-binding domain of HMG-CoA reductase {Human (Homo sapiens)}
LSDAEIIQLVNAKHIPAYKLETLIETHERGVSIRRQLLSKKLSEPSSLQYLPYRDYNYSLVM
GACCENVIGYMPIPVGVAGPLCLDEKEFQVPMATTEGCLVASTNRGCRAIGLGGGASSRVLA
DXKSVVCEAVIPAKVVREVLKTTTEAMIEVNINKNLVGSAMAGSIGGYNAHAANIVTAIYIA
CGQDAAQNVGSSNCITLMEASGPTNEDLYISCTMPSIEIGTVGGGTNLLPQQACLQMLGVQG
ACKDNPGENARQLARIVCGTVMAGELSLMAALAAGHLVKSHMIHN
>d1hw8a2 d.179.1.1 (A:441-586,A:704-861) Substrate-binding domain of HMG-CoA reductase {Human (Homo sapiens)}
EPRPNEECLQILGNAEKGAKFLSDAEIIQLVNAKHIPAYKLETLIETHERGVSIRRQLLSKK
LSEPSSLQYLPYRDYNYSLVMGACCENVIGYMPIPVGVAGPLCLDEKEFQVPMATTEGCLVA
STNRGCRAIGLGGGASSRVLADXKSVVCEAVIPAKVVREVLKTTTEAMIEVNINKNLVGSAM
AGSIGGYNAHAANIVTAIYIACGQDAAQNVGSSNCITLMEASGPTNEDLYISCTMPSIEIGT
VGGGTNLLPQQACLQMLGVQGACKDNPGENARQLARIVCGTVMAGELSLMAALAAGH
>d1hw8c2 d.179.1.1 (C:488-586,C:704-860) Substrate-binding domain of HMG-CoA reductase {Human (Homo sapiens)}
HERGVSIRRQLLSKKLSEPSSLQYLPYRDYNYSLVMGACCENVIGYMPIPVGVAGPLCLDEK

EFQVPMATTEGCLVASTNRGCRAIGLGGGASSRVLADXKSVVCEAVIPAKVVREVLKTTTEA
MIEVNINKNLVGSAMAGSIGGYNAHAANIVTAIYIACGQDAAQNVGSSNCITLMEASGPTNE
DLYISCTMPSIEIGTVGGGTNLLPQQACLQMLGVQGACKDNPGENARQLARIVCGTVMAGEL
SLMAALAAG
>d1qaxa2 d.179.1.1 (A:4-110,A:221-428) Substrate-binding domain of HMG-CoA reductase {Pseudomonas mevalonii}
DSRLPAFRNLSPAARLDHIGQLLGLSHDDVSLLANAGALPMDIANGMIENVIGTFELPYAVA
SNFQINGRDVLVPLVVEEPSIVAAASYMAKLARANGGFTTSSSAPXRLARAQVRITPQQLET
AEFSGEAVIEGILDAYAFAAVDPYRAATHNKGIMNGIDPLIVATGNDWRAVEAGAHAYACRS
GHYGSLTTWEKDNNGHLVGTLEMPMPVGLVGGATKTHPLAQLSLRILGVKTAQALAEIAVAV
GLAQNLGAMRALATEGIQRGHMALHARNIAVVAGARGDEVDWVARQLVEYHDVRADRAVALL
KQKRGQ
>d1qaxb2 d.179.1.1 (B:504-610,B:721-875) Substrate-binding domain of HMG-CoA reductase {Pseudomonas mevalonii}
DSRLPAFRNLSPAARLDHIGQLLGLSHDDVSLLANAGALPMDIANGMIENVIGTFELPYAVA
SNFQINGRDVLVPLVVEEPSIVAAASYMAKLARANGGFTTSSSAPXRLARAQVRITPQQLET
AEFSGEAVIEGILDAYAFAAVDPYRAATHNKGIMNGIDPLIVATGNDWRAVEAGAHAYACRS
GHYGSLTTWEKDNNGHLVGTLEMPMPVGLVGGATKTHPLAQLSLRILGVKTAQALAEIAVAV
GLAQNLGAMRALATE
>d1k92a2 d.210.1.1 (A:189-444) Argininosuccinate synthetase, C-terminal domain {Escherichia coli}
AYSTDSNMLGATHEAKDLEYLNSSVKIVNPIMGVKFWDESVKIPAEEVTVRFEQGHPVALNG
KTFSDDVEMMLEANRIGGRHGLGMSDQIENRIIEAKSRGIYEAPGMALLHIAYERLLTGIHN
EDTIEQYHAHGRQLGRLLYQGRWFDSQALMLRDSLQRWVASQITGEVTLELRRGNDYSILNT
VSENLTYKPERLTMEKGDSVFSPDDRIGQLTMRNLDITDTREKLFGYAKTGLLSSSAASGVP
QVENLENK
>d16vpa_ d.180.1.1 (A:) Conserved core of transcriptional regulatory protein vp16 {Herpes simplex virus type 1}
SRMPSPPMPVPPAALFNRLLDDLGFSAGPALCTMLDTWNEDLFSALPTNADLYRECKFLSTL
PSDVVEWGDAYVPERTQIDIRAHGDVAFPTLPATRDGLGLYYEALSRFFHAELRAREESYRT
VLANFCSALYRYLRASVRQLHRQAHMRGRDRDLGEMLRATIADRYYRETARLARVLFLHLYL
FLTREILWAAYAEQMMRPDLFDCLCCDLESWRQLAGLFQPFMFVNGALTVRGVPIEARRLRE
LNHIREHLNLPLVRSAATEEPGAPLTTPPTLHGNQARASGYFMVLIRAKLDSYSSFTTSPSE
AVMREHAYSRAPTKNNYGSTIEGLLDLPDDDAPEEAGLAAPRLSFL
>d1bdfa2 d.181.1.1 (A:53-178) RNA polymerase alpha subunit {Escherichia coli}
GCAVTEVEIDGVLHEYSTKEGVQEDILEILLNLKGLAVRVQGKDEVILTLNKSGIGPVTAAD
ITHDGDVEIVKPQHVICHLTDENASISMRIKVQRGRGYVPASTRIHSEEDERPIGRLLVDAC
YS
>d1i6va2 d.181.1.1 (A:50-172) RNA polymerase alpha subunit {Thermus aquaticus}
GTAVTSVYIEDVLHEFSTIPGVKEDVVEIILNLKELVVRFLDPKMASTTLILRAEGPKEVRA
VDFTPSADVEIMNPDLHIATLEEGGKLYMEVRVDRGVGYVPAERHGIKDRINAIPVDAIFS
>d1i50c2 d.181.1.1 (C:42-172) RPB3 {Baker's yeast (Saccharomyces

cerevisiae)}
PTLAIDSVEVETNTTVLADEFIAHRLGLIPLQSMDIEQLEYSRDCFCEDHCDKCSVVLTLQA
FGESESTTNVYSKDLVIVSNLMGRNIGHPIIQDKEGNGVLICKLRKGQELKLTCVAKKGIAK
EHAKWGP
>d1el6a_ d.182.1.1 (A:) Baseplate structural protein gp11 {Bacteriophage T4}
SRLADFLGFRPKTGDIDVMNRQSVGSVTISQLAKGFYEPNIESAINDVHNFSIKDVGTIITN
KTGVSPEGVSQTDYWAFSGTVTDDSLPPGSPITVLVFGLPVSATTGMTAIEFVAKVRVALQE
AIASFTAINSYKDHPTDGSKLEVTYLDNQKHVLSTYSTYGITISQEIISESKPGYGTWNLLG
AQTVTLDNQQTPTVFYHFERTA
>d1fh6a_ d.183.1.1 (A:) Major capsid protein gp5 {Bacteriophage HK97}
SLGSDADSAGSLIQPMQIPGIIMPGLRRLTIRDLLAQGRTSSNALEYVREEVFTNNADVVAE
KALKPESDITFSKQTANVKTIAHWVQASRQVMDDAPMLQSYINNRLMYGLALKEEGQLLNGD
GTGDNLEGLNKVATAYDTSLNATGDTRADIIAHAIYQVTESEFSASGIVLNPRDWHNIALLK
DNEGRYIFGGPQAFTSNIMWGLPVVPTKAQAAGTFTVGGFDMASQVWDRMDATVEVSREDRD
NFVKNMLTILCEERLALAHYRPTAIIKGTFSS
>d1fn9a_ d.196.1.1 (A:) Outer capsid protein sigma 3 {Reovirus}
MEVCLPNGHQVVDLINNAFEGRVSIYSAQEGWDKTISAQPDMMVCGGAVVCMHCLGVVGSLQ
RKLKHLPHHRCNQQIRHQDYVDVQFADRVTAHWKRGMLSFVAQMHEMMNDVSPDDLDRVRTE
GGSLVELNWLQVDPNSMFRSIHSSWTDPLQVVDDLDTKLDQYWTALNLMIDSSDLIPNFMMR
DPSHAFNGVKLGGDARQTQFSRTFDSRSSLEWGVMVYDYSELEHDPSKGRAYRKELVTPARD
FGHFGLSHYSRATTPILGKMPAVFSGMLTGNCKMYPFIKGTAKLKTVRKLVEAVNHAWGVEK
IRYALGPGGMTGWYNRTMQQAPIVLTPAALTMFPDTIKFGDLNYPVMIGDPMILG
>d1qgwa_ d.184.1.1 (A:) Phycoerythrin 545 alpha-subunits {Cryptophyte (Rhodomonas sp.), cs24}
AMDKSAKAPQITIFDHRGCSRAPKESTGGKAGGQDDEMMVKVASTKVTVSESDAAKKLQEFI
TFEKGIDGPFTSKN
>d1qgwb_ d.184.1.1 (B:) Phycoerythrin 545 alpha-subunits {Cryptophyte (Rhodomonas sp.), cs24}
AMDKSAKAPVITIFDHRGCSRAPKEYTGAKAGGKDDEMMVKAQSVKIEVSTGTAEGVLATSL
AKMTK
>d1jyoe_ d.184.1.2 (E:) Virulence effector SptP domain {Salmonella typhimurium}
DKAYVAPEKFSSKVLTWLGKMPLFKNTEVVQKHTENIRVQDQKILQTFLHALTEKYGETAVN
DALLMSRINMNKPLTQRLAVQITECVKAADEGFINLIKSK
>g1hle.1 e.1.1.1 (A:,B:) Elastase inhibitor {Horse (Equus caballus)}
MEQLSTANTHFAVDLFRALNESDPTGNIFISPLSISSALAMIFLGTRGNTAAQVSKALYFDT
VEDIHSRFQSLNADINKPGAPYILKLANRLYGEKTYNFLADFLASTQKMYGAELASVDFQQA
PEDARKEINEWVKGQTEGKIPELLVKGMVDNMTKLVLVNAIYFKGNWQQKFMKEATRDAPFR
LNKKDTKTVKMMYQKKKFPYNYIEDLKCRVLELPYQGKELSMIILLPDDIEDESTGLEKIEK
QLTLDKLREWTKPENLYLAEVNVHLPRFKLEESYDLTSHLARLGVQDLFNRGKADLSGMSGA
RDLFVSKIIHKSFVDLNEEGTEAAAATAGTILLAXEENFNADHPFIFFIRHNPSANILFLGR

FSSP
>d1ovaa_ e.1.1.1 (A:) Ovalbumin {Hen (Gallus gallus)}
GSIGAASMEFCFDVFKELKVHHANENIFYCPIAIMSALAMVYLGAKDSTRTQINKVVRFDKL
PGFGDSIEAQCGTSVNVHSSLRDILNQITKPNDVYSFSLASRLYAEERYPILPEYLQCVKEL
YRGGLEPINFQTAADQARELINSWVESQTNGIIRNVLQPSSVDSQTAMVLVNAIVFKGLWEK
AFKDEDTQAMPFRVTEQESKPVQMMYQIGLFRVASMASEKMKILELPFASGTMSMLVLLPDE
VSGLEQLESIINFEKLTEWTSSNVMEERKIKVYLPRMKMEEKYNLTSVLMAMGITDVFSSSA
NLSGISSAESLKISQAVHAAHAEINEAGREVVGSAEAGVDAASVSEEFRADHPFLFCIKHIA
TNAVLFFGRCVSP
>g1as4.1 e.1.1.1 (A:,B:) Antichymotrypsin, alpha-1 {Human (Homo sapiens)}
GLASANVDFAFSLYKQLVLKAPDKNVIFSPLSISTALAFLSLGAHNTTLTEILKGLKFNLTE
TSEAEIHQSFQHLLRTLNQSSDELQLSMGNAMFVKEQLSLLDRFTEDAKRLYGSEAFATDFQ
DSAAAKKLINDYVKNGTRGKITDLIKDLDSQTMMVLVNYIFFKAKWEMPFDPQDTHQSRFYL
SKKKWVMVPMMSLHHLTIPYFRDEELSCTVVELKYTGNASALFILPDQDKMEEVEAMLLPET
LKRWRDSLEFREIGELYLPKFSISRDYNLNDILLQLGIEEAFTSKADLSGITGARNLAVSQV
VHKAVLDVFEEGTEASRATAVKITLLXGTIVRFNRPFLMIIVPTDTQNIFFMSKVTNPKQ
>d1qlpa_ e.1.1.1 (A:) Antitrypsin, alpha-1 {Human (Homo sapiens)}
FNKITPNLAEFAFSLYRQLAHQSNSTNIFFSPVSIATAFAMLSLGTKADTHDEILEGLNFNL
TEIPEAQIHEGFQELLRTLNQPDSQLQLTTGNGLFLSEGLKLVDKFLEDVKKLYHSEAFTVN
FGDTEEAKKQINDYVEKGTQGKIVDLVKELDRDTVFALVNYIFFKGKWERPFEVKDTEEEDF
HVDQVTTVKVPMMKRLGMFNIQHCKKLSSWVLLMKYLGNATAIFFLPDEGKLQHLENELTHD
IITKFLENEDRRSASLHLPKLSITGTYDLKSVLGQLGITKVFSNGADLSGVTEEAPLKLSKA
VHKAVLTIDEKGTEAAGAMFLEAIPMSIPPEVKFNKPFVFLMIEQNTKSPLFMGKVVNPTQK
>d1atta_ e.1.1.1 (A:) Antithrombin {Cow (Bos taurus)}
VEDVCTAKPRDIPVNPMCIYRATEGQGSEQKIPGATNRRVWELSKANSHFATAFYQHLADSK
NNNDNIFLSPLSISTAFAMTKLGACNNTLTQLMEVFKFDTISEKTSDQIHFFFAKLNCRLYR
KANKSSELVSANRLFGDKSITFNETYQDISEVVYGAKLQPLDFKGNAEQSRLTINQWISNKT
EGRITDVIPPQAINEFTVLVLVNTIYFKGLWKSKFSPENTRKELFYKADGESCSVLMMYQES
KFRYRRVAESTQVLELPFKGDDITMVLILPKLEKTLAKVEQELTPDMLQEWLDELTETLLVV
HMPRFRIEDSFSVKEQLQDMGLEDLFSPEKSRLPGIVAEGRSDLYVSDAFHKAFLEVNEEGS
EAAASTVISIAGRSLRVTFKANRPFLVLIREVALNTIIFMGRVANPCVD
>d1e05i_ e.1.1.1 (I:) Antithrombin {Human (Homo sapiens)}
SPVDICTAKPRDIPMNPMCIYRSPEKKATEDEGSEQKIPEATNRRVWELSKANSRFATTFYQ
HLADSKNDNDNIFLSPLSISTAFAMTKLGACNDTLQQLMEVFKFDTISEKTSDQIHFFFAKL
NCRLYRKANKSSKLVSANRLFGDKSLTFNETYQDISELVYGAKLQPLDFKENAEQSRAAINK
WVSNKTEGRITDVIPSEAINELTVLVLVNTIYFKGLWKSKFSPENTRKELFYKADGESCSAS
MMYQEGKFRYRRVAEGTQVLELPFKGDDITMVLILPKPEKSLAKVEKELTPEVLQEWLDELE
EMMLVVHMPRFRIEDGFSLKEQLQDMGLVDLFSPEKSKLPGIVAEGRDDLYVSDAFHKAFLE
VNEEGSEAAASTAVVIAGRSLNPNRVTFKANRPFLVFIREVPLNTIIFMGRVANPCV
>d1a7ca_ e.1.1.1 (A:) Plasminogen activator inhibitor-1 {Human (Homo sapiens)}
HHPPSYVAHLASDFGVRVFQQVAQASKDRNVVFSPYGVASVLAMLQLTTGGETQQQIQAAMG
FKIDDKGMAPALRHLYKELMGPWNKDEISTTDAIFVQRDLKLVQGFMPHFFRLFRSTVKQVD

FSEVERARFIINDWVKTHTKGMISNLLGKGAVDQLTRLVLVNALYFNGQWKTPFPDSSTHRR
LFHKSDGSTVSVPMMAQTNKFNYTEFTTPDGHYYDILELPYHGDTLSMFIAAPYEKEVPLSA
LTNILSAQLISHWKGNMTRLPRLLVLPKFSLETEVDLRKPLENLGMTDMFRQFQADFTSLSD
QEPLHVAQALQKVKIEVNESGTVESSSTAVIVSARMAPEEIIMDRPFLFVVRHNPTGTVLFM
GQVMEP
>d1by7a_ e.1.1.1 (A:) Plasminogen activator inhibitor-2 {Human (Homo sapiens)}
EDLCVANTLFALNLFKHLAKASPTQNLFLSPWSISSTMAMVYMGSRGSTEDQMAKVLQFNEV
GAAADKIHSSFRSLSSAINASTGNYLLESVNKLFGEKSASFREEYIRLCQKYYSSEPQAVDF
LECAEEARKKINSWVKTQTKGKIPNLLPEGSVDGDTRMVLVNAVYFKGKWKTPFEKKLNGLY
PFRVNSAQRTPVQMMYLREKLNIGYIEDLKAQILELPYAGDVSMFLLLPDEIADVSTGLELL
ESEITYDKLNKWTSKDKMAEDEVEVYIPQFKLEEHYELRSILRSMGMEDAFNKGRANFSGMS
ERNDLFLSEVFHQAMVDVNEEGTEAAAGTGGVMTGRTGHGGPQFVADHPFLFLIMHKITNCI
LFFGRFSSP
>g1jjo.1 e.1.1.1 (A:,C:,E:) Neuroserpin {Mouse (Mus musculus)}
TITEWSVNMYNHLRGTGEDENILFSPLSIALAMGMMELGAXENQYVMKLANSLFVQNGFHVN
EEFLQMLKMYFNAEVNHVDFSQNVAVANSINKWVENYTNSLLKDLVSPEDFDGVTNLALINA
VYFKGNWKSQFRPENTRTFSFTKDDESEVQIPMMYQQGEFYYGEFSDGSNEAGGIYQVLEIP
YEGDEISMMLALSRQEVPLATLEPLLKAQLIEEWANSVKKQKVEVYLPRFTVEQEIDLKDIL
KALGVTEIFIKDANLTAMSDKKELFLSKAVHKSCIEVNEEGSEAAAASGMIAISXYPQVIVD
HPFLYLIRNRKSGIILFMGRVMNPHH
>d1sek__ e.1.1.1 (-) Serpin K {Tobacco hawkmoth (Manduca sexta)}
GETDLQKILRESNDQFTAQMFSEVVKANPGQNVVLSAFSVLPPLGQLALASVGESHDELLRA
LALPNDNVTKDVFADLNRGVRAVKGVDLKMASKIYVAKGLELNDDFAAVSRDVFGSEVQNVD
FVKSVEAAGAINKWVEDQTNNRIKNLVDPDALDETTRSVLVNAIYFKGSWKDKFNKERTMDR
DFHVSKDKTIKVPTMIGKKDVRYADVPELDAKMIEMSYEGDQASMIIILPNQVDGITALEQK
LKDPKALSRAEERLYNTEVEIYLPKFKIETTTDLKEVLSNMNIKKLFTPGAARLENLLKTKE
SLYVDAAIQKAFIEVNEEGAEAAAANAFKITTYSFHFVPKVEINKPFFFSLKYNRNSMFSGV
CVQP
>d1k9oi_ e.1.1.1 (I:) Alaserpin (serpin 1) {Tobacco hornworm (Manduca sexta)}
GETDLQKILRESNDQFTAQMFSEVVKANPGQNVVLSAFSVLPPLGQLALASVGESHDELLRA
LALPNDNVTKDVFADLNRGVRAVKGVDLKMASKIYVAKGLELNDDFAAVSRDVFGSEVQNVD
FVKSVEAAGAINKWVEDQTNNRIKNLVDPDALDETTRSVLVNAIYFKGSWKDKFVKERTMDR
DFHVSKDKTIKVPTMIGKKDVRYADVPELDAKMIEMSYEGDQASMIIILPNQVDGITALEQK
LKDPKALSRAEERLYNTEVEITLPKFKIETTTDLKEVLSNMNIKKLFTPGAARLENLLKTKE
SLTVDAAIQKAFIEVNEEGAEAAAANAFGIVPKSLILYPEVHIDRPFYFELKIDGIPMFNGK
VIEP
>g1f0c.1 e.1.1.1 (A:,B:) Viral serpin crmA (cytokine response modifier protein) {Cowpox virus}
MDIFREIASSMKGENVFISPPSISSVLTILYYGANGSTAEQLSKYVEKEADKNKDDISFKSM
NKVYGRYSAVFKDSFLRKIGDNFQTVDFTDCRTVDAINKCVDIFTEGKINPLLDEPLSPDTC
LLAISAVYFKAKWLMPFEKEFTSDYPFYVSPTEMVDVSMMSMYGEAFNHASVKESFGNFSII
ELPYVGDTSMVVILPDNIDGLESIEQNLTDTNFKKWCDSMDAMFIDVHIPKFKVTGSYNLVD

ALVKLGLTEVFGSTGDYSNMCNSDVSVDAMIHKTYIDVNEEYTEAAAATCALVADCAXSTVT
NEFCADHPFIYVIRHVDGKILFVGRYCSPTTN
>d1imva_ e.1.1.1 (A:) Rigment epithelium-derived factor, PEDF
{Human (Homo sapiens)}
TGALVEEEDPFFKVPVNKLAAAVSNFGYDLYRVRSSMSPTTNVLLSPLSVATALSALSLGAD
ERTESIIHRALYYDLISSPDIHGTYKELLDTVTAPQKNLKSASRIVFEKKLRIKSSFVAPLE
KSYGTRPRVLTGNPRLDLQEINNWVQAQMKGKLARSTKEIPDEISILLLGVAHFKGQWVTKF
DSRKTSLEDFYLDEERTVRVPMMSDPKAVLRYGLDSDLSCKIAQLPLTGSMSIIFFLPLKVT
QNLTLIEESLTSEFIHDIDRELKTVQAVLTVPKLKLSYEGEVTKSLQEMKLQSLFDSPDFSK
ITGKPIKLTQVEHRAGFEWNEDGAGTTPSPGLQPAHLTFPLDYHLNQPFIFVLRDTDTGALL
FIGKILDPRGP
>d1ecra_ e.2.1.1 (A:) Replication terminator protein (Tus)
{Escherichia coli}
DLVDRLNTTFRQMEQELAIFAAHLEQHKLLVARVFSLPEVKKEDEHNPLNRIEVKQHLGNDA
QSLALRHFRHLFIQQQSENRSSKAAVRLPGVLCYQVDNLSQAALVSHIQHINKLKTTFEHIV
TVESELPTAARFEWVHRHLPGLITLNAYRTLTVLHDPATLRFGWANKHIIKNLHRDEVLAQL
EKSLKSPRSVAPWTREEWQRKLEREYQDIAALPQNAKLKIKRPVKVQPIARVWYKGDQKQVQ
HACPTPLIALINRDNGAGVPDVGELLNYDADNVQHRYKPQAQPLRLIIPRLHLYVAD
>d1es5a_ e.3.1.1 (A:) D-ala carboxypeptidase/transpeptidase
{Streptomyces sp., K15}
KPTIAAVGGYAMNNGTGTTLYTKAADTRRSTGSTTKIMTAKVVLAQSNLNLDAKVTIQKAYS
DYVVANNASQAHLIVGDKVTVRQLLYGLMLPSGCDAAYALADKYGSGSTRAARVKSFIGKMN
TAATNLGLHNTHFDSFDGIGNGANYSTPRDLTKIASSAMKNSTFRTVVKTKAYTAKTVTKTG
SIRTMDTWKNTNGLLSSYSGAIGVKTGAGPEAKYCLVFAATRGGKTVIGTVLASTSIPARES
DATKIMNYGFAL
>d1hvba_ e.3.1.1 (A:) D-ala carboxypeptidase/transpeptidase
{Streptomyces sp., R61}
DLPAPDDTGLQAVLHTALSQGAPGAMVRVDDNGTIHQLSEGVADRATGRAITTTDRFRVGSV
TKSFSAVVLLQLVDEGKLDLDASVNTYLPGLLPDDRITVRQVMSHRSGLYDYTNDMFAQTVP
GFESVRNKVFSYQDLITLSLKHGVTNAPGAAYSYSNTNFVVAGMLIEKLTGHSVATEYQNRI
FTPLNLTDTFYVHPDTVIPGTHANGYLTPDEAGGALVDSTEQTVSWAQSAGAVISSTQDLDT
FFSALMSGQLMSAAQLAQMQQWTTVNSTQGYGLGLRRRDLSCGISVYGHTGTVQGYYTYAFA
SKDGKRSVTALANTSNNVNVLNTMARTLESAFCGKP
>d1ci9a_ e.3.1.1 (A:) Esterase EstB {Burkholderia gladioli}
AASLAARLDAVFDQALRERRLVGAVAIVARHGEILYRRAQGLADREAGRPMREDTLFRLASV
TKPIVALAVLRLVARGELALDAPVTRWLPEFRPRLADGSEPLVTIHHLLTHTSGLGYWLLEG
AGSVYDRLGISDGIDLRDFDLDENLRRLASAPLSFAPGSGWQYSLALDVLGAVVERATGQPL
AAAVDALVAQPLGMRDCGFVSAEPERFAVPYHDGQPEPVRMRDGIEVPLPEGHGAAVRFAPS
RVFEPGAYPSGGAGMYGSADDVLRALEAIRANPGFLPETLADAARRDQAGVGAETRGPGWGF
GYLSAVLDDPAAAGTPQHAGTLQWGGVYGHSWFVDRALGLSVLLLTNTAYEGMSGPLTIALR
DAVYA
>d1btl__ e.3.1.1 (-) beta-Lactamase, class A {Escherichia coli,
TEM-1}
HPETLVKVKDAEDQLGARVGYIELDLNSGKILESFRPEERFPMMSTFKVLLCGAVLSRIDAG

QEQLGRRIHYSQNDLVEYSPVTEKHLTDGMTVRELCSAAITMSDNTAANLLLTTIGGPKELT
AFLHNMGDHVTRLDRWEPELNEAIPNDERDTTMPVAMATTLRKLLTGELLTLASRQQLIDWM
EADKVAGPLLRSALPAGWFIADKSGAGERGSRGIIAALGPDGKPSRIVVIYTTGSQATMDER
NRQIAEIGASLIKHW
>d1bza__ e.3.1.1 (-) beta-Lactamase, class A {Escherichia coli, TOHO-1}
SVQQQLEALEKSSGGRLGVALINTADNSQILYRADERFAMCSTSKVMAAAAVLKQSESDKHL
LNQRVEIKKSDLVNYNPIAEKHVNGTMTLAELGAAALQYSDNTAMNKLIAHLGGPDKVTAFA
RSLGDETFRLDRTAPTLNTAIPGDPRDTTTPLAMAQTLKNLTLGKALAETQRAQLVTWLKGN
TTGSASIRAGLPKSWVVGDKTGSGDYGTTNDIAVIWPENHAPLVLVTYFTQPEQKAERRRDI
LAAAAKIVT
>d1g56a_ e.3.1.1 (A:) beta-Lactamase, class A {Klebsiella pneumoniae, SHV-1}
SPQPLEQIKLSESQLSGRVGMIEMDLASGRTLTAWRADERFPMMSTFKVVLCGAVLARVDAG
DEQLERKIHYRQQDLVDYSPVSEKHLADGMTVGELCAAAITMSDNSAANLLLATVGGPAGLT
AFLRQIGDNVTRLDRWETELNEALPGDARDTTTPASMAATLRKLLTSQRLSARSQRQLLQWM
VDDRVAGPLIRSVLPAGWFIADKTGAGERGARGIVALLGPNNKAERIVVIYLRDTPASMAER
NQQIAGIGAALIEHWQR
>d1g6aa_ e.3.1.1 (A:) beta-Lactamase, class A {Pseudomonas aeruginosa, PSE-4 carbenicillinase}
SKFQQVEQDVKAIEVSLSARIGVSVLDTQNGEYWDYNGNQRFPLTSTFKTIACAKLLYDAEQ
GKVNPNSTVEIKKADLVTYSPVIEKQVGQAITLDDACFATMTTSDNTAANIILSAVGGPKGV
TDFLRQIGDKETRLDRIEPDLNEGKLGDLRDTTTPKAIASTLNKFLFGSALSEMNQKKLESW
MVNNQVTGNLLRSVLPAGWNIADKSGAGGFGARSITAVVWSEHQAPIIVSIYLAQTQASMEE
RNDAIVKIGHSIFDVYTS
>d1alq__ e.3.1.1 (-) beta-Lactamase, class A {Staphylococcus aureus}
SEPIVLVIFTNKDNKSDKPNDKLISETAKSVMKEFAAGSKNAAKELNDLEKKYNAHIGVYAL
DTKSGKEVKFNSDKRFAYASTSKAINSAILLEQVPYNKLNKKVHINKDDIVAYSPILEKYVG
KDITLKALIEASMTYSDNTANNKIIKEIGGIKKVKQRLKELGDKVTNPVRYEIELNYYSPKS
KKDTSTPAAFGKTLNKLIANGKLSKENKKFLLDLMLNNKSGDTLIKDGVPKDYKVADKSGQA
ITYASRNDVAFVYPK
>d1ghpa_ e.3.1.1 (A:) beta-Lactamase, class A {Staphylococcus aureus}
KELNDLEKKYNAHIGVYALDTKSGKEVKFNSDKRFAYASTSKAINSAILLEQVPYNKLNKKV
HINKDDIVAYSPILEKYVGKDITLKALIEASMTYSDNTANNKIIKEIGGIKKVKQRLKELGD
KVTNPVRYDIELQYYSPKSKKDTSTPAAFGKTLNKLIANGKLSKENKKFLLDLMLNNKSGDT
LIKDGVPKDYKVADKSGQAITYASRNDVAFVYPKGQSEPIVLVIFTNKDNKSDKPNDKLISE
TAKSVMKEF
>d4blma_ e.3.1.1 (A:) beta-Lactamase, class A {Bacillus licheniformis}
DDFAKLEEQFDAKLGIFALDTGTNRTVAYRPDERFAFASTIKALTVGVLLQQKSIEDLNQRI
TYTRDDLVNYNPITEKHVDTGMTLKELADASLRYSDNAAQNLILKQIGGPESLKKELRKIGD
EVTNPERFEPELNEVNPGETQDTSTARALVTSLRAFALEDKLPSEKRELLIDWMKRNTTGDA

LIRAGVPDGWEVADKTGAASYGTRNDIAIIWPPKGDPVVLAVLSSRDKKDAKYDDKLIAEAT
KVVMKALN
>d1buea_ e.3.1.1 (A:) beta-Lactamase, class A {Enterobacter cloacae, NMC-A carbapenemase}
NTKGIDEIKNLETDFNGRIGVYALDTGSGKSFSYRANERFPLCSSFKGFLAAAVLKGSQDNR
LNLNQIVNYNTRSLEFHSPITTKYKDNGMSLGDMAAAALQYSDNGATNIILERYIGGPEGMT
KFMRSIGDEDFRLDRWELDLNTAIPGDERDTSTPAAVAKSLKTLALGNILSEHEKETYQTWL
KGNTTGAARIRASVPSDWVVGDKTGSCGAYGTANDYAVVWPKNRAPLIISVYTTKNEKEAKH
EDKVIAEASRIAIDNLK
>d1bsg__ e.3.1.1 (-) beta-Lactamase, class A {Streptomyces albus G}
SDAERRLAGLERASGARLGVYAYDTGSGRTVAYRADELFPMCSVFKTLSSAAVLRDLDRNGE
FLSRRILYTQDDVEQADGAPETGKPQNLANGMTVEELCEVSITASDNCAANLMLRELGGPAA
VTRFVRSLGDRVTRLDRWEPELNSAEPGRVTDTTSPRAITRTYGRLVLGDALNPRDRRLLTS
WLLANTTSGDRFRAGLPDDWTLGDKTGAGRYGTNNDAGVTWPPGRAPIVLTVLTAKTEQDAA
RDDGLVADAARVLAETLG
>d1mfoa_ e.3.1.1 (A:) beta-Lactamase, class A {Mycobacterium fortuitum}
APIDDQLAELERRDNVLIGLYAANLQSGRRITHRPDEMFAMCSTFKGYVAARVLQMAEHGEI
SLDNRVFVDADALVPNSPVTEARAGAEMTLAELCQAALQRSDNTAANLLLKTIGGPAAVTAF
ARSVGDERTRLDRWEVELNSAIPGDPRDTSTPAALAVGYRAILAGDALSPPQRGLLEDWMRA
NQTSSMRAGLPEGWTTADKTGSGDYGSTNDAGIAFGPDGQRLLLVMMTRSQAHDPKAENLRP
LIGELTALVLPSLL
>d1e25a_ e.3.1.1 (A:) beta-Lactamase, class A {Pseudomonas aeruginosa, PER-1}
SPLLKEQIESIVIGKKATVGVAVWGPDDLEPLLINPFEKFPMQSVFKLHLAMLVLHQVDQGK
LDLNQTVIVNRAKVLQNTWAPIMKAYQGDEFSVPVQQLLQYSVSHSDNVACDLLFELVGGPA
ALHDYIQSMGIKETAVVANEAQMHADDQVQYQNWTSMKGAAEILKKFEQKTQLSETSQALLW
KWMVETTTGPERLKGLLPAGTVVAHKTGTSQIKAGKTAATNDLGIILLPDGRPLLVAVFVKD
SAESSRTNEAIIAQVAQTAYQFELKKLSAL
>d1dy6a_ e.3.1.1 (A:) beta-Lactamase, class A {Serratia marcescens, Sme-1}
NKSDAAAKQIKKLEEDFDGRIGVFAIDTGSGNTFGYRSDERFPLCSSFKGFLAAAVLERVQQ
KKLDINQKVKYESRDLEYHSPITTKYKGSGMTLGDMASAALQYSDNGATNIIMERFLGGPEG
MTKFMRSIGDNEFRLDRWELELNTAIPGDKRDTSTPKAVANSLNKLALGNVLNAKVKAIYQN
WLKGNTTGDARIRASVPADWVVGDKTGSCGAYGTANDYAVIWPKNRAPLIVSIYTTRKSKDD
KHSDKTIAEASRIAIQAID
>d1fr1a_ e.3.1.1 (A:) AMPC beta-Lactamase, class C {Citrobacter freundii}
AAKTEQQIADIVNRTITPLMQEQAIPGMAVAIIYQGKPYYFTWGKADIANNRPVTQQTLFEL
GSVSKTFNGVLGGDAIARGEIKLSDPVTQYWPELTGKQWQGISLLHLATYTAGGLPLQVPDD
VTDKAALLRFYQNWQPQWAPGAKRLYANSSIGLFGALAVKPSGMSYEEAMSKRVLHPLKLAH
TWITVPQSEQKDYAWGYREGKPVHVSPGQLDAEAYGVKSSVIDMTRWVQANMDASQVQEKTL
QQGIELAQSRYWRIGDMYQGLGWEMLNWPVKADSIISGSDSKVALAALPAVEVNPPAPAVKA

SWVHKTGSTGGFGSYVAFVPEKNLGIVMLANKSYPNPVRVEAAWRILEKLQ
>d1ga0a_ e.3.1.1 (A:) AMPC beta-Lactamase, class C {Enterobacter cloacae, P99, cephalosporinase}
PVSEKQLAEVVANTVTPLMKAQSVPGMAVAVIYQGKPHYYTFGKADIAANKPVTPQTLFELG
SISKTFTGVLGGDAIARGEISLDDPVTRYWPQLTGKQWQGIRMLDLATYTAGGLPLQVPDEV
TDNASLLRFYQNWQPQWKPGTTRLYANASIGLFGALAVKPSGMPYEQAMTTRVLKPLKLDHT
WINVPKAEEAHYAWGYRDGKAVRAVRVSPGMLDAQAYGVKTNVQDMANWVMANMAPENVADA
SLKQGIALAQSRYWRIGSMYQGLGWEMLNWPVEANTVVEGSDSKVALAPLPVAEVNPPAPPV
KASWVHKTGSTGGFGSYVAFIPEKQIGIVMLANTSYPNPARVEAAYHILEALQ
>d1i5qa_ e.3.1.1 (A:) AMPC beta-Lactamase, class C {Escherichia coli, cephalosporinase}
APQQINDIVHRTITPLIEQQKIPGMAVAVIYQGKPYYFTWGYADIAKKQPVTQQTLFELGSV
SKTFTGVLGGDAIARGEIKLSDPTTKYWPELTAKQWNGITLLHLATYTAGGLPLQVPDEVKS
SSDLLRFYQNWQPAWAPGTQRLYAASSIGLFGALAVKPSGLSFEQAMQTRVFQPLKLNHTWI
NVPPAEEKNYAWGYREGKAVHVSPGALDAEAYGVKSTIEDMARWVQSNLKPLDINEKTLQQG
IQLAQSRYWQTGDMYQGLGWEMLDWPVNPDSIINGSDNKIALAARPVKAITPPTPAVRASWV
HKTGATGGFGSYVAFIPEKELGIVMLANKNYPNPARVDAAWQILNALQ
>d1k55a_ e.3.1.1 (A:) Class D beta-lactamase {Pseudomonas aeruginosa, OXA-10}
SITENTSWNKEFSAEAVNGVFVLCKSSSKSCATNDLARASKEYLPASTFKIPNAIIGLETGV
IKNEHQVFKWDGKPRAMKQWERDLTLRGAIQVSAVPVFQQIAREVGEVRMQKYLKKFSYGNQ
NISGGIDKFWLEGQLRISAVNQVEFLESLYLNKLSASKENQLIVKEALVTEAAPEYLVHSKT
GFSGVGTESNPGVAWWVGWVEKETEVYFFAFNMDIDNESKLPLRKSIPTKIMESEGIIG
>d1k25a4 e.3.1.1 (A:264-631) Penicillin-binding protein 2x (pbp-2x), transpeptidase domain {Streptococcus pneumoniae}
TLSSPLQSFMETQMDAFLEKVKGKYMTATLVSAKTGEILATTQRPTFNADTKEGITEDFVWR
DILYQSNYEPGSAMKVMTLASSIDNNTFPSGEYFNSSELKIADATTRDWDVNEGLTTGGMMT
FLQGFAHSSNVGMSLLEQKMGDATWLDYLKRFKFGVPTRFGLTDEYAGQLPADNIVSIAQSS
FGQGISVTQTQMLRAFTAIANDGVMLEPKFISAIYDTNNQSVRKSQKEIVGNPVSKEAASTT
RNHMILVGTDPLYGTMYNHYTGKPIITVPGQNVAVKSGTAQIADEKNGGYLVGSTNYIFSAV
TMNPAENPDFILYVTVQQPEHYSGIQLGEFATPILERASAMKESLNLQSPAKNLDKVT
>d1qmea4 e.3.1.1 (A:264-620) Penicillin-binding protein 2x (pbp-2x), transpeptidase domain {Streptococcus pneumoniae}
TISSPLQSFMETQMDAFQEKVKGKYMTATLVSAKTGEILATTQRPTFDADTKEGITEDFVWR
DILYQSNYEPGSTMKVMMLAAAIDNNTFPGGEVFNSSELKIADATIRDWDVNEGLTGGRMMT
FSQGFAHSSNVGMTLLEQKMGDATWLDYLNRFKFGVPTRFGLTDEYAGQLPADNIVNIAQSS
FGQGISVTQTQMIRAFTAIANDGVMLEPKFISAIYDPNDQTARKSQKEIVGNPVSKDAASLT
RTNMVLVGTDPVYGTMYNHSTGKPTVTVPGQNVALKSGTAQIADEKNGGYLVGLTDYIFSAV
SMSPAENPDFILYVTVQQPEHYSGIQLGEFANPILERASAMKDSLNL
>d1hd8a2 e.3.1.1 (A:3-262) Penicillin-binding protein 5, N-terminal domain {Escherichia coli}
LNIKTMIPGVPQIDAESYILIDYNSGKVLAEQNADVRRDPASLTKMMTSYVIGQAMKAGKFK
ETDLVTIGNDAWATGNPVFKGSSLMFLKPGMQVPVSQLIRDINLQSGNDACVAMADFAAGSQ
DAFVGLMNSYVNALGLKNTHFQTVHGLDADGQYSSARDMALIGQALIRDVPNEYSIYKEKEF

TFNGIRQLNRNGLLWDNSLNVDGIKTGHTDKAGYNLVASATEGQMRLISAVMGGRTFKGREA
ESKKLLTWGFRF
>d1ei5a3 e.3.1.1 (A:3-335) D-aminopeptidase, N-terminal domain {Ochrobactrum anthropi}
KFDTSALEAFVRHIPQNYKGPGGVVAVVKDGEVVLQHAWGFADLRTRTPMTLDTRMPICSVS
KQFTCAVLLDAVGEPELLDDALEAYLDKFEDERPAVRDLCNNQSGLRDYWALSVLCGADPEG
VFLPAQAQSLLRRLKTTHFEPGSHYSYCNGNFRILADLIEAHTGRTLVDILSERIFAPAGMK
RAELISDTALFDECTGYEGDTVRGFLPATNRIQWMGDAGICASLNDMIAWEQFIDATRDDES
GLYRRLSGPQTFKDGVAAPYGFGLNLHETGGKRLTGHGGALRGWRCQRWHCADERLSTIAMF
NFEGGASEVAFKLMNIALGVSSS
>d1lbea_ e.4.1.1 (A:) ADP ribosyl cyclase {Sea hare (Aplysia californica)}
IVPTRELENVFLGRCKDYEITRYLDILPRVRSDCSALWKDFFKAFSFKNPCDLDLGSYKDFF
TSAQQQLPKNKVMFWSGVYDEAHDYANTGRKYITLEDTLPGYMLNSLVWCGQRANPGFNEKV
CPDFKTCPVQARESFWGMASSSYAHSAEGEVTYMVDGSNPKVPAYRPDSFFGKYELPNLTNK
VTRVKVIVLHRLGEKIIEKCGAGSLLDLEKLVKAKHFAFDCVENPRAVLFLLCSDNPNAREC
RL
>d4blca_ e.5.1.1 (A:) Catalase I {Cow (Bos taurus)}
NRDPASDQMKHWKEQRAAQKPDVLTTGGGNPVGDKLNSLTVGPRGPLLVQDVVFTDEMAHFD
RERIPERVVHAKGAGAFGYFEVTHDITRYSKAKVFEHIGKRTPIAVRFSTVAGESGSADTVR
DPRGFAVKFYTEDGNWDLVGNNTPIFFIRDALLFPSFIHSQKRNPQTHLKDPDMVWDFWSLR
PESLHQVSFLFSDRGIPDGHRHMDGYGSHTFKLVNADGEAVYCKFHYKTDQGIKNLSVEDAA
RLAHEDPDYGLRDLFNAIATGNYPSWTLYIQVMTFSEAEIFPFNPFDLTKVWPHGDYPLIPV
GKLVLNRNPVNYFAEVEQLAFDPSNMPPGIEPSPDKMLQGRLFAYPDTHRHRLGPNYLQIPV
NCPYRARVANYQRDGPMCMMDNQGGAPNYYPNSFSAPEHQPSALEHRTHFSGDVQRFNSAND
DNVTQVRTFYLKVLNEEQRKRLCENIAGHLKDAQLFIQKKAVKNFSDVHPEYGSRIQALLDK
YNE
>d1dgfa_ e.5.1.1 (A:) Catalase I {Human (Homo sapiens)}
RDPASDQMQHWKEQRAAQKADVLTTGAGNPVGDKLNVITVGPRGPLLVQDVVFTDEMAHFDR
ERIPERVVHAKGAGAFGYFEVTHDITKYSKAKVFEHIGKKTPIAVRFSTVAGESGSADTVRD
PRGFAVKFYTEDGNWDLVGNNTPIFFIRDPILFPSFIHSQKRNPQTHLKDPDMVWDFWSLRP
ESLHQVSFLFSDRGIPDGHRHMNGYGSHTFKLVNANGEAVYCKFHYKTDQGIKNLSVEDAAR
LSQEDPDYGIRDLFNAIATGKYPSWTFYIQVMTFNQAETFPFNPFDLTKVWPHKDYPLIPVG
KLVLNRNPVNYFAEVEQIAFDPSNMPPGIEASPDKMLQGRLFAYPDTHRHRLGPNYLHIPVN
CPYRARVANYQRDGPMCMQDNQGGAPNYYPNSFGAPEQQPSALEHSIQYSGEVRRFNTANDD
NVTQVRAFYVNVLNEEQRKRLCENIAGHLKDAQIFIQKKAVKNFTEVHPDYGSHIQALLDKY
N
>d1a4ea_ e.5.1.1 (A:) Catalase I {Baker's yeast (Saccharomyces cerevisiae)}
DVREDRVVTNSTGNPINEPFVTQRIGEHGPLLLQDYNLIDSLAHFNRENIPQRNPHAHGSGA
FGYFEVTDDITDICGSAMFSKIGKRTKCLTRFSTVGGDKGSADTVRDPRGFATKFYTEEGNL
DWVYNNTPVFFIRDPSKFPHFIHTQKRNPQTNLRDADMFWDFLTTPENQVAIHQVMILFSDR
GTPANYRSMHGYSGHTYKWSNKNGDWHYVQVHIKTDQGIKNLTIEEATKIAGSNPDYCQQDL
FEAIQNGNYPSWTVYIQTMTERDAKKLPFSVFDLTKVWPQGQFPLRRVGKIVLNENPLNFFA

QVEQAAFAPSTTVPYQEASADPVLQARLFSYADAHRYRLGPNFHQIPVNCPYASKFFNPAIR
DGPMNVNGNFGSEPTYLANDKSYTYIQQDRPIQQHQEVWNGPAIPYHWATSPGDVDFVQARN
LYRVLGKQPGQQKNLAYNIGIHVEGACPQIQQRVYDMFARVDKGLSEAIKKVAE
>d1e93a_ e.5.1.1 (A:) Catalase I {Proteus mirabilis}
KKLTTAAGAPVVDNNNVITAGPRGPMLLQDVWFLEKLAHFDREVIPERRMHAKGSGAFGTFT
VTHDITKYTRAKIFSEVGKKTEMFARFSTVAGERGAADAERDIRGFALKFYTEEGNWDMVGN
NTPVFYLRDPLKFPDLNHIVKRDPRTNMRNMAYKWDFFSHLPESLHQLTIDMSDRGLPLSYR
FVHGFGSHTYSFINKDNERFWVKFHFRCQQGIKNLMDDEAEALVGKDRESSQRDLFEAIKRG
DYPRWKLQIQIMPEKEASTVPYNPFDLTKVWPHADYPLMDVGYFELNRNPDNYFSDVEQAAF
SPANIVPGISFSPDKMLQGRLFSYGDAHRYRLGVNHHQIPVNAPKCPFHNYHRDGAMRVDGN
SGNGITYEPNSGGVFQEQPDFKEPPLSIEGAADHWNHREDEDYFSQPRALYELLSDDEHQRM
FARIAGELSQASKETQQRQIDLFTKVHPEYGAGVEKAIKVLE
>d1hbza_ e.5.1.1 (A:) Catalase I {Micrococcus lysodeikticus}
TTPHATGSTRQNGAPAVSDRQSLTVGSEGPIVLHDTHLLETHQHFNRMNIPERRPHAKGSGA
FGEFEVTEDVSKYTKALVFQPGTKTETLLRFSTVAGELGSPDTWRDVRGFALRFYTEEGNYD
LVGNNTPIFFLRDPMKFTHFIRSQKRLPDSGLRDATMQWDFWTNNPESAHQVTYLMGPRGLP
RTWREMNGYGSHTYLWVNAQGEKHWVKYHFISQQGVHNLSNDEATKIAGENADFHRQDLFES
IAKGDHPKWDLYIQAIPYEEGKTYRFNPFDLTKTISQKDYPRIKVGTLTLNRNPKNHFAQIE
SAAFSPSNTVPGIGLSPDRMLLGRAFAYHDAQLYRVGAHVNQLPVNRPKNAVHNYAFEGQMW
YDHTGDRSTYVPNSNGDSWSDETGPVDDGWEADGTLTREAQALRADDDDFGQAGTLVREVFS
DQERDDFVETVAGALKGVRQDVQARAFEYWKNVDATIGQRIEDEVKRHEGDGIPGVEAGGEA
RI
>d1cf9a2 e.5.1.1 (A:27-597) Catalase II {Escherichia coli, HPII}
DSLAPEDGSHRPAAEPTPPGAQPTAPGSLKAPDTRNEKLNSLEDVRKGSENYALTTNQGVRI
ADDQNSLRAGSRGPTLLEDFILREKITHFDHERIPERIVHARGSAAHGYFQPYKSLSDITKA
DFLSDPNKITPVFVRFSTCQGGAGSADTVRDIRGFATKFYTEEGIFDLVGNNTPIFFIQDAH
KFPDFVHAVKPEPHWAIPQGQSAHDTFWDYVSLQPETLHNVMWAMSDRGIPRSYRTMEGFGI
HTFRLINAEGKATFVRFHWKPLAGKASLVWDEAQKLTGRDPDFHRRELWEAIEAGDFPEYEL
GFQLIPEEDEFKFDFDLLDPTKLIPEELVPVQRVGKMVLNRNPDNFFAENEQAAFHPGHIVP
GLDFTNDPLLQGRLFSYTDTQISRLGGPNFHEIPINRPTCPYHNFQRDGMHRMGIDTNPANY
EPNSINDNWPRETPPGPKRGGFESYQERVEGNKVRERSPSFGEYYSHPRLFWLSQTPFEQRH
IVDGFSFELSKVVRPYIRERVVDQLAHIDLTLAQAVAKNLGIELTDDQLNITPPPDVNGLKK
DPSLSLYAIPDGD
>d1buca2 e.6.1.1 (A:1-232) Butyryl-CoA dehydrogenase {Megasphaera elsdenii}
MDFNLTDIQQDFLKLAHDFGEKKLAPTVTERDHKGIYDKELIDELLSLGITGAYFEEKYGGS
GDDGGDVLSYILAVEELAKYDAGVAITLSATVSLCANPIWQFGTEAQKEKFLVPLVEGTKLG
AFGLTEPNAGTDASGQQTIATKNDDGTYTLNGSKIFITNGGAADIYIVFAMTDKSKGNHGIT
AFILEDGTPGFTYGKKEDKMGIHTSQTMELVFQDVKVPAENMLGEE
>d1jqia2 e.6.1.1 (A:4-234) Butyryl-CoA dehydrogenase {Rat (Rattus norvegicus)}
VYQSVELPETHQMLRQTCRDFAEKELVPIAAQLDKEHLFPTSQVKKMGELGLLAMDVPEELS
GAGLDYLAYSIALEEISRGCASTGVIMSVNNSLYLGPILKFGSSQQKQQWITPFTNGDKIGC
FALSEPGNGSDAGAASTTAREEGDSWVLNGTKAWITNSWEASATVVFASTDRSRQNKGISAF

LVPMPTPGLTLGKKEDKLGIRASSTANLIFEDCRIPKENLLGEPG
>d3mdda2 e.6.1.1 (A:11-241) Medium chain acyl-CoA dehydrogenase {Pig (Sus scrofa)}
GFSFELTEQQKEFQATARKFAREEIIPVAAEYDRTGEYPVPLLKRAWELGLMNTHIPESFGG
LGLGIIDSCLITEELAYGCTGVQTAIEANTLGQVPLIIGGNYQQQKKYLGRMTEEPLMCAYC
VTEPGAGSDVAGIKTKAEKKGDEYIINGQKMWITNGGKANWYFLLARSDPDPKAPASKAFTG
FIVEADTPGVQIGRKEINMGQRCSDTRGIVFEDVRVPKENVLTGE
>d1egda2 e.6.1.1 (A:10-241) Medium chain acyl-CoA dehydrogenase {Human (Homo sapiens)}
LGFSFEFTEQQKEFQATARKFAREEIIPVAAEYDKTGEYPVPLIRRAWELGLMNTHIPENCG
GLGLGTFDACLISEELAYGCTGVQTAIEGNSLGQMPIIIAGNDQQKKKYLGRMTEEPLMCAY
CVTEPGAGSDVAGIKTKAEKKGDEYIINGQKMWITNGGKANWYFLLARSDPDPKAPANKAFT
GFIVEADTPGIQIGRKELNMGQRCSDTRGIVFEDVKVPKENVLIGD
>d1ivha2 e.6.1.1 (A:6-241) Isovaleryl-coa dehydrogenase {Human (Homo sapiens)}
VDDAINGLSEEQRQLRQTMAKFLQEHLAPKAQEIDRSNEFKNLREFWKQLGNLGVLGITAPV
QYGGSGLGYLEHVLVMEEISRASGAVGLSYGAHSNLCINQLVRNGNEAQKEKYLPKLISGEY
IGALAMSEPNAGSDVVSMKLKAEKKGNHYILNGNKFWITNGPDADVLIVYAKTDLAAVPASR
GITAFIVEKGMPGFSTSKKLDKLGMRGSNTCELIFEDCKIPAANILGHEN
>d1frpa_ e.7.1.1 (A:) Fructose-1,6-bisphosphatase {Pig (Sus scrofa)}
NIVTLTRFVMEQGRKARGTGEMTQLLNSLCTAVKAISTAVRKAGIAHLYGIAGATNVTGDQV
KKLDVLSNDLVINVLKSSFATCVLVTEEDKNAIIVEPEKRGKYVVCFDPLDGSSNIDCLVSI
GTIFGIYRKNSTDEPSEKDALQPGRNLVAAGYALYGSATMLVLAMVNGVNCFMLDPAIGEFI
LVDRNVKIKKKGSIYSINEGYAKEFDPAITEYIQRKKFPPDNSAPYGARYVGSMVADVHRTL
VYGGIFMYPANKKSPKGKLRLLYECNPMAYVMEKAGGLATTGKEAVLDIVPTDIHQRAPIIL
GSPEDVTELLEIYQKHA
>d1ftaa_ e.7.1.1 (A:) Fructose-1,6-bisphosphatase {Human (Homo sapiens)}
DVVTLTRFVMEEGRKARGTGELTQLLNSLCTAVKAISSAVRKAGIAHLYGIAGSTNVTGDQV
KKLDVLSNDLVMNMLKSSFATCVLVSEEDKHAIIVEPEKRGKYVVCFDPLDGSSNIDCLVSV
GTIFGIYRKKSTDEPSEKDALQPGRNLVAAGYALYGSATMLVLAMDCGVNCFMLDPAIGEFI
LVDKDVKIKKKGKIYSLNEAYAKDFDPAVTEYIQRKKFPPDNSAPYGARYVGSMVADVHRTL
VYGGIFLYPANKKSPNGKLRLLYECNPMAYVMEKAGGMATTGKEAVLDVIPTDIHQRAPIIL
GSPDDVLEFLKVYEKHS
>d1bk4a_ e.7.1.1 (A:) Fructose-1,6-bisphosphatase {Rabbit (Oryctolagus cuniculus)}
FDTDISTMTRFVMEEGRKAGGTGEMTQLLNSLCTAVKAISTAVRKAGIAHLYGIAGSTNVTG
DQVKKLDVLSNDLVMNMLKSSFATCVLVSEEDKNAIIVEPEKRGKYVVCFDPLDGSSNIDCL
VSIGTIFGIYRKKSTDEPSTKDALQPGRNLVAAGYALYGSATMLVLAGGSGVNSFMLDPAIG
EFILVDKNVKIKKKGNIYSLNEGYAKDFDPAVTEYIQKKKFPPDNSSPYGARYVGSMVADVH
RTLVYGGIFLYPANKKSPDGKLRLLYECNPMAFIMEKAGGMATTGKEAILDIVPTDIHQRAP
VILGSPDDVQEFLEIYKKHAVK
>d1spia_ e.7.1.1 (A:) Fructose-1,6-bisphosphatase {Spinach

(Spinacia oleracea)}
AATQTKARTRSKYEIETLTGWLLKQPMAGVIDAELTIVLSSISLACKQIASLVQRAGISNLT
GIQGAVNIQGEDQKKLDVVSNEVFSSCLRSSGRTGIIASEEEDVPVAVEESYSGNYIVVFDP
LDGSSNIDAAVSTGSIFGIYSPNDECIVDSDHDDESQLSAEEQRCVVNVCQPGDNLLAAGYC
MYSSSVIFVLTIGKGVYAFTLDPMYGEFVLTSEKIQIPKAGKIYSFNEGNYKMWPDKLKKYM
DDLKEPGESQKPYSSRYIGSLVGDFHRTLLYGGIYGYPRDAKSKNGKLRLLYECAPMSFIVE
QAGGKGSDGHQRILDIQPTEIHQRVPLYIGSVEEVEKLEKYLA
>d1dcua_ e.7.1.1 (A:) Fructose-1,6-bisphosphatase {Garden pea
(Pisum sativum)}
KRSGYEIITLTSWLLQQEQKGIIDAELTIVLSSISMACKQIASLVQRANISNLTGTQGAVNI
QGEDQKKLDVISNEVFSNCLRSSGRTGIIASEEEDVPVAVEESYSGNYIVVFDPLDGSSNLD
AAVSTGSIFGIYSPNDECLPDFGDDSDDNTLGTEEQRCIVNVCQPGSNLLAAGYCMYSSSVI
FVLTIGKGVFVFTLDPLYGEFVLTQENLQIPKSGKIYSFNEGNYKLWDENLKKYIDDLKEPG
PSGKPYSARYIGSLVGDFHRTLLYGGIYGYPRDKKSKNGKLRLLYECAPMSFIVEQAGGKGS
DGHQRVLDIQPTEIHQRVPLYIGSTEEVEKVEKYLA
>d2hhma_ e.7.1.1 (A:) Inositol monophosphatase {Human (Homo
sapiens)}
WQECMDYAVTLARQAGEVVCEAIKNEMNVMLKSSPVDLVTATDQKVEKMLISSIKEKYPSHS
FIGEESVAAGEKSILTDNPTWIIDPIDGTTNFVHRFPFVAVSIGFAVNKKIEFGVVYSCVEG
KMYTARKGKGAFCNGQKLQVSQQEDITKSLLVTELGSSRTPETVRMVLSNMEKLFCIPVHGI
RSVGTAAVNMCLVATGGADAYYEMGIHCWDVAGAGIIVTEAGGVLMDVTGGPFDLMSRRVIA
ANNRILAERIAKEIQVIPLQRDDE
>d1g0ha_ e.7.1.1 (A:) Archaeal inositol
monophosphatase/fructose-1,6-bisphosphatase {Archaeon
Methanococcus jannaschii, MJ0109}
MKWDEIGKNIAKEIEKEILPYFGRKDKSYVVGTSPSGDETEIFDKISEDIALKYLKSLNVNI
VSEELGVIDNSSEWTVVIDPIDGSFNFINGIPFFAFCFGVFKNNEPYYGLTYEFLTKSFYEA
YKGKGAYLNGRKIKVKDFNPNNIVISYYPSKKIDLEKLRNKVKRVRIFGAFGLEMCYVAKGT
LDAVFDVRPKVRAVDIASSYIICKEAGALITDENGDELKFDLNATDRLNIIVANSKEMLDII
LDLL
>d1inp__ e.7.1.1 (-) Inositol polyphosphate 1-phosphatase {Cow
(Bos taurus), brain}
MSDILQELLRVSEKAANIARACRQQETLFQLLIEEKKEGEKNKKFAVDFKTLADVLVQEVIK
ENMENKFPGLGKKIFGEESNELTNDLGEKIIMRLGPTEEETVALLSKVLNGNKLASEALAKV
VHQDVFFSDPALDSVEINIPQDILGIWVDPIDSTYQYIKGSADITPNQGIFPSGLQCVTVLI
GVYDIQTGVPLMGVINQPFVSQDLHTRRWKGQCYWGLSYLGTNIHSLLPPVSTRSNSEAQSQ
GTQNPSSEGSCRFSVVISTSEKETIKGALSHVCGERIFRAAGAGYKSLCVILGLADIYIFSE
DTTFKWDSCAAHAILRAMGGGMVDLKECLERNPDTGLDLPQLVYHVGNEGAAGVDQWANKGG
LIAYRSEKQLETFLSRLLQHLAPVATHT
>d1ka1a_ e.7.1.1 (A:) 3';5'-adenosine bisphosphatase, PAP
phosphatase {Baker's yeast (Saccharomyces cerevisiae)}
ALERELLVATQAVRKASLLTKRIQSEVISHKDSTTITKNDNSPVTTGDYAAQTIIINAIKSN
FPDDKVVGEESSSGLSDAFVSGILNEIKANDEVYNKNYKKDDFLFTNDQFPLKSLEDVRQII
DFGNYEGGRKGRFWCLDPIDGTKGFLRGEQFAVCLALIVDGVVQLGCIGCPNLVLSSYGAQD

LKGHESFGYIFRAVRGLGAFYSPSSDAESWTKIHVRHLKDTKDMITLEGVEKGHSSHDEQTA
IKNKLNISKSLHLDSQAKYCLLALGLADVYLRLPIKLSYQEKIWDHAAGNVIVHEAGGIHTD
AMEDVPLDFGNGRTLATKGVIASSGPRELHDLVVSTSCDVIQSR
>d1jp4a_ e.7.1.1 (A:) PIPase {Rat (Rattus norvegicus)}
HNVLMRLVASAYSIAQKAGTIVRCVIAEGDLGIVQKTSATDLQTKADRMVQMSICSSLSRKF
PKLTIIGEEDLPPGEVDQELIEDGQSEEILKQPCPSQYSAIKEEDLVVWVDPVDGTKEYTEG
LLDNVTVLIGIAYEGKAIAGIINQPYYNYQAGPDAVLGRTIWGVLGLGAFGFQLKEAPAGKH
IITTTRSHSNKLVTDCIAAMNPDNVLRVGGAGNKIIQLIEGKASAYVFASPGCKKWDTCAPE
VILHAVGGKLTDIHGNPLQYDKEVKHMNSAGVLAALRNYEYYASRVPESVKSALIP
>d1kfsa2 e.8.1.1 (A:519-928) DNA polymerase I (Klenow fragment)
{Escherichia coli}
HKGPLNVFENIEMPLVPVLSRIERNGVKIDPKVLHNHSEELTLRLAELEKKAHEIAGEEFNL
SSTKQLQTILFEKQGIKPLKKTPGGAPSTSEEVLEELALDYPLPKVILEYRGLAKLKSTYTD
KLPLMINPKTGRVHTSYHQAVTATGRLSSTDPNLQNIPVRNEEGRRIRQAFIAPEDYVIVSA
DYSQIELRIMAHLSRDKGLLTAFAEGKDIHRATAAEVFGLPLETVTSEQRRSAKAINFGLIY
GMSAFGLARQLNIPRKEAQKYMDLYFERYPGVLEYMERTRAQAKEQGYVETLDGRRLYLPDI
KSSNGARRAAAERAAINAPMQGTAADIIKRAMIAVDAWLQAEQPRVRMIMQVHDELVFEVHK
DDVDAVAKQIHQLMENCTRLDVPLLVEVGSGENWDQAH
>d1qtma2 e.8.1.1 (A:423-831) DNA polymerase I (Klenow fragment)
{Thermus aquaticus}
EERLLWLYREVERPLSAVLAHMEATGVRLDVAYLRALSLEVAEEIARLEAEVFRLAGHPFNL
NSRDQLERVLFDELGLPAIGKTEKTGKRSTSAAVLEALREAHPIVEKILQYRELTKLKSTYI
DPLPDLIHPRTGRLHTRFNQTATATGRLSSSDPNLQNIPVRTPLGQRIRRAFIAEEGWLLVA
LDYSQIELRVLAHLSGDENLIRVFQEGRDIHTETASWMFGVPREAVDPLMRRAAKTINFGVL
YGMSAHRLSQELAIPYEEAQAFIERYFQSFPKVRAWIEKTLEEGRRRGYVETLFGRRRYVPD
LEARVKSVREAAERMAFNMPVQGTAADLMKLAMVKLFPRLEEMGARMLLQVHDELVLEAPKE
RAEAVARLAKEVMEGVYPLAVPLEVEVGIGEDWLSAK
>d1xwl_2 e.8.1.1 (469-876) DNA polymerase I (Klenow fragment)
{Bacillus stearothermophilus, newly identified strain as yet
unnamed}
EQDRLLVELEQPLSSILAEMEFAGVKVDTKRLEQMGKELAEQLGTVEQRIYELAGQEFNINS
PKQLGVILFEKLQLPVLKKTKTGYSTSADVLEKLAPYHEIVENILHYRQLGKLQSTYIEGLL
KVVRPDTKKVHTIFNQALTQTGRLSSTEPNLQNIPIRLEEGRKIRQAFVPSESDWLIFAADY
SQIELRVLAHIAEDDNLMEAFRRDLDIHTKTAMDIFQVSEDEVTPNMRRQAKAVNFGIVYGI
SDYGLAQNLNISRKEAAEFIERYFESFPGVKRYMENIVQEAKQKGYVTTLLHRRRYLPDITS
RNFNVRSFAERMAMNTPIQGSAADIIKKAMIDLNARLKEERLQAHLLLQVHDELILEAPKEE
MERLCRLVPEVMEQAVTLRVPLKVDYHYGSTWYDAK
>d1t7pa2 e.8.1.1 (A:211-704) T7 phage DNA polymerase
{Bacteriophage T7}
LEAVDIEHRAAWLLAKQERNGFPFDTKAIEELYVELAARRSELLRKLTETFGSWYQPKGGTE
MFCHPRTGKPLPKYPRIKTPKVGGIFKKPKNKAQREGREPCELDTREYVAGAPYTPVEHVVF
NPSSRDHIQKKLQEAGWVPTKYTDKGAPVVDDEVLEGVRVDDPEKQAAIDLIKEYLMIQKRI
GQSAEGDKAWLRYVAEDGKIHGSVNPNGAVTGRATHAFPNLAQIPGVRSPYGEQCRAAFGAE
HHLDGITGKPWVQAGIDASGLELRCLAHFMARFDNGEYAHEILNGDIHTKNQIAAELPTRDN

AKTFIYGFLYGAGDEKIGQIVGAGKERGKELKKKFLENTPAIAALRESIQQTLVESSQWVAG
EQQVKWKRRWIKGLDGRKVHVRSPHAALNTLLQSAGALICKLWIIKTEEMLVEKGLKHGWDG
DFAYMAWVHDEIQVGCRTEEIAQVVIETAQEAMRWVGDHWNFRCLLDTEGKMGPNWAICH
>d1ih7a2 e.8.1.1 (A:376-903) T4-like DNA polymerase {Bacteriophage RB69}
QNKVIPQGRSHPVQPYPGAFVKEPIPNRYKYVMSFDLTSLYPSIIRQVNISPETIAGTFKVA
PLHDYINAVAERPSDVYSCSPNGMMYYKDRDGVVPTEITKVFNQRKEHKGYMLAAQRNGEII
KEALHNPNLSVDEPLDVDYRFDFSDEIKEKIKKLSAKSLNEMLFRAQRTEVAGMTAQINRKL
LINSLYGALGNVWFRYYDLRNATAITTFGQMALQWIERKVNEYLNEVCGTEGEAFVLYGDTD
SIYVSADKIIDKVGESKFRDTNHWVDFLDKFARERMEPAIDRGFREMCEYMNNKQHLMFMDR
EAIAGPPLGSKGIGGFWTGKKRYALNVWDMEGTRYAEPKLKIMGLETQKSSTPKAVQKALKE
CIRRMLQEGEESLQEYFKEFEKEFRQLNYISIASVSSANNIAKYDVGGFPGPKCPFHIRGIL
TYNRAIKGNIDAPQVVEGEKVYVLPLREGNPFGDKCIAWPSGTEITDLIKDDVLHWMDYTVL
LEKTFIKPLEGFTSAAKLDYEKKASLFDMFDF
>d1tgoa2 e.8.1.1 (A:348-773) T4-like DNA polymerase {Archaeon Thermococcus gorgonarius}
STGNLVEWFLLRKAYERNELAPNKPDERELARRRESYAGGYVKEPERGLWENIVYLDFRSLY
PSIIITHNVSPDTLNREGCEEYDVAPQVGHKFCKDFPGFIPSLLGDLLEERQKVKKKMKATI
DPIEKKLLDYRQRAIKILANSFYGYYGYAKARWYCKECAESVTAWGRQYIETTIREIEEKFG
FKVLYADTDGFFATIPGADAETVKKKAKEFLDYINAKLPGLLELEYEGFYKRGFFVTKKKYA
VIDEEDKITTRGLEIVRRDWSEIAKETQARVLEAILKHGDVEEAVRIVKEVTEKLSKYEVPP
EKLVIYEQITRDLKDYKATGPHVAVAKRLAARGIKIRPGTVISYIVLKGSGRIGDRAIPFDE
FDPAKHKYDAEYYIENQVLPAVERILRAFGYRKEDLRYQKTRQVGLGAWLKPKT
>d1qhta2 e.8.1.1 (A:348-750) T4-like DNA polymerase {Archaeon Thermococcus sp., 9on-7}
STGNLVEWFLLRKAYKRNELAPNKPDERELARRRGGYAGGYVKEPERGLWDNIVYLDFRSLY
PSIIITHNVSPDTLNREGCKEYDVAPEVGHKFCKDFPGFIPSLLGDLLEERQKIKRKMKATV
DPLEKKLLDYRQRAIKILANSFYGYYGYAKARWYCKECAESVTAWGREYIEMVIRELEEKFG
FKVLYADTDGLHATIPGADAETVKKKAKEFLKYINPKLPGLLELEYEGFYVRGFFVTKKKYA
VIDEEGKITTRGLEIVRRDWSEIAKETQARVLEAILKHGDVEEAVRIVKEVTEKLSKYEVPP
EKLVIHEQITRDLRDYKATGPHVAVAKRLAARGVKIRPGTVISYIVLKGSGRIGDRAIPADE
FDPTKHRYDAEYYIENQVLPAVERILKAFGY
>d1d5aa2 e.8.1.1 (A:348-756) T4-like DNA polymerase {Archaeon Desulfurococcus tok}
STGNLVEWFLLRKAYERNDVAPNKPDERELARRTESYAGGYVKEPEKGLWENIVYLDYKSLY
PSIIITHNVSPDTLNREGCREYDVAPQVGHRFCKDFPGFIPSLLGDLLEERQKVKKKMKATV
DPIERKLLDYRQRAIKILANSYYGYYAYANARWYCRECAESVTAWGRQYIETTMREIEEKFG
FKVLYADTDGFFATIPGADAETVKNKAKEFLNYINPRLPGLLELEYEGFYRRGFFVTKKKYA
VIDEEDKITTRGLEIVRRDWSEIAKETQARVLEAILKHGDVEEAVRIVKEVTEKLSRHEVPP
EKLVIYEAGPHVAAAATVISYIVLKGPGRVGDRAIPFDEFDPAKHRYDAEYYIENQVLPAVE
RILRAFGYRKEDLR
>d1gcxa2 e.8.1.1 (A:348-758) T4-like DNA polymerase {Archaeon Pyrococcus kodakaraensis}
STGNLVEWFLLRKAYERNELAPNKPDEKELARRRQSYEGGYVKEPERGLWENIVYLDFRSLY

PSIIITHNVSPDTLNREGCKEYDVAPQVGHRFCKDFPGFIPSLLGDLLEERQKIKKKMKATI
DPIERKLLDYRQRAIKILANSYYGYYGYARARWYCKECAESVTAWGREYITMTIKEIEEKYG
FKVIYSDTDGFFATIPGADAETVKKKAMEFLKYINAKLPGALELEYEGFYKRGFFVTKKKYA
VIDEEGKITTRGLEIVRRDWSEIAKETQARVLEALLKDGDVEKAVRIVKEVTEKLSKYEVPP
EKLVIHEQITRDLKDYKATGPHVAVAKRLAARGVKIRPGTVISYIVLKGSGRIGDRAIPFDE
FDPTKHKYDAEYYIENQVLPAVERILRAFGYRKEDLRYQ
>d1jx4a_ e.8.1.5 (A:) DinB homolog (DBH) {Archaeon Sulfolobus solfataricus, DNA polymerase IV}
MIVLFVDFDYFYAQVEEVLNPSLKGKPVVVCVFSGRFEDSGAVATANYEARKFGVKAGIPIV
EAKKILPNAVYLPMRKEVYQQVSSRIMNLLREYSEKIEIASIDEAYLDISDKVRDYREAYNL
GLEIKNKILEKEKITVTVGISKNKVFAKIAADMAKPNGIKVIDDEEVKRLIRELDIADVPGI
GNITAEKLKKLGINKLVDTLSIEFDKLKGMIGEAKAKYLISLARDEYNEPIRTRVRKSIGRI
VTMKRNSRNLEEIKPYLFRAIEESYYKLDKRIPKAIHVVAVTEDLDIVSRGRTFPHGISKET
AYSESVKLLQKILEEDERKIRRIGVRFSKFI
>d1im4a_ e.8.1.5 (A:) DinB homolog (DBH) {Archaeon Sulfolobus solfataricus}
HHHHHIVIFVDFDYFFAQVEEVLNPQYKGKPLVVCVYSGRTKTSGAVATANYEARKLGVKAG
MPIIKAMQIAPSAIYVPMRKPIYEAFSNRIMNLLNKHADKIEVASIDEAYLDVTNKVEGNFE
NGIELARKIKQEILEKEKITVTVGVAPNKILAKIIADKSKPNGLGVIRPTEVQDFLNELDID
EIPGIGSVLARRLNELGIQKLRD
>d1k1sa_ e.8.1.5 (A:) DinB homolog (DBH) {Archaeon Sulfolobus solfataricus}
MIVIFVDFDYFFAQVEEVLNPQYKGKPLVVSVYSGRTKTSGAVATANYEARKLGVKAGMPII
KAMQIAPSAIYVPMRKPIYEAFSNRIMNLLNKHADKIEVASIDEAYLDVTNKVEGNFENGIE
LARKIKQEILEKEKITVTVGVAPNKILAKIIADKSKPNGLGVIRPTEVQDFLNELDIDEIPG
IGSVLARRLNELGIQKLRDILSKNYNELEKITGKAKALYLLKLAQNKYSEPVENKSKIPHGR
YLTLPYNTRDVKVILPYLKKAINEAYNKVNGIPMRITVIAIMEDLDILSKGKKFKHGISIDN
AYKVAEDLLRELLVRDKRRNVRRIGVKLDNIIIN
>d1jiha_ e.8.1.5 (A:) DNA polymerase eta {Baker's yeast (Saccharomyces cerevisiae)}
MSKFTWKELIQLGSPSKAYESSLACIAHIDMNAFFAQVEQMRCGLSKEDPVVCVQWNSIIAV
SYAARKYGISRMDTIQEALKKCSNLIPIHTAVFKKGEDFWQYHDGCGSWVQDPAKQISVEDH
KVSLEPYRRESRKALKIFKSACDLVERASIDEVFLDLGRICFNMLMFDNEYELTGDLKLKDA
LSNIREAFIGGNYDINSHLPLIPEKIKSLKFEGDVFNPEGRDLITDWDDVILALGSQVCKGI
RDSIKDILGYTTSCGLSSTKNVCKLASNYKKPDAQTIVKNDCLLDFLDCGKFEITSFWTLGG
VLGKELIDVLDLPHENSIKHIRETWPDNAGQLKEFLDAKVKQSDYDRSTSNIDPLKTADLAE
KLFKLSRGRYGLPLSSRPVVKSMMSNKNLRGKSCNSIVDCISWLEVFCAELTSRIQDLEQEY
NKIVIPRTVSISLKTKSYEVYRKSGPVAYKGINFQSHELLKVGIKFVTDLDIKGKNKSYYPL
TKLSMTITNFDII
>d1mml__ e.8.1.2 (-) MMLV reverse transcriptase {Moloney murine leukemia virus}
TWLSDFPQAWAETGGMGLAVRQAPLIIPLKATSTPVSIKQYPMSQEARLGIKPHIQRLLDQG
ILVPCQSPWNTPLLPVKKPGTNDYRPVQDLREVNKRVEDIHPTVPNPYNLLSGLPPSHQWYT
VLDLKDAFFCLRLHPTSQPLFAFEWRDPEMGISGQLTWTRLPQGFKNSPTLFDEALHRDLAD

FRIQHPDLILLQYVDDLLLAATSELDCQQGTRALLQTLGNLGYRASAKKAQICQKQVKYLGY
LLK
>d1c9ra2 e.8.1.2 (A:1-429) HIV-1 reverse transcriptase {Human immunodeficiency virus type 1}
PISPIETVPVKLAPGMDGPKVKQWPLTAEAIAALVAICTAMEAEGKISKIGPENPYNTPVFA
IKKAAAAAAAALVDFRELNKRTQDFWEVQLGIPHPAGLKKKKSVTVLDVGDAYFSVPLDEDF
RKYTAFTIPSIANATPGIRYQYNVLPQGWKGSPAIFQSSMTKILEPFKKQNPDIVIYQYIDD
LYVGSDLEIGQHATKIAELRQHLLAWGLTTPDKKHAAEPPFLWMGYELHPDAWTVAPAALAA
AASAAVNDIQKLVGKLNWASQIYPGIAVRALSAAAAGTKAAAEAAAATAAAALALAAAREAL
AAPVHGVYYDPSKDLIAEIQAQGQGQWTYQIYQEPFKNLKTGKYARMRGAHTNDVKQLTEAV
QKITTESIVIWGKTPKFKLPIQKETWETWWTEYWQATWIPEWEFVNTPPLVKLWYQL
>d1c9rb1 e.8.1.2 (B:) HIV-1 reverse transcriptase {Human immunodeficiency virus type 1}
PISPIETVPVKLAPGMDGPKVKQWPLTEEKIKALVEICTEMEKEGKISKIGPENPYNTPVFA
IKKKDSTKWRKLVDFRELNKRTQDFWEAALGIPHPAGLKKKKSVTVLDVGDAYFSVPLDEDF
RKYTAFTIPSINNETPGIRYQYNVLPQGWKGSPAIFQSSMTKILEPFKAQNPDIVIYQYIDD
LYVGSDLEIGQHRTKIEELRQHLLRWGLTTPDKKHQKEPPFLWMGYELHPDKWAAAAAAAAA
AAAATVNDIQKLVGKLNWAAQIYPGIAAAALSAALAGTKALTAAAPLTAAAALELAANRAAA
AAAAAGVYYDPSKDLIAEIQKQGQGQWTYQIYQEPFKNLKTGKYARMRGAHTNDVKQLTEAV
QKITTESIVIWGKTPKFKLPIQKETWETWWTEYWQATWIPEWEFVNTPPLVALWYALE
>d1har__ e.8.1.2 (-) HIV-1 reverse transcriptase {Human immunodeficiency virus type 1}
PISPIETVPVKLKPGMDGPKVAQWPLTAAKIAALVAICTEMEKEGKISKIGPENPYNTPVFA
IKKKDSTKWAKLVDFRELNKRTQDFWEVQLGIPHPAGLKKKKSVTVLDVGDAYFSVPLDEDF
RKYTAFTIPSINNETPGIRYQYNVLPQGWKGSPAIFQSSMTKILAPFKAANPDIVIYQYMDD
LYVGSDLAIGAHRTKIEELRQHLLRWGLTT
>d1vrta2 e.8.1.2 (A:4-429) HIV-1 reverse transcriptase {Human immunodeficiency virus type 1}
PIETVPVKLKPGMDGPKVKQWPLTEEKIKALVEICTEMEKEGKISKIGPENPYNTPVFAIKK
KDSTKWRKLVDFRELNKRTQDFWEVQLGIPHPAGLKKKKSVTVLDVGDAYFSVPLDEDFRKY
TAFTIPSINNETPGIRYQYNVLPQGWKGSPAIFQSSMTKILEPFRKQNPDIVIYQYMDDLYV
GSDLEIGQHRTKIEELRQHLLRWGLTTPDKKHQKEPPFLWMGYELHPDKWTVQPIVLPEKDS
WTVNDIQKLVGKLNWASQIYPGIKVRQLCKLLRGTKALTEVIPLTEEAELELAENREILKEP
VHGVYYDPSKDLIAEIQKQGQGQWTYQIYQEPFKNLKTGKYARMRGAHTNDVKQLTEAVQKI
TTESIVIWGKTPKFKLPIQKETWETWWTEYWQATWIPEWEFVNTPPLVKLWYQL
>d1ceza_ e.8.1.3 (A:) T7 RNA polymerase {Bacteriophage T7}
IAKNDFSDIELAAIPFNTLADHYGERLAREQLALEHESYEMGEARFRKMFERQLKAGEVADN
AAAKPLITTLLPKMIARINDWFEEVKAKRGKRPTAFQFLQEIKPEAVAYITIKTTLACLTSA
DNTTVQAVASAIGRAIEDEARFGRIRDLEAKHFKKNVEEQLNKRVGHVYKKAFMQVVEADML
SKGLLGGEAWSSWHKEDSIHVGVRCIEMLIESTGMVSLHRQNAGVVGQDSETIELAPEYAEA
IATRAGALAGISPMFQPCVVPPKPWTGITGGGYWANGRRPLALVRTHSKKALMRYEDVYMPE
VYKAINIAQNTAWKINKKVLAVANVITKWKHCPVEDIPAIEREELPMKPEDIDMNPEALTAW
KRAAAAVYRKDKARKSRRISLEFMLEQANKFANHKAIWFPYNMDWRGRVYAVSMFNPQGNDM
TKGLLTLAKGKPIGKEGYYWLKIHGANCAGVDKVPFPERIKFIEENHENIMACAKSPLENTW

WAEQDSPFCFLAFCFEYAGVQHHGLSYNCSLPLAFDGSCSGIQHFSAMLRDEVGGRAVNLLP
SETVQDIYGIVAKKVNEILQADAINGTDNEVVTVTDENTGEISEKVKLGTKALAGQWLAYGV
TRSVTKRSVMTLAYGSKEFGFRQQVLEDTIQPAIDSGKGLMFTQPNQAAGYMAKLIWESVSV
TVVAAVEAMNWLKSAAKLLAAEVKDKKTGEILRKRCAVHWVTPDGFPVWQEYKKPIQTRLNL
MFLGQFRLQPTINTNKDSEIDAHKQESGIAPNFVHSQDGSHLRKTVVWAHEKYGIESFALIH
DSFGTIPADAANLFKAVRETMVDTYESCDVLADFYDQFADQLHESQLDKMPALPAKGNLNLR
DILESDFAFA
>d1rdr__ e.8.1.4 (-) Viral RNA-dependent RNA-polymerase
{Poliovirus type 1, strain Mahoney}
VGYPIINAPSKTKLEPSAFHYVFEGVKEPAVLTKNDPRLKTDFEEAIFSKYVGNKITEVDEY
MKEAVDHYAGQLMSLDINTEQMCLEDAMYGTDGLEALDLSTSAGYPYVAMGKKKRDILNKQT
RDTKEMQKLLDTYGINLPLVTYVKDELRSKTKVEQGKSRLIEASSLNDSVAMRMAFGNLYAA
FHKNPGVITGSAVGCDPDLFWSKIPVLMEEKLFAFDYTGYDASLSPAWFEALKMVLEKIGFG
DRVDYIDYLNHSHHLYKNKTYCVKGGMPSGCSGTSIFNSMINNLIIRTLLLKTYKGIDLDHL
KMIAYGDDVIASYPHEVDASLLAQSGKDYGLTMTPADKSATFETVTWENVTFLKRFFRADEK
YPFLIHPVMPMKEIHESIRWTKDPRNTQDHVRSLCLLAWHNGEEEYNKFLAKIRSVPIGRAL
LLPEYSTLYRRWLDSF
>d1c2pa_ e.8.1.4 (A:) Viral RNA-dependent RNA-polymerase
{Hepatitis C virus}
HHSYTWTGALITPCAAEESKLPINALSNSLLRHHNMVYATTSRSAGLRQKKVTFDRLQVLDD
HYRDVLKEMKAKASTVKAKLLSVEEACKLTPPHSAKSKFGYGAKDVRNLSSKAVNHIHSVWK
DLLEDTVTPIDTTIMAKNEVFCVQPEKGGRKPARLIVFPDLGVRVCEKMALYDVVSTLPQVV
MGSSYGFQYSPGQRVEFLVNTWKSKKNPMGFSYDTRCFDSTVTENDIRVEESIYQCCDLAPE
ARQAIKSLTERLYIGGPLTNSKGQNCGYRRCRASGVLTTSCGNTLTCYLKASAACRAAKLQD
CTMLVNGDDLVVICESAGVQEDAASLRAFTEAMTRYSAPPGDPPQPEYDLELITSCSSNVSV
AHDASGKRVYYLTRDPTTPLARAAWETARHTPVNSWLGNIIMYAPTLWARMILMTHFFSILL
AQEQLEKALDCQIYGACYSIEPLDLPQIIERLHGLSAFSLHSYSPGEINRVASCLRKLGVPP
LRVWRHRARSVRARLLSQGGRAATCGKYLFNWAVKTKLKLTPIPAASQLDLSGWFVAGYSGG
DIYHS
>d1khva_ e.8.1.4 (A:) Viral RNA-dependent RNA-polymerase {Rabbit
hemorrhagic disease virus}
FCGEPIDYRGITAHRLVGAEPRPPVSGTRYAKVPGVPDEYKTGYRPANLGRSDPDSDKSLMN
IAVKNLQVYQQEPKLDKVDEFIERAAADVLGYLRFLTKGERQANLNFKAAFNTLDLSTSCGP
FVPGKKIDHVKDGVMDQVLAKHLYKCWSVANSGKALHHIYACGLKDELRPLDKVKEGKKRLL
WGCDVGVAVCAAAVFHNICYKLKMVARFGPIAVGVDMTSRDVDVIINNLTSKASDFLCLDYS
KWDSTMSPCVVRLAIDILADCCEQTELTKSVVLTLKSHPMTILDAMIVQTKRGLPSGMPFTS
VINSICHWLLWSAAVYKSCAEIGLHCSNLYEDAPFYTYGDDGVYAMTPMMVSLLPAIIENLR
DYGLSPTAADKTEFIDVCPLNKISFLKRTFELTDIGWVSKLDKSSILRQLEWSKTTSRHMVI
EETYDLAKEERGVQLEELQVAAAAHGQEFFNFVCRELERQQAYTQFSVYSYDAARKILADRK
R
>d1hhsa_ e.8.1.6 (A:) dsRNA phage RNA-dependent RNA-polymerase
{Bacteriophage PHI-6}
PRRAPAFPLSDIKAQMLFANNIKAQQASKRSFKEGAIETYEGLLSVDPRFLSFKNELSRYLT
DHFPANVDEYGRVYGNGVRTNFFGMRHMNGFPMIPATWPLASNLKKRADADLADGPVSERDN

LLFRAAVRLMFSDLEPVPLKIRKGSSTCIPYFSNDMGTKIEIAERALEKAEEAGNLMLQGKF
DDAYQLHQMGGAYYVVYRAQSTDAITLDPKTGKFVSKDRMVADFEYAVTGGEQGSLFAASKD
ASRLKEQYGIDVPDGFFCERRRTAMGGPFALNAPIMAVAQPVRNKIYSKYAYTFHHTTRLNK
EEKVKEWSLCVATDVSDHDTFWPGWLRDLICDELLNMGYAPWWVKLFETSLKLPVYVGAPAP
EQGHTLLGDPSNPDLEVGLSSGQGATDLMGTLLMSITYLVMQLDHTAPHLNSRIKDMPSACR
FLDSYWQGHEEIRQISKSDDAMLGWTKGRALVGGHRLFEMLKEGKVNPSPYMKISYEHGGAF
LGDILLYDSRREPGSAIFVGNINSMLNNQFSPEYGVQSGVRDRSKRKRPFPGLAWASMKDTY
GACPIYSDVLEAIERCWWNAFGESYRAYREDMLKRDTLELSRYVASMARQAGLAELTPIDLE
VLADPNKLQYKWTEADVSANIHEVLMHGVSVEKTERFLRSVMPR
>d1i50a_ e.29.1.1 (A:) RBP1 {Baker's yeast (Saccharomyces
cerevisiae)}
VGQQYSSAPLRTVKEVQFGLFSPEEVRAISVAKIRFPETMDETQTRAKIGGLNDPRLGSIDR
NLKCQTCQEGMNECPGHFGHIDLAKPVFHVGFIAKIKKVCECVCMHCGKLLLDEHNELMRQA
LAIKDSKKRFAAIWTLCKTKMVCETDVPSEDDPTQLVSRGGCGNTQPTIRKDGLKLVGSWKK
DRATGDADEPELRVLSTEEILNIFKHISVKDFTSLGFNEVFSRPEWMILTCLPVPPPPVRPS
ISFNESQRGEDDLTFKLADILKANISLETLEHNGAPHHAIEEAESLLQFHVATYMDNDIAGQ
PQALQKSGRPVKSIRARLKGKEGRIRGNLMGKRVDFSARTVISGDPNLELDQVGVPKSIAKT
LTYPEVVTPYNIDRLTQLVRNGPNEHPGAKYVIRDSGDRIDLRYSKRAGDIQLQYGWKVERH
IMDNDPVLFNRQPSLHKMSMMAHRVKVIPYSTFRLNLSVTSPYNADFDGDEMNLHVPQSEET
RAELSQLCAVPLQIVSPQSNKPCMGIVQDTLCGIRKLTLRDTFIELDQVLNMLYWVPDWDGV
IPTPAIIKPKPLWSGKQILSVAIPNGIHLQRFDEGTTLLSPKDNGMLIIDGQIIFGVVEKKT
VGSSNGGLIHVVTREKGPQVCAKLFGNIQKVVNFWLLHNGFSTGIGDTIADGPTMREITETI
AEAKKKVLDVTKEAQANLLTAKHGMTLRESFEDNVVRFLNEARDKAGRLAEVNLKDLNNVKQ
MVMAGSKGSFINIAQMSACVGQQSVEGKRIAFGFVDRTLPHFSKDDYSPESKGFVENSYLRG
LTPQEFFFHAMGGREGLIDTAVKTAETGYIQRRLVKALEDIMVHYDNTTRNSLGNVIQFIYG
EDGMDAAHIEKQSLDTIGGSDAAFEKRYRVDLLNTDHTLDPSLLESGSEILGDLKLQVLLDE
EYKQLVKDRKFLREVFVDGEANWPLPVNIRRIIQNAQQTFHIDHTKPSDLTIKDIVLGVKDL
QENLLVLRGKNEIIQNAQRDAVTLFCCLLRSRLATRRVLQEYRLTKQAFDWVLSNIEAQFLR
SVVHPGEMVGVLAAQSIGEPATQMTLNTFHFAGVASKKVTSGVPRLKEILNVAKNMKTPSLT
VYLEPGHAADQEQAKLIRSAIEHTTLKSVTIASEIYYDPDPRSTVIPEDEEIIQLHFSLLDE
EAEQSFDQQSPWLLRLELDRAAMNDKDLTMGQVGERIKQTFKNDLFVIWSEDNDEKLIIRCR
VVRPKSLDAETEAEEDHMLKKIENTMLENITLRGVENIERVVMMKYDRKVPSPTGEYVKEPE
WVLETDGVNLSEVMTVPGIDPTRIYTNSFIDIMEVLGIEAGRAALYKEVYNVIASDGSYVNY
RHMALLVDVMTTQGGLTSVTRHGFNRSNTGALMRCSFEETVEILFEAGASAELDDCRGVSEN
VILGQMAPIGTGAFDVMIDEESL
>d1i6vc_ e.29.1.1 (C:) RNA-polymerase beta {Thermus aquaticus}
KIKRFGRIREVIPLPPLTEIQVESYKKALQADVPPEKRENVGIQAAFKETFPIEEGDKGKGG
LVLDFLEYRIGDPPFSQDECREKDLTYQAPLYARLQLIHKDTGLIKEDEVFLGHLPLMTEDG
SFIINGADRVIVSQIHRSPGVYFTPDPARPGRYIASIIPLPKRGPWIDLEVEASGVVTMKVN
KRKFPLVLLLRVLGYDQETLVRELSAYGDLVQGLLDEAVLAMRPEEAMVRLFTLLRPGDPPK
KDKALAYLFGLLADPKRYDLGEAGRYKAEEKLGVGLSGRTLVRFEDGEFKDEVFLPTLRYLF
ALTAGVPGHEVDDIDHLGNRRIRTVGELMADQFRVGLARLARGVRERMVMGSPDTLTPAKLV
NSRPLEAALREFFSRSQLSQFKDETNPLSSLRHKRRISALGPGGLTRERAGFDVRDVHRTHY
GRICPVETPEGANIGLITSLAAYARVDALGFIRTPYRRVKNGVVTEEVVYMTASEEDRYTIA

QANTPLEGDRIATDRVVARRRGEPVIVAPEEVEFMDVSPKQVFSLNTNLIPFLEHDDANRAL
MGSNMQTQAVPLIRAQAPVVMTGLEERVVRDSLAALYAEEDGEVVKVDGTRIAVRYEDGRLV
HPLRRYARSNQGTAFDQRPRVRVGQRVKKGDLLADGPASEEGFLALGQNVLVAIMPFDGYNF
EDAIVISEELLKRDFYTSIHIERYEIEARDTKLGPERITRDIPHLSEAALRDLDEEGIVRIG
AEVKPGDILVGRTSFKGEQEPSPEERLLRSIFGEKARDVKDTSLRVPPGEGGIVVGRLRLRR
GDPGVELKPGVREVVRVFVAQKRKLQVGDKLANRHGNKGVVAKILPVEDMPHLPDGTPVDVI
LNPLGVPSRMNLGQILETHLGLAGYFLGQRYISPVFDGATEPEIKELLAEAFNLYFGKRQGE
GFGVDKREKEVLARAEKLGLVSPGKSPEEQLKELFDLGKVVLYDGRTGEPFEGPIVVGQMFI
MKLYHMVEDKMHARSTGPYSLITQQPLGGKAQFGGQRFGEMEVWALEAYGAAHTLQEMLTIK
SDDIEGRNAAYQAIIKGEDVPEPSVPESFRVLVKELQALALDVQTLDEKDNPVDVFEGL
>d1i50b_ e.29.1.2 (B:) RBP2 {Baker's yeast (Saccharomyces cerevisiae)}
FEDESAPITAEDSWAVISAFFREKGLVSQQLDSFNQFVDYTLQDIICEDSTLILEQLAQHTT
ESDNISRKYEISFGKIYVTKPMVNESDGVTHALYPQEARLRNLTYSSGLFVDVKKRTYEAID
VPGRELKYELIAEESEDDSESGKVFIGRLPIMLRSKNCYLSEATESDLYKLKECPFDMGGYF
IINGSEKVLIAQERSAGNIVQVFKKAAPSPISHVAEIRSALEKGSRFISTLQVKLYGREGSS
ARTIKATLPYIKQDIPIVIIFRALGIIPDGEILEHICYDVNDWQMLEMLKPCVEDGFVIQDR
ETALDFIGRRGTALGIKKEKRIQYAKDILQKEFLPHITQLEGFESRKAFFLGYMINRLLLCA
LDRKDQDDRDHFGKKRLDLAGPLLAQLFKTLFKKLTKDIFRYMQRTVEEAHDFNMKLAINAK
TITSGLKYALATGNWGEQKKAMSSRAGVSQVLNRYTYSSTLSHLRRTNTPIGRDGKLAKPRQ
LHNTHWGLVCPAETPEGQACGLVKNLSLMSCISVGTDPMPIITFLSEWGMEPLEDYVPHQSP
DATRVFVNGVWHGVHRNPARLMETLRTLRRKGDINPEVSMIRDIREKELKIFTDAGRVYRPL
FIVEDDESLGHKELKVRKGHIAKLMATEYQDIEGGFEDVEEYTWSSLLNEGLVEYIDAEEEE
SILIAMQPEDLEPAEANEENDLDVDPAKRIRVSHHATTFTHCEIHPSMILGVAASIIPFPDH
NQSPRNTYQSAMGKQAMGVFLTNYNVRMDTMANILYYPQKPLGTTRAMEYLKFRELPAGQNA
IVAIACYSGYNQEDSMIMNQSSIDRGLFRSLFFRSYMDQEKKYGMSITETFEKPQRTNTLRM
KHGTYDKLDDDGLIAPGVRVSGEDVIIGKTTPISPDEEELGQRTAYHSKRDASTPLRSTENG
IVDQVLVTTNQDGLKFVKVRVRTTKIPQIGDKFASRHGQKGTIGITYRREDMPFTAEGIVPD
LIINPHAIPSRMTVAHLIECLLSKVAALSGNEGDASPFTDITVEGISKLLREHGYQSRGFEV
MYNGHTGKKLMAQIFFGPTYYQRLRHMVDDKIHARARGPMQVLTRQPVEGRSRDGGLRFGEM
ERDCMIAHGAASFLKERLMEASDAFRVHICGICGLMTVIAKLNHNQFECKGCDNKIDIYQIH
IPYAAKLLFQELMAMNITPRLYTDRSRDF
>d1i6vd_ e.29.1.2 (D:) RNA-polymerase beta-prime {Thermus aquaticus}
KEVRKVRIALASPEKIRSWSYGEVEKPETINYRTLKPERDGLFDERIFGPIKDYECACGKYK
RQRFEAKVCERCAVEVTRSIVRRYRMAHIELATPAAHIWFVKDVPSKIATLLDLSATELEQV
LYFNKYIVLDPKAAVLDAVPVEKRQLLTDXXXXXXXXXXXXXXXXXXXXXXXXXXXXXXXXX
XXXIDARMGAEAIQELLKELDLEKLERELLEEMKHPSRARRAKARKRLEVVRAFLDSGNRPE
WMILEAVPVLPPDLRPMVQVDGGRFATSDLNDLYRRLINRNNRLKKLLAQGAPEIIIRNEKR
MLQEAVDAVIDNGRRGSPVTNPGSERPLRSLTDILSGKQGRFRQNLLGKRVDYSGRSVIVVG
PQLKLHQCGLPKRMALELFKPFLLKKMEEKAFAPNVKAARRMLERQRDIKDEVWDALEEVIH
GKVVLLNRAPTLHRLGIQAFQPVLVEGQSIQLHPLVCEAFNADFDGDQMAVHVPLSSFAQAE
ARIQMLSAHNLLSPASGEPLAKPSRDIILGLYYITQVRKEKKGAGMAFATPEEALAAYERGE
VALNAPIVVAGRETSVGRLKFVFANPDEALLAVAHGLLDLQDTVTTRYLGRRLETSPGRILF

ARIVGEAVGDEKVAQELIQMDVPQEKNSLKDLVYQAFLRLGMEKTARLLDALKYYGFTLSTT
SGITIGIDDAVIPEEKQRYLEEADRKLRQIEQAYEMGFLTDRERYDQVIQLWTETTEKVTQA
VFNNFEENYPFNPLYVMAQSGARGNPQQIRQLCGMRGLMQKPSGETFEVPVRSSFREGLTVL
EYFISSHGARKGGADTALRTADSGYLTRKLVDVAHEIVVREADCGTTNYISVPLFQMDEVTR
TLRLRKRSDIESGLYGRVLAREVEALGRRLEEGRYLSLEDVHFLIKAAEAGEVREVPVRSPL
TCQTRYGVCQKCYGYDLSMARPVSIGEAVGVVAAESIGEPGTQLTMRTFHTGGVAVGTDITQ
GLPRVIELFEARRPKAKAVISEIDGVVRIEEGEDRLSVFVESEGFSKEYKLPKDARLLVKDG
DYVEAGQPLTRGAIDPHQLLEAKGPEAVERYLVDEIQKVYRAQGVKLHDKHIEIVVRQMLKY
VEVTDPGDSRLLEGQVLEKWDVEALNERLIAEGKVPVAWKPLLMGVTKSALSTKSWLSAASF
QNTTHVLTEAAIAGKKDELIGLKENVILGRLIPAGTGSDFVRFTQVVDQRTLKAIE
>d1bpya2 e.9.1.1 (A:92-335) DNA polymerase beta, catalytic (31 kD) fragment {Human (Homo sapiens)}
DTSSSINFLTRVSGIGPSAARKFVDEGIKTLEDLRKNEDKLNHHQRIGLKYFGDFEKRIPRE
EMLQMQDIVLNEVKKVDSEYIATVCGSFRRGAESSGDMDVLLTHPSFTSESTKQPKLLHQVV
EQLQKVHFITDTLSKGETKFMGVCQLPSKNDEKEYPHRRIDIRLIPKDQYYCGVLYFTGSDI
FNKNMRAHALEKGFTINEYTIRPLGVTGVAGEPLPVDSEKDIFDYIQWKYREPKDRSE
>d1jn3a_ e.9.1.1 (A:) DNA polymerase beta, catalytic (31 kD) fragment {Rat (Rattus norvegicus)}
DDTSSSINFLTRVTGIGPSAARKLVDEGIKTLEDLRKNEDKLNHHQRIGLKYFEDFEKRIPR
EEMLQMQDIVLNEVKKLDPEYIATVCGSFRRGAESSGDMDVLLTHPNFTSESSKQPKLLHRV
VEQLQKVRFITDTLSKGETKFMGVCQLPSENDENEYPHRRIDIRLIPKDQYYCGVLYFTGSD
IFNKNLRAHALEKGFTINEYTIRPLGVTGVAGEPLPVDSEQDIFDYIQWRYREPKDRSE
>d1jmsa2 e.9.1.1 (A:243-510) Terminal deoxynucleotidyl transferase {Mouse (Mus musculus)}
DERYKSFKLFTSVFGVGLKTAEKWFRMGFRTLSKIQSDKSLRFTQMQKAGFLYYEDLVSCVN
RPEAEAVSMLVKEAVVTFLPDALVTMTGGFRRGKMTGHDVDFLITSPEATEDEEQQLLHKVT
DFWKQQGLLLYCDILESTFEKFKQPSRKVDALDHFQKCFLILKLDHGRVHSEKSGQQEGKGW
KAIRVDLVMCPYDRRAFALLGWTGSRQFERDLRRYATHERKMMLDNHALYDRTKRVFLEAES
EEEIFAHLGLDYIEPWERNA
>d1jaja_ e.9.1.1 (A:) DNA polymerase X {African swine fever virus}
MLTLIQGKKIVNHLRSRLAFEYNGQLIKILSKNIVAVGSLRREEKMLNDVDLLIIVPEKKLL
KHVLPNIRIKGLSFSVKVCGERKCVLFIEWEKKTYQLDLFTALAEEKPYAIFHFTGPVSYLI
RIRAALKKKNYKLNQYGLFKNQTLVPLKITTEKELIKELGFTYRIPKKRL
>d1fa0a2 e.9.1.2 (A:3-351) Poly(A) polymerase, catalytic domain {Baker's yeast (Saccharomyces cerevisiae)}
SQKVFGITGPVSTVGATAAENKLNDSLIQELKKEGSFETEQETANRVQVLKILQELAQRFVY
EVSKKKNMSDGMARDAGGKIFTYGSYRLGVHGPGSDIDTLVVVPKHVTREDFFTVFDSLLRE
RKELDEIAPVPDAFVPIIKIKFSGISIDLICARLDQPQVPLSLTLSDKNLLRNLDEKDLRAL
NGTRVTDEILELVPKPNVFRIALRAIKLWAQRRAVYANIFGFPGGVAWAMLVARICQLYPNA
CSAVILNRFFIILSEWNWPQPVILKPIEDGPLQVRVWNPKIYAQDRSHRMPVITPAYPSMCA
THNITESTKKVILQEFVRGVQITNDIFSNKKSWANLFEK
>d1f5aa2 e.9.1.2 (A:20-364) Poly(A) polymerase, catalytic domain {Cow (Bos taurus)}
YGITSPISLAAPKETDCLLTQKLVETLKPFGVFEEEEELQRRILILGKLNNLVKEWIREISE

SKNLPQSVIENVGGKIFTFGSYRLGVHTKGADIDALCVAPRHVDRSDFFTSFYDKLKLQEEV
KDLRAVEEAFVPVIKLCFDGIEIDILFARLALQTIPEDLDLRDDSLLKNLDIRCIRSLNGCR
VTDEILHLVPNIDNFRLTLRAIKLWAKRHNIYSNILGFLGGVSWAMLVARTCQLYPNAIAST
LVHKFFLVFSKWEWPNPVLLKQPEECNLNLPVWDPRVNPSDRYHLMPIITPAYPQQNSTYNV
SVSTRMVMVEEFKQGLAITDEILLSKAEWSKLFEA
>d1knya_ e.9.1.3 (A:) Kanamycin nucleotidyltransferase (KNTase)
{Staphylococcus aureus}
MNGPIIMTREERMKIVHEIKERILDKYGDDVKAIGVYGSLGRQTDGPYSDIEMMCVMSTEEA
EFSHEWTTGEWKVEVNFYSEEILLDYASQVESDWPLTHGQFFSILPIYDSGGYLEKVYQTAK
SVEAQTFHDAICALIVEELFEYAGKWRNIRVQGPTTFLPSLTVQVAMAGAMLIGLHHRICYT
TSASVLTEAVKQSDLPSGYDHLCQFVMSGQLSDSEKLLESLENFWNGIQEWTERHGYIVDVS
KRIPF
>d1k8ta_ e.9.1.4 (A:) Adenylylcyclase toxin (the edema factor)
{Bacillus anthracis}
DRIDVLKGEKALKASGLVPEHADAFKKIARELNTYILFRPVNKLATNLIKSGVATKGLNVHG
KSSDWGPVAGYIPFDQDLSKKHGQQLAVEKGNLENKKSITEHEGEIGKIPLKLDHLRIEELK
ENGIILKGKKEIDNGKKYYLLESNNQVYEFRISDENNEVQYKTKEGKITVLGEKFNWRNIEV
MAKNVEGVLKPLTADYDLFALAPSLTEIKKQIPQKEWDKVVNTPNSLEKQKGVTNLLIKYGI
ERKPDSTKGTLSNWQKQMLDRLNEAVKYTGYTGGDVVNHGTEQDNEEFPEKDNEIFIINPEG
EFILTKNWEMTGRFIEKNITGKDYLYYFNRSYNKIAPGNKAYIEWTDPITKAKINTIPTSAE
FIKNLSSIRRSSNVGVYKDSGDKDEFAKKESVKKIAGYLSDYYNSANHIFSQEKKRKISIFR
GIQAYNEIENVLKSKQIAPEYKNYFQYLKERITNQVQLLLTHQKSNIEFKLLYKQLNFTENE
TDNFEVFQKIIDE
>d1cy9a_ e.10.1.1 (A:) DNA topoisomerase I, 67K N-terminal domain
{Escherichia coli}
FVPEEFWEVDASTTTPSGEALALQVTHQNDKPFRPVNKEQTQAAVSLLEKARYSVLEREDKP
TTSKPGAPFITSTLQQAASTRLGFGVKKTMMMAQRLYEAGYITYMRTDSTNLSQDAVNMVRG
YISDNFGKKYLPESPNQYASKENSQEAHEAIRPSDVNVMAESLKDMEADAQKLYQLIWRQFV
ACQMTPAKYDSTTLTVGAGDFRLKARGRILRFDGWTKVMPALRKGDEDRILPAVNKGDALTL
VELTPAQHFTKP
>d1ecl__ e.10.1.1 (-) DNA topoisomerase I, 67K N-terminal domain
{Escherichia coli}
GKALVIVESPAKAKTINKYLGSDYVVKSSVGHIRDLTSGSAAKKSADSTSTKTAKKPKKPDE
RGALVNRMGVDPWHNWEAHYEVLPGKEKVVSELKQLAEKADHIYLATDLDREGEAIAWHLRE
VIGGDDARYSRVVFNEITKNAIRQAFNKPGELNIDRVNAQQARRFMDRVVGYMVSPLLWKKI
ARGLSAGRVQSVAVRLVVEREREIKAFVPEEFWEVDASTTTPSGEALALQVTHQNDKPFRPV
NKEQTQAAVSLLEKARYSVLEREDKPTTSKPGAPFITSTLQQAASTRLGFGVKKTMMMAQRL
YEAGYITYMRTDSTNLSQDAVNMVRGYISDNFGKKYLPESPNQYASKGNSQEAHEAIRPSDV
NVMAESLKDMEADAQKLYQLIWRQFVACQMTPAKYDSTTLTVGAGDFRLKARGRILRFDGWT
KVMPALRKGDEDRILPAVNKGDALTLVELTPAQHFTKPPARFSEASLVKELEKRGIGRPSTY
ASIISTIQDRGYVRVENRRFYAEKMGEIVTDRLEENFRELMNYDFTAQMENSLDQVANHEAE
WKAVLDHFFSDFTQQLDKAEKDPEEGGMRPN
>d1i7da_ e.10.1.1 (A:) DNA topoisomerase III {Escherichia coli}
MRLFIAEKPSLARAIADVLPKPHRKGDGFIECGNGQVVTWCIGHLLEQAQPDAYDSRYARWN

LADLPIVPEKWQLQPRPSVTKQLNVIKRFLHEASEIVHAGDPDREGQLLVDEVLDYLQLAPE
KRQQVQRCLINDLNPQAVERAIDRLRSNSEFVPLCVSALARARADWLYGINMTRAYTILGRN
AGYQGVLSVGRVQTPVLGLVVRRDEEIENFVAKDFFEVKAHIVTPADERFTAIWQPSEACEP
YQDEEGRLLHRPLAEHVVNRISGQPAIVTSYNDKRESESAPLPFSLSALQIEAAKRFGLSAQ
NVLDICQKLYETHKLITFPRSDCRYLPEEHFAGRHAVMNAISVHAPDLLPQPVVDPDIRNRC
WDDKKVDAHHAIIPTARSSAINLTENEAKVYNLIARQYLMQFCPDAVFRKCVIELDIAKGKF
VAKARFLAEAGWRTLLGSKERDEENDGTPLPVVAKGDELLCEKGEVVERQTQPPRHFTDATL
LSAMTGIARFVQDKDLKKILRATDGLGTEATRAGIIELLFKRGFLTKKGRYIHSTDAGKALF
HSLPEMATRPDMTAHWESVLTQISEKQCRYQDFMQPLVGTLYQLIDQAKRTPVRQFRGIVAP
>d1gkub3 e.10.1.1 (B:499-1054) Topoisomerase "domain" of reverse gyrase {Archaeon Archaeoglobus fulgidus}
QEFDLIKPALFIVESPTKARQISRFFGKPSVKVLDGAVVYEIPMQKYVLMVTASIGHVVDLI
TNRGFHGVLVNGRFVPVYASIKRCRDCGYQFTEDRESCPKCGSENVDNSRSRIEALRKLAHD
AEFVIVGTDPDTEGEKIAWDLKNLLSGCGAVKRAEFHEVTRRAILEALESLRDVDENLVKAQ
VVRRIEDRWIGFVLSQKLWERFNNRNLSAGRAQTLVLGWIIDRFQESRERRKIAIVRDFDLV
LEHDEEEFDLTIKLVEEREELRTPLPPYTTETMLSDANRILKFSVKQTMQIAQELFENGLIT
YHRTDSTRVSDVGQRIAKEYLGDDFVGREWGESGAHECIRPTRPLTRDDVQRLIQEGVLVVE
GLRWEHFALYDLIFRRFMASQCRPFKVVVKKYSIEFDGKTAEEERIVRAEGRAYELYRAVWV
KNELPTGTFRVKAEVKSVPKVLPFTQSEIIQMMKERGIGRPSTYATIVDRLFMRNYVVEKYG
RMIPTKLGIDVFRFLVRRYAKFVSEDRTRDLESRMDAIERGELDYLKALEDMYAEIKSID
>d1bjt__ e.11.1.1 (-) DNA topoisomerase II, C-terminal fragment (residues 410-1202) {Baker's yeast (Saccharomyces cerevisiae)}
RKSRITNYPKLEDANKAGTKEGYKCTLVLTEGDSALSLAVAGLAVVGRDYYGCYPLRGKMLN
VREASADQILKNAEIQAIKKIMGLQHRKKYEDTKSLRYGHLMIMTDQDHDGSHIKGLIINFL
ESSFLGLLDIQGFLLEFITPIIKVSITKPTKNTIAFYNMPDYEKWREEESHKFTWKQKYYKG
LGTSLAQEVREYFSNLDRHLKIFHSLQGNDKDYIDLAFSKKKADDRKEWLRQYEPGTVLDPT
LKEIPISDFINKELILFSLADNIRSIPNVLDGFKPGQRKVLYGCFKKNLKSELKVAQLAPYV
SECTAYHHGEQSLAQTIIGLAQNFVGSNNIYLLLPNGAFGTRATGGKDAAAARYIYTELNKL
TRKIFHPADDPLYKYIQEDEKTVEPEWYLPILPMILVNGAEGIGTGWSTYIPPFNPLEIIKN
IRHLMNDEELEQMHPWFRGWTGTIEEIEPLRYRMYGRIEQIGDNVLEITELPARTWTSTIKE
YLLLGLSGNDKIKPWIKDMEEQHDDNIKFIITLSPEEMAKTRKIGFYERFKLISPISLMNMV
AFDPHGKIKKYNSVNEILSEFYYVRLEYYQKRKDHMSERLQWEVEKYSFQVKFIKMIIEKEL
TVTNKPRNAIIQELENLGFPRFNKEGKPYYGSPNDEIAEQINDVKGATSDEEDEESSHEDTE
NVINGPEELYGTYEYLLGMRIWSLTKERYQKLLKQKQEKETELENLLKLSAKDIWNTDLKAF
EVGYQEFLQRDAEARG
>d1ab4__ e.11.1.1 (-) DNA Gyrase A {Escherichia coli}
VGRALPDVRDGLKPVHRRVLYAMNVLGNDWNKAYKKSARVVGDVIGKYHPHGDSAVYDTIVR
MAQPFSLRYMLVDGQGNFGSIDGDSAAAMRYTEIRLAKIAHELMADLEKETVDFVDNYDGTE
KIPDVMPTKIPNLLVNGSSGIAVGMATNIPPHNLTEVINGCLAYIDDEDISIEGLMEHIPGP
DFPTAAIINGRRGIEEAYRTGRGKVYIRARAEVEVETIIVHEIPYQVNKARLIEKIAELVKE
KRVEGISALRDESDKDGMRIVIEGEVVLNNLYSQTQLQVSFGINMVALHHGQPKIMNLKDII
AAFVRHRREVVTRRTIFELRKARDRAHILEALAVALANIDPIIELIRHAPTPAEAKTALVAN
PWQLGNVAAMLEDAARPEWLEPEFGVRDGLYYLTEQQAQAILDLRLQKLTGLEHEKLLDEYK
ELLDQIAELLRILGSADRLMEVIREELELVREQFGDKRRTEIT

>d1d3ya_ e.12.1.1 (A:) DNA topoisomerase IV, alpha subunit {Archaeon Methanococcus jannaschii}
QAKIFAQTTKMLEFAKQLLETDDFSTLREAYYVSKNWGEARFDDQQASNNVIEDLEAALGVL
REHLGFIPEEDGSSVVGPLKIIEETPEGELVVDCTKLGTGAYNIPNDVTKLNLETDADFILA
IETSGMFARLNAERFWDKHNCILVSLKGVPARATRRFIKRLHEEHDLPVLVFTDGDPYGYLN
IYRTLKVGSGKAIHLADKLSIPAARLIGVTPQDIIDYDLPTHPLKEQDIKRIKDGLKNDDFV
RSFPEWQKALKQMLDMGVRAEQQSLAKYGLKYVVNTYLPEKIKDESTWLP
>d1dd9a_ e.13.1.1 (A:) DNA primase DnaG catalytic core {Escherichia coli}
TLYQLMDGLNTFYQQSLQQPVATSARQYLEKRGLSHEVIARFAIGFAPPGWDNVLKRFGGNP
ENRQSLIDAGMLVTNDQGRSYDRFRERVMFPIRDKRGRVIGFGGRVLGNDTPKYLNSPETDI
FHKGRQLYGLYEAQQDNAEPNRLLVVEGYMDVVALAQYGINYAVASLGTSTTADHIQLLFRA
TNNVICCYDGDRAGRDAAWRALETALPYMTDGRQLRFMFLPDGEDPDTLVRKEGKEAFEARM
EQAMPLSAFLFNSLMPQVDLSTPDGRARLSTLALPLISQVPGETLRIYLRQELGNKLGILDD
SQLE
>d1ee8a_ e.14.1.1 (A:) DNA repair protein MutM (Fpg) {Thermus thermophilus}
PELPEVETTRRRLRPLVLGQTLRQVVHRDPARYRNTALAEGRRILEVDRRGKFLLFALEGGV
ELVAHLGMTGGFRLEPTPHTRAALVLEGRTLYFHDPRRFGRLFGVRRGDYREIPLLLRLGPE
PLSEAFAFPGFFRGLKESARPLKALLLDQRLAAGVGNIYADEALFRARLSPFRPARSLTEEE
ARRLYRALREVLAEAVELGGSTLSDQSYRQPDGLPGGFQTRHAVYGREGLPCPACGRPVERR
VVAGRGTHFCPTCQGEGP
>d1i3ja_ e.30.1.1 (A:) DNA-binding domain of intron endonuclease I-TevI {Bacteriophage T4}
KFCKCGVRIQTSAYTCSKCRNRSGENNSFFNHKHSDITKSKISEKMKGKKPSNIKKISCDGV
IFDCAADAARHFKISSGLVTYRVKSDKWNWFYIN
>d1a31a2 e.15.1.1 (A:215-430) Eukaryotic DNA topoisomerase I, N-terminal DNA-binding fragment {Human (Homo sapiens)}
IKWKFLEHKGPVFAPPYEPLPENVKFYYDGKVMKLSPKAEEVATFFAKMLDHEYTTKEIFRK
NFFKDWRKEMTNEEKNIITNLSKCDFTQMSQYFKAQTEARKQMSKEEKLKIKEENEKLLKEY
GFCIMDNHKERIANFKIEPPGLFRGRGNHPKMGMLKRRIMPEDIIINCSKDAKVPSPPPGHK
WKEVRHDNKVTWLVSWTENIQGSIKYIMLN
>d1ois__ e.15.1.1 (-) Eukaryotic DNA topoisomerase I, N-terminal DNA-binding fragment {Baker's yeast (Saccharomyces cerevisiae)}
DTIKWVTLKHNGVIFPPPYQPLPSHIKLYYDGKPVDLPPQAEEVAGFFAALLESDHAKNPVF
QKNFFNDFLQVLKESGGPLNGIEIKEFSRCDFTKMFDYFQLQKEQKKQLTSQEKKQIRLERE
KFEEDYKFCELDGRREQVGNFKVEPPDLFRGRGAHPKTGKLKRRVNPEDIVLNLSKDAPVPP
APEGHKWGEIRHDNTVQWLAMWRENIFNSFKYVRLAA
>d1g71a_ e.16.1.1 (A:) DNA primase {Archaeon Pyrococcus furiosus}
MLMREVTKEERSEFYSKEWSAKKIPKFIVDTLESREFGFDHNGEGPSDRKNQYSDIRDLEDY
IRATSPYAVYSSVAFYENPREMEGWRGAELVFDIDAKDLPLKRCNHEPGTVCPICLEDAKEL
AKDTLIILREELGFENIHVVYSGRGYHIRILDEWALQLDSKSRERILAFISASEIENVEEFR
RFLLEKRGWFVLKHGYPRVFRLRLGYFILRVNVPHLLSIGIRRNIAKKILDHKEEIYEGFVR
KAILASFPEGVGIESMAKLFALSTRFSKAYFDGRVTVDIKRILRLPSTLHSKVGLIATYVGT

KEREVMKFNPFRHAVPKFRKKEVREAYKLWRESL
>d1jeya_ e.31.1.1 (A:) Ku70 subunit {Human (Homo sapiens)}
GRDSLIFLVDASKAMFESQSEDELTPFDMSIQCIQSVYISKIISSDRDLLAVVFYGTEKDKN
SVNFKNIYVLQELDNPGAKRILELDQFKGQQGQKRFQDMMGHGSDYSLSEVLWVCANLFSDV
QFKMSHKRIMLFTNEDNPHGNDSAKASRARTKAGDLRDTGIFLDLMHLKKPGGFDISLFYRD
IISIAEDEDLRVHFEESSKLEDLLRKVRAKETRKRALSRLKLKLNKDIVISVGIYNLVQKAL
KPPPIKLYRETNEPVKTKTRTFNTSTGGLLLPSDTKRSQIYGSRQIILEKEETEELKRFDDP
GLMLMGFKPLVLLKKHHYLRPSLFVYPEESLVIGSSTLFSALLIKCLEKEVAALCRYTPRRN
IPPYFVALVPQEEELDDQKIQVTPPGFQLVFLPFADDKRKMPFTEKIMATPEQVGKMKAIVE
KLRFTYRSDSFENPVLQQHFRNLEALALDLMEPEQAVDLTLPKVEAMNKRLGSLVDEFKELV
YPPDY
>d1jeyb_ e.31.1.2 (B:) Ku80 subunit {Human (Homo sapiens)}
NKAAVVLCMDVGFTMSNSIPGIESPFEQAKKVITMFVQRQVFAENKDEIALVLFGTDGTDNP
LSGGDQYQNITVHRHLMLPDFDLLEDIESKIQPGSQQADFLDALIVSMDVIQHETIGKKFEK
RHIEIFTDLSSRFSKSQLDIIIHSLKKCDISLQFFLPFSLGKEDGSGDRGDGPFRLGGHGPS
FPLKGITEQQKEGLEIVKMVMISLEGEDGLDEIYSFSESLRKLCVFKKIERHSIHWPCRLTI
GSNLSIRIAAYKSILQERVKKTWTVVDAKTLKKEDIQKETVYCLNDDDETEVLKEDIIQGFR
YGSDIVPFSKVDEEQMKYKSEGKCFSVLGFCKSSQVQRRFFMGNQVLKVFAARDDEAAAVAL
SSLIHALDDLDMVAIVRYAYDKRANPQVGVAFPHIKHNYECLVYVQLPFMEDLRQYMFSSLK
NSKKYAPTEAQLNAVDALIDSMSLAKKDEKTDTLEDLFPTTKIPNPRFQRLFQCLLHRALHP
REPLPPIQQHIWNMLNPPAEVTTKSQIPLSKIKTLFPLIEAKKK
>d1daaa_ e.17.1.1 (A:) D-amino acid aminotransferase {Bacillus sp., strain YM-1}
GYTLWNDQIVKDEEVKIDKEDRGYQFGDGVYEVVKVYNGEMFTVNEHIDRLYASAEKIRITI
PYTKDKFHQLLHELVEKNELNTGHIYFQVTRGTSPRAHQFPENTVKPVIIGYTKENPRPLEN
LEKGVKATFVEDIRWLRCDIKSLNLLGAVLAKQEAHEKGCYEAILHRNNTVTEGSSSNVFGI
KDGILYTHPANNMILKGITRDVVIACANEINMPVKEIPFTTHEALKMDELFVTSTTSEITPV
IEIDGKLIRDGKVGEWTRKLQKQFETKIP
>d1i1ka_ e.17.1.1 (A:) Branched-chain amino acid aminotransferase {Escherichia coli}
KADYIWFNGEMVRWEDAKVHVMSHALHYGTSVFEGIRCYDSHKGPVVFRHREHMQRLHDSAK
IYRFPVSQSIDELMEACRDVIRKNNLTSAYIRPLIFVGDVGMGVNPPAGYSTDVIIAAFPWG
AYLGAEALEQGIDAMVSSWNRAAPNTIPTAAKAGGNYLSSLLVGSEARRHGYQEGIALDVNG
YISEGAGENLFEVKDGVLFTPPFTSSALPGITRDAIIKLAKELGIEVREQVLSRESLYLADE
VFMSGTAAEITPVRSVDGIQVGEGRCGPVTKRIQQAFFGLFTGETEDKWGWLDQVNQ
>d1ekfa_ e.17.1.1 (A:) Branched-chain amino acid aminotransferase {Human (Homo sapiens), mitochondrial}
ASSSFKAADLQLEMTQKPHKKPGPGEPLVFGKTFTDHMLMVEWNDKGWGQPRIQPFQNLTLH
PASSSLHYSLQLFEGMKAFKGKDQQVRLFRPWLNMDRMLRSAMRLCLPSFDKLELLECIRRL
IEVDKDWVPDAAGTSLYVRPVLIGNEPSLGVSQPRRALLFVILCPVGAYFPGGSVTPVSLLA
DPAFIRAWVGGVGNYKLGGNYGPTVLVQQEALKRGCEQVLWLYGPDHQLTEVGTMNIFVYWT
HEDGVLELVTPPLNGVILPGVVRQSLLDMAQTWGEFRVVERTITMKQLLRALEEGRVREVFG
SGTACQVCPVHRILYKDRNLHIPTMENGPELILRFQKELKEIQYGIRAHEWMFPV
>d1et0a_ e.17.1.1 (A:) Aminodeoxychorismate lyase {Escherichia

coli}
MFLINGHKQESLAVSDRATQFGDGCFTTARVIDGKVSLLSAHIQRLQDACQRLMISCDFWPQ
LEQEMKTLAAEQQNGVLKVVISRGSGGRGYSTLNSGPATRILSVTAYPAHYDRLRNEGITLA
LSPVRLGRNPHLAGIKHLNRLEQVLIRSHLEQTNADEALVLDSEGWVTECCAANLFWRKGNV
VYTPRLDQAGVNGIMRQFCIRLLAQSSYQLVEVQASLEESLQADEMVICNALMPVMPVCACG
DVSFSSATLYEYLAPLCE
>d2frvb_ e.18.1.1 (B:) Nickel-iron hydrogenase, large subunit
{Desulfovibrio gigas}
NKIVVDPITRIEGHLRIEVEVEGGKIKNAWSMSTLFRGLEMILKGRDPRDAQHFTQRACGVC
TYVHALASVRAVDNCVGVKIPENATLMRNLTMGAQYMHDHLVHFYHLHALDWVNVANALNAD
PAKAARLANDLSPKKTTTESLKAVQAKVKALVESGQLGIFTNAYFLGGHPAYVLPAEVDLIA
TAHYLEALRVQVKAARAMAIFGAKNPHTQFTVVGGCTNYDSLRPERIAEFRKLYKEVREFIE
QVYITDLLAVAGFYKNWAGIGKTSNFLTCGEFPTDEYDLNSRYTPQGVIWGNDLSKVDDFNP
DLIEEHVKYSWYEGAGAHHPYKGVTKPKWTEFHGEDRYSWMKAPRYKGEAFEVGPLASVLVA
YAKKHEPTVKAVDLVLKTLGVGPEALFSTLGRTAARGIQCLTAAQEVEVWLDKLEANVKAGK
DDLYTDWQYPTESQGVGFVNAPRGMLSHWIVQRGGKIENFQLVVPSTWNLGPRCAEGKLSAV
EQALIGTPIADPKRPVEILRTVHSYDPCIACGVH
>d1h2rl_ e.18.1.1 (L:) Nickel-iron hydrogenase, large subunit
{Desulfovibrio vulgaris}
SSYSGPIVVDPVTRIEGHLRIEVEVENGKVKNAYSSSTLFRGLEIILKGRDPRDAQHFTQRT
CGVCTYTHALASTRCVDNAVGVHIPKNATYIRNLVLGAQYLHDHIVHFYHLHALDFVDVTAA
LKADPAKAAKVASSISPRKTTAADLKAVQDKLKTFVETGQLGPFTNAYFLGGHPAYYLDPET
NLIATAHYLEALRLQVKAARAMAVFGAKNPHTQFTVVGGVTCYDALTPQRIAEFEALWKETK
AFVDEVYIPDLLVVAAAYKDWTQYGGTDNFITFGEFPKDEYDLNSRFFKPGVVFKRDFKNIK
PFDKMQIEEHVRHSWYEGAEARHPWKGQTQPKYTDLHGDDRYSWMKAPRYMGEPMETGPLAQ
VLIAYSQGHPKVKAVTDAVLAKLGVGPEALFSTLGRTAARGIETAVIAEYVGVMLQEYKDNI
AKGDNVICAPWEMPKQAEGVGFVNAPRGGLSHWIRIEDGKIGNFQLVVPSTWTLGPRCDKNN
VSPVEASLIGTPVADAKRPVEILRTVHSFDPCIACGVH
>d1frfl_ e.18.1.1 (L:) Nickel-iron hydrogenase, large subunit
{Desulfovibrio fructosovorans}
TPQSTFTGPIVVDPITRIEGHLRIMVEVENGKVKDAWSSSQLFRGLEIILKGRDPRDAQHFT
QRACGVCTYVHALASSRCVDDAVKVSIPANARMMRNLVMASQYLHDHLVHFYHLHALDWVDV
TAALKADPNKAAKLAASIDTARTGNSEKALKAVQDKLKAFVESGQLGIFTNAYFLGGHKAYY
LPPEVNLIATAHYLEALHMQVKAASAMAILGGKNPHTQFTVVGGCSNYQGLTKDPLANYLAL
SKEVCQFVNECYIPDLLAVAGFYKDWGGIGGTSNYLAFGEFATDDSSPEKHLATSQFPSGVI
TGRDLGKVDNVDLGAIYEDVKYSWYAPGGDGKHPYDGVTDPKYTKLDDKDHYSWMKAPRYKG
KAMEVGPLARTFIAYAKGQPDFKKVVDMVLGKLSVPATALHSTLGRTAARGIETAIVCANME
KWIKEMADSGAKDNTLCAKWEMPEESKGVGLADAPRGSLSHWIRIKGKKIDNFQLVVPSTWN
LGPRGPQGDKSPVEEALIGTPIADPKRPVEILRTVHAFDPCIACGVH
>d1cc1l_ e.18.1.1 (L:) Nickel-iron hydrogenase, large subunit
{Desulfomicrobium baculatum}
VKISIDPLTRVEGHLKIEVEVKDGKVVDAKCSGGMFRGFEQILRGRDPRDSSQIVQRICGVC
PTAHCTASVMAQDDAFGVKVTTNGRITRNLIFGANYLQSHILHFYHLAALDYVKGPDVSPFV
PRYANADLLTDRIKDGAKADATNTYGLNQYLKALEIRRICHEMVAMFGGRMPHVQGMVVGGA

TEIPTADKVAEYAARFKEVQKFVIEEYLPLIYTLGSVYTDLFETGIGWKNVIAFGVFPEDDD
YKTFLLKPGVYIDGKDEEFDSKLVKEYVGHSFFDHSAPGGLHYSVGETNPNPDKPGAYSFVK
APRYKDKPCEVGPLARMWVQNPELSPVGQKLLKELYGIEAKKFRDLGDKAFSIMGRHVLRAE
ETWLTAVAVEKWLKQVQPGAETYVKSEIPDAAEGTGFTEAPRGALLHYLKIKDKKIENYQIV
SATLWNANPRDDMGQRGPIEEALIGVPVPDIKNPVNVGRLVRSYDPXLGCAVH
>d1e3db_ e.18.1.1 (B:) Nickel-iron hydrogenase, large subunit
{Desulfovibrio desulfuricans}
TPRSNYTGPIVVDPLTRIEGHLRIEVEVEGGVIKEARSCATLFRGIETILKGRDPRDAQHFT
QRTCGVCTYTHALASTRCLEDAINKPIPANATYIRNLVLGNQFMHDHLVHFYHLHALDFVDV
TSALLADPAKAAKLANSISPRKATTEEFAAVQAKLKTFVASGQLGPFTNAYFLGGHEGYYMD
PEANLVCTAHYLQALRAQVEVAKGMAVFGAKNPHTQFTVAGGVTCYEALTPERIKQFRELYV
KARAFIEEVYIPDLLLVASYYKDWGKIGGTNNFMAFGEFPAPGGERDLNSRWYKPGVIYDRK
VGSVQPFDPSKIEEHVRHSWYEGKARAPFEGETNPHFTFMGDTDKYSWNKAPRYDGHAVETG
PLAQMLVAYGHNHKTIKPTIDAVLGKLNLGPEALFSTLGRTAARGIQTLVIAQQMENWLNEY
ENNIVKDKQIVEDYAVPTSARGVGFADVSRGGLSHWMTIEDGKIDNFQLVVPTTWNLGPRDD
KGVPSAAEAALVGTPVADPKRPVEILRTIHSFDPCIACSTH
>d2frva_ e.19.1.1 (A:) Nickel-iron hydrogenase, small subunit
{Desulfovibrio gigas}
KKRPSVVYLHNAECTGCSESVLRTVDPYVDELILDVISMDYHETLMAGAGHAVEEALHEAIK
GDFVCVIEGGIPMGDGGYWGKVGGRNMYDICAEVAPKAKAVIAIGTCATYGGVQAAKPNPTG
TVGVNEALGKLGVKAINIAGCPPNPMNFVGTVVHLLTKGMPELDKQGRPVMFFGETVHDNCP
RLKHFEAGEFATSFGSPEAKKGYCLYELGCKGPDTYNNCPKQLFNQVNWPVQAGHPCIACSE
PNFWDLYSPFYSA
>d1h2rs_ e.19.1.1 (S:) Nickel-iron hydrogenase, small subunit
{Desulfovibrio vulgaris}
LMGPRRPSVVYLHNAECTGCSESVLRAFEPYIDTLILDTLSLDYHETIMAAAGDAAEAALEQ
AVNSPHGFIAVVEGGIPTAANGIYGKVANHTMLDICSRILPKAQAVIAYGTCATFGGVQAAK
PNPTGAKGVNDALKHLGVKAINIAGCPPNPYNLVGTIVYYLKNKAAPELDSLNRPTMFFGQT
VHEQCPRLPHFDAGEFAPSFESEEARKGWCLYELGCKGPVTMNNCPKIKFNQTNWPVDAGHP
CIGCSEPDFWDAMTPFYQN
>d1frfs_ e.19.1.1 (S:) Nickel-iron hydrogenase, small subunit
{Desulfovibrio fructosovorans}
KHRPSVVWLHNAECTGCTEAAIRTIKPYIDALILDTISLDYQETIMAAAGETSEAALHEALE
GKDGYYLVVEGGLPTIDGGQWGMVAGHPMIETCKKAAAKAKGIICIGTCSPYGGVQKAKPNP
SQAKGVSEALGVKTINIPGCPPNPINFVGAVVHVLTKGIPDLDENGRPKLFYGELVHDNCPR
LPHFEASEFAPSFDSEEAKKGFCLYELGCKGPVTYNNCPKVLFNQVNWPVQAGHPCLGCSEP
DFWDTMTPFYEQG
>d1cc1s_ e.19.1.1 (S:) Nickel-iron hydrogenase, small subunit
{Desulfomicrobium baculatum}
KKAPVIWVQGQGCTGCSVSLLNAVHPRIKEILLDVISLEFHPTVMASEGEMALAHMYEIAEK
FNGNFFLLVEGAIPTAKEGRYCIVGETLDAKAHHHEVTMMELIRDLAPKSLATVAVGTCSAY
GGIPAAEGNVTGSKSVRDFFADEKIEKLLVNVPGCPPHPDWMVGTLVAAWSHVLNPTEHPLP
ELDDDGRPLLFFGDNIHENCPYLDKYDNSEFAETFTKPGCKAELGCKGPSTYADCAKRRWNN
GINWCVENAVCIGCVEPDFPDGKSPFFYVAE

>d1e3da_ e.19.1.1 (A:) Nickel-iron hydrogenase, small subunit {Desulfovibrio desulfuricans}
SRPSVVYLHAAECTGCSEALLRTYQPFIDTLILDTISLDYHETIMAAAGEAAEEALQAAVNG
PDGFICLVEGAIPTGMDNKYGYIAGHTMYDICKNILPKAKAVVSIGTCACYGGIQAAKPNPT
AAKGINDCYADLGVKAINVPGCPPNPLNMVGTLVAFLKGQKIELDEVGRPVMFFGQSVHDLC
ERRKHFDAGEFAPSFNSEEARKGWCLYDVGCKGPETYNNCPKVLFNETNWPVAAGHPCIGCS
EPNFWDDMTPFYQN
>d1dg4a_ e.20.1.1 (A:) DnaK {Escherichia coli}
LSLGIETMGGVMTTLIAKNTTIPTKHSQVFSTAEDNQSAVTIHVLQGERKRAADNKSLGQFN
LDGINPAPRGMPQIEVTFDIDADGILHVSAKDKNSGKEQKITIKASSGL
>d1dkza_ e.20.1.1 (A:) DnaK {Escherichia coli}
VLLLDVTPLSLGIETMGGVMTTLIAKNTTIPTKHSQVFSTAEDNQSAVSIHVLQGERKRAAD
NKSLGQFNLDGINPAPRGMPQIEVTFDIDADGILHVSAKDKNSGKEQKITIKASSGLNEDEI
QKMVRDAEANAEADRKFEELVQTRNQGDHLLHSTRKQVEEAGDKLPADDKTAIESALTALET
ALKGEDKAAIEAKMQELAQVSQKLMEIAQ
>d2bpr__ e.20.1.1 (-) DnaK {Escherichia coli}
SIEGRVKDVLLLDVTPLSLGIETMGGVMTTLIAKNTTIPTKHSQVFSTAEDNQSAVTIHVLQ
GERKRAADNKSLGQFNLDGINPAPRGMPQIEVTFDIDADGILHVSAKDKNSGKEQKITIKAS
SGLNEDEIQKMVRDAEANAEADRKFEELVQTRNQGDHLLHSTRKQVEEA
>d1ckra_ e.20.1.1 (A:) DnaK {Rat (Rattus norvegicus)}
SENVQDLLLLDVTPLSLGIETAGGVMTVLIKRNTTIPTKQTQTFTTYSDNQPGVLIQVYEGE
RAMTKDNNLLGKFELTGIPPAPRGVPQIEVTFDIDANGILNVSAVDKSTGKENKITITNDKG
RLSKEDIERMVQEAEKYKAEDEKQRDKVSSKNSLE
>d1dqsa_ e.22.1.1 (A:) Dehydroquinate synthase, DHQS {Aspergillus nidulans}
PTKISILGRESIIADFGLWRNYVAKDLISDCSSTTYVLVTDTNIGSIYTPSFEEAFRKRAAE
ITPSPRLLIYNRPPGEVSKSRQTKADIEDWMLSQNPPCGRDTVVIALGGGVIGDLTGFVAST
YMRGVRYVQVPTTLLAMVDSSIGGKTAIDTPLGKNLIGAIWQPTKIYIDLEFLETLPVREFI
NGMAEVIKTAAISSEEEFTALEENAETILKAVRREVTPGEHRFEGTEEILKARILASARHKA
YVVSADEREGGLRNLLNWGHSIGHAIEAILTPQILHGECVAIGMVKEAELARHLGILKGVAV
SRIVKCLAAYGLPTSLKDARIRKLTAGKHCSVDQLMFNMALDKKNDGPKKKIVLLSAIGTPY
ETRASVVANEDIRVVL
>d1jq5a_ e.22.1.2 (A:) Glycerol dehydrogenase {Bacillus stearothermophilus}
AAERVFISPAKYVQGKNVITKIANYLEGIGNKTVVIADEIVWKIAGHTIVNELKKGNIAAEE
VVFSGEASRNEVERIANIARKAEAAIVIGVGGGKTLDTAKAVADELDAYIVIVPTAASTDAP
TSALSVIYSDDGVFESYRFYKKNPDLVLVDTKIIANAPPRLLASGIADALATWVEARSVIKS
GGKTMAGGIPTIAAEAIAEKCEQTLFKYGKLAYESVKAKVVTPALEAVVEANTLLSGLGFES
GGLAAAHAIHNGFTALEGEIHHLTHGEKVAFGTLVQLALEEHSQQEIERYIELYLCLDLPVT
LEDIKLKDASREDILKVAKAATAEGETIHNAFNVTADDVADAIFAADQYAKAYKEK
>d1kq3a_ e.22.1.2 (A:) Glycerol dehydrogenase {Thermotoga maritima, TM0423}
HMITTTIFPGRYVQGAGAINILEEELSRFGERAFVVIDDFVDKNVLGENFFSSFTKVRVNKQ
IFGGECSDEEIERLSGLVEEETDVVVGIGGGKTLDTAKAVAYKLKKPVVIVPTIASTDAPCS

ALSVIYTPNGEFKRYLFLPRNPDVVLVDTEIVAKAPARFLVAGMGDALATWFEAESCKQKYA
PNMTGRLGSMTAYALARLCYETLLEYGVLAKRSVEEKSVTPALEKIVEANTLLSGLGFESGG
LAAAHAIHNGLTVLENTHKYLHGEKVAIGVLASLFLTDKPRKMIEEVYSFCEEVGLPTTLAE
IGLDGVSDEDLMKVAEKACDKNETIHNEPQPVTSKDVFFALKAADRYGRMRKNL
>d1lci__ e.23.1.1 (-) Luciferase {Firefly (Photinus pyralis)}
AKNIKKGPAPFYPLEDGTAGEQLHKAMKRYALVPGTIAFTDAHIEVNITYAEYFEMSVRLAE
AMKRYGLNTNHRIVVCSENSLQFFMPVLGALFIGVAVAPANDIYNERELLNSMNISQPTVVF
VSKKGLQKILNVQKKLPIIQKIIIMDSKTDYQGFQSMYTFVTSHLPPGFNEYDFVPESFDRD
KTIALIMNSSGSTGLPKGVALPHRTACVRFSHARDPIFGNQIIPDTAILSVVPFHHGFGMFT
TLGYLICGFRVVLMYRFEEELFLRSLQDYKIQSALLVPTLFSFFAKSTLIDKYDLSNLHEIA
SGGAPLSKEVGEAVAKRFHLPGIRQGYGLTETTSAILITPEGDDKPGAVGKVVPFFEAKVVD
LDTGKTLGVNQRGELCVRGPMIMSGYVNNPEATNALIDKDGWLHSGDIAYWDEDEHFFIVDR
LKSLIKYKGYQVAPAELESILLQHPNIFDAGVAGLPDDDAGELPAAVVVLEHGKTMTEKEIV
DYVASQVTTAKKLRGGVVFVDEVPKGLTGKLDARKIREILIKAKK
>d1amua_ e.23.1.1 (A:) Phenylalanine activating domain of gramicidin synthetase 1 {Bacillus brevis}
GTHEEEQYLFAVNNTKAEYPRDKTIHQLFEEQVSKRPNNVAIVCENEQLTYHELNVKANQLA
RIFIEKGIGKDTLVGIMMEKSIDLFIGILAVLKAGGAYVPIDIEYPKERIQYILDDSQARML
LTQKHLVHLIHNIQFNGQVEIFEEDTIKIREGTNLHVPSKSTDLAYVIYTSGTTGNPKGTML
EHKGISNLKVFFENSLNVTEKDRIGQFASISFDASVWEMFMALLTGASLYIILKDTINDFVK
FEQYINQKEITVITLPPTYVVHLDPERILSIQTLITAGSATSPSLVNKWKEKVTYINAYGPT
ETTICATTWVATKETIGHSVPIGAPIQNTQIYIVDENLQLKSVGEAGELCIGGEGLARGYWK
RPELTSQKFVDNPFVPGEKLYKTGDQARWLSDGNIEYLGRIDNQVKIRGHRVELEEVESILL
KHMYISETAVSVHKDHQEQPYLCAYFVSEKHIPLEQLRQFSSEELPTYMIPSYFIQLDKMPL
TSNGKIDRKQLPEPDLTF
>d1ad2__ e.24.1.1 (-) Ribosomal protein L1 {Thermus thermophilus}
KRYRALLEKVDPNKIYTIDEAAHLVKELATAKFDETVEVHAKLGIDPRRSDQNVRGTVSLPH
GLGKQVRVLAIAKGEKIKEAEEAGADYVGGEEIIQKILDGWMDFDAVVATPDVMGAVGSKLG
RILGPRGLLPNPKAGTVGFNIGEIIREIKAGRIEFRNDKTGAIHAPVGKACFPPEKLADNIR
AFIRALEAHKPEGAKGTFLRSVYVTTTMGPSVRINPHS
>d1cjsa_ e.24.1.1 (A:) Ribosomal protein L1 {Archaeon Methanococcus jannaschii}
MDREALLQAVKEARELAKPRNFTQSFEFIATLKEIDMRKPENRIKTEVVLPHGRGKEAKIAV
IGTGDLAKQAEELGLTVIRKEEIEELGKNKRKLRKIAKAHDFFIAQADLMPLIGRYMGVILG
PRGKMPKPVPANANIKPLVERLKKTVVINTRDKPYFQVLVGNEKMTDEQIVDNIEAVLNVVA
KKYEKGLYHIKDAYVKLTMGPAVKVKK
>d1dwua_ e.24.1.1 (A:) Ribosomal protein L1 {Archaeon Methanococcus thermolithotrophicus}
MDRENILKAVKEARSLAKPRNFTQSLDLIINLKELDLSRPENRLKEQVVLPNGRGKEPKIAV
IAKGDLAAQAEEMGLTVIRQDELEELGKNKKMAKKIANEHDFFIAQADMMPLVGKTLGPVLG
PRGKMPQPVPANANLTPLVERLKKTVLINTRDKPLFHVLVGNEKMSDEELAENIEAILNTVS
RKYEKGLYHVKSAYTKLTMGPPAQIEK
>d1dn1a_ e.25.1.1 (A:) Neuronal Sec1, NSec1 {Rat (Rattus norvegicus)}

IGLKAVVGEKIMHDVIKKVKKKGEWKVLVVDQLSMRMLSSCCKMTDIMTEGITIVEDINKRR
EPLPSLEAVYLITPSEKSVHSLISDFKDPPTAKYRAAHVFFTDSCPDALFNELVKSRAAKVI
KTLTEINIAFLPYESQVYSLDSADSFQSFYSPHKAQMKNPILERLAEQIATLCATLKEYPAV
RYRGEYKDNALLAQLIQDKLDAYKADDPTMGEGPDKARSQLLILDRGFDPSSPVLHELTFQA
MSYDLLPIENDVYKYETSGIGEARVKEVLLDEDDDLWIALRHKHIAEVSQEVTRSLKDFSSS
KRMNTGEKTTMRDLSQMLKKMPQYQKELSKYSTHLHLAEDCMKHYQGTVDKLCRVEQDLAMG
TDAEGEKIKDPMRAIVPILLDANVSTYDKIRIILLYIFLKNGITEENLNKLIQHAQIPPEDS
EIITNMAHLGVPIVTDSTLRRRSKPERKERISEQTYQLSRWTPIIKDIMEDTIEDKLDTKHY
PYISTRSSASFSTTAVSARYGHWHKNKAPGEYRSGPRLIIFILGGVSLNEMRCAYEVTQANG
KWEVLIGSTHILTPQKLLDTLKKLNKTDEEI
>d1epua_ e.25.1.1 (A:) Neuronal Sec1, NSec1 {Longfin inshore squid (Loligo pealei)}
ALKTAVHEKIMNDVVLAVKKNAEWKVLIVDQLSMRMVSACCKMHEIMSEGITLVEDINRRRE
PLPLLEAVYLITPTEESVKCLMADFQNPDNPQYRGAHIFFTEACPEELFKELCKSTTARFIK
TLKEINIAFLPYESQIFSLDSPDTFQVYYNPSRAQGGIPNKERCAEQIATLCATLGEYPSVR
YRSDFDENASFAQLVQQKLDAYRADDPTMGEGPQKDRSQLLILDRGFDPISPLLHELTFQAM
AYDLLPIENDVYKYVNTGGNEVPEKEVLLDEKDDLWVEMRHQHIAVVSQNVTKKLKQFADEK
RMGTAADKAGIKDLSQMLKKMPQYQKELSKYSTHLHLAEDCMKQYQQHVDKLCKVEQDLAMG
TDADGEKIRDHMRNIVPILLDQKISAYDKIRIILLYIIHKGGISEENLAKLVQHAHIPAEEK
WIINDMQNLGVPIIQDGGRRKIPQPYHTHNRKERQADHTYQMSRWTPYMKDIMEAAVEDKLD
TRHYPFLNGGGPRPSCQQPVSVRYGHWHKDKGQASYKSGPRLIIFVVGGISYSEMRSAYEVT
QTAKNNWEVILGSTHILTPEGLLRDLRKISNP
>d1e2ua_ e.26.1.1 (A:) Hybrid cluster protein (prismane protein) {Desulfovibrio vulgaris}
MFCFQCQETAKNTGCTVKGMCGKPEETANLQDLLIFVLRGIAIYGEKLKELGQPDRSNDDFV
LQGLFATITNANWDDARFEAMISEGLARRDKLRNAFLAVYKAKNGKDFSEPLPEAATWTGDS
TAFAEKAKSVGILATENEDVRSLRELLIIGLKGVAAYAEHAAVLGFRKTEIDEFMLEALAST
TKDLSVDEMVALVMKAGGMAVTTMALLDEANTTTYGNPEITQVNIGVGKNPGILISGHDLKD
MAELLKQTEGTGVDVYTHGEMLPANYYPAFKKYPHFVGNYGGSWWQQNPEFESFNGPILLTT
NCLVPLKKENTYLDRLYTTGVVGYEGAKHIADRPAGGAKDFSALIAQAKKCPPPVEIETGSI
VGGFAHHQVLALADKVVEAVKSGAIKRFVVMAGCDGRQKSRSYYTEVAENLPKDTVILTAGC
AKYRYNKLNLGDIGGIPRVLDAGQCNDSYSLAVIALKLKEVFGLDDINDLPVSYDIAWYEQK
AVAVLLALLFLGVKGIRLGPTLPAFLSPNVAKVLVENFNIKPIGTVQDDIAAMMAGK
>d1jjya_ e.26.1.2 (A:) Ni-containing carbon monoxide dehydrogenase {Carboxydothermus hydrogenoformans}
QNLKSTDRAVQQMLDKAKREGIQTVWDRYEAMKPQCGFGETGLCCRHCLQGPCRINPFGDEP
KVGICGATAEVIVARGLDRSIAAGAAGHSGHAKHLAHTLKKAVQGKAASYMIKDRTKLHSIA
KRLGIPTEGQKDEDIALEVAKAALADFHEKDTPVLWVTTVLPPSRVKVLSAHGLIPAGIDHE
IAEIMHRTSMGCDADAQNLLLGGLRCSLADLAGCYMGTDLADILFGTPAPVVTESNLGVLKA
DAVNVAVHGHNPVLSDIIVSVSKEMENEARAAGATGINVVGICCTGNEVLMRHGIPACTHSV
SQEMAMITGALDAMILDYQCIQPSVATIAECTGTTVITTMEMSKITGATHVNFAEEAAVENA
KQILRLAIDTFKRRKGKPVEIPNIKTKVVAGFSTEAIINALSKLNANDPLKPLIDNVVNGNI
RGVCLFAGCNNVKVPQDQNFTTIARKLLKQNVLVVATGCGAGALMRHGFMDPANVDELCGDG
LKAVLTAIGEANGLGGPLPPVLHMGSCVDNSRSVALVAALANRLGVDMDRLPVVASAAQAMH

EKAVAIGTWAVTIGLPTHIGVFPPITGSLPVTQILTSSVKDITGGYFIVELDPQVAADKLLA
AINERRAGLGLPR
>d1jqka_ e.26.1.2 (A:) Ni-containing carbon monoxide dehydrogenase {Rhodospirillum rubrum}
ETAWHRYEKQQPQCGFGSAGLCCRICLKGPCRIDPFGEGPKYGVCGADRDTIVARHLVRMIA
AGTAAHSEHGRHIALAMQHISQGELHDYSIRDEAKLYAIAKTLGVATEGRGLLAIVGDLAAI
TLGDFQNQDYDKPCAWLAASLTPRRVKRLGDLGLLPHNIDASVAQTMSRTHVGCDADPTNLI
LGGLRVAMADLDGSMLATELSDALFGTPQPVVSAANLGVMKRGAVNIAVNGHNPMLSDIICD
VAADLRDEAIAAGAAEGINIIGICCTGHEVMMRHGVPLATNYLSQELPILTGALEAMVVDVQ
CIMPSLPRIAECFHTQIITTDKHNKISGATHVPFDEHKAVETAKTIIRMAIAAFGRRDPNRV
AIPAFKQKSIVGFSAEAVVAALAKVNADDPLKPLVDNVVNGNIQGIVLFVGCNTTKVQQDSA
YVDLAKSLAKRNVLVLATGCAAGAFAKAGLMTSEATTQYAGEGLKGVLSAIGTAAGLGGPLP
LVMHMGSCVDNSRAVALATALANKLGVDLSDLPLVASAPECMSEKALAIGSWAVTIGLPTHV
GSVPPVIGSQIVTKLVTETAKDLVGGYFIVDTDPKSAGDKLYAAIQERRAGL
>d1h5wa_ e.27.1.1 (A:) Upper collar protein gp10 (connector protein) {Bacteriophage PHI29}
RQKRNRWFIHYLNYLQSLAYQLFEWENLPPTINPSFLEKSIHQFGYVGFYKDPVISYIACNG
ALSGQRDVYNQATVFRAASPVYQKEFKLYNYRDMKEEDMGVVIYNNDMAFPTTPTLELFAAE
LAELKEIISVNQNAQKTPVLIRANDNNQLSLKQVYNQYEGNAPVIFAHEALDSDSIEVFKTD
APYVVDKLNAQKNAVWNEMMTFLGIKNANLEKKERMVTDEVSSNDEQIESSGTVFLKSREEA
CEKINELYGLNVKVKFRYDI
>d1io1a_ e.32.1.1 (A:) F41 fragment of flagellin {Salmonella typhimurium}
NIKGLTQASRNANDGISIAQTTEGALNEINNNLQRVRELAVQSANSTNSQSDLDSIQAEITQ
RLNEIDRVSGQTQFNGVKVLAQDNTLTIQVGANDGETIDIDLKQINSQTLGLDTLNVQQKYK
VSDTAATVTGYADTTIALDNSTFKASATGLGGTDQKIDGDLKFDDTTGKYYAKVTVTGGTGK
DGYYEVSVDKTNGEVTLAGGATSPLTGGLPATATEDVKNVQVANADLTEAKAALTAAGVTGT
ASVVKMSYTDNNGKTIDGGLAVKVGDDYYSATQNKDGSISINTTKYTADDGTSKTALNKLGG
ADGKTEVVSIGGKTYAASKAEGHNFKAQPDLAEAAATTTENPLQKIDAALAQVDTLRSDLAA
VQNRFNSAITNLGNTVNNLTSAR
>d1htya_ e.33.1.1 (A:) Golgi alpha-mannosidase II {Fruit fly (Drosophila melanogaster)}
CQDVVQDVPNVDVQMLELYDRMSFKDIDGGVWKQGWNIKYDPLKYNAHHKLKVFVVPHSHND
PGWIQTFEEYYQHDTKHILSNALRHLHDNPEMKFIWAEISYFARFYHDLGENKKLQMKSIVK
NGQLEFVTGGWVMPDEANSHWRNVLLQLTEGQTWLKQFMNVTPTASWAIDPFGHSPTMPYIL
QKSGFKNMLIQRTHYSVKKELAQQRQLEFLWRQIWDNKGDTALFTHMMPFYSYDIPHTCGPD
PKVCCQFDFKRMGSFGLSCPWKVPPRTISDQNVAARSDLLVDQWKKKAELYRTNVLLIPLGD
DFRFKQNTEWDVQRVNYERLFEHINSQAHFNVQAQFGTLQEYFDAVHQAERAGQAEFPTLSG
DFFTYADRSDNYWSGYYTSRPYHKRMDRVLMHYVRAAEMLSAWHSWDGMARIEERLEQARRE
LSLFQHHDGITGTAKTHVVVDYEQRMQEALKACQMVMQQSVYRLLTKPSIYSPDFSFSYFTL
DDSRWPGSGVEDSRTTIILGEDILPSKHVVMHNTLPHWREQLVDFYVSSPFVSVTDLANNPV
EAQVSPVWSWHHDTLTKTIHPQGSTTKYRIIFKARVPPMGLATYVLTISDSKPEHTSYASNL
LLRKNPTSLPLGQYPEDVKFGDPREISLRVGNGPTLAFSEQGLLKSIQLTQDSPHVPVHFKF
LKYGVRSHGDRSGAYLFLPNGPASPVELGQPVVLVTKGKLESSVSVGLPSVVHQTIMRGGAP

EIRNLVDIGSLDNTEIVMRLETHIDSGDIFYTDLNGLQFIKRRRLDKLPLQANYYPIPSGMF
IEDANTRLTLLTGQPLGGSSLASGELEIMQDRRLASDDERGLGQGVLDNKPVLHIYRLVLEK
VNNCVRPSKLHPAGYLTSAAHKASQSLLDPLDKFIFAENEWIGAQGQFGGDHPSAREDLDVS
VMRRLTKSSAKTQRVGYVLHRTNLMQCGTPEEHTQKLDVCHLLPNVARCERTTLTFLQNLEH
LDGMVAPEVCPMETAAYVSSHS
>d1knza_ e.34.1.1 (A:) NSP3 homodimer {Simian 11 rotavirus}
TQQMAVSIINSSFEAAVVAATSALENMGIEYDYQDIYSRVKNKFDFVMDDSGVKNNPIGKAI
TIDQALNNKFGSAIRNRNWLADTSRPAKLDEDVNKLRMMLSSKGIDQKMRVLNACFSVKRIP
GKSSSIIKCTKLMRDKLERGEVEVDDSFVDEKM
>g1jmu.1 e.35.1.1 (A:,B:) Membrane penetration protein mu1
{Reovirus}
TINVTGDGNVFKPSAETSSTAVPSLSLSPGMLNXPGGVPWIAIGDETSVTSPGALRRMTSKD
IPETAIINTDNSSGAVPSESALVPYNDEPLVVVTEHAIANFTKAEMALEFNREFLDKLRVLS
VSPKYSDLLTYVDCYVGVSARQALNNFQKQVPVITPTRQTMYVDSIQAALKALEKWEIDLRV
AQTLLPTNVPIGEVSCPMQSVVKLLDDQLPDDSLIRRYPKEAAVALAKRNGGIQWMDVSEGT
VMNEAVNAVAASALAPSASAPPLEEKSKLTEQAMDLVTAAEPEIIASLVPVPAPVFAIPPKP
ADYNVRTLKIDEATWLRMIPKTMGTLFQIQVTDNTGTNWHFNLRGGTRVVNLDQIAPMRFVL
DLGGKSYKETSWDPNGKKVGFIVFQSKIPFELWTAASQIGQATVVNYVQLYAEDSSFTAQSI
IATTSLAYNYEPEQLNKTDPEMNYYLLATFIDSAAITPTNMTQPDVWDALLTMSPLSAGEVT
VKGAVVSEVVPAELIGSYTPESLNASLPNDAARCMIDRASKIAEAIKIDDDAGPDEYSPNSV
PIQGQLAISQLETGYGVRIFNPKGILSKIASRAMQAFIGDPSTIITQAAPVLSDKNNWIALA
QGVKTSLRTKSLSAGVKTAVSKLSSSESIQNWTQGFLDKVSTHFPAP
>d2btva_ e.28.1.1 (A:) BTV inner layer core protein vp3 {Bluetongue
virus, strain 1}
VDFTVPDVQQILDDIKALAAEQVYKIVKVPSTSFRHIVTQSRDRVLRVDTYYEEMSQVGDVI
TEDEPEKFYSTIIKKVRFIRGKGSFILHDIPARDHRGMEVAEPEVLGVEFKNVLPVLTAEHR
AMIQNALDGSIIENGNVATRDVDVFIGACSEPIYRIYNRLQGYIEAVQLQELRNSIGWLERL
GQRKRITYSQEVLTDFRRQDMIWVLALQLPVNPQVVWDVPRSSIANLIMNIATCLPTGEYIA
PNPRISSITLTQRITTTGPFAILTGSTPTAQQLNDVRKIYLALMFPGQIILDLKIDPGERMD
PAVRMVAGVVGHLLFTAGGRFTNLTQNMARQLDIALNDYLLYMYNTRVQVNYGPTGEPLDFQ
IGRNQYDCNVFRADFATGTGYNGWATIDVEYRDPAPYVHAQRYIRYCGIDSRELINPTTYGI
GMTYHCYNEMLRMLVAAGKDSEAAYFRSMLPFHMVRFARINQIINEDLHSVFSLPDDMFNAL
LPDLIAGAHQNADPVVLDVSWISLWFAFNRSFEPTHRNEMLEIAPLIESVYASELSVMKVDM
RHLSLMQRRFPDVLIQARPSHFWKAVLNDSPEAVKAVMNLSHSHNFINIRDMMRWVLLPSLQ
PSLKLVLEEEAWAAANDFEDLMLTDQVYMHRDMLPEPRLDDIERFRQEGFYYTNMLEAPPEI
DRVVQYTYEIARLQANMGQFRAALRRIMDDDDWVRFGGVLRTVRVKFFDARPPDDILQGLPF
SYDTNEKGGLSYATIKYATETTIFYLIYNVEFSNTPDSLVLINPTYTMTKVFINKRIVERVR
VGQILAVLNRRFVAYKGKMRIMDITQSLKMGTKLAAPTV
>d1cola_ f.1.1.1 (A:) Colicin A {Escherichia coli}
AKDERELLEKTSELIAGMGDKIGEHLGDKYKAIAKDIADNIKNFQGKTIRSFDDAMASLNKI
TANPAMKINKADRDALVNAWKHVDAQDMANKLGNLSKAFKVADVVMKVEKVREKSIEGYETG
NWGPLMLEVESWVLSGIASSVALGIFSATLGAYALSLGVPAIAVGIAGILLAAVVGALIDDK
FADALNNEIIR
>d1a87__ f.1.1.1 (-) Colicin N {Escherichia coli}

SAKVGEITITPDNSKPGRYISSNPEYSLLAKLIDAESIKGTEVYTFHTRKGQYVKVTVPDSN
IDKMRVDYVNWKGPKYNNKLVKRFVSQFLLFRKEEKEKNEKEALLKASELVSGMGDKLGEYL
GVKYKNVAKEVANDIKNFHGRNIRSYNEAMASLNKVLANPKMKVNKSDKDAIVNAWKQVNAK
DMANKIGNLGKAFKVADLAIKVEKIREKSIEGYNTGNWGPLLLEVESWIIGGVVAGVAISLF
GAVLSFLPISGLAVTALGVIGIMTISYLSSFIDANRVSNINNIISSVIR
>d1cii_1 f.1.1.1 (451-624) Colicin Ia {Escherichia coli}
DAINFTTEFLKSVSEKYGAKAEQLAREMAGQAKGKKIRNVEEALKTYEKYRADINKKINAKD
RAAIAAALESVKLSDISSNLNRFSRGLGYAGKFTSLADWITEFGKAVRTENWRPLFVKTETI
IAGNAATALVALVFSILTGSALGIIGYGLLMAVTGALIDESLVEKANKFW
>d1f0la3 f.1.2.1 (A:201-380) Diphtheria toxin, middle domain
{Corynebacterium diphtheriae}
CINLDWDVIRDKTKTKIESLKEHGPIKNKMSESPNKTVSEEKAKQYLEEFHQTALEHPELSE
LKTVTGTNPVFAGANYAAWAVNVAQVIDSETADNLEKTTAALSILPGIGSVMGIADGAVHHN
TEEIVAQSIALSSLMVAQAIPLVGELVDIGFAAYNFVESIINLFQVVHNSYNRPAY
>d1dlc_3 f.1.3.1 (61-289) delta-Endotoxin (insectocide),
N-terminal domain {Bacillus thuringiensis tenebrionis, CRYIIIA
(BT13)}
TTKDVIQKGISVVGDLLGVVGFPFGGALVSFYTNFLNTIWPSEDPWKAFMEQVEALMDQKIA
DYAKNKALAELQGLQNNVEDYVSALSSWQKNPVSSRNPHSQGRIRELFSQAESHFRNSMPSF
AISGYEVLFLTTYAQAANTHLFLLKDAQIYGEEWGYEKEDIAEFYKRQLKLTQEYTDHCVKW
YNVGLDKLRGSSYESWVNFNRYRREMTLTVLDLIALFPLYDVR
>d1ji6a3 f.1.3.1 (A:64-290) delta-Endotoxin (insectocide),
N-terminal domain {Bacillus thuringiensis, CRY3bb1}
DAVGTGISVVGQILGVVGVPFAGALTSFYQSFLNTIWPSDADPWKAFMAQVEVLIDKKIEEY
AKSKALAELQGLQNNFEDYVNALNSWKKTPLSLRSKRSQDRIRELFSQAESHFRNSMPSFAV
SKFEVLFLPTYAQAANTHLLLLKDAQVFGEEWGYSSEDVAEFYHRQLKLTQQYTDHCVNWYN
VGLNGLRGSTYDAWVKFNRFRREMTLTVLDLIVLFPFYDIR
>d1ciy_3 f.1.3.1 (33-255) delta-Endotoxin (insectocide),
N-terminal domain {Bacillus thuringiensis, CRYIA (A)}
YTPIDISLSLTQFLLSEFVPGAGFVLGLVDIIWGIFGPSQWDAFLVQIEQLINQRIEEFARN
QAISRLEGLSNLYQIYAESFREWEADPTNPALREEMRIQFNDMNSALTTAIPLLAVQNYQVP
LLSVYVQAANLHLSVLRDVSVFGQRWGFDAATINSRYNDLTRLIGNYTDYAVRWYNTGLERV
WGPDSRDWVRYNQFRRELTLTVLDIVALFSNYDSRRY
>d1i5pa3 f.1.3.1 (A:1-263) delta-Endotoxin (insectocide),
N-terminal domain {Bacillus thuringiensis subsp. kurstaki, CRY2AA}
MNNVLNSGRTTICDAYNVVAHDPFSFEHKSLDTIQKEWMEWKRTDHSLYVAPVVGTVSSFLL
KKVGSLIGKRILSELWGIIFPSGSTNLMQDILRETEQFLNQRLNTDTLARVNAELIGLQANI
REFNQQVDNFLNPTQNPVPLSITSSVNTMQQLFLNRLPQFQIQGYQLLLLPLFAQAANMHLS
FIRDVILNADEWGISAATLRTYRDYLRNYTRDYSNYCINTYQTAFRGLNTRLHDMLEFRTYM
FLNVFEYVSIWSLFK
>d1g5ma_ f.1.4.1 (A:) Bcl-2 {Human (Homo sapiens)}
HAGRTGYDNREIVMKYIHYKLSQRGYEWDAGDDVEENRTEAPEGTESEVVHLALRQAGDDFS
RRYRGDFAEMSSQLHLTPFTARGRFATVVEELFRDGVNWGRIVAFFEFGGVMCVESVNREMS
PLVDNIALWMTEYLNRHLHTWIQDNGGWDAFVELYGPSMR

>d1bxla_ f.1.4.1 (A:) Apoptosis regulator Bcl-xL {Human (Homo sapiens)}
MSMAMSQSNRELVVDFLSYKLSQKGYSWSQFSDVEENRTEAPEGTESEAVKQALREAGDEFE
LRYRRAFSDLTSQLHITPGTAYQSFEQVVNELFRDGVNWGRIVAFFSFGGALCVESVDKEMQ
VLVSRIAAWMATYLNDHLEPWIQENGGWDTFVELYGNNAAAESRKGQERLEHHHHHH
>d1lxl__ f.1.4.1 (-) Apoptosis regulator Bcl-xL {Human (Homo sapiens)}
MSMAMSQSNRELVVDFLSYKLSQKGYSWSQFSDVEENRTEAPEGTESEMETPSAINGNPSWH
LADSPAVNGATGHSSSLDAREVIPMAAVKQALREAGDEFELRYRRAFSDLTSQLHITPGTAY
QSFEQVVNELFRDGVNWGRIVAFFSFGGALCVESVDKEMQVLVSRIAAWMATYLNDHLEPWI
QENGGWDTFVELYGNNAAAESRKGQERLEHHHHHH
>d1maz__ f.1.4.1 (-) Apoptosis regulator Bcl-xL {Human (Homo sapiens)}
MSQSNRELVVDFLSYKLSQKGYSWSQFSDVEENRTEAPEGTESEMETPSAINGNPSWHLADS
PAVNGATGHSSSLDAREVIPMAAVKQALREAGDEFELRYRRAFSDLTSQLHITPGTAYQSFE
QVVNELFRDGVNWGRIVAFFSFGGALCVESVDKEMQVLVSRIAAWMATYLNDHLEPWIQENG
GWDTFVELYG
>d2bida_ f.1.4.1 (A:) Proapoptotic molecule Bid {Human (Homo sapiens)}
GSMDCEVNNGSSLRDECITNLLVFGFLQSCSDNSFRRELDALGHELPVLAPQWEGYDELQTD
GNRSSHSRLGRIEADSESQEDIIRNIARHLAQVGDSMDRSIPPGLVNGLALQLRNTSRSEED
RNRDLATALEQLLQAYPRDMEKEKTMLVLALLLAKKVASHTPSLLRDVFHTTVNFINQNLRT
YVRSLARNGMD
>d1ddba_ f.1.4.1 (A:) Proapoptotic molecule Bid {Mouse (Mus musculus)}
MDSEVSNGSGLGAKHITDLLVFGFLQSSGCTRQELEVLGRELPVQAYWEADLEDELQTDGSQ
ASRSFNQGRIEPDSESQEEIIHNIARHLAQIGDEMDHNIQPTLVRQLAAQFMNGSLSEEDKR
NCLAKALDEVKTAFPRDMENDKAMLIMTMLLAKKVASHAPSLLRDVFHTTVNFINQNLFSYV
RNLVRNEMD
>d1f16a_ f.1.4.1 (A:) Proapoptotic molecule Bax {Human (Homo sapiens)}
MDGSGEQPRGGGPTSSEQIMKTGALLLQGFIQDRAGRMGGEAPELALDPVPQDASTKKLSEC
LKRIGDELDSNMELQRMIAAVDTDSPREVFFRVAADMFSDGNFNWGRVVALFYFASKLVLKA
LCTKVPELIRTIMGWTLDFLRERLLGWIQDQGGWDGLLSYFGTPTWQTVTIFVAGVLTASLT
IWKKMG
>d1ikpa3 f.1.5.1 (A:252-394) Exotoxin A, middle domain {Pseudomonas aeruginosa}
EGGSLAALTAHQACHLPLETFTRHRQPRGAEQLEQCGYPVQRLVALYLAARLSWNQVDQVIR
NALASPGSGGDLGEAIREQPEQARLALTLAAAESERFVRQGTGNDEAGAANADVVSLTCPVA
AGECAGPADSGDALLERNY
>d1c3wa_ f.2.1.1 (A:) Bacteriorhodopsin {Halobacterium salinarum}
TGRPEWIWLALGTALMGLGTLYFLVKGMGVSDPDAKKFYAITTLVPAIAFTMYLSMLLGYGL
TMVPFGGEQNPIYWARYADWLFTTPLLLLDLALLVDADQGTILALVGADGIMIGTGLVGALT
KVYSYRFVWWAISTAAMLYILYVLFFGFSMRPEVASTFKVLRNVTVVLWSAYPVVWLIGSEG

AGIVPLNIETLLFMVLDVSAKVGFGLILLRSRAIFG
>d1c8sa_ f.2.1.1 (A:) Bacteriorhodopsin {Halobacterium salinarum}
TGRPEWIWLALGTALMGLGTLYFLVKGMGVSDPDAKKFYAITTLVPAIAFTMYLSMLLGYGL
TMVPFGGEQNPIYWARYADWLFTTPLLLLNLALLVDADQGTILALVGADGIMIGTGLVGALT
KVYSYRFVWWAISTAAMLYILYVLFNVTVVLWSAYPVVWLIGSEGAGIVPLNIETLLFMVLD
VSAKVGFGLI
>d1e12a_ f.2.1.1 (A:) Halorhodopsin {Halobacterium salinarum}
RENALLSSSLWVNVALAGIAILVFVYMGRTIRPGRPRLIWGATLMIPLVSISSYLGLLSGLT
VGMIEMPAGHALAGEMVRSQWGRYLTWALSTPMILLALGLLADVDLGSLFTVIAADIGMCVT
GLAAAMTTSALLFRWAFYAISCAFFVVVLSALVTDWAASASSAGTAEIFDTLRVLTVVLWLG
YPIVWAVGVEGLALVQSVGATSWAYSVLDVFAKYVFAFILLRWVANNERTVAV
>d1h68a_ f.2.1.1 (A:) Sensory rhodopsin II {Natronobacterium pharaonis}
VGLTTLFWLGAIGMLVGTLAFAWAGRDAGSGERRYYVTLVGISGIAAVAYVVMALGVGWVPV
AERTVFAPRYIDWILTTPLIVYFLGLLAGLDSREFGIVITLNTVVMLAGFAGAMVPGIERYA
LFGMGAVAFLGLVYYLVGPMTESASQRSSGIKSLYVRLRNLTVILWAIYPFIWLLGPPGVAL
LTPTVDVALIVYLDLVTKVGFGFIALDAAATL
>d1hzxa_ f.2.1.1 (A:) Rhodopsin {Cow (Bos taurus)}
MNGTEGPNFYVPFSNKTGVVRSPFEAPQYYLAEPWQFSMLAAYMFLLIMLGFPINFLTLYVT
VQHKKLRTPLNYILLNLAVADLFMVFGGFTTTLYTSLHGYFVFGPTGCNLEGFFATLGGEIA
LWSLVVLAIERYVVVCKPMSNFRFGENHAIMGVAFTWVMALACAAPPLVGWSRYIPEGMQCS
CGIDYYTPHEETNNESFVIYMFVVHFIIPLIVIFFCYGQLVFTVKEAAAQQQESATTQKAEK
EVTRMVIIMVIAFLICWLPYAGVAFYIFTHQGSDFGPIFMTIPAFFAKTSAVYNPVIYIMMN
KQFRNCMVTTLCCGKNPLGDDEASTTVSKTETSQVAPA
>d1jfpa_ f.2.1.1 (A:) Rhodopsin {Cow (Bos taurus)}
LAAYMFLLIMLGFPINFLTLYVTVQHKKLRTPLNYILLNLAVADLFMVFGGFTTTLYTSLHG
YFVFGPTGCNLEGFFATLGGEIALWSLVVLAIERYVVVCKPMSNFRFGENHAIMGVAFTWVM
ALACAAPPLVGWSRYIPEGMQCSCGIDYYTPHEETNNESFVIYMFVVHFIIPLIVIFFCYGQ
LVFTVKEAAAQQQESATTQKAEKEVTRMVIIMVIAFLICWLPYAGVAFYIFTHQGSDFGPIF
MTIPAFFAKTSAVYNPVIYIMMNKQFRNCMVTTLCCGKNPLGDDEASTTVSKTETSQVAPA
>d1dxrh2 f.2.1.2 (H:1-36) Photosynthetic reaction centre, L-, M- and H-chains {Rhodopseudomonas viridis}
MYHGALAQHLDIAQLVWYAQWLVIWTVVLLYLRRED
>d1dxrl1 f.2.1.2 (L:) Photosynthetic reaction centre, L-, M- and H-chains {Rhodopseudomonas viridis}
ALLSFERKYRVRGGTLIGGDLFDFWVGPYFVGFFGVSAIFFIFLGVSLIGYAASQGPTWDPF
AISINPPDLKYGLGAAPLLEGGFWQAITVCALGAFISWMLREVEISRKLGIGWHVPLAFCVP
IFMFCVLQVFRPLLLGSWGHAFPYGILSHLDWVNNFGYQYLNWFYNPGHMSSVSFLFVNAMA
LGLHGGLILSVANPGDGDKVKTAEHENQYFRDVVGYSIGALSIHRLGLFLASNIFLTGAFGT
IASGPFWTRGWPEWWGWWLDIPFWS
>d1dxrm1 f.2.1.2 (M:) Photosynthetic reaction centre, L-, M- and H-chains {Rhodopseudomonas viridis}
ADYQTIYTQIQARGPHITVSGEWGDNDRVGKPFYSYWLGKIGDAQIGPIYLGASGIAAFAFG
STAILIIILFNMAAEVHFDPLQFFRQFFWLGLYPPKAQYGMGIPPLHDGGWWLMAGLFMTLSL

GSWWIRVYSRARALGLGTHIAWNFAAAIFFVLCIGCIHPTLVGSWSEGVPFGIWPHIDWLTA
FSIRYGNFYYCPWHGFSIGFAYGCGLLFAAHGATILAVARFGGDREIEQITDRGTAVERAAL
FWRWTIGFNATIESVHRWGWFFSLMVMVSASVGILLTGTFVDNWYLWCVKHGAAPDYPAYLP
ATPDPASLPGAPK
>d1qovh2 f.2.1.2 (H:11-35) Photosynthetic reaction centre, L-, M- and H-chains {Rhodobacter sphaeroides}
DLASLAIYSFWIFLAGLIYYLQTEN
>d1qovl1 f.2.1.2 (L:) Photosynthetic reaction centre, L-, M- and H-chains {Rhodobacter sphaeroides}
ALLSFERKYRVPGGTLVGGNLFDFWVGPFYVGFFGVATFFFAALGIILIAWSAVLQGTWNPQ
LISVYPPALEYGLGGAPLAKGGLWQIITICATGAFVSWALREVEICRKLGIGYHIPFAFAFA
ILAYLTLVLFRPVMMGAWGYAFPYGIWTHLDWVSNTGYTYGNFHYNPAHMIAISFFFTNALA
LALHGALVLSAANPEKGKEMRTPDHEDTFFRDLVGYSIGTLGIHRLGLLLSLSAVFFSALCM
IITGTIWFDQWVDWWQWWVKLPWWANIPGGING
>d1qovm1 f.2.1.2 (M:) Photosynthetic reaction centre, L-, M- and H-chains {Rhodobacter sphaeroides}
AEYQNIFSQVQVRGPADLGMTEDVNLANRSGVGPFSTLLGWFGNAQLGPIYLGSLGVLSLFS
GLMWFFTIGIWFWYQAGWNPAVFLRDLFFFSLEPPAPEYGLSFAAPLKEGGLWLIASFFMFV
AVWSWWGRTYLRAQALGMGKHTAWAFLSAIWLWMVLGFIRPILMGSWSEAVPYGIFSHLDWT
NNFSLVHGNLFYNPFHGLSIAFLYGSALLFAMHGATILAVSRFGGERELEQIADRGTAAERA
ALFWRWTMGFNWTMEGIHRWAIWMAVLVTLTGGIGILLSGTVVDNWYVWGQNHG
>d2rcrh2 f.2.1.2 (H:1-35) Photosynthetic reaction centre, L-, M- and H-chains {Rhodobacter sphaeroides}
MVGVTAFGNFDLASLAIYSFWIFLAGLIYYLQTEN
>d1eysh2 f.2.1.2 (H:7-43) Photosynthetic reaction centre, L-, M- and H-chains {Thermochromatium tepidum}
HYIDAAQITIWAFWLFFFGLIIYLRREDKREGYPLDS
>d1eysl1 f.2.1.2 (L:) Photosynthetic reaction centre, L-, M- and H-chains {Thermochromatium tepidum}
AMLSFEKKYRVRGGTLIGGDLFDFWVGPFYVGFFGVVGFCFTLLGVLLIVWGATIGPTGPTS
DLQTYNLWRISIAPPDLSYGLRMAPLTEGGLWQIITICAAGAFISWALREVEICRKLGIGFH
VPFAFSFAIGAYLVLVFVRPLLMGAWGHGFPYGILSHLDWVSNVGYQFLHFHYNPAHMLAIS
FFFTNCLALSMHGSLILSVTNPQRGEPVKTSEHENTFFRDIVGYSIGALAIHRLGLFLALSA
AFWSAVCILISGPFWTRGWPEWWNWWLELPLW
>d1eysm1 f.2.1.2 (M:) Photosynthetic reaction centre, L-, M- and H-chains {Thermochromatium tepidum}
PEYQNIFTAVQVRAPAYPGVPLPKGNLPRIGRPIFSYWLGKIGDAQIGPIYLGLTGTLSIFF
GLVAISIIGFNMLASVHWDVFQFLKHFFWLGLEPPPPQYGLRIPPLSEGGWWLIAGLFLTLS
ILLWWVRTYKRAEALGMSQHLSWAFAAAIFFYLVLGFIRPVMMGSWAKAVPFGIFPHLDWTA
AFSIRYGNLYYNPFHMLSIAFLYGSALLFAMHGATILSVSRFGGDREIDQITHRGTAAEGAA
LFWRWTMGFNATMESIHRWAWWCAVLTVITAGIGILLSGTVVDNWYLWAVKHGMAPAYPEVV
TAVNPYET
>d1ocra1 f.2.1.3 (A:) Cytochrome c oxidase {Cow (Bos taurus)}
MFINRWLFSTNHKDIGTLYLLFGAWAGMVGTALSLLIRAELGQPGTLLGDDQIYNVVVTAHA

FVMIFFMVMPIMIGGFGNWLVPLMIGAPDMAFPRMNNMSFWLLPPSFLLLLASSMVEAGAGT
GWTVYPPLAGNLAHAGASVDLTIFSLHLAGVSSILGAINFITTIINMKPPAMSQYQTPLFVW
SVMITAVLLLLSLPVLAAGITMLLTDRNLNTTFFDPAGGGDPILYQHLFWFFGHPEVYILIL
PGFGMISHIVTYYSGKKEPFGYMGMVWAMMSIGFLGFIVWAHHMFTVGMDVDTRAYFTSATM
IIAIPTGVKVFSWLATLHGGNIKWSPAMMWALGFIFLFTVGGLTGIVLANSSLDIVLHDTYY
VVAHFHYVLSMGAVFAIMGGFVHWFPLFSGYTLNDTWAKIHFAIMFVGVNMTFFPQHFLGLS
GMPRRYSDYPDAYTMWNTISSMGSFISLTAVMLMVFIIWEAFASKREVLTVDLTTTNLEWLN
GCPPPYHTFEEPTYVNLK
>d1ocrb2 f.2.1.3 (B:1-90) Cytochrome c oxidase {Cow (Bos taurus)}
MAYPMQLGFQDATSPIMEELLHFHDHTLMIVFLISSLVLYIISLMLTTKLTHTSTMDAQEVE
TIWTILPAIILILIALPSLRILYMMDEI
>d1ocrc1 f.2.1.3 (C:) Cytochrome c oxidase {Cow (Bos taurus)}
MTHQTHAYHMVNPSPWPLTGALSALLMTSGLTMWFHFNSMTLLMIGLTTNMLTMYQWWRDVI
RESTFQGHHTPAVQKGLRYGMILFIISEVLFFTGFFWAFYHSSLAPTPELGGCWPPTGIHPL
NPLEVPLLNTSVLLASGVSITWAHHSLMEGDRKHMLQALFITITLGVYFTLLQASEYYEAPF
TISDGVYGSTFFVATGFHGLHVIIGSTFLIVCFFRQLKFHFTSNHHFGFEAGAWYWHFVDVV
WLFLYVSIYWWGS
>d1ocrd1 f.2.1.3 (D:) Cytochrome c oxidase {Cow (Bos taurus)}
SVVKSEDYALPSYVDRRDYPLPDVAHVKNLSASQKALKEKEKASWSSLSIDEKVELYRLKFK
ESFAEMNRSTNEWKTVVGAAMFFIGFTALLLIWEKHYVYGPIPHTFEEEWVAKQTKRMLDMK
VAPIQGFSAKWDYDKNEWKK
>d1ocrg1 f.2.1.3 (G:) Cytochrome c oxidase {Cow (Bos taurus)}
ASAAKGDHGGTGARTWRFLTFGLALPSVALCTLNSWLHSGHRERPAFIPYHHLRIRTKPFSW
GDGNHTFFHNPRVNPLPTGYEK
>d1ocri1 f.2.1.3 (I:) Cytochrome c oxidase {Cow (Bos taurus)}
STALAKPQMRGLLARRLRFHIVGAFMVSLGFATFYKFAVAEKRKKAYADFYRNYDSMKDFEE
MRKAGIFQSAK
>d1ocrj1 f.2.1.3 (J:) Cytochrome c oxidase {Cow (Bos taurus)}
FENRVAEKQKLFQEDNGLPVHLKGGATDNILYRVTMTLCLGGTLYSLYCLGWASFPHK
>d1ocrk1 f.2.1.3 (K:) Cytochrome c oxidase {Cow (Bos taurus)}
APDFHDKYGNAVLASGATFCVAVWVYMATQIGIEWNPSPVGRVTPKEWR
>d1ocrl1 f.2.1.3 (L:) Cytochrome c oxidase {Cow (Bos taurus)}
SHYEEGPGKNIPFSVENKWRLLAMMTLFFGSGFAAPFFIVRHQLLKK
>d1ocrm1 f.2.1.3 (M:) Cytochrome c oxidase {Cow (Bos taurus)}
ITAKPAKTPTSPKEQAIGLSVTFLSFLLPAGWVLYHLDNYKKS
>d1ar1a1 f.2.1.3 (A:) Cytochrome c oxidase {Paracoccus
denitrificans}
GFFTRWFMSTNHKDIGILYLFTAGIVGLISVCFTVYMRMELQHPGVQYMCLEGARLIADASA
ECTPNGHLWNVMITYHGVLMMFFVVIPALFGGFGNYFMPLHIGAPDMAFPRLNNLSYWMYVC
GVALGVASLLAPGGNDQMGSGVGWVLYPPLSTTEAGYSMDLAIFAVHVSGASSILGAINIIT
TFLNMRAPGMTLFKVPLFAWSVFITAWLILLSLPVLAGAITMLLMDRNFGTQFFDPAGGGDP
VLYQHILWFFGHPEVYIIILPGFGIISHVISTFAKKPIFGYLPMVLAMAAIGILGFVVWAHH
MYTAGMSLTQQAYFMLATMTIAVPTGIKVFSWIATMWGGSIEFKTPMLWAFGFLFLFTVGGV
TGVVLSQAPLDRVYHDTYYVVAHFHYVMSLGAVFGIFAGVYYWIGKMSGRQYPEWAGQLHFW

MMFIGSNLIFFPQHFLGRQGMPRRYIDYPVEFAYWNNISSIGAYISFASFLFFIGIVFYTLF
AGKRVNVPNYWNEHADTLEWTLPSPPPEHTFET
>d1ar1b2 f.2.1.3 (B:1-107) Cytochrome c oxidase {Paracoccus denitrificans}
QDVLGDLPVIGKPVNGGMNFQPASSPLAHDQQWLDHFVLYIITAVTIFVCLLLLICIVRFNR
RANPVPARFTHNTPIEVIWTLVPVLILVAIGAFSLPILFRSQEMP
>d1qlec1 f.2.1.3 (C:) Cytochrome c oxidase {Paracoccus denitrificans}
AHVKNHDYQILPPSIWPFFGAIGAFVMLTGAVAWMKGITFFGLPVEGPWMFLIGLVGVLYVM
FGWWADVVNEGETGEHTPVVRIGLQYGFILFIMSEVMFFVAWFWAFIKNALYPMGPDSPIKD
GVWPPEGIVTFDPWHLPLINTLILLLSGVAVTWAHHAFVLEGDRKTTINGLIVAVILGVCFT
GLQAYEYSHAAFGLADTVYAGAFYMATGFHGAHVIIGTIFLFVCLIRLLKGQMTQKQHVGFE
AAAWYWHFVDVVWLFLFVVIYIWGR
>d1qled1 f.2.1.3 (D:) Cytochrome c oxidase {Paracoccus denitrificans}
TDHKHGEMDIRHQQATFAGFIKGATWVSILSIAVLVFLALANS
>d1ehka1 f.2.1.3 (A:) Cytochrome c oxidase {Thermus thermophilus, ba3 type}
AYPEKKATLYFLVLGFLALIVGSLFGPFQALNYGNVDAYPLLKRLLPFVQSYYQGLTLHGVL
NAIVFTQLFAQAIMVYLPARELNMRPNMGLMWLSWWMAFIGLVVAALPLLANEATVLYTFYP
PLKGHWAFYLGASVFVLSTWVSIYIVLDLWRRWKAANPGKVTPLVTYMAVVFWLMWFLASLG
LVLEAVLFLLPWSFGLVEGVDPLVARTLFWWTGHPIVYFWLLPAYAIIYTILPKQAGGKLVS
DPMARLAFLLFLLLSTPVGFHHQFADPGIDPTWKMIHSVLTLFVAVPSLMTAFTVAASLEFA
GRLRGGRGLFGWIRALPWDNPAFVAPVLGLLGFIPGGAGGIVNASFTLDYVVHNTAWVPGHF
HLQVASLVTLTAMGSLYWLLPNLTGKPISDAQRRLGLAVVWLWFLGMMIMAVGLHWAGLLNV
PRRAYIAQVPDAYPHAAVPMVFNVLAGIVLLVALLLFIYGLFSVLLSRERKPELAEAPLPFA
EVISGPEDRRLVLAMDRIGFWFAVAAILVVLAYGPTLVQLFGHLNPVPGWRLW
>d1ehkb2 f.2.1.3 (B:3-40) Cytochrome c oxidase {Thermus thermophilus, ba3 type}
DEHKAHKAILAYEKGWLAFSLAMLFVFIALIAYTLATH
>d1ehkc1 f.2.1.3 (C:) Cytochrome c oxidase {Thermus thermophilus, ba3 type}
EEKPKGALAVILVLTLTILVFWLGVYAVFFARG
>d1ffta1 f.2.1.3 (A:) Ubiquinol oxidase {Escherichia coli}
VDHKRLGIMYIIVAIVMLLRGFADAIMMRSQQALASAGEAGFLPPHHYDQIFTAHGVIMIFF
VAMPFVIGLMNLVVPLQIGARDVAFPFLNNLSFWFTVVGVILVNVSLGVGEFAQTGWLAYPP
LSGIEYSPGVGVDYWIWSLQLSGIGTTLTGINFFVTILKMRAPGMTMFKMPVFTWASLCANV
LIIASFPILTVTVALLTLDRYLGTHFFTNDMGGNMMMYINLIWAWGHPEVYILILPVFGVFS
EIAATFSRKRLFGYTSLVWATVCITVLSFIVWLHHFFTMGAGANVNAFFGITTMIIAIPTGV
KIFNWLFTMYQGRIVFHSAMLWTIGFIVTFSVGGMTGVLLAVPGADFVLHNSLFLIAHFHNV
IIGGVVFGCFAGMTYWWPKAFGFKLNETWGKRAFWFWIIGFFVAFMPLYALGFMGMTRRLSQ
QIDPQFHTMLMIAASGAVLIALGILCLVIQMYVSIRDRDQNRDLTGDPWGGRTLEWATSSPP
PFYNF
>d1fftb2 f.2.1.3 (B:27-117) Ubiquinol oxidase {Escherichia coli}

SALLDPKGQIGLEQRSLILTAFGLMLIVVIPAILMAVGFAWKYRASNKDAKYSPNWSHSNKV
EAVVWTVPILIIIFLAVLTWKTTHALEPS
>d1fftc1 f.2.1.3 (C:) Ubiquinol oxidase {Escherichia coli}
HDAGGTKIFGFWIYLMSDCILFSILFATYAVLVNGTAGGPTGKDIFELPFVLVETFLLLFSS
ITYGMAAIAMYKNNKSQVISWLALTWLFGAGFIGMEIYEFHHLIVNGMGPDRSGFLSAFFAL
VGTHGLHVTSGLIWMAVLMVQIARRGLTSTNRTRIMCLSLFWHFLDVVWICVFTVVYLMGA
>d1c0va_ f.2.1.4 (A:) Subunit C {Escherichia coli}
MENLNMDLLYMAAAVMMGLAAIGAAIGIGILGGKFLEGAARQPDLIPLLRTQFFIVMGLVDA
IPMIAVGLGLYVMFAVA
>d1c17m_ f.2.1.4 (M:) Subunit A {Escherichia coli}
HGKSKLIAPLALTIFVWVFLMNLMDLLPIDLLPYIAEHVLGLPALRVVPSADVNVTLSMALG
VFILILFYSIKMKGIGGFTKELTLQPFNHWAFIPVNLILEGVSLLSKPVSLGLRLFGNMYAG
ELIFILIAGLLPWWSQWILNVPWAIFHILIITLQAFIFMVLTIVYLS
>d1h6ia_ f.2.1.5 (A:) Aquaporin-1 {Human (Homo sapiens)}
LFWRAVVAEFLATTLFVFISIGSALGFKYPVGNNQTAVQDNVKVSLAFGLSIATLAQSVGHI
SGAHLNPAVTLGLLLSCQISIFRALMYIIAQCVGAIVATAILSGITSSLTGNSLGRNDLADG
VNSGQGLGIEIIGTLQLVLCVLATTDRRRRDLGGSAPLAIGLSVALGHLLAIDYTGCGINPA
RSFGSAVITHNFSNHWIFWVGPFIGGALAVLIYDFILAP
>d1fx8a_ f.2.1.5 (A:) Glycerol uptake facilitator protein GlpF
{Escherichia coli}
TLKGQCIAEFLGTGLLIFFGVGCVAALKVAGASFGQWEISVIWGLGVAMAIYLTAGVSGAHL
NPAVTIALWLFACFDKRKVIPFIVSQVAGAFCAAALVYGLYYNLFFDFEQTHHIVRGSVESV
DLAGTFSTYPNPHINFVQAFAVEMVITAILMGLILALTDDGNGVPRGPLAPLLIGLLIAVIG
ASMGPLTGFAMNPARDFGPKVFAWLAGWGNVAFTGGRDIPYFLVPLFGPIVGAIVGAFAYRK
LIGRHL
>d1kpka_ f.2.1.13 (A:) Clc chloride channel {Escherichia coli}
QAARLRRRQLIRQLLERDKTPLAILFMAAVVGTLVGLAAVAFDKGVAWLQNQRMGALVHTAD
NYPLLLTVAFLCSAVLAMFGYFLVRKYAPEAGGSGIPEIEGALEDQRPVRWWRVLPVKFFGG
LGTLGGGMVLGREGPTVQIGGNIGRMVLDIFRLKGDEARHTLLATGAAAGLAAAFNAPLAGI
LFIIEEMRPQFRYTLISIKAVFIGVIMSTIMYRIFNHEVALIDVGKLSDAPLNTLWLYLILG
IIFGIFGPIFNKWVLGMQDLLHRVHGGNITKWVLMGGAIGGLCGLLGFVAPATSGGGFNLIP
IATAGNFSMGMLVFIFVARVITTLLCFSSGAPGGIFAPMLALGTVLGTAFGMVAVELFPQYH
LEAGTFAIAGMGALLAASIRAPLTGIILVLEMTDNYQLILPMIITGLGATLLAQFTGGKPLY
SAILARTLAKQEAEQL
>d1kpla_ f.2.1.13 (A:) Clc chloride channel {Salmonella
typhimurium}
TPLAILFMAAVVGTLTGLVGVAFEKAVSWVQNMRIGALVQVADHAFLLWPLAFILSALLAMV
GYFLVRKFAPEAGGSGIPEIEGALEELRPVRWWRVLPVKFIGGMGTLGAGMVLGREGPTVQI
GGNLGRMVLDVFRMRSAEARHTLLATGAAAGLSAAFNAPLAGILFIIEEMRPQFRYNLISIK
AVFTGVIMSSIVFRIFNGEAPIIEVGKLSDAPVNTLWLYLILGIIFGVVGPVFNSLVLRTQD
MFQRFHGGEIKKWVLMGGAIGGLCGILGLIEPAAAGGGFNLIPIAAAGNFSVGLLLFIFITR
VVTTLLCFSSGAPGGIFAPMLALGTLLGTAFGMAAAVLFPQYHLEAGTFAIAGMGALMAASV
RAPLTGIVLVLEMTDNYQLILPMIITCLGATLLAQFLGGKPLYSTILARTLAKQDAEQ
>d1f6ga_ f.2.1.11 (A:) Potassium chanel protein {Streptomyces

lividans}
MPPMLSGLLARLVKLLLGRHGSALHWAAAGAATVLLVIVLLAGSYLAVLAERGAPGAQLITY
PAALWWSVETATTVGYGDLYPVTLWGRCVAVVVMVAGITSFGLVTAALATWFVGREQERRGH
FVRHSEKAAEEAYTRTTRALHERFDRLERMLDDNRR
>d1jq2a_ f.2.1.11 (A:) Potassium chanel protein {Streptomyces lividans}
LWGRCVAVVVMVAGITSFGLVTAALATWFVGREQ
>d1k4cc_ f.2.1.11 (C:) Potassium chanel protein {Streptomyces lividans}
SALHWRAAGAATVLLVIVLLAGSYLAVLAERGAPGAQLITYPRALWWSVETATTVGYGDLYP
VTLWGRCVAVVVMVAGITSFGLVTAALATWFVGREQERRGH
>d1g4yb_ f.2.1.11 (B:) Small conductance potassium channel {Rat (Rattus norvegicus)}
DTQLTKRVKNAAANVLRETWLIYKNTKLVKKIDHAKVRKHQRKFLQAIHQLRSVKMEQRKLN
DQANTLVDLAKTQLEHHHHH
>d1kkda_ f.2.1.11 (A:) Small conductance potassium channel {Rat (Rattus norvegicus)}
RKLELTKAEKHVHNFMMDTQLTKRVKNAAANVLRETWLIYKNTKLVKKIDHAKVRKHQRKFL
QAIHQLRSVKMEQRKLNDQANTLVDLAKTQ
>d1msla_ f.2.1.11 (A:) Gated mechanosensitive channel {Mycobacterium tuberculosis}
ARGNIVDLAVAVVIGTAFTALVTKFTDSIITPLINRIGVNAQSDVGILRIGIGGGQTIDLNV
LLSAAINFFLIAFAVYFLVVLPYNTLRKKGEVEQPGDTQVVLLTEIR
>d1be3c1 f.2.1.8 (C:) Cytochrome bc1 transmembrane subunits {Cow (Bos taurus)}
MTNIRKSHPLMKIVNNAFIDLPAPSNISSWWNFGSLLGICLILQILTGLFLAMHYTSDTTTA
FSSVTHICRDVNYGWIIRYMHANGASMFFICLYMHVGRGLYYGSYTFLETWNIGVILLLTVM
ATAFMGYVLPWGQMSFWGATVITNLLSAIPYIGTNLVEWIWGGFSVDKATLTRFFAFHFILP
FIIMAIAMVHLLFLHETGSNNPTGISSDVDKIPFHPYYTIKDILGALLLILALMLLVLFAPD
LLGDPDNYTPANPLNTPPHIKPEWYFLFAYAILRSIPNKLGGVLALAFSILILALIPLLHTS
KQRSMMFRPLSQCLFWALVADLLTLTWIGGQPVEHPYITIGQLASVLYFLLILVLMPTAGTI
ENKLLKW
>d1be3e2 f.2.1.8 (E:1-69) Cytochrome bc1 transmembrane subunits {Cow (Bos taurus)}
SHTDIKVPDFSDYRRPEVLDSTKSSKESSEARKGFSYLVTATTTVGVAYAAKNVVSQFVSSM
SASADVL
>d1be3f1 f.2.1.8 (F:) Cytochrome bc1 transmembrane subunits {Cow (Bos taurus)}
AVSASSRWLEGIRKWYYNAAGFNKLGLMRDDTIHENDDVKEAIRRLPENLYDDRVFRIKRAL
DLSMRQQILPKEQWTKYEEDKSYLEPYLKEVIRERKEREEWAKK
>d1be3g1 f.2.1.8 (G:) Cytochrome bc1 transmembrane subunits {Cow (Bos taurus)}
GRQFGHLTRVRHVITYSLSPFEQRAFPHYFSKGIPNVLRRTRACILRVAPPFVAFYLVYTWG
TQEFEKSKRKNPAAYENDR

>d1be3k1 f.2.1.8 (K:) Cytochrome bc1 transmembrane subunits {Cow (Bos taurus)}
RNWVPTAQLWGAVGAVGLVSAT
>d1qcrg1 f.2.1.8 (G:) Cytochrome bc1 transmembrane subunits {Cow (Bos taurus)}
GRQFGHLTRVRHVITYSLSPFEQRAFPHYFSKGIPNVLRRTRACILRVAPPFVAFYLVYTWG
TQEFEKSK
>d1qcrh1 f.2.1.8 (H:) Cytochrome bc1 transmembrane subunits {Cow (Bos taurus)}
TTVREQCEQLEKCVKARERLELCDERVSSRSQTEEDCTEELLDFLHARDHCVAHKLFNSL
>d1qcrk1 f.2.1.8 (K:) Cytochrome bc1 transmembrane subunits {Cow (Bos taurus)}
MLTRFLGPRYRQLARNWVPTAQLWGAVGAVGLVSATDSRLILDWV
>d1bccc1 f.2.1.8 (C:) Cytochrome bc1 transmembrane subunits {Chicken (Gallus gallus)}
APNIRKSHPLLKMINNSLIDLPAPSNISAWWNFGSLLAVCLMTQILTGLLLAMHYTADTSLA
FSSVAHTCRNVQYGWLIRNLHANGASFFFICIFLHIGRGLYYGSYLYKETWNTGVILLLTLM
ATAFVGYVLPWGQMSFWGATVITNLFSAIPYIGHTLVEWAWGGFSVDNPTLTRFFALHFLLP
FAIAGITIIHLTFLHESGSNNPLGISSDSDKIPFHPYYSFKDILGLTLMLTPFLTLALFSPN
LLGDPENFTPANPLVTPPHIKPEWYFLFAYAILRSIPNKLGGVLALAASVLILFLIPFLHKS
KQRTMTFRPLSQTLFWLLVANLLILTWIGSQPVEHPFIIIGQMASLSYFTILLILFPTIGTL
ENKMLNY
>d1bccd3 f.2.1.8 (D:196-241) Cytochrome bc1 transmembrane subunits {Chicken (Gallus gallus)}
PEHDHRKRMGLKMLLMMGLLVPLVYYMKRHKWSVLKSRKLAYRPPK
>d1bcce2 f.2.1.8 (E:1-69) Cytochrome bc1 transmembrane subunits {Chicken (Gallus gallus)}
SHTDIKVPNFSDYRRPPDDYSTKSSRESDPSRKGFSYLVTAVTTLGVAYAAKNVVTQFVSSM
SASADVL
>d1bccf1 f.2.1.8 (F:) Cytochrome bc1 transmembrane subunits {Chicken (Gallus gallus)}
SRWLEGIRKWYYNAAGFNKYGLMRDDTIYENDDVKEAIRRLPENLYDDRMFRIKRALDLNMR
QQILPKEQWTKYEEDVPYLEPYLKEVIRERKEREEWDK
>d1bccg1 f.2.1.8 (G:) Cytochrome bc1 transmembrane subunits {Chicken (Gallus gallus)}
RQFGHLTRVRHLITYSLSPFEQRPFPHYFSKGVPNVWRRLRACILRVAPPFLAFYLLYTWGT
QEFEKSKRKNPAAYVN
>d1bcch1 f.2.1.8 (H:) Cytochrome bc1 transmembrane subunits {Chicken (Gallus gallus)}
LVDPLTTVREQCEQLEKCVKARERLELCDERVSSRSQTEEDCTEELFDFLHARDHCVAHKLF
NSLK
>d1bccj1 f.2.1.8 (J:) Cytochrome bc1 transmembrane subunits {Chicken (Gallus gallus)}
TLTARLYSLLFRRTSTFALTIVVGALLFERAFDQGADAIYEHINEGKLWKHIKHKYENK

>d1ezvc1 f.2.1.8 (C:) Cytochrome bc1 transmembrane subunits {Baker's yeast (Saccharomyces cerevisiae)}
MAFRKSNVYLSLVNSYIIDSPQPSSINYWWNMGSLLGLCLVIQIVTGIFMAMHYSSNIELAF
SSVEHIMRDVHNGYILRYLHANGASFFFMVMFMHMAKGLYYGSYRSPRVTLWNVGVIIFTLT
IATAFLGYCCVYGQMSHWGATVITNLFSAIPFVGNDIVSWLWGGFSVSNPTIQRFFALHYLV
PFIIAAMVIMHLMALHIHGSSNPLGITGNLDRIPMHSYFIFKDLVTVFLFMLILALFVFYSP
NTLGHPDNYIPGNPLVTPASIVPEWYLLPFYAILRSIPDKLLGVITMFAAILVLLVLPFTDR
SVVRGNTFKVLSKFFFFIFVFNFVLLGQIGACHVEVPYVLMGQIATFIYFAYFLIIVPVIST
IENVLFYIGRVNK
>d1ezvd2 f.2.1.8 (D:261-306) Cytochrome bc1 transmembrane subunits {Baker's yeast (Saccharomyces cerevisiae)}
PEHDERKRLGLKTVIILSSLYLLSIWVKKFKWAGIKTRKFVFNPPK
>d1ezve2 f.2.1.8 (E:31-86) Cytochrome bc1 transmembrane subunits {Baker's yeast (Saccharomyces cerevisiae)}
KSTYRTPNFDDVLKENNDADKGRSYAYFMVGAMGLLSSAGAKSTVETFISSMTATA
>d1ezvf1 f.2.1.8 (F:) Cytochrome bc1 transmembrane subunits {Baker's yeast (Saccharomyces cerevisiae)}
QSFTSIARIGDYILKSPVLSKLCVPVANQFINLAGYKKLGLKFDDLIAEENPIMQTALRRLP
EDESYARAYRIIRAHQTELTHHLLPRNEWIKAQEDVPYLLPYILEAEAAAKEKDELDNIEVS
K
>d1ezvg1 f.2.1.8 (G:) Cytochrome bc1 transmembrane subunits {Baker's yeast (Saccharomyces cerevisiae)}
GPPSGKTYMGWWGHMGGPKQKGITSYAVSPYAQKPLQGIFHNAVFNSFRRFKSQFLYVLIPA
GIYWYWWKNGNEYNEFLYSKAGREELERVNV
>d1ezvh1 f.2.1.8 (H:) Cytochrome bc1 transmembrane subunits {Baker's yeast (Saccharomyces cerevisiae)}
VTDQLEDLREHFKNTEEGKALVHHYEECAERVKIQQQQPGYADLEHKEDCVEEFFHLQHYLD
TATAPRLFDKLK
>d1ezvi1 f.2.1.8 (I:) Cytochrome bc1 transmembrane subunits {Baker's yeast (Saccharomyces cerevisiae)}
SSLYKTFFKRNAVFVGTIFAGAFVFQTVFDTAITSWYENHNKGKLWKDVKARIAA
>d1fumc_ f.2.1.9 (C:) Fumarate reductase respiratory complex transmembrane subunits {Escherichia coli}
TTKRKPYVRPMTSTWWKKLPFYRFYMLREGTAVPAVWFSIELIFGLFALKNGPEAWAGFVDF
LQNPVIVIINLITLAAALLHTKTWFELAPKAANIIVKDEKMGPEPIIKSLWAVTVVATIVIL
FVALYW
>d1fumd_ f.2.1.9 (D:) Fumarate reductase respiratory complex transmembrane subunits {Escherichia coli}
INPNPKRSDEPVFWGLFGAGGMWSAIIAPVMILLVGILLPLGLFPGDALSYERVLAFAQSFI
GRVFLFLMIVLPLWCGLHRMHHAMHDLKIHVPAGKWVFYGLAAILTVVTLIGVVTI
>d1qlac_ f.2.1.9 (C:) Fumarate reductase respiratory complex transmembrane subunits {Wolinella succinogenes}
MTNESILESYSGVTPERKKSRMPAKLDWWQSATGLFLGLFMIGHMFFVSTILLGDNVMLWVT
KKFELDFIFEGGKPIVVSFLAAFVFAVFIAHAFLAMRKFPINYRQYLTFKTHKDLMRHGDTT

LWWIQAMTGFAMFFLGSVHLYIMMTQPQTIGPVSSSFRMVSEWMWPLYLVLLFAVELHGSVG
LYRLAVKWGWFDGETPDKTRANLKKLKTLMSAFLIVLGLLTFGAYVKKGLEQTDPNIDYKYF
DYKRTH
>d1jb0a_ f.2.1.12 (A:) Photosystem I {Synechococcus elongatus}
RVVVDNDPVPTSFEKWAKPGHFDRTLARGPQTTTWIWNLHALAHDFDTHTSDLEDISRKIFS
AHFGHLAVVFIWLSGMYFHGAKFSNYEAWLADPTGIKPSAQVVWPIVGQGILNGDVGGGFHG
IQITSGLFQLWRASGITNEFQLYCTAIGGLVMAGLMLFAGWFHYHKRAPKLEWFQNVESMLN
HHLAGLLGLGSLAWAGHQIHVSLPINKLLDAGVAAKDIPLPHEFILNPSLMAELYPKVDWGF
FSGVIPFFTFNWAAYSDFLTFNGGLNPVTGGLWLSDTAHHHLAIAVLFIIAGHMYRTNWGIG
HSLKEILEAHKGPFTGAGHKGLYEVLTTSWHAQLAINLAMMGSLSIIVAQHMYAMPPYPYLA
TDYPTQLSLFTHHMWIGGFLVVGGAAHGAIFMVRDYDPAMNQNNVLDRVLRHRDAIISHLNW
VCIFLGFHSFGLYVHNDTMRAFGRPQDMFSDTGIQLQPVFAQWVQNLHTLAPGGTAPNAAAT
ASVAFGGDVVAVGGKVAMMPIVLGTADFMVHHIHAFTIHVTVLILLKGVLFARSSRLIPDKA
NLGFRFPCDGPGRGGTCQVSGWDHVFLGLFWMYNCISVVIFHFSWKMQSDVWGTVAPDGTVS
HITGGNFAQSAITINGWLRDFLWAQASQVIGSYGSALSAYGLLFLGAHFIWAFSLMFLFSGR
GYWQELIESIVWAHNKLKVAPAIQPRALSIIQGRAVGVAHYLLGGIATTWAFFLARIISVG
>d1jb0b_ f.2.1.12 (B:) Photosystem I {Synechococcus elongatus}
ATKFPKFSQDLAQDPTTRRIWYAIAMAHDFESHDGMTEENLYQKIFASHFGHLAIIFLWVSG
SLFHVAWQGNFEQWVQDPVNTRPIAHAIWDPQFGKAAVDAFTQAGASNPVDIAYSGVYHWWY
TIGMRTNGDLYQGAIFLLILASLALFAGWLHLQPKFRPSLSWFKNAESRLNHHLAGLFGVSS
LAWAGHLIHVAIPESRGQHVGWDNFLSTMPHPAGLAPFFTGNWGVYAQNPDTASHVFGTAQG
AGTAILTFLGGFHPQTESLWLTDMAHHHLAIAVLFIVAGHMYRTQFGIGHSIKEMMDAKDFF
GTKVEGPFNMPHQGIYETYNNSLHFQLGWHLACLGVITSLVAQHMYSLPPYAFIAQDHTTMA
ALYTHHQYIAGFLMVGAFAHGAIFLVRDYDPAQNKGNVLDRVLQHKEAIISHLSWVSLFLGF
HTLGLYVHNDVVVAFGTPEKQILIEPVFAQFIQAAHGKLLYGFDTLLSNPDSIASTAWPNYG
NVWLPGWLDAINSGTNSLFLTIGPGDFLVHHAIALGLHTTTLILVKGALDARGSKLMPDKKD
FGYAFPCDGPGRGGTCDISAWDAFYLAMFWMLNTIGWVTFYWHWKHLGVWEGNVAQFNESST
YLMGWLRDYLWLNSSQLINGYNPFGTNNLSVWAWMFLFGHLVWATGFMFLISWRGYWQELIE
TLVWAHERTPLANLVRWKDKPVALSIVQARLVGLAHFSVGYILTYAAFLIASTAAKF
>d1jb0f_ f.2.1.12 (F:) Photosystem I {Synechococcus elongatus}
DVAGLVPCKDSPAFQKRAAAAVNTTADPASGQKRFERYSQALCGEDGLPHLVVDGRLSRAGD
FLIPSVLFLYIAGWIGWVGRAYLIAVRNSGEANEKEIIIDVPLAIKCMLTGFAWPLAALKEL
ASGELTAKDNEITVSPR
>d1jb0i_ f.2.1.12 (I:) Photosystem I {Synechococcus elongatus}
MMGSYAASFLPWIFIPVVCWLMPTVVMGLLFLYIEGEA
>d1jb0j_ f.2.1.12 (J:) Photosystem I {Synechococcus elongatus}
MKHFLTYLSTAPVLAAIWMTITAGILIEFNRFYPDLLFHPL
>d1jb0k_ f.2.1.12 (K:) Photosystem I {Synechococcus elongatus}
ILCNLFAIALGRYAIQSRGKGPGLPIALPALFEGFGLPELLATTSFGHLLAAGVVSGL
>d1jb0l_ f.2.1.12 (L:) Photosystem I {Synechococcus elongatus}
LVKPYNGDPFVGHLSTPISDSGLVKTFIGNLPAYRQGLSPILRGLEVGMAHGYFLIGPWVKL
GPLRDSDVANLGGLISGIALILVATACLAAYGLVSFQKGGSSSDPLKTSEGWSQFTAGFFVG
AMGSAFVAFFLLENFLVVDGIMTGLFN
>d1jb0m_ f.2.1.12 (M:) Photosystem I {Synechococcus elongatus}

MALTDTQVYYVALVIALLPAVLAFRLSTELYK
>d1jb0x_ f.2.1.12 (X:) Photosystem I {Synechococcus elongatus}
PTYAFRTFWAVLLLAINFLVAAYYFAAAA
>d1eula_ f.2.1.10 (A:) Calcium ATPase {Rabbit (Oryctolagus cuniculus)}
MEAAHSKSTEECLAYFGVSETTGLTPDQVKRHLEKYGHNELPAEEGKSLWELVIEQFEDLLV
RILLLAACISFVLAWFEEGEETITAFVEPFVILLILIANAIVGVWQERNAENAIEALKEYEP
EMGKVYRADRKSVQRIKARDIVPGDIVEVAVGDKVPADIRILSIKSTTLRVDQSILTGESVS
VIKHTEPVPDPRAVNQDKKNMLFSGTNIAAGKALGIVATTGVSTEIGKIRDQMAATEQDKTP
LQQKLDEFGEQLSKVISLICVAVWLINIGHFNDPVHGGSWIRGAIYYFKIAVALAVAAIPEG
LPAVITTCLALGTRRMAKKNAIVRSLPSVETLGCTSVICSDKTGTLTTNQMSVCKMFIIDKV
DGDFCSLNEFSITGSTYAPEGEVLKNDKPIRSGQFDGLVELATICALCNDSSLDFNETKGVY
EKVGEATETALTTLVEKMNVFNTEVRNLSKVERANACNSVIRQLMKKEFTLEFSRDRKSMSV
YCSPAKSSRAAVGNKMFVKGAPEGVIDRCNYVRVGTTRVPMTGPVKEKILSVIKEWGTGRDT
LRCLALATRDTPPKREEMVLDDSSRFMEYETDLTFVGVVGMLDPPRKEVMGSIQLCRDAGIR
VIMITGDNKGTAIAICRRIGIFGENEEVADRAYTGREFDDLPLAEQREACRRACCFARVEPS
HKSKIVEYLQSYDEITAMTGDGVNDAPALKKAEIGIAMGSGTAVAKTASEMVLADDNFSTIV
AAVEEGRAIYNNMKQFIRYLISSNVGEVVCIFLTAALGLPEALIPVQLLWVNLVTDGLPATA
LGFNPPDLDIMDRPPRSPKEPLISGWLFFRYMAIGGYVGAATVGAAAWWFMYAEDGPGVTYH
QLTHFMQCTEDHPHFEGLDCEIFEAPEPMTMALSVLVTIEMCNALNSLSENQSLMRMPPWVN
IWLLGSICLSMSLHFLILYVDPLPMIFKLKALDLTQWLMVLKISLPVIGLDEILKFIARNYL
EG
>d1kzua_ f.3.1.1 (A:) Light-harvesting complex subunits {Purple bacterium (Rhodopseudomonas acidophila)}
MNQGKIWTVVNPAIGIPALLGSVTVIAILVHLAILSHTTWFPAYWQGGV
>d1kzub_ f.3.1.1 (B:) Light-harvesting complex subunits {Purple bacterium (Rhodopseudomonas acidophila)}
ATLTAEQSEELHKYVIDGTRVFLGLALVAHFLAFSATPWLH
>d1lgha_ f.3.1.1 (A:) Light-harvesting complex subunits {Rhodospirillum molischianum}
SNPKDDYKIWLVINPSTWLPVIWIVATVVAIAVHAAVLAAPGFNWIALGAAKSAAK
>d1lghb_ f.3.1.1 (B:) Light-harvesting complex subunits {Rhodospirillum molischianum}
RSLSGLTEEEAIAVHDQFKTTFSAFIILAAVAHVLVWVWKPWF
>d1jo5a_ f.3.1.1 (A:) Light-harvesting complex subunits {Rhodobacter sphaeroides}
ADKSDLGYTGLTDEQAQELHSVYMSGLWLFSAVAIVAHLAVYIWRPWF
>d1ijda_ f.3.1.1 (A:) Light-harvesting complex subunits {Rhodopseudomonas acidophila}
MNQGKIWTVVPPAFGLPLMLGAVAITALLVHAAVLTHTTWYAAFLQ
>d1ijdb_ f.3.1.1 (B:) Light-harvesting complex subunits {Rhodopseudomonas acidophila}
AEVLTSEQAEELHKHVIDGTRVFLVIAAIAHFLAFTLTPW
>d1g90a_ f.4.1.1 (A:) Outer membrane protein A (OMPA) transmembrane

domain {Escherichia coli}
APKDNTWYTGAKLGFSQYHDTGFINNNGPTHENQLGAGAFGGYQVNPYVGFEMGYDFLGRMP
YKGSVENGAYKAQGVQLTAKLGYPITDDLDIYTRLGGMVFRADTKSNVYGKNHDTGVSPVFA
GGVEYAITPEIATRLEYQFTNNIGDAHTIGTRPDNGMLSLGVSYRFGQGEAA
>d1qjpa_ f.4.1.1 (A:) Outer membrane protein A (OMPA) transmembrane domain {Escherichia coli}
APKDNTWYTGAKLGWSQYHDTGLINNNGPTHENKLGAGAFGGYQVNPYVGFEMGYDWLGRMP
YKGSVENGAYKAQGVQLTAKLGYPITDDLDIYTRLGGMVWRADTYSNVYGKNHDTGVSPVFA
GGVEYAITPEIATRLEYQWTNNIGDAHTIGTRPDNGMLSLGVSYRFG
>d1qj8a_ f.4.1.1 (A:) Outer membrane protein X (OMPX) {Escherichia coli}
ATSTVTGGYAQSDAQGQMNKMGGFNLKYRYEEDNSPLGVIGSFTYTEKSRTASSGDYNKNQY
YGITAGPAYRINDWASIYGVVGVGYGKFQTTEYPTYKNDTSDYGFSYGAGLQFNPMENVALD
FSYEQSRIRSVDVGTWIAGVGYRF
>d1i78a_ f.4.4.1 (A:) Outer membrane protease OMPT {Escherichia coli}
STETLSFTPDNINADISLGTLSGKTKERVYLAEEGGRKVSQLDWKFNNAAIIKGAINWDLMP
QISIGAAGWTTLGSRGGNMVDQDWMDSSNPGTWTDEARHPDTQLNYANEFDLNIKGWLLNEP
NYRLGLMAGYQESRYSFTARGGSYIYSSEEGFRDDIGSFPNGERAIGYKQRFKMPYIGLTGS
YRYEDFELGGTFKYSGWVESSDNDEHYDPKGRITYRSKVKDQNYYSVAVNAGYYVTPNAKVY
VEGAWNRVTNKKGNTSLYDHNNNTSDYSKNGAGIENYNFITTAGLKYTF
>g1qd6.1 f.4.2.1 (A:,C:) Outer membrane phospholipase A (OMPLA) {Escherichia coli}
AVRGSIIANMLQEXFTLYPYDTNYLIYTQTSDLNKEAIASYDWAENARKDEVKFQLSLAFPL
WRGILGPNSVLGASYTQKSWWQLSNSEESSPFRETNYEPQLFLGFATDYRFAGWTLRDVEMG
YNHDSNGRSDPTSRSWNRLYTRLMAENGNWLVEVKPWYVVGNTDDNPDITKYMGYYQLKIGY
HLGDAVLSAKGQYNWNTGYGGAELGLSYPITKHVRLYTQVYSGYGESLIDYNFNQTRVGVGV
MLNDLF
>d1hxxa_ f.4.3.1 (A:) Porin {Escherichia coli, different sequences}
AEIYNKDGNKVDLYGKAVGLHYFSKGNGENSYGGNGDMTYARLGFKGETQINSDLTGYGQWE
YNFQGNNSEGADAQTGNKTRLAFAGLKYADVGSFDYGRNYGVVFDALGYTDMLPEFGGDTAY
SDDFFVGRVGGVATYRNSNFFGLVDGLNFAVQYLGKNERDTARRSNGDGVGGSISYEYEGFG
IVGAYGAADRTNLQEAQPLGNGKKAEQWATGLKYDANNIYLAANYGETRNATPITNKFTNTS
GFANKTQDVLLVAQYQFDFGLRPSIAYTKSKAKDVEGIGDVDLVNYFEVGATYYFNKNMSTY
VDYIINQIDSDNKLGVGSDDTVAVGIVYQF
>d1pho__ f.4.3.1 (-) Porin {Escherichia coli, different sequences}
AEIYNKDGNKLDVYGKVKAMHYMSDNASKDGDQSYIRFGFKGETQINDQLTGYGRWEAEFAG
NKAESDTAQQKTRLAFAGLKYKDLGSFDYGRNLGALYDVEAWTDMFPEFGGDSSAQTDNFMT
KRASGLATYRNTDFFGVIDGLNLTLQYQGKNENRDVKKQNGDGFGTSLTYDFGGSDFAISGA
YTNSDRTNEQNLQSRGTGKRAEAWATGLKYDANNIYLATFYSETRKMTPITGGFANKTQNFE
AVAQYQFDFGLRPSLGYVLSKGKDIEGIGDEDLVNYIDVGATYYFNKNMSAFVDYKINQLDS
DNKLNINNDDIVAVGMTYQF
>d2por__ f.4.3.1 (-) Porin {Rhodobacter capsulatus}

EVKLSGDARMGVMYNGDDWNFSSRSRVLFTMSGTTDSGLEFGASFKAHESVGAETGEDGTVF
LSGAFGKIEMGDALGASEALFGDLYEVGYTDLDDRGGNDIPYLTGDERLTAEDNPVLLYTYS
AGAFSVAASMSDGKVGETSEDDAQEMAVAAAYTFGNYTVGLGYEKIDSPDTALMADMEQLEL
AAIAKFGATNVKAYYADGELDRDFARAVFDLTPVAAAATAVDHKAYGLSVDSTFGATTVGGY
VQVLDIDTIDDVTYYGLGASYDLGGGASIVGGIADNDLPNSDMVADLGVKFKF
>d3prn__ f.4.3.1 (-) Porin {Rhodopseudomonas blastica, strain DSM2131}
MISLNGYGRFGLQYVEDRGVGLEDTIISSRLRINIVGTTETDQGVTFGAKLRMQWDDGDAFA
GTAGNAAQFWTSYNGVTVSVGNVDTAFDSVALTYDSEMGYEWSSFGDAQSSFFAYNSKYDAS
GALDNYNGIAVTYSISGVNLYLSYVDPDQTVDSSLVTEEFGIAADWSNDMISLAAAYTTDAG
GIVDNDIAFVGAAYKFNDAGTVGLNWYDNGLSTAGDQVTLYGNYAFGATTVRAYVSDIDRAG
ADTAYGIGADYQFAEGVKVSGSVQSGFANETVADVGVRFDF
>d1osma_ f.4.3.1 (A:) Porin {Klebsiella pneumoniae}
AEIYNKDGNKLDLYGKIDGLHYFSDDKDVDGDQTYMRLGVKGETQINDQLTGYGQWEYNVQA
NNTESSSDQAWTRLAFAGLKFGDAGSFDYGRNYGVVYDVTSWTDVLPEFGGDTYGSDNFLQS
RANGVATYRNSDFFGLVDGLNFALQYQGKNGSVSGEGATNNGRGALKQNGDGFGTSVTYDIF
DGISAGFAYANSKRTDDQNQLLLGEGDHAETYTGGLKYDANNIYLATQYTQTYNATRAGSLG
FANKAQNFEVAAQYQFDFGLRPSVAYLQSKGKDLNGYGDQDILKYVDVGATYYFNKNMSTYV
DYKINLLDDNSFTRSAGISTDDVVALGLVYQF
>d1e54a_ f.4.3.1 (A:) Porin {Comamonas acidovorans}
ESSVTLFGIVDTNVAYVNKDAAGDSRYGLGTSGASTSRLGLRGTEDLGGGLKAGFWLEGEIF
GDDGNASGFNFKRRSTVSLSGNFGEVRLGRDLVPTSQKLTSYDLFSATGIGPFMGFRNWAAG
QGADDNGIRANNLISYYTPNFGGFNAGFGYAFDEKQTIGTADSVGRYIGGYVAYDNGPLSAS
LGLAQQKTAVGGLATDRDEITLGASYNFGVAKLSGLLQQTKFKRDIGGDIKTNSYMLGASAP
VGGVGEVKLQYALYDQKAIDSKAHQITLGYVHNLSKRTALYGNLAFLKNKDASTLGLQAKGV
YAGGVQAGESQTGVQVGIRHAF
>d1af6a_ f.4.3.2 (A:) Maltoporin (also LamB protein) {Escherichia coli}
VDFHGYARSGIGWTGSGGEQQCFQTTGAQSKYRLGNECETYAELKLGQEVWKEGDKSFYFDT
NVAYSVAQQNDWEATDPAFREANVQGKNLIEWLPGSTIWAGKRFYQRHDVHMIDFYYWDISG
PGAGLENIDVGFGKLSLAATRSSEAGGSSSFASNNIYDYTNETANDVFDVRLAQMEINPGGT
LELGVDYGRANLRDNYRLVDGASKDGWLFTAEHTQSVLKGFNKFVVQYATDSMTSQGKGLSQ
GSGVAFDNEKFAYNINNNGHMLRILDHGAISMGDNWDMMYVGMYQDINWDNDNGTKWWTVGI
RPMYKWTPIMSTVMEIGYDNVESQRTGDKNNQYKITLAQQWQAGDSIWSRPAIRVFATYAKW
DEKWGYDYTGNADNNANFGKAVPADFNGGSFGRGDSDEWTFGAQMEIWW
>d2mpra_ f.4.3.2 (A:) Maltoporin (also LamB protein) {Salmonella typhimurium}
VDFHGYARSGIGWTGSGGEQQCFQATGAQSKYRLGNECETYAELKLGQEVWKEGDKSFYFDT
NVAYSVNQQNDWESTDPAFREANVQGKNLIEWLPGSTIWAGKRFYQRHDVHMIDFYYWDISG
PGAGIENIDLGFGKLSLAATRSTEAGGSYTFSSQNIYDEVKDTANDVFDVRLAGLQTNPDGV
LELGVDYGRANTTDGYKLADGASKDGWMFTAEHTQSMLKGYNKFVVQYATDAMTTQGKGQAR
GSDGSSSFTEKINYANKVINNNGNMWRILDHGAISLGDKWDLMYVGMYQNIDWDNNLGTEWW
TVGVRPMYKWTPIMSTLLEVGYDNVKSQQTGDRNNQYKITLAQQWQAGDSIWSRPAIRIFAT
YAKWDEKWGYIKDGDNISRYAAATNSGISTNSRGDSDEWTFGAQMEIWW

>d1a0tp_ f.4.3.2 (P:) Sucrose-specific porin {Enterobacterium (Salmonella typhimurium)}
SGFEFHGYARSGVIMNDSGASTKSGAYITPAGETGGAIGRLGNQADTYVEMNLEHKQTLDNG
ATTRFKVMVADGQTSYNDWTASTSDLNVRQAFVELGNLPTFAGPFKGSTLWAGKRFDRDNFD
IHWIDSDVVFLAGTGGGIYDVKWNDGLRSNFSLYGRNFGDIDDSSNSVQNYILTMNHFAGPL
QMMVSGLRAKDNDERKDSNGNLAKGDAANTGVHALLGLHNDSFYGLRDGSSKTALLYGHGLG
AEVKGIGSDGALRPGADTWRIASYGTTPLSENWSVAPAMLAQRSKDRYADGDSYQWATFNLR
LIQAINQNFALAYEGSYQYMDLKPEGYNDRQAVNGSFYKLTFAPTFKVGSIGDFFSRPEIRF
YTSWMDWSKKLNNYASDDALGSDGFNSGGEWSFGVQMETWF
>d1by5a_ f.4.3.3 (A:) Ferric hydroxamate uptake receptor FhuA {Escherichia coli}
QESAWGPAATIAARQSATGTKTDTPIQKVPQSISVVTAEEMALHQPKSVKEALSYTPGVSVG
TRGASNTYDHLIIRGFAAEGQSQNNYLNGLKLQGNFYNDAVIDPYMLERAEIMRGPVSVLYG
KSSPGGLLNMVSKRPTTEPLKEVQFKAGTDSLFQTGFDFSDSLDDDGVYSYRLTGLARSANA
QQKGSEEQRYAIAPAFTWRPDDKTNFTFLSYFQNEPETGYYGWLPKEGTVEPLPNGKRLPTD
FNEGAKNNTYSRNEKMVGYSFDHEFNDTFTVRQNLRFAENKTSQNSVYGYGVCSDPANAYSK
QCAALAPADKGHYLARKYVVDDEKLQNFSVDTQLQSKFATGDIDHTLLTGVDFMRMRNDINA
WFGYDDSVPLLNLYNPVNTDFDFNAKDPANSGPYRILNKQKQTGVYVQDQAQWDKVLVTLGG
RYDWADQESLNRVAGTTDKRDDKQFTWRGGVNYLFDNGVTPYFSYSESFEPSSQVGKDGNIF
APSKGKQYEVGVKYVPEDRPIVVTGAVYNLTKTNNLMADPEGSFFSVEGGEIRARGVEIEAK
RPLSASVNVVGSYTYTDAEYTTDTTYKGNTPAQVPKHMASLWADYTFFDGPLSGLTLGTGGR
YTGSSYGDPANSFKVGSYTVVDALVRYDLARVGMAGSNVALHVNNLFDREYVASCFNTYGCF
WGAERQVVATATFRF
>d1fepa_ f.4.3.3 (A:) Ferric enterobactin receptor FepA {Escherichia coli}
DDTIVVTAAEQNLQAPGVSTITADEIRKNPVARDVSKIIRTMPGVNLTGNSTSGQRGNNRQI
DIRGMGPENTLILIDGKPVSSRNSVRQGWRGERDTRGDTSWVPPEMIERIEVLRGPAAARYG
NGAAGGVVNIITKKGSGEWHGSWDAYFNAPEHKEEGATKRTNFSLTGPLGDEFSFRLYGNLD
KTQADAWDINQGHQSARAGTYATTLPAGREGVINKDINGVVRWDFAPLQSLELEAGYSRQGN
LYAGDTQNTNSDSYTRSKYGDETNRLYRQNYALTWNGGWDNGVTTSNWVQYEHTRNSRIPEG
LAGGTEGKFNEKATQDFVDIDLDDVMLHSEVNLPIDFLVNQTLTLGTEWNQQRMKDLSSNTQ
ALTGTNTGGAIDGVSTTDRSPYSKAEIFSLFAENNMELTDSTIVTPGLRFDHHSIVGNNWSP
ALNISQGLGDDFTLKMGIARAYKAPSLYQTNPNYILYSKGQGCYASAGGCYLQGNDDLKAET
SINKEIGLEFKRDGWLAGVTWFRNDYRNKIEAGYVAVGQNAVGTDLYQWDNVPKAVVEGLEG
SLNVPVSETVMWTNNITYMLKSENKTTGDRLSIIPEYTLNSTLSWQAREDLSMQTTFTWYGK
QQPKKYNYKGQPAVGPETKEISPYSIVGLSATWDVTKNVSLTGGVDNLFDKRLWRAGNAQTT
GDLAGANYIAGAGAYTYNEPGRTWYMSVNTHF
>d1ek9a_ f.5.1.1 (A:) Integral outer membrane protein TolC, efflux pump component {Escherichia coli}
ENLMQVYQQARLSNPELRKSAADRDAAFEKINEARSPLLPQLGLGADYTYSNGYRDANGINS
NATSASLQLTQSIFDMSKWRALTLQEKAAGIQDVTYQTDQQTLILNTATAYFNVLNAIDVLS
YTQAQKEAIYRQLDQTTQRFNVGLVAITDVQNARAQYDTVLANELTARNNLDNAVEQLRQIT
GNYYPELAALNVENFKTDKPQPVNALLKEAEKRNLSLLQARLSQDLAREQIRQAQDGHLPTL
DLTASTGISDTSYSGSKTRGAAGTQYDDSNMGQNKVGLSFSLPIYQGGMVNSQVKQAQYNFV

GASEQLESAHRSVVQTVRSSFNNINASISSINAYKQAVVSAQSSLDAMEAGYSVGTRTIVDV
LDATTTLYNAKQELANARYNYLINQLNIKSALGTLNEQDLLALNNALSKPVSTNPE
>d7ahla_ f.6.1.1 (A:) Alpha-hemolysin {Staphylococcus aureus}
ADSDINIKTGTTDIGSNTTVKTGDLVTYDKENGMHKKVFYSFIDDKNHNKKLLVIRTKGTIA
GQYRVYSEEGANKSGLAWPSAFKVQLQLPDNEVAQISDYYPRNSIDTKEYMSTLTYGFNGNV
TGDDTGKIGGLIGANVSIGHTLKYVQPDFKTILESPTDKKVGWKVIFNNMVNQNWGPYDRDS
WNPVYGNQLFMKTRNGSMKAADNFLDPNKASSLLSSGFSPDFATVITMDRKASKQQTNIDVI
YERVRDDYQLHWTSTNWKGTNTKDKWTDRSSERYKIDWEKEEMTN
>d1pvl__ f.6.1.1 (-) Leucocidin K component LukF-PV
{Staphylococcus aureus}
AQHITPVSEKKVDDKITLYKTTATSDSDKLKISQILTFNFIKDKSYDKDTLILKAAGNIYSG
YTKPNPKDTISSQFYWGSKYNISINSDSNDSVNVVDYAPKNQNEEFQVQQTVGYSYGGDINI
SNGLSGGGNGSKSFSETINYKQESYRTSLDKRTNFKKIGWDVEAHKIMNNGWGPYGRDSYHS
TYGNEMFLGSRQSNLNAGQNFLEYHKMPVLSRGNFNPEFIGVLSRKQNAAKKSKITVTYQRE
MDRYTNFWNQLHWIGNNYKDENRATHTSIYEVDWENHTVKLIDTQSKEKNPMS
>d3lkfa_ f.6.1.1 (A:) Leukocidin F (HlgB) {Staphylococcus aureus}
EGKITPVSVKKVDDKVTLYKTTATADSDKFKISQILTFNFIKDKSYDKDTLVLKATGNINSG
FVKPNPNDYDFSKLYWGAKYNVSISSQSNDSVNVVDYAPKNQNEEFQVQNTLGYTFGGDISI
SNGLSGGLNGNTAFSETINYKQESYRTTLSRNTNYKNVGWGVEAHKIMNNGWGPYGRDSFHP
TYGNELFLAGRQSSAYAGQNFIAQHQMPLLSRSNFNPEFLSVLSHRQDGAKKSKITVTYQRE
MDLYQIRWNGFYWAGANYKNFKTRTFKSTYEIDWENHKVKLLDTKETENNK
>d1prea2 f.8.1.1 (A:85-470) (Pro)aerolysin, pore-forming lobe
{Aeromonas hydrophila}
IPTLSALDIPDGDEVDVQWRLVHDSANFIKPTSYLAHYLGYAWVGGNHSQYVGEDMDVTRDG
DGWVIRGNNDGGCDGYRCGDKTAIKVSNFAYNLDPDSFKHGDVTQSDRQLVKTVVGWAVNDS
DTPQSGYDVTLRYDTATNWSKTNTYGLSEKVTTKNKFKWPLVGETELSIEIAANQSWASQNG
GSTTTSLSQSVRPTVPARSKIPVKIELYKADISYPYEFKADVSYDLTLSGFLRWGGNAWYTH
PDNRPNWNHTFVIGPYKDKASSIRYQWDKRYIPGEVKWWDWNWTIQQNGLSTMQNNLARVLR
PVRAGITGDFSAESQFAGNIEIGAPVPLAADSKVRRARSVDGAGQGLRLEIPLDAQELSGLG
FNNVSLSVTPAANQ
>d1pfo__ f.9.1.1 (-) Perfringolysin {Clostridium perfringens}
DITDKNQSIDSGISSLSYNRNEVLASNGDKIESFVPKEGKKAGNKFIVVERQKRSLTTSPVD
ISIIDSVNDRTYPGALQLADKALVENRPTILMVKRKPININIDLPGLKGENSIKVDDPTYGK
VSGAIDELVSKWNEKYSSTHTLPARTQYSESMVYSKSQISSALNVNAKVLENSLGVDFNAVA
NNEKKVMILAYKQIFYTVSADLPKNPSDLFDDSVTFNDLKQKGVSNEAPPLMVSNVAYGRTI
YVKLETTSSSKDVQAAFKALIKNTDIKNSQQYKDIYENSSFTAVVLGGDAQEHNKVVTKDFD
EIRKVIKDNATFSTKNPAYPISYTSVFLKDNSVAAVHNKTDYIETTSTEYSKGKINLDHSGA
YVAQFEVAWDEVSYDKEGNEVLTHKTWDGNYQDKTAHYSTVIPLEANARNIRIKARECTGLA
WEWWRDVISEYDVPLTNNINVSIWGTTLYPGSSITYN
>d1svb_2 f.10.1.1 (1-302) Envelope glycoprotein, central and
dimerisation domains {Tick-borne encephalitis virus}
SRCTHLENRDFVTGTQGTTRVTLVLELGGCVTITAEGKPSMDVWLDAIYQENPAKTREYCLH
AKLSDTKVAARCPTMGPATLAEEHQGGTVCKRDQSDRGWGNHCGLFGKGSIVACVKAACEAK
KKATGHVYDANKIVYTVKVEPHTGDYVAANETHSGRKTASFTISSEKTILTMGEYGDVSLLC

RVASGVDLAQTVILELDKTVEHLPTAWQVHRDWFNDLALPWKHEGAQNWNNAERLVEFGAPH
AVKMDVYNLGDQTGVLLKALAGVPVAHIEGTKYHLKSGHVTCEVGLEKLKMKGL
>d1g5ga1 f.12.1.1 (A:33-66,A:224-454) Head and neck region of the ectodomain of NDV fusion glycoprotein {Newcastle disease virus}
DGRPLAAAGIVVTGDKAVNIYTSSQTGSIIIKLLXQITSPALTQLTIQALYNLAGGNMDYLL
TKLGVGNNQLSSLISSGLITGNPILYDSQTQLLGIQVTLPSVGNLNNMRATYLETLSVSTTK
GFASALVPKVVTQVGSVIEELDTSYCIETDLDLYCTRIVTFPMSPGIYSCLSGNTSACMYSK
TEGALTTPYMTLKGSVIANCKMTTCRCADPPGIISQNYGEAVSLIDRQSCNILSLDGITLRL
SGEFDATYQKNISIQDSQ
>d1acc__ f.11.1.1 (-) Anthrax protective antigen {Anthrax bacillus (Bacillus anthracis)}
SSSQGLLGYYFSDLNFQAPMVVTSSTTGDLSIPSSELENIPSENQYFQSAIWSGFIKVKKSD
EYTFATSADNHVTMWVDDQEVINKASNSNKIRLEKGRLYQIKIQYQRENPTEKGLDFKLYWT
DSQNKKEVISSDNLQLPELKQKSSNSRKKRSTSAGPTVPDRDNDGIPDSLEVEGYTVDVKNK
RTFLSPWISNIHEKKGLTKYKSSPEKWSTASDPYSDFEKVTGRIDKNVSPEARHPLVAAYPI
VHVDMENIILSKNEDQSTQNTDSETRTISKNTSTSRTHTSEVHGNAEVHASFFDIGGSVSAG
FSNSNSSTVAIDHSLSLAGERTWAETMGLNTADTARLNANIRYVNTGTAPIYNVLPTTSLVL
GKNQTLATIKAKENQLSQILAPNNYYPSKNLAPIALNAQDDFSSTPITMNYNQFLELEKTKQ
LRLDTDQVYGNIATYNFENGRVRVDTGSNWSEVLPQIQETTARIIFNGKDLNLVERRIAAVN
PSDPLETTKPDMTLKEALKIAFGFNEPNGNLQYQGKDITEFDFNFDQQTSQNIKNQLAELNA
TNIYTVLDKIKLNAKMNILIRDKRFHYDRNNIAVGADESVVKEAHREVINSSTEGLLLNIDK
DIRKILSGYIVEIEDTEGLKEVINDRYDMLNISSLRQDGKTFIDFKKYNDKLPLYISNPNYK
VNVYAVTKENTIINPSENGDTSTNGIKKILIFSKKGYEIG
>g1cph.1 g.1.1.1 (B:,A:) Insulin {Cow (Bos taurus)}
FVNQHLCGSHLVEALYLVCGERGFFYTPKAXGIVEQCCASVCSLYQLENYCN
>g1pid.1 g.1.1.1 (B:,A:) Insulin {Cow (Bos taurus)}
FVNQHLCGSHLVEALYLVCGERGFFXGIVEQCCASVCSLYQLENYCN
>d1efea_ g.1.1.1 (A:) Insulin {Human (Homo sapiens)}
FVNQHLCGSHLVEALYLVCGERGFFYTPKTRRYPGDVKRGIVEQCCTSICSLYQLENYCN
>g1a7f.1 g.1.1.1 (B:,A:) Insulin {Human (Homo sapiens)}
FVNQHLCGSHLVEALELVCGERGGFYTPKXGIVEQCCTSICSLYQLENYCN
>g1g7a.1 g.1.1.1 (B:,A:) Insulin {Human (Homo sapiens)}
FVNQHLCGSHLVEALYLVCGERGFFYTPKTXGIVEQCCTSICSLYQLENYCN
>g1hui.1 g.1.1.1 (B:,A:) Insulin {Human (Homo sapiens)}
EVNQHLCGSELVEALELVCGERGFFYEPKXGIVEQCCTSICSLYQLENYCN
>g1qj0.1 g.1.1.1 (B:,A:) Insulin {Human (Homo sapiens)}
VNQYLCGSHLVEALYLVCGERGFFYTPKXGIVEQCCTSICSLYQLENYCN
>g1sjt.1 g.1.1.1 (B:,A:) Insulin {Human (Homo sapiens)}
FVNQHLCGSDLVEALYLVCGERGFFYTDKXGIVEQCCTSICSLYQLENYCN
>g1vks.1 g.1.1.1 (B:,A:) Insulin {Human (Homo sapiens)}
FVNQHLCGSDLVEALYLVCGERGFFYTKPTXGIVEQCCTSICSLYQLENYCN
>d1zeia_ g.1.1.1 (A:) Insulin {Pig (Sus scrofa)}
FVNQHLCGSHLVEALYLVCGERGFFYTDKAAKGIVEQCCTSICSLYQLENYCN
>g1dei.1 g.1.1.1 (B:,A:) Insulin {Pig (Sus scrofa)}

FVNQHLCGSHLVEALYLVCGERGXGIVEQCCTSICSLYQLENYCN
>g1sdb.1 g.1.1.1 (B:,A:) Insulin {Pig (Sus scrofa)}
NQHLCGSHLVEALYLVCGERGFFXGIVEQCCTSICSLYQLENYCN
>g6rlx.1 g.1.1.1 (B:,A:) Relaxin {Human (Homo sapiens)}
SWMEEVIKLCGRELVRAQIAICGMSTWXELYSALANKCCHVGCTKRSLARFC
>d1b9ga_ g.1.1.1 (A:) Insulin-like growth factor {Human (Homo sapiens)}
GPETLCGAELVDALQFVCGDRGFYFNKPGIVDECCFRSCDLRRLEMYCAPLKPAKSA
>d1igl__ g.1.1.1 (-) Insulin-like growth factor {Human (Homo sapiens)}
AYRPSETLCGGELVDTLQFVCGDRGFYFSRPASRVSRRSRGIVEECCFRSCDLALLETYCAT
PAKSE
>d1imxa_ g.1.1.1 (A:) Insulin-like growth factor {Human (Homo sapiens)}
ETLCGAELVDALQFVCGDRGFYFNKPTGYGSSSRRAPQTGIVDECCFRSCDLRRLEMYCAPL
>d2gf1__ g.1.1.1 (-) Insulin-like growth factor {Human (Homo sapiens)}
GPETLCGAELVDALQFVCGDRGFYFNKPTGYGSSSRRAPQTGIVDECCFRSCDLRRLEMYCA
PLKPAKSA
>d3lria_ g.1.1.1 (A:) Insulin-like growth factor {Human (Homo sapiens)}
MFPAMPLSSLFVNGPRTLCGAELVDALQFVCGDRGFYFNKPTGYGSSSRRACQTGIVDECCF
RSCDLRRLEMYCAPLKPAKSA
>g1bom.1 g.1.1.1 (B:,A:) Bombyxin-II {Silkworm (Bombyx mori)}
EQPQAVHTYCGRHLARTLADLCWEAGVDXGIVDECCLRPCSVDVLLSYC
>d1ehs__ g.2.1.1 (-) Heat-stable enterotoxin B {Escherichia coli}
STQSNKKDLCEHYRQIAKESCKKGFLGVRDGTAGACFGAQIMVAAKGC
>d1vib__ g.2.2.1 (-) Neurotoxin B-IV {Milky ribbon worm (Cerebratulus lacteus)}
ASATWGAAYPACENNCRKKYDLCIRCQGKWAGKRGKCAAHCIIQKNNCKGKCKKE
>d1wgta3 g.3.1.1 (A:87-129) Wheat germ agglutinin (WGA) {Wheat (Triticum vulgaris)}
IKCGSQAGGKLCPNNLCCSQWGYCGLGSEFCGEGCQNGACSTD
>d2cwga2 g.3.1.1 (A:53-86) Wheat germ agglutinin (WGA) {Wheat (Triticum vulgaris)}
ATCTNNQCCSQYGYCGFGAEYCGAGCQGGPCRAD
>d9wgaa1 g.3.1.1 (A:1-52) Wheat germ agglutinin (WGA) {Wheat (Triticum vulgaris)}
ERCGEQGSNMECPNNLCCSQYGYCGMGGDYCGKGCQNGACWTSKRCGSQAGG
>d9wgaa2 g.3.1.1 (A:53-86) Wheat germ agglutinin (WGA) {Wheat (Triticum vulgaris)}
ATCPNNHCCSQYGHCGFGAEYCGAGCQGGPCRAD
>d9wgaa3 g.3.1.1 (A:87-129) Wheat germ agglutinin (WGA) {Wheat (Triticum vulgaris)}

IKCGSQSGGKLCPNNLCCSQWGFCGLGSEFCGGGCQSGACSTD
>d9wgaa4 g.3.1.1 (A:130-171) Wheat germ agglutinin (WGA) {Wheat (Triticum vulgaris)}
KPCGKDAGGRVCTNNYCCSKWGSCGIGPGYCGAGCQSGGCDA
>d1ehda1 g.3.1.1 (A:1-45) Isolectin VI {Stinging nettle (Urtica dioica), UDA}
ERCGSQGGGATCPGLRCCSIWGWCGDSEPYCGRTCENKCWSGERS
>d1ehda2 g.3.1.1 (A:46-89) Isolectin VI {Stinging nettle (Urtica dioica), UDA}
DHRCGAAVGNPPCGQDRCCSVHGWCGGGNDYCSGGKCQYRCSSS
>d1en2a1 g.3.1.1 (A:1-45) Isolectin VI {Stinging nettle (Urtica dioica), UDA}
ERCGSQGGGGTCPALWCCSIWGWCGDSEPYCGRTCENKCWSGERS
>d1en2a2 g.3.1.1 (A:46-86) Isolectin VI {Stinging nettle (Urtica dioica), UDA}
DHRCGAAVGNPPCGQDRCCSVHGWCGGGNDYCSGSKCQYRC
>d1hev__ g.3.1.1 (-) Hevein {Hevea brasiliensis}
EQCGRQAGGKLCPNNLCCSQWGWCGSTDEYCSPDHNCQSNCKD
>d1mmc__ g.3.1.2 (-) Antimicrobial peptide 2, AC-AMP2 {Tassel (Amaranthus caudatus)}
VGECVRGRCPSGMCCSQFGYCGKGPKYCGR
>d1f2si_ g.3.2.1 (I:) Trypsin inhibitor {Bitter gourd (Momordica charantia), linn. Cucurbitaceae, seed}
RICPRIWMECKRDSDCMAECICVMGHCG
>d1mcti_ g.3.2.1 (I:) Trypsin inhibitor {Bitter gourd (Momordica charantia), linn. Cucurbitaceae, seed}
RICPRIWMECTRDSDCMAKCICVAGHCG
>d2stai_ g.3.2.1 (I:) Trypsin inhibitor {Squash (Cucurbita maxima)}
RVCPRILMECKKDSDCLAECVCLEHGYCG
>d1ha9a_ g.3.2.1 (A:) Trypsin inhibitor {Spiny bitter cucumber (Momordica cochinchinensis), MCOTI-II}
SGSDGGVCPKILKKCRRDSDCPGACICRGNGYCG
>d2btci_ g.3.2.1 (I:) Trypsin inhibitor {Vegetable marrow (Cucurbita pepo)}
RVCPKILMECKKDSDCLAECICLEHGYCG
>d2let__ g.3.2.1 (-) Trypsin inhibitor {Jumping cucumber (Ecballium elaterium)}
GCPRLLMRCKQDSDCLAGCVCGPNGFCG
>d4cpai_ g.3.2.1 (I:) Carboxypeptidase A inhibitor {Potato}
ZHADPICNKPCKTHDDCSGAWFCQACWNSARTCGPYV
>d1clvi_ g.3.2.1 (I:) alpha-amylase inhibitor (AAI) {Prince's feather (Amaranthus hypochondriacus)}
CIPKWNRCGPKMDGVPCCEPYTCTSDYYGNCS

>d1kal__ g.3.3.1 (-) Kalata B1 {African plant (Oldenlandia affinis dc)}
SWPVCTRNGLPVCGETCVGGTCNTPGCTC
>d1df6a_ g.3.3.2 (A:) Cycloviolacin O1 {Plant (Viola odorata)}
SCVYIPCTVTALLGCSCSNRVCYNGIPCAE
>d1bh4__ g.3.3.3 (-) Circulin A {Chassalia parviflora}
CGESCVWIPCISAALGCSCKNKVCYRNGIP
>d1c4ea_ g.3.4.1 (A:) Gurmarin, a sweet taste-suppressing polypeptide {Gymnema sylvestre}
EQCVKKDELCIPYYLDCCEPLECKKVNWWDHKCIG
>d1dkca_ g.3.4.2 (A:) Antifungal peptide PAFP-S {Pokeweed (Phytolacca americana)}
AGCIKNGGRCNASAGPPYCCSSYCFQIAGQSYGVCKNR
>d1hyka_ g.3.5.1 (A:) Agouti-related protein {Synthetic, based on Homo sapiens sequence}
CVRLHESCLGQQVPCCDPCATCYCRFFNAFCYCRKLGTAMNPCSRT
>d1omc__ g.3.6.1 (-) Conotoxin {Sea snail (Conus geographus), G IVa}
CKSPGSSCSPTSYNCCRSCNPYTKRCY
>d1ag7__ g.3.6.1 (-) Conotoxin {Synthetic, based on Conus geographus, GS}
ACSGRGSRCPPQCCMGLRCGRGNPQKCIGAHEDV
>d1cnna_ g.3.6.1 (A:) Conotoxin {Sea snail (Conus magus), M VIIc}
CKGKGAPCRKTMYDCCSGSCGRRGKC
>d1omg__ g.3.6.1 (-) Conotoxin {Sea snail (Conus magus), M VIIa}
CKGKGAKCSRLMYDCCTGSCRSGKC
>d1mvj__ g.3.6.1 (-) Conotoxin {Conus striatus, S VIb}
CKLKGQSCRKTSYDCCSGSCGRSGKC
>d1fyga_ g.3.6.1 (A:) Conotoxin {Conus striatus, SO3}
CKAAGKPCSRIAYNCCTGSCRSGKC
>d1kcp__ g.3.6.1 (-) Conotoxin {Conus purpurascens, kappa-pVIIa}
CRIPNQKCFQHLDDCCSRKCNRFNKCV
>d1eyoa_ g.3.6.1 (A:) Conotoxin {Conus tulipa, T VIIa}
SCSGRDSRCPPVCCMGLMCSRGKCVSIYGE
>d1g1za_ g.3.6.1 (A:) Conotoxin {Conus ermineus, E VIa}
DDCIKPYGFCSLPILKNGLCCSGACVGVCADL
>d1f3ka_ g.3.6.1 (A:) Conotoxin {Conus textile, Tx VII}
CKQADEPCDVFSLDCCTGICLGVCMW
>d1agg__ g.3.6.2 (-) omega-Agatoxin IV, IVa, IVb {Funnel web spider (Agelenopsis aperta)}
EDNCIAEDYGKCTWGGTKCCRGRPCRCSMIGTNCECTPRLIMEGLSFA
>d1oav__ g.3.6.2 (-) omega-Agatoxin IV, IVa, IVb {Funnel web spider (Agelenopsis aperta)}
KKKCIAKDYGRCKWGGTPCCRGRGCICSIMGTNCECKPRLIMEGLGLA

>d1omb__ g.3.6.2 (-) omega-Agatoxin IV, IVa, IVb {Funnel web spider (Agelenopsis aperta)}
CIAEDYGKCTWGGTKCCRGRPCRCSMIGTNCECTP
>d1eit__ g.3.6.2 (-) mu-Agatoxin-I {Funnel web spider (Agelenopsis aperta)}
ECVPENGHCRDWYDECCEGFYCSCRQPPKCICRNNN
>d1kqha_ g.3.6.2 (A:) ACTX-HI:OB4219 {Funnel-web spider (Hadronyche infensa)}
KCLAEAADCSPWSGDSCCKPYLCSCIFFYPCSCRPKGW
>d1axh__ g.3.6.2 (-) Atracotoxin-hvI (versutoxin) {Australian funnel-web spider (Hadronyche versuta)}
SPTCIPSGQPCPYNENCCSQSCTFKENENGNTVKRCD
>d1hvwa_ g.3.6.2 (A:) Atracotoxin-hvI (versutoxin) {Australian funnel-web spider (Hadronyche versuta)}
CIPSGQPCPYNENCCSQSCTGGRCD
>d1vtx__ g.3.6.2 (-) Atracotoxin-hvI (versutoxin) {Australian funnel-web spider (Hadronyche versuta)}
CAKKRNWCGKTEDCCCPMKCVYAWYNEQGSCQSTISALWKKC
>d1dl0a_ g.3.6.2 (A:) J-atracotoxin-hv1c {Australian funnel-web spider (Hadronyche versuta)}
AICTGADRPCAACCPCCPGTSCKAESNGVSYCRKDEP
>d1g9pa_ g.3.6.2 (A:) Atracotoxin-hv2a {Funnel-web spider (Hadronyche versuta)}
LLACLFGNGRCSSNRDCCELTPVCKRGSCVSSGPGLVGGILGGIL
>d1qdp__ g.3.6.2 (-) Robustoxin {Funnel-web spider (Atrax robustus)}
CAKKRNWCGKNEDCCCPMKCIYAWYNQQGSCQTTITGLFKKC
>d1qk6a_ g.3.6.2 (A:) Huwentoxin-I {Chinese bird spider (Selenocosmia huwena)}
ACKGVFDACTPGKNECCPNRVCSDKHKWCKWKL
>d1i25a_ g.3.6.2 (A:) Huwentoxin-II {Chinese bird spider (Selenocosmia huwena)}
LFECSFSCEIEKEGDKPCKKKKCKGGWKCKFNMCVKV
>d1qk7a_ g.3.6.2 (A:) Lectin SHL-I {Chinese bird spider (Selenocosmia huwena)}
GCLGDKCDYNNGCCSGYVCSRTWKWCVLAGPW
>d1d1ha_ g.3.6.2 (A:) Hanatoxin 1 {Tarantula (Grammostola spatulata)}
ECRYLFGGCKTTSDCCKHLGCKFRDKYCAWDFTFS
>d1emxa_ g.3.6.2 (A:) Heteropdatoxin 2, hptx2 {Spider (Heteropodidae venatoria)}
DDCGKLFSGCDTNADCCEGYVCRLWCKLDW
>d1c6wa_ g.3.6.2 (A:) Maurocalcin {Scorpio maurus}
GDCLPHLKLCKENKDCCSKKCKRRGTNIEKRCR

>d1i26a_ g.3.6.3 (A:) PTU-1 {Assassin bug (Peirates turpis)}
AEKDCIAPGAPCFGTDKPCCNPRAWCSSYANKCL
>d1jzaa_ g.3.7.1 (A:) Scorpion toxin {Centruroides sculpturatus ewing, variant 2}
KEGYLVNKSTGCKYGCLKLGENEGCDKECKAKNQGGSYGYCYAFACWCEGLPESTPTYPLPN
KSCS
>d2sn3__ g.3.7.1 (-) Scorpion toxin {Centruroides sculpturatus ewing, variant 3}
KEGYLVKKSDGCKYGCLKLGENEGCDTECKAKNQGGSYGYCYAFACWCEGLPESTPTYPLPN
KSC
>d1b3ca_ g.3.7.1 (A:) Scorpion toxin {Centruroides sculpturatus ewing, beta}
KDGYLVEKTGCKKTCYKLGENDFCNRECKWKHIGGSYGYCYGFGCYCEGLPDSTQTWPLPNK
TC
>d1vnb__ g.3.7.1 (-) Scorpion toxin {Centruroides sculpturatus ewing, variant 1}
KEGYLVKKSDGCKYDCFWLGKNEHCNTECKAKNQGGSYGYCYAFACWCEGLPESTPTYPLPN
KSC
>d1nrb__ g.3.7.1 (-) Scorpion toxin {Centruroides sculpturatus ewing, variant V}
KKDGYPVDSGNCKYECLKDDYCNDLCLERKADKGYCYWGKVSCYCYGLPDNSPTKTSGKCNP
A
>d1aho__ g.3.7.1 (-) Scorpion toxin {Scorpion (Androctonus australis hector), Toxin II}
VKDGYIVDDVNCTYFCGRNAYCNEECTKLKGESGYCQWASPYGNACYCYKLPDHVRTKGPGR
CH
>d1cn2__ g.3.7.1 (-) Scorpion toxin {Mexican scorpion (Centruroides noxius hoffmann), toxin II}
KEGYLVDKNTGCKYECLKLGDNDYCLRECKQQYGKGAGGYCYAFACWCTHLYEQAIVWPLPN
KRCS
>d1bcg__ g.3.7.1 (-) Scorpion toxin {Scorpion (Buthotus judaicus), BJXTR-IT}
MKKNGYPLDRNGKTTECSGVNAIAPHYCNSECTKVYYAESGYCCWGACYCFGLEDDKPIGPM
KDITKKYCDVQI
>d1snb__ g.3.7.1 (-) Scorpion toxin {Scorpion (Buthus martensii), toxin m8}
GRDAYIADSENCTYFCGSNPYCNDVCTENGAKSGYCQWAGRYGNACYCIDLPASERIKEGGR
CG
>d1djta_ g.3.7.1 (A:) Scorpion toxin {Chinese scorpion (Buthus martensii), toxin m1}
VRDAYIAKPHNCVYECARNEYCNDLCTKNGAKSGYCQWVGKYGNGCWCIELPDNVPIRVPGK
CH
>d1sn4a_ g.3.7.1 (A:) Scorpion toxin {Chinese scorpion (Buthus martensii), toxin m4}

VRDAYIAKPENCVYHCAGNEGCNKLCTDNGAESGYCQWGGRYGNACWCIKLPDDVPIRVPGK
CH
>d1dq7a_ g.3.7.1 (A:) Scorpion toxin {Indian red scorpion (Buthus tamulus), neurotoxin}
GEDGYIADGDNCTYICTFNNYCHALCTDKKGDSGACDWWVPYGVVCWCEDLPTPVPIRGSGK
CR
>d1b7da_ g.3.7.1 (A:) Scorpion toxin {Scorpion (Tityus serrulatus)}
KEGYLMDHEGCKLSCFIRPSGYCGRECGIKKGSSGYCAWPACYCYGLPNWVKVWDRATNKC
>d1i6fa_ g.3.7.1 (A:) Scorpion toxin {Bark scorpion (Centruroides sculpturatus), cse-v5}
KDGYPVDSKGCKLSCVANNYCDNQCKMKKASGGHCYAMSCYCEGLPENAKVSDSATNICG
>d1lqq__ g.3.7.1 (-) alpha toxin {Leiurus quinquestriatus quinquestriatus, LQQIII}
VRDAYIAKNYNCVYECFRDSYCNDLCTKNGASSGYCQWAGKYGNACWCYALPDNVPIRVPGK
CH
>d1lqi__ g.3.7.1 (-) alpha toxin {Scorpion (Leiurus quinquestriatus hebraeus)}
MVRDAYIAKNYNCVYECFRDAYCNELCTKNGASSGYCQWAGKYGNACWCYALPDNVPIRVPG
KCR
>d1bmr__ g.3.7.1 (-) LQH III alpha-like toxin, LQH {Hebraei scorpion (Leiurus quinquestriatus hebraeus)}
VRDGYIAQPENCVYHCFPGSSGCDTLCKEKGGTSGHCGFKVGHGLACWCNALPDNVGIIVEG
EKCHS
>d1big__ g.3.7.2 (-) Bmtx1 {Buthus martensii}
EFTDVKCTGSKQCWPVCKQMFGKPNGKCMNGKCRCYS
>d1bkt__ g.3.7.2 (-) Bmktx {Buthus martensii}
VGINVKCKHSGQCLKPCKDAGMRFGKCINGKCDCTPK
>d2bmt__ g.3.7.2 (-) Bmtx2 {Buthus martensii}
EFTNVSCSASSQCWPVCKKLFGTYRGKCMNSKCRCYS
>d1du9a_ g.3.7.2 (A:) Bmp02 neurotoxin {Chinese scorpion (Buthus martensii)}
VGCEECPMHCKGKNAKPTCDDGVCNCNV
>d1hp2a_ g.3.7.2 (A:) alpha-KTX, K+-channel blocker {Brazilian scorpion (Tityus serrulatus), Tstx-k alpha}
VFINAKCRGSPECLPKCKEAIGKAAGKCMNGKCKCYP
>d1jlza_ g.3.7.2 (A:) alpha-KTX, K+-channel blocker {Scorpion (Tityus cambridgei)}
ACGSCRKKCKGSGKCINGRCKCY
>d1mtx__ g.3.7.2 (-) Margatoxin {Scorpion (Centruroides margaritatus)}
TIINVKCTSPKQCLPPCKAQFGQSAGAKCMNGKCKCYPH
>d1sxm__ g.3.7.2 (-) Noxiustoxin {Scorpion (Centruroides noxius hoffmann)}

TIINVKCTSPKQCSKPCKELYGSSAGAKCMNGKCKCYNN
>d1txm__ g.3.7.2 (-) Maurotoxin {Scorpion (Scorpio maurus)}
VSCTGSKDCYAPCRKQTGCPNAKCINKSCKCYGC
>d1cmr__ g.3.7.2 (-) Charybdotoxin {Scorpion (Leiurus quinquestriatus hebraeus)}
CTTSKECWSVCQRLHNTSKGWCDHRGCICES
>d2crd__ g.3.7.2 (-) Charybdotoxin {Scorpion (Leiurus quinquestriatus hebraeus)}
EFTNVSCTTSKECWSVCQRLHNTSRGKCMNKKCRCYS
>d1scy__ g.3.7.2 (-) Scyllatoxin {Scorpion (Leiurus quinquestriatus hebraeus)}
AFCNLRMCQLSCRSLGLLGKCIGDKCECVKH
>d1agt__ g.3.7.2 (-) Agitoxin {Scorpion (Leiurus quinquestriatus hebraeus)}
GVPINVSCTGSPQCIKPCKDAGMRFGKCMNRKCHCTPK
>d1chl__ g.3.7.2 (-) Chlorootoxin {Scorpion (Leiurus quinquestriatus), venom}
MCMPCFTTDHQMARKCDDCCGGKGRGKCYGPQCLCR
>d1c55a_ g.3.7.2 (A:) Butantoxin {Brazilian scorpion (Tityus serrulatus)}
WCSTCLDLACGASRECYDPCFKAFGRAHGKCMNNKCRCYT
>d1tsk__ g.3.7.2 (-) Toxin ts kappa {Scorpion (Tityus serrulatus)}
VVIGQRCYRSPDCYSACKKLVGKATGKCTNGRCDC
>d1sis__ g.3.7.2 (-) Toxin I5a {Scorpion (Buthus eupeus)}
MCMPCFTTDPNMAKKCRDCCGGNGKCFGPQCLCNR
>d1pnh__ g.3.7.2 (-) Toxin analog {Scorpion (Androctonus mauretanicus mauretanicus)}
TVCNLRRCQLSCRSLGLLGKCIGVKCECVKH
>d1acw__ g.3.7.2 (-) Toxin analog P01 {Scorpion (Androctonus mauretanicus mauretanicus)}
VSCEDCPEHCSTQKAQAKCDNDKCVCEPI
>d1sco__ g.3.7.2 (-) OSK1 TOXIN {Central asian scorpion (Orthochirus scrobiculosus)}
GVIINVKCKISRQCLEPCKKAGMRFGKCMNGKCHCTPK
>d2ktx__ g.3.7.2 (-) Kaliotoxin (KTX) {Scorpion (Androctonus mauretanicus mauretanicus)}
GVEINVKCSGSPQCLKPCKDAGMRFGKCMNRKCHCTPK
>d1lir__ g.3.7.2 (-) LQ2 toxin {Scorpion (Leiurus quinquestriatus hebraeus)}
EFTQESCTASNQCWSICKRLHNTNRGKCMNKKCRCYS
>d1c49a_ g.3.7.2 (A:) Pandinus toxin {Emperor scorpion (Pandinus imperator), PITX-Kb}
TISCTNEKQCYPHCKKETGYPNAKCMNRKCKCFGR
>d1qkya_ g.3.7.2 (A:) PI7 {Scorpion (Pandinus imperator)}

DEAIRCTGTKDCYIPCRYITGCFNSRCINKSCKCYGCT
>d1fjna_ g.3.7.3 (A:) Defensin MGD-1 {Mediterranean mussel (Mytilus galloprovincialis)}
GFGCPNNYQCHRHCKSIPGRCGGYCGGWHRLRCTCYRCG
>d1i2ua_ g.3.7.4 (A:) Heliomicin {Tobacco budworm (Heliothis virescens)}
DKLIGSCVWGAVNYTSDCNGECKRRGYKGGHCGSFANVNCWCET
>d1myn__ g.3.7.4 (-) Drosomycin {Fruit fly (Drosophila melanogaster)}
DCLSGRYKGPCAVWDNETCRRVCKEEGRSSGHCSPSLKCWCEGC
>d1ica__ g.3.7.4 (-) Defensin A {Flesh fly (Phormia terranovae), larva}
ATCDLLSGTGINHSACAAHCLLRGNRGGYCNGKGVCVCRN
>d1gpt__ g.3.7.5 (-) gamma-Thionin {Barley (Hordeum vulgare)}
RICRRRSAGFKGPCVSNKNCAQVCMQEGWGGGNCDGPLRRCKCMRRC
>d1gps__ g.3.7.5 (-) gamma-Thionin {Wheat (Triticum turgidum)}
KICRRRSAGFKGPCMSNKNCAQVCQQEGWGGGNCDGPFRRCKCIRQC
>d1ayj__ g.3.7.5 (-) Antifungal protein 1 (RS-AFP1) {Radish (Raphanus sativus)}
EKLCERPSGTWSGVCGNNNACKNQCINLEKARHGSCNYVFPAHKCICYFPC
>d1bk8__ g.3.7.5 (-) Antimicrobial protein 1 (AH-AMP1) {Horse chestnut (Aesculus hippocastanum)}
LCNERPSQTWSGNCGNTAHCDKQCQDWEKASHGACHKRENHWKCFCYFNC
>d1jkza_ g.3.7.5 (A:) Defensin 1 (PSD1) {Pea (Pisum sativum)}
KTCEHLADTYRGVCFTNASCDDHCKNKAHLISGTCHNWKCFCTQNC
>d1brz__ g.3.7.5 (-) Brazzein {J'oublie (Pentadiplandra brazzeana)}
EDKCKKVYENYPVSKCQLANQCNYDCKLDKHARSGECFYDEKRNLQCICDYCEY
>d2cbh__ g.3.8.1 (-) Cellobiohydrolase I {Trichoderma reesei, ct-cbh I}
TQSHYGQCGGIGYSGPTVCASGTTCQVLNPYYSQCL
>d1boea_ g.3.9.1 (A:) Insulin-like growth factor-binding protein-5 (IGFBP-5) {Human (Homo sapiens)}
ALAEGQSCGVYTERCAQGLRCLPRQDEEKPLHALLHGRGVCLNEKS
>d1igra3 g.3.9.1 (A:150-299) Cys-rich domain of the type 1 insulin-like growth factor receptor {Human (Homo sapiens)}
DLCPGTMEEKPMCEKTTINNEYNYRCWTTNRCQKMCPSTCGKRACTENNECCHPECLGSCSA
PDNDTACVACRHYYYAGVCVPACPPNTYRFEGWRCVDRDFCANILSAESSDSEGFVIHDGEC
MQECPSGFIRNGSQSMYCIPCEGPCP
>d1lpba1 g.3.10.1 (A:6-44) (Pro)colipase {Pig (Sus scrofa)}
GIIINLDEGELCLNSAQCKSNCCQHDTILSLSRCALKAR
>d1lpba2 g.3.10.1 (A:45-90) (Pro)colipase {Pig (Sus scrofa)}
ENSECSAFTLYGVYYKCPCERGLTCEGDKSLVGSITNTNFGICHNV
>d1pco_1 g.3.10.1 (1-44) (Pro)colipase {Pig (Sus scrofa)}

VPDPRGIIINLDEGELCLNSAQCKSNCCQHDTILSLSRCALKAR
>d1imt_1 g.3.10.1 (1-36) Intestinal toxin 1 {Black mamba (Dendroaspis polylepis polylepis)}
AVITGACERDLQCGKGTCCAVSLWIKSVRVCTPVGT
>d1imt_2 g.3.10.1 (37-80) Intestinal toxin 1 {Black mamba (Dendroaspis polylepis polylepis)}
SGEDCHPASHKIPFSGQRMHHTCPCAPNLACVQTSPKKFKCLSK
>d1edmb_ g.3.11.1 (B:) Factor IX (IXa) {Human (Homo sapiens)}
VDGDQCESNPCLNGGSCKDDINSYECWCPFGFEGKNCEL
>d1rfnb_ g.3.11.1 (B:) Factor IX (IXa) {Human (Homo sapiens)}
MTCNIKNGRCEQFCKNSADNKVVCSCTEGYRLAENQKSCEPAVPFPCGRVSVSQTSK
>d1pfxl1 g.3.11.1 (L:47-86) Factor IX (IXa) {Pig (Sus scrofa)}
DGDQCEPNPCLNGGLCKXDINSYECWCQVGFEGKNCELDA
>d1pfxl2 g.3.11.1 (L:87-146) Factor IX (IXa) {Pig (Sus scrofa)}
TCNIKNGRCKQFCKTGADSKVLCSCTTGYRLAPDQKSCKPAVPFPCGRVSVSHSPTTLTR
>d1danl1 g.3.11.1 (L:49-86) Coagulation factor VIIa {Human (Homo sapiens)}
QCASSPCQNGGSCKDQLQSYICFCLPAFEGRNCETHKD
>d1danl2 g.3.11.1 (L:87-142) Coagulation factor VIIa {Human (Homo sapiens)}
DQLICVNENGGCEQYCSDHTGTKRSCRCHEGYSLLADGVSCTPTVEYPCGKIPILE
>d1dval1 g.3.11.1 (L:42-86) Coagulation factor VIIa {Human (Homo sapiens)}
ISYSDGDQCASSPCQNGGSCKDQLQSYICFCLPAFEGRNCETHKD
>d1f7ea_ g.3.11.1 (A:) Coagulation factor VIIa {Human (Homo sapiens)}
SDGDQCASSPCQNGGSCKDQLQSYICFCLPAFEGRNCETHKDDGSA
>d1g1sa2 g.3.11.1 (A:119-158) E-selectin, EGF-domain {Human (Homo sapiens)}
TASCQDMSCSKQGECLETIGNYTCSCYPGFYGPECEYVRD
>d1g1ta2 g.3.11.1 (A:119-157) E-selectin, EGF-domain {Human (Homo sapiens)}
TAACTNTSCSGHGECVETINNYTCKCDPGFSGLKCEQIV
>d1fjsl_ g.3.11.1 (L:) Factor X, N-terminal module {Human (Homo sapiens)}
KLCSLDNGDCDQFCHEEQNSVVCSCARGYTLADNGKACIPTGPYPCGKQTLE
>d1xkba1 g.3.11.1 (A:48-86) Factor X, N-terminal module {Human (Homo sapiens)}
DQCETSPCQNQGKCKDGLGEYTCTCLEGFEGKNCELFTR
>d1apo__ g.3.11.1 (-) Factor X, N-terminal module {Cow (Bos taurus)}
KDGDQCEGHPCLNQGHCKDGIGDYTCTCAEGFEGKNCEFSTR
>d1kigl_ g.3.11.1 (L:) Factor X, N-terminal module {Cow (Bos taurus)}
CSLDNGGCDQFCREERSEVRCSCAHGYVLGDDSKSCVSTERFPCGKFTQGR

>d1autl1 g.3.11.1 (L:49-96) Activated protein c (autoprothrombin IIa) {Human (Homo sapiens)}
QCLVLPLEHPCASLCCGHGTCIDGIGSFSCDCRSGWEGRFCQREVSFL
>d1autl2 g.3.11.1 (L:97-146) Activated protein c (autoprothrombin IIa) {Human (Homo sapiens)}
NCSLDNGGCTHYCLEEVGWRRCSCAPGYKLGDDLLQCHPAVKFPCGRPWK
>d1eqga2 g.3.11.1 (A:33-73) Prostaglandin H2 synthase-1, EGF-like module {Sheep (Ovis aries)}
VNPCCYYPCQHQGICVRFGLDRYQCDCTRTGYSGPNCTIPE
>d1cvua2 g.3.11.1 (A:33-73) Prostaglandin H2 synthase-1, EGF-like module {Mouse (Mus musculus)}
ANPCCSNPCQNRGECMSTGFDQYKCDCTRTGFYGENCTTPE
>d1a3p__ g.3.11.1 (-) Epidermal growth factor, EGF {Mouse (Mus musculus)}
PGXPSSYDGYCLNGGVXMHIESLDSYTCNCVIGYSGDRCQTRDLR
>d3egf__ g.3.11.1 (-) Epidermal growth factor, EGF {Mouse (Mus musculus)}
NSYPGCPSSYDGYCLNGGVCMHIESLDSYTCNCVIGYSGDRCQTRDLRWWELR
>d1jl9a_ g.3.11.1 (A:) Epidermal growth factor, EGF {Human (Homo sapiens)}
CPLSHDGYCLHDGVCMYIEALDKYACNCVVGYIGERCQYRDL
>d3tgf__ g.3.11.1 (-) Transforming growth factor alpha {Human (Homo sapiens)}
VVSHFNDCPDSHTQFCFHGTCRFLVQEDKPACVCHSGYVGARCEHADLLA
>d1xdtr_ g.3.11.1 (R:) Heparin-binding epidermal growth factor, HBEGF {Human (Homo sapiens)}
PCLRKYKDFCIHGECKYVKELRAPSCICHPGYHGERCHGLS
>d1urk_1 g.3.11.1 (6-49) Plasminogen activator (urokinase-type) {Human (Homo sapiens)}
QVPSNCDCLNGGTCVSNKYFSNIHWCNCPKKFGGQHCEIDKSKT
>d1hae__ g.3.11.1 (-) Heregulin-alpha, EGF-like domain {Human (Homo sapiens)}
SHLVKCAEKEKTFCVNGGECFMVKDLSNPSRYLCKCQPGFTGARCTENVPMKVQNQEKAEEL
Y
>d1adx__ g.3.11.1 (-) Thrombomodulin, different EGF-like domains {Human (Homo sapiens)}
QMFCNQTACPADCDPNTQASCECPEGYILDDGFICTDIDE
>d1dx5i1 g.3.11.1 (I:345-387) Thrombomodulin, different EGF-like domains {Human (Homo sapiens)}
VEPVDPCFRANCEYQCQPLDQTSYLCVCAEGFAPIPHEPHRCQ
>d1dx5i2 g.3.11.1 (I:388-422) Thrombomodulin, different EGF-like domains {Human (Homo sapiens)}
MFCNQTACPADCDPNTQASCECPEGYILDDGFICT
>d1dx5i3 g.3.11.1 (I:423-462) Thrombomodulin, different EGF-like

domains {Human (Homo sapiens)}
DIDECENGGFCSGVCHNLPGTFECICGPDSALAGQIGTDC
>d1zaq__ g.3.11.1 (-) Thrombomodulin, different EGF-like domains {Human (Homo sapiens)}
EPVDPCFRANCEYQCQPLNQTSYLCVCAEGFAPIPHEPHRCQMF
>d1emo_1 g.3.11.1 (2124-2166) Fibrillin-1 fragment (residues 2124-2205) {Human (Homo sapiens)}
SAVDMDECKEPDVCKHGQCINTDGSYRCECPFGYILAGNECVD
>d1emo_2 g.3.11.1 (2167-2205) Fibrillin-1 fragment (residues 2124-2205) {Human (Homo sapiens)}
TDECSVGNPCGNGTCKNVIGGFECTCEEGFEPGPMMTCE
>d1apq__ g.3.11.1 (-) Complement protease C1R {Human (Homo sapiens)}
AVDLDECASRSKSGEEDPQPQCQHLCHNYVGGYFCSCRPGYELQEDRHSCQAE
>d1tpg_1 g.3.11.1 (51-91) Plasminogen activator (tissue-type), t-PA {Human (Homo sapiens)}
CSEPRCFNGGTCQQALYFSDFVCQCPEGFAGKSCEIDTRAT
>d1hz8a1 g.3.11.1 (A:1-41) Low density lipoprotein (LDL) receptor, different EGF domains {Human (Homo sapiens)}
GTNECLDNNGGCSHVCNDLKIGYECLCPDGFQLVAQRRCED
>d1hz8a2 g.3.11.1 (A:42-82) Low density lipoprotein (LDL) receptor, different EGF domains {Human (Homo sapiens)}
IDECQDPDTCSQLCVNLEGGYKCQCEEGFQLDPHTKACKAV
>d1ijqa2 g.3.11.1 (A:643-692) Low density lipoprotein (LDL) receptor, different EGF domains {Human (Homo sapiens)}
VNWCERTTLSNGGCQYLCLPAPQINPHSPKFTCACPDGMLLARDMRSCLT
>d1jv2b4 g.3.11.6 (B:532-562) Integrin beta EGF-like domains {Human (Homo sapiens)}
KGEMCSGHGQCSCGDCLCDSDWTGYYCNCTT
>d1jv2b5 g.3.11.6 (B:563-605) Integrin beta EGF-like domains {Human (Homo sapiens)}
RTDTCMSSNGLLCSGRGKCECGSCVCIQPGSYGDTCEKCPTCP
>d1gl4a2 g.3.11.5 (A:359-398) EGF-like domain of nidogen-1 {Mouse (Mus musculus)}
TCANNRHQCSVHAECRDYATGFCCRCVANYTGNGRQCVAE
>d1h4ua2 g.3.11.5 (A:367-398) EGF-like domain of nidogen-1 {Mouse (Mus musculus)}
CSVHAECRDYATGFCCRCVANYTGNGRQCVAE
>d1klo_1 g.3.11.2 (11-65) Laminin gamma1 chain {Mouse (Mus musculus)}
CPCPGGSSCAIVPKTKEVVCTHCPTGTAGKRCELCDDGYFGDPLGSNGPVRLCRP
>d1klo_2 g.3.11.2 (66-121) Laminin gamma1 chain {Mouse (Mus musculus)}
CQCNDNIDPNAVGNCNRLTGECLKCIYNTAGFYCDRCKEGFFGNPLAPNPADKCKA

>d1klo_3 g.3.11.2 (122-172) Laminin gamma1 chain {Mouse (Mus musculus)}
CACNPYGTVQQQSSCNPVTGQCQCLPHVSGRDCGTCDPGYYNLQSGQGCER
>d1nuba2 g.3.11.3 (A:53-77) Domain of BM-40/SPARC/osteonectin {Human (Homo sapiens)}
APCQNHHCKHGKVCELDENNTPMCV
>d1b9wa1 g.3.11.4 (A:1-45) Merozoite surface protein 1 (MSP-1) {Plasmodium cynomolgi}
MSSEHRCIDTNVPENAACYRYLDGTEEWRCLLYFKEDAGKCVPAP
>d1b9wa2 g.3.11.4 (A:46-89) Merozoite surface protein 1 (MSP-1) {Plasmodium cynomolgi}
NMTCKDKNGGCAPEAECKMNDKNEIVCKCTKEGSEPLFEGVFCS
>d1ceja1 g.3.11.4 (A:1-45) Merozoite surface protein 1 (MSP-1) {Plasmodium falciparum}
NISQHQCVKKQCPQNSGCFRHLDEREECKCLLNYKQEGDKCVENP
>d1ceja2 g.3.11.4 (A:46-96) Merozoite surface protein 1 (MSP-1) {Plasmodium falciparum}
NPTCNENNGGCDADAKCTEEDSGSNGKKITCECTKPDSYPLFDGIFCSSSN
>d2bi6h1 g.3.12.1 (H:8-31) Bromelain inhibitor VI (cysteine protease inhibitor) {Pineapple (Ananas comosus)}
TDTYSDCPGFCKTCKAEFGKYICL
>g2bi6.2 g.3.12.1 (L:,H:1-7,H:32-41) Bromelain inhibitor VI (cysteine protease inhibitor) {Pineapple (Ananas comosus)}
TACSECVCPLRXEEYKCYCXDLISPNDCVK
>d1pi2__ g.3.13.1 (-) Bowman-Birk inhibitor, BBI {Soybean (Glycine max), PI-II}
YSKPCCDLCMCTRSMPPQCSCEDRINSCHSDCKSCMCTRSQPGQCRCLDTNDFCYKPCKSR
>d1d6ri_ g.3.13.1 (I:) Bowman-Birk inhibitor, BBI {Soybean (Glycine max)}
KPCCDQCACTKSNPPQCRCSDMRLNSCHSACKSCICALSYPAQCFCVDITDFCYEPCK
>d2bbi__ g.3.13.1 (-) Bowman-Birk inhibitor, BBI {Soybean (Glycine max)}
DDESSKPCCDQCACTKSNPPQCRCSDMRLNSCHSACKSCICALSYPAQCFCVDITDFCYEPC
KPSEDDKEN
>d1pbia_ g.3.13.1 (A:) Bowman-Birk inhibitor, BBI {Winter pea (Pisum sativum)}
KSACCDTCLCTKSNPPTCRCVDVGETCHSACLSCICAYSNPPKCQCFDTQKFCYKQCHNSEL
EEVIKN
>d1df9c_ g.3.13.1 (C:) Bowman-Birk inhibitor, BBI {Mung bean (Vigna radiata)}
SHDEPSESSEPCCDSCDCTKSIPPQCHCANIRLNSCHSACKSCICTRSMPGKCRCLDTDDFC
YKPCESMDKD
>d1c2aa1 g.3.13.1 (A:4-64) Bowman-Birk inhibitor, BBI {Barley (Hordeum vulgare)}

KRPWKCCDEAVCTRSIPPICTCMDEVFECPKTCKSCGPSMGDPSRRICQDQYVGDPGPICR
>d1c2aa2 g.3.13.1 (A:65-123) Bowman-Birk inhibitor, BBI {Barley (Hordeum vulgare)}
PWECCDKAICTRSNPPTCRCVDEVKKCAPTCKTCLPSRSRPSRRVCIDSYFGPVPPRCT
>d1tabi_ g.3.13.1 (I:) Bowman-Birk inhibitor, BBI {Adzuki bean (Phaseolus angularis)}
SESSKPCCDQCSCTKSMPPKCRCSDIRLNSCHSACKSCACTYSIPAKCFCTDINDFCYEPCK
>d1flei_ g.3.14.1 (I:) Elafin, elastase-specific inhibitor {Human (Homo sapiens)}
TKPGSCPIILIRCAMLNPPNRCLKDTDCPGIKKCCEGSCGMACFVPQ
>d2rel__ g.3.14.1 (-) Elafin, elastase-specific inhibitor {Human (Homo sapiens)}
AQEPVKGPVSTKPGSCPIILIRCAMLNPPNRCLKDTDCPGIKKCCEGSCGMACFVPQ
>d1bx7__ g.3.15.1 (-) Hirustasin {Medicinal leech (Hirudo medicinalis)}
GNTCGGETCSAAQVCLKGKCVCNEVHCRIRCKYGLKKDENGCEYPCSCAKA
>d1ejab_ g.3.15.1 (B:) Bdellastasin {Medicinal leech (Hirudo medicinalis)}
TTPCGPVTCSGAQMCEVDKCVCSDLHCKVKCEHGFKKDDNGCEYACICADAPQ
>d1skz_1 g.3.15.1 (7-58) Factor Xa inhibitor antistasin {Mexican leech (Haementeria officinalis)}
GCEEAGCPEGSACNIITDRCTCSGVRCRVHCPHGFQRSRYGCEFCKCRLEPM
>d1skz_2 g.3.15.1 (59-110) Factor Xa inhibitor antistasin {Mexican leech (Haementeria officinalis)}
KATCDISECPEGMMCSRLTNKCDCKIDINCRKTCPNGLKRDKLGCEYCECRP
>d1hic__ g.3.15.2 (-) Hirudin {Leech (Hirudo medicinalis)}
VVYTDCTESGQNLCLCEGSNVCGQGNKCILGSDGEKNQCVTGEGTPKPQSH
>d1hrti_ g.3.15.2 (I:) Hirudin {Leech (Hirudo medicinalis)}
VVYTDCTESGQNLCLCEGSNVCGQGNKCILGSDGEKNQCVTGEGTPKPQSHNDGDFEEIPEE
YLQ
>d4htci_ g.3.15.2 (I:) Hirudin {Leech (Hirudo medicinalis)}
ITYTDCTESGQNLCLCEGSNVCGKGNKCILGSNGKGNQCVTGEGTPKPESHNNGDFEEIPEE
YLQ
>d1dec__ g.3.15.2 (-) Decorsin {North american leech (Macrobdella decora)}
APRLPQCQGDDQEKCLCNKDECPPGQCRFPRGDADPYCE
>d1e0fi_ g.3.15.2 (I:) Haemadin {Indian leech (Haemadipsa sylvestris)}
IRFGMGKVPCPDGEVGYTCDCGEKICLYGQSCNDGQCSGDPKPSSEFEEFEIDEEEK
>d1qgma_ g.3.16.1 (A:) N-terminal domain of granulin-1 {Carp (Cyprinus carpio)}
VIHCDAATICPDGTTCSLSPYGVWYCSPFS
>d1g26a_ g.3.16.1 (A:) N-terminal domain of granulin-1 {Human (Homo sapiens)}

VVHCDMEVICPDGYTCCRLPSGAWGCCPFTQ
>d1fwoa_ g.3.16.1 (A:) Oryzain beta chain {Rice (Oryza sativa)}
DHVCDDNFSCPAGSTCSSAFGFRNLSLVWGCSPVE
>d1hy9a_ g.3.17.1 (A:) Satiety factor CART (cocaine and amphetamine regulated transcript) {Human (Homo sapiens)}
YGQVPMCDAGEQCAVRKGARIGKLCDCPRGTSCNSFLLKCL
>d1gl1i_ g.4.1.1 (I:) Protease inhibitor PMP-C {Migratory locust (Locusta migratoria)}
ISCEPGKTFKDKCNTCRCGADGKSAACTLKACPN
>d1gl0i_ g.4.1.1 (I:) Protease inhibitor PMP-D2V {Migratory locust (Locusta migratoria)}
KCTPGQVKQQDCNTCTCTPTGVWGCTLMGCQP
>d1kgma_ g.4.1.1 (A:) Protease inhibitor SGCI {Desert locust (Schistocerca gregaria)}
EVTCEPGTTFKDKCNTCRCGSDGKSAACTLKACPQ
>d1kioa_ g.4.1.1 (A:) Protease inhibitor SGCI {Desert locust (Schistocerca gregaria)}
EVTCEPGTTFKDKCNTCRCGSDGKSAACTRMACPQ
>d1kj0a_ g.4.1.1 (A:) Protease inhibitor SGTI {Desert locust (Schistocerca gregaria)}
EQECTPGQTKKQDCNTCNCTPTGVWACTRKGCPPH
>d1mkna_ g.5.1.1 (A:) Midkine, a heparin-binding growth factor, N-terminal domain {Synthetic}
KKKDKVKKGGPGSECAEWAWGPCTPSSKDCGVGFREGTCGAQTQRIRCRVPCNWKKEFG
>d1mkca_ g.5.1.2 (A:) Midkine, a heparin-binding growth factor, C-terminal domain {Synthetic}
CKYKFENWGACDGGTGTKVRQGTLKKARYNAQCQETIRVTKPC
>d2bbg__ g.6.1.1 (-) Amb V allergen {Giant ragweed (Ambrosia trifida), pollen}
DDGLCYEGTNCGKVGKYCCSPIGKYCVCYDSKAICNKNCT
>d3ebx__ g.7.1.1 (-) Erabutoxin B (also neurotoxin B) {Sea snake (Laticauda semifasciata)}
RICFNHQSSQPQTTKTCSPGESSCYHKQWSDFRGTIIERGCGCPTVKPGIKLSCCESEVCNN
>d1tgxa_ g.7.1.1 (A:) gamma-Cardiotoxin {Snake (Naja nigricollis)}
LKCNQLIPPFWKTCPKGKNLCYKMTMRAAPMVPVKRGCIDVCPKSSLLIKYMCCNTDKCN
>d1fas__ g.7.1.1 (-) Fasciculin {Green mamba (Dendroaspis angusticeps)}
TMCYSHTTTSRAILTNCGENSCYRKSRRHPPKMVLGRGCGCPPGDDYLEVKCCTSPDKCNY
>d1qm7a_ g.7.1.1 (A:) Fasciculin {Green mamba (Dendroaspis angusticeps)}
TMCYSHTTTSRAILTNCPGETNCYKKSRRHPPKMVLGRGCGCPTVAPGIKLNCCTTDKCNY
>d1ntn__ g.7.1.1 (-) Neurotoxin I {Snake (Naja naja oxiana)}
ITCYKTPIITSETCAPGQNLCYTKTWCDAWCGSRGKVIELGCAATCPTVESYQDIKCCSTDN
CNPHPKQKRP

>d1cdta_ g.7.1.1 (A:) Cardiotoxin V4II (Toxin III) {Naja mossambica mossambica}
LKCNKLIPIAYKTCPEGKNLCYKMMLASKKMVPVKRGCINVCPKNSALVKYVCCSTDRCN
>d1kxia_ g.7.1.1 (A:) Cardiotoxin V {Taiwan cobra (Naja naja atra)}
LKCHNTQLPFIYKTCPEGKNLCFKATLKKFPLKFPVKRGCADNCPKNSALLKYVCCSTDKCN
>d2ctx__ g.7.1.1 (-) alpha-Cobratoxin {Cobra (Naja naja siamensis)}
IRCFITPDITSKDCPNGHVCYTKTWCDAFCSIRGKRVDLGCAATCPTVKTGVDIQCCSTDNC
NPFPTRKRP
>d1lsi__ g.7.1.1 (-) Long neurotoxin 1 (component LSIII) {Sea snake (Laticauda semifasciata)}
RECYLNPHDTQTCPSGQEICYVKSWCNAWCSSRGKVLEFGCAATCPSVNTGTEIKCCSADKC
NTYP
>d1tfs__ g.7.1.1 (-) FS2 toxin {Black mamba (Dendroaspis polylepis polylepis)}
RICYSHKASLPRATKTCVENTCYKMFIRTHREYISERGCGCPTAMWPYQTECCKGDRCNK
>d1hc9a_ g.7.1.1 (A:) Bungarotoxin {Many-banded krait (Bungarus multicinctus), Alpha-bungarotoxin}
IVCHTTATSPISAVTCPPGENLCYRKMWCDVFCSSRGKVVELGCAATCPSKKPYEEVTCCST
DKCNPHPKQRPG
>d2abxa_ g.7.1.1 (A:) Bungarotoxin {Many-banded krait (Bungarus multicinctus), Alpha-bungarotoxin}
IVCHTTATIPSSAVTCPPGENLCYRKMWCDAFCSSRGKVVELGCAATCPSKKPYEEVTCCST
DKCNHPPKRQPG
>d1kbaa_ g.7.1.1 (A:) Bungarotoxin {Many-banded krait (Bungarus multicinctus), kappa-bungarotoxin}
RTCLISPSSTPQTCPNGQDICFLKAQCDKFCSIRGPVIEQGCVATCPQFRSNYRSLLCCTTD
NCNH
>d1f94a_ g.7.1.1 (A:) Bucandin {Malayan krait (Bungarus candidus)}
MECYRCGVSGCHLKITCSAEETFCYKWLNKISNERWLGCAKTCTEIDTWNVYNKCCTTNLCN
T
>d2cdx__ g.7.1.1 (-) Cardiotoxin CTXI {Taiwan cobra (Naja naja atra)}
LKCNKLIPIASKTCPAGKNLCYKMFMMSDLTIPVKRGCIDVCPKNSLLVKYVCCNTDRCN
>d1chvs_ g.7.1.1 (S:) Cardiotoxin II {Taiwan cobra (Naja naja atra)}
LKCNKLVPLFYKTCPAGKNLCYKMFMVSNKMVPVKRGCIDVCPKSSLLVKYVCCNTDRCN
>d1cb9a_ g.7.1.1 (A:) Cardiotoxin II {Central asian cobra (Naja naja oxiana)}
LKCKKLVPLFSKTCPAGKNLCYKMFMVAAPHVPVKRGCIDVCPKSSLLVKYVCCNTDKCN
>d1i02a_ g.7.1.1 (A:) Cardiotoxin III {Taiwan cobra (Naja naja atra)}
LKCNKLVPLFYKTCPAGKNLCYKMFMVATPKVPVKRGCIDVCPKSSLLVKYVCCNTDRCN
>d1kbs__ g.7.1.1 (-) Cardiotoxin IV {Taiwan cobra (Naja naja atra)}
RKCNKLVPLFYKTCPAGKNLCYKMFMVSNLTVPVKRGCIDVCPKNSALVKYVCCNTDRCN

>d1cod__ g.7.1.1 (-) Cobrotoxin II (ct2) {Taiwan cobra (Naja naja atra)}
LECHNQQSSQTPTTTGCSGGETNCYKKRWRDHRGYRTERGCGCPSVKNGIEINCCTTDRCNN
>d1g6ma_ g.7.1.1 (A:) Cobrotoxin II (ct2) {Monocled cobra (Naja kaouthia)}
LECHNQQSSQTPTTTGCSGGENNCYKKEWRDNRGYRTERGCGCPSVKKGIGINCCTTDRCNN
>d1nea__ g.7.1.1 (-) alpha-Toxin {Snake (Naja nigricollis)}
LECHNQQSSQPPTTKTCPGETNCYKKVWRDHRGTIIERGCGCPTVKPGIKLNCCTTDKCNN
>d1ntx__ g.7.1.1 (-) alpha-Toxin {Black mamba (Dendroaspis polylepis polylepis)}
RICYNHQSTTRATTKSCEENSCYKKYWRDHRGTIIERGCGCPKVKPGVGIHCCQSDKCNY
>d1nor__ g.7.1.1 (-) Neurotoxin II (Nt2) {Central asian cobra (Naja naja oxiana)}
LECHNQQSSQPPTTKTCSGETNCYKKWWSDHRGTIIERGCGCPKVKPGVNLNCCRTDRCNN
>d1txb__ g.7.1.1 (-) Toxin B (long neurotoxin) {King cobra (Ophiophagus hannah)}
TKCYVTPDATSQTCPDGQDICYTKTWCDGFCSSRGKRIDLGCAATCPKVKPGVDIKCCSTDN
CNPFPTWKRKH
>d1jgka_ g.7.1.1 (A:) Candoxin {Malayan krait (Bungarus candidus)}
MKCKICNFDTCRAGELKVCASGEKYCFKESWREARGTRIERGCAATCPKGSVYGLYVLCCTT
DDCN
>d1drs__ g.7.1.2 (-) Dendroaspin {Dendroaspis jamesoni kaimosae}
RICYNHLGTKPPTTETCQEDSCYKNIWTFDNIIRRGCGCFTPRGDMPGPYCCESDKCNL
>d1erh__ g.7.1.3 (-) CD59 {Human (Homo sapiens)}
LQCYNCPNPTADCKTAVNCSSDFDACLITKAGLQVYNKCWKFEHCNFNDVTTRLRENELTYY
CCKKDLCN
>d1btea_ g.7.1.3 (A:) Type II activin receptor {Mouse (Mus musculus)}
ETQECLFFNANWERDRTNQTGVEPCYGDKDKRRHCFATWKNISGSIEIVKQGCWLDDINCYD
RTDCIEKKDSPEVYFCCCEGNMCNEKFSYFPEME
>d1es7b_ g.7.1.3 (B:) BMP receptor Ia ectodomain {Human (Homo sapiens)}
TLPFLKCYCSGHCPDDAINNTCITNGHCFAIIEEDDQGETTLASGCMKYEGSDFQCKDSPKA
QLRRTIECCRTNLCNQYLQPTLPP
>d1ktzb_ g.7.1.3 (B:) TGF-beta type II receptor extracellular domain {Human (Homo sapiens)}
PQLCKFCDVRFSTCDNQKSCMSNCSITSICEKPQEVCVAVWRKNDENITLETVCHDPKLPYH
DFILEDAASPKCIMKEKKKPGETFFMCSCSSDECNDNIIFSEEY
>d1brbi_ g.8.1.1 (I:) Pancreatic trypsin inhibitor, BPTI {Cow (Bos taurus)}
AGEPPYTGPCKARIIRYFYNAKAGLCQTFVYGGCRAKRNNFKSAEDCMRTA
>d1ejmb_ g.8.1.1 (B:) Pancreatic trypsin inhibitor, BPTI {Cow (Bos taurus)}
RPDFCLEPPYTGPCRLRIIRYFYNAKAGLCQTFVYGGCRAKRNNFKSAEDCLRTCGGA

>d1faki_ g.8.1.1 (I:) Pancreatic trypsin inhibitor, BPTI {Cow (Bos taurus)}
APDFCLEPPYDGPCRALHLRYFYNAKAGLCQTFYYGGCLAKRNNFESAEDCMRTC
>d1g6xa_ g.8.1.1 (A:) Pancreatic trypsin inhibitor, BPTI {Cow (Bos taurus)}
RPDFCLEPPYAGACRARIIRYFYNAKAGLCQTFVYGGCRAKRNNFKSAEDCLRTCGGA
>d3bthi_ g.8.1.1 (I:) Pancreatic trypsin inhibitor, BPTI {Cow (Bos taurus)}
DFCLEPPYTGPCHARIIRYFYNAKAGLCQTFVYGGCRAKRNNFKSAEDCLRTCGGA
>d5pti__ g.8.1.1 (-) Pancreatic trypsin inhibitor, BPTI {Cow (Bos taurus)}
RPDFCLEPPYTGPCKARIIRYFYNAKAGLCQTFVYGGCRAKRNNFKSAEDCMRTCGGA
>d1ktha_ g.8.1.1 (A:) Collagen type VI (domain C5 from alpha 3 chain) {Human (Homo sapiens)}
ETDICKLPKDEGTCRDFILKWYYDPNTKSCARFWYGGCGGNENKFGSQKECEKVCAPV
>d1adz__ g.8.1.1 (-) Tissue factor pathway inhibitor {Human (Homo sapiens)}
DYKDDDDKLKPDFCFLEEDPGICRGYITRYFYNNQTKQCERFKYGGCLGNMNNFETLEECKN
ICEDGPNGF
>d1irha_ g.8.1.1 (A:) Tissue factor pathway inhibitor {Human (Homo sapiens)}
EFHGPSWCLTPADRGLCRANENRFYYNSVIGKCRPFKYSGCGGNENNFTSKQECLRACKKG
>d1tfxc_ g.8.1.1 (C:) Tissue factor pathway inhibitor {Human (Homo sapiens)}
KPDFCFLEEDPGICRGYITRYFYNNQTKQCERFKYGGCLGNMNNFETLEECKNICEDG
>d1aapa_ g.8.1.1 (A:) Alzheimer's amyloid B-protein precursor, APPI {Human (Homo sapiens)}
VREVCSEQAETGPCRAMISRWYFDVTEGKCAPFFYGGCGGNRNNFDTEEYCMAVCG
>d1bik_1 g.8.1.1 (25-78) Bikunin from inter-alpha-inhibitor complex {Human (Homo sapiens)}
SCQLGYSAGPCMGMTSRYFYNGTSMACETFQYGGCMGNGNNFVTEKECLQTCRT
>d1bik_2 g.8.1.1 (79-134) Bikunin from inter-alpha-inhibitor complex {Human (Homo sapiens)}
VAACNLPIVRGPCRAFIQLWAFDAVKGKCVLFPYGGCQGNGNKFYSEKECREYCGV
>d1dtx__ g.8.1.1 (-) alpha-Dendrotoxin {Green mamba (Dendroaspis angusticeps)}
EPRRKLCILHRNPGRCYDKIPAFYYNQKKKQCERFDWSGCGGNSNRFKTIEECRRTCIG
>d1bunb_ g.8.1.1 (B:) beta2-bungarotoxin, neurotoxin chain {Many-banded krait (elapid) (Bungarus multicinctus)}
RKRHPDCDKPPDTKICQTVVRAFYYKPSAKRCVQFRYGGCNGNGNHFKSDHLCRCECLEYR
>d1shp__ g.8.1.1 (-) Trypsin inhibitor {Sea anemone (Stichodactyla helianthus)}
SICSEPKKVGRCKGYFPRFYFDSETGKCTPFIYGGCGGNGNNFETLHQCRAICRA
>d1dtk__ g.8.1.1 (-) Dendrotoxin K {Black mamba (Dendroaspis

polylepis polylepis)}
AAKYCKLPLRIGPCKRKIPSFYYKWKAKQCLPFDYSGCGGNANRFKTIEECRRTCVG
>d1den__ g.8.1.1 (-) Dendrotoxin I {African elapid snake (Dendroaspis polylepis polylepis)}
QPLRKLCILHRNPGRCYQKIPAFYYNQKKKQCEGFTWSGCGGNSNRFKTIEECRRTCIRK
>d1bf0__ g.8.1.1 (-) Calcicludine (cac) {Green mamba (Dendroaspis angusticeps)}
WQPPWYCKEPVRIGSCKKQFSSFYFKWTAKKCLPFLFSGCGGNANRFQTIGECRKKCLGK
>d1tocr1 g.8.1.2 (R:1A-56) Ornithodorin {Soft tick (Ornithodoros moubata)}
SLNVLCNNPHTADCNNDAQVDRYFREGTTCLMSPACTSEGYASQHECQQACFVGGED
>d1tocr2 g.8.1.2 (R:57-119) Ornithodorin {Soft tick (Ornithodoros moubata)}
HSSEMHSSCLGDPPTSCAEGTDITYYDSDSKTCKVLAASCPSGENTFESEVECQVACGAPIE
G
>d1d0da_ g.8.1.2 (A:) Anticoagulant protein, factor Xa inhibitor {Soft tick (Ornithodoros moubata)}
YNRLCIKPRDWIDECDSNEGGERAYFRNGKGGCDSFWICPEDHTGADYYSSYRDCFNACI
>d1dfna_ g.9.1.1 (A:) Defensin HNP-3 {Human (Homo sapiens)}
DCYCRIPACIAGERRYGTCIYQGRLWAFCC
>d1ijva_ g.9.1.1 (A:) Beta-defensin, BD {Human (Homo sapiens), HBD1}
DHYNCVSSGGQCLYSACPIFTKIQGTCYRGKAKCCK
>d1e4qa_ g.9.1.1 (A:) Beta-defensin, BD {Human (Homo sapiens), HBD2}
PVTCLKSGAICHPVFCPRRYKQIGTCGLPGTKCCKKP
>d1fd3a_ g.9.1.1 (A:) Beta-defensin, BD {Human (Homo sapiens), HBD2}
GIGDPVTCLKSGAICHPVFCPRRYKQIGTCGLPGTKCCKKP
>d1e4ta_ g.9.1.1 (A:) Beta-defensin, BD {Mouse (Mus musculus), MBD5}
NSKRACYREGGECLQRCIGLFHKIGTCNFRFKCCKFQ
>d1e4ra_ g.9.1.1 (A:) Beta-defensin, BD {Mouse (Mus musculus), MBD6}
NEPVSCIRNGGICQYRCIGLRHKIGTCGSPFKCCK
>d1bnb__ g.9.1.1 (-) Beta-defensin, BD {Cow (Bos taurus), BD12}
APLSCGRNGGVCIPIRCPVPMRQIGTCFGRPVKCCRSW
>d1ewsa_ g.9.1.1 (A:) Alpha-defensin rk-1 {Rabbit (Oryctolagus cuniculus)}
MPCSCKKYCDPWEVIDGSCGLFNSKYICCREK
>d1b8wa_ g.9.1.1 (A:) Defensin-like peptide, DLP {Duckbilled platypus (Ornithorhynchus anatinus), DLP-1}
FVQHRPRDCESINGVCRHKDTVNCREIFLADCYNDGQKCCRK
>d1d6ba_ g.9.1.1 (A:) Defensin-like peptide, DLP {Duckbilled

platypus (Ornithorhynchus anatinus), DLP-2}
IMFFEMQACWSHSGVCRDKSERNCKPMAWTYCENRNQKCCEY
>d2bds__ g.9.1.1 (-) BDs-I defensin {Sea anemone (Anemonia sulcata)}
AAPCFCSGKPGRGDLWILRGTCPGGYGYTSNCYKWPNICCYPH
>d1sh1__ g.9.1.1 (-) Sea anemone neurotoxin-1 {Sea anemone (Stichodactyla helianthus)}
AACKCDDEGPDIRTAPLTGTVDLGSCNAGWEKCASYYTIIADCCRKKK
>d1atx__ g.9.1.1 (-) Sea anemone toxin IA {Sea anemone (Anemonia sulcata)}
GAACLCKSDGPNTRGNSMSGTIWVFGCPSGWNNCEGRAIIGYCCKQ
>d1ahl__ g.9.1.1 (-) Anthopleurin-A {Giant green sea anemone (Anthopleura xanthogrammica)}
GVSCLCDSDGPSVRGNTLSGTLWLYPSGCPSGWHNCKAHGPTIGWCCKQ
>d1apf__ g.9.1.1 (-) Anthopleurin-B {Giant green sea anemone (Anthopleura xanthogrammica)}
GVPCLCDSDGPRPRGNTLSGILWFYPSGCPSGWHNCKAHGPNIGWCCKK
>d1bhta1 g.10.1.1 (A:35-126) Hepatocyte growth factor {Human (Homo sapiens)}
RRNTIHEFKKSAKTTLIKIDPALKIKTKKVNTADQCANRCTRNKGLPFTCKAFVFDKARKQC
LWFPFNSMSSGVKKEFGHEFDLYENKDYIR
>d1gmnb1 g.10.1.1 (B:42-125) Hepatocyte growth factor {Human (Homo sapiens)}
FKKSAKTTLIKIDPALKIKTKKVNTADQCADRCTRNKGLPFTCKAFVFDKARKQCLWFPFNS
MSSGVKKEFGHEFDLYENKDYI
>d1i8na_ g.10.1.2 (A:) Anti-platelet protein {Leech (Haementeria officinalis)}
ETITAGNEDCWSKRPGWKLPDNLLTKTEFTSVDECRKMCEESAVEPSCYILQINTETNECYR
NNEGDVTWSSLQYDQPNVVQWHLHACS
>d1ans__ g.11.1.1 (-) Neurotoxin III (ATX III) {Sea anemone (Anemonia sulcata)}
RSCCPCYWGGCPWGQNCYPEGCSGPKV
>d1ajj__ g.12.1.1 (-) Ligand-binding domain of low-density lipoprotein receptor {Human (Homo sapiens)}
PCSAFEFHCLSGECIHSSWRCDGGPDCKDKSDEENCA
>d1cr8a_ g.12.1.1 (A:) Ligand-binding domain of low-density lipoprotein receptor {Human (Homo sapiens)}
PGGCHTDEFQCRLDGLCIPLRWRCDGDTDCMDSSDEKSCEGV
>d1d2la_ g.12.1.1 (A:) Ligand-binding domain of low-density lipoprotein receptor {Human (Homo sapiens)}
GSPPQCQPGEFACANSRCIQERWKCDGDNDCLDNSDEAPALCHQH
>d1f5ya1 g.12.1.1 (A:1-44) Ligand-binding domain of low-density lipoprotein receptor {Human (Homo sapiens)}
GSAVGDRCERNEFQCQDGKCISYKWVCDGSAECQDGSDESQETC

>d1f5ya2 g.12.1.1 (A:45-85) Ligand-binding domain of low-density lipoprotein receptor {Human (Homo sapiens)}
LSVTCKSGDFSCGGRVNRCIPQFWRCDGQVDCDNGSDEQGC
>d1f8za_ g.12.1.1 (A:) Ligand-binding domain of low-density lipoprotein receptor {Human (Homo sapiens)}
ATCRPDEFQCSDGNCIHGSRQCDREYDCKDMSDEVGCVN
>d1j8ea_ g.12.1.1 (A:) Ligand-binding domain of low-density lipoprotein receptor {Human (Homo sapiens)}
GSHSCSSTQFKCNSGRCIPEHWTCDGDNDCGDYSDETHANCTNQ
>d1ldl__ g.12.1.1 (-) Ligand-binding domain of low-density lipoprotein receptor {Human (Homo sapiens)}
AVGDRCERNEFQCQDGKCISYKWVCDGSAECQDGSDESQETCLSVT
>d1k7ba_ g.12.1.1 (A:) soluble Tva ectodomain, sTva47 {Quail (Coturnix coturnix)}
SCPPGQFRCSEPPGAHGECYPQDWLCDGHPDCDDGRDEWGCG
>d1ejga_ g.13.1.1 (A:) Crambin {Abyssinian cabbage (Crambe abyssinica)}
TTCCPSIVARSNFNVCRLPGTPEALCATYTGCIIIPGATCPGDYAN
>d1bhp__ g.13.1.1 (-) beta-Purothionin {Wheat (Triticum aestivum)}
KSCCKSTLGRNCYNLCRARGAQKLCANVCRCKLTSGLSCPKDFPK
>d2plh__ g.13.1.1 (-) alpha-1-Purothionin {Wheat (Triticum aestivum)}
KSCCRSTLGRNCYNLCRARGAQKLCAGVCRCKISSGLSCPKGFPK
>d1ed0a_ g.13.1.1 (A:) Viscotoxin a3 {European mistletoe (Viscum album)}
KSCCPNTTGRNIYNACRLTGAPRPTCAKLSGCKIISGSTCPSDYPK
>d1i5ka_ g.14.1.1 (A:) Plasminogen kringles {Human (Homo sapiens)}
ECMHGSGENYDGKISKTMSGLECQAWDSQSPHAHGYIPSKFPNKNLKKNYCRNPDRDLRPWC
FTTDPNKRWEYCDIPRC
>d1krn__ g.14.1.1 (-) Plasminogen kringles {Human (Homo sapiens)}
DCYHGDGQSYRGTSSTTTTGKKCQSWSSMTPHRHQKTPENYPNAGLTMNYCRNPDADKGPWC
FTTDPSVRWEYCNLKKC
>d1pmla_ g.14.1.1 (A:) Plasminogen kringles {Human (Homo sapiens)}
SDCYFGNGSAYRGTHSLTESGASCLPWNSMILIGKVYTAQNPSAQALGLGKHNYCRNPDGDA
KPWCHVLKNRRLTWEYCDVPSCST
>d1ceaa_ g.14.1.1 (A:) Plasminogen kringle domains {Human (Homo sapiens)}
ECKTGNGKNYRGTMSKTKNGITCQKWSSTSPHRPRFSPATHPSEGLEENYCRNPDNDPQGPW
CYTTDPEKRYDYCDILEC
>d5hpga_ g.14.1.1 (A:) Plasminogen kringle domains {Human (Homo sapiens)}
DCMFGNGKGYRGKRVTTVTGTPCQDWAAQEPHRHSIFTPETNPRAGLEKNYCRNPDGDVGGP
WCYTTNPRKLYDYCDVPQCAAP
>d2pf1_1 g.14.1.1 (66-156) Prothrombin kringle domain {Cow (Bos

taurus)}
CAEGVGMNYRGNVSVTRSGIECQLWRSRYPHKPEINSTTHPGADLRENFCRNPDGSITGPWC
YTTSPTLRREECSVPVCGQDRVTVEVIPR
>d2pf2_1 g.14.1.1 (66-146) Prothrombin kringle domain {Cow (Bos taurus)}
CAEGVGMNYRGNVSVTRSGIECQLWRSRYPHKPEINSTTHPGADLRENFCRNPDGSITGPWC
YTTSPTLRREECSVPVCGQ
>d1a0ha1 g.14.1.1 (A:164-270) Meizothrombin kringle domain {Cow (Bos taurus)}
SPLLETCVPDRGREYRGRLAVTTHGSRCLAWSSEQAKALSKDQDFNPAVPLAENFCRNPDGD
EEGAWCYVADQPGDFEYCDLNYCEEPVDGDLGDRLGEDPDPDAAIEG
>d2hppp_ g.14.1.1 (P:) Meizothrombin kringle domain {Cow (Bos taurus)}
CVPDRGREYRGRLAVTTSGSRCLAWSSEQAKALSKDQDFNPAVPLAENFCRNPDGDEEGAWC
YVADQPGDFEYCNLNYC
>d2hpqp_ g.14.1.1 (P:) Meizothrombin kringle domain {Human (Homo sapiens)}
CVPDRGQQYQGRLAVTTHGLPCLAWASAQAKALSKHQDFNSAVQLVENFCRNPDGDEEGVWC
YVAGKPGDFGYCDLNYC
>d1kdu__ g.14.1.1 (-) Urokinase-type plasminogen activator kringle domain {Human (Homo sapiens)}
TCYEGNGHFYRGKASTDTMGRPCLPWNSATVLQQTYHAHRSDALQLGLGKHNYCRNPDNRRR
PWCYVQVGLKPLVQECMVHDCAD
>d3kiv__ g.14.1.1 (-) Apolipoprotein A kringle domain {Human (Homo sapiens), IV-10/M66 variant}
QCYHGNGQSYRGTFSTTVTGRTCQSWSSMTPHRHQRTPENYPNDGLTMNYCRNPDADTGPWC
FTTDPSIRWEYCNLTRC
>d1i71a_ g.14.1.1 (A:) Apolipoprotein A kringle domain {Human (Homo sapiens), IV-7 variant}
DCYHGDGQSYRGSFSTTVTGRTCQSWSSMTPHWHQRTTEYYPNGGLTRNYCRNPDAEIRPWC
YTMDPSVRWEYCNLTQCPVME
>d1bhta2 g.14.1.1 (A:127-210) NK1 fragment of hepatocyte growth factor {Human (Homo sapiens)}
NCIIGKGRSYKGTVSITKSGIKCQPWSSMIPHEHSFLPSSYRGKDLQENYCRNPRGEEGGPW
CFTSNPEVRYEVCDIPQCSEVE
>d1pdc__ g.14.1.2 (-) PDC-109, collagen-binding type II domain {Cow (Bos taurus)}
DYAKCVFPFIYGGKKYETCTKIGSMWMSWCSLSPNYDKDRAWKYC
>d1e88a1 g.14.1.2 (A:42-101) Fibronectin {Human (Homo sapiens)}
AVTQTYGGNSNGEPCVLPFTYNGRTFYSCTTEGRQDGHLWCSTTSNYEQDQKYSFCTDHT
>d1e88a2 g.14.1.2 (A:102-160) Fibronectin {Human (Homo sapiens)}
VLVQTRGGNSNGALCHFPFLYNNHNYTDCTSEGRRDNMKWCGTTQNYDADQKFGFCPMA
>d1ck7a3 g.14.1.2 (A:217-277) Gelatinase A (MMP-2) type II modules {Human (Homo sapiens)}

EGQVVRVKYGNADGEYCKFPFLFNGKEYNSCTDTGRSDGFLWCSTTYNFEKDGKYGFCPHE
>d1ck7a4 g.14.1.2 (A:278-335) Gelatinase A (MMP-2) type II modules {Human (Homo sapiens)}
ALFTMGGNAEGQPCKFPFRFQGTSYDSCTTEGRTDGYRWCGTTEDYDRDKKYGFCPET
>d1ck7a5 g.14.1.2 (A:336-393) Gelatinase A (MMP-2) type II modules {Human (Homo sapiens)}
AMSTVGGNSEGAPCVFPFTFLGNKYESCTSAGRSDGKMWCATTANYDDDRKWGFCPDQ
>d1j7ma_ g.14.1.2 (A:) Gelatinase A (MMP-2) type II modules {Human (Homo sapiens)}
SWMSTVGGNSGGAPCVFPFTFLGNKYESCTSAGRSDGKMWCATTANYDDDRKWGFCPDQG
>d1ks0a_ g.14.1.2 (A:) Gelatinase A (MMP-2) type II modules {Human (Homo sapiens)}
RIPVKYGNADGEYCKFPFLFNGKEYNSCTDTGRSDGFLWCSTTYNFEKDGKYGFCPHEA
>d1sgpi_ g.15.1.1 (I:) Ovomucoid III domain {Turkey (Meleagris gallopavo)}
VDCSEYPKPACTAEYRPLCGSDNKTYGNKCNFCNAVVESNGTLTLSHFGKC
>d3ovo__ g.15.1.1 (-) Ovomucoid III domain {Japanese quail (Coturnix coturnix japonica)}
LAAVSVDCSEYPKPACPKDYRPVCGSDNKTYSNKCNFCNAVVESNGTLTLNHFGKC
>d2ovo__ g.15.1.1 (-) Ovomucoid III domain {Silver pheasant (Lophura nycthemera)}
LAAVSVDCSEYPKPACTMEYRPLCGSDNKTYGNKCNFCNAVVESNGTLTLSHFGKC
>d1hpt__ g.15.1.1 (-) Secretory trypsin inhibitor {Human (Homo sapiens)}
DSLGREAKCYNELNGCTYEYRPVCGTDGDTYPNECVLCFENRKRQTSILIQKSGPC
>d1tgsi_ g.15.1.1 (I:) Secretory trypsin inhibitor {Pig (Sus scrofa)}
TSPQREATCTSEVSGCPKIYNPVCGTDGITYSNECVLCSENKKRQTPVLIQKSGPC
>d1tbrr1 g.15.1.1 (R:1-51) Rhodniin {Bug (Rhodnius prolixus)}
EGGEPCACPHALHRVCGSDGETYSNPCTLNCAKFNGKPELVKVHDGPCEPD
>d1tbrr2 g.15.1.1 (R:52-103) Rhodniin {Bug (Rhodnius prolixus)}
EDEDVCQECDGDEYKPVCGSDDITYDNNCRLECASISSSPGVELKHEGPCRT
>d1nuba3 g.15.1.1 (A:78-135) Domain of BM-40/SPARC/osteonectin {Human (Homo sapiens)}
CQDPTSCPAPIGEFEKVCSNDNKTFDSSCHFFATKCTLEGTKKGHKLHLDYIGPCKYI
>d2bus__ g.15.1.1 (-) Seminal plasma inhibitor IIa {Cow (Bos taurus)}
EGAQVDCAEFKDPKVYCTRESNPHCGSNGETYGNKCAFCKAVMKSGGKINLKHRGKC
>d1pce__ g.15.1.1 (-) PEC-60 peptide {Pig (Sus scrofa)}
EKQVFSRMPICEHMTESPDCSRIYDPVCGTDGVTYESECKLCLARIENKQDIQIVKDGEC
>d1an1i_ g.15.1.1 (I:) Leech derived tryptase inhibitor (LDTI-C) {Medicinal leech (Hirudo medicinalis)}
KVCACPKILKPVCGSDGRTYANSCIARCNGVSIKSEGSCP
>d1ldtl_ g.15.1.1 (L:) Leech derived tryptase inhibitor (LDTI-C)

{Medicinal leech (Hirudo medicinalis)}
KKVCACPKILKPVCGSDGRTYANSCIARCNGVSIKSEGSCPTGILN
>d4sgbi_ g.15.1.2 (I:) Plant chymotrypsin inhibitor {Potato tuber (Solanum tuberosum)}
PICTNCCAGYKGCNYYSANGAFICEGQSDPKKPKACPLNCDPHIAYSKCPR
>d1ce3a_ g.15.1.2 (A:) Multidomain proteinase inhibitor {Winged tobacco (Nicotiana alata)}
MKACTLNCDPRIAYGVCPRSEEKKNDRICTNCCAGTKGCKYFSDDGTFVCEGES
>d1fyba1 g.15.1.2 (A:1-55) Multidomain proteinase inhibitor {Winged tobacco (Nicotiana alata)}
DRICTNCCAGTKGCKYFSDDGTFVCEGESDPRNPKACTLNCDPRIAYGVCPRSEE
>d1tih__ g.15.1.2 (-) Multidomain proteinase inhibitor {Winged tobacco (Nicotiana alata)}
DRICTNCCAGTKGCKYFSDDGTFVCEGESDPRNPKACPRNCDPRIAYGICPLA
>g1qh2.1 g.15.1.2 (B:,A:) Multidomain proteinase inhibitor {Winged tobacco (Nicotiana alata)}
RICTNCCAGKKGCKYFSDDGTFICEGESXKACTLNCDPRIAYGVCPR
>d2pspa1 g.16.1.1 (A:1-53) Pancreatic spasmolytic polypeptide {Pig (Sus scrofa)}
EKPAACRCSRQDPKNRVNCGFPGITSDQCFTSGCCFDSQVPGVPWCFKPLPAQ
>d2pspa2 g.16.1.1 (A:54-106) Pancreatic spasmolytic polypeptide {Pig (Sus scrofa)}
ESEECVMQVSARKNCGYPGISPEDCAARNCCFSDTIPEVPWCFFPMSVEDCHY
>d1hi7a_ g.16.1.1 (A:) PNR-2/PS2, TFF1 {Human (Homo sapiens)}
EAQTETCTVAPRERQNCGFPGVTPSQCANKGCCFDDTVRGVPWCFYPNTIDVPPEEECEF
>d1e9ta_ g.16.1.1 (A:) Intestinal trefoil factor {Human (Homo sapiens)}
EEYVGLSANQCAVPAKDRVDCGYPHVTPKECNNRGCCFDSRIPGVPWCFKPLQEAECTF
>d1pdga_ g.17.1.1 (A:) Platelet-derived growth factor BB {Human (Homo sapiens)}
EPAMIAECKTRTEVFEISRRLIDRTNANFLVWPPCVEVQRCSGCCNNRNVQCRPTQVQLRPV
QVRKIEIVRKKPIFKKATVTLEDHLACKCETVAA
>d1fltv_ g.17.1.1 (V:) Vascular endothelial growth factor, VEGF {Human (Homo sapiens)}
EVVKFMDVYQRSYCHPIETLVDIFQEYPDEIEYIFKPSCVPLMRCGGCCNDEGLECVPTEES
NITMQIMRIKPHQGQHIGEMSFLQHNKCECRPK
>d1fzva_ g.17.1.1 (A:) Placenta growth factor-1, PLGF-1 {Human (Homo sapiens)}
SSEVEVVPFQEVWGRSYCRALERLVDVVSEYPSEVEHMFSPSCVSLLRCTGCCGDENLHCVP
VETANVTMQLLKIRSGDRPSYVELTFSQHVRCECRPLR
>d1ktza_ g.17.1.2 (A:) TGF-beta3 {Human (Homo sapiens)}
ENCCVRPLYIDFRQDLGWKWVHEPKGYYANFCSGPCPYLRSADTTHSTVLGLYNTLNPEASA
SPCCVPQDLEPLTILYYVGRTPKVEQLSNMVVKSCKCS
>d1tgj__ g.17.1.2 (-) TGF-beta3 {Human (Homo sapiens)}

ALDTNYCFRNLEENCCVRPLYIDFRQDLGWKWVHEPKGYYANFCSGPCPYLRSADTTHSTVL
GLYNTLNPEASASPCCVPQDLEPLTILYYVGRTPKVEQLSNMVVKSCKCS
>d2tgi__ g.17.1.2 (-) TGF-beta2 {Human (Homo sapiens)}
ALDAAYCFRNVQDNCCLRPLYIDFKRDLGWKWIHEPKGYNANFCAGACPYLWSSDTQHSRVL
SLYNTINPEASASPCCVSQDLEPLTILYYIGKTPKIEQLSNMIVKSCKCS
>d1klaa_ g.17.1.2 (A:) TGF-beta1 {Human (Homo sapiens)}
ALDTNYCFSSTEKNCCVRQLYIDFRKDLGWKWIHEPKGYHANFCLGPCPYIWSLDTQYSKVL
ALYNQHNPGASAAPCCVPQALEPLPIVYYVGRKPKVEQLSNMIVRSCKCS
>d1bmp__ g.17.1.2 (-) Bone morphogenetic protein-7 (BMP-7) {Human (Homo sapiens)}
QACKKHELYVSFRDLGWQDWIIAPEGYAAYYCEGECAFPLNSYMNATNHAIVQTLVHFINPE
TVPKPCCAPTQLNAISVLYFDDSSNVILKKYRNMVVRACGCH
>d1es7a_ g.17.1.2 (A:) Bone morphogenetic protein-2 (BMP-2) {Human (Homo sapiens)}
KSSCKRHPLYVDFSDVGWNDWIVAPPGYHAFYCHGECPFPLADHLNSTNHAIVQTLVNSVNS
KIPKACCVPTELSAISMLYLDENEKVVLKNYQDMVVEGCGCR
>d1agqa_ g.17.1.2 (A:) Glial cell-derived neurotrophic factor, GDNF {Rat (Rattus norvegicus)}
NRGCVLTAIHLNVTDLGLGYETKEELIFRYCSGSCEAAETMYDKILKNLSRSRRLTSDKVGQ
ACCRPVAFDDDLSFLDDSLVYHILRKHSAKRCGCI
>d1bnda_ g.17.1.3 (A:) Brain-derived neurotrophic factor/neurotrophin 3 heterodimer, BDNF/NT3 {Human (Homo sapiens)}
GQLSVCDSISEWVTAADKKTAVDMSGGTVTVLEKVPVSKGQLKQYFYETKCNPMGYTKEGCR
GIDKRHWNSQCRTTQSYVRALTMDSKKRIGWRFIRIDTSCVCTLTIK
>d1bndb_ g.17.1.3 (B:) Brain-derived neurotrophic factor/neurotrophin 3 heterodimer, BDNF/NT3 {Human (Homo sapiens)}
RGEVSVCDSESLWVTDKSSAIDIRGHQVTVLGEIKTQNSPVKQYFYETRCKEARPVKNGCRG
IDDKHWNSQCKTSQTYVRALTSENNKLVGWRWIRIDTSCVCALSRK
>d1b8mb_ g.17.1.3 (B:) Neurotrophin 4 {Human (Homo sapiens)}
GELAVCDAVSGWVTDRRTAVDLRGREVEVLGEVPAAGGSPLRQYFFETRCKADNAEEGGPGA
GGGGCRGVDRRHWVSECKAKQSYVRALTADAQGRVGWRWIRIDTACVCTLLSRTGRA
>d1hcfa_ g.17.1.3 (A:) Neurotrophin 4 {Human (Homo sapiens)}
GVSETAPASRRGELAVCDAVSGWVTDRRTAVDLRGREVEVLGEVPAAGGSPLRQYFFETRCK
ADNAEEGGPGAGGGGCRGVDRRHWVSECKAKQSYVRALTADAQGRVGWRWIRIDTACVCTLL
SRT
>d1bet__ g.17.1.3 (-) beta-Nerve growth factor {Mouse (Mus musculus)}
GEFSVCDSVSVWVGDKTTATDIKGKEVTVLAEVNINNSVFRQYFFETKCRASNPVESGCRGI
DSKHWNSYCTTTHTFVKALTTDEKQAAWRFIRIDTACVCVLSRKA
>d1wwwv_ g.17.1.3 (V:) beta-Nerve growth factor {Human (Homo sapiens)}
SSHPIFHRGEFSVCDSVSVWVGDKTTATDIKGKEVMVLGEVNINNSVFKQYFFETKCRDPNP
VDSGCRGIDSKHWNSYCTTTHTFVKALTMDGKQAAWRFIRIDTACVCVLSRK
>d1hcna_ g.17.1.4 (A:) Glycoprotein hormones alpha chain

(Gonadotropin A, Follitropin alpha) {Human (Homo sapiens)}
QDCPECTLQENPFFSQPGAPILQCMGCCFSRAYPTPLRSKKTMLVQKNVTSESTCCVAKSYN
RVTVMGGFKVENHTACHCSTCYY
>d1hcnb_ g.17.1.4 (B:) Gonadotropin B chain {Human (Homo sapiens)}
KEPLRPRCRPINATLAVEKEGCPVCITVNTTICAGYCPTMTRVLQGVLPALPQVVCNYRDVR
FESIRLPGCPRGVNPVVSYAVALSCQCALCRRSTTDCGGPKDHPLTCD
>d1fl7b_ g.17.1.4 (B:) Follicle stimulating hormone, follitropin, beta chain {Human (Homo sapiens)}
CELTNITIAIEKEECRFCISINTAWCAGYCYTRDLVYKDPARPKIQKTCTFKELVYETVRVP
GCAHHADSLYTYPVATQCHCGKCDSDSTDCTVRGLGPSYCSFGEM
>d1jpya_ g.17.1.6 (A:) Interleukin 17F, IL-17F {Human (Homo sapiens)}
HTFFQKPESCPPVPGGSMKLDIGIINENQRVSMSRNIESRSTSPWNYTVTWDPNRYPSEVVQ
AQCRNLGCINAQGKEDISMNSVPIQQETLVVRRKHQGCSVSFQLEKVLVTVGCTCVTPV
>d1aoca_ g.17.1.5 (A:) Coagulogen {Japanese horseshoe crab (Tachypleus tridentatus)}
ADTNAPICLCDEPGVLGRTQIVTTEIKDKIEKAVEAVAQESGVSGRGFSIFSHHPVFRECGK
YECRTVRPEHSRCYNFPPFTHFKSECPVSTRDCEPVFGYTVAGEFRVIVQAPRAGFRQCVWQ
HKCRFGSNSCGYNGRCTQQRSVVRLVTYNLEKDGFLCESFRTCCGCPCRSF
>d1hcc__ g.18.1.1 (-) Factor H, 15th and 16th modules {Human (Homo sapiens)}
EGLPCKSPPEISHGVVAHMSDSYQYGEEVTYKCFEGFGIDGPAIAKCLGEKWSHPPSCI
>d1hfi__ g.18.1.1 (-) Factor H, 15th and 16th modules {Human (Homo sapiens)}
EKIPCSQPPQIEHGTINSSRSSQESYAHGTKLSYTCEGGFRISEENETTCYMGKWSSPPQCE
>d1g40a1 g.18.1.1 (A:1-64) Complement control protein {Vaccinia virus}
CCTIPSRPINMKFKNSVETDANANYNIGDTIEYLCLPGYRKQKMGPIYAKCTGTGWTLFNQC
IK
>d1g40a2 g.18.1.1 (A:65-126) Complement control protein {Vaccinia virus}
RRCPSPRDIDNGQLDIGGVDFGSSITYSCNSGYHLIGESKSYCELGSTGSMVWNPEAPICES
>d1g40a3 g.18.1.1 (A:127-184) Complement control protein {Vaccinia virus}
VKCQSPPSISNGRHNGYEDFYTDGSVVTYSCNSGYSLIGNSGVLCSGGEWSDPPTCQI
>d1g40a4 g.18.1.1 (A:185-243) Complement control protein {Vaccinia virus}
VKCPHPTISNGYLSSGFKRSYSYNDNVDFKCKYGYKLSGSSSSTCSPGNTWKPELPKCV
>d1ckla1 g.18.1.1 (A:1-62) CD46 (membrane cofactor protein, MCP) {Human (Homo sapiens)}
CEEPPTFEAMELIGKPKPYYEIGERVDYKCKKGYFYIPPLATHTICDRNHTWLPVSDDACYR
>d1ckla2 g.18.1.1 (A:63-126) CD46 (membrane cofactor protein, MCP) {Human (Homo sapiens)}
ETCPYIRDPLNGQAVPANGTYEFGYQMHFICNEGYYLIGEEILYCELKGSVAIWSGKPPICE

KV
>d1c1za5 g.18.1.1 (A:244-326) beta2-glycoprotein I {Human (Homo sapiens)}
SCKLPVKKATVVYQGERVKIQEKFKNGMLHGDKVSFFCKNKEKKCSYTEDAQCIDGTIEVPK
CFKEHSSLAFWKTDASDVKPC
>d1quba1 g.18.1.1 (A:1-62) beta2-glycoprotein I {Human (Homo sapiens)}
GRTCPKPDDLPFSTVVPLKTFYEPGEEITYSCKPGYVSRGGMRKFICPLTGLWPINTLKCTP
>d1quba2 g.18.1.1 (A:63-120) beta2-glycoprotein I {Human (Homo sapiens)}
RVCPFAGILENGAVRYTTFEYPNTISFSCNTGFYLNGADSAKCTEEGKWSPELPVCAP
>d1quba3 g.18.1.1 (A:121-183) beta2-glycoprotein I {Human (Homo sapiens)}
IICPPPSIPTFATLRVYKPSAGNNSLYRDTAVFECLPQHAMFGNDTITCTTHGNWTKLPECR
E
>d1quba4 g.18.1.1 (A:184-243) beta2-glycoprotein I {Human (Homo sapiens)}
VKCPFPSRPDNGFVNYPAKPTLYYKDKATFGCHDGYSLDGPEEIECTKLGNWSAMPSCKA
>d1quba5 g.18.1.1 (A:244-326) beta2-glycoprotein I {Human (Homo sapiens)}
SCKVPVKKATVVYQGERVKIQEKFKNGMLHGDKVSFFCKNKEKKCSYTEDAQCIDGTIEVPK
CFKEHTDASDVKPC
>d1ghqb1 g.18.1.1 (B:1-66) Complement receptor 2, cr2 {Human (Homo sapiens)}
AISCGSPPPILNGRISYYSTPIAVGTVIRYSCSGTFRLIGEKSLLCITKDKVDGTWDKPAPK
CEYF
>d1ghqb2 g.18.1.1 (B:67-129) Complement receptor 2, cr2 {Human (Homo sapiens)}
NKYSSCPEPIVPGGYKIRGSTPYRHGDSVTFACKTNFSMNGNKSVWCQANNMWGPTRLPTCV
S
>d1elva2 g.18.1.1 (A:342-409) Complement C1S protease domain {Human (Homo sapiens)}
LDCGIPESIENGKVEDPESTLFGSVIRYTCEEPYYYMENGGGGEYHCAGNGSWVNEVLGPEL
PKCVPV
>d1bgk__ g.19.1.1 (-) Sea anemone toxin k {Sea anemone (Bunodosoma granulifera), BGK}
VCRDWFKETACRHAKSLGNCRTSQKYRANCAKTCELC
>d1c2ua_ g.19.1.1 (A:) Sea anemone toxin k {Sun anemone (Stichodactyla helianthus), SHK}
RSXIDTIPKSRCTAFQCKHSAKYRLSFCRKTCGTX
>d1roo__ g.19.1.1 (-) Sea anemone toxin k {Sun anemone (Stichodactyla helianthus), SHK}
RSCIDTIPKSRCTAFQCKHSMKYRLSFCRKTCGTC
>d2ech__ g.20.1.1 (-) Echistatin {Echis carinatus}

ECESGPCCRNCKFLKEGTICKRARGDDMDDYCNGKTCDCPRNPHKGPAT
>d1fvl__ g.20.1.1 (-) Flavoridin {Snake (Trimeresurus flavoviridis)}
GEECDCGSPSNPCCDAATCKLRPGAQCADGLCCDQCRFKKKRTICRIARGDFPDDRCTGLSN
DCPRWNDL
>d1kst__ g.20.1.1 (-) Kistrin {Agkistrodon rhodostoma}
GKECDCSSPENPCCDAATCKLRPGAQCGEGLCCEQCKFSRAGKICRIPRGDMPDDRCTGQSA
DCPRYH
>d1mdal_ g.21.1.1 (L:) Methylamine dehydrogenase {Paracoccus denitrificans}
VDPRAKWQPQDNDIQACDYWRHCSIAGNICDCSAGSLTSCPPGTLVASGSWVGSCYNPPDPN
KYITAYRDCCGYNVSGRCACLNTEGELPVYNKDANDIIWCFGGEDGMTYHCSISPVSGA
>d2bbkl_ g.21.1.1 (L:) Methylamine dehydrogenase {Paracoccus denitrificans}
TDPRAKWVPQDNDIQACDYWRHCSIDGNICDCSGGSLTNCPPGTKLATASWVASCYNPTDGQ
SYLIAYRDCCGYNVSGRCPCLNTEGELPVYRPEFANDIIWCFGAEDDAMTYHCTISPIVGKA
S
>d1atb__ g.22.1.1 (-) Ascaris trypsin inhibitor, ATI {Pig roundworm (Ascaris Lumbricoides), variant suum}
EAEKCTKPNEQWTKCGGCEGTCAQKIVPCTRECKPPRCECIASAGFVRDAQGNCIKFEDCPK
>d1eaic_ g.22.1.1 (C:) Ascaris elastase inhibitor {Pig roundworm (Ascaris suum)}
GQESCGPNEVWTECTGCEMKCGPDENTPCPLMCRRPSCECSPGRGMRRTNDGKCIPASQCP
>d1coua_ g.22.1.1 (A:) Anticoagulant protein {Dog hookworm (Ancylostoma caninum)}
KATMQCGENEKYDSCGSKECDKKCKYDGVEEEDDEEPNVPCLVRVCHQDCVCEEGFYRNKDD
KCVSAEDCELDNMDFIYPGTRNP
>d1ccva_ g.22.1.1 (A:) Chymotrypsin inhibitor AMCI {Honeybee (Apis mellifera)}
EECGPNEVFNTCGSACAPTCAQPKTRICTMQCRIGCQCQEGFLRNGEGACVLPENC
>d1hx2a_ g.22.1.2 (A:) BSTI {Fire-bellied toad (Bombina bombina)}
NFVCPPGQTFQTCASSCPKTCETRNKLVLCDKKCNQRCGCISGTVLKSKDSSECVHPSKC
>d1apj__ g.23.1.1 (-) Fibrillin {Human (Homo sapiens)}
SAQDLRMSYCYAKFEGGKCSSPKSRNHSKQECCCALKGEGWGDPCELCPTEPDEAFRQICPY
GSGIIVGPDDSA
>d1exta1 g.24.1.1 (A:13-71) Tumor necrosis factor (TNF) receptor {Human (Homo sapiens)}
SVCPQGKYIHPQNNSICCTKCHKGTYLYNDCPGPGQDTDCRECESGSFTASENHLRHCL
>d1exta2 g.24.1.1 (A:72-115) Tumor necrosis factor (TNF) receptor {Human (Homo sapiens)}
SCSKCRKEMGQVEISSCTVDRDTVCGCRKNQYRHYWSENLFQCF
>d1exta3 g.24.1.1 (A:116-172) Tumor necrosis factor (TNF) receptor {Human (Homo sapiens)}
NCSLCLNGTVHLSCQEKQNTVCTCHAGFFLRENECVSCSNCKKSLECTKLCLPQIEN

>d1ncfa3 g.24.1.1 (A:116-150) Tumor necrosis factor (TNF) receptor {Human (Homo sapiens)}
NCSLCLNGTVHLSCQEKQNTVCTCHAGFFLRENEC
>d1ncfb3 g.24.1.1 (B:116-155) Tumor necrosis factor (TNF) receptor {Human (Homo sapiens)}
NCSLCLNGTVHLSCQEKQNTVCTCHAGFFLRENECVSCSN
>d1d0gr1 g.24.1.1 (R:21-61) Death receptor-5 (dr5) fragment {Human (Homo sapiens)}
SSPSEGLCPPGHHISEDGRDCISCKYGQDYSTHWNDLLFCL
>d1d0gr3 g.24.1.1 (R:102-128) Death receptor-5 (dr5) fragment {Human (Homo sapiens)}
KCRTGCPRGMVKVGDCTPWSDIECVHK
>d1d4va1 g.24.1.1 (A:69-114) Death receptor-5 (dr5) fragment {Human (Homo sapiens)}
PQQKRSSPSEGLCPPGHHISEDGRDCISCKYGQDYSTHWNDLLFCL
>d1d4va2 g.24.1.1 (A:115-154) Death receptor-5 (dr5) fragment {Human (Homo sapiens)}
RCTRCDSGEVELSPCTTTRNTVCQCEEGTFREEDSPEMCR
>d1d4va3 g.24.1.1 (A:155-185) Death receptor-5 (dr5) fragment {Human (Homo sapiens)}
KCRTGCPRGMVKVGDCTPWSDIECVHKESGD
>d1du3a3 g.24.1.1 (A:102-123) Death receptor-5 (dr5) fragment {Human (Homo sapiens)}
KCRTGCPRGMVKVGDCTPWSDI
>d1jmab1 g.24.1.1 (B:4-59) Cellular receptor HveA {Human (Homo sapiens)}
CKEDEYPVGSECCPKCSPGYRVKEACGELTGTVCEPCPPGTYIAHLNGLSKCLQCQ
>d1jmab2 g.24.1.1 (B:60-105) Cellular receptor HveA {Human (Homo sapiens)}
MCDPAMGLRASRNCSRTENAVCGCSPGHFCIVQDGDHCAACRAYAT
>d1vgh__ g.25.1.1 (-) Heparin-binding domain from vascular endothelial growth factor {Human (Homo sapiens)}
ARQENPCGPCSERRKHLFVQDPQTCKCSCKNTDSRCKARQLELNERTCRCDKPRR
>d1afp__ g.26.1.1 (-) Antifungal protein (AGAFP) {Mold (Aspergillus giganteus)}
ATYNGKCYKKDNICKYKAQSGKTAICKCYVKKCPRDGAKCEFDSYKGKCYC
>d1e88a3 g.27.1.1 (A:1-41) Fibronectin {Human (Homo sapiens)}
YGHCVTDSGVVYSVGMQWLKTQGNKQMLCTCLGNGVSCQET
>d1fbr_1 g.27.1.1 (1-46) Fibronectin {Human (Homo sapiens)}
AEKCFDHAAGTSYVVGETWEKPYQGWMMVDCTCLGEGSGRITCTSR
>d1fbr_2 g.27.1.1 (47-93) Fibronectin {Human (Homo sapiens)}
NRCNDQDTRTSYRIGDTWSKKDNRGNLLQCICTGNGRGEWKCERHTS
>d1qgba1 g.27.1.1 (A:17-60) Fibronectin {Human (Homo sapiens)}
SKPGCYDNGKHYQINQQWERTYLGNALVCTCYGGSRGFNCESKP

>d1qgba2 g.27.1.1 (A:61-109) Fibronectin {Human (Homo sapiens)}
EAEETCFDKYTGNTYRVGDTYERPKDSMIWDCTCIGAGRGRISCTIANR
>d1tpg_2 g.27.1.1 (1-50) Tissue-type plasminogen activator, t-PA {Human (Homo sapiens)}
SYQVICRDEKTQMIYQQHQSWLRPVLRSNRVEYCWCNSGRAQCHSVPVKS
>d1icfi_ g.28.1.1 (I:) MHC class II associated p41 invariant chain fragment {Human (Homo sapiens)}
LTKCQEEVSHIPAVHPGSFRPKCDENGNYLPLQCYGSIGYCWCVFPNGTEVPNTRSRGHHNC
SES
>d1e8ra_ g.29.1.1 (A:) Endo-1;4-beta-xylanase A CBDX {Pseudomonas fluorescens, subsp. cellulosa}
MGNQQCNWYGTLYPLCVTTTNGWGWEDQRSCIARSTCAAQPAPFGIVGSG
>d1e8qa_ g.55.1.1 (A:) Cellulose docking domain, dockering {Piromyces equi}
ASCWAQSQGYNCCNNPSSTKVEYTDASGQWGVQNGQWCGIDYSYGQ
>d1dtdb_ g.30.1.1 (B:) Carboxypeptidase inhibitor {Medicinal leech (Hirudo medicinalis)}
DESFLCYQPDQVCCFICRGAAPLPSEGECNPHPTAPWCREGAVEWVPYSTGQCRTTCIPYV
>d1hdla_ g.57.1.1 (A:) Serine proteinase inhibitor lekti, domain one {Human (Homo sapiens)}
KNEDQEMCHEFQAFMKNGKLFCPQDKKFFQSLDGIMFINKCATCKMILEKEAKSQ
>d1dqca_ g.31.1.1 (A:) Tachycitin {Horseshoe crab (Tachypleus tridentatus)}
YLAFRCGRYSPCLDDGPNVNLYSCCSFYNCHKCLARLENCPKGLHYNAYLKVCDWPSKAGCT
SVNKECHLWKT
>d1ha8a_ g.58.1.1 (A:) Pheromone ER-23 {Euplotes raikovi}
GECEQCFSDGGDCTTCFNNGTGPCANCLAGYPAGCSNSDCTAFLSQCYGGC
>d1danl3 g.32.1.1 (L:1-48) Coagulation factor VIIa {Human (Homo sapiens)}
ANAFLEELRPGSLERECKEEQCSFEEAREIFKDAERTKLFWISYSDGD
>d2pf1_2 g.32.1.1 (36-65) Prothrombin {Cow (Bos taurus)}
SATDAFWAKYTACESARNPREKLNECLEGN
>d2pf2_2 g.32.1.1 (1-65) Prothrombin {Cow (Bos taurus)}
ANKGFLEEVRKGNLREECLEEPCSREEAFEALESLSATDAFWAKYTACESARNPREKLNECL
EGN
>d1cfi__ g.32.1.1 (-) Coagulation factor IX (IXa) {Human (Homo sapiens)}
YNSGKLEEFVQGNLERECMEEKCSFEEAREVFENTERTTEFWKQYVD
>d1pfxl3 g.32.1.1 (L:1-46) Coagulation factor IX (IXa) {Pig (Sus scrofa)}
YNSGKLEEFVRGNLERECIEEKCSFEEAREVFENTEKTNEFWKQYV
>d1iodg_ g.32.1.1 (G:) Coagulation factor X {Cow (Bos taurus)}
ANSFLEEVKQGNLERECLEEACSLEEAREVFEDAEQTDEFWSKY
>d1d6ga_ g.33.1.1 (A:) Cholecystokinin A receptor, N-domain {Human

(Homo sapiens)}
MDVVDSLLVNGSNITPPCELGLENETLFCLDQPRPSKEWQPAQVILL
>d1vpu__ g.34.1.1 (-) HIV-1 VPU cytoplasmic domain {Human immunodeficiency virus type 1}
LQIDRLIDRITERAEDSGNESEGDQEELSALVERGHLAPWDVDDL
>d1isua_ g.35.1.1 (A:) HIPIP (high potential iron protein) {Rhodocyclus tenuis}
GTNAAMRKAFNYQDTAKNGKKCSGCAQFVPGASPTAAGGCKVIPGDNQIAPGGYCDAFIVKK
>d1b0ya_ g.35.1.1 (A:) HIPIP (high potential iron protein) {Allochromatium vinosum, (formerly Chromatium vinosum)}
SAPANAVAADNATAIALKYNQDATKSERVAAARPGLPPEEQQCANCQFMQADAAGATDEWKG
CQLFPGKLINVNGWCASWTLKAG
>d1js2a_ g.35.1.1 (A:) HIPIP (high potential iron protein) {Allochromatium vinosum, (formerly Chromatium vinosum)}
MEFMSAPANAVAADDATAIALKYNQDATKSERVAAARPGLPPEEQHCANCQFMQADAAGATD
EWKGCQLFPGKLINVNGWSASWTLKAG
>d3hipa_ g.35.1.1 (A:) HIPIP (high potential iron protein) {Chromatium purpuratum}
VPANAVTESDPAAVALKYHRDAASSERVAAARPGLPPEEQHCENCQFMNPDSAAADWKGCQL
FPGKLINLSGWCASWTLRAG
>d2hipa_ g.35.1.1 (A:) HIPIP (high potential iron protein) {Ectothiorhodospira halophila}
EPRAEDGHAHDYVNEAADASGHPRYQEGQLCENCAFWGEAVQDGWGRCTHPDFDEVLVKAEG
WCSVYAPAS
>d1hpi__ g.35.1.1 (-) HIPIP (high potential iron protein) {Ectothiorhodospira vacuolata}
MERLSEDDPAAQALEYRHDASSVQHPAYEEGQTCLNCLLYTDASAQDWGPCSVFPGKLVSAN
GWCTAWVAR
>d1eyta_ g.35.1.1 (A:) HIPIP (high potential iron protein) {Thermochromatium tepidum}
AAPANAVTADDPTAIALKYNQDATKSERVAAARPGLPPEEQHCANCQFMQANVGEGDWKGCQ
LFPGKLINVNGWCASWTLKAG
>d1dj7a_ g.36.1.1 (A:) Ferredoxin thioredoxin reductase (FTR), catalytic beta chain {Synechocystis sp.}
NNKTLAAMKNFAEQYAKRTDTYFCSDLSVTAVVIEGLARHKEELGSPLCPCRHYEDKEAEVK
NTFWNCPCVPMRERKECHCMLFLTPDNDFAGDAQDIPMETLEEVKAS
>d1a1ga1 g.37.1.1 (A:103-131) ZIF268 {Mouse (Mus musculus)}
RPYACPVESCDRRFSDSSNLTRHIRIHTG
>d1a1ha1 g.37.1.1 (A:103-131) ZIF268 {Mouse (Mus musculus)}
RPYACPVESCDRRFSQSGSLTRHIRIHTG
>d1a1ia1 g.37.1.1 (A:103-131) ZIF268 {Mouse (Mus musculus)}
RPYACPVESCDRRFSRSADLTRHIRIHTG
>d1a1ia2 g.37.1.1 (A:132-159) ZIF268 {Mouse (Mus musculus)}
QKPFQCRICMRNFSRSDHLTTHIRTHTG

>d1a1ia3 g.37.1.1 (A:160-187) ZIF268 {Mouse (Mus musculus)}
EKPFACDICGRKFARSDERKRHTKIHLR
>d1aaya1 g.37.1.1 (A:103-131) ZIF268 {Mouse (Mus musculus)}
RPYACPVESCDRRFSRSDELTRHIRIHTG
>d1f2ig1 g.37.1.1 (G:1093-1131) ZIF268 {Mouse (Mus musculus)}
NLLNYVVPKMRPYACPVESCDRRFSRSDELTRHIRIHTG
>d1rmd_1 g.37.1.1 (87-116) V(D)J recombination activating protein 1 (RAG1), dimerization domain {Mouse (Mus musculus)}
LMVKCPAQDCNEEVSLEKYNHHVSSHKESK
>d2drpa1 g.37.1.1 (A:103-139) Tramtrack protein (two zinc-finger peptide) {Drosophila melanogaster}
FTKEGEHTYRCKVCSRVYTHISNFCRHYVTSHKRNVK
>d2drpa2 g.37.1.1 (A:140-165) Tramtrack protein (two zinc-finger peptide) {Drosophila melanogaster}
VYPCPFCFKEFTRKDNMTAHVKIIHK
>d1paa__ g.37.1.1 (-) ADR1 {Synthetic, based on Saccharomyces cerevisiae sequence}
KAYACGLCNRAFTRRDLLIRHAQKIHSGNL
>d2adr_1 g.37.1.1 (102-130) ADR1 {Synthetic, based on Saccharomyces cerevisiae sequence}
RSFVCEVCTRAFARQEHLKRHYRSHTNEK
>d2adr_2 g.37.1.1 (131-161) ADR1 {Synthetic, based on Saccharomyces cerevisiae sequence}
PYPCGLCNRAFTRRDLLIRHAQKIHSGNLGE
>d1znf__ g.37.1.1 (-) XFIN, third domain {Xenopus laevis}
YKCGLCERSFVEKSALSRHQRVHKN
>d5znf__ g.37.1.1 (-) ZFY {Human (Homo sapiens)}
KTYQCQYCEYRSADSSNLKTHIKTKHSKEK
>d7znf__ g.37.1.1 (-) ZFY {Human (Homo sapiens)}
KTYQCQYCEKRFADSSNLKTHIKTKHSKEK
>d1ncs__ g.37.1.1 (-) SWI5 zinc-finger domains {Baker's yeast (Saccharomyces cerevisiae)}
TLPRGSIDKYVKEMPDKTFECLFPGCTKTFKRRYNIRSHIQTHLEDR
>d1zfd__ g.37.1.1 (-) SWI5 zinc-finger domains {Baker's yeast (Saccharomyces cerevisiae)}
DRPYSCDHPGCDKAFVRNHDLIRHKKSHQEKA
>d2glia1 g.37.1.1 (A:103-134) Five-finger GLI1 {Human (Homo sapiens)}
ETDCRWDGCSQEFDSQEQLVHHINSEHIHGER
>d2glia2 g.37.1.1 (A:135-167) Five-finger GLI1 {Human (Homo sapiens)}
KEFVCHWGGCSRELRPFKAQYMLVVHMRRHTGE
>d2glia3 g.37.1.1 (A:168-197) Five-finger GLI1 {Human (Homo sapiens)}

KPHKCTFEGCRKSYSRLENLKTHLRSHTGE
>d2glia4 g.37.1.1 (A:198-228) Five-finger GLI1 {Human (Homo sapiens)}
KPYMCEHEGCSKAFSNASDRAKHQNRTHSNE
>d2glia5 g.37.1.1 (A:229-257) Five-finger GLI1 {Human (Homo sapiens)}
KPYVCKLPGCTKRYTDPSSLRKHVKTVHG
>d1bbo_1 g.37.1.1 (1-28) Enhancer binding protein {Human (Homo sapiens)}
KYICEECGIRXKKPSMLKKHIRTHTDVR
>d1bbo_2 g.37.1.1 (29-57) Enhancer binding protein {Human (Homo sapiens)}
PYHCTYCNFSFKTKGNLTKHMKSKAHSKK
>d4znf__ g.37.1.1 (-) Enhancer binding protein {Human (Homo sapiens)}
RPYHCSYCNFSFKTKGNLTKHMKSKAHSKK
>d1sp1__ g.37.1.1 (-) Transcription factor sp1 {Human (Homo sapiens)}
KKFACPECPKRFMRSDHLSKHIKTHQNKK
>d1sp2__ g.37.1.1 (-) Transcription factor sp1 {Human (Homo sapiens)}
RPFMCTWSYCGKRFTRSDELQRHKRTHTGEK
>d1bhi__ g.37.1.1 (-) Transactivation domain of cre-bp1/atf-2 {Human (Homo sapiens)}
MSDDKPFLCTAPGCGQRFTNEDHLAVHKHKHEMTLKFG
>d1ubdc1 g.37.1.1 (C:295-322) Ying-yang 1 (yy1, zinc finger domain) {Human (Homo sapiens)}
TIACPHKGCTKMFRDNSAMRKHLHTHGP
>d1ubdc2 g.37.1.1 (C:323-350) Ying-yang 1 (yy1, zinc finger domain) {Human (Homo sapiens)}
RVHVCAECGKAFVESSKLKRHQLVHTGE
>d1ubdc3 g.37.1.1 (C:351-380) Ying-yang 1 (yy1, zinc finger domain) {Human (Homo sapiens)}
KPFQCTFEGCGKRFSLDFNLRTHVRIHTGD
>d1ubdc4 g.37.1.1 (C:381-408) Ying-yang 1 (yy1, zinc finger domain) {Human (Homo sapiens)}
RPYVCPFDGCNKKFAQSTNLKSHILTHA
>d1tf3a1 g.37.1.1 (A:1-40) Transcription factor IIIA, TFIIIA {Xenopus laevis}
MKRYICSFADCGAAYNKNWKLQAHLSKHTGE
>d1tf3a2 g.37.1.1 (A:41-70) Transcription factor IIIA, TFIIIA {Xenopus laevis}
KPFPCKEEGCEKGFTSLHHLTRHSLTHTGE
>d1tf3a3 g.37.1.1 (A:71-101) Transcription factor IIIA, TFIIIA

{Xenopus laevis}
KNFTCDSDGCDLRFTTKANMKKHFNRFHNIK
>d1tf6a1 g.37.1.1 (A:10-40) Transcription factor IIIA, TFIIIA
{Xenopus laevis}
YKRYICSFADCGAAYNKNWKLQAHLCKHTGE
>d1tf6a4 g.37.1.1 (A:101-131) Transcription factor IIIA, TFIIIA
{Xenopus laevis}
KICVYVCHFENCGKAFKKHNQLKVHQFSHTQ
>d1tf6a5 g.37.1.1 (A:132-160) Transcription factor IIIA, TFIIIA
{Xenopus laevis}
QLPYECPHEGCDKRFSLPSRLKRHEKVHA
>d1tf6a6 g.37.1.1 (A:161-188) Transcription factor IIIA, TFIIIA
{Xenopus laevis}
GYPCKKDDSCSFVGKTWTLYLKHVAECH
>d1yuja_ g.37.1.1 (A:) GAGA factor {Drosophila melanogaster}
PKAKRAKHPPGTEKPRSRSQSEQPATCPICYAVIRQSRNLRRHLELRHFAKPGV
>d1fu9a_ g.37.1.2 (A:) U-shaped transcription factor, different
fingers {Fruit fly (Drosophila melanogaster)}
GSAAEVMKKYCSTCDISFNYVKTYLAHKQFYCKNKP
>d1fv5a_ g.37.1.2 (A:) U-shaped transcription factor, different
fingers {Fruit fly (Drosophila melanogaster)}
GSLLKPARFMCLPCGIAFSSPSTLEAHQAYYCSHRI
>d1aw6__ g.38.1.1 (-) Gal4 {Baker's yeast (Saccharomyces
cerevisiae)}
MKLLSSIEQACDICRLKKLKCSKEKPKCAKCLKNNWECRYSPK
>d1d66a1 g.38.1.1 (A:8-48) Gal4 {Baker's yeast (Saccharomyces
cerevisiae)}
EQACDICRLKKLKCSKEKPKCAKCLKNNWECRYSPKTKRSP
>d1pyia1 g.38.1.1 (A:30-71) PPR1 {Baker's yeast (Saccharomyces
cerevisiae)}
SRTACKRCRLKKIKCDQEFPSCKRCAKLEVPCVSLDPATGKD
>d1zmec1 g.38.1.1 (C:31-66) PUT3 {Baker's yeast (Saccharomyces
cerevisiae)}
SVACLSCRKRHIKCPGGNPCQKCVTSNAICEYLEPS
>d1hwtc1 g.38.1.1 (C:59-97) Hap1 (Cyp1) {Baker's yeast
(Saccharomyces cerevisiae)}
RIPLSCTICRKRKVKCDKLRPHCQQCTKTGVAHLCHYME
>d2hapc1 g.38.1.1 (C:55-97) Hap1 (Cyp1) {Baker's yeast
(Saccharomyces cerevisiae)}
RKRNRIPLRCTICRKRKVKCDKLRPHCQQCTKTGVAHLCHYME
>d1cld__ g.38.1.1 (-) CD2-Lac9 {Milk yeast (Kluyveromyces lactis)}
QACDACRKKKWKCSKTVPTCTNCLKYNLDCVYS
>d2alca_ g.38.1.1 (A:) Ethanol regulon transcriptional activator
ALCR DNA-binding domain {Aspergillus nidulans and Emericella

nidulans}
GSMADTRRRQNHSCDPCRKGKRRCDAPENRNEANENGWVSCSNCKRWNKDCTFNWLSSQRSK
NSS
>d3gata_ g.39.1.1 (A:) Erythroid transcription factor GATA-1 {Chicken (Gallus gallus)}
KRAGTVCSNCQTSTTTLWRRSPMGDPVCNACGLYYKLHQVNRPLTMRKDGIQTRNRKVSSKG
KKRR
>d7gata_ g.39.1.1 (A:) Erythroid transcription factor GATA-1 {Chicken (Gallus gallus)}
MKNGEQNGPTTCTNCFTQTTPVWRRNPEGQPLCNACGLFLKLHGVVRPLSLKTDVIKKRNRN
SANS
>d1gnf__ g.39.1.1 (-) Erythroid transcription factor GATA-1 {Mouse (Mus musculus)}
GSEARECVNCGATATPLWRRDRTGHYLCNACGLYHKMNGQNRPLIR
>d1dszb_ g.39.1.2 (B:) Retinoid X receptor (RXR-alpha) DNA-binding domain {Human (Homo sapiens)}
GSFTKHICAICGDRSSGKHYGVYSCEGCKGFFKRTVRKDLTYTCRDNKDCLIDKRQRNRCQY
CRYQKCLAMGMKREAVQEERQR
>d2nlla_ g.39.1.2 (A:) Retinoid X receptor (RXR-alpha) DNA-binding domain {Human (Homo sapiens)}
CAICGDRSSGKHYGVYSCEGCKGFFKRTVRKDLTYTCRDNKDCLIDKRQRNRCQYCRYQKCL
AMGM
>d2nllb_ g.39.1.2 (B:) Thyroid hormone receptor (TR-beta) DNA-binding domain {Human (Homo sapiens)}
DELCVVCGDKATGYHYRCITCEGCKGFFRRTIQKNLHPSYSCKYEGKCVIDKVTRNQCQECR
FKKCIYVGMATDLVLDDSKRLAKRKLIEENREKRRREELEK
>d1cita_ g.39.1.2 (A:) Orphan nuclear receptor NGFI-B {Rat (Rattus norvegicus)}
GRCAVCGDNASCQHYGVRTCEGCKGFFKRTVQKSAKYICLANKDCPVDKRRRNRCQFCRFQK
CLAVGMVKEVVRTDSLKGRRGRLPSKP
>d1hcqa_ g.39.1.2 (A:) Estrogen receptor DNA-binding domain {Human and chicken (Homo sapiens) and (Gallus gallus)}
MKETRYCAVCNDYASGYHYGVWSCEGCKAFFKRSIQGHNDYMCPATNQCTIDKNRRKSCQAC
RLRKCYEVGMMK
>d1glua_ g.39.1.2 (A:) Glucocorticoid receptor DNA-binding domain {Rat (Rattus norvegicus)}
MKPARPCLVCSDEASGCHYGVLTCGSCKVFFKRAVEGQHNYLCAGRNDCIIDKIRRKNCPAC
RYRKCLQAGMNLEARKTKK
>d1lata_ g.39.1.2 (A:) Glucocorticoid receptor DNA-binding domain {Rat (Rattus norvegicus)}
RPCLVCSDEASGCHYGVLTCEGCKAFFKRAVEGQHNYLCKYEGKCIIDKIRRKNCPACRYRK
CLQAGMNLE
>d2gda__ g.39.1.2 (-) Glucocorticoid receptor DNA-binding domain {Rat (Rattus norvegicus)}

LCLVCSDEASGCHYGVLTCGSCKVFFKRAVEGQHNYLCAGRNDCIIDKIRRKNCPACRYRKC
LQAGMNLEAR
>d1dsza_ g.39.1.2 (A:) Retinoic acid receptor DNA-binding domain {Human (Homo sapiens)}
PCFVCQDKSSGYHYGVSACEGCKGFFRRSIQKNMVYTCHRDKNCIINKVTRNRCQYCRLQKC
FEVGMSKESVRND
>d1hra__ g.39.1.2 (-) Retinoic acid receptor DNA-binding domain {Human (Homo sapiens)}
PPRVYKPCFVCQDKSSGYHYGVSACEGCKGFFRRSIQKNMIYTCHRDKNCVINKVTRNRCQY
CRLQKCFEVGMSKESVRN
>d1a6ya_ g.39.1.2 (A:) Orphan nuclear receptor reverb {Human (Homo sapiens)}
LLCKVCGDVASGFHYGVHACEGCKGFFRRSIQQNIQYKRCLKNENCSIVRINRNRCQQCRFK
KCLSVGMSRDAVRFGR
>d1b8ta1 g.39.1.3 (A:1-35) Cysteine-rich (intestinal) protein, CRP, CRIP {Chicken (Gallus gallus)}
MPNWGGGKKCGVCQKAVYFAEEVQCEGSSFHKSCF
>d1b8ta2 g.39.1.3 (A:36-100) Cysteine-rich (intestinal) protein, CRP, CRIP {Chicken (Gallus gallus)}
LCMVCKKNLDSTTVAVHGDEIYCKSCYGKKYGPKGKGKGMGAGTLSTDKGESLGIKYEEGQS
HRP
>d1b8ta3 g.39.1.3 (A:101-143) Cysteine-rich (intestinal) protein, CRP, CRIP {Chicken (Gallus gallus)}
TNPNASRMAQKVGGSDGCPRCGQAVYAAEKVIGAGKSWHKSCF
>d1b8ta4 g.39.1.3 (A:144-192) Cysteine-rich (intestinal) protein, CRP, CRIP {Chicken (Gallus gallus)}
RCAKCGKSLESTTLADKDGEIYCKGCYAKNFGPKGFGFGQGAGALIHSQ
>d1ctl_1 g.39.1.3 (1-35) Cysteine-rich (intestinal) protein, CRP, CRIP {Chicken (Gallus gallus)}
MAQKVGGSDGCPRCGQAVYAAEKVIGAGKSWHKSC
>d1a7i_1 g.39.1.3 (8-35) Cysteine-rich (intestinal) protein, CRP, CRIP {Japanese quail (Coturnix coturnix japonica), CRP2}
NKCGACGRTVYHAEEVQCDGRSFHRCCF
>d1a7i_2 g.39.1.3 (36-67) Cysteine-rich (intestinal) protein, CRP, CRIP {Japanese quail (Coturnix coturnix japonica), CRP2}
LCMVCRKNLDSTTVAIHDAEVYCKSCYGKKYG
>d1ibia1 g.39.1.3 (A:117-144) Cysteine-rich (intestinal) protein, CRP, CRIP {Japanese quail (Coturnix coturnix japonica), CRP2}
AEKCSRCGDSVYAAEKVIGAGKPWHKNC
>d1ibia2 g.39.1.3 (A:145-175) Cysteine-rich (intestinal) protein, CRP, CRIP {Japanese quail (Coturnix coturnix japonica), CRP2}
FRCAKCGKSLESTTLTEKEGEIYCKGCYAKN
>d1iml_1 g.39.1.3 (1-28) Cysteine-rich (intestinal) protein, CRP, CRIP {Rat (Rattus rattus)}

PKCPKCDKEVYFAERVTSLGKDWHRPCL
>d1iml_2 g.39.1.3 (29-76) Cysteine-rich (intestinal) protein, CRP, CRIP {Rat (Rattus rattus)}
KCEKCGKTLTSGGHAEHEGKPYCNHPCYSAMFGPKGFGRGGAESHTFK
>d1g47a1 g.39.1.3 (A:1-35) Pinch (particularly interesting new Cys-His) protein {Human (Homo sapiens)}
MANALASATCERCKGGFAPAEKIVNSNGELYHEQC
>d1g47a2 g.39.1.3 (A:36-70) Pinch (particularly interesting new Cys-His) protein {Human (Homo sapiens)}
FVCAQCFQQFPEGLFYEFEGRKYCEHDFQMLFAPC
>d1zfo__ g.39.1.4 (-) LASP-1 {Pig (Sus scrofa)}
MNPNCARCGKIVYPTEKVNCLDKFWHKACF
>d1d4ua2 g.39.1.5 (A:1-36) DNA repair factor XPA DNA- and RPA-binding domain, N-terminal subdomain {Human (Homo sapiens)}
MEFDYVICEECGKEFMDSYLMDHFDLPTCDDCRDAD
>d1xpa_2 g.39.1.5 (98-133) DNA repair factor XPA DNA- and RPA-binding domain, N-terminal subdomain {Human (Homo sapiens)}
MEFDYVICEECGKEFMDSYLMNHFDLPTCDNCRDAD
>d1jj2t_ g.39.1.6 (T:) Ribosomal protein L24e {Archaeon Haloarcula marismortui}
RECDYCGTDIEPGTGTMFVHKDGATTHFCSSKCENNADLGREARNLEWTDTAR
>d1fjgn_ g.39.1.7 (N:) Ribosomal protein S14 {Thermus thermophilus}
ARKALIEKAKRTPKFKVRAYTRCVRCGRARSVYRFFGLCRICLRELAHKGQLPGVRKASW
>d1aaf__ g.40.1.1 (-) HIV nucleocapsid {Human immunodeficiency virus type 1, different isolates}
MQRGNFRNQRKIIKCFNCGKEGHIAKNCRAPRKRGCWKCGKEGHQMKDCTERQAN
>d1eska_ g.40.1.1 (A:) HIV nucleocapsid {Human immunodeficiency virus type 1, different isolates}
NVKCFNCGKEGHTARNCRAPRKKGCWKCGKEGHQMKDCTERQ
>d1f6ua_ g.40.1.1 (A:) HIV nucleocapsid {Human immunodeficiency virus type 1, different isolates}
MQKGNFRNQRKTVKCFNCGKEGHIAKNCRAPRKKGCWKCGKEGHQMKDCTERQAN
>d1nc8__ g.40.1.1 (-) HIV nucleocapsid {Human immunodeficiency virus type 2}
AQQRKVIRCWNCGKEGHSARQCRAPRRQG
>d1cl4a_ g.40.1.1 (A:) Nucleocapsid protein from mason-pfizer monkey virus (MPMV) {Mason-pfizer monkey virus}
VPGLCPRCKRGKHWANECKSKTDNQGNPIPPH
>d1a6bb_ g.40.1.1 (B:) Zinc finger protein ncp10 {Moloney murine leukemia virus}
GERRRSQLDRDQCAYCKEKGHWAKDCPKKPRGPRGPRPQT
>d1dsqa_ g.40.1.1 (A:) Nucleic acid binding protein p14 {Mouse mammary tumor virus}

KGPVCFSCGKTGHIKRDCKEE
>d1dsva_ g.40.1.1 (A:) Nucleic acid binding protein p14 {Mouse mammary tumor virus}
PPGLCPRCKKGYHWKSECKSKFDKDGNPLPP
>d1f4la3 g.41.1.1 (A:141-175) Methionyl-tRNA synthetase (MetRS), Zn-domain {Escherichia coli}
VKGTCPKCKSPDQYGDNCEVCGATYSPTELIEPKS
>d1mea__ g.41.1.1 (-) Methionyl-tRNA synthetase (MetRS), Zn-domain {Escherichia coli}
GSDRFVKGTCPKCKSPDQYGDNCEVCGA
>d1zin_2 g.41.2.1 (126-160) Microbial and mitochondrial ADK, insert "zinc finger" domain {Bacillus stearothermophilus}
GRRICRNCGATYHLIFHPPAKPGVCDKCGGELYQR
>d1e4ya2 g.41.2.1 (A:122-156) Microbial and mitochondrial ADK, insert "zinc finger" domain {Escherichia coli}
GRRVHAPSGRVYHVKFNPPKVEGKDDVTGEELTTR
>d2ak3a2 g.41.2.1 (A:125-161) Microbial and mitochondrial ADK, insert "zinc finger" domain {Cow (Bos taurus), mitochondrial izozyme-3}
ARWIHPGSGRVYNIEFNPPKTMGIDDLTGEPLVQRED
>d1ak2_2 g.41.2.1 (147-176) Microbial and mitochondrial ADK, insert "zinc finger" domain {Cow (Bos taurus), mitochondrial izozyme-2}
PQSGRSYHEEFNPPKEPMKDDITGEPLIRR
>d1aky_2 g.41.2.1 (131-168) Microbial and mitochondrial ADK, insert "zinc finger" domain {Baker's yeast (Saccharomyces cerevisiae)}
GRLIHPASGRSYHKIFNPPKEDMKDDVTGEALVQRSDD
>d1zaka2 g.41.2.1 (A:128-158) Microbial and mitochondrial ADK, insert "zinc finger" domain {Maize (Zea mays)}
GRRLDPVTGKIYHLKYSPPENEEIASRLTQR
>d1tfi__ g.41.3.1 (-) Transcriptional factor SII, C-terminal domain {Human (Homo sapiens)}
KTGGTQTDLFTCGKCKKKNCTYTQVQTRSADEPMTTFVVCNECGNRWKFC
>d1pft__ g.41.3.1 (-) Transcription inititiation factor TFIIB, N-terminal domain {Archaeon Pyrococcus furiosus}
MVNKQKVCPACESAELIYDPERGEIVCAKCGYVIEENIIDMGPEWRAFDA
>d1dl6a_ g.41.3.1 (A:) Transcription inititiation factor TFIIB, N-terminal domain {Human (Homo sapiens)}
ASTSRLDALPRVTCPNHPDAILVEDYRAGDMICPECGLVVGDRVIDVGSEWRTFSNDK
>d1d0qa_ g.41.3.2 (A:) Zinc-binding domain of DNA primase {Bacillus stearothermophilus}
GHRIPEETIEAIRRGVDIVDVIGEYVQLKRQGRNYFGLCPFHGEKTPSFSVSPEKQIFHCFG
CGAGGNAFTFLMDIEGIPFVEAAKRLAAKAGVDLSVYELD

>d1yua_1 g.41.3.3 (1-65) Prokariotic DNA topoisomerase I, a C-terminal fragment {Escherichia coli}
MNGEVAPPKEDPVPLPELPCEKSDAYFVLRDGAAGVFLAANTFPKSRETRAPLVEELYRFRD
RLP
>d1yua_2 g.41.3.3 (66-122) Prokariotic DNA topoisomerase I, a C-terminal fragment {Escherichia coli}
EKLRYLADAPQQDPEGNKTMVRFSRKTKQQYVSSEKDGKATGWSAFYVDGKWVEGKK
>d1qf8a_ g.41.4.1 (A:) Casein kinase II beta subunit {Human (Homo sapiens)}
VSWISWFCGLRGNEFFCEVDEDYIQDKFNLTGLNEQVPHYRQALDMILDLEPDEELEDNPNQ
SDLIEQAAEMLYGLIHARYILTNRGIAQMLEKYQQGDFGYCPRVYCENQPMLPIGLSDIPGE
AMVKLYCPKCMDVYTPKSSRHHHTDGAYFGTGFPHMLFMVHPEYRPKRP
>d1rb9__ g.41.5.1 (-) Rubredoxin {Desulfovibrio vulgaris}
MKKYVCTVCGYEYDPAEGDPDNGVKPGTSFDDLPADWVCPVCGAPKSEFEAA
>d2rdva_ g.41.5.1 (A:) Rubredoxin {Desulfovibrio vulgaris}
MKKYVCTVCGYEYDPAEGDPDNGVKPGTAFEDVPADWVCPICGAPKSEFEPA
>d1rdg__ g.41.5.1 (-) Rubredoxin {Desulfovibrio gigas}
MDIYVCTVCGYEYDPAKGDPDSGIKPGTKFEDLPDDWACPVCGASKDAFEKQ
>d6rxn__ g.41.5.1 (-) Rubredoxin {Desulfovibrio desulfuricans, strain 27774}
MQKYVCNVCGYEYDPAEHDNVPFDQLPDDWCCPVCGVSKDQFSPA
>d1iro__ g.41.5.1 (-) Rubredoxin {Clostridium pasteurianum}
MKKYTCTVCGYIYNPEDGDPDNGVNPGTDFKDIPDDWVCPLCGVGKDQFEEVE
>d5rxn__ g.41.5.1 (-) Rubredoxin {Clostridium pasteurianum}
MKKYTCTVCGYIYDPEDGDPDDGVNPGTDFKDIPDDWVCPLCGVGKDEFEEVEE
>d1brfa_ g.41.5.1 (A:) Rubredoxin {Archaeon Pyrococcus furiosus}
AKWVCKICGYIYDEDAGDPDNGISPGTKFEELPDDWVCPICGAPKSEFEKLED
>d1qcva_ g.41.5.1 (A:) Rubredoxin {Archaeon Pyrococcus furiosus}
AKWVLKITGYIYDEDAGDPDNGISPGTKFEELPDDWVAPITGAPKSEFEKLED
>d1dx8a_ g.41.5.1 (A:) Rubredoxin {Guillardia theta}
MEIDEGKYECEACGYIYEPEKGDKFAGIPPGTPFVDLSDSFMCPACRSPKNQFKSIKKVIAG
FAENQKYG
>d1h7va_ g.41.5.1 (A:) Rubredoxin {Guillardia theta}
MEIDEGKYECEACGYIYEPEKGDKFAGIPPGTPFVDLSDSFMCPACRSPKNQFKSIKKVI
>d1dvba2 g.41.5.1 (A:148-191) Rubrerythrin, C-terminal domain {Desulfovibrio vulgaris}
FLREQATKWRCRNCGYVHEGTGAPELCPACAHPKAHFELLGINW
>d1dxga_ g.41.5.2 (A:) Desulforedoxin {Desulfovibrio gigas}
ANEGDVYKCELCGQVVKVLEEGGGTLVCCGEDMVKQ
>d1dfx_2 g.41.5.2 (1-36) Desulfoferrodoxin N-terminal domain {Desulfovibrio desulfuricans}
PKHLEVYKCTHCGNIVEVLHGGGAELVCCGEPMKHM
>d1ocrf_ g.41.5.3 (F:) Cytochrome c oxidase Subunit F {Cow (Bos taurus)}

ASGGGVPTDEEQATGLEREVMLAARKGQDPYNILAPKATSGTKEDPNLVPSITNKRIVGCIC
EEDNSTVIWFWLHKGEAQRCPSCGTHYKLVPHQLAH
>d1gh9a_ g.41.6.1 (A:) Hypothetical protein MTH1184 {Archaeon Methanobacterium thermoautotrophicum}
MYIIFRCDCGRALYSREGAKTRKCVCGRTVNVKDRRIFGRADDFEEASELVRKLQEEKYGSC
HFTNPSKRE
>d1d09b2 g.41.7.1 (B:101-153) Aspartate carbamoyltransferase, Regulatory-chain, C-terminal domain {Escherichia coli}
ERIDNVLVCPNSNCISHAEPVSSSFAVRKRANDIALKCKYCEKEFSHNVVLAN
>d2atcb2 g.41.7.1 (B:101-152) Aspartate carbamoyltransferase, Regulatory-chain, C-terminal domain {Escherichia coli}
ERNIDVLVCPDSNCISHAEPVSSSFAVRRADDIALKCKYCEKEFSHNVVLAN
>d1ffkw_ g.41.8.1 (W:) Ribosomal protein L37ae {Archaeon Haloarcula marismortui}
PTGRFGPRYGLKIRVRVRDVEIKHKKKYKCPVCGFPKLKRASTSIWVCGHCGYKIAGGAYTP
ETVAGKAVMKA
>d1jj2y_ g.41.8.1 (Y:) Ribosomal protein L37ae {Archaeon Haloarcula marismortui}
RTGRFGPRYGLKIRVRVADVEIKHKKKHKCPVCGFKKLKRAGTGIWMCGHCGYKIAGGCYQP
ETVAGKAVMKA
>d1jj2z_ g.41.8.2 (Z:) Ribosomal protein L37e {Archaeon Haloarcula marismortui}
TGAGTPSQGKKNTTTHTKCRRCGEKSYHTKKKVCSSCGFGKSAKRRDYEWQSKAGE
>d1jj22_ g.41.8.3 (2:) Ribosomal protein L44e {Archaeon Haloarcula marismortui}
MQMPRRFNTYCPHCNEHQEHEVEKVRSGRQTGMKWIDRQRERNSGIGNDGKFSKVPGGDKPT
KKTDLKYRCGECGKAHLREGWRAGRLEFQE
>d1qyp__ g.41.9.1 (-) RBP9 subunit of RNA polymerase II {Archaeon Thermococcus celer}
GSHMEQDLKTLPTTKITCPKCGNDTAYWWEMQTRAGDEPSTIFYKCTKCGHTWRSYE
>d1i50i1 g.41.9.1 (I:1-49) RBP9 subunit of RNA polymerase II {Baker's yeast (Saccharomyces cerevisiae)}
MTTFRFCRDCNNMLYPREDKENNRLLFECRTCSYVEEAGSPLVYRHELI
>d1i50i2 g.41.9.1 (I:50-122) RBP9 subunit of RNA polymerase II {Baker's yeast (Saccharomyces cerevisiae)}
TNIGETAGVVQDIGSDPTLPRSDRECPKCHSRENVFFQSQQRRKDTSMVLFFVCLSCSHIFT
SDQKNKRTQFS
>d1i50l_ g.41.9.2 (L:) RBP12 subunit of RNA polymerase II {Baker's yeast (Saccharomyces cerevisiae)}
ATLKYICAECSSKLSLSRTDAVRCKDCGHRILLKARTKRLVQFEAR
>d1dfea_ g.42.1.1 (A:) Ribosomal protein L36 {Thermus thermophilus}
MKVRASVKRICDKCKVIRRHGRVYVICENPKHKQRQG
>d1fre__ g.43.1.1 (-) Nuclear factor XNF7 {African clawed frog

(Xenopus laevis)}
EKCSEHDERLKLYCKDDGTLSCVICRDSLKHASHNFLPI
>d1fbva4 g.44.1.1 (A:356-434) CBL {Human (Homo sapiens)}
TPQDHIKVTQEQYELYCEMGSTFQLCKICAENDKDVKIEPCGHLMCTSCLTSWQESEGQGCP
FCRCEIKGTEPIVVDPF
>d1rmd_2 g.44.1.1 (1-86) V(D)J recombination activating protein 1 (RAG1), dimerization domain {Mouse (Mus musculus)}
NCSKIHLSTKLLAVDFPAHFVKSISCQICEHILADPVETSCKHLFCRICILRCLKVMGSYCP
SCRYPCFPTDLESPVKSFLNILNS
>d1chc__ g.44.1.1 (-) Immediate early protein, IEEHV {Equine herpes virus type 1}
MATVAERCPICLEDPSNYSMALPCLHAFCYVCITRWIRQNPTCPLCKVPVESVVHTIESDSE
FGDQLI
>d1bor__ g.44.1.1 (-) Acute promyelocytic leukaemia proto-onkoprotein PML {Human (Homo sapiens)}
EEEFQFLRCQQCQAEAKCPKLLPCLHTLCSGCLEASGMQCPICQAPWPLGADTPAL
>d1g25a_ g.44.1.1 (A:) TFIIH Mat1 subunit {Human (Homo sapiens)}
MDDQGCPRCKTTKYRNPSLKLMVNVCGHTLCESCVDLLFVRGAGNCPECGTPLRKSNFRVQL
FED
>d1e4ua_ g.44.1.1 (A:) Not-4 N-terminal RING finger domain {Human (Homo sapiens)}
MSRSPDAKEDPVECPLCMEPLEIDDINFFPCTCGYQICRFCWHRIRTDENGLCPACRKPYPE
DPAVYKPLSQEELQRI
>d1jm7a_ g.44.1.1 (A:) brca1 RING domain {Human (Homo sapiens)}
MDLSALRVEEVQNVINAMQKILECPICLELIKEPVSTKCDHIFCKFCMLKLLNQKKGPSQCP
LCKNDITKRSLQESTRFSQLVEELLKIICAFQLDTGLEYAN
>d1jm7b_ g.44.1.1 (B:) bard1 RING domain {Human (Homo sapiens)}
MEPDGRGAWAHSRAALDRLEKLLRCSRCTNILREPVCLGGCEHIFCSNCVSDCIGTGCPVCY
TPAWIQDLKINRQLDSMIQLCSKLRNLLHDNELSD
>d1dcqa2 g.45.1.1 (A:247-368) Pyk2-associated protein beta ARF-GAP domain {Mouse (Mus musculus)}
LTKEIISEVQRMTGNDVCCDCGAPDPTWLSTNLGILTCIECSGIHRELGVHYSRMQSLTLDV
LGTSELLLAKNIGNAGFNEIMECCLPSEDPVKPNPGSDMIARKDYITAKYMERRYARKKH
>d1mhu__ g.46.1.1 (-) Metallothionein {Human (Homo sapiens)}
KSCCSCCPVGCAKCAQGCICKGASDKCSCCA
>d2mhu__ g.46.1.1 (-) Metallothionein {Human (Homo sapiens)}
MDPNCSCAAGDSCTCAGSCKCKECKCTSCK
>d2mrb__ g.46.1.1 (-) Metallothionein {Rabbit (Oryctolagus cuniculus)}
MDPNCSCAAAGDSCTCANSCTCKACKCTSCK
>d1mrt__ g.46.1.1 (-) Metallothionein {Rat (Rattus rattus)}
KSCCSCCPVGCAKCSQGCICKEASDKCSCCA
>d2mrt__ g.46.1.1 (-) Metallothionein {Rat (Rattus rattus)}
MDPNCSCATDGSCSCAGSCKCKQCKCTSCK

>d4mt2__ g.46.1.1 (-) Metallothionein {Rat (Rattus rattus)}
MDPNCSCATDGSCSCAGSCKCKQCKCTSCKKSCCSCCPVGCAKCSQGCICKEASDKCSCCA
>d1dfsa_ g.46.1.1 (A:) Metallothionein {Mouse (Mus musculus)}
KSCCSCCPVGCSKCAQGCVCKGAADKCTCCA
>d1dfta_ g.46.1.1 (A:) Metallothionein {Mouse (Mus musculus)}
MDPNCSCSTGGSCTCTSSCACKNCKCTSCK
>d1ji9a_ g.46.1.1 (A:) Metallothionein {Mouse (Mus musculus)}
KSCCSCCPAGCEKCAKDCVCKGEEGAKAEAEKCSCCQ
>d1dmc__ g.46.1.1 (-) Metallothionein {Crab (Callinectes sapidus), alpha and beta domains}
SPCQKCTSGCKCATKEECSKTCTKPCSCCPK
>d1dme__ g.46.1.1 (-) Metallothionein {Crab (Callinectes sapidus), alpha and beta domains}
PGPCCNDKCVCQEGGCKAGCQCTSCRCS
>d1fmya_ g.46.1.1 (A:) Metallothionein {Baker's yeast (Saccharomyces cerevisiae)}
QNEGHECQCQCGSCKNNEQCQKSCSCPTGCNSDDKCPCGN
>d1qjka_ g.46.1.1 (A:) Metallothionein {Purple sea urchin (Strongylocentrotus purpuratus)}
PDVKCVCCTEGKECACFGQDCCVTGECCKDGTCCGI
>d1qjla_ g.46.1.1 (A:) Metallothionein {Purple sea urchin (Strongylocentrotus purpuratus)}
ICTNAACKCANGCKCGSGCSCTEGNCAC
>d1jjda_ g.46.1.1 (A:) Cyanobacterial metallothionein SmtA {Synechococcus sp., PCC 7942}
TLVKCACEPCLCNVDPSKAIDRNGLYYCSEACADGHTGGSKGCGHTGCNCHG
>d1co4a_ g.47.1.1 (A:) Zinc domain conserved in yeast copper-regulated transcription factors {Synthetic}
MVVINGVKYACDSCIKSHKAAQCEHNDRPLKILKPRGRPPTT
>d1adn__ g.48.1.1 (-) Ada DNA repair protein, N-terminal domain (N-Ada 10) {Escherichia coli}
MKKATCLTDDQRWQSVLARDPNADGEFVFAVRTTGIFCRPSCRARHALRENVSFYANASEAL
AAGFRPCKRCQPDKANPRQHRLDKITHACR
>d1ptq__ g.49.1.1 (-) Protein kinase C-delta (PKCdelta) {Mouse (Mus musculus)}
HRFKVYNYMSPTFCDHCGSLLWGLVKQGLKCEDCGMNVHHKCREKVANLC
>d1faq__ g.49.1.1 (-) RAF-1 {Human (Homo sapiens)}
LTTHNFARKTFLKLAFCDICQKFLLNGFRCQTCGYKFHEHCSTKVPTMCVDW
>d1tbo__ g.49.1.1 (-) Protein kinase c-gamma {Rat (Rattus rattus)}
QTDDPRNKHKFRLHSYSSPTFCDHCGSLLYGLVHQGMKCSCCEMNVHRRCVRSVPSLCGVDH
TERR
>d1kbea_ g.49.1.1 (A:) Kinase suppressor of Ras, Ksr {Mouse (Mus musculus)}
GSVTHRFSTKSWLSQVCNVCQKSMIFGVKCKHCRLKCHNKCTKEAPACR

>d1e53a_ g.49.1.2 (A:) TFIIH p44 subunit cysteine-rich domain {Human (Homo sapiens)}
LDAFQEIPLEEYNGERFCYGCQGELKDQHVYVCAVCQNVFCVDCDVFVHDSLHCCPGCI
>d1vfya_ g.50.1.1 (A:) vps27p protein {Baker's yeast (Saccharomyces cerevisiae)}
DWIDSDACMICSKKFSLLNRKHHCRSCGGVFCQEHSSNSIPLPDLGIYEPVRVCDSCFEDYE
FIVTD
>d1joca1 g.50.1.1 (A:1348-1411) Eea1 {Human (Homo sapiens)}
KWAEDNEVQNCMACGKGFSVTVRRHHCRQCGNIFCAECSAKNALTPSSKKPVRVCDACFNDL
QG
>d1dvpa2 g.50.1.1 (A:149-220) Hrs {Fruit fly (Drosophila melanogaster)}
MFTADTAPNWADGRVCHRCRVEFTFTNRKHHCRNCGQVFCGQCTAKQCPLPKYGIEKEVRVC
DGCFAALQRG
>d1zbdb_ g.50.1.1 (B:) Effector domain of rabphilin-3a {Rat (Rattus norvegicus)}
EELTDEEKEIINRVIARAEKMETMEQERIGRLVDRLETMRKNVAGDGVNRCILCGEQLGMLG
SASVVCEDCKKNVCTKCGVETSNNRPHPVWLCKICLEQREVWKRSGAWFFKGFPKQVLPQPM
>d1f62a_ g.50.1.2 (A:) Williams-Beuren syndrome transcription factor, WSTF {Human (Homo sapiens)}
ARCKVCRKKGEDDKLILCDECNKAFHLFCLRPALYEVPDGEWQCPACQPAT
>d1fp0a1 g.50.1.2 (A:19-88) Nuclear corepressor KAP-1 (TIF-1beta) {Human (Homo sapiens)}
GTLDDSATICRVCQKPGDLVMCNQCEFCFHLDCHLPALQDVPGEEWSCSLCHVLPDLKEEDV
DLQACKLN
>d1adt_2 g.51.1.1 (266-385) First Zn-domain of early E2A DNA-binding protein, ADDBP {Human adenovirus type 5}
TGCALWLHRCAEIEGELKCLHGSIMINKEHVIEMDVTSENGQRALKEQSSKAKIVKNRWGRN
VVQISNTDARCCVHDAACPANQFSGKSCGMFFSEGAKAQVAFKQIKAFMQALYPNAQT
>d1adt_3 g.51.1.1 (386-529) Second Zn-domain of early E2A DNA-binding protein, ADDBP {Human adenovirus type 5}
GHGHLLMPLRCECNSKPGHAPFLGRQLPKLTPFALSNAEDLDADLISDKSVLASVHHPALIV
FQCCNPVYRNSRAQGGGPNCDFKISAPDLLNALVMVRSLWSENFTELPRMVVPQFKWSTKHQ
YRNVSLPVAHSDARQNPFDF
>d1qbha_ g.52.1.1 (A:) 2MIHB/C-IAP-1 {Human (Homo sapiens)}
GSHMQTHAARMRTFMYWPSSVPVQPEQLASAGFYYVGRNDDVKCFCCDGGLRCWESGDDPWV
EHAKWFPRCEFLIRMKGQEFVDEIQGRYPHLLEQLLSTS
>d1f9xa_ g.52.1.1 (A:) BIR domains of XIAP {Human (Homo sapiens)}
MSDAVSSDRNFPNSTNLPRNPSMADYEARIFTFGTWIYSVNKEQLARAGFYALGEGDKVKCF
HCGGGLTDWKPSEDPWEQHAKWYPGCKYLLEQKGQEYINNIHLTHSLEECLVRTT
>d1g73c_ g.52.1.1 (C:) BIR domains of XIAP {Human (Homo sapiens)}
LPRNPSMADYEARIFTFGTWIYSVNKEQLARAGFYALGEGDKVKCFHCGGGLTDWKPSEDPW
EQHAKWYPGCKYLLEQKGQEYINNIHL
>d1g73d_ g.52.1.1 (D:) BIR domains of XIAP {Human (Homo sapiens)}

LPRNPSMADYEARIFTFGTWIYSVNKEQLARAGFYALGEGDKVKCFHCGGGLTDWKPSEDPW
EQHAKWYPGCKYLLEQKGQEYINNIHLTHSLEECLVRTTE
>d1i3oe_ g.52.1.1 (E:) BIR domains of XIAP {Human (Homo sapiens)}
FALDRPSETHADYLLRTGQVVDISDTIYPRNPAMYSEEARLKSFQNWPDYAHLTPRELASAG
LYYTGIGDQVQCFACGGKLKNWEPGDRAWSEHRRHFPNCFFVLGRNLNI
>d1jd5a_ g.52.1.1 (A:) BIR2 domain of DIAP1 {Fruit fly (Drosophila melanogaster)}
GNYFPQYPEYAIETARLRTFEAWPRNLKQKPHQLAEAGFFYTGVGDRVRCFSCGGGLMDWND
NDEPWEQHALWLSQCRFVKLMKGQLYIDTVAAKPVLAEEKEES
>d1e31a_ g.52.1.1 (A:) Anti-apoptotic protein survivin {Human (Homo sapiens)}
TLPPAWQPFLKDHRISTFKNWPFLEGCACTPERMAEAGFIHCPTENEPDLAQCFFCFKELEG
WEPDDDPIEEHKKHSSGCAFLSVKKQFEELTLGEFLKLDRERAKNKIAKETNNKKKEFEETA
KKVRRAIEQLAA
>d1f81a_ g.53.1.1 (A:) CREB-binding transcriptional adaptor protein CBP {Mouse (Mus musculus)}
SPQESRRLSIQRCIQSLVHACQCRNANCSLPSCQKMKRVVQHTKGCKRKTNGGCPVCKQLIA
LCCYHAKHCQENKCPVPFCLNIKHK
>d1hc7a3 g.56.1.1 (A:404-477) C-terminal domain of ProRS {Thermus thermophilus}
TRKVDTYEAFKEAVQEGFALAFHCGDKACERLIQEETTATTRCVPFEAEPEEGFCVRCGRPS
AYGKRVVFAKAY
>d1exka_ g.54.1.1 (A:) Cysteine-rich domain of the chaperone protein DnaJ. {Escherichia coli}
GVTKEIRIPTLEECDVCHGSGAKPGTQPQTCPTCHGSGQVQMRQGFFAVQQTCPHCQGRGTL
IKDPCNKCHGHGRVERS
